# Supplementary material for: The challenges arising from the COVID-19 pandemic and the way people deal with them. A qualitative longitudinal study
Source: PLoS One. 2021 Oct 11;16(10):e0258133. doi: 10.1371/journal.pone.0258133 (PMC8504766; doi:10.1371/journal.pone.0258133)
Supplement: S1 Table — (DOCX) [file pone.0258133.s001.docx]

**S1 Table. A full description of the changes occurring in Poland at the time of the study.**

| **Before the study:  04.03-29.03.2020** | **4.03.2020** - first patient diagnosed with COVID-19 in Poland. **11.03.2020** - governmental decision to close educational and cultural facilities. Recommendation to switch to remote work if possible (**31 confirmed cases, no deaths**). **13.03.2020** - the state of epidemic threat is introduced to Poland. Restoration of border control. Ban on gatherings of over 50 people. The obligation to quarantine after returning from abroad (**68 confirmed cases, 1 death**).  **20.03.2020** - the state of epidemic is introduced to Poland (**425 confirmed cases, 5 deaths**). **24.03.2020** - decision to tighten the self-isolation rules: ban on gatherings of more than 2 people, limiting exits to the absolute minimum (**901 confirmed cases, 10 deaths**). |
| --- | --- |
| **1st wave of the study:  30.03-2.04.2020** | **Confirmed cases for 30.03.2020: 1984 and 26 deaths.** **31.03.2020** - The government is introducing further quarantine restrictions, including the rule to keep min. 2 meters distance from each other on the street. Changes in the trade rules: senior hours - stores and pharmacies are open from 10-12 only for people over 65 years of age, the obligation to wear gloves in stores, the rule of maximum 3 people for one cash register in the store. Minors may leave the house only under adult supervision. Parks and forests are closed. |
| **2nd wave of the study:  6.04-10.04.2020** | **Confirmed cases for 6.04.2020: 4412 and 107 deaths.** **9.04.2020** - The government is extending the existing restrictions until April 26. Shopping malls, cultural facilities, hairdressers closed until April 19. The government also makes decision to postpone state exams, including high school-leaving exams. The obligation to cover nose and mouth for everyone, who leaves the house is introduced from April 16. |
| **3rd wave of the study:  20.04-24.04.2020** | **Confirmed cases for 20.04.2020: 9593 and 380 deaths.** **20.04.2020** - the first stage of lifting the restrictions. Restoration of access to forests and parks, as well as increasing the number of people in the store to 4 for cash. 1 person per 15m2 will be able to stay in the church. Persons under 18 years of age and over 13 years of age will be able to leave the house on their own. |
| **4th wave of the study:  4.05-8.05.2020** | **Confirmed cases for 04.05.2020: 14 006 and 698 deaths.** **4.05.2020** - the second stage of lifting the restrictions. Restrictions on trade partly lifted: shopping centers and large stores open, but with a limited number of people and with no food zones opened. Construction stores open on weekends. Opening of the hotels but without swimming pools, gyms and restaurants. Cultural institutions will be gradually opened after consultation with the Sanitary Inspector. Children of working parents will be able to return to nurseries or kindergartens. |
| **Break before the 5th wave of the study: 9.05-31.05.2020** | **10.05.2020 -** governmental decision not to hold presidential elections planned for May 10 (**15 366 confirmed cases and 776 deaths**). **18.05.2020** - the third stage of lifting the restrictions. Restaurants, cafes, bars and sports facilities can be open in line with the safety rules. Beauty and hair studios can operate in a way to ensure the safety of service providers and clients. The passengers limit in public transport: half of the seats (**17 204 confirmed cases and 861 deaths**). **30.05.2020** - the fourth stage of lifting the restrictions. Lifting the obligation to cover the mouth and nose in open space (if 2-meters social distance is maintained), at work (if an employer provides a relevant distance between the working stations) and in bars/restaurants while sitting. It is allowed to organize assemblies in open spaces and outdoor concerts up to 150 people. Abolish of the limits of people in the trade and catering industry, worship places and post office. Under certain sanitary conditions the cultural and sports facilities, such as gyms and swimming pools are able to resume their activities from 6th June. Obligation to cover mouth and nose is still in the effect in closed spaces. 2 meters social distance is still in the effect outdoors (**23 571 confirmed cases and 1061 deaths**). |
| **5th wave of the study:  1.06-12.06.2020** | **Confirmed cases for 01.06.2020: 24 165 and 1074 deaths.** **08.06.2020** - record daily increase in infections - 599 cases (in total: **27 160 confirmed cases and 1166 deaths**). |
| **Break before the 6th wave of the study: 12.06-26.10.2020** | **28.06.2020** - the first round of presidential elections (**33 980 confirmed cases and 1438 deaths**) **21.07.2020** - further lifting the restrictions: shortening the social distance in public space from 2 meters up to 1.5; swimming pools no longer have the limit of the number of users (**40 782 confirmed cases and 1636 deaths**) **5.08.2020** - governmental decision that students will return to schools from September (**48 789 confirmed cases and 1756 deaths**) **08.08.2020** - record daily increase in infections - 809  **9.08.2020** - the government introduces new regulations related to pandemic prevention. There are "red" and "yellow" counties with stricter rules than in the "green" ones (**51 791 confirmed cases and 1807 deaths**) **10.10.2020** - the whole country (besides counties, which were declared "red" zones) is declared the "yellow" zone with new restrictions: the obligation to cover nose and mouth for everyone, who leaves the house; restaurants with limited opening hours; limiting the number of people in events to 75; cultural events with the participation of 25% of the audience (**125 816 confirmed cases and 3004 deaths**). **24.10.2020** - the whole country is declared the "red" zone with new restrictions: distance learning for students from grade 4; closure of restaurants with the exception of take-away orders; ban on gatherings of more than 5 people (**241 946 confirmed cases and 4351 deaths**). |
| **6th wave of the study:  27.10-5.11.2020** | **Confirmed cases for 27.10.2020: 280 229 and 4615 deaths. 5.11.2020** - record daily increase in infections - 27 143**;** introducing further restrictions: shopping malls and cultural facilities closed (**466 679 confirmed cases and 6842 deaths**). |
| Sources:  1. JHU CSEE. COVID-19 Data Repository by the Center for Systems Science and Engineering (CSSE) at Johns Hopkins University. 2020 [cited 2021 Jun 1]. Available from: https://github.com/CSSEGISandData/COVID-19#covid-19-data-repository-by-the-center-for-systems-science-and-engineering-csse-at-johns-hopkins-university. 2. Polish Academy of Sciences. Understanding COVID-19. Report by the COVID-19 team at the President of the Polish Academy of Sciences. 2020 Sep 14. [Cited 2021 Jun 1]. Available from: https://informacje.pan.pl/images/2020/opracowanie-covid19-14-09-2020/ZrozumiecCovid19_opracowanie_PAN.pdf | |
