# Supplementary material for: The challenges arising from the COVID-19 pandemic and the way people deal with them. A qualitative longitudinal study
Source: PLoS One. 2021 Oct 11;16(10):e0258133. doi: 10.1371/journal.pone.0258133 (PMC8504766; doi:10.1371/journal.pone.0258133)
Supplement: S2 Table — (DOCX) [file pone.0258133.s002.docx]

**S2 Table. Characteristics of study participants.**

| **No.** | **Gender (*)** | **Age** | **Family status** | **Education**  **(**)** | **Vocational circumstance** | **How did the coronavirus pandemic affect their vocational circumstance?** |
| --- | --- | --- | --- | --- | --- | --- |
| 1 | F | 25 | Single | H | Unemployed | Trouble finding a job. |
| 2 | F | 27 | Single | H | Administrative assistant | Partly remote work; 80% of salary. |
| 3 | F | 54 | Single | H | Volunteer work | No volunteer work during pandemic; no income changes. |
| 4 | M | 32 | Couple, no children | H | Unemployed | Job loss was not related to the pandemic; no income changes. |
| 5 | M | 39 | Single | H | Specialist in a fuel company | Remote work, no changes in salary. |
| 6 | M | 24 | Couple, no children | H | Barista in a coffee shop | Not working during lockdown; substantial income decrease. |
| 7 | M | 28 | Couple, no children | H | Communication specialist | Remote work (also before the pandemic); no changes in salary. |
| 8 | M | 30 | Couple, no children | H | PhD candidate | Remote work; no changes in salary. |
| 9 | F | 25 | Couple, no children | H | Accountant | Remote work, no changes in respondent’s salary; husband’s income decrease. |
| 10 | F | 55) | Couple, no children | H | Cosmetic saleswoman | Less work; substantial income decrease. |
| 11 | M | 35 | Two adults and child | S | Gas station and shop owner | Income increase. |
| 12 | M | 33 | Two adults and children | H | Engineer | Remote work; no changes in salary. |
| 13 | M | 46 | Two adults and child | H | Surgeon | Less work; substantial income decrease. |
| 14 | M | 55 | Two adults and children | H | Private kindergarten owner | Remote work (also before the pandemic); substantial income decrease. |
| 15 | M | 43 | Two adults and child | H | Psychologist, entrepreneur | Less work, partly remote; slight income decrease. |
| 16 | F | 36 | Two adults and children | H | Finance specialist; maternity leave | No changes in the way of working and income. |
| 17 | F | 35 | Adult and child | H | Beautician | Not working during lockdown; substantial income decrease. |
| 18 | F | 48 | Two adults and children | S | Beauty salon owner | Change of job; slight income decrease. |
| 19 | F | 39 | Two adults and children | H | Accountant | Remote work; no changes in salary. |
| 20 | F | 25 | Two adults and child | S | Math tutor | Not working during lockdown; substantial income decrease for respondent; no changes in husband’s income. |

(*) F – Female; M – Male

(**) S – Secondary; H – Higher
