# Supplementary material for: The challenges arising from the COVID-19 pandemic and the way people deal with them. A qualitative longitudinal study
Source: PLoS One. 2021 Oct 11;16(10):e0258133. doi: 10.1371/journal.pone.0258133 (PMC8504766; doi:10.1371/journal.pone.0258133)
Supplement: S1 Dataset — (ZIP) [file pone.0258133.s003.zip › Transcriptions/stage 4/10.4_F_55_couple, no children.docx]

**10.4_F_55_couple no children**

**Co się działo przez ostatnie 2 tygodnie?**

Jak to się mówi po angielsku: so, so. Jako tako. Jestem już zmęczona tymi wszystkimi niedogodnościami, szczególnie, że mam tutaj różnych takich na głowie spraw bezpośrednio związanych z tym koronawirusem, jak np. wakacje nad morzem i nie wiadomo, czy te kempingi będą/ nie będą. Teoretycznie już mają być otwarte, ale tam różne z tego powodu są trudności. Bardzo to przeżywam po prostu i jest to już bardzo męczące.

**Jak wyglądała majówka? Coś się działo innego?**

Zupełnie innego, bo byłam w domu i w zasadzie można powiedzieć, że odpoczywałam w domu. Nic nie robiłam specjalnego. W piątek to w ogóle byłam w domu, w sobotę robiłam jakieś zakupy, a w niedzielę znowu byłam w domu.

**Jak pani spędzała czas przez ostatnie 2 tygodnie? Coś się zmieniło?**

Zmieniło się o tyle, że zostałam poproszona na ostatnim spotkaniu, żeby zanotować różne rzeczy. Nie wiem, czy ta rozmowa tak mnie pobudziła bardzo, w każdym razie pamiętam, że tego dnia miałam bardzo dużo różnych spraw jeszcze do pozałatwiania, byłam u ojca, zakupy, ale wieczorem po prostu totalnie się rozpadłam i wpadłam w taką jakąś rozpacz i w taki płacz, wręcz w szloch taki nie do...W ogóle coś okropnego. Dzwoniłam do jednego syna, drugiego...Załamka totalna już nastąpiła i mój syn starszy powiedział, nie ma nawet 2 zdań, w sobotę przywozimy ci Zuzię. No i w piątek cały dzień tutaj w związku z tym jak chora sprzątałam, podłogi, wszystko, bo to przecież małe dziecko i faktycznie od soboty do wtorku miałam wnuczkę.

**Pomogło?**

Bardzo. Było cudownie. Była ładna pogoda dodatkowo i poza tym ta mała jest...Ja już nie widziałam jej ponad miesiąc przecież. Już syn z synową weszli do środka, to dziecko, więc tak w zasadzie można powiedzieć, że nasza izolacja już się zakończyła. Ja już przedtem miałam tak wszystkiego dosyć...Nie wiem, może oni też czekali na moment, kiedy coś takiego się wydarzy, bo już też chcieli odpocząć.

**Jak się pani z tym czuje, że spotkała się pani z synem?**

Dobrze się czuję, tym bardziej, że następnego dnia były pierwsze urodziny mojej wnuczki. We wtorek ją zabrali, no wiadomo, w środę chcieli mieć ją u siebie w domu, bo to urodzinki. Ja akurat wtedy nie mogłam przyjechać, bo miałam ostatni dzień swojego katalogu, więc zawsze muszę to podopinać, natomiast w czwartek pojechałam do nich, poszłyśmy z synową do Pepco, żeby kupić jakieś prezenty, bo tak się umówiłyśmy. Ubranka na lato w dużej ilości dla Zuzi, bo nic tam nie miała na lato. Widziałam się i z Zuzią, i z Zosią - tą starszą córką partnerki syna. Ja tam byłam, w międzyczasie przyjechał też ojciec mojej synowej. Siedzieliśmy w maseczkach, ale z tego co wiem, to wcześniej też się z nimi nie kontaktował, a wtedy też jednak przyjechał. Stwierdziłam, że teraz to już co będzie to będzie.

**Państwo wszyscy siedzieli w maseczkach też w domu?**

Nie, tylko jak był ten dziadek, a potem, jak on wyszedł, to ja zdjęłam to.

Czy pojawiło się coś nowego, jeśli chodzi o codzienne czynności, zajęcia?

Nie, do wtorku miałam dziecko, więc byłam jakby totalnie wyłączona z tego, w środę byłam zajęta w pracy mocno, czwartek też cały dzień jeździłam i w piątek już był dzień wolny, więc sobie odpoczywałam, w sobotę robiłam zakupy. Mąż pojechał z teściową na 1 dzień na działkę, zobaczyć co i jak, natomiast ja się śmiałam, że musi być obiad majówkowy, więc była kiełbasa i kaszanka z grilla domowego i sałatka ziemniaczana. Tradycja majówkowa zachowana. W niedzielę byłam też z domu i w zasadzie w ogóle nie wychodziłam. Dzisiaj mamy czwartek. Tak te dni...Wtorek miałam taki dosyć intensywny, bo musiałam do klientek pochodzić, pozanosić zamówienia. Wczoraj miałam też śmieszną sytuację, bo miałam się spotkać z koleżanką, bo przez moje zamówienia szły jej zamówienia, bo nie opłacało się kupować na ileś kont i musiałyśmy się wczoraj spotkać. Umówiłyśmy się w połowie drogi, Ja miałam jej zawieźć to zamówienie, w międzyczasie odwieźć swoje dokumenty do księgowej, a ona miała dojechać autobusem i miałyśmy się spotkać na przystanku, tyle tylko, że wcześniej wniknęła taka sytuacja, czy przypadkiem dzisiaj nie jechałabym do mamy swojej synowej zrobić różne analizy, badania. Wysłałam do swojej koleżanki sms, żeby się wstrzymała jeszcze z wyjazdem, bo może miało się okazać, że jadę w tamtą stronę i wszystko bym jej podwiozła. Potem zadzwoniła, powiedziałam, że to jednak nieaktualne i umawiamy się tak jak było na 16. Tyle tylko, że ona tego smsa, żeby się wstrzymała z wyjazdem, odczytała potem i myślała, że to już była po naszej rozmowie. Ja pojechałam, czekałam na nią, jak zadzwoniła z pytaniem jak długo ma się wstrzymywać. Ja już byłam na miejscu, więc musiałam czekać aż ona przyjedzie, dojedzie, ale to jeszcze jest nic, bo 4 maja były jej imieniny, więc ja też torebeczkę naszykowałam dla niej - jakieś drobiazgi w ramach prezentu i wychodząc, wzięłam też psa ze sobą, złapałam te 2 siateczki i jak się potem okazało ja nie wzięłam tego zamówienia, więc już  w ogóle był totalny galimatias, bo ona się spóźniła, nie odczytała smsa, ja byłam na miejscu, czekałam na nią, a tak na prawdę nie miałam dla niej tego  zamówienia. Efekt był taki, że ona przyjechała tutaj do domu razem ze mną i tu sobie jeszcze siedziałyśmy i gadałyśmy. No więc też już się spotkałam z nią. Siedziałyśmy w domu bez maseczek, więc...

**To spotkanie też Pani poprawiło humor?**

Śmiałyśmy się z całej tej sytuacji, że to najpierw ja byłam zła na nią, że czekałam, a potem ja ją przepraszałam, że nie mam tego zamówienia. Oczywiście tak trochę...Całować to żeśmy się nie całowały jak zawsze na powitanie, ale gdzieś tam jakaś we mnie taka troszeczkę myśl była, że może źle robimy, ale...Ona siedziała z jednej strony, ja z drugiej. Dystans trochę był zachowany, ale jechałyśmy razem samochodem pół godziny bez maseczek.

**Co stanowi teraz dla pani największe wyzwanie?**

Generalnie nic mi się nie chce i to jest okropne, bo nie mogę się do niczego tak naprawdę...Muszę się przymuszać do tych rzeczy, które już naprawdę muszę zrobić i je robię, natomiast tak, jak zawsze ma się ten swój cykl zajęć i wszystkiego co się robi, co tak naturalnie wychodzi, tak teraz nie. Dzisiaj już nawet z siostrą rozmawiałam, że Boże, żeby mi się tak chciało chcieć, jak mi się nie chce. Po prostu nie chce mi się, nic mi się nie chce. Poza tym zaczynam się znowu denerwować, że mnóstwo tu spraw zaczyna wchodzić mi na głowę i nie wiem, jak to mam wszystko pogodzić, i się tym denerwuję, boli mnie żołądek z boku...Nie wiem, czy to jest żołądek, czy to jest trzustka, czy to jest śledziona, bo to z lewej strony, ale generalnie już w przyszłym tygodniu mam mieć zrobione wszystkie badania przez mamę mojej synowej.

**A te inne rzeczy, to głównie ta kwestia z tą przyczepą kempingową?**

Tak.

**Z tej przyczepy państwo też zarabiają?**

To jest na wynajem, ale ja z niej nie zarabiam, ponieważ to nie wychodzi na zarobek. Ja ją trzymam i tylko po to ją wynajmuję, żeby móc tam przyjeżdżać, bo nie stać nas, żeby zapłacić 16 tysięcy za trzymanie przyczepy. Mamy tę przyczepę już ponad 20 lat i zdaje się, że to będzie ostatni sezon w ogóle w tym roku, bo już wiemy, że od przyszłego roku ma kosztować 22 tysiące, co już jest dla mnie w ogóle kompletnym...I oczywiście kemping też cały czas, że nie, że w tym roku już absolutnie żadnego wynajmowania. No tak, tylko to już od 2 lat jest, że żadnego wynajmowania, ale na zasadzie, że rodzina może przyjeżdżać, więc kto mi powie, że to nie jest rodzina? To trudne wszystko takie jest do wychwycenia. Właściciele kempingów najchętniej by chcieli przyjąć pieniądze i najchętniej, żeby tam nikt nie przyjechał i nikt nie korzystał. Teraz o tyle to jest groźne, że obieg tych ludzi będzie znowu duży na tych kempingach. Z drugiej strony mój mąż mówi, żeby zwrócić te zaliczki i nie stawiajmy tej przyczepy w tym roku, ale ja oczywiście się poorientowałam ze wszystkimi znajomymi, którzy tam jeżdżą i wszyscy stawiają, więc ja znowu nie chcę wyjść na takiego osła, że ja nie postawię, nie wyjadę, nie zrobię nic, a być może już w przyszłym roku jej w ogóle nie będę mogła postawić. Nie ma żadnej pewności, bo gdyby nie koronawirus, to zapowiadałby mi się cudowny sezon, bo już w styczniu miałam wszystkie rezerwacje porobione i żeby było śmieszniej, to wczoraj jeszcze jakaś osoba dzwoniła. Mam nadzieję, że nie podstawiona z kempingu i pani też mnie mam nadzieję nie wyda. W każdym razie, że koniecznie chce, że w zeszłym roku widziała moją przyczepę i że bardzo jej się podobała i ktoś jej udostępnił numer. Dzwoni i chce w tym czasie, kiedy ja jeszcze mam ją wolną, więc to już w ogóle jest takie...Ja teraz chcę tam pojechać w czerwcu, bo w czerwcu ma być piękna pogoda i owszem, ja się tam zawsze strasznie napracuję, ale to taki jest reset też. I naprawdę to jest na łonie natury, bo ja wychodzę i mam 10 kroków do zatoki, po drugiej stronie 200 m do morza. To jest kosmos. Ja chcę tam sobie w czerwcu pobyć i może też przyjadą też dzieci troszeczkę i razem pobędziemy. Od 13 marca moja siostra w ogóle się nie włącza w pomoc dla mojego ojca i ja już jestem tym nie tyle zmęczona, bo ja tam raz na tydzień tylko jeżdżę i robię zakupy, ale z drugiej strony już słyszę, że wychodzi do Lidla, że wychodzi, tutaj, tam, czyli też się styka, więc nie wiem, kogo ona w tym momencie chce chronić, czy ojca, czy siebie, bo skoro sama już gdzieś wychodzi to też się naraża. Ja przychodząc do ojca też jego narażam, bo też wszędzie chodzę. Jak pojadę nad tą zatokę, to siostra siłą rzeczy będzie musiała już przejąć opiekę. Pani Diano, pani mnie rozumie, tak?

**Tak, jak najbardziej.**

Aczkolwiek znowu jest problematyczne, bo to jest dokładnie połowa czerwca, kiedy ja bym chciała tam pojechać, natomiast do połowy czerwca ma wizę ta nasza opiekunka i jak to będzie dalej, i w ogóle...Dużo jest takich rzeczy, które mnie tak. W normalnych warunkach, gdyby nie było tego Covida, to byłyby coroczne sprawy, które w tym czasie się odbywają i już. Natomiast teraz, to jest takie, a co będzie, a co będzie? A jak się jakieś ogniska chorobowe zrobią na tych kempingach? To jednak je pozamykają. A co z płatnościami? Takie to jest wszystko okropne. Z drugiej strony staram się oczywiście nie czytać, natomiast, że my musimy się jakoś nauczyć żyć z tym wirusem, że on w ogóle będzie z nami jakoś przez najbliższe te miesiące. Niewykluczone, że i lata tak jak grypa, tak i to. Mama mojej synowej mówi, że w 2008 r na tzw. świńską grypę w Polsce zmarło 800 osób, o czym nikt nie mówił, tego się tak nie rozdmuchiwało, a tak samo się to wszystko działo.

**Wracając do kempingu, ludzie też planują przyjeżdżać w tym roku? Udostępnia pani w tym roku ludziom na wynajem?**

Ja mam wpłacone już wszystkie zaliczki, ja mam cały sezon zajęty, nikt jeszcze nie odwołał. Już nie mówiąc, że wczoraj ta osoba była zupełnie nowa, która też chce i koniecznie, żebym jej zrobiła rezerwację na sierpień.

**Ta podwyżka płatności pola namiotowego wynika z sytuacji koronawirusowej?**

Na pewno podejrzewam, że też, a po drugie owszem, na tym naszym kempingu, w porównaniu do pozostałych było jeszcze tak troszeczkę taniej, bo u nas jest taki kemping rodzinny, a te inne już od dawna mają dużo wyższe ceny. To w tej chwili się zrobił sposób spędzania dla naprawdę bogatych ludzi, którzy mają wypasione przyczepy i dla nich to jest wszystko jedno, czy oni zapłacą 20 czy 30 tysięcy za sezon stania przyczepy. I właściciele kempingu w sumie do tego dążą, żeby tzw. mydłków jak my ze starymi 30-letnimi przyczepami się po prostu pozbyć. Tamci postawią, przyjadą sobie na weekend, posiedzą, wyjadą, przyczepa stoi, pieniądze wchodzą i koniec.

**Powiedziała pani, że nic się pani nie chce. Jak pani myśli, z czego to wynika?**

Z takiego ogólnego spowolnienia wszystkiego. Nie ma obowiązków, nie ma rutyny. Cały czas mnie zżerają takie wyrzuty sumienia od środka wobec męża. On tak jakby...Nie to, że się poddał, bo on jest taki bardzo zadaniowy i na nim wszystko...Na jego plecach wszystko stoi. Ta moja działalność w tej chwili, to...Dopóki jeszcze pracowałam w (miejsce pracy), to się wszystko świetnie zaczęło i miało to jakieś...Myślałam, że przynajmniej jakoś do tej 60-tki jakoś to się będzie toczyło, a teraz to naprawdę jest bardzo słabo i w tej chwili niewiele tam zarabiam. Jednocześnie też, oczywiście on mi podsuwa różne pomysły, nawet i związane z (miejscem pracy), co bym mogła tam zrobić, ale ja tego nie robię. On teraz wznowił normalnie działalność tej szkółki, już zaczynają przychodzić, cały czas tego pilnuje. Ja nie wychodzę z psem ani rano, ani wieczorem, bo tam kiedyś w jakiejś awanturze powiedziałam, że to nie mój pies i nie będę z nim wychodzić i on w zasadzie wszystko to sam robi, rano wychodzi, ja śpię, jak on się kładzie, to ja siedzę i do drugiej oglądam filmy...Jedno, co teraz rzeczywiście się zmieniło, to zawsze przygotowuję obiad, że to ma. Tak, poza tym, to taka czuję się trochę bezużyteczna, taka trochę jak taki pasożyt, a z drugiej strony nic z tym nie robię.

**Znalazła pani jakieś obrazki obrazujące pani emocje?**

Nie. Jedyne, co zapisałam, to ten wieczór taki, ten stan emocjonalny. Wzmocniony był jeszcze, bo wypiłam jakiegoś drinka czy coś, no więc już wtedy rozłam był kompletny i w ogóle.

**Emocje - zdjęcia**

Jeśli chodzi o ten stan sprzed tego czwartku...Wtedy po tej naszej rozmowie potem jeszcze cały dzień miałam mnóstwo zajęć i to wszystko zrobiłam, natomiast byłam taka bardzo wyczerpana. Zresztą chyba powiedziałam, że "wymęczyła mnie dzisiaj pani". I to gdzieś się musiało jakoś skumulować. Ten stan wieczorny, to bym dała 9 - te błyskawice, ta trąba powietrzna, która właśnie mnie gdzieś wciągnęła w taki wir tej takiej niemocy, rozpaczy. Ja dawno się tak nie rozkleiłam. Moja siostra, jak ze mną rozmawiała, to ja po prostu wyłam. Nawet teraz mi się oczy szklą. Że w jakim ja żyję kraju, że co to za ludzie. Najbardziej chyba tak te wszystkie hejty, ta ciemnota tego narodu, no i dlatego ta 9. Potem natomiast, jak dostałam tę moją wnusię, to jest takie słoneczko i takie kochane, to 6 i 13. Wtedy było bardzo dużo radości i to jest po prostu miłość moja największa teraz. Ja mówiłam, że mam taką bliską przyjaciółkę w Kanadzie i ona tego samego dnia, właśnie we czwartek też zadzwoniła. Akurat tak, jakby wyczuła, że coś się dzieje, tyle tylko, że ja już byłam po tych płaczach wszystkich, ale jej tak mówiłam i się tak skarżyłam, że te hejty, a ona mówi, że to nie tylko u nas, że wszędzie tak jest, że w Kanadzie jest tak samo. Strach przed tym wirusem powoduje u wszystkich takie zachowania. Nie wiem, czy to mnie trochę uspokoiło, czy nie, ale zdałam sobie sprawę, że to jest taki mechanizm obronny ludzi. Tyle tylko, że to świadczy dla mnie jednak w jakimś tam stopniu o takim jednak prymitywizmie ludzi, bo nikt inteligentny, mądry, rozsądny nie będzie podchodził w ten sposób, że prawie na taczkach wywiozą lekarza, bo przychodzi i zaraża osiedle.

**Teraz też utrzymuje się ten stan radości z 6 i 13 po tej wizycie wnuczki?**

Nie, nie utrzymuje się. Ten weekend majowy był taki spokojny, nic się nie działo, odpoczęłam naprawdę, natomiast w zasadzie już w czasie weekendu zaczęły się znowu jakieś historie z moją synową, że ona jakieś depresyjne stany ma, coś tam, a przelewa to bardzo na mnie, bo swojej mamie tego nie mówi tylko mi, co między innymi też jest związane z moim synem. No ale co ja na to poradzę? I to znowu gdzieś zaczęło do mnie wracać, bo ja też mam w związku z tym takie lęki. To są bardzo takie duże lęki, że oni nie przetrwają, że to się zakończy, że tu jest takie cudne dziecko jedno i drugie, i co to będzie, i w ogóle. Potem dołączyła do tego natychmiast od poniedziałku ta cała sprawa z tą przyczepą, więc też były te nerwy. Wszystko się skumulowało. Wczoraj też były takie sytuacje, że ona chciała się zapisać do lekarza, ale nie ma miejsca...Bo to do takiego terapeuty, do którego też mój syn chodzi. Potem ja oczywiście rano zadzwoniłam i okazało się, że mnie się udało ją zapisać na następny dzień i teraz się śmiejemy, że wszyscy już teraz będziemy na antydepresantach i witaj w klubie, i w ogóle. W tym przypadku myślę, że to nie jest wpływ koronawirusa, tylko tam z nią były wcześniej inne rzeczy.

**Jak pani obserwuje innych, to jak oni sobie radzą? Obserwuje pani u bliskich jakieś zmiany w nastrojach?**

Moja siostra jest w ogóle osobą dosyć mocno stojącą na nogach i ona nie przejmuje się tak wszystkim, jak ja. Chociażby to, że od 2 miesięcy nie tyle nie wychodzi z domu, bo wiem, że wychodzi i ze swoją córką i wnukami też zaczęła się na spacerach spotykać, ale ona nie to, że się nie przejmuje, ale tak jakoś nie pokazuje tego. Koleżanka, która wczoraj tutaj była, to jest zachwycona z całej sytuacji, bo do pracy ma daleko i musi codziennie o 5 wstawać, a w tym momencie nie musi, bo pracuje zdalnie w domu od 7 do 15, kończy pracę i nie musi 2 godz. wracać, więc cała happy. Dla niej to bardzo pozytywne. Moja teściowa z kolei już też powoli głupieje i jak była ta moja wnusia tutaj, to oczywiście też poszłam z nią na spacer i teściowa była. Też szła bez maseczki i jak ludzie szli to zakładała, a jak nie było to zdejmowała, czyli też z jednej strony nie pozwala do siebie wejść do mieszkania, ale jak jesteśmy na zewnątrz, to też kompletnie się już nic...Zresztą pojechała z mężem na tę działkę i pytałam się, czy siedzieli w maseczkach. "No coś ty?" Żeby nie było, to ja jutro z nią jadę na działkę na 3 dni. To jest taki wyjazd przymuszony. Nie bardzo mi to pasuje, bo uważam, że tam nie ma co w tej chwili robić na tej działce, a teściowa po prostu się zaparła, że trzeba. Na zasadzie wyrzucania starych butów. Mnie się nie chce jechać na 3 dni i wyrzucać stare buty z szaf, gdzie wszystko tam jest już tak czy tak posprzątane, bo ktoś tam posprzątał, zostało za to zapłacone. Stwierdziłam, że już zrobię to dla niej, a ponieważ ja się bardzo boję zimna na tych...Wiadomo, że po zimie...To jest taki duży dosyć dom, ale tam jest zimno. Wiem, że te 3 dni teraz mają być w miarę takie ciepłe, więc już pojadę. Tak bardziej dla świętego spokoju.

**Jak u pani teraz wyglądają zakupy? Czy coś się zmieniło?**

Nic się nie zmieniło, oprócz tego, że staram się robić zakupy raz na tydzień. W ten weekend majowy pojechałam sobie w sobotę. W czwartek robiłam zakupy, ale to głównie dla taty, więc już swoimi zakupami się nie zajmowałam. W sobotę sobie pojechałam. Wtedy robię takie duże zakupy, śmieje się, że jak bym na wojnę się szykowała, ale nadal są to tylko zakupu jakby codziennego użytku + żywieniowe. Nic więcej. nadal z listą, bo bez listy to ja masła nie kupię, jak nie będę miała na liście. Teraz przez epidemię jest tak, że staram się chodzić raz, aczkolwiek to często też nie wychodzi, bo była też taka sytuacja z tą moją wnusią jak miałam ją tutaj. Oczywiście synowa przyniosła mi wszystkie słoiczki, ale ja tego nie uznaję, tak? Uznaję w momencie, kiedy jest jakaś sytuacja kryzysowa, ale jak człowiek jest w domu, to jak to? Nie ugotuję jej jedzenia? Zrobiłyśmy taką akcję z moją teściową, że ja na piechotę poszłam z wózkiem i z wnusią pod Biedronkę, teściowa tam dojechała samochodem i ona tam przez 10 minut chwilę pochodziła, a ja w tym czasie zrobiłam wszystkie takie zakupy potrzebne dla tej małej, żeby jej ugotować zupkę. I w związku z tym wcale nie wyszło tak, że wyszłam raz w tygodniu, tylko jednak dużo więcej, bo też w tym tygodniu musiałam kupić też maseczki dla męża do szkółki jazdy i musiałam 2 razy w ciągu dnia pójść do tej samej żabki, bo za jednym razem mi nie chcieli sprzedać 2 kompletów. Więc też częściej, ale nadal to są takie...Jak już wyszła sprawa z tą przyczepą, to w Biedronce pojawiły się takie lampki solarne i już mam pół bagażnika tych lampek na kemping, jakieś grabie, coś.

**Zdarzyły się pani jakieś zakupy tak typowo dla przyjemności?**

z racji tego, że muszę te zamówienia do (pracy) robić też na swoje konto, to wybrałam tam sobie jakąś torebkę. Trochę jest za mała, ale ok. Jest jaka jest. Sandałki też za małe, ale postaram się je jutro sprzedać mojej pani, bo od jutra przychodzi do mnie z powrotem moja pani Ukrainka do sprzątania, bo ja nie mam siły sprzątać już, a nie mogę już tak zarosnąć w brudzie, więc już wracamy.

**Ta torebka i sandałki to było tak trochę na poprawę humoru?**

Nie, to na tej zasadzie, że musiałam dobić do jakiegoś poziomu, więc musiałam sobie coś wybrać i musiałam wybrać coś, co ewentualnie mi się przyda. Potem jak się okazało, wcale te zakupy nie były konieczne, bo ten poziom został jednak przekroczony przez inne zamówienia, no ale już to zostało zakupione.

**W poniedziałek otworzono GH. Co pani o tym sądzi?**

Z jednej strony, to ja sama nie wiem, jak to powinno być. Jak czytam, że w ogóle nie powinno być tych obostrzeń, że tak i tak wszyscy się musimy z tym wirusem spotkać jakoś...To, że zostały wprowadzone te obostrzenia, to nie wiem, czy to było dobre z racji tej, że to się tylko przeciągnie, ale na pewno było dobre, bo służba zdrowia na pewno nie była przygotowana na to, żeby ten pik był taki duży od razu, więc tu jest ok. Ale w tym momencie uważam, że jak już zostały wprowadzone, to mogłyby dalej trwać, tyle tylko, że ta gospodarka idzie w dół totalnie, wszyscy na tym rzeczywiście tracą. Mnie się wydaje, że te takie uchylenia tych obostrzeń w tej chwili, to jest czysto taka zagrywka polityczna przed tymi wyborami. Skoro można już tu iść, tu iść, tu iść, to dlaczego nie można iść na wybory? Takie przepychanki teraz są. ja na razie nie wybieram się do żadnej galerii, bo na razie nie mam po co.

**Ktoś z pani otoczenia planuje takie zakupy albo już zrobił?**

Nie wiem. Wg mnie to, że są otwarte galerie to nic nie zmienia, bo skoro są od dawna otwarte Biedronki i inne sklepy, to tak czy tak ludzie tam chodzą. Tym bardziej, że skoro nadal nie są czynne te kawiarnie i wszystko, to same te zakupy w galerii niewiele się różnią od wyjścia do Biedronki, gdzie wiem, że już dawno nie są przestrzegane ilości osób, bo nie raz jest więcej.

**Teraz są kolejki w ogóle?**

Do kas oczywiście, że są, ale przed wejściem to się nie spotkałam. Kolejki są takie same jak przed epidemią. Był taki moment, że były bardzo duże kolejki, a teraz jest są takie, jak sprzed epidemii.

**Łatwość wydawania pieniędzy - skala**

Tak chyba jestem w połowie, ok. 5. U mnie przede wszystkim jest to spowodowane jakimś limitem finansowym. Taką mamy umowę z mężem, że ja dostaję jakieś pieniądze na tzw. życie, czyli zakupy, jedzenie, środki czystości, kwiatki na balkon. Muszę się zmieścić w tym, co dostaję, aczkolwiek to też nie jest tak, że ja to dostaję na początku miesiąca, tylko tak mi mąż dozuje trochę. Potem w międzyczasie ja się go pytam, ile ja tam mam jeszcze pieniędzy, ile tam mi jeszcze zostało. Za to kupuję to, co jest najpotrzebniejsze. Staram się nie wydawać na jakieś ekstrawagancje, więc raczej jest to z reguły to samo - nabiał, białe sery dla męża, bo ja tego nie jadam a powinnam, bo może bym trochę schudła. U mnie myślę, że to też jest z zupełnie innych powodów ta moja otyłość czy nadwaga. Niestety w stosunku do mojej wagi z dużych dzieci to jestem starsza i grubsza o 25 kg, więc to jest sporo. Owszem, jak jest nowy miesiąc, to pozwalam sobie na zakup do domu jakiejś szynki parmeńskiej czy coś tam, ale nie za każdym razem. Zawsze coś tam mam w zamrażalniku, żeby dołożyć w razie czego, więc to są zakupy bez szaleństwa, takie normalne zakupy. Wiem, że jeśli coś sobie wynajdę i to kupię, i wyjdę poza to, to wiadomo, że muszę dołożyć z tej kasy od siebie. Ponieważ ostatnio jest bardzo z tym ciężko, bo prawie nie zarabiam od stycznia, naprawdę nie duże były to pieniądze, a ten dziadek nas kosztuje nas naprawdę dużo, to staram się po prostu oszczędzać. Teraz na pewno przez fakt, że te zakupy się robi tak rzadziej, a poza tum od 1.5 miesiąca nie mieszkają z nami syn z dziećmi i synową, to te finanse jakby trochę lepiej w sensie, że nie ma takich braków, bo tak, to pod koniec miesiąca już były braki. Teraz też ten limit został jakby zmniejszony z racji koronawirusa w sensie, że nie ma pieniędzy po prostu.

**Patrząc na wydatki ogólnie, to jak kupuje pani na początku miesiąca taki lepszy produkt, to jak się pani z tym czuje? Jest z tego zadowolenie i przyjemność?**

Tak, tak. Nie mam z tego powodu wyrzutów sumienia.

**Jak to wygląda, jeśli chodzi o większe zakupy - meble, wakacje, etc. Jak tutaj podejmuje pani decyzje?**

Większe do domu, to dawno nie było takich zakupów, za którymi ja bym optowała. W ty roku miała u nas zostać wymieniona kuchenka i piekarnik, płyta ceramiczna, ale chwilowo to zostało odłożone, no bo to...Natomiast mój mąż poczynił jakieś tu zakupy, ale to on decyduje o tym, ja w to nie wnikam. W tygodniu przed zamknięciem wszystkiego u nas zakupił i monterzy zamontowali klimatyzację w domu. To był taki zakup, można powiedzieć z wyższej półki. Zmienił, ale to on u siebie - kupił sobie taką nową kanapę do swojego jakby biura małego, tam, gdzie dzieci śpią, jak przyjeżdżają. Teraz, żeby mieć więcej miejsca, to jest taka składana kanapa, a takie łóżko, które tam stało, to on od razu chciał wyrzucić. Nie wiem, czy o tym łóżku była tutaj mowa, ale ono bardzo długo stało na korytarzu u nas. Ja się oczywiście nie zgodziłam na to, żeby to wyrzucić i znalazłam kogoś, kto to łóżko w końcu zabrał. Stało 3 tygodnie, męża to strasznie denerwowało, ale stało na klatce i nikomu nie przeszkadzało, a potem przyjaciółka powiedziała, że ma wreszcie na czym spać na działce i pierwszy raz nie bolą jej plecy. itp. Ja nie lubię tak iść i wyrzucić. Ktoś mógł jeszcze z tego skorzystać. Jeśli chodzi o wakacje, to moje wyglądają tak, jak mówiłam w sensie wyjazdów nad morze, natomiast my od lat jeździmy na wakacje oddzielnie. Może inaczej - nie jeździmy razem. Czy to dobrze, czy to źle, nie wiem. Ja bardzo się przez wiele lat wściekałam z tego powodu, ale z drugiej strony już się poddałam i jeśli to ma być dla mnie jakaś wielka łaska, że on zamierza ze mną jechać na wakacje i mamy się tam kłócić tylko, to niech sobie każdy jedzie sam. Szczególnie, że ja od ponad 10 lat mam np. te wyjazdy chociażby do Kanady, tyle tylko, że one są co 2 lata. Tak do tej pory się udawało. Czy za rok pojadę? Nie wiem, bo nie wiadomo jak to będzie. Ostatni raz byłam rok temu.

**A taka sytuacja, kiedy pani wydawała jakieś większe pieniądze na coś?**

Była taka sytuacja w tym roku w styczniu, bo pojechałam do Indii przecież. Ja byłam u koleżanki w tych Indiach, zresztą byłej konsultantki (miejsce pracy), którą poznałam tu w Polsce. Mieszkała w tym samym bloku co ja i się bardzo, bardzo zaprzyjaźniłyśmy, tyle tylko, że ona już 2 lata temu wyjechała z Polski do Indii. To bardzo skomplikowana sytuacja, bo na skutek jakichś małżeńskich niesnasek i... Nieważne. W każdym razie my cały czas mamy z nią kontakt bardzo dobry. My razem. I jakoś tak jesienią rok temu taki pomysł padł, żebym do niej przyjechała, a ponieważ ja bardzo lubię sobie podróżować, to stwierdziłam, że muszę się nad tym zastanowić i decyzja zapadła szybko, że jadę, szczególnie że też byłam zaproszona na ślub i wesele jej rodzonego brata.

**Jak szukała pani biletu lotniczego, to jak to wyglądało? Szukała pani jak najkorzystniejszych ofert?**

Tak, szukałam oczywiście. Ja szukałam i moja koleżanka z Kanady też sprawdzała tam u siebie i mówiła mi co i jak, które najlepsze. Potem musiałam oczywiście ustalić z tą moją Snehą, jakie terminy wchodzą w grę i oczywiście brana była pod uwagę cena, ale tam wchodziły w grę też inne rzeczy - nie tylko cena, ale też konkretne terminy.

**Jak się pani czuła z tym, że trzeba było wydać większą kwotę na ten bilet?**

Bardzo dobrze się czułam. bo akurat pod koniec roku dobrze zarabiałam wtedy. Nie było problemu. Na tym polega ten nasz układ domowy, że jeżeli ja pracuję i coś tam sobie zarabiam, to mogę z tego spokojnie sobie korzystać. Do tego stopnia, że tam nawet mąż mi trochę dołożył do tego mojego wyjazdu do Indii. Chyba tylko i wyłącznie po to, żebym go się nie czepiała, że razem gdzieś nie jedziemy. Miałam bardzo przykrą przygodę wracając z tych Indii i jeszcze musiałam 2000 dopłacić do tego wyjazdu, bo spóźniłam się na samolot w Istambule i musiałam kupić 2 bilet. To w ogóle był absurd, w życiu mi się coś takiego nie zdarzyło. Spędziłam na lotnisku 22 godziny i dobrze, że miałam kartę kredytową, żeby kupić ten drugi bilet. Przeżycie było masakryczne.

**Uznałaby się pani raczej za osobę oszczędną czy rozrzutną?**

Oszczędną. Może inaczej. Każdy ma swoje priorytety, na które wydaje pieniądze. Ja w życiu sobie nie kupię drogiej torebki czy drogich butów, bo po prostu to jest mi niepotrzebne. Ani drogich ciuchów, ani nic takiego. Nie cierpię tego. Może nie tyle nie cierpię, co nie ma to dla mnie znaczenia, że ktoś coś takiego nosi czy kupuje. Moja teściowa długo ze mną walczyła, że ja nie jestem elegancka, ale już jej powiedziałam, żeby się ode mnie odwaliła i że będę chodzić tak ubrana, jak chcę. Już nie raz dochodziło do scysji, że ktoś był nieodpowiednio ubrany na jakichś świętach czy coś. Ubrania nie są dla mnie priorytetem ani tym bardziej jakieś biżuterie czy tego typu dobra, natomiast ja z wielką chęcią wydam na podroż, samolot. Oczywiście to też są duże sumy, bo każdy taki wyjazd nawet do Kanady to mnie kosztuje ok. 6000. Poza tym ja zawsze przywożę i prezenty, i w ogóle.  Na podróż jestem w stanie wydać większą kwotę i się tym cieszyć.

**Wspomniała pani, że u pani te zarobki teraz się znacznie zmniejszyły?**

Ale one nie zmniejszyły się z okazji koronawirusa. Ja w (miejscu pracy) jestem już od 12 lat - to po śmierci mojej mamy się zaczęło. Ktoś mnie wciągnął, jak ja byłam w takiej depresji, żebym została konsultantką, natomiast potem ta moja droga zawodowa przebiegała w rożny sposób. Zajmowałam się różnym sprawami, piekłam, to tamto w domu, potem się opiekowałam dziećmi - byłam nianią, a potem, ponieważ złamałam nogę to nie mogłam się już się zajmować i stąd ten (miejsce pracy), ale na zasadzie takiej, że zostałam tym liderem, zaczęłam budować swój zespół. To było 2.5 roku temu. Po pierwsze jakoś tak ambitnie do tego podeszłam i wciągnęłam się, a dodatkowo od stycznia właśnie zaczęłam pracować w tym (miejsce pracy), co było dla mnie zupełną nowością, bo ja raczej jestem jednak taka trochę nieśmiała, a to jednak była taka praca na stoisku w sklepie, jako taki doradca, konsultant i w ogóle. Jak się okazało to było dla mnie po prostu wymarzone, było super. To były najfajniejsze 2 lata i najdłużej w ogóle, jeśli chodzi o jakąkolwiek moją pracę taką czysto zawodową. Ja strasznie rozpaczałam, że oni to zamykają i stąd też cały ten rok mój jest teraz dużo gorszy, ponieważ ja pracując tam miałam jednak dużo większe możliwości, chociażby z tego względu, że...Już nie chce mi się wnikać. Ja miałam połowę jakby sprzedaży swojego całego zespołu z (miejsce pracy) i połowę ze swoich konsultantów, a teraz tego po prostu nie ma. To nie są te sumy, ale tak dla przykładu - jeśli miałam sprzedaż 50 tysięcy w danym katalogu, to wtedy 25 było z (miejsce pracy), a 25 z mojego zespołu. W tym momencie zostaje mi tylko te 25. Żeby mieć o ileś % więcej te wszystkie progi, za które mamy pieniądze, jeśli chodzi o prowizje, to my musimy rok do roku robić przyrost sprzedaży przynajmniej o złotówkę, więc skoro mi brakuje połowy sprzedaży, to ja jestem ciągle w tym dole. Przyrost sprzedaży mnie się może dopiero zacząć od stycznia, więc cały ten rok idzie na takiej...Jest słaby. Teraz sprzedaż też przeszła w działania online, których ja nienawidzę i dlatego może też nie mam dalej...Albo też może nie zajmuję się tym odpowiednio dobrze. Ja już powinnam dawno, jak mi mój mąż mówił, że siedzę w domu teraz, nie wychodzę, to zrobić stronę internetową, że on mi pomoże, a ja tego nie zrobiłam. No i błąd, bo być może coś bym miała z tego.

**Ma pani wrażenie, że to wróci do normy w styczniu przyszłego roku?**

Nie wiem, czy to wróci do normy, trudno powiedzieć, ale wtedy jest szansa, że będę robić te przyrosty, natomiast jeśli ja też nie podejdę do tego tak, że chcę to rozwijać w inny sposób...W tym (miejsce pracy) było super, bo tam wiadomo, że miałam sprzedaż i tam pozyskiwałam ambasadorki. To się skończyło i do marca jeszcze były takie inne dyżury na Słowiczej, ale tam też się chodziło i tam też coś się działo. Teraz już niby to otwierają z powrotem, ale to oczywiście nie ma porównania. Ja oczywiście zamierzałam dalej się tu rozwijać, ale ta sytuacja tak jakoś zaczęła się zmieniać - z tym koronawirusem, z tymi dodatkowymi odczuciami i wszystkim, że ja tak jakbym osiadła w tej chwili...Może nie na laurach, bo nie mam laurów, ale tak jakbym przestała działać i to mnie bardzo denerwuje.

**Czy to, że pani zarobki spadły to jest zagrożenie dla budżetu domowego?**

Nie, bo one nigdy się nie wliczały do budżetu domowego. To jest obok. Oczywiście jest to bardzo...Mój maż by bardzo chciał, żeby to się wliczało i dlatego ja mam takie gdzieś tam wyrzuty. Teraz zawsze ma obiad, a już w sobotę, niedzielę to staram się, żeby to było coś bardzo fajnego. Nie musi być wykwintne, ale jakieś fajne rzeczy. On zawsze usiłuje mnie jakoś tak dźgać, że ja bym krocie zarobiła na tym jedzeniu, gdybym robiła catering czy coś, a ja nie chcę tego robić. Nie chcę, nie umiem. Ja już raz przez pięć lat gotowałam i sprzedawałam, miałam gdzie, ale nie wiem, jak bym to miała teraz robić. I on za każdym razem mi to mówi, jak coś nie daj Boże zrobię dobrego, że jaką ja bym miała kasę na tym. Ja się już w ogóle nie odzywam, bo wiem, że tego nie będę robić. Kiedyś teściowa mnie też męczyła, że załóżmy przedszkole, a ja się nie nadaję do takich biznesów. Nie wiem, czy to wynika ze strachu, z lenistwa, nie wiem, z czego. Z ograniczenia jakiejś mojej wolności? I dlatego ten (miejsce pracy) dla mnie i nawet ta praca w (miejsce pracy) to było dla mnie coś cudownego, bo ja tam sama ustalałam, kiedy ja przyjdę do pracy, sama wysyłałam grafik. Jak wiedziałam, że muszę jechać przygotować przyczepę, to się w ten grafik nie wpisywałam i to było takie decydowanie. Jak pracowałam w młodości w holenderskich liniach lotniczych, to ta praca się skończyła, bo ja nie mogłam się dogadać z kierownictwem choćby odnośnie głupich 5 dni urlopu, który miałam wziąć za pół roku. Ktoś może spojrzeć, że całe życie miałam wygodne, ale to też nie jest tak, bo ja całe życie prawie i wyłącznie tylko dom, dzieci. To było tylko i wyłącznie na mojej głowie. Do 12 lat mieszkaliśmy na 4 piętrze bez windy i ja tam przeżyłam 2 ciąże, wszystkie wózki, wszystkie zakupy, a ja teraz słyszę, że córka mojej siostry nie może wózka wnieść na 1 piętro, więc mnie po prostu szlag trafia. I ci ludzie śmią narzekać, że im jest ciężko.

**Czy teraz podjęła pani jakieś kroki, żeby ograniczać wydatki?**

Tak. Nie kupuję żadnych tzw. zbytków, nic. Kupiłam sandałki za 50 zł i torebkę dlatego, że musiałam coś kupić w tym (miejsce pracy). Oczywiście mogę to zwrócić, ale to już nie chce mi się wnikać w te procedury.

**Pojawia się szukanie jakichś tańszych zamienników, przecen, okazji?**

Ja od dawna zakupy robię w dyskontach, czyli Biedronka i Lidl. Jeszcze tańszych nie wynajduję.

**Jak się pani czuje z tą zmianą, że trzeba było zrezygnować z tych zbytków? czy to było trudne w jakiś sposób?**

Ale ja nigdy tego nie miałam.

**Kontroluje pani jakoś swój budżet, coś pani np. spisuje?**

Nie, właśnie powinnam. Spisuję i kontroluję na pewno wydatki dla mojego taty, ponieważ na koniec miesiąca my się z siostrą rozliczamy, więc każda z nas pilnuje swoich wydatków.

**Teraz w czasie epidemii powinno się ograniczać wydatki?**

Nie mam zdania na ten temat, nie wiem.

**Jak jest z oszczędzaniem pieniędzy u pani w domu?**

Ja nie mam nawet najmniejszego pojęcia, jakie są zasoby zaoszczędzone w naszym...No bo mąż mi tego nie mówi, ja nie pytam.

**Czyli, jakbym spytała, na ile miesięcy państwu wystarczy oszczędności, to nie wiedziałaby pani tego?**
Wiedziałabym, bo się zapytałam. Akurat tutaj się zapytałam, więc na 2-3 miesiące. To mąż tą częścią zarządza.

**A pani oszczędności?**

Jeśli mam jakiś cel, ostatnio był to wyjazd do tych Indii, to tam sobie liczyłam, ile bilet, ile to, ile tamto i na to po prostu. Potem musiałam z tego, co zarobiłam pospłacać tą kartę kredytową jedną, drugą, więc to też i był moment, że nie miałam po prostu żadnych oszczędności. do tego stopnia, że musiałam akurat w międzyczasie zapłacić za zimowanie przyczepy i za serwis przyczepy. Nie miałam w ogóle pieniędzy a nie chciałam pożyczać od męża, więc zaczęłam wtedy pobierać już te zaliczki na sezon. Coś tam zarobiłam, bo oprócz gołej pensji za katalog, to tam są jeszcze jakieś bonusy, to powiedzmy ze 4000 zarobiłam w bonusach, ale to dopiero teraz odłożyłam tę całą sumę zaliczek, żeby to było żelazne - czy ja to będę musiała zwrócić tym ludziom, czy to pójdzie na opłatę kempingową. Dopiero w tym tygodniu już jestem spokojna, że to mam.

**Czy pani sama odkłada te pieniądze, czy schodzi jakaś suma na inne konto?**

Ja mam 4 konta. Jedno to jest moje osobiste, drugie jest zakupowe, trzecie (związane z działalnością zawodową), na które wpływają mi wpłaty od klientów, a czwarte firmowe. Sama lawiruję pomiędzy tymi kontami. Dzisiaj miałam właśnie na jednym koncie odłożyć tę całą sumę, żeby to już zostało. U nas nie ma miesięcznej pensji, bo to katalogowo się rozlicza i wtedy, kiedy ja wystawię fakturę, to (pracodawca) mi wypłaca i wtedy jakoś tam sobie staram się ustalić policzyć co na co. Czasami oczywiście okazuje się, że muszę iść po pieluchy dla ojca i się poprzesuwają te płatności, oszczędności.

**Dlaczego pani oszczędza?**

To trudno nawet nazwać oszczędzaniem, ale żeby była ta jakby rezerwa jak coś się dzieje. W tej chwili nie tyle ja oszczędzałam, co ja dążyłam do tego, żeby z powrotem zebrać całą tę kwotę zaliczek, z której musiałam skorzystać. To nie jest takie oszczędzanie, tylko takie układanie co na co, że tego już nie mogę ruszyć, a reszta to...Szczególnie, że z tej reszty to głównie są opłaty na ojca, bo emerytura taty nie pokrywa tego i to jest połowa tego, co my wydajemy na tatę, bo musimy dopłacić do opiekunki + musimy opłacić wszystkie opłaty i wszystkie zakupy. Miesięcznie każda z nas ok 1000 zł do tego dopłaca. Ja na razie jeszcze się jakoś wyrabiałam do tej pory. Na razie przez 3 miesiące nie muszę płacić ZUS-u, odebrałam jedno postojowe, więc to też jest jakiś zastrzyk. Ja jeszcze przez 1,5 roku płacę ten średni ZUS, bo ten najmniejszy już się skończył, ale jeśli nic się nie zmieni od stycznia, to ja będę musiała zrezygnować z tej swojej działalności i przejść na umowę zlecenie, bo nie będę miała z czego zapłacić ZUS-u. Gdybym wiedziała, że tak to będzie, to bym pewnie do tych Indii nie poleciała, no ale nikt tego nie wie jak by było, co by było.

**Czy w takich sytuacjach jak epidemia warto mieć oszczędności?**

Oczywiście. Jak oszczędności są...To przede wszystkim chodzi o pracę, że jak się traci pracę albo coś, to można za to żyć, zanim coś nowego.

**Czy pani myśli o tym, kiedy ta sytuacja się skończy?**

Oczywiście, że myślę, tylko nikt nie umie dać na to odpowiedzi. Mama mojej synowej, która jest szefem laboratorium w szpitalu mówi, że o ni też nic nie wiedzą, że oni dopiero badają. To, co się ukazuje w mediach to jest takie szukanie po ciemku. Nikt nie wie co będzie, jak będzie, na ile będzie i na jak długo.

**A ma pani jakieś przeczucie?**
Przeczucia nie mam żadnego, natomiast bardzo bym chciała, żeby to się skończyło w czerwcu i żeby ta przyczepa normalnie działała, żeby można było wrócić do normalnego życia. Grypa też jest co roku sezonowa i 2 razy się pojawia. Nikt nie wie, czy to zostanie z nami, czy to się po 2 latach zupełnie wyciszy i odejdzie. Na pewno jedno co chcę zrobić, to jak będę robiła cały ten panel badań, to się dowiem...Od wczoraj można robić badania na przeciwciała Covid. Teraz te badania, które mają być dostępne, to mają być głównie w tej klasie IGG, czyli późne zakażenie, czyli że dużo czasu od tego zakażenia minęło. Nadal mogą być nieścisłości i jeżeli były ewidentne objawy i te przeciwciała wyjdą, to nie ma co dyskutować, że się to przeszło. Jeśli się przechodziło to bezobjawowo to nadal są 2 możliwości - że organizm nie musiał wytwarzać tych przeciwciał albo one były w tak małej ilości, że one są niewykrywalne i nadal nic nie wiemy, lub że z kolei są wykryte i można to interpretować znowu na 2 sposoby. Mogło być bezobjawowe i w porządku, ale wszyscy właściciele psów, kotów, mogą mieć również dodatni, bo zwierzęta mają mnóstwo innych koronawirusów. Taki wynik też może być błędny. Wynik może być dodatni i wcale nie oznaczać, że się to przeszliśmy. To nadal jest niepewne.

**Co najbardziej zaprząta pani uwagę w kwestii przyszłości?**

Ja bym bardzo chciała już wiedzieć, jak my mamy z tym wirusem żyć. Czy mamy już z nim żyć normalnie i to już tak jest, i wiadomo, że niektórzy się zarażą, niektórzy wyzdrowieją, niektórzy umrą i koniec, czy po prostu epidemia minie. tego nie jestem w stanie się dowiedzieć.

**Plany wakacyjne?**

Na pewno chcę trochę tam pobyć nad morzem w czerwcu, na pewno bym chciała, żeby tam były moje dzieci też, może uda mi się pojechać na tydzień też w sierpniu razem z mężem, bo żeby było śmieszniej, mąż wynajął ode mnie przyczepę. Ja mam jeszcze w ramach bonusów z (miejsca pracy) bon na wakacje 1000 zł. Wakacje pl. - mogę go wykorzystać do stycznia 2021. Oczywiście zamierzam z niego skorzystać tylko jeszcze nie wiem kiedy, nie wiem dokąd. Oczywiście w lipcu, jeśli wszystko będzie szło tak jak trzeba, czy nawet niezależnie, to jeszcze jest ta działka, na której też będę chciała pobyć.
