# Supplementary material for: The challenges arising from the COVID-19 pandemic and the way people deal with them. A qualitative longitudinal study
Source: PLoS One. 2021 Oct 11;16(10):e0258133. doi: 10.1371/journal.pone.0258133 (PMC8504766; doi:10.1371/journal.pone.0258133)
Supplement: S1 Dataset — (ZIP) [file pone.0258133.s003.zip › Transcriptions/stage 4/4.4_M_32_couple, no children.docx]

**4.4_M_32_couple no children**

**Wydaje mi się, że zmieniłeś fryzurę?**

A może tak, może się tak dawno nie widzieliśmy, że brodę zgoliłem trochę. No tak, tak. Bo już za długą miałem. Ja tam przycinałem raz czy drugi sobie sam, ale to trochę już tam krzywo było. I w pewnym momencie też jakoś tak było, już nie pamiętam dlaczego, ale stwierdziłem, że… Być może, gdyby właśnie nie było całej epidemii, to poszedł bym do fryzjera, czy barbera czy do kogoś. Ale tak, to stwierdziłem, że już sobie… Ja co jakiś czas skracam brodę, mniej więcej tak, nie wiem, pół roku zapuszczam, przycinam, coś tam. I co pół roku sobie tak… No nie golę się na zupełnie, ale sobie tak strzygę.

**Czyli sobie sam ostrzygłeś?**

Tak, tak, tak. Ale właśnie mówię, bo to głównie opowiadam dlatego, że jeżeli wyszukujesz takich rzeczy, które się zmieniają, no to ja czasem chodzę do fryzjera albo barbera. Znaczy do fryzjera, który mi strzyże brodę. To nie jest barbershop, golibroda z ciepłym ręcznikiem itd. Tylko taki mniej trochę fancy punkt, gdzie pani mi ładnie tą brodę przycina. A teraz zrobiłem to sam. Ale to nie jest gigantyczne odstępstwo od reguły, bo czasami też to robię sobie sam.

**Ja mam takie wyobrażenie, że to wygląda tak, że bierzesz maszynkę i sobie golisz tę brodę. Ale rozumiem, że taka usługa u fryzjera jest lepsza niż sobie sam strzyżesz?**

To zależy. Po pierwsze przy długiej, długiej brodzie, jak ktoś chce mieć coś takiego, jak ja teraz, to właściwie nie ma sensu chodzić do jakiegoś tam barbera, wystarczy sobie kupić maszynkę. Być może są mężczyźni, którzy nie mają w ogóle elektrycznych maszynek w domu i golili się tam jednorazówką. Pewnie brzytwą to nie. Jednorazówką czy jakąś wielorazową taką z żyletkami maszynką na zero się golili po prostu. Chcieli zapuścić brodę, nie wiedzą, jak do tego podejść i zaczynają chodzić do fryzjerów, barberów może tak. Ale tak zasadniczo jak chcesz mieć taką do centymetra, dwóch, to naprawdę można sobie po prostu… Ustawiasz na maszynce odpowiednią długość i tam jedziesz. To nie żaden problem. Jak chcesz mieć długą, to musisz sobie… To tak jak długimi włosami. To musisz sobie jakoś tam przycinać, żeby było równo itd. I część osób, mężczyzn raczej, chociaż może kobiety też są takie, które sobie zapuszczają, nie wiem, część mężczyzn z tych długobrodych sobie samemu strzyże te brody. Ale to jest trudne. Znaczy to nie jest rocket science, ale to wymaga jakiegoś tam… umiejętności. I część chodzi do barberów też po to, żeby mieć jakąś wiesz, już zupełnie wystylizowaną, elegancką, super fajną itd. A jeśli chodzi o to, czy to się bardzo różni, no to… Jest trochę tak, że strzyżenie jak strzyżenie. Możesz zrobić w domu samemu, można pójść do fryzjera, a można pójść, są ci golibroda, barber shop, czy tam barberzy, to z angielskiego. I to jest takie… To się zrobiła jakaś taka moda, z zachodu przyszła, że to są takie wiesz, takie miejsca, nie wiem, jak je nazwać, żeby ich nie obśmiać zupełnie. Bo to jest coś przyjemnego. Takie tam, jak pójdziesz sobie do takiego fajnego miejsca, gdzie tam płacisz stówę czy 2 stówy za zrobienie tej brody. I zwykle tam są też dobrzy fachowcy od tych bród. To są takie trochę, nie wiem, jak kobiety mają spa i tam jest pachnące mydełko i ręczniczek i tam miła pani i szlafroczek. Nie byłem w spa, ale tak sobie wyobrażam. I że to jest takie miejsce, gdzie jest tak w ogóle miło, przyjemnie, leci muzyka w tle itd., to tamte miejsca są takie… No trochę są takie, taki maczyzm tam jest. Ale to są takie miejsca męskie. Wystylizowane. Tak, że wchodzisz i np. nie wiem, są jakieś strzelby, poroża albo harley stoi. I w ogóle jakieś takie rzeczy. I właśnie tam, nie wiem, są jakieś retro, jak to się nazywa, no taki… jak się nazywa ten taki blat, gdzie są umywalka i tam… U normalnego fryzjera też jest takie coś, nie? Jest po prostu taka część jak w łazience ze zlewem, jakimś tam lustrem itd. I to jest na przykład jakoś super wystylizowane, zrobione. I też, nie wiem, pan zanim ci tam ogoli tą brodę, to ci tam gorący ręcznik kładzie na twarz i jakieś takie są bajery. A, no i whisky ci nalewają do szklanki. Ja nie lubię whisky. Whisky ci tam nalewają do szklanki albo jakiegoś piwa dobrego. Więc to jest jakby takie trochę… Niektórzy faceci, to jest trochę metroseksualne, chodzą tam na przykład w kilku. To jest takie wydarzenie czasami towarzyskie dla niektórych. Niektórzy przychodzą tylko ogolić sobie ładnie brodę. A inni to tam przychodzą wręcz trochę pobyć chwilę. Wiem, że są np. tak, że faceci na imprezy takie, np. jakiś tam wieczór kawalerski, to najpierw idą do golibrody. No, jak mają długie i jest to modne w Warszawie. A potem idą gdzieś tam na miasto.

**Ale ty nie chodzisz do takich barberów.**

Nie. Znaczy zdarzyło mi się, bo gdzieś tam w pracy, jak byliśmy, to jakieś tam spotkanie z klientem, takie trochę integracyjne. Czy tam z jakimś partnerem biznesowym. No tak. I rzeczywiście tam piętnastu nas szło. I to było na początku trochę dziwne dla mnie. Bo jednak to jest jakiś rodzaj salony beauty. I on jest przybrany w męskie piórka, ale to są nadal piórka. I to jest takie nadal trochę dziwne, że się faceci umówili do fryzjera, żeby razem pójść. To brzmi trochę dziwnie co najmniej. Ale było nawet przyjemnie. Bo tam 10 czy 15 osób, siedzimy, gadamy, jemy jakieś kanapeczki z sushi czy inne jakieś takie rzeczy, pijemy sobie… No ja mówię, ja piję piwo, ale chłopaki pili whisky. Jakieś rozmowy o dupie Maryni albo o biznesie i jakichś takich rzeczach. I nie wiem, tam po kolei każdy, jak ma ochotę, to sobie siada u tych tam, było 3 czy 4 tych barberów było, chyba 4 fotele. I nie wiem, byli tacy, co jakby nie noszą długiej brody. I właściwie tam ich tylko trochę opędzlowali, równo pan przyciął i o. Ale na przykład chłopaki, którzy noszą długie brody, czy nosili w tych zespołach, w których pracowałem, to traktowali to jako też fajną okazję do tego, żeby sobie dobrze, przyjemnie zrobić, porządnie, ładnie wystylizowaną brodę. Która tam porządnie wygląda, jak na jakimś spotkaniu.

**Ale to jest tak, że teraz miałbyś ochotę pójść do takiego barbera?**

No teraz to w ogóle nie widziałbym potrzeby. Więc ja doceniałem to, że tam byli goście, którzy się w tych brodach specjalizują. I zrobili naprawdę raz mnie, łącznie z głową, z fryzurą, fajnie gość tam zrobił. Też właśnie trochę tam miałem taki etap, że ja zawsze nosiłem długie włosy, potem się ogoliłem raz na zero. A potem zacząłem odpuszczać. I jak miałem taką szopę, to on mnie tam wystylizował całkiem fajnie. Jakoś tak lekko podgolił, coś tam zrobił. I to wyglądało spoko. Natomiast teraz nie miałbym jakiejś wielkiej potrzeby. A za to się sporo płaci. W sensie też, płaci się trochę za ten cały… Nie tylko za to, że to jest gość, który dobrze potrafi zrobić brodę, czyli za jego umiejętności. Ale też za tą całą otoczkę. One są często w jakiejś dobrej lokalizacji. Nie zawsze, ale w dobrej lokalizacji i jacyś tam, jakby… Wystylizowane całe miejsce, dostaniesz jeszcze browara czy coś tam innego i właśnie… A nie mam takiej wielkiej potrzeby. A chwilowo nie szastam kasą. I to nawet nie mówię o koronawirusie, tylko o tym, że też nie pracuję w tej chwili. Więc… To jest miłe i fajne, ale daleko na mojej liście rzeczy, których ja potrzebuję w życiu.

**A masz w ogóle takie rzeczy, może to nie jest ten barber, ale takie, że wiesz, że jakby płacisz za coś więcej niż za samą tę rzecz, którą kupujesz?**

Wiem, wiem, że to nie jest optimum ceny do jakości, że ja wiem, że to już jest taki segment premium. Nie, rzadko. Bo ja właściwie rzadko. Bo ja pewnie bym wymyślił taką rzecz. Ale ja staram się właśnie tego unikać w życiu. Bo mam wrażenie, że to są rzeczy, w których „system” trochę jest tak skonstruowany, że… Nie wiem, jak to powiedzieć. Jest mnóstwo miejsc, w których płacisz za coś… Właśnie nie za stricte, za ten rdzeń, za to meritum sprawy, tylko za tą otoczkę. Czyli, nie wiem, pójdziesz sobie do Biedronki, to jest dyskont i możesz tam sobie kupić coś, jakieś jedzenie za ileś tam pieniędzy. Ale możesz to też kupić w Piotrze i Pawle albo w jakimś… Nawet nie umiem wymienić, Bomi kiedyś było, odpowiednik droższy. I właściwie to jest trochę to samo. Ale jest tam ładniej ubrana pani albo ładniejsze opakowania itd. I trochę jakby ja czuję, że ja tego nie potrzebuję. Więc staram się też, mam wrażenie, że to jest tak, że jak by człowiek nie myślał o tym, to te rzeczy są tak skonstruowane, żeby się na nie łapać. Są zwykle wygodne, łatwe, proste. I mam wrażenie, że w ten sposób… Może, gdybym był bardzo zamożny, to bym w takie rzeczy wchodził. Ale tak to czuję, że będę wydawał pieniądze na rzeczy, których… Wydam na przykład na to, żeby pójść na imprezę, kupić sobie drinka czy wódkę w knajpie, chwilę potańczę, wrócę taksówką. Nie wiem, potem zrobię zakupy w Żabce, a nie w Biedronce czy czymś innym. Bo blisko i tam nie będę łaził. A poza tym to nie pamiętałem, żeby to zrobić przed 20, tylko robię to o 23. Potem jeszcze, nie wiem, co musiałbym wymyślić, co jest z takich drogich zachowań według mnie. O, nie ugotuję sobie obiadu, tylko zamówię sobie coś z knajpy. Albo do pracy nie będzie mi się chciało dzień wcześniej ugotować, tylko zamówię potem z ludźmi w firmie. I dużo takich mikro zachowań powoduje, że właściwie na te podstawowe rzeczy mógłbym wydawać spokojnie całą pensję. Nawet zakładając, że pracuję i mamy z żoną dwie dość przyzwoite pensje, no to moglibyśmy spokojnie wtedy… Całość jesteś w stanie bardzo łatwo wypultać. Bo co to jest stówa czy dwie na flaszkę wódki w knajpie. No właśnie, a to szybko schodzi. Więc ja się pilnuję z takimi rzeczami. I uważam, że można to robić taniej. I wtedy nie musisz sobie rezygnować z tego rdzenia, czyli nadal możesz pójść się spotkać z kolegami, tylko w mniej luksusowym miejscu. Nadal możesz zrobić coś przyjemnego i zdrowo zjeść, tylko musisz sobie to zrobić samemu. Nie jedziesz taksówką, tylko poprosisz kogoś, żeby cię podwiózł, jeśli mówimy o lotnisku albo o czymś takim. Albo na imprezę pojedziesz sobie nad Wisłę rowerem. I jak się dba w takich mikro zachowaniach. Znaczy staram się nie przeginać. Zwłaszcza jak pracowaliśmy we dwójkę, to starałem się nie przeginać. Żeby to nie było tak, że non stop oszczędzamy wszędzie na każdej możliwej rzeczy. Ale w ten sposób można żyć, robić wszystko to, co wcześniej, a jeszcze ci zostaną pieniądze. I możesz zaoszczędzić, zainwestować, coś zrobić.

**A masz coś takiego, na czym nie oszczędzasz?**

Nie. Chyba nie. Znaczy na pewno jest coś, na co jestem w stanie nie dowolną ilość pieniędzy, ale większą wydać. Ale… No czasami na przykład, jak w zeszłym roku jechaliśmy w podróż dużą i wyposażaliśmy sobie busa, żeby tam był fajny. To miałem takie momenty, w związku z tym, że kupowaliśmy dużo i chciałem, żeby to było fajne, funkcjonalne, niektóre rzeczy trochę lepsze. To tam trochę miałem tak, że już nie optymalizowałem wszystkiego. Tylko o, fajne, podoba mi się. Nie wiem, ładny dywanik albo jakieś tam firanki. No dobra, 100 zł, a nie 80, ale przyjdzie do mnie, wiem, że to jest dobre. Tak. No to tam trochę na zasadzie takiego *raise fiber*. Czyli wakacje są moją taką trochę no nie pietą achillesową. Ale na wakacje lubię wydawać pieniądze. Tylko to nie znaczy, że lubię luksusy. Tylko lubię na przykład… No właśnie, zrobić je sobie sam po swojemu. Nie umiem tego do końca wyjaśnić. Ale tak, to jest takie miejsce, gdzie na przykład czuję, że mogę wydać jakąś tam… 100 zł ekstra na coś.

**Jak byś tak ogólnie sobie pomyślał o swoim podejściu do pieniędzy i takiego właśnie wydawania (skala 1 – 10 trudność w wydawaniu pieniędzy).**

No to pewnie takie mocne 3-. No wiesz, być może ktoś inny powiedziałby, że nawet 2 czy coś takiego. Ale nie, chyba bez przesady. Znaczy nie obracam aż każdej złotówki w dłoni 2 razy. Znam osobę, chociaż chyba tylko jedną, która jest bardziej, tak wyraźnie bardziej skąpa niż ja. Ale tak, no ja jestem tak mocno oszczędny i zdyscyplinowany, jeśli chodzi o kasę.

**No, ale właśnie, czy to jest skąpstwo?**

Musielibyśmy zdefiniować sobie, co to znaczy być skąpym dokładnie. Bo to jest… Bo jeżeli skąpiec nam się kojarzy ze Sknerusem McKwaczem, który jest bogaty i ma skarbiec pełny złota, no to po pierwsze ani nie moja liga, to raz. A dwa, nie mam takiej potrzeby, żeby te pieniądze mi leżały tak, że ja się chcę jak Sknerus McKwacz tak pławić. Że ja sobie na materac rzucam i sobie w nich leżę. Tylko raczej widzę w nich środek do czegoś. Więc w tym sensie nie czuję się sknerą. Raczej czuję się skrupulatny, dokładny, taki niefolgujący sobie. W tą stronę. Chociaż trochę nad tym pracuję też, żeby właśnie wypośrodkować to bardziej. W moim, też się sporo zmieniło, jak przestaliśmy spłacać kredyt na mieszkanie, to moja taka wewnętrzna presja trochę zmalała. Natomiast wśród moich znajomych jest pewnie poczucie takie, że jestem tą osobą taką właśnie skąpą. Ale ja jestem też po prostu osobą często optymalizującą. I mnie denerwuje, nie wiem, gdzieś jedziemy i denerwuje mnie, że śpimy w pierwszym lepszym miejscu, które ktoś znalazł na bookingu zamiast przejrzeć, zamiast przedzwonić w 5 miejsc albo tam przeszukać. No nauczyłem się tego, że zauważyłam, że jak gdzieś jeździmy, bo to wtedy są te miejsca, kiedy wydaję, jakby porównuję swój schemat wydawania pieniędzy ze schematami innych osób wśród znajomych. To zauważyłem, to też mam z domu pewnie wyniesione, że ja mocno optymalizuję i cierpliwie to robię. W związku z tym w miarę znajdę tańszy hotel, tańszą łódkę do czarteru, tańszy… Nie wiem, zoptymalizuję jakąś trasę, wymyślę, żeby tam jechała nas odpowiednia ilość osób, żeby się samochód wypełnił. Nie wiem, przeczytam, jeżeli jedziemy do jakiegoś drogiego kraju, to co należy wziąć z domu, bo jest drogie tam i niesmaczne na przykład, a co można śmiało olać. I tak dalej, mnóstwo tego typu rzeczy.

**Tylko mam wrażenie, że wakacje są u ciebie bardzo specyficznym obszarem do wydawania pieniędzy. Czy to jest tak, że na co dzień, jak podejmujesz decyzje przy wydawaniu jakichś większych pieniędzy, to też sobie tak optymalizujesz?**

Tak, tak, tak.

**Jak powiedzmy kupujesz jakieś meble czy coś takiego.**

Tak. To jest też tak, że jeżeli chodzi o meble, to ja nie lubię, ja jestem chyba dość mocno specyficzny. Ludzie lubią sobie zmienić, nie? Coś przestawić, coś przełożyć, kupić coś nowego, ładnego na ścianę itd. Ja takich rzeczy lubię robić raczej mało. W związku z tym… Ale lubię, gdzieś tam, no nie mam najładniejszego pewnie, najlepiej wydesignowanego pomieszczenia w Warszawie, czy domu. Natomiast lubię, żeby to było ładne, schludne, czyste, wyremontowane. Na przykład nie lubię robić remontów na raty. W związku z tym, jak żeśmy się wprowadzali teraz do naszego mieszkania kilka lat temu… No pierwszego tak nie było, bo to pierwsze mieszkanie to nie mieliśmy ani takich pieniędzy ani tam powiedzmy, że tak trochę inaczej to wyglądało. Tam już część mebli było, więc było takie zastane. Teraz żeśmy kupili puste mieszkanie, zrobiliśmy generalny remont. I dbałem bardzo o to, żebyśmy skończyli wszystko zanim się wprowadzimy. No, może nie mówię o składaniu ostatniej szafki z Ikei. Ale całkowity remont, wszystko odnowione, pomalowane, zrobione. Żebym już się nie musiał do tego dotykać, żebym już nie musiał o tym myśleć, żebym nie żył w remoncie itd. I wtedy żeśmy zastanawiali się, jakie meble. I mam takie poczucie, że owszem, wybieraliśmy pewnie rzeczy głównie z katalogu Ikei, czasem coś tam innego mamy pewnie. Raczej nie szukamy jakichś bardzo drogich rzeczy albo bardzo pięknych. Lubimy takie też proste rzeczy, więc to idzie akurat w parze z Ikeą, która jest relatywnie tania. Ale nie mam takiego czegoś, że ojojoj, musimy kupić najtańszą szafkę i to musi być taka za 300 zł. Nie, mamy też nie wiem, kredens, który nie jest ze sklejki czy jakiejś płyty robiony, tylko z drewna. I on wtedy kosztuje nie jak szafka w Ikei 4 stówy, tylko tysiąc. Ale to nie zmienia faktu, że patrzyliśmy na to, na zasadzie takiej: aha, a to w białym kolorze są za 300, a w szarym za 400. OK, to może białe. No to zobaczymy. Dobra, to może białe. Jak nam pasowało. Ale też tutaj nie było tak. Jak się meblowaliśmy to nie miałem takiego problemu, że to musi być najtańsze. Nie. Tylko, że wtedy byłem gotów przeszukać ten katalog… Usiąść. Nie lubię kupować rzeczy, a już mebli to już w ogóle. Byłem gotów usiąść, przeszukać te katalogi, Black Red White, Ikea, JYSK, coś tam jeszcze. Obejrzeć, przeszukać, porównać sobie itd. I wtedy mogliśmy wszystko kupić. Nie kupiliśmy chyba tylko stołu z takich rzeczy… Stół mamy gdzieś tam z odzysku, który jest trochę nie pasujący. I to było tam, że… Bo stwierdziliśmy, że ładny stół kosztuje 1500 zł, a wtedy nie mieliśmy trochę na to kasy. Ale teraz, jak już mamy taki stół, który dostaliśmy od kogoś, kto ma stół z PRL-u, to się przyzwyczailiśmy, już jakoś nie mamy potrzeby, żeby wymieniać. Więc dobijając do brzegu, ja po prostu nie lubię często podejmować tych decyzji. W sensie nie lubię się tym zajmować. Meblami albo jakimiś tam innymi rzeczami. W związku z tym często wolę nie podjąć tej decyzji i czegoś nie kupić niż kupić impulsywnie. I na zasadzie o, fajne, podoba mi się, kupuję. Chociaż robię pewnie tego trochę więcej niż kiedyś. O, to też, czuję, że to się trochę zmienia. Ale nadal jestem trójką. Nadal raczej nie wydaję pieniędzy niż…

**Rozumiem, że te meble to było już jakiś czas temu, prawda?**

No, 3 lata temu, tak.

**A pamiętasz jakiś ostatni większy zakup?**

Tak. 2 czy 3 tygodnie temu kupiliśmy łóżko.

**A możesz opowiedzieć o tym, jak to łóżko kupowałeś?**

To jest tak, mieliśmy łóżko z żoną, które kupiliśmy przed ślubem, czyli lat temu chyba prawie 10. No tak, 9 lat czy coś takiego.

**A to ten materac? Bo ty coś mówiłeś o materacu.**

No tak, bo to łóżko, które jest materacem na… Mieliśmy wcześniej łóżko, które było takim materacem na nóżkach. To się nazywa formalnie materac, ale to tak naprawdę wygląda jak łóżko, tylko nie ma ramy, nie ma… O, materac jest obudowany drewnianą ramą, która jest jeszcze obszyta materiałem. I to stanowi jedność. I to było takie na nóżkach. I to było też takie proste w formie i mi się to podobało i w ogóle fajnie. To też nie było jakieś bardzo drogie, jak kupowałem to 10 lat temu, bo wydałem chyba na to 9 stów? Może 8. Coś takiego. Na pewno poniżej tysiąca. Za łóżko takie 1,20 na 2,10. A ono jeszcze było 2,10, bo ja lubię długie, żeby było. I ono było na wymiar robione. Więc w ogóle fajnie. No i już teraz po tych kilku latach żona zaczęła narzekać, że jej się trochę niewygodnie, że tam już jakieś sprężyny czuć itd. Mi się wydaje, że my po prostu się starzejemy, a nie tam, że te sprężyny było czuć. Po prostu dużo siedzimy, mało się ruszamy i mamy po 30 lat, więc zaczynają nas plecy boleć. No, ale ona twierdziła, że się nie wysypia. Więc ja powiedziałem dobra, znaczy ja mogę właśnie oszczędzać na różnych rzeczach, ale nie żebyś mi potem marudziła to raz, a dwa, żebym miał poczucie, że oszczędzam na twoim zdrowiu. No to tego nie. No to mówię kup sobie materac jaki tam chcesz. Ja bym był kupić znowu to samo, tylko nowe. Bo mi to sprężynowe się podobało. Ale ona wymyśliła, że gąbkowy. Pojechaliśmy do tego samego sklepu, w którym kupowaliśmy 10 lat temu, to jest taki sklep w Radzyminie. Który robi łóżka na zamówienie, dowolny typ, rodzaj, materiał, wszystko. Bardzo polecam. Nazywają się New Yorker. I no pojechaliśmy tam, żeby ona mogła sobie obejrzeć. I potem pojechaliśmy do Ikei, żeby ona mogła sobie jeszcze posiedzieć na materacach. Bo tam robią tylko sprężynowe materace, a ona chciała też gąbkowy zobaczyć, czy jej się podoba itd. Bo taki sobie kupiła jej siostra. Więc tutaj na przykład, to jest też ciekawa opowieść w kwestii mojego niewydawania pieniędzy. Bo ja powiedziałem, że tak, mi się śpi dobrze na tamtym, to jest coś, co ja lubiłem. Ja bym tego nie zmieniał. A jak już musimy zmienić, to bym kupił drugi raz to samo albo podobne. Ale ona chce inny materac. Więc ona musi kupić ramę łóżka… A, jeszcze wymyśliliśmy jedno, to było ważne. Że tamto miało nóżki i mieliśmy pojemnik pod spód wsuwany, a wymyśliliśmy, że kupimy takie łóżko, które ma ramę. I wiesz, podnosi się całe do góry, cały materac. I w środku jest wielka skrzynia. Że to jest świetne, żeby tam wrzucać różne graty takie. I jest całkiem dobry dostęp, niż po szafach to… podnosisz, wszystko widzisz. To mnie cieszyło akurat, bo to porządkuje przestrzeń, nie leżą różne pierdoły na zewnątrz albo nie piętrzą się w szafkach. No dobra. No to pojechaliśmy, wybraliśmy, ale jej nie pasował ten ze sprężynami. No to pojechaliśmy do Ikei, to usiadła na dziesięciu, spodobał się jakiś. Ale potem jeszcze poczytała i oczywiście tak: można było kupić sprężynowy materac z ramą za 1600 zł w tym New Yorkerze, zrobiony na wymiar, obity kolorem jaki sobie chcesz i w ogóle wszystko fantastycznie. Ale jej nie pasował ten materac. Więc kupiliśmy chyba za 1500 samą ramę, taką specjalną deską, która jeździ do góry. I tam jeszcze na nią się nakłada materac, bo to jest dodatkowa konstrukcja. No i że ona sobie dokupi do tego materac. Ja mówię dobra, były takie materace tam, powiedziałem jej, które dla mnie były OK, większość była w porządku. Nie chciałem tylko takiego, co zapamiętuje kształt twojego ciała. Jest tak, że są teraz takie materace, ja to się śmieję, że ja czuję jakbym opowiadał po prostu tutaj skecze. Ale jest tak, że idziesz do Ikei i są materace opisane tak, że jest materac, który się składa z kilku warstw. I ta górna warstwa jest termicznie jakaś tam. Pod wpływem ciepła ciała, czyli innego ugniecenia, ona się powoli zapada. Więc ty kładziesz się na twardy materac, po czym tak delikatnie zaczynasz osiadać, jak byś w piasku się położyła i jak cię fale obmywają, to tak człowiek cię powolutku się zapada. To tam jest tak samo.

**Ale to brzmi świetnie, a ty nie chciałeś tego, tak?**

Nie, to jest strasznie niewygodne. Znaczy strasznie takie dziwne. I to koleżanka jedna mówiła nam o tym, że jej mama miała taki materac. Jak pojechała i były nowe łóżka czy coś, to spała na tym. Mówi, że pierwsze noc jest tragiczna, nie możesz się ułożyć. Bo się przewracasz i wydaje ci się, że jest twardo. A potem zaczynasz się powoli, jak w ruchomych piaskach właśnie zapadać. I powiedziała, że to jest tragedia. Ale powiedziała, że za którymś razem człowiek się przyzwyczaja i to jest bardzo fajne. Więc nie wiem, może dłużej byłoby OK. Powiedziałem, że to mi średnio leży. Odrzuciłem chyba jakieś za miękkie i jakieś tam. Takie najtańsze też tam były, ale to już od razu powiedzieliśmy sobie, że nie. Bo rzeczywiście były niewygodne. I pojechaliśmy do domu. I ona tam w końcu znalazła jakąś firmę, obczytała się, jakiś dobry materac. No i oczywiście co, no są 2. Jest materac zwykły, zwykły materac 9 stów. To nawet nie jest materac sprężynowy, który ma 10 lat gwarancji czy 20, to jest pianka. To za 3 lata będzie do wyrzucenia, za 9 stów. Nie, nie, nie, to nie jest jeszcze produkt premium. Moja żona lubi produkty premium. Był produkt premium z dodatkową warstwą materaca na to. I jakąś taką podwójną… Coś tam było zakombinowane. Nie śpi się na tym jakoś bardzo źle. Ale nie powiem, żeby dużo lepiej niż na poprzednim. 1400. Więc generalnie, jak się tam zsumowaliśmy to, transport, to łóżko mieliśmy za 3 tysiące chyba z hakiem jakimś. Więc generalnie zamienił stryjek siekierkę na kijek, zapłaciłem 3 tysiące, moim zdaniem śpi się tak samo. Nie pytałem jej jeszcze, mamy to łóżko 2 tygodnie, nie pytałem jej, czy ból pleców jej przeszedł. Ale chyba nie. Więc moim zdaniem, było tak jak się spodziewałem, to znaczy to jest starość, a nie materac. I tutaj powiedziałem jej, że nie chcę, potem będzie tak, że podejmę decyzję, że słuchaj, ale jednak to jest dużo pieniędzy, weźmy jakieś tańsze rozwiązanie, pójdźmy w jakieś półśrodki, zróbmy jakiś kompromis itd., to jak ją nadal będą plecy bolały, to będzie, że to jest moja wina do końca życia i ja właściwie zrobiłem zamach na jej dobrostan, tak? Więc ja powiedziałem, że nie, w takim razie już wydajmy te pieniądze, miejmy to z głowy. Więc to jest historia naszego ostatniego mebla. Ale to ten kupiliśmy, ale przez ostatnie 3 lata żadnego. Więc wszystko zrobiliśmy te 3 lata temu, jak się wprowadziliśmy. No, 3 lata temu, wiadomo, to chwilę trwa, to jest proces, umeblowanie, tam powiedzmy 3 miesiące czy 4 kupowaliśmy jakieś rzeczy. I kombinowaliśmy, co tu powstawiać po kolei. A potem długo nic. I teraz to łóżko. I myślę, że teraz długo nic. Chyba, że tam jeszcze dojrzymy do tego, żeby jedną rzecz zmienić. Bo jedna nas denerwuje, mamy taki mebel, który nam nie pasuje zupełnie. Ale nie mamy alternatywy wobec niego, żeby funkcję spełniało. Bo to jest z takim koszem na bieliznę. Półka z takim koszem na bieliznę. Znaczy na bieliznę, w sensie na pranie. Wysuwany kosz na prawie. W sypialni to stoi i chcemy to jakoś wywalić. Ale to musimy to przemyśleć. I zastąpić to wtedy meblami z Ikei. Więc tak będzie. Ale to wymaga, znowu, optymalizacji przestrzeni tym razem.

**A jak podejmowaliście tę decyzję o zakupie tego łóżka.**

Ja nie podejmowałem. Ja powiedziałem, że ona ma podjąć, bo mi jest wszystko jedno. Ja się zgadzam na wszystko, żeby nie było potem, że ja przyoszczędziłem na jej zdrowiu. Więc to nie ja podejmowałem, to na podejmowała.

**No tak, ale uczestniczyłeś w tym procesie.**

No musiałem. Musiałem wnieść to, potem złożyć, wynieść stare łóżko.

**Chodzi mi o to, jak się czułeś, jak kupowaliście to łóżko, jakie emocje ci towarzyszyły?**

Ojejku, no wiesz co? Trochę tak, jak przyszło, to się ucieszyłem, bo jak już przełknąłem to, że wydaliśmy tyle kasy, której mi trochę było szkoda. To się ucieszyłem, bo mamy nowe, ładne, pachnące. Jest czyste i przyjemne. Takie rzeczy jak materace, to trochę jak ubrania. To jest jednak tkanina. Jak jest nowa, to jest przyjemna. Bo jak coś jest drewniane, to w ogóle nie mam takiego problemu, mogę wytrzeć to, umyć, polakierować ewentualnie, jak trzeba. I może być stuletnie krzesło, super. Natomiast takie rzeczy, które są z tkaniną, to fajnie, jest gąbka, to miło jest po 10 latach jednak mieć świeżą. Strasznie mi się podoba ten kufer wielki. Którego jeszcze nie zagospodarowaliśmy całego. Bo to też jest proces, bo musimy kupić kolejne pojemniki, które nam dobrze wypełnią tą przestrzeń na dole. Są takie plastikowe pojemniki na pościel, znaczy takie… Plastikowe w sensie no foliowe. Żeby nie wrzucać tych wszystkich kocy po prostu do środka, tylko żeby zorganizować tam. Więc musimy to sobie jeszcze tam przemyśleć. Fakt epidemii nam troszkę utrudnia, bo nie można sobie po prostu pójść do Ikei i popatrzeć. W sensie nawet już teraz chyba można, ale nie chcemy jeszcze tego robić. Więc emocje, jakie były. Cieszyłem się, irytowałem się, że ona tak długo wybiera materac. Bo ona wybierała materac… W związku z tym, że ja się wycofałem z tej odpowiedzialności i z tego wybierania i powiedziałem, że niech zrobi, co chce, to się zaczęła miotać długo. Bo tak, to by narzekała, że jej nie chce pozwolić na wydanie, coś tam. Więc chwilę jej to zajęło. 3 miesiące wybierała. I to też było dobre, bo pojechaliśmy do tego sklepu i zrobiliśmy całe to zamówienie. I potem powiedzieliśmy panu, że jeszcze jedziemy wybrać materac. I że nie potwierdzamy, bo jeszcze nie zmierzyliśmy dwóch rzeczy. Pierwsza to była wysokość tego zagłówka, który jest z tyłu za głową w łóżku. Bo nie wiedzieliśmy, jakiej może być wysokości, bo mamy lampkę w kinkiecie nad głową. I trzeba było, żeby nie była czasem, żeby nie zawadziła. Więc trzeba było to sprawdzić. I jakiś drugi wymiar był jeszcze do określenia. Więc pan na formularzu zgłoszenia miał jeszcze jakieś 2 rzeczy do dookreślenia, więc nie wpłaciliśmy zaliczki, więc to wisiało. Takie zamówienie bez zamówienia. Więc pojechaliśmy do Ikei. Oczywiście ona się nie mogła zdecydować. Miała jeszcze z siostrą przegadać jej materac i jakąś koleżanką, to trwało. Po dwóch miesiącach zdążyła pandemia na świecie wybuchnąć. I koronawirus, ludzie zamknięci, tutaj w maseczkach. W ogóle po dwóch i pół miesiącach, dzwonię do faceta. I mówię dzień dobry, to ja i tutaj taka nietypowa sprawa, bo myśmy kiedyś łóżko zamawiali. I ten facet mówi: a, pan Mikołaj, to ja wszystko pamiętam! Metr czterdzieści na dwa, niebieskie obicie. I zaczyna mi opowiadać dokładnie, jakie to łóżko. Ja nie pamiętałem jakie to łóżko było. A on mi pikowany materiał, z tyłu gładki coś tam, skrzynia wysokość 40. I chcieliście, żeby były tam cienkie… Bo rzeczywiście chodziło o to, że chcemy mieć szerszy materac, 1,40m ale potrzebujemy bardzo cienkie obramówki. BO mamy niedużą sypialnię. Więc, żeby to nie było wielkie, szeroka rama, to ta rama ma chyba 3 centymetry. To jest centymetr deski i 2 centymetry najcieńszej tam gąbeczki. I facet wszystko zaczyna opowiadać. Więc to też było, rozbawiło mnie to strasznie.

**Ja rozumiem, że generalnie to jest tak, że jesteś osobą oszczędną, tak byś się nazwał, tak mi się wydaje.**

Tak. No, no, no. Oszczędność na granicy skąpstwa.

**Dlatego chciałabym zrozumieć, czy ty w ogóle jak wydajesz pieniądze, jak coś kupujesz, to czy ty się wtedy z tym czujesz dobrze? To znaczy czy tam jakieś pozytywne emocje towarzyszą. Czy raczej czujesz się z tym źle.**

To zależy. Jeżeli wiem, że wybrałem optymalnie… To u mnie to jest, rzeczywiście. Być może mógłbym chodzić do psychoterapeuty jakiegoś z tym uczuciem. Bo nawet nie chodzi o to, czy coś jest drogie albo tanie. Tylko czy to jest dobry wybór. Jeśli wiem, że to jest dobry wybór, to się czuję z tym dobrze. I wtedy tak. Ale natomiast nie lubię mieć poczucia takiego, że przepłaciłem albo nie wybrałem najwyższej jakości. Więc tak. Więc bardzo często mnie zakupy w obszarach… Ten, o, no właśnie moja żona tutaj pokazuje tak. Że kręci pejsa żydowskiego. Bo moi znajomi się zawsze śmieją, że właśnie nagroda złotego pejsa. Tak. Więc są obszary, w których ja się czuję pewnie, to ja zwykle wiem, co kupuję. Zresztą łatwiej mi osiągnąć zadowolenie z takich zakupów, tak? Natomiast, jeśli są obszary, na których się nie znam, to mnie to męczy, bo muszę dużo przeczytać i poznać i się dowiedzieć. A jak tego nie zrobię, to wtedy jestem niezadowolony. Ale zwykle nie mam czasu ani chęci się wgryzać w jakieś… Na przykład optymalizacja, tam właśnie jaki najlepszy wybrać garnek. Nie wiem tego, więc…

**A pamiętasz ostatni zakup, z którego byłeś niezadowolony albo czułeś, że przepłaciłeś albo te wszystkie rzeczy, tak jak mi powiedziałeś, że jesteś niezadowolony z zakupu.**

Bardzo często to ma miejsce przy samochodowych rzeczach. Tylko niedotyczących kempingowej części wyposażenia, bo tam mam rozeznanie, jest mi to łatwiej ogarnąć. Tylko takich związanych z mechaniką albo pół mechaniką. Na przykład kupowałem przez AliExpress, to była duża decyzja, która, trochę nie wiedziałem jak… Była stresująca właśnie. I do dzisiaj nie wiem, czy jestem zadowolony z tego właśnie. Kupowaliśmy ogrzewanie postojowe suche do samochodu. I można to zrobić w kilku wariantach. To znaczy można pojechać do serwisu… Te ogrzewania postojowe są popularnie nazywane webasto, ponieważ tak się nazywa. I to się na to mówi trochę tak jak na adidasy w Polsce. Że mamy adidasy, bo to są… Ale możemy mieć adidasy Reeboka w Polsce. Więc u nas można mieć np. webasto Planara. Bo Webasto to jest największa niemiecka firma, która robi to ogrzewanie postojowe. Więc możesz pojechać do autoryzowanego serwisu Webasto, tego niemieckiego. I tam ci założą za 3 tysiąca, od 2 do 4 tysięcy możesz kupić sobie Webasto. Montaż tysiaka. I wszystko jest na niemieckich częściach, serwisowalne, najlepsze. I w ogóle super, od ręki. I się nie martwisz itd., tylko wydajesz na to ze 4 tysiące. OK. Możesz pojechać i kupić… A, jeszcze jest drugi, troszeczkę tańszy, Eberspacher, też niemiecki, jest jeszcze firma Planar rosyjska. I te Planary na przykład montują ludzie, którzy jeżdżą na wschód, ponieważ tam się daje naprawić, tirowcy albo jacyś tam w tranzycie. Ale też osoby, które jeżdżą na 4x4 na Syberię, to sobie właśnie te ruskie wrzucają. Bo tam jest szansa znalezienia części albo serwisu albo czegokolwiek. I ten ruski jest 2x tańszy. Ale można kupić jeszcze taką chińską podróbę. One są produkowane w identyczny, to jest zżyłka z tego Ebersprachera, podobno to się nie różni niczym. Tylko nie ma kontroli jakości takiej, bo kupujesz od Chińczyka, no name. I wtedy zamawiasz od 500 do 800 zł, ściągasz sobie paczkę z Chin. Tylko to, co ci przyjdzie, jest wielką niespodzianką. Bo jeden działa, drugi nie działa. Kwestia jest, żeby to zamontować. W związku z tym, że to jest tańsze i bez tej kontroli jakości, w dodatku być może ma jakiś szacher macher, to serwisy renomowane nie chcą się dotykać. Więc trzeba szukać pana Mietka, który to zainstaluje, nie wiadomo, czy pan Mietek zrobi to dobrze. Całe są fora, opisy, ten. Jest grupa na Facebooku poświęcona tym chińskim webasto cała. Nie jestem członkiem tej grupy, ale wiem, że istnieje taka. I to jest na przykład właśnie taki moment, kiedy kupujesz coś, co ma sens cenowy, bo płacisz za to 5 stów zamiast trzech tysięcy. Ale masz taki kłopot, że nie wiesz, co ci przyjdzie, nie wiesz, czy to będzie dobrze działało. Jak nie będzie dobrze działało to prawdopodobnie nie znajdziesz serwisu, który ci z tym pomoże. Więc ja znalazłem jakiegoś gościa, który mi to założył. I zrobił to chyba dobrze. I to działa. I działa już cały rok. Natomiast ma problem taki, że trochę podśmierduje jak wypuszcza powietrze, takim plastikiem trochę wali. My tego rzadko używamy. Być może trzeba byłoby to kiedyś zostawić na dobę na przykład, żeby się przepaliło solidnie. Bo być może gdzieś tam jest jakaś część obudowy czy czegoś, która musi się po prostu wypalić i przestać śmierdzieć. Bo to jest silnik po prostu, w sensie komora spalania tam jest. I jak to założyliśmy, to tam też miałem perypetie, że działało, raz nie działało. A potem, jak się przygotowaliśmy do wyprawy i wymienialiśmy akumulator i coś tam jeszcze, to potem nagle przestało działać. No i ja panika, bo tu tydzień do wyjazdu, jedziemy do Norwegii, tam jest zimno i pada deszcz i w ogóle, moja żona, która wiecznie marznie, powiedziała, zgodziła się pojechać w tą podróż pod warunkiem, że będziemy mieli dodatkowe ogrzewanie. I ja mówię, słuchaj, to nie działa. A to szukaj, no to jest… No i się robi dramatycznie. No i ja jadę do tego serwisu, który mam najbliżej, z którym czasami współpracuję. No i oni rozłożyli ręce, że nie wiedzą, słabo. No i pojechałem do tego gościa, który mi montował to. Ale on jest po drugiej stronie Warszawy, 30 kilometrów. Ja jestem z północnej części, on jest z południowej. Więc w ogóle jadę tam do niego. On mówi, że on ma robotę na najbliższy tydzień. Ja mówię to nie, za tydzień to ja już powinienem być w ogóle w drodze. To ja już… A on mówi, to pan przyjedzie, tylko rano, spróbujemy się jakoś tam wcisnąć i zobaczymy. Pół dnia ze mną spędzili. I znaleźli skubańcy. Okazało się, że z innego serwisu, w którym mi coś naprawiali, przecięli przewód, znaczy przytrzasnęli, uziemili przewód od pompki paliwa w tym urządzeniu. I wywalało bezpiecznik za każdym razem, jak się uruchamiało. Ale nie samo urządzenie, tylko jakiś tryb w urządzeniu. W związku z tym myśmy już podejrzewali, że to elektronika w środku. I wiedziałem, że jak to będzie elektronika w środku, to właśnie dlatego, że to jest podróba, to wtedy nie jesteś w stanie tego naprawić. I to był taki moment, kiedy ja sobie myślałem, no to świetnie, chciałem zaoszczędzić, chciałem zrobić dobrze. Ale wylądowałem w miejscu, że już wydałem 5 stów na zakup urządzenia, 5 stów na montaż. Teraz mam to właśnie niedziałające. Mam wyjeżdżać, no to całkowicie do dupy. Zamontowałem to, to jest tam wiesz, nawiercanie samochodu na wylot od spodu, robienie dziur przy zbiorniku paliwa, bo to musisz tam wprowadzić coś, bla, bla, bla. I w związku z tym ja sobie myślałem, no, nie chcę przeklinać teraz, ale naprawdę cisnęły mi już, gorąco mi się robiło na myśl o tym, że nie dość, że to jest taki… Jakby że źle podjęta decyzja, to jeszcze się zaraz okaże, że to w ogóle zmarnowane pieniądze. I jeszcze nawet nie jestem w stanie tego naprawić, zrobić tego przed wyprawą. Więc to jest taka na przykład. I w samochodowych rzeczach tak miewam, że nie jestem w stanie określić, czy coś jest sensowne czy nie. Bo się słabo na tym znam, na mechanice, na samochodach. Ja być może brzmię teraz jak bym się na tym znał. Ale tak nie jest. Ja po prostu obczytuję się tyle, ile mogę, bo muszę sobie jakoś z tym radzić.

**A mógłbyś mi jeszcze opowiedzieć jakąś taką historię o twoim oszczędzaniu, z której się śmieją twoi znajomi?**

Nie wiem, bo to są sytuacyjne bardziej. Nie wiem, czy jakąś konkretną był w stanie. Bo to są o mnie historie, więc na pewno moja żona sypnęłaby jak z rękawa takimi historiami. Ale kurcze, trudno mi jest powiedzieć.

**Chodzi mi o taki flagowy przykład twojego oszczędzania. Tego skąpstwa, jak to nazwałeś.**

No nie wiem, nie wiem. Bardzo staram się nie robić głupich oszczędności. To znaczy nie robić takich rzeczy. Żeby nie oszczędzać na rzeczach, które się odbijają na tobie. To znaczy pojechać skrótem, który ma ci zaoszczędzić 100 km, bo to jest 30 zł na paliwo. A potem się okazuje, że urwało ci zawieszenie, bo były dziury. Albo kupowania najtańszej żarówki za to 3 razy, a nie energooszczędnej. Więc takich rzeczy staram się nie robić. Nie, raczej z takiej skrupulatności się ze mnie czasami nabijają, że ja na przykład tam rozliczam różne rzeczy. Ale to też działa w dwie strony. Bo ja na przykład nie lubię, czuję się niekomfortowo i uważam, że jest nie fair, jak czasami korzystamy z… Nie wiem, jedziemy do kogoś na działkę powiedzmy i korzystamy przez tydzień z czyjegoś domku na Mazurach. I zużywamy mu prąd, wodę, śmiecimy, szambo, cokolwiek. No i potem wszyscy sobie, tu ktoś przyjedzie w poniedziałek, tu ktoś we wtorek, tutaj ten, tu jest impreza, tutaj wakacje, miło, sympatycznie, wyjeżdżamy, tam sprzątniemy oczywiście. No ale dla mnie wypada się jakoś rozliczyć z tych kosztów. A ludzie na przykład nie mają takiego poczucia. No ktoś zaprosił do domku… Albo jest tak, że nawet nie zaprosił, bądźcie moimi gośćmi, tylko powiedział, że możemy przyjechać, bo to już jest jakaś cykliczna rzecz, miejsce, do którego czasami ze znajomymi jeździmy. No i ja uważam, że takie rzeczy należy rozliczać, jakoś jednak zrzucać koszty. W związku z tym wtedy pytam, słuchajcie, chcecie to rozliczyć, co chcecie rozliczyć. Znaczy tego gospodarza zapytam, jakie poniósł koszty. Oni zwykle nie chcą mówić. Bo u nas jest w kulturze tak, że nie można takich… Postaw się, a zastaw się. Więc ja tam dopytam. I potem mówię, słuchajcie, no to nie wiem, chcecie po równo się tym rozliczyć, czy kto, ile nocy był, czy jak to zrobimy? Bo jakoś, żeby to zrobić. Więc z takich rzeczy wtedy ludzie właśnie… Trochę ich to irytuje powiedzmy… Bo część dlatego, że po prostu chciałaby pewnie nie zapłacić, a część dlatego, że nie chce się tym zajmować. Też ludzie mają różne priorytety. Więc to jest na przykład taka rzecz, która… Że ja na przykład potem chodzę i oni się trochę śmieją. Bo ja mówię, co chcecie, albo po równo, tylko to głupio wyjdzie, bo ktoś przyjechał na 1 weekend, reszta siedziała 7 dni. I wszyscy zapłacą na przykład po stówce albo tam po 200 zł i co? Tam za jedzenie, za coś tam. I wyjdzie na to, że ta osoba przyjechała na 2 dni i zapłaci 2 stówy a inni siedzieli 7 dni i też zapłacili 2 stówy? No trochę nie bardzo. No i potem zaczyna się wyliczanie, kto był, kto nie był. Więc na przykład.

**W twoich dochodach rozumiem, że teraz w trakcie tej całej pandemii nic się nie zmieniło, bo nie pracowałeś przed pandemią i teraz też nie pracujesz?**

Znaczy może akurat nie dotyczyło to stycznia i lutego, ale jeszcze w 2019 roku październik, listopad, w grudniu to już mniej, bo tam święta były, to jakieś dorywcze rzeczy robiłem. Jakieś zlecenia miałem. Więc tak, ta pandemia, pewnie by coś funkcjonowało i pewnie byłoby więcej… Trochę mi trudniej zacząć coś robić też.

**A na ile to jest tak, że to jest jakieś zagrożenie dla waszego budżetu, to że teraz tych dorywczych rzeczy nie robisz?**

Nie jest. Nie, ja dorabiałem rzędu powiedzmy w miesiącu 1000 zł. Więc to raczej taki, raczej chodzi o to, żeby to były rzeczy, z których na przykład czasami miałem… Chociaż nie, miałem też 1 projekt, z którego 3 tysiące zarobiłem. No tak, dodatkowe pieniądze. A traktowaliśmy to jako dodatkowy budżet, to możemy właśnie, nie wiem, odłożyć te pieniądze albo gdzieś pojechać.

**A czy w związku z tym, że tego dodatkowego budżetu u was nie ma, to wy podjęliście teraz w trakcie tej całej pandemii jakieś działania w celu ograniczenia tego waszego wydawania?**

Nie. Nie. I tak wydajemy mniej niż na co dzień. Znaczy nie dramatycznie mniej. Bo myślę, że ludzie, którzy wydają właśnie, nie wiem, na co dzień w klubach, w knajpach, chodzą na obiady, chodzą na zakupy do galerii handlowych i dużo w ogóle konsumują, no to one poczują taką, że o, ale dużo mniej wydaję. No my robimy takich rzeczy niewiele. Jeszcze było, ten, dla nas to trochę taki sam okres jak… No, troszkę mniej wydajemy niż… ale to trochę, 15%, może 20% mniej niż na co dzień. Myślę, że 15 raczej niż na co dzień. To jest dla nas tryb trochę tak jak zwykle tylko bez wakacji. Bo u nas właściwie, dosyć dużo na wyjazdach wydajemy.

**Ale rozumiem, że to, że nie planujecie wakacji to nie wynika jakby z ograniczeń waszego budżetu, tylko z tego, że po prostu nie można wyjechać nigdzie.**

Teraz tak, z tego, że nie można wyjechać, są ograniczenia itd. Trochę w tym roku rozmawialiśmy o tym, to wakacje, które będziemy mieli, nie będą takie bardzo jakieś fancy. Nie będą jakieś super wybajerzone. Ale byliśmy już w tym roku w styczniu na tydzień na Sycylii. Więc tak naprawdę…

**Bardzo mi przykro, że to powiedziałeś, bo miałam właśnie na Wielkanoc lecieć na Sycylię.**

No to niech ci będzie smutno, bo bardzo polecam. I wydaje mi się, nie byłem tam latem nigdy w południowych Włoszech, ale byłem w środkowych latem. Nie do zniesienia jest. W związku z tym wydaje mi się, że południe musi być jeszcze bardziej nie do zniesienia. I Wielkanoc to jest chyba ostatni moment, kiedy tam warto lecieć. Myśmy byli w styczniu. I to było… Rzeczywiście oni mówili, że już jest strasznie zimno i chodzili tam w kurtkach itd. Więc to znaczy, że nie każdego dnia było 20 stopni. Wieczorem było 10. I oni uważali, że to jest bardzo zimno. I rzeczywiście u nich to był jakiś taki okres ferii zimowych. Więc pod tym względem trochę źle trafiliśmy, bo niektóre miejsca były takie wymierające. O 15 się wszystko tam zamykało i czasami nie otwierały się te rzeczy w tym trybie popołudniowym. Więc trochę było rzeczywiście tak, że to bardzo nie sezon był. Więc polecałbym jechać tam 2 tygodnie później. Bo oni mówili, że dla nich końcówka stycznia to jest takie zamknięcie, zamknięcie. Ale luty – super. Nie jest gorąco, jest puściutko, nie ma tłumów.

**A jeszcze wracając do tego budżetu i tego, jak teraz wasz budżet wygląda. To czy na zakupach, jak robicie jakieś zakupy, to szukasz w ogóle jakichś przecen?**

Teraz właściwie, właściwie to jest też ciekawe, bo nie ma przecen. W sensie, że bardzo mała ilość promek jest teraz w Biedronce czy w jakimś… Bo teraz to jeżdżę tylko do Biedronki. Raz w tygodniu, raz na 7-10 dni po prostu jadę do Biedronki i robię na te 7 do 10 dni zakupy. Robię też, poza tym chodzę do piekarni. Też raz na tydzień. Bo nie kupuję pieczywa w Biedronce, bo uważam, że jest niesmaczne. I jeszcze czasami chodzę do takiego innego sklepu, u nas pod domem dokupić... To tam mówiłem ci, że herbatę tam kupujemy i coś jeszcze. Tam dokupuję rzeczy, których nie jestem w stanie kupić w Biedronce. I tam mieliśmy taki dylemat, żeby na początku nie chodzić. Teraz też tam już raz byłem. Dokupiłem herbaty, 400 torebek herbaty kupiłem.

**Ale to jest tak, że teraz nie ma promocji, ale zwykle jest tak, że ty szukasz jakichś takich przecen, promocji?**

Nie. Znaczy raczej staram się nie kupować rzeczy, których bym nie kupił… No chyba, że to będzie tak, że nie wiem, powiedzmy kupuję sobie, załóżmy orzechy… O, powiedzmy, że kupuję sobie orzeszki laskowe, czyli te ziemne, bo są dobre, lubię je, smaczne itd. Ale moimi ulubionymi orzechami będą pistacje. No to orzeszki laskowe to jest 10 zł za kilo, pistacje są 60. No to rzeczywiście, gdyby była taka promka, że pistacje są po 25 zł za kilogram, to bym pewnie powiedział, no dobra, no to sobie kupię pistacje, które lubię. Ale to, że będzie przecena na… Nie wiem na co. Na coś, co nie jestem jakimś wielkim... Jakby to, że będzie przecena na prażone orzechy w miodzie, które są też smaczne, ale to nadal będzie drogi produkt, którego niespecjalnie jestem jakimś gigantycznym fanem, no to już pewnie go nie kupię, tylko zostanę przy tych swoich orzeszkach ziemnych. Natomiast na pewno zwracam uwagę na produkty tego typu, które kupujemy, ten rodzaj, które kupuję. Jeśli to, co kupuję, jest w promocji albo coś klasy wyżej jest w promocji i jest w podobnej cenie, a jest na przykład lepsze, smaczniejsze itd., to wtedy kupuję tą lepszą rzecz. Albo jeżeli to, co kupuję zwykle jest w promce, to kupuję często na zapas. I korzystam dosyć… Jeżeli coś nie jest szybko psującą się rzeczą, to bardzo często kupuję w tych tam… Kup dwie, zapłać za jedną, czy tam trzecia gratis itd. I wtedy kupuję, nie wiem… I 20 sztuk mogę kupić. Jak jest sos pomidorowy jak jest w dobrej przecenie, no to ja 2 palety do domu przyniosę.

**Te pistacje to pamiętam, że na Wielkanoc kupowałeś chyba sobie. A czy w ciągu tych ostatnich 2 tygodni kupiłeś sobie coś takiego dla przyjemności?**

No, dla przyjemności sobie kupiłem jakieś słodycze. Ale nadal kupuję takie tanie słodycze. Tak. To słodycze i przekąski typu chipsy to jest taka moja pięta achillesowa. No tak. Ja dosyć dużo podjadam łakoci, za dużo.

**A w takim razie jak teraz wygląda takie kontrolowanie twojego budżetu? Jakoś spisujesz wydatki?**

Ja zawsze mniej więcej wiem, co potrzebuję kupić, zawsze chodzę z listą do sklepu. Dość powtarzalny jest nasz koszyk zakupowy. Większą jego część staramy się, żeby jednak zajmowały owoce i warzywa. Nie jadamy właściwie mięsa, bardzo rzadko. I mniej więcej kupujemy, mu kupujemy w kółko te same produkty. Za wyjątkiem, mówię, jak coś jest w dużej promocji albo jak są warzywa sezonowe, no to to dostosowujemy. I jest tak, że ja zawsze mniej więcej wiem. Przestałem spisywać, kiedyś robiłem sobie takie listy, w sensie spisywałem wydatki. Ale właśnie, żeby nie wpadać w paranoję i nie patrzeć, to w pewnym momencie z tego zrezygnowaliśmy. Ale to nie spowodowało, że my jakoś wydajemy niesamowicie dużo. Ja raczej mam listę, wiem mniej więcej, czego potrzebuję. Teraz robię dokładne listy, bo jak by co, to nie pójdę drugi raz. W sensie staram się, żeby nie było tak, że chodzę 3 razy w tygodniu do sklepu. Tylko raz na tam 7-10 dni. Tyle załapaliśmy, że na tyle jesteśmy w stanie kupić świeżych warzyw i owoców. Gdyby nie warzywa i owoce, to moglibyśmy chodzić raz na miesiąc do sklepu bez problemu.

**Rozumiem, że zawsze planowaliście te zakupy i pilnowaliście tego budżetu domowego. Ale czy tobie się wydaje tak ogólnie, że w tej sytuacji obecnej to jest dobrze ograniczać wydatki czy nie?**

No, to zależy jakie wydatki. Jeżeli mówimy o wydatkach konsumpcyjnych, to zawsze jestem za tym, żeby w rozsądny sposób je ograniczać. Natomiast, jeśli chodzi o to, że chcesz teraz inwestować w coś, to może to jest dobry moment. Jeżeli chcesz wydawać… Nie ma teraz takiego dramatu jeszcze na razie. Znaczy być może są ludzie, którzy już go przeżywają. Ale nie wygląda, żeby… Czytałem, ale ja nie miałem potrzeby, być może będę musiał zaraz pójść po jakieś ciuchy. Ale czytałem, że dużo odzieżówek miało online’owo przeceny, dużo przecen. Ponieważ ubrań ludzie, no długofalowo muszą kupować, ale są w stanie się powstrzymać na miesiąc albo 2 albo nawet 3. W związku z tym, jak wybuchła ta epidemia czy pandemia i był ten lockdown i ludzie schowali się do domów itd. to podobno ta branża odzieżowa to po prostu spadły im niemalże do zera te obroty. Więc oni zrobili jakieś straszne promocje w sklepach online’owych swoich. No jakieś marki odzieżowe i obuwnicze. I dlaczego ja to mówię?

**Bo pytałam, czy warto teraz ograniczać wydatki.**

Tak. Więc jeśli na przykład teraz chcesz sobie dokupić, nie wiem, stwierdzasz, że masz jakiś model dżinsów albo spodni i będziesz w takich chodzić jeszcze, zawsze w takich chodzisz i właściwie potrzebujesz nowej pary, to może warto sobie kupić 3 pary. Tak? Więc w tym sensie nie warto być może teraz oszczędzać. Tylko warto sobie zrobić zapasy, jeżeli coś jest taniej. Albo jeżeli potrzebujesz kupić, nie wiem, mieszkanie teraz albo samochód, może będą tańsze zaraz z rynku wtórnego.

**A ty oszczędzasz w takim znaczeniu, że posiadasz jakieś oszczędności?**

Tak. Staram się mieć podwójnie. Bo pierwsze na zasadzie takiej, że na czarną godzinę i jak by co, żeby mieć pieniądze, gdyby się coś wydarzyło itd. Albo gdyby na przykład właśnie pieniądze też w jakimś… Jeżeli bym żył tak zupełnie od pierwszego do pierwszego, to mogłoby być tak, że nawet bym nie mógł optymalizować poprzez robienie odpowiednich zapasów. To znaczy, jeśli masz małą pensję i nie masz oszczędności, no to na przykład, jeśli wiesz, że zużywasz, będziesz potrzebowała dżinsy albo bluzkę jakąś, nawet do pracy i właśnie byś ją sobie kupiła, bo jest przecena 50% w jakiejś twojej ulubionej sieci. I byś kupiła sobie 3 bluzki, bo by ci się przydały, ale nie masz pieniędzy na koncie, to jest bez sensu. Bo jeśli ty wiesz, że to jest dobra okazja. Np. one ze 100 zł do 30 zł, bo jest koniec kolekcji, a już w tym miesiącu skończył ci się hajs. I mówisz, kurde, ja właściwie bym potrzebowała tą bluzkę a na pewno w lipcu będę potrzebowała drugiej. No ale nie mam pieniędzy. No to znaczy, że ty kupisz tą bluzkę prawdopodobnie w lipcu i w maju, tylko już nie za te 30 czy 40 zł, bo jest końcówka kolekcji tylko z nowej kolekcji kupisz za stówę. Więc w tym sensie czasami brak płynności jakiejś tam nie pozwala ci korzystać z okazji. Czy mówimy o… O jedzeniu to już są śmieszne kwoty, nie wiem, 50 zł. O ciuchach, no to być może czasem warto się na przecenach obkupić. Ja tego nie robię, ale rozumiem ludzi, którzy to robią. W sensie rozumiem, jeżeli to jest rozsądne, a nie takie szaleńcze, to rozumiem, że można pójść i kupić sobie do pracy nowe koszule, garnitury, sukienki, żakiety, cokolwiek. I wydać na to 4 stówy czy 8 stów raz do roku. Dla mnie to jest całkiem rozsądne wydać osiem stów w jednym miesięcy, zamiast każdego miesiąca po dwie stówy na jedną rzecz przez pół roku. I wydać generalnie 1200. A już nie mówiąc o tym, że to w większych rzeczach to ma jeszcze większe znaczenie. To znaczy kwestia samochodu. Albo mieszkania. To znaczy znam przypadek osób, których nie było stać na to, żeby wykupić mieszkanie od urzędu gminy, państwa czy czegoś takiego, jak one były z dobrym rabatem. Bo kiedyś taka była sytuacja. I nie mieli tam 50 czy 100 tysięcy czy iluś jak gmina to oferowała, czy w Warszawie to są dzielnice. I po paru latach mieli te pieniądze, ale zmieniła się polityka miasta i skończyła się wyprzedaż tych nieruchomości. W sensie to nie jest tak sprzedaż na wolnym rynku, tylko że tam najemcy wieloletni nabywali prawo do tego, żeby właśnie złożyć taki wniosek o przejęcie na własność z jakąś tam bonifikatą czy tam rabatem. I jak ktoś nie miał akurat w kieszeni 50 czy 100 tysięcy, no to nadal mieszkał w nie swoim mieszkaniu, w którym nic nie jest w stanie zrobić. Może je teraz od miasta wykupić, tylko pewnie za pełną kwotę. Czyli zamiast 100 tysięcy za 400. No to w przypadku bułki to jest śmieszna rzecz albo dżinsów. Ale móc kupić w odpowiednim momencie swojego życia samochód, mieszkanie albo nie wiem, co jeszcze dużego można kupować takiego. Działkę rolną czy cokolwiek. Dlatego w tym sensie uważam, że warto mieć pieniądze, żeby korzystać z różnych opcji. Nie wiem, móc sobie to optymalizować znowu i korzystać sensownie. A dwa, na czarną godzinę. Czyli, nie wiem, połamię nogę, nie chcę się martwić, że nie mam pieniędzy. Albo teraz jesteśmy na jednej pensji. Jak byśmy nie mieli oszczędności, to byśmy się stresowali, a co to będzie jak koronawirus uderzy też w branże mojej żony i ją zwolnią na przykład. Albo cokolwiek się zdarzy losowego.

**Rozumiem, jaką funkcję to oszczędzanie u ciebie pełni. Ale w jaki sposób ty oszczędzasz, tak jakby konkretnie?**

No w ten sposób, że kiedyś mieliśmy tak, że mieliśmy z góry ustaloną kwotę, którą co miesiąc... Nie, odwrotnie. Kiedyś było tak, że wydawaliśmy pieniądze i pod koniec miesiąca patrzyliśmy, ile nam zostało. I wrzucaliśmy to na jakieś lokaty albo jakiś rachunek oszczędnościowy, jakieś takie rzeczy. Odkładaliśmy to jakby na osobną kupkę, która jest nie do korzystania. I patrzyliśmy, jak tam idzie, zostawialiśmy sobie tam parę stów. No i rozpoczynał się nowy miesiąc, jakieś tam pensje czy wypłaty. Ja wtedy pracowałem w takim systemie jeszcze nie jednej pensji tylko od zlecenia do zlecenia. I tam sobie patrzyliśmy. I to powodowało taki trochę stresujący układ, że zawsze staraliśmy się zoptymalizować, żeby na koniec miesiąca jak najwięcej zostało. I wtedy właśnie było takie to oszczędzanie idące w skąpstwo. Więc zmieniliśmy strategię po roku czy dwóch małżeństwa. Zmieniliśmy strategię i zrobiliśmy tak. Mieliśmy estymację, ile mniej więcej zarabiamy. U mojej żony to było łatwiej, bo ona pracuje na etacie prawie od zawsze. W sensie miała tam jakieś za czasów studenckich nieetatowe prace, ale potem na etacie. Ja różnie. I mniej więcej byliśmy w stanie dokładnie albo mniej więcej oszacować, ile mamy pieniędzy i na przykład z tego ustalaliśmy sobie, jaką kwotę oszczędzamy. Tam nie wiem, 2, 3, 4 tysiące. Znaczy tyle chcemy, żeby nasza kupka miesięcznie przyrastała. I przychodziły tam pensje czy wypłaty czy jakieś inne rzeczy. I robiliśmy, tak jak trochę robisz na mieszkanie, znaczy w sensie tam płacisz rachunki: czynsz, telefon, prąd, gaz, coś tam jeszcze, oszczędności. Pyk, odpowiednia kwota ląduje na koncie. A reszta jest do wydania. Z tym, że te oszczędności miały tylko i wyłącznie charakter, to były oszczędności na nadpłatę kredytu, na czarną godzinę. Na ewentualnie jakieś takie, może samochód albo jakieś takie duże… Samochód albo takie np. jak by trzeba było, nie wiem, w coś zainwestować, coś takiego. Takie rzeczy, które raczej nie służyły konsumpcji, zużywaniu rzeczy. I to sobie oszczędzaliśmy tak. A wakacje staraliśmy się robić z tych bieżących środków.

**Czyli teraz jest tak, że trzymacie swoje oszczędności na koncie oszczędnościowym?**

Na lokacie. Ale właściwie te lokaty są w tej chwili tak… My, jak kupiliśmy, mieliśmy trochę więcej pieniędzy przed zakupem mieszkania. Teraz, jak kupiliśmy mieszkanie, no to się wypłukaliśmy dosyć mocno. Odrabialiśmy to częściowo, potem nadpłacaliśmy kredyt. Jak tu robiliśmy jakąś górkę, no to przehulaliśmy w tej Norwegii sporo kasy. Nie wyczyściliśmy się do zera, ale jednak wydaliśmy sporo. I teraz nie dysponujemy jakąś niesamowitą ilością oszczędności. Właściwie to powinniśmy z nimi robić coś mądrzejszego. Ale one tak trochę siedzą na lokatach, które są beznadziejne. W sensie na lokacie w tej chwili, nam to służy trochę jako rodzaj zablokowania środków. Takie, żeby one sobie po prostu były w innej przegródce. I ewentualnie, gdybyśmy kartę zgubili, to żeby ktoś nam nie ściągnął łatwo wszystkiego. Ale to prawda, myślałem już o tym, że powinniśmy mieć konta oszczędnościowe osobne założone. To chyba zwiększa bezpieczeństwo.

**Czyli żadnego inwestowania tych środków u was nie ma.**

Chcielibyśmy, ale do tej pory naszym inwestowaniem było nadpłacanie kredytu.

**Bo rozumiem, że to jest tak, że macie jedno konto, na którym oboje...**

Nie. Po prostu mieliśmy konta, zanim zostaliśmy małżeństwem, każde z nas miało swoje. I po prostu tak zostało. Robiliśmy tak, że przerzucamy tylko czasami, teraz to już dawno, na początku, przerzucaliśmy pieniądze między jednym a drugim kontem, w zależności od tego, gdzie były jakie promocje na lokaty jakieś. Choć teraz, trochę może mało to ostatnio śledzę, ale mówię, te kwoty też nie są takie duże. Jak mieliśmy więcej pieniędzy, to bardziej na to zwracaliśmy uwagę. Było trochę tak, że w tej chwili stwierdziliśmy, że tak jakby cyklicznie patrząc, że cały czas u mojej żony na koncie były lepsze warunki. W związku z tym de facto ona trzyma wszystkie pieniądze. A teraz jak ja nie zarabiam, to już w ogóle. To u mnie tylko maleje, a u niej rośnie. Więc teraz właściwie tak.

**A patrząc na te oszczędności. Też nie wiem, czy patrzeć ja te twoje, czy twojej żony, czy wasze, jak na to patrzeć, ale to zaraz mi powiesz. To jak byś teraz utracił jakiekolwiek źródło dochodu, to…**

Wiem, o co chodzi. Ile jestem w stanie żyć, gdybyśmy oboje stracili pracę i przychody. Gdybyśmy bardzo… Nie zmieniając naszego sposobu życia, to myślę, że tak rok. Przycinając różne rzeczy, to myślę, że… Rok nawet nie, 10 miesięcy. Przycinając trochę różne rzeczy, to myślę, że półtora roku mniej więcej bylibyśmy w stanie żyć z samych oszczędności. Tylko u nas w takich codziennych rzeczach, tak jak teraz w koronawirusie to już w ogóle, nie bardzo byłoby z czego zejść. Znaczy moglibyśmy schodzić ewentualnie… Nie wiem, w związku z tym, że żyjemy oszczędnie no to moglibyśmy jeszcze tańsze jedzenie, ale to jest pytanie, czy byśmy zdrowiem tego nie przypłacali. Bo to jednak już co? Byśmy ziemniaki w kółko jedli albo tam ryż czy coś? No nie, żeby jednak te jedzenie było pełnowartościowe. Moglibyśmy pewnie z niektórych rzeczy… O, właśnie ubrania można przyciąć na chwilę, łóżka sobie nie kupować. Takich rzeczy to na pewno. Więc, myślę tak, rok na spokojnie, półtora roku oszczędzając. Sprzedając nasz samochód, który jest głównie narzędziem turystycznym i ma jakąś tam wartość i generuje koszty, gdybyśmy jego się pozbyli, to byśmy byli w stanie z samych oszczędności, bo on generuje kosz, to byśmy pewnie z 2 lata. A gdyby jeszcze doliczyć pieniądze z niego, to pewnie ze 3 lata bylibyśmy w stanie żyć tak bidując. A jak byśmy się wyprowadzili z mieszkania, co jest naszym planem takim awaryjnym i wprowadzili do, mamy w rodzinie miejsce, gdzie moglibyśmy przycupnąć, gdyby nam się podwinęła noga, no to jeszcze taki mamy plan, że gdyby coś się wydarzyło, to możemy się wyprowadzić 3 bloki dalej do rodziny na chwilę, na pół roku czy coś. I wynająć nasze. No to wtedy w ogóle…

**Czyli całkiem sporo byście dali radę.**

Ale to już mówię, do roku to jest tak, że na zasadzie, że OK. Potem to już by się zaczęło takie mocne przycinanie i z takim bólem w końcu. Więc tego wolałbym uniknąć.

**A to wasze oszczędzanie to jest tak, że czujesz, że oboje tak samo podejmujecie, inicjujecie tematy oszczędzania, czy to jest tak, że ktoś bardziej?**

Teraz już jest takie automatyczne. Ale myśmy dużo dyskutowali na ten temat. Mamy trochę inne wzorce wyniesione z domu. Ale rozmawialiśmy o tym wiele razy przed ślubem, jeszcze na takiej zasadzie ogólnej. A później nam się ucierały różne rzeczy. Ja na początku rzeczywiście mocno… Mieliśmy mało pieniędzy i właśnie ja tak dosyć, na początku sam jak mieszkałem, to tak dosyć spartańsko różne rzeczy robiłem, żeby ten budżet wytrzymywał itd. Jak zaczęliśmy żyć razem, to się trochę bałem, czy właśnie nie będzie tak, żebyśmy w sposób taki rozbuchany. I tam kontrolowałem, sam sobie kontrolowałem koszty. Jak wzięliśmy ślub, to nadal je tam zliczałem itd. I potem stwierdziłem, że właściwie, jak nam się już zrobiło trochę więcej pieniędzy, a ja nadal mocno liczę, to właśnie zmieniliśmy ten system. To znaczy zmieniliśmy system na taki, ile mniej więcej chcemy oszczędzić, a resztę możemy wydać. Żeby też mieć coś, możliwość pójścia do knajpy czasem. A nie duszenia każdego grosza. Tylko pójścia na imprezę, pójścia sobie do kina, pojechania gdzieś itd. I wtedy żeśmy też rozmawiali dużo w ogóle o tym, jak oszczędzać, ile oszczędzać, gdzie chcemy dojść. Czy w przyszłości chcemy, że jak kiedyś będziemy kupować mieszkanie to trochę… Wydaje mi się, że też ja tutaj inicjowałem te tematy i rozmawiałem z moją żoną dużo na ten temat. Że jak się ma więcej na wkład własny, to są lepsze warunki. Albo że można wtedy próbować jakoś ten kredyt obchodzić albo właśnie nadpłacać. I to bardzo mocno maleje całkowity koszt kredytu. I różne tego typu rzeczy. I myśmy się trochę na to przygotowywali. Tak żeby w perspektywie 20-30 lat, żeby… jak to powiedzieć. Pieniądze trochę zaczęły, może nie gigantyczne, ale żeby to one na nas pracowały albo chociaż żebyśmy my byli górką, a nie one nad nami górką i nam ciążyły. Tak jak ludzie mają duże kredyty i się przez to spinają. No to my właśnie… I to było gdzieś tam przegadane kilka lat temu. I teraz już nam weszło w krew i nie musimy tego bardzo… I wydaje mi się, że tutaj też moja żona długofalowo zgodziła się ze mną, że taki styl życia jest… dobry to złe słowo, bo to wtedy znaczy, że inne są niedobre. Ale że ma wiele korzyści. Że żyjemy spokojnie. Nie mamy czegoś takiego, że własne jak jedno z nas straci pracę albo coś, to tam nie wiem, jest dramat i nie zepną nam się rachunki albo strasznie nas to boli. Bo na co dzień żyjemy dość skromnie. Ale mówię, jesteśmy w stanie wyjechać na wakacje dużo. Lubimy wyjazdy i…

**A myślisz jakoś tak o swojej przyszłości zawodowej w tym momencie?**

Bardzo dużo. Niestety nie jestem, nadal nie mam jasności trochę na co się zdecydować. Bo jestem trochę rozdarty między rzeczami, które wiem, że mógłbym robić i monetyzować szybko swoje umiejętności w czymś, czego nie lubię. I wróciłbym trochę do tego, co już robiłem. Mogę pójść do miejsca, które wydaje mi się, że jest czymś, co bym bardziej lubił i co robiłem jeszcze wcześniej, związane z edukacją, ale to są słabe pieniądze i tam są inne wady. I jeszcze zastanawiam się, czy byłbym w stanie się przebranżowić do jednej rzeczy. I trochę mi tu koronawirus w pewnym momencie trochę popsuł myślenie. Bo już byłem zdecydowany na jakieś tam ruchy i próbowałem się ogarnąć w jakimś kierunku, ale tak mnie trochę wybił z tego tematu. I zastanawiam się, czy jeszcze nie iść w stronę czegoś, co mogę bardziej zdalnie w przyszłości wykonywać i bardziej tak elastycznie.

**Czyli bliżej którego z tych rozwiązań byłeś wcześniej? Bo podałeś 3.**

Tak, żeby w projektach edukacyjnych się zahaczyć gdzieś.

**Czyli rozumiem, że to jest coś, co robiłeś wcześniej i jest gorzej płatne?**

Tak. Bo najpierw pracowałem w edukacji, potem w marketingu. I już właśnie trochę nie chcę pracować w marketingu albo przynajmniej w jakimś innym. W sensie w innej roli niż do tej pory. Długo by tam tłumaczyć. W każdym bądź razie już nie wgłębiając się w to dokładnie. Myślałem, żeby może wrócić do tej edukacji. A jeszcze się zastanawiałem nad tym, czy nie zająć się programowaniem. Ja kiedyś trochę programowałem, ale słabo. I miałem się trochę tego poduczyć, ale to mi tam słabo szło. I właściwie to tak trochę bez sukcesu, to się nieprzyjemnie nawet o tym mówi. I tutaj już gadałem, że ewentualnie w tej edukacji jakiś projekt z dziećmi, tam wracałem trochę do tego tematu. I się zastanawiałem, jak to pokierować też, żeby nie wylądować po prostu jako nauczyciel w szkole, bo bardzo bym tego nie chciał. A przynajmniej nie, że to tylko bym robił, bo to strasznie jest takie… No, obniżające morale niesamowicie. Zniechęcające i nużące. W ogóle publiczna edukacja jest strasznie przykra w tej chwili. I tak kombinowałem, żeby tam nie zostać. A teraz trochę wszystko właściwie się tak zamknęło i trochę nie wiadomo, jak to będzie. A jeszcze będą jakieś kryzysy itd. I zastanawiam się, czy jednak nie próbować trochę w tym programowaniu. Robiłem tam jakieś rzeczy związane z automatyzacją rzeczy w Excelu. I taki projekt właśnie wszedł w listopadzie. I trochę to była też, gdzieś tam gałąź, o której myślałem. Ale to są nadal niewiadome dla mnie.

**Trochę poszliśmy w tej rozmowie od końca. Chciałam cię zapytać, czy coś się zmieniło u ciebie w ogóle w ciągu tych ostatnich 2 tygodni.**

Nie. Jak tak myślę o tym. Znaczy zmieniło się w tym, co robimy, w samych działaniach pewnie niedużo. Bo po świętach już rozmawialiśmy, tak, między świętami a majówką. Chyba niedużo. Natomiast zmieniło się trochę to, że… Już wiem, rozmawialiśmy ostatnio zaraz po tym, jak otworzyli, była większa możliwość przemieszczania się, otworzyli parki i lasy. Tak, to zmieniło się tyle, że mamy właśnie swobodę z korzystania z tych…. Z jeżdżenia sobie gdzieś tam na łąkę, lasy, parki. To jest dla nas bardzo istotne. Bo to jest nasz sposób spędzania czasu wolnego. Więc na przykład zaczęliśmy się umawiać na spacery, teraz dużo robimy spacerów. Bo jeżdżę gdzieś na łąkę z moim psem. Ale też umawiamy się i czy ja sam czy z żona, jeździmy też spotykać się z moją szwagierką, z siostrą mojej żony, bo ona psa sobie zaadoptowała. I chodzi o to, żebyśmy, bo ten pies jest ze schroniska, jest jakiś wylękniony itd. Chodzi o to, żeby go powoli, na spokojnie poznawać. Tak, żeby poznawał nas i się już nas nie bał. I będziemy powoli próbować go połączyć, nasz pies też nie ma łatwego charakteru, żeby te 2 psy się powoli poznały i polubiły. Więc na przykład to jest rzecz, która się zmieniła.

**Czyli spotykacie się z ludźmi już teraz?**

Właśnie to jest u nas trudny temat. W sensie może nie trudny, co skomplikowany. Ponieważ jest tak, że nie wiadomo, co z tą epidemią. Właściwie już nie wiadomo trochę, na czym stoimy i o co chodzi. Rząd z jednej strony… To aż mi się nie chce już o tym rozmawiać…

**Możesz nie wchodzić w rządowo-polityczne kwestie.**

Nie, ale wiesz, bo to jest ważne. Dla mnie problemem jest to, że ja nie wiem, na czym stoję. W sensie nikt nie wie, gdzie to wszystko zmierza, co my robimy, dlaczego tak a nie inaczej. Czyli, nie wiem, zamknęli te parki i lasy, co było jakąś bzdurą całkowicie, te tereny zielone. Przy tam x zachorowaniach dziennie. Jak ich było 2x, 2 razy więcej, to je otworzyli. Teraz jest niezmienna tendencja i otwierają jakieś kolejne rzeczy, w ogóle przedszkola. Znaczy ja rozumiem, wiadomo, wszyscy wiedzą, dlaczego. Chodzi o to, żeby ludzie mogli wrócić do pracy, bo nie chcą już wypłacać tego świadczenia opiekuńczego. A chcą odmrażać gospodarkę, bo to zaraz wszystko pierdzielnie. No, ale te dzieci w jakimś chaosie zupełnym wrzucają do szkół. I to jest tak, my byśmy nie chcieli… Ja nie chciałbym tego, żebyśmy zupełnie działali, że to wszystko olać. I tak naprawdę to nie ważne, olejmy tam te wszystkie restrykcje, maseczki i to w ogóle cała epidemia to jest bzdura. A z drugiej stronu nie chciałbym też tak, że strasznie się bać i nic już nie robić. Ale mam wrażenie, że to idzie od złej strony. To znaczy uruchamiamy jakieś właśnie przedszkola i…

**No galerie handlowe na przykład teraz się otworzyły.**

No tak, dla mnie to znak, że to już nic jakby… Jeszcze przy tym, że ludzie właściwie w ogóle przestają respektować to noszenie maseczek czy czegokolwiek, to tak trochę… A nie mam poczucia, żeby się szpitale i jakby świat naokoło nas nauczył dobrze działać. Po to miał być też ten lockdown, żeby dać wszystkim czas, mieliśmy dać sprzęt medyczny, zorganizować się, Sanepid powinien wypracować procedury itd. I ja mam wrażenie, że to słabo było zrobione. Jak czytałem gdzieś jakieś relacje. W ogóle też mniej czytam, bo już ten temat, tam niewiele się zmienia. Z tymi wyborami teraz czytamy, ale to się dopiero dzisiaj i jutro będzie okazywać. Będą jakieś śmieszne rzeczy. Ale też już jestem tym zniechęcony, bo już tak się zakręciło, że już nikt nie wie, o co tam chodzi, jak to będzie. I co robić, co ma sens, co nie ma sensu. Jest to w tak dużym chaosie, że człowiek jest już trochę zagubiony. Więc tu jest trochę tak, że… Pytałaś, dlaczego ja tak teraz zrzędzę, bo pytałaś czy my się spotykamy z ludźmi. Nie wiem. To znaczy niby nie, bo nic nie zmieniliśmy. Bo też już kilka raz z żoną się o to sprzeczaliśmy. Ja mówię, słuchaj, bo wychodzi tak, że my nic nie zmieniliśmy, bo nie podjęliśmy żadnych decyzji, nie wypracowaliśmy sobie żadnych reguł naszego działania. Poza tym, że nie zmieniamy tych konsumpcyjnych, tego chodzenia do sklepu itd. Bo to nawet nam odpowiada, to jest OK, ja nadal robię zakupy dla połowy rodziny. Natomiast moja siostra zapowiedziała już, że ona… Co prawda nie do końca wiem, na czym to polega, bo wiem, że tam trochę dezynfekuje klamki, ręce, jakieś takie rzeczy. Ale z drugiej strony z dziećmi wychodzi na zewnątrz. I tak dosyć normalnie funkcjonuje. I mówi, że jakoś bardzo się nie boi. I jak będą przedszkola i szkoły, to chce dzieci puścić, bo one też już się nudzą. I jakby tak… Wydaje mi się, że ona dość racjonalnie do tego podchodzi. Moi rodzice też już pytali, czy nie przyjdziemy do nich na planszówki. Ktoś tam jeszcze czy też coś nie zrobimy. I tak jest, że my… tak właśnie trochę głupio jest teraz, my tego nie robimy. Ale doraźnie, jak coś, np. przyszła ostatnio do mnie nasza szwagierka coś odebrać. I kiedyś to brała tą rzecz i wychodziła, bo epidemia, żeby nie być w pomieszczeniu z nami, bo to się chucha na siebie nawzajem. A teraz właściwie weszła na herbatę i została z nami godzinę. Bo tak się odmraża to wszystko i tak o.

**Właśnie chciałam się zapytać, co się stało, jak ci się wydaje, że ci ludzie dookoła ciebie nagle zmienili to podejście? Bo rozumiem, że wy nie zmieniliście za specjalnie, ty i twoja żona?**

Wiesz co, ja bym chciał zmienić. Tylko zmienić w sposób sensowny, w tych miejscach, które mnie interesują. Tam, gdzie uważam, że ryzyko jest akceptowalne. Albo nieakceptowalne jest takie funkcjonowanie przez 2 lata czy ileś, jakby w czasie nieokreślonym. I na przykład chciałbym zmienić widywanie się z rodzicami, albo coś takiego, żeby było częstsze. Albo jakieś takie reguły. Bo na przykład, no nie wiem, byliśmy u znajomych, którzy też mocno byli zaizolowani. Zrobiliśmy sobie taki spacer. Spacer po Żoliborzu. Że mamy różnych znajomych w różnych miejscach, już długo różnych ludzi nie widzieliśmy. Więc stwierdziliśmy, chodź, zamiast pójść sobie do parku, pójdziemy tym razem po mieście. I będziemy iść do ludzi pod okno, gdzieś tam pod blok, pod kamienicę. I dzwonić do nich, żeby nam pomachali, pogadamy sobie tam przez okno jak ktoś na parterze. A jak ktoś gdzieś wysoko, to przez telefon. I to zawsze jakiś taki rodzaj kontaktu i taka śmiechostka. No dobra. Zrobiliśmy. To jednego kolegę spotkaliśmy akurat u niego pod blokiem. Szedł do sklepu, to z nim poszliśmy. Potem następnych nie zastaliśmy. Potem jeden z nami wyszedł na balkon i gadał. I to było takie śmieszne gadanie przez balkon jak w latach 90-tych. A do następnych jak przyszliśmy, to jak nas zobaczyli przez okno, to powiedzieli, o Jezu i zbiegli na dół. I powiedzieli, że idą z nami na spacer, bo od miesiąca nikogo nie widzieli. Wzięli psa i poszliśmy gdzieś. W związku z tym nie podjęliśmy tej decyzji. Ale się z nimi spotykaliśmy. To nie są tacy znajomi, z którymi się widywaliśmy non stop i teraz też pragniemy. Więc my czasami ich też nie widywaliśmy miesiąc albo 2. Bo oni są bardzo zajęci. Natomiast oni są towarzyscy i takie aktywne życie prowadzą. Jak oni przesiedzieli miesiąc w domu, to już wytrzymać nie mogli. Powiedzieli, że jak kogoś zobaczyli, to od razu wzięli psa na smycz i poszli z nami. I 2 godziny z nami chodzili po Żoliborzu. A to nie tak, że 2 metry od siebie szliśmy. 4 osoby na chodniku to się ledwo mieszczą, jeszcze 2 psy ganiające na smyczach wokół. To myśmy się tam mijali co chwilę itd. Potem poszliśmy gdzieś… Właśnie szwagierka przyszła, to została na godzinę. Poszliśmy gdzieś indziej, to też ktoś mówi, ale to już wejdźcie, to tam coś. I to wygląda tak, że nie ma reguły. I niby przestrzegamy, ale przestajemy przestrzegać. I to jest tak bez sensu, bo tak się trochę z losowymi osobami spotykamy.

**Ale co się zmieniło w takim razie? Bo jeszcze pamiętam, jak przed Wielkanocą rozmawialiśmy o tym, że nie spotykaliście się z rodziną, tylko rozmawialiście na Skype?**

No tak. Ja byłem nawet za tym, nie wiem, czy pamiętasz, że ja byłem za tym, żeby się spotkać. W sensie, żeby uznać, że jesteśmy tak zaizolowani, że prawdopodobieństwo, że jesteśmy chorzy jest małe. I po prostu, żeby normalnie spędzić te święta. Dziewczyny nie chciały. No i OK. I co się zmieniło? Ja myślę, że zmęczenie materiału wśród ludzi. Brak takiego planu, horyzontu, ta rzecz się wydaje bezkresna. W sensie nie wygląda, żebyśmy, że jak doczekasz do… To odroczenie jest po prostu bezterminowe. W związku z tym to bardzo męczy. Po trzecie nie wygląda, żeby niebezpieczeństwo narastało, ta epidemia i tam zgony itd. I temat stał się oklepany. Myślę, że po prostu oswoiliśmy ryzyko. Przyzwyczailiśmy się do tego i wszyscy tak stwierdzili, że OK.

**A co to znaczy, że nie narasta ta epidemia?**

W takim znaczeniu, że nie ma tak, żeby na przykład właśnie były doniesienia o tym, że tych chorych i umierających jest coraz więcej. Że powoli zaczynają tam szpitale być przepełnione. Nie ma też dramatycznych obrazków z ludźmi pod respiratorami. Albo tam lekarze tacy w maskach, zapoceni. Tak mało dramatycznie się zrobiło po prostu. Jest po prostu, aha, 100 osób i 4 umarły, OK. 200 nowych osób i 7 umarło. OK, dobra. Już żeśmy się z tym oswoili. Mam wrażenie, że po prostu się oswaja ryzyko. Że po prostu człowiek do wszystkiego jest w stanie przywyknąć. Jak była wojna i bombardowania to myślę, że na początku ludzie działali po prostu w szoku, tutaj zamykali się itd. Myślę, że w takiej Anglii, po pół roku, jak było bombardowanie, jak była bitwa o Anglię, to ludzie po prostu jak słyszeli syreny, to schodzili na dół, tylko gazetę brali do poczytania. Z tym się oswaja człowiek, nawet z największym ryzykiem, jeśli ono jest permanentne. On musi funkcjonować. Myślę, że to jest jakiś psychologiczny mechanizm.

**Czyli w tym momencie jest tak, że wy jakby nie planujecie tych spotkań z ludźmi, tylko one jakoś tak wychodzą czasem.**

No, ja też już bym chciał się spotykać bardziej i normalnie funkcjonować. Ale no mówię, trochę pofolgowaliśmy tam sobie, nie wiem, czasem pójdziemy do tego sklepu dodatkowego, tam poszedłem raz. Drugi raz, ostatnio czegoś nie było, to też kupiłem na bazarze jakieś warzywa i owoce, bo były lepsze niż w Biedronce. Ustaliliśmy też tak, że… Bo to też głupio wyszło. Zaprosiła nas znajoma, która mieszka w dziwnym systemie, bo czasami mieszka w Warszawie, czasami pod Warszawą. A jest lekarzem, żeby było śmieszniej. Ona w tej chwili skończyła kwarantannę, bo miała podejrzenie koronawirusa, jest wynik ujemny. Ale ona teraz tam tydzień siedzi w domu gdzieś. I ona nas na majówkę zaprosiła do siebie. I było, czy do niej jedziemy czy nie. No i tam jeszcze z jakąś ekipą. Bo tam jakaś ekipa chciała jechać, my mamy duże auto, więc jedźmy wszyscy i w ogóle. I moja żona mówi, że właśnie, że nie bardzo. Że to jest jednak niebezpieczne, że 7 osób w samochodzie, potem tego grilla, jeszcze mamy tam nocować. Że to jednak jest nieodpowiedzialne, że na pewno zachorujemy a potem na pewno to przywleczemy mojej teściowej albo babci i one umrą. Ja mówię, słuchaj, no to właściwie wygląda na to, że i tak się spotykamy w różnych momentach. Że wszyscy się spotykają, twoja mama też już wychodzi z domu, do mojej babci też ktoś w odwiedziny wpada. I to tak trochę kurcze… No jest jakieś ryzyko, ale nie wiadomo, co, 2 lata nie będziemy… I nie ma jakiejś reguły, zasady, jak tu działać, jak tu żyć z tym. I jednak długa była debata, ale nie. No i dobra. To potem żeśmy stwierdzili, no to może tak, że jak się na dworze z kimś spotykamy, no to jest OK. Że to jednak małe pomieszczenie, tą drogą kropelkową, to największa szansa na tą transmisję jest wtedy, kiedy jest długa ekspozycja. No dobra. I z tą ekipą, ta koleżanka nas zaprosiła, skrzyknęliśmy się tam na Facebooku, że może się spotkajmy, było rozmawianie. Okazało się, że nie jedziemy, no to nie jedziemy. Ale następnego dnia, tylko bez niej, bo ona jest 70 km od Warszawy, umówiliśmy się z tymi samymi ludźmi na rowery. Więc trochę wyszło, że ją olaliśmy. Ale pojechaliśmy tam gdzieś do lasku Młocińskiego czy gdzieś tam na piwo i mini grilla w kilka osób. I właśnie, ja uważam, że to, no dla mojej żony to było akceptowalne ryzyko. Ja uważam, że to trochę nie było dużej różnicy, niż jak byśmy tam pojechali do niej, do tej koleżanki. No nie wiem. I właśnie nie wiadomo, jak tu funkcjonować. Zdaje się, że podjęliśmy w tej chwili taką decyzję, że jeszcze niepełną, ale będziemy się spotykać z najbliższą rodziną po prostu normalnie. A z innymi ludźmi będziemy się spotykać na dworze. W sensie spacery, piwo nad Wisłą itd. Że to jest mniejsza szansa na tą transmisję. Ale musimy się zdecydować, że podejmiemy to ryzyko, będziemy je podejmować. Bo inaczej, to wygląda na to, że przez 2 lata będziemy nikogo nie widzieć.

**Czyli rozumiem, że mimo, że to robicie, to uważasz nadal, że podejmujecie jakieś ryzyko.**

Znaczy tak, podejmujemy ryzyko. Czy ono jest uzasadnione czy nie – nie wiem. Nie mam pojęcia. Powiem ci, że jestem po prostu zagubiony w tym. W sensie spodziewałem się też… Po pierwsze spodziewałem się tego, że ta epidemia będzie większa, gwałtowniejsza itd. I w związku z tym mniej przyjemna, ale krótkotrwała. Znaczy trochę jak wyciąganie tam, co to się wyciąga? Drzazgę czy...? Nie, plaster się odrywa. Jak odrywanie plastra. Mam wrażenie, że on jest teraz tak powoli odrywany, tak włosek po włosku, zamiast jednym, szybkim ruchem. I raz, że to tak trochę nie wiem, jak z tym, bo inne miałem wyobrażenie. A dwa, że już trochę nie umiem sobie budować wyobrażenia na temat tego, co się dzieje. Ponieważ jestem zmęczony, żeby czytać ciągle o tych rzeczach. Bo ich jest dużo, nie zmieniają się. I są często nierzetelne. W sensie, że są… No nie wiem, no. Na przykład nie mogłem nigdzie znaleźć statystyki. Nie, żebym bardzo szukał, ale wszyscy podają, ile osób zachorowało, ile osób umiera od początku epidemii oraz dnia wczorajszego. To są najczęstsze statystyki. I jeszcze czasami wykresy w czasie. To są statystyki, które znajdziesz na każdej, TVN24, gazeta, rzeczpospolita, polityka, to są głównie takie dane. Natomiast nigdzie nie widziałem żadnego rzetelnego artykułu, na przykład porównanie całkowitej śmiertelności w marcu i w kwietniu rok do roku. I ze średnią z ostatnich 10 lat. Żeby sobie na przykład zobaczyć, czy to nie jest czasem tak, że oczywiście, umarło 100 osób czy tam 500. Nie wiem, ile już teraz umarło w Polsce osób na ten COVID-19. Że umarło 500 osób, ale to znaczy, że umarło, nie wiem, w tym roku zmarło w marcu w Polsce 7 tysięcy osób, a w zeszłym roku 6800. To by oznaczało mniej więcej te 300 ekstra. Tak? Albo tylko 200 ekstra. To znaczy połowa być może tych osób się pokrywa z osobami, które są starsze i i tak by umarły. Bo i tak mają raka albo coś. I na przykład takich analiz jakichś ciekawszych nie widać. Tylko jest takie, mam wrażenie strasznie pustymi liczbami. Tylko dramatyczne, to jest mój częsty zarzut do dziennikarstwa. Tylko emocje, tylko dramaty. Rząd nie wiadomo właściwie na czym jest skoncentrowany i co chce robić. Robi jakieś dziwne ruchy, niezrozumiałe dla mnie, nie mówi, na jakiej zasadzie działa ani… Trochę wygląda, że z palca bierze te różne obostrzenia i odwoływania tych obostrzeń.

**Dopytam o te statystyki. Rozumiem, że taka potrzeba w tobie, żeby znaleźć te statystyki np. właśnie z ostatnich 10 lat, jest dlatego, że masz takie podejrzenie, że ta sytuacja teraz nie jest tak dramatyczna, jak mogłoby się wydawać wcześniej?**

Nie, chodzi o to bardziej, że… Znaczy mam takie podejrzenie, że nie jest tak dramatyczna, jak miało być albo jak wydawało się, że będzie. Natomiast bardziej chodzi mi o coś takiego, że nie umiem sobie w tej chwili zbudować obrazu świata. Tak? Jakby nie potrafię sobie wyobrazić, czy to znaczy, że jest źle, czy to znaczy, że nie jest źle. Na przykład, to może nie jest najlepszy przykład, ale ta koleżanka, która jest lekarzem i pracuje w szpitalu i właśnie miała styczność z tymi osobami chorymi na COVID, w związku z tym… U niej na oddziale się pojawił i w związku z tym została wycofana z pracy, na kwarantannie i tam robiono jej 2 razy testy i coś tam jeszcze. To ona w ogóle mówi, że w szpitalach jest, nie przymierzając, burdel, chaos. I w ogóle mówi, że osoby z oddziału zakaźnego chodzą tym samym korytarzem co z innego. I w ogóle jak jest osoba, którą się z izolatki wiezie do tego MSWiA, który jest teraz szpitalem zakaźnym wywożą pacjenta, to wywożą go przez windę, tą samą, którą jeżdżą na jakiś blok operacyjny z innym pacjentem. Ona mówi, że w ogóle pierdzielnik jest totalny. I że szpitale się bardzo wolno uczą tych procedur i bezpiecznych rzeczy. A z drugiej strony się absolutnie nie przejmuje. Ona mówi, no dobra, mocne zapalenie płuc, trochę nas umrze, ale trudno.

**Pamiętam, że jak na samym początku rozmawialiśmy, to dla ciebie właśnie to, że ktoś, kto ma jakąś wiedzę, autorytet coś mówi, to nadaje takiej...**

Ja nie wiem, czy ona jest… To jest specyficzny człowiek. W związku z tym ja nie wiem, czy ja bym ją traktował jako autorytet. Bardziej chodzi o to, że to jest osoba, która jest gdzieś na tej pierwszej linii frontu i w dużym ryzyku itd. I w jednej wypowiedzi mówiąca o tym, że jest właściwie chaos, beznadzieja i nic się nie poprawia. Ona myślę, że może trochę przesadzać tutaj. Ale że generalnie to syf. A z drugiej strony mimo tego syfu, ona się nie przejmuje. Nie wiem, czy na zasadzie takiej, że już trudno, bo i tak… Chyba na zasadzie takiej, że mix dwóch rzeczy. Że to nie jest tak tragiczne, tak złe i taka wielka śmiertelność i raczej przeżyje ona. Plus to, że i tak nie może z tym nic zrobić. Czyli takie znowu, oswojenie ryzyka.

**Czyli podsumowując, to jest tak, że w jakimś tam stopniu podjęliście decyzję, żeby się spotykać z pewnymi ludźmi pod pewnymi warunkami. To nie jest jeszcze przypieczętowane, że będziecie tak a nie inaczej robić. To jest gdzieś tam w jakiejś dyskusji powiedzmy. Twoja żona ma bardziej takie stanowisko, żeby mimo wszystko jednak trochę jeszcze unikać pewnych osób.**

Tak. Ona się trochę boi. Ona ma taki lęk chyba głównie… Trochę się boi o siebie i zachorowania i o swoich najbliższych tak bardziej. A ja chyba jestem trochę w takiej fazie, że też się boję, ale mam poczucie małej sprawczości. To znaczy mam wrażenie, że i tak musimy wszyscy zachorować. I wygląda na to, że po prostu trzeba się nie przejmować.

**Więc to, co wpłynęło trochę na to, że teraz zaczęliście się inaczej zachowywać, to jest to, że nie wiesz już totalnie co robić. Bo nie wiesz, na czym stoisz, są podejmowane jakieś decyzje, one są oparte nie wiadomo o co. Plus ta sytuacja nie wydaje się tak tragiczna jak mogłaby być. Plus nie wiesz, ile jeszcze to miałoby potrwać. I nie wyobrażasz sobie tego, że przez 2 lata siedzieć w domu i żeby było tak, jak jest teraz. Tak podsumowując to.**

No tak, tak. Znaczy wyobrażam sobie przez 2 lata siedzieć w domu. Ale nie wyobrażam sobie, że… Nie zmienimy wszyscy tak naszego życia. To znaczy społeczeństwo się tak nie przetransformuje. Więc trochę, jak nie jestem w jakiejś grupie ryzyka, to trochę, wiesz, siedzieć i izolować się dla innych, jak oni się nie izolują, bez sensu, nie?

**Ale właśnie masz też trochę poczucie, że to, że ludzie zaczęli się nie izolować, wychodzić z domu i spotykać z innymi ludźmi, to sprawiło, że ty też zacząłeś to robić?**

No przede wszystkim tak, bo żeby się z kimś spotkać, to ta druga osoba musi chcieć się z tobą spotkać. Więc w tym sensie trochę tak.

**To chciałam porozmawiać z tobą o tych różnych emocjach. A nie wiem, czy się udało te obrazki znaleźć.**

Nawet był taki moment, że się nad tym zastanawiałem. Ale nie. Znaczy ostatnio jakoś przestało mnie to interesować. Ale tak ze 2 razy rozmawialiśmy. Trochę mam złość na zarządzanie tym wszystkim i ten chaos informacyjny. I mam wrażenie, że jeszcze jest tak, że niesamowity przeskok jest między po prostu wystawianiem mandatów przez policję za totalne bzdury jakieś na początku tej… Za drobne przewinienia gigantyczne kary, nieproporcjonalne zupełnie. I takie mam wrażenie, że policja się zrobiła, coś dziwnego w nich wstąpiło i to był jakiś przykaz odgórny plus poczucie władzy. Na początku tego lockdownu, że tak się ciskali o różne rzeczy. W dodatku czasami nie ciskali się, w innym przypadku się ciskali, że ktoś rowerem jechał, to już jest dla przyjemności i robili jakieś mandaty gigantyczne. A teraz jest właściwie tak, że jest totalny luz. I ludzie robią cokolwiek, ludzie chodzą bez maseczek, które są nakazane po ulicach. I policja obok przejeżdża i w ogóle wywalone mają na to. I mam wrażenie, że to jest też odgórny przykaz przed wyborami. I to mnie trochę irytuje. Bo mam wrażenie, że właśnie czuję, że interes polityczny spowodował, że tylko to jest dyskusją w tej chwili w Polsce. W dodatku też w chaosie ta dyskusja jest w i bałaganie. I tam czy będą te wybory, czy nie będą, a kiedy, a jakie. No przecież to jest… Jednak ważna sprawa zupełnie chaotyczna. I mam wrażenie, że to jest dużo ważniejsze dla zarządzających państwem niż jak z tą epidemią tak naprawdę. Więc ja wiedzę w to, że minister zdrowia się zajmuję teraz wyborami, listonoszami i innymi rzeczami, a nie na przykład właśnie maseczkami czy respiratorami. Więc tutaj mam taką irytację, złość. Myślę sobie, że… Że na zachód.

**Emocje – zdjęcia. Pokażę ci te obrazki jeszcze raz. Jest tu coś takiego, co do ciebie teraz przemawiało?**

Właśnie już nie pamiętam. Pamiętam, że wskazywałem na początku 12. Na pewno 13, 15 i 16 kiedyś.

**A jakie teraz?**

Nie wiem. Na pewno nie jest to 12. Bo też na przykład w związku z tym, że się budzi z powrotem do życia wszystko, to też właśnie bardziej tak jak mówisz o tym zawodowym, myślę o tym tak naprawdę. Nawet, jeżeli wydaje się, że nic tam nie działam z tym. To gdzieś tam myślę. To już wtedy nie łączy się z kamieniami zen, nie łączy się właśnie taka potrzeba zadziałania. I myślenie o tym, co będę robił. I w którym kierunku iść. Nie wiem. Ale na pewno coś z 1 związane. Ona mnie korci, żeby dzisiaj ją wskazać. Bo ta 1, to jest ten korek, w którym... Jak stoisz w korku, to się właśnie irytujesz. On jest z taką ciasnotą, taką dusznością związany. I to jest takie uczucie, które ja gdzieś tam czasami teraz odczuwa. W związku z tą właśnie sytuacją, o której ci mówię, tą niepewnością polityczno – społeczną, jak to wszystko będzie wyglądało. Czy nasz świat się w tej chwili zmienia i jak bardzo. I tak dalej. Ale to jest taka niepewność z taką irytacją powiązana. To taka. I ta irytacja mi się z tym korkiem kojarzy.

**A jakieś zagrożenia w tym momencie odczuwasz? Czymś, czymkolwiek?**

No w jakimś stopniu tym koronawirusem. Znaczy w sensie możliwością zakażenia się. Ale nawet nie się, co zagrożeniem dla rodziny. Ale nie jest tak, że jak byśmy mieli skalę w procentach wyrażoną, gdzie 1 to jest właściwie takie totalne zero bania się jakiegokolwiek, to jest właściwie takie bycie na jakimś prozacu czy innym takim, a 100% to jest taka, właściwie patrzysz w oczy śmierci i lecą bomby. To powiedzmy, że jak to poczucie niebezpieczeństwa i zagrożenia gdzieś w normalnym moim życiu jest na poziomie 10%, 15 może. Bo jednak masz świadomość, że coś się może zdarzyć cały czas, pożar, potrąci cię samochód, a co będzie jutro, a jak ktoś umrze na zawał, albo zachoruje. No to zawsze człowiek się tam gdzieś… Właśnie po to są oszczędności też. Ja o tym myślę, więc staram się być w miarę przygotowanym. To teraz czuję, że tak, nie że to ten koronawirus winduje mnie z tych 10 do 80, tylko tak no do 17. To jest taki… Że po prostu tak trochę się martwię, że coś może się zdarzyć. Raczej się pewnie nie zdarzy nic takiego dramatycznego. Szanse są małe. Ale trochę się denerwuję. Ale tak trochę bardziej.

**W takim razie masz jakieś takie przewidywania co do tego, kiedy twoje życie wróci do jakby takiego stanu sprzed tej pandemii?**

Trochę nie. Bo kiedy wróci? Ja myślę, że ono nie wróci nigdy dokładnie. Albo mam nadzieję też taką. Ponieważ ja jestem w trakcie długofalowej zmiany. W związku z tym moje życie powinno się zmienić, czy jest koronawirus czy nie. W sensie co najmniej zawodowym. Ale też na przykład mam nadzieję, że nie wiem, mam jakieś pomysły na to, żeby wykorzystać być może też jedną rzecz związaną z tym koronawirusem i kryzysem na rynku, który się pojawi, że coś tam bym chciał zrobić. I to na przykład też będzie coś, co by zmieniło trochę nasze życie. To znaczy być może, gdyby ten koronawirus był dużo mocniejszy i świat się zmienił bardzo na online, to moja żona już w ogóle przejdzie na pracę zdalną. I wtedy na przykład dla nas się otwiera… Jeżeli ja też bym był w stanie, jeżeli marketing online się przeniesie całkowicie, będzie pracą zdalną, to ja na przykład wracając do tego albo robiąc to programowanie, ale już nie edukację, też będę w stanie na przykład… Być może byśmy się przeprowadzili z miasta. Bo nie będzie potrzeby siedzenia tutaj. I może zaczniemy spędzać czas, wieść życie trochę gdzieś bardziej na uboczu. Więc ja mam wrażenie, że różne są scenariusze. Ale szansa, że on zupełnie wróci do punktu wyjścia jest mała. A nawet to jest niepożądany scenariusz. Ja wolałbym… Dostosowując się do tego koronawirusa i wszystkich zmian, ja chciałbym pójść naprzód. No, to byłoby wskazane. Czy nam się uda, zobaczymy.

**Masz jakieś takie obawy związane z najbliższą przyszłością? Mówię o perspektywie kilku tygodni.**

Nie, nie, nie. Ale teraz też jestem w zawieszeniu, bo jestem bardzo ciekawy, co się zdarzy przez najbliższe 5 dni. Bo może być tak, że nic z tej awantury nie wyniknie. I nic w naszym kraju poza dalszym, powolnym takim rozpadem i degrengoladą i marnowaniem publicznych pieniędzy się nie zmieni. Ale może się zmienić dużo. Bo możemy skręcić nagle, może upaść, istnieje jakiś scenariusz upadku rządu. Bo zdaje się, że w tej chwili… Nie mam pojęcia, bo właśnie przestałem trochę śledzić, bo to jest strasznie tam… Nie jestem aż, żebym tak aktywnie, zawodowo potrzebował to śledzić, a w związku z tym trochę to sobie olewam. Jest szansa, że nie będzie już większościowego rządu za parę dni. Ale jest szansa, że się skonsoliduje pięknie Prawo i Sprawiedliwość i będzie partią silniejszą za chwilę z nowo wybranym prezydentem. A być może będą musieli łamać kolejne reguły, żeby później umotywować mandat Dudy w tych dziwnych wyborach. I może się okazać, że będziemy zmierzać w kierunku jakiegoś właśnie dziwnego tworu państwa jakiegoś autorytarno-totalitarnego, dziwnego jakiegoś takiego. Trochę jak Białoruś czy coś podobnego. Nie? Że możemy w tą stronę się staczać. Więc na razie nie wyrokuję, ale zapytaj mnie za 2 tygodnie. Bo to jest duże pytanie tak naprawdę, co to się stanie w tych wyborach. Czy na przykład okaże się, że będą skandaliczne wybory z prezydentem, wybrany zostanie Duda i rząd powie tak, to jest nowy prezydent. Wszyscy się będą nie zgadzać, to mogą być jakieś większe niż w obronie sądów, mogą być jakieś zamieszki, ruchy. Nie wiem, może Unia Europejska nam coś powie i wtedy nas wywalą? Raczej nie, mała szansa. Ale może? A może właśnie za chwilę będzie tak, że za chwilę upadnie rząd i będą przedterminowe wybory. I się skończy PiS i nie wiem, kto będzie następny. Ale może znowu skręcimy ku zachodowi. Wszystko może być.

**Czy jeśli te wybory się odbędą, to ty zamierzasz w nich głosować?**

(śmiech) My rozmawialiśmy już o tym 2 razy z żoną po godzinie. Z moimi rodzicami, którzy też polityką się interesują, nie są obojętni na takie tematy. I nikt nie potrafił z nas dojść do tego, co należałoby zrobić. Ale nie pomijając idealistyczne scenariusze, jak byłoby najlepiej dla wszystkich, gdyby się wszyscy dogadali albo zwolennicy czy przeciwnicy jakiejś opcji. Bo to są niemożliwe rzeczy. Tylko w tym chaosie właśnie, co należy zrobić, żeby było rozsądnie. Nikt nie ma pojęcia. Więc ja powiem ci, że naprawdę nie wiem. A być może nie przyjdzie w ogóle do mnie ta karta do głosowania, to się nie będę miał nad czym zastanawiać. Ja nie mieszkam w miejscu zameldowania. Ale mam niedaleko, bo wystarczy, że się 20 minut spacerem przejdę do miejsca zameldowania. W tej samej dzielnicy jestem zameldowany, więc mogę pójść odebrać swoją kartę u kogoś. Więc naprawdę nie wiem. Chyba będę za tym, żeby jednak zagłosować. Widzę tylko dwie opcje. Pierwsza jest taka, że jeśli… Zakładam, że nie jesteś zwolennikiem PiS-u i masz jakąś inną opcję polityczną. Nie wiem, czy na zasadzie: nie chcę ich, ale nie wiem, co innego. Albo na zasadzie: tak, ja wiem, że Biedroń jest moim ulubionym kandydatem i muszę na niego zagłosować, to będzie najlepszy prezydent, albo kochasz Bosaka, wszystko jedno. Jest tak, że jeśli nie chcesz głosować na PiS i Dudę, chcesz, żeby Duda nie był prezydentem, to wydaje mi się, że są tylko 2 rozsądne opcje. Albo zagłosuj na innego kandydata i to na takiego, który się wydaje, że ma duże szanse w drugiej turze. Czyli nie na Bosaka, sorry, jeżeli to jest twój ulubiony kandydat. Ty nie możesz nic mówić?

**To nie jest ważne, co ja uważam (śmiech). Czyli ty nie chcesz, żeby Duda był prezydentem?**

Ja bym nie chciał, tak, tak, tak. Ale chociaż, to też nie jest właśnie takie do końca. Bo na 5 lat bym go nie chciał prezydentem. Ale gdyby była taka opcja, że wybory są za pół roku, to na przykład byłoby spoko, żeby on jeszcze przez te pół roku był prezydentem. To był wolał. Żeby pół roku albo rok on pobył. Bez zmieniania konstytucji oczywiście. To bym wolał, żeby on został z tym prezydentem. Bo wolałbym wybory w spokojniejszych warunkach, gdzie można się zastanowić itd. I ludzie podejmą sensowniejszą decyzję. I będzie taka mocniej umotywowana, to znaczy z mocniejszym mandatem. Wszyscy będą rozumieć, że tak wybraliśmy i się będą gotowi z tym pogodzić. Bo to, co teraz nastąpi nie ma znaczenia. Zawsze druga opcja powie, że to było oszustwo. I wolałbym tego uniknąć. A poza tym uważam, że niech się ta zaraz wypali do końca. Niech ludzie poczują, że nie wiem… co prawda będą tutaj na epidemię zwalać. Niech poczują, że oni nie robili rezerw budżetowych albo państwo nie jest przygotowane na nic. I są tylko dwie możliwości moim zdaniem sensowne. Pierwsza to jest taka: zagłosuj na innego kandydata, który jest sensowny. W sensie, który ma jakieś szanse na elekcję. Nie wiem, kto to ma być, nie czytałem tych sondaży różnych. To się tak zmienia i tak jest niepewne, że… Zdaje się, że nie jest to, żeby było śmieszniej PO, czy Koalicja Obywatelska, która jest największym ugrupowaniem opozycyjnym. Ma w tej chwili kandydata, która ma prawie najniższe poparcie. Czyli ta Kidawa-Błońska, chyba nikt na nią… Ona tak dziwaczne ruchy zrobiła, że nie. Trzeba sobie wybrać kogoś. Może ten Kosiniak-Kamysz, może ten Hołownia, któryś z tych dwóch chyba się najsensowniejszy wydaje. I na niego zagłosować. Nawet jak go nie lubisz jakoś strasznie. Liczyć, że Duda nie wygra w pierwszej turze i ten będzie w drugiej. I to jest taka opcja, że no trudno, bawisz się w tą grę, w którą nie chcesz, te złe wybory itd. A druga opcja to jest taka, że nie głosuj. Zrób coś takiego, nie wiem, zachować tą kartę, odeślij ją, ale tak wtedy aktywnie, zgłoś protest wyborczy. Coś, żeby spowodować, żeby te wybory były w jakimś sensie nieważne. Albo mandat przyszłego prezydenta w wyniku tych wyborów był mniejszy. Żeby można było potem powiedzieć, nie wiem, na przykład wybory w Polsce się odbyły, ale było półtora miliona protestów wyborczych. Bo nie doszło prawdopodobnie 7 milionów kart do głosowania. A dodatkowo jeszcze 500 tysięcy się zgubiło, nie dotarło z powrotem. A jeszcze ludzie tam nie mogli czegoś zrobić. No to wtedy zrób tak, żebyś nie wiem, żeby naprawdę spowodować, że będzie przyczynek jakiś. Nie znam się na prawie na tyle dobrze, żebym mógł powiedzieć co. Ale wiem, że tam Giertych i ten Dubois, ten jego kolega prawnik, to oni mieli jakąś koncepcję, jak należy zrobić tak, że właśnie nie można oddać głosów w tych wyborach, bo one chyba są nieważne. I ta karta nie jest przecież zgodna, zatwierdzona przez PKW zgodnie z kodeksem wyborczym. W związku z tym należy… Ale nie można za bardzo jej wyrzucić do śmieci. Bo oczywiście to jest chyba karane. Więc należy ją odesłać do jakiegoś tam ministerstwa, które odpowiada za te wybory. I chyba tam, żeby to wysłać poleconym, czy z jakimś doręczeniem. I jak się uzyska to doręczenie, jakąś adnotację tam zrobić. I zęby to załączyć do wniosku potem, do skargi wyborczej do PKW, że się nie miało możliwości zagłosować w wyborach.

**I ty jeszcze nie wiesz, co ty zrobisz z tych dwóch opcji, które są.**

No, to są według mnie dwie sensowe. Mówię o tym, żeby nie robić tak, że to mnie skłania do tego, żeby nie robić tak, jak niektórzy mówią, że oni na przykład nie wyślą albo podrą kartę albo zablokują skrzynkę. Zablokowanie skrzynki jest bardzo spoko moim zdaniem, żeby listonosz nie mógł wrzucić ci pakietu. Pod warunkiem, że chcesz potem… To jest trochę prowokatorstwo. Ale blokujesz skrzynkę i wtedy piszesz, że nie dostałeś karty, albo że nie miałeś możliwości zagłosowania. I wtedy to jest sensowne. Natomiast robienie takiego umycia rąk jest bez sensu. To jak tak, to już lepiej wtedy zagłosować przeciwko Dudzie.

**(fragment rozmowy niezwiązanej z badaniem? – mniej więcej ostatnie 3 minuty pominęłam)**

**Koniec nagrania.**
