# Supplementary material for: The challenges arising from the COVID-19 pandemic and the way people deal with them. A qualitative longitudinal study
Source: PLoS One. 2021 Oct 11;16(10):e0258133. doi: 10.1371/journal.pone.0258133 (PMC8504766; doi:10.1371/journal.pone.0258133)
Supplement: S1 Dataset — (ZIP) [file pone.0258133.s003.zip › Transcriptions/stage 4/9.4_F_25_couple, no children.docx]

**9.4_F_25_couple no children**

**Jak wyglądały u ciebie te ostatnie 2 tygodnie?**

Tak naprawdę wyglądały tak samo. Nic się nie zmieniło zupełnie. Jedyne może, wydarzyło się coś innego, to w majówkę spotkaliśmy się ze znajomymi. Ale to też nie było tak, że gdzieś wychodziliśmy… W sensie wychodziliśmy, ale pojechaliśmy w sumie do lasu pod Siechnicami*.*

**To jest gdzieś tam w okolicach…**

Wrocławia. Tam, gdzie są pomidory, które dają łunę. Jeżeli słyszałaś kiedyś o magicznych pomidorach. Takie memy są z Króla Lwa, świt tam na Sawannie i mój synu to Siechnice. To właśnie tam pojechaliśmy. Tam jest dużo… Znaczy tam płynie rzeka i jest bardzo zielono. Tak że pochodziliśmy sobie kilka godzin, powiedziałabym, że troszeczkę w dziczy. A jak już się spotkaliśmy i już rozmawialiśmy i już pluliśmy na siebie, to stwierdziliśmy, że możemy wypić piwko razem i zjeść pizzę. Więc poszliśmy do nas i tak w sumie spędziliśmy już w sumie do nocy czas razem.

**Ale to było to, że się spotkacie, to jak wyglądało planowanie tego?**

Nie było w ogóle planowane. W sensie wcześniej umawialiśmy się… Bo jakoś jeszcze w poprzedni weekend tak z nudów chciałam, żeby mnie mąż zawiózł, pokazał, gdzie pracuje. Bo on zmienił kilka miesięcy temu pracę, ale nie pracuje we Wrocławiu tylko w Jelczu. No i mówię, to chodź pojedziemy sobie na przejażdżkę. I pojechaliśmy. I akurat zadzwonił do nas znajomy. I mówił, wiecie co, no nie możemy się spotkać, ale może umówmy się wszyscy wspólnie na Skypie na piwko. Ja mówię, no tak możemy zrobić. Co najśmieszniejsze, jeździ do rodziców co tydzień, ale z nami się nie chciał spotkać. A jeszcze pracuje jako fizjoterapeuta i ma normalnie klientów. Ale OK, z nami nie chciał. No i mieliśmy się, najpierw spotkać na to piwo. I myśmy byli naszykowani na to, że w majówkę właśnie coś takiego się odbędzie w szóstkę. A zamiast tego jakoś po południu siedziałam sobie, robiłam pranie, właśnie włączyłam pralkę. I nagle dzwoni nasz najlepszy przyjaciel, czy nie chcemy z nim i jego dziewczyną właśnie pojechać do lasu, bo oni się wybierają. No to my dobra, daj nam pół godziny. No i od razu odwirowywanie, żeby nam pralka nie wylała, bo się woda wraca z niej. No i przebraliśmy się i pojechaliśmy. Trochę jak wsioki. Ale pojechaliśmy do tych Siechnic. Tak że zupełnie to było nieplanowane. Wzięłam jakąś wodę, ciasteczka i tak sobie chodziliśmy po prostu. A potem tak wracaliśmy do Wrocławia i ja z mężem taka krótka wymiana SMS-owa, bo się chciałam zapytać, czy się ze mną zgadza. I dawaj, zaprosimy ich na pizzę. No i pytamy się, czy jedliście obiad? Nie. No to jedziemy do nas na piwo i na pizzę. Tak że tak w sumie wszystko spontanicznie wynikło.

**A nie mieliście żadnych wątpliwości, żeby się spotkać z tymi znajomymi?**

Powiem szczerze, bo my się i tak bardzo długo nie widzieliśmy, 2 miesiące no to… Znaczy w sumie mąż cały czas z przyjacielem jest na… Jakimś tam TeamSpeaku, czy co oni tam mają, jakieś te… No, już nie pamiętam. W każdym razie byliśmy cały czas w kontakcie. I rzeczywiście trochę się obawialiśmy tego kontaktu. Ale jak już wyszedł ten las i tak naprawdę… Może jakoś drastycznie nie spadła ilość zachorowań, ale… No, wcześniej czy później myślę, że bardziej prawdopodobne jest to, że zachorujemy lub już zachorowaliśmy… Zresztą, tak jak mówiłam, ja się bardziej obawiam o osoby, które są chore. W sensie, które mają jakieś dodatkowe schorzenia i które są starsze. A ja wiem, że oni są zdrowi. Wiem, że nie chorowali, że też się pilnują. Więc w końcu stwierdziliśmy, że nie ma co się dziczyć samemu.

**Bo zastanawia mnie właśnie to podejście, że mówiłaś wcześniej, że najbardziej te osoby starsze mogą zachorować itd. Ale jeszcze jakiś czas temu nie chciałaś się spotykać.**

No nie chciałam. Natomiast… Nie wiem, czy to jest, czy to tak podświadomie człowiek wręcz musi, czy sobie właśnie wmawia, że już wszystko jest w porządku. Ale tak jak patrzę na ulice. Zresztą samo to, że już gospodarka odmraża się, no widzę, że już wszystko zaczyna wracać do normy. I tak naprawdę jedynie w miejscach publicznych te zaostrzenia są widoczne. Tak jak dzisiaj na przykład przyszedł do nas właściciel, bo zalaliśmy sąsiadkę. Albo wczoraj sąsiadka do nas przyszła, bo ją zalaliśmy. I ani ona ani właściciel nie mieli maseczek. I nie bali się kontaktu z nami. Tak że wydaje mi się, że już jakby najgorsze mamy za sobą.

**Ale czy to jest tak, że to spotkanie się z tymi znajomymi jakby otworzyło ci już taką furtkę, że teraz normalnie się będziesz spotykać z ludźmi?**

Na pewno będę się spotykać z tymi znajomymi. Już właśnie planuję w weekend ich zaprosić. Wiadomo, że nie będziemy wychodzili nigdzie do żadnego baru, ani no kina, nie da się. Na pewno nie planujemy nigdzie wychodzić. Natomiast wydaje mi się, że takie spotkanie w czwórkę, oczywiście dbając o zasady higieny, czyli oni przychodzą, myją porządnie ręce. Wydaje mi się, że teraz to nie jest nic złego. Nie czuję się winna.

**A do rodziców myślałaś, żeby pojechać?**

Tak. I chciałam jechać albo w ten weekend albo w kolejny. Ale chyba pojadę w kolejny, bo mój tata ma urodziny. No i chcę pojechać na tydzień. I na przykład mój mąż jest bardzo przeciwko. Bo on uważa, że nie powinniśmy jeździć do rodziców jeszcze. I on na pewno też nie może jechać do swoich rodziców. Bo jego tata opiekuje się jego dziadkiem, który ma raka płuca. Więc istnieje bezpośrednie zagrożenie, że coś tam się stanie. I on jakby nie chce potem wiedzieć, że się do czegoś mógł przyczynić. Znowuż, jesteśmy z jednej miejscowości. Więc no głupio, jak ja pojadę do rodziców, on mnie zawiezie. Pójdziemy do jego teściów a do jego rodziców nie zajrzymy. I wtedy będzie im przykro. Więc po prostu myślimy teraz, jak to rozegrać. Bo znowuż też, żebym ja jechała tam pociągiem, to myślę, że w obecnej sytuacji nie jest to najrozsądniejsze rozwiązanie. Jeszcze staram się unikać takich miejsc zbiorowej użyteczności. Natomiast tak, myślę o tym, rozmawiałam też z rodzicami. Tata to już po prostu nie może się doczekać, bo on sam tam dziczeje w tym domu. Mama na początku się ucieszyła, mówi tak, tak, przyjedź na weekend. A jak powiedziałam, że chcę zostać tydzień, że mogę pracować z domu, to mama nagle: no jak chcesz. I nie bardzo wiem, o co jej chodzi (śmiech). I wczoraj właśnie rozmawiałam z nią. I mówię: mamo, no nie chcesz, żebym przyjeżdżała? Czy się martwisz o swoje zdrowie, czy może już mnie nie kochasz? A mama mówi: nie, po prostu tak myślałam sobie, że będziesz miała problemy z internetem w domu. Ogólnie się wybieram, babcia już o mnie pyta, więc… Nie wiem, czy spotkanie z babcią to dobry pomysł, jeszcze o tym pomyślę. Ale z drugiej strony moja mama cały czas się spotyka z chorymi pacjentami, więc… Tak naprawdę to nie wiem, czy jest jakieś lepsze wyjście. Nie wiem, czy jest sens dłużej czekać, o to mi chodzi.

**Powiedziałaś, że starasz się unikać takich dużych skupisk, czyli np. jeżdżenia pociągiem, czegoś jeszcze unikasz teraz?**

No tramwajami nie jeżdżę, od kiedy to się wszystko zaczęło. Wiadomo, że też, jak robimy zakupy, to staramy się nadal zrobić jedne duże zakupy w tygodniu i tylko jedno z nas je robi. I mimo, że otworzyli galerie handlowe to też… Na pewno nie będę się udzielała tam. Natomiast wiem, że muszę pójść. Bo mam wciąż kartę podarunkową, której ważność się kończy. Więc jakby trochę z przymusu muszę się wybrać.

**To jest ta karta podarunkowa, której nie możesz przez internet zrealizować?**

Tak, dokładnie, dokładnie.

**W ogóle uważasz, że to otwarcie galerii to jest dobry pomysł?**

Właśnie po naszej ostatniej rozmowie 2 tygodnie temu rozmawiałam o tym z mężem i mówię mu, że uważam, że to jest zły pomysł. I on mi dopiero uświadomił, ilu ludzi tam pracuje. Ja nie wiem, dlaczego ja o tym zupełnie nie pomyślałam, przecież to jest takie oczywiste. I pomyślałam sobie, kurcze, faktycznie, kupa ludzi. I stwierdziłam, że rzeczywiście ma to sens, pod względem ekonomicznym bardzo dużo. I zmieniłam zdanie.

**A z twoją pracą jak teraz to wygląda?**

U mnie cały czas jest tak samo. Tak że pracuję z domu. Tak jak mówiłam wcześniej, strasznie się u nas wszystkie procesy komplikują przez to. I mamy bardzo dużo pracy ogólnie.

**Macie jakieś takie perspektywy powrotu do pracy normalnej?**

Dostaliśmy tydzień temu maila od jakiegoś pana, nie wiem, kim on tam jest. Jakiś tam szef, którego nigdy nie poznałam i nie poznam. Ale z jego maila wynikało, że oni planują pracę z domu do 7 lipca. Natomiast ja właśnie nie wiem, czy on jest dyrektorem regionalnym, czy on jest ogólnym dyrektorem tej firmy. Tak jak mówiłam, nie wiem, co to za gość, po prostu wysłał nam maila. Wiem, że jest dyrektorem.

**Czyli wysłał maila, że on planuje pracować do 7 lipca.**

Tak, że ogólnie firma tak planuje. Tylko teraz ja nie wiem, czy on jest regionalnym na Polskę, czy on jest ogólnie dyrektorem tej korporacji i mówi ogólnie o wszystkich krajach. Myślę, że to wszystko będzie rozpatrywane indywidualnie. Pytaliśmy się naszej supervisorki też na takiej telekonferencji, co ona o tym wie. I mówiła, że to na pewno nie jest perspektywa najbliższych dni, tylko raczej tygodni. Dlatego, że nawet w biurze nie mają możliwości zorganizowania tych odstępów co 2 metry. Więc nawet, jeżeli będziemy wracać przed 7 lipca, to na pewno będą to takie powroty tylko częściowe.

**A ktoś z twojego otoczenia już zaczął wracać do pracy?**

Na przykład ten przyjaciel, z którym byliśmy w lesie, to on cały czas tak naprawdę pracował normalnie z pracy. I on bardzo nie lubi pracować w domu. I właśnie bardzo się denerwował, bo oni przenosili swoją placówkę w inne miejsce. I tak naprawdę pracował z domu nie dlatego, że był koronawirus, tylko dlatego, że to miejsce, do którego się przeprowadzili, nie zostało przez koronawirusa wykończone. I on miał bardzo utrudnione warunki pracy tam.

**Ale w ogóle myślisz, że to już jest dobry moment, żeby ludzie zaczynali wracać do pracy?**

Myślę, że pod względem takim epidemiologicznym to chyba nie za bardzo. Obiektywnie, jak byśmy zupełnie się odizolowali od wszystkich takich bodźców zewnętrznych, no to… Najlepszym sposobem, żeby wyglądać z wirusem, jest po prostu odizolowanie się w domu, ograniczenie kontaktów, wszystkiego, żeby się po ludziach jakby… Żeby nie było wektorów. Natomiast nie jesteśmy odseparowani od rzeczywistości. I myślę, że pod względem gospodarczym jest to niezbędne. Że to już i tak jest dość późno. Na przykład zaczęłam myśleć o tych ludziach, nie wiem, pracowniku takiego *Medicin*. Ktoś sobie pracuje, pracuje i nagle ma 2 miesiące takiej niechcianej, nieplanowane przerwy. I nie wie tak naprawdę, co z tą pracą. Bo nie jest w stanie tej pracy wykonywać.

**Ale to rozumiem, że to właśnie ci uświadomiła ta rozmowa z…**

Tak, bo zaczęłam się zastanawiać.

**Ale czy w takim razie, że ty właściwie no jesteś w domu, nie masz strasznej perspektywy tego, że możesz stracić pracę, że już sobie zaczęłaś wychodzić, spotykać się ze znajomymi. Czy mimo wszystko jest tak, że coś ci przeszkadza teraz?**

Ja sobie rozmawiałam z koleżanką i tak doszłyśmy do wniosku, że byłoby idealnie pracować z domu, ale gdyby wszystko na zewnątrz było w porządku. Czyli na przykład pracować z domu i wychodzić do kina. Albo wychodzić na zakupy potem po pracy. No idealnie. Natomiast teraz tak naprawdę czuję, że jestem cały czas trochę w zawieszeniu. I trochę zaczynam się bać tego powrotu do biura. Bo już się odzwyczaiłam od tego, już zupełnie zmieniłam tą rutynę swoją. I no… Ja tak lubię w domu, no nie wiem.

**Ale jak myślisz, czego ci będzie najbardziej brakować z tej pracy w domu?**

Myślę, że takiej powiedzmy niezależności. W sensie w biurze jest głośno, cały czas ktoś chodzi, gada, zaczepia. Fajnie sobie przerwać pracę i poplotkować. Ale tak naprawdę to też zmniejszało moją produktywność. A tutaj mam szanse, mam wrażenie, że robić coś szybciej. Chociaż z drugiej strony jest też właśnie ta bariera komunikacyjna. Że jak czegoś nie wiem, to piszę, piszę na tym Skypie. I jest to też czasochłonne. I w domu przede wszystkim jest komfortowo, mogę sobie usiąść w dresie, spodnie z wysokim stanem mi nie obciskają brzucha. Nie musze się niczym przejmować. Tak że to na pewno będzie…

**Bo też ostatnio mówiłaś, że planujesz obejrzeć jakieś sztuki teatralne online?**

Tak.

**To nie wiem, czy ci się udało w końcu.**

To było tak, że jedną miałam obejrzeć i zapomniałam. I zorientowałam się dokładnie dzień po. Co więcej, zorientowałam się, że oni puszczali 3 razy tą samą sztukę teatralną na streamingu, ja jej 3 razy nie obejrzałam. I było mi tym bardziej szkoda. Co więcej, okazało się, że nastroje tego teatru, bo to teatr warszawski był. Na stronie teatru są dostępne online różne sztuki, ale akurat nie ta, którą chciałam obejrzeć. Natomiast pamiętałam o jednym koncercie. Obejrzałam w majówkę The Dumplings na żywo. I to był ostatni koncert z serii w ogóle. Ale czekałam na niego, nastawiłam sobie specjalnie budzik. I też na przykład wczoraj dowiedziałam się, że do dzisiaj można chyba zobaczyć za darmo spektakl Frankenstein z, nie wymówię tego, *kankamberbaczem.*

**A wiem, Cumberbatch.**

I w ogóle jest spektakl, w której on ma dwie odsłony. W jednej odsłonie ten *Kakamber* gra Frankensteina, a w drugiej odsłonie gra potwora. I że można obejrzeć ten spektakl na dwa różne sposoby. Ale akurat ten teatr udostępniał tylko jedną wersję, z *Kakamberem* jako potworem. Ale oczywiście to było do dzisiaj i już chyba nie zdążę tego obejrzeć.

**Ale to jest tak, że jak się ogląda te sztuki, to też trzeba za nie płacić, za to, że je się ogląda?**

Właśnie akurat ja o tej sztuce usłyszałam z *filmowanych* wczoraj. I z tego, co zrozumiałam, jest to darmowe do dzisiaj.

**A w tym teatrze, co mówiłaś, że któryś warszawski?**

Też miała być darmowa. Bo to była Śpiąca Królewna. Może to trochę infantylne, ale stwierdziłam, a, pocieszę się Śpiącą Królewną. I ona była normalnie darmowa. I są tam inne sztuki, ale to… Tam były chyba Damy i huzary. No tak nie przepadam.

**Ale myślisz, że jak by była jakaś sztuka, którą byś chciała obejrzeć, to byłabyś gotowa w ogóle zapłacić za takie oglądanie?**

Tak, tak.

**Chciałam z tobą porozmawiać, jak się trochę czujesz. Nie wiem, czy udało ci się jakieś obrazki znaleźć.**

Ja w ciągu tych dwóch tygodni prawie w ogóle się nie internetowałam.

**Ale to tak celowo?**

Powiem szczerze, że ja nie przepadam. Na przykład często mam problemy z Facebookiem. To znaczy trudno się ze mną skontaktować przez Facebooka. Bo ja bardzo rzadko wchodzę. I potem mam straszne zaległości. I wchodzę w wiadomości i widzę, że od 2 tygodni nie odpisałam. I jest mi tak strasznie głupio. I nie wiem, czy mam odpisywać czy nie. Więc w tym tygodniu stwierdziłam, że robię sobie znowu detoks. I też starałam się 9gaga nie przeglądać, bo to jest taki zapychacz czasu. Więc obrazków nie ma.

**Ale ten detoks był jakoś związany z pandemią, czy to tak w ogóle oddzielnie?**

Chyba stwierdziłam, że zauważyłam, że wcześniej, jak się zaczęła pandemia, bardzo dużo czytałam. A teraz czytam trochę mniej. I to chyba przez to, że tak siądę do komputera i coś zaczynam przeglądać. I potem nagle zejdzie mi na tym 2 godziny. Więc stwierdziłam, że w tym tygodniu czytam, nie przeglądam.

**To w takim razie pokażę ci te obrazki, które ja mam przygotowane. Czy jest jakiś tutaj, który oddaje to, jak się czujesz?**

Myślę, że 12. Dlatego, że kojarzy mi się z naszą majówką w lesie. I dodatkowo po tej wycieczce zyskałam taką równowagę, tak jak te kamienie.

**Ale co ci to dało w tej wycieczce?**

Przede wszystkim spotkałam się z przyjaciółmi. I to był pierwszy kontakt, który nie był moim mężem albo sprzedawczynią. To było naprawdę bardzo dużo. Po drugie miałam w końcu okazję się odciąć od pracy, od takiego trochę bezproduktywnego siedzenia w domu. I tak jak człowiek styka się z naturą i widzi ten jej ogrom i to piękno, no to wszystkie problemy stają się takie małe i nieważne. Tak topnieją w obliczu tej przyrody.

**A jak byś właśnie nazwała te emocje, które teraz ci towarzyszą, na tym obrazku?**

Wtedy, w tym lesie to był spokój. Spokój i taka beztroska. A teraz już po tygodniu ciężkiej pracy (śmiech), znaczy ciężkiej, wzmożonej, bo naprawdę mamy bardzo dużo pracy, no to ja jestem już trochę zmęczona i czekam na piątkowe wino.

**Czyli zmęczenie w związku z tym, że masz dużo pracy. A jest w tobie jeszcze jakiś strach czy lęk?**

Już nie. I właśnie trochę się o to martwię. Dlatego, że mam takie wrażenie, jak by już wszystko wracało do normy. A tak naprawdę wciąż nic nie jest normalnie. I trochę się właśnie tego boję. No na przykład czuję, że mogę już bez problemu pojechać do rodziców, że już przecież wszystko jest za nami, że już wracamy do normalności. No, ale ten koronawirus dalej jest. I nadal są chorzy. I ta liczba zachorowań nawet się może zwiększyć. Jakby nie wiem, czy ten czas wpłynął na to, że już tak nabrałam wielkiego dystansu do koronawirusa, czy to jest może związane właśnie z tym odmrażaniem gospodarki. Że tak człowiek sobie myśli, a, to jednak wszystko idzie ku dobremu. Czy może tak zobojętniałam, nie wiem właśnie.

**OK, czyli to jest jakby… Ale czy w takim razie ty z tym strachem w ogóle robisz? Podejmujesz jakieś działania, żeby tak się nie czuć, nie bać się?**

Myślę, że to nie jest taki paraliżujący strach, tylko taka obawa. O której czasem myślę. Ale tak naprawdę towarzyszyła mi przez cały ten, ostatnie 2 miesiące. Więc tak naprawdę co mogę tutaj zrobić? Myślę, że takie właśnie przełamanie, gdybym na przykład pojechała teraz do domu. I zobaczyła, że nic złego się nie stanie. No to to może wpłynęłoby na to, że przestałabym taką obawę czuć.

**Bo rozumiem, że mówisz, że ta obawa ci towarzyszyła przez cały czas.**

Tak.

**I teraz ona jest taka sama, jak 2 tygodnie temu? Czy ona się zmniejszyła, czy ona się zwiększyła przez to, że trochę jest rozluźnienie?**

Bardzo się zmniejszyła. Ale myślę, że to może być też związane z tym, że ja się po prostu przyzwyczaiłam do tej sytuacji. Że to już nie jest nic dziwnego.

**I rozumiem, że tak jakby czujesz, że to jest nie do końca dobre, że się przyzwyczaiłaś?**

Tak. Bo jak się człowiek przyzwyczaja, to się traci czujność. A jednak w obliczu choroby, której nie znamy, nadal powinniśmy pozostać czujni.

**A zauważyłaś, że w twoich jakichś zachowaniach jakoś ta utrata czujności się objawia?**

Jak wtedy pojechaliśmy do lasu, to potem pojechaliśmy szybko do Lidla. Bo była promocja na lody (śmiech). Czyli po pierwsze wysłałam męża po lody. A jeszcze miesiąc temu nie wysłałabym go tak bez potrzeby. I jak wchodziliśmy do mieszkania, to nawet zapomniałam o tym, że pierwsze, co musi zrobić to umyć ręce, bo on był w tym sklepie. Zapomniałam o tym po prostu. Niby siedziałam w aucie i czekałam, aż on kupi te lody. Ale wydawało się to takie naturalne jakoś. Może też dlatego, że byliśmy wtedy z przyjaciółmi, więc też jakoś tak inaczej to wyglądało. Jakoś tak przypominały nam się te wszystkie nasze majówki, które gdzieś tam spędzaliśmy na wyjazdach.

**A czy to, jak w ogóle robicie teraz zakupy się zmieniło?**

Nie. Cały czas jest tak samo, czyli mąż ubiera maseczkę, rękawiczki. Ja robię bardzo dokładną listę, bo muszę pisać, ile jabłek ma kupić. No i go wysyłam.

**A w ogóle w ciągu ostatnich 2 tygodnie kupiłaś sobie coś takiego dla przyjemności, żeby sobie poprawić humor?**

Mam otwarte 2 koszyki. Jeden na Minti Shopie. A drugi na księgarni Znak. I planuję sobie coś. A, jeszcze, była promocja na e-obuwie. Ja przypomniałam mężowi, że miał mi kupić perfumy na dzień kobiet. I że potem zaczęli zamykać centra handlowe i że mi nie kupił tych perfum. Więc czekam na to, aż mi kupi buty, to po pierwsze. Ale jeszcze nie wybrałam dokładnie tak… Rzuciłam mu raz, on powiedział, dobrze, dobrze, wybierz sobie, to ja ci zafunduję. Ale ja teraz czekam, aż on przejmie inicjatywę i powie: pokaż mi, które buty chcesz. Żeby nie było, że ja go zmuszam (śmiech). Sama mogę sobie kupić, no przecież. Natomiast moje koszyki na Minti Shopie i Znaku, jest problem. Dlatego, że wrzuciłam wszystkie potrzebne rzeczy i wyszło mi 700 zł (śmiech). I od tygodnia siedzę i codziennie uszczuplam listę. Czyli codziennie rano wstaję i staram się takim trzeźwym spojrzeniem spojrzeć na to, czego naprawdę potrzebuję, żeby zmniejszyć tą listę tak do 150 zł. Natomiast, jeżeli chodzi o Znak, tam było tak samo, wyszło mi około 600 zł. No i wczoraj zaczęłam przeglądać te książki pod kątem, które postaram się zdobyć nielegalnie jako e-booki. I wywalam z tej listy tych książek, które chcę kupić te, które mogę dostać jako e-booki. I staram się wyselekcjonować tylko te książki, o których będę mogła za 20 lat powiedzieć: tą książkę musiałam mieć w swojej biblioteczce. Tak że też tak planuję do 200 zł zmniejszyć tą wartość. A pozwalam sobie na takie zakupy, bo mam urodziny w maju i uważam, że mi się należy. Choć raz.

**Ale na tym Minti Shopie to powiedziałaś, że patrzysz na to, co ci jest potrzebne. Czyli właśnie to są rzeczy, które sobie kupujesz tak dla przyjemności, czy dlatego, że są ci potrzebne?**

Trochę tak, trochę tak. Najpierw zawsze robię tak, że przeglądam całego Minti Shopa. I wrzucam sobie do koszyka te rzeczy, które bym chciała. Potem te rzeczy, które są za drogie i nie są niezbędne wpisuję na listę. I zazwyczaj potem tą listę daję mężowi, jak się zbliżają jakieś święta czy coś takiego i ludzie chcą wiedzieć… Znaczy w sensie moi rodzice czy mój mąż, co mi kupić. To ja wtedy daję te rzeczy, które bardzo chciałam, ale stwierdziłam, że nie są niezbędne. Potem dokonuję kolejnej selekcji. Czyli wywalam z koszyka te rzeczy, które dodałam niepotrzebnie. Na przykład, podam jakiś…

**No właśnie, ja teraz patrzę, co tam jest w ogóle, bo nie znałam tego sklepu.**

Ja mam wielki problem, jestem wielką fanką paletek Nabli. I mam już, no, może już, mam dwie paletki Nabli, które uwielbiam. I weszła nowa linia takich malutkich paleteczek Nabli, są 3 czy 4. No i ja strasznie, strasznie chciałam. I wiem, że bardzo, bardzo podobają mi się kolory we wszystkich tych. Więc jak mąż się pytał, co chcę na urodziny, no to wypisałam mu te 4 małe paletki, z nadzieją, że którąś z nich dostanę. Nie są takie drogie, więc myślę, że może mi wpadnie któraś. Tak że paletki zostały wyrzucone. Wyrzuciłam też na przykład takie rzeczy jak… Stwierdziłam, że zamówię sobie hydrolaty. Żeby sobie lać na głowę, na włosy w sensie. Bo strasznie mam wysuszoną skórę. I może nie widać, ale ja się z tym źle czuję. I normalnie kupiłabym w Rossmanie za 9 zł wodę brzozową. Ale stwierdziłam, zamówię sobie hydrolat z oczaru. No, ale wywaliłam go z koszyka, stwierdziłam, że kupię wodę brzozową za 9 zł. Tak że to była decyzja selekcyjna po prostu.

**OK, ale czyli on był jakiś droższy?**

Kosztował 30 zł. I miał chyba tylko 200 ml. A woda brzozowa ma pół litra i kosztuje 9 zł.

**Ale to on jest jakiś lepszy? Co w nim jest takiego, że chciałaś go kupić?**

Ja myślę, że on o wiele lepiej by na tą głowę zrobił. Natomiast, żeby ona była dobrze nawilżona, to zauważyłam, że muszę tego troszeczkę więcej polać. I bardzo szybko by mi się zużył. Więc uznałam, że to jest nieekonomiczne po prostu.

**A jak byś tak sobie pomyślała o takiej skali 1 – 10 (łatwość wydawania pieniędzy), to gdzie byś siebie tutaj umieściła?**

7.

**I na czym polega u ciebie ta łatwość wydawania pieniędzy?**

Ja od zawsze wiedziałam, że biorę ślub z moim mężem, bo on jest tą stroną oszczędzającą. To był jedyny powód, żebym nie popadła w długi (śmiech). To znaczy to jest tak, że oczywiście nie dokonuję nieprzemyślanych zakupów. Nigdy nie kupiłam czegoś, czego bym żałowała, że kupiłam. Natomiast, jak już robię zakupy, to uważam, że porządne. Czyli jak już robię zamówienie z Minti Shopu, to robię je co kwartał i płacę 300 zł. Chociaż w sumie kwartał, no to stówa na miesiąc na kosmetyki, to wydaje mi się, że to nie jest dużo. Ale nie blokuję się za bardzo. I to samo mam z ubraniami. Czyli na przykład często przeglądam strony, często sobie dodaję rzeczy do koszyka. Ale zawsze staram się odczekać tydzień. I sprawdzam czy po tygodniu ta rzecz mi się bardzo podoba i czy nadal chcę ją mieć. I czy mi do wszystkiego pasuje. Więc powiedziałabym, że dokonuję zawsze zakupów przemyślanych. Nie mam tak, że żałuję tych wydanych pieniędzy. Po prostu. Bo na przykład mój mąż no to będzie przeżywał buty długo. Bo on wie, że je musi kupić, ale zastanawia się, no takie pieniądze?

**OK, ale jak robisz zakupy na tym Minti Shopie, to jakie emocje ci towarzyszą?**

Najfajniejsze jest wybieranie. I takie myślenie sobie, o, ale by było fajnie, gdybym mogła kupić sobie te wszystkie rzeczy, które bym chciała. I potem jest ten ból, kiedy trzeba selekcjonować to wszystko. I kiedy przychodzi do takiego racjonalnego oceniania tego, co potrzebuję, a czego nie potrzebuję. To powiedziałabym, że pod tym względem nie jest wesoło. Bo to jednak przykre, trzeba sobie czegoś odmawiać.

**A jak już zamówisz?**

O, to najpiękniejsze jest czekanie na to, co przyjdzie (śmiech). Jeszcze w Minti Shopie wszystko wysyłają w takich papierowych torebeczkach z cukierkami w środku. Fajnie.

**A jak ustalasz sobie tę kwotę, którą możesz wydać na te kosmetyki?**

Jak ustalam. W sumie no to zazwyczaj, jak robię takie bardzo, bardzo duże zamówienie, tak jak już mówiłam, staram się wniknąć w głąb siebie i stwierdzić, czego naprawę potrzebuję. I nie dodawać rzeczy typu paletki.

**No tak. Ale właśnie mówiłaś teraz, że chciałabyś tam chyba do 150 zł wydać.**

No tak, ale to ktoś mi ją kupi, nie ja sobie. Dlatego te rzeczy, które bardzo chcę, ale uważam, że są zbytkiem, to wtedy dołączam je do listy i zazwyczaj z jakichś okazji takie rzeczy dostaję. Bo trochę głupio mi… Może nie, że wydawać na własne przyjemności, bo książki to co innego. Ale staram się po prostu do tego rozsądnie podchodzić. I na przykład ta paletka to nie jest rzecz, którą muszę mieć teraz. Jest to po prostu coś przyjemnego. Nie jest niezbędne, ale gdybym dostała taki prezent, bardzo bym się cieszyła.

**Pamiętasz jeszcze jakąś taką sytuację, kiedy kupowałaś coś takiego większego, poza codziennymi zakupami?**

Ale, że drogiego, tak? Czy większe zamówienie?

**Na przykład wakacje czy coś do domu większego. Coś poza spożywczymi zakupami.**

Ja przyznaję, że… A, teraz miałam zamawiać z Ikei. Bo ostatnio powiedziałam mężowi, wiesz co, my nie mamy talerzy. On mówi co ty, daj spokój, mamy tyle talerzy. Ale przyszli znajomi na pizzę i co? Nie mamy talerzy. I jedliśmy na głębokich. I chciałam zamówić najtańsze talerze, te, co są różowe i szare. Bo uważam, że kiedyś będę miała porcelanę. Ale jak będę już może po trzydziestce, to sobie jakąś tam bolesławiecką fundnę, taki zestawik. Teraz, my wciąż żyjemy tak trochę powiedzmy półstudencko. W sensie nie w swoim mieszkaniu. Bo nie będę inwestowała w nieswoje. A znowuż nie chcę też żyć w brzydkim mieszkaniu. No wiadomo, jak to jest. Więc stwierdziłam, że kupię ładne talerze z Ikea. Nie będzie szkoda, jak się potłuczą. Ale nie będą… Ale trochę pocieszą wzrok. Bo to jednak ładne, powiedzmy różowe będą, coś innego. Ale jak pododawałam te wszystkie rzeczy do koszyka, no znowuż wyszło mi 200 zł. I znowu redukcja. Czy ja tego potrzebuję czy nie.

**A bo rozumiem, że dodałaś coś oprócz tych talerzy jeszcze?**

Tak, dodałam szafkę. Bo była szafka po 50 zł chyba. I tak pomyślałam sobie, ale to super opcja na przechowywanie. Bardzo by mi się przydała. Ale zaczęłam się zastanawiać, gdzie ja tą szafkę wstawię? I ja ogólnie miałabym pomysł na to, ale… No, nie widzę sensu, żeby inwestować w to mieszkanie po prostu. Tak że czekam, bo zastanawiam się, czy nie podjechać do tej Ikei. Bo zastanawiam się, czy talerze przyjdą całe. Z drugiej strony widziałam ostatnio jakiś artykuł, że tylko otworzyli Ikeę 4 maja i ludzie po prostu dostali gorączki. I uważam, że na razie trzeba odczekać tydzień, jak się uspokoi ten ruch w Ikei i może wtedy dopiero pojechać.

**Ale myślisz, że jak już tam pojedziesz, to kupisz tylko te talerze?**

Oj nie, nie. Ikea tak nie działa niestety (śmiech). Ale na pewno nie kupię wielu niepotrzebnych… No pewnie tam… Zbiłam ostatnio na przykład zaparzaczkę. Więc muszę kupić zaparzaczkę. Może kupię sobie jakąś pościel przy okazji (śmiech).

**Rozumiem. Czyli coś tam musisz kupić, że jest ci potrzebne, ale może coś jeszcze się okaże potrzebne, jak to zobaczysz?**

Tak, tak. Natomiast, tak jak mówię, nie żałuję nigdy swoich zakupów. Uważam, że wszystkie są zawsze trafne. Często jest tak, że mój mąż mówi: co? Takie buty chcesz sobie kupić? Ale jak kupuję, mówi ooo, ale fajne. Albo wpadam na pomysł, na przykład kupiłam kiedyś, ja mam często takie wizje, w sensie nie prorocze, tylko mam dekoracyjne wizje. Tak jak mówiłam, mieszkamy w wynajmowanym mieszkaniu, mamy tylko 2 pokoiki. No i osobą kuchnię. I widziałam kiedyś w Aldim, są takie, nie wiem, jak to nazwać, takie koszyki, które się zakłada w szafie na półkę. One są tak jakby wklikiwane w tą półkę. I ja wiedziałam, że mamy za grube półki i nigdzie nie założę tej szuflady. Ale wiedziałam, że nie mamy w kuchni miejsca na toster. Więc kupiłam tą szufladę, podwiesiłam ją na ramie okiennej, tak akurat przy ścianie. I mamy półeczkę na toster, wiszącą.

**Czyli najpierw kupiłaś tę półkę, a potem… Czy już mieliście toster?**

Już mieliśmy toster. I on zawsze stał na blacie i mnie denerwował. I ja zobaczyła tą półeczkę i stwierdziłam, tak zrobię, podwieszę na oknie. No i jest. Albo byliśmy w Biedronce i były hamaki. To było 2 lata temu. Po 40 zł. I ja mówię, boże, muszę mieć ten hamak. I Kuba mówi: gdzie ty go w ogóle dasz. I już mi tutaj piłeczka na główce, a, ja wiem, gdzie go dam. I potem przez następny rok naprawdę powiesiłam sobie na kratach na balkonie hamak. Wtedy mieliśmy balkon jeszcze. I normalnie sobie siedziałam na hamaku. A tak mi wszyscy mówili, a gdzie ty go powiesisz. Teraz z kolei nie mam gdzie powiesić hamaka, więc zamierzam sobie zamówić stelaż na hamak.

**Jak to działa?**

No, są takie po prostu, bardzo proste tak naprawdę, ja myślę, że mój tata by to zrobił za 40 zł. Jest kawałek drewna i w to drewno są wbite takie jakieś… Podłużne, metalowe coś. I do tego są jeszcze jakieś takie metalowe podpórki. I na tym można powiesić hamak. I chciałam to kupić, bo nie mamy teraz balkonu. I stwierdziłam, że nie będzie to zły wydatek. Bo ja myślę perspektywicznie i wiem, że jak sobie kupimy własne lokum, w którym na pewno będzie balkon, to przyda mi się taki stelaż na hamak. Ale niestety zaczęłam się zapuszczać w czeluść internetu. I okazało się, że są składane hamaki ze stelażami, które ważą tylko 3kg. Więc jest to teraz moje marzenie i dodałam do to wish listy urodzinowej. Może ktoś mi kupi stelaż na hamak.

**A w ogóle ta twoja wish lista urodzinowa, to ile ona ma pozycji?**

To znaczy to jest tak, że w pewnym momencie, jak się zbliżają święta albo urodziny, a ja… Powiem szczerze, że ja bardzo lubię robić prezenty, ale nie lubię dostawać nietrafionych prezentów. Wolę, żeby ktoś mnie zapytał, jak nie wie. Albo u nas na przykład w domu jest tak, że… Ja mówiłam już chyba, że się wychowałam z kuzynką jako małe dziecko. Mieszkałyśmy w jednym domu ogólnie. I zawsze się dostawało takie większe prezenty pod choinkę od rodziców. I zawsze były dwie wigilie. Nasza, tak powiedzmy ja i rodzice. I potem się schodziło na dużą wigilię, gdzie zjeżdżali się już wszyscy z okolicy. I to już była ta wigilia właśnie z kuzynką i wujostwem. I u nas się robiło tak, że robiło się takie malutkie listy do Mikołaja, nazwałabym to. Teraz to się nazywają wish listy już. I mimo, że jesteśmy stare krowy, to nadal wysyłamy wish listy. Bo pytają się. Więc no wpisuję jakieś tam drobne rzeczy, powiedzmy do 50 zł, które potrzebuję. A to nie wiem, może jakąś szminkę. Tak, żeby nie dostawać czegoś, czego się nie potrzebuje, albo czegoś, co się nie będzie używało. Więc robię tak samo, mąż mnie zawsze prosi właśnie przed świętami, przed urodzinami, robię listę tego, co bym chciała. I jakby nie kontroluję się tutaj. Po prostu robię kategorie, tam powiedzmy do 50 zł, do 100 zł i trochę droższe. No i nie wiem, no on jakby… Jak ktoś się go pyta, co chce, no to on wie, jaką rzecz może rozdysponować. Czy ma powiedzieć, że nic, że czekoladę, czy może powiedzieć na przykład, że skarpetki. A ja mam wieczny niedobór skarpetek stópek. W tamtym roku dostałam bardzo dużo skarpetek. Czy może na przykład powiedzieć paletkę. Także na mojej liście znalazł się taki mały, ręczny odkurzacz za 150 zł.

**To też jest moje marzenie, taki odkurzacz.**

Praktyczne. I nawet ma dobre oceny, powiem szczerze.

**A to masz już jakiś konkretny model?**

Tak. Znalazłam sobie na Morelach. Wprawdzie dostaliśmy z okazji ślubu taką… Takiego i-robota, taką podróbkę roomby. Mamy bardzo małe mieszkanie i to się nie sprawdza w ogóle. Bo ten robot to nie ma tutaj w ogóle, gdzie sprzątać.

**Ale co to jest? Bo ja nie wiem, jak takie roboty działają.**

No, taka okrągła, mała… Takie okrągłe coś, jeździ i zamiata.

**A, dobra, kojarzę. A w ogóle to jest tak, że nazwałabyś się tak ogólnie osobą raczej oszczędną czy raczej rozrzutną?**

Raczej rozrzutną. Ale nie mam wyrzutów sumienia, bo tak naprawdę największe kwoty wydaję nie na siebie, tylko na innych. Nie, żeby mnie to miało jakoś oczyścić czy coś. Ale przyznaję, że lubię robić prezenty i nigdy na tym nie oszczędzam. Zresztą ja uważam, że lepiej kupić droższą rzecz niż jedną tanią, która mi się zaraz zepsuje.

**A masz jakiś taki przykład swojego zachowania, które świadczy o tym, że jesteś raczej rozrzutna niż oszczędna?**

No ja też nie oceniam tego jako… Jakby nie żyję ponad stan. Staram się zawsze odkładać jakąś sumę pieniędzy. Rzeczywiście często mam z tym problem. Ale to jest też tak, że no nie wiem, powiedzmy przed świętami było… Do stycznia było tak, że tak naprawdę nie miałam żadnych własnych oszczędności. Ale to się wiąże z tym, że ja wiele rzeczy biorę na siebie, takich z domu. Czyli idę do Rossmana i kupuję, nie wiem, płyn do płukania, kapsułki do prania, wiesz o co chodzi. I to często są drogie zakupy. Jesteś w stanie jednorazowo wydać w Rossmanie 200 zł. Tylko dlatego, że jest promocja na Persila. I wiesz, że jak teraz kupisz więcej, to nie będziesz musiała kupować przez najbliższy czas. I nie wiem, dlaczego płacę zawsze swoją kartą. Powinno iść to z karty mojej i męża wspólnej. I potem dochodzi do takich sytuacji, że OK, może odłożyliśmy z tego wspólnego konta, no bo ja zamiast zapłacić za coś ze wspólnego, zapłaciłam z mojego. I tak naprawdę to, co odłożył mój mąż, a mój mąż bardzo dużo odkłada, to ja uważam za nasze wspólne oszczędności. Dlatego, że on odłożył to dlatego, że ja za coś zapłaciłam ze swoich pieniędzy, a nie z naszych. I po drugie ja też, od kiedy skończyłam studia, cały czas staram się robić jakieś kursy… Jakieś kursy językowe. Teraz zrobiłam kurs księgowości. I na to naprawdę schodzi dużo pieniędzy. I tak naprawdę wszystkie oszczędności w ciągu ostatnich dwóch lat wydawałam na to. Teraz z powodu koronawirusa nie chodzę na żadne kursy. Skończyłam ostatnio kurs księgowości, kurs językowy. Więc jakby dopiero teraz jestem w stanie odkładać te pieniądze.

**A właśnie w ogóle jak u ciebie to oszczędzanie wygląda? W sensie w jaki sposób oszczędzasz?**

Dostaję wypłatę, staram się tak około 1200 odkładać na oszczędnościówkę. No chyba, że np. muszę zapłacić za kurs. Powiedzmy 1000 odkładam na oszczędnościowe. Biorę też pod uwagę, zrobiliśmy sobie z mężem listę urodzin i wydarzeń. Czyli ile musimy wydać pieniędzy w tym roku. A jest tego dużo, bo mój tata ma teraz sześćdziesiąte urodziny, więc okrągłe. Potem jest osiemdziesiąte urodziny mojej babci, więc okrągłe. 25 urodziny mojej kuzynki, tej, z którą się wychowywałam, no to jak siostry, no to jak mam jej nie kupić czegoś ładnego. Potem jest trzydziesta rocznica ślubu moich rodziców. I jeszcze potem są urodziny mojego męża. I jeszcze po drodze są urodziny przyjaciół. A nie jesteśmy już studentami, już mogę kupić coś porządniejszego teraz. Więc zawsze biorę pod uwagę to, ile muszę dodatkowo pieniędzy wydać na takie okolicznościowe sprawy w miesiącu. I ewentualnie dorzucam do tej oszczędnościówki z taką myślą, że to wypłacę. Więc na przykład zamiast 1000 złotych na oszczędnościówkę wrzucam 1500. Dodatkowo zawsze 1500 przelewam ja i mąż na konto wspólne. Z tego konta płacimy za mieszkanie i płacimy za jedzenie. Chociaż nie wiem, jak to się stało, ale od 3 miesięcy strasznie nam skoczył poziom wydatków. I jak kiedyś zostawało nam około 300 zł, to w tej chwili musimy dopłacać. Myślę, że to jest związane z tym, że robimy jedne, bardzo duże zakupy. Właśnie przez koronawirusa. Więc… Człowiek wie, że będzie miał utrudniony dostęp i bierze rzecz, która niekoniecznie jest potrzebna. I to, co mi zostaje, no to powiedzmy, to są pieniądze takie, które gdzieś tam wydaję na takie… No nie wiem, muszę kupić coś dla siebie, to są pieniądze moje. Czyli kupię sobie spodnie, kupię sobie nowe buty, kupię sobie kosmetyki. Akurat mąż mi się dorzuca do tych na przykład kwartalnych, dużych zakupów na Minti Shopie. Albo odłożę, bo nie wiem, bo muszę kupić płaszcz. Także to powiedziałabym, że to są już takie sprawy indywidualne.

**Czyli to konto oszczędnościowe to jest twoje konto oszczędnościowe czy też wspólne?**

Mam swoje konto oszczędnościowe. I mamy też wspólne konto oszczędnościowe. I tak jak mówię, mąż jest osobą, która odkłada. I jest w tym bardzo dobry. Ale dlatego ja się nie czuję winna, bo odkładam mniej. Dlatego, że wiem, że jakby mąż odkłada za nas oboje. No tak jak ci tłumaczyłam, że ja często przejmuję jakieś tak funkcje dużych wydatków.

**A jak sobie myślisz o tych oszczędnościach, czy tych twoich osobisty czy takich waszych wspólnych, jak byś miała powiedzieć, gdyby nagle wam obojgu się skończyły dochody, to ile byście byli w stanie z tych oszczędności przeżyć? Tak mniej więcej.**

Tak zupełnie bez pracy? No myślę, że dałabym sobie max rok. I nie więcej. Nawet… Bo teraz zaczęłam liczyć, że muszę za mieszkanie zapłacić. Powiedzmy, że wydajemy, za mieszkanie płacimy 2 tysiące. Razem się jakby na te… Liczymy, że 3 tysiące wydajemy na wszystko, razem z wydatkami na jedzenie. No to nie, to krócej (śmiech), to krócej byśmy wytrzymali. W sensie… Bo to jest tak, mamy na przykład odłożone pieniądze z wesela. Mamy też pieniądze, które sami odkładamy na ileś lat, ale powiedzmy są to pieniądze tak zupełnie nie do dotknięcia. Ja ich nie wliczam tutaj. Nie chciałabym z nich żyć. Na pewno. Nie dałabym sobie tak… Bo bardzo się boję stracić płynność finansową…

**OK, czyli to jest tak, że masz takie oszczędności, z których możesz skorzystać w razie, gdybyś miała potrzebę. I masz takie oszczędności, które…**

Które są nie do ruszenia.

**A co ci dają te oszczędności nie do ruszenia? Po co one są?**

To znaczy, my liczymy, że jest to takie… bo szykujemy się do zakupu, do kredytu i do zakupu jakiegoś lokum. Więc liczymy, że jest to taka, to są takie oszczędności na taki wypadek, kiedy będziemy już obciążeni kredytem.

**Czyli gdyby była taka sytuacja, że byście stracili pracę, to po prostu nie chciałabyś ruszać tamtych oszczędności, tylko byś żyła z tych takich, właśnie tych, które możesz wyciągnąć w razie czego.**

Tak.

**To jeszcze mi powiedz, czy wy coś robicie z tymi oszczędnościami, czy po prostu je trzymacie na koncie?**

Akurat oszczędnościami zajmuje się mąż, bo tak jak mówiłam, nie jestem w tym dobra. To znaczy trzymamy je normalnie na lokacie. Ja mam jedną lokatę, nie, mam dwie lokaty na koncie. I mąż też ma jedną. Jedną mamy na koncie wspólnym. No tak naprawdę to staramy się nie pobierać z tego. Wydaje mi się, że teraz… Znaczy wydaje mi się, teraz mąż kupował nowy komputer. Ja uważam, że jest to inwestycja, no bo on pracuje, przecież potrzebuje mieć dobry sprzęt. I właśnie zaproponowałam mu, żeby wziął pieniądze z tej naszej puli… O której ci mówiłam, że gdybym teraz straciła pracę, to bym z niej żyła.

**Właśnie, bo też chciałam o inwestowanie wypytać. Czy w ogóle inwestujecie w jakiś sposób?**

Mąż coś kiedyś robił na tych krypto walutach. Ale szybko mu się to znudziło. Mamy kolegę, który bardzo długo na tym operował. I nie zarobił wielkich pieniędzy, ale tam z 1000 zł zarobił na tym. Jeżeli chodzi o inwestowanie, to ja nie umiem. Ja tylko na lokatę wrzucam pieniądze i patrzę, jak procentuje. Jeżeli chodzi o inwestowanie w siebie, to inwestuję w siebie te pieniądze, które mam na lokacie (śmiech).

**Jak w tej obecnej sytuacji teraz widzisz oszczędzanie?**

Wydaje mi się, że jest łatwiej. Ja widzę, że o wiele mniej pieniędzy wydaję. Dlatego też pozwalam sobie w tym miesiącu na… Normalnie, nie robiłabym… Znaczy zrobiłabym te zakupy na Minti Shopie. Ale normalnie nie kupowałabym książek. Ja chyba mówiłam gdzieś na początku, że dopiero podczas koronawirusa zaczęłam kupować książki. Dlatego, że… Oczywiście wcześniej też miałam itd. Ale chodzi o to, że kupowałam może 2-3 co jakiś czas. A teraz robię naprawdę duże zamówienia. Bo to jest taka namiastka, czy takie trochę pocieszenie. Właśnie przez to, że nie mogę wychodzić z domu, jest taka sytuacja. A jeżeli chodzi o oszczędzanie. Powiedzmy, że idę do pracy. Żeby wytłumaczyć, jak mi się pieniądze rozchodziły. Pracowałam, moje biuro było w galerii handlowej. Czyli teraz tak, po pracy, jakby rzadko robiliśmy, znaczy staraliśmy się robić jedne, jakieś tam większe zakupy w soboty. Ale to wyglądało tak, że kupowaliśmy po prostu wodę i mleko. A ja w tygodniu chodziłam na takie mniejsze zakupy do Biedronki. I kupowałam to, co potrzebujemy na bieżąco. Chleb tostowy, zupę, jakieś tam owoce. I okazuje się, że te małe zakupki najwięcej nam ściągały kasy. Bo były nieprzemyślane. A teraz, jak wyprawiam męża na zakupy, robię mu listę. I on rzeczywiście nie kupi nic poza tą listę. No to okazuje się, że o wiele mniej wydaje. Zresztą, jak ja bym poszła na zakupy do Biedronki i zobaczyłabym ładne skarpety, to bym je kupiła. Jak mąż widzi, to ich nie kupuje. Tak jest. Tak że ja widzę, że bardzo dużo oszczędziłam. Też myślę, że dużo oszczędziliśmy przez to, że się nie spotykaliśmy ze znajomymi. Bo wino. No nie będę piła sikacza za 10 zł, to już kupię sobie takiego za 19 chociaż. I już 19 zł. Mąż kupi 3 piwa. Powiedzmy, że dobra, nie będzie kupował kraftowych, kupi sobie 3 piwa gryczane. No i już mamy 30 zł. Muszę kupić przekąskę, przecież idę do ludzi. I wydaję na jedno spotkanie 50 zł. A teraz nie wychodzimy, nie ma takiej potrzeby (śmiech).

**Ale właśnie, myślisz, że to się równoważy mimo wszystko w tym budżecie? Bo powiedziałaś, że mniej wydajecie przez to, że nie ma wyjść i nie ma takich spontanicznych zakupów. Ale też powiedziałaś, że robisz większe zakupy takie dla siebie. To czujesz, że wychodzi na to samo, czy mimo wszystko…**

Wychodzi na to samo. Chociaż to też dla mnie trochę nowa sytuacja, bo ja pierwszy raz jestem w takiej sytuacji, że ja nie płacę nikomu. W sensie nie mam żadnego kursu, nie chodzę na język. Dlatego to jest dla mnie nowa sytuacja, że ja mam nagle oszczędności. Natomiast jak tylko trochę sytuacja się zmieni, no to wiem, że muszę iść do dentysty. I już liczę dużo pieniążków na to. Wiem, że muszę w końcu się zapisać znowu na język, więc już też liczę dodatkowo, że to wszystko zacznie znowu topnieć.

**A propos tych zakupów, czy w ogóle myślisz, że taki sposób zakupów, jaki macie teraz, to on zostanie przez was utrzymany po pandemii, skoro mniej wydajecie dzięki temu?**

Zastanawiam się i myślę, że może być. Tak. To też trochę było związane z tym, że… mąż miał 2 wypadki samochodowe pod rząd w tamtym roku. Nie, 2 lata temu. I w pewnym momencie bardzo ograniczyliśmy wyjazdy na zakupy, takie duże. Żeby się na nowo przyzwyczaił do samochodu. I teraz trochę też się sytuacja zmieniła, bo po prostu w pewnym momencie był zmuszony znowu wsiąść do auta, żeby jeździć do pracy. Więc też się przełamał. I wcześniej na przykład wolał nie jechać w sobotę na zakupy autem do Lidla. Bo wiadomo, tam jest trudny zjazd, dużo ludzi itd. Natomiast teraz wydaje mi się, że poczuł się pewniej. I możemy znowu wrócić do tej opcji robienia takich jednych, dużych zakupów. Może też dlatego ostatnio mieliśmy takie problemy z tym, że musieliśmy dopłacać jeszcze do tego wspólnego konta. Bo ja robiłam zakupy i po prostu wydawałam za dużo pieniędzy.

**Czyli jest lepiej, jak on robi zakupy niż jak ty robisz?**

Tak.

**Ale w ogóle ta wasza sytuacja finansowa jakoś się zmieniła teraz w związku z pandemią?**

Tak. I powiedziałabym, że o połowę. Bo mąż jest od kwietnia chyba, chyba tak, jest na postojowym. Czyli zarabia tylko połowę. Znaczy tam 64%. No i jeszcze od któregoś kwietnia. Tak że jeszcze tą wypłatę za kwiecień dostał taką powiedzmy w porządku. Ale już liczymy na to, że tą majową to dostanie o połowę obciętą. A jednak zarabia o wiele więcej niż ja i…

**Czyli to się jakby dopiero zacznie teraz od przyszłego miesiąca.**

Tak myślę. Też jeszcze, dlaczego oszczędziliśmy. Bo mąż nie jeździł do pracy. A miesięcznie wydaje bardzo dużo pieniędzy na paliwo, musi dojechać do Jelcza.

**To jest daleko?**

Około pół godziny, 40 minut się jedzie tam.

**Czy to postojowe to jest tak, że są jakieś perspektywy, ile to może trwać u niego?**

Miał 1 maja wrócić do pracy. I dzwonił do niego jego szef kilka dni przed. I okazało się, że oni specjalnie dla niego przygotowywali materiały, w sensie specjalnie ciułali jakieś tam zadania, żeby miał do czego wrócić. Bo teraz, chodzi po prostu o to, że tną jak najbardziej… Znaczy zwiększają wszystkim zakresy obowiązków. I wszyscy, czegokolwiek by nie robili, czy byliby menadżerami, czymkolwiek, wszyscy, którzy zostali zatrudnieni tam chyba od tego roku, zostali wysłani na postojowe. A że mąż został zatrudniony w tym roku, też został wysłany na postojowe. A najgorsze jest to, że jego jakby współpracowniczka ma bardzo, bardzo dużo pracy. I oni wcześniej ledwo wyrabiali razem. A teraz ona wszystko musiała wziąć na siebie. Więc oni napisali jakieś specjalne pismo do jakby tych władz wyższych firmy, wymieniając obowiązki, do których Kuba jest im potrzebny. Ale okazało się, że nie mogli nawet tego pisma przedstawić, dlatego że przyszło kilka dni przed powrotem Kuby takie odgórne zalecenie, że te wszystkie obowiązki mają być przejmowane przez osoby… Nie wiem, coś mi się wydaje, że tam coś innymi krajami jeszcze, bo to jest jakaś sieć. No nie wiem, o co tam chodziło. W każdym razie okazało się, że on nie może wrócić jeszcze. No i czekamy. Natomiast pracy jest bardzo dużo, więc prędzej czy później będą musieli go wziąć. Tak myślę

**Czyli nie wiadomo, kiedy może to wrócić do normy.**

Tak naprawdę oni zaczęli, bo oni się zajmują produkcją poduszek powietrznych. I no mąż jest inżynierem, więc współpracuje bezpośrednio z produkcją. A produkcja ruszyła w ogóle od maja. Więc myślę, że na razie się gromadzą sprawy, do których od za, myślę, że góra 2 tygodnie będzie potrzebny.

**Czyli myślisz, że to będzie 1 wypłata, która będzie niższa?**

Tak myślę, tak liczę na to.

**A skąd takie przekonanie, aha, właśnie powiedziałaś, że właśnie jest potrzebny.**

Bardzo dużo pracy jest. Bardzo dużo pracy i wydaje mi się, że jak na nowego pracownika też sobie całkiem nieźle radził. Samo to, że napisali ten wniosek, żeby go tam przywrócili szybciej. Więc mam nadzieję, że przywrócą. Na razie mam służącego, więc ja nie będę tutaj narzekała (śmiech)

**A w ogóle jak wam się żyje, zamknięci we dwójkę cały czas?**

No lubię go, przyznaję (śmiech).

**Za to, że dzięki niemu nie bankrutujesz, jest oszczędną osobą.**

Oczywiście (śmiech). Nie, muszę powiedzieć, że bardzo dobrze, mniej się kłócimy.

**Mniej? Bo właśnie myślałam, że to w drugą stronę.**

Prawie w ogóle tak naprawdę. Myślę, że to trochę dlatego, że człowiek nie przenosi stresów z pracy do domu. W sumie się pracuje z domu, ale jakoś to inaczej, jak ta druga osoba jest i można tam sobie wyciszyć słuchawki, powiedzieć, ja nie mogę, co za cioł. W pracy tego nie powiem. Tak że myślę, że jest lepiej. Po drugie teraz ja uważam, że skoro ja pracuję, mam prawo wydawać polecenia. Więc wydaje polecenia, mam posprzątane, ugotowane. Dostaję kawę o 11 i ciastko. Nie narzekam.

**Jeszcze wracając do tego budżetu. Mimo tego, że uważasz, że to jest taka sytuacja, która nie potrwa długo, to czy wy podjęliście jakieś działania w celu ograniczenia waszego budżetu czy czegokolwiek?**

Ograniczenia, żeby wydawać mniej pieniędzy?

**No właśnie nie wiem, czy w ogóle jakieś zmiany wprowadziliście, jakiekolwiek.**

To znaczy, jeżeli chodzi… Zmiany nie. Natomiast, tak jak na przykład wyszła sprawa z tym komputerem. I on już gadał o tym komputerze od dłuższego czasu. I ja nie mogę takich rzeczy słuchać. Bo potrzebujesz? Kup, no kurde, no proste. I zaczął się koronawirus i się okazało, że ceny komponentów bardzo idą w górę. No i mąż nie chciał kupować komputera ze względu na to, że nie wiadomo, jaka będzie sytuacja z pracą. A ja mu kazałam kupić z oszczędności. Dlatego, że uważam, że jeżeli coś by się miało stać, to dobry komputer jest podstawą do tego, żeby on zaczął… Bardzo nie lubi programować, ale gdyby coś się stało, to jest to pewna praca. A do tego, zakładam, albo gdyby miał coś zacząć projektować, te programy wszystkie, jakieś ilustratory, to potrzebuje strasznie dobrego przebiegu i dobrej grafiki. A komputer już był bardzo stary. Ja nie widzę sensu, żeby się męczyć z takim gratem. Dlatego stwierdziłam, że poświęcę te pieniądze z oszczędności, niech sobie kupi nowy komputer, bo jest to inwestycja taka długofalowa. I uważam, że mogłoby nam pomóc, gdyby rzeczywiście coś tam się z pracą potoczyło nie tak.

**Czyli rozumiem, że to, że jest taka opcja, że powiedzmy w przyszłym miesiącu ten wasz budżet będzie niższy, to nie uważasz, że powinniście coś zmienić w tym, jak wydajecie, jak robicie zakupy czy cokolwiek takiego.**

Moim zdaniem ten budżet jest nie… Jest to znaczny spadek, ale też nie ukrywam, że ja po studiach tyle zarabiałam, mniej, znacznie mniej i jakoś dawaliśmy sobie radę. Więc myślę, że teraz też sobie poradzimy.

**A w ogóle w jakiś sposób ten swój budżet kontrolujesz, spisujesz, planujesz?**

Na pewno jak poszłam na studia, to było mi trudno. Pierwszy rok naprawdę, co miesiąc dostawałam SMS-y od banku albo dzwonili do mnie, czy chcę sobie zwiększyć debet. Bo ja cały czas miałam coś na minusie. To nie były duże kwoty. To było 20 zł, 50. I ja zawsze chodziłam do bankomatu i uzupełniałam w gotówce te pieniądze. Ale samo to, że doprowadzałam do takiego stanu. Chociaż to też trochę inaczej, jak poszłam na studia i nagle dostawałam określoną kwotę pieniędzy, która wcale nie była jakoś duża. Na pierwszym roku jeszcze nie miałam czasu, żeby pracować. Bo to zazwyczaj jest największy odsiew, najwięcej nauki na pierwszym roku. Więc taką trochę szkołę życia przeszłam. Ale Kuba mi już wtedy zaczął pomagać z finansami. W sensie rozsądnie jakoś nimi operować. I wydaje mi się, że po prostu się jakoś tak nauczyłam kontrolować. Czyli robię tak samo jak on. Dostaję wypłatę, wpłacam pewną kwotę na oszczędnościówkę rozsądną. Pewną kwotę przelewam na mieszkanie, pewną kwotę odkładam na jedzenie. I jeżeli coś mi zostanie pod koniec miesiąca, no to wpłacam to znowu na oszczędnościówkę.

**Czyli tak jakby od niego się nauczyłaś takiego oszczędzania właściwie?**

Tak. Znaczy moi rodzice też bardzo oszczędzali, ale ja tego nigdy nie czułam. Bo to nie były moje pieniądze, no to wiadomo.

**Czy w ogóle myślisz, że w tej sytuacji, jaka jest teraz, to dobrze jest te wydatki ograniczać?**

Myślę, że jest rozsądnie. Dlatego, że nie wiemy tak naprawdę, czego się spodziewać. I dla mnie trochę abstrakcyjne jest mówienie… Znaczy trochę to surrealistycznie, kiedy słucham, że tylu ludzi straciło pracę. Dlatego, że ja na szczęście jeszcze się z tym nie spotkałam. Ale nie bagatelizuję tego problemu. I wiem, że nic nie jest pewne. I że mnie to też może spotkać tak naprawdę. Dlatego w razie czego myślę o alternatywach. Ja się zawsze pocieszałam. Jak miałam jakiś ciężki czas w pracy i naprawdę miałam ochotę rzucić to w (niezrozumiałe), to myślałam sobie tak: boże, pójdę kurde do odzieżówki. No każdego przyjmą w odzieżówce. A teraz nagle pojawił się problem. Bo przecież zamknięte są galerie. Znaczy zamknięte, ograniczony handel w galeriach handlowych. I nagle wyjście nr 2 w razie czego, takie naiwne wmawianie sobie już… No nie jest tak wprost realizowalne. Znaczy ja nie mówię, że to moja aspiracja, ale ja tak zawsze się pocieszam. Pocieszałam się tym, że trudno w dzisiejszych czasach nie mieć pracy. Bo naprawdę, cały czas, nie musi to być jakaś superopłacalna praca, ale zawsze jakiś dochód jest. A teraz nagle się okazuje, że może być z tym problem, to jest niepokojące.

**A masz jakiś nowy plan b w takim razie?**

Na razie nie.

**Na ten czas pandemii.**

Gdyby coś się stało ewentualnie? Nie wiem, myślę, że może coś… Ale ja się nie nadaję do gastro. Ja próbowałam kilka razy, byłam najgorszą kelnerką na świecie, przysięgam. Jak bym miała coś robić w jedzeniu, to…

**Nie wiem, jaką masz też sytuację z rodzicami, ale czy czujesz, że w razie czego też twoi rodzice by cię wspierali finansowo, gdyby była taka potrzeba?**

Myślę, że tak, ale myślę, że byłoby mi głupio. Ja cały czas mam taki dług wdzięczności za te studia. No i nie czuję, że próżnowałam, bo dużo też pracowałam i uważam, że dobrze spędziłam ten czas. Ale mimo wszystko czuję się taka nieodpłacona. W sensie… Mama mi kiedyś powiedziała, że jak będę miała swoje dziecko, to zrobię tak samo. Ja zakładam, że pewnie tak. Natomiast wiem, że wiele osób nie miało tak prostej sytuacji i… Czasem jest mi po prostu z tego powodu głupio. I jakby teraz często staram się robić rodzicom jakieś droższe prezenty czy… Nie wiem, jakoś zwrócić im to dobro. Bo po prostu ciężko mi się żyje z tym, że… No wiesz, o co chodzi. Takie może nie, że dług niespłacony, ale… No na przykład mąż się cały czas utrzymywał sam. W sensie przez pierwsze pół roku dostawał pieniądze od rodziców, ale potem już tak naprawdę miał stypendium i tylko z tego stypendium sobie tam funkcjonował. I tym bardziej jest mi głupio (śmiech). Tak że myślę, że by mnie bez problemu wsparli, ale ja tego nie chcę.

**Chciałabym na koniec jeszcze trochę o przyszłości z tobą porozmawiać. Czy ty w ogóle myślisz o tym, kiedy to się skończy wszystko?**

Zastanawiam się. I myślę, że w czerwcu, pod koniec czerwca albo w połowie będę wracała do pracy, tak mi się wydaje, do biura. A jeżeli chodzi o to, kiedy się skończy, no to pewnie wtedy, kiedy wynajdą szczepionkę, która będzie działająca. Tak naprawdę, żeby sprawdzić to, czy ona działa, no to jest potrzebny kolejny jakiś rok. I myślę, że nawet, jeżeli tutaj ta pandemia przycichnie, no to nie będzie koniec, tylko początek. Dlatego, że myślę, że w dzisiejszych czasach nie powinniśmy się obawiać wojen. A jeśli już, to właśnie takie. Czyli ekologiczne albo biologiczne. I ta tendencja do tych pandemii rośnie z roku na rok. My cały czas na to patrzymy, ja nie pamiętam tych epidemii SARS, jakoś nie byłam wtedy tak bardzo świadoma. Pamiętam, że nie było tak na pewno. Natomiast wydaje mi się, że to jest tylko początek. Szczególnie, że teraz, po tej pandemii widać, jak ludzie są nierozsądni i że się nie potrafią często podporządkować. Mimo, że wydaje mi się, że ta sytuacja w Polsce była akurat całkiem dobrze rozegrana, no ale na przykład Włochy. Wiesz co, no epidemie tak naprawdę cały czas się zdarzają. Tylko nie w takiej skali, powiedziałabym. I raczej chorób, które my już znamy i potrafimy opanować. I myślę, że w kolejnych latach wirus się będzie po prostu, może nawet ten sam, będzie się mutował. I trudno powiedzieć, co z tego wyniknie. Mnie się wydaje, że ja przeszłam tego koronawirusa, jestem prawie na 100% pewna, w jakimś tam bardzo lekkim chyba stopniu. Ale objawy, wszystko wskazuje na tego koronawirusa, czas. Ale na przykład chciałabym sprawdzić to, ale testy są bardzo drogie. A gdybym wiedziała, to na przykład nie bałabym się tak jeździć do rodziców czy spotykać się ze znajomymi. Bo wiedziałabym, że przynajmniej tym pierwotnym koronawirusem nie zarażam.

**Czyli mówisz o tej sytuacji na samym początku tej pandemii, tak?**

Tak.

**Czy to jest tak, że jak byś się raz zaraziła i przeszła tę chorobę, to potem już nie zaraża się.**

Podobno tak. Natomiast wirusy się mutują. I wystarczy się zarazić mutacją no i koniec. Na przykład moja mama… u mnie zawsze było w domu tak, że tata miał końskie zdrowie, ja jako dziecko cały czas miałam anginę. A mama nic. I w pewnym momencie się zaczęło. A przecież była pielęgniarką i cały czas wszyscy na nią tam kaszlali itd. I nie wiem, wydaje mi się, że chyba po czterdziestce zaczęła nagle wszystko łapać. Natomiast nie tak, żeby się rozchorowała na tydzień, nie, nie. Mama przychodziła do domu w piątek, rozkładało ją, gorączkowała piątek, sobotę. A w niedzielę się okazywało, że jest zdrowa i musi iść do pracy. I bardzo dużo chorowała. I w pewnym momencie już miała takie grypy, że się zaszczepiła. Skończyło się to tak, że w kolejnym roku pochorowała się jeszcze bardziej, bo złapała jakąś mutację. I ja nie wiem co ten, układ chyba immunologiczny tak zareagował, po prostu tak ją rozłożyło, że ją przywozili do domu. Tak że trudno mi powiedzieć, co by mi dało, gdyby się okazało, że rzeczywiście byłam chora. Nie wydaje mi się, żeby to jakoś mi szczególnie pomogło w kolejnych mutacjach.

**Czyli mówisz, że tak naprawdę to się dopiero zacznie. I będą jakieś takie pandemie, nie wiadomo czy tego wirusa, czy może jakiegoś innego wirusa. Ale myślisz, że w takim razie my się jakoś powinniśmy przygotowywać na to, coś robić?**

Tak mi się wydaje. Teraz może trochę tak katastroficznie myślę, dlatego że sytuacja wygląda tak, a nie inaczej. Natomiast samo to, że taka sytuacja się pojawiła… Nie wydaje mi się, żeby to był taki fenomen, który zabłyśnie i zniknie. Tylko to jest coś, co będzie się powtarzać. Bez względu na to, co dzieje się z planeta, jak my żyjemy, ile nas jest, jak wygląda transport, komunikacja, jak wygląda higiena. Myślę, że z tych wszystkich powodów. I rok temu pracowałam w jeszcze innej firmie. I miałam koleżankę, nazywała się Gosia i ja wiem, ona była tak pod sześćdziesiątkę. Ale bardzo otwarta kobieta, świeży umysł, bardzo racjonalna. I za każdym razem jak ja z koleżanka, czyli te kobiety od niej młodsze o 30 czy tam 40 lat, rozmawiałyśmy sobie o jakiejś przyszłości, tak mówiłyśmy, no, jak ja będę miała dziecko gdzieś tam w przyszłości… To ona się śmiała i mówi: no dziewczyny, ja was nie chcę martwić, ale wasze dzieci tego nie dożyją. I my tak mówiłyśmy, Gosiu, o czym ty mówisz? I nagle, równo rok po tym, jak ona nam to powiedziała, nagle wybucha koronawirus (śmiech).

**Ale czy uważasz, że tak będzie? To znaczy nie wiem, czy planujesz dzieci w ogóle.**

Znaczy nie spieszy mi się. W ogóle ostatnio tak miałam, usiadłam sobie z winem, z paluszkami, oglądałam serial i stwierdziłam: ale mi dobrze (śmiech). To znaczy… Ja myślę, że ja jeszcze dożyję starości. Z kolei zaczynam się rzeczywiście mocno zastanawiać, czy moje dzieci dożyją. Jeszcze ostatnio całkiem dużo czytałam takiej fantastyki, dużo Philippa Dicka i trochę „Jestem Legendą”. I wydaje mi się, że jesteśmy na dobrej drodze, żeby… No, może jeszcze pokolenie moich dzieci. Ale nie wiem, czy tak wnuków doczekam, chyba już nie bardzo. Chociaż mi się wydaje, że wcześniej czy później jakaś bomba atomowa i…

**Ale to myślisz, że co trzeba by było robić, jak się przygotowywać teraz?**

Szczerze powiedziawszy, to nawet nie wiem, czy w tym dzisiejszym sklepie jest możliwość cokolwiek zmienić, czy to już nie poszło za daleko. Bo tak naprawdę tylko jakieś skrajne wydarzenie, takie naprawdę skrajne mogłoby chyba nas trochę z tej drogi odwrócić. Ale to nie wiem, co by się musiało stać, jakaś naprawdę straszna klęska żywiołowa albo jakaś straszna epidemia. Natomiast mam wrażenie, że dostajemy takie trochę znaki od wszechświata, że powinniśmy uważać i tyle.

**Czyli myślisz, że ta pandemia nie wystarczyła do tego, żeby się zmieniło coś tak globalnie?**

Nie.

**Ale to, skoro tak nic nie wiadomo, to może nie warto w ogóle oszczędzać?**

Zastanawiałam się nad tym ostatnio (śmiech).

**Bo i tak wszystko jest poza naszą kontrolą, nic nie wiadomo, no to może nie warto?**

Zastanawiałam się nawet… Chyba właśnie czytałam takie krótkie opowiadanie, już nie pamiętam. W każdym razie zaczęłam się zastanawiać, no kto mi da gwarancję, że ja się w ogóle jutro obudzę. No ale równie dobrze mogłam myśleć o czymś takim przed pandemią. To nie musi być wcale jakąś śmierć z powodu choroby, to może być właśnie ta bomba atomowa. I stwierdziłam, że… Ja nie jestem chyba zbyt spontaniczna, ja bym chyba tak nie potrafiła. Ja nie oszczędzam... Znaczy trochę oszczędzam. Znaczy jestem rozrzutna, ale powiedziałabym, że tak racjonalnie. Natomiast bez przesady, nie potrafiłabym się tak zupełnie odciąć od świata. I też wydaje mi się, że ja lubię planować, lubię sobie usystematyzować wszystko, lubię mieć wszystko poukładane. Lubię sobie docierać do pewnych celów. Więc dla mnie to jest sama przyjemność, kiedy ja sobie coś tam planuję w przyszłości. Czy to właśnie zakup mieszkania czy jakieś dzieci w przyszłości. Lubię mieć tą wizję tego, że coś jest przyjemnego przede mną. Nie chcę tak pesymistycznie myśleć, że to mój ostatni dzień.

**A jakie masz w takim razie plany na najbliższe tygodnie?**

Najbliższe tygodnie. Wyselekcjonować koszyki na Minti Shop i na Znaku. I zamówić. Zastanawiałam się, czy… Na pewno pojechać do domu. Myślę, że na pewno będę chciała jeszcze wybrać się… Marzy mi się piknik. No i mam urodziny niedługo, więc tak się zastanawiałam, czy nie pojechać ze znajomymi, niedużą ilością, w czwórkę, max w szóstkę właśnie nad rzekę może, w jakieś takie miejsce, gdzie nikogo nie ma. I wypić piwko, zjeść sobie coś dobrego. Taki mam plan. Tak naprawdę dużo planów związanych z przyszłością, czyli ewentualna zmiana pracy czy zakup mieszkania, wszystko stanęło w miejscu. Też, no mam nadzieję, że długo nie potrwa to postojowe męża, bo to będzie źle wyglądało w historii… W sensie historii przychodów.

**Ale to nie sądzisz, że to będzie wtedy usprawiedliwione, ze względu na to, że była pandemia?**

No właśnie trudno mi powiedzieć. Szczególnie, że bank odrzucił prośbę o kredyt moich znajomych. A była już pandemia. Czyli jakby oni odrzucili niby z jakichś tam przyczyn, coś im tam w przychodach nie pasowało. Ale wydaje mi się, że odrzucili dlatego, że się wtedy zaczynała pandemia, oni starali się ograniczać. Natomiast myślę, że jak już sytuacja zacznie się poprawiać, to może to świadczyć na naszą niekorzyść. Bo sporo osób będzie chciało wrócić do żywych finansowo i sporo będzie się decydowało na inwestycje. I jest plotka, że cena nieruchomości ma spaść. Ale trudno powiedzieć, czy ona spadnie teraz, czy ona spadnie za 2 lata i czy ja się w ogóle na tą zniżkę załapię.

**Bo wy kiedy planowaliście kupić mieszkanie?**

Ja we wrześniu miałam dostać umowę na czas nieokreślony i mąż też. I wtedy jakby mielibyśmy najlepszą sytuację kredytową. Widzieliśmy się z doradcą kredytowym i on nam powiedział, że jak będziemy mieli oboje na czas nieokreślony, to tak naprawdę każdy bank nam udzieli pożyczki na bardzo dobrych zasadach. Ja też nie chciałabym właśnie ze względu na koronawirusa pochopnie do tego podchodzić. W sensie nie wiem, czy nie wolałabym poczekać troszeczkę niż ryzykować, że dostanę strasznie nieprzyjemny kredyt, który będę musiała spłacać przez najbliższe kilkanaście lat.

**A w tym momencie myślisz, że te plany na wrzesień będą utrzymane?**

Myślę, że nie. To znaczy zobaczymy, jak sytuacja będzie wyglądać. Ale nie wydaje mi się.

**Dziękuję.**
