# Supplementary material for: The challenges arising from the COVID-19 pandemic and the way people deal with them. A qualitative longitudinal study
Source: PLoS One. 2021 Oct 11;16(10):e0258133. doi: 10.1371/journal.pone.0258133 (PMC8504766; doi:10.1371/journal.pone.0258133)
Supplement: S1 Dataset — (ZIP) [file pone.0258133.s003.zip › Transcriptions/stage 4/16.4_F_36_couple, with children.docx]

**16.4_F_36_couple with children**

**Co się działo przez te 2 tygodnie w twoim życiu?**

No co się działo. Znaczy tak generalnie to niewiele się zmieniło, znaczy nie zmieniło się tak jakoś radykalnie. Tak ogólnie to jakichś wielkich zmian nie było. Jedyne, co się tak zmieniło bardziej, no to to, że w końcu mój mąż stwierdził, że możemy przerwać izolację, jeśli chodzi o najbliższą rodzinę. I żeśmy się widzieli z taką najbliższą rodziną.

**I jak to wyglądało?**

Mieliśmy jechać na działkę z rodzicami.

**Na majówkę?**

Nie, nie, teraz, ten weekend co był, jeszcze później.

**To była majówka.**

A, dobra, to była majówka, sorry, już te daty mi się tam mylą. Tak, na 1 dzień tylko, bo tam rodzice nie nocują jeszcze. Ale w końcu pogoda jeszcze była kiepska. Po drugie moja mama się jakoś źle czuła w ten weekend i w końcu żeśmy nie pojechali. Oni też nie pojechali. I zamiast tego pojechaliśmy do mojej siostry. Więc widzieliśmy się z moją siostrą. No a z dziadkami się właśnie widzieliśmy wczoraj, byliśmy u dziadków na tam parę godzinek. Tak, no głównie dzieci (śmiech), im też głównie zależało na dzieciach.

**Użyłaś takiego sformułowania „przełamaliśmy się”. Co się stało, że się przełamaliście, jak do tego doszło?**

Znaczy, szczerze mówiąc, to mój mąż podejmuje takie decyzje. Na początku był, jeszcze tydzień temu był przeciwny, stwierdził, że nie. A potem zmienił decyzję i stwierdził, że tak. Więc… Ja się ogólnie ucieszyłam.

**A gdyby to od ciebie zależało, to by wcześniej już było to przełamanie?**

Właśnie ja nie wiem. Bo ponieważ wiem, że to nie ode mnie nie zależy, więc ja się tam specjalnie nie interesowałam tematem, czy… Znaczy musiałabym bardziej zgłębić temat. Więc nie wiem, trudno mi powiedzieć.

**Musiałabyś zgłębić temat. Jaki, żeby coś więcej wiedzieć?**

No nie wiem, jakoś… No nie wiem, nie wiem dokładnie. Musiałabym się zgłębić, żeby wiedzieć, jakie pytania w ogóle sobie zadać. A ja nie zadawałam sobie pytań, więc trudno mi nawet odpowiedzieć. Nie wiem w tym momencie.

**Czyli to jest troszkę tak, że zdałaś się w tych decyzjach po prostu na twojego męża i co on zdecyduje, to będzie?**

No tak.

**To jest dla ciebie wygodne, to jest dla ci potrzebne, jak to jest? Czy w ogóle tak jest, że…**

Nie, w ogóle. Znaczy generalnie mamy taki układ, że jak coś tam jest takiego tego, to mój mąż podejmuje ostateczną decyzję.

**Czyli taka nowość w postaci odzyskania kontaktów z rodziną. Jak się z tym czułaś?**

No fajnie, fajnie, bardzo pozytywnie. Fajnie, że… No cieszyłam się bardzo, że można się w końcu zobaczyć, jakoś tak porozmawiać z ludźmi w 4 oczy. Nie przez ten komputer, cały czas gapienie się w ten ekran. Mimo, że to też jest… Znaczy fajnie, bo zrobiłyśmy sobie… Znaczy ja tam spotykam się ze znajomymi trochę na Skypie. Ale też właśnie z takimi moimi przyjaciółkami sobie zrobiłyśmy spotkanie na właśnie Skypie. I też bardzo fajnie, że żeśmy się spotkały, pogadały, bo to trochę inaczej. Natomiast no takie spotkania w 4 oczy to zupełnie co innego. Żeśmy sobie usiedli tak przy stole razem, dzieciaki tam sobie ganiały. To przecież cała radość. Nic tego nie zastąpi. To już bardziej o dzieci chodzi. Bo myślę, że ja bym to wytrzymała jeszcze bardziej. No przecież były takie, że gdzieś tam człowiek wyjeżdża na dłużej i też się nie widzi, pisze do siebie, dzwoni itd. I to jest jakoś do wytrzymania. Ale dzieci jakby mniej są w stanie wytrzymać. Więc bardziej się cieszyłam ze względu na nich. I po prostu mój syn był wniebowzięty.

**A towarzyszyły ci jakieś obawy przed tym spotkaniem, w trakcie tego spotkania?**

Nie, niespecjalnie. Nie.

**Wyglądało to tak jak przed pandemią, czy wyglądało to jednak inaczej?**

Nie, tak samo.

**Ale co, było całowanie się na przywitanie, były takie rzeczy?**

Szczerze mówiąc to uważam, że nie ma to różnicy, jeżeli ma się małe dzieci. Bo je i tak się nie da odgrodzić. Czy ja się tam uściskam z moją mamą czy nie na 5 sekund, to i tak nie ma żadnego znaczenia, jak one i tak będą się ściskać z moimi dziećmi przez 3 godziny. Jak się ma coś przenieść, to i tak się przeniesie.

**Więc ty się też możesz wyściskać?**

No tak.

**I co się jeszcze zdarzyło przez te 2 tygodnie? Co pamiętasz?**

No to nie, moje życie chyba monotonne jest. Znaczy jestem z siebie dumna, że skończyłam czytać dwie książki. Bo dawno nie czytałam jakoś książek, bo nie wiem, nie chciało mi się, bardziej słuchałam jakieś podcasty itd. niż czytałam książki. A właśnie ostatnio się wzięłam znowu za książki, przeczytałam dwie książki i jestem z tego bardzo szczęśliwa.

**Coś ciekawego przeczytałaś?**

Nie, ja takie kryminały dwa przeczytałam, które mi gdzieś tam leżały. No, ja bardzo lubię takie fajne, czytam czasami. A też tak zawodowo, właśnie odezwał się mój taki szef, promotor, który mnie namawia tam do powrotu na uczelnię. I tak nie wiem za bardzo jaką podjąć decyzję w tym względzie. I tak rozmawiamy trochę o tym z mężem. Jakby co dalej robić. I tak nie wiem. Nie wiem, jeszcze nie podjęłam decyzji. Bo nie wiemy.

**Czyli pojawiły się nowe spotkania, pojawiły się myśli zawodowe, bo są nowe opcje, które się pojawiły. Co jeszcze się zdarzyło?**

Nie, no chyba nic tak szczególnie…

**A czy z czegoś zrezygnowałaś w ciągu ostatnich dwóch tygodni, ograniczyłaś jakieś zachowania, coś się zmieniło na co dzień?**

Nie, ograniczyć to chyba nie. Ucieszyłam się, że znieśli te godziny dla seniorów, bo już mogę normalnie z dziećmi wyjść na zakupy w ciągu dnia. W moich ulubionych porach.

**Bo dotychczas nie chodziłaś z dziećmi na zakupy?**

Na zakupy to może za dużo powiedziane. Bo nadal nie biorę dzieci do takich większych sklepów. Ale jak idę na bazarek, to po prostu tam i tak jedna osoba zazwyczaj może wejść do takiej budeczki tam. To dzieci stoją te 2 metry ode mnie za budeczką i po prostu czekają na mnie. Ale to nie jest jakieś… Znaczy no staram się, żeby nie wchodziły do sklepu ze mną.

**I co robisz wtedy z nimi? Idziesz na bazarek i wchodzisz do sklepu, który nie jest zamknięty, tak?**

Znaczy no nie, bo na bazarku, jak są np. warzywa, to pani wychodzi i podaje itd. To nawet nie trzeba wchodzić. Jakby do sklepu nie trzeba wchodzić. A jak wchodzę, no to po prostu oni czekają, przy otwartych drzwiach czekają po prostu na zewnątrz. I tyle.

**Czy coś się zmieniło, jeśli chodzi o wasze miejsca, gdzie chodzicie na spacery?**

No ostatnio to w ogóle nie chodzimy… No tak, bo właśnie czy już na bazarek z nimi wyjdę. Ja tak właśnie lubię jakiś cel sobie obrać spaceru. Więc już tak bez celu się tak nie włóczymy. Jak zazwyczaj gdzieś idziemy, to właśnie ja mówię, a to pójdę po pieczywo, a to przy okazji kupię owoce. Ja wolę mieć jakiś cel.

**Zoo otworzyli już czy jeszcze nie?**

No nie, właśnie jeszcze nie otworzyli zoo. Właśnie jeszcze takich wszystkich… Ani plac zabaw ani takich rzeczy nie otworzyli, więc no pod tym względem akurat słabo.

**A jeśli chodzi o środki ostrożności, które zachowujesz, to jak to w tej chwili wygląda?**

No, w sklepie rękawiczki. I noszę tą maseczkę, zakładam. Jak nikogo nie ma w pobliżu i jak mi się ciężko już oddycha, to trochę tam zdejmuję. Ale no… Czy gdzieś tam, jak nie ma ludzi, to zdejmuję trochę. Bo to się jednak źle w tym oddycha.

**Dla własnego komfortu rozumiem.**

Tak. Znaczy ograniczam swój komfort, jak widzę ludzi. Bo rozumiem, że może to być dla nich niekomfortowe.

**A czy pojawiły się takie rzeczy, które ci zaczęły przeszkadzać?**

Przeszkadzać… Z takich codziennych rzeczy? Nie, nie, nie. Nie, chyba nie, nic mi nie przychodzi do głowy.

**Bo rozmawiałyśmy o tym, że przeszkadza ci izolacja, to zamknięcie. Że przeszkadzają ci te maski. Że przeszkadza ci to, że nie możesz dalej pójść. Że czujesz się taka, no właśnie troszkę odizolowana z tymi dziećmi w domu.**

To cały czas jest to samo. Trochę mi to przeszkadza, no wiadomo. Cały czas to samo.

**Jakieś kontakty towarzyskie poza rodzinne ci się uruchomiły? Albo myślisz o tym, żeby je uruchomić?**

Znaczy uruchomiły… Znaczy no nie spotykamy się z nikim jeszcze poza tą najbliższą rodziną. Nie wiem, jaka jest decyzja jeszcze co do dalszych. Nie, nie wiem, co tam dalej. Natomiast no właśnie mam tam parę, to właśnie taka dalsza rodzina, koleżanki, które, po prostu mam ciuchy dziecięce dla nich. I po prostu muszę im podrzucić te rzeczy. Bo mi zalegają, a one potrzebują, bo już dzieci rosną. Takie maluchy, nie? Bo ja mam dużo właśnie takich rzeczy i muszę podrzucić im. I właśnie tak zastanawiamy się, czy zrobimy ten przekaz bezkontaktowy czy kontaktowy. Ale jeszcze tam decyzja nie zapadła wspólna, więc…

**Jeszcze rozważacie. A jakie są za i przeciw jak myślicie o tej decyzji, o czym wtedy rozmawiacie, co bierzecie pod uwagę?**

Tak w sumie to nie rozdrabiamy się na ten temat. Bo ja mówię, że nie wiem, czy my się już spotykamy ze znajomymi, czy jeszcze nie. Więc tylko tyle, jakby wszyscy to akceptują. Tak jak mówię, ja w ten temat, nie chce mi się w to wchodzić już i zastanawiać się nad tym. Nawet mi się nie chce o tym myśleć, czy już się można spotykać czy też nie. Dlatego po prostu z chęcią, że tak powiem, zostawiam to mojemu mężowi.

**A jeśli chodzi o doniesienia, prasowe, medialne, jakiekolwiek, śledzisz je w jakiś sposób?**

Śledzę, śledzę.

**I co śledzisz, czego szukasz?**

Ja śledzę głownie teraz ten cały bajzel polityczny, który jest teraz zrobiony. I to jest takie ciekawe, jak to wszystko się tam rozpada, te wszystkie plany, wychodzą. I tu ktoś wymyśla jakąś większą głupotę, tamten coś wymyśla innego. Tak że tak śledzę takie bardziej polityczne niż te takie typowo… te chorobowe, może tak.

**Wiesz, ile w tej chwili jest diagnozowanych w Polsce osób, zarażonych?**

To się wszędzie wyświetla na tych stronach. To nawet już mi te cyfry tam gdzieś… Ale tak, no jak przeglądam wiadomości, to zawsze tam jest w tych nagłówkach, bo przecież media tym żyją, ile osób umarło. Więc gdzieś mi się to przejawia.

**I twoje subiektywne odczucie jest jakie? Że Polska jest w trochę lepszej sytuacji niż inne państwa europejskie? Gorszej, takiej samej?**

Pod względem czego, zachorowań?

**W tej sytuacji koronawirusowej.**

Znaczy, bo czym innym jest jakby sytuacja zachorowań. A czym innym są konsekwencje tego, na przykład gospodarcze. Nie wiem tak do końca. Znaczy mamy mniej tych zachorowań jak Niemcy na przykład czy te wszystkie kraje zachodnie. Ale, no właśnie. Ale to też jest dużym kosztem, jakby gospodarczo jest bardzo duży koszt. Bo jesteśmy już, no 2 miesiące jesteśmy zamknięci. I dużo ludzi nie chodzi do pracy, dzieci nie chodzą do szkoły itd. Więc myślę, że ten koszt gospodarczy może być bardzo duży. I zobaczymy jakby jak sobie z tym poradzimy dopiero. A te wszystkie pomysły tego fantastycznego rządu naszego no są po prostu jak kulą w płot. Dla mnie to jest bez sensu to wszystko, co oni… No może nie wszystko, bo nie powiem tak.

**Co jest bez sensu?**

No przede wszystkim to, że produkują jakieś tarcze antykryzysowe, już chyba trzecia wersja powstała. I ja się pytam jakby no… No nie można tego zrobić raz a porządnie? Tylko trzeba ludziom co 2 tygodnie dawać nową stertę papierów do wertowania? I jeszcze jak tam w tych przemycają jakieś podwyższenie podatków w tej tarczy kryzysowej, no to przecież no nie, no po prostu…

**A zrobili coś takiego?**

Bo zdaje się, że nałożyli podatek na Netflixa i te wszystkie VOD-y. No właśnie jak teraz ludzie siedzą i to oglądają, bo nie wychodzą do kina czy gdzieś tam. To przecież to jest śmiech na sali. No oceniam to tragicznie. Więc uważam, że nie wróżę temu przyszłości wielkiej i sukcesu. No, zobaczymy, jak będzie. Bo teraz jest taki bajzel polityczny, że wszystko może się rypnąć. Więc mam nadzieję, że się rypnie.

**A jeśli chodzi o twoje uczucia, emocje, w ciągu tych ostatnich dwóch tygodni. Udało ci się coś zanotować, zapisać, zrobić zdjęcie?**

No tych zdjęć nie. Ale to są właśnie te rzeczy, o których już rozmawiałyśmy, to co sobie specjalnie zanotowałam w pamięci. To właśnie te spotkania, to było najwięcej, właśnie ta niepewność w związku z tą sytuacją zawodową. Natomiast jeszcze właśnie, jak 2 tygodnie temu, poprzedni weekend właśnie mój mąż powiedział, że jeszcze się nie spotykamy, oj, to było mi strasznie ciężko, bo wtedy moja siostra pojechała właśnie na działkę z rodzicami. Więc oni się spotkali. A my się nie spotkaliśmy. Więc, oj, no strasznie to przeżyłam, strasznie smutno mi było z tego powodu.

**Było ci smutno i jak się jeszcze czułaś wtedy? Możesz to opisać dookoła, to nie musi być jedno słowo, żeby nazwać ten stan. Tylko wiesz, co sobie myślałaś…**

Miałam nadzieję, że już gdzieś wyjdziemy na to świeże powietrze, że już tego, że będzie fajnie. I taka zawiedziona trochę byłam, że mój mąż był taki stanowczy w tym. Nie naciskałam go, bo mówię, to jest twoja decyzja i podporządkujemy się. Ale zawiedziona byłam trochę. I smutno mi było, byłam pełna nadziei, że to już zaraz, już zaraz…. Ale jakoś tam przetrwaliśmy.

**A czułaś się rozczarowana, rozżalona? Stęskniona? Co to było?**

No to wszystko, to wszystko naraz. Nie wiem, tak mi trudno zdefiniować te uczucia. No tak, ja byłam stęskniona właśnie za takim wyjściem gdzieś. Jakby już opuszczeniem tego domu i pojechaniem właśnie… Właśnie nie do lasu jako takiego, tylko do mojego konkretnego lasu tam, tego lasu, który znam, do tej rzeki, którą znam, na te łąki, które znam, a nie tam byle jakie łąki. Więc no bardzo, bardzo… Znaczy ja się czuję tak emocjonalnie związana z tym miejscem. Więc jak nie mogę z jakiegoś powodu tam pojechać raz na jakiś czas to jest mi w ogóle ciężko.

**Ja ci pokażę jednak te zdjęcia, które pokazywałam ci zwykle. Bo może coś z tego ci ułatwi i będzie pasowało do tego, jak się czułaś. Bo mówiłaś, że to były 2 stany. Jeden był taki, który opisywałaś jako to oczekiwanie na to spotkanie i czułaś się taka smutna i zawiedziona, że ono nie doszło do skutku. Potem to spotkanie doszło do skutku, więc rozumiem, że emocje były inne.**

No nie wiem, teraz jak patrzę na tą 13 gdzie jest to słońce i to… To jest takie dotykanie tych miejsc. Właśnie z takim ciepłem mi się to kojarzy. Takim dotykaniem tych znajomych, takich mi bliskich sercu miejsc.

**I tak się poczułaś, jak z nimi się spotkałaś już?**

Nie, tak się chciałam poczuć, jak chciałam pojechać na działkę.

**Właśnie o to mi chodziło, to jest twoja tęsknota, tego ci brakowało?**

Tak.

**Czy jest tutaj jakieś zdjęcie, które pokazuje, jak się czułaś, kiedy się okazało, że nie możesz tego zrealizować?**

To myślę, że to jest 11.

**Czemu?**

Dlatego, że no to jest właśnie takie oglądanie świata zza szyby. Gdzieś tam za tą szybą jest ta tęcza, ta nadzieja. Ale jednocześnie jest, nie można się tam dostać, bo jest deszcz, jest szaro i jest szyba.

**A ten moment, kiedy się spotkaliście, czy on tutaj gdzieś jest? Czy tego zdjęcia brakuje i powinno być jakieś inne?**

Nie, nie, nie. To żadne mi tu nie pasuje.

**A co powinno być na ten moment, kiedy się spotkaliście?**

Nie, nie wiem. Dla mnie to nie… Nie wiem, czy to takie. Znaczy ja się czułam radośnie. Ale to nie jakieś takie… Znaczy czułam radość, cieszyłam się z tego, że się spotkaliśmy w końcu. Ale nie wiem, czy to takie emocjonalne. Nie wiem. Dla mnie to nie jest, że to jakiś pik emocjonalny po prostu.

**Byłoby inaczej, gdyby się udało wyjechać na tą działkę?**

Nie wiem, może tak, trudno mi powiedzieć.

**A jak myślisz, czemu?**

Bo to miejsce jest takie sentymentalne dla mnie. Może dlatego. Ale nie wiem, trudno mi powiedzieć, czy tak by było, czy tak by nie było. Znaczy ja się czułam cały czas jakby blisko moich rodziców czy mojej siostry, bo żeśmy cały czas rozmawiali, cały czas byliśmy w kontakcie. Natomiast fajnie… Znaczy wiadomo, że takie spotkanie w 4 oczy jest bardziej… Jest lepsze, jest głębsze. I też ze względu na dzieci to jest w ogóle… Natomiast no tak bez przesady (śmiech).

**Czy coś jeszcze wpływa na twoje emocje, na to jak się czujesz w obecnej sytuacji?**

Czy coś jeszcze wpływa? No bardzo mnie rozgrzewa ta sytuacja polityczna. Naprawdę, po prostu do czerwoności mnie rozgrzewa. Znaczy tak bardzo mnie to wkręciło w ogóle. Czytanie tych wszystkich rzeczy i trochę dyskusje na ten temat właśnie z ludźmi. No, tak że jakoś mnie to tam…

**A powiedz, co czytasz i czego słuchasz na ten temat i jakie to są emocje?**

No właśnie takie… Czego słucham… Nie, właśnie ostatnio nie słucham. Chociaż może trochę słucham. Nie wiem, trochę chyba słuchałam, coś chyba w radiu słuchałam. Znaczy w Radiu w net tam słuchałam coś. A tak, to jeśli chodzi o czytanie, to ja zaglądam, to co zawsze, coś nowego, zaglądam na TVN24, zaglądam na wykop, trochę czytam komentarze na wykopie, bo one też czasami są takie, że ktoś na coś zwrócił uwagę i można się… Wyłapać jakieś takie manipulacje czy jakieś sugestie, którymi media rzucają.

**A jakie to są uczucia, które się w tobie budzą, jak angażujesz się w ten temat? Co on ci robi, ten temat.**

Co on mi robi? Nie wiem, co on mi robi. Po prostu jakoś tak ciekawie… Uważam, że taka jest sytuacja nietypowa. Bo właściwie jest coś, co się u nas w historii tej najnowszej nie wydarzyło. I uważam, że to ciekawe obserwowanie tego. I potem może jakieś ciekawe rzeczy z tego wynikną.

**Tak, ja rozumiem ciekawość. Ale ciekawość też może mieć swoje konotacje. I może to być ciekawość podszyta smutkiem. Może być ciekawość podszyta złością, obojętnością. Tam może być cała gama różnych uczuć, które się wiążą z zaciekawieniem czy ekscytacją tematem.**

No część rzeczy połączona jest na pewno ze złością. Bo… No tak jak czytam niektóre te właśnie pomysły i tak dalej, to mnie to denerwuje strasznie. Próbuję znaleźć w tym jakiś sens i logikę, ale nie widzę i mnie to wkurza. Natomiast… No jakieś takie właśnie ułożenie sobie w głowie, co teraz się dzieje w tym temacie.

**A jak sobie z tym radzisz teraz jak cię coś wkurza i złości?**

To jest takie chwilowe, jakby mnie… Albo gadam czasami z moim mężem o tym, trochę się po… Pogadamy sobie trochę o tym, poemocjonujemy się. I wystarczy

**Czyli nie znalazłaś jakiegoś zajęcia, które cię odrywa, nic tutaj się nie zmieniło.**

Nie, bo to nie jest taki poziom wściekłości. Nie wpływa to jakoś tak dogłębnie na moje życie. Tak raczej powierzchownie.

**Nie jest to taki poziom wściekłości jak co? Co miałaś na myśli?**

No nie wiem, potrafię się bardziej wkurzyć na ludzi w moim otoczeniu, jak mi coś zrobią przykrego niż… Bardziej się wkurzam na mojego męża, jak mnie zdenerwuje niż na polityków.

**A denerwuje cię teraz twój mąż?**

No czasami mnie denerwuje (śmiech).

**A jest jakaś różnica w twoich nerwach takich domowych w tej chwili, w czasach koronawirusowych?**

Różnica? Nie, nie. Chyba na dzieci bardziej się denerwuję niż na mojego męża. Na mojego męża to standardowo, no co jakiś czas muszę się na coś zdenerwować, bo jakby wszyscy są ludźmi, popełniają błędy.

**A na siebie się denerwujesz?**

Na siebie się denerwuję, tak. Trochę się czasami denerwuję na siebie. Denerwuję się, jak mi nie starczy cierpliwości na dzieci. A nie starcza mi ostatnio, też się wkurzam na to. Wkurzam się na siebie, że się wkurzam na dzieci.

**Bardziej się wkurzasz na dzieci niż dawniej, jak siebie pamiętasz? Sprzed dwóch tygodni, 4 tygodni, 3 miesięcy?**

No właśnie nie wiem, czy to jest kwestia… Ja nie wiem, czy więcej, czy to jest kwestia tego, że oni też wariują i dostają głupawki. I albo wchodzą w taki po prostu etap, że… Bo dzieci tak mają etapami trochę. Że mają taki czas, że po prostu się nie słuchają i są takie straszne, wszystko jest na nie. A potem wchodzą w taki czas, że wszystko jest OK itd. I mam wrażenie, że znowu trochę weszli w taką fazę upartości, niesłuchania się.

**Takie zmęczenie materiału nastąpiło w tej chwili?**

No może tak, może to też wynika z tego, że jakby no już chcieliby bardziej uczestniczyć w życiu poza domem. A nie da się tego zrobić.

**Powiedz, a jak obserwujesz swoich znajomych, swoje otoczenie, swojego męża, czy w ich zachowaniu, w ich emocjach, masz wrażenie, że nastąpiła jakaś zmiana?**

No mój mąż jest bardzo szczęśliwy. Bardzo szczęśliwy z koronawirusem. Doszedł do wniosku, że dla niego to jest super. Bo on pracuje online. On jest takim introwertykiem i on nie musi się spotykać teraz z ludźmi. Nie lubi takich small talków, takich pogaduszek między tam tego. I on jest bardzo, właśnie dzisiaj przy obiedzie, tam przy stole oświadczył, że on jest bardzo zadowolony z tego, on może tak pracować. Nie musi się z ludźmi spotykać, nie musi wychodzić w ogóle z domu, to jest najlepiej dla niego (śmiech). Także, no ja mówię dobrze, to cieszę się, że się tak ucieszyłeś.

**A co ty na to? Jak ty się poczułaś, jak on tak powiedział?**

Nie, no mnie to śmieszy, bo jakby znam go. I wiem, że on by najlepiej z domu nie wychodził. Ale no... No ma to swoje dobre i złe strony. Znaczy rozumiem, że dla niego to jest OK. Ale chyba on też zdaje sobie sprawę, że potrzebuje też z normalnymi ludźmi trochę poprzebywać.

**Masz trochę wrażenie, że to, że jemu się podoba to bycie w domu i home office, to może on troszkę przedłuża tą sytuację koronawirusową u was w domu?**

Czy dlatego… Nie wiem, może tak. Może tak. Nie myślałam o tym w ten sposób.

**A ma jakiś problem w ogóle z tym, co się dzieje czy nie? Coś mu przeszkadza, uważa, że już ma dosyć, chciałby, żeby wróciło do normy. Czy są takie rzeczy, tak jak ciebie drażnią te maski, jest ci w nich niewygodnie.**

Nie, mój mąż się czuje jak ryba w wodzie. Jedyne, co go drażni, to chyba to, że nie wiadomo, co będzie z wakacjami. Bo na wakacje to lubi wyjechać. Więc chyba to jest jedyne takie.

**A inne osoby z twojego otoczenia? Jak się czują z tym, co się dzieje, jakie są ich emocje. Czy coś zmieniło się w ich zachowaniu.**

No, to zależy. Moja siostra jest sfrustrowana już szkolnym… Domową edukacją. I już ma po dziurki w nosie tego. Szwagier znowu jest zestresowany chyba bardziej, bo biznes mu się wali ze względu na tego koronawirusa. No i rodzice, to tam… No moja mama to tam siedzi w domu. Też się wkurza, bo ona lubi… Ona jest taką dosyć aktywną osobą. I lubi sobie pochodzić, połazić, pogadać z ludźmi. A teraz siedzi w domu. Znaczy już zaczęła też wychodzić trochę tam do sklepu czy coś w tym stylu. Znaczy no takie małe raczej rzeczy, krótkie te spacery. A mój tata też pracuje z domu. Ale nie wiem, mój tata to w ogóle jest taki, że on to w ogóle lepiej by się nie odzywał. On też jest takim introwertykiem i nie mówi za wiele.

**A zaobserwowałaś jakieś nowe sposoby radzenia sobie z trudną sytuacją u ludzi ostatnio?**

Nowe sposoby radzenia sobie z sytuacją. Nie. Nie, nic mi nie przychodzi do głowy.

**Twoja siostra, twój szwagier, mówisz, że ona jest wykończona lekcjami online, jemu się biznes coś tam chwieje. Więc coś wymyślili takiego, żeby…**

No nie, na razie chyba nic nie wymyślili z tą edukacją. Coś tam próbują wymyślać jakieś ten. Ale nie słyszałam, żeby coś jakiegoś nowego…

**Chodzi mi o to, czy wymyślili coś, żeby lepiej sobie radzić z własnymi emocjami, żeby mieć jakąś odskocznię. Coś sobie takiego wymyślić, co im pomoże lepiej funkcjonować w trudnej sytuacji.**

Nie, nie, nie odpowiem ci na to pytanie, bo nie wiem. Nic mi nie przychodzi do głowy. A może coś zrobili, ale nie informował nas nikt o czymś takim. No, jak żeśmy się spotkali, to też jakby nic chyba się specjalnie nie zmieniło u nich.

**A czy w tobie w tej chwili jest jakiś lęk, niepokój, obawa?**

No ja mam tylko obawy… Znaczy mam tak, obawy w związku z tym, że na przykład… No właśnie, mamy jeszcze problem z wynajęciem mieszkania naszego. No i pytanie brzmi, czy je wynajmiemy czy nie. No i to jest taka trudna sytuacja faktycznie dla nas. Bo szukamy kogoś i zobaczymy, jak to będzie.

**Mówiłaś, że lokatorka wam wymówiła.**

Tak, tak. Więc jedna została, jedna się wyprowadziła. I szukamy jakby jednej osoby. Więc zobaczymy. No i z tą moją sytuacją zawodową. Chociaż to nie jest tak do końca związane z tym koronawirusem, bo i tak trzeba by podjąć tą decyzję i tak. Bo mi się zbliża, że tak powiem, koniec mojego… Zamkną mi przewód po prostu, jak nie napiszę tego doktoratu, no i tyle.

**Ile ci czasu zostało?**

Chyba do końca 2021, więc musiałabym się za to zabrać teraz.

**A takie obawy dotyczące samego koronawirusa, zdrowia twojego, twoich najbliższych, jak to wygląda w tej chwili u ciebie?**

No nie wiem, ja się jakoś… Myślę, że izolujemy się nadal, jakby nie łazimy nigdzie w jakichś takich… Z tego, co się orientuję, nie słyszałam o czymś takim, żeby ktoś się zaraził gdzieś… Żeby były jakieś ogniska zarażenia w sklepach, na bazarkach, czyli tam, gdzie powiedzmy przebywamy. Więc jakoś tak myślę, że nie jest to jakieś wysokie ryzyko teraz.

**Rozumiem, że nikt z twojego otoczenia nie jest chory. To jest takie gdzieś…**

Nie znam nikogo, kto byłby chory. Nie znam nikogo, kto by znał kogoś, kto byłby chory.

**A jak się teraz sprawa zakupów u was rozgrywa?**

No ja sama jeżdżę. I jak już właśnie, tak jak ci mówiłam, z dziećmi jak sobie pójdę na bazarek, to tam sobie coś przy okazji kupię.

**Bo rezygnowałaś z tych twoich ulubionych godzin, które zajęli seniorzy, więc jeździłaś wcześnie rano ostatnio.**

Jeździłam rano i zaczęłam teraz, właściwie już mi się nie chce, nie lubię wstawać rano. Więc już… Znaczy bałam się, że jak pojadę wieczorem, to nic nie będzie po prostu. I zaczęłam jeździć trochę wieczorem i okazało się, że nie jest tak tragicznie. Więc w sumie ostatnio jeżdżę popołudniami. Znaczy, jak dzieci pójdą spać, o 20. Chyba, że mąż ich położy spać, to tam koło 19. Więc okazało się, ostatnio byłam i nie było tak tragicznie. Więc stwierdziłam, że może to jest lepsze rozwiązanie, bo nie muszę wcześnie wstawać. No i tak właśnie, jak wyjdę gdzieś z dziećmi na spacer, to przy okazji jakąś piekarnię zaliczę albo jakiś warzywniak, albo sklep z rybkami.

**A czy te zakupy robisz teraz częściej niż jeszcze 2 tygodnie temu?**

Tak.

**Masz takie poczucie powracania do czasów sprzed pandemii przy zakupach?**

Takie malutkie. Bo już na przykład, jak sobie pomyślę, że jak nie będę miała chleba, no to sobie wyskoczę na bazarek z dziećmi. A wcześniej tak jakoś nie wychodziłam z nimi do sklepu. Więc… Ale i tak, no jeszcze staram się kupować na kilka dni. Żeby nie musieć, że tak powiem iść. Ale jak przy okazji będę, no to jakby kupić świeże pieczywo.

**Planujesz nadal zakupy czy przestałaś planować?**

A czy ja planowałam w ogóle kiedykolwiek?

**Znaczy niekoniecznie z listą planowałaś, ale rozmyślałaś, kiedy pojadę, jak to zrobić, która to będzie godzina, gdzie mi się opłaca jechać. Takie rzeczy brałaś pod uwagę, pamiętam.**

To teraz cały czas biorę pod uwagę, gdzie pojadę. Więc trochę tam sprawdzam, gdzie akurat promocja na pieluchy, bo to mnie głównie motywuje do pojechania do Biedronki czy do Lidla (śmiech). Ale no też, właśnie do Carrefoura czasami jadę, bo mój mąż ma upodobanie w różnych takich fikuśnych piwach, których nie ma w normalnych sklepach. Więc muszę jechać do Carrefoura.

**Rozumiem, że dzieci do supermarketu jeszcze nie zabierasz.**

Nie, nie, dzieci do supermarketu nie. Zresztą nawet nie widziałam, żeby ktoś przychodził z dziećmi. I są nawet te takie komunikaty na niektórych sklepach, że prosimy o zakupy, żeby dzieci nie przychodziły.

**Czy te zakupy to jest w tej chwili jakiś element dla ciebie przyjemności? Zaczął być znowu? Bo mówiłaś o tych kolejkach, które ci tak…**

No nie ma już kolejek. Przyjemność, czy ja wiem? Przyjemność jest taka, że wychodzę sobie sama, mam czas tylko dla siebie. No tak, tak, jest większa przyjemność niż jeszcze jakiś czas temu. Tak, zdecydowanie.

**I z czego ona wynika?**

Że może jestem sama na przykład.

**Ale 2 tygodnie temu też robiłaś sama te zakupy.**

No wiem, ale jakaś taka byłam bardziej zestresowana, że jak czegoś zapomnę, to już kurde na amen zapomnę. Że kolejny raz, jak będę musiała wyjść, to po prostu znowu będę musiała zostawić dzieci albo coś. A teraz taki mam większy luz. Że nawet jak czegoś zapomnę, to tam wyskoczę sobie z nimi na bazarek. O każdej godzinie, o której będę chciała.

**I to nie oznacza, że znowu się ustawisz w tej kolejce do chłodni w Selgrosie. Pamiętam, że jakiś koszmar dla ciebie, ta kolejka. Że stałaś i jak czegoś zapomniałaś, to musiałabyś stać jeszcze raz. Tak?**

Tak. No, te kolejki to były fatalne. Teraz już nie ma szczęście.

**A powiedz, bo też opowiadałaś, że tak lubisz przejść przez różne półki, regały, popatrzeć, co jest w tym sklepie. W ciągu ostatnich 2 tygodni kupiłaś coś dla siebie dla przyjemności po prostu?**

Nie. Nie, nie, nie. Z takich… Znaczy poza spożywcze to nie.

**Nic kompletnie?**

Dla siebie nie. Dla siebie nie.

**A dla kogoś?**

No dla mojego syna kupuję gazety z zadaniami, bo on bardzo lubi. To bardziej dla dzieci, jakieś kolorowanki, gazety z zadaniami. Kredki im kupiłam, bo już wykończyli jakieś tam kredki.

**A korciło cię, żeby coś sobie kupić dla poprawy humoru, dla przyjemności po prostu?**

Nie. Znaczy z takich rzeczy jakichś takich… Ja jestem niestety człowiekiem, któremu za dużo przyjemności sprawia na przykład zjedzenie lodów.

**A słodycze też sobie można kupić dla przyjemności.**

No właśnie. Dlatego ja, jak chcę sobie zrobić przyjemność, to właśnie byliśmy na lodach. To takie rzeczy to tak.

**Byłaś na lodach i co jeszcze zrobiłaś dla swojej przyjemności?**

To chyba tylko to. Co ja jeszcze robiłam dla swojej przyjemności? Nie wiem. Z takich rzeczy zakupowych to nie, to chyba nic takiego nie robiłam.

**Gdzie byliście na tych lodach?**

Byliśmy w naszej lodziarni, którą mamy tutaj niedaleko. Także zdjęliśmy maseczki i jedliśmy lody. Tak jak duża ilość osób, która tam była. Ale każdy się trzymał z dala od siebie.

**Rozumiem, że tam można zjeść na zewnątrz, nie można nigdzie usiąść nadal.**

To jest taka budeczka tylko. Oni tam nie mają gdzie usiąść, jest tylko takie okienko w budce. Znaczy oni mają tam taką ławeczkę, która jest przy tym. I tam zazwyczaj ludzie siedzą i jedzą. Teraz napisali, żeby tam nie siadać. I tak ludzie odchodzą i tam jedzą… Znaczy my, ponieważ byliśmy z dziećmi, to też taka ławeczka zwykła, osiedlowa, to tam sobie usiedliśmy i tam zjedliśmy te lody. I tyle.

**Był to taki powrót do normalności trochę dla ciebie?**

No, trochę tak. Fajnie było. Fajnie, cieszę się, że otworzyli te lody. Już mieli otworzyć je wcześniej, ale nie otworzyli wcześniej, dopiero teraz. Tak że fajnie, że to zrobili.

**A w poniedziałek otworzyli galerie handlowe. Co ty o tym myślisz?**

No co ja o tym myślę. No OK. Znaczy można to otwierać powoli. Bo po prostu no nie można w nieskończoność zamykać biznesów. I trzeba powoli próbować to robić. I dobrze, jak tam są jakieś ograniczenia. Znaczy ja nie byłam w galeriach żadnych teraz. Więc nie wiem, jak to wygląda. Uważam, że no trzeba otwierać. Ale chętnych jakoś super dużo nie będzie. Tak jak zwyczajnie. Pewnie tam trochę ludzi przyjdzie, ale nie będzie takich tłumów jak zawsze.

**Trzeba to otwierać, żeby biznes ruszył, to o to chodzi?**

Myślę, że tak, trzeba otwierać.

**A uważasz, że to jest bezpieczne rozwiązanie?**

Znaczy, jak się zachowa jakieś tam środki bezpieczeństwa, to myślę, że może nie być tragedii. Sklepy spożywcze są otwarte i z powodu spożywczych sklepów nie było jakichś tragedii. Tak jak mówię, no nie słyszałam o tym, żeby gdzieś jakiś sklep był… Żeby jacyś kasjerzy byli chorzy i roznosili tego. No nie wiem, chyba że ukrywają przed nami takie informacje. Nie wiem. Ale nie słyszałam o tym, żeby jakiś sklep był ogniskiem zachorowań. Więc myślę, że to nie jest…

**To nie jest takie straszne, tak?**

To nie jest takie straszne.

**Myślisz o tym, żeby się wybrać do jakiejś galerii albo jakiegoś innego stacjonarnego sklepu, który został otwarty?**

Nie, na razie nie mam takiej potrzeby.

**A znasz kogoś, kto ma potrzebę, był albo się wybiera?**

Nie, chyba nie. Wiesz, jak moje koleżanki mają dzieci w takim szkolnym wieku albo coś w tym stylu, to kto ma czas na takie rzeczy, żeby chodzić po sklepach?

**Tak, siedząc z dziećmi na lekcjach online, jak rozumiem. Chciałam trochę z tobą jeszcze pogadać o kwestii wydawania pieniędzy i w ogóle twoim stosunku do pieniędzy. Skala 1 – 10 (łatwość wydawania pieniędzy). Gdzie byś siebie umieściła?**

Na 3.

**Dlaczego tak? Podaj mi trochę przykładów.**

Bo ja jestem bardzo oszczędną osobą, lubię oszczędzać.

**Podaj mi przykłady, takie sytuacje, które pokazują to, że właśnie jesteś osobą, która lubi oszczędzać, która raczej z trudem niż łatwością wydaje pieniądze.**

Ja po prostu zanim sobie coś kupię, już mąż się na mnie wkurza, że chodzę w jakichś starych, brzydkich swetrach. Ale ja po prostu nie lubię wydawać na takie rzeczy pieniędzy. Uważam zawsze, że to jest zbędne. Że jak mam ciuchy, które jeszcze się nadają do chodzenia, to ich nie wyrzucam. Tylko zajeżdżam, że tak powiem, dopóki nie będą dziurawe albo cokolwiek. Więc no mówię, już mój mąż czasami się wkurza za to (śmiech). I ja jakby po prostu czasami potrafię przymierzyć bardzo dużo rzeczy i powiedzieć nie. Bo jednak może nie jest mi to potrzebne.

**Czyli to jest taka kategoria ciuchy, donosisz do końca po prostu, wydrzesz i wtedy wyrzucisz. Na co jeszcze? Jaka jest jeszcze taka sytuacja, która pokazuje, że ty z trudem wydajesz pieniądze.**

No nie wiem, no co jeszcze. No nie wiem, wydaje mi się, że w ogóle staramy się jakoś tak… Nie wiem, no na przykład staram się kupować dużo rzeczy, jak się da kupić używane, to kupujemy używane. Typu nawet sprzęty, ostatnio meble na przykład kupujemy używane. W dobrym stanie, sprawdzamy to. Ale na przykład używane, żeby zaoszczędzić trochę kasy.

**A gdybyś kupiła te dżinsy, które przymierzyłaś. To jak byś się z tym czuła? Jeszcze masz niewydarte, jeszcze możesz pochodzić z pół roku, a jednak kupujesz. To co?**

(śmiech) o matko, nie wiem. Znaczy czasami sobie kupię tam jakąś rzecz. I się przekonuję wewnętrznie, że jest mi to potrzebne. Znaczy kupuję tylko jak się przekonam, jakby samą siebie, że jest mi to potrzebne. Że mi się to przyda, że nie jest to zbędny wydatek. Więc jakby…

**Jesteś w stanie sobie przypomnieć ostatnią taką sytuację, kiedy przekonałaś siebie, że jednak tak, miałaś dylemat, ale przekonałaś siebie, że jest ci to potrzebne i to sobie kupisz.**

Ja zawsze mam dylemat. Zawsze (śmiech). Nawet jakby to była jakaś pierdoła za ileś tam, to zawsze mam dylemat, czy na pewno potrzebuję. Jakaś rzecz, którą sobie kupiłam… No nie, no mówię, ja zawsze mam dylemat. I zawsze muszę się jakoś przekonać. I czasami, jak ponoszę jakiś ciuch, to czasami jestem zadowolona ze swojej decyzji, ale czasami jestem niezadowolona ze swojej decyzji. Bo okazuje się, że ten ciuch się nie sprawdził, bo się tam sprał, bo się rozciągnął, bo coś tam.

**A czy jak już przekonasz siebie, żeby kupić ten ciuch, to raczej się z tego cieszysz, że go masz, czy raczej masz takie poczucie winy, że jednak go kupiłaś i nie przekonałaś się, że nie powinnaś go była kupować.**

Dopóki spełnia swoje funkcje i jest OK, to jestem zadowolona bardzo. Tak, to jestem bardzo zadowolona. Natomiast jestem właśnie zawiedziona, jeśli właśnie się okazuje, że właśnie kupiłam coś, co mi potem przestaje pasować. Albo się właśnie jakoś tam, nie wiem, zmechaci, rozwali. Albo mężowi się nie podoba. Wtedy jestem niezadowolona.

**Konsultujesz, wolisz skonsultować z nim takie zakupy?**

Tak, tak, wolę skonsultować.

**Jak byś tak popatrzyła retrospektywnie, to częściej jesteś zadowolona jak już coś kupisz, czy częściej się okazuje, że masz takie uczucie, że trzeba było tego nie kupować?**

Nie, no częściej jestem zadowolona, tak, częściej jest zadowolona.

**Czyli to są te przemyślane decyzje. Musisz się przespać tą myślą, że coś kupisz, zobaczysz, potem wyjdziesz, potem wrócisz, czy to się na bieżąco dzieje? Jak to wygląda?**

Nie, nie. Zazwyczaj nie. Nie, no jak już coś przymierzę i mi pasuje i pobiję się z myślami w sklepie. I jeszcze mąż powie, że jest OK, no to wtedy od razu już, nie ma co.

**Od razu, czy wyjdziesz, jeszcze pójdziesz do innego sklepu?**

A nie, no to, żeby obejść wszystkie sklepy, no to tak, wiadomo. Chociaż ostatnio na przykład kupowałam sobie taką kurtkę taką na wiosnę i po prostu żeśmy obeszli kilka sklepów. I nic nie było po prostu. Jak przymierzyłam jedną, która mi po prostu bardzo pasowała, to stwierdziłam, że już mi się nie chce po kolejnych sklepach i wracać. Więc już mówię, dobra, bierzemy od razu. Tak że no to różnie bywa. To zależy od zmęczenia materiału i tego, ile trzeba pilnować dzieci w sklepie.

**Ale też rozumiem, że musisz czuć, że to ci jest potrzebne. Że nie kupujesz tych rzeczy jako przedmiot, jako kolejną pięćsetną bluzkę.**

Nie.

**A jak byś sobie spróbowała przypomnieć takie sytuacje, kiedy wydałaś subiektywnie duże pieniądze na coś niespożywczego. To mogą być wakacje, to mogą być meble, coś do domu. Coś takiego innego niż takie typowe, codzienne zakupy.**

No.

**Jakie emocje temu towarzyszyły?**

Nie, no ja generalnie lubię nowe rzeczy czasami… To znaczy lubię, jak się pojawiają nowe rzeczy. Jak coś kupię takiego, że… Znaczy no zazwyczaj, właśnie staramy się, żeby to było przemyślane i nie kupować jakichś bezsensownych rzeczy.

**Ale to powiedz mi na przykładach. Co takiego to było, jak się z tym czułaś, jak wyglądała decyzja, żeby to kupić? Czy też się biłaś z myślami, czy potrzebne, czy niepotrzebne.**

No właśnie żeśmy kupili, to dawno, niedawno ekspres na przykład nowy kupiliśmy. Ale to tylko dlatego, że na przykład stary nam się zepsuł. I był bardzo długo w naprawie, żeśmy go oddali do naprawy. I kupiliśmy w sumie nowy, a nie używany, chociaż tamten kupiliśmy używany. No i tak żeśmy długo podejmowali decyzję. Tak zastanawialiśmy się, ile to czekać. Znaczy już mój mąż nie mógł wytrzymać bez tej kawy, tak po prostu chodził wściekły (śmiech), że tyle bez kawy po prostu. I w końcu tam znalazł, mówi dobra, może jakiś inny kupimy, a ten sprzedamy. Ja mówię dobra, to kupmy. On mówi dobra, to jedź kup. Ja mówię dobra, jadę, kupię. No i kupiłam.

**On cię przekonał. Ty gdybyś miała kupić sama, to kupiłabyś używany?**

No właśnie problem polega na tym, że nie jestem w stanie odpowiedzieć na to pytanie, bo nie kupiłabym sama. Bo takie większe zakupy podejmujemy wspólnie decyzje. I zawsze jak się nie zgadzamy, no to on podejmuje tą ostateczną decyzję.

**No tak, a wyobraź sobie taką sytuację, twój mąż mówi: wiesz co, tym razem ty zdecyduj, jaki ekspres kupujemy.**

No nie. Nie, to tak nie działa.

**A czy przychodzi ci jakiś zakup do głowy, gdzie to ty byłaś inicjatorką, decydentem, to ty namawiałaś do tego?**

Chyba, że ja chcę i on na mnie sceduje to. Bo ja na przykład, ja zazwyczaj wybieram, gdzie pojedziemy na wakacje. Bo ja strasznie lubię siedzieć i szukać tych wszystkich miejsc, jak tam dojechać, co będziemy zwiedzać, co będziemy robić, gdzie będziemy mieszkać itd. No to ja zazwyczaj szukam takich miejsc. Przedstawiam mu propozycje i tam razem sobie próbujemy zadecydować jakby gdzie, co wybierzemy.

**Łatwiej się ci wydać pieniądze na wakacje czy na ekspres do kawy?**

No łatwiej na wakacje. Bo ja zdecydowanie bardziej wolę na wakacje jeździć niż pić kawę (śmiech).

**Co jest takiego, że łatwiej ci jest wydać na wyjazd niż na rzeczy? Dlaczego?**

No nie wiem, bardziej mnie cieszą po prostu. Bardziej mnie cieszą wakacje niż wypicie tej kawy codziennie.

**A czy jak jesteś na wakacjach to też łatwiej ci się wydaje na co dzień pieniądze niż jak jesteś nie na wakacjach w domu?**

No tak. Bo jakby ja wakacje, dla mnie to są też wakacje od takich domowych obowiązków typu gotowanie na przykład. Więc na przykład my zawsze jemy gdzieś tam na mieście. Ja nie gotuję w wakacje i jestem z tego powodu, właśnie odpoczywam. Dla mnie to jest odpoczynek.

**A jak zobaczysz na wakacjach ładny ciuch w sklepie, to też ci jest go łatwiej sobie kupić?**

Nie.

**Czyli to dotyczy jedzenia, ale nie dotyczy nadal rzeczy materialnych.**

Tak, tak.

**Też miejsc, do których pójdziesz, zapłacisz za bilet, takich rzeczy? Ale nie kupowania fizycznych przedmiotów, jak rozumiem?**

Tak, tak.

**Są takie 2 określenia, że ktoś jest oszczędny albo rozrzutny, a ty jaka jesteś?**

No ja jestem zdecydowanie oszczędna.

**I co to znaczy? W czym się przejawia twoja oszczędność, jeszcze, poza tym, o czym mówiłyśmy.**

Znaczy ja lubię odkładać pieniądze. Uważam, że trzeba odkładać pieniądze. I właśnie nie wydawać na rzeczy, które mi są niepotrzebne tak naprawdę. Jakby wolę się zadowolić mniejszym telewizorem, gorszym ekspresem do kawy, nie najnowszym telefonem. W ogóle uważam, że takie rzeczy materialne, jak spełniają swoją funkcję, to jakby nie trzeba mieć jakichś takich topowych rzeczy.

**Dopóki samochód jeździ to jeździ i nie trzeba go wymienić co roku na najnowszy model tylko dlatego, że wyszedł, tak?**

To tak, tak. Tak.

**A w tej sytuacji z koronawirusem, która teraz jest, czy w waszym budżecie domowym, waszej sytuacji coś się zmieniło?**

No trochę tak. Bo mąż trochę mniej zarabia niż zarabiał wcześniej.

**Mąż zarabia mniej, lokatorka zrezygnowała. Coś jeszcze się zmieniło w waszych dochodach?**

Nie, chyba nie.

**Na ile według ciebie duży jest ten spadek waszych przychodów w tej chwili?**

No, taki średni. Ja bym nie powiedziała, że jest jakiś super duży.

**A masz wrażenie, że to wróci do normy?**

No chyba nieprędko. Znaczy nie sądzę, żeby to w tym roku wróciło do takiego, jak było wcześniej.

**A dlaczego tak myślisz?**

Bo myślę, że ludzie będą tracić pracę i będą mniej wydawać pieniędzy. Ale też no… Jakby liczę się z tym, że jakby niektóre rzeczy stanieją powiedzmy. Chociaż niektóre też zdrożeją, więc to też trudno… Myślę, że będzie gorzej, bo przyszedł ten kryzys gospodarczy i będzie gorzej.

**Ale to znaczy, że myślisz, że pensja twojego męża nie wróci do poprzedniej normy? Albo trzeba będzie taniej wynajmować mieszkanie? Czy jeszcze jakieś rzeczy tutaj wchodzą w grę?**

Wchodzi w grę to, że my możemy płacić mniej za mieszkanie i to się jakoś tam wyrówna. Ale też no mogą ceny pójść w górę, takie żywnościowe, takie pierwsze potrzeby. Liczę się z tym, że to może pójść w górę.

**A czy w związku z takim twoim przekonaniem, że to nieprędko wróci do normy, podjęłaś jakieś kroki minimalizujące wasze wydatki albo inaczej kontrolujesz wasze wydatki, coś tutaj się zmieniło?**

Na razie nie. Bo tak jak mówiłam, my byliśmy trochę na to przygotowani, na ten kryzys taki finansowy. Więc nie wydaje mi się, żebym jakoś tak drastycznie, że tak powiem zmieniła jakieś zakupowe moje rzeczy. Chyba nie.

**Bo mówisz, pojadę do takiego sklepu, gdzie jest promocja na pieluchy, bo na to zawsze patrzę i zwracam uwagę. Ale w ogóle masz taką skłonność do tego, żeby jeździć tam, gdzie są promocje, wynajdujesz te promocje?**

Tak, zawsze jeżdżę tam, gdzie są najtańsze pieluchy. To się nie zmieniło. Kiedyś jeszcze bardziej na to zwracałam uwagę. Znaczy nawet teraz mniej, bo nie jeżdżę do… wiesz, że tutaj na przykład pojadę po pieluchy, a potem skoczę jeszcze, bo tu są w promocji pomarańcze. A jeszcze zajrzę przy okazji do tego sklepu, bo tu jest fajniejsze pieczywo. Właśnie nie robię tak.

**Bo jedziesz do jednego sklepu, to o to chodzi?**

No tak, tak, tak. Bo jadę tylko do jednego sklepu. No ja zawsze tak staram się celować w te promocje. Jak już one są, to chcę je wykorzystać.

**Czy to jest dla ciebie kłopot, że właśnie teraz nie możesz tak w różnych miejscach znaleźć tych korzystnych dla ciebie ofert. Czy to ci przeszkadza?**

Trochę mi przeszkadza. Wolałabym żyć tak, jak żyłam, nie chciałabym tego zmieniać. Ale no trudno.

**Te obostrzenia się zmieniły, poluzowało się sklepach, kolejek nie ma. Czemu tego nie robisz?**

Dobre pytanie (śmiech). Nie wiem, szczerze mówiąc. Znaczy wydaje mi się, że robię to dlatego, że rzadziej jeżdżę do sklepów. Bo to też na przykład właśnie… Nie było tak, że jednego dnia robiłam taki rajd po wszystkich sklepach, tylko właśnie tak, jednego dnia tu, po te rzeczy, następnego dnia po te rzeczy do tego sklepu. Bardziej tak. A teraz już właśnie, jak kupuję rzeczy na 2-3 dni, no to wiem, że już po prostu… Inaczej po prostu.

**A skąd wiedziałaś, gdzie pojechać? Wyszukiwałaś w internecie, brałaś gazetki ze sklepu, skąd to wiedziałaś, gdzie co będzie?**

Znaczy tak, jeśli chodzi o takie właśnie, to przeglądam jakieś tam… W Selgrosie właśnie przeglądam czasami, jakie są promocje, czy są na niektóre rzeczy. W Lidlu też przeglądam. W Biedronce to bardziej… Chociaż czasami też przeglądam. Nie zawsze, ale przeglądam. Gazetki przeglądam. Znaczy przez internet.

**W czasie pierwszej naszej rozmowy powiedziałaś, że wyście byli przygotowani, że ten kryzys kiedyś nastąpi. Może niekoniecznie myśleliście, że on będzie spowodowany wirusem, ale na kryzys finansowy byliście przygotowani. I dlatego się zabezpieczyliście. Powiedz, ta wasza poduszka finansowa, którą sobie zapewniliście, to gdybyście przestali mieć w tej chwili w ogóle dochody, to ile byście byli w stanie w ogóle z tego żyć?**

To zależy na jakim poziomie i gdzie.

**Na takim, żeby nie obniżać poziomu życia to ile czasu?**

Ojejku, nie wiem (śmiech). Bo pytasz o liczby, tym się mąż zajmuje, więc ja musiałabym go zapytać.

**Ale tak na twoją intuicję. Bo nie chodzi mi, żebyś mi dokładnie wyliczyła, ile wydajecie miesięcznie i na ile to starczy. Tylko tak na twoje przeczucie i rozeznanie, czy to jest raczej na miesiąc, raczej na kwartał, czy to jest na pół roku? Na więcej niż rok. Wiesz, dzisiaj mi ktoś powiedział miesiąc to góra.**

Nie, no to my tak z rok to co najmniej. Myślę, że spokojnie.

**Bez obniżania poziomu życia?**

Tak.

**I bez sprzedawania dobytku.**

Tak.

**A to oszczędzanie, które mówisz, że jest dla ciebie ważne. Razem oszczędzacie, macie jakiś wypracowany sposób na to, żeby oszczędzać?**

Znaczy generalnie chyba ja mojego męża trochę zaraziłam takim pomysłem na życie, typu właśnie oszczędzamy. I kupujemy dopiero rzeczy, na które nas stać, nie żyjemy na kredyt. I nie wiem, czy… Bo mam wrażenie, że jak żeśmy rozmawiali o tym, to żeśmy wynieśli z domu trochę inne pomysły takie. Że moi rodzice właśnie byli tacy bardziej oszczędni, a jego nie. I myślę, że… Nie wiem, czy on by się ze mną zgodził, ale myślę, że on bardziej przejął tutaj moją filozofię. I on się zajmuje finansami. Tak że ja tam tego nie… Co on robi z tymi pieniędzmi…

**W jakim sensie on się zajmuje?**

W sensie takim, że no on tam patrzy, ile tego jest, ile powinniśmy odkładać, na ile możemy sobie pozwolić, żeby właśnie wydać np. na wakacje. Gdzie te pieniądze lądują, na jakichś lokatach. To on to ogarnia.

**A system jest taki, że odkładacie, jak coś zostanie, po miesiącu? Czy raczej jest taki, że odkładacie, zanim wydacie, niezależnie od tego, zawsze jakąś na początku miesiąca…**

Nie wiem, wiesz? Nie wiem, naprawdę jak on to robi. Nigdy mi nie powiedział, przestań wydawać albo coś w tym stylu. Jakby ja sama się bardziej ograniczam. Mówię, że już więcej nie będę wydawać, bo mam wrażenie, że jednego dnia w sklepie wydałam za dużo (śmiech). On nigdy mi nie powiedział, dobra, przestań już wydawać.

**Kontrolujesz jakoś wydatki, zbierasz kwitki?**

Nie, tego nie. Jakoś w głowie, nie wiem.

**Jakiś taki system, który pomaga oszczędzać, typu, że niektóre tam opcje bankowe są takie, że jak coś wydasz, to ci końcówkę zaokrąglają i ta końcówka ląduje na jakimś koncie?**

Nie. Pierwsze słyszę w ogóle o czymś takim.

**A jakiś system kontrolowania budżetu w tej chwili, jak ten koronawirus i trochę wam spadły przychody, czy coś nowego się pojawiło w waszych zachowaniach takich domowo-budżetowych.**

Nie, chyba nie. Znaczy oprócz tego, że nie wyjeżdżamy nigdzie, to nie, chyba nie.

**Ale masz takie poczucie, że trochę się bardziej trzymasz za kieszeń w tej chwili niż przed tym marcem, zanim to wybuchło?**

Znaczy ja zawsze mam takie falowo, wiesz, że jednego dnia po prostu idę na zakupy, a, to, to, to, idę na zakupy, jeszcze sobie kupię jakieś fajne pierożki, albo kupię jakieś fajne, nie wiem, coś innego. A potem następnego dnia, jak idę na zakupy to już mówię: o, tego nie kupię, bo wczoraj wydałam za dużo pieniędzy. Więc u mnie to jest constans. Tak jak było, tak nadal jest.

**Rozumiem, że masz takiego kontrolera wewnętrznego.**

Tak.

**Jak wczoraj zaszalałam, kupiłam pierożki, to dzisiaj kupię tylko biały, chudy ser.**

No, coś w tym stylu. To dzisiaj kupię to, co zawsze kupuję i nie będę szaleć.

**A te oszczędności i to zabezpieczenie, które macie czy inwestujecie te pieniądze, trzymacie je na lokacie, co robicie?**

Nie wiem. Ja nie wiem.

**A twoim zdaniem to w ogóle jest dobry czas w tej chwili, żeby inwestować?**

Nie wiem, ja się na tym nie znam kompletnie. Po prostu nie wiem. Ja naprawdę się na tym kompletnie nie znam, nie wiem, czy teraz jest dobry czas czy nie jest dobry czas. Bo niektóry powiedzą, że jest dobry, niektórzy powiedzą, że jest niedobry. Nie wiem, naprawdę. Ale też, żeby inwestować, kurcze, to trzeba mieć odwagę. Jakby ja nie czuję się odważna w tych kwestiach.

**Odwagę, żeby nie stracić, to o to chodzi?**

Tak. Bo w inwestowanie jest włączone ryzyko, nie?

**Gdyby to od ciebie zależało, to lepiej tego ryzyka nie podejmować? Gdybyś to ty miała decydować.**

Jeśli chodzi o finanse ja nie lubię ryzyka. Nie lubię ryzykować.

**Mieć zabezpieczenie, nawet niech nie urośnie, niech się nie potroi, tylko niech ono będzie. Na tej zasadzie?**

Tak. Ja nie lubię ryzykować, jeśli chodzi o pieniądze. Chociaż wiem, że czasami ryzyko popłaca. Ale ja osobiście nie lubię, nie czuję się z tym komfortowo.

**Nie nadajesz się na hazardzistkę.**

Nie, nie nadaję się kompletnie. No na takiego nawet biznesmena, takiego własnego też się nie nadaję, bo to też trzeba by zainwestować swoje pieniądze. A ja jestem taka po prostu zbyt zachowawcza w tej kwestii.

**Ostatnia rzecz, to taka rozmowa o przyszłości. Czy w ogóle się zastanawiasz, kiedy to się skończy, co się teraz dzieje? Czy są takie momenty, w których zauważasz, że się nad tym zastawiasz? Czy są takie momenty, kiedy bardziej o tym myślisz niż w innych momentach?**

Czy się zastanawiam nad tym, kiedy to się skończy? No zastanawiam się. Ale nie wiem, wydaje mi się, że mam… Znaczy tych zmiennych jest tyle. Że to zastanawianie się moje jest takie trochę, znaczy do niczego nie prowadzi tak naprawdę. Że to jest takie trochę wróżenie z fusów. Nie wiem, nie wiem.

**Kiedy takie myśli pojawiają ci się w głowie? Czy masz je ciągle gdzieś z tyłu głowy, czy jak właśnie idziesz na spacer, czy przed zaśnięciem, czy jak się budzisz?**

Nie, nie mam tego z tyłu głowy na pewno. Na pewno nie zaprzątam sobie tym za często głowy. Bardziej się nad tym zastanawiam wobec jakichś takich planów, które dotyczą naszej rodziny, właśnie wakacji, czy właśnie tego, że planuję dziecko starsze posłać do przedszkola już od września. Właśnie tego, czy wrócić do pracy czy nie. To wtedy jakby się zastanawiam, czy to wróci do normalności czy nie wróci.

**Ta normalność, to jest dla ciebie co? Jakie tutaj są najważniejsze rzeczy?**

Normalność? No, że nie będzie już żadnych ograniczeń, jeśli chodzi o prowadzenie biznesów czy będą działały szkoły, przedszkola. To jest dla mnie normalność. Komunikacja na przykład będzie normalnie działać, a nie tam, że 5 osób kurcze w tramwaju. No, że będą otwarte granice. To też jest dla mnie, już dla mnie to jest normalność. No i właśnie te, znaczy wydaje mi się te obostrzenia wszystkie, typu 5 osób na kasę, 2 metry od siebie. To też nie jest normalne dla naszej kultury i dla mnie. To nie jest normalne. Więc no to też jest takie narzucone, więc chciałabym, żeby tego nie było.

**Czyli żeby w ogóle wszystko było tak jak przedtem.**

No tak. Albo lepiej nawet. Żeby było lepiej niż przedtem (śmiech).

**A jak myślisz, jak to będzie, jak to się wszystko potoczy?**

Oj, nie wiem, nie wiem, nie wiem. Kurczę, no nie wiem, bo jest tyle zmiennych, tyle jakby rzeczy, jak to wszystko… Nie, nie wiem naprawdę, trudno mi powiedzieć. Nie, nie wiem.

**A masz jakieś takie przewidywania, oczekiwania, wyobrażasz sobie? Czy coś zasadniczego się w ciągu najbliższych kilku tygodni może zmienić?**

W związku z czym? Z taką…

**W stosunku do tego, jak mamy teraz, jak teraz żyjemy.**

No może się zmienić dużo ze względu na ten cały bajzel polityczny, który mamy. Bo może zostać wprowadzony ten stan klęski żywiołowej. Znaczy no właściwie kurcze, trudno cokolwiek powiedzieć. Bo po prostu jak czytam te, no nie wiem, te medialne informacje, to z jednej strony chcą rozluźniać już, z drugiej nagle minister mówi, że nagle się może okazać, że znowu przywrócone zostaną te zakazy tego poruszania się czy coś tam. No to ja już nie wiem w ogóle, czego się spodziewać. Już przestałam oczekiwać czegokolwiek, bo… No nie da się.

**(problemy techniczne). Mówiłaś, że mogą stan wyjątkowy wprowadzić.**

No właśnie, mogą wprowadzić ten stan klęski żywiołowej, więc znowu mogą jakieś ograniczenia zrobić. Już minister mówi, że ta górka tych zachorowań, czy jak to się tam mówi, szczyt tej epidemii, to jeszcze przed nami jest. Więc w ogóle, tak jak mówię, to się u nas zmienia jak w kalejdoskopie wszystko. Jednego dnia mówią jedno, drugiego drugie. I nie wiadomo.

**Czujesz się zdezorientowana tym wszystkim, co się dzieje?**

Znaczy może nie tyle zdezorientowana, co czuję się po prostu… Nie wiem, czy to jest dobre słowo. Czuję się przede wszystkim traktowana… No trochę traktują mnie jak kretyna. W sensie takim, że nie powiedzą w sumie, na czym się opierają, te swoje wszystkie założenia. Tylko tak wyciągają z rękawa jakieś tam… A to już teraz nie będzie tego. A nie wiadomo, co będzie. I w sumie to my nic nie wiemy, a podejmujemy jakieś decyzje. A nie wiemy, czy już koniec tej epidemii czy nie. W ogóle nie ma jakiegoś takiego… Znaczy ja w tym nie widzę sensu i czuję się po prostu traktowana jak idiota.

**To, że chcą otworzyć przedszkola, otworzyli galerie a nie otwierają fryzjerów. Czy to jest ten element taki, że czujesz, że nie wiesz, dlaczego tak się dzieje? Czy to o coś innego chodzi?**

Nie, to chodzi o coś innego. Bardziej mi chodzi o to, na jakiej podstawie jakby, jak oni przewidują rozwój tej epidemii w Polsce. Tam nie ma… Wypowiadają się politycy i jakby właściwie oni nie mówią, na jakiej podstawie podejmują te decyzje. Czy oni sobie to wymyślają, rzucają kostką. Czy mają jakiegoś eksperta, który im mówi, jak będzie wyglądać ta epidemia, czy robią jakieś badania. No jakby nie wiemy kompletnie, na jakiej podstawie otwarta jest galeria a nie fryzjer. Czy to był rzut kostką?

**A ty jak myślisz, czy to jest rzut kostką, czy oni robią jakieś analizy?**

Znaczy to wygląda, jakby był rzut kostką. Bo oni nie przedstawiają, znaczy ja nie widziałam przynajmniej, żeby były jakieś analizy ekspertów, którzy wypowiadają się, że to jest spłaszczenie tej krzywej tych zachorowań. Jakby ile to ma jeszcze trwać? Jak spłaszczyliśmy tą krzywą, OK, fajnie, ile jeszcze będziemy spłaszczać tą krzywą. I jakby no nie wiem. Pan minister zdrowia po prostu za każdym razem mówi co innego. Że jeszcze ten szczyt zachorowań będzie nagle w maju, potem mówi, że we wrześniu, potem mówi, że w czerwcu. No i kurde (śmiech)

**A ty jak myślisz, jak długo to wszystko potrwa. Ta huśtawka i te rzuty kostką…**

No ja myślę, że oni będą tak cały czas robić. Znaczy myślę, że będą tak cały czas robić, bo nagle nie obudzą się jednego dnia i powiedzą: proszę państwa, to teraz będziemy kompetentnie państwa informować o wszystkim, na jakiej podstawie podejmujemy decyzje. I proszę, tu jest jeden od epidemii, tu jest drugi ekspert od gospodarki, a tu jest trzeci ekspert od czegoś. No jakby nie wierzę w to, że się to zmieni. Bo nie ma w społeczeństwie w ogóle takiej też… Takich głosów, głośnych przynajmniej, żeby coś takiego było.

**Brakuje ci takiego eksperckiego panelu, który stanie, wszystko opowie?**

Może nawet nie panelu, ale takiej dyskusji takiej naprawdę na poziomie. Bo naprawdę są różne głosy w tej sprawie. I brakuje mi takiej merytorycznej dyskusji na ten temat. I wśród polityków i wśród ekspertów. I też media są trochę za to odpowiedzialne, że nie zainteresują nikogo czymś takim. Takiej faktycznie, no takiej dyskusji na ten temat. Na poziomie. Bez jakichś kurcze głupich odzywek, głupich pytań itd.

**Gdybyś miała obstawiać, to według ciebie, ile to jeszcze potrwa? Kilka miesięcy, rok, dwa?**

Ja myślę, że rok. Myślę, że rok. Chyba, że ta władza, że tak powiem, odejdzie w niepamięć. Co jest możliwe, jeśli teraz ten cały bałagan polityczny im nie wyjdzie z tymi wyborami itd. No to może się pozmieniać, jak będzie zmiana, że tak powiem na stołkach, tam w rządzie itd. No zobaczymy.

**I wtedy jest szansa, że to się szybciej skończy?**

Znaczy będzie szansa… Nie wiem. Kurde, nie wiadomo tak naprawdę, co druga strona, jakie ma pomysły. Bo na razie ma tylko pomysły takie, żeby krytykować rząd itd. No zobaczymy. Nie wiem. Mam niskie, nisko oceniam naszą klasę rządzącą. Zarówno rządzącą jak i o opozycyjną.

**OK, trudno prognozować, bo się nic nie wie.**

Ale jeśli chodzi o epidemię, to wydaje mi się, że jeszcze z rok tak. Znaczy, jeśli chodzi o samą chorobę. Bo jeśli chodzi o te wszystkie obostrzenia itd., no to mówię, to może się zmieniać.

**Dziękuję bardzo.**
