# Supplementary material for: The challenges arising from the COVID-19 pandemic and the way people deal with them. A qualitative longitudinal study
Source: PLoS One. 2021 Oct 11;16(10):e0258133. doi: 10.1371/journal.pone.0258133 (PMC8504766; doi:10.1371/journal.pone.0258133)
Supplement: S1 Dataset — (ZIP) [file pone.0258133.s003.zip › Transcriptions/stage 4/17.4_F_35_single, with child.docx]

**17.4_K_35_single with child**

**Jak minęły Ci te ostatnie dwa tygodnie?**

Co to było? Chyba od majówki się nie widziałyśmy. Nie jeszcze wcześniej.

Łamiemy kwarantannę, mamy ogólny bunt, wychodzimy z domu. Nawet działa podziemie i chodzę do pracy, więc jest naprawdę wewnętrzny bunt przeciwko wszystkiemu.

Więc wszystko się zmieniło.

**Ale normalnie w salonie chodzisz do pracy czy podziemie inaczej funkcjonuje?**

Normalnie w salonie. Zamykamy bramę, klientki wchodzą na telefon, zamykamy drzwi. Jest dramat, jest to śmieszne, ale powiem ci, że już klientki nie wytrzymywały. One już po prostu naciskały na nas, żeby wracać do pracy. I w momencie, kiedy otworzyli markety, to każdy po prostu taki był wściekły. Ja mówię, a w nosie to mam, wracamy do roboty. I normalnie od poniedziałku

**To podziemie jest podobno coraz bardziej popularne?**

Tak. Wszyscy pracują. I najlepsze jest to, że nawet rozmawiałam z policjantem, który stwierdził, że oni nawet nie będą nas szukać. Że oni nie mają na celu wyłapywanie pracujących salonów, bo oni mają świadomość, że wszyscy pracują. Że jest to głupota. Więc tak

**A oprócz tego, że wróciłaś do pracy, to co jeszcze? Dlaczego postanowiliście wychodzić, łamać kwarantannę?**

Bo mam chyba już dosyć. Znaczy na pewno mam już dosyć. Mam dosyć siedzenia, mam dosyć tego, że okej jest jakiś wirus, niby on gdzieś jest, niby znam 2 osoby, które były zarażone, ale wszyscy żyją, nikomu nic się nie dzieje. Te osoby które umierają to jest jakiś promil. I czy to jest naprawdę aż taka tragedia, że trzeba nas zamknąć w domu? Czy to nie jest gdzieś drugie dno?

Jeszcze na początku to się naprawdę tego wirusa obawiałam, że rzeczywiście tych rodziców mogę zarazić bo była taka nagonka, tak teraz mówię "no halo". Przecież ludzie chodzą do Lidla, do marketów, do urzędów do pracy i są wszyscy zdrowi. Więc nie wiem, gdzie jest ten wirus.

**Sprawdzasz teraz statystyki?**

Coraz mniej. Tak jak na początku je przeglądałam tak porządnie, tak teraz rzucę okiem i szczerze to już nawet nie pamiętam, co tam było. Takie jest okej. Mam wrażenie, że przeszłam już na porządku dziennym do tego koronawirusa. Że on jest. Ale jest jak grypa, angina, jak wszystko po kolei.

**A czy poza pracą pojawiły się nowe rzeczy, które zaczęłaś teraz robić?**

Nie, wróciłam do pracy więc już nie mam czasu. Wiem, mówiłam, że jak wrócę do pracy, to pewnie będę ograniczać pracę, ale pracowałam wczoraj od 8 do 8. Dzisiaj będzie to samo.

**Czyli pewnie będziesz pracowała takim trybem jak przed epidemią?**

Tak. Tak czuję, że to tak zostanie. Chyba że się trochę uspokoi, bo to może być taki boom teraz, że te kobiety są jakieś wyposzczone, one potrzebują tych paznokci, rzęs, brwi i jak dzwonią i zadajesz pytanie na kiedy, to na już. I nie masz sumienia odmówić. Może to się zaraz trochę uspokoi, może wrócimy do normalności. Chociaż nie wiem, bo jest jakiś szał.

**A w takim razie czy z czegoś zrezygnowałaś?**

Na pewno jest mi teraz trudniej gotować. Więc muszę to po nocach. Nie miałam czasu ćwiczyć dwa dni. Więc już mi tego brakuje. Bo wracam z pracy i myślę "dobra, to potrenuję", ale nie mam czasu. Tak jakby wracam do normalności. Niekoniecznie mi się do końca to podoba, ale tak jakoś to będzie teraz chyba wyglądać.

**A przed czy po majówce wróciłaś?**

Po majówce.

**A majówka?**

Poszliśmy do znajomych na grilla. Złamaliśmy kwarantannę. Zadzwonił do mnie kolega i mówi "mam już dość, wpadajcie na grilla". Ja w pierwszym momencie mówię "nie, Sebastian, chyba nie przyjdziemy, bo ja mam tych dziadków w domu, trochę się obawiam. Daj mi chwilę, ja to przemyślę i do Ciebie oddzwonię". I ta chwila trwała 5 minut. Mówię "walę to, pakujemy się i jedziemy". Tam byli znajomi i dzieciaki z klasy od syna. Mój Michał był cały tydzień na służbie, natomiast się wyrwał i uwierz mi dzieci nasze - tam było 5 dzieci - to jak wypuszczone z lasu. Ja nawet nie wiem, co się z nimi działo. 6 godzin oni byli tak zafascynowani sami sobą, że tylko pytałam czy zjedli, a my jak dzieci małe. Po prostu się cieszyliśmy. To była jakaś głupawa. Starzy ludzie, a mieliśmy głupawę, że wreszcie się spotkaliśmy. W ogóle było krzyczenie do sąsiadów przez płot "ej, nie podajcie nas nigdzie, że łamiemy kwarantannę", a sąsiedzi "my też łamiemy, nie przejmujcie się". Całe osiedle się darło, że wszyscy łamią kwarantannę. Jakiś odlot, naprawdę, totalny odlot.

**To było pierwszy raz dla Was, a dla znajomych?**

Tak, to pierwszy raz. Bo tak to się spotykaliśmy przelotem, ale nigdy na żadnych grillach. Ewentualnie przy furtce, bo coś trzeba było sobie przekazać. Ale nigdzie u siebie nie siedzieliśmy. A teraz na oficjalu rozpaliliśmy grilla, usiedliśmy, otworzyliśmy wino. Jakbyśmy mieli 15 lat się cieszyliśmy, że możemy spędzić ze sobą czas. Naprawdę, niesamowite.

**To ile Was tam było?**

5 dorosłych i 5 dzieci.

**Czyli ta majówka to był moment, gdy stwierdziliście, że już wystarczy? Dosyć?**

Tak. Każdy miał już dosyć. I to z kim nie rozmawiam, czy to są osoby, które wierzyły w koronawirusa, czy nie wierzyły, te które się pilnowały i te które się nie pilnowały - wszyscy mają dosyć. Mają dosyć, bo to zakrawa... Można zwariować. Zobacz co się stało z nami po miesiącu - my po miesiącu, gdzie też się w normalnych warunkach nie widujemy co tydzień, czuliśmy się jak wypuszczeni z lasu, że siebie nie widzieliśmy - po prostu trzeba by to nagrać. Mój Michał, który musiał jechać na służbę - więc tylko mógł zdezerterować, ja zgodą przełożonego wyszedł - to przyjeżdżał na godzinę, jechał się odmeldować i wracał na godzinę. Ja mówię "stary, to w ogóle jest bez sensu", a on "nieważne, ja już mam dosyć tego zamknięcia". I tak naprawdę krążył. Normalnie to by tak nie przyjechał, by mu się nie chciało.

**Ale on rozumiem, że normalnie pracował?**

Tak. Znaczy 2 tygodnie był na urlopie, tydzień był w pracy zdalnej. Potem pracowali na zmiany. Więc ta praca nie była tak do końca normalna. Te 8 godzin to on pracuje ze 3 tygodnie, chyba. Tak to było kombinowane wcześniej.

**A tam miał normalną styczność z ludźmi czy też ograniczali?**

Ograniczyli. On jest przedstawicielem handlowym, więc mu zabronili się poruszać po świecie, a że pracuje w firmie, która handluje węglem, to w ogóle firma  zablokowała sprzedaż dla osób indywidualnych. Tylko mogły kupić firmy. Więc wjeżdżał ciężarowy samochód, i odjeżdżał. Nikt nie mógł wejść do biura. A teraz to nie wiem jak. I oni się też nie widywali.

**Czy coś ci zaczęło jeszcze przeszkadzać?**

Chyba nie.

Znaczy ja nadal nie mogę się oswoić z widokiem ludzi w maseczkach. Może nie tyle co mi przeszkadza, ale nie jest to komfortowy widok. A tak to ok.

Zakupy się już super robi, bo znieśli godziny dla seniorów, więc wchodzisz do sklepu normalnie. Nie odczuwa się tego. Zauważyłam, że ludzi jest więcej na mieście. Chyba już wszyscy zaczynają wracać do normalności.

A tak to już nic mi nie przeszkadza

**A nosisz maseczkę?**

Noszę, bo się boję mandatu. Wiem, bo już wyczytaliśmy, że nie potrzebne zaświadczenie, można wcisnąć kit, że ma się astmę, cokolwiek. Ale nie chce mi się dyskutować z policją, więc noszę.

**Ale nosisz jak należy?**

Noszę normalnie, bo mi ona nie przeszkadza. Jak już ją założę, to jest ok.

**Emocje.**

Generalnie miałam z tego ubaw. Jeszcze ci wyślę coś, gdzie jest dużo prawdy i daje do myślenia o co tu chodzi z tym całym koronawirusem.

(1) To było, co ja wiedziałam,   że to jest to o co mi chodzi. I moim klientkom. Które naprawdę doszło do tego, że one mnie błagały, że one wejdą tyłem, żebym otworzyła tylne wejście.

Nie wybierałam, ale mi się rzucił w oczy. I się zaczęłam strasznie śmiać. Bo to jest prawda. Był ten moment, jak otworzyli lasy, żebyśmy dla zdrowia psychicznego mogli jechać do lasu, a moje milion wiadomości w telefonie wtedy "kiedy otwieramy salon?". Ja nie chcę iść do lasu, ja chcę iść do pracy. To z jednej strony było śmieszne, a z drugiej strony prawdziwe. Że rzeczywiście nikt nie chciał do tego lasu, miał to w nosie, każdy chciał zacząć funkcjonować normalnie.

**Ale temu że miał w nosie towarzyszyła złość czy coś innego?**

To była złość przez śmiech. Bo się wszyscy śmiali, po co oni będą szli do tego lasu, ale z drugiej strony byli źli, że ktoś im ogranicza wolność. Że ok, nawet jak ja chcę przyjść na te paznokcie przysłowiowe, to ja ryzykuję. I ja mam zaufanie do dziewczyn, która mi robi paznokcie. Albo ja mam zaufanie do klientki, czy mnie zarazi czy nie. I nie chcę, żeby decydował o tym, ktokolwiek inny. Jaka jest różnica czy ktoś przyjdzie na paznokcie, czy spotka obcego człowieka w lesie, który będzie miał koronawirusa? No żadna. I była złość, bo pozwalają do lasu iść, a nie pozwalają mi zarabiać pieniędzy. Teraz to już jest ubaw. I każdy ma śmiechu co niemiara z tej całej sytuacji.

**Teraz funkcjonujecie tak jak przed epidemią czy jeszcze nie?**

Kryjemy się. Ok, pracujemy ale nikogo nie informujemy. Jeżeli ktoś nam zada pytanie, to każda w swojej głowie analizuje czy tej klientce powiedzieć, czy nie. Czy jest na tyle zaufana, że nas nigdzie nie sprzeda. Bo wiesz, to są jednak obce osoby dla nas. Więc musi to być zaufane. Przychodzą obce klientki, ale ja wiem, że to są od moich zaufanych osób, więc mam wewnętrzny spokój, że jednak nigdzie mnie nie podadzą i nikt nie przyjdzie nałożyć mi kary. Brama jest zamknięta. Otwieramy bramę na telefon. Zamykamy bramę, drzwi. Żeby nikt nie zapukał nam do drzwi. Niby samochody stoją pod salonem, ale udajemy, że nas tu nie ma.

**Jak się z tym czujesz, że musisz udawać?**

Teraz już mnie  to bawi. Na początku się trochę stresowałam. Bardzo mnie stresowało, jak sąsiadka przez płot mnie zapytała "czy pracujecie?". Ja nie wiedziałam, co jej odpowiedzieć i mówię, że "nie, nie pracujemy". Po czym za 3 dni mówię, "no dobra Ola pracujemy". Nie chcę się tym chwalić. Bo jak by mnie ktoś zapytał na ulicy, to powiem, że nie pracuję, absolutnie.

**Towarzyszy temu złość, lęk?**

Bardziej lęk, chociaż trochę mnie uspokoił ten znajomy policjant, który powiedział, że "nie martw się, nie mamy odgórnego nakazu szukania podziemia w kosmetyce". Ten lęk trochę zszedł. Skoro nie szukają, to jest szansa, że jak nikt nie nakabluje na nas, to nikt nie przyjdzie i przeczekamy do oficjalnego otwarcia.

**(2) Teraz o tym powiedz**

To przedstawia to co, ja już myślę o tym koronawirusie. Skoro on jest taki śmiercionośny i zaraźliwy i po prostu przejdziesz obok kogoś 5 metrów i on mocno kichnie, wpadnie na Ciebie jeden wirus to będziesz chory i zarazisz całą rodzinę, to czemu kurde panie w Lidlu są wszystkie zdrowe? Czemu ja mając kontakt z 2 osobami zarażonymi, z pozytywnym wynikiem, nikt od nas nie jest chory?

**A kiedy miałaś kontakt?**

Z miesiąc temu, albo lepiej.

**To była ta pani z przychodni, a druga?**

To był znajomy, a ja się z nim na klatce widziałam. Jego tata był zarażony i oni całą rodziną się zakazili i byli na kwarantannie. I my się minęliśmy. Jechaliśmy jedną windą, dotykaliśmy jednych guzików. I tak opowiadają w tej telewizji, że trzeba wszystko dezynfekować, klamki, nie dotykać, łokciami się witać. A ja mając bardzo bezpośredni kontakt, bo to sąsiad. Mogło być że wtedy nie był zarażony. Natomiast  z tą kobietą to się widziałam tydzień przed tym jak ona miała pozytywny wynik, objawy już miała. Byłyśmy 3 metry od siebie, nawet nie. Nie jestem ja zarażona, nikt ode mnie nie jest zarażony z rodziny. Nikt nie jest zarażony kto miał z nią kontakt. Mąż jej nie jest. To coś mi tu nie pasuje. Nie ma nikt umierać, ale nawet kataru ani kaszlu?

**A jakie emocje to wywołuje?**

Złoszczę się. Mam wrażenie, że nam kit sprzedają w tej telewizji. Że teraz każą nam nosić maseczki, tak się chronić, a nikt nie mówi o tym, jak zasmarkani chodziliśmy do pracy. Nie mówię, że grypa jest tak samo śmiercionośna, bo może rzeczywiście jest mniejsza umieralność, ale jest równie zaraźliwa. I nikt nie mówił, że jak dzieciaki chodziły zasmarkane do szkoły to było nie ok. Ludzie przychodzili do pracy po prostu pluli na nas, zasmarkani, i było ok. A teraz? Kichniesz i strach przejść, żeby w łeb nie dostać.

Popatrz na Lidl, czy jakikolwiek market. Przecież tam setki ludzi przechodzi. I te panie nie są tam godzinę tylko są 8 godzin. I nikt ich nie zaraził? Składając te puzzle to coś mi tu nie gra.

**A na ile się czujesz w takim razie zagrożona sytuacją? Samym koronawirusem?**

Nie, bo do momentu kiedy on był  gdzieś daleko, to mówię "dobra, nikt w otoczeniu nie jest zarażony więc się nie zarazimy". Ale jak on był ramię w ramię, to ja byłam dosyć poddenerwowana tym, czy ja nikogo nie zarażę. I nagle się okazuje, że nikt nie ma objawów. Jakichkolwiek. Nikt się nawet słabiej nie czuje. Nikt. Nikt nie kichnął, nie miał stanu podgorączkowego.

Absolutnie nic się nie działo. No to gdzie ta zaraźliwość? Coś tu mi nie pyka. Fakty mi się nie zgadzają.

**A czy jest coś co pozwala radzić sobie z tą złością?**

Dużo mi pomaga praca. Jak ja wyszłam do pracy, to tak jakby się oderwałam od telewizji, Internetu, od tego natłoku informacji. Odłączyłam się od tego. I mam wrażenie, że wróciłam do normalności, a gdzieś tam z tyłu jest jakiś koronawirus i muszę założyć maseczkę jak idę do sklepu.

**A śledzisz teraz jakiekolwiek informacje?**

Tak, te co zawsze. Na Instagramie. Ale naprawdę je już przerzucam. Nie analizuję już ich. Patrzę tylko, czy nikt nie umarł w Radomiu. Bo jak ktoś szedł do szpitala w Radomiu, to prawie wszyscy poumierali. Więc tylko to patrzę, czy jest ktoś z Radomia i czy nie ma kogoś mega młodego. A nawet już nie wiem, co się dzieje we Włoszech, co się dzieje w Hiszpanii. I co najdziwniejsze, chłopak którego obserwuję, taki Łukasz Cośtam - sam odszedł od raportowania, bo on dosłownie raportował dane, czyli wrzucał suche dane, informacje, kto umarł, ile było ozdrowieńców, ile zachorowań, ile zrobionych testów - każde państwo po kolei. I on  okolicach majówki sam od tego odszedł. Powiedział, że odchodzi od raportowania, bardziej się skupi na tym, co robią teraz państwa - jak rozluźniają gospodarkę, odmrażają. Więc nawet informacje, które ja obserwuję, już się zmieniają. Już się je inaczej czyta

**Jak odcięłaś się od informacji - to od koronawirusa, czy od wszystkiego?**

W ogóle się odcięłam. Nic nie czytałam. Jak mi się coś rzuciło w oczy, to mogłam jest przerzucić, ale nie skupiłam się w ogóle na tym.

**Polityka?**

W ogóle. Odcięłam się od informacji jakichkolwiek.

**I teraz powoli wracasz... Jak?**

Ja zawsze selekcjonowałam, bo ja lubię wiedzieć. Wracam, bo de facto było mi niedobrze z tą niewiedzą. Wracam, ale selekcjonuję - szukam tylko tego, co chcę wiedzieć. Czyli skupiam się na tych 2-3 profilach, które są dla mnie wiarygodne i przerzucam informacje. Coś zapamiętam, coś nie i przerzucam dalej.

**Ale tam są też inne informacje?**

Tak. Ale te profile się skupiły na koronawirusie. Więc jak przedstawiają sytuację polityczną w jakimś kraju to i tak ona jest oparta na koronawirusie. Bo on zdominował wszystkich po kolei. I każda informacja,  nawet o tym z Korei Północnej Kim dzong czy jak on się nazywa, to też było w świetle koronawirusa - czy przez przypadek to nie ma związku. Każda teraz informacja, ma gdzieś podtekst koronawirusa. Dramat.

**A jak to wygląda w przypadku osób z Twoim otoczeniu?**

Zaczynają wracać do normalności. Wszyscy po kolei. Jest to jeszcze takie ograniczone, z taką dozą niepewności, ale już to zaczyna małymi krokami. Np. mój tata ma 30 kwietnia urodziny, więc już moja siostra do nas przyjechała. Posiedziała normalnie. Jej córka została u nas na noc, na cały tydzień, a nie widzieliśmy się od połowy marca. Więc pozwoliła, żebym ja zabrała moją siostrzenicę na grilla. I to idzie takimi małymi krokami, że na coraz więcej sobie pozwalamy.

**Czyli jest takie rozluźnienie?**

Dokładnie. Ale małymi krokami. Nikt się nie rzuca na głęboką wodę, nie lata teraz wszędzie. Tylko to jest tak, na coś sobie pozwolimy, gdzieś pójdziemy i poczekamy trochę. I potem znowu gdzieś pójdziemy. Jest taka doza niepewności, ale to już nie jest chyba strach.

**Zakupy.**

Wróciliśmy do normalności. Czyli jedziemy na zakupy, jak jest taka potrzeba. To już nie jest wyprawa. Ale zauważyłam, że nauczyło nas to robienia zakupów potrzebnych. Już nie wpadamy do sklepu i nie kupujemy na żywioł, tylko jest to zaplanowane, np. że dzisiaj kupimy kurczaka, bo jutro zrobimy to i żeby się nie zmarnowało. Więc to jest plus, który wyniosłam z tego koronawirusa. Bo zazwyczaj to były zakupy, że wpadałam do sklepu głodna, że coś zrobimy, to jeszcze kupię to, to i to, i połowa lądowała niestety w koszu. Ale każdy już robi tak, że jak jest potrzeba to jadę do sklepu. Już nie ma kolejek. Jest luz. W sklepach jest już normalność. Nie odczułam absolutnie żadnego jakiegoś... Czuję się tak, jak się czułam przed koronawirusem w sklepie oprócz tego, że mam maseczkę.

**Czy coś dla przyjemności?**

Nie. Nawet śmiałam się, że myślałam, że jak otworzą markety, to wpadniemy zaraz na buszowanie po sklepach. Ale nawet nie mam potrzeby.

**W sensie galerie handlowe?**

Tak, tak.

**Co sądzisz o tym, że otworzyli GH?**

Ja w ogóle nie pojmuję.. Galeria handlowa może być otwarta, do Ikei mogą być kolejki, a ja mam nie pracować. I co jest najbardziej zabawne, czemu w galeriach handlowych wszystkie sklepy mogą być otwarte, oprócz salonów kosmetycznych i wysp. Jak mi ktoś wytłumaczy o co tu chodzi, to może to pojmę. Tak wyczytałam. Nie wiem, nie byłam.

Jeżeli ktoś mi powie, jak jest różnica jak ja będę stała przy kasie z Zarze u pani, a będę robiła pani paznokcie, to ja się może z tym zgodzę. Bo ja nie widzę różnicy. Żadnej. Nie ogarniam tego.

I to jest taki bunt od poniedziałku, po prostu jestem zła na to, że ktoś tak wymyślił. Wciskają nam najprościej mówiąc kit, że my mamy bliski kontakt z klientem, a pani w Zarze czy gdziekolwiek indziej nie ma. Hello?

**Raczej się nie wybierasz na zakupy?**

Znaczy potrzebuję kilku rzeczy, ale w ogóle mnie tam nie ciągnie. Nie chce mi się tam iść. Wiem, że potrzebuję, muszę młodemu kupić kilka ciuchów, bo po prostu przyszła wiosna i wyrósł, ale nie chce mi się. Nie jest to takie, że muszę iść bo nie mam w czym chodzić.

**I nie brakuje Ci chodzenia tam?**

No nie. Ja ogólnie nie lubiłam

**Czy ktoś się z Twojego otoczenia wybiera?**

Nie. Wysłała mi tylko koleżanka, bo ktoś jej wysłał zdjęcia od nas z galerii z Radomia, to była pusta. Puściutka podobno. I nawet moja mama rozmawiała ze swoją koleżanką, która pracuje w galerii, bo była ciekawa, czy to nie będzie nagle milion ludzi i będą tłumy niczym w czarny piątek. To mówi, że absolutnie nie było nikogo. A jeżeli ktoś przychodził, to przychodził celowo. Czyli wchodziła pani do sklepu - ta koleżanka pracuje  w sklepie z ubraniami dla kobiet - i wiedziała co chce. Nie było klientów chodzących i szukających, grzebiących. Jak wszedł klient, to już kupił.

Ale to był początek. Ciekawa jestem co będzie na weekendzie.

**Styl wydawania pieniędzy. Określ się na skali.**

Dosyć nisko. Między 2 a 3. Bo jednak dwa razy obracam złotówkę i się zastanawiam, czy  naprawdę tego potrzebuję. Może to być też to dlaczego nie idę na zakupy. Bo wiem, że nie mam żadnej pilnej potrzeby. Niby dobra wróciłam do pracy, ale ja nie wiem, jak to się będzie działo. Gdzieś z tyłu głowy mam nadzieję, że koło połowy maja nas oficjalnie otworzą i będzie można z ulgą pracować. No i trzeba te dwa miesiące nierobienia odbić. Więc gdzieś ta doza niepewności finansowej gdzieś jeszcze jest. Myślę, że jeszcze chwilę potrwa zanim zacznę wydawać, może nie wolną ręką, ale bardziej swobodnie te pieniądze.

**A to że nisko na skali byś się umiejscowiła, to jest związane z czasem pandemii? Przed pandemią było inaczej?**

Wcześniej nie szastałam i nie wydawał 1000 zł na głupoty absolutnie. Ale było to bardziej swobodne. Jak poczułam potrzebę, że nie mam spodni, to za 2 dni jechałam je kupić. Natomiast teraz się zastanawiam, czy dobra, może poczekam tydzień lub dwa. Jak już będę pewniejsza tej pracy. Więcej zarobię. Te oszczędności się gdzieś rozeszły przez 2 miesiące, więc trzeba tą kupkę na nowo uzbierać. Myślę, że wtedy będzie bardziej swobodne wydawanie pieniędzy.

**A co było przed pandemią?**

Chociażby teraz chodzi o fryzjera. Pofarbowałam sobie i mogłabym teraz skorzystać z fryzjera, bo podziemie hula. Ale stwierdziłam, że nie będę wydawać pieniędzy, że pofarbuję sobie sama. A kiedyś to było normalne, że co miesiąc byłam u fryzjera i zostawiałam 150zł. I nie było to dla nie szastanie pieniędzmi, tylko to była norma. Po prostu szłam co miesiąc. A teraz się już zastanawiam

I tak było ze wszystkim. Jak widziałam, że mój syn nie ma butów, to ok, jedziemy, kupujemy buty. A teraz patrzę i mówię, że jeszcze przełazisz z tymi, poczekamy ten miesiąc i wtedy kupimy. I teraz rzeczywiście się zastanawiam dwa razy.

Wcześniej też nie wydawałam na jakieś głupoty, np. 17 bluzkę kupuję tylko po to, żeby ją mieć, ale bardziej to było, że fajna kurtka, nie mam takiej, to sobie kupię bo stara jest zajechana. A teraz to myślę, że no dobra przechodzę chwilę i dopiero kupię.

**A jakie emocje towarzyszyły zakupom przed epidemią?**

Ja lubiłam jak miałam coś nowego. Zawsze lepiej się czułam. Czy jak włosy zrobione. Może to nie było, że chodziłam przeszczęśliwa, ale było to fajne, jak człowiek miał coś nowego, świeżego na sobie.

**Większe wydatki.**

Na początku roku sobie umeblowaliśmy cały salon. To mega się czułam. Bo pojechaliśmy, wybraliśmy piękne meble i stoją piękne. Oczywiście dużo lepiej się potem leżało na sofie, niż ze starymi meblami. To fajne było.

Ale to nigdy nie był zakup spontaniczny. Jeżeli chodzi o duże zakupy, to nigdy nie było tak "dobra, jedziemy, kupujemy". To było zaplanowane. Czekałam na to, np. że w styczniu jedziemy i kupujemy meble. Czekałam na to jak dziecko i się cieszyłam jak dziecko jak były skręcane meble.

**To planowanie z czym się wiązało? Na czym polegało?**

Na pewno robiłam research jeśli chodzi o meble czy wyjazdy. I budżet trzeba było zaplanować. Że np. meble to nas będą kosztować tyle i tyle, to tyle mamy a tyle trzeba dołożyć. Tak planować budżet domowy trzeba było.

**A przed epidemią - rozrzutna czy oszczędna?**

Pośrodku.

**A co to dla Ciebie znaczy?**

Na pewno nie jestem mega oszczędna. Taka, że grosik do grosika, ale nie szaleję. Nie mam fanaberii, takich że "ach, dzisiaj zaszaleję, kupię sobie", że jadę i kupuję bluzę Calvina Kleina za 700zł, bo mam taki kaprys. To było tak zawsze pośrodku. Ani jestem Żydem, ani szastam kasą, żeby się potem zastanawiać, czy ja mam żeby samochód zatankować. Wydaje mi się, że tak normalnie.

To co zarobiłam i trzeba było opłacić, to się opłacało. To co zostało i można było wydać, to się wydawało. A to co zostało, to się zostawiało w oszczędnościach. I jakoś to tak funkcjonowało.

**Czy ta zmiana finansowa u Ciebie, to była duża zmiana?**

Dla mnie to było duże zagrożenie, bo ja zostałam totalnie bez pracy. I żadne świadczenia mi się nie należały. Żadna pomoc od państwa, nic, nic. Więc ja zostałam bez grosza przy tyłku. I o ile te 2 tygodnie w marcu, to mówię "dobra super, urlop", kwiecień już był bardzo niepokojący. [*klientka dzwoni :)*]. Bo ja zawsze mówiłam, że potrzebuję mieć stabilizację finansową. Wolę trochę mniej, ale co miesiąc, niż raz na 3 miesiące większą kwotę. Tak mam. I zawsze tak miałam, a tu nagle w kwietniu ja wiem, że mi nic na konto nie wejdzie. To takie, wiesz, kurczę. I myślę, że to też było spowodowane, że ja wróciłam do pracy, tak po prostu tupnęłam nogą i mówię "wracam, bo ja za chwilę nie będę miała na chleb".

 U mnie to była bardzo.. dołek.

**Myślisz, że wróci do normy?**

Tak. Ja myślę, że to wróci, no miesiąc takiego... No chyba to powinno wrócić do normalnego, jak nas oficjalnie otworzą. Bo nadal teraz jest strach trochę. Niby jest ok, niby się śmiejemy, niby przyjdę do pracy i "haha, hihi, chodźcie do podziemia", ale gdzieś z tyłu głowy, to ja nie wiem, czy za tydzień te klientki też będą dzwonić. Więc stabilizacja przyjdzie w momencie, kiedy nam powiedzą "ok, otwieramy salony kosmetyczne". Ale myślę, że nawet tak jak teraz wróciłam do pracy, to już się uspokoiłam. Już widzę pieniądze, które mi się zaczynają zbierać.

**Rozumiem, że selekcjonujecie klientki i macie ograniczone możliwości przez to?**

Tak.

**Czy w związku z tym, że miałaś ograniczony budżet, podejmowałaś jakieś działania, które miałyby zabezpieczyć ten budżet?**

No w ogóle nie robiłam zakupów. Wiesz, tak jakby, w ogóle odcięłam się od takich wydatków które nie są potrzebne. Doszło do tego, że na lody z młodym nie łaziliśmy co 2 dzień jak normalnie, tylko raz na miesiąc poszliśmy. Zresztą mu też wytłumaczyłam, że "synu, musimy oszczędzać, bo ja nie wiem, co będzie jutro". Tak jakby żyliśmy z dnia na dzień, bez żadnych dodatkowych wydatków. Absolutnie. To było zminimalizowane do zera. Nic nie kupiliśmy takiego, co wpadło w oko. Nawet za 5 zł. Mówiłam sobie, że jestem w stanie bez tego przeżyć.

**Tańsze produkty niż zwykle?**

Może tańszych nie, bo ja zawsze kupowałam te same produkty. Raczej już wygrzebane, z tej niższej półki. Ale na pewno te zakupy były takie zaplanowane. Że ja nic nie kupowałam ponad. Nie kupowałam 'bo się przyda', 'bo na później', 'bo teraz kupię, bo coś'. Nie robiłam zapasów, chociażby chemii. Kiedyś jak był Domestos na promocji to brałam 3, a teraz  mówię dobra, wystarczy mi 1. I pojedzie się za 2 tygodnie po kolejny.

Często to było, że brałam wcześniej zapasy. A teraz nie. I zauważyłam, że bardziej zaczęłam zwracać uwagę na cenę. Bo ja się mniej więcej orientowałam, ale nie wiem, czy coś kosztowało 15 czy 17 zł to nie było dla mnie różnicy. A teraz już była różnica i się już zastanawiałam.

**Jak wcześniej robiłaś zapasy, to jak się czułaś?**

To było tak, że jak coś kosztuje normalnie 10zł, a ja mogę kupić za 5zł to biorę. I tak tego potrzebuję, będę musiała to kupić - więc tak jakby z oszczędności to brałam.

**Czy ta zmiana była dla Ciebie trudna?**

Ona weszła naturalnie. Prawdopodobnie gdybym z Tobą nie rozmawiała, to bym się nad tym nie zastanowiła.

To po prostu przyszło, tak się zadziało. Nie planowałam tego, nie zastanawiałam się nad tym. Nie usiadłam ani razu, nie zastanowiłam się, co zrobić, żeby zaoszczędzić kasę, albo jak tu się zabezpieczyć. To były po prostu takie naturalne kroki, jak to zrobić

**A czy teraz oprócz ograniczeń zakupów zbędnych, robisz coś jeszcze aby kontrolować budżet?**

Nie. Nie jestem w tym dobra, więc nie. W przeszłości też nie.

**A czy teraz jest dobrze ograniczać wydatki?**

W ogóle, to mi się wydaje, że ta sytuacja dobrze na nas wpłynie, bo my się staliśmy bardzo konsumpcyjni i bardzo roszczeniowi. Ludzie wydawali i ja zresztą też. Ja rozumiem, że gdzieś jest bieda na pewno, ale ja zauważyłam nawet teraz to analizując, że tej biedy jest coraz mniej. Nawet w szkołach coraz mniej widać takie naprawdę biedne dzieci, co nie mają co jeść i chodzą w podartych ciuchach. Jednak ludzie mieli dobrobyt. I absolutnie nikt się nie zastanawiał nad tym, że to się może kiedyś skończyć. Wszyscy mieli takie poczucie, że "przecież zarabiam, mam pracę to będę miała kasę". I to było takie normalne. Ten koronawirus, nawet jak ze znajomymi gdzieś rozmawiam, spowodował to, że każdy się zatrzymał na chwilę i zastanowił "o o, ja mogę nie mieć tej pracy". Ja na przykład rzadko kiedy byłam bezrobotna, raz w życiu po urodzeniu Miłosza. Zawsze pracowałam, jak nie jedna praca, to druga. Nie było problemu z pracą - lepsza, gorsza ale ta praca była. A w tej sytuacji jeżeli ktoś by został zwolniony, to ja nie wiem, czy on tak szybko znajdzie pracę. I myślę, że to spowodowało taką refleksję. Że ludzie zaczną po pierwsze szanować pracę, a po drugie myśleć nad tym przyszłościowo nad planowaniem budżetu. Bo żyło się od pierwszego, do pierwszego.

**A co sądzisz o posiadaniu oszczędności? Ważne do było dla Ciebie?**

Tak, ja musiałam mieć zawsze jakiś zapas gotówki. Ale to nie były kwoty, które pozwolą mi przez trzy lata żyć bez pracy. To była kwota na miesiąc, dwa, która da mi spokój psychiczny, że ja przeżyję, opłacę rachunki. Natomiast ja nigdy nie rozmyślałam nad tym, że ja mogę nie mieć pracy dłużej niż miesiąc. Gdzieś tam oszczędności były. Czasami się z nich coś zabrało na poczet wakacji, mebli czy czegokolwiek, ale człowiek wiedział, że za 2-3 miesiące znowu tam dołoży. Ale absolutnie nic nie rozmyślałam w kwestii, że za 5 lat jak uzbieram ileśdziesiąt tysięcy to coś kupię. To było wszystko na krótkoterminowe. Żeby mieć na dzisiaj. Na góra dwa miesiące do przodu.

**Oszczędności Twoje to 1-2 miesiące były tak?**

Tak

**A jeżeli oszczędzasz, to jak to robisz?**

Staram się co miesiąc wrzucać niewielką kwotę, która musi być. Kilkaset złotych. Jak mam więcej to też tam przerzucam. Natomiast to jest zawsze tak, że to jest niewielka kwota.

Dla mnie oszczędzanie było bardzo naturalne. Nie wiem, skąd się wzięło. Po prostu było od zawsze.

**A co robisz z oszczędnościami?**

Trzymam na koncie. Mam takie subkonto przy koncie osobistym i one sobie tam leżą. Może są jakoś śmiesznie oprocentowane, jak to na koncie oszczędnościowym. Ale leżą tam. Kiedyś jak trzymałam w domu, to widziałam, że ta kupka się czasami uszczuplała niepotrzebnie. Więc trzymam na koncie, do którego nie mam dostępu. W sensie że jeśli nie przerzucę tych pieniędzy, to one sobie tam są. Jeszcze mam taki myk, że to konto jest ukryte, więc jak wchodzę na konto to nie widzę stanu tego konta.

Oszczędzam tez, bo jak wypadnie coś takiego nagłego. Te oszczędności, to ja nie myślę w kwestii oszczędzam, bo potem wyjadę na wakacje. Tylko raczej, bo potem mogę nie mieć pracy, będę miała wypadek i będę musiała coś zrobić taki nagły wydatek, który nie będzie się kojarzył koniecznie z przyjemnością - wręcz w tą drugą stronę. Żebym ja się nie martwiła, np. na coś takiego o czym na co dzień nie myślę. Np. jak rozwalę komuś samochód, to żebym miała skąd mu to naprawić. Na przyjemności staram się bardziej wydawać na bieżąco. A to zło, które może mi się przydarzyć to pójdzie w oszczędności

**Czy teraz warto oszczędzać?**

Bardzo warto oszczędzać. Oczywiście, że warto. Czy dużo? Na ile kto może sobie pozwolić, bo niektórzy 10zł miesięcznie, drudzy 100zł, a inny 10 tys. Natomiast warto. Bo sytuacja jest tak niestabilna, że nie wiesz, co będzie jutro.

**A może warto by teraz wydać skoro wszystko poza kontrolą?**

Nie! Mi by strach nie pozwolił. Ja się zawsze bałam tego, że zostaniemy bez pieniędzy, więc mi by strach nie pozwolił.

**A jak myślisz, czy teraz jest dobry czas na inwestycje?**

Tak, zawsze  w dołku jest najlepszy. Ale ludzie się boją. Natomiast ci inwestorzy wyższej klasy, to oni kupę kasy teraz pozarabiają.

**A Tobie zdarzało się inwestować?**

Nie. Ja nie mam kupy kasy

**Inwestowanie wiąże się z tym, że trzeba mieć większą kwotę?**

Kiedyś się zastanawiałam na inwestowaniem. To gdybym miała taką wolną gotówkę, po prostu że ktoś mi daje, ciocia z ameryki mi przysyła spadek, to bym mogła. Ale te wszystkie swoje zarobione pieniądze - ja nie chcę ich stracić. Ja wiem, ile na to pracowałam.

[telefon] Właśnie dzwonił mój tata, że moje dziecko łamie kwarantannę. Bo ja jestem w  pracy, a tu moje koleżanki dzieci są i on przyjedzie i będzie z nimi siedział. Rozumiesz? Tu są dzieciaki teraz - jeden chłopiec jest młodszy od Miłosza o rok, albo dwa. Jeszcze są dziewczynki i łamiemy kwarantannę.  Ja miałam po niego jechać, ale tata zadzwonił że go przywiezie więc muszę otworzyć bramę.

**Przyszłość. Kiedy to się skończy?**

Długo się nie skończy. Ludzie przejdą na porządku dziennym do tego. I zaczną funkcjonować. Natomiast  świecie politycznym, gospodarczym będzie to cały czas. Ja się cały czas zastanawiam, co się będzie działo od września czy października. Bo wtedy pojawią się wirusy i pytanie, czy głowy państwa, bo ja nie wiem, kto tak naprawdę zawładną umysłami ludzi - to jest dla mnie fenomen - czy we wrześniu, październiku przejdziemy na porządku dziennym i po prostu jest sezon grypowo-koronawirusowy i proszę na siebie uważać. Czy znowu będzie powtórka z rozrywki ze zdwojoną siłą. Natomiast nie jestem w stanie tego przewidzieć. Nie wiem jak myśleć o tym. Która opcja będzie dobra.

**A kiedy zdarza Ci się myśleć?**

Co chwilę.

Jedyny nawet temat z klientkami nawet, to jest koronawirus. Cały czas to jest miętolone, gdybane, przewijane i każdy się zastanawia. I każdy ma coś w pracy. I coś jest nie tak. I w tej pracy, do tej pory nie wiedzą  w którym momencie wrócić do normalności. Chociażby wczoraj miałam pielęgniarki od nas ze szpitala. I one chwilę temu były spokojne, bo nie przyjmowały nowych pacjentów, od tego tygodnia są nowi pacjenci. I też nie wiedzą, czy to jest dobry moment, żeby do tego wrócić. Więc tak jakby każdy nie wie, który dzień będzie tym dobrym, żeby wrócić do normalności. Więc ja nie wiem, czy to się kiedykolwiek... Mi się wydaje, że nam się bardzo zmieni podejście do życia. Ta sytuacja poprzestawia ludziom w głowach - nie wiem, czy na dobre, czy na złe, ale będziemy inaczej na wszystko patrzeć.

**Co najbardziej zaprząta Twoją uwagę?**

Bardziej zastanawiam się teraz, co będzie ze szkołami. Bo mi się wydaje, że jeżeli nas nawet nie otworzą oficjalnie to podziemie będzie bardzo sprawnie działać. I jakoś my będziemy pracować, może trochę mniej, mniej zarobimy, ale będziemy pracować. A co będzie ze szkołami? Wczoraj dostałam taka dziwną informację od klientki, że podobno matur i tak ma nie być. Że ona ma od nas z kuratorium informacje, że ma nie być. Że to jest taka informacja na uspokojenie.  I wiesz - niby sobie myślę, co ona za głupoty gada, ale z drugiej strony ja już nie wiem, może rzeczywiście ktoś puścił plotę na zasadzie "uspokoimy naród do czerwca, w czerwcu powiemy, że nie będzie matur". Nie wiem. Dziwne to jest.

**Jak dalej potoczy się sytuacja?**

Według mnie to wszystko pootwierają, przeprowadzą wybory, a potem nie wiem, co będzie bo nie wiem, kto wygra. Jest to bardzo polityczna rozgrywka w tej chwili. Więc to jest moja opinia. Natomiast jak kampanie wyborcze są nieprzeprowadzone, ja obejrzałam ostatnią debatę - część - bo nie chciało mi się dalej słuchać, to ja połowy kandydatów nie znam. Nie wiem, kto to jest. Czuję się jak 18 letni dzieciak, który dopiero wchodzi w życie polityczne i zaczyna się orientować polityką. I jest jakiś pan, który chce być kandydatem. Ja nic o nich nie wiem. Nic totalnie. I to jest dziwne. Bo się starałam uczestniczyć. Może nie byłam jakaś bardzo polityczna i taka, że ja wierzę w daną partię i znam na 100% jej poglądy. Ale gdzieś kojarzyłam kto, po co, kto ma jakie postulaty, itd. A tu nie wiem nic

**Jak to z tym jest teraz?**

Źle. Bo gdzieś zawsze mi się wydawało, że jeżeli dużo wiem, na temat kto rządzi krajem, czy to jest mój pogląd czy nie mój, ale ja o nim wiem, to tak jakby - nawet jeżeli to jest wróg - to znam swojego wroga. A w tej chwili mam taką dezinformację. Nie wiem, co się dzieje w kraju. Chociaż jak ja rozmawiam z ludźmi, to dużo osób tak żyje. Że "ja się nie interesuję polityką, mnie to nie dotyczy". A to nieprawda! To nas strasznie dotyczy. Ja nie wiem, jak te osoby żyją, bo ja mam takie... Nawet jak pójdę na wybory, albo mi przyślą list wyborczy, bo nie wiem, co zrobią - to znaczy jak wyślą list, to głosy są jakby już policzone. To jest moja opinia. Więc może nie będę im tego odsyłać - po co im moja papierkologia. Natomiast jak pójdę to będę się grubo zastanawiać. Bo nie wiem.

**A to się wiąże z wrażeniem braku poczucia wpływu czy to jest co innego?**

Wiesz, że ja nie lubię niewiedzy. Nie lubię, nie wiedzieć. Nie lubię rozmawiać na tematy gdzie nie wiem. Lubię wiedzieć. Jeżeli czegoś nie wiem, to nie rozmawiam, ale idę do domu i doczytam, dopytam, dowiem się. Męczy mnie to tak w środku.

**A co Ci daje to, że wiesz?**

Nie wiem, co mi to daje. Może poczucie bezpieczeństwa, że ja to wiem. I że jak z kimś rozmawiam, to nikt mi kitu nie będzie... Nie lubię jak ktoś mi wali ściemę. Ja nie mówię, że to co ja wiem, to jest święta prawda. Bo jeśli ktoś przyjdzie do mnie i mnie argumentami przekona, to mogę to zaakceptować: spoko, masz rację. Już na studiach jak słyszałam " a bo wszyscy Polacy to są tacy, a uchodźcy to są tacy..." Jacy wszyscy? Nie ma wszyscy. I mnie to zawsze drażniło. Nie uogólniajmy ludzi. Ja muszę się dowiedzieć, że ten jest taki, ten jest taki, a ten taki. I to że ja coś wiem, to mi daje poczucie... No musze wiedzieć. Ze wszystkim tak mam. W pracy też. Nie mogę naściemniać klientce. Od strony biologicznej nawet.

**Jakie masz obawy związane z najbliższą przyszłością?**

Teraz to tak naprawdę moim punktem będzie informacja czy otwierają salony oficjalnie. Jeżeli to się stanie, to ja odetchnę z ulgą, bo nie będę musiała zamykać bramy itd. i zacznę normalnie pracować. I normalnie odbierać telefony. I to jest punkt na który czekam. Myślę, że w graniach tygodnia premier, czy minister powinien się wypowiedzieć, czy nas otworzą. Wcześniej czekałam na otwarcie szkół, ale to wiem, że się nie wydarzy. Więc niech siedzi w domu - jest babcia, więc my możemy pracować. A potem nie wiem. Jak nas otworzą, to będę się zastanawiać, co dalej. Wakacji nie planujemy. Wyjazdów nie planujemy. Myślę, że to będzie takie przeczekanie do września. Wakacje też nam umkną - nie zrobimy sobie wakacji, bo będziemy myśleć, że przecież w kwietniu mieliśmy wakacje.

**Na razie nie wiadomo jak będzie?**

Tak.
