# Supplementary material for: The challenges arising from the COVID-19 pandemic and the way people deal with them. A qualitative longitudinal study
Source: PLoS One. 2021 Oct 11;16(10):e0258133. doi: 10.1371/journal.pone.0258133 (PMC8504766; doi:10.1371/journal.pone.0258133)
Supplement: S1 Dataset — (ZIP) [file pone.0258133.s003.zip › Transcriptions/stage 4/8.4_M_30_couple, no children.docx]

**8.4._M_30_couple no children**

**Co działo się u ciebie w ciągu ostatnich 2 tygodni?**

W zasadzie, to nic się nie wydarzyło albo wydarzyło się bardzo niewiele, bo mam od jakiegoś czasu wrażenie, że dni w tej izolacji koronawirusowej zlewają się w jedno. Wszystkie są praktycznie takie same. I tak, jak w poprzednich tygodniach, siedzę dalej na *home office.* Próbuję pisać pracę. I w zasadzie do tego to się sprowadza. Co jakiś czas wychodzę z psem, przygotowuję obiad, zjadam obiad. I tak mijają kolejne dni.

**Jak majówka? Ona była inna w porównaniu do tych sprzed epidemii?**

Była znacząco inna, bo w poprzednie majówki starałem się gdzieś wyjechać, gdzieś się ruszyć z domu. A teraz to był zwykły 3-dniowy weekend spędzony w domu. Tak samo, jak wszystkie poprzednie weekendy od 1,5-2 miesięcy. A więc nie traktowałem tego jako majówkę.

**Nic się nie zmieniło, nie ma żadnych nowych rzeczy w zachowaniu?**

Nie.

**Są jakieś rzeczy, które zaczynają bardziej doskwierać?**

Chyba nie. Może trochę zaczyna być coraz bardziej nużące to, że wszystkie dni wyglądają tak samo. Zwykłe znużenie, zmęczenie siedzeniem w domu i powtarzaniem tych samych czynności o tych samych porach. Taką monotonię widzę w tym wszystkim. Ale jakoś nie cierpię strasznie.

**Jeżeli chodzi o ostatnie 2 tygodnie, to co według ciebie jest największym wyzwaniem? To znużenie czy coś innego?**

Myślę, że to może być w zasadzie jeden z największych problemów, bo to znużenie chyba już się wszystkim daje we znaki i może to utrudniać utrzymywanie w izolacji innych ludzi, a przez to też rozprzestrzenianie się wirusa.

**W jaki sposób starasz się sobie radzić z tym znużeniem?**

Nie wiem, czy mógłbym powiedzieć, że staram się sobie radzić z tym, bo tak w zasadzie, to staram się sobie trwać i do tego to się sprowadza. Nie podejmuję kroków, żeby sobie z tym radzić specjalnie.

**Dlaczego nie podejmujesz tych kroków?**

To znużenie nie osiąga jakiegoś krytycznego nasilenia, które by mnie skłaniało do jakichś ruchów związanych z tym.

**Czy udało ci się znaleźć obrazek/zdjęcie, który przedstawia emocje?**

Nie. Nawet się zastanawiałem nad tym i miałem to w głowie, ale taką jedną miałem konstatację, że w ostatnim czasie nawet tych memów koronawirusowych jest mniej i przestałem na to trafiać. Chyba nawet wszyscy tym tematem się znudzili.

**Obrazki. Wybierz jeden lub 2, który najlepiej oddaje twoje emocje.**

No to może 9 i dodatkowo 7.

**9**

Na tym zdjęciu, to tutaj nie powiem nic nowego, dalej mi się kojarzy z jakimś zagrożeniem, które nadciąga, jest względnie blisko, jest w pewien sposób nieuchronne i nie bardzo da się przed nim w pełni zabezpieczyć. To chyba tyle, co mi się kojarzy z tym obrazkiem.

**Poczucie zagrożenia, przed którym nie da się zabezpieczyć, to jest coś, co się utrzymuje już od jakiegoś czasu u ciebie?**

Tak. Może nie nazwałbym tego, że się utrzymuje to uczucie u mnie. Ja sam tego lęku nie odczuwam, ale wszystko mi się kojarzy z nadciągającym zagrożeniem.

**7**

A ten obrazek to tak wybrałem do skojarzenia z tą wszechogarniającą nudą, marazmem, znużeniem. I to było główne moje skojarzenie.

**Nuda, marazm pojawiły się w ciągu ostatnich 2 tygodni, bo wcześniej doskwierały mniej?**

Tak. Wcześniej się pojawiały, ale teraz powiedzmy, że troszkę to narasta.

**Co się utrzymuje, jeśli chodzi o twoje emocje, a co jest czymś nowym, co teraz zaczyna się pojawiać?**

Ciężko mi jest dokładnie określić, kiedy to znużenie zaczęło się pojawiać. Bo pewnie zaczęło się pojawiać 3-4 tygodnie, może dawniej. Tylko tak stopniowo cały czas narasta pomału. Nie wiem, czy coś zupełnie nowego, jakiekolwiek zupełnie nowe emocje się pojawiły w związku z sytuacją. Nie wydaje mi się.

**A oprócz znużenia, czy coś jeszcze jest teraz?**

Jakieś może wątpliwości cały czas związane z sytuacją polityczną i gospodarczą. To się cały czas utrzymuje na podobnym poziomie - ani nie rośnie, ani nie maleje.

**Czy mógłbyś jeszcze w jakiś sposób opisać to, jak się czujesz?**

Czy coś jeszcze innego? Ja bym to nazwał jakimś takim marazmem, w który też popadam z powodu tej otaczającej monotonii, który sprawia, że jeszcze ciężej mi się zabrać do jakiejkolwiek pracy czy zrobienia czegoś konstruktywnego, czy do jakichś ćwiczeń. Im mniej się dzieje, tym mniej ja mam energii do tego, żeby się zabrać do czegoś paradoksalnie.

**Czy to może być, że nie bardzo robisz coś, żeby przeciwdziałać tym emocjom? Czy nie jest to powiązane?**

Tak. Niewykluczone. Myślę, że może być takie błędne koło.

**Czy chciałbyś sam wygenerować taki obrazek, który idealnie opisywałby twoje emocje?**

Szczerze mówiąc, nie mam takiej potrzeby. Nie czuję przelewania swoich emocji na jakieś obrazki.

**Na ile teraz czujesz się zagrożony sytuacją?**

W minimalnym stopniu zupełnie.

**Co się na to składa?**

Po pierwsze to, że dalej uważam, że nie jestem osobą specjalnie zagrożoną zachorowaniem czy jakimś cięższym przechodzeniem koronawirusa. Po drugie, że moja sytuacja, jeśli chodzi o zatrudnienie też jest stabilna i nie przewiduję, żeby w ciągu najbliższych 2 lat się cokolwiek w niej zmieniło, a dalej w przyszłość na razie nie wybiegam, jeśli chodzi o swoją przyszłość, więc czuję się bezpiecznie.

**Jakie emocje pojawiają się w twoim otoczeniu?**

Na pewno narasta już nie tylko znużenie, ale irytacja związana z izolacją i brakiem kontaktów towarzyskich czy nawet u niektórych, z tego co słyszę, brakiem kontaktów, nie tylko bezpośrednich, ale takich, żeby po prostu byli dookoła jacyś ludzie, np. w pracy. Nawet bez konieczności odzywania się do nich, tylko po prostu, żeby byli. Z takich może nie najbliższego otoczenia, to chyba maleje jakieś poczucie zagrożenia, związanego z wirusem, bo jak na początku większość osób się stosowało do noszenia maseczek, tak teraz widzę, że coraz mniej. Chyba nic poza tym nie zaobserwowałem.

**Jak oceniasz te działania?**

To samo, co wcześniej parokrotnie mówiłem, jeśli chodzi o przebywanie w otwartej przestrzeni, gdzie nie ma się bliskiego bezpośredniego kontaktu z innymi ludźmi, to uważam, że te maseczki są do niczego niepotrzebne. I raczej uważam to za racjonalne podejście, to nienoszenie maseczki w takiej sytuacji. Ale samo to, że zauważyłem, że wcześniej tych osób w maseczkach było więcej niż teraz, to odbieram jako to, że chyba to poczucie zagrożenia też maleje.

**To uczucie znużenia - dla ciebie to jest naturalne, że się pojawia, bo u twoich bliskich występuje podobnie?**

Tak, oczywiście. Wydaje mi się, że wszyscy mają prawo być już tym zmęczeni.

**Czy obserwujesz jakieś działania, które podejmują twoi bliscy, żeby sobie radzić ze znużeniem?**

Niektórzy po prostu wrócili do pracy normalnej już. Nie w trybie *home office*. A niektórzy tak częściowo, że kilka dni są w pracy, a kilka dni pracują z domu.

**To była ich decyzja czy odgórna?**

Nie, to była we wszystkich przypadkach ich decyzja.

**Jak wyglądają teraz u ciebie zakupy?**

Na razie do zachowań zakupowych sprzed epidemii nie wróciłem i utrzymują się dokładnie te same zachowania, co w poprzednich kilku tygodniach. Robię zakupy z taką samą regularnością i kupuję, wydaje mi się, te same produkty i tutaj nic się nie zmieniło.

**Robiliście listę zakupów?**

Tak.

**Dlaczego nadal planujecie te zakupy?**

Lista zakupów to jest raczej takie po prostu ułatwienie dla mnie, żeby niczego nie zapomnieć ze sklepu. Przed epidemią też korzystałem z takiej listy. Jedyna różnica jest taka, że po prostu częściej zdarzało mi się spontanicznie, np. wracając z pracy, pojechać do sklepu i zrobić jakieś małe zakupy bez listy. Ale jak jechałem na większe zakupy, to starałem się mieć taką listę przygotowaną.

**Dlaczego nie wracasz do zachowań sprzed epidemii?**

Raczej nie ma takich sytuacji, żebym gdzieś jechał i wstępował do sklepu przy okazji. Jak wychodzę z domu w ogóle, to jest to właśnie na spacer z psem albo specjalnie po to, żeby zrobić zakupy. To jest chyba główna różnica, jeśli chodzi o zachowania zakupowe. Wiadomo, że wcześniej też czasem się zdarzało przy okazji zakupów spożywczych wejść też do jakiegoś sklepu z ubraniami. Teraz te sklepy są zamknięte, więc po prostu nie wchodzę, ale też nie mam specjalnej potrzeby, żeby do nich wchodzić.

**Czy ostatnio kupiłeś jakieś rzeczy dla przyjemności? Na poprawę humoru?**

Tak. Kupiłem pudełko lodów np. dla przyjemności. Jakieś różne słodycze czy chipsy z rzeczy spożywczych. Z rzeczy niespożywczych, to ostatnio kupiłem telefon, ale to była decyzja przemyślana, do której się zbierałem od pół roku pewnie. Kupiłem plecak turystyczny, ale to też dlatego, że przyda mi się w niedługim czasie - mam nadzieję, a był w bardzo korzystnej promocji. Więc nie traktowałbym tego jako taki zakup dla przyjemności, bardziej z rozsądku. Nic więcej nie przychodzi mi do głowy.

**Teraz tych słodyczy jest więcej w twojej diecie?**

Słodyczy może nie. Chociaż, jeśli by wliczać lody jako słodycze, to pewnie tak. Dobra, powiedzmy, że trochę więcej. Chipsów też w ostatnim czasie jem trochę więcej. Nie są to duże ilości, ale jeszcze parę miesięcy temu prawie w ogóle przez dłuższy okres nie jadałem takich rzeczy.

**To jest tak na poprawę humoru, czy "teraz to mi wolno"?**

Nigdy nie miałem takiego poczucia, że mi nie wolno. Myślę, że to jakiś sposób na zabicie nudy dodatkowy.

**W poniedziałek otworzyli galerie handlowe - co o tym sądzisz?**

Nie wiem, dlaczego akurat galerie handlowe, bo wydaje mi się, że przed galeriami handlowymi np. sensowniej byłoby otworzyć jakieś salony fryzjerskie czy kosmetyczne, bo zdecydowanie łatwiej tam zachować warunki higieny i wszelkie środki profilaktyczne, jeśli chodzi o koronawirusa. Galerie handlowe myślę, że w dalszej kolejności powinny być otwierane. A jeśli chodzi w ogóle o otwieranie teraz nagle galerii handlowych i luzowanie tych wszystkich obostrzeń, to trochę taką widzę niekonsekwencję w działaniach rządu, bo w momencie, gdy było kilkadziesiąt zachorowań na koronawirusa, to wszystko było zamykane i były wprowadzane bardzo ostre restrykcje. A teraz, gdy codziennie mamy dobrych kilkaset zachorowań... Ostatnio patrzyłem, to było ok. 300 zachorowań dziennie. To teraz nagle postanawiają wszystko otwierać. Trochę się obawiam, że skutkiem tego może być kolejny wzrost i jeszcze większa zachorowalność za 2 tygodnie.

**Dlaczego otworzono galerie?**

Myślę, że po pierwsze mógł być to sposób jakoś na łagodzenie skutków gospodarczych tej epidemii, żeby przynajmniej za sprawą tych sklepów jakoś wzrost gospodarczy próbować ciągnąć w górę. Po drugie, nie wykluczam też takiego działania, że mogło być to w jakiś sposób powiązane z planowaniem organizacji wyborów, tak, żeby pokazać społeczeństwu, że już jesteśmy coraz bezpieczniejsi, już mamy mniejsze powody do obaw przed wirusem, więc nie ma tak samo powodów do obaw przed organizacją wyborów prezydenckich.

**Wydaje ci się, że to działa? Że społeczeństwo czuje się bezpieczniej?**

Wydaje mi się, że wiele osób może czuć się bezpieczniej i wielu osobom może się wydawać, że to zagrożenie mija.

**Wybierasz się w najbliższym czasie na zakupy stacjonarne?**

W tym tygodniu będę musiał się wybrać, bo skończą mi się produkty spożywcze.

**Mam na myśli nie spożywcze, tylko może do galerii?**

Nie, nie planowałem takich zakupów.

**Może znasz kogoś, kto planuje taką wyprawę?**

Być może kogoś takiego znam, ale nie wiem na pewno o takich jego planach.

**Podejście do pieniędzy. Skala.**

Myślę, że gdzieś w okolicy 4.

**Na czym to polega? Podaj jakiś przykład.**

Powiedzmy tak... Umieściłem siebie mniej więcej blisko środka tej skali, bo są sytuacje, gdzie nie przejmuję się tymi pieniędzmi, które wydaję, bo wiem, że muszę je wydać i nie ma co tego rozważać. Typu "muszę zatankować samochód" albo "muszę zrobić konkretne produkty spożywcze". Ale są też sytuacje, że właśnie muszę kupić coś droższego albo co nie jest mi jakoś bardzo potrzebne, tylko jest to sposób na sprawienie sobie przyjemności albo jest to większy wydatek. Wtedy zwykle staram się, żeby to był mocno przemyślany zakup. Z większą ilością myśli wiąże się ten proces zakupowy i ciężej mi te pieniądze wydać.

**Jakie emocje wiążą się z wydawaniem pieniędzy, kiedy musisz iść i kupić rzeczy spożywcze? A jakie, jak kupujesz rzeczy, które niekoniecznie są potrzebne?**

Wydaje mi się, że takim codziennym zakupom, nad którymi się nie zastanawiam, to z tego powodu, że się nie zastanawiam, to niespecjalnie mi jakiekolwiek emocje towarzyszą. Może czasem mi towarzyszy jakieś zmęczenie albo znużenie, albo irytacja, że muszę być w tym sklepie i to zrobić. A jeśli chodzi o inne zakupy, które wiążą się z większym procesem myślowym, to mogę mieć wątpliwości, czy na pewno to są dobrze wydane pieniądze. Jeśli z kolei mam poczucie, że są to dobrze wydane pieniądze, to mogę czuć jakiś rodzaj satysfakcji czy radości, jeśli to jest coś, co chciałem kupić i mam poczucie, że zrobiłem dobry interes na tym zakupie.

**Pojawia się jakieś poczucie winy ze względu na wydaną sumę?**

Myślę, że może się w jakimś niewielkim stopniu co jakiś czas pojawiać, jeśli mam poczucie, że to jest coś, co niekoniecznie mi było potrzebne, a kupiłem to tylko po prostu z czystej chęci posiadania. To wtedy może jakieś poczucie winy się pojawiać, że można było te pieniądze przeznaczyć na coś innego. Ale to nie jest jakieś znaczne.

**To poczucie winy jest silniejsze niż radość z zakupu?**

Nie potrafię określić, bo mogą być bardzo różne rozbieżności pomiędzy różnymi zakupami. Dlatego też tak umieściłem siebie z dala od jednej i drugiej skrajności. Trochę bliżej tylko tej, w której ciężko przychodzi wydawanie pieniędzy.

**Kiedy ostatnio wydałeś większą kwotę - opowiedz o tej sytuacji. Jak się czułeś?**

Z ostatnich sytuacji, to kupiłem telefon, nad którym się zastanawiałem od dłuższego czasu, bo poprzedni zaczynał się coraz gorzej sprawować. Dużo czasu spędziłem, szukając alternatyw dla tego konkretnego modelu albo jakichś lepszych ofert cenowych. Aż w końcu znalazłem taką, która mi się w danej sytuacji wydawała najsensowniejsza. Po paru miesiącach przyglądania się cenom, stwierdziłem, że już raczej ta cena nie spadnie i teraz jest najlepszy moment na zakup.

**Nazwałbyś siebie osobą rozrzutną czy oszczędną?**

Raczej oszczędną.

**I co to dla ciebie znaczy?**

Że staram się mimo wszystko nie wydawać pieniędzy na bzdety. Staram się regularnie jakiś procent ze swojej pensji odkładać, żeby mieć jakieś finansowe zabezpieczenie.

**Z sytuacjami, kiedy wydajesz pieniądze, wiążą się pozytywne czy negatywne emocje?**

Nie jestem w stanie uogólnić. Czasem są pozytywne, czasem negatywne.

**Może mógłbyś podać jakiś przykład, kiedy odczuwasz pozytywne, a kiedy negatywne emocje?**

Może to być sytuacja, jak kupuję sprzęt typu pralka. Muszę to kupić, bo jest to potrzebny sprzęt, natomiast samo posiadanie pralki nie sprawia, że ja jestem jakiś szczęśliwszy. Oczywiście, jakbym nie miał pralki, musiał chodzić w brudnych rzeczach, to byłbym nieszczęśliwy, ale sama pralka nie jest jakimś urządzeniem, które mi dostarcza szczęścia. Więc raczej jestem niezadowolony, że musiałem wydać te pieniądze na nową palkę i że stara pralka nie mogła podziałać trochę dłużej.

**Zakupy jakiego typu sprawiają ci dużo przyjemności i frajdy?**

Przyjemność to mogą być pieniądze wydane na telefon, a w jeszcze większym stopniu na jakiś wyjazd. Kiedy mam poczucie, że te pieniądze są pożytkowane w fajny sposób.

**Czy coś się zmieniło w twoich perspektywach finansowych na przyszłość? Bo wspominałeś, że w dochodach nie było zmian.**

Nie, pod tym względem też się nic nie zmieniło.

**Czyli obecna sytuacja nie jest zagrożeniem dla budżetu domowego?**

Nie. Zagrożeniem na pewno nie. Co najwyżej trochę więcej jestem w stanie oszczędzić.

**Czy ten sposób oszczędzania się zmienił w stosunku do tego, jak przed epidemią?**

Sam sposób oszczędzania się nie zmienił, tylko zmniejszyła mi się liczba stałych wydatków, typu wydatki na transport i dojazdy do pracy czy wydatki na lunch w pracy. To odchodzi, więc siłą rzeczy trochę więcej pieniędzy na koniec miesiąca.

**Oszczędzasz w ten sposób, że dobrowolnie odkładasz to, co zostanie na koniec miesiąca?**

To znaczy, zwykle na początku miesiąca, jak mi wpłynie pensja, to staram się mniej więcej, tak tylko z grubsza oszacować, ile pieniędzy w tym miesiącu wydam z jakimś dużym zapasem. To, co po odjęciu tego zostaje, przelewam na konto oszczędnościowe. A oprócz tego mam ustawione automatyczne przelewy co miesiąc, gdzie idzie mi określona stała kwota na konto emerytalne.

**Poszukujesz teraz tańszych produktów niż zazwyczaj, jak robisz zakupy?**

Nie.

**Poszukujesz przecen, okazji?**

Nie. W takim samy stopniu jak przed epidemią.

**A jak już ich poszukujesz, to jak się z tym czujesz?**

Nie mam poczucia, że muszę znaleźć, bo rzadko raczej mam tak duże wydatki, gdzie mam poczucie, że jeśli nie znajdę tego *dealu*, to nie będę w stanie temu sprostać Myślę, że jest to takie poczucie właśnie, że zrobiłem dobry interes, czy dobry *deal* i właśnie dzięki temu udało mi się zaoszczędzić i np. w następnym miesiącu będę mógł trochę więcej na to konto oszczędnościowe przelać.

**W obecnej sytuacji dobrze jest ograniczać wydatki?**

Wydaję mi się, że zawsze jest dobrze ograniczać wydatki, jeśli można to zrobić. Oczywiście wszystko w jakiś rozsądnych granicach. Ograniczanie takich zbędnych wydatków.

**Dlaczego?**

Ponieważ, wydaję mi się, że zawsze warto mieć jakąś, choćby niewielką poduszkę finansową. Nie możemy być pewni przyszłości i ona [poduszka] może czasem uratować skórę. A po drugie potem posiadanie takich oszczędności procentuje tym, że można je wydać w jakiś sensowny sposób - czy na remont, czy na wyjazd.

**Jakiej wielkości są twoje oszczędności? Na ile by starczyły?**

Spokojnie kilka dobrych miesięcy.

**W waszym gospodarstwie domowym, kto inicjuje oszczędzanie?**

Ona też się stara oszczędzać. Nawet powiedziałbym, że w bardziej zorganizowany sposób niż ja, bo ja w jakiś zgrubny sposób szacuję, ile planuję wydać. Natomiast ona nieraz nawet spisuje sobie paragony, robi jakieś kalkulacje wydatków, rachunków, więc jest to zdecydowanie bardziej zorganizowane i lepiej przygotowane niż w moim przypadku.

**Można powiedzieć, że to jej inicjatywa czy oboje uważacie, że warto oszczędzać?**

Każde z nas ma podobny pogląd, że jest to sensowne działanie.

**Co robisz ze swoimi oszczędnościami? Inwestujesz je w jakiś sposób?**

Nie, raczej nie. Wydaję mi się, że żebym mógł inwestować, to musiałbym mieć trochę większe przychody albo znacznie większe oszczędności. A tak małe środki, jakimi ja na razie dysponuję niestety, to raczej dużo większego pożytku niż na koncie oszczędnościowym nie zrobię.

**Myślisz, że w obecnej sytuacji mamy kontrolę nad oszczędnościami?**

Nie no, jak najbardziej to, co się dzieje, ma wpływ na oszczędności. Bo ostatnio NBP zmniejszył stopy procentowe, więc automatycznie oprocentowanie na wszystkich albo prawie wszystkich kontach oszczędnościowych i lokatach poszło w dół. Też rośnie inflacja, więc te nasze oszczędności, gdzie by ich nie trzymać, to z czasem będą traciły na wartości. Ale jak nie ma się sensownych sposobów na zainwestowanie ich, to lepszym rozwiązaniem mimo wszystko jest moim zdaniem trzymanie ich na koncie oszczędnościowym czy lokacie z minimalnym oprocentowaniem niż trzymanie ich w skarpecie.

**Teraz jest dobry czas na inwestycje?**

Nie umiem powiedzieć. Podejrzewam, że jeśli ktoś się dobrze na tym zna, to potrafiłby wybrać jakieś dobre kierunki inwestowania pieniędzy, które przyniosłyby zyski. Natomiast ja się tym nie interesowałem ani na rynkach finansowych się nie znam, więc nie umiem powiedzieć. Aczkolwiek, podejrzewam, że teraz może być taki trudny okres na inwestowanie i wymagający większych umiejętności.

**Prawdopodobnie, gdybyś inwestował, mógłbyś mieć większe opory?**

Jeszcze dokładniej bym to przemyślał.

**Kiedy obecna sytuacja się skończy?**

Tak obstawiłbym widełki może 5-12 miesięcy.

**W jakich momentach o tym myślisz? Myślisz o tym spontanicznie?**

Nie. Teraz daję ci taką odpowiedź, bo pytasz, ale odpowiedź nie jest tak zupełnie stworzona na poczekaniu, bo opieram się na artykułach i analizach, które czytałem.

**Na podstawie jakich informacji twierdzisz, że tak to będzie?**

Są wyliczenia konkretne, jak szybko przy różnych założeniach ta epidemia się będzie rozwijała. Więc to są opublikowane w czasopismach naukowych wyliczenia czy różne modele matematyczne, czy modele bardziej logistyczne, opierające się na cały czas spływających danych. W zależności od założeń początkowych tych modeli, ja bym takie widełki obstawił.

**5-12 miesięcy to według ciebie czas, kiedy nabędziemy jakąś odporność? Czy wynalezienie szczepionki?**

To jest czas, kiedy będzie można odejść całkowicie od tych obostrzeń sanitarnych. I raczej bym stawiał na wykształcenie się już jakiegoś poziomu odporności zbiorowej niż szczepionkę. Jeśli chodzi o szczepionkę, to spekulowanie, kiedy ona się pojawi i czy się pojawi, to na razie bym traktował jako wróżenie z fusów. Jest zbyt wiele niewiadomych jeszcze.

**Uważasz, że może w ogóle się nie pojawić?**

Może się okazać, że nie będzie potrzebna, bo już samoistnie ta epidemia wygaśnie. Słyszałem takie spekulacje, aczkolwiek nie zagłębiałem się w nie dokładnie, więc nie wiem, na ile to jest wiarygodne, że tak może być. Sytuacja ze szczepionką na koronawirusa może być taka jak ze szczepionką na grypę, że co sezon trzeba będzie ją aktualizować.

**Czy zdarza ci się może myśleć o końcu tej sytuacji, kiedy odczuwasz to znużenie?**

Na pewno mi się zdarza, bo zastanawiałem się, czy w okresie wakacyjnym mi się uda wyjechać czy nie. Czy na jakąś konferencję naukową w perspektywie kilku miesięcy mi się uda wyjechać czy nie. Głównie w takich momentach się nad tym zastanawiam.

**Co najbardziej zaprząta twoją uwagę przy myśleniu o przyszłości?**

Najczęściej są to takie przyziemne rzeczy, jak wakacje. Aczkolwiek też czasem myślę o kwestii, związanej z kontaktami rodzinnymi, które są teraz mocno utrudnione. Że fajnie by było wrócić do sytuacji sprzed epidemii, kiedy nie trzeba było się niczego obawiać.

**Jak dalej potoczy się sytuacja?**

Podejrzewam, że pomału będą zdejmowane kolejne ograniczenia. O ile oczywiście nie okaże się nagle w perspektywie najbliższych kilku tygodni, że po zdjęciu tych pierwszych ograniczeń, nagle jest lawina zachorowań i nagle nasz cały system służby zdrowia upada i wtedy trzeba znów będzie wrócić do punktu wyjścia i wprowadzenia tych restrykcji. Jeśli do takiego załamania nie dojdzie, to myślę, że powoli będą zdejmowane te ograniczenia.

**To są twoje obawy, że tak mogłoby się zadziać?**

Wydaję mi się, że jest takie prawdopodobieństwo. Raz w związku z otwieraniem galerii handlowych, a dwa, wydaję mi się, że jeszcze większa liczba zakażeń może się wiązać z otwarciem przedszkoli. Dzieciom się nie wytłumaczy, że mają się nie bawić, nie zbliżać do siebie - to jest oczywiste. Nie ma też mowy o jakichkolwiek rękawiczkach czy maseczkach w przypadku małych dzieci. A one, nawet jak będą przechodzić koronawirusa bezobjawowo czy podobnie do innych infekcji, to te dzieci mogą bardzo szybko pozarażać całą rodzinę i to może sprzyjać bardzo szybkiemu rozprzestrzenianiu się nawrotów wirusa. Trzeba pamiętać, że liczba zachorowań nie maleje, a jednak cały czas narasta.

**Czego się najbardziej obawiasz?**

W krótkoterminowej perspektywie, to przede wszystkim jakieś takie po prostu mogą być niedogodności dla nas, związane z jakimiś ograniczeniami wolności czy swobodami obywatelskimi. W dłuższej perspektywie, to oczywiście jeszcze większe pogłębienie recesji gospodarczej i to może być taki skutek, który się będzie ciągnął przez kolejne lata i będzie widoczny we wszystkich gałęziach gospodarki.

**Myślisz, że wtedy to też wpłynie na ciebie bezpośrednio?**

W jakiś sposób na pewno na mnie wpłynie. Nawet, jeśli nie wpłynie na moje zatrudnienie, to może wpłynąć na moje oszczędności, na wartość mojego mieszkania, która może zmaleć. Trudno teraz przewidywać, jak to może wpłynąć, bo spektrum tych efektów, może być niezwykle szerokie.

**W całej tej sytuacji czujesz, że masz kontrolę nad tym, co się dzieje czy jest brak kontroli?**

Wydaje mi się, że ja jako jednostka, to nie mam nad tą sytuacją najmniejszej kontroli. I mogę tylko się spróbować dostosowywać do tej rzeczywistości panującej i starać się jakoś trwać w tym.

**Ten brak kontroli ci przeszkadza?**

Nie wiem, czy przeszkadza, bo chyba nigdy nie aspirowałem do sprawowania jakiejkolwiek kontroli, związanej z zarządzaniem państwem. Aczkolwiek jest to po prostu dla mnie osobiście męczące, że jest taka sytuacja, a nie inna.

**Miałam na myśli kontrolę nad tym, co ty możesz w danej sytuacji.**

Na pewno trochę mi przeszkadza, że nie mogę w tym momencie, np. wyjechać na majówkę ze znajomymi czy w wakacje nie będę mógł pojechać na żaden festiwal, czy przy okazji świąt nie spotkać się z rodziną. Więc to na pewno jest męczące, irytujące.

**Ale nie jest bardzo przytłaczające?**

Nie. Przytłaczającym bym tego nie nazwał.
