# Supplementary material for: The challenges arising from the COVID-19 pandemic and the way people deal with them. A qualitative longitudinal study
Source: PLoS One. 2021 Oct 11;16(10):e0258133. doi: 10.1371/journal.pone.0258133 (PMC8504766; doi:10.1371/journal.pone.0258133)
Supplement: S1 Dataset — (ZIP) [file pone.0258133.s003.zip › Transcriptions/stage 4/11.4_M_35_couple, with child.docx]

**11.4_M_35_coule with child**

**Co tam u was było na majóweczce?**

A nic, nudziliśmy się. Żadna majóweczka. tak samo, jak żadne Święta Wielkanocne, to żadna majóweczka. W ogóle żadna majóweczka. Kiedy było ładnie? Sobota chyba?

**To zależy, gdzie byłeś.**

Byłem w Warszawie i teraz też jestem w Warszawie. Można powiedzieć, że jesteśmy w świecie teraz. Pokłóciłem się z teściem i wróciłem do Warszawy. Powiedziałem, że mam w dupie tam siedzieć.

**Ale nie mieszkacie z teściem?**

Nie, ale się z nim pokłóciłem i po co ja tam będę siedział, jak mogę siedzieć tutaj? I już, i przyjechałem.

**Wszyscy przyjechaliście?**

Nie, no wszyscy. Ostatnio ustaliliśmy, że jestem tyranem.

**Co robiliście przez ostatnie 2 tygodnie?**

Dopóki tam byliśmy, to standardowo cały czas. Zajęliśmy się stacją, a teraz ja działam z powrotem w swoim marketingu, zrobiłem kilka stron internetowych, które były zaległe no i tyle. Czekamy tylko na decyzję, kiedy Marek do szkoły wróci. Wczoraj czy przedwczoraj rozmawiałem już z osobą, która coś na ten temat może powiedzieć. Jakby wewnątrz problemu jest i faktycznie się szykują, żeby po 24 to wszystko ruszyło z powrotem, ale są tam jakieś kłopoty. Jeśli otworzą je, to muszą dzieciaki chodzić, natomiast robili badanie i jest cześć rodziców, która nie chce puszczać dzieci do szkoły. Mi się wydaje, że jak już szkoły otworzą, otworzą wszystkie firmy, to myślę, że to automatycznie już idziemy do pracy normalnie i dzieciaki do szkoły. To będzie chyba wymuszone. Marek jeździ do Jedlińska czasem z mamą, czasem jest tu i tyle. Trochę jest inaczej, bo jak jesteśmy w Warszawie, to trochę inaczej spoglądamy, jest jakby większa komunikacja międzyludzka i więcej się widzi. Faktycznie, jak byśmy mieli tu siedzieć te 6 czy 8 tygodni, to można doła złapać.

**Czemu?**

My sobie poszliśmy na spacer przedwczoraj...W Galerii byli wczoraj. jak byliśmy na spacerze i byłą ładna pogoda, to troszkę inaczej jest. Fajnie, bo ludzi dużo jest w parku i widać, że tam ludzie uciekli, chodzą w tych maskach faktycznie. fajne jest to, że więcej ojców się chyba teraz zajmuje dzieciakami, więcej ojców spędza z nimi czas. Zazwyczaj było tak, że to mamy się widziało z dziećmi, a teraz będąc w parku widziałem, że to jednak ci ojcowie...Jest ich przeważająca większość, więc chyba fajnie. Nie wiem czym to jest spowodowane, bo jak jest parę dzieciaków w domu, to pewnie też jest to rozbite, ale to widać i fajne to jest. Poszliśmy sobie do naszej miejscówki, gdzie często chodzimy sobie na pizzę i piwo. Dobra, idę z nimi pogadać, żeby nam otworzyli tak dla nas, żebyśmy sobie usiedli. Jest trochę inaczej. Ci ludzie są jednak pochowani...

**Otworzyli wam?**

Nie, bo 5000 kary to jest dużo jak za takie coś. Z tego, co słyszę, to jednak policjanci zwracają na to uwagę. Byliśmy gdzieś tam na kebabie, usiedliśmy sobie na zewnątrz przy stolikach...Jakaś para czekała też na kebaba, zaczęli jeść tego kebaba i właściciel podszedł, i poprosił, żeby wyszli, bo niestety są takie a nie inne kary i faktycznie są te lokale sprawdzane z tych wytycznych. Dół? Bo dół, bo jednak jest pozamykane. Pojechaliśmy do Galerii, bo Marek telefon rozwalił, a ja jestem osobą, która lubi dotknąć zanim kupi, więc pojechaliśmy do Galerii. Ja jestem w szoku. Myślałem, że będzie zupełnie inny efekt, a tu jednak powiem ci, że chyba ludzie są przerażeni. Chyba faktycznie ten lęk i ten strach coś jednak w ludziach pozostawił, bo nie było boomu. To jest porażka.

**Nie było ludzi?**

Nie. Fakt faktem, że z punktu klienta, mając jakieś biznesy albo będąc w biznesie, patrząc na te sklepy w tych marketach...Oni nie wiedzą, co mają robić. To widać. Albo część jest zamknięta, część się w ogóle nie opłaca. Komputronik są pozamykane nadal, bo przy tej sprzedaży, którą mieli normalnie, to pewnie im się teraz nie opłaca otwierać sklepu. Sklepy z ciuchami nie wchodzą w nowy sezon z ciuchami jeszcze i mają tylko wyprzedaże. Ewidentnie widać, że przedsiębiorcy jeszcze nie wiedzą jak się mają zachowywać. Nic nie jest pewne. Sklepy z elektroniką użytkową? Daj sobie spokój, nawet nie ma sensu jeździć. Tylko to, co mają na półkach mają do sprzedania a wszytko, reszta jest w głównych magazynach. W momencie zamknięcia galerii i tych kompleksów, to wszystkie wartościowe rzeczy były z powrotem wysyłane do central i nie ma co kupić. Zostaje ci de facto ten internet cały czas. Jest to takie przybijające. Doła z tego powodu nie mam, natomiast już będąc tutaj zaczyna się widzieć więcej tych problemów, które faktycznie są związane z tym koronawirusem niż będąc sielsko sobie na wsi i zajmując się po prostu codziennością i działalnością. To zupełnie inny świat naprawdę. Jest mega różnica.

**Te jednodniowe wizyty nie pokazywały aż tak tej różnicy?**

Nie.

**Co jeszcze cię uderzyło i zdołowało trochę?**

Ja cały czas w tego całego koronawirusa nie wierzę ogólnie. Może nie w to, że go nie ma, tylko nie wierzę w tak wielki wyolbrzymiony jego skutek i efekt. Nawet mogę co wczorajszą statystykę podać, bo cały czas w Polsce się mówi o sztukach osób, a tutaj dają procentowo i trzeba dopiero to sobie przeliczać, więc śmiech na sali, bo my jesteśmy na koronawirusie 5%, a grypa to 8%, a o grypie nic nie mówią. Gdzieś cały czas mi się to wszystko nie skleja, tym bardziej, że już wiemy, że za granicą niektóre rządy zablokowały firmy albo wspierają firmy, które bankrutują, te które są na giełdzie, albo nie pozwalają innym firmom wykupywać tych firm. To jest bardzo mądre posunięcie, bo nie wiedzą czy np. to...Cały czas chyba też nie do końca mają wiarę w to, co się dzieje, bo wiadomo, że to jest naturalne w biznesie, że tak miało być, natomiast to znaczy, że oprócz tego, że ja wierzę w jakieś kwestie…Jak to się mówi...Ogólnie, że to nie do końca jest prawda. Same ruchy rządów państw, które zaczynają pompować własne pieniądze w firmy, które bankrutują, co w teorii nie ma sensu, oznacza, że nie do końca wiedzą, co tak dokładnie jest z tym koronawirusem. Wracając do doła...Dołujące jest mimo wszystko to, że ja nie będąc tutaj i nie myśląc o tym, złapałem się na sytuacji, że podszedłem...My lubimy, jak idziemy na spacer pójść sobie pod jakieś Veturilo, wziąć rowerek i przejechać kawałek. Ostatnio wyciągnąłem rower Marka, bo chciałem, żeby też trochę pojeździł swoim, podszedłem do stacji Veturilo, bo nie mam pompki do roweru, kurde, i wiesz, że rękaw zakładałem na rękę, żeby wziąć pompkę do ręki. Tak sobie myślę, że Boże, co się ze mną stało? To też gdzieś tam jednak w głowie, nawet w takiej mojej głowie, gdzie śmieję się z tego wszystkiego, to też jest taka...Nieufność? Powiem ci więcej. Byłem w jakimś sklepie małym i ja kaszlę bardzo, bo ja kaszlę od papierosów. Ilość papierosów przepalanych przeze mnie w dniach, kiedy jestem zły albo coś się dzieje nie tak, to jestem w stanie wypalić 2 paczki. Ten kaszel palacza czasem mi się gdzieś odezwie i powiem ci, że jak się tak kaszlnie, fakt faktem, że w tych maskach nieszczęsnych, ale jak się kaszlnie, to ci ludzie patrzą na ciebie.

**Jak patrzą?**

To jest lęk? brak takiego zaufania? Nie wiem, ale to jest widoczne.

**A jak myślisz, co oni sobie pomyśleli?**

Nic nie pomyśleli, bo ja zawszę wtedy mówię: ten cholerny koronawirus. Także nie wiem, co sobie pomyśleli. Jedni pewnie poszli do domu i zaczęli bluźnić na mnie, drudzy...Nie wiem. Zaczyna być to dziwne, tak? Na ludzi jednak bardzo mocno gdzieś tam to wpłynęło. To nie tylko się o tym mówiło, ale to były jakieś konkretne podejmowane kroki zmieniające życie, biorąc pod uwagę dzieciaki nie do szkół, firmy zwalniające, więc piętno jest duże tego naszego koronawirusa.

**Co jeszcze cię uderzyło po powrocie?**

Cały czas się zastanawialiśmy będąc tam, że to wszystko jest takie napompowane, ale tu jak jestem, to...O sobie nie myślę, myślę o tym co mnie otacza, jeśli mówię o koronawirusie i jest to dziwne. Pozamykane, te ulice są mimo wszystko puściejsze. To jest smutne takie ogólnie. Nie ma takiego życia jak było. Nie byłem na Nowym Świecie i nie wiem, jak to wygląda, ale podejrzewam, że przeszedłbym przez Nowy Świat i dół - wszystko zamknięte, wszystkie lokale. Ja mam też taką Café, do której lubię często chodzić na kawę rano i ona jest taka zawsze w światełka przybrana, tak zawsze jest przyjemnie, jak patrzysz na nią. To jest w takim betonowym naszym świecie warszawskim coś przyjemnego, a tu wiesz, nic, nie ma życia.

**Zamknięte?**

Tak.

**A moja kawiarnia otwarta.**

I normalnie można tam chodzić?

**Można na wynos wziąć. Chciałbyś, żeby otworzyli się chociaż na wynos?**

Nawet nie wiem, czy nie mają na wynos. Nie wiem, nie sprawdzałem. Miasto po prostu nie żyje, nawet nie wiem, czy żyje w 50% i jeśli miałbym wybierać życie w takim mieście a życie na wsi, to bym wybrał życie na wsi. To miasto nie jest miastem w prawdziwym tego słowa znaczeniu. Czuję się jakbym był gdzieś w mieście daleko na Śląsku albo daleko pod ukraińską granicą - miasto, które upada. Jak jakiś Białogard Szczeciński czy inne dziury, w których byłem i oprócz tego, że mają "miasto" w swoim statucie, to nie wygląda to jak miasto. Nie ma nic, to miasto nie funkcjonuje w 40%. Podejrzewam, że jak jeszcze poszedłbym na Nowe Miasto, przeszedł się wzdłuż Wisły...Chociaż nie wiem, jak jest na bulwarach. Pozwalają spacerować?

**Nie wiem.**

No, ale jak przeszedłbym po tych restauracjach wieczorem, wszystko pozamykane, to człowiek by po prostu...Shit, po co tu siedzieć?

**Jak ty się czujesz, jak jesteś teraz w takim mieście?**

Ja to się zajebiście czuję, bo to wynika też z mojego charakteru, że pokłóciłem się z teściem i pierwsze to odpływam tutaj i nie będę nic tam robił. Teraz to ja funkcjonuję na swoim charakterze i swojej upartości. Ale powiem ci, że to nie jest to miasto, do którego ja jestem przyzwyczajony. Jak przyjechałem ze wsi kilkanaście lat temu do tego miasta, to miasto mi się podobało, bo mogłem zrobić, kiedy i co chcę, o której godzinie chcę i z kim chcę, nikt mnie na tym nie napiętnował. To miasto to nie jest to miasto, które ja znam.

**Teraz byś do niego nie przyjechał?**

Teraz dużo większym komfortem jest mieszkanie na wsi. Zajebiście jest mieć dom na wsi w takiej sytuacji i móc do takiego domu na wsi sobie wyjeżdżać. Spakować się, zamknąć drzwi w bloku i sobie tam żyć i mieszkać.

**Jak sobie teraz radzicie, kiedy jesteście w mieszkaniu, nie macie ogrodu, nie możecie Marka wypuścić przed blok?**

Chciałbym tak zrobić...Dalej się pracuje, więc dalej rodzina wyjeżdża, a z Markiem 2 dni temu byliśmy na spacerze. Chodzi się w tych maskach przeklętych i siedzimy w domu.

**Jak wyglądał dzisiaj wasz dzień?**

Ja spałem do 15, bo o 5 chyba poszedłem spać. Rano tylko wstałem i zjedliśmy śniadanie razem, i poszedłem dalej spać. Ewelinka zrobiła śniadanie, więc zjadłem parę łyżek i poszedłem spać dalej. Pojechała na stację, a ja spałem dalej. Obudziłem się w momencie, jak Ewelinka przyjechała. Marek był z nią.

**Zdarza się, że Marek zostaje z tobą w Warszawie?**

Tak.

**I co wtedy robicie?**

Nie wiem, co robimy. Różne chyba rzeczy. Na pewno nie czytam z nim książek.

**Czego jeszcze nie robisz z nim?**

Może tak będzie łatwiej...Lekcje mi się zdarza z nim odrabiać, ale to jest bardzo rzadko, bo on nie chce ze mną odrabiać i ty już wiesz, dlaczego nie chce...

**Tak, jesteś tyranem, pamiętam.**

Nie wiem, gramy, robimy sobie tortilki i tak...Nic specjalnego.

**Czy to jest dla ciebie ważne, żeby ta szkoła wróciła?**

Dla mnie osobiście nie, ale powrót do szkoły, to zaraz zacznie funkcjonować to w miarę wszystko normalnie. Będzie wracało do normalności, więc to by było dobre. Ministerstwo zastanawia się czy w maskach, czy nie w maskach, czy w szkołach te płyny dezynfekujące. Jest bardzo od groma takich jeszcze pytań i nie wiem, jak to rozwiązać. Nie mam pojęcia, ale ja uważam, że niech wracają i jeśli ten koronawirus jest naprawdę i jest naprawdę typem ciężkim, to i tak będzie drugi rzut choroby. Jeśli okaże się, że wszystko wróci do normy i to będzie na poziomie, który jest albo na poziomie grypy, tzn., że to po prostu jest kolejna mutacja grypy. No ale niech to wszystko funkcjonuje z powrotem. Ostatnio powiedziałaś, że ty masz gorzej, jeśli chodzi o sprawy służbowe. Do nas to nie docierało, bo nam jest dobrze. Mieliśmy świadomość, że taka sytuacja jest, natomiast teraz poszliśmy na spacer, chodzimy wokół tych małych lokali i ludzi, których znamy i wszystko jest pozamykane. Jest jedna dziewczyna, która ma 60 m wynajęte i nie prowadzi tej działalności, 3 lokale dalej kolejna taka sytuacja. Tak idzie ten człowiek chodnikiem i się zastanawia, że jednak trochę tych ludzi ma kłopoty. To są pewnie dla niektórych życiowe problemy, już tak mocno. To jest kolejny taki "dół", że jak się idzie tymi osiedlami, które znamy zawsze stąd, że to tętniło życiem, że te lokale pootwierane były na oścież, żeby wchodźcie i tylko zostawiajcie pieniądze, a tu tego nie ma. I teraz też zastanawiasz się nad tymi ludźmi, tak?

**Zobaczyłeś to dopiero w Warszawie?**

Tak, taka jest prawda. To dopiero do mnie teraz dotarło. Cały czas to było gdzieś obok nas, tym bardziej, że my naprawdę na tym skorzystaliśmy. I są też firmy, które na koronawirusie skorzystały. Mam znajomego, który kolejny lokal chce kupić, bo jedni bankrutują a on poszedł mocno w wysyłkę i jest taki rozwój, że jest w stanie w tej dobie...I remontuje, zaczął remontować. Jest grubo, tylko pewnie większość firm dostało jednak po tyłku. Jak jesteś na wsi, robi ci się obrót, wszystko jest zajebiście, jeszcze większa sprzedaż...Dostosowujesz się oczywiście do tych nakazów rządu, ale wszystko ci jest ok, natomiast tu przyjeżdżasz, idziesz na ten spacer...Kurde, faktycznie nie ma tego życia. Gdzie są ci ludzie, te lokale?  Mamy takiego fryzjera, nie naszego, ale z naszych rejonów pochodzi. Dobrze strzyże, wiecznie miał kolejki, wiecznie trzeba było na telefon i nie można się było do niego dostać z ulicy. Chłopak ma wynajęte, sam strzygł i zamknięte, i smutno, bo naprawdę robił kokosy, a teraz 10 tysięcy miesięcznie, to jest drugi miesiąc, więc już 20 tysięcy. Przecież nie każdy ma taki komfort, żeby pieniądze sobie odkładać, nawet jak ma firmę. Nie oszukujmy się, myślę, że większość nie ma takiego komfortu. To jest tragedia, to na pewno są tragedie w domach i jeszcze najgorsze jest to, że przez to, że siedzą ci ludzie razem w domach może się to przekładać na relacje między nimi, bo oboje zaczynają być zestresowani, jeśli chodzi o finanse. Ogólnie nie jest tak ładnie, kolorowo i ciekawie, jak to u nas na wsi. Ale o takich rzeczach się pomyślało faktycznie dopiero, kiedy się przyjechało, przeszło się, zobaczyło się, jaki świat nie jest kolorowy, co daje jedną konkluzję - żyj, pracuj całe życie w mieście, na starość kup sobie domek na wsi.

**To jest takie przygnębienie u ciebie, jak zobaczyłeś to wszystko?**

Nie tyle przygnębienie, co realia. Zobaczyłem, jakie są realia spowodowane koronawirusem i my tego nie odczuwaliśmy na wsi. U nas było sielsko i jak do nas dzwonili, to byli w szoku, że my jesteśmy tacy "ho, ho, ho"

**Teraz już ci nie jest sielsko?**

Nie jest.

**To jak jest teraz?**

Odczuwam piętno koronawirusa, tak. Tak to mogę nazwać. Wychodzę na balkon wieczorami, no to jest fajnie, bo widać, że sąsiedzi, którzy się dawniej pewnie nawet nie znali, to zaczynają gadać między blokami. Jest to przyjemne, natomiast od 8 rano do 17-18 ta Warszawa nie żyje tym życiem, którym powinna żyć. Jest to przygnębiające. Może to jest dobre też słowo. Na pewno dotarła do nas skala tego problemu i zaczął się człowiek zastanawiać nad tymi osobami i nad tym, co się dzieje i jakie to miało znaczenie.

**Co teraz jest dla ciebie największym wyzwaniem?**

Nie mam żadnych wyzwań. Jestem tylko nieogarnięty i wychodzę czasem bez maski. No tak, wychodzę czasem bez maski do sklepu. Nie jestem typem, żeby ktoś czy coś mógł mnie...Ubezwłasnowolnić? Zastopować? Nie wiem.

**A Marek jak się odnajduje w Warszawie?**

On jest teraz w normalnym świecie - internet. Tam to 50 razy musiał się łączyć ze światem w ciągu dnia. Poza tym u niego nie ma takiej skali różnorodności, bo jeśli wychodził na dwór na wsi, to tylko wychodził po to, żeby nas zawołać, a nie po to, żeby się samemu pobawić. Jest na etapie takim, że dajcie mi Play Station, telewizor i telefon komórkowy i może zapomnieć, że jest jakaś epidemia. On tego nie odczuwa aż tak bardzo, tym bardziej, że nawet na wsi nie miał roweru swojego. Tutaj mamy swoją przechowalnię i ma swój rower, to mu go wyciągnąłem, no ale powietrza akurat nie było i muszę kupić pompkę.

**Kolejna wyprawa do sklepu cię czeka?**

Ogólnie wczoraj byliśmy w sklepie, ale to szukaliśmy laptopa dla niego i pamiętałem, żeby kupić, ale gdzieś tam w tym czasie wyleciało z głowy. Trzeba jechać i kupić po prostu.

**A czemu laptop? Telefon kupiliście w końcu?**

Tak, kupiliśmy. To była 5-ta galeria, w której mieli 2 sztuki. Laptop, bo jakiś tam pomysł na prezent był taki, że już by mu się przydał. Już wiesz, mają te swoje konta w tych szkołach, mają swoje własne aplikacje, mogą się sami komunikować ze sobą, to dobrze by było, żeby już to robił na swoim, a już nie zabierał czyjegoś. Będzie miał tam wszystko zalogowane, będzie się z automatu ze wszystkimi łączył bez żadnych problemów. Tym bardziej, że to połączyliśmy z prezentem, bo miał urodziny. No ale powiem ci, że jest porażka, z elektroniką jest porażka. Telefon na szczęście udało nam się kupić, bo znaleźliśmy gdzieś tę sztukę i jeszcze nawet szukaliśmy poza Warszawą, bo wyobraź sobie, że w Warszawie nie było. W Babicach przy Kampinosie było więcej niż jedna sztuka. W ogóle iść na zakupy się nie opłaca. Jeszcze żyjemy internetem, zajebiście to funkcjonuje podobno, rozwój jest ogromny.

**A jedzeniowe zakupy jakieś robicie czy przywozicie ze stacji?**

I tak, i tak. Nie chodzimy po jakichś marketach. My chodzimy na ogół to takich małych sklepików lokalnych.

**Jak tam sytuacja wygląda. Widać tego koronawirusa?**

Pierwszy raz byłem w sklepie od 6-7 tygodni. W moim przypadku zmiana była taka zmiana, że "przyjmiecie mnie bez maski? Dobra, niech pan wchodzi."  Dwa, to były zmiany w kasach. Tylko tyle, że pozmieniało się, jeśli chodzi o kasy - inny rozstaw, uzbrojone dziewczyny w pleksy nie w pleksy, brak komfortu wykładania pieniędzy dla mnie, bo wszystko za szybą, więc tylko to. Ja na nic więcej nie zwracałem uwagi. Byłem przestraszony, bo wyszedłem bez maski i nie wiedziałem czy mnie gdziekolwiek wpuszczą, a że jestem z natury leniwy, to mi się nie chciało wrócić do domu po tę maskę. Wolałem być wyganiany ze sklepów i iść do tego momentu, aż mnie przyjmą w którymś niż wrócić do domu.

**Czujesz taką presję, żeby chodzić w masce po ulicy?**

Nie czuję presji, ale jeśli wszyscy się do czegoś zobowiązują, to chodźmy.

**Ale łaziłeś bez maski?**

A tam, na dół do sklepiku i z powrotem. Presji nie czuję. Po prostu zapominam o tym, nawet przez 2 dni nie miałem swojej maski, która mi już dobrze leży na twarzy i mi nie dokucza. Miałem nową jakąś maskę, Ewelinka mi dała, bo są 3 takie rezerwowe i tragedia, uszy mnie już bolały. Próbowałem trzymać maskę...Nieee. Z tymi maskami to jest...

**Te maski przeżywasz jakoś, poprzednio też przeżywałeś.**

Ja nie lubię rzeczy, które ograniczają i masek też.

**Jeszcze jakieś plany zakupowe macie?**

Nie mamy planów zakupowych.

**A te dresy do chodzenia po domu?**

Kupiłem przez internet. Jak chodziliśmy za elektroniką, to ja się przyglądałem w formie jakby biznesu. Przyglądałem się na te sklepy odzieżowe duże. Zaczyna Reserved wypowiadać umowy, Empik też zaczyna wypowiadać umowy galeriom, więc byłem ciekawy jak to wygląda na dzień dzisiejszy i powiem ci, że pomimo tego, że otworzyli te galerie, to te wielkie sklepy dalej będą miały ogromne straty. Pracowników dalej, nie wiem, dlaczego, to dla mnie było śmieszne, że wchodzimy do Xcom'u, pracowników jest dalej 11, a 3 ludzi i oni liczą ilu ludzi ma wejść. To trochę zaczyna być głupie, tak? To dalej będą mega straty dla tych firm. Do ciuchowych nie wchodziłem, ale powiem ci, dlaczego - część albo zamkniętych i chyba tylko Zara nie miał plakatów z wyprzedażami. Nie wiem, dlaczego. Czy faktycznie mają już nowe ciuchy z nowym sezonem? Ale, to by się reklamowali, a tu nic. Jest stagnacja, stagnacja i nie wiadomo co jutro będzie. Chyba kupowanie ciuchów w sklepie, żeby iść to będzie wtopą, bo nie kupisz świeżych ciuchów, jeśli chodzi o modę i oni chyba mają tylko to, co im zostało i koniec. I oni chyba dalej nie zamawiają, nie produkują. Nie jest ciekawie.

**Dobrze, że rząd otworzył te galerie?**

Nie wiem, czy był czas na zamknięcie galerii, nie wiem, kiedy był czas na otwarcie galerii. Pomijając fakt, że dla mnie to robią to stricte pod wybory, to też z drugiej strony jak patrzę na tę sytuację w galeriach, to chyba dobrze, że to otworzyli. Ja się bałem, że to pójdzie moim tokiem myślenia - że otworzą te galerie i będzie boom. Pewnie to było związane z tym, że ja dalej sobie żyłem sielsko na tej wsi i nie widziałem skali problemu, skali sytuacji. Nam było dobrze albo jeszcze lepiej niż normalnie. Będąc tu nie zmieniło się moje podejście, natomiast skalę widzę, widzę jakie będą kłopoty i jakie pewnie są teraz kłopoty w domach, w niektórych pewnie i tragedie...Wtedy myślałem, że otwierają galerie i jest boom, bo idioci się rzucą na to, a teraz otworzyli galerie i jest dalej to samo. Galerie mogłyby być zamknięte i mogą być otwarte, i nie ma tej różnicy. Tych osób naprawdę jest do policzenia na jednej ręce.

**A widziałeś, co się przy Ikeach działo?**

Nie. Pewnie dużo osób, tak?

**Podobno kolejka była wokół budynku.**

Myślę, że to jest spowodowane tym, że ludzie siedzą w domach, bo identyczna sytuacja jest z wszystkimi sklepami budowlanymi. Kolejki są, taśmy są, ja nawet byłem w szoku. Taśmy są ponaklejane na parkingach, gdzie ludzie mogą stać. To są kolejki na 200-300 m. Ludzie siedząc w domach faktycznie pewnie robią remonty sobie, kupują meble, robią sobie ogrody. Chcą coś zrobić, wykorzystać ten czas. Tych facetów jest chyba więcej niepracujących teraz. Można porównać, ile jest panów pracujących na kasach a ile pań, więc dlatego tych panów jest teraz więcej z dzieciakami i ze względu na to, że tych mężczyzn jest trochę więcej teraz w domach, to ich kobiety ganiają do tych remontów. Byliśmy wczoraj w Wola Parku. Galeria pusta prawie, a tam kolejki i to kolejki takie, że ludzie stoją. Nie wiem, co mam ci powiedzieć. Czy to dobrze? Myślę, że dobrze.

**A czemu?**

Wracamy do jakiegoś normalnego życia. Ja nie wiem, jak jest z tymi małymi sklepikami i ja nie wiem, czy one były otwarte cały czas mimo wszystko, czy galerie były całe zamknięte na klucz. Jak to było? Ja nie mam pojęcia.

**Były zamknięte całe.**

No to dobrze chociażby dla tych małych kiosków, dla tych działalności jednoosobowych, które najczęściej są we franczyzie, mega uzależnieni od swojego franczyzodawcy, więc to chociażby dla nich dobrze, aczkolwiek musiałbym przeliczyć moje siedzenie tam 12 godzin, czy pracownika + prąd na ilość osób. która przyjdzie do mojego sklepu teraz. Czytałem w jednym z takich magazynów online - to jest Business Insider albo NY Business, to są 2, które lubię i naprawdę z ich obliczeń wynika, że jest szansa, że z powrotem dzięki temu do łask wrócą te małe sklepiki na osiedlach. I teraz dla sklepów typu mała Żabka to jest maga wzrost. To są sklepy, które teraz przegoniły wszystkich w łeb, jeśli chodzi o sprzedaż szybkiej żywności. Był taki okres, że zaczęły się zamykać sklepiki osiedlowe i to postawili normalni ludzie, którzy mieli swoje sklepy i podeszli do tego biznesowo. Francja trochę im pomogła, bo jak zwykle kapitał musiał być zewnętrzny, żeby takie pieniądze zebrać i to zaczyna wygrywać.

**Jeszcze chciałam chwilę o usługach fryzjerskich...**

Wczoraj słyszałem, że fryzjerzy są nieczynni, ale fryzjerzy dla zwierząt są czynni. Usługi fryzjerskie dla zwierząt są. Kuriozalne, bo z tego co czytałem kiedyś tam, to podobno szczury, koty i psy roznoszą koronawirusa.

**Mówiłeś, że by ci się fryzjer przydał. Byłeś?**

Nie. Ewelinka mi proponowała jakiegoś na telefon. Chrzanię to kurde. I tak jestem brzydki i nie muszę być ładny. Mężczyzna powinien być tylko ładniejszy od diabła.

**Ewelina robi paznokcie? Była 2 raz?**

Powiem najbardziej trafną odpowiedź - nic mi o tym nie wiadomo. Ale nie sądzę, bo bym pewnie zauważył. Myślę, że ja jestem akurat takim typem, że niestety, ale widzę takie rzeczy.

**Odwiedziła was już pani do sprzątania?**

Nie, nie dzwoniliśmy. Wczoraj chyba nawet rozmawialiśmy o niej, że trzeba by ją wziąć faktycznie, ale chyba nawet nie dzwoniliśmy.

**Nie przeszkadza wam teraz, że musicie sami sprzątać?**

Ja ogólnie lubię porządek, więc dla mnie nie ma problemu posprzątać. Lubię też sprzątanie, bo można się troszeczkę odstresować. Tak samo lubię pracować w ogrodzie. Ostatnio co prawda dość szybko mi się motywacja kończy, bo dochodzę do odkurzania i...Nie lubię odkurzać chyba albo muszę sobie kupić jakiś fajny sprzęt do odkurzania, bo nie lubię odkurzać takim odkurzaczem.

**Może taki, który sam odkurza?**

A oglądałem przedwczoraj i stwierdziłem, że przy naszym bałaganie to on będzie miał dużo kłopotów, ale to jest dobry pomysł na mieszkanie. Do domu bym tego na pewno nie kupił, bo musiałbym chyba z 10 mieć, bo dom mamy obszerny, natomiast tu...Jak bym miał mieć psa tu w domu, to pierwsze co bym zrobił, to bym kupił ten sprzęt.

**Łatwość wydawania pieniędzy - skala**

5, bo ja czasem tak [gwiżdże], a czasem jestem sknera. Ja nie lubię, nie lubię, nie lubię, natomiast jak coś idę kupić, to lubię kupować mądrze i kupuję rzeczy drogie z natury, np., nie wiem, jak to wytłumaczyć. Ja nie lubię kupować 2 razy tego samego.

**Co to znaczy mądrze kupować?**

Jak np. mam śrubokręt do kupienia. Standardowy człowiek idzie do Brickman'a i kupuje śrubokręt, który kosztuje 10 zł. Ja kupując śrubokręt idę do sklepu profesjonalnego i kupuję go za 90 zł, gdzie, kurwa, 90% ludzi nie kupiłaby tego za Chiny. Tylko że ja ten śrubokręt mam podpisane, że jak mi się ułamie, czy coś, to ja mam dożywotnią gwarancję i dla mnie to jest mądre wydawanie tych pieniędzy, aczkolwiek ktoś mi kiedyś zarzucił, że za tę kwotę ja bym miał 10 sztuk i zanim te moje 10 sztuk się wyłamie, to już minie trochę lat albo ja umrę. Dobrze, tylko ty masz opcję, że ci się na 99% wyłamie, a ja mam opcję, że mi się na 10% wyłamie. Ja nie lubię wydawać pieniędzy, ale jak już wydaję, to nie daj Boże. Ewelinka jest skłonna, żeby było, a ja, żeby to było cacy.

**Jakie są kategorie, gdzie nigdy się nie zgodzisz na tani zakup?**

Ogólnie nie lubię kupować rzeczy...To nie jest chorobą, że co drogie, to lepsze. Nie w ten sposób, bo ja lubię sprawdzać rzeczy, naprawdę lubię porównywać i czytać. To nie jest tak, że ja idę i wiem, że jak coś kosztuje 200 zł drożej to jest lepsze. To nie do końca tak jest. To też psychologia biznesu mówi, wręcz przeciwnie. Ja porównuję rzeczy, ja sprawdzam co mi daje taka funkcjonalność i dlaczego mam za to płacić takie pieniądze. Jest bardzo często tak, że jak ktoś mi mówi o sytuacji, dlaczego ja wolałem wydać 3 x więcej, to ja mu mówię, skąd się bierze ta cena i skąd taka moja decyzja. To nie jest tak, że stoją 2 rzeczy, jedna za 100, druga za 1000 i ja biorę tę za 1000, bo na pewno jest lepsza.

**A może jest kategoria produktów, w której aż tak bardzo się na tym nie skupiasz i możesz kupić coś tańszego bez porównywania?**

Mogę kupić tańszą ziemię do kwiatów. Wszystko porównuję, chemię do domu porównuję, wszystko, elektronikę. Jak ja stoję w sklepie, to ja już wiem, co kupić. Ja nienawidzę stać w sklepie.

**Gdzie ty to porównujesz?**

No tak, że jak siedzę sobie teraz z tobą tutaj, to teraz czytam i porównuję.

**A jak sprawdzasz tę chemię? W domu?**

Biorę droższe produkty i sprawdzam, jak się będą zachowywały, mam jakieś wybrane swoje lepsze produkty i wiem, że będzie mi pachniało ubranie 2 tygodnie, więc to jest dobre.

**A jak jedziecie na wakacje, to jakie wakacje lubisz? jak jest z wydawaniem pieniędzy na wakacje?**

My żyjemy na wakacjach tak samo, jak żyjemy w Warszawie. U nas wakacje to bardziej jest to, że spacerujemy, że jest mniej telefonów, jesteśmy w innym miejscu, możemy iść w góry, możemy iść na plażę. My nie idziemy, że kupujemy pamiątki czy coś.

**Gdzie byliście ostatnio na większych wakacjach?**

Nie wiem, nie pamiętam. Chyba w Karpaczu.

**Sami sobie szukacie hotelu, śpicie w agroturystyce?**

Tylko hotele. Niestety tak.

**Czemu niestety?**

Bo to kupę pieniędzy kosztuje.

**To można zmienić na coś tańszego?**

Ale my nie chcemy zmieniać, bo nie myślimy wtedy o podstawowych rzeczach i wtedy odpoczywamy. Jest skala różnic, bo czasem się spotykamy ze znajomymi czy z siostrami Eweliny i porównujemy ich wyjazd gdzieś tam i wydatki. Aż miło słucha się, ale my po prostu jedziemy i chcemy mieć wszystko w dupie, rozumiesz?  Nie myślimy o rzeczach codziennych.

**Czyli właściwie nie ma znaczenia, ile to kosztuje, byle było wygodnie i przyjemnie?**

To było może perfidne, ale prawdziwe. Tak to niestety na tę chwilę wygląda. Jak będziemy mieli 4-5 dzieci, to będziemy się nad tym zastanawiać. Może.

**Nazwałbyś siebie osobą oszczędną czy rozrzutną w codziennych wydatkach?**

Rozrzutną. Idę sobie np. nie mając planu coś kupić, chciałbym, zrobić, żeby się młody ucieszył, albo młody zasłużył, to dawaj młody, kupimy ci coś i coś kupuję. Nie mam takich hamulców. Ostatnio schodziliśmy z jakiejś góry, są takie budki, młody zobaczył jakąś zabawkę i Boże, po co kupować ten badziew. Pobawi się godzinę, rozwali i już. I nie kupujemy, ale np. pójdziemy gdzieś do sklepu z młodym i zobaczę, że jest rzecz, którą wiem, że się interesuję bądź lubi, a był dobry, to pójdę i mu będę kupował.

**Co ostatnio, poza tym, że telefon?**

Karty Pokemon, bo on jest zbieraczem i ja mu często kupuję, no teraz w tej dobie koronawirusa, to nieczęsto. Ostatnio to V-dolce. Do Play Station je kupujesz, żebyś mogła je później w tych grach wydawać. Wirtualne punkty i on sobie może kupować postaci, ubrania, ble, ble, ble. Mega świetny pomysł biznesowy, bo tam najmniejsze pieniądze, jakie wydajesz to jest 50 zł i wielokrotność tego, ale to też jest wydatek, nie oszukujmy się.

**Ty wiesz, ile wydajesz miesięcznie?**

Nie mam zielonego pojęcia. Przed koronawirusem się zastanawialiśmy, czy by tego nie obliczyć, bo można by było gdzieś coś zaoszczędzić. Z 2 miesiące temu zrobiłem nawet research naszych bezsensownych wydatków, bo co telefon, to nam pobierało z konta - a to Netflix, HBO, wszystkie te subskrypcje. Okazało się, że wszystkie te subskrypcje powielone iluśkrotnie. I powiem ci, że pousuwaliśmy sobie. Usiedliśmy z Eweliną, z wszystkich usunęliśmy, zrobiliśmy takie większe, żeby było na kilka urządzeń albo na kilka kont, ale w skali miesiąca to w naszym przypadku jest pewnie oszczędność 200-300 zł, bo jak mieliśmy subskrypcje na kilku telefonach czy telewizorach? I taka konsternacja, bo ja mam takie konto szybkiego wydawania pieniędzy, pod które mam podłączony telefon i wiem, że mam tam 100 zł. Idę do sklepu, kupuję coś za 100 zł., bo wiem, że mam. Podchodzę do kasy, pyk, pyk, nie ma. What's the fuck? A to jest takie konto właśnie do subskrypcji. Były takie momenty, więc zaczęliśmy liczyć. Kiedyś żeśmy usiedli i powiem ci, że pomyśleliśmy jak oszczędni ludzie, racjonalnie pomyśleliśmy i pousuwaliśmy te konta faktycznie. To było racjonalne myślenie w naszym przypadku, a jest to rzadkie.

**Dobrze wam z tym, jak funkcjonujecie finansowo?**

Bardzo nam dobrze jest, tylko czasem się pojawiają takie momenty...Wystarczyłoby np. w jednym roku nie pojechać do tych hoteli, do których jedziemy i mielibyśmy to albo tamto. To są tylko takie momenty, jak faktycznie nam się coś ubzdura, ale tak no to nie. Moja miłość jest jeszcze lepsza w tym wszystkim, bo jak się dorwie do pieniążków...Oj, mogłaby obkupić cały świat, zapominając nawet o sobie.

**Wam teraz jest lepiej finansowo?**

Tak. U nas by był rzeczywiście mega płacz i wtopa, jak by kazali zamknąć produkcję, nie produkcję, czy coś. Pewnie są teraz takie przypadki, że są jakieś tragedie życiowe, ale w naszym przypadku nie ma to żadnego znaczenia.

**Teraz siłą rzeczy górka rośnie, robicie coś z tym?**

My to na bieżąco wydajemy.

**Nie myśleliście, żeby coś odłożyć?**

Nie. My wzięliśmy automatycznie trochę modernizacji stacji benzynowej. W ogóle zmiana logistyczna, magazyn z tyłu, więc od razu te pieniądze skierowaliśmy w inne rzeczy. Ja też ma agencję reklamową i był taki motyw, żeby szybko postawić sklep internetowy, żeby zacząć się bawić w wysyłki pożywienia albo dostarczanie własnymi autami, ale to jakoś upadło. Na szczęście upadło, bo...Ale to był dobry czas, żeby zacząć. To nie jest tak, że to się gdzieś odkłada, tylko od razu puk, puk, puk, to modernizacja, to coś nowego. Od groma wydatków i to na bieżąco by można wydawać. Akurat to nie ma znaczenia. Jest po prostu lepiej, tak? Pozwoliło to nam x, y, z rzeczy zrobić.

**W ogóle macie poduszkę finansową? Trzeba oszczędzać i mieć jakieś oszczędności?**

Nie w tym wieku.

**A gdyby urwał wam się teraz dochód, to ile czasu moglibyście żyć?**

1 dzień.

**Żartujesz?**

Pół dnia, dobrze.

**Ale serio. Jesteście w stanie się utrzymać bez bieżącego dochodu?**

My jesteśmy ogólnie osobami obrotnymi, więc dalibyśmy radę bez większego problemu. Nawet siedząc i rozmawiając z tobą ja bym się w ogóle nie zastanawiał nad zabezpieczeniem swojej osoby, bardziej bym się zastanawiał nad zabezpieczeniem dzieciaka, tak? To już jest dobry motyw, żeby choćby kupić mieszkanie jedno, żeby zabezpieczyć Marka. Za 10 lat Marek będzie sobie mógł w nim mieszkać i iść na studia albo brać z czynszu na życie dla siebie. Za 10 lat, to by już było troszeczkę spłacone, więc takie rzeczy bardziej.

**Ty byś to mieszkanie kupił na kredyt teraz?**

To kwestia zastanowienia się. Ja nie jestem kredytowcem, wolałbym brać z innych rzeczy, żeby to były koszty moje. Kupować coś za gotówkę...Ja też jestem zdania, że jak bierzesz coś w leasing, to znaczy, że cię stać na kupno gotówką, bo w Polsce jest takie myślenie, że biorę coś w leasing, bo mi to koszty rozbije na raty, ale są sytuacje takie, że przychodzi koronawirus albo inna sytuacja, rozwiązuje z tobą firma kontrakt i nie masz pieniędzy na comiesięczną spłatę i jesteś w dupie i jeszcze ci bank robi koło w niej. Sorry, nakręcam się. Jak nie masz gotówki, żebyś mogła iść do sklepu i kupić tę rzecz, to lepiej nie brać tego w leasingu, bo zrobisz sobie jeszcze więcej biedy.

**Ale to mieszkanie, jak ty sobie wyobrażasz to mieszkanie? Da się je w leasing wziąć?**

Da się. Nawet w tamtym roku bracia nie mieli pieniędzy, żeby kupić mieszkanie - 10 lat mieszkali w mieszkaniu, facet chciał sprzedać i oni mieli pierwokup. Powiedziałem im, żeby zrobili to sobie w leasingu. Księgowa im powiedziała, że się nie da, udowodniłem im, że się da, dzisiaj mieszkają i są właścicielami. Na firmę. Wszystko się da, w Polsce żyjemy.

**Zdarzyła ci się kiedyś taka sytuacja w miesiącu, że nagle się kasa skończyła?**

Miałem taką sytuację i trzeba było sobie radzić. O moim życiu to można książki pisać. Miałem taką sytuację, wiem co to znaczy liczyć makaron na ileś dni następnych.

**Ale to nie skłania cię w żaden sposób, żeby mieć oszczędności?**

Nie skłania mnie. A po co mieć oszczędności? Nie lepiej inwestować? Nie nazywajmy oszczędnością tego, że mam w kieszeni te pieniądze. Ja wolałbym inwestować. Ileś pieniędzy się nie uda, bo inwestycje zazwyczaj się...Są ludzie, którzy mają nosa do inwestycji, inwestują i wszystko im się udaje, są ludzie, którzy inwestują i im się udaje część rzeczy i są ludzie, którzy inwestują i im się nic nie udaje. Nie wiem...Ja mam jakiś tam plan z Ewelinka i...Zrobić jeszcze parę rzeczy, które będą przynosiły...W planie będą przynosiły pieniądze i dla mnie to są oszczędności. No bo co ci da ta oszczędność? Do banku dać pieniądze na przecinek 1, przecinek coś? To jest żadna oszczędność. To wolę te pieniądze wydać. Można to wydać na bitcoiny i zarobić 10 razy tyle, albo stracić. Można iść na giełdę z tymi pieniędzmi, można w coś zainwestować. Nie uda się...Kupuję jakiś lokal, nie uda się, sprzedam, to zostanie mi chociaż z tego lokalu. Oszczędnością myślę, że jest inwestycja. I teraz tak - masz te oszczędności, coś ci się dzieje w życiu teraz powiedz mi, bierzesz z tych oszczędności pieniądze? Daj Panie Boże i tobie życzę, i wszystkim na świecie życzę, żeby mieli tyle oszczędności, żeby do końca życia im starczyło, natomiast te oszczędności się kurczą pewnie wszystkim, jakkolwiek by nie było, więc chyba takie inwestowanie pieniędzy...No dobra, umówmy się, warto mieć gdzieś w kieszenie gdzieś tam 20, 30 czy 100 tysięcy zł na wszelkie W. To ok. 20 tysięcy zł nie możemy nazywać oszczędnością, bo to nie jest oszczędność. To jest na czarny piątek.

**Na taką czarną, najczarniejszą godzinę, to jaka kwota powinna być, żebyś sobie myślał, że to jest ok. Ile ty musisz mieć?**

Żebym powiedział, że mam oszczędności, to chyba mi się wydaje, że zadaję sobie pytanie, za ile mogę postawić kolejny biznes. To jest minimum 100 tysięcy. to jest 200-300 tysięcy, żeby postawić jakiś normalnie funkcjonujący biznes. 100 tysięcy minimum to znaczy, że mam jakąś oszczędność na koncie. 20 tysięcy to jest właśnie na taki czarny piątek, gdyby coś się działo. Zamykają nam stację i za te 20 tysięcy możemy skrupulatnie...2 miesiące za to jesteśmy w stanie przeżyć, ale nie uważam, że 20 tysięcy to są oszczędności, bo ja z tymi oszczędnościami nic nie zrobię tylko je wydam. Nie jestem w stanie z nich zainwestować, chyba że ryzyk fizyk w bitcoina, bo mój kolega za 5000 bitcoina kupił, zarobione jest 100 tysięcy ponad, ale miał farta. Pięciu jego kolegów nie miało tego farta.

**Masz np. ubezpieczenie na życie?**

Teraz nie mam. Miałem, nie mam, bo mi się skończyło, ja nie przedłużyłem, bo była opcja, że zrobimy wszyscy razem sobie z Markiem też. To się wiązało z tym, że kiedyś szkoła narzucała ci ubezpieczenie i od tego roku nie było już tego. Ja miałem w życiu kilka wypadków i to takich poważnych. Miałem 99%, że będę kaleką i będę dożywotnio na wózku jeździł i ja korzystałem z takich ubezpieczeń. Jestem osobą, która faktycznie wzięła pieniądze i to grube pieniądze za operację i przebywanie w szpitalach, ale nam się to ucięło. My rozmawialiśmy o tym ostatnio z Ewelinką, ale to nam ucieka. Nam dużo rzeczy ucieka takich papierologicznych, które mamy zrobić. Jedyne, to mamy mieszkanie ubezpieczone i tyle. Musimy zacząć się zastanawiać...Dom, to ubezpieczenia teraz nie mam faktycznie, natomiast to jest związane z moim brakiem odpowiedzialności pewnie i roztrzepaniem jakimś, bo wiem, że to jest konieczne, pomocne i...Faktycznie przypomniałaś mi o tym, bo temat ruszyłem z Eweliną jakieś 2 tygodnie temu, żeby mi przypomniała, jak to jest z Markiem, bo trzeba wszystko do kupy zebrać. Tym bardziej, że chcę też szukać oferty na stację benzynową, żeby to ubezpieczyć...Zaczęło się od tego i ble, ble, ble, ja też nie jestem, a jestem osobą, która naprawdę skorzystała z tego. Mówimy o ubezpieczeniu na życie a nie o jakimś bezsensownym, bo jak ja sobie sam nie zarobię i nie dam rady, to na pewno żadna firma x mi tego nie da, ale zdrowotne tak. Obecnie nie mam, ale jest to tylko i wyłącznie moje zaniedbanie i mój błąd. Najgorsze jest to, że przez moje zaniedbanie i Ewelinka nie ma, i co najważniejsze Marek. Jest koniec prawie roku szkolnego i Marek cały rok był bez ubezpieczenia. Wszystko fajnie jest, tfu, tfu, tfu, do momentu, żeby nie upadł głupio, nie złamał ręki, tak? Ale to jest de facto tylko moje zaniedbanie i brak pamięci.

Jak byś siebie jeszcze opisał? Jesteś rozrzutny, trochę roztrzepany, papierologia trochę ci nie wychodzi. Jaki jeszcze jesteś?

**Jeśli chodzi o finanse?**

Tak.

**Chodzi o moje wydawanie pieniędzy?**

O podejście do wydawania pieniędzy.

Ja tak siebie odbieram, że jestem osobą rozrzutną. Ja bym powiedział, że nie jestem rozrzutny, gdybym...Ja uwielbiam i najczęściej wymagam, Ewelinka się złości czy nie złości, w firmach i gdziekolwiek moja osoba istnieje, to tabelki, tabelki, statystyki. Wszędzie. Mówiąc o sobie, że nie jestem rozrzutny, to musiałbym - portfel, aplikacja jest taka na telefonie i musiałbym każdy swój wydatek analizować, wpisywać, żeby na koniec miesiąca zobaczyć. Podejrzewam, że gdybym usiadł i tak zrobił, to bym mógł bardzo dużo oszczędzić pieniędzy, bardzo dużo. Siedząc na wsi oszczędziliśmy kilka, kilkanaście tysięcy zł, bo u nas się skończyło np. jedzenie na wynos codziennie.

**Ile wydajecie miesięcznie na takie żarcie?**

Nie mam zielonego pojęcia, bo nie robię takich statystyk. Na paliwo, gdzie przez cały czas samochód w jedną i drugą stronę jeżdżący codziennie to jest 100 km w jedną stronę. 200 km dziennie, to sobie wyobraź, jakie są koszta paliwa miesięcznie. Bardzo dużo zaoszczędziliśmy.

**Ale to takie oszczędzanie z przymusu było. Wróciliście do zwyczaju jedzenia na wynos codziennie?**

Przed rozmową z tobą już była debata, co zamawiamy dzisiaj.

**I co dzisiaj będziecie jedli?**

Nie wiem. Kebab? Nie wiadomo, jeszcze nie było decyzji. Mi się powoli już to przesyca. Nawet chyba parę dni temu rzuciłem temat, że może jakaś firma by nam przygotowała, jakąś dietę wypisała, może takie posiłki, bo chyba brzuch mnie zaczyna męczyć, już coraz starszy jestem. Ostatni tydzień moje kolacje - pizza, kebab, pizza, kebab, pizza, pizza, kebab.

**Monotonnie dość?**

No ja lubię. Wiem, że to może monotonnie brzmi, ale mnie to zupełnie nie przeszkadza, ale chyba już zaczyna przeszkadzać mojemu organizmowi, bo jakby to dodatkowo się witaminizuje sztucznie. Maksymalnie niezdrowy tryb życia i jedzenia, ale powiem ci, że chodzi mi koło głowy, żebyśmy jakąś firmę cateringową...[niezrozumiałe] w ich przypadku okazało się oszczędnością wykupienie od firmy cateringowej posiłków. Aż byłem w szoku, bo jak oni zaczęli nam mówić, przekalkulowywać, jak oni jedli na co dzień i przygotowywali jedzenie, a to co płacą miesięcznie i mają gotowca, to u nich to była oszczędność, to u nas to już w ogóle szał ciał, ale to chodzi bardziej o zdrowie. Fajnie też Marka by było nauczyć jeść inne rzeczy, bo na razie Marek to mięso, mięso, mięso, a jak zobaczy pomidora, to zaczyna wymiotować.

**Jak myślisz, kiedy się skończy ta sytuacja? Kiedy sobie pomyślisz, że to już jest normalnie?**

Wiem, że tego nie powiem. Myślę, że będzie normalnie, ale wiem, że tego nie powiem, bo się już sparzyłem na galeriach. Ja nie wiedziałem, że tak spiętnowało to ludzi. To nie są żarty. Ja myślałem, że ludzie są poważniejsi, ale ludzie nie są. Ludziom można naprawdę wszystko w tą głowę wrzucić. No nie, nie powiem, kiedy się to skończy. Jak byś mnie na wsi spytała, to pewnie bym powiedział, że jak otworzą restauracje i lokale. Teraz jestem pewny, że otworzą i dalej będzie tak samo. Otworzą, a ludzie dalej do tych restauracji nie będą chodzić, będą się bali.

**Chyba w maju mają już otwierać?**

Nie wiem. Akurat ci moi znajomi, którzy działają mocno w gastronomii warszawskiej to wolą, żeby to było zamknięte. Dla nich biznesowo lepiej, jak się okazuje. Nie wiem, czy będzie normalnie. Wczoraj w galerii patrzę i Multikino. Wiesz, kiedy kina otwierają?

**Nie wiem.**

Otworzą mi kino, to poszedłbym mimo wszystko. Pewnie wynika to też z mojego charakteru, że nie pozwoliliście mi, to pierwsze co zrobię...Ale poszedłbym. I otworzą te kina i myślisz, że te sale będą pełne? Absolutnie. Może się okazać, że trzeba będzie zdeformować w ogóle formę kina i będzie tak jak kina kiedyś - 30osobowe. Ja miałem taki pomysł, żeby tam zrobić puszczanie filmów nocą, telebim i może się okazać, że to będzie kolejne pójście w ruch. Widziałem co się dzieje przy otwarciu GH i z tego względu nie jestem w stanie nazwać punktu, miejsca, w którym powiemy, że to już jest koniec tego.

**Jak to będzie wyglądać w najbliższych tygodniach?**

Boję się jednego. Czekam z utęsknieniem na efekt otwarcia wszystkiego. Jest wszystko z powrotem z punktu widzenia biznesowego, kulturowego, wszystko jest otwarte i czekam. Czekam, czy się pojawi 2 fala, bo każdy normalny wirus, pandemia, ma zawsze 2 fale. Nie wiem, czekam. Jeśli będzie 2 fala, to powiem, że ale głupi byłem, był jednak ten koronawirus mocny. Boję się, że będzie 2 fala, boję się, że te dzieciaki pójdą do tych szkół, ci rodzice wrócą i jeżeli faktycznie on był, to będzie już porażka społeczna. To już będzie taki lęk międzyludzki...Wyobraź sobie, że wszystko otwierają, wszystko wraca do normy, jest 2 rzut i 2/3 więcej niż teraz osób umiera, bo nie było przestrzegane. No to powiem ci, że w wielu kwestiach będzie tragedia. Tego się boję ogólnie. Nie wiem, kiedy będzie punkt, że powiem, że tego już nie ma, patrząc na dzień dzisiejszy na Warszawę, bo ja na wsi tego nie odczułem, inny świat zupełnie. Ja nie wiem, czy w takim mieście to tak szybko wróci do normy. Nawet może to trwać ładne kilka...Tym bardziej, że będzie też susza, w przyszłym tygodniu mają oficjalnie podpisać suszę w naszym kraju, więc będziemy mieli ciężki rok.

**W wakacje będzie normalnie, na jesień?**

Myślę, że w wakacje, jeśli się wszystko otworzy, to wakacje to będzie taki moment, kiedy te wszystkie firmy będą to chciały sobie odbić, więc my będziemy mieli wszystko bardzo drogie. Mówimy o hotelach, ale mówimy też o jabłku. Będzie przerażająco droższe, bo pożywienie będzie droższe, prąd będzie droższy, skoro będzie susza, bo już są problemy z produkcją. Będzie porażka.

**Macie plany wakacyjne?**

W ogóle nie mamy nigdy. My jesteśmy tak, że w czwartek mówimy, że jedziemy gdzieś, bo trzeba odpocząć i jedziemy w piątek. Był kiedyś plan wyjazdu za granicę, chyba samochodem mieliśmy jechać do Szwajcarii, ale ten plan się skończył na wyrobieniu przeze mnie paszportu.

**Masz jeszcze jakieś przemyślenia, jeśli chodzi o przyszłość?**

Nie wiem, chciałbym, żeby to już się skończyło. To nie chodzi nawet o mnie tylko o tych ludzi. Szkoda mi tych ludzi. Ja wszystko mam. Mam na szczęście pieniądze, mam dziecko, które mogę torturować, mam ukochaną kobietę. Ja mam wszystko na swoim miejscu. Mi jest dobrze. Ogranicza mnie tylko moja wolność przebywania w różnych miejscach. To jest ograniczenie jedyne, chociaż hotele otworzyli. Śmieliśmy się wczoraj, że w Sopocie przy deptaku mają jedną rezerwację. Czad, jedziesz do hotelu i masz cały hotel dla siebie.

**Ale restauracji nie otworzyli.**

Wiem, bo mieliśmy opcję, że sobie wyjedziemy na kilka dni, pewnie w góry, ale mógłby być kłopot na początku z organizacją tych posiłków. Musielibyśmy obdzwonić po prostu tam lokale. W Warszawie jest inaczej, 80 km dalej jest inaczej, a ja nie mam pojęcia, jak jest w górach. Im jest bliżej myślenia wsiom niż miastom i może się okazać, że do niektórych lokali po cichu można wejść, można zjeść. Nie dzwoniliśmy, nie wiemy. Niech się naprawdę już skończy, niech dzieciak wróci do szkoły. Kiedyś musimy wrócić do normalnego, codziennego aparatu społecznego i życia. To musi działać i musi się trybić. Tak czy siak musimy to zrobić, bo im dłużej siedzimy w tych domach, tym gorzej jest dla nas samych i dla naszych głów. Na wsiach się w domach nie siedzi, ci ludzie pracują na polu. Oni mają inne podejście, to widać, jest ogromna różnica. Ludzie w mieście wcale nie chcieliby siedzieć w tych ścianach, chcieliby iść do pracy. Brak tego piętnuje ludzi. Nie jest dobrze psychologicznie, emocjonalnie, jak patrzę. Chcę, żeby ludzie się uśmiechali, żeby wrócili do pracy. Czekam, aż otworzą restaurację i jedziemy.
