# Supplementary material for: The challenges arising from the COVID-19 pandemic and the way people deal with them. A qualitative longitudinal study
Source: PLoS One. 2021 Oct 11;16(10):e0258133. doi: 10.1371/journal.pone.0258133 (PMC8504766; doi:10.1371/journal.pone.0258133)
Supplement: S1 Dataset — (ZIP) [file pone.0258133.s003.zip › Transcriptions/stage 4/18.4_F_48_couple, with children.docx]

**18.4_F_48_couple with children**

**Co u ciebie słychać, co się działo przez te 2 tygodnie?**

No, takie intensywne tygodnie dwa.

**Bo?**

2 tygodnie intensywne, dużo takich działo się zawodowych spraw. Dużo takich i w sklepie i ogólnie z naszą działalnością. Takich z koronawirusem wiążących się. Więc jakieś takie finansowe sprawy, góra, dół, te tarcze antykryzysowe. Więc dużo takich rzeczy, którymi się nigdy nie zajmowałam, ale… I musiałam się zmierzyć i to wszystko się skumulowało.

**Ale opowiedz troszkę o tym, co się działo, co to były za nowe rzeczy, co się zmieniło?**

To jakieś tam, nie wiem, z najemcami, że jakieś obniżenia czynszów, negocjacje, jakieś sprawy podatkowe. Więc też musiałam korzystać z pomocy doradcy podatkowego. Więc też to jakby na szybko. I tak, no nigdy nie korzystałam z takich usług, a musiałam, więc też jakieś wyzwanie. To też w całym takim…. Właśnie, to chyba taki lęk i taka nagła niepewność wszystkiego. Łącznie z tym, że i z porady adwokata skorzystałam. Co prawda to chyba było tak bardziej dla mojego uspokojenia, bo mąż mówił, że nie trzeba, że nie. Ale mówi dobrze, to idź, żebyś się lepiej czuła. I jak usłyszałam od fachowca to, co wiedziałam, to poczułam się pewniej.

**Z czym to się wiązało? Co takiego się stało, że tak się zmieniła sytuacja i z tego w miarę spokojnego funkcjonowania sklepu nagle tutaj tarcze, porady, co się zadziało?**

No tak, bo jeszcze dodatkowo, że mamy w tym budynku, jeszcze wynajmujemy pomieszczenia. Więc tam część… No też najemcy poprosili o zmianę. No to jakby… Znaczy zrozumiałe i ja przed tym się nie broniłam, tylko jakby żeby to wszystko było… Bo to wszystko na szybko, tu coś niepewnego. I nagle ta cała taka płynność nasza finansowa została narażona. Może też to więcej było takiej paniki mojej niż… Ale ja lubię mieć poukładane. I jak nagle coś mi się tak rozchwieje, to ja już tak tracę grunt pod nogami i właśnie szukam… Ale szukam takich, żeby upewnić się, żeby to było tak fachowo. I mówię, i to się zebrało jednego dnia. Więc ja stwierdziłam, że to był jakiś taki czarny dzień. Gdzie z jednymi też rozwiązywałam umowę, ponieważ tam zupełnie… Tego się najbardziej nie spodziewałam, bo zostały zamrożone pieniądze z urzędu. To znaczy taki chaos, bo nawet… No mówię, prawnik no to swoje mi powiedział, że może chcą naciągnąć sytuację itd. Ja mówię nie, to młodzi ludzie, którzy tak zainwestowali swoje pieniądze i nagle zostali odcięci od tego. Więc nie widziałam w tym jakiegoś drugiego dna. Natomiast im się zawaliło, no mi mniej, ale bardziej im się zawaliło. Ale też, żeby z tego jakoś wybrnąć no tak, dla wszystkich w miarę dogodnych warunkach.

**Czyli mnóstwo takiego załatwiania w związku z tym właśnie, że macie te lokale?**

Tak. I żeby to tak no też… No z jednej strony spokój by było taki, że dobrze, no jakoś tak też finansowo, żeby się rozliczyć. Ale też nie tak, żeby do ostatniej kropli krwi wypić, żeby każdy był… No jakby rozumiem sytuację. Więc… No mówię, jakieś takie przepisy i księgowe sprawy. Takie, żeby to dobrze rozliczyć, dobrze napisać. Bo bardziej im musiałam to tłumaczyć, że to nie jest moja zła wola, że ja zwlekam z tym. Tylko po prostu, no jest taki czas, że i księgowy pod tym się nie podpisze, ja też się nie podpiszę. Bo jakby te finansowe sprawy z Urzędem Skarbowym… Mówię, no wy jesteście jeszcze młodzi, jeszcze nie robiliście takich rzeczy. A to zupełnie inaczej wygląda w teorii, a inaczej… Fiskus nie patrzy na takie rzeczy. Tam są czyste cyfry i tego się trzeba trzymać.

**I to teraz wynikło dlatego, że jest kwiecień, maj i te rozliczenia były konieczne? Czy z jakiegoś innego powodu?**

Znaczy to też. Ale jakby no przepisy, jak się rozwiązuje umowę, to też zupełnie inaczej. Bo takich rzeczy nikt nie przewidział. W umowach takich siła wyższa jakby… No to dopiero, gdy na drodze sądowej to można dociekać. Natomiast w takim momencie przy takich zwykłych umowach nie było tego. Bo nikt tego nie przewidział. Jakby z każdej strony, a tu nagle coś takiego, gdzie… No nie. Więc też nowa sytuacja dla wszystkich. Ja też nie lubię w takim pośpiechu, chaosie, że szybko, szybko, bo komuś się grunt pod nogami wali. No mi też się wali. No może nie na taką skalę, ale też jakby chcę zadbać o swoje interesy. Ja nie jestem taka biznesowa, więc mnie to dużo kosztuje energii. Natomiast, w efekcie, jak już wszystko załatwiłam i rzeczywiście dużo rzeczy sama zrobiłam, to byłam z siebie dumna. Że jakby no dałam radę. I to, co ja założyłam na początku i o czym wiedziałam, wszystko się potwierdziło. Natomiast no to było takie stresujące. Też złożyło się, że ta jedna z moich pracownic, która jest na tym zwolnieniu, okazuje się dalej będzie dłużej na tym zwolnieniu. Znaczy ja też jakby to przewidywałam. Natomiast nie przewidywałam, że będzie wirus w tym czasie. Czyli, że to będzie wszystko inne, że teściowej nie będzie, że te całe warunki sprzedaży będą inne. Więc też tu dalej się coś tam blokuje. Też jedna z dziewczyn dostała jakiegoś ataku histerii, paniki, nie wiem, jakoś tak zaczęła dziwnie się zachowywać. Znaczy taka była i w stosunku do klientów. I widziałam, że jakoś tak… Więc też musiałam jakoś ją dyscyplinować. I szukać pokładów spokoju i myśleć, jak to rozwiązać, żeby jakby…

**A to było związane z koronawirusem, jak ona się zachowywała, to co się z nią działo? Coś wiesz więcej na ten temat?**

W efekcie potem, jak już na spokojnie i opanowałam i jakby jej wyjaśniłam, że takiego zachowania nie będę tolerować i niech ona się zastanowi, czy… Ale tak jakby łagodnie, bo też coś nagle się zadziało, więc no też… Ja nie jestem takim typem, że nie wiem, nie podoba mi się, do widzenia itd. Więc no nie, ona w końcu stwierdziłam, że tak reaguje, że ma jakąś tam nerwicę, jakieś takie rzeczy, więc…

**A co zrobiła takiego, że to było dla ciebie dziwne?**

Ona po prostu… Znaczy taka prosta sytuacja w sklepie, gdzie obsługuje się, nie wiem, 3 kasy, są klienci. I przyszła kobieta, która chciała wyjaśnić na paragonie, że jej się nie zgadzał makaron. I grzecznie, spokojnie zapytała się, dlaczego ma tu, nie wiem, 4 makarony a dostała tylko 3, czy tam już nie pamiętam. I ona po prostu zaczęła mówić, że nieprawda, że tam jest wycofane. I po prostu zaczęła tak krzyczeć na tą kobietę, takiego dostała ataku w ogóle… no tak nieproporcjonalne do tego, gdzie… No mówię, starsza osoba, grzecznie pytająca się. Która tak naprawdę chyba nie rozumiała, co tam jest na tym paragonie. No my też nie mogłyśmy jej opanować, każda była zajęta swoją pracą, swoim klientem. Więc też… No i że spokojniej. Ona nie dała sobie wytłumaczyć w tym momencie. Ja w efekcie stwierdziłam, jak czytałam ten paragon, to nie wiedziałam, co czytam, więc stwierdziłam, że dobrze, dam ten makaron, którego tej pani brakuje i przepraszam i w ogóle. Więc ona była jeszcze bardziej oburzona, że dlaczego ja nie stanęłam po jej stronie. Że to wychodzi, że ona oszukuje. W ogóle dostała jakiejś takiej histerii. Ja mówię… Ale mówię, musiałam odczekać, żeby przemyśleć to, porozmawiać z nią. Że jej przecież nikt nie atakował. To, że ja, nie wiem, tej kobiecie dałam tej makaron za dużo, to… Ja mówię, pani zachowanie było tak nieadekwatne i tak w ogóle… Nie warte było w ogóle nawet tej sztuki makaronu. Ja nie potrafię w takim chaosie, w tym krzyku funkcjonować. Ale mówię, nikt nie oskarżył pani, nie padło słowo, że jest pani winna. No w ogóle krzyczeć... Ja nie krzyczałam. Ja mówię: nie, krzyczała pani, wszyscy to słyszeli. To nie jest to, że ktoś wymyślił, bo było więcej osób. I nawet klienci pytali, dlaczego jest u was głośno. A tak naprawdę tylko ona krzyczała.

**A jak myślisz, z czego wynikało takie jej inne zachowanie, dziwne?**

Znaczy nie, ona w końcu stwierdziła, że ona rzeczywiście tak reaguje. Że ona ma nerwicę i ona po prostu, jeśli… Ona od razu odbiera to… No nawet nikt jej nie mówi, że pani mnie oszukała. Bo nikt tak nie powiedział, że oszukała, tylko że dlaczego tu jest, chciałam wyjaśnić sprawę. A ona to odbiera bardzo do siebie, że od razu czuje się bardzo oskarżona. I taki błąd, że to jest nieprawda, od razu przekłada się w krzyk. Ona mówi, że tak funkcjonuje. Ja mówię, no ja wszystko rozumiem, ale musi pani nad tym panować, pracować nad tym. No coś z tym zrobić. Bo dla mnie w ogóle taka sytuacja nie ma prawa mieć miejsca. Więc to wszystko takie zebrane w jedno…

**I te 2 tygodnie były pełne różnych takich napięć?**

Jeden tydzień. To tak się skumulowało w jeden tydzień taki bardzo intensywny. Ale potem to tak się wyprostowało, dałam radę. I to też jakby… Czy to jest sytuacja wirus? Często się zdarzały takie sytuacje, że w jednym momencie. No jak to się mówi, że nieszczęścia chodzą parami, czy jak to tam się nazywa. Natomiast no mówię, dużo, dużo tych napięć. I coś takiego, jak się dzieje, to jeszcze bardziej tak potęguje.

**A sama majówka? Jak ci minęła majówka?**

Majówka jak minęła? Bardzo sympatycznie. Bardzo. Pogrzebałam w ogródku, kwiatki sobie posadziłam. W sobotę pracowaliśmy. Bo stwierdziliśmy, że będziemy pracować. Że właściwie tak naprawdę nie wiadomo, co nas czeka. Mamy i tak towar, dziewczyny były chętne. Tam jedna chciała urlop, więc miała urlop. Więc stwierdziliśmy, że idziemy swoim rytmem. Ale to tam było tylko pół dnia, więc… Więc całkiem fajnie. Posiedziałam na tarasie, kwiatki posadziłam, więc…

**Pojawiły się jakieś nowe rzeczy, nowe aktywności w twoim życiu, coś zaczęłaś nowego robić?**

No ruszyliśmy na spacery. Ruszyłam, wróciłam do swoich kijków (śmiech). I to było po prostu coś fantastycznego. Poszliśmy na pierwszy spacer, zrobiliśmy swoją taką rundkę z mężem. I zawsze przechodzimy koło kuzynki. I ja mówię, no to pomachamy im. No i oni wejdźcie, wejdźcie. Ja mówię, że nie, że nie wejdziemy, w ogóle absolutnie nie. No, ale chodźcie, nie wygłupiajcie się. Ja mówię, nie, no to, że możemy wychodzić na dwór, no to nie znaczy, że od razu będziemy korzystać i wizytować się. Ja mówię, no możemy posiedzieć na tarasie. Bo ja w ogóle potrzebuję powietrza. Ale ponieważ kuzynka miała mokre włosy, powiedziała, że nie, że nie usiądzie, żebyśmy weszli do domu. I weszliśmy. Usiedliśmy w czwórkę w jakichś odstępach, nie ściskaliśmy się, nie całowaliśmy. Ale ja miałam takie poczucie, że robię coś złego.

**To była pierwsza sytuacja takiego spotkania?**

Tak. Bo o ile do mamy jechałam i też na podwórku widziałam się z nią, chwilę rozmawiałam. To tutaj było coś takiego… Tak dziwnie się z tym czułam. Mówię, tak jak bym jakieś przestępstwo robiła. Coś takiego… No to było takie nowe.

**Gdybyś miała wrócić do tej sytuacji, powtórzyłabyś ją, weszłabyś tam? Czy teraz myślisz, że to był błąd, trzeba było…**

Nie, weszłabym. Weszłabym. Weszłabym, bo tak naprawdę patrząc… Nie, to w ogóle jakiś… Patrząc, co się dzieje i jak ludzie się zachowują, to naprawdę nie robiłam nic… A poza tym za ich przyzwoleniem, nie narzucałam się, więc…

**W dużej odległości od siebie siedzieliście. W maseczkach czy bez?**

Bez.

**I byłaś z mężem czy sama?**

Z mężem.

**Mąż też miał poczucie winy?**

Nie (śmiech).

**A oni?**

Oni nie.

**Tylko ty, tak?**

W ogóle oni to o tej całej sytuacji, że to jest przesada. I żeby się nie wygłupiać. I tak każdy z nas zachoruje kiedyś, będzie miał tego wirusa. Więc w ogóle… Bo jakby ja się tłumaczyłam, że nie chcę wejść, bo jakby ja jestem bardziej aktywna od nich. Chociaż, no nie wiem. Ale oni też chodzą do sklepu, chodzą gdzieś na zakupy. Więc też nie są, że całkiem siedzą, tak jak moja mama odizolowana. Ale miałam takie przemyślenia, że jest to coś takiego nie…

**Ale jak byś miała tak zanalizować to swoje poczucie winy i ten swój dyskomfort, który wtedy czułaś, to jakie tam były składowe? Czułaś się źle, bo?**

Bo jednak nie jest zalecane spotykanie się. Jakby z jednej strony, że jest jakiś przepis, zakaz. Przepisy albo się łamie albo się do nich stosuje.

**I poczułaś się niepraworządnym obywatelem?**

Tak, tak. Potem stwierdziłam, że… No, ale jeśli ja mam więcej styczności ludźmi, jeśli tego wirusa mam. I nie wiem, gdyby oni zachorowali, to pierwsze bym pomyślała, że może to jednak ode mnie, że jednak ja im przyniosłam do domu. Więc takie poczucie winy, że mogę coś komuś źle zrobić. Nie sobie, ale komuś. Takie zupełnie, nie wiem. Jakby stosowałam się do wszystkiego i to była pierwsza rzecz, którą zrobiłam wbrew jakimś tam zasadom, ustaleniom.

**Masz wrażenie, że to przetarło tobie jakieś szlaki do spotykania się z ludźmi? Że, gdyby koło waszego domu ktoś przechodził znajomy, zaprosiłabyś go do siebie?**

Przez grzeczność tak, ale nie do końca, żebym to tak… Zaprosiłabym i gdyby skorzystał fajnie, a jeśli nie, to też bym jakoś nie naciskała.

**Ale jak rozumiem jakieś takie przyzwolenie w tobie większe na to, żeby się spotykać już jest?**

Tak. Powiedzmy 4 osoby, a nie 10, to zdecydowanie. Takie rozgraniczenie, żeby robić to powoli.

**Coś jeszcze ci się nowego pojawiło w ciągu tych 2 tygodni? Bo to jest nowość ewidentna po czasach izolacji.**

Coś nowego? No poszłam też z koleżanką na spacer, umówiłyśmy się. No i to było fantastyczne, bo… Wyszłyśmy w maseczkach, no tam nie było 2 metrów, bo chciałyśmy pogadać, więc to trudno żebyśmy krzyczały do siebie. Ale poszłyśmy na spacer do parku. No i to było coś fantastycznego. Bo to nawet chyba był pierwszy, wcześniej niż z mężem wyszłam. Bo czekając na nią w ogóle spojrzałam, że kwitną bzy. I w ogóle to mi jakoś umknęło. I po prostu widok tych kwitnących bzów to tak mnie rozczulił, że to było tak piękne, tak przyjemne. A z drugiej strony stwierdziłam, że ile rzeczy mi umyka. Ilu rzeczy ja nie widzę takich, które w ogóle… No mówię jejku, to już kwitną bzy. Po prostu. To było dla mnie takie odkrycie, że to już jest ten czas. I mi to umyka.

**Co ci jeszcze umknęło?**

No właśnie nie wiem, ta zielona trawa, że… Kaczki, łabędzie. Nie, łabędzi nie było, kaczki. Jakieś tu kwitnące… Park, który znam i po którym zawsze chodziłam i był tak blisko, nagle ja go odkrywam. Że jest dla mnie taką nowością, takim czymś wow. Z jednej strony to było bardzo przyjemne i to był fantastyczny czas. A z drugiej tak mi się smutno zrobiło. Że przez ten natłok pracy, to odizolowanie, to niewychodzenie, no takie… Niby proste, drobne rzeczy, a mi umykają. Tak szkoda mi tego. No, tak mi się smutno zrobiło. Z jednej strony taka wielka radość i tak po prostu super. A z drugiej no taki smutek, że… No, ale cóż no. Ale zdrowa jestem (śmiech).

**A to spotkanie z koleżanką to była twoja inicjatywa czy jej?**

Moja. Moja, bo ja pojechałam tam do pracy po swoje rzeczy. Potrzebowałam jeszcze jakichś rękawiczek, maseczek. I ona blisko mieszka. I tak po prostu, że jestem w pobliżu, czy ma ochotę. Bo wiem, że ona wychodzi, bo jest po operacji kręgosłupa i musi chodzić. I ona bardzo chętnie. Gdzie ona tak bardzo przestrzegała. Że ona nawet, jak do apteki wychodziła, to mówi, wiesz, ja się tak bałam, że idę do tej apteki. Ona jest w ogóle taka bardzo strachliwa i taka przepisowa. Więc mówi, że nawet jak szła po chleb, to myślała, że jak ją ktoś zatrzyma, to ona powie, że idzie do apteki (śmiech). No i jak ona się zgodziła, to już było fajnie, że… I była mi bardzo wdzięczna, dziękowała mi później, że właśnie ten czas dla niej znalazłam. I właśnie, że na spokojnie sobie przeszłyśmy.

**I tu nie miałaś poczucia, że robisz coś złego?**

Nie, tutaj nie.

**Pamiętam, jak mówiłaś 2 tygodnie temu, że ta izolacja zaczyna ci doskwierać i przeszkadzać. Czy jest coś, co w tej chwili jest dla Ciebie takim wyzwaniem, co ci przeszkadza, co się pojawiło jako taka rzecz drażniąca, irytująca, doskwierająca?**

Co mi doskwiera? To rozprężenie i zachowanie ludzi. To mi ewidentnie doskwiera. Takie… Ja mam wrażenie, że od 4 maja to się wszystko zmieniło. I to po prostu tak się zmieniło, że nagle ludzie zaczęli wychodzić, spotykać się. Bo to widać. Stoją w grupkach. Te maski noszą gdzieś tu na brodzie. Nie wiem, to otwarcie tych galerii, że… Ja nie byłam w takich budowlanych miejscach, ale podobno tam jest zupełnie tak jak było. Że tam nikt nie przestrzega żadnych procedur, odstępów, ilości osób. No właśnie. I tak teraz… Zresztą słyszy się więcej takich głosów, że w ogóle ten wirus to naciągane, to prowokacja. No to mi przeszkadza. Że nagle, z dnia na dzień coś się zmieniło w ludziach, takie podejście. A statystyki mówią, że więcej jest zachorowań. I to mi się zupełnie się to nie zbiega. Tu już nie wiem, o co chodzi.

**Co ci to robi? Bo mówisz, że to cię drażni, ale jakie to są uczucia, jakie to są emocje?**

Jest taki żal do ludzi. Że no było niebezpieczeństwo, wpadli w panikę. A nagle nie, tamto to było nieważne, to była bzdura, to było oszukaństwo, robimy teraz tak jak my uważamy. No i właśnie to takie już, że… Nawet ja zauważyłam dzisiaj rano w sklepie, że tak pilnowali, że po 3 osoby… Owszem, no jednak większość pilnuje. Ale teraz częściej się zdarza, że ludzie wchodzą, już nie patrzą, ile osób jest, na to ogłoszenie, że 3 osoby mogą przebywać. Jakby to już nie jest ważne. To już się nie liczy. I że trzeba przypominać o tym. Prosić, że jednak prosimy, żeby były 3 osoby. I jest takie spojrzenie, że właściwie, no taka może nie arogancja, ale ignoracja, że zaraz, no to byłem w Leroy, tam mogłem sobie chodzić swobodnie. A tu nagle w jakimś sklepiku ktoś zwraca mi uwagę. Tak niepoważnie już się nas traktuje, takim komunikatem.

**Nie wiadomo, o co tu chodzi w ogóle.**

Tak. A jakby ja się chcę trzymać dalej tego, co było. Bo ja uważam, że nawet, jeśli ten wirus jest wymyślony, nawet jeśli nam nie zagraża, to też takie zachowanie odstępu, przestrzeganie, że będzie mniej osób w sklepie niż 3 nie zaszkodzi. Nie jest czymś, co jakoś bardzo utrudni komuś życie.

**Czyli to byś jeszcze utrzymała. To jest taki wentyl bezpieczeństwa?**

Maseczki też bym utrzymała.

**Coś jeszcze cię drażni poza zachowaniem innych ludzi? I tym, że właściwie nie wiadomo, o co chodzi?**

Co mnie jeszcze drażni? Rozdrażniło mnie, rozmawiałam z moją koleżanką kosmetyczką, z którą razem pracujemy. Ona wyjechała z dziećmi. Ona ma rodzinę na Zamojszczyźnie i tam wyjechała z dziećmi. I ona jest wdową, ma dwoje małych dzieci. I tam po prostu wyjechała do rodziców. I mówi, że teraz pojawiły się telefony, zresztą ja też mam takie SMS-y, takie niby grzeczne, co słychać. Ale z takim podtekstem, czy bym może kogoś nie przyjęła, czy bym nie zrobiła jakiegoś… A do niej wręcz oficjalnie klientki dzwonią i że teraz chciałyby zrobić jakieś tam zabiegi. I to takie zabiegi no na twarz, kosmetyczne, inwazyjne. Gdzie ona mówi, no tak naprawdę raz, że ja dotykam skóry. No nie ma możliwości nawet tak jak ja przy manicure zachowania odstępu. No nie wiem, są jakieś nakłuwania, jest ingerencja taka, gdzie ja się po prostu boję, żeby potem mi ktoś nie powiedział, że się u mnie zaraził. Bo on zarazi się gdzie indziej. I wręcz z taką, ona też tak to odebrana, że są oburzone, że dlaczego na nie chce ich przyjąć, zarobić. No przecież ona jest w trudnej sytuacji finansowej. No i ona mówi, no i tak, jak ja byłam w domu, sama z dziećmi, gdzie jeszcze mogłyśmy pracować i wszyscy się wypisali, bo panika. I ja siedziałam i rzeczywiście miałam wolny czas. I mogłam to robić legalnie, zgodnie z przepisami, to nikt się nie przejmował moją sytuacją finansową. A teraz nagle po miesiącu, półtora, gdzie już zaczął wygląd właśnie a propos tego wyglądu doskwierać, mam czas i coś bym dla siebie zrobiła, to jest oburzenie, że dlaczego ja nie chcę, jakby gardzę taką super ofertą. Przyjdę i zapłacę. I mówi całe szczęście, że ja mam wytłumaczenie, że jestem daleko, że jestem z dziećmi. To jeszcze jakoś tak daje jej usprawiedliwienie, że ona odmawia. Więc mnie to przeraża. Bo, no, ja też dostałam propozycję, że przyjedzie klientka do mnie do domu, zabierze do swojego i mnie odwiezie, w ogóle jakiś taki kosmos robienia. Żebym tylko właśnie zrobiła jej manicure, pedicure. No więc wytłumaczyłam się, że nie, że nie ma takiej możliwości. Bo nie mam dostępu do narzędzi i nie pracuję. Ale to mnie zadziwia. Zadziwia, tak. Bo inaczej jest, jak ktoś ma problem taki, no rzeczywiście, czy jakieś chore paznokcie, że trzeba to leczenie kontynuować, czy jakieś wrastające, czy jakieś takie schorzenia pięt. To rozumiem, bo jest to problem. Zresztą tak naprawdę od poniedziałku mogłabym zabiegi podologiczne wykonywać. Natomiast jak pomyślałam, że muszę przechodzić tę drogę, takiej właśnie izolacji, tak jak w sklepie, pleksy, kolejne miejsca do sterylizacji, to nie chce mi się.

**To poczekasz, aż będzie bardziej można?**

Tak. Tak, tak, tak, bo to jest za dużo… I tłumaczenie się, dlaczego robię ten zabieg, a nie ten. Więc ja już odpuściłam totalnie, nie bawię się w to, że tak powiem. Natomiast mówię, no takie zachowania i to, jak właśnie można kogoś, trochę też nękać takimi prośbami, no to… Ale w ciągu dwóch tygodni na przykład też korzystałam z dentysty. Ale to już zaczęłam się z tego chyba trochę śmiać. Znaczy zaczęłam się śmiać, jak się okazało, że mój dentysta przyjmuje. Bo na początku nie było mi do śmiechu. Bo chyba z 5 lat nie miałam nic robione przy zębach, oczywiście oprócz takich kontroli, bo to nie tak, że nie chodzę. Ale na standardowe czyszczenie, sprawdzanie, wszystko super. I poczułam taki dyskomfort, a ja jestem taka zapobiegliwa, że nie może mnie boleć nic. I tak zobaczyłam, że mają dyżur i poprosiłam o wizytę. Powiedziałam, że no tak trochę czuję, że mnie boli, żeby nie było. I okazało się, że… Pan doktor mówi: nie, to nieszczelna plomba, to jak coś pani czuje, wyczyścimy. Ale okazało się, że to leczenie kanałowe. Więc mówię, tak z 5 lat miałam spokój, gdzie mogłam w komfortowych warunkach bez…Ale tak się zdarzyło, ja cieszę się, że przyjmują, że jestem zaopiekowana i mam poczucie bezpieczeństwa. Chociaż to było takie, no, mówię, co to będzie.

**Ale czułaś taki niepokój, idąc tam? O swoje bezpieczeństwo?**

Nie, tam już nie, bo… No nie.

**Chciałabym trochę porozmawiać o emocjach. Znalazłaś jakieś swoje zdjęcie, swój obrazek czy wracamy do naszego zestawu?**

Nie zrobiłam zdjęcia tego pięknego bzu. Ale po prostu kwiat bzu, który… Bo potem, jak już odeszłam i mówię, to było idealne, to co czułam przy tym bzie. To była po prostu taka radość. I takie rozczulenie w ogóle, taka przyjemność. No wszystko, co dobre to było przy tych kwiatkach fioletowego bzu. To w ogóle była niesamowita radość. A z kolei z tych naszych obrazków to przy tych całych moich problemach, przy tym całym, co się działo i czytaniu tych maili, tych przepisów, to ja cały czas widziałam ten obrazek tych… lin takich powiązanych.

**Tak, 14.**

Tak. To ja widziałam ten obrazek. To byłam cała ja, po prostu ściśnięta, skręcona, pozwijana. Taka uziemiona, uwikłana we wszystko.

**Jak sobie radziłaś z tymi uczuciami? Co z tym robiłaś? Gdzie to czułaś, jak to było, co ci to robiło i jak sobie z tym radziłaś?**

Znaczy, no z tymi dobrymi, to ja czułam całą sobą. To w ogóle rozłożyło się na wszystko, błogość niesamowita. Natomiast z tym ściśnięciem… jak sobie radziłam? No ja gadałam o tym. Ja gadałam, po prostu chodziłam, gadałam, kto był, to gadałam. Znaczy kto był – mąż, syn, nie wiem, mama. Do mamy dzwoniłam. I musiałam o tym gadać, że jest mi źle, że jestem zła, że dlaczego znowu muszę z czymś się mierzyć. Taki niepokój, lęk, wszystko razem. Ale ja sobie po prostu gadałam głośno. Albo sobie po cichu gadałam do siebie w myślach, że przecież nic się nie dzieje, że nie jest to tragedia. Trochę mój mąż musiał mnie tak… Znaczy to jest dobry sposób na mnie, że weź się już uspokój, ręki nikomu nie urwało. No, jak ja tak usłyszę, ręki nikomu nie urwało, no to tak, no to już sobie pogadałam, że dobrze, no przecież nic się nie dzieje, to nie są rzeczy tragiczne. Tylko ich jest za dużo. I muszę sobie z tym poradzić.

**Czyli to była i złość, i irytacja, i ściśnięcie, i zdenerwowanie. Same negatywne, takie napięciowe rzeczy?**

Tak. Ale był też taki smutek. Że dlaczego ciągle coś mnie… Jak już się prostuje, to coś nowego mnie spotyka. Więc to… No, ale tak jak w życiu. Tylko, że mówię, dużo tego się zebrało i w takich innych warunkach. Więc to było takie bardziej dołujące i bardziej takie właśnie plączące.

**Miałaś wrażenie, że w nadmiarze na to reagujesz? Czy nie? Czy to było adekwatne do tej sytuacji?**

Nie, myślę, że było adekwatne. Bo… Tak, tak. Bo gdyby to mi się rozłożyło na dni, czy jakoś tak bardziej rozciągnęło. Ale po prostu takie 3, tak naprawdę to były takie 3 mocne dni. No to było tego za dużo. A poza tym to też było adekwatne, bo jakby widziałam zachowanie męża jak tak chodzi koło mnie. I tak to, jak już mnie zdyscyplinował, że uspokój się, ręki ci nie urwało, to też był inny ton, taki przemawiający. No też inaczej. Poza tym też i zachowanie, jak moja mama o tym mówiła, jak syn się zachowywał wobec mnie, to wszystkie sygnały mówiły, że nie przesadzam.

**Właśnie, jak twoje otoczenie w ogóle sobie radzi? Czy zauważasz jakieś nowe emocje, nowe zachowania, nowe sposoby radzenia sobie z nimi? Czy coś tutaj się zmienia w Twoim najbliższym otoczeniu?**

Znaczy no myślę, że chyba większych zmian nie ma. Moja mama powoli sobie skorzystała z tych godzin dla emeryta, że sobie tam wyszła do sklepu po jakieś takie drobne zakupy, gdzieś tam sobie pojechali. Że też byli, pojechała do siostry, ale też na podwórku, nie wchodzili do mieszkania. I lepiej się poczuła i teraz z powrotem osiadła w domu. I mówi, tak raz w tygodniu, jak sobie zrobi taką „wycieczkę”, to sobie radzi. No i jest ogródek, więc do tego ogródka się… No korzystają z telefonów, więc dużo, tak jak teściowa słyszę, bo słychać, no to dużo właśnie dzwoni, do niej dzwonią, więc tak bardzo przeszło na ten kontakt telefoniczny. I coraz częstszy.

**Ale masz takie wrażenie, że się ludzie wokół ciebie ogólnie uruchamiają społecznie w tej chwili?**

Tak, tak, tak. Nawet czy ktoś rowerem gdzieś jedzie, to nawet to dużo dało. Jednak taka swoboda poruszania się, że właśnie można na spacer, można rowerem, to… Nawet mój syn się przełamał, spotkał się z kolegą. Co prawda siedzieli w samochodach.

**Każdy w swoim?**

Tak, tak. Ale to też dla niego, bo on jest… On przepisowo, tak jak trzeba się zachowywać. No on ma internet, oni się tam cały czas spotykają wirtualnie. Więc to jest też to pokolenie, gdzie tak funkcjonują na co dzień. (niezrozumiałe) spotkają się na boisku, pograją w piłkę, że tego mu brakuje, więc planują iść na boisko, pograć w piłkę.

**I co ty na to? To jest bezpieczne zachowanie czy nie?**

No myślę, że jak… Czy jest bezpieczne? Myślę, że tak. Bo są to koledzy, których zna. Z których nikt nie był na jakiejś kwarantannie, nie miał jakiegoś chorego. Planują być w maseczkach, w sześciu maksymalnie.

**Czyli zgodnie z przepisami.**

Tak.

**A jeśli chodzi o taki twój poziom niepokoju, obaw, zagrożenia, jest coś takiego w tej chwili?**

No właśnie nawet czasem się zastanawiałam. Bo nie pamiętam dokładnie, jaki był przy naszym pierwszym spotkaniu mój poziom lęku i takiego…

**Mówiłaś, że miałaś te ataki paniki i wtedy to było prawie 80. A jak rozmawiałyśmy, to tak się zatrzymywało w okolicach 60.**

I ja tak sobie myślę, że ten mój taki poziom takiego lęku, niepokoju, to jest cały czas te 60. Jakby tutaj ja… To mi się nie zmieniło. Nie mam tych ataków takiego lęku i że coś się dzieje, tego nie ma. Natomiast niepokój i taki lęk o sam wirus, ewentualnie zachorowanie jest. I przy całym tym rozprężeniu, dużej ilości zachorowań w porównaniu do tego pierwszego tygodnia, to tak, ta obawa jest cały czas. Ale bez takiej paniki.

**Ale to jest tak, że sprawdzasz wiadomości, patrzysz na te statystyki?**

Nie. To bardziej jest na takim poziomie takiej świadomości, że to jednak jest. I to, że wyszłam na spacer, że się z kimś spotkałam… Dla mnie to nie zniknęło w ciągu jednego weekendu. To po prostu jest, bardziej się do tego przyzwyczajamy, oswajamy. Natomiast ja bym w takie rozluźnienie nie szła, że to już jest lepiej.

**A czy to rozluźnienie jest jednocześnie dla ciebie nowym źródłem niepokoju i lęku?**

Tak. To jest takie, że jednak ci ludzie chętniej do tych sklepów chodzą. Że jednak się bardziej chętnie spotykają. Zresztą ja słyszałam, że spotykają się na jakichś tam grillach. No spotykają się. Więc gdzieś z tyłu głowy mam, że może ta osoba, która była na takim spotkaniu, gdzie było, nie wiem, 12 osób, a nie 4, gdzie też nie ma gwarancji, że wśród czterech się nie zarazimy, ale podobieństwo jest mniejsze. Czy to nie spowoduje większego przenoszenia się i… No mówię, gdzieś, że jednak to jest. Nie udawajmy, że tego nie ma, że się polepszyło. I tak, ja rozumiem i zgadzam się z tym, że więcej ludzi umiera na zawał serca, bo zawsze tak było. Ale jakby nie prowokujmy czegoś więcej. To tak nie poniosę się chyba szybko takiej fali, że jest wszystko w porządku.

**W twoim najbliższym otoczeniu jest dużo osób, które masz wrażenie, że właśnie uważają, że tego wirusa nie ma, że to jest wymyślone, że to jest manipulacja, jakiś spisek?**

Nie. Wśród takich najbliższych to nie. Jakaś może z jedna osoba taka, właśnie ten, u którego byliśmy, bo on tak stwierdził, że to jest w ogóle… Nie, co to tam będzie itd., więc… Ale nie, raczej większość tak z dystansem podchodzi i ostrożnie.

**A zakupy? Czy coś się zmieniło u ciebie w tej chwili z zakupami? Częściej, rzadziej, jak to jest?**

Kupiłam bluzkę sobie. Kupiłam sobie bluzkę (śmiech), śmiałam się, że po prostu zakup kwartału (śmiech). Ale to też pojechałam do sklepu po maseczki dla moich chłopaków, takie z materiału. Bo miałam tylko w jakieś kropeczki i gwiazdeczki, a oni chcieli jakieś ciemne. Więc pojechałam do takiego zaprzyjaźnionego sklepu z odzieżą, bo wiedziałam, że… Wyczytałam na Facebooku, skorzystałam z dobrodziejstwa mediów, że mają w sprzedaży te maseczki. No i jak już byłam u tych dziewczyn i one takie całe, no w ciężkiej sytuacji, bo jakby towar… Ona mówi, że 2 tygodnie przed całym tym, jak się zaczęło, po prostu zamówiła, zapłaciła za towar na wiosnę-lato. To po prostu… To jest, pieniądze ma zamrożone, a sprzedaje maseczki po 6 zł. Więc no tak przygnębiająco się zrobiło. I mówi, no starają się coś tam robić, jakieś zdjęcia. Ale to też nie jest taki towar, który się kupuje w internecie. I tak stałam i tak stwierdziłam, że właściwie kupię sobie bluzeczkę, bo to będą 2 korzyści. Zrobię sobie przyjemność po takim czasie. A też miałam takie poczucie, że je wspieram. Że robię coś dobrego dla kogoś i dla siebie.

**To był legalnie otwarty sklep?**

Tak, tak.

**One dopiero teraz się otworzyły jak rozumiem?**

To jest mały sklep, taki butikowy, to nie jest w galerii, to jest wolnostojący. I one mogą pracować.

**A i ta sprzedaż im, jak rozumiem, niespecjalnie idzie, jak mówisz.**

Nie. A poza tym też takie ubrania, to jest taki sezon, nie wiem, nie ma imprez, oni dużo mają takich, jakieś tam komunijne rzeczy, te wszystkie stroje. Czy nawet, tak jak ona mówi, że sporo jej klientek pracuje w biurach. A teraz pracują online, więc tak naprawdę chodzą w dresach a nie w żakietach, koszulowych bluzkach.

**A coś jeszcze kupiłaś dla przyjemności? Sobie albo komuś?**

Nie (śmiech). Sobie kupiłam kwiatki do doniczek, do ogródka. Zdecydowanie, to była przyjemność dla wszystkich, bo wszystkim się podobały. Więc tak.

**A planujesz coś kupować jeszcze, tak dla poprawy humoru, przyjemności? Z takich rzeczy niespożywczych.**

Tak, w końcu kupię sobie suszarko lokówkę, którą już kupuję chyba rok czasu. I w końcu stwierdziłam, że to jest ten moment, że ja ją kupię.

**Dlaczego to jest ten moment?**

Dlaczego? Bo tyle czasu się zbierałam. Nie miałam czasu obejrzeć. Że aż się zebrałam, pojechałam do sklepu ją obejrzeć z zamiarem kupienia. Ale otóż nie było tego modelu, pan nie miał opakowania.

**Jeszcze coś? Jak tak pogrzebiemy, to się cała lista zrobi (śmiech).**

Nie, chyba więcej nie. Nie mam planów.

**Zakupy spożywcze tak jak zwykle, jak ostatnio? Czy już wracasz troszkę sprzed zwyczajów sprzed pandemii.**

A dobra. To tak, jak tak pogrzebiemy, to faktycznie (śmiech). Jak przejeżdżałam, tam akurat właśnie, jak jeździłam w tych sprawach swoich i przejeżdżałam obok sklepu, tam w niedalekiej okolicy jest taki duży sklep z włoskimi rzeczami, w ogóle z winami, z makronami. Mnóstwo rzeczy. I też to zawsze, że ja w biegu i pędzę, i nie miałam czasu tam skręcić. I jak przejeżdżałam, mówię, ojej, jest otwarty. Mówię, jak będę wracać i jeszcze będzie otwarty, to ja sobie pójdę i zrobię zakupy. Więc tak, kupiłam sobie butelkę wina, takiego nalewanego. Oliwki i szynkę parmeńską. Tak po prostu, dla takiej przyjemności, żeby… No właśnie, to było coś innego, czego przez ten czas… Jakby robiłam zakupy w takich sklepach, ale w tym konkretnym, do którego się wybierałam dłuższy czas, to teraz dotarłam.

**I powiedz mi, bo to jest bardzo ciekawa historia, którą opowiedziałaś. Czy to jest tak, że miałaś takie poczucie, że robisz sobie przyjemność, ale też się nagradzasz w jakiś sposób?**

Tak. Tak, bo to był ten moment, gdzie ja już załatwiłam wszystkie te sprawy, już miałam czystą kartę. I miałam taki luz, swobodę, nie miałam już nic na głowie. Znaczy wszystko pozałatwiałam, to co miałam załatwić. I to był ten moment, gdzie już ten ciężar ze mnie zszedł, te supły się rozwiązały. To było takie, że tak ze spokojem, nie mam nic na głowie i mogę spokojnie po prostu sobie wybrać wino.

**W momencie tych supłów, które miałaś, w ogóle by ci przyszło do głowy, żeby kupić sobie bluzkę albo…**

Nie, absolutnie nie. Nie. Ja mam zadanie, ja mam rzeczy do wykonania.

**Czyli na to potrzeba luzu i takiego odprężenia troszkę?**

Tak, właśnie. Ja zresztą tak funkcjonuję. Że ja, żeby… Znaczy nie wiem, to nie jest dobre… Bo ja w ogóle, jak coś robię takiego, że przyjemność, że dla siebie… Znaczy ja się staram, ale nie dbam o siebie tak do końca. To ja lubię mieć taki spokój, że nie mam nic takiego do załatwienia, do uregulowania, do… I wtedy jestem w stanie sobie spokojnie, nie wiem, nawet jak dietę sobie planowałam. Czy nawet dieta, czy zdrowe żywienie, już ja nie mówię o jakichś takich… To lubię w takich komfortowych warunkach. Że to jest tylko to i na tym się skupiam. Natomiast, jak mam coś ważnego do załatwienia, coś takiego poważnego, to takie już przyjemności odchodzą na bok.

**Czyli dopóki to się dzieje, dopóki załatwiasz, to ewentualnie o tym możesz gadać, rozmawiać, na części rozdzielać, ale takie rzeczy, żebyś miała sobie to przerwać, uprzyjemniając sobie butelką świetnego wina albo oliwkami to niekoniecznie?**

No niekoniecznie.

**Czy to też mogłoby pomóc w takiej sytuacji?**

No chyba nie. No nie. Nie, bo… Nie, bo nie (śmiech).

**Czyli nic z tego by nie było, gdyby to było w tamtym momencie, to jedzenie tych oliwek i picie tego wina ze ściśniętym supłem to nie jesteś ty.**

Nie, to w ogóle nie.

**A gdzie ten świetny sklep jest?**

To jest w Ursusie. To jest Ursus przy… To jest na dawnym terenie zakładów w Ursusie. Bo jest dużo takich małych sklepików. A ten rzeczywiście, mówię, pierwszy raz, nawet stałam w kolejce grzecznie. Jeszcze spotkałam fantastycznego chłopaka, młody chłopak, który kroił tą wędlinę i w ogóle rozmawiał z klientami. Więc w ogóle… Strasznie taki miły czas, stojąc w kolejce. Było widać, że chłopak robił to z pasją. On w ogóle o tych szynkach opowiadał. Ja strasznie lubię, jak ludzie coś robią i widać, że cieszą się tym. Więc no taki (śmiech)

**Mówisz, że cię te rozluźnienia, które wprowadzają, drażnią. A otwarcie galerii handlowych, co o tym myślisz?**

Co o tym myślę? To znaczy na takich zasadach, jak to jest… To znaczy mówię, to jest niespójne. Bo w Castoramie można sobie chodzić, jak się chce, a tutaj są jakieś takie obostrzenia… Znaczy na tych zasadach no ja myślę, że tak jak mój sklepi funkcjonuje. Jeśli to jest właśnie z zachowaniem wszystkich tych odstępów, jakichś takich zasad… No myślę, że dobrze. A poza tym ludzie wrócą do pracy też. Będą mieli jakiś cel, jakieś takie poczucie, że coś robią. Że nawet jak za mniejsze pieniądze, bo to tak będzie wyglądało, to będą czuli się potrzebni, będą coś robić. Ja myślę, że to będzie dobre dla tych, którzy pracują, bo jednak takie galerie zatrudniają mnóstwo ludzi. Więc to zdecydowanie… No myślę, że tak stopniowo…

**A jak myślisz, dlaczego w ogóle je otworzono? Jaki był powód?**

No, że… (śmiech) Znaczy ja w to nie wnikałam. Bo jakby to są tak różne i dziwne zachowana rządów, że ja tam logiki nie widzę. Znaczy to jest pewnie jakiś cel, ale jakoś tego nie analizuję. Nie wiem, czy to trochę związane z wyborami, żeby jednak społeczeństwo tak trochę ugłaskać, że jednak te galerie są bardzo ważne w naszym społeczeństwie. Że ludzie lubią przebywać w takich miejscach i spędzać czas. No, bo to, żeby odmrażać gospodarkę, po części może tak. Ale jakoś tak mi to… (śmiech).

**Bardziej takie igrzyska niż dobro ludzi?**

Tak, tak, tak. No, a otwarcie żłobków zupełnie mnie zaskoczyło. Zupełnie, bo no nie wiem, ja bym bardziej chyba takie starsze dzieci. Na takich właśnie zasadach, że nie wiem, w maseczkach, że się tam nie… Nie wyobrażam sobie, że małe dzieci, żłobkowe, półtoraroczne, dwuletnie, że nie wiem, nie bawią się, nie dotykają, a pani ich nie bierze na ręce i nie przytula. W ogóle ja tego nie widzę. Tym bardziej, że samorządy mają się tym zająć, samorządy są nieprzystosowane. Zresztą tak jak nasz pracownik też do przedszkola dziecka nie posłał, bo tak naprawdę burmistrz się nie zgodził do końca na otwarcie. Te miejsca, które są otwarte i są dostępne w pierwszej kolejności mają dzieci pracowników służby zdrowia. I tam jest chyba 12 osób. Więc on automatycznie tego dziecka nie może wysłać do przedszkola. Więc to zupełnie… Na jakiej zasadzie te dzieci mają być przyjmowane?

**Jak rozumiem, jesteś przeciwna, żeby to otwierano. Czy nie?**

Myślę, że tak. No nie wiem. A z drugiej strony słyszę takie głosy, że na przykład już rodzice z takimi małymi dziećmi są już zmęczeni. I podejrzewam, że gdybym miała małe dziecko, może bym była chętna. Że niech otworzą, że ja w końcu pozbędę się, odpocznę. Albo nawet dzisiaj w sklepie jakaś mama z dziewczynką powiedziała, że niech już wróci ta opiekunka, bo ja mam już dosyć. Więc punkt widzenia od punktu siedzenia (śmiech). Ale nie widzę w tym jakiejś takiej logiki. Że dlaczego żłobki, a nie na przykład klasy 1-3? Gdzie i nauka jest i jakoś bardzie można te dzieci, nie wiem, wytłumaczyć im, zdyscyplinować.

**A to otwarcie galerii, jeszcze wracając do sklepów, uważasz, że to jest bezpieczne rozwiązanie teraz?**

Czy jest bezpieczne? Znaczy ja widziałam jakieś urywki w telewizji, jak to wygląda. Nie wiem, czy jest bezpieczne. Tak naprawdę to nie mamy gwarancji, co jest bezpieczne, co nie. Ale w porównaniu do tego, co… Mówię, przejeżdżałam koło Leroy i tak jak kuzynka mi opowiadała, tak jak pojechała w ciągu dnia, bo akurat miała urlop, mówi, to w ciągu dnia nie będzie ludzi. To ona się przeraziła tym tłumem. Gdzie siedziała miesiąc w domu, pojechała z myślą właśnie, że z zachowaniem odstępu wejdzie do sklepu, kupi jakiś tam nawóz do kwiatków. A ona mówi, po prostu znalazłam się w jakimś epicentrum. No to taka galeria, gdzie są odstępy, gdzie są czyszczone klamki, to jest chyba mniejszym zagrożeniem.

**Wybierasz się w najbliższym czasie?**

Nie.

**A znasz kogoś, kto się wybiera?**

Nie. Chociaż podejrzewam jedną koleżankę, która myślę, że może się wybrać, bo ona jest uzależniona od galerii. I na pewno jej tego brakuje. Więc ona skorzysta. Ale też, jak usłyszałam jakieś takie dwie opinie, urywki, bo ja te wiadomości to naprawdę tak powierzchownie oglądam i słucham. Jak usłyszałam, że właściwie… Przyszłam z ciekawości, zobaczyć, no to byłam zdziwiona, trochę przerażona. Bo jak zaczniemy chodzić w różne miejsca z ciekawości, to zaraz pójdziemy w to miejsce, w tamto miejsce dla samej ciekawości. Albo starsza pani, która mówi, no już siedziałam miesiąc czasu to tak sobie przyszłam.

**Próbuję zrozumieć. Bo tak, jak ty poszłaś po to wino i po te oliwki, to było przy okazji. Bo byłaś tam i stwierdziłaś, że jak będziesz wracała, to zrobisz sobie przyjemność. A po tę bluzkę pojechałaś specjalnie dla przyjemności? Czy też to było przy jakiejś okazji?**

Nie, przy okazji. Bo ja pojechałam po maseczki.

**Aha, prawda, przy okazji. Czyli takiego wyjścia dla przyjemności specjalnie po zakupy stacjonarne, takie niespożywcze jeszcze sobie nie robisz?**

Nie.

**A planujesz w najbliższym czasie, żeby zacząć to robić?**

Nie, nie planuję.

**To jaki byłby moment, w którym ty byś powiedziała, a już jest pora, żeby sobie pochodzić. Tak jak sobie zawsze chodziłam, żeby sobie coś kupić.**

No jest problem, bo ja tak nie lubię chodzić (śmiech).

**Ale mówiłaś, że jakieś rzeczy, że gdyby można było dotknąć, to byś kupiła?**

Tylko, że to nie są rzeczy takiej pierwszej potrzeby. Więc nie mam takiego… Nie, raczej nie.

**I w ten sposób doszłyśmy do tematu wydawania pieniędzy. Gdybyś miała o sobie pomyśleć i na skali od 1 do 10 siebie gdzieś się umieścić (łatwość wydawania pieniędzy – skala) to gdzie byś była?**

Ale w obecnej sytuacji czy w ogóle?

**Najlepiej i w obecnej i w ogóle jak siebie widzisz?**

Jak siebie widzę (śmiech). To też zależy na co wydaję te pieniądze, bo to ma znaczenie. Ale generalnie to wydawania pieniędzy taka jestem bliżej 1 niż 10.

**Czyli z dużym trudem?**

Tak. Ja nie jestem takim z łatwością wydającym pieniądze. Ja muszę przemyśleć. To nie, to nie jest tak, że…

**To opowiedz, na czym to polega. Jakichś kilka przykładów, na czym polega to, że jesteś bliżej 1?**

Mój mąż by opowiedział o tym bardzo długą historię i wiele przykładów przytoczył. Ale to chociażby z tą moją suszarko-lokówką. Gdzie właśnie powiedziałam, wiesz co, to podjedziemy do sklepu, ja bym sobie zobaczyła. A mój mąż mówi: ale ty już dawno miałaś kupić, to ty jeszcze nie kupiłaś tego? Ja mówię nie, bo wiesz, tak się zastanawiałam, czy ona mi jest tak do końca potrzebna, czy mi pasuje. A właściwie którą chcę i dlaczego. Tak że mi to zajęło tyle czasu. Ostatnio też coś było, że… No tutaj akurat, kupując tą bluzkę, to jakby byłam, wiedziałam mniej więcej, która mi się przyda, bo to musi być też praktyczne. Ale też nie na takiej zasadzie, że kupuję 4. Tylko muszę się zastanowić, czy ona mi jest potrzebna czy nie. Albo się zastanawiam długo nad czymś, to kupuję. Na przykład kupowałam to wino. To też nie było tak, że weszłam, dobra, kupuję wino, szaleję, biorę to. No nie wiem, generalnie mogłam kupić 3 butelki, bo były 3 rodzaje wina. A ja dalej się zastanawiałam… To nawet nie jest kwestia, czy ja wydam tyle czy tyle. Tylko… No tak nie mam takiej łatwości wydawania.

**Ale co czujesz, co się z tobą dzieje, nad czym się zastanawiasz… Właśnie masz wybraną tę suszarkę, a jednak jej nie kupujesz w tym momencie ani w zaraz następnym, ani jeszcze w następnym, jak twierdzi twój mąż. To co się dzieje w twojej głowie, jakie to są myśli, jakie to są uczucia? Że wiesz, że właściwie masz ją wybraną, a jednak jej nie kupujesz?**

No właśnie ja sama się zastanawiam, dlaczego ja mam taki opór. Bo to nie jest to, że ja nie mam tych pieniędzy. Bo jest inaczej, gdybym rzeczywiście musiała odkładać, jakoś bardzo sobie z czegoś rezygnować. Ale zawsze myślę, czy na pewno jest mi to potrzebne? Że może bez tego też, przecież sobie daję radę. Nie popsuła mi się stara, to dlaczego mam kupować nową? To tak jak ta bluzka. No przecież jeszcze mam dobre, nowe, kilka, to po co mi kolejna? No to tak… Tak w sumie nie do końca, znaczy nie wiem, skąd. Mój mąż mówi, że to mam po swoim tatusiu. Że on 4 razy przemyśli, że po co to wydawać i na co to wydawać.

**Trzeba się przespać z tą myślą koniecznie, zanim się wyda pieniądze? A gdybyś sobie spróbowała przypomnieć jakieś swoje większe zakupy niespożywcze. Typu, że wydałaś na wakacje, na meble, na coś subiektywnie dużego. Co to było, co to była za sytuacja? Taka poza codziennymi zakupami.**

No to tak, wydać na wycieczkę, na wyjazd. To tak, to tu się nie zastanawiam. To tu mam łatwość (śmiech). Takie wyjazdy to tak.

**Co jeszcze?**

Nie mam też trudności z wydawaniem pieniędzy, kupowaniem rzeczy dla kogoś.

**Coś dużego dla kogoś kupiłaś? Taki subiektywnie większy wydatek.**

Znaczy powiedzmy tak. Gdy mam do wyboru, nie wiem, niech to będzie odkurzacz. I mam sobie kupić odkurzacz, a mam kupić córce, mamie. To jeśli mam dla którejś z nich kupić, nie ma problemu, ja pójdę teraz i kupię. Natomiast dla siebie, to się pozastanawiam.

**Bo może jeszcze twój się nadaje?**

Tak, dokładnie (śmiech).

**Coś jeszcze, jakiś przykład ci przychodzi do głowy z takich rzeczy właśnie niecodziennych, większych?**

Kosmetyki bardzo… Nie, na przykład, jeśli potrzebuję jakiś kosmetyk taki droższy, dobry, to wolę kupić i nie mam problemu, że kupię jeden droższy niż mnóstwo kupować, ciągle jakichś nowych i eksperymentować. To tu też.

**A czemu tak? Z czego to wynika?**

Wolę kupić jedną, dobrą rzecz, którą lubię, o której wiem, że nie dostanę alergii, że jest sprawdzona. To wtedy tak. To wolę mieć jedną, porządną rzecz niż kilka. I tu mogę kupić od razu. Kończy mi się dobry krem, to kupuję czy potrzebuję drugiego, żeby sprawdzić, to tu mam większą łatwość. No i na kwiaty jestem w stanie wydać też. Ja bardzo lubię mieć świeże kwiaty w wazonie. I też było tak, że tutaj w trakcie takich tych zawirowań, gdzie… No kwiaty najczęściej na targu, jakimś tam bazarku kupuję. Albo mąż mi gdzieś tam kupuje. No i nie kupił, bo nie było. To pojechałam specjalnie do kwiaciarni, kupiłam kwiatki. I w ogóle nie patrząc… Tak, bo potrzebowałam mieć kwiatki.

**Powiedziałaś tak spontanicznie o tych wyjazdach, wycieczkach, że na to tak, na to bez problemu. Dlaczego na to bez problemu, co ci dają te wycieczki? Co masz za te pieniądze, które wydajesz?**

Co mam? Mam spokój, mam to, że spełniam jakieś swoje marzenia, że poznaję nowe miejsca, nowych ludzi, zmieniam otoczenie. Nie wiem… Oglądam to, o czym czytałam czy oglądałam. Takie… Zaspokaja taką moją ciekawość świata.

**Czyli, jeśli dobrze to rozumiem, łatwiej ci jest wydać pieniądze na doświadczanie, doznawanie nowych rzeczy. Bo z tymi kosmetykami mam wrażenie, że to też jest jakiś rodzaj doznawania i z kwiatami. Łatwiej niż na rzeczy materialne typu bluzka, suszarka czy cokolwiek takiego.**

Tak, tak. Zdecydowanie.

**To jest taka inna kategoria potrzeb, zaspokajasz sobie inną potrzebę? I tutaj mogłabyś powiedzieć, że na tej skali to jest jak, gdzie jesteś?**

W takich potrzebach niematerialnych?

**Tak, na te wycieczki, te kwiaty.**

O, i na masaże. O, na masaż mogę też, tutaj sprawdzanie, tak. O, to jednak trochę (śmiech). To rzeczywiście tak. I na takie doznania to tu już jestem taka bliżej 8. 8-9. Tu mam mniejsze opory i uważam…

**A gdybyś miała powiedzieć, czy jesteś osobą rozrzutną czy oszczędną, to jak byś siebie nazwała?**

Oszczędną.

**I co to znaczy dla ciebie? Oszczędna to znaczy jaka?**

To znaczy, że właśnie zastanawiam się nad tym, co mam kupić, że planuję wydatki. Że staram się mieć kontrolę nad tym, nad swoimi finansami. Że nie jest to takie lekkomyślne, że jakby mam taki podział rzeczy ważnych, typu opłaty, rzeczy, które muszę jakby no wszystkie opłacić, rachunki. I tak, żeby właśnie zostało jeszcze na te swoje przyjemności. I że nie jest to takie pochopne. I że takie dzielenie, że rzeczy bardziej potrzebne, mniej potrzebne.

**A ta kontrola wydatków na czym u ciebie polega? Co robisz?**

Znaczy kontrola wydatków, no może to źle powiedziane. To jest, no właśnie, że nie kupuję sobie czegoś ekstra, a za prąd jak przyjdzie, to będę myślała. Że najpierw zapłacę za prąd. Jakby no wiem, ile mniej więcej w miesiącu muszę zapłacić za telefon, za prąd, za jakiś tam gaz. Pomijając te służbowe rzeczy, ZUS itd. I jakby to jest priorytetem. I właśnie, czy mogę w danym miesiącu odłożyć sobie na wyjazd czy nie. Albo tak kalkulować, żeby jeszcze zostało na coś. Albo mieć, też inaczej, no takie zabezpieczenie, że wypada coś ekstra, jakaś potrzeba, właśnie typu, że muszę znaleźć się u dentysty. I nie jest to, że ojejku, z czego zapłacę. Tylko, że na takie rzeczy muszę mieć takie… No nie ma dyskusji powiedzmy.

**Jak patrzysz na waszą obecną sytuację, to jak to, co się dzieje, zmieniło waszą sytuację finansową?**

Jak zmieniło?

**Czy zmieniły się wasze dochody, jak to teraz wygląda?**

Tak naprawdę minimalnie się obniżyły.

**Minimalnie?**

Tak. Bo jakby, no to jest taka składowa kilku działalności. I jakby jedne poszły w dół, czyli tak jak u mnie, mam zero. Powiedzmy te spożywcze poszły w górę, ale jakieś tam pozostałe… I to się wyrównało tak naprawdę.

**Czyli to nie jest jakieś zagrożenie budżetu domowego w tej chwili?**

Nie. Nie, nie, nie. Nie jest aż tak… Znaczy zagrożenie by było, gdybyśmy na przykład musieli zamknąć sklep. No to tu jest zagrożenie, bo mamy kredyt. Więc to jest takie największe obciążenie. Natomiast na tą chwilę jest to taka stabilna sytuacja.

**Na ile masz wrażenie, że to wróci do normy sprzed pandemii? Jak myślisz?**

Czy to wróci do normy… No nie wiem. To trudne jest pytanie, bo to jest niewiadoma. I tak z jednej strony ja wcześniej zakładałam, że nie wróci. Bo nawet, jeśli ja wrócę do swojej pracy, to takie miałam przemyślenia, że właściwie czy ja będę miała do czego wracać? Ale tu wychodzi takie moje subiektywne spojrzenie. Że powiedzmy ja, w momencie takiego zagrożenia, niestabilnej sytuacji, że właśnie tutaj czy praca taka czy taka, czy nie wiem, obniżka pensji, no ja tak funkcjonuję, że powiedzmy z tych przyjemności dla siebie, no to tak bym ograniczyła do minimum. Ale z drugiej strony patrzę, że jednak chyba większość działa tak, że no dobrze, siedzę tyle czasu, to ja pójdę sobie zrobię ten manicure. Niech to będą moje ostatnie pieniądze, ale ja zrobię sobie przyjemność. I tutaj, no mówię, to jakby to jak ja to widzę, a jak to funkcjonuje, czy jak ludzie zachowują się, to są dwie różne rzeczy.

**Czyli trudno ci jest przewidywać, czy to wróci do normy i kiedy.**

Tak. Bo to jest… Mówię, bo patrząc po sobie, wróci do normy, ale stopniowo. Natomiast patrząc, jak się ludzie zachowują, jak właśnie same te telefony klientek, no to jakby – zaczynajmy od jutra!

**Jakby już wróciło?**

Prawda, spotykajmy się, wszystko będzie takie… No to trudno mi powiedzieć.

**A czy w ogóle w związku z tym, że jednak ten jakiś minimalny spadek przychodów zanotowaliście, ty podjęłaś jakieś działania ograniczające wasze wydatki?**

Tak naprawdę to ograniczyło się samo (śmiech). Ograniczyło się samo, bo, no cóż, nie wychodzimy do restauracji. Wyjazdy odpadły. Więc… No to już jest jakby, to się samo ograniczyło.

**Ale na poziomie zakupów codziennych, nie kupię teraz takiej herbaty, tylko kupię tą tańszą. Albo poczekam, aż coś będzie w promocji. Czy coś takiego też się zadziało czy nie?**

Nie, nie, nie. No nie. Na szczęście nie muszę tego w takich kategoriach rozpatrywać. I nigdy nie byłam taka skora do promocji. Zawsze lubiłam, że kupię sobie mniej, ale to, co lubię.

**Nie byłaś łowcą promocji jak rozumiem.**

Nie.

**A czy kontrola wydatków jakaś jest inna w tej chwili niż była przed koronawirusem?**

Czy jest inna? Nie, myślę, że my cały czas staramy się to kontrolować, żeby… Znaczy nawet takie wydatki, no właśnie, taka sytuacja, której nikt nie przewidział, akurat, że koronawirus. Natomiast mówię, przy kredycie i przy takim, gdzie się zatrudnia pracowników, gdzie się ma stałe opłaty, tak naprawdę my zawsze funkcjonowaliśmy z takim zapleczem finansowym na jakiś okres czasu, gdyby się coś wydarzyło.

**Na jaki okres czasu? Jeżeli macie jakieś oszczędności, jakąś tą poduszkę, to na jaki czas by wam to wystarczyło? Nagle nie macie przychodów. To ile czasu możecie wytrzymać na tych oszczędnościach waszych?**

To znaczy to ja… Ja bardziej patrzę tak globalnie z całą firmą, z pracownikami. Że taki no kwartał mamy takiego zabezpieczenia. Że spokojnie z płaceniem świadczeń, to jest taki…

**Powiedziałaś, że zawsze staraliście się mieć to zabezpieczenie.**

Tak.

**Czy to jest tak, że ty albo twój mąż bardziej o tym myślicie? Jak to u was w domu wygląda, czy oboje o tym myślicie?**

No tu zdecydowanie mój mąż jest bardziej taki cyferkowy. Tu on bardziej… Znaczy przy moich sugestiach. Bo jakby on ma jakieś tam swoje takie wyliczenia, a ja dokładam… Znaczy na zasadzie uzupełnienia, ale zdecydowanie mój mąż jest bardziej w tym…

**Bardziej cyferkowy. A jeśli chodzi o taką skłonność do oszczędzania? Też on jest bardziej czy nie?**

Nie (śmiech). To ja jestem. Ja muszę trzymać rękę na pulsie i…

**Właśnie, miałam wrażenie, że on tutaj bardziej zarządza tymi oszczędnościami, ale to ty jesteś taką osobą, tak mi się właśnie wydawało, że powiesz, że to ty. A zawsze tak miałaś, czy to jest coś, czego się nauczyłaś? Jak to jest z tą taką skłonnością do odkładania, oszczędzania u ciebie?**

Znaczy to zawsze chyba tak miałam. Zawsze tak.

**Ale tak było w domu, tak wyniosłaś z domu czy sama tak wymyśliłaś?**

No chyba sama, tak mi się… No mówię, może trochę mój tata taki właśnie, trochę mam po tacie, że właśnie, żeby było odłożone, żeby nie zaszaleć za bardzo. No to tak chyba tutaj bym takiej inspiracji szukała.

**A jakie są twoje metody na oszczędzanie? Bo ludzie to w różny sposób robią. Niektórzy na początku miesiąca odkładają, zanim wydadzą, niektórzy to, co im zostanie. Niektórzy mają jakieś zaokrąglanie na kontach, żeby im ta reszta szła na jakieś inne rzeczy. Metod jest mnóstwo.**

Mówię, to z określonego dochodu, po odliczeniu tych rzeczy, tych podstawowych, to co muszę, żeby nie robić zaległości, jakiegoś zadłużenia, jakiegoś opóźnienia. To na zasadzie, że dobrze, to co nam zostaje, mamy na swoje takie potrzeby na bieżąco. A jak coś zostaje, to jest wtedy odkładane.

**I odkładane jest gdzie? Macie konto oszczędnościowe, macie specjalne pudełko?**

Na konto. Tak, bo to z takich rozrachunków po prostu wychodzi. I to, co pozostaje, to jest… Chociaż mój mąż lubi mieć trochę gotówki, to on ma taki swój… Ale to jest takie naprawdę na…

**A właśnie, poza tym, że macie to konto, to jest wasze wspólne, to oszczędnościowe? Oszczędzacie razem?**

Razem, tak.

**A osobno każde też?**

Nie. My jesteśmy taką patologiczną rodziną teraz, wszystko mamy wspólne (śmiech).

**Powiedziałaś, bo kredyt, bo dentysta, bo się ma pracowników. Są jeszcze jakieś powody, dla których masz potrzebę, żeby mieć tą poduszkę finansową, te oszczędności?**

To znaczy ja myślę, że taki na przykład… taki komfort… Znaczy ja bardziej myślę o takich zdarzeniach losowych, że coś się dzieje. I po prostu… Mam ten komfort, że nie wiem, nie czekam na lekarza 3 miesiące, tylko po prostu idę, nie wiem, robię badania. I nie oglądam się. Bo to chyba jest takie najbardziej… No, takie namacalne, gdzie mi to pomaga. Bo to, czy ja kupię sobie lepszy czy gorszy samochód, no to jakby to… To nie jest dla mnie. Ale nie wiem, no chociażby jak mój tata zachorował. I miałam ten komfort, że po prostu zanim doczekał się na rehabilitację w NFZ, ja miałam ten komfort, że miałam te pieniądze na super rehabilitację na dobre zabezpieczenie, żeby on wyzdrowiał.

**A nie po to, żeby zaoszczędzić i wydać na super furę i nią podjechać pod sklep albo pod salon kosmetyczny.**

Nie, nie, w ogóle to nie (śmiech). To bardziej, żeby odłożyć, że wystarczy mi na fajny wyjazd gdzieś egzotyczny. Albo że pojadę do jakiegoś fajnego spa. I tam będę po prostu mogła korzystać. A nie oszczędzać, że no dobrze, to teraz nie pójdę na kawę, bo ona kosztuje tyle i tyle, albo nie zjem kolacji, bo pójdę sobie na zapiekankę. To mi wtedy daje taki komfort, wtedy korzystam z tych pieniędzy tak naprawdę dla swojej przyjemności.

**No właśnie. Miałam zapytać od razu przy tych wyjazdach. Czy jak już wyjeżdżasz i jesteś na wakacjach, to wtedy będziesz się zastanawiała też i przesypiała z myślą, czy kupić taką suszarkę, jeżeli ją zobaczysz gdzieś w sklepie, czy ją po prostu tam kupisz?**

Może suszarkę nie, ale wtedy korzystam ze wszystkich dobrodziejstw, jakie tam mam. Czyli powiedzmy, nie wiem, gdzieś chcę dojechać dodatkowo, czy wejść do jakiegoś muzeum, to wtedy już nie zastanawiam się. Jestem tam i ze wszystkiego… No, w miarę budżetu, jaki mam, prawda? Ale nie przeliczam tego.

**Tak, ale to się tak jakoś łączy z większą łatwością wydawania na wyjeździe, jak rozumiem.**

No chyba tak.

**W ramach rozsądku, to wszystko rozumiem.**

Tak. Ale jest taki komfort, że nie jadę, nie wiem, wysupłałam te pieniądze i będę teraz tam korzystać tylko z takiego minimum. Nie, już wtedy jakby… Albo z ubezpieczeniem, nie oszczędzam na takich rzeczach. Nie wiem, ubezpieczenie, jakieś dodatkowe rzeczy. Wtedy mam ten komfort i już wtedy nie oszczędzam.

**Czy w twoim podejściu do oszczędzania coś się zmieniło w związku z tym, że mamy tę sytuację z koronawirusem?**

Czy coś się zmieniło? Znaczy no… Nie, no bardziej, no nawet te sytuacje ostatnie, że właśnie takie… Oszczędzanie, oszczędzanie. Znaczy ja jeszcze bardziej nad tym myślę. No właśnie, wchodząc po taką maseczkę, nie zastanawiałabym się, dlaczego kupuję tą bluzkę, czy dla przyjemności, czy komuś pomagam. To takie rzeczy się zmieniły. Ale czy oszczędzam?

**Znaczy, czy jeszcze bardziej jesteś przekonana, że warto oszczędzać? Czy może poszło ci w tą stronę, że kurcze, tyle oszczędzałam, teraz wszystko szlag trafi, nie będę miała na co wydać i tak trzeba było używać tych pieniędzy…**

Ja myślę, że na tyle, jakie są moje potrzeby, to korzystałam. I z tego się cieszę, bo nawet a propos tych wyjazdów, to też nie mam takiego, no po prostu, one się nie odbędą. I właśnie, to, że korzystałam z nich wcześniej, to mam ten komfort, że superfajnie, że korzystałam. Teraz jest czas na niekorzystanie. Będzie na korzystanie z innych rzeczy. I działka na Mazurach jest super, ekstra i też jest pięknie. Dalej jestem właśnie przekonana, że warto oszczędzać, żeby mieć ten komfort, że właśnie jak coś się zadziało, że mogłam sobie pozwolić na to, żeby dać pracownikom dodatkową premię za to, że przychodziły do pracy. Że to nie było na granicy. Że jak trzeba było zapłacić więcej za rękawiczki, to ja miałam na to pieniądze. Że ja miałam na zabezpieczenie. I nie zastanawiałam się, nie, no oszczędzę, niech chodzą cały dzień w jednej parze, żeby nie wydać. I to daje poczucie bezpieczeństwa. I uważam, że dla takich rzeczy warto nie kupować sobie 10 bluzek w miesiącu (śmiech).

**Ale gdybyś nie korzystała do tej pory, nie wyjeżdżała, tylko cały czas czekała na te wyjazdy, to jakoś masz wrażenie, że inaczej byś do tego podeszła? Powiedziałaś: no na szczęście zdążyłam troszkę powyjeżdżać za te oszczędności.**

Że na szczęście zdążyłam? Nie, ja jestem wdzięczna, że mogłam, to bardziej tak.

**Rozumiem, tylko chciałam się upewnić, czy jest w tym jakieś drugie dno, że trzeba jednak używać tych pieniędzy na przykład.**

Nie. Trzeba korzystać, ale bez szaleństwa. Myślę, że umiar…

**A jeśli chodzi o inwestowanie, macie taką skłonność, żeby te oszczędności inwestować w coś? Czy raczej to jest trzymanie na koncie?**

No nie, bardziej inwestować czy rozbudowywać to, co mamy. Więc to jest bardziej inwestycja i stąd kredyty, no bo…

**I właśnie tutaj jest mąż od tych cyferek i kombinowania, jak rozumiem. Czy to są wspólne decyzje?**

Nie, no to są wspólne decyzje. Ale to jest takie… My się uzupełniamy. Bo on się nakręca, ja tam troszkę stopuję. I idziemy na kompromis.

**Czyli on ma większy rozmach w tych pomysłach swoich na inwestycje?**

Jest bardziej odważny może też. Bo ja jestem taka troszkę ostrożna i tak chciałabym, ale się boję. Ale jak już tak dostanę takie wsparcie, że razem i on mi to przedstawi, uargumentuje, wytłumaczy. I ja tu nie widzę dużego zagrożenia, no to jestem… Właśnie tak jak nawet z takim kredytem, to ja właśnie muszę mieć takie poczucie, że w razie czego mi starczy na te raty, a nie że mi wszystko zabiorą. Bo to właśnie nie tak, że kredyt za kredytem i wiecznie na debecie, to nie. Ja na długach to nie.

**Rozumiem. A teraz czy masz wrażenie, czy w ogóle pojawił się temat, żeby inwestować teraz, w tych czasach pandemicznych?**

Jeśli się ma co, jeśli się ma fundusze, to można inwestować.

**A to jest dobry czas na inwestycje?**

Myślę, że jeśli się znajdzie odpowiednią, nie wiem, dziedzinę, w którą inwestować. Bo wiem, że sporo osób kupowało złoto. Niektórzy inwestowali w waluty, tam na początku. No to myślę, że gdyby były fundusze, to zawsze znajdzie się jakieś źródło, w które można zainwestować. To myślę, że tak.

**Koronawirus nie przekreśla tego, że może to być dobry czas na inwestycje, jak rozumiem.**

No to tak trochę jak na wojnach. Jedni tracą, drudzy zarabiają. To na tym polega, taki jest *rynek(?)*

**A twoje myślenie o przyszłości, o tym co będzie. Jakie są twoje przewidywania na temat przyszłości? Tej bliższej i tej dalszej.**

Ale przyszłości…

**Już nie tylko w kontekście finansowym. W ogóle. Tego koronawirusa, tego jak to długo będzie wszystko trwało, czy wrócimy do normy sprzed, czy nie wrócimy.**

No to się zmienia. Bo na początku myślałam, że w ogóle świat się zmieni, ludzie się czegoś nauczą, wyciągną wnioski. Ale myślę, że na taką dużą skalę to nie ma szansy. Bo jakby… No nie. Nie. Część pewnie coś tam przemyśli, coś zrozumie, część nie. Więc tu już moje nadzieje, że świat się zmieni, to już legły w gruzach. Myślę, że ekonomicznie odczujemy to. Ogólnie jako kraj, jako społeczeństwo. Odczujemy, bo to są takie… No mówię, z różnymi ludźmi się spotykam, z różnymi zawodami. I to są takie mniejsze i większe dramaty. I to się dzieje i może w takiej skali, jak ktoś ładnie opowiada, to nie. Ale to pozmienia się. A wiadomo, za tą strefą ekonomiczną idzie cała reszta. No nie wiem. My się w takich trudnych sytuacjach jako rodzina czy jako małżeństwo zbliżamy. Ale widzę, że dla wielu jest to w drugą stronę. Więc trudno jest to przewidywać. Bo to wszystko jest tak dynamiczne, tak zmienne, że to chyba nie ma na to mądrych.

**Ale zdarza ci się o tym myśleć, zastanawiać się, kiedy to się wreszcie skończy?**

Tak. I nie mam odniesienia… Ja sobie bardziej założyłam taki wrzesień. Nie wiem, dlaczego. Że jednak te wakacje, to lato, że to będzie inne. Że jednak ta ostrożność, że to musi potrwać, żeby… No nie wiem, czy nabrać odporności na tego wirusa, czy się z nim oswoić. No jakiś taki, ja zakładam dłuższy czas.

**A czy są jakieś sytuacje, w których takie myślenie o tym, kiedy to się wreszcie skończy, pojawia się częściej niż w innych sytuacjach? Jakieś takie momenty? Da się to jakoś tak określić? Kiedy ty o tym myślisz?**

Znaczy, tak jak na przykład w sklepie. Jak przychodzą klienci, jak zaczyna się to rozluźnienie, to czuję takie zmęczenie sytuacją i takie: niech to się już skończy. Bo nie wiem, kolejny raz zwracanie komuś uwagi, niech założy maseczkę albo że tu ma płyn do dezynfekcji. Albo że niech nie wchodzi kolejna osoba. I to jest męczące, bo to jest taka walka z wiatrakami. I jakby to, co powiedziałam na początku. Że mam wrażenie, że nie wszyscy to poważnie traktują. I to jest męczące. No i niech to się skończy.

**Uczucie zmęczenia, takiego już znużenia tym?**

Tak, takiej bezradności, gdzie nie mamy wpływu na inną osobę, na sytuację, a chcemy się chronić, czy swoich pracowników. Więc tutaj już taka niemoc i niech to się skończy. I wtedy myślę o tym, kiedy to się skończy, kiedy będzie taka normalność-normalność. Bo na razie to jest udawana normalność.

**Takie twoje obawy na kilka tygodni do przodu to dotyczą jakich rzeczy? Gdybyś miała powiedzieć, czego się obawiasz w najbliższych kilku tygodniach.**

Tego, żeby nie zarazić się wirusem. Mówię, to jest jedyna rzecz, której tak naprawdę się obawiam. Jakiejś takiej choroby. I to nawet chyba też nie wirusa. Bo ja cały czas mówię, nie wiem, dla mnie to, że zamknięte przychodnie, czy tam ograniczony dostęp do przychodni, do lekarzy, to jest taka moja największa obawa, że… Bo powiedzmy, nie wiem, czy kupię to czy to, czy ubiorę się w to czy w to, to jakby no nie ma takiego znaczenia. Ale już to nawet z tym dentystą, czy ja się do niego dostanę? No dobrze, jak mnie zacznie boleć, pójdę do innego. Ale nie chciałabym iść do innego, przypadkowego. I to są takie obawy w takich sytuacjach, na które nie mam wpływu. A jednak decydują o naszym zdrowiu i życiu.

**I to jest ta perspektywa kilku tygodni twoich obaw? Czy to jest w ogóle perspektywa ogólna na kilka miesięcy?**

No to w zależności jak sytuacja będzie postępować. I jak, nie wiem, służba zdrowia będzie funkcjonować, jak ta liczba będzie malała, rosła? No to jest taka największa niepewność.

**A twoje największe nadzieje na najbliższy czas są jakie?**

Że będzie ciepło i będę więcej korzystać ze świeżego powietrza.

**Podobno ma być nawet bardzo ciepło, nawet upalnie.**

Że pojadę na Mazury albo że będę się cieszyła swoim ogródkiem. Więc tak bardzo nie wybiegam (śmiech).

**Założyłaś, że do września powinno się rozwikłać.**

Tak. No tak sobie, taką przyjęłam.

**Chcesz jeszcze coś dodać od siebie, o co nie zapytałam?**

Czy ja coś jeszcze? Ja sobie nawet takie notatki zrobiłam. No tak, ale to właściwie wszystko omówiłyśmy (śmiech).

**A co to za notatki, co zapisałaś?**

Odnośnie takich, to co mówiłaś, żeby zwrócić uwagę na emocje, z czym się wiązały. No to te finansowe, tu rzeczywiście z tym lękiem. I właściwie to, o czym mówiłyśmy. Mówię, więcej było tych takich spięć i tych trudnych rzeczy. Ale mówię, te spacery i ten bez, który… I to, co w ogóle w całej tej sytuacji, co cały czas ja sobie… Ja się rano uśmiecham do tego i z tego. Jak wychodzę o 5 rano, pięknie śpiewają ptaki. I po prostu jak już wstaję taka, o Jezu, znowu muszę iść, muszę wstać. I one tak śpiewają. I po prostu ja się tak do nich uśmiecham. I mówię do męża, wiesz co, nie wiedziałam… Znaczy wiedziałam, bo powiedzmy, jak jesteśmy na działce, to te ptaki śpiewają, ale tu w domu… Ja mówię, wiesz co, jak one pięknie o 5 rano śpiewają. A on mówi tak, no o 7 też śpiewają. Ale ja mówię nie, o 5 inaczej śpiewają. I to mnie chyba jakoś tak… Po tym takim, jak wstanę z tego łóżka i idę taka nie do końca szczęśliwa, że wstałam i idę, to po prostu jak wychodzę i to świeże powietrze… I to w tej całej sytuacji pokręconej, to mnie tak… To daje mi taki strzał na dzień dobry.

**W tej chwili przeważa w tobie bardziej ta natura, te ptaki, ten bez, czy jeszcze ten supeł w tobie siedzi?**

Nie, już supła nie ma. To zamknęłam ten rozdział. I już teraz korzystam i cieszę się z tych małych rzeczy.

**A czy to była w ogóle taka sytuacja, że ona was zmusiła do naruszania oszczędności, poduszki finansowej czy nie?**

Znaczy zachwiała. Na pewno zachwiała, bo jakby nasz plan był… Zachwiała, natomiast nie… Mówię, ten nasz dystans do tego, żeby nie być na granicy się opłacał.

**Czyli trzeba było pomyśleć trochę o innych ruchach, ale nic się strasznego nie stało.**

Tak. Jest mniejszy komfort, ale nadal jest.

**Dziękuję bardzo.**
