# Supplementary material for: The challenges arising from the COVID-19 pandemic and the way people deal with them. A qualitative longitudinal study
Source: PLoS One. 2021 Oct 11;16(10):e0258133. doi: 10.1371/journal.pone.0258133 (PMC8504766; doi:10.1371/journal.pone.0258133)
Supplement: S1 Dataset — (ZIP) [file pone.0258133.s003.zip › Transcriptions/stage 4/20.4_F_25_couple, with child.docx]

**20.4_F_25_couple with child**

**Jak minęły ci ostatnie 2 tygodni?**

Co robiłam w majówkę? Szykowałam ten pokój i wysłałam ci zdjęcia efektów i parapetówkę z dziadkami. Wysłałam ci 2 zdjęcia z mojej działki, żebyś wiedziała, dlaczego wybieram te rzeczy zielone. W majówkę byliśmy u znajomych wreszcie na grillu. Nic więcej się nie działo.

**Czy coś się zmieniło?**

No właśnie to spotkanie ze znajomymi i chyba to było jedyne, co się zmieniło. No oprócz całej sytuacji, która się zmieniła w tym sensie, że więcej rzeczy jest otwartych. Galerie są pootwierane, ale jeszcze nie korzystałam oprócz Castoramy i OBI ze względu na ten pokój, które już wcześniej były otwarte.

**Jak to się stało, że postanowiliście się spotkać ze znajomymi?**

Po prostu zadzwonili do mnie i nas zaprosili. Stwierdzili, że chyba już można. Zawsze jak jest okazja, święto, nie święto, to się spotykamy wszyscy i tak teraz też się spotkaliśmy wszyscy. Ludzie już sobie chyba dali spokój z tą całą ostrożnością. Już nie wiadomo, co o tym myśleć.

**W jak dużym gronie się spotkaliście?**

Było może 12 osób. 6 par. Całkiem sporo, ale stwierdziliśmy, że skoro już wszystko jest otwarte to tak samo, jak idę do sklepu, gdzie jest 30 osób, których nie znam albo 100 osób w Castoramie, których nie znam, więc chyba już nie ma problemu, żeby się spotkać.

**Zachowywaliście jakieś środki bezpieczeństwa?**

Nie. Nie mieliśmy maseczek, nie mieliśmy rękawiczek. Tak normalnie. To było u znajomych w domu na działce. To nie była ich działka, tylko tam, gdzie mieszkają, ale mają duże podwórko, duży teren.

**Pojawiło się coś, co ograniczyłaś, z czego zrezygnowałaś?**

Nie. Raczej z niczego nie zrezygnowałam. Raczej wszystko jest bez zmian.

**A coś stało się wyzwaniem?**

Nie. Już teraz jest mniej wyzwań z tego względu, że na początku było ciężko przestawić się i przyzwyczaić się do pewnych rzeczy, a teraz raczej jest normalnie. Wręcz przeciwnie, wszystko się odwraca. W takim sensie, że wraca do tej niby normalności. Jest już o wiele łatwiej. W sklepie nie ma już takich kolejek. Oprócz tej maseczki i rękawiczek, ale to było już jakiś czas temu i do tego jestem przyzwyczajona, to normalnie się robi zakupy. Wiele rzeczy się jakby uluźniło.

**Mówisz niby normalności. Czego jeszcze brakuje, żeby było normalnie?**

Ja ciągle mówię, że to jest niby normalność, bo ta normalność wcale nie jest taka normalna. Jest taki dr Mateusz, pewnie go znasz?

**Nie kojarzę.**

On różne takie swoje przemyślenia wrzuca na FB. Ogólnie bardzo mądra osoba, z częścią jego przemyśleń się zgadzam, z częścią nie, ale właśnie wrzucił ostatnio posty na temat tej kwarantanny. On jest znaną osobą, ale ja dopiero pól roku temu zaczęłam go obserwować. Bardzo fajny facet, bardzo fajnie często mówi o wielu rzeczach i nie boi się mówić o wielu rzeczach. W 100% się zgadzam z nim w tym co napisał o kwarantannie i zachowaniach ludzi. To jest chyba najgorsze i dla mnie było takie uderzające, że wszystko robimy niestety pod ludzi, się okazuje, bo jak jesteśmy w domu, to tak jak ja - nie maluję paznokci, nie muszę się ładnie ubierać, chodzimy w dresach. A co się stanie za jakiś miesiąc? Znowu trzeba będzie się fryzować, cudować, ale po co? Już nie dla siebie, tylko dla ludzi, bo gdybyśmy robili coś dla siebie to byśmy robili to i teraz. A tu wszystkie takie rzeczy materialne stały się bardzo mało ważne. Naprawdę bardzo mało ważne. To znaczy, że wszystko, 99% rzeczy, które robimy, to robimy po to, żeby podobać się komuś tam z zewnątrz, a nie sobie. No i właśnie on o tym napisał parę rzeczy.

**Czy pojawiły się jakieś rzeczy, które bardzo zaczęły ci doskwierać?**

Nie, raczej wszystko wraca do życia sprzed 3 miesięcy.

**To spotkanie ze znajomymi. Stwierdziliście, że już dłużej nie wytrzymacie i musicie się spotkać?**

Nie, po prostu. Zadzwonił znajomy, że jest majówka organizuje grilla, czy przyjdziemy. Nikt się za bardzo nie zastanawiał. Wszyscy z naszego towarzystwa, gdzie jedni są w Szczecinie, drudzy w Warszawie, trzeci gdzieś tam, zjechali akurat na majówkę do domu i się spotkaliśmy, ale w ogóle nie poruszaliśmy tematu obecnej sytuacji. Jakoś tak było bardzo normalnie.

**Porozmawiajmy o zdjęciach, które mi wysłałaś.  Najpierw jest zdjęcie pokoju twojej córeczki.**

Miałam zbierać informacje z ostatnich 3 tygodni i to są akurat zdjęcia z majówki.

**Jakie emocje wam towarzyszyły?**

Była bardzo pracowita i nie myślałam za bardzo o tym, że jest majówka, bo mieliśmy bardzo dużo pracy w domu, bo babcia robiła remont w pokoju i po prostu mieliśmy dużo pracy. Jedyne, co było takiego fajnego, to ten grill, to wyjście. Zawsze na majówkę to mnie nie ma w domu zazwyczaj, zazwyczaj gdzieś wyjeżdżamy, a w tym roku nie wyjechaliśmy i tak bardzo nie odczulam, że to jest w ogóle majówka. Raczej jakieś tam dni wolne ze względu na to, że mój mąż był w domu, bo dni wolnych to jest już bardzo dużo od jakiegoś czasu, ale mój mąż był w domu, więc to bardziej taki przedłużony weekend a nie majówka.

**Jakie emocje towarzyszyły ci w ciągu ostatnich 2 tygodni? Jak się czułaś?**

Ja to mam problem w ogóle z nazywaniem uczuć, ale jak się mogłam czuć...Naprawdę bardzo normalnie. Jakoś nie miałam takich emocji w sensie góra, dół. Mozę stabilizacja, nie wiem...Może jak mi podpowiesz jakieś słowa?

**Nie miałaś zmian nastrojów?**

Nie miałam.

**Udało się też odpocząć?**

Tak, ten grill był taką rozrywką, której bardzo dawno już nie było. Nie spotykaliśmy się ze znajomymi wcześniej. Szczęście, że się udało skończyć ten pokój. Córka się bardzo cieszyła. Pewnie na porządku dziennym nie mielibyśmy tej możliwości i pewnie, gdyby nie ta kwarantanna, to byśmy w ogóle nie wpadli na ten pomysł, żeby zrobić ten pokój. Więc fajnie, że się udało. Teraz korzysta i jest zadowolona. Jakiś pozytywny aspekt tej majówki.

Jak się czułaś, jak spotkałaś się pierwszy raz od tak długiego czasu ze znajomymi? **Pamiętam, że mówiłaś, że dla ciebie jest trudne się nie spotykać, a jeszcze trudniejsze jest to dla córki.**

Córka to nadal tam…Jedyne co, to byliśmy 2 razy u mojej siostry ciotecznej. Ona ma też dwójkę dzieci i właśnie na tę majówkę musiałam ją zawieźć na jeden dzień, jak malowaliśmy, bo przecież by nam nie dała malować w ogóle. Ona też się garnie, bo ona bardzo lubi dzieci, więc ona się strasznie cieszy, ale to tylko i wyłącznie widziała się z dziećmi tej siostry i taka tam jest jeszcze dziewczynka na zdjęciu z działki. Od strony męża rok starsza od niej dziewczynka, ale to widzieliśmy się parę razy. Tutaj w okolicy niestety jeszcze nie było spotkań rodzinnych.

**Jak spotkaliście się w tym większym gronie, to czułaś, że to spotkanie jest jakieś inne?**

To było dziwnie w ogóle zobaczyć wszystkich w jednym miejscu i po tak długim czasie, ale po jakichś 15 minutach to w ogóle już...To tak jak byśmy się spotkali normalnie tydzień po tygodniu. Nawet nie zwracaliśmy uwagi, że faktycznie nie widzieliśmy się tyle tygodni. Nawet rozmowa się toczyła tak, jak byśmy rozmawiali dopiero co ze sobą. Na początku nie było wiadomo, czy się przywitać, nie przywitać, ale oczywiście jakoś tam każdy...

**Wysłałaś mi jeszcze screen z maseczkami ślubnymi. Dlaczego?**

Mówiłaś, żeby wysłać, jak się trafi coś, co zaintryguje. Te maseczki to masakra w ogóle. Wysłałam to też Dianie. To już jest paranoja jakaś. Nie wiem, kto to kupuje, a jak ktoś to kupuje to jest mega głupi. Ciekawa jestem, ile to kosztuje.

**Tu są ceny. Maseczki brokatowe, tiulowe 60 zł za komplet, z kryształkami 90 zł.**

O, nawet nie doczytałam. Chodziło mi o sam fakt, że w ogóle są takie ogłoszenia. To było pierwsze, na jakie trafiłam, ale od paru dni na tablicy na FB mi się wyświetla mega dużo tych koronkowych maseczek do ślubu. Paranoja. To po prostu głupota. Ci, co to robią to nie są głupi tylko głupi są ludzie, którzy to w ogóle kupują. Nie wyobrażam sobie wystąpić na ślubie w maseczce. Po pierwsze bym w życiu nie zrobiła teraz wesela, a po drugie, jak bym chciała zrobić wesele, to bym zrobiła wesele dla najbliższych. Jakiś ślub i tyle, gdyby mi bardzo zależało, żeby się pobrać, ale na pewno bym teraz nie robiła ślubu w maseczkach. Nawet nie wyobrażam sobie, jak by to miało wyglądać. Jak w tych maseczkach? Ja po 40 minutach już mam dosyć, nie da się w nich wytrzymać. I powieś sobie zdjęcie w maseczce na ścianie. Nie, to już idioci, idioci, idioci.

**Ostatnie zdjęcie to ten post dr Mateusza. Dlaczego zgadzasz się z tym, co tu jest napisane?**

On o tym pisze, że dziewczyny, które zazwyczaj się stroją, żeby ładnie wyglądać, zakładają szpilki nagle mają kapcie przez 2 miesiące. Jest im bardzo wygodnie i w ogóle szpilki nie są im potrzebne. A dlaczego? Bo są w domu i nie muszą się nikomu podobać i nie muszą się pokazywać. To jest najgorsze, że dopiero teraz zdajemy sobie sprawę, że wszystko robimy pod ludzi, żeby się ludziom podobać a nie sobie. A to jest bez sensu. I on pisze, że nagle dresy za 50 zł są 100 razy lepsze niż te komplety za 5000 markowe. Bo taka jest prawda. Cenimy sobie teraz wygodę i luz i okazuje się, że te dresy są 100 razy bardziej przydatne niż te wszystkie luksusowe ubrania, na które ludzie wydają ogromne pieniądze. Pisze też o tym, że oczywiście sam fakt zamknięcia, gdzie mąż i żona muszą ze sobą spędzić sporo czasu, więc dużo rzeczy o sobie się dowiadują. On też tam pisze o sobie i też pisze o sobie negatywnie, nie ubarwia. Tak jak on, gdzie ma dużą pozycję, milion tytułów, jest znany na całym świecie, ma mnóstwo pieniędzy i okazuje się, że to mu wcale nie jest potrzebne, jeżeli jest z żoną sam, bo się okazuje, że np. nie umią ze sobą rozmawiać, że zwykła komunikacja międzyludzka jest 100 razy ważniejsza od milionów tytułów, które się posiada.

**Dlaczego wydało ci się to ważne. Czy u ciebie teraz też coś się zmieniło, coś się zmieni?**

Chodzi mi o zdawanie sobie sprawy z tego, jak żyjemy i gdzie dla nas ta normalność, o której mówię, która wcale nie jest normalna...Bo to nie jest normalne, tak? I tak jak on też tam napisał, że przed kwarantanną było tysiące telefonów, itd., bo ktoś coś od ciebie potrzebował i różne rzeczy. I jest kwarantanna i nagle urywa się telefon, nagle ludzie już nie dzwonią, bo nie mają po co, bo nie mają potrzeby. I się okazuje, że te osoby, które są w domu są dla ciebie najważniejsze na całym świecie, i że powinno się budować te relacje z tymi domownikami. W takim sensie, żeby starać się o ich względy, a nie o względy reszty ludzi, którzy dzwonią do ciebie tylko jak czegoś chcą. Chodzi tam też o jakieś sfery biznesowe. Chodzi o to, że najważniejsze w życiu jest to, żeby dbać o tych najbliższych, których ma się w domu przy sobie i się okazuje, że jeżeli jest dobra relacja z tymi najbliższymi, to w ogóle inni ludzie nie są kompletnie potrzebni.

**To jest taka refleksja, która tobie też towarzyszyła?**

Tak, jak najbardziej. Ja zawsze rodzinę stawiałam bardzo, bardzo wysoko w moich wartościach, może nawet i najwyżej, albo na równi z czymś innym, ale nigdy nie było czasu, żeby to sprawdzić, żeby się o tym przekonać, itd. A to był czas teraz taki, że można było wyciągnąć jakieś odpowiednie wnioski.

**Czy czujesz się obecnie zagrożona sytuacją?**

Nie czuję się już zagrożona. Stwierdziłam, że jeżeli do tej pory nie zachorowałam, to już nie zachoruję. Poza tym ja się nie znam na polityce, ale czytam dużo różnych artykułów i piszą, wypowiadają się też doktorzy. Pewnie ty też czytałaś czy słyszałaś, że to jest jedna wielka ściema. Może, że to jest jedna wielka ściema to nie uważam, ale to, że rząd zastraszył tak społeczeństwo okropnie. Być może to było potrzebne, żeby ci ludzie zostali w domu, ale z drugiej strony ja myślę, że 90% dostało paranoi po prostu i tyle.

**Był taki moment, że przestałaś śledzić wiadomości?**

Teraz też już nie śledzę. Nie śledziłam żadnych wiadomości. Mieszkam z rodzicami, więc te wiadomości zawsze gdzieś tam w tle leciały, ale ja się kompletnie nie interesuję polityką. Kompletnie. Teraz, jak była debata prezydencka to też tam na chwilę przyleciałam, ale cóż mi z tego, jak ja kompletnie się na tym nie znam i nawet nie wiem, o czym oni mówią. Nie jest mi to w ogóle do szczęścia potrzebne i mnie nawet nie interesuje, kto zostanie tym prezydentem, Naprawdę, bo tak czy tak, ja nadal będę miała pracę, jaką mam i mój mąż będzie miał pracę, jaką ma, jakoś sobie w życiu damy radę. Czy będzie 500+, czy nie będzie, trzeba myśleć o sobie i polegać na sobie, a nie na tym, co rząd nowego zaoferuje albo co nam zabierze. Ja się ogólnie nie interesuję polityką. Koronawirus, jak najbardziej. Na początku każdy był ciekawy - a ten pierwszy przypadek, a potem co się stanie, ale teraz od dłuższego czasu w ogóle już nie oglądam. czasami mi na WP, bo się loguję na pocztę, wyskoczy mi, ile było przypadków, czasami jakieś statystyki sobie zobaczę, jak wygląda sytuacja w innych krajach, czy te zachorowania się zmniejszają, czy zwiększają, liczba zgonów, itd. To tak po prostu z czystej ciekawości to zobaczę i tyle.

**Jak to wszystko wygląda teraz wśród twoich bliskich? Co jest dla nich teraz ważne?**

U nas nic się nie zmieniło. Na początku, wiadomo, każdy był jakiś wystraszony...Może nie nawet wystraszony, ale każdy był ciekawy jak to będzie dalej. Po tygodniu czy dwóch, jak mama wróciła z babcią do domu, to jest normalna szara codzienność. Tak jak było, tak jest teraz. Nie rozmawiamy już o tym koronawirusie i wszystko jest tak, jak było. Nie zauważyłam nic szczególnego w tym, co mówią.

**Jak teraz wyglądają u was zakupy. Czy coś się zmieniło?**

Robimy zakupy co 2-3 dni tak jak robiliśmy wcześniej i też nic się nie zmieniło przez te 2 tygodnie. Wczoraj właśnie, jak byłam z mamą w Radomiu, to zrobiłyśmy zakupy w większym supermarkecie. Przy okazji tego, że jakieś sprawy urzędowe załatwiałyśmy. Nic się raczej nie zmieniło.

**Jakieś planowanie wam towarzyszy? Robicie jakąś większą listę/ bez listy?**

Jeżeli wybieramy się na większe zakupy, to zazwyczaj mamy jakąś tam listę, ale wczoraj akurat nie było czasu na to, więc po prostu szybko wzięłyśmy z półki to, co po prostu było potrzebne. Jak idziemy do tych małych sklepów tutaj u nas, to po prostu otwieramy lodówkę i patrzymy co jest potrzebne. Raczej tego nie spisujemy, ale nie spisywaliśmy tego też wcześniej. Jakieś nowe nawyki na pewno nie weszły. Może wcześniej, na początku tej epidemii, to myślałyśmy, co takiego ważnego trzeba kupić. Teraz jakoś te sklepy są bardziej dostępne, w sensie, że szybko się robi te zakupu i w każdej chwili znowu można podjechać, więc długo się nad tym nie zastanawiamy. Jak się czegoś zapomni, to podjedzie się następnym razem. Wcześniej to w tej kolejce trzeba się było wystać i nie opłacało się jechać 2 razy, bo zakupy zajmowały 2-3 godziny nawet. Teraz jest tak, jak było.

**Kupiłaś coś tak typowo dla przyjemności w ostatnich 2 tygodniach?**

Ja oczywiście chipsy ciągle kupuję dla przyjemności. Córce, jak jesteśmy na zakupach, to trzeba kupować coś, bo jak wraca się ze sklepu, to ona "mamo, kupiłaś coś?", więc jej ciągle kupujemy coś dla przyjemności a sobie raczej nie. Chipsy ja zawsze kupowałam dla przyjemności i dlatego mówię, że nic się nie zmieniło. Zakupy wyglądają tak, jak wyglądały. Jak mieliśmy na coś ochotę, to zawsze to kupowaliśmy, więc nie weszła jakaś nowa rzecz do naszych zakupów.

**W poniedziałek otworzyli GH. Co o tym sądzisz?**

Sądzę, że mogliby otworzyć kino, ale no nie wiem. Nie mam na ten temat zdania, bo już sama jestem zmieszana wszystkimi informacjami, które napływają. Nie wiem, które są prawdziwe, które nie, więc przestałam się nad tym zastanawiać. Ja w galerii jeszcze nie byłam i nie wiem, kiedy pojadę. Może w przyszłym tygodniu? Może, jak coś tam będzie potrzebne? Na razie nie jest mi nic potrzebne, ponieważ zamawiałam to, co było potrzebne na wiosnę przez internet. Chyba się teraz trochę odzwyczailiśmy od chodzenia po tych sklepach też. Kiedyś bardzo często bywałam. Nawet jak miałam jakąś przerwę między zajęciami, to zawsze się weszło, coś się kupiło, coś obejrzało, coś tam zawsze było potrzebne. Teraz tak naprawdę nie ma jeszcze potrzeby jechać do tej galerii. Nie pytałam znajomych, czy byli, ale z tego co wiem, to też jeszcze nikt nie był. Chyba są pustki, za dużo osób nie ma, jeszcze osoby się boją, ale no co? Otworzyli, tak rząd zdecydował i tyle.

**Myślisz, że to bezpieczne?**

Przecież nie możemy siedzieć całe życie zamknięci w domu, bo nie wiadomo, kiedy to się skończy, nie wiadomo, kiedy będzie znowu nawrót. Trzeba się nauczyć jakoś z tym żyć. Czy to bezpieczne? No to nic nie byłoby bezpieczne, tak? Ci, co mają potrzebę, to pójdą, ale jest tak zastraszone społeczeństwo, że myślę, że jeszcze przez długi czas mało osób z tej galerii skorzysta. Ale też ludzie muszą zacząć sprzedawać te rzeczy. No ileż może być zamrożona ta gospodarka? No nie wiem...Ja na polityce się nie znam, powtarzam po raz setny i nie wiem. Jeżeli tak zdecydowali, to niech tak będzie. To jest indywidualna sprawa i przecież nikt nie musi tam chodzić. Jeżeli ktoś uważa, że to jest niebezpieczne, to po prostu tam nie pójdzie i tyle. Mają ludzie wolny wybór.

**Łatwość wydawania pieniędzy - skala**

Ja bym siebie umiejscowiła na 3, a mój mąż mnie na 10.

**Na czym to polega?**

Ja ogólnie szanuję pieniądze i kupuję to, co jest mi potrzebne. Mój mąż uważa, że tak nie jest, że kupuję za dużo rzeczy, ale naprawdę ja szanuję pieniądze. Ja od zawsze pracowałam, pomagałam rodzicom, więc jeżeli mam pieniądze i wiem, że mogę je wydać, to je wydam, jeżeli nie mam tych pieniędzy, to ich po prostu nie wydaję, więc uważam, że racjonalnie z nich korzystam, ale mój mąż był w trochę innym domu wychowany i u nich dużą wartość w domu ma oszczędność, czyli pieniądze wkłada się do skarpety i się je zamraża. Ja uważam, że po to się pracuje, po to się zarabia, żeby je właśnie wydawać na przyjemności i tutaj się różnimy. Dlatego mój mąż by powiedział, że ja z łatwością wydaję pieniądze, a ja uważam, że po prostu wydaję je logicznie i po to wkładam jakiś trud we wszystko co robię, żeby potem mieć na przyjemności. Mogę umrzeć za tydzień, za 5 lat, mogę nagle stać się niepełnosprawna i nie móc nigdzie więcej pojechać. Poza tym uważam, że trzeba żyć po to, żeby być szczęśliwym. Jeżeli mi sprawia przyjemność wyjechanie na wakacje za granicę i mnie na to stać, to po prostu jadę a nie myślę o tym, żeby odłożyć te pieniądze i nie wiem...Na co odłożyć te pieniądze? Nie wiem po co w ogóle jest...Wiadomo, jakieś zabezpieczenie finansowe trzeba mieć, ale bez przesady, żeby teraz odkładać pieniądze i nie kupić sobie czegoś fajnego, nie pojechać gdzieś, nie zjeść czegoś dobrego, nie wyjść do kina, nie iść do restauracji. To bez sensu, to po co żyć i po co się tyle trudzić? Na pewno przecież nie będziemy chodzić do kina, jak będziemy mieć 70 lat, prawda? Wtedy będzie czas na co innego. Wtedy będziemy wydawać na wnuki a nie na siebie. Ja mam takie zdanie.

**Kiedy ostatnio wydałaś jakieś większe pieniądze na coś poza codziennymi zakupami?**

Ostatnio właśnie wydaliśmy pieniądze na meble do córki do pokoju, np. na szafę. To był spory wydatek...Ale jakie emocje odczuwałam? W sensie, czy mi było szkoda? Nie było mi szkoda, bo mi się ta szafa bardzo podoba i skoro już robiłam dziecku pokój, i mąż się na to zdecydował, to bez sensu kupować coś, co się nie podoba. Mi się podobała, więc taką też kupiłam, chociaż jak mnie tata zapytał, ile kosztowała, to mówi "o Boże, nie!".

**Wydałaś i była radość z tego?**

Tak, oczywiście. Bardzo się cieszę. Za każdym razem jak wchodzę do tego pokoju to mi się bardzo podoba i nie wyobrażam sobie, żeby stała tam jakaś inna szafa. Ta już była obmyślona. Było dużo czasu przez kwarantannę, więc siedziałam na tych stronach, szukałam. Ta była najładniejsza ze wszystkich, które przejrzałam i cieszę się, że jest u mnie i w ogóle nie żałuję tych pieniędzy, które wydałam.

**Wracając do przeszłości, nazwałabyś siebie rozrzutną czy oszczędną?**

Nie, no chyba bardziej rozrzutną, bo ja zazwyczaj oszczędzam pieniądze na to, żeby mieć na przyjemności. To zazwyczaj ja oszczędzam na wakacje. Mąż oszczędza, bo oszczędza. Nie wiadomo na co, a ja zawsze np. na jakieś fajne wyjazdy. Przez rok zbieram pieniądze na wakacje i po roku je wydaje. Potem znowu zbieram, ale znowu na wakacje, więc ja nie chowam pieniędzy gdzieś tam, żeby sobie było. To mój mąż to robi i dlatego to ja muszę finansować takie właśnie przyjemności.

**Czy coś się zmieniło w twoich dochodach albo perspektywach finansowych na przyszłość?**

Zmieniły się moje dochody, ponieważ ja teraz nie zarabiam z tego względu, że nie udzielam korepetycji, a miałam ich bardzo dużo. To była moja wypłata. Dobra wypłata. W sumie dobre pieniądze a nie za dużo pracy, no to lubiłam i to było mało męczące. Teraz niestety już nie mam korepetycji przez 3 miesiące. Mój mąż nadal pracuje i jedyne co mnie denerwuje, to że nie mam jakichś tam swoich pieniędzy, chociaż on nie jest człowiekiem, który zabrania albo nie daje mi jak tam niektórzy mężowie. Ja zawsze lubiłam mieć swoje pieniądze i wtedy nie tłumaczyć się, nie informować, tylko na swoje jakieś tam. Mąż pracuje cały czas, więc za bardzo nic się nie zmieniło u nas.

**Jak myślisz, kiedy to wróci do normy?**

Maturzyści się właśnie do mnie odezwali, że od przyszłego tygodnia, bo oni piszą maturę na początku czerwca. Napisali do mnie czy przyjadę na te korepetycje od przyszłego tygodnia i się zgodziłam, więc myślę, że powoli wróci wszystko do normy już od przyszłego tygodnia. Z innymi uczniami się jeszcze nie kontaktowałam i nie mam pojęcia. Czekam aż oni wrócą do szkoły.

**Czy w związku z tym, że ten dochód trochę spadł, to starałaś się jakoś ograniczać wydatki, poszukiwać np. tańszych produktów?**

No tak. Może nie tańszych produktów, bo ja uważam, że lepiej kupić coś jednego a dobrego niż 5 rzeczy bez sensu, ale na pewno było mniej tych rzeczy. Jak mam swoje pieniądze, to raczej wtedy za bardzo się z mężem nie rozliczam z tego co kupiłam. Mamy tak podzielone. To nie jest tak, że to są moje a to są jego pieniądze, absolutnie. Ale sama wiesz - jak są twoje pieniądze, jedziesz do sklepu, bierzesz co chcesz bez tłumaczeń. A tu daj mi na to, bo muszę zrobić takie zakupy, a przyjdzie taka paczka, inna paczka. A faceci są jacy są. A po ci kolejne te buty? A po co to? A po co, na co? A tak, to się nie muszę chociaż tłumaczyć, tak? My się nie rozliczamy, więc jeżeli ja jestem na zakupach, to płacę ze swoich pieniędzy, które mam w portfelu, jeżeli on jest, on płaci. My się wymieniamy, więc to są wspólne pieniądze, ale tutaj jednak jemu wpływa wypłata na koto i muszę się go pytać o te pieniądze albo, żeby mi wypłacił i to jest bardzo męczące, bo ja jestem nauczona tego, że zawsze są jakieś tam...Mam swoje i nikogo nie muszę o nic się prosić.

**Czy poszukujesz przecen i okazji?**

Ubraniowych tak, ale to zawsze ich poszukiwałam i np. kupuję z zalando lounge a nie na zalando. Codziennie praktycznie obserwuję, co tam jest na wyprzedaży, bo wiesz pewnie co to jest zalando lounge?

**Tak.**

Ale to ja już wcześniej tak robiłam, więc to się nie zmieniło. Może teraz, ze względu na to, że nie mam tych swoich pieniędzy, więc zamiast kupić córce 5 par spodni, kupiłam 2 pary. Ja promocji szukałam zawsze w ubraniach. Jakieś tam dobre buty, torebki - na to mogę sporo wydać, na wakacje mogę sporo wydać, ale na jakieś tam bluzki, koszulki, spodnie i takie rzeczy do chodzenia, które się szybko niszczą, to na to ja zawsze szukam promocji.

**Na te lepsze jakościowo rzeczy, bo uważasz, że tak trzeba, czy to jest związane z takim dobrym dealem?**

Często kupuję mojej córce ubranka z River Island, gdzie na zalando są bardzo drogie, ale np. co jakiś czas na zalando lounge wrzucają parę rzeczy i się cieszę, że komplet, który kosztował 150 zł mogę kupić za 49. I wtedy bluzeczka ze spodniami - no super, opłaca się. A gdybym miała wydać 150 zł? Jak tak dużo coś kosztuje, to do mojej mamy zawsze idę i mówię "zobacz babciu, jaki tutaj ładny zestawik" i te droższe rzeczy to moja mama kupuje Helence, a ja to zawsze szukam okazji, bo opłaca się. A jak coś, co mi się podoba, to się cieszę, że mogłam zapłacić tak mało.

**Wydaje ci się, że w obecnej sytuacji, tak ogólnie, dobrze jest ograniczyć wydatki?**

Teraz to rozumiem ludzi, którzy wydają mniej. Ja też wydaję teraz mniej. Jak ktoś nie wie co będzie potem i ja nie mówię o odległości rok, pięć lat, ale kiedy wróci do pracy, kiedy będzie miał znowu wypłatę...Ja mam ten komfort, że mąż pracuje i to główny dochód to był od niego i nic się nie zmieniło, więc ok, mamy jakąś tam stabilizację. Tym bardziej, że on jest przewoźnikiem, wozi mięso i to się nie zmieni, bo nie zamkną spożywczych sklepów, ale dużo osób straciło pracę, dużo osób nie wie, kiedy wróci do pracy, tak jak fryzjerki, kosmetyczki, więc ja jak najbardziej uważam, że powinny oszczędzać. Nie wiadomo, kiedy to ruszy i było milka miesięcy bez dochodów, a jeszcze jak ktoś zatrudniał pracowników, to nawet jak jakieś dofinansowanie z ZUS-u dostał, to jednak ciężko.

**Wprowadziliście jakąś kontrolę budżetu w tym czasie?**

Nie, nawet o tym nie pomyślałam. Dobrze, że mój mąż tego nie słyszy.

A co sądzisz o posiadaniu oszczędności? Warto posiadać oszczędności takie, poza zbieraniem na wakacje?

Warto, ale też, żeby to nie było bez sensu, bo nie rozumiem, a znam dużo, naprawdę dużo takich osób, które nie pojadą na wakacje, nie kupią sobie nic fajnego, tego nie mogą zjeść, do restauracji nie wyjdą, a na końcu mają np. 30000, bo oszczędzają. Ale na co ty oszczędzasz człowieku? Jeżeli nie oszczędzasz na dom, masz dorosłe dzieci, to na co tyle tych pieniędzy oszczędzać, skoro nie korzystają z życia tu i teraz. Rozumiem, że trzeba mieć coś na zaś, gdyby właśnie straciło się pracę i na czas poszukiwania kolejnej. Na 2-3 miesiące życia, bo nie uważam, że dłużej trzeba szukać pracy, bo pracy jest bardzo dużo, tylko ludzie są mega leniwi i im się nie chce. Nie boję się żadnych kryzysów gospodarczych z tego względu, że ja wszędzie się odnajdę. Ja mogę iść do każdej pracy. Mogę iść sprzątać, mogę iść opiekować się dziećmi a mogę iść pracować w biurze. Mi to nie przeszkadza, bo praca to praca i jeżeli ktoś potrzebuje pieniędzy, to niech idzie do pracy jakiejkolwiek. Tym bardziej jest teraz taka minimalna krajowa, że na spokojnie na życie starczy. Chodzi mi o takie kryzysowe momenty. Jeżeli ktoś ma dom postawiony, ma samochód i wszystko jest ok, to nie rozumiem tego oszczędzania, tego odkładania pieniędzy. Nie wiem po co. Ja zawsze odkładam na coś. Teraz się stawiam, więc jakieś pieniądze są odłożone tylko na budowę domu, ale to mam jakiś cel. Nie rozumiem odkładania bez celu na czarną godzinę. Ale kiedy ta czarna godzina przyjdzie? Jak będziesz miał 80 lat albo zakopią cię z tymi pieniędzmi? Żeby porozdawać? Całe życie sobie odmawiać, żeby potem...No nie rozumiem.

**A masz takie oszczędności na 2-3 miesiące?**

Tak, mam, ale nie mam ich jakoś dużo. Mam odłożone jakieś na mieszkanie, na coś, na coś. Teraz mój mąż dokupił kolejnego busa i w razie kryzysu ja mogę go sprzedać, więc te pieniądze są też w jakiś sposób ulokowane. Jeżeli ja kupiłam działkę, to ja tę działkę też będę mogła sprzedać.

**A gdyby nie liczyć tego, co można sprzedać, to gdybyście nie mieli dochodu, to jak długo moglibyście żyć z oszczędności?**

To zależy, bo pieniądze, które mam na mieszkanie przeznaczyłabym np. na życie. Są pieniądze na mieszkanie, na tegoroczne wakacje, które musieliśmy odwołać i dostaliśmy zwrot kosztów za rezerwację, ale takich typowo wolnych pieniędzy, to tak myślę, że mam na 3 miesiące. Zawsze mogę też ruszyć te pieniądze odłożone na jakiś cel, ale to nie wiem, co musiałoby się stać. Jakaś bomba atomowa chyba by musiała spaść na nas, żeby wszystko było zniszczone, ale wtedy to chyba bym też umarła. Nie wiem, co takiego musiałoby się stać, żeby był taki mega ciężki czas. Jest możliwość wyjechania za granicę...Żyjemy w takim świecie, że możemy wszystko, tylko trzeba trochę chcieć.

**Dla męża ważne jest posiadanie oszczędności?**

Tak, bardzo ważne. To jest jego pierwszorzędna potrzeba. To, żeby mieć. Jak on ma, to...Tylko, żeby nie wydawać. On sobie odkłada i nie wydaje.

**Te pieniądze, które odkładasz, to raczej wolisz inwestować?**

Ja za mało zarabiam, żeby ze swoich pieniędzy inwestować, więc ja raczej odkładam na przyjemności. Mój mąż ma ten główny dochód. Oczywiście, ja bym wolała, ale on woli mieć niż je wydać.

**W jaki sposób odkładasz na przyjemności?**

Ja na koncie nie mam praktycznie w ogóle pieniędzy, tylko jeżeli mi są potrzebne, żeby zrobić przelew i wtedy je wpłacam. To był dla mnie problem przy zakupach, że ja musiałam wpłacać pieniądze, żeby skorzystać z karty. To co mam, to ja zawsze sobie gdzieś chowam. Osobną mamy też skarbonkę. Skarbonka jest rozsądna, bo wtedy nie wyciąga się tak pieniędzy. Mama nas tak nauczyła. Moja mama ma skarbonkę, córka ma skarbonkę i co jakiś czas tam naprawdę bardzo duże sumy jesteśmy w stanie odłożyć nawet przez takie głupie skarbonki.

**W obecnej sytuacji warto oszczędzać czy właśnie wydawać, bo nie mamy kontroli nad tym co się dzieje?**

Nie można sobie jakoś ograniczać tych przyjemności, bo życie jest po to, żeby te przyjemności mieć. Jeżeli dla kogoś przyjemnością jest wyjazd a nie fajne ciuchy, to niech sobie wyjedzie, a ubiera się skromnie. Jeśli ważne jest, żeby fajnie wyglądać, to niech fajnie wygląda, itd. Trochę to stawiasz między hulaszczym wydawaniem

 a oszczędzaniem, a można też tak pośrodku, racjonalnie do tego podchodzić. W obecnej sytuacji nie warto sobie odmawiać tych przyjemności. U nas za bardzo nic się nie zmieniło, ale są osoby, które mają tak, że i jedno, i drugie straciło pracę. Bratowa mojego męża jest protetykiem i ona była zatrudniona na jakąś ciulową umowę na 1/4 etatu, czyli bardzo mała podstawa, a od każdych zębów, które zrobiła, dostawała mega duże pieniądze i zarabiała naprawdę dużo, bo ok.6-7 tysięcy. Nagle ma wypłatę 1000 zł i to jest ogromny przeskok. Ona dopiero teraz wróciła do pracy, a wiadomo, że też mają jakiś kredyt mieszkaniowy, itd. Jej mąż zarabia o wiele mniej niż ona, więc to jej dochód był tam głównym utrzymaniem, ale też uważam, że te osoby mają teraz nauczkę na przyszłość, że jednak ta umowa o pracę jest bardzo ważna i żeby tyle, ile się dostaje, było na umowie. Tata zamawia tiry z towarem i ona zawsze do nas przyjeżdża i coś sobie wybiera. Dzisiaj właśnie ma być ten tir i ona zadzwoniła, że nie przyjedzie, bo nie ma kasy. Ona boi się o swoją sytuację i ją rozumiem. To jest podejście bardzo racjonalne i ok., ale jeżeli ktoś nadal pracuje, jak pracował i nie zapowiada się, żeby tę pracę stracił, jak np. mój mąż, to dlaczego ma sobie czegoś żałować i żyć w strachu?

**Jak myślisz, kiedy ta cała sytuacja się skończy?**

Myślę, że już powoli dobiega końca, że w wakacje będzie już ten 4-5 etap, nie wiem, ile jest tych etapów powrotu do normalności, bo już zapomniałam. Chyba 4, więc myślę, że będzie koniec. Ja nawet teraz widziałam, bo mieliśmy akurat zarezerwowane wakacje we Włoszech i mieliśmy jechać na taki Eurocamp. Ja i tak wykupywałam ubezpieczenie od kwoty, w razie czego, gdyby dziecko zachorowało i chciałam poczekać z rezygnacją do wakacji, do ostatniego dnia, ale zostałam przymuszona przez siostrę, która też miała jechać, żeby odwołać, bo we Włoszech i przecież tam jest największe ognisko i w ogóle nie ma opcji. No dobra, zrezygnowałam z tego, a teraz patrzę i ludzie piszą z campu, że jest już otwarty od któregoś mają. A mówili, że gospodarka turystyczna będzie wstrzymana do września i nagle już w maju się otwierają wszystkie campy. I tak właśnie jest słuchać informacji. Nigdy nie wiadomo. teraz trochę żałuję, bo mieliśmy jechać w lipcu i być może okaże się, że wszystko będzie ok. Bo co? Teraz nagle przestaniemy wyjeżdżać za granicę? Przez ile? Przez rok, 2, 3, 5? Nie wiem. Nie mam bladego pojęcia, jak to będzie.

**W jakich momentach myślisz o tym, kiedy to się skończy?**

Bardziej myślę, co zrobić z wakacjami, bo ja bardzo lubię wyjeżdżać i byliśmy też ciekawi, kiedy otworzą hotele i już otwierają. Już ludzie zaczynają wyjeżdżać do Polski. Może lotniska to nie za bardzo, ale naprawdę można się zarazić wszędzie. Wszędzie jesteśmy narażeni na to niebezpieczeństwo, więc nie rozumiem, czemu teraz mielibyśmy nie podróżować. Skoro w Polsce mogę skorzystać z różnych hoteli, to dlaczego nie mogę skorzystać z hoteli we Włoszech za 2 miesiące, gdzie słychać, że tam już jest sytuacja w jakiś sposób opanowana i że też tam dobiega końca. Ja myślę teraz tylko o tych wakacjach, bo nie wiem, co zrobić. Teraz każdy się wybierze w lasy na Mazurach? Będzie tam przecież takie zagęszczenie, że będzie bardziej niebezpiecznie niż nad morzem.

**Jak myślisz, jak dalej potoczy się ta sytuacja?**

Ludzie zaczną wyjeżdżać w takie spokojne miejsca, ale wtedy znowu będzie cyrk. Tak samo jak zaczęła się kwarantanna i wtedy ludzie zbiorowo jechali do lasu. Mój brat zabrał dziewczynę do lasu, gdzie u nas na wsi tam naprawdę nikogo nie ma, podjechał do wejścia do lasu i tam stało ze 20 samochodów z Radomia. I wejdź teraz do lasu. Niby taki bezpieczny, ale jak wszyscy będą tam jeździć, to już nie będzie taki bezpieczny. Ciężko powiedzieć, ale myślę, że w tym roku ludzie wybiorą takie spokojniejsze miejsca jak Mazury. I to nie będzie Mrągowo ani Mikołajki tylko jakieś mniejsze miejscowości.

**A masz jakieś obawy związane z najbliższą i przyszłością i taką za parę miesięcy?**

Parę miesięcy, to tylko te wakacje, ale obawy, to jedynie ze szkołą, bo ja teraz się bronię i wczoraj dostałam informację od mojej sympatycznej pani doktor, która powiedziała, że mam się bronić 29 czerwca, czyli za 1.5 miesiąca, a jeszcze nie wiadomo nawet jak to będzie wyglądać, tzn., że mogę nawet mieć obronę online. Mają być jeszcze praktyki i mają być odbywane zdalnie. Już nie będziemy wolontariuszami i teraz będziemy pracować zdalnie, czyli mam sobie zorganizować grupę dzieci np., którym zaoferuję naukę online, czyli zorganizuję im zajęcia online, wyślę im z tego nagrania, filmy, zdjęcia, materiały, przygotuję konspekty zajęć. To jest paranoja. Ja jeszcze ciągle czekam i mam nadzieję, że jednak odbędę te praktyki normalnie w więzieniu. Praktyki są przewidziane na 60 godzin, ale jak jestem w jakimś ośrodku, to przygotowuję z tego jakieś 2 konspekty zajęć i to jest ok, a tu będzie siedzenie i robienie czegoś bez sensu. Dzieci ledwo wyrabiają na zajęciach online ze szkoły i nie mam pojęcia jak teraz miałabym przygotować zajęcia tym dzieciom i ich zachęcić, jeszcze konspekty im przygotować...Masakra ogólnie.

**Obrona online wywołuje jakiś niepokój w tobie?**

Nie wiem, jak to w ogóle miałoby wyglądać, ale wiadomo, że to jest jakieś ułatwienie, bo zmniejszenie stresu, a to jest bardzo ważne, można mieć materiały obok siebie, może mama trzymać odpowiedzi na pytania, ale chyba nie tak to powinno wyglądać. Poza tym nie rozumiem, dlaczego po jednej osobie nie miałoby być wpuszczane. Komisja to są 3 osoby i to chyba nie jest problem, żeby w jednej sali przebywały 4 osoby. Online to jest ułatwienie, ale jak przerwie, jak odetnie internet?
