# Supplementary material for: The challenges arising from the COVID-19 pandemic and the way people deal with them. A qualitative longitudinal study
Source: PLoS One. 2021 Oct 11;16(10):e0258133. doi: 10.1371/journal.pone.0258133 (PMC8504766; doi:10.1371/journal.pone.0258133)
Supplement: S1 Dataset — (ZIP) [file pone.0258133.s003.zip › Transcriptions/stage 4/5.4_M_39_single.docx]

**5.4_M_39_single**

**Co się działo przez ostatnie 3 tygodnie?**

To jest pokłosie moich starych...Mam zdiagnozowaną nerwicę zaawansowaną, z objawami somatycznymi. Myślę, że izolacja miała bardzo duży wpływ na to, że aktywowało się to w taki mocny dość sposób + jakieś prywatne sprawy. Dostawałem jakiegoś wysokiego ciśnienia, wylądowałem na pogotowiu + silny stres na głowie, którego nie mogłem w ogóle zmniejszyć + dostawałem ataków paniki nie wiadomo skąd. Kiedyś one były po prostu rzadko, ale się nasiliły. Przez pierwsze 2 tygodnie, jak byłem chory, to miewałem właściwie codziennie małe lub większe ataki paniki związane z tym, czy ja czasem tego koronawirusa nie mam. To się od tego chyba takie domino zaczęło.

**Ostatnio, jak rozmawialiśmy, to miałeś plany wyjazdowe, plany towarzyskie?**

Nie pomogło to na dłuższą metę. Byłem w lesie, byłem nad morzem...Nad morze pojechałem *[sprawdza datę w telefonie]* ...To było jeszcze w kwietniu. 26 pojechałem i już wtedy jakieś ciśnienie, nie mogłem sypiać...Pojechałem tam, tak trochę niby pomogło, ale rozpędziło się to w taki sposób, że już sam nie mogłem sobie z tym poradzić i zatrzymać tego. Nawet ta rozmowa, którą mieliśmy przeprowadzić, to ja czułem, że dam radę, ale jak się rozłączyłem po naszej rozmowie, to ja dostałem jakiegoś ataku paniki, że kurde mogę nie dać rady i odpuściłem to, bo jednak pomyślałem, że zdrowie moje jest ważniejsze i jak się ustabilizuję, to wtedy będę mógł z tobą pogadać.

**Oczywiście, że tak. Jak myślisz, czy to, co się z tobą działo ma związek z tym wirusem, który jest albo go nie ma, jak mówiłeś, czy bardziej z tym, że byłeś zamknięty i odizolowany od ludzi, czy może jest to coś, do czego miałeś skłonność i czasem to na ciebie nachodzi?**

Ja po 2 tygodniach i podtrzymuję to teraz, ja zamknąłem temat koronawirusa, natomiast się to wszystko nawarstwiło. W dniu, kiedy została wprowadzona kwarantanna ja się rozchorowałem, ale do dzisiaj tak jest, że ja mam w dupie tego koronawirusa, bo ja muszę żyć. Myślę, że takich jak ja...Zresztą taka pani doktor, która ze mną rozmawiała też powiedziała, że nie jestem pierwszy i ostatni, u którego przez izolację i przez stresy związane z tym uaktywniły się rzeczy, które pewnie by się uaktywniły później. U ludzi choroby się zaczną wcześniej uaktywniać przez tę właśnie sytuację.

**Jak teraz spędzasz czas?**

Przez dziesięć dni mieszkałem u rodziców, żeby się poczuć bezpieczniej, żeby mieć kogoś jakby co, jestem na lekach wyciszających. Nie są to jakieś psychotropy oczywiście, ale pomagają, bo czuję się coraz lepiej, sypiam, więc jest to dużo. Jestem teraz na długim urlopie i od 3 dni jestem już u siebie w domu. Wczoraj skończyłem 40 lat i myślę, że to też miałem z tyłu w głowie i to taki rykoszet był tego, że człowiek myślał o tym dużo, że kurde stary, kończysz 4 dychy, czas na jakieś ruchy. Ostatni gwizdek, nie?

**Podsumowania sobie robiłeś, rozumiem?**

No.

**Jak spędziłeś te urodziny, jak było?**

Zaczęły się już w sobotę właściwie, bo znajomi mieli wolne, więc przyszli i mi tutaj niespodziankę zrobili z tortem. Ja nie piję, bo jestem na lekach, ale oni chętnie pili, posiedzieliśmy i potem pojechałem do rodziców. Wczorajszy dzień, to pół dnia przygotowywałem tutaj taki uroczysty obiad dla rodziców i brata, więc to zajęło też moje myśli. Przyszli rodzice, trochę pojedliśmy, trochę się pośmialiśmy. Ogólnie cały dzień z rodzicami.

**Coś się zmieniło w twoich zachowaniach, twojej aktywności, w spotkaniach z ludźmi?**

Niestety zrobiłem krok w tył, bo się tak źle czułem, że nie chciałem z nikim rozmawiać, widzieć się. Potrzebowałem się wyciszyć, spokoju dla siebie. Na pewno zacząłem dużo spacerować. Na siłę wręcz spacerowałem, bo się bardzo źle czułem. Te leki na początku bardzo źle wchodziły, teraz już jest tak, że się organizm przyzwyczaił, ale początek miałem bardzo trudny. Właściwie się cofnąłem z tym wszystkim, nie? Aktywności w stylu rower trochę odpuściłem, bo po tych lekach człowiek jest taki trochę...No różnie. Wiadomo - filmy, muzyka, spacery.

**A czy coś ci teraz szczególnie doskwiera w codziennym życiu?**

Nie, staram się...Skróciły się, spłyciły się te moje myśli w sensie do mojego samopoczucia i do mojego zdrowia. Myślę tylko o tym, żeby szybko dojść do siebie i znowu cieszyć się życiem. Z tego, co lekarz mówił, to po 3 tygodniach te leki będą działały już w pełni, więc człowiek się z dnia na dzień czuje, że jest jakiś bardziej chroniony. Potem będziemy schodzić z tych leków i wracam do normalności. Musiałem się po prostu zatrzymać. Zaciągnąłem hamulec ręczny, bo było już nieciekawie. Moja była dziewczyna bardzo mi w tym pomogła i pomaga, bo też miała w życiu traumę. To jest w ogóle lekarz polecony przez nią, świetnie dobrał leki, bo ja na spotkaniu mówiłem, że ja absolutnie nie chcę psychotropów, bo miałem kiedyś epizod i to nie dla mnie jest. To za ciężkie + chyba moje objawy nie są chyba takie, że aż tak trzeba głęboko się tutaj...No i nie mogę się w pracy ujawnić tej informacji. Jestem na razie na długim urlopie, potem ewentualnie wezmę jakieś chorobowe, ale nie od psychiatry, bo mam zbyt odpowiedzialną pracę.

**Mógłbyś zostać zwolniony z pracy przez to?**

Być niedopuszczony do pracy. Urlop mam do 25 maja i zobaczymy co później. Ja do końca nie jestem w stanie zrozumieć tego, co się ze mną działo. Rozumiem mechanizm, że jak człowiek się stresuje` dużymi rzeczami...Inaczej. Jak jest człowiek młody to stresuje się, ale te rzeczy nie wywierają wielkiego wpływu. Poza tym człowiek się nie przejmuje zbyt wieloma rzeczami, tylko istotnymi, a u mnie ta bariera...No nie wiem, ta nerwica się u mnie tak rozwinęła, że po prostu z ma łych rzeczy się robiły już duże i nie mogłem niestety już tego powstrzymać w ogóle. Choćbym nie chciał, nie myślał o tym, to i tak to samo przychodziło, nie miałem już na to wpływu.

**Majówka już była popsuta przez to co się działo?**

Weekend majowy to w ogóle apogeum było. Ja z pracy się zwolniłem i pojechałem na pogotowie, bo już było źle ze mną.

**Jak jest teraz ze środkami ostrożności, które zachowujesz?**

Raczej tylko do pomieszczeń wchodzę w maseczce. Ze znajomymi, jak gdzieś jesteśmy to niekoniecznie. Teraz to ja nawet nie wiem, ilu jest zarażonych, nic nie wiem. Odpuściłem to wszystko, nie interesuje mnie to i czekam aż się to wszystko odmrozi i człowiek będzie wracał do normalności. Ten wirus będzie i tyle.

**Czujesz się w ogóle w jakimś stopniu zagrożony tym koronawirusem teraz?**

Nie. Może na 99% nie.

**A twoje otoczenie? Znajomi, rodzina?**

Coraz więcej ludzi mięknie w końcu i wyluzowują.

**Wracają do zachować sprzed pandemii, czy jednak ich zachowanie czymś się różni? Jak to teraz wygląda?**

Wszyscy mniej więcej idą w tę stronę, w która ja poszedłem. Starają się wracać do normalności, żyć normalnie, zachowywać się normalnie tylko na takim stand by ‘u, wiadomo. Umyć te ręce, w pomieszczeniach założyć tę maseczkę jak się gdzieś idzie i tyle, nie?

**A jak się spotykacie, to zachowuje się większy dystans niż dawniej, inaczej się wita?**

Myślę, że wraca też to. Ja się staram zachowywać normalnie. Z tymi moimi najbliższymi przyjaciółmi, z którymi się widuję na co dzień, to nie obściskujemy się jakoś czy coś, ale żółwika przybijamy, do moich przyjaciółek jakoś się tam człowiek może i przytulił, ale tak to...Ludzie po prostu są jeszcze na takim stand by ‘u, że spoko, może i jesteśmy obok siebie bez maseczek, ale może nie pijemy z tych samych kubków.

**Spotykacie się w domu czy na dworze?**

To zależy u kogo. Mam moją przyjaciółkę, do której przychodzę i przyjeżdżam do niej na ogródek, bo jej mama nie pozwala, żeby jej znajomi wchodzili do domu. Moi znajomi przyszli do mnie do domu. 2 osoby przyszły. Grono osób, które chcą już wrócić do spotykania się powiększa się cały czas. Już po prostu mają dość. Bardziej tego mają dość niż strachu przed, kurwa, jakąś pandemią.

**Czego ludzie mają najbardziej dosyć, co im najbardziej przeszkadza? O czym mówią twoi znajomi?**

Brak pracy. Moje najbliższe otoczenie to jest duży rozstrzał wiekowy, ale ci, co mają po dwadzieścia kilka lat, to potracili pracę, nie? Niektórzy wrócili do rodziców, niektórzy czekają na te zasiłki, co to od państwa mają być, siedzą w domach, starają się nie wydawać. No nie jest kolorowo. Jak te poluźnienia wszystkie będą, to ludzie może gdzieś wrócą do pracy. To są głównie branże knajpiane i imprezowe wśród moich znajomych.

**Jak z twoją muzyką w tej chwili? Nadal się nią zajmujesz?**
Nie, odpoczywam. Staram się w ogóle odpoczywać. Dobrze mi to robi i nie będzie lepszej okazji.

**Dawno nie rozmawialiśmy, jak teraz wyglądają u ciebie zakupy?**

Idę raczej z listą w głowie lub w telefonie, dezynfekuję ręce na wejściu, robię zakupy, wracam do domu, myję ręce. Jakoś nie ma większego już...Już nie zwracam uwagi na porę, nie idę tuż przed zamknięciem. Nie, rozładowało się już to wszystko, ten cały taki korek i ja się śmieję, że już znowu w sklepach są makarony.

**A nie było?**

No raczej były półki poczyszczone trochę. Wygląda, jakby wszystko wracało do normy w tych sklepach.

**Kupowałeś sobie jakieś rzeczy dla przyjemności w ostatnim czasie?**

Czekam na zakup butów, ale w sklepie, który uruchomił się w galerii jeszcze nie doszły. Już 2 tygodnie na nie czekam. Bardzo by mi to poprawiło humor. No i kupiłem sobie na urodziny moje ulubione perfumy, które idealnie się akurat 2 dni przed urodzinami skończyły.

**Coś jeszcze?**

Nie. Na nic nie wydaję, wziąłem się za wiosenne ogarnianie samochodu, czyli wymieniłem opony na letnie, jakiś tam przegląd, gaśnica, czyli to, co normalnie się robi na wiosnę.

**Perfumy to prezent na urodziny, a te buty?**

Pół na pół pomiędzy prezentem a tym, że przydałyby mi się już nowe.

**Co myślisz o tym, że otworzyły się GH?**

To jest to odmrażanie. Byłem w galerii po te perfumy. Tam są duże środki ostrożności. Te laski w maskach i rękawiczkach, trzeba się zdezynfekować na wejściu, założyć rękawiczki, wszystkie te półki z kosmetykami, wszystko to poprzykrywane, więc nie wiem, jak się odbywa ten handel. Ja tam wszedłem, wziąłem swoje ulubione perfumy z półki, zapłaciłem i wyszedłem z galerii. Więcej nie łaziłem. Wcześniej to coś tam może bym tam zerknął, a teraz to raczej wszedłem, załatwiłem sprawę i...Z tego co widziałem, to sklepy z ciuchami chyba w ogóle były zamknięte, niektóre się w ogóle likwidują. Widziałem, że Reserved chyba się zamyka. Po prostu stwierdziłem, że nie jest to dobry moment + niedługo czeka mnie trochę wydatków, bo ubezpieczenie samochodu...Mówię, dobra, na razie poopłacam to, co mam i zobaczymy, jak będzie, to ewentualnie sobie coś kupię.

**To nie był dobry moment, bo nie chciałeś wydawać pieniędzy, czy może to jeszcze nie pora, żeby tę galerię otwierać?**

Ja myślę, że teraz to w ogóle nie są takie czasy, żeby wydawać tak lekką ręką pieniądze. Jednak z tyłu głowy człowiek ma, że dzisiaj jest tak, a za chwilę może być gorzej. Ceny produktów rosną, a...Umowę teraz przedłużałem na telefon i nie wziąłem sobie nowego telefonu, po prostu tylko abonament przedłużyłem. Stwierdziłem, że nie będę się wiązał kolejnym kredytem na telefon, żeby go spłacać, bo ten jest ok i nie jest to dobry czas, żebym miał wydać kilka tysięcy zł na telefon.

**W innych czasach byś wziął?**

Myślę, że bym wziął jakiegoś nowego iPhone’a kolejnego, ale nie odczuwam w tym momencie potrzeby, że musi być.

**Zaraz jeszcze wrócimy do wątku wydawania pieniędzy, ale jeszcze powiedz, czy uważasz, że otwarcie GH teraz jest ok? To jest bezpieczne?**

Myślę, że jeszcze chwilę dłużej i wrócimy do epoki kamienia łupanego. Nie, no trzeba ratować gospodarkę. Ja uważam, że jeśli ten wirus jest, to jest tylko bardziej zaraźliwy niż wirus grypy, ale nie jest bardziej śmiertelny, więc o czym w ogóle rozmawiamy? Dla mnie to jest za mocno rozdmuchane przez co w ogóle wszystko wróci do lat 90-tych. Będzie trudno o wszystko, myślę, że się łapówkarstwo z powrotem odrodzi, bo ludzie będą chcieli mieć łatwiej i lepiej gdzieś zarobić, mieć w ogóle z czego żyć, załatwić w ogóle pracę, jakiś handel wymienny, no nie wiem. Wrócimy do czasów z 90-tych lat. Trzeba będzie wszystko załatwiać przez znajomych. Znajomości będą nr 1, a nie to, co sobą reprezentujemy. Rynek będzie znowu pracodawcy a nie pracownika. Ja uważam, że i tak każdy z tyłu głowy ma, że spoko, trzeba GH otworzyć, ale jakieś środki bezpieczeństwa niech będą, a nie że nagle tysiące ludzi w galeriach. Załatwiajmy potrzebne rzeczy a nie...Nie wiem, co z tego będzie. Mówi się, że na jesieni trzeba będzie znowu jakieś obostrzenia...Ja nie wiem w ogóle, jak ten świat będzie wyglądał w kolejną zimę. Nie mam pojęcia.

**Łatwość wydawania pieniędzy - skala**

Zależy też od pory roku, bo wiadomo, że w lato człowiek tak chce się wyluzować bardziej. Miedzy 6 a 7 przed koronawirusem, a teraz może jakieś 3.

**Na czym polega to 6-7?**

Pewnie już dzisiaj bym sobie szukał jakichś fajnych ciuchów, ale w tej chwili mam tak, że mam z czego wystartować w tę wiosnę. Bardziej już bym pewnie teraz kupił sobie w sieci ze 3 nowe koszulki i jedną nową bluzę, ale na razie to odpuszczam.

**Jak to było poprzednio, zanim to 3?**

Jak mi coś wpadło w oko, to kupiłem. Nie wiem, jak to określić. Raczej np. szedłem po coś, ale coś innego mi wpadło w oko, przymierzyłem, było fajne, to kupowałem, a dziś na razie nie chcę wchodzić w ogóle do sklepu.

**Były takie rzeczy, na które łatwiej ci było wydać pieniądze i takie, na które trudniej?**

Wiadomo, wcześniej też człowiek miał lżejszą rękę do tego, żeby iść do restauracji i zjeść obiad, a teraz więcej gotuje się w domu. Na pewno taki plus zauważyłem, że jakaś mała górka została pieniędzy, bo człowiek nie wydawał na imprezach, nie jeździł tak po kraju, nie wydawał w restauracjach, nie wydawał na alkohol. Przed koronawirusem wszystko to było na porządku dziennym, że poszedłem do restauracji, że chciałem, to sobie wsiadałem w samochód i gdzieś pojechałem do przyjaciół na weekend, podobała mi się jakaś koszulka, to sobie ją kupowałem.

**Ale jednak powiedziałeś, że 6-7 a nie 10. Gdzie były te hamulce?**

A kto ma 10? Kto przepieprza tak pieniądze? No chyba, że ktoś ma ich tak bardzo dużo. Trzeba mieć jakiś balans z w życiu. Jakieś są bariery. Powiedziałem sobie np., że nigdy w życiu nie wydam więcej na sportowe buty niż 350 zł. Mam taką barierę jakąś psychiczną. Nie wydam w restauracji na danie, które kosztuje 80 zł. Gdzieś są jakieś w głowie...50 zł mogę wydać na obiad z piwem. No jeszcze, ale jeśli danie kosztuje 50 zł, to dla mnie to już jest przeginka. Mam w głowie takie swoje granice przyzwoitości cenowej. Na takiej zasadzie to działa, a nie, że jakieś działy konsumenckie odpuszczam. Działam w każdym sektorze, jako konsument, ale wszędzie właśnie mam jakieś granice.

**A ma dla ciebie znaczenie marka?**

Tak, ale to się chyba z wiekiem zawęża albo odkrywam nowe, bo często marki z czasem tracą na jakości i idą na ilość. Czasami odnajduję nową markę, która jest świetna jakościowo i wolę wydać na bluzę trochę więcej, ale jakościowo będzie świetna i będę w niej chodził 2-3 lata niż co roku nową kupować, bo po sezonie będzie się do śmieci nadawała i w efekcie wydam więcej niż na tę jedną. Tak samo z głupimi skarpetkami. Wolę zapłacić za oryginalne firmy, bo testowałem i mogę je nosić latami, a takie gówniane, to po kilku praniach pójdą do śmieci i to jest żadna oszczędność.

**A jeśli chodzi o trendy? To miało/ ma dla ciebie znaczenie?**

Bardziej ja sam wyznaczam, w sensie, że swój styl mam taki. Nie nazwałbym siebie gadżeciarzem i bardziej wolałbym kupić jakąś fajną płytę czy bluzę niż jakiś gadżet.

**Przypomnij sobie jakiś swój większy wydatek na coś niecodziennego?**

Takie wydatki, to raczej długo mi się schodzi nad decyzją. I nawet moment, kiedy muszę to wydać, zapłacić, to jeszcze się 10 razy zastanowię.

**Podaj mi jakiś przykład takiej sytuacji?**

W tamtym roku do Azji leciałem i wiedziałem, że będzie to kosztowało kilka tysięcy złotych. Bardzo się cieszyłem i zawsze chciałem, ale jednak myśl, ile to może być pieniędzy, chociaż wiedzieliśmy, że to się rozłoży na raty w jakiś sposób, to do samego końca czekałem z tym i dopiero mnie ponaglili, że mam zrobić przelew za samoloty. Kurde, do końca byłem jakiś wstrzymany do tego i jak już przelałem, to już od tego momentu zacząłem dopiero myśleć o tym wylocie, bo to znaczyło, że klamka zapadła. Jak już przelałem, to i z następnymi kosztami się muszę pogodzić.

**Co sobie myślałeś - że może nie warto, może lepiej wydać na coś innego? Jak to było?**

Postawiłem wtedy na priorytet i już nic nie kupowałem. Jak postanowiłem, że lecę do Azji na 2.5 tygodnia, to reszta zeszła na dalszy plan.

**Warto było wydać te pieniądze na wyjazd?**

Oczywiście. Podróże poszerzają horyzonty. To chyba kwestia charakteru i natury ludzkiej. Myślę, że jestem typowym bykiem i jestem hedonistą. Podróże, dobre jedzenie, przyjemności - to zawsze tak u mnie było. Wydawać na rzeczy też lubię. Myślę, że tu i tu się jakoś odnajdę. Na podróże nigdy nie żałowałem.

**A na rzeczy?**

Przy ciuchach, przy konsumenckich rzeczach jakaś ta bariera jest, ale jeśli czegoś przy podróży chcę, to wiem, że trzeba wydać i w ogóle nie myślę o tym. Przykładowe buty, na które nie wydaję powyżej 350, to wiem, że jeżeli polecę gdzieś za granicę i trzeba będzie dołożyć...Bo to się tak mówi, że przeliczy sobie człowiek w głowie, że samolot tyle, hotel tyle, coś tam tyle, to trzeba i tak dołożyć z 1/3, że będzie więcej kosztów. Zawsze tak było. Totalnie się z tym liczę.

**A takie buty za 350 to jest przemyślany wydatek, czy zdarzało się spontanicznie?**

Buty to zawsze jest przemyślany wydatek. Potrzebuję, to kupuję.

**A ciuchy?**

To już naprawdę coś musi mi wpaść w oko. Raczej przemyślane, że potrzebowałbym tego, to szukam.

**Miałeś kiedyś wyrzuty sumienia, żałowałeś, że coś kupiłeś? Że po co, że dałeś się ponieść chwili i to było bez sensu?**

Rzadko. Chyba jak każdemu, sprawia mi przyjemność kupienie sobie czegoś fajnego. Nie jestem w jakiś sposób próżny w tym wszystkim, więc żeby było fajnie, ale najlepiej, żeby też było jakoś pragmatycznie tutaj. Bardziej wtedy przekonuje mnie zakup. Nie dość, że coś jest fajne, to jeszcze będzie to tak uniwersalne, że...

**Masz wrażenie, że jesteś osobą oszczędną niż rozrzutną?**

Bardziej rozrzutną. Wolę dobrze zjeść w restauracji niż samemu sobie gotować. Myślę, że to też wynika z lenistwa. Wolę gdzieś wyjść ze znajomymi niż siedzieć w domu, bo będzie taniej. Myślę, że to się zmieni, jeśli mi się w życiu prywatnym pozmienia, bo inne priorytety będą - żona, dzieci, dom. Pewnie będę wolał dla dziecka wydać na zieloną szkołę niż dla siebie na nowe buty.

**Teraz straciłeś dodatkowy dochód z grania. Czy twoja sytuacja finansowa pogorszyła się w zauważalny sposób?**

Te pieniądze, które ja zarabiałem dodatkowo, to ja inwestowałem dalej w muzykę. Mogę kontynuować to co robiłem i robię bez tego dodatkowego przychodu. Trochę z własnej kieszeni muszę wydać na nową muzykę, ale na razie nie odczuwam tego, że to jest jakaś tragedia.

**Masz wrażenie, że to wróci do normy sprzed koronawirusa w jakimś możliwym do określenia czasie?**

Nie sądzę. Myślę, że nasz rząd tak zrobi, że to będzie ciężko, żeby to wróciło do takich rozmiarów, jakie były. Pewnie to będzie, ale pewnie nie w takim wydaniu jak było. Nie wiem.

**Wspomniałeś, że starasz się teraz oszczędniej żyć. Jak kontrolujesz swoje wydatki? Miałeś jakieś sposoby? Jakie masz teraz?**

Nie, nic.

**Na czym polega, że teraz starasz się być mniej rozrzutny?**

Nie kupuję nic, co wybiega poza funkcjonowanie, czyli jeść i spać. Nic nie wydaję na nic, kompletnie. Odciąłem wszelkie jakieś wydatki. Czasami sobie zamówię i wezmę na wynos pizzę. Po 18-tym będą odmrożone bary, ale że ja nie piję, więc nie zrobi to na mnie wrażenia i nie będę wydawał też na alkohol.

**Jakie wydatki jeszcze odciąłeś w tej chwili?**

Same się odcięły. Nie wydaję na podróże, co się wiąże z ilością paliwa, nie wydaję na razie jeszcze na ciuchy, na restauracje, na alkohol. To głównie chyba o to chodzi.

**Ale to raczej zostało odcięte za ciebie, a nie, że sam to sobie odciąłeś?**

No tak.

**A sam z czegoś rezygnujesz albo bierzesz np. tańszy odpowiednik czegoś, co zwykle kupowałeś, albo czegoś nie kupujesz w ogóle, co zwykle kupowałeś, traktując to jako przedmiot zbytku?**

Nie, nie. Kupuję nadal to, co lubiłem.

**Czyli to się po prostu samo zmieniło, bo tych innych potrzeb poza podstawowymi nie masz jak realizować w tej chwili?**

Tak.

**Czy to jest trudna zmiana? To jest dla ciebie problem?**

Nie jestem w takim miejscu, że jestem załamany, bo ktoś mi coś odciął, bo doskonale sobie z tym radzę na zasadzie, że chcę pizzę, to sobie po nią pojadę. Nie muszę tam siedzieć w środku w tej pizzerii.

**Były takie sytuacje, że miałeś ochotę na pizzę, ale stwierdziłeś, że nie, że to nie pora na takie wydawanie pieniędzy?**

Ja teraz dużo siedziałem u rodziców i miałem po prostu podsunięty talerz, więc ostatnio o tym nawet w takich kategoriach nie myślałem, bo nie robiłem jedzenia, nie kupowałem, nie zamawiałem, bo jadłem u rodziców. Myślę, że jak będzie cieplej, jak będzie można gdzieś posiedzieć, zjeść, to pewnie wrócę do tych nawyków.

**Co myślisz o oszczędzaniu i o posiadaniu oszczędności?**

Pewnie, że powinno się jakoś oszczędzać, ale ja nie potrafię. One się same stworzyły i w jakiś sposób coś zostało przez to, że człowiek nie wydawał na codzienne życie, bo się nie ruszał z domu. Ale, żeby oszczędzać w jakiś sposób, to ja nie umiem, nie potrafię.

**Gdybyś został bez przychodów w momencie, gdy zaczął się koronawirus, to na ile czasu by ci wystarczyło tego co masz?**

Po wypłacie?

**Powiedzmy, że ostatnia wypłata w lutym, a od marca 0?**

Nie, no na miesiąc. Nie mam zaskórniaków. Teraz samoistnie pieniędzy szło mniej, bo nic nie robiąc człowiek nie wydaje.

**Próbowałeś kiedyś w jakiś sposób oszczędzać?**

Myślę, że jak z kimś będę, jak już z kimś zamieszkam, to wtedy będzie to miało miejsce, bo trzeba będzie w jakiś sposób, nie? Na razie żyję raczej chwilą.

**A jak chcesz na coś odłożyć pieniądze - jakiś wyjazd, jakaś droga rzecz, to jak to robisz?**

Wtedy przycinam kurek do rzeczy, które są mi zbędne i przestaję być rozrzutny. Na chwilę przykręcam kurek. Wiadomo, że wyjazdy się zawsze jakoś rozkładają finansowo w czasie, czyli z jednej wypłaty człowiek kupi bilety, z następnej będzie miał na pobyt.

**Czyli zostają ci te pieniądze na koncie, ale nie przekładasz ich na inne konto, nie wyjmujesz i nie odkładasz, tylko po prostu starasz się mniej wydawać na bieżąco?**

No tak.

**Inwestowanie w czasach takich jak teraz. Czy to jest sensowne? Gdybyś wygrał pieniądze, to co byś zrobił?**

Jak zaczął się koronawirus, to na pewno bym zainwestował na giełdzie, bo mnóstwo akcji spadało na łeb, na szyję. Jak to mówił Warren Buffet - gdy krew się leje na ulicach, trzeba inwestować. Z pewnych rzeczy trzeba uciekać, a w pewne inwestować. Akcje jakichś spółek za chwilę znowu wrócą. Myślę, że nie ma sensu w jedną spółkę inwestować. Przemyślałbym to. Nie myślałem o tym wcale, bo nie było oszczędności. W dobrym momencie myślę rynek mieszkań się zatrzyma, myślę, że będą spadać, bo ludzie nie będą mieli pieniędzy na te mieszkania. Myślę, że chwilę jeszcze trzeba poczekać, ale tak będzie, że będzie regres i nie będą iść w górę te mieszkania. Pewnie bym zainwestował w jakieś mieszkanie pod wynajem. Po takim koronawirusie życie pokazało, że dobrze mieć działkę i może bym kupił kawałek ziemi. Lokaty w banku bym nie zakładał, konta oszczędnościowego też nie, bo teraz przy tym rządzie, to nie wiadomo co oni tam zrobią, inflacje, nie inflacje. Nie, nie, nie. Raczej nieruchomości i akcje.

**Jak myślisz, kiedy ta cała sytuacja się skończy?**

Uważam, że ten koronawirus będzie. Był, jest i będzie. Nie wiem, co nasi rządzący będą szyli na bieżąco, nie mam zielonego pojęcia, co oni wymyślą. Zmieniają te koncepcje co chwilę. Sytuacja na świecie będzie miała też wpływ. Będą nasi patrzeć na innych, bo sami nic kurwa nie wymyślą. Nie wiem. To jest nowy porządek świata niestety. Bez naszego udziału w sensie, że to nie my podejmowaliśmy decyzje, tylko za nas to zrobiono. Trzeba się będzie od nowa nauczyć żyć, zarabiać, a Polacy są narodem kombinatorów, więc będą bardzo kombinować. Nasz rząd będzie udawać, że pomaga, jednocześnie rozkradając ciężko zarobione pieniądze ludzkie.

**Często się zastanawiasz nad tym, jak to się potoczy i jak będzie?**

Nie wiem. Inaczej bym podchodził pewnie do sprawy, gdybym miał inną pracę, ale moja jest tak stała, że ona się nie zamknie z dnia na dzień, więc nie zaprzątam sobie tym za mocno głowy. Pewnie za klubami będę trochę tęsknił, bo pewnie będzie różnie albo nie będzie wcale, albo nie wiadomo jak. W codziennym życiu praca będzie. Myślę, że jest za wcześnie na jakieś wnioski. Bardziej na koniec lata możemy o czymś rozmawiać, bo będziemy wiedzieli, co oni wymyślili po drodze i co będzie na jesień, zimę, bo uważają, że to wszystko wróci. To nie wróci, to jest po prostu. Ja podchodzę do tego, że nie umrę, że to jest jak grypa. Szczepionki nic nie dadzą wg mnie, bo na grypę się ludzie szczepią i chorują, i umierają.

**Masz jakieś obawy dotyczące najbliższych kilku tygodni, związane z sytuacją?**

Nie.

**A w perspektywie kilku miesięcy?**
Nie wie, za wcześnie jeszcze. Zobaczymy co będą szyli.

**Masz jakieś swoje przemyślenia?**

Jesteśmy chyba stadni i chyba każdy już tęskni za przebywaniem ze sobą, w ogóle za tym, że człowiek się zobaczy z kimś. Jest jeszcze jedna kwestia, o której z moimi przyjaciółmi też rozmawiam, że ci, którzy są sami, będą mieli ekstremalnie ciężko poznać kogoś ciekawego, bo wszyscy chodzimy w maskach. Ludzie, szczególnie młodzi, bo tu niestety z roku na rok jest coraz gorzej. Młodzież coraz gorzej odczytuje ludzkie emocje, bo rozmawiają poprzez wiadomości tekstowe, a teraz nie będą widzieli nawet mimiki nawet mimiki twarzy. Jak oni potem będą tworzyć rodziny? To będzie tak straszne w skutkach, że jakaś taka empatia ludzka w ogóle tak pójdzie w dół. I tak już idzie. Niedojrzali ludzie tworzą rodziny. Moja ostatnia relacja...To była właśnie osoba, która tak nie do końca potrafiła to czytać, która potrafi się uzewnętrznić. My się emocji uczyliśmy na trzepaku, w parku. Człowiek dostał w dziób, człowiek się przewrócił, człowiek powiedział, co myśli, człowiek się pośmiał, człowiek się nauczył śmiać z samego siebie. To będą emocjonalnie ludzie niepełnosprawni. W tym momencie zawód psychologa, psychiatry, to będzie zawód nr 1, żeby to wszystko dźwigać. Ja czekam na dofinansowanie państwa na wizyty u psychologa. Ja chętnie skorzystam.
