# Supplementary material for: The challenges arising from the COVID-19 pandemic and the way people deal with them. A qualitative longitudinal study
Source: PLoS One. 2021 Oct 11;16(10):e0258133. doi: 10.1371/journal.pone.0258133 (PMC8504766; doi:10.1371/journal.pone.0258133)
Supplement: S1 Dataset — (ZIP) [file pone.0258133.s003.zip › Transcriptions/stage 4/7.4_M_28_couple, no children.docx]

**7.4_M_28_couple no children**

**Co było przez dwa tygodnie?**

W skrócie, nic ważnego się nie wydarzyło. Dwa tygodnie minęły dosyć spokojnie, bez większych fajerwerków. Te zdjęcia, które ci wysłałem, oddają właściwie różne moje nastroje i jakoś obrazują wszystko, co w miarę czuję. Pod takim kątem starałem się je dobrać. Żeby wiedzieć, co powiedzieć.

**Co robiliście? Jak rozmawialiśmy ostatnio, miałeś dużo pracy.**

Teraz bez zmian, cały czas mam dużo pracy, cały czas tkwię w takim stanie, że muszę wymyślać nowe rzeczy, nowe pomysły na jakieś aktywności w sieci - głównie dla sponsorów, żeby trochę wyciągnąć od nich kasy na lato, żebyśmy mogli w miarę przetrwać. Także pracy mam sporo i w różnych momentach - niezależnie czy jest weekend czy wieczór.

**Czujesz się przepracowany?**

Mam wrażenie, że pomimo tego, że już wcześniej pracowałem w domu, to jednak cały czas była dość wyraźna granica tego, kiedy zaczyna się weekend. Teraz ta granica trochę się zatarła, niekoniecznie przez to, ze akurat szef czegoś potrzebuje, tylko sam akurat tak zauważam. Czasami pytam się Dominiki, słuchaj, dzisiaj jest wtorek? No tak. I zaczynam się zastanawiać, gdzie podział się mój poniedziałek. Nie potrafię wskazać jakichś konkretnych etapów, moment, czynności. Tak strasznie zlewa mi się czas.

**Wspominałeś na pierwszym spotkaniu, że dlatego, że pracujesz z domu, masz swój wypracowany rytm. Nadal jest tak samo, czy on się rozjechał?**

Pod tym kątem to akurat się nie zmieniło, rano zawsze wygląda podobnie. Zmieniło się to, że czasami wieczorem siadam do pracy, albo siadam do niej w weekend. Przez przynajmniej ostatnie pół roku zdarzało mi się to rzadziej. Może przez to, ze sytuacja jest inna, a może przez to, że nie wychodzę wieczorem, czy w weekend, więc mogę popracować.

**Dobrze czujesz się z tym, że jak wieczorem nie wychodzisz, to możesz popracować?**

No właśnie nie koniecznie. Jednak bardzo ceniłem sobie to, że - nawet pomimo tego, że pracowałem w domu już wcześniej i przyzwyczaiłem się do tego, nie mam problemów z efektywnością, czy zabraniem się do pracy - ceniłem sobie, że mogłem wieczorem pójść i odpocząć. To znaczy, moja głowa mogła odpocząć, w końcu. Na spacerze, spotkaniu z kimś, w kinie, czy gdziekolwiek.

**Technicznie rzecz ujmując, kiedy jest ładna pogoda, mógłbyś chyba iść na spacer, zamiast pracować. Dlaczego wybierasz pracę?**

Wyszedłem na ten spacer chyba raz, ale to tak przypadkowo, bo akurat odbierałem paczkę z paczkomatu. Nie wróciłem wtedy bezpośrednio do domu, tylko pochodziłem sobie jeszcze 15 min po okolicznych miejscach. Właściwie nie potrafię wskazać, dlaczego zamiast tego pracuję. Niektóre rzeczy faktycznie mógłbym zrobić rano następnego dnia, ale stwierdzam, że skoro mam chwilę wolnego czasu i nie mam innych rzeczy na głowie, mogę to zrobić teraz. I wtedy zabieram się za pracę następnego dnia pół godziny później. Nie nazwałbym tego jakąś zmianą rytmu, bo zawsze zaczynam między 9 a 10. Więc jak zacznę o 9:50, a nie o 9, to taki czas, że mogę sobie dłużej poleżeć w łóżku. Ale nie jest to jakaś zmiana rytmu, aż tak.

**Co jeszcze robiliście?**

Była majówka, w weekend, więc też średnio zauważalna. Udało nam się wyjechać na kilka godzin do lasu. To była chyba majówkowa sobota. To było fajne, można było troszeczkę odpocząć. Był akurat taki fragment tej soboty, że była fajna pogoda - 2-3 godziny. Co my jeszcze robiliśmy? <intensywnie myśli> No właściwie dużo się nie zmieniło. Była jeszcze jakaś jedna telekonferencja ze znajomymi. Jeszcze w poprzednim tygodniu. Dużo w pracy się działo. Troszkę jestem też bardziej w tematach rodzinnych ostatnio. Jeszcze bardziej, bo trochę złe rzeczy dzieją się z moją babcią, która już jest bardzo bardzo starą i schorowaną osobą. A przez to, ze mieszka z rodzicami, jestem troszkę bardziej na bieżąco. Przez to jutro pojadę do rodziców, choć mam mieszane uczucia - czy to już jest dobry moment na spotkania, czy nie jest? Sam w ogóle nie wychodzę, poza tym sklepem i poza tym jednym spacerem. Tych wyjazdów do lasu nie liczę, bo tam naprawdę nikogo nie spotykamy, schodzimy na dół do samochodu i jedziemy. Po rozmowie z rodzicami, którzy mają dokładnie taką samą sytuację, bo nie chodzą do pracy, tylko siedzą w domu, to stwierdziłem, że pojadę. To może być ostatni moment, żeby zobaczyć się z babcią, czy jakoś rodziców oderwać od różnych rzeczy, pomóc im rozmową. Powiedzieli, że bardzo cieszą się, że przyjadę.

**Bierzesz ze sobą Dominikę?**

No nie, jadę sam. Mamy też taką zasadę, że nawet kiedy jeździmy do niej do rodziców, po cośtam przy okazji, to ona wchodzi, ja nie. Jest analogicznie u moich rodziców tak samo, na wszelki wypadek ograniczamy to ryzyko. Wiem, że kiedy ona wróci od rodziców, może zarazić mnie i na odwrót, ale staramy się to ograniczyć tak czy tak. Jest to zawsze jakiś dodatkowy krok.

**Długo już chyba nie widziałeś swoich rodziców?**

Tak, jest maj, a ja widziałem się z nimi raz w tym roku kalendarzowym. Zazwyczaj widzę się z nimi raz na miesiąc/półtora. Teraz też właściwie nie wiem, kiedy pojadę następnym razem. Myślę, że to nie będzie prędko. Też pewnie nie pojechałbym, gdyby nie ta sytuacja, że dzieje się coś złego, teraz to bardziej sytuacja losowa.

**Poza wyjazdem masz jakieś plany na najbliższe dni? Dalej praca, czy coś jeszcze?**

Ciężko mi coś zaplanować. Myślę, że jeśli coś wystąpi, będzie to jakaś okazyjna przejażdżka do lasu. Jeśli pojawi się pogoda i czas u mnie i u Dominiki. A tak, raczej niczego nie planuję. Zastanawiam się, kiedy będę mógł spotkać się z kimś z przyjaciół. Ale wydaje mi się, że w maju to jeszcze nie nastąpi.

**Nie korci cię, żeby to zrobić?**

Korci mnie bardzo. I nawet słyszę, że niektórzy już się tam wyłamują, jakieś pojedyncze spotkania już się odbywają. Ale chyba jeszcze poczekam. Myślę, że to może już nastąpić w czerwcu, ale czy w maju to nie wiem.

**Masz na myśli takie spotkanie w stylu jak przed epidemią?**

Tak, raczej w tym wypadku na sto procent u kogoś w domu, też raczej w małym gronie. 3-4 osoby maksymalnie. Jakieś winko, piwko, rozmowa, tego typu spotkanie. To pewnie będzie inaczej wyglądać, jak spotkamy się na żywo, czy jakiś Messenger, czy coś.

**Czy poza tym, że więcej pracujesz, pojawiło się jeszcze coś nowego?**

Nie, w zasadzie cały czas towarzyszą mi podobne emocje do tych, o których już rozmawialiśmy. To też jest na tych zdjęciach. Na pewno coraz bardziej narasta chęć, żeby już wyjść, spotkać się z kimś. Widzę, że coraz więcej osób to robi, obluzowania jednak działają na ludzi. To też jest z jednej strony denerwujące w taki sposób, że może oni nie powinni jeszcze tego robić, ale kurczę, skoro to robią, to może faktycznie już można. Ale nie znamy odpowiedzi na to pytanie. Najbliższy weekend zapowiada się tak, że Dominika będzie miała bardzo dużo pracy przy różnych rzeczach. Wiem, że nie będziemy mieć za bardzo czasu, żeby coś zrobić. Pojadę akurat wtedy do rodziców. Ale gdyby nie to, to nie wiem kompletnie, co bym robił. Byłem trochę źle nastawiony do tego weekendu. Myślałem, że pomimo tego, że jest weekend, znowu będę siedzieć, będę musiał znaleźć sobie jakieś zajęcie, bo ona fizycznie nie będzie mogła. Ale sprawa rozwiązała się przez ten wyjazd i mam nadzieję, że do czerwca już powoli, że do czerwca nastąpi to przełamanie.

**Myślisz, że to kwestia czasu, czy rzeczy, które muszą się zadziać, żebyś ty się przełamał?**

W tym momencie wydaje mi się, że to raczej kwestia czasu. Chyba że nagle okaże się, że 1 tys. osób dziennie jest zarażonych, albo wzrośnie liczba zgonów, albo wprowadzą kolejne obostrzenia, albo coś jeszcze innego, co by mną mocno wstrząsnęło i zniechęciło mnie.

**Śledzisz na bieżąco statystyki?**

Nie za bardzo, tak jak ostatnio rozmawialiśmy, to się nie zmieniło. Dalej są zachorowania i zgony, więc skupiam się na sobie i tym, co robi Dominika, czy inni moi najbliżsi, czy z nimi ok, czy na siebie uważają. Raczej to się trochę zawęziło.

**Masz w ogóle poczucie, jaka to jest teraz liczba chorych?**

Zatrzymałem się na tym, że to jest kilkanaście tysięcy osób. Nie wiem, kiedy to było. Czy tydzień temu? O liczbie zgonów nawet nie jestem w stanie ci powiedzieć. Nie wiem, ile to jest teraz, zatrzymałem się na tym, jak było ponad 10 tys., później trochę więcej. W tym momencie, zupełnie nie wiem.

**A skąd docierają do ciebie te liczby?**

Jak sprawdzam informacje dot. sportu i kultury, wchodząc na Onet, mam pasek na górze.

**Mówisz, że widać luzowanie u ludzi. Co dokładnie widać?**

A, właśnie, nie powiedziałem ci o jednej rzeczy, która wydarzyła się wczoraj. Pojechaliśmy do centrum handlowego - Galerii Młociny. A późnej byłem przez chwilkę w Arkadii, już sam. To, co zobaczyłem w Arkadii, to byłem załamany, zły, i nie wiem jeszcze jak inaczej to wyrazić. To, że galerie zostały otwarte, a nadal obowiązują inne obostrzenia, to jest dla mnie totalnie nie zrozumiałą rzecz. To się totalnie wyklucza, zaprzecza i nie powinno mieć moim zdaniem miejsca. Obserwując to, co działo się w Arkadii, kiedy byłem tam dosłownie 15 min, po jedną rzecz i uciekłem stamtąd, to poza tym, że większość ludzi miała maseczki, to jakiś dystans i unikanie zagrożenia - to w ogóle nie istniało. To przypominało normalny ruch w centrach handlowych. Był tłum, każdy chodził własnymi drogami, niemal ocierając się o drugą osobę. Ludzie byli bardzo blisko siebie, większość w ogóle wydawała się być tym w ogóle nie przejęta, nie zwracała uwagi na to, czy jest jakiś dystans, czy nie. Mieli te maseczki, ale też nie wszyscy - niektórzy mieli je opuszczone, jak ostatnio opowiadałaś. Nie wiem ,jaki to jest sens.

**A po co pojechałeś?**

Akurat szukaliśmy jednej rzeczy w sklepie Kuchnie świata. Byłem więc zmuszony, aby akurat tam, do Arkadii się udać. I myślę, że drugi raz tego nie zrobię.

**Nie spodziewałam się po tobie, że pojedziesz do centrum handlowego.**

Ja też się nie spodziewałem, ale stwierdziliśmy tak z biegu, że ok, może spróbujmy. Też w sumie byłem ciekaw, jak to wygląda Spodziewałem się trochę czegoś innego. Dlatego drugi raz bym już tego nie zrobił. W Galerii Młociny było dużo spokojniej, tam nie czułem takiego zagrożenia. Ale to też jest takie centrum, które jest od niedawna i nawet jak bywałem tam wcześniej ,nie było aż takiej liczby ludzi. Więc tam ok, w jakimś tam przypadku, w mniej obleganej godzinie - może nie miałbym nic przeciwko. Ale Arkadia absolutnie nie.

**Ktokolwiek w ogóle sprawdzał liczbę osób?**

Są jakieś limity, zależne chyba od powierzchni, ale nie widziałem, aby ktoś to sprawdzał. Wychodząc z parkingu podziemnego widziałem, że stał pan ochroniarz, który sprawdzał, czy ludzie faktycznie mają maseczki. Ale z drugiej strony widziałem, że obok niego przechodziły dwie czy trzy osoby bez niej i nie zareagował. Więc może to wcale nie była jego rola. Próbuję sobie przypomnieć, skojarzyć, ale nie widziałem, żeby ktoś tego pilnował, liczył, pilnował jakiegoś dystansu.

**Byłeś zły, źle się czułeś z tym, że tak to wygląda?**

No tak. Widziałem na przykład artykuł po otwarciu Ikei. To też było o tym dosyć głośno, że ustawiały się ogromne kolejki do tego sklepu Też zastanawiałem się o co chodzi. Wiem, że są różne potrzeby, itd., ale Ikea też jest sklepem, w którym można coś zamówić internetowo, jeśli ktoś ma coś super pilnego. Dlatego troszkę nie rozumiem tego, że pierwszy dzień otwarcia, a tam jest kilka tysięcy osób. Trochę mi się to gryzie.

**Na co/ na kogo byłeś najbardziej zły w Arkadii?**

To jest dobre pytanie. Ni wiem, czy na to, że odgórnie już można, czy bardziej na to, że ludzie są nieuważni. To chyba tak równomiernie się rozkłada. Z drugiej strony, skoro można, dlaczego by tam nie pójść? Dlatego mam wrażenie, że nasz rząd zakłada dosyć optymistyczne scenariusze. Że może faktycznie w tym centrum handlowym pojawią się ludzie, którzy mają potrzebę, albo będą uważać. Z drugiej strony nie mogę tego tak jasno ocenić. Ale widząc grupkę osób, która spacerowała sobie z kawą, nigdzie się nie spiesząc, po prostu spacerując sobie, a później się zatrzymując - nie widać było, że im się spieszy. Faktycznie, było widać kilka osób, które zmierzają do konkretnego celu - dosyć szybko. Później widziałem, jak gdzieś wracają. To rozumiem. Ale nie rozumiem takiego typowego udania się do centrum handlowego po prostu po to, aby spędzić tam czas.

**Jak myślisz, z czego wynikało to, jak postępowali ci ludzie?**

Wydaje mi się, że chyba się nie boją, skoro tak robią. Może stwierdzili, że już można i dlaczego nie skorzystać. Rozumiem, że może niektórym też tego brakuje. Jeśli ktoś to robił dosyć często, albo ktoś w końcu chciał wyrwać się z domu, zrobić coś innego, niż wcześniej. Ale wydaje mi się, że to jest trochę przełamanie zdrowego rozsądku. Tego typu akcje. Tak mi się wydaje.

**Pojechaliście najpierw na Młociny, a później do Arkadii?**

Tak, tylko w GM nie znaleźliśmy tej rzeczy, dlatego pojechaliśmy jeszcze do Arkadii, a Dominika została już wtedy w samochodzie, a ja poszedłem szybko na górę, tylko na 15 min i wróciłem.

**A byłeś zły na siebie, że pojechałeś, że podjąłeś tę decyzję?**

Właściwie chyba nie. Nie miałem czegoś takiego. Plusem było to, że zobaczyłem, że tak to wygląda i można to sobie wybić z głowy na jakiś czas, jeśli nie będzie jakiejś super ważnej potrzeby. Ale nie byłem tak jakoś zły. Starałem się zrobić to szybko, przede wszystkim uważnie. I tak, żeby jak najszybciej stamtąd wyjść, po prostu.

**Nadal kupujesz w Carrefourze obok, czy poszerzyłeś listę tych sklepów?**

Poszerzyłem. W tym tygodniu, zamiast pójść 2 czy 3 razy d Carrefoura na zakupy żywnościowe, pojechaliśmy do Lidla i tam zrobiliśmy dużo większe zakupy na raz, żeby mając pełną lodówkę na jakiś tydzień, nie wychodzić już do sklepu. Chyba, że w takim przypadku, że czegoś nam braknie, albo o czymś zapomnieliśmy. Ale raczej nie przewidujemy.

**To były zakupy, żeby nie wychodzić, czy chcieliście zmienić sobie sklep?**

Myślę, że jedno i drugie. Bo jednak w Lidlu dla Dominiki produktów wegańskich jest dużo więcej, niż w Carrefourze. Więc mogliśmy kupić dodatkowe rzeczy. A przy okazji to, że zrobiliśmy jedne duże zakupy to też jest to, ze nie musimy do tego Carrefoura chodzić więcej razy, więc to też taki komfort. Mnie co prawda nie będzie, ale nie sądzę, żeby Dominika miała w tym czasie konieczność, żeby pójść po coś. Raczej myślę, że do kolejnego wtorku tych zakupów nam wystarczy.

**Myślisz, że te jedne większe zakupy, to lepsza strategia?**

To zależy. Byliśmy w tym Lidlu, ale tam nie było aż tak wielu osób. Z drugiej strony, dlatego, że to jest większy sklep i może być tam więcej osób, to jest tam więcej miejsca. Nasz Carrefour jest taki, że dwie osoby wszerz między alejkami się nie zmieszczą. Zawsze ktoś musi wyjść, żeby zrobić miejsce, albo tak maksymalnie się ścieśniać i przepuszczać kogoś. W Lidlu tego nie było, było więcej przestrzeni, wydaje mi się, ze czułem się tam bardziej bezpiecznie, luźno. Tam łatwiej zachować dystans.

**Widzisz różnice w zachowaniu ludzi porównując mniejsze i większe sklepy?**

Szczerze mówiąc, w zachowaniu ludzi nie widziałem różnic. Myślę, że po prostu fakt większej przestrzeni pomaga, że ludzie mogą się zmieścić, mogą jakoś tak się przemieścić, że jest to bardziej bezpieczne.

**Masz/ miałeś produkty, które kupiłeś dla przyjemności?**

Kupiliśmy lody z myślą o sobie. Ja akurat nie jestem super pożeraczem lodów, ale akurat tutaj miałem ochotę. I kupiłem sobie whisky. Bo kupowałem też dla taty, który miał urodziny, a przez to, że jutro będę u niego, dam mu jako prezent. I wziąłem sobie też dla siebie, bo lubię mieć po prostu, usiąść sobie czasami wieczorem. Taka butelka mi starcza na 2-3 miesiące. Na jakieś takie okazyjne rzeczy.

**To taki twój zwyczaj, żeby ją mieć?**

Trochę tak. Czasem coś nieoczekiwanie wypada, albo jest jakiś trudny dzień. Nie zdarza mi się to często, że po trudnym dniu piję whisky, raczej to jest weekend. To taki moment, że nawet do jakiegoś filmu, czy bywały nawet te telekonferencje ze znajomymi. To tak żeby mieć w domu, przy okazji.

**A masz teraz coś takiego, że skoro siedzisz w domu, chętnie kupiłbyś sobie coś fajnego?**

Tak, mam tak czasem. Szczególnie właśnie w weekend. Pomimo tego siedzenia w domu, lubię poczuć weekend. W jakiejś formie. Mieliśmy taki rytuał od początku całej tej sytuacji, że co tydzień gotowałem jakąś kolację w tematyce jakiejś innej kuchni. I do tego sobie siadaliśmy, czasami napiliśmy się wina, czasami piwa lub jakiegoś innego drinka. Staraliśmy się tak w miarę beztrosko spędzić czas, żeby choć odrobinę poczuć ten weekend. Z takich rzeczy dla siebie, zakupy internetowe są jakieś tak w miarę obecne. Teraz zamówiłem sobie jakieś nowe ubrania... Pewnie i tak bym je zamówił, niezależnie od sytuacji, po prostu potrzebowałem, ale no, czy jakieś jedzenie na dowóz. To też jest zawsze jakaś taka forma sprawienia sobie przyjemności. Zjedzenie sobie czegoś nawet.

**Odkrywacie jakieś nowe knajpy?**

Ostatnio mieliśmy pierwszą przerwę od tego. Była kuchnia izraelska, meksykańska, wcześniej robiłem bao - bułeczki azjatyckie. Było włoskie, później robiłem pizzę domową, więc tak na razie nie ma nowych pomysłów. W ten weekend też nie będzie, przez ten wyjazd. Ale w sumie dalej pomysły się znajdą, mamy trochę rzeczy, które chcielibyśmy zjeść, albo po jakiejś przerwie przyjdzie nam ochota. Pod tym kątem na brak pomysłów raczej nie narzekamy. Raczej staramy się zorganizować pod tym kątem, żeby w miarę możliwości czasowych, robić sobie namiastkę tego weekendu.

**Nadal traktujesz wysyłkę do paczkomatu jako dobry powód do wyjścia na spacer?**

Przez to, że teraz jest dużo tych paczek, jest krótszy czas na odbiór. Koszty nie opłacają się aż tak, nie są dużo tańsze, niż kurier, więc przerzuciliśmy się na kuriera. Poza tym, jak ostatnio byłem w paczkomacie, była do niego kolejka, to znaczy do kuriera w samochodzie, przy paczkomacie. I to była taka kolejka na 7-8 osób. To też zabierało jakiś czas, albo musieliśmy zmieniać swoje plany, żeby szybko pójść do paczkomatu, aby zdążyć w określonym na odbiór czasie. I ostatnie dwa razy tak wyglądały. Ale przez to, że jest teraz ładna pogoda, pewnie coraz częściej będziemy starać się wyjeżdżać gdzieś na te kilka godzin.

**Zdjęcia**

Właściwie kolejność jest dowolna. 1 to substytut mojej miłości do gotowania. To jedna z nielicznych rzeczy, podczas których czuję teraz, że się rozwijam, czegoś uczę i mogę połączyć przyjemne z pożytecznym. 2 - starałem się znaleźć jakieś takie zdjęcie, które odzwierciedli mój nastrój, który pojawia się, kiedy się budzę lub idę spać. Akurat padło na scenę z filmu, który bardzo lubię. Kiedy kładę się spać lub wstaję rano, zastanawiam się, czy coś się dzisiaj lub jutro zmieni. To czasami taki nostalgiczny moment, że nic się nie zmieni, że to znowu będzie wyglądać tak samo i to jest takie, nie chcę powiedzieć przygnębiające, ale nie jest to miłe uczucie. Nie czuję się wtedy jakoś super. Nr 3 to jest mój komentarz wobec tej sytuacji, która wydarzyła się w Arkadii - to moja złość na to, kiedy weszliśmy do domu i uświadomiłem sobie, co się tam działo. Nr 4 bardzo łączy się z nr 2 - to moje znużenie i zmęczenie. Czasami kończą mi się pomysły na nowe rzeczy. Nie zawsze mam w sobie tyle energii, aby iść do kuchni na 2-3 godziny i wymyślać nowe rzeczy. Bo jestem bardziej lub mniej zmęczony - to takie znużenie, że mam po prostu już dosyć tej sytuacji. To się pojawia. Nr 5 - właściwie codziennie, kiedy patrzę za okno, mam wrażenie, że to życie się toczy. Tzn., że czasami mam wrażenie, że bardzo mało osób stosuje się do tych obostrzeń, patrząc na jakiś widok za oknem. Myślę sobie, kurczę, no cały czas tam jest to życie i może jednak nie powinno tak być, że to wszystko, co się tam dzieje, powinno być teraz. Może jednak ludzie powinni mniej wychodzić, bardziej uważać, to jednak jest pandemia, itd. Z drugiej strony to zdjęcie też obrazuje to, że mieszkamy w dużym mieście, a mam wrażenie, że ten dom jest gdzieś z dala od tego miasta. Przez to, że siedzę w domu, jestem gdzieś z dala, to jest jakby już inny świat. Mimo tego, że wielkie miasto jest obok, ten pęd, ruch, to jest takie dość odległe ode mnie. 6 - to jest obraz tego, do czego już nawiązaliśmy. Czasami okrutnie miesza mi się czas. Łączy mi się, czasami tracę rozeznanie co, kiedy się dzieje. Bywają takie dni, że mam wrażenie, że jest godzina 19, a jest 23. Nie czuję upływu czasu, a później uświadamiam sobie, że on mi ucieka. Ostatnio jak spojrzałem za okno, kiedy było chłodno, deszczowo i wietrznie. Uświadomiłem sobie, że mamy 4 czy 5 maja, a zarazem to mógłby być 4 marca. Minęły dwa miesiące kompletnie gdzieś. Takich momentów, które pamiętam, im dalej, im później, jest coraz mniej. Ten czas gdzieś uciekł. Jednocześnie czasami jest dość rozlazły, a czasami w ogóle gdzieś znika. I dlatego przyszedł mi do głowy Dali i zegary.

**Masz poczucie, że to stracony czas, czy to nie jest dobre określenie?**

Myślę, ze trochę tak, to trochę czas stracony. Kiedyś miałem taki czas, że pomimo, ze pracowałem rano, to bardzo późno kładłem się spać, bo uważałem, że lepiej korzystać z czasu, nawet robiąc jakieś rzeczy. Nawet nie spać, tylko korzystać z życia. Tutaj trochę faktycznie tak czuję, że można było ten czas lepiej spożytkować. Nie mówię pod kątem jakichś ambicji, tylko nawet tak jeśli chodzi o czas z najbliższymi, wrażenia, doświadczenia. Biorąc też pod uwagę to, że w najbliższym czasie mieliśmy zaplanowane dwa wyjazdy, nie odwołałbym festiwalu, miały dziać się różne rzeczy. Dlatego jest takie poczucie, że szkoda, że to się tak potoczyło. Obrazek 7...

**Oj, jak ty lubisz ten film.**

Tak, z nim przyszły mi do głowy różne stany emocjonalne. Chodzi o to tutaj, że przez ten czas spędzony w domu korci mnie, nosi, żeby zrobić coś... szalonego, to może złe słowo, ale coś, żeby totalnie się wyluzować. Z drugiej strony, jak mieliśmy dwie telekonferencje z przyjaciółmi, były jakieś tańce, śpiewy. To takie rzeczy, których normalnie bym nie zrobił, a widziałem, że to wpłynęło na mnie tak, że po prostu potrzebowałem jakiegoś takiego mocnego wyluzowania i odpoczynku dla głowy. Dlatego to zdjęcie to jest też taka tęsknota, objaw tego, że jak to się niedługo skończy, to bardzo chętnie zrobiłbym coś takiego, może nie szalonego, ale nietypowego dla mnie. O tak.

**Masz pomysł, co to by było, czy to będzie coś totalnie spontanicznego?**

To nawet nie musi być coś super odkrywczego i wymyślnego. To może być to, że spotkam się z przyjacielem i pójdziemy na nocne piwo, gdzieś w jakimś plenerze. I posiedzimy sobie, pogadamy, będzie jak za dawnych lat. Już to jest dla mnie objaw czegoś nietypowego w tej sytuacji, że będę mógł wyjść na to przysłowiowe piwo. Nie wiem, co to będzie. Na pewno coś mocno towarzyskiego, bo to najbardziej doskwiera. No i nr 8 to takie zdjęcie, z którym niekoniecznie czuję się jakoś mono związany, ale jednak cały czas mam z tyłu głowy to, że ta długa droga wreszcie nas do czegoś doprowadzi, albo wreszcie się zakończy i dojdziemy do celu i w końcu wróci jakiś rodzaj normalności.

**Myślisz, że takim celem, do którego dążymy, jest normalność?**

Tak, to znaczy, jeśli chodzi o mnie, nie mam tego typu przemyśleń, że jak to się skończy, nagle będę chciał zmienić swoje życie, przyzwyczajenia, rzeczy, które wcześniej robiłem. Raczej chciałbym wrócić do tego, co było przedtem. To dla mnie zupełnie ok pod względem wrażeń, mocji, doświadczeń.

**Chciałabym wrócić do zdjęcia nr 4, tam, gdzie Brad Pitt ziewa, jest zmęczony, znudzony tą sytuacją. Masz sposoby, żeby rozładować swoje znudzenie i zmęczenie?**

Z jednej strony, przydaje mi się rowerek stacjonarny do ćwiczeń, z którego częściej korzystam. I dłużej, jeśli mogę. Też czuję potem jakieś rozładowanie. Z drugiej strony, super ważny jest dla mnie ten moment w weekend. Kolacja, kieliszek wina, czy telekonferencja z kimś Żeby odczuć, że można odpocząć. A inne sposoby, myślę, że przebywanie z moim kotem to też jest sposób. Kiedy jestem zły i czuję dosyć silne emocje, to przychodzi czasami kot i potrafi je we mnie rozładować. Jakąś taką swoją kocią miłością. Rozmawialiśmy z Dominiką nawet na ten temat, że w tej sytuacji kot jest nawet bardzo pomocny. Nie jest szkodliwy pod takim względem, że szaleje, rozrabia i mam  go dosyć. Czasem są takie momenty, że zrzuci kwiatek, ale z innej strony ta jego obecność, jakaś miłość, kiedy siedzi obok, czy leży w łóżku, to naprawdę pomaga. Jest takim dodatkowym domownikiem, pod takim względem, że jak ktoś mieszka sam, czy potrzebuje kogoś, to wydaje mi się, że takie zwierze może pomóc.

**Czy jest jeszcze coś takiego, co w ciągu ostatnich dwóch tygodni zapadło ci w pamięć? Pozytywnie albo negatywnie.**

Wydaje mi się, że nie. Negatywnie to te centra handlowe i obraz ludzi. Sytuacja rodzinna też nie jest ok, ale to już jest poza tematem, sytuacją. Wydaje mi się, że nie.

**Śledzisz pomysły na kolejne luzowania?**

Nie śledzę. Interesowałem się bardziej kwestią dotyczącą wyborów. Poluzowań nie śledziłem i nie wiem, czy one nastąpią w kolejny poniedziałek?

**Teraz otworzyli przedszkola. Dali możliwość. Można otworzyć przedszkola i żłobki.**

Ach, właśnie. Ciężko wyobrazić sobie małe dzieci w maseczkach. Czytałem o takich pomysłach, aby dezynfekować zabawkę po każdym dziecku. Wydaje mi się, że trzeba by  było wtedy mieć jednego opiekuna przypisanego na jedno dziecko, żeby pilnował tego cały czas. Wydaje mi się, że dzieci bawią się raczej różnymi zabawkami i bawią się blisko siebie. Więc w przypadku takich małych osób, to chyba jest dość problematyczne. Na miejscu rodziców chyba bym się nie pokusił, chyba wolałbym mimo wszystko poczekać. Różne osoby mają różne sytuacje zawodowe - może niektórzy musieli już wracać do pracy, ale nie wiem, wydaje mi się to dosyć szalone. A czy wiadomo coś na temat szkół?

**Do 29 maja na razie jest edukacja zdalna. Co do reszty, nie wiadomo.**

A później byłby obowiązek powrotu do szkoły, czy byłby wybór?

**Nie wiem. A jak uważasz, że powinno być?**

Nie wiem, jak to miałoby przebiegać pod względem organizacyjnym. Że nauczyciel najpierw prowadzi lekcje normalnie, od 8-13, a później online? Nie wiem tego. Pod takim względem, żeby nie brać pod uwagę żadnych innych aspektów, uważam, że powinien być wybór. Tzn., że rodzice mają prawo bać się o własne dzieci, tak po prostu. Ale nie wiem, jak to fizycznie, organizacyjnie mogłoby przebiegać. Nie wiem czy też czy prezydent Trzaskowski przypadkiem nie zgodził się na te żłobki i przedszkola?

**Państwowe są na razie zamknięte (...), bo na razie nie są w stanie spełnić wytycznych. Otworzyły się prywatne. To właściciel przedszkola decyduje, czy się otwierają.**

Ciężko mi to sobie wyobrazić, jak miałoby to przebiegać w miarę bezpieczny sposób.

**1-10 (1 = z dużym trudem, 10 = z dużą łatwością wydaję pieniądze)**

Myślę, że dałbym sobie... Zastanawiam się, czy 6, czy 7. Myślę, że dałbym sobie 6. Bo jednak mam tak, że na razie jestem w dość komfortowej sytuacji, że nie muszę aż tak, wchodząc do sklepu, zwracać uwagę na ceny, czy później wyjmować z koszyka. Z drugiej strony odkładam pieniądze. Takie duże zakupy, typu ubrania, to nie jest u mnie coś, co występuje co miesiąc, tylko raz na 3-4 miesiące. Czasami raz na pół roku. Więc dałbym sobie 6 - z czymś takim, że jeśli czegoś potrzebuję, mogę sobie na to pozwolić i wystarcza mi na to, ale z takim zachowaniem rozsądku i oszczędnościami, jakimś tam rozsądnym gospodarowaniem tymi pieniędzmi.

**A jakie masz kategorie produktów, na które nie lubisz wydawać, masz z tym większą trudność?**

Hm. <długo myśli>

**To może na odwrót, na co łatwo wydajesz pieniądze?**

Rzeczy, na które najczęściej wydaję, to takie rzeczy konsumpcyjne, ale pod takim względem, że jeśli chcę iść do kina, jeśli chcę iść do restauracji, albo jeśli myślę o jakimś wyjeździe, to na te rzeczy najłatwiej wydawać pieniądze. To takie rzeczy typowo wrażeniowe, doświadczeniowe. Taka kultura spędzania czasu, co bym śmiesznie określił. Dłużej zastanawiam się nad wydawaniem pieniędzy na ubrania. Nie mam czegoś takiego, że nagle pojawia mi się ochota na kupienie czegoś do ubrania, tak po prostu. Raczej nie mam wypchanej szafy, pełnej rzeczy, które zakładam raz. To są takie rzeczy przemyślane, że jeśli coś kupuję, to faktycznie jest taka potrzeba. Myślę, że trochę inną kategorią są też podróże. To nie jest tak, że na każdą podróż jestem w stanie z miejsca wydać duże pieniądze. Jeszcze aż tak mnie na to nie stać, to są w miarę przemyślane rzeczy. Z przygotowaniem, rozeznaniem. Jak był jakiś drogi lot, nie decydowaliśmy się od razu, tylko czekaliśmy. Jesteśmy w takim momencie, że nie planujemy wieloletnich inwestycji, typu kredyt na mieszkanie, samochód, itd. To jeszcze sprawa odległa. Więc może to taka rzecz, na którą najtrudniej wydać mi pieniądze. Pewnie moglibyśmy sobie na to pozwolić, ale nie chcemy - jeszcze. Te sprawy przeżyciowe [wrażenia] i spędzanie czasu górują nad wszystkim innym. Jeśli miałbym sobie kupić jakieś drogie buty, ale nie wychodzić przez miesiąc, to nawet nie ma takiej mowy. Idąc dalej - gdybym miał płacić pieniądze na kredyt, pewnie nie wydawałbym dużo większych niż na wynajem, to jednak wolę te sprawy przeżyciowe, od swojego komfortu.

**Kiedy przychodzi ci wydać pieniądze na wakacje, jak się z tym czujesz?**

Raczej ok. Ale też pod takim kątem, że jak jadę na wakacje, nie wydaję wszystkich swoich pieniędzy. Staram się tak tym gospodarować, że to są troszkę pieniądze zbierane, troszkę odkładane, ale też nie na zasadzie, że jadąc na wakacje wydaję wszystkie swoje oszczędności. Zawsze staram się mieć jakiś plan w głowie, zabezpieczenie. Jestem raczej z takich osób, które planują wydatki. Jeśli muszę kupić sobie coś większego, zazwyczaj jestem na to przygotowany wcześniej.

**Gdybyś miał wybierać pomiędzy rozrzutnym a oszczędnym, to jesteś raczej oszczędny?**

Nie wiem, czy można powiedzieć o oszczędnym to, że wychodzi się kilka razy w miesiącu gdzieś. To są rzeczy, bez których kilka razy w miesiącu teoretycznie można się obyć. Gdybym czuł się z czystym sumieniem oszczędny, nie wydawałbym pieniędzy czy w restauracji, czy kinie, barze. Stawiałbym się raczej pośrodku.

**Kiedy myślisz na zakupy spożywcze, masz coś takiego, że, a, to mam ochotę na to, czy wezmę sobie tamto?**

Wtedy nie ma przeciwwskazań. Na przykład to whisky. To też nie było coś, co planowałem kupić. Widziałem, że skończyła się jakieś 2-3 tygodnie temu, ale też nie jechałem na zakupy z myślą o niej. Wpadło mi to w oko. Zdarzają mi się rzeczy, żeby wrzucać do koszyka to, na co mam ochotę.

**W obecnej sytuacji odczułeś jakieś zmiany w dochodach?**

Z festiwalem odbyło się bez cięć. Nie wiem, jak to przeliczyć... Straciłem jakoś 1/8 dochodów przez to, że współpracowałem z jednym klubem w Warszawie. Z jednej strony promocyjnie, z drugiej - grałem u nich jako DJ. Przez to, że jest zamknięte, straciłem te dochody. Ale to jest taka sytuacja, że mam mniejsze oszczędności co miesiąc.

**Odczuwasz to realnie na co dzień?**

Raczej nie. Pieniądze trafiałyby na konto, więc w codziennym życiu, kupowaniu różnych rzeczy nie mam tak, że muszę sobie czegoś odmawiać. To była taka nadwyżka, która szła na kupkę oszczędności.

**Czy w związku z epidemią zmieniłeś swój sposób wydawania?**

Część pieniędzy szła na te przeżyciowo-wyjściowe rzeczy, więc to zauważyłem, że o ile wspólne wydawania pieniędzy się nie zmieniło, tak mam wrażenie, że troszkę więcej pieniędzy zostaje mi na koncie własnym. Przez te wyjścia [ich brak teraz] wydajemy troszkę mniej. W tym miesiącu uda nam się pojechać na Podlasie, na 4 dni do chatki na wsi, przez to, że wrócił najem krótkoterminowy. To jest ta rzecz, która wraca, jeśli chodzi o przeżyciowe rzeczy. Ale ostatnio było tego mało. Może też częściej zamawiamy jedzenie, jeśli nie mamy czasu, ale to też nie są takie super znaczące w dłuższej skali.

**Jesteś osobą, która zwraca uwagę na promocje?**

Nie sprawdzam jakichś promocji w internecie, czy gazetek wcześniej. Ale jeśli na zakupach coś wpadnie mi w oko, zdarza mi się kupić. Nie zawsze, ale od czasu do czasu.

**A zdarza ci się stać przed półką i wybrać tańszy produkt z dwóch?**

Czasami. Ale to chyba wynika nie tylko z ceny. To czasami tak, że produkty są podobne, ja nie czuję różnicy w smaku, czy w czymś. Wtedy biorę tańsze.

**Czy w związku z epidemią przywiązujesz większa wagę do planowania budżetu?**

Myślę, że nie. To wygląda tak samo. Zazwyczaj wiem, na co przeznaczę pieniądze po kolejnej wypłacie. Jestem z tych osób, które planują wydatki, więc wiem, ile wydajemy na co dzień, wiem, jeśli pojawi się coś, co muszę kupić dodatkowo - rowerek, ubrania, czy wyjazd. To się chyba nie zmieniło.

**Jesteś człowiekiem posiadającym oszczędności?**

Mam oszczędności. Nie wiem, jak je oceniać, ale staram się oszczędzać regularnie. Specjalnie odkładam na konto. Po wypłacie, pieniądze są od razu grupowane. Pieniądze na życie trafiają na wspólne konto, pieniądze oszczędnościowe trafiają na oszczędnościowe, a te, które zostają na nadprogramowe wydatki, zostają na tym koncie. Mam stałą kwotę, jaką przeznaczam na oszczędności, ale staram się ją zwiększać. Nawet nieznacznie, ale powiększać, jeśli się udaje.

**Gdybyście stracili dochód, jak długo byłbyś w stanie się utrzymać z tego, co masz odłożone?**

Biorąc pod uwagę całość, wydaje mi się, że 2-3 miesiące. Od 3 miesięcy nie dostaję dodatkowych pieniędzy, z których byłby pewnie dodatkowy miesiąc. Więc tak bym oceniał.

**To jest poziom, który daje ci poczucie bezpieczeństwa finansowego?**

Wydaje mi się, że nie. Te oszczędności byłyby większe, ale pod koniec roku planowaliśmy różne rzeczy i te pieniądze trochę się rozeszły. Akurat na początku roku dostałem też podwyżkę, więc oszczędności w tym roku miały się pojawić. W tym roku miałem uzbierać taką kwotę, która mogłaby dać mi poczucie komfortu na jakiś czas.

**Na ile miesięcy chciałbyś mieć odłożone, aby mieć poczucie komfortu?**

Minimalnie na pół roku. To byłyby takie środki, przestrzeń, że gdyby coś się stało, nie spanikowałbym.

**Robisz coś z oszczędnościami, czy one po prostu sobie leżą?**

Na ten moment to tylko konto oszczędnościowe. Nigdy nie inwestowałem, ale też nigdy nie brałem kredytu. Nie jestem nim przeciwny, ale uważam, że to stresujące i obciążające. Nie miałbym nic przeciwko nim, gdybym miał faktycznie takie wynagrodzenie, że czułbym się bezpiecznie. Uważam, że nie mam jeszcze aż takiego komfortu. Mimo, że na ratę kredytu wydawałbym tyle samo, co na wynajem, to jakoś nie czułbym się tak komfortowo, jak w tym momencie. Wydaje mi się, że musiałbym mieć dwa razy większe wynagrodzenie.

**To wynika z tego, że z wynajmu możesz się wycofać, a z kredytu nie?**

Tak, w takiej sytuacji, gdzie straciłbym pracę, nie wiedziałbym, jak zachować się z kredytem. Czułbym duże obciążenie, że mam  na sobie jeszcze spłatę dużego kredytu i nie wiem. Dlatego się cieszę, że w tym momencie tego nie mam, że zawsze jest jakaś furtka. Bez zobowiązań czuję się teraz dobrze, aczkolwiek w skali 5-10 lat, to może być już nieekonomiczne. Musi przyjść moment, kiedy będę mógł sobie na to pozwolić przy zachowanym komforcie finansowym. Kiedy to nastąpi - nie wiem. Być może nastąpi.

**W czasach pandemii, ważność posiadania oszczędności wzrasta?**

Teraz chyba to podziałało też na mnie, myślę o tym bardziej. Chciałbym znaleźć nawet kilkaset złotych więcej, to zawsze jest jakiś krok zabezpieczający. Te sprawy przeżyciowe, na które nie wydaję - staram się przynajmniej te pieniądze dokładać do kwoty oszczędności, skoro nie dostaję pieniędzy z klubu. Jako zamiennik.

**Myślisz, że ludzie ogólnie mają oszczędności? Jak to finansowo wpłynęło na twoich znajomych?**

Jeśli chodzi o moich przyjaciół, znajomych, rówieśników - wydaje mi się, że większość nie ma tych oszczędności. Ale oni jakoś funkcjonują, bez większych problemów. Nie stracili swoich prac, ale nie wiem co by było, gdyby stracili. Ten temat pieniędzy - nie rozmawialiśmy o tym. To nie jest taka kwestia, którą się omawia. Niezależnie od tego, czy jest pandemia, czy nie.

**Myślisz jednak, ze raczej nie są to ludzie, którzy mają oszczędności i w razie utraty dochodu, mogłoby być gorzej?**

Tak, bo czasami nawet w żartach jedna czy dwie osoby wspominały, że wypadły czyjeś urodziny, a one już jadą na karcie kredytowej, czy tam debecie, czyli już pod kreską. To pojawiło się niby w żartach, ale jednak zapamiętałem ten moment.

**Rozumiem, że tobie nie zdarzyło się jechać pod kreską. Przynajmniej w ostatnim czasie.**

W ostatnim na pewno nie.

**Doprowadziłbyś do takiej sytuacji? Żeby na przykład żyć z kart kredytowej przez miesiąc, albo dwa tygodnie miesiąca?**

Nie wiem, mam ten komfort, ze nie jestem w takiej sytuacji. Ale na pewno nie czułbym się z tym dobrze. To musiałaby być chyba konieczność. Bardzo bym tego nie chciał.

**Myślisz, że obecny czas jest dobrym na inwestycje?**

Zastanawiałem się nad tym ostatnio. Na przykład jeśli chodzi o mieszkania. To są jakieś gdybania, ale wydaje mi się, że skoro mnóstwo ludzi traci pracę, czy ma cięcia finansowe, itd., wydaje mi się, że ten rynek musi stanieć, mieszkania muszą być tańsze. Żeby po prostu mogły się sprzedać. Ale jakoś mocno nie interesowałem się tym. Wydaje mi się, że to może być taki moment, że akurat komuś uda się kupić mieszkanie ileś % taniej, czy coś innego. Nawet patrząc na wyprzedaże w sklepach internetowych. Szukając butów widziałem, że niektóre modele potrafiły kosztować 600 zł, a teraz na wyprzedaży kosztują 370 zł. To spora obniżka.

**Jak myślisz, jak będzie dalej? Myślisz o tym?**

Wydaje mi się, że nawet kosztem jakiegoś bezpieczeństwa sytuacja będzie rozwijać się tak, że ludzie będą dążyć do tego, żeby żyć jak najbardziej normalnie. Korzystać z lata, różnych możliwości. Trochę sam bym chciał, żeby tak było. Z tyłu głowy planuję, że jak przyjdzie czerwiec, to może będzie przynajmniej można spotkać się w małym gronie. Aczkolwiek to są jakieś małe rzeczy. Ja pracuję w branży imprez masowych i kultury - nie wiem, czy tam coś się zmieni do końca lata. Znowu jesienią ma być kolejna fala zachorowań, więc zupełnie nie wiem. Jak to się może skończyć.

**Na co dzień myślisz o tym?**

No tak, myślę sobie "czy coś się jutro zmieni". Jak stwierdzam, że nie, staram się ulokować w czasie ten moment, kiedy się może zmienić. Dlatego na ten moment jest to powiedzmy czerwiec. Ale nie wykluczam, że tydzień przed końcem maja zmienię to myślenie, jeśli tak odczuję. Trudno mi to wytłumaczyć, jakoś określić, jak to może wyglądać za jakiś czas.

**Jeśli chodzi o najbliższe tygodnie, masz jakieś obawy?**

To są bardziej takie pracowe rzeczy. Praca, którą wykonuję teraz, tj. wymyślić różne rzeczy, rozpisywać projekty, znaleźć różne pomysły, po to, że kiedy jako festiwal nie pozyskamy pieniędzy na lato, to nie będzie tak, że firma się przez to zamknie, czy zbankrutuje. Taka praca da mi jakiś komfort. Pomimo, że festiwal się nie odbędzie, będę czuł, że troszkę zapracowałem na to, żeby firma miała dochody. I będę czuł się lepiej pod tym względem, że dostaję pieniądze bez zmian. Trochę obawiam się też sytuacji rodzinnej. Moja babcia, jak mówiłem, jest już umierająca. Nie wiem, co się wydarzy w przypadku, kiedy będzie musiał być zorganizowany pogrzeb. Podobno w kościołach i na cmentarzach też poluzowano ograniczenia, ale wiem, że to taka kwestia, która moich rodziców też może stresować. Że nie będą mieć gdzie zorganizować obiadu po pogrzebie, albo że nie wszyscy przyjdą do kościoła, albo, że będzie trzeba komuś powiedzieć, że nie może teraz przyjść/ przyjechać na ten pogrzeb. To mnie też tak stresuje.

**A masz jakieś pozytywne myśli związane z najbliższym czasem?**

Będzie fajnie, kiedy już będę mógł się z kimś spotkać. To na pewno będzie fajny czas. Poczujemy tęsknotę. Pod względem zawodowym życzę sobie i myślę życzeniowo o przyszłym roku, czy jesieni - może przez to, że ludzie tak długo siedzieli w domu, może później będą mieli większy apetyt? Może to pomoże firmie i się odkujemy? Kultura w ogóle? Trudno mi o tym rozmawiać. Mój znajomy, który od niedawna mieszka w Berlinie, bo chciał się rozwijać, występować, robić muzykę i  mieszka tam od pół roku, czy nawet roku, on już dostaje taką pomoc od rządu, dzięki której jest zupełnie spokojny. On i jego żona nie muszą pracować, a mają małe dziecko. Z dotacji od rządu wystarczy im na wszystko. Rozmawiałem też ze znajomym ze Szwajcarii. Tam jego firma płaci mu 20%, a rząd 80%. Jestem zły, że w Polsce nie możemy liczyć na coś takiego. Wiele krajów w Europie znajduje na to pieniądze. Już nie mówiąc o kulturze, ale o wszystkich, na jakieś dotacje. Kultura to już w ogóle inny temat w Europie, a inny w Polsce.
