# Supplementary material for: The challenges arising from the COVID-19 pandemic and the way people deal with them. A qualitative longitudinal study
Source: PLoS One. 2021 Oct 11;16(10):e0258133. doi: 10.1371/journal.pone.0258133 (PMC8504766; doi:10.1371/journal.pone.0258133)
Supplement: S1 Dataset — (ZIP) [file pone.0258133.s003.zip › Transcriptions/stage 4/2.4_F_27_single.docx]

**2.4_F_27_single**

**Chcę pogadać z tobą o dwóch rzeczach: o tym, co się wydarzyło przez ostatnie dwa tygodnie, jakie miałaś przemyślenia oraz o wydawaniu pieniędzy. Opowiesz mi, jak minęły ci ostatnie dwa tygodnie?**

Mówiłaś, żebym znajdowała jakieś zdjęcia, ale nie znalazłam żadnych zdjęć, dlatego że jak wychodziłam do pracy, widziałam ciągle to samo i nic nie pokazywało, jak się czułam. Ale wymyśliłam sobie, jak się czułam, tylko to będę musiała opowiedzieć, bo to jest taki obrazek, na przykład w Tokio, w metrze, jak jest bardzo dużo ludzi, w jakiejś takiej codziennej sytuacji, typu metro albo na Times Square, jak jest bardzo dużo ludzi. Jakby rozumiem, że nie ma tych ludzi na ulicach, ale generalnie w moim przeświadczeniu już teraz wszyscy żyją względnie normalnie. To jest taka metafora, wiadomo, że tych ludzi nie ma, ale w tym świecie koronawirusa już jest dość normalnie. Nie normalnie pod względem choroby, ale wydaje mi się (mówię tylko o swoich doświadczeniach, ewentualnie moich znajomych), że już jakoś tak to stało się codziennością.

**Czyli to nie jest tak normalnie, jak było wcześniej, tylko to już taka nowa normalność?**

Wydaje mi się, że tak. Teraz, wydaje mi się, że więcej ludzi jakoś lepiej sobie z tym radzi niż wcześniej.

**A w czym się przejawia to, że oni sobie lepiej radzą?**

Już mniej widzę w internecie jakieś takie narzekania... I tyle.

**Ok, czyli mniej ludzie narzekają na sytuację, tak?**

Tak i nie jest to już nowością w ogóle. Dzisiaj policzyliśmy z Filipem, że jesteśmy w domu, znaczy on jest w domu, bo ja wychodzę często, 55. dzień czy coś takiego, więc stało się to po prostu codziennością, takie mam wrażenie. I nie jest to zaskoczeniem, nie jest to coś, o czym trzeba mówić codziennie, bo to już jest, nic nowego.

**Skoro przywołałaś metaforę pełnego metra i Times Square i mówisz, że to wraca do rzeczywistości, to jak jest z twoimi emocjami? Jakie emocje towarzyszyły ci przez ostatni czas?**

To też sobie przemyślałam, co ci powiem, dlatego że od poniedziałku do piątku czuję się absolutnie normalnie, nie ma żadnej emocji, ale tak jak mówiłyśmy, nie jest to obojętność i nie jest to coś negatywnego ani zmęczenie, tylko po prostu klasyczne życie. Więc uznałam, że nie mam co o tym opowiadać, znaczy mogę opowiadać, co robiłam, oczywiście, ale nie ma żadnych specjalnych emocji, które mi towarzyszą, po prostu tak sobie żyję. Nie robiłam absolutnie nic specjalnego w tamtym tygodniu. Byliśmy na zakupach może raz, nie, nawet nie, nic zupełnie nie robiliśmy. I dopiero w weekend miałam akurat jakieś emocje już i uświadomiłam sobie, że nie przeszkadza mi absolutnie siedzenie w domu i bardzo lubię siedzieć w domu, o ile widzę, że jest na to takie społeczne przyzwolenie, żeby siedzieć w domu i nic nie robić. I wtedy super się czuję, naprawdę, że mogę to robić, ale kiedy widzę już, że moi znajomi wychodzą i robią coś, to czuję się może nie zła, ale trochę zazdroszczę, czuję, że ja już też może powinnam coś robić. Mimo że zupełnie nie przeszkadza mi to, że siedzę w domu cały weekend i nie spotykam się z ludźmi, nie robię nic, i fajnie siedzieliśmy z Filipem, i zupełnie nie czułam potrzeby wyjścia gdzieś, to jak widziałam, że ktoś tam pojechał do lasu ze znajomymi, ktoś się spotkał, tam dziewczyny mówiły, że z kimś się spotkały, ktoś z kimś, to już raczej tak smutno mi było.

**Ale smutno ci było, bo chciałabyś jeszcze móc posiedzieć w domu bez takiej presji społecznej?**

Tak, wydaje mi się, że tak. Zupełnie nie potrzebowałam robić tych rzeczy. Pewnie jakbym potrzebowała, to pewnie jakoś bym sobie załatwiła, a może właśnie przez to, że nikt nie zaproponował mi robienia czegoś, to ja się też sama nie zgłosiłam do robienia czegoś, więc może było mi smutno, że mnie nikt nie zaprosił, a może po prostu było mi smutno, dlatego że miałam poczucie, że może coś powinnam robić.

**Czyli takie może-może, nie wiadomo, co było przyczyną.**

Tak, to mnie uderzyło na pewno w weekend bardzo.

**Jak poprzednim razem się widziałyśmy, to powiedziałaś coś takiego, że zaczynasz się właśnie w tym odnajdywać, że to takie wakacje, które mogłyby jeszcze potrwać.**

Tak.

**Czy to jest tak, że ktoś ci mówi, że to koniec wakacji?**

Trochę tak. A z drugiej strony nie, bo te rzeczy, które robią ludzie, to są typowo wakacyjne rzeczy, w sensie nie spotykają się na pracę czy wspólne uczenie się, tylko raczej na spacer, coś, co jest naprawdę fajne i też mogłabym to robić, ale po prostu inne niż to, co robili do tej pory.

**Ale to jak teraz te możliwości się poszerzyły i przychodzi weekend, to ty w końcu chciałabyś czy nie chciałabyś wyjść? Myślałaś sobie: "Kurczę, pojechałabym na tę majówkę" czy "Poleżałabym do góry brzuchem"?**

Nie, "Kurczę, wszyscy pojechali". I to było jedyne. Absolutnie nie zorganizowałabym wyjazdu, bo nie miałam na to ochoty, ale z drugiej strony: "Kurczę, ja nie pojechałam, to trochę szkoda, że nikt nie chciał tego robić ze mną". Nie wiem, to raczej taka kwestia.

**Czyli fajnie by było, gdyby ktoś zrobił ten pierwszy krok?**

Tak! Albo też żebym mogła odmówić, coś tego typu raczej. I to było dość może wkurzające nawet. I nie dlatego, że ktoś tam łamie przepisy czy naciąga te przepisy, że nie można wychodzić i wychodzi albo że jest nieostrożny, tylko raczej kwestia tego, że fajnie było mieć społeczne przyzwolenie na robienie zupełnie niczego.

**Myślisz, że już nie ma tego przyzwolenia?**

W weekend wydaje mi się, że jest dużo mniejsze.

**Ok, czyli już jest takie oczekiwanie, że ludzie zaczną aktywności?**

Po prostu zaczynają chyba i przez to mi narzucają jakieś takie poczucie, że może już trzeba.

**A ty sama nie czujesz, że trzeba?**

Jakby mnie ktoś zaprosił, tobym rozważyła. Nie miałabym problemu, żeby odmówić, jeżeliby mi się naprawdę nie chciało, ale mogłabym też pójść.

**Rozumiem, że to nie jest tak, że ty się boisz wyjść, bo ten wirus krąży.**

Nie, nie boję się wyjść, ale nie chcę wyjść, dlatego że mi się podoba niewychodzenie generalnie. Zresztą dobrze wiesz, że przez ten cały czas ja dużo wychodziłam, więc nie miałam z tym za dużego problemu, żeby wychodzić, tylko raczej chodziło o to, żeby coś z ludźmi robić, jakieś coś nowego.

**Teraz mieliśmy te trzy dni majówki - co miałaś ochotę robić?**

Ja ci mogę powiedzieć, co robiłam, bo właściwie robiłam to, na co miałam ochotę. W zeszłym roku zrobiliśmy z Filipem grilla w mieszkaniu, bo dostaliśmy patelnię grillową i w tym roku spytałam się, czy robimy to samo w piątek, i będziemy pić piwo z puszki, które będzie mrożone w misce, generalnie tak jak na grillu, tylko że to po prostu jest w mieszkaniu. Tylko że w tym roku we dwójkę. W sumie nawet myśleliśmy o tym, czy kogoś zaprosić, ale Filip powiedział coś takiego, że może lepiej nie, bo potem ci ludzie będą się nas czepiać i w sumie faktycznie, nie potrzebowałam tych innych ludzi, dobrze moglibyśmy się bawić sami, nie chce mi się tego słuchać, że ktoś nie powinien do nas przychodzić. Ale przyszła do nas Ola, koleżanka Filipa, bo Filip ją zaprosił, dlatego że ja zaprosiłam Magdę, ale Magda miała jakiś zły dzień czy coś tam, powiedziała, że nie ma ochoty i przyszła tylko Ola. I było naprawdę fajnie. Nie było w ogóle tematu koronawirusa. Ale jeszcze warto tutaj zwrócić uwagę na jedną rzecz, bo to otworzy drzwi do mojego dalszego tygodnia, czyli to, że znalazłam na śmietniku szafkę. Poszliśmy wyrzucić śmieci i znalazłam szafkę. Już ci ją pokazuję, bo naprawdę warto ją zobaczyć, tak wygląda.

**Stylowa i dobrze zachowana.**

Tak. Właśnie sobie ją znaleźliśmy, jak szliśmy ze śmieciami i wnieśliśmy ją na trzecie piętro do nas. I to był piątek. Przez większość czasu chyba sprzątałam, robiłam jakieś takie typowo sobotnie, oprócz zakupów, bo zrobiliśmy je chyba wcześniej... Tak, ja byłam w Lidlu na piechotę. To jest daleko dość...

**To o zakupach porozmawiamy później. Opowiedz mi o tym swoim piątku. Zrobiłaś taką małą sobotę w piątek...?**

Tak. Ta szafka też nam dużo czasu zajęła. Bo poszliśmy potem do sklepu, więc wrócić z tego sklepu, przynieść tę szafkę, nie było szybko, potem ją wymyć, zdezynfekować jednak na wszelki wypadek tym płynem antybakteryjnym, skoro go mam, to mogę nim przetrzeć tę szafkę. A potem to zaczął się ten grill. Marynowałam warzywa, jakieś takie rzeczy robiłam. Potem Ola przyszła, więc zaczęło się to wszystko robić, siedzieliśmy, gadaliśmy. Tak wyglądał piątek. W sobotę umówiliśmy się z Filipem, że coś będziemy razem oglądać albo nawet nie umówiliśmy, tylko coś oglądaliśmy. I też w piątek albo nawet w czwartek zaczęłam robić ćwiczenia, stretching na You Tubie. Nie wiem, co mi przyszło do głowy, ale jakoś tak byłam ostała czy coś, więc zaczęłam to robić i wyglądało to tak przez pierwsze dwa dni, że Filip leżał i oglądał telewizję, a ja przynosiłam sobie wyciszonego laptopa i robiłam te ćwiczenia przed nim na podłodze. I w sobotę Filip się zapytał, albo w niedzielę, nie wiem dokładnie, czy może robić ze mną. I od tej pory robimy ćwiczenia codziennie. Już wyewoluowało to, co robiliśmy na początku, bo na początku było 10 minut stretchingu, a dzisiaj już robiliśmy 50 minut czy nawet 60 workout całego ciała. Więc na pewno robiłam te ćwiczenia w sobotę i jeszcze coś tam na pewno, a potem zdarzyła się dziwna rzecz, bo Filip powiedział, że idzie na randkę. I tutaj kolejne negatywne emocje, które mnie spotkały, to były takie, że znowu jest kolejny weekend, kiedy ludzie robią coś z innymi ludźmi, a Filip już nawet dalej, bo z jakąś nieznajomą osobą, którą poznał w internecie, a ja nie robię nic. To było podobne uczucie, jak w zeszły weekend, ale w dalszym ciągu świetnie się czułam, jak siedziałam sama w domu. A potem Filip wrócił z tej randki i powiedział, że było tak fajnie, że idzie kolejnego dnia na tę samą randkę. Wtedy trochę mu zazdrościłam w sumie, właśnie wtedy akurat się czułam źle, dlatego że nie miałam potrzeby poznawać kogoś nowego, ale fajnie, że mu się tak udało i coś nowego sobie zrobi, więc to jest fajne, ale nie umiałam się z tego cieszyć. W niedzielę rano wstaliśmy, ale zanim się zebraliśmy, to on poszedł na tę randkę, więc siedziałam sama w domu i oglądałam telewizję.

**On tak wcześniej nie wychodził, tak bardzo siedział w domu, poza wychodzeniem do sklepu i wyjazdem na Wielkanoc do rodziców.**

Tak.

**A co go nagle tak natchnęło i to nie na spacer, a od razu na randkę?**

Ale to była randka-spacer.

**No tak, ale on nie wychodził, a teraz od razu z obcą osobą. Co się wydarzyło?**

Wiesz co, on w normalnym życiu dość często chodził na randki. Teraz już mu się znudziło i ściągnął sobie Tindera, i szukał tam jakichś chłopaków, ale zepsuł mu się ten Tinder. Nie wie, jak mu się to zepsuło, ale zepsuło mu się tak, że nikt mu się nie pokazywał w telefonie i nie działała mu ta aplikacja, pisał do supportu, nie może usunąć konto... - długo mi o tym mówił, ale nie rozumiem, w każdym razie nie działał mu ten Tinder i to nie działał mu przez kilka dni, więc ściągnął sobie inną aplikację randkową. Tam poznał takiego chłopaka i coś tam sobie pisali. Nie wiem, może ten chłopak był jakoś wyjątkowo fajny, nie wiem, pisali ze sobą co najmniej tydzień. Może po prostu ten chłopak okazał się wyjątkowo fajny, bo Filip też nie wydaje mi się, żeby teraz się bał, po prostu nie miał co robić za bardzo. Szczególnie, że przychodziła do nas jego koleżanka Ola przez ten cały czas, więc nie bał się spotkać z ludźmi. Nie wiem, po prostu poznał fajną osobę i chciał się z nią spotkać, wydaje mi się, że to jest kwestia tego.

**W niedzielę to już mu trochę zazdrościłaś, co?**

Tak, bo w niedzielę był z 5 godzin!

**To długo sama posiedziałaś. Co robiłaś?**

No... Oglądałam telewizję. Nie wiem, czy w ogóle wstałam przez ten cały czas, jak on był, chyba raczej nie.

**Taka piżamowa niedziela.**

Tak, zdecydowanie tak. Oprócz ćwiczeń, które robiliśmy rano.

**Czyli od poniedziałku do piątku jest w miarę ok, bo pracujesz, życie toczy się trochę swoim rytmem, przychodzi weekend i trochę się gorzej czujesz, bo ludzi zaczynają robić różne rzeczy.**

Tak, najgorzej, że byli beze mnie, ale to już akurat nie jest kwestia koronawirusa absolutnie. A z drugiej strony mam jeszcze taki problem, że Magda... Generalnie u nas w pracy jest teraz dość słabo, dlatego że trzeba... Z tej całej przeprowadzki, o której ci opowiadałam, jak były ogromne problemy z tą przeprowadzką, to zrobił się nowy problem, czyli taki, że trzeba wypowiedzieć jedno piętro biura i zrobić na nowo ekipę, wszystko porozstawiać tak, żeby się ci ludzie zmieścili. Więc wszystko, co już było i na co poświęciłam strasznie dużo czasu, trzeba robić teraz jeszcze raz. I o ile mnie to aż tak mocno nie zabolało, to wiem, że Magdę, moją szefową, bardzo zabolało. A że jest też jedną z moich bliższych koleżanek, może najbliższą, to ona też za bardzo nie chce ze mną spędzać czasu po pracy, dlatego że nie chce myśleć o pracy, a siłą rzeczy kojarzę się jej z tą pracą. Więc to też może mi też troszeczkę przeszkadzać ewentualnie.

**Z nowych rzeczy, które się u ciebie pojawiły, to te ćwiczenia.**

Tak, i jeszcze jedno, o czym ci nie powiedziałam. Ta szafka nie była w takim stanie, tylko ja ją sobie odrestaurowałam, kupiłam bejcę. Bo wczoraj byłam też w Leroy Merlin, o czym też nie mówiłam jeszcze, bo opowiadałam tylko o weekendzie. Kupiłam kwiatki, które tutaj sobie poustawiałam, zrobiłam przemeblowanie pokoju.

**To bardzo aktywnie! Dotychczas to najwięcej zmian!**

Tak, ale naprawdę nie jestem pewna, czy to jest kwestia tego, co się dzieje wokół czy po prostu ta szafka mnie pobudziła do tego - znalazłam szafkę, więc uznałam, że muszę zmienić wszystko pod tę szafkę. Nie pojechałam do Leroy Merlin akurat specjalnie po szafkę, tylko Magda odebrała wczoraj klucze od swojego nowego mieszkania i spytała się, czy pojadę z nią kupić jakieś tam rzeczy albo obejrzeć. Przy okazji kupiłam sobie bejcę, więc od razu wieczorem papierem ściernym przetarłam tę szafkę, umyłam. Zobaczyłam, że tak fajnie mi się to zrobiło, że jeszcze regał tak sobie zrobiłam i zaczęłam szafę, ale szafa nie jest z drewna, więc nie wyszło tak, jakbym chciała, ale nie jest źle. Kupiłam sobie też kwiatki, które postawiłam, więc zrobiłam coś nowego w pokoju i zamierzam kupić dywan.

**A skąd te zmiany, z tej szafki?**

Wydaje mi się, że tak.

**Ale to źle się czułaś w tym swoim pokoju?**

Nie, ja wiem, że ty byś mogła pomyśleć, że tutaj siedzę przez cały czas, patrzę i jednak sobie zmieniam, tak jak większość ludzi w domu robi, że coś im nie pasuje, większość czasu spędzają w domu, więc teraz chcą zrobić jakby przytulniej czy coś takiego - nie mam tak, zupełnie nie. Ten pokój, tak jak było wcześniej tutaj, podobało mi się prawie tak samo, tylko jak już zrobiłam sobie tę szafkę, to uznałam, że coś sobie zmienię. To było przypadkowe tak naprawdę: ustawiłam tę szafkę, potem coś niechcący przestawiłam i tak wyszło.

**A robiłaś porządki przy okazji?**

Nie.

**A kwiatki...**

Dobra, akurat z kwiatkami mogę powiedzieć...

**To jest taka totalna nowość?**

Wzięłam jednego kwiatka z pracy. Skoro rezygnujemy z jednego piętra, to mogłam sobie wziąć jednego, który mi się podobał, bo i tak nie będzie miejsca na niego. No i wzięłam tego kwiatka, i mi się spodobał, więc zamówiłam sobie inne.

**Z kwiaciarni, Leroy Merlin?**

Nie, przez internet zamówiłam.

**A dlaczego przez internet?**

Bo wydaje mi się, że to miejsce, z którego chciałam kupić, jest zamknięte, bo to jest miejsce, w którym zawsze kupuję kwiatki... Znaczy nie zawsze, bo ja zawsze nie kupuję kwiatków za bardzo, ale jakbym chciała kupować, to właśnie z tego miejsca, dlatego że jak Basia, moja szefowa, potrzebuje, żebym kupiła jakiś prezent jej mamie, babci czy coś takiego, zawsze jadę do tego sklepu i tam wybieram coś dla niej. Więc wiedziałam, że jeżeli będę chciała jakiś kupić... Długo o tym myślała, tylko jakoś tak nie miałam ochoty tego zrobić, ale skoro już ten jeden kwiatek napędził spiralę kwiatków w moim pokoju, to uznałam, że zamówię tam.

**Czyli dużo zmian u ciebie było. A czy jest jakaś zmiana związana z tą sytuacją, którą mamy teraz? Może nie z tym, że się boisz, ale z tym, że zmieniły się obostrzenia, coś się zadziało?**

Akurat muszę ci powiedzieć, że trochę się boję. Nie boję się oczywiście ani choroby, ani bycia samej w domu, tylko właśnie wręcz przeciwnie - powrotu do biura albo właściwie nie tylko do biura, co do normalnego życia, dlatego że to będą nowe rzeczy, których się będzie trzeba nauczyć i o których będzie trzeba pamiętać. Na przykład o tym, żeby w dalszym ciągu nie podawać sobie ręki. To przykład, nie wiem, czy robię to na co dzień, co mi będzie przeszkadzało, ale generalnie chodzi o to, że łatwo jest pamiętać o zasadach typu utrzymanie odległości od siebie czy coś, jeżeli jestem z ludźmi, których widzę na co dzień, więc przyzwyczailiśmy się do tego, że stoimy od siebie trochę dalej. A jeżeli to będą nowe osoby, z którymi się nie widziałam i będą teraz w biurze albo w jakimś życiu, to będzie to znowu nienaturalne dla mnie.

**Czyli trochę nie wiadomo, co będzie, jak się za chwilę zachowywać?**

Tak.

**I boisz tego?**

Wolałabym trochę to odwlec.

**Masz poczucie, że coś nowego związanego z koronawirusem się pojawiło albo coś jeszcze ograniczyłaś?**

Ograniczyłam, nie ograniczyłam, ale mam przemyślenia na temat obostrzeń, a właściwie zniesienia tych obostrzeń.

**Masz dużo przemyśleń dzisiaj, jesteś bardzo przygotowana.**

Tak, dlatego że ja... Powiedziałaś mi, żebym robiła zdjęcia, a ja oczywiście nie robiłam tych zdjęć, na początku, żebym zapisywała i wiedziałam, że mi się nie chce tego zapisywać. Nawet na studiach niczego nie zapisywałam, a w liceum w zeszycie to ja robiłam długopisem nad kartką, żeby tylko nic nie zapisywać. Nienawidzę pisać, nie wiem, jaki mam problem z tym, dlatego powtarzałam w głowie, żeby ci powiedzieć.

**To opowiedz mi o swoich przemyśleniach związanych z obostrzeniami.**

Takie mam nowe przemyślenia, że nie wiem, na ile zostały słusznie zniesione, ale strasznie mnie zastanawia, dlaczego zostało zniesione te godziny dla seniorów. Nie rozumiem, dlaczego to zostało zniesione, co to komu przeszkadza. Ja to nawet sobie pomyślałam, że to w sumie całe życie by tak mogło być, zupełnie to nikomu nie szkodzi, bo 10-12 to są takie godziny, kiedy i tak nikogo tak naprawdę nie ma na ulicach, jakichś takich młodszych. Wiadomo, że są, ale nie mają powodu, żeby być, to jest po tym czasie pójścia do pracy, a przed lunchem.

**A wiesz, jak rząd to argumentuje?**

Nie wiem, nie interesowałam się. Jak? Rozmawiałam o tym z Filipem i Filip powiedział, że to jest dlatego, że w tym czasie sprzedawcy sprzedają mniej. Ale z drugiej strony, skoro do sklepu może wejść tyle samo osób, czyli powiedzmy te trzy na kasę czy coś takiego, to... Nie wiem, czy jest dużo więcej starszych ludzi czy normalnie w tym czasie. Pewnie ktoś, kto się tym zajmuje, wie. Ja mogę sobie gdybać, że dla mnie to jest bez sensu, ale dla mnie to jest dziwne, że coś takiego zostało zniesione. Zresztą na tych starszych ludzi w sklepach w dalszym ciągu trzeba uważać, więc to akurat jeszcze by mogło spokojnie zostać.

**A inne obostrzenia? Kojarzysz, co się wydarzyło?**

Otwarcie sklepów budowlanych w sobotę.

**Bo byłaś w Leroy Merlin, to wiesz.**

Ale właśnie nie, bo one były otwarte chyba od poniedziałku do piątku, tylko w sobotę było zamknięte. A wiem to tylko dlatego, że bardzo chciałam iść do sklepu od razu, jak znalazłam tę szafkę, czyli w sobotę chciałam już pojechać po bejcę, ale nie mogłam, dlatego że był zamknięty ten sklep. Przepraszam, zapomniałam ci powiedzieć, byli u mnie rodzice. Byli u mnie rodzice, dlatego że... To stało się spontanicznie, dlatego że mój tata kupił nowy telewizor i lodówkę. O, to już sobie możesz zapisać, ile moi rodzice kupują teraz rzeczy! Zupełnie odwrotnie niż ja. I mój tata kupił sobie telewizor i lodówkę. I miał taki jeden stary telewizor, który... Przenosił telewizory z jednego pokoju do innego pokoju, w sensie jakby updatował telewizor w danym pokoju. W jednym pokoju, w sypialni chyba, ma taki telewizor, który nie był smart tv, a ja mam takiego chromecasta, który się podłącza do telewizora i robi z każdego telewizora smart tv. Mój tata chciał zobaczyć, czy u niego w tym telewizorze to będzie działać, więc coś tam gadał, czy może przyjechać, pożyczyć. I przy okazji powiedział, że jedzie na bazarek wcześniej i spytał, czy mi coś kupić. Więc przyjechał z zakupami, wziął to, a przy okazji dałam mu prezent dla mojej mamy, bo mama miała w niedzielę urodziny. Ale przez to, że nie działał mu ten chromecast albo już po prostu mu się znudziło, jeszcze raz pojechali w sobotę na zakupy, to przyjechali do mnie dać nowe zakupy i oddać tego chromecasta. I już wtedy była mama, i już wtedy weszli na górę, i mama zaczęła sprzątać, tata się tam rozsiadł, tak było normalnie.

**W sensie weszła i zaczęła sprzątać?**

Mhm. Blat. "Co wy macie tutaj tak brudno?" A ja mówię, że na czarnym blacie zawsze się tak brudzi. A ona, że nie wierzy, że pewnie jakieś słabe płyny masz, pokaż, jakie masz płyny. Więc wyciągnęłam jej i zaczęła sprzątać. Moja babcia robi dokładnie to samo u moich rodziców, więc w ogóle mnie to nie zdziwiło.

**Twoi rodzice mieli jakiś problem z tym, żeby cię odwiedzić?**

Nie, wydaje mi się, że się nawet cieszyli, wydaje mi się, że nawet tak chętnie. Bo tacie się nie chciało wchodzić w piątek na górę, ja zeszłam do niego na dół. A w sobotę już w ogóle bez żadnej dyskusji weszli tutaj po prostu.

**Myślisz, że trochę się za tobą stęsknili i dlatego przyjechali?**

Tak, bo w piątek, jak się widziałam z tatą na dole, to mówił: "Ale kiedy do nas przyjedziesz?", a ja na to: "Nie mogę, nie można wychodzić jeszcze, nie mogę teraz przyjeżdżać" - Można, można, przyjedziesz?" - "Nie, nie przyjadę, nie można". Ale dlatego że mi się nie chciało, a nie dlatego że nie mogę wychodzić. Ale on też wiedział doskonale, dlaczego mu tak mówię.

**Więc przyjechali, skoro ty nie chciałaś.**

Myślę, że tak. Szczególnie, że mama miała urodziny, a skoro ma urodziny w majówkę, to zawsze jednak mimo wszystko ktoś u nas był albo byliśmy na jakiejś działce, albo na jakimś wyjeździe, no a teraz w tym roku tego nie było, może to też chcieli zrobić.

**Mamy bardzo dużo nowości w tym tygodniu.**

Ale nie było mojego brata.

**Ale mamy wizytę rodziców, przemeblowanie, odnawianie szafki, zamówienie kwiatów...**

Odkurzacz kupiliśmy.

**Przez internet czy pojechaliście stacjonarnie?**

Przez internet.

**Ale zepsuł się wam?**

Problem z odkurzaczem u nas w domu był taki, że tylko ja sprzątałam, dlatego że ten odkurzacz u nas był schowany w szafce i trzeba było za każdym razem go rozkładać, tę rurę wyciągać i Filipowi się chyba nie chciało tak robić. On też nie lubi odkurzać, więc on tego nie robił zupełnie, a ja się wkurzałam. Nie powiedziałam oczywiście o tym, co mnie wkurza, ale ja wiem, że Filip wiedział doskonale, o co mi chodzi, ale jakby nie mógł się przemóc może. A mnie też wkurzało wyciąganie tego odkurzacza w jedną i drugą stronę. No i chyba pojawiła się Filipowi reklama odkurzaczy, nie wiem dokładnie, ale pokazał mi zdjęcie w telefonie i powiedział: "Ej, kupimy ten odkurzacz?", ja powiedziałam: "Dobra, spoko". Spytaliśmy właściciela naszego mieszkania, co on na ten temat myśli i na ten temat myślał, że on nam kupi ten odkurzacz, więc żebyśmy sobie znaleźli. Filip znalazł ten odkurzacz, nawet dwa, spytałam tatę, który kupić, zamówiłam i przyszedł chyba kolejnego dnia.

**Ten jest taki, że nie trzeba go teraz chować?**

Ten jest taki pionowy.

**Jakie jeszcze nowości sobie planujesz na najbliższy czas?**

Dywan. Koniecznie muszę sobie kupić. A nowości, to chyba powoli będzie trzeba wracać do biura, już mniej będzie czasu na robienie takich rzeczy.

**A czy w związku z powrotem do biura planujesz kupować jakieś ciuchy?**

Kupiłam sobie jakieś ubranie. A, majtki sobie kupiłam.

**Bo jak rozmawiałyśmy, to mówiłaś, że jakiejś tam bluzki potrzebujesz...**

Tak, parę razy wchodziłam na Zalando, ale nic mnie nie zainteresowało. A jak kupowałam te majtki (bo na H&M było -20% i darmowa dostawa), to weszłam na bluzki i sukienki, ale nic mi się nie podobało, tak żeby kupić, więc zostałam przy samych majtkach.

**Poza dywanem będzie coś z nowości zakupowych w najbliższym czasie?**

Nie. Jedne buty widziałam, które chciałabym kupić, ale też bez spiny, tak że muszę sobie je kupić. Podobają mi się, ale nie potrzebuję. Chciałabym kupić jeszcze więcej rzeczy takich budowlanych, żeby sobie albo coś pobejcować, albo coś, tylko nie mam czego pobejcować, ale myślę właśnie, żeby jakąś bejcę kupić, takie rzeczy, żeby porobić w domu.

**Ale tu chodzi o to, żeby stworzyć ładne otoczenie czy fajnie spędzić czas? Co jest ważniejsze dla ciebie?**

Fajnie spędzić czas. Bardzo lubię takie rzeczy robić, bo wtedy nawet nie mam ochoty patrzeć na telefon ani nic, po prostu jestem totalnie wyłączona i robię sobie to. Tak samo jak maluję. To jest też fajne, bo widać początek i koniec, to jest proces, który jest zamknięty, więc wiem, ile czasu... Znaczy nie wiem, ile czasu mi się zejdzie, ale wiem, że nie przestanę nie myśleć ani nie przejmować się niczym, dopóki nie skończę tego robić. Chyba że się zmęczę, no to na przykład na dwa dni, ale wiem, że to jest taki moment, kiedy mogę się odciąć i odpocząć.

**Czyli chodzi bardziej o to robienie, żeby mieć ten czas wypełniony jakąś przyjemnością?**

Tak.

**Czy coś jeszcze sprawia ci przyjemność?**

Chyba teraz te ćwiczenia, bo o ile są strasznie męczące i się czujemy okropnie, jak je robimy, to robimy je we dwójkę, więc trochę się wspieramy, razem nienawidzimy tej baby, która coś tam każe robić, razem się obijamy, jak nam się nie chce. I naprawdę chce nam się to robić. Dzisiaj robiliśmy to piąty dzień czy coś takiego, więc nie jest to superdługo, ale ani razu nie było takiej myśli, że może dzisiaj nie - nie, po prostu robimy to. A jutro umówiliśmy się na 7:30.

**Przed pracą?**

Niestety chyba tak musimy, dlatego że idę jutro do Magdy, do tego nowego mieszkania po pracy i się boimy, właśnie to aż niesamowite, że się boimy, ale boimy się, że nie będzie czasu. Jeżeli prosto po pracy pójdę do Magdy, wtedy nie będziemy tego robić w nocy na pewno, nie wiem, o której wrócę. A podczas pracy to nie da się trochę. Szczególnie w mojej pracy, bo ktoś coś chce nagle i jak to przerwiemy, to potem raczej już nie wrócimy do tego. A żeby zgrać dwie prace, to się nie da, dlatego musimy po prostu zrobić rano.

**Te ćwiczenia są dla przyjemności, a są jakieś zakupy dla przyjemności?**

Nie, właśnie nie, dla mnie kupowanie to jest raczej taki smutny obowiązek niż jakaś przyjemność. Lubię kupować ludziom różne rzeczy, na przykład nie mam problemu, jeżeli oglądam coś na Zalando (nie stało się tak od początku tej kwarantanny, a może się stało), ale jak sobie coś oglądam do kupienia i wiem, że komuś by się to spodobało albo chciałabym to komuś dać, to kupiłabym to dużo szybciej, niż jakbym to miała kupić sobie. Znaczy jakąś inną rzecz, wiadomo, bo nie muszą nam się te same podobać.

**A mamie co kupiłaś w prezencie?**

Takie krem Estée Lauder. Kupiłam jej go, dlatego że ona chciała taki krem. Niedokładnie taki, ale lubi takie kremy, które są lepszej marki i wtedy ona ma wrażenie, że one działają lepiej. Wmawia sobie może, może naprawdę działają, nie wiem, ale lubi jakieś takie odmładzające czy coś takiego, dlatego jej to po prostu kupiłam, taki wybrałam.

**Teraz, jak zaczęliście już ćwiczyć, to masz potrzebę pójść na siłownię, fitness itp.?**

Jak już będzie można? Nie, bo fajne jest to ćwiczenie w domu pod tym względem, że robimy to razem i się trochę obijamy razem, nikt na nas nie patrzy. Trochę jak na wf-ie w szkole, bo tu też jest tak, że baba gada, a my przestajemy nagle to robić. Zresztą też oglądamy telewizję przy tym, więc to jest tak na luźno bardziej. Wiadomo, chcemy osiągnąć jakieś efekty, ale raczej chodzi o coś takiego innego i nowego.

**Na początku jeździłaś codziennie do pracy, potem pojawiła się praca zdalna, a jak jest teraz?**

Dzisiaj nie byłam, ale wczoraj byłam. Ale jeżdżenie do biura samemu to jest zupełnie co innego, niż jak będą inni ludzie. Czasami zdarza mi się, że jadę do biura nawet nie po to, żeby się lepiej skupić, tylko żeby jakoś tak mieć wrażenie, że coś robię, nawet jeżeli akurat nie mam nic do roboty w pracy. No bo czasem tak się zdarza, że na przykład... Ja zdaję sobie sprawę, że jakbyśmy byli w biurze i bym przez 20 minut nic nie robiła, tylko z kimś gadała, to nikt by nie miał z tym problemu, ale jak jestem w domu 20 minut i już nic nie robię, to jest tak, że głupio trochę, szczególnie, że wiem, że inni ludzie w tym czasie pracują. Wiadomo, ta praca rozkłada się różnie, więc to nie jest tak, że powinnam się obwiniać czy coś, ale jak jestem w biurze i te 20 minut nic nie robię, to mam poczucie, że przynajmniej byłam w biurze.

**A robisz coś, żeby się przygotować do tego powrotu do normalnego życia? Jakoś mentalnie przygotowujesz się?**

No tak, myślę sobie.

**Myślisz sobie i trochę się boisz. Robisz coś z tym strachem?**

Nie, dlatego że to jest taki strach z tyłu głowy, no ale nie wyobrażam sobie sytuacji, w której sobie nie radzę z powrotem do normalnego życia, więc raczej to jest tak, jak na przykład stresowanie się przed kartkówką, nie raczej nie...

**Ale przed kartkówką możesz się uczyć.**

Tak, właśnie wiem, dlatego powiedziałam, że to jest zły przykład. A może to jest taki stres w ostatniej chwili przed kartkówką, czyli ostatnie pół godziny, jak się stoi pod klasą. Albo na przykład przed jakąś prezentacją, może to bardziej, przed prezentacją, ale właśnie też tak ostatni czas przed tą prezentacją. I tak już nic nie zapamiętam, pomiesza mi się to jeszcze bardziej, więc tylko stoję i się stresuję, ale wiem, że zaraz będzie ta sytuacja i nie będzie to jakoś wyjątkowo straszne, nie umrę od tego, tylko po prostu trzeba będzie to zrobić i dalej sobie coś innego.

**A myślisz, że w ogóle da się jakoś przygotować na tę sytuację powrotu?**

Wiesz co, przypomniało mi się, że akurat to robię, czyli wstaję troszeczkę wcześniej niż wstawałam, dlatego że teraz wstaję o 8:50, 55 nawet, a będę musiała wstawać trochę wcześniej, myślę, że przed 8. Ale w dalszym ciągu to jest taka godzina i to też nie będzie tak wolne ruszanie się rano, tylko raczej takie ruszanie się normalne. Więc staram się naprawdę, już teraz myję się przed 9 i ubieram. Właśnie, coś czego nie robiłam, czyli się nie myłam i nie ubierałam, to teraz to robię, żeby tak jakoś przywyknąć do tego powoli.

**A czy to jest tak, że dokładasz takie małe cegiełki do powrotu do rzeczywistości?**

Chyba tak. Nie wiem, w jakim stopniu to jest świadome tak naprawdę, bo już zaczęłam myć się wcześniej w zeszłym tygodniu, przed 9, bo nawet jak wychodziłam, to się myłam oczywiście, ale teraz zaczęłam się myć przed 9, tak, żeby na 9 być już gotową albo w szlafroku (ale ubiorę się w 3 minuty). Stopniowo, powoli, wydaje mi się, że zaczęłam to wdrażać.

**Jakieś następne pomysły, co jeszcze przybliży cię do tej normalności?**

Nie mam kolejnych pomysłów, ale jeszcze jedna rzecz, która mnie przeraża - zastanawiam się, jak ja w ogóle jadłam w pracy. Nie wiem, trzeba będzie coś przygotowywać dzień wcześniej, czy już na nowo będzie można zamawiać? No nie wiem.

**Ale na wynos chyba można cały czas?**

Można, ale skoro już teraz wszyscy sobie raczej gotują... Bo ja dużo rzadziej już teraz zamawiam jedzenie, niż ci mówiłam na początku.

**Czyli to nie chodzi o to, że knajpy będą zamknięte, tylko że zamawialiście sobie grupowo?**

Tak, ale ja raczej codziennie sobie gotuję, nie pamiętam, kiedy ostatni raz zamówiłam, moim zdaniem ponad tydzień nie zamawiałam albo nawet może odkąd rozmawiałyśmy jeszcze wcześniej. Nawet przez weekend nie zamówiliśmy nic z Filipem, mimo że trochę planowaliśmy.

**Myślisz, że będziesz gotować wieczorami na następny dzień?**

No właśnie nie wiem! Nie wyobrażam sobie takiej sytuacji.

**Masz trochę przerażenie na twarzy.**

No bo nie chce mi się tego robić po prostu.

**No ale nie musisz, będziesz mogła zamówić tak jak wcześniej, dlaczego nie?**

Bo to jednak się sporo pieniędzy oszczędza, jak się gotuje. A właściwie może nie do końca, dlatego że ja zdaję sobie sprawę, ile ja wyrzucam tego jedzenia, teraz jeszcze więcej. I to nawet nie chodzi o to, że ja za dużo kupię, tylko ja za dużo zrobię tego jedzenia. Po prostu nie umiem zrobić obiadu na jedną osobę na jeden dzień, a ja już nie chcę kolejnego dnia tego jeść, kolejnego to już zmuszę się i zjem to, a już kolejnego to absolutnie nie mam ochoty w ogóle. Nie wiem, jak to będzie wyglądało po prostu. A jeżeli już nawet drugiego nie chcę tego jeść, to już to wyrzucę, więc równie dobrze za te całe pieniądze wyrzucone mogłam zamówić i wydać może nawet trochę więcej, ale nie wyrzucić tego jedzenia.

**Myślisz, że inni też będą sobie gotować? Myślisz, że wam to gotowanie zostanie?**

Myślę, że tak, dlatego że my przygotowywaliśmy się już do gotowania sobie i robiliśmy sobie challenge, na przykład: "Cały tydzień nie zamawiamy, ok?" i udawało się, czasem się nie udawało, ale mieliśmy z tyłu głowy, że fajnie by było jednak nie zamawiać codziennie jedzenia, tylko gotować. Więc jeżeli już teraz w domu przywykliśmy do tego, że się gotuje, to wydaje mi się, że będziemy więcej przynosić sobie jedzenia.

**Czy jeszcze jakieś rzeczy kołaczą ci się w głowie związane z sytuacją wokół koronowirusa?**

Tak jak mówiłam, to, że nie wiem, jak się zachowywać z ludźmi, żeby chociaż pozornie zachować jakieś bezpieczeństwo. No to coś takiego ewentualnie jeszcze.

**A masz poczucie, że ludzie z twojego otoczenia są wrażliwi na kwestie bezpieczeństwa i że jest takie oczekiwanie, że będziemy się tak zachowywać?**

Moi znajomi może nie są podatni na to, ale są podatni na to, że wymagane jest od nas, żebyśmy się tak zachowywali. Wydaje mi się, że większość rzeczy, które robię z ludźmi z pracy, to nie są rzeczy, na których nam zależy, żebyśmy robili albo w jakiś sposób się zachowywali, tylko po prostu jest to presja społeczeństwa. Nie wiem, co może być takim przykładem. Nie wiem, czy mówię o sobie czy o nich, ale wydaje mi się, że mają wrażenie, że są pewnymi ludźmi, więc w jakiś określony sposób muszą się zachowywać, kulturalnie, tak jak trzeba po prostu, dlatego że jednak są wyedukowani albo coś takiego.

**Masz jakieś emocje związane z koronawirusem? Coś się wydarzyło i ty się poczułaś jakoś?**

Szczerze, to na początku, w pierwszym tygodniu, jak się widziałyśmy, to w ogóle nie pamiętałam, że jest koronawirus. Można z tego pożartować ewentualnie, ale nie żeby się bać na serio. Nie wiem, ilu tych ludzi jest chorych. Coś dzisiaj słyszałam, że TVP powiedziało, że Polska jest jednym z najlepszych krajów, które wychodzą z tej pandemii, ale to były jakieś źródła opłacane przez TVP w Wielkiej Brytanii, a się okazało, że to jednak są cztery kraje, w których właśnie nic się nie zmieniło, ani nie jest lepiej, ani gorzej i jesteśmy jednym z tych krajów. Więc raczej tak neutralnie podchodziłam do kwestii koronawirusa, ale jak znieśli te obostrzenia, trochę się przestraszyłam, poczułam troszeczkę niepokój w dwóch aspektach. Pierwszy to jest taki, że trzeba wrócić do pracy, do takiego normalnego życia i to mi się nie podobało. A druga kwestia jest taka, że tak naprawdę nie wiadomo i może się zdarzyć tak, jak we Włoszech, że to jest jednak za szybko i ludzie za bardzo będą żyć normalnie, i może faktycznie ta pandemia się rozrosnąć do monstrualnych rozmiarów.

**Czyli trochę za szybko pozwalamy sobie na różne rzeczy?**

Tak, to nie jest tak, że się boję tak personalnie, ale przeszło mi przez myśl, że może coś takiego się zdarzyć.

**Masz w swoim otoczeniu kogoś, kto się tak realnie boi?**

Nie, chyba nie.

**A czy twoim znajomi, rodzina czy Filip boją się czegoś poza zarażeniem? Czy jest coś, czego się nadal boją?**

Wydaje mi się, że boją się reakcji ludzi, w sumie ja trochę też, ale ktoś tam mi to powiedział, może Magda, nie wiem, że podczas tej pandemii ludzie dużo bardziej irracjonalnie reagują na różne rzeczy, dużo szybciej się denerwują, dużo bardziej się stresują i panikują trochę. I nawet nie chodzi o kwestie zdrowia i bezpieczeństwa, ale raczej wszystkiego wokół. Swoje decyzje podejmują dużo bardziej gwałtownie. I to jest rzecz, której można się bać. Nie że się pochopnie swoje decyzje podejmie, ale że ktoś podejmie zbyt pochopnie decyzje, które wpływają na ciebie.

**Pogadajmy jeszcze chwilę o zakupach. Jak wyglądają u was teraz zakupy?**

Nie planowaliśmy, ale przez to, że robiliśmy tego grilla, no to trzeba kupić na grilla specjalne rzeczy, których nie mieliśmy w domu. W środę spytałam się Filipa, czy nie ma ochoty pojechać, bo wiem, że w czwartek byłyby kolejki, a nie chciałabym w nich stać na pewno, ale Filip powiedział, że nie ma ochoty, a ja akurat, to był taki dzień, kiedy miałam bardzo dużo energii i bardzo dużo ochoty. Mój kolega z pracy, odkąd ten koronawirus jest taki powszechny, dużo wychodzi na spacery, znaczy odkąd można wychodzić i odkąd pracujemy zdalnie. Teraz za radą coacha wprowadzili coś takiego, może nie wprowadzili, tylko ci headzi, którzy chcą, robią coś takiego jak półgodzinne spotkanie - nierozmawianie o pracy w czasie pracy. Chodzi o to, żeby zbudować zaufanie między headem a pracownikiem, żeby można było swobodnie sobie porozmawiać, troszeczkę odpocząć od pracy, zrobić jakąś przerwę. Przez to, że w dziale prawnym są tylko dwie osoby, oprócz tej ich szefowej, a w starym biurze siedzieliśmy bardzo blisko siebie i właściwie cały czas spędzaliśmy ze sobą czas i żartowaliśmy, i się naprawdę lubimy, no to ta Zuza dodała też mnie i Magdę do tych spotkań. Oczywiście nie są obowiązkowe i nikt nie woła na nie, ale jeżeli ktoś akurat ma te pół godziny w czasie, kiedy to się odbywa, to dołączamy. I właśnie Maciek powiedział, że on bardzo dużo chodzi. Akurat stało się tak, że wszyscy już poszli, a my zostaliśmy tylko we dwójkę i spytałam się go o te spacery, jak on to robi, czy mu się chce, czy nie chce, bo ja też kiedyś lubiłam spacerować, ale teraz mi się nie chce. Opowiadał, że sobie rozmawia z ludźmi przez telefon, że tak sobie chodzi bez celu. Pomyślałam, że to jest bardzo fajne i wróciłam, miałam dużo energii, a Filip nie chciał iść do tego sklepu, więc uznałam, że pójdę pieszo do Lidla i poszłam. I rozmawiałam całą drogę przez telefon z mamą.

**Na ile masz poczucie, że twój styl zakupów jest taki, jak przed epidemią? Czym najbardziej się różni?**

Ilością, przede wszystkim ilością. Te rzeczy, które mam w lodówce, to nie są rzeczy, których ja nie miałam nigdy w lodówce, ale miałam ich mniej, bo częściej je kupowałam, wiedziałam, że zawsze będę mogła iść po nie. Teraz w sumie też zawsze mogę, ale mam takie przeświadczenie, że nie mogę albo że będzie kolejka, albo coś takiego.

**Czyli większe zapasy?**

Tak. No i raczej nie zdarza mi się wychodzić na większe zakupy, tak jak wcześniej, gdzieś blisko, ale tak blisko, że się idzie na przykład 10 minut. Bo nie doniosę tego. Już wolimy pojechać gdzieś dalej i wrócić taksówką. Bardziej taksówkowe zakupy niż piesze-chodzone, ale to wynika z tego, że po prostu jest ich więcej. Trochę więcej też dlatego, że więcej gotuję i też więcej jestem w domu, więc częściej mam ochotę coś zjeść.

**Jak byłaś w tym Lidlu, kupiłaś coś tak po prostu dla przyjemności?**

Piwo bezalkoholowe, ale zawsze je kupuję, jak jestem w sklepie.

**A coś takiego, co pomyślałaś sobie: "Dobra, dawno nie było, nie wyjeżdżam na majówkę, kupię sobie!"?**

Nie.

**Od poniedziałku otworzyli galerie handlowe - co o tym sądzisz?**

Z tego, co wiem, to jest wyliczone, ile osób może wejść do konkretnego sklepu, ale nie na korytarzach, w sensie na korytarzach nikt nie sprawdza, ile jest osób. To nie jest potwierdzona informacja, ale tak słyszałam, więc tak powtarzam. Jeżeli tak to jest, to nie jest to zbytnio przemyślane, w sensie naprawdę dużo osób jest w galeriach handlowych, na przykład przed świętami. Wiem, że to nie jest przed świętami. Teraz, z tego co słyszałam, jest mało osób, ale w Leroy Merlin było strasznie dużo. Koszmarna była ilość osób. To było na Targówku i tam było wszędzie bardzo dużo osób, samochody wszędzie, ludzie wszędzie.

**Tam byłaś wczoraj, tak?**

Tak. Uważam, że powinno się uważać, że to jest niebezpieczne. Ale nie wiem... Tak jak ci mówiłam, w ogóle do mnie nie dochodzi, że mogłabym się zarazić. Czy ludzie się zarażą w tych galeriach handlowych? Nie wiem, możliwe. W dalszym ciągu uważam, mimo że uważam, że się nie zarażę.

**Masz ochotę wybrać się na takie zakupy?**
Nigdy nie miałam na to ochoty, nigdy, chyba odkąd się urodziłam. Jak to otworzyli, to tym bardziej nie mam, ale Filip ma ochotę, więc obiecałam mu, że się niestety wybierzemy. Już dzisiaj się dopytywał, kiedy pójdziemy, ale na razie powiedziałam, że nieprędko, bo się boję.

**Co go tak ciągnie?**

On lubi sobie po prostu pochodzić, popatrzeć.

**Chodzi o to, żeby to było wyjście dla przyjemności, czy żeby było tak jak kiedyś?**

Dla przyjemności. Filip, tak jak ja, też nie tęskni za takim normalnym życiem, żeby gdzieś pójść, coś robić. On też lubi siedzieć w domu.

**Przecież można siedzieć w domu, nikt nie zmusza do tego, żeby wychodzić, poza tym, że musisz iść do pracy.**

Tak, ale wiemy, że ludzie robią coś.

**Jest bardzo dużo ludzi, którzy spędzają życie na kanapie.**

Ale to chyba nikt nie chce być taką osobą, która spędza życie na kanapie.

**Czyli to chodzi o to, jak ludzie cię postrzegają?**

Nie, nawet jak ja sama siebie postrzegam. Jak jest takie przyzwolenie albo nawet taki obowiązek siedzenia w domu, to nie mam wyrzutów sumienia, że siedzę w domu i że nic nie robię. Wiadomo, mogłabym skończyć pracę magisterską i robić masę innych rzecz, których nie robię, ale trzeba siedzieć w domu, więc siedzę w domu. Ale jeżeli już widzę, że można wychodzić i można coś robić, a ja tego nie robię, to już jest mi troszeczkę głupio samej ze sobą.

**Czyli te aktywności dobrze widziane społecznie...**

Albo jakieś te kulturalne czy coś. Jakieś podlewanie lasów.

**Jakie podlewanie lasu?**

Jak jest susza, to właśnie czytałam, że można się umawiać na podlewanie lasów.

**Chciałabym pogadać z tobą o tym, jak wydajesz pieniądze, ale cofnijmy się do czasu sprzed pandemii. Gdzie umieściłabyś się na skali wydawania pieniędzy?**

Pośrodku. Wyjaśnię może i może dojdę do odpowiedzi. Generalnie uważam, że jestem bardzo skąpa i naprawdę ciężko jest mi wydawać pieniądze, boli mnie zamawianie sobie rzeczy, nie lubię, jak mi znikają te pieniądze z konta. Jeśli chodzi o kupowanie droższych rzeczy albo nie wiem, czy droższych, ale może jakichś takich większych... nie, takich, które zostają na zawsze, które są przedmiotami, które ma się w domu przez lata czy przez jakiś czas, to nie lubię na to wydawać pieniędzy. Natomiast jeśli chodzi o takie rzeczy, które ułatwiają mi życie w danym momencie, to bardzo łatwo. Na przykład nie mam problemu, żeby jeździć taksówką za swoje pieniądze, w sensie nie służbowo, wszędzie jeżdżę taksówką, nie mam z tym żadnego problemu. Żeby zamówić sobie jedzenie, też nie. Jeżeli zamawiam jedzenie i właśnie jem z Filipem, i zamówię też jemu, to na pewno nie proszę go, żeby mi oddał pieniądze i w ogóle nie rozpaczam nad tymi pieniędzmi. On też mi czasem zamawia. Ale chodzi o to, że zupełnie nie myślę o tym, że wydałam 100 zł na jeden obiad, bo nam się nudziło, więc zamówiliśmy. Albo zamówiliśmy też Bubble Tea. Nie wiem, czy wiesz, co to jest, to jest takie picie chińskie/japońskie/azjatyckie, z takimi kulkami. Wydaliśmy 50 zł na dwa takie picia i w ogóle nie martwiłam się, że wydałam te 50 zł. Ale jeżeli 50 zł, może 100 zł, miałabym wydać na spodnie, to raczej byłby to przykry obowiązek i bym myślała o tym, że wydałam te pieniądze.

**Jak powiedziałaś, że duże wydatki przychodzą ci z trudem, to pomyślałam o meblach...**

Nie, ja nie potrzebuję mieć dużo rzeczy.

**Czym się różnią spodnie od obiadu?**

Nie wiem, nie rozumiem tego właśnie, ale dużo ciężej jest mi, nie wiem. Uważam, że cieszyłabym się ze spodni tak samo jak z obiadu albo może nawet bardziej.

**Na jakie jeszcze kategorie produktów ciężej ci wydawać pieniądze?**

Ubrania i buty. Jakieś rzeczy do domu, na przykład świeczki. Lubię mieć te świeczki, ale troszeczkę nie chcę ich kupować. Takie kategorie: do domu, ubrania. Nie wiem, co jeszcze się kupuje. Elektronika, ale to się kupuje raz na jakiś czas.

**Z elektroniką też jest ci ciężej?**

Ostatnie, co kupiłam sobie, to telefon i nie było mi w ogóle ciężko, bardzo chciałam sobie kupić ten telefon.

**Czyli to właściwie było prostsze?**

Tak, ale kupiłam go na raty. A, tak! Bo nie chciałam na raz wydawać.

**Rzeczy, na które łatwo się wydaje, to jest jedzenie, taksówka...?**

Wyjście do klubu, piwko alkoholowe albo nie, papierosy.

**A na wakacje jest ci łatwo wydawać?**

Pół na... Nie, nie pół na pół! Dużo łatwiej. Dużo łatwiej niż na spodnie.

**Jak masz kupić spodnie i trzeba wydać 100-150 zł, jak się wtedy czujesz?**

Nie doprowadziłam do sytuacji, w której muszę kupić spodnie, tylko raczej to jest tak, że widzę jakieś spodnie, które mi się podobają i uczucie jest takie, że chodzę przez 3 dni, do tygodnia i myślę o tym, czy to kupić czy nie kupić. Ale w końcu ktoś mnie przekonuje, żeby jednak kupić, więc kupuję sobie.

**Ale zobacz, spodnie kosztują stówę, obiad dla waszej dwójki też...**

To ja bym mogła kupić trzy obiady w tym czasie, ale bym nie kupiła spodni.

**Co sprawia, że jedno jest ot tak, a drugie jest trudno?**

Jeśli chodzi o obiad, to może to jest kwestia tego, że jemy go z Filipem i to jest też trochę dla niego. I trochę jest to rzecz, którą robimy razem, więc w sumie jest to bardziej płacenie za atrakcję niż za jedzenie.

**Powiedziałaś, że są momenty, kiedy jesteś bardzo skąpa - tak byś siebie nazwała?**

No nie wiem, jak mi szkoda 100 zł wydać na spodnie... Nie, właśnie to nie chodzi o to, że byłam skąpa, tylko że bardzo długo myślę o pieniądzach, które muszę wydać na rzeczy, które są rzeczami.

**Czyli łatwiej jest wydawać na przeżycia?**

Mhm, jakieś takie małe przyjemności. Te małe przyjemności trochę poprawiają mój komfort życia. A spodnie? Ani mnie grzeją, ani mnie ziębią, po prostu mam te spodnie.

**A to by znaczyło, że przy tych rzeczach, które poprawiają ci komfort życia, to jesteś wręcz rozrzutna?**

Dużo mniej deliberuję nad tymi pieniędzmi. Jesteśmy w stanie pójść do Żabki i wydać 50 zł na picie, samo picie w butelce, różnego rodzaju i nie myślę ani przez sekundę o tych pieniądzach, które wydałam. Po prostu idę, kupuję wszystko, na co mam ochotę i sobie to piję. A jeśli chodzi... Nie wiem, nie umiem tego wyjaśnić. Nie wiem, co wpływa na to, że nie mam ochoty kupić spodni za 100 zł. Wiadomo, oczywiście kupuję sobie te ubrania, tylko że raczej to jest tak z przymusu.

**A jak trzeba jakiś większy wydatek ponieść? Kupowałaś w ciągu ostatniego pół roku coś większego?**

To ten telefon. I nie chciałam go kupić na całość, kupiłam go na raty z tego powodu.

**Ile w sumie on będzie cię kosztował?**

Wydaje mi się, że płacę 180 zł przez 20 miesięcy. 3600 zł, coś koło 4000 zł.

**I tu miałaś problem, żeby tak na raz wydać?**

Tak, mimo że miałam te pieniądze. I to nie jest tak, że ja je sobie odłożę czy coś, nie, ja miałam te pieniądze i mogłam je wydać, ale pomyślałam, że nie chcę ich tak dużo tracić na raz.

**Jeżeli można by było wybrać tylko jedno określenie: rozrzutna lub oszczędna, to które bardziej do ciebie pasuje?**

Rozrzutna. Ale jak jest to 10-stopniowa skala i rozrzutna jest na 10, no to 6.

**Czy to jest tak, że ty jesteś na 6 czy jesteś raz tu, raz tu?**

A nie wiedziałam, że tak można, bo kazałaś wybrać jedną.

**Bo chciałam cię zmusić, żebyś coś wybrała, ale widać się nie da.**

Nie no, wybrałam 6, bardziej rozrzutna.

**A w obecnej sytuacji? Mówiłaś, że zmniejszyli wam pensje.**

Teraz bardziej myślę o tym, że nie powinnam wydawać pieniędzy, ale czy je wydaję jakąś cięższa ręką, to nie wiem.

**Dlaczego myślisz, że nie powinnaś teraz wydawać pieniędzy?**

Bo tak naprawdę nie wiem, przez jaki czas będzie ta pensja zmniejszona. Są ustalenia, że przez trzy miesiące, ale może się to zmniejszyć albo zwiększyć, w zależności od sytuacji. Szczerze mówiąc, wątpię, że skróci się ten czas i nie dlatego, że firma nie zarabia, tylko dlatego, że po prostu wygodnym jest, jak się umówiło z ludźmi na trzy miesiące, żeby jednak... Znając moich szefów, raczej to tak by to wyglądało. Zdaję sobie sprawę, że to nie jest personalnie we mnie i akurat ja, wydaje mi się, że dostawałabym pełną pensję, gdybym powiedziała, że na przykład nie przyjmuję tych trzech miesięcy ze zmniejszoną pensją. Więc nie wiem, może trzeba bardziej odkładać pieniądze. Mam takie poczucie, że skoro mam mniej pieniędzy, to może powinnam ich mniej wydawać.

**Masz poczucie, że powinnaś. A w jakiś sposób starasz się kontrolować to wydawanie?**

Nie zamawiam jedzenia, jak jestem w domu ani w biurze chyba też nie zamawiałyśmy, bo nie widziałyśmy się w biurze. Więc nie zamawiam jedzenia, co jest pewną formą oszczędności mimo wszystko. Te kwiatki kupiłam troszeczkę z bolącym sercem, ale jak już zaczęłam... Nie, wydałam pieniądze z oszczędności na te kwiatki, żeby nie widzieć w bieżącym funduszu, że wydałam. Czy jeszcze się jakoś staram? Nie no, nie będę popadała w paranoję. Nie kupuję jakichś najtańszych produktów, dlatego że kosztują trochę mniej niż to, co zawsze kupuję.

**Coś jeszcze robisz, żeby mniej wydawać? Traktujesz coś jako oszczędność?**

Nie kupiłam butów, mimo że chciałam je kupić, dlatego że uznałam, że powinnam mniej wydawać. Dużo form wydawania pieniędzy odpadło przez to, że zamknięte są miejsca, w których można wydawać pieniądze.

**Nie można pójść na piwo, do knajpy ze znajomymi.**

Nie można, tak.

**Mają niedługo otworzyć te knajpy.**

To nie wiedziałam.

**18.05. mają otworzyć ogródki dla mniejszej liczby osób. Myślisz, że to sprawi, że będziesz więcej wydawać?**

Na początku na pewno wydam przez to więcej pieniędzy, bo jestem pewna, że się umówię z kimś w takim miejscu po to, żeby być w tym miejscu, bo akurat coś takiego chętnie bym zrobiła.

**Wiesz, ile wydajesz miesięcznie na swoje utrzymanie? Kontrolujesz to w jakiś sposób, żeby wiedzieć, czy teraz wydajesz mniej?**

Bardzo nie lubię tego kontrolować, ale wiem, że teraz nie wiem, ile wydaję. W sensie nie wiem, ile wydaję, bo teraz specjalnie nie chcę wiedzieć, ile wydaję. Staram się wydawać mniej. Jeśli dowiem się, że wydałam więcej, to trudno, wezmę pieniądze z oszczędności, jeżeli naprawdę wydam wszystko, ale wątpię, że coś takiego się zdarzy. Więc nie, nie jestem w stanie tego porównać. Nie no, na pewno wydaję mniej.

**Dlaczego nie chcesz wiedzieć, ile wydajesz teraz?**

Nigdy nie chcę wiedzieć, ile wydaję.

**Bo?**

Nie wiem, wydaje mi się, że to jest jakiś taki niepotrzebny stres wiedzieć, ile ma się pieniędzy.

**To oznacza, że nie wiesz też, ile masz oszczędności?**

Nie, wiem, ile mam oszczędności.

**Czyli nie wiesz, ile masz na tym koncie bieżącym, tak?**

Tak. Ale wiesz, oszczędności to już jest większa kwota, więc na przykład nie muszę się nad nią zastanawiać, tylko to po prostu są pieniądze, które zbierałam przez jakiś czas. Wiem, że to jest dużo pieniędzy, więc jestem spokojna o to. A jak się na przykład okaże, że bardzo dużo wydałam w tym miesiącu, mam mało pieniędzy na koncie, no to już mnie to zmartwi.

**A zdarza ci się taka sytuacja...**

Że mi odrzuca kartę? Nie, nie, nie.

**Do tego nie doprowadzasz?**

Trochę jednak kontroluję, ile mam pieniędzy.

**Ale mówisz, że nie sprawdzasz.**

Tak, ale czasami mi się zdarza przez przypadek, że sprawdzę. W sensie przez przypadek, że sprawdzę - nie, ale po prostu muszę na przykład zrobić przelew z aplikacji na telefon, więc muszę wejść i zobaczyć, ile mam pieniędzy. Tego typu rzeczy, że nawet jak się nie chce wiedzieć, ile ma się pieniędzy (co jest absurdem nie chcieć wiedzieć, ile ma się pieniędzy), no to jednak... Czasami mi się zdarza sprawdzić jednak z ciekawości albo ze strachu.

**Boisz się takich sytuacji, że ci odrzuci kartę? Że sobie myślisz: "Kurczę, tyle już wydałam w tym miesiącu"?**

Nie, jeżeli tak myślę, to sprawdzam. Ale ja i tak w taki sposób swoimi pieniędzmi dysponuję, że ja bardzo dużo, trzy czwarte powiedzmy, przelewam na swoje konto oszczędnościowe. I z tego opłacam czynsz. Staram się mieć na swoim koncie jak najmniej tak naprawdę i nie robić z normalnego konta wydatków większych niż 100 zł. Właśnie zapomniałam, że tak robiłam, ale faktycznie tak robiłam, mniej więcej 500 zł, może 400 zł miałam zawsze na koncie. Nie pamiętam, jak to robiłam. Może całość wypłaty przelewałam na to oszczędnościowe i tylko zostawiałam sobie 500 zł? Coś tego typu. Jakoś tak robiłam, żeby nie mieć tak dużo pieniędzy, żeby nie wydać na raz.

**Czyli przychodzi pensja, wrzucasz ją na oszczędnościowe i potem zasilasz to normalne konto co jakiś czas?**

Jakoś tak, myślę, że tak robię.

**Przelewasz dodatkowe sumy, jak się kończą pieniądze na koncie?**

Albo jak mi się wydaje, że jest mało.

**Mówiłaś, że teraz trzeba trochę oszczędzać, ale to nie jest tak, że będziesz szukała bardzo tanich produktów. A myślisz sobie w ten sposób: "Dobra, to w tej Żabce zamiast 50 zł, to wydam 30 zł na te napoje"?**

Nie no, to już nie kupuję tych napoi. Kupię jeden, dwa, ale nie kupię za 50 zł.

**Czyli ograniczasz trochę ilość?**

Mhm i staram się też w ogóle nie kupować w Żabce.

**Bo?**

Jest tam chyba drożej niż w jakichś takich większych sklepach, a skoro już do nich jeździmy i wracamy taksówką, to mogę sobie przywieźć wszystko, co potrzebuję.

**Czyli zmniejszasz ilość, kupujesz w innych sklepach - jaką jeszcze masz strategię, żeby trochę mniej wydać?**

Mówiłam rodzicom, żeby mi przywieźli warzywa i owoce.

**Oni nie wymagają od ciebie opłacenia paragonu?**

Nie, no jeszcze tego by brakowało, absolutnie.

**Myślałaś o tym, żeby spisywać swoje wydatki teraz?**

Nie chcę tego robić.

**To byłoby dużym stresem?**

Mhm.

**Jeszcze jakieś niefajne emocje by ci towarzyszyły?**

Stres, napięcie, trochę też takie przebicie bańki. Jednak wolę siebie troszeczkę oszukiwać i nawet jak przeleję sobie tę wypłatę na konto oszczędnościowe, a potem będę z niej pobierać więcej niż sobie założyłam, to w dalszym ciągu oszukuję sama siebie, ale nie zauważam tego. Bo jednak zrobiłam to dla siebie i to była przyjemność, a jeżeli bym miała to spisywać i myśleć o każdej złotówce, którą wydałam - nie mam na to ochoty po prostu, to jest dla mnie nieprzyjemne i nie jestem w takiej sytuacji finansowej, żebym musiała to robić. Wiem, że fajnie by było oszczędzić więcej pieniędzy, zawsze lepiej mieć więcej pieniędzy niż mniej, nie wydawać na głupoty, ale jednak fajnie jest też wydawać te pieniądze na głupoty i nie myśleć w ogóle o tym, że nie mogę tego robić albo że mogłabym za to mieć coś innego.

**Gdyby zdarzyło się teraz, że twoja firma pada, ile byłabyś w stanie się utrzymać z tego, co masz?**

Na takim poziomie jak teraz? 8 miesięcy może? 10? Nie wiem, ile wydaję pieniędzy teraz podczas tej kwarantanny przez to, że ta pensja jest obcięta o te 15%, a ja nawet nie wiem, ile zarabiałam wcześniej. Mnie więcej mam jakieś pojęcie, ile zarabiam, ale jest to z dokładnością do 500 zł, może 300 zł.

**A po co ty w ogóle oszczędzasz?**

Bo lubię mieć dużo pieniędzy na koncie oszczędnościowym.

**Chodzi o sumę, które widnieje w aplikacji?**

Mhm.

**Ale to jest oszczędzanie na coś?**

Nie, absolutnie to nie jest na żadną konkretną rzecz, może troszeczkę na wszelki wypadek. No na przykład w takiej sytuacji, że nie mam pracy, to wolałabym jednak nie musieć prosić rodziców o pomoc. Pewnie chciałabym to zrobić, ale wolałabym tego nie robić.

**Rozumiem, że w krytycznej sytuacji, poprosiłabyś o wsparcie?**

Tak.

**Natomiast lepiej się czujesz, gdy wiesz, że nie jesteś w takiej sytuacji?**

Mhm. Albo zepsuje mi się coś, czego nie będę mogła kupić na raty albo będę czegoś bardzo potrzebowała, albo odbije mi coś i będę chciała gdzieś wyjechać na zawsze, i będę musiała kupić bilet i wszystko tam. Po prostu na wszelki wypadek albo też, żeby spełnić swoją zachciankę, jeżeli się taka pojawi.

**Czy masz chociaż w swojej głowie jakiś limit, ile chcesz maksymalnie ze swojej miesięcznej pensji wydawać?**

Myślę, że mam limit. W ogóle kiedyś wymyśliłam, że będę wydawała tylko 100 zł na tydzień, ale mi nie wyszło.

**Trudne to bywa.**

Trudno. Kurczę, nie pamiętam za bardzo, jak ja wydawałam pieniądze, ale tak, raczej mam z tyłu głowy to, że już powinnam na przykład przestać wydawać pieniądze. Albo nie kupię sobie... Wiesz co, powiedziałam ci, że na buty mi szkoda pieniędzy, ale nie do końca, dość lubię sobie kupować buty. I na przykład nie kupię sobie butów, bo już wydałam za dużo pieniędzy. Albo nie zamówię czegoś do jedzenia.

**Czyli przychodzi taki moment w miesiącu, kiedy sobie myślisz: "Kurczę, już dużo, wystarczy"?**

Nie, nie zawsze przychodzi, ale jeżeli sobie tak pomyślę, to przestaję po prostu.

**Oprócz trzymania oszczędności na koncie oszczędnościowym cokolwiek innego z nimi robisz czy one sobie tam leżą?**

Nie, leżą. Leżą, dlatego że ja obracam tymi pieniędzmi tak naprawdę. Jeżeli kupuję coś droższego (najczęściej nie jest to dla mnie, tylko komuś kupuję prezent), to wydaję z tamtego konta. Jeżeli to jest oczywiście większa rzecz. Więc chyba to by się nie opłacało, bo to jakieś chyba kary się ponosi. Trzeba płacić za to, że się wypłaca te pieniądze non stop na takim koncie, które zarabia. Może to byłoby słuszne, może bym wtedy jakoś inaczej to robiła, ale no właśnie ponownie - nie chcę po prostu o tym myśleć.

**Czyli ty się w ogóle nie lubisz zajmować pieniędzmi?**

Nie.

**Fajnie jest mieć, ale żeby się nimi w ogóle nie zajmować.**

Mhm.

**A myślałaś o tym, żeby te pieniądze na jakiś czas gdzieś ulokować albo w coś zainwestować?**

Nie znam się na takich rzeczach za bardzo, na pewno nie będę inwestować, bo w ogóle nie umiem, nie wiem. A jeśli chodzi o takie konto z oprocentowaniem, no w sumie mogłabym, ale nie myślałam o tym nigdy. To konto, na którym zbieram pieniądze, to jest po prostu konto, które kiedyś miałam, a potem zrezygnowałam z karty, nie pamiętam z jakiego powodu, ale ciągle mam to konto, więc po prostu... A, nie, to było tak, że moja mama mi przelewała na to kieszonkowe. Ona cały czas mi przelewała, mimo że już... Nie, po prostu ma stałe przelewy, więc one ciągle się tam już wtedy zbierały. A teraz po prostu korzystam sobie z tego konta jako drugiego.

**A myślisz, że w obecnej sytuacji warto oszczędzać?**

Tak, bo nie wiadomo, co się stanie. Nikt nie ma pewności, czy nie straci pracy. Nawet może nie tyle że on, ale może trzeba będzie też komuś pomóc? Albo na przykład coś się zepsuje, teraz nie do końca wszystko działa tak i trzeba zapłacić więcej za jakąś usługę.

**Żyjemy teraz w dużej niepewności, nie wiemy, co będzie za chwilę i są takie teorie, że nie warto mieć oszczędności, bo nam je zabiorą. Myślisz, że to tak jest?**

No może tak jest. Ja słyszałam taką teorię, że starają się wycofać transakcje gotówkowe, żeby rząd wiedział, ile mamy pieniędzy. No i może faktycznie tak jest. Tylko że akurat wątpię, że ktoś mi zabierze pieniądze, jest tyle osób, które mają więcej pieniędzy, że raczej moimi się nie będą interesować. Nie ma takiej rzeczy, którą chciałabym kupić za te pieniądze, wolę je mieć, niż mieć rzeczy za te pieniądze. Oczywiście jeżeliby się pojawiło coś, co bardzo chciałabym mieć, to... Chciałam powiedzieć, że pewnie bym kupiła, ale nie, raczej bym nie kupiła, ale bym myślała sobie o tym. W ogóle nie chcę pozbyć się tych pieniędzy, nawet jeżeli ktoś powiedział, że tak byłoby lepiej.

**Myślisz, że teraz jest dobry czas na inwestowanie tych pieniędzy?**

Nie wiem. W ogóle nic na ten temat nie powiem, bo nie wiem. Jeżeli ktoś ma tyle pieniędzy, żeby na przykład kupić sobie mieszkanie, no to może... Chyba teraz są tańsze mieszkania. Ciężej jest dostać kredyt, ale chyba tańsze są mieszkania, więc może warto byłoby zainwestować w mieszkanie.

**Czyli dla ciebie twoje oszczędności to jest poduszka na wszelki wypadek?**

Mhm.

**I lepiej, żeby była, niż żeby jej nie było, i żeby była większa niż mniejsza.**

Mhm.

**A jest taka suma, poniżej której ty byś się źle czuła, gdyby twoje oszczędności spadły?**

Pewnie tak, ale nie wiem, jaka to jest kwota.

**Czyli nie wydałaś nigdy z tamtego konta tyle, żeby się tak poczuć?**

Nie, absolutnie, bo na tym koncie bardzo dużo odkładam, generalnie co miesiąc i bardzo długo to odkładam. Zresztą zanim się przeprowadziłam od rodziców, a już pracowałam, to właściwie 80% można było odłożyć, no bo na co tu wydawać, skoro nie trzeba nic kupować oprócz przyjemności dla siebie. A zresztą, jak się mieszkało z rodzicami, to rodzice też tam dali czasem jakieś pieniądze albo babcia, często się widywało z tymi ludźmi, którzy dają pieniądze, więc siłą rzeczy się tych pieniędzy miało więcej. I ja je odkładałam, dlatego że, tak jak już mówiłam, ja za bardzo nie lubię kupować sobie rzeczy. Szkoda mi jest pieniędzy na te rzeczy, więc ich po prostu nie kupowałam, więc miałam te pieniądze, one mi się odkładają, dlatego nigdy nie było tak, żebym miała ich jakoś mało albo żebym się martwiła o te oszczędności.

**Myślisz, że teraz ogólnie ludzie lub ludzie w twoim otoczeniu mają problem z pieniędzmi, oszczędnościami?**

Wiesz co, nie wiem. Często ludzie mówią, że nie mają pieniędzy, tak się mówi, ale nie jestem pewna, czy to naprawdę oznacza, że oni nie mają pieniędzy, raczej nie. Tylko kończą się im pieniądze, bo jest koniec miesiąca, ale to nie znaczy, że nie będą mieli co jeść, tylko że mają mało, ale na przykład wezmą z oszczędności. Ale w ogóle nie rozmawiam z moimi znajomymi o pieniądzach.

**A nie pojawia się coś takiego, że ktoś mówi: "Kurczę, nie zamówię, bo nie mam"?**

No jeżeli to jest przyjemność, to zdarzało mi się coś takiego słyszeć: "Dobra, no to kupię po wypłacie", ale nie zdarzyło mi się coś takiego, żeby ktoś naprawdę z moich znajomych nie miał pieniędzy.

**Masz takie wrażenie, że wszyscy wokół ciebie jednak mają jakieś oszczędności?**

Wiesz co, no nie wiem, naprawdę nie wiem, dlatego że Magda, która doskonale wie, ile zarabiam, dlatego że jest moją szefową i co miesiąc mi akceptuje wypłatę, ona do tej pory mi nie powiedziała, ile ona zarabia. Nie mam żadnego pojęcia, mogę mniej więcej zgadywać, ale do tego stopnia nie rozmawiam z nią o pieniądzach, że mogłam zobaczyć gdzieś czy coś, ale to tyle. Nie wiem. I nikt ze mną za bardzo nie rozmawia o pieniądzach. Nie wiem, czy mają oszczędności czy nie. Jakieś pewnie tak, ale jakiego rzędu są to oszczędności, to nie wiem.

**Teraz macie na trzy miesiące zmniejszoną tę pensję, ale mogą to przedłużyć. Jak długo jesteś w stanie akceptować tę niższą pensję?**

No właśnie zastanawiałam się nad tym, dlatego że rozmawiałam z Magdą i Magda powiedziała, że jeżeli tak będzie do sierpnia, to ona zamierza znaleźć sobie nową pracę, bo generalnie wkurza ją ta praca tak czy inaczej, a jeżeli ma zarabiać mniej pieniędzy niż dotychczas, to nie chce tego robić. Zresztą ona za każdą zatrudnioną osobę miała bonus jeszcze po trzech miesiącach, a te bonusy się skończą, odkąd przestała zatrudniać nowe osoby. Wydaje mi się, że mi by to nie przeszkadzało, nie wiem, do końca mogę akceptować.

**Jeżeli by ci powiedzieli, że nie wracacie do poprzednich pensji?**

Zależy, jak by było w innych pracach. Zależy, czy to byłaby po prostu norma, że nikt nie wraca, czy to byłoby tylko u mnie. Bo jeżeliby to była norma, tobym to przyjęła, nie brakuje mi pieniędzy. Wiadomo, pewnie byłabym zła i trochę bym się zmartwiła. A jeżeli to by było tak, że inni by mieli taką sytuację, że oni by wrócili do normalnych pensji albo większość, to mogłoby mi się to przestać podobać.

**Ale do tego stopnia, że szukałabyś nowej pracy?**

Mhm.

**Jak myślisz, kiedy to się skończy, kiedy będzie ten powrót do normalności?**

Normalne funkcjonowanie to nie wydaje mi się, że prędko. W poniedziałek chyba będzie u nas można wrócić, kto chce. I dopiero po tym poniedziałku będę mogła ci powiedzieć tak naprawdę, na ile to jest normalne. Bo na przykład, jeżeli wróci 5 osób, to nie będzie to w ogóle normalne i nie będzie się różniło od tego, co było do tej pory. No ale jeżeli z tych 60 osób to będzie 40 albo nawet 20... nie, 20 bym pewnie nie zauważyła, ale 30-40 to już pewnie byłoby coś normalnego bardziej.

**Czyli to zależy u ciebie trochę od tego, jak szybko zaczną ludzie wracać, tak? W poniedziałek *możecie*?**

To nie jest jeszcze oficjalnie potwierdzone, możliwe, że będzie tak, że w poniedziałek będziemy mogli wrócić.

**A mówi się w ogóle o tym, kiedy będzie *trzeba*wrócić.**

Nie. W ogóle generalnie u nas w pracy nie ma żadnych bardzo oficjalnych informacji na ten temat, tylko mi po prostu powiedziała Magda.

**A jeśli chodzi o funkcjonowanie społeczeństwa, jak myślisz, kiedy jako społeczeństwo wrócimy do czegoś bliższego normalności, że ty już poczujesz, że jest normalnie?**

Właśnie zastanawiam się, jakie są objawy tego, że jest normalnie i ciężko mi tak naprawdę cokolwiek wymyślić, bo jak sobie tak myślę, to większość sektorów społeczeństwa zachowuje się dość normalnie tak naprawdę. Nie widziałam żadnych kolejek do sklepów, ludzie chodzą w tych maseczkach, ale oprócz tego wydaje mi się, że jest dość normalnie i tak jak było, tylko w głowach może... Nie, właśnie nie w głowach, bo jakby było w głowach, toby ludzie nie wychodzili. Nie wiem, jaki jest wyznacznik tego, że jest normalnie, no chyba po prostu jak nie będzie trzeba chodzić w maseczkach.

**Myślisz, że ważnym wyznacznikiem jest otwarcie tych różnych punktów usługowych, szkół?**

A, no tak. Przez to, że nie chodzę do szkoły ani nie mam dzieci, to w ogóle nie przyszły mi na myśl. Tak, to jest ważne, bo jak zamknęli szkoły, to potem też zamknęło się zaraz od razu wszystko.

**A teraz się wszystko otwiera, a szkoły się nie otwierają.**

Ale otwierają się przedszkola. I żłobki.

**W sensie mogą się otworzyć.**

Mogą.

**Bo Warszawa się nie otwiera.**

Bo właśnie chyba w Warszawie nie mogą.

**W Warszawie nie mogą, we Wrocławiu, w Poznaniu.**

No to może jak się otworzą szkoły...?

**Zastanawiam się właśnie, jakie są dla ciebie wyznaczniki tego, że już będzie normalnie. A na przykład to, że będziesz mogła oficjalnie pójść na paznokcie? Byłaś na paznokciach przez ostatnie dwa tygodnie?**

Nie pamiętam, kiedy się widziałyśmy, ale idę w czwartek, a dwa i pół tygodnia się zapisałam.

**To chyba miałaś już nowe wtedy.**

Tak.

**Ale teraz nadal idziesz tak nieoficjalnie.**

Tak, ale teraz już ludzie chodzą do fryzjerów, korzystają z usług fryzjerów - może tak. I to nie jest tak, jak ja chodziłam na paznokcie, kiedy nikt inny nie chodził nigdzie. Zwiększała się ta liczba z czasem, ale teraz to już większość chyba chodzi. Znaczy większość, no nie wiem, jak bardzo potrzebuje się tego fryzjera, ale widziałam, że moja szefowa była, Maciek z pracy był i słyszy się o tym, że ludzie chodzą do fryzjerów.

**I nie mają z tym problemu?**

Chyba problem jest taki, że nie zawsze to są ich fryzjerzy, tylko jacyś tam poleceni. I to jest chyba ich największy problem, że to nie jest ich fryzjer.

**Czyli właściwie już jest ta możliwość korzystania, więc to nie jest wyznacznik normalności, konieczny element?**

Wiesz co, ale jest konieczny generalnie do takiego odmrożenia gospodarki i takiego poczucia, że już się coś zmienia, bo ci ludzie jednak w ogóle nie zarabiają pieniędzy, jeżeli nie robią tego w podziemiu fryzjerskim. Więc to akurat jest ważny krok, żeby to otworzyć, nawet dla takiego poczucia, że już jest lepiej. Mimo że można sobie korzystać z tych usług, to oficjalnie nie można.

**Coś jeszcze jest takim wyznacznikiem normalności?**

Imprezy masowe, jakieś zgromadzenia

**A masz jakieś obawy związane z najbliższą przyszłością?**

Tak jak już mówiłam, że może wrócić ten wirus ze zdwojoną siłą, jeżeli otworzymy to wszystko za wcześnie. Nie jestem w stanie powiedzieć, czy to jest za wcześnie, tylko że jeżeli tak jest, no to może się tak wydarzyć. Jeszcze słyszałam takie głosy, że to wszystko znowu zostanie zamknięte, jak będzie już po wyborach. Czy to jest obawa? Raczej przed takim mieszaniem po prostu, już dajmy spokój, posiedźmy w tych domach i wyjdźmy, jak będzie pora na to, a nie takie mieszanie. Raczej to są takie moje nawet nie tyle obawy, co jakieś przemyślenia, które mam i czuję, że to takie niekomfortowe.

**Że nastąpi taki powrót, tak?**

Tak i nie dlatego, że nie lubię żyć w ten sposób czy źle się w tym czuję, tylko raczej po prostu to jest takie kolejne zamieszanie, robienie czegoś po to, żeby robić coś na nowo innego. I nie podoba mi się takie coś.

**Jeszcze jakieś refleksje o przyszłości?**

Refleksje mam takie, że mimo wszystko uważam, że to jest troszeczkę za wcześnie na wprowadzanie tych etapów. Ustalenie tych etapów - spoko pomysł, ale trochę za szybko, mam wrażenie.

**Za szybko te galerie handlowe?**

Wiesz co, nawet nie chodzi o to, że coś konkretnego, tylko raczej taki nastrój wśród ludzi, że już jest poprawa, mimo że patrząc na przykład na statystyki, to wcale nie. I też część biur już wraca do pracy, rotacyjnie czy coś tam, ale jednak już tak się szykują do normalnego życia. Ja wiem, że to jest uciążliwe dla firm i wszystkich ludzi, żeby żyć zdalnie, ale można ponieść większy koszt niż niewygoda pracowania zdalnie.

**Czyli warto przemęczyć się, pracując w domu, bo konsekwencje pracy w biurze mogłyby być większe?**

Wydaje mi się, że tak, tak sobie myślę, ale nie wiem.

**Myślisz, że będą dalej otwierać, realizować kolejne etapy?**

Myślę, że tak. Niestety myślę, że tak.

**Tak pesymistycznie trochę zakończyłyśmy.**

No tak, ale jak już mówiłam, ja się bardzo komfortowo czuję jednak w tej sytuacji, więc dlatego to jest taka obawa.
