# Supplementary material for: The challenges arising from the COVID-19 pandemic and the way people deal with them. A qualitative longitudinal study
Source: PLoS One. 2021 Oct 11;16(10):e0258133. doi: 10.1371/journal.pone.0258133 (PMC8504766; doi:10.1371/journal.pone.0258133)
Supplement: S1 Dataset — (ZIP) [file pone.0258133.s003.zip › Transcriptions/stage 4/14.4_M_55_couple, with children.docx]

**14.4_M_55_couple with children**

**Co się u Ciebie ostatnio wydarzyło?**

To, co najważniejsze to to, że musimy otworzyć przedszkola. Teoretycznie musimy zrobić to dzisiaj, ale tego nie robimy. Głównie po to, żeby zobaczyć, co się będzie działo w ogóle. Czy inne przedszkola będą się otwierać, czy nie. Sytuacja jest taka, że pan prezydent Trzaskowski rekomenduje - bo nakazać nie może - aby nie otwierać przedszkoli publicznych na terenie Warszawy. Z kolei Kuratorium - wszystkie władze zależne od rządu - mówią coś zupełnie odwrotnego. I my jesteśmy postawieni troszkę pomiędzy młotem a kowadłem. Nie wiemy, co robić. Poza tym, wprowadzono bardzo dużo obostrzeń w działalności przedszkola. Te przepisy są takie, że macie otworzyć, ale macie dostosować się do wytycznych. A te wytyczne, w praktyce są niemożliwe do spełnienia.

**Co jest w nich niemożliwe?**

<zdenerwowanym głosem> Proste, w salach mogą być tylko rzeczy, powierzchnie, które nadają się do dezynfekcji. A my na przykład mamy w salach wykładzinę dywanową. To nie jest taki dywan, który można zabrać, tylko wykładzina, która jest na stałe przymocowana, od kiedy na etapie budowy została położona. Ona nie nadaje się do dezynfekcji, bo nie można zlać jej spirytusem, ani niczym innym. Tym bardziej nie można tego robić co godzinę. Bez niej z kolei, dzieci będą się przewracać i mieć porozbijane głowy. To, co możemy teraz zrobić, a co robią wszystkie przedszkola, to opracować takie procedury, aby zabezpieczyć nie dzieci, tylko żeby zabezpieczyć siebie, przed odpowiedzialnością cywilną i karną. Odpowiedzialność karna za zaniedbania mogące prowadzić do zakażenia, to jest do trzech lat w pierdlu. Tak. Już nie mówię o odpowiedzialności cywilnej, którą będą nam zarzucać rodzice za to, że dziecko zaraziło się koronawirusem w przedszkolu.

**A jak jest z chęcią rodziców, aby wysyłać dzieci do przedszkola?**

Nie wiem. My na 50 dzieci w jednym, mamy 7 czy 8 chętnych. W drugim, też na 50 parę, mamy też coś koło 8. Czyli to jest kilkanaście procent. Trzaskowski powiedział, że sam od 18 [maja] otworzy publiczne przedszkola. Ale data tak naprawdę nie ma znaczenia. Czy otworzymy 11 - bo tak na razie myślimy, czy 18 - ryzyko zostaje dokładnie takie samo. Istnieje tylko prawdopodobieństwo, że jak nie dojdzie do wyborów i nie daj boże zostaną one przeniesione na sierpień - to oni przywrócą zakaz działalności przedszkoli i tyle. No bo po co im to było? Po to, żeby stworzyć wrażenie, że wszystko idzie ku normalności, tak? Możecie chodzić do galerii handlowych, mieszkać w hotelu. Możecie posłać dzieci do przedszkola, to możecie pójść na wybory. Jakie to jest ryzyko? <parska cynicznym śmiechem>

**Gdyby to od Ciebie zależało, otwierałbyś przedszkole?**

Otwierałbym, pod jednym warunkiem. Że państwo weźmie na siebie odpowiedzialność za wszelkie ryzyka związane z zakażeniami w przedszkolu. Państwo może to zrobić. Powiedzieć, my, jako państwo, bierzemy wszelką odpowiedzialność za to. Tymczasem oni zrobili w ten sposób, że "możecie otwierać", ale za wszystko "wy jesteście odpowiedzialni". Wy - przedszkola, dyrektorzy, organy założycielskie. Wy jesteście za wszystko odpowiedzialni.

**Ale możecie, czy musicie otwierać?**

Przepis nam mówi, że musimy. Tylko jest w tej ustawie furtka, że jeżeli otwarcie miałoby wiązać się z ryzykami sanitarnymi, możemy nie otwierać. Więc tak naprawdę otworzyć w ogóle się nie możemy. Możemy na przykład powiedzieć, że wytyczne są na tyle skomplikowane, ze potrzebujemy więcej czasu, który sobie dajemy. Uważam, że jeżeli damy sobie trzy dni, to nikt nam głowy nie urwie. A otwierać się dzisiaj... Wczoraj mieliśmy bardzo intensywną dyskusję na ten temat z dyrektorami naszych przedszkoli. Doszliśmy do wniosku, że najbezpieczniej będzie otworzyć się 11. Dlaczego? Zobaczymy, co przez te trzy dni będzie się teraz działo. Dlaczego mamy być pierwsi, narażać się jako pierwsi? Tym bardziej, że wątpię, że w Warszawie otworzy się jakiekolwiek przedszkole. No, może będzie kilka.

**Jakie nastroje są wśród pracowników waszych przedszkoli?**

Mamy kilku pracowników, którzy poszli na zwolnienia lekarskie. W tej chwili dzwoni się do lekarza rodzinnego i ma się takie zwolnienie - nawet bez oglądania. Ale ogólnie rzecz biorąc, większość ma ochotę wrócić do pracy, bo mają już zwyczajnie dosyć tego siedzenia w domu. Więc większość by chętnie wróciła.

**Myślisz, że te zwolnienia lekarskie są zwolnieniami lękowymi?**

Myślę, że to mogą być osoby, które mają jakieś tam drobne schorzenia, na które normalnie nie zwróciliby uwagi, a teraz się trochę boją.

**A poza sytuacją zawodową, co się u Ciebie ostatnio działo?**

Na długi weekend wyjechaliśmy sobie na Mazury i było bardzo fajnie. Pojechaliśmy do znajomych, do ich domku letniskowego. Postanowiliśmy, że postąpimy niezgodnie z przepisami, bo ci znajomi tam byli. To była w miarę spontaniczna, podjęta z dnia na dzień decyzja. Były jakieś tam obawy, bo zawsze jest jakaś tam obawa, istnieje jakieś prawdopodobieństwo zarażenia się, ale postanowiliśmy pojechać, bo mieliśmy ochotę na wyjazd. Skoro znajomi nas zaprosili, to znaczy, że oni się nie obawiają. Tak po prostu na chama byśmy się do nich nie wprosili. Poza tym spotkaniem, nie mieliśmy ostatnio innych kontaktów towarzyskich.

**Co z tym znudzeniem, które towarzyszyło Ci ostatnio?**

Może jest go już teraz mniej. Bardziej się człowiek zajmuje pracą, tym, co się dzieje. Ta obecna sytuacja raczej powodów do nudy nie daje. Czekamy ze zniecierpliwieniem na rozwój sytuacji, pewnie dużo rzeczy będzie działo się jeszcze dziś. Zobaczymy, jak to dalej będzie w tym naszym kraju. Czy będziemy mieli ustrój typu putinowskiego, na co się zanosi, czy jednak nie. Może.

**Co działo się jeszcze?**

W zasadzie nic poza tym.

**Pojawiły się jakieś nowe rzeczy/**

Przez dwa tygodnie byłem na diecie ketogenicznej. Miałem nadzieję trochę schudnąć. Nie schudłem nic. Od dzisiaj już na niej nie jestem, to było od początku założenie, że będę na niej dwa tygodnie. Ani nie schudłem, ani nie utyłem, kompletnie nic się nie zmieniło. Będąc na niej, czułem się normalnie. Nie brakowało mi wina, brakowało mi mojego chleba, który piekę. W tym czasie piekłem go nadal, ale po to, aby rozdać go znajomym. Robiłem to dlatego, że co jakiś czas trzeba odświeżać zakwas. Jeśli co jakiś czas się na nim, nie piecze, on może się zestarzeć. Do niego dodaje się ciasta z chleba pieczonego na bieżąco, więc trzeba to robić.

**Są jakieś rzeczy, które ograniczyłeś?**

Ze względu na dietę nie piłem alkoholu. Ale prawdopodobnie, możliwe, że ograniczę w jakiś sposób jego konsumpcję na dłużej. Bardzo dobrze się czułem nie pijąc. Lepiej mi się spało.

**Czy pojawiło się coś nowego, co zaczęło ci doskwierać?**

Nie. To znaczy, coraz bardziej przeraża mnie perspektywa tego, że przez bardzo długi czas nie będzie można wyjechać za granicę. Tym bardziej, że już nasze władze to zapowiadają. Minister zdrowia zapowiedział, że granice zostaną otwarte najpóźniej ze wszystkich tych ograniczeń. To mi bardzo przeszkadza. Ta świadomość, że nie mogę wyjechać. No, wyjechać mogę, tylko jak wrócę, będę musiał siedzieć dwa tygodnie na jakiejś tam kwarantannie.

**Byłbyś gotów pojechać i odsiedzieć później te dwa tygodnie?**

Nie, jeśli będzie trzeba siedzieć dwa tygodnie na kwarantannie, to nie pojadę nigdzie. Tym bardziej, że nie mam gdzie siedzieć, wtedy cała rodzina musiałaby siedzieć na kwarantannie. Poza tym, na razie nie ma nawet gdzie wyjechać, bo sąsiedzi jeszcze nie wpuszczają. Samoloty nie latają. Teoretycznie są kraje, które wpuszczają bez ograniczeń, tj. Szwecja i Holandia. Ale trzeba tam polecieć samolotem, albo przejechać tranzytem przez kraje, które nie wpuszczają. Więc...

**Czy wobec tego pojawia ci się jakieś alternatywne rozwiązanie co do tego, co można zrobić, skoro nie można pojechać za granicę?**

Być może będziemy podróżować po Polsce. Na pewno będziemy podróżować po Polsce. Choć nie mamy na razie żadnych planów. Być może będą to jakieś wycieczki 1-2 dniowe. Jakoś tak specjalnie na wyjazd kilkutygodniowy w Polsce to nie mam żadnej ochoty.

**Znalazłeś jakieś swoje zdjęcia/ obrazki obrazujące twoje emocje w ciągu ostatnich dwóch tygodni?**

Nie, nie, absolutnie nie myślałem na temat swoich emocji. Sorry, powinienem był, ale jakoś tak.

**To jest dla ciebie zwykły stan, że nie myślisz o swoich emocjach?**

Tak, tak.

**Obrazki**

1 i 14. 1 to jest jakiś taki totalny chaos znów - się robi. A 14 to jest jakiś taki węzeł - węzeł gordyjski, który nie wiadomo, jak rozwiązać. Czyli to, co ci mówiłem, że jesteśmy między młotem a kowadłem i czego byśmy nie zrobili, może być uznane za złe. Czyli, jakiej decyzji byśmy nie podjęli, to zawsze będzie się mógł ktoś przypieprzyć. Ten chaos to jest taka niepewność i zdenerwowanie, a czasami takie poczucie bezradności, bo niezależnie... Zaczynam mieć poczucie, że przestaję mieć kontrolę nad, znaczy nie, kontrolę mam, ale... Zaczynam mieć poczucie takie, że każda decyzja, będzie złą decyzją. Zaczynam łapać się na tym, że robię się asekurancki. Tak, jak teraz. Możemy otworzyć dziś oba przedszkola, ale tego nie robimy, aby popatrzeć, co robią inni. To jest dla mnie coś nowego. Ja zwykle nie miałem obaw w podejmowaniu decyzji. Człowiek żył w jakiejś w miarę jasnej sytuacji prawnej. A tutaj mamy taką sytuację, że jeden organ państwowy mówi jeden, a drugi organ państwowy mówi nawet nie 0, tylko "-1". Czyli mamy coś zupełnie przeciwstawnego. Poza tym, jak zadajemy pytania tymże organom, jak mamy postępować, to oni robią wszystko, aby nam nie udzielić odpowiedzi. Tzn., udzielić nam odpowiedzi takiej, która będzie wygodna dla nich. Pomyślałem sobie, że skoro oni w tej sytuacji tak postępują, dają nam odpowiedzi, które są wygodne dla nich, to ja też podejmę decyzję, która jest wygodna dla nas. Nie będziemy szli do przodu, po prostu nie.

**Jakie emocje to u ciebie wywołuje?**

To mnie smuci i złości. Bardziej złości nawet.

**A ten węzeł, jakie to uczucia?**

Nie wiem, jak postąpić. Do tej pory nie było czegoś takiego. Do tej pory, we wszystkich decyzjach, które podejmowałem, byłem w miarę pewien tego, co robię. A tutaj, jest totalna niepewność, w którą wpędzili nas - niestety - rządzący nami. Nawet wczoraj oglądałem jakąś konferencję prasową dla właścicieli przedszkoli. Były tam jakieś panie z kuratorium, był pan z Inspekcji Sanitarnej. Nie pozwolono zadawać żadnych pytań. Odpowiadali tylko na pytania, które zostały zadane wcześniej. Tylko że trudno było... Nie wiedziałem w ogóle, że jest taka możliwość, aby wcześniej zadawać te pytania. Pytania były bardzo grzeczne, a jak były jakieś z kategorii zaczepnych, jak stwierdził ten pan - choć ja uważam, że to były normalne, logiczne pytania, to te panie odpowiadały, że przecież nie będą nas wyręczać w podejmowaniu decyzji. Bo przecież mamy wszelkie kompetencje, aby podejmować decyzje. I że my wiemy lepiej, jak postąpić w takiej sytuacji. Naprawdę. Człowiekowi nóż w kieszeni się otwierał. Urzędnicy robią wszystko, aby nie podjąć żadnej konkretnej decyzji, zrzucają ją jedynie na nas. Wytyczne, które zrobili, to taki program maksimum, aby nikt nie zarzucił im, że czegoś nie przewidzieli. Przecież jeśli dziecko pobawi się zabawką, to zanim inne dziecko będzie mogło się nią pobawić, będziemy ją musieli zdezynfekować. No wyobrażasz to sobie?

**Nie.**

Ale taki jest, k, przepis! No sorry, za mój francuski, no!

**Rozumiem, że to cię po prostu wkurza.**

Oni zrobili tak, oni wprowadzili przepisy, umyli rączki, proszę bardzo, tu są przepisy. Ale wy macie otworzyć przedszkola - bo rozporządzenie mówi, że mamy obowiązek otworzyć - tylko musimy dostosować się do tych wytycznych, które są nie do zrealizowania. Poza tym jeszcze, są jeszcze inne wytyczne. Że na jedno dziecko w sali i nauczyciela, jest 4 m2. To jest praktycznie 3 razy więcej powierzchni, która była do tej pory. W sali może znajdować się 1/3 dzieci, które znajdowały się tam do tej pory. Jeśli mieliśmy przedszkole do którego jest zapisane 60 osób - a mieliśmy 100 miejsc - to teraz może być w nim 30 dzieci. Po pierwsze, gdyby ten przepis miał zostać na dłużej, to prowadzenie przedszkola jest totalnie nieopłacalne. Koszty stałe mamy dokładnie takie same, a przy połowie dzieci, zarabiamy tylko połowę dotychczasowego dochodu. W tej chwili całe szczęście, że tylko te kilkanaście procent rodziców chce w ogóle oddać dzieci, ale gdyby wszyscy chcieli oddać dzieci, to nie jesteśmy w stanie wywiązać się z podpisanych umów. Wyobraź sobie, do czego te gnoje doprowadziły. Postawili nas pod ścianą i oczywiście mówią, że cała odpowiedzialność jest na nas. A na nich kurwa nic. No sorry, no, ale inaczej nie jestem już po prostu w stanie się wyrażać.

**A jak sobie z tym radzisz, że oni postawili cię w takiej sytuacji?**

No, tak, jak mówię. My też wprowadzamy zasady, które są maksymalnie asekuranckie. Rodzic, kiedy przyprowadzi dziecko, będzie musiał wypełnić kartkę w 4 egzemplarzach - oświadczeń i różnych intercyz, że on oddaje dziecko na własną odpowiedzialność, że my jesteśmy zwolnieni ze wszelkiej odpowiedzialności, że dziecko jest zdrowe, że nie ma chorób ukrytych... Musimy coś takiego mieć. I codziennie będzie coś takiego wypełniał. Więc przyprowadzając dziecko, codziennie będzie spędzał pół godziny na wypełnianiu papierów. Ja nie jestem w stanie tego zorganizować inaczej.

**A na takim poziomie osobistym?**

Nic, ja się staram odłączać od tego. Wczoraj się denerwowałem, dziś już może trochę mniej. Czasami nawet już popadam w taka konstatację, że co będzie, to będzie.

**Jak starasz się odłączać?**

Nie wiem, nic nie robię specjalnie. Dochodzę czasami do wniosku, że może po prostu przestanę się przejmować. Co będzie, to będzie. A w ogóle to ja mam nadzieję, że może jeszcze do końca tygodnia odwołają ten cały cyrk z otwarciem przedszkoli.

**Śledzisz wiadomości, statystyki?**

Statystyki - widzę, że jest wzrost zachorowań. Więc może oni się zorientują, że skoro jest wzrost zachorowań, to przedszkoli nie otworzymy. Może galerie handlowe też się z powrotem zamkną? Ja nie wiem, no. Szumowski zapowiadał przecież, że zawsze można cofnąć te wszystkie poluzowania. Powiedział wczoraj, w jakimśtam wywiadzie.

**Jak rozumiesz ten wzrost zachorowań?**

Nie wiem, może wszyscy zaczynają się zachowywać tak, jak ja. Spotykać się, wyjeżdżać. Ja nie mówię, że jestem bez winy - też pojechałem. Ale na pewno otwarcie przedszkoli, czy galerii handlowych nie spowodują, że ilość zakażeń będzie się zmniejszała. Wręcz przeciwnie.

**Jak rozumiesz te zakażenia - to ludzie będący w szpitalach, czy...**

Nie wiem, nie analizuję tego tak mocno. Widzę tylko, że wczoraj było 200 parę, a dziś już jest 400 parę. Możliwe, że w weekend nie raportowano tych ilości i część zachorowań, które były 2 czy 3 maja, dopiero zaraportowano teraz. Albo w weekend robiono mniej testów - bo weekend, czy święto narodowe. I tych, których mieli przetestować wtedy, przetestowano dopiero teraz, dlatego statystyka wzrosła. To wszystko jest granica błędu statystycznego, to znaczy, mam na myśli, że nie codziennie jest robiona taka sama ilość testów, a urzędnicy pracują bardziej w ciągu tygodnia, niż w weekendy.

**Jakie są twoje obserwacje dotyczące twojego otoczenia?**

Wczoraj przejechałem się przez miasto. Jednak wszyscy chodzą w tych maseczkach. Zauważyłem też, że zaczynają się robić korki w mieście, coraz więcej ludzi chodzi do pracy, zwiększa swoją aktywność. Co też może skutkować tym, ze ludzie mają ze sobą więcej kontaktu. Trudno, żeby ludzie chodzili do pracy po to, żeby tam samemu siedzieć, bo jeżeli ma siedzieć sam, to równie dobrze może siedzieć w domu. Te korki, większa ilość samochodów na drogach, zaczęły się już pojawiać w zeszłym tygodniu. Na pierwszy rzut oka widać też większą liczbę ludzi chodzących po chodnikach. Teraz, kiedy ludzie zaczną odwozić dzieci do przedszkola, znów zwiększy to jakiś tam ruch. I to widać.

**A o czym teraz ludzie mówią, czym się martwią?**

Nie rozmawiam z ludźmi. Poza tymi, z którymi rozmawiałem zawsze. Jakoś tak ze znajomymi dużo rozmawialiśmy, ale to był taki krąg ludzi podróżujących lub utrzymujących się z podróżowania czy turystyki. To rozmawialiśmy o tym, kiedy w końcu będzie można wyjeżdżać i kiedy do nas będą przyjeżdżać. Są znajomi, którzy w Polsce pracują w branży turystycznej, hotelarze. Ich ta cała sytuacja bardzo mocno uderzyła.

**To ich zmartwienie utrzymuje się na tym samym etapie, czy ulega zmianom?**

Tak samo, jak było. W tej chwili zastanawiają się, jakie powództwa, przeciwko komu... Mają zamiar sądzić się ze Skarbem Państwa. Ciekaw jestem, jak teraz będzie wyglądało jakiekolwiek sądzenie się ze skarbem Państwa w sytuacji kiedy Sąd Najwyższy został już przejęty przez PiS. Czy są jakiekolwiek szanse na wygranie jakiegokolwiek procesu ze Skarbem Państwa w Polsce. Jeszcze do niedawna była taka szansa. A teraz, nie widzę jej. Tak samo jak w Rosji nie można wygrać procesu ze Skarbem Państwa, tak w Polsce prawdopodobnie nie da się tego zrobić.

**Nie masz takiego wrażenia, że w twoim otoczeniu następuje w ludziach jakiś wzrost optymizmu?**

Wiesz co, może niewielki. Ale raczej nie, raczej nie ma tego. Raczej jest takie poczucie, że żyjemy w takim Matrixie, że mówią nam, że jest lepiej, a tak naprawdę lepie nie jest. Na każdym kroku próbuje się nam wciskać ciemnotę. Z drugiej strony, to też ludzie mówią, nie sposób żyć zbyt długo w takim zamknięciu. Prędzej czy później gospodarka musi ruszyć i nawet jeżeli nie ma medycznych powodów, żeby poluzować, to jednak trzeba poluzować. Bo przecież trzeba z czegoś te wszystkie zasiłki, dopłaty, sfinansować. Inaczej, niż ruszeniem gospodarki, nie da się tego zrobić. Ja też jestem zdania, że gospodarkę trzeba ruszać, rozruszać. Jestem też zdania, że szkoły i przedszkola powinny ruszyć. Tylko jestem też zdania, że nie można wprowadzać wytycznych i ograniczeń, które czynią to albo nieopłacalnym, albo niemożliwym, albo w ogóle nierealnym. Jeżeli rząd chce rozruszać gospodarkę, to niech weźmie, kurwa. mać., za to odpowiedzialność. Niech nie wprowadza durnych przepisów, tylko niech powie, dobra, przedszkola ruszają, a wy zwróćcie zdroworozsądkowo uwagę, aby dzieci częściej myły ręce. Ale jeżeli wprowadza się przepis, który mówi, że pani z kuchni nie może kontaktować się ani z nauczycielami, ani z dziećmi, to jak ten posiłek, który jest zrobiony w kuchni, ma znaleźć się w sali, gdzie dzieci jedzą? On ma się zdematerializować w kuchni i zmaterializować w sali? Jeżeli mówi się, że toalety muszą być ciągle dezynfekowane, a w sali, oprócz dzieci mogą znajdować się tylko nauczyciele przypisani do grupy, to w jaki sposób pani sprzątaczka przejdzie przez salę do toalety? No w jaki sposób? Też zdematerializuje się w jednym miejscu i zmaterializuje w drugim? Przepisy są absurdalne, ale jeśli nie będziemy się do nich stosować, grożą nam pozwy, procesy sądowe, sprawy karne.

**Czy twoje obawy osobiste dotyczą jeszcze jakichś innych sfer?**

Boję się tego, że Polska jako kraj, stanie się ustrojowo państwem zbliżonym do Uzbekistanu lub Kazachstanu. I że przestaniemy być w Europie odbierani jako kraj europejski, tylko będziemy postrzegani, jako jakaś marionetkowa dyktaturka, czy zabawna dyktaturka, na którą nikt nie zwraca uwagi. To jest dla mnie bardzo ważne, bo ja przeżyłem okres, kiedy my, jako Polacy, byliśmy, że tak powiem, pariasami Europy. Kiedy wszędzie, na każdej granicy, traktowano nas jak podejrzanych, jak takich gorszych. Byłą granica Austrii, Niemiec. Ja pamiętam, że jak człowiek jechał przez Niemcy, to był dwa razy zatrzymywany i dwa razy sprawdzano, czy samochód nie jest kradziony. To mnie osobiście dotykało. Pamiętam czasy, kiedy na austriackiej granicy była osobna kolejka dla Polaków, gdzie trzepali nas jak podejrzanych. I boję się, żeby to nie wróciło. Tak, jak w tej chwili, człowiek z polskim paszportem jest traktowany jak bia... jak każdy, jak Portugalczyk, Włoch, każdy inny, tak znów wyjdzie na to, że będziemy krajem drugiej, a nawet trzeciej kategorii. I właśnie, to nie jest najlepiej. Mam całe mnóstwo znajomych za granicą i nie chcę spotykając się z nimi, czy spotykając się z nimi, być traktowany paternalistycznie, z przymrużeniem oka.

**Co w tej chwili dzieje się z zakupami u was? Coś się zmieniło?**

W zasadzie... Mam ochotę robić zakupy. Myślę sobie na przykład o tym, żeby kupić jakieś fajne, drogie słuchawki... Mam ochotę sobie kupić jakieś takie, no nie wiem, czasem... Takie rzeczy dla przyjemności. Kupiłem sobie bardzo fajną, drogą kosiarkę do trawnika, żeby się nie męczyć z tym, tylko taką fajną, akumulatorową, z napędem, żeby to się szybciutko robiło. Koszę cały trawnik przez 15 minut, a wcześniej mi to zajmowało prawie godzinę. Taka, powiedzmy, przyjemność. Bo strasznie drogo ta kosiarka kosztowała. Ale pomyślałem sobie, a dlaczego nie? Na podróże pieniędzy nie wydaję, a wydawałem dużo, to przynajmniej na takie pierdoły sobie wydaję!

**Gdzie ją kupiłeś?**

A, w takim sklepie tutaj, ogrodniczym. Niedaleko od razu mają serwis, to nie będzie trzeba daleko jeździć. Tak po prostu, pomyślałem sobie, że można by było sobie kupić. Dlaczego mam szukać gdzieś dalej, skoro tutaj obok jest sklep. Gdzie mam jeździć, zastanawiać się? Kosztuje 2 000 zł, to zapłacę 2 000 zł. No proszę bardzooo, nie ma problemuu. No.

**Co jeszcze dla przyjemności kupiłeś?**

No właśnie mówię, chyba sobie zaraz kliknę i zamówię słuchawki. Podobają mi się, to sobie zamówię.

**Zamówisz, czy pojedziesz kupić stacjonarnie?**

Nie no, zamówię, no. Nie chce mi się. Teraz to przywiozą, albo do Paczkomatu wrzucą. Kosiarkę to trzeba obejrzeć, to jest troszkę co innego. Poza tym, nie wiem, co jeszcze kupię, wymyślam sobie. Mówiłem ci ostatnio, że zaczęliśmy też kupować towary lepszej jakości. Jakieś droższe sery, tak, czemu nie. To nadal się utrzymuje. Jeśli chodzi o sposób robienia zakupów - jak byłem dwa tygodnie na tej diecie, to nie robiłem zakupów spożywczych - miałem catering. Teraz pewnie znów zacznę - jakiś Selgros najbliższy. Teraz nie ma już takiego tematu, że trzeba stać w kolejce. W tych sklepach jest raczej luźno i nie ma kolejek, jak było jeszcze dwa tygodnie temu. Można swobodnie wejść do sklepu. Teraz raczej już bez listy... Choć może czasem z listą, bo to łatwiej. Ale nie mam jakoś specjalnych oporów przed chodzeniem do sklepów. Wracam do poprzednich zwyczajów. Wcześniej to, co mnie odstraszało, to te kolejki przed sklepami. Teraz już tego nie ma, więc wczoraj nawet - przypadkowo zresztą, bo musiałem pójść do apteki - pojechałem do apteki w galerii handlowej i się przeszedłem po galerii, jak wygląda. Niczego nie chciałem kupić, ale chciałem się przejść, z ciekawości zobaczyć, jak teraz wyglądają galerie handlowe. Połowa sklepów jest zamknięta, połowa otwarta. Może nawet więcej, niż połowa jest otwarta. No, ale bardzo dużo jest jeszcze zamkniętych. Nie wiem, dlatego, że nie mają pracowników, czy dlatego, że nie chcą. Albo może nie mają, czego sprzedawać, może za szybko im pozwolili? Bo skoro w środę ogłasza się decyzję i masz jeden dzień roboczy [była majówka] na przygotowanie się do otwarcia, to podejrzewam, że wiele tych placówek może nie być gotowe do otwarcia. Skoro pozwalniali pracowników, kto ma tam pracować? Muszą najpierw albo przyjąć ludzi z powrotem, albo nie wiem co.

**Co myślisz o tym, że galerie zostały otwarte?**

Uważam, że to był taki ruch przedwyborczy, żeby pokazać ludziom, że proszę, można pójść do galerii handlowej, to możecie głosować w wyborach. Wszystko robi się lepiej. Było to posunięcie czysto propagandowe. Poza tym jest jeszcze inna sprawa, została wcześniej wprowadzona ustawa, czy rozporządzenie dotyczące tego, ze najemcy w galeriach handlowych nie muszą płacić czynszu. Co prawdopodobnie skutkowałoby pozwami ze strony właścicieli galerii handlowych, przeciwko Skarbowi Państwa. Więc chcą te pozwy ograniczyć, mówią, proszę bardzo, galerie handlowe są otwarte. Więc oni ograniczają sobie ten okres, za jaki ewentualnie musieliby dopłacać, czy zwracać te niezapłacone czynsze. Ja jestem przekonany, że wszyscy ci galernicy będą składać pozwy. Bo, dlaczego nie? Druga rzecz, otwarcie przedszkoli jest po to, aby nie płacić zasiłków opiekuńczych. Rząd po prostu widzi, że zaczynają mu się kończyć pieniądze. Oczywiście znów ta decyzja to może być taki ruch przedwyborczy, a z drugiej strony widać, że im się kończą pieniądze. Z tym, że z tymi przedszkolami powiedzieli, że otwierają, jest opcja, aby się nie otworzyć, ale trzeba zawiadomić kuratorium, itd. A najpierw ogłosili, że zasiłki będą dalej przysługiwać, ale tylko tam, gdzie przedszkola będą dalej zamknięte. Potem zaczęli mówić, że nawet, jak przedszkole będzie otwarte, ale sam postanowisz nie posłać dziecka do przedszkola, będziesz dostawał zasiłek. Tylko to jest nieweryfikowalne, więc ja nie wiem, jak oni z tego wybrną. A prawnie sytuacja jest już totalnie skomplikowana, bo z jednej strony jest rozporządzenie, ale ono jest niezgodne z ustawą - na temat tych zasiłków. Więc bardzo możliwe, ze nawet ci, którym wypłacą te zasiłki, to później będą musieli te zasiłki zwracać. Taka jest sytuacja prawna. No bo skoro jest niezgodne z ustawą to sorry, no, ale ono jest nieważne.

**Wybierasz się w najbliższym czasie do galerii raz jeszcze?**

Nie, nie mam takiej potrzeby. Chyba, że będę musiał pojechać do apteki. Lubię tam chodzić, bo tam jest apteka, którą lubię i tam na ogół kupuję to, co muszę, oni raczej są dobrze zaopatrzeni. Tam lubię.

**Miałeś poczucie zagrożenia, będąc w galerii?**

Nie.

**Coś cię zaskoczyło, zdziwiło?**

Nie, po prostu przyjąłem do wiadomości, że połowa sklepów jest jeszcze zamknięta.

**Znasz ludzi, którzy czekali na otwarcie galerii?**

Nie, nie znam takich ludzi osobiście, ale wiem, że jest ich mnóstwo, bo na przykład w poniedziałek widziałem korki przed outletem na Annopolu. Pomyślałem, że jednak da większości brak galerii handlowych to jak brak powietrza. Nie mam dla tego zrozumienia, ale skoro tak jest, muszę to przyjąć do wiadomości. Takie mamy społeczeństwo.

**Gdybyś miał siebie określić 1-10 (1 = z trudem, 10 = z dużą łatwością wydaję pieniądze).**

Może 8 lub 9? Coś takiego. 8. Wcześniej, przed tą całą epidemią, jak miałem kupić jakąś drogą rzecz, zastanawiałem się długo, czy warto, czy dam sobie radę bez niej. A teraz mam ochotę, to kupuję. Zaczynam dochodzić do wniosku, że co mam sobie żałować.

**A gdybyś miał określić siebie przed pandemią?**

Wtedy powiedzmy 5-6, coś takiego.

**Opowiedz o tej zmianie, na czym ona polega.**

Wcześniej nigdy nie miałem oporów przed wydawaniem pieniędzy na przykład na podróże. Uważałem, że... Teraz nie mogę na to wydawać. To jest dla mnie jakiś taki Ersatz [surogat, namiastka]. I tyle.

**Dlaczego wcześniej zastanawiałeś się nad wydawaniem pieniędzy na rzeczy, choć na podróże wydawałeś chętnie?**

No bo taki jestem. Do takich rzeczy materialnych... Zawsze dyskutowaliśmy z żoną, ona na przykład chciała sobie kupić do domu jakąś kanapę. A ja mówiłem, że ta kanapa kosztuje tyle, co bilet lotniczy do Australii. Ja wolę wydać pieniądze na ten bilet, niż kupować kanapę. Teraz na bilet lotniczy do Australii i tak nie wydam, to już niech będzie ta kanapa. To nie jest tak, że my chcemy teraz kupić tę kanapę, to jest po prostu taki przykład. To jest takie, podaruj sobie odrobinę rozkoszy. Bardziej chodzi o to, żeby doświadczać czegoś.

**Czy to się tyczy też jedzenia? Czy to tak, że prędzej kupisz jakiś dobry ser, czy pójdziesz do knajpy, niż kupisz coś drogiego do ubrania, albo lampę?**

Tak, prędzej to pierwsze. Ja do ubrań nigdy nie przywiązywałem jakiejś większej wagi. Ale też nie będę kupował sobie gry, czy zmieniał telefonu, który mam od półtora roku i spełnia swoją funkcję. Słuchawki kupuję tylko dlatego, że mi przeszkadza trochę noszenie takich słuchawek dousznych, więc kupię nauszne, bo jest wygodniej.

**Rozrzutny, czy oszczędny?**

Raczej oszczędny. Staram się jednak jakoś kontrolować to, co wydaję. Staram się nie wydawać pieniędzy na skutek emocji. Tylko jednak bardzo długo zawsze przemyślę każdy zakup. Nie jest tak, żebym na przykład wszedł do galerii handlowej i zrobił zakupy, nie planując ich. Jeśli już ide do galerii to po to, aby coś kupić, bo wiem, że czegoś mi brakuje - koszuli, swetra, bo uważam, że nie mam, czy zniszczyło się. Zakupy pod wpływem impulsu to nie moja bajka.

**A jak było z tą kosiarką?**

W piątek przed długim weekendem chciałem odpalić kosiarkę, którą już mamy i po prostu jej nie odpaliłem. Mówię sobie, jest długi weekend, chciałoby się skosić trawnik. Która jest godzina? 16. Do 17 jest czynny sklep ogrodniczy, tutaj niedaleko. Wsiadłem w samochód i kupiłem kosiarkę. Planowałem ją kupić, ale zastanawiałem się, a może jeszcze nie trzeba, a może ta stara jeszcze pochodzi. Od roku chciałem ją kupić. A teraz ta stara nie działa. I tak naprawdę pomyślałem sobie z taką lekką ulgą "o, nie działa, teraz nie mam absolutnie żadnych obiekcji, aby kupić nową". Starą dałoby się naprawić, ale nie wiem, kiedy by się ją naprawiło, a trzeba skosić trawnik. Mógłbym pożyczyć od sąsiada, no, ale.. Ale kupię. Mogłem kupić kosiarkę za 600 zł. Ale kupiłem za 2 000 zł.

**Jak wygląda obecnie wasza sytuacja finansowa?**

Ona się absolutnie... Ona się pogorszyła, jeśli chodzi o firmę, bo przychodów mamy mniej, ale to nie jest tak, że nagle widzimy, ze musimy zacząć oszczędzać na takich drobnych rzeczach życiowych. Poza tym obawiam się też o dłuższą perspektywę dochodów. Jeżeli te głupie wytyczne pozostaną na dłużej, będziemy mieli problem z ty, jak będzie miało funkcjonować przedszkole. Z jeszcze innej strony, zdaję sobie sprawę z tego, że te wytyczne dotyczą nie tylko nas, a wszystkich przedszkoli. Więc, jeżeli dotyczą wszystkich przedszkoli, nawet publicznych, to nagle w Polsce pojawi się 1/3, czy 1/2 tych miejsc w przedszkolach, które były do tej pory, a co z resztą dzieci? One będą musiały gdzieś się podziać, czyli zwiększy się popyt na miejsca w przedszkolu. Czyli może będziemy mogli podnieść czesne? Bo skoro mogliśmy przyjąć X dzieci, a teraz będziemy mogli przyjąć 0,5 X, przy takich samych ponoszonych przez nas kosztach, to oczywiście czesne, które płaci jedno dziecko będzie musiało być dwukrotnie wyższe.

**Czyli jest pewien spadek przychodów związany z obecną sytuacją, ale on nie dotyczy was na bieżąco?**

Może nas dotyczyć, bo to wszystko nie zdarzy się od razu. Jeśli będziemy żądać jakiejś ceny, to muszą pojawić się tacy, którzy będą gotowi taką cenę zapłacić. Pytanie, czy tacy będą. Być może alternatywą dla wielu będzie po prostu zostanie w domu i nie pójście do pracy. Bo w przedszkolach publicznych tych miejsc też będzie mniej. Ja nie wiem, co się będzie działo. Uważam, że w tej chwili wprowadzili te durne wytyczne, a już teraz zastanawiają się, jak się z nich wycofać. Najpierw wprowadzili je zupełnie od czapy, nie myśląc, jakie będą skutki dla całego systemu oświaty przedszkolnej.

**Jeśli chodzi o wasz domowy budżet, czy coś się zmieniło?**

Absolutnie nie. Żyjemy, jak wcześniej. Chociaż, dwa dni temu usiedliśmy z żoną - przyszedł wyciąg z karty kredytowej. Skąd ta suma? Nigdzie nie wyjeżdżaliśmy, nigdzie specjalnie nie wydaliśmy więcej pieniędzy - wydaliśmy tyle, co zazwyczaj, to się nie zmniejszyło. Stwierdziliśmy, że tak jest i już. To było takie "skąd to się wzięło? O, uhm.".

**Jakie jest twoje podejście do oszczędzania?**

Uważam, że trzeba mieć oszczędności zawsze. I je mam.

**Macie jakiś system? Jak je gromadzicie?**

Mamy różne instrumenty. Ogólnie jest to gotówka, czasami w jakichś lokatach, czasami w walutach, czasami w jakichś funduszach. Staramy się to robić na różne sposoby. Odkładamy to, czego nie wydamy. To nie jest tak, że od razu zakładamy sobie, że coś odłożymy. Odkładamy to, co zostanie nam po wydaniu. To nie tak, że wydajemy tyle, ile nam zostanie po zaoszczędzeniu - raczej odwrotnie.

**Robicie to wspólnie, czy któreś z was ma większe skłonności do oszczędzania?**

Ja mam większe skłonności do oszczędzania i ja kontroluję te zasoby.

**Gdyby było tak, że zostajecie bez dochodów (...), jak długo moglibyście z takich oszczędności żyć?**

Hm, nie wiem. Długo. Kilka lat.

**Zdarzało wam się naruszać te oszczędności?**

Jeżeli tak, były to wydatki inwestycyjne, typu samochód, remont domu, czy postanowiliśmy na przykład zainwestować więcej w biznes. Ostatnio raczej nie było takich rzeczy.

**Tę skłonność do oszczędzania wypracowałeś, czy ona zawsze w tobie była?**

Chyba zawsze taką skłonność miałem. Od czasów, kiedy mogę sobie pozwolić na to, aby zaoszczędzić. Był okres, kiedy byliśmy na dorobku, kiedy miałem 30 parę lat. Wtedy budowaliśmy dom, otwieraliśmy firmę - wtedy wszystko szło na bieżąco, jakichś większych oszczędności nie było. Ale też starałem się unikać kredytu - mam do tego bardzo dużą awersję. I to zostaje. Mogliśmy wybudować trzy razy większy dom na kredyt, ale myślę, po co? Teraz też, moglibyśmy mieć piękniejszy dom, na większej działce, ale zastanawiam się, ale po co? Ten, który mamy, wystarcza nam w zupełności.

**W kontekście pandemii koronawirusa - warto oszczędzać?**

Wiesz co, nie wiem. To trudne pytanie. Nie myślałem o tym. Pytanie, jeżeli wydawać, to na co? Jak mówiłem, nie mamy w tej chwili żadnych ograniczeń, jeżeli chodzi o wydatki bieżące. To, na co moglibyśmy wydać, to wydatki typu inwestycyjnego - działkę kupić, czy coś. Cały czas mamy na przykład pieniądze, które są, bo chcemy rozwijać biznes. Cały czas myślimy o tym, żeby kupić działkę, zbudować nowy budynek szkoły, itd. To, że tego do tej pory nie zrobiliśmy to dlatego, że szukamy pasującej nieruchomości. Gdyby się taka trafiła, na pewno wydalibyśmy pieniądze. Czasami myślę o tym, że nie pojechałem gdzieś, gdzie mogłem, a teraz nie wiadomo, czy będę mógł pojechać. Ale to nie dlatego, że nie było mnie stać. Tak nigdy nie było. Jeśli gdzieś nie pojechałem to raczej dlatego, że nie miałem czasu. I w tym kontekście o tym myślę, a nie w kontekście, że mogłem wydać pieniądze na podróże, nie wydałem, a teraz te pieniądze nie wiem, kiedy będę mógł je wydać.

**Teraz jest dobry czas na inwestowanie?**

Uważam, że tak, że dobrze by było w tej chwili zainwestować. Chociaż jestem, jak to mówiła nauczycielka na polskim, bohaterem literackim rozdartym wewnętrznie. I nie wiem, czy to dobry czas na inwestycje. Wszystko zależy, jakie inwestycje. Nieruchomości być może tak, chociaż one być może stanieją  - nie wiadomo. Być może ludzi nie będzie stać na nieruchomości. A mogą też i zdrożeć, bo będzie inflacja, czy coś takiego. Też nie wiadomo. Wszystko bardziej zależy od tego. Jak mówię, gdyby trafiła się działka na budowę szkoły, to byśmy ją kupili. Ale to zupełnie abstrahując od tego czy to dobry, czy zły czas na inwestycje. To nie tak, że ja teraz zacząłem myśleć, aaa, może byśmy kupili dom na mazurach, bo może to być taniej, tak nie.

**Czyli trochę nie wiadomo, czy to dobry, czy zły czas na inwestycje?**

Uważam, że nie należy teraz zmieniać swoich planów, jeśli chodzi o inwestycje. Uważam, że w jakiś tam sposób ograniczanie, czy nagła chęć do inwestowania nie są dobre. Po prostu uważam, że nie powinno się zmieniać swoich jakiś tam celów.

**Jeśli chodzi o przyszłość, jak myślisz, ile to jeszcze potrwa?**

Dwa lata. Wszystko zależy od tego, jak będzie wyglądał poziom zachorowań. Prawdopodobnie trzeba poczekać, aż będzie jakaś szczepionka i wystarczająco dużo osób zostanie nią zaszczepiona. Dopiero wtedy będzie można myśleć o tym, żeby znów swobodnie podróżować, w miarę swobodnie się poruszać. Dlaczego mówię o podróżowaniu? To nie chodzi tylko o moją swobodę podróżowania. Wszystko, całe podróżowanie, to bardzo duża część gospodarki - weźmy pod uwagę hotele. Są całe kraje, których GBP zależy od turystyki. Dopóki nie będzie można podróżować, gospodarka światowa nie stanie na nogach. A będzie można podróżować dopiero wtedy, kiedy ludzie będą zdrowi, bezpieczni, nie będą przenosić chorób. Bardzo możliwe, że zostanie wprowadzone coś, co w tej chwili jest wprowadzone dla żółtej febry - jeśli chcesz podróżować do jakiegoś kraju, musisz mieć pieczątkę, że jesteś zaszczepiona przeciwko febrze. Bardzo możliwe, że zostanie wprowadzony paszport epidemiologiczny, że albo masz przeciwciała, albo szczepionkę. I dopiero wtedy wpuszczą cię do takiego, czy innego kraju. Ale to może zająć kilka lat. A nie wiadomo, czy w tym czasie wirus nie zmutuje. Czy to nie jest wirus taki, jak grypa, że co roku jest inny. Z drugiej strony też, w tej chwili ilość osób, które szczepią się na grypę, to 5%. Ja się szczepię co roku, ale szczepi się 5 czy 10%. A jeśli szczepi się tak mało osób, nie jesteśmy w stanie tej grypy zwalczyć. Jakby zaszczepiło się 100% osób, to bardzo możliwe, że grypa przestałaby istnieć. Może byłoby jak z ospą - ona istniała, dopóki nie zaszczepiono 100% populacji. To jest jedyny sposób, aby ospa zniknęła. Jeśli wszyscy będziemy się szczepić, ten wirus nie będzie mógł zagnieździć się w ciele człowieka. A że to jest cząstka DNA, nie będzie się dalej multiplikowało.

**A jeśli chodzi o twoje myśli o przyszłości, w perspektywie kilku tygodni?**

To ten biznes - przedszkole, szkoła. Kiedy będziemy mogli się normalnie otworzyć, zarabiać pieniądze. I czy będziemy mogli normalnie zarabiać pieniądze. To jest podstawowa rzecz, którą się zajmujemy. Poza tym, mam w tej chwili córkę maturzystkę. Nie wiem, jak będzie wyglądała rekrutacja na studia, czy ona będzie w ogóle mogła na te studia pójść, kiedy będzie mogła zacząć studiować. Mam syna, który pracuje na lotnisku. Też nie wiem, kiedy będzie mógł pójść do pracy, czy będzie w ogóle mógł pójść do pracy.

**On już rozgląda się za przebranżowieniem, czy czeka, co będzie?**

Czeka, co będzie. Zastanawia się nad tym, czy od października nie wrócić na studia. Ale tutaj też bardzo dużo zależy od tego, jak będzie wyglądała sytuacja, co się będzie działo, kiedy zaczną rekrutację. Uczelnie miały zacząć ją w maju, ale nie zaczęły, bo nie ma matur. Popatrzymy, jak dalej będzie. Syn co prawda mam maturę sprzed kilku lat, mógłby się rekrutować, złożyć gdzieś papiery, ale na razie nie ma jeszcze takiej możliwości.

**A córka jak odbiera sytuację?**

Mam wrażenie, że jest zadowolona, że nie musi już teraz pisać matury. Ale też zastanawiam się... Ja to byłbym wkurzony, chciałbym mieć to już z głowy. Ale jakoś tak ona chyba nie. Nie wiem.

**Masz wrażenie, że twoje dzieci radzą sobie dobrze?**

Mam wrażenie, że są lekko zdezorientowane i przygaszone. Rozmawiamy czasem na te temat, no ale. Mam wrażenie, że oni jakoś tak bardziej cool do tego podchodzą, niż ja. Do wszystkiego. Nawet nie śledzą tych informacji, tego, co się dzieje. Żyją trochę w oderwaniu od bieżącej sytuacji. Takie mam wrażenie. Co będzie, to będzie. Oni są w domu, jest komfort. To nie jest tak, że są na swoim własnym utrzymaniu. Gdyby Maurycy [syn] miał się sam utrzymać, gdyby kwestia zarabiania pieniędzy była dla niego kwestią przeżycia, pewnie inaczej by do tego podchodził. Tymczasem on pracował i wszystkie pieniądze praktycznie zostawały mu w kieszeni. Miał gdzie spać, lodówka była zawsze pełna. Nie było problemu. Zupełnie inaczej byłoby, gdyby on się z tego utrzymywał.
