# Supplementary material for: The challenges arising from the COVID-19 pandemic and the way people deal with them. A qualitative longitudinal study
Source: PLoS One. 2021 Oct 11;16(10):e0258133. doi: 10.1371/journal.pone.0258133 (PMC8504766; doi:10.1371/journal.pone.0258133)
Supplement: S1 Dataset — (ZIP) [file pone.0258133.s003.zip › Transcriptions/stage 4/6.4_M_24_couple, no children.docx]

**6.4_M_24_couple no children**

**Co się u ciebie działo przez ostatnie 2 tygodnie?**

W zasadzie tak samo. Cały czas nic się nie zmieniło. Nic za wiele nowego się nie wydarzyło oprócz tego, że urządziliśmy sypialnię do końca z dziewczyną. Także mieszkanie jest już w pełni do życia i w sypialni już można normalnie spać. Wcześniej mieliśmy salon i w salonie kanapę. Ta kanapa była wygodna przez pierwszy miesiąc, a później już była konieczność kupienia łóżka. Zamówiliśmy sobie nowe łóżko, materac i już jest fajnie.

**Właśnie widzę, że zmieniłeś lokalizację.**

Tak. Jestem w domu teraz rodzinnym.

**Coś robiliście specjalnego w majówkę?**

Byliśmy u znajomych. Na piwie tylko, ale nic konkretnego. W 1 dzień tylko - w niedzielę.

**To spotkanie ze znajomymi - nie mieliście wątpliwości, obaw?**

Nie. Już szczerze mówiąc po prostu wyszliśmy, wzięliśmy maseczki ze sobą, które i tak nic nie dają w zasadzie. Kolega zapewnił mnie, że on pracuje, ale ma swój warsztat, w którym jest sam, to jest jego firma. To jest kolega, o którym mówiłem, że nie wie, czy będzie zamykał tą firmę. Zapewnił mnie, że nie spotyka się ani z klientami ani nic, tylko siedzi w tym warsztacie, robi to, co trzeba, jeździ samochodem do domu bezpośrednio, więc funkcjonuje w ten sam sposób, co ja, chodząc do sklepu. Jego dziewczyna pracuje zdalnie, więc też siedzi w domu. Więc stwierdziłem, że ok, chodźmy i tyle.

**Oni byli inicjatorami tego?**

To chyba było tak, że chcieliśmy się przejść jakoś. Ja wcześniej byłem z tym kolegą nad Wisłą się przejść i się umawialiśmy na jakieś spotkanie w 4. Chcieliśmy się przejść nad Wisłę w 4, ale już było za późno, już było zimno i oni nas zaprosili do siebie, więc to wybraliśmy.

**Co się takiego wydarzyło, że stwierdziłeś, że możesz się spotykać ze znajomymi?**

Nie stwierdziłem, że mogę, ale nie wiem... Szczerze mówiąc, nie myślałem nad tym jakoś bardziej. Zdaję sobie sprawę, że to jest jakieś złamanie tego, co jest narzucone i może tego, co sam wcześniej mówiłem, ale nie wiem...

**To jest złamanie tych zasad w ogóle?**

Nie wiem w sumie. Nie jest to zgromadzenie, jakby nie patrzeć... Też nie wiem, jak jest zgromadzenie publiczne definiowane. Ale też jest mówione, żeby raczej zostawać w domach, nie wychodzić. A myśmy wyszli, poszliśmy do znajomych, bawiliśmy się dobrze.

**Planujesz teraz z większą częstotliwością się spotykać ze znajomymi?**

Nie, to było dość spontaniczne. Nie, nic takiego nie planuję. Ewentualnie może z tym kolegą jakieś piwo też, ale na pewno nie prędko.

**Obserwujesz u ludzi w otoczeniu większą chęć spotykania się?**

Tak. Ja teraz jestem w domu. Generalnie powiedział mi to tato, że on - jest lekarzem, więc myślę, że mogę mu zaufać - że jakoś bardzo nie widzi przeciwskazań, żebym przyjechał. Tata przyjechał po mnie do Warszawy samochodem. Nie jechałem transportem publicznym. Przyjechał tutaj ze mną też do Przemyśla. Powiedział, że tak naprawdę większe prawdopodobieństwo jest, że jeżeli miałbym zachorować, to zachorowałbym w Warszawie, bo tam jest więcej zachorowań niż w Przemyślu. Generalnie mówił, że nie widzi przeciwskazań i że śmiało mogę przyjechać.

**Przez jaki czas planujesz być u rodziców?**

Myślę, że ok. 1-1,5 tygodnia.

**Traktujesz to jako wakacje?**

Nie, po prostu spotkanie się z rodziną. Też trochę skorzystałem z propozycji. Bo to nie był mój pomysł. To była propozycja z ich strony. I w zasadzie, to skorzystałem.

**Twoja dziewczyna dalej pracuje z domu?**

Tak.

**A ona ma jakieś obawy przed spotykaniem się z ludźmi?**

Nie no, w zasadzie wtedy byłem z nią na tym spotkaniu. Także nie ma obaw. Miała iść do pracy 5 maja, miał być powrót do biur. Ale odwołali to i jeszcze ma miesiąc na *home office* spędzić. Mówiła, że to by nie było raczej... Że dobrze, że jest to *home office* i że jeszcze zostanie.

**A ty co o tym myślisz, że ludzie zaczynają wracać do firm?**

Myślę, że to trochę jednak za wcześnie. Na pewno takie otwarcie pełne kawiarni, teatrów, kin to jest za wcześnie.

**Tobie coś w tym momencie przeszkadza w tej sytuacji?**

Tak. To, że nie ma pełnej swobody. Mimo tego, że możemy wychodzić z domów, tylko musimy zachowywać odległość między sobą, nosić maseczki, co na pewno nie jest skuteczne do końca, to jakby są to jakieś ograniczenia dalej i dalej w pełni nie mamy swobody. Ja mimo wszystko nie czuję się w pełni wolny, że tak powiem. I nawet te obrazki, co ci wysyłałem, to miałem takie odniesienia do tego. Niby jest takie rozluźnienie sytuacji, ludzie się przyzwyczaili, że jest ten koronawirus, ale nie budzi to takiej grozy, jak na początku. Natomiast dalej nie ma w pełni wszystkich przywilejów, co były wcześniej i ja się osobiście jeszcze super swobodnie i wolno nie czuję. Np. wychodząc biegać ostatnio też wiedziałem, że muszę mieć maseczkę na twarzy, bo mogę dostać mandat, jakbym jej nie miał. Więc biegało mi się dość krępująco.

**Te maseczki nosisz ze względu na obawę przed mandatem?**

Teraz przed dostaniem kary, tak. Tak na dobrą sprawę, idąc do sklepu, chcąc zachować te 2 metry, mijając ludzi gdzieś w korytarzach, ja mogę to robić, ale nie zawsze inni ludzie się do tego stosują. I tak przechodzą nieraz obok mnie, ocierając się nawet. Więc moim zdanie mając tę maseczkę, czy jej nie mając i tak nic nie da. To jest takie pozorne działanie.

**To karanie ludzi jest słuszne?**

Słuszne z punktu widzenia prawa jest. Ale czy z moralnego punktu widzenia, to nie wiem. Chyba nie do końca. Nie wiem, jakie są kary za nienoszenie maseczek, ale myślę, że wlepianie komuś mandatu na parę tysięcy złotych za nienoszenie maseczki to jest przesada.

**Te obostrzenia powinny być już zdjęte?**

No w sumie z drugiej strony nie, jeszcze uważam, że nie. Bo mimo wszystko to jednak jest rozluźnienie, ale dalej nie ma tych zgromadzeń publicznych, wianków nad Wisłą, ludzie się nie gromadzą w jednym miejscu, nie ma protestów. Więc powiedzmy, że pod tym względem to jakoś działa. Ale... Także nie, jeszcze nie powinny być zdejmowane te ograniczenia.

**A kiedy?**

Nie wiem. Jeszcze nie, po prostu. Jeszcze za wcześnie.

**Obrazki. Znaleziony przez respondenta - "Cattle Decapitation. The atlas".**

Tu miałem taką myśl, że tam jest ten kościotrup z kosą i to może symbolizować śmierć. I ta płonąca planeta. Od razu mi się nasunęło na myśl, że śmierć zbiera żniwa, a świat płonie i dosłownie, bo mieliśmy te pożary. To była ta myśl i obrazek wydał mi się adekwatny do tych sytuacji.

**Jakie to budzi w tobie emocje, myśli?**

Że żyjemy w dość niespokojnych czasach i że człowiek ma ogromny wpływ na planetę, na środowisko i na to, co się dzieje dookoła niego. I że trzeba podejmować odpowiedzialne decyzje, żeby ograniczyć takie sytuacje. Bo tak naprawdę, te pożary są często przez głupotę ludzką.

**Czyli to się bardziej tyczy tych pożarów?**

Bardziej tak.

**A co można robić, żeby temu zapobiegać?**

Tym pożarom?

**Tej całej sytuacji, o której opowiadasz.**

Nie wiem szczerze mówiąc. Edukować chyba.

**Powiedziałeś, że człowiek ma wpływ. To można jakoś zapobiegać?**

Rzuciłem to bardziej ogólnie szczerze mówiąc. Nie przychodzi mi teraz do głowy, jak można globalnie działać, żeby zapobiec wszystkiemu.

**Następny obrazek - z leżącymi trupami.**

Ten sam zespół to jest. To jest album wcześniejszy. Ogólnie ten obrazek jest taki aż odpychający. Ta okładka jest obrzydliwa bym powiedział. Przynajmniej takie można mieć uczucie, jak się pierwszy raz na to patrzy. I takie obrzydzenie np. - może nie takie - ale obrzydzenie generalnie we mnie wywołuje to, że idąc biegać, czy gdzieś w ogóle idąc i to się też tyczy koronawirusa, że widzę, ile tych śmieci leży po rowach, krzakach, laskach - znacznie więcej niż przed pandemią. Mnie to naprawdę brzydzi i ja nigdy nie śmieciłem, więc wiem, że da się tego nie robić. To była ta myśl i emocja konkretna wywołana tym, czyli obrzydzenie.

**Czy zazwyczaj ty próbujesz ograniczać plastik czy inne działania prośrodowiskowe?**

Generalnie z tym plastikiem... To jest trudny temat troszkę. To nie jest czarno-białe i na to wpływa bardzo dużo czynników. Nie wiem, jakby ci to powiedzieć... Bo np. czy szkło jest lepsze niż plastik? Generalnie może być tak, że szkło da się łatwiej potem przetworzyć. Ale z drugiej strony, patrząc na to, jak się wytwarza szkło, to szkło potrzebuje znacznie więcej energii do jego wytworzenia. Żeby przewieźć szkło, trzeba zużyć więcej paliwa, a przez to emituje się więcej dwutlenku węgla. Jest dużo czynników, które wpływają na to wszystko i ciężko mi szczerze mówiąc...

**Rzuciłam tym, bo plastik wydaję mi się najbardziej kojarzący się z takimi zachowaniami.**

Ok. Ja ze swojej strony staram się robić, co mogę ograniczając śmieci. Znaczy, ograniczając śmieci... Segreguję śmieci, to na pewno. Jeśli chodzi o puste opakowania plastikowe, np. po jogurtach czy coś, to zawsze do zmywarki staram się dorzucić w wolne miejsce, żeby one się umyły, bo taki umyty plastik jest znacznie łatwiej przetworzyć. On nie musi być myty w tym miejscu przetwarzania. Jeżeli ja mam możliwość zrobienia tego, to próbuję to robić. Tak samo szkło, butelki po keczupie, słoiki, butelki staram się w zmywarce umieścić tak, żeby były czyste i oddać je do... Nie wiem, czy to w ogóle ma jakiś wpływ na to, czy jakby to ratuje jakoś środowisko, ale mam wrażenie, że może to jakoś pomóc. Więc, jeżeli mogę tak robić, to tak robię.

**Skoro jest pandemia, to może nie powinniśmy się przejmować tymi śmieciami, które leżą gdzieś tam? Może to ważniejsze, żeby ludzie używali jednorazowych rękawiczek, maseczek.**

No właśnie, no w tym momencie, ale patrząc tak ogólnie, to oczywiście, można tak postępować, że wyrzucasz śmieci gdzieś wokół domu, zakopujesz je i teraz to nie jest ważne. Ale ważne to będzie w ciągu tych iluś set lat, jak to się będzie rozkładało. To będzie zalegało i szczerze mówiąc, obserwując, ile tych śmieci jest, to ja się dziwie, jak myśmy jeszcze nie zatonęli i też nie wiem do końca, jak te przerabiarnie śmieci funkcjonują. Ale po sobie wiem, że produkując tyle odpadów na tydzień, wynoszę ileś tam worków - staram się oczywiście robić tego jak najmniej. Odpady bio do bio, itd. Dziwie się, że jak to jest możliwe, że z mojego jednego domu wychodzi tyle odpadów, od innych ludzi musi ich wychodzić jeszcze więcej i ja to wszystko jest przerabialne. Także uważam, że trzeba właśnie działać w ten sposób, żeby ograniczyć zużywanie energii.

**Na początku pamiętam, że mówiłeś, że skutki ekologiczne pandemii mogą być pozytywne.**

Tak. Bo wtedy nie było tylu tych śmieci szczerze mówiąc. Nie widziałem tego w ogóle, po prostu było normalnie. Mówiłem wtedy o samochodach, że nie wiem, czy ludzie będą używali mniej czy więcej samochodów. Teraz jest inaczej i to zdecydowanie widać, że tych śmieci trochę jest. Środowisko trochę cierpi. Jeśli chodzi o plastikowe rzeczy, to one degradują środowisko. Bardzo długo to zajmuje, ale główny problem jest taki, że to zalega. Nie mówię już o petach papierosów wyrzucanych gdzieś w trawę, bo tego totalnie nie rozumiem. Przecież to ma i metale ciężkie i inne związki. Za to się powinno karać moim zdaniem, bo to ma bardzo negatywne skutki.

**Następny obrazek - Autumn.**

Ok, to tu ten obrazek mi pasował w sumie do tego, że jesteśmy tacy bezsilni, bezradni wobec tego. jedyne, co możemy zrobić, to podejmować jakieś działań i czekać aż to przeminie albo jak ludzie to przechorują. Szczepionka to też jakieś działanie. A na razie jesteśmy jakoś ograniczenie.

**A to jest tak, że jak się zachoruje, to później się jest bardziej odpornym?**

Nie wiem. Czytałem, że chyba nie jest to potwierdzone naukowo, że osoba, która zachorowała wytwarza przeciwciała i nie zachoruje. Ale nie jest to udowodnione. Tak rzuciłem w sumie. Ale w kościach czuję, że jak bym zachorował, to mam mniejsze ryzyko zachorowania ponownie, tak samo jak na grypę.

**Czyli ta okładka to taka bezradność?**

Tak, bezradność, bezsilność. Nawet mógłbym to w kontekście trochę politycznym też powiedzieć. Te wybory, których ma nie być ostatecznie w niedzielę, to do końca chyba ludzie 2 dni temu nie wiedzieli na czym stoją i nic nie mogli zrobić tak naprawdę. Więc też taka bezradność, bezsilność wobec tego, co rządzący robią. Także myślę, że jest to adekwatne. A ten obrazek wybrałem, bo tam jest jakaś taka postać, która się tak skula, tak łapie za głowę, jakby nie wiedziała, co ma robić.

**Ty też jesteś w takim stanie, że nie wiesz, co masz robić?**

Nie, nie. Ja absolutnie. Aż tak nie.

**Kolejny obrazek - mózg w klatce.**

To mi się trochę skojarzyło z ograniczeniem swobody. Bo jak mózg jest ograniczony klatką, to ma ograniczoną swobodę działania, myślenia. Od razu mi się to skojarzyło z ograniczeniem swobody, wolności. Ale też może nakazywanie myślenia w konkretny sposób i tu odniesienie, że rząd pokazuje, że odmrażamy gospodarkę, więc jest bezpiecznie. Tak robimy, więc macie myśleć w ten sposób. Także tak mi się to skojarzyło. Mózg ograniczony może myśleć tylko w jeden sposób.

**Ty uważasz, że działania rządu są celowe - po to, żeby ludzie myśleli, że wszystko jest w porządku?**

Nie mówię, że tak jest. Ale dopuszczam to do możliwości. Ponieważ już często rząd stosował takie zagrywki, że np. nagłaśniał jakąś sprawę medialnie po to, żeby przepchnąć coś innego po cichu. Np. jakąś ustawę, o której nikt nie wie, którą się nikt nie będzie interesował, bo głośno jest np. o czarnym piątku. Także o to mi bardziej chodziło. Że w ten sposób mogą manipulować.

**To działa?**

Nie wiem szczerze mówiąc. Ja wśród swoich znajomych mam większość ludzi, którzy są zdecydowanie wkurzeni na to, jak Polska przez ostatnie lata wyglądała pod rządami PiSu i chcieliby to zmienić. Nie mam znajomych, którzy są przychylni partii rządzącej, więc ciężko mi powiedzieć. Nie wiem też, co myśli społeczeństwo. Nie śledzę statystyk i sondaży.

**Planowałeś iść na wybory?**

Tak. Jeżeli by się odbyły, to bym poszedł.

**Gdyby się odbyły w formie korespondencyjnej?**

To też, jak najbardziej bym wziął udział.

**Były różnego rodzaju obawy, jeśli chodzi o te korespondencyjne.**

Tak. O korespondencyjne, to chyba obawy były takie, że ludzie się bali fałszowania. Bo to będzie przechodziło z rąk do rąk, te koperty. No pewnie. Też mówiłem chyba, żeby się zabezpieczyć przed taką możliwością, to można byłoby sobie zrobić zdjęcie dowodu osobistego obok karty to głosowania. Ale też, no wybory mają być tajne. A samo to, że będą przechodzić przez tyle rąk, to zaprzecza jednej z tych 3 zasad, że wybory mają być powszechne, tajne i nie pamiętam jakie jeszcze.

**Następny obrazek. Weedpecker 2.**

Tu mi chodziło, że mamy taką perspektywę skierowaną ewidentnie na drogę jakąś, ścieżkę, która prowadzi dokądś i tam jest jakieś światło. Nie wiem, może jakaś nadzieja, ale generalnie nie wiemy, co jest na końcu tej drogi i jak to będzie wyglądało. Też taka niepewność, niewiedza, jak to się skończy, ta cała pandemia, ile osób zginie, ile osób zachoruje. Tak mi się nasunęło.

**Teraz śledzisz te statystyki zachorowań?**

Nie.

**Czemu?**

Nie wiem. Nie odczuwam potrzeby. Myślę, że to nic dodatkowego nie wniesie do mojego życia. Jeżeli byłby news jakiś, że pojawiła się szczepionka, to na pewno bym się tym zainteresował i dalej tego szukał. Ale nie odczuwam potrzeby, żeby tracić czas na to, żeby sprawdzać, ile osób zginęło. To są takie wiadomości, które nie napawają optymizmem. Prędzej już takie wiadomości, które mówią, że ileś osób wyzdrowiało. I gdzieś mi się pojawiają informacje, że ileś tysięcy osób wyzdrowiało w Polsce i to jest świetna wiadomość.

**Kolejny obrazek. Persistence of time.**

Tu chodziło mi o to, chyba też to połączyłem trochę z tytułem tego albumu - persistence of time, trwałość czasu - chyba tak to można przetłumaczyć. Tu mi chodziło o to, że mamy już 8 maja. Od początku pandemii w Polsce minęły już prawie 2 miesiące. I ten czas w sumie tak leci niby szybko, niby wolno. Z jednej strony szybko, bo mamy maj, a mam wrażenie, że dopiero co był marzec i początek tego wszystkiego. Mało tego, mam wrażenie, że dopiero się obroniłem, skończyłem studia, a to było w lutym, także ten czas leci bardzo szybko. A z drugiej, że leci wolno, dlatego, że mi się to trochę zlewało w taki ciąg. W jeden dzień - nie wiem, jakby to określić. I bywało monotonnie bardzo przez ten okres pandemii. I czy szybko, czy wolno, tak czy inaczej, wiedziałem, że ten czas jest, istnieje, daje o sobie znać, a nasze życie idzie do przodu, starzejemy się, itd. Dlatego może trwałość czasu. Jest tutaj zegar. A nawiasem mówiąc, teraz wpadłem na to, że anthrax to jest wąglik. Także taka niefortunna nazwa zespołu akurat.

**Masz poczucie tracenia tego czasu?**

Miewałem takie poczucie. Szczególnie, jeżeli chodzi o kwestie związane z pracą, że coraz więcej czasu mija od skończenia studiów, ja dalej nie mam pracy w zawodzie i zapominam coraz więcej po tych studiach. Chociażby w ten sposób tracę ten czas. Ale też poczucie, że nie chodzę do pracy, nie zarabiam pieniędzy, więc tracę czas. Ale z drugiej strony jestem produktywny na innych płaszczyznach w domu, czyli gram na gitarze, uczę się programowania, czytam książki, artykuły.

**Czyli ten czas dziwnie, inaczej płynął?**

Tak, dokładnie. A jeszcze tam ten jeden obrazek, ten z bezsilnością, to często miałem tak - łapałem się nieraz na tym podczas dni pandemii, że czułem taką bezsilność, bezradność, że nic mi się nie chce. Nie wiem, co tu ze sobą zrobić, czy się położyć spać, czy coś poczytać. Z jednej strony miałem ochotę coś robić, a z drugiej nie. I też dlatego się czułem bezsilnie, bezradnie, że nie wiedziałem co ze sobą zrobić. Trochę mogę to porównać do takiego uczucia, że czasem się budzisz w nocy i nie chce ci się spać, ale też nie chce ci się robić nic innego. Nie chce ci się oglądać, czytać. Tylko leżysz i się przewracasz z boku na bok. Mniej więcej takie uczucie mi towarzyszyło, miewałem takie dni.

**Teraz też tak miewasz?**

Ostatni raz takie coś czułem w zeszłym tygodniu na weekend, a teraz obecnie nie.

**Jak sobie próbujesz z tym radzić?**

Zazwyczaj chyba tracąc czas właśnie, czyli biorąc telefon do ręki i bezmyślnie *scrollując*, nie zwracając uwagi na to, co tam jest po to, żeby jakoś się zająć. Często mnie łapał taki stan wieczorem, więc zazwyczaj już się kładłem spać wtedy i zaśnięcie przychodziło mi dość łatwo z tego, co pamiętam. I już czekałem na następny dzień, więc tak sobie radziłem.

**Następny obrazek.**

To jest bardzo podobne do tego Weedpecker 2. Też jakaś droga, też gdzieś tam za górami się chowa ta ścieżka, czy nie wiem co to jest, rzeka? Też można podążać za tą rzeką i też nie wiadomo co tam do końca jest. Ten obrazek jest bardzo podobny do tamtego. One są zbiorcze.

**Następny obrazek.**

To nie jest okładka. To znalazłem po prostu w Internecie, wpisując chyba teorie spiskowe. Bo śmieszy mnie... Zastanawia mnie, że ludzie jeszcze w dobie... Chodzi mi generalnie o teorie spiskowe i dorabianie sobie różnych takich historii, że np. koronawirus jest dlatego, żeby Chińczycy stawiali maszty 5G, żeby wpłynąć na umysły. To jest takie straszne wymyślanie moim zdaniem.

**Gdzie to usłyszałeś?**

Gdzieś na YouTube, ktoś udostępnił coś na Facebooku też.

**Skąd ludzie coś takiego biorą?**

Z łańcuszków internetowych głównie. I z takich grup szemranych na Facebooku, bo tam się dużo takich rzeczy rodzi. Sam jestem w grupie na Facebooku Chemtrails i coś tam porozmawiajmy normalnie. Ktoś mnie tam dodał kiedyś, jakaś znajoma i tam jestem. Nie jest to jakoś bardzo aktywna grupa, ale czasami coś wrzucają.

**To jest grupa osób, które wierzą w Chemtrails?**

Powiem ci, że kompletnie się tym nie interesowałem. Dosłownie raz obejrzałem filmik, że ktoś wrzucił, że tutaj są dowody na Chemtrails, oczywiście filmik z YouTube. Że ktoś tam pokazał sadzę na samochodzie, że gdzieś przeleciał samolot i to są w zasadzie całe dowody.

**Czemu ludzie w to wierzą z tym koronawirusem?**

Myślę, że ludzie lubią teorie spiskowe, bo to jest ciekawe. Jest aura tajemniczości i lubią sobie podyskutować na takie tematy. Bo łatwiej sobie różne rzeczy dopowiadać, wymyślać może, zamiast skupiać się na twardych faktach, na dowodach naukowych, który często są przedstawione w sposób naukowy raczej trudny w odbiorze dla takiego zjadacza chleba.

**Czyli to jest łatwiejsze i ciekawsze?**

Tak. Dlatego ludzie w to wierzą.

Na tym obrazku była chyba piramida z tym okiem - Illuminati i reptilianie. To też jest ciekawa teoria spiskowa, że światem rządzą reptilianie.

**Jest jakaś teoria reptilian i koronawirusa?**

Chyba nie.

**Może, jakby poszukać, to udałoby się znaleźć.**

Może tak. Chociaż coś chyba widziałem, że się dogadują z Chińczykami, ale nie chcę też wymyślać tutaj przykładów.

**Jakie emocje widzisz wśród bliskich?**

Już takie trochę znudzenie. Nie wiem, czy mogę to za znudzenie uznać. Wczoraj rozmawiałem trochę z ojcem podczas drogi, to mówił właśnie a propos maseczek, że to jest kretyński pomysł, bo one nic nie dają i mało tego stwarzają środowisko dla patogenów i ułatwiają rozwój dla jakichś chorób. Plus, te maseczki mogą stwarzać takie pozorne poczucie bezpieczeństwa, dzięki któremu zaniedbujemy inne rzeczy, jak np. mycie rąk. To też trochę było tak, że Polska nie jest pierwszym krajem, która wprowadziła te maseczki. I to jest trochę tak, że na arenie międzynarodowej chcieliśmy być równi. W innych krajach jest wprowadzone, to u nas też, bo będą coś tam mówić. Mówię, nie że tak jest, tylko dopuszczam możliwość z jakimś prawdopodobieństwem.

**Jak u ciebie wyglądają zakupy obecnie? Coś się zmieniło?**

Nie. Cały czas tak samo, z listą do sklepu. Zakupy mniej więcej na 4 dni. Te zakupy na 4 dni i tak wychodzą na co tydzień, więc chodziliśmy zazwyczaj co tydzień. Oprócz tego dość sporo zamawiałem ostatnio z Allegro, więc...

**Co zamawiałeś?**

Łóżko, lakier bezbarwny do łóżka, pędzel, termometr do pokoju, karmę dla kotów. Coś jeszcze zamawiałem, ale takie rzeczy do domu raczej.

**Kupiłeś coś ostatnio dla przyjemności?**

Nie. Jedną rzecz kupiłem, ale to już wcześniej mówiłem. Książkę Dawida Myśliwca "Przepis na człowieka". Ale to tak po prostu, zadziałałem pod wpływem chwili, że chyba obejrzałem jakiś jego film, on polecił tę książkę i wtedy typowo dla siebie ją kupiłem.

**Co sądzisz o otwarciu galerii handlowych?**

Nie wiem w sumie. Nie wyobrażam sobie, żeby w galeriach handlowych sklepy miały działać tak, że coś podają na wynos, jak McDonald ‘s przez okienko albo w kiosku. Myślę, że to jest jeszcze za wcześnie.

**Planujesz zakupy stacjonarne inne niż dotychczas?**

Tak. Chciałbym sobie kupić buty do biegania, więc może będę musiał się wybrać do takiego sklepu, żeby kupić te buty. Myślę, że problem tu leży w tym, że taki sklep sobie działa przez 8 godzin, to może tam wchodzić wielu ludzi, dotykać przedmiotów i zostawiać tam swój ślad. I w ten sposób uważam, że nie jest to do końca dobre, żeby sobie działało bez ograniczeń. Bo wątpię, żeby kasjer, sprzedawca latał ze szmatką i płynem do dezynfekcji po każdym człowieku, który wszedł do sklepu. Więc pod tym względem myślę, że dotykanie takich rzeczy może nie być dobre po prostu.

**Przedszkola i żłobki mają się też otworzyć.**

Tak. I do żłobków można zapisywać swoje dzieci, ale dobrowolnie, to nie jest przymus. Ok, przyjmuję to na klatę, że tak powiem, ale też uważam, że jeszcze za wcześnie. Bo właśnie przedszkola i żłobki to są wylęgarnie różnych chorób. Bardzo często dziecko coś przynosi do domu i od tego choruje cała rodzina, więc nie wiem czy to dobry pomysł.

**To bieganie też się u ciebie jakoś teraz pojawiło?**

Tak. No zacząłem w końcu ćwiczyć. Ale okazało się właśnie, że mam złe buty do biegania. Dzisiaj mi to mama powiedziała i dlatego chcę kupić nowe. Ale byłem, póki co, dwa razy, a poza tym, to robię jeszcze te treningi brzuszków, pompek, więc jest fajnie. Lepiej się czuję.

**Maseczka przeszkadza podczas biegania, ale mimo wszystko będziesz w niej biegał?**

To jest tak. Ja tam sobie wychodzę na taki wał przy Wiśle. On jest na Białołęce, tam jest ścieżka rowerowa. On jest bardzo długi, można długo pobiec. To, jak widzę, że nikogo nie ma w pobliżu mnie, to zazwyczaj staram się ją odsunąć tak, żeby chociażby nos było mi widać, bo wtedy się łatwiej oddycha. Ale jak np. dobiegam do jakiegoś miejsca, które może być bardziej zaludnione, to zakładam. Albo, jak widzę kogoś, że się minę, to na wszelki wypadek ją zakładam. Po prostu jakoś. też to nic nie daje oczywiście, ale może też, żeby tej osobie zapewnić większy komfort. Może trochę odruchowo, nie wiem.

**Skala wydawania pieniędzy.**

Nie wiem w sumie, bo zależy na co. Jak np. jestem na festiwalach albo koncertach i wtedy zazwyczaj mam jakiś budżet, który wiem, że mogę wydać. Kilkaset zł powiedzmy. Nastawiam się, że kupię sobie jakąś koszulkę albo będę pił piwa i nie będę miał żadnego umiaru, to wydaję dość łatwo. Ale jeżeli chodzi np. o jedzenie, to zdarzało mi się robić tak, że też dość łatwo wydawałem, np. takie zamawiane. Często nie miałem czasu nic zrobić, bo miałem tyle nauki, że nie było fizycznie jak tego zrobić. Więc zamawiałem sobie coś w 1,2,3 dzień. Łatwo mi to przychodziło i jednocześnie też z trudem, bo miałem wyrzuty sumienia, że wydaję 3-ci dzień z rzędu na takie zamawiane. Ale z drugiej strony usprawiedliwiałem się tym, że jestem głodny i nie mam, jak zrobić jedzenia, więc z drugiej strony łatwo. Na ubrania aż tak w sumie nie wydaję pieniędzy. Muszę przyznać, że dużo ubrań finansowała mi albo mama, albo babcia. Aż tak sam z siebie często nie kupowałem. Bardziej takie koszulki zespołowe, czy coś. Na takie, które mi się podobają, to łatwo. Ale już na takie, które mi się podobają, ale kosztują 200 ileś zł, trudno. Na co jeszcze? Hmm... Na sprzęt komputerowy teraz mi się będzie łatwo wydawało, bo coś tam mam odłożone. Ale część dostanę dofinansowania od mamy za moją obronę. Ale tą moją część mi się łatwo wyda, bo wiem, że komputer mi jest potrzebny, mój własny, nowy.

**Póki co na wszystko całkiem łatwo.**

No... Jeszcze tak sobie myślę. Teraz generalnie na jedzenie zamawiane jest mi trudno wydać. Dużo gotujemy. Bardzo dużo, praktycznie cały czas. Tam, jak kiedyś rozmawialiśmy, to mówiłem, że zamawiałem pizzę tydzień temu. Teraz w ogóle nie zamawiamy praktycznie. Może raz w ciągu miesiąca. Teraz trudno by mi się wydawało na jedzenie zamawiane, bo chodzimy na te zakupy co tydzień, robimy sobie specjalnie listy, żeby jakoś to funkcjonowało i nie odczuwam takiej potrzeby. Chyba tyle.

**Pamiętasz jakiś ostatni większy zakup?**

No tak, na łóżko. Trudno mi się trochę wydawało tyle pieniędzy na to łóżko, bo kosztowało nas łącznie 1200 zł. Ja od siebie musiałem dodać 600 zł. Wydało mi się je z ciężkim sercem, bo wiem, że można by było znaleźć taniej. Ale z drugiej strony pomyślałem, że na takich rzeczach się raczej nie oszczędza, bo to też ma służyć temu, żebym się wysypiał, miał zdrowe plecy, itd. Na początku, jak moja dziewczyna mi zaproponowała, że może taki materac, taki stelaż, to powiedziałem, że może coś innego. Ale ostatecznie dogadaliśmy się i tak.

**Ty chciałeś kupić coś tańszego?**

Tak.

**A czemu się zdecydowaliście na droższe?**

Bo chyba przeczytaliśmy jakąś opinię dobrą. Poza tym, myśmy na początku chcieli zrobić... O, wiem, dlaczego. Bo ja na początku chciałem zrobić łóżko z europalet, takich tanich, jak możesz kupić na skupie za kilka złotych prawdopodobnie. I wiedziałem, że by mnie to wyniosło już nie 470 za samo łóżko, tylko góra stówę. I dlatego mi się ciężko wydało tą kasę na to łóżko. Ale potem moja dziewczyna uświadomiła mi, że żeby zrobić takie łóżko z tanich europalet, to trzeba by było kupić sprzęt, żeby to pomalować, oszlifować, stracić na to czas, pomalować to. Trochę roboty by przy tym było i uświadomiła mi, że lepiej wziąć już to łóżko, na które się zdecydowaliśmy, ale wydać więcej.

**Czyli ty chciałeś to zrobić z europalet dlatego, że tak było taniej?**

Tak. I jak mi pokazała to łóżko droższe, to wtedy stwierdziłem, że trudno mi by było wydać te pieniądze. Ale jak mi uświadomiła, że trzeba by było te europalety ze skupu jakoś ogarnąć, to stwierdziłem, że ok, że lepiej w ten sposób to zrobić.

**Coś się zmieniło w kwestii twojej pracy?**

Zmieniło się, tak. Jeszcze nie, ale dostałem zaproszenie na rozmowę rekrutacyjną w przyszłym tygodniu w czwartek do oczyszczalni ścieków "Czajka". Jako laborant, analityk w laboratorium. Pani zadzwoniła do mnie 2 dni temu, więc bardzo się cieszyłem z tego, że w końcu coś ruszyło i mam nadzieję, że się uda, bo bardzo chciałbym już się zawodowo jakoś... Zobaczyć przynajmniej czy warto było studiować ten kierunek. I czy chce w tym dalej trwać. I po prostu chciałbym już mieć jakąś pracę i dochód. Jestem na tym przestoju teraz i dostaję pieniądze, ale nie za wiele. Lepszy rydz niż nic, ale...

**O ile ci obniżono pensję?**

To nie jest tak, że mi obniżono, tylko ja niestety podpisałem umowę taką, że pracuję na 3/8 etatu, bo jeszcze studiowałem wtedy i nie zdążyłem tej umowy przepisać. W sumie nawet nie wiem, czy chciałem ją przepisać na pełny etat, bo po prostu stwierdziłem, że umowę mam taką, ale chodzę do pracy więcej i zarabiam więcej pieniędzy. Więc na umowie mam tyle. Nie przypuszczałem, że będzie koronawirus, pandemia i jest jak jest. Gdybym taką umowę podpisał, to bym dostawał pełen etat i dostawałbym te pieniądze, nie chodząc do pracy.

**Czyli oficjalnie na umowie masz tyle samo, ale dostajesz mniej pieniędzy?**

Chociaż faktycznie chyba jest tak, że dostaje 20% mniej. Tak, bo dzisiaj dostałem 100 zł mniej wypłaty. Więc jest też zmiana.

**Ta zmiana w zarobkach jest zagrożeniem dla waszego budżetu domowego?**

W zasadzie teraz nie, bo nie mamy aż tyle wydatków. Nie chodzimy gdzieś, nie chodzimy do kina, do restauracji, na koncerty, itd. Nie wyjeżdżamy na zorganizowane wyjazdy, więc nie mamy aż tyle wydatków. Chociaż trochę też jest na to łóżko np. czy inne takie potrzebne rzeczy. Ja też dostaję to wsparcie od rodziców i też dlatego bardzo chciałbym już pracować, żeby już nie mieć tego wsparcia, żeby się uniezależnić. Generalnie, jeżeli chodzi o sytuację finansową, to jest cały czas tak samo. Do tej pory, jak studiowałem, to przynosiłem pieniądze z pracy, tyle, ile mogłem.

**Wiem, że pewnie nie traktujesz pieniędzy od rodziców jako dochodu...**

Nie, absolutnie.

**Ale jednak zasila to twój budżet. Czy ty ten budżet kontrolujesz? Spisujesz wydatki?**

Odkładam sobie pieniądze na konto oszczędnościowe. Oprócz tego... Czy spisuje wydatki? W jednym miesiącu pamiętam, że obliczałem wydatki i doszła mi tam jakaś kwota, ale często właśnie coś dokupywaliśmy, jakaś roleta, itd. i to ten budżet jakoś zaburzało, więc przestałem te wydatki spisywać.

**To jest tak, że od rodziców dostajesz jakąś stałą kwotę?**

Tak, stałą kwotę.

**Nazwałbyś się osobą raczej oszczędną czy rozrzutną?**

Oj nie, rozrzutną to na pewno nie. Bo jeżeli przychodzi do kupowania chociażby kosmetyków czy perfum, to ja nie odczuwam potrzeby, żeby brać coś z najwyższej półki. Czy jakieś inne rzeczy, np. narzędzia, tabletki do zmywarki np. Jak wiem, że są jakieś tabletki za 50 zł i są jakieś za 15, to raczej wezmę te za 15, bo też będą działały, myły i w ten sposób. Raczej umiarkowanie wydającą pieniądze. Normalnie. Ale też, czy oszczędnie? No nie jem makaronu z keczupem. A wiem, że takie osoby są, które funkcjonowały w ten sposób wśród moich znajomych na studiach np. Że cały czas kupowały coś z Biedronki, jakieś *lasagne*, itd. byle tylko po taniości. No ok, ma się wtedy więcej pieniędzy, ale kosztem zdrowia, bo takie jedzenie jest bardzo przetworzone chemicznie i nie chcę oszczędzać w ten sposób np. Miałem na pewno momenty rozrzutności, chociażby z takim jedzeniem zamawianym. Ale mogłem je usprawiedliwić czasami tym, że nie miałem czasu czegoś przygotować.

Wychodzę z założenia, że coś ma być przede wszystkim praktyczne i ma mi się podobać po prostu. Na szczęście nie jestem osobą, która przepada za jakimiś markami Gucci czy coś takiego. Nie odczuwam potrzeby, żeby się tak ubierać, żeby nosić złote zegarki, łańcuszki, no nie, po prostu nie.

**To dotyczy wszystkich obszarów zakupowych? Masz jakieś rzeczy, na które możesz wydać więcej pieniędzy?**

Na okulary wydałem trochę więcej. Miałem możliwość kupienia tańszych, ale wybrałem akurat takie. Te oprawki były dość drogie, bo 600 zł. Są z *Emporio Armani* i jak moja mama zobaczyła tą markę to się bardzo zaśmiała. A ja po prostu kupiłem, bo mi się bardzo podobały. Nie miałem potrzeby akurat tutaj oszczędzania. Wziąłem, bo mi się podobały, ale też nie z myślą, że to jest świetna marka i dlatego ją biorę. A i wyjazdy. Na wyjazdach też mam jakieś pieniądze odłożone i wiadomo, w ramach zdrowego rozsądku, trzeba przeznaczyć tyle pieniędzy na 1 dzień, żeby w następnym było. Ale generalnie tyle tych pieniędzy przeznaczam, że nie jest tak, że muszę nie pójść, nie wejść gdzieś, bo nie mam pieniędzy. Jeżeli chodzi o wyjazdy, to też zazwyczaj... Ale też nie wiem, czy to można nazwać rozrzutnością, a nie takim wiedzeniem, że mogę sobie na to pozwolić.

**Na wyjazdach wydajesz inaczej niż na co dzień?**

Tak, inaczej. Nie mam na pewno wyrzutów sumienia, nawet jak zdarza się za coś przepłacić, bo pójdę na drinka w centrum miasta, w centrum Budapesztu, to nie będzie mnie to bolało, że wydam ileś. Byle mi starczyło do dalszego funkcjonowania. A tak to nie.

**A na co dzień, nie na wakacjach, zdarza ci się, że masz wyrzuty sumienia, jak zapłacisz więcej?**

Zdarza mi się, jak przepłacę za coś, a nie musiałem tego robić, a zdarzało mi się też tak, że zamówiłem coś przez Internet z jednej strony, a potem znalazłem na innej taniej. I wtedy mam pretensje do siebie, że mogłem poszukać trochę dłużej i zapłacić mniej. Jak coś mi się zdarza zniszczyć bardzo szybko, np. przez przypadek, to wtedy mam wyrzuty sumienia.

**Wspomniałeś trochę o tym oszczędzaniu, że masz konto oszczędnościowe. Też nie wiem, czy z dziewczyną macie wspólny budżet.**

Tak, mamy.

**I to konto macie wspólne czy to jest indywidualne twoje?**

Ja mam jedno swoje oszczędnościowe, mamy też wspólne oszczędnościowe i mamy wspólne, z którego korzystamy na co dzień. Co miesiąc przelewamy na to wspólne i z niego opłacamy rzeczy typu jakaś chemia do domu, zakupy, jakieś pizzę, burgery, itd. Wspólne konto oszczędnościowe działa od niedawna, bo jakoś pół roku i też umówiliśmy się, że przelewamy co miesiąc jakąś kwotę i już się tam uzbierało i powiedzieliśmy sobie, że z tego konta skorzystamy, jak trzeba będzie iść na jakieś wesele. Bo miały być 3 wesela w tym roku, a wesele też zazwyczaj trochę kosztuje. Tzn. wypada przynajmniej kilkaset zł komuś dać. A że nie będzie wesel, mam nadzieję, to te pieniądze tam są i jeszcze będą na przyszłość.

**Masz jakiś pomysł po co ci mogłyby być oszczędności?**

Tak. Na nowy komputer, jakbym chciał sobie kiedyś zmodernizować. Na jakiś kurs językowy. Bardzo bym chciał nauczyć się niemieckiego dobrze, rosyjskiego i wiem, że najefektywniej jest, jak się idzie na taki kurs i ma się takiego "bata" nad sobą. Także na jakieś egzaminy, tego typu rzeczy, to te pieniądze są. Chciałbym mieć też tatuaż nowy. Gitarę chciałbym sobie kupić kiedyś elektryczną taką swoją. Nie muszę zaczynać od jakiejś wyższej półki, byleby była na początku. Ale to też jest wydatek. Samochód, zdecydowanie samochód też. Ale to samochód byłby bardziej wspólny, więc powiedzmy, że ze wspólnego konta oszczędnościowego.

**Te oszczędności są po to, żeby spełnić jakieś marzenia?**

Tak, takie zachcianki.

**Czy masz taką myśl, że potrzebowałbyś oszczędności po to, żeby mieć większe poczucie bezpieczeństwa?**

Jasne, też myślę o tym w ten sposób. Tylko, że jeszcze nie zarabiam własnych pieniędzy. Wiem, że takie oszczędności będą potrzebne, jak najbardziej. Zacznę się tym interesować, jak już będę miał swoje pieniądze konkretne.

**Ile trzeba by zarabiać, żeby mieć zabezpieczenie?**

Zabezpieczenie plus, żeby odkładać na coś dla siebie i jeszcze mieć na wydatki codzienne?

**Tak.**

W Warszawie myślę, że miesięcznie trzeba by było zarabiać 6 tys zł na rękę, żeby mieć godne warunki życia, czyli takie, jak teraz określiłem, bo uważam, że to są takie naprawdę godne. Możesz sobie pozwolić na kino, na teatr, na restaurację, na odłożenie tych pieniędzy, na kupienie jakiegoś ubrania niekoniecznie już z tej niższej półki, tylko to, co ci się podoba i jest droższe i nie masz takiego wyrzutu sumienia, że muszę tyle wydać, to wezmę coś tańszego. Uważam to za naprawdę godne życie. Jakiś wyjazd też na narty, na deskę.

**Wy wynajmujecie to mieszkanie, w którym teraz mieszkacie?**

Tak.

**Myślicie o tym, żeby mieć własne kiedyś?**

Tak, myślimy, ale nie w Warszawie.

**Dlaczego?**

W Trójmieście, tam byśmy chcieli się udać, ale zobaczymy, jak wyjdzie. Moja dziewczyna jest bardzo zafascynowana statkami, portem, itd. Ma wykształcenie logistyczno-celne i chciałaby się załapać gdzieś do pracy w porcie, tak, żeby się pracować z transportem morskim. A jeżeli ma to robić, to właśnie tam najlepiej. Plus mi się bardzo Trójmiasto podoba i jej też. Ja np. jeżeli chodzi o moje hobby, czyli windsurfing, to chciałbym to hobby rozwijać i wiem, że jak miałbym to rozwijać, to najlepiej właśnie nad morzem. A jeżeli chodzi o sprawy zawodowe, to widzę po Warszawie, że nie ma aż tak dużo ofert wcale, jeżeli chodzi o moją branżę. I myślę, że w Trójmieście byłoby troszkę łatwiej.

**Skoro pojawia się możliwość pracy w zawodzie, to jest tak, że odkładasz ten plan z przebranżowieniem się?**

Nie. To jest tak. Plan był taki, żebym znalazł pracę, zdobył doświadczenie tutaj tak właśnie z rok i wtedy się przenieść nad morze. I pracując, zdecydowałbym na co chcę iść dalej na studia. Tak aż nie myślałem o tym programowaniu. Myślałem, że bardziej bym poszedł do pracy w zawodzie i zobaczył, gdzie są braki na rynku. Ktoś by mi coś podpowiedział, że może idź w tym kierunku albo ta gałęź chemii jest najbardziej opłacalna. Mam pomysł, jaka może być najbardziej opłacalna, ale... Znaczy pożądana, do której warto iść, ale nie widzę ofert tutaj w Warszawie. Chodzi o przetwórstwo tworzyw sztucznych. Czyli właśnie wytwarzanie opakowań, folii, tego typu rzeczy. Bo jest bardzo dużo innych, mnóstwo tego jest. I polimery właśnie są potrzebne, wszędzie dookoła siebie mamy polimery. Myślałem, żeby tam uderzać, ale z drugiej strony tak sobie pomyślałem, że chyba nie chce w tą chemię tak brnąć mocno, bo nie chcę ani na uczelni zostać, ani nie pracuję w zawodzie, więc nie wiem, czy mi się to spodoba. Pomyślałem, że jak pójdę do pracy w zawodzie, to zobaczę, gdzie bym chciał iść na studia. Tak pomyślałem o tym programowaniu, bo z tego są pieniądze i najlepiej, żeby to połączyć jakoś z tym, co miałem na studiach. Czyli jakieś modelowanie syntez chemicznych. Nie wiem, muszę się jeszcze rozejrzeć.

**Czy w waszym związku jest tak, że któreś z was jest bardziej inicjujące oszczędzanie?**

Nie, myślę, że nie. Po prostu umówiliśmy się jakoś i obydwoje się tego trzymamy. Jesteśmy systematyczni, przelewamy na to wspólne konto. Więc nie trzeba nic inicjować, to jakoś wyszło tak samo z siebie. Często moja dziewczyna jest inicjatorem różnych wydatków, takich, jeżeli chodzi o dom. Jakieś rolety np. to ona przeważnie o tym myśli. Znaczy ja też o tym myślę, ale ona wybiera zazwyczaj pod kątem wizualnym i przy tym zazwyczaj zostajemy. Ale jeżeli chodzi o oszczędności, to się wspólnie dogadujemy.

**Czy ty myślisz o tym, kiedy ta sytuacja się skończy?**

Nie. Nie wiem, kiedy ta pandemia ustanie, nie mam pojęcia. Myślę, że będzie coraz większe rozluźnienie, bo nadchodzi lato i późniejszy etap wiosny. I ja sam temu rozluźnieniu uległem trochę też. Nawet nie ze swojej inicjatywy, tylko moich rodziców. Na początku ja sam stwierdziłem, że lepiej zostać w Warszawie, ale jak już mi tam zaczęli mówić, no przyjedź, to powiedziałem no dobra. Mam wrażenie, że takie rozluźnienie będzie postępowało, ale kiedy to się skończy, to nie mam pojęcia.

**Twoja mama ma też podejście, jak ty i tata?**

Też, luźne raczej. Ona już mówiła, że aż tak się tym nie przejmuje. I też, nie wiem, co o tym myśleć do końca. Miałem takie różne wyrzuty sumienia, że to jest niestosowanie się do zasad, że możemy tym komuś zaszkodzić. Ale z drugiej strony przedsięwzięliśmy wszelkie kroki, żeby nie spotykać się z innymi ludźmi faktycznie, żeby to jakoś ograniczyć. Więc zaczyna się kombinowanie generalnie, żeby to troszkę obchodzić i jakoś zacząć normalnie funkcjonować i myślę, że tak będzie coraz bardziej. Moja mama raczej luźno podchodzi do tego wszystkiego, mój tato też.

**Masz jakieś obawy, związane z przyszłością?**

Obawy mogę mieć takie, że ewentualnie umrze więcej ludzi. Jeżeli chodzi o moje zachorowanie, to nie wiem. Myślę, że jakbym zachorował, to nie przebiegało by to super trudno. Myślę, że bym przechorował coś takiego. Nigdy nie chorowałem jakoś specjalnie i nie mam żadnych chorób poważnych współtowarzyszących. Myślę, że ja sam bym to przebył bezboleśnie. Jeżeli chodzi o przyszłość, to o swoją zawodową - jest trochę jaśniejsza, bo bardzo mnie ta rozmowa... No, jeszcze jej nie było, ale samo to, że dostałem zaproszenie, to jest fajnie. A jak to będzie dalej wyglądało, to ciężko powiedzieć. Bo wiem, że w historii ludzkości choroby trwały nawet latami. Hiszpanka trwała 2 lata, SARS w Japonii kilka miesięcy. Ciężko powiedzieć. To jest ta niepewna droga, o której mówiłem a propos obrazków. Nie wiadomo, jaka jest ta przyszłość, jaka będzie, kiedy będzie koniec.

**Masz jeszcze jakieś przemyślenia?**

Takie, że już chciałbym, żeby było normalnie.
