# Supplementary material for: The challenges arising from the COVID-19 pandemic and the way people deal with them. A qualitative longitudinal study
Source: PLoS One. 2021 Oct 11;16(10):e0258133. doi: 10.1371/journal.pone.0258133 (PMC8504766; doi:10.1371/journal.pone.0258133)
Supplement: S1 Dataset — (ZIP) [file pone.0258133.s003.zip › Transcriptions/stage 4/1.4_F_25_single.docx]

**1.4_F_25_single**

**Co się działo przez ostatnie 2 tygodnie?**

Niewiele zmian. Jedna dobra wiadomość - zwolnił się u moich przyjaciółek ten pokój, o którym ja z nimi rozmawiałam. Ta ich współlokatorka pojechała do domu i albo straciła pracę, albo po 2 miesiącach niepracowania już nie stać jej na ten pokój, więc napisała dziewczynom, że musi się wyprowadzić i teraz w majówkę zabrała rzeczy. Rozmawiałyśmy, że ja się chętnie do nich wprowadzę, bo to jest fajne cenowo, jest w dobrej miejscówce w Warszawie. Dobrze je znam i dużo przyjemniej jest mieszkać z kimś, z kim naprawdę jestem blisko niż z obcymi osobami, bo tak, to patrzyłabym raczej na kawalerki, które pewnie kosztowałyby więcej, więc to jest super wiadomość. Jutro jadę zobaczyć, jak tam wszystko w pokoju wygląda, co potrzebuję dokupić. O tyle to jest podnoszące na duchu, że tam praca pracą, ale mam te oszczędności, więc mogę bez pracy się wyprowadzić, a mam termin, do którego mogę siedzieć u rodziców, a potem wiem, że mam jakieś fajne miejsce w Warszawie. To jest 15 min tramwajem od Centralnego i do metra też jest blisko i to mnie cieszy. Teraz tylko pozostała jakaś praca. Teraz tylko czekam na te staże. Jeszcze czekam na odpowiedź. Oni mieli w tym terminarzu, że między 6 maja a 12 czerwca robią spotkania z ludźmi, jako kolejny etap. W najbliższym czasie powinni dawać znać, kto przeszedł dalej, kto nie. Jeszcze znalazłam aplikację w międzyczasie na jeden staż w jakimś banku, więc czekam na różne odpowiedzi, gdzieś tam aplikuję, ale raczej się na razie nie odzywają. Nie wiem, czy firmy już planują wracanie do biur, czy jeszcze czekają na ostatnie etapy rozluźniania.

**Ten termin, do którego będziesz u rodziców? To jakoś cię podnosi na duchu, że masz konkretną datę, po której coś się zmieni?**

Tak. Generalnie przeprowadzanie się, zanim będę miała pracę też jest o tyle bezsensowne, że będę wtedy siedzieć zamknięta w tym mieszkaniu. Dopóki nie muszę nigdzie chodzić, to siedzenie u rodziców jest przyjemniejsze, bo tu mam las bliżej, wczoraj na spacerze pies pogonił sarnę. Tu jest przyjemniej jednak spędzać kwarantannę czy izolację i ten brak obowiązków, ale ta data jakby bardziej mnie motywuje do tego, żeby jakąś pracę już sobie, nawet taką tymczasową, ale żeby już coś się działo i być już w mieście, i szukać dalej. To pomaga dać koniec tej całej sytuacji. Byłam tu ostatni tydzień marca, cały kwiecień, teraz leci już maj, więc jest to już drugi miesiąc siedzenia i nicnierobienia, i jest to już długo. Są te myśli, kiedy to się wreszcie skończy. Nawet nie tego, kiedy cała sytuacja się skończy, bo kwestia podróżowania i tego wszystkiego to pewnie będzie dłużej, ale tak kiedy to się skończy na tyle, że już można mniej więcej wrócić do takiego życia, że chodzimy do pracy, ludzie wychodzą, tylko wiadomo, że są te zasady typu maseczki, odkażanie czy co tam jeszcze. Dodaje to takie wrażenie, że już ten koniec widać, majaczy gdzieś tam na horyzoncie.

**Jak spędziłaś majówkę?**

Nie różniła się niczym od reszty czasu. Nikt nie przyjeżdżał, my też nigdzie nie jeździliśmy, bo też wszystko jest zamknięte, sklepy nawet są zamknięte tak naprawdę. Byliśmy w domu, w ogrodzie, na spacerze z psem. Normalnie, tak jak ostatni miesiąc.

**W twoim zachowaniu, spędzaniu czasu coś się zmieniło?**

Nie, dalej jest tak samo. Dalej jest mi ciężko wyjść, bo mam znowu teraz problemy z zasypianiem, a ostatnio z piątku na sobotę jakaś taka rzecz się wydarzyła, że całą noc nie zasnęłam. Tak leżałam do ranka, o 6 wstałam i wyszłam na poranny spacer z psem. Myślałam, że to pomoże mi jakoś to przełamać, ale też nie wyszło i cały czas jest tak. Wstaję 9-10 i ciężko jest mi zasnąć przed północą czy 1-szą i tak to trwa. dzisiaj próbowałam mimo wszystko wstać wcześniej i nastawiłam sobie budzik na 8, żeby mieć więcej czasu przed naszą rozmową, ale nie dałam rady. Jestem z rana tak zmęczona i jeszcze potrzebuję tej godziny snu, żeby mieć te moje 9 i nie wstałam o tej 8. Dopiero oczy otworzyłam 9.20. Ja myślę, że to też jest kwestia tego, że jakbym wiedziała, że muszę wstać, bo gdzieś się spóźnię, to wstałabym pomimo zmęczenia, ale fakt, że ten budzik dzwoni, a ja wiem, że ja nic nie muszę, jest tak rozbrajający, że ja nie jestem w stanie się zmusić z rana do wstania. Poproszę chyba mamę, żeby mnie zimną wodą wyrwała z tego łóżka któregoś dnia wcześnie, to może będę mogła wrócić do takiego normalnego spania, żebym mogła zasnąć wieczorem. Ja bym chciała iść spać wcześniej. Wczoraj wyłączyłam komputer 21.30, zgasiłam światło i kręciłam się do północy jak nie dłużej. Standardowo starałam się być o 22 w łóżku, jeśli o 7 wstaję i między 7 a 22 jest 15 godzin, czyli w normalnym dniu 15-16 godzin jestem na chodzie. Jeżeli ja otwieram oczy o 10, to nie ma siły, żebym przed północą albo 1-szą była zmęczona na tyle, żeby zasnąć. I dodatkowo w ciągu dnia siedzę z laptopem na fotelu, ale nie pracuję, nie wytężam myślenia. Teraz oglądam filmy, wyjdę na spacer z psem, porozmawiam z rodzicami, coś razem zjemy...Nie jestem zmęczona dniem. 2 dni temu poszłam pobiegać, bo myślałam, że może wieczorne bieganie mnie zmęczy, ale też nic nie dało. Już bym chciała wrócić do jakiejś rutyny.

**Coś jeszcze teraz mocniej ci zaczęło przeszkadzać?**

Nie chyba.

**Co teraz jest dla ciebie największym wyzwaniem?**

Nie ma chyba nic takiego. Dalej jest to martwienie się. Pomijając sytuację, aj generalnie mam problem, jaka praca byłaby taka, żebym ja była usatysfakcjonowana i co szukać dla siebie. Ze znalezieniem odpowiedniej drogi to już od dłuższego czasu mam problem, a sytuacja nie ułatwia. Martwienie się, jaka praca się trafi, jakie będą, czy to jakoś mogę się już starać...Jeżeli te staże się udadzą, to byłoby super, bo to będą rzeczy, które mnie będą pchały w kierunku, w którym ja chcę, a nie w jakimkolwiek. Więc to szukanie pracy może, ale tak, to w sumie przyzwyczaiłam się już do tego. Nawet zaczynam się martwić, jak to będzie, jak ta rutyna przyjdzie, bo wiadomo, że ona pomoże z tym spaniem, ale przestawienie się na to, że coś trzeba będzie na pewno niewygodne. Jest pewna przyjemność z nicnierobienia, chociaż to z czym się zmagam, to taki brak motywacji. Tam leży sterta rzeczy do prasowania i deska, którą już rozłożyłam, ale już od tygodnia czy nawet dłużej nie jestem w stanie się zebrać do tego. Mam tyle czasu...Im mniej czasu na coś masz, tym lepiej się zorganizujesz, żeby wszystko zrobić. A jak ja mam dzisiaj cały dzień, jutro i następny tydzień albo miesiąc, to w głowie masz takie "dobra, zdążę".

**Podejmujesz teraz jakieś nowe działania, żeby sobie radzić w tej sytuacji?**

Ostatnio Netfix poszedł w odstawkę. To są ostatnie 3 dni...Natrafiłam na stronę, która zrzesza i ma taką wyszukiwarkę miejsc odludnych na wakacje - jakichś leśniczówek, dom wynajmowany w górach, namioty, jurty w środku lasu. Ludzie bardzo się odcinają od miasta. Oni tam też mają kategorię vanów i ileś tam ludzi ma swoje przerobione vany na campery i wynajmują je. Zaczęłam oglądać. Już kiedyś myślałam o tym, że kiedyś coś takiego chciałabym zrobić, ale teraz tak mi się wkręciło mocniej z tym, że chciałabym sobie przerobić vana. Od 3 dni siedzę, odkryłam cały ten camperowy van life na YT i siedzę i oglądam tych wszystkich ludzi w Polsce i nie w Polsce, którzy poprzerabiali sobie vany na mieszkania do życia, jeżdżą i podróżują. To jest droga impreza, więc miałam taki pomysł, że może bym je robiła i sprzedawała, żeby na tym jeszcze zarabiać. Mamie powiedziałam. Moja mama zawsze się emocjonalnie zapala do takich rzeczy, ale mając takie zaplecze, żeby sprawdzić, czy na to jest popyt, czy to się opłaca, czy to nie jest większy koszt niż by się zarobiło, bo taka była reakcja mojego taty. Dyskutowałam o tym wczoraj z rodzicami, bo tata ma jeden taki większy samochód, który stoi i nie używamy go teraz. Może z niego by coś zrobić? To może nie jest ścieżka zawodowa, ale taki plan. To mi się wzięło też stąd, że muszę sobie spisać te różne moje cele, uporządkować pewne rzeczy na następną terapię, bo mamy o tych celach tez rozmawiać. To jest dalekosiężny plan na kilka następnych lat, bo zanim będę miała pieniądze, żeby taki van kupić i go przerobić, a potem jeszcze podróżować...Jest to też taka opcja na mój plan, bo jeśli ja chcę kiedyś gdzieś pracować zdalnie, to miałoby sens, tylko wtedy też pewnie potrzebowałabym partnera, który jeździłby ze mną. Podróżować z kimś a nie samemu. Jest to też dobra opcja później na wakacje, na lato w Europie, w Polsce, nawet z dziećmi pewnie będzie można jeździć. To jest taki projekt, który by mnie na maxa ekscytował i to jest też do robienia po pracy, co byłoby bardzo pewnie napędzające i ładujące baterie. To było ostatnio a przedtem dużo spacerowałam z psem i jakoś wciąż nie zabrałam się do czytania książek.

**Jeżeli masz jakiś plan przed sobą, to jest ci łatwiej funkcjonować?**

Ja jestem " to do list" osobą. Jedna lista leży na biurku, ale w takich dniach, kiedy ma się dużo czasu, to się odbywa tak, że lista do zrobienia z niedzieli przesuwa się na poniedziałek, ta z poniedziałku na wtorek. A, jeszcze co się zaczęło w ostatnich 3 tygodniach...Moja mama jest nauczycielem i tłumaczem języka migowego a babcia moja nie słyszy, dziadek też był niesłyszący. Z racji tego, że moja babcia bardzo dobrze mówi - miała 5 lat, jak straciła słuch. Trochę się jej nie chciało, trochę to było wygodniejsze do nas mówić...To też był taki strach, jak byliśmy dziećmi, ponieważ u nas jest to rodzinne...Taki strach, czy my na pewno słyszymy. Babcia do nas nie migała, tylko mówiła, sama czyta z ruchu warg bardzo dobrze. Mama też do nas nie migała, a jak się dorasta, to już jest jak uczenie się obcego języka. I my od lat z bratem powtarzamy, że chcielibyśmy się nauczyć, bo znamy tylko alfabet i kilka podstawowych znaków. Z racji tego, że siedzimy w domu i jest mnóstwo czasu, to pierwszy raz od nie wiem jakiego czasu udało mi się namówić mamę na te lekcje. Mama oczywiście zawsze była chętna, tylko z tego nigdy nic nie wychodziło, a teraz mniej więcej codziennie siadamy z mamą na godzinę i robimy taką lekcję. To wzięło się z tego, że parę razy poszłam z babcią do sklepu. Kiedy ja mam maseczkę...Niektóre z ekspedientek mają tylko te przyłbice, więc babcia może widzieć, co one mówią, ale jak ja z nią idę, to często jest tak, że ekspedienci coś do niej mówią odwracając się i ona nie widzi. Zaczęło mnie irytować, że muszę ściągać tę maseczkę, żeby ją o coś zapytać. W takiej sytuacji język migowy byłby pomocny. To mnie tak najbardziej popchnęło do tego. Z hiszpańskim to była tylko moja kwestia, a tu była mi potrzebna mama i jest to pierwsza sytuacja, kiedy ona też ma tak dużo czasu. To jest taka fajna rzecz.

**Jak się czułaś przez ostatnie 2 tygodnie? Jakiś własny obrazek?**

Nie zapisywałam. Niedużo się zmieniło w moich odczuciach.

**Emocje - zdjęcia**

6 i 12. Tak bardziej spokojnie się czuję w ostatnich 2 tygodniach. Trochę wynika to z tego, że to mieszkanie mam i tym już nie muszę się martwić, nie muszę szukać. Wiem już kiedy, za ile, wszystko samo się rozwiązało. Praca jest już teraz kwestią, na jaką się zdecyduję, bo jakieś prace są. Codziennie przychodzi ileś tam ofert pracy i to jest bardziej teraz kwestia zdecydowania, czy muszę już jakąkolwiek, czy jeszcze czekam i szukam czegoś bardziej odpowiedniego.

**Przeszkadzało ci poczucie uwięzienia. Czy dzięki tej sytuacji mieszkaniowej to też jakoś...?**

Tak. Teraz też od poniedziałku kolejny etap wprowadzili. Nawet nie byłam nigdzie, nawet nie jechałam sama nigdzie, ale wiedząc, że mogę pojechać do miasta po tym ostatnim wypadzie i w sumie nic się takiego nie działo, nikt mnie nie zatrzymywał...

**Nie mówiłaś, że byłaś u przyjaciółki?**

Już mi się wszystko tak zlewa...Rozmawiałyśmy 21.04 i ja byłam u Magdy chyba w piątek następny. To jest ta moja przyjaciółka, do której się przeprowadzam i ja byłam u niej w Warszawie. Potem ona przyjechała do mnie. Sam fakt bycia w mieście, zobaczenia, że nic się nie dzieje i jak bym chciała, to mogę pojechać...Jechałam komunikacją miejską i nic złego się nie stało. To też jest uspokajające. Teraz jeszcze, jak otworzyli muzea, to znaczy, że można wyjść na ulicę i jak się policja zapyta, to możesz powiedzieć, że do muzeum. Można wychodzić już więcej, zaczynają odmrażać to wszystko powoli i to napawa też takim pozytywnym podejściem do sytuacji. Dzięki temu siedzenie tutaj stało się przyjemniejsze, bo wiem, że jakbym chciała to mogę pojechać do miasta albo do kogoś, z kimś się zobaczyć. Sama świadomość, że mogę jest pozytywna i jadę jutro też.

**Jeszcze jakieś emocje wynikają z tych obrazków poza spokojem i pozytywną energią?**

Może trochę nuda czasami, chociaż mam dużo rzeczy do zrobienia i po prostu ich nie robię. Wychodzę do ogrodu, tata tam różne rzeczy robi, więc czasem pomożemy tacie, innym razem coś tam mamie posadziłam, chodzimy dużo z psem do lasu, co jest przyjemne. Będąc w tym lesie ja się staram tak przyswajać ten las, bo wiem, że jak potem wrócę do miasta, to tego nie będzie tak na co dzień. Teraz jest przepięknie w lesie, ta wiosenna aura lasu jest przyjemna i mogę codziennie do niego wyjść. Staram się doceniać, że teraz mam to na co dzień.

**Ostatnio mówiłaś o poczuciu niepewności. Ta niepewność nadal jest?**

Na pewno jest trochę lepiej, bo to szukanie mieszkania byłoby stresujące nawet bez tej obecnej sytuacji. To mieszkanie na pewno jest dużą ulgą i zostaje ta niepewność z pracą, ale to jest też to, co ja miałam już wcześniej - gdzie pójść, żeby to było to, co ja chcę i żeby to mnie pchało w kierunku rozwoju, który byłby dla mnie dobry. To mam pomijając sytuację.

**Pojawia się jakaś irytacja, złość na sytuację?**

Jest na fakt braku podróżowania, bo teraz siedząc w tych wszystkich kanałach YT...Lato w Warszawie teraz mnie cieszy, ale jak przyjdzie zima, to wiem, że będę marzyła, żeby móc gdzieś pojechać albo móc to zaplanować. To jest irytujące, że prawdopodobnie cała zima w Warszawie i to mnie nie cieszy w ogóle i bardzo mnie irytuje. Złość na sytuację o tyle, że 2 lata siedziałam w domu, wymyśliłam przeprowadzkę na 1.5 roku a jestem z powrotem tutaj chociaż chciałam uciec i coś zmienić. Ja potrzebuję zmian i lubię jak rzeczy się zmieniają, bo inaczej szybko się nudzę.

**Ja sobie radzą inni w twoim otoczeniu?**

Inni to jest moja rodzina i kilka przyjaciółek. Większość znajomych pracuje zdalnie. Oni muszą sobie poradzić z sytuacją siedzenia w jednym miejscu tyle czasu, ale ta przyjaciółka, która siedzi w swoim mieszkaniu to przyjechała do mnie, żeby mieć jeden dzień inny. Poszłyśmy na spacer i posiedziała sobie ze mną na wsi pod Warszawą. Druga przyjaciółka ma rodziców tutaj niedaleko i ona przyjeżdża do rodziców i jest to zawsze jakaś odmiana. Przyjaciel, który był u mnie jest w OSP, więc on weekendami może wyjść z domu albo ma całe weekendy zajęte przy okazji OSP. Mam znajomych introwertyków, którym odpowiada to, że siedzą w domu. Ludzie tymi spacerami i krótkimi wyjazdami starają się poradzić sobie z tym siedzeniem cały czas w jednym miejscu. Większość moich znajomych pracuje, więc nie ma tego problemu, że straciło pracę. Nie przychodzi mi do głowy nikt, kto by stracił pracę. A, jeszcze w sobotę moja przyjaciółka, której ślub miał być w kwietniu - ja miałam lot z Londynu na jej ślub i potem z powrotem. Oni przesunęli ten ślub na 2 tygodnie później, na ostatnią sobotę, bo chyba teraz mogli mieć więcej niż te 5 osób w kościele. Byli rodzice i rodzeństwo i mieli transmisję, więc oglądałam, a wesele jest przeniesione na lipiec.

**Mieli w kościele maseczki?**

Nikt nie miała maseczek. Byli rozstawieni, nie siedzieli blisko siebie w rzędach, ale komunię ksiądz podawał normalnie. Robiłam zdjęcia transmisji i mamy i psa, jak ze mną oglądają. Szkoda, bo na pewno wszyscy bardzo chcieliśmy tam być z nimi, ale ja się cieszę, bo wiem, że jej to było potrzebne, żeby to już się odbyło. Oni już mają mieszkanie od marca wynajęte, tylko obie rodziny są takie mocno religijne i wierzące...On jest z Krakowa, to mieszkanie jest w Krakowie i ona się tam wyprowadziła, ale nie mieszkali tam na stałe. Teraz już będą mogli sobie normalnie razem zamieszkać. Ona też ma problem z pracą, bo właśnie miała szukać po tej przeprowadzce, ale ma wsparcie rodziców i teraz ma męża, więc nie jest sama sobie pozostawiona. To taka śmieszna rzecz - pandemiczny ślub.

**Widzisz jakieś zmiany w emocjach u bliskich osób?**

Nie. Moim rodzicom się niewiele zmienia, brat przed komputerem non stop, ale studia i pracę ma zdalne, więc jemu nie zaburza się ciąg planów życiowych tylko jest ta niewygoda, że jest to nieprzyjemne i uciążliwe siedzieć w jednym pokoju 7 dni w tygodniu, bo 5 dni pracował a potem ma 2 dni studiów. Przy zjeździe siedzi ciurkiem 12 dni przed komputerem w domu i może wyjść tylko po pracy, więc jest to męczące. Tak ogólnie to nie ma za dużo zmian.

**Czy coś się zmieniło u ciebie w kwestii zakupów?**

Staram się ograniczyć, bo wydatki lecą a zarabiania nie ma, ale już dzisiaj przyszła jedna paczka, czeka w paczkomacie i jeszcze 2 idą. Znowu to są kosmetyki, jak ostatnio - olejki, podgrzewacz do olejku do masażu, bo to mi się też marzyło od jakiegoś czasu i są ciuchy. Są klapki i jedna koszula, a to wzięło się z tego, że inPost zrobił taką promocję, że jak się w przeciągu 2 h odbierze przesyłkę w paczkomacie, to rozdawali bony do Reserved. W sumie cieszę się, bo chciałam takie klapki i pewnie kupiłabym je drożej, gdybym nie kupiła ich w Reserved. To są takie letnie rzeczy jak już będzie można wychodzić.

A jak z zakupami spożywczymi?

W sumie byłam przedwczoraj na takich małych z bratem, bo wymyśliłam sobie jakiś konkretny obiad i potrzebowałam kilku składników. W ostatnich dniach staram się sobie robić takie zdrowsze rzeczy, bo chyba we wtorek tydzień temu robiłam sobie detoks sokowy. Zamówiłam sobie [ns] takich soczków i cały dzień piłam tylko te soki, żeby sobie zmniejszyć ilość jedzenie i rzeczywiście od tamtego wtorku trochę mniej jem, nie najadałam się na wieczór. Chciałam zmniejszyć, bo ja też zajadam nudę zazwyczaj, więc dużo jadłam wcześniej. Tydzień temu byłam też na takich większych zakupach w Biedronce z rodzicami, bo oni jechali i zabrałam się z nimi, żeby sobie kupić kilka rzeczy. I tak na tydzień te moje zakupy + ich zakupy mi wystarczyły.

**Robiłaś sobie listę?**

Tak, bo parę wegańskich potraw sobie robiłam w tym tygodniu, więc spisałam, co potrzebuję do tych przepisów. Chciałam zrobić coś zdrowszego po tym detoksie sokowym. Wczoraj się za mocno najadłam i właśnie to czuję już dzisiaj, ale to też wynikało z tego, że na obiad zjadłam tylko zupę i zdążyłam zgłodnieć tak późno i to było już za późno, żeby się tak najadać, więc muszę raczej jeść coś większego w porze obiadowej, żeby potem do wieczora już nie być głodną.

**Co cię skłoniło, żeby zmienić trochę swoje nawyki, zrobić ten detoks?**

Waga, która idzie w górę. Może nie idzie w górę, ale jest najwyższa od jakiegoś czasu. To, że się mało ruszam przez problemy z kolanami. Staram się biegać, ale potem zazwyczaj jest z tymi kolanami problem i mnie bolą. Na tyle sobie dawałam upust, żeby jeść cokolwiek na co mam ochotę przez pierwszy czas przez tę sytuację, a teraz czuje się lepiej, poprawił się mój nastrój z powodu mieszkania i łatwiej mi się zmobilizować teraz, żeby jeść zdrowiej, bo trzeba skurczyć żołądek i trochę ograniczyć objadanie się i jakoś zdrowiej zacząć jeść.

**Twoje zakupy spożywcze teraz wyglądają podobnie do tego, jak było przed epidemią? Też robiłaś sobie listę, sprawdzałaś przepisy?**

Różnie, bo w sumie to zależy, kiedy mi się zebrało, żeby zrobić konkretny przepis, a kiedy miałam tak, że kupie chleb, coś do chleba i jakieś warzywo. Miałam coś takiego w głowie, szłam i o, jest ta pasta wegańska, którą lubię, o, jest ogórek, mam ochotę na ogórka. Ja rzadko gotowałam. Jadłam w pracy w bufecie albo jakieś gotowe rzeczy, nie miałam czasu, żeby gotować, a teraz jest go więcej. W piątek, jak robiłam wegańską tartę, to pół dnia mi to gotowanie zajęło tak na prawdę. Jeżeli miałam tak, że wymyśliłam sobie co chcę zrobić z jakiegoś przepisu, to spisywałam sobie listę, żeby niczego nie pominąć, ale jeżeli akurat nie gotuję tylko jej jakieś kanapki, jakieś płatki, to pamiętam takie pojedyncze rzeczy w głowie i bardziej to jest na zasadzie, że pamiętam to, co jest najbardziej potrzebne a reszta jest na zasadzie, na co mam ochotę.

**W ostatnich 2 tygodniach zdarzyły ci się zakupy typowo dla przyjemności?**

Nie, chyba to nie jest na poprawę humoru.

**A spożywcze?**

Wczoraj byłam 1-szy raz od tygodnia, bo kupiliśmy sporo rzeczy przed weekendem majowym. Prócz rzeczy na obiad kupiłam sobie coś słodkiego i to też jest powód, dla którego chciałabym chodzić do tego sklepu mniej, bo im częściej idę do sklepu, tym większa jest pokusa, żeby sobie coś słodkiego dokupić. A jak nie kupię, jestem w domu i tego nie mam, to po prostu nie jem. Przed majówką byłam z rodzicami w Biedronce i mój tata zawsze jak widzi, że kupiłam chleb, to jest zawsze, że o, ile chleba kupiłam - taki niezdrowy, taki tuczący. Tak samo jest ze słodyczami, więc będąc z nimi nie kupiłam słodyczy chcąc uniknąć przytyków taty. To była pozytywna część tych zakupów. No, ale jak już byłam z bratem sama, to kupiłam sobie jakieś żelki.

**Patrzysz trochę na wyjście do sklepu spożywczego jak na formę rozrywki?**

W kwarantannie tak było, ale jako, że już byłam w mieście, to teraz wyjazd do miasta jest większą rozrywką. Nie, już mi przeszło. Teraz to jest uciążliwe, bo trzeba pamiętać o tej maseczce, o rękawiczkach. Wczoraj byliśmy pod wieczór i w naszym sklepie mają koszyczek z rękawiczkami i ja wzięłam ostatnią parę jak weszliśmy, więc trzeba pamiętać o nich, bo czasem może ich nie być w niektórych sklepach. I to kupowanie w tych rękawiczkach, odblokowywanie mojego telefonu z rozpoznawaniem twarzy z maseczką na twarzy...To jest uciążliwe.

**Co sądzisz o otwarciu GH?**

Z tego co słyszałam, to one mają być otwarte, ale z jakimiś obostrzeniami, co do ilości ludzi. Niektóre miejsca mają być jeszcze zamknięte w galeriach. Na pewno kina...

**I jeszcze restauracje.**

No właśnie, więc ciekawa jestem jak będą pilnować ilości. Nie wiem, jak jest w innych sklepach, ale w naszym wydaje mi się, że nikt nie pilnuje ilości ludzi na cały sklep. Dla mojego brata nie starczyło wczoraj rękawiczek i nikt mu nie zwrócił uwagi, że nie ma rękawiczek. Mam wrażenie, że ludzie są też już zmęczeni tym wszystkim. Maseczkę to widać, czy ktoś ma czy nie ma...Myślę, że to wynika z tego, że nikt z nas, albo większość z nas w takim sklepie nie ma np. przypadku koronawirusa w rodzinie albo wśród znajomych. Dla osób, które siedzą zamknięte, nie mają nikogo takiego w rodzinie i wśród znajomych, a już tym bardziej śmierci z tego powodu, to problem jest taki trochę fikcyjny i wirtualny wciąż. Nikt z nas nie choruje, nikt nie czuje się gorzej, ja nie znam nikogo takiego. Dla osoby, która dodatkowo może być sceptyczna co do zasadności wszystkiego to jest to wszystko uciążliwe a nie na zasadzie, że jest to realny problem i stosujemy się do tego, bo trzeba, ale myślę, że w ludziach też już luzuje ta ostrożność, bo jakby nic takiego się nie dzieje wokół nich tak naprawdę, a jest długotrwała dość uciążliwa zmiana życia codziennego.

**Pójście do GH jest bezpieczne teraz?**

Myślę, że nie różni się od pójścia do sklepu, do którego chodzę tu blisko. Nawet powiem, że jeżeli pilnują ilości ludzi w galerii, ktoś stoi i liczy, to myślę, że skoro ona jest powierzchniowo większa...Pewnie nie chodzi do nich jakaś ogromna ilość ludzi, bo ulice są pustsze, co oznacza, że część ludzi siedzi ze strachu w domu. Podejrzewam, że przy takiej dużej przestrzeni jest nawet mniejsze prawdopodobieństwo coś od kogoś złapać niż w takim moim sklepie, który jest mniejszy i przy kasach ma dość mało miejsca i ludzie tam się tłoczą.

**Planujesz iść na zakupy do GH albo do jakiegoś sklepu stacjonarnego z ubraniami np.?**

Na razie nie miałam takich planów. Z ubraniami nie, bo jakieś nowe sobie pozamawiałam, mam sporo takich letnich nowych ubrań i butów, więc obecnie nie mam nic takiego, co koniecznie musiałabym przymierzać w sklepie. O jednym sklepie myślałam, tylko nie wiem, czy one są czynne. Potrzebuję szamponu. mam taki specjalistyczny szampon, który kupuję u trychologa w Warszawie na Świętokrzyskiej. Do galerii? Może poszłabym sprawdzić, jak to działa wszystko i czy ktoś pilnuje, ktoś liczy, jakaś ochrona. Bardziej z ciekawości bym poszła zobaczyć. Może by mi przyszło do głowy wejść do któregoś ze sklepów, ale nie mam jakiejś ogromnej potrzeby iść do GH.

**A ktoś z twojej rodziny, znajomych planuje takie zakupy? Słyszałaś coś o tym?**

Nie. Moja znajoma wrzuciła wczoraj na Instagrama...Chciała kupić zwyczajne ikeowe szklanki - takie jak te po 2 zł chyba i pokazywała koszyk z zamówienia online z Ikei. 3 takie szklanki 7 coś złotych całe zamówienie, a w podsumowaniu dostała 49 zł. Zdaje się, że Ikea ma takie drogie dostawy, bo tam są większe gabarytowo zwykle rzeczy. Może jak bym chciała iść do Ikei, to bym się zastanawiała nad pójściem do niej stacjonarnie, ale na razie nie mam takiej potrzeby.

**Łatwość wydawania pieniędzy - skala**

Ja myślę, że takie solidne 9. To jest zabawne, bo to jest tak, że mój tata...My go nazywamy czasami sknerą, bo wyciągnięcie od niego pieniędzy niejednokrotnie jest trudne i to nas w dzieciństwie z bratem nauczyło takich bardzo uargumentowanych dyskusji. Jak potrzebowaliśmy, to nie wystarczyło przyjść i powiedzieć, że potrzebuję to i to, albo chcę to i to. Trzeba było przemyśleć całą strategię argumentowania, dlaczego ja tego potrzebuję i dopiero szliśmy z tatą rozmawiać. Z jednej strony jest to czasami upierdliwe i czasami bywało upierdliwe, z drugiej, dzięki temu cały budżet domowy się spina. Tata zarządza tym budżetem finansowym w taki sposób, że mamy ten dom po 20 latach. Teraz już będzie 20 lat, jak tutaj rodzice mieszkają, 3 samochody i jakby wszystko się kręci i jakoś wszyscy tutaj funkcjonujemy., rodzice jakoś funkcjonują. Tata się często wkurza, że jaką kwotę by nie wymyślił...Na wiosnę ma np. zawsze w budżecie nowe roślinki i nowe kwiatki do ogródka. Zawsze w budżecie jest kwota na te wiosenne roślinki, porządki. Ostatnio, jak z tego sklepu z nimi wracałam, to się kłócili, bo tata nie ważne, jaką kwotę - co roku większą przeznaczy mamie na te roślinki, to ona zawsze wyda więcej, albo wyda wszystko. Mama ma rzeczywiście tak, że ona, jakby miała się tym budżetem zajmować, to może niekoniecznie to by tak wszystko dobrze wyglądało, bo mama potrafi wydać wszystkie pieniądze. Zawsze potrafi wymyślić, na co je przeznaczyć i niekoniecznie ma takie hamulce. Jej podejście jest takie, że pieniądze są po to, żeby je wydawać a nie po to, żeby były i je trzymać. Poniekąd racja, ale to trzeba też z umiarem. Rodzice tak balansują trochę w tym aspekcie, ale ja chyba mam to mocniej od mamy i stąd moje umiejętności oszczędzania nie są jakieś wybitne, mimo tego, że budżet swój prowadzę i jestem w stanie monitorować te wydatki na tyle, żeby nie zostać w połowie miesiąca z zerem na koncie. Ale jeżeli tylko czuję, że mam jakąś nadwyżkę, to leci to szybko. Teraz mam dużo, bo mam te wszystkie oszczędności, zwrócili mi za mieszkanie w Londynie, więc ta nadwyżka jest duża, mam duży zapas i jak mówię - tutaj jest bon do Reserved, to kupiłam coś i ci chwilę coś takiego.

**Podaj jakieś przykłady, kiedy z taką łatwością zdarzyło ci się coś kupić?**

Na pewno z tyłu głowy jest takie coś, że kurde, za dużo tych pieniędzy wychodzi, za szybko to się wszystko rozchodzi, ale ja sobie jednak tak tłumaczę, że pieniądze są od tego, żeby je wydawać. W końcu mam na to np., żeby kupić sobie to i to, o czym myślałam już od tylu i tylu tygodni. Jak byłam w szkole, miałam kieszonkowe i nie pracowałam, to była to naprawdę mała kwota, więc zawsze trzeba było odkładać tygodniami albo miesiącami na coś większego. Nawet na jakiś porządniejszy ciuch, który sama chciałam sobie kupić. W momencie, kiedy tych pieniędzy jest trochę więcej...Uczucie, że wchodzę do sklepu, wybieram coś, co mi się podoba i mogę to kupić jest bardzo przyjemne i takie jakby inne. Ciuchy myślę to jest coś, na co wydaję chyba sporo. Im większy mam zapas, mimo tego wydatku, tym lepiej się czuję w związku z tym wydatkiem.

**A takie zakupy inne niż codzienne - jakaś elektronika, jakieś meble, wakacje? Jak u ciebie przebiega ten proces decyzyjny?**

Np. komputer mam na raty i jeszcze zostały mi chyba 3 miesiące tych rat. Dlatego, że w międzyczasie ja też nadpłaciłam trochę więcej. Założyłam sobie, że ok., będę miała trochę więcej niż ta rata wynosi, ale ta rata jest bezpieczna - to takie minimum, które co miesiąc muszę zapłacić. Komputer już potrzebowałam nowy, bo mój był stary, ważył 3 kg, itd. Bardzo potrzebowałam nowego, lżejszego laptopa i nie żałuję, bo ten komputer jest mi bardzo przydatny i jest też bardzo wygodny do jeżdżenia czy przenoszenia się z nim. Kupiłam go na te raty Allegro i to jest fajne, bo to są raty 0%, więc nie mam poczucia, że przepłacam i te raty nie są takie uciążliwe co miesiąc.

**A jak wybierałaś komputer, to porównywałaś jakieś oferty?**

Chciałam sobie spróbować z Mac'iem, więc wiedziałam, że Mac'a. Oferty sprawdzałam cenowo.

**Nazwałabyś siebie osobą rozrzutną czy oszczędną?**

Chyba bardziej w kierunku rozrzutnej niż oszczędnej.

**Co o tym świadczy?**

Ja lubię wydać na przyjemności. Ciuchy są jeszcze czymś takim, co zastaje i ileś służy, czy komputer. Komputer jest narzędziem pracy, więc to też można, jako inwestycję uważać. Wydawanie na jedzenie jest już taką inną przyjemnością. Idę wydać na jakieś lepsze jedzenie, bo chcę sobie zrobić przyjemność albo wyjść z kimś znajomym i to jest przyjemność, że z tym kimś idę na jakieś dobre jedzenie. Jeżeli zamawiam pizzę...Teraz te ceny rzeczy zamawianych w ogóle poszły w górę, ale nawet wcześniej zamówienie pizzy w Warszawie to było 30 zł z dowozem. To jest dużo droższe niż planowanie sobie posiłków samemu, a wynikało to z mojego lenistwa, niechęci do gotowania codziennie i też z łakomstwa na zasadzie, że strasznie miałam ochotę na pizzę, więc powody, dla których ja tę pizzę zamawiam są na tyle negatywne i lepiej byłoby zrobić sobie samemu coś tańszego, zdrowszego, nie obejść się pizzą... To jest taka rozrzutność z dużą negatywną jeszcze dodatkowo...Z innymi skutkami p przyczynami.

**Te negatywne skutki ci wtedy doskwierają?**

No przytycie tak, ale przyczyny są w głowie - że nie chciało mi się, miałam ochotę. Potem się objem i powiem sobie, że o nie, już więcej nie będę...[śmiech] To jest też kwestia, że ja wiem, że to jest droższe, że są opcje tańsze, żeby to zrobić, tylko trzeba się trochę wysilić.

**Wiesz, że to jest droższe, ale i tak ci to sprawiło przyjemność?**

Podczas jedzenia tak.

**Jak zmieniła się twoja sytuacja przez epidemię? To twoje zabezpieczenie - na ile ono by ci starczyło bez przychodów?**

Od czerwca mam zapłacić za mieszkanie. Zaraz spojrzę w budżet, to ci powiem, bo wszystko spisałam. Mam w Excelu budżet na parę miesięcy w przód i tylko sobie zmieniam pozycję - dopisuję, co, ile kosztuje, więc mam wszystkie formułki i widzę cały miesięczny koszt. Z tego, co tutaj wychodzi...Tak z 5 miesięcy bez pracowania z mieszkaniem w tym mieszkaniu.

**Teraz od jakiegoś czasu nie masz dochodu...**

Od marca nie mam, bo do końca lutego pracowałam.

**Jak wyglądają perspektywy na zmianę tej sytuacji? Czy to wróci do normy? Kiedy?**

Jak już mam się do tego mieszkania wyprowadzać w czerwcu, to siedzenie na tyłku i nicnierobienie w nim nie ma sensu, więc liczę na to, że znajdą się takie prace, których szukam, o które mi chodzi. Jeżeli do końca maja nic takiego się nie znajdzie, to będę patrzeć na cokolwiek od początku czerwca. Liczę na to, że coś tam się pchnie. Mam jedną taką rozmowę u kuzyna w pracy, gdzie oni mi po rozmowie parę tygodni temu powiedzieli, że wszystko fajnie, chcemy kolejny etap rozmów, tylko dopiero, jak będzie można wrócić do biura. Teraz w maju odpuszczają obostrzenia, więc podejrzewam, że do końca maja będzie można w firmach już wrócić do biura. Liczę na to, że do czerwca oni to zrobią. Napiszę do nich pewnie maila w tym tygodniu, żeby zapytać, jak oni to planują, czy u nich coś już wiadomo. Jeżeli mi nie z tych prac się nie ruszy, to będę próbowała cokolwiek z takich opcji bardziej dostępnych, ale też z takich, które łatwo jest rzucić szybko. Będę pracowała gdziekolwiek i szukała dalej tego, co mnie interesuje. Gdyby te staże się zaczynały, to one wszystkie zaczynają się od początku lipca, ale też dobrze by było gdybym w czerwcu coś tam przepracowała przez miesiąc, coś zarobiła, a potem zaczynają się płatne staże.

**Podjęłaś jakieś kroki, żeby ograniczyć wydatki na co dzień?**

Mam ten limit, że jak go dotknę, to nie powinnam już ruszać nic. Sama go sobie ustaliłam, ale teraz nie wydaję nic na życie, na bycie w domu, żadne rachunki, bilety, to tylko jak pojechałam do Warszawy, jedzenie generalnie kupują rodzice a ja tylko jak chcę dokupić coś dla siebie. Czasem też coś do domu, bo rodzice też jedzą, jak coś ugotuję. czasami babcia mnie zabiera na zakupy i mówi, za ja ci kupię, ja ci kupię i kupuje mi jedzenie. Te moje zakupy ekstra, które robię, to są właściwie moje jedyne wydatki teraz. No i jakieś opłaty za telefon, komputer - opłaty stałe, które schodzą z konta.

**Jak robisz zakupy, to szukasz jakichś specjalnych ofert, promocji, tańszych zamienników?**

Ten sklep, który mamy obok jest dość drogim supermarketem - to jest coś pomiędzy dawną Almą a zwykłym supermarketem, ale ten sklep jest fajny, bo mamy dostęp do różnych produktów, których gdzie indziej nie ma.

Mądrzej by było w ogóle nie wydawać i oszczędzać maksymalnie, ile się da w momencie, kiedy nie wiem, kiedy znajdę pracę, za ile j znajdę i kiedy wciąż mam też inne plany, na które pieniądze by mi się przydały. Część z tych zakupów na pewno jest takim sposobem na polepszenie sobie nastroju w tej całej sytuacji. Teraz ostatnio może już nie odczuwam tego polepszenia nastroju, ale to poniekąd też miało na celu chyba to.

**Ten arkusz w Excelu miałaś też wcześniej, przed epidemią?**

Tak, tak. Mój pierwszy był chyba w 2018 roku jak wróciłam z podróży.

**Uważasz, że w kontekście pandemii dobrze jest ograniczać wydatki?**

W kontekście gospodarczym to lepiej, żebyśmy wydawali pieniądze, żeby one krążyły, tylko dużo ludzi nie ma dochodu albo u nich się ten dochód skurczył, albo ich najzwyczajniej nie stać. Pewne priorytety wychodzą teraz na początek - mieszkanie, jedzenie, to, co jest najbardziej potrzebne. Sporo ludzi na pewno nie pojedzie na wakacje, nie wyda na wakacje i jest to słabe w sensie rozkręcania gospodarki, bo nie będziemy mieli turystów przyjeżdżających do kraju, więc dobrze by było, żebyśmy to my jeździli na wakacje gdzieś po kraju i wydali pieniądze w tych miejscach. To zależy, kto jaką ma sytuację finansową. Z jednej strony tak sobie mówię robiąc te zakupy, że kupując w polskich firmach, a Reserved jest polską sieciówką, mogę trochę się przyczynić do rozkręcenia gospodarki, ale też trzeba to gdzieś trzymać w ryzach, żeby nie zaszaleć za mocno.

**W jaki sposób oszczędzałaś?**

To nie są oszczędności. Ja to tak nazywam, bo to jest wszystko co mam. Brałam pożyczkę przed tymi studiami. Policzyłam sobie, że pożyczka w banku polskim jest tańsza niż w brytyjskim. Wiedziałam, że muszę mieć w Londynie na początek jakieś pieniądze na "w razie czego" i potem te pieniądze miały być na studia. Jest to inwestycja w siebie, więc nie brałam pożyczki na wydanie jej na wakacje, jak ludzie robią czy na remont, gdzie potem nic się z tego nie ma poza podwyższeniem standardu życia. Jedna z moich stałych rat to jest teraz rata pożyczki. Mam jeszcze taki plan, że jeśli nie wydam na wakacje, a ustabilizuje mi się praca, to część tej kwoty, którą mam oddam, żeby sobie zmniejszyć ratę pożyczki, zmniejszyć odsetki. Na razie chcę to trzymać, żeby mieć to zabezpieczenie, dopóki praca się nie wyklaruje.

**A przed pożyczką jak u ciebie wyglądało z oszczędnościami?**

Te oszczędności moje były takie nieduże, bo też z racji małej pensji miałam małe możliwości przy mieszkaniu przez parę miesięcy w Warszawie. Zazwyczaj, jeśli jakąś kwotę chciałam oszczędzić w danym miesiącu, to wpisywałam ją. Jedyny problem jest taki, że teraz mam konto oszczędnościowe i ta pożyczka jest na koncie oszczędnościowym - ostatnio zarobiła 20 zł. Oszczędności wcześniej miałam na jednym wspólnym koncie i to niestety...Jak się widzi taką sumę na koncie, to o, jeszcze mam, mogę wydać np. Mimo tego, ze wiem, że to w budżecie jest rozpisane na oszczędności, to to nie pomaga i chyba pozostawię sobie to konto oszczędnościowe i ono będzie dobrym miejscem, żeby przelewać na nie te kwoty, które sobie zaznaczyłam, żeby ich nie było na koncie wspólnym, żeby one znikały stamtąd. Czasami udawało się zostawić na tym koncie wspólnym to, co sobie zaplanowałam w budżecie a czasami było to uszczknięte albo nic z tego nie zostawało. Czasami były takie niezaplanowane wydatki konieczne w miesiącu a czasami zwyczajnie zaszalałam. Ja nie jestem osobą, która pójdzie na imprezę i wyda jakąś nie wiem jaką kwotę. Takie rzeczy mi się nie zdarzają i nawet nie próbuję sobie wyobrażać, co by było, gdybym była taką osobą, bo pewnie byłabym na minusie, na debecie z powodu imprez. Ale może być tak, że chcę z kimś wyjść i wyjdę, i zaszaleję, i zjem ten obiad za 30-50 zł, który jest drogim posiłkiem jak na polskie warunki. Czasami mam bardzo ochotę to zrobić, czasami chce się wyjść w to dobre miejsce, zjeść. Pomimo tego, że wiem, że nie powinnam i to jest zjadanie oszczędności, to zdarza się, że to robię.

**Dlaczego oszczędzałaś?**

Bo już od 2 lat miałam w głowie pomysł ze studiami i wyjazdem. To było na konkretny cel. Jeżeli to nie jest ka konkretny cel, na konkretny wyjazd, to zawsze i tak jest to na jakiś wyjazd. Zawsze wolałabym mieć jakiś budżet na wyjazdy niż go nie mieć.

**Czy teraz, w kontekście epidemii, warto jest oszczędzać?**

Tak, bo każdy kto ma poduszkę finansową jest w lepszej sytuacji niż ci, którzy nie mają. Jakiś czas temu czytałam książkę Michała Szafrańskiego - tego blogera od finansów. Finansowy Ninja, gdzie on pisze o tym jak wychodzić z długów, itd. On tam pisze, że poduszka finansowa jest istotna, bo są rzeczywiście sytuacje nieprzewidziane, takie jak ta i każdy, kto ma poduszkę finansową w stylu - pieniądze na 3-6 miesięcy życia bez pracy jest teraz w bardziej komfortowej sytuacji i się nie stresuje aż tak bardzo. Szczególnie, jak ludzie mają dzieci, rodzinę. Inaczej jest ze mną, kiedy ja się stresuję, że co ja zrobię, nie będę mogła sobie pojechać tam i tu i sobie gdzieś mieszkać, tylko będę musiała wrócić do rodziców, bo wolałabym inaczej, ale nie wyobrażam sobie ludzi, którzy mają w tym momencie dzieci i to nie jest tylko stres o to, gdzie ja będę mieszkał, tylko gdzie ja będę mieszkał z całą rodziną przy utracie pracy, gdzie nie będę miał na następny miesiąc. Logicznym jest mieć poduszkę na kilka miesięcy życia, bo albo jest to sytuacja taka, jak ta, albo jest to taki też komfort psychiczny na zasadzie, że coś przestaje mi odpowiadać w pracy, chcę zmienić pracę. Nie musisz siedzieć i czekać aż z jednej pracy przejdziesz płynnie w drugą tylko masz zabezpieczenie na kilka miesięcy, żeby zrobić to jakoś inaczej i założyć sobie miesiąc na szukanie nowej pracy, ale już mając dużo czasu na to. To jest logiczne bardzo i daje na pewno dużo plusów, żeby mieć taką poduszkę. Nie udało mi się jeszcze mieć takiej z samych oszczędności, ale na pewno będę kiedyś dążyć do takich rozwiązań.

**Czy teraz może być dobry czas na inwestycje?**

Zależy w co. Może w maseczki albo żele? Nie wiem, nie jestem ani ekspertem...Musiałabym się porozumieć z kimś, kto się zna na ekonomii i gospodarce i potrafi się jakoś rozeznać. Z kimś, kto może zajmuje się jakimiś trendami ekonomicznymi i jest w stanie przewidzieć jakie mogą zajść scenariusze. Szczerze mówiąc przy tak niestabilnej sytuacji, to raczej inwestycja brzmi dla mnie niestabilnie w tym momencie. Mam znajomego, który już rok temu inwestował sobie w Teslę i jeszcze w coś. Miał taką większą sumę, bardzo chciał zainwestować i zainwestował. Ciekawa jestem, bo jak wyjeżdżałam, to jakieś pierwsze zarobki mu się zrobiły na tych akcjach, ale nie wiem, jak wygląda ta sytuacja teraz. Czy to jest tak, że stracił coś, czy to się wyrównało? Totalnie nie wiem. Nie śledzę giełdy, ale jakieś tam urywki z wiadomości, że leci na łeb, na szyję i jest jakaś tragedia to były. Nie wiem. Ktoś mi ostatnio mówił, że ceny wynajmu mieszkań i kupna powinny teraz spaść, ale pytanie, czy tak będzie. Teoretycznie, jeśli spadłyby ceny mieszkań, a ktoś ma, to może zainwestowanie teraz w mieszkanie, które po ty wszystkim zacznie znowu zyskiwać na wartości może miałoby sens. Tylko pytanie czy rzeczywiście te ceny teraz spadną. Na mój obecny stan wiedzy i na samo słowo inwestycja, to do inwestycji raczej chciałoby się mieć stabilną sytuację gospodarczą, to nie wiem. Mam za mało wiedzy i rozeznania w rynkach, bo na pewno są teraz rynki, gdzie inwestycja miałaby sens, bo coś staniało a wiadomo, że podrożeje po pandemii. Tylko, że to dotyczy osób, które wciąż mają środki na takie rzeczy pomimo pandemii.

**Czy myślisz o tym, kiedy ta sytuacja się skończy?**

Pewnie, bo najbardziej frustruje mnie i doskwiera mi brak możliwości zabookowania lotu i polecenia gdzieś. Trochę zastanawiam się, na ile jest racja z tą kolejną falą i co ta kolejna fala przyniesie. Czy rządom przyjdzie znowu do głowy...Mniej niż samego wirusa w różnych miejscach, ja się boję teraz gdzieś polecieć, nawet, jeżeli byłyby tam loty. Nawet, jeżeli będzie można gdzieś polecieć, nawet, jeżeli to będzie w miarę bezpiecznie wyglądało, to jeżeli na jesieni ma powrócić znowu fala i zacznie się nagle rejestrowanie znowu większej ilości zachorowań, znowu jakaś panika wróci, to pytanie, co rządy zrobią, które rządy zrobią co, bo też nie wiemy. U nas zamknięte były granice po jakimś czasie, a Izrael zamknął się od samego początku do końca maja. Nie wiadomo, jak byłoby z tym wracaniem. Nie chciałabym po raz kolejny być zmuszona do kupowania tego Lotu do Domu, który kosztował dużo, bo ponad 600 zł.

**Ta myśl, kiedy to się skończy jest w tobie cały czas, czy ona się pojawia w jakichś konkretnych momentach?**

Jest gdzieś tam cały czas. jak coś oglądam o podróżach, to kiedy ja znowu będę mogła, w sklepach - kiedy wreszcie przestaniemy używać tych maseczek i rękawiczek i czy będziemy te maseczki nosić przez następne 2 lata może. jest to cały czas myślę. Jak szukam pracy, to myślę, kiedy ten rynek wreszcie wróci. Widać, że są takie specyficzne oferty teraz - jest sporo tych zdalnych. Zawsze było duże zapotrzebowanie na programistów, ale teraz są głównie te.

**Co najbardziej ci zaprząta głowę - te podróże czy coś innego?**

Szukanie pracy na razie. Podróże też, ale jednak to jest jakby luksus. Rynek pracy, bo ja już od jakiegoś czasu mam problem, żeby znaleźć coś odpowiedniego dla siebie, a jak teraz jeszcze są` ograniczone możliwości, bo część miejsc musiała ograniczyć rekrutację, okroić jakieś wydatki. Ogromna korporacja, w której pracuje moja przyjaciółka zaczęła im obcinać pensje. To podobno ma być taka poduszka i jak wszystko wróci do normy, to ostanie im to zwrócone po jakimś czasie, więc to nie` jest tak, że ograbiają ich z tych pieniędzy. Bardziej rezerwują je na chwilę. Gigantyczne korporacje robią takie rzeczy, więc jest to takie destabilizujące.

**Jak sądzisz, jak się potoczy sytuacja?**

To zależy od tego, czy będzie nawrót i kolejna fala. Też na przykładzie poprzednich pandemii. Z hiszpanką było tak, że groźniejsza i bardziej śmiertelna była druga fala. Pytanie, kiedy ta szczepionka będzie albo kiedy lek wymyślą? Czy to rzeczywiście będzie działało? Zastanawiam się też, jakie będzie podejście rządu do drugiej fali i czy będzie zawieszanie gospodarki po raz kolejny. Mam nadzieję, że nie. Gospodarka jest już i tak w fatalnym stanie. Teraz będzie powoli napędzanie jej znowu i jak za kilka miesięcy miałoby nastąpić kolejne zamknięcie, to dla niektórych miejsc to będzie już gwóźdź do trumny.

**Masz jakiś taki punkt, kiedy myślisz, że to się skończy? Jaka to jest perspektywa?**

Ja myślę, że w przeciągu roku. Taką mam nadzieję. Myślę, że kiedy szczepionka zostanie wynaleziona i ludzie zaczną się szczepić...Pytanie, czy będą szczepić wszystkich przymusowo czy nie, ale jak ta szczepionka się pojawi to, czy to będzie iluzoryczne czy nie, nieważne, ale jakiś spokój w społeczeństwie się pojawi - wymyśliliśmy szczepionkę, jesteśmy bezpieczni. panika społeczna wtedy trochę się zmieni. Taka panika rządów. Rządy reagują też tak trochę na pokaz, żeby część społeczeństwa, ta bardziej przerażona problemem nie obwiniała ich za to, że nic nie robią. Czy to wszystko było lepszym pomysłem niż po prostu noszenie maseczek i wprowadzenie samych obostrzeń typu odpowiednia odległość, może mniejsza ilość ludzi w sklepach, ale nie zamykanie ich kompletnie. Nie wiadomo. To pewnie będzie można wyliczyć, jak już się wszyściutko za 2 lata całkowicie skończy. Hiszpanka była na przestrzeni 2 lat bodajże, ale mam nadzieję, że przy obecnych technologiach to będzie krócej niż 2 lata, że maksymalnie rok. Pytanie, kiedy pojawi się szczepionka, ilu ludzi zaszczepią i jak to zadziała na społeczeństwo.

**Czego najbardziej się obawiasz w perspektywie najbliższych kilku tygodni?**

To, kiedy i jaką pracę zacznę. Też dla swojego zdrowia psychicznego chciałabym już zacząć robić coś, że wstaję po coś rano a nie na kolejny dzień takiego snucia się i robienia czegokolwiek.

**A w perspektywie kilku miesięcy?**

Też praca, tylko w kontekście, gdzie ta praca mnie zabierze, bo już jestem zmęczona taką pracą tylko po to, żeby zarabiać. Chciałabym, żeby ta praca mnie rozwijała albo była tak fantastyczna finansowo, żebym miała pieniądze na takie rzeczy interesujące mnie po pracy. To jest do osiągnięcia trudniejsze niż praca płatna normalnie, ale rozwijająca mnie.
