# Supplementary material for: The challenges arising from the COVID-19 pandemic and the way people deal with them. A qualitative longitudinal study
Source: PLoS One. 2021 Oct 11;16(10):e0258133. doi: 10.1371/journal.pone.0258133 (PMC8504766; doi:10.1371/journal.pone.0258133)
Supplement: S1 Dataset — (ZIP) [file pone.0258133.s003.zip › Transcriptions/stage 4/19.4_F_39_couple, with children.docx]

**19.4_F_39_couple with children**

**Co się działo przez ostatnie 2 tygodnie?**

Jestem cały czas na tej opiece, jeszcze ten tydzień. Lżej tak psychicznie, bo nie muszę godzić tego co w pracy i tego co w domu. Trochę sobie odpoczywam. Nic konkretnego nie robiłam, po prostu się leniłam. Podstawowe obowiązki domowe, trochę poczytałam książki, więcej na podwórku i wszystko tak na spokojnie. Nie było tego skakania pomiędzy komputerem a kuchnią.

**Jeszcze tydzień i wracasz do pracy?**

Nie wiem jeszcze, co będzie dalej. Wiem, że przedłużyli tę opiekę do 24.05., więc raczej jest cały czas taka prośba, żeby jedna osoba z działu nie pracowała, tylko, żeby była na urlopie czy na takiej opiece ze względu na to, że są niższe obroty i prezes twierdzi, że jest też mniej pracy. Na księgowość nie bardzo się to przekłada, bo u nas jest zawsze dużo tych papierów i wcale to nie maleje. Jak rozmawiam z dziewczynami, to mówią, że dużo tego jest, na moim biurku też jakaś mała sterta leży. Zarząd inaczej na to patrzy. Myślą, że jak obroty mniejsze, to wszystkiego mniej. Teraz ja jestem tą osobą, która nie pracuje, bo mam małe dzieci i mogę iść na opiekę.

**Jak to odbierasz, że szefostwo tak postanowiło?**

ten poprzedni tydzień, to raczej jeszcze byłam spokojna, bo potrzebowałam tego wytchnienia. W tym tygodniu to już zaczęłam mieć takie myśli, że może jak wrócę, to okaże się, że nie mam po co tam wracać. Wiadomo, że inaczej jest, jak się jest na miejscu i widzisz, co się dzieje, bo różne są też osoby w pracy i każdy dba o swój interes. Jak jesteś na miejscu, to możesz się wykazywać przed pracodawcą, a jak jesteś poza biurem, to wiadomo, że ktoś może myśleć, że nie chce ci się pracować czy coś. Bałam się, że wrócę do pracy i ta główna księgowa, która przejęła moje obowiązki, to przez to, że jest mniej pracy, to ona będzie to kontynuować, a dla mnie tym samym nie będzie co robić i stwierdzą, że jestem niepotrzebna. Zapewniła mnie, bo rozmawiałam z nią o tym i powiedziałam, że się boję...Mimo, że ona mnie uspokaja, to wiadomo, że są jakieś czarne myśli. To jest trudna sytuacja i wszyscy się boja o pracę. Zapewniła mnie, że to, że ktoś jest na opiece czy na urlopie, to nie znaczy, że jest do zwolnienia. Oni starają się zrobić wszystko, żeby nie zwalniać i dlatego zmniejszyli te wynagrodzenia i jeśli już, to powiedziała mi w zaufaniu, że jeśli sytuacja dalej będzie tak trwała, to będą się skłaniać, żeby jeszcze trochę zmniejszyć, tak do 20% obniżyć wynagrodzenia, ale nie chcą nikogo zwalniać na tę chwilę. I żebym się nie bała.

**I uspokaja cię to?**

Trochę mnie uspokoiło, ale mimo wszystko chciałabym już wrócić i powiedziałam jej, że myślę o tym, żeby wrócić i żeby być tam na miejscu. Też chcę, żeby to normalnie wszystko zaczęło funkcjonować.

**Maja się otwierać przedszkola i chyba te młodsze klasy też?**

Tak, wychowawczyni dzwoniła i robiła taką ankietę kto będzie chętny. ja pytałam, jak to będzie wyglądało - czy to będą zajęcia opiekuńcze, czy dydaktyczne. jak dydaktyczne, to jeszcze mogę to rozważać, a jak opiekuńcze to raczej nie. Powiedziała, że to nie będzie taka normalna nauka, że będzie to zupełnie inaczej wyglądało, ale muszę odpowiedzieć czy tak, czy nie, bo oni muszą wiedzieć, jak się zorganizować. Powiedziałam, że do 24 maja może zostać w domu, bo mamy dla niej zapewnioną opiekę. Mój mąż jest na pewno zdalnie do 24 maja, więc wiadomo, że jest w domu. A później nie wiem, ale powiedziała, że muszę już podjąć tę decyzję, więc powiedziałam, że nie zgadzam się, żeby wracała i chyba wielu rodziców tak odpowiedziało. Po tych ankietach, po podjęciu decyzji następnego dnia u nas ruszyły videolekcje w zerówce. Dużo rodziców pyta o te zajęcia dydaktyczne, bo chcemy, żeby się jeszcze pouczyli, więc pani 2 x w tygodniu będzie z nimi prowadzić półgodzinne videolekcje. Wczoraj o 10 było pierwszy raz i jutro mają być drugie zajęcia. Mają sobie zarezerwować więcej miejsca w pokoju, bo pani będzie takie ruchowe też robić z nimi zadania i trochę pracy w książkach. Asia bardzo przeżywała te zajęcia. Dzień wcześniej bała się, że co będzie jak nie będzie umiała odpowiedzieć, a jak nie zdąży zjeść śniadania rano, a jak się zaśpi i nie wstanie...Potem widziałam, że jej się bardzo podobało, umiała skoordynować ten mikrofon, że jak nie chciała czegoś powiedzieć, to go wyłączała i później już powiedziała w ogóle, żebym wyszła z pokoju, że ona sama. Widziałam, jak odpowiadała, przyklaskiwała. Już to jakoś ogarnęła. Na początku widziałam, że te dzieci się przekrzykiwały i ją to denerwowało, bo nie mogła nic powiedzieć. Ona chyba myślała, że będzie cisza i ona wtedy odpowie, a oni wszyscy na hura, ale potem widziałam, że się przestawiła.

**Czemu nie chciałaś, żeby wracała na lekcje?**

Dlatego, że jest to jednak tylko opieka, a nie będzie żadnych zajęć dydaktycznych. Dla mnie to nie jest jakieś wartościowe to przebywanie w tej szkole, a wręcz jest dużym zagrożeniem, że te dzieci jakoś poroznoszą tego koronawirusa, jeśli ktoś tam się zarazi. jak ma to być sama opieka, to nie ma to sensu. Gdyby to był inny etap roku szkolnego, ale jak do końca roku zostało niewiele czasu, to też to zaważyło.

**Co jest teraz dla ciebie wyzwaniem?**

Nie wiem, trudno powiedzieć. Uspokoiłam się tak ogólnie wewnętrznie i jakoś tak wszystko ogarniam. Więcej wolności jest, bo więcej się już wychodzi poza miejsce zamieszkania.

**Nadal ci czegoś brakuje?**

Może takiej radości na ulicach, tak tych ludzi więcej, bo jak się wyjdzie, to jednak widać, że są pustki, że ludzie się stosują. W majówkę kilka razy wychodziliśmy. Raz pojechaliśmy wszyscy nad jezioro w ciągu dnia, a po południu tylko we dwoje gdzieś sobie poszliśmy na spacer i widać, że nie ma ludzi nigdzie, naprawdę. Na spacerach, gdzie są te tereny zielone tak w ciągu dnia, to się trochę znalazło osób na rowerach czy spacerujących, ale na ulicach są pustki.

**Udało ci się znaleźć jakieś obrazki?**

Nie, jakoś nic mi nie wpadło w oko.

**Emocje - zdjęcia**

Może 12? Takie pozytywne kolory, ja czuję się tak pozytywnie raczej, ale nie takie wyostrzone te barwy...Taka stagnacja bym powiedziała i to mi się kojarzy z taką stagnacją.

Raczej pozytywne emocje, tylko takie delikatne, bez euforii. Tak neutralnie bym powiedziała, ale w kierunku pozytywnego.

**Ta stagnacja czym się objawia?**

No taką nudą, że cały czas siedzimy w tych domach i nic się tak za bardzo nie dzieje.

**Jest w tobie jeszcze jakiś strach, lęk?**

Nie, na tę chwilę chyba nie. Już widzę, że tak każdy akceptuje sytuację, wszyscy tak się dostosowują do jakichś zmian, przystosowują wszystkie te miejsca jakoś tak pod względem bezpieczeństwa - sklepy, biura. Ja pozytywnie myślę, że nawet, jak by to miało jakoś dłużej trwać, to jakoś to wszystko może ogarniemy.

**Czujesz, że ty też się przystosowałaś?**

Tak, myślę, że tak. Wczoraj mieliśmy takie spotkanie firmowe, też videorozmowę z prezesem i tłumaczył nam, jak właśnie biura zostały przystosowane u nas, bo od dzisiaj wracają pracownicy powoli do firmy. Wiem, ze u mnie w pokoju, gdzie mamy 4 osoby, to między mną a koleżanką taka pleksa została zamontowana i między kolejnymi koleżankami też, więc widzę, że mają obmyślone to wszystko, że starają się wprowadzić te względy bezpieczeństwa. Wszystko zostało przemyślane - jak ma wyglądać kontakt z kimś z zewnątrz. jakieś ściśle określone zasady są, więc myślę, że jakoś to po prostu ogarniemy. No i spokojną głowę mam o to, że ta młodsza córka nie wraca już, że ta starsza też ma te lekcje, więc jakoś się nie boję o nie, a u mojego męża mają na razie decyzję, że do 24 nie wracają, ale to jest tak duża firma, że raczej jeszcze pewnie dłużej to potrwa i raczej to będzie zmianami, że jedni jeden tydzień, inni drugi tydzień. Ludzie po prostu nie chcą nikogo narażać i starają się, żeby to jakoś płynnie przechodziło, żeby wracać do normalności, ale tak stopniowo. Można to jakoś poodgarniać.

**Spotykacie się teraz z jakimiś ludźmi?**

Z rodziną tak, z sąsiadami na ulicy też wiadomo, że się zamieni parę słów. Ktoś znajomy przyjechał nad jezioro, więc też się widzieliśmy, ale każdy zachowuje dystans, wszyscy są w maseczkach i każdy tego pilnuje. Nie ma jakichś takich spotkań, że długo razem przebywamy w pomieszczeniu. Raczej tak na powietrzu. Nie umawiamy się ze znajomymi na kolacje i nie siedzimy z nimi w domu.

**Czy coś się zmieniło w tym, jak robicie zakupy?**

Teraz to już częściej niż raz w tygodniu i ja też już często chodzę do sklepu albo jadę gdzieś tam coś kupić. jakieś kwiatki kupowałam na balkon...No często wychodzę, naprawdę. Nie cierpię teraz z tego powodu, że tak siedzę w tym domu. Może to też takiej normalności nadało w ciągu dnia jak się wychodzi.

**Otworzyli galerie. Byłaś już tam na zakupach?**

Nie, nie byłam, ale zamówiłam jeszcze trochę ubrań przez internet w międzyczasie dla dziewczyn, więc nie mam potrzeby iść. Słyszałam, moja mama coś wspominała, że są otwarte galerie, ale przymierzalnie są zamknięte. Dla mnie to jest takie trochę, jak zamawianie przez internet. Tyle, że dotkniesz materiału i widzisz mniej więcej, jaki to fason, ale ja lubię przymierzyć.

**Kupiłaś w ciągu ostatnich 2 tygodni coś dla siebie, dla przyjemności?**

Nie, chyba sobie nic na razie nie kupowałam. Przed kwarantanną jakieś nowe rzeczy sobie kupiłam, ale nie było okazji założyć, więc czekam, że może teraz jak wrócę do biura, to coś tam założę z tych rzeczy. Może jak już się pogoda wyklaruje, jak się cieplej zrobi, to może coś sobie kupię. Na tę chwilę aż takiej potrzeby nie mam, bo nie mam gdzie tego założyć.

**A te kwiatki?**

To takie doniczkowe na taras. Kupowałam je w takich sklepach naszych lokalnych. Tam mają jakieś sadzoneczki w promocyjnych cenach.

**Łatwość wydawania - skala**

No nie wiem...6-7?

**Dlaczego?**

Bo jeśli coś potrzebuję, to...Może 6 bardziej, bo ja raczej nie jestem rozrzutna i lubię oszczędzić. No ale też czasami mam takie podejście, że dla przyjemności też warto coś sobie kupić. W końcu po to zarabiam, żebym coś też z tego życia miała, a nie tylko gromadziła. Uważam, że na pewno warto tak funkcjonować i żyć, żeby sobie odłożyć na wakacje. Dla mnie wakacje to jest priorytet, bo cały rok pracuję po to, żeby jakoś tak nałapać tej energii w lato i zawsze 2 razy jedziemy gdzieś. Wiadomo, że jak dziewczyny potrzebują jakieś ubrania to też im się kupuje. Podstawowe rzeczy - jakieś buty, kurtki, a resztę, to żeby tak ładnie je wystroić. Ja praktycznie na takie rzeczy wydaję - jakieś kosmetyki, ubrania.

**Czy są takie rzeczy, na których nie chcesz oszczędzać?**

Nie wiem, chyba nie. Nie umiem określić nic takiego. Jak coś jest potrzebne, to wiadomo, że trzeba to kupić. Nie lubię kupować jakichś najtańszych butów. Są osoby, które pójdą, kupią jakieś ze sztucznej skóry, wydadzą poniżej 100 zł i kupią sobie częściej. ja nie lubię tak kupować. Ja wolę kupić buty tak do 300 zł, ale, żeby były skórzane i wiem, że w nich 3-4 lata pochodzę, a modele kupuję takie, że wiem, że pasują do większości ubrań i nie znudzą mi się jakoś szybko. Na tym może jakoś nie oszczędzam, ale też nie jest to też jakieś bardzo drogie. No to może to.

**A przykład, kiedy na czymś próbowałaś oszczędzić albo oszczędziłaś?**

Nie wiem...No może, jak jedzie się na jakieś sporty typu zimowe, to jeśli są to produkty dotyczące dzieci i dzieci rosną, to wtedy wolę kupić używane. Kupiliśmy narty, buty, kijki dla młodszej córki, jak jechaliśmy na narty i zapłaciliśmy za to 100 zł, bo ktoś sprzedawał po młodszym dziecku. Na tym wiadomo, że można oszczędzić, bo za rok to może już być za małe i w ogóle niepotrzebne. Tak samo jakieś maski do nurkowania - to też mogę kupić używane. Dużo ludzi kupuje na jeden raz, a potem jest im to niepotrzebne. Tylko patrzę, w jakim to jest stanie, bo nie kupię byle czego i zniszczonego, zużytego, bo dla mnie to też jest ważne, żeby to jakoś higienicznie ładnie wyglądało.

**Lubisz kupować rzeczy na jakichś promocjach, okazjach?**

Zawsze jak kupuję, to porównuję między sklepami, staram się do jakichś newsletterów zapisywać. Nawet jak mam jakieś 2 konta prywatne i jedno służbowe, to czasem, jak już podałam jeden mail, to czasem podam i inny, żeby ta zniżka przyszła. Na to patrzę, tak. Na koszty przesyłki albo jak wiem, że może będę to zwracała, to czy jest to darmowe, czy nie.

**Pamiętasz ostatnią sytuację, kiedy wydałaś jakieś większe pieniądze poza codziennymi zakupami? Wakacje, meble?**

Wakacje to wiadomo, ale to już wiadomo, że one będą, one są na horyzoncie i już się po trochu na nie odkłada. Ostatnio sobie jakieś buty kupiłam przed kwarantanną i już nie mogę się doczekać, aż je założę. Sportowe takie, trochę droższe. Na sportowe staram się dużo nie wydawać. Wolę na eleganckie trochę więcej wydać. Teraz sobie kupiłam, 300 coś zł zapłaciłam. Zastanawiałam się długo, no ale stwierdziłam, że skoro już tak długo o nich myślę. To nie jest tak, że to jest kaprys, tylko ja czasami długo myślę, że akurat pasowałyby mi takie buty, one są w sumie uniwersalne, kolor i tak dalej...Więc tak myślę, myślę jakiś czas, a [potem akurat nadarzy się okazja albo spotkam coś fajnego, no to` kupuję. Tak samo z ubraniami. Pasowałaby mi jakaś beżowa fajna spódnica, później za parę miesięcy coś takiego się pojawi w sklepie, więc wiem, że o tym myślałam, że też jest takie uniwersalne, ponadczasowe, więc kupuję. No i właśnie te buty...Leżą w szafie jeszcze nie rozpakowane.

**Kupowałaś je stacjonarnie?**

Nie, przez internet.

**Pamiętasz, jak się czułaś, jak je kupowałaś?**

Do momentu sfinalizowania to jeszcze wahanie czy nie za drogie, czy na pewno ich potrzebuję, ale później jak je kupiłam, to wszystko to odeszło gdzieś w niepamięć. Założyłam, okazało się, że bardzo wygodne, fajne. Zakup pozytywny. Nie miałam wyrzutów sumienia. Jak wiadomo, że one są wygodne i będą pasowały do wielu rzeczy, to nie. Wiem, że wykorzystam.

**A zdarzają się takie zakupy, że żałujesz, że to kupiłaś?**

Czasami się na pewno zdarzają. To też jakieś babskie zakupy - właśnie typu buty, sukienka. W ogóle z ubraniami jest tak, że często idziemy do sklepu, kupujemy to, a potem się okazuje, że masz 5 takich podobnych bluzek. Fakt, że to jest nowe - to chyba tak działa, że się w euforii było, jak się to kupowało.

**Często ci się tak zdarza?**

Raz na jakiś czas na pewno. Nie wiem, może jak się ma jakiś gorszy dzień? czasami mam tak, że mam pieniądze i idę do sklepu, żeby coś kupić czy na jakąś okazję, czy po prostu sezon się zmienił i chciałabym coś nowszego, a okazuje się, że nic w tych sklepach nie ma. A czasami przechodzę gdzieś idąc po spożywcze zakupy i zobaczę coś fajnego a nie mam za bardzo pieniędzy. Wtedy staram się zawsze to kupić, bo wiem, że później mi się już taka okazja nie trafi i to są zawsze najlepsze zakupy. Takie niespodziewane, jak coś wpadnie w oko. Wtedy to jest zawsze trafione na pewno. Jeszcze mi się nie zdarzyło, żebym żałowała.

**Raczej jesteś oszczędna czy rozrzutna?**

Raczej oszczędna chyba, mimo wszystko. Są te momenty takie, że wiadomo...Ale to każdy tak ma, że coś tam kupi takiego nie planowo, no ale ja raczej w kierunku oszczędnej.

**Jakie masz podejście do oszczędzania pieniędzy?**

No nie wiem...Ja mam mieszane uczucia. Na pewno nie wydałabym tak wszystkiego na bieżąco, jak się zarabia, żeby wychodzić na zero. Uważam, że trzeba mieć jakieś pieniądze, trzeba coś odkładać. Tym bardziej jak są dzieci i też jakaś czarna godzina. Różnie to bywa, ktoś zachoruje i nie wiadomo wtedy, skąd wziąć pieniądze. Ale też czasami sobie tak myślę, że tak gromadzimy te pieniądze, każdy jakieś ma te oszczędności, a później w sumie i tak one są na przyszłość tych dzieci naszych i my tak za bardzo z nich nie skorzystamy, mimo, że tak gromadziliśmy. Te dzieciaki też muszą i tak w sumie oszczędzać nadal i w sumie nikt tak w pełni nie poszaleje za te pieniądze. Normalnie, rozsądnie żyjesz, ale nie ma jakiegoś takiego szaleństwa, mimo, że coś tam masz odłożone.

**Jak u was to oszczędzanie wygląda?**

Staramy się jakieś lokaty sobie zakładać, jak już się coś tam więcej uzbiera. Mój mąż regularnie co miesiąc coś tam odkłada, a ja tak raczej, jak już coś się więcej uzbiera, to coś tam sobie blokuję. I na tej zasadzie. Tylko tak.

**Ile czasu moglibyście przeżyć z tych oszczędności bez przychodów?**

Nie wiem, trudno mi powiedzieć, ale na pewno byśmy szukali jakiejkolwiek pracy i nie patrzylibyśmy na oszczędności, żeby z nich żyć, bo raczej nikt nie chciałby tego wszystkiego stracić. Nawet, jak bym musiała zdjąć koronę i iść do sklepu spożywczego w takiej sytuacji, to musiałabym to zrobić. Nie bazowałabym na oszczędnościach i nie chciałabym ich zużywać. Myślimy też o dzieciach. Zuzia ma 12 lat, więc za jakiś czas pójdzie na studia i nie wiadomo, czy będzie to gdzieś w okolicy, czy gdzieś dalej. Ona jest bardzo samodzielna, więc na pewno będzie chciała szybko zacząć dorosłość, więc myślimy o tym, że kiedyś to będzie potrzebne i żeby ona miała jakiś łatwiejszy start, to trzeba będzie jakoś tam pomóc.

**Jak teraz widzisz oszczędzanie? Warto jest oszczędzać?**

Zawsze warto jest oszczędzać. To jest takie bezpieczeństwo psychiczne, że jednak coś tam masz, a tym bardziej, że teraz tak drożeje ta żywność, widać. I słychać, że będzie to drożało. Widać, że jest susza, więc może z warzywami też będzie tak, że te ceny będą wyższe. Jakieś zakazy podlewania...Słyszałam, że a Piasecznie jakieś mandaty nakładają na ludzi, jak ktoś z wodociągów podlewa ogródki. mandat w wysokości 5000. Cały czas jest takie ryzyko, że to wszystko będzie drożało i jeszcze przez tę kwarantannę, gdzie dużo zakładów na pewno przystopowało z jakąś produkcją. nie w tym momencie, ale za jakiś czas to się na nas odbije. Dlatego warto oszczędzać.

**Inwestujecie jakoś pieniądze, czy tylko te lokaty?**

Na razie nie inwestujemy, ale zastanawiamy się nad tym, bo nasza gospodarka wygląda tak jak wygląda i ta wartość pieniądza też się zmienia, i może się okazać za chwilę, że to, co mamy na lokacie, to już nie jest tyle warte. Zastanawiamy się, czy może w jakąś działkę zainwestować?

**Teraz jest dobry czas na inwestycje?**

Myślę, że tak... Jeśli chodzi o działki, to myślę, że na pewno. Nawet słyszę, że jest bardzo duże zainteresowanie Polaków teraz działkami. Patrząc na to, że wszyscy jesteśmy w jednym miejscu na tym podwórku, to nawet możliwość gdzieś sobie pojechać na tę działkę, coś tam porobić, zmienić otoczenie. Też pod tym względem.

**Któreś z was bardziej inicjuje te tematy oszczędzania, inwestowania?**

Może bardziej mój mąż, bo on się może tak bardziej orientuje, ale zawsze mnie pyta o zdanie, razem ustalamy, oglądamy, porównujemy. On może się tak bardziej angażuje, że on więcej ogląda, sprawdza. Nawet ostatnio zaczął tak trochę te działki oglądać z ludźmi, trochę się kontaktować. Przez to, że rozmawia z tymi wszystkimi ludźmi to też widzi jakie oni mają podejście do tematu, co tam kto zatai, wie o co pytać. Już tak bada rynek jakby i tak trochę sobie gadamy na te tematy, więc on tak głównie.

**Czy w twoich/ waszych dochodach dużo się zmieniło od strony perspektywy finansowej na przyszłość?**

Trochę się zmieniło, ale nie jakoś tak kolosalnie. Wiadomo, ja mam tę obniżkę, ale przez to, że nigdzie jakoś nie wydajemy tych pieniędzy, nie idziemy gdzieś jeść na miasto, nie pójdziemy do kina, do teatru, nie pojedziemy nigdzie na weekend, to jakby nie wydajemy aż tyle pieniędzy i nie jest to może tak bardzo odczuwalne. Głównie wydajemy na jedzenie.

**Masz myśli o tym, czy to wróci w ogóle do normy? Ten wasz budżet?**

Mam nadzieję taką, że wróci, ale wiem, że może być tak, że przez najbliższe kilka miesięcy nie. Nie wiadomo i musimy się liczyć z różnymi scenariuszami, ale myślę, że też trzeba myśleć pozytywnie. ja mam taki zawód, że księgowi są wszędzie potrzebni, więc wiadomo, że czy trochę mniej zarobić, czy trochę więcej...Różne są zarobki, ale zawsze gdzieś tę pracę się znajdzie. Trzeba myśleć pozytywnie.

**A sytuacja twojego męża?**

U nich to jest wielka korporacja. Oni co roku mają cele do wykonania i trzeba zwolnić ileś tam osób co roku. I w tym roku też były jakieś zwolnienia. On zmienił trochę strukturę, przeszedł w inne miejsce wewnątrz firmy i został jakby uratowany, ale nie wiadomo, czy za parę miesięcy ktoś nie powie, że małe są obroty i znowu trzeba kogoś tam zwolnić. Niby na ten rok te osoby już zostały wyznaczone, bo to tak jest, że na początek zgłaszają się chętni. Ktoś ileś lat pracował, wie, że mu się odprawa będzie należała i dla niego może być korzystniej się zwolnić, a może coś tam ma na horyzoncie. Potem te miejsca, które zostały do zwolnienia to już muszą wybrać sami przełożeni.

**Podjęliście jakieś działania, żeby ograniczyć wydatki?**

Może ja tak nie oglądam już tych ciuchów, nie kupuje tak nic dla siebie, ale to są takie delikatne, bo to nie jest jakieś szaleństwo, jeśli kupujesz kilka ubrań. jakoś bardzo nie ograniczaliśmy. Na paliwie się zaoszczędzi, bo w ogóle nie jeździmy samochodem, ale z kolei nam się akumulator rozładował i rozsypał i trzeba kupić nowy. Na jednym zaoszczędzisz a coś innego musisz kupić. Tak to wygląda. Nie było takiej rozmowy i rozważań, na czym by można zaoszczędzić.

**Jesteś zła na to, że pewnych rzeczy teraz nie kupujesz?**

Nie, ja po prostu się pogodziłam z tą sytuacją, zaakceptowałam to, dostosowałam się.

**Masz wrażenie, że masz takie optymistyczne podejście do życia?**

Chyba tak. Mój mąż bardziej jest taki, że się szybciej denerwuje i ona raczej ma takie negatywne...Ciągle marudzi na wszystko, a ja chyba tak inaczej. No i tak samo on narzeka, że już nie może na nas patrzeć, bo ciągle w tym domu się mijamy, jest hałas i w ogóle, to ja mu powiedziałam, że w sumie nie jest tak źle i jakoś się dogadujemy w tym domu, że już się przyzwyczaiłam do tego i że on w sumie też nie ma tak źle i że doceni to dopiero, jak będzie stał w korkach do pracy.

**Mówiłaś, że teraz możesz robić takie różne rzeczy, których normalnie byś nie doceniała albo byś ich w ogóle nie robiła?**

No tak i nawet ostatnio zaczęłam myśleć, że chyba mi będzie tego brakować i zaczęłam się bać tego, że ja zaraz wrócę do pracy. Nie masz takiej możliwości normalnie, żeby tak siedzieć w domu 2 miesiące. A tutaj wszystkim nagle spadła taka możliwość, że mogą sobie z tą rodziną tak częściej poprzebywać.

**Największym pozytywem jest to, że możesz spędzać czas z rodziną?**

Tak, tak. I poznać ich lepiej, bo tak, to się większość dnia spędza gdzieś w szkole, w pracy, poza domem, a te wieczory to takie krótkie, szybko mijają. Teraz praktycznie taka ciągłość tych 2 miesięcy, gdzie my non stop wszyscy razem jesteśmy.

**Co byś musiała jeszcze dodać do tej sytuacji, która jest teraz, żeby dla ciebie było w porządku? Co ci jeszcze teraz przeszkadza i co by trzeba było zabrać, żeby było optymalnie dla ciebie teraz?**

No jakieś takie wyjścia masowe. To też jest fajne. Niedługo będzie czerwiec i będą jakieś wianki nad Wisłą albo jedziesz na fontanny i sobie chodzisz, i wszędzie tak dużo ludzi. To, że takie skupiska są i tak widać radość wszędzie.

**Teraz ludzie są ogólnie mniej radośni?**
Chyba tak i w ogóle tak jakoś się unikają, ten dystans zachowują. Kilka tygodni temu to niektórzy może też tak panicznie reagowali, że ktoś się zbliżył. Teraz może już aż tak nie reagują, ale widzę, że wszyscy w tych maseczkach. To też w nas może budzić takie przeświadczenie, że bezpieczniej się czujemy jak widzimy kogoś innego w tej maseczce.

**Teraz nie obawiasz się, że jak wychodzisz, to możesz się od kogoś zarazić?**

Jak sama pilnuję tego mycia rąk, żeby nie dotykać oczu, nosa, twarzy, to myślę, że nie boję się jakoś tak. Ważne, żeby tego pilnować i już każdy ma te nawyki wyrobione. Jak będę tego pilnować, to raczej chyba się nie zarażę. Kiedyś może się zarażę, nie wiem. Boję się, że jak wrócę do pracy, to może tak być, bo wtedy zmienia się to otoczenie i jednak każda z tych osób jest z jeszcze innego otoczenia, i ma z wieloma innymi osobami kontakt, więc nie wiadomo, jak tam w pracy będzie. To jest już większe ryzyko.

**A ludzie dookoła ciebie, twoi bliscy? Jak oni się teraz zachowują?**

Wszyscy przestrzegają. Jeśli do sklepu, to każdy bierze swoje rękawiczki, maseczkę i każdy myje ręce, dezynfekuje, czy klamki przy drzwiach...My też wszyscy, jak przychodzimy, to myjemy mydłem, dezynfekujemy tymi płynami.

**A obserwujesz jakieś zachowania w twoim otoczeniu, że ktoś się inaczej zachowuje niż wy teraz?**

Nie, chyba aż tak za bardzo kontaktu nie mam. Nie rozmawiamy też tak dużo na ten temat. Widzę, że zachowują te podstawowe reguły, ale nie wdajemy się w rozmowy szczegółowe. Nie mam rozeznania.

**Kiedyś mówiłaś, że w pracy cały czas wysyłaliście sobie memy o koronawirusie?**

A, to na początku tak było, tak. Teraz już to się uspokoiło i nikt tak chyba już nie wysyła. To chyba trochę tak spowszedniało i każdy już chyba przyzwyczaił się do tej sytuacji. Każdy się przystosowuje. tak się życie zmieniło i trzeba jakoś funkcjonować w tym wszystkim.

**Myślisz w ogóle o tym, kiedy to się skończy?**

Myślę, myślę, ale to chyba parę miesięcy jeszcze potrwa. Z tego co czytam i jak naukowcy się wypowiadają, to chyba to się tak szybko nie skończy. W tych maseczkach też słyszę, że trochę pochodzimy. Jakoś trzeba się przyzwyczaić. Jak jeździliśmy rowerami gdzieś, gdzie ludzie nie byli tak blisko, to tam widziałam, że większość ściągała te maseczki albo zdejmowała, żeby pooddychać. My, jak wychodzimy na ulicę z dziewczynami, żeby sobie pospacerowały czy na deskorolce pojeździły, to też tu przy domu to bez maseczek. Dopiero jak gdzieś poza ten teren się idzie, to wtedy wiadomo, że...

**Masz takie momenty, że szczególnie o tym myślisz i się zastanawiasz, kiedy będzie koniec?**

Teraz przestałam tak myśleć. Już się tak przyzwyczaiłam może do tej całej sytuacji. Wcześniej może, jak widziałam, że nie wiadomo, jak to dalej będzie, a teraz, jak widzę jak to powoli wraca do normy, w sensie, że powroty do biur, to jakoś tak sobie ogarniam.

**Masz jakieś obawy związane z najbliższą przyszłością?**

Może takie, że jak wrócimy do tych biur, to żeby nie wyszło, że ktoś zachoruje, bo nagle wszyscy ze wszystkimi będą mieli styczność. Może to? Wczoraj prezes prosił, żeby teraz jak idziemy do kuchni na kawę, to żeby nie robić takich spotkań jak kiedyś, żeby jedna osoba poszła i zrobiła sobie coś do picia i tak pojedynczo, żeby w tej kuchni przebywać. Tam, gdzie jedliśmy posiłki był taki stół, gdzie w kilka osób jadło się obiad i też powiedział, że ten stół będzie zlikwidowany teraz i też prosi, żeby jeść przy swoich komputerach. Pomyślałam sobie, że w sumie fajnie, bo zastanawiałam się, jak on to rozwiąże i dobrze zrobił, moim zdaniem. Ale co z tego, że on teraz to mówi, jak ja wiem, że jedna koleżanka tydzień temu wróciła do biura i podobno sobie je normalnie obiady ze wszystkimi, w kuchni normalnie z ludźmi gada, w kilka osób sobie stoją...No jedni mają takie podejście, inni takie. Boję się, że się zarażę przez nieuwagę innych.

**Gdybyś się dowiedziała, że ktoś w twojej pracy ma koronawirusa i się zaraziłaś od niego, to co?**

Wtedy to już chyba trzeba postępować tak, jak w tych wszystkich procedurach. Pewnie jakaś kwarantanna czy coś. To zależy, czy byłyby jakieś objawy, czy nie. Mamy opracowane procedury. Dawniej było wejście do biura na odcisk, teraz jest już to zdjęte i jest urządzenie termowizyjne. Stajesz tam i mierzy ci temperaturę. Jeżeli u kogoś wykaże temperaturę, to będzie ta osoba proszona o opuszczenie biura i kontakt z lekarzem albo videowizytę. jakieś procedury już są opracowane, jak postępować. Czujesz się bezpieczniej, bo widzisz, że pracodawcy zależy na tym, żeby wszyscy byli zdrowi i nie chce nikogo narażać. To tak pozytywnie odbieram.

**A masz jakieś obawy w perspektywie kilku miesięcy?**

Obaw może na razie żadnych nie mam, bo nawet zaplanowaliśmy sobie wakacje już. Znaleźliśmy takie miejsce trochę odludne. Tam jest chyba z 5 domków, ci ludzie mają dużą posesję i jak z nimi rozmawiałam, to się pytałam, co będzie, jak się okaże, że któryś z gości zachorował albo jesteśmy na kwarantannie i ta pani powiedziała, że zwracają zaliczkę...Albo jeśli znowu nałożą obostrzenia. Powiedziała, że spokojnie, że to dla wszystkich jest sytuacja nowa, ale że zwracają zaliczkę i żeby się nie denerwować. Poza tym u nich od domku do domku jest spora odległość, teren jest duży, więc będą zachowywali środki bezpieczeństwa, będą wszystko dezynfekować. Nad morzem sobie zarezerwowaliśmy i od nich jest dojście na plażę akurat w takim miejscu, że tłumów raczej nie będzie, bo daleko od głównych wejść na plażę.

**Przyjmujesz taką możliwość, że obostrzenia mogą jeszcze wrócić?**

Tak, tak, bo jak teraz wszyscy powracamy do pracy, to może tak być. Zresztą tak uprzedzali naukowcy i lekarze, że nagle może zacząć przyrastać tych zakażeń.
