# Supplementary material for: The challenges arising from the COVID-19 pandemic and the way people deal with them. A qualitative longitudinal study
Source: PLoS One. 2021 Oct 11;16(10):e0258133. doi: 10.1371/journal.pone.0258133 (PMC8504766; doi:10.1371/journal.pone.0258133)
Supplement: S1 Dataset — (ZIP) [file pone.0258133.s003.zip › Transcriptions/stage 4/13.4_M_46_couple, with children.docx]

**13.4_M_46_couple with children**

**Jak wyglądały Pana ostatnie 2 tygodnie?**

Dokładnie tak samo, jak wcześniejsze, czyli praca co 2 dzień, trochę pomoc w nauce syna i praca przy domu. I tyle właściwie. Z jedną różnicą. Razem z żoną i synem pojechaliśmy w Bieszczady w końcu na jakiś spacer po górach.

**Z okazji majówki?**

Nie. Niezależnie od majówki.

**A jak wyglądała ta wycieczka? Była z noclegiem?**

W góry, bez noclegu. Zaparkowaliśmy samochód na parkingu, wyjechaliśmy rano, weszliśmy na Połoninę Wetlińską, w dół i do domu.

**A jak wygląda spędzanie czasu na co dzień obecnie? Coś nowego się pojawiło?**

Nie, nic nowego się nie pojawiło. Od momentu, jak otwarto las byliśmy 2 razy na rowerze rodzinnie też. Już nie tylko wirtualne jeżdżenie na rowerze, tylko też normalnie.

**Czy podczas jazdy na rowerze nosili państwo maseczki?**

Nie, na rowerze nie. A może inaczej. Na terenie wsi tak, ale zaraz za wsią, jak wjeżdżamy do lasu, to zdejmujemy.

**Są jakieś rzeczy, z których pan zrezygnował?**

Nie. Nic nowego nie przybyło i nic nie ubyło.

**Co jest obecnie największym wyzwaniem? Nadal konieczność odpowiadania na pytania w kwestii wirusa?**

Mniej pytań. Ludzie się uodpornili na sytuację. Tak podejrzewam. Przyzwyczaili się do tego, co jest i mam wrażenie, że uważają, że nie jest tak groźnie. Bo początek był taki, naprawdę - bombardowanie pytaniami. Przez powiedzmy ponad miesiąc. Teraz jest tego mniej. Oczywiście też się zdarzają różne pytania, ale nie aż tyle. Nawet 2 tygodnie temu to jeszcze było dużo więcej.

**Teraz coś innego stanowi wyzwanie?**

Myślę, że nie. Te same zadania przede mną. Myślę, że w tej chwili największym wyzwaniem jest prowadzenie lekcji z synem. Bo jeżeli mam do zrobienia z nim tylko zadanie domowe, to jest to sprawa prosta. Natomiast poprowadzenie z nim lekcji od początku do końca sprawia mi trudność. Nie jestem nauczycielem, nie wiem, czy to dobrze robię. Nie wiem, czy on wynosi z tego tyle, co powinien.

**Syn ma lekcje zdalne czy nie ma ich wcale?**

Ma lekcje zdalne. Ma w programie, który akurat używa szkoła, podane linki do różnego rodzaju zadań. Ma wypisane, co ma zrobić w podręczniku, które rozdziały z płyt z nauką języka wysłuchać. Natomiast wiadomo, że to nie jest... Ja nie potrafię poprowadzić właściwie lekcji, ale to, co potrafię, to robię z nim. Ale to się nie zbliża do właściwej lekcji.

**Czyli te lekcje wymagają dużego uczestnictwa z państwa strony?**

Oczywiście. Zaangażowania rodziców. I to dużego. Na szczęście żona jest nauczycielem. Akademickim, ale ma przygotowanie do nauczania, więc jej to idzie dużo lepiej niż mi.

**Czy coś jeszcze się pojawiło, co zaczęło bardziej przeszkadzać niż wcześniej?**

Nie.

**Na koniec ostatniego spotkania poprosiłam pana o znalezienie obrazka lub zapisanie towarzyszących panu emocji. Czy coś takiego się pojawiło?**

Nie. Zupełnie nic.

**W takim razie wyświetlę te same obrazki, co poprzednio. Które z nich najlepiej oddają pana emocje?**

Zostaje to samo - 1 i 4. Nic się nie zmienia.

**Mógłby pan opowiedzieć o swoich emocjach na podstawie tych obrazków?**

Dalej tkwimy w jakimś takim... Trudno powiedzieć stagnacji, ale niewiele się chyba zmienia w obecnej sytuacji, więc czekanie, czekanie... Oczywiście pozorowane jakieś tam ruchy ze strony władz tak naprawdę w mojej świadomości niewiele zmieniają. I ludzie, którzy starają się sobie jakoś radzić w sytuacji i pomagają sobie nawzajem. I zaczynają sobie dobrze radzić.

**Widzi pan po innych, że zaczynają sobie coraz lepiej radzić z sytuacją?**

Tak. To, co było na początku takie uderzające, to w pierwszym momencie, ludzie, którzy prowadzili jakąś działalność z obszaru gastronomicznej czy różnych usług, to oni byli przerażeni tym, co się dzieje i jakie mają perspektywy. Ale stopniowo to... I oczywiście oni kompletnie - jakieś tam kawiarnie, czy małe piekarnie, cukiernie, oni nie mieli w pierwszej chwili klientów, ale poprzestawiali troszkę sposób funkcjonowania, pozmieniali to. W trochę inny sposób zaczęli zmieniać klientów, a ludzie dobrze na to zareagowali i myślę, że jest nieźle. Pytam ludzi w restauracjach, jak czasem odbieram jedzenie, jak funkcjonują. I jednak się to powtarza, że na początku było trudno, ale troszkę zmieniliśmy sposób funkcjonowania i jest dużo lepiej. Nie jest tak, jak było przed, musimy się starać, ale jest lepiej niż na początku.

**Czy u siebie też pan widzi to, że teraz sobie pan lepiej radzi z tą sytuacją ogólnie?**

Nie.

**U pana to jest tak jak na początku?**

Myślę, że tak. Ja w moim funkcjonowaniu zawodowym i w domu nic się tak w ciągu tego czasu - od dużych zmian, czyli od zmiany rytmu pracy, się niewiele zmieniło u mnie i jest tak samo. Może w tej chwili mamy więcej pacjentów na oddziale, bo się zaczynają pojawiać skutki ograniczeń wprowadzonych przez władzę. I zaczynają trafiać pacjenci, którzy się bali wcześniej przyjść do szpitala z objawami, które być może rozpoczynały jakiś ciąg niekorzystnych zdarzeń i czekali z tym. A w tej chwili już trafiają w takim stanie mocno zaawansowanej choroby, gdzie wymagają bezwzględnie szybkiej pomocy i ta pomoc często nie jest wystarczająca.

**To jest to, o czym pan wspominał wcześniej, że ludzie nie przestali nagle chorować na inne choroby i są to skutki uboczne koronawirusa?**

Tak. Jest to niezwykle ważne, bo wszyscy mówią o koronawirusie i przestało się nagle mówić o chorobach, które tak naprawdę przynoszą dużo większe żniwo śmiertelnych ofiar niż koronawirus. Koronawirus nie jest główną przyczyną śmierci w Polsce. Ci pacjenci się boją po prostu. Boją się przychodzić do szpitala, boją się, żeby się nie zarazić. Natomiast w tym czasie postępuje dalej ich choroba podstawowa, czy to choroba wieńcowa, czy niedokrwienie kończyn, cukrzyca, niewydolność nerek. I ci chorzy w końcu trafiają do szpitala w tak ciężkim stanie, że czasami trudno im już pomóc skutecznie. Trzeba im amputować nogi albo trafiają z przechodzonym zawałem czy rozchwianą cukrzycą w takim stanie, że często trudno ich z tego wyprowadzić. Jest tych pacjentów coraz więcej. To wyraźnie zauważalne od powiedzmy 10 dni- 2 tygodni. My mamy na oddziale dużo więcej chorych. Tak, jak mieliśmy 4-5 osób na 15 łóżkach, to mamy w tej chwili 10-12 osób. I to są ludzie ciężko chorzy, którzy bezwzględnie wymagają leczenia natychmiastowego.

**Czy to oznacza, że teraz ma pan więcej pracy i jest ona trudniejsza?**

Nie. To jest i tak 30% tego, co było przed epidemią. Dla mnie dalej to jest jeszcze luźny charakter pracy. Taki dużo spokojniejszy i wolniejszy niż wcześniej. Natomiast rzeczywiście jest więcej pacjentów. Ja czekam na normalny tryb pracy, kiedy operujemy 6-8 zabiegów dziennie, a nawet więcej, a nie 3-4. Dla mnie tamta sytuacja jest normalniejsza.

**Czy w związku z obecną sytuacją pojawiają się w panu jeszcze jakieś emocje? Wcześniej pojawiała się też złość wynikająca z działań rządu.**

W tej chwili myślę, że złość to nawet jest za mało powiedziane. To, co obecna władza wyrabia już się zaczyna odbijać na echem w Europie. I zarzuty ze strony innych państw członków Unii się pojawiają, że w Polsce się demokrację burzy, niszczy. I trudno temu nie przyznać racji. Bo obecna władza, wprowadzając tarczę antykryzysową, przemyca tam różnego rodzaju zapisy, które ułatwiają im funkcjonowanie i tworzą państwo dyktatury, a nie państwo demokratyczne. I to mnie doprowadza do wściekłości. Nie mogę tego tolerować. W zestawieniu jeszcze... Internet jest taki medium, gdzie nie zostaje nic... Nie da się usunąć skutecznie tego, co byśmy chcieli i zostają różnego rodzaju wypowiedzi polityków sprzed lat. Można oszaleć, jak się słucha tego. Ludzie, którzy w tej chwili nie mają żadnych zasad w rządzeniu, opartych na demokracji, na poszanowaniu konstytucji, prawa, urabiają sobie prawo na własne potrzeby, a 10 lat temu mówili zupełnie coś innego. Jak byli w opozycji oczywiście. To jest przykre. Oczywiście wszyscy wiedzą... Może inaczej, nie wszyscy, ale ludzie myślący wiedzą, że politycy mamią ludzi obietnicami po to, żeby się dorwać do władzy i jak ktoś ma ochotę porządzić, to czasem po trupach do celu. Ale jak się widzi, to w aż tak bardzo wyraźny sposób, to to boli. I tyle mam do powiedzenia.

**Ta wściekłość jest cały czas czy pojawia się w jakichś konkretnych momentach?**

Nie, ona jest cały czas. Bo mnie to cały czas otacza. Codziennie. Nie oglądam wiadomości. Jak mi się zdarzy raz na jakiś czas oglądać jakież reżimowe programy, to przecież można... Z resztą nie oglądam ich w całości, tylko gdzieś raz na jakiś czas, albo zaglądając do dyżurki pielęgniarek albo pacjenci czasem oglądają i jak wchodzę, to słyszę parę zdań. Nie da się tego wytrzymać.

**Uważa pan, że odcięcie się od tych informacji mogłoby być w jakiś sposób pomocne, czy i tak czułby pan, że to jest?**

Oczywiście, że czuję, że to otacza. Nie da się inaczej, jeżeli np. w szpitalu rządzą ludzie, którzy nie mają o tym zielonego pojęcia, co robią, a mimo to, mają poparcie polityków. Bo żadne inne, bo cały personel jest przeciwny, żeby ta osoba była dyrektorem, a ta kobieta, która jest umocowana w różnego rodzaju układach politycznych, nie ma skrupułów do tego, żeby podejmować beznadziejne decyzje ze szkodą dla wszystkich. Dla pacjentów przede wszystkim i później też dla personelu.

**Czyli ze względu na to, że decyzje polityków odbijają się na pana pracy, to nie ma pan możliwości się od tego odciąć?**

Nie ma możliwości, bo jest to szpital publiczny. W jakimkolwiek prywatnym przedsiębiorstwie, firmie - czy małym, czy dużym, wygląda to zupełnie inaczej. Miałem szanse to porównać. Robiłem specjalizację 2,5 roku z chirurgii naczyniowej w ośrodku komercyjnym. Prywatna sieć szpitali ogólnopolska i tam polityka nie stanowiła tak dużego problemu. Tam na pierwszy plan wysuwały się pieniądze, a polityka była gdzieś w tle - daleko, daleko. A w placówkach publicznych - bo to nie tylko w naszym szpitalu, ale też w innych, nr 1 to jest układ polityczny, a cała reszta gdzieś daleko za tym. A na ostatnim miejscu jest pacjent, który jest tak naprawdę przeszkodą dla tych udających zarządzanie szpitalem. I to, że gdzieś tam jeszcze jest ten pacjent, to same kłopoty.

**Czuje się pan zagrożony obecnie sytuacją?**

W pewnym sensie tak, ponieważ mój ordynator, nie mogąc już wytrzymać głupich i niebezpiecznych decyzji dyrekcji, złożył wypowiedzenie z okresem miesięcznym. I jak dyrekcja przyjmie to wypowiedzenie i nie uda się dojść do kompromisów, to ja też będę musiał złożyć wypowiedzenie. To znaczy, chcę złożyć wypowiedzenie razem z kolegami. Więc coś, co budowaliśmy przez 5 lat - nasz oddział, i zbudowaliśmy bardzo dobrze działającą jednostkę, niestety trzeba będzie to zatopić, opuścić - nie wiem, jak jeszcze można to określić. I to jest przykre. Nie jest w jakimś bezpośrednim sensie zagrożenie dla mnie, bo ja wiem, że pracę znajdę, natomiast ucierpi na tym mój komfort, mój wolny czas. W dalszym ciągu będzie to półśrodek. Bo nie o to chodzi, żeby utrudniać sobie życie, bo im człowiek jest starszy, tym chciałby mieć łatwiej.

**To budzi w panu lęk, niepewność?**

Lęk nie, niepewność tak.

**Jak pan obserwuje otoczenie, to jak sobie radzą w sytuacji epidemii inni?**

Myślę, że ludzie są - to przy poprzednim spotkaniu mówiłem - że ludzie nie są tak czujni i nie mają jeszcze takiego odczucia zagrożenia, jak miesiąc temu. Uważam, że czujność się zmniejszyła, że ludzie tak podchodzą do tego, "a, tak naprawdę nic się nie dzieje i chyba nie jest to duży problem". Patrząc na ilość ludzi i na ulicy i czasem w rozmowach różne rzeczy wychodzą, to chyba nie czują takiego zagrożenia, jak 2 miesiące temu.

**Widzi pan, że ludzie przestrzegają ograniczeń?**

Myślę, że w większości tak.

**Wracając jeszcze do pana perspektywy. Jak sobie pan radzi z tymi emocjami. Czy nadal hobby pomaga, czy coś innego?**

Tak. To jest najlepsze, co można chyba zastosować. Przynajmniej dla mnie. Czyli hobby plus jakaś praca, która jest też może w pewnym sensie hobby albo czymś co lubię robić. Jeśli chodzi o sytuację w pracy, to staramy się pomóc ordynatorowi w takim zakresie, w jakim możemy - uruchamiając różnego rodzaju znajomości, pisząc pisma i starając się, żeby jednak ktoś zwrócił uwagę na bałagan, który się dzieje. Postanowiliśmy się nie poddawać i nie rezygnować bez walki. Więc to też jest jakaś forma reakcji na sytuację w pracy.

**Ta pomoc ordynatorowi buduje to poczucie solidarności, o której pan wspominał?**

Na pewno. To jest to, co gdzieś tam u mnie wychodzi w formie tego obrazka, gdzie jest 5 czy 6 rąk splecionych. To chyba też to reprezentuje. A ponieważ jednomyślnie mamy podejście do sytuację w pracy - wszyscy zatrudnieni lekarze tak samo postrzegają, co się dzieje, więc chyba ten obrazek jest adekwatny.

**Z pana perspektywy, nawet w tym scenariuszu, jeśli musiałby pan złożyć wypowiedzenie, czuje się spokojny o znalezienie pracy?**

Dość spokojny. Jest to kwestia tylko odległości, jaką będę musiał pokonywać. I sposobu, w jaki będę pracował. Czyli, jeśli będę miał daleko do pracy, to nie będzie to codzienna jazda, tylko będę jechał na 2 dni do pracy, później jakiś jeden dzień plus dyżur.

**Jak u pana teraz wyglądają zakupy?**

Nic się nie zmieniło. Muszę tylko uważać na godziny, w których robię zakupy w sklepach spożywczych. Czyli ten przedział 10-12, który jest zarezerwowany dla starszych osób. Zapominam o tym czasami i czasami mi brakuje czasu na to, żeby skończyć te zakupy na spokojnie. Jeżeli za późno wyjadę z domu, czy wracając z pracy zapomnę, że od 10 sklepy są otwarte dla seniorów, to czasem muszę zrezygnować z odwiedzenia niektórych sklepów, robiąc np. w sklepie spożywczym zakupy, a później chcę jeszcze podjechać do jakiegoś sklepu z materiałami budowlanymi, to później jednak rezygnuję z tego sklepu budowlanego, bo przez godziny dla seniorów już nie zdążę. Tyle się zmieniło. Dalej robię zakupy w Internecie. Te same produkty. Może doszły jakież rzeczy, które są związane z aktualnymi pracami. Odnowiłem meble ogrodowe, idzie sezon letni, więc trzeba będzie tam co nieco ponaprawiać, więc trochę rzeczy też zakupiłem związanych z życiem w ogrodzie.

**A jeśli chodzi o zakupy spożywcze, to robi pan je z taką samą częstotliwością, czy coś się zmieniło?**

Raczej tak. Staramy się zrobić większe zakupy a rzadziej chodzić do sklepu niż częściej mniejsze ilości.

**A jak często teraz pan robi zakupy?**

2 razy w tygodnie, czasem rzadziej. Czasem raz w tygodniu.

**To są zakupy z listą?**

Tak, z listą.

**Państwo zawsze robili zakupy z listą?**

Na pewno teraz częściej się ta lista pojawia, ale wcześniej też ta lista dość często była.

**A ona się pojawia, żeby ograniczyć czas spędzany w sklepie?**

Dla mnie tak. I częstotliwość.

**Pojawiły się jakieś zakupy dla przyjemności w ostatnim czasie? Na poprawę humoru?**

Nic mi do głowy takiego nie przychodzi.*[śmiech]*

**Wczoraj otworzyli galerie handlowe. Co pan o tym sądzi?**

Będzie wzrost liczby zachorowań. I myślę, że dość radykalny, dość duży.

**Dlaczego otworzyli te galerie?**

Żeby uspokoić nastroje przed nadchodzącymi wyborami.

**To decyzja polityczna?**

Uważam, że czysto polityczna decyzja.

**Czy pan planuje robić zakupy w galerii?**

Nie. Nie mam nic takiego, żebym musiał tam iść. Nic do kupienia. Także absolutnie nie planuję i dla mnie otwarcie galerii nie jest jakimś tam... Jak to ładnie ująć? Nie spadł mi kamień z serca, że były zamknięte i wreszcie są otwarte, więc szybko pędzę do sklepu, bo muszę pochodzić po sklepach i kupić jakieś rzeczy, które do tej pory były dostępne tylko przez Internet. Nie mam takiego odczucia.

**Czyli to otwarcie galerii nie jest pana zdaniem bezpieczne?**

Moim zdaniem nie, bo szczyt zachorowań w Polsce to właśnie w tej chwili się nam objawił. A jak ktoś się zastanowi sensownie nad przebiegiem epidemii i porówna Polskę, Włochy - kiedy był tam szczyt zachorowań, kiedy jest u nas, to widać, że to wszystko sztucznie jest u nas przeprowadzone. Albo się decydujemy na taki model, jak jest w Szwecji i kto umrze, to umrze, albo staramy się jeszcze ograniczyć, wtedy cały przebieg epidemii w Polsce będzie się wydłużał, ale nie będzie on tak tragiczny w skutkach. Łączna ilość ofiar będzie taka sama, ale nie będzie tak boleć, bo będzie rozciągnięta w czasie.

**Dlaczego zakupy budowlane robi pan czasem stacjonarnie, a nie w Internecie?**

Niektóre rzeczy muszę pooglądać. Jakieś takie elementy drobne, metalowe - muszę je po prostu zobaczyć. Nie wszystko jest dość dobrze opisane, a samo zdjęcie nie zawsze przynosi wystarczającą ilość informacji.

**Czy słyszał pan wśród otoczenia o osobach, które planują robić zakupy w galeriach handlowych?**

Na razie nie. Ale rozmawiam z kolegami z pracy, z koleżankami i z najbliższą rodziną. Wśród nich nie. Wśród sąsiadów, tych najbliższych, z którymi rozmawiamy, to też nie. Nie jest dużo tych osób, więc to może nie jest bardzo adekwatne do nastrojów społecznych, ale wśród mojego otoczenia nie.

**W skali 1-10 na ile łatwo wydaje się panu pieniądze?**

Odpowiedź nie jest łatwa. Myślę, że mniej więcej 4-6 w zależności od tego, co kupuję.

**Mógłby pan podać jakieś przykłady, kiedy jest łatwiej, a kiedy trudniej?**

Łatwiej mi się wydaje pieniądze w sklepie spożywczym, a trudniej mi się wydaje pieniądze na produkty typu RTV/AGD, jakieś większe wydatki. Może łatwiej na ubrania. Aczkolwiek nie są to zakupy, że tak powiem znienacka. Muszę pójść po konkretny produkt. I jeśli szukam np. marynarki i znajduję ją, to nie jest jakiś duży problem. Jeśli mi się spodoba, to wydam większe pieniądze, a jeśli mi się nie podoba, to po prostu nie kupuję na siłę. Wtedy, wydawanie pieniędzy na rzeczy, które mi się mniej podobają, jest dużo trudniejsze. A na te, które mi się podobają, łatwiej się wydaje.

**To, że jest trudniejsze, wynika z tego, że jest konieczność kupienia czegoś...**

Tak. A nie ma przekonania, że to właśnie to, co mi w pełni odpowiada.

**Trochę łatwiej się wydaje na zakupy spożywcze. Z czego to wynika? Z tego, że są to mniejsze kwoty?**

Chyba nie, że mniejsze kwoty. Czasami jest pomysł na jakiś tam smakołyk. Nie zastanawiam się, że jest to mniejsza kwota, to mogę więcej wydać. Bo też można produkt o połowę tańszy, ale powiedzmy decyduję się na coś innego. Nie chodzi mi o bezwzględną wartość, ale na jakiś rząd wielkości.

**A jak pan wydaje pieniądze w sklepie spożywczym na smakołyki, to jakie emocje się w panu pojawiają?**

[Śmiech]. Bardzo dziwne pytanie. W ogóle się nie zastanawiam nad emocjami. Po prostu mam ochotę np. przygotować jakąś potrawę i szukam odpowiednich produktów. Jeśli je znajduję, to jestem zadowolony, że udało mi się kupić np. polędwicę wołową albo rybę określoną. I to mi sprawia przyjemność. Nie mam żadnych przemyśleń, jeśli chodzi o wydawanie pieniędzy wtedy. Nie myślę o tym, że wydaję pieniądze. Jestem zadowolony, że kupiłem to, co chciałem.

**Jakby się pan miał zastanowić nad tymi niecodziennymi zakupami, jak np. RTV, meble, wakacje - jak pan tutaj podejmuje decyzję?**

To są produkty np. typu telefon. Wtedy się zastanawiam czy muszę kupić telefon... Porównuję ceny najnowszej generacji, a może poprzedniej, a może 2 generacje w tył - jakie mają możliwości parametryczne. Czy to, co jest mi potrzebne jest tylko w tym produkcie najnowszym, który jest zdecydowanie najdroższy. I często 2 razy droższy albo nawet więcej niż ten, który był sprzedawany 2 lata temu, a wciąż można kupić jako nowy. Na to patrzę. Patrzę na to, czy rzeczywiście muszę mieć poduszkę w paski czy może być gładka. Czy rzeczywiście zapłacić 200 zł za poduszkę, czy 600 zł za poduszkę - ja podaję takie może idiotyczne przykłady, ale chodzi o podejście. Czy muszę kupić poduszkę w paski czy może być gładka. A cena jest znacząco różna. Zastanawiam się, czy rzeczywiście ten produkt jest mi potrzebny. Czasami rezygnuje z zakupów, myśląc, że nie jest to towar pierwszej potrzeby i może to poczekać na inny moment, bo teraz są inne wydatki. I te produkty kupowane bardzo rzadko, okazjonalnie, które są formą sprawienia sobie przyjemności, są takie chyba mocno przemyślane. I też może nie chodzi o to, czy ja wydam duże pieniądze, tylko czy te rzeczy są rzeczywiście konieczne i czy ja je muszę kupić. Może w ten sposób. A jeśli już się decyduję, to szukam produktu, który może nie jest ani najtańszy, ani najdroższy, tylko gdzieś tam się mieści w tych wartościach średnich - cena produktu. Natomiast szukam opinii, informacji na temat jakości produktu. Nie musi to być produkt jakiejś znanej marki, natomiast jeśli on jest często kupowany, ma opinię wśród ludzi dobrego produktu, to wolę kupić coś takiego.

**Czyli szuka pan rozwiązań jak najbardziej dla pana optymalnych?**

Tak. I tu zdecydowanie, jakby pani na tej skali umieściła łatwość wydawania pieniędzy, to przy tych produktach będzie to 4.

**Przy wydaniu pieniędzy np. na telefon, pojawia się zadowolenie z samego zakupu, tak?**

Tak, to zdecydowanie tak.

**Jak to jest z wakacjami? Jak wygląda podejmowanie decyzji w kwestii wakacji?**

Od wielu lat jeździmy z żoną na wyjazdy, które sami sobie organizujemy. Więc wygląda to w ten sposób, że kupujemy - jeśli lecimy gdzieś dalej, to szukamy biletów. I wiadomo, że bilety na samolot - były do tej pory, a teraz zobaczymy, jak będzie - okresowo są promocje, zniżki i wiedzieliśmy, że kupując bilet na lot do Azji jesienią warto zwrócić uwagę na ceny biletów styczeń/luty, bo wtedy były dość spore obniżki. Przygotowując się do dalszego wyjazdu, patrzymy na to. Lecąc na Sardynię, wiemy, że warto popatrzeć, jak się kształtuje cena biletów, bo często jest różnica rzędu 25-30 % ceny biletu lotniczego. Więc też sprawdzamy ofertę, powiedzmy, 6-8 miesięcy przed wakacjami. Nie jest to w ostatniej chwili planowanie. Szukając noclegów też wybieramy. Oczywiście, że nie jest to pierwszy lepszy nocleg, który nam się trafi, tylko przeglądamy sobie oferty i wyszukujemy, co jest dla nas atrakcyjne - położenie, wyposażenie obiektu. Położenie wiąże się z wieloma ważnymi rzeczami, czy dostępność do plaży, jeśli jedziemy nad morze, czy do stoków narciarskich, jeśli to są wyjazdy zimowe. Dostępność wypożyczalni, miejsce, gdzie możemy wykupić lekcje windsurfingu itp. - rzeczy, które są dla nas ważne.

**Czyli Państwo mają jakieś założenia dot. wakacji, a następnie porównują państwo ceny biletów, itp., żeby nie wydać niepotrzebnie za dużo?**

Oczywiście. Można przeznaczyć pieniądze... Zamiast zapłacić 1000 zł za bilet lotniczy można zapłacić 800 zł, a te 200 zł można przeznaczyć już na miejscu na różnego rodzaju wydatki, związane z codziennym funkcjonowaniem - jedzenie, czy dodatkowe atrakcje. Więc warto zwrócić na to uwagę.

**Nazwałby się pan osobą oszczędną czy rozrzutną?**

Myślę, że oszczędną. Żona mówi, że ja jestem niemiecki księgowy.

**Jakie sytuacje świadczą o tej oszczędności?**

Myślę, że porównywanie cen. I to, że zwracam uwagę, czy można coś naprawić, odnowić, a nie od razu wyrzucać i kupować nowe. Mogę zaszyć leżak, jeśli się rozpruł na szwach, to wolę wziąć igłę z nitką i to przyszyć i to wygląda, jak wcześniej, a nie kupować nowe, a to wyrzucać. I przeglądam oferty. Ta oszczędność też pojawia się w momencie, kiedy ja przeglądam w Internecie produkty. Np. szukając materiału na poduszki do ogrodu, przeglądam materiały, a nie pierwszy lepszy, który wpadnie mi w oko od razu kupuję. Chyba też to, co powiedziałem o telefonach, o sprzęcie RTV. Nie oszczędzam pieniędzy na wakacjach, bo uważam, że ciężko pracuję przez cały rok, a wakacje są po to, żeby odpocząć. To oczywiście też nie polega na tym, że wyrzucam te pieniądze na prawo i lewo, ale jak już jedziemy gdzieś, to idąc na kolację, czy jak wypożyczamy sprzęt, jak rower, windsurfing, narty, buty, to wtedy już nie jest to moment, w którym ja mówię "oj nie, nie mogę sobie na to pozwolić, nie chcę, zrezygnuję z jednego dnia wypożyczenia nart dla syna, bo to za drogo". Nie, w takich momentach to tak nie funkcjonuje. Jeżeli chcę, siedząc przy kolacji, wypić jeszcze jeden więcej kieliszek wina, to już się wtedy nie zastanawiam nad tym, że "o, to 4 czy 10 euro za kolejny kieliszek wina, to już nie, bo to za dużo". Tylko chcę tą kolację zjeść tak, żeby mi było przyjemnie. I wtedy te pieniądze nie są nr 1.

**Wspominał pan, że musiał zaprzestać praktykę prywatną w gabinecie i to zmieniło sytuację finansową. Jak ona teraz wygląda u Państwa? To się wiąże ze spadkiem dochodów?**

Tak. Dość znacznym.

**Ten znaczny spadek tworzy zagrożenie dla państwa?**

Jeszcze nie. Dochód, który mam z pracy w szpitalu, wystarcza na to, żeby nie czuć zagrożenia na razie. Jeszcze nam się budżet dopina i na codzienne funkcjonowanie, ponieważ jest sytuacja, w której nie planujemy wakacji, wyjazdów, więc te finanse, którymi w tej chwili dysponuję, pozwalają na to, żeby sobie żyć. Ale gdybym musiał zaplanować wakacje i jakiś wyjazd zimowy, to by trzeba było się zastanowić wystarczy.

**Myśli pan, że ta sytuacja finansowa wróci do normy po epidemii?**

Wróci. Tak.

**Dlaczego pan tak sądzi?**

Ludzie chorują. Mam taki zawód, który jest potrzebny. Ludzie się cały czas wypytują, kiedy będę miał otwarty gabinet i wydzwaniają do pani z rejestracji. Nic tu się chyba nie zmieni, bo ludzie będą dalej chorować. Jeżeli ja nie zrobię czegoś takiego, że ludzie nie będą chcieli do mnie przychodzić, to będą przychodzić, bo potrzebują pomocy, a ja tej pomocy udzielam. Z racji zawodu. Tak samo, jak piekarz dalej będzie potrzebny i fryzjer - co widać w tej chwili na ulicach i wśród moich kolegów, że przydałoby się, żeby ten sektor funkcjonował normalnie [śmiech]. Lekarze też będą potrzebni i pielęgniarki też. I ratownicy medyczni i cała branża medyczna wróci do funkcjonowania, a ludzie czekają, żeby było normalnie.

**Czy w związku ze zmianą dochodów podjął pan jakieś działania w celu ograniczenia budżetu?**

Myślę, że tak. Pewne przyjemności zakupowe, które mógłbym sobie sprawić, zeszły na plan dalszy. Ale też miałem w planach zrobić remont... Mam taki swój pokój muzyczny, w którym stoi sprzęt. Chciałem tam zrobić remont, kupić meble - i to musi zejść na dalszy plan.

**To jest rezygnacja z takich rzeczy "ekstra"?**

Tak. To są rzeczy, które nie muszą być, nie są najważniejsze.

**Pojawia się szukanie tańszych zamienników czy specjalnych okazji?**

Jeszcze nie. Ponieważ ograniczyłem zakupy dla przyjemności od końca stycznia, to nie szukam, bo na razie nie mam w planach kupować. Tylko te rzeczy potrzebne.

**A wśród rzeczy potrzebnych to się nie pojawia?**

Nie, bo nie zmieniłem podejścia do zakupów. Uważam, że wyszukiwanie produktów jak najtańszych niekoniecznie jest dobre. Wolę rzeczy, które są pewne, swoją jakością gwarantują dobrą jakość użytkowania. Dla mnie produkt powinien być taki, żebym ja za chwilę nie musiał kupować kolejnego egzemplarza, bo tamten się zepsuł, bo był tani. Więc to bez sensu.

**To ograniczenie tych dodatkowych wydatków było dla pana trudne?**

Nie. Jestem niemieckim księgowym, więc podejmuję racjonalne decyzje. Jak trzeba to trzeba.

**Ma pan jakieś sposoby na kontrolowanie budżetu, jak np. spisywanie wydatków?**

Nie.

**I to się wcześniej też nie pojawiało?**

Nie. Ja tego nie robiłem.

**Patrząc ogólnie na tę sytuację, czy teraz generalnie dobrze jest ograniczać wydatki?**

Tak, oczywiście?

**Dlaczego?**

Bo ludzie stracili bardzo dużą część dochodów. Niektórzy całkowicie stracili dochód.

**A u ludzi, u których się nic nie zmieniło - oni powinni ograniczać wydatki?**

Jest to ich decyzja. Myślę, że nie. Nawet powiem pani, że uważam, że dla ludzi, u których sytuacja ekonomiczna się nie zmieniła i nie ma perspektyw, że się pogorszy, to jest dobry okres do robienia różnego rodzaju inwestycji, zakupów. Bo część firm obniża ceny na swoje produkty po to, żeby zachować ciągłość funkcjonowania, więc są przeceny, promocje, itd. Można sporo rzeczy kupić fajnych, jeśli ktoś ma na to fundusze, możliwości w niższych cenach.

**Czy pana zdaniem obecny czas jest dobry na inwestycje?**

Nie jestem ekonomistą i nie chciałbym takich opinii wydawać. Czy jest dobry na inwestycje - nie wiem. Mam wrażenie, że może być. Na pewno się pojawi trochę okazji w nieruchomościach. Ludzie, którzy tracą przychód z racji konieczności zamknięcia hoteli, hosteli, małych noclegowni i nie są w stanie utrzymać obiektu, będzie musiała go sprzedać. To wymusi różnego rodzaju zmiany cen na rynku. Nie jestem w stanie powiedzieć jak, bo nie jest to moja branża. Ale podejrzewam, że może coś takiego być. Wśród moich znajomych niektórzy mają ten problem i będą sobie musieli jakoś poradzić. A sprzedanie w tej chwili obiektu, który był przygotowany *strcite* pod hostel na pewno będzie powodowało bardzo utrudnioną sytuację. Bo też ktoś, kto będzie chciał kupić taki obiekt, to będzie osoba, która kupi go z przeznaczeniem na taki cel albo chcąc go przebudować, zmodyfikować, będzie próbowała obniżyć cenę. Tak mi się wydaje, a nie wiem, jak jest faktycznie. Może niektóre rzeczy będzie można kupić taniej i niektóre inwestycje będą korzystne. Ludzie, którzy będą musieli ratować budżet, sprzedadzą nieruchomość, ziemię taniej po to tylko, żeby uzyskać środki na przetrwanie w trudnym okresie.

**W jaki sposób pan oszczędza?**

Nie mam konto oszczędnościowego. Mam takie programy emerytalne, w których się oszczędza, wpłaca się pieniądze. Nie jest to konto w banku.

**Pan ma ustalony procent z konta, który schodzi co miesiąc?**

Określoną sumę. nie jest to procent, tylko suma.

**Na jak długo wystarczyłyby Państwa oszczędności?**

Raczej krótki okres. Rzędu powiedzmy 2-3 miesięcy.

**Pojawia się też takie oszczędzanie na co dzień, jak np. schodzenie reszt z zakupów na konto?**

Nie, tego nie stosujemy z żoną.

**Co zazwyczaj pan robi z oszczędnościami?**

Nie da się uniknąć sytuacji, kiedy trzeba przeznaczyć jakąś gotówkę na remont, związany z domem i jakieś nowe pomysły. Nowe meble, ogród, remont garażu i różne tego typu rzeczy. To pochłania sporą część oszczędności. Plus z takiego oszczędzania na stałe są też wyjazdy wakacyjne, bo to pochłania sporą część oszczędności.

**Dlaczego pan oszczędza?**

Żeby mieć jakieś minimalne zabezpieczenie w razie, gdybym się znalazł w sytuacji np. bez pracy albo pracodawca nie wypłacał mi pieniędzy, co już niejednokrotnie się zdarzało - z dużym opóźnieniem, np. 3-miesięcznym dostałem pieniądze za wykonaną pracę. I po to, żeby w razie pomysłu na jakiś remont mieć z czego zapłacić. Nie tylko remont, ale pomysł, że kupimy sofę, stół, meble do ogrodu, żeby móc z tych oszczędności skorzystać.

**W takich sytuacjach, jak epidemia, dobrze jest mieć oszczędności?**

Oczywiście.

**Co te oszczędności dają ludziom?**

Możliwość funkcjonowania. Czyli, jeśli ktoś ma kredyty, to nie zawsze uda się zawiesić spłatę kredytu. Jeśli tracimy możliwość uzyskiwania dochodu, to pozwala nam to na jakiś czas w miarę normalnego życia, tak, żeby mieć pieniądze na jedzenie, na opłaty za prąd, gaz i znalezienie sobie innego źródła dochodu. Czy dopasowania się do warunków jakie panują. I można byłoby spróbować wtedy uzyskiwać dochód z tego samego źródła, ale po jakiejś zmianie funkcjonowania, żeby się udało. Może niekoniecznie to musi być w pełni dotychczasowego przychodu, ale jednak pozwalającego na łatwiejsze życie.

**Czy myśli pan o tym, kiedy obecna sytuacja się może zakończyć?**

Myślę. Wydaję mi się, że to potrwa parę lat zanim wróci do sytuacji sprzed tej pandemii, ale mam wrażenie, że niektóre rzeczy w ogóle nie wrócą.

**Jakie rzeczy nie wrócą?**

Beztroska przy podróżowaniu. To chyba się już nie pojawi. Myślę, że podróżując, będziemy poddani różnego rodzaju mechanizmom, które do tej pory nie funkcjonowały. Sprawdzania, czy jesteśmy chorzy, czy nie przenosimy z sobą infekcji, zarazków. Oczywiście nie w dosłownej formie sprawdzani, ale różnego rodzaju mechanizmy, typu ankiet w samolocie, sprawdzania na lotnisku temperatury. Te rzeczy funkcjonowały w wielu regionach i podróżując myśmy się z tym stykali, ale była to dla nas egzotyka. Ale myślę, że teraz to się stanie bardziej popularne i może będzie codziennością, a nie egzotyką. Myślę, że takie masowe przemieszczanie się ludzi w celach wakacyjnych, wypoczynkowych też nie będzie już tak popularne. Ludzie nie będą tak chętnie jeździli po całym świecie.

**Czyli z jednej strony będą to zmiany narzucone z zewnątrz, a z drugiej strony ludzie w sobie też mogą czuć taką zmianę?**

Myślę, że mogą. Z resztą, myślę, że ta cała sytuacja spowoduje wzrost cen i za bilety lotnicze i wzrost cen w kurortach, noclegowniach. Podróżowanie będzie mniej popularne przez parę lat.

**Ta myśl o tym, kiedy to się zakończy się pojawia, ale ona towarzyszy cały czas, czy jest w konkretnych momentach?**

Czasami rozmawiając z kolegami albo znajomymi sąsiadami, w pracy. Czasem ktoś zapyta: jak myślicie, kiedy życie wróci do normalności? No to sobie tam rozmawiamy na ten temat. Ja uważam, że w ogóle myśmy się pierwszy raz zetknęli z pandemią spowodowaną patogenem, który od wielu, wielu lat już powoduje jakieś lokalne małe epidemie. I nikt do tej pory nie zwracał na to uwagi, bo te infekcje nie miały globalnego zasięgu. A w tej chwili się pojawiła pandemia, no więc wszyscy się nagle zorientowali, że jest coś takiego, jak koronawirus. To jest w ogóle śmieszne - ludzie wysyłają w Internecie zdjęcia etykiet ze środków odkażających sprzed 5 lat, gdzie jest napisane, że niszczy koronawirusa. I tu jest pytanie: dlaczego nas oszukiwali? Takie niezrozumienie tematu, że to jest pandemia spowodowana patogenem, który od wielu lat ludzi gdzieś tam od czasu do czasu zakaża i te infekcje były. Tylko nie o takim zasięgu.

**To, że ta pandemia jest inna od poprzednich, powoduje, że trudno przewidywać przyszłość pana zdaniem?**

Trudno. Moim zdaniem trudniej przez to. Bo ja sobie zadaję pytanie, jak szybko pojawi się następna tego typu pandemia, ile czasu minie. Czy to jest początek już takich dużych zmian w funkcjonowaniu świata, czy będzie to jednorazowo i będziemy mieli spokój na 50 lat.

**Czy pojawiają się jeszcze jakieś myśli w związku z przyszłością po pandemii się pojawiają?**

Nic mi do głowy nie przychodzi. Myślałem o tym, czy zmieni się podejście Europejczyków i pewnie też Amerykanów, którzy do tej pory tak bardzo chętnie kupowali produkty w Chinach. Bo wie pani, jak to wszystko funkcjonuje. To, co my kupujemy pod marką Bosh, Siemens czy jakieś inne jeszcze europejskie znaczki, jest produkowane w Chinach i sprzedawane z odpowiednim nadrukiem i często nie jest to nawet pomysł Siemensa czy Philipsa, tylko jest to pomysł Chińczyków, który jest kupowany na jakimś przetargu przez koncern i wybierany z iluś propozycji. Jeśli jest dostatecznie dobry, to później się pojawia na rynku. Czy dalej to będzie funkcjonowało w ten sposób? Może niektóre firmy zastanowią się nad tym, żeby jednak wrócić z produkcją do Europy czy Stanów i sprzedawać te produkty rzeczywiście wyprodukowane w Europie. Może za trochę wyższą cenę, ale wyprodukowane w Europie. Zastanawiam się, czy to zmieni jakoś funkcjonowanie koncernów. Ale tak naprawdę nie tylko koncernów, bo i małe firmy sobie w ten sposób z tym radzą - przenoszą produkcję do Azji.

**Jak pana zdaniem sytuacja może się dalej potoczyć?**

Mam nadzieję, że w ciągu kilku miesięcy. Może do końca roku, do pierwszego kwartału przyszłego roku, gospodarka wróci do normalnego funkcjonowania. Czyli, że firmy, które nie działają, zaczną funkcjonować w takim wymiarze, jak przed epidemią. Połączenia lotnicze, że wrócą do normalnego funkcjonowania. Linie lotnicze - nie wiem, może część upadnie, ale te o globalnym zasięgu i te, które wykonywały dużą ilość lotów, że one w dalszym ciągu będą funkcjonowały tak, jak przed epidemią. Mam nadzieję, że tak będzie. Boję się, że... I pewnie też tak będzie. Że upadnie mnóstwo drobnych firm. Ludzie, którzy sobie funkcjonowali w firmie jakiejś rodzinnej, usługowej i to im wystarczało, żeby przeżyć i w miarę normalnie funkcjonować, to po tej epidemii, z powodu różnego rodzaju obostrzeń, oni stracą te firmy, nie będą mogli funkcjonować i będzie im trudno wrócić do tego, co było. Tak to postrzegam. I zastanawiam się, kiedy się pojawi następna epidemia i z kolejnymi takimi dużymi obostrzeniami, które dobiją tych, którzy właśnie się podnoszą z upadku. No i zastanawiam się jeszcze nad tym, jak bardzo obecna władza zniszczy kraj decyzjami, które podejmie w cieniu epidemii. Bo do tej pory tak to dla mnie wygląda, że jest to jakaś forma legalizacji różnych barbarzyńskich działań obecnej władzy. Epidemia jest przykrywką do potężnych zmian, których może nie udałoby się wprowadzić, gdyby nie było epidemii. Jak długo nasz kraj będzie wracał do w miarę normalnego, demokratycznego funkcjonowania. Bo to, co teraz jest, to nie jest demokracja.

**Ma pan wrażenie, że kolejna epidemia mogłaby się pojawić w perspektywie kilku lat?**

Tak. Myślę, że tak.

**Co stoi za tym przekonaniem?**

Nie mam jakiś popartymi dowodami naukowymi przemyśleń na temat nawrotu epidemii. Natomiast mam wrażenie, że, ponieważ doszło do ewolucji koronawirusa i w dalszym ciągu nie potrafię powiedzieć, jak pewnie większość nawet ludzi, którzy się zajmują tym tematem nie jest w stanie powiedzieć czy jest to sztuczny twór, akurat ten szczep koronawirusa. Czy był stworzony w laboratorium, czy jest to naturalna mutacja patogenu żyjącego na zwierzętach. Jeśli dochodzi do takiej mutacji w sposób naturalny, to należy się spodziewać, że będą kolejne. A przede wszystkim to, czego możemy się spodziewać i co jest bardzo prawdopodobne, to jest jakby druga fala. Bo ta pandemia nie wystąpiła równocześnie we wszystkich miejscach na świecie, tylko to jest fala, która się rozprzestrzenia. Ludzie w dalszym ciągu nie izolują się całkowicie. I dalej ci zakażeni podróżują, nie wiedząc o tym, że mają infekcję i przenosząc ją z miejsca w miejsce. Więc myślę, że należy się spodziewać drugiej fali. Tak się o tym mówi też w mediach. W Chinach tam się to pojawiło już w innych prowincjach niż ta, w której do tej pory epidemia poczyniła największe spustoszenie. Więc należy się spodziewać, że tak może też być na świecie.

**Czy jest coś, czego się pan obawia w perspektywie kilku tygodni?**

Ja się obawiam, że będzie duży wzrost zachorowań teraz po otwarciu galerii handlowych i takim rozluźnieniu troszkę rygoru epidemicznego. Można by było w jakiś sposób spróbować, żeby funkcjonowały jakieś fabryki, przedsiębiorstwa, żeby można było kupić przez Internet z jakąś sprzedażą wysyłkową. Ale otwieranie takich miejsc, jak galerie handlowe moim zdaniem przyspieszy i zwiększy ilość zachorowań. Ale może to też jest sposób, żeby społeczeństwo uzyskało odporność na wirusa. Więc trudno mi powiedzieć. Może jest to dobre rozwiązanie.
