# Supplementary material for: The challenges arising from the COVID-19 pandemic and the way people deal with them. A qualitative longitudinal study
Source: PLoS One. 2021 Oct 11;16(10):e0258133. doi: 10.1371/journal.pone.0258133 (PMC8504766; doi:10.1371/journal.pone.0258133)
Supplement: S1 Dataset — (ZIP) [file pone.0258133.s003.zip › Transcriptions/stage 4/15.4_M_43_couple, with children.docx]

**15.4_M_43_couple with children**

**Jak wyglądały ostatnie 2 tygodnie?**

Nie różniły się znacząco od poprzedniego okresu.

**W pracy obowiązki są z takim samym natężeniem?**

Z takim samym. Miałem trochę takich terminowych rzeczy - musiałem pojechać do pracy na jedną z sobót, co nieczęsto się ostatnio zdarzało...A, były zmiany jednak, ponieważ opuściłem miejsce zamieszkania. Wyjechaliśmy na 3 dni. To był wyjazd roboczo-wypoczynkowy jeszcze przed majówką. Pojechaliśmy do Świnoujścia nad morze, ponieważ właśnie rozpoczyna się sezon, powiedzmy, że sezon, turystyczny, a jesteśmy współwłaścicielami takiego małego mieszkania nad morzem i do moich obowiązków co roku należy przejrzenie całego mieszkania po zimie i w związku z tym była to okazja, żeby spędzić trochę czasu tej izolacji czy kwarantanny...Nie kwarantanny, odosobnienia w innym miejscu. Z zachowaniem reżimu oczywiście.

**Taka zmiana otoczenia jakoś poprawiła państwa nastrój? Coś dała?**

Te obostrzenia w dalszym ciągu obowiązują, więc nie było możliwości pójść do restauracji chociażby. Była możliwość wyjścia na plażę, ponieważ nie jest to zakazane i w maseczkach dało się to zrobić, byliśmy w lesie, ale tutaj w Poznaniu też bywamy w lesie, bo jest koło nas. Zabraliśmy cały sprzęt, więc praca zdalna i tak się odbywała. Oprócz fizycznej zmiany miejsca, to jakby nie było istotnych różnic. W domu prowadzę i remonty i tam też musiałem przeprowadzić jakieś drobne remonty i usprawnienia.

**Remonty w domu nadal trwają?**

Szczęśliwie nie, już zakończyłem i na razie nie rozpoczynam kolejnych prac, ponieważ zakopałem się w przednich i na razie muszę sobie zrobić chwilę przerwy. Natomiast wchodzi mi teraz ekipa remontowa. Ja nie będę tego robił fizycznie, ale ktoś jednak to będzie robił. Mam nadzieję, że to będą krótkie akcje, maksymalnie dwudniowe.

**Jakoś planuje pan sobie inaczej zapełniać czas?**

Nie. Teraz będę musiał doglądać ekipy remontowe, więc wyjdzie czasowo na to samo, ale bez mojego zaangażowania fizycznego.

**Jest coś, co pan ograniczył, z czegoś zrezygnował?**

Tak, chyba trochę bardziej zrezygnowałem z folgowania sobie w jedzeniu, czyli wracam do jakichś starych nawyków, bo faktycznie odczuwam obciążenie fizyczne, mniejszą wydolność po prostu. Czas powrócić do jakiegoś reżimu żywieniowego, żeby nie tracić tego zysku, który kiedyś wypracowałem. To przede wszystkim wynika ze zdrowego rozsądku i jednak przy takim dość ograniczonym, stabilnym a nie mobilnym trybie życia, to faktycznie ta wydolność organizmu spada bardzo szybko. Wystarczą 2 tygodnie i człowiek traci tę wydolność, którą miał wcześniej i stąd też pomysł na to, żeby w mniejszym zakresie żywić się węglowodanami czy tłuszczami.

**Zaczęło coś panu ostatnio mocniej przeszkadzać w tej autoizolacji?**

Nie.

**Co jest teraz największym wyzwaniem, co sprawia trudność?**

Chyba nie ma niczego takiego. Na tyle się zaadoptowaliśmy do tych warunków - podział obowiązków, opieka nad dzieckiem, te remonty, sprawy domowe, chodzenie do pracy. To jest po prostu poukładane i to nie stanowi wyzwania. Można powiedzieć, że przyzwyczailiśmy się do tej sytuacji.

**Obserwuje pan jakieś zmiany w zachowaniach ludzi w pana otoczeniu?**

Tak społecznie nie zauważam większych zmian, natomiast wiem, że mój teść, który jest lekarzem został przymuszony do powrotu do pracy do poradni, więc teraz 3-4 dni po miesiącu czy nawet więcej takiej autoizolacji, którą sobie sami włączyli, od tygodnia na 4 dni w tygodniu jeździ już do pracy. Zgłasza jakieś obawy z tym związane, bo jest w wieku i w grupie ryzyka, jednak podjął taką decyzję, że wyraża zgodę na propozycję pracodawcy i powrócił do pracy.

**W pana otoczeniu jest jeszcze ktoś, kto nie mógł pracować przez ograniczenia, a już może?**

W gronie znajomych, to nie widzę, tylko co znaczy, że nie może? Że miejsce pracy było zamknięte?

**Tak.**

Nie, nie. Zauważam inny problem, że być może część ludzi może nie tyle chce już wrócić do pracy, co jest zmuszana już do powrotu, tak jak już częściowo uczelnie są otwierane, przynajmniej w tej części naukowo-technicznej. Ktoś z mojej rodziny pracujący na uczelni ma w dalszym ciągu pod opieką dziecko poniżej lat 8 i jest przymuszany przez przełożonego, żeby powracać do pracy, a nie ma możliwości zaopiekowania się tym dzieckiem w żaden sposób, oprócz tego formalnego wniosku o opiekę, czego oczywiście nie chce robić, ze względu na ograniczenia finansowe. To jest problem, że nadal te realia życiowe są takie, że nie ma co zrobić z dziećmi, bo nie wszystkie przedszkola są otwarte akurat i w tym przypadku przedszkole nie będzie otwarte do końca roku szkolnego.

**Czy zapamiętał pan jakieś szczególne emocje z ostatniego okresu? Było coś takiego?**

Zauważyłem coś takiego. Zauważyłem, że zaczyna mnie uspokajać i wprowadzać w dobry nastrój muzyka, która jest w formie dosyć ostra i wyrazista. Bardzo dawno tego nie robiłem, ale powróciłem do słuchania zespołów, których bardzo dawno nie słuchałem i to wprowadzało mnie naprawdę w dobry nastrój, chociaż ta muzyka sama w sobie w formie podania jest dla większości ludzi niestrawna. Mówię u np. o muzyce black metalowej, która kojarzy się raczej przeciętnemu odbiorcy z wartościami negatywnymi, natomiast mi daje spory zastrzyk energii i ci jest też zaskakujące, że zacząłem też w tym dostrzegać jakąś harmonię i piękno. To jest dosyć ciekawe w mojej opinii. Nie ma czegoś takiego, jak obraz czy piosenka, ale pewien trend, który się pojawił, że coś, co obiektywnie jest nieatrakcyjne, nieładne, wstrętne, trudne w odbiorze, nagle zaczęło sprawiać mi przyjemność. Z jednej strony dodaje energii, a z drugiej też poprawia nastrój.

**Wcześniej też pan lubił taką muzykę, tylko niekoniecznie ona wprowadzała w spokojny nastrój?**

To może jeszcze inaczej...W spectrum muzyki rockowej jest bardzo wiele gatunków. Ja się zawsze poruszałem w obrębie muzyki rockowej, a teraz sięgam po te odmiany najbardziej ekstremalne, po które sięgałem chyba ostatni raz w liceum, co było jakieś 170 lat temu i zaczęło mi to ponownie sprawiać jakąś radość, przyjemność. Wcześniej nigdy nie miałem ochoty po to sięgać, od tego czasu licealnego. Raczej poruszałem się w obrębie muzyki cięższej, ale niekoniecznie tak ekstremalnie ciężkiej. Jako przykład - może bardziej zespół Slayer niż zespoły typu Dimmu Borgir czy Behemoth z wcześniejszego okresu.

**Coś konkretnego za tym stoi, że sięgnął pan po tę muzykę?**

To był przypadek i zupełnie przypadkiem to odkryłem, ponieważ pracowałem malując pokój dla syna z włączonym przypadkowym zestawem utworów z YT i okazało się, że play lista sama zaproponowała taką muzykę i już przy niej pozostałem po prostu. Wchłonęła mnie.

**Jest tak, że teraz specjalnie pan włącza tę muzykę, żeby się wprawić w lepszy nastrój, czy słucha pan tej muzyki i tak, i ona przy okazji poprawia ten nastrój?**

Celowo, żeby sobie włączać tę muzykę, to takich zachowań nie rejestruję, ale to jest też tak, że nie zawsze mam możliwość włączenia sobie takiej muzyki wtedy, kiedy bym chciał, ze względu na jakieś różne obowiązki zawodowe czy rodzinne, natomiast w momencie, kiedy mam możliwość posłuchania, to włączam ją. Chciałbym oczywiście mieć taką sytuację, że mam gorszy nastrój i siadam sobie, i chcę sobie coś włączyć, i w ten sposób sobie poprawiam nastrój, ale to tak niestety nie działa. Zresztą powiedzmy sobie uczciwie, że ten nastrój nie jest wcale taki najgorszy, ale ta muzyka akurat ma taką wartość bardzo mocno energetyzującą. Ona nie tonizuje tylko energetyzuje.

**Emocje - zdjęcia**

Pozostałbym przy 6 i 13, bo to jest wciąż to wyglądanie słońca, które będzie i ogrom nadziei, który płynie z tych 2 zdjęć, ale dołożyłbym też 1.

6 i 13 są w formie i wymowie bardzo zbliżone. One wciąż dają poczucie nadziei, że ten element pandemiczny kiedyś dobiegnie końca, że będzie większe poczucie wolności, ale to jest wciąż w obrębie nadziei. Z drugiej strony tak sobie teraz myślę, że to był, jest też czas jakiegoś zysku i teraz pytanie, co będzie po nim. Zysk może niekoniecznie potraumatycznego wzrostu, bo o tym jeszcze nie mówimy, ale udało mi się w tym czasie zrealizować wiele rzeczy, których nie udało mi się wcześniej zrealizować. I trzeba będzie powrócić do dawnej aktywności w tej formie poprzedniej, co chociażby ze względu na to, że byłem w stanie wykonywać dwie czynności zawodowe pracując zdalnie w domu, to teraz, kiedy fizycznie będę musiał być gdzieś tam obecny, to będzie to trudniejsze. Człowiek się szybko przyzwyczaja do tego, co jest dobre i co daje dużo większe możliwości rozwoju. Ja bym chyba tu jeszcze dołożył element 9-tki, tzn., to co nas czeka będzie pewnego rodzaju burzą w życiu, bo na ten moment jest wszystko fajnie poukładane, udaje się zrobić więcej rzeczy i zarobić więcej pieniędzy niż wcześniej, bo są takie techniczne możliwości, a też część zleceniodawców czy pracodawców działa w mniejszym zakresie, dając większą możliwość pracy czy plastyczności pracownikom. A jak będzie powrót do starych reguł, to będzie to taka burza, nie? Ta niewiadoma dotyczy przyszłości po okresie pandemicznym.

**A ta przyszłość to jest rodzaj niepewności? Jakie jeszcze emocje to w panu budzi?**

Niepewność na pewno, ale też taka forma ciekawości jak to będzie wszystko wyglądało, bo wiem, że to będą zadania, które trzeba będzie zrealizować i które trzeba będzie zaplanować. Być może zmodyfikować ponownie swoje podejście do aktywności zawodowej, więc to jest czas oczekiwania w obliczu wyzwania. Tak bym określił obrazek nr 9. Nie mam za bardzo niepokoju czy lęku z tym związanego, raczej ciekawość w takim bardziej pozytywnym sensie.

1 ja wiążę z takim stanem rozprężenia, które się pojawia w społeczeństwie, co już widać i tak w zasadzie, to ta jedynka może nie jest zbyt symboliczna, ale zarówno wczoraj jak i dzisiaj, jadąc do pracy, zauważyłem, że ponownie stoję w korkach. To jest jakiś powrót do stanu wcześniejszego, którego przez długo czas nie dawało się zarejestrować. 1 wyraża zaskoczenie, że tak to wygląda. Obostrzenia zostały częściowo zniesione, ale stan epidemiczno-pandemiczny nie uległ znaczącej zmianie, tzn. wciąż rośnie nam liczba osób zakażonych i ta ogólna liczba zakażonych też w dalszym ciągu, a mimo wszystko coś się zmieniło. Są jakieś elementy normalności zauważalne.

**To zaskoczenie jest raczej pozytywne czy negatywne?**

Nie przypisywałbym tutaj żadnej wartości. W zależności od kontekstu spojrzenie, to jest to w jakimś zakresie pozytywne, bo ok, bo gdzieś wraca ta normalność, z drugiej strony to zaskoczenie ma jednak wymiar negatywny, bo po pierwsze skończył się dość ciekawy okres, gdzie więcej można było zrobić i druga sprawa, że jednak te wszystkie obostrzenia, które są ogłaszane, że one do końca nie są jednak przestrzegane. Pozytywne jednak może być to, że nie mamy mimo rozluźnień obostrzeń, nie mamy jakiegoś nagłego wzrostu zakażeń. Aktualnie w moim miejscu pracy mamy więcej problemów z ludźmi, których trzeba dowozić na dializy w specjalnych warunkach, niż z transportem ludzi zakażonych. Są też jakieś pozytywy. Na dwoje oceniam to zdjęcie - z jednej strony zaskoczenie pozytywne, że można, że się da, a z drugiej strony kończy się coś ciekawego i też jednocześnie nie przestrzegamy tych wszystkich obostrzeń teraz.

**Czy podejmuje pan jakieś działania, żeby mieć w sobie te pozytywne emocje?**

Nie, nic ponad to...Chociaż nie. W zasadzie jedna z możliwych czynności zastępczych, z takich strategii unikowych...Stwierdziłem, że zacznę się przygotowywać do powrotu do pracy i pozwoliłem sobie na trochę inwestycji finansowych ukierunkowanych na narzędzia pracy. To jest taki mechanizm, który de facto nie jest do końca biznesowy, mimo tego, że jestem w pierwszym okoleniu Poznaniakiem, a oni słyną z oszczędności...Zauważam, że zdarza mi się dokonać kilku zakupów, które mi sprawiają satysfakcję, ale to jest tak, że ja sobie układam jakieś plany dotyczące tego, co chciałbym mieć i co bym potrzebował i później kupowanie tego sprawia mi radość. Nie robiłem tego w czasie pandemicznym i we wcześniejszym okresie też nie przypominam sobie, kiedy ostatnio to robiłem, a teraz faktycznie kupiłem trochę sprzętu, musiałem uzupełnić trochę narzędzi diagnostycznych, gdzieś w ferworze walki ktoś mi nie oddał podręcznika do Wechslera i skali słownej, więc musiałem to sobie zakupić i teraz od poniedziałku prawdopodobnie już zaczynamy powrót do pracy w szpitalu, więc też potrzebowałem jakieś proste rzeczy typu głośnik do laptopa, żebym był słyszalny. Oczywiście poradziłbym sobie i bez tego głośnika, ale zakup tego głośnika sprawił mi ogromną radość, tym bardziej, że znalazłem dla niego szereg zastosowań. To było trochę takie poszukiwanie potrzeby na siłę i wydawanie pieniędzy, ale taki mechanizm kiedyś rejestrowałem i to w jakimś zakresie powróciło.

**Jak teraz wyglądają pana zakupy spożywcze?**

Z racji tego, że najczęściej robiliśmy duże zakupy raz na 2 tygodnie, to mniej więcej ten tryb jest utrzymany nawet mimo wejścia tych obostrzeń. Nie zmieniło się zbyt wiele od czasów przedpandemicznych. W zasadzie zakupiłem wszystko co było mi potrzebne do domu do remontu, więc w zasadzie nie kupuję już tych rzeczy. Teraz faktycznie pojawiły się te zakupy ukierunkowane na aktywność bardziej zawodową, aczkolwiek jeszcze jakąś lampkę dla dziecka kupiłem na biurko, żarówki lodowe, ale to już jest ta końcówka remontu i to z tym jest powiązane. Jeśli chodzi o codzienne zakupy to ten trend jest utrzymany i tylko pewnego rodzaju nowością było kupienie tych kilku rzeczy do aktywności zawodowej.

**A sam sposób kupowania? Nadal jest lista i zaplanowane zakupy?**

Tak. Nie stałem już ostatnio w kolejce, bo faktycznie raz mi się zdarzyło wcześniej stać w 7-8 osobowej kolejce do Lidla i teraz ostatnio udało się wejść bez kolejki.

**Ma pan poczucie, że do tych zakupów spożywczych w czasach pandemii trzeba się jakoś specjalnie przygotować? Że to musi być zaplanowane, odpowiednia pora, itp.?**

Nie miałem możliwości zaplanowania tego w inny sposób. Po prostu musiałem zrealizować zakupy w tym czasie, który był mi dany, więc nie byłem w stanie dobrać innych godzin, żeby uniknąć kolejek, aczkolwiek wiem, że większość sklepów jest otwarta całą dobę teraz, więc pewnie dałoby się to zrealizować bezkolejkowo. Nigdy nie przyszło mi do głowy, żeby planować zakupy akurat w ten sposób. Oczywiście trzeba pamiętać, żeby się zabezpieczyć i zabezpieczyć otoczenie w tym podstawowym zakresie typu maseczka i rękawiczki. Lista była zawsze.

**Czy pojawiły się zakupy dla przyjemności?**

Tak, część narzędzi do pracy, ale powiedzmy sobie uczciwie, że ja byłbym w stanie też bez nich się obejść. Kupowałem je właśnie częściowo też z tego powodu, że czasami lubię to zrobić. Czasami mam taki dzień, kiedy wiem, że muszę sobie poszukać różnych rzeczy i zamówić je hurtem. Ten moment kupowania sprawiał mi jakąś przyjemność i radość.

**A ze spożywczych było coś typowo na poprawę humoru?**

Ja nie, ale chyba żona przywiozła kawałek jagnięciny. Jagnięcinę jadamy stosunkowo rzadko i to są może nie tyle specjalne okazje, ale rzadko jest dostępna i też częściowo cena. Tak, to nie był mój zakup, ale zjadłem to z przyjemnością i to zjedzenie kawałka martwego jagnięcia sprawiło mi dużą przyjemność. Ja sam nie przypominam sobie, żebym kupował ze spożywczych rzeczy coś, czego zjedzenie sprawi mi przyjemność, a powiedziałbym nawet, że kupuję mniej pistacji niż kupowałem kiedyś. Jestem gorącym zwolennikiem pistacji i nie pamiętam...Na pewno w tym okresie epidemicznym nie jadłem ani razu pistacji.

**W poniedziałek otworzono GH. Co pan o tym sądzi?**

Zakładam, że ze względów gospodarczych musimy to zrobić pod pewnymi uwarunkowaniami i obserwować to, co się będzie działo, więc oceniam to pozytywnie. Musimy przede wszystkim nauczyć się w najbliższym czasie żyć z koronawirusem i tyle. To może być etap powrotu do normalności albo testowania rzeczywistości, co się wydarzy.

**Teraz to jest bezpieczne, żeby iść do GH na zakupy?**

Poziom ryzyka jest wciąż ten sam od początku pandemii. To jest tak samo, jak idziemy do supermarketu. Te same przestrzenie mniej więcej, te same odległości, więc tu się nic w tym zakresie nie zmienia. Jest to bezpieczne, a czy to jest racjonalne? Może bardziej nad tym bym się zastanowił, ale to bardziej chodzi o model spędzania wolnego czasu i czy chodzenie po GH może być formą przyjemności. Dla mnie wątpliwą. Dla mnie nie. Nie planuję tam iść, chyba, że w jakimś zakresie sytuacja mnie do tego zmusi - konieczność zakupu czegoś nagle bez możliwości zakupu online, to może tak, ale nie dla przyjemności. Nie dlatego, żeby poczuć ponownie, że żyjemy, bo to zupełnie nie ten trop.

**W pana otoczeniu słyszał pan o takich osobach, które planują teraz zakupy w GH?**

Może nie tyle, że to było wyczekiwane, ale moja teściowa pojechała do jakiegoś sklepów w GH po to, żeby dokonać jakichś zakupów bieliźnianych. Być może to była po prostu potrzeba zakupu czegoś, co jest niezbędne, ale wiem, że do takiej sytuacji doszło. Trudno jest prawdopodobnie kupić biustonosz przez internet. Ale nie było tam na pewno elementu wyczekiwania, że minuta po północy, wsiadamy w auta i tłumnie jedziemy. Nie słyszałem, żeby ktoś z moich znajomych stał w korkach do Ikei.

**Łatwość wydawania pieniędzy - skala**

Co do zasady powiedziałbym, że najczęściej znajduję się w okolicach 3. Nie jestem zwolennikiem wydawania pieniędzy na zasadzie konsumpcjonizmu i konsumeryzmu, wydawania, bo coś jest zgodne z obowiązującym trendem, modą czy moją zachcianką, aczkolwiek po zaplanowaniu pewnych działań, chociażby remontów, to powiedziałbym, że w ostatnim czasie pozwoliłem sobie na wszystko, na co chciałem sobie pozwolić, jeśli chodzi o remont domu. Przy tym remoncie, to powiedziałbym, że to było 10 dla budżetu domowego. To było zaplanowane pod kątem realizacji pewnych zadań, a nie np. ograniczeń budżetowych. Np. chciałem wydać na panele fotowoltaniczne 15000, a finalnie podrapałem się po głowie i stwierdziłem, że można wziąć nieco większą moc i wydałem 22000 i te 7000 zł wydałem z ogromną łatwością. To jest jednak dosyć duża kwota i nie zastanawiałem się za bardzo nad tym. Kupując teraz okno mogłem wybrać takie albo siakie, ale wybrałem droższe, wydając na nie więcej pieniędzy. Szykując się do remontu tarasu mogłem wybrać płytki, ale stwierdziłem, że jednak wezmę duże płytki granitowe, tzw. pływające za dużo większe pieniądze niż wstępnie planowałem, więc w tym zakresie 10 i żadnych skrupułów dla budżetu domowego. Gdybym miał pół miliona, to też bym pewnie pół miliona wydał na rzeczy takie powiązane z remontami.

**Czuł pan zadowolenie z tych zakupów? Jakie emocje były wokół tego?**

Trudno chyba odnaleźć emocje pozytywne w samym momencie wydawania pieniędzy, ale dałem sobie przyzwolenie na to, żeby nie myśleć o tym. To jest jakiś tam też plus w przypadku utrzymywania pewnego poziomu pozytywnych emocji. Nie zastanawiałem się, że co teraz będzie i czy to było dobrze. Po prostu powiedziałem, że ok., są te pieniądze wydane, więc teraz trzeba zobaczyć w jaki sposób one będą kwitły w domu pod względem tych zmian, które następują. Same zmiany, to co widzę po remoncie...Zmiany wizualne działają na mnie bardzo pozytywnie. Jestem zadowolony z efektów pracy, czyli pośrednio z wydawania pieniędzy.

**Jakie to są sytuacje, kiedy jest to 3?**

Jak np. w Lidlu nie było wina przecenionego o 50% i musiałem je kupić w normalnej cenie. To jest straszna rzecz, bo ja zawsze bardzo lubię kupować...Taka moja stara zasada, że lubię sobie kupić rzecz ekstremalnie drogą, czasami nawet luksusową, pod warunkiem, że jest przeceniona o 90%. Zawsze szukam takich możliwości, szukam tańszych zamienników albo rezygnuję z czegoś, jeśli nie znajdę czegoś w dobrej cenie, w przecenie albo w okazji.

**Zdarza się, że kupi pan coś w regularnej cenie?**

Zdarza się, ale generalnie szukam alternatyw. Nie spełniam sobie zachcianek, bo wie pani, Poznaniacy jednak ponoć są trochę inni pod tym względem. Najczęściej jednak hamuje się z wydawaniem pieniędzy. W czasach normalnych najczęściej kupowałem w TK Max i zawsze patrzyłem na metki, żeby to było jak najbardziej przecenione, bo chcąc kupić dobrą marynarkę mogłem pójść do regularnego sklepu i zapłacić 1500 zł, czego nie zrobiłbym w życiu, nawet posiadając taką kwotę, bo to jest po prostu kwota za duża. Uważam, że jest za duża i wolę kupić 1500 - 70%, zakładając, że nie jest to manipulacja, przy jakiejś okazji. Przy tym remoncie takiego myślenia nie było. Było wręcz odwrotnie. Nie wiem, czy w czasach...No właśnie...Czy ja bym tak samo postąpił w czasach przed epidemią? Myślę, że miałbym mniej czasu na myślenie o remontach i pewnie bym skorzystał wg swojego starego schematu ze sprawdzonych, najtańszych rozwiązań i wcale bym nie wydawał tych pieniędzy więcej, czyli to jest jakaś zmiana jednak, jak obserwuję swój typ postępowania. Ale czy to się wiązało z podnoszeniem nastroju ogólnego? Nie wiem właśnie i ciężko mi na to odpowiedzieć. To chyba psycholog by musiał odpowiedzieć.

**A takie większe wydatki typu meble, elektronika, już nie w kontekście pandemii, jak przebiega u pana podejmowanie decyzji nad wydaniem takiej większej kwoty? Na co pan zwraca uwagę?**

Wszystko zaczyna się od planowania i pytania, czy ten wydatek jest niezbędny, a jeśli jest niezbędny, to co on mi da w sensie usprawnienia życia, podniesienia jakości życia, ewentualnego wymiernego zysku. Na początku to jest na pewno kwestia ceny i zasadności zakupu. Później od ogółu do szczegółu, czyli najpierw szukam...Przykład nie z mojego życia, ale taki ogólny - kupić kanapę rozkładaną czy sofę nie rozkładaną. To jest taka ogólna kategoria i dopiero później, jak już jest decyzja, co funkcjonalnie będzie potrzebne, jest etap oglądania modeli i producentów. Jeśli uznaję, że coś pasuje do wizji, którą mam, czyli mam konkretnego producenta i model, to staram się szukać okazji. Porównuję ceny. Jeśli chodzi o marki, to też nie mogę powiedzieć tak, że ja się poruszam tylko w obrębie marek lowcostowych albo tylko i wyłącznie luksusowych. To bardzo mocno zależy. Na co dzień nie mam czasu na zajmowanie się remontami i nie kupuję najdroższych narzędzi, tylko kupuję te najtańsze. Ja wiem, że one i tak mi starczą na bardzo długo, ale pewnie gdybym był majsterkowiczem czy zajmował się mechaniką samochodową dla przyjemności, to pewnie te narzędzia miałbym dużo lepsze. Z drugiej strony nie jeżdżę samochodem marki Dacia, która jest uznawana za markę, która ma być tania i ma jeździć i taka jest. W tym zakresie kupiłem coś bardziej sprawdzonego, może bardziej zaawansowanego technologicznie czy trwalszego. To wszystko zależy.

**Postrzega pan siebie jako osobę oszczędną?**

Tak, zdecydowanie. Oszczędzającą i niewydającą pieniędzy. powiedziałbym nawet, że to jest kolejny atut, który widzę w tej sytuacji, ponieważ zauważyłem, że pomimo nawet zmniejszonego przychodu, te rozchody są dużo niższe. Jest dużo mniej kosztów życia na ten moment. Odpadały takie rzeczy jak ochlejstwo i wyżerka w restauracjach, wyjścia do znajomych, więc kupowanie jakichś prezentów, kupowanie wina, karnety na siłownię, odpłatności za aktywności dodatkowe dziecka, paliwo. Tych wydatków tak kwotowo było mniej, chociaż dużo rzeczy udało nam się zrobić i też wydawaliśmy na to pieniądze. To pokazuje, jak wiele kosztuje nas takie codzienne życie. Ja jestem zaskoczony bardzo pozytywnie stanem swoich finansów przez ostatnie 6 tygodni.

**Czyli nie było intencjonalnego obniżenia wydatków?**

Nie było. To była niezamierzona oszczędność.

**Wspomniał pan, że teraz przychód jest nieco mniejszy?**

Tak. Nie prowadziłem działalności gospodarczej przez okres ostatnich 6-7 tygodni.

**Ale to nie jest zagrożenie dla państwa budżetu domowego?**

Nie, w żaden sposób. W odwodzie zawsze mamy oszczędności, które można gdzieś tam na takie cele wykorzystać. Stara biznesowa zasada - zawsze musi być ta minimalna rezerwa na 3 miesiące życia na wysokim poziomie bez jakiegokolwiek źródła przychodu.

**Ma pan takie oszczędności?**

Tak, zdecydowanie tak.

**A na jaki mniej więcej okres?**

Wydaje mi się, że na pół roku. Około 6-7 miesięcy byłbym w stanie żyć.

**Czy w czasie epidemii podjął pan jakieś kroki, żeby ograniczyć wydatki?**

Nie, w żaden sposób. To, co wynikło, wynika po prostu ze zmiany funkcjonowania codziennego typu paliwo i brak wydatków na rzeczy, które stanowiły naszą codzienność. Intencjonalnie nie, wręcz w tych planach pojawiło się więcej inwestycji domowych. Można powiedzieć, że tych pieniędzy do wydania jest więcej.

Czy generalnie w takich sytuacjach jak epidemia dobrze jest ograniczać swoje wydatki?

Jest to jedna ze strategii. Tak, dobrze. Nie ma nigdy jednoznacznej odpowiedzi, bo z drugiej strony, skoro to życie się zmienia w taki sposób, to można też wydać sobie trochę pieniędzy z tego tytułu, że jest taki specyficzny okres. Jakaś forma świętowania. Zrozumiem takie podejście, że ktoś powie, że nie musi prowadzić firmy, ma 5 mln zł oszczędności, to sobie po prostu pofolguję i ok. Jeśli ktoś powie, że ojej, żyję z dnia na dzień, zarabiam 4500 na rękę, ledwo mi starcza na życie, moja żona zarabia podobnie, więc jesteśmy stosunkowo biedni, więc ograniczmy wydatki, ponieważ wiadomo, że nie mamy oszczędności i żyjemy wychodząc na zero, nie wiadomo co będzie później, stracimy pieniądze. Tak, rozumiem, że ktoś może podejść też w ten sposób. Ograniczmy pewne wydatki, ponieważ ta przyszłość jest nieznana. Scenariuszy jest kilka. Patrząc z takiego ogólnospołecznego poziomu, rozsądnie jest ograniczać wydatki, aczkolwiek stałą rezerwę finansową ja mam od wielu lat, tzn. są to takie formy oszczędności, których ja nigdy nie ruszam. Plus do tego mam oszczędności ruchome, które albo idą gdzieś na inwestycje albo, które czasami można uszczknąć, jak się robi coś większego, czy jak zaczyna brakować, ale później oczywiście się je uzupełnia czy nawet powiększa.

**Jaką ma pan metodę na oszczędzanie?**

Sam oddzielam pieniądze do oszczędzenia. Najczęściej oddzielam pieniądze, które są pieniędzmi dodatkowymi z działalności gospodarczej. To najczęściej idzie jako forma oszczędności, natomiast to, co wypracowuję z etatu jest tą częścią, która idzie na życie.

**Oszczędności są na koncie, na lokatach?**

One są zdywersyfikowane. Część jest w walucie obcej, część w walutach nieuznawanych oficjalnie za waluty, część mam na kontach oszczędnościowych, część mam na lokatach, część mam w polisach inwestycyjnych.

**Zdarza się panu też inwestować?**

W ostatnim czasie nie uczestniczyłem w działaniach giełdowych. Obligacji nigdy nie miałem, miewałem akcje, ale na tym moment mam waluty i czekamy na odbicie. Nie doinwestowuję już tych obszarów, bo to wymaga jakiegoś tam zaangażowania. Na ten moment tracę z każdą złotówką ulokowaną na lokacie albo na koncie oszczędnościowym.

**Dlaczego pan oszczędza?**

Raz, że zebranie większej kwoty umożliwia dokonywanie większych zakupów. Mam przed sobą wymianę samochodu, kończy się leasing i muszę wziąć kolejny, to wymaga to chociaż takiego pierwszego wkładu. Remontuję też dom etapami, ponieważ jestem właścicielem domu, który ma prawie 40 lat i etapowo on wymaga pewnych inwestycji, które są zaplanowane i nie da się tego zrobić za 5 czy 7 tysięcy zł zebranych doraźnie. Druga sprawa to zabezpieczenie, bo z mojej filozofii życiowej wynika, że muszę mieć pieniądze na przynajmniej 3 miesiące życia.

**W tym momencie dobrze jest mieć oszczędności?**

Tak, dobrze jest je mieć. Ich rolą jest dodatkowe zabezpieczenie. Jeśli się je ma, to część zmartwień może nie być tak bardzo istotna.

**Czy to może być też dobry czas na inwestycje?**

To jest na pewno dobry czas na to, żeby patrzeć kto upada i wykupować udziały albo przejmować całą firmę; na pewno robi się ciekawy rynek na rynku mieszkaniowym i tam też jest sporo możliwości; myślę, że to jest też świetny moment, żeby kupić samochód. Pod tym względem to jest bardzo dobry i bardzo ciekawy okres. Też z mojej perspektywy, bo wziąłem udział w przetargu i okazało się, że byłem jedynym startującym w przetargu. Nikt nie był zainteresowany poza mną. Nie wiem, czy ludzie odpuścili sobie w jakimś zakresie taką działalność jakby przyjmując, że skoro działalność jest zawieszona to znaczy jest zawieszona i być może trzeba to przetrwać, a jak się okazuje, wiele okazji może przejść koło nosa.

**Czy myśli pan o tym, kiedy pandemia się skończy?**

Nie, nie myślę. Wiem, że elementem końca będzie wynalezienie szczepionki albo lekarstwa, które będzie nas zabezpieczało. Czy ja tego oczekuję? Jestem adaptacyjny, więc kiedykolwiek ten moment nastąpi, to ok, ale nie myślę, kiedy to będzie. Można to oczywiście starać się po rożnych przesłankach przewidzieć ten termin, możemy powiedzieć, że rok, półtora roku, ale nie myślę o tym w żaden sposób. Zaczynam myśleć trochę bardziej długofalowo, bo do tej pory planowałem najbardziej 2-3 dni do przodu i stąd ten szybki wyjazd do Świnoujścia, który nie był wcześniej planowany. Teraz już wiem, że przetarg będę musiał realizować od poniedziałku, więc już myślę o tym, co będzie w przyszłym tygodniu od strony zawodowej. Narzędzia do pracy też już kupowałem w poprzednim tygodniu, więc myślałem z dwutygodniowym wyprzedzaniem, jak się zorganizować w czasie ograniczeń, żeby jednak realizować te zadania. Ta moja perspektywa czasowa jednak ulega wydłużeniu w przód.

**Zastanawia się pan nad tym co będzie po epidemii?**

Nie, w ogóle nie mam takich przemyśleń. Zakładam, że ten okres to jest okres przejściowy i że on będzie trwał, będą jakieś ograniczenia, ale że finalnie prawdopodobnie albo powrócimy do normalnego funkcjonowania, albo znajdziemy sobie alternatywne formy życia. Ja być może nigdy już nie będę trenował z 30 osobami na siłowni, bo może będzie to niemożliwe. Może trzeba będzie sobie ustawić w piwnicy swoją własną siłownię? Nie myślę o tym w ogóle. Przyjdzie czas, będzie wyzwanie i trzeba będzie znaleźć rozwiązanie.

**Jak pan widzi perspektywy w pana obszarze działalności gospodarczej. To będzie tak jak wcześniej? Jeśli tak, to kiedy?**

Zakładam, że to wróci do poniedziałku. Z prognoz, które mam z obserwacji rynku, ale też z obserwacji moich własnych możliwości czy tego, co się zapowiada tutaj w otoczeniu, powiedziałbym, że to mogą być najlepsze 3 lata finansowe w moim życiu od poniedziałku.

To jest też z obserwacji takiego rynku lokalnego - co jest realizowane, co jest zlecane, ale też z rozmów z ludźmi, co będzie do zrobienia i w jakim zakresie. Tutaj jest dosyć duży ruch i dosyć duże zainteresowanie.

**Jak dalej potoczy się sytuacja w Polsce?**

Paradoksalnie myślę, że nie będzie już dużego skoku zakażeń, że ten trend się utrzyma, lato wcale nie obniży, a jesień wcale nie podniesie. Dochodzi coraz więcej głosów, że to, co robiliśmy w czasie obostrzeń to był też taki element bardzo szeroko zakrojonej prewencji, ale może niekoniecznie było to niezbędne i bardziej potrzebowaliśmy tego okresu silnych obostrzeń, żeby przygotować opiekę medyczną. Przy niewielkiej liczbie zakażonych uruchomić ten system pomocy, żebyśmy nie zostali zaskoczeni tak, jak zostały zaskoczone Włochy czy Hiszpania. Zakładam, że już nie będzie dramatycznego przebiegu i teraz prawdopodobnie rozpocznie się normalne życie z obostrzeniami, które są i które będą, ale możemy zaczynać wracać do normalnego funkcjonowania. Wiadomo, że część instytucji, uczelnie być może do października nie ruszą, ale to wynika też ze struktury roku akademickiego. Wydaje mi się, że my dosyć szybko gospodarczo powrócimy do ładu. Może będą przetasowania i zmieni się rynek pracy, tzn. dużo ludzi zmieni miejsca pracy i zmieni branżę, bo to wszystko było bardzo mocno ustabilizowane, brakowało rąk do pracy, więc mieliśmy rynek pracownika a nie rynek pracodawcy, ale to teraz zostało nagle zburzone i nie ma czegoś takiego. Wszystko trzeba będzie poukładać od początku i w tym zakresie będziemy mieli duże zmiany. Wydaje mi się, że dużo więcej pokory będzie, że już nie będzie tak, że będzie podział na stanowiska i pracę dla ludzi spoza naszego kraju i Polaków. Będziemy znowu starali się doceniać tę pracę, która jest. O stronie politycznej się nie wypowiadam, bo muszę zachować neutralność polityczną, ale sądzę, że tu będziemy się musieli odbudowywać z gruzów państwowości. Będziemy żyli prostszymi wartościami i one będą nas cieszyły. Taka jest przyszłość społeczna. Myślę, że przez jakiś czas to się utrzyma tak, jak po tragedii smoleńskiej, zawaleniem się WTC czy śmierci św. Jana Pawła II. Będzie "tadam, jesteśmy Polakami", a później znowu wyciągniemy nożyki i będziemy sobie podrzynać gardła.

**W perspektywie najbliższych paru tygodni ma pan jakieś obawy dotyczące pana przyszłości?**

Oczywiście, że tak. Mam zarezerwowane wakacje, na które wpłaciłem jakąś tam niewielką kwotę, ale wpłaciłem. Nawet tych pieniędzy nie jest mi żal, tylko żal mi jest rodziny, która nie będzie mogła skorzystać z tej oferty. Trochę moich obaw wzbudza realizacja umowy przetargowej, którą już podpisałem a też nie wiem, jak to będzie wyglądało. To jest jednak praca z ludźmi, jest pytanie, czy będzie chęć tych potencjalnych współpracujących, czy będzie większa chęć do współpracy czy też nie, czy nie będę miał jakiejś obawy związanej z zakażeniem, bo jednak jakiś element ekspozycji jest. Coś jednak zaprząta moje myśli i jest w polu uwagi. Jak to wszystko teraz zorganizować, czy może szukać wersji realizacji przetargu online. Zobaczymy. Myślę, że w piątek będę wiedział więcej i będę miał cały weekend na to, żeby podjąć pewnego rodzaju działania.
