# Supplementary material for: The challenges arising from the COVID-19 pandemic and the way people deal with them. A qualitative longitudinal study
Source: PLoS One. 2021 Oct 11;16(10):e0258133. doi: 10.1371/journal.pone.0258133 (PMC8504766; doi:10.1371/journal.pone.0258133)
Supplement: S1 Dataset — (ZIP) [file pone.0258133.s003.zip › Transcriptions/stage 4/12.4_M_33_couple, with children.docx]

**12.4_M_33_couple with children**

**Jak u ciebie wyglądały ostatnie dwa tygodnie?**

Tak ogólnie, bardzo dużo się działo, trochę się zmieniło podejście do całej sytuacji. Mogę powiedzieć, że od ostatniego spotkania, które mieliśmy też w środę, już praktycznie od piątku była duża zmiana. I jakoś powoli, cały czas te zmiany następują.

**Na czym polegała ta zmiana?**

Ogólnie otworzyliśmy się na spotkania rodzinne w takim większym gronie, udało się nam wyjechać na majówkę poza dom, w zupełnie inne klimaty. W pracy też się pozmieniało, to znaczy, od jutra już działam w terenie. Tak ogólnie, porównując do tego, co było poprzednim razem, kiedy rozmawialiśmy, można powiedzieć, że sporo się pozmieniało.

**W większym gronie, czyli jak dużym?**

To spotkania rodzinne na poziomie kuzynostwa, czy też brat-siostra. Nawet bardziej w tym kierunku, brat-siostra i dzieciaki. Dużo się tak naprawdę zmieniło dla dzieciaków, ponieważ poprzednio dosyć długi okres dzieciaki się nie widziały, tyle, co przez Messengera. Tęskniły już za sobą i taka duża zmiana nastąpiła w piątek dwa tygodnie temu. Postanowiliśmy, że zaczniemy do siebie przyjeżdżać i rozszerzymy to jeszcze wtedy zamknięte grono do większej ilości osób. Na razie to jest tylko rodzina i to też jeszcze w okrojonym składzie, nadal nie spotykamy się ze znajomymi. Mieliśmy jakieś dawno temu zaplanowane spotkania [ze znajomymi] i one zostały przeniesione na jakiś inny, bardziej sprzyjający termin.

**Z czego wynikała decyzja, że postanowiliście spotkać się w większym gronie?**

Ogólnie też pod kątem dzieci, tego, że zbliżała się majówka. Każdy ma już chęć wyjść z domu, jakoś coś zrobić ze sobą. Doszło do tego też to, że ta cała sytuacja coraz bardziej blaknie. Jeśli chodzi o alerty, zagrożenia. To nie znaczy, że nie będziemy się teraz całkowicie pilnować, ale stwierdziliśmy ogólnie, mniej więcej w jednym czasie, bo decyzja musiała zapaść po obu stronach, że już jest czas na to, że można się powoli otwierać na spotkania towarzyskie.

**Co masz na myśli, mówiąc, że sytuacja blaknie jeśli chodzi o alerty? Sprawdzasz statystyki?**

Nie opieram się w ogóle na statystykach. Opieram się tylko i wyłącznie na takich własnych odczuciach, gdzie obserwując osoby z otoczenia, które teoretycznie mają dużo większe ryzyko zachorowania, jak na przykład sąsiedzi, gdzie jeden jest kierowcą autobusu, nie zachorowali. Choć być może zachorowali i przeszli to bezobjawowo. To też pomogło mi podjąć decyzję, że może nie będę od razu w ogóle nie myślał o koronawirusie i zachowywał się jak przed, ale to już chyba taki czas, że można sobie troszeczkę zwiększyć jakieś swobody.

**Czyli na ten moment nie czujecie się zagrożeni sytuacją?**

Nie, ja wręcz mam już takie podejście, że może nawet nie myślę w kategoriach co ma być, to będzie, tylko po prostu bezpośrednio nie czuję tego zagrożenia, jak to było wcześniej. Nadal mamy tę barierę, granicę, z kim się widujemy, a z kim nie, ale została ona powiększona o jakiś tam obszar.

**A ten wyjazd?**

Pojechaliśmy na działkę, przez pół Polski, nad morze. Tam spędziliśmy sobie kilka, 5, dni. Od piątku do wczoraj [wtorku]. Wczoraj w nocy wróciliśmy. Ogólnie to też było zetknięcie się z zupełnie innym myśleniem ludzi. Tam też mamy jakichś sąsiadów. Tam widzę, że ludzie jakby w ogóle inaczej ludzie podchodzą do sprawy niż tutaj, w centrum Polski. Tam noszą te maseczki, ale tak bardziej bo trzeba. Nie czują tej potrzeby, że to jest konieczność, dla wspólnego dobra. Widziałem taką sytuację, gdzie dwie sprzedawczynie stały sobie na zewnątrz i jedna śmiała się z drugiej "no załóż tę maseczkę, załóż", a tamta mówiła "niee, nie potrzebna". Tam być może oni tego jakoś tak nie odczuli. To złagodziło u mnie nastroje. Przecież ludzie wszędzie pracują - kasjerki w sklepach spożywczych, poczta, itd. Ci ludzie pracują, mają zwiększone ryzyko. Patrzyłem tak z przymrużeniem oka, że oni kiedyś pewnie zachorują, że to nie da się tak, żeby nie zachorowali, skoro widzą się z tyloma osobami. Natomiast wygląda na to, że środki zapobiegawcze, które stosują - już nie mówię o tym wyjątku z dwoma sprzedawczyniami, a o ludziach, którzy rzeczywiście stosują maski, rękawiczki, żele dezynfekujące - w jakimś stopniu musi to zapewniać im ochronę. Stwierdziłem, że jeśli dalej będę utrzymywał takie środki, to jakiś kontakt z ludźmi może zostać zwiększony.

**Na ten wyjazd pojechaliście sami, czy też z rodziną?**

Pojechaliśmy w dwie rodziny, do domu, który jest niewynajmowany, tylko własny. Tutaj też pod kątem bezpieczeństwa. Stwierdziliśmy, że skoro widzimy się tutaj, nie ma problemu, żebyśmy nie widzieli się tam. Byliśmy my z dzieciakami, siostra żony, jej mąż i ich dzieciaki.

**A praca? Powiedziałeś, ze tam też dużo się zadziało.**

Przeczuwałem, że przyjdzie moment w maju, gdzie trzeba będzie pojechać, zobaczyć się z klientem, coś skonfigurować, coś zrobić. Na ten moment mam już zaplanowane wyjazdy na jutro, piątek, poniedziałek i wtorek. W różnych lokalizacjach. Także ruszyło to pełną parą, ze strony PKP też już otworzyli trochę swoje granice. Wiadomo, będziemy musieli zachować środki bezpieczeństwa, natomiast nie obawiam się tego wyjazdu. Myślę, że po prostu trzeba jechać i zrobić. Jak mówię, każdy pracuje w jakiejś dziedzinie i trzeba sobie radzić.

**To będzie już taki system pracy, jak przed epidemią, poza tymi maskami i rękawiczkami?**

Trochę inaczej są zorganizowane odbiory, na przykład klient jest podzielony na dwie grupy. Kiedy wychodzi jedna, pomieszczenie jest dezynfekowane i dopiero przychodzą następni. Jest system rotacyjny. Udało nam się uruchomić też taki system, który pozwala na zdalne przeprowadzanie odbiorów. Mieliśmy dosyć silny nacisk ze strony zarządu, aby to domknąć, aby to się udało zrobić. I się udało, więc część odbiorów jest przeprowadzana zdalnie. Część z jakimiś dodatkowymi obostrzeniami. Mam tu na myśli, że jak na przykład jadę do budynków PKP, poprosiłem, aby wyizolowali mi jeden pokój. Tylko dla mnie, po to, abym ewentualnie mógł pojawiać się jedynie na czas ewentualnych pytań, czy mini szkolenia w tej grupie osób odbierających produkt. Pozostały czas będę mógł przebywać sobie poza nimi. Takie odbiory trwają dwa tygodnie i dłużej. Jest też w związku z tym takie zarządzenie, że początek rusza ze wsparciem bezpośrednim, ale jest plan, żeby od trzeciego dnia to wsparcie było tylko pośrednie - na zasadzie wdzwaniania się dwa razy dziennie, omawiania jakichś problemów. Interwencyjnie zaś, tylko w celu omawiania problemów technicznych.

**Czy w ciągu ostatnich dwóch tygodni pojawiły się u Ciebie jakieś nowe zachowania?**

Bardzo dużo zmienił nasz wyjazd. Nie wiem, czy na dobre, być może na złe. Skutków może - mam nadzieję - nie odczujemy. Natomiast zmieniło się całkowicie myślenie. W jedną stronę jechaliśmy w nocy, na jednym baku. Kiedy się zatrzymywaliśmy, to poza stacjami benzynowymi, bez wchodzenia na nie. A wracaliśmy już normalnie. Wiadomo, dzieci zostały w samochodzie, natomiast ja wszedłem sobie na stację, kupiłem tę kawę, jakieś przekąski, itd. Można powiedzieć, że złapaliśmy trochę dystansu do sytuacji, ten wyjazd na pewno pomógł. Tam jechaliśmy z taką myślą, aby nigdzie nie stawać, a jeśli już to tak, aby nie korzystać z jakichś publicznych toalet, itd., a z powrotem, mieliśmy już troszkę inne myślenie.

**A czy są jakieś rzeczy, które teraz ograniczyłeś?**

Nie, to raczej idzie w drugą stronę. Po tych ograniczeniach, jakie były, teraz już zmniejsza się te ograniczenia. To takie poszerzanie granic co do tego, co jest akceptowalne, a co nie. One się zmieniły, więcej rzeczy akceptuję, większe ryzyko. Bo tak naprawdę, gdybyśmy chcieli wyeliminować ryzyko całkowicie, musielibyśmy siedzieć w domu, kurier powinien dostarczać posiłki, czy też produkty, które stałyby dwa dni [kwarantanna], itd. Natomiast tutaj jest taka kwestia, że poluzowaliśmy, szczerze mówiąc.

**Czy jest coś, co jest w tym momencie dla ciebie największym wyzwaniem?**

W tym momencie, to pogodzenie trybu, który wypracowaliśmy ostatnio z nowymi realiami, jeśli chodzi o pracę. Więc głównie praca. Od kilku miesięcy siedzę w domu. Siedzę za drzwiami, ale jestem. Natomiast u nas decyzja poszła, że przedszkole nie będzie jeszcze funkcjonowało. Jutro rano wstanę, toaleta, śniadanie i mnie nie ma. Wrócę pewnie koło 18-19. Tuta bardziej wyzwanie dla żony, niż dla mnie ale ja też na pewno będę o tym myślał, jak sobie poradzi. Na pewno sobie poradzi, ale jak wrócę, też będzie zamiana. Ona pojedzie gdzieś do swojej pracy, nawet wieczorem, czy w nocy, ja przejmę dzieciaki i pewnie się miniemy. A w piątek znowu to samo, bo będę jechał do innej lokalizacji i nie wiem, ile mi zejdzie, choć pewnie sporo. Ale dobrze, że niedługo weekend.

**To mijanie się będzie znów przeszkadzać?**

Do dobrego można się szybko przyzwyczaić. Na razie możliwość pracy zdalnej mamy do 8 maja. Ale wiem, że oni się zastanawiają, czy nie przedłużyć tej pracy zdalnej. Rozmawiałem już ze swoim szefem i stwierdziłem, zapowiedziałem mu, że ja chcę mieć trzy dni pracy zdalnej jako standard. Jakoś krytycznie na to nie zareagował, także mam nadzieję, że uda się to później jakoś przeprocedować. Myślę, że w długotrwałej perspektywie jest na to szansa i nawet nie tyle dla mnie, co dla całego zespołu chciałbym to kontynuować. Wskaźniki są dosyć dobre, nawet powiedziałbym, że lepsze, niż takiej pracy stacjonarnej. Mamy tutaj możliwość oparcia w doświadczeniu, które teraz łapiemy. Więc będę celował na pewno w tym kierunku. Aczkolwiek to też będzie inaczej, bo żona będzie wtedy normalnie jeździła do pracy, dzieci do przedszkola, a ja będę siedział w domu i pracował.

**Czy jest coś jeszcze, co zaczyna przeszkadzać?**

Chyba tak. Ogólnie to są tak naprawdę rzeczy, które doskwierały do tej pory. Choć bardziej, niż wyjazdu do pracy, cieszę się na spotkanie ze znajomymi. Może wcześniej nie myślałem jeszcze w tych kategoriach, że fajnie by było spotkać kolegów i koleżanki z pracy, bo jednak się myśli o kolegach czy koleżankach, a przyjaciołach na innym poziomie. Teraz jest tak, że spotkanie znajomych w pracy sprawia mi raczej przyjemność, niż napędza strachu wizją zarażenia.

**Emocje, obrazki. Czy udało się znaleźć coś własnego?**

Tak, mam, ale to jest bardzo prostolinijny przekaz.


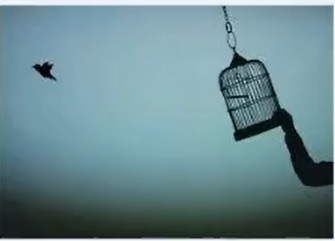


Ogólnie powiem tak, wybierałem to na szybko, ale chyba te na szybko wybory też są dobre. Nie wybrałem pierwszego lepszego z brzegu. Tutaj ta ręka jest moim zdaniem taka emanująca takim...Bo to opuszczenie klatki, to wiadomo. Nagle jest wolność. Tylko że większość takich obrazków przedstawia samą klatkę i samą wolność. Tutaj ta ręka jest dosyć taka, ja nie wiem, czy mi się zmieniły poglądy, czy nie, ale gdzieś tutaj czuję, że ktoś trzyma jednak tą ręką całą sytuację - czy to gospodarczą, czy inną. To mogą być rządy, jakieś teorie spiskowe. Natomiast jest ta ręka. Zakazów, nakazów, narzuconych przez kogoś. Ten obrazek moim zdaniem jest bardziej w ten deseń, że ktoś to trzyma, a mimo to udało się uciec. W ten sposób, może. Samo opuszczenie klatki wiąże się z takim przekazem, że człowiek był zamknięty, a teraz nie jest. Natomiast tutaj ta ręka dużo zmienia. Ktoś jednak jeszcze jest w grze, nie tylko klatka i ta uwięziona w środku istota. Ktoś tę klatkę trzyma. Można to zinterpretować różnie. Na przykład, że ta osoba właśnie wypuściła tego ptaka. Można też zinterpretować, że ten ptak po prostu uciekł. Ja interpretuję to raczej w drugą stronę, ten drugi przykład. Że mimo tego, że ktoś próbuje ukierunkować mnie, zapanować nad tym, co powinienem robić, a co nie, czuję się trochę jako taki uciekinier. Pojechałem w końcu nad morze, pomimo tej całej kampanii zostań w domu.

**Jakie emocje wiążą się z tym obrazkiem?**

Takie ogólnie mieszane trochę, ale zdecydowanie z przewagą pozytywnych emocji. Wiesz, być może nie powinniśmy i tak dalej, ale pojechaliśmy i było naprawdę fajnie, każdy jest mega zadowolony. I też widzieliśmy ludzi na plaży i spacerujących na deptakach, jakieś otwarte restauracje, bo to właśnie 4 chyba znieśli ten zakaz dotyczący restauracji. Na pewno pomógł też ten poniedziałkowy przełom zniesienia ograniczeń. Taki symbol tego był też wtedy, kiedy po drodze na ryby, nad jezioro, musieliśmy przejść przez bramę restauracji, która była zamknięta. Nie poszliśmy więc na ryby, musieliśmy pocałować klamkę i wrócić. Dzieciaki były z tego powodu bardzo złe. Nastawiły się na wędkowanie. Ale już w poniedziałek brama byłą otwarta. To był znak, że się odpuszcza.

**Jak czułeś się w ciągu ostatnich dwóch tygodni?**

Czuję się swobodny, bardziej elastyczny, mniej ograniczony. Nie powiedziałbym, ze to szczęście, bo to kojarzy mi się z zupełnie czymś innym, ale taki właśnie bardziej naturalny.

**Czego najbardziej się teraz boisz?**

To taki standardowy lęk, który jeszcze mi pozostał. Mniej boję się o siebie i rodzinę. Z tych wszystkich doniesień można wywnioskować, że osoby młode lepiej sobie radzą z tego typu wirusem. Bardziej martwimy się o dziadków, którzy z nami mieszkają. I chyba tylko pod tym względem są jakieś obawy, żeby się jakoś bardziej pilnować.

**Robisz coś, aby radzić sobie z tymi negatywnymi emocjami, które czasami się jeszcze pojawiają?**

To takie odsuwanie ich. I sposób tłumaczenia - może to zły sposób, ale - dlaczego ja mam być aż tak narażony, skoro sąsiad kierowca tyle czasu wozi po 30 osób z różnych miejsc i nie jest zarażony - całe szczęście, najlepiej mu życzę. Z drugiej strony, taki pracownik stacji benzynowej, który też codziennie przychodzi do pracy i mnóstwo osób się z nim widzi, nie jest zarażony. W takim razie noszenie maseczek, dezynfekowanie rąk - to wszystko musi pomagać. Ciężko mi uwierzyć, że na stacji benzynowej, na ruchliwej trasie, nie trafiła się żadna osoba z wirusem. Być może była, ale nikogo nie zaraziła.

**Jak zachowują się teraz twoi bliscy?**

Druga część rodziny, kiedy dowiedziała się, że spotkaliśmy się z jedną, już nas do siebie zaprosiła. Chyba każdy już tak troszeczkę powiększa te granice i to nie tak, że jesteśmy takimi pionierami, że my zaczęliśmy je powiększać, a za nami reszta, tylko bardziej mniej więcej wszyscy w jednym momencie. Moja mama, kiedy jeszcze nie wiedziała, że z kimś się widzimy i gdzieś jedziemy, proponowała, żebyśmy w majówkę przyjechali do nich. Więc chyba każdy ma już dużo luźniejszy stosunek do sytuacji. Wydaje mi się, że to bardzo naturalne. Z takiego na początku śmiechu, później takiego "ojej, może coś z tego będzie", następnie szoku, trwania w napięciu, poprzez luzowanie. To naturalna rzecz, że te emocje się tak objawiają. Każdy jest w miarę elastyczny i uczy się żyć. Nawet z wizją wirusa, gdzieś tam, z tyłu.

**Mówisz o elastyczności. Jak myślisz, z czego jeszcze wynika to, jak inni podchodzą teraz do sytuacji?**

Wziąłem na siebie część obowiązków służbowych związanych z wyjazdem, bo myślę, że to już czas na to, aby pokazać, że jakoś dajemy radę. Często dzwoniłem do ludzi, aby przyszli do biura, coś sprawdzili, załatwili, spotkali się z kimś. Czuję, że już za długo siedzę w domu, to może wpłynąć na innych, jeśli chodzi o pracę.

**Z czego wynika to poczucie, że to już czas?**

Źle bym się czuł z taką myślą, że siedziałbym w domu, a byłem wcześniej na majówce. Podjąłem takie największe ryzyko, że jedziemy na wyjazd. Ktoś tam o tym wie, bo byłem w kontakcie z kolegami z pracy. Wydaje mi się, że nie byłbym fair, gdybym znów się tu zaszył i tylko wysyłał innych po całej Polsce. Wydaje mi się, że powinienem postępować konsekwentnie, czy to przyjemność, czy praca. Granica została już poszerzona, więc nie widzę sensu, aby siedzieć w domu i bronić się, rękami i nogami przed tym, aby nigdzie nie jechać.

**Dlaczego ludzie w twoim otoczeniu też poszerzają granice, o których mówisz?**

Myślę, że każdy ze swoich powodów. I tak bardzo długo się nie widzieliśmy, wytrzymaliśmy w jakiejśtam izolacji. W nieskończoność nie będziemy ciągnąc tego stanu. Każdy chce poszerzyć sobie granice. Nie spotykamy się ze znajomymi, nie spotykamy się towarzysko z kolegami, natomiast wydaje mi się, że każdy już czuje tę potrzebę, aby spotkać się po prostu z rodziną. Matka z synem, brat z siostrą, kuzynostwo, itd. Każdy tak naprawdę patrzy, co dzieje się wokół niego. Do mnie też doszły słuchy, że osoba, która mieszka dość blisko moich rodziców, zachorowała. Przebywała w domu na kwarantannie z całą rodziną i tylko ona miała wynik pozytywny - pozostali członkowie rodziny nie. Dziwne trochę, bo sądziłem, że to wirus, że wystarczy przejść obok kogoś metr i już na pewno jestem zainfekowany. Natomiast tutaj widać, że nie. W grę pewnie chodzi jakieś szczęście, ostrożność, itd.

**Zakupy - jak zapatrujesz się na otwarcie galerii handlowych?**

Ogólnie uważam, że gospodarka powinna iść dalej, do przodu. Każdy indywidualnie powinien sobie stosować jakieś bariery - zabezpieczenia, itd. Był w gospodarce ten oddech, pauza, uważam jednak, że to powinno już ruszyć. Od marca, cały kwiecień - ludzie też chcą zarabiać, mieć jakieś stałe dochody. Ta niepewna sytuacja na pewno nie pomaga. Wydaje mi się, że na tyle zrobiono pauzę wszystkiego, że ten boom zachorowań nie powinien nam grozić, taki ogromny. Że te ograniczenia były wprowadzone w miarę dobrym momencie i wydaje mi się, że to w miarę dobry czas, aby odświeżać tę gospodarkę, łącznie właśnie z galeriami, sklepami, jakimiś drobnymi przedsiębiorstwami. Ja też mam takie myślenie, patrząc na siebie nawet. Był ten czas ścisłej kwarantanny, a teraz możemy trochę zacząć wszystko odbudowywać.

**Myślisz, że to bezpieczne?**

Myślę, że jest obarczone pewnym - średnim - ryzykiem. Nikt nie zapewni bezpieczeństwa w 100%. Możemy tylko mówić o ewentualnych akceptowalnych stopniach zagrożenia. Jak miałbym sobie kupić T-shirt, bo potrzebuję, to bym sobie kupił, poszedłbym do galerii. Być może bez przymierzania, być może któryś z kolei z wieszaka - niedotykany, ale bym sobie kupił. Zresztą, miałem taką sytuację na wyjeździe, jeszcze przed otwarciem galerii handlowych. Potrzebowałem czapki, bo zorientowałem się, że żadnej nie wziąłem i ją sobie kupiłem - w sklepie stacjonarnym. Też się trochę zapomniałem, bo byłem już w takim myśleniu, że wziąłem, przymierzyłem - dobra, kupuję. Wyszedłem, jadę sobie w tej czapce. I sobie myślę, kurczę, ile było przede mną osób, które ją przymierzały. Aaale, stwierdziłem, że już dobra, akceptuję to ryzyko. Także jakbym coś musiał kupić, to bym kupił. Strach by mnie przed tym nie wstrzymał. Gdybym miał potrzebę, to bym ją zrealizował. Natomiast jeśli miałbym sobie iść ot tak na zakupy, to raczej bym nie poszedł, mogę poczekać.

**To realizowanie potrzeb sprawia, że jest normalniej?**

Takie realizowanie potrzeb działa w drugą stronę - przypomina o całym zagrożeniu. Tak, jak mówię, byłem dobrej myśli, dopóki nie kupiłem czapki. Myślałem wtedy, że może powinienem ją odstawić na dwa dni, wrzucić do foliowej torebki, położyć gdzieś. Ale wtedy ten zakup nie miałby sensu. To mnie bardziej ściągnęło z normalności do tej nienormalności, wizji wirusa i tego, co się dzieje, przypomniało powagę sytuacji. Dlatego każde takie zakupy, czy wizyta u fryzjera, mogą ściągać uczucia niepokoju, myślenia o tym, że jest taka sytuacja, że ciągle gdzieś tam jest ten wirus.

**Rozumiem, że chwilowo nie planujesz wizyty w galerii, chyba, że będziesz miał jakąś pilną potrzebę?**

Tak, nie mam potrzeby na siłę jechać do tej galerii. Nie ciągnie mnie żeby zobaczyć, jak tam jest, jak to teraz wygląda. Chociaż koledzy wspominają mi, że jest dużo różnych promocji - trochę kuszą. Ale to bardziej mowa o elektronice na internecie, niż zakupach w galeriach. Na razie się nie wybieram i pewnie nie będę tego planował przez jakiś tydzień, dwa.

**A później?**

Mam nadzieję, że pogoda się poprawi i może będę miał potrzebę, żeby coś sobie kupić. Krótkie spodnie, czy coś w ten deseń. Natomiast na razie nie czuję takiej potrzeby i nie wybieram się, nie planuję.

**A czy ktoś z twojego bliskiego otoczenia wspominał, że się tam wybiera?**

Nie słyszałem. Nikt nie chwalił mi się, że będzie wyjeżdżał na zakupy do galerii.

**Jak wyglądają teraz u ciebie zakupy spożywcze?**

Nic się nie zmieniło, bo zazwyczaj, jak ci mówiłem, robiliśmy je raz w tygodniu z listą. Ostatnio zauważyłem, że w sklepach nie ma już kolejek przed wejściem, jest trochę więcej ludzi w sklepie, zachowywali się oni też bardziej swobodnie. Przed wyjazdem tak samo zaplanowaliśmy jadłospis, ale zaopatrzyliśmy się w rodzinnych sklepach, nie miałem potrzeby i czasu, żeby jechać do marketu – to jakieś 30 km w jedną stronę. W dniu wyjazdu jeszcze pracowaliśmy. Musieliśmy przygotować mieszkanie, zorganizować opiekę dziadkom, spakować potrzebne rzeczy. Żona robiła te zakupy w nocy, koło 22 i w nocy pojechaliśmy na działkę. Wzięliśmy całe jedzenie ze sobą i na wyjeździe nie musieliśmy robić takich zakupów. Zazwyczaj kupujemy te same produkty, co przed epidemią. Raz tylko słyszałem, że tonik pomaga na koronawirusa, to chciałem kupić gin z tonikiem. Kupiłem gin, a toniku zapomniałem. <śmiech>

**Czy w ciągu ostatnich dwóch tygodni kupiłeś coś, co poprawiło ci humor?**

Ogólnie czapka podoba mi się, chodzę w niej cały czas, prawie cały czas. <śmiech> Ale rzeczywiście, wyjeżdżając, zmieniając środowisko, zmieniłem swoje restrykcje dotyczące jedzenia, diety. Humor na pewno poprawiła mi taka paczka karkówki - zdecydowanie. I ogólnie takie grillowe rzeczy. Tam można było grillować sobie co dziennie, a normalnie tego nie robię. Czyli taki wakacyjny zestaw - grill, piwko, itd. Ale to było spowodowane wyjazdem, a nie jakąś melancholią związaną z wirusem.

**1-10 (1 = z dużym trudem, 10 = z dużą łatwością wydaję pieniądze)**

Wiesz co, myślę, że 8. Zawsze starałem się nie wydawać niepotrzebnie pieniędzy, ale będąc na wyjeździe, jak mieliśmy już coś obejrzeć, to zawsze wybierałem takie oferty VOD, żeby rzeczywiście obejrzeć sobie coś ciekawego, jak jest okazja. Jakoś nie patrzyłem na koszty, że tu 14 zł, tam 14 zł. Druga rzecz, przez tą całą sytuację, przez siedzenie 2 miesiące w domu, zrobiło się, wygenerowało trochę oszczędności. Odpadło nam trochę aktywności związanych z wydawaniem pieniędzy - takich, że tam się gdzieś pojedzie, tam się coś kupi. Teraz łatwiej z tego korzystać.

**Przed epidemią było podobnie? Kiedy miałeś oszczędności, łatwo je wydawałeś?**

Ogólnie myślę że kiedy są pieniądze, zaczyna się myśleć, co z nimi zrobić. I zawsze znajdowałem jakiś sposób, żeby je wydać. Czy to były jakieś inwestycje związane z domem, czy na przyjemności. To też chyba taki mój charakter. Teraz się zastanawiam, co zrobić dalej z oszczędnościami. U mnie jest tak, że ciężko, żeby te pieniądze gdzieś tam sobie leżały gdzieś tam na koncie. Mam zrobioną jakąś barierę bezpieczeństwa, żeby nic mnie nie zaskoczyło. Natomiast wszelkie nadwyżki próbuję sobie lokować - czy to w rzeczy przydomowe, czy inne tego typu.

**Jakie emocje towarzyszą twoim zakupom?**

Raczej nie myślę, że mogłem nie wydawać pieniędzy. Raczej się cieszę. Człowiek pracuje, zarabia, stać mnie, to wydaję. Ma to sprawiać przyjemność.

**Kiedy ostatnio wydałeś większą kwotę na niecodzienny zakup (...). Opowiedz o tym.**

To możemy pogadać o tym w kontekście wakacyjnym. W tym roku miałem z całą rodziną zaplanowane wakacje w Turcji. Bądź co bądź, to już 5 osób. Mieliśmy wybrać się na 10 dni i zapłaciłem zaliczkę w październiku. Kiedy układałem sobie taki plan, wiedziałem, że w maju - bo wtedy mieliśmy lecieć - będzie mnie na nią stać. To jest ciekawe, że wszystko sobie tak poukładałem, że wiedziałem, że będę miał pieniądze na ten wyjazd. Tak sobie myślałem, że to jest kupa pieniędzy i można za to dużo rzeczy zrobić, natomiast też można za te pieniądze zabrać gdzieś rodzinę. To była też decyzja wspólna z żoną. Nie byliśmy już chyba 2 lata na zagranicznej wycieczce, zw na różne inwestycje w poprzednich latach, to w tym roku sobie pozwolimy. Aczkolwiek sytuacja potoczyła się inaczej, nie jedziemy na tę wycieczkę [koronawirus]. Ale nie mam problemów, żeby jakąś większą sumę pieniędzy wydać, mimo że nie jest to kwota, która robiłaby jakąś większą różnicę w domowym budżecie. Dlatego stwierdziłem, dobrze, jedziemy - z żoną podjęliśmy decyzję - jedziemy, będzie fajnie. Natomiast koszty całej wycieczki były dosyć wysokie. [w związku z obecną sytuacją] Przeciągali nas do samego końca, do kwietnia. Praktycznie do 24 kwietnia, gdzie lecieć mieliśmy 8 maja, była wciąż mowa, że może jeszcze polecimy. Ale tego 24 dostaliśmy wiadomość, że do 31 maja wszystkie wycieczki są odwołane i mieliśmy do wyboru trzy opcje - zwrot kosztów w formie vouchera na przyszły rok, zmianę terminu wycieczki, czy zwrot pieniędzy w ciągu pół roku. Stwierdziliśmy, że zdecydujemy się na ostatnią, bo gdybyśmy mieli gdzieś wybierać się za rok, nie będziemy ograniczeni tym biurem.

**Czyli ten wyjazd na działkę był swego rodzaju alternatywą?**

Pewnie gdybyśmy mieli lecieć, na działkę byśmy już nie pojechali. Musiałbym wziąć więcej urlopu. Pewnie po prostu przygotowalibyśmy się do wyjazdu zagranicznego.

**(...) Rozrzutny czy oszczędny?**

A jest coś pomiędzy? Kwestia jest taka, że jakoś łatwo mi przychodzi - to chyba mi zostało z akademika - takie kombinowanie pieniędzmi. W sensie, wiem, że coś mi wpadnie, tyle mogę wydać - i wydaję, mam z tego jakąś korzyść. Natomiast łatwo mi przychodzi planowanie. Po takich większych inwestycjach w ubiegłym roku, to był chyba listopad, czy październik, gdzie w pewnym momencie wypłukałem się totalnie - kupiłem samochód i takie tam różne rzeczy - pokazałem żonie, że w maju będziemy mieć kasę na wycieczkę. Nie powiedziałbym, że jestem bardzo oszczędny, ponieważ wydaję te pieniądze. Natomiast z jakimś takim rozsądkiem i planowaniem. Rozrzutny i skąpy to dwie skrajności. Ja pieniądze wydaję i lubię wydawać, natomiast z buforem bezpieczeństwa i planem na to.

**Czy obecnie coś się zmieniło w twoich dochodach/ perspektywach finansowych na przyszłość?**

Ogólnie jestem w takim momencie, gdzie oszczędności zaczęły mi tak fajnie rosnąć. To nie jest do końca spowodowane tym, że siedzimy w domu, choć ileś pieniędzy zostało przez to, że mieliśmy jechać, a nie pojedziemy. Ale to nie o to chodzi. Bardziej o to, że duże inwestycje zostały zrealizowane i w tym momencie mam świadomość, na ile jestem elastyczny, jeśli chodzi o to, ile mogę odłożyć, na ile mogę sobie pozwolić. W tym momencie mam dosyć dobrą kondycję finansową i w myślę, że w tamtym roku na ten moment - po dużych inwestycjach - kiepską sytuację finansową. W tym momencie jestem wręcz trochę zaskoczony i zadowolony. Kredyty, czy pożyczki, które ciążyły na mnie rok temu, zostały odpracowane w 100% i praktycznie drugie tyle zostało wypracowane na oszczędności. Daje mi to większe perspektywy na przyszłość. Jeśli chodzi o pracę, co roku coś nam dorzucają, już wiem, że w tym też tak będzie. Gorzej nie będzie, chyba tylko lepiej. Tym bardziej, że mieliśmy wystąpienie zarządu i dostaliśmy zapewnienie, że jako forma jesteśmy w bardzo dobrej kondycji finansowej. Podobno w tej sytuacji radzimy sobie nawet najlepiej w Polsce z tej grupy korporacyjnej. Biznes nie ucierpiał, wręcz przeciwnie, jak już wspominałem, nadgoniliśmy z terminami. O podwyżkę nie prosiłem, a ją dostałem, więc to ogólnie też jest budujące.

**Mówisz o lepszej sytuacji finansowej, o tym, że możesz sobie na więcej pozwolić. Czy kupujesz w związku z tym więcej droższych produktów?**

Tak, mogę to porównać choćby do tego, że do tej pory korzystałem z HBO Go. Teraz w zakup poszedł też Netflix. Wydaję więcej. Wiem, że mógłbym zaspokoić się samym HBO Go - i tak bym tego nie obejrzał od deski do deski, a zawsze znalazłbym coś ciekawego. Mimo wszystko, nie przeszkodziło mi to w tym, aby dokupić sobie jeszcze Netflix na miesiąc, czy dwa, żeby zobaczyć sobie choćby Wiedźmina.

**Poszukujesz przecen/ okazji?**

Nie szukam. Robię zakupy za potrzebą, takie szybkie. Czasami nie mam czasu na to, żeby pozwolić sobie na szukanie jakichś okazji, patrzenie, wyszukiwanie. Nie poluję na promocje. Koledzy mówią mi, że są fajne na elektronikę. Ale jak w pierwszym odruchu wszedłem na jakąkolwiek stronę, stwierdziłem, że nie mam co kupić. Bo na siłę nie będę wybierał. I dałem sobie spokój.

**A jeśli promocja jest na coś, czego akurat potrzebujesz?**

To jest wtedy fajne, jak najbardziej. One też pomagają w zakupach. Jeśli tylko jest więcej czasu, to myślę, że każdy zrobi rozeznanie rynku. One przyciągają wzrok, choć może nie zawsze nimi [promocjami] są tak do końca.

**W jaki sposób kontrolujesz swój budżet? Czy w ogóle to robisz?**

Tak, kontroluję go dosyć szczegółowo. Są granice. Duże kwoty - operuję sobie nimi tak, że tu odcinam, tu odkładam na różne rzeczy. Mam kilka kont oszczędnościowych i to idzie w różnych kierunkach. Natomiast wydatków stricte w miesiącu, nie kontroluję za bardzo. Na początku miesiąca wiem, jakie będę miał opłaty. Kwotę, która zostaje, dzielę na pół. Pół od razu idzie na konto oszczędnościowe, a z drugiej żyję. Więc przydzielam sobie kwotę do wydania i jak coś zostanie, fajnie, też odkładam na oszczędnościowe i jest fajnie. A jeśli brakuje, wyciągam sobie z tego oszczędnościowego, ale bez jakichś bóli. Po prostu tak jest, brakło.

**Wydaje ci się, że w obecnej sytuacji warto oszczędzać?**

Tak, ja w ogóle twierdzę, że warto oszczędzać. Jeśli ktoś ma taką możliwość, warto się zabezpieczyć. Żeby przynajmniej można było przez pół roku przeżyć bez jakichś tam dochodów. To by była też taka bariera bezpieczeństwa i człowiek byłby bardziej wyluzowany, spokojniejszy. Są pewne kwoty, których się nie rusza po to, że kiedy cokolwiek się wydarzy z domem, autem - aby bez żadnych komplikacji sięgnąć po tę kwotę. A resztą można sobie obracać - inwestować, umieszczać, pożyczać, cokolwiek. Ja rok temu musiałem ruszyć taką kwotę nie do ruszenia - to żyłem w stresie. Takim naprawdę stresie, poczułem, co to znaczy. Mając wtedy dwójkę dzieci i żonę w ciąży, wypłukać się do cna i nie mieć tego zapasu, to przez trzy miesiące chodziłem w takim stresie, który towarzyszył mi codziennie, bo nie miałem płynności finansowej. Wiadomo, mógłbym wziąć kredyt, ale swoje gotówka, to swoja. I dopiero kiedy zauważyłem ten moment - na kartce, czy w Excelu, że są warunki, aby to odpracować, dopiero wtedy poczułem ulgę. Natomiast mówię, bardzo fajnie się to odpracowało i teraz czuję się znów komfortowo.

**Ten bufor, o którym mówisz, to kwoty pozwalające przeżyć jak długi czas, będąc pozbawionym dochodów? To te pół roku, o którym wspomniałeś?**

Dla mnie to kwoty rzędu 20-25 tysięcy zł. Żeby to było nieruszone i to by pewnie pozwalało nam przeżyć pół roku, gdybyśmy rozważyli sytuację, że nagle nikt nie pracuje i nie mamy gotówki.

**Zdarza ci się inwestować?**

Na razie nie, choć o tym myślę. Ale to plany przyszłościowe. Na pewno będę szukał inwestycji na przyszłość. Nie chciałbym tego po prostu składować na kontach, bo różnie może być, dlatego chciałbym zainwestować. Dotychczas tego nie zrobiłem, bo miałem dużo potrzeb inwestycyjnych wokół siebie. Zrealizowałem je - to był dom, ogrodzenie, samochód, tego typu wydatki. W momencie, kiedy czuję, że kolejne inwestycje w moim otoczeniu nie są już tak pilne, mogę pomyśleć o innych inwestycjach - lokowaniu pieniędzy.

**Wiele osób twierdzi, że obecna sytuacja jest poza kontrolą. Może w takim razie nie warto teraz oszczędzać?**

To zależy, jaką formę oszczędzania wybierzemy. Sam miałem ostatnio problem z wypłaceniem w oddziale większej ilości gotówki, musiałem radzić sobie innymi sposobami - blikiem, kartą - poza oddziałem. To było słabe. Jednak wydaje mi się, że są też inne formy oszczędzania. To może być dobry czas na inwestycje, przez kryzys, którego widmo istnieje. Pewne usługi, czy dobra, można kupić taniej, niż przy normalnym stanie rzeczy. Ktoś, kto ma pieniądze, może teraz to kupić taniej, bo ludzie będą potrzebować jakiejś deski.

**Jak myślisz, kiedy obecna sytuacja się skończy?**

To zależy, jak ją definiować, bo ona się dynamicznie zmienia. Pojawiają się i znikają obostrzenia. Natomiast dla mnie wykładnikiem końca epidemii jest powrót imprez masowych. To jest taki największy kontakt ludzi, środowiska są wymieszane, ryzyko zarażenia jest największe na koncercie czy innej imprezie masowej. Tam nikt nie patrzy na to, że 10 tys. osób dotknęło przed nim klamki i 10 tys. osób dotknie jej za nim, wchodząc gdziekolwiek. Myślę, że takie imprezy zaczną wracać gdzieś od stycznia 2021. Natomiast restrykcje będą poluzowane dużo bardziej już z końcem wakacji, tak mi się wydaje.

**Często zdarza ci się o tym myśleć?**

Nie tak często. Ogólnie, mówię, jeśli ktoś może pracować z domu, pracować codziennie - i dobrze mi z tym, że pracuję z domu - to nie ma takiej obsesji na tym punkcie, kiedy to się skończy. Natomiast czasami o tym myślę, lecz nie jest to tak często.

**W jakich momentach ci się to zdarza?**

Kiedy myślę o wakacjach. Pod kątem wyjazdów. Mamy ze znajomymi zaplanowany wyjazd we wrześniu. Cicho się zastanawiam, czy to będzie zrealizowane, czy nie. Czy nawet, jak będą zdjęte restrykcje, to czy ludzie będą chcieli jechać. Czy ja będę chciał jechać? Bardziej pod tym kątem. Nie biorę tu pod uwagę tak naprawdę jakiejś pracy, czy przeszkód z nią związaną - jakoś sobie poradzę. Wiem, że wesela, chrzciny są przełożone na wrzesień**.**Trochę się zastanawiam, czy to się uda. Ale to też jakiś wyznaczony punkt w czasie. Skoro masa osób przekłada takie imprezy z maja na wrzesień, może faktycznie będzie już można je wtedy zorganizować.

**Co najbardziej zaprząta twoją uwagę, kiedy myślisz o przyszłości?**

Jak słyszę hasło "myśleć o przyszłości", myślę o przyszłości za 15 lat. To będzie czas, kiedy moje dzieci będą wchodzić w dorosłość, dlatego na tym punkcie skupiona jest moja uwaga, jako rodzica. To jest taki mój deadline, aby do tego czasu dokonać odpowiednich inwestycji. To, co będzie w perspektywie kilku tygodni/ miesięcy, jest jeszcze przed przyszłością. Ale teraz najbardziej myślę o takiej perspektywie pięcioletniej, związanej z różnymi inwestycjami. Ten temat z pieniędzmi był akurat pod tym kątem bardzo trafiony, bo ostatnio dużo myślę o inwestycjach różnych, żeby coś zrealizować, zrobić, poobracać pieniędzmi, żeby mieć korzyść. Nie mam obaw, jeśli chodzi o najbliższy czas - tygodnie, miesiące. Mam wrażenie, w perspektywie mojej rodziny, że gorzej już nie będzie. Mieliśmy ten czas, że trzeba było siedzieć w domu, nigdzie nie wyjeżdżać dalej. Skoro z tym sobie poradziliśmy, poradzimy sobie także dalej w niedalekiej przyszłości. Tym bardziej, że całość sytuacji jest pozytywnie nastrajająca - idzie lato, można sobie zorganizować coraz więcej aktywności na zewnątrz. Można pojechać na ryby, postać przy grillu. To może być oderwane od koronawirusa, bo można to robić też we własnym gronie, nawet nie tyle najbliższej rodziny, a nawet troszkę większym. Nie myślę więc w kategoriach obaw. O wrześniu myślę raczej z ciekawością - czy wyjazd wypali, czy te imprezy, na które jesteśmy zaproszeni, się odbędą - natomiast bez jakichkolwiek obaw.
