# Supplementary material for: The challenges arising from the COVID-19 pandemic and the way people deal with them. A qualitative longitudinal study
Source: PLoS One. 2021 Oct 11;16(10):e0258133. doi: 10.1371/journal.pone.0258133 (PMC8504766; doi:10.1371/journal.pone.0258133)
Supplement: S1 Dataset — (ZIP) [file pone.0258133.s003.zip › Transcriptions/stage 4/3.4_F_54_single.docx]

**3.4_F_54_single**

**Co się u ciebie działo przez ostatnie 2 tygodnie?**

U nas jest super *[śmiech].* Mnóstwo rzeczy na świeżym powietrzu. Co prawda od 3 dni pada, ponieważ jest bardzo sucho. Więc ogólnie rzecz biorąc u nas jest bomba. Takie mamy nieustające wakacje jeszcze. Chociaż ostatnio Julka miała niby w byłej pracy, wezwali ich do uczestnictwa w spotkaniu na Skype, a potem się okazało, że część tych projektów być może będzie ruszać, więc ci, którzy by chcieli ewentualnie powrócić, to może część by już mogła, a reszta być może w jakimś następnym terminie, więc ich trzymają trochę na takim stand by. Ale to oznacza tylko tyle, że nasza sielanka może się skończyć. Tzn. ja jestem przygotowana na to, że ona się kiedyś skończy i że to nie będzie trwało wiecznie. Tak musi być po prostu.

**Lista się nie skończyła jeszcze?**

Nie ma szansy w ogóle. Praca na roli ma to do siebie, że ta lista się odnawia cyklicznie.

**Jak w tym roku spędziłyście majówkę?**

Spędziłyśmy ją tak, jak ostatni miesiąc, czyli siedziałyśmy. Nawet się nie dało grillować, bo pogoda, myślę, że pomogła nam wszystkim - może nam akurat najmniej, ale w ogóle, że nie prowokowała do jakichś szczególnych wycieczek, bo było po prostu mokro, brzydko i chłodno. Ale nie robiłyśmy nic specjalnego. Dni jak co dzień. Ponieważ my nie pracujemy zawodowo, to mamy takie dni, jak co dzień. Julka długo śpi, potem schodzi na dół, coś jemy, a potem decydujemy, co będziemy robić, w zależności od pogody. Także, żebym pamiętała, co robiłam 1,2,3 maja...

**A wczoraj jak dzień wyglądał?**

Wczoraj jeszcze pamiętam. Wczoraj np. posadziłyśmy pelargonie, które kupiłam w poniedziałek. Ponieważ nasz pan wójt odblokował nam taki "rynek" - na kawałku placu rolnicy i nie tylko sprzedają płody rolne i jakieś pierdoły. I kupiłam właśnie w poniedziałek pelargonie oraz pory i selery. Było dosyć paskudnie, ale pory i selery musiałyśmy posadzić w poniedziałek od razu, żeby nie zdechły nam, a pelargonie poczekały do wczoraj. Znajomy mi przywiózł wiaderko torfu. Bo mamy takie nieopodal olszyny, tzn. takie miejsca, gdzie rosną olszyny i płyną strumyczki, które bobry wykorzystują na miejsce zamieszkania. Jak są tam bobry, to jest żyzna ziemia, ale też rośnie torf. Więc znajomy przywiózł mi torf, zmieszaliśmy go z ziemią, także... A taka ziemia, nie kupiona w sklepie, tylko pojeździłyśmy po sadzie, zebrałyśmy trochę z kretowisk licznych. Posadziłyśmy pelargonie, a później czytałyśmy. Ostatnio mamy dni czytelnicze w związku z tym, bo jest pogoda taka mniej robotna, a bardziej czytelnicza. Także udało nam się trochę wyższej kultury. Chociaż nie wiem, czy zważywszy na to, jakie to książki, trudno to nazwać wyższą kulturą, ale powiedzmy, że trochę kultury wprowadzić w ten rolniczy krajobraz.

**Jak to jest ze spotykaniem się u was z ludźmi?**

Ja to tak sobie myślę, że ja to korzystam z tego, że nie należy się z nimi spotykać i się nie spotykam. Mam takie *good excuse*, że nie jeżdżę. Chociaż no tutaj mam taką starszą sąsiadkę, której czasem coś kupuję i przez ten miesiąc ostatni, czy półtora - bo już nie wiem, ile to trwa, całkiem już zagubiłam się w obliczeniach... Nie wiem, od kiedy to trwa, od połowy marca?

**Jedna respondentka policzyła, że to 54 dni już.**

To ja właśnie jestem absolutnie zagubiona, ale to być może. Więc te kontakty są bardzo ograniczone, nawet jak na moje nieliczne kontakty. Więc nawet byłam ostatnio. Zwykle to wrzucam jej jakieś zakupy i spadam. Ale posiedziałam u niej chwilę i wypiłam kawę, bo już biedni są, bo potrzebują pogadać z kimś. Także nie no, w dalszym ciągu... Teraz się pojawia coś takie, że no, powinnam pojechać gdzieś albo odwiedzić kogoś, ale to odsuwamy to tak. My w sensie tu społeczność. Nie są wyrywni jacyś bardzo do odwiedzania się. Chociaż myślę, że jak ktoś potrzebuje, to po prostu jedzie. Niby się nie spotyka ze znajomymi, ale jak potrzebuje coś ze sklepu czy kupić jakieś gwoździe, czy rozrusznik, część do naprawy ciągnika, to wsiada i jedzie i kupuje. Tu nigdy nie było jakichś wycieczek. Ta moja miejscowość to jest raczej kolonia, więc te domy są oddalone od siebie, a ja najbliższy to mam z 500 metrów. Także do mnie przypadkiem nikt nie może zajrzeć ani po drodze do niczego, bo ja jestem na samym końcu i już nie ma drogi żadnej. Więc po drodze do mnie też nikt nie zagląda.

**Więc musi być celowy wyjazd?**

Żeby ktoś do mnie przyjechał, to tak. A ja mam lepiej. Bo ja mogę po drodze wpadać i regulować sobie to wpadania, ale też nie za bardzo jestem taka wyrywna do tego. W ogóle nie jestem.

**Nie jest pani wyrywna, bo na co dzień pani nie lubi?**

Tak, na co dzień tak raczej oszczędnie. Ja się najlepiej czuję we własnym sosie chyba, a teraz mamy taki sos już w ogóle na bogato, bo jestem z Julką, więc potrzebę spotykania się mam wyczerpaną. Znaczy zaspokojoną. Trudno mi powiedzieć, jak oni się kontaktuję, ale myślę, że zdecydowanie... Chociaż do tej mojej sąsiadki przyjechały w niedzielę jej siostra, też bardzo mocno starsza od niej ze swoją córką z Płocka w odwiedziny. Ale zważywszy na to, że w Płocku od początku tego zajścia przykrego, teraz to nie wiem, naprawdę, ale... Jeszcze 2 tygodnie temu w całym powiecie Płockim mieliśmy 6 zachorowań, z czego 3 wyzdrowiały, 1 osoba zmarła, 1 osoba przebywała w szpitalu, a jedna w domu chora. Ale ten pan, który zmarł, to zmarł u nas. Znaczy dokładnie 3-4 km ode mnie. Więc nastąpiło ogólne poruszenie. I to się stało jakoś 1,5 tygodnia temu był pogrzeb. A stało się to tak, że to był pan chory na nowotwór i przyjmował taką paliatywną chemię, która poprawia jakość życia. Różnie to trwa, może trwać i parę lat nawet. To był pan 60 parę lat i on jeździł nawet o własnych siłach do Warszawy na Szaserów na tę chemię raz na jakiś czas. I pojechał właśnie i tam go w tym szpitalu zarazili koronawirusem. Już stamtąd nie wyszedł i niestety zmarł. Także tutaj nikogo nie zaraził. Ale mówię to dlatego, żeby pokazać, jaka jest skala zjawiska u nas. Jest 6 osób, z których to nie jest tak, że 6 jest chorych i cały czas zaraża, tylko one najprawdopodobniej żadna z nich nic nie ma. Jak były podawane informacje o naszym sąsiedzie, to zostały podane bardziej ogólne, dotyczące powiatu. Wziąwszy pod uwagę, że on zmarł w Warszawie, to nie wiem czemu obciąża powiat płocki. Ale skala zjawiska jest taka, że ci ludzie niby to wszystko wiedzą, ale to ich nie dotyka ani bezpośrednio, ani pośrednio. No może ci żałobnicy, którzy, jak wzięliśmy udział w pogrzebie... Słyszałam, że może być 50 osób. Rzeczywiście w kościele było tak, że on był ze zdiagnozowanym koronawirusem, więc odbyło się to w kościele, przy czym w kościele była tylko najbliższa rodzina, a reszta stała przed kościołem. Trumna nie została wprowadzona do kościoła w ogóle, przyjechała specjalnym karawanem z Warszawy i później została już tylko wyprowadzona na cmentarz. Ale było nas z 80 sztuk śmiało. Myślę, że tak 50 osób na cmentarzu, to nie wiem... Może w Warszawie ktoś odlicza. Nie wiem, kto tego powinien pilnować. Ksiądz, kościelny? Nie wiem, jak to wygląda od strony zadań przydzielonych, bo co z tego, że my sobie powiemy, że 50 osób. To co, sami się mamy pilnować, odliczać? Kto wszedł, kto nie wszedł. W każdym razie tyle, ile przyszło, tyle nas było. Także np. to nie jest w ogóle... No, może jest, jeden przypadek to żadna próbka w ogóle, żeby cokolwiek stwierdzić.

**Myśli pani, że okolica jest poruszona tym, że to koronawirus?**

Nie, myślę, że nie. Jego tu nie było w ogóle. Owszem, pan wójt się wypowiedział na Facebooku. Bo ja wiem, że ja tego nie mogę oglądać, więc na Facebooku to było, bo ja to mam z trzeciej ręki. Pan wójt się wypowiedział, że informuje, że niestety zmarł, itd. oczywiście bez wytykania imienia i nazwiska, ale tak to napisał, że wzbudził zainteresowanie, niepokój. Bo patrzyłam na jakieś komentarze pod spodem i widziałam np. - myślę, że słusznie, bo ja wiedziałam, że on tam został zarażony w szpitalu i w tym szpitalu został. Natomiast, jak ludzie się dowiedzieli, że zmarł na koronawirusa sąsiad najbliższy, to chcieli się dowiedzieć, w jakich okolicznościach. Byli zaniepokojeni, przynajmniej te pierwsze komentarze, które zobaczyłam. Nie wiem, czy pan wójt to jakoś sprostował czy nie. Ale więcej tutaj nie słyszałam żadnych. Ale też ja nie jestem takim odbiorcą plotek. Nikt tu do mnie w takich sprawach nie dzwoni ani się nie zwraca. Chociaż jedna z sąsiadek mnie przepytała, bo wie, że mam znajomego w tej wsi, co zmarł ten pan i spytała, czy to prawda, bo tak ludzie mówią. Więc ja powiedziałam, że to prawda. Ale w ogóle nikogo nie zaraził, bo nie przyjechał z Warszawy i tak. Więc myślę, że w sumie to super, że to jest od nas tak daleko. Ale myślę, że młodzi to... Jak już mówiłam, że widziałam kogoś bez maski. To był mój sąsiad, młody chłopak, który powątpiewa w ogóle w to, że ten koronawirus w ogóle jest. Ale zasadniczo wszyscy na tym pogrzebie w maskach. Te maski mają służyć głównie temu, żeby osoba, nosząca maskę nie zaraziła wszystkich wokół. Różnie to jest. Niektórzy noszą takie szpitalne maseczki, niektórzy sobie szyją maseczki i tu mamy takie kolory, kwiatuszki i inne i super, tylko, że ona wtedy nie służy, bo nie ma tylu warstw i coś tam, ale dobra, tu i tak nikt nie jest chory. Jeszcze. Na tym pogrzebie, to ja byłam zaskoczona, ale część osób miała czarne maski.

**Czyli przygotowani byli?**

Tak. Normalnie, regularnie mieli czarne maski. Bo ci panowie z zakładu pogrzebowego z Warszawy, jakiegoś ĄĘ, który najprawdopodobniej może jest certyfikowany np. do tego, żeby chować tych ludzi z koronawirusem. Bo to na pewno są jakieś dość ostre... Np. nie można takiej osoby pochować - nie wiem, dlaczego - w istniejącej mogile murowanej. Tylko musi być grób ziemny. To mnie najbardziej ze wszystkiego zaskoczyło, bo wydawało mi się, że to odwrotnie by trzeba było zrobić. Jeżeli już, to w jakimś murowanym. A tutaj jest jakiś przepis, że można chować takie osoby... Musieli dokupić kwaterę ziemną, pomimo tego, że rodzinnie mają kwaterę murowaną. Nie chce mi się zastanawiać nad tym, bo po co. Straciłam wątek - o czym rozmawiałyśmy?

**Że sytuacja z koronawirusem nie wpływa na poziom lęku otoczenia?**

Na poziom lęku myślę, że nie. Że ten poziom lęku spada i zastępuje go taka... Na razie może nie frustracja, bo nie słyszałam, że ktoś jest bez pracy i nie wie, co ma robić, itd. Nie wiem, czy w ogóle nam to tutaj grozi, bo na wsi głównie brakuje ludzi do pracy. Ale nie, poziomu lęku nam to nie podniosło.

**Na ile ludzie idą w stronę normalnego życia, jak przed epidemią?**

Tutaj to ja myślę, że można powiedzieć, że to życie bardzo się nie różni. Oprócz ograniczeń w sklepach, które przybrały taki, że jest absolutnie zasadnicza różnica. W sensie takim, że przestały występować kolejki przed sklepami. W tych godzinach, w których ja się przemieszczam, to nie widziałam nigdzie kolejki. Po raz pierwszy zdecydowałam się pojechać do Płocka w sprawie jakichś zakupów i czegoś, bo czegoś mi brakowało - nie pamiętam czego - czegoś, czego tutaj nie mogę dostać. Więc w Lidlu, gdzie byłam przygotowana, że spędzę z pół dnia przed tym sklepem, po prostu płynnie wchodzimy, wychodzimy. Jest część wózków zablokowana i każdy musi być z wózkiem. I wszystko na ten temat, ale te powierzchnie są takie, że tych klientów jest tam tryliard. Nie jakoś, żeby jeden na drugim siedział, bo nigdy tak nie było. Po prostu, jak teraz bym weszła do takiego sklepu, gdybym nie wiedziała, co się dzieje, to bym się nie dowiedziała. U nas, w naszym sklepie, takim markecie, pan raz na jakiś czas puszcza komunikat. W sprawie ewentualnych braków na półkach, że personel robi wszystko, co może. Żeby dezynfekować te ręce, że w ramach wspólnego dobra oraz... Także mamy taką... To bym się zorientowała. Ale np. w Lidlu bym się nie zorientowała. Tak mi się wydaje, że na pewno więcej płacę w sklepie, więc na pewno jest drożej. Ja nie kupuję więcej, ale widzę, że ludzie wywożą takie jakieś.... Myślę, że dużo rzadziej robią zakupy. I w związku z tym, wożą w takich koszach... Wcześniej, to się wchodziło do takiego sklepu po 3 rzeczy. A teraz, jak parę razy byłam ostatnio w Biedronce i w Lidlu, to te kosze... Ale myślę, że to nie jest tak, że cały czas robią zapasy, tylko po prostu rzadziej chodzą i dlatego walą do tych koszy wiadro straszne tego towaru. Ale teraz już bym powiedziała, że nie za bardzo to widać. Byłam w Leroy Merlin np. po jakieś śrubki, to oprócz tego, że wszyscy chodzą w tych maseczkach, to nie ma... Byśmy się może nie zorientowali.

**A pani zakupy - nie myślała pani o tym, żeby robić je rzadziej?**

Nie no, dużo rzadziej je robię.

**Jak jest potrzeba, to pani jedzie?**

Tzn. nie, jakoś je tak zbieram. Ja w ogóle nie robię bardzo często zakupów, bo jestem przyzwyczajona do tego, ponieważ 1 w tygodniu jeździłam do Płocka. To mam taki nawyk, że nauczyłam się tak robić te zakupy, żeby nie wyjeżdżać częściej po byle gówno. Zawsze mam w pobliżu ten sklep 5 km, jak mi czegoś zabraknie albo jak zapomnę, to mogę dokupić. Ale po pierwsze mam metodę karteczkową, więc sobie zapisuję, co mi brakuje. I jestem przyzwyczajona do robienia tego raz w tygodniu i to się zasadniczo nie zmieniło. Tylko teraz to nie jest poniedziałek np. tylko jakiś inny losowo wybrany dzień, w który mi się nie chcę pojechać, ale muszę pojechać. I tyle. Bo jak ja mówię, że i do Lidla, i do Leroy, i coś, to ja to wszystko skomasowałam w jednym dniu.

**Dzień wycieczek do sklepu?**

Tak. Dzień sklepowy taki. Wcześniej to był dzień seniorów, a później jechałam do sklepów i wracałam później. A teraz jadę do sklepu tylko. Komunikuję się z tymi moimi seniorami przez telefon tylko. Nie, więcej to ja nie jeżdżę zdecydowanie. Czasem mi się zdarza pojechać, jak jest Julka. Nie kupuję czasem różnych rzeczy, o których nie pamiętam, że trzeba je kupić. Bo moje dziecko lubi Danonki. Więc jak nie pamiętam, to muszę któregoś dnia. Specjalnie po te Danonki nie jadę, ale jak uzbieram sobie na tej karteczce kilka rzeczy, to wtedy jadę.

**Czyli taki dodatkowy się zdarza?**

Zdarza się zwłaszcza teraz, jak nas jest 2.

**Jest coś, czego pani bardzo brakuje?**

Oprócz fryzjera? [śmiech].

**Nie było podziemnego fryzjera?**

Nie, nie było fryzjera. Julka mówi: "Mamo, to ja może ci pofarbuję te włosy". A ja mówię, że nie, mi to tak bardzo nie przeszkadza. Rzadko w lustro patrzę, to jak tobie nie przeszkadza, to mnie tym bardziej. Ja już poczekam na tego fryzjera, kupiłam spineczki, wiec jak coś robię, to sobie wpinam takie wsuwki celem, aby mi to nie wpadło do talerza. No brakuje mi np. trochę tych seniorów już. Może nawet nie wiem, czy mnie ich brakuje, ale na pewno im brakuje mnie. I to jest takie... Szkoda mi ich po prostu. Ja siedzę sobie na tzw. łonie natury, już nie wspominając, że w przemiłym towarzystwie. Ale w ogóle, mogę wyjść, mogę się przejść, mogę zrobić coś takiego innego niż sprzątanie. A oni, którzy i tak nie mają jakoś szczególnie dużo aktywności i są takie dni, kiedy siedzą, to może nie mogą za bardzo czytać, bo nie widzą i jest to kłopot. Siedzą w tych domach i jest mi strasznie ich szkoda, naprawdę. I jak z nimi rozmawiam, to oni już tęsknią i by pograli, i by się zobaczyli i że to raz w tygodniu wychodzili na tego brydża, ale że to było takie fajne i stanowią też między sobą taką zżytą grupę. I już to by im się przydało... Ja nie wiem, czy jak ruszą przedszkola, to ruszą też te starsze osoby. Myślę, że nie, bo one są chyba... Chociaż nie wiem. Patrząc na okolice powiatu płockiego, to jak tu nikt tego do cholery nie przywiózł, jakoś trzeba zacząć powoli wracać do normalności. Oni do sklepów na pewno wychodzą. Po pierwsze muszą coś jeść. Nawet, jak na początku byli mocniej przestraszeni i ktoś im robił te zakupy. Gdzieś muszą wyjść, bo to jest nieludzkie, żeby oni siedzieli w tej chałupie. Nie wiem, ile oni tam mają, 40-50 metrów kwadratowych? To już przesadzam nawet, bo mieszkania w tym Płocku... Znam panią, co mieszka w 24. I mieszkała tam z mężem, ma mnóstwo mebli jakichś i w ogóle się nie da przejść. Więc ja myślę, że można oszaleć. Moglibyśmy chyba zacząć jakoś powoli się spotykać. Ale nic się nie przebąkuje o tych osobach. Może nawet zadzwonię do MOPSu i się dowiem, jak to wygląda, czy oni jakoś ruszają z tymi spotkaniami. Bo jak puszczą dzieci do szkoły, to będą musieli uruchomić świetlice te środowiskowe najprawdopodobniej, bo te dzieci gdzieś będą się musiały podziać, jak rodzice pójdą do pracy. Nie wiem w ogóle, jak to ruszy. Trudno mi powiedzieć.

**Myśli pani, że z punktu widzenia wirusowego, to już jest czas, żeby zacząć otwierać szkoły, przedszkola, różne społeczne rzeczy?**

A skąd ja mam to wiedzieć? [śmiech]. Z punktu widzenia wirusowego nie mam bladego pojęcia. Natomiast z punktu widzenia psychologicznego, myślę, że czas by był troszkę poluzować. I tę gospodarkę troszkę wzmocnić. Myślę, że jakieś koszty są zawsze. Największe wątpliwości mam wokół tych osób starszych. Ale jeżeli trochę puścimy dzieci do szkół, czyli zwolnimy rodziców, którzy będą mogli wrócić do części pracy... Tutaj w Płocku, to w ogóle nie wiem, jak to się odbywa. Te dzieci pewnie są z dziadkami w tej chwili, bo u nas nie ma dużo takiej zdalnej pracy, a największym pracodawcą jest Orlen, a tam zdalnie się nie pracuje, ten Orlen normalnie pracuje. Mamy tu jeszcze New Holland - fabrykę maszyn rolniczych, więc tutaj nie ma dużo zdalnej pracy. Więc jak otworzą jakieś zakłady i trochę więcej ludzi pójdzie do pracy i ruszą dzieci w szkole, no to ci dziadkowie znowu będą potrzebni, żeby odebrać dziecko ze szkoły, itd. Więc siłą rzeczy zostaną włączeni w ten obieg. Myślę, że dla nich na pewno jest najmniej bezpiecznie z tego tytułu, że oni są w grupie ryzyka. Myśląc kategoriami lokalnymi, myślę, że w Płocku nie będzie dużych zmian. Ale myśląc w kategoriach krajowych, to może to mieć jakieś znaczenie. Zwłaszcza, jak te masy ludzkie, które teraz są pochowane po domach, zaczną znowu przez Warszawę się przewijać. Bo oni muszą przyjechać, wyjechać, wywieźć siebie na weekend - w jakimś trybie pracują. To na pewno zwiększy ruch. Być może zwiększy też te zachorowania. Chociaż ostatnio już parę dni w ogóle nie śledziłam tych wiadomości, bo już nie mogłam. Ostatnio, co prawda wczoraj, dostaliśmy dobrą wiadomość. Ale do tej pory, ponieważ wzmogły się wszystkie komunikaty wyborcze, że świetnie, że możemy, że to jest tak zdrowo, itd., to już nie mogłam nawet oglądać, bo co chwila pokazywał mi się któryś z tych durniów i już nawet nie mogłam wiadomości oglądać.

**To już lepiej wyłączyć wtedy telewizor i poświęcić wszystkie wiadomości.**

Tak. Chrzanić to. Włączam Animal Planet albo cokolwiek, bo nie jestem w stanie towarzyszyć tym osobom. Na szczęście osoby, z którymi rozmawiam, jestem w kontakcie telefonicznym, nie mają takiego pierdzielca na punkcie tych wyborów. Chociaż jedna znajoma ze Śląska się pytała, czy ja będę głosować. Ja mówię, kochana, na razie, to ja wysłałam list do mojego pana wójta mailem, że sobie nie życzę, żeby moje dane były przekazywane Poczcie Polskiej, chyba, że on ma jakąś podstawę prawną, o której ja nie wiem i chciałabym się zapoznać z nią. Nie zamierzam nigdzie iść. Ona mówi, wiesz, bo to ludzie różnie mówią, jedni idą, drudzy nie idą... Ja mówię, każdy ma swój rozum. Ja na pewno nie idę ani nie będę wysyłać listów.

**Poza seniorami, to dzieciakom z 5 klasy pani pomaga - jak oni sobie teraz radzą?**

Wie pani co, oni sobie radzą tak, że oceny mają bardzo dobre, poprawiły się bardzo oceny. Bo większość rzeczy u tych moich uczniów np. odrabiają rodzice lub opiekunowie. Żeby kogoś czegoś nauczyć, to trzeba mieć jakąś wiedzę metodologiczną, bo to, że się samemu coś umie, to jeszcze nic nie oznacza. Ale jak nie umiemy czegoś nauczyć, to najłatwiej jest coś za kogoś zrobić, jeżeli jesteśmy w stanie to wykonać. Ja mówię o ty, którzy są. Akurat ci moi uczniowie są w takiej dobrej sytuacji, że w jednym miejscu jest mama, która jak poczyta książkę... No, może z matematyką jest największy problem, ale teraz przerabiają figury, to ogarnia jakoś. A w drugim przypadku rodzeństwo starsze, które w całości jest kumate, więc... Najłatwiej zrobić za kogoś. Poświęcają trochę pracy albo i nie i wykonują te rzeczy. W tych przypadkach akurat oceny są lepsze niż były. A najlepsze, u jednego chłopca, najlepsze ma oceny ze wszystkich przedmiotów... Ta mama jest u kresu wytrzymałości, bo on ma wyrąbane na wszystko i bardzo go trudno skłonić do tego, żeby się czymś zajął i że on głównie by chciał spędzać czas na dworze i dużo bardziej go interesuje sadzenie, pielenie, zabawy w lesie, itd. Ale jedyna rzecz, o którą się biją, kto to zrobi, to są wszystkie prace plastyczne, techniczne - wtedy obydwoje chcą to zrobić. Ogólnie oceny ma lepsze, ale w sprawie poziomu wiedzy, to umówmy się, że to jest nieporozumienie towarzyskie. Być może licealiści np. są w stanie nauczyć się czegoś w taki sposób. Dzieci, które nie rozumieją... Moje dzieci, te tutaj, które nie czytają biegle, w szczególności ze zrozumieniem, którym trzeba wytłumaczyć jak krowie na rowie, którym trzeba naprawdę jakieś metodologiczne cuda czynić, żeby one w ogóle coś pojęły, to szansa, że one to zrozumieją online... Pani, która zada zadania, nie tłumacząc im, tylko po prostu, one mają sobie przeczytać rozdział z książki. Te książki, które teraz są za darmo wydawane tym dzieciom, to poziom... Ja nie wiem, kto takie książki wymyślił. To są książki, z których w zasadzie... Mówię o podręcznikach do klas 4-8 - one są katastrofalne. Tam nie ma żadnego omówienia. Może są zgodne z nowoczesnym stylem nauczania pod warunkiem, że on się odbywa za pomocą nauczyciela, a nie za pomocą własnej pracy. To są książki, w których nie ma teorii, tylko tam samemu analizując poszczególne przypadki, musimy wysnuć teorię, którą później można zastosować. Takie analityczne myślenie jest dużo bardziej skomplikowane i te dzieci, ani nawet rodzic nie jest w stanie nauczyć dziecka, bo nie jest w stanie z tej książki sobie przypomnieć. Myślę, że jeśli chodzi o nauczanie podstawowe, to jest beznadziejna sytuacja. Ale też nie sądzę, żeby on jakoś, przynajmniej tutaj, w naszych okolicznościach, żeby zaważyła na czymś bardzo. Tak naprawdę, to jaki to jest problem? Zróbmy z konkursu świadectw, niech te dzieci pójdą do szkół. Tyle lat funkcjonowaliśmy... Ja nie mówię, że to najlepszy system, ale w takich okolicznościach jakie mamy, robienie na siłę w połowie wakacji jakiegoś zamieszania... I tak będzie zamieszania od cholery. jakieś praktyki zawodowe, których ci młodzi ludzie nie mogli odbyć... Mamy tutaj dużo techników i szkół zawodowych. Oni byli w trakcie praktyk, jak to się zaczęło i teraz nie mogą odbyć egzaminu zawodowego. I tak mamy zamieszania od cholery, to po co komplikować jeszcze tym biednym dzieciom, ten egzamin ósmoklasisty... Tyle lat robiliśmy to z konkursu świadectw, zmieniliśmy to i teraz nagle mamy jakiś straszny kłopot. Tu akurat myślę, że nie mamy kłopotu. Kłopot mają maturzyści, którzy nie wiadomo, kiedy maturę będą zdawać, nie wiadomo, na kiedy się uczyć. Ale wszyscy jakoś damy radę. Myślę, że bardziej się powinniśmy przejmować gospodarką niż egzaminem ósmoklasisty. A jak jakiś medialny temat się znajdzie, to łatwiej się o tym rozmawia, a to temat zastępczy. Naprawdę, nic tym dzieciom nie przybędzie z tej nauki i też chyba niewiele ubędzie. No cóż, po prostu w przyszłym roku powtórzą. *[Refleksje nt. programu nauczania].* Dadzą radę i bez tego.

**Jak się pani czuje w obecnej sytuacji? W kontekście informacji z zewnątrz, ale poza wyborami.**

Tak naprawdę, to ja w zasadzie, to jestem odcięta do tej wiadomości. Dopóki ja sobie nie otworzę dostępu do tej wiadomości, to on do mnie - to może jest trudne do wyobrażenia dla was - ale ja się mało spotykam, a poza tym nikt prywatnie nie rozmawia o gospodarce, nawet o wyborach. Ja sama też nie mam żadnej potrzeby o tym rozmawiać, w zasadzie mi się to nie zdarza. Moja córka też nie jest szczególnie do rozstrząsania historii gospodarczo-koronawirusowych. Nie siada mi tu i nie każe oglądać tych wiadomości. Wręcz przeciwnie. Od razu mi wyłącza, bo wie, że ja się denerwuję tym.

**Czyli pojawia się złość?**

No denerwuję się, ale miałyśmy nie mówić o wyborach.

**Tylko na te wybory się pani denerwuje?**

Denerwuję się na tych polityków, którzy pieprzą bez sensu.

**Czyli całokształt polityki?**

Tak. To jest paskudna... Ja im nawet współczuję, że oni są politykami. Może bym im współczuła, gdyby nie to, że oni sami tego chcą. Mam swoje zdanie na ten temat i go nie zmienię pewnie. Chociaż nigdy nie wiadomo. Jeżeli chodzi o politykę, to denerwuje mnie poziom naszej klasy politycznej i to, co się dzieje. Uważam, że problemy są gdzie indziej, a oni mają jeden podstawowy problem, żeby wybrać pana Dudę. Uważam, że niech on sobie będzie prezydentem jeszcze parę lat, będziemy mieli wszyscy spokój, temat się utnie od razu. I tak go nie włączam i go nie oglądam. Nie no, to oczywiście żartem. Ale jeżeli chodzi o ten wirus, to się sama odcinam od tych wiadomości, już prześledziłam. Sama pani mówi, że to 54 dni, to... Jestem odcięta nawet od wiadomości z gazet, bo nie byłam w Warszawie, więc nie pobrałam od mojej koleżanki Gazety Wyborczej zużytej. Więc nawet teraz, jak palę w piecu, to palę w piecu gazetami z początku pandemii. To jest śmieszne, bo palę w piecu tymi gazetami, w których pojawiały się pierwsze przypadki i to jest taki styczeń, grudzień. I nawet przykucnęłam sobie i te nagłówki wyczytałam. Pojedyncze przypadki gdzieś w Chinach, coś tam. Wtedy tak jak o grypie pisali, że to może taka grypa, tylko może taka bardziej grypa i ciekawe co to będzie, że świat stoi przed wielkim wyzwaniem. I teraz rzeczywiście, to wyzwanie jest potężne.

**Co pani sądzi o otwarciu galerii handlowych?**

Co ja mogę o tym sądzić... Wyczytałam gdzieś, że - bo mi się pojawiają takie w telefonie, że któraś z tych firm sieci sklepów Reserved i coś jeszcze postanowili nie otworzyć sklepów, bo stwierdzili, że ten ograniczony dostęp do galerii i wszystkie obostrzenia spowodują to, że oni nie wyjdą na swoje. A co ja myślę o otwarciu? No, skoro jest otwarty Auchan i dajemy radę z ograniczeniem kontaktów i ograniczeniem osób i możemy to w miarę kontrolować, to otwórzmy galerie. Rozumiem, że te miejsca, gdzie się spotyka więcej osób, czyli kino np. nie zostaje otwarte. To jest takie miejsce, gdzie wszyscy na siebie oddychają, a jeszcze jak idą na komedię, to się śmieją. I jedzą ten popcorn, wszystko się wala po podłodze. To to już by była przesada. Ale myślę, że wszyscy musimy się uczyć nowych zasad bezpieczeństwa, które epidemiolodzy i ludzie mądrzejsi od nas uznają za niezbędne i może niewystarczające, ale niezbędne w tej sytuacji, to musimy jakoś... Najpierw nie chodziliśmy do sklepów, potem zaczęliśmy do nich chodzić w mało osób, teraz chodzimy w więcej osób. To nauczmy się funkcjonować w półzamkniętej przestrzeni po to, żebyśmy mogli potem funkcjonować w zamkniętej przestrzeni. Skoro to jest jakaś sytuacja, która, jak rozumiem się nie zmieni diametralnie. To nie jest tak, że wszyscy się wychorują i będzie dziękujemy państwu bardzo i nie ma już dżumy, trądu, czegoś. Rozumiem, że te wirusy będą z nami, będą się mutować. ten konkretny jest jakiś paskudy i ileś lat to potrwa, więc jakoś musimy się z tym nauczyć żyć. To może i dobrze, że powoli stopniowo się będziemy z tym oswajać. Stopniowo musimy wejść do jakiegoś większego pomieszczenia wszyscy. Jeżeli możemy być w Lidlu, to nie wiem, dlaczego nie możemy być w czymś innym... Np. nie wiem, jak to wygląda z tym mierzeniem rzeczy, itd. Ale tak samo wygląda, że może pani wziąć paczkę cukru, popatrzeć na nią i potem ją odłożyć i weźmie ją ktoś inny. I co? Musimy się z tym nauczyć żyć. Część z na sto przechoruje i cześć, a część przechorowała i nawet o tym nie wie.

**A pani się wybiera z Julką do galerii?**

Nie. Na razie się nie wybieram. Julka nie zgłasza żadnych potrzeb, a ja sama też nie potrzebuję nic. Więc to nie jest tak, że ja czekam, przestępuje z nogi na nogę, kiedy pójdę do Zary czy do Media Markt. Także nie wybieramy się.

**Okolica też raczej nie jeździ?**

Nie, tutaj to w ogóle w zasadzie. W tych galeriach jest drogo przede wszystkim. Dla większości z tych ludzi to są takie rzeczy... Oni jeżdżą na bazar, na rynek. W Płocku mamy jakiś rynek, ale on podobno jest cały czas jest otwarty, nie był zamknięty, tylko były jakieś obostrzenia, jeśli chodzi o ilość osób przy stoisku. Mało sobie to wyobrażam, jak by można to było skontrolować. Tutaj nie mamy. Tutaj to w zasadzie, oprócz maseczek... Na rynku, jak byłam w poniedziałek, to też nie widziałam... No, może starają się stać w jakiejś odległości od sąsiada. Ale sąsiad za nim stanie bliżej niego. Gdyby nie maseczki, to nie dałoby się zauważyć u nas, że coś jest inaczej. Chyba, że są jakieś obostrzenia, czy... Na pewno u nas sytuacja jest absolutnie inna. Z resztą zawsze jest inna. W normalnych warunkach różnice między miastem, a wsią, są niewyobrażalne i dla jednych i dla drugich. Np. co zaobserwowałam - tutaj są cmentarze takie malutkie. Zabrałam ostatnio znajomego, który pomaga wykonywać nam prace remontowe przy ławeczkach na cmentarz północny. To po prostu, dla niego to jest tak, jakby on... Nie trzeba jechać za granicę, wystarczyło 100 km od Warszawy. Jak on wszedł na ten cmentarz, to oniemiał ze zdumienia. Po pierwsze, że to jest takie wielkie, ale przede wszystkim na tych cmentarzach małych, te groby są jedne na drugich. U nas te aleje... Wyobrażenie wiejskie o życiu miejskim i odwrotnie nie dorasta do pięt rzeczywistości. Niewiele osób będąc i tu, i tu widzi te różnice, a jak ktoś nie widzi, to nie może sobie dobrze wyobrazić. Cały czas ludzie ze wsi, rolnicy, mierzą miasto swoją miarą czy swoimi możliwościami wyobrażenia sobie. A to wyobrażenie nie dosięga do rzeczywistości. Bo nawet mieszkając w mieście, ja, która znam realia miejskie, jak nie ma mnie dłuższy czas w jakiejś okolicy i ja przejeżdżam i tam jest pobudowane coś... Dla nich to jest niewyobrażalne.

**Wydawanie pieniędzy - skala.**

Myślę, że byłabym bliżej krańca, że nie sprawia mi to kłopotów. Bliżej 7. Że łatwo, bo je mam. Nie jestem skąpa na pewno. Oczywiście, nie jest tak, że mam ich nieograniczoną ilość, zwłaszcza w tej chwili, muszę jakoś racjonalnie nimi gospodarować, ale nie mam kłopotów z wydaniem, jak np. mam kupić coś Julce albo nawet sobie, jeśli uważam, że to jest fajne albo potrzebne albo coś takiego. Nie jestem myślę rozrzutna, ale nie mam kłopotów z wydawaniem pieniędzy.

**Na co jeszcze łatwo wydać pieniądze?**

Np. mogę sobie kupić nasionka, roślinki, książki, sprzęt, który mi się przydaje w gospodarstwie. Ostatnio kupiłam sobie ręczną maszynę do pielenia, którą zobaczyłam u sąsiada i mi się spodobała. Ale np. był też taki moment, że bez kłopotu wydawałam pieniądze na rzeczy związane z moim hobby, np. do haftowania albo jakieś plastyczne historie. Jeździłam do różnych hurtowni i tam kupowałam takie do ozdabiania jajek, bombek, takie rzeczy. Ostatnio mniej tego kupuję, bo mniej tego robię i trochę się wysyciłam, jeśli chodzi o ten rodzaj działalności. Takie rzeczy nie sprawiają... Nie, żebym nie miała granic. Np. jeśli chodzi o książki, to było dość bolesne finansowo, więc w tej chwili... Jak mieszkałam w Warszawie, to te książki kupowałam, zdecydowanie. W tej chwili, jestem zapisana do biblioteki w Płocku, do biblioteki gminnej i po pierwsze nie mam tyle miejsca na książki, ile miałam w Warszawie i jednak znakomitej większości z tych książek nie muszę mieć. Przeczytam je i owszem, czasem bym zajrzała do którejś książki, żeby sobie coś przypomnieć, ale żeby ją kupić w tym celu, to nie. Rzadko się zdarza w tej chwili, ale zdarza się, że pożyczę coś z biblioteki, a potem kupuję to, bo uważam, że chciałabym mieć taką książkę w domu. Z książkami już trochę się ogarnęłam i to udało mi się przestać ich tyle kupować, więcej ich wypożyczam.

**Jak się pani czuła z tym, że trzeba było ograniczyć kupowanie książek?**

Kiepsko. Na początku mi się wydawało... Tak, to jak kupowałam książki, a jak wchodziłam do księgarni, to już katastrofa po prostu. Najlepiej mi się książki kupowało przez Internet, bo łatwiej mi się było ograniczyć. W księgarni wszystkie tytuły leżące, powykładane, to kłopot był duży. Jest z resztą. Także staram się nie wchodzić do księgarni w ogóle. Ale nie zawsze mi się uda. Na początku było ciężko, ale później się przekonałam, że jak przeczytam tę książkę, a jej nie mam, to się nic takiego nie dzieje. Większość książek, które mam na półce, to są książki do których nie sięgnęłam drugi raz. Chętnie je komuś pożyczam. Nie ma wiele takich, ale zdarzają się takie, które chciałabym mieć.

**Jak to jest z wydawaniem na ogrodnicze rzeczy - jest to miłe, przyjemne?**

W sensie wydawanie pieniędzy czy posiadanie tych rzeczy?

**Ten moment, kiedy się to kupuje.**

To jest raczej fajne, bo jak się chce mieć takie coś, to fajnie, że można to kupić.

**A spożywcze rzeczy?**

Nie zastanawiam się nad tym za bardzo. Kupuję te rzeczy, których używam i wtedy je kupuję. Mam taki komfort, że przy takich rzeczach nie muszę patrzeć, ile kosztują. Nie porównuję jakoś bardzo cen. Nie kupuję najdroższej rzeczy w danej kategorii. Ale np. jeśli używam jakieś masło, to je kupuję, nie patrząc na to, że obok leży tańsze. No po prostu. Nawet się nie zastanawiam nad tym, że mogłabym je kupić taniej. Po prostu biorę to, którego używam. Masło, śmietanę, rzeczy spożywcze. Podobnie ma się sytuacja, jak takie drogeryjne rzeczy, proszki do prania, szampon, itd. Używam jakieś i po prostu używam. I się nawet nie zastanawiam. Jest coś nowego, nie biorę, są tańsze rzeczy, też nie biorę. Natomiast zdaję sobie sprawę z tego, że mnie po prostu na to stać. Bo większość osób tutaj w szczególności kupuje rzeczy najtańsze.

**A jak jest teraz? Alimenty od męża spływają, jak rozumiem?**

No mam nadzieję. Nawet nie sprawdziłam ostatnio, ale mam nadzieję, że tak.

**Myśli pani, że może być problem finansowy?**

Myślę, że... No mój mąż prowadzi firmę. Jest to firma zaopatrzenia medycznego. Więc ona w tej chwili, ponieważ tylko koronawirus... Jest to firma, związana z zaopatrzeniem miejsc, które oferują operacje, zabiegi planowe. W związku z czym, ta działalność w tej chwili jest wstrzymana. Mam nadzieję, że ta sytuacja się... No ona się musi poprawić. To nie jest np. biuro podróży, gdzie obawy byłyby dużo większe. Na pewno takie operacje planowe ruszą i będą musiały ruszyć, bo nie przestaliśmy chorować jako społeczeństwo, tylko dlatego, że jest koronawirus. Może część z nas umrze i nie będzie potrzebowała operacji planowych, ale jednakowoż większość z nas zostaje. Więc, oczywiście, że się martwię. Znaczy, martwię... Może martwię to złe słowo. Mam to gdzieś z tyłu głowy, że taka sytuacja może się stać. Mam nadzieję, że jeśli się stanie, to będzie to sytuacja przejściowa. Na razie, póki mnie to nie dotknęło, to nie muszę się tym za bardzo przejmować, jakoś dam radę. Oczywiście, żartem, na pewno się wyżywię. W sensie takim, że mogę przestać jeść śmietanę i masło, a pietruszkę i pietruszkę będę miała. I ziemniaki od sąsiada. Daje to takie poczucie zabezpieczenia. jestem w miejscu, w którym jednak jest dużo łatwiej sobie poradzić od takiej strony czysto egzystencjalnej, żeby przeżyć. Mam nadzieję, że do tego nie dojdzie, żeby to się dramatycznie posypało i że mąż były i firma, będą się po tych przejściowych kłopotach odbudowywać. Choć oczywiście nie mam takiej pewności. A ponieważ kontakty mamy w zasadzie zerowe, to też nie mam... Mogłabym się od dzieci dowiedzieć, gdybym chciała. Na pewno od syna, który bardziej uczestniczy w sytuacjach biznesowych byłego męża. Ale na razie, póki się nic szczególnego nie dzieje, biorę to pod uwagę. Sama jestem w pewien sposób dotknięta tą sytuacją, bo mam mieszkanie w Warszawie, w którym w części mieszka Julia, a część jest wynajmowana. I np. nie mam teraz najemcy. Więc mam taki kłopot, że te wydatki związane z mieszkaniem w Warszawie nie są niskie i muszę brać z tego, co mam na życie, więc ta pula się zmniejszyła. Na pewno nie mam takiego luzu, jaki miałam jakiś czas temu. W dalszym ciągu nie czuję się jeszcze zagrożona.

**Czy brak tego luzu spowodował zmiany w zachowaniu zakupowym?**

Być może tak. Np. wpadłam na taki świetny pomysł, że chciałam sobie kupić takie duże krosno, taką dużą ramę, żeby tkać coś. Jak sobie podsumowałam, ile ja muszę wydać na taką ramę, to wcale nie jest tak dużo, ale na taką ramę z czółenkiem, osnową, żeby sobie tkać, to co zamierzałam, to zrezygnowałam z tego. Myślę, że w dużej mierze z tego powodu. Mam jakiś kłopot z rurą kanalizacyjną, czekam na pana, który przyjdzie. Chciałabym kupić sobie nowy piec, który by spełniał wymogi spalania - w tej chwili to przesuwam, odkładam. To są wydatki rzędu paru tysięcy. Nawet, jeśli bym znalazła teraz na to pieniądze, to one będą mi potrzebne, np. żeby płacić czynsz w Warszawie, a i tak muszę płacić te opłaty. Więc odkładam to. Cały czas mam wrażenie, że to tylko odsuwam w czasie. Pewnie tak. Oczywiście ci ludzie jakoś wrócą do tej Warszawy. To nie jest tak, że przestaną tu pracować. Pewnie wręcz przeciwnie będzie. Ale jest tak, że ta sytuacja w pewnym sensie mnie dotyka. Nawet powiedziałabym, że bezpośrednio, bo najemca się wykruszy.

**Mamy przesunięcie w czasie dużych wydatków, jak ten piec i wydatków przyjemnościowych, jak krosno. A w codziennych wydatkach są jakieś zmiany?**

Myślę, że nie. Chociaż to tak jest, że ja nie jestem jakby specjalnie chętna do chodzenia... Trudno mi jest odpowiedzieć z całą pewnością, bo ja nie chodzę bardzo chętnie do sklepu. Ale jak już idę do tego sklepu, to kupuję. Więc np. mamy skrzynię plastikową na poduchy, na pierdoły i pękła ta skrzynie, więc pomyślałam, że kupię sobie nową. Żeby sobie kupić tą nową, musiałabym wyjść. Bo ja jednak nie jestem pokoleniem kupowania przez Internet. Kupuję, ale rzeczy, których dokładnie wiem, którą chcę. Książkę śmiało mogę kupić, bo na pewno się nie pomylę. Ale taką skrzynię, to nie chcę jej tu mieć, a później ją odsyłać, więc chrzanię to. Być może jest tak, że jej nie kupuję, bo nie idę do tego sklepu, bo on jest zamknięty albo nie zamknięty, ale mam dobry pretekst, żeby do niego nie pójść, bo teraz się po sklepach nie chodzi. Nie wiem czy to dlatego, czy dlatego ta sytuacja jest bardziej niepewna. Nie mogę powiedzieć z całą pewnością, dlaczego tej skrzyni nie kupuję. Na pewno w tej pękniętej też te rzeczy mogę trzymać. Chociaż nie lubię tego. Nie wyrzucam rzeczy, które można naprawić, nawet jeśli mnie stać na nowe. Ale pod warunkiem, że to nie wygląda, jak skrajne dziadostwo. Jakbym mogła na to nakleić jakąś kwiecistą łatę i zakleić to wporzo. A jak mam to zakleić albo związać sznurkiem, to bym to wyrzuciła jednak. Trudno jest mi powiedzieć, na ile... Trochę się tego obawiam, że nastąpi taka trochę degradacja tych rzeczy, że się nie odnawia tych rzeczy z powodu, że nas nie stać na nowe. A potem, to jest ciężko. Bo, jak na bieżąco się wymienia takie uszkodzone rzeczy, których nie da się naprawić, to jest łatwiej. A jak się ich nazbiera w kupie, to później już zaczyna być duży problem i tego się po prostu nie robi.

**To, że człowiek na bieżąco dba o rzeczy wokół, sprawia, że nie wpada w stan takiego zdziadzienia?**

Tak. No tak mi się wydaję. Że to jest takie... Na pewno ja nie wpadłam jeszcze w jakiś dół. Ale widzę taką sytuację, że to się może w tę stronę rozwinąć.

**Czyli trzeba zapobiegać temu?**

No tak. Byłoby dobrze. Ale z drugiej strony, nie jest to jakoś szczególnie ważne. Tak naprawdę ja mogę tę skrzynię wyrzucić i nie mieć nic w zamian. Bo ja te rzeczy gdzieś upchnę, do jakiejś szafy, czy... Chociaż niechętnie, bo jak mam tę skrzynię, to jest wygodne. Trochę się tego obawiam właśnie, że to jest łatanie starej opony łatką zamiast kupienia nowej opony, bo ją trzeba kupić.

**Na co dzień kontroluje pani wydatki?**

Tak, kontroluję. Wiem, ile mnie potrzeba miesięcznie. Wiem też, że są ludzie, którym potrzeba wiele mniej. Wiem, ile potrzebuję, ile mam wydatków stałych, ile wydaję na bieżące rzeczy. Nie rozróżniam czy to szampon, czy inne, ale tak ogólnie, bieżące.

**Czy pani zdaniem w obecnej sytuacji ludzie powinni ograniczać wydatki?**

Myślę, że trzeba by racjonalnie oszacować każdy sobie czy powinien to robić czy nie. W zależności od tego, z jakiego źródła ma dochody i na ile to źródło jest zagrożone. Nie mówiąc o tym, że te osoby, które straciły pracę, nie mają wyjścia. Jeżeli ktoś ma np., mówiąc w przenośni, zakład pogrzebowy, to może nie musi ograniczyć tych wydatków. Nawet może przejściowo jest w lepszej sytuacji. Ale jeżeli ktoś prowadzi działalność taką... Na pewno ta sytuacja ekonomiczna się wielu osobom znacznie pogarsza. Wszyscy, którzy pracują w sferach gospodarki wrażliwych na tę sytuację, to na pewno się muszą grubo zastanowić. Nawet, jeśli mają jakiś zapas. Bo jeśli nie będzie dopływu, to ten zapas będzie malał. To z całą pewnością.

**A co pani sądzi generalnie o oszczędzaniu? Na ile to jest ważne?**

Myślę, że posiadanie oszczędności jest ważne. Być może nie takich, które rosną i ich nie ruszam, ale posiadanie takiego zaplecza, które pozwala na to, że w sytuacji konieczności albo podjęcia decyzji życiowej, że coś trzeba by było zmienić, dają taki nazwijmy to luz. Czyli taką możliwość, że np. mogę odłożyć tysiąc zł miesięcznie, to np. jeżeli będę musiała kupić piec za 6 tys., to za pół roku będę mogła to zrobić. A jeszcze lepiej, jeśli zrobię to za rok, żeby mi trochę zostało. Żeby mieć taką sytuację, że jeżeli ten piec pieprznie... Oczywiście każdy ma jakiś swój piec. Mieszkając w mieście nie macie pieca, ale ma pani np. pralkę, bez której przy dzieciach życie jest trudne. Nie mówię, że niemożliwe, ale zdecydowanie utrudnione. Każdy z nas ma taki swój piec, czyli taką rzecz, bez której życie jest trudne albo niemożliwe prawie. I myślę, że warto mieć taką sytuację, żeby chociaż mieć takie zabezpieczenie. Ale np. ja teraz żyję w środowisku ludzi, którzy w ogóle nie mają oszczędności. Którzy żyją z dnia na dzień i powiem pani, że oni w ogóle nie myślą.... Może to nawet i lepiej, patrząc na nich. Bo oni w ogóle nie myślą o takim zabezpieczeniu, a co za tym idzie, w ogóle nie czują się niekomfortowo jak go nie mają.

**Pani ma takie zabezpieczenie?**

Tak, ja mam takie zabezpieczenie.

**Gdyby nagle się okazało, że nie ma dopływu pieniędzy, to ile jest pani w stanie tak na spokojnie przeżyć?**

Na spokojnie, to nie, ale myślę, że ze 4 miesiące bym dała radę. Na pewno nie spokojnie, bo poziom lęku na pewno by wzrósł. Bo jak trzeba sięgnąć po taką rezerwą, to na pewno nic miłego. Ale przez te 4 miesiące dałoby się żyć. W związku z tym, że ta rezerwa by się kurczyła to i poziom potrzeb by malał. Na pewno poziom potrzeb by rósł, tylko możliwość ich zaspokojenia by malała, więc na pewno część rzeczy dałoby się wyeliminować, jeśli chodzi o zakupy, więc może starczyłoby na więcej. Zawsze mam jeszcze takie zaplecze, że mam dzieci, z czego syn pracuje i zarabia. Więc na pewno z głodu bym nie umarła.

**To jest taki poziom bez tego, że to mieszkanie np. trzeba ruszyć?**

Tak.

**Te pieniądze sobie leżą czy pani coś z nimi robi?**

Nie, ja ich nie lokuję. Mam je po prostu w banku. Co prawda na jakimś koncie oszczędnościowym, ale to są takie procenty jak inflacja.

**To jest tak, że co miesiąc pani celowo odkłada coś, czy to jest na zasadzie reszty?**

Ja jestem w takiej sytuacji, że i tak muszę celowo odkładać, ponieważ muszę od tego zapłacić podatek. Nie mam obowiązku odprowadzania miesięcznie podatku od alimentów, tylko kiedy się chce do 30 kwietnia. Więc odkładam o 1000 zł więcej niż muszę. Z taką świadomością, że jak będę miała jakiś wydatek, to one tam są. Ich jest widokowo więcej, bo mam odkładane na ten nieszczęsny podatek. Więc to nie jest tak, że czekam co zostanie, bo tak mi by było dużo trudniej. Chociaż i tak myślę, że jestem zdyscyplinowana, bo raczej nie pobieram stamtąd tych pieniędzy. Zdarzyło mi się raz - już nie pamiętam co to było - że to, co odłożyłam, musiałam wziąć.

**Warto obecnie oszczędzać?**

Myślę, że zawsze warto. Nie jestem skąpa, myślę, że wręcz przeciwnie. Ale nie jestem rozrzutna też. Chociaż gdyby ktoś tu przyjechał i zobaczył, ile ja mam jakichś rzeczy takich zbędnych, to myślę, że może by miał inne zdanie na temat rozrzutności. Ale ja staram się myśleć o sobie, że nie jestem rozrzutna. Więc z mojego punktu widzenia warto. Mi to daje takie poczucie, że gdyby np. coś się stało, że ja ten piec mogę kupić. Albo jak mi się rozpadną te okulary, co mam na oczach, też mogę pójść i kupić. Tutaj się funkcjonuje zupełnie inaczej. Tu, jak trzeba coś sobie kupić ekstra, to się bierze kredyt czy pożyczkę. I potem się martwimy z czego to spłacimy. Na razie bierzemy pieniądze, załatwiamy, co trzeba, a potem się martwimy. Ja mam inne nawyki i myślę, że one wynikają z innych doświadczeń. Bo jakbym tutaj się urodziła, to na pewno bym nie miała ani oszczędności ani takich historii, bo skąd.

**Czy w takiej niepewnej sytuacji ci ludzie zaczynają myśleć o tym, że trzeba oszczędzać?**

Tutaj na pewno nie. Bo oni nie mają z czego oszczędzać. Jeżeli tutaj renta osoby samotnej, renta KRUS, to jest w granicach 1000 zł miesięcznie. To proszę sobie tam to przeliczyć, ale nawet na minimalnym poziomie potrzeb, oni muszą zapłacić prąd, podatek od ziemi raz do roku, jakieś leki na absolutnie minimalnym poziomie. Apteka w mieście i apteka na wsi to są dwa różne światy, ponieważ oni tu kupują absolutne minimum. Nie mają w domu żadnych zapasów leków, nic takiego. A plaster? Niech pani zapomni. Jak sobie palec przetną, to zawiązują papierem toaletowym, jeśli jest. Więc myślenie o oszczędzaniu, jak się ma 900 zł na wydatki, to jest abstrakcja. Z czego tu odłożyć, jak tu wystarcza na podstawowe artykuły wszystko, co najtańsze i kupowane w tzw. promocji.

**A myśli pani, że w dużych miastach, ludzie się orientują, że te oszczędności są potrzebne?**

Myślę, że nie mam pojęcia dużego. Może to jest funkcja wieku jednak trochę. Jak byliśmy młodzi, to może nie myśleliśmy tak o oszczędzaniu, jak o tym, żeby żyć na tyle, na ile można. Też nie były to takie pieniądze, żeby oszczędzać, bo też nie było bardzo z czego. Myślę, że może z wiekiem to przychodzi. Trudno mi powiedzieć, co teraz myślą ludzie w mieście. To pani lepiej wie, jak osoby miastowe widzą tą sytuację. Być może ci chcą oszczędzać, którzy uważają, że oszczędności dałyby im poczucie komfortu.

**A np. Hubert? Czy on jest osobą oszczędzającą?**

On jest osobą niewydającą dużo pieniędzy. Wygląda mi tak, no... Nie wydaje dużo pieniędzy. Nie tak, że jest skąpy, ale to, co ma mu wystarcza. Nie jest tak, że jak ma więcej, to wydaje więcej. Więc rozumiem, że część odkłada z tych pieniędzy, które ma. Nie wiem, na ile jest dobrym przykładem dla swoich rówieśników. Myślę, że nie jest i nigdy nie był, ale może pod tym względem jest taki, jak inni. To jeszcze nie oznacza, że młodzi ludzie nie oszczędzają.

**A ogólnie, obecna sytuacja gospodarcza, to dobry moment na inwestowanie? Jak się ma kasę?**

Zależy w co pewnie. Jeżeli ktoś ma i nie martwi się o to, że mu to źródełko wyschnie, to znaczy, że ma te pieniądze ulokowane w takich dziedzinach, które dają mu zabezpieczenie. Myślę, że to jest dobry moment, bo to znaczy, że on wie, w co inwestować i te inwestycje są prawidłowe. Za to inwestowanie dla samego inwestowania... Nie wiem, zależy od mnóstwa czynników. Jak szybko można np. upłynnić taką inwestycję. Ja jestem może teoretycznie dużo bardziej zaawansowana niż praktycznie, bo nie jestem w stanie np. śledzić i wymyślić i badać takie możliwości inwestycji. Nigdy mnie to nie pociągało i uważam, że skala moich oszczędności jest absolutnie... W zasadzie obojętne, czy ja te pieniądze zainwestuję czy nie. Bo zwrot z takich inwestycji nie poprawi bardzo tych finansów. Myślę, że to wszystko kwestia skali. Myślę, że jak ktoś ma miliony, to zarówno dobre, jak i złe inwestowanie ma kolosalne konsekwencje. A jak ktoś ma 10 tysięcy, to jest to zasadniczo za jedno, czy je zainwestuje czy nie. Ewentualność utraty jest dużo bardziej bolesna niż ewentualny zysk, który jest znikomy. Myślę, że to jest zmartwienie osób, które... Banki oferują bezustannie jakieś fundusze, czy coś tam. I chyba tylko banki na tym zarabiają. Myśmy należeli do takiej wyższej klasy średniej, jeśli chodzi o majętność. Stać nas było, żeby nasze dzieci chodziły do niepublicznej szkoły. I mąż, bardzo ostrożny i bardzo racjonalny, próbował inwestować za pomocą doradców i przez te 30 lat wspólnego życia, nie udało nam się zarobić. Udało nam się nie stracić, bo były ulokowane w różnych funduszach. Czyli w sumie wychodziło na 0, bo te pieniądze, leżące na zwykłym rachunku oszczędnościowym przyniosłyby jakiś procent. A tak to wyszło na 0, ale uważam, że dość szczęśliwie, bo ten, który musi zarobić, to zarobił, czyli bank. Myślę, że to nie jest w ogóle skala, żeby myśleć o inwestycjach. Myślę, że to jest pic na wodę, ale ja młoda i piękna byłam, a teraz jestem mądra i mogę sobie takie rzeczy mówić. A młodzi - i słusznie, bo byśmy żyli w zacofaniu przez setki lat, gdyby życie się toczyło według tego, co myślą starsi.

**Kiedy ta cała sytuacja się skończy?**

Ja myślę, że ona się de facto nie skończy. Tylko, że my sobie jakoś z nią poradzimy w sensie takim, że się z nią oswoimy. Oczywiście, ta liczba zachorowań będzie maleć z różnych powodów. Część osób przechoruje, jakieś tam się zaszczepimy, ale zaraz coś zmutuje. I będzie tak, jak z grypą, jak z innymi wirusami, że one jakoś sobie są, my się nauczymy z tym żyć, że część osób będzie miała jakąś odporność i coś tam, więc myślę, że ona się nie skończy, tylko my się oswoimy. To nie jest jakaś magiczna historia, że my pstrykniemy i zrobimy coś z tym wirusem, powiemy mu, że ma sobie pójść i w ogóle.

**My się nauczymy z tym żyć?**

Nie będziemy mieli wyboru. To jest tak, że coś nas w życiu spotyka. Nam się wydaje, ze to nie nastąpi, ale one następują. Chorujemy, odchodzą najbliżsi, rozwodzimy się, rozstajemy się. Różne rzeczy się dzieją. Gdzieś nas los rzuca. Pomimo tego, że nam się to nie podoba, chcielibyśmy to odczynić, to to się nie staje i musimy oswoić się z tą sytuacją, która następuje. I takie magiczne myślenie, że to przestanie być, że cofniemy tą sytuację do startu, to po prostu nie nastąpi moim zdaniem. Po prostu ją oswoimy i przestaniemy ją zauważać. Przystosujemy się do niej, bo na tym polega moim zdaniem ewolucja, że musimy się przystosować do tych zmian, które mamy. Oczywiście, możemy z tym walczyć, to nie znaczy, że mamy się poddać. I tak, jak do zmian klimatu się musimy jakoś przyzwyczaić, choć sobie tu stękamy, bo te zmiany klimatu to też jest taka pandemia, tylko myśmy ją sami sobie zrobili i po prostu musimy... Trochę te skutki będziemy łagodzić, a trochę się musimy do tego przyzwyczaić, oswoić i zacząć tak działać, żeby w tych nowych warunkach móc funkcjonować. Tak myślę.

**Ma pani jakieś obawy związane z tym, co będzie dla ludzi najtrudniejsze w tych nowych warunkach?**

Nie, myślę, że to nie ma takiego... Motorem do tego, żeby to oswoić będą pieniądze, bo żeby żyć, trzeba mieć za co żyć. I będziemy musieli wrócić do jakiejś rzeczywistości, żeby te osoby, które nie pracują, zaczęły pracować. A te osoby, które korzystają z zasobów, które mają, to żeby one im się nie skończyły. Myślę, że to jest klucz do tego zajścia. To ten motor, który nas popchnie do oswojenia tej sytuacji. Nie możemy się odizolować na nie wiadomo jak czas. Musimy to jakoś ogarnąć. Więc, mówiąc szczerze, może zupełnie się mylę, a może to świadczy o tym, że te 4 miesiące, które mam zapasu jakoś mi dodają otuchy, ale myślę, że nie boję się, że to się pogorszy, czy że ta sytuacja się nie rozwiąże.
