# Supplementary material for: The challenges arising from the COVID-19 pandemic and the way people deal with them. A qualitative longitudinal study
Source: PLoS One. 2021 Oct 11;16(10):e0258133. doi: 10.1371/journal.pone.0258133 (PMC8504766; doi:10.1371/journal.pone.0258133)
Supplement: S1 Dataset — (ZIP) [file pone.0258133.s003.zip › Transcriptions/stage 3/19.3_F_39_couple, with children.docx]

**19.3_F_39_couple with children**

**Co robiłaś przez ostatnie 2 tyg.? Co się działo w twoim życiu?**

No standardowo pracowałam, cały czas pracowałam i dzisiaj mam dopiero 1-szy dzień opieki, więc dzisiaj trochę luźniej. Święta jakoś tam spędziliśmy sobie w swoim gronie, na jakichś zakupach byłam, na wycieczki rowerowe zaczęliśmy jeździć. Byliśmy w pn, wt., jak otworzyli te lasy, parki. No i to tyle właściwie z takich zmian.

**Jak w końcu ta Wielkanoc u was wyglądała?**

Przygotowaliśmy trochę potraw świątecznych. Sama Wielkanoc przy stole głównie - przy takim świątecznym stole zastawionym. Zdzwanialiśmy się z rodziną na video, byliśmy na podwórku - akurat było ciepło w 1-szy dzień świąt. Byłą moja mama, siostra, nakryliśmy sobie stół świątecznym obrusem w ogrodzie i sobie tam siedzieliśmy. Zdzwoniliśmy się z babcią na videorozmowę, pogadaliśmy trochę. No i takie świętowanie. Sporo na powietrzu.

**Spotkaliście się z rodziną, ale nie ze wszystkimi?**

To rodzina, która mieszka na dole, także widzimy się z nimi codziennie. Z pozostałymi nie widzieliśmy się. Nikt się nie ruszał z domu i tylko takie videorozmowy, no i z tymi, co zazwyczaj się spotykamy, robiliśmy takie grupowe videorozmowy - z kilku domów się łączyliśmy.

**Dzisiaj jest 1-szy dzień, kiedy przeszłaś na opiekę. Masz już plan dnia, czy najbliższych dni?**

Nie, na razie nie mam. Wczoraj miałam ciężki dzień, bo praktycznie cały dzień byłam zapłakana i nie wiem, co się ze mną działo. Dziś też trochę mi się głos łamie. Nie wiem, mnie już przytłacza to wszystko chyba. Nie umiem wyjaśnić. Wczoraj to był taki wewnętrzny niepokój w ogóle, wszystko mnie denerwowało. Jakoś miałam jeszcze...Nie wiem, może akurat taka presja, że muszę zrobić VAT, a VAT robimy do 25-go i początkowo opcja byłą taka, że od pn mam iść na opiekę - przedwczoraj, ale w piątek zadzwoniła do mnie moja szefowa i powiedziała: "No to tak, jak się umawiałyśmy, jeszcze cały przyszły tydzień pracujesz i dopiero później sobie pójdziesz na opiekę". Ja mówię, że przecież ja już rano pojechałam do pracy, wypisałam wniosek i miałam zaczynać od poniedziałku.  I ona mówi, że jej się wydaje, że my tego nie ustalałyśmy, że umawiałyśmy się, że ona do mnie zadzwoni i da mi odpowiedź, więc to było takie w ostatniej chwili, gdzie ja się już nastawiłam, że będę miała cały tydzień już po prostu bez pracy. I powiedziała, po kilku godzinach zadzwoniła, że przeprasza, że mi popsuła plany, że wiedziała, że chciałam z moimi córkami trochę spędzić czas i trochę szkoły nadrobić, i że jak uporam się z tą deklaracją do środy, to czwartek, piątek, będę sobie mogła wziąć wolne. To byłą taka presja, że muszę to zrobić w tą środę i nie wiem, bo jeszcze coś mi tam nie działało, i jeszcze hałas był, bo mój mąż miał cały czas telekonferencję, więc on rozmawia na głośniku i wszystko słychać, dzieci podchodziły. Ogólnie byłam tak zdołowana wczoraj, że cały dzień płakałam. Po prostu poszłam do sypialni w trakcie pracy, położyłam się pod kołdrę i musiałam się wypłakać.

**Udało ci się koniec końców to skończyć?**

Tak. Jak skończył pracę, też dłużej siedzi, oczywiście, więc koło 17-tej skończył tę telekonferencję, przyszedł do mnie, chwilę posiedzieliśmy i powiedziałam, że jak wszyscy wyjdą z domu, to ja dopiero sobie pójdę i w tej ciszy sobie usiądę, i to zrobię. Ja nie lubię, jak jest hałas, bo ja muszę myśleć intensywnie, bo nie umiem się skupić i najprostsze rzeczy wydają się najtrudniejsze. Dopiero, jak wyszli na dwór, to ja się z tym uporałam i nie było to wszystko takie trudne, i jakoś dałam radę.
**Wcześniej też miałaś taki problem z pracą w domu przez to, że był taki hałas?**

Nie, nie. Wcześniej było inaczej. Normalnie było. Po prostu może to już długo trwa. Nie wiem, nie umiem tego wyjaśnić.

**Powiedziałaś, że masz dosyć tego wszystkiego, ale to w kontekście pracy czy jeszcze czegoś?**

No nie wiem. mnie to męczy chyba ogólnie, że nie ma takiego rozdzielenia tej...Może natłok wszystkiego jest. Jak wychodzisz rano do pracy, siadasz sobie przy biurku, siedzisz w ciszy. No przyjdzie od czasu do czasu ktoś, z którym się porozmawia, ale robisz tę pracę, możesz się na tym skupić i tyle. A jak siedzisz w domu, to podchodzi do ciebie dziecko i chce coś tam z tobą zrobić. Ty mówisz, że nie możesz w tym momencie, no to jest mu przykro i nawet ci to powie, że jest mu przykro, bo ty ciągle jesteś zajęta. I już czujesz tę presję, że kurczę, nie wywiązujesz się z tego. W międzyczasie jeszcze z tyłu głowy myśl, że zajęć nie zrobiłyśmy z 3 ostatnich dni. Mamy wydrukowane, jeszcze nie zrobiłyśmy. I tu druga sprawa - ta praca. Tu ktoś maila wysyła, trzeba odpowiedzieć, tu jakiś błąd, wyszła jakaś dziwna sytuacja, bo klient przyniósł faktury i trzeba to wyjaśniać, dzwonić, tu jeszcze w domu jakiś obiad zrobić czy coś. I po prostu jest tego tak strasznie dużo naraz, nie ma takiego czasu poświęconego na daną rzecz w tych godzinach, tylko po prostu wszystko naraz i to mnie już też przytłacza. Taki chaos, a ja nie lubię chaosu.

**To musi być rzeczywiście trudne tak pracować w domu i jednocześnie w pracy.**

Jak raz na jakiś czas sobie popracujesz w domu, to nawet fajnie, ale jak jest to takim ciągiem długim to jest trudno.

**Czyli ten wczorajszy dzień był jakiś taki specjalnie trudny dla ciebie. Potrafisz jakoś określić, dlaczego wczoraj było bardziej trudno niż zwykle?**

Nie, nie umiem tego określić.

**Masz informację od szefowej, kiedy będziesz mogła wrócić do pracy?**

Na razie umawiałyśmy się, że dzisiaj, jutro nie pracuję i na przyszły tydzień się umawiałyśmy, ale szkoły są na razie oficjalnie zamknięte do 26, czyli do niedzieli. Nie ogłosili jeszcze, że w przyszłym tygodniu będą zamknięte, no ale raczej będą, więc ja na wszelki wypadek wypisałam taki wniosek w pracy na przyszły tydzień i to sobie po prostu leży, żebym już nie musiała jeździć i wypisywać 2 raz. Później, po majówce zobaczymy. Nie wiemy, co tam się dalej będzie działo. Wszystko jest też tak ogłaszane w ostatniej chwili.

**Z tego, co pamiętam, mówiłaś, że na taką opiekę możesz iść maksymalnie na 3 miesiące czy 3 tygodnie?**

Z tą opieką okazało się, że to jest zupełnie inna opieka niż ta, którą się bierze na dziecko chore. Jak dzieci chorują, to idziesz do lekarza i bierzesz takie zwolnienie/ opiekę na dziecko to jest [ns] dni w roku. Jeśli chodzi o tę na szkoły, to jest takie dodatkowe jakby. I to jest w ogóle inny wniosek wypisywany, i nie możesz tego wziąć na wyrost, na zapas. Jak ogłoszą, że szkoły zamknięte są do któregoś dnia, to tylko do tego dnia możesz, nie możesz poza. Dopiero, jak dadzą ci kolejny limit, bo znowu te szkoły zamknięte, to możesz na te kolejne dni.

**Powiedz, skoro teraz chwilowo nie pracujesz, to czy pojawiły się jakieś takie nowe rzeczy, które ci jakoś specjalnie w tej sytuacji przeszkadzają, coś ci zaczęło nowego doskwierać?**

Nie wiem. Nie przychodzi mi nic na myśl w tym momencie.

**A co jest dla ciebie w ogóle największym problemem w tym momencie, związanym jakby z całą tą sytuacją epidemii, pandemii?**

Taka swoboda, że ci ludzie mają kontakt wszędzie. Byłam w pracy, to też tam kilka osób na krzyż siedziało, wszyscy w odstępach. Na górze 2 osoby, na dole ze 3 osoby, każdy zachowuje dystans i nie ma już takiej swobody. To już takie nawyki po prostu, bo są plotki, że to chyba będzie długo trwało.

**Problemem dla ciebie jest to, że jakby nie ma takiej większej bliskości z ludźmi czy chodzi o to, że to jest jakieś nienaturalne, dziwne?**

Znaczy, że nie ma takich spotkań w grupach większych. Zazwyczaj w pracy szliśmy sobie wszyscy rano na kawę i to było w małym pomieszczeniu 3 na 3 m i stało 10 osób. Teraz już tak nie będzie. Nawet jak pojechałam i mówię do koleżanki, że napiję się tej naszej dobrej kawy z ekspresu, to mówi: ok, dobra, to wiesz co, ja się przejdę do kuchni, umyję ekspres, to poczekaj, a potem sobie zrobisz. To już taka wymiana, żeby tylko jedna osoba tam poszła, a reszta sobie tutaj przy biurkach siedzi, więc już takie pilnowanie się.

**Powiedziałaś, że są plotki, że taka sytuacja może potrwać długo?**

No tak. Wg tego, co piszą wszędzie, co minister się wypowiada, to wygląda na to, że trochę to potrwa. Jeszcze słyszałam, że jesienią ma wrócić taka druga fala. Dla mnie najgorsze to jest to, że te szkoły będą pozamykane. Też sobie zdaję sprawę, że te dzieci przecież chyba muszą się uczyć, tak? No wszystko tracą - i kontakt z rówieśnikami, a tak jak u tej małej, to się wszystko dopiero rozwija, te znajomości, to wszystko, te umiejętności społeczne. Ta starsza zaniedbuje pływanie, bo tego się nie da inaczej zrobić. Jakiś profesor się wypowiadał, że jak tak dalej będzie, to do publicznego basenu najwcześniej się wejdzie za 2 lata. To też takie jest przerażające, no bo to jest strata wszystkiego, co do tej pory się uzyskało. Formy, techniki, wszystkiego. Nie wyobrażam sobie, naprawdę. A szkoda, bo jednak myśli się o przyszłości tych dzieci.

**Czyli myślisz, że to, że one teraz będą miały przerwę nie wiadomo na ile, to jakoś może negatywnie wpłynąć też na ich przyszłość?**

Myślę, że tak. W tamtym roku był strajk nauczycieli, w tym roku to się przedłuża, te lekcje, które są videolekcjami, to nie jest taka lekcja z prawdziwego zdarzenia. jest krótsza, bo trwa 30 min i jeżeli mają 2 historie w tyg., to tylko jedna jest video, a druga, to pan przysyła materiały i trzeba samemu sobie jakoś opracować. Poza tym są takie zgrzyty, że ktoś z klasy wysyła link do jakiejś patologii, tak to nazwę i np. włączają się na lekcji i puszczają film pornograficzny. Taka sytuacja była 2 dni temu. Córka się zalogowała na lekcję i usłyszała jakieś przekleństwa. Kamerka była wyłączona u tych osób. Usłyszała jakieś przekleństwa, więc szybko się rozłączyła, bo pomyślała, że się pomyliła, że jakiś zły link kliknęła albo pani zły link przesłała, a było jeszcze 5 min przed lekcją. No i o równej godzinie się zalogowała i nadal to było i jakieś przekleństwa, wyzywanie pani i jeszcze oni dużo wiedzieli o tej pani, i gdzie mąż pracuje - mąż jest policjantem, więc w ogóle takie obelgo związane z policją. Ona się wyłączyła z lekcji i potem właśnie słyszała, że i film pornograficzny ktoś włączył. No a pani nie może takiej osoby zablokować. Akurat te przeglądarki...No nie wie, ale tam, gdzie odbywają się te lekcje nie można wyłączyć nikogo, a jeśli pani sama się wyłączy, to ta lekcja jest stracona, bo nie wróci już na ten link. Musiałaby wysyłać drugi i informować i jakimś spotkaniu w innym miejscu - i hasło. i ID musiałaby na nowo wysyłać, więc nie ma też jak nad tym zapanować. Wszyscy dostaliśmy wiadomość od wychowawcy i od wicedyrektorki, że będą jakieś podejmowane kroki w tym kierunku, żeby wyjaśnić tę sprawę i żeby te osoby poniosły konsekwencje. No i apelują o to, żeby ktoś się przyznał do tego. Przez to, że ktoś się nie przyznaje, to traci cała klasa, bo w tym momencie jest zawieszona matematyka. Pani powiedziała, że dopóki nie zostanie przeproszona, nie będzie zajęć z matematyki. A jeszcze dyrektor się zastanawiał, czy w ogóle w naszej klasie nie zlikwidować wszystkich videolekcji. To jest trochę przerażające. Przez to, że klasa jest taka, że jest więcej chłopców i jest dużo takich łobuzów, to te dzieci, które chcą się uczyć tracą. No niestety.

**Bo rozumiem, że to było tak, że to musiał ktoś z uczniów wysłać ten link komuś tam, kto...Czyli w tym momencie w ogóle ta matematyka jest zawieszona i nie wiadomo, co dalej?**

No i videolekcji nie ma. Są tylko materiały przesyłane. Dzisiaj właśnie rano miała mieć matematykę, ale nie miała.

**I kiedy się ta sytuacja wydarzyła?**

2 dni temu.

**Ty byłaś przy tym, jak te lekcje się odbywały, czy po prostu opowiedziała ci później twoja córka?**

Nie, ona mi to opowiedziała i potem dostaliśmy wiadomość od wychowawcy, bo ona też była zszokowana.

**A ty jak na to zareagowałaś?**

No jestem oburzona. Nie wiem...To już jest chyba jakaś druga sytuacja. Poprzednia była jakaś lżejsza, nie było żadnych filmów, ale ktoś tam przeklinał. Cały czas wychowawca apeluje, żeby porozmawiać z dziećmi. Nie zdaje sobie sprawy, że nie wszyscy rodzice mają super kontakt z dziećmi i pewnie nie wszystkich interesuje, co oni robią na tych komputerach. Nie wiem, czy rozmawiali, czy nie. Poza tym niektórzy rodzice, to widzę, że mają zaburzony obraz tych swoich dzieci, bo myślą, że to jest grzeczny dzieciak, a tak naprawdę to widzę, co wypisują na klawiaturach i jakich słów używają. Drugi raz wychowawca zaapelował, żeby porozmawiać z dziećmi i żeby się tym zainteresować. Nie wiem, jak to będzie dalej. Jeden chłopak, który jest podejrzany, to z nim w ogóle podobno są problemy, on podobno ma kuratora i już miał ileś uwag, żeby po prostu go wyrzucili. I jest wyrzucany ze szkół cały czas, więc myślę, że ta matka sobie może zdawać z tego sprawę, ale może udaje, że nic nie widzi. Nie wiem.

**Ale to wychowawca zaapelował, żeby porozmawiać z dziećmi o czym?**

No o tej sytuacji.

**Ok., dobra, bo właśnie nie wiem, czy to miało być porozmawianie o tym, jak używać internetu, czy...**

Nie, o tej sytuacji, żeby to się nie powtarzało no i żeby też ktoś się przyznał.

A macie jakieś plany, jeśli się okaże, że te lekcje w ogóle się nie będą odbywały?

No, jeśli tak będzie, to na pewno będę chciała napisać do wychowawcy, może nawet do dyrektora. Nie wiem. To jest taka trudna sytuacja. Wiadomo, że jest jakieś domniemanie niewinności i nikogo nie można oskarżyć. Są to tylko jakieś podejrzenia. Nie wiem, na jakiej zasadzie zbiera się dowody kto tam włączył się na tę lekcję albo kto przesłał link. No nie wiem, coś muszą zrobić, ale w tej trudnej sytuacji, to te lekcje są ważne. Wszystkie, jakie by nie były.

**Tak, ja też jestem bardzo zdziwiona, bo to rzeczywiście jest takie szokujące, że ktoś coś takiego w ogóle zrobił. I mówisz, że to też nie była pierwsza sytuacja, że już coś podobnego się zdarzyło wcześniej?**

Tak, że 2 tyg. temu na innej lekcji, ale to pani chyba jakoś sobie poradziła z tym i nie było to aż tak dramatyczne, bo teraz to więcej się działo no i w ogóle obelgi były pod adresem pani. Nikt by tego nie wytrzymał.

**Myślisz, że to, że takie rzeczy się dzieją, to wynika właśnie z tego, że te zajęcia są prowadzone online? Czy po prostu ta klasa taka jest?**

Ta klasa taka jest. Jest ciężka, ja wiem, że jest ciężka. Tam jest po prostu tak, że jest 22 chłopców i 5 dziewczyn, i połowa klasy to są piłkarze, a druga połowa pływacy. U pływaków są dziewczyny i chłopcy. Pływacy są spokojniejsi, bo mamy 2 trenerów - 1 od pływaków, 1 od piłkarzy i nawet ten od pływaków mówi, że z nimi nie ma żadnych problemów, że z nimi się można dogadać i są obowiązkowi, zdyscyplinowani, dobrzy uczniowie. A ci piłkarze są tacy bardzo nadpobudliwi, pełni energii, łobuziarscy trochę też i z nimi są problemy. Chciałam Zuzię, proponowałam jej, że może przeniosę ją do innej klasy, żeby ona na tym nie traciła, bo ona jest wzorową uczennicą, ale ona powiedziała, że kocha pływanie i że ona sobie nie wyobraża, żeby z tego zrezygnować, więc stwierdziliśmy, że może ta podstawówka nie jest aż tak ważna, że skoro to kocha, to niech tę pasję sobie rozwija, a nauka, to niech się uczy tyle, ile się uczy, a już tam to liceum jest ważniejsze. Wiadomo, że może później trzeba się będzie przygotować do tych egzaminów ósmoklasistów bardziej, ale niech sobie pływa, jak to lubi. To też jest taka jej druga rodzina. Trener jest świetny, ma super podejście no i te dzieciaki są dla siebie takie fajne, zżyte, więc to też jest ważne. Może nawet ważniejsze niż ta nauka, to liceum.

**Jak ona sobie radzi z tym, że nie ma teraz tego pływania?**

Widzę, że dzieci mają o wiele więcej energii. Obydwie. Wieczorem nawet jest tak, że trochę się tak biją. Tak chyba chcą z siebie wyrzucić to. Ja już ich nawet nie rozdzielam, bo widzę, że chyba jakoś trzeba wytracić tę energię. Do tej pory się coś robiło. Ogólnie Zuzia ma tak, że dostaje jakiś harmonogram od trenera i ćwiczenia takie na lądzie robią, więc muszą co jakiś czas nawet wysłać filmik z nagraniem, że robią jakieś konkretne ćwiczenia. No i na tej zasadzie. Wypełniają jakiś tam harmonogram. Ona mówi, że jako jedyna z dziewczyn wysłała, tam kilku chłopców wysłało filmik, no to dostała 6, a trener rzadko stawia 6, więc myślę, że ją docenił przez to i że pamięta o tym, że się angażuje, że coś robi.

**Zaczęłaś opowiadać, jak się czułaś przez ostatnie 2 tyg., ale chciałabym, żebyśmy wróciły do tych obrazków, które ci pokazywałam[...]**

**Emocje - zdjęcia**

Może 11? Ogólnie sytuacja nie jest dla mnie taka pozytywna, no ale szukam jakichś dobrych stron w tym, co się dzieje i ta tęcza na horyzoncie może tak obrazować to, że staram się szukać dobrych stron. No i te krople deszczu to był ten mój nastrój momentami. Takie wahania, tak?

**Ten twój nastrój jest teraz taki, że raz jest lepszy a raz jest gorszy, tak?**

No chyba tak.

**A dzisiaj?**

Dzisiaj jeszcze trudno mi powiedzieć. Jestem taka trochę przygaszona rano. Nie chcę sobie nic planować po prostu. Chcę się cieszyć tym, że mam wolne i że nie mam już tej presji pracy. Tego podchodzenia do komputera i ciągle w takiej gotowości, żeby być. Dlatego się umówiłam dzisiaj na rano, żeby już tak mieć to za sobą i już dalej sobie luźno spędzić ten dzień. Jakkolwiek, już bez żadnych planów, żeby tylko ten obiad jakiś tam był dla dzieciaków i wyjdę chyba na podwórko, bo słońce świeci, więc tak trochę na słońcu spędzę czas. A jutro może posprzątam, zobaczymy. Nie chcę też po prostu z jednej pracy wchodzić w drugą, że jak skończyłam tę służbową to sobie teraz nagle zacznę dom sprzątać. Chcę się wyciszyć, uspokoić, jakoś tak.

**Mówiłaś, że starasz się szukać jakichś pozytywów. Teraz jakie pozytywy widzisz?**

To, że mogę z dziećmi spędzić trochę czasu, że tak lepiej się można poznać, jak się jest tyle czasu. Bo nawet jak się na urlop wyjedzie, to zawsze to jest jakiś ograniczony czas, a tutaj praktycznie...No i wiem, na czym polega praca mojego męża, jak te moje dzieciaki się zachowują w tych rożnych sytuacjach. Myślę, że to jest takie pozytywne. Że w sumie nie jest aż tak źle, w tych relacjach naszych. Wiadomo, że czasem ktoś na siebie wybuchnie, ale tak, jak niektórzy mówią, że będzie więcej rozwodów po tej całej pandemii i tej kwarantannie, no to myślę, że my się jakoś dogadujemy w miarę. Jak ktoś jest w kuchni i widzi, że to drugie pracuje, to przygotuje coś do obiadu, to to drugie później, jak jest sterta naczyń, to pozmywa. Nie ma napisanych zasad, ale każdy widzi, że coś tam musi od siebie dać.

**I też powiedziałaś, że wczoraj też było tak, że jak powiedziałaś swojemu mężowi i dzieciom, żeby wyszli i cię zostawili, to zrobili to i ci to jakoś pomogło skończyć tę pracę?**

Tak. No są takie momenty, że wybuchamy na siebie, coś tam krzykniemy i coś się posprzeczamy, ale to jest chyba po to, żeby tę całą skumulowaną energię wywalić, ale nikt się o to nie obraża i za chwilę każdy podchodzi do siebie z uśmiechem, coś zażartuje i to się wszystko zniweluje, rozproszy i jest normalnie. Dużo jest takich sytuacji, ale to nie jest, że ktoś do kogoś pała agresją i nienawiścią, tylko to wszystko takie jest z miłości wszystko i jedno o drugie się troszczy. I tak każdy chce iść na rękę tej drugiej osobie, więc nie ma co narzekać.

[instrukcja moderatora do następnego spotkania - zapisywanie uczuć, znajdowanie adekwatnych obrazków, zdjęć]

**Ten obrazek, jak zrozumiałam to jest bardziej takie zawieszenie, trochę takie mieszane emocje - raz pozytywne, raz negatywne, ale też szukasz pozytywów w tej sytuacji. Czy ty w ogóle teraz czujesz się jeszcze jakkolwiek zagrożona tą sytuacją?**

Nie wiem, czy czuję się zagrożona. Jeśli chodzi o zdrowie, to nie dotknęłam tego koronawirusa, więc nie czuję, że on jest, bo wokół mnie nic takiego się nie dzieje. To jest tylko to, co gdzieś słychać w tv. Są czasami takie myśli, też ze znajomymi w pracy rozmawialiśmy, że może to ktoś wymyślił? No są takie, bo dziwne rzeczy się dzieją. Np. w Szwecji nie ma żadnych ograniczeń. Oni nadal pracują i cała gospodarka, wszystko funkcjonuje. A tu wszędzie wokół są...No nie wiem, jedni tracą, a inni na tym korzystają.

**To, że to jest wymyślone? Skąd takie myśli się u was pojawiają?**

No bo nie ma aż takiego może natężenia tych chorób. Tak jakby trochę taka stagnacja nastała. Na początku tak straszyli, teraz też powoli wraca do życia ta gospodarka. Odmrażają trochę to wszystko i znoszą zakazy. Nie wiem. To jest właśnie dziwne dla mnie, że...Tak jakby jakaś zmowa była.

**Co konkretnie w tym jest takie dziwne?**

Że tak można zablokować wszystko naraz. Niby nigdy nie można było, a nagle teraz wszyscy się zmobilizowali i na całym świecie każdy siedzi w domu i w ogóle nic się nie dzieje. A z kolei w innych krajach nagle mogą żyć i normalnie prowadzić tam to wszystko. Nie wiem, o co tu chodzi.

**Jest w tobie takie niezrozumienie tego, że w niektórych krajach trzeba tak wszystko zamykać i zamrażać, a w innych nie trzeba...**

No jest dziwne i tam, gdzie nie zamrożą tego i normalnie funkcjonują okazuje się, że można i że wcale aż tak źle nie jest i tych zachorowań wcale aż tak dużo nie ma. No to? Dziwne.

**Jak oceniasz takie podejście, jak w Szwecji?**

Teraz to już się zastanawiam, co jest właściwe. Może rzeczywiście nie trzeba aż takich obostrzeń wprowadzać? Może u nas to zrobili, bo się bali tej fali, że sobie nie poradzą, że tych sprzętów będzie za mało, ale że ludzie tak się też zmobilizowali i to szycie maseczek też było - te akcje społeczne różne. Może to też tak zaplusowało i tych sprzętów, tego wszystkiego było więcej, bo i te respiratory przecież drukowali na tych drukarkach 3D. Może zobaczyli, że nie jest tak źle, że jakoś da się to ogarnąć, że ludzie się słuchają i może dlatego też teraz wszystko tak znoszą? Nie wiem, co o tym myśleć. To jest wszystko takie dziwne.

**Myślisz, że w Polsce taka sytuacje, że nie byłoby żadnych zakazów? W Szwecji to jest wszystko na poziomie rekomendacji [...] Takie podejście sprawdziłoby się w Polsce?**

Nie, w sumie nie. Wydaje mi się, że może właśnie musieli postraszyć. Tak. I jak postraszyli, to ludzie to sobie bardziej poważnie potraktowali. Na pewno są jednostki, które się nie boją, ale jak postraszyli, to większość jednak się zastosowała, a jakby może tak na luzie, to wszyscy by może wyśmiewali to i każdy by to traktował niepoważnie. Może to o to chodziło, nie wiem.

**O jakich zmianach w ograniczeniach ostatnio słyszałaś?**

Noszenie maseczek. To jest uciążliwe, bo trzeba w słońcu ich używać i nie jest to fajne. Tak samo, jak jechaliśmy na rowery i też wszyscy w maseczkach. Zamiast oddychać świeżym powietrzem, bo wiosna się zrobiła, to musisz się zakrywać i dusić się pod tym materiałem. To jest uciążliwe bardzo.

**Jak myślisz, po co jest takie ograniczenie?**

Może po to, żeby nie było tego kontaktu, tego powietrza wydychanego. Może wtedy bardziej poluzują to wychodzenie na ulicę i takie kontakty z ludźmi i przebywanie w miejscach publicznych, jeśli wszyscy będą w tych maseczkach? To pewnie po to. Rozumiem to, ale nie jest to dla mnie przyjemne. Też koleżanka z pracy, która jeździ komunikacją mówiła, że prawie jej się odparzyło tutaj na twarzy, bo stała w słońcu a te gumki zawsze coś tam dociskają, to powietrze nie dochodzi. No jest to kłopotliwe. Teraz mamy jeszcze taką umiarkowaną temperaturę, ale jak będzie bardzo ciepło, a przecież może być w Polsce bardzo ciepło, to będzie bardzo uciążliwe. Czy w autobusie, czy nawet gdzieś się w wakacje pojedzie troszkę...Nie wyobrażam sobie na plaży leżeć w maseczce.

**Wy nosicie maseczki całą rodziną?**

Tak. jak wychodzimy na ulicę też zakładamy maseczki. Widzę, że wszyscy sąsiedzi się stosują. Teraz tak więcej ludzi widać na ulicy, ale widzę, że wszyscy się już od 1 dnia stosowali - każdy w maseczce albo chociaż jakiś tam szal, który zakrywa nos, usta.

**A jakie wy macie maseczki?**

My mamy takie wielokrotnego użytku z materiału. Młodszej córce założyłam taka jednorazową, bo nie miałam takich małych. Dostałam w pracy 10, bo dostawaliśmy wszyscy takie pakiety, więc jej związałam te gumeczki, bo to wiadomo, że jej potrzebny mniejszy rozmiar, żeby to się trzymało i ona właśnie zakładała te jednorazowe.

**Ona rozumie, dlaczego tę maseczkę musi nosić?**

Myślę, że rozumie. Poza tym widzi, że wszyscy je noszą, że tak trzeba. To, że wszyscy noszą to chyba ma największy wpływ, że widać, że to jest takie powszechne. To wszystko można wytłumaczyć.

**Jeszcze coś się zmieniło w ograniczeniach w ciągu ostatnich 2 tyg.?**

W ograniczeniach, to nie kojarzę, bardziej to, że poluzowali. Otworzyli te parki, lasy, że można chodzić, więc więcej osób wyszło na ulicę już 1-go dnia, bo od poniedziałku to było. Pojechaliśmy na rowery, to widać było, że dużo ludzi na rowerach, na spacerach, naprawdę dużo. Pojechaliśmy na wał nad Wisłę i tam do Wisły sobie podeszliśmy i wałem jechaliśmy. tam mamy jezioro takie małe i dużo zieleni, stadniny koni, więc tam zawsze jeździmy na wycieczki rowerowe. Tak ok. 10 km.

**Czy to jest popularne miejsce i tam zazwyczaj dużo ludzi przyjeżdża, czy teraz jakoś więcej?**

Ludzie są tam rozproszeni tak ogólnie, jak w lato się tam jeździ i nie ma tam aż takich tłumów, ale teraz to zauważyłam, że dużo było i chyba ludzie szukali takich miejsc właśnie ustronnych. I nawet na tym wale, czy ktoś z psem na spacer, czy ojciec z dziećmi, to widziałam, że ludzie szli w te miejsca ustronne i nawet niektórzy zdjęli sobie maseczki, żeby tak pooddychać bardziej tym świeżym powietrzem, więc wychodzili, dużo wychodziło i dużo było w tych miejscach takich, gdzie spodziewali się, że nie będzie ludzi.

**Słyszałaś o planach na przyszłość luzowania tych ograniczeń?**

Nic więcej nie wiem. Wiem jeszcze, że teraz może być więcej osób w sklepach. To słyszałam. A o obostrzeniach, to że te maseczki mamy tak długo nosić.

**To jest uciążliwe już teraz, ale boisz się, że w lato będzie jeszcze gorzej?**

No będzie. Wyobrażasz sobie w 30-stopniowym upale iść z zakrytymi ustami i nosem?

**A jeśli chodzi o to zdejmowanie obostrzeń? Jest kilka takich etapów w planach [...]?**

Tak, coś słyszałam i widziałam tę tabelkę.

**Jak oceniasz to, że są takie plany?**

To jest chyba taka kolej rzeczy, że muszą to kiedyś poluzować, tylko jeszcze jest kwestia tego, w jakim to będzie czasie, bo oni mogą mówić, co będzie w tym i w tym etapie, ale nie wiadomo, ile to będzie trwało. A może coś w międzyczasie się zmieni, że zachorowań będzie więcej i w ogóle poprzeciągają te etapy? Nie wiem. Wszyscy chyba najbardziej czekają na 3-ci - na kosmetyczki i fryzjerów, ale to chyba poczekamy jeszcze trochę. Tak mi się wydaje.

**Czyli uważasz, że to jest naturalna kolej rzeczy, że tak należy robić, ale dlaczego tak uważasz?**

No, żeby to wreszcie wracało do normalności, bo ile to będzie trwać? Chociaż każdy mówi, że to będzie taki rok najtrudniejszy i że w tym roku raczej nic nie będzie normalne. I chyba tak już trzeba zakładać. Wakacji nie będzie normalnych i jeśli będzie ta kolejna tura zachorowań, co przewidują, to we wrześniu może też szkoły nie będzie. Nie wiadomo.

Na początku mówiłaś, że nie planujecie wakacji, a teraz już pojawiły się jakieś plany?

Urlopy mamy zaakceptowane i pewnie będą chcieli, żeby je wykorzystać, bo teraz nikt nie chce tworzyć rezerw na urlopy, tylko żeby je wykorzystywać. No i mój mąż zaczął ostatnio mówić, żeby może zarezerwować jakiś domek nad morzem? Mamy 1-sze 2 tyg. lipca, więc...No nie wiem. Ja mówię, że nie wiem jeszcze czy rezerwować, bo może to się jakoś poprzeciąga, może nie będzie można nigdzie jeździć, a poza tym, jeśli będzie dużo ludzi to też...Bo wiesz, jak już w krew ci wejdzie takie unikanie ludzi, to dziwnie będzie tak pomiędzy nimi przebywać. I nie wiem, nie wiem. Fajnie by było gdzieś pojechać na jakieś takie ubocze i pochodzić po plaży, ale wiem, że to działa też tak, że jak coś pozwolą robić, to nagle się tłumy tam zwalą i nie będzie ubocza żadnego, bo nagle tam, gdzie nie było ludzi zazwyczaj, to będą dziesiątki.

**Czyli obawiasz się, że nawet, jeśli będzie można gdzieś jeździć, to nie będzie to takie do końca przyjemne, bo tam, gdzie normalnie ludzi nie będzie, to nagle będą te tłumy?**

Tak, a pewnie trzeba będzie na siebie uważać jednak w dłuższym czasie.

**Czyli masz takie poczucie, że nawet jeśli te ograniczenia zaczną być zdejmowane, to nadal jakieś tam zostaną, tak?**

No to chyba już tak wejdzie w krew, żeby może bardziej dbać i higienę i uważać na siebie.

**Z tych różnych ograniczeń, które są teraz, to które powinny zostać dłużej?**

No ta dezynfekcja rąk myślę. W miarę możliwości, jeśli da się tak zorganizować pracę, to ten dystans między ludźmi. Myślę, że to. Żeby takich skupisk nie było. Wiem, że na pewno powoli będą ludzie wracać do firm i na pewno teraz wszystkie zarządy się zastanawiają, jak to rozwiązać. Wiem, że u nas w pracy też nasza przełożona myśli o tym, że jak ktoś jest w naszym pokoju, to jak ktoś drugi przyjdzie, to żeby siedział po prostu w takim odstępie. My pracujemy 4 w pokoju, więc jak byśmy były 4, to już by było złamanie tej zasady. U mojego męża też chyba pracują nad tym co tu zrobić, żeby ludzie jakoś etapami może wracali, wiem, że mierzą odstępy, bo oni siedzą w takich puszkach, że są 3 biurka obok siebie i naprzeciwko 3 kolejne, potem jakiś odstęp i znowu takie segmenciki, więc między tymi 3 osobami nie ma 2 m, ale jakby na jednym końcu biurka posadzić jedną i na końcu tego 3-go drugą, to już jest odstęp, ale z kolei naprzeciwko, jak ktoś usiądzie, to tam nie ma tego odstępu. I zastanawiają się jak to zrobić, że może jedni będą w jednym tyg. przychodzić a inna część firmy w drugim tyg. Tak na zmianę ze zdalną pracą. Myślę, że każdy tak racjonalnie chce podchodzić, nikogo nie narażać, ale żeby jakoś to życie wracało.

**Myślisz, że te ograniczenie, żeby trzymać dystans, to do kiedy one powinny trwać?**

Nie umiem tego powiedzieć.

**Co musiałoby się stać, żebyśmy nie musieli już tego robić?**

Może jak jakoś zacznie spadać ta liczba zarażonych i jakoś będzie to widoczne, że to naprawdę spada, ale nie dlatego, że robią mniej testów.

**Teraz jest tak, że tych testów jest robione więcej czy mniej?**

Akurat na dzień dzisiejszy nie wiem, ale wiem, że były momenty takie, że gdzieś w kraju sąsiednim przyrastało, a u nas nie, u nas w bardzo małym stopniu przyrastało, a okazało się, że testów było o połowę mniej robione niż 3 dni wcześniej.

**A czy z tych różnych ograniczeń są takie, które uważasz, że powinny zacząć szybciej być zdejmowane?**

Nie wiem, chyba nie. Nie. Może jeszcze trzeba poczekać.

**A np. otwarcie szkół?**

Ale ja sobie tego nie wyobrażam, bo też słyszałam o takim pomyśle, że mają być odmrażane dla tych młodszych dzieci, dla klas 1-3 i że będzie mniej dzieci w salach, ale to jak oni to sobie wyobrażają?  Że pięcioro dzieci w wieku 6 lat będzie sobie w sali siedzieć jedno w jednym kącie, drugie w drugim, a reszta gdzieś tam porozmieszczana? Przecież ich plan dnia wygląda tak, że oni i bawią się razem. Nie wiem, jak mieliby to zorganizować. Albo co, w maseczkach mają być cały dzień? Na pewno któreś dziecko się zapomni i czy dotknie innego, czy zdejmie maseczkę. Nie ma szans, żeby uniknąć tego przy takich małych dzieciach. W przedszkolach tym bardziej. Dezynfekować zabawki? To chyba po jednym dziecku w sali by musiało być.

**Nie chcesz, żeby te szkoły były otwierane, czy chcesz, ale nie wiesz, jak by to miało wyglądać?**

Ja bym chciała, żeby były otwarte, ale nie wyobrażam sobie jak by mieli to rozwiązać.

**Te wątpliwości dotyczą tylko tych młodszych dzieci?**

Mi się wydaje, że oni próbują otwierać dla tych młodszych, bo ta opieka jest przeznaczona tylko dla dzieci do 8 r.ż. i to o to chodzi. A starsze mogą mieć nadal te videolekcje i nie trzeba ich aż tak pilnować. Jakoś to sobie ogarniają. U tych młodszych rodzice są też uziemieni, bo muszą w tym domu jakoś się nimi zaopiekować i też na tą szkołą czuwać, nad wszystkim, więc nie mogą normalnie wracać do pracy. Może o to chodzi. To jest wszystko takie błędne koło trochę.

**A restauracje? Powinny zacząć być niedługo otwierane?**

Nie, restauracje to akurat nie jest jakieś dobro tak bardzo potrzebne, że muszą być otwarte i można sobie bez tego poradzić. Takie najpotrzebniejsze rzeczy to ta szkoła i praca. Lekarza to też można teraz załatwić widzę, bo e-recepty i te videowizyty są.

**A te inne miejsca, które nie są niezbędne? Kiedy na nie będzie czas?**

Może jak spadnie tak definitywnie ta liczba zachorowań. Jak będzie widać, że ta fala przechodzi jakoś, ale ludzie będą nadal zachowywali te środki ostrożności.

Czy te miejsca powinny mieć jakieś nowe zasady funkcjonowania niż przed epidemią?

Na początku to chyba też powinny być jakieś ograniczenia, żeby ta liczba osób była mniejsza, żeby może te stoliki nie były ściśnięte, żeby te odstępy też jednak były.

**A są chyba jakieś ograniczenia dotyczące dzieci - że mogą/ nie mogą się same przemieszczać. Jak to wygląda teraz?**

Do 13 lat muszą z osobą dorosłą. Myślę, że to ma sens, że to jest dobre ograniczenie, bo te młodsze dzieci są takie raczej jeszcze nieodpowiedzialne, więc mogą się i gromadzić w grupach, i łamać to obostrzenie, żeby ten dystans zachować.

Jak teraz wygląda u ciebie takie dbanie o siebie?

Ja standardowo maluję się codziennie, jakieś kąpiele, dbanie o włosy. Paznokcie sobie też na święta zrobiłam i położyłam hybrydę, bo hybrydę sobie robię sama i tyle. Nic się jakoś nie zmieniło za wiele. Dbam o siebie, staram się. To jest dla lepszego samopoczucia.

**Nie zrezygnowałaś z niczego?**

Z fryzjera jedynie, ale to jest też taka opcja, że moja fryzjerka zaproponowała, że może, jak zadzwonię i będę chciała sobie farbę położyć, to umawiam się z nią i...To wiesz, o co chodzi, bo mówiłam ostatnio. Jeszcze się nie umówiłam, ale już myślę o tym i jeśli będę miała w przyszłym tyg. wolne, to chyba się już umówię.

**Jak się na co dzień teraz ubierasz?**

Jakieś dżinsy, bluzka. jak siedzimy sobie przy komputerach, to muszę trochę cieplej się ubrać, jakieś skarpety ciepłe, bo jednak jak się siedzi, to jest zimno. jak jest taki cieplejszy dzień, to i sukienkę sobie założę, i wyjdę na słońce.

**Pytam dlatego, bo może np. czasem chodzisz w piżamie, w której spałaś przez cały dzień?**

No może ze 2 razy tak się zdarzyło, że byłam w piżamie, ale ogólnie nie lubię tak chodzić, bo jakoś taki dzień jest wtedy niepoukładany. Nie wiesz, czy wstałeś, czy jeszcze śpisz...Nie, ja lubię się już ubrać normalnie i wtedy wiem, że już jakoś funkcjonuję. Takie poczucie uporządkowania tego dnia i takiej energii, i że to jest normalny dzień, a nie taki rozmemłany. Od czasu do czasu ma się tak, że lubi się w piżamie, ale to rzadko.

**Mówiłaś, że do włosów używasz specjalistycznych kosmetyków. Czy jest tak, że na teraz zrezygnowałaś z jakichś kosmetyków? Z jakichkolwiek?**

W sumie nie, chyba wszystko tak normalnie raczej. Tak, żebym się czuła dobrze ze sobą, więc wszystkiego chyba używam tak, jak dotychczas. Paznokci tylko tak nie maluję, żeby sobie odpoczęły i tylko na święta sobie zrobiłam. To akurat jest na plus, że w tym momencie nie czuję się jakoś źle, bo wiem, że mogę sobie sama to wszystko zorganizować z paznokciami.

**Na co dzień też je sobie sama robisz?**

Tak. I jak krążyły te memy, że po kwarantannie wszyscy wyjdziemy z odrostami i brzydkimi paznokciami, to stwierdziłam, że mnie akurat to nie grozi, bo większość umiem przy sobie zrobić, więc nie będę się jakoś źle czuła, ale zdaję sobie sprawę, że muszą cierpieć bardzo te panie, które regularnie wszędzie gdzieś tam chodziły.

A jeśli chodzi o zaopatrzenie do tej pielęgnacji. Kupujesz teraz takie rzeczy?

Te do włosów to przez internet, bo jest taniej, a jakieś balsamy i te rzeczy podstawowe, to mam Rossmanna i tam jest kasa samoobsługowa, więc idę, sama sobie ponaliczam to wszystko i też widzę, że mało ludzi tam jest. Już byłam ze 2 razy. Do fryzjera bym chętnie poszła. Mam długie włosy i grube, więc zawsze mi schodziło tak do 3 godz. z farbowaniem i strzyżeniem. To też było dla mnie takie fajne, bo taki relaks - wszystko przy tobie zrobią, ufasz im, wiesz, że nic nie popsują, więc możesz spokojnie zamknąć oczy i obudzić się za 3 godz. Brakuje mi tego, bo to jest takie fajne, przyjemne.

**Powiedziałaś, że już niedługo będzie ten czas, że powinnaś pójść do fryzjera. Jak to oceniasz, że już jest ten moment?**

Już odrost się jakiś na włosach, więc normalnie bym poszła, położyliby mi tę farbę i przy okazji jakieś strzyżenie by było, więc te włosy by fajniej układały. Teraz będę się musiała zadowolić tym, że po prostu tylko tę farbę położę sobie sama, mam nadzieję, że jakoś dam radę, no i tyle. Jak ma się te dłuższe włosy, to wiesz, że można przechodzić, one urosną i one jeszcze tak źle nie wyglądają. Gorzej mają ci, co mają krótkie i jak tylko urośnie cm, to już źle się układa i wygląda brzydko.

**Wprowadziłaś jakieś nowe rzeczy do swojej pielęgnacji?**

Nie, chyba nie. Raczej nic się nie zmieniło.

**Brakuje ci kosmetyczki?**

No fajnie by było sobie pójść i jako taki bonus za to wszystko sobie coś zrobić. Chętnie sobie bym gdzieś poszła, może jakieś rzęsy zrobiła czy coś. Tak, żeby się lepiej poczuć, kobieco. Albo ubrać się ładnie. Zazwyczaj, jak się chodzi do pracy, ja lubię w spódnicach chodzić. W lato to nawet na rower takie luźniejsze spódnice do kolan s=zakładam i sobie jadę. No lubię, a teraz, jeśli będziemy siedzieć w domu, to nie będzie się nikt ubierał i stroił, a to fajnie tak się ubrać i sobie wyjść.

**Kupujesz teraz ubrania na wiosnę, na lato?**

Nie, na razie nie kupuję. Chciałabym pójść do sklepu, przymierzyć sobie i dopasować. Nawet dziewczynom moim to też miałam nadzieję, że coś będzie otwarte i że można będzie coś kupić...Zobaczyć i dotknąć to, żeby widzieć, jaki to materiał, jak to wygląda. W internecie to mam wrażenie, że albo zaraz będę musiała oddawać i nawet nie chce mi się sobie głowy zawracać tym szukaniem, przeglądaniem.

**Ubrań na cieplejsze momenty nie kupujesz, bo chciałabyś je kupić stacjonarnie?**

Tak, ale jak to się będzie przeciągało, to będę musiała zamówić coś. Może być tak, że maj i nagle się zrobi upał. Najbardziej to buty by chciała jakieś. Sandałki. Z tym też niełatwo przez internet.

**Jak myślisz, do kiedy będziesz czekać?**

Nie wiem, chyba muszę zacząć już powoli oglądać, żeby tak w maju coś kupić, bo się momentalnie zrobi ciepło na pewno. Bez butów, no...Ubranka to jeszcze jakieś są z tamtego roku, ale butów to już nie, bo zniszczone czy za małe, bo przecież ta stopa rośnie. Ja mam jakieś tam swoje rzeczy, więc ze mną nie ma problemu, tylko z dzieciakami, bo u nich to się najbardziej zmienia.

**Teoretycznie będąc w domu mogłabyś się zupełnie nie malować, ale robisz to. Dlaczego?**

Bo lubię, bo się dobrze czuję wtedy. Czuję się zadbana. Facet jest w domu, więc też niech widzi mnie z takiej dobrej strony a nie z tej złej. Mówią: idziesz do pracy - ubierasz się ładnie, wracasz z pracy - ubierasz się w dres. U mnie też często tak było, że do pracy się wystroiłam, widzieliśmy się rano 5 min, po południu wracałam, to się już w luźniejsze ubrania ubrałam, wygodniejsze, no to co on mnie widział? Zawsze normalna, więc jak teraz jesteśmy wszyscy w domu, to też nie mogę tak spocząć na laurach i byle jak. Myślę, że to jest ważne.

**Z punktu bycia konsumentem, czego ci teraz najbardziej brakuje?**

Tak, jak na takie zakupy stacjonarne odzieżowe chodziłam raz na jakiś czas, bo nie lubiłam tłumów. Ja nie lubię tłumów i jak jest tak gęsto nawieszane na tych wieszakach, to nie umiem nic znaleźć i od razu wychodziłam, bo mnie to denerwuje, ale teraz, to naprawdę chętnie bym poszła. Nawet tak przejść się i popatrzeć, co jest. A może coś wpadnie w oko? Taka potrzeba, żeby coś kupić, coś nowego mieć. Idzie ta wiosna, no to każdy lubi tak odświeżyć się. Chętnie bym poszła i pochodziła. Bardzo chętnie.

**A takie: siłownia, basen, fitness?**

No też. Na basen na pewno przyjemnie byłoby pójść.

**To wszystko są takie miejsca bardziej dla przyjemności?**

Tak.

**Skoro tych wszystkich miejsc nie ma, to co ty robisz dla siebie, dla przyjemności?**

Tak, jak ci mówiłam, że ćwiczę trochę i teraz na rowerze pojeździliśmy. To też jest fajne, bo można tak sobie wrócić do tych starych nawyków. Czasami kupię jakiś kosmetyk, jakieś balsamy czy jakiś olejek do opalania sobie kupiłam, bo już teraz słońce, to może trochę wyjdę się poopalać, bo ja lubię sobie na słońcu poleżeć. No i teraz, jak będę miała wolne, to zamierzam sobie trochę poczytać. Ojej, chociaż trochę. Tak sobie, wiesz, na luzie poleżeć, poczytać. Bardzo bym chciała. No a tak, to czasami sobie coś zjemy dobrego, jakieś lody, takie rzeczy. Brakuje mi, żeby pójść gdzieś na jakieś lody czy do cukierni. Tak kupić sobie jakieś ciastko, usiąść.

**Z tym czytaniem, czemu to jest takie…?**

No bo ciągle nie mam czasu. Ja lubię takie książki w wersji papierowej najbardziej, a ostatnio nie mam kiedy tak usiąść. Jak już mam chwilę, to wolę już wyjść na podwórko z dzieciakami coś porobić niż usiąść z tą książką. Teraz te 8 godz., które nie przeznaczę na pracę, to sobie może coś wygospodaruję, żeby sobie usiąść i po prostu poczytać.

**Na ile czujesz się z tym dobrze, że nie będziesz teraz pracować, a na ile będzie ci tego brakowało?**

Teraz to chcę odpocząć, więc czuję się dobrze z tym, już czekałam na to i już się nastawiłam, że będę miała to wolno. Już pal licho, że to będzie mniej trochę zapłacone, ale naprawdę już potrzebowałam, bo to już jest takie napięcie, że jesteś w gotowości. W salonie i pracujesz, i odpoczywasz. To jest strasznie przytłaczające, bo nie masz możliwości zapomnieć o tej pracy. Jak z pracy wyjdziesz o 16, to już nie myślisz do następnego dnia. Nie ma szans, żeby jakaś myśl przypłynęła o pracy. Tutaj nie, nie da się od tego odciąć i to mnie strasznie przytłacza, męczy. Ja muszę mieć oddzielone te sfery życia, muszę.

**Teraz, jak jest ta epidemia, o której zaczynałaś dzień pracy, o której kończyłaś?**

Praktycznie wstawaliśmy po 7, jakieś śniadanie, więc już koło 8 włączałam komputer i powinnam pracować do 16, ale jak tam w ciągu dnia coś wyszło takiego, że ktoś coś ode mnie chciał i wiedziałam, że odezwie się po 16, to bywało, że nie wyłączałam komputera i jeszcze tam był w gotowości. Pocztę też czasami sprawdzałam jeszcze po południu, czy na telefonie się jeszcze logowałam, albo tak, jak wczoraj mi nie działało, to koło 19 dopiero skończyłam. W międzyczasie miałam tę przerwę, bo sobie popłakałam, poleżałam, ale ogólnie to bywa, że po 16 się jeszcze coś zrobi.
