# Supplementary material for: The challenges arising from the COVID-19 pandemic and the way people deal with them. A qualitative longitudinal study
Source: PLoS One. 2021 Oct 11;16(10):e0258133. doi: 10.1371/journal.pone.0258133 (PMC8504766; doi:10.1371/journal.pone.0258133)
Supplement: S1 Dataset — (ZIP) [file pone.0258133.s003.zip › Transcriptions/stage 3/8.3_M_30_couple, no children.docx]

**8.3._M_30_couple noc children**

**Jak wyglądały twoje ostatnie 2 tyg.?**

W zasadzie bez zmian, dalej siedzę na home office. Tyle, że w czasie świąt odwiedziłem rodziców, którzy mieszkają niedaleko. Żadnych innych zmian. Wszystko tak samo, jak w poprzednich tygodniach.

**Spotkałeś się z kimś jeszcze? Jak spędzałeś czas?**

Spędzałem czas w domu poza koniecznością spacerów z psem i zrobienia zakupów.

**Jak wyglądała Wielkanoc?**

W okrojonym składzie 4-osobowym. Byliśmy z dziewczyną u moich rodziców i posiedzieliśmy u nich w niedzielę i w poniedziałek. I już.

**W okrojonym składzie, ze względu na?**

Ze względu na zagrożenie epidemiologiczne wywołane koronawirusem. Jest to bardziej odpowiedzialne ze względu na starszą część rodziny, jako, że oni są w grupie podwyższonego ryzyka. tak samo ograniczyliśmy kontakty nawet z tą młodszą częścią rodziny, ale tą dalszą częścią rodziny, żeby po prostu niwelować szanse na rozsiewanie tego wirusa. A z moimi rodzicami i ta się co jakiś czas musiałem widzieć, żeby podrzucić psa na przechowanie, także nie uważam, żebyśmy tym spotkaniem świątecznym jakoś zwiększyli ryzyko czy zagrożenie dla kogokolwiek,

**Dopytuję, bo słyszałam, że pojawiają się takie głosy, że w sumie od dłuższego czasu wszyscy jesteśmy w jakiejś tam izolacji, więc spotkanie wielkanocne nawet w większym gronie nie niesie ze sobą ryzyka. Jak ty to widzisz?**

Niesie, ponieważ każdy jednak musi co jakiś czas zrobić zakupy i może stać się nawet tym bezobjawowym nosicielem i roznosić tę chorobę?

**Jak reagowali inni członkowie rodziny?**

Ze spokojem to przyjęli, bo wszyscy wiedzą, jaka jest sytuacja, wszyscy śledzą jakieś doniesienia medialne na pewno, więc myślę, że dla wszystkich to było zrozumiałe, że takie postępowanie w tym roku jest jedynym rozsądnym.

**Wspominałeś, że raczej nie pojawiły się nowe rzeczy. A może z czegoś zrezygnowałeś?**

Z niczego więcej nie zrezygnowałem, a jeśli chodzi o nowe zachowania, to jak jadę do sklepu, to biorę maseczkę, bo wszedł ten przepis, ale poza tym chyba nic się nie zmieniło.

**Czy pojawiły się jakieś nowe rzeczy, które ci przeszkadzają?**

Przeszkadzałby mi pewnie nakaz odgórny stosowania maseczki wszędzie, jak się tylko wychodzi z domu, ale obecnie mi niespecjalnie przeszkadza, ponieważ się do niego zwyczajnie nie stosuję, jak wychodzę np. z psem. Stosuję się do niego tylko jak jestem w sklepie i to jest oczywiste, ale jak wychodzę z psem na łąkę, to się do niego nie stosuję, bo uważam to za idiotyczne. Z nikim się tam nie spotykam, nie mam szans nikogo zarazić.

**A w drodze na tę łąkę?**

W drodze przechodzę przez swoją małą osiedlową uliczkę pod blokiem. Mam ze 20 m do tej łąki, więc maseczki nie zakładam. Mam taką bawełnianą chustkę na szyi w razie czego, jakby przypadkiem się pojawił patrol policji, żeby ją naciągnąć na nos. To tak na wszelki wypadek.

**Widziałeś w okolicy patrole policji?**

Nie widziałem, ale dostałem ostatnio ostrzeżenie od sąsiada, że się pojawili rzeczywiście w okolicach tej łąki.

**Na tej łące przyczepiliby się do ciebie, gdybyś był bez zasłoniętej twarzy, czy raczej nie?**

Myślę, że by się przyczepili, bo wiem, że się do innych przyczepiali. Do spacerujących tam z psami. To jest miejsce, gdzie wiele osób wychodzi z psami na spacer. Nie wiem, czy kończy się to mandatami/ upomnieniami. Nie wnikałem.

**Jakie działania podejmujesz, żeby radzić sobie z całą sytuacją? Jak jest teraz, a jak było 2 tyg. temu. Czy są jakieś zmiany?**

Nie, wydaje mi się, że żadnych więcej działań ja nie mogę podjąć. Tak samo dbam o higienę jak dbałem i nic w moim postępowaniu ani na plus, ani na minus się nie zmienia, wydaje mi się.

**A jakie emocje odczuwasz w związku z epidemią i jakie towarzyszyły ci w ciągu ostatnich 2 tygodni?**

Pojawia się na pewno zmęczenie całą tą sytuacją przedłużającą się, jakieś takie uczucie niepewności, co do tego, co będzie dalej, zwłaszcza z sytuacją gospodarczą, z wyborami prezydenckimi, które będą albo nie będą. Tak jak wcześniej, nie zacząłem odczuwać jakiegoś lęku związanego z samym ryzykiem zachorowania. Chyba tyle.

**Powiedziałeś: zmęczenie. Czy robisz coś, żeby minimalizować to zmęczenie?**

Nie wydaje mi się. [śmiech]

**A ta niepewność? Są jakieś sposoby, które pozwalają ci radzić sobie z tymi emocjami?**

To nie są emocje, z którymi ja mam jakiś duży problem, które by wywoływały we mnie jakiekolwiek napięcie utrudniające mi funkcjonowanie czy powiększające mój poziom stresu tak, żebym czuł jakikolwiek negatywny wpływ na moje życie tych uczuć. To są raczej uczucia, które się pojawiają po prostu, jak myślę o tej sytuacji i o tym, co będzie dalej, ale nie jest to coś co wymagałoby ode mnie jakiegoś zaangażowania w radzenie sobie z tym.

**Emocje - zdjęcia**

Znowu mi ta 9 najbardziej pasuje, jeśli mogę ją wybrać ponownie. Przedstawia burzę z trąbą powietrzną, jakiś rodzaj kataklizmu, a to, co mi się kojarzy z obecną sytuacją, to pustka na tym obrazku i właśnie jakieś takie zagrożenie związane z nadchodzącym żywiołem, na który nie bardzo mamy wpływ i jedyne co możemy zrobić, to spróbować się jakoś uchronić przed nim.

**Pustka i zagrożenie. Jeszcze jakieś emocje się z tym wiążą?**

To nie były emocje za bardzo, ale jeśli chodzi o emocje, to strach albo lęk, zdenerwowanie.

**Co ma wpływ na te emocje?**

Obecność zagrożenia, na które nie do końca mamy wpływ. To zagrożenie rzutuje na wiele sfer w naszym życiu.

**To zagrożenie towarzyszy nam już od jakiegoś czasu. Czy pojawiają się jakieś czynniki zewnętrzne, które mają wpływ na to, jak bardzo odczuwasz te emocje? Czy to się jakoś zmienia?**

Zmienia się trochę, jeśli chodzi o to co słyszę o sytuacji gospodarczej. Widać, że jesteśmy gospodarczo na coraz gorszej drodze i skutki tego mogą być bardzo długofalowe.

**To są informacje, wiadomości, które do ciebie docierają czy coś jeszcze?**

Przede wszystkim doniesienia medialne + informacje od rodziny czy od znajomych o sytuacji na rynku pracy, dosłownie braku tej pracy.

**Jak powinien wyglądać obrazek, który idealnie opisywałby twoje emocje z ostatnich 2 tygodni?**

Myślę, że byłby znacznie spokojniejszy. Może jakaś plaża i lekko zachmurzone niebo, ale poza tym spokój.

**Na ile czujesz się obecnie zagrożony sytuacją?**

2 na 10 powiedzmy.

**Czy robisz coś, żeby mniej odczuwać te wszystkie emocje, ten lęk, czy to jest tak samo, jak mówiłeś wcześniej, to są emocje, na które nie bardzo masz wpływ i które nie do końca ci jakoś bardzo przeszkadzają?**

Tak, to są emocje, które mnie bezpośrednio jakoś mocno nie dotykają, nie wpływają specjalnie na mój dobrostan, jeśli tak to mogę nazwać. Ja sam, jeśli chodzi o moje zdrowie nie czuję się specjalnie zagrożony, moja sytuacja zawodowa też jest aktualnie dosyć stabilna, więc na mnie to bezpośrednio nie wpływa jakoś mocno.

**A jak radzą sobie inni w twoim otoczeniu?**

W większości radzą sobie dobrze, aczkolwiek u wielu osób jest to powód do podwyższonego stresu. U niektórych znajomych z bardziej trywialnych powodów, typu po prostu brak kontaktów ze znajomymi - tych takich bardziej bezpośrednich. U innych to już są poważniejsze przyczyny związane z odwołaniem planów takich, jak ślub i wesele, a jeszcze innych to jest też spory stres związany np. z brakiem przychodów.

**Ten motyw odwołanego ślubu i wesela już się pojawiał. Czy te emocje i działania, które obserwujesz u swoich znajomych zmieniły się jakoś w ciągu ostatnich 2 tygodni?**

Nie mam okazji ich bardzo dobrze obserwować - tych zachowań, tych reakcji, no bo tyle, co tak naprawdę porozmawiamy przez jakiegoś Messengera czy Skype'a raz na jakiś czas. Specjalnych zmian nie zauważyłem. Zmiana może być taka, że może już trochę bardziej się oswoili z tą myślą, że niektóre plany muszą zrewidować no i jakoś na swój sposób na pewno próbują sobie z tym radzić, ale dokładnie jak, to nie umiem powiedzieć.

**Wspominałeś, że ostatnio widziałeś się z rodzicami. W ich zachowaniach i tym jak sobie radzą coś się zmieniło na przestrzeni ostatnich 2 tygodni?**

Myślę, że podobna jest u nich sytuacja. Tęż mają zmniejszony przychód spowodowany np. brakiem zleceń. Ale czy w ich podejściu coś się zmieniło? Wydaje mi się, że nie. Też już zdają sobie od dłuższego czasu sprawę z tej sytuacji, więc tylko mogą czekać aż to się trochę uspokoi i unormuje.

**Wg ciebie, jeśli chodzi o twoich rodziców, to jest sytuacja jakoś bardzo odmienna od tego jak reagowali i zachowywali się na samym początku tej całej sytuacji, czy to jest w miarę podobne?**

Jeśli chodzi o to jak mi dają odczuć jak reagują, to wydaje mi się, że bardzo podobnie, aczkolwiek nie wiem, jak reagują, jak mnie nie ma w pobliżu. Być może część stresu jest ukrywana, ale mam nadzieję, że nie.

**A gdybyś miał porównać swoje emocje i zachowania - ten moment teraz i moment, jak wszystko się zaczynało?**

Na pewno nie sądziłem wcześniej, że to tak długo potrwa i tak się będzie rozwijało. Myślę, że trochę ta niepewność i obawa związana z gospodarką we mnie narosła.

**Na razie nie masz za bardzo obaw co do swojej pracy i zatrudnienia. Czy myślisz, że w jakiejś dłuższej perspektywie to się może zmienić?**

W dłuższej może się zmienić. nikt z nas chyba nie może przewidzieć, czym się będzie zajmować za dobrych kilka lat, a na pewno to może rzutować na wiele, wiele lat, jeśli ta sytuacja wywoła jakiś poważny kryzys gospodarczy. Nie wiem np., czy jak za 2 lata obronię doktorat, to czy nie będę musiał szukać pracy w jakimś innym miejscu, czy to w prywatnej firmie, czy w innej instytucji. Są różne przewidywania ekspertów, że ten rynek pracy może się zmieniać z tzw. rynku pracownika, który przeważał ostatnio w stronę rynku pracodawcy, więc może być i ciężej znaleźć zatrudnienie, i oczywiście ciężej znaleźć zatrudnienie na dobrych warunkach, powrotu do zatrudnienia na śmieciówkach.

**Myślisz, że w tym sektorze, w którym jesteś umowy śmieciowe są zagrożeniem?**

W moim sektorze nie ma wielu umów śmieciowych. Jednak w PANie większość osób pracuje na umowach o pracę. To jest państwowa instytucja, więc nie sądzę, żeby to się zmieniło, ale w sektorze prywatnym wydaje mi się to bardzo prawdopodobne. Bardziej bym się spodziewał dużej redukcji stanowisk w instytucjach państwowych niż śmieciówek.

**O jakich zmianach w obostrzeniach ostatnio słyszałeś?**

Ostatnie zmiany, to było najpierw wprowadzenie noszenia maseczek wszędzie, we wszystkich miejscach publicznych poza lasami, które znowu zostały otwarte. I to chyba wszytko, nie wiem, czy jakieś jeszcze zmiany pamiętam...Nie wiem, czy nie mają być wprowadzane zmiany, jeśli chodzi o liczbę kupujących w sklepach...Chyba ma być przelicznik nie osoby na liczbę kas, ale osoby na powierzchnię sklepu, ale nie śledziłem tego dokładnie.

**Jak odnosisz się do tego, że znowu można chodzić do lasu, że tam nie trzeba tej maseczki?**

Uważam, że jest to sensowna zmiana, bo ograniczanie wstępu do lasu nikomu nie służyło. To jest akurat miejsce, gdzie łatwo zachować szeroką bardzo przestrzeń osobistą, tak, żeby nie stwarzać dla nikogo zagrożenia ani żeby też samemu nie czuć się zagrożonym. Uważam, że jak ktoś ma do wyboru przespacerować się po lesie a przespacerować się po ulicy, to lepiej, żeby wybrał las.

**Jak ci się wydaje, na ile nakaz noszenia maseczek ma realny wpływ na ograniczenie epidemii?**

Sam nakaz noszenia maseczek w miejscach publicznych jest ok, aczkolwiek moim zdaniem jest to za szeroko wprowadzone. Jest to zasadne np. w sklepach, w miejscach, gdzie to zagęszczenie ludzi jest większe, ale właśnie w jakichś parkach, lasach, na łąkach uważam, że jest to zupełnie niepotrzebne.

**A jak się czujesz z tym, że jest ten nakaz noszenia maseczki nawet w parku?**

No ja uważam, że jest to idiotyczne i niczemu to nie służy. W niektórych przypadkach może być nawet szkodliwe, bo jeżeli się dłużej taką maseczkę nosi, a ona wilgnie, to może zbierać całą masę innych drobnoustrojów. Wirusy grypy, które normalnie nie wywołałyby choroby w takiej ilości, a jak się zbiorą, to może ta ich liczba wzrosnąć na tyle, żeby jakieś zakażenie wywołać, więc uważam, że jest to zwyczajnie głupie.

**Rozumiem, że widzisz zagrożenie dla osoby, która nosi taką maseczkę, a dla otoczenia?**

Dla otoczenia, jeśli się zachowuje odpowiednią odległość pomiędzy ludźmi to też większego zagrożenia przy braku maseczki nie ma i ta maseczka w żaden sposób tego bezpieczeństwa nie zwiększa.

**Jakie emocje to w tobie wywołuje, że postanawiasz nie nosić maseczki?**

Ogólnie z natury nie lubię robić rzeczy, które uważam za bezsensowne, więc z tą maseczką to też podejrzewam, że być może z czystej przekory. Jak idę na wspomnianą łąkę to jej nie zakładam. Poza tym, że byłoby to niewygodne, to oczywiście jakiegoś prawdopodobnie negatywnego wpływu by to na mnie nie miało, ale jako, że uważam, że jest to idiotyczne to tego nie robię. Może trochę kontestuję nie nosząc maseczki.

**Powiedziałeś: Niewygodne. Co jest w tym najbardziej niewygodnego?**

Gorąco się robi pod tą maseczką, jak dłużej się ją nosi, te gumki się w uszy wpijają. mam doświadczenie z maseczkami w pracy, jak je muszę zakładać do jakiejś operacji i jak przez godzinę, dwie w czasie operacji mam ją na twarzy, to oczywiście jest to konieczne, ale jest to bardzo nieprzyjemne i niewygodne. Jak nie muszę tego zakładać i nie czuję, że jest to potrzebne, to wolę tego nie robić.

**Powiedziałeś, że jeśli zachowujemy odstęp, to otoczenie jest bezpieczne, a co w miejscach publicznych?**

Nie wiem, jaka jest dokładnie definicja prawna miejsca publicznego, więc nie wiem na ile dokładnie można to w przepisach zdefiniować, ale uważam, że zasadny może być nakaz noszenia maseczek właśnie we wszelkich sklepach, w komunikacji miejskiej, być może właśnie na ulicach. Na pewno bym z tego wyłączył wszelkie parki, lasy, bulwary, tereny niezagospodarowane.

**Dlaczego w tych miejscach noszenie maseczki byłoby sensowne, jeśli założyć, że wszyscy zachowują od siebie odstęp?**

W tych miejscach czasem jest niemożliwe zachowanie tego odstępu i czasem nie mamy na to wpływu, że zagęszczenie ludzi jest zbyt duże. Jako, że nie mamy na to wpływu, nie możemy być tego pewni, to ten nakaz noszenia maseczki może być sensowny wtedy. Gdyby można było wszędzie zachować odpowiednią odległość, to wtedy maseczki byłyby niepotrzebne.

**Czy słyszałeś coś na temat nowych zasad w handlu, liczby osób w sklepie?**

Wcześniej był przepis dotyczący dozwolonej liczny osób w stosunku do liczby kas i to się miało albo ma się zmienić na przeliczenie względem powierzchni sklepu. Ogólnie to ograniczenie liczby osób w sklepie jest całkiem sensowne, bo jest to działanie ograniczające zagęszczenie ludzi, więc tym samym zmniejsza ryzyko rozprzestrzeniania się wirusa. Czy ta zmiana liczby kas na powierzchnię sklepu coś zmienia? Nie potrafię powiedzieć, jak to wpłynie na liczbę ludzi w sklepie, bo nie widziałem takich wyliczeń, a nie potrafię sobie w głowie wyobrazić, jaka może być powierzchnia sklepu, a ile musi być tam kas. Nie wiem, na ile to jest sensowna zmiana.

**Możliwość przemieszczania się w celach rekreacyjnych przy zachowaniu zasad typu maseczka. Coś jeszcze na ten temat przychodzi ci do głowy?**

Ogólnie uważam, że przy zachowaniu tych odstępów nie powinno być ograniczeń, jeśli chodzi o przemieszczanie się w celach rekreacyjnych, a trzeba to robić, bo ma się psa czy po prostu chce się wyjść na spacer dla poprawy samopoczucia. Czy na spacer, czy na rower, czy na rolki.

**Cały czas wyłączone są place zabaw. Co sądzisz na ten temat?**

Plac zabaw może być całkiem sensownym wyłączeniem, bo zazwyczaj place zabaw mają dosyć ograniczoną powierzchnię, dzieci może być tam jeszcze dosyć sporo, a wytłumacz dzieciom, żeby się do siebie nie zbliżały, a one oczywiście mogą być [ns] tego wirusa.

**Siłownie zewnętrzne też były pozamykane. Wiesz może, czy to się zmieniło?**

Nie wiem.

Gdyby były dalej zamknięte, to jest to dobra decyzja?

Nie, wydaje mi się, że jednak dorosłym ludziom można wytłumaczyć jak bezpiecznie korzystać z takiej siłowni, bo przed użyciem danego sprzętu można go czymś po prostu odkazić tak samo jak się odkaża ręce. Oczywiście przed skorzystaniem z tego też należałoby odkazić ręce i wtedy wydaje się to dosyć bezpieczne.

**W czyjej gestii powinno być to odkażanie? Każdy powinien mieć swój płyn czy państwo powinno o to zadbać?**

Idealnie by było oczywiście, gdyby państwo o to dbało, ale w Polsce jest to zupełnie nierealne, więc myślę, że raczej każdy musiałby samodzielnie o to dbać. Patrząc na to, jaki jest popyt na te płyny do dezynfekcji rąk i jak ludzie rzeczywiście przejmują się tym zagrożeniem epidemicznym, to wydaje mi się, że rzeczywiście w zdecydowanej większości dbaliby o to.

**1 osoba na 15 m w kościele. Jak do tego mógłbyś się odnieść?**

Teoretycznie, jeśli byłoby to przestrzegane, to uważam, że jest to w miarę bezpieczne. Warto by było wszystkie ławy i miejsca, których się dotyka rękami odkażać po każdej mszy czy po każdej osobie, która tam siedziała. Oczywiście należałoby zlikwidować w takiej sytuacji wodę święconą, bo to jest dobre miejsce na roznoszenie się wirusa - takie naczynko z wodą, w której każdy macza rękę i potem tą ręką dotyka sobie do twarzy. No i oczywiście wszelkie środki higieny związane z podawaniem opłatka wiernym. Mimo mojego dosyć antyklerykalnego podejścia, to myślę, że to byłoby w miarę bezpieczne.

**Wydaje ci się, że to jest realne, żeby zachować te wszystkie zasady, stosować się do nich w takim miejscu?**

W 100% nigdy nie będzie realne, bo już przy wcześniejszych obostrzeniach były przypadki dosyć ostrego łamania zakazów, ale wydaje mi się, że w większości kościołów dałoby się to jakoś przeprowadzić.

**A jeżeli chodzi o możliwość samodzielnego przemieszczania się osób powyżej 13 lat?**

Wydaje mi się, że to jest sensowna zmiana, zapomniałem o niej. Jak najbardziej uważam, że osoby powyżej 13 r.ż. są już, przynajmniej zazwyczaj w miarę rozsądne i są w stanie przestrzegać tych wszystkich przykazów związanych z profilaktyką rozprzestrzeniania się wirusa.

**Czy może do któregoś jeszcze z nakazów masz zastrzeżenie i nie wiesz, czy chciałbyś się do niego stosować lub nie stosujesz się?**

Chyba cały czas jest utrzymany ten nakaz utrzymania odległości 2 m pomiędzy spacerującymi, niezależnie od tego, czy mieszkają razem, czy są rodziną, czy nie. To też jest idiotyczne. Jeśli nawet z kimś mieszkam, to przebywając z nim na zewnątrz mam zachować od tej osoby odstęp.

**A przemieszczanie się w celach rekreacyjnych w maseczce. Czy zdarza ci się teraz wychodzić na rower, pobiegać? Czy wtedy zakładasz maseczkę?**

Do tej pory mi się nie zdarzyło. Akurat nie naszła mnie ochota na jazdę na rowerze, a za bieganiem nie przepadam, ale zarówno, jeśli chodzi o rower czy o bieganie, to w tej sytuacji nie zakładałbym maseczki. Wziąłbym pewnie na wszelki wypadek znowu chustkę na szyję. Na wypadek zobaczenia gdzieś na horyzoncie patrolu policji, ale nie miałbym jej na twarzy cały czas, bo nie jest to do niczego potrzebne, a zwłaszcza przy zwiększonym wysiłku to jednak utrudnia oddychanie i myślę, że dawałoby się we znaki.

Czy te zasady, przepisy determinowałyby wybór miejsca, do którego planowałbyś się wybrać, żeby uprawiać sport?

Na pewno bym preferował miejsca, w których bym się nie spodziewał ludzi albo spodziewałbym się ich jak najmniejszej liczby, ale niezależnie od zagrożenia koronawirusem tak samo bym w normalnej sytuacji preferował takie miejsca na ten rodzaj aktywności.

**Słyszałeś o planach luzowania ograniczeń?**

Słyszałem, że są przymiarki do tego, żeby te ograniczenia stopniowo zdejmować.

**Na które zwróciłeś uwagę?**

Wiem, że były plany, żeby zdejmować ograniczenia z kolejnych sklepów, np. odzieżowych, z jakichś zakładów fryzjerskich czy kosmetycznych. To chyba nie zostało jeszcze zapowiedziane, ale wiem, że są petycje wielu klubów sportowych, żeby z nich też ograniczenia zdejmować albo przynajmniej poluzować. Chyba o tych słyszałem, póki co.

**Jak oceniasz te plany?**

Żeby to móc w pełni racjonalnie ocenić, to uważam, że ktoś musiałby mieć dostęp do trochę większej wiedzy na temat aktualnego stanu polskiej służby zdrowia, jej wydolności i tego, na ile ona sobie może poradzić z jakimś wzrostem liczby zachorowań. Jeśli jest taki zapas wydolności służby zdrowia, to uważam, że te obostrzenia można luzować. Jeśli jesteśmy w momencie, że nasza służba zdrowia nie wyrabia, to zdejmowanie tych ograniczeń może być bardzo złym pomysłem.

**Jak to powinno wyglądać? Co powinno w 1-szym etapie być otworzone, co powinno dłużej być zamknięte?**

Najtrudniej jest wyważyć te potrzeby, które są całego społeczeństwa, a też wziąć pod uwagę potrzeby np. osób prowadzących restauracje, kawiarnie, itd. Dla ogółu społeczeństwa uważam, że nie jest niezbędne to, żeby wejść do restauracji, kawiarni, tam usiąść i coś zjeść, jeśli można skorzystać z opcji dostawy do domu albo wziąć coś na wynos, ale wiem, że to się wiąże ze znacznie mniejszymi przychodami dla właścicieli tych miejsc i ich pracowników, z ryzykiem zwolnień, bankructw czy zamykania ich działalności. Wydaje mi się, że to jest bardzo ciężko wyważyć, w którym momencie zdjąć te obostrzenia. Tak samo z jakimiś sklepami odzieżowymi, GH. Z jednej strony przez jakiś czas nie są to niezbędne rzeczy, aczkolwiek z czasem coraz częściej będą się pojawiały sytuacje, że ktoś rzeczywiście potrzebuje kupić coś do ubrania i już nie może dalej chodzić w jakichś zniszczonych rzeczach. Tu też powoli trzeba te ograniczenia zdejmować i też brać pod uwagę sytuację właścicieli i pracowników tych firm.

**Powiedziałeś wcześniej, że należałoby wziąć pod uwagę stan służby zdrowia i oprócz tego jeszcze sytuację gospodarczą?**

Sytuacja gospodarcza jest coraz bardziej paląca, ale podstawą jest ochrona zdrowia, bo jeśli to padnie, to czekają nas potem ponowne obostrzenia, pewnie jeszcze większe i tylko pogłębianie się tego kryzysu.

**Czyli, jeżeli za szybko zdejmiemy ograniczenia, to może to spowodować, że później jeszcze dłużej będziemy musieli pracować nad tym, żeby gospodarczo było lepiej?**

Tak.

**Które z obecnych ograniczeń powinny zostać dłużej, a które najpierw powinny być znoszone?**

Oczywiście w pierwszej kolejności zniósłbym obowiązek noszenia maseczek na terenach niezagospodarowanych, w lasach parkach i bezwzględny obowiązek zachowania 2 m odległości. Wydaje mi się, że mniej więcej jednocześnie można by było znieść ograniczenia dotyczące sklepów, zakładów fryzjerskich, kosmetycznych czy klubów sportowych. Może nie całkowite zniesienie, ale przynajmniej zmniejszyć te obostrzenia z zachowaniem np. jakiejś maksymalnej liczby osób w takich miejscach. 2 osoby na zakład fryzjerski i jakaś liczba osób na powierzchnię klubu sportowego. Pomału, jeszcze z jakimiś ograniczeniami, ale już pomału je otwierać, żeby jednak tę sytuację nieprzyjemną finansowo dla osób związanych z tymi branżami niwelować.

**Te 2 m odstępu, to masz na myśli osoby mieszkające razem, czy w ogóle zniesienie tych 2 m?**

Nie, wobec osób, które mieszkają razem.

**Które obostrzenia powinny zostać najdłużej?**

Wydaje mi się, że wszystkie, które dotyczą zmniejszania zagęszczenie ludzi, czy to w komunikacji miejskiej, czy właśnie w sklepach. Trudno mi powiedzieć, w którym momencie i które z nich powinny być znoszone i czy wszystkie naraz czy nie, bo do określenia tego na pewno jest potrzebne grono specjalistów z różnych dziedzin. Dopiero wtedy można racjonalnie taką decyzję podjąć. Ja się nie czuję kompetentny, żeby powiedzieć więcej na ten temat.

**A jak odnosisz się do otwierania szkół?**

To jest trudna bardzo sytuacja. Może dobrym rozwiązaniem byłoby wprowadzenie na okres przejściowy jakiegoś systemu zmianowego w szkołach, żeby jakoś ograniczyć zagęszczenie uczniów, ale wydaje mi się, że jest to, póki co nierealne, bo wiem, że już w wielu szkołach konieczne było prowadzenie lekcji w systemie dwu lub nawet trzyzmianowym.  Wydaje mi się, że do wakacji przynajmniej może być zasadne utrzymanie zamknięcia szkół. Jest to fatalna sytuacja dla uczniów, ale może być konieczna.

**Do wakacji, a co później?**

To zależy wszystko od wskaźników. Od tego, jak ta epidemia będzie się rozwijała - czy ta liczba zachorowań będzie rosła, w jakim tempie będzie rosła, jak duży procent społeczeństwa miał już okazję zetknąć się z tym wirusem, bo to też można policzyć, jaki jest poziom odporności zbiorowej. On z czasem będzie narastał. Być może w oparciu o te dane sensownym by było np. skrócenie teraz roku szkolnego i wcześniejsze rozpoczęcie go.

**Co wg ciebie powinno być ważniejsze - otworzenie sektora związanego z gospodarką, czyli sklepy, usługi, czy jednak szkoły?**

Patrząc długofalowo, to oczywiście otwarcie szkół, ale ze względu na to przeludnienie, które jest w ogromnej liczbie szkól, jest to o wiele trudniejsze do zrealizowania. Od dobrego szkolnictwa, gdybyśmy takie mieli oczywiście, długofalowo zależy rozwój całego państwa. To kolejne pokolenia, które będą musiały się zarządzaniem państwem zajmować, czy wszelkimi branżami związanymi z gospodarką czy nauką, więc ważne jest zapewnienie jak najlepszej edukacji. Wiem, że teraz, mimo wszelkich starań do nauki zdalnej, wielu uczniów nie ma dostępu, albo nie ma dobrego dostępu do tej zdalnej edukacji i jest to fatalna sytuacja.

**Jeżeli uczniowie będą fizycznie w szkole, to jest szansa, że jakość tego kształcenia będzie wyższa?**

Wydaje mi się, że tak. Przede wszystkim ze względu na ten obecnie nierówny dostęp do tej zdalnej edukacji. Jednak nie wszyscy mają dostęp do komputerów, do wystarczająco szybkiego internetu, w niektórych domach jeden komputer musi być dzielony pomiędzy wiele osób czy dzieci. Dlatego nie wszyscy mają zapewniony równy dostęp do tej edukacji.

Słyszałeś, w jaki sposób Szwecja radzi sobie z pandemią?

Słyszałem, że Szwecja podjęła decyzję, żeby zminimalizować wszelkie restrykcje, aczkolwiek to jest chyba też kwestia tego, jak funkcjonuje szwedzkie społeczeństwo, ponieważ oni mimo braku wprowadzenia jakichś prawnych obostrzeń są, z tego, co słyszałem, bardzo skrupulatni, jeśli chodzi o wszelkie środki profilaktyki. Same zalecenia, w przypadku ich społeczeństwa wystarczą do tego, żeby w naprawdę istotny sposób ograniczyć ryzyko zakażenia właśnie przez ograniczenie spotkań ze znajomymi, rodziną, home office. Wszystkie te rzeczy, które my robimy. Mimo to wskaźniki zachorowań są tam wyższe znacznie niż w Polsce, ale ponieważ mają znacznie lepszą opiekę zdrowotną, mogą sobie z tym też dużo lepiej radzić. Uważam, że ich podejście jest całkiem racjonalne, dość sensowne, ale w polskich warunkach nie do zrealizowania.

**Dobre jest to pozostawanie na poziomie rekomendacji zamiast zakazów?**

W przypadku takiego społeczeństwa, jak stawiane często za wzór społeczeństwa skandynawskie, to jest dobra droga. Pozwala to zminimalizować wszelkie negatywne skutki przede wszystkim gospodarcze. Wiąże się to z trochę wyższymi wskaźnikami zachorowań, ale radzi sobie z tym znacznie lepsza opieka zdrowotna.

**Byłoby to do wykonania w Polsce?**

Nie. Raz, że polskie społeczeństwo wydaje mi się, że w tym stopniu nie byłoby w stanie podporządkować się rekomendacjom, a nawet gdyby było, to w przypadku tak fatalnej opieki zdrowotnej, jaka jest w Polsce, to każdy wzrost tych wskaźników zachorowań mógłby być tragiczny. W takim państwie jak Polska szczególnie ważne jest trzymanie tych wskaźników zachorowań na jak najniższym poziomie.

**Gdyby u nas były tylko rekomendacje, to nikt by sobie z tego niczego nie robił?**

Nie, uważam, że wiele osób by się do tych rekomendacji dostosowało, ale istotnie mniej niż w przypadku, gdy jest to zakaz.

**A czy wiesz, jaka jest różnica pomiędzy kwarantanną a izolacją?**

Wydaje mi się, że kwarantanna jest związana z obowiązkiem pozostania w domu pod groźbą jakichś represji finansowych i kwarantanna jest nakładana przez Sanepid. Nie wiem, czy jest jakiś prawny zapis związany z izolacją, czy jest to bardziej pojęcie potoczne w tym przypadku.

**Hasło "zostań w domu" to jest?**

Jest to izolacja.

**Jak w obecnym czasie wygląda u ciebie dbanie o siebie?**

Zmieniło się to, że zamiast pójść do fryzjera musiałem poradzić sobie ze swoimi włosami samodzielnie za pomocą maszynki, czego rezultaty były znacznie gorsze niż by były w przypadku wizyty u fryzjera. Nie chodzę do pracy, więc mniej regularnie się golę. Jest to dla mnie pozytywna zmiana, że nie muszę tego robić tak często. Poza tym większość czasu spędzam w ubraniach, które może są mniej wyjściowe, ale za to są wygodne.

**Kupiłeś maszynkę czy miałeś ją w domu?**

Miałem. Dawno, dawno temu zdarzało mi się przycinać sobie włosy samodzielnie, aczkolwiek już od paru lat tego nie robiłem. Zawsze to znacznie gorzej wychodzi niż jak zrobi to profesjonalista. Zawsze są gdzieś nierówno ścięte.

**A jak w przypadku twojej dziewczyny. Coś się zmieniło?**

Nie wydaje mi się, żeby w jej przypadku to się jakoś istotnie zmieniło. Nie korzystała z tego jakoś specjalnie często. Z racji tego, że też siedzi na home office, to nie musi się rano malować przed pracą, ale jeśli chodzi o resztę, to się to nie zmieniło. W dalszym ciągu dba o siebie.

**A jeśli chodzi o ubiór? Ty powiedziałeś, że chodzisz w trochę wygodniejszych ubraniach?**

Ona tak samo.

**Zdarza się teraz chodzić dłużej albo częściej w piżamie?**

W piżamie akurat nie, ale w takich ubraniach przeznaczonych tylko do domu i na wyjścia z psem.

**Coś się zmieniło, jeśli chodzi o kosmetyki?**

Nie. Wydaje mi się, że kupujemy dokładnie te same kosmetyki, tak samo często i tutaj nic się nie zmieniło. Jeśli chodzi o zmianę, to z racji, że siedzimy na home office, to w warunkach domowych nie używamy perfum, ale jeszcze i tak nie minęłoby w tej naszej izolacji tyle czasu, żeby w normalnej sytuacji mogły nam się te perfumy skończyć, więc tak czy siak nie mielibyśmy potrzeby ich kupowania. Ale być może zaoszczędziliśmy trochę w ten sposób.

**Gdzie zazwyczaj kupowaliście kosmetyki?**

Albo w sklepie typu Rossmann albo przy okazji zakupów spożywczych w dowolnym supermarkecie. Te miejsca się też nie zmieniły.

**Zacząłeś sam przycinać włosy. Czy jeszcze coś nowego w zwyczajach się u was pojawiło?**

Nic mi nie przychodzi do głowy.

**A coś, z czego zrezygnowaliście?**

Nic, poza tym, o czym mówiłem.

**A jeśli chodzi o paznokcie, bo wiele kobiet korzystało z profesjonalnych usług?**

Moja dziewczyna akurat nie malowała paznokci i nie korzystała z takich usług, więc to się nie zmieniło.

**Chodzenie w wygodniejszych ubraniach - czy poza aspektem wygody, przemawia za tym coś jeszcze?**

Wydaje mi się, że chodzi w 100% o wygodę.

**Czy brakuje ci tych wizyt u fryzjera. Czy poza tym, że było ładniej, to coś jeszcze stało za takimi wizytami?**

Nie, raczej dla mnie to była zawsze konieczność. Raczej niedogodność, że musiałem pójść do tego fryzjera niż to, że jakoś chciałem, albo czerpałem z tego przyjemność. Na szczęście włosy muszę przycinać dosyć rzadko, więc nie jest to problem.

**Czy teraz kupujecie jakieś ubrania w związku z nową porą roku?**

Czy ja coś kupowałem? Nie wiem, czy w związku z porą roku, ale kupiliśmy przez internet buty, bo akurat były w promocji no i wiem, że Daria się przymierza do zakupu jakiejś kurtki bardziej wiosennej. To chyba tyle.

**Wcześniej też kupilibyście te rzeczy przez internet czy stacjonarnie?**

Pewnie przez internet, bo tu akurat chodziło o atrakcyjną cenę.

Brakuje ci stacjonarnych sklepów, jeśli chodzi o ubrania?

Niespecjalnie.

**Internet jest w stanie zupełnie zastąpić takie sklepy?**

Zupełnie nie, ponieważ jednak wiele rzeczy...Może w dobie tego, że możemy wszystko zwracać bezpłatnie i nie ma konieczności wizyty w sklepie i przymierzania, to w wielu przypadkach jednaj kupienie w sklepie jest po prostu o wiele wygodniejsze, więc wydaje mi się, że jednak internet nie jest w stanie całkiem zastąpić sklepów.

**Kupiliście, bo atrakcyjna cena, ale czy coś jeszcze stało za decyzją tego zakupu?**

Uzasadnienie było takie, że akurat nam obojgu zwyczajnie przydałyby się tego typu buty, bo to akurat trampki były i to jest całe uzasadnienie.

**Z punktu widzenie bycia konsumentem, czego ci teraz najbardziej brakuje?**

Brakuje mi na pewno wyjść do kina, możliwości wyjścia do pubu ze znajomymi i brakuje mi dostępności siłowni.

Jaką funkcję pełniły te 3 miejsca dla ciebie?

Jeśli chodzi o kino, to po prostu czysta rozrywka i jeden ze sposobów spędzania wolnego czasu przede wszystkim w weekendy. Puby to możliwość spotkania się ze znajomymi i czasem też możliwość spróbowania jakiegoś nowego piwa. Siłownia czy klub sportowy to po prostu miejsce, do którego chodziłem, żeby jakoś dbać o swoją kondycję i o swoje zdrowie.

**Czym się różni wyjście do kina od obejrzenia filmu w domu?**

To nie jest to samo jednak. Trochę innych emocji dostarcza jednak wizyta w kinie i obejrzenie tego filmu np. niedługo po premierze, na wielkim ekranie w ciemnej dobrze nagłośnionej sali. I jednak obejrzenie tego samego filmu w domu nawet na dużym i dobrym telewizorze jakoś...Nie umiem nawet nazwać tych emocji, których nie ma, ale ich nie ma.

**Oglądasz teraz więcej filmów niż przed epidemią?**

Nie, wydaje mi się, że podobną ilość porównując do tego, co oglądałem wcześniej i w domu, i w kinie.

Teraz jakoś starasz się jakoś kompensować sobie brak kina oglądając filmy w domu?

W pewien sposób jest to na pewno kompensowane. Nie chodziłem do tego kina jakoś bardzo często, ze 2 x w miesiącu, więc nie jest trudno to nadrobić.

**Wspomniałeś wcześniej o aspekcie premiery. Zdarzało ci się często chodzić na pokazy premierowe? To było dla ciebie ważne?**

Premierowe nie, bo nie lubię tłumów, ale zdarzało mi się chodzić w miarę niedługo po premierze i po prostu czekać na premierę i żeby ten film wreszcie zobaczyć. Zwykle tak czekałem kilka dni od premiery i wtedy planowałem to wyjście do kina.

Teraz rozumiem, że obejrzenie filmu szybko od premiery jest utrudnione?

Zdecydowanie, ale zauważyłem, że już wielu dystrybutorów filmowych wychodzi temu naprzeciw i bardzo szybko te nowe filmy są wydawane w jakimś systemie on demend, więc jakoś staje się to w miarę dostępne. Oprócz tego mamy Netflix czy HBO Go, z których korzystamy.

**Te platformy były już u was przed epidemią, czy to jest coś nowego?**

Były dużo wcześniej i już od długiego czasu z nich korzystamy.

**A czy może pojawiły się jakieś inne pakiety?**

My telewizji zwykłej nie mamy i nie mieliśmy żadnej możliwości rozszerzania. Gdybym miał możliwość, to bym polepszył internet, ale niestety nie mam takiej możliwości.

Brakuje ci wyjść do pubu ze znajomymi. Czy zdarzają ci się takie spotkania online?

Tak, zdarzyło mi się ze 3 razy do tej pory. Jest to jakiś przyjemny sposób na spotkanie w takiej sposób, ale nie można tego porównywać do spotkania na żywo. Jest to jednak zupełnie inny rodzaj kontaktu i raczej te spotkania trwają np. 1.5 godz. a nie 3-4, jak to by miało miejsce w przypadku spotkania się gdzieś na mieście.

**W jak dużym gronie zdarzało wam się spotykać fizycznie i czy to grono różni się teraz?**

Online się spotykam w trochę innym gronie...Tzn. w tym gronie, z którym się spotykam online, też zdarzało nam się spotykać na żywo, aczkolwiek na żywo częściej się spotykałem z innymi osobami, z którymi nie spotykamy się jednocześnie online.

**Brakuje ci tych spotkań?**

Trochę tak.

**Siłownia to był aspekt dbania o siebie. Jakoś to sobie rekompensujesz?**

Zdarza mi się ćwiczyć w domu, aczkolwiek jest to znacznie mniej regularne, znacznie mniej intensywne i jest to niestety tylko taki słaby substytut.
