# Supplementary material for: The challenges arising from the COVID-19 pandemic and the way people deal with them. A qualitative longitudinal study
Source: PLoS One. 2021 Oct 11;16(10):e0258133. doi: 10.1371/journal.pone.0258133 (PMC8504766; doi:10.1371/journal.pone.0258133)
Supplement: S1 Dataset — (ZIP) [file pone.0258133.s003.zip › Transcriptions/stage 3/4.3_M_32_couple, no children.docx]

**4.3_M_32_couple no children**

**Jak wyglądały te 2 tygodnie ostatnie u ciebie, co się działo?**

Niedużo właściwie chyba. Muszę sobie przypomnieć. Ostatni raz widzieliśmy się chyba w poniedziałek 6 kwietnia, tak? Albo we wtorek, siódmego.

**To było przed Wielkanocą jeszcze.**

No tak, tak. No to dużo się nie działo. Wielkanoc spędziliśmy w domu z żoną. Nie spotykaliśmy się z rodziną, poszliśmy tylko do teściowej. I właściwie, ona ma ogródek, też mieszka w bloku, ale ma ogródek, to tak spotkaliśmy się, że tak powiem, trochę tak w ogródku przez balkon. Znaczy ona wyszła do nas, my weszliśmy do ogródka, ona wyszła na balkon. I chwilę porozmawialiśmy, tak, żeby sobie złożyć życzenia świątecznie. Ale to było całe nasze spotkanie. I zdzwanialiśmy się na Messengerze przy śniadaniu Wielkanocnym. I to chyba wszystko. I tak dziać to się dużo nie działo u mnie, z takich rzeczy.

**A jeśli chodzi o Wielkanoc, pamiętam, że mówiłeś, że nie za bardzo ci się podoba ten pomysł, żeby się jednak nie spotykać.**

Tak. W sensie, że ja byłem za tym, żeby… No nie tak, że zupełnie stwierdzić, że olać wszystko i tam normalnie je przeprowadzić całkowicie. Ale żeby się z tymi najbliższymi jednak spotkać, co najmniej w takim mikro gronie. I po prostu z tych zaizolowanych fragmentów po prostu połączyć kilka tych kręgów, tych klastrów. Ale tutaj dziewczyny zdecydowały inaczej. W sensie moja żona, teściowa i szwagierka. No bo moi rodzice są we trójkę z moją siostrą młodszą i babcią, spędzali czas. A moja siostra starsza ze swoim mężem i dziećmi. To ich jest też czwórka, no to też jest to już jakieś grono. I są z jednego gospodarstwa. A my tak siedzieliśmy trochę sami. A jeszcze bardziej myślałem o tym, że jeszcze bardziej same będą siedzieć moja szwagierka i teściowa. I dlatego tak myślałem, że może jednak warto się spotkać. Zwłaszcza, że my okazyjnie tam przy wymianie zakupów itd. też mamy jakiś ze sobą śladowy kontakt. Więc uznawałem, że to nie ma takiego znaczenia. I głosowałem za tym, żeby, namawiałem je, żebyśmy się po prostu spotkali przy tym śniadaniu. Już uznając, że jest to jakieś ryzyko, ale ponieśmy je i trudno. No, ale one się nie zdecydowały na to. Więc spędzaliśmy, tak jak spędzaliśmy, tak jak przed chwilą mówiłem.

**A jak wyglądało to łączenie się? Znaczy w którym momencie się połączyliście?**

No tam mamy jakieś godziny umówione, że 9 czy 10 jak zaczynaliśmy śniadanie, to po prostu… No, chcieliśmy, żeby była właśnie jakaś odświętność, żeby to był taki trochę dzień inny niż zwykle. Bo jak się nie wychodzi, to też człowiek by cały czas biegał w dresie i też zjadł sobie tylko tosty na śniadanie. Więc, no powiedzmy, że nie szliśmy do kościoła, nie ma tego spotkania z rodziną, więc już niedużo jest tych świątecznych elementów, znacznie mniej. Więc chociaż żeśmy zadbali o to, żeby te… My zwykle nie przygotowujemy wigilii, Wielkanocy, trochę pasożytujemy na innych częściach rodziny. Bo rzadko się u nas odbywa coś i nie ma jakichś większych możliwości. Poza tym chyba to jest też takie typowe, że się starszych członków rodziny zwykle odwiedza i do nich przychodzi, oni mają większą potrzebę robienia różnych rzeczy. No a w tym roku na nas przypadło to, więc żeśmy ugotowali parę takich typowych wielkanocnych potraw tradycyjnych, jakiś tam obrus położyli na stole, komputer. Połączyliśmy się na Messengerze. I gadaliśmy, jedząc jakieś tam jajko i rzeżuchę itd.

**Czyli jakoś wam się udało mimo wszystko tą odświętność Wielkanocy zachować w taki sposób, że powiedziałeś, że się po prostu ubraliście w coś innego niż dres, tak?**

No tak w cudzysłowie. Bo też się ubieramy w inne rzeczy niż dres. Staramy się, bo to już człowiek całkowicie był, fatalnie by było, gdyby tak chodził cały czas w piżamie. Ale tak, ubraliśmy się trochę odświętnie. Trochę też żartując sobie, że to są właśnie święta przez Skype, więc ja ubrałem koszulę i krótkie spodnie. Bo, tak jak są te memy albo jakieś inne, że tam rozmowa o pracę, nie? Przez Skype, to że człowiek tutaj garnitur, marynarka, krawat i wygląda profesjonalnie. I dół ma od piżamy. Bo tego już nie widać. No i na takiej samej zasadzie ja, śmiejąc się, że właśnie to są takie święta przez Skype, więc też się tam ubrałem. Ale to w ramach raczej dowcipu i gagu itd. Natomiast jakiś rodzaj odświętności był.

**Powiedz mi jeszcze, czy w ciągu tych ostatnich 2 tygodni coś się w twoim takim codziennym życiu zmieniło?**

No od wczoraj się zmieniło to, że poluzowali nam troszeczkę, te przepisy się delikatnie zmieniły. I są te poluzowania w kwestii bycia na zewnątrz, przemieszczania się itd. Więc od wczoraj nastąpiła ta zmiana, z której skwapliwie korzystamy. Właśnie teraz wróciliśmy, staraliśmy się zmieścić jeszcze przed tym wywiadem, na długim spacerze byliśmy. I to jest dla nas bardzo pozytywna, przyjemna zmiana, bo pogoda jest sprzyjająca. I fajnie jest pójść do parku. To zupełnie jest inaczej niż cały czas siedzieć trochę przytłoczonym w domu. A wczoraj byłem, moja żona pracowała, więc nie mogła, ale ja pojechałem sobie na godzinę tak w tereny zielone poza Warszawę. I to super jest takie przewietrzenie się. I bardzo przyjemne. No to to jest ta zmiana. Jeśli chodzi o wcześniej to nie. Mam jedną taką zmianę, którą pewnie sobie chętnie odhaczysz, bo zmieniłem w końcu tą rzecz, że nie pojechałem do Leroy po jakieś narzędzia, których potrzebowałem, tylko sobie zamówiłem przez internet. To rzeczywiście było na takiej zasadzie, żeby uniknąć kolejnego pojechania gdzieś i kolejnych kontaktów itd. A mniej więcej wiedziałem, czego potrzebuję, więc zamówiłem sobie zszywacz tapicerski przez Allegro zamiast pojechać do Leroy.

**Czyli żeby uniknąć, powiedziałeś, że dlatego to zrobiłeś, żeby uniknąć kolejek.**

Nawet nie kolejek, tylko wizyty w sklepie. Kolejnych kontaktów. Kolejnego takiego chodzenia do jakiegoś publicznego miejsca, gdzie jest dużo ludzi na zamkniętej przestrzeni, gdzie jest jakieś prawdopodobieństwo transmisji. No, jest też w tym element lenistwa jakiegoś. Bo ten, mamy teraz ten, to się nazywa smart, dostawy smart czy jakieś tam smart plus, czy smart dom czy coś takiego. Allegro smart, o. Na Allegro jest ten… Że tam przesyłki do iluś, od iluś. Wykupuje się taki, jak to nazwać, taką opcję darmowych przesyłek.

**Ale to jest taka jakaś subskrypcja?**

No coś takiego. Że płacisz na Allegro, 40 czy 50 zł czy coś takiego i dzięki temu przez jakiś okres czasu masz darmowe przesyłki. Rozumiem, że Allegro wtedy pokrywa koszt tych przesyłek temu wysyłającemu. Tak ja rozumiem. A teraz z okazji… Myśmy czasami z tego korzystali w momencie, jak mieliśmy bardzo dużo jakichś zakupów, taki okres w zeszłym roku mieliśmy bardzo gorący. I z tego korzystaliśmy. I to nam się wtedy bardzo opłaciło. A teraz z okazji tego koronawirusa i różnych akcji społecznych Allegro wprowadziło, że to jest za darmo na jakiś tam okres czasu. Nie wiem, czy dla wszystkich czy akurat dla nas. My mamy tą ofertę jako jacyś tam użytkownicy Allegro. Bo tak jak powiedziałem, moja żona różne rzeczy zamawia zasadniczo online. I korzystamy z tego Allegro co najmniej co jakiś czas. Więc teraz mamy ten Allegro Smart. Więc uznałem, że jak mi i tak to za darmo dowiozą do domu, to już właściwie wręcz ekonomicznie jest uzasadnione, bo nie muszę jechać sam, tam samochodem podjeżdżać.

**To ciekawe, właśnie nie słyszałam o takiej opcji na Allegro. Sprawdzę to też.**

Jeżeli się dużo korzysta, to ten… Albo jeżeli jest tak, że na przykład… Bo wiadomo jak jest, czasami kupujemy rzeczy, bo już są nam potrzebne, bo coś się skończyło albo ja chcę w tym tygodniu zrobić coś, więc zamawiam i jeszcze sprawdzam, żeby ta przesyłka była za 3 dni. A są takie rzeczy, że trochę jest nam wszystko jedno. Ważne, żeby to zrobić do świąt, do sylwestra, do przyszłego roku. Nie wiem, przed wiosną. To wtedy można sobie zakumulować ileś tych zakupów, kupić ten pakiet tych darmowych przesyłek. No i wtedy wszystko pozamawiać.

**A jak oceniasz to doświadczenie tych zakupów ostatnich online? Bo mówiłeś wcześniej, że lubisz jednak takie rzeczy do majsterkowania kupować sobie, po prostu pochodzić po sklepie.**

Tak, tak, tak. Ale uznałem, że to jest na tyle drobna rzecz, że mam nadzieję, że jak wtopię, to nie będzie takie duże wtopienie. To nie jest bardzo kluczowe. Trochę się bałem, czy nie kupię jakiegoś tam plastikowego szajsu. Ale wśród kilku tam najtańszych, posortowaliśmy po cenie, pośród kilku najtańszych był zszywacz tapicerski Topexa. Więc to jest jakaś tam marka. Uznałem, że… Chodzi o to, że to nie no name, tylko jakiś ten. Było opisane, że jest metalowy. Uznałem, że jest szansa, że to nie będzie taki plastik, co to użyję 3 razy i mi się rozpadnie w rękach. A nie było też tak, że kupiłem go w związku z tym 3 razy drożej. Bo to wiadomo, że czasami różne rzeczy, które są markowe i premium itd. no jakby cena prześciga jakość. W sensie, że szybciej rośnie niż jakość. No to staram się nie wchodzić w ten zakres, bo to jest bezsensowne przepłacanie dla mnie. W związku z tym tutaj się ucieszyłem, bo był w sensownej cenie. I przyszedł rzeczywiście elegancki, wygląda na to, że będzie dość trwały, więc jestem zadowolony.

**Powiedziałeś, że też zaingurowałeś sezon wczoraj wychodzenia z domu.**

Tak, tak, tak.

**Ale powiedz mi jeszcze, jakby co teraz jest dla ciebie w tym momencie największym wyzwaniem, największym problemem?**

Ale w ogóle czy związanym z koronawirusem?

**Może być i to, i to.**

Bo mam taki problem, że tak jak ci na początku mówiłem, że ja w tej chwili nie pracuję. I to jest trochę zamierzone, a trochę nie było zamierzone, żeby tak długo ten stan trwał. Jestem trochę w zawieszeniu i to wynika też trochę z mojego niezdecydowania się, w którym kierunku teraz chciałbym iść. I trochę jest tak, że może to jest moje wewnętrzne zahamowanie, może jakieś lenistwo, nie wiem. Natomiast ta sytuacja trochę nie pomaga mi podjąć tej decyzji, bo nie wszystkie możliwości w tej chwili są możliwe. Trochę nie wiem, jak będzie w przyszłości wyglądało. Tak jakby rynek i świat się teraz trochę zmieniły. I to mi też nie ułatwia podjęcia decyzji, jest kolejną tam składową. I nadal nie mogę sobie wymyślić, co bym chciał robić. A jak bym nawet wymyślił, to nie wiem, czy bym mógł łatwo, w związku z tym, że u mnie to będzie wymagało jakichś przetarcia szlaków, pewnie zaczęcia czegoś od nowa, poszukania, znalezienia, być może przejścia… No być może są teraz jakieś rekrutacje online w tej chwili. Patrzyliśmy, że coś tam się dzieje, że są firmy, które jednak mimo tego całego zamrożenia, krachu itd., jakoś tam niektóre firmy rekrutują. Może być teraz trochę trudniej pewnie, jest więcej konkurencji. W każdym bądź razie jest tak, że tutaj jakby wyjście z tego stanu, w którym jestem, trochę mam utrudnione. To jest mój taki największy kłopot. Chyba. Nie widzę innych dużych. Jak bym miał zajęcie jakieś, najlepiej takie online albo takie, które nie narażałoby mnie na jakieś, nie było dla mnie dużym kłopotem, czyli miałbym pracę albo de facto bym jej nie miał albo się martwił, że ją stracę, bo jest zamrożenie gospodarki i nie ma zamówień, zleceń albo coś. Tylko jak bym miał normalną pracę, taką, która funkcjonuje i działa. Nie wiem, byłaby w segmencie, którego na razie kryzys nie dotknął i te obostrzenia, to byłoby mi pewnie dużo łatwiej.

**Czyli to jest taki problem tego, że nie masz po prostu co robić w ciągu dnia? Czy bardziej takiego niezdecydowania i niepewności co do przyszłości?**

Chyba to drugie. Znaczy, no. Wiesz, bo to jest tak, że… Znaczy nie mam co robić…

**Nie, bo chodzi mi o to, że powiedziałeś, że łatwiej by ci było, gdyby to była taka praca, która rzeczywiście coś robisz w ciągu dnia. A nie taka, że po prostu masz zatrudnienie.**

No tak, bo wtedy, gdyby było tak, że mam zatrudnienie, ale się zastanawiam, że być może je zaraz stracę, to byłbym trochę w tej samej sytuacji, nie? Bo to nie jest tak, że ja w tej chwili nie mam co do garnka włożyć. Natomiast myślę trochę o przyszłości. Na takiej zasadzie, że jednak warto byłoby, żebyśmy mieli zdywersyfikowane przychody, tak? Albo żebyśmy, nie wiem, szybciej generowali oszczędności. Albo coś w tym stylu.

**Czyli trochę teraz nie szukasz tak aktywnie tej pracy, bo też nie do końca wiesz, w którym kierunku pójść, bo nie wiesz, jak będzie wyglądała przyszłość tak jakby…**

To jest dodatkowo. Ja sam nie wiem, co chcę robić. A dodatkowo jeszcze nie wiem, jak będzie wyglądała przyszłość. To nie jest tylko tak, że ja wiem, co bym chciał robić, tylko nie wiem, czy to ma sens. Bo to jest innego typu zagadnienie. Ja nie wiem, czy to, co bym chciał robić, z jakichś moich pomysłów czy to by miało sens.

**Czyli jesteś w tym momencie na takim etapie życia i jesteś taką osobą, że nie jesteś zdecydowany. A ta sytuacja związana z koronawirusem jeszcze bardziej ci utrudnia podjęcie tej decyzji.**

Tak.

**Ale to nie jest tak, że ta sytuacja jest bezpośrednim powodem tego, że w tym momencie nie masz podjętej decyzji co do pracy.**

Dokładnie tak.

**Rozumiem, że to jest jakiś taki stan, który trwa u ciebie od jakiegoś czasu?**

Tak, tak, tak.

**A czy jest tak, że coś zaczęło ci teraz przeszkadzać, coś zaczęło ci doskwierać?**

Nie, chyba w taki wyraźny sposób nie. Znaczy nie, żeby coś się tak bardzo zmieniło. Czyli te rzeczy, które były wcześniej to tak, ale…

**Czyli nie czujesz, żeby w ciągu tych ostatnich dwóch tygodni coś się specjalnie u ciebie zmieniło. Poza tym, że wczoraj mogłeś sobie wyjść i dzisiaj też wyszliście z domu na spacer. Więc robicie więcej rzeczy niż wcześniej.**

No tak, robimy trochę więcej rzeczy niż wcześniej. Ale one są nawet nie tyle, bo to ilościowo godzina spaceru, to niby tak dużo nie zmienia. Ale wydaje mi się, że to zmniejsza takie poczucie uwięzienia i takiego braku możliwości poruszania się. Więc my się nie pchamy do publicznych miejsc. Nie jest nam bardzo tęskno do takich… I tak nigdy nie chodziliśmy specjalnie po galeriach handlowych. Czasem pewnie chodziliśmy do jakiegoś kina, ale to też rzadko. I to jest rzecz, z której jesteśmy w stanie… Kino, teatr na pół roku to jesteśmy w stanie zrezygnować bez bólu. O, nadal nie spotykamy się ze znajomymi i rodziną, to jest przeszkadzające. Ale jeżeli chodzi o takie miejsca publiczne, to nie mamy wielkiego bólu. Jesteśmy w stanie odpuścić kina, teatry, rzadko korzystaliśmy, do restauracji trochę chodziliśmy, ale też odpuścimy bez większego bólu. Nie wiem, puby, knajpy spoko, dla mnie nie ma problemu.

**Emocje – zdjęcia.**

Do dzisiejszego i wczorajszego dnia dobrze pasuje ta 13. Bo jest taka wiosna, spacer. To jest dobra, dojrzewające zboża, takie kłosy to już raczej takie późne lato. Ale zasadniczo ten stan taki. I już zaczęliśmy też planować trochę nieśmiało, że może byśmy sobie wyskoczyli na ten weekend albo na jakiś następny. Bo to już majówka chyba się nawet zaczyna. Czekaj, zerkam w kalendarz. Tak. W ten albo następny weekend może byśmy gdzieś wyskoczyli sobie nad właśnie jakąś łąkę, jezioro. No nawet jak sami się jeszcze nie decydujemy na grupowanie i trzymamy się tej izolacji, no to wyskoczyć sobie gdzieś we dwójkę nad jakieś jezioro, rzekę, łąkę tym naszym pseudo kamperkiem.

**Czyli mielibyście gdzie spać, tak?**

Tak, tak, tak. My mamy taki, że mamy rozkładane łóżko. Wyjmujemy jeszcze 2 fotele i wkładamy tam szafkę z kuchenką. Więc możemy sobie coś ugotować. W takim turystycznym powiedzmy wydaniu. Nie, że piekarnik i jakieś lepienie pierogów. Ale można sobie ugotować posiłek, mamy gdzie spać. Mamy jakiś rozkładany tam przedsionek albo daszek. O, składane krzesło czy fotel, czy hamak, jak się do lasu wjedzie, to można ten. I nawet mamy też takie bardziej… Bo tak jak mówię, tak jak te wakacje spędziliśmy właściwie 2 miesiące w tym samochodzie. Więc mamy też takie opatentowane rzeczy jak jakiś prysznic, kwestię toalety rozwiązaną jakoś prowizorycznie. I te rzeczy nie są bardzo konieczne, jak się na weekend wyjeżdża gdzieś, na jedną noc. Tą jedną noc można przeżyć bez prysznica albo się w jeziorze przekąpać, tak? Popływać. Ale o, tak jak mówię, dzisiaj i jutro to jest ta 13.

**Jeśli chodzi o tę 13, to jak być nazwał te emocje czy uczucia, które ci właśnie towarzyszyły?**

No nie wiem, taki trochę spokój, relaks, takie… Nie wiem, jak to nazwać, szukam dobrego słowa, mam to na końcu języka. Kurcze. Jak to nazwać? To jest taki, taki, taki… Taki właśnie wewnętrzny jakiś, mówię, kurcze, nie uspokojenie, nie relaks, ale zbliżone słowo. Mam na końcu języka. Dobra, nie wymyślę teraz.

**Jak ci się przypomni to możesz powiedzieć później.**

Tak. I to jest właśnie chyba to. Taka przyjemność po prostu takiego wyjścia gdzieś. Trochę jak takie mini wyjście w góry. Że tam ci nikt nie przeszkadza, chodzisz sobie, jest ładna pogoda, po prostu jest przyjemnie.

**I zacząłeś też mówić, że jeszcze jakiś inny obrazek chciałeś wybrać?**

Chciałem wybrać do wcześniejszego tygodnia, który nie był aż taki zen, bo tam było takie trochę obniżenie nastroju, trochę zirytowanie tym siedzeniem w domu. I teraz mam wrażenie, że to troszkę się zmieni, bo była momentami ładna pogoda. Jak jest brzydka, to denerwowało człowieka, że jest brzydka pogoda. Jak była ładna, to był jeszcze bardziej zdenerwowany, że jest ładnie, a nie można wyjść nigdzie. I tu nie wiem, czy jest taki obrazek. Może 14 jako taki ten węzeł, że on jest w jakimś takim napięciu. Ale też trochę taki rodzaj marazmu. Tutaj nie ma takiego, że takiego snucia się po domu, z lewa na prawo, bez takiej większej przyjemności. To to byłby taki zeszły tydzień. Trochę to było usprawiedliwione, bo były święta i poświątecznie. Więc te święta zawsze są takim czasem trochę leniwym i że można sobie trochę pozwolić na nierobienie niektórych rzeczy. Ale potem już to zaczynało tak pod koniec tygodnia trochę wkurzać. A, w piątek jeszcze nielegalnie byłem na rowerze z kolegą. To jeszcze nie wolno było jeździć na rowerze wtedy. A przynajmniej nie wiadomo było, czy nie było to czasami niedozwolone. Bo to też nie było jasne, na co te przepisy pozwalały, na co nie. I to też było właśnie fajne, takie przeżycie takiego po prostu wyrwania się na chwilę, na godzinkę, pojeżdżenia sobie gdzieś po ścieżkach rowerowych Warszawy, było bardzo fajnie.

**A nie bałeś się tego, że nie do końca wiesz, czy możesz to robić?**

Trochę tak, trochę nie. Bo po pierwsze tak, różne interpretacje pojawiały się w różnych miejscach, więc po pierwsze nie było to całkowicie zakazane. Ale się pojawiały doniesienia, że policja czasami mandat wystawiała za takie rzeczy. Ja takiego mandatu bym nie przyjął i próbował kierować sprawę do sądu i jakoś się potem wybronić, co nawet byłoby ciekawym… Mogłoby być kosztownym, ale ciekawą nauką, bo nigdy w sądzie nie byłem. I może człowiek by się czegoś nauczył, dowiedział, otrzaskał w tym, nabrał jakieś pewności. A poza mieliśmy też takie dobre wytłumaczenie. Bo stwierdziliśmy, że jedziemy sobie razem, jak by co, to się nie znamy. Jak policja jednego zatrzyma, to drugi jedzie i w ogóle rżnie głupa, że w ogóle nie jedziemy przecież razem i o co chodzi. I zawsze mieliśmy wytłumaczenie, że jak by co, to jedziemy do sklepu po jakieś zakupy, bo nie wiem, jedziemy w kierunku Auchan, po drugiej stronie Wisły najtańszy sklep w okolicy i po prostu jedziemy tam po tanie produkty i niech się odwalą. Miałem taki moment rzeczywiście takiej niepewności, bo żeśmy niechcący tam pobłądzili gdzieś i zjechaliśmy na Żeraniu, tam są takie tereny należące do kolei, do PKP. I tam nawet był jakiś znak, że zakaz wjazdu, myśmy tam przejechali. I tak nagle nas jakoś… Myślałem, że zaraz zjedziemy gdzieś przy kanałku Żerańskim, okazało się, że tam już nie ma, jest jakaś siatka, płot i wjechaliśmy na jakieś tereny przemysłowe. I między tymi wagonami tam jeździliśmy. I tam właściwie nie wytłumaczylibyśmy się, co robimy, bo tam chyba całkowicie nielegalnie przebywaliśmy. I tam spotkaliśmy radiowóz, ale na szczęście szybko skręciliśmy i nie wiem, czy nas nie zauważyli czy na nas machnęli ręką. No, ale to był taki moment troszkę delikatnego stresu. Ale tak poza tym to jednak nie.

**Czyli trochę było takiego jakiegoś zdenerwowania i powiedziałeś takiego marazmu, lenistwa takiego nieprzyjemnego w tamtym tygodniu. A potem to już był taki spokój, który trwa do teraz?**

Tak. Tak jest po prostu jest przyjemnie. Jest taka wiosna, można wyjść, jest piękna pogoda, świeże powietrze. Jest po prostu fajnie.

**To chciałam ci powiedzieć, że jeśli w ciągu tych przyszłych 2 tygodni znajdziesz jakiś obrazek, który oddaje to, jak się czujesz w danym momencie, to chciałabym, żebyś to gdzieś zapisał i mi to pokazał [instrukcje do zadania].**

No trudno się chyba koncentrować na swoich emocjach w taki świadomy sposób. Ja mam bardzo dużo też, ja teraz ci tu dałem takie lenistwo, marazm, troszkę irytacji a potem taki spokój, o takich mówiłem. Ale we mnie, wiesz, każdego dnia jest dużo złości. Tak jak robię sobie prasóweczkę rano. To albo rozmawiamy o tym z żoną, jak się wymieniamy spostrzeżeniami, kto co przeczytał. No to wtedy się pojawia, to każdego dnia mógłbym wskazać jakieś zdjęcie z granatem. Albo miotaczek ognia.

**Ale to super, to wiem, czego nam brakuje w tych obrazkach.**

Takiego kadru z… Czekaj, jak się nazywa ten… Znowu wyleciało mi nazwisko. Ja mam chyba jakiegoś wczesnego Alzheimera początkującego. No, ten gość, który nakręcił Django, Nienawistną ósemkę, Wściekłe psy. Tarantino, o! To z filmów Tarantino masz takie ujęcia krwawe i soczyste.

**Ok, to jest fajny pomysł. Możesz mi to pokazać. Możesz mi przesłać link na chacie.**

Ale to niewiele na tym widać. Ale to by też pasowało do naszej sytuacji. Bo tutaj taki luz i… Czekaj, to będzie dobre, zaraz dostaniesz.

**Rzeczywiście jest to miotacz ognia.**

No, to tam właśnie Leonardo DiCaprio przed chwilą się relaksuje z książką i uczy się roli jakiejś, czy tam z drinkiem w tym basenie. I tam mu przez okno wpada do niego jakiś bandyta. Czy chyba to jest nawet ona, przestępczyni, która okrada jego mieszkanie. I on biegnie po miotacz ognia i ją właśnie spala, przysmaża ją w tym basenie. Wtedy to jest pięknie do tej sytuacji. Czyli jestem ja, relaksujący się z moją wiosną. I utożsamienie sytuacji politycznej, to jest ta osoba, która zaburza nam ten spokój. Wpada nam do basenu i musimy ją tym miotaczem ognia, mielibyśmy ochotę wszystkich tych… To jest bardzo takie niepohamowane i krwawe. Ja wiem. Ja jestem spokojnym człowiekiem, ja nie życzę nikomu śmierci. Ale są pewne osoby, które jak by im się kopyrtnęło, to trudno by mi było po nich płakać.

**Czyli twój miotacz ognia jest skierowany nie w stronę koronawirusa tylko sytuacji politycznej, tak?**

Tak. No wiesz, koronawirus jest jakby częścią… Znaczy mam takie wrażenie, że to jest coś takiego jak w naturze. To jest przykre strasznie itd., ale jakby no nie wiem, trudno mi się na koronawirusa denerwować, że on jest. Tak jak trudno mi się zdenerwować, że jest pożar w budynku. Znaczy mogę się zdenerwować, że się nie ubezpieczyłem albo ktoś zrobić źle instalację elektryczną albo nie było piorunochronu. Na to się mogę bardziej denerwować niż sam pożar. Więc na koronawirusa w tym sensie się nie denerwuję, że on jest. Zaraz takie zawsze były i są przykre. I tak teraz wydaje się, że to nie jest najgorsza na świecie, jaka była w historii ludzkości albo nawet historii Polski. Ale to, co się dzieje i to jak my reagujemy jako poszczególni ludzie, jako państwo, jako kraj, no to mnie wkurza strasznie.

**Czyli bardziej reakcja ludzi na tę sytuację niż…**

No rządu. Znaczy Prawa i Sprawiedliwości jako partii rządzącej.

**A powiedz mi jeszcze, czy ty tak na teraz, czy ty się boisz?**

Ale czego?

**Właśnie nie wiem, czego. Czegokolwiek. Czegoś.**

Nie, no wiesz, cały czas się boję. Każdego dnia się boję różnych rzeczy. Znaczy, nie wiem, śmierci się boję, albo że coś się stanie moim bliskim itd. I teraz powiedzmy, że widzę trochę większe prawdopodobieństwo. Więc tak, trochę się boję. Ale nie w taki sposób chyba paniczny i taki, który odbiera mi zdolność jakiegoś takiego logicznego działania itd.

**A masz takie wrażenie, że ten strach, czy to jest tak, że to słabnie czy rośnie z czasem?**

To tak falami. Tak sinusoidalnie. Czasami, jak myślę o tym, że… Na przykład dzisiaj, wczoraj, jak pójdę sobie na dwór, jest piękna pogoda. Właściwie tak doraźnie się nikomu nic nie dzieje, bo nie mamy takich sytuacji. Opowiadałem tam któregoś razu, że mamy trochę znajomych w służbie zdrowia. To tam kilka osób trafiło na kwarantannę, taką wiesz, Sanepidu zarządzoną. I mieli te testy, wyszły ujemnie, więc nic im nie jest, póki co. Ta koleżanka, o której mówiłem, że się trochę martwimy, jak to będzie, bo jest w ciąży, urodziła tydzień temu? Tak? W zeszłą środę urodziła zdrowego chłopczyka i są już w domu. Więc no mówiła, że przykre to było, że jej mąż nie mógł być z nią i tak trochę to było gorzej. Ale poza tym… No i że poród jest trudny. Ale tak w ogóle, to poza tym, to mówi, że rodziła w szpitalu, który jest w 100% szpitalem położniczym. Nie ma tam w ogóle jakby innych chorób, więc tych… No oczywiście, że zawsze ktoś się może przypałętać chory, ale tam na razie nie ma dużo przypadków. I raczej była spokojna. I wyszła już do domu, więc nie musi tam być hospitalizowana jakoś długo z jakichś powodów. Więc jest w domu, raczej też są zdrowi, nic się nie dzieje, więc też jest fajnie. No i takie różne sygnały. Z rodziny nie mam żadnych chorych, żadnych osób, które są chore albo potrzebują jakiejś hospitalizacji itd. Więc no nie mam jakiegoś takiego stresu dużego, bezpośredniego. Więc taki miły dzień odgania takie różne, że coś się kiedyś być może w przyszłości pojawi. To taki miły dzień pozwala ci nie myśleć o takich rzeczach. Ale jak sobie czytam artykuły, że nie wiem, że właśnie a może będzie kryzys, a może służba zdrowia się zaraz złoży, a za miesiąc może nie będzie działała albo coś takiego, bo się tak zapadnie wszystko. I sobie o tym myślę, że może by tak było, że akurat, nie wiem, będzie straszny wzrost przypadków do góry. I oddziały szpitalne nie będą funkcjonować albo karetki przestaną jeździć, bo nie będzie komu na nich jeździć. A wtedy akurat w mojej rodzinie, w naszym otoczeniu, pojawi się tutaj na warszawskich Bielanach koronawirus, nie wiem, bo w lokalnym sklepie albo bazarku był ktoś, kto kaszlnął na wszystkich i pół osiedla jest chore. No tak, to wtedy to może… Ale to jest na razie dalekie, to jest mgliste. To jest na zasadzie, że zawsze może się zdarzyć coś złego. Gdziekolwiek lecimy, to samolot może spaść. Jedziesz na wakacje, możesz się… To jest taki wiesz, to jest strach taki, nie wiem, niskiego prawdopodobieństwa. Albo taki, który można lekceważyć, bo jest, nie wiem, taki nienaoczny.

**Czyli ja zrozumiałam, że u ciebie taki strach czy niepokój jest głównie powodowany tym, że po prostu coś przeczytasz akurat w internecie czy jakieś doniesienia medialne do ciebie dochodzą.**

No bardziej, że czytanie o jakichś rzeczach albo myślenie, albo rozmawianie o nich na przykład, wymienianie się poglądami ze znajomymi, rodziną, powoduje, że po prostu sobie myślę o tym. I to wtedy wchodzi na pierwszą… No myślę o tym. I jeżeli na co dzień o tym nie myślę, to wtedy nie ma tego strachu. Bo to nie jest coś, co mnie dotyczy w tym momencie bezpośrednio. To nie jest tak, że ja w tej chwili mam, nie wiem, właśnie umierającą bliską osobę.

**A czy w związku z tym, że jesteś świadom tego, że kiedy o tym nie myślisz, no to się po prostu nie denerwujesz tą sytuacją i nie boisz się, to czy jest tak, że ty podejmujesz jakieś działania, żeby właśnie unikać tego myślenia o tym, czy tam nie czytać o tym? Czy niespecjalnie?**

Trochę może mniej zacząłem czytać. Ale też niespecjalnie. Bo nie chcę działać na zasadzie takiej, że… Jak zamknę oczy, to zła nie ma. To jest bez sensu. To dzieci tak mogą zrobić, tak? Oczywiście, jak by mnie to już przytłaczało strasznie itd. to tak, warto sobie robić jakiś rodzaj… Tak jak nie wiem, jak ktoś jest lekarzem i widzi mnóstwo cierpienia, to warto, żeby jednak czasem jechał na urlop, znaczy na rajską wyspę albo w Bieszczady, gdzieś, gdzie spędza miło czas z rodziną i łowi ryby. I nie ma tego stresu. Bo ważne jest, żeby, jak na co dzień podejmuje trudne decyzje, żeby sobie dawać ten wentyl jakiś. Ale też nie chcę robić tak, że nie będę o tym myślał, czytał itd.

**Jak pewnie wiesz, w ostatnim czasie w Polsce zmieniły się różne ograniczenia. I chciałabym o tym trochę porozmawiać. Ale najpierw chciałabym, żebyś ty mi powiedział, o jakich ty zmianach słyszałeś ostatnio?**

Znaczy zmiany, jakie były największe, to chyba że od zeszłego czwartku, zapowiedziane zaraz przed świętami albo w święta, że od czwartku zeszłego, czyli 16 kwietnia mamy wszyscy zacząć nosić maseczki. I to wyglądało, że to jest takie 4 dni, że te zmiany wyprzedzały kolejne zmiany. Że mamy zacząć nosić te maseczki i się do nich przyzwyczajać po to, żeby od wczoraj, czyli od 20 kwietnia było ten, rząd przedstawił… Bo pytałaś, o których wiem. No to przedstawił ten czterostopniowy plan odmrażania gospodarki czy w ogóle przywracania jakiegoś rodzaju normalności. I wczoraj nastąpił ten pierwszy krok. Czyli, że można z powrotem wychodzić z domu w celach rekreacyjnych, do wszystkich tych parków itd. To jako odmrażanie gospodarki to ma niewiele z gospodarką wspólnego. Ale powiedzmy jako w ogóle regulacje życia społecznego. Znaczy, że wolno rekreację na świeżym powietrzu uprawiać. W sposób taki, że nadal trzeba mieć maseczkę, nadal trzeba zachowywać odległość. Ale wolno to już robić, to jest legalne i policja nie powinna się do tego przyczepiać. Oraz że będą chyba, tam jest więcej osób, jeżeli chodzi o gospodarkę, więcej osób w sklepach. Zmieniła się ta regulacja, że nie na liczbę kas, tylko na powierzchnię jest limit osób. I ten limit jest taki trochę mniej ograniczający duże sklepy. Więc w tych dużych sklepach jest trochę więcej… Będzie mniej tych kolejek i chyba zagęszczenie osób w środku trochę może być większe. I jakieś działalności, nie wiem jakie, już nie pamiętam co. Któreś, musiałbym pomyśleć. Nie wczytywałem się w to, bo nic z tego nie było dla mnie takie superinteresujące. Jakieś rodzaje działalności zostały przywrócone znowu, że zmniejszono ograniczenia albo w ogóle przywrócono do działania. Ale nie pamiętam już które. Jeszcze kolejną rzeczą, która nie ma daty, czyli to będzie powiedzmy za tydzień, 2 lub 3. Bo na przykład teraz chyba markety budowlane w weekend nie działają? Co rozumiem, że ma trochę spowodować, że ludzie, którzy robią remonty, tak ja to rozumiem. I materiały budowlane kupują, to mają je kupować w dzień tygodnia. A jak ktoś wykańcza wnętrza i sobie szuka lampy albo dywanu, to ma tego nie robić. Bo to dziwna taka interpretacja byłą, że w sobotę, niedzielę Leroy i Castoramy są zamknięte. To jakoś było dziwne. To teraz to jakoś ma być tam poluzowane. Więc rozumiem, że to są jakieś takie kolejne rzeczy, które są zmieniane.

**I powiedziałeś, że też się jakoś tak bardzo dokładnie nie wczytywałeś, bo nic specjalnie ważnego tutaj dla ciebie nie było.**

Nie.

**A z tych różnych poluzowań, które dla ciebie jest najważniejsze?**

Dla mnie, w tej chwili 2 rzeczy, które mi doskwierają bądź doskwierały to nie wyjeżdżanie w tereny zielone. Czy to na jednodniowe czy to na spacer do parku czy to na spacer do lasu pod Warszawą. Czy to nawet takie, tak jak planujemy sobie wyskoczyć na weekend nad jakieś jezioro, rzekę. To jest coś, co mi doskwiera lub doskwierało, bo teraz już wolno to robić. A druga rzecz, to kwestia spotykania się z rodziną i znajomymi. To nie jest twardo zabronione, bo nikt mi nie zabroni, a już na pewno mnie z tego nie zweryfikuje, że ja wsiądę do samochodu i pojadę odwiedzić znajomych. Moglibyśmy przecież to robić z żoną, nikt by nie był w stanie tego wyłapać, że ja wchodzę do bloku, gdzie nie mieszkam. Natomiast tego nie robimy. I to mi doskwiera. Resztę rzeczy, czyli kina, teatry, kręgielnie, restauracje i nie wiem, co tam jeszcze, znaczy galerie handlowe to tak nieszczególnie. To jest rzecz, która dla mnie jest drugo, trzecioplanowa.

**A jeśli chodzi o to, że nie możesz jeździć do rodziny i do znajomych, to powiedziałeś, że nie robicie tego, chociaż wiesz, że nikt by tego nie skontrolował. Ale na ile to jest tak, że ty w ogóle rozumiesz, czemu tego nie możesz robić?**

Nie wiem, czy dokładnie, do końca, w 100% rozumiem. W sensie mam jakieś wyobrażenie. Czy nasze wyobrażenia są słuszne to jest trudno powiedzieć na pewno. Czy ty dokładnie wiesz, co ty czujesz? To czy ja dokładnie wiem… Jakby mam pewne wyobrażenie. Mam takie wyobrażenie, że ilość tych transmisji jest jakoś tam proporcjonalna do naszej aktywności społecznej. Czyli jeżeli wirus się przenosi od osoby do osoby, w związku z tym, jeśli się spotykamy w społeczeństwie mniej, to chronimy siebie, chronimy swoich bliskich oraz społeczeństwo jako całość. Więc no tak to rozumiem.

**Bardziej chodziło mi o to, jak ty to rozumiesz, a nie jak jest w rzeczywistości.**

Ja to rozumiem tak, że chodzi o to, żeby tych transmisji było na tyle mało, żebyśmy byli w stanie przechorowywać tych… Znaczy, żeby pomagać tym, którzy w tej chwili potrzebują pomocy. Mamy nie chorować wszyscy na raz, to jest pierwsza rzecz. Bo się ten system załamie i nie będzie możliwości pomocy medycznej dla wszystkich, którzy są chorzy. Ja to tak rozumiem i rozumiałem. A dwa, wirus jest nowy, nie wiemy wszystkiego, ale będziemy się powoli dowiadywać. Więc jest szansa, że jeśli jakiś czas nie pochorujemy to: a, mało realistyczne, ale powstanie szczepionka. B, powstanie lek albo jakaś kuracja, która zmniejszy śmiertelność. C, po prostu zwiększymy pojemność służby zdrowia. Co niestety w Polsce i tu jest właśnie ten element mojej złości, w Polsce zdaje się, że trochę nie ma miejsca. Nie słyszałem, żebyśmy jakoś właśnie zwiększyli wydatnie możliwości przyjęcia pacjentów itd. Ale to są moje 2, pierwsza to ta przyczyna, że siedzimy w domu, żeby wolno zachorowywać, b, kupujemy czas, bo może się coś zmieni.

**A jeśli chodzi o te maseczki. Jak oceniasz obowiązek chodzenia w maseczkach?**

Ja trochę nie czuję się ekspertem. Znaczy muszę komuś zaufać, bo ja… Ja, nie wiem, jak ty, ja nie widziałem nigdy na oczy żadnego wirusa. Są ludzie, którzy widzieli pod mikroskopami i kolekcjonują, katalogują je. Coś tam o nich wiedzą, badają ich wpływ. Wiedzą, jaką one mają średnicę i jak przechodzą przez różne materiały. Trochę nie wiem, trudno mi to jest ocenić. Wydaje mi się, że tak, jak patrzę na takie… Wydaje mi się, że jakiś to ma sens. Ale to jest taki sens statystyczny. Nie traktowałbym, że jak masz coś na twarzy, to chronisz się stuprocentowo, tylko może, ewentualnie zmniejszasz… Jeżeli na przykład jest tak, że na aerozolu, czyli na tych drobnych kropelkach płynów, które w ramach oddychania z naszych ust wylatują, osadza się ten wirus, a te kropelki dobrze się skraplają na jakichś warstwach materiału, to być może usta i nos zatkane jakimiś warstwami szmatek nam rzeczywiście coś pomagają. I powodują, że tego wirusa jest mniej. Oraz, że na przykład strumień jest mniejszy. Czyli, że jak kaszlniesz albo coś powiesz, to ten aerozol nie leci na 4 metry, tylko na pół metra. Nie wiem, na ile się zmniejsza. No to może rzeczywiście tam trochę spowoduje, że przechodząc obok innej albo stojąc koło innej, losowej osoby w kolejce po chleb albo w metrze się nie pozarażamy. Ale wydaje mi się, że z drugiej strony musielibyśmy, żeby to miało duży sens, to musielibyśmy mieć tych masek dużo więcej. Trochę bardziej, tam chyba tego wyższego rzędu ochrony. Być trochę bardziej konsekwentni w ich stosowaniu, wymienianiu albo praniu. Sami dla siebie powinniśmy uważać, żeby tam, nie wiem, nie dotykać tej maseczki potencjalnie zainfekowanymi rękoma itd. Wydaje mi się, że… Po prostu mam takie poczucie, że to ma sens. Ale to, jak to robimy zmniejsza jego sens. To znaczy widzę, że ludzie tak chodzą z tym maseczkami, czy starsi czy młodsi z lekko odsuniętymi. Albo nos odsłonięty. Albo zupełnie ta maseczka do twarzy nie przystaje. I wtedy tak widzę, że to jest taka mniejsza sensowność. Wczoraj jechałem samochodem, to widziałem robotników, pracujących na budowie. Wszyscy mieli maseczki, każdy jeden. I wszyscy mieli zsunięte na szyję. Więc wydaje mi się, że to jest trochę tak, że oni je mają, bo przepisy wymagają itd. Natomiast oni tam ciężko, fizycznie pracowali, utrudnia im to oddychanie, więc oni je wszyscy zdejmą.

**I co myślisz o tym, że oni tak robią?**

Nie wiem, trudno mi powiedzieć. Wydaje mi się, że… W ich przypadku to w ogóle mi się wydaje, że dziwne. Bo oni tam kuli jakąś, część z nich kuła asfalt a część robiła jakieś tam wylewki. W takich oparach z takiego rozgrzanego asfaltu, ja nie wiem, jak to się tam dokładnie nazywa, takim żużlem tam sypali drogę. To wydaje mi się, że idealnie byłoby, jak oni to robią rzeczywiście codziennie po 8 godzin w takiej ekipie, żeby oni w ogóle na co dzień mieli jakieś maseczki z takim filtrem chemicznym, bo ja nie wiem, czy oni się nie podtruwają cały czas trochę tym. Ale nie wiem. Z drugiej strony są w swoim otoczeniu, jeżdżą tymi samochodami, jest ich dziesięciu czy tam siedmiu. Mają ze sobą kontakt nawzajem cały czas. Więc chyba, czy mają maseczki czy nie, to i tak by się pozarażali. A z kolei naokoło nich, bo oni przy drodze pracowali, koło nich nikogo nie ma. W związku z tym mi jest trudno powiedzieć. Jest jedna rzecz, którą na pewno dobrze z tymi maseczkami, nawet krzywo pozakładanymi itd., trochę… Uważam, że jest takim dobrym skutkiem ubocznym. Że ludzie, zakładając tą cholerną maseczkę albo tą szmatkę na twarz, która chyba nie ma żadnego znaczenia w sensie transmisji, przypomina wszystkim, że jesteśmy w jakimś stanie specjalnej sytuacji. Jest to, mam wrażenie, rodzaj takiego markera, którym jesteśmy teraz wszyscy oznaczeni, że to nie jest zwykły czas. Że to jest czas trochę wyjątkowy. W związku z tym, że jak znowu, nie wiem, byłby bardzo szybko wzrost zachorowań i rząd by znowu nam bardzo restrykcyjnie zakazał spotykania się ze sobą i znowu ograniczył ilość rzeczy, żeby wyhamować trochę tą epidemię, bo szpitale by się zapchały. I już byłyby pierwsze doniesienia o tym, że nie wiem, tam przed szpitalem 1000 trumien leży gotowych na przykład i takie obrazki byłyby w mediach. To te maseczki, mam wrażenie, że trochę nam przypominają o tym specjalnym czasie i ułatwią nam samym psychologicznie oraz rządowi w sposób skuteczny nas z powrotem zagonić do domów. To jest taka trochę przypominajka. Dzisiaj, jak właśnie byliśmy na spacerze w parku, to… Bo byłem wczoraj tam na odludziu, to przez godzinę spotkałem 2 osoby, zresztą na odludziu, więc tam mieli ludzie pozsuwane maseczki i tam jeden rowerzysta mnie minął bez maseczki, jacyś młodzi chłopaczkowie sobie szli, to też nie mieli. Ale tam to i ja miałem ściągniętą. Bo po prostu mówię, przez godzinę spaceru spotkałem 2 osoby, to… A dzisiaj w parku też było dużo osób i właśnie miało, nie wiem, trochę krzywo, trochę tam zdjęte, część bardzo dobrze jest zasłonięta. I jest tutaj duże rozwarstwienie. I widzę dużo przykładów, że to jest tam… A ludzie to trochę lekceważą albo jest po macoszemu traktowane. Więc wydaje mi się, że to właśnie, to jest taki przykład, wtedy zacząłem myśleć, czy to w ogóle ma sens. I pomyślałem sobie o takim ubocznym skutku, że przynajmniej taka dodatkowa rzecz jest. Że ludzie pamiętają, że to jednak nie jest do końca zwyczajny stan teraz.

**Czyli to tak bardziej psychologicznie działa, tak jak powiedziałeś. A powiedz mi jeszcze, czy ty nosisz maseczkę tak… Jak ją nosisz? Czy w ogóle ją nosisz?**

Wiesz co, zastanawialiśmy się nad tym. W maseczki dopiero późno się wyposażyliśmy, takie profesjonalne. Jeszcze zanim był nakaz chodzenia w maseczce, to jak już ją miałem, to chodziłem w niej, mam taką FPP2 tą taką… Skuteczna i naprawdę profesjonalna jest ta trójka. Ale to nawet tam chyba WHO zaleca, żeby te trójki, żeby zwykli ludzie ich nie używali, żeby one były dla medyków. Ja miałem dostęp do tych dwójek. I to jest taka w pełni zasłaniająca, z takim filterkiem itd. Ona powinna być jednorazowego użytku. To ja jej tak nie używam. To znaczy używam jej kilkakrotnie, pewnie niezgodnie z przeznaczeniem. Ale nie mam do nich dostępu tyle, żebym mógł wymieniać codziennie na nową. Ja jej używam tylko jak chodzę po zakupy. Czyli 2 razy w tygodniu. Raz jak idę po chleb. I potem, jak jadę do Biedronki. A na spacer z psem zakładam sobie po prostu przewidzianą zgodnie z prawem, a chyba nie mającą dużo wpływu na roznoszenie tego wirusa, taką chustkę typu bandankę na twarz.

**Powiedziałeś, że są takie ograniczenia, które działają tak bardziej psychologicznie. Ale czy z tych różnych ograniczeń, które cały czas obowiązują, które z tych obostrzeń ty uważasz za takie, które realnie mają wpływ na ograniczenie tej epidemii?**

Na pewno te, które powodują, że nie gromadzi się w jednym miejscu dużo osób. Czyli jednak ten brak koncertów, jakichś dużych spotkań itd. To na pewno. Mi się wydaje, że to są takie cegiełki, które razem dobudowują… bo to mówimy o statystyce. Czyli coś zmniejsza o 20%, następna o 15, a potem któraś o 30 itd. I to razem one dają efekt, nie? Więc wydaje mi się, że te duże spotkania gremialne są dość ważne. Dwa, wydaje mi się, że to, że część osób nie pracuje lub pracuje zdalnie, to jest kolejna ważna rzecz, że szkoły i uczelnie są poodwoływane. To są takie, które moim zdaniem mają duże znaczenie. Znaczy szkoły, garnizony wojskowe to są zawsze miejsca, gdzie epidemia najszybciej wybuchała. Najwięcej epidemii w takich miejscach i roznoszenia różnych zarazków było. Co tam jeszcze? Nie wiem, czy coś jeszcze takiego uważam za strasznie słuszne albo strasznie dobrze działające. Nie, bo chyba nie mamy takich… Najlepiej byłoby, gdybyśmy jeszcze działali w ten sposób, że… Ale to w naszym społeczeństwie jest to niemożliwe, to może jacyś Chińczycy mogą to zrobić albo jacyś tacy… Na przykład społeczeństwa, które w jakiś sposób inaczej funkcjonują. Bo byłoby najlepiej, gdybyśmy na przykład życiowo i geograficznie się podzielili. Na przykład zakaz podróżowania między dzielnicami. Tak jak kraje pozamykały swoje dzielnice. To gdybyśmy podzielili się na jakieś gminy, osiedla itd. I byłby zakaz podróżowania pomiędzy jednymi a drugimi. Tylko to gospodarkę by zabijało całkowicie. Bo teraz jesteśmy nawet nie tylko globalni, to już nam utrudniło zamknięcie granic. Ale dla większości biznesów, jak akurat świnie hodujesz w jednej, a ktoś paszę trzyma w magazynie w drugim powiecie, to już byłaby katastrofa dla tych ludzi. Ale gdyby te granice byłyby jakoś łatwiej, gdyby ta sieć była mniej gęsta i podzielenie było łatwiejsze, mówię, w jakimś innym świecie, no to na przykład dzielenie się geograficzne byłoby dla nas pewnie sensowne.

**A czy któreś z tych ograniczeń oprócz noszenia maseczek, czujesz, że mają tylko takie działanie psychologiczne, uspokajające dla ludzi, ale nie są efektywne w rzeczywisty sposób?**

Nie, nie wydaje mi się. Wydaje mi się, że one mają swoje znaczenie. Znaczy inna kwestia jest, że każdy z nich ma koszt. Jest jakiś rodzaj kosztów ponoszony. I jest pytanie, czy ten koszt jest uzasadniony. Natomiast nie jest tak, żebym uważał, że… Te lasy, chodzenie do tych lasów i terenów zielonych, szczególnie pozamiejskich. Bo to, że się zamknie Pola Mokotowskie czy coś takiego, to mogę dyskutować. Natomiast jak ktoś zamyka, wiesz, las w Suwałkach, żeby ktoś tam nie ten. No to dla mieszkańców Augustowa to jest jakiś poroniony pomysł. Znaczy tam mieszka 10 tysięcy osób, tam każdy ma hektar lasu dla siebie. To jest w ogóle, zamykanie lasów w całej Polsce to jest w ogóle jakiś obłęd dla mnie. To było głupie. Tu się zgadzam, że to… I tego na szczęście nie ma. Bo to było bez sensu. Natomiast w innych kwestiach no koszty są duże. Znaczy ludzie, nie wiem, nie pracują, nie mają przychodów itd. To możemy się pytać, czy lepiej jest, żeby tam dodatkowo ileś procent osób zachorowało, ale jednak, nie wiem ileś osób dzięki temu ma pracę, tak? I nie żyje teraz, właściwie głód im w oczy nie zagląda, tak?

**W takim razie co myślisz o tych różnych planach luzowania tych ograniczeń? Wspomniałeś, że są jakieś etapy.**

Tak, tak. Te etapy są… To znaczy tak, po pierwsze wydaje mi się, że fajnie, że w ogóle coś w końcu przedstawili. Natomiast wygląda na to, że… Bo to jest bardzo niekonkretne. To znaczy nie ma żadnych dat. Było mniej więcej, co będą robić, ale bez żadnych dat i bez żadnych wyznaczników. To znaczy nie powiedzieli, jeśli osiągniemy to, to zrobimy to. Jeżeli na przykład, nie wiem, śmiertelność spadnie poniżej jakiegoś progu, jeżeli śmiertelność będzie powyżej jakiegoś progu, jeżeli liczba łóżek szpitalnych będzie zajęta więcej niż 70%, to będziemy robić to, będziemy robić to, będziemy robić to. Żadne tego typu rzeczy nie padły. Znaczy były tylko takie: kiedyś zrobimy to, kiedyś zrobimy tamto. No to jesteś moją dziewczyną, no to kiedyś się zaręczymy, kiedyś weźmiemy ślub i kiedyś zbudujemy dom. To jest zupełnie co innego niż zaręczymy się, jak skończymy studia, weźmiemy ślub, jak będziemy mieli pracę. I nie wiem, no rozumiesz? To jest zupełnie inaczej. To było takie bardzo niekonkretne, bardzo mgliste dla mnie. I nadal nie wiadomo, czym się ten rząd kieruje. W sensie jaki jest model, jakieś… No są jakieś modele epidemiologiczne, różne przyjęte taktyki działania, uzasadniane różnymi eksperckimi opiniami. Znaczy rząd wychodzi na konferencji prasowej i coś przedstawia, coś mówi, ale nie wiadomo na jakiej podstawie te działania są podjęte dla mnie. Brakuje tam jakiegoś uzasadnienia. Bo oni powiedzą, że otworzą za jakiś czas kawiarnie albo fryzjerów, albo szkoły. I kiedy to będzie albo dlaczego to będzie. To jest zupełnie niewyjaśnione. Wygląda na to, że któregoś dnia Morawiecki i Szumowski zamkną się w dużym, owalnym gabinecie i zrobią tak, no słuchaj, chyba już. No chyba już, dobra, to otwieramy to. Ale stacje benzynowe? Stacje też otwórzmy tam. To trochę tak wygląda. Może tam jest jakiś plan. Ale ten plan, jeśli jest, to jest głęboko przed obywatelami ukryty.

**Czyli ty potrzebowałbyś jakiegoś takiego wyjaśnienia, dlaczego tak a nie inaczej.**

Znowu, ja to jestem co najwyżej, ewentualnie, żeby się uspokoić lub dla komfortu psychicznego. Albo takiego przyjemnego poczucia, że żyję w cywilizowanym państwie. To dla mnie. Natomiast jak bym był przedsiębiorcą, to bym tak… To może życie by może od tego zależało całe. Znaczy, czy ja utrzymam swój biznes. Bo masz koszty stałe. Czy ja już mam zwalniać ludzi albo czy ja już mam wypowiadać umowę najmu lokalu. Albo czy… Różne ludzie mogą podejmować kroki. Czy ja mam już ogłaszać upadłość, cokolwiek. W związku z tym tak, jak bym był takim przedsiębiorcą, jak bym był fryzjerką i zastanawiał się, kiedy otworzę swój gabinet, to chciałbym wiedzieć, czy to nastąpi w kwietniu, maju, czerwcu, lipcu, sierpniu, czy może dopiero po grudniu. Albo co musi się stać. Jakby, no rozumiesz. To wtedy byłbym dużo bardziej zaniepokojony tym. Bo ta grupa rzemieślnicza właśnie, ta najmniejsza, jest chyba w najgorszej sytuacji. Bo oni są chyba w najgorszym, w tym 4 etapie. Tam są fryzjerzy, makijażyści, szewcy. Chociaż szewcy to nie wiem. Ale tam salony tatuażu. Wszystko, gdzie jest bliski kontakt, jest odsunięte do 4 etapu. I cholera wie, kiedy to nastąpi. Ci ludzie mogą czuć bardzo dużą niepewność. Zwłaszcza, że to są często mali wyrobnicy, nie? To znaczy małe biznesy. Oni nie mają, oni naprawdę są w stanie stracić źródło swojego zarobkowania. Np. salon fryzjerski, którego pozycję na rynku budowali od 10 lat.

**A czy ty masz jakieś takie poczucie, wiedzę, instynkt, kiedy te ograniczenia dotyczące właśnie tej grupy, o której teraz powiedziałeś, co jest granicą, kiedy te ograniczenia powinny przestać obowiązywać?**

Nie wiem. To zależy od tego, jaki model przyjmiemy walki z tą sytuacją. A tutaj ja trochę nie wiem. Bo po pierwsze jest dla mnie dużo znaków zapytania, nie mam dostępu do wszystkich informacji. I też na szczęście odpowiedzialność i decyzje nie są na mnie. Ja wcale nie chciałbym być teraz wiesz, premierem czy prezydentem czy czymkolwiek. A, prezydentem to właściwie… Prezydentem to smutne, bo by mnie już drugi raz nie wybrali. Bo idzie kryzys i to wiadomo, że się władzy, jaka by nie była, dostanie. Ale nie chciałbym teraz podejmować decyzji. Bo to musi być trudne i przykre. Bo to jest trochę na przykład sterowanie, nie wiem… Przesuwasz cyferki, liczby i mówisz, dobra, wzrost, tam podejmiemy działania typu x, ale to wzrost zakażeń szacowany jest o 15%, śmiertelność wzrośnie dwukrotnie od tych 15%. To znaczy, że uratujemy 200 tysięcy miejsc pracy i PKB skurczy się nie o 7% tylko o 5 w tym roku, ale umrze 1000 osób więcej. Kurcze, podjąć taką decyzję? Chciałoby się powiedzieć nie, podejmuję taką, że nikt nigdy nie umiera. Ale jak nikt nigdy nie umiera, to znaczy, że za jakiś czas umrą ci, którzy będą żyli w gorszych warunkach bytowych. Bo nie będą mieli pieniędzy, nie będziemy mieli pieniędzy na coś innego, tak? Nie wiem, na wykrywanie gruźlicy wśród studentów. To się chyba jeszcze robi cały czas przesiewowo. No właśnie. Nie miałaś badania, rentgena klatki piersiowej na pierwszym roku studiów? To jest na gruźlicę.

**Miałam to na studiach doktoranckich dopiero.**

O, to ciekawe. I to jest, zdaje się, przesiewówka na gruźlicę. Nie rozumiem, dlaczego i czy sensownie się ją robi, ale ktoś, państwo za to płaci. I jest ileś takich programów, które państwo robi. I jak PKB będzie słabe, to w przyszłości będą ludzie, którzy nie będą objęci jakimiś programami. Albo nie będą chodzić na… No rozumiemy, że ludzie w przyszłości, jak są biedniejsi, to krócej żyją. Tylko to jest pytanie, czy poświęcimy teraz ileś osób, poświęcimy to jest trochę złe słowo, jak taki master of puppets. Bo nie wszystko jest takie łatwe do zdecydowania. Ale tak, w którą stronę idziemy. Czy na przykład mówimy, no trudno, teraz jest ciężko, ale nie poświęcamy całej gospodarki. Albo no.

**A powiedz mi w takim razie, czy masz jakieś takie przekonanie co do tego, które z tych obecnie obowiązujących ograniczeń powinno zostać dłużej? Którego się nie powinno znosić w najbliższym czasie?**

Hmm, zastanawiam się nad tym. Na pewno bym, o, to jest przykre dla pewnej grupy, ale ja na przykład bez bólu całkowicie, ale to jest być może tylko moje, osobiste, ja bez bólu bym wyciął całkowicie spotkania typu sportowe. I koncerty. W sensie to uważam, że dostęp do kultury i sportu jest jakąś fajnością, bez której jesteśmy naprawdę spoko w stanie żyć. I być może nie ma powodu, żeby się tam 10 tysięcy osób spotykało na jakimś stadionie dużym w Warszawie. To na pewno. Ja bym też był w stanie, ale to myślę, że dla wielu osób to jest nie do przejścia w naszym społeczeństwie, to jest ogromna potrzeba. Bardzo dużo ludzi regularnie spotyka się dużymi grupami w kościele. Co tydzień po kilkadziesiąt, kilkaset osób, tak? Spotykają się całe wioski, całe miasteczka, grupują się na wspólnych spotkaniach. Nie wiem, jaki procent ludzie teraz chodzi w Polsce do kościoła, 30-40% co tydzień. Tak myślę, nie wiem tego. Wiesz ile?

**Nie, nie mam pojęcia.**

Ja też nie wiem. Ale myślę, że w latach 90-tych to było z 70%, a teraz myślę, że jest jakieś tam, nie wiem, 40, 30? Plus minus 20%. Strzelam. To znaczy, że jedna trzecia do połowy kraju grupuje się co tydzień w kościele. To kościół gdyby wyciąć, pozamykać kościoły. Ale wszystkie. Rzymskokatolickie, zbory, meczety itd. To myślę, że to na przykład też mogłoby mieć pod względem epidemii sens. Ja bym w sumie też pozamykał w sumie galerie handlowe, po których ludzie się tak włóczą. Są trochę bez sensu pod względem epidemicznym. Być może małe sklepiki na Nowym Świecie czy jakieś takie są, więcej mają tam świeżego powietrza, to też miałoby większy sens. Nie? Że butiki wolnostojące są zdrowsze niż galerie handlowe. Tak mi się wydaje.

**A jeśli chodzi o te miejsca. Bo powiedziałeś, że mógłbyś je wyciąć. Ale czy to oznacza, że mógłbyś je wyciąć na zawsze?**

Nie, nie, w sensie z powodów epidemii.

**Czyli potem mogłyby wrócić.**

Tak, tak. Nie, no dlaczego nie? To są rzeczy, z których, wydaje mi się, że moglibyśmy łatwo zrezygnować. Zwłaszcza, że w przypadku koncertów i sportu to mam wrażenie, że… No wiadomo, że zawsze są ludzie, którzy, nie wiem, żyją z gastronomii na koncertach. I to nie muszą być wcale bogacze, których mi nie szkoda. Ale na przykład samych piłkarzy albo działaczy sportowych to mi mniej szkoda. Mam wrażenie, że to jest grupa, która jest dosyć zamożna i sobie tam odbije za jakiś czas. I że to nie ma wpływu na inne części gospodarki tak duże. Przy koncertach mam trochę poczucie, że artyści to pal licho, jacyś tam muzycy i nagłośnieniowcy. Gorzej dla tych właśnie, którzy są na dole. Czyli jakaś gastronomia, ochroniarze. To tutaj jest taki dramat ludzki, że oni tracą pracę. A czy tam jakoś gospodarka się mocno blokuje? Chyba nie, tylko taki ludzki dramat. Bo gorzej, jak się takie rzeczy sypią, które w przyszłości byśmy chcieli mieć funkcjonujące. A długofalowo… Na przykład nie wiem, że jest fabryka, ta fabryka się zamknie. To jak ona się zamknie i ją ktoś sprzeda i maszyny zostaną sprzedane i rozwiezione, to ona się w 2 miesiące nie otworzy później. To jest problem też. To są trudne decyzje, ja nie potrafię tego wszystkiego powiedzieć. Bardzo ciekawy jest ten model szwedzki.

**Właśnie to jest kolejne pytanie, które chciałam ci zadać. Bo rozumiem, że słyszałeś o tym modelu, skoro sam go teraz wspomniałeś.**

Tak, tak, tak. Z dużą ciekawością obserwuję. Oni tam mieli… Ich jest niecałe 10 milionów, zdaje się, tych Szwedów. I oni mają tam dość dużą liczbę zarażeń. Relatywnie wysoką śmiertelność. Chyba nie taką, jak w tych Włoszech w tych najgorszych regionach, ale wydaje się, że dosyć dużą. Ale mają bardzo dużą śmiertelność u osób starszych. Wysoką, bo też się im posypało, tak jak u nas w tych domach pomocy społecznej. Oni mają ten zwyczaj chyba jako społeczeństwo, że tam są, sposobem spędzania na starość jest bycie w domach starców. I u nich po tych domach starców, trochę jak w szkołach i po garnizonach im tam poleciało. I tam mają dużą śmiertelność. I duże skupiska tej choroby w chyba źle zaizolowanych albo źle opiekowanych tych domach starości. I to tam było duże oburzenie. Ale słyszałem, taki artykuł wczoraj właśnie czytałem, że wygląda na to, że okolice Sztokholmu, które są najgorsze. I tam było już właśnie na granicy załamania tego systemu zdrowotnego. Że tam wygląda na to, że możliwe, że się zaraz zaczną już im, poziomowanie tej ilości chorych, może nawet potem spadek. I to by oznaczało, że ta odporność zbiorowa się zaczyna budować powoli. Czy to aż to by szybko poszło i dobrze? Jeśli u nich by to tak zadziałało, to znaczy, że naprawdę trzeba byłoby u nas emerytów, noworodki schować wszystkich do mieszkania na 2 tygodnie. A reszta, po prostu reszta na dużą imprezę w centrum. Zarażamy się, 80% bezobjawowo i już. I jesteśmy uodpornieni populacyjnie. Będą jeszcze przypadki cały czas pojedyncze, ale to sobie już służba zdrowia poradzi z tym.

**Myślisz, że to by się sprawdziło w Polsce, taki model?**

Trudno mi powiedzieć. Wydaje mi się, że nie. Obawiam się, że wyjściowo jesteśmy w trochę innym, że tamten kraj jest jednak zamożniejszy. Wydaje mi się, że Szwecja ma dużo atutów w tej sytuacji. Ich własnych, zapracowanych oraz niekoniecznie. To znaczy są krajem zimnym o kulturze takiej nie południowej. To znaczy nie mają w zwyczaju się tyle dotykać, przytulać, jest mniej tego kontaktu fizycznego w kulturze w ogóle. Wydaje mi się, że mają bardzo niską gęstość populacji na swoim terenie. Poza kilkoma ośrodkami. W tym Sztokholmie mieli ciężko, bo to duża aglomeracja. I tam właśnie było pytanie, czy im się to nie załamie, że tam też młodzi ludzie chorowali itd. I mają dosyć dobrą służbę zdrowia. Znaczy są zamożnym krajem z dosyć dobrze rozwiniętą tą służbą zdrowia. Takim też egalitarnym, to znaczy spłaszczonym bardzo. Czyli nie mają bardzo dużego rozwarstwienia społecznego. I to mi się wydaje, że to powoduje wszystko, że u nich mają, jakby na wejściu byli w dobrej sytuacji, tak? Znaczy niewielki kraj o małej gęstości, ludzi, którzy się nie spotykają gremialnie i nie całują ciągle jak Włosi czy Hiszpanie. Tak. I dla nich pozamykanie się chyba w domach było też w miarę łatwe. I naturalnie im przyszło. Jeżeli rząd nie wymusił tego, tylko zarekomendował, to dla nich pewnie było to dosyć proste. Więc myślę, że tu, jak pytałaś mnie, czy w Polsce by to się sprawdziło… A, Szwedzi mają jeszcze jeden, może nie tak, że super, super, bo jednak mają dużo imigrantów, którzy nie są tak zdyscyplinowani, ale Szwedzi są jednak narodem skandynawskim i zdyscyplinowanym. I jak rząd zasugeruje coś, to duża część społeczeństwa tak zrobi, bez nakazu. U nas jest dużo więcej takiego… No to tam mówili, no to dobrze nawet, ale to niech inni tak robią, a nie ja. Albo, a co mi zrobi, kto mnie złapie za rękę, tak? A tam jest dużo takiej też, państwa obywatelskiego, takiego poczucia, że coś jest wspólne. Więc oni są dużo łatwiej sterowalni też nie takimi nakazami i pałą jak u nas. Że tam właśnie policja musiała mandaty zacząć wystawiać, bo by się ludzie nie słuchali. Tylko nawet grzeczna prośba dużo u nich zrobi, nie?

**Czyli to jest po prostu inne społeczeństwo, więc taki model u nich…**

Plus te inne rzeczy. To znaczy lepsza służba zdrowia, mniejsza gęstość populacji. Ja nawet, mówię, cały czas nie dotarłem do tego, bo mi się to nie przebija gdzieś w tych, ja jestem ciekawy, co się dzieje w jakichś Indiach albo miejscach, które słyną ze swojej gęstości, z dużego zaludnienia. Takie Indie są mega ciekawe. Znaczy były tylko te sceny, jak policja biła ludzi pałkami na ulicach, żeby szli do domu. Ale to był rodzaj tylko takiego… Że państwo jest opresyjne. Ale nie słyszałem na przykład, czy tam jest jakaś ogromna śmiertelność.

**Jedna rzecz mi się przypomniała. Czym jest dla ciebie ta kwarantanna? To znaczy czy ty w ogóle odróżniasz izolacja, kwarantanna?**

Tak, tak, tak. Ja uważam, że słowo kwarantanna jest nadużywane. W sensie, że jest źle używane. I czasami rozumiem jako skrót myślowy. Natomiast to jest… To jest problem często w ogóle takiego jak w języku, czy użyć słów, które są powszechnie rozumiane, ale trochę błędne i nieprecyzyjne, ale wszyscy mnie zrozumieją. Tylko, że może nadejść taki przypadek, że ten błąd semantyczny, który popełniamy, będzie istotny. Więc ludzie tak mówią, że a, kwarantanna, kwarantanna coś tam. Znaczy nie, jesteśmy w tej izolacji społecznej. I że jak mylimy to z kwarantanną, to właśnie też nielogiczność się wkrada. Ja uważam, że z tym hasłem, z tym hasztagiem, jak tam było? Stay at home, stay home czy jakoś tak? Też on miał ten psychologiczny zły skutek moim zdaniem. Ponieważ ludzie z jakiegoś powodu zrozumieli, znaczy dlatego, że ten wydźwięk był taki, że masz zostać w domu. I w związku z tym były takie, że tam właśnie ktoś siedział na krawężniku pod blokiem z dzieckiem i dziecko rysowało kredą po asfalcie. I ludzie prawie że wrzucali na Facebooka, że patrzcie, jakie to są złamasy, jak siedzą na zewnątrz i po prostu są aspołeczni, bo się tam zarażają. Pojedyncze osoby bez kontaktu z innymi w terenie jakimś tam zielonym albo na podwórku nie powodują rozprzestrzeniani się epidemii. Więc to nie jest właśnie takie negatywne. I to siedzenie w domu, to hasło stay home, tak silnie, że ten dom jest jakiś taki, te budynki nas chronią, to był ten błąd moim zdaniem. W sensie, że to nie budynki nas chronią, tylko brak kontaktu z innymi. Ty masz się nie spotykać z innymi, a nie siedzieć w domu. O właśnie. Więc tak, zapytałaś mnie czy ta izolacja versus kwarantanna. Dlatego ja bym to jednak rozróżniał. Bo kwarantanna jest ścisła, mocna, twardo zarysowana, masz nie wychodzić z domu w ogóle. Bo jesteś bardzo podejrzany. A izolacja ma tylko znaczenie takie statystyczne. Ma znaczenie takie, starajmy się, żeby tych okazji do transmisji choroby było mniej. Czyli właśnie na przykład… Bo mam wrażenie, że ludziom idzie w takie głupie, takie nielogiczne, niemądre zachowania. Czyli na przykład rząd napisał, że wolno chodzić do sklepu, bo trudno tego zakazać, bo ludzie nie będą mieli co jeść. Więc wolno chodzić do sklepu, więc oni będą codziennie chodzić do sklepu, bo wolno. Ale na przykład powstrzymają się przed podlaniem kwiatków w ogródku. Bo to jest poza domem, a to nie jest konieczne, żeby oni przeżyli. Bo to nie jest konieczna rzecz do przeżycia. Więc to się powstrzymają. Ale bułki są koniecznością. A nie są przecież, bo można bułki kupić jedne raz na tydzień, zamrozić albo cokolwiek. I tutaj ta nielogiczność nam się wkradła właśnie. Ale to trudno od ludzi takiego dużego zrozumienia wymagać.

**To chciałabym zapytać cię w takim razie, jak w ogóle u ciebie w obecnym czasie wygląda dbanie o siebie? Chodzi mi o różne zabiegi pielęgnacyjne, ubieranie się.**

Z ubierania się, to też, wydaje mi się, że tak nie ustaliliśmy tego, ale z żoną obydwoje przeszliśmy do tego, że jednak staramy się cały dzień w piżamie albo w dresie. Wiadomo, że chodzimy w jakichś tam domowych ubraniach, ale jednak, żeby ten moment wstanięcia, takiego rozpoczęcia dnia następował. Czasem z opóźnieniem jakimś, czyli ubieram się o 11. Ale jednak, żeby to ten. Tak, tu moja żona jeszcze stoi i się ze mnie śmieje, tu nie widać. Dlatego powinny być właśnie fokusownie, te badania. Bo ja muszę cały czas, wiesz, muszę uważać, co mówię. Bo ona się ze mnie śmieje, że ja do 11 albo 12 to śpię. Tak, to prawda, czasami tak, jak długo oglądamy filmy, albo mi się nie chce wstawać, to rzeczywiście czasami długo. Ale staram się tego nie robić, żeby to nie było takie aż strasznie rozmemłane. Więc tak, staram się wstawać, ubierać, robić różne rzeczy.

**A co ci to daje, że to robisz?**

No, żeby to nie było wszystko takie już strasznie rozmemłane, takie rozmyte. Takie, żeby był jakikolwiek rodzaj reżimu czy rygoru. Jednak człowiek potrzebuje tego. Tak samo jak niektórzy, którzy nawet pracują z… Niektórzy mówią, że nie są w stanie pracować z domu, tak na co dzień, abstrahując od epidemii, bo potrzebują tego wyjścia z domu. Albo nawet potrzebują tego wyjścia z pracy. Że już są w domu i już nie pracują. To jest jakby, wyjście z budynku, przemieszczenie się, zmiana otoczenia powoduje, że już umysł inaczej pracuje i oni przestają zajmować się tamtymi problemami i zaczynają zajmować się innymi rzeczami. Są w stanie wtedy odpocząć, złapać dystans itd. Więc człowiek potrzebuje tych reżimów, tych takich rzeczy, które mu rytm nadają itd. W związku z tym też staram sobie się jakiś tutaj nadawać, choćby minimalny jakiś rytm. A jeśli chodzi o inne rzeczy, takie higieniczno-wizerunkowe, nie wiem, jak je nazwać. To tu jest taki kłopot, bo ja jestem złym modelem do zadawania pytań. Bo ja nie chcę powiedzieć, że nie dbam o siebie. Ale trochę tak. W sensie, że ja nie jestem ani specjalnie nie chodzę do fryzjera. To się też zmieniło trochę…

**Ale zmieniło się teraz?**

Nie, nie, nie. Kiedyś. W sensie, że ja przez 15 lat nie byłem ani razu u fryzjera. Bo miałem długie włosy i tylko przycinałem końcówki. I to nie wymagało żadnej zmiany. Teraz trochę tam czasem chodzę albo tam jakąś brodę. Ale też tak trochę… Parę razy byłem u jakiegoś golibrody. Tak, to mnie trochę denerwuje i w końcu może po prostu obetnę na krótko brodę, bo jest bardzo krzywa już itd. Ale na razie nic z tym jeszcze nie robiłem. A inne rzeczy są takie… Ja nigdy się nie stroiłem specjalnie ani nie jestem jakimś elegantem. W związku z tym tutaj też nie ma jakiegoś dużego wpływu. Bo to jakby nie poszło ani w dół, ani w górę. Znaczy nie wiem, dlaczego ktoś miałby się bardziej stroić z powodu epidemii. Ale nie poszło, nie zmieniło się bardzo, bo zawsze było na niskim poziomie. Tu nie ma z czego schodzić trochę. Więc już musiałbym się przestać myć chyba. A to nie, myję się. Myję głowę, golę się czasem.

**A może w takim razie u swojej żony zauważyłeś, że jakieś pielęgnacyjne zabiegi nowe weszły do jej życia, a może coś przestała robić?**

Nie, znaczy przestała tak. Myślę, że ona by pewnie chętnie poszła do fryzjera, albo gdzieś tam. Też jest tak, że moja żona nie jest takim typowym strojnisiem. Dużo się nie maluje. Raczej maluje się, nie wiem, 4 razy w roku pewnie na jakieś imprezy albo jakieś święta, albo coś takiego, wyjścia na zewnątrz. Ona mówi, że jej to nie przeszkadza, że ma nawet jakiegoś umówionego fryzjera na maj, ale jak się przełoży na czerwiec, to nawet nie będzie jej przeszkadzało. Bo też wiesz, nie wychodzimy, nie widujemy się z ludźmi, nie chodzi do pracy. Więc też jakoś bardzo jej to nie robi, nie przeszkadza. Bo my nie jesteśmy jakimiś strojnisiami. My jesteśmy raczej takimi, styl taki, nie wiem jak to nazwać. Nawet nie, że casualowy. Tylko taki dżinsy plus T-shirt. Tego typu. Ewentualnie moja żona zamiast torebki, jak to eleganckie panie nosi raczej ten plecak do chodzenia po górach czy coś takiego.

**A jakieś kwestie ubraniowe? Bo nie wiem jak często robicie zakupy ubraniowe?**

Jak najrzadziej. Ja jak najrzadziej, moja żona też. Znaczy, nie wiem, ja kupiłem w zeszłym roku… W sensie kupiłem to jest duże słowo, żona mi kupiła, bądźmy szczerzy, choć to bardzo źle brzmi. Żona mi kupiła… Jedną czy dwie pary spodni? Teraz jedną. No właśnie, jedną mi parę spodni kupiła. Żebym miał, jak bym dostał pracę, żebym miał w czym chodzić do biura, bo poprzednie biurowe rzeczy już się nie nadawały. I przeszły w taki stan… Bo to jest gradacja. Są rzeczy nowe, w nich się chodzi do ludzi i do biura, potem są takie gorsze i w nich się chodzi do sklepu, z psem wokół bloku itd. I potem są takie najgorsze i to się używa do bycia na działce albo do jakichś prac w ogródku albo coś takiego.

**Czyli teraz w domu chodzisz w których?**

No właśnie, żeby już nie chodzić w takich zupełnie podartych i piżamach, to w takich codziennych.

**Czyli te środkowe?**

No tak, tak. Jak przedwczoraj sprzątałem piwnicę, bo właśnie stwierdziłem, że zrobię porządek, tak trochę wiosennie a trochę dlatego, że mam czas, to mamy suszarnię w piwnicy, w której suszymy pranie, to żeby to miejsce było czyste, to tam odkurzyłem, umyłem okno w piwnicy, bo było strasznie zasyfione. I jakieś chwasty, którymi to okno, bo to jest w takim obniżeniu, zarosło, jakimi krzakami, to też to wyciąłem itd. To ubrałem takie rzeczy typu najgorszy dres i brudna bluza.

**Pytam się dlatego, że niektórzy ludzie na przykład, jak przychodzi nowa pora roku, to kupują nowe ubrania. Ale rozumiem, że ty nie należysz do tej grupy osób?**

Nie, nie, nie. Absolutnie nie. Nie, ja myślę, że są… To się zmieniło w którymś momencie, ze względu głównie takich zawodo-pracowych. Że poszedłem i kupiłem kiedyś 4 koszule w kratę, 10 nowych T-shirtów. I 2 pary spodni. Zawsze z butami mam kłopot, bo mam bardzo dużą stopę i nie ma… Kiedyś próbowałem kupić buty skórzane na jesień. Bo też żona się denerwowana, że weź wreszcie porządne buty, a nie chodzisz tam w szmaciakach, zawsze ci noga potem marznie albo coś tam. I tam masz mokre itd. Poszedłem kiedyś, właśnie chciałem takie buty, jak masz dżinsy i koszulę, żeby pasowały. Nie lakiery jakieś eleganckie do garnituru, tylko takie po środku, żeby były też trochę wygodne, żeby dało radę wytrzymać w nich 8 godzin itd. Skórzane może jakieś buty. Poszedłem do Arkadii i przeszedłem, żeby sobie udowodnić, że to nie jest tylko moje takie lenistwo i niechęć do zrobienia zakupów. I przeszedłem po wszystkich sklepach, żeby zobaczyć, czy mają na mnie buty. No to ja wchodzę do jakiegoś tam CCC i jest ściana… Wchodziłem nawet do takich sklepów, w których bym nie kupił butów, bo bym nie chciał tyle pieniędzy wydać na nie, jakieś tam typu Hugo Boss czy coś. I mówiłem: dzień dobry, potrzebuję takie i takie buty, skórzane mogą być, lekko ocieplane, coś tam. I ściana po prostu butów jest. I ja mówię, tylko wie pani co, jest taki problem, ja mam numer 47. A pani mówi aha, no to z tego to ten, ten i tutaj brązowe na przykład. I z tysiąca pudełek, które tam stoi są 4 pudełka. No to ja przymierzam. Większość jest albo brzydka albo nie pasuje. I tyle. Nie byłem sobie w stanie kupić. Więc ja kupuję sobie buty w Decathlonie. Decathlon ma różne, sportowe obuwie i tam są, jest moja rozmiarówka. I się znajduje jakąś parę, która jest w miarę wygodna. A poza tym to kupiłem 3 lata temu czy 4… Ze 4 lata temu właśnie takie zakupy, T-shirty, parę koszul, dwie pary długich spodni. I to mi na 4 lata wystarczyło. To były rzeczy do biura. Ja w biurze też w T-shircie chodziłem. Tylko na jakieś tam powiedzmy spotkania to… Nawet miałem tak, że w biurze chodziłem w T-shircie, ale w biurku, w szafce miałem poprasowane, poskładane ubrania takie lepsze pochowane. Że jak na przykład trzeba było pójść na spotkanie, to szybko do łazienki, koszula, jakieś, nie wiem, spodnie w kancik. I już idę na spotkanie. I potem z powrotem do biurka i spokój.

**Zaczęłam o tych ubraniach mówić, bo chciałam się dowiedzieć, czy teraz kupujesz jakieś ubrania.**

Nie, nie, nie. Nie, mówię, mam jedne, eleganckie spodnie, w których do tej pory… Znaczy eleganckie, nowe, w których byłem 2 razy na rozmowie rekrutacyjnej. Bo pasują do koszuli i są w dobrym stanie. I to mi wystarczy na najbliższy… Jak nie znajdę pracy, to na najbliższe 5 lat. A jak znajdę pracę to na najbliższe półtora roku.

**Powiedz mi jeszcze tak jakby z punktu widzenia bycia konsumentem, to czego ci teraz najbardziej brakuje? Bo mówiłeś na przykład, że chodzenie po galeriach to nie jest coś, co wy normalnie robicie. Ale takie inne aktywności, zachowania. Nie wiem, siłownia, basen, fitness.**

No tak, tak. zapomniałem, że to właśnie też się konsumuje w jakimś sensie. No tak, na basen czasami chodzę. To trochę tak, trochę szkoda. Konsumenckim to wiosna sprzyja piciu alkoholu w plenerze czy to w knajpkach czy to gdzieś na bulwarach itd. To tego konsumencko mi najbardziej brakuje. Będzie mi za chwilę brakowało. Takiego wiosennego bycia gdzieś w ogródkach piwnych albo jakichś takich spotkań. Basenu też. Nie wiem, czy… Co ja konsumuję i czego… Wiesz co, czasami takiego po prostu wstąpienia sobie do takiego… Właśnie trochę pilnuję się, żeby nie robić mikro zakupów. I takiego czasem, że a, to pójdę 3 rzeczy, co nam brakuje, albo na co mam ochotę, takiego impulsowego kupowania. Że właśnie, nie wiem, z kolegą byłem na rowerze. I pojeździliśmy sobie na rowerze, uznając, że jedziemy sobie razem, coś tam gadamy i właściwie mamy jakąś odległość między sobą metra do dwóch. I właściwie możemy spoko chwilami pogadać, jest to jakiś rodzaj kontaktu. Ale już na przykład na piwo się nie zatrzymaliśmy. A wiem, że gdyby nie było epidemii, to pewnie byśmy sobie obrali, że jedziemy do jakiegoś miejsca, gdzie można usiąść i wypić sobie piwo. Nie wiem, nad Wisłą albo pod jakimś sklepem czy coś takiego.

**I mówisz, że to właśnie ze względu na porę roku, pogodę ci tego brakuje?**

Tak, tak, tak. No, wiosna, lato to są fajne takie outdoorowe rzeczy. O, teraz na przykład tak. Kupiłem sobie, co potrzebowałem, bo naprawiałem łóżko, bo nam się tam rozwaliło i potrzebowałem ten zszywacz tapicerski, to sobie kupiłem. A teraz przed kolejną decyzją konsumencką, to jest śmieszne o tym elaborat opowiadać, ale pożyczyła ode mnie i nie zwróciła koleżanka i wiem z plotek, że zepsuła. Miałem spasowane takie pudełka w tym naszym kamperze. I ona pożyczyła, bo ona organizowała kolonie z dziećmi, bo ona jest tam pedagogiem. I potrzebowała skrzynek, które miałem w samochodzie. Takich, które da się jeszcze pionizować. I pożyczyła to ode mnie na 2 tygodnie 3 miesiące temu na ferie. I mi wszystkie narzędzia, jakieś graty turystyczne po samochodzie latają. Już wiem, że od niego tego chyba nie odzyskam z powrotem, zwłaszcza, że teraz jest epidemia, a to zostało w jakimś samochodzie jej fundacji, coś tam. Ja muszę odkupić sobie te pudełka chyba po prostu, już nie wiem, czy one do mnie wrócą kiedykolwiek. No i teraz zastanawiam się, jak to zrobić. Bo właśnie muszę wymyślić, jakie one powinny być. A normalnie bym sobie np. do Leroy i pooglądał, co mają. Albo po jakichś sklepach. I tutaj nie wiem, pewnie będę musiał stronę internetową jakąś przeszukać i pomyśleć, co bym chciał. I zakupić pewnie online. To to jest taka różnica. Że nie mogę sobie pójść powybierać do sklepu.

**Dziękuję bardzo.**
