# Supplementary material for: The challenges arising from the COVID-19 pandemic and the way people deal with them. A qualitative longitudinal study
Source: PLoS One. 2021 Oct 11;16(10):e0258133. doi: 10.1371/journal.pone.0258133 (PMC8504766; doi:10.1371/journal.pone.0258133)
Supplement: S1 Dataset — (ZIP) [file pone.0258133.s003.zip › Transcriptions/stage 3/10.3_F_55_couple, no children.docx]

**10.3_F_55_couple no children**

**Co się u Pani działo przez ostatnie dwa tygodnie?**

My się chyba widziałyśmy tuż przed świętami.

**To był piątek przed świętami.**

Tak. Ja potem spędziłam 5 godzin w sklepach po zakupy, bo stałam bardzo długo - 2 godziny po to, żeby wejść do sklepu, później odpowiednio w sklepie długo. Przy kasie były jakieś problemy, bo miałam bardzo duże zakupy. Potem je rozwoziłam do swojego taty, do teściowej. Byłam bardzo zła ogólnie. No a potem, w sobotę, już przed świętami, to kończyłam jeszcze jakieś porządki. Wpadłam nagle na pomysł, że jeszcze zrobię ciasto, które wypatrzyłam w telewizji. Pobiegłam do sklepu i mnie nie wpuścili, bo akurat pobiegłam w tym czasie dla seniorów - zapomniałam o tym *[śmiech]*. Ale w efekcie, jednak zrobiłam to ciasto. A potem, to już były święta i były takie bardzo nietypowe. Miałam powiedzieć, czy zrobiłam ten pasztet, co wtedy rozmawiałyśmy. No zrobiłam, ale nie, nie będę tego powtarzać. Bardziej udane było to nowatorskie ciasto. To ciasto było ok. A tak, no to z mężem po prostu usiedliśmy do śniadania. Przedtem trochę przez Messengera porozmawialiśmy z synem, z synową i tyle. Takie bardzo spokojne były te święta.

**A ten spokój to dobrze dla pani?**

Nie było smutno. Jakoś tak - ok. Ja po prostu odpoczywałam. Nic się takiego nie działo. Pierwszy dzień, to po tym śniadaniu chyba się zdrzemnęłam, tak na spokojnie. A drugiego dnia, to z kolei cały dzień szyłam. Na łóżku, tak jak teraz siedzę, bo uwielbiam tu na tym łóżku. Najpierw zaczęłam szyć jakieś kolorowe maseczki dla mnie i dla męża, jeszcze muszę je dokończyć. Potem miałam jakieś prace ręczne jeszcze do szycia i oglądałam filmy jeden po drugim.

**Pani wcześniej też szyła?**

Ja mam takie 2 torby, w które zawsze obie wkładam to, co trzeba naprawić w domu do szycia. I jak przychodzi ten dzień, to zabieram się za to i to robię.

**Szyła Pani wcześniej też rzeczy od zera?**

Tak, to od zawsze.

**Jak jeszcze spędzała pani czas, już po świętach?**

Po świętach wróciłam trochę do pracy. Nawet tak się coś niecoś ruszyło. I w zasadzie, no trochę w domu, trochę w pracy. Był taki moment, że już kompletnie zwariowałam, że już chciałam wziąć do siebie wnuczkę małą. Że już miałam dosyć. Ale jednak odpuściłam od tego, więc na razie nadal jesteśmy w izolacji. Aczkolwiek różne opinie n temat dochodzą, że jak najbardziej mogę się z tą wnuczką zobaczyć i to są słowa lekarza - nie to, że tam... Innego z kolei, że nie. I tak w końcu nie zdecydowałam się.

**Co to był za moment, że pani pomyślała, żeby wziąć wnuczkę do siebie?**

Po pierwsze, to już strasznie za nią tęsknię. Po drugie, chciałam tak trochę... No bo, ja wiem, że ta moja wnuczka to jest niezły gagatek i ona mocno dokazuje synowi i synowej. Ponieważ oni... Znaczy nie to, że nie są cierpliwi, to nie o to chodzi, ale tak chciałam po prostu im trochę ulżyć, żeby oni mogli sobie odpocząć przez 3 dni, a ja żebym się pocieszyła nią sama. Ale tak, pomijając, że w ogóle się nie zdecydowałam, to byłyby też inne trudności. My mamy psa, on jest uratowany przez mojego syna i ten pies jest po przejściach, był źle traktowany i on bardzo nie lubi dzieci nadal. Ja wymyśliłam, że nawet, jak wezmę wnuczkę do siebie, to oddam na ten czas psa synowi. Nieraz tak było, że on psa zabierał do siebie, bo to w końcu jego pies. Natomiast w międzyczasie mój mąż kompletnie zwariował na punkcie tego psa i uważa, że nikt się tak dobrze nie opiekuje tym psem, jak on. I że jak on nie jest z nami, to dzieje mu się jakaś straszna krzywda. W związku z czym, nie zgodził się, żebym zamieniła na 3 dni wnuczkę z psem. Jak oni byli tu wszyscy, to każdy uwagę zwracał. A jak byłabym i ja i pies i Zuzia - a Zuzia jest taka, że wszędzie łazi i tego psa najchętniej by wytarmosiła. Już raz ją ugryzł. Więc nie chcemy doprowadzać, żeby... Więc bałabym się, że musiałabym mieć oczy dookoła głowy. Ale tak czy owak, do tego w ogóle nie doszło, bo ani pies nie pojechał, ani Zuzia nie przyjechała.

**Pojawiły się jakieś nowe czynności? Szycie nie pojawiało się wcześniej w trakcie epidemii?**

Nie, ale u mnie szycie pojawia się co jakiś czas, kiedy gdzieś się to zbiera, to ja siadam i sobie szyję. Maszyna stoi na biurku, jest nieschowana, bo mam zamiar uszyć pościel dla Zuzi.

**A są jakieś czynności, z których pani zrezygnowała?**

Nie.

**A jak z pani pracą? Ja wyglądają zamówienia?**

Tak, tuż przed świętami dostałam jakieś zamówienia. Wczoraj też spotkałam się z taką klientką, która z resztą została ambasadorką i dzisiaj między innymi wysyłałam jeszcze te zamówienia. No tak po prostu z kilka osób się zgłosiło.

**To są kosmetyki do makijażu czy raczej pielęgnacyjne?**

I takie, i takie. Wszystko. To jest moje źródło pracy - katalog. Tu jest wszystko, nawet ubrania, które bardzo sobie chwalę.

**Czyli nie pojawiły się jakieś zmiany w trendach zamówień?**

Nie, nie.

**A ubrania ludzie teraz zamawiają?**

Tak, oczywiście. I ubrania, i bielizna, i piżamki. Generalnie wszystko.

**W tym czasie epidemii nie ma zmian, jeśli chodzi o zamówienia ubrań?**

Nie ma.

**Pojawiły się jakieś rzeczy, które zaczęły pani przeszkadzać?**

Oczywiście, no dzisiaj taki dzień jest może nie tyle lżejszy, co taki zaplanowany, więc się tak nie zastanawiam. Natomiast na samym początku tygodnia bardzo się źle czułam. Miałam już dosyć tej izolacji, tego koronawirusa, no po prostu... Płakać mi się strasznie chciało. Że nie wiadomo, jak to długo potrwa. Ja mam przyczepę kempingową nad morzem, z której sami korzystamy po części, a po części też przyjeżdżają tam ludzie. I zupełnie nie mam pojęcia, jak to będzie w tym roku. Czy w ogóle do tego dojdzie, czy nie. Jestem taka strasznie tym... Poza tym, nie mogę już czytać tych rzeczy w Internecie. Tak, jak kiedyś wszyscy bili brawo dla medyków, a teraz jak się słyszy o tym hejcie... Ja się aż popłakałam chyba w poniedziałek czy wtorek, jak czytałam, jak ci lekarze muszą... No pod osłoną nocy, wracają, czy jak ludzie wylewają na nich pomyje. W głowie mi się to nie mieści po prostu.

**Podjęła pani jakieś działania, żeby sobie radzić z pogorszeniem nastroju?**

Tak, jak mówię, jak mam sporo zajęć różnych, to wiadomo, że muszę się tym zająć, robię. To związane było i z pracą, i z aptecznymi zakupami dla mojego taty, musiałam tam jeździć odbierać i potem znowu zawozić do niego. A, już wiem, co jeszcze się działo! Ja w poniedziałek miałam prowadzić kolejne szkolenie, ale strasznie się źle poczułam dodatkowo, bo mam zapalenie pęcherza i to mnie tak też wyprowadziło z równowagi. Menadżerka nade mną przejęła to ode mnie. Ja byłam na tym szkoleniu, ale nie prowadziłam tego sama, tylko ona, bo ja bardzo się źle czułam. Teraz też biorę te leki. Dlatego siedzę na łóżku, w kocu.

**Obrazki. Który z obrazków najlepiej oddaje pani emocje teraz i w ciągu ostatnich 2 tygodni?**

Pierwsze, co mi się od razu nasuwa, co było związane z początkiem tygodnia, kiedy tak się źle czułam i miałam wszystkiego dosyć, to jest 14. Że byłam taka po prostu... Nie wiem, jak to określić. No taka zaplątana. Że już miałam tego szczerze dosyć. Że nie widziałam, jak tą sytuację rozwiązać. Jak rozwiązać z tą Zuzią. Wszędzie oczywiście też są skrajnie różne opinie. Niektórzy mówią, że jednak powinniśmy wychodzić, że te maseczki nic nie dają. Oczywiście, teraz przecież jeszcze chodzimy w tych maskach, co jest uciążliwe bardzo. Więc taka byłam zaplątana. Po prostu zła.

**Tak, że nie widać rozwiązania?**

Dokładnie. Nie widać rozwiązania. Nie widać, kiedy to nastąpi, jak to nastąpi.

**Jeszcze jakiś z obrazków pasuje? To był początek tygodnia. A później?**

Potem, oczywiście, tutaj nasuwa mi się 16, ale to w związku z pożarami w Biebrzańskim Parku. Jeszcze dodatkowo jest tak sucho wszędzie. Ale to mi się skojarzyło na zasadzie, że tam się to dzieje, natomiast może nie odnośnie mojego odczuwania.

**Czyli ta 14 oddaje pani emocje najlepiej?**

Tak.

**Skąd się wziął ten stan zaplątania?**

Według mnie to już trwa trochę długo. Poza tym, z tej niepewności, że nikt tak naprawdę nie wie, jak to ma dalej wyglądać, jak to długo będzie trwało. Poza tym takie zmienne, jakby najpierw nasz minister mówił ze śmiechem, że maseczki do niczego są nieprzydatne, później nagle całą populację ubrał w maseczki, że musimy je nosić. To jest dla mnie wszystko takie, tak naprawdę, nie wiadomo, co tu jest prawdą i...

**Nie wiadomo, w co wierzyć?**

Tak, nie wiadomo, w co wierzyć. I takie...

**Z jednej strony to, że to trwa długo i nie wiadomo, ile jeszcze, a z drugiej, że są sprzeczne informacje?**

Dokładnie.

**A jakieś jeszcze emocje się pojawiły? Np. zmęczenie sytuacją?**

To nie jest zmęczenie. To jest taka wściekłość, bezradność, taka... Złość, no złość. Nie wiem, no. W poniedziałek, jak rozmawiałam z siostrą i jak ja mam te swoje stany, kiedy się gorzej czuję, to we mnie gdzieś tak to buzuje, że ja sobie z tym nie radzę i czasami muszę sobie pokrzyczeć. I właśnie mówiłam do siostry, że wstanę i będę krzyczeć, bo inaczej, to tego nie wyładuję z siebie.

**Udało się pani to wyładować w inny sposób, czy pokrzyczała sobie pani?**

Nie pokrzyczałam, ale po prostu jakoś się już, nie pamiętam, tak krok po kroku czymś zajęłam.

**Dla pani to zajęcie się czymś, to sposób radzenia sobie. z tymi emocjami?**

Tak.

**Wspomniała pani o bezradności. Czuje pani, że nie wpływu na sytuację?**

No, czuję, że ewidentnie nie mam wpływu i to mnie tak właśnie denerwuje. Już pomijając, bo te ostatnie tygodnie były pod kątem wysyłania wniosków do ZUSu o niepłacenie tych składek, wystąpienie o postojowe, no różne takie... To musiałam sobie pozakładać te różne strony, to PUE w ZUSie i ogóle, to też takie...

**Może przychodzi pani do głowy jakiś lepszy obrazek niż te, które ja pokazuję, który oddaje pani ogólny stan emocjonalny w związku z sytuacją?**

Znaczy, obrazek żaden mi nie przychodzi, ale czasami tak sobie myślę i tak się zastanawiam, że jeszcze nigdy w życiu nie znalazłam się w takiej dziwnej sytuacji. Nie mówię, że tylko ja - ogólnie, my tu wszyscy. Ja się np., tak jak zawsze się bałam, że zachoruję na nowotwór i będę powielać historię kobiet w naszej rodzinie, tak teraz najbardziej się boję tego koronawirusa. Że jeśli coś się stanie, to ja go mogę nie przeżyć np. I nie tylko ja. Staram się teraz nie czytać o tym, bo im więcej czytam, tym gorzej to na mnie wpływa. Ale teraz tam gdzieś, że ludzie z grupą krwi A są bardziej podatni na te zakażenia. I w tym momencie panika straszna, bo mój mąż i moje dzieci mają grupę krwi A. Ja mam B. I w związku z tym, co to będzie, jeśli im się coś stanie. I takie to wszystko...

**Pojawia się lęk nie tylko o panią, ale też o bliskich?**

Oczywiście. Jak się pytałam, bo mam znajomych lekarzy, to jedna powiedziała, że to nie ja jestem zagrożeniem dla wnuczki, tylko wnuczka jest dla mnie, żebym to ja nie zachorowała. Ale... Nie wiem, nie wiem. Najchętniej to bym chciała się dowiedzieć, że ja już to przechodziłam. Teraz oczywiście też od początku tygodnia wszędzie piszą o tym, że zaczęły się pojawiać te testy molekularne. Nie molekularne, tylko serologiczne na wykrycie tych przeciwciał, czy się miało, czy nie miało. Ale jedni twierdzą, że one wskazują te przeciwciała do dwóch tygodni, a jeśli się miało wcześniej, to nie wykażą. Inni twierdzą, że wykażą i nadal nikt nic nie wie, jedni przed drugimi...  Że te testy są niemiarodajne, a inni twierdzą, że jak najbardziej, że należy robić te testy. Bo to już nawet nie jest kwestia ceny, ale że one mają się pojawić w aptekach. Z resztą nawet dzisiaj rano czytałam jakiś wywiad z lekarzem ze szpitala na Wołoskiej i on też pisał o tym, że za chwilę będziemy mieli już dostęp do testów serologicznych, które dadzą nam odpowiedź, czy to przechorowaliśmy czy nie. Ja np. bardzo bym chciała taki test móc zrobić i zobaczyć.

**To by panią uspokoiło?**

Tak. Gdybym się dowiedziała, że to przeszłam.

**Ten lęk się zmienia czy jest taki sam, jak na początku?**

To są takie... On nie jest przez cały czas. To są momenty, kiedy... Generalnie może słabnie, tylko bardziej złości, że nie wiadomo, co dalej z tym. Natomiast oczywiście zdarzają się chwile jakiejś paniki, że jednak jest to bardzo groźne i że może się coś stać.

**W jakich momentach pojawia się ta panika?**

Np. jeśli oglądam jakieś wiadomości, jeśli coś kolejny raz czytam o wynikach. Ile np. osób się zaraziło, ile osób zmarło. I wtedy to jest takie dobitne, że to jest, że to cały czas się dzieje, że to cały czas nas dotyczy. Że to, jak tego nie oglądam, to nie znaczy, że to nie ma miejsca.

**Moment zetknięcia się z wiadomościami powoduje gorsze samopoczucie?**

Dlatego staram się jak najmniej. No niestety. Znaczy nie niestety, tylko tak nie chcę ciągle oglądać tych wiadomości, bo to... Już nawet nie chcę wiedzieć, co tam się dzieje w tych Stanach.

**Mówiła pani o swojej wizycie z sklepie przed świętami. Jak się wtedy pani czuła?**

Byłam zła... Że te ograniczenia, to ja to rozumiem. Natomiast czułam się trochę zła i do tej pory się czuję trochę wykorzystywana przez moją rodzinę. Bo musiałam robić duże zakupy dla mojej teściowej, podczas gdy moja teściowa ma syna - mojego męża, i ma córkę. I oni nie robią jej zakupów, tylko robię ja. A oni przychodzą i chodzą z nią na spacerek. Z drugiej strony, ojciec, ja i siostra. Moja siostra jest ode mnie starsza i już nie przyjeżdża, bo twierdzi, że nie wchodzi do tramwaju. Ale ja chodzę do sklepu. Z drugiej strony, jak sobie pomyślę, że by im się miało coś stać dlatego, bo ja stwierdziłam, że więcej tego robić nie będę, to nie mogłabym też tego sobie podarować. Z drugiej strony, ja się cały czas narażam. I w ten piątek ja byłam taka już wściekła.

*[Odczytała wiadomość od synowej]*No właśnie, a propos tych testów. Że te testy paskowe, czy tam jakieś, że są niemiarodajne. Ona mi właśnie to pisze. Bo cały czas się zastanawiamy, czy tych testów nie kupić, nie robić.

**Czyli złość podczas wizyty w sklepie wynikała z tego, że musiała pani pomóc rodzinie i to panią obciążało?**

Tak. W którymś momencie doszło do jakiejś scysji między mną a mężem. Wykrzyczałam mu, że są lepsi i gorsi. Że ja jestem tego gorszego sortu, bo muszę robić te zakupy teściowej, a inni są do celów wyższych.

**Jak pani bliscy znoszą teraz tę sytuację?**

Mój mąż cały czas wychodzi do pracy. Mimo, że na razie nie może prowadzić zajęć takich, jak zwykle. Aczkolwiek powoli już się ludzie zaczynają zgłaszać. Bo mój mąż prowadzi kursy nauki jazdy. Więc już się zaczyna przygotowywać, zakupił różne maseczki, przyłbice, płyny. Bo wiadomo, że musi być ileś osób na jednej przestrzeni, żeby powrócić do wykładów. Więc on nie daje za wygraną, on się przygotowuje. Teściowa cały czas siedzi w domu i... A, jeszcze taka sytuacja była - to są teki głupoty, że chciała doniczki na balkon. Do tej pory przez wiele lat ja zawsze z nią jeździłam na giełdę i jej tam wszystko robiłam. W tym roku, ona chyba nie chciała mnie obciążać przez to wszystko i poprosiła mojego męża. I proszę sobie wyobrazić, że on sobie nie poradził z tymi doniczkami. Nie potrafił ich ani zamówić, ani coś, mimo, że on jest taki najmądrzejszy i wszystko wiedzący i w ogóle. I gdzieś tam nawet pojechał po te doniczki, ale jak zobaczył tłum ludzi, no to przecież on nie będzie stał do wejścia do Castoramy. I efekt był taki, że teściowa już mówiła: *nie, nie, to zostawcie te doniczki,*ale oczywiście w międzyczasie to kółko wraca do mnie. *Eluniu, a może jednak byś...?*Efekt był taki, że oczywiście ja zamówiłam te doniczki. Już przyszły i udało się i zamówić, i wszystko kurierem. Jej córka - ta wyższego sortu, to przyjeżdża tylko właśnie na spacer z nią wychodzi. A też mogłaby matce kupić doniczki. I tak mnie to... Że ja niby jestem taka wspaniała, bo... Ale to, ja już mam dosyć być taką wspaniałą. Niech sobie sami zaczną radzić. Więc, jak się okazało, niekoniecznie.

*[Dzwoni kurier]*O, między innymi właśnie kolejna rzecz zamówiona dla teściowej. Bo nikt nie może jej zamówić. Bo jakby powiedziała córce albo synowi, to oni by na nią nawrzeszczeli, że po co to potrzebne. A Elunia weźmie i zamówi.

**Czuje pani obciążenie z tego powodu, że to zawsze pani musi pomagać?**

No tak. Ja nie mogę powiedzieć, bo ja bardzo kocham swoją teściową i my bardzo dobrze żyjemy ze sobą. Ona tam czasem gada te swoje głupoty, ale to już inna historia. Natomiast nikt tam do niej nie ma cierpliwości pokazać w komputerze czy telefonie, wszyscy ją zbywają. I w końcu zawsze spada to na mnie. Ja od lat robię teściowej przelewy przez Internet, bo ona sama się boi, nie jest w stanie przejść tych wszystkich kroków. Nikt się nie zastanawia... Ale ja nie narzekam i robię to od lat, miesiąc w miesiąc.

**Zauważyła pani jakieś zmiany w emocjach wśród pani bliskich?**

Nie no, moja teściową jest pełna takiej, że będzie lepiej. Ona nawet, jak wyczuje, że ja jestem gorsza czy słabsza, to ona mnie pociesza. Mój mąż dzisiaj stwierdził, że no tak, jeśli będzie wszystko wracało, to znowu się zrobi problem z transportem, że dużo więcej osób będzie chciało jeździć transportem publicznym, a przecież te ograniczenia są. To może w związku z tym dużo więcej osób będzie chciało robić prawo jazdy. *[śmiech]* I tym sobie tak... Ja mówię, no, oby tak było.

**To jest jakaś szansa dla jego biznesu?**

Tak, powrotu do biznesu. Moja siostra jakoś nie narzeka. Mój tata nie może uwierzyć w tę całą sytuację. Ma 87 lat, więc dla niego to jest nie do uwierzenia. Ale on jest taki trochę... Nie, że nie dochodzi do niego coś, tylko... *[Kurier dzwoni do drzwi]*Tata jest trochę oderwany od rzeczywistości - po pierwsze chory, po drugie ma mocną sklerozę. Najbardziej go złości, że nie możemy tak przyjść i posiedzieć. Że jak przychodzimy, to i wychodzimy zaraz. On ma oczywiście swoje teorie, że to na pewno nie ma żadnego wirusa, że wszyscy kłamią - no, takie tam.

**Pani tata namawia panią, żeby pani jednak przyszła i posiedziała dłużej?**

Tak. On nie mówi, że nie ma czym się stresować, tylko, że jest to niesamowite i nie wie, czy w to wierzyć czy nie.

**Jeśli chodzi o dalsze otoczenie, to zauważyła pani jakieś zmiany w emocjach czy zachowaniach?**

Nie. Tylko takie zdziwienie. Że jak się kontaktujemy, to na odległość. Że nie możemy się ani przywitać... Bardziej takie zdziwienie, że w jakich nam przyszło żyć czasach. Jak to się świat zmienił.

**Ma pani wrażenie, że to jest takie nierzeczywiste, nierealne?**

Tak.

**O jakich zmianach w ograniczeniach pani słyszała?**

O tych, że możemy z powrotem lasy, parki. Co prawda place zabaw nadal zamknięte dla dzieci. Że może być już więcej osób w sklepach. Natomiast, ja taką miałam... Kiedy to było? A, już wiem! W pierwszą sobotę po świętach pojechałam rano na takie duże zakupy, żeby zdążyć przed seniorami. Co prawda, teraz już w sobotę nie będzie tego, szczęśliwie. Natomiast byłam nie tyle oburzona, tylko taka zdziwiona i zaskoczona. Bo nie chciało mi się liczyć tam wszystkich tych ludzi w Biedronce, ale było zdecydowanie więcej niż do tej pory miało być, czyli że 3 osoby na kasę w sklepie. Stały normalnie długie takie ogonki do kas. Cały sklep był ludzi. Większość w maseczkach, ale zdarzali się też ludzie bez maseczek.

**Wtedy już obowiązywał nakaz noszenia maseczek?**

Tak. A nie wszyscy mieli. Wtedy obowiązywały 3 osoby na jedną kasę, a na pewno było znacznie więcej. Dlatego mówię, że już nie chciało mi się stać i liczyć ludzi w sklepie, ale... Bo jeszcze przedtem jak tam robiłam zakupy, to ktoś stał przed wejściem i kontrolował sytuację. Z resztą, tak samo, jak ja przed świętami robiłam, to 2 godziny stałam, żeby wejść do sklepu w ogóle. Na ulicy.

**Teraz już nie było tej kontroli?**

Nie.

**A ludzie trzymali odległość od siebie?**

Nie, nic.

**Jak pani się z tym czuła, że ludzie nie przestrzegali tych zasad?**

No właśnie i to mnie tak wtedy zastanawia, czy mamy tego przestrzegać, czy nie, czy to jest tylko robienie nas w konia. Dla mnie już absurdalny jest w ogóle ten wymóg noszenia maseczki na zasadzie, że to nie musi być maseczka, tylko niech będzie chustka, czy cokolwiek. To jest dla mnie głupie, bo to... Nie wiem, kogo ma ochronić - czy tych, co noszą, czy tych, którzy...

**Jak to jest z tymi maseczkami? Kogo one chronią?**

Generalnie maseczka chroni osobę, nie tą, która maseczkę nosi, tylko ma chronić następną osobę, żeby ta, co ma maseczkę jej nie zaraziła. Wiadomo, że inaczej wygląda sytuacja w szpitalach, gdzie cały personel medyczny musi nosić maseczki. Tutaj jakby w drugą stronę to ma zadziałać. Ale tyle się mówi o tym, że jeśli te maseczki będą wilgotne, to więcej zbiorą na sobie tych drobnoustrojów. Już pomijając, że nawet, jak oddychamy, kaszlemy i ten sprej, który wydychamy, że przez taką maseczkę bawełnianą wielokrotnego użytku, to to wszystko nadal przechodzi. Ja uszyłam takie bawełniane maseczki, ale w każdą wkładam warstwę takiej, nie wiem, jak to się nazywa. Nie płótno, tylko...

**Flizelina?**

Coś takiego. Takie na zasadzie filtra jakby.

**Pani zdaniem nakaz noszenia maseczek jest realnym zabezpieczeniem? Może ograniczyć rozprzestrzenianie się epidemii?**

Hmm... Znaczy, na pewno, jeśli byłabym na miejscu rządu albo miała nosić albo nie nosić, to też bym raczej wolała, żeby noszono te maseczki.

**Dlaczego?**

Chociażby dlatego, że to jednak jakaś tam bariera... Nawet, jak 2 osoby się zetkną i każda z nich ma maseczkę, to w tym momencie jest mniejsze prawdopodobieństwo, że jedna zadrugą naplują czy nakichają, niż jakby postawić te dwie osoby bez maseczek.

**Jak pani wspomniała, na początku minister mówił, że nie trzeba nosić tych maseczek, a później zmienił decyzję. Jak pani myśli, dlaczego?**

Cały czas się mówiło, że zdrowy nie powinien nosić maseczki, że maseczka jest dla osoby chorej. I nawet WHO było na stanowisku, że nie. Teraz ostatnio czytałam, ze stwierdzili, że można nosić. Więc tak naprawdę każda osoba mówi co innego na temat tych maseczek.

**Pani zdaniem lepiej jednak nosić?**

Lepiej jednak nosić.

**Jak pani ocenia decyzję, że teraz można wchodzić do lasów i parków?**

Dla mnie w ogóle zamknięcie tego bym takie troszeczkę bezsensowne. Bo skoro ludzie nadal chodzili po ulicach, jeździli na rowerach, to dlaczego mieliby nie wchodzić do lasów. Dla mnie ten las jest tylko groźniejszy z tego względu, że jest tak strasznie sucho, ale to jest już zupełnie inna kwestia.

**Zakaz w momencie wprowadzenia nie miał sensu, więc teraz dobrze, że jest zniesiony?**

Tak jest.

**A możliwość przemieszczania się w celach rekreacyjnych?**

Uważam, że powinno tak być. Znaczy powinno... Tak, co mnie najbardziej w tym wszystkim napawa takim, no tak mnie zastanawia, to że nikt tak naprawdę nie wie, jak to jest z tym koronawirusem, w jakim stopniu jesteśmy narażeni. I te wszystkie obostrzenia są takim ograniczeniem naszych typowych praw. Tylko, że tak naprawdę właśnie do końca nie wiadomo, jak to jest. Ja tak się zastanawiałam, że dlaczego tak strasznie padają te domy opieki społecznej. Dlaczego tam tyle jest tych zakażeń? No pewnie, że z reguły tam są ludzie słabsi, że już mają słabszą odporność, choroby współistniejące, itp. Ale ja sobie tego jakoś nie potrafię wyobrazić, dlaczego w takim ogrodu jest tyle osób chorych, a nie słyszy się, że, tle sklepów jest otwartych, a że w sklepach nikt nie choruje? Wszystkie kasjerki siedzą frontem do wszystkich ludzi i że nic takiego się nie dzieje.

**To jest zastanawiające?**

Dokładnie.

**A liczba osób w sklepie i w kościele - jak pani ocenia zmiany w tej kwestii?**

Dla mnie to już jest w ogóle... Ja nie jestem osobą... Tak, jak już rozmawiałyśmy. Akurat ten rząd w tym kierunku jest specyficzny. Uważam, że to nie jest dobrze, bo akurat tam w tych kościołach może być najwięcej osób teraz.

**Dlaczego to nie jest dobrze?**

Bo rzeczywiście mogą się bardziej pozarażać.

**Dlaczego rząd podjął taką decyzję?**

Bo jest rządem i celuje, żeby go jak najbardziej popierano, a wiadomo, że jest prokościelny i szuka wsparcia u tych wszystkich.

**Czyli to mogła być decyzja polityczna?**

Ależ oczywiście!

**A jak pani ocenia kwestię tego, że osoby pow. 13 r.ż. mogą się same przemieszczać?**

To znowu jest dla mnie takie... Bo z drugiej strony wiadomo, że mnóstwo i młodzieży ma jakieś tam zajęcia. Może nie takie stricte, jak są normalnie, bo ich nie ma, ale... Więc powinni się przemieszczać. To znowu jest nałożenie ograniczenia takiej wolności, że nie możesz nic zrobić.

**Ten zakaz był od początku niepotrzebny pani zdaniem?**

Znaczy tak, ja wtedy chyba nawet opowiadałam pani o tym filmiku, co widziałam o nastolatkach, co na tą panią pluli. W tym momencie, to było dla mnie jak najbardziej na tak, że powinno się tych gówniarzy i smarkaczy zamknąć w domu, a nie żeby się pałętali. Więc to też są takie skrajne odczucia. Nikt tego nie wie, czy to... No ja nie mam powyżej 13 lat, ale wiem, że gdybym miała, to bym się czuła osaczona. I tak się czuję, że nie możemy tego robić, tego... W sensie, nie wiem, co to będzie z tym latem, z tymi wakacjami. Dla mnie to jest strasznie frustrujące.

**Teraz mówi pani, że jednak powinni się przemieszczać. Co się zmieniło?**

Nie każdy z tej młodzieży jest jakimś, za przeproszeniem, gnojkiem, który będzie pluł na kogoś. Wtedy to było akurat podyktowane takim, że byłam zła na tamtą sytuację i bym skwitowała:*a bardzo dobrze, niech siedzą w domu.*

**Teraz po przemyśleniu doszła pani do wniosku, że nie wszyscy powinni być wrzucani do jednego worka?**

Dokładnie.

**Które z tych ograniczeń mają realny wpływ na ograniczenie epidemii?**

Głównie to, że nadal są zamknięte wszystkie centra handlowe, kina - te miejsca, gdzie najbardziej jest przemieszana ludność.

**Coś jeszcze?**

To, że w tej chwili są zamknięte granice, że nie można... W tym sensie, że kto przyjeżdża, to przechodzi tą kwarantannę.

**Te ograniczenia mają realny wpływ, bo ograniczają kontakty?**

Tak, według mnie tak.

**Czy są jakieś ograniczenia, które pani zdaniem zostały wprowadzone z innych powodów? Np. uspokojenie lub przestraszenie społeczeństwa?**

To, co było jeszcze przed świętami - zakaz przemieszczania się, że nie można było pojechać na cmentarz, że... To takie było... Też kiedyś oglądałam jakiś filmik, gdzie jakiś przedsiębiorca prywatny jechał i był 50 km dalej od miejsca zamieszkania i był w tendencyjny sposób przesłuchiwany przez policję. I to było okropne. On sam jeden w samochodzie jechał i miał jakieś ważne sprawy. A oni go tak po prostu osaczali, że co on tu robił, że dlaczego wyruszył z miejsca zamieszkania. Dla mnie to było straszne. Już nie mówiąc, co było dalej, że niektórzy i byli na cmentarzach i odbyły się rocznice, jakie się odbyły. I że to właśnie takie szczucie przeciętnego obywatela, a wszyscy inni mogą robić to, co chcą. A jeszcze się straszy mandatami i w ogóle.

**Niektóre z decyzji mogły powodować szczucie obywateli?**

Oczywiście.

**Pani przestrzega wszystkich ograniczeń?**

Przestrzegam, ponieważ ja nic takiego nie robię. Noszę maseczkę, wychodzę tylko do sklepu albo z psem.

**Dlaczego pani ich przestrzega?**

Maseczkę to tak, jak mówię, z obawy, żeby się jednak nie zarazić. Natomiast, nawet jak jestem gdzieś na dużej przestrzeni i miałabym nie mieć tej maseczki, a mają mi wlepić jakiś durny mandat za to, to oczywiście wolałabym go nie zapłacić. Więc to też jest takie wymuszenie na mnie, że się dostosuję do tego. Nawet, jeśli się nie zgadzam z tym, to nie znaczy, że mam zapłacić 5 tysięcy, za to, że nie będę miała maseczki.

**A kwestia tego, że miała do pani przyjechać wnuczka. Planuje pani, żeby jednak przyjechała?**

Na razie odpuściłam.

**Tylko ze względu na te komplikacje z psem, itd.?**

Odpuściłam, ponieważ tak naprawdę nie wiem, co tutaj jest dobre i nie chcę po prostu ryzykować. Chociażby z tego względu, że muszę chodzić do starego ojca i nie wiem, czy czegoś tam nie przyniosę.

**Czyli też ze względów bezpieczeństwa?**

Tak. Tylko cały czas gdzieś nie do końca wierzę, że to tak... Nikt nie wie, czy się zarażę, czy się nie zarażę. Gdyby można było zrobicie testy, żeby one były wiarygodne i żeby było wiadomo, kto już to przeszedł, to człowiek nie miałby już tego strachu przed tym. Skoro to jest tak zaraźliwy i skoro tyle jest tzw. bezobjawowych zarażeń, to my nie jesteśmy sobie w stanie wyobrazić, ile już osób mogło to przejść. I to mnie tak złości, ponieważ nic nie wiemy, tych testów nie ma i... Jesteśmy tacy uziemieni, tacy nadal... I wiadomo, że gdybym ja wiedziała, że, tak jak kiedyś pani opowiadałam, że my wszyscy na przełomie lutego i marca byliśmy przeziębieni i chorzy i gdyby się okazało, że my to już wszyscy przeszliśmy, to już byłaby zupełnie inna jakość życia.

**Jaka byłaby ta jakość życia?**

Że po pierwsze my nie zachorujemy i nie jesteśmy zagrożeniem dla kogoś innego.

**Widzi pani jakąś różnicę między kwarantanną a izolacją?**

Izolacja to jest to, co staramy się stosować, czyli nie kontaktować się z ludźmi, nawet z rodziną bez potrzeby. A kwarantanna, to jeśli ktoś wraca z jakichś terenów albo w rodzinie kogoś miał, to te 2 tygodnie musi być w takiej izolacji zamkniętej. Żeby po prostu się nie okazało, że on może być chory i w związku z tym, że zaraża dalej.

**Słyszała pani o planach łagodzenia restrykcji?**

Że mają być jakieś 4 etapy. Że ten pierwszy teraz, a 3 następne mają być jednak rozciągnięte w czasie i takie tam rzeczy.

**Co pani sądzi o łagodzeniu restrykcji?**

Oczywiście ze względu na stan ogólny firm, gospodarki, no to ja nie wiem, czy to się uda powrócić do stanu sprzed. Ale ludzie z czegoś muszą żyć. My też przecież musimy... Jedno wynika z drugiego. Natomiast jedna z moich znajomych lekarek powiedziała mi coś takiego, że te wszystkie obostrzenia, to były tylko i wyłącznie po to, żeby dać służbom medycznym więcej czasu. Że i tak wszyscy musimy się tym zarazić, przejść, natomiast to jedynie wpłynie na takie rozciągnięcie w czasie, że nie jesteśmy przygotowani na to, żeby to był nagły wzrost zachorowań. I że to w tym celu były te wszystkie obostrzenia. I może uznali, że to już na tyle się będzie rozciągało... A zadowolenie społeczeństwa jest na tyle, że już się wszyscy wściekają, co dalej, to powoli będą wdrażać nowe, kolejne...

**Te etapy mogą częściowo służyć temu, żeby uspokoić tych, którzy się wściekają?**

Myślę, że tak.

**Które z ograniczeń powinny zostać dłużej, a które krócej?**

Trudno mi jest na to pytanie odpowiedzieć. Chociażby z tego względu, że tak naprawdę my nie wiemy... Nie wiemy, ile z nas jest chorych, ile przeszło tą chorobę, ile jeszcze się zarazi. Dla mnie to wszystko jest taką niewiadomą. No może z kolei wszystko zaczną otwierać tak powoli, ale to nadal nie wróci do stanu sprzed epidemii. Może się okazać, że nagle ten wzrost zachorowań zacznie znów rosnąć - wtedy nie mam pojęcia, co oni zrobią. Czy znowu zaczną się wycofywać... Ja nie wiem, ja się boję całej tej sytuacji. Jedni twierdzą, że w lecie to wygaśnie, a potem inni, że absolutnie nie. Że nawet, jak się teraz zmniejszy, to potem będzie fala jesienią. Inni, że przynajmniej przez 2 lata ten wirus będzie krążył albo, jak się całe społeczeństwo uodporni i przejdzie albo, jak wprowadzą szczepionki. Nie wiem, ja już po prostu... Ja tego już nie ogarniam. Złoszczę się na to, że nic nie wiem.

**Co jest tą granicą, kiedy ograniczenia powinny przestać obowiązywać?**

Może musiałaby zacząć maleć liczba zarażonych, co wskazywałoby na to, że ta pandemia powoli się wycisza.

**Słyszała Pani o rozwiązaniu, które jest stosowane w Szwecji?**

Tak.

**Co pani o tym słyszała?**

Że jedynie wyższe uczelnie mają zawieszone, a normalnie mają otwarte kina, restauracje, kawiarnie, że mogą się normalnie spotykać.

**Co pani o tym sądzi?**

Nie wiem. Ja po prostu też nie wiem, co jest w tym momencie... Bo usiłowałam sobie porównywać te... Ta zachorowalność tam też jest wysoka i też dużo osób zmarło i ogóle, więc... Ja nie wiem, na ile te dane, które my otrzymujemy, które są u nas, na ile one są prawdziwe.

**Tu też nie wiadomo w co wierzyć?**

Tak.

**Jak wygląda teraz pani dbanie o siebie?**

Niekoniecznie się zmieniło na dobre.

**To znaczy?**

To znaczy, obiecywałam sobie, że tam sobie porobię te różne spa takie i nic z tego nie wyszło. Natomiast wręcz w święta, w przypływie jakiegoś stresu i w ogóle, po prostu złapałam za włosy i tak obcięłam*[śmiech].*Bo już po prostu...

**Dlaczego się pani zdecydowała, żeby obciąć te włosy?**

Bo było mi gorąco, było mi niewygodnie, źle. I czekam, kiedy wreszcie otworzą mojego fryzjera i będę mogła spokojnie pójść i zrobić porządek taki, jak chcę.

**Czyli fryzjera brakuje?**

Brakuje bardzo.

**Jak się pani czuje z tym, że ta fryzura nie jest taka, jak by pani chciała?**

Generalnie, mam to gdzieś w tym momencie. Do tego stopnia, że podjęłam też decyzję, że przestaję farbować włosy. Ja już raz taką próbę zrobiłam. Na swoje 50 urodziny stwierdziłam, że... I rzeczywiście miałam króciusieńkie włosy swoje, siwe. Ponieważ się uparłam, to nikt nic nie mówił, ale jednak potem się przekonałam i zaczęłam z powrotem farbować. Natomiast w tym momencie, w dzisiejszych czasach tej pandemii, stwierdziłam, że to nie ma najmniejszego znaczenia. A ponieważ ja mam bardzo gęste włosy, grube, jest mi w nich ciężko... I w tej chwili stwierdziłam, że mam to w nosie, nie będę się dalej męczyć, znów je będę ścinać na krótko i znów dojdę do swoich naturalnych. Żeby ich nie farbować, żeby one nie były suche, bo potem, to ja nie mogę sobie poradzić. Oczywiście to się może zmienić, bo ktoś mi powie, że wyglądam ohydnie i znów zacznę farbować. Ale to jest u mnie taki etap, że ja mam długie włosy, potem coraz krótsze, potem na zapałkę i zapuszczam od początku. Na ile lat mi jeszcze to wystarczy w życiu, tych cykli, zobaczymy.

**Te włosy mają małe znaczenie, bo są ważniejsze rzeczy czy ma małe znaczenie, bo nie wychodzi pani z domu?**

Nie to, że nie wychodzę z domu, tylko czy jakbym się miała zarazić koronawirusem, to czy będę miała piękne włosy, czy krótkie, to nie ma żadnego znaczenia.

**Dlaczego nie zrobiła pani w końcu tego spa przed świętami?**

Ja tego nie lubię. Ja do dzisiaj nie stosuję żadnych kremów ani nic. Jedynie, jeśli muszę zrobić jakiś makijaż, co jest sporadyczne, to wiadomo, że pod podkład muszę nałożyć jakiś krem. A tak, ja się nie maluję, aj tego bardzo nie lubię. Jedyne, co mam na stałe, to mam permanentny makijaż oczu i brwi, bo bym sobie sama nawet nie pomalowała. I to jest tyle. Natomiast ja nie lubię, jak mi się coś lepi. W związku z tym borykam się z tym, że mam zawsze suche ręce, suche stopy, które czasami bolą, pękają, ale to wszystko wynika z tej niechęci, że nie lubię czegoś mieć na sobie. Wręcz odwrotnie, jeśli chodzi o mojego męża, bo mnie aż ciarki przechodzą, jak on się tak nabalsamuje, a potem zakłada ubranie. To mnie aż wszystko trzeszczy, że on tak się ubiera. Ja kiedyś tak chciałam sobie zrobić takie domowe spa, takie peelingi, kąpanie, taka skóra piękna, jędrna, potem się jeszcze jakimś balsamem nasmarowałam, po czym posiedziałam 10 minut i musiałam pójść to wszystko zmyć.

**A makijaż permanentny - potrzebuje już pani wizyty u kosmetyczki? Brakuje tego?**

Nie. Regulacja brwi, to wiadomo, bo brwi odrastają... *[telefon od synowej].*A tak, to nie brakuje, bo ja nie chodzę do kosmetyczki. Moja przyjaciółka bliska jest fryzjerką i ma swój zakład kosmetyczny i nie może zdzierżyć tego, że ja nie lubię tych zabiegów. Więc czasami mnie tam czymś uszczęśliwi tak na siłę w postaci prezentu. I wtedy pójdę do niej mi tam coś zrobi. Ale obecnie też nie, bo kosmetyczki nie ma jako takiej. A tak normalnie, to ja już nie mam cery młodzieńczej z wypryskami czy coś, więc czyszczenie niekoniecznie. Nie mam suchej cery, więc nawet, jak nie nałożę kremu, to mi się ta skóra nie ściągnie i nic się z nią nie dzieje. Więc uważam, że stosowne do swojego wieku to jest w bardzo dobrej kondycji, nie używając żadnych kremów.

**W obszarze tej pielęgnacji nie za wiele się zmieniło, tylko kwestia włosów. A są jakieś czynności, z których pani całkiem zrezygnowała?**

Nie.

**Pojawiły się jakieś nowe czynności?**

Nie.

**A jak to jest z kosmetykami? Kupuje pani teraz jakieś kosmetyki?**

Ja jako ambasadorka Avon od 12 lat nie muszę kupować kosmetyków, bo mam ich 2 szafy w domu. Mam stały do nich dostęp i z nich nie korzystam. Oprócz perfum, które ja bardzo lubię, natomiast mój mąż nie cierpi perfum. Więc też, jak jestem w domu, to nie za bardzo mogę się otulać perfumami, bo on zaraz Wrzeszcz, że mu to przeszkadza. Ponieważ rzadko, kiedy wychodzę teraz, to mało używam. Tak patrzę na moją półkę, to mam 15 butelek perfum.

**W domu pani nie stosuje perfum?**

Nie.

**Jak to jest z ubieraniem się, kiedy jest pani w domu?**

Zawsze tak bardziej wygodnie. Czyli niestety u mnie na porządku dziennym są jakieś dresiki - to, czego nie może znieść moja teściowa, a ja się zawsze z nią o to kłócę, że ja nie będę... Nie mówię, kto ma racje, czy ona, czy ja. Ona mówi, że w domu należy też być elegancką kobietą. Ja nigdy elegancką kobietą nie byłam, nie cierpię tego stylu. Dla mnie nawet tak zwana elegancja to musi być daleko idąca prostota i takie coś... Nigdy nie założę szpilek itp. Ani spódniczek i takich nie. Raczej niech to będzie sweter i biała koszula.

**To jest zgodne z pani stylem, żeby w domu się czuć wygodnie?**

Tak.

**Czy teraz czuje się z tym pani lepiej, że może być pani więcej w domu i więcej chodzi w tych luźnych ubraniach?**

Nie no, zawsze tak było. Zawsze, jak jestem w domu, to jestem w luźnych rzeczach, które mnie nie krępują. A ponieważ jestem nie tylko starsza w latach, ale starsza o 25 kg, to nie wygodnie mi w jakiś spodniach. Ja natomiast jestem też taką osobą, że jak potrzebuje szybko polecieć do sklepu, a jestem w dresie, to chwycę klucz i pójdę. Natomiast znam osoby, które choćby miały ze śmieciami wyjść, zjechać windą, to od razu cały rynsztunek muszą od początku zrobić. Ja nie, ja do tego nie należę.

**Teraz pani kupuje ubrania?**

Nie.

**Z czego to wynika?**

Po pierwsze z tego, że mam całą szafę ubrań, których jeszcze nie założyłam nigdy. Po drugie z oszczędności. Bo mam tutaj różne, np. tą przyczepę i klienci powpłacali mi zaliczki na przyczepę, a ja musiałam je z różnych powodów już sprzeniewierzyć, to teraz muszę je szybko odrobić te straty. Bo może się okazać, że za chwilę będę je musiała oddać. Zaliczki zwykle się traci, ale nie u mnie, bo ja mam takich zaufanych przyjaciół i ludzi, którzy do mnie przyjeżdżają i wiadomo, że im będę musiała zwrócić. A to jest już nie mała kwota. Więc nie kupuję żadnych rzeczy w tej chwili.

**Brakuje pani chodzenia po sklepach?**

Nie. Ja tak po sklepach to dawno nie chodziłam. W takim sensie, żeby tak sobie coś kupić, to uwielbiałam chodzić na ciuchy, ale ostatnio tak bardziej dla wnuczki, a nie dla siebie. Teraz tego nie ma, aczkolwiek wczoraj spotkałam się z klientką i mówiła, że robią porządki w sklepach, bo mają je niedługo już otworzyć, więc zobaczymy.

**A jak otworzą, to pani tam pójdzie?**

Myślę, że tak. Szczególnie, że to jest obok apteki, w której zawsze realizuję zamówienia dla mojego taty, a to jest obok drzwi w drzwi.

**Czego pani najbardziej brakuje z punktu widzenia konsumenta?**

Hmm... Na pewno ten fryzjer tu jest taki... zdecydowanie. Ja uważam, że życie powinno się toczyć i kawiarnie, restauracje powinny być czynne i w ogóle, natomiast ja strasznie nad tym nie ubolewam. Miałyśmy się spotkać z koleżankami z pracy i nie zdążyłyśmy, bo już wszystko zostało zamknięte i teraz cały czas piszemy, kiedy się wreszcie się będziemy mogły spotkać. Ale ja za tym tak nie tęsknię bardzo. Bardziej mnie ta niepewność odnośnie zachorowania przytłacza. A to, że akurat są nieczynne kawiarnie... No nie wiem.

**Dlaczego brakuje fryzjera? Bo mówiła pani, że teraz nie jest to ważne...**

Nie tyle, żeby o włosy dbać, tylko, żeby ja miała wreszcie wygodę, żeby je ściąć na krótko tak, jak zamierzyłam. Ja cały czas mam uderzenia gorąca związane z menopauzą i mi to bardzo przeszkadza. Jak miałam długa włosy to nienawidziłam ich myć, bo trzeba było długo suszyć, itd. Teraz marzy mi się takie, że mogę codziennie umyć głowę i nie muszę na to poświęcać nie wiadomo, jakiego czasu.

**Czyli to bardziej dla wygody?**

Tylko i wyłącznie.

**Dziękuję. Z mojej strony to wszystko.**
