# Supplementary material for: The challenges arising from the COVID-19 pandemic and the way people deal with them. A qualitative longitudinal study
Source: PLoS One. 2021 Oct 11;16(10):e0258133. doi: 10.1371/journal.pone.0258133 (PMC8504766; doi:10.1371/journal.pone.0258133)
Supplement: S1 Dataset — (ZIP) [file pone.0258133.s003.zip › Transcriptions/stage 3/3.3_F_54_single.docx]

**3.3_F_54_single**

**Co się działo w ciągu ostatnich 2 tygodni? Co się działo, jak spędziłyście czas, co robiłyście?**

A myśmy się widziały przed świętami? No, wie pani co. No, święta nam minęły super oraz te 2 tygodnie. To znaczy jesteśmy na wielkich rzymskich wakacjach obecnie. Robimy sporo rzeczy na zewnątrz. I tak naprawdę nie bardzo wiemy, co się dzieje na świecie. To znaczy może tak, ja wiem, a Julka też wie na pewno, ale ja oglądam wiadomości bez niej tylko rano. Bo się zadowalam po prostu jakąś suchą dawką takich czystych statystyk powiedzmy. Staram się nie słuchać całej tej wojny medialnej. No i tyle. Martwimy się trochę pożarami, ale to wszystko tak trochę z daleka.

**No tak, ale pożary to jest zupełnie inna kwestia. To w ogóle nie ma związku z…**

No tak, tak. Ale po prostu życie toczy się dalej. Mamy pożary, mamy suszę, mamy trochę więcej niż koronawirus.

**To jak taki dzień wygląda? Wczoraj co na przykład robiłyście?**

No, wczoraj to był szczególny dzień. Ale tak normalnie to ja rano tutaj coś ogarniam w domu i czekam, aż moja córka wstanie. To bywa w godzinach późno przedpołudniowych. A później planujemy jakieś czynności, na przykład ostatnie te 2 tygodnie spędziłyśmy na przygotowaniu poletka pod warzywa, na wysianie tych warzyw, na ogrodzenie tego poletka takim ogrodzeniem tymczasowym w postaci palików i sznurka i takich fladr, żeby nie wchodziły tam zwierzęta. W szczególności psy oczywiście, bo koty sobie nic nie robią z czegoś takiego. Ale też szkody powodowane przez koty w zasiewach są daleko mniejsze niż przez psy. Przygotowałyśmy szklarnię, wysiałyśmy sałatę, rzodkiewki i takie rzeczy, takie warzywka, nowalijki nazwijmy je. No, po prostu bezustannie coś robimy. No a później, w międzyczasie coś jemy. W międzyczasie albo po. A wieczorem sobie siedzimy i oglądamy jakieś w telewizji niezobowiązujące filmy. Głównie używamy tych programów takich przyrodniczych. I oglądamy albo niezwykły doktor Pol, to taki pan weterynarz z Minnesoty chyba albo Michigan pewnie. No wszystko jedno. I tak nam mija dzień. Wieczorem jeszcze coś jemy tutaj wspólnie. I potem się oddalamy do czynności sennych. Myjemy się. Ja palę w piecu w międzyczasie, bo to nie mamy tutaj centralnego ani nic takiego, więc… Tak że generalnie takie naprawdę miłe dni. Bo to takie wiosenne dni, wie pani, przyroda się budzi do życia. I my też tutaj mamy, to jest taki okres, powiedziałabym wzmożonej aktywności w ogóle. A w tym okresie, jak jest Julka, no to te prace są dużo przyjemniejsze. Bo oprócz tego, że one są, to jeszcze jak się je robi wspólnie, to jest wspólny czas spędzamy. I motywacja lepsza do pracy. Są 2 osoby to się lepiej robi niż jednej.

**A Wielkanoc jak w końcu spędziłyście?**

A super. Spędziłyśmy jak każdy inny dzień. To znaczy tak, jakby jej nie było zasadniczo. Tym się różniło, że mamy na stole taki koszyczek z paroma jajkami, które ja wykonałam wcześniej, takimi tam wydmuszkami. A poza tym to jadłyśmy, co chciałyśmy, bez żadnych napinek, żadnych nie robiłyśmy nawet potraw takich świątecznych. I bardzo nam to dobrze posłużyło, bo po co? No nie będziemy we dwie sobie szykować, żeby usiąść przy stole. Zwłaszcza, że dla nas nie ma to takiego emocjonalnego znaczenia, tylko takie czysto, bym powiedziała…. Trochę nam tylko brakuje no takiej większej gromady przy tym stole. Ale to po prostu takie święta. Już kiedyś nam się zdarzyło mieć takie okrojone święta, jak mój brat wyjechał z bratową i z dziećmi do Hiszpanii na Boże Narodzenie. I spędzili je gdzieś tam na wyspach, gdzieś na Teneryfie pewnie, na plaży. Takie mieliśmy wtedy też tu mniejsze grono. Ale wtedy było jakoś… No wtedy oni pojechali tam z wyboru. A teraz nie ma wyboru, tylko jest jak jest. Więc jakoś łatwiej się z tym pogodzić pewnie. Tak musi być i tak jest.

**A była ta kaczka?**

Nie, nie było kaczki, był indyk.

**Czyli coś było (śmiech).**

No tak, było. Kaczki nie było, bo to wymagałoby pojechania do Płocka i zakupienia jeszcze ze dwóch nóg tej kaczki. A tymczasem nie było takich okoliczności jednak. Pomyślałam, że może nie będę stała nie wiadomo ile czasu przez tym Lidlem w Płocku, żeby kupić albo nie kupić. A ja nie jestem takim fanem zakupów, żeby… A kaczki jestem fanem, ale nie aż takim, więc zrobiłam, miałam jakiś… Moja córka za to jest fanem kończenia różnych rzeczy albo wykorzystywania różnych rzeczy, które są. A ja mam duże zapasy. Więc wykorzystałyśmy na przykład udziec z indyka, który znalazłam w lodówce. I zrobiłyśmy indyka takiego duszonego z jabłkiem. I był pyszny. I nie był kaczką, ale też był ptakiem.

**Rozumiem, że Hubert nie przyjechał na święta.**

Nie, Hubert nie.

**Oni zostali w domu, czy pojechali tam do rodziców, nie pamiętam imienia dziewczyny. Bo mieli tam ewentualnie jechać do rodziców.**

Magda. Nie, z jakiegoś powodu nie pojechali. Ale zostali zaprowiantowani przez rodziców. Zostali sami w domu. Oni są specyficzną podgrupą, czy małą grupką dwuosobową, bo im dużo do szczęścia jest niepotrzebne. Znaczy, jeśli chodzi o ludzi i o kontakty. Oni są obydwoje tak skonstruowani, że potrzebują siebie. No i najbliższych członków rodziny. Ale sobie świetnie radzą we dwójkę. No i nie drążyłam tematu, bo to żaden dramat, że nie pojechali. No skoro podjęli taką decyzję, no to zostali. Nie wiem czy dla bezpieczeństwa tamtych rodziców czy wszystko jedno. Tak czy inaczej zostali w domu. I bardzo sobie chwalili. Bo Hubert na przykład się zapoznał z jakimiś nowymi potrawami wielkanocnymi, których w życiu nie widział, a które łączą… Bo nie widział ich głównie dlatego, że są rzeczy, których nie je. On nie je. A między innymi są to grzyby oraz jajka na przykład. Jajka to już chyba zjadł wszystkie, co miał zjeść w początkowej fazie życia i już mu na całe życie wystarczy. Bo był fanem jajek. A grzybów nie je, bo nie je. I na przykład był w dużym szoku, że można, bo faszerowane jajka to u nas się robi. On ich nie je, ale widział taki wynalazek. Ale jajko faszerowane grzybami to jest coś, to przekracza jego… Taki, wie pani, zamiast żółtka się robi taki farsz z pieczarek i z czego. No to on rzeczywiście tego nie widział. A Magda z dużym uwielbieniem to jadła, bo ma taką domową tradycję. No i poczynił różne obserwacje, że można jeść różne rzeczy, których on nie zna na przykład w ogóle, że może istnieć coś takiego. Więc myślę, że miło spędzili ten czas.

**Czyli szkoda, że ich nie było, ale do przeżycia, zrozumiałe w obecnej sytuacji.**

No do przeżycia. Rozmawiamy, Julka tu organizują w Hubertem takie spotkania, w telefonie się oglądamy. Ja nie jestem fanem takich… Znaczy jak bym tak miała porozmawiać z kimś przez telefon, to z nim rozmawiam, ale nie patrzę na niego. Ale jak Julka tu organizuje z Hubertem takie rozmowy i widzimy się. I jego, i Magdę, i on nas. I jeszcze na dodatek jakieś potrafią robić takie… Nie wiem, jak to nazwać w ogóle, modyfikują nasze wizerunki. Nam jakieś uszy królicze dorobił. A Julka jemu dorobiła. I w ogóle to taka jest zabawa w pewnym sensie. Jeśli kogoś to bawi oczywiście, no ale powiedzmy, że jakoś to urozmaica ten przekaz. Tak że było całkiem śmiesznie.

**A czy w związku z koronawirusem, tą całą sytuacją, cokolwiek się zmieniło w pani życiu w ostatnich dwóch tygodniach? Jakkolwiek zmiana?**

Zmieniło się to, że teraz mam oprócz rękawiczek mam jeszcze maski w samochodzie. Ja jestem dość nieogarnięta, jeśli chodzi o… Jestem bardzo zadaniowa chyba i sobie bardzo dobrze radzę i sobie na kartce piszę itd. Ale takie rzeczy, bezustannie wszystko gdzieś tracę, gubię przez przerwy takie rzeczy. A już teraz, jak muszę pilnować i rękawiczek, i maski. Mam 3 maski w samochodzie, parę par rękawiczek i pudełko. A i tak są takie sytuacje jak wczoraj, kiedy po prostu miałam dwie maski, jak wyruszałam. A później już potraciłam je w różnych miejscach. To znaczy one wszystkie gdzieś są. Tylko ja muszę wtedy wykonywać jakieś operacje intelektualne, gdzie ja ostatnio ją miałam i co ja wtedy miałam w ręku itd., żeby to wszystko poodnajdować. I to zawsze mnie to drażniło, że mi się wszystko gdzieś traci. A teraz po prostu widzę to potrójnie, bo mam 3 razy tyle rzeczy do pilnowania. No to się zmieniło. No to maska jest do dupy, a w szczególności jak ktoś ma okulary, jak pani dobrze wie, bo sobie chuchamy… Co prawda mam taką maskę, która ma ten drucik, który dopasowuje tą maskę do nosa trochę. To trochę pomaga, bo jak się ją uszczelni, no to to powietrze bardziej leci powiedzmy bokami i przez samą maskę, a mniej pod okulary. Ale i tak jest do dupy, no ale dobra.

**Tą maskę wyżej trzeba założyć, tak pod okulary i okulary włożyć na maskę. To mniej dmucha. To już sprawdziłam.**

No tak, a to trzeba zmienić okulary zaraz. No sama pani dobrze wie, bo ma pani okularki. No ja mam takie okularki troszkę już zdezelowane. W sensie takim, że luźne. Musiałabym pójść do optyka, żeby troszkę mi poprzyginał te wszystkie uszka. Do dupy to jest, bo tu mi się zsuwa, tutaj coś tam, tu mi paruje. Ale my okularnicy jesteśmy do tego przyzwyczajeni. Zimą nam paruje i bez maski, więc jakoś sobie dajemy radę. Mój syn też się skarży.

**O maskach sobie jeszcze za chwilę pogadamy. Ale czy coś jeszcze poza tym się zmieniło?**

Nie. Chyba nie. Przez 2 tygodnie na pewno nie.

**Emocje – zdjęcia.**

**6.**

**Co jest na tej 6?**

Myślę, że to jest taki spokój i takie… Ja wiem, nadzieja? No po prostu dobre samopoczucie. Jest zieleń, jest wiosna, tak mi się wydaje. Jest taka dobra atmosfera. W moim bezpośrednim oczywiście otoczeniu.

**Bo u pani właściwie nie ma takich negatywnych w tej chwili emocji, związanych z tą całą sytuacją?**

Nie. Wie pani, na rozum, jak to bym sobie chciała rozłożyć, to może nie ma dużo pocieszających informacji. Ale tak, jeśli chodzi o emocje takie bezpośrednie, związane z taką sytuacją tu i teraz. Mam przy sobie dziecko, mam piękną pogodę, mam wiosnę, wszystko się budzi do życia. I ta przyroda sobie jakoś radzi pomimo wszystko. No to od strony emocji takich, to myślę, że one są zdecydowanie pozytywne. A jak usiądę i się nastawię i zacznę myśleć o zagrożeniach, no to oczywiście można zwariować nawet. Bo jest ich dużo. Począwszy od tego nieszczęsnego wirusa a skończywszy na wszystkim wokół. Na polityce, na ociepleniu klimatu i różnych takich historiach. Ale staram się nie funkcjonować w ten sposób, bo to…. Jakby staram się pracować nad tym, na co mam wpływ. Czyli nad sobą. I tym się zastanawiać. No nie mam wpływu na ocieplenie klimatu, a jeśli go mam, to umiarkowany. I staram się postępować zgodnie z zasadami, które rządzą tym procesem i więcej nie mogę zrobić. A jak usiądę i będę strasznie się martwić, to nic to dobrego nie przyniesie na pewno. Staram się uśmiechać, jestem no umiarkowaną optymistką. Ale żyjąc tu i teraz, i ciesząc się tym, co mam, no to generalnie myślę, że to dobre są emocje.

**To, żeby nie wpaść ten stan takich negatywnych emocji, ale jednak pogadać o tym koronawirusie, jak pani sobie ogląda te wiadomości rano, czy tam nie wiem, co jest w internecie, telewizji, wszystko jest. To co tak zapada w pamięć, na czym jest… W ogóle po co pani to włącza?**

No włączam, bo mam takie poczucie, że jednak dobrze jest być poinformowanym. Że nie mam na to wpływu, ale dobrze jest się orientować jakby w sytuacji. Że taki poziom powiedzmy wiedzy, pewnej wiedzy też nas… Po pierwsze mniej się tego boimy, jak to znamy. Możemy jakoś się zachować w miarę poprawnie, nie wiem, zrezygnować z jakichś zachowań, które są absurdalne, a wprowadzić jakieś zachowania, które mogą pomóc nam, naszym bliskim czy otoczeniu. Tak że myślę, że jakiś poziom wiedzy jest potrzebny. Skądś go muszę czerpać. Włączam tą telewizję nieszczęsną, bo… Ponieważ ja tu głównie, teraz nie jestem sama, ale głównie jestem sama, więc ta telewizja mi zapewnia taką obecność czyjąś zwykle. Więc taki mam nawyk. To nie jest tak, że ona mi się pali na okrągło. Ale jak jestem w pokoju, to nawet sobie wyciszam wszystkie głosy itd. Ale sam ten, taka obecność jakiegoś obrazu czy czegoś. No widocznie coś mi daje, skoro czasem mam ją włączoną. No włączam ją i się wkurzam prawie za każdym razem, bo widzę te mordy durne w tej telewizji. No i oprócz koronawirusa, oni też się nudzą w tej telewizji tym koronawirusem już powoli, więc coraz częściej mamy, teraz zbliżając się do tego nieszczęsnego maja, informacje o wyborach itd. Więc myślę, że tyle mi daje. Jak już widzę, że mi nie daje, tylko powoduje wzrost ciśnienia i poziomu agresji to po prostu wyłączam to.

**A jak tak pani ogląda, to myśli pani, że już jesteśmy na dobrej drodze czy jeszcze taka górka przed nami?**

Nie, no gdzie tam na dobrej drodze. Ja myślę, że my mamy po prostu pierdyliard osób zarażonych czy też nosicieli tego wirusa. Czy nie wiem, jak to nazwać, bo to nie jest tak, że… Są nosicielami, tylko po prostu go mają i tylko bezobjawowo go przenoszą. I w ogóle nie jesteśmy w stanie wykryć. Może nawet i dobrze. Myślę, że jesteśmy mniej więcej tak samo jak świat. Jeszcze idziemy do góry moim zdaniem. Ale tak naprawdę to co ja mogę mieć za zdanie? Ja mogę mieć takie zdanie jak mi tam jakiś matoł w telewizji pokazuje. Wczoraj tam było lepiej, dzisiaj jest gorzej, czy tam odwrotnie. Bo tam było, że był jakiś dzień, gdzie było 500, a teraz jest 300. I to się wszyscy strasznie ucieszyli. Ale to było to wczoraj. I trzeba poczekać chwilę, aż się ucieszymy. Myślę, że jesteśmy cały czas na wznoszącym się poziomie. No są jakieś takie informacje… Nie wiem, jak jest jakiś dom pomocy, który dzisiaj ewakuują, to jest jakiś taki dom dla osób poudarowych, leżących, gdzie są wszyscy pacjenci chorzy. Na 48 jest 48. Z czego tam już 11 wywiezionych a resztę wywożą dzisiaj. Plus 17 osób personelu, z których chore są wszystkie. Znaczy posiadają tego koronawirusa. No to nie mam złudzeń co do tego, że to jest bardzo zakaźne. Myślę, że część z nas przeszła już to. Przeszła albo przechodzi tego wirusa. Młodzi ludzie najczęściej bezobjawowo, dopóki nie zrobimy testów na przeciwciała, to nie będziemy wiedzieć, kto to przeszedł. I tak to chyba wygląda po prostu. Wszyscy się z nim prędzej czy później spotkamy. Pytanie, jak tam to będzie wyglądać, jeśli chodzi o odporność. Więc, no… Wie pani, czy ja wiem? Myślę, że jesteśmy na wznoszącej. Chociaż myślę, że te dane, które nam podają w telewizji to po prostu… Podają, bo coś muszą podawać. Bo robią ileś tam testów, ileś mają wyników. Przecież też nikt o tym nie mówi, że jak wczoraj zrobili tam, nie wiem, 14 tysięcy testów, to wyniki, które podają dzisiaj, to nie są z tych wczorajszych 14 tysięcy. Tylko sprzed paru dni. Bo przecież zanim im to wszystko spłynie. I nie wiem, ile to trwa, no ileś tam godzin. No, tak naprawdę myślę, że nie ma to większego znaczenia. No po prostu musimy jakoś to przeżyć. Niestety… Znaczy, dopóki nas to nie dotyczy bezpośrednio, to wszystko to jest dalekie. Ale tak jest ze wszystkim. Jak nas nie dotyczy, to nam się o tym dobrze rozmawia.

**A co tam słychać u sąsiadów?**

U sąsiadów.

**Koronawirus to jest temat tutaj w okolicy?**

Nie, zasadniczo to nie jest temat moim zdaniem tutaj. Już powoli się przyzwyczają po prostu. Może nawet mniej kontestują. W maseczkach – no to w maseczkach, to kupili sobie maseczki. Ja już nawet im nie mówię, że te maseczki, co trzeba z nimi robić. Albo tak jak te rękawiczki, jak oni noszą. To już nawet myślę, że to nie ma sensu większego, żeby to jakby naprawiać czy uświadamiać im, po co są te rękawiczki. I na której części tych rękawiczek co się osadza i co trzeba z nimi zrobić po wyjściu ze sklepu. No, przystosowują się do kolejnych tam obostrzeń albo do znoszenia tych obostrzeń. Pan policjant jeździ i zbiera… Ostatnio słyuszalam, że gdzieś tam w naszej tu najbliższej okolicy są te wiejskie sklepiki takie, co to głównie handlują alkoholem. I sprzedają wyłącznie osobom nietrzeźwym, bo nikt trzeźwy nie kupuje alkoholu w takim miejscu. I tam panowie policjanci przyjeżdżają i na przykład zbierają 8 takich panów i tam ładują im jakieś… Bo oni stoją w ośmiu na przykład. Jak zwykle stoją i piją. A mogą się spotykać tylko po dwóch. No i dostają jakieś mandaty i coś tam. To są takie atrakcyjne wtedy informacje, przekazywane przez tubylczą ludność, że taką miała okoliczność sytuacja, że kogoś tam spisali albo coś. A tak, poza tym to się niewiele dzieje.

**A daje to coś, że oni spisują? Następnego dnia nie jest tak samo, że oni znowu w tych ośmiu stoją?**

Nie. Oni się gdzieś tam schowają, oni się u kogoś za płotem następnego dnia. A za 3 dni już nie pamiętają, że ich spisali. Bo przecież tam mózgu, wie pani, te komórki nerwowe nie występują albo w zaniku. No, może komórki są, ale połączeń już nie ma. I zapomną za chwilę. I ich spisują. Nawet potem dostają jakieś mandaty na przykład. Oczywiście ich nie płacą, to w ogóle nie ulega wątpliwości. I mając ileś ich, nawet raz na jakiś czas któryś trafia, no teraz to nie, ale będzie trafiał do, jak to tu mówią, na wczasy. Czyli do więzienia.

**A są jakieś negatywne emocje wśród sąsiadów?**

Ale z powodu koronawirusa?

**Nie wiem, no tej sytuacji całej.**

Wie pani co. Może tak. Ale ja, jak idę gdzieś do kogoś, to ponieważ się zasadniczo uśmiecham i ich pocieszam. Albo im coś tłumaczę, ale z uśmiechem, oni sobie poprawiają wtedy nastrój. Więc staram się nie… Ja nie powoduję, jak sądzę, negatywnych emocji. I jak ktoś ma jakąś obawę czy coś, to staram się mu to wytłumaczyć, dlaczego tak jest. Zawsze, nie tylko związane jest to z koronawirusem. Tylko staram się, jakby służę za taką, no nie chcę tu użyć wielkiego słowa, ale za taką chodzącą encyklopedię, w sensie tłumaczenia rzeczywistości. Bo oni mają bardzo często taką wiedzę, no że na przykład, nie wiem, te kredyty frankowe… bo ostatnio tłumaczyłam komuś, co to się stało z tymi kredytami frankowymi i kiedy, że ci, co mają te franki, to mają przesrane. Ale dlaczego na przykład i co to się stało, że ci frankowicze, to oni nie wnikają w ogóle w taką sytuację. Przekazują jakieś…No to im tłumaczę, żeby nie gadali głupot na przykład albo w ogóle, żeby wiedzieli, o czym mówią. Bo mi się wydaje, że jak wiemy coś, to jest fajniej niż jak nie wiemy. Ja sama jak czegoś nie wiem, to pierwsze co to bach do wujka Googla teraz. Kiedyś do encyklopedii, teraz do internetu. I na przykład ostatnio oglądałam coś, nie wiem, o wodospadzie Królowej Wiktorii. I myślę sobie kurde, gdzie jest ten wodospad? Czy to jakaś Ameryka Południowa, bo ten wodospad taki wielki. Ale wygooglałam sobie, że to tam Wielkie Rowy Afrykańskie, Mozambik. I już proszę bardzo, wszystko wiem. Tak że być może mają jakieś emocje negatywne. Ale ja, jak już przychodzę do nich, to oni się rozpromieniają zasadniczo. Do tych, co chodzę. A to im jakąś zupę dobrą przyniosę. Na przykład tym moim dziewczynom tutaj starszym ostatnio zrobiłam. Bo ja bardzo dobrze gotuję. I robię pyszną dupę dyniową. I nie potrafię jej zrobić mało. Bo dynia to w ogóle takie obszerne warzywo jest, jeśli chodzi o… I mam jakieś jeszcze pomrożone wory z tą dynią. I robię zupę i rozwożę to. I jak przywożę taką zupę, to wie pani, od razu jest uśmiech na buzi.

**A czy w ogóle to jest tak, że tam jakoś widać na przykład problemy z pracą, związane z tym, że jest koronawirus? Bo w Warszawie dość dużo się mówi o tym, że ludzie mają albo mniejsze pensje albo tracą pracę. Bo firmy zmniejszają zatrudnienie. U was to w ogóle jest widoczne?**

U nas to w ogóle nie jest widoczne. A powód po pierwsze jest taki, że tutaj generalnie brakuje rąk do pracy. Wszyscy, którzy potrzebują kogoś do pomocy, borykają się z tym, że nie mają takich osób. Praca tutaj, to jest tylko praca jakby… albo na czarno albo za minimum, za tą płacę minimalną. I zasadniczo tutaj wszyscy poszukują kogoś do pracy. I to się nie zmienia. Oczywiście, no ci ludzie, którzy… Nikt nie szuka takiej pracy ciężkiej. Bo to jest praca fizyczna w rolnictwie. Albo przy hodowli bydła albo gdzieś tam na polu. Zasadniczo to są prace ciężkie. Więc brakuje ludzi. I tu nie tracą, nie ma tych ludzi do pracy. A ci, którzy ewentualnie są, to dostają 500 plus, robią dzieci. I chrzanią, nie chodzą do pracy.

**A ci, którzy na jakichś TIR-ach jeżdżą albo z zagranicy przyjechali, to oni dalej siedzą? Czy już gdzieś się ruszyli.**

Nie, ci co jeżdżą na TIR-ach, to teraz jeżdżą. Ponieważ teraz znieśli im tą kwarantannę, tak że oni jeżdżą. Taka jest sytuacja niewiadoma trochę z tymi, którzy pracują za granicą na stałe. Tu jest różnie. Są tacy, którzy wrócili z zagranicy i… No, tylko że nie mogą jakby… Poza tym to nie jest taki ruch transgraniczny dosłowny. To nie jest tak, że oni jadą rano, a wracają wieczorem na drugą stronę i nie mogą teraz tego robić. Tak że część z tych ludzi stąd pojechała. No i nie będą mogli wrócić teraz po tygodniu jak zwykle, tylko no muszą tam zostać aż wytrzymają. A jak wrócą, no to nie wiem, na 2 tygodnie znowu będą musieli wrócić czy coś. Tu się nie orientuję dokładnie, jak to wygląda. Ale wiem, że część z nich wraca, a część się jeszcze waha czy wracać. Zwłaszcza, jak tutaj no mam takiego jednego sąsiada, który widzę, że nie jedzie do tej pracy. Ale nie wiem, czy on szuka… Tutaj mu coś drukowałam, jakieś CV. Więc może szuka czegoś tutaj bliżej. Ale też ma półtoraroczne dziecko i żonę na wylocie ciąży.

**To może nie chce jechać, bo może się boi, że nie wróci.**

Tak myślę. Ale drugi, którego żona będzie rodzić tam w lipcu, pojechał. Bo mówi, on pojedzie teraz, popracuje jeszcze 3 miesiące. Bo tak, to by nie pracował pół roku. Bo jak by teraz został do porodu, a potem po porodzie coś tam jeszcze, no to… Tak że no, cześć osób pracuje dalej. Mam tutaj też takiego sąsiada, co pracuje w Holandii, to nie ma go. Więc rozumiem, że jest tam i że pojechał i pracuje. Tak że może się trochę to, zwłaszcza tutaj, być może jest tak, że się trochę oswajają z tą sytuacją. I każdy myśli, że za coś trzeba jeść. A ponieważ tutaj nikt nie ma zapasów, bo to nie są bardzo duże pieniądze, które oni zarabiają. Tutaj nie ma tak, że oni mają na kontach poodkładane pieniądze. Nie mają oszczędności. Więc no po prostu musza pracować. Bo inaczej nie będą mieli za co jeść. Więc może się trochę boją, ale jakieś decyzje życiowe podejmują.

**No dobra, bo zaczęła pani mówić o tych maseczkach, że to do kitu wszystko. To co z tymi maseczkami? One działają według pani, nie działają?**

Na pewno one działają. Pytanie jak i na co (śmiech). Jak rozumiem, sens działania maseczki jest taki, żeby osoba, która… Zawsze tak było. Azjaci na przykład mają świra na tym punkcie. Ale tam maseczki zawsze nosili ci, którzy byli chorzy. Żeby nie zarazić zdrowych. Czyli żeby swoim tym oddechem i parą wydychaną i drobinkami tam wody z wirusami nie zarażać innych. I taki jest sens działania maseczki. Po to ją nosimy. Nie, żeby ktoś na nas nie nakichał, tylko żeby, bo jak ktoś kicha, to kicha wszędzie, nie tylko nam na twarz, ale też na ręce, na coś tam, na zakupy, na wszystko. Natomiast po to ona jest, żeby nie zarazić innych. No i dobrze. No oczywiście one są bardziej lub mniej szczelne. Ale też, żeby zachorować, to trzeba jakąś ekspozycję… To nie jest tak, że przejdziemy koło kogoś, kto jest chory i w tą sekundę czy mijania, nawet jak on na nas odetchnie, to my od razu już zachorujemy. No to nie jest ospa, tylko COVID-19. Więc aż tak to nie działa szybko. I nosimy. Najbardziej to jest, myślę, sensowne u tych osób, które są chore i o tym nie wiedzą. To to jest jakby to źródło zakażeń, które jest realne i które z całą pewnością w części eliminujemy. Aczkolwiek sądzę, że te maseczki, które są robione w domach, z chustki albo jakiegoś kolorowego materiału, bo teraz będziemy modni. I to nawet fajnie wygląda. Mam taki filmik, bo teraz się uaktywnili wszyscy ci znajomi, którzy przesyłają filmiki w internecie, takie jakieś śmieszne. Mam taką panią, chyba jakąś taką arabskiego pochodzenia, bo taka uroda orientalna, która wykonuje makijaż na tej maseczce. Rewelacja. Po jakimś czasie to po prostu wygląda, jakby jej nie miała na sobie. No, będziemy je nosić. Myślę, że powinniśmy się przyzwyczaić, że jeżeli mamy troszkę uwolnić i więcej osób spotykać, no to szansa, że spotkamy kogoś zarażonego, kto o tym nie wiem, no jest dużo większa. Zwłaszcza wy w mieście. Bo my tutaj może nie. No tutaj część osób jest taka, oni tego nie widzą. Oni tego nie widzą, to oni tak nie bardzo wierzą w to, że to tak jest. Ale noszą karnie. Tu może bardziej karnie nawet niż… No nie wiem, jak jest w mieście, czy wszyscy noszą.

**Różnie. Znaczy tak z moich obserwacji.**

Na pewno z moich obserwacji wczorajszych wynika, że wszyscy grzecznie w Płocku, dużo więcej osób jest. Bo wczoraj oprócz tego, że miałam urodziny, to jeszcze byliśmy u weterynarza z psem. I spotkałam w związku z tym sporo ludzi. A ponieważ była kolejka do tego weterynarza, więc Julka została w kolejce z psem, a ja pojechałam kupić jakieś śrubki do ławeczki na cmentarz. I w ogóle do czegoś i do Biedronki po jedzenie dla kota. Więc tak. Noszą, wszyscy w maseczkach. Tylko jest dużo więcej osób. Ja rozumiem, że od tego poniedziałku. Bo jak byłam poprzednio w Płocku, to po prostu pustki absolutne. A tak, to jakby… No nie wiem, trudno mi powiedzieć, bo i tak ruch w tym Płocku jest dużo mniejszy niż w Warszawie. Ale… No może nie było korków jeszcze. Ale ten ruch taki był zdecydowanie wzmożony.

**A co w ten poniedziałek, że ten ruch większy?**

No zostały zniesione te obostrzenia. Można tam wychodzić z domu nie tylko z pracy i do pracy. I myślę, że takie troszkę, taki sygnał, no to dobra, huzia na Józia, jedziemy.

**Tak, jest ta możliwość przemieszczania się w celach rekreacyjnych. Tak to ładnie nazwali.**

Tak. Ale myślę, że też trochę, a to sklepach może być więcej osób. Więc troszkę to zostało poluźnione. Więc myślę, że ludzie tak się poczuli trochę bezpieczniej. Zupełnie bezpodstawnie, myślę, ale tak się stało. No to troszkę trzeba tą gospodarkę gdzieś tam ożywić. A tu w Płocku trzeba pamiętać, że… No, oczywiście część osób pracuje zdalnie, ale tutaj my mamy petrochemię. My mamy rafinerię, czyli Orlen. Czyli jak to mówią tutejsi, mazowieckie. Bo to kiedyś były tam, aż nie chce mi się szukać w Googlach jak to się nazywało, ale tu się mówi: pracują na mazowieckich. Czyli tam jakieś rafinerie pewnie coś tam, mazowieckie coś tam. I oni nie mogą pracować online. I są te godziny takie, akurat to była godzina weterynaryjna, bo myśmy były umówione na 15:40. I to jest taki moment, że ci pracownicy wychodzą o 15. Oni pracują od 7 do 15. No i rzeczywiście był wzmożony ruch. Ale kolejki przed sklepami, więcej osób może być w sklepie, no więc nie było kolejki już przed sklepem. Bo to jest po prostu… O, przyszła moja córka. Więc rzeczywiście więcej osób. Ale wszyscy grzecznie w tych maseczka i rękawiczkach.

**A zachowują ten odstęp, dystans zalecamy? Czy tak już trochę, bo maseczkę mam, więc już bez przesady?**

Tak. Myślę, że trochę tak. Jest więcej tych osób w sklepie, no niby jak byśmy mieli zachować ten odstęp? Ja, to jak idę z tym wózkiem, to prowadzę go przed sobą. A jak idę do kasy, to mam go za sobą. Żeby sobie jakby zapewnić ten dystans od… Tam, gdzie ja reguluję z przodu, to mogę nie podejść. A za mną, jak ktoś może do mnie dobić. Więc sobie ustawiam ten wózek. Staram się wyregulować ten obszar wokół siebie. A ludzie, no wie pani, jak ktoś jest na przykład razem, we dwójkę idzie do tego sklepu, no to idzie we dwójkę. To przecież nie będą stać półtora metra od siebie, bo za chwilę wsiądą do samochodu i będą koło siebie.

**Ale zgodnie z przepisami powinni stać półtora metra od siebie.**

Ale to jest absolutnie absurdalne. Ja tego na przykład też nie przestrzegam. Bo wczoraj byłam w sklepie ze Stasiem. Bo pojechaliśmy we trójkę, bo potem wracaliśmy, pod drodze kupowaliśmy… No i jak byliśmy razem w sklepie, to jak mamy chodzić, bez sensu, tak? Nie, utrzymuję dystans od osób, z którymi zwykle się nie spotykam. A z tą osobą, z którą mieszkam czy się z nią widuję codziennie, tak jak z Julką. Jak weszłyśmy do weterynarza to też ona stała przy psie. A pan mówi, o, tylko jedna osoba. Ja mówię, ale my razem występujemy. To ja mogę od pana mieć 2 metry, usiądę sobie na krześle. Ale to jakiś absurd. No tak, tak, tak, pan powiedział dobrze, to proszę usiąść.

**A to dobrze, że zwiększają liczbę ludzi w sklepie, która może być?**

Ja myślę, że powoli się dostosowujemy. Tak jak już kiedyś o tym rozmawiałyśmy. Trudno jest postawić taką granicę. Nie wszędzie możemy postawić taką granicę i nie wszędzie ta granica… Jakby nie każdy przypadek możemy rozpatrywać indywidualnie. Jeżeli mamy duży sklep i są tam 3 kasy czy 4. I mogą na nich 12 osób i po prostu nie ma pani opcji… Na przykład tak jak w Leroy Merlin. Jak tam wejdzie 12 osób, to tam personelu jest 10 razy więcej pewnie. Spotkanie kogoś w tym sklepie, jak jest 12 osób na takiej powierzchni jest w zasadzie niemożliwe. Chyba, że kupujecie to samo, to może pani chwilę poczekać. Także… A z drugiej strony jest sklep osiedlowy, w którym są 3 kasy i jak tam jest 12 osób, to jest tłok. Więc myślę, że powoli. Też ten rząd się nie może wszystkim zajmować czy ci ludzie odpowiedzialni za takie rzeczy, epidemiolodzy czy tam ktokolwiek jest do tego zatrudniony. Czy powinien być. Ale powoli to jakoś… W niedzielę już teraz na przykład seniorzy nie będą uprzywilejowani. Bo widocznie może to jest bez sensu, bo w niedzielę więcej osób ma czas. I niech sobie też pójdą na te zakupy. No dochodzi do jakichś absurdów, no ale trudno, no musi. Myślę, że przeliczanie powiedzmy na metry kwadratowe powierzchni sklepowej jest zasadniczo bardziej sensowne. A z drugiej strony oczekiwanie od jakiegoś sklepikarza, który ma 2 metry kwadratowe czy 4 powierzchni, że sobie będzie 1 osobę wpuszczał, czy będzie to przeliczał na przykład, no to jest za dużym wyzwaniem. Jakieś regulacje muszą być, powoli się dopasowujemy do tego. Już znieśliśmy w sobotę i w niedzielę, seniorzy nie są uprzywilejowani od 10 do 12. Ale u nas dochodzi do absurdów. Na przykład ktoś poszedł do banku. I nie mógł wejść, bo była 11 a on nie miał 65+. Nie było nikogo w tym banku. Ale panie go wyprosiły. Czy tam nie, nawet jakiś pan policjant chyba. Bo u nas nawet jakiś patrol policji mamy jeden, który no co ma robić? Nudzi się, to jeździ. I zapytali go, czy on ma 65. A on mówi, że nie. No to do widzenia, proszę wyjść. No i te panie siedzą i czekają. Ja rozumiem, to ma jakieś uzasadnienie. Bo jak przyjdzie ten senior, no to dobra, niech już sobie załatwi są sprawę od tej 10 do 12. No wie pani, czy to dobrze? No myślę, że powoli w tym bałaganie czy w tym… nawet może nie bałaganie, tylko w takiej nowej sytuacji czasem się podejmuje decyzje, które nie są za bardzo trafione. Zdarza się. I dobrze, że się potrafią z tego wycofać. I przeliczyć to na metry kwadratowe, że ktoś jednak nad tym siedzi i myśli. A nie, że już jak tak zarządzili, to już niech tak będzie, żeby nie mieszać ludziom w głowach.

**A są jeszcze jakieś takie obostrzenia, które były totalnie bezsensowne?**

Nie chyba. Ja to jestem ostrożna w tym bezsensowne, bo to zależy dla kogo bezsensowne. Dla mnie bezsensowny zakaz wyjścia do lasu. Z mojej perspektywy jest absurdalny, bo ja mam 60 hektarów lasu, do którego nikt nie wchodzi. To znaczy nie ja mam go osobiście, bo ja mam hektar czy tam półtora. Ale obok mnie jest 60 hektarów i też tam nikt nie chodzi. Ale dla was w mieście to jest bardzo sensowne. Bo macie jakiś tam lasek, Kampinos, czy nie wiem, jakiś Lasek Bielański na przykład. No to jak pół Bielan pojedzie. Ja nie mówię o Warszawie całej, ale część Bielan pójdzie na spacer do lasu, to będzie tam jak w ulu. Na parkingach i w takich miejscach zacznie się tłok. Do kibla na przykład czy do czegoś. I zacznie się problem.

**No dobra, ale to znieśli w poniedziałek. Już wolno do lasów i parków chodzić.**

No i dobrze. W maseczce. Znieśli, ale.

**Nie, bo lasu można bez maseczki. Na parkingu przy lesie trzeba w maseczce, a do lasu już można bez maseczki.**

No widzi pani. Trochę jednak ktoś tam weryfikuje to. Ma jakieś przemyślenia na temat sensu i bezsensu jakichś rozwiązań. I ktoś tam… Wie pani, ci ludzie też w tych domach dostają świra. Trudno mi sobie w ogóle wyobrazić, jak funkcjonujecie w mieście. W czterech ścianach i to jeszcze… Nie, no w ogóle wszyscy muszą jakoś funkcjonować, pracować, dzieci się uczyć, bawić się i gdzieś tam realizować jakąś potrzebę ruchu czy coś takiego. No to już w ogóle mi się wydaje to absurdalnie niemożliwe w takich 4 ścianach, jak nie można gdzieś wyjść. Więc się nie dziwię, że ludzie sobie psy pożyczają.

**Jeszcze jedną rzecz, którą teraz zmienili w poniedziałek to jest to, że teraz nastolatki takie starsze, powyżej 13 roku życia mogą chodzić same po ulicy.**

No znowu, gdzieś ta granica musi być, tej odpowiedzialności. No myślę, że może trochę dobrze, a trochę niedobrze, zawsze trudno powiedzieć. Te młodsze dzieci na pewno nie mogą o siebie zadbać. Bo one nie są w stanie jakby świadomie czy na dłuższą metę przestrzegać, nie mają tyle wyobraźni, żeby przestrzegać zasad. Gdzieś tą granicę trzeba postawić. Myślę, że 18 lat to jednak była gruba przesada. Czy 16 to było?

**18. Tak jak dorosłość.**

No właśnie. To z mamusią i z tatusiem wychodzić, no to było mało realne. Ale czy 13 to dobrze? Trudno mi powiedzieć. Ja ostatnio mam małą styczność z dziećmi takimi… Większą z taką młodzieżą młodszą do 10-11 roku życia. Więc trudno mi powiedzieć, na ile trzynastolatek jest rozsądny. Ale myślę, że jak w każdej grupie, można znaleźć dwudziestolatka, który jest absolutnie nieodpowiedzialny i dziesięciolatka, który… No gdzieś tą granicę trzeba postawić. Myślę, że przesunięcie jej w dół było z kimś konsultowane, tak? Pewnie z jakimiś psychologami, pedagogami, którzy widzieli problem taki, że ci młodzi ludzie nie mogą nagle chodzić z mamusią i z tatusiem. Gdzieś tam palą papieroski czy coś tam. No muszą móc sobie jakoś to życie ogarnąć. Oczywiście muszą przestrzegać również zasad, muszą chodzić w maskach itd., są rodzice w dalszym ciągu za nich odpowiedzialni. Ale myślę, że dobrze. Bo 18 lat było dość, no takim, no… Tak jak rozmawiałyśmy, tak jak zakaz wejścia do lasu. Trzeba było to jakoś poprawić jakimś…

**A słyszała pani o tych wszystkich planach luzowania? Tego, co ma być zdjęte.**

Julka mi tu czytała któregoś dnia, ale to było dawno.

**Coś zapadło w pamięć?**

No ja to potrzebuję fryzjera (śmiech).

**I kiedy ten fryzjer? Bo tam są te etapy, prawda?**

Chyba w trzecim dopiero, w trzecim. Ale ja myślę, że fryzjer to taki jest potrzebny zawód bardzo obecnie. Bo nawet dzisiaj właśnie w tych porannych wiadomościach polsatowskich był wywiad z jakimś panem stylistą fryzur. Bo fryzjer to teraz się nie mówi o nich, tylko stylista fryzur. I była rozmowa z nim i z panem doktorem jakimś. To ten pan miał propozycje a ten pan doktor miał je skomentować, te propozycje. Ochrony i jak to w ogóle zorganizować. Żeby taki salon fryzjerski otworzyć. Studio fryzur. No jest to wyzwanie na pewno. Więc stąd wiem, że w trzecim etapie.

**Bo jest potrzeba duża (śmiech).**

No na pewno tak. No ludzie potrzebują… Wie pani, jak ja tutaj patrzę, tak patrząc jakby ze strony rynku pracy w Płocku, to jest taki trudny rynek, bo dla młodych ludzi w zasadzie nie ma dobrej pracy. Dobrej w sensie dobrze płatnej i takiej ambitnej. Ci młodzi ludzie muszą jechać gdzieś do Warszawy chyba, żeby pracować w takich sensownych miejscach. Natomiast to, co tutaj jest i nie bankrutuje to są sklepy spożywcze. Apteki w mniejszym stopniu, bo tu w Płocku mamy taką sieć bardzo tanich aptek. I w związku z powyższym pozostałe apteki nie bardzo sobie radzą. Ale powiedzmy banki. I właśnie fryzjerzy i zakłady kosmetyczne. I to są takie miejsca, które funkcjonują. I nie bankrutują. A reszta, jak się tam coś otworzy, nawet taki gówniany, no to tam jest parę, ale już nowe, jak się otwierają, to już widzę, że się zamykają. Nawet kawiarni nie ma, takich rzeczy. W ogóle takiej usługi małej gastronomii, to jest naprawdę bardzo mało. Tak że myślę, że ci fryzjerzy… No pokazuje to tak naprawdę, że jest duży rynek. I że głównie kobiety, ale nie tylko, potrzebują się ostrzyc. Mężczyźni też potrzebują. Może mniej jakby od strony upiększenia się, a dla odmiany od strony jakiejś tam wygody i potrzebują skorzystać, potrzebują się ostrzyć. Kobiety sobie potrzebują pofarbować włosy, żeby się lepiej poczuć. A w tej sytuacji może nawet szczególnie. Na pewno jakieś szczególne zasady bezpieczeństwa trzeba wprowadzić w te miejsca.

**A jakie to mogłyby być zasady? Żeby umożliwić funkcjonowanie tych miejsc, fryzjera, kosmetyczki, nie wiem.**

No dzisiaj ten pan stylista fryzur się wypowiadał. Że to trzeba… On opracował jakiś tam taki zestaw zachowań. A jeszcze tam gdzieś usłyszał, że właśnie rozszerzył swoje zasady o to, żeby na wstępie, w ogóle na dzień dobry zmierzyć klientce temperaturę, jeszcze zanim wejdzie w ogóle do tego warsztatu, żeby jej zmierzyć temperaturę. A potem, jak już przejdzie pierwsze… A potem pani recepcjonistka ma z nią przeprowadzić wywiad, jak się czuje, czy dobrze. W każdym razie a potem, żeby rozsunąć siedzenia. Żeby i klientki, i personel takiego zakładu czuli się bezpiecznie. Bo to ma być takie miejsce w końcu, gdzie się dba o własną urodę. I to jest taki czas poświęcony dla tej klientki czy tego klienta. No, ale żeby on się czuł bezpiecznie i dobrze, to ten personel też musi się czuć bezpiecznie i dobrze. No więc trudno mi sobie wyobrazić strzyżenie w maseczce, ale może jakoś sobie poradzimy z tym. No na chwilę tam zza tego ucha trzeba to wyjąć (śmiech).

**To fryzjera pani zapamiętała. Co jeszcze z takich ograniczeń padło?**

Nie, no to teraz wiem, że w tym drugim etapie hotele. Nie wiem po co zasadniczo, ale rozumiem, że hotelarze mają trudne życie, bo nikt teraz nie jeździ. Mało sobie wyobrażam, żeby ktoś zaczął jeździć. Ale może służbowo na przykład. Bo nie wiem w jakiej inne sprawie by to miało być. I już nie pamiętam, co tam jeszcze więcej mieliśmy zluzować w drugim etapie.

**Instytucje kultury. Muzea, galerie sztuki.**

A, właśnie. Ja mam wystawę, muzeum w Płocku, które jest zamknięte, obrazów Stryjeńskiej. I bym chętnie poszła. Bo my w ogóle mamy Muzeum Secesji w Płocku. I naprawdę bym bardzo chętnie poszła do muzeum. I jeszcze na dodatek drażnią, znaczy drażnią, przypominają mi o tym plakaty na mieście, że jest wystawa. Ale nie można na nią pójść. To tak, to dobry pomysł. Ale co, kina też?

**Nie, kina i teatry dopiero w ostatnim etapie. To takie biblioteki, muzea. Tam, gdzie nie siedzi się koło siebie.**

Tak, tak. No biblioteki to myślę, że dobrze. Bo co prawda ja jeszcze nie przeczytałam wszystkich książek, które mam w domu z biblioteki, bo już nie mówię o innych, bo nie mam kiedy. Bo teraz jest taki moment wiosenny, więc teraz jest dużo innych rzeczy do robienia. Ale biblioteki no to dobrze, żeby otworzyli. Zwłaszcza dla tych, wie pani, dla mieszkańców miast. Bo ja korzystam z biblioteki w Płocku, to tam bardzo dużo przychodzi takich seniorów właśnie. Starszych osób, które wypożyczają, bo książki są jednak bardzo drogie w kupnie. I czytają mnóstwo. I z tamtych książek, na przykład ja porównuję bibliotekę swoją gminną tutaj i bibliotekę w Płocku. No to już tam nie mówię, że jest mniejszy księgozbiór oczywiście. Ale stan tych książek, w sensie takim, ile osób je czyta, to w Płocku to one są tak sczytane, że bardzo często są potem tak spinane takimi kotwami. I tego się zasadniczo nie da czytać, bo trudno jest rozchylić kartki nawet do końca jakoś skutecznie. A tutaj u mnie czasem pożyczam coś dla tych moich sąsiadek, to te książki tutaj są w dużo lepszym stanie. Po prostu dużo mniej osób czyta. W miastach to myślę, że to jest… Dla osób miastowych to będzie ważne, że będą mogli pójść do biblioteki.

**Coś jeszcze z tych etapów pamięta pani?**

Nie, bo chyba nic mi więcej oprócz fryzjera nie bardzo potrzebne jest.

**Bo na przykład w trzecim etapie jest otwarcie gastronomii.**

No może.

**Otwarcie sklepów w galeriach handlowych.**

No to ja jestem w ogóle niezainteresowana tymi tematami (śmiech).

**I jeszcze widzę, że w trzecim etapie jest organizacja opieki nad dziećmi w przedszkolach, żłobkach i klasach szkolnych 1-3.**

Ale jak rozumiem ten etap na razie nie nastąpi.

**No jeszcze nie. Bo jesteśmy w pierwszym chyba.**

No, na pewno jesteśmy w pierwszym. A dzisiaj ma pan, dzisiaj? Nie, do końca tygodnia mają się wypowiedzieć, no to może dzisiaj albo jutro, kiedy ten drugi etap ewentualnie mają wdrożyć. Tak dzisiaj pani mówiła w telewizji. No, zobaczymy. Wie pani co, z mojej perspektywy fryzjer i biblioteka. To są takie 2 miejsca, z których ja korzystam z tych etapów, które tam są wymienione.

**A co według pani powinno się zadziać, żeby ten następny etap otworzyć, uruchomić?**

No tak, żeby on miał nastąpić, jakieś kryteria. No ja nie mam bladego pojęcia, jakimi kryteriami się nasi politycy kierują. Myślę, że jakimiś kryteriami na pewno bezpieczeństwa z jednej strony. A z drugiej strony ruszenia gospodarki, czyli takie teraz mamy modne słowo, odmrażania.

**Ale gdyby pani miała podejmować decyzje, to jakie byłyby te kryteria? Co by pani brała pod uwagę? Wiem, że na szczęście nie musimy my tego podejmować.**

Nie musimy, no tak, tak. Myślę, że tutaj trzeba by było to ważyć jakoś. To znaczy ważyć jakby zagrożenia i zyski płynące. Nawet nie tak, że właśnie tam liczby zachorowań czy spadek czy coś. Tylko ważyć to, na ile na przykład, jak wygląda, trudno to zmierzyć na pewno, ale jak się kształtuje na przykład zdrowie psychiczne tych ludzi, którzy siedzą w odosobnieniach teraz. Bo myślę, że to jest duży problem. Ja się tu komunikuję z tymi moimi seniorami za pomocą SMS-ów głownie. No to oni tęsknią i siedzą w tych domach. I są tacy… Myślę, że nastrój mają obniżony. Więc być może takie wyjście, nie wiem, do parku czy do biblioteki czy gdzieś na pewno bym im dobrze zrobiło. Nie wiem, na ile oni na przykład… No, ale myślę, że tak, że gdyby te miejsca zostały uwolnione i na przykład nie trzeba by było wnosić opłaty, wchodząc do muzeum, to myślę, że na przykład część z tych ludzi, w takich mniejszych miastach zwłaszcza… Jakoś tych starszych właśnie albo na przykład dla tych seniorów znieść te finansowe ograniczenia. Myślę, że może oni by też skorzystali. Bo ten ich nastrój na pewno ma też wpływ na ich odporność. A z drugiej strony być może, jak wyjdą na ulice, to zaczną się zarażać. No też trudno powiedzieć. Też, nie wiem, być może trzeba by było wprowadzić jakieś zróżnicowanie. Na przykład w Warszawie czy w tych dużych skupiskach miejskich, tam, gdzie jest najwięcej zachorowań, rozluźniać taką sytuację bardziej powoli. Albo może właśnie się zarażajmy. Nie wiem. Musiałabym długo myśleć nad tym, żeby… I się jeszcze na tym znać w ogóle. Jakieś mieć dane epidemiologiczne. Wolałabym nie podejmować takich decyzji, na szczęście nie muszę.

**No dobra, a na przykład te maseczki. Jak pani myśli, do kiedy powinniśmy je nosić?**

No te maseczki to myślę, że powinniśmy je nosić ho, ho, ho. Biorąc pod uwagę, że powinniśmy je nosić wtedy, kiedy my zarażamy, a o tym nie wiemy, to myślę, że powinniśmy je nosić długo.

**Ale długo to jest pół roku? Rok? 2 lata? Ile to jest długo?**

Długo to jest tyle, ile będziemy chorować. Aż będziemy mieć na przykład takie testy na przeciwciała na przykład. Że jeżeli już zachorowaliśmy to na przykład nie musimy ich nosić, jeżeli jesteśmy odporni. I już jesteśmy po jakby. Przeszliśmy to, możemy nie nosić. A dopóki nie mamy takiej pewności, że większość społeczeństwa jest w miarę odporna na to, albo może przeszczepiona na przykład. Ja myślę, że te szczepienia to na pewno jest dobry moment, żeby to zdjąć. Jak sobie wszyscy zafundujemy jakieś przeciwciała. Czy wyprodukujemy tak naprawdę przeciwciała. Skoro już je… Jeżeli będziemy chodzić po ulicach i będziemy mniej… Im więcej nas będzie na ulicach, tym bardziej powinniśmy nosić te maseczki na pewno. To z całą pewnością.

**A na przykład kiedy szkoły powinniśmy otwierać?**

Ja myślę, że nie ma się co spieszyć z tymi dziećmi, chociaż one w ogóle przechodzą to bezobjawowo prawie. I są w zasadzie bezpieczne. Pokazują to wszystkie wyniki. Myślę, że już jest teraz taka próbka i tych chorych i ofiar, i osób pod respiratorami, że można już w tej chwili określić, jakie jest niebezpieczeństwo. Chociaż jednak dzieci bardzo wyizolowaliśmy. Bo nie dość, że one zostały w domach, to jeszcze nie mogą chodzić do sklepów żadnych. Więc nie spotykają się w zasadzie. Ewentualnie między sobą. Ale jako że są izolowane od większości osób, to… Ale myślę, że nie ma się co spieszyć. Jak pójdą we wrześniu, będzie super.

**A to dobrze, że tak te dzieci izolujemy od wszystkiego teraz, skoro one nie chorują?**

Nie wiem, czy dobrze. Ja wiem, czy dobrze? Myślę, że one sobie przechorują to tak czy inaczej. Nie wiem. Może niedobrze. Ale jeżeli byśmy puścili te dzieci do szkoły, to musimy całą armię ludzi puścić, która obsługuje te dzieci. I to już są ludzie dorośli. Więc ten ruch, bym powiedziała, osób, który jest związany z tym, że dzieci idą do szkoły jest dużo większy niż tylko dzieci. Więc może pod tym względem, może dlatego jakby się nie przemieszczamy tak bardzo, bo jak dzieci mamy w domu, to i my jesteśmy w domu więcej. I ta sytuacja jest może bardziej opanowana. A tak jak puścimy, może im by się akurat najmniej stało. Nie wiadomo, co by się stało ze wszystkimi dookoła. Może jesteśmy w stanie to przewidzieć? No, myślę akurat, że takie dane już mamy bardziej. Nie wiem, możemy patrzeć na kraje azjatyckie, co tam się dzieje. Tam jednak oni wracają do życia. Chociaż na pewno są bardziej karnym społeczeństwem.

**Czytałam, że w Holandii otwierają od poniedziałku szkoły. Że już dzieci wracają w Holandii do szkół.**

Wie pani co, no może i dobrze. Ja mam tych znajomych na Śląsku lekarzy. I oni na przykład uważają, że ich wnuki i dorosłe dzieci przeszły już tego koronawirusa. Bo jak tam byli w styczniu we Włoszech i wracali, bo to Śląskie województwo miało ferie jako pierwsze. I wszyscy wrócili bardzo tacy właśnie przeziębieni, z gorączką. I dorośli, czyli powiedzmy 30+ te osoby, czyli rodzice tych dzieci łącznie z jakimiś… Mieli takie bardzo kiepskie samopoczucie. Łącznie z jakimiś kłopotami takimi oddechowymi, że… Ale to jeszcze w ogóle nie było wtedy mowy, to były pierwsze przypadki w Chinach. No i te dzieci wszystkie przechorowały też, zostali tydzień w domach po powrocie. I wszystkie te dzieci potem wróciły… Oni uważają, że oni już tego koronawirusa mieli. I że już są… No być może powinny te dzieci wrócić do szkoły.

**A czy słyszała pani jak Szwecja podchodzi? Bo Szwecja trochę inaczej niż inne kraje europejskie się zachowuje.**

Szwecja to się chyba tak zachowuje trochę, jak się zachowywał Boris Johnson, jak Wielka Brytania.

**No tak. Oni tam formalnie prawie że restrykcji nie mają. Nie mają takich oficjalnych zakazów, tylko zalecenia, rekomendacje. Ale mają otwarte sklepy, restauracje, fryzjerów mają otwartych.**

A jakieś dane? Bo jakoś danych się nie przekazuje z tych krajów.

**Nie wiem, nie mam danych.**

No właśnie. Bo jak pokazują, wie pani, te paski na dole, co chwilę tam się pokazują jakieś, no to zdają relację głównie z krajów Europy południowej czy Europy zachodniej, Francja, Włochy, Hiszpania, tam, gdzie tych zachorowań jest najwięcej. Niemcy jako sąsiedzi. No i Stany Zjednoczone, które teraz są jakąś po prostu masakrą. Ale tych skandynawskich krajów nie pokazują. Nie wiem, jak to tam wygląda.

**Nie wiem. Tego nie mam, nie wynotowałam sobie.**

Może trzeba by to przeanalizować. Nie wiem, jak tam wygląda zachorowalność. I też pytanie jest o poziom służby zdrowia i takich instytucji opiekuńczych, jak to wygląda. Bo jeżeli, oni mają na pewno tą służbę zdrowia na dużo wyższym poziomie. Bo Szwecja akurat to kraj, gdzie są podatki 50%. I na pewno tam nie zabraknie respiratorów. Więc nie wiem, nie mam bladego pojęcia, jak to tam wygląda.

**Ale co pani sądzi w ogóle o takim podejściu luźnym? Oni tam na przykład też mówią, jeżeli czujesz się chory, no to zostań w domu. Po prostu.**

No tak. Ale to też jest taki kraj, gdzie jak jesteś chory i zostajesz w domu, nie musisz pójść do lekarza, żeby powiedzieć w pracy, że nie idziesz. Po prostu dzwonisz i mówisz, że się źle czujesz i nie idziesz. I ci się to liczy jako chorobowe czy coś. No ma to jakieś tam pewnie korzenie w funkcjonowaniu społeczeństwa, w jakichś nawykach. Jak oni, nie podejrzewaniu każdego o to, że nadużywa takich sytuacji, żeby uniknąć pójścia do pracy, tak? Nie zakładamy, że każdy z nich jest oszustem. Myślę, że jeżeli społeczeństwo jest gotowe do takiego… Gotowe, odpowiedzialne i bardziej wyedukowane, no to, skoro im się to sprawdza, to super. Nie wiem, czy to by się u nas sprawdziło. Myślę, że nie. Myślę, że u nas to by było zdecydowanie nadużywane. Ale my jesteśmy, moim zdaniem, w szczególnej sytuacji narodu, który ma szczególne dość cechy. Głównie takie, że jak ktoś coś nam powie i jest u władzy, to my, nawet za zaszczyt sobie poczytujemy to, że się temu sprzeciwiamy. Myślę, że tutaj lata pod zaborami i to, jak trzeba było funkcjonować no ma kluczowe znaczenie. I potrzeba setek lat, żeby to zmienić. Szwedzi nie mieli zaborców. I są moim zdaniem, te zachodnie społeczeństwa są dużo bardziej dojrzałe. Bo oni są po prostu dorośli, a my jesteśmy dziećmi.

**A jeszcze dzisiaj wyczytałam rano, że totalnie na drugim krańcu jest Nowa Zelandia, która na 5 tygodni zamknęła wszystko. Taki totalny lockdown kraju zrobiła. Zamknęła wszystko. Jakby zostały tylko elektrownie, żeby był prąd, wodociągi, żeby była woda. Takie tylko podstawowe, a wszystko zostało zamknięte.**

Mam kolegę w Nowej Zelandii, ale nie utrzymuję z nim jakichś szczególnych kontaktów.

**Co pani sądzi o takim podejściu, że tak wszystko, wszystko zamykają?**

Ale jak, no wszystko, wszystko się nie da. A jak jedzą, co jedzą?

**Mieli zrobić zakupy. Mieli 48 godzin na zatowarowanie się na 4 tygodnie.**

No to pojechali się zarazi wszyscy od wszystkich. Nie wiem, no wie pani co, no każdy robi tak… Moim zdaniem pokazuje to daleko posuniętą bezradność. Bo nie mamy… Bezradność czy brak takich procedur. Bo jak jedni otwierają wszystko i mówią, dobra, zachowujcie się rozsądnie ludzie, bo się możecie pozarażać, no to… A drudzy zamykają wszystko. No to bardzo zależy. No, zależy od zwyczajów, od karności takiego społeczeństwa, od dojrzałości, od nawyków. No dlaczego te kraje Europy zachodniej mają takie duże zachorowania na początku? Bo oni i się witają i się całują wszyscy, i się ściskają. I spędzają czas z rodzinami, jedzą posiłki w gronie 20 osób a nie dwóch czy pięciu. Chodzą do knajpy, w domach czajnika nawet nie mają. Bo na kawę rano idą na dół do jakiejś kafejki. No, to jak im powiemy, słuchajcie, miarkujcie się i zarekomendujemy im, to co? Pójdą sobie kupić czajniki wszyscy? No nie. A w takiej Szwecji czy w Norwegii być może to inaczej wygląda. Na Islandii nie trzeba im mówić, żeby się izolowali, bo oni są i tak wyizolowani.

**To jeszcze mam ostatni temat. I to jest ten temat, który już się pojawił, czyli takiego dbania o siebie. Pojawiła się potrzeba fryzjera. Jak to w ogóle wygląda u pani z tym, nie wiem, miała pani taki pomysł, żeby sobie samemu nożyczkami? Albo farbę samemu albo coś?**

Wie pani co, mnie stosunkowo to mało przeszkadza, że ja mam siwe. Chociaż nie tak do końca, bo maluję włosy. Farbuję. Ale tak, jestem w takiej szczególnej sytuacji, że ścięłam sobie włosy z długich, bardzo długich, na krótkie w wieku lat 30. I do tej pory chodzę, to było 24 lata temu. I do tej pory chodzę do tej samej fryzjerki. Do jednej. Więc tak, nie mam takiej pokusy, żeby gdzieś pójść i na przykład, poprosić kogoś, żeby mi coś podciął. Nawet Julka mi mówiła, mamo, to ja ci coś podetnę. Nie. Kupiłam sobie takie spineczki. Więc jak już to mi przeszkadza, jak coś robię i mi wpada w oczy, to sobie podpinam. No bym sobie pomalowała te włosy. No, jak są długie, to po prostu są dłuższe. Bo jak by były długie, to ja bym sobie spięła tak jak przez 30 lat życia w kucyk i bym miała z czaszki. Ale od kiedy podjęłam tę decyzję, że mam je krótkie, no to one wymagają po prostu ciągłej kontroli długości przede wszystkim. Ale nie mam takiej pokusy, wie pani, ja nie jestem obcięta na pazia, więc nie mogę sobie tak podciąć ich. No jakoś sobie poradzę. Nie spędza mi to snu z powiek, chociaż to jest jakby taka rzecz, której mi najbardziej brakuje. W sensie takim, że myślę, że pierwsze kroki skieruję w tamtą stronę właśnie (śmiech). Jak ta moja dziewczyna wróci do pracy. Co tam. No, jeżeli chodzi o paznokcie na przykład, które również już od wielu lat robię, ale też u tej samej pani. Należę do wiernych po prostu.

**Lojalna pani jest strasznie, tak.**

Tak, tak, jestem wierna, zawsze byłam wierna. Więc chodzę do tej samej pani. Ale tutaj, na przykład no… I jeszcze do Warszawy w tej sprawie w ogóle dodam, przemierzam 100 km w jedną stronę do tej pani od paznokci. Ale tutaj mam taką sąsiadkę, która mi… W takiej jednej z rodzin, którym tu pomagam. I tam jedno z dzieci w tej rodzinie wielodzietnej jest pełnoletnie. Dziewczyna skończyła szkołę kosmetyczną. I tam jeszcze jakieś inne szkoły. I taka jest, czasem mi pomaga. I ona mi już ze 2 razy zrobiła paznokcie, przyszła. Bo i tak przychodzi i tak. Sama ma kontakt tutaj w okolicy, no, mieszka 6 kilometrów ode mnie. I ja się opiekuję i tą rodziną i jej babcią właśnie. I jej najmłodszym rodzeństwem, najmłodszym bratem. Któremu pomagałam w szkole. Więc ten kontakt mamy. I ona mi tutaj przyszła z 2 razy te paznokcie zrobiła. I jak tak dalej pójdzie, no to skorzystam. Bo jednak tutaj na tej roli. I, że się tak wyrażę, ja mam je bez przerwy po prostu… Ja mam żel na nich, bo jest mi wygodniej zdecydowanie i one wyglądają estetyczniej niż takie bez. No, ale jak bym ich nie miała, to Julka na przykład też korzysta i nie ma, w tej chwili ma zdjęty ten żel, bo jej zlazł cały. I na przykład nie ma takiej potrzeby, żeby… Stwierdziła, że będzie odpoczywać od takiej funkcji. No to takich rzeczy mi brakuje.

**A poza fryzjerem i paznokciami coś w obszarze takiego dbania o siebie u pani się zmieniło jeszcze? Bo do fryzjera się nie da teraz po prostu.**

No nie da się, nie da. Nie da się, to nie. Ale wie pani, by się dało. Bo Julka mówi, mamo, to ja ci pofarbuję. A ja mówię, e tam, będziemy farbować. Jak już może będę miała siwe do pasa, no to może się zastanowię nad tym, nie mówię nie. Ale bardziej mi przeszkadza, że mi w oczy wpadają. Ale jak się przemęczę jakiś czas z tymi spineczkami, to może będę mogła je sobie w jakiś tam kucyk spiąć.

**Dla pani pewnie wiele się nie zmieniło. Nie wiem, nie jest tak, że teraz pani nie chodzi do pracy, więc jest inny ubiór, albo inaczej dni wyglądają, bo one właściwie wyglądają prawie tak samo.**

Tak samo, tak.

**A u Julki? Jak to jest, jak ona teraz nie chodzi do pracy. Widzi pani u niej zmianę tych nawyków dbania o siebie?**

Ja nie widzę, ale ona na pewno się nie maluje. Ale to też jest tak, że jak przyjeżdżała tutaj, to rezygnowała z makijażu. Nigdy nie był on… Ja się w ogóle nigdy nie malowałam w życiu, więc nie mam takich w ogóle problemów. Nie miałam też, jak pracowałam. A ona ma tam, używa jakiegoś podkładu i coś tam. I nie wiem co, rzęsy chyba czy… Dobra, coś tam używa. Rzęsy chyba sobie pomalowała. No to teraz tego nie robi. Ale też ja o tyle to obserwuję, że o tym wiem. Bo ona jak przyjeżdżała do mnie to i tak tego nie robiła, dawała tej buzi odpocząć. To nie było, z mojego punktu widzenia to nie jest jakby bardzo widoczne. Znaczy jak jest umalowana i ją widzę umalowaną, bo gdzieś wychodzi, czy widywałam, no to widzę różnicę. Ale to nie jest taka różnica, że nie wiem, anioły klękajcie albo coś takiego. No więc nie maluje się teraz.

**A jak jedziecie do Płocka to też nie?**

Nie, też nie. Ale też tego nie robiła chyba, jak tu była. Ale nie wiem. Bo zawsze ten makijaż taki dyskretny był, więc… Ale chyba nie robiła nigdy, jak przyjeżdżała na wieś.

**A ubrania takie na wiosnę, teraz idzie nowa pora roku?**

To kiepsko pani trafiła, bo ja, jeśli chodzi o ubrania, to… No, to w zasadzie ich nie kupuję. Nie zmieniam. Raczej, raczej dostaję jakieś tam ciuchy, tu głównie dostawałam z Warszawy od znajomych. No takie, wie pani, jak to robimy porządki w szafie i inne jakieś, i pościel, i ręczniki dla tych moich podopiecznych i jakieś ubrania. To czasem tam sobie coś wzięłam. Szczególnie od Inki, jak tam z tego śląska coś dostaję. Bo ona ma podobne do mnie gabaryty. Więc jakąś sukienkę, ona ma ich za dużo, to mi oddaje i ja w niej chodzę później. Nie, nie, nie mam jakichś zmian zwyczajów.

**Ale mówiła też pani, że jak Julka jeszcze wcześniej przyjeżdżała, to czasami jeździłyście sobie do Płocka, bo tam jednak w tych galeriach mniej ludzi, lepiej się chodzi.**

Tak, to Julce kupowałam.

**Tego wam nie brakuje? Znaczy wam albo Julce, żeby sobie wyjść, pochodzić po sklepach.**

Wie pani co, nie, ja myślę, że to była bardzo dobra okazja, żeby sobie tam coś nowego kupić. Ale chyba nam nie brakuje, bo ona też nie jest jakoś szczególnie zakupowa. No nie wiem, trudno mi powiedzieć. Może jej brakuje. Muszę ją zapytać. Mnie na pewno nie brakuje. Bo ja jak już muszę sobie coś kupić, no to cierpię, nawet bym powiedziała, z tego powodu. Ale idę sobie coś kupić, bo tam na przykład, nie wiem, jakiś bal był karnawałowy, jakąś sukienkę trzeba było kupić… To ciężko. Ale daję radę, czasem sobie coś kupię. Ale z rzadka.

**Czyli z takich rzeczy do dbania o siebie to właściwie zostaje tylko nam ten fryzjer i te paznokcie.**

Tak.

**Dziękuję.**
