# Supplementary material for: The challenges arising from the COVID-19 pandemic and the way people deal with them. A qualitative longitudinal study
Source: PLoS One. 2021 Oct 11;16(10):e0258133. doi: 10.1371/journal.pone.0258133 (PMC8504766; doi:10.1371/journal.pone.0258133)
Supplement: S1 Dataset — (ZIP) [file pone.0258133.s003.zip › Transcriptions/stage 3/14.3_M_55_couple, with children.docx]

**14.3_M_55_couple with children**

**Powiedz Przemek, co się wydarzyło w ciągu ostatnich 2 tygodni u ciebie, jaki to był czas?**

No cóż, trochę mieliśmy trochę roboty. Bo walczyliśmy trochę z rodzicami, którzy nam próbują nie płacić czesnego. Udało nam się na przykład też wynegocjować pewne ustępstwa ze strony wynajmujących nam budynek. Wiesz, wszystko się działo wokół tego całego wirusa, wokół tej dziwnej sytuacji, którą teraz mamy.

**Ja pamiętam, że mówiłeś, że jeszcze nie byłeś pewny, czy nie będziesz musiał przedszkola otwierać, jak rozmawialiśmy ostatnio.**

Bo nie było wiadomo. Dopiero to się okazało kolejnego dnia. Wszystko oficjalnie zamknięte, nie wiem, do chyba dwudziestego… Nie wiem nawet do którego. Czy do 29 czy do 24 kwietnia?

**Jakoś tak.**

Mamy taką sytuację, że nie wiadomo, co będzie u nas za tydzień. Różne są plotki, że zaraz będą otwierać, bo już po prostu nie mają na zasiłki te opiekuńcze (śmiech).

**Czyli wymuszone otwarcie sytuacją?**

No tak. No i teraz pytanie właśnie, jak się do tego odniosą rodzice, jak się odniosą dzieci. Czy nie będą wprowadzone jakieś ograniczenia, typu, że dzieci będą musiały chodzić po przedszkolu w maseczka. Co by było w ogóle debilne, no ale bardzo możliwe, że coś takiego wprowadzą. Dzieci, nauczyciele itd. Chodzą różne słuchy. Tam jakaś data chodziła, 15 maja czy coś takiego. No nie wiadomo.

**Ogólnie nadal nic nie wiadomo?**

Nic nie wiadomo, nikt nic nam nie mówi. Zresztą wszyscy, wiesz, też mieszkasz tu i widzisz jak…

**Ale co, ten tydzień przed świętami to była właśnie taka nerwówka związana z załatwianiem…**

To była bardzo duża nerwówka właśnie z tym związana. I ten tydzień po świętach, ten ostatni to był troszkę spokojniejszy. Ale teraz znów już czekaliśmy na drugą tarczę, żeby wiedzieć, co możemy z niej uszczknąć ewentualnie. Teraz już wiemy, co możemy uszczknąć. I wiemy, ile z tego, co możemy uszczknąć, możemy dalej przekazać rodzicom po prostu. To wszystko, co nie zapłacimy w ramach tej tarczy po prostu przeznaczymy na obniżkę czesnego i tyle.

**A sama Wielkanoc? Co się działo w Wielkanoc?**

Mówię, do dekoracji jakoś tam specjalnie uczuciami nie pałam.

**No tak, mówiłeś, że to jakaś ulga, że właśnie nic nie trzeba, nie ma tego przymusu.**

No właśnie. I to było fajnie pod tym względem.

**Nie było tego zaskoczenia, że czegoś brakuje, że jednak ta Wielkanoc jest inna niż zwykle?**

Absolutnie. Dla mnie właśnie to, że trzeba obchodzić jakąś Wielkanoc, jeździć do rodziny coś to… Z tej okazji. Bo ja nie mam nic przeciwko jeżdżeniu do rodziny, tylko ja mam przeciwko jeżdżeniu z okazji Wielkanocy. I robienie z tego jakiegoś takiego cyrku. Nigdy jakoś mi się to nie podobało. A to, że teraz nie trzeba było jechać, nie było żadnego przymusu, nawet wręcz przeciwnie to fajnie.

**I co? Siedzieliście sami w domu?**

Tak. W domu. Spotkaliśmy się z rodziną na jakimś tam Zoomie czy innym Googlu, o takie były jakieś rzeczy.

**I ogrodowych spotkań żadnych też nie odbywaliście? Bo często osoby, które mają ogród, była ładna pogoda, to mówią, a, to sobie usiądziemy… Nie?**

Nie. Absolutnie, nic takiego.

**Jakieś nowe rzeczy się pojawiły u ciebie w życiu w ciągu ostatnich dwóch tygodni?**

Nie powiedziałbym, że się coś pojawiło.

**A z czegoś, z jakichś typów aktywności, zachowań zrezygnowałeś może? Coś robiłeś, miałeś jakiś zwyczaj, a teraz już nie.**

Nie, nie. Absolutnie nie. Kiedy będę mógł sobie pojechać już nawet na jakąś dłuższą wycieczkę rowerową. Chociaż nie jestem pewien do końca, czy to jest zgodne z prawem. Ale nawet, jak nie jest zgodne z prawem, no to co.

**No to co wtedy, jak cię złapią?**

No przecież na Sybir mnie za to nie ześlą.

**A jakieś rzeczy, które ci jakoś bardziej zaczęły przeszkadzać? Mówiłeś, że cię sporo rzeczy takich biurokratycznych bardzo złościło, irytowało. Jak jest teraz? Czy jest coś takiego, co zauważyłeś, że ci bardziej przeszkadza? Co cię już tak męczy, doskwiera ci?**

Wiesz co, doskwiera mi to, że… No, w ogóle ta cała sytuacja już mnie po prostu zaczyna nudzić lekko, wiesz? Bo ciągle jest ten sam temat. Włączasz telewizję – koronawirus. Włączasz radio – koronawirus, czytasz gazetę – koronawirus. Tak jakby poza tym nic na świecie się nie działo. Nudno się zaczyna robić. Wcześniej codziennie się starałem śledzić informacje, jak to wygląda. W tej chwili to już nawet nie śledzę. Bardziej mnie interesuje sytuacja w innych krajach niż w Polsce. Bo po prostu wszystko mnie tutaj zaczyna nudzić. Jeszcze chrzanienie ciągłe o tych wyborach, będą, nie będą. Głosować, nie głosować, wiesz, jakieś takie dyskusje… Nie wiem. Nie wiem, czy będę głosować, decyzję podejmę w ostatniej chwili, w zależności od tego, jak to będzie wyglądało. Nie mam zamiaru uczestniczyć w czymś, co będzie totalnie niezgodne z prawem. Bo nie będę popierał jakiejś hucpy. A jeżeli będzie to zorganizowane w sposób, nie wiem, w jakiś sposób przynajmniej zbliżony do tego, czym powinny być wybory, no to będę uczestniczyć. A jeżeli będzie to jakaś totalna hucpa bez możliwości sprawdzenia, bez możliwości weryfikacji wyników, jakiejś tam społecznej, to będzie jedna wielka lipa i nie ma sensu w tym uczestniczyć.

**Ale to, że nie sprawdzasz już tak tych wiadomości, nie śledzisz tego tak pilnie, jak śledziłeś, to jest zmiana jednak. Bo jak się spotykaliśmy, to mówiłeś, że na bieżąco wszystko wiesz, patrzysz w te wszystkie…**

Nie, bo już po prostu mnie to nudzi zwyczajnie. Wolę się zająć czymś innym, wolę przeczytać jakąś książkę o czymś innym niż obecna sytuacja. Wolę obejrzeć jakiś serial niż ciągle śledzić wiadomości. Znaczy człowieka i tak ciągle coś dochodzi. Bo trudno, żeby to nie dochodziło. Bo rano sobie przejrzę gazetę, tak? Bo ma prenumeratę Rzeczpospolitej elektroniczną, więc sprawy jakieś tam służbowe, firmowe, chcę być na bieżąco, jak tam kwestie podatkowe, zusowskie itd., jak to się tutaj rozwija, z czego można skorzystać. Po prostu z punktu widzenia pracy, dlatego śledzę. I przy okazji się przejrzy tę gazetę, żeby wiedzieć, co się dzieje. Czy jakąś tam Wyborczą się jeszcze przejrzy. Bo mam dwie prenumeraty. Gazet papierowych nie kupuję już od dawna. Ale jakąś tam prenumeratę mam i to oglądam.

**Tą prasówkę sobie robisz. Ale mówisz, że bardziej cię interesują teraz informacje z zagranicy niż z Polski.**

Tak.

**Które informacje?**

Interesują mnie informacje z Rosji, ponieważ w Rosji bardzo długo mieszkałem. I ten kraj jest mi bliski, tam mam znajomych, z którymi jestem w ciągłym kontakcie, więc rozmawiamy ze sobą. Oni mi opowiadają, jak tam jest w tej Moskwie, ja im opowiadam, jak jest w Polsce i się dzielimy wrażeniami. Interesuje mnie też sytuacja w Niemczech, w Stanach. Tam, gdzie mam znajomych, gdzie mam przyjaciół, z którymi od czasu do czasu porozmawiam i… Mało ciekawsze jest to, co się w Polsce dzieje.

**A czy to, co czytasz o Rosji w polskich mediach, w polskich gazetach to jest zgodne z tym, co słyszysz od swoich znajomych? U nas są wiarygodne informacje na temat tego, co się dzieje w Rosji?**

No to, co ja czytam, czyli *Nadziwinowicz* ciekawie pisze, Tomek *Kułakowski* ciekawie pisze, no to, mimo że on w tej chwili nie jest w Moskwie, tylko w Polsce, no ale on też śledzi to, co się tam dzieje. No to są bardzo ciekawe rzeczy. I one są mniej więcej zgodne z tym, jaka jest moja opinia, jakie jest moje doświadczenie. Ale nie wiem, bo gdzie indziej to ja nic nie czytam, nie wiem, kto jeszcze o Rosji pisze.

**Nie wiem, ja statystyki jakieś ostatnio oglądałam i mnie zdziwiły. Wydawały mi się jakieś takie mało realistyczne. Ale nie znam się.**

Te rosyjskie?

**Nie, u nas w Polsce podane, jakie tam są zachorowania, zgony, wyzdrowienia, takie tam rzeczy.**

Nie, no tam jest w tej chwili ogromny problem. Bo na przykład wczoraj rozmawiałem z prawnikiem, znaczy takim moim znajomym, kolegą byłym z pracy w Rosji, który jest szefem działu prawnego w firmie. I on mówi, że jest to duży problem. Ponieważ, to jest akurat Selgros, czyli firma handel detaliczny. Więc już w jednym zakładzie wykryto koronawirusa, w jednym sklepie, no to się zastanawiają, czy nie zamknąć, na razie zrobili dezynfekcję. O ile w Polsce w takiej sytuacji są w miarę konkretne, z tego co się zorientowałem, wskazówki Sanepidu, to tam po prostu oni są zostawieni totalnie sami sobie. Nie wiedzą, czy mają kogoś izolować. Nie śledzi się na przykład kontaktów osoby zarażonej, z kim się spotykała itd. Nikogo to nie interesuje.

**W tym kontekście uważasz, że u nas jest to jakoś bardzie uporządkowane i lepiej zarządzane?**

Z tego, co słyszę na temat Rosji, to tam jest po prostu totalny burdel. Na przykład doszło do takiej sytuacji, że wprowadzono jakieś takie elektroniczne przepustki. Że trzeba, jak się gdzieś jedzie, na przykład do pracy czy załatwić jakieś sprawy, albo SMS-em albo przez internet napisać, gdzie się chce jechać. I na telefon przychodzi jakiś taki QR kod, który potem sprawdza policja. I w pierwszym dniu obowiązywania tych przepustek policja wszystkim, którzy wchodzili do metra, sprawdzała to. Co doprowadziło do tego, że się ludzie zaczęli tłoczyć przy wejściach. Czyli po prostu cała strategia, żeby się ludzie nie kontaktowali ze sobą, wzięła w łeb. Specjaliści zaczęli mówić, no to tydzień kwarantanny po prostu, tego całego, izolacji w Moskwie po prostu zwyczajnie wziął w łeb. Bo ludzie nie mieli innego wyjścia. Po prostu miasto, które ma 15 milionów mieszkańców, no nie można zwęzić wejścia do metra i każdemu sprawdzać przepustki, bo to po prostu doprowadza do takiej sytuacji. Mimo, że i tak jeździ, powiedzmy, jedna piąta tej ilości osób, która jeździła kiedyś. W mieście, które słynęło z największych korków w Europie, zniknęły korki.

**Tak, tam to jest rzeczywiście…**

A zaczęli sprawdzać przepustki wszystkim, którzy wjeżdżają do Moskwy, na granicy miasta się ustawili, co doprowadziło do ogromnych korków, kilkugodzinnych, żeby wjechać do centrum. Co też nie było zamierzone. I na przykład ci, którzy zwykle pracują w innej dzielnicy, jeździli na przykład obwodnicą moskiewską, no to jak wjechali na tę obwodnicę, to później nie mogli z niej zjechać, bo wszędzie były bramki i (śmiech).

**I sprawdzanie.**

I sprawdzanie, tak. To przecież jakaś totalna bzdura. Po jednym dniu, oczywiście następnego dnia już było wszystko w porządku. Ale to jest światełko, totalna indolencja władz. Więc można narzekać na indolencję władz w Polsce, ale to, co pokazały władze rosyjskie to po prostu był debilizm to entej potęgi.

**Może być gorzej, jednym słowem.**

To mnie nie pociesza, ale, ale (śmiech).

**Wspomniałeś o książkach. Pamiętam, że mówiłeś, że chciałbyś poczytać, ale nie możesz się skupić nad książkami. To się jakoś zmieniło?**

Jeżeli chodzi o czytanie, to nie. Ale audiobooki, słucham audiobooka, jak z psem wychodzę na przykład, czy coś takiego. Zacząłem jakąś tam książkę czytać do snu. Nie, teraz… Też nie bardzo się mogę skoncentrować, bo od razu zasypiam, jak czytam.

**Jak myślisz, a to jest takie zasypianie, bo jesteś znudzony? Czy to jest takie zasypianie… Bo co? Co się dzieje poza tym?**

Po prostu zasypiam.

**Masz wrażenie, że więcej śpisz teraz niż spałeś?**

Raczej tak.

**Odpoczywasz w czasie tego snu czy nie bardzo?**

Tak, tak, mimo problemów, tak.

**Jeszcze pamiętam, że mówiłeś, że zostawiasz telefon w domu, jak idziesz do lasu, na rower.**

Ale to z uwagi na ten bzdurny zakaz chodzenia do lasów. Jakaś mania mi się prześladowcza pojawiła, że będą śledzić, którędy ludzie chodzą itd. 2 czy 3 razy to zrobiłem i potem już przestałem to robić, bo doszedłem do wniosku, że nie będę się poddawał paranoi. A w tej chwili to już się w ogóle nie przejmuję.

**A jakieś sposoby spędzania czasu, które by, nie wiem, właśnie jak jesteś znudzony, żeby czymś się zająć. Jakieś nie wiem, może jakieś hobby nowe ci wpadło?**

Nic takiego. Nie, z żoną seriale oglądamy. Jakiś tam zaczęliśmy oglądać, „Opowieści podręcznej” na przykład.

**A to jest zmiana? Dawniej też oglądaliście razem seriale czy nie?**

Tak, tak. Tylko że dłuższy czas już nie oglądaliśmy. To znaczy ona oglądała swoje, ja jakieś tam swoje. A teraz oglądamy razem. O „Opowieści podręcznej” to jest serial, który się zaczyna robić aktualny. Nie wiem, czy ty znasz.

**Tak, tak, obejrzałam.**

No właśnie.

**Zgroza. Nie chcę myśleć, że to się zaczyna robić aktualne, ale rzeczywiście coś w tym jest, co mówisz.**

No a tendencja, tendencja.

**Tak i maseczki jeszcze pogłębiają to wrażenie (śmiech).**

No właśnie tak.

**Emocje – zdjęcia.**

**12.**

**Może tutaj jakiegoś zdjęcia brak, brakuje ci czegoś, co powinno być na tym zdjęciu.**

Brakuje mi jakiegoś takiego, nie wiem, nudnego, pustego krajobrazu.

**Nudny, pusty, krajobraz, czarno-biały, kolorowy, opisz.**

Może czarno-biały. Coś w rodzaju dziesiątki.

**Które wydaje ci się, częściej ci towarzyszyło? To coś w rodzaju dziesiątki, tego pustego krajobrazu czy 12?**

Po prostu oba te rysunki, te obrazki są takie nudne.

**Czym się różni 12 i 10?**

I tu i tu są jakieś tam drzewa w tle, tylko jedne są bardziej wyraźne, drugie mniej. One są takie nudne, bez akcji, takie nic się nie dzieje. A czym się różnią? Jedno jest czarno-białe, drugie kolorowe. No to kolorowe takie może… Nie wiem, trudno powiedzieć. Nie wiem, ja w te skojarzenia naprawdę nie jestem dobry.

**To poza tym, że to jest znudzenie, to jakie jeszcze uczucia ci przychodzą do głowy, które by pasowały do tego, jak się czułeś w ciągu ostatnich dwóch tygodni. Wiesz, bo to może być znudzenie, ale jednocześnie irytacja, znudzenie i złość, znudzenie i smutek. I w ogóle co jeszcze.**

Wiesz co, bardziej znudzenie i jakiś taki smutek. No właśnie. Smutek, bo mnie zawsze emocjonowano robienie jakichś planów na kolejne miesiące. A tutaj… Wiesz co, sytuacja jest taka, że za bardzo nie ma co robić jakichś planów. Robiłem sobie plany dotyczące jakichś podróży, dotyczące tego, czym się będę zajmował. A tutaj jest jakoś tak no… Człowiek taki jest trochę, zresztą już mówiłem o tym, czuje się taki jakiś trochę zamknięty w tej sytuacji całej. I już nawet mnie to nie irytuje, tylko raczej właśnie smuci. I coraz bardziej mnie to zniechęca.

**Masz jakieś sposoby radzenia sobie ze smutkiem i zniechęceniem?**

Nie, no coś ciekawego obejrzeć, jakiś film, jakąś książkę. To wiesz, w taki sposób sobie człowiek radzi (niezrozumiałe) smucił i zniechęcał. Od czasu do czasu można sobie jakieś spotkanie ze znajomymi przez Zooma zorganizować i pożartować, o, to takie coś.

**No właśnie, mówiłeś 2 tygodnie temu, że byłeś po pierwszym takim spotkaniu.**

No to gdzieś było kolejne i jeszcze kolejne. I teraz na piątek znów czy na sobotę jakieś tam kolejne planujemy. Poza tym postanowiliśmy z żoną Anią, skoro już jesteśmy na miejscu i nie planujemy żadnych podróży, żadnych przerw, to sobie zamówiliśmy na 2 tygodnie dietę pudełkową jakąś tam. Bo chcemy schudnąć na przykład. Ale i tak w domu jesteśmy, zawsze problem był z tym pójściem na dietę, bo się coś działo. Zawsze gdzieś wyjeżdżaliśmy. A jak się jest na tej diecie, to trzeba się cały czas ściśle jej się trzymać. A tutaj wiem, że nigdzie nie wyjedziemy przez najbliższe 2 tygodnie, więc spokojnie możemy się na tym skoncentrować i skorzystać z tego, że na tyłku siedzimy.

**To wprowadza jakiś rodzaj takiego wyzwania w wasze życie, jakiejś odmiany? Czy to jest raczej…**

No tak. Jest to jakaś tam odmiana, są to nowe doznania, bo to jest dieta, nie jakaś tam głodząca, tylko to jest dieta ketogeniczna (niezrozumiałe). Jakie w ogóle będą efekty, no coś nowego. Nie mówimy o dietach pudełkowych jakichś tam owocowo-warzywnych typu Dąbrowska nie Dąbrowska, ale to niesmaczne takie było, już na sam zapach tej kapusty to mi się niedobrze robiło. A to coś innego, jakieś takie ciekawsze doznania. Urozmaicenie.

**Ketogeniczna, nie będziesz głodny, bo na ketogenicznej nie powinno się odczuwać głodu.**

No właśnie. Dzisiaj właśnie jest pierwszy dzień.

**To jestem ciekawa, co mi powiesz za 2 tygodnie o tej diecie, jak jest. A mówiłeś, że wino ci pomagało i potem to wino od Kondrata, które zamówiłeś. Sprawdziło się wino od Kondrata?**

Tak, bardzo dobre. Ale teraz 2 tygodnie nie będzie wina. Bo na tej diecie ketogenicznej nie powinno się pić. Więc robimy sobie przerwę, zobaczymy, jak będzie bez wina. Czy będzie nam tego brakowało. Pewnie tak, ale może nie. Zobaczymy.

**Jeszcze coś wymyśliliście, żeby sobie w tym czasie jakoś odmienić?**

Nie wiem, no cały czas coś wymyślamy. Zobaczymy, może się znów coś pojawi, jakaś ciekawa rzecz.

**A jak byś popatrzył na swoje najbliższe otoczenie, na swoich znajomych, to jak oni sobie radzą? Czy jest jakaś zmiana w tym, jak oni się czują, co mówią, co robią?**

Też trudno powiedzieć. Bo każdy jest w innej sytuacji. I mamy znajomych, którzy są mocno zdołowani, głównie dlatego, że po prostu stracili biznes, który budowali przez kilkanaście lat.

**Mówisz o tych hostelach?**

Tak, bo to był biznes hotelowy. I nagle po prostu świat się zawalił. Zostali bez przychodów i z mnóstwem kosztów stałych. I nie wiedzą, na ile im pieniędzy starczy. To też tragedia. Jak z nimi rozmawiam, to ja jestem tak naprawdę pełen podziwu, że jeszcze mają trochę poczucia humoru w tym wszystkim. Bo ja nie wiem, jak ja bym był w takiej sytuacji, czy ja bym to wytrzymał. Bo my jakoś sobie damy radę, tak? My w sumie, aż tak mocno w porównaniu z innymi nasz biznes nie ucierpiał. No jakieś tam, powiedzmy będziemy mieli, nie wiem, przez jakiś tam okres będziemy ponosić straty. Ale w ciągu roku no to nawet (niezrozumiałe) to wszystko zakończy. Albo może wyjdziemy na zero. No jeżeli człowiek wychodzi na zero w obecnej sytuacji, to jest, ja uważam super.

**To prawda. A poza tymi znajomymi, którzy są w takiej rzeczywiście trudnej sytuacji biznesowej, to jak, nie wiem, jak twoja żona sobie radzi, jak twój syn sobie radzi, jak znajomi sobie inni radzą?**

No nie wiem. Jakoś tam też… Tak to chodził do pracy i miał tam jakieś tam zajęcia. A w tej chwili siedzi w domu, z nikim się nie spotyka. Tam czasem zajmuje, gra sobie w jakieś gry komputerowe, tak spędza czas.

**Nadal odkurza, czy już przestał?**

Może z mniejszą intensywnością. Córka jest w klasie maturalnej, więc cały czas ma jakieś tam zajęcia. Ciągle też ze znajomymi jakimiś tam jest na chatach, na jakichś tam spotkaniach video, czy coś takiego.

**A co w związku z maturą? Co ona mówi na ten temat? To jest dla niej źródło stresu, czy co będzie, to będzie? Jak to wygląda?**

Ona chyba uważa, że co będzie, to będzie.

**A w ogóle nie wspomniałeś o swoich obawach, lękach. Jak to z tym jest w tej chwili?**

Właśnie obawy mam takie tylko, że nie wiadomo, jak to długo potrwa. I obawy mam takie też, że się do tego, do tej sytuacji przyzwyczaję za bardzo. Wiesz? Że… No właśnie, że to wszystko jakoś tak oklapnie, że to się stanie dla nas jakąś taką rutyną. I się przyzwyczaimy, że to tak już będzie teraz zawsze. Co zresztą nie jest do wykluczenia, bo świat się bardzo mocno zmieni po tej pandemii, tak? Nie wiadomo, czy w ogóle kiedyś będziemy mogli swobodnie podróżować po całym świecie. Czy nie będzie jakichś ograniczeń ogromnych, które poszczególne kraje będą wprowadzały.

**A dlaczego się boisz, że się przyzwyczaisz? Co ci się zmieni w życiu, jeżeli się przyzwyczaisz do tego?**

No co się zmieni. Po prostu za bardzo nie mam, to, co pozwalało mi się nie nudzić to to, że byłem ciągle w ruchu. A jeżeli człowiek nie będzie ciągle w ruchu, no to za bardzo nie mam jeszcze pomysłów na to, czym się zająć. Ja nie potrafię, bardzo mnie tak jakoś denerwuje rutyna. Czyli codziennie, każdy dzień taki sam. Wkurza mnie coś takiego. I właśnie (niezrozumiałe), że codziennie to samo.

**Jest to jakaś pustka, którą nie wiadomo, czym wypełnić, jak rozumiem?**

No właśnie. Tak. I samo zamknięcie, to nie jest tak, że ja jestem zamknięty w jakimś małym mieszkanku na piętrze, z albo bez balkonu. Mamy dom, ogród, obok las. Więc to nie jest tak, że my się dusimy w czterech ścianach. Ale to, że się właśnie nic nie dzieje. No my co miesiąc gdzieś wyjeżdżaliśmy praktycznie. Czy w Polsce czy zagranicę. A czasem nawet kilka razy w miesiącu się gdzieś wyjeżdżało. A teraz nagle… My naprawdę mieliśmy bardzo intensywne życie. Tak jak ci mówiłem, nie było czasu na 2 tygodnie diety, bo ciągle gdzieś byliśmy w ruchu. Albo był plan jakiegoś jednego wyjazdu, drugiego, trzeciego albo… No cokolwiek, coś się zawsze działo. A teraz po prostu nie ma nic. Codziennie praktycznie jest to samo.

**Stagnacja, brak nowych doznań, takie rzeczy, które po prostu… Ile można oglądać seriale, tak?**

Tak. No właśnie.

**Gdyby tak miało być, to myślisz, że jesteś osobą, która będzie sobie wynajdowała jakieś nowe pasje tutaj wokół siebie, w domu, w ogrodzie, w lesie?**

Nie wiem. Na razie jeszcze… Jestem wystarczająco zajęty tym, co się dzieje w firmie, że… To nie jest tak, że ja się nudzę, bo nie mam co robić przez cały dzień, tak? Tylko się nudzę, bo to jest codziennie to samo.

**Ale słuchaj, bo jak rozmawialiśmy 2 tygodnie temu, to mówiłeś, że poziom obaw lęku bardzo wzrasta, jeśli chodzi o kwestie gospodarcze i że czujesz się bardziej zagrożony tą sytuacją koronawirusową niż poprzednio.**

Tak, ale wtedy to była nowość. A w tej chwili już się przyzwyczaiłem do tego, że to jest. I że ta niepewność jest. I nauczyłem się może z tą niepewnością żyć w jakiś tam sposób.

**Czy to jest dla ciebie dobrze, że tak się stało?**

Może tak, bo jestem może trochę spokojniejszy. Poza tym jestem też przekonany, że jednak w jakiś sposób będzie luzowane, że te ograniczenia będą zdejmowane. Że za kilka tygodniu część już jakiejś działalności gospodarczej w Polsce zacznie wracać. Ale nie dlatego, że zacznie się zmniejszać zagrożenie epidemiologiczne. Po prostu państwu brakuje pieniędzy. No i po prostu zacznie to wszystko być przywracane. Co się będzie wiązało z dużymi kłopotami. Bo tak, jeżeli na przykład… Jeden z naszych biznesów to jest przedszkole, szkoła. Dzieci wrócą i co potem? Dzieci będą musiały w maseczkach chodzić? Nie wiadomo. A co się stanie, jeżeli w sytuacji działającego przedszkola pojawi się, nie wiem, nauczyciel z koronawirusem? Bo w tej chwili to jest sytuacja dość jasna. Nie my zamknęliśmy przedszkole, tylko zamknięte zostało odgórną decyzją, tak? A jeżeli otworzą oficjalnie przedszkola i pojawi się w indywidualnym przypadku zagrożenie, no to w tym momencie my będziemy musieli podjąć tę decyzję. I to już będzie zupełnie inna sytuacja, jeżeli chodzi o rozmowy z rodzicami. Już nie będziemy mogli się powoływać na to, że no oni postanowili, bo to my postanowiliśmy. No to od razu się zacznie wyciąganie: no, ale to wy jesteście winni, bo wy dopuściliście do tego, że… I tak dalej, i tak dalej. Na pewno tak będzie. I może być, że z punktu widzenia, problemów związanych z prowadzeniem biznesu może być więcej, a nie mniej. Tak? Bo w tej chwili po prostu nie działa, lekcje są online. A jak przywrócą przedszkola, no to… A przywrócą, bo już po prostu państwa nie będzie stać na te zasiłki w pewnym momencie.

**A te obostrzenia, które są w tej chwili, jak ty się z nimi czujesz, na ile się stosujesz, co w ogóle myślisz o tym?**

A jakie są obostrzenia w tej chwili?

**No jakie są?**

No ja nie widzę, żeby były jakieś obostrzenia. Do lasu mogę pójść, na rowerze można jeździć.

**A ta maseczka, którą musisz nosić?**

Ta maseczka? Wiesz co, ja nie wychodzę za bardzo z domu. Do sklepu to ja i tak, zanim pojawił się ten obowiązek noszenia maseczki, to ja i tak ją zakładałem, maseczkę, rękawiczki. Po ulicach nie spaceruję za bardzo. Bo jeżeli się gdzieś poruszam, to jadę samochodem. W samochodzie nie trzeba nosić, jak wyjdę, to idę sklepu czy chodzę do lekarza. To i tak trzeba było założyć. Nie wiem, mnie to jakoś specjalnie nie… A tutaj po okolicach nie zakładam, jak wychodzę z domu u mnie na wsi, to w ogóle… Znaczy mam ze sobą na wszelki wypadek. Ale jej nie noszę.

**Jasne. Obostrzenia mamy cały czas pewne stałe, czyli te zamknięte knajpy, kina, usługi, szkoły, przedszkola.**

Chciałbym pójść do fryzjera, a nie mogę. To jest jedyna rzecz, bo zacząłem obrastać. Ale tak, to…

**Ale z punktu widzenia takiej sensowności dla powstrzymania, zahamowania epidemii, to które z tych przepisów, zakazów, nakazów wydają ci się sensowne, a które nie?**

Wiesz co, to co mi się wydaje sensowne, to zalecenie pracy zdalnej. To jest bardzo sensowne ograniczenie. Czyli to, żeby ludzie jak najmniej się poruszali do pracy i z pracy. Czyli zmniejszenie zagęszczenia w transporcie publicznym. I wychodzi na to, że to są też sensowne, że… Wiesz, to co mi się teraz spodobało w tym wszystkim to to, że wiele firm doszło do wniosku, że oni tak naprawdę mogą funkcjonować w ten sposób dłużej. Że to, wiesz, ściąganie ludzi do jakiegoś tam jednego pomieszczenia czy tam do jednego biura, żeby razem pracowali, to w sytuacji takiej, jaka jest teraz, że pracujemy w większości elektronicznie, korespondencja jest elektroniczna, to być może wcale wiesz, być może firmy nie wrócą do takiej pracy stacjonarnej, że wszystkich spędzają w jedno miejsce. Bo nie ma konieczności. Co też w sposób niejako naturalny zmniejszy obciążenie transportu publicznego. I w ogóle wiesz, mniej się będziemy poruszać po drogach, do pracy, z pracy. Nie będzie się jeździć do urzędów, żeby załatwiać sprawy, ponieważ można bardzo dużo rzeczy, okazuje się, załatwić (niezrozumiałe). Wcześniej to trzeba było zawieźć papier w zębach, a teraz okazuje się, że wszystko można prawie załatwić online.

**(niezrozumiałe) możemy robić wszystko z pozycji komputera, albo dużo.**

I to jest bardzo dobre ograniczenie. Drugie dobre ograniczenie, związane z tą pandemią, to… chociaż nie, to nie jest dobre. W zasadzie tamto ograniczenie, to jest, takie zachęcenie do pracy online to jest jedyne ograniczenie, które ja widzę, że jest dobre. Poza tym ja też nie jestem specjalistą, jeżeli chodzi o to, jakie zagrożenie tak naprawdę stanowi ten wirus. Jeżeli do tej pory w Polsce umarło kilkaset osób, czyli tyle, co dziennie umiera na raka, no to nie wiem, czy to jest coś, co rzeczywiście stanowi, wiesz, istotne jakieś zagrożenie, jeśli chodzi o każdego z nas. Wychodzi na to, że coraz bardziej może, wszyscy zaczynają dochodzić do wniosku, nie tylko w Polsce, ale w innych krajach, że nie taki diabeł straszny. Zaczynamy się uczyć tego wirusa, zaczynamy rozumieć, jakie on tak naprawdę może mieć skutki. I może właśnie z uwagi na to, że specjaliści coraz bardziej go poznają, zaczynają rekomendować jakieś luzowanie jednak. Zacznijmy pracować, zarabiać pieniądze, bo nie ma sensu wariować.

**Bo być może wirus będzie z nami dłużej?**

Niezależnie od tego, że to było wszystko nowe, nie wiadomo co, to na wszelki wypadek zamknijmy. Poza tym to ten czas, żeby się do tego przygotować, żeby wyposażyć szpitale, żeby te respiratory kupić i wyposażyć, to wszystko. Żeby wygrać czas trochę w tej chwili.

**A do kiedy powinniśmy nosić twoim zdaniem te maseczki?**

Ja nie jestem pewien, czy je w ogóle trzeba nosić, na ulicy na przykład, spacerując. Ja uważam, że je trzeba nosić na przykład, jeżeli się wchodzi do sklepu, jeżeli trzeba wchodzić do jakiegoś środka komunikacji publicznej, tak? Tam, gdzie mamy bliski kontakt z innymi ludźmi. Ale żeby spacerując po ulicy, trzeba było nosić maseczkę, to ja uważam, że to jest bzdura.

**A teraz zakładasz, jak wychodzisz z domu na spacer?**

Tutaj w okolicy mojej nie. Znaczy ja je mam ze sobą. Bo jak na przykład może kogoś spotkam i będę chciał z nim porozmawiać, no to wtedy jak on zakłada, to i ja zakładam. Taka grzeczność wobec tej innej osoby. Bo też nie wiem, jaką ta osoba ma, czy ona się boi, czy się nie boi. Nie chcę u nikogo powodować, jakiejś takiej sytuacji, że on się będzie czuł zagrożony dlatego, że ja tej maseczki nie mam.

**A jeżeli spotykasz znajomego i on mówi: słuchaj, nie zakładaj, ja się nie boję. To wtedy założysz?**

Zakładam, ja i tak zakładam. Nie, ja uważam, że to nawet należy założyć. Bo jeżeli stoimy obok się nawet te 2 metry, załóżmy…

**Ale masz też poczucie, że to chroni ciebie także?**

Nie, nie uważam, że to chroni mnie. To jest taka, można powiedzieć, grzeczność wobec innej osoby. To łatwo zrobić taki gest uprzejmości wobec innej osoby. Spotykasz, załóż tą maseczkę. Każdy z nas mówi inaczej, jedni mówiąc w sposób bardziej plujący, inni mówią w sposób mniej plujący. Ja też nie wiem, w jaki ja sposób mówię. Czasami mówi człowiek głośno, no to coś tam z tych ust wyleci. Załóżmy to. To jest żaden problem. Ale żeby z tym po mieście paradować? No bez sensu trochę.

**Czyli sklep, kwestia rozmowy, przebywania bliżej, czy w pomieszczeniu też…**

No ostatnio jedyne pomieszczenia, do których chodzę, to są sklepy. Albo byłem wczoraj u lekarza. Bo lekarz też miał, zdezynfekowałem ręce.

**Są obostrzenia zupełnie bezsensowne w tej chwili?**

No te, co były bezsensowne, to całe szczęście się ktoś opanował i zdjął, prawda?

**Czyli ten las i co jeszcze?**

Las i to, że nie można sobie swobodnie wyjść do parku i pojeździć na rowerze. W ogóle ograniczenie totalnie bezsensowne. I widać, że to, że oni to zdjęli, to nie jest znak jakiegoś tam luzowania. Tylko po prostu oni doszli do wniosku, że to jest bzdura. I nie ma sensu ludzi obarczać tego rodzaju ograniczeniami. Bo może doprowadzić do tego, że ten rząd się po prostu stanie bardzo niepopularny. I w obawie po prostu o swój własny rating (śmiech). Poza tym oni to spróbowali w jakiś tam sposób wygrać. Ja widziałam w telewizji publicznej, bo sobie włączyłem w poniedziałek, no to cały czas leciało, jacy to Polacy są szczęśliwi, bo mogą sobie pójść do lasu. No znów, to dowcip o kozie i rabinie się przypomina. Co zrobić, żeby ludzie byli szczęśliwi? Najpierw im zakazać czegoś, a potem pozwolić. To jest najlepsze.

**A te etapy poluzowania, bo już zostały przedstawione takie 4 etapy, o ile pamiętam. To co ty o tym myślisz, na co zwróciłeś uwagę?**

Słuchaj, wiem, że jakieś tam 4 etapy są, bo oglądałem, ale nawet już nie pamiętam, jakie to etapy są. Poza tym co mi z tej pięknej prezentacji, skoro nie ma tam żadnych dat?

**Oni tam w drugim etapie planują otworzenie sklepów budowlanych w weekendy, mam ściągę…**

Nie, teraz chyba są otwarte sklepy budowlane w weekendy. Czy nie?

**Nie, jeszcze nie.**

Nie? Jeszcze nie?

**Nie, to dopiero jest drugi etap. Otwarcie w drugim etapie hoteli i innych miejsc noclegowych. Otwarcie niektórych instytucji kultury jak biblioteki, muzea i galerie sztuki. To jest etap drugi, teraz jesteśmy w pierwszym. Potem jest etap 3, to jest gastronomia, fryzjerzy, salony kosmetyczne, sklepy w galeriach. I na końcu jest masaż, solaria, kluby fitness.**

A edukacja tam gdzieś jest?

**Edukacja jest w etapie 3. Żłobki i przedszkola w klasach 1-3 ale ustalona ma być maksymalna liczba dzieci w sali.**

No to dobrze, no to możliwe, że ten pierwszy etap wprowadzą od przyszłego tygodnia albo od 1 maja czy coś. Ten drugi etap znaczy.

**Ten drugi.**

Co tam ma być oprócz…

**Tam mają być hotele i miejsca noclegowe. To dla twoich przyjaciół byłoby dobrze.**

No tak, ale co z tego. Skoro oni nawet, jak im pozwolą otworzyć, no to, jeżeli u nich 95% gości to byli obcokrajowcy, bez otwarcia granic, a otwarcia granic tam nigdzie nie było w tym planie.

**Nie było.**

No właśnie. Bez otwarcia granic to można wiesz, o jedno miejsce potłuc wszystko. (niezrozumiałe) że będą otwarte hotele, no to co? No to będę sobie mógł pojechać do Wrocławia, przenocować we Wrocławiu i wrócić.

**Słuchaj, a jak myślisz, czy to jest tak, że jakieś obostrzenia powinny zostać na dłużej niż tak etapami? Czy to są maseczki, czy to są obostrzenia w sklepach, ta liczba osób na metr. Czy to są zgromadzenia, czy to są spotkania, kina, teatry, imprezy?**

Wiesz, imprezy masowe na pewno. Jakieś takie większe imprezy. Nie wiem, imprezy sportowe. Tam, gdzie się setki czy tysiące osób w jednym miejscu skupiają. I jeszcze na dodatek powiedzmy w jakichś tam klubach razem sobie tańczą. Czyli jest pot, wszelkiego rodzaju wydzieliny cielesne. Czyli tam wszędzie, gdzie się skupiają w jednym miejscu. Tymi miejscami nie są restauracje. Bo tutaj jednak ta odległość jest większa. Jak ludzie siedzą przy jednym stoliku, no to siedzi kilka osób, 2-3. Co jeszcze. Czy zgromadzenia publiczne takie, które są na zewnątrz, trudno powiedzieć. Boję się, że mogą tego też nie luzować, bo rządzącym obecnie nie jest jednak na rękę, żeby się ludzie gromadzili. Więc pod pretekstem walki z koronawirusem to ograniczenie zostawić na dłużej. Ale na przykład, bardzo był fajny przykład w Izraelu. Ludzie się zebrali na jakąś tam demonstrację, ale wszyscy od siebie, demonstrujący trzymali odległość 2 metrów.

**Tak, niesamowicie to wyglądało.**

Tylko boję się, że jednak tutaj czegoś takiego w Polsce nie wprowadzą. No, może izraelskie społeczeństwo jest bardziej zdyscyplinowane. Jednak to, że oni wszyscy przeszli przez armię i jednak potrafią się stosować do jakichś tam rozsądnych nakazów i zakazów, to doprowadziło do tego, że… No tak, wiemy, jesteśmy świadomi, trzymamy od siebie tę odległość półtora metra czy dwóch metrów, tak? Inny sposób wychowania społeczeństwa. Gdyby w Polsce było społeczeństwo w taki sposób wychowane, że każdy bez wyjątku przechodziłby wojsko, przy czym normalne wojsko, nie takie, w którym ja byłem, to też byłoby zupełnie inaczej.

**A jak już o tym mowa, ten model szwedzki, który jest. Wiesz jak to tam wygląda?**

Tak.

**I co myślisz o modelu opartym o to, że rekomendują jakieś zachowania, ale w zasadzie niczego nie zakazują? Tam wszystko działa.**

I widzisz. Ale to też jest bardzo mocno kulturowe. Po pierwsze w Szwecji jest bardzo wysokie zaufanie do władz wszelakich. Niezależnie od opcji, która akurat rządzi. Bo twierdzą, że ci, którzy nami rządzą… Tym bardziej, że tam, zwróć uwagę, też dużo na ten temat czytałem, tam, jeżeli jest jakaś konferencja prasowa, gdzie się mówi o sytuacji związanej z epidemią, tam nie występują politycy. Tam nie występuje premier. Tam występuje główny epidemiolog. Czyli specjalista w tej dziedzinie. I to jest jego decyzja, jakie wprowadzać ograniczenia. Czyli oni niejako polityków od tej decyzji odsunęli. A w Polsce te wszystkie decyzje podejmują politycy. Którzy, co jest charakterystyczne, co jest cechą immanentną tego zawodu, oni się nie zawsze kierują dobre obywateli, ale raczej własnymi, partykularnymi interesami. A gdyby to wprowadzali niezależni specjaliści, niebędący wybranymi politykami, to też być może byłoby inaczej. A jeżeli mamy 2 polityków czy 3 polityków, premier, minister edukacji i minister zdrowia, no to są politycy, którzy się PR-ują po prostu na tych konferencjach. I nie ma na przykład… W Polsce te decyzje nie zostały oddane niezależnym ekspertom. Tylko one zostały oddane politykom.

**Ale właśnie, gdyby zostały oddane takim ekspertom, gdyby się politycy w ogóle nie mieszali, to myślisz, że taki system właśnie proponowania zachowań a nie zakazywania zachowań, to by się sprawdziło?**

Mogłoby zadziałać. Tak, mogłoby w Polsce też zadziałać. I tak w Polsce w tej chwili większość ludzi się dostosowuje do tych ograniczeń. Przynajmniej to, co widać, że się ludzie dostosowują. Jak przejechałem się samochodem po mieście, to widziałem, wszyscy chodzą w maskach. Ja to mówię, że to są namordniki, wszyscy chodzą w namordnikach. Po rosyjsku słowo namordnik to jest kaganiec, więc to tak trochę fajnie brzmi. No i ja uważam, że gdyby niezależny ekspert, który by, że tak powiem, siłą swojej wiedzy i doświadczenia decydował o tych zakazach, czy o w ogóle całym tym programie zwalczania wirusa, to też byłoby inaczej. Druga rzecz, bo rozmawialiśmy o Szwecji. To tam jest sytuacja, że Szwedzi, oni nie są tacy znów rodzinni, że oni się ciągle spotykają. Oni są indywidualistami. Bardzo dużo gospodarstw domowych to jest jednoosobowych, dwuosobowych. Tam nie ma wielopokoleniowych rodzin, które razem mieszkają na kupie. Poza tym nie ma takiej gęstości zaludnienia. No właśnie, czyli… Więc są takie cechy kulturowe. Dlaczego we Włoszech się ta epidemia tak rozprzestrzeniła? Bo oni, jak się spotykają, to się obejmują, całują, prawda? Nie ma też tego takiego dystansu. Jak Włoch z tobą rozmawia, to rozmawia z tobą na metr. Szwed, jak z tobą rozmawia, to stoi i tak 2 metry dalej. Bo ten obszar prywatności, który każdy ma, jest w tych krajach południowych dużo mniejszy niż w krajach Europy Północnej. I to też ma duży wpływ na to, jak to się rozprzestrzenia. I jak popatrzymy też, gdzie się te przypadki zachorowań w Szwecji są, one są w środowiskach imigranckich, Somalijczyków itd., oni wszyscy mieszkają na kupie. Więc tam są większe ryzyka rozprzestrzeniania się. No i w domach dla seniorów. Którzy też mieszkają na kupie w jednym miejscu. I tam jest bardzo wysoka śmiertelność. Ale to wynika z tego, że tam jest bardzo wysoka ilość ludzi starych, w Szwecji w ogóle. I to czysto matematycznie, procentowo, stąd ta…

**Wysoka zachorowalność i śmiertelność może wynika z nadreprezentacji ludzi starszych i schorowanych?**

Tak, tak, tak. Po prostu ze struktury wiekowej społeczeństwa.

**A ten dystans między ludźmi, trzymanie się w odległości. Czy to jest coś, co uważasz, że powinno zostać na dłużej?**

Tak. Mi to osobiście nie przeszkadza. Ja jestem osobą, która też woli od kogoś być dalej niż bliżej.

**Rozumiem, że nie najlepiej byś się czuł, tak jak wyglądał kiedyś Stadion X-lecia i były te wszystkie stragany tam i nie można było szpilki wcisnąć.**

Ja mam klaustrofobię. I tak nie wiem, jak się jeszcze ta fobia nazywa, ja się boję dużych skupisk ludzkich. Jak na przykład czasami w jakiś weekend wchodzę do zatłoczonej galerii handlowej, to ja się tam źle czuję. Ja zaczynam się bać i chcę stamtąd wyjść. Jak jest dużo ludzi, to ja… Moja żona to już o tym wie i nawet mnie nie namawia na wchodzenie do takich miejsc. Rzadko kiedy chodzę na jakieś takie imprezy zamknięte, jakieś kluby, koncerty, bo ja tam się źle czuję po prostu.

**I rozumiem, że metro w godzinach szczytu to też nie jest twoja bajka.**

Nienawidzę tego. Nienawidzę jechania komunikacją publiczną zatłoczoną. Nie lubię tego.

**Czy myślisz, że ludzie się tak zmienią, że to tak będzie, że ludzie zaczną trzymać dystans od siebie?**

Tak, oczywiście. Na pewno tak będzie. I to jest bardzo dobry efekt tego, co się w tej chwili dzieje. Może też być odwrotna reakcja. Bo o, wreszcie można, to zaczniemy się teraz masowo spotykać na jakichś tam imprezach itd. Ale mi się wydaje, że nie będzie odreagowania raczej. Że ta obawa o… Bo my żeśmy od wielu lat jako społeczeństwo żyli bez w zasadzie obaw o jakąś tam chorobę zakaźną. Ja jeszcze pamiętam ze swojego dzieciństwa, jak chodziłem do szkoły, to ciągle jacyś tam koledzy w klasie chorowali, to na koklusz, to na jakąś tam szkarlatynę itd. To jest coś, coś się nie pojawia już w tej chwili. Poza tym było bardzo dużo lekarzy, którzy byli specjalistami chorób zakaźnych, był szpital przeciwgruźliczy. Ja jeszcze widywałem na ulicy ludzi chorych na Heine-Medina, czy tych, którzy przeszli chorobę Heinego-Medina. I kiedyś myśmy żyli z taką dużo większą świadomością tego, że te choroby nam zagrażają i mogą potencjalnie być śmiertelne. I podejrzewam, że gdyby się w tamtych czasach pojawił ten koronawirus, to na niego w ogóle by nikt nie zwrócił uwagi. Bo w porównaniu z innymi zagrożeniami, które były wówczas epidemiologicznymi… Chociażby sama gruźlica, która jeszcze wtedy zbierała żniwo, mimo że były już antybiotyki, które ją leczyły. One były wszędzie, przecież szpitale przeciwgruźlicze, które niby zostały, ale chorych na gruźlicę w Polsce praktycznie nie ma. Teraz jak byłem w Afryce, w Botswanie, to się dowiedziałem, że tam największym zagrożeniem nie jest AIDS wcale, tylko gruźlica. I widziałem wszędzie plakaty na temat gruźlicy, żeby zwracali uwagę, żeby się zgłaszali do lekarza itd. Po prostu myśmy się zapomnieli o tym, że jest takie zagrożenie. A teraz to się pojawiło. I mi się wydaje, że jednak będziemy żyć z tą świadomością, że istnieje coś takiego jak choroba zakaźna. I zwracać uwagę na to, żeby jednak się przed nią jakoś chronić. Nie wiem, co będzie z tymi wszystkimi antyszczepionkowcami, których się teraz namnożyło mnóstwo, którzy twierdzą, że… Już w tej chwili nawet słyszę, że… Bo to nawet, wydawałoby się, mądrzy, inteligentni ludzie, których znam i szanuję, zaczynają mówić takie rzeczy, że cały ten koronawirus to jest spisek firm farmaceutycznych, które potem chcą nam wszystkim sprzedawać szczepionki, które nas tam, wszczepią nam te szczepionki jakieś chipy. Jak ja słyszę takie bzdury, to mi się po prostu aż w głowie przewraca. I to mówią inteligentni ludzie.

**To prawda, to jest niesamowite. Mówisz o takich dobrych zmianach, które mogą wyniknąć z tego, co się teraz dzieje. Widzisz jeszcze jakieś dobre, długofalowe i złe, długofalowe skutki tego, co się dzieje, tej sytuacji?**

Ja uważam, że to zmniejszenie ruchu samochodowego i to, że ludzie będą mniej jeździć do pracy z pracy, to bardzo będzie miało pozytywny wpływ na środowisko naturalne. Będzie miało pozytywny wpływ na cenę na przykład ropy.

**Już ma.**

I uważam, że też zmniejszy się ilość podróżujących, latających samolotami. Bo się ludzie będą bali. Przynajmniej na samym początku, przez kilka lat będzie tak, że ludzie nie będą latać samolotami, mniej latać. Może dlatego, że jeszcze różne kraje wprowadzały różne ograniczenia. Na przykład będzie coś takiego, że żeby podróżować trzeba będzie mieć żółtą książeczkę szczepień i będzie musiał być jakiś tam wpis, że się jest albo zaszczepionym na tego koronawirusa albo że się go przeszło. Będzie się po prostu jeździło ze świadectwami szczepień.

**Czyli uważasz, że taka ostrożność i obawa zostaną na dłużej?**

Tak, to na pewno zostaną na dłużej.

**Coś jeszcze ci się wydaje takiego znaczącego w tych zmianach, takich społecznych głównie, bo gospodarcze to też mówiliśmy sporo i właściwie nie wiadomo, co tutaj będzie.**

Nie wiadomo, wszystko zależy od tego, jak to długo będzie trwało. Tylko, wiesz, jeszcze jaki jest problem? Każdy kraj stosuje różną politykę, jeżeli chodzi o wspieranie gospodarki. I o ile na przykład w Niemczech oni mogą być spokojni, że większość firm nie upadnie, bo tam te programy pomocowe są bardzo mocne, o tyle na przykład w takiej Rosji programów pomocowych nie ma żadnych dla biznesu. Wszyscy ludzie, którzy prowadzą jakieś biznesy, zostali zostawieni totalnie sami sobie. W Polsce jakieś tam te programy pomocowe są, jakieś tam te zwolnienia ze składek. Można na ten temat mówić, czy to jest wystarczające czy nie. Oczywiście, że to nie jest wystarczające.

**Ale z twojego punktu widzenia, to ci pomogło?**

Oczywiście, że to pomogło. Bo jak sobie policzyłem, ile dostaniemy zwolnienia ze składek ZUS, no to po prostu byśmy to przełożyli na wysokość czesnego i my to wszystko oddajemy rodzicom. My nic na tym nie zarabiamy. Tylko po prostu nawet jeszcze dokładamy z naszego zysku, żeby rodzicom dać jakiś tam rabat na tym czesnym.

**Ale to jakoś uspokoiło sytuację?**

Tak, tak, tak. W szkole na pewno uspokoiło. Bo wczoraj właśnie wysłaliśmy maile, że obniżamy o 20% czesne za kwiecień. Bo my to robimy, nie dajemy, nie mówimy, ile będzie w następnym miesiącu, bo my cały czas pracujemy. A widzimy, że z tego, co zdołaliśmy uzbierać, to możemy czesne w szkole obniżyć o 20%. To 20% to coś jest. To jest tam, czesne w szkole wynosi 1400 zł, no to 20% to już jest te 280 zł. To już każdemu 280 zł w miesiącu, no zawsze coś jest.

**A z twojego punktu widzenia, kiedy powinny ruszyć szkoły i przedszkola?**

Ja uważam, że zdrowe byłoby, gdyby dzieci już nie wracały do szkół i przedszkoli w tym roku.

**Ale ta spodziewana fala na jesieni kolejna?**

No nie wiadomo, czy ona będzie. Może będzie, może nie będzie. Jeżeli wierzyć temu, co się mówi, że tak naprawdę tych ukrytych zachorowań to jest, nie wiem, 20 razy więcej niż tych, które są objawowe, czy tam jeszcze więcej, słyszałem, że (niezrozumiałe) razy więcej czy coś. Nawet czytałem jakieś tam badania z jakiejś gminy w Niemczech, że tam było zachorowań sporo, ale 10 razy więcej osób miało już przeciwciała. Tak że świadczy to, że jakoś tam, w ukryty sposób przeszli, uodpornili się. Nie mieli objawów, bo mieli wysoki (niezrozumiałe), tak że może się uodpornili. Może nastąpi za te 4 miesiące jakieś tam uodpornienie społeczeństwa na tego wirusa i to nie będzie już takie groźne. A poza tym, nawet jeżeli się pojawi ten wirus, to specjaliści nauczą się, jak go leczyć, jak traktować ludzi, jak ich izolować. Poza tym też będzie dużo lepsze wyposażenie placówek medycznych w niezbędny sprzęt. Bo te ograniczenia są wprowadzone dlatego, żeby po prostu wygrać z czasem. Żeby nie było takiej dużej ilości zachorowań na samym początku, tak jak we Włoszech. Oni totalnie byli nieprzygotowani, tam brakowało po prostu tych wentylatorów czy respiratorów. Ale w Polsce wychodzi na to, że zajętych respiratorów jest 10% z tego, co jest. Czy mniej niż 10%. Więc duży zapas jest w Polsce.

**Używasz słowa izolacja, to tak przykuło moją uwagę. Bo jestem ciekawa, widzę, że to jest zamiennie często traktowane, kwarantanna i izolacja. Jakie jest dla ciebie rozróżnienie tych dwóch terminów?**

Wiesz co, ja może źle użyłem słowa. Bo to nie jest izolacja. Bo my się nie izolujemy, raczej takie dystansowanie bardziej społeczne. Izolacja… Może rzeczywiście izolacja, ale w jakichś takich… Częściowa izolacja, unikanie kontaktów. A jakie rozróżnienie izolacji od kwarantanny? No nie, to jest w zasadzie synonim. Tylko, że kwarantanna to jest w momencie, kiedy jest jakieś podejrzenie, żeby… Izolacja to jest prewencyjna taka. A kwarantanna to w momencie, kiedy już jest jakieś podejrzenie i czekamy, czy się coś z tego rozwinie czy nie. Na przykład w Rosji jest używany termin samoizolacja. I to w zasadzie jest to, co ja miałem na myśli. Że człowiek się sam izoluje, unikając nadmiernej ilości takich fizycznych kontaktów z innymi ludźmi.

**A jak jest kwarantanna to w ogóle nie ma kontaktów z innymi ludźmi?**

Kwarantanna to w ogóle nie ma kontaktów z innymi ludźmi, bo… Kwarantanna to jest coś narzuconego. A ta izolacja czy powiedzmy samoizolacja to jest coś, co my robimy dobrowolnie. Bo ja na przykład jak bym chciał, to mógłbym się spotkać, no nie wiem, pojechać do mojej mamy do Koszalina. No mógłbym pojechać. Bo zgodnie z przepisami mi wolno. Ale ja tego nie robię. Bo wiem, że moja mama ma 90 lat. I ja też nie wiem, czy ja nie jestem czasem na przykład nosicielem. Więc ja wolę nie pojechać. Mimo, że nikt mnie do tego nie zmusza, ale ja tę decyzję podejmuję sam.

**A testowanie, czego ty byś się spodziewał? Żeby był dostęp do testów, żebyś ty mógł robić testy przesiewowe swoim nauczycielom w szkole, w przedszkolu? Jak to wygląda?**

To by było dobre. To by było dobre. Zresztą ja uważam, że powinno być tak, że każdy ma prawo do tego, żeby raz na jakiś czas, żeby przynajmniej za własne pieniądze mógł się przetestować.

**Teraz można. Na Ursynowie można zrobić za 500 czy 600 zł sobie test.**

Podobno można. No nie wiem, nie wiem.

**Pojechałbyś zrobić sobie taki test?**

Nie. Znaczy ja bym może sobie zrobił test ten drugi, czy ja mam już odporność czy nie. Bo to mi da większą wiedzę o sobie. Bo przynajmniej bym wiedział, czy mam te przeciwciała czy nie, czy ja to przeszedłem czy nie przeszedłem. No to wtedy bym już wiedział, że na przykład mogę więcej. Miałbym mniej obaw. A to, czy ja akurat w tej chwili… Chociaż też nie wiem, jak to będzie, jeżeli pojawią mi się objawy bliskie temu, co się uważa za objawy tego koronawirusa, nie wiem, jak ja bym się zachował.

**A jak myślisz, jak byś się zachował?**

Nie wiem. Wiesz, czego ja się najbardziej boję? Ja boję się trafić do szpitala po prostu. Wiem, że szpitale są miejscami, są w tej chwili ogniskami zakażenia. To są najbardziej niebezpieczne miejsca… Więc ja robię wszystko, żeby tam nie trafić. No musiałem wczoraj pójść do lekarza, bo okazuje się, że mam zapalenie ucha. I musiałem pójść do lekarza, bo mnie po prostu ucho bolało, inaczej się tego nie dało zrobić, musiała mnie lekarka fizycznie zbadać. Ale też bardzo niechętnie to zrobiłem. Na początku zadzwoniłem, na początku to była teleporada. I pani doktor powiedziała, nie, no proszę przyjechać. Więc pojechałem.

**Bałeś się bardziej niż idąc do sklepu?**

Tak. No tam na każdym kroku co 2 metry stoi jakieś tam urządzenie, że sobie można ręce zdezynfekować. Nie, to był Medicover. Medicover jest przygotowany, wchodzisz, wypełniasz ankietę, są czyste, brudne długopisy do wypełnienia tej ankiety. Mnie to troszkę też dawało takiego poczucia bezpieczeństwa, że jednak tam jest OK. Te maseczki, rękawiczki, mycie rąk, no widziałem też, bo pani doktor też to wszystko robiła tak jak trzeba.

**Czyli to pleksi w sklepach to też jest coś takiego, co niech sobie będzie.**

No tak, tak, oczywiście.

**Napomknąłeś o diecie, napomknąłeś, że do fryzjera byś się wybrał, ale nie możesz. Czy tutaj coś się zmieniło u ciebie? Zmieniłeś jakieś swoje zwyczaje?**

Nie, nie, nie. Nic się nie zmieniło pod tym względem, bo ja i tak się w garnitury nie ubierałem. Od czasu jak przestałem pracować w korporacji, to nie zakładam marynarki, krawata itd. Nie wiem, nie pamiętam, kiedy ostatnio zakładałem, chyba w listopadzie. Więc to nie jest coś, co ja stosowałem. I tak pracując z domu raczej byłem na luzie. Czasami, jak tam jakieś miałem bardziej oficjalne spotkanie, no to może założyłem jakąś marynarkę, ale bez krawata. Więc to nie jest tak, że ja bardziej o siebie dbam. Chociaż nie, kiedyś na przykład nie biegałem tak systematycznie, a teraz codziennie, czy tam co drugi dzień idę się przebiec rano.

**Jak myślisz, z czego to wynika, że teraz to robisz codziennie?**

Z czego to wynika? Bo zauważyłem, że ogólnie mam mniej ruchu. Bo jednak jak człowiek gdzieś wychodził i chodził chociażby po ulicy czy tam gdzieś, to jakiś tam ruch był. To takie siedzenie w domu i ta generalnie mniejsza ilość ruchu, no jednak ja potrzebuję. Przynajmniej sobie idę rano pobiegać, to jakieś tam 3-4 kilometry sobie przebiegnę, tam z przerwami. Ale zawsze jakaś tam przebieżka jest.

**Coś jeszcze takiego robisz dla ciała i ducha?**

Na pewno jak mi to ucho przejdzie, to zacznę jeździć na rowerze. Na razie tego nie robię, bo jednak mnie to ucho boli i w ogóle nie chcę ryzykować. No i na pewno będę jeździł na rowerze. I pojadę sobie, nie wiem, nad Zalew Zegrzyński i z powrotem, coś takiego.

**A jak obserwujesz swoją żonę, to u niej coś się zmieniło?**

Też zaczęła właśnie chodzić do lasu. Ona nie biega, bo ona ma tam jakieś problemy z kostkami. Ale na przykład sobie chodzi z kijami.

**A w kontekście robienia makijażu, zabiegów pielęgnacyjnych.**

Jest wkurzona, że nie może sobie pójść paluchów zrobić. Jest wkurzona, ale i widzę to u niej, taka lekka frustracja. Ale jakoś, nie wiem, funkcjonujemy.

**A farbowanie włosów, obcinanie włosów.**

No właśnie mówię, że w tym kontekście chciałaby pójść do fryzjera, a nie może. A sama sobie nie zrobi. Znaczy ona mi tam jeszcze zaoferowała, że ona mnie ostrzyże. Ale mi to specjalnie nie przeszkadza, nie muszę być piękny. Ale ja raczej bym się nie podjął zrobienia czegoś.

**A kupowanie ubrań? Czy to jest coś, co nie wiem, mieliście taki zwyczaj na wiosnę? Jak to u was wygląda z tą sferą?**

Kupowaliśmy sobie, ale jakoś brak tego mi specjalnie nie przeszkadza.

**Ale przychodzi wam do głowy, żeby kupić przez internet, wobec tego?**

Nie myślałem o tym.

**A żona?**

Nie zauważyłem.

**Z takiego punktu widzenia konsumenckiego, mam na myśli i tych fryzjerów i sklepy z ubraniami, baseny i siłownie i knajpy, czego ci najbardziej brakuje w tej chwili?**

Kina, teatru. Jeszcze… No może siłowni rzeczywiście, bo ja chodziłem, przynajmniej raz w tygodniu się starałem tam pójść. Trochę mi tego brakuje i dlatego może zacząłem częściej też biegać. Co jeszcze? No to chyba to tylko.

**Restauracje?**

No wiesz, można zamówić do domu. I zamawialiśmy już. A co się zmieniło u nas, zauważyłem, że zaczęliśmy kupować lepsze jedzenie.

**Co to znaczy lepsze jedzenie?**

Jakieś takie wyszukane, bardzo dobre sery, drogie, zaczęliśmy sobie kupować. Zauważyłem, że zacząłem kupować lepsze wino i droższe.

**Z czego to wynika?**

To takie podaruj sobie odrobinę rozkoszy. Jeżeli nie mogę robić czegoś… To jest takie rekompensowanie jednego braku czymś innym.

**A to jest tak, że wyszukujecie te rzeczy w sklepie, czy zamawiacie to przez internet?**

Przez internet na ogół.

**I siadacie i oboje tam wygrzebujecie, co chcecie?**

Nie, wino to ja kupuję. A na przykład jedzenie, tam jakieś fajne sery, no to w sklepie, jak widzę, że jest coś takiego fajnego, rzadkiego, a, może byśmy spróbowali. I teraz przy tej diecie, to też było takie wyszukiwanie, no bo ta dieta tania nie jest. A dlaczego nie. A to jest naprawdę fajne i smaczne. I dlaczego nie spróbować.

**Sami wpadliście na pomysł tej diety, czy ktoś was zainspirował?**

Nie, Dominika nam podpowiedziała.

**Ok, że jesteście razem na diecie.**

Tak.

**Namawiała was, czy to po prostu tak…**

Nie, to wyszło przypadkiem, bo mieliśmy jakiś tam na video, ona akurat coś jadła. Co jesz Dominika? A jem właśnie to i to. A co to takiego? To jest moja dieta. A co to za dieta? A skąd? Z jakiej firmy? Następnego dnia zamówiliśmy.

**Ale była między wami jakaś rozmowa, czy to dobry moment, dlaczego teraz, a może nie teraz.**

Nie, no Dominika powiedziała, że dla niej to jest fajne, bo ona nie musi chodzić zakupów robić żadnych. Wszystko przywiozą, to akurat fajna rzecz.

**No tak, rzeczywiście teraz po te zakupy nie musicie chodzić. Powiedziałeś o kinie i teatrze na pierwszym miejscu. Co ci dawało kino, czego nie może ci dać oglądanie filmów w domu?**

To znaczy wiesz, w tej chwili jest tak. Było kiedyś i teraz. I filmowych kiedyś nie można było obejrzeć w domu, teraz już można. Więc kiedyś, żeby zobaczyć jakiś fajny film, to trzeba było pójść do tego Elektronika czy to jakiegoś innego fajnego kina. A teraz można zobaczyć na platformach cyfrowych. Więc to się niejako zastąpiło. Bo ci producenci filmowi też to muszą gdzieś sprzedawać. Im też spadły obroty. Więc sprzedają filmy na platformy cyfrowe, to przynajmniej trochę zarobią na tym, zamiast… Chociaż też nie wiem, czy oni tak mniej zarabiają na tym platformach. Bo to też jest mniej kosztowe. I pewnie muszą więcej zapłacić niż płacą platformy cyfrowe. Chociaż nie wiem, jakie tam są rozliczenia. Ale bardzo dużo fajnych filmów można zobaczyć na tych platformach cyfrowych. Z tym, że nowości filmowych przez jakiś czas pewnie nie będzie, bo też nikt nic nie kręci teraz.

**Ale jednak powiedziałeś, że brakuje ci kina.**

Brakuje mi kina. Teatru też mi brakuje, bo czasami się chodziło do tego teatru, można było gdzieś wyjść, zaplanować. Nawet, jak my… Zaczęliśmy czasami latać sobie do Londynu, żeby pójść do jakiegoś tam teatru.

**Opowiadałeś, że jeszcze w marcu byliście.**

Jeszcze w marcu byliśmy. Tego mi trochę brakowało. Ale najbardziej mi jednak brakuje podróżowania.

**Ale ja jeszcze nie rozumiem, dlaczego kino. Jaką funkcję pełni kino, że jest fajniejsze niż obejrzenie tej samej nowości w domu.**

Tam jest taka atmosfera. Konkretna godzina i ja wiem, że przez tę godzinę nikt mi nie będzie przeszkadzał. A jak oglądasz nawet na tej platformie, no to zadzwoni jakiś telefon, no to się przerywa oglądanie i się rozmawia. A tam się wchodziło i te 2 godziny człowiek był zamknięty dla całego świata, sobie oglądał film.

**Jesteś tylko ty i film.**

No właśnie.

**Kina, teatry, siłownia jeszcze powiedziałeś. Gdybyś miał sprzęt do ćwiczeń taki w domu, to też byś wolał chodzić do siłowni, czy to jest w domu do zastąpienia?**

No pewnie byłoby do zastąpienia, ale ja jakoś tak nie mam miejsca na to.

**Ale czy też jest w tym pójściu do siłowni coś takiego innego dla ciebie, niż gdybyś miał to miejsce i mógł ćwiczyć w domu?**

Ja się nie socjalizuję w siłowni, więc… Bo dla niektórych jest to jakiś tam element socjalizowania się z innymi, tak? A ja to traktuję czysto indywidualnie.

**Czy coś jeszcze przychodzi ci do głowy, czego nie poruszyliśmy?**

Nie, jakoś tak do głowy mi nic nie przychodzi.

**A jeszcze tak, bo mówisz, że ubierasz się, jak ubierałeś, ale chodzi mi o zachowanie żony. Mówisz, że brakuje jej paznokci, że sobie nie może zrobić. A coś się zmieniło u niej, np. z tym, że się malowała codziennie, a teraz się nie maluje? Albo się maluje bardziej, bo ma więcej czasu?**

Mniej się maluje. Bo ona się malowała, jak gdzieś wychodziła. A teraz wychodzi mniej. Chociaż ona czasami woli na przykład pójść sobie do pracy popracować, bo tam i tak jest sama. I tam się bardziej może skupić na pracy. Więc wychodzi sobie. I dla niej to jest jakaś taka odskocznia. A jak i tak w domu pracowałem.

**No właśnie, ale chodzi o to, czy się jakoś inaczej ubiera niż jak jest w domu? Mimo, że tam też jest sama?**

Chyba tak, chyba tak.

**Czyli to takie rozluźnienie domowe dla niej, jak rozumiem… Ona potrzebuje też wyjścia, żeby mieć trochę tego, jak było kiedyś?**

Tak.

**Jest tak, że mniej się chce o takich rzeczach myśleć, jak się siedzi w domu? Żeby się uczesać, żeby się…**

No może tak. To byłoby logiczne, rzeczywiście, żeby mniej o tym… Jak siedzisz w domu, to mniej się… Chociaż żona zawsze raczej dbała o swój wygląda. Nawet w domu dba. Tylko jakoś tak może, jak wychodzi, to dba bardziej.

**Ale zauważyłeś, że na przykład, skoro teraz więcej jest w domu, to wprowadziła jakieś nowe zabiegi pielęgnacyjne, nie wiem, robi sobie spa w łazience czy jakieś takie historie?**

Nie, nie zauważyłem.

**Czyli w zasadzie u was jest, jak było, jeżeli chodzi o takie wellness wasze.**

No tak, w zasadzie tak.
