# Supplementary material for: The challenges arising from the COVID-19 pandemic and the way people deal with them. A qualitative longitudinal study
Source: PLoS One. 2021 Oct 11;16(10):e0258133. doi: 10.1371/journal.pone.0258133 (PMC8504766; doi:10.1371/journal.pone.0258133)
Supplement: S1 Dataset — (ZIP) [file pone.0258133.s003.zip › Transcriptions/stage 3/9.3_F_25_couple, no children.docx]

**9.03_F_25_couple no children**

**Ja ci minęły te 2 tygodnie?**

Nic ciekawego. Robiłam to samo cały czas, czyli pracowałam, trochę czytałam, trochę oglądałam Netflixa. Miałam 2 dni wolnego, bo były święta i głównie wypoczywałam. Głównie siedzę w domu, ale jednak psychicznie trzeba trochę odpocząć. Jedyne urozmaicenie - byłam na spacerze w poniedziałek i się przeziębiłam, więc ten tydzień trochę umierałam, ale już się czuję lepiej.

**Jak wygląda teraz wypoczywanie w domu?**
Czytam, oglądam seriale, sprzątam, bo to jest jednak odprężające, ale na pewno będę chciała w przyszłym tygodniu wychodzić na spacery. Codziennie może nawet. Tak jeszcze czekałam, aż się wyklaruje ta sytuacja, tak naprawdę nowa sytuacja, że można wychodzić. Chciałam zobaczyć, jak ludzie zareagują. Bałam się, że wyjdę i zaczną do mnie z okien zrzucać coś: "dlaczego wychodzisz!?", ale zorientowałam się, że wszyscy teraz wychodzą, wszyscy chodzą w parach, ale mają maseczki, więc przymykam na to oko i również stwierdziłam, że już można wychodzić w takim razie.

**W parach można chodzić?**

Nie, ale chodzą. Głupio tak samemu iść na spacer i też jak wyszłam z mężem na spacer, to też nie było tak, że sobie łaziliśmy gdzieś przez centrum, tylko rzeczywiście wyszliśmy do parku, tak jak teraz zalecają i unikaliśmy innych. Wszyscy tak robią. Zdecydowanie więcej ludzi teraz wychodzi. Były takie momenty, jeszcze w poprzednim tygodniu, kiedy naprawdę ulice były puste, a teraz mam wrażenie, że miasto zaczęło odżywać. Pary chodziły bardzo rzadko, a w tej chwili wszyscy chodzą w parach. Przynajmniej tak widzę przez okno.

**A jak wyglądała Wielkanoc?**

Oglądałam sobie mszę online, nie doczekałam się Rezurekcji, bo zasnęłam. Raczej nie czuć było w ogóle Wlk. Piątku, Wlk. Soboty, ale w sobotę kupiliśmy świeże pieczywo, jakąś sałatkę zrobiłam, oczywiście jajka, więc w niedzielę zjedliśmy rano śniadanie z rodzicami męża i z jego siostrą. Wszyscy byliśmy połączeni - w 3 rożnych miejscach byliśmy i byliśmy połączeni na Skype. Potem mieliśmy wypić kawę o 12 z moimi rodzicami, ale...Ja pochodzę z Karpacza i tam zawsze Orange się psuje i zepsuło się im w Wielkanoc. Zepsuło się też rodzicom męża, więc nie mieli internetu, tv i telefonu do godz. 18 i dopiero w poniedziałek wypiliśmy tę kawę o 12. W poniedziałek też kawę z rodzicami męża i tak minęły ze 3 godz. Ogólnie było ciepło, odpoczywaliśmy sobie, ja czytałam.

**To łączenie się przez Skype. jaki to jest doświadczenie dla ciebie?**

Akurat moi rodzice mają to do siebie, że jak im się nie powie, co mają robić, to będą kombinowali jak koń pod górę, ale dadzą w końcu radę i się połączą. Z nimi nie było problemu, ale żeby rodzice męża pościągali Skype, żeby potworzyli konta, żeby im to wszystko poustawiać, potem jeszcze odkrycie taty, że może to mieć na telefonie i że może dziadkom się pokaże. I zaczęło się kombinowanie, żeby mógł hot spot zrobić z tego telefonu na tablet, żeby mieli większy obraz...Naprawdę kilka godzin to zajęło i w poniedziałek, i w niedzielę, ale udało się ostatecznie wytłumaczyć. Wiadomo, że to jednak nie zastępuje kontaktu twarzą w twarz, ale zawsze jakaś rozmowa. Moi rodzice byli zażenowani - siedzieli przy tym komputerze w pracowni u taty, bo tam jest kamerka i mama oczywiście swobodnie, ale tata, to widzę, że tak dziwnie mu trochę było. Rozmowa była naturalna, ale wyczuwało się tę barierę techniczną.

**Będziecie się jeszcze w taki sposób łączyć?**

Mówiliśmy sobie, że na pewno jeszcze porozmawiamy, ale to raczej też jest tak, że chyba nie ma takiej potrzeby, żeby się zobaczyć, kiedy się słyszy. Lepiej się zobaczyć na żywo niż online. Tu chodziło raczej o to, że jakiś posiłek będziemy celebrować razem, a i tak zarówno rodzice męża jaki moi cały czas się kontaktują, więc jesteśmy w stałym kontakcie.

**Myślicie o tym, kiedy będziecie mogli się spotkać?**

Myślę, i bardzo bym chciałam zrobić tak, że mam w piątek wyjątkowo wolne i okazało się, że Niemcy też mają wolne (ja teraz dla niemieckiego zespołu pracuję). Tak niefajnie ten weekend majowy wypada w tym roku i skoro mam 1 maja wolny to zastanawiałam się...Chciałam pojechać w czwartek po pracy do domu i wziąć sobie jeszcze poniedziałek wolny. Chciałam tak w domu zostać na tydzień, może na 2 tygodnie, bo tak naprawdę nic mnie tu nie trzyma, wystarczy, że wezmę laptop. Rozmawialiśmy z mężem o tym i doszliśmy do wniosku, że złamię główną zasadę, którą teraz starami się piastować, czyli żeby oddzielać się od ludzi, bo nie jesteśmy pewni czy jesteśmy zdrowi, czy nie. I w związku z tym ostatecznie odłożyłam swoje plany na bok, chociaż jest mi przykro, bo jedna z koleżanek jedzie już w tym tygodniu do rodziny na wieś i też nie wychodzili przez tydzień z domu, żeby się sprawdzić i dopiero teraz jadą. Myślałam o tym, ale to chyba jeszcze troszeczkę za szybko. Obawiam się, że mogę kogoś zarazić, tym bardziej, że w tym samym domu mieszka babcia, która miała nowotwór i moi rodzice też nie są już pierwszej świeżości. Tata mnie bardzo namawia, ale mama jakby rozumie to, że się trochę obawiamy, szczególnie, że ona jest pielęgniarką i jednak w tym szpitalu jest, i ona chyba też za bardzo nie chce nas narażać.

**A spotkania ze znajomymi?**

Nie. Jako, że mąż jest teraz na postojowym do maja, to jest w stałym kontakcie ze znajomymi i ja jestem też włączona w te rozmowy, więc powiedziałabym, że nawet kontaktujemy się częściej niż normalnie, ale takie spotkanie nie. Chcieliśmy zaprosić znajomych na Wielkanoc i moją kuzynkę, ale też stwierdziliśmy, że to mija się z celem i że my możemy ich pozarażać.

**Czy coś jeszcze się zmieniło w ciągu ostatnich 2 tygodni?**

Chyba nie, chociaż przed świętami już byłam bardzo, bardzo zmęczona pracą i już naprawdę bardzo emocjonalnie do tego podchodziłam, a wydaje mi się, że ta przerwa taka dłuższa, dała mi jakiś taki dystans + jeszcze wiem, że mogę teraz normalnie pójść sobie do tego parku, pojechać do tego lasku i nawet sama ta myśl mnie podnosi na duchu. Planujemy w sobotę może pojechać sobie gdzieś nad jakąś Ślężę. Tam raczej nie będzie ludzi. Gdzieś po prostu połazić na uboczu. Koleżanka, która jedzie do rodziny, to oni jutro śpią pod namiotem tu przy Wrocławiu, ale gdzieś tam nad Odrą w takim dzikim miejscu, żeby sobie jakieś urozmaicenie wprowadzić.

**Coś ci przeszkadza teraz?**

Na pewno nadal to odseparowanie, bo teraz jest już taki moment, że zaczęłam się zastanawiać, jak ja mam wrócić do biura? Przecież to jest takie nienormalne. I co? Będę musiała się ubrać, umalować, wcześniej wstać? Nie chce mi się. Trudno mi się o tym myśli. Rozmawiałam o tym wczoraj z moją mamą i powiedziałam, że bardzo dobrze mi się pracuje z domu, ale mama mi mówi, że dziczeję, że ojciec mi już zdziczał, na zakupy do Stokrotki go wysyła, bo dziwnie się zachowuje już. [śmiech] Trudny będzie powrót. Teraz jestem w domu i jak ktoś mnie zdenerwuje, to ja sobie popsioczę i mogę głośno to zrobić, i nikt mnie z tego nie rozlicza, ale jak mnie w pracy ktoś zdenerwuje i będą różni ludzie dookoła, i każdy będzie chciał ode mnie coś osobiście, to myślę, że to będzie ciężkie do przeskoczenia. Trochę mi się też nie chce wstawać, malować ubierać. Z jednej strony marzę o tym - tak patrzę na moje ubrania w szafie i myślę, Boże, takie biedne, dopiero co kupione niektóre, a nie mogę ich włożyć, ale z drugiej strony dobrze się z tego domu pracuje jednaj, wygodnie.

**Było ci trudno cały czas przy mailu, telefonie. Ta przerwa świąteczna ci tak pomogła?**

Tak myślę. jakoś te 2 dni takie dodatkowe, prawie tydzień i rzeczywiście odpoczęłam i stwierdziłam, że nie ma co, muszę korzystać teraz, szczególnie, że te wakacje stoją pod znakiem zapytania.

**No właśnie. Planujecie?**

Planowaliśmy. Ten rok to miał być rok moich city break'ów i jak ja sobie wszystko rozplanowałam...Już wyliczyłam, że pojedziemy do Oslo i nawet znalazłam (to był chyba początek marca), że była jakaś bardzo ciekawa opera i nawet nie była taka droga i już wszystko zaplanowałam, no ale się okazało, że jest koronawirus i zaczęły się schodki. Dlatego ja jestem bardzo taka wyposzczona i jakby te city break'i zupełnie odłożyliśmy, więc zaczęłam się w takim razie zastanawiać nad wakacjami. Mówiłam mężowi, że dawaj, dawaj, teraz szybko na sierpień kupimy. Mąż mówi, że przecież nie wiadomo, co będzie w sierpniu. Ja na to, że daj spokój, na pewno wszystko będzie w porządku. No a teraz się okazuje, że ten stan rzeczy może się utrzymać nawet i z rok. Na pewno nie tak restrykcyjnie, jak teraz, ale nie wiadomo, co będzie z tymi granicami i szczerze mówiąc jestem bardzo rozczarowana, ale myślę, że będzie niezdrowo nigdzie nie wyjechać i tak zastanawiałam się, że może Mazury? No bo nie ma ludzi, nikomu nie będę szkodziła, jak sobie wsiądę w auto we Wrocławiu i wysiądę na Mazurach przy jakiejś chałupce. Myślę nad tą opcją.

**O jakim czasie myślisz?**

Lipiec, sierpień. Zobaczę, jak sytuacja się rozwinie.

**Emocje - zdjęcia**

Ja bym powiedziała, że dzisiaj jestem 15-tką w ostatnim czasie. Z jednej strony chodzi o tę siłę żywiołu czy natury, bo teraz też się z tym mierzymy, a z drugiej strony nie wiem dlaczego, ale takie oczyszczające jest to zdjęcie. Jakoś mi się dobrze kojarzy. Może właśnie te klimaty wakacyjne? Ja wiem, że tam wielka fala w tle, ale też morze. My jesteśmy pod taką falą siły natury, czyli tego wirusa, ale z drugiej strony jesteśmy cały czas na granicy, że z jednej strony spokojna woda i takie spokojne życie, trwanie cały czas w jednym miejscu, a z drugiej ten świat, na którym panuje ten...

**Twoje życie teraz jest spokojne?**

Tak.

**A czegoś się boisz?**

Zaczęłam się zastanawiać w ostatnich dniach nad takimi, ale to raczej nie są sprawy związane z koronawirusem, tylko może nad przyszłością. podam bardzo głupi przykład, ale stąd się to wzięło. Kończymy "Jak poznawała waszą matkę" po raz któryś tam i jest moment, jak Lilly i Marshall wyjeżdżają na rok do Rzymu. Tam nagle wszystkim bohaterom zaczyna się układać i wszyscy dostają wspaniałe propozycje. I ja sobie zdałam sprawę, że ja stoję w miejscu i się z tym źle czuję. Zastanawiam się, czy dostanę kiedyś taką wspaniałą propozycję, która odmieni moje życie, czy zawsze będę w tym jednym punkcie.

**To dotyczy życia zawodowego czy szerzej?**

Chyba raczej zawodowego. Zastanawiałam się jakie to jest szczęście móc pracować i przy okazji realizować się i jeszcze zarabiać dobre pieniądze. To już w ogóle...

**Myślisz o tym, żeby zmienić pracę?**

Trochę narzekam, ale obiektywnie jak patrzę, to myślę, że to jest całkiem dobra praca i chyba nie chciałabym się przebranżowić. Myślę raczej o tym, że zawsze chciałam - to tako mrzonka, utopia, ale zawsze chciałam być pisarką i się tak zastanawiam, że gdyby się zdarzyła taka cudowna oferta...Gdyby mój mąż, bo on, jako inżynier ma największe pole manewru, dostał propozycję wyjazdu na rok do jakiegoś innego państwa, to ja teraz już będąc doświadczona tą pracą w domu wiem, że jest możliwe normalnie pracować z domu, zarabiać pieniądze i spełniać jakieś tam swoje dodatkowe aspiracje. Zaczęłam się zastanawiać nad taką opcją. Tak się martwię tym moim niemieckim i wiadomo, że jak używam tylko zestawu słów dotyczącego księgowości, to ta wiedza mi spada i ja zapominam różne rzeczy, ale dlaczego nie wyjechać do Niemiec? Nie mówię na całe życie, ale na rok. Nie zaszkodziłoby mi to w ogóle i się zastanawiam nad tą kwestią. To przemyślenie wynikło tej sytuacji z koronawirusem.

**Zastanawiasz się czy podejmujesz już jakieś działania?**

Ja już kiedyś chciałam podjąć jakieś działania, ale męża głównie trzymają rodzice. Zastanawiam się, bo ten rok nie byłby głupi. Wiem, że w męża firmie ludzie często wyjeżdżają do Szwecji właśnie na takie kontrakty, bo ta ich firma jest szwedzka. No to się zaczęłam zastanawiać, że w sumie można by popracować nad tym. Mąż na razie się śmieje, ale myślę, że dlaczego by nie zobaczyć, czy jest taka możliwość w ogóle, bo właśnie teraz okazało się, że pewne drzwi się otworzyły. Myślałam zawsze, że jestem uwiązana. Bałam się, że gdybyśmy się zdecydowali na wyjazd, to jest to bardzo niepewne, że jedna osoba pewnie nie miałaby pracy, bo po filologii polskiej, to jak bym skończyła? Na magazynie. Chyba, że w jakimś niemieckojęzycznym kraju. A teraz okazuje się, że mogę spokojnie wykonywać pracę, którą już znam i być gdzie indziej.

**Wydaje ci się, że ta praca zdalna stanie się bardziej powszechna?**

Myślę, że tak będzie. U mnie w firmie wydaje mi się, że wszystko już załatwiono. Są jeszcze jakieś drobne sprawy, które trzeba załatwiać, ale wydaje mi się, że wszystko funkcjonuje bardzo dobrze. Spełniamy swoją funkcję i nie mamy zbytnich przestojów. Oglądałam ostatnio wywiad z wirusologiem na YT. (Kanał Karola Paciorka) To był wywiad z końca marca i oni rozmawiali o tym, jak sytuacja może się potoczyć. Oczywiście gdybali i nie wiedzieli, że skończy się tak, jak teraz, ale ten naukowiec stwierdził, że teraz takie epidemie będą coraz bardziej powszechne i że on wróży już kolejne takie fale - albo nawrotu tego wirusa, albo jakiegoś innego w najbliższym czasie i że jest to dla nas takie realne zagrożenie. Powinniśmy się zacząć przyzwyczajać, że będą takie rzeczy się zdarzały i raczej nie umrzemy ze starości tylko przez epidemię albo przez bombę atomową.

**Co taka myśl, perspektywa u ciebie powoduje?**

Na razie to jest takie gdybanie, jak się nie ma dowodów popierających. Dopóki to są tylko przypuszczenia, to tak naprawdę nie napawa mnie to żadnym lękiem, ale jest to jakaś ciekawa wizja czy bliższej, czy dalszej przyszłości.

**Ja ty byś nazwała swoje emocje, uczucia teraz?**

Może odprężona? Myślę, że tak.

**A na ile czujesz się obecnie zagrożona całą sytuacją?**

Odprężona czuję się w tej chwili i nie jest to globalne uczucie. Powiedziałabym, że wynika z chwili. Jeżeli chodzi o sytuację chorobową, to prawie w ogóle nie czuję się zagrożona. Ja raczej nie przejmuję się swoim zdrowiem. Nie mam problemów ze zdrowiem, więc wydaje mi się, że lekko bym przeszła tego koronawirusa, tylko raczej o zdrowie innych. Jeżeli chodzi o pracę też raczej nie czuję się zagrożona. Myślę, że jest to czas i miejsce, żeby zacząć myśleć o jakiejś dalszej przyszłości - gdzie chcę się widzieć za 5 lat. Takie najgłupsze pytanie z rozmowy kwalifikacyjnej - oczywiście, że w waszej firmie. [śmiech]

**Jak byś odpowiedziała w tym momencie?**

Mam takie wymarzone...Mamy takiego team leadera, który narwany jest ogólnie i chyba mu coś powiedzieli, bo 3 tyg. przed koronawirusem on powiedział, że zrobi zabawę integracyjną. Nienawidzę takich rzeczy, ale ok, trzeba się dostosować i udawać, że się podoba...I on zadał właśnie takie pytanie. I tak siedzę i myślę, że co ja im powiem? Przecież oni chcą usłyszeć jedną informację, ale on mówi, żeby puścić wodze fantazji. Patrzę, a dziewczyny malują palmy i stwierdziłam, że nikt mi nie zakaże myśleć abstrakcyjnie i ja za 5 lat widzę się w pięknym, przytulnym domu w Szwajcarii nad jeziorem i żeby były też góry i jestem znaną pisarką, ale nikt o mnie nie wie. Podpisuję się pseudonimem, mam fanów, moje książki są czytane, ale nikt nie wie, że to ja i nikt się mnie nie czepia.

**Czy czujesz, że koronawirus na ten twój plan jakoś wpłynął?**

Na razie nie, ale obserwując postępującą sytuację, będę dochodziła do wniosku, że albo trzeba robić coś wcześniej...Teraz jestem w tym punkcie, że taki dzwoneczek mi zadzwonił: dzyń, dzyń, Ania, może lepiej weź się za to teraz? Cały czas odkładałam swoje rzeczy na później. Myślę, że wpłynie, ale czy przyspieszy, czy oddali, to jeszcze nie wiem. Jestem pewna, że te plany się nigdy nie ziszczą, ale trzeba się jakąś piękną wizją na duchu wspierać.

**Czy widzisz jakieś zmiany w odczuwaniu tej sytuacji przez twoich bliskich?**

Wydaje mi się, że mój mąż chyba za bardzo się zacietrzewił. Nic by się chyba nie stało, gdybyśmy pojechali do tego domu na kilka dni, szczególnie, że z jednej strony mama nie chce mnie zarazić, a z drugiej chyba zdaje sobie sprawę, że mam 26 lat i w sumie nic nie powinno mi się stać. Cieszę się, że jest taki konsekwentny, ale wydaje mi się, że można by było już powoli chodzić na jakieś ustępstwa. Nie zamierzam tam chodzić po mieście. Tam jest zielono, można gdzieś wyjść na podwórko i zupełnie być odgrodzonym od ludzi. Może troszeczkę powinien się dostosować do tej zmieniającej się już sytuacji, do tego odmrażania. Moi rodzice są trochę młodsi od jego rodziców (oboje jesteśmy z Karpacza) i możemy być spokojnie u mnie, nie ma problemu. Babcia ma osobne mieszkanie i możemy do niej nie chodzić, tylko że on wie, że nie możemy pojechać do jego rodziców, bo oni utrzymują kontakt bezpośredni z dziadkami. Kilka razy dziennie jego tata ich odwiedza. On wie, że wystarczy, żeby ta babcia się tylko delikatnie zaziębiła i umrze. I ona, i dziadek. Są już bardzo mocno zaawansowani wiekiem i schorowani. Myślę, że u niego jest taka blokada, że on ma po prostu realną możliwość, że coś się stanie.

**A inni bliscy?**

Moi rodzice musieli wypracować sobie pewne systemy. Mamy dom, który ma 3 pietra. Na jednym jest babcia, na samej górze moi rodzice, a na samym dole mieszka moja ciocia z wujkiem. Wiem, że nie mogą się kontaktować teraz. Mają oddzielne klatki schodowe, ale starają się nie wychodzić, szczególnie, że ciocia i wujek są też od nich starsi. Moja mama, poza tym jest zarzewiem, tak naprawdę - nie wiadomo, co przyniesie z pracy. Ten koronawirus cały czas gdzieś tam obok niej oscyluje, bo już jacyś tam lekarze, z którymi ona pracowała są chorzy. Pielęgniarki się wymieniają między szpitalami, lekarze też dojeżdżają, więc jest ten transfer. Realnie, naprawdę ma szansę coś jej się przytrafić niemiłego i wiem, że martwi się o to i dlatego właśnie ograniczyli kontakty w domu. Wiadomo, że musiała też jakoś tatę przygotować...No co mogą robić? Noszą maseczki, oczywiście nie w domu, mama stara się jak najbardziej prewencyjnie w pracy uważać, żeby przypadkiem czegoś nie przynieść. W strachu chyba raczej nie żyją. Mama się śmieje, że umyła ostatnio auto, że jakby coś się stało, zamknęliby szpital i musiała w nim zostać, to jakby mieli jej coś przynieść z auta, to przynajmniej wie, że czysto. [śmiech] Mama już chyba odpuszcza i stwierdziła, że tata powinien już zacząć wychodzić na zakupy. Tata jest na emeryturze. Chodzi o to, żeby zaczął wychodzić do ludzi, bo dziczeje. U n ich chyba też już jest odwilż.

**A wśród twoich znajomych też taką odwilż obserwujesz?**

To zależy, u kogo. Mamy przyjaciela, który tak naprawdę jest trochę zły, że musi pracować z domu, bo zajmuje się drukiem 3D i drukują teraz te przyłbice. kazali im się z firmy przenieść do domu. Po drugie w ciągu ostatnich dni okazało się, że mieli zacząć pracę w nowym biurze, ale ze względu na koronawirusa jest nie wykończone i nie mogą. A te maszyny naprawdę hałasują i przeszkadzają w domu. Siedzi, drukuje, denerwuje się i chciałby wrócić do biura. Mam też przyjaciółki, które są badaczkami jak ty i pracują na uniwersytecie, i teraz ta kwestia naukowa, akademicka...Nie wiem, jak to działa, ale wiem, że one zupełnie pracują z domu, jakoś tam musiały sprawy ze studentami pozałatwiać i to dla nich też jest trudne. Jedna, to w ogóle miała mieć wesele za miesiąc. Wróciła ze Stanów i w ogóle po kwarantannie została w domu. Ja jestem pewna, że ona od lat nie była tak długo w domu i nie wiem, jak sobie z tym radzi. Myślę, że średnio, że niedługo wróci. Mam wrażenie, że u wielu znajomych całe życie zaczyna już odmarzać. Nawet moi znajomi, którzy są fizjoterapeutami już zaczynają znowu chodzić do ludzi. Wszystko ruszyło, ale widzę, że nadal wszyscy stosują ograniczenia. Starają się nie spotykać ze znajomymi jednak i robić tylko to, co muszą.

**O jakich zmianach w ograniczeniach słyszałaś?**

Ja w ogóle (pamiętam, że to było w niedzielę 19-go) od razu weszłam na gov i sprawdzałam jakie są nowe ograniczenia, bo było info, że zostaną wprowadzone. Trochę mnie to zdziwiło, dlatego że jakby wszystkie miejsca, które zostały zablokowane - centra handlowe, kosmetyczki, itd., przy wszystkich były informacje, że to zamknięcie trwa do 19.04. I ja byłam pewna, że w poniedziałek powinni odmrozić wszystko i byłam bardzo zdziwiona, bo to przecież jest nielogiczne. Okazało się, że w poniedziałek wprowadzają tylko ten 1 etap -parki, skwery, lasy i zdaje się, że to jest tylko plan odmrażania i że oni to mają jakoś etapami robić. Cieszę się, że to się dzieje. Myślę, że to się dzieje ze względu na wybory. [śmiech] Chcą na tyle odmrozić sytuację, żebyśmy mogli głosować. Myślę, że to jest głupota, no ale...

**Nie powinny być jeszcze odmrażane te różne miejsca?**

Powinny. Te lasy, parki to było bardzo wskazane, bo ludzie dostają wścieklizny w domu, tylko zastanawiam się, co będzie z kolejnymi etapami. Czy to będą etapy odmrażania dla nas, dla ludzi, żebyśmy nie powariowali, dla naszej wygody i bezpieczeństwa, czy to będą etapy odmrażane sztucznie tylko dlatego, żebyśmy mogli zagłosować.

**Które z ograniczeń mają realny wpływ na ograniczanie epidemii?**

Najbardziej zamknięcie miejsc handlu, takiego w jednym miejscu. Tam wszyscy się mieszali w jednym sosie. No i wprowadzenie maseczek. Jakie by tam nie były, bo wiadomo, że niektóre nie działają zupełnie, większość działa wybiórczo...Podobno jest taki test, że trzeba włożyć maseczkę i spróbować zdmuchnąć zapałkę. Jeżeli zdmuchniemy, to znaczy, że ta maseczka...Nie bardzo, ale jak nie zdmuchniemy (a przez swoją nie zdmuchnęłam), to podobno jest nie najgorsza. Moja mama mówiła, że ogólnie chodzi o to, żeby nie przepuszczała za bardzo wody, żeby te płyny się nie przedostawały.

**Jaką ty masz maseczkę?**

Jakąś...Nie mam pojęcia co to jest. Nie mieliśmy żadnej, aż pewnego dnia zamówiłam na "zajączka" mężowi trampki z Big Star i co dołączyli? Maseczkę. Przed świętami mąż poszedł do takiego blaszaka, gdzie jest dużo stoisk i okazało się, że jeden sklep, ale taki elektroniczny dla dziadków, ci takie radyjka są...Ten sklep zaczął sprzedawać maseczki, żele antybakteryjne, ręczniki kuchenne. Poszłam ostatnio i okazało się, że tam kolejki się ustawiają. Sprzedają rękawiczki nielegalnie na sztuki - kupili wielkie opakowanie i sprzedają parę za 3 zł, ale ludzie kupują, bo trudno jest teraz dostać te rzeczy. Maseczki też sprzedają i tam je kupiliśmy. To jest po prostu jakiś kawałek materiału. Mąż też kupił żel antybakteryjny za 26 zł tylko. [śmiech]. Mama też mówiła mi, że chce mi jakieś wysłać, ale te, które mamy, można prać, więc na razie się jeszcze nie wysłużyły. Moja mama bardzo długo nie miała maseczek, aż pewnego dnia przysłali im materiał, przyszła pani z maszyną do szycia i pielęgniarki szyły maseczki. Mama ma jeszcze trochę tego materiału, uszyła dla siebie, dla cioci, wujka, babci i mówiła, że może nam trochę wysłać. Moja koleżanka z pracy, która również zajmuje się szyciem, to sprzedaje teraz te maseczki i robi dobry interes, tylko trochę się zdenerwowała, jak jej powiedziałam o sprawdzaniu z zapałką.

**Otwieranie miejsc, żebyśmy nie powariowali? Powiedz coś więcej.**

Wiadomo, że każde wyjście z domu w dobie epidemii jest jakimś ryzykiem i wychodząc na zakupy świadomie to ryzyko podejmujemy, że możemy zarazić siebie i kogoś. Wydaje mi się, że tutaj wybrano mniejsze zło i myślę, że to dobra decyzja, bo spacer po parku lub po lesie ma to do siebie...Jakby nacechowany od razu jest tą samotnością, bo jest to duży teren i w parku się po prostu mija ludzi w oddali. Taki spacer po zieleni ma nam ładować akumulatory i z definicji, raczej nie udajemy się dużą grupą na taki spacer. Jakiś kompromis tutaj osiągnięto. My zawsze chodziliśmy na spacery, natomiast po Wrocławiu raczej mało. Ja tutaj w ogóle nie znam okolicy. Zawsze, jak chcieliśmy odpocząć, to się jechało do domu, po prostu. Tu zawsze był zgiełk i ruch, a w Karpaczu zawsze było cicho, góry i raczej tam chodziliśmy. Teraz zaczynam odkrywać te wrocławskie parki, skwery i te okolice.

**Które ograniczenia powinny zostać na dłużej?**

Centra handlowe. Moim zdaniem nie przeszkadza, że są zamknięte. Jak człowiek coś chce sobie kupić, to ma możliwość online. Firmy przystosowały się teraz do tego, bo te wszystkie darmowe zwroty. Nie ma problemu z wymianą, chociaż ja mam pewien problem, bo dostałam kartę podarunkową i nie mogę jej zrealizować, bo sklep online nie przyjmuje mojej karty. To mnie zdenerwowało. To jest sklep Medicine - ubrania. Bardzo go lubię, dostałam kartę podarunkową na święta i cały czas czekałam, aż się zrobi ciepło i wrzucą skarpetki-stopki, bo są to najlepsze stopki na świecie. Już pisałam do nich, żeby mi powiedzieli, ile w ogóle mam czasu na to i czekam na odpowiedź.  Myślę, że powinny też pozostać te zaostrzenia dotyczące liczby ludzi w jednym momencie w sklepie. Ludzie się już teraz przyzwyczaili i ja tutaj nie widzę żadnych kolejek. Wydaje mi się, że na początku ludzie nie potrafili sobie zorganizować tych zakupów, a teraz wiadomo, że wysyła się jedną osobę, która robi większe zakupy na tydzień. Wydaje mi się, że tak się teraz funkcjonuje i zdaje to chyba egzamin. Na pewno ograniczenie imprez masowych i jakichś tam zgromadzeń. Nie mam pojęcia, jak funkcjonują teraz jakieś kawiarnie. Nie wiem, jestem ciekawa, bo myślę, że to też jest potencjalne miejsce, w którym można się albo kogoś zarazić. Gastronomia...Uważam, że te dowozy są świetne, bo zaczynają dowozić z miejsc, które nigdy nie dowoziły. Ja się czuję wspaniale z tym. Gdyby jeszcze nie było takiej groźby w powietrzu...

**Czy w ogóle to kiedyś powinno wrócić w takim funkcjonowaniu jak kiedyś?**

Jak najbardziej. Centra handlowe muszą wrócić przed upływem mojej karty podarunkowej, a imprezy masowe przed którymś sierpnia, bo mełliśmy jechać na koncert Slipknota. Już któryś raz się wybieraliśmy i ja jestem tak zdenerwowana, że akurat jak kupiliśmy bilety, to akurat koronawirus. Ja mam nadzieję, że wszystko do lipca/ sierpnia zacznie się normować. Myślę, że te centra mogą spokojnie z jakimś tam ograniczeniem funkcjonować. A, jeszcze jedno mi się bardzo podoba - te godziny dla seniorów.

**Co jest taką granicą, do której te ograniczenia powinny obowiązywać?**

To zależy od ilości ludzi zakażonych, takich stwierdzonych zarażeń, od ilości osób hospitalizowanych i od prognoz, bo wiem, że ma być potem jeszcze jakaś 2 fala, jakby nawrotu. Jak się skończy pierwsza fala, to jeszcze jacyś tam będą chorzy i oni też jeszcze potem będą zarażali. Jeżeli to drastycznie spadnie i powrócimy do tych 1-szych wskaźników, bo na takie całkowite, to nie ma szans, myślę przez rok co najmniej, dopóki szczepionki nie zrobią...To wtedy można się zająć takim odmrażaniem.

**Są jakieś ograniczenia, które już powinny być zdejmowane?**

Myślę, że teraz jest bardzo dobrze i że nie można za dużo ludziom zaproponować, bo wystarczyło, że wprowadzono wyjścia do lasu i do parku, a ja cały czas tu widzę spacerujących pod blokiem. Nie mówię, że to jest złe, bo rzeczywiście wszyscy mają maseczki. Nawet ostatnio widziałam takiego menelka, który wyciągał pety i miał maseczkę. Wszyscy mają maseczki i tak jak teraz jest dobrze, bo spokojnie wychodzimy, mamy wszystko zapewnione, możemy sobie zrobić zakupy a tak naprawdę rozrywkę możemy mieć online. Ja już odwołałam 2 wyjścia do teatru. Miałam być w teatrze jutro i tydzień temu i tylko cały czas czekam na te zwroty. Koleżanka mi poleciła, że Teatr Narodowy warszawski Śpiącą Królewnę...Nie wiem, co to będzie, ale zobaczę. Monika Brodka jakiś koncert daje, więc na razie spokojnie można się powstrzymać i oglądać online.

**Słyszałaś o tym, jak Szwecja podeszła do pandemii?**

Nie, nie wiem. Przyznaję, że czytałam ostatnio o eugenice szwedzkiej.

[moderator wyjaśnia]

**Co o tym myślisz?**

A orientujesz się, jaka tam jest zachorowalność?

**Nie mam pojęcia. Jak ci się wydaje?**

Mi się wydaje, że może być spora. Z drugiej strony te kraje skandynawskie takie odcięte są, jak by nie patrzeć. Inny był temat tej książki, ale dowiedziałam się sporo na temat rządu w Szwecji. Nie zdawałam sobie sprawy, jak ten rząd funkcjonuje. Teraz wiedząc to i wiedząc, jak zachowują się ludzie...Oni są bardzo karni i wydaje mi się też, że bardzo podporządkowani państwu. Tam wszystko jest podporządkowane władzy, nawet kościół chyba jest państwowy. To jest zupełnie inna mentalność i myślę, że tam to może zdać egzamin, bo rzeczywiście, jeżeli oni tak polegają na zarządzeniach, to wystarczy, że większość się podporządkuje i znacznie ograniczają tę zachorowalność. Wszystko się wiąże z tą mentalnością, bo my Polacy, to tak nie lubimy za bardzo...Oni są bardziej usystematyzowani i bardziej zawierzają rządowi, to może im tylko takie zalecenia wystarczą.

**W Polsce by takie coś przeszło?**

Nie, na pewno nie. Ja bardzo szanuję Polskę i uważam, że jesteśmy ogólnie dobrym narodem, ale też prawdą jest, że te archetypy nie biorą się znikąd. Gdzieś w ten stereotyp wpisana jest jakaś prawda i myślę, że byśmy się nie za bardzo podporządkowali.

**Jaki stereotyp?**

Nie, że jesteśmy nieposłuszni, ale lubimy negować różne rzeczy tylko ze względu na to, żeby coś móc zanegować. Ja tak samo na początku podchodziłam - że ja przecież jestem mądrzejsza, że oni przesadzają, przecież nic się nie dzieje. A się działo.

**Może trzeba by było, żeby wszyscy się zarazili?**

Jak te imprezy z ospą. [śmiech] Na początku, jak koronawirus się zaczął, to ja słyszałam, że ponad 70% populacji go przejdzie, tylko nie będą o tym wiedzieli lub będą wiedzieli. Być może ja już przeszłam, być może ty też i o tym nie wiesz. W sumie nie jesteśmy w stanie powiedzieć, ile osób w tej chwili tak realnie jest zarażonych lub przeszło tę chorobę. A wracając jeszcze do Szwecji, to oni tam przecież zupełnie inaczej patrzą na drugiego człowieka. Nie mówię, że są całkowicie wyalienowani, ale chyba są bardziej zdystansowani. Te ludy skandynawskie mają to do siebie. Stoją na przystanku metr od siebie normalnie, a 1.5 m przy koronawirusie.

**Czy ty odróżniasz bycie na kwarantannie od izolacji?**

Ja zawsze mówię kwarantanna, chociaż wiem, że tak technicznie biorąc nie jest to kwarantanna tylko bardziej izolacja. Nazywam to kwarantanną i wydaje mi się, że zespół znaczeń, z którymi utożsamiam to siedzenie w domu teraz, czyli to, żeby nie zarażać innych, to jest tak naprawdę tożsame z kwarantanną. Normalnie kwarantanna to też jest izolacja, ale przymusowa, a ja tutaj nie jestem przymusowo.

**Jak u ciebie obecnie wygląda takie dbanie o siebie?**

Przyznam się, że pierwszy tydzień nie myłam włosów 5 dni. Ja wiem, jak to brzmi, brzmi to okropnie, ale jak już raz związałam w kitkę i zakręciłam, to mąż mówił, że wygląda, jakbym myła włosy wczoraj.  Na początku stwierdziłam, że to może być dobre dla włosów, żeby tak troszeczkę...Jest taka metoda, że myje się włosy samą wodą i moja koleżanka próbowała myć włosy samą wodą. Wytrzymała 3 dni i powiedziała, że nie była w stanie patrzeć na siebie w lustrze. Ja przyznaję, że już też jakoś tak bardziej...Jednak już normalnie myje te włosy, chociaż nie tak często. Teraz np. nie suszę ich w ogóle, a wcześniej musiałam umyć wieczorem, wysuszyć. Teraz sobie plotę warkocz, kładę się i w sumie mogę nawet pracować w mokrych. Nie suszę, bo nigdzie nie wychodzę i nie muszą mi się układać. Nie maluję się - nie nakładam żadnych kolorów, podkładów, ale przyznaję się, że mam teraz taki swój zabieg. Wstaję sobie rano, myję twarz, nakładam aloes, na to nakładam krem nawilżający i bardzo nie lubię się świecić, więc delikatnie oprószam pudrem ryżowym. To wprowadziłam na kwarantannie, bo normalnie, jak nakładam makijaż, to staram się nie dawać za dużo warstw, więc daję tylko krem i podkład i ten puder. Teraz stwierdziłam, że mogę się normalnie nawilżyć, ale powiem szczerze, że zaczęłam się zastanawiać i jednak ta skóra jak oddycha i nie jest niczym przeciążona, to wygląda lepiej, więc zamówiłam sobie krem BB i będę go sprawdzała. Normalnie wysypywałoby mnie strasznie po czekoladzie, bo cały czas ją jem, bo mogę - nikt mnie nie widzi, ale zauważyłam, że teraz wysypuje mnie mniej. Jest zauważalna różnica. Normalnie pewnie zjadłabym ją w pracy i miałabym makijaż, i podkład, i ta skóra by nie oddychała, a teraz zjem, wysypie mnie, ale szybko znika.

**Zamieniłaś część rzeczy na inne?**

Na takie lżejsze, tak. Wcześniej dbałam bardziej o wygląd, a teraz dbam bardziej o skórę. Jedno mnie boli - nie potrafię zająć się paznokciami, nie umiem. Mam tak poobgryzane, ale no cóż...Normalnie bym miała takie swoje, ładne, hybrydkę. Miałam hybrydę i moje odrosły tak długie, i miałam nadzieję, że mi się może powyłamują, ale nie chciały. Powinnam tę hybrydę specjalnie acetonem zawinąć, jakieś sreberko, a ja ją po prosu zerwałam razem z płytką, ale już mi odchodziła, więc...

**Planujesz pójść te paznokcie zrobić?**

Nie wiem, czy teraz jest sens, bo i tak mnie nikt nie widzi. Normalnie chodzi o to, żeby moje ręce wyglądały estetycznie, a teraz jak jestem w domu, to nikt mi nie patrzy na te ręce, chociaż...Zawsze dobrze się poczuć, że są ładne, zrobione, no ale...

**Te rzeczy pielęgnacyjne robisz, bo?**

Żeby jednak cały czas dbać o tę skórę, żeby jej nie zapuścić, bo potem trudno ją odpuścić. Zaczęłam się zastanawiać jeszcze z tymi paznokciami. Myślę, że jest jeden powód, żeby coś sobie z nimi zrobić, bo ja inaczej je obgryzam, a jak mam hybrydę, to nie mogę jej obgryźć. Robię to dla siebie, żeby się dobrze czuć. Mama powiedziała mi, że powinno się w domu wyglądać ładnie i pamiętam ja brałam ślub, to mama mi mówiła (a już wiele lat mieszkaliśmy razem), że bielizna i skarpetki zawsze muszą być ładne, nie może być dziur. Może o to chodzi, że bielizna, skarpety i twarz powinny zawsze być zadbane.

**A jeśli chodzi w ogóle o ubranie?**

Chodzę normalnie w dresach teraz. Wiem, że ludzie się dzielą na tych, którzy mają ciuchy po domu i którzy chodzą w tych ciuchach, w które mieli od rana. Ja mam zawsze ciuchy po domu i cały czas w nich chodzę. Czyli w dresach. Jak na zakupy idę, to się ubiorę, podmaluję się czasem. Teraz maseczka - nikt nic nie widzi. Okulary, maseczka i nie trzeba w ogóle nic robić. Przyznaję.

**Jak często chodzisz do fryzjera?**

Nie farbuję włosów, więc ostatni raz byłam we wrześniu/ październiku a poprzedni to nie pamiętam. Zapuszczam włosy, więc tylko końcówki podcinam. Nie brakuje mi fryzjera.

**Jakieś wyjścia na basen, siłownię?**

Bardzo nie lubię zimnej wody, więc baseny odpadają. jestem tym typem osoby, który ogólnie ćwiczy, ma Multisporta, ale na siłowni bywam raz na 2 miesiące. Przyjęłam z pewną ulgą fakt, że moją kartę zawieszono. To jest taki wentyl bezpieczeństwa - ta karta jest, ja z niej nie korzystam, ale nie chcę jej odwoływać, bo jestem taka fit, prawda? Ćwiczę w domu i tu się nic nie zmieniło. Bardzo nie lubię Ewy Chodakowskiej, ale ćwiczę Ewę Chodakowską. Wyłączam ją, zmniejszam jak najbardziej, daję na bok ekranu i puszczam sobie serial. Ona takie bzdury gada i tak mnie demotywuje...Zawsze z nią ćwiczyłam i ogólnie to nie potrafię tak za bardzo. Wiem, że pewnie niedobrze robię te ćwiczenia, ale ogólnie sprawiają mi radość, więc...To, że nie przynoszą efektu...Ja się dziwię, bo ja ćwiczę a nie mam sześciopaku. Ta czekoladka i te złe ćwiczenie - to chyba o to chodzi.

**Robisz teraz jakieś zakupy na nową porę roku?**

Robię i zbliżają się moje urodziny i mąż mi powiedział wczoraj, że mam 24h na zrobienie listy, bo jego siostra coś tam chce, czy coś. Ja uważam, że najlepsze, jak już muszą mi coś dać, to jest karta podarunkowa i właśnie zrobiłam sobie listę sklepów, do których chciałabym ją dostać. Tylko jeżeli nie będzie jej możne wykorzystać online? Mam nadzieję, że będzie na tyle długa, że będę ją mogła wykorzystać, jak już się skończy ten okres zamknięcia. Oglądam sobie cały czas, poluję na wyprzedaże. Zdarzyło mi się już kupić, ale zazwyczaj zakupy robię na początku miesiąca, więc teraz tylko przeglądam strony i liczę na uśmiech wszechświata, który wygląda tak: przez tydzień otwieram różne karty i jak dostanę wypłaty, a te rzeczy będą dalej w moim rozmiarze, to może je kupię wtedy. Zazwyczaj już w trakcie tygodnia się kończą i zostają już tylko te, które są rzeczywiście mi przeznaczone. Bardzo mocno teraz odkrywam Aliexpress. Na początku byłam bardzo negatywnie nastawiona, ale jak się dokładnie poczyta skład ciuchów i obejrzy zdjęcia, to można naprawdę kupić za dobre ceny bez tego narzutu w sieciówkach. Myślę, że sobie coś tam kupię, zwłaszcza, że jestem w stanie wydać więcej pieniędzy, jeżeli wiem, że jakaś rzecz będzie ponadczasowa, że zawsze mi się przyda i raczej nie wyjdzie z mody. Są takie rzeczy. Jak coś jest modne tylko w tym sezonie, to wolę to zamówić na Aliexpress, bo jeżeli to będzie bubel, to w sumie nie wydałam dużo pieniędzy. Teraz przychodzą wszystkie moje paczki z Aliexpress, które zamówiłam w lutym i nie mogę nic z tym zrobić, bo zamówiłam sobie jakieś apaszki, opaski, no i nie mam gdzie w tym wyjść teraz. Ja wtedy w lutym bałam się zamawiać, bo już była epidemia w Wuhan, ale kolega mi powiedział, że jego żona zamawiała i przyszły jej w ciągu tygodnia. Przyszła jedna paczka wtedy, jedna jak się zaczął koronawirus, to w ogóle bałam się to otwierać i teraz ta reszta dochodzi.

**Czego się bałaś wtedy?**

Nie wiem...Te chińskie rączki. Nie mam pojęcia. Jakaś taka blokada. Niby wiem, że obiektywnie nic się nie powinno stać, ale mimo wszystko z rezerwą podeszłam do tej paczki. Teraz już tego nie mam.

**Z punktu widzenia konsumenta, czego ci najbardziej brakuje?**

Myślę, że kino i kawa. Wychodzimy czasem coś zjeść, ale ja wolę zamówić sobie do domu. Kawa bardzo, szczególnie, że mamy taki zwyczaj z koleżankami, że chodzimy sobie raz w tygodniu na kawę i dobre ciasto, jakąś bezę...Tego i bardzo brakuje, żeby sobie gdzieś usiąść na spokojnie, pogadać. Kina, teatry. Mieliśmy teraz iść na 2 spektakle, na które się bardzo nastawiałam. Bilety kupowaliśmy w styczniu czy w lutym. To jest bardzo przykre. Kino, to wiadomo, że można sobie wypożyczyć filmy teraz, ale to jednak nie zastąpi takiego wyjścia do kina ze znajomymi, potem na piwo. Dawno nie byłam na piwie...To też jednak boli. Bardzo boli też ta rozłąka ze znajomymi. Zastanawialiśmy się ostatnio, czy nie zorganizować takich planszówek online, ale jak się jest razem w jednym pomieszczeniu, to nie nudzi się ta gra, bo cały czas jest ten kontakt, a tak online, to myślę, że byłoby to po prostu nudne. To też był nasz taki zwyczaj, że co 2 tyg. spotykaliśmy się na planszówkach. Centra handlowe to nie bardzo...Raczej taka szeroko pojęta kultura.

**Próbujesz to jakoś sobie zastępować w domu?**

Raczej tak. Tak, jak mówiłam o tych platformach streamingowych. Wykupiliśmy dostęp do jakichś filmów. akurat ostatnio oglądaliśmy Parasite, ale rzeczywiście skorzystaliśmy z tej opcji video na życzenie i planujemy dalsze takie przedsięwzięcia, jak już niektóre tytuły stanieją. Nigdy wcześniej tak nie oglądaliśmy, bo zawsze woleliśmy iść do kina. Dawno temu w Hollywood obejrzeliśmy sobie na HBO, bo też nie zdążyliśmy pójść na to do kina.

**Z jakich platform korzystacie?**

Ja jestem serialowa, więc mamy 3 - Netflix, HBO i Amazon i w sumie między tymi trzema operujemy, a jak czegoś nie ma, to rzeczywiście można wypożyczyć. Ja się szykuję na Małe Kobietki, szczególnie, że zamówiłam sobie książki i je przeczytałam. Czytałem Dobre Żony w święta i bardzo chciałabym obejrzeć.
