# Supplementary material for: The challenges arising from the COVID-19 pandemic and the way people deal with them. A qualitative longitudinal study
Source: PLoS One. 2021 Oct 11;16(10):e0258133. doi: 10.1371/journal.pone.0258133 (PMC8504766; doi:10.1371/journal.pone.0258133)
Supplement: S1 Dataset — (ZIP) [file pone.0258133.s003.zip › Transcriptions/stage 3/1.3_F_25_single.docx]

**1.3_F_25_single**

**Jak wyglądały Twoje ostatnie 2 tygodnie?**

Ten tydzień wielkanocny, ta Wielkanoc tak zleciała szybko. Nic specjalnego, bo dla nas nic się nie zmienia. Tak, czy siak w takim gronie zawsze Wielkanoc obchodzimy. Wszyscy byliśmy w domu, brat już przyjechał tydzień przed Wielkanocą. I siedzi na razie, i pracuje tutaj od rodziców.

W zeszłym tygodniu większy dół mnie dopadł i zaczęły mi się problemy ze spaniem. Nie mogłam zasnąć parę nocy pod rząd. Teraz jest w miarę lepiej. Dzisiaj się w ogóle obudziłam przed świtem. Bo wczoraj zasnęłam wcześniej. Mam nadzieję, że teraz wróci do normy. Ale miałam taki moment i dołka większego, i spać nie mogłam. Ale co? Nic się nie zmienia. Taka regularność się zrobiła chodzenia na spacery z psem. Zaczęłam go zabierać regularnie na południowy, dłuższy spacer i parę razy wyszliśmy z naszymi sąsiadami, żeby on się wybiegał z psem sąsiada. A tak poza tym, to nic się nie zmieniło.

**Czyli w takim spędzaniu czasu tylko te spacery z psem się pojawiły? A coś jeszcze? Bo jak ostatnio rozmawiałyśmy, to byłaś w sklepie po raz pierwszy. A od tamtej pory chodziłaś do sklepów?**

Tak, byłam parę razy. Czy sama coś sobie kupić, czy wczoraj byłam na większych zakupach z babcią. Ja zabrałam i pojechałyśmy też do innych sklepów. Byłyśmy w aptece wykupić jej leki. I niektórzy ludzie jakoś strasznie panikują. Wszyscy, a przynajmniej większość, są w maseczkach ale w tej aptece są jakieś obostrzenia ile osób może wejść, ile przy okienku może stać. Przed okienkiem postawili w ogóle takie pudełka, w których przychodzi im dostawa, żeby ludzie nie podchodzili do tego okienka. Żeby stali metr od okienka. Nie pozwoliły te panie mojej babci wejść do środka, miała czekać na zewnątrz. Gdzie ja tą receptę musiałam z nią skonsultować, co to są za leki, co ona jeszcze chce. Ja zapłaciłam jej. No ale ja muszą ją obsłużyć, muszę iść jej to wykupić - po pierwsze dlatego, że ona nie wie, jak działają e-recepty, a po drugie dlatego, że wszyscy ludzie mają teraz zasłonięte usta, a moja babcia jest niesłysząca, więc zawsze czytała z ruchu warg. I po prostu jak ludzie nie wiedzą, to tego nie robią. Więc ona albo nie reaguje, albo musi powiedzieć jak nie wie, co do niej mówią, żeby mówili do niej wprost. Bo jeżeli patrzą na nią to ona zrozumie, co ktoś mówi. Ale w tym momencie nie ma takiej możliwości w ogóle, więc ja muszę po to z nią pojechać. To się panie przyczepiły, że skoro ja jestem w środku, to po co ona w środku?

Te panie co tam są też miały maseczki, oprócz mnie była jeszcze jedna pani. Nie było żadnego tłumu. Babcia stała w dużej odległości ode mnie, od okienek i od drugiej osoby. Ale nie  wiem, kto to - chyba właściciel apteki, bo bocznym wejściem wchodził, jakieś dostawy ogarniał - chodził jakiś mężczyzna bez maseczki, bez niczego do środka i na zewnątrz. Więc skoro on wchodząc i wychodząc nie ma maseczki, a one się przyczepiają o moją babcię, która w maseczce stała w bezpiecznej odległości... Ludzie się przyczepiają do tych, co robią coś innego,   ale jak ktoś od nich wewnątrz jest bez maseczki, to już nie jest nagle problem.

Zaczęły do mnie docierać, też różne historie. Widziałam wywiad z ratownikiem medycznym, który mówił,   że sąsiedzi patrzą na niego krzywo i że są takie historie gdzie na różnych osiedlach ludzie - to mi mama opowiadała, że słyszała - poszli do rady spółdzielni mieszkańcy zapytać, gdzie mieszkają lekarze i żądali przekwaterowania tych lekarzy na czas epidemii, z ich domów. Więc z jednej strony jest to klaskanie i to dziękowanie wszystkim lekarzom, a z drugiej niektórym strasznie odwala.

My wszyscy z domu mamy takie podejście, że trzeba się dostosować, wiadomo, ale jest to trochę wszystko rozdmuchana panika. I oczywiście może noszenie tych maseczek pomoże i to że szkoły są zamknięte, i że większe skupiska ludzi są zamknięte. To ok. Ale ludziom się paranoja włącza w niektórych momentach. A i tak ciężko jest się ustrzec jak wychodzimy a musimy wychodzić - niektórzy do pracy, do sklepów itd. To wszystko jest narażające. No ale nie wiem. Jest to takie, że nie ma się ochoty wychodzić do tych ludzi, bo... Też z tego co ja zrozumiałam z tymi maseczkami, to jest obowiązek noszenia ich w miejscach, w których niemożliwym jest trzymanie 2 metrowego dystansu od siebie. Co oznacza, że po moim osiedlu, czy jak odę z psem do lasu na łąkę gdzie mijam jedną osobę z drugim psem ileś metrów ode mnie, to nie mam tam obowiązku noszenia maseczki na spacerze. No bo to ma dotyczyć takich przestrzeni, gdzie trudno od siebie być [dalej]. Albo pomieszczeń, tak jak sklepy.

**Wróćmy jeszcze na chwilę do Twoich sposób spędzania czasu. Czy coś się zmieniło? Jak spędzałaś czas przez ostatnie dwa tygodnie?**

Nic się nie zmieniło. Kolejny mi się włączył serial hiszpański - nie wiem, czy mówiłam.

**Mówiłaś, że oglądałaś "Dom z papieru" i się uczyłaś hiszpańskiego dzięki niemu.**

Tak, i to skończyłam. Ale przerzuciłam się na następny, gdzie były 3 sezony więc też obejrzałam po hiszpańsku. Zamówiłam sobie podręczniki do pierwszego poziomu, żeby trochę też gramatyki sobie poćwiczyć. Więc sobie czasami w tych podręcznikach robię. Do czytania jakoś wciąż nie mogę się zmobilizować. Nie wiem, dlaczego nie mogę się skupić do tego. Ale tak miałam cały czas teraz.

Spacery z psem są takim stałym elementem. I raz na jakiś czas jest to wyjście do paczkomatu. I właśnie przed chwilą wróciłam. Więc jak coś do paczkomatu przychodzi, to te wyjścia są taką dzienną rozrywką.

A tak to nic się nie pozmieniało.

**A co Cię skłoniło, żeby tego hiszpańskiego się zacząć uczyć?**

Miałam taką chęć już od jakiegoś czasu. Ja - nie pamiętam kiedy to było - miałam taki czas że prawie rok na duolingo siedziałam i codziennie tą lekcję hiszpańskiego robiłam. I to było na tyle długo, że zaczęłam rozumieć słowa, pamiętać je, i byłam proste Więc też oglądając te seriale rozumiałam część słów. Albo zwroty. Więc już miałam raz zaczątek tego. A z racji dużej ilości czasu chciałam do tego wrócić.

Aha, i chyba o tym nie mówiłyśmy. I 2go dostałam zadanie stażowe - nie wiem, czy mówiłyśmy

**Tak. Wiem, że dostałaś, ale nie wiem, co się dalej działo**

Miałam do czwartku zeszłego termin, więc je robiłam. Nie wiedziałam do końca o co im chodzi, co chcą sprawdzić i ono było dość problematyczne. Więc cały czas świat i po świętach zanim odesłałam zadanie, odsuwałam robienie go od siebie. Tak jest, jak mamy coś co nas przerasta, albo zadanie jest na tyle duże że przytłacza ogrom, to odsuwamy to. Ale w końcu zrobiłam i wysłałam. Nie wiem, chyba 6go maja się dowiem najwcześniej, czy idę na następny etap. Tam potem jest rozmowa i dopiero potem decydują kto jest przyjęty. To też mi psuło humor. Nie mogłam się w pełni relaksować, bo cały czas miałam z tyłu głowy, że muszę do tego zdania usiąść. Strasznie mnie to stresowało. Irytował mnie ten czynnik zadania stażowego. Ale jak odesłałam, to mi przeszło. I zaraz potem jakoś mi wjechał spadek nastroju mocny.

W sobotę mam co tydzień sesję z terapeutą online i mieliśmy pracować nad ustaleniem dalszego planu i celami, ale w sumie doszłam do wniosku, że nie będę na razie nic planować konkretnie bo to trochę nie ma sensu. Z pracą to będzie tak, że coś się uda i co się trafi fajnego i mnie interesuje i są pieniądze ok, to jest taka loteria i nie do końca to można zaplanować jak w normalnych okolicznościach. Bo zależy jak która branża została dotknięta. Z tą pracą  to nie jest tak, że ja mogę sobie stwierdzić, że chcę dostać tą i ta pracę. I ją dostać. Bo to będzie zależało od tego, jak gospodarka się będzie trzymała. Bo ja mam wrażenie, że to nie jest kwestia tylko tych branż których dotknęło całe to zamknięcie nas, ale większość firm wstrzymała rekrutacje, po ograniczała. Dopóki nie będzie można wrócić do biur, to też nie mają jak przeprowadzać rekrutacji czy nowych ludzi wprowadzać do firm.

**Jak ostatnio rozmawiałyśmy, to mówiłaś że jednak robisz te plany, że to Ci pomaga jakoś radzić sobie z sytuacją. Kwestie pracy, czy jakieś wyjazdy. A teraz mówisz, że nie ma po co tego planować. Co się stało, że tak stwierdziłaś?**

Nie wiem. Takie mam wahania nastroju i myślenie o tym, co zrobić, jak zrobić.

Takie moje ciągłe rozmyślania jak ja bym chciała, jaką mogę dostać pracę, jaka jest możliwość dostania tej pracy. Ale właśnie doszłam do wniosku przy okazji mojej terapii i powiedziałam terapeucie, że ja sama nie wiem, co ja bym chciała itd. Bo mam sprzeczne chęci życiowe, sprzeczne ze sobą oczekiwania jak ta rzeczywistość będzie wyglądała, w sensie pomijając pandemię - tak ogólnie. Że z jednej strony chciałabym to, a z drugiej, coś co się wyklucza. I doszłam do wniosku, że dopóki nie wiem, kiedy będzie można wyjść, kiedy jakiekolwiek możliwości pracy się odblokują. Teraz to najłatwiej byłoby pójść do Biedronki, czy innego sklepu na kasę - bo to jest teraz wciąż pewnie potrzebne. Ale ja wiem, że ja nie chcę czegoś takiego robić. Nie jest tak źle na ten moment, żebym musiała szukać jakiejkolwiek pracy. Więc ja chcę znaleźć pracę, która będzie coś tam mi dawała  czy to jest coś, że się rozwijam w kierunku który mnie interesującym kierunku, albo dobra kasa. Teraz z dobrą kasą to raczej nie, bo w moim przypadku jest problem z tym, że nie mam doświadczenia w dziedzinach, które bym chciała, żeby to była dobra kasa. A jak ktoś nie ma doświadczenia, no to jest to kosztem wypłaty. A to nie jest tak, że ja chcę byle jakiej pracy, a obecna sytuacja nie poprawia tego. Nie wiadomo, kiedy to się wszystko skończy. Czy już z początkiem maja będzie można wracać do biur, czy w połowie maja. Czy w ogóle z końcem maja.

**Czy to znaczy, że miałaś poczucie wcześniej, że miałaś wrażenie kiedy to się skończy, jakieś przeczucie i to Ci pozwalało planować, a teraz już tego nie ma?**

Może tak być. Nie jestem w stanie do końca wyjaśnić. Może wcześniej mi się wydawało, że  ta data taka jest, no do końca kwietnia powinniśmy wyjść, albo że maj to już się powinno odblokowywać. A teraz nie wiadomo. Bo są kraje w których cały czas zaostrzają, u nas niby robią te luzowania i te etapy wyglądają dobrze - ja już się nie mogę doczekać na 3 etap. W drugim chyba już galerie otwierają, więc skoro otwierają galerie to znaczy że będzie można wyjść.

Jak będzie można wyjść i nie jest tak, że będą cię pytać czy idziesz do pracy czy na zakupy. Bo jak otwierają galerie, to równie dobrze mogę powiedzieć, że jadę do muzeum. Skoro jest otworzyli. Więc sobie  myślę, że wtedy wyjdę się spotkać z przyjaciółkami i pojadę do Warszawy. Dzisiaj jest wtorek, a w niedzielę albo w sobotę przyjechał przyjaciel mnie odwiedzić. Więc już przynajmniej z jedną osobą się zobaczyłam fizycznie.

**Do Was, do rodziców przyjechał?**

No on ma samochód i wtedy to jest prostsze, niż jechać komunikacją.

Nie wiem, skąd mi się wzięło, że wcześniej tak mi się wydawało, że to planowanie ma sens, a potem przestało. Mam takie coraz większe - zabrzmi śmiesznie - ja chciałam iść do psychologa na terapię, ale moja mama twierdzi, że to jest mi niepotrzebne, że psychologowie to jest bez sensu bo to są ludzie, którzy sami mają problemy, a mówią mi co ja mam robić z moimi problemami. I wysłała mnie w październiku do astrologa. Więc poszłam na tarota. Stwierdziłam - mama płaci, to sobie zobaczę jak to wygląda. No i powiedział różne rzeczy. I on mi właśnie przepowiedział, że w drugiej połowie kwietnia - bo tam coś mówiłam o stanach depresyjnych, o takich dołkach które miewałam od dwóch lat - on powiedział od czego to może być zależne i że "praca to najwcześniej w drugiej połowie kwietnia". Ale tak trochę czuję, że też te problemy ze spaniem - to są takie rzeczy wskazujące. Ciężko jest teraz stwierdzać, kto ma depresję, bo nie można wychodzić z domu i w łóżku można siedzieć cały dzień, więc tak jakby... Nie wiem, czy depresja jest teraz prostsza do stwierdzenia. Śmieję się z ta depresją i tarotem, ale jest do tego podłoże jak najbardziej.

Nastroje i te wahania są coraz mocniejsze, bo jest to strasznie męczące. Wróciłam już miesiąc temu, i od miesiąca nie wychodzę poza naszą miejscowość. Jak w samochód wczoraj z babcią wsiadłam i pojechałyśmy do sklepu który jest dalej po drugiej stronie wsi, ale to jest 5 minut w samochodzie w jedną stronę. Nigdzie daleko nie jechałam. Nie byłam w ogóle w mieście, w Warszawie od powrotu z lotniska.

**A to jest tak, że to Ci zaczęło teraz mocniej przeszkadzać?**

Coraz bardziej jest uczucie jak uwięzienie. Bo bywały okresy, kiedy ja w ostatnim czasie siedziałam dużo tutaj u rodziców. I to jest przyjemne, bo jak jest ładna pogoda - teraz coraz ładniej się robi - to i w naszym ogrodzie jest przyjemnie, parę rzeczy do zrobienia jest, wczoraj odmalowywałam coś rodzicom bo mnie poprosili. Jest gdzie wyjść z psem. Mamy do około dużo więcej zieleni. A to nie są takie parki, żeby policja zabraniała tam wchodzić. Dużo bardziej współczuję ludziom w mieście, w blokach bez balkonu i z dziećmi. Ale coraz bardziej jest takie uczucie uwięzienia w tej mojej miejscowości. Bo wsiadanie teraz do komunikacji i jechanie do miasta, to się można natknąć na policję która będzie się pytała gdzie jedziemy. Nie wiem, jak to jest z tym wlepianiem mandatów - to pewnie zależy od policjanta. Nie wiem, w jaki sposób sprawdzają, kto mówi, że rzeczywiście jedzie do pracy, a kto nie, do lekarza czy do apteki. To jest takie poczucie, że nie mogę tak swobodnie wyjechać, pojechać do sklepu, załatwić czegoś czy po prostu umówić się z kimś i spotkać. Moi znajomi nie są tacy jacyś przewrażliwieni, żeby nie chcieć się spotkać bo się boją, tylko jest to bardziej kwestia tego że można spotkać policję, która będzie zadawała pytania.

Zagrożenie - w ogóle go nie czuję, no bo ja ani osobiście nie znam nikogo kto jest zarażony, albo przynajmniej nikt mi nie powiedział. Może w sumie ludzie nie dzielą się takimi rzeczami. Ale o nikim, kto był oddzielony i miał stwierdzone zakażenie nie słyszałam wśród znajomych. O śmierci tym bardziej. Wciąż mam wrażenie - z tego co w wiadomościach widzę, że te śmierci to są ci ludzie starsi, bardziej osłabieni. Może trochę mam strach  o babcię. Ale z drugiej strony byłam z nią wczoraj w sklepie nie w tych godzinach dla starszych, bo mnie by nie wpuszczono z nią. I jakby ona nie ma z tym problemu. Ale u niej to jest tekst w stylu "ja się wirusa nie boję, na coś trzeba umrzeć" - babcia jest już na tym stadium, że ma takie rzeczy w nosie.

**A co jest dla Ciebie największym wyzwaniem teraz w tej codzienności?**

Już przy tej długości trwania tego - bo ja miałam tydzień kwarantanny jeszcze w Londynie, kiedy nic się nie działo, a dwa tygodnie przedtem byłam tam sama, mogłam wychodzić, spotykałam się z ludźmi, szukałam pracy, ale nie miałam tego normalnego trybu, że byłam cały dzień w pracy, to dużo spędzałam czasu sama. Więc taką półkwarantannę miałam pierwsze 2 tygodnie. Potem od powrotu już 4 tygodnie ponad. Więc to się robi mi co najmniej 5 tygodni. I to już jest długi okres, bez rutyny, bez możliwości zaplanowania czegoś konkretnie. Bo z jednej strony myślałam o mieszkaniu, patrzyłam na mieszkania i myślałam, że z początkiem maja się będę mogła wyprowadzić. Ale teraz na razie wciąż nie można wychodzić poza tymi pracą, apteką, sklepem, więc oglądanie mieszkania to będzie problem. Poza tym stwierdziłam, że to się  tak wydłuża i wciąż może być problem z tą pracą, to dobrze byłoby najpierw wiedzieć, kiedy to się wszystko kończy, można wracać do biur, kiedy jakaś praca wpadnie. I jak ta praca będzie, to wynajmować mieszkanie. Bo po co mam wynająć mieszkanie na maj, jak się okaże że zacznę pracę dopiero w czerwcu. Dopóki nie będę musiała jeździć do pracy, to dużo wygodniej jest mi siedzieć w domu tutaj niż w mieszkaniu w Warszawie. W Warszawie ma sens jak codziennie chodzę do pracy i chcę się wieczorami, swobodnie spotkać ze znajomymi i nie musieć tutaj wracać. Bo tu wracanie wieczorem jest problematyczne.

A jak mam siedzieć w Warszawie w mieszkaniu sama, bo nie będzie można swobodnie wychodzić, gdzie to będzie kosztowniejsze, i nie mogę się spotykać ani iść do pracy, to to nie ma sensu. Ja bym chciała się stąd wyrwać ale nie można.

**Powiedziałaś o uczuciu uwięzienia, o rutynie - że ciężko z tym wytrzymać. To jakie działania podejmujesz, żeby sobie radzić z tą sytuacją?**

To co mi poprawiło humor, to jak wyszłyśmy z mamą na spacer ten popołudniowy z psem ją wyciągnęłam. Bo wtedy jak się rozdzielimy to on biega między nami i sobie trochę energii mu uchodzi. Poszłyśmy ścieżkami, którymi ja normalnie nie chodzę. I to była taka nowość. Poszłam na spacer w miejsca, gdzie nigdy nie chodziłam. Taki długi spacer. To jest sposób na ruch, a dwa że to jest jedyna forma podróżowania którą można obecnie uskutecznić. To mnie trochę - może nie podnosi na duchu - ale urozmaica dzień.

Ale brzmi to smutno (śmiech). Te seriale trochę. Bo kiedy się je ogląda to ten czas leci dużo szybciej. To jest taka ucieczka. Ale jest to o tyle niefajne, że ja mam takie poczucie, że czas ucieka a ja nie zrobiłam nic pożytecznego. Trochę sobie wmawiam, że to jest pożyteczne z tym hiszpańskim, ale już skończyłam te sezony. Teraz nie wiem co znajdę - hiszpański, czy coś innego.

Dużo podcastów słucham. Z nimi jest o tyle fajnie, że ja mogę robić coś innego. Jeśli chodzi o gry na telefon, to nie przepadam za takimi normalnymi grami. Mam takie, które są takimi grami stricte rozwojowymi dla mózgu (opis dwóch gier). Strasznie się wkręciłam w te gry. Mam takie ciągi słuchania podcastu całego, grając na telefonie w kółko w jedną grę.

**A poza tym, że to Ci sprawia przyjemność i czas szybciej mija, to jeszcze spełnia to jakieś funkcje? Ma to jakieś znaczenie?**

Nie, chyba nie.

**A czy to jest przyjemność? Poprawa humoru?**

To taki sposób, żeby czas szybciej przeleciał.

**Emocje.**

Tym razem to 2 albo 14. Czyli albo to przyklejenie się - uwięzienie w domu, w mojej miejscowości - albo ten węzeł, takie przywiązanie do jednego miejsca i niemożność wyrwania się gdzieś indziej.

**To opowiedz mi więcej o tych obrazkach.**

Tu chyba chodzi o ten węzeł głównie. Z tą gumą to też jest chyba taki rodzaj przyklejenia, taki niesmaczny - w sensie, nikt nie lubi wejść w czyjąś wyzutą gumę. To tak obrazuje dobrze niechciane, czy nie z mojej winy przymocowanie się do jednego miejsca. Jedno to jest że nie mogę się wyrwać do Warszawy za bardzo, bo ta policja. No i nie ma po co tam jechać. Bo mam pewnie takich znajomych, którzy by się nie chcieli spotkać ze względu na wirusa, a ci co by się chcieli to nie mają za dużo czasu, bo pracują. Sam fakt tego, że podróżować nie będzie można jeszcze dłużej, to mnie irytuje. Jest dość nie na rękę. Bo ja zawsze miałam pociąg do podróży, do przemieszczania się. Sam fakt wyprowadzki do Wielkiej Brytanii miał być czymś takim innym na najbliższe 1.5 Roku. 2 Lata siedziałam w Warszawie i było mi z tym źle, to było dla mnie za długo siedzenia w jednym miejscu. Trochę było urozmaicenia jak się wyniosłam do mieszkania w Warszawie. Ale wcześniej mieszkałam u rodziców. To mi już ciążyło. Bo ja lubię zmieniać miejsca, być w nowych miejscach dopóki się nie ustatkuję to bym chciała pomieszkać w różnych miejscach na świecie. A teraz to się wzięło - jak się wyprowadziłam - i skończyło w 3 tygodnie. I jestem zmuszona wrócić do rodziców, zmuszona siedzieć w Polsce, zmuszona szukać pracy w Warszawie, bo zwyczajnie nie będzie możliwości, żebym teraz - myślę do końca lata, jak nie do końca roku - szukała pracy w innym kraju, gdziekolwiek leciała. Nawet gdyby była taka możliwość, to z drugiej strony nie wiadomo ile będzie fal powrotnych tego wirusa. Bo o tym tez się mówi. I tak jak czytałam o poprzednich pandemiach, to miały one kilka fal zakażeń. Więc to że w lecie  coś się poluzuje i wrócimy to jakiejś tam czegoś bliższego normie, nie oznacza, że potem znów się coś nie zacznie. A zawsze pierwszych obostrzeniem, które będą kraje wprowadzały będzie zamykanie granic albo ograniczanie lotów.

**A ten węzeł?**

To oba tak samo.

**Powiedziałaś, ze czujesz irytację [...]. Czy jakieś jeszcze emocje się pojawiają?**

Nie wiem. Poczucie niesprawiedliwości trochę (śmiech). O tyle mnie to podminowało, że te ostatnie dwa lata miałam takie kiepskie. I to był taki gorszy czas w życiu, pewne rozczarowania po podróży odnośnie życia, nie do końca moje określone co ja chcę robić, gdzie jest to moje miejsce. Jeszcze sobie tego nie znalazłam. I to był długi okres mojego niezadowolenia. Praca ok, ale bez szału. Sam fakt wyprowadzki - pomijając studia, pracowania w anglojęzycznym środowisku, a ja lubię mówić, pracować i żyć po angielsku. I miałam nadzieję, że sobie wrócę do lepszego akcentu, słownictwo dopracuję.  W Londynie były też studia, które wnoszą język na wyższy poziom. To mi dało poczucie jakiegoś rozwoju.

**To poczucie niesprawiedliwości wynika z tego, że [...] były nieprzyjemne i teraz jeszcze to, tak?**

W momencie jak chciałam zmienić moją sytuację, która mi nie odpowiadała. Zrobiłam kroki, planowałam to przez pół roku, to wszystko się rypło. I można wiadomo się doszukiwać pozytywów, wnioski może mi z tego by wyszły, że to będą lepsze zmiany, może w dłuższej perspektywie czasu będę widziała pozytywne rezultaty tej sytuacji tego jak to się później potoczyło. Ale na dany moment, nie wiem, jak to się wszystko potoczy, kiedy będzie można wracać do normalnego funkcjonowania, planowania - to raczej mnie to irytuje, podminowuje, wprowadza w depresyjny stan i nastrój. Ja chciałam zmienić tą sytuację moją i to nie wyszło. I nie z mojej winy w ogóle.

Z tym bym się nawet pogodziła, że nie ten plan, to inny. Rzecz w tym, że nic nie mogę w tym momencie. Muszę czekać aż sytuacja się jakkolwiek rozluźni. Teraz działaniem byłoby podjęcie pracy, czy szukanie jej w kraju czy za granicą. Za granicą w ogóle nie ma sensu, bo nigdzie nie polecę. Pomijając to czy ja się boję wirusa czy nie, bardziej bym się bała czy przy nawrocie fal zakażeń czy nie pozamykają granic. A nie chciałabym być w innym kraju z taką niestabilną sytuacją - bo to zawsze kilka miesięcy zanim człowiek się ustabilizuje w nowym miejscu. Na razie samo wylatywanie gdziekolwiek wątpię, żeby było rozsądne. Dopóki sytuacja się nie uspokoi całkowicie, a to może potrwać rok, a nawet półtora czy dwa lata.

**A te dołki, problemy ze snem. To wtedy czułaś smutek, czy jakie to były emocje?**

I trochę złości. To jest związane z tym, że ja lubię działać. Nie jestem osobą, która jak coś jest do zrobienia, to może siedzieć i czekać. Nie jestem w stanie się odprężyć, dobrze odpoczywać, dlatego odpoczywanie się kiedy miałam do zrobienia to zadanie stażowe, było męką a nie odpoczywaniem.  Ja musze działać i mieć poczucie, że zrobiłam wszystko z mojej strony polepszyć. A teraz nic nie mogę. Ani pracy. Ani mieszkania. Jestem skazana na siedzenie z rodzicami, zajmowanie się psem. Tyle mogę.

Ale też to jest dodatkowa kwestia, która w nie najlepszym czasie się dzieje, ale tez ostatecznie zakończyłam  skomplikowaną, ale związkową relację po dwóch latach. Takiego przepychania się w relacji. Więc to tez jest ciężkie i nie na rękę w tym całym odosobnieniu od przyjaciół i jakichkolwiek zajęć. Bo jest  jeszcze smutek towarzyszący rozstaniom i zrywaniom relacji.

**A to zerwanie relacji miało w jakikolwiek sposób związek z tym, że jesteś zamknięta w domu?**

Nie. To by się działo niezależnie od sytuacji.

Tylko sytuacja nie pomaga sobie z tym poradzić,

**A jak sobie z tym poradziłaś - z tym dołkiem, problemy ze snem. Co zrobiłaś, żeby się poczuć trochę lepiej?**

Nic szczerze mówiąc. Bo ja mam tendencję raczej do tego, żeby wpadać w takie dołki i kule śnieżne zamartwiania się nad sobą i umartwiania. Więc to tak potrwało dwie noce, że naprawdę nie mogłam spać, bo ten sen mi się rozregulował na dłużej. Ale to samo przechodzi. Więc na razie mam takie wahania. To najczęściej mi się robi wieczorem i w nocy, więc dlatego ja w nocy nie mogłam spać. Bo to że jest ciemno, że robi się cicho to automatycznie człowiek się czuje bardziej sam, jak wszyscy idą spać. Jestem sama w sowim pokoju. A jak się zaczyna  nowy dzień, zaczynam spędzać trochę czasu z psem, to tak samoistnie mnie wyciąga z tych dołków. Ja sama nie podejmuję żadnych działań, żeby z tego wyjść. Tym bardziej, że gdybym miała motywację w stylu "dobra, trzeba zrobić to i to bo mam iść do pracy, albo mam zadanie", to jeszcze bym się motywowała. Ale w sytuacji, w której ja nic nie muszę, nic się nie dzieje - myślę, że dla kogoś z depresją to jest idealne podłoże, żeby tylko siedzieć w tym łóżku i tą depresję bardziej rozwijać. To jest takie wchodzenie w tą kulę umartwiania się i smutku. I takie siedzenie w tym. Samo przychodzi i przechodzi.

**A jeśli chodzi o obawy? Bo nie czujesz zagrożenia wirusem [tu przytoczenie poprzedniego wywiadu]. Czy tu coś się zmieniło?**

Finansowo to na razie jest ok. Bo dzięki temu, że miałam te oszczędności na studia to powiedzmy, że na parę miesięcy jest ok. Nawet lepiej niż ok. Ale taka obawa jaka będzie ta praca, jak będzie finansowo potem. Za parę miesięcy wrócę do sytuacji, kiedy to będzie problem, itd. Na razie myślę mocno w przód. Jak będzie?

Poza tym, to finansowo się nie stresuję.

Bardziej co będzie można? Czego nie będzie można?

**Taka niepewność tak?**

Tak. No i to, że nie będzie można podróżować. A o tym długo myślałam. To jest coś, co bym chciała robić, dopóki nie będę zakładać rodziny. A teraz trochę dłuższy niż same obostrzenia w kraju, będzie to niemożliwe. Albo bardzo drogie. Każdy kraj będzie też miał swoje obostrzenia.

**A jak z Twoimi bliskimi? Jak Twoje otoczenie sobie radzi? Widzisz w nich jakieś zmiany?**

Brat tez już wariuje. On ma też ten problem, że on w tygodniu siedzi przed komputerem w pracy. A teraz miał zjadł w weekend online. I normalnie te jego zjazdy są dość męczące, bo oni siedzą na uczelni od 9 do 21 w sobotę. A tym razem miał taki długi ciąg w domu przed komputerem. I miał te wykłady online. Więc on tez od tego siedzenia przed komputerem wariuje. A też może tylko wyjść z psem do ogrodu, albo na spacer. Albo się na rowerze przejechać. Więc też mu ciężko. Ale jemu jest ciężko tylko psychicznie i społecznie, bo on i pracuje, dostaje normalną wypłatę, kontynuuje studia, więc jemu się plany życiowe nie zmieniły.

Ten pies, to jest duży plus w tym wszystkim. Bo to trochę takie małe dziecko, które potrzebuje uwagi. Więc jest to coś, co mobilizuje że trzeba się nim zająć. Myślę, że bez psa byłoby jeszcze ciężej nam.

Czasami brat coś ugotuje, pomoże mamie. Ale poza tym, chyba nie [ma rozwiązań na radzenie sobie].

**A jak inne osoby?**

Tu się niewiele zmieniło. Tata zajmuje się ogrodem, albo pisaniem swoich rzeczy ja do tej pory. Tylko na basen nie może, ale to jest do przeżycia. Babci też się niewiele zmieniło. Na początku jej do sklepu nie puszczaliśmy, ale teraz już z nią jeździmy do sklepu. A wcześniej też mało wychodziła, bo ma problem z biodrem. I znajomi jej poumierali co po niektórzy, więc to co jej poprawiło ostatnimi czasy humor, to kupiliśmy jej na święta smartfona, bo się dowiedziała, że znajomi mają. I dzięki temu może sobie na WhatsAppie na video rozmawiać z nimi, migać. Więc tak jak wcześniej mogła tylko smsy, to tak teraz się z nimi łączy na video i może migać ze znajomymi. Ale to jeszcze przed pandemią widziałam, że jej polepszyło humor. Bo ma poczucie, że jest w kontakcie, widzi tych swoich znajomych. Ale babcia to jest już w trybie "ja już bym umarła". Bo jej się nudzi. Marudzi, że jakby mieszkała w mieście to by mogła do znajomych chodzić, czy wyjść gdzie jej się podoba - ale to nie prawda, bo chodzenie po schodach ją męczy przez to biodro.

Mama ma przymusowe wakacje, z którymi jest tak dziwnie. Ale też znajduje sobie, czy prace w ogrodzie, bo wiosna się zaczęła. To jest taki czas kiedy rodzice zawsze mieli dużo prac ogrodowych. Zajmuje się też gotowaniem, sprzątaniem, swoimi rzeczami zapomnianymi. Ona ma dużo rzeczy, które może dzięki temu zrobić, bo ma czas.

Więc ich to tak bardzo nie dotyka. Tylko mnie to pokrzyżowało plany. I wróci im do normy, jak będzie można wrócić do szkół.

**Ograniczenia. O jakich zmianach słyszałaś?**

Wprowadzili obowiązek noszenia maseczek. Najpierw to miało być - tak rozumiałam na początku - wszędzie na zewnątrz. Potem w wiadomościach to było określone - tylko ja nie wchodziłam na żadne strony rządowe, więc tylko mówię na podstawie wiadomości czy telewizji. Później słyszałam, że to głównie w sklepach i miejscach, gdzie to ciężko jest się mijać na odległość 2 metrów. Otworzyli te parki i lasy wczoraj. Ale to mi niewiele zmienia, bo myśmy i tak do lasu chodzili. Nas las jest na tyle mały, że ciężko nazwać to lasem. I dużo mniej policji już teraz jest. Na kwarantannie przyjeżdżała. I raz po kwarantannie widziałam. Tu tak nie patrolują na wsi. Może na rynku, ale tam też jest mniej ludzi z racji zamkniętych różnych miejsc.

Sklepy się uspokoiły. Były kolejki przed Wielkanocą. Ale to wiadomo święta. I ludzie normalnie więcej kupują a przy tych wszystkich obostrzeniach i mniejszej ilości ludzi, to się robiły kolejki.

Ale teraz już nie ma kolejek. Wczoraj byłyśmy w Lidlu. Tylko w tej aptece te panie były przewrażliwione. Taka mała apteka, więc też mało miejsca.

**Jak Ty się czujesz z tymi ograniczeniami, np. że trzeba nosić maseczki?**

Jest to uciążliwe. Przyszły do mnie soczewki, więc o tyle dobrze. Jak miałam okulary, to sobie je parowałam oddychając w maseczkę. Bo ona w tym miejscu ma otwór, który dmucha ci pod okulary. Ja mam też małe uszy, więc te maseczki wyginają mi uszy albo mi z nich spadają. Jest to wkurzające. Ale jest to do przeżycia, przy tym że może rzeczywiście wspomaga, szczególnie personel. Ale byłam w sklepie, większość personelu miała, ludzie mieli, a widziałam pracownika sklepu, który nie miał.

**Jak one twoim zdaniem działają? Kogo chronią?**

One zapobiegają temu rozpylaniu się drobinek z naszych ust. Przy oddychaniu i mówieniu. Tego nie widać, ale przy każdym słowie trochę wypluwamy śliny. Mniemam, ze taka maseczka stopuje to. Ale tego przenoszenia na rękach, na rękawiczkach to myślę, że to już nie działa. Z tego co ja słuchałam wywiadów mniej się trzeba bać o to co się przeniesie na powierzchniach, bardziej o to co jest w powietrzu. Nie wiem tego do końca.

Mówią, że maseczka po jakimś czasie przestaje działać i trzeba by ją wymieniać co chwilę. Nikt tego nie robi, bo nikogo nie stać. Kupowałyśmy z babcią maseczki w aptece. Jedna kosztuje 3.50.  Nie wiem, ile one kosztowały przed pandemią, ale podejrzewam, że grosze. Więcej niż 50gr podejrzewam, że nie kosztowała. Więc przebitka jest ogromna w tym momencie. Maseczki takie bawełniane są 18-20zł. Ja mam taką maseczkę jeszcze z Nepalu, bo tak ją nosiłam na pył. Dostaliśmy 3 maseczki od policji jak nas raz kontrolowali. I kupiłyśmy wczoraj. Ale jak się ich dotyka, jak się zdejmuje. Ja np. byłam z babcią w sklepie - i moja mama zna język migowy - ale ponieważ moja babcia czyta z ruchu warg i bardzo dobrze mówi, to nie uczyła nas języka migowego. Babcia nas rozumie, my nie jesteśmy biegli w języku migowym. A jak mam maseczkę to ona nie może mi czytać z ruchu warg. Więc ja będą z sklepie i chcąc ją o coś zapytać, to musiałam co chwila zdejmować. Więc to jest uciążliwe. I babcia nie może sama pójść do sklepu czy apteki, bo nie dogada się z nikim, kto ma zasłonięte usta.

**To jest realne zabezpieczenie Twoim zdaniem?**

Nie mam zielonego pojęcia. I nie wiem, jakiego specjalisty posłuchać, żeby to było prawdziwe. Mój tata znalazł jakieś wykresy, nie wiem, czy tam był podział na kraje, czy na co - że ta epidemia się wolniej rozwija w miejscach, czy krajach w których się używało maseczek. A w tych co się nie używało, to szybciej. Nie wiem.

Jestem w stanie w to uwierzyć, że skoro to jest przenoszone drogą kropelkową i tym oddychaniem, to może to ograniczyć wdychanie tych drobinek albo wypluwanie ich do powietrza. Na pewno nie całkowicie. Z tego co lekarze mówią, to najlepsze są te całkowicie przylegające, że do całego obszaru przylegają i mają filtr. Bo te chirurgiczne, one mają na celu jak lekarz mówi podczas operacji, żeby nie napluł w  ranę. Albo żeby jemu do ust i nosa nie wleciało. Ale  w przypadku tego wirusa ponoć te dziury sprawiają, że mimo wszystko mogą się wydostać te drobinki. Trochę to pomaga, ale nie jest całkowicie zabezpieczające.

**Czy to ma w takim razie inną funkcję, może uspokajać ludzi?**

Może część tak, ale mnie to nie uspokaja.

**A może Twoim zdaniem wpędzać w panikę, albo w złość?**

Może co poniektórych w złość tak, ale myślę, że  to swój wygląd jakiegoś rodzaju. Chociażby Ci ludzie, którzy tych ratowników medycznych, czy lekarzy chcą przesiedlać. Albo ludzie krzywo patrzą na ratownika medycznego, który wraca do domu. No jest to jakiegoś rodzaju bariera, to robi poczucie, bariery i tego, że musimy się odseparowywać od innych. Albo tego, że trzeba się bać innych ludzi.

Nie wiem, jak teraz będzie w sytuacjach, kiedy się kichnie. Ja już miałam parę razy miałam taką sytuację, że szłam i chciało mi się kichnąć, albo kichałam w tą maseczkę - bo weszłam do miejsca chłodniejszego niż byłam, albo jakiś zapach zakręcił mi w nosie. Od przyjazdu w ogóle nie czułam się chora, nie bolało mnie gardło, nic mi nie jest. Więc teraz pytanie, jak ludzie będą reagowali na to, że kto kicha albo kaszlnie?

**Czyli te maseczki to może być taki element, który ludzi jakoś do siebie źle nastawia?**

Nie same maseczki. Ale całokształt tego. Niektórych też dezinformacja - jak to się niby przenosi. Na pewno pokazuje powagę sytuacji, i myślę, że jest jakimś takim tworzeniem bariery między ludźmi. Ale też np. - i to jest śmieszne - widzę ludzi, którzy ściągają tą maseczkę z nosa. Ja wiem, że to jest łatwiej czasami mieć ją tylko na ustach niż na nosie, bo kiedy się ma i tu i tu to robi się bardzo ciepło od tego wdychania powietrza. Ja mam też taki komin, który mogę zaciągać na noc i usta do biegania w zimie. Wtedy wdychanie zimnego powietrza bywa bolesne [tu o bieganiu]. I czasami jak jest cieplej, to mam go tylko na ustach, a nie tez na nosie. Bo robi się gorąco. I tak samo widzę ludzi, którzy ściągają maseczkę z nosa i mają tylko na ustach. A to całkowicie wypacza sens noszenia maseczki. Albo co chwila widzę ludzi, którzy jak są dalej od skupiska, nie są w sklepie, to mają tą maseczkę na uszach, ale ściągają ją na brodę całkowicie. To jest ciągłe dotykanie tej maseczki wtedy. I często to jest takie "noszę bo trzeba" albo "daje mi to poczucie, że coś robię" , ale nie zawsze to spełnia swoje zadanie.

Takie pozorne poczucie bezpieczeństwa.

**A inne ograniczenia - które z nich Twoim zdaniem są potrzebne i dają realne zabezpieczenie?**

Myślę, że realnym jest brak zgromadzeń i zamknięte szkoły. Bo ludzie przestali się przemieszczać i między sobą tak nadmiernie roznosić. Nie mam poczucia, że to wyeliminuje wirusa. Jeżeli ktokolwiek mówi, że to zakończy pandemię i będzie koniec, to nie. Bo jak tylko wrócimy to może być następna fala.

Nie wiem, jak jest w innych krajach, ale u nas tych testów też się mało robi. To że my wciąż mamy mniej niż 10 tysięcy zakażeń to jest dziwne w porównaniu do krajów dookoła, które mają ich dużo więcej. Jakim cudem u Niemców jest już tyle zakażeń, a u nas nie ma jeszcze 10 tysięcy? Więc to pewnie kwestia zakłamanych danych, przy małej ilości testów robionych. I tego, że ci którzy mają bezobjawowe przechodzenie tego, to w ogóle nie są badani.

**W tym ograniczeniu zgromadzeń, czy dla Ciebie też to wchodzi to że jest mniej osób w sklepach?**

Nie. Ja myślę, że sam fakt tego, że ludzie chodzą do sklepu, to że oni są w tym sklepie nie wszyscy na raz, tylko później, to nie wiem, czy to coś zmienia. Może trochę. Aczkolwiek to czy siak wciąż różni ludzie wchodzą do tego sklepu i mogą coś tam zostawić i wyjść. A ktoś kto wejdzie po nich może wdychać to powietrze, dotknąć czegoś. Na logikę myśląc, to nie wiem, czy to działa 100%. Może trochę pomaga, ale nie jestem pewna.

Znaczy to jest strasznie dużo gdybania, bo nigdy nie było takiej sytuacji. Oni wciąż nie do końca wiedzą, jak ten wirus rzeczywiście się przenosi. Z tego co słuchałam od wirusologów, to w ogóle nie wiadomo z tymi wirusami na jakie odległości i jak długo one się mogą utrzymywać. I teorie były że 5 dni na plastiku, a na papierze 2 godziny. Wszystko brzmi wciąż jak takie pytania bardziej niż odpowiedzi. Jeżeli on się utrzymuje na powierzchniach, to co za różnica czy ludzie wejdą do sklepu wszyscy na raz, czy po kolei. Jak i tak ta jedna osoba będzie miała kontakt z tą półką w sklepie.

**To po co Twoich zdaniem to ograniczenie ludzi w sklepie?**

Myślę, że wszystko co rząd robi, czy to jest uzasadnione czy nieuzasadnione naukowo, czy to ma znaczenie czy nie, to rząd robi pewne rzeczy, żeby nikt nie zarzucił, że "nic nie robiliśmy". Ale myślę, że my się nigdy nie dowiemy na ile tamto czy inne ograniczenie miało sens.

**A jak oceniasz w ogóle to decyzje z przywróceniem wstępów do lasów i parków? I przywróceniem możliwości przemieszczania się w celach rekreacyjnych?**

Myślę, że to dobrze bo inaczej byśmy mieli więcej ludzi z samobójstwami i w psychiatrykach niż tych zabitych przez pandemię. Zastanawiam się, czy to jest kwestia tych ograniczeń i tego, że rząd szybko zadziałał, że mamy tylko 380 zgonów. Bo to nie jest dużo. Oczywiście, że lepiej byłoby jakby nikt nie umarł, ale ... Na zwykłą grypę umarło pewnie w zeszłym roku więcej ludzi niż teraz w tych statystykach. Pytanie, na ile te statystyki są prawidłowe. Bo we Włoszech nagle były super wysokie, gdzie mówiono potem ze to jest kwestia bardzo starego społeczeństwa i tego, że  w te statystyki wpisywali wszystkie zgony, a nie tylko te od wirusa.

Myślę, że dobrze. Ja mam to szczęście, że mogę wyjść do ogrodu, ale ludziom w blokach to nie zazdroszczę. I sama sytuacja tego, że niektórzy potracili prace, nie mogą znaleźć nowej jest pewnie problematyczna. Więc sam fakt tego, że nie można wyjść bez mandatu na dwór, to jest  w ogóle myślę dobijające. Dlatego dobrze, bo to ludziom na pewno pomoże przetrwać.

Wprowadzili to po to, żeby się ludzie nie gromadzili. Te matki z dziećmi. Ale tak na dobrą sprawę, to nie są w stanie tego przypilnować do końca. Bo policja nie ma takiej siły przerobowej, żeby wszystkie miejsca, gdzie ktoś może się spotkać w grupie większej niż 2 osoby, żeby to wszystko pilnować. Te lasy i parki były dla mnie trochę bez sensu.

**A kwestie tego, że teraz osoby powyżej 13 roku życia mogą same się przemieszczać. To jest Twoim zdaniem dobra decyzja?**

To też nie wiem dlaczego zabronili nastolatkom się samym przemieszczać. Może zakładając, że nastolatkowie są głupsi niż dorośli-  a co nie jest wcale dobrym założeniem - i będą się bardziej gromadzić niż dorośli. Ma się też takich dorosłych, którzy będą mieli przepisy w dupie i będą się gromadzili tak żeby ich nie było widać. Tak samo nastolatkowie, którzy się nudzą. W tym momencie myślę, że wielu dorosłych jest w takim stanie psychicznym, że zachowują się tak samo jak nastolatkowie, bo mają już dosyć sytuacji.

nie wiem, jaki sens miało ograniczanie tym nastolatkom wychodzenia samym.

**A msze święte?**

Przy tym, że zwiększyli też te limity w sklepach to ok. Skoro w sklepach zwiększają to w kościele też mogą. Ale sam fakt tego, że w ogóle były dopuszczone msze przy tych 5 osobach, to było dla mnie śmieszne. Bo po pierwsze co to jest 5 osób? A po drugie, nie jest to niezbędne do przeżycia, żeby do tego kościoła chodzić. Znaczy pewnie niektórzy by się spierali, ale umówmy się, że z logicznego punktu widzenia, to nie jest to niezbędne. A mam przyjaciela zakonnika, którego pracą w niedzielę było odsyłanie ludzie sprzed kościoła do domu.

Jest to przerażające, jak niektóre przyzwyczajenia, takie kultowe, i takie rytualne są ważniejsze niż logiczne bezpieczeństwo. Czy przepisy. Jeżeli nie można chodzić do szkoły - no czym się różni kościół od szkoły? Tym bardziej, że w tych kościołach właśnie są ci starsi ludzie. Głównie ci starsi się strasznie bulwersowali na brak możliwości wejścia do kościoła. Albo mi ten przyjaciel opowiadał o kobiecie, z którą dyskutował na temat tego, że ona nie mogła przeżyć, że ona nie może przyjąć komunii od księdza.

**A Ty generalnie przestrzegasz ograniczeń, które są narzucane?**

Poza tym, że przyjechał do mnie przyjaciel, więc pewnie złamaliśmy jakieś tam zalecane, żeby się nie spotykać. Ale przyjechał samochodem. Mieszka sam. Więc jest mało narażony na cokolwiek.

**A dlaczego zdecydowaliście się, żeby się spotkać?**

Bo i jemu się rzuca na głowę siedzenie samemu i dla mnie to też było urozmaicenie, że w końcu można się było spotkać z kimś. Ja pracowałam jako masażystka, z zawodu jestem. Mam stół do masażu. On nie raz przyjeżdżał do mnie na masaże jak mieszkałam u rodziców. Powiedział, że od siedzenia przed komputerem już nie może z bólu pleców, więc przyjechał na masaż. A tego się nie da zrobić zdalnie. Pogadać mogliśmy przez telefon.

Raz, że to jest podnoszące na duchu. No nie jest to powrót do normalności, ale jakkolwiek jest to czymś innym. Widziałam kogoś innego niż rodzinę.

**Czy to była taka namiastka normalności?**

Dla mnie ważniejsze byłoby gdybym ja się mogła gdzieś przemieścić. Bo dla mnie zmiana otoczenia jest ważna. To że on przyjechał, to wciąż nie dało poczucia zmiany. Bardziej urozmaicenie niż namiastka normalności.

**A kwestia tego chodzenia do lasu. Wcześniej chodziłaś też jak było zakazane. Dlaczego się zdecydowałaś tam chodzić?**

Nie wiem, czy to było zakazane. Bo nasz las chyba nie jest lasem państwowym. Pewnie to wymyślili, bo to są miejsca gdzie można się zgromadzić i nie być zauważonym. Nie widzę innego wytłumaczenia. Ale w naszym lesie nikt się nie gromadzi. I ja też nie idę tam w celu gromadzenia się tylko wybiegać psa. A nie mogę go puścić przy ulicy, bo mógłby wbiec pod samochód lub skakać na ludzi. Nie mam poczucia, że na tej łące za lasem z psem robiłam coś niebezpiecznego. Bo nie idę tam  w 10 osób się gromadzić i chuchać sobie nawzajem w nosy, tylko wyprowadzić psa.

**A pozostałych ograniczeń przestrzegasz?**

Maseczki. Rękawiczki w sklepach tak.

**A dlaczego przestrzegasz tych ograniczeń?**

Dla świętego spokoju z jednej strony, bo uważam, że rękawiczki są w ogóle zbędne. Z maseczką niech im będzie, że coś to daje. I też założenie maseczki na czas wejścia do sklepu nie jest dla mnie tak uciążliwe. Rękawiczki są. Jak pierwszy raz poszłam do sklepu, to nie zauważyłam, że te rękawiczki są. Wiem, że personel się upomni jak mniejszy sklep. Jak nie personel, to ktoś inny upomni. Więc dla świętego spokoju.

**A kwestie bezpieczeństwa do Ciebie przemawiają?**

Rękawiczki nie. Bo to jest bezsensowne. I tak dotykamy ich z zewnątrz, żeby je założyć. Mam teoretycznie nie przenieść tego co dotknęłam w sklepie na siebie, czy do domu? I tak wkładam tyle opakowań z tego sklepu do torby, które przywożę do domu i rozpakowuje zakupy rękami bez rękawiczek. Że jakby to była kwestia przenoszenia na powierzchniach [wirusa] to musiałabym rozpakować wszystkie rzeczy w rękawiczkach w domu, zdezynfekować samochód, którym jechałam w tych rękawiczkach, torbę zakupową i umyć każde z opakowań, które przywiozłam. Jakby iść takim tokiem myślenia, to trzeba by wszystko dezynfekować. Skoro tego nie robimy, to co mi te rękawiczki zdjęte po wyjściu ze sklepu dają.

**A kwestia innych ograniczeń? Niespotykanie się w większych gronach. Zostawanie w domu. Tego przestrzegasz. Z czego to wynika?**

Z wyjściem z domu to jest kwestia, że wolałabym nie dostać mandatu. Od nadgorliwego policjanta, bo ja nie wiem, jak oni sprawdzają, czy ktoś mówi prawdę z tym, gdzie idzie.

No i nie mamy za bardzo gdzie jechać. Mogłabym odwiedzić jedną czy drugą przyjaciółkę, ale to kwestia taka, że się zastanawiam jak na drodze by to wyglądało. Teraz czekam jak otworzą te galerie i muzea, to teoretycznie można do nich chodzić. I nie ma już obostrzeń wychodzenia tylko do pracy, apteki  i sklepu. Więc żeby się z kimś zobaczyć to poczekam do tego. Oni z resztę pracują, więc ja bym mogła tylko po południu czy wieczorem pojechać, a taki wyjazd na krótko do miasta tylko po to żeby się z kimś zobaczyć...

**A czym dla Ciebie się różni kwarantanna od izolacji? Czy to się w ogóle różni?**

Zależy jaką to ma definicję. Kwarantanna obowiązkowa to oznaczała dla mnie niewychodzenie z domu. Izolowanie się jest tym samym teoretycznie, z tym że teraz mogę wyjść na spacer z psem, mogę wyjść poza obręb działki i już policja mnie nie sprawdza. To jest różnica. Mogę pójść do sklepu. Ale innej różnicy nie ma - dla mnie to to samo.

Wyjście do sklepu daje to, że widzę ludzi innych niż moja rodzina. To jest taka namiastka normalności. Świat istnieje. Ale to wciąż nie jest to samo co normalne życie społeczne, czyli nie spotykanie się w większej grupie, niepójście na imprezę gdzie chcę poznać nowych ludzi, spotkanie się ze znajomymi. Chodzenie do sklepu samej nie różni się  zbyt wiele od tego, że siedzę zamknięta w domu i nie widzę się ze znajomymi. Bo to nam społecznie poprawia humor, nastrój. I nas stymuluje. To są spotkania z ludźmi dla nas ważnymi, a nie z panią w sklepie.

**Wspomniałaś, że czekasz na etap 3 luzowania ograniczeń. Co w ogóle wiesz na ten temat?**

Ustawiłam sobie stronę z tym opisem nawet, żeby móc to sprawdzać. A tutaj.. [ Szuka]. Ta rządowa, gov.pl - z tego smsa co przysłali w niedzielę. Wiem to co jest tutaj napisane [tu czyta].

**A dlaczego czekasz na ten etap 3?**

Na 2. Otwarcie bibliotek, muzeów i galerii sztuki. [potem czyta 3]. O bardzo bym chciała zrobić sobie paznokcie, więc etap trzeci to bym chciała. Ale pytanie jak ciężko będzie się zapisać, bo tyle osób na raz będzie chciało się zapisać na raz do fryzjera i na paznokcie, że to będą zapisy na 6 miesięcy w przód.

**Jak oceniasz w ogóle to, że rząd w tym momencie planuje już łagodzenie restrykcji?**

Ja to oceniam jako to, że widzą, że gospodarczo jest źle. I oglądałam program amerykański w którym bardzo jadą po Trumpie i Trump cytował jakiegoś dziennikarza  stronniczych mediów, w których oni gadają, że "the cure shouldn't be worse than the problem" - coś takiego, że rozwiązanie nie może być gorsze niż sam problem. Czyli zamknięcie ludzi, co skutkuje zapaścią gospodarki będzie za chwilę gorszym problemem, niosącym więcej złego i więcej problemów niż sama pandemia. Tego to się dowiemy po wszystkim. I ciężko będzie powiedzieć, co było gorszym rozwiązaniem. Czy większą śmiertelność przyniosłoby życie normalnie kiedy jest pandemia, czy będziemy w totalnej dupie jak cała gospodarka teraz mocno zwolniła i będzie spory kryzys.

**Co ci daje to, że sobie sprawdzasz te etapy (zapisałaś stronę)?**

Wiem, że to są rzetelne informacje odnośnie tego, co można. Bo to jest rządowa strona. A tu nie chodzi o badania i naukowców, tylko kwestia co pozwalają. A tu będą wszystkie informacje, które trzeba. Brakuje mi tam jeszcze informacji, kiedy będzie można wrócić do biur i nie pracować zdalnie. A tego tam nie ma. Bo to mnie interesuje pod tym kątem, że mam tą jedną rozmowę o pracę - że powiedzieli, że następny etap i ewentualne rozpoczęcie pracy, to jak będzie się można w biurze spotkać.

**A które z tych ograniczeń, które teraz są, powinny obowiązywać dłużej a które krócej?**

Nie wiem. Myślę, że to co zadziałałoby to machnięcie czarodziejska różdżką i zamrożenie ludzi w miejscu. Ale wiadomo, że to nierealne. W tym momencie pytanie, jak duże będą koszta kryzysu gospodarczego, i czy nie będzie to gorsze dla nas wszystkich niż sama pandemia. Widać, że rząd wprowadza etapy, bo musi odmrozić gospodarkę. Bo będzie bardzo źle, jak tego nie zrobią za chwilę. Myślę, że sporo to też takie działanie PRowe. Potem nikt nie zarzuci, że nic nie robili. Bo zamknęli ludzi w domach, bez możliwości wyjścia na spacer w pewnym momencie. A pytanie ile z tego działa? Czy to było przemyślane, że cokolwiek da - to inne pytanie. Robią to bo trzeba - jest coraz gorzej, nie można trzymać kraju zamkniętego kolejny miesiąc, bo każdy dzień tego zamknięcia, zamknięcia biznesów to wydłużanie tego procesu wychodzenia z kryzysu.

Nie wiem, co powinno być dłużej, a co krócej. Dla mnie jak się zniesie część np. dzieci wrócą do szkół, to już rozwala cały system. Bo system zamknięcia działa, jak wszystko jest zamknięte i prawie nikt się nie przemieszcza, tylko ci co muszą. Ale wciąż jest mnóstwo ludzi, którzy do pracy jadą, do sklepu idą, do apteki. Teraz innych rozrywek nie ma, więc sporo ludzi chodzi do sklepów, bo nic innego nie mogą robić.

**Co w takim razie powinno być granicą, kiedy te ograniczenia powinny przestać obowiązywać? Czy to wynalezienie szczepionki? Spadek zachorowań? Czy coś innego? A może kryzys gospodarczy?**

A myślę, że będzie to teraz coraz bardziej podupadająca, zamrożona gospodarka. Najlepiej by było, gdyby to była szczepionka. Albo lek. Albo wynalezienie sposobu jakiegoś. Słyszałam, że u nas mają testować leczenie ludzi zarażonych osoczem ludzi, którzy wyzdrowieli. Najlepiej byłoby jakiś sposób. Bo wtedy można będzie leczyć. Największy sens w tym zamknięciu, to ja widzę w tym, że to spłaszczanie tego wykresu zachorowań. Chodziło o to, żeby nie obciążać szpitali tak bardzo. Nie wiem, czy one są tak strasznie obciążone w kwestii ilości chorych bo mamy zaledwie niecałe 10 tys. chorych rozłożonych równomiernie po kraju, bo w całym kraju są w zależności od wielkości województwa. 10 tys. na prawi 39 milionów ludzi w kraju to nie jest dużo. Więc nie pewnie problemem jest bardziej to, że te szpitale są zamykane, ludzie muszą być odseparowywani, lekarze muszą być w kwarantannach w tych szpitalach, w których był ktoś zarażony. Bo każdy kto miał kontakt, jest potencjalnym roznosicielem. Więc to bardziej kwestia zamykania tych szpitali z tego powodu niż to że jest za dużo ludzi. 380 zgonów to też nie jest dużo.

Szczepionka to nie wiem, bo ja mam takie podejście.. Szczególnie do tych szczepionek na grypę, czy tego typu rzeczy - na szczepionkę jest trochę za późno jak już mamy pandemię. Bo szczepionka musi być ileś czasu przed wdrożona. Ile ludzi mówi, że ktoś kto jest zaszczepiony powinien siedzieć miesiąc, dwa w domu bo to jest moment w którym jego odporność jest osłabiona tą szczepionką. Szczepionka będąca lekiem na pandemię jest dla mnie odwróconym myśleniem. Bo szczepimy się jako dzieci, żeby na choroby nie zapaść w przyszłości. Z wyprzedzeniem. A nie kiedy populacja choruje. Więc bardziej widziałabym coś co leczy.

Ale czekanie roku, to za dużo dla gospodarki.

To jest myślenie w kwestii teorii spiskowych, albo podważania statystyk, ale ja bym się nie zdziwiła gdyby czy rząd czy odpowiednie media, czy kto tam tym manipuluje czy może manipulować, nie zrobiono tak, żeby tak te statystyki przedstawić, że rząd w dobrym momencie zaczął ograniczać i rozwiązywać to wszystko. Ponieważ ja nie znam nikogo kto umarł, nie jestem w stanie stwierdzić jak z tą umieralnością jest - czy te 380 to jest zawyżone, zaniżone czy o co chodzi? Nie widzę jakiś wielkich połaci worków foliowych leżących na zewnątrz szpitali tak jak to we Włoszech było. Więc nie mam jak zweryfikować prawdziwości jakichkolwiek statystyk.

Wystarczy zmanipulować statystyki tak, żeby wyglądało, że rząd robi wszystko w odpowiednim czasie, żebyśmy wszyscy nie zostali bez pracy, itd, itd.

**A jak już te miejsca zostaną otwarte - kina, restauracje, szkoły - czy one powinny funkcjonować w jakiś inny sposób, czy tak samo jak wcześniej?**

Na tych kolejnych etapach jest powiedziane, że z nowymi zasadami sanitarnymi, czyli dezynfekowanie wszystkiego, inne godziny otwarcia, wymuszone przerwy w których będzie musiał wejść personel, który zdezynfekuje fotele w kinie i wszystkie rzeczy, których się dotyka.

**Uważasz, że to jest ok?**

[myśli] Wolę, żeby było tak i żeby były miejsca otwarte i gospodarka się odmroziła niż żebyśmy mieli siedzieć w domu

Czy to działa nie wiem i nigdy się nie dowiem. Nie jestem specjalistą. I myślę, że specjaliści sami nie są w stanie tego stwierdzić bez badań. Badaniami będziemy my testujący sytuację, czy wrócenie do normalnego życia ale spryskując wszystko dezynfektantem, który jest chemiczna substancją, więc też nie jest super najlepszy dla naszego zdrowia i naszej odporności. I noszenie maseczek wszędzie. Czujemy się pewnie bezpieczniej, niektórzy są uspokojeni bardziej, rząd może powiedzieć, że coś jest zarządzone. A czy to będzie powstrzymywało ludzi od chorowania i umierania, to się przekonamy sami.

**Szwecja.**

Nie czytałam. Nie sprawdzałam. Oni chyba w ogóle sobie nic z tego nie robią. Nie mają obostrzeń.

**[opis]**

Nie lubię się wypowiadać, jak nie mam w czymś wiedzy. Mogę mówić, co mi się wydaje. Dowiemy się, kto miał większy sens w działaniach jak ileś czasu minie. ZA dwa lata będą statystyki pokazujące te wszystkie zgony, ileś fal, nawrotów zakażeń.

Z drugiej strony jak można podchodzić do statystyk zakażeń, czy ilości zarażonych kiedy nie robiliśmy odpowiedniej ilości testów co w innych krajach. A jak wyglądają ich statystyki?  Mi się tylko obiło o uszy, że mają te zalecenia i nie wychodzi im na dobre, bo mają dużo zachorowań i umieralność mają.

**Nie mam dokładnych statystyk. Ale jeszcze jeden pan mi mówił, że zachorowań jest sporo i zgonów jest sporo.**

Jeżeli mają więcej zgonów niż my, a Szwecja to jest mniejsza populacja, to oznacza, że gorzej im idzie.  To co jest proste do zmanipulowania i pokazania ludziom "o patrzcie, jak im źle idzie" i pokazanie tylko liczby zarażonych i zgonów u nas i u nich. Tylko że trzeba przyjąć to w kwestii procentowej. Trzeba mieć na względzie ilość ludzi w kraju i powierzchnię kraju. Bo jeśli u nas zagęszczenie ludności jest większe na jakimś terenie, to tez ma wpływ, że to się szybciej roznosi. Jeżeli w Szwecji mają ludzi mniej i mają mniejszą powierzchnię kraju, ale oni tam pewnie na północy mają bardzo małe zagęszczenie ludności, a na południu mają większe, to na pewno na południu zachorowalność jest inna niż na północy. I też trzeba patrzeć inaczej.

Z tego co słyszałam, to ktoś się wypowiadał, że te zalecenia były, a Szwedzi są takim narodem, który bardziej się słucha takich zaleceń. Nie wiem, czy tak jest bo nie znam wielu Szwedów, może tak. Ale myślę, że jakiś odsetek się słucha, bardziej niż Polacy by się słuchali. Brytyjczycy mieli na początku podobne podejście, dużo później wprowadzili ograniczenia, ale to dlatego że wszyscy mieli w nosie te zalecenia. W Londynie w ogóle nie było widać żadnych zmian na początku jak u nas zaczęło się dział. I Johnson tylko zalecał, a nie zakazywał. I ludzie siedzieli w restauracjach, pubach i zachowywali się normalnie. Jeździli metrem. Wszędzie były wciąż tłumy, jakby nic się nie zmieniało. Poza sklepami, w których kupowali jak najęci.

Niezależnie od mentalności w Szwecji, to pewnie wciąż mnóstwo ludzi wychodziło, jeśli można było wyjść. Ja teraz nie wychodzę, bo się boję mandatu. Ale gdybym nie miała takiego strachu, że policja może mi dać mandat za to, że nie idę do pracy tylko albo do sklepu, to myślę, że już bym się spotykała ze znajomymi i bym wyjeżdżała z domu. Spotykałabym się z 1-2 osobami naraz, to nie byłoby wciąż duże zgromadzenie w którym jest to bardziej groźne do rozniesienia.

Nie było nigdy takiej sytuacji. Jest bezprecedensowa. Nawet w przypadku hiszpanki, która się rozniosła - czytałam, że miała kilka fal  na wszystkich kontynentach prawie, to w tamtych czasach nie podróżowało się tak swobodnie jak teraz. Wiadomo, ze w XXI wieku to na inną skalę może funkcjonować i się roznosić, bo na inną skalę teraz żyjemy.

**Dbanie o siebie. Jak wygląda u Ciebie?**

Nie wyglądam. Jestem bardzo zła na siebie i zdegustowana brakiem motywacji do wszelkiego ruchu. Tak jak normalnie bardziej mi się nie chce niż chce, to jednak chodziłam na siłownię,   miałam multisporta. Z bieganiem mam o tyle problem - a to była taka moja rzecz, którą zawsze robiłam, że mam teraz problem z kolanami. I jest to dodatkowa wymówka, która mówi "ech, bolą cię kolana, nie ćwicz". Mam naprawdę na kiepskim poziomie motywację wszelaką.

**Z czego to wynika?**

Brak jakiejkolwiek rutyny. Tego, że ja cokolwiek robię. To co dodaje energii, to jest jakiś plan, coś robię - że jakoś tam się funkcjonuje, a danym momencie kiedy jest marazm, że ja siedzę i ani nie wiem, co będzie, ani nie wiem, co mam zrobić, ani nie wiem, jak dalej będę mogła planować, to jest to bardzo demotywujące do czegokolwiek. Bo mi się ciężko zmusić do czytania książek. Do czegokolwiek pożytecznego.

**Czyli ten ruch powinien być częścią rutyny. [...]**

A dodatkowo zajadanie nudy i stresu. Ja tak mam, że jak się nudzę, to zajadam słodyczami. Więc siedzę i zajadam słodyczami rzeczywiście. Nie ma dnia, żebym nie zjadła czegoś słodkiego. I to jest bardzo ciężkie do przerwania u mnie czasami. Jak jestem zajęta to jest mi łatwiej czasami nie jeść, albo jeść mniej. A teraz mi okropnie trudno pilnować

**A jakieś rytuały pielęgnacyjne?**

Nie częściej niż normalnie. Zawsze pielęgnacja włosów to jest dużo czasu, ale to się nie zmieniło. Ale teraz na przykład ich nie suszę, bo mogą się suszyć same. Nie obchodzi mnie, że się pokręciły, albo spuszyły bo i tak siedzę w domu.

**Mniejszą wagę przywiązujesz do fryzury?**

Nie mam potrzeby ich suszyć. Nie muszą być wyprostowane, tak jak zazwyczaj na szczotce je suszę. Bo wszystko mi jedno.

Byłam w paczkomacie właśnie bo odebrałam sobie rozjaśniacz, tylko zastanawiam się, czy to nie będzie za mocne. Bo ja mam takie odrost zimowy. Ja nigdy nie farbowałam, więc nie wiem, czy zrobię. Trochę się boję

**A co Cię skłoniło, żeby kupić?**

Już od jakiegoś czasu myślałam o rozjaśnieniu włosów, ale chciałam naturalnie. Więc nie wiem. To jednak chemia.  No i jak to będzie wyglądać. I jak będą odrastać

**A skoro nie wychodzisz, to dobry moment, żeby to sprawdzić?**

Tak. Pod tym kątem o tym myślę, że nawet jak nie wyjdzie tak jak chciałam, to albo będę mogła wymyśleć jakieś ratowanie tego. Może zrobię tak, że nałożę na super krótki czas, niż jak w instrukcji piszą. I zobaczę. Nie było to drogie, więc zawsze można kupić następny.

**A jak wygląda Twoje ubieranie się?**

Chodzę w dresowych bluzach. Dodatkowo nasz pies gubił włosy tak czy siak, a teraz zaczął linieć na wiosnę, więc kłaki są wszędzie. Wszystko z czym chodzę, to jak on się przytula, to jestem w jego kłaczkach. Więc ubieram się z ciuchy, których nie szkoda. Tak na maksa wygodnie.

**Makijaż?**

Nie w ogóle. Znaczy tylko wychodząc do pracy, choć on zawsze był ograniczony do minimum - jak bym mogła to bym podkładu nie nakładała. Ale to kwestia wyrównania kolorytu, więc nakłada podkład  z korektorem, tusz do rzęs i w minutę jestem w stanie zrobić sobie brwi cieniem. Eyeliner nakładam od wielkiego wyjścia.

**A dlaczego teraz bez makijażu?**

BO nigdzie nie chodzę. Jeżeli teraz nie wychodzę, nie widzę się ze znajomymi, to nie mam takiej potrzeby. Będąc tu u rodziców nawet jak wychodzę do sklepu, to też się nie maluję. Nie widzę nikogo znajomego, więc to nie jest powód do malowania. Jak miałam rozmowy o pracę, takie video, to się pomalowałam, żeby wyglądać lepiej. Taka kwestia pierwszego wrażenia.

Wyjście do pracy, czy jak się widzę z ludźmi, to się lubię czuć lepiej, bo wiem, że wyglądam lepiej w makijażu.

**A nowe czynności pielęgnacyjne?**

Wkręciłam się w hydrolaty. Od powrotu z Indii siedziałam sporo i mam kolekcję. I mam sklep gdzie kupuję olejki eteryczne. Hydrolat to jest produkt uboczny produkcji olejków [tu jak to się robi]. To jakby woda kwiatowa albo ziołowa. Mam to w butelce ze sprayem i mogę używać jako toniku na twarz i włosy. Używałam teraz z krwawnika i  czystka - śmierdzą jak cholera, ale nie muszę teraz ładnie pachnieć. I jakość polepszył mi się stan włosów.

Teraz zamówiłam cynamonowy do włosów. Będzie ładnie pachnieć, a ma dobre działanie na wzrost włosów. A ponieważ chciałam je rozjaśnić, to stwierdziłam, że dobrze żeby były wzmocnione.

To jedyne w co się wkręciłam i pozamawiałam ich strasznie dużo.

Poza tym nic nowego.

**To kupujesz przez Internet?**

Tak

**A coś jeszcze z kosmetyków kupiłaś ostatnio?**

Poza rozjaśniaczem, to jeszcze płyn do soczewek.

**Na paznokcie byś się chciała zapisać, jak to się skończy...**

No teraz właśnie je obcięłam bo były dłuższe, ale długie bez hybrydy to się zaczynają łamać. A że miał przyjechać mój przyjaciel i mama jęczała o masaż, to długimi paznokciami niewygodnie. Teraz mam mega krótkie, więc musiałabym poczekać aż odrosną.

Marzy mi się od jakiego czasu, żeby je mieć zrobione.

**U fryzjera też?**

Fryzjera miałam zaplanowanego na ostatni piątek, bo w sobotę miał być ślub mojej przyjaciółki. Więc miałam bilet z Londynu na piątek, wczoraj miałam lecieć z powrotem do Londynu wedle planu. Loty są oczywiście odwołane przy czym LOT nie zwraca kasy tylko dostałam maila, że do końca roku mogę sobie zabukować inny bilet  na tej trasie. Tylko nie wiem po cholerę mi teraz bilet do Londynu i z powrotem jak w najbliższym czasie nie mam tam po co lecieć.

I miałam tą wizytę na botoks, takie odżywienie włosów. Ale że fryzjerzy są zamknięci, to wizyty nie było. Za to oni uruchomili bony u tego fryzjera - że można kupić teraz, żeby oni kasę dostali a bon wykorzystać później do końca roku, przy przez rok. Normalnie ten zabieg 5 stów, a z bonem 3 stówy. Więc stwierdziłam, że skoro i tak chciałam to zrobić i sprawdzić jak to działa, to tak oszczędzę. Więc kupiłam ten bon.

**A jeśli chodzi o fryzjera to nie czujesz takiej konieczności, że już powinnaś iść?**

Nie. Mi najlepiej rosną włosy jak podcinam końcówki w miarę regularnie i chciałam to robić co 2 miesiące. Podcinałam przed wyjazdem - w połowę lutego. Więc połowa kwietnia to miał być botoks z podcięciem końcówek. Ale w dobrym stanie są, więc - znaczy nie mam wyjścia - przedłużę że co 3 miesiące. Mam nadziejże, że za miesiąc będę mogła iść, chociażby na podcięcie końcówek. Ale nie jest tragicznie. Fryzjer nie jest mi niezbędny. Bardziej tęsknię za paznokciami.

**A jak się z tym czujesz, że na co dzień chodzisz ubrana tak, żeby bardziej było wygodnie, że się nie malujesz, nie suszysz włosów? To ci doskwiera, że nie ma tych różnych rzeczy związanych z dbaniem o wygląd, które były wcześniej?**

To jest akurat wygodne i przyjemne dość.

**To rodzaj ulgi, że nie trzeba?**

Nie, nie ma tak że ulgi. Wiadomo, że jak trzeba umyć włosy czy coś , to zabiera czas. Szczególnie, jak czasu jest mniej, a teraz mam mnóstwo czasu więc nie muszę się niczym przejmować. To co jest fajne to to, że na np. nie lubię myc włosów zbyt często. Generalnie myję maksymalnie co dwa dni. A jeden z tych hydrolatów służy do tego, żeby się mniej przetłuszczały i rzeczywiście działa. A teraz czasami 3 dni, albo i dłużej trzymam. Bo nie wychodzę, i nie muszą być super świeże. Jak wychodzę, to lubię mieć świeże.

A teraz im rzadziej je myję, tym skóra głowy jest w lepszym stanie. To że teraz tak robię, to wydłuży czas w którym one będą się przetłuszczały, więc nie będę musiała wracać do mycia co dwa dni, bo się zdążą przyzwyczaić.

**A kwestia zakupów ubraniowych? Wcześniej kupiłaś sukienki...**

Zrobiłam jeszcze dwa zakupy. Kupiłam dwie spódnice w H&M i buty. Takie bardziej sportowe,  ale też że do sukienki będę mogła założyć.

**A czemu teraz te zakupy?**

To dobre pytanie. Trochę dlatego, że cały czas mam duży zapas pieniędzy, a u mnie to zawsze jest tak, że jak mam duży zapas pieniędzy to daje poczucie bezpieczeństwa, że "teraz możesz wydać". A ja mam tak po mamie, że pieniądze zawsze przyjdą. Tata się śmieje, że nie ważne jaką kwotę da, to mama zawsze ją zagospodaruje i wyda. Więc jakiś taki luz w podchodzeniu do wydawania pieniędzy mam po mamie i ograniczam się tylko kiedy mam bardzo limitowaną ilość pieniędzy. A mój budżet w tym momencie nie jest taki zapchany. Jestem w domu, nie płacę za jedzenie, tylko jak sobie idę raz na jakiś czas kupić coś dla siebie. Ale to nie są normalne zakupy jedzeniowe. Nigdzie nie jeżdżę, więc to nie ma biletów, benzyny. Więc to jedyne rzeczy, na które teraz wydaję. Więc sobie trochę pozwalam. Plus jest to poprawienie humoru. Chociaż to jest drugorzędne, to nie jest sam cel.

Spódnice były mi potrzebne. Buty może nie były mi potrzebne, ale lubię buty, więc na buty zawsze się znajdzie miejsce w szafie.

**Ale potrzebne po epidemii?**

No teraz ich nie noszę. Bo bym się bała, że mi pies je właśnie poszarpie.

**Z perspektywy konsumenta, czego Ci najbardziej brakuje?**

Brakuje mi wyjść na imprezy. Nie to, że w klubie, tylko jakiekolwiek możliwości miejsc, w których można poznać nowych ludzi. I wyjść do znajomych.

Kino jest do przeżycia, że go nie ma. Lubię czasami iść, ale nie doskwiera mi jego brak.

Galerie, muzea też nie, bo się nie chodzi tak często.

To że mogłabym pojechać do miasta. Np. szampon, którego używam mogę kupić tylko u trychologa, do którego chodziłam. Oni nie mają strony, nie mają wysyłki. Nie wiem, czy działają nawet. Musiałabym pojechać do Warszawy po ten szampon. I chyba najbardziej to te paznokcie mi brakuje. Ale to jest moje widzi-misie, to nie jest niezbędna rzecz. Bo jak są długie, to się łamią bez hybrydy. A jak mam krótkie to mi się skórki robią. Więc przydałoby się, żeby ktoś mi te skórki doprowadził do porządku, bo są okropne i suche.

Mam też suche ręce od tego mycia i tych płynów, ale to staram się kremem nawilżać.

Ja się po prostu lepiej czuję jak patrzę na swoje ładne paznokcie. Bardziej niż makijaż na twarzy, bo na nie patrzę sama.
