# Supplementary material for: The challenges arising from the COVID-19 pandemic and the way people deal with them. A qualitative longitudinal study
Source: PLoS One. 2021 Oct 11;16(10):e0258133. doi: 10.1371/journal.pone.0258133 (PMC8504766; doi:10.1371/journal.pone.0258133)
Supplement: S1 Dataset — (ZIP) [file pone.0258133.s003.zip › Transcriptions/stage 3/13.3_M_46_couple, with children.docx]

**13.3_M_46_couple with children**

**Jak wyglądały pana ostatnie 2 tygodnie?**

Bardzo dobrze, świetnie, ponieważ pracuję w takim systemie co drugi dzień, więc co 2-gi dzień miałem czas, żeby go poświęcić na pracę wokół domu, a to mi sprawia dużo przyjemności.

**A Wielkanoc?**

Wielkanoc to wyzwanie, zwłaszcza dla rodziców, bo zostali sami w domu - i teściowa, i rodzice, w związku z czym myśmy się łączyli przez internet. Nie widzieliśmy się bezpośrednio, nie było żadnych rodzinnych spotkań. Niektórym się łza w oku zakręciła i większość osób stwierdziło, że to najgorsze święta do tej pory w życiu.

**Pan miał podobne przemyślenia?**

Myślę, że to nie były normalne święta.

**To połączenie przez internet trochę rekompensowało?**

Tak, ale słabo.

**Jakieś tradycje świąteczne, ozdoby, o których wcześniej pan wspominał?**

Oczywiście były, ale to są powierzchowności. Dla mnie święta to bardziej spotkanie z bliskimi, a nie ozdoby. Tej esencji świąt brakowało.

**Coś jeszcze się zmieniło w ostatnich 2 tyg.?**

Wciągnąłem żonę w świat wirtualnej jazdy na rowerze. To uważam za ciekawy trend. Coś, co z pozoru myślałem, że mi się nie uda, to mi się udało. Jej się spodobało, sama ma chęć i teraz jeździ. Ja jeździłem trochę wcześniej, a teraz jeździmy wspólnie. Wcześniej jeździliśmy na zewnątrz.

**Pana zachowanie w jakiś sposób się zmieniło?**

Nie. Myślę, że nie, ale to by musiał odpowiedzieć ktoś z zewnątrz. Ja zmian nie widzę.

**Jakieś nowe rzeczy się pojawiły, nowe hobby np.?**

Mam dość hobby, żeby niczego sobie nie szukać. Takie jest moje podejście do życia. Nie mam ochoty na szukanie sobie nowych rzeczy. Zajmowanie ogrodem się nasiliło i są fajne efekty, ponieważ jest ładna pogoda i można tego czasu więcej przeznaczyć. Widać pracę i to cieszy oko i głowę. Jak się robi większe rzeczy, to sprawia to większą przyjemność.

**A zmieniło się coś w pracy, w codzienności zawodowej?**

Postępuje arogancja niestety i głupota nie wywietrzała. W pracy się pojawiają komunikaty skierowane do nie wiadomo kogo, podpisane przez dyrekcję. Dokumenty, które mają nazwę, która w ogóle nie powinna się znaleźć w dokumentach szpitalnych kierowanych do oddziałów, typu: obwieszczenie albo informacja. Nie wiadomo, do kogo to jest skierowane i tam są jakieś dziwne rzeczy pisane. Takie dokumenty śmieci. To psuje ogólnie nastrój i niestety nie zwiększa w nas chęci do pracy. Arogancja władz szpitala postępuje.

**Jeśli chodzi o codzienność, to jest coś, jakieś czynności, z których pan ostatnio zrezygnował?**

Może hobby. Pojechałbym już pewnie z synem na ryby, gdyby nie sytuacja. Oczywiście jakieś tam wyjazdy weekendowe w góry, w Bieszczady. To jest nie do zrobienia w tym momencie, ale poza tym chyba nic. Oczywiście moja praca, bo normalnie chodziłbym jeszcze do swojego gabinetu prywatnego, ale to już od początku epidemii.

**Odczuwa pan mocno brak wyjazdów weekendowych?**

Staram się wypełniać ten czas właśnie pracą wokół domu, ale zważywszy na ładną pogodę i dobre warunki, to mi trochę brakuje, bo wiem, że pewnie byłoby pięknie w Bieszczadach w tej chwili i chętnie bym pojechał, a nie mogę. Staram się racjonalnie i pragmatycznie podchodzić do życia, ale wiadomo, że gdzieś jakaś tęsknota jest. Chodzenie po górach to jest jedna z aktywności, którą bardzo lubię i staram się ją uskuteczniać.

**Co jest dla pana największym wyzwaniem obecnie? Ostatnio mówił pan, że ludzie pytają pana o epidemię. Coś tu się zmieniło, pojawiają się nowe wyzwania?**

Nie. Jest taki sam poziom trudności, skomplikowania życia, jak był. Tu się nic nie zmieniło i w moich osobistych odczuciach nic się nie pozmieniało.

**A nowe rzeczy, które przeszkadzają?**

Dla mnie ostatnie zalecenia kompletnie sprzeczne. Najpierw się ludziom nakazuje nosić maseczki, później się otwiera parki, lasy. To mi przeszkadza, bo to uważam za kompletny idiotyzm. Noszenie maseczek w sytuacjach, kiedy wokół mnie nie ma ludzi albo są daleko to są żarty. I jeszcze rodzaj tych maseczek...Nie chodzi o faktyczne zabezpieczenie tylko chodzi o jakiś rytuał, czy nie wiem, jak to nazwać, bo z medycznego punktu widzenia to jest bez sensu.

**Czy podejmuje pan jakieś działania, żeby radzić sobie z codzienną sytuacją, poza tymi, o których mówiliśmy?**

Nie, to wystarcza. To, co chciałbym mieć dla siebie, to niestety cały mój plan storpedowany przez epidemię, więc nic z tego. Chciałem jeszcze trochę pochodzić na lekcje gitary i miałem na to środę wolną. Zredukowałem sobie ilość dni w szpitalu do 4 i miał być 1 dzień w tygodniu taki dla mnie. I teraz go nie mam, bo jest epidemia. Miałem mieć tę jedną środę, a teraz pracuję co 2-gi dzień, więc co 2 tygodnie mam inny tryb pracy. Albo pon., środa, piątek, albo wtorek, czwartek. To mi troszkę plany rozbiło, ale...Poczekamy. Fajne są kursy online, zresztą zapisany jestem na jednej ze stron i tam się można doskonalić. Są bardzo fajne, ale w mojej opinii to nie zastąpi, przynajmniej dla początkujących, kontaktu z nauczycielami. Dużo widać i oczywiście można jakoś mechanicznie powtarzając pewne rzeczy się uczyć, ale łatwo wtedy też o błędy i przynajmniej ten początkowy etap nauki jest fajniejszy bezpośrednio z nauczycielem. Online tak - doszkalanie, żeby się nauczyć jakichś utworów, zagrywek, improwizacji, ale mnie to nie zastąpi. Mnie o te lekcje chodziło, bo ja też mam czasami różne pytania, a tak przez komputer...Ja korzystałem z takich lekcji ogólnodostępnych i nie jest to spotkanie live z nauczycielem. Może są takie, ale nie wiem, jak to w przypadku instrumentów wychodzi. To nie zastąpi kontaktu personalnego, bo w trakcie rozmowy wychodzą różne fajne rzeczy i sama nauka gry na instrumencie to nie wszystko, bo dla mnie ważne są też różne inne rzeczy, które w trakcie tych lekcji gdzieś wychodzą. Miałem bardzo fajnego nauczyciela, który opowiadał mi też o historii muzyki i różne ciekawostki wywlekał. To dodatkowa wartość, ogromna zresztą i stawiałbym ją na równi z samą nauką gry. Ta gra na gitarze to takie kolejne hobby, od liceum jeszcze.

**To jakoś pana odstresowuje?**

Ostatnio nie siadam do gitary od paru tygodni, więc w tej chwili nie, ale tak generalnie, to jak najbardziej. To jest bardzo fajny sposób, żeby troszkę oderwać się od codzienności.

**Nie siadał pan ostatnio do gitary. Coś konkretnego za tym stoi?**

Nie, myślę, że nie. Są takie okresy, kiedy chętniej sięgam po gitarę, a czasami ona zostaje zupełnie nie ruszana.

**Emocje - zdjęcia**

1 i 4, a 7 gdzieś troszkę dalej.

**1 i 4 pojawiały się u pana już wcześniej?**

Cały czas. 1 to jest tkwienie w miejscu, brak możliwości postępu. Nie posuwamy się naprzód i to jest sytuacja epidemii w naszym kraju i na świecie. I to, co dotyczy mnie w pracy. Dokładnie to samo. A 4 - ludzie, którzy mnie otaczają to jest takie dla mnie zaufane grono, z którym wiem, że mogę robić różne fajne rzeczy, pomagają sobie wszyscy i tyle. To mi się nie zmienia.

**To zaufane grono to pana bliscy?**

Tak, ale też i koledzy z pracy. Część z nich to tacy prawie, że przyjaciele. To jest chęć pomocy, wsparcie wzajemne, siła.

**To daje poczucie siły?**

Tak. Kontroli nad sytuacją nie, ale wysiłek ludzi pozwala na to, żeby było trochę lepiej i wzajemna pomoc pozwala jakoś łatwiej znosić to, co jest.

**To daje też trochę nadzieję?**

Może tak. Bez nadziei można by zakończyć to funkcjonowanie.

**Jakie emocje budzi 1? To, że stoimy w miejscu, że nie ma postępu?**

Na pewno gniew. Chyba głównie gniew. To jest zdecydowanie negatywne. 4 jest pozytywne, 1 negatywne.

**Robi pan coś, poza tym, o czym mówiliśmy, żeby radzić sobie z gniewem z powodu sytuacji w pracy?**

Nie. Hobby pomaga też w tym.

**Jak jeszcze by pan opisał to, jak się pan czuje? Czy np. odczuwa pan irytację, smutek?**

Tak. Irytację, sfrustrowanie sytuacją w szpitalu i w kraju. Chyba tyle. Jestem zadowolony bardzo tak prywatnie, niezależnie od wszystkiego, bo wczoraj sobie porozmawiałem z kolegą, z którym się nie słyszałem od 6-7 lat. To kiedyś był mój bliski przyjaciel, potem nam się drogi rozeszły, wczoraj mi się udało z nim porozmawiać i było jak przed laty. Bardzo mnie to dobrze nastroiło do życia.

Był jakiś konkretny powód rozmowy z tym kolegą?

Nie wiem. On się odezwał po wielu latach braku kontaktu z jego strony kompletnie, poza tym, że wysyłał jakieś życzenia świąteczne. Myślę, że automatyczne do wielu ludzi równocześnie, nie odpowiadał na jakieś moje próby kontaktu, ale ostatnio się parę dni temu odezwał przez FB. Zaproponowałem rozmowę, chętnie się zgodził i zadzwonił.

**To ciekawe, bo często spotykam się z tym, że ludzie zaczynają odbudowywać kontakty, które gdzieś były zapomniane?**

Ma pani na myśli czas epidemii? Uważam, że inna przyczyna była, ale może taki trend jest. Jak ludzie zwolnili troszkę tempo codziennego życia, to przychodzi troszkę różnego rodzaju przemyśleń, co uważam akurat za korzystne następstwo tego stanu, który mamy. Ludzie troszkę wolniej działają i bardziej zastanawiają się nad tym, co ich otacza, nad kontaktami z ludźmi. Może zaczynają bardziej doceniać to, czego kiedyś nie zauważali, czyli kontakty z bliskimi, możliwość wyjścia na spacer. To kiedyś było oczywiste i w ogóle niezauważane, ale zamiast pójść na spacer ludzie gnali od sklepu do sklepu i zwiedzali Galerie zamiast sobie pójść nad rzekę. Może zwalniając ludzie zaczynają bardziej zdawać sobie sprawę z tego, co nas otacza i może zwartości pewnych rzeczy, sytuacji, stanów, a nie poświęcają czas na gonitwę za nowym.

**Myśli pan, że to będzie trwała zmiana?**

Nie. jak tylko otworzą GH, to tłum znowu tam ruszy. Zadowolony, że wreszcie może zrobić zakupy. Myślę, że u części osób może zostaną te przemyślenia i refleksje i troszkę sobie życie pozmieniają i przewartościują niektóre pozycje, ale u większości osób wróci to, co było.

**Czuje pan niepokój, niepewność co będzie?**

Tak, trochę tak. Myślę, że to bardziej wynika z sytuacji w kraju niż z sytuacji zawodowej. Boję się, że coraz większa arogancja władzy doprowadzi do tego, że ten kraj rzeczywiście legnie w gruzach, co jest chyba niechybnym następstwem idiotycznych działań, które w tej chwili są podejmowane. Ciężko będzie się z tego wygrzebać i znowu, pomimo osiągnięcia pewnego poziomu rozwoju gospodarki, jakiejś kultury politycznej, znowu zejdziemy do poziomu troglodytów. Kurczę, będziemy to odbudowywać latami. Już były okresy w historii, które niszczyły dorobek państwa i teraz mam wrażenie, że to się powtarza. Z powodu ambicji kilku małych, tak naprawdę ludzi...Oni są może inteligentni, przebiegli, ale z racji tego, że myślą głównie o własnym interesie i swoich najbliższych, niszczą dorobek kilkudziesięciu lat. I to mnie martwi, bo się niszczy demokrację. Coś, co ciężko jest zbudować w naszym kraju. Kraj jest stary, tradycję mamy bardzo długą i z demokracją bywało pod górkę. Teraz wydawało mi się, że te ostatnie 20 lat zmierza w kierunku stabilizacji i normalizacji sytuacji w kraju i też na arenie międzynarodowej też jakiegoś w miarę przyzwoitego poziomu życia, że złotówka będzie w miarę stabilna, a tu się znów zaczyna robić huśtawka. Męczy mnie to trochę, ponieważ wiadomo, że jesteśmy zależni od całego świata i nie ma co udawać, że jesteśmy narodem wybranym. To trzeba wieszczom zostawić. Troszkę realizmu. Obecna władza niszczy poczucie stabilizacji i normalizacji w kraju i też na arenie międzynarodowej. Zostawią nas boso, ale w ostrogach.

**Czy czuje się pan też w jakiś sposób bezsilny?**

Oczywiście, że tak. Przecież ta władza pozmieniała konstytucję, przejęli sądownictwo, mają wpływ na różne aspekty naszego życia i tak naprawdę nawet nie można się pójść poskarżyć do sądu na to, że ktoś coś złego zrobił, bo jak się okazuje, że to jest kolega wójta, starosty, wojewody, to można od razu zapomnieć, że będziemy mieli jakikolwiek wpływ na przebieg sprawy. nawet, jeśli to jest najgorsza kanalia i drań, to w logiczny dla mediów sposób to zostanie wytłumaczone. Właściwie nie ma się do kogo odwołać i nie ma jak sobie poradzić z takimi rzeczami. Mam przykład w szpitalu i jest to nie do pomyślenia.

**Obserwując to ma pan też takie poczucie odrealnienia?**

Nie, to są 2 różne światy. My jesteśmy specjalistami i znamy zasady funkcjonowania naszego oddziału, naszej specjalności, wiemy co powinno być, jak powinno być. Wiemy, że w ramach tego finansowania, które szpital przeznacza na działanie np. naszego oddziału jesteśmy w stanie to zrobić na bardzo wysokim poziomie. Decyzje dyrekcji szpitala powodują, że ten poziom jest co najwyżej średni. My nadrabiamy naszym oddaniem, sercem, zaangażowaniem w pracę. Mówię my, jako zespół, bo nie tylko lekarze, ale tak samo pielęgniarki, bo w ogromnym stopniu właśnie one to robią, chociaż mogłyby powiedzieć, żeby dyrekcja się pocałowała w nos, bo za te pieniądze, które im daje i za te możliwości, i za traktowanie ich w sposób przedmiotowy, to nie powinni się spodziewać nic więcej niż bardzo, bardzo niska średnia krajowa. A jednak ci ludzie dają z siebie wszystko i to są 2 światy. Idiotyczne decyzje i propozycje dyrekcji - rzeczy, które dobrze funkcjonują dyrekcja zmienia, bo ma właśnie taki pomysł, żeby sobie porządzić i nieważne, jakie będą konsekwencje, a my próbujemy łatać dziury spowodowane tymi decyzjami. Nie jest to normalne, nie jest to poważne, nie ma się komu poskarżyć. A na samym szczycie tego jest przecież dobro pacjenta, o którym się mówi. Logo szpitala - wielkie litery przy wejściu mówi, żebyśmy pomagali ludziom z radosnym entuzjazmem. Nie wiem, co tam jeszcze Dyrektor wymyślił, natomiast jej działanie nie ma nic wspólnego z dobrem pacjenta. To jest indywidualna chęć pokazania się, niekoniecznie w dobrym świetle. Nie rozumiem tego. To są zupełnie nienormalne rzeczy dla mnie i to powoduje, że myślę, że jesteśmy w światach równoległych - władze szpitala i pracownicy. Ja pracuję w tym szpitalu 20 lat, dyrektorów było już przynajmniej 10-ciu. Każdy robi bałagan, zostawia po sobie zgliszcza. Przychodzi następny, udaje, że naprawia, a robi jeszcze gorzej i my się zastanawiamy, czy może być coś jeszcze gorsze, chociaż doświadczenie nam pokazuje, że każdy następny jest jeszcze gorszy. Już w tej chwili poziom jest tak niski, tak żenujący u nowo przychodzących dyrektorów...Poprzedni dyrektor był chyba gangsterem, bo razem ze swoimi doradcami działał jak jakaś rodzina mafijna. Teraz pani dyrektor kompletnie nie wie, z czym ma do czynienia i podejmuje takie decyzje, że rozwala szpital. I tyle. Nie mam nic do powiedzenia więcej, bo się zaczynam denerwować.

**A jak jest z pana poczuciem zagrożenia sytuacją związaną z koronawirusem w tej chwili?**

Tak, jak do tej pory. Z rzeczy nowych, to pojawiło mi się wrażenie, że ludzie, w związku z dość długim okresem trwania różnego rodzaju obostrzeń, mają troszkę przytępioną uwagę i wydaje im się, że nie ma takiego zagrożenia jak było 2-3 tyg. temu. Trochę chyba uśpili czujność, mimo tego, że trzeba nosić maseczki. Coraz więcej ludzi jest na ulicy. Wczoraj wieczorem jechałem ulicą, która biegnie wzdłuż deptaka nad rzeką i było z 10 x więcej ludzi niż tydzień temu. Gdzieś zaczyna się ludziom zmieniać w głowach podejście, stają się mniej ostrożni i ta uwaga już nie jest tak napięta jak kiedyś. Myślę, że część ludzi uważa, że jak do tej pory nic się nie działo, to już pewnie nić się nie będzie działo więcej i mają wrażenie, że chyba jest mniejsze zagrożenie. Ja tak nie uważam i myślę, że zagrożenie jest takie samo cały czas. My akurat mieszkamy w takim regionie, gdzie tych zachorowań może nie jest tak dużo, a właściwie nie ma tak dużo stwierdzonych zachorowań czy nosicielstwa. Robi się mało testów i ludzie sobie trochę nie zdają sprawy. Dodatkowo jeszcze otwarcie lasów, parków od wczoraj, pogrzeby do 50 osób. Wydaje mi się, że chyba można też do kościoła iść w grupie 50 osób. Wydaje mi się, że ludzie mają wrażenie większego bezpieczeństwa, jeżeli się takie decyzje podejmuje. Ludzie zaczynają wychodzić z domów na jakieś zajęcia rekreacyjne w parku czy w lesie, a nie wolno było tego robić i w ich głowach będzie myśl, że chyba jest lepiej, skoro nam pozwolili. Dla mnie zagrożenie się nie zmieniło, ale mam wrażenie i boję się, że ludzie to traktują inaczej.

**To, że ludzie są mniej ostrożni budzi pana obawy, że może to pogorszyć sytuację?**

Tak...Pozorny wzrost bezpieczeństwa, wrażenie poprawy sytuacji spowoduje, że wzrośnie liczba zakażeń, bo ludzie chętniej wyjdą, chętniej się spotkają z innymi. Cały czas się pojawia pytanie, czy można już pójść do rodziców/ nie można? A może rodzice do nas? A dziecko się nie widziało z babcią/ dziadkiem. Takie różne pytania - cały czas to jest, że może już można, bo my zdrowi, oni zdrowi, itd. Temat jest nie do określenia jednoznacznie - czy można, czy nie można, kiedy można. To są cały czas za trudne pytania.

**Czy poza tym coś jeszcze budzi pana obawy?**

To, co do tej pory, czyli obawiam się rozpadu państwa i dramatycznej sytuacji ekonomicznej w kraju. To zostaje cały czas. Jak w filmie Hitchcocka - zaczęło się trzęsieniem ziemi i zobaczymy, jak się skończy, ale na pewno nie jest to optymistyczny wariant. Jakikolwiek wariant zakończenia. Nie ma optymistycznego. Państwo się zajmuje innymi rzeczami, zamiast zatroszczyć się o obywatela. Ja to mówiłem wielokrotnie i to mi się nie zmieniło. Cały czas jest wałkowanie tematu wyborów, różne dziwne ruchy władz. Ludzie są gdzieś tam na szarym końcu. Ci, którzy troszczyli się o siebie i dawali państwu wytchnienie i nie brali od państwa pieniędzy, tylko płacili podatki, utrzymywali innych płacąc składki na ZUS, teraz dostaną po głowie za to, że byli prywatnymi przedsiębiorcami, małymi przedsiębiorcami. Nikt im w tej chwili nie pomoże, jak sami sobie nie pomogą. Niektórzy mają takie branże, że mogą się w miarę dobrze przystosować po to, żeby nie umrzeć z głodu i nie rozsypać się całkowicie, jako firmy. Jakieś tam próbują podjąć działania, ale większość nie ma takiej możliwości i oni dostaną bardzo mocno w kość. To mnie martwi i to się nie zmieni.

**Widzi pan podobne emocje, zmartwienia wśród pana bliskich?**

Tak. Myślę, że jest tak samo. Ci, z którymi spotykam się na co dzień w pracy mają dokładnie takie samo podejście do tematu. W rodzinie częściej się pojawiają pytania, że może już nie trzeba takiej izolacji, może już zacząć spotkania. To nie jest większe poczucie bezpieczeństwa, ale właśnie takie zmniejszenie czujności.

**A emocje, nastroje w pana otoczeniu? Widzi pan jakieś zmiany?**

Teraz władza pozwoliła wychodzić do parków, więc ludzie trochę odetchnęli, bo narastała frustracja tym, że trzeba siedzieć w domu i nie można nigdzie wyjść i się poruszać. Teraz trochę emocje negatywne się zmniejszą - wściekłość i jakaś tam... Głównie wściekłość, jak obserwowałem wśród bliskich.

**Widzi pan jakieś nowe sposoby radzenia sobie z sytuacją w pana otoczeniu?**

Nie. Oczywiście pojawiły się, ale to już dawno, spotkania internetowe i my też z tego korzystamy. Kamera i kilka osób się widzi jednocześnie na ekranie. To chyba całkiem skuteczny sposób, żeby się zobaczyć z ludźmi, z którymi może wcześniej byśmy tego nie robili tak często. Teraz zmieniło się podejście i częściej się widujemy w ten sposób. To pozwala podtrzymać relacje, ale wcześniej przecież też były takie możliwości i jakoś z tego nie korzystaliśmy. To jest konsekwencja troszkę wolniejszego trybu życia.

Zmieniają się kwestie ograniczeń. O czym pan słyszał? Jak to wygląda teraz?

Prawdę mówiąc nie mam jasności, co do możliwości opuszczania domu, bo kiedyś przepis był dość jasno sformułowany - zakaz opuszczania domów, poza trzema sytuacjami, a w tej chwili nagle...Skoro możemy wychodzić do parku na rekreację, to...Nie wiem, nie przeglądałem jeszcze strony MZ, bo tam dość często sprawdzam, jak się zmieniają te zalecenia. Zaskoczyły mnie wczoraj informacje, że może być 50 osób na pogrzebie nie wliczając w to obsługi ceremonii, czyli księdza, ministrantów i grabarzy. Dziwne. Koszmarna sytuacja z udziałem pana Kaczyńskiego i jego pochlebców z wizytą na cmentarzu, ze składaniem kwiatów, itd. To było pokazanie obywatelom naszego kraju, że my jesteśmy władza, a wy macie nas słuchać i tyle. Obostrzenia są, ale nie dla wszystkich. Ostatnio była propozycja ustawy mającej jakoś uregulować badania personelu medycznego i to nie przeszło. Głosował przeciwko tej ustawie człowiek, który niedawno był chory, członek rządu...I nie ma problemu...Obostrzenia są w tej chwili na usługach władzy. Nie podoba mi się to, co się ostatnio stało, czyli nakaz noszenia maseczek przez wszystkich, a wczoraj otwarcie parków, lasów. Uważam, że to jest działanie niespójne. Są to rzeczy, które się wykluczają - albo zaostrzamy rygor, bo tym dla mnie jest nakaz noszenia maseczek przez wszystkich, albo go troszkę rozluźniamy, puszczając ludzi do parków. Jedno z drugim jakoś nie idzie w parze.

**Jak to jest z tymi maseczkami? Jak się pan na to zapatruje?**

Po co nosimy maseczki? My wszyscy wiemy, że maseczki są dla osób chorych, zakażonych. Są miejsca, gdzie tych infekcji jest rzeczywiście bardzo dużo i miejsca, gdzie jest ich mało. ja uważam, że noszenie maseczki jest to tylko i wyłącznie działanie na psychikę człowieka - tego typu nakaż, że wszyscy noszą i prawie w każdym miejscu. Z punktu widzenia medycznego to jest bezsensowne. Wiadomo, że te maseczki to jest wolna twórczość, każdy robi co chce. Ludzie się prześcigają - niektórzy mają prawie pancerne, a niektórzy mają szmatkę. Przepis mówi, że trzeba zasłaniać twarz, więc jeśli ktoś to zrobi przy pomocy fragmentu odzieży, jak golf czy szalik...Przecież to bzdury są, kompletne bzdury. jakie to ma znaczenie z punktu widzenia medycznego? To nic dobrego nie robi. Uważam, że jest to czysta manipulacja społeczeństwem a nie chęć ograniczenia epidemii.

**Jak to działa psychologicznie na społeczeństwo?**

Uspokaja. Jak zaczęła się epidemia, to zaczął się ruch na rynku maseczek, rękawiczek i środków odkażających. Już brakowało wszystkiego. W tej chwili środki odkażające stoją na półkach na stacjach benzynowych i nie ma problemu, żeby kupić duże opakowanie 5l. fakt, że ta cena jest kilkukrotnie wyższa niż przed epidemią, ale one są dostępne. Ludzie przedtem zapisywali się nawet na zakup takich środków, w tej chili to jest. Rękawiczki też można kupić, maseczki też. Te maseczki dają ludziom takie złudne poczucie bezpieczeństwa, że jak ubiorę maseczkę, to pewnie się nie zakażę. Mogę chodzić, mogę być bliżej ludzi, a nic mi się nie stanie, bo przecież mam maseczkę. Moje odczucie jest takie, że tak myśli większość ludzi, którzy noszą maseczki, a z drugiej strony manipulacja społeczeństwem, bo nakażemy nosić maseczki, oni się poczują bezpieczni i nikt nam nie zarzuci, że byliśmy opieszali, nic nie zrobiliśmy. No przecież wydaliśmy nakaz, wszyscy mają nosić maseczki. A właściwie nie nosić maseczki, tylko zasłaniać twarz. To jest istotna różnica, bo zupełnie inną sytuacją byłoby, gdyby nasz rząd zobowiązał nas do noszenia maseczek konkretnego typu, zapewnił te maski, nawet, żeby ludzie je kupili, ale byłby obowiązek noszenia określonej maski, która rzeczywiście zmniejsza ryzyko infekcji. Zasłanianie twarzy golfem nie zmniejsza ryzyka i tu się nie ma co łudzić, a mnóstwo ludzi tak chodzi.

**Które z tych ograniczeń mają realny wpływ na ograniczenie epidemii?**

Ograniczenie kontaktów.

**Ograniczenie liczby osób w sklepie?**

Oczywiście. Mniejsza liczba osób, określone godziny dla seniorów, jednak to pozostanie w domach i to ma rzeczywiście sens.

**A kwestia możliwości przemieszczania się rekreacyjnego?**

Trzeba by było się zastanowić, bo dla mnie to też nie jest sprawa jednoznaczna. Wyjście do lasu to dla różnych ludzi jest zupełnie inny czynnik ryzyka infekcji, bo jeżeli ktoś mieszka w 10 piętrowym bliku i zejdzie po schodach z 8 piętra, wsiądzie do samochodu i pojedzie do lasu, to będzie zupełnie czymś innym niż wyjście do lasu dla osoby, która mieszka na wsi, wyjdzie ze swojego domu i po drodze do lasu nie spotka nikogo, albo się minie z innymi osobami w odległości 10 m. W dużym mieście przechodzi się przez klatkę schodową, przez osiedle, spotyka się z ludźmi na parkingu...Myślę, że jest to nie do określenia, czy jest to dobry pomysł, czy niedobry pomysł. Są ludzie, dla których aktywność fizyczna, spacery, bieganie jest częścią życia. Ja w zupełności to rozumiem, prawie jest to uzależnienie i ludzie, jak nie mogą tej aktywności uprawiać, to czują się źle. Jest to ważny element życia. Bardzo ważny, niezbędny dla niektórych i dla nich będzie bardzo istotne, że mogą do tego lasy wyjść. Jak to zrobią, czy to będzie bezpieczne, to jest całkiem inna sprawa.

**To powinno być uregulowane na poziomie decyzji państwa?**

Raczej tak i jednak raczej w kierunku ograniczenia. Jeżeli chcemy mówić o zmniejszeniu ryzyka infekcji, to po prostu izolacja i ograniczenie. Tutaj nie jesteśmy w stanie dopasować przepisu do różnych możliwości. Polacy są takim narodem, który szuka, jak ominąć prawo i jak to skutecznie zrobić. Świetnie nam się to udaje i każdy będzie sobie szukał, że to niekonstytucyjne, tamto bezprawne, inne rzeczy godzą w godność, burzą porządek myślenia i będą usprawiedliwiać swoje różne odstępstwa od zaleceń. Takiego przepisu, który będzie sprawiedliwy, mądry, dobry dla wszystkich nie da się stworzyć u nas.

**A decyzja o pozwoleniu na wychodzenie do parków, lasów też miała funkcję psychologiczną, żeby uspokoić społeczeństwo?**

Oczywiście. Dla mnie to jest etap przygotowywania społeczeństwa do nadchodzących wyborów, które zdaniem prezesa PiS muszą się odbyć i koniec. Jest na to mnóstwo memów i filmów w internecie, chociaż to taki śmiech przez łzy. To jest element gry politycznej - uspokojenie i powiedzenie, że przecież nie jest tak źle - wychodzicie do lasu, idziecie do parku, można pójść na pogrzeb, czyli sytuacja się stabilizuje. A ja w pracy dostaję wczoraj dokument, gdzie w 2 zdaniu jest napisane, że "w związku z pogarszającą się sytuacją epidemiologiczną..." Podpisane przez dyrektora naczelnego. Jeden wielki chaos i bałagan, brak porozumienia między różnymi formami i poziomami władzy. Nie ma spójnej linii, jednego kierunku działania.

**Teraz 1 osoba może być na 15 m2 w kościele. Co pan o tym myśli?**

Pan Bóg nie potrzebuje kościołów. Jak ktoś chce się pomodlić, to może to zrobić w domu, w ogródku, w szafie, w piwnicy, gdzie chce. Miejsca kultu są ważne, ale mamy sytuację taką, która myślę, że usprawiedliwia, że ludzie nie pójdą do kościoła, tylko pomodlą się w domu. Nie widzę potrzeby stwarzania zagrożeń. Większość chodzących do kościoła to są ludzie starsi, czyli bardziej podatni na powikłania i niekorzystny przebieg. Pomieszanie religii z instytucją w Polsce stworzyło bardzo złe warunki do czczenia Boga. To jest przykre, jak się na to patrzy i tłumaczenie sobie, że 1 osoba na 15 m...Ok, ale niech siądą wszyscy w jednej ławce...Uważam, że nie będziemy wpuszczać policji do kościołów, żeby rozganiali ludzi. Ludzie mogą zostać w domu. Nie chodzimy do kina, nie chodzimy do teatru, można oglądać nabożeństwa online, a Bogu to nie przeszkadza, w jakiej formie go czcimy.

**A kwestia tego, że osoby po 13 r.ż. mogą się same przemieszczać?**

Nie wiem, czy to jest dobrze. W pewnych sytuacjach tak, w większości nie. Dla mnie najważniejszym czynnikiem ograniczenia epidemii jest izolacja.

**Pan przestrzega ograniczeń?**

Tak. Myślę, że wszystkich. Chodzę jedynie do rodziców z zakupami i jest to kwestia podania zakupów przez próg. Przestrzegam, bo pracuję w szpitalu i to jest pierwsze najważniejsze takie moje...Myślę, że ma to merytoryczne uzasadnienie, bo mam większe ryzyko kontaktu z nosicielem wirusa i mogę to przenieść na inne osoby, a poza tym mam taką naturę, że jeżeli są jakieś nakazy prawa, przepisy, to staram się to respektować.

**Z racji zawodu czuje pan też odpowiedzialność za to, żeby dawać przykład innym?**

Tak. Nie zawsze się zgadzam z wszystkimi rzeczami, ale myślę, że nie ma przepisu idealnego i my czasami musimy wybrać mniejsze zło. Nawet, jeśli pewne rzeczy nie są całkowicie logiczne, to ogólne korzyści z poszanowania tego przepisu są większe niż te niedogodności. Nie z każdym można porozmawiać i tłumaczenie każdemu z osobna w jakich warunkach powinien nosić maseczkę, po co, itd. zostało załatwione jednym przepisem - zasłaniajmy usta, zasłaniajmy twarz. Ja się z tym może nie do końca zgadzam, ale szanuję to. Też mam swoje maseczki i ich używam, jak wychodzę.

**Słyszał pan o planach luzowania ograniczeń?**

Tak, ale jeszcze nie przeanalizowałem tego dokładnie. Jest to trochę dziwne, bo w Polsce nie mieliśmy jeszcze szczytu zachorowań i myślę, że znowu to służy uspokajaniu sytuacji przed wyborami. Pokażmy ludziom, że już mamy plan, że już będzie lepiej. Dla mnie to jest tandetne, ale większość społeczeństwa na to pójdzie. Zwłaszcza ci, którzy nie mają własnych firm i są na garnuszku. Im to nie przeszkadza - czekają na kolejny zasiłek, na jakieś tam pieniądze dane. To jest bardzo ważne dla niektórych ludzi w Polsce, że im się coś da. Jak ktoś daje to jest dobry. Poprzedni rząd nie dał i był niedobry. Ten jest dobry. I tyle. To przykre. Te plany wybudzania gospodarki, to chyba tak na wyrost na razie. Należałoby się zastanowić troszkę nad sensownością takich ruchów. Oczywiście trzeba pomyśleć o tym, że ludzie muszą powoli wracać do funkcjonowania, chociaż mam wrażenie, że zaraz będziemy mieli eksplozję, jak się zacznie luzowanie ograniczeń. Należałoby zacząć od tego, że się robi więcej testów, że się traktuje poważnie obywateli, a nie, że dochodzi do takich sytuacji, że testy wykonane przez inną firmę niż jedyną uznaną przez nasze państwo, nie są zaliczane do statystyki, chociaż wynik jest pozytywny. Jest firma w Warszawie, która robi testy lekarzom, pielęgniarkom, pracownikom UW też i te wyniki pozytywne nie są wliczane do statystyki zachorowań. Ręce opadają. Mówienie o wybudzaniu gospodarki, o poprawie sytuacji jest śmieszne. Dla mnie na luzowanie jest za wcześnie.

**Które z ograniczeń powinny zostać dłużej?**

Ograniczenie kontaktów i wszystko, co z tego wynika. Niestety zakaz przemieszczania się, turystyki, itd. Ograniczenie tego do niezbędnych tylko sytuacji.

**Kiedy te ograniczenia powinny zacząć być luzowane?**

Myślę, że taki widoczny, bardzo istotny spadek liczby zachorować przy utrzymaniu na stałym poziomie diagnostyki, czyli wykonywaniu testów. Kiedy zobaczymy, że nam spada liczba zachorowań, liczba zgonów, rośnie liczba osób, które zdrowieją, a my cały czas wykonujemy tyle samo testów na dobę. Musi to być rzetelnie i naukowo udowodnione, że liczba tych zachorowań spada, zastanowić się w jakich rejonach. Może zacząć od tego, żeby w obszarach, gdzie tych zachorowań jest znacznie mniej, gdzie się nie stwierdza nowych. Tam zacząć wybudzanie gospodarki, bo nie wszystkie zakłady mięsne muszą ruszyć na raz, nie wszystkie fabryki. Nie jest to moja działka, nie chcę się wymądrzać, ale takie rzeczy mi do głowy przyszły.

**Podobnie z restauracjami, kinami, itd.?**

Tak, jak będzie wyraźny spadek.

**A kwestia otwarcia szkół?**

Ten sam problem. Myślę, że nie powinny być otwarte do wakacji. Jest to ogromna uciążliwość dla osób, które mają dzieci, które wymagają wspólnej nauki. Nie każdy rodzić jest do tego przygotowany. Po to są nauczyciele, żeby uczyć. Rodzice pomagają, ale uczenie dziecka, prowadzenie lekcji na co dzień jest trudne i rodzice nie mają do tego przygotowania. Dzieci na tym cierpią. W szkole mają równe szanse, bo jest jeden nauczyciel, który reprezentuje sobą jakiś poziom, najczęściej wyższy niż mogą go zaproponować rodzice.

**Są jakieś ograniczenia, które powinny obowiązywać krócej, być już znoszone lub za moment?**

Myślę, że na razie nic. Jeżeli nie mamy jeszcze szczytu zachorowań, to powinniśmy siedzieć spokojnie w domu i tyle. Zagryźć zęby, trudno. Mamy balkony, mamy ogródki. takie warunki są i jeżeli to uszanujemy, to mamy szansę trochę zmniejszyć koszty tej epidemii. Jak nie uszanujemy, to będzie coraz więcej.

**Kiedy będzie już spadek zachorowań, zacznie się luzować ograniczenia, to restauracje, kina urzędy powinny funkcjonować tak samo, jak przed epidemią?**

Nie wiem jak do tego podejść. Myślę, że ktoś merytorycznie bardziej przygotowany do tego, epidemiolodzy...Pewnie się zastanowią nad tym. Te obszary, gdzie stwierdza się mniej zachorowań - gdzie jest spadek, albo w ogóle nie ma nowych...Może trzeba by się było zastanowić i uzgodnić jakiś okres, w czasie którego cały czas badamy społeczeństwo, albo badamy te osoby, które z różnych względów są bardziej narażone na infekcję. Przy stałej liczbie testów wykonywanej w społeczeństwie, w tych grupach ludzi - nie mamy nowych zachorowań przez okres 2-3 tyg. i na jakimś terenie dopuszczamy do działania restauracje, kina. Może znowu z ograniczeniem liczby osób przebywających. Może nie takie drastyczne, jak są w tej chwili w sklepach, ale trochę mniej osób. Nie potrafię odpowiedzieć, jak to miałoby wyglądać.

**Słyszał pan o rozwiązaniach stosowanych w Szwecji?**

Oni przyjęli taki model, żeby wytworzyć odporność stada, czyli eliminujemy słabsze jednostki, ludzie nabywają naturalnej odporności i co będzie, to będzie. Nie chciałbym, żeby moi rodzice zachorowali dlatego, że nie ma żadnych obostrzeń w kraju, chyba że mogliby uniknąć ciężkiego przebiegu choroby albo liczyć na to, jeżeli się rozchorują, że będą mogli być leczeni. Nie chciałbym odbierać im tych możliwości dlatego, że ktoś traktuje nas jak stado. To tak trochę z przekąsem, ale jest to oczywiście mądre i my w większości chorób w ten sposób funkcjonujemy, jako populacja ludzka. Nabieramy odporności w sposób naturalny. Przecież nie wszyscy się szczepią, mało kto się szczepi na grypę i jakby przebadać całe społeczeństwo, to by się okazało, że jakiś nieduży % tylko się szczepi, a nabieramy przecież odporności. Model szwedzki jest trochę taki, powiedziałbym, że arogancki w stosunku do osób, które mogą przebyć tę chorobę w taki ciężki sposób. Akurat zakażenie tym wirusem powoduje dość ciężki przebieg zapalenia płuc i statystycznie często są wymagane respiratory, żeby pomóc tym ludziom, niezależnie od tego, że dość dużo ich umiera. Jak będziemy mieli zmasowane zachorowania i tych ludzi będzie naraz bardzo dużo, to wiadomo, że system sobie z tym nie poradzi. Nie chodzi o to, żeby wprowadzić takie ograniczenia, żeby w ogóle uniknąć zakażeń i kontaktu z wirusem, bo jest to niemożliwe, bo i tak wszyscy w pewnym momencie to przechorują, tylko nie doprowadzać do sytuacji, kiedy będzie np. 10000 osób w ciężkim stanie, bo sobie z tym nie poradzimy systemowo. Nie mamy tyle sprzętu i nasz system zdrowotny nie jest przygotowany na tak masowe zachorowania o ciężkim przebiegu. Nie wiem, jak jest w Szwecji z opieką zdrowotną, czy ich stać na to, żeby tych wszystkich ludzi, którzy mają ciężki przebieg choroby hospitalizować i mają na tyle sprzętu. Patrzyłem wczoraj na statystyki i dużo zgonów jest w Szwecji, więc chyba sobie nie radzą z tym tak dobrze. Nie wiem jak się do tego obywatele Szwecji ustosunkowali - czy są bierni, czy może się zaczynają jakieś niepokoje. W Szwecji jest dużo uchodźców. Wiadomo, że element napływowy też inaczej funkcjonuje i na pewno nie stać ich na pewne rzeczy, na opiekę zdrowotną w pełnym wymiarze.

**Rozumiem, że ten model nie sprawdziłby się w Polsce?**

Myślę, że nie. Mielibyśmy duże problemy. Polacy też nie są narodem biernym, od razu by się zaczęły niepokoje. Mieliśmy podobną sytuację przecież w Anglii, gdzie początek epidemii był czasem beztroski dla Anglików i oni przyjęli właśnie taki model, że się nie przejmujemy i społeczeństwo samo nabierze odporności. No i nabrało przy tak gwałtowny wzroście liczby zachorowań, że się rząd nagle obudził z potrzebą zmiany jednak postępowania.

**Chciałabym porozmawiać, jak obecnie wygląda pana dbanie o siebie. Czy coś się zmieniło?**

Tak. Zmierzam w stronę bycia hippisem. Jeszcze dłużej jak się utrzymają zakazy funkcjonowania zakładów fryzjerskich, to będę miał coraz dłuższe włosy. Reszta rzeczy się nie zmieniła.

**A używanie kosmetyków pielęgnacyjnych?**

Nie, to jest stały poziom. Mam swoje ulubione kosmetyki i korzystam z nich niezależnie od tego czy jest epidemia, czy nie. Ponieważ chodzę do pracy regularnie, właściwie nic się nie zmienia. Mam możliwość kupienia kosmetyków czy w sklepie, czy przez internet. Tu problemu nie ma. Te podstawowe środki higieny osobistej i sprzęty są do kupienia w każdym sklepie i nie potrzebuję żadnych specjalnych sklepów. A resztę bardziej wykwintnych rzeczy można kupić przez internet.

**A kwestia dbania o zarost?**

Ran na jakiś czas się golę.

**A jeśli chodzi o ubieranie się do pracy, w domu?**

Nic się nie zmieniło w tej kwestii. W domu ubiór jest zdecydowanie bardziej swobodny, chociaż moja praca wymusza na mnie stosowanie ubioru roboczego do chodzenia po oddziale, na blok operacyjny przebieramy się w inne ubrania, więc mogę do pracy chodzić też ubrany luźno. Nie jest to problem.

**Ale rozumiem, że w domu nie chodzi pan np. w piżamie?**

Bardzo bym chciał, ale to mi się zdarza 2-3 razy w roku. Fantastyczne dni, ale tak na co dzień ubieram się luźno w taka odzież, którą lubię - żeby nie krępowała ruchów i żebym się dobrze w niej czuł.

**Kupił pan jakieś rzeczy w związku ze zmieniającą się porą roku?**

Nie. Zrobiłem duże zakupy jesienią. Byliśmy na wyjeździe urlopowym w lutym, więc też przygotowując się do wyjazdu...Mam trochę tej odzieży, którą zakupiłam na wyjazd i ona się przydaje na co dzień, przyda się teraz, jak będzie trochę cieplej. Nie lubię robić często zakupów i zwykle rzeczy wystarczają mi na trochę dłużej. Nie kupuję co sezon ubrań, bo jest nowy sezon. Nienawidzę tego. Kupowanie ubrań to konieczność a nie przyjemność.

**Brakuje panu chodzenia po sklepach?**

Nie. Ja idę do sklepu po coś. Nie chodzę po sklepach. Jak mi potrzeba spodni, to idę do sklepu ze spodniami po spodnie, nie do GH, żeby ją całą przejść. Po buty do sklepu z butami. W ten sam sposób robię zakupy dla syna. Jak mam coś kupić, to idę tylko do tych sklepów, gdzie mogę tę konkretną rzecz kupić.

**Brakuje panu takich wyjść eleganckich, gdzie trzeba trochę inaczej się ubrać?**

Pewnie, że tak. Bardzo chętnie poszedłbym gdzieś do restauracji. My zamawiamy jedzenie z restauracji przynajmniej raz w tygodniu, ale chętnie bym poszedł do tej restauracji. Spędzenie tam czasu to jest inny sposób funkcjonowania. To jest przyjemne i tego mi brakuje. Poszedłbym też do kina, do teatru. Rzadko się zdarza teatr, bo mieszkamy w Przemyślu i musimy sobie pojechać gdzieś, aczkolwiek, jak to się zdarza, to jest to przyjemne.

**To jest przyjemność płynąca z samego tego doświadczenia, czy też z przygotowań do niego, bo np. można wyglądać trochę ładniej, bardziej o siebie zadbać?**

[śmiech] Marynarka nie sprawia mi przyjemności. Nie chodzi o przygotowywanie się na wyjście, tylko o samo wyjście i uczestniczenie w zdarzeniu.

**A jak to wygląda u pana żony?**

Musiałaby pani z nią porozmawiać.

**A obserwuje pan jakieś zmiany u niej, jeśli chodzi o rytuały pielęgnacyjne, o makijaż?**

Nie, żona utrzymuje ten sam stały wysoki poziom, więc tu się nie zmienia. Dba o siebie. Myślę, że to naturalna konsekwencja tego, że nam ograniczono możliwość aktywności fizycznej i musimy sobie jakoś radzić, więc żona dba o formę w domu. Kiedyś biegała, teraz musi to zrobić w domu, więc widać to częściej. Dba bardzo.

**Słyszał pan od żony, że brakuje jej np. fryzjera, kosmetyczki?**

Nie przypominam sobie, ale może tak być. Pewne takie stałe, powtarzalne elementy jak wizyta u fryzjera raz na jakiś czas. Myślę, że jest to naturalność i każdemu tego brakuje. Ja też nie lubię, jak zaczynam mieć problemy z fryzurą, bo się robi nie wiadomo co. Moja żona mnie nie strzyże, więc muszę czekać na fryzjera.

**A basen, siłownia? Tego brakuje?**

Nie. Nie lubię siłowni, basen jest ok, chociaż dawno nie byłem. Nie chodzę na basen. Myślę, że żonie może brakować siłowni, bo ona chodziła, miała tam jakieś swoje schematy ćwiczeniowe, zresztą całkiem fajne. Ja mam swoją siłownię wokół domu. Całkiem skuteczną. I rower na nogi. To działa.

**Czego najbardziej panu teraz brakuje z punktu widzenia konsumenta?**

Myślę, że tego życia towarzyskiego - wyjścia do restauracji, do pubu. Tego, że nie można sobie siąść w ogródku w jakiejś kawiarni i napić się kawy z grupą znajomych, pójść na lody. Lubię widok ulicy w weekend, w święta, gdzie ludzie chodzą spokojnie, leniwie jedzą lody i sobie spacerują. To jest przyjemne. Lubię to robić, bo sprawia mi to przyjemność. Wygląd ulicy, gdzie widać życie też jest przyjemny, ale życie takie spokojne. Dla mnie ważny jest spokój a nie gonitwa. Bardzo nie lubię miasta zatłoczonego, gdzie ludzie pędzą.
