# Supplementary material for: The challenges arising from the COVID-19 pandemic and the way people deal with them. A qualitative longitudinal study
Source: PLoS One. 2021 Oct 11;16(10):e0258133. doi: 10.1371/journal.pone.0258133 (PMC8504766; doi:10.1371/journal.pone.0258133)
Supplement: S1 Dataset — (ZIP) [file pone.0258133.s003.zip › Transcriptions/stage 3/15.3_M_43_couple, with children.docx]

**15.3_M_43_couple with children**

**Co się działo przez ostatnie dwa tygodnie?**

Chyba wszyscy zaczynamy mieć problemy z datami. Wszystkie dni stają się coraz bardziej podobne, kalendarz staje się coraz mniej potrzebny. Ostatnie dwa tygodnie, czyli od 9 kwietnia, to w zasadzie od okresu przedświątecznego. Były święta. Po nich niezwłocznie wróciliśmy do aktywności zawodowych. I to chyba z grubsza tyle. Nie było ani dramatycznych sytuacji, ani dużych zmian życiowych w trakcie tych ostatnich dwóch tygodni. Raczej kontynuacja tego, co było wcześniej. Święta spędziliśmy w sposób tradycyjny, ale w okrojonym gronie. Z rozmowami telefonicznymi i bez uczestnictwa w obrzędach religijnych, w kościołach. Nabożeństwa online, tak samo święcenie pokarmów. Poza tym wiadomo, w poprzednich latach wyjeżdżaliśmy do jednych i drugich rodziców po jednym dniu, tym razem byliśmy w domu i chyba nawet z korzyścią. Stwierdziliśmy, że święta spędzone w domu są dużo lepsze, niż te wyjazdowe, które bywają bardziej męczące. Tak, było bardziej stonowanie. Było więcej spokoju. Moja żona drugi dzień świąt spędziła w łóżku, stwierdziła, że tak jest fajnie. Wychodziła tylko na świąteczne posiłki i było ok. W sensie fizycznym te święta były mniej angażujące.

**Obrzędom online udało się z powodzeniem zastąpić tradycyjne?**

Ja akurat nie mogę się wypowiedzieć, bo w nich nie uczestniczyłem. Małżonka uczestniczyła, sama stwierdziła, że z dużą korzyścią. Idąc do kościoła parafialnego jest skazana na określony tok prowadzenia kazania. W internecie mogła wybrać sobie ulubionego księdza, który zawsze ma coś ciekawego do powiedzenia i stwierdziła, że w sumie szkoda, że częściej nie można uczestniczyć w wersji online, nawet w czasach pełnego zdrowia. Korzyść intelektualna jest większa jeśli wiesz, że będzie do ciebie mówił ktoś, co ma coś ciekawego do przekazania.

**Jeśli chodzi o kontakt z rodziną - to były rozmowy telefoniczne, czy wideo rozmowy?**

Wideo rozmowy. Wiadomo, to nigdy nie zastąpi spotkania face to face. Ale z powodzeniem spełniło swoją rolę złożenia życzeń i pogadania zupełnie o niczym.

**Jak jeszcze spędzał Pan czas, poza świętami?**

Zajmuję się modernizacją i remontami w domu. W aktywności zawodowej trochę się pozmieniało - w centrum powiadamiania ratunkowego dostaliśmy informację, że musimy ruszyć ze szkoleniami, co już nastąpiło. Tę informację dostałem wczoraj rano. Ale to jest taka jedyna zmiana, nowa, sprzed dwóch dni. Może się w związku z tym okazać, że będę miał więcej pracy. Na przykład będę musiał iść do pracy w sobotę, gdzie w te dni nigdy dotychczas nie dyżurowałem. Z drugiej strony, w przyszłym tygodniu będę miał ten dzień gdzieś do odebrania. Cykl szkoleniowy zamykamy w piątek, w weekend będą egzaminy. Od przyszłego tygodnia wrócimy do dotychczasowego trybu pracy.

**Czy w zachowaniu Pana lub Pana najbliższych coś się zmieniło?**

Nie, nie wydaje mi się, żeby to się w jakikolwiek sposób zmieniało. Po tym początkowym efekcie wow, ostatnie dwa tygodnie były już ustabilizowane. Można powiedzieć, że przyzwyczailiśmy się do tej sytuacji. Jeśli chodzi o syna, też nie widzę jakichś dużych wahań. Nadal nie chce mu się odrabiać zadań domowych. Z racji zniesienia części obostrzeń, mamy teraz więcej możliwości. Spotykamy się więc na odległość w lesie, nieopodal którego mieszkam, z jakimiś ludźmi, żeby przynajmniej zamienić kilka słów. Wiadomo, to nie są jakieś wspólne zabawy dzieci, jest to jednak jakiś inny człowiek w zasięgu wzroku, niż najbliższa rodzina. Więc jest ta okazja, można wyjść z dziecięcym rowerkiem i trochę pospacerować. O, dziecko zmieniło nawyki żywieniowe. Zaczęło jeść więcej rzeczy, których nie jadało wcześniej. Wiadomo, że dzieci mają swoje preferencje, ale syn zaczyna teraz próbować nowych rzeczy. Wcześniej nigdy nie zjadł w domu tradycyjnej kanapki, na którą składa się jakieś pieczywo, smarowidło – masło w tym przypadku i jakaś wędlina, warzywo. W ostatnim czasie stwierdził, że chce kanapkę. Oczywiście my to trochę motywujemy tym, że teraz jest koronawirus i nie ma wszystkiego dostępnego, trzeba jeść to, co jest. I gdzieś być może taka argumentacja do dziecka trafiła. Wróciliśmy też do treningów karate, w wersji online. Syn trenował karate i na ten moment szkoła karate uruchomiła zamknięte szkolenia wideo. Dziecko powróciło do ćwiczeń. Ja nigdy nie trenowałem z synem. Normalnie są to zajęcia na sali sportowej, dla dzieci. Nie było takiej możliwości. Teraz tak przez chwilę o tym myślałem. Musiałbym się porozumieć jedynie z trenerami. Wiadomo, że ja płacę tylko za lekcje syna, więc już też nie chciałem ładować się z butami w założenia biznesowe kogośtam. Ale rozpatrywałem taką koncepcję i być może do niej wrócę.

**Pojawiło się coś, co stanowi wyzwanie codzienności, stanowi trudność, przeszkadza?**

Nie. Może takie rzeczy będą, ale one wynikają z moich własnych decyzji, działań. Jak wspomniałem, zacząłem prowadzić prace modernizacyjne, remontowe. To, co zaczyna mi w sumie przeszkadzać to to, że jestem sam jeden z remontami. Gdzieś tam pojawił się bałagan z tym powiązany. Robię remont generalny pokoju syna, więc on musi spać teraz gdzieś indziej, a wszystkie jego rzeczy są w moim gabinecie. To mi przeszkadza. Założyłem sobie, że trochę szybciej sobie z tym poradzę, a to się jednak ciągnie, tej pracy jest jednak trochę więcej. Przeszkadzają mi więc teraz rzeczy syna w moim własnym pokoju.

**Obrazki**

Myślę, że to będzie 6 i 13. One wskazują jakieś dążenie do celu, ale też myślę, że mogę powiedzieć, że z mojej perspektywy to jest jakaś forma oczekiwania na zakończenie. Czegokolwiek. Zakończenie po prostu. Jest jakiś punkt świetlny - Słońce - i podążanie ku czemuś. Dobrze by było już powrócić do pełnosprawnego funkcjonowania społecznego. To oczekiwanie jest spokojne, bazuje na wiedzy, którą posiadam - w zasadzie służbowo. Wiem, że tego czekania będzie jeszcze trochę. Jakiekolwiek spinanie się, reagowanie w sposób niekontrolowany emocjonalnie, nie ma żadnego sensu. Jest to okres, który trzeba przeczekać i tyle. Jest to stan oczekiwania, ale nie nachalnego, a raczej spokojnego, z nadzieją.

**Tym źródłem, skąd biorą się emocje, ostatnio była Pana osobowość. Czy teraz pojawiły się jeszcze jakieś nowe źródła tych emocji? Poza tymi informacjami, które Pan ma z pracy, że tego czekania będzie jeszcze trochę?**

Nie, nie sądzę. Szukam nawet czegoś mało istotnego, ale chyba nie. Jestem zmotywowany w taki sam sposób i tymi samymi czynnikami, którymi byłem motywowany dwa tygodnie temu.

**Czy pojawiają się jeszcze jakieś inne emocje? Może w zestawie powinien znaleźć się zupełnie inny obrazek?**

Na pewno widzę, że po tym pierwszym okresie, w którym wszystko było bardzo nowe - ale w sensie pozytywnym - widzę teraz takie spłycenie emocjonalne. Funkcjonuję normalnie. Wcześniej, próbując odnaleźć się w nowej sytuacji, człowiek starał się odnaleźć w nowej sytuacji, ale w taki sposób radośniejszy, robiąc wiele różnych rzeczy. Teraz afekt trochę się spłycił, nie ma już takiego hurraoptymizmu, że można robić coś nowego. Jest normalnie. Amplituda nie wychyla się ani w stronę emocji pozytywnych, ani też negatywnych. Na samym początku byłem bardziej radosny, niż na co dzień. Teraz jestem mniej radosny, niż w pierwszym okresie, ale tak samo, jak przed wybuchem epidemii.

**A czy pojawia się w Panu jakaś złość, związana z epidemią?**

Nie, nie mam takich odczuć związanych z epidemią. Frustrujące jest to, że zrobiłem sobie bałagan w połowie domu i teraz muszę to posprzątać.

**A pojawia się w Panu jakieś poczucie odrealnienia, czy nie?**

Nie, ta sytuacja nie jest w żaden sposób dziwna. Ona jest naturalna, normalna. Mamy ogromne szczęście, że mamy możliwość przeżyć taką sytuację i poznać siebie od tej strony izolacyjnej.

**Co nam może to dać?**

Oprócz całego spektrum zaburzeń z kategorii F, z ICD 10, myślę, że wiele osób inaczej spojrzy na życie. W tym sensie, że przynajmniej na jakiś czas po zamknięciu tego,  dostrzeże inne wartości, będzie bardziej doceniała to, co ma. To taka radość z prostych czynności, typu pójście do lasu na spacer, czy podróżowania komunikacją miejską, na którą do tej pory narzekaliśmy.

**Wydaje się Panu, że to będzie trwała zmiana?**

Wydaje mi się, że zaczniemy powracać do nawyków, schematów i wartości, które mieliśmy przed okresem epidemii. Ale na pewno będzie jakiś czas refleksji. Ona będzie, choć będzie gasła. To trochę jak z przykładem śmierci Jana Pawła II, czy katastrofy smoleńskiej, po których to wydarzeniach byliśmy bardzo solidarni. Ale to wszystko z czasem wygasa i zaczyna wracać do normy, Legia Warszawa nadal będzie nienawidziła Lecha Poznań, prawica lewicy, lewica prawicy, itd. Wszystko się ustabilizuje, spokojnie. :)

**Jak się Panu wydaje, na ile Pana zawód i informacje, do których ma Pan dostęp nieco wcześniej, niż opinia publiczna , sprawiają, że jest w Panu ten spokój, nie pojawia się lęk?**

Nie wiem, czy to ma jakikolwiek wpływ. To nie jest tak, że my mamy informacje dwa tygodnie przed wprowadzeniem czegoś. Te decyzje wtedy jeszcze nie istnieją, one nie są podejmowane z takim wyprzedzeniem. Ale to jest tak, że na przykład dwie godziny przed ogłoszeniem czegoś przez premiera, posiadamy jakieś informacje cząstkowe. Czy to ma jakikolwiek wpływ? Nie wiem, to są bardzo ogólnodostępne informacje. Ja wiem to po prostu dwie godziny wcześniej, a równie dobrze mógłbym to samo obejrzeć dwie godziny później na jakimś kanale informacyjnym w telewizji. W żaden sposób to raczej nie będzie na mnie wpływało.

**Jak zmieniają się emocje w Pana najbliższym otoczeniu?**

Widzę, że zarówno teściowie, jak i rodzice izolują się w dalszym ciągu. Starają się być aktywni zawodowo w tych obszarach, które nadal, już jako prawie emeryci, nadal są w stanie realizować. Wiadomo, że nie znają wszystkich możliwych technologii, ale widzę, że teść, który jest lekarzem, przegląda jakieś materiały, coś czyta, czegoś się jeszcze uczy, choć de facto nie musi. Przygotowuje sobie jakieś prezentacje. Takie rzeczy techniczne. Izolują się z życia społecznego - trochę na zasadzie autoizolacji, bo nie mają ani kwarantanny, ani podejrzenia. Szukają takich zabezpieczeń stricte fizjologicznych - typu jedzenie - kupcie nam coś, a oni sobie przygotują obiad. Nie są aktywni społecznie. Starają się chyba skoncentrować na tym, aby przetrwać. Nie zauważyłem u nich żadnych nowych sposobów radzenia sobie z sytuacją. Tak, jak mniej więcej dwa tygodnie temu, był u nich pewien poziom niezadowolenia, wyrażany również werbalnie - że to jest już nudne. Oni byli zawsze bardzo aktywni, zawsze wyjeżdżali na wakacje, a teraz tych wakacji nie będzie - ta forma narzekania się utrzymuje, mniej więcej od tych nawet trzech tygodni już.

**A jakieś dalsze otoczenie, obserwacje społeczne?**

Zwiększył się ruch w mieście. Poza tym nie widzę niczego w bliższym otoczeniu społecznym. Przemieszczając się samochodem do pracy i z powrotem widzę, że jest trochę więcej korków. Chyba są też mniejsze kolejki do sklepów. Poza wygłupami rodzicielskimi mojego kolegi, który wstawił do mediów społecznościowych swoje zdjęcie, na którym siedzi z rodziną przy stole, mając na głowie karton z wyciętymi otworami, jak się domyślam imitujący zapewne głowę z Minecrafta, nie widziałem w zasadzie niczego więcej, co mogłoby być niecodziennym sposobem radzenia sobie z sytuacją. Mieliśmy zgłoszenie na numer 112, takie, na które też zwróciłem uwagę, że w miejscowości Krotoszyn, w woj. Wielkopolskim, ktoś chodził z megafonem po osiedlu i nawoływał ludzi do zarzucenia wszelkich obostrzeń. To taka forma nie wiadomo w zasadzie, czy buntu? Nie wiem, jak to wszystko się skończyło, czy był to człowiek z zaburzeniami, czy po prostu może przedstawiciel nurtu antyszczepionkowego, któremu zaczęło się nudzić i teraz jest przedstawicielem nurtu antyobostrzeniowego. Nie mam pojęcia. Bo oprócz tego, że mieliśmy zgłoszenie, dalsze losy tego człowieka pozostają mi obce. Choć to na pewno była dość nietypowa forma, powiązana ze sporą odwagą cywilną, gdzie to było wiadome, że na to osiedle policja przyjedzie w ciągu trzech minut. No, może 15, bo tam wszyscy policjanci są w kwarantannie, więc policja penie musiała dojechać z pobliskiej miejscowości. Ale pewnie przyjechali i go zgarnęli. Jeśli ktoś jest z natury antykonformistą, być może musiał sobie znaleźć nowy obiekt do negowania. Ale nie mam pojęcia, z czym ostatecznie wiązać zachowanie tego człowieka. Ale to jest tylko luźna interpretacja faktu, który został przekazany na 112 – nic więcej.

**Jeśli chodzi o nastroje społeczne - ma Pan jakieś nowe obserwacje? Coś się Pana zdaniem zmienia w ludziach, ich nastawieniu?**

Wydaje mi się, że zluzowanie obostrzeń trochę odwróciło uwagę od koronawirusa. Chociaż sama sytuacja epidemiologiczna nie zmieniła się w żaden sposób, a nawet być może będzie się w jakimś tam zakresie pogarszać. Być może, że ta uwaga poszła bardziej w kierunku jakiejś ograniczonej rekreacji. Ludzie chyba mniej śledzą doniesienia medialne. Owszem, łapią jakieś informacje, ale to już nie jest tak, że ktoś siedzi i ogląda TVP Info przez 12 godzin, żeby dowiedzieć się, czy kolejna osoba zmarła, czy nie. Jest teraz więcej możliwości.

**Wydaje się Panu, że to uspokaja społeczeństwo?**

Myślę, że tak. Jak cofnięcie części zakazów. Na pewno jest poczucie większej wolności.

**Ograniczenia. O jakich zmianach Pan słyszał?**

Zniesiono między innymi zakaz przemieszczania się w celach rekreacyjnych. Oczywiście, to wiąże się teraz z maseczkami. Wiadomo, że obostrzenia w dalszym ciągu jakieś są, nie ma pełnego poluzowania. Zmiany sklepowe wpłynęły na zmniejszenie kolejek. Część urzędów będzie chyba wracała do pełnej sprawności, patrząc na informacje płynące od ludzi pracujących choćby w Prokuraturze, czy sądownictwie. Choć w maju chyba jeszcze nie będzie wszystkich rozpraw - będą tylko tzw. rozprawy niejawne. Ale takie informacje docierają. Możliwe, że część przedszkoli będzie otwarta już maju. Część pewnie dopiero we wrześniu - to pewnie będzie zależało od formy własności danego przedszkola, tego, czy to placówka publiczna, czy prywatna. Takie informacje są i te zmiany na pewno w jakimś zakresie będą następowały. To chyba przyniesie najbliższa przyszłość.

**Obowiązkowe noszenie maseczek - jak Pan postrzega ten nakaz?**

Daje on możliwość jakiegoś podstawowego zabezpieczenia innych i jeszcze mniej podstawowego zabezpieczenia siebie. Myślę, że mogą one realnie ograniczyć i spowolnić rozprzestrzenianie się epidemii. Możliwe, że kolejna fala zakażeń, która nadejdzie, może nie będzie dzięki nim ostrym peakiem w górę i nie zabraknie miejsc w szpitalach. Na ten moment nie mamy chyba żadnych innych możliwości zabezpieczenia się, oprócz pełnej izolacji w domu. Są więc one niezbędne i z nich korzystam. Mam nawet w samochodzie paczkę 10 sztuk jednorazowych. Z drugiej strony, też o tym już wspominałem, oprócz motywacji wewnętrznej, trudno byłoby mi nie przestrzegać przepisów i dać się złapać w sposób głupi. Gdzie teoretycznie powinienem być też wzorcem zachowań etycznych, przestrzegania norm prawnych. No więc także z tego tytułu stosuję się do nakazu.

**Czy mógłby Pan rozwinąć temat tego, kogo chronią maseczki?**

Te, z których korzystamy - dwuwarstwowe z podstawowym, bardzo cienkim filtrem HEPA, one chronią tych, którzy są wokół mnie, bo zatrzymują mój materiał biologiczny. Ale nie koniecznie bezpośrednio chronią mnie. Oczywiście, jeśli ktoś na mnie kichnie i to trafi na moją maseczkę, którą szybko ściągnę - owszem, w tym znaczeniu też oddalę od siebie zagrożenie. Ale te maseczki nie filtrują powietrza wdychanego. Mamy też te maseczki bardziej profesjonalne, które faktycznie filtrują powietrze docierające do człowieka oddychającego. Ale znowuż w powietrzu wydychanym wylatuje wtedy wszystko, w 100%, więc one są nawet mniej bezpieczne dla otoczenia. Część ludzi korzysta też z nich. Założenie jest takie, aby chronić innych, ale nie jest prawnie zabronione chronienie tylko siebie. Jest tylko mowa o szczelnym zakrywaniu ust i nosa.

**Jak postrzega Pan zniesienie ograniczeń w rekreacyjnym przemieszczaniu się?**

Z osobistego punktu widzenia bardzo pozytywnie. To zwiększa też moje możliwości spędzania wolnego czasu, czy jakiejkolwiek, podstawowej aktywności fizycznej. Z punktu zarządzania krajem i społeczeństwem też to rozumiem. Przetrwaliśmy pierwszą falę zakażeń, zapoznaliśmy się z nią, mniej więcej wiemy, jak przebiegają te zakażenia, jaką mamy wydajność i wydolność opieki medycznej. Mając dotychczas 20% miejsc w szpitalach przeznaczonych pod hospitalizację osób z koronawirusem, możliwe, że można by tę liczbę zwiększyć teraz do połowy, aby zwiększyć nasz kontakt z patogenem, abyśmy choć częściowo sami się na niego uodpornili - bez pomocy medycznej, być może przeszli go bezobjawowo. Istnieje założenie, że jeśli będzie mutacja to ktoś, kto wcześniej chorował, lżej przejdzie infekcję. Choć na razie wszystko to jest pisane patykiem po wodzie. Nie mamy jeszcze zbyt wielu informacji na temat samego wirusa, ale jakąś drogę trzeba obrać. Ten sposób zakażania, zarażania większej części społeczeństwa poprzez luzowanie ograniczeń, zwiększając przy tym liczbę miejsc w szpitalach dla osób zarażonych, jest konieczny. Nie sposób żyć przez lata w pełnej izolacji domowej - tak się po prostu nie da, trzeba w jakimś zakresie zaryzykować. To będzie po części też uspokajać społeczeństwo, bo zniesie nam część zakazów, ale nie mamy jeszcze tej największej fali zachorowań za sobą. Pytanie, czy kiedykolwiek będziemy mieli szczepionkę na tego wirusa. Więc coś trzeba robić. Dlatego w pełni rozumiem takie działania.

**A jeśli chodzi o zwiększenie liczby osób w sklepach i kościołach?**

Z punktu widzenia użytkownika sklepu - bardzo pozytywnie. Z punktu widzenia strategii rządowej oswajania ludzi z zakażeniami - też pozytywnie. Nie będę krytyczny w tym zakresie. To są decyzje, które podejmują osoby w pewnych obszarach dużo bardziej doświadczone, mądrzejsze nawet życiowo, strategicznie i politycznie od nas. Dlatego mi nic tu do krytykowania. Może nie tyle chodzi tu tyle o zaufanie do polityków, lecz o wybór jakiejś strategii. Czy politycznie, gospodarczo, społecznie, czy prywatnie. Nie mamy zbyt wielkiego wyboru. Możemy się albo w pełni izolować przez lata, dramatycznie zmienić swoje życie, albo próbować alternatyw. Sam nawet bym się nie zastanawiał, czy im ufam, czy nie. Po prostu taka decyzja jest i tyle, idziemy dalej. Wątpliwości jakieś zawsze będą, nie wiemy jeszcze zbyt wiele na temat koronawirusa. Być może okaże się, że w najbliższym otoczeniu będą straty z jego powodu. Być może w mojej rodzinie nawet już są. Prawdopodobieństwo jest prawie zerowe, ale jakąś drogę trzeba wybrać. To jakiś sposób radzenia sobie państwa, rządu, z zaistniałą sytuacją. Gdybyśmy mieli więcej niż dwie możliwości działania, być może można by było różnie wybierać. Trzeba zaakceptować tę rzeczywistość, która jest i starać się dalej żyć normalnie.

**Co sądzi Pan o możliwości samodzielnego przemieszczania się osób powyżej 13 r.ż.?**

To z mojej perspektywy zupełnie bez znaczenia. Nie dotyczy mnie osobiście. W pewnym zakresie okresowe ograniczenie możliwości było dobre, ponieważ mieliśmy nawet zgłoszenia związane z tym, że podczas nieobecności dorosłych, którzy mimo nałożonej kwarantanny poszli do pracy, dzieci - młodzież - biegały po podwórku. Nikt nie miał kontroli nad osobami w okresie adolescencji. Teraz zostało to jasno doprecyzowane, że wyjścia są możliwe tylko w towarzystwie osoby dorosłej, i koniec. Zejście w ostatnim czasie do 13 r.ż. pewnie jest ok, bo w końcu dziecko będzie mogło samo wyjść z psem na spacer, a nie w towarzystwie rodzica. Podejrzewam, że rodzicom też już się pewnie nie chciało.

**Które z ograniczeń obecnie obowiązujących mają według Pana realny wpływ na ograniczanie epidemii, a które z nich mają bardziej funkcję psychologiczną dla społeczeństwa?**

Myślę, że wprowadzone ograniczenia mają realny wpływ. Weźmy pod uwagę hipotetyczną sytuację dużego koncertu, kiedy okazałoby się, że w zamkniętej sali jest 10 czy 12 tysięcy osób. Wystarczyłoby kilka chorych osób i to rozprzestrzenianie byłoby dużo szybsze. Dlatego takie odwoływanie, czy ograniczanie imprez masowych na pewno będzie miało znaczenie. Nie jestem ekspertem z tej dziedziny, ale wydaje mi się, że tak, to ma znaczenie. Te obostrzenia też są trochę dobierane na zasadzie innych państw. Nie mieliśmy zbyt dużego własnego doświadczenia w tym zakresie. Rządzący wzięli więc zapewne jakąś średnią rozwiązań zastosowanych w innych krajach. Zastosowali je pewnie do końca nie wiedząc, w jakim kierunku to wszystko pójdzie, ale dzięki takim działaniom ograniczaliśmy ryzyko. Ma to sens, żeby nie okazało się, że nagle w skali tygodnia mamy dużo więcej chorych, niż miejsc w szpitalach, którymi dla nich dysponujemy.

**Myśli Pan, że maseczki uspokajają społeczeństwo, czy wręcz przeciwnie?**

Myślę, że krytycznym momentem były początki, kiedy widok ludzi w maseczkach był czymś nowym. To mogło zapadać w pamięć. Natomiast teraz jest to pewnego rodzaju standard. Ludzie mogą narzekać na to, że się gorzej oddycha, ktoś może się hiperwentylować, ktoś poddusić. Nie jest już tak przyjemnie, jest za gorąco. Ale po prostu je nosimy. Stało się to rzeczą powszechną i raczej chyba bardziej przykuwa uwagę ktoś, kto idzie bez, aniżeli osoba, która ją nosi. Czy to uspakaja? Nie wiem. Może dawać jakieś poczucie kontroli. Jeśli ktoś na mnie kichnie, może to dać możliwość podjęcia działań minimalizujących zarażenie. Można to jakoś racjonalizować. Ale czy sama maseczka uspokaja? Poddusza raczej.

**Jak wyglądało to spotkanie na odległość w lesie, o którym mówił Pan na początku?**

To jest las miejski, więc po obu stronach duktu są ławeczki w odległości ok. 3 m od siebie. Można więc siąść naprzeciw siebie i rozejść się w przeciwnych kierunkach. I to tyle. W ten sposób zamieniliśmy kilka słów. Ale to raczej jakaś ciekawostka - i tak mamy spory kontakt poprzez komunikatory. Nie mieliśmy sobie de facto zbyt wiele nowego do powiedzenia, ale zobaczyliśmy się.

**Dlaczego postąpił Pan zgodnie z nakazem? Czy nie kusiło, aby podejść bliżej, zmniejszyć ten dystans?**

Ze względu na bezpieczeństwo. Nie wiemy jaki kontakt miały te osoby, z którymi rozmawialiśmy. To chęć zabezpieczenia się, motywacja wewnętrzna.

**Jaka jest różnica pomiędzy izolacją a kwarantanną?**

Kwarantanna jest stanem dookreślonym przez instytucję, która ją nakłada. Izolacja to odosobnienie, które może być wynikiem swojej własnej decyzji. Zastanawiałem się jeszcze nad różnicą między ekspozycją na koronawirusa a jej podejrzeniem - mamy izolatoria z MSW, do których trafiają osoby podejrzane o kontakt z patogenem. Jest też prawne rozróżnienie w naszym regulaminie wewnętrznym na izolację i kwarantannę.

**Plany łagodzenia restrykcji**

Mamy przed sobą jeszcze trzy etapy z ostatnim, który będzie dopuszczał najszersze formy komunikacji z innymi osobami, czyli między innymi imprezy sportowe. I stopniowe uruchamianie na wcześniejszych etapach instytucji kultury, szkolnictwa, części usług - na ograniczonych zasadach - sądownictwa, urzędów, ale też punktów usługowych - barberów, fryzjerów, salonów kosmetycznych. Wyglądamy zresztą coraz ciekawiej. Te ramy, plany - oceniam pozytywnie. Myślą nad tym dużo sprawniejsi intelektualnie ode mnie. A jeśli chodzi o etapy - to trochę jest związane z ty, co jest nam potrzebne, jak ten fryzjer. Z drugiej strony to na pewno jest też powiązane ze strony gospodarczej - jak długo można wypłacać całemu społeczeństwu cały szereg świadczeń, jeśli najprawdopodobniej środki kurczą się w zatrważającym tempie i może będziemy musieli sięgnąć do rezerw walutowych. Rozumiem więc, że to musi być wprowadzone, bo inaczej ciężko by było funkcjonować. Należy pod kontrolą przywracać kolejne obszary funkcjonowania społecznego - patrzeć, co będzie się działo - nie mamy specjalnie innego wyboru. Więc ogólnie postrzegam to pozytywnie. Co do poszczególnych etapów - nie wiem, może wolałbym zgodnie z moimi osobistymi preferencjami uruchomili w pierwszej kolejności imprezy masowe. Ale to jest moja osobista preferencja, nie mające nic wspólnego ze zdrowym rozsądkiem, a jedynie z moją rozrywką.

**Które z ograniczeń obecnie obowiązujących, Pana zdaniem powinny zostać na dłużej, a które na krócej?**

O, zgrozo. Mogłaby zostać wprowadzona liberalizacja zamknięcia żłobków i przedszkoli, z tego powodu, że mi samemu byłoby lżej. Wolałbym, aby nie powróciły na razie zajęcia uczelniane w pełnej formie - stacjonarnej, jak seminaria dla 26 osób. To spotkania z dorosłymi ludźmi, którzy mają nieco większe możliwości urozmaicenia kontaktów społecznych, przez co ryzyko rozprzestrzeniania może być wyższe, niż choćby w przypadku dzieci. Druga sprawa, że jakbym miał wrócić teraz do prowadzenia takich zajęć, musiałbym pozmieniać inne plany zawodowe, które w międzyczasie się pojawiły i spowodowały wzrost zarobków. Wolałbym więc, żeby zajęcia pozostały w takiej formie, jak teraz, online. Najlepiej do października. To takie bardzo egocentryczne, moje własne podejście.

**Co powinno być granicą, kiedy obostrzenia powinny być łagodzone?**

Potwierdzona odporność większej części społeczeństwa - czy po szczepionce, czy przejściu infekcji, wszystko jedno. Kiedy będziemy pewni, że społeczeństwu nic już nie grozi, to powinien być moment, kiedy ostatnie obostrzenia powinny zostać zdjęte.

**A co powinno być ostatnimi obostrzeniami?**

Zakaz imprez masowych. Może nawet nie tyle masowych - bo to są liczby około 1000 osób, co zakaz zgromadzeń tak powyżej 50 osób.

**Model szwedzki**

Słyszałem. Oni zdecydowali się na zdecydowanie bardziej naturalny proces przechodzenia społeczeństwa przez tę fazę ostrą epidemii. To jest ich własna decyzja. Być może będzie ona wiązała się z większymi stratami w populacji ogólnej. Ale przyjęli taki model, ok. Czy lepiej na tym wyjdą, ciężko stwierdzić. Przebieg wydarzeń może być jakoś symulowany, ale my tego do końca nie wiemy - to tylko i wyłącznie analizy. Czy model szwedzki jest lepszy od ograniczeń - nie mam pojęcia, to dopiero będziemy mogli stwierdzić po jakimś czasie, monitorując sytuację.

**Czy model szwedzki sprawdziłby się w Polsce?**

Prawdopodobnie daleko przekroczylibyśmy możliwości wydajności opieki medycznej. To też był jeden z powodów, dla którego nie wprowadzono tego u nas. Szwecja jest jednak krajem zdecydowanie mniej licznym o zdecydowanie wyższym poziomie usług medycznych - nie w znaczeniu wykształcenia kadr medycznych, a możliwości technologicznych. Możliwe, że oni mogą sobie pozwolić na to, aby od razu hospitalizować pół miliona ludzi. Możliwe, choć nie znam szczegółów. My natomiast nie mamy i tyle. Siłą rzeczy, ktoś musiałby podejmować naprawdę trudne decyzje przy tak liberalnym modelu. Ktoś musiałby umrzeć. Ale jeśli oni są w stanie zapewnić podczas największej fali zachorowań respiratory dla osób potrzebujących, to ok. To pewnie decyzja, która wynika z posiadanych zasobów, po prostu.

**Czy kiedy ograniczenia zostaną zniesione, po tym momencie granicznym, o którym mówiliśmy, miejsca takie jak szkoły, uniwersytety itp., powinny funkcjonować na jakichś odmiennych zasadach, niż przed epidemią?**

Myślę, że nie, ale powinniśmy systemowo - jako państwo - być gotowi na tego typu zdarzenia. Zebrać te doświadczenia, jakie teraz mamy w sensie technologicznym, personalnym, kadrowym, strategicznym - powinniśmy być pod tymi aspektami gotowi na ewentualne zaistnienie podobnych sytuacji w przyszłości. Ale poza tym, jeśli będziemy już po wszystkim, będziemy mieć pewien czas spokoju. Będziemy bogatsi o pewne doświadczenia i miejmy nadzieję, że będziemy czekać bardzo długo na kolejną tego typu szansę i okazję przeżycia epidemii. Mam nadzieję, że będziemy liczyć to w latach, czy dziesiątkach lat. Ale jeśli sytuacja będzie bezpieczna i stabilna, nie będzie sensu wprowadzać jakichś dodatkowych obostrzeń, typu koncerty z maseczkami na ustach. Po co?

**Jak wygląda u Pana teraz dbanie o siebie?**

Od 12 czy 13 marca, czyli od czasu, kiedy zamknęli przedszkola, odbywa się to bez aktywności sportowych typu siłownia czy basen. Ten element zdroworozsądkowej dbałości o siebie, nie istnieje u mnie na ten moment. Jeśli chodzi o czynności związane z pielęgnacją, nie ma u mnie w tym aspekcie większych zmian. Jak wspominałem ostatnio, zamówiłem sobie nawet większą ilość kremów, z których uprzednio korzystałem. Staram się misternie ułożyć moje trzy włosy, które pozostały mi na czubku głowy, a są nieco dłuższe, niż były. Tak, żeby wyglądały jeszcze jakoś w miarę normalnie. Ale też żona, pierwszy raz w życiu, weszła w rolę fryzjera męskiego i coś tam nawet z tego wyszło. W formie żartu, powstał u mnie ostatnio szatański plan. Remontując, nie goliłem się trzy dni. Postanowiłem z tego zarostu wykroić coś, więc wykroiłem sobie wąsy w stylu wczesnego Wałęsy. Zastanawiałem się, czy to zostawić w formie żartu i zostawiłem, może będę je zapuszczać jeszcze kilka dni. Więc wczesny Wałęsa, zwany też wąsami kierowcy PKSu. Ale takie zachowania zdarzały mi się też wcześniej, więc nie wynikają one z czasów, tylko nadarzyła się ku temu okazja, bo dłużej się nie goliłem.

**Ubrania**

Przebywając w domu, w mniejszym stopniu chodzę też w marynarce, czy garniturze. Wpływa to więc trochę na ubieranie się. Jest teraz trochę więcej porozciąganych dresów, ale z drugiej strony - ciężko jest przecież malować dziecięcy pokój w marynarce. Trochę jedno wynika z drugiego. Staram się jednak trzymać pewien poziom wychodząc do pracy - może nie jest to poziom krawatu, ale mimo wszystko. W tych samych rozciągniętych dresiczkach, dresach, czy ortalioniku no nie pójdę do pracy, w której przemieszczam się po ogrodzie domowym.

**A kiedy zostaje pan w domu?**

Strój zawsze jest uzależniony od planów i tego, co robię, ale nie chodzę w piżamie. Piżama, to piżama. Druga sprawa jest taka, że nie wszyscy sypiają w piżamach, nie sposób też chodzić nago przez cały dzień, trzeba się jakoś ubrać.

**Pojawiły się jakieś nowe czynności pielęgnacyjne, kosmetyki?**

Nie, używam tych samych kosmetyków, co wcześniej. A, korzystam z masażera wodnego do stóp, ale korzystałem z niego okazjonalnie wcześniej. Teraz z racji tego, że jest trochę więcej czasu, korzystam z niego częściej.

**Jak wygląda kwestia dbania o siebie u żony?**

Żona maluje się rzadziej, ale to nie tak, że nie robi tego w ogóle. Kiedy wyjeżdża do pracy, nakłada tak zwaną urodę na twarz, czyli makijaż. Farbowała sobie w ostatnim czasie samodzielnie włosy. Kolor wyszedł nieco inny od dotychczasowego i może bez pasemek, bo nie znalazła farby w swoim ulubionym kolorze i pasemek też nie była sobie sama zrobić. Ale podjęła taką próbę. Do prac domowych, typu sprzątanie ekstremalnych zakamarków ubiera się w strój roboczy, natomiast na co dzień nie koniecznie w smart casualu, ale takich normalnych ubraniach, typu jeansy i koszulka, czy jakaś bluza.

**Żonie brakuje fryzjera, czy kosmetyczki? Skarżyła się na takie rzeczy?**

Nie skarżyła się, choć korzystała z takich usług stosunkowo często, więc pewnie w jakimś zakresie brakuje jej tych starych nawyków. Ale raz, że nie zwracam na to uwagi, a dwa, że ona nie skarży się z tego tytułu.

**Brakuje Panu teraz wyjść, okazji do eleganckiego ubrania się?**

Nie. Zresztą, podczas świąt Wielkanocnych ubraliśmy się w sposób zbliżony do eleganckiego. Nie zakładałem pełnego garnituru, ale do ciemnych spodni założyłem białą koszulę, marynarkę i krawat. Żona też podobnie. Więc jakaś forma odświętnego ubioru była. Marynarkę i koszulę też zakładam zazwyczaj do kontaktów online ze studentami. Trzymamy się tych ogólnie przyjętych kanonów.

**Czy kupował Pan kolejne ubrania, w związku ze zmieniającą się porą roku?**

Dokupiłem kolejne koszulki krakowsko-tarnowskiego zespołu Mgła, o czym już kiedyś wspominałem. To wynika ze zmienności pór roku. Pierwszy raz będę nosił zespół Mgła, ale wcześniej nosiłem koszulki innych zespołów - w sytuacjach mniej formalnych.

**Brakuje Panu chodzenia po sklepach z ubraniami?**

Z ubraniami nie, absolutnie.

**A po innych sklepach, tak dla przyjemności?**

Dalej mi się zdarza bywać w innych sklepach. Ostatnio pojechałem na zakupy po dwóch, czy nawet trzech tygodniach. Większe takie, z pełnym wózkiem. Teraz zabrakło mi farby, której już nie będę zamawiał z dowozem, jak robiłem to ostatnio, bo koszty dowozu przekroczą koszt zakupu farby, więc dzisiaj w godzinach późnowieczornych, kiedy zakończę pracę o 20, pojadę jeszcze do Castoramy dokupić farbę. Ale nie brakuje mi tego, ja nigdy nie byłem gorącym zwolennikiem zakupów. Oprócz wołowiny i wina - czyli zakupów normalnych, codziennych - nie spędzałem czasu w galeriach, nie szukałem okazji, aby outfit był określony, czy nie. Jestem raczej tradycyjnym dziadem, który nie przywiązuje do tego aż takiej uwagi.

**Czy te zakupy budowlane sprawiają Panu przyjemność?**

Nie, jaką przyjemność można czerpać w Castoramie? To jest po prostu sklep, gdzie kupuje się farbę i się wychodzi.

**Z punktu bycia konsumentem, czemu Panu najbardziej brakuje?**

To usługi, które były częścią mojego życia - typu aktywność fizyczna na siłowni czy basenie. To było ze względów zdroworozsądkowych, zdrowotnych. Jeszcze 10 czy 12 lat temu byłem o 20 kg cięższy i byłem osobą dość otyłą. Teraz jestem zdecydowanie mniej otyły. Staram się to podtrzymywać, więc aktywność fizyczna pomagała mi w tym. Aktywność sportowa była też czasem, kiedy ja odgradzałem się od natłoku informacji zewnętrznych, ale też nadmiaru ludzi. Pracując w takich miejscach jak ja, prowadząc działalność naukową, dydaktyczną, pracując w urzędzie, tych interakcji było całe mnóstwo, więc też siłownia dawała mi możliwość odcięcia się od tego - tylko i wyłącznie z muzyką. Choć teraz relacji jest mniej, więc jest też mniejsza potrzeba odcinania się. Powiedzmy sobie, że ta równowaga jest gdzieś utrzymana. Brakuje mi też koncertów - okazjonalnie, ale jednak one były. To była rozrywka i okazja do spotkania się. Mamy takie grono pracowników, kumpli, przyjaciół. Na koncerty wybieraliśmy się większą, zorganizowaną grupą. Większą, niż trzy osoby. Najpierw mieliśmy spotkanie organizacyjne, później jakieś spotkanie, gdzie dosłuchiwaliśmy sobie płyt, kilka dni później byliśmy na koncercie. Przed koncertem wiadomo, trzeba było jeszcze jakieś tanie wino wypić, czy cośtam. Najczęściej chodziliśmy już na wybrane koncerty, preferowaliśmy, jak emeryci, miejsca siedzące. Często kupowaliśmy bilety VIPowskie, żeby siedzieć blisko, żeby móc faktycznie dostrzec czy samo instrumentarium, czy to, jak muzyk się zachowuje, jakich technik w grze  na instrumentach używa, żeby też obejrzeć instrumenty. Interesowały nas takie technikalia. To była forma rekreacji, spędzania wolnego czasu, ale też była powiązana z jakimiś zainteresowaniami muzycznymi, nieco bardziej rozbudowanymi, niż tylko słuchanie muzyki z YouTubea. Już jak się na początku przedstawiałem, mówiłem, że mam całe mnóstwo płyt i instrumentów, trochę grywam, pomimo braku wykształcenia muzycznego. Te koncerty stanowiły jakąś część mojego osobistego życia. Więc rozrywka, rekreacja, możliwość spotkania się, spożycia alkoholu z kumplami, przedyskutowania pewnych spraw muzycznych, ale też realizacji swojej własnej pasji muzycznej - patrząc, jak inni potrafią grać, a ja nie. Ale też nie jest to ogromna część mojego życia. Wybieraliśmy sobie 3-4 koncerty rocznie, więc na ten moment nie brakuje mi tego jeszcze bardzo, na ten moment nie odczuwam jeszcze aż tak dużej potrzeby. Tym bardziej, że byłem na koncercie jeszcze tuż przed ogłoszeniem restrykcji. Na koncercie Waldemara Malickiego - czyli pojawiła się jakaś forma kabaretowa jeszcze. To był luty, więc te dwa miesiące nie stanowią jeszcze żadnego problemu. Choć już ostatnio znalazłem na HBO GO koncerty do oglądania. Oglądałem sobie koncert U2, którego nigdy na żywo nie widziałem, bo nie jestem ich jakimś wielkim pasjonatem. Ale był do obejrzenia koncert z Berlina, więc go sobie zobaczyłem. Nie są to może zespoły, które chciałbym obejrzeć, ale jak się okazuje, nawet U2 można obejrzeć na żywo i jakoś to brzmi i wygląda. Może brakuje mi też moich własnych konsumentów, na których zarabiałem prowadząc własną działalność gospodarczą. Działań szkoleniowych, projektów unijnych - tego na ten moment nie ma i też zauważam tu pewne braki. Poza tym, może raz w miesiącu wyjścia do restauracji. Ale też nie koniecznie. Wie Pani, my jednak jesteśmy z Poznania, tu jednak jest trochę inaczej.
