# Supplementary material for: The challenges arising from the COVID-19 pandemic and the way people deal with them. A qualitative longitudinal study
Source: PLoS One. 2021 Oct 11;16(10):e0258133. doi: 10.1371/journal.pone.0258133 (PMC8504766; doi:10.1371/journal.pone.0258133)
Supplement: S1 Dataset — (ZIP) [file pone.0258133.s003.zip › Transcriptions/stage 3/12.3_M_33_couple, with children.docx]

**12.3._M_33_couple with children**

**Opowiedz, co działo się u ciebie w ciągu ostatnich 2 tygodni.**

Nie zmieniło się dużo. Tydzień roboczy wygląda u mnie dość monotonnie, codziennie praca do 17 - 17:30 - 18, różnie, później zajmuję się dziećmi. Żona ma czas na swoje obowiązki w pracy. Weekendy się wyróżniają, ten był akurat bardzo fajny. Poprzedni to były święta, inna atmosfera, myślenie świąteczne. Dość szybko to wszystko minęło. Potem znowu w wir pracy. Ubiegły weekend mieliśmy dosyć ciekawy, bo wspólnie pracowaliśmy przy domu, robiliśmy ogródek, sialiśmy warzywa. Sobota fajna, w niedzielę troszeczkę... też było ciepło. Niedziela i święta przypominają bardziej wakacje, gdzie staramy się wypocząć jak najbardziej wspólnie. Poleżenie na leżaku, obejrzenie filmu i tak dalej. Dużo się nie zmieniło. Idziemy tym trybem.

**Jak spędzaliście święta?**

Całe przygotowania trochę się różniły od normy. Wszystko szykowaliśmy dla mniejszej liczby osób. Jednak odwiedziła nas rodzina, tylko w mniejszym gronie. Symboliczne spotkanie wielkanocne. Bardzo pozytywne, każdy już tęsknił za spotkaniem, żeby usiąść razem przy stole. Obiad już trochę inaczej wyglądał, dziadkowie gdzieś na dole zawsze sami jedzą, a my na górze sami jemy. Tutaj różnica była w tym, że wspólnie usiedliśmy przy stole. Dużo rozmów na messengerze z rodziną, rodzicami, teściami i tak dalej. To, co można zauważyć w mediach. Komputer na stole albo duży telewizor podpięty pod komputer. Dziadkowie też mogli pogadać, napić się było jak. Jak zawsze inna atmosfera, ale też nie było jakoś strasznie, żeby była jakaś przygnębiająca aura, żebyśmy szybko zrobili rzeczy związane z tradycją i wrócili do normalności. Ten dzień był nawet wesoły. Udało się odpocząć, była fajna pogoda, z żoną po obiedzie przenieśliśmy się do ogrodu na leżaki. Co chwila ktoś dzwonił na messenger i tak dzień zleciał.

**Znalazłeś jakieś nowe zajęcia?**

Można tak powiedzieć. Zacząłem oglądać filmy na raty, na przykład jak coś robię w kuchni. Obejrzałem 1/3, za dwa dni wróciłem do kuchni i obejrzałem dalej. Mam zajawkę, żeby kończyć filmy i powiększać listę filmów, które obejrzałem. Jeżeli chodzi o coś zupełnie nowego to nie, nie mam czasu. Praca zajmuje dużo czasu w ciągu dnia. To, co mi zostaje, już mam zarezerwowane na kilka tygodni do przodu.

**Co tobą kieruje, gdy wybierasz filmy?**

Ogólnie uwielbiamy z żoną oglądać TV, filmy czy seriale, tutaj idziemy kompromisowo. Na przykład żona nigdy ze mną horroru nie obejrzy, a mnie nie interesują filmy obyczajowe. Ja wybieram sobie takie filmy, które spełniają 100% moich oczekiwań, typu mocne sci-fi, na które żonę musiałbym bardzo namawiać. Szukam produkcji, które ja mam ochotę obejrzeć. Biegając na bieżni wybrałem taki serial, którego żona by nie wybrała, jakieś odjechane akcje.

**Korzystasz z jakiejś platformy streamingowej czy TV?**

Oglądam na telefonie. Mam aplikację NC+ Go, mam dostęp do masy filmów, które są w miarę świeże. Mam dostęp do VOD, ogromny wybór i wybieram takie bardziej nowości. Ostatnio oglądałem Tarantino - Hollywood, potem Zimną Wojnę. Takie produkcje. Miałem to już przed epidemią, jestem mega zadowolony. Od sierpnia ubiegłego roku odciąłem się od poprzedniego dostawcy telewizji, przerzuciłem się na NC+ z pakietem "mistrzowska piłka". Mają fajny rozbudowany pakiet, HBO Go do tego, NC+ Go w pakiecie, czy to jest komputer czy telefon. Można oglądać treści na żywo, te same co w TV z dekodera plus ogromna baza bajek, filmów.

**Z jakich powodów oglądasz te filmy?**

Staram się nie marnować czasu, tylko wyciągać z niego jak najwięcej. Kiedy wracam z pracy albo szykuję opał do domu, to słucham audiobooków. Żeby się rozwijać, zaspokoić potrzeby. Później są tematy do rozmów i tak dalej.

**Wcześniej miałeś do tego mniej okazji?**

Tak, ale jest taka różnica, że wtedy otoczenie się dynamicznie zmieniało. Byłem w pracy w Warszawie, w biurze, nie potrzebowałem urozmaicać sobie czasu. Bywają dni, kiedy popracuję i odbieram dzieciaki, za chwilę trzeba myśleć o kolacji. Żona pracuje. Więcej z tego korzystam, bo przebywam w czterech ścianach dłużej niż kiedyś. To urozmaicenie, jest taka świadomość, że się robi coś dla siebie. Brakuje czasu, tak jak mówię, przy takim małym dziecku to jednak od rana do wieczora trzeba się zająć.

**Czy są jakieś rzeczy, które od ostatnich 2 tygodni ograniczyłeś?**

Raczej chyba nie. Staram się mieć wszystko zaplanowane. Ciężko mi zrobić jakiś spontan. Jak gram codziennie z dziećmi w kosza i bym zrezygnował, to można powiedzieć, że tak, ale ciężko mi znaleźć przykład.

**Czy są jakieś rzeczy, które zaczęły ci ostatnio przeszkadzać?**

Chyba zwracam większą uwagę na czystość w domu. Ganiam dzieci na każdym kroku i to może wydawać się trochę dziwne. Muszę nieraz przystopować, bo co będą mnie widzieć, to będę im zwracał uwagę. Trochę mi to przeszkadza. Mija trzeci czy czwarty tydzień, co chwila zawieram z nimi umowę, a tu dwa dni posprzątają i trzeciego zapominają. To się cały czas powtarza. Trochę już odpuszczam i idę na kompromisy, na przykład sprzątanie pokoju w piątek. Ostatni weekend dzieciaki odczuły, bo im powiedziałem, że jak mamy umowę, to potrzymałem je dłużej w weekend, aż posprzątały pokój. Ale nie boli ich to za bardzo. Traci się jakieś argumenty, jakieś zaplecze do dyskusji. Teraz mają fajnie, bo co chwilę wybiegają na zewnątrz i nie potrzebują (mediów) tak jak zimą. Tutaj trzeba iść na kompromis, ale też utrzymywać konsekwencje. To mnie trochę wkurza.

**Czy jest to dla ciebie wyzwaniem?**

Tak, trzeba znaleźć złoty środek. Tak wywierać presję. Widać, że się starają, po prostu nie pamiętają o tym, jak się umawialiśmy. Nieraz oszukują, innym razem robią nie wszystko, o co ich proszę. Nie czytałem żadnej książki jak wychować dzieci. Może metodą prób i błędów.

**Co było największym wyzwaniem ostatnich dwóch tygodni?**

To chyba tego typu rzeczy, odpowiednie relacje z dziećmi i żoną. Siedzimy już któryś tydzień i są różne spięcia. Gdyby można było gdzieś pojechać, na wakacje, to daje dużo luzu. Jednak w domu działamy sobie na nerwy trochę. To jest chyba największe wyzwanie. Bardziej niż dom, ogród. Ja i tak trochę się odcinam w tym pokoju na 8 godzin, gdy pracuję. Oni się tam bardziej żrą ze sobą. Staram się jakoś rozładować poczucie zamknięcia i ograniczenia. Dzisiaj rzuciłem pomysł, żeby się gdzieś wybrać w teren, nad staw, może wędki naszykuję. Substytut wycieczki.

**Czy poczucie odizolowania w pokoju pracy ci doskwiera?**

Trochę tak. Może żona mi trochę zazdrości tej pracy, że mam coś, czym mogę się zająć. Ona ma teraz ze dwie godzinki pracy i wraca. Od rana do wieczora z dzieciakami, które biją się i wrzeszczą. Ja też się nie obijam tylko siedzę w pokoju i mam pracę. Później schodzę na dół i też zajmuję się dziećmi, ale ciężko wejść w cudzą skórę. Na pewno byłoby łatwiej układać relację, gdybyśmy mieli wszystkiego po równo.

**To wiąże się z poczuciem sprawiedliwości czy zrozumienia?**

Ja zawsze bym chciał dla żony jak najlepiej. Tyle ile mogę to pomagam przy dzieciach. Widzę różnicę, nawet jak na chwilę pojedzie do pracy to zadowolona, inny człowiek. A tutaj to przytłaczające, cały czas dzieci, coś im ugotować. Bardziej pod tym kątem, żeby mogła sobie odpocząć od tego, pracując nawet. Praca potrafi oderwać od codzienności.

**Które obrazki najlepiej oddają twoje emocje z ostatnich dwóch tygodni?**

Piątka mnie najbardziej zaciekawiła. Ogólnie utożsamiam się z tym obrazkiem. Wiele razy nocą patrzyłem w to niebo i wydaje mi się to coś odległego i nieograniczonego. Patrzę się trochę w ten sposób, że my jesteśmy ograniczeni tu na dole, a tam na górze jest przestrzeń. Trochę zaczyna doskwierać to uwięzienie. Trochę uprzykrzać. To niebo przykuwało moją uwagę ostatnio. Lubię na nie popatrzeć, komfort się robi, że jest to niebo takie nieograniczone. Wcześniej tego nie robiłem. Jednak jesteśmy ograniczeni w relacjach z innymi i to się przeciąga. Można usłyszeć, że to może jeszcze potrwać. Ale kiedyś to znowu wróci do normalności i człowiek nie będzie ograniczony. To, co teraz jest, jest tylko tymczasowe, ale jest gdzieś ten świat, który czeka. To raczej pewność. To jest i będzie, tylko czeka. Kiedyś wróci do normy ten świat plaż, jezior, McDonaldów. Gdzieś to tam jest, będzie można sobie pojechać do restauracji, usiąść z rodziną.

**Czy uwięzieniu towarzyszą jeszcze jakieś emocje?**

Święta zrobiły swoje, później chce się tego kontaktu. Chciałoby się z kimś spotkać, gdzieś pojechać razem. W poprzednich tygodniach gdzieś tam pojechaliśmy, ale w tym momencie już zaczyna być rutyna. Mam wyjazd jeden gdzieś na zakupy, wracam, sąsiadów widzę przez okno. Wcześniej zamienialiśmy kilka słów, ale gdzieś to powoli zniknęło.

**Wyobrażasz sobie obraz, który opisuje idealnie to co czujesz?**

Nie wiem, może jakaś bezludna wyspa? Może nie bezludna, ale jakaś wyspa. Mamy tu dużo plusów w okolicy, które w porównaniu z innymi ludźmi dają zupełnie inny poziom postrzegania. Mamy ogród, leżaki, kosz, cokolwiek. Dużo, ale to ciągle wyspa. Od czasu do czasu przypłynie jakiś prom. Wyspa, ale nie skromna, bo jak rozmawiam ze znajomymi z Warszawy to zdaję sobie sprawę, że w porównaniu z nimi mamy fajnie.

**Czy czujesz się zagrożony sytuacją?**

Tak, jak najbardziej. Co jakiś czas śledzę, co się dzieje. Widzę, ze jest problem globalny. Każdy z nas może się zarazić. Pytanie na ile to wyrządzi szkód. Jest takie zagrożenie i gdzieś człowiek o tym myśli.

**Na ile silne jest to poczucie zagrożenia w ciągu ostatnich 2 tygodni?**

No, 30% bym powiedział. Czyli trójka w skali dziesięciostopniowej. Nie czuję się zagrożony, ale ciekawe co będzie, gdy będę musiał regularnie jeździć do pracy. Wszystko wskazuje, że to nastąpi w maju. W domu nie daję się porywać złym emocjom. Ludzie w pracy jeżdżą tam codziennie, w porządku, jak każdy będzie dbał o swoje bezpieczeństwo, higienę, środki dodatkowe, żele, rękawiczki. Myślę, że nie ma takiego wysokiego ryzyka, żeby coś się wydarzyło. Ludzie są uświadomieni. Mając objawy gorączki wstrzymają się od kontaktów z innymi, to daje komfort psychiczny.

**Myślisz, że koniec pracy zdalnej zwiększy zagrożenie?**

Na pewno. Będę się spotykał z obcymi ludźmi. W tym momencie z osobami mieszkającymi po sąsiedzku zachowuję dystans, nie pozwalam podchodzić dzieciom. Jak raz sąsiad przyszedł, to postawiłem mu piwo na słupku. Ze znajomymi trzyma się dystans i myśli o tym, że można się zarazić. Z obcymi tym bardziej. Zagrożenie na pewno się zwiększy.

**Czy zauważyłeś jakieś zmiany w zachowaniu ludzi z otoczenia?**

Myślę, że ogólnie sytuacja spowszedniała. Dlatego zamknięcie zaczyna doskwierać i wydaje się, że możemy zwiększyć te granice. Tak naprawdę nie zauważyłem, żeby coś się zmieniło jeśli chodzi o strach. Nie ogląda się już kanałów informacyjnych z emocjami. To się zdążyło utrzeć w świadomości. Przyjęliśmy, że to może potrwać. Nikt nie jest w stanie przewidzieć zmian.

**Czy to spowszednienie napędza do działania?**

Wydaje mi się, że to jest właśnie powiązane z tym, że izolacja doskwiera. Ta niewiadoma. Może tak będzie przez najbliższe 5 lat? Może to będzie wracało cyklicznie? Może jakieś restrykcje zostaną zniesione, ale wirus będzie krążył. Ta niewiadoma jest wkurzająca. Już ludzie przywykli, żeby chodzić w maskach, trzymać dystans. To już standard. Może to krok do tego, aby na więcej sobie pozwolić, nie zabudowywać się tak bardzo.

**Ale czy to spowszednienie wywołuje bezsilność, czy raczej jakieś pozytywne reakcje?**

Ciężko powiedzieć czy to pozytywne w ogólnym sensie. Najlepiej, gdyby to się po prostu skończyło i wróciło do normy. Wydaje mi się, że to jest własne dostosowanie się do sytuacji. Gdyby to miało potrwać przez dwa lata, to nie wyobrażam sobie siedzenia w domu. Musimy chodzić do sklepów, gdzieś pojechać z dziećmi i tak dalej. Pewnie będą jakieś restrykcje lub zachowania, ale ja czuję, że to szybko się nie skończy i jakoś będzie trzeba z tym żyć. Jakoś się dostosować. Zaczyna troszkę denerwować.

**Ostatnio zmieniły się pewne ograniczenia. Czy wiesz jakie i jak je oceniasz?**

Tak, zostały otwarte jakieś parki, lasy. Możliwość spacerów, nieletni mogą przebywać na ulicach bez opieki, znowu restrykcje związane z noszeniem masek. Jestem na bieżąco. Oceniam to dobrze, w porządku. Nie wyobrażam sobie, żeby nie można było się w bezpiecznej odległości przespacerować. Las to nie ciasne pomieszczenie. Nakaz noszenia masek to był dla mnie standard jeszcze wcześniej. Teraz jak jadę samochodem sam, to też jej nie noszę, to bez sensu. W kontaktach z innymi ją zakładam. Jechałem do rodziców przed świętami, maseczkę miałem założoną. Ogólnie ostatnie zmiany są dobre.

**Jak postrzegasz skuteczność maseczek?**

Maseczka bardziej chroni osoby, które ja mogę zarazić. Mogę przechodzić to bezobjawowo. Kiedy coś mówię, ta maseczka na pewno blokuje wyrzut jakichś wirusów. To jest ochrona bardziej kogoś, niż siebie. Jak noszę maseczkę to przeważnie do 2 godzin. Później ją dezynfekuję. Mam miejsce na filtr, ale go nie używam - używałbym gdybym nosił ją dłużej niż 2 godziny. Materiał jest ważny, czy to maseczki bawełniane czy inne chirurgiczne, czy zawierają filtry i tak dalej. Myślę, że to dobre, że ludzie noszą maseczki. Ja zwiększam ich bezpieczeństwo, a oni nosząc zwiększają moje.

**Co sądzisz o nowych zasadach dotyczących rekreacji i sportu z koniecznością zakrywania twarzy?**

To bez sensu ogólnie. Sport wymaga dostarczania więcej tlenu. O to chodzi w sporcie. W maskach bieganie? Nie widzę w ogóle takiej opcji.

**Co myślisz o nowych zasadach w handlu?**

Hmm, a jak to się zmieniło? Bo w tym momencie nie wiem.

**Wcześniej maksymalna liczba klientów była liczona względem kas, teraz względem powierzchni sklepu.**

Nie wiem, ja nie przykładam do tego wielkiej wagi. Jak jestem w sklepie, to też zachowuję dystans. To chyba od poniedziałku weszło? Jeszcze na zakupach nie byłem, zobaczę jak to będzie wyglądało jutro. Każdy może sam o ten dystans zadbać. Nie odczułem różnicy, bo byłem na zakupach w zeszłym tygodniu.

**A jeżeli chodzi o kult religijny? Dopuszcza się jedną osobę na 15 metrów kwadratowych kościoła.**

Dopóki to się nie skończy, nie będziemy chodzić do kościoła. To zwiększa zagrożenie, ale potrafię to zrozumieć. Osoby w kościele powinny o tym wiedzieć, że to zwiększa ryzyko zakażenia. Ja na pewno nie będę do kościoła jeździł ani zabierał dzieci, dopóki nie będzie zielonego światła. Dopóki sam nie powiem, że jest okey, że sprawdziliśmy to i nie ma przypadków od pewnego czasu. Do tego momentu będziemy uczestniczyć we mszy przez transmisję telewizyjną.

**Co myślisz o możliwość przemieszczania się osób powyżej 13 roku życia bez opieki osoby dorosłej?**

To rozsądne, moim zdaniem te ostatnie obostrzenia były trochę bezsensowne. 16 i 17-latki to prawie osoby dorosłe. To śmieszne, że miały być pod opieką.

**Czyli nie ma obostrzeń, z którymi się nie zgadzasz?**

No na pewno bieganie w maskach, to dla mnie bez sensu.

**Biegasz podczas epidemii?**

Nie chce mi się. Nie wiem, przed epidemią jeździłem na basen i biegałem na bieżni. W tym momencie zrobiłem pierwszy krok i ją rozłożyłem. Planuję, że pobiegam, aczkolwiek kontroluję wagę. Teraz trzymam dietę, kontroluję wagę i jestem zadowolony. Siedzę w domu i mam wszystko na wyciągnięcie ręki, ale potrafię się powstrzymać i trzymam standard.

**Gdyby przyszła chęć na bieganie na zewnątrz to nakaz biegania w masce by cię powstrzymał?**

Nie, absolutnie. Maskę mogę mieć przy sobie w razie czego. Jak widzę, że nikt za mną nie biegnie ani przede mną. Też się nie chce biegać na bieżni bo jest fajna pogoda na zewnątrz. Myślałem, żeby się przestawić chociaż raz w tygodniu na rower lub pobiegać na zewnątrz. Na razie bieżnię rozłożyłem, aczkolwiek prędzej zdecyduję się zrobić coś na powietrzu. Na pewno nakaz biegania w masce mnie nie powstrzyma. Nie będę biegał w masce. Będę wybierał takie tereny, żeby nie spotykać ludzi. Mieszkam na wsi i mam ten komfort, że mogę zrobić 10km rowerem i wybrać taką trasę, żeby do nikogo się nie zbliżyć.

**Czy słyszałeś o planach zdejmowania ograniczeń?**

Tak. Słyszałem o sklepach odzieżowych i punktach usługowych typu fryzjer i tak dalej. Myślę, że to dobry pomysł ogólnie. Ten pierwszy boom, żeby się nie rozpowszechniało, to było dobre posunięcie. Teraz ludzie mają większą świadomość i sami będą się zachowywać bardziej odpowiedzialnie. Jednak biznesy muszą prosperować, ludzie potrzebują takie rzeczy.

**Od czego powinniśmy uzależniać otwieranie kolejnych instytucji?**

To chyba powinno być uzależnione od jakichś obserwacji, wskaźników zachorowań może. Pierwszeństwo powinny mieć jakieś, nie wiem, trudno wytypować, zostawiam to ekspertom, nie zastanawiałem się, ciężko mi się wypowiedzieć. Jak będą otwarte sklepy odzieżowe to któregoś dnia się wybiorę. Jak otworzą zakłady fryzjerskie to się nie wybiorę, bo mam maszynkę i mnie żona strzyże, a ja dzieci.

**Które z ograniczeń powinny twoim zdaniem zostać na dłużej, a które można szybciej znieść?**

Na pewno jakieś kluby, restauracje, koncerty, imprezy masowe, mecze jak najbardziej jeszcze zabronione. Natomiast wszystkie small biznesy, punkty usługowe powinny być otwarte. Każdy pewnie zmieni te zasady u siebie - mniej osób w sklepie, jakaś dezynfekcja. Baseny nie powinny funkcjonować, siłownie też nie. To jednak nie są najczystsze miejsca i jedna osoba może tam zarazić wiele innych. Natomiast sklepy i punkty usługowe typu "wymień oponę" powinny działać.

**Mniejsze punkty powinny działać tak samo, jak przed epidemią, czy coś powinno się zmienić?**

Oczywiście powinno się zmienić. Wszystko z zachowaniem bezpieczeństwa. Nakaz noszenia maseczek pewnie będzie jeszcze pewnie przez długi czas i ja tak uważam.

**Czy słyszałeś o tym, jak Szwecja podeszła do epidemii?**

Chyba nie.

**Nie wprowadzono wielu ograniczeń. Zakazano zgromadzeń powyżej 50 osób, szkoły i przedszkola są wciąż otwarte, lecz szkoły średnie i uniwersytety działają w trybie zdalnym.** **Władze apelują o to, żeby osoby chore zostały w domu. Jak oceniasz taki system, który bazuje na rekomendacjach, a nie zakazach i nakazach?**

Mieszkałem przez pół roku w Szwecji. Tam ludzie mają ogólnie większą świadomość. Oni sami zadbają, żeby było w porządku. Ale to, że przedszkola są otwarte, to nie jest zbyt dobrze. Tam zawsze były rządy luzackie, mniej restrykcyjne. Społeczeństwo jest bardziej wyluzowane moim zdaniem. W Polsce to było konieczne, ale w Szwecji powinni bardziej stanowczo zareagować. U nas cała polityka jest brutalna i stanowcza, pełna obrazów władzy totalnej i tak dalej, ale musieli tak zareagować. Tam powinni zareagować trochę mocniej i porządniej.

**Rozwiniesz, dlaczego Szwedzi powinni mocniej zareagować?**

Mi rozum podpowiada, że szkoły, żłobki i przedszkola powinny być zamknięte, bo dzieci mogą przechodzić bezobjawowo i zarażać. Widzę po swoich dzieciach, że nie chodzą do przedszkola i nie chorują. Nawet kataru nie miały. W przedszkolach dzieci wszystkiego dotykają i szybko roznoszą się tam choroby.

**Czy dobrze rozumiem, że rząd szwedzki jest twoim zdaniem nieodpowiedzialny?**

No dokładnie. Dobry rząd powinien w pewnym momencie wziąć na siebie głosy krytyki i poparcia, natomiast sami powinni wziąć tą odpowiedzialność. Najważniejsze jest zawsze zdrowie.

**Kiedy żłobki i przedszkola powinny zostać otworzone w Polsce?**

To zależy, kiedy sytuacja zacznie się polepszać. Ja widzę, że od września wszystko wróci do normy jeśli chodzi o szkolnictwo, nie wcześniej, jeśli w czerwcu spadnie liczba zakażonych. W lipcu zobaczymy, że to już niedużo osób i restrykcje zaczną być zdejmowane. Sierpień na obserwację, czy nie idzie druga fala. Po takim miesiącu czy dwóch powinno szkolnictwo ruszyć.

**Myślisz, że polityka rekomendacji mogłaby sprawdzić się w Polsce?**

Nie, u nas to był mus, żeby zdecydowanie zareagować. Można porównać do tego, że głowa rodziny musi pokazywać, że działamy stanowczo, bez wahania i oznak słabości. Rząd też musi, żeby dać ludziom poczucie, że tam są zdecydowani i odpowiedzialni ludzie. Ja jestem wyznawcą zasady, że decyzja powinna być szybka. Im dłużej trwa, tym bardziej okazujemy słabość i to działa na niekorzyść. Nieraz gorsze wyjście podjęte szybko i dynamicznie daje lepszy efekt. Chociaż opozycja szaleje, nie chce mi się tego oglądać, ale trzeba wziąć tą krytykę, bo nigdy nie będzie tak, że wszyscy będą zadowoleni.

**Co daje nam ta stanowczość decyzji?**

To jest takie poczucie, że ludzie podejmujący te decyzje podejmują je stanowczo i są utwierdzeni w tych decyzjach. Poza tym można teraz zauważyć, że podjęte stanowczo decyzje można wycofać. Jak zamknięcie lasów. Dając zakaz okazujemy zdecydowanie, a po 2 tygodniach zdejmując go też okazujemy zdecydowanie. Taki model mi się bardziej podoba niż dyskusja, gdy trzeba podjąć decyzję.

**Jakie korzyści z tak podejmowanych decyzji odnoszą obywatele?**

Poczucie satysfakcji, że rząd potrafi podejmować szybkie decyzje, szybko zareagować. Że w sytuacji kryzysowej szybko wychodzą ustawy, szybko działa rząd.

**Czy to ma związek z poczuciem bezpieczeństwa?**

Nie no, dokładnie. Ci ludzie tutaj wybrani do tego rządu będą zdeterminowani, żeby o to bezpieczeństwo zadbać. Można im zaufać. Łatwiej jest szaremu człowiekowi zaufać osobie która mówi zdecydowanie "robimy tak" niż osobie, która będzie się wahała. Wszędzie tak jest.

**Jak dbasz o siebie w ostatnim czasie?**

Rzadziej się golę, mniej robię zakupów bo przeważnie kupuje ubrania w sklepach, a nie internecie. Jeśli chodzi o strzyżenie, poradziłem sobie z żoną trochę. Ja przynajmniej jakieś takie podstawy, standard utrzymuję. Jak wiem, że nikt mnie nie będzie widział cały dzień, ani czuł, ale i tak się zawsze wodą toaletową popsikam i włosy ułożę. Jeśli chodzi o brodę, to jak z kimś mam się widzieć to ogolę, ale tak nie wczuwam się.

**Co daje ci dbanie o siebie?**

Ktoś dał mi taką radę. Mam taki rytuał, który się sprawdza i nie wyobrażam sobie z niego zrezygnować. Ktoś mi powiedział, że jak chcę dobrze pracować w domu, to trzeba do tego podchodzić tak, jakbym chodził do pracy.

**Czy to działa?**

Tak, zdecydowanie. Przykładowo, dla mnie to taki wyznacznik dres-jeansy. Nie przesadzam, już nie będę siedział w koszuli. Lepiej się czuje w t-shirtach. Jak zakładam jeansy to lepiej mi się pracuje, niż w dresie. Dres jest do domu, gotowania, oglądania telewizji. Czyli ubieram się tak, jakbym chodził do pracy. Koszulę do pracy rzadko zakładam, tylko od czasu do czasu, jednak gdzieś tam pracuję na stanowisku kierowniczym. Mam młode osoby w zespole, nieraz przyjdę w śmiesznym t-shircie i jest śmiesznie.

**Czy u żony coś się zmieniło jeśli chodzi o pielęgnację?**

Widzę, że z córką bardziej sobie tak eksperymentuje - paznokcie, maseczki. Myślę, że ten czas, kiedy córka jest non stop w domu, to sobie wymyślają jakieś takie damskie zabiegi. Chyba jest tego więcej niż wcześniej.

**Jeżeli chodzi o kosmetyki to zmieniłeś może przyzwyczajenia?**

Nie, raczej nie, raczej trzymam standard. Jedyne co, to może rzadziej się golę. Tego teraz nie widać jak rozmawiamy. Zawsze miałem mniejszy zarost, jak miałem jakieś spotkania w pracy, to jest jedyna różnica, jeśli mogę tak powiedzieć.

**Czy w sferze dbania o siebie pojawiło się coś poza samodzielnym strzyżeniem i rozłożeniem bieżni?**

Walka trwa jeśli chodzi o odżywanie. Już miałem taki okres, kiedy obiady kończyłem deserami, ale teraz codziennie się ważę. Wolę trzymać rękę na pulsie, pilnuję się, aby nie poszaleć z tymi kaloriami. Natomiast co do ćwiczeń mam dzień, że na przykład co jakiś czas idę na drążek albo zrobię 30 pompek. Natomiast nie jest to regularne takie, jak było przed tą całą sytuacją. Przed operacją, którą miałem pod koniec lutego, to też mnie wybiło. Miałem zakaz cięższych ćwiczeń do początku kwietnia. To też mnie wybiło z rytmu. Wcześniej raz w tygodniu jeździłem na basen, jeden bieżnia plus dieta plus jakieś takie różne. Kółeczko kupiłem do trenowania mięśni brzucha. Nie wytrenowałem sposobu, żeby ustabilizować sobie rytm. Jak sobie ustabilizuję, że w czwartki jeżdżę na rowerze, a się okaże, że za miesiąc wrócę w czwartki basen, to ta niepewność wpływa na to, żeby ułożyć sobie rytm. Na razie kontroluję wagę.

**Dlaczego kontrola wagi jest dla ciebie ważna?**

Dlatego, bo włożyłem dużo pracy, aby tę wagę osiągnąć. W tym momencie dużo osób mi mówiło, że dużo zrzuciłem, ale zobaczymy jak to będzie. Trzymam rękę na pulsie, żeby z tych 20kg nagle 10kg mi nie wróciło. Brakuje mi teraz możliwości pójścia na basen. To coś, co naprawdę robię dla siebie. Nieraz pojechałem i na półtorej godziny popływałem pół godziny, a godzinę posiedziałem w saunie, ale to było takie dla mnie. Odciąć się, odstresować po tygodniu pracy.

**Czego ci najbardziej brakuje?**

Fryzjera na pewno mi brakuje. Będę musiał coś pomyśleć niedługo, albo ogolę się na łyso jak sąsiad. No i tego basenu. Sporty to ja tak trochę inaczej. Basen mógłbym sobie czymś innym zastąpić, natomiast fryzjera... ale ostatnio byłem zadowolony jak mnie żona ostrzygła. Ale basen jest dla mnie zdecydowanie wyżej niż fryzjer. Basen to jest wyprawa. Po przemyśleniu jednak wybrałbym basen. Na basen rzadko jeździłem sam, przeważnie ze znajomymi, rodziną, szwagrem, z sąsiadem. Z sąsiadami regularnie co tydzień, we dwa samochody. To nie tylko sport, ale też rekreacja, sauna, relaks. Nie brakuje mi chodzenia po sklepach, bo często mnie to wkurzało. Raczej robię zakupy automatycznie. Nie wczuwam się w zakupy.

**Czy internet byłby w stanie zastąpić takie zakupy?**

Raczej nie, nieraz już tak się przejechałem na internecie. Bluzę mam niby L-kę, ale wisi na mnie. Buty jeszcze tak, ale ubrania nie.

**Czego ci najbardziej brakuje z punktu widzenia konsumenta?**

Brakuje mi możliwości pojechania gdzieś, zabrania dzieci. Bardziej tego mi brakuje, żeby można było sobie bez problemów zabrać rodzinę na majówkę nad morze na przykład. Już mieliśmy plany na majówkę, teraz musimy je zmienić. Ogólnie miejsca, gdzie moglibyśmy się udać - wyjazd, kino, zoo, restauracja. Tego mi brakuje.
