# Supplementary material for: The challenges arising from the COVID-19 pandemic and the way people deal with them. A qualitative longitudinal study
Source: PLoS One. 2021 Oct 11;16(10):e0258133. doi: 10.1371/journal.pone.0258133 (PMC8504766; doi:10.1371/journal.pone.0258133)
Supplement: S1 Dataset — (ZIP) [file pone.0258133.s003.zip › Transcriptions/stage 3/7.3_M_28_couple, no children.docx]

**7.3_M_28_couple no chidlren**

**Co się wydarzyło przez ostatnie 2 tygodnie?**

W sumie dużo się wydarzyło. Święta minęły tak, jak zapowiadałem, że wyjechaliśmy sobie do domu dziadków Dominiki i tam sobie posiedzieliśmy 2 dni. To były fajne 2 dni, bo można było w końcu posiedzieć na powietrzu, trochę filmy i ogólnie relaks. Później było trochę mniej przyjemnie, bo miałem taki dosyć intensywny tydzień pracy - musieliśmy niestety odwołać nasz festiwal, nad którym pracujemy cały rok. Ja w sumie jestem jednoosobowym działem komunikacji tego festiwalu, więc wszystko spadło na mnie i miałem sporo pracy. No i ze względu na to jakiś smutek, zdenerwowanie, trochę też taki lęk co będzie dalej z pracą i z przychodami, ale ostatecznie jakby nic się nie zmieniło. Żyjemy z moim szefem na takiej dosyć specyficznej stopie kumpelsko - zawodowej i powiedział mi szczerze i szczerze mogłem z nim porozmawiać, więc ten lęk przynajmniej chwilowo minął, no ale też jakby musiałem trochę odreagować, więc w piątek tuz po odwołaniu zrobiliśmy sobie skype'ową małą imprezę ze znajomymi. W tym tygodniu jest dalsze zamykanie spraw festiwalowych w pracy i przymierzanie się do edycji za rok. Zastanawialiśmy się nad przeniesieniem na później, ale to jest dosyć trudne. 100 koncertów zaplanowanych na 3 dni - dosyć trudno byłoby wszystkich przenieść na tę samą datę, poza tym wydaje mi się, że w tym roku raczej już żaden festiwal się nie odbędzie. Przynajmniej w takiej formule wielotysięcznej. Przymierzamy się do tego za rok, aczkolwiek to też nie jest nic pewnego, że za rok będziemy mogli to zrobić.

**To, że w tym roku nie ma festiwalu, to nie znaczy, że ty nie będziesz miał co robić teraz?**

Z jednej strony będę szykował teraz kolejny festiwal, z drugiej, pod katem takim czysto finansowym, to ze względu na to, że mamy sponsorów, to i tak ta komunikacja i to, że sponsorzy nas wspierają, trwa dużo dłużej. Kończymy jedną edycję i za miesiąc, dwa ogłaszamy kolejną. Już prowadzimy rozmowy, że dostaniemy cześć środków obiecanych za promocję, za cześć świadczeń, które im obiecaliśmy. Z drugiej strony troszeczkę w innej formie niż dotychczas, z innym planem, harmonogramem zaczynamy dużo wcześniej ogłaszać tę edycję za rok i może uda nam się coś zrobić w jakiejś dużo, dużo, dużo mniejszej formie - transmisje online, czy jakieś tam inne akcje w lato.

**Teraz to jest dość modne. Nie myśleliście, żeby przynajmniej kilka zostawić w takiej formie w tym roku?**

Właśnie dokładnie taki jest plam. jak uda nam się pozyskać jakieś środki od sponsorów i też całą zeszłą niedzielę pisałem wniosek do ministerstwa, bo minister uruchomił środki na wsparcie kultury. Wysłaliśmy i zobaczymy, co z tego wyjdzie.

**To dla ciebie taki ciężki okres?**

Ciężki. Ciężki też pod takim względem, że z jednej strony obawy o pracę, tzn. spodziewałem się raczej tego, że nie zostanę bez pracy, być może z jakimś tam ucięciem małym kosztów, bo tej pracy nie ma aż tyle, ale też byłem pełen nadziei i z tego co rozmawiałem z moim szefem, to raczej się na to nie zapowiadało...Odbyliśmy szczerą rozmowę na ten temat i ten lęk w dużej mierze minął, aczkolwiek sytuacja, którą mamy - teraz chodzi mi głównie o stanowisko rządu. Festiwal odwoływaliśmy w piątek, a już spodziewaliśmy się i nastawialiśmy się na to od kilku dni...Czekaliśmy na jakieś konkretne stanowisko premiera w kontekście imprez masowych i w ogóle kultury. W czwartek jeszcze tego stanowiska nie było. Było tylko pytanie zadane przez jednego z dziennikarzy, na które on powiedział, że ta branża nie wpływa aż tak znacząco na gospodarkę, co w naszej branży też odbiło się takim smutnym echem, że kultura jest tak nieważna i imprezy masowe. Angela Merkel powiedziała, że w Niemczech kultura jest teraz jedną z najważniejszych rzeczy, żeby dać ludziom odpocząć. To był taki dosyć trudny czas i zmiana duża. Powoli się z różnych rzeczy wygrzebujemy i planujemy inne rzeczy, więc mam nadzieję, że to się jakoś w miarę bezboleśnie ułoży.

**A jak wygląda takie twoje codzienne funkcjonowanie?**

Właściwie to się nie zmieniło. Wręcz powiedziałbym, że mam więcej pracy niż mniej na ten moment. Właśnie ta ostatnia niedziela, czy teraz te ostatnie dni. Zmieniło się moje samopoczucie na pewno na początku, bo jednak pracujemy nad czymś przez cały rok, to jest praca wielu ludzi. My jesteśmy w ogóle takim festiwalem, który jest dosyć chałupniczo robionym, bo nad całym festiwalem pracują tylko 4 osoby przez cały rok. Później ta liczba się zwiększa i na samym festiwalu jest mnóstwo osób pracujących, ale to też jest nasz taki rodzinny, kumpelski projekt. Ja przy tym festiwalu pracuję od 7 lat, a festiwal ma 15. Byłem za mały, żeby pracować przy nim wcześniej. To szybko przerodziło się w taką relację dosyć bliską i też tak bardzo osobiście traktowaliśmy ten projekt. Nie czysto zarobkowo, ale też tak poważnie. W moim codziennym funkcjonowaniu na razie nic się nie zmieniło.

**Dalej jest taki trochę inny styl, że trochę więcej gotowania, więcej czasu we dwójkę na jakieś filmy i takie rzeczy?**

W sumie więcej to nie, dalej to gotowanie jest i to spędzanie czasu we dwójkę, aczkolwiek ja miałem też sporo pracy i Dominika też cały czas jest zapracowana, więc tego czasu we dwójkę jest w sumie niewiele. Tak od 20-21 dopiero, jak zdążymy już wszystko zrobić do pracy. Może jutro uda nam się wreszcie wyjechać za miasto.

**Na działkę?**

Celujemy, żeby jechać gdzieś indziej, trochę inny kierunek. Jeszcze nie wiem dokładnie, gdzie pojedziemy, dzisiaj pewnie to ustalimy. Mam nadzieję, że tego czasu wolnego będzie teraz trochę więcej. Może to dobrze? W sumie sam nie wiem, czy lepiej więcej pracować i nie przejmować się niewychodzeniem z domu, czy odwrotnie.

**Ile czasu dla ciebie trwa ta pandemia? Jak ty to czujesz?**

Dla mnie tak od początku marca, czyli już prawie 2 miesiące.

**Co jest w tej chwili największym dla ciebie wyzwaniem w tej sytuacji?**

Hmm...Wydaje mi się, że oswoiłem się już z tym, że są ograniczenia, tzn. nawet z noszeniem maseczek i rękawiczek, z dużo rzadszym wychodzeniem, ale mimo wszystko brakuje mi takiego odświeżenia, zrobienia czegoś innego w końcu po tych 2 miesiącach, może jakiegoś wyjazdu, może w końcu spotkania z kimś, może takiego powrotu do starych aktywności. Sam nie wiem. Czuję taką stagnację, która mi już zaczyna coraz bardziej doskwierać.

**Stagnacja, takie zawieszenie?**

Tak, dokładnie.

**Coś jeszcze?**

To jest dosyć specyficzna sytuacja, bo wcześniejszy, pewnie troszeczkę większy lęk przed zarażeniem bardziej przerodził się już teraz w zmęczenie tym wszystkim. Zmęczenie informacjami na ten temat, zmęczenie niewychodzeniem z domu, zmęczenie w takim kontekście, że praktycznie robimy cały czas te same rzeczy. To jest dla mnie taka mała zmiana, że właśnie mniej odczuwam ten lęk.

**A jesteś zły na coś w tej całej sytuacji?**

Myślę, że w pewien sposób tak, ale to jest raczej taka złość w formie bezradności. Jestem zły, ale bardziej z tego powodu, że nic się nie da z tym zrobić, co jest nadal złością na to, że coś takiego się przydarzyło i dalej trwa.

**Jesteś zły na sytuację?**

Tak.

**A na ludzi?**

Nie, nie, nie.

**Emocje - zdjęcia**

**Obrazki na poprzedni tydzień i na ten moment**

6 i 8

8 to moment, kiedy dowiaduję się o tym, że już jest przesądzone, że odwołujemy festiwal. Okazało się, że ta 6-miesięczna praca to była droga do nikąd, która była długa autostradą, jak na tym zdjęciu, ale ostatecznie nie ma żadnych perspektyw, czyli nic nie widać. Wszystko trafiło do kosza.

6 dlatego, że od dłuższego czasu mi ten lęk doskwierał, że ten festiwal się nie odbędzie i z drugiej strony jest to pewien rodzaj ulgi, że w końcu ta decyzja została podjęta, że już się nie stresujemy tym, że już możemy podjąć jakieś kroki.

**Przestałeś być w takim zawieszeniu z tym lękiem swoim o ten festiwal? Już dobrze, że wiem?**

Myślę, że tak. To naprawdę od końcówki marca było już dosyć stresujące, słysząc różne głosy, a to WHO, a to różne kraje różne kroki podejmowały w tym kierunku. U nas ciągle nie było żadnego klarownego stanowiska w tym względzie, więc to było naprawdę takie stresujące, martwiące. Jakoś cały czas miałem to z tyłu głowy prowadząc tę komunikację festiwalu, że kurczę, staramy się cały czas, staramy się myśleć pozytywnie, ale z biegiem czasu ta nadzieja malała.

**Jak już się okazało, że nie będzie i był ten smutek, to jaką miałeś strategię poradzenia sobie z tym?**

Od razu w sumie poczułem taką ulgę, oddech, coś w środku mnie puściło, a z drugiej strony musiałem też odreagować w taki sposób, że napiłem się trochę alkoholu, że zrobiliśmy małą imprezę. Po prostu taki wieczór, żeby o tym nie myśleć. To nie była jakaś wielka balanga i wielkie picie, ale takie symboliczne poświęcenie kilku godzin, żeby odciąć się chociaż na chwilę. To byli moi przyjaciele, którzy co prawda jeżdżą na ten festiwal, więc znają go doskonale, też dużo wiedzą, jaki ja mam stosunek do niego, ale to nie są ludzie bezpośrednio związani i rozmów na temat tego festiwalu praktycznie nie było za dużo, żeby nie psuć atmosfery.

**Ten lęk odszedł, a ten lęk o bliskich, o zarażenie?**

Mam wrażenie, że zmalał, ale być może zmalał dlatego, że ta sytuacja trwa już jakiś czas, przedłuża się i tym bliskim nadal nic się nie stało, i być może to jest takie naiwne myślenie, że skoro nadal nic się nie stało, to już pewnie się nie wydarzy. Mniej mnie jakoś to martwi, ale to może być złudne. Psychicznie jest mi z tym lepiej, mam w ogóle mniej myśli na ten temat. Rozmawialiśmy ostatnio, jak bardzo się tym interesuje i czytam na ten temat, i to mocno zmalało. Już nie jest tak, że wstaję rano i patrzę, co się wydarzyło w nocy, jakie nowe informacje wypłynęły. Już raczej nie. Jeśli zrobię to raz dziennie, to w ogóle często przypadkowo. Odpuściłem to śledzenie danych, bo to jednak zostawało mi w głowie i tak na dłuższą metę stwierdziłem, że chyba nie ma sensu tak robić, nie ma sensu się tym tak martwić, tylko trzeba się z tym raczej oswoić i może coś zmienić właśnie. Może mniej się interesować, mniej czytać, mniej brać to do siebie i tak, czuję się lżej dzięki temu.

**A jak wygląda to u twoich bliskich? Jak twoi rodzice sobie radzą?**

Moi rodzice radzą sobie dosyć nieźle i też czuję różnicę w rozmowie z nimi. Też chyba oswoili się trochę z tą sytuacją. Z tego co wiem, to nie odczuwają jakiegoś wielkiego lęku, jeśli chodzi o siebie, bardziej chyba odczuwają lęk o moją siostrę, która mieszka w Hiszpanii. Tam jest sytuacja trochę poważniejsza i ona też została właściwie bez środków do życia, bo wszystkie firmy stoją. Kierunek ich zmartwień przeniósł się właśnie tam. Zobaczyli, że ja sobie tutaj radzę razem z Dominiką, mój brat też sobie radzi i bardziej w tamtą stronę się skierowali.

**Masz kogoś w swoim otoczeniu, kto tak psychicznie źle znosi tę sytuację?**

Mam i to są w sumie wszyscy moi przyjaciele, to też jest Dominika i to też jestem ja, tylko, że to nie jest stałe uczucie. Po prostu przychodzą takie dni, kiedy co raz jedna osoba mówi, że już ma dosyć, że już chce wyjść, że już nie chce o tym myśleć, że ma dosyć tej sytuacji. Obserwuję to i słucham o tym już od kilku tygodni. Dosłownie każdy, kogo znam o tym mówi.

**Oni są zmęczeni tą sytuacją, są źli, wkurzeni już strasznie. Jaki to jest rodzaj emocji?**

Wydaje mi się, że to jest złość przede wszystkim. Złość na tę sytuację i z drugiej strony też bezsilność. Niektóre osoby wspominają też, że są złe na ludzi, którzy nie stosują się do obostrzeń, którzy cały czas stwarzają zagrożenie i dzięki temu też może sytuacja się przedłuża. Raczej to jest taka złość na sytuację, która zdarzyła się bez niczyjej specjalnie winy.

**Pytałam się poprzednio o takie dziwne zachowania. Czy pojawiło się coś takiego?**

Usłyszałem ostatnio, że ktoś...Nie wiem, czy to jest dziwne, ale ktoś mówił, że otrzymał jakąś paczkę i musiał ją zdezynfekować, tzn. umył ją całą z tego wirusa, a z drugiej strony poszedł za chwilę do sklepu i kupił jakąś rzecz, i otworzył ją i zjadł ją od razu bez mycia rąk. Nie na ulicy, już w domu. Bardzo dziwne i ja bym znacznie szybciej pomyślał, żeby umyć ręce w ogóle po wyjściu, a przed zjedzeniem czegoś tymi rękoma, to podejrzewam, że mógłbym umyć nawet 2 razy. Innych dziwnych zachowań nie kojarzę.

**Przez ostatnie 2 tyg. chodziłeś do sklepów?**

Tak, wydaje mi się, że wychodziłem 3-4 razy.

**Zauważyłeś jakieś różnice w zachowaniu ludzi?**

No właśnie przez to, że już nie ma takich obostrzeń, to zauważyłem, że ludzie już są mniej uważni. Wcześniej przynajmniej widać było to, że ludzie starają się przechodzić obok siebie z jakimś dystansem. W tym tygodniu widziałem, że już niekoniecznie, widziałem osoby, które już nie noszą rękawiczek. Są mniej uważni. Poza tym widzę teraz mnóstwo ludzi na ulicach znowu, też przez poluzowania i pewnie też przez pogodę.

**Co myślisz, jak widzisz tak dużo ludzi?**

Z jednej strony jestem zły w sumie na nich, że stwarzają zagrożenie w sumie, że idą po ulicy obok siebie, że nie noszą rękawiczek. Z drugiej strony zastanawiam się, czy dobrze rozumiem ostatnie obluzowania obostrzeń.

**A jak je rozumiesz? Co poluzowali?**

Oczywiście otwarto lasy i parki, co akurat bardzo cieszy, że można w końcu wyjść w takie bardziej ciche miejsce, ale to też stwarza zagrożenie, więc mam takie mieszane uczucia. Nie wiem, czy nadal osoby mieszkające ze sobą...Czy mogą wyjść razem, bo wcześniej była taka wersje, że nie.

**Wydaje mi się, że nie.**

No właśnie. Też tak obstawiałem i sami tego nie robimy, ale widziałem, że pojawiło się już trochę takich par, grupek, rodziców z dzieciakami idących całą grupką.

**Mama, tata, 2 dzieci. Czy z twojego punktu widzenia, powinni razem wychodzić na spacer?**

Wydaje mi się, że do miejsca, w którym mogą być inne osoby nie, ale jeśli chodzi o wyjazd za miasto do lasu czy gdzieś tam, to nie widzę problemu, skoro nadal razem mieszkają, to nic to nie zmieni, jeśli pojawią się w miejscu, gdzie nie ma innych osób. A jeśli idą w grupce w miejscach publicznych, to chyba tak nie do końca.

**Wiesz, jakie zmiany się zadziały od poniedziałku? Czytałeś, sprawdzałeś?**

Tak, tzn. już nie z taką uwagą jak wcześniej. Wiem o lasach i o parkach, wiem o zniesieniu obostrzeń w sklepach, wiem, że można jeździć na rowerach, ale nie miejskich, że przedłużono teraz i szkoły są zamknięte i ustalono w końcu egzaminy, matury, itd. I właściwie tyle. Nie interesowałem się tym mocniej.

**A to, że teraz może być więcej ludzi w sklepie? Co o tym sądzisz?**

No właśnie to chyba nie jest dobrze. Nie wiem, po co to zostało zrobione. Uważam, że zagrożenie nie minęło aż tak, żeby to robić. poprzednie zasady lepiej zapobiegały rozprzestrzenianiu się wirusa, bo przynajmniej, jak wychodziłem w tamtym okresie do sklepu, to kolejki do sklepów, odstęp między osobami to faktycznie były te 2 m. To było widać i ludzi było znacznie mniej w sklepach, więc był ten dystans. Teraz wchodząc do sklepu tak naprawdę czuję, że cały czas jest ktoś obok mnie. Cały czas ktoś przechodzi, ktoś jest blisko, ludzie już aż tak nie unikają tego kontaktu i to jest nie ok., bo to nadal stwarza zagrożenie.

**Parki i lasy są ok?**

Tak, pod warunkiem, jeśli faktycznie są to miejsca odosobnienia, gdzie nie spotyka się innych osób, albo przynajmniej w dużym dystansie, bezpiecznym. Jeśli to jest park w mieście, czy jakieś bardzo oblegane miejsce, to dla mnie to nie ma różnicy, że to jest otwarte. Nadal uważam i nadal tam nie chodzę.

**Teraz już można się przemieszczać w celach rekreacyjnych. Co o tym sądzisz?**

Nie wiem, jak rozumieć cele rekreacyjne. Można sobie pobiegać, pojeździć na rowerze? No właśnie co do samych poluzowań, to nic do nich nie mam, jeśli to dotyczy miejsc, które nie stwarzają zagrożenia dla innych osób. Ok., fajnie, że możemy w końcu wyjść na powietrze, ale nadal uważajmy. Część osób ostatnio tego nie przestrzega, mam wrażenie.

**Coś jeszcze ci się zapamiętało z tych zmian?**

Szczerze mówiąc nie. To był taki okres, że miałem dużo na głowie i w sumie nie czytałem więcej.

**1 osoba na 15 m w kościołach?**

Staram się myśleć z perspektywy tych osób, które faktycznie potrzebują pójść do kościoła, bo pewnie są takie osoby. Dla nich pewnie to jest ok, tylko też zastanawiam się...Wydaje mi się, że z punktu widzenia społeczeństwa to jednak nie, że powinniśmy jeszcze poczekać. Ciężko mi jest w to uwierzyć, że wszyscy zachowają ten odpowiedni odstęp. Są kościoły, które są ogromne i takie naprawdę małe, gdzie ciężko uniknąć tego kontaktu. Można by było z tym poczekać, tym bardziej, że już przez chwilę dało się do tego przyzwyczaić, że msze były w tv czy w internecie. Nie byłoby wielkiej szkody, gdyby z tym jeszcze poczekać.

**Od 13 r.ż. można być samemu na ulicy?**

Będąc 13-latkiem, to radość z przebywania na zewnątrz, to głównie radość z przebywania z przyjaciółmi, więc też nie wiem, czy to bezpieczne. Wiadomo, że czasem może chodzić o wyjście do sklepu czy do paczkomatu, ale jednak obawiałbym się nadal, że jak spotka się grupa 5 dzieci w tym wieku, to w sumie mogą poprzenosić tego wirusa na kilkadziesiąt osób.

**Co sądzisz o obowiązku noszenia maseczek?**

Wydaje mi się, że wszystko, co może pomóc w tej sytuacji, czyli te maseczki, większa uwaga i powiedzmy mniejsze zagrożenie, jak najbardziej. Jest to męczące oczywiście, bo nawet idąc na zakupy inaczej się w tym oddycha. Jest to trochę męczące, ale wydaje mi się, że nie na tyle, żeby z tego rezygnować.

**One realnie zmniejszają zagrożenie?**

Wydaje mi się, że tak. czasami komuś zdarza się kichnąć, czy nawet rozmawiając przypadkowo plunąć małą kropelka i jest to jakieś zagrożenie. Maseczka to jest zawsze jakieś dodatkowe zabezpieczenie.

**Kogo to zabezpiecza?**

Tę drugą osobę. Maseczka ma chronić inne osoby a nie nas, tzn. nas chroni też. Działa to w dwie strony w sumie.

**Działa fizycznie, czy bardziej psychologicznie, bo ludzie czują się bezpieczniej, że mają?**

Myślę, że to jest dodatkowa zapora, ale nie przywiązywałbym do tego takiej wago, że to jest coś, co w każdej sytuacji nas ochroni. To jest dodatkowy środek zapobiegawczy, ale nie aż tak.

**Obserwujesz, że ludzie noszą te maseczki?**

Raczej tak, ale też zdarzają się ludzie, którzy w każdej wolnej chwili, jak już oddalą się od innych...To zauważyłem, patrząc na przystanek autobusowy niedaleko sklepu. Jak nikogo nie ma, to ludzie ściągają te maseczki wtedy albo je sobie opuszczają.

**To jest nie ok, jak stoją 20 m od innych?**

Wydaje mi się, że to traci sens w momencie, gdy wychodzimy ze sklepu, gdzie dotykaliśmy tymi rękawiczkami różnych rzeczy, a później tymi samymi ściągamy sobie tę maseczkę.

**Ściągasz maseczkę rękawiczkami?**

Widziałem, że tak robili ludzie właśnie. Mimo tego, że maja rękawiczki w jakimś celu, to potem dotykają nimi swojej twarzy.

Jak ty wychodzisz z domu, to masz rękawiczki przez cały czas?

Tak. Od razu zakładam w domu. Myślę, że jest szybciej wtedy. Wolę je od razu założyć i już o tym nie myśleć niż stać przed sklepem i robić to na szybko.

**Kiedy co zdejmujesz?**

najpierw zdejmuje rękawiczki i wyrzucam je do kosza w domu w torebce. W domu, bo niestety nie mam żadnego kosza po drodze, więc muszę to zrobić w domu, ale te rękawiczki od razu pakuję, zawijam i zawiązuję i wyrzucam je, żeby nie zostawiały śladu w śmietniku. Później delikatnie zdejmuję maseczkę i od razu wyrzucam ją do prania.

**Ile masz maseczek?**

Mamy po 3, więc cały czas jedna czy dwie mogą być w praniu i jedna jest do wyjścia. To nam na razie wystarcza.

**Obserwujesz, że ludzie mają w prawidłowy sposób założone te maseczki?**

Wydaje mi się, że tak, ale nie przyglądałem się temu aż tak bardzo. Raczej tak. Jak ktoś ma tę maseczkę, to chyba raczej dobrze założoną.

**Plany luzowania. Kojarzysz, co będą zdejmować?**

Tak. Siłą rzeczy, przez tę sytuacje z festiwalem bardzo mocno się wczytywałem w ten plan. Imprezy masowe i kulturalne nie były w żadnym etapie tego planu, więc w sumie przez to.

**Pamiętasz, co tam było?**

Właśnie próbuję sobie przypomnieć, co jest w tym 2 etapie. Chyba otwarcie marketów budowlanych?

W weekendy, bo one są teraz otwarte pn-pt.

**Właśnie, tak, to zapamiętałem. Kurczę, zapomniałem.**

[moderator przypomina 2 etap]

A, taki najem krótkoterminowy, tak.

**Ten etap jest znaczący dla ciebie, dla ludzi?**

Myślę, że w sumie tak, jeśli faktycznie będzie można pójść sobie do muzeum na fajną wystawę. Oczywiście pewnie będą obostrzenia, ale zazwyczaj w muzeum jest tyle przestrzeni, żeby mogły się tam znaleźć ze 3 osoby To już jest jakaś forma wyjścia i spędzenia czasu. Do marketów budowlanych jeżdżę bardzo rzadko, więc mnie to nie do końca dotyczy. Hotele to też jest miła rzecz pod tym względem, że można gdzieś sobie wyjechać.

**To nie skłoni ludzi do tego, żeby więcej się przemieszczać i tego wirusa rozprzestrzeniać?**

No właśnie, wydaje mi się, że chyba tak. Fajnie jest gdzieś sobie wyjechać, ale mówię ze swojego punktu widzenia. Jeśli ja gdzieś wyjadę w tym momencie, powiedzmy za miesiąc, to raczej wybiorę chatkę na Mazurach albo w innym miejscu, gdzie jest jednak mniej osób. Ale co do tego, że to stwarza zagrożenie, to zgadzam się całkowicie.

**Kojarzysz coś z etapu 3?**

Chodzi o lokale usługowe?

**Tak, fryzjerzy, kosmetyczki, sklepy w GH [...] Co o tym sądzisz?**

Dalej niesie ze sobą duże zagrożenia. Może ja jestem zbyt ostrożny, bo z drugiej strony musimy w końcu jakoś odnaleźć się w tej sytuacji i powoli różne rzeczy uruchamiać i też dawać ludziom zarobić, czy dawać ludziom większy komfort i wolność w tym, co robią, no ale to nadal jest zagrożenie. Nadal nie potrafię stwierdzić, że komfort psychiczny ludzi jest ważniejszy niż zagrożenie koronawirusa.

**Gdybyś ty podejmował decyzje, jakie wskaźniki brałbyś pod uwagę? Kiedy byłoby można uruchamiać np. fryzjerów i kosmetyczki?**

Wydaje mi się, że nałożyłbym większą granicę czasową niż mniejszą, tzn. ten okres poluzowań...Coś trwa 2 tygodnie, jest weryfikowane przez ministra czy możemy wprowadzać kolejny etap. Wydaje mi się, że to jest dosyć krótko. Teoretycznie mówi się o tym, że 14 dni to jest taki główny okres, kiedy się ten wirus rozwija, ale wydaje mi się, że to jest dosyć krótko mimo wszystko. Ja bym wydłużył te okresy, bo wydaje mi się, że potencjalnie dłuższy okres może bardziej pomóc niż zaszkodzić. Jak trwamy już w tej sytuacji jakiś czas, to wolałbym się przemęczyć, ale poradzić sobie z tym lepiej, niż bardziej ryzykować i stwarzać zagrożenie, że to się przedłuży albo powróci.

**Czyli bierzesz pod uwagę, głównie aspekt ilości zachorowań? A bierzesz pod uwagę, że to ma też skutki gospodarcze?**

Tak, tylko cały czas w mojej głowie istnieje idealny świat, kiedy te cierpiące branże i ludzie z tego powodu, otrzymują jakaś pomoc, a w sumie to jest dziwne, bo ta branża, w której ja pracuję nie dostaję żadnego wsparcia, więc nie wiem w sumie, dlaczego tak myślę. Chciałbym, żeby tak było.

**Myślisz, że Polskę stać, żeby wspierać te branże, które są najbardziej dotknięte?**

Skoro stać nas na zorganizowanie wyborów, to stać nas na pomoc. [śmiech]

**Do kiedy powinniśmy chodzić w maseczkach obowiązkowo?**

Pewnie procent nowych zachorowań, a przynajmniej taki procent zachorowań, który jest stały i potencjalnie jest opanowany, kontrolowany w jakiś rozsądny sposób. Nic innego nie przychodzi mi do głowy.

**Minister powiedział, że aż będzie szczepionka. To dobry pomysł?**

Jeśli to ma stworzyć...Jeśli faktycznie to jest jedyny krok, być może jest, to tak. Liczę się z tym, że może się tak wydarzyć.

**Wyobrażasz sobie funkcjonowanie przez rok z maseczką we wszystkich miejscach publicznych?**

Nie wyobrażam sobie, ale dlatego, że nigdy czegoś takiego nie musiałem przeżyć. Nie wiem, jak to będzie, ale staram się...Jeśli trzeba będzie, to tak, to dam radę.

**Myślisz, że ludzie dadzą radę?**

Wydaje mi się, że nie.

**To jest fizycznie możliwe, żeby ludzie chodzili w maseczce we wszystkich miejscach publicznych?**

Wydaje mi się, że to może być trudne, szczególnie, jak ktoś pracuje w firmie, gdzie jest dużo osób, osoby, które pracują w Call Center i używają aparatu mowy do swojej pracy. Nie wiem, czy ten glos tak samo dobrze brzmi? Większość osób używa telefonów w pracy, więc byłoby to trudne dosyć. Ja może nie jestem realistą w tym, o czym ci mówię, znaczy, że uważam, że powinno być dłużej, że powinniśmy bardziej uważać, itd., tylko że z drugiej strony część osób nie jest w stanie przetrwać przez te wszystkie ograniczenia zawodowo czy finansowo, więc jest to trudne. Chciałbym, żeby może Polska była Szwecją, powiedzmy.

**Dlaczego Szwecją? Tam nie ma zakazów?**

No właśnie nie ma zakazów, ale na to sporo czynników wpływa. Tam żyje zupełnie inne społeczeństwo, ale też ma warunki do tego, żeby żyć i postępować zupełnie inaczej, mając super opiekę zdrowotną, mając super świadczenia socjalne, itd. Mam wrażenie, że tam ludzie zachowują się trochę inaczej. Czytałem o tym ostatnio i tam ludzie bezpośrednio po wystąpieniu rządu wypełniali ankietę - było jakieś badanie, czy będą się stosować i tam znaczna większość powiedziała, że tak. tam nie ma nigdy problemu, żeby płacić ludziom, jak są na zwolnieniach, są zachęcani, żeby korzystać z tego. Zasady dotyczące opieki zdrowotnej, która tam jest, stwarzają jednak inne możliwości życia. To wszystko powoduje duże zaufanie do rządzących. Wydaje mi się, że bardzo dużo rzeczy zależy od nas i pod tym względem zazdroszczę Szwecji, że tam ludzie mogą nie mieć zakazów, a jednak zachowywać się w porządku, bo są odpowiedzialni. Mogą być odpowiedzialni, bo kiedy nie pracują, to też dostają pieniądze.

**Sprawdzałeś, jak im idzie z ta liczbą zakażonych?**

Idzie im dobrze, podobno.

**Wiesz, ile w Polsce teraz jest zgonów?**

Nie, zupełnie przestałem się tym interesować.

**Właśnie sprawdziłam, że w Szwecji mają już 2000 zgonów, a ich jest dużo mniej.**

Tak, ale też czytałem, że oni mają konkretny plan na to, tzn., że wiedzą, kiedy będzie pik tych zachorowań, ilu ludzi może zachorować. Planują, że 1% zachoruje, czyli to chyba jest 100000. Zachorują, ale chyba nie wszyscy muszą być w szpitalu. Niektórzy to przechodzą dosyć bezboleśnie, czy bezobjawowo.

**Czytam teraz, że są szacunki, że 1.05. w Sztokholmie 26% już będzie po. Dla ciebie chory to jest taki, który przeszedł test i ma pozytywny wynik, czy taki, który musi mieć pomoc lekarską, czy taki, który przeszedł w różnym stopniu zakażenie?**

Wydaje mi się, że to ostatnie. Nawet jak ktoś ozdrowiał albo przeszedł bezboleśnie, to w sumie miał wirusa. Jeśli sam przeszedł to bezboleśnie, to i tak mógł podać go komuś dalej.

**Interesowałeś się, na ile w porównaniu z innymi chorobami, koronawirus jest zakaźny, śmiertelny?**

Z tego, co się interesowałem, to jednak najbardziej zagrożone są osoby starsze albo takie, które mają obniżoną odporność. Początkowo w ogóle znajdowałem informacje na temat jakichś mitów, że u młodych osób to nie dotyka, albo, że jak będzie ciepło, to temperatura zabije tego wirusa, ale to oczywiście były mity.

**A co sądzisz o tym, żeby dzieci wróciły do przedszkoli i szkół?**

Sam nie wiem.

Kiedyś muszą wrócić. Nie wiem, jak to będzie wyglądało w środku, jakie środki bezpieczeństwa będą podjęte, jakie zmiany.

**Jakie sobie wyobrażasz?**
Zastanawiałem się, czy wszyscy będą siedzieć w maseczkach.

**To jest do zrobienia?**

To pewnie byłoby trudne, ale zastanawiałem się, czy może klasy mogłyby być podzielone, itd., ale często nie ma infrastruktury, żeby coś takiego zrobić i nauczycieli pewnie nie ma aż tylu. Kurczę, sam nie wiem, to trudne. Czy dzieciaki nadal będą siedzieć obok siebie w ławkach?

**Jesteś za tym, żeby te dzieci tak szybko wracały, czy dopiero od września?**

Normalnie powiedziałbym, że byłbym za tym, żeby nie wracały, ale nie wiem, jak można by było rozwiązać kwestię matur czy egzaminów, rekrutacji na studia. Matury mają być w 2 połowie czerwca.

**Czy w ogóle wyobrażasz sobie, że bezpiecznie byłoby puszczać dzieci w tym roku do szkoły?**

Nie do końca wciąż. Ta sytuacja jest jeszcze nieobliczalna i to może być niebezpieczne.

Wolę założyć najgorszy scenariusz, więc uważam, że nie powinny wracać.

**Czytałam wczoraj, że Nowa Zelandia zamyka wszystko na 5 tyg. i ludzie mają 48 godz. na przygotowanie się do zamknięcia kraju i się zamknęli? Co o tym sądzisz?**

48 godz. to trochę mało, biorąc pod uwagę reakcje ludzi, olejki do sklepów, wykupienie wszystkiego, ale generalnie uważam, że to nie jest zły pomysł. Przy naszej 1-szej rozmowie powiedziałem, że uważam, że te zakazy powinny być jak największe i wolałbym się przemęczyć, żeby trwało to wszystko krócej i bezboleśnie niż tak to rozkładać.

**Oni zamknęli tam wszystko., poza wodociągami i prądem...**

Wolałbym chyba taki scenariusz. Wiem, że u nas ten lock down nie jest taki mocny, bo nadal można sobie zamówić np. jedzenie do domu, coś kupić internetowo, wyjść do sklepu, ale to też powoduje taki chaos informacyjny i takie różne podejście ludzi, bo gdyby faktycznie trzeba było siedzieć w domu 5 tyg. bezkompromisowo, to jednak trzeba by było i może jednak wtedy to by się zatrzymało

**Masz jeszcze jakieś przemyślenia na temat obostrzeń/ luzowania?**

To, jaki mam problem z tym wszystkim teraz, to że to wszytko jest takie jakby ruchome. Patrząc też na Czechy czy Niemcy. Tam są jakieś konkretne plany, daty. Ok., one się mogą zmienić, ale już można coś sobie pod to planować, coś robić, coś wiedzieć, co też jest ważne dla samego samopoczucia, że jest jakaś konkretna data, że do tego czasu nie możemy tego i tego. A tutaj jest tak, że z jednej strony są duże obostrzenia, ale jednak tutaj jest obluzowanie w czymś. Mam wrażenie, że czasami nie ma jakiejś logiki w tym wszystkim i z tym mam problem.

**A najbardziej nielogiczna rzecz?**

Nawet te parki i lasy. Zamykamy, jak jest 2000 zachorowań, a otwieramy, kiedy jest 10000 i ta krzywa zachorowań, z tego co mi się wydaje, mocno nie spada. Może to jest po to, żeby dać ludziom jakąś przyjemność w końcu? Nie kupuję tego.

**Widzę zmianę w twojej fryzurze?**

Z jednej strony to zamkniecie fryzjerów dało mi się we znaki, bo było to już irytujące, kiedy włosy rosną w każdą stronę i nie można sobie z nimi poradzić, mimo tego, że się siedzi w domu. Poza tym, kiedy jak nie teraz? Spotkałem się z tym, że część ludzi planuje sobie różne rzeczy, bo kiedy, jak nie teraz - ścięcie włosów, zmiana koloru włosów i jakieś tam takie zmiany estetyczne.

**Jest u ciebie jakaś zmiana w dbaniu o siebie na co dzień?**

Ciężko mi powiedzieć. Golę się raz na 2-3 tyg. i akurat teraz trafiliśmy na taki moment, jak jestem przed goleniem i to się jakoś nie zmienia. Nie podchodzę do tego tak, że wreszcie mogę się zapuścić, bo w sumie nikt mnie nie widzi.

**Nie kusiło cię?**

Nie.

**A żeby w piżamie cały dzień?**

Nie chyba. Jakoś źle bym się z tym czuł.

**I nie korci cię, żeby tak poluzować sobie?**

Nie mówię, że to nie nastąpi. Jeśli będę chciał tak zrobić, czy tak sobie wymyślimy, to czemu nie, ale jeśli chodzi o taki rytm dnia, to wolę tego nie zmieniać. Ja od kilku lat już pracuję w domu, więc to aż tak nie zmieniło mojego rytmu dnia, że wstaje o innej godzinie, bo nie idę do pracy. I dlatego tym bardziej staram się tego trzymać, nie zmieniać tego, nie zapuszczać włosów, brody i stwarzać takiego...Nie chcę tego zmieniać po prostu.

**A myślałeś, żeby coś dodać do dbania o siebie?**

Zacząłem ćwiczyć niedawno, dzięki temu, że mamy rowerek i te gumy do ćwiczeń. To tak, to jest ważne i staram się tego nie opuszczać, 2-3 x w tyg. poćwiczyć. A tak, to niewiele się chyba zmieniło.

**Dzisiaj ktoś mi powiedział rano, że pierwszy raz zrobił sobie takie skarpetki peelingujące na stopy. Nie kusiło cię coś takiego nowego?**

Ja już kiedyś takie miałem. Czasami Dominika mnie potrafi przekonać do zrobienia jakiejś maseczki, czy tego typu rzeczy i nie mam z tym problemu.

**Ale tak sam z siebie to nie za bardzo?**

Różnie, w sumie mam tak czasem, że widzę, że skóra mi się pogorszyła i zapytam ją co można zrobić, ale to też nie jest coś, co występuje z powodu czasu koronawirusa.

**A jak otworzą siłownie?**

Myślę, że wrócę mimo wszystko. Chciałbym wrócić, bo teraz to jest takie uproszczone ćwiczenie. Wolałbym wrócić.

**A za fryzjerem będziesz bardzo tęsknił, jak odrośniesz?**

Rozmawialiśmy o tym z Dominiką i mam nadzieję, że w ciągu 2 mies. się otworzą te salony, bo nie chciałbym powtarzać tego.

**Słyszałam dzisiaj, że 18 maja mają otwierać GH. Myślisz, że to jest bezpieczna data? Czy ty 18 maja poszedłbyś już do fryzjera?**

Myślę, że jeszcze nie. Muszę się sam dobrze poczuć z takim momentem, że już będę mógł. Nie potrafię sobie ustalić jakiegoś konkretnego zdarzenia czy konkretnej daty. To musi być taka sytuacja, że w końcu sam dobrze się z tym poczuję. Tak samo, jak mówiliśmy dzisiaj o tym, że teraz się mniej boję. Ja nie wiedziałem, że to nastąpi i nie uzależniłbym tego od jakiejś daty czy zdarzenia. Po prostu to nastąpiło, oswoiłem się z tym. I tak samo będzie z tym fryzjerem pewnie.

**A jakieś zakupy ubraniowe, bo wiosna?**
Przymierzam się trochę. Myślałem o tym, żeby sobie kupić kilka rzeczy na lato, które by mi się przydały, więc pewnie internetowo to się odbędzie.

**A nie myślisz, żeby poczekać, aż otworzą galerie?**

Nie, ale my przeważnie robimy zakupy ubraniowe przez internet, uciekając od tłumu. Zdarzało się też w galeriach, ale na razie nie wiem. bardziej się nastawiam na internet, ale nie mówię stanowczo, że na pewno nie udam się do galerii w czerwcu, czy coś.

**Masz potrzebę, żeby sobie kupić ubrania, bo już nie masz w czym chodzić, czy masz ochotę na coś nowego?**

Jedno i drugie. Część rzeczy się niszczy, część przestaje się podobać i fajnie kupić sobie coś świeżego. Tak dla siebie, dla własnego samopoczucia.

**Jak myślisz o sobie, jako o konsumencie, to czego ci najbardziej brakuje?**

Na pewno restauracje, bo to jednak nie jest to samo, jak się zamawia jedzenie a nie je się w danym miejscu. Nie robię tego codziennie, ale raz na 2 tygodnie jednak fajnie było wyjść sobie gdzieś. Kino na pewno. Bardzo sobie plujemy w brodę, bo styczeń/ luty mieliśmy takie, że było dużo rzeczy, które chcieliśmy obejrzeć, ale uważaliśmy, że jeszcze zdążymy. Myślę, że w ogóle otwarcie miejsc, gdzie można spotkać się ze znajomymi - bary, puby, restauracje.
