# Supplementary material for: The challenges arising from the COVID-19 pandemic and the way people deal with them. A qualitative longitudinal study
Source: PLoS One. 2021 Oct 11;16(10):e0258133. doi: 10.1371/journal.pone.0258133 (PMC8504766; doi:10.1371/journal.pone.0258133)
Supplement: S1 Dataset — (ZIP) [file pone.0258133.s003.zip › Transcriptions/stage 3/16.3_F_36_couple, with children.docx]

**16.3_F_36_couple with children**

**Co się działo w ostatnich 2 tygodniach?**

No, w sumie to generalnie to chyba nic się nie działo (śmiech). Codziennie to samo w sumie.

**Masz wrażenie, że to codziennie to samo od tygodni?**

Tak. Mam takie wrażenie, że jest cały czas to samo, że się niewiele zmienia. Jakieś tam drobne drobiazgi. No wiadomo, no święta były, trochę było takiego innego czasu.

**Co było innego?**

No mąż na przykład nie pracuje w święta w ogóle. No to te 2 dni takie, że miał dobre. Więc to akurat. Myśmy święta tak bardziej uroczyście spędzili, w sensie żeśmy się ładnie ubrali, żeśmy wszystko… wszystkie posiłki razem żeśmy jedli. Bo tak, to w ciągu dnia różnie bywa.

**A co to znaczy, że się ładnie ubraliście? Jak to wyglądało?**

No my sukienki, koszule panowie. No święta w końcu, tak?

**Byliście sami, czy jednak ktoś do was dołączył?**

Nie, sami byliśmy, sami.

**I co, ładnie się ubraliście, usiedliście do śniadania, opowiedz o tej Wielkanocy, jak to wyglądało?**

Znaczy w ogóle fajnie, znaczy też właśnie tak stwierdziłam, że dzięki temu, że właśnie wszystkie wydarzenia były online, całe te kościelne, że tak powiem, to uczestniczyłam we wszystkich, w których się dało uczestniczyć (śmiech). Bo normalnie to bym chyba nie uczestniczyła w tym wszystkim, bo tak bym nie wyszła po prostu po południu czy wieczorem do kościoła z dziećmi. A tak, to włączyłam sobie telewizor, sobie słuchałam, tyle, ile oczywiście byłam w stanie, jak oni tam nie przeszkadzali itd. Ale nawet nieźle poszło. A w niedzielę zrobiliśmy sobie śniadanie. Jeszcze w sobotę tam żeśmy malowali jajka z Julianem, bo on bardzo, bardzo mu się to podobało. Zrobiliśmy koszyczek. A w niedzielę, no to właśnie śniadanko razem zjedliśmy, takie fajniejsze, że tak powiem, nie takie zwykłe, tylko tam coś, no takie inne śniadanko było, uroczyste.

**Co zrobiliście na to uroczyste śniadanie?**

O rany, co my tam jedliśmy? Nie pamiętam. No jajka przede wszystkim były, trzeba było zjeść te kolorowe jajka, to była atrakcja. No, tam jakiegoś łososia kupiłam, śledzia, jakieś, już nie pamiętam, co myśmy tam jedli jeszcze takiego ekstra. Szczerze mówiąc to już nie pamiętam, co żeśmy jedli. No właśnie prócz tych jajek i jakichś tam łososiów i coś tam, to już nie pamiętam.

**A co ze święconką? W końcu było to święcenie telewizyjne czy nie było?**

Nie, nie, nie było telewizyjnego święcenia. W ogóle chyba nie można używać wody święconej teraz w ogóle w kościele, z tego co tam się orientuję. Więc… Nie, no zrobiliśmy koszyczek, wsadziliśmy czekoladowego króliczka, jajka, jakiś tam kawałek tego. I stało na stole. A żeśmy potem zjedli po prostu na śniadanie to, co tam było w tym koszyczku. I tyle.

**Kontaktowaliście się jakoś z rodziną w czasie świąt?**

No tak, tak. Właśnie po śniadaniu to była cała seria telefonów, Skype’ów. Do wszystkich żeśmy dzwonili i rozmawiali.

**Jakie to były święta? Mówisz, że przeszłaś jak nigdy przez wszystkie te kościelne obowiązki, bo mogłaś. One ci coś dały? To ma dla ciebie znaczenie?**

Znaczy nie, fajne to było, mądre niektóre rzeczy. Niektóre były takie mądrzejsze, niektóre mniej. Żeśmy właśnie tak… W sobotę to żeśmy razem z mężem oglądali. To też był taki fajny czas, bo w czwartek i w piątek nie, sama oglądałam, bo on jeszcze pracował. A w sobotę już oglądaliśmy razem. No to też taki fajny czas, żeśmy sobie tak pogadali trochę. Niektóre były fajniejsze niż inne. Więc te, te liturgie. Bo to zależy, ja na przykład najbardziej lubię kazania, jak są mądre kazania. I niektóre były mądre, a niektóre były takie…

**Została ci jakaś myśl, która zrobiła na tobie wrażenie?**

Nie, że jakaś jedna, po prostu… Po prostu miło posłuchać mądrych ludzi czasami. I akurat, bo ja słuchałam z tej (niezrozumiałe) warszawskiej. I miał kazanie właśnie jednego dnia biskup, którego znam osobiście, bardzo sympatyczny człowiek. I po prostu bardzo mądry. I powiedział fajne kazanie. Tak miło się zrobiło.

**Miło, bo co?**

Bo znam człowieka i powiedział mądre rzeczy i tak no…

**Co się jeszcze działo? Co się zmieniło może przez te 2 tygodnie, czy cokolwiek nowego zauważyłaś?**

Co się zmieniło oprócz tego, że już można wychodzić na spacer teraz?

**To ważne?**

No tak. Bo przecież tak, to trochę żeśmy się tak… Znaczy ja chodziłam z dziećmi tak tutaj po osiedlu. Ale i tak do końca… Znaczy, bo nie było wiadomo za bardzo, czy można wychodzić na te spacery, czy nie można wychodzić na te spacery. Tak w sumie nie wiadomo było, czy się trafi na kogoś, kto… I kto coś powie na ten temat, czy nie powie. I tak żeśmy nie wychodzili w sumie za osiedle. Ale rozumiem, że teraz jak już są otwarte te wszystkie parki i lasy, to jakby nikt nie może się przyczepić. Więc wychodzimy sobie. Tak, dla mnie to jest ważne, żeby z dziećmi trochę wyjść, żeby tego świeżego powietrza nabrały, żeby obejrzały kwiatki, roślinki, które rosną, zwierzątka. Zwierzątka, w sensie jakieś ptaszek śpiewa, robaczek chodzi.

**Gdzie byliście już?**

No wczoraj byliśmy, tutaj akurat za oknem mamy taki, no, jest to parczek taki… Dziki park, że tak powiem. A dzisiaj pojechaliśmy już wszyscy razem do parku na Moczydło. I Moczydło i tam park Sowińskiego jest.

**Pamiętam, że mówiłaś o tym zoo, że tak planowałaś na wiosnę.**

Zamknięte jest zoo.

**Zamknięte cały czas?**

Zoo jest zamknięte, tak. Bo tam to jednak…

**A zrezygnowałaś z jakichś zachowań, coś robisz inaczej, coś ograniczyłaś?**

Nie, no ograniczyć to chyba nie. Każą nosić maseczki teraz, więc zakrywam usta. Aczkolwiek muszę sobie zrobić jakąś swoją tą, bo na razie nie mam. I po prostu mi się źle w tym oddycha strasznie, jest mi niedobrze w tym. Zakrywanie takie usta i nosa, nie jest to dla mnie komfortowa, że tak powiem, sytuacja.

**A do tego 16-tego, kiedy wprowadzili ten obowiązek do tego momentu nie zakrywałaś, nie nosiłaś maseczek?**

Nie, nie, nie, nie. Ja nie lubię. Znaczy w ogóle mi się źle… Ja noszę w ogóle okulary na zewnątrz. Więc jak to zakrywam, to mi wszystko paruje. To jest po pierwsze niewygodne. A po drugie mi się źle po prostu oddycha. I ja nie lubię tego robić, nie lubię zakrywać nosa.

**W związku z tym wychodzisz mniej?**

Nie, no nie. Nie, no i tak jakby nie wychodzę często jakoś. Teraz no codziennie do tego parku czy tam coś. No to jak, szczerze mówiąc, to jak byłam wczoraj sama w tym parku z dwójką moich dzieci i nikogo nie widziałam, no to sobie tam ten nos odsłoniłam. Myślę, że nikt mi (śmiech) nie pozwie o to. Nie wyślą mnie do więzienia za to. Bo po prostu źle mi się oddycha. A w sklepie po prostu, to naprawdę muszę się czasami zatrzymać, bo jest mi po prostu niedobrze aż. Mam jakieś takie…

**Jest jeszcze coś, co bardzo teraz przeszkadza? Poza tym obowiązkiem maseczki, zasłanianiem nosa i ust?**

Ja wiem, czy coś mi przeszkadza? Znaczy no, na przykład nie lubię nosić tych plastikowych rękawiczek. Strasznie mi się ręce w tym pocą. To jest tak niewygodne. I w ogóle nie jest to jakaś komfortowa sytuacja. Rozumiem, że można tam na chwilę to założyć i jakby korona mi z głowy nie spadnie, ale to nie jest jakieś takie fajne.

**Tak podpytuję, bo różne ograniczenia nam wprowadzają. I się zastanawiam, czy jest teraz coś takiego, co w ciągu tych dwóch tygodni, co się zmieniło, że przeszkadza ci bardziej albo doskwiera ci bardziej? Czy jest coś, co ci teraz tak jakoś w czasie tych dwóch tygodni doskwiera, przeszkadza?**

Coś nowego to nie. Cały czas mi doskwiera to, co mi cały czas doskwierało (śmiech).

**Czyli to, że nie możesz wyjść tam, gdzie chcesz. (niezrozumiałe) pamiętam, że ci strasznie doskwierały. Co jeszcze ci doskwiera?**

No to, że nie spotykamy się z rodziną i znajomymi. No, to też jest takie doskwierające. Też mi to przeszkadza.

**Czujesz się odizolowana od innych ludzi?**

Znaczy może nie aż tak bardzo, znaczy bez przesady. Jakiś tam kontakt cały czas mamy. A to przez Skype a to rozmawiamy czy to przez telefon, czy sobie piszemy do siebie. Jakby nie… Znaczy były też takie sytuacje w naszych relacjach, że też człowiek się nie spotykał z innych względów. Miałam takie miesiące, po prostu miesiąc cały, gdzie dzieci były chore. A to jedno, a to drugie. I też nie spotykałam się ze znajomymi, siedziałam w domu. Albo nie wiem, miałam dziecko małe i też nie wychodziłam z domu przez jakiś tam czas, nie spotykałam się ze znajomymi, bo miałam noworodka w domu. Więc nie jest to jakieś coś, czego nie doświadczyłam w życiu. Natomiast… Coś chciałam powiedzieć i zapomniałam.

**To było a propos tego, czy czujesz się odizolowana.**

No, no, no, no to tak. A, właśnie! Jest jedna pozytywna rzecz tego (śmiech). Bo mam takich znajomych, którzy spotykają się na spotkania właśnie, tak na żywo, że tak powiem, o godzinie 20 w ciągu tygodnia. I ja nie mogę na te spotkania przychodzić, bo mój mąż właśnie w tym czasie pracuje zawsze. I no nie mogę dzieci samych zostawić w domu. A dzięki temu, że teraz są spotkania przez internet, no to jakby ja uczestniczę też (śmiech).

**A, czyli zyskałaś nawet spotkanie towarzyskie.**

Zyskałam, tak.

**Emocje – zdjęcia.**

Teraz do mnie najbardziej przemawia ten piękny las.

**Który?**

Nr 6 (śmiech).

**6. Coś jeszcze?**

Nie, nie. Znaczy no reszta jest trochę…

**To mogą być skrajnie różne rzeczy, które się zdarzyły przez te 2 tygodnie. Dlatego się pytam, czy coś jeszcze jest takiego, co myślisz, że pasuje do tego, jak się czułaś.**

Niespecjalnie.

**Dobra, czyli 6.**

To jest jedyna wolność na razie (śmiech). Pusty las. To jest jedyne miejsce, gdzie można teraz poczuć się, trochę wyjść i zaznać wolności.

**Czyli to jest takie poczucie wolności, jakie tu są jeszcze uczucia, emocje, jak patrzysz na to?**

No słoneczko, wiosenka, no po prostu ja już bym chciała… I chciałabym gdzieś wyjechać z tego betonowego świata na razie.

**Czy to jest tak, że to jest wyraz twojej tęsknoty bardziej niż emocji, które w tobie są? Czy masz takie emocje w sobie?**

A tęsknota też może być emocją (śmiech).

**Tęsknota może być uczuciem, tak, oczywiście. Ale czy to jest coś, co już zdarzało ci się już poczuć, to co widzisz na tym zdjęciu?**

No nie, jeszcze nie. Bo ten kawałek lasku, który tutaj mamy, czy tego parczku, powiedzmy, trudno to nazwać lasem i trudno nazwać takim… Wyjście na godzinę to nie jest to samo, co taki wyjazd do takiego lasku i połażenie tam. I taka całodniowa wycieczka to jest zupełnie co innego. A tu zupełnie co innego.

**To powiedz mi, bo mam wrażenie, że słyszę, do czego tęsknisz. Ale cały czas nie potrafię do końca poczuć, jak ty się czujesz teraz. Jakie są twoje emocje teraz, co w tobie siedzi. Wiesz, tam mogło nie być takiego obrazka, który ci pasuje. Więc może potrafisz wytworzyć albo opowiedzieć, co na takim obrazku powinno być.**

No nie wiem, czy ja potrafię do końca to nazwać. Myślę, że ogólnie mój stan nie różniłby się jakoś od tego, co by było, gdyby nie było tej całej sytuacji. W sensie też bym chciała już gdzieś wyjechać, bo mam taką potrzebę raz na jakiś czas. Wyrwać się, gdzieś zmienić otoczenie i zmienić swoje tutaj, no, takie… no otoczenie.

**Ale to chodzi o to, o czym mówiłaś, jak zaczęłyśmy się spotykać, że rodzice ci pomagali i miałaś ten czas dla siebie, którego teraz ci brakuje? Czy to coś innego?**

Nie, nie, nie. Znaczy to też, ale bardziej tak. Znaczy no mam coś takiego, co się jakby… Mam często (śmiech). Znaczy to nie jest tak, że teraz nagle i tego nigdy nie doświadczyłam w życiu, tylko po prostu ja lubię wyjeżdżać. Lubię gdzieś pojechać, do innego miasta, gdzieś wyjechać. Na kilka dni, tak? Połazić gdzieś, gdzieś w inne miejsce, iść do muzeum, iść na spacer do lasu, iść gdzieś tam. No lubię wycieczki. To nie muszą być jakieś nie wiadomo jakie wycieczki. Ale takie nawet małe.

**Czyli czujesz się przymknięta.**

No… Trochę tak, znaczy nie, nie wiem, czy to jest dobre słowo. Nie wiem, musiałabym się zastanowić. Znaczy, bo na przykład planowaliśmy na święta wyjechać, tylko do Krakowa tak naprawdę, ale zawsze to jest jakaś taka fajna wycieczka, ja takie rzeczy lubię. Na parę dni. A teraz nie wiadomo, kiedy w ogóle wyjdziemy z domu (śmiech).

**Czy jest jakiś element, gdybyś miała wymyślić tak na szybko, zdjęcie, które by oddało to, jak się czujesz, czy coś ci przychodzi do głowy?**

Nie wiem tak naprawdę. Znaczy, bo… Bo mam takie, znaczy nie, znaczy jak ja się czuję (śmiech). Czasem dobrze, czasem źle, nie? To jest takie, no czasem słońce, czasem deszcz. O, coś takiego.

**No tak, a ja właśnie chcę od ciebie się dowiedzieć, czy to jest tak, że… Dlatego mówiłam, mogą być skrajności. Tak mi się wydaje, że to nie jest tak, że człowiek się czuje constans cały czas. Ma lepiej, gorzej, czasem ma tak płasko. Doświadcza się też takich rzeczy dosyć silnych często. I nie wiem, czy to u ciebie na przykład to jest gniew, złość, czy to jest bezsilność, której doświadczasz, czy to jest smutek?**

Ja myślę, że wszystkiego po trochu. Znaczy staram się być osobą, którą nie targają emocje (śmiech). W takim sensie, że staram się panować nad emocjami, a nie żeby emocje panowały nade mną. Tak że gdzieś one tam są, ja doświadczam jakiegoś tam smutku, tego, że na przykład widzę moich rodziców cały czas na tym Skypie i nie mogę się z nimi spotkać. Wkurza mnie, jak czytam jakieś wiadomości itd. Ale też cieszę się z takich małych rzeczy, typu, moja córka dzisiaj chyba pierwszy raz powiedziała swoje imię. I to było po prostu super. I byliśmy na spacerze i też było fajnie. I się wygłupiamy z moim mężem i też jest fajnie. Więc no, tak jak mówię.

**A co robisz, żeby zapanować nad emocjami, które ci nie pasują, których nie chcesz czuć?**

Co robię, żeby nad nimi zapanować?

**Tak. Jakie masz sposoby na to.**

No, czasami są dobre sposoby, czasami złe. Znaczy no nie wiem, tak generalnie staram się, żeby… Myślę, że dobrym sposobem jest nazwanie tego, nazwanie tej emocji, tego, że jestem wkurzona. Tak, to mnie wkurza. I jak już nazwę tą emocję, to już przynajmniej wiem, z czym mam do czynienia. I jakby wiem, na co się wkurzam, zastanawiam się, czy mogę coś z tym zrobić, czy nie mogę z tym nic zrobić. I zazwyczaj mi przechodzi po jakimś czasie. Po prostu zaczyna się myśleć o czymś innym, bo trzeba zrobić coś innego. Albo przeczytam coś innego i to minie. A czasami, jeżeli faktycznie jest coś takiego, nad czym nie jestem w stanie zapanować i mnie tak bardzo denerwuje i jestem bardzo wkurzona, to zazwyczaj się modlę. I no myślę, że to jest dobra rzecz.

**Czyli to jest albo racjonalne pójście w wytłumaczenie sobie albo poszukanie odskoczni takiej duchowej, tak?**

No tak, tak.

**Przypominasz sobie taką szczególnie trudną jakąś sytuację, która by się zdarzyła tobie emocjonalnie w ciągu ostatniego czasu, tych dwóch tygodni?**

Nie, no bez przesady. Czasami się wkurzam na jakichś ludzi, z którymi dyskutuję (śmiech).

**Raczej się wkurzasz, bo widzisz, ja próbuję dotrzeć do tego, co się z tobą dzieje. Raczej się wkurzasz niż smucisz?**

Nie, nie, smucę raczej nie, raczej się wkurzam.

**Wkurzasz się na ludzi za co?**

Za to, że nie myślą logicznie. No, głównie za to.

**Na przykład? Co to znaczy, nie myślą logicznie. Sytuacja.**

Bo na przykład próbuję z nim dyskutować i przedstawiam jakiś argument A, a on mi pisze, że ale B. Ja mówię, ale ja nie pisałam nic o B, ja piszę o A. No i ten człowiek odpisuje znowu, ale B, przecież B! No i taka rozmowa. Jakby no…

**A w sytuacji tej koronawirusowej, coś cię szczególnie wkurza?**

Wkurza mnie, generalnie wkurzają mnie wszystkie decyzje naszego rządu (śmiech).

**Tak, a czemu? Co cię wkurza?**

Wkurza mnie, strasznie mnie wkurzyło, jak wydali to rozporządzenie o tym maseczkach. Bo napisali, znaczy… Nie rozumiem tego, jakąś decyzję podejmują, która jest… Jak się ją czyta, to widać, że to jest bez sensu. Bo było pierwsze rozporządzenie o maseczkach, że będzie obowiązek noszenia maseczek poza miejscem zamieszkania. I nie było żadnych wyjątków od tego. No i jakby… Przecież no wystarczy 3 minuty o tym pomyśleć i każdy człowiek zdaje sobie sprawę, że to jest bez sensu. No i jakby po co coś takiego robić, nie można od razu jakby przemyśleć sprawy i napisać od razu coś, co ma ręce i nogi? Tylko takie, rzucanie takich haseł, tak? No i dobrze, wydali to, tak? Potem, dzień wcześniej chyba doprecyzowali to, że dzieci do drugiego roku życia i tam coś tam, że w samochodzie nie trzeba. Myślę sobie, no dobrze, no drugi rok życia, to przynajmniej jedno moje dziecko się nie załapie. Drugie, nie wiem, co zrobię, chyba zamknę w domu, bo pewnie nie będzie chciało nosić tej maseczki.

**Bo na razie jak wychodzicie, to mu nie zakładasz?**

Nie, bo zmienili rozporządzenie, że to czwartego roku życia. Więc po prostu już trzecie jakby, tak? Przecież po prostu ja już myślałam, że jak bym spotkała tego człowieka, to ja bym mu po prostu, no nie wiem. No nie, no to mnie wkurzyło. Bo jakby no ile… Nie wiem, może jakieś konsultacje zanim się wyda jakieś rozporządzenie, a nie kurcze wydają, a potem piszą na Tweeterze, żeby konsultacje jakieś, czy to jest w porządku czy nie. No to kurcze, nie mogli zrobić tego wcześniej? A potem jeszcze raz zmienili to rozporządzenie (śmiech), więc po prostu… No więc, na szczęście się okazało, że jest do czwartego roku życia. Więc dzieci nie zakładają.

**Ale to nie jest jedyna zmiana w tych obostrzeniach, z którymi się spotykamy. Cały czas, coś tam się dzieje. Powiedz, jak ty się do tego odnosisz, jak to postrzegasz, co o tym myślisz? Maseczki już słyszę, że cię wkurzają maksymalnie.**

Trochę tak (śmiech).

**Co jeszcze?**

Nie wiem, co jeszcze tam… Może ja nie wiem, jakie, ale coś masz na myśli takiego konkretnego?

**Np. o tych zasadach w handlu?**

Teraz chyba zluzowali te obostrzenia.

**Teraz zluzowali. Nie wiem, czy już byłaś w sklepie po tym, jak to zluzowali?**

No byłam. Ale ja tak właśnie, ponieważ nie lubię tych kolejek, to jeżdżę do sklepu o 7 rano. I o 7 rano nie ma w ogóle ludzi w sklepie.

**Omijasz kolejki. Otworzyli nam ten wstęp do parków i możliwość przemieszczania się. Można wejść do kościoła, jest teraz 15…**

Tak, na metr kwadratowy.

**1 osoba na 15 metrów.**

No wiem, ale to jeszcze do kościoła, to nie byliśmy. Zresztą my i tak nie pójdziemy, no mamy małe dzieci, no to przecież…

**Ale tak sobie myślę, czy są wśród tych nakazów, zakazów, obostrzeń takie, które uważasz, że są sensowne, dają ci poczucie bezpieczeństwa? Uważasz, że rzeczywiście działające na zahamowanie rozprzestrzeniania się, czy zatrzymanie epidemii. I takie, które uważasz, że są zupełnie od czapy, bez sensu.**

No to chodzenie do tego lasu to było od czapy totalnie.

**Zakaz chodzenia do lasu?**

Tak. No był od czapy, przecież bez sensu totalnie. Zwłaszcza… Jeszcze rozumiem, że w takiej Warszawie czy coś, to tych ludzi w tych parkach może było więcej. Ale no kurde, całą Polskę od razu zamykać? Przecież to jest taka głupota, że już nie wiem. Zwłaszcza, że no tutaj akurat w Warszawie jest dosyć sporo tych zachorowań. Nie wiem, tak naprawdę trudno jest mi to wszystko ocenić, bo jakby nie jestem ekspertem w temacie.

**Ale chodzi mi o to, czy na przykład któraś z tych rzeczy sprawia, że ty się czujesz bezpieczniej, pewniej, wychodząc na ulicę, idąc do sklepu?**

No nie zastanawiałam się nad tym, szczerze mówiąc, czy ja się czuję pewniej… Nie, nie wiem. Generalnie chyba ten pomysł z noszeniem tych maseczek nie jest zły. I uważam, że trochę lepiej, że ludzie nie chuchają na siebie i nie dmuchają. Ale czy ja się czuję bezpieczniej? Nie wiem.

**A w ogóle jaki jest poziom twojego lęku w tej chwili? Czego się teraz obawiasz?**

Myślę, że poziom mojego lęku się nie zmniejszył ani nie zwiększył w ogóle w żaden sposób. Raczej się mniej obawiam nawet niż się obawiałam (śmiech).

**A jak myślisz, czemu?**

Myślę, że dlatego, że… Znaczy, mimo że tych zachorowań jest powiedzmy więcej i więcej tych chorych… Chociaż tak naprawdę pewnie chorych jest dużo więcej niż tych oficjalnych danych, to jednak na przykład z tego, co te oficjalne dane pokazują, to główne zachorowania to są te ośrodki medyczne wszelakie. Tam jest wysyp tych wszystkich przypadków. Tak że jakby wydaje mi się, że może być mniejsze ryzyko wśród właśnie ludzi, powiedzmy… Znaczy ja nie mówię, żeby od razu wracać do normalnego funkcjonowania, nie wiem, czy to by było dobre. Ale wydaje mi się, że jest mniejsze ryzyko teraz.

**Niż było 2 tygodnie temu?**

Mam takie wrażenie, może to jest mylne wrażenie. Że jakby ta choroba gdzieś tam się rozprzestrzenia właśnie w tych kręgach tych szpitali, DPS-ów, tam są te ogniska zachorowań. I jakby tam się to dzieje bardziej niż jakby, że ci ludzie tak latają wszędzie i ta choroba jakby jest wszędzie.

**W kręgach, w które ciebie nie dotyczą w tym momencie?**

Tak. No nie wiem, tak jak mówię, może to jest mylne. Może to jest takie mylne poczucie bezpieczeństwa i takie złudne, o, może to jest lepsze słowo. Ale takie mam wrażenie teraz.

**Ale w ogóle śledzisz te doniesienia, statystyki i tak dalej?**

O ja uwielbiam statystyki w ogóle, uwielbiam (śmiech). Uwielbiam te cyferki, mimo że ja w ogóle nie jestem z wykształcenia matematykiem ani nic nie mam wspólnego, że tak powiem. Ale uwielbiam te cyferki, ja cały czas patrzę na te cyferki. Co prawda one i tak są pewnie nie do końca właściwe, w sensie, że mogą dawać złudne poczucie tego, że wiemy, ile jest tych zakażeń itd. Ale ja lubię tam sobie porównywać. Patrzę, cały czas patrzę (śmiech).

**I co ci wychodzi z tych porównań? Że jak jest u nas? Jak jest na tle innych?**

Tak naprawdę nikt nie wie, ile jest u nas. To jest właśnie… Znaczy, ja lubię tak sobie patrzeć na to, ale tak niekoniecznie da się wyciągnąć z tego jakiś wniosek, ile jest tych zakażeń itd. Więc sobie tak patrzę, porównuję sobie. Patrzę, jak tam rośnie na przykład w Stanach Zjednoczonych czy tam we Włoszech już trochę mniej rośnie. Porównuję sobie na przykład z Chinami, gdzie tam jest, w Chinach to tam stabilnie w miarę od dłuższego czasu. No więc sobie tak patrzę na te cyferki, ale staram się nie wyciągać jakichś wniosków, bo wiem, że statystyka się nijak ma do pojedynczych przypadków.

**A ta cała kwestia, która się pojawia, tego żebyśmy się poddawali kwarantannie albo izolacji. Czy dla ciebie jest jasne, co oznacza kwarantanna, co oznacza izolacja?**

No kwarantanna to jest jak ktoś jest chory albo ma podejrzenie zarażenia. A izolacja to jest taka… Prewencyjna.

**Trochę tak sama sobie sterujesz tą izolacją?**

No tak. Nie jest jakby narzucona. Znaczy no rozumiem, że trochę jest narzucona ta izolacja. Ale no nie jest taka… To zachowanie tylko prewencyjne.

**W sensie możesz iść do sklepu, ale nie możesz przyjmować znajomych, to o to chodzi?**

Znaczy, no to już są takie płynne, bym powiedziała bardzo.

**Czyli kwarantanna jest restrykcyjna, izolacja jest taka trochę nie wiadomo jaka.**

No tak. Znaczy, bo kwarantanna to rozumiem, że to jest taki termin… Nie wiem, bo ja się też nie znam, na medycynie, ale rozumiem, że to jest taki medyczny termin, ta kwarantanna. W sensie…

**Nie, ale one się przeplatają. I mam wrażenie, że w tej chwili ludzie zatracili trochę rozróżnianie tego. I dlatego się pytam, co dla ciebie to znaczy.**

No nie, no dla mnie kwarantanna to już jest takie typowe, że jestem pod… Miałam styczność z kimś, kto jest chory, albo sama jestem chora. I wtedy faktycznie muszę być pod kwarantanną, żeby sprawdzić faktycznie, czy jestem chora czy nie, czy roznoszę chorobę czy nie roznoszę. I to dotyczy każdej choroby zakaźnej, nie tylko tej. Natomiast ta izolacja, to jest tylko takie prewencyjne działanie ku temu, żeby nie rozprzestrzeniało się to wśród ludzi, którzy nie wiedzą, że są chorzy, nie mieli styczności itd. Natomiast zasady tej izolacji są cały czas płynne. Tak jak teraz zmieniają się rozporządzenia, można wyjść do lasu, nie można, można kurcze biegać, nie można biegać. Tak że to się zmienia co drugi dzień.

**Pilnujesz tego, co wolno a czego nie wolno?**

Trochę tak, no trochę pilnuję. Znaczy no patrzę, bo jakby nie chcę potem zostać, że tak powiem, zaskoczona.

**Już mówiłaś, że rozejrzałaś się w parku, nikt nie widział, to ściągnęłaś maseczkę.**

No tak, ale mi się źle oddycha.

**Co jeszcze ci się zdarza tak zrobić?**

W sensie, że co?

**Że nie wolno, ale rozejrzysz się i a, zrobię.**

Nie, to chyba nie ma nic takiego… Znaczy rozumiem, że z tymi maseczkami, to też jest tak, że w sensie na przykład, osoby, które mają problemy z oddychaniem jakby nie muszą ich nosić. I nawet nie trzeba mieć jakby potwierdzenia tego, że nie możesz oddychać.

**No właśnie, a twoja maseczka, to jak myślisz, czy ona ma chronić ciebie, czy ona ma chronić innych od ciebie? W ogóle po co to zrobiono?**

Znaczy z tego, co ja rozumiem, nie słyszałam tego od naszych, że tak powiem, rządzących, to maseczka chroni innych ludzi przed osobami, które są zakażone, a nie mają objawów i nie wiedzą o tym, że są zarażone. Że one nie rozpylają tego aerozolu, że tak powiem, z wirusem, tak? Więc one mają chronić jakby mnie, rozumiem. Tylko że… No tak, tak to rozumiem.

**A co myślisz o tych planowanych turach luzowania?**

Ja myślę, że jakie tury w ogóle, o czym my mówimy? Przecież w ogóle oni co 2 dni zmieniają rozporządzenia. Te tury w ogóle, nie ma żadnych dat, nie ma żadnego jakby przedstawionego… Jakby takiego, no, naukowego podejścia do tego, jak ta epidemia będzie się rozwijać w tym kraju. To w ogóle o czym my mówimy? Dla mnie to jest śmiech na sali, te punkty kolejne. Bo tak naprawdę za 2 dni może się okazać, że będzie zupełnie co innego. Jakby nie ma żadnych… To jest wymyślone, w ogóle nie wiem, kto to wymyślił. Napisali sobie coś takiego, mogą to zmienić w każdej chwili. Nie ma żadnych podstaw ku temu, jakby dlaczego akurat to, tak? W takim tym. Nie ma żadnego w czasie tego rozpisanego, no to jest śmiech. A pierwszy ten etap, to co? Otworzyli lasy, które zamknęli 2 tygodnie wcześniej. No wow. Nie, no po prostu (śmiech). To jest żenujące.

**Coś się szczególnie zażenowało w tym planie?**

Co mnie najbardziej zażenowało?

**No tak, czy jeszcze coś poza tym, że oni nagle wow, otworzyli lasy i wielkie halo.**

No nie, no po prostu nie ma w ogóle co o tym… Ja nie mam nic do powiedzenia na ten temat. Uważam, że to jest w ogóle… No nie ma o czym w ogóle mówić.

**To może inaczej. Czy są któreś z tych obostrzeń, tych zakazów, nakazów, które nas dotyczą w tej chwili, które twoim zdaniem powinny zostać jak najdłużej? Bo rzeczywiście są sensowne. Jeżeli tak, to które to są?**

No nie wiem do końca, tak jak mówię, nie mam jakby danych, żeby w ogóle…

**Ale chodzi mi o to, czy mamy np. chodzić w nieskończoność w maseczkach, czy mamy się izolować, nie spotykać z ludźmi w domach. Czy mamy nie otwierać szkół. Wiesz, co można poluzować twoim zdaniem, a co jak najdłużej powinno się trzymać w obostrzeniu?**

No nie wiem, tak naprawdę. Zawsze uważałam, że na przykład to, że granice są zamknięte, to jest dobrze. Aczkolwiek czy one są tak do końca zamknięte i czy są szczelne to też nie do końca wiadomo. Co do tych szkół... Nie wiem, tak naprawdę akurat mnie to w ogóle nie dotyczy. Niektórym wyjdzie to na lepiej, niektórym wyjdzie to na gorzej i tak naprawdę nie wiadomo. To jest taki eksperyment z tymi wszystkimi rzeczami, że…

**A komu to wyjdzie na lepiej, komu na gorzej?**

No dzieciom, nie? Myślę, że niektóre dzieci na tym skorzystają.

**W jaki sposób?**

W jaki sposób? Znaczy wydaje mi się, że indywidualne nauczanie jest bardziej efektowne niż takie nauczanie w klasie. I to, że… Wydaje mi się, że większości rodziców jakby zależy na tym, żeby swoje dzieci uczyć. Bo większość ludzi kocha swoje dzieci i chce ich tam czegoś nauczyć. I no jakby, no zawsze indywidualne nauczanie jest bardziej efektywne niż takie w klasie trzydziestoosobowej. Więc myślę, że może niektórym dzieciom to wyjdzie na dobre. Może niektórym na gorsze. To już jakby…

**To zamknięcie sklepów, wszystkich poza spożywczymi. To powinno być jak najdłużej czy nie?**

Nie wiem tak naprawdę. Jakby… Nie chciałabym takiego zdania opierać w jakichś moich emocjach i widzimisię mojego. Wolałabym, żeby to było oparte na jakichś faktycznie prognozach, jakichś opiniach ekspertów, na których ja bym mogła się oprzeć na tym. Uważam generalnie, że… Chciałbym, żeby jak najszybciej zostało otwarte, żeby ludzie mogli pracować normalnie. Natomiast kiedy to wszystko zrobić, jak to zrobić, no to ja naprawdę, no nie wiem.

**A to zdjęcie maseczek, jakie jest twoje zdanie, kiedy powinno się zdjąć maseczki?**

No tego też do końca nie wiem. Na przykład no myślę, że to jest rzecz, która może wejść do naszej kultury w ogóle na stałe. Być może.

**Ale to będziemy już zawsze chodzić w maskach na twarzy?**

Znaczy no nie wiem, czy zawsze. Na przykład no w krajach azjatyckich jest to dosyć powszechna rzecz, że oni tam chodzą w maseczkach, bardzo dużo ich, w komunikacjach takich miejskich itd. To jest taki… Nie wiem, dla mnie to jest taki obraz, który w ogóle mnie nie dziwi, jak patrzę gdzieś tam na te kraje azjatyckie. I być może to zawita do nas na stałe. I ludzie, nie tak, że będzie rozporządzenie, wszyscy będą musieli, tylko ludzie będą chcieli. Bo do tego przywykną, będą się czuli bezpieczniej. Bo będą widzieli, że to przynosi jakieś efekty. Nawet nie tylko przed tym koronawirusem, ale też przed grypą, jakąś tam… No, różnymi rzeczami.

**Przy całej twojej niechęci do tej maseczki i tego, że masz ją na twarzy, źle ci się oddycha, czujesz się bezpieczniej, jak jesteś w niej w sklepie?**

Znaczy, tak jak mówię, że mnie bardziej jakby to, że inni ludzie noszą, to ja się czuję bardziej bezpieczna niż to, że ja noszę. Ja mogę zabezpieczać w ten sposób ludzi, jak ja jestem chora. Tak to rozumiem. Bo jeżeli ja na przykład czuję, że nie jestem chora, to mam mniejszą potrzebę zasłaniania i chowania (śmiech).

**Ale mogłabyś być bezobjawowa.**

No mogłabym. Ale no jakby nie do końca w to wierzę, że miałabym się gdzieś zarazić i… Znaczy biorąc pod uwagę, że się z nikim nie spotykam, wychodzę do sklepu raz w tygodniu.

**To niech inni noszą, a tobie niech pozwolą nie nosić, bo to jest… To troszkę tak jest?**

Bo to jest tak, że każdy może ufać tylko sobie, a nie wszystkim innym. I rozumiem, że ludzie też będą się czuć lepiej, kiedy wszyscy inni będą nosić. Ale też, no ja na przykład, jeżeli widzę kogoś 50 metrów ode mnie i ten ktoś ma niezakryty nos, to ja też nie robię afery z tego powodu. Jakby też daję trochę ludziom wolności w tym. A nie jakby uważam, że przepisy przepisami, ale człowiek człowiekiem, nie?

**A ten model szwedzki. Czytałaś o tym, jak tam oni podeszli do sprawy?**

No czytałam, tak, słyszałam o tym. Że tam są dużo mniejsze te restrykcje. Natomiast właśnie wdałam się w niejedną dyskusję na ten temat na Facebooku.

**To wdaj się ze mną teraz (śmiech).**

Uważam, że nie można porównywać tak, to nie jest takie proste porównanie dwóch krajów pod jakimś tam względem. Bo my mamy różne przyzwyczajenia, różne takie kulturowe rzeczy, które wynikają jakby z naszej kultury właśnie. Czego się nie da do końca porównać. Wydaje mi się, że tutaj Szwedzi akurat po pierwsze są w ogóle takim narodem, który się dużo bardziej izoluje od innych ludzi. Znaczy oni mieszkają podobno, dużo ludzi mieszka samych, dużo starszych ludzi mieszka samych, że młodzi nie mieszkają z… Natomiast, no to Włosi na przykład są rodzinami wielopokoleniowymi. Dlatego tak dużo ludzi się pozarażało, że ci młodzi przynosili do domu, zarażali swoich rodziców, dziadków, bo mieszkają z nimi. Tak że no to są takie kwestie kulturowe. I niuanse takie, które no jakby nie do końca da się wyłapać. I myślę, że to porównywanie się z innymi krajami jest… Jest bardzo trudne. I nie zawsze się tak da.

**Oprócz tego, że oni nie zamknęli restauracji, działają niektóre kina, działają podstawówki, żłobki, przedszkola itd. i sklepy, i fryzjerzy, to wszystko działa. To poza tym dla mnie coś takiego jest interesujące, że oni nie nakazują, nie zakazują, tylko rekomendują, żeby czegoś nie robić.**

No tak, tak, tak. Ale tak jak mówię, no to jest inny… To jest też kwestia kulturowa. Że u nich wystarczy rekomendować coś i ludzie się słuchają itd. A u nas po prostu powiedzą, że rekomendują i wszyscy by to mieli w czterech literach.

**U nas to by nie przeszło, rekomendacja?**

Wydaje mi się, że nie. W niektórych przypadkach naprawdę. Jeszcze wydaje mi się, że młodsi ludzie jakoś tak trochę inaczej na to patrzą. Natomiast to starsze pokolenie jest takie, że no ciężko… Oni tam wiedzą swoje i wirusa się nie boją, bo na coś muszą umrzeć, tak? No nie wiem, no wydaje mi się, że to są takie niuanse... Trzeba by to sprawdzić, u nas to chyba nie… Obawiam się, że mogłoby to nie zadziałać. Aczkolwiek, tak jak mówię, trzeba by zrobić jakiś eksperyment chyba.

**Wiesz, bo tak mi się skojarzyło i stąd przeszłam do tej Szwecji, bo powiedziałaś, że niedługo to może u nas tak będzie jak w krajach azjatyckich, że każdy włoży tą maseczkę, bo uzna, że to jest bezpieczniej. Ale z drugiej strony nie widzisz, żeby w tym momencie była taka możliwość, żeby rekomendacja wystarczyła, żeby ludzie sami siebie ograniczyli.**

Bo są takie rzeczy, które są dla nas obce, tak jak noszenie tej maseczki, tak? Znaczy my tego nie mamy. W krajach azjatyckich to się rozwijało przez ileś tam lat. I to nie tak, że teraz… Znaczy ja nie wiem, może tam też jest jakaś rekomendacja, żeby to zakrywać, nie wiem, jak jest dokładnie. Natomiast mi się kojarzy taki obraz z Azją, że oni zakrywają te usta. I jakby to jest u nich takie powszechne.

**Ale to nie było bardziej z powodu smogu?**

Chyba też, chyba też, tak, tak. Natomiast u nas to jest taka jeszcze nowinka, taka ten… I faktycznie, jeżeli coś jest takie niezrozumiałe dla ludzi itd., to rozumiem, że dlatego jest rozporządzenie i nakaz, tak? Natomiast, jeżeli coś jest bardziej takie naturalne dla ludzi, to nie trzeba jakby nakazów robić. Tak jak mówię, mamy różną kulturę i…

**A czy to jest trochę tak, że Szwedzi mają ograniczenie prędkości i go przestrzegają a Polacy mają i nie przestrzegają, chyba, że mają duży mandat, to też o to chodzi?**

No właśnie to też jest takie ciekawe. Bo, no właśnie to jest, właśnie podejście do prawa też jest taką kulturą rzeczą, nie? Im dalej na północ tym bardziej ludzie są tacy bardziej zachowawczy, jeżeli chodzi o prawo. A im bardziej na południe tym mniej, mam wrażenie, jeśli chodzi nawet o samą jazdę samochodem. Po prostu jak się wsiądzie gdzieś na południu Europy do samochodu, to strach się bać, bo oni tak jeżdżą, że to jest masakra. Jakby no my jesteśmy gdzieś po środku. Z jednej strony uważamy, a z drugiej u nas nawalone tych znaków jest tak, że czasami człowiek po prostu widzi, że pewne są przepisy, które są bez sensu totalnie. I jakby nie przestrzega, bo wie, że jakby nie ma takiej uzasadnionej jakby potrzeby na to ograniczenie powiedzmy.

**Ale gdybyś miała zagłosować, czy robimy w Polsce tak jak w Szwecji, rekomendujemy i niech każdy robi, co chce, on sam dba o swoje bezpieczeństwo. Albo jest tak, jak mamy teraz. To co byś wybrała, którą wersję?**

No ja zdecydowanie wybrałabym opcję bardziej wolnościową.

**Czyli tak jak w Szwecji.**

Tak. Aczkolwiek nie wiem, czy by się to sprawdziło. Ale jak bym miała wybierać, to tak.

**Czy coś byś zmieniła w tym - tam są otwarte żłobki, przedszkola, szkoły podstawowe. Średnie i wyższe są zamknięte, działają online. Tam jest tak, że komunikacja działa. Parki są otwarte, niektóre kina, restauracje, puby, sklepy. No, nie można mieć zgromadzeń dużych, powyżej 50 osób, nakazu maseczek chyba nie ma, z tego co wiem.**

No co, dobrze.

**Przyjęłabyś ten model szwedzki? Chciałabyś, żeby u nas tak było?**

No ja bym chciała, żeby było więcej wolności. Tylko, że wtedy trzeba jakby, moim zdaniem troszeczkę musi być inna, no jednak inna trochę polityka. Znaczy w takim sensie, że ludzie powinni być bardziej informowani o tym wszystkim, co się dzieje. Więcej, bardziej testowani, tak? No generalnie musiałoby to iść w taką większą, moim zdaniem, przezroczystością ze strony władz.

**Jakich informacji ci brakuje? Gdzie jesteś niedoinformowana?**

No… Znaczy wydaje mi się, że jednak… Uważam, że chorych… Znaczy chyba to wszystkie te modele pokazują, że więcej jest chorych niż jest robionych, tych oficjalnych chorych niż tych w ogóle chorych. I generalnie tutaj powinno być więcej tego testowania. A jak ja słyszę dzisiaj, że po co marnować testy na zdrowych ludzi, no to…

**Tak? Dzisiaj tak ktoś powiedział? Nie słyszałam.**

No tak, jakiś tam wiceminister. Bo na przykład nie chcą badać tych… Większość zachorowań to są te ośrodki zdrowia. A nie chcą testować tych medyków. Bo mówią, bo po co marnować na zdrowych ludzi. No kurcze, no jakby… No tak, jakby zaoszczędźmy pieniądze na tym, to na pewno po prostu może będziemy mogli wydać potem na nie wiadomo co. Na respiratory (śmiech). Przecież to jest bez sensu totalnie. Jakby… no to jest to. Tak jak mówiłam, nie wiem, jakimi oni tam modelami tych zachorowań się posługują, bo jakby nikt nie mówi. Żadni eksperci się nie wypowiadają od tej strony rządowej. No jeden minister. On chyba jest lekarzem, ale nie jest jakim epidemiologiem, tak? On zresztą powiedział, że on apeluje do lekarzy, żeby wykonywać więcej testów. Przecież to jest jakieś, śmiech na sali, przecież on jest odpowiedzialny za to wszystko. To niech on nie apeluje, tylko niech on wydaje rozporządzenie. Jakby każdemu w Polsce jest w stanie wydać rozporządzenie, że ma nosić maski, a nad lekarzami nie panuje. Mimo, że to jest jego resort. A do lekarzy apeluje, żeby robili więcej testów. To kurde, no on jest szefem.

**Ale to on powiedział właśnie, że maski zdejmiemy wtedy, jak znajdzie się szczepionka. Co ty na to?**

No tak. Pan minister już mówił różne rzeczy. I jeszcze 2 miesiące temu mówił, że on nie wie, po co ludzie w ogóle noszą maski, one są niepotrzebne. Tak że… Dzisiaj powie to, jutro powie co innego.

**Jak ty to słyszysz i sobie wyobrażasz, że będziesz miała nakaz noszenia maseczki aż do czasu wynalezienia szczepionki, to co sobie myślisz? Że co to będzie z tobą w takiej sytuacji?**

Ze mną. No, że nie będzie fajnie. Znaczy to nie jest jakieś fajne, tak? Zwłaszcza jak będzie lato. I co? Wszędzie trzeba będzie nosić. No przecież człowiek się po prostu ugotuje w tym, nie będzie miał czym oddychać. Nie wiem, jak będą wyglądały wakacje wtedy (śmiech). Nie wiem.

**Kiedy powinno się zdjąć te maski? Kiedy powinno być tak, że zakładasz albo nie zakładasz, dowolnie?**

Dla mnie sensowne byłoby, nie wiem, czy da się coś takiego zrobić, bo to by, znowu jakby… Że no jak nie jesteśmy gdzieś w pobliżu jakichś ludzi, nie widzimy ich z bliska, to ta maska jest, jakby nie ma żadnego sensu. W sensie idę na spacer na plażę czy do lasu. No już do tego lasu powiedzieli, że nie trzeba nosić masek. No to to jest, jakby no… I co, wyjdzie policjant i wystawi mandat? Przecież to jest w ogóle jakieś nieporozumienie, tak? Znaczy w ogóle… Nie da się tego prawnie, że tak powiem, opisać każdej sytuacji w życiu, kiedy maseczki trzeba nosić, kiedy nie nosić. Jak ma się kontakt z drugim człowiekiem, to ja rozumiem. Ale też, nie w każdej sytuacji się tego nie da opisać.

**Przejdźmy do ostatniego punktu spotkania, dbanie o siebie. Jak to jest w tej chwili w stosunku do tego, jak było kiedyś. Czy w ogóle coś się u ciebie zmieniło? Mam na myśli i fryzjera, i kosmetyczkę, i chodzenie na paznokcie, kupowanie sobie ciuchów, wiesz, masaże, spa, baseny, wszystko, co tam sobie wymyślisz. Jak to było przedtem, jak jest teraz. Czy jest jakaś różnica.**

(śmiech) Nie chodzę do fryzjera, nie chodzę w takich… Nie, paznokcie sobie robię tylko latem, na lato, więc na razie nie.

**Ale robisz sobie sama, czy robiłaś sobie gdzieś?**

Nie, robiłam sobie gdzieś. Ale to tylko w lato, jak wyjeżdżam gdzieś na wakacje, bo wtedy lubię mieć zrobione. A tak to w ciągu roku to sobie nie robię. Nie, nie, nie, ja nic takiego, no nie chodzę, na żadne siłownie nie chodzę, bo mam małe dzieci, tak? No nie chodzę na takie rzeczy.

**Ale coś się zmieniło w ogóle w związku z sytuacją koronawirusową dla ciebie? Że robisz czegoś mniej albo czegoś więcej? Bo niektórzy teraz sobie wykorzystują na przykład ten czas na to, żeby się obalsamować i żeby im się skóra nawilżała, bo jak chodzili do pracy, to nie mogli. Albo maski na włosy albo jakieś oleje na włosy i oleje na paznokcie. Wiesz, no różne rzeczy ludzie robią.**

Nie wiem, no moje życie się jakoś tak za bardzo nie zmieniło w tej sytuacji. I tak codziennie opiekowałam się dziećmi, tak samo teraz codziennie opiekuję się dziećmi. Więc tak na co dzień to mi się nic… Nie mam więcej czasu na nic z okazji tego, że jest koronawirus. Raczej mam mniej czasu niż więcej, bo nie mogę z nikim dzieci zostawić i sama sobie gdzieś tam wyjść na zakupy czy coś. Więc jakby no…

**A sposób ubierania się, coś się zmieniło?**

Nie, ja w domu cały czas chodzę w dresie, tak jak chodziłam.

**A makijaż?**

No w domu się nie maluję. Znaczy malowałam się na święta. Ale w domu nigdy się nie malowałam i nie maluję się w domu.

**Czyli to też się za bardzo nie zmieniło. A jak teraz wychodzisz do sklepu, malujesz się?**

Nie, do sklepu też się nigdy nie maluję.

**Ogólnie malujesz się rzadziej niż malowałaś się przedtem?**

Znaczy, maluję się, jak wychodzę, nie wiem, żeby się spotkać z takimi obcymi ludźmi, że tak powiem (śmiech). A teraz nie wychodzę się spotkać z obcymi ludźmi.

**Ostatnio na święta, a przedtem, kiedy się malowałaś?**

No chyba właśnie jak… Gdzie ja byłam? Nie wiem, gdzieś wychodziłam z mężem chyba. A, bo były ferie i żeśmy tak częściej chodzili gdzieś tam.

**Czyli jeszcze w lutym jakoś tak to było pewnie.**

No tak, tak, tak. Bo wtedy żeśmy dużo wychodzili właśnie z domu. A potem, nie wiem, już nie pamiętam.

**Brakuje ci tego? Pomalować się, wyjść.**

Nie. Znaczy no wyjść to tak, no wiadomo. Ale bez przesady z tym makijażem. Ja nie lubię tak na co dzień. Nie lubię tego na co dzień robić.

**A jeśli chodzi o takie rzeczy, których ci brakuje z takiego dobrostanu, tu wyjść gdzieś, tu wyjść na basen, pójść gdzieś coś zrobić dla siebie. Do fryzjera nie chodzisz, ale może na zakupy, kupić sobie ciuchy wiosenne nowe, cokolwiek, buty nowe.**

No tak. Znaczy no lubię tam sobie połazić po sklepach. To każdy tak lubi, myślę, każda kobieta lubi sobie tam pochodzić, połazić, popatrzeć. Ale ja też nie kupuję za dużo takich rzeczy. Bo do pracy nie chodzę, tylko tyle, co tam po domu.

**A co byś chciała, gdyby był koncert życzeń i masz sobie wybrać pierwsze 3 miejsca z kategorii zadbania o siebie samą, takiego dobrego samopoczucia, to co byś chciała, żeby ci udostępniono?**

Ja bym chętnie poszła z mężem na randkę. W sensie, że bym mogła zostawić dzieci gdzieś z kimś. I poszła sobie z mężem do jakiejś restauracji, do pubu, na lody.

**Co jeszcze? Bo 3 rzeczy miały być. Jedna to jest wyjście na randkę. Bo przepraszam, bo mówiłaś, że randki sobie robicie w niedzielę w domu.**

W domu to tak, ale to w domu to… Bo randki domowe to trochę taki przymus ze względu na to, że mieliśmy małe dzieci i nie mogliśmy ich zostawić. Znaczy ja nie mogłam zostawić. Bo jak jeszcze karmiłam itd. to w ogóle nie mogłam ich zostawić. Ale już właśnie, ponieważ ja dopiero niedawno odstawiłam moje dziecko najmłodsze, no to właśnie dopiero żeśmy zaznali jakby taką możliwość, że możemy gdzieś ich zostawić i wyjść sami.

**Co ci daje takie wyjście z mężem, zostawienie dzieci pod opieką i wyjście?**

To jest piękny czas (śmiech). No to jest taki czas tylko dla nas. I można spokojnie porozmawiać, nikt nie przerywa nam w pół zdania. I można się napić alkoholu, bo przy dzieciach raczej nie pijemy, w sensie jak jesteśmy z dziećmi.

**I nie musisz być taka na stand by cały czas, tak?**

Tak, tak. Że trzeba cały czas pilnować jedno, drugie, co tam robi. Nie da się tak być ze sobą nawzajem cały czas, tylko jesteśmy we czwórkę, a nie we dwójkę.

**Czyli mamy pierwszą rzecz, otworzyć wam jakieś knajpki, bary, nawet na dworze, jak rozumiem, bo będzie ciepło, żeby wyjść. Co jeszcze? Co dla ciebie można by było zrobić, żebyś się poczuła lepiej? Czego ci brakuje?**

Właśnie teraz sobie zdaję sprawę, że nie robię nic dla siebie (śmiech). Nie, no to jest takie… Znaczy jeszcze ja nie wyszłam z tego trybu, że po prostu… Że cały czas z tymi małymi dziećmi. Jakby nie do końca ja jestem w stanie ich tak zostawić, znaczy dopiero zaczynam ich zostawiać na dłużej gdzieś. Jeśli chodzi o mojego starszego syna, no to już bardziej, natomiast ta młodsza to jeszcze tak… No właśnie, jeszcze do niedawna w ogóle jej nie zostawiałam z nikim. Więc nie robię w ogóle tak na co dzień takich rzeczy, że sama coś tam robię.

**No OK, czyli trochę to tak dla mnie wynika z tego, co mówisz, że bardziej byś się ucieszyła, gdyby ci otworzyli sklepy z ciuchami i butami niż koniecznie tych fryzjerów, kosmetyczki, spa i takie…**

Tak, tak, tak. Ja takich rzeczy w ogóle nie robię. Lubię pochodzić, nawet z dziećmi łażę po tych sklepach czy… Najbardziej bym się ucieszyła, gdyby otworzyli to zoo. Nawet niż te sklepy. Ja już naprawdę nie muszę mieć tych butów, naprawdę nie muszę tej sukienki kupić. Zoo mi otwórzcie, bardziej mi to zależy.

**A dlaczego zoo? Bo jak już masz parki, co jest takiego w tym zoo, co cię…**

Kurcze, fajnie, ja lubię do tego zoo chodzić, nawet ja lubię do tego zoo chodzić. I widzę, jak te dzieci się cieszą z tego zoo. Tam jest zawsze fajnie, zawsze te zwierzątka oglądamy razem, już z tym starszym synem tam sobie gadamy o tych zwierzątkach, opowiadamy sobie różne historie. On się już pyta o różne rzeczy. Więc już możemy sobie porozmawiać. To jest jedna rzecz. A druga, na której mi naprawdę zależy, to żeby jak najszybciej, żeby zostały otwarte hotele. Bo ja chcę wyjechać gdzieś na wakacje. Żebym mogła wyjechać. Bo już mam zarezerwowane i chcę wyjechać (śmiech).

**I masz zarezerwowane, kiedy?**

Na czerwiec mamy zarezerwowane.

**Dowiadywałaś się, czy jest jakaś, jak oni tam to widzą, gdzie macie rezerwację?**

Znaczy rozumiem, że na razie rezerwacja jeszcze jest utrzymana, więc jeszcze tam to działa. No zobaczymy. Bo z tego, co widziałam, to następny ten etap z tych wszystkich niby, co napisali do tej pory, co nie wiadomo i tak, czy będzie, czy nie zmienią tego, to w następnym etapie mają już być otwarte te hotele i te miejsca, tam powiedzmy… no te mieszkaniowe. Bo tam rekreacyjne to… Szczerze mówiąc, to bardziej mi chodzi o to, żeby można było wyjechać. Bo mamy zarezerwowane nad morzem i po prostu… tak naprawdę jest mi wszystko jedno, ja już powiedziałam, że ja mogę tam codziennie jechać i mogę nawet gotować codziennie, byleby tam jechać i codziennie z tymi dziećmi iść na tą plażę.

**W maseczce na plażę pójdziesz?**

Wszystko mi jedno (śmiech).

**Nawet w maseczce na plażę?**

Tak. Znaczy myślę, jak nie będzie dużo ludzi, chociaż nie wiem, czy będzie dużo ludzi, bo… Bo jak teraz dzieci nie mają teoretycznie szkoły, to może być więcej ludzi tak naprawdę, jak otworzą te wszystkie hotele itd., kto wie. Ale jak nie będzie ludzi, no to kurcze, mam nadzieję, że mnie nikt helikopterem tam nie dojrzy czy jakimś dronem, że ja nie mam maseczki na plaży.

**Czy jest coś, o co nie zapytałam cię - zwykle cię pytam na koniec o takie twoje refleksje. Czy w ostatnich dwóch tygodniach coś się zadziało jeszcze?**

No nie wiem, chyba nic mi nie przychodzi do głowy. Chyba wszystkie moje frustracje powiedziałam, co mnie denerwuje (śmiech).

**Mam do ciebie jeszcze taką prośbę, bo będziemy się spotykać za 2 tygodnie. Chciałabym, żebyś spróbowała, gdyby cię dopadły jakieś emocje, które są takie większe niż mniejsze, jakieś znaczące rzeczy, które ci się przytrafiają w czasie tych dwóch tygodni, zrób sobie jakąś notatkę, albo zrób zdjęcie miejsca, które ci to przypomina [instrukcje do zadania].**

Dobrze, chociaż ja nie lubię się skupiać na moich emocjach (śmiech).

**Dziękuję.**
