# Supplementary material for: The challenges arising from the COVID-19 pandemic and the way people deal with them. A qualitative longitudinal study
Source: PLoS One. 2021 Oct 11;16(10):e0258133. doi: 10.1371/journal.pone.0258133 (PMC8504766; doi:10.1371/journal.pone.0258133)
Supplement: S1 Dataset — (ZIP) [file pone.0258133.s003.zip › Transcriptions/stage 3/18.3_F_48_couple, with children.docx]

**18.3_F_48_couple with children**

**Co działo się przez ostatnie dwa tygodnie?**

Nic bardzo szczególnego, ale był taki kryzys przemęczenia. Ten tydzień przed świętami był ciężki, pojawiło się było dużo pracy, chaosu, zmęczenie fizyczne, dodatkowo jeszcze nasiliły się zachowania ludzi związane z frustracją. Wszystko się skumulowało. W dodatku z tyłu głowy miałam to, że nie wiadomo, jak będą wyglądać święta. Było wiele rzeczy dających do myślenia, ogólne było mi ciężko. Dotychczas po powrocie do domu jeszcze coś robiłam. W tygodniu poprzedzającym święta najchętniej nie robiłabym nic - nawet nie interesowało mnie przeglądanie gazet, czy oglądanie filmów. Najchętniej bym usiadła i siedziała, nie robiąc nic. To był chyba taki moment, kiedy wszystkich nas dopadła niemoc. Na przykład teściowej było ciężko - zastanawiała się, jak długo ma jeszcze siedzieć w tym domu. Mama stwierdziła, że lubi być w domu, ale teraz jest już tego za dużo. W dodatku wpędzała się w jakieś poczucie winy mówiąc, że muszę się nimi zajmować, pomimo, że mam bardzo dużo swoich spraw. Choć odpowiadałam jej, że to akurat najmniejszy - o ile w ogóle można to traktować w tej kategorii - problem. Zebrało się dużo rzeczy. Ale później przyszły święta i odpoczynek. Kiedy odcięłam etap pracy, nie było już ludzi i nawału wszystkich obowiązków, zrobienie ciasta i całe przygotowania świąteczne sprawiły mi przyjemność. To było nawet formą relaksu. Poza tym, udało mi się poczytać, skorzystać z tarasu, ze względu na ładną pogodę. I to jakoś wróciło do normy.

**Poczułaś się trochę oderwana od sytuacji w momencie przygotowań i samych świąt?**

Tak, oderwałam się od sytuacji i miałam taką namiastkę normalności. To nie były duże przygotowania, ale pozwalały zająć głowę innymi sprawami. Kupiłam stroik, postawiłam jakieś kwiaty - to dało namiastkę świątecznego nastroju. To wszystko dało mi złapać oddech.

**Czy kiedy byłaś zmęczona pracą, myślałaś o tym, żeby nie zrobić w tym roku świąt?**

Myślałam, że one muszą być. Ze względu na odpoczynek, ale też taką funkcję odcięcia się w rodzinnym gronie od wydarzeń mających miejsce na zewnątrz. Najbardziej potrzebowałam chyba spokoju w domowym zaciszu.

**Jak wyglądały te święta?**

To było minimum minimum. Najpierw wyjęłam koszyczek, ale stwierdziłam, że to bez sensu, skoro i tak nie będę z nim szła do kościoła. Stwierdziłam wtedy, że skoro będzie w domu, przygotuję to inaczej - zrobię tylko to, co ma być na talerzu. Mąż symbolicznie pobłogosławił jedzenie. Podzieliliśmy się jajkiem, nie było jakichś większych zmian. Troszeczkę się bałam tego, że nie było moich rodziców - byliśmy tylko we czwórkę. W tygodniu poprzedzającym święta  sporo o tym myślałam - nie wiedziałam, jak to będzie, puszczałam wodze fantazji. Jestem bardzo związana z tatą. Jak myślałam o tym, że przy stole świątecznym nie będzie rodziców, to trochę tak, jakby już miało ich nigdy nie być. Miałam jakieś takie dziwne myśli. No, nie wiem, czy dziwne, ale tak mnie jakoś nachodziły. Wydawało mi się, że to będzie takie straszne i że będzie smutno. Tak, jakby rodzice umarli - takie miałam wyobrażenia. Ale oni zadzwonili, porozmawialiśmy przez telefon - oni nie są raczej w stanie połączyć się na wideo rozmowie, trzeba by było im wszystko ustawić - było całkiem spokojnie. Przed świętami, podczas tych rozmyślań, przypomniało mi się jeszcze dzieciństwo. Zawsze spędzaliśmy Wigilię u dziadków. Dziadkowie mieszkali w Sochaczewie, a my w Pruszkowie. Jak wprowadzono stan wojenny, miałam siedem lat. Pamiętam, że wtedy nie pojechaliśmy na Boże Narodzenie, Wigilię. Mieliśmy w domu wszystko - choinkę, prezenty. Z perspektywy dziecka naprawdę niczego mi nie brakowało. Pomimo tego, zawsze wspominałam te święta jako, może nie najgorsze, ale tak inne, niefajne święta, że wszystko było nie takie - i ta choinka, wszystko. Tak myślałam sobie teraz przed Wielkanocą, czy to nie będą właśnie takie święta, jak te w dzieciństwie. Ale nie były takie złe. Było przyjemnie, spokojnie, choć ta obawa przed nimi towarzyszyła. Z akcentów religijnych, wysłuchałam orędzia papieża. Nie interesowały mnie żadne msze, ale tego miałam potrzebę posłuchać. Chyba szukałam w tym pewnego rodzaju wsparcia, opieki, otuchy. Mąż na początku mówił, że pewnie papież powie to, co zawsze. Ale ja chciałam posłuchać, więc to zrobiliśmy. Padło na przykład takie zdanie, żeby czerpać siłę od kobiet, które mają siłę, aby brnąć do przodu. To jakoś szczególnie zapadło mi w pamięć z tych wielu mądrych i w sumie prostych, normalnych myśli, z którymi się zgadzałam. To przemówienie dało mi spokój, wrażenie wsparcia dla kobiet i takie poczucie opieki nad wszystkimi - różnymi państwami, w różnych sytuacjach. W tych krótkich wypowiedziach miałam wrażenie podzielania z nim pewnych opinii.

**Uważasz, że kobiety potrzebują więcej wsparcia, niż mają?**

Myślę, że tak, zdecydowanie. Zawsze mogłoby być lepiej. Tak, jak obserwuję w swojej pracy, w branży kosmetycznej głównie są to kobiety, choć to zaczyna się zmieniać i panowie też bywają aktywni. Ja mam to wsparcie, nie muszę się pytać, prosić. Mam jednak wrażenie, że wiele kobiet jest zostawiona sama sobie - z dziećmi, zakupami. Nawet, jeśli są czynne zawodowo i zajmują wysokie stanowiska, potrafią ukrywać przed mężem koszty związane z choćby zrobieniem paznokci. Co mnie zastanawia, bo przyrównując do zarobków tych pań, nie są to duże kwoty. Przyzwyczaiłam się do tego, ale zupełnie tego nie rozumiem. Nigdy nie miałam takich sytuacji w swoim domu, ani w najbliższym otoczeniu, ale wiem, że takie sytuacje mają miejsce. Mówi się, że głową rodziny nadal jest facet - pomimo tego, że to często kobiety są od ogarniania wszystkiego. I to widać. Na przykładzie pielęgniarki, która po dyżurze robi jeszcze zakupy, myśląc dodatkowo o potrzebach sąsiadki. Panowie z kolei myślą jednym torem - wykonują konkretne zadanie i nic więcej.

**Czy coś zmieniło się w Twoim życiu w ciągu ostatnich dwóch tygodni?**

Nie zaobserwowałam wielkich zmian. W weekend po świętach zmęczenie wciąż jeszcze odpuszczało. Nadal wstaję wcześnie rano, co jest związane z pracą w sklepie, ale ogólnie na pewno więcej śpię. Kiedy mam czas wolny, organizm dopomina się odpoczynku. Mąż ma podobnie. Nawet ostatnio śmiał się z nas nasz syn. Oglądaliśmy film i ciągle na zmianę, któreś z nas przysypiało. Syn stwierdził wtedy, że to bez sensu, że lepiej sobie odpuścić film i żebyśmy poszli z mężem spać. Poza tym nic się nie zmieniło. W sklepie jest trochę mniej pracy - otwarcie całodobowych dyskontów odciążyło nas. Praca ciągle jest, choć jest spokojniejsza.

**Co Ci teraz najbardziej doskwiera?**

Potrzebuję już kontaktu ze znajomymi, rodziną. Chciałabym usiąść z rodzicami w pomieszczeniu - obecnie spotykamy się i rozmawiamy tylko przez chwilę na podwórku, kiedy przywożę im jakieś rzeczy. Na początku nawet trochę cieszyłam się, że tych spotkań towarzyskich będzie mniej. Teraz odpoczęłam, zobaczyłam, jak to jest i zaczyna mi tego brakować. Nawet nie tyle brakuje mi imprez, co tego, żeby sobie po prostu usiąść ze znajomymi i porozmawiać - nie telefonicznie, tylko tak w realu.

**Czy zamknięcie w domu z rodziną zmieniło wasze relacje?**

Postrzegam to jako plus. Jakieś 20 lat temu pracowaliśmy z mężem razem, więc byliśmy ze sobą prawie cały czas. Później oddzieliłam się i podzieliliśmy zajęcia. Teraz wracamy do korzeni. Dzielimy większą ilość czasu i to mi przypomina tamte lata, jest fajne. To jest teraz inny rodzaj kontaktu. Wieczorami rozmawiamy więcej o wspólnych doświadczeniach, tematach, zamiast opowiadać sobie jak komu indywidualnie minął dzień. Choć zdarza się też, że szukam przestrzeni dla siebie, ale to raczej nie jest związane z sytuacją koronawirusa. Jakiś czas temu zamknęłam się z gazetą w łazience, leżąc w wannie. Myślałam co prawda, że spędzę tam nie wiadomo jak dużo czasu, a okazało się, że wcale nie. To równie dobrze mogłoby zdarzyć się też wcześniej. Zapewniam sobie więc jakieś minimum prywatności, ale to nie jest tak, żebym musiała jakoś uciekać. Może nawet wcześniej bardziej szukałabym okazji do oddzielenia się. Teraz ta bliskość faktycznie jest większa. Przebywanie razem sprawia, że mam większe poczucie bezpieczeństwa. Kiedyś oglądałam filmy sama. Teraz wybieramy wspólnie taki, który każdemu może jakoś odpowiadać i oglądamy razem. Wcześniej nie przeszkadzało mi spędzanie takiego czasu osobno - teraz mam większy komfort, kiedy robimy to wspólnie.

**Obrazki**

4, ale też 9 z 10 są całkiem niezłe. 4 - tu właśnie są poprzeplatane dłonie. To dla mnie oznacza wspólnotę, to, że wszyscy trzymają się razem. To najbardziej odzwierciedla te nasze wspólne posiedzenia w wieczory i weekendy, to, że trzymamy się razem. To również duże pokłady miłości - nie tylko wobec syna i męża, z którymi jestem fizycznie, lecz też telefoniczne wsparcie rodziców. Poczucie troski, wsparcia. Jest to może trochę wzruszające, ale i radosne. Mam się kogo chwycić. Ktoś chwyta mnie, ale i ja kogoś też.

9 - burza, tornado. To był ten tydzień przedświąteczny, w którym pojawił się ogrom spraw, w dodatku jakichś kwestii urzędowych. To był ten pęd, w którym na pewno pojawił się też lęk przed tym, czy podołam fizycznie. Nie miałam pewności co do tego, czy jestem w stanie cały czas funkcjonować na tak dużych obrotach. Poza tym pojawiła się wtedy większa ilość zachorowań, ogniska pojawiły się też w pobliżu - ośrodku pomocy społecznej, czy naszych sąsiednich szpitalach, gdzie dwa oddziały zostały zamknięte. Towarzyszyła mi obawa przed tym, że to się zbliża, zwiększył się strach przed zarażeniem. Obawiałam się tego, czy w tym chaosie czegoś nie przeoczę, na przykład założenia rękawiczek, czy wystarczająco dobrej dezynfekcji. Dodatkowo zbliżały się święta, które nie wiedziałam jak będą wyglądały. Te wszystkie emocje chyba zatrzymywały mnie w miejscu. Na początku miałam taki moment, kiedy to wszystko mnie napędzało. Ale tamten czas przed świętami zatrzymał mnie w zawieszeniu, poczuciu bezsilności.

10 - to by się pokrywało z tym, co już powiedziałam. Chodzi o lęk, stan zagrożenia. Ten wirus to wróg, którego nie widać. Obrazek jest ponury, jest na nim burza. Nie ma tu kolorowych elementów, jest pozbawiony optymizmu. Dla mnie to oznacza wewnętrzne zamknięcie się w sobie, zatrzymanie - w przeciwieństwie do obrazka 9, który symbolizował bardziej taki zewnętrzny pęd. Te negatywne emocje wiążą się jednak zdecydowanie z tygodniem przedświątecznym. Później miałam czas na wypoczynek, złapanie oddechu. Teraz czuję się spokojniejsza, bardziej zmotywowana do działania. Zresztą, wydaje mi się, że duży wpływ na to miało wprowadzenie nakazu noszenia maseczek. Od początku byłam za taką formą ochrony i dziwiłam się, że nie zostało to wprowadzone wcześniej. W innych państwach, jak Czechy, stosowano takie działania znacznie wcześniej. U nas komunikaty były sprzeczne, niejasne. Jak ludzie zaczęli nosić maseczki, naprawdę dużo to dało. My co prawda już na początku zamontowaliśmy sobie zabezpieczenie z pleksi - ono też dało dużo. Poczucie bezpieczeństwa wzrosło od kiedy my i klienci nosimy maseczki. Widzę to nawet po dziewczynach w pracy - teraz czują się swobodniej. O ile na początku mówiłam, że staram się je otaczać opieką, teraz stały się samodzielne. Widać, że czują się pewniej, nie potrzebują wsparcia - że mówimy to, zrobimy to. Bariera mechaniczna zdecydowanie ułatwiła funkcjonowanie psychiczne. Choć funkcjonowanie fizyczne jest utrudnione - na przykład przez maseczki. Teraz zakupiłam im przyłbice, więc są zachwycone, bo mogą oddychać - przyłbica wystarcza, jest zgodna z przepisami, co sprawdziłam. Ale ogólnie tak, jak mówiłam, ruch zmalał, wydaje mi się, że jest tak, jakby powoli wszystko wracało do normalności.

**Jak radziłaś sobie przed świętami z poczuciem, że zagrożenie się zbliża?**

W sklepie, ale i wychodząc, używałam rękawiczek. Kiedy byłam zmuszona do przebywania w miejscu z większą liczbą osób, używałam też maseczki. Bardzo pilnujemy ograniczania kontaktów i przebywania w grupie nie więcej, jak trzyosobowej. Nawet, kiedy teraz odgórne zasady uległy zmianie, my dalej trzymamy się swoich zasad, nie powiększamy tej grupy. Nie mogłam komuś nakazać, żeby założył maseczkę, czy odsunął się. Teraz myślę, że ludzie są bardziej zdyscyplinowani, pilnują siebie - noszą maseczki, ale też pilnują siebie nawzajem.

**W jednej z wcześniejszych rozmów wspominałaś o atakach paniki, mierzeniu temperatury. To nadal się u Ciebie pojawiało?**

Teraz już nie. Jeśli chodzi za to o wiadomości, ich już nie da się oglądać, żadnych. Wolę przeczytać - oficjalny komunikat na tych gov.pl, albo jakąś wzmiankę - o tendencji zachorowań - czy to maleje, wzrasta, utrzymuje się. Ale o zachorowaniach, nie zgonach. I wystarczy mi, że sprawdzę to raz na dwa dni, nie muszę codziennie.

**Jak czujesz się teraz z obecną liczbą zachorowań?**

To, że jest większa liczba zachorowań mnie nie dziwi. Byłam świadoma, że to będzie rosło. Nie wiedziałam tylko w jakim tempie. Choć też nie wiem, na ile to są prawdziwe informacje, testów robi się jednak mało. To jest raczej orientacyjne. Nie przywiązuję się raczej do tej cyfry, nie twierdzę, że tak jest i to jedyna prawda. Sprawdzam te dane, żeby mieć taki ogólny zarys.

**Jak radzą sobie ludzie w Twoim otoczeniu?**

Każdy jakoś sobie radzi <śmiech>. Teściowa wymyśla różne gotowania, zaczyna rozpieszczać nas kulinarnie, przekłada w szafie po raz 10. Ona dotychczas z braku czasu nie gotowała pewnych rzeczy. Teraz może zrobić potrawy bardziej czasochłonne - jakieś pierożki w różnych odsłonach. Moja mama, tak jak mówiłam, lubi siedzieć w domu i nawet z nią rozmawiałam o tym. Powiedziała, że tak, lubi, ale ona zawsze jednak gdzieś bywała, gdzieś podjechała. Nie chodziła na jakieś wielkie spacery, ale jakiś kontakt z otoczeniem miała. Dziś z nią rozmawiałam, bo była w przychodni. Powiedziała mi, że ma wrażenie, że zapomniała, jak się chodzi. Ona praktycznie od początku [pandemii] była w domu, nigdzie nie wychodziła. Jak teraz wyszła na ten świat zewnętrzny, powiedziała, że ciężko było jej się odnaleźć. Wszystko było tak nowe. Te same miejsca wydawały się zupełnie inne. Przychodnia - samo wejście sprawiało jej problem, ze względu na mnogość procedur - dezynfekcję rąk, zakładanie rękawiczek, pomiar temperatury. Powiedziała, że gdyby codziennie miała tak funkcjonować, to jest to dla niej strasznie męczące. Kiedy po takim czasie weszła do sklepu, była oszołomiona – a wydawałoby się, że zakupy to taka prosta rzecz. Mama nie wiedziała, jak ma się zachować, co kupić. Jakby ta izolacja ją uwsteczniła. To było takie bardzo na nie. Natomiast też wiem, że jeśli ona na chwilę wyszła, kogoś spotkała - jak choćby panią w przychodni - to już na jakiś czas wystarczy jej tego kontaktu.

**Co dla Ciebie oznaczają terminy izolacja vs. kwarantanna?**

Ostatnio nawet w wiadomościach było nadużywane słowo kwarantanna. Dla mnie kwarantanna jest wtedy, kiedy ktoś już miał ewidentną styczność z zakażonym, chorym, wrócił z jakiegoś kraju, tak, jak było wcześniej. Natomiast izolacja jest wtedy, kiedy to ja izoluję się od tych wszystkich zagrożeń. Kwarantanna jest narzucona - takie są jej założenia, że nie można by w ogóle wychodzić. Izolacja jest dobrowolna. Ale rozmawiałam z mężem, bo miałam nieścisłość, dlaczego o jakimś artyście mówiono, że jest na kwarantannie, skoro nie miał styczności. I mąż doszedł do wniosku, że teraz w telewizji o właściwie o wszystkich, którzy siedzą w domu mówią, że są na kwarantannie.

Wcześniej było tak, że jak ktoś wrócił z zagranicy, musiał być na dwutygodniowej kwarantannie. Tak samo, jak pielęgniarka, jeśli na oddziale był ktoś zakażony. To osoba, która nie chodzi do pracy, bo miała styczność. Tak to sobie zakodowałam. Natomiast izolacja to ktoś, kto nie miał styczności lub o tym nie wie, ale sam decyduje, że w ten sposób będzie się chronić. Tak to sobie wytłumaczyłam. Choć słowo kwarantanna stało się popularne i używa się go w odniesieniu do każdego, kto siedzi w domu.

**Co myślisz o kolejnych obostrzeniach, które nastąpiły po naszych rozmowach?**

Był zakaz wstępu do lasu, później chyba myjnie. Później godziny handlu dla seniorów i limity klientów w sklepie, ale o tym rozmawiałyśmy. Jeśli chodzi o wprowadzenie maseczek, byłam za tym od samego początku. Ostatnio widziałam jakieś archiwalne -  ze stycznia, czy lutego – wypowiedzi naszego ministra zdrowia na temat tego, że maseczki nic nie dają. A teraz okazuje się, że dają. Tak naprawdę, taka bariera mechaniczna, co by nie mówić, daje jakiś procent zabezpieczenia. To wynika nawet z tych naukowych potwierdzeń. Szczerze mówiąc, one by nie zaszkodziły, gdybyśmy musieli je nosić trzy tygodnie wcześniej. Natomiast mogłyby - skoro chodzi o drogę kropelkową - może bardziej zabezpieczyć, niż zakaz wstępu do lasu, parku, czy wyjścia na spacer.

**Kogo chroni maseczka?**

Teraz to już chyba dla wszystkich <śmiech>. Jeśli używałam maseczki podczas swojej pracy, chroniłam siebie. Bo ta maseczka, której używałam w kosmetyce, służyła temu, abym nie wdychała tego całego pyłu, który unosił się podczas zabiegu. Natomiast tutaj, kiedy mówimy o przenoszeniu drogą kropelkową, chronię kogoś. Nie wiem, czy jestem zarażona, czy nie, więc cały ten aerozol zostanie wokół mnie.

**Które z obostrzeń uważasz za słusznie wprowadzone?**

Kwarantannę dla osób, które miały kontakt, maseczki, liczbę osób w sklepie. To były dobre posunięcia. Natomiast to, co teraz zostało wprowadzone - nie wiem. W momencie, kiedy rośnie liczba zakażeń i rozluźnia się tego typu obostrzenia, no to nie wiem, czy to w tym momencie ma sens. Te wszystkie zgromadzenia, skupiska - ich zakazanie miało sens. Natomiast, kiedy idzie razem rodzina, to jest dla mnie totalnym nieporozumieniem, żeby mieli zachować między sobą 1,5 metra. Kolega opowiadał mi, że dostali ostatnio za to mandat w Pruszkowie, bo nie utrzymali odstępu. To jest dla mnie wyłudzenie, to niczemu nie służy. Oni byli w czwórkę, ale to rodzina z dziećmi. Ja rozumiem, jakby to szli koledzy. Ale w momencie, kiedy to rodzina, to dla mnie robienie sztucznego tworu. To mnie wkurza. Tak samo, jak w godzinach dla seniorów sklep obsłuży kogoś z innej kategorii wiekowej. I to nie są kary rzędu 500 zł, 1000 zł. To są 3000 zł, 5000 zł. Gdyby to było tak, że te dwie godziny będą tylko dla seniorów i oni tylko wtedy mogą robić zakupy - uważam, że to by było super, miało rację bytu, zabezpieczało ich. Tym bardziej, że oni czasami trochę nie myślą o tym, że mogą przenieść tego wirusa na kogoś. Myślą "ja mam już tyle lat, to co mi będzie".  Poza tym, to byłoby dobre z takich fizycznych powodów - oni często mają problem z założeniem tych rękawiczek, kładą tę maseczkę... Mają ograniczenia ruchowe. Nawet płyn do dezynfekcji - nie wiedzą jak, ile. Wtedy, gdyby oni mieli tylko te dwie godziny, to by była taka izolacja seniorów od reszty społeczeństwa - młodszych, starszych, pracujących, niepracujących. Natomiast ci seniorzy chodzą cały dzień. W ciągu tych dwóch godzin przychodzą - bo część zrozumiała, że to im się należy, że to ich godziny. I oni myślą, że nadal tak jest. Jak w sobotę usłyszałam awanturę - kto przed kim, za kim stał - przypomniało mi się moje dzieciństwo. Ale jest jakaś równowaga - przyszła też do nas przesympatyczna staruszka i zaczęła zakupy od tego, że ona bardzo mnie przeprasza, że nie robi ich między 10 a 12. Wytłumaczyła się tym, że ma 83 lata i nie może dogadać się ze swoimi rówieśnikami, którzy ciągle się przepychają, kłócą, spieszą nie wiadomo gdzie. I ona tego nie rozumie, a sama nie potrafi tak funkcjonować. I podobno tłumaczy im, że w tym wieku, to można się tylko do śmierci spieszyć. To była taka gwiazdka. Ale takich osób jest bardzo mało.

Ja trochę też buntowałam się przeciw tym obostrzeniom, kiedy na przykład wchodził pan z budowy - ewidentnie po bułkę, bo w danym momencie ma przerwę na śniadanie. Więc dlaczego ja miałabym mu czegoś nie sprzedać, skoro i tak nie ma nikogo innego w sklepie? Wiem też, że są osoby, które pomimo, że pracują zdalnie, muszą być przy komputerze w określonych godzinach. Czasami nie mogą nawet od niego odejść. Poza tym, jak mam sprawdzać wiek tych ludzi? Przecież ja nie mogę każdego legitymować. 80 latka może wyglądać jakby miała 70 lat i mniej, i na odwrót. I tak się zastanawiałam, jak to wygląda, dopóki syna kolegi rodzice, którzy prowadzą sklep monopolowy, mieli kontrolę.  Choć dla mnie to nie jest kontrola, dla mnie to jest takie wyszukiwanie, wyłudzanie. W mniejszej społeczności łatwiej wyłapać osoby obce. Ci znajomi zauważyli, że w zaparkowanym pod sklepem samochodzie siedzą jakieś obce im osoby. Te zasady z godzinami dla seniorów, dotyczą każdego punktu handlowego. Po godzinie 10 weszła pani - młoda, na pewno nie miała 60, czy tam 65 lat - i bardzo prosiła o sprzedanie wody, bo ona właśnie szybko wybiegła z pracy. To jest już takie na siłę prowokowanie tej sytuacji. To nie jest, że ktoś wchodzi i sprawdza, pyta, dlaczego ktoś właśnie obsługuje młodzieńca. To jest na siłę szukanie pieniędzy. Ja się czymś takim brzydzę. I tak, jak dotychczas myślałam, że nikt nie będzie mi dyktował czegoś, co jest bez sensu, tak od poniedziałku - co prawda dopiero - zdecydowałam się, że nie obsługujemy tych młodszych osób i grzecznie wypraszamy, przepraszamy za coś, co nie jest naszym pomysłem. Natomiast płacenie 5000 zł za coś takiego.. Ja nawet złotówki bym nie dała do tego budżetu, czy nie wiem, kto to sobie bierze te pieniądze! Takie rzeczy mnie wkurzają i nie ma na to mojej zgody. Natomiast uległam, bo nie będę zbawiać świata, a nie stać mnie na płacenie takich kar dla kogoś, kto sobie w środku nocy wymyślił jakiś przepis, który naprawdę niczemu nie służy.

**Coś jeszcze wkurza Cię w tych obostrzeniach?**

Te spacery, ten las był taki nieszczęsny. Niedawno było tak, że otwierało się bazary, a nie można było chodzić po lesie. Ludzie tłoczyli się w alejkach - i to mogą, a w lesie, gdzie jest wolna przestrzeń - nie mogą?

**Teraz już można. Również dzieci od 13 roku życia mogą wychodzić same. Co o tym myślisz?**

Nie zastanawiałam się nad tym, to mnie nie dotyczy.

**Etapy luzowania ograniczeń**

To ma być odmrażanie gospodarki. Nie wiem, w jaki sposób ona ma być odmrożona, jeśli otworzy się lasy i parki. Słyszałam, że sklepy budowlane i z meblami mają być otwarte w weekendy. Zastanawiam się, dlaczego akurat te, a nie inne? Jest w tym dla mnie mało logiki. Z kolei fryzjer, czy kosmetyka - to nawet nie chodzi o to, że mam parcie, żeby wrócić od razu do swojej pracy - tylko o to, że to przecież miejsce, gdzie te wszystkie zasady były przestrzegane już wcześniej. Nosiłyśmy maseczki, jest dezynfekcja, sterylizacja - te procesy są od lat. I te miejsca są zamknięte, a nagle otwiera się jakiś bazar, czy market budowlany, gdzie ludzie są w dużo większej liczbie. W salonie jest 1 na 1. Łatwiej jest zadbać o higienę, bezpieczeństwo, niż w miejscu, gdzie są zupełnie przypadkowe osoby, grupują się. Rodzina nie może być razem na spacerze, a nagle w markecie budowlanym przy kasie mogą stać cztery osoby. No nie wiem.

**Gdybyś miała mieć wpływ na to odmrażanie, luzowanie - co byś zrobiła?**

Co bym poluzowała jako pierwsze? To jest trudne. To takie przeskakiwanie. Tak, jak mówiłam, przez pierwsze dwa tygodnie, zamknęłabym wszystkie sklepy - zostawiłabym piekarnie i apteki. I wtedy sukcesywnie otwierała. Też oczywiście najpierw żywnościowe, potem usługi typu szewc, wulkanizacja, mechaniczne. Takie rzeczy, które są ludziom potrzebne, żeby coś na przykład naprawić. Serwisy, sklepy budowlane, ale stopniowo. I nie w niedzielę. Dlaczego w niedzielę, a nie wystarczy tydzień, kiedy większość ludzi i tak nie pracuje? Trudno mi jest powiedzieć, jakie kryteria bym przyjęła. Nie chciałabym tak chaotycznie, jak rząd robi - raz to, raz tamto - takie skakanie z kwiatka na kwiatek.

**Uważasz, że otwierając, należałoby pozostawić jakieś obostrzenia w tych miejscach, czy powinny one działać na dawnych zasadach?**

Ilość osób, która może znajdować się w tych zamkniętych pomieszczeniach, bo to tak naprawdę najbardziej chodzi. Czy to sklepy, czy zakłady usługowe. Zachowanie odległości, maseczki też bym zostawiła.

**Co dla Ciebie jest granicą, do której należy nosić maseczki?**

Gdyby nie odnotowano żadnego zakażenia. Ale to jest nierealne, bo tych testów się nie robi. To nie jest stuprocentowa gwarancja, że nie ma tego zakażenia, czy tego wirusa. Ciężko jest podać datę.

**Szumowski powiedział, że wszyscy będziemy chodzić w maseczkach do wynalezienia szczepionki. Co Ty na to?**

To długi czas. Mi by to nie przeszkadzało, nie jest to rzecz uciążliwa. Może nie dla wszystkich, bo wiem, że osoby, które mają problemy z oddychaniem, jacyś astmatycy, mają z tym problem. Z kolei nie muszą w nich chodzić cały czas, jak my w sklepie. Jest taka zmiana, która chodzi w maseczce czy przyłbicy 8 godzin. Natomiast nie jest to coś bardzo uciążliwego, czego nie można by było zrealizować. Do momentu wynalezienia szczepionki - jeszcze jakieś badania - to musi potrwać. Minimum rok chyba.

**Model szwedzki**

Nie słyszałam, to mi umknęło.

**<przedstawienie modelu szwedzkiego> Co o tym sądzisz?**

Nie znam szwedzkiego społeczeństwa, tego, jak ono jest zdyscyplinowane. To chyba zależy od określonej nacji. Jak jest zdyscyplinowana, na ile ma poczucie wspólnoty, odpowiedzialności za innych. To też ma znacznie.

**Myślisz, że to by się sprawdziło w Polsce?**

Ja nie wierzę w ten naród. To nawet pokazuje na moim przykładzie. Dopiero kiedy kara zrobiła się realnie duża i namacalna - nie ważne już czy nakaz jest słuszny, czy nie, to jest kwestia indywidualna - postanowiłam dostosować się. Dlatego jednak system nakazów uważam za lepszy. Po wprowadzeniu nakazu nie wiedziałam ludzi bez maseczek.

**Można u was kupić maseczki?**

Nie, ja sama szukam, gdzie je kupić. To jest towar deficytowy.

**Chodzisz po ulicy w przyłbicy, czy maseczce?**

Według tego, co wyczytałam z mężem, w przyłbicy można chodzić w sklepie, jeśli jest dodatkowa pleksi. Na ulicy jest mowa o maseczkach, więc noszę maseczkę, choć widziałam też ludzi w przyłbicach. Nawet mężowi, kiedy pojechał do hurtowni w przyłbicy, zwrócono uwagę, że to ma być maseczka. Ja jestem przyzwyczajona do maseczek, więc nie robi to dla mnie różnicy. Oby tylko można było je kupić.

**Powinny być rozdawane, skoro jest nakaz, czy wystarczyłoby, aby cena była przystępna?**

Fajnie by było, gdyby były rozdawane. To podobało mi się w Austrii, tam rzeczywiście państwo je rozdawało. Natomiast wydaje mi się, że gdyby one były w takich cenach, jak dotychczas, nie musiały by być rozdawane. Wystarczyłoby, aby była większa kontrola nad ceną, jakie one na wolnym rynku osiągają. Ja wiem, że na każdej wojnie i nieszczęściu jedni tracą, inni zarabiają. Tu jest sprawa życia i śmierci. No cóż, gdyby ta cena była lepsza. Ale mam rodzinę w Sochaczewie i tam podobno gmina zadbała o to i wrzucano im po dwie maseczki do skrzynek pocztowych. Uważam, że to przynajmniej rozwiązanie na początek, że to bardzo dobre zachowanie, zapewnienie takiego minimum. U nas burmistrz ma dopiero teraz je rozdawać.

**Dbanie o siebie**

Staram się do tego podchodzić na zasadzie, że są rzeczy ważne i ważniejsze. Nie robię nic z paznokciami - to jest kwestia regeneracji - stwierdziłam, że to jest dobry czas, żeby odpoczęły. Jest tylko odżywka, piłowanie na krótko. Też wcześniej nie miałam nie wiadomo jakich, ale to jest czas, kiedy nie ma eksperymentów, kolorowych lakierów, itd. Mam oliwkę, którą sobie wsmarowuję wieczorem, czego nie robiłam wcześniej. Natomiast to jest ta pielęgnacja, to minimum, które sobie zapewniam. Fryzura - jakby nie mogę poszaleć, bo jest przyłbica. Ja też nie jestem typem, który musi mieć wszystko wypielęgnowane. Fajnie, jak fajnie wyglądam, ale też nie mam czegoś takiego, ze jak się nie pomaluję i nie zrobię pełnego makijażu, to nie wyjdę z domu. Mam takie koleżanki, które nie wyjdą. Ale zaczyna teraz przeszkadzać mi grzywka. Nie wiem, co mam z nią teraz zrobić. Ostatnio zadzwoniłam do mojej koleżanki fryzjerki, ze może byśmy zrobiły takie spotkanie online i ona by mnie pokierowała, co mam zrobić z tymi nożyczkami i bym obcięła tą grzywkę. I ona powiedziała, że może jakoś podjadę do niej, to mi ją obetnie. Ale powiedziałam, że aż bardzo to nie potrzebuję, tak tylko myślałam, jak to rozwiązać. Śmieję się do dziewczyn, że skoro nosimy maseczki, oszczędzamy też na kosmetykach, bo się nie malujemy, bo to bez sensu. Nie używamy szminek, bo to się wszystko wymaże, więc podchodzę do tego na luzie.

**Malujesz się teraz mniej?**

Tak, wcześniej malowałam się delikatnie, teraz ograniczam to do minimum. Z takich zabiegów kosmetycznych, które regularnie robię, to jest henna brwi i rzęs. Wtedy nie muszę ich nawet malować. Teraz tego nie mam, więc muszę używać tuszu. Ale też czasami [przed epidemią] jak nie miałam czasu, czy przegapiłam i nie miałam tej henny zrobionej to nie było tragedii, wtedy też używałam tuszu. Włosy, jeśli mi się nie układają, po prostu je podpinam i to też nie jest tragedia. Myślę, że z odrostem też da się żyć. Miałam już wcześniej takie samo podejście, jest mi dzięki temu łatwiej, że fajnie jak zrobię sobie makijaż, ładną fryzurę - jest fajnie, przyjemnie - ale jak tego nie ma, to da się żyć. Nie odczuwam z powodu tego braku jakiegoś dużego dyskomfortu. Choć ta grzywka to mi przeszkadza, denerwuje. Miałam jednak SMSy od klientek, czy może potajemnie bym ich gdzieś nie przyjęła. Dla niektórych jest to problem, może nie wszyscy są w stanie zrezygnować z przyjemności, jak wygląd. Zresztą wspominałam, że kuzyn był załamany, że nie poleci gdzieś na majówkę.

**A co myślisz o osobach, które tego potrzebują?**

Znając swoje klientki wiem, że dla niektórych to naprawdę bardzo ważne. Są takie osoby, które potrafią wydać ostatnie pieniądze na to, że musi być ten fryzjer raz w tygodniu, musi być zrobiony manicure, jakieś maseczki, zabiegi - dla nich to jest naprawdę ważne. I psychicznie źle znoszą taki brak, pomimo, że wyglądają fajnie z mojej perspektywy. Więc wiem, że to dla niektórych psychicznie duży dyskomfort.

**Chodzi Ci o taką grupę, która nie wyrzuci śmieci bez makijażu?**

Ta, która ich nie wyrzuci bez makijażu, to mniejsza grupa. Ta, o której mówię, to nie wiem, czy to nie są jakieś osoby, które mają kompleksy, coś sobie tym wyglądem nadrabiają? To jest trochę szersza grupa.

**Robisz coś jeszcze dla siebie?**

Jakąś kąpiel, tak, jak mówiłam, gdzie miałam zamiar siedzieć dwie godziny w łazience. Wcześniej szłam do koleżanki, ona nałożyła mi jakąś maseczkę i miałam załatwione. Teraz sama muszę skupić się na sobie. W sobotę stwierdziłam, że opiłuję sobie paznokcie. Przypomniały mi się rytuały z dzieciństwa. W sobotę zawsze się wtedy sprzątało, robiło zakupy, a wieczorem robiłyśmy sobie z mamą manicure. To był już taki schemat, że sobota po południu to było takie dbanie o siebie. I tu znów szczerze mówiąc, wracam do korzeni.

**Kupiłaś dodatkowe kosmetyki?**

Nie, chociaż już zaczęłam się przymierzać. Że może coś więcej mi się przyda.

**Co daje Ci dbanie o siebie?**

To na pewno sprawia, że skupiam się na sobie. Wtedy jestem ja - tu i teraz. O niczym innym nie myślę, skupiam się na sobie. To jest takie minimalne, ale to jest mój czas, daje mi taki relaks, jest przyjemniej. To jest przyjemność. Nie myślę wtedy o świecie zewnętrznym. Tworzę sobie taki mój mały świat, włączam jakąś muzyczkę, nawet zapaliłam sobie świeczkę, która się może w końcu wypali, bo kupiłam super świeczkę, która się pali i pali.

**Chciałabyś zachować te sobotnie rytuały?**

Myślę, że fajnie by było. Dlatego, że takie sobotnie szykowanie się na imprezy jest szybkie. Ja zazwyczaj jeszcze pracuję w soboty - tu fryzjer, tam pomaluje mnie córka lub koleżanka. Tak naprawdę teraz to dbanie o siebie to takie wyhamowanie. To dzieje się tak na spokojnie, to takie celebrowanie tego wszystkiego.

**Co najbardziej przeszkadza Ci z rzeczy, z których nie możesz skorzystać na zewnątrz? Czego Ci najbardziej brakuje?**

To właśnie ta grzywka, ten fryzjer. Już nie muszę robić żadnego koloru - chcę ją po prostu obciąć.

**A jeśli chodzi o to, jak się teraz ubierasz? Czy normalnie kupowałabyś ubrania, sprawdzała kolekcje?**

Sprawdzać kolekcje - aż tak to nie, bo aż tak nie relaksuję się na zakupach. Ale na pewno bym sobie sprawiła przyjemność i coś nowego zakupiła. Bo kiedy jest słońce, robi się tak wiosennie - zawsze coś nowego sprawiłoby mi przyjemność. Szukałam w internecie, ale stwierdziłam, że to nie jest ta przyjemność. Jak nie dotknę, nie spojrzę, nie ma frajdy. W internecie muszę sobie kupić blender, bo mi się popsuł - to takie praktyczne rzeczy, które muszę kupić przez internet, bo nie mam jak zrobić tego inaczej, a potrzebuję go. Muszę tu złamać swoje zasady. Gdyby popsuła mi się kurtka, albo nie miałabym spodni, musiałabym je kupić przez internet. Natomiast to tak czysto praktycznie, ale dla przyjemności nie.

**A zmieniło się coś w tym, co teraz nosisz?**

Ja mam raczej styl swobodny, nie muszę chodzić w szpilkach. Natomiast to, co noszę teraz to bardzo swobodne, wygodne ubieranie się. Kiedyś było tak, że mogłam sobie pozwolić na sukienkę. Teraz to jest zupełnie niepraktyczne. Żadne buty na obcasie, to by mnie spowolniło. Nie robi mi to wielkiej różnicy, bo ubranie się w coś eleganckiego, fajnego, to były te moje wyjścia na imprezy. Ja jestem strasznie wygodna i praktyczna. Dla mnie wygoda przede wszystkim, więc z wielu rzeczy tak naprawdę nie zrezygnowałam. To, że noszę teraz buty sportowe, a nie jakieś na koturnach, ale też swobodne, nie robi mi jakiejś większej różnicy. Chociaż, dlaczego miałabym nie założyć w sobotę sukienki i siedzieć w niej w domu?

**A jak nie musisz iść do pracy, jak się ubierasz?**

Chodzę w dresie. Ale właśnie dlaczego ta sobota, po manicurze, nie miałaby być w sukience? No właśnie, za bardzo się wyluzowałam. Pewnie gdyby nie izolacja, nie chodziłabym po domu w dresie. Gdzieś bym wychodziła, ktoś przychodziłby do nas. Chociaż w niedziele chodziłam w szlafroku. Mam taki szlafroczek i stwierdzałam, że ta niedziela może nie tyle będzie piżamowa, co taka w szlafroczku. Tak mi dobrze z tym było. Ale to tak mi się ta jedna niedziela w tygodniu trafiła, poza nią raczej się ubieram.

**Dlaczego twierdzisz, że może trochę za bardzo wyluzowałaś?**

Cały tydzień na sportowo, a czasami coś takiego kobiecego, ładnego, jak sukienka, przydałoby się od czasu do czasu. W sumie mam dwóch mężczyzn w domu. O to chodziło, że albo styl sportowy, albo w dresie po domu. Tak, muszę to zmienić! Tak.

**Jakie są Twoje obserwacje, jeśli chodzi o potrzeby innych związane z dbaniem o siebie?**

Najbardziej ten fryzjer doskwiera. Mama też stwierdziła, że już ma odrosty siwe i w ogóle, ale jednak do fryzjera nie pójdzie, bo się nigdzie nie chodzi, to jej tak bardzo nie przeszkadza i ona poczeka. Znajoma mówiła, że na początku myślała, że bez fryzjera i kosmetyczki da się żyć, to nie jest nic takiego. A teraz podobno, jak spojrzała na swoje odrosty, to ma ombre, sombre i wszystko na tej głowie i przydałoby się to zmienić, no ale mówi „dobrze”. Ta sama osoba ma problem z wrastającymi paznokciami, więc potrzebuje systematycznych wizyt. Zadzwoniła do swojej kosmetyczki, ale ona powiedziała, że nie będzie ryzykowała, bo właśnie te mandaty, że nie można. Poradziła jej telefonicznie, co ma robić, czego unikać, aby przetrwać ten moment. Jeśli chodzi o paznokcie jeszcze, obserwuję wśród klientek w sklepie - duże odrosty, zdjęte nie do końca dobrze te paznokcie. One zaczęły sobie jakoś radzić same. No, ale ten fryzjer to jest taka rzecz. Jedna z moich pracownic stwierdziła, że poradzi sobie sama. Córka pomalowała jej włosy, wyszły jej jakieś kolorowanki. Więc ona musiała wysuszyć włosy, założyć kaptur, odstać w kolejce do Rossmanna, żeby kupić kolejną farbę. Jakoś wybrnęła z sytuacji.

**A co z wychodzeniem do kina, knajpy, na basen?**

Kina mi nie brakuje, to chyba najmniej. Ostatnio tak pomyślałam właśnie, że bardzo chętnie bym już poszła na basen. On bardzo dobrze robi na kręgosłup. Ja nie ćwiczę teraz, choć przed epidemią chodziłam z kijami, uczęszczałam na jogę. Gdyby to wszystko było online, nie mogę się jakoś zebrać. Ta moja dziewczyna prowadzi tak właśnie zajęcia, ale jeszcze do tego nie dorosłam. Brakuje mi ruchu, takiego fizycznego zmęczenia. Jak się przejdę z tymi kijami - zmęczę, spocę - czuję, że jestem taka zaopiekowana, wszystko się rozluźnia. Ruch w domu u mnie w ogóle się nie sprawdza. Podziwiam wszystkich, którzy ćwiczą w domu - rozkładają maty, robią jakieś przysiady. Ja raz skusiłam się na Chodakowską, rozstawiłam wszystkie gadżety - ale to było jeszcze lata temu, jak ona zaczynała - 10 minut poćwiczyłam, potem usiadłam. Świetnie się to ogląda. Ja jestem właśnie takim typem, który musi wyjść. I brakuje mi już jakichś spotkań na zewnątrz. Mam co prawda ciasto, kawę, usiądziemy z teściową i wypijemy tę kawę. Ale brakuje mi trochę powrotu do normalnego życia, choć minimalnie. To nie jest coś, za czym tak tęsknię. Przyjemnie by było, ale.

**Czy coś jeszcze przyszło Ci do głowy?**

Przypomniało mi się odnośnie ostatniej rozmowy. Chodzi o pytanie, czy kupiłabym coś, czego normalnie bym nie kupiła. To był litr spirytusu. Bo jak nie mogłam kupić środków do dezynfekcji, poradziłam sobie w ten sposób. Normalnie w życiu bym tego nie kupiła, bo ani nie robię nalewek, ani nic.

**Kiedy ostatnio rozmawiałyśmy, mam wrażenie że byłaś nieco bardziej przygaszona, w gorszej kondycji psychicznej.**

Tak, masz rację. Dobrze, że tydzień temu się nie widziałyśmy, w tym tygodniu poprzedzającym święta, bo to był największy dół. Wtedy było najgorzej, najciężej. Teraz czuję zdecydowaną poprawę.

**Spodziewasz się kolejnych luzowań obostrzeń?**

Myślę, że tak, bo trzeba iść na wybory. One będą. Ale nie cieszy mnie to luzowanie, utrzymałabym restrykcje.
