# Supplementary material for: The challenges arising from the COVID-19 pandemic and the way people deal with them. A qualitative longitudinal study
Source: PLoS One. 2021 Oct 11;16(10):e0258133. doi: 10.1371/journal.pone.0258133 (PMC8504766; doi:10.1371/journal.pone.0258133)
Supplement: S1 Dataset — (ZIP) [file pone.0258133.s003.zip › Transcriptions/stage 3/17.3_F_35_single, with child.docx]

**17.3_F_35_single with child**

**Opowiedz mi o tym, jak minęły Ci ostatnie dwa tygodnie.**

Bez zmian od miesiąca. Nic się nie zmienia, siedzimy w domu. Nawet się ostatnio zastanawiałam - ktoś mnie zapytał, czy jest mi bardzo źle, siedząc w domu - wcale nie jest mi źle. Tak zaczęłam sobie uświadamiać, że ja lubię siedzieć w domu. Jedyny problem jest z tym, że nie można pracować. A tak, to siedzimy, odpoczywamy, ogarniamy mieszkanie. Dzień, jak co dzień, tylko nie muszę wychodzić do pracy.

**Co Ci się w tym podoba?**

Wreszcie mam dużo czasu na wszystko. I dom jest ogarnięty, wszystko jest takie, jakie być powinno. A nie wiecznie biegiem, poranne wstawanie, biegiem do szkoły, ze szkoły, biegiem do pracy. Cały czas było biegiem. A tak, jakoś spokojnie się żyje.

**A jak święta?**

Było mi strasznie przykro, bo podjechałam do mojej babci, która mieszka z moją ciocią. Tylko dałam cioci tulipany przez furtkę, spłakałyśmy się. Mamy świadomość, że babcia ma 89 lat i nie wiadomo, które to są święta, czy nie są ostatnie. Tak poza tym, święta, jak wiadomo. Tylko tej babci brakowało. Ja spędziłam Wielkanoc z rodzicami, z którymi mieszkam. Moja siostra mieszka na tej samej ulicy, więc pomachałyśmy sobie. Wiem, że moich rodziców zabolało to, że nie spotkali się ze swoimi przyjaciółmi. Oni od 35 lat zawsze w Poniedziałek Wielkanocny jedzą śniadanie. W tym roku pierwszy raz nie zjedli i widziałam, że ich to zabolało. Dla mnie tak poza tym, to święta święta i po świętach. No, było mniej  jedzenia. Według mnie, w ogóle nie było też przygotowań. Zjedliśmy barszcz i trochę to było sztuczne. My zawsze spędzamy święta w co najmniej 20 osób, a teraz byliśmy w piątkę. To było takie, no, podzielmy się jajeczkiem. Bez koszyczka, bez tego wszystkiego. Ubierzmy się odświętnie. Ja pytałam - po co? To obiad jak obiad. No, ale się ubraliśmy na tę chwilę, żeby nie było. To było takie nie świąteczne na pewno. Nie poczułam, że to są święta jako takie. Nie było tej całej atmosfery - absolutnie. W ogóle jej nie było. Nawet żeby pójść z tą święconką - gdzie całą rodziną się szło do kościoła. A teraz - jak, mówię, dzień, jak co dzień. Wstaliśmy na śniadanie - śniadanie, jak śniadanie - nic nadzwyczajnego.

**Pojawiły się u Ciebie jakieś nowe rzeczy, czynności, których wcześniej nie wykonywałaś?**

Tak, ćwiczę. To jest po prostu coś niebywałego. Ja, która z ćwiczeń uznaję tylko leżenie na kanapie. Nienawidzę ćwiczyć. Nie chodziłam na WF. Naprawdę, to jest jakiś dramat. Stwierdziłam, że jestem kolejną ofiarą koronawirusa. Normalnie, codziennie ćwiczę. Nie wiem. Jeszcze tego nie ogarnęłam, co się ze mną stało. Nigdy w życiu nie ćwiczyłam, słowo, nigdy. Kupowałam tylko karnety na siłownię. I byłam tam raz, może dwa. Nigdy w życiu nie zmobilizowałam się, żeby ćwiczyć w domu. Ale tak mówię, kurczę, może zrobię coś dla siebie. Nie mam teraz przynajmniej wymówki, że nie mam czasu. Drugą rzecz, którą robię, to szkolę się do pracy - ja robię paznokcie w kosmetyce. Jest okazja, dziewczyny, które szkolą, wystawiły świetną ofertę. Stwierdziłam, że skoro jest szansa skorzystać, będzie trochę taniej, mam dużo czasu - nic nie stracę. Więc co dwa, trzy dni mam jakieś szkolenie online. I super, zobaczymy, czy to mi się przyda. Ale.

**To szkolenie wydaje Ci się być na równie dobrym poziomie, jak stacjonarne?**

Wiesz, wydaje mi się, że jest lepsze. Myślałam, że to będzie takie po łebkach. Jeśli idę na szkolenie grupowe, jest jak w klasie. Ta dziewczyna musi podejść do każdej kursantki i wiadomo, że pewnych rzeczy nie zauważy, czasami nie podejdzie - dokładnie, jak w przypadku dzieci w klasie. Tutaj mam kontakt 1 na 1. Połączę się z nią kamerką i ona wszystko widzi. Jest zdecydowanie lepiej.

**Jeśli chodzi o te ćwiczenia, jak to wygląda?**

Nie ćwiczę sama, bo nie umiem. Gdzieś pojawiła mi się strona Moniki Kołakowskiej - było tam jakieś wyzwanie 30 dni. I oczywiście ja oglądałam. Od oglądania nic się nie dzieje. I w Lany Poniedziałek tak siedziałam, była paskudna pogoda. Stwierdziłam, dobra. Powiedziałam do swojego syna, ubieraj się w dresy, będziemy ćwiczyć. On popatrzył na mnie, jak na wariatkę. Powiedziałam, Miłosz, siedzimy w domu, ty nie masz treningów [piłka nożna, basen], ja siedzę w domu, nawet nie wychodzę nigdzie. Będziemy tacy sflaczali. No, i ćwiczymy codziennie we dwoje. On nie ma teraz ruchu, jemu to też się przyda trochę. To jest jakieś pół godziny ćwiczeń. Jakieś przysiady, wymachy nogami w prawo, w lewo. Niby nic trudnego, a można się zmęczyć.

**A czy są rzeczy, z których zrezygnowałaś lub je ograniczyłaś?**

Nie wychodzę nigdzie, nie robię zakupów. Mój tata się zbuntował, powiedział, że on będzie chodził na zakupy. Mnie strasznie frustrowało to stanie w tych kolejkach. I jechałam tam naprawdę prawie o północy, żeby nie stać w kolejce, bo to był po prostu jakiś dramat. Jak za komuny, albo i gorzej. I mój tata się w końcu zbuntował, powiedział, że znalazł jakiś sklep, w którym nie ma kolejek i on sobie jedzie i mówi, że jest zadowolony. Myślę, że też potraktował to trochę jako spacer, bo oni też już wariują w tym domu. Ale ja ogólnie nie kupuję teraz żadnych rzeczy. W ogóle nic nie jest mi potrzebne do szczęścia, oprócz jedzenia. Poza tym, życie cały czas toczy się tak samo.

**A jak radzą sobie Twoi bliscy, czy zauważyłaś u nich jakieś zmiany, jak na przykład te zakupy u taty?**

Moja mama cały czas robi dokładnie to samo. Mój Michał siedzi na działce i ciągle coś sobie wynajduje, remontuje - następny. Wynajduje czyszczenie kosiarek, płotu, bóg wie czego, byleby tylko w domu nie siedzieć. Moja siostra całymi dniami szyje maski. Dwudziestoletnia siostrzenica całymi dniami śpi - chyba tylko z przerwami na jakieś wykłady. Mój szwagier przestał biegać, a biegał maratony. Nie wiem, czy teraz do tego wróci. Wiem, że tego mu bardzo brakowało, dla niego to było nawet rozładowanie emocji. Jak ktoś miesiąc nie biega, to może być to dla niego trudne. Zaczął za to układać puzzle ileś tysięcy. Już chyba trzecie układa z nudów. Nie wiem, kiedy dokładnie zaczął je układać, wydaje mi się, że jakoś teraz, niedawno. Wysłał mi nawet  ostatnio zdjęcie, jakiś kosmos. Chyba, że to są już następne, a ja nie wiedziałam. Myślę, że każdy sobie szuka zajęcia. Może to jakiś pomysł, na zabicie czasu. <wchodzi syn, ale stwierdza, że nie będzie przeszkadzał> O, młody musi odrabiać lekcje i ciągle jest z tym foch. Bo za wcześnie, bo za późno, bo nie w ciągu. Zawsze jest zły dzień na odrobienie lekcji. On naprawdę nie ma z nimi problemów pod tym kątem, że potrafi zrobić to sam - pomimo, że już miesiąc nie chodzą do szkoły. Odrobi to naprawdę w pół godziny, więc mówię mu, żeby zrobił to teraz, bo mają później jechać z tatą pograć w piłkę, skoro już można wychodzić. Niech idzie, pobiega trochę na zewnątrz. Ale nie, teraz jest zły czas na odrabianie lekcji. Za pół godziny, za pięć minut, za chwilę, i tak w kółko.

**Pojawiły się jakieś rzeczy, które Ci przeszkadzają w obecnej sytuacji?**

No to te zakupy. Ja w ogóle nie lubię robić zakupów. Powinnam wpaść do sklepu i z niego wypaść. Bardzo mnie denerwuje, że jak chcę kupić mleko, to się zastanawiam, czy będę tam stała godzinę w kolejce, czy uda mi się trafić bez kolejki. I żeby to nie było między 10 a 12, bo mnie nie wpuszczą do sklepu. Te zakupy nie mogą być spontaniczne, ja muszę je zaplanować. Z jednej strony jest to ok, bo robię listę zakupów. Wtedy robi się porządne zakupy i ok. Ale jeśli mi czegoś zabraknie, to jest dla mnie stresujące. Czy jak pójdę do osiedlowego sklepu, będę tam stała, czy nie. Jak to będzie działać. To mnie najbardziej chyba drażni. Poza tym, chyba się już pogodziłam z tą sytuacją. Nie ma się na kogo, na co złościć. Trzeba to przetrwać i tyle.

**Jeśli jest kolejka w sklepie osiedlowym, czekasz w niej?**

Raczej nie, wtedy po prostu zawracam do domu i nie kupuję tego, rezygnuję i radzę sobie jakoś bez tej rzeczy.

**Takie ćwiczenia, o których wcześniej wspominałaś, pomagają poczuć się lepiej, czy jest to typowo sposób na zabicie czasu?**

Na pewno lepiej czuję się sama ze sobą. Wiedziałam, że potrzebuję zrobić coś ze swoim ciałem. To był dobry moment, żeby się za to wziąć. Wydaje mi się, że naprawdę, w miarę pogodziłam się z sytuacją i przyzwyczaiłam się do niej. Nie walczę z nią. Tak jest, tak trzeba żyć, funkcjonować. Coś tam sobie znajdę, w domu zawsze jest co robić. Młody ma te lekcje. Teraz można już wychodzić na spacery, więc korzystamy z okazji i wychodzimy. Wczoraj nie było nas pół dnia w domu. Najpierw Miłosz grał z tatą w piłkę ze trzy godziny, na jakimś tam boisku. Później pojechaliśmy na działkę - pod Radomiem, ale w samym lesie. Zawsze tam jeździmy, nawet, kiedy nie można było wychodzić do lasu. Wiesz, z samochodu od razu na działkę. Żeby chociaż na chwilę posiedzieć na tej działce, choć tam nie ma co robić. To zawsze było jakieś oderwanie od codzienności. Tylko jak nie można było chodzić po lesie, Miłoszowi się tam nudziło. Jeździliśmy wtedy trochę rzadziej. A teraz, kiedy już można chodzić po lesie, idziemy na spacer po nim. Myślę, że gdyby to była zima, byłoby gorzej. A że jest wiosna, trochę wykorzystuję ten czas, żeby zrobić coś dla siebie, odpocząć. Posiedzieć z rodziną, spędzić czas z dzieckiem.

**Mówiłaś, że pogodziłaś się z sytuacją. Jakie to wywołuje u Cebie emocje?**

Trochę obojętności. W sensie takim, że to jest, bo jest i nie mam na to większego wpływu. Nie wiem, komu wierzyć. Ile w tym jest prawdy. Już są na świecie takie teorie spiskowe, że głowa mała. Chyba nie ma co tam się złościć na to i walczyć z tym. Trzeba to jakoś przetrwać. Nic nie da, że będę tupać nóżką i się denerwować. To się sama wykończę, sama ze sobą. Więc jakoś tak, wydaje mi się, zobojętniałam do tego. Nawet zauważyłam ostatnio, że te dane, które wypuszcza ministerstwo, przykładowo, ze siedem osób zmarło - reaguję na nie w stylu "ok, no, zmarło". Rusza mnie dopiero, kiedy jest to ktoś młody, jak ostatnio ten medyk z Radomia, rehabilitant zmarł. To mnie poruszyło, młody facet, jeszcze okazało się, że to znajomy znajomego. Więc wiesz, jak to jest, tak się to bierze bardziej osobiście, do siebie. Ale poza tym to jakoś tak - co ma być, to będzie.

**Czy pojawiają się jeszcze jakieś emocje w tym kontekście?**

Nie, na pewno zła, smutna, ani też szczęśliwa nie jestem. Generalnie źle mi w domu nie jest, ale poszłabym do pracy. Choć też myślę, że poszłabym do pracy, ale nie chciałabym już pracować tyle, co pracowałam. Chciałabym na pewno trochę ten system ogarnąć, na tyle, żeby móc więcej być w domu. To na pewno. Dobrze jest mi w domu, jest mi fajnie spędzać czas z rodziną, jest mi fajnie zjeść obiad o 16, jak biały człowiek, a nie o 21. Nie mam tego poczucia wiecznego biegu, że wracając z pracy muszę podjechać do domu, zabrać Miłosza, zawieźć go na trening, w międzyczasie zrobić zakupy. Tylko jakoś tak wszystko jest spokojniej. Wiesz, wstaję rano, spokojnie. O widzisz, spokój. Ja mam po prostu spokój w sobie. Ja się teraz nigdzie nie spieszę, nigdzie. Mam wrażenie, że ostatnie kilka lat u mnie to był wieczny pęd. A już jak młody poszedł do szkoły, to była wieczna bieganina. Do tego wożenie go na treningi. Całe życie jest podporządkowane dziecku. Ja musiałam ogarnąć to, żeby jego jakoś zawieźć, te treningi były dwa razy w tygodniu. Jeszcze gdzieś tam basen, a jeszcze trzeba lekcje zrobić w międzyczasie. Szkołę miał w jakichś durnych godzinach. To było takie wszystko szybko, biegiem, żeby zdążyć, nic nie nawalić, żeby wszystko było. A jeszcze trzeba w międzyczasie pomyśleć, żeby było coś na obiad i ten obiad ugotować. A teraz jest tak wiesz, tak fajnie. Ja chętnie zawiozę go na trening, spoko. Ale tak, żebym nie musiała biegać. Więc moja decyzja jest taka, że pierwsze co zrobię, jak wrócę do swojej pracy, to ogarnę ten system godzin tak, żebym nie musiała pracować do 21.

**Mówiłaś, że trochę byś już do tej pracy wróciła. To jest związane z kwestiami czysto gospodarczymi, czy coś jeszcze Cię do niej ciągnie?**

Wiadomo, że kasa by się przydała, bo oszczędności się kurczą, jak nic nie przychodzi. Natomiast też ja lubię kontakt z ludźmi, więc brakuje mi moich klientek, z którymi mogłam sobie pogadać, porobić coś innego, niż siedzenie w domu. Porozmawiać z kimś innym, niż dziewięciolatek czy chłop w domu. Czasami są potrzebni inni ludzie. Po to bym poszła.

**Obrazki**

Waham się między 12 a 13. Wiesz, chyba 13. Mi jest tak dobrze, jak na tej 13. Wybrałam go, bo na nim się nic nie dzieje. Jest na nim spokój, ktoś sobie idzie i macha rączką po polu. I tak mi się wydaje, że ja się tak teraz czuję, jakbym przechadzała się po polu w spokoju, na spacerze. Zero stresu. Bez żadnych problemów, bez niczego, po łąkach. Na zewnątrz zrobiło się słonecznie, więc to może mieć wpływ. A wewnętrznie, jeszcze do niedawna próbowałam się uspokoić. Byłam poddenerwowana tą całą sytuacją. Że nas zamknęli, nie pozwalają mi iść do pracy, nie wiadomo, jak będzie z tarczą antykryzysową. Czy dadzą jakieś pieniądze, czy nie. Czy wrócimy do pracy, czy nie. Byłam takim kłębkiem nerwów. Ale któregoś dnia stwierdziłam, że co mi to da, kiedy będę się denerwować. Absolutnie nic mi to nie da, a powodowało tylko, że chodziłam po domu i się wściekałam, denerwowałam, na wszystkie pytania odpowiadałam z wielkim fochem. Wylewałam te złe emocje na wszystkich członków rodziny. Stwierdziłam, że po co. Przecież totalnie nie mam na to wpływu. Przecież nie pójdę do pana ministra i nie powiem, hej, chłopaku, może byś zrobił coś żebyście pomogli naszej branży. Takich osób jak ja, jest teraz pewnie setki tysięcy. Stwierdziłam, że trzeba się uspokoić, przetrwać to i potem będzie już tylko lepiej. Każdy dzień zbliża nas do tego. Czekam cierpliwie na rozwój sytuacji.

**Gdybyś mogła coś zmienić w obrazku, coś byś zmieniła?**

Trochę bym schowała to słońce. Ono mi się tu kojarzy z tym, że patrzę na coś i będzie cudownie. A tak naprawdę nie wiem, jak będzie. I dołożyłabym tu swojego syna. Ja to jestem prawdziwa matka Polka i wychowuję mamisynka. <śmiech> Nie no, żartuję. Ale jesteśmy mega związani. No wiesz, jak ma się syna, to tak jest, że zawsze z nim. Tak jakoś we dwoje jest fajniej.

**Jakie emocje obserwujesz u swoich bliskich?**

Zauważyłam, że moi rodzice zaczęli się trochę mniej bać koronawirusa. Tak, jakby nadal mają obawy, ale to już nie jest panika. Oni w pewnym momencie już naprawdę panikowali. Ja wchodziłam do domu, to mówili, idź do siebie, umyj ręce, nie podchodź do nas, bo nas zarazisz. Albo jak Miłosz wstawał i szedł do nich, dziadek wystawiał nogę, że nie będą się witać. Mówiłam, boże, przecież i tak mieszkamy w jednym domu, więc bez sensu. Teraz widzę, że już trochę zluzowali. Nadal pilnują siebie i nadal nie latają nie wiadomo gdzie. Ale sam fakt, że tata wyszedł do sklepu i wiem, że już gdzieś planują, żeby wyjść na spacer. Oczywiście nie polecą do marketu na zakupy, tylko gdzieś tam sobie wybiorą miejsce ustronne, żeby się przejść na chwilę. Dużo moich bliskich, którzy trochę śmieszkowali z koronawirusa, już przestali to robić. To jest teraz takie no rzeczywiście, może to jednak istnieje. Mają jakieś kontrargumenty, że jest to wymysł ludzi z trzeciego świata, ale jednak jest zagrożenie. No i na przykład moja siostra jest chodzącą panikarą, która uważa, że już nie pamiętam kto wypuścił tego wirusa, bo już się pogubiłam. Ona wierzy w teorię spiskową. To jest ta siostra, która szyje maseczki.

**Jak myślisz, jest coś, co pomaga Twoim bliskim radzić sobie? Na przykład uspokoić się Twoim rodzicom.**

Może to, że mniej czytają już wiadomości. Na początku zarzucali mnie informacjami, że ktoś gdzieś był chory, umarł, różne takie dziwne. Teraz widzę, że nawet nie poruszają tego tematu. Wydaje mi się, że zaczęliśmy przechodzić do tego na początku dziennym. Że ten wirus jest, będzie i musimy się z tym pogodzić. Nie jest to już temat pierwszoplanowy. Wirus jest, no i jest. Trzeba z tym jakoś żyć. Poza tym, wydaje mi się, że poza tym, że przestali czytać wiadomości, to nic innego. Nie wiem dokładnie, jak to jest w przypadku na przykład mojej siostry. Myślę, że to szycie maseczek faktycznie pomaga jej to znieść, bo ona czuje się dzięki temu potrzebna. Ona pomaga. Z drugiej strony, wydaje mi się, że ona się bardzo w to wkręciła. Fajnie, że pomaga, szyje, ale z drugiej strony jest takie, jakbyś z nią porozmawiała, ona by ci powiedziała, że i tak wszyscy umrzemy na koronawirusa. Że wszystkie dane są sfałszowane, wszyscy, którzy umarli, mieli koronawirusa i to jest jakaś bomba biologiczna. Ja nie mówię, że nie, bo nie mam pojęcia, jak jest naprawdę. Ale naprawdę, dochodzi już do takich sytuacji, że ja nie jestem w stanie z nią rozmawiać, więc na te tematy na razie wcale nie rozmawiamy. Rozmawiamy tylko o jakichś pierdołach. A kiedy wchodzi temat koronawirusa, ja ucinam go, kończę rozmowę. Bo to jest takie nakręcanie się, że boże kochany, mówię do niej, po co ci to? Kobieto!

**Myślisz, że w jej przypadku to lęk, czy coś zupełnie innego?**

Myślę, że trochę lęk. Przez to, że ona szyje te maseczki, ma duży kontakt ze służbą zdrowia. A wiesz, jak to jest. Bo ten powiedział temu, bo ma znajomą, która jest położną w szpitalu i ona powiedziała, że ktośtam gdzieś powiedział. I mniej więcej takie plotki ona przekazuje i w nie wierzy. Ja też słucham takich informacji, nie mówię, że nie. Tylko staram się usłyszeć je, przeanalizować i nie przeżywać tak tego, nie brać każdej informacji do siebie, bo byśmy zwariowali. Wiesz, jak szerzy się plotka. Ktoś coś przekręci, ktoś inny dopowie. I ona w to wszystko wierzy, co mnie osobiście strasznie męczy, dlatego z nią nie rozmawiam na ten temat.

**A na ile Ty czujesz się zagrożona sytuacją?**

Osobiście czekam na testy, żebym mogła zbadać, czy przeszłam koronawirusa. Mam prawie pewność, że ja go przeszłam. Jakoś od początku grudnia, nawet końca listopada, miałam objawy. Na początku dziewczyny leczyły mnie gęsim smalcem i propolisem - to były takie krople na alkoholu. Rzeczywiście, w miarę mi to przechodziło, ale ciągle byłam podziębiona. I wtedy pojechałam na jakieś ostatki. I dosłownie tydzień później rozłożyło mnie tak, że miałam 40,2 stopni gorączki i błagałam, żeby mnie dobili. Jadąc od lekarza płakałam, przysięgam, myślałam, że umieram. Gorączka nie schodziła mi w ogóle przez 5 dni. Ewentualnie do 38,5 i to na równe trzy godziny z zegarkiem w ręku. Ja, mój Michał, jego syn i potem w pracy jeszcze jednej dziewczyny dzieciaki to przeszły. Mieliśmy identyczne objawy i wtedy lekarz powiedział, że jest taki jakiś dziwny wirus i nie wiemy, co z tym zrobić. Mój Michał miał to wszystko dwa tygodnie przede mną, a jego syn był z tym w szpitalu. Mi przeszło trochę przed świętami, pamiętam, bo zastanawiałam się, czy dam radę pracować. Nie zaraziłam nikogo w domu, więc prawdopodobnie oni są odporni. Później dzieci znajomej chorowały, a od połowy stycznia w szkole była masakra. Wszystkie dzieci były chore dokładnie na to samo, siedziały w domu po dwa-trzy tygodnie, miały po 40 stopni gorączki, niemiłosierny kaszel, gorączka nie do zbicia, antybiotyki nie działały. Już myślałam, że to ja sobie wymyśliłam, że zaczęłam podciągać swoją chorobę pod coś. Ale zaczęłam rozmawiać z koleżankami i mówiły, że rzeczywiście, lekarze mówili o jakimś dziwnym wirusie, który wywoływał coś jak zapalenie płuc, ale nie wiadomo było, o co chodzi. Gdybym zrobiła takie testy na przeciwciała, mogłabym mieć poczucie, że ok, byłam już chora, więc może tak szybko nie zarażę się ponownie. Z chęcią dowiem się, czy to było to. Gdybym miała świadomość, że to przeszłam i nie zaraziłam nikogo w domu, to jest szansa, że oni są odporni i się nie zarażą. Tak mi się wydaje. Ale najbardziej boję się o pracę, czy nie przyjdzie taki moment, że będę musiała się przekwalifikować. A już bym tego nie chciała, bo robiłam to już trzy razy w swoim życiu. Naprawdę, myślałam już, że znalazłam zawód swojego życia, a tu okazuje się, że może być niespodzianka. Bardziej boję się tego, niż samego zarażenia. To uczucie dopada mnie tak od czasu do czasu, zwłaszcza, jak wejdę na fora z branży beauty. Gdzieś tam słyszę, że salon się zamknął, że ktoś musi sprzedać jakiś sprzęt, przekwalifikować się. Wtedy mam takie, o boże, może i ja będę musiała. Ale później rozmawiam z dziewczynami z pracy i dochodzimy do wniosku, że zamkną się najsłabsi, a najmocniejsi, czyli my, zostaniemy. I to jest tak co chwilę. Taki niepokój, ale za moment mówię: "dobra, zamkną się najsłabsi - oni będą musieli się przekwalifikować, potem zostaniemy my i to dla nas będzie tak naprawdę szansa, żeby się wybić". To nie jest natomiast takie uczucie, żeby stresowało mnie to do tego stopnia, żebym nie mogła spać po nocach, czy miała rozmyślać o tym całymi dniami. To przekwalifikowanie się - nie chcę do siebie dopuszczać do siebie tej myśli. Wolę znaleźć wytłumaczenie, dlaczego nie będę musiała tego robić.

**Zmiany ograniczeń. O czym słyszałaś?**

O tym, że lasy nam otworzyli. Co mi się strasznie podobało, na jakimś forum przedsiębiorców - okrutne to będzie, przepraszam! - że otworzyli lasy, żeby właściciele firm mieli gdzie pójść się powiesić. Trochę mnie to bawiło, naprawdę. No ale dobra. No, i w tych sklepach jest na pewno mniejsze ograniczenie co do ilości osób na sklepie. A, i w weekendy nie ma godzin dla emerytów. To tyle kojarzę, nie wiem, czy coś jeszcze było.

**Z tych, które pamiętasz, jak je oceniasz?**

Te lasy - spoko. Nadal nie rozumiem, dlaczego je zamknęli. Rozumiem zamknięcie placów zabaw, żeby dzieciaki się nagle nie zbiegły, ale lasów? No nie, to dla mnie było głupotą. A, i jeszcze wypuścili małolatów na miasto! W sensie, że poniżej 18 r.ż. mogą już wychodzić samodzielnie na miasto, na dwór. To jest paranoja, bo u nas na dzielnicy już chodzą całe grupy młodzieży. Równie dobrze mogliby otworzyć szkoły i puścić dzieci do szkoły. Byłby ten sam efekt. Są tłumy dzieciaków - nie będących z jednej rodziny - chodzących po osiedlu. Nie wiem, jak należałoby to ugryźć, czy do 18 r.ż. Ale chyba przynajmniej gimnazjaliści, czy do 8 klasy SP tak. Żeby się nie grupowali - to w sumie przecież było po to wprowadzone. Te dzieciaki, nie idąc do szkoły, grupowały się w parkach i pod klatkami. I tak jakby wróciliśmy do punktu wyjścia. Chyba jednak lepiej by było, gdyby siedzieli w domach, przynajmniej taka jest moja opinia. Zagrożenie koronawirusem wzrosło. Cały psikus polega na tym, aby jak najmniej mieć kontakt z ludźmi. Co innego, jeśli to byliby sąsiedzi i przychodzili do siebie do domów, cały czas widywaliby się tylko w swoim towarzystwie, w tym samym pomieszczeniu. A tutaj w tej chwili oni latają po całym mieście. Jest możliwość, że oni rzeczywiście gdzieś tam będą nosić tego wirusa. Rząd z jednej strony ogranicza ludziom możliwości pracy, wejścia do sklepów, a z drugiej strony nie pilnują dzieciaków. Jeszcze tydzień temu samotny rowerzysta mógł zgarnąć mandat za to, że jechał - tak było u nas w Radomiu. Na dróżce rowerowej stał patrol i wlepiał mandaty dla zasady. Gdzie jeden człowiek jadący samotnie dróżką jest małym zagrożeniem, mniejszym na pewno - bo potencjalnie każdy z nas jest jakimś zagrożeniem - niż dziesięcioro młodzieży, które lata po całym osiedlu. Nie ma co prawda złotej rady. Nawet jak taki rowerzysta jedzie i gdzieś to poleci [aerozol], to można się zarazić. Natomiast ta młodzież to bezsens. Albo wypuśćmy wszystkich z domu, albo... no.

Co do tego, że jest trochę więcej osób na sklepie - nie wiem, nie byłam. Nie wiem, co się teraz dzieje. Widziałam tylko gdzieś tam w internecie, że są teraz straszne kolejki - zamiast na zewnątrz, to w środku. Natomiast nie wiem, nie byłam, ciężko mi się do tego odnieść.

**A jak odnosisz się do tego, że można już przemieszczać się w celach rekreacyjnych, jak choćby na rowerze, ale jednak należy przy tym zachować odpowiedni odstęp oraz mieć zakryty nos i usta?**

Jeśli każdy by do tego racjonalnie podszedł. Nie szedł tam, gdzie wszyscy jeżdżą na rowerze. Albo szedł pobiegać, ale nie tam, gdzie robi to całe miasto - bo są takie trasy. To może wtedy będzie miało to jakiś sens. Natomiast jeśli zaczniemy wszyscy wychodzić biegać, czy jeździć na rowerze do jednego miejsca, to będzie to powtórka z rozrywki. Zrobi się to, co było na samym początku. Jak pod Tatrami na tej polanie, gdzie było 500 samochodów. To będzie to samo. Będzie ciepło i wszyscy wpadną na pomysł, żeby zrobić grilla. I nie zrobią go u siebie na działce, żeby choć trochę ograniczyć kontakt z obcymi osobami. Wszyscy pojadą na polanę, gdzie będzie setka ludzi i wszyscy będziemy palić grilla, bo nam wolno. Trzeba do tego trochę zdroworozsądkowo podejść, to taka moja opinia. Nawet tak, jak my. Przecież mogę wziąć młodego i iść na rower. Ale nadal tego nie robimy. Idziemy na tą działkę, do lasu - gdzie wiemy, że nikogo nie ma. Tam nawet nie ma takiej wydeptanej dróżki, bo chodzą tam sami swoi. A mogłabym pojechać z nim do lasu, gdzie wiem, że będzie piękna trasa rowerowa. Ale wiem, że tam będzie kilkadziesiąt osób. Mam jednak gdzieś te obawy, że mogę się zarazić i nie chcę mieć poczucia, że naraziłam się na to sama, świadomie. Z jednej strony wydaje mi się, że się nie zarażę, bo byłam już chora, z drugiej strony nie mam pewności, bo nie robiłam testu na przeciwciała. Świadome narażanie się na wirusa nie jest rozsądne. Jeszcze gdybym ja się zaraziła, może bym jakoś to przebolała. Ale gdybym się zaraziła i nie daj boże moje dziecko zostało samo, nie wiem, co by było wtedy. Ewentualnie gdybym ja była zdrowa, a on był chory, bo jego naraziłam, po prostu bym sobie tego nie darowała. Racjonalne podejście, takie egoistyczne nawet trochę. Ja nie myślę o tym, żeby nie zarazić innych. Myślę o tym, żeby siebie nie zarazić.

**Jak odnosisz się do nakazu chodzenia w maseczkach?**

Powiem ci tak. Nie rozumiem rządu. Najpierw mówili, że absolutnie, maseczki nie są nam potrzebne. Teraz jest nakaz. Dla mnie to jest schizofrenia i rozdwojenie jaźni. Najpierw wmawiali całemu narodowi, że maseczka to zło i bez sensu jest jej noszenie, a teraz możesz mieć karę za to, że jej nie założysz. Uważam, że ok, bardziej chodzi o to, żeby zasłaniać twarz, w sensie nie kichnąć na kogoś. Bo to chyba bardziej o to chodzi. Natomiast ludzie nie potrafią nosić tych maseczek i to jest bez sensu, bo macają je, noszą w kieszeni, nie piorą ich, nie prasują. Tygodniami noszą tą samą, więc jest to siedlisko bakterii. Nie uważam, że nas to ochroni. Nie wiem, jak inni, ja natomiast nie oswoiłam się jeszcze z widokiem ludzi w maseczkach. Jest to dla mnie takie dziwne i  takie wiesz, to coś nienaturalnego.

**Co rozumiesz przez dziwne? Nienaturalne, coś jeszcze?**

Przede wszystkim nienaturalne. Nie widzę ich twarzy, tylko same oczy. Nie poznaję ich na ulicy. Takie to wiesz, trochę - ja wiem, że to jest nadinterpretacja, ale to jest trochę tak, jak my zamknęliśmy zwierzęta i założyliśmy im kagańce. Teraz nam ktoś zakłada coś na twarz. Ani w tym nie można rozmawiać, ani oddychać. Wiem, że wiele ludzi naprawdę ma z tym problem. Ja co prawda jestem przyzwyczajona, bo w pracy używałam maseczek na co dzień. Dla mnie to nie jest jakaś nowość, ale wiem, że mnóstwo ludzi się w tym poddusza. Moja znajoma, starszawa kobitka, mówi, że ma atak paniki, jak to zakłada, że czuje, że się udusi. Gdyby ludzie naprawdę nosili to jak należy. Dla mnie to bez sensu, kiedy widzę ludzi w samochodzie w maseczce. To nie o to chodzi. Albo wyciągają ją z kieszeni. Nie na tym wic polega.

**Porównałaś maseczkę do kagańca. Czy masz na myśli, że to jest ograniczenie naszej wolności?**

Oczywiście, że tak. W ogóle cała ta sytuacja powoduje, że nam ktoś mówi, jak mamy żyć. Oczywiście, że to jest taki kaganiec, bo tak, jak mówiłam, ani w tym mówić, ani oddychać. Nie wiem, może się do tego człowiek przyzwyczai i za parę miesięcy będzie to dla nas naturalne, natomiast w tej chwili naprawdę szokuje mnie na mieście widok ludzi w maseczkach. Co wydawało mi się, że nie będzie to dla mnie nic dziwnego. Przecież ja pracuję z ludźmi w maseczkach - wszystkie w nich biegamy. Swego czasu pracowałam jeszcze w przychodni, gdzie też wszyscy je nosiliśmy. A teraz, jak widzę to na mieście, wydaje mi się to takie dziwne.

**To jak to jest, przeszkadza Ci ich noszenie?**

W pracy nie. Na mieście tak. W pracy jestem w pracy, to część mojego stroju służbowego - muszę mieć fartuch, maseczkę i rękawiczki. Kiedy przychodzi nowa klientka, zmieniam rękawiczki. Wychodząc z pracy, ściągałam z siebie te elementy. Teraz tego nie robię. Niby wiem, po co jest ta maseczka, jednak wewnętrznie gdzieś się przed nią buntuję. I ciągle kogoś upominam. Po co zakładasz maseczkę do samochodu? Weź ją uprasuj! Bez sensu! Taka jest, walka. To nawet nie tyle jest związane z czasem, przez który mam ją nosić, a z tym, że ktoś każe mi coś robić. Z jednej strony rozumiem motyw maseczki, po co ona jest. Natomiast ludzie nie używają ich poprawnie i one nie będą działać tak, jak być powinno. To jest siedlisko miliona innych bakterii i wirusów. To wszystko mi razem nie działa. Z jednej strony ona chroni, z drugiej wiem, że ona nie chroni. Wiem, że to nie są wyprane i wyprasowane maseczki, a wyciągnięte z kieszeni. To trochę jakby lekarz miał rękawiczki do operacji, ale przedtem, przed daną operacją, operowałby kogoś innego w tych samych rękawiczkach. To takie złudne bezpieczeństwo. Niby ma rękawiczki, ale w ogóle bez sensu, że je ma. Tutaj też to tak trochę działa.

**A po co ona jest, według założenia? Kogo chroni?**

Dobrze używana, chroni innych, nie mnie. Jeśli kichnę, a jestem zarażona, nie zarażę innych. Może te chirurgiczne, porządne, mogą chronić i mnie, ale te zwykłe bawełniane -nie. Równie dobrze można wokół nosa zakręcić szalik. A jest to bez sensu, bo ludzie totalnie nie wiedzą, jak to nosić. I wydaje mi się, że wszyscy się buntują, nie tylko ja. Dużo osób nie chce przyjąć do wiadomości, że trzeba mieć coś na twarzy.

**Jak myślisz, czym to jest  spowodowane, że ludzie nie potrafią lub nie chcą nosić maseczek?**

Z jednej strony to jakiś bunt. Nakładają na nas jakieś ograniczenia, poza tym wyglądamy w tym, jak w kagańcach. Dwa, że zrobili nam mętlik w głowie. Jeżeli ktoś nie miał nigdy do czynienia z maseczką - nie wie, na czym to polega i tak dalej - nie wie, że zwykła bawełniana różni się totalnie od chirurgicznej bądź od tej Fcośtamcośtam. W telewizji od początku krzyczeli, że maski nie są nam potrzebne, że to bzdura i absolutnie przed niczym nie chroni. Nawet wczoraj przywoływali wypowiedź ministra zdrowia, że maseczki to śmiech na sali. On jeszcze naprawdę z takim podśmiewaniem się wypowiadał. Dwa tygodnie później, każą je nosić. Zalatuje to schizofrenią, jakimś po prostu rozdwojeniem jaźni, robieniem głupków z ludzi. Skąd taki przeciętny Kowalski ma wiedzieć, czy mu ta maska pomoże, czy nie? On nie wie, on kogoś posłucha. A już abstrahując od poglądów politycznych - kogo ma słuchać?  Albo lekarzy, albo ministra zdrowia. A w takiej sytuacji, kiedy w krótkim czasie podawane są sprzeczne informacje, co taki człowiek ma myśleć? Ma po prostu mętlik w głowie i się buntuje. Jeden może powie, że stwierdzili, że jednak maseczka chroni, a drugi powie - halo, nie kazali nosić, to dlaczego teraz mi każą? Wydaje mi się, że to stąd.

**Co myślisz o nowych przepisach obowiązujących w kościele, dotyczących przypadających na osobę 15 metrów kwadratowych?**

Uważam, że kościoły powinny być zamknięte. Naprawdę, w dobie internetu można wysłuchać mszy w telewizji. Skoro papież może to robić, u nas też daliby radę, nic by się nie zadziało. Tym bardziej, że do kościoła, nie oszukujmy się, przychodzą przede wszystkim osoby starsze. Rozumiem, że wiara jest dla niektórych jedyną deską ratunku, żeby jakoś radzić sobie w tych ciężkich czasach, ale jest tyle alternatyw, że jakoś daliby sobie radę. U nas na przykład była taka sytuacja, że ksiądz odprawił mszę, jakiś tam sąsiad zadzwonił po policję i okazało się, że jest więcej osób niż mogło - to było w czasie, kiedy dopuszczano maksymalnie 5 osób. Ksiądz dostał 30 000 zł mandatu. Tłumaczył się, że nie był w stanie wyprosić nadprogramowych osób, że na jakiej zasadzie miał je wybrać. Wcale mu się zresztą nie dziwię. Powinni albo zakazać wszystkim, albo nikomu. Natomiast na koniec okazało się jeszcze, że ksiądz jest zarażony koronawirusem. Myślę, że bóg nie obraziłby się, jakby nikt nie poszedł do kościoła, naprawdę. Tak zresztą tłumaczę mojej mamie, która przeżywa, że nie może iść do kościoła. Mówię jej, że bóg się na ciebie nie obrazi, nawet będzie z ciebie dumny, że zostałaś w domu, a nie narażasz siebie i innych na zarażenie.

**Stosujesz się do zasad?**

Tak, noszę maseczkę, żeby nie zarobić mandatu. Nawet w weekend chcieliśmy gdzieś iść, ale powiedziałam, że nie idziemy, bo dopiero od poniedziałku oficjalnie będzie można. Nie ryzykuję, nie będę się tłumaczyć policjantowi. Nie mam ochoty nikomu się tłumaczyć, dlaczego wychodzę. Dlatego stwierdziłam, że jak zostaniemy jeden dzień dłużej w domu, nic nam nie będzie. Noszę maseczkę, bo ludzie zabijają wzrokiem, jak jej nie masz. Więc noszę, taką ładną, w potworki. Z tym, że ja rzeczywiście mam ich dużo, więc piorę je, prasuję. A że mało chodzę po sklepach, i mieście, noszę je rzadko. Staram się wybierać takie miejsca do spacerów, żeby nie spotkać drugiego człowieka.

**Słyszałaś o planach luzowania ograniczeń? Jakich?**

Będą chyba otwierać hotele i biblioteki. Nie znam drugiego etapu, bo trzymam się trzeciego, który mnie bezpośrednio dotyczy. Czekam na ten trzeci, kiedy to nastąpi, bo nikt nie ma pojęcia.

**Jak oceniasz te plany?**

To, że oni chcą zluzować, to z jednej strony jest ok. Ale od razu otwierać hotele? Biblioteki w porządku. Ale co, otworzą teraz kurorty, gdzie będzie kilkadziesiąt osób w jednym hotelu, a na przykład nie chcą otworzyć fryzjera i kosmetyczki, bo uważają, że to bliski kontakt. Myślę, że nie ma na to do końca pomysłu i to jest takie - otwierają, bo otwierają, ale tak naprawdę nie ma ku temu logicznego wyjaśnienia. Według mnie niczym nie różni się otworzenie hotelu czy marketów od otworzenia salonów kosmetycznych. Jest na przykład bunt tatuażystów, któremu się wcale nie dziwię. Oni są w IV etapie, razem z masażystami. Rząd tłumaczy, że tatuażysta ma najbliższy kontakt z klientem. A czym różnią się w takim razie kosmetyczki, które nakłuwają skórę? Robią zabiegi na twarz z igłą, brwi permanentne, depilacje. Mam wrażenie, że oni tego trochę nie przemyśleli. Strzelili na pałę, że będą otwierać. Robiąc paznokcie, czy będąc fryzjerem naprawdę jest się w stanie ograniczyć kontakt z klientem. Można nawet założyć klientce dodatkowo przyłbicę do maseczki, jeśli będzie trzeba. Mam kontakt 1 na 1 i to dla rządu jest większe zagrożenie, niż markety? Ja jestem w szoku. Oni ciągle wmawiają, że branża beauty to za bliski kontakt klientem. Ja rozumiem, że to bliski kontakt, ale naprawdę, nie widzę logicznego wyjaśnienia - zwłaszcza w przypadku masażystów i tatuażystów, których już w ogóle potraktowali po macoszemu. Wydaje mi się, że nikt nie ma na to pomysłu, a kończy się kasa. Zawiesić tyle składek ZUS, dopłacać do pracowników, są jeszcze jakieś pożyczki - państwo nie ma swoich pieniędzy, to są nasze pieniądze. Więc jeżeli nie ma wpływów, oni nie mają skąd brać. Więc to otwieranie to czysty biznes.

**Które z ograniczeń powinny według Ciebie pozostać na dłużej, a które należałoby znieść w pierwszej kolejności?**

Wydaje mi się, że można otworzyć hotele, jeśli nie będzie tam SPA. Jeśli wszyscy spotkają się na basenie, to naprawdę nie będzie dobry pomysł. Wydaje mi się też, że markety wielkopowierzchniowe - galerie handlowe, też powinny pozostać pozamykane. Tam jest zamknięta przestrzeń. Co innego, jak w parku spotka się 50 osób, a co innego w markecie, gdzie nie ma okien i nie można nawet tego wywietrzyć. Z drugiej strony rozumiem sklepy, które muszą na siebie zarabiać, ale ja bym tych wielkopowierzchniowych jeszcze nie otwierała. Rozumiem, że ludzie muszą jeść, dlatego sklepy spożywcze, to co innego. Ich nie mogliby zamknąć. Natomiast Castoramy i im podobne - dla mnie to bez sensu, że to nadal jest otwarte. Ja bym się tu zastanawiała, bo oni, jeśli dobrze pamiętam, chcą te sklepy otwierać w drugim etapie. Wydaje mi się, że to za szybko. Chcą najpierw otwierać molochy, choć chorych nie ubywa.

**Co otworzyłabyś w pierwszej kolejności?**

Wiadomo, swój biznes. To jest egoistyczne, ale mam na myśli te małe biznesy, gdzie skupiska ludzi są małe. Wiadomo, nie od razu wszystkie salony beauty. Ale jakby się dziewczyny ogarnęły, jesteśmy w stanie w miarę zachować bezpieczeństwo. Da się zrobić dużo rzeczy - ograniczyć liczbę ludzi w salonie, pozakładać pleksi, żeby to jakoś działało. Zazwyczaj też znamy swoje klientki i ja na przykład mogę wiedzieć, że ona nie była ostatnio we Włoszech. To też pewien rodzaj dbania o bezpieczeństwo. Nasz salon jest w budynku, w którym jest też przychodnia. Mogłam mieć kontakt z położną tam pracującą, która miała koronawirusa. Ona nie wiedziała, gdzie się zaraziła - nie była w szpitalu. Kiedy źle się poczuła, od razu poszła na L4. Ale obracając się w wąskim gronie wiedziała, kogo powiadomić o zaistniałej sytuacji. W markecie budowlanym mam styczność z obcymi ludźmi, którzy w razie czego nie będą wiedzieli, jaką grupę zawiadomić o ewentualnym ryzyku. Dlatego powinno się w pierwszej kolejności otwierać małe biznesy, gdzie kontakt międzyludzki jest w miarę ograniczony i w miarę pod kontrolą.

**Jaka jest różnica między kwarantanną a izolacją?**

Kwarantannę ma chyba ten zarażony? Czy on ma izolację? Jak to jest? Wydaje mi się, że jedno ma ktoś, kto jest zarażony, a drugie ma ktoś z domniemaniem zarażenia. Wydaje mi się, że na kwarantannę idą osoby, które mogły się zarazić, a izolację ma osoba zarażona. Natomiast wszyscy mówią, że są na kwarantannie. Gdziekolwiek, kogokolwiek słyszę. Choć logiczne by było, że mamy izolację, bo mamy się odizolować od innych ludzi. Ale wszędzie słyszę kwarantanna. Wiesz, że nie wiem? Ja też używam raczej słowa kwarantanna w odniesieniu do siedzenia w domu osób zdrowych. Wszyscy są na kwarantannie.

**Co jest granicą, kiedy dane ograniczenia powinny przestać obowiązywać?**

Kiedy umieralność jest mała. Wydaje mi się, że jeśli ktoś rozchoruje się już tak, że będzie mu potrzebny respirator, to dla niego już nie ma ratunku. Może to nie są w setkach liczone osoby, ale nie wszyscy mają też robione testy. Statystyka jest na tyle dziwna, że w zależności, co będziesz brała pod uwagę, taki będziesz miała wynik. Ale nie wiem, sama gubię się już w tym, co by było dobrym momentem. Ale może gdyby to się trochę uspokoiło, gdybyśmy zauważyli, że inne kraje już stopują, że tam się nic nie dzieje. W Hiszpanii i Włoszech ludzie nadal umierają w setkach, chociaż krzywa się w miarę wypłaszczyła. Natomiast nie jest to stabilizacja. Choć powoli powinniśmy wracać do życia. Ale zobacz, dzieciakom pewnie nie otworzą już szkół w tym roku szkolnym, ale wymyślili, że będzie opieka 1-3. Zamiast zrobić im lekcje, będziemy dawać dzieci na świetlicę? Gdzie jest dziesięcioro dzieci, tłum. Mam wrażenie, że nasz rząd idzie trochę w tym kierunku, żeby nie troszczyć się o nasze zdrowie, tylko przestać nam dodatkowe pieniądze - na opiekę, zwieszenie ZUSu, itp. To ich zabolało, aniżeli same zachorowania.

**A co sądzisz o takich miejscach jak siłownie, baseny, kluby sportowe? Kiedy te miejsca powinny zostać otwarte i na jakich zasadach?**

Mocno zastanawiałabym się nad siłowniami, gdzie ludzie się przecież pocą. Tak samo z basenami. Te wszystkie wydzieliny będą gdzieś roznoszone. Jednak są to miejsca, gdzie w ogóle można zarazić się różnymi dziwnymi rzeczami, bo jesteśmy tam rozebrani, chodzimy na bosaka. Na siłowni się spocimy, tu przetrze się czoło, tam nos, później dotknie się hantla i to się roznosi. Wiem, że to nóż na gardle dla przedsiębiorców, natomiast siłownie można w dużym stopniu przenieść do internetu. Widziałam mnóstwo różnych profili, na których można znaleźć naprawdę profesjonalne treningi - nie tylko machanie nóżką, które praktykuję. Jeśli zamówisz sobie na internecie hantle, czy gumy, spokojnie możesz wykonać taki sam trening w domu, jak na siłowni. Dlatego one powinny zostać otwarte dopiero, jak to się ustabilizuje. A kiedy to nastąpi, myślę, że może dowiemy się jesienią.

**A co z zasadami, jakie powinny obowiązywać w takich miejscach? Czy warto by coś zmienić?**

Nie wiem, ile osób wchodzi na siłownię. O ile w autobusie, czy kościele jesteś w stanie wyznaczyć miejsca, o tyle w siłowni nie wiem, jak miałoby to wyglądać. Może powinni wprowadzić jakieś ograniczenia, ale nie mam pojęcia, jakie. To powinny się już wypowiedzieć osoby, które zarządzają takimi miejscami. Ja nie mam pojęcia, jak to działa. Choć ogólnie wydaje mi się, że jak to ucichnie - bo to raczej nie tyle skończy się, co ucichnie - my będziemy się dystansować. Nie będziemy stać ramię w ramię, przestaniemy się witać, cmokać w policzki. Nawet podczas rozmowy, co widzę już sama po sobie, nie staję zbyt blisko drugiego człowieka, jest takie coś, że staję krok do tyłu. To nam chyba już zostanie.

**A czy słyszałaś, w jaki sposób Szwedzi podeszli do tematu epidemii?**

Obiło mi się o uszy, że oni nie bardzo podeszli do tej epidemii. Ona jest, ale chyba nie mają narzuconych żadnych ograniczeń. Przynajmniej tak chyba było.

**<przedstawiony model szwedzki> Co o tym sądzisz?**

Szwedzi mają inną mentalność. Oni pewnie się posłuchali i w miarę dbają o to. U nas, gdyby tak było, byłoby pewnie to samo, co we Włoszech. Hulaj dusza, piekła nie ma. Gdyby nasza mentalność była taka, że my się posłuchamy - super. Ale to jest inny kraj. Dlaczego we Włoszech zadziało się tak, a nie inaczej? Bo oni nic nie zrobili sobie z rekomendacji. Szwedzi, kiedy usłyszeli, że mają zostać w domach, zostali w domach. Dali dzieci do szkoły i tyle. Wydaje mi się, że to jest kwestia mentalności, a my takiej nie mamy, my będziemy się buntować. W Polsce jest to absolutnie nie do zrobienia. Jeśli Polak słyszy, że jest zakaz, to dla niego oznacza, że trzeba go złamać. Głupi przykład z ograniczeniami prędkości - jak jest 50, mogę jechać 70. To jest cały Polak.

**Ok, skoro chętnie łamiemy zakazy, może lepiej byłoby pozostać w sferze rekomendacji? Co jest lepsze?**

Ja bym wolała rekomendacje, ale chyba na społeczeństwo by to nie podziałało. U nas działa tylko zasada kary. Ludzie się burzą, denerwują, ale jak usłyszą, że mogą dostać 30 tys. mandatu, to jednak nie robią. Na tym to polega. Dopóki była rekomendacja, żeby dzieci nie wychodziły się, nie grupowały po zamknięciu szkół, i tak było ich wszędzie pełno. Dopiero, kiedy zaczęła grozić za to kara, podwórka opustoszały, dzieci nagle zniknęły w domach. Kiedy zamknęli szkoły, koledzy Miłosza przychodzili po niego jeszcze tydzień. Uważam, że skoro mówią, aby nie wychodzić, to gdybym pozwoliła mu wyjść z nimi, równie dobrze mógłby iść do szkoły. Po coś to jest.

**A chciał wyjść?**

Oczywiście, że chciał, ale on też jest w miarę mądrym dzieciakiem. Rozmawiam z nim, jak z dorosłym. Wytłumaczyłam mu, o co chodzi w koronawirusie. Przez parę kolejnych dni, kiedy przychodzili po niego koledzy, powiedział im jeszcze kilka razy, że nie wychodzi, a w końcu zrobił im wykład na temat tego, że mogą pozarażać wszystkich, łącznie ze swoimi dziadkami, co jest bardzo nieodpowiedzialne. Więcej już nie przyszli. Dlatego niestety, lepsze są w naszym kraju zakazy.

**Jak w obecnym czasie wygląda u Ciebie dbanie o siebie?**

Bez żadnych istotnych zmian. Mniej się maluję, bo nie mam potrzeby, tylko jak wiem, że będę gdzieś wychodzić. Trochę się już do tego przyzwyczaiłam, że nie trzeba się malować - spoko. Fryzjera nie ma, więc sama musiałam sobie zrobić włosy. Wyszły czerwone, bo pomyliłam farby, ale już je zafarbowałam drugi raz, więc ok. Przekręciłam numer farby i wyszły mi brązowo-czerwone. Leciałam więc do sklepu z jeszcze lekko wilgotnymi po drugą farbę. To taka jedna wpadka, ofiara koronawirusa. A tak, nie korzystam z usług swoich koleżanek. Nie spotykamy się, żeby nie ryzykować. Stwierdziłam, że nigdzie nie wychodzę, nie jest mi potrzebne zrobienie brwi, czy czegoś. Paznokci nie maiłam zrobionych z miesiąc - szewc bez butów chodzi. Dopiero ostatnio je zrobiłam na szkoleniu. Trochę odpuściłam - w ten sposób.

**Malujesz się teraz, kiedy wychodzisz. A zdarza Ci się malować, kiedy wiesz, że zostaniesz w domu?**

Nie, kiedy wiem, że nie wychodzę, nie chce mi się. Jak gdzieś wychodzę, trochę się podmaluję, żeby trochę lepiej się czuć - taką naszykowaną. W domu nie zdarzało mi się. Widziałam gdzieś nawet hasztagi na internecie #wyszykujsię #wymalujsię #robimyimprezęonline - mi to się nawet nie chce. Zawsze tak miałam, że przychodząc do domu zakładałam wygodne dresy i zmywałam makijaż.

**A jeśli chodzi o ubieranie się, coś się zmieniło?**

Tak, noszę dresy lub getry. Coś bardziej wyjściowego zakładam tylko kiedy jadę do sklepu, to czasami sobie założę. Rzeczywiście noszę mniej takich ubrań, jakie nosiłam na co dzień. Nie kupuję też na razie żadnych ubrań, jak mówiłam już wcześniej. Stwierdziłam, że mam pełną szafę i nie potrzebuję nowych. Jedyne co bym musiała w tej chwili, to kupić parę rzeczy, bo udało mi się trochę schudnąć i nie mam ubrań, które są na mnie dobre. Nie mam na przykład spodni i to naprawdę. Mogłabym je zamówić przez internet, ale stwierdziłam, że i tak nigdzie nie wychodzę, więc coś tam w tej szafie się znajdzie. Jak otworzą sklepy, pewnie wybiorę się na zakupy na zasadzie kupić coś nowego na wiosnę, w swoim rozmiarze. Wolałabym to zrobić stacjonarnie - zwłaszcza jeśli chodzi o spodnie. Całą resztę mogę kupić przez internet, ale spodnie tylko stacjonarnie, bo mam dziwną figurę i nie umiem ich kupić przez internet. A ogólnie nie lubię chodzić po sklepach, dlatego muszę mieć cel - jak kupić spodnie. Wtedy kupię, wyjdę i dziękuję.

**Wyjścia do sklepu wiążą się teraz z tym, że zwracasz większą uwagę na swój wygląd podczas zakupów, niż było to przed epidemią?**

Zależy. Zazwyczaj przed epidemią szłam do sklepu między pracą a jakimiś zajęciami. Zawsze byłam więc naszykowana tak, jak miałam funkcjonować przez cały dzień. W ogóle, kiedyś wyjście z domu było takie normalne. Trzeba było wstać, umyć włosy, pomalować się i nie ważne, czy szło się do sklepu, czy pracy lub znajomych - mniej więcej rytuał był taki sam. Teraz wyjście do sklepu po chleb to jest takie wiesz, uuu, wychodzimy. I zależy, jak mi się chce, to się ogarnę. A jak nie, zrobię cebulę na głowie i zakładam bluzę. Na pewno teraz mniej przejmuję się swoim wyglądem. Kiedyś wydawało mi się, że wyjście bez makijażu to coś nie do pomyślenia. A teraz? Idę.

**Jak Ci z tym jest?**

Dobrze mi z tym.

**Myślisz, że to będzie miało na Ciebie wpływ, kiedy epidemia się skończy?**

Myślę, że to będzie miało wpływ na postrzeganie mnie samej przez siebie samą. Nie będę się już widzieć tylko przez sukienkę i makijaż. Nie ma to różnicy, czy ja założę tę, czy inną bluzkę - nadal będę tą samą Martyną. Kiedyś - może nie tyle przed zakupami, ale bardziej przed spotkaniem ze znajomymi myślałam, że lepiej, aby nie widzieli mnie bez makijażu. Teraz mam to w nosie, w ogóle się tym nie przejmuję.

**A jakie czynności pielęgnacyjne utrzymujesz?**

Cały czas te same. Ja zawsze dbałam o buzię, nic się absolutnie nie zmieniło. Nadal tak samo ją myję, pielęgnuję, nakładam maseczki raz w tygodniu. Tu się nic nie zmieniło.

**Zrezygnowałaś z jakichś kosmetyków, lub coś dołożyłaś?**

Nie. To pewnie kwestia też tego, gdzie pracuję. Mam już swoje sprawdzone kosmetyki, używam ich od lat. Nie muszę tu eksperymentować, bo nie narzekam na cerę. W kółko to samo. Na razie wszystko mam, ale jak mi się skończą, dokupię je. Niektóre, te które jestem w stanie kupić stacjonarnie - w Rossmannie. Niektóre zaś są dostępne tylko w salonach, więc wtedy je zamówię.

**Jak teraz wygląda Twój makijaż, kiedy już się malujesz?**

Ja nigdy nie malowałam się bardzo mocno. Rzęsy, trochę pudru, różu i tyle. Nigdy nie jest to nic więcej. Nie umiem profesjonalnie malować oczu, więc tego nie robię. Poza tym, ja jestem trochę nudna. Jak się do czegoś przyzwyczaję, to w kółko robię to samo. To samo jest z ubraniami, życiem. Jak mi jest z czymś dobrze, to już tak zostaje na stałe.

**Brakuje Ci wizyt w profesjonalnych salonach?**

Niektórych tak, niektórych nie. Włosy spoko, przeżyję. Brakuje mi trochę brwi, bo mam zarośnięte chaszcze, a sama tego nie umiem zrobić. Chętnie bym już sobie poszła, ale jeszcze wytrzymam. Natomiast da się bez tego żyć. Nie mam ciśnienia, żeby mnie gdzieś w podziemiach przyjęły, bo muszę mieć zrobione. Jestem w stanie z tym przetrwać, bo mam świadomość, że to się za chwilę skończy i wszystko wróci do w miarę normalności. Inaczej by było, gdyby mi to na zawsze zabrali i miałabym już zawsze radzić sobie sama. To może bym to jakoś odczuła. A tak, to.

**Czego brakuje Ci w tej sytuacji najbardziej? To są te kosmetyczki, czy coś zupełnie innego?**

Najbardziej brakuje mi takiego poczucia wolności, swobody. Tego mi brakuje. Że jak będę chciała, to pójdę sobie do kosmetyczki. A jak będę chciała, to pojadę sobie do znajomych. Albo pójdę na spacer. Taki wiesz, chcesz, to idziesz. Nie brakuje mi żadnej konkretnej rzeczy, nie brakuje mi konkretnych znajomych. Ja też nie jestem z tych, że co tydzień imprezowałam i co tydzień się spotykaliśmy. W dobie internetu mamy ten kontakt. Można zadzwonić, pogadać. Bardziej brakuje mi tego poczucia wolności, swobody. Mi to zabrano i tego mi brakuje. Poza tym wszystkie usługi są pozamykane. Działają tylko sklepy spożywcze i Rossmanny. Zepsuł mi się komputer i nie mam nawet gdzie go oddać na gwarancję. Więc zostaliśmy bez komputera. Jeśli będę miała teraz jakąkolwiek potrzebę inną niż jedzenie i chemia, nie mam gdzie jej zrealizować.
