# Supplementary material for: The challenges arising from the COVID-19 pandemic and the way people deal with them. A qualitative longitudinal study
Source: PLoS One. 2021 Oct 11;16(10):e0258133. doi: 10.1371/journal.pone.0258133 (PMC8504766; doi:10.1371/journal.pone.0258133)
Supplement: S1 Dataset — (ZIP) [file pone.0258133.s003.zip › Transcriptions/stage 3/5.3_M_39_single.docx]

**5.3_M_39_single**

**Co się działo przez ostatnie 2 tyg.? Jak spędzałeś czas?**

Właściwie to aż tak dużo to się nie wydarzyło. No niestety. Takie jest życie teraz. Trochę jest takie bezbarwne. Otworzyli lasy, ale dopiero chyba jutro zaliczę, bo pracowałem.

**To opowiedz najpierw co się działo w tygodniu przed świętami? Pamiętam, że byłeś zdegustowany powrotem do pracy. Jak teraz jest w pracy?**

Nadal są przytyki takie trochę, że człowiek był na tym chorobowym, więc to irytuje mnie. Zawodowo część tematów miała już wrócić na swoje tory w tym tygodniu. Teraz mam wolne, więc pewnie jutro jak wrócę, to już będzie większość rzeczy po staremu?

**To znaczy jak?**

My tam takie analizy robimy. one były okrojone ze względu na małą ilość laborantek, bo część jest na urlopach, na chorobowych, na opiekach, albo się podzielili inaczej na zmianach, żeby ich było tam mniej, więc mniej rąk do pracy jest, żeby robić te analizy.

**Mówiłeś poprzednio, że szykujesz się do swojego streamu?**

Tak, były już 2 nawet. Zawsze to taka fajna odskocznia, nie? Na chwilę. Ludzie trochę komentowali, na 1-szym streamie mnie oglądało prawie 70 osób, więc to też miło. Przy tym graniu, to wiadomo, jakieś winko wypiłem. No i tak trochę taka chwilowa odskocznia.

**Raz przed Wielkanocą, raz po?**

Boże...Już mi się zlewają te dni...Robiłem w piątek i tydzień później w piątek.

**A jak było w święta?**

W Wielkanoc pracowałem. Po pracy na godzinę wpadłem do rodziców, zjadłem, posiedziałem i poszedłem do domu, bo na drugi dzień znowu do pracy szedłem.

**Jak wspominasz tę Wielkanoc?**

No, bez przytulania, wiadomo...Tak bez jakichś oklasków w ogóle. Taka słaba ta atmosfera świąteczna.

**Inaczej niż zwykle?**

No taka okrojona, nie? Tym bardziej, że ja zawsze miałem wtedy jakieś wolne, a tym razem nie dało się, więc to też tak podwójnie było dziwnie.

**Pojawił się stream, co nowego jeszcze się pojawiło w ciągu tych 2 tygodni?**

Większość to taki Dzień Świstaka jest. Mi to się tak te dni zlewają, że nawet nie wiem jaki jest czasami dzień tygodnia. Jedynie co, to moja kumpela, która ma psa, mieszka w domku, ma ogródek, do niej zaglądam. Wczoraj byłem u niej na ogródku na jej urodzinach, ale to w gronie, że tylko ja, ona i jej mama. Trochę się pośmialiśmy, coś tam wypiliśmy. A parę dni wcześniej byliśmy sobie na parowie z psem, bo z psami można wychodzić i uznaliśmy, że jak ktoś by nas zatrzymał, to powiedzielibyśmy, że jesteśmy rodziną i tyle.

**Ktoś was zatrzymał?**

Nie, ale takich jak my to było trochę. No ludzie nie wytrzymują, nie? Nie wysiedzą. Ludzie nie przywykli do takiej izolacji.

**A ty nie wytrzymujesz, już nie możesz wysiedzieć?**

No.

**Widziałeś się z kimś jeszcze?**

Właściwie to nie. Kolega na chwilę podjechał w jakiejś tam sprawie. Z daleka pogadaliśmy. Dzisiaj wsiadłem na rower, więc jak skończymy, to dalej jadę pojeździć na rowerze.

**Jeździłeś też przedtem w czasie tych 2 tygodni?**

Tak.

**Co jeszcze się działo?**

No jak już tak się otwieramy i gadamy szczerze, trochę byłaś na bieżąco...Pojawiła się jakiś czas temu jakaś osoba, myślałem, że to idzie w dobrą stronę i ktoś zaczął robić jakieś zwroty akcji. Początkowo musiałem sobie odpowiedzieć, czy to jest, tak jak ci mówiłem...Ja muszę wiedzieć, czy to ja tęsknię, czy jestem samotny, bo to są dwie różne rzeczy. No i im dłużej się nie widzieliśmy, tym coraz bardziej o niej myślałem, więc uznałem, że to jest jednak to, że jednak o niej myślę. No i ona jakieś zwroty akcji zaczęła robić, nie wiem, takie sprzeczne sygnały, więc...Najpierw chciała, potem ja nie byłem jeszcze pewien, jak ja chciałem, to ona nagle zrobiła jakiś zwrot akcji, więc chyba to już jest koniec. Dopadło mnie...Dopadł mnie w tym momencie zawód, ale i podwójna samotność w takim układzie. Trochę się zawiodłem na wszystkim, nie? Ja się śmieję, że w jakiś sposób mnie podpuściła. Ja się otworzyłem i nagle...Nie do końca nawet to wyjaśniła. W ogóle tak ogólnikowo. Wydawała się bardzo mądra i dojrzała, ale taka ta końcówka była, że mam co do tego wątpliwości.

**Spotkałeś się z nią, czy to wszystko było online/ przez telefon?**

Widziałem, że przestała mieć trochę ochotę...Ja ją trochę zagadywałem, ona niby trochę też, ale te spotkania w ogóle jakoś nie dochodziły do skutku. Gdzieś tam na chwilę się złapaliśmy na parkingu, pogadaliśmy chwilę o niczym. I myślę, kurde, coś tu nie gra. W końcu się wkurzyłem i napisałem tekst, że mam wolny weekend, cały i może masz ochotę ze mną go spędzić. Stwierdziła, że coś praca, że coś tam się źle czuje, coś...Jakieś takie bzdury w ogóle. I napisałem w końcu, że albo w lewo, albo w prawo, bo ja w tym momencie się męczę i to jest bez sensu, że mnie tak zwodzisz. Starałaś się, dopuściłem cię do siebie i nagle, kiedy ja chciałem coś, ja się postarałem, to ty zrobiłaś zwrot jakiś. Ja mam tego dość, mówię i albo robimy coś z tym dalej, albo...Bo źle się z tym czułem. Człowiek i gorzej sypia po nocach przez to, bo za dużo o tym myślał... No i takie ogólnikowe było, że niby nic nie...Ja mówię, nie, to nie ma sensu, jeżeli ktoś nie jest konkretny i nie wie, czego chce. Ja nie mam na to czasu. Coś tu nie zagrało, ale ja nie będę już dopytywał. Zakończyłem to wszystko i tyle.

**Jak jeszcze było w czasie tych 2 tyg.?**

Właściwie to skupiałem się na wszystkich tych samych rzeczach, które były wcześniej. Zmieniłem pomysł na produkcję muzyki. Stwierdziłem, że skoro nie będą kluby otwierane, odwołują festiwale, odwołali największy festiwal elektroniczny, na którym pewnie miałem grać i inne też...Stwierdziłem, że nie ma teraz takiego ciśnienia na to, żebym się promował, jako producent elektroniczny i zacząłem robić muzykę taką instrumentalną, bo nie będzie lepszej okazji. Za 2 tyg. jestem umówiony i będziemy żywe instrumenty nagrywać. mam na to pewien pomysł. Chodzi o to, żeby zmienić coś w swojej głowie, żeby się coś wydarzyło, bo już zaczęło mnie to trochę nudzić i chciałem jakiegoś nowego impulsu. Postanowiłem, że żeby zająć myśli, to muszę coś zmienić. To stwierdziłem, że muzyka mnie zawsze wyciąga, ale ta ostatnio mnie nie wyciągała, to muszę zmienić po prostu muzykę. Tu jest jakaś zmiana.

**A jak z tą jogą online, którą planowałeś zacząć?**

Kurde, to jakoś siadło, nie wiem...Może wrócę jeszcze do tego. Nie myślałem jakoś...

**Jeszcze z czegoś zrezygnowałeś, co zwykle robiłeś? Mówiłeś np. o puzzlach, które układasz?**

Puzzle przestałem układać, bo też mi się już znudziły. Było to dobre na początku. To jest taka stagnacja, że głowa pęka. Człowiek jak by w klatce był zamknięty. Tym bardziej, że jeszcze ta samotność człowieka dopadła po tym, jak ktoś się odwrócił i trochę taki zawód. Nie wiedziałem, że to tak odczuję. Myślę, że właśnie ta samotność przez tę izolację i to było spotęgowane.

**Słaby moment na takie rzeczy?**

Bardzo słaby.

**Wychodzenie do pracy coś ci daje?**

Nie, totalnie nie.

Jest coś, co daje ci teraz przyjemność, radość?

Nie wiem...Nowa muzyka i czasami człowiek docenia te małe przyjemności. Wiadomo, jak nie ma dużych, to człowiek docenia te małe. Dzisiaj zjadłem pizzę, którą lubię, wczoraj napiłem się alkoholu, który lubię. To są takie małe rzeczy, których człowiek nie zauważa na co dzień może?

**Co jeszcze robisz, żeby sobie radzić?**

Myślę, że nic więcej niż wcześniej. Myślę, że teraz będzie można już trochę więcej wychodzić, więc teraz trochę pozmieniam, w sensie, że ten rower, ten las. Myślę, że za chwilę ludzie się będą jakoś tam się częściej widywać. Jest taka szansa, że może pojadę do kogoś nad morze w weekend. Co za różnica, czy będę spacerował w lesie, czy nad morzem, ale jednak człowiek zmieni miejsce.

**Do lasu i tak wychodziłeś?**

No, byłem 2 razy nielegalnie.

**Emocje – zdjęcia**

Nadal po staremu, nie? Czyli 2 i 11, ale z przyszłością 6.

**Jakiegoś zdjęcia, elementu na zdjęciu tu brakuje?**

Mam nadzieję, że się taki pojawi w mojej głowie, jak pojadę nad morze i to będzie morze i fale, i piasek. Takie raczej spokojne morze.

**2**

Człowiek już rusza, ale jeszcze chwila, jeszcze ta guma jest przyklejona.

**Co sprawia, że czujesz, że już się rusza?**

No jak otworzyli te lasy, to człowiek już wie, że już może jakieś ruchy wykonać i nie będą go tam szarpać, nie?

**Co powoduje to uczucie uwiązania?**

Ludzie nadal są zdystansowani, generalnie nie chcą się widywać. Na pewno jakieś zdjęcie z rowerem bym dołożył, bo lubię jeździć. Dzisiaj nawet taką wiadomość dałem na Instastory - zdjęcie mojego roweru z podpisem, że jak ktoś się chce ze mną zobaczyć, to podjadę na chwilę rowerem. W sensie, żeby zachować odległość, ale chociaż przez chwilę pogadać. Na dzisiaj dodałbym zdjęcie roweru.

**Jakie to są emocje, uczucia?**

Hmm...To jest samotność, ale dzięki tej 6, dzięki zdjęciu z rowerem, dzięki temu morzu, będę w stanie i pomoże mi to wyjść z tego, żeby się zająć i zająć czymś myśli.

**Tu jest taki element nadziei?**

Tak, tak. Zawsze trochę mi to pomagało. Ja zawsze mówiłem na takie rzeczy, że to są antydepresanty. Rower, las, jezioro, morze, natura, same chmury gdzieś tam...

**11**

Liczę, że 11 zaraz zniknie.

**Czyli, co zniknie?**

Za chwilę przestanie padać ten deszcz, będzie bardziej widać tę tęczę, to co jest za oknem i może bym się skierował w stronę 5. 5 jest jakimś kosmosem, jakąś rzeczą, która też jest jakimś elementem natury. Człowiek patrzy w nocy w gwiazdy i myśli o różnych rzeczach. W tym momencie nawet marzyć nie trzeba, bo marzenia są odłożone. Marzenia są na razie odłożone, nie można ich spełniać, bo są te obostrzenia, blokady. 5, ten las, morze, są rzeczami, gdzie człowiek się może uspokaja i zaczyna może uruchamiać jednak gdzieś tam, że w jakiejś przyszłości zacznie człowiek realizować te malutkie marzenie, które kiedyś człowiek pewnie by nawet ich nie nazwał marzeniami, tylko życiem codziennym. Różnica jest w wielkości tych marzeń i w formie tych marzeń teraz, bo kiedyś to, o czym człowiek myślał, to były raczej rzeczy, które ma sobie człowiek zrobić, na które ma ochotę. Marzenia były czymś większym, czymś bardziej górnolotnym albo czymś bardziej skomplikowanym, albo czymś, w co trzeba włożyć więcej wysiłku. Dzisiaj one w ogóle nie są w zasięgu, więc człowiek myśli o prostszych rzeczach, czyli np. o, chciałabym zobaczyć morze. Takie po prostu i to jest ta różnica. Dawniej człowiek tylko myślał, kiedy?

**Jakie tego typu pragnienia przychodzą ci do głowy?**

Na tę chwilę, to głównie te związane z naturą. Bardzo chciałbym, żeby życie towarzyskie wróciło, bo jeżeli to tak się wydarzyło z kimś, to chyba chwila oddechu i chyba miałbym ochotę kogoś poznać, a nie mam możliwości teraz, bo nie ma gdzie. Mało tego, na piękne oczy człowiek nie podejmie decyzji, bo nie widać twarzy. Nie dość, że nie ma gdzie, to nawet tego człowieka nie zobaczysz, bo ma maskę. Byłem w aptece teraz, gdzie zawsze chodzę i jest taka aptekarka, której wcześniej nie widziałem. Miała coś w sobie, te oczy i wizualnie, fizycznie też mi odpowiadała, natomiast miała maseczkę. Wszedłem i zacząłem się śmiać i mówię do niej, że wydaje mi się, że się do mnie uśmiecha, ale nie wiem na pewno, bo ma maskę. I na sam koniec, kiedy płaciłem, zdjąłem tę maskę. Liczyłem, że też ją zdejmie na chwilę.

**I jak zareagowała?**

Popatrzyła na mnie. Nic więcej. Zerknąłem, czy nie ma pierścionka i myślę, że po wit.C jeszcze tam wrócę.

**A jak jest teraz z twoimi obawami związanymi z koronawirusem?**

Dużo myślę o tym, że wszelkie ruchy medyczne będą przełożone, odłożone w czasie. Jakieś badanie zrobić, to teraz bardzo ciężko będzie, zatka się służba zdrowia w tym roku. Czas mnie nagli kurde, kończę w tym roku 40 i chyba chciałbym się związać, więc bardzo mi ten koronawirus mi to utrudnił w tym momencie i z tego powodu jestem wkurzony. I dlatego też, że ktoś zrobił taki ruch, którego ja nie rozumiem do końca, a może ktoś sobie uzmysłowił właśnie i to dlatego, że jestem tak dużo starszy.

**A obawy związane z epidemią?**

Nie wiem...Coraz bardziej nie wierzę w ten wirus. To jest za mocno napędzane przez media, ludzie żyją w strachu, władza to wykorzystuje do podejmowania decyzji bez wiedzy obywateli i bez ich zgody. Na siłę chcą, kurna, zrobić wybory. To jest w ogóle jakiś idiotyzm. Skoro tak ludziom napędzili strachu i tak pozamykali to wszystko...Ale wybory chcą zrobić. Albo zamykają las przy 4000 zakażonych, a przy 9000 otwierają. To jest w ogóle jakaś...

**Mówiłeś, że śledzisz statystyki?**

Już nie. Jeszcze z tydzień temu zerknąłem, a teraz już wcale. Nie chcę już w ogóle o tym...Dla mnie to nie ma znaczenia, czy w Stanach jest pół miliona zakażonych, czy u nas jest 10, czy 20000. Myślę, że się liczy tu i teraz. Jestem zdrowy, świeci słońce i w tym momencie moje samopoczucie psychiczne jest ważniejsze i fizyczne niż strach, który paraliżuje i przez który moje zdrowie jest tylko gorsze. Ja bez względu na to, co te gadające głowy, czyli władza wymyśli, to dla mnie priorytetem jest mój stan psychiczny, fizyczny i moje życie. Oni nie będą mi dyktować. Jeżeli tak, to ja poproszę o pieniądze potem na psychologów, bo ja sobie nie poradzę na dłuższą metę z tym, chyba, że będzie jakiś program, to ja poproszę, chętnie porozmawiam.

**Było w tobie poprzednio sporo obaw na temat twojego zdrowia, zdrowia twoich bliskich. To w tej chwili też ci się spłaszczyło?**

Tak.

**Czy to wpływa na to, jakie środki ostrożności zachowujesz?**

Idę i zachowuję takie, żeby się ludzie bezpieczniej poczuli, Jeśli chodzi o samotne przebywanie gdzieś na drodze, chodniku, to mam to gdzieś, w dupie. Omijam trochę ludzi, ale nie jestem jakiś zeschizowany z tym wszystkim, żeby jakoś panicznie...Zauważyłem, że ludzie wpadają ze skrajności w skrajność z moich znajomych, albo są tacy, o których w życiu bym nie pomyślał, że tak się w to wkręcą. Tacy, że zawsze tak wszystko rozsądnie, itd. A teraz to w ogóle nie możemy porozmawiać, bo ktoś się boi. Nie spodziewałbym się po pewnych osobach.

**Co obserwujesz w otoczeniu? Jakie to są emocje? Jakie zachowania?**

Są 3 grupy. Albo ktoś totalnie ma to wszystko w dupie, bo znam też takich.

**Opowiedz o ej grupie - jak się zachowują, co robią, co mówią?**

Opowiadają, że tego koronawirusa nie ma, że to jest wszystko ściema. To są też tacy ludzie, którzy uważają, że to jest jak kolejna grypa i na inne choroby więcej ludzi umiera i w tym samym czasie będzie więcej zawałów, bo ludzie się będą stresować tym koronawirusem albo, że majątek stracą i więcej będzie samobójstw, rozwodów, zawałów. To są ludzie, którzy nie chcą w ogóle o tym myśleć, chcą jak najbardziej żyć po swojemu, mają to gdzieś.

**Dużo znasz takich osób?**

Co jakiś czas się zdarzają takie osoby. 2 grupa to są tacy, którzy są takimi pośrodku, którzy na samym początku, do tego momentu, przechodzili albo w lewo, albo w prawo. Z takiego czegoś, że nie uważali wcale, a potem zaczęli uważać już na wszystko, a są też tacy, którzy uważali na wszystko, a teraz przechodzą w stan tej pierwszej grupy, czyli mają to wszystko w dupie. Ja na to mówię grupy przejściowe i ja jestem w tej grupie przejściowej.

**Tak, pamiętam, że odkażałeś paczkę...**

Właśnie, a dzisiaj to w ogóle przestaję o tym myśleć. Czytałem jeszcze...Mało tego czytam, ale czasami coś ciekawego, jak jakiś pulmonolog z Włoch, z Bolonii napisał...Oczywiście państwo wytoczyło mu już jakiś proces, bo był przeciwny władzy i miał swój pogląd na to wszystko. On powiedział o tym, że nasze maseczki, kurwa nie chronią. Kurwa, mało tego, rękawiczki to wcale nie jest dobry pomysł, ponieważ jak każde inne bakterie, to co jest na naszych rękach, to w jakiś sposób nasza skóra i nasz organizm powoli się przyzwyczaja do tych bakterii, wirusów, itd. Ja zawsze mówiłem, że brudne dziecko to szczęśliwe i zdrowe dziecko. I tak samo jest z tymi bakteriami. Jak będziemy sterylni, to będziemy, kurwa, chorować na wszystko. Rękawiczki zakładam już tylko do Biedronki i jakbym poszedł do innego supermarketu, a tak to mam w...

**Dlaczego tam zakładasz?**

Bo widzę, że ludzie tam tak dosyć przestrzegają + na wejściu są.

**Chodzi o spojrzenia innych ludzi?**

Tak, to jest dla innych. jak wsiadam do samochodu, to dezynfekuję ręce, wchodzę do domu, wrzucam zakupy i dezynfekuję te ręce. Rękawiczki idą do kosza. No i 3 grupa to taka, którzy się totalnie nadal bardzo izolują i którzy są nadal pełni obaw. Oni chyba do końca roku w ogóle z tego nie wyjdą, bo będą się wiecznie bali.

Izolują się, nie chcą się spotykać. Co jeszcze obserwujesz u tych ludzi?

Wiesz, zwracają innym uwagę, nie chcą się witać. U mnie jest zwyczaj tzw. żółwika w pracy. Ten, co ma totalnie wszystko w dupie się normalnie ze mną wita. Ci ludzie nie chcą kontaktów i to głównie do tego się sprowadza + zwracają innym uwagę. Myślę, że tv ich tak nakręciła.

**Oddzieliłeś się od statystyk, a co z innymi wiadomościami?**

Nie mam telewizora, przestaję czytać artykuły, zająłem się muzyką i zaczynam powoli wracać do większych relacji ze znajomymi. Tzn. większych...W ogóle jakichkolwiek.

Zaraz jadę na rower i kilka osób odwiedzę.

**Tak z żółwikiem czy jednak na dystans?**

Może bez żółwika. Po prostu sobie staniemy, pogadamy.

**W jakiej odległości - gdybyś to ty miał decydować?**

2 m.

**Czyli jednak ten dystans byś zachował?**

Dla nich.

**Wiesz, jakie w tej chwili są obostrzenia? Orientujesz się w tym?**

Otworzyli lasy i parki. Nic więcej. Dla mnie to głupota - odmrażanie gospodarki od otwierania lasów. To są idioci.

**Do tego dojdziemy, a na razie powiedz, jak ty się czujesz z obostrzeniami, które są teraz?**

Ja je coraz bardziej mam gdzieś. Ja jestem w tym etapie przejściowym i za chwilę w ogóle nie będę myślał o tym, że ktoś mi zwróci uwagę, że gdzieś idę czy coś, bo ja mam cały czas przygotowane w głowie kilka wersji tej historii - albo jadę po leki, albo jadę po zakupy, albo w tym momencie prowadzę swój biznes i muszę mieć na przeżycie, więc proszę mnie puścić i tyle.

**A maseczka?**

No...Tam, gdzie trzeba mam ją założoną, a tak to...Dla innych. W sklepach tak. W pracy, to dojadę, zdejmuję i...Musimy nosić w drodze do i z pracy, ale w pracy nie musimy nosić.

**Kogo te maseczki mają chronić?**

Nikogo. To jest tylko i wyłącznie...Oglądałaś serial Czarnobyl?

**Nie.**

Świetny, polecam. Tam, ci ludzie, którzy pracowali, górnicy, mieli pracować w kombinezonach i ktoś im zwrócił uwagę, czemu nie pracują w kombinezonach, a ten szef górników w pewnym momencie powiedział: "Rozmawiajmy szczerze. Przecież te kombinezony nie chronią". I odpowiedź była: „No tak." "No to dajcie nam pracować bez." I tak samo jest z tymi maseczkami. Każą w nich chodzić tylko i wyłącznie dla poczucia społecznego bezpieczeństwa i żeby nie było, że nic państwo nie zrobiło. Zawsze znajdą się tacy, którzy będą uważali, że te maseczki chronią. A ten Włoch, który pisał artykuł, to powiedział, że maseczka to jest tak, jakby - cytuję: jakby w płocie zainstalować furtkę i miałaby chronić przed komarami.

**A jest coś, co nas chroni? Czy któryś z tych nakazów/ zakazów działa na rzecz przeciwdziałania epidemii?**

No, jeżeli faktycznie jest ten wirus i on jest taki śmiercionośny, jest taki...Zaraźliwy podobno jest bardziej niż grypa. No dobrze, mógłbym się z tym zgodzić. To tak: kwarantanna owszem, natomiast jest jeszcze tzw. społeczna odporność na wirusa, czyli przebywanie w tym wszystkim, że szybciej się może uodpornimy. Szczepionki...Ja nie wiem, czy szczepionki, to w ogóle będzie dobry pomysł. Ludzie nie będą się chcieli szczepić. Niektórzy. Myślę, że bardziej potrzebne jest teraz lekarstwo.

**Kwarantanna jest sensowna, coś jeszcze?**

Jestem tym przejściowym teraz, który idzie do grupy 1 i za chwilę będę miał to wszystko w dupie, więc ja nie wiem, czy to wszystko w ogóle ma sens. Zaraz dojdziemy do gospodarki...Dla gospodarki to jest dramat, bo to są błędne decyzje w jakiś sposób.

**Jak to się stało, że przechodzisz do tej grupy? Co spowodowało taką zmianę w twoim myśleniu?**

To jest tak, jak trzymanie ręki w garnku, który jest postawiony na palniku i ma się gotować. Człowiek trzyma tę rękę, trzyma, trzyma, dopóki może wytrzymać, a potem po prostu ją wyjmuje, więc to jest proces. Powoli zaczynam po prostu wyciągać tę rękę.

**A te nowe zasady w sklepach?**

A jakie są nowe? Nie zauważyłem różnicy. Rzadko chodzę do sklepu, bo raz na parę dni, jadam już co niedzielę u rodziców i tyle. Chodzę o 11 wieczorem zawsze po zakupy i tyle.

**1 osoba na 15 m w kościele?**

Nie, w ogóle nie chodzę do kościoła i to mnie nie obchodzi.

**Mogą się przemieszczać bez opieki dzieci powyżej 13 lat?**

Żeby jakiekolwiek dziecko się zaraziło, to jest jeszcze mniejsze prawdopodobieństwo niż jak ten starszy. Odporność jest inna i dzieci nie umierają, tak? Dla mnie to jest w ogóle bezsens.

**Które ograniczenia są wg ciebie sensowne? Maseczki?**

Już nie.

**Ograniczona liczba osób w sklepie?**

Dla mnie to jest bezsens. Przecież ktoś, kto jest zarażony, to te bakterie i wirusy zostają w powietrzu. Przecież to jest...[śmiech]

**Jak myślisz, jaki był cel nakazu noszenia maseczek prawie wszędzie? Co to ma dać ludziom?**

Tylko uspokojenie społeczne i nic więcej. Dobrze wiedzą, że one nic nie dają. Jeśli na 100 osób, dzięki maseczkom, jedna się uchroni, no to ok, to może miało to sens. Nie wiem.

**To co mówiłeś zabrzmiało, jakbyś uważał, że to jest manipulacja nastawiona na efekt psychologiczny?**

Tak, trochę tak.

**Plan luzowania. Co z niego pamiętasz i co o nim myślisz?**

Dla mnie to w ogóle wszystkie ich decyzje są idiotyczne. Oni na szybko to...Jakby coś mieli szyć. Fryzjerzy nie, ale coś tam, coś tam. Albo place zabaw. Co to ma za znaczenie, kurde, czy to jest park, czy to jest plac zabaw? Kto jest na placu zabaw? Przecież to są dzieci.

**A mówisz, że one nie chorują, albo chorują lżej. A koncepcja, że może nie chorują, ale jednak roznoszą?**

Nie wierzę w żadne te koncepcje już. Na prawdę. Przechodzę na poważnie do grupy 1. Mam już też tego wszystkiego dość. Znasz kogoś, kto się zaraził? Bo ja nie.

**A ten pomysł, żeby był nakaz noszenia maseczek do czasu wynalezienia szczepionki?**

A jeżeli nigdy nie wynajdą? Nie, nie ma mowy.

**Bardzo jestem ciekawa twojego podejścia do modelu szwedzkiego. Wiesz, na czym on polega?**

Tak. Sugerowali a nie działali i nie ubili gospodarki. Tam ci ludzie i tak się izolują. Ja tam byłem. To jest inna mentalność niż my i południowcy. W Szwecji to...Za gościnni to oni nie są. Tam jest trochę prohibicja, to jest takie trochę inwigilowane państwo. Ja myślę, że oni na tym wyjdą podobnie jak my, bo tam się ludzie izolują na co dzień.

**Używasz pojęć kwarantanna i izolacja. Jaka jest różnica między nimi? Jest jakaś?**

Kwarantanna jest przymusowa, a izolacja jest nakazem władzy. Dla własnego dobra i dla innych to jest kwarantanna. Izolacja to...Są zbliżone, ale, kurde...Izolacja to, że ludzie chcą się od innych izolować.

**Chodzi o to, że izolacja jest mniej narzucona?**

Tak, ale chyba też można tego używać zamiennie.

**Jak chorowałeś i obawiałeś się, że to może być koronawirus, to byłeś w kwarantannie czy w izolacji?**

Nie wiem. Chyba w izolacji, w samoizolacji.

**Czy model szwedzki mógłby się u nas sprawdzić?**

U nas są inni ludzie. Tam przestrzegają reguł, zasad, a u nas...Polacy to jest tak ciężki naród. Nic by sobie z tego nie robili. Jeśli faktycznie ten wirus jest, jaki jest, to faktycznie na naszą nację to była dobra decyzja, porównując do Szwecji. Tylko, że oni się boją o władzę. Nie o zdrowie, tylko o władzę, żeby im potem nie wypomniano, że przez to, że nie zrobili izolacji, jest tyle zachorowań.

**Jeżeli założymy, że ten wirus jest i jest taki zaraźliwy, to kiedy powinno się luzować ograniczenia i które może zostawić na dłużej?**

Jak najszybciej trzeba wracać z gospodarką. Uważam, że nawet, jak by się w tej chili GH pootwierały, to i tak ludzie będą sami na siebie wpływać, żeby ograniczyć te wizyty, ale ruszy to wszystko powoli. Każdy będzie czuł, że może teraz trochę tu, bo tu się nic nie dzieje, może będzie można to porobić, tamto. Zacznie się ten ruch pieniądza, który został zatrzymany w ogóle. Zostawiłbym zamknięte kościoły. Szkoły podstawowe, żłobki, przedszkola na pewno bym uruchomił.

**Z powodu przekonania, że dzieci nie chorują?**

Tak.

**Siłownie, baseny, kina?**

Z tym może jeszcze nie, jeżeli bierzemy to na poważnie. Tam jest większe takie wydalanie z siebie wszystkiego pod wpływem wysiłku i w zamkniętych pomieszczeniach, ale takie siłownie na powietrzu. które też są otaśmowane. Dzisiaj widziałem. To jest bezsens. Myjnie samochodowe to jest też nieporozumienie. Nie było można i nie wiem, czy dzisiaj można. mam samochód zasrany i nie wiem właśnie. Ty mi powiedz.

**Podobno można. Tak samo, jak można wymieniać opony.**

No tak, bo to służy, jako dojazd do pracy.

**Restauracje, kluby?**

Restauracje, które mają ogródki, to ja bym od razu to puścił. To jest na świeżym powietrzu, w weekend będzie podobno 20 st. Ja bym to odpalił. Jakieś lody na świeżym powietrzu.

**Ograniczenia liczby ludzi w sklepach?**

Ludzie sami się będą bali i nawet jak to będzie otwarte, to nie będzie takiego ruchu.

**Rękawiczki i płyny w sklepach?**

Myślę, że zostaną. Dezynfekuję tym płynem.

**Przestajesz wierzyć w wirusa, ale jednak dezynfekujesz?**

Tak na wszelki wypadek. Maseczki można zostawić, żeby ludzie się czuli bezpieczniej, ale ja bym nie karał, jak ktoś nie ma. Zostawiłbym to tak jak jest, otwarcie bym o tym nie mówił, że nie będzie się za to ścigać. Powiedzieć, że nakazujemy i to wystarczy w tym momencie, ale nie karać ludzi.

**Boisz się, że zostaniesz ukarany za łamanie jakiegoś zakazu?**

Nie, bo mam na wszystko wymówkę.

**Znasz kogoś, kto został ukarany?**

Nie.

**Jaką byś miał wymówkę na zdjęcie maski?**

Obsunęła mi się, nie miałem wolnej ręki, żeby założyć. Ja na ulicy mam ją założoną, nie?

**Co z klubami, koncertami, muzyką?**

Kluby to się na pewno do jesieni nie otworzą. W lato ewentualnie miejsca na wolnym powietrzu będą działały i myślę, że tam będzie obłożenie. Masowe imprezy, powiedzmy, że jeszcze nie.

**Turystyka, hotele?**

**Do tego jeszcze jest daleko, bo myślę, że ludzie się będą bali, ale domek w lesie...**

**A jak teraz jest u ciebie z podejściem do dbania o siebie?**

Byłem na nielegalnym fryzjerze właśnie. U mojej sąsiadki, bo miałem już takie długie włosy, że rano ich w ogóle nie mogłem przyklepać do pracy. Poszedłem do niej - nie pracuje na razie, ma zamknięty zakład, nie zarabia, więc...Zapytała, czy gdzieś nie łażę, nie jeżdżę. Powiedziałem, że chodzę tylko do pracy i nic więcej. Nie była w maseczce i ja też nie musiałem.

**A dbanie o zarost?**

Mam swoją maszynkę i trochę przycinam. 2 tygodnie to w ogóle nic nie robiłem przy sobie. Nie myślałem o fryzjerze i przycinaniu brody, bo byłem chory i leżałem.

**A jak wyszedłeś z choroby?**

No poszedłem do fryzjera i obciąłem się. Podciąłem sobie sam trochę brodę i nic więcej.

**Jak jest z ubieraniem się? Coś się zmieniło?**

Mam dres do domu i mam dres na dwór. Nie przejmuję się wyglądem. Wszyscy wyglądają tak samo w tym momencie. Założone mają maseczki i każdy jest takim no name.

**Zrezygnowałeś z jakichś elementów dbania o siebie?**

No do golibrody bym poszedł takiego profesjonalnego, bo raz na parę miesięcy chodzę. Nie byłem już dawno.

**A kupowanie ubrań. Czy normalnie o tej porze roku szukałbyś sobie czegoś nowego?**

Coś bym dokupił. Teraz nie chodzę do GH, bo są zamknięte.

**Ale jest internet?**

Jakoś przez to wszystko przestałem o tym...Na razie nie przywiązuję do tego wagi. Coś tam zacząłem przeglądać, ale bez jakiejś dużej aktywności. Myślę, że niedługo kupię sobie jakąś bluzę, parę koszulek i to mi wystarczy.

**Czego ci najbardziej brakuje, jako konsumentowi? Fryzjer, golibroda, sklep, siłownia, basen, restauracja, coś innego?**

Myślę, że przez to, że nie mogę się z nikim spotykać + ten może jakiś związek, który się nie wydarzył, więc myślę, że - jestem dorosły, brakuje mi seksu, a będzie o to trudniej może, bo będzie ciężej kogoś poznać teraz. Brakuje mi restauracji, klubów, barów. To jest rzecz, która by przywróciła trochę mój naturalny styl bycia.

**Jaką funkcję te miejsca pełniły w twoim życiu?**

Człowiek jest zwierzęciem stadnym i lubi być w towarzystwie. Może jestem bardziej towarzyski.

**Te imprezy towarzyskie online - nadal się odbywają?**

Z kilkoma jestem cały czas na takiej linii. Wczoraj też gadałem, jakieś piwko wypiliśmy przez telefon. On się wziął za sadzenie. ż nudów posadził sobie warzywa na balkonie, pomalował sobie wczoraj mieszkanie, kupił sobie jakieś Play Station, na którym gra. Też jest sam i też nie chce zwariować. Jesteśmy w tym samym momencie, w identycznym. Też tam coś z kimś chciał, nie wyszło. 2 krople wody jesteśmy.

**Wymieniacie się pomysłami, co robić w tym trudnym czasie?**

Tak, ale jednak trochę mamy na siebie inne pomysły. Obaj muzyką się zajmujemy, ale...

**Jak on sobie radzi?**

Ogląda filmy, też robi streamy i nowością jest to, że posadził sobie warzywa na balkonie i wymalował pokój.

**To jest dziwne zachowanie?**

No sadzenie warzyw, na pewno.

**Mówił czemu to zrobił?**

Wielu ludzi tak teraz robi i nie wiadomo czemu tak robią. Żeby zabić tę nudę, samotność, znaleźć sobie zajęcie, a może warzywa to też jest jakaś natura? Taka jakaś metafora natury. Każdy teraz zajmuje się takimi rzeczami, na które normalnie albo nie miał czasu, albo...Nagle taki człowiek, którego bym nigdy nie posądził, że będzie sadził warzywa...

**A tobie się pojawiło coś takiego, o co byś siebie nie podejrzewał?**

Ta joga była może taka, ale byłem tak słaby, że potem to sobie odpuściłem, a potem tak jakoś może zapomniałem o tym. takiego bardzo nietypowego czegoś to chyba nie.

**Mówisz, że teraz będzie cieplej i ludzie tak czy inaczej zaczną się spotykać? Ty masz już takie plany towarzyskie?**

Ja myślę, że te zakazy już nie będą takie restrykcyjne. Skoro tak wszystko władza odmraża, to już tak nie będą za to ganiać, chyba, że ktoś faktycznie będzie przeginał i zrobi imprezę na 20 osób w ogródku i całe miasto będzie słyszało, jak będą śpiewać 100 lat.

**Jaką imprezę można zrobić?**

Nie obnosić się po prostu.

**Jak ty byś to zrobił?**

U rodziców za domkiem w ogródku na działce, 5 osób.

**Zebrałbyś teraz te 5 osób, żeby przyszły?**

To jest dobre pytanie, bo jeszcze o tym z nimi nie gadałem, a za 3 tyg. mam urodziny.

**No tak, te 40-te?**

Straszna depresja. Chyba zbiorę te 5 osób, które przyjdą. Nie wiem, zobaczymy, co będzie za te 3 tyg., co się wydarzy.

**Jakieś twoje przemyślenia na koniec?**

Niech to wszystko wraca, bo psychologowie będą mieli pełne ręce roboty. U mnie ta cała izolacja podbiła to wszystko, o czym myślałem, że muszę coś ze sobą zrobić. Zauważyłem, że moja pani psycholog przyjmuje teraz tylko online, więc ja zaczekam aż skończy się to szaleństwo i będę mógł nie tylko online. Sesja z psychologiem online, to jak lizanie cukierka przez papierek. Jednak gesty ciała, oczu, rąk. W szczerej rozmowie chyba to jest najważniejsze. Z moimi przyjaciółmi staram się, żeby przynajmniej online były te rozmowy, bo wtedy jest sympatyczniej. Z tobą bym nie dał rady tyle czasu rozmawiać przez telefon.
