# Supplementary material for: The challenges arising from the COVID-19 pandemic and the way people deal with them. A qualitative longitudinal study
Source: PLoS One. 2021 Oct 11;16(10):e0258133. doi: 10.1371/journal.pone.0258133 (PMC8504766; doi:10.1371/journal.pone.0258133)
Supplement: S1 Dataset — (ZIP) [file pone.0258133.s003.zip › Transcriptions/stage 3/2.3_F_27_single.docx]

**2.3_F_27_single**

**Co się u ciebie działo przez ostatnie dwa tygodnie?**

Od Świąt chyba?

**Widziałyśmy się w środę przed świętami.**

W tygodniu pewnie jakoś klasycznie praca - nic specjalnego, na co mogłabym zwrócić uwagę. Potem były Święta. Filip pojechał do domu, ja zostałam. W sobotę nie robiłam zupełnie nic. On poszedł po 13 jakoś chyba, więc ja obejrzałam jakiś film... Już dawno nie spędzałam czasu sama, więc po prostu sobie tak pochodziłam w ciszy. Nie było mi ani przykro, ani nic takiego. Tak właściwie neutralnie. A w niedzielę pojechałam do Magdy - mojej koleżanki. Rano zadzwonił do mnie tata i z nim chwilkę rozmawiałam, poprosił, żebym zadzwoniła do babci, więc do babci też zadzwoniłam. A potem poszłam do dziewczyn i tam siedziałam cały dzień. I w poniedziałek już przyjechał Filip. Więc zleciało jak każdy weekend, poza tym, że nie można było wychodzić.

**Czemu pojechałaś do koleżanek, a do rodziców nie?**

Bo mnie nie zaprosili.

**Bo jak rozmawiałyśmy ostatnio, to mówiłaś, że spędzisz sama, to poleżysz, odpoczniesz...**

Tak. I tak by było, ale dziewczyny napisały, czy nie chcę przyjechać i powiedziałam, że chcę. Tylko tyle.

**A co robiłyście?**

Chyba oglądałyśmy telewizję, rozmawiałyśmy, nic specjalnego. Grałyśmy trochę w jakieś gry. Jakieś takie rzeczy, co się robi w domu, jak jest ktoś więcej niż domownicy.

**Uczciłaś jakoś tą Wielkanoc?**

Nie. W ogóle jej nie uczciłam. I wydaje mi się, że nie chciałabym jej nigdy czcić tak naprawdę. Cieszyłam się z tego, że tak jest. Mogłam robić, co mi się podoba. I nie wiem, może to znaczy już, że będę tak mogła robić do końca życia.

**Że to będzie taki precedens?**

Może tak.

**A myślisz, że jak nie będzie koronawirusa, to uda się tak robić?**

Mam nadzieję, że tak, skoro raz się udało, to troszeczkę się tradycja zerwała i może już nie będzie to tak egzekwowane u mnie w domu.

**To chodzi głównie o to, że rodzice...**

Może nie tyle rodzice, ale jest jakiś taki społeczny obowiązek, żeby spędzać święta z rodziną. Szczerze mówiąc, wątpię, żeby babcia albo rodzice się na mnie obrazili, gdybym nie przyjechała na święta, ale głupio byłoby im powiedzieć o tym albo coś takiego. A tak może to się rozmyje. No nie wiem, nie będę cierpiała, jeśli będę musiała tam pojechać i spędzę miło czas z rodziną na pewno. Ale podobało mi się, że nie musiałam tego robić. Ale też nie musiałam się wstydzić za to, że tego nie robię.

**Czyli to było też wygodne, komfortowe psychicznie, że nie trzeba było się tłumaczyć?**

Tak.

**A jak wrócił Filip w poniedziałek, to co robiliście?**

Nie pamiętam, bo albo robiliśmy coś razem albo właśnie wręcz przeciwnie. Jeden z kolejnych dni był taki, kiedy nic nie robiliśmy razem. Poniedziałek jeszcze spędziliśmy razem, ale wtorek środa to już w samotności, każdy sam. Czasem też musimy od siebie odpocząć. Znaczy właściwie Filip musi odpocząć ode mnie, więc wtedy ja zajmuję się sobą, a on robi to, na co ma ochotę.

**A pracowo jak wyglądały te dwa tygodnie?**

Cały czas jest intensywnie, ale w taki dziwny sposób intensywnie. Nie jest to normalna praca i te ramy czasowe są dość mocno zatarte. To też mi się tak naprawdę bardzo podoba, bo to, że mogę się obudzić o 9... Nawet nie miałam nigdy potrzeby budzenia się później, ale jeżeli mogę się obudzić o 9, to jest to bardzo fajne. I wiem, że będę musiała coś po południu, czy wieczorem robić, ale tak jakoś fajniej. W pracy też dużo osób zaczęło przychodzić do biura, więc się widziałam z większą ilością osób niż wcześniej.

**A to nie jest tak, że mieliście pracować zdalnie?**

Pracujemy zdalnie. Ale to nie jest tak, że ktoś każe komuś pracować z domu i jak dział finansów musi się spotkać, to po prostu musi. Wydaje mi się, że to nie jest do końca kwestia tego, że naprawdę musi, bo nie ma rozwiązań Internetowych, ale raczej kwestia tego, że tam dyrektorem jest taki starszy pan i on może nie ogarniać za bardzo i woli się spotkać z tymi ludźmi niż zrobić to na komputerze. I też pewnie mu się nudzi, a on jest na tyle ważny w firmie, że nikt mu nie może zabronić albo powiedzieć, że nie chce się spotykać.

**A jak ludzie to przyjmują?**

U na w pracy jest tak, że każdy ma dużą świadomość tego, że musi wypełnić swoje obowiązki - nieważne o której, nieważne jak. Też nie ma takich sztywnych, że trzeba pracować od 8 do 16 - każdy pracuje jak mu się podoba. Jak ktoś chce pracować w niedzielę, żeby skończyć jakieś rzeczy, to nie zauważyłam, żeby ktoś miał z tym problem. Często widzę, jak ktoś pracuje w niedzielę i nie wydaje mi się, żeby ktoś był zły - wie, że musi, zostaje to wynagrodzenie. Na tym statusie finansowym nie było jednego chłopaka. Nie wiem, czy nie chciał, czy się boi, czy mu się nie chciało, ale jego faktycznie nie było.

**Kto jeszcze przychodzi do biura?**

Tylko finanse i jedna dziewczyna z działu prawnego, ale to dlatego, że ona teraz się będzie zajmowała *payrollem*, a to jest bardzo odpowiedzialna rzecz i ona musiała przyjść i się podszkolić w tej kwestii. Chłopaki z działu komputerowego przez to, że było dużo zwolnień, muszą przyjść i odbierać sprzęt od ludzi, którzy go odsyłają i sprawdzać. I tyle.

**Jak to było z tymi zwolnieniami? Bo wcześniej mówiłaś, że nie było w zasadzie zwolnień.**

Było. Chyba, że cały czas one dochodzą albo coś powiedziałam nie tak. W każdym razie było dużo zwolnień. Myślę, że ze 12 osób na 80, czy nawet 65 było w Warszawie samej... Nie wiem, może ze 20 tak. Nie wszyscy zostali zwolnieni. Niektórzy nie podpisali aneksu o zmniejszeniu wypłaty.

**Wszystkich ten aneks obowiązuje teraz u was?**

Tak.

**To też jakaś nowość?**

Może tego nie mówiłam, ale jakoś nie wiem, zignorowałam to jakoś. Nie wywarło to na mnie żadnych emocji. Nie wiem, wydaję mi się, że mówiłam. Było coś takiego.

**A jak ludzie na to reagują?**

2 albo 3 osoby nie podpisały. Właśnie ciężko mówić o nastrojach, jak nie widzę ludzi i nie wiem, jak to wygląda. Mogę powiedzieć ci, jeśli chodzi o ludzi z działu prawnego, bo z nimi akurat rozmawiałam, to nic. Coś z tą dziewczyną, która się zajmuje *payrollem* się śmiałyśmy, jak pakowałyśmy rzeczy przed tą przeprowadzką, to ja mówię: trzeba pakować na 100%, a ona, że teraz to na 85%. Czyli tyle, ile dostajemy wypłaty teraz. Coś w stylu takie żarty. Jej wiem, że na pewno jest ciężko, bo jej chłopak stracił pracę i ona zarabia mniej. Ale generalnie nie zauważyłam, żeby ktoś był oburzony. Ja wiem, że jest ciężko, ale to chyba zrozumiałe jest po prostu. Każdy rozumie. Właściwie no można się buntować, ale zupełnie nie ma, co zrobić w tej sprawie.

**Nie ma co zrobić, ale ludzie mogą być smutni albo jakoś to przeżywać.**

Może przeżywają i są może źli, ale ja po prostu o tym nie wiem.

**A ty jak podeszłaś do tego, że ci zmniejszyli pensję?**

Znaczy no... Okej. Było mi smutno, no jasne, wolałabym zarabiać więcej niż mniej. Ale jak jest taka sytuacja, to nie mam co narzekać. Mogłabym też stracić pracę np. To nie jest tak, że nie mam żadnych oszczędności, albo nie mam, jak się utrzymać. To jest tak, że albo nie odłożę sobie pieniędzy albo nie kupię czegoś, albo odmówię sobie jakichś drobiazgów, przyjemności, które kupuję po prostu, bo wejdę do sklepu i coś zauważę, a nie, że będę sobie odejmować od ust.

**Co jeszcze się zmieniło w ciągu ostatnich 2 tygodni?**

Jakoś tak już mi było w sumie ciężko. Chciałam, żeby to się skończyło. Ale nie do końca umiałabym powiedzieć co - czy ta przeprowadzka z tej pracy... W sumie już się skończyła. Ale ciągle to nie jest bycie tam na 100%, bo nie ma tych ludzi jednak. Czy ten koronawirus, żeby się skończył, czy może to, jak się czułam. Nie wiem, ale po prostu chodziłam tak ze 2-3 dni i miałam taką myśl, że chciałabym, żeby to się wszystko skończyło. Ale nie do końca umiałam powiedzieć co tak naprawdę, bo zastanawiałam się, żeby sobie jakoś zaradzić.

**Pamiętasz jaka to była sytuacja, co wtedy innego się zdarzyło?**

Że Filip chciał ze mną mniej spędzać czasu albo właściwie ktoś tam nie chciał ze mną pisać, bo robił coś innego. Nie wydaję mi się, żeby to było coś dużego i ważnego, tylko po prostu, jakoś tak i trochę mi się nudziło i chciałam już porobić coś innego, a nie bardzo mogłam coś innego porobić. Tego typu sytuacja.

**Pojawiły się coś z czego zrezygnowałaś, co ograniczyłaś?**

Nie. W ogóle wydaje mi się, że te 2 tygodnie miałam jakieś takie ciężkie, ale przez to, że nie rozmawiałyśmy w zeszłym tygodniu, to tego aż tak nie pamiętam, dlatego, że mi się po prostu zmieniło. No jakoś tak bardzo ciężko mi było generalnie i wstawać w sumie i funkcjonować. Nie umiałam powiedzieć, czemu, ale że jednak chciałabym coś innego.

**Pamiętam, jak mówiłaś przed świętami, że jest trochę lepiej, bo jest słońce. Teraz jest trochę gorzej?**

Nie, właśnie nie, też jest dobrze. Zwracam uwagę na to, że w zeszłym tygodniu było troszkę gorzej faktycznie, a tym już jest w porządku. Szczerze mówiąc, jak było tam jakieś ogłoszenie premiera czy coś, o tych etapach, to poczułam, że to już się może niedługo kończyć i zrobiło mi się strasznie przykro i nie chciałam i mówiłam: "Filip, to się nie może jeszcze skończyć, przynajmniej miesiąc jeszcze", a on mówił: "Weź, cicho siedź".

**Dlaczego?**

Bo to jest trochę jak życie na wakacjach. Nawet to, że jest praca i trzeba robić różne rzeczy i w dalszym ciągu ponosić odpowiedzialność, ale jednak to jest na tyle luźniej, że można się właśnie wyspać, nie umyć się do tej pracy, podczas pracy wstawić pranie albo coś ugotować albo wyjść do sklepu. Jestem pewna, że w pracy przez dużą ilość czasu robię inne rzeczy niż praca, więc to nie jest tak, że jakoś wyjątkowo oszukuję. Ale po prostu i troszkę mam takie poczucie, że oszukuję i trochę swobodniej się czuję.

**Teraz już możesz pracować zdalnie? Bo wcześniej jeździłaś do biura.**

Teraz jeżdżę rzadziej. Są takie rzeczy, które trzeba zrobić z biura, bo nie mam pieczątki czy jakiś takich rzeczy. Raczej rzadziej. Byłam tylko wczoraj, a dziś jest środa.

**A w zeszłym tygodniu?**

Bardzo mało. 2 razy chyba. Ale w zeszłym tygodniu skończyłam tę przeprowadzkę tak, że teraz przychodzę tam i się bardziej szwendam.

**A co jest dla ciebie teraz największym wyzwaniem?**

Nie, nie ma chyba żadnego wyzwania, żadnego sobie nie postawiłam.

**A jakieś problemy, niefajne myśli?**

Nie. No wiadomo, poszłabym sobie na imprezę. Chętnie bym to zrobiła, ale znowu to nie jest coś takiego, co... Oczywiście, jeśli ktoś by dał taką 24-godzinną przepustkę, że można robić co się chce, to bym pewnie poszła na imprezę. Ale wiem, że nie można, więc... No nie może być dla mnie problemem to, że nie mogę iść na imprezę - no bez przesady. Tak naprawdę możemy sobie zrobić imprezę we 2 i wyjdzie na to samo albo możemy zrobić imprezę z ludźmi z pracy, będąc w tej pracy. Raczej brakuje mi czegoś nowego po prostu i tyle, ale nic konkretnego.

**A jak spędziłaś ten weekend ostatni?**

W ten weekend się bardzo słabo czułam, ale wydaję mi się, że to jest kwestia tego, że jestem przed okresem i ciężko znoszę ten PMS albo sobie wmawiam, że to kwestia tego i pozwalam sobie odpuścić na te parę dni. W każdym razie jest tak, że chodzę i jęczę strasznie. Byłam na paznokciach.

**U tej koleżanki?**

Znaczy, wiesz, to jest koleżanka, ale ona normalnie biznesy normalnie robi te paznokcie. Ma wynajęte mieszkanie. Właśnie z nią rozmawiałam o tym, bo tam są 3 dziewczyny w tym mieszkaniu i tylko ona pracowała, a teraz w niektóre dni przychodzi jeszcze taka jedna.

Gotowałam, zrobiłam dwa różne obiady w sobotę i niedzielę, umyłam okna w niedzielę.

**No to nie powiem, żebyś sobie odpuściła ten weekend.**

Tak, bo wiedziałam, że jak będę leżeć i oglądać albo czytać coś mało pochłaniającego, to będę jeszcze bardziej się nad sobą użalała. A jak coś zacznę robić, to może nie poprawi mi się, ale przynajmniej zapomnę i coś. Więc zleciał mi ten weekend, ale chciałam w sumie zrobić coś innego. Chciałam np. pojechać do Magdy, ale nie powiedziała nic na ten temat. Zaproponowała mi w sobotę, żebym w niedzielę przyjechała, ale się skrzywiłam, bo nie miałam wtedy ochoty. A w niedzielę już nie ponowiła zaproszenia, więc nie proponowałam, ale w sumie chciałam tam pojechać. Więc była trochę rozczarowana. Ale z drugiej strony, wiem, że nie można się komuś wpraszać do domu w tym okresie, bo może ktoś naprawdę się bać.

**Ale byłaś u niej tydzień temu?**

Tak. Byłam też w poniedziałek. Ale wydaję mi się, że wpraszać się nie można wtedy, kiedy jest koronawirus.

**Nie myślałaś o tym, żeby ją do siebie zaprosić?**

Myślałam, ale jej się nie chciało. Spytałam, ale powiedziała, że jej się nie chce.

**Miałaś domowo pracowity weekend?**

Zrobiłam dużo, ale nie dlatego, że musiałam, ale dlatego, że trochę mi się nudziło.

**A masz takie poczucie, że już ci się trochę nudzi?**

Hmm... Bardzo ciężko, nie wiem, nie umiem powiedzieć. Np. mogłoby się tak zdarzyć, że w sobotę poszłabym na imprezę, miałabym kaca i w niedzielę nie mogłabym wstać, źle bym się czuła i to byłoby porównywalne do tej nudy. Tylko teraz dobrze się czułam, więc mogłam umyć okna, a tak, to był nic nie robiła. Wydaję mi się, że nie ma więcej tej nudy. Wydaję mi się, że może być mniej, jeśli chodzi o nasze życie we 2 w domu, dlatego, że staramy się wynajdować sobie jakieś zajęcia, bo wiemy, że nie ma nic do roboty. Więc wydaje mi się, że przez to jest mniej tej nudy u nas w domu, ale jeśli chodzi o inne aspekty, to może być trochę więcej.

**Są takie rzeczy, za którymi tak naprawdę już tęsknisz?**

Nie, bo akurat tak mi się udało, że w ostatnim tygodniu, kiedy można było wyjeżdżać, to byłam na wakacjach. I wiem, że teraz byłoby mi bardzo ciężko, gdybym nie była na tych wakacjach, bo na nich naprawdę wyjątkowo odpoczęłam. To były pierwsze wakacje, z których wróciłam wypoczęta. Nie wyobrażam sobie, jak bym się czuła, gdybym na tych wakacjach nie była. Wiem, że na pewno okropnie, bo pracą byłabym zmęczona, wtedy bym wiedziała, że mniej zarabiam i to by mnie denerwowało. A teraz, po prostu wydałam na te wakacje te pieniądze, co miałam, a nawet mniej niż planowałam, więc nie. Mogłoby mi brakować wakacji, ale byłam, więc nie.

**A czegoś innego ci brakuje?**

Brakuje mi codziennego spotykania z ludźmi z pracy - tego mi naprawdę brakuje. I nie z jakimiś ludźmi z działu finansów, których widywałam czasem i mówiłam cześć, tylko takich, z którymi spędzałam nawet 20 minut dziennie, rozmawiając.

**Brakuje ci pełnego biura, że ci ludzie się przewijali?**

Na przykład. Ale też, żeby z tymi ludźmi porozmawiać na żywo, stać obok nich i coś do nich mówić.

**A teraz nadal bywacie w te same dni z tą koleżanką w biurze, z którą sobie zamawiałyście jedzenie, itd.?**

Ona jest moją szefową i ma tez dużo obowiązków w tym biurze innych niż bycie w tym biurze i dużo ma pracy strasznie. Czasami staram się tam jeździć sama, po to, żeby mówić jej, że ona nie musi, przez to, żeby robiła inne rzeczy, które musi robić. Bo naprawdę ma dużo pracy, dużo więcej niż ja. W zeszłym tygodniu starałam się tak, żeby w ogóle nie była i żebym tylko ja była. Bo tylko my we 2 mamy karty. Jej nie było w zeszłym tygodniu. W tym była, ale tylko dlatego, że nie mogła się skupić w domu.

**A macie tak, że są też inne osoby, które wpadają do biura?**

Finanse wpadają, więc trzeba im zostawić kartę albo być wtedy, żeby ich wpuścić. I nikt inny.

**Czyli nie jest tak, że jest dowolność w pracy zdalnej/niezdalnej?**

Kwestia tego, że oni nie wiedzą, gdzie jest to nowe biuro. Znają adres, ale nie byli tam jeszcze, nie wiedzą, gdzie siedzą, nie mają swoich miejsc. Więc jakby to jest chyba troszkę inna sytuacja. Gdybyśmy byli w starym biurze, to częściej by ktoś przychodził, mówił, że czegoś zapomniał czy coś. A teraz nie ma takiej możliwości. Więc po prostu oni nie przychodzą.

**Macie jakiś plan w firmie, kiedy będziecie fizycznie wracać do biura?**

Nic nie wiem o tym. Mogłoby coś być i może nawet pani Magda wiedzieć, ale nie pytałam jej. Bo w sumie nawet nie chcę wiedzieć. Ja bym chciała, żeby jak najdłużej.

**Jakie są największe plusy tych wakacji, które teraz odczuwasz?**

To, że mogę wstawać bez budzika. Znaczy pewnie, jakby spała jakoś długo to nie, ale na szczęście wstaję tak, że budzę się przed budzikiem 10 minut, więc to jest idealnie. To, że nie muszę się ubierać. Ani właściwie myć włosów tak cała. Kąpać się też nie muszę. Mogę skończyć też robienie tej pracy wcześniej. Chociaż nie dzieje się to często, ale nie mogłabym... Wydaje mi się, że bym mogła, ale byłoby mi głupio, nie wypadałoby, żeby wychodzić wcześniej codziennie 20 minut z pracy. A teraz mogłabym to robić. I też dużym plusem jest to, że nie zabiera dnia - mimo, że nie mam i tak, co robić, na dojazdy do pracy.

**A jakie są minusy takich "wakacji"?**

Chyba to, że nie ma takich ludzi, z którymi można rozmawiać nie o pracy.

**Obrazki. Który z obrazków oddaje twoje emocje w tej chwili i te emocje sprzed tygodnia, kiedy miałaś spadek?**

W zeszłym tygodniu może 1. A w tym tygodniu może 3.

**1**

Taki zastój mojego życia, nie podobało mi się nic. Chciałam, żeby już wszystko ruszyło - nie ruszyło. Takie czekanie w sumie na coś. Nuda. Rozgoryczenie. Irytacja.

**Dlaczego w takim razie zmienił się ten nastrój, skoro mówisz, że nic się nie ruszyło?**

Nie wiem. Wydaję mi się, że po prostu zaczęliśmy z Filipem więcej czasu ze sobą spędzać albo... Nie wiem. Albo z innymi osobami rozmawiałam niż rozmawiałam dotychczas.

**Masz poczucie, że ten zastój był spowodowany tym, że musieliście z Filipem trochę od siebie odpocząć?**

Możliwe. Zdarza mu się to i mi się za bardzo to nie podoba, bo to nie jest mój wybór, a Filipa. I trochę mi się to nie podoba. A potem jest tak, że już się z tym godzę i jest ok, a potem zaczynamy znów spędzać czas razem.

**A 3?**

Wymyśliłam sobie coś, ale jak patrzę na to, to nie wygląda tak, jak myślałam. Bo ja na początku myślałam, że to taka gra w klasy. I dlatego powiedziałam, że ta gra w klasy, ta 8, jako taki kolejny etap tego, przez co przechodzimy. Widać tam w rogu, że to jest koniec, ale ja tego nie widziałam i myślałam, że jakiś numer z kolei.

**Czyli to jest jakiś numer z kolei i już jesteśmy na kolejnym etapie?**

Tak.

**A jak myślisz, jaki etap przed nami?**

No właśnie nie mogę myśleć, bo tutaj nie ma na tym obrazku.

**A jakiego etapu byś chciała?**

Dziwnie pewnie to słyszeć, ale nie wiem, taki sam mógłby być co najmniej przez miesiąc. Ja się całkiem dobrze w tym odnajduję.

**Jesteś pierwszą osobą, która mówi, że chciałaby, żeby jeszcze to trwało.**

Naprawdę nie żyje się tak źle. Można powiedzieć, że są kolejki do sklepów. Ale nie - nie ma. Jakaś jest., faktycznie, ale te zakupy się robi w bardziej komfortowy sposób. Czy czegoś w sklepie nie ma? Nie, wszystko jest. Dodatkowo są jeszcze jakieś promocje cały czas, można kupić coś taniej. Ja wiem, że dlatego, że ludziom upadają biznesy i oni starają się jakoś zachęcić klientów - zdaję sobie z tego sprawę. Ale z mojego punktu widzenia to są cały czas jakieś promocje, darmowa dostawa, różne rzeczy zachęcające do kupowania. Nie kupuję tego, ale czuję, że można coś fajnego kupić sobie. Zdaję sobie sprawę z kryzysu ekonomicznego i tego wszystkiego, co się dzieje złego. Ale jest to naprawdę wygodne życie. Nie trzeba świętować urodzin ludzi, których się nie chce świętować. Nie trzeba chodzić na imprezy, nie trzeba znajdować wymówek, tylko się po prostu na nich nie jest. Po prostu ich nie ma. Ale jeszcze mam takie przemyślenie - to właśnie z Filipem rozmawialiśmy, że ludzie mają jakieś takie wrażenie, że o każdej porze dnia i nocy - no nocy może nie, ale po pracy, tak 17-23, jeżeli napiszą: *ej zdzwonimy się?,*to liczą, że ja albo Filip się z nimi zdzwonimy. Nie, przecież to jest porównywalne do jakiegoś wyjścia. Nikt o zdrowych zmysłach z moich znajomych w środę o 22 by nie pytał czy gdzieś z nimi wyjdę. Więc nie wiem, skąd pomysł, że o 22 bym chciała się z nimi zdzwaniać i rozmawiać, jeżeli bym byli w pracy to nikt by nie myślał o tym, że bym z nimi rozmawiała. I jest takie - ale przecież i tak nic nie robisz. No, ale właśnie coś robię. Jakbym nie robiła, to może faktycznie, ale właśnie coś robię.

**Masz wrażenie, że ludziom się nudzi i łakną kontaktu? I mają wrażenie, że wszyscy mają tyle czasu wolnego?**

Tak, wydaje mi się, że tak. I tak zakładają, że ja na pewno mogę z nimi rozmawiać, bo nie mam nic innego do roboty. A prawda jest taka, że my naprawdę bardzo ładnie sobie żyjemy z Filipem, bardzo dobrze sobie organizujemy czas we 2, nawet jeżeli to jest oglądanie telewizji, to oglądamy od początku do końca razem. Więc nie mam potrzeby tego przerywać tylko po to, żeby z kimś porozmawiać o tym, o czym bym mogła porozmawiać z Filipem. Wydaje mi się, że jesteśmy bardzo samowystarczalni, jeśli chodzi o tą kwarantannę. Wiadomo, fajnie jest porozmawiać z innymi ludźmi, ale nie potrzebuję się z nimi zdzwaniać, wtedy, kiedy oni tego potrzebują. Filip jeszcze z nikim nie rozmawiał. Mi się zdarzało, nie, że byłam pomysłodawcą tego. A Filip raczej odrzucał te zaproszenia - mówił, że mu się nie chce, że nie może.

**Czyli on unika kontaktów towarzyskich poza tymi z tobą?**

Tak, wydaje mi się, że tak.

**A masz poczucie, że jesteście trochę zależni od siebie, jeśli chodzi o wspieranie się? Że samemu byłoby dużo ciężej?**

Filipowi nie. Mi na pewno.

**Myślisz, że dlatego, że on. jest taki samowystarczalny?**

Wydaje mi się, że nie jest, ale ma takie momenty, kiedy bardzo potrzebuje być sam. I tak ja już mu to bardzo zmieniam i bardzo zmieniam jego formę spędzania czasu samemu. Bo nawet, jak siedzi sam i ja mam ochotę posiedzieć z nim, to mówię, że będę cicho i robię coś na telefonie albo czytam, albo robię coś. A on robi zupełnie coś innego. I nie rozmawiamy, ale po prostu tam siedzę. I on też mi już na to przyzwala. Ignoruje moją obecność i robi to, co by robił, jakby był sam.

**Jak byś nazwała swoje emocje w tym momencie?**

Nie wiem, ale jeszcze muszę uzupełnić. Wiem, że w ogóle nie odpowiem na twoje pytanie. Ale to nie jest tak do końca, że Filip nie chce ze mną spędzać czasu. Tylko to jest tak, że albo on mówi, że nie chce ze mną spędzać czasu albo powie coś, co mnie tak zdenerwuje, że się obrażam, bo ja się bardzo też obrażam. I ja potrafię do niego przez tydzień nic nie mówić. Albo mówić tylko te rzeczy, które muszę. Ja wiem, że to jest okropne, ale czuję, że jeszcze nie. Jak mi odpuści, to po prostu sama przyjdę i już będzie wszystko w porządku, lepiej niż gdyby chodził za mną. Możliwe, że w zeszłym tygodniu się za coś obraziłam. Możliwe, że za to, że nie chciał ze mną spędzać czasu. Tak to mniej więcej wygląda, że on nie chce, ja się obrażam i ja nie chcę i później musi to wracać do normy.

**A wracając do tych emocji...**

Nie wiem, ja w ogóle zapominam, że jest ten koronawirus. Jak na początku zapominałam, że to jest choroba, tak teraz zapominam, że coś takiego jest, jakiegoś innego niż normalne.

**Odczuwasz spokój?**

Tak, jak najbardziej. Spokój to jest coś mam wrażenie bardziej wyjątkowego niż normalny stan rzeczy. A obojętność to jest jakaś ignorancja, więc tego też nie czuję. Czuję się po prostu całkowicie normalnie.

**Na pierwszym spotkaniu mówiłaś o tym, że czujesz, jakby to był odrealniony świat. Na ile masz dalej takie poczucie?**

Miałam tak na początku, jak wszyscy zaczęli chodzić w maseczkach, ale przed tym, jak trzeba było chodzić w maseczkach. Tak tuż przed tym, jak była taka akcja "nie czekam do czwartku" i ludzie chodzili w maseczkach wcześniej. W sensie, ja też chodziłam. I to było dla mnie takie trochę dziwne, że ci wszyscy ludzie chodzą w maseczkach. I rozmawiałam Magdą w sobotę i Magda powiedziała, że dla niej to jest dziwne, a ja powiedziałam, że już to miałam, że już to przechodziłam. I nie dziwi już mnie to, wygląda całkowicie normalnie.

**Nadal Nowy Świat jest tak pusty?**

Nie, nie jest.

**Co się zaczęło pojawiać? Ludzie, kwiaciarki?**

Kwiaciarek chyba nie ma, ale ludzie są. Też nie przechodziłam tamtędy dawno w sumie, ale jak przejeżdżałam dzisiaj, to widziałam, że byli. Jakoś tak generalnie jest dużo ludzi na ulicy.

**I to powoduje, że mniej czujesz się jak w grze?**

Tak.

**Co jeszcze zmniejsza to poczucie odrealnienia?**

Takie przywyknięcie wydaje mi się. Bo nie wiem, trzeba umyć ręce, jak się wchodzi do sklepu, faktycznie ciągle zapominam to robić i pani mi raz zwróciła uwagę, raz Filip. A raz, zanim założyłam rękawiczki, to już byłam przy kasie. W sensie wzięłam już przez rękawiczkę tę rzecz, którą kupowałam, ale nie zdążyłam jej założyć. To mnie troszeczkę, spowalnia mnie ten koronawirus. Co jeszcze? Może też pogoda, w sensie to, że świeci słońce i jest mniej szaro. Na początku była jeszcze taka szara pogoda, a teraz dużo jest słoneczniej. Przede wszystkim to, że jest dużo więcej osób i jest troszeczkę głośniej. Albo nie jest już tak cicho albo po prostu cisza to jest też rzecz, do której już przywykłam. Tego nie jest pewna.

**A masz jakieś obserwacje, czy u ludzi się pojawiają nowe emocje, nowe zachowania?**

Chyba takie bardziej ryzykowne. W sensie, ludzie teraz, np. te finanse spotykają się w biurze, czego nie robili wcześniej. Może ze względu na to, że to biuro nie było gotowe, a może na to, że teraz już się trochę uspokoiło. Ale właśnie nie wiem, co się uspokoiło. Bo czy ten wirus się uspokaja? No niekoniecznie. A jednak wśród ludzi panuje większy - nawet nie tyle spokój, co pewność siebie może.

**A oni jak się spotykają, to mają maseczki, rękawiczki czy już tak normalnie?**

Nie, normalnie. Wszyscy ludzie w pracy nie mają rękawiczek, jak są w biurze. I maseczek też nie. Ale przychodzą w rękawiczkach i maseczkach.

**Co sądzisz o tym, że teraz trzeba chodzić w maseczkach?**

Nie wiem, dlatego, że... Czytałam, ale właściwie, jak to wygląda, jeśli chodzi o czytanie i moją osobę - że czytam nagłówki więcej niż artykuły. Generalnie, słyszałam coś takiego, że nie powinno się chodzić w maseczce. A raczej nie tyle, że nie powinno, tylko ta maseczka starcza na krótko, więc trzeba ją prać i dezynfekować. Wydaje mi się, że ludzie tego nie robią. Wydaje mi się, że chodzi w maseczce jednorazowej więcej niż raz. Więc, czy to ma jakiś sens? Nie do końca wiem. Ale może to też wzbudzać strach np. Co uważam, że w tej sytuacji jest dobre. I odwołanie tych festiwali wszystkich muzycznych w wakacje, to jest generalnie dobra decyzja. Otwarcie lasów - nikt nie chciał, żeby lasy były zamknięte, nikt nie chce, żeby były otwarte. To jest w ogóle jakiś absurd.

**Absurd to jest zamknięcie czy otwarcie?**

Jedno i drugie. To jest jakaś rzecz, którą zrobili i potem się chwalą tym, że wszyscy się cieszą z powodu otwarcia tych lasów. Nie wiem. Wydaje mi się, że ludzi to ani grzeje, ani ziębi.

**Wracając do maseczek, ty wierzysz, że ta maseczka ma szanse zapobiegać zachorowaniom?**

Na pewno zapobiega pod tym względem, że mniej się dotyka mimo wszystko brudnymi rękami ust, twarzy i oczu. W moim przypadku to bardzo działa, bo dotykam dużo swojej twarzy. A jak mam i rękawiczki, i maseczkę, to robię to zdecydowanie mniej. Więc pod tym względem na pewno.

**A jak chodzisz po ulicy, to idziesz w rękawiczkach?**

Tak. Bo ja oprócz tego, że dotykam twarzy, to dotykam też wszystkiego innego, co widzę na swojej drodze.

**Z maseczkami mówisz, że ok. Z lasami właściwie wszystko jedno...**

Tak, bo ja jestem takiej sytuacji, że nie mam blisko lasu, ani nie mam zwierząt, ani dzieci, z którymi miałabym potrzebę pójścia tam. I nie robiłam tego przed koronawirusem, nie chodziłam do lasu na spacer, więc nie będę udawać, że na tym mi zależy.

**A jak obserwujesz, to ludzie przestrzegają nakazu noszenia maseczek?**

Tak.

**A te nowe zasady w sklepach?**

Że teraz więcej osób może wejść.

**Byłaś w sklepie od tej zmiany?**

Tak. Byliśmy przedwczoraj w Kauflandzie - w sklepie, w którym jeszcze nie byliśmy nigdy w życiu. Było super. No właśnie to jest też fajna sytuacja, bo można robić takie zakupy, których nigdy nie robiliśmy. Wczoraj tak otworzyliśmy tą lodówkę i pomyśleliśmy - boże, dlaczego my mamy tak dużo jedzenia? I teraz właśnie przez koronawirusa mamy coś takiego, że może nawet nie planujemy, ale zawsze musimy mieć dużo jedzenia w domu. Nie wiem, dlaczego. Ale pójście do takiego sklepu to jest bardzo fajna forma spędzania czasu.

**A czemu wybraliście Kaufland?**

Ja byłam w Kauflandzie z dziewczynami w zeszłym tygodniu - a nie, nie w zeszłym tygodniu. Ale muszę ci powiedzieć, że jak byłam dwa razy w sklepie, to jeszcze od tamtej pory nie byłam ani razu, dopiero w Kauflandzie w poniedziałek. I w tym Kauflandzie nie byliśmy nigdy w całym naszym życiu. W żadnym Kauflandzie. Ja nie lubię chodzić już do jednego sklepu, jaki mamy blisko, czyli do Biedronki, bo nie podoba mi się tam. Mamy też Carrefour w Domach Centrum i chciałam do niego iść i sama bym pewnie poszła, ale zobaczyłam natężenie ruchu w Internecie i zobaczyłam, że jest cały czas na czerwono do 22. Ale i tak bym tam pewnie poszła, gdyby nie to, że Filip chciał iść ze mną i stwierdziliśmy, że skoro we dwójkę, to może do tego Kauflanda. A to jest oblegany Kaufland, bo to ten na Stalowej obok Tesco.

**To kawał drogi?**

Nie, bo metrem 5 stacji.

**Co kupiliście? Czemu ci się tak podobało w tym Kauflandzie?**

Bo moim wspomnieniem z dzieciństwa jest to, jak jeździliśmy raz na jakiś czas - nie wiem, jak często, ale myślę, że dosyć rzadko, bo pamiętam, że to były jakieś ogromne rachunki 600-700 zł, a jak byliśmy we 3, to nie wydaje mi się, żeby to było na tydzień. Więc rzadko. Ale jeździliśmy i uwielbiałam chodzić po - wtedy to było Auchan na Modlińskiej, to jest super sklep, bardzo mi się tam podobało. I przez długi czas marzyłam, jak byłam dzieckiem, żeby mieszkać w supermarkecie. Wydawało mi się tam super. Może dlatego tak mi się podobało. Nie wiem, duży był ten sklep, można sobie pochodzić, na wszystko popatrzeć, wszystko jest. Co kupiliśmy? Na pewno kupiliśmy rzeczy do *Mac and Cheese*, bo będziemy to robić dzisiaj. Jakieś rzeczy na śniadanie typu twarożek albo serek wiejski - takie do kanapek. Na pewno picie. Filip kupił wino. Warzywa jakieś, ale były bardzo średnie, więc tylko to, co było. I rzeczy do sprzątania, bo akurat kończy się papier toaletowy.

**A ile czasu spędzacie w takim Kauflandzie?**

Już ci mówię, bo sprawdziliśmy. Od wyjścia z domu do spojrzenia na zegarek pod sklepem minęło równe 2 godziny. Skoro z 15 minut jechaliśmy, no to...

**To trochę żeście tam pochodzili.**

No tak, to jest ogromny sklep. Ale naprawdę, byliśmy w każdej alejce.

**Ale poczekaj, bo pojechaliście po kilka rzeczy...**

Nie, pojechaliśmy po zakupy, ale nie robimy listy. Może w głowie mamy jakieś rzeczy, które trzeba kupić, ale generalnie, to chodzimy po sklepie i patrzymy, które z tych rzeczy może potrzebujemy.

**I teraz taką samą metodą, chodzenia po alejkach?**

Tak. Ale było ciężko w tej maseczce wytrzymać te 2 godziny. A w ogóle, tego jeszcze nie powiedziałam, zrobiłam nam maseczki ze starej sukienki bawełnianej. Nie potrzebowaliśmy maseczek, mamy maseczki, ale zobaczyłam taki fajny wzór - mam to nawet przed oczami, taki wzór, co trzeba wyciąć z materiału, żeby zrobić fajną maseczkę. Filip go przerobił na trochę większy, a ja zrobiłam wykrój, zrobiłam nam maseczki i byliśmy w tych maseczkach.

**I ciężko się chodzi w tej maseczce?**

Jak wróciliśmy do domu, to już bardzo ciężko mi było oddychać.

**Masz wrażenie, że już rozluźniło się w tych sklepach po tej zmianie?**

Tak, wydaje mi się, że tak. Ale ten Kaufland jest ogromny, jest dużo kas, dużo osób może wejść, więc nie wiem. Nie wpadałam na żadnych ludzi. Nie przeszkadzali mi na pewno.

**A jakaś jeszcze zmiana tych ograniczeń?**

Wydaje mi się, że od poniedziałku dzieje się więcej, jest więcej wszystkiego - ludzi, otwierają się powoli różne miejsca, które nie były otwarte. Może trochę za szybko. I nie mówię tego dlatego, że mam nadzieję, że to będzie trwało jak najdłużej, tylko z obawy generalnie.

**A jak myślisz, co wpłynęło na to, że jest więcej ludzi?**

Wydaje mi się, że taka pewność siebie, że skoro widać koniec... Mi też się wydawało, jak przeczytałam o tych 4 etapach wracania do normalności, to wydawało mi się, że te etapy nastąpią dzień po dniu. Zdawałam sobie sprawę z tego, że to są miesiące, ale jak to czytałam, to było dla mnie takie, że już widać koniec tak naprawdę. Dopiero, jak sobie usiądę i pomyślę, to faktycznie, wcale nie. Ale to jest taka świadomość moim zdaniem, że już tak widać koniec, że można powoli wracać do normalności. Z resztą, te etapy są wracaniem do normalności, więc dla tych ludzi może ty być wychodzenie z domu jako normalność.

**Teraz też się można w celach rekreacyjnych. Może to też wpłynęło?**

Może tak. Jest tych ludzi więcej na ulicach mam wrażenie.

**A to, że znieśli ten zakaz dla ludzi poniżej 18 r.ż, żeby byli sami na ulicy?**

Nie myślałam o tym. Jak teraz się pytasz...

**Ale to dobrze czy źle? Pierwsze skojarzenie.**

Nie wiem. Żadna różnica. Mogłyby te dzieci siedzieć w domu - co mają robić. Taką mam prostą odpowiedź. Osoby od 13 do 18 r.ż. to są takie osoby, które za bardzo nic nie muszą robić. Nie muszą być na dworze. Dla przyjemności raczej, ale to nie jest pilna potrzeba. A właściwie raczej taka niepotrzebna potrzeba, czyli chodzenie, szwendanie się, nastoletnie formy spędzania czasu. Ja też coś takiego robiłam, że wychodziłam i gdzieś chodziłam ze znajomymi. A czy jest to potrzebne im? Może dla rozwoju w sumie tak. Ale nie wiem, wydaje mi się, że mogą mieć jakieś głupie pomysły i robić coś głupiego. Nie wiem, co w tej sytuacji może być głupie, ale coś w stylu kasłanie na starszych ludzi czy coś takiego. Zdarzają się takie pomysły, jakieś filmiki w Internecie, więc wydaje mi się, że coś takiego może się dziać.

**Mówiłaś, że słuchałaś tego planu...**

Czytałam tylko, nie słuchałam. Przestałam się tak interesować jak wcześniej.

**A co z tego planu najbardziej zapamiętałaś?**

Nic nie pamiętałam, bo się martwiłam o to, że zaraz trzeba będzie wrócić do pracy.

**Ile jest tych etapów?**

4. I wiem, że w 3 etapie na pewno wracają paznokcie, solarium, fryzjerzy i takie rzeczy. Ale wiem to dlatego, że byłam na paznokciach i właśnie Kasia mówiła, że ta jedna dziewczyna, co z nimi pracuje, się boi, więc ona wróci w 3 etapie przynajmniej. Więc to wiem, bo mi o tym mówiła. Skoro jesteśmy w pierwszym, to musi też być jakiś drugi, ale nie wiem co tam jest.

**A wiesz tak cokolwiek? Otwarcie sklepów budowlanych w weekendy, otwarcie hoteli i niektórych instytucji kultury, jak biblioteki, muzea, galerie sztuki.**

Okej. No to właśnie nie wybierałam się do żadnego z tych miejsc.

**A w trzecim zapamiętałaś, ale to od koleżanki wiesz, że te paznokcie?**

Tak. A w czwartym to chyba jakieś imprezy do 50 osób.

**Już w trzecim.**

Acha, to czwartym nie mam pojęcia, co jest.

**W trzecim jest jeszcze otwarcie sklepów w galeriach handlowych.**

A no, bo Filip się zastanawiał, kiedy te galerie handlowe będą otwarte.

**A jak myślisz, co się musi zadziać, żeby kolejne etapy się pojawiały?**

Nie wiem, zupełnie nie wiem. Też się nad tym zastanawiałam, jak to będzie w ogóle. Ja nawet teraz nie wiem, ile osób jest chorych, a wydaje mi się, że to według tego się powinno mierzyć. Albo nawet może nie tyle, ile osób jest chorych, ale stosunek chorych do wyleczonych albo coś takiego. Ale nie wiem. Wydaje mi się, że przez to, że to tyle już trwa, to jak mówiłam, że na początku się interesowałam i ile jest osób chorych, tak teraz te osoby się dla mnie stały bardziej anonimowe i nawet nie wiem, ile osób jest chorych.

**A masz pojęcie, czy teraz dziennie przybywa więcej niż mniej?**

Nie mam pojęcia. Moim zdaniem więcej.

**Powiedziałaś, że trzeba liczyć te osoby, które są wyleczone, a nie wyzdrowiały.**

Wydaje mi się, że się nie zdrowieje samemu na koronawirusa. Nie wiem. Mam o tym małą wiedzę i jak kiedyś się tym szczerze interesowałam, że wiedziałam, co się dzieje, to teraz już nie za bardzo wiem. Wydaje mi się, że jakoś to trzeba leczyć, bo jakby się samo zdrowiało, to by się siedziało w domu, a ludzie są w szpitalach. Więc coś tam, jakieś leki się podaje.

**Masz poczucie, ile osób, które mają koronawirusa, nie ląduje w szpitalu, czy nie do końca się orientujesz?**

Jestem świadoma, że to jest bardzo dużo osób, ale nie wiem, ile. Nie no, ale przecież nikt nie wie, ile to jest osób. Na pewno bardzo dużo. Z resztą w ogóle - to nie jest moja teoria, ja ją usłyszałam i mogę się z nią zgodzić, że bardzo możliwe, że większość z nas już miała koronawirusa, np. się słabo czuło przez weekend, przez parę dni, tylko przeszło to jako przeziębienie albo bezobjawowo. Bo chyba 80%... A no właśnie, to jednak wiem - 80% przechodzi bezobjawowo.

**Pamiętasz, gdzie słyszałaś tą teorię?**

Przeczytałam na Instagramie.

**Co dla ciebie powinno być kamieniami milowymi w znoszeniu ograniczeń? Np. otwieramy fryzjerów w momencie, gdy...**

Nie wiem, właśnie nie wiem. Dla mnie, to w ogóle, kiedy by nikt nie był chory, to powinno się otworzyć, ale wiem, że jest tyle milionów osób w Polsce, że jest to niemożliwe, żeby... No nie wiem, nie wiem. Cały czas choruje dużo osób, wydaje mi się, że więcej niż mniej, więc nie powinno się otwierać tych fryzjerów. Ale z drugiej strony słyszałam, że bardzo prężnie działa podziemie usługowe, *beauty*. Ja chodzę na paznokcie, więc wiem, że działa i na pewno fryzjerzy też pracują. Tylko, że w domach. I to też właśnie trochę jest niebezpieczne, bo salony nie mogą być otwarte, więc robią to w domach. I w domu jest też łatwiej się zarazić, mniejsze są te mieszkania. ten pył z tych paznokci... Wiem, że muszą to robić, ale wiem, że jest to trochę bardziej niebezpieczne niż gdyby to było w salonie.

**A ty czemu chodzisz?**

Bo ona robi w mieszkaniu, które jest salonem. To nie jest jej mieszkanie, tylko mieszkanie, w którym zawsze robi paznokcie. Może nie jest salon, nie ma przeszklonych drzwi, tylko jest to mieszkanie w bloku, więc nikt nie wie, że tam są robione paznokcie. To jest właścicielki, legalnie zarejestrowane, itd.

**Nie masz obawy, jak tam chodzisz?**

Zawsze tam robią 3 dziewczyny paznokcie, a teraz tylko ta jedna. Ma jedną asystentkę. My tam byłyśmy we 3, a później przyszła Magda, więc we 4. Z Magdą spędzam czas w pracy i po pracy, więc... A one spędzają cały czas razem. Więc jakieś ryzyko, ale... Ta Kasia, która robiła paznokcie, siedziała w masce cały czas.

**Ale jednak oficjalnie nie wolno robić tych paznokci.**

No tak, tak.

**A kiedy powinni znieść zakaz noszenia maseczek?**

Jeżeli te maseczki będą noszone w poprawny sposób, prane, itd., a jednorazowe jako jednorazowe, to nie wiem, kiedy powinny być, ale wydaje mi się, że zostaną zniesione za długi czas dopiero.

**A długi to znaczy ile - rok, półtora, dwa?**

Nie wiem. Rok, półtora, dwa to są dla mnie abstrakcyjne okresy czasu. Może i tak, ale to jest bardzo długo.

**Co miałaś na myśli, mówiąc, że dług czas?**

Nie wiedziałam. Wiedziałam, że długo, ale rok, to się wydaje strasznie długo. Ale z drugiej strony jest już ponad miesiąc.

**Teraz mówią, że mniej więcej półtora roku.**

To to jest bardzo długo dla mnie,

**Myślisz, że to ma sens, noszenie masek przez półtora roku?**

Nie wiem, na ile działają te maseczki. Ale generalnie bycie ostrożnym przez półtora roku, tak ostrożnym jak teraz, to uważam, że jest w porządku. Ale nie wiem, nie umiem powiedzieć, ile powinniśmy nosić te maseczki.

**A myślisz, że powinniśmy czekać tak długo aż będzie szczepionka?**

Nie wiem, czy jest gwarancja, że będzie szczepionka.

**Pracują nad tym. Gwarancji to chyba nigdy nie ma.**

No właśnie... Nie możemy czekać aż tak długo, jak nie mamy gwarancji, że to się stanie kiedykolwiek. Pewnie się stanie, ale nie wiemy, kiedy. Może... No nie wiem, bardzo dziwne to jest dla mnie w ogóle. Właśnie takie rzeczy, jak mówisz, to jest jak gra komputerowa. Bo jak się żyje w tej sytuacji, jak się chodzi w tych maseczkach, to się o tym nie myśli. Ale jak mówisz o tym, że jeszcze przez półtora roku będziemy chodzili w maseczkach, to to już jest jakaś dziwna sytuacja.

**A słyszałaś, jak Szwecja podeszła do tej sytuacji?**

Nie.

**Przytoczenie opisu rozwiązań w Szwecji. Co ty na to?**

Są jakieś wyniki. Mają jakieś wyniki w koronawirusie?

**Nie wiem. Co sądzisz o takim podejściu?**

Wydaje mi się, że w Polsce też na początku mieliśmy takie podejście, ale potem ten koronawirus nie zwalniał i... Nie wiem, tak naprawdę w Polsce akurat koronawirus jest świetną okazją dla rządu do robienia tego, co im się podoba. Więc nie wiem, czy Polska jest dobrym przykładem, żeby pokazywać, jak działa nasza ochrona przed koronawirusem. Ale wydaje mi się, że w innych krajach, np. we Włoszech, tak szybko nie wprowadzili tych restrykcji, jak w Polsce i tam dużo więcej osób jest chorych. Ciekawe, ile w Szwecji jest, ale nie wydaje mi się, żeby było dużo, bo bym słyszała o tym.

**Ile jest, to nie wiem, ale przeczytałam wypowiedź jakiegoś wirusologa Szwedzkiego, że oni przeszli ten *peak* zarażeń i szacują, że 60% Sztokholmu już przeszło.**

Ale w ogóle te skandynawskie kraje działają inaczej niż inni i oni są tacy... Nie wiem, jacy, ale może bardziej tacy zgrani albo zdyscyplinowani. Jakoś tak wynajdują rozwiązanie, bo jestem pewna, że ludzie w Polsce by oszukiwali. Z resztą oszukiwali, bo wiem o takiej sytuacji, nie jakiejś głośnej, że ktoś miał koronawirusa czy podejrzenie korona wirusa, ale oszukiwał i chodził do pracy. Albo takie przeświadczenie, że mnie to nie dotyczy, więc - to ja jestem przykładem, że cały czas tak myślę, że mnie to nie dotyczy, ja nie będę chora, więc będę sobie normalnie chodził i normalnie żył. Wydaje mi się, że w Polsce by coś takiego nie bardzo przeszło, bo Polacy lubią oszukiwać w różnych kwestiach.

**Myślisz, że dobre jest na poziomie społecznym, że to jest rekomendacja, żeby coś robić, a nie zakazy?**

Mówiłam ci już, że moim zdaniem dobrze działa takie straszenie ludzi. Na przykładzie Polski. A na przykładzie Szwecji, to może coś takiego już ich przestraszy albo może nie przestraszy, ale posłuchają się tej rekomendacji jako dobrej rady.

**I to wynika z tego, jacy są Szwedzi?**

Wydaje mi się, że tak. Nie mogę się dużo wypowiedzieć o Szwedach. Może są narody, w których by to zadziałało, ale w Polsce to by nie zadziałało.

**Polaków trzeba bardziej zakazami, nie rekomendacjami?**

Tak mi się wydaje.

**Czy dbanie o siebie się u ciebie jakoś zmieniło od sytuacji z koronawirusem?**

Nie, nie, powtórzę to setny raz - nie zmieniło się w moim życiu nic, oprócz tego, że mogę później wstawać. Nie zmieniło mi się to, mam ochotę iść do fryzjera, ale tylko dlatego, że chodzi do fryzjera raz na rok i po pół roku jest coś takiego, że te włosy są za krótki i za długie w tym samym czasie. Pewnie, gdyby można było chodzić do fryzjera, to nawet bym nie poszła, ale przez to, że nie można, to mam ochotę prosić ludzi, żeby mi obcięli włosy. Filip wie, że tego nie zrobi, bo nie umie tego robić i wie, jaka będzie awantura, jak mi zrobi źle. Bo akurat włosy to jest coś takiego, co musi być zrobione naprawdę dobrze. I prosiłam innych ludzi, ale ludzie się ostrzegli, że nie, nikt mi nie obetnie tych włosów, więc nie. Chciałam zrobić sobie to sama, ale wiem, że będę zła tylko na siebie i wiem, że nie pójdę do nikogo tego naprawić, bo nie mam stałego fryzjera, tak jak mam tą dziewczynę od paznokci. A właśnie, Kasia mi mówiła, że piszą do niej ludzie, których ona nie zna. Ale ona takich w ogóle nie przyjmuje, których nie zna. Nie przyjmuje wszystkich, tylko 4-5 klientek na dzień. A przyjmowała, no nie wiem, strasznie dużo, od 9 do 22 i godzinę na klientkę.

**Więc o paznokcie dalej dbasz. Do fryzjera chodzisz na tyle rzadko, że...**

Wiesz co, jeśli chodzi o paznokcie, to jest tak, że... Bo ja na początku myślałam, że ona odwołała te paznokcie wszystkim i do niej się nie chodzi. Przyjęłabym to. Okej, bym sobie obcięła te paznokcie i tyle. Ale przez to, że do mnie nic nie napisała, że generalnie odwołuje, ale żebym ja przyszła - nie. Po prostu napisała SMS dzień wcześniej: przypominam o wizycie. Jakby odwołała mi te paznokcie, to nie miałabym z tym dużego problemu. Tym bardziej, że moja szefowa też do niej chodzi i jej odwołała, a ona mnie złapała na zrobionych paznokciach. A ona mówi, że w sumie się cieszy, bo jej odpoczną te paznokcie. Ale jakbym nie robiła tych paznokci, to bym też nie cierpiała z tego powodu wydaje mi się. Bo z tą dziewczyną to jest tak, że ona trochę zmusza do chodzenia do niej.

**To znaczy?**

Ciężko jej odmówić. Tak, do niej od razu się zapisujesz nawet na 3 miesiące wcześniej. Się otwiera kalendarz i tam zapisuje od razu. Ona jest taką trochę toksyczną osobą i tak pochłania.  I już potem głupio jest. Wiadomo, jak się pójdzie do kogoś innego, to będzie patrzyła krzywo, bo strasznie obgaduje. To jest taka pani od paznokci, której nie można przesunąć. Bo wiem, że ona będzie kręciła nosem, itd.

**Mówiłaś, że jak nie jedziesz do biura, to nie musisz myś głowy, szykować się. Co jeszcze odpuszczasz?**

Tak. Ja się nigdy nie maluję, więc nie maluję się też teraz. Nie robię ze sobą więcej nic. Miałam raz ochotę zrobić maseczkę na włosy, ale tego nie zrobiłam.

**A jak siedzisz więcej w domu, to robisz sobie jakieś peelingi, maski na twarz?**

Ja za bardzo nie lubię robić takich rzeczy. Filip się czasem moimi kosmetykami maluje. Ale on to robi, bo jemu się nudzi. Ja się nie maluję. A z resztą, on to też robi dlatego, że mnie te kosmetyki uczulają i mogę je właściwie wyrzucić, bo ja ich nigdy nie użyję.

**A są jakieś kosmetyki, z których zrezygnowałaś?**

Ja korzystam z bardzo małej ilości rzeczy i one są sprawdzone i to są rzeczy, z których korzystam zawsze i to jest opracowany zestaw rzeczy, których używam.

**Opowiesz mi kilka słów o swojej pielęgnacji?**

Bardzo jest mała ta pielęgnacja. Myję twarz żelem pod prysznic z Yope, on jest taki naturalny, więc wydaje mi się, że można nim myć twarz. Jeżeli wyskoczyły mi jakieś krosty albo zaczerwienienia, to przecieram płynem micelarnym albo takim olejkiem z Lirene z jakimś kwasem, ale nie wiem do końca jakim, mama mi to dała. I to jest wszystko. Jeśli chodzi o włosy, to używam szamponu niebieskiego z Yope i odżywki takiej samej. I myję się żelem pod prysznic tym samym, którym myję twarz. I to wszystko.

**I nie masz potrzeby, żeby sobie coś więcej wprowadzać?**

Czasem faktycznie, ale nie teraz, tylko tak ogólnie w życiu, mam ochotę sobie coś zrobić, to jakieś naturalne sobie coś... Spytam się kogoś albo jak gdzieś przeczytam, że jak połączę 15 dziwnych składników, to wychodzi coś fajnego. Jakiś takich z domu. Czy np. siemię lniane ugotować i nałożyć taką maskę na włosy. To chętnie zrobię. Tylko problem jest taki, że mam bardzo małą umywalkę tutaj, brudzi mi się wszystko.

**A jak z tym ubieraniem się? Jak zostajesz w domu, to jak się ubierasz?**

Piżama.

**Czyli jak nie idziesz do pracy, to w piżamie cały dzień?**

Nie no, cały dzień koniecznie.

**A dlaczego koniecznie?**

Bo ja uwielbiam chodzić w piżamie. Dzisiaj wyszłam z domu. Nie byłam w biurze, ale byłam u Basi po jakieś podpisy, dlatego jestem ubrana. Jeśli chodzi o ubieranie się, to wtedy, kiedy miałam taki cięższy okres, to Filip mi kazał się ubrać i umyć.

**A ty chciałaś w piżamie siedzieć?**

To było tak, że on przygotował obiad na święta. Mówiłam ci, że nic nie robiliśmy na święta - sorry, faktycznie, robiliśmy, ale to nie były moje świętowanie, nie chciałam tego robić, to chciał robić Filip. I ja byłam w piżamie, on zaczął to robić i ja powiedziałam dobra, możesz to robić. Chociaż nie miałam na to ochoty zupełnie. I się spytał, czy ja się umyje do tego, a ja powiedziałam, że nie. Bardzo mnie prosił, a ja bardzo nie chciałam. Bo to był pierwszy dzień, kiedy nie byłam w biurze od dawna i miałam nadzieję, że uda mi się prześlizgnąć i nie myć i być w piżamie cały dzień. Może bym to zrobiła, ale kurczę to było o 19:30 i tak wytrzymałam cały dzień, to na koniec, niepotrzebnie się myć w ogóle? Ale zrobiłam to, ale naprawdę płakałam. Nie dlatego, że musiałam się ubrać, tylko po prostu. To była taka bezsilność, nie miałam ochoty tego robić i musiałam. Wtedy poczułam pierwszy raz, że chcę, żeby to się skończyło, kiedy musiałam się umyć.

**A jak ubierasz się w piżamę i pracujesz, to nie masz poczucia, że to nie taka praca do końca?**

No mam, dlatego to są wakacje. Ale jeśli chodzi o jakość mojej pracy, to nie zmienia się.

**Masz jakieś plany zakupów ubraniowych?**

Dobrze, że pytasz, bo mam plany. Nie mam nic konkretnego, ale już parę razy myślałam, że chciałabym zamówić jakieś ubrania, więc bardzo możliwe, że to zrobię. Ale przymierzam się dopiero. Na pewno to zrobię na Zalando. Ale nie wiem, może po wypłacie? Nie, to raczej nie zależy od wypłaty, tylko od tego, kiedy coś mi się spodoba.

**To ma być coś konkretnego?**

Na pewno jakieś bluzki na krótki rękaw. Może jakąś sukienkę taką na teraz, czyli, żeby miała długi rękaw, ale była sukienką i nie trzeba było rajstop. Jakieś może luźne spodnie. Nic konkretnego. Żadnej kurtki ani butów.

**Czemu akurat teraz naszło cię na te zakupy?**

Zmienia się pogoda, ale też jest taka w kratkę. Wygląda, jakby było ciepło, ale jest chłodno. A w zeszłym tygodniu było duszno np. I już dawno sobie nic nie kupowałam.

**To kupowanie, to będzie przyjemność czy z potrzeby?**

Wiesz co, chyba nie robię tego tak, jak sobie wyobraziłaś, że to robię, bo jak zaczęłam szukać odpowiedzi, to nie umiałam. Najczęściej jest tak, że w sekundę wpada mi pomysł, że sobie kupię ubrania, to wchodzę na Zalando, wpisuję T-shirt i ustalam od najtańszego. I patrzę np. "o nie mam takiego białego" i kupię od razu ze 3 bluzki. Cały proces trwa maksymalnie 5 minut i nie myślę o tym. Potem to przychodzi i się zastanawiam, czy mi się to podoba czy nie. Raczej nie no, na pewno mi się to podoba, jak to sobie wybrałam, ale czy pasuje, itd. Nie mam dużych przymiarek do takich rzeczy na Zalando. Jeśli są jakieś promocje, to 20 zł kosztuje bluzka. Jeśli chodzi o takie rzeczy, to po prostu je kupuję. To, że na Zalando można za darmo oddać i za darmo to przychodzi, to pozwala, że można sobie rzeczy bezmyślnie kupować, ale przez to dużo rzeczy mam takich, których bym nie kupiła, gdybym o tym dłużej myślała.

**A to są takie rzeczy pracowe, czy po domu, które chcesz kupić?**

Nie, ja nie mam żadnych pracowych ubrań. U mnie w domu nie trzeba się ubierać w ogóle w żaden sposób elegancko, ani w żaden sposób nawet przyzwoicie. Można wchodzić nawet w *crop topach*. Nie mam rozróżnienia na ubrania po biurze i na weekend np. Po domu chodzę raczej w tym, w czym śpię. Może są to dwa różne, ale zwykle jest to taka sukienka do połowy uda. Mam takich z 7 różne kolory i to są z sieciówek i one się szybko przecierają. Więc teraz są takie średnie. Kiedyś chodziłam w nich normalnie, a teraz uznałam, że są trochę za krótkie. Nie mam problemu, żeby wyjść w tym do sklepu.

**Czyli to nie jest tak, że musisz się przebrać z tego, jak idziesz po pomidora?**

Oj nie, absolutnie. Znaczy do Kauflandu bym tak nie pojechała na pewno, ale na dół tak.

**A dlaczego do Kauflandu nie?**

Bo to jest bardzo krótkie.

**Brakuje ci możliwości pójścia na siłownię, na basen?**

Mam ochotę pójść na basen, ale mam ochotę od roku pójść na basen.

**Czego ci najbardziej brakuje z punktu widzenia bycia konsumentem? Bo teraz mamy te ograniczone możliwości...**

Nie mamy ograniczonych możliwości. Mamy dużo możliwości.

**Ale nie możesz pójść do fryzjera tak z ulicy.**

Dobra, nie. Tego faktycznie nie możemy. Ale możemy sobie bardzo dużo rzeczy kupić, nie wychodząc z domu. Moim zdaniem te możliwości są wręcz rozszerzone.

**Czego nie było wcześniej?**

Wydaje mi się, że szybciej działają teraz takie rzeczy wysyłkowe. Tańsze są te dostawy. Jedzenie, którego nie można było zamówić, da się zamówić. Nagle się okazało, że jednak da się zamówić. Teraz na Volcie można sobie nawet jakieś gazety zamawiać. Nikomu nie przyszło do głowy, że to jest potrzebne, dopóki nie było tej kwarantanny i zaczynają wymyślać jakieś głupoty, które mogą być ludziom potrzebne.

**Jak rozumiesz słowo kwarantanna?**

Że rekomenduje się ludziom siedzenie w domu.

**I to jest dla ciebie kwarantanna?**

W skrócie tak. Ja wiem, co to znaczy kwarantanna i wiem, że kwarantanna to taki okres, kiedy naprawdę nie można wychodzić z domu, bo się wróciło z zagranicy albo z jakiegoś innego powodu, ale to jest właśnie takie pojęcie, które się przyjęło wśród użytkowników Internetu i tak się mówi o tym okresie, który teraz jest. Od pierwszego dnia pracy zdalnej.

**Jak byś nazwała to, że ktoś został wysłany do domu i nie może wychodzić na dwa tygodnie?**

To jest ta prawdziwa kwarantanna.

**A ta, co ty jesteś?**

Ta to jest właśnie takie określenie tego, w którym są ludzie, tacy *millenialsi* powiedzmy, którzy siedzą w domu, pracują zdalnie, zamiast pracować z biura. I nie ma imprez. W dużym skrócie, ale raczej tak. Nie ma żadnych spotkań towarzyskich.

**Czyli brak spotkań towarzyskich to ważny element kwarantanny?**

Tak. I ta rekomendacja siedzenia w domu. Że można wychodzić z domu, ale właśnie się rekomenduje, że trzeba siedzieć.
