# Supplementary material for: The challenges arising from the COVID-19 pandemic and the way people deal with them. A qualitative longitudinal study
Source: PLoS One. 2021 Oct 11;16(10):e0258133. doi: 10.1371/journal.pone.0258133 (PMC8504766; doi:10.1371/journal.pone.0258133)
Supplement: S1 Dataset — (ZIP) [file pone.0258133.s003.zip › Transcriptions/stage 3/6.3_M_24_couple, no children.docx]

**6.3_M_24_couple no children**

**Jak ci minęły ostatnie 2 tygodnie?**

W zasadzie cały czas to samo, o czym mówiłem na samym początku - nauka programowania, gra na gitarze, oglądanie filmów, czytanie książek, sporadyczne wychodzenie do sklepu, teraz, jak jest ładna pogoda to trochę siedzenie na balkonie i w zasadzie tyle. Minęło szybko. Mogę powiedzieć, że szybko, bo w zasadzie dni się już teraz trochę zlewają w jedną całość. Szybko i trochę właściwie monotonnie.

**Zlewają się w całość, ale czujesz, że jest weekend albo, że jest tydzień?**

Czuję, że jest weekend, bo Karolina ma wolne. Dla mnie w zasadzie sobota jest takim samym dniem jak środa, gdyby nie to. W tygodniu, to ostatnio mi się pomyliły dni. Dzisiaj mamy piątek a ja w środę myślałem, że właśnie mamy piątek.

**Jak myślisz, czemu tak jest?**

Nie wiem. Jakoś...Straciłem rachubę czasu po prostu siedząc w domu i nie przykładam też do tego aż takiej wagi, nie sprawdzam kalendarza, nie mam jakichś zaplanowanych konkretnych czynności, spotkań czy pracy i pewnie z tego to wynika.

**Czy mimo wszystko zacząłeś coś nowego robić, czego nie robiłeś wcześniej?**

Nie.

**A jest coś teraz, co jest specjalnie dla ciebie męczące, coś co cię martwi, denerwuje?**

Tak, jest. Ta perspektywa, że nie wiadomo, kiedy to się skończy i właśnie to, że te dni idą tak monotonnie i niby człowiek się może rozwijać, jak chce, ale jednak brakuje mu tej swobody troszkę, że może wyjść sobie z domu, kiedy ma na to ochotę bez żadnych masek, maseczek, bez uważania specjalnie, żeby nie chodzić blisko z kimś. To chyba ta opcja mnie denerwuje. Wcześniej jeszcze mówiłem o wydarzeniach muzycznych, że mam nadzieję, że w lecie się uda coś zorganizować, ale ostatnimi czasy doszedłem do wniosku, że jednak chyba w tym roku w ogóle żadnych wydarzeń muzycznych nie będzie, ani kulturalnych. Nie zdziwiłbym się, gdyby tak było w każdym razie.

**To są twoje przypuszczenia?**

Tak. W kościach czuję, że nic z tego nie będzie.

**Powiedziałeś, że mimo wszystko można się rozwijać. Ty się rozwijasz teraz?**

Na pewno nie stoję w miejscu, bo to w sumie niekoniecznie jest równoznaczne. Nie czuję, że stoję w miejscu, bo gdybym stał w miejscu, to powiedzmy codziennie przez ten miesiąc bym tylko oglądał seriale i grał na komputerze albo tylko leżał. Czuję, że ja chcę się uczyć tego oprogramowania i gry na gitarze i czuję, że to jest jakieś rozwijające na tyle, ile mogę. Oczywiście bardziej bym się rozwijał mając możliwość pójścia na jakiś kurs czy lekcje do kogoś, ale trzeba sobie radzić jak można.

**Największym problemem jest, że nie wiesz, jaka będzie przyszłość i jak to wszystko będzie wyglądało?**

Tak, myślę, że tak.

**O jakie obszary swojego życia się martwisz najbardziej?**

O pracę w zawodzie na pewno. Nie wiem, jak to będzie, jak to się skończy, kiedy to się skończy i już w tym momencie biorę pod uwagę, że we wrześniu czy w październiku będę się rekrutował na inne studia - właśnie na informatykę prawdopodobnie i to, że będę się musiał przebranżowić jest jakby coraz bardziej namacalne dla mnie.

Mówiłeś, że szukałeś pracy w swoim zawodzie i nie mogłeś jej znaleźć, a teraz przerwałeś te poszukiwania, ale myślisz o tym, żeby zmienić zupełnie?

Też nie zupełnie, bo to co chciałem zrobić, to jakoś połączyć swój kierunek z tym czym się obecnie interesuję. Nie jest to łatwe, bo patrzyłem też po kierunkach studiów 2 stopnia i nie ma za bardzo czegoś, co by wiązało technologię chemiczną z informatyką, więc myślałem, że po prostu pójdę na drugie studia i będę miał 2 dyplom, a potem będę już jakoś kombinował dalej.

**Czy na to, że chcesz się przebranżowić wpływa też pandemia, która jest teraz?**

To jest chyba niezależne. Na pewno inaczej bym na to spoglądał, gdybym już znalazł pracę w zawodzie albo gdybym miał trochę ofert, gdyby zaczęły do mnie spływać oferty albo zaproszenia na rozmowy rekrutacyjne, ale jako, że tak nie jest i nie było przez pierwsze 2 tygodnie...Dostałem tylko jedną ofertę, tzn. zaproszenie na jedną rozmowę, to zacząłem właśnie myśleć w ten sposób. Poza tym myślałem o tym już w zasadzie trochę wcześniej, bo niektórzy znajomi, którzy już skończyli ten kierunek mówili, że generalnie to nie jest zbyt opłacalna praca, w laboratorium nie dostaje się za wiele pieniędzy, no ale myślałem, że jak nie laboratorium, to może coś innego. Jakiś przemysł czy coś, ale w Warszawie chyba nie ma za wiele takich placówek przemysłowych, bardziej pod Warszawą, a ja bym jednak chciał coś w mieście znaleźć, żeby wygodnie dojeżdżać.

**A jeśli chodzi o tę pracę, która masz teraz, to coś się zmieniło?**

Nie, dalej mam okres przestoju, dalej dostaję wynagrodzenie za ten okres i żadnych informacji nie dostawałem, żeby coś miało się zmieniać, żebym miał ruszać w teren.

**Jeszcze są jakieś obszary twojego życia, co do których czujesz jakąś niepewność?**

W zasadzie ogólnie czuję niepewność, może też ciekawość, jak to wszystko będzie wyglądało, jak to się skończy, jak to wszystko będzie stawało na nogi. Chodzi mi o te koncerty, o gospodarkę, o różne wydarzenia kulturalne, o sklepy, o studia tatuażu, o wszystko. To nie tylko dotyczy sfery mojego życia, ale ogólnie świata.

**Jakie są twoje przewidywania?**

Koncerty, to myślę, że najprędzej w przyszłym roku, chociażby dlatego, żeby zachować bezpieczny dystans. Powiedzmy, kończy się pandemia, ale na wszelki wypadek nie organizujmy niczego przez najbliższe parę miesięcy. Wydaje mi się, że coś takiego może mieć miejsce. W tym roku największy otwarty plenerowy festiwal, czyli Pol&Rock absolutnie nie wyobrażam sobie, żeby miał być zorganizowany, a nawet jeżeli by miał, to myślę, że ludzie sami z siebie będą na tyle zaniepokojeni albo niepewni, żeby nie udawać się na taki festiwal. Sam bym sobie odpuścił. Zdecydowanie. Nawet jeżeli byłoby oficjalnie powiedziane, że pandemia kończy się w lipcu, a to jest organizowane w sierpniu, to ja i tak miałbym obawy, żeby się na to wybierać. Jeżeli chodzi o resztę sektorów gospodarki i usług, to nie wiem. Za dużo tego, żebym mógł to swoim umysłem ogarnąć w tej chwili. Nie wiem, jak to będzie wstawało z kolan. Jestem ciekaw, bo na pewno będzie to wszystko inaczej wyglądało i funkcjonowało niż tak, jak to znaliśmy do tej pory. Właściwie, to już tak jest.

**Emocje - zdjęcia**

Wcześniej na pewno nie wybierałem 4, ale teraz mógłby być dobry, bo pokazuje pewną wspólnotę, jakąś więź, może współpracę między ludźmi i w zasadzie to musimy współpracować teraz wszyscy ze sobą, żeby to się szybciej skończyło. Mieć głowę na karku, starać się nie gromadzić w większych grupach, itd. Myślę, że podtrzymuję też te poprzednie obrazki, czyli 10, 12, 13, bo jest słonecznie. Chyba jeszcze 7.

**Czy ty się czujesz teraz blisko innych ludzi w czasie tej pandemii?**

No nie, absolutnie nie. Chodzi mi raczej o taką współpracę, o koordynację działań.

**Mówiłeś jeszcze o 7?**

Mówiłem, że podtrzymuję, ale nie wiem, czy teraz bym go wybrał. to nie jest tak, że go skreślam, bo wtedy mogłem mieć coś na myśli, że go wybierałem... Mogłabyś powiększyć? O, myślę, że ten obrazek również mógłby pasować.

**Dlaczego?**

Chyba weszły jakieś przepisy niedawno pozwalające na przechadzki po lasach, parkach, więc myślę, że byłby adekwatny. Ciekawe, że te obrazki zostały do brane właściwie po kolei do tego, co się dzieje. Mówię teraz o 6. Teraz jak najbardziej jeszcze wybrałbym 6. Słońce też tam pasuje, bo mamy ładną pogodę. 7 w sumie też może być, bo taka droga, nie wiadomo, dokąd prowadząca, zwiastująca niepewność, co tam jest dalej. dalej są jakieś chmury, taka pogoda niezbyt ciekawa, więc też to może być odniesienie do naszej sytuacji. Nie wiadomo jak to będzie wszystko wyglądało i w sumie ciekawe, co będzie na końcu.

**Jesteś ciekaw, ale masz raczej przewidywania pozytywne/ negatywne?**

Nie myślałem o tym w ten sposób. Po prostu będzie inaczej. Na pewno dla niektórych będzie zdecydowanie trudno. Mówię o przedsiębiorcach i właścicielach jakichś interesów, ale pewnie będą też ludzie, którzy już mają interes teraz w tej pandemii i im to jest na rękę, że ona jest, więc to jest względne dosyć.

**Komu to może być na rękę?**

Myślę, że politykom chociażby albo rządowi. Jeśli chodzi o przeprowadzenie wyborów, to jest im na rękę, że jest pandemia teraz. Wydaje mi się, że mogą mieć zwiększone szanse wygrania tych wyborów. Podtrzymuję 7, a właściwie wybieram ją ponownie, bo wtedy nie pamiętam czemu ją wybrałem, ale chyba kolorystyka mi się taka wydawała szara i smutna, i tamta rzeczywistość taka była też niepewna. Teraz zdecydowanie niepewna droga i dlatego 7.

**Smutek teraz jeszcze odczuwasz?**

Nie, nie. W sumie tak tylko powiedziałem. Wtedy jak wybierałem ten obrazek, to też nie smutek, tylko może taka nostalgia, że jest jakaś pandemia, źle się dzieje na świecie i to mi się skojarzyło.

**Mówiłeś jeszcze o 10?**

Tu pamiętam, że mówiłem, że świat postapokaliptyczny mi się trochę kojarzy i właściwie dalej mi się kojarzy ten obrazek z takim światem, ale kolorystyka jest tutaj szara, taka zimna, a obecnie mamy słoneczną pogodę, jest kolorowo, więc akurat pod tym względem bym go nie wybierał.

**Jeszcze 12?**

To też mogę podtrzymywać, bo symbolizuje takie odosobnienie, ale z kimś bliskim, czyli np. rodziny zamknięte w domach, albo ja z Karoliną i z kotami, albo ty ze swoim chłopakiem i też z kotem.

**A 13?**

Na to akurat miała wpływ pogoda i teraz jeszcze wybrałbym 16, bo ostatnio płonął Biebrzański PN i lasy wokół Czarnobyla też, także ten obrazek też będzie adekwatny. Aż ciekawe, że te obrazki tak idealnie pasują do tego, co się dzieje teraz. Przypadek?

**Jak ty się czujesz z tym, że te lasy płoną?**

No cóż...Nie wiem, też nie myślałem jakoś o tym bardziej. Ubolewam nad tym, że tak się dzieje, bo ubolewam nad ludzką głupotą przede wszystkim, bo z tego to się wzięło. Takich rzeczy spokojnie można uniknąć. Pożar w Czarnobylu był zaprószony przez przypadek przez jakiegoś chłopaka. Ubolewam, że przez takie głupie czyny musi cierpieć fauna i flora. To też powoduje jakieś komplikacje, jeśli chodzi o zanieczyszczenie powietrza, chociaż niektórzy się bali o skażenie radioaktywne.  Do tego jeszcze strażacy są niepotrzebnie narażani na niebezpieczeństwo, a BPN chyba też został ten pożar zaprószony przez przypadek + jeszcze na dodatek mamy suchą porę teraz, ale więcej o tym nie wiem, poza tym, że chyba zostało opanowany ten pożar wczoraj, ale też nie jestem pewien.

**Czy czujesz jeszcze jakikolwiek strach/ lęk?**

Trochę dalej lęk. Na pewno odczuwam lęk i gdyby ktoś zaproponował mi teraz imprezę na 20 osób, to odczuwałbym lęk przed pójściem na tę imprezę, ale też niepewność jednocześnie, ale w sumie też strach. Lęk, jeżeli chodzi też o sprawy zawodowe, bo lękam się o tę swoją przyszłość troszkę, ale mam wrażenie, że robię wszystko, co w mojej mocy, żeby to jakoś miało ręce i nogi kiedyś. Jest stabilnie.

**Czy u osób dookoła ciebie widzisz jakąś zmianę w tym, jak oni odczuwają tę sytuację?**

Tak, na pewno czują się już troszkę zamknięci, zapuszkowani. Moja babcia, z tego co mi wiadomo, wychodzi z domu co jakiś czas starając się zachować wszystkie...Robi wszystko, żeby było jak najbezpieczniej, ale mówi, że ciężko jej siedzieć w domu, więc ostatnio była gdzieś w lesie. Pojechała tam z takim swoim znajomym. Moi rodzice chyba sporo jeżdżą na rowerze teraz, chociaż ostatnimi czasy mówili, że chcieliby pójść na rower, ale się boją, że mogą dostać mandat. To było chyba zanim otworzyli parki i lasy. Generalnie czują się mocno zamknięci, mimo tego, że mają spory dom i sporą działkę, więc mogą sobie wyjść i posiedzieć na zewnątrz, ale i tak odczuwają tę kwarantannę i to, że muszą siedzieć w jednym miejscu. Głównie moja mama, bo ojciec jest lekarzem i chodzi do pracy.

**Czy jakoś go to specjalnie dotyka, ponieważ jest lekarzem?**

Aż tak z nim nie rozmawiałem na ten temat, ale tak, na pewno, bo jednak musi się pojawiać w szpitalu. Z tego co wiem, w szpitalu w Przemyślu też już były chyba 2 zachorowania na koronawirusa, ale te osoby zostały przetransportowane do szpitala w Łańcucie. Nie wiem, szczerze mówiąc nie rozmawiałem z nim.

**Czy wy też czujecie się tacy zapuszkowani?**

Trochę tak. Ja się trochę czuję. Tak.

**Wychodzisz na zakupy i jeszcze gdzieś wychodzisz?**

Ostatnio byłem ze znajomym nad Wisłą, też starając się zachować bezpieczny odstęp, itd., maska na twarzy. Poszliśmy się przejść. To potrwało z 1.5 godz. Tak, poza tym nigdzie nie byłem.

**O jakich zmianach w ograniczeniach słyszałeś?**

Szczerze mówiąc, to ostatnimi czasy tylko o tych, że można wchodzić do parku i do lasu. Dalej się nie interesowałem, nie czytałem, nie słuchałem, bo ostatni raz wiadomości oglądałem chyba 2 tyg. temu i nie odczuwam ich braku, ani potrzeby, żeby być cały czas na bieżąco z tym, co się dzieje. Gdzieś tam do mnie docierają takie szczątkowe informacje, zazwyczaj coś mi się wyświetla na FB, albo jakiś znajomy mi coś podeśle, albo do konwersacji ktoś co wrzuci i czasem to otworzę. Tak, żebym sam z siebie miał szukać informacji, to nie bardzo i dlatego nie jestem aż tak obeznany z tymi ograniczeniami.

**Chodzisz w maseczce?**

Chodzę.

**Na ile uważasz, że to jest słuszne, że trzeba w nich chodzić?**

Ciężko powiedzieć. Słyszałem różne fakty. Z jednej strony noszenie maseczki nie zapobiega temu, że ja się zarażę, tylko zapobiega wydzielaniu przeze mnie kropelek, którymi przenosi się wirus, więc...Na pewno nie mogę traktować jej, jako 100% zabezpieczenia, Bardziej traktuję ją, jako taką prewencję, że ewentualnie, gdybym ja był zarażony jakimś cudem, to żeby ograniczyć wydzielanie tych kropelek. Słyszałem też i to jest logiczne, że noszenie takiej maseczki przez dłuższy czas sprzyja rozwijaniu się patogenów w obrębie jamy ustnej, bo jest wilgotne środowisko itd. To może nawet sprzyjać zarażeniu się. I w zasadzie nie wiem, co mam o tym myśleć.

**Jaką masz maseczkę?**

Bawełnianą, szytą na zamówienie. Tata mi wysłał pocztą. Słyszałem jeszcze, że niektórzy ludzie zaniedbują inne standardy bezpieczeństwa, bo właśnie ta maseczka daje im takie złudne poczucie bezpieczeństwa i np. zapominają myć ręce częściej, albo tych maseczek nie piorą tak często, jak powinni. Też można pod tym kątem patrzeć na te maseczki, że one same w sobie ograniczać wydzielanie tych kropelek, ale dzięki nim ludzie zapominają się w innych kwestiach.

**Które ograniczenia mają realny wpływ na ograniczenie rozprzestrzeniania się epidemii, a które tak bardziej psychicznie coś ludziom dają?**

Mam wrażenie, że noszenie maseczek to jest bardziej na zasadzie uspokojenia sumienia, może właśnie dania ludziom takiego fałszywego poczucia bezpieczeństwa. Tak naprawdę myślę, że trzeba by było przeprowadzić jakieś badania i sprawdzić na jakiejś grupie osób bez maseczek i z maseczkami. Sprawdzić, czy to w ogóle coś daje i też nie wiem, czy to można by było odnosić do całej populacji. Noszę, bo trzeba nosić i tyle. Gdyby nie trzeba było, to pewnie bym nie nosił, bo myślę, że jednak to nie daje aż tyle.

**Które ograniczenia dają coś rzeczywiście?**

Myślę, że gęstość zaludnienia w sklepach - to na pewno coś daje. Limit osób do 24 w tym Auchan, w którym robię zakupy. Nie wiem, jak teraz, bo może coś się zmieniło. Chyba jakieś luzowanie miało być, ale nie wiem, czy to dotyczy sklepów. To jest skuteczne w ograniczaniu rozprzestrzeniania się koronawirusa a nie skuteczne w leczeniu. Poza tym chyba nic innego mi nie przychodzi do głowy.

**Co słyszałeś o luzowaniu?**

Że coś ma być luzowane i że odmrażanie gospodarki i w zasadzie tyle.

**Co o tym myślisz?**
Nie wiem, czy to nie jest za wcześnie. Myślę, że jest za wcześnie, choć ludziom, którzy tracą na tej pandemii na pewno jest to na rękę. To może mieć też podłoże polityczne, czyli rząd chce pokazać, że już może się robić trochę lepiej i dlatego wprowadza te poluzowania, żeby zachęcić ludzi do głosowania w wyborach.

**Za wcześnie, ale czy mimo wszystko uważasz, że któreś z tych obostrzeń powinny być luzowane?**

Nie, myślę, że nie. Nie śledzę statystyk i tych różnych wykresów, na których może być widać, czy wzrost zachorowań spada, czy może utrzymuje się na stabilnym poziomie, czy wzrasta. Gdyby wzrost zachorowań spadał, odsetek wyleczonych wzrastał, albo są oznaki na temat pierwszej szczepionki, to myślę, że to byłby odpowiedni czas, żeby wprowadzać jakieś zmiany luzujące. Wątpię, że coś takiego teraz może mieć miejsce, więc...Myślę, że trochę jest za wcześnie. Musi być jakaś widoczna zmiana, a póki co nie ma żadnych doniesień chyba, że ma być lepiej.

**Czy te miejsca, które są cały czas zamknięte powinny funkcjonować tak samo jak przed pandemią po ich otwarciu?**

To jest trudne pytanie. Nie wyobrażam sobie nawet końca tej pandemii w sensie momentu, w którym ktoś mówi, że już jest bezpiecznie i możemy wrócić do dawnego życia. Nie wiem, czy to byłby też dobry moment na otwieranie nagle wszystkich studiów tatuażu i salonów fryzjerskich, ale jeżeli miałyby takie miejsca działać, to raczej na zasadzie takiej, jak działały wcześniej. Nie wyobrażam sobie strzyżenia kogoś w odległości 2 m.

**Co byś najbardziej chciał, żeby zostało otwarte? Czego ci najbardziej brakuje?**

Jakichś imprez, chociażby na działce. Najbardziej imprez, wydarzeń muzycznych.

**Imprezy powinny działać w jakiś inny sposób po wznowieniu?**

Hmm...Nie wiem, chyba nie. Jak będą działały to będzie to oznaczało, że kryzys jest totalnie zażegnany i nie istnieje w ogóle ryzyko powrotu tego, bo to by się nikomu nie opłacało. Nie wiem.

**Myślisz, że w najbliższym czasie to jest realne, żebyś mógł się spotkać ze znajomymi na działce?**

Chyba nie, Na tych wakacjach to jeszcze nie będzie realne. Być może planuję jakieś spotkania, ale nie w formie imprezy, jakiegoś zgromadzenia, gdzie każdy będzie miał bliski kontakt ze sobą. Jak już, to takie spotkania w gronie dosłownie 2-3 osób. To biorę pod uwagę.

**Słyszałeś, jak Szwecja podeszła do tematu pandemii?**

Nie.

[wyjaśnienie]

**Co myślisz o takim podejściu?**

Ciekawe to jest. To znaczy, że rząd ma chyba duże zaufanie do swoich obywateli i pokłada w nich nadzieje, że to są ludzie, którzy potrafią używać mózgu i sami to jakby wezmą w swoje ręce. Sami będą wiedzieli co robić, żeby to się nie rozprzestrzeniało. Ciekawe podejście, ale chyba za luźne, wg mnie i też nie wiem, jaka tam jest statystyka zachorowań i zgonów, więc nie wiem, czy to się sprawdza w ogóle u nich.  Ciekawe, ale jak dla mnie zbyt luźne podejście.

**W Polsce coś takiego by się sprawdziło?**

Nie. Każdy byłby zadowolony, gdyby mógł sobie otworzyć salon, ale myślę, że to by raczej nie pomogło. Czasami widzę, że ludzie nie noszą maseczek, więc nie stosują się do tych nakazów, nie stosują się do rekomendacji, więc gdyby u nas coś takiego weszło, to nie funkcjonowałoby to tak, jak powinno. Może w Szwecji też tak jest, nie wiem.

**Ludzie nie noszą maseczek w Polsce?**

Tak, czasami widzę, że idą bez maseczki i z papierosem, albo idą w ogóle bez maseczki i tylko jak SM jest, to ją zakładają, albo nie utrzymują odstępu 2 m. Niby wiedzą o tych zakazach wszystkich, ale nie traktują ich do końca poważnie.

**Jak ci się wydaje, dlaczego?**

Nie wiem. Bo są lekkomyślni, bo im się nie chce. Często im się nie chce nosić maseczki, bo jest im ciężko oddychać albo nie chce im się czekać w kolejce do sklepu, czekać aż ktoś wyjdzie, więc wchodzą i stwarzają większe zgromadzenie.

**Co myślisz o tym, że mają być wybory?**

Uważam, że to dosyć ryzykowne. Ja już sam nie wiem, czy one mają być takie, że idziemy do urn, czy mają być korespondencyjne. Pójście do urn uważam za szczyt głupoty, bo to jest zaprzeczanie temu wszystkiemu, co było wprowadzane do tej pory - te wszystkie zakazy o zgromadzeniach, itd. Z tego co wiem, to też brakowało chętnych do komisji wyborczych. Jeżeli miałyby być korespondencyjne, to chyba będą niejawne i uważam, że wtedy są bardziej podatne na sfałszowanie tych wyborów, bo mogą przechodzić przez więcej rąk zanim trafią do miejsca, gdzie się to ostatecznie zlicza. Najrozsądniej byłoby ich nie przeprowadzać jeszcze teraz.

**Będziesz brał udział w wyborach?**

W korespondencyjnych myślę, że na pewno bym brał udział i biorąc udział w takich wyborach, próbowałbym pewnie podjąć jakieś środki, żeby ograniczyć to, co mówiłem... Może próbowałbym zrobić jakieś zdjęcie mojej karty do głosowania z dowodem osobistym, żeby potwierdzić, że ja zagłosowałem na tego kandydata. Jeżeli byłyby w trybie zwykłym, to jeszcze nie wiem. Nie zastanawiałem się, chociaż już coraz bliżej. No, będzie ciężko. Nie wiem, czy będę chciał ryzykować, ale chciałbym, żeby się coś zmieniło już.

**Jakiego określenie używasz na siedzenie w domu? To jest jakaś kwarantanna?**

Tak, w zasadzie to kwarantanna i...Odosobnienie nie. Wiem, że kwarantanna to jest określenie dla ludzi, którzy są chorzy i wtedy oni muszą się całkowicie odciąć od środowiska i od członków swojej rodziny nawet, chociaż nie wiem, jak można się odciąć od członków rodziny mieszkając w tym samym domu, ale nie mogą nigdzie wychodzić przez 2 tyg., nawet do sklepu ani nic. Kwarantanna jest jeszcze poważniejszym hasłem niż to, jak ja funkcjonuję. Ja nazywam to kwarantanną, ale jak to powinno się nazywać, to nie wiem. W zasadzie nie wiem, czy jestem chory, bo nie przeprowadzałem testów, ale czuję się dobrze. W zasadzie byłem chory w marcu i bałem się wtedy, że to może być to, ale to była zwykła grypa i przeszła po chyba tygodniu. Mama wtedy mi mówiła, żebym lepiej zadzwonił do Sanepidu, bo wtedy to się zaczynało wszystko. Raz, że bałem się tam dzwonić, bo bałem się, że mnie zabiorą z domu i będę musiał odbywać prawdziwą kwarantannę. Druga sprawa, że czułem się na tyle dobrze, że nie chciałem dzwonić i zajmować linii, bo wiedziałem, że jak ktoś naprawdę jest chory, jak ktoś np. wrócił z Włoch, to taka osoba powinna mieć pierwszeństwo. Odczekałem i wyzdrowiałem.

**Jak u ciebie teraz wygląda dbanie o siebie? Czy się np. ubierasz rano, czy chodzisz w piżamie przez cały dzień?**

A wiesz, to różnie. Myję się codziennie, jeżeli o to chodzi, ale pewnie są niektórzy, którzy myją się co 2-3 dzień. Zakładam, że tak może być. Mi się może zdarzyło z raz w życiu, że 2 dni się nie myłem. Ubieram się w takie ciuchy po domu, jak zawsze. Nie perfumuję się, bo uważam, że nie ma potrzeby. Golenie się tak, co jakiś czas się golę, ale ogólnie wolę zarost i golenie się przysparza mi skórnych, niefajnych rzeczy - swędzi mnie, piecze, więc golę się jak najrzadziej - tylko wtedy, kiedy muszę. Jeśli chodzi o włosy, to stwierdziłem, że na razie spróbuję zapuszczać. W sumie to jest dobra okazja ku temu, bo za wiele nie wychodzę i tak nie mam jak ich obciąć, bo maszynkę elektryczną mam niesprawną, a nożyczkami stwierdziłem, że nie będę się strzygł. Mógłbym się ogolić na łyso, bo mógłbym pożyczyć maszynkę od kogoś, ale chyba za dużo zachodu, więc zapuszczam włosy.

**Normalnie chodzisz do fryzjera czy sam sobie włosy obcinasz?**
Ostatnio przed pandemią chodziłem do fryzjera przez jakiś rok, ale zdarzało mi się samemu golić na łyso zazwyczaj. Lubiłem chodzić na łyso i absolutnie mi to nie przeszkadzało. Wiem, że niektórzy teraz golą się na łyso, bo widziałem zdjęcia i ten kolega, z którym się spotkałem, też ogolił się na łyso.

**Dlaczego teraz ludzie coś takiego robią?**

Dlatego, że nie ma ich kto ostrzyc tak, jak by chcieli, profesjonalnie. Mój kolega chciał, żeby go tata ostrzygł, ale zrobił to na tyle nieudolnie, że kolega stwierdził, że woli być łysy. Poza tym myślę, że ludziom to już teraz nie przeszkadza. Ja akurat nigdy nie miałem obaw, żeby być łysym, mnie się to podoba i lubię chodzić na łyso czasami, ale są niektórzy, którzy nie lubią, bo np. mówią, że mają kształt głowy nie taki, że nie, bo będą wyglądali jak dresiarz, a teraz myślę, że przyszły takie czasy, że ludziom aż tak nie przeszkadza, jak będą wyglądali, bo wiedzą, że inni ludzie nie będą ich też oceniać tak surowo, bo sami też tak funkcjonują.

**Czy w ogóle czujesz, że trochę sobie odpuściłeś teraz?**

Nie, chyba nie, ale na pewno nie mam jakichś oporów, żeby chodzić w samej bieliźnie po domu przy odsłoniętych oknach. Chyba nigdy za bardzo nie miałem, a teraz jeszcze mniej.

**Te ciuchy po domu, to jakie to są ubrania?**

Przeważnie krótkie spodenki, koszulki takie, w których chodziłem kiedyś i albo są już sprane, albo kilka mam takich, które mają dziury po kocich pazurach i w sumie miałem je zanieść do krawca, ale nie zaniosłem i stwierdziłem, że niech będą na po domu.

**Jak idziesz na zakupy to się przebierasz?**

Mam też parę dziurawych spodni, w których nie mam problemu, żeby chodzić po domu, ale jak bym miał w nich wyjść na zewnątrz, to tak. Jeżeli mam takie spodnie na sobie, to je przebieram, ale jeśli mam np. dresy, w których zazwyczaj nie chodzę na zewnątrz, bo zazwyczaj chodzę w bojówkach, to często wychodzę w dresach. W jakiejś koszulce domowej też mi się zdarza wychodzić. Zazwyczaj zakładałem bluzę, teraz...No, zależy też od koszulki. Bywa, że się przebieram i bywa, że się nie przebieram albo, że się przebieram niecałkowicie. To zależy od stanu tych ubrań.

**A u twojej dziewczyny zauważyłeś jakaś zmianę rytuałów pielęgnacyjnych?**

Nie, dalej jest tak samo. Ona zawsze lubiła różne kremy, nie kremy i dalej z tego dużo korzysta. Jest tak samo.

**Kupujesz teraz jakieś ubrania?**

Nie, nic kompletnie. Ostatnim razem kupiłem jakąś koszulkę w zeszłym roku. Ja nie kupuję praktycznie ubrań - kupuję wtedy, kiedy mi potrzeba, jak mi coś brakuje. Najczęściej kupowałem spodnie. Spodnie jakoś najczęściej zużywałem. Koszulek mam dość sporo takich nawet starszych, po 5-6 lat, ale to też są takie koszulki, do których jestem przywiązany bardzo, bo są z modami zespołów metalowych. Ta akurat ma rok dopiero, ale do niej też jestem przywiązany i myślę, że będę ją miał dość długo.

**Mówiłeś, że planujesz zacząć ćwiczyć?**

No jeszcze nie zacząłem, ale coś tam już zacząłem czytać, szukać, patrzeć na ten temat. Ja zawsze myślę sobie, co będę robił w dany dzień i zazwyczaj jest to ta gra na gitarze, programowanie, obejrzenie czegoś, ale zazwyczaj wpada jeszcze, że coś trzeba posprzątać, zrobić jedzenie, bo ja teraz jestem odpowiedzialny za robienie jedzenia głównie, więc jakoś mi ten dzień mija naprawdę szybko, że nie mam...No, że czasu nie mam to jest źle powiedziane, ale po prostu robię te inne aktywności i na tę już brakuje czasu, więc muszę ją zamienić z czymś albo okresowo sobie zamieniać - w jeden dzień gram na gitarze i ćwiczę, a w inny nie ćwiczę, tylko gram na gitarze i uczę się programowania.

**Czemu ty jesteś odpowiedzialny teraz za gotowanie?**

Bo Karolina ma home office i ona od rana do 17-18 siedzi przy komputerze i nie ma się jak oderwać w ogóle, bo ma na bieżąco cały czas jakieś zlecenia, zadania, więc musi to robić. Ma jedną przerwę półgodzinną w ciągu 8 godz., ale to zazwyczaj jest za mało, żeby zrobić coś do jedzenia.

**I jak sobie z tym radzisz?**
Chyba w porządku. Trochę nabrałem pewności w kuchni, bo kiedyś czułek się jakbym miał 2 lewe ręce w tej kuchni, zero intuicji, ale teraz trochę lepiej, choć i tak zazwyczaj pracuje z jakimiś przepisami, ale zdarza mi się je modyfikować, coś dodać, albo w większym stopniu zmodyfikować i też wychodzi całkiem nieźle.

**Czyli masz jakieś pozytywne skutki pandemii?**

Jak najbardziej.

**Wcześniej chodziłeś na jakiś basen, siłownię?**

Na basen chodziłem, chociaż nie jakoś często - może raz w miesiącu. To jest bardzo mało, chciałbym częściej, ale studia, praca, czasem mi się już nie chciało albo nie miałem siły. Byłem 2 razy na siłowni w lutym. Kiedyś byłem bardzo aktywny fizycznie, ale to jeszcze zanim poszedłem na studia.

**Brakuje ci tego?**

Tak, trochę mi brakuje, bo nie mogę się zmobilizować w domu, bo jestem w domu. Trochę tak na to zwalam bezsensownie zamiast zacząć to robić. Siłownia i basen to jest taki bodziec, że już wyszedłem z domu, mam ten cel, więc już idę i to robię. W domu zawsze znajdę sobie jakaś wymówkę albo inne zajęcie, które stwierdzę, że jest ważniejsze.

Dlaczego myślisz, żeby zacząć ćwiczyć?

Przybrałem na wadze, dostałem brzucha, którego kiedyś nie miałem. Zawsze chciałem trochę rozbudować klatkę piersiową, ale nigdy nie byłem fanem siłowni. Ja zawsze wolałem grać w tenisa, pływać na desce i o siłowni nigdy nie myślałem. Teraz wzięło mnie jakoś na tę siłownię. Mam znajomego, który mieszka niedaleko i on był kiedyś "zajawiony" siłownia mocno, więc nawet się umówiliśmy jeszcze przed pandemią, że będziemy chodzili, kupimy sobie karnet, tylko w przyszłym miesiącu, bo finansowo on nie da rady w marcu, ale przyszła pandemia, więc nic z tego. Miałem żal trochę do tego koronawirusa, że ten plan mi też pokrzyżował. No i miałem też żal z tą pracą.

**To przytycie, to teraz podczas siedzenia w domu?**

Nie, ja podczas studiów przytyłem, ale ja też sobie zdaję sprawę, że ja lubię słodycze i sporo tych słodyczy zdarzało mi się jeść, więc to jest uzasadnione.

**Teraz też jesz więcej słodyczy?**

Nie, akurat w tej chwili nie. Od 1.5-2 tygodni nic takiego poważniejszego nie zjadłem, ale czasem mam taką ochotę, że najdzie mnie, że mam ochotę zjeść batona albo nawet tabliczkę czekolady całą i kupuję.

**A jak w ogóle tę Wielkanoc w końcu spędziłeś?**

Kupiliśmy sobie pewną ilość trawy i się zjaraliśmy. To było zaplanowane.

**Jak oceniasz to doświadczenie?**

Jak najbardziej pozytywnie, bardzo mi się podobało. To były pierwsze święta spędzone w ten sposób. Nie były to święta spędzone z rodziną, ale z moją partnerką i było bardzo fajnie. Ja nie lubię za specjalnie świąt i tej atmosfery takiej. Bardziej o Boże Narodzenie chodzi, ale trzeba łazić do kościoła z jakąś palmą, z czymś tam, dzielić się jajkiem - nie cierpię tego, a teraz spędziliśmy święta tak, jak my chcieliśmy. Minus jest taki, że nie zobaczyłem się z rodziną. Rozmawiałem z rodzicami przez Zooma i chyba 1.5 godz. trwała rozmowa po tym śniadaniu.

**Jak ci się rozmawia przez takie komunikatory?**

Fajnie jest ze wszystkimi się zobaczyć, ale...Trochę na siłę...Znaczy nie na siłę...Już po pewnym czasie, jak się z kimś tak siedzi...Między nami jest inna rozmowa teraz, bo mamy konkretne tematy do omówienia, ale tak spontanicznie, żeby się z kimś połączyć na tym Zoomie, to zdarzyło nam się chyba raz tak ze znajomymi rozmawiać. Potrwało z godzinę i się rozłączyliśmy, bo jest zupełnie inaczej niż rozmowa z bliska. Ciężko mi powiedzieć, jak to oceniam. Na pewno nie za często bym mógł tak gadać i nie odczuwam jakiejś potrzeby specjalnie. Chyba wole zadzwonić przez telefon, ale też miło jest się z kimś zobaczyć czasami na żywo, ale nie na za długo.

**Byliście w jakimś parku, lesie?**

Tylko z tym kolegą nad Wisłą. Jest tam taki lasek i przeszliśmy się między drzewami.

**A na majówkę coś planujecie?**

Nie, w zasadzie nie.
