# Supplementary material for: The challenges arising from the COVID-19 pandemic and the way people deal with them. A qualitative longitudinal study
Source: PLoS One. 2021 Oct 11;16(10):e0258133. doi: 10.1371/journal.pone.0258133 (PMC8504766; doi:10.1371/journal.pone.0258133)
Supplement: S1 Dataset — (ZIP) [file pone.0258133.s003.zip › Transcriptions/stage 3/11.3_M_35_couple, with child.docx]

**11.3_M_35_couple with child**

**Co słychać?**

Nic, jest pięknie, lato się robi. Niedługo będziemy sprzedawać lody gałkowe i już będzie fajnie. Poza tym, bez zmian. Wstaję rano - praca, kładę się spać - myślę o pracy, wstaję rano - praca. I tak w koło Macieju.

**A coś miłego się dzieje?**

A ja nie wiem, czy to jest niemiłe. Ze względu na to, że jest ta praca, nie ma jakoś zmian. A, Maciek po raz pierwszy w życiu pomył podłogi w całym domu. Przed koronawirusem on wykorzystywał mnie, to teraz ja jego. :)

**A w weekendy też pracujesz?**

Tak, ostatnio nie byliśmy na weekend w Warszawie, zostaliśmy tutaj. Wczoraj pierwszy raz od koronawirusa pojechaliśmy pod Warszawę. Byliśmy po prezent i podjechaliśmy do Zalesia, pod Piaseczno. Tam jest taki duży staw i poszliśmy się przespacerować. Rzuciłem temat, czy nie chcą się przejść, chcieli, to poszliśmy.

**U was nie da się spacerować?**

W sumie da się, ale tutaj tego nie robimy. Można by było, bo i rzeczka jest - z 200-300 m, ale my nie chodzimy. Jesteśmy sfokusowani na pracę.

**Dlaczego nie pojechaliście do Warszawy odpocząć w weekend, przynajmniej na jeden dzień?**

A nie wiem. Coś musieliśmy zrobić? Nie wiem, nie pamiętam. Ale powiem ci, że dużo pracujemy.

**Chcecie, czy musicie?**

Ja chcę. Jak się nakręcę, ciężko mnie zatrzymać. Dla Ewelinki to jest codzienność tutaj. Natomiast jak ja już zacznę, to ciężko nawet zrobić sobie ze mną rozmowę przez internet.

**Wczoraj jednak zrobiłeś przerwę.**

Jak już tam pojechaliśmy, byliśmy nawet na kebabie. Poszliśmy zjeść go w parku. W sumie nawet nie wiem, czy można. Można?

**Nie wiem, trzeba mieć maseczkę. Jak to zrobiliście?**

Zdjęliśmy maseczki. Nawet się tak zastanawialiśmy, bo byliśmy pod kamerą i nie wiedzieliśmy, czy ktoś do nas przyjdzie, czy nie. Ale udało się. Zjedliśmy w parku, w którym ja też nie byłem już parę lat, więc przypominałem sobie jaki był, a jak teraz wygląda. Po zjedzeniu, jadąc już w stronę tej wsi, zapytałem, czy jeszcze się nie przejdziemy i pojechaliśmy sobie nad ten staw.

**Dlaczego pojechaliście na zakupy aż do Warszawy?**

Bo moja teściowa i jej dzieci - córki - nie mają już pomysłu, co kupować mężowi i ojcu na prezent.

**Co wymyśliliście?**

Ja wymyśliłem praktyczny prezent. To jest taki a'la majsterkowicz, który ma bardzo dużo narzędzi - zresztą dużo robi, po godzinach i w trakcie pracy, kiedy musi coś szybko naprawić u siebie w firmie. Bardzo często pracuje jednym narzędziem, więc kupiłem mu narzędzie - to samo, tylko akumulatorowe. Żeby się nie męczył, bo ręcznie to narzędzie wymaga, żeby używać sporo siły, a jeśli pracuje nim długo, jest w stanie się zmęczyć. Więc wymyśliłem, że kupcie mu ten sprzęt na baterie elektryczne. A że to jest sprzęt niespotykany w Polsce, nieznany, bo to amerykańska firma, tych sklepów jest mało, a jeden z trzech jest w Warszawie. Baliśmy się, że kurierem nie zdążyło by to dojść, bo to na dzisiaj, czy jutro. Ofertę zrobili nam dopiero po 14:00, więc już jeden dzień odpadłby z kurierem. Tym bardziej, że tu na wsi z tym kurierem jest tak, że on może będzie, a może nie. Jakby nie załapał się na piątek, mielibyśmy to dopiero w poniedziałek, to by już na pewno było po imieninach. Chciałem wsiąść w samochód i pojechać tam i z powrotem, ale jak wsiadłem, to wszyscy inni zrobili to samo.

**Jak myślisz, co ich skłoniło, aby z tobą pojechać?**

Cudowne przebywanie ze mną i czas spędzony ze mną w samochodzie.

**Ale przecież cały czas jesteście razem, masz tę stację za płotem podobno.**

Ale Marek nie widzi mnie przez cały dzień, nie ma mnie w domu. Ja przychodzę tylko na chwilę, zapytam, co tam słychać. On czasami przyleci do mnie na podwórko i tyle. Chociaż była awantura, że nie mogą ze mną jechać. Powiedziałem Markowi, że jak będę rozmawiał z tobą przez telefon, to żeby się nie odzywał, żeby mi nie przeszkadzał. Na jego szczęście udało się nie rozmawiać z tobą. On nie lubi, jak z Tobą rozmawiam, bo rozmawiam za długo. Jak rozmawiałem z tobą pierwszy raz, leciał co trzy minuty do mamy i mówił, że rozmawiam z jakąś dziewczyną przez komputer - i to długo.

**Udało się zrobić coś jeszcze razem?**

Filmy to się staramy przez większość wieczorów razem oglądać. Może dwa wieczory nie oglądaliśmy. To jest naturalne. Z Marem trochę się tłuczemy tutaj. On mi dokucza tutaj trochę na dworze, nie może wejść na stację benzynową. Ustaliliśmy, że ma tutaj tylko swoje podwórko i nie przechodzi na stację. Czasem to wygląda paradoksalnie, bo stoi za furtką i krzyczy "Oskar chodź, Oskar chodź". No, ale tego się jakoś trzymamy, nie chcemy, żeby on tam się za bardzo pojawiał. Ogólnie znowu standardowo - ognisko sobie zrobiliśmy wieczorem. Już prawie nie mam drzewa. Spaliłem przez ten czas dwie kupki drzewa. Trochę pobawiliśmy się na dole, obmyślamy, żeby zrobić mu tutaj - z nim oczywiście - duży salon gier. Taki na wypasie, żeby mógł na weekend przyjeżdżać tu z kolegami i żeby mogli, jak to nazywam, ciupać w te gry. Czy coś robiliśmy tu razem poza tym? Nie. My po prostu spędzamy czas. Do tego stopnia, że jak palę papierosa, to chcę siedzieć i siedzieć, gadać. A gadamy o różnych rzeczach - głupich i niegłupich. Więc spoko.

**A jak szkoła? Udaje ci się angażować? Miałeś mu pomagać?**

Nie udaje mi się angażować i coraz mniej angażuje się też sam Marek. Ma chyba zaległości z trzech dni. Będzie dziś pewnie nadrabiał.

**Przejmujesz się tym, że ma zaległości?**

Zupełnie się tym nie przejmuję. On udaje, że nic nie umie, nie rozumie. Wiem o tym, że pamięć ma dobrą, więc sobie poradzi. Nie ma co się spinać. Wystarczy mu powiedzieć, że nie będzie grał przez najbliższy tydzień, jeśli tego nie zrobi i będzie siedział, aż to zrobi, więc spoko.

**Czyli będzie szantaż?**

Tak, on mówi na mnie zresztą "terrorysta"? Nie pamiętam. Mamusia jest "tygrysica", a Oskar jest.. <krzyczy> Marek, jak ty na mnie mówisz? <nie dostaje odpowiedzi> No, nie pamiętam.

**Co jest teraz dla ciebie największym wyzwaniem?**

Chodzenie w masce. To jest tragedia. Na stacji też muszę w niej chodzić. To jest bez sensu, dusimy się. <przychodzi Marek, Oskar ponawia pytanie> O, „tyran”. To wygląda tak, że wchodzę tam na trzy minuty w masce, a kiedy wychodzę, zdejmuję ją, bo  to jest porażka. Nie mogę się do tego przyzwyczaić. To chyba jakiś dyskomfort jest, który odczuwam osobiście, fizycznie, jeśli chodzi o koronawirusa. Uszy mnie już od tego bolą. Pierwszą maskę jaką miałem, to trochę małą. Później Eweliny siostra poszyła nam, dla całej rodziny, maski, więc ta już była bardziej przystosowana do mojej wielkiej głowy - bo mam ogromną. No ale, jest ogromny dyskomfort z tymi maskami. Wczoraj nawet patrzyliśmy w tym parku. Wiesz, pierwszy raz od półtora tygodnia byłem w Warszawie. Patrzyliśmy po ludziach, pierwszy raz poszliśmy do takiego parku, co jest prawdziwym parkiem i się spaceruje. Ludzie ostro jednak chodzą w tych maskach - wszyscy. Niektórzy mają rękawiczki, niektórzy nie. Ja na przykład nie noszę w ogóle rękawiczek, dopóki nie idę do sklepu jakiegośtam. Ewelinka nosi non stop. Odkaża nawet te rękawiczki. Ja nie lubię tak. Czuję się wtedy ubezwłasnowolniony. I z tymi rękawiczkami, i z tą maską.

**Poza maską, coś jeszcze cię wkurza, jest trudne?**

Nie, chyba nie mam takich rzeczy. Nic mnie nie wkurza. Nie przeszkadza mi, że dziecko jest w domu. Prywatnie nie ma co mnie wkurzać. Radzimy sobie we trójkę i nie wkurzam się - poza tą maską. Przez ostatnie trzy tygodnie zrobiłem fizycznie więcej rzeczy, niż przez ostatnie kilka lat. Jemy więcej. Na tej stacji przez ostatnie cztery tygodnie zrobiliśmy więcej, niż przez ostatnie dwa lata. Ja nie widzę minusów, dla mnie koronawirus nie jest minusem.

**A to, że nie ma was w Warszawie?**

Ja już się chyba do tego przyzwyczajam i może dlatego nie pojechaliśmy w ten weekend? Chociaż to może błąd, bo już w poniedziałek, czy wtorek była taka sytuacja, że głupio, że nie pojechaliśmy, bo ten reset by się przydał. Więc wczoraj, jak to u nas bywa - spontanicznie - ale trzeba nam było jednak tego spaceru. Miało nam zejść tylko tam i z powrotem, nie pojechaliśmy nawet do mieszkania. A tu kebab, zaczęło nam się coraz mniej chcieć wracać, spacer. Wrzuciłem na autostradzie tempomat na 100 km/h, więc sobie tak toczyliśmy się - żeby nie za szybko.

**A czemu nie zostaliście choć na jedną noc w tym mieszkaniu, w Warszawie?**

Są jakieś rzeczy do zrobienia, które sobie założyliśmy i trzeba działać. Wiesz, ja lubię pracować w ogrodzie, a nie wiem, kiedy ostatnio to robiłem, chyba ze dwa lata temu, jak tu więcej czasu byliśmy. W tym roku ubzdurało mi się, że zrobię automatyczne podlewanie, zraszanie w całym ogrodzie. Zaczynamy kupować, na trawie pełno rurek. Nasz ten podstawowy ogród ma gdzieś ponad 1000 m2, więc wiesz. Oprócz tego, że robimy na stacji, ja jeszcze coś w tym domu działam. Chyba z nudów. Choć może nie tyle nudów, co potrzeby odizolowania się od tej stacji, tych problemów tej stacji, wykorzystania czasu, że jest się na - teoretycznie nazywam to - wolnym. Nie jestem w Warszawie, to jestem na wolnym. Nawet, jak sporo pracuję, jestem na wolnym.

**Jakie macie plany na nadchodzący weekend? Jedziecie do Warszawy?**

Najprawdopodobniej jutro rano. Myślałem, żeby dzisiaj, ale mam jeszcze kilka rzeczy do zrobienia, więc coś chyba porobię. Jutro się po prostu spakujemy w południe i pojedziemy. Widzisz, w Warszawie jest tak, że tam spacerując już było, "ale ciepło, to może byśmy sobie wyszli do parku, na przykład Górczewska, coś byśmy sobie tam pooglądali". Już pewnie w domu też nie posiedzimy. Dzieciak mógłby iść na rower, widzę, że dzieciaki już tymi rowerami - w maskach, bo w maskach - ale jeżdżą. Nie mam pojęcia, nawet nie sprawdziliśmy, kiedy otwierają jakieś galerie, sklepy. Mamy plan kupić sobie rolki - ja z Eweliną. Do celów rekreacyjno-sportowych. A Marek na rowerze. Ewelina uwielbia jeździć na rolkach. Ja będę się przewracał, a Marek będzie uciekał na rowerze. Więc jest fajnie. Tylko muszą nam otworzyć te galerie. Natomiast jak ci już wcześniej mówiłem, jak otworzą galerie, wypierniczymy sobie na tydzień gdzieś dalej. Pewnie góry, bo kocham góry i wolę je, zamiast morza.

**A te imieniny teścia?**

Ja nie jadę, bo mi się nie chce. Marek sobie zarządził, że on jedzie. Nie wiem, jak sobie to załatwi, chyba, że załatwi sobie transport. Ja nie jestem osobą, której da się coś narzucić, więc nie. Jeśli nie zrobię czegoś z własnej chęci, to nie jest zrobione. Ewelina pewnie pojedzie ze mną do Warszawy.

**Czyli odstawicie Marka do dziadków?**

Nie wiem, to trzy minuty decyzji, więc nie zastanawiamy się nad tym, to nie problem.

**A to nie jest problem, że jak on się teraz z nikim nie widuje, to pojechałby tam?**

Tak, dlatego ciągnie go, żeby pojechać tam do dzieciaków. Pewnie mu się za nimi trochę tęskni. Natomiast jadąc do Warszawy też jestem mu w stanie załatwić kolegów - bo jak szukaliśmy swojego lokum, to tak, żeby było blisko jego szkoły, więc też jego znajomych. On w Warszawie czuje się lepiej, niż tutaj.

**Myślisz, że to bezpieczne, żeby spotykał się z kolegami?**

Nie wiem, wszystko zależy, co robi ten jego kolega.

**Teraz go izolujecie, jest u was, nie może wychodzić poza obręb podwórka, w parku też był z wami. Pozwolicie mu się teraz spotkać z kolegami, gdyby chciał, w weekend?**

Tak, myślę, że z jednym tak. On ma 8 lat, więc to wiąże się z tym, że jeśli rodzice się znają, chłopaki zajmują się sobą cały dzień i nie trzeba ich ciągle pilnować. Jeśli byłoby więcej dzieci, zaraz byłyby spotkania rodziców, ble ble ble, nie... Nie myślałem o tym. Ale nie byłoby problemu. Natomiast mamy dużo wspólnych rzeczy do zrobienia! Mówię ci, rowery, wyjść, przespacerować się, trochę porzucać się w tym parku po trawie. Jest co robić.

**A za czym teraz najbardziej tęsknisz? Jak pojedziesz do Warszawy, co będzie takim musem?**

No dla mnie to spacer. Oprócz tego, mój mały balkonik. Bo będąc tu, porównując, tamten to jest balkonik. To są sentymenty - siadam sobie tam rano z kawą. Trzecia rzecz, to obiecane kilka godzin gry z małym - na jakimś PlayStation, czy czymś takim. Tu w ogóle z nim nie gram, nie interesuję się tym, nie mam czasu. Siedzę do wieczora. Więc pewnie pogramy sobie w niedzielę. Mama będzie robiła coś jeść, a my będziemy, jak to ja mówię, ciupać.

**Które ze zdjęć najlepiej oddaje to, jak czułeś się w ciągu ostatniego tygodnia?**

12 - pomyślałem sobie, że jestem taki, że cały czas coś mnie gania, że muszę coś zrobić. Zakładam coś sobie i tak kamyczek do kamyczka, tak sobie robię.

**Jak czujesz się z tym, że tak dokładasz kamyczek do kamyczka?**

Dobrze.

**A nie brakuje ci czegoś w tym dokładaniu kamyczków?**

Warszawy.

**Co dałaby ci Warszawa, poza tym, że nie byłbyś na wsi?**

Nie byłbym tu.

**Normalnie mieszkacie w Warszawie, bo młody chodzi do szkoły. Minimum 5 dni jesteście normalnie tam. To przecież wasza decyzja, że mieszkacie teraz na wsi.**

No tak, ale.

**Dlaczego nie możecie nadal mieszkać tam, a na weekendy przyjeżdżać tu?**

Mimo wszystko mamy tu więcej przestrzeni. Czas tej sytuacji, że wszystko jest pozamykane i ta przestrzeń swobody jest nam trochę narzucana, to chyba lepiej jednak na tych 2000 m siedzieć sobie na łące, niż wiesz, w 100, w Warszawie. Nie oszukujmy się.

**Korzystacie z tej działki, czy ty głównie pracujesz?**

No nie, jeszcze znajduję czas na te ogniska chociażby. Poganiam się z tym dzieciakiem dookoła domu. Ogródek zaczęliśmy robić. Taki dla swoich warzyw. Płotek zrobiłem do ogródka - no, naprawiłem, bo był.

**No tak, ale nadal nie rozumiem, dlaczego tęsknicie za Warszawą, a tego nie zmienicie.**

Ta Warszawa nie jest teraz tą prawdziwą Warszawą. Nie jest ona tym miastem, w którym żyje się normalnie, na co dzień. Nie ma rowerków Veturilo. Tak, to sobie szliśmy i mówiliśmy, a chodź, podjedziemy rowerkiem. I pyk, 200 m rowerkiem do następnej stacji. I już. Często tak robiliśmy. Zresztą, ja jestem rekordzistą, jeśli chodzi o Warszawę, nieoddania roweru. Chyba 3 tygodnie. Czy 2,5. Nie pamiętam. Ta normalna Warszawa to jest miejsce, w którym na tę chwilę, w moim wieku, czuję się najlepiej. Nie jest wcale powiedziane, że za 10 lat, kiedy już będę stary i brzydki, nie będę wolał siedzieć tutaj.

**A dlaczego tych rowerków według ciebie nie ma, to jest słuszna decyzja?**

Przez koronawirusa. Dla mnie ogólnie, wszystkie decyzje o zamknięciu tego wszystkiego były słuszne. Dla mnie niesłuszne są teraz decyzje, że to wszystko otwierają. Powinno być cały czas tak samo, jak jest. Jeśli były obostrzenia, to one dalej powinny być, bo z jednej strony pozwalanie na to ludziom, przyzwyczajanie ich tylko po to, żeby ten 10.05. był ich upragnionym dniem wyborów, z mojej perspektywy może okazać się strzałem w kolano dla partii rządzącej. Nawet, gdyby obecny prezydent wygrał wybory, a okazałoby się, że wirus zrobił sobie drugi etap takiego ataku na populację, to rząd się z tego nie wywinie. Jest to głupie.

**Które złagodzenia uważasz za niesłuszne?**

Nie wiem nic na temat stanu, co tak naprawdę wie świat na temat koronawirusa. Z tego, co wiem, nie ma ani szczepionki, ani tabletki na to, żeby to wstrzymać, albo coś. Więc dalej jesteśmy za przeproszeniem w tyłku. Więc skoro się dało w te obostrzenia, wydaje mi się, że one nadal powinny być, dopóki nie będzie jakiegoś rozwiązania. No ja nie widzę na razie rozwiązania. Nie sądzę, że wirus się na tyle zmutował, że nie ma go w populacji zagęszczonej. Gdyby była szczepionka, można by wszystko powoli otwierać.

**Mówi się, że do wynalezienia szczepionki jeszcze jakiś rok. Myślisz, że można by tak funkcjonować do tego czasu?**

Myślę, że przez rok zniknęłoby wiele państw. Może nie z mapy, ale gospodarczo. To jest dużo czasu. Polska myślę, że mogłaby przez ten czas funkcjonować. Mamy wszystkie swoje złoża, więc myślę, że tak. W tym roku szkolnym dzieci chyba nie pójdą do szkoły. Co będzie, kiedy otworzą wszystko przed wyborami? Tylko po to, żeby je przeprowadzić. Co w sytuacji, kiedy okaże się, że znów będzie jakiś horrendalny wzrost tych zachorowań?

**Nie wiem, a Ty myślisz, że będzie?**

Nie mam zielonego pojęcia.

**A wiesz, ilu jest teraz chorych w Polsce?**

Nie. Wiem, że 24 wczoraj umarło. Że to wcale się nie zmniejsza, a idzie do góry. Może dojdzie do sytuacji, że będziemy traktować koronawirusa jako grypę. Że ludzie umierają  - pewnie więcej ludzi umarło wczoraj z powodu powikłań grypowych, niż koronawirusa. Ale jest boom na tego koronawirusa, już państwa na świecie zaczynają się tym zasłaniać, widać, kto na tym zarabia. Dochodzi do tego, że mądrzejsze kraje zaczynają patrzeć, jakie firmy wykupują drugie firmy. Mówimy o tych globalnych firmach. Patrzą, które firmy mają spadki na giełdzie, a kto je kupuje. Zaczynają szukać chyba już tzw. teorii spiskowych. Więc jest ciekawie. Ja nie mam co narzekać, moi znajomi nie mają co narzekać. Moi koledzy, którzy mają restaurację, potrafili się dostosować, zrobić jedzenie na zamówienie. Kumpel dzwonił już do mnie dwa razy w ciągu koronawirusa, czy nie chcę kupić z nim kolejnej restauracji, bo mówi, że to, co się dzieje, to jest czad. Pomimo tego, że są zamknięte. Nie ma ani w przyrodzie, ani w biznesie pustych przestrzeni. Ludzie umierają, będą następni. U nas na sklepie prezerwatywy schodzą jak świeże bułki. Ludzie się kochają, miłują, jest spoko. Jedyne co, to te obostrzenia. No powiedz mi sama, niech je zwolnią, ale pójdziesz od razu, polecisz do kina? Ja nie. Natomiast podejrzewam, że jak otworzą te galerie, to tam przez pierwszy tydzień nie będzie miejsca na parkingu.

**Mówiłeś, że sam na to czekasz.**

Ja na to czekam po to, żeby wsiąść w samochód i uciec do hoteli. Ewelinka mi powiedziała parę dni temu, że już zaczynają w drugim etapie otwarcia się na świat, otwierać hotele.

**Dlaczego uważasz, że hotele są ok, ale galerie handlowe nie?**

Ja nie uważam, że hotele są ok.

**Ale mówisz, że będziesz z nich korzystał?**

Tak. Nie wiem, ale tak bym zrobił.

**A dlaczego tak zrobisz?**

Żeby uciec od pędu.

**A nie myślisz, że wszyscy uciekną?**

Nie, absolutnie. Ci, którzy mają swoje biznesy, będą chyba chcieli sprawić, aby one odżyły. Są rodziny, które nie wyobrażały sobie weekendu bez galerii handlowej. Jest większość takich ludzi. Myślę, że taka Warszawa będzie bardzo zapchana. To jest moja wizja tego, co będzie, jak rząd odpuści.

**A ty wiesz, jakie teraz obostrzenia znieśli?**

Mówiłem ci, że zastanawiałem się nawet, pytałem Ewelinki, czy my możemy w parku jeść, czy nie dostaniemy mandatu. Nie wiem, co oni w poniedziałek zrobili. Ja mam tylko styczność z rzeczami dotyczącymi handlu. U nas są teraz dwie osoby więcej na kasę. Tyle mnie interesuje. Więcej mnie nie interesuje, nie mam pojęcia. Ja i nasza trójka ma to szczęście, że mega jesteśmy wyłączeni z tego koronawirusa. My się nie fokusowaliśmy w ogóle, nie czytamy tego. Ja, jak biorę telefon i zaczynam czytać pierwszą wiadomość o koronawirusie, drugą wiadomość o koronawirusie, mówię - booże, jakie to wszystko smutne, chrzanić to. I od razu się z tego wszystkiego wyłączam.

**Teraz można przemieszczać się w celach rekreacyjnych, na przykład wyjść na rower. To dobrze?**

Widzę większe zagrożenie w 8 osobach w sklepie, niż 2 jadących rowerem. Jeszcze w masce. To jest akurat ok.

**Otworzyli wstęp do lasów i parków.**

W lasach pod Warszawą był spęd ludzi. Tego było od groma. Teraz chociaż nie będą dostawali mandatów. Przy Bemowie, w Kampinosie, nie było gdzie postawić samochodu. Ja mam Park Kampinoski po drugiej stronie ulicy. Jak przejeżdżamy sobie samochodem, pojadę inną drogą i wiem już, co się dzieje.

**Co sądzisz o tym, że teraz już można chodzić do lasów i parków?**

Jak wczoraj byłem w parku, to nie było tak, że było jakoś nie wiadomo ile ludzi. Ludzie chyba też uważają. Może nie jest tak, że wszyscy się na to rzucą. Dla mnie, to źle, że oni znieśli ten zakaz, bo ja myślę, że zrobili to tylko na potrzebę wyborów.

**Ale poszedłeś wczoraj do tego parku.**

No, super sprawa. Ktoś rozwiązuje mi skrzydła, które mi gdzieś tam związuje. To akurat przyjemne. Przyjemnie jest posiedzieć 5 minut. A my faktycznie, zjedliśmy i poszliśmy, nie siedzieliśmy nie wiadomo ile. To nie jest jeszcze taki odpoczynek. Czy to jest dobre, trudno mi powiedzieć. Nie wiem, jaką oni mają faktyczną wiedzę na temat tego wirusa. W jakich momentach on się rozprzestrzenia. Przeczytałem jakichś naukowców, że on ginie po kilku godzinach w temperaturze 26 stopni. To coś jest nie tak, skoro w krajach tropikalnych też się pojawił i żyje sobie w tych ukropach. No nie wiem, to wszystko jest takie, nie wiem.

**Zmienili też liczbę osób dopuszczalnych w kościele, co o tym sądzisz?**

Cały czas jestem negatywny.

**A dzieciaki od 13 r.ż., które mogą same wychodzić?**

Negatywny. Dzieciaków się nie skontroluje. Wszystko dla mnie jest negatywne, bo jest robione celowo.

**A większa ilość ludzi na stacji - to też jest negatywne?**

Negatywne.

**A co wiesz o tych planach otwierania?**

Drugi poziom to chyba galerie i chyba hotele.

**Poczekaj, przeczytam ci <czyta>. Nie ma galerii handlowych.**

Jeśli chodzi o biznes, to jest spoko. Ale jeśli chodzi o ogół, to jest dla mnie hipokryzja przed 10.05., przed wyborami.

**Widzisz sens otwierania tego, jeśli chodzi o gospodarkę?**

Absolutnie nie.

**Dlaczego? Myślisz, że ona da sobie radę?**

Absolutnie, że tak. Nie ma pustych przestrzeni w biznesie. Jak padnie facet, który przez 30 lat prowadził biznes, to za tego faceta powstaną dwie nowe firmy. Nie ma takiego czegoś. Dziś wszedłem na stronę i mogę odkupić od gościa nowe BMW za 5 tys. zł, bo gościa nie stać na to, żeby płacił leasing. Wyobraź sobie, że facet sam dorobił się 3 tirów - to firma transportowa. A 20 tirów w jego firmie jest w leasingu. I gość rozkłada ręce i płacze. Ja mu zadaję proste pytanie. Słuchaj, skoro bierzesz coś w leasing, to znaczy, że mogłeś to kupić. A bierzesz w leasing tylko po to, żeby mieć koszty. Czyli tak naprawdę pompujesz swoją firmę, która de facto nie istnieje, bo po miesiącu musisz się zamykać. To wszystko zależy. Ktoś chce się rozwijać, bierze kredyty, a nie stać go, aby to kupić. Czy to jest odpowiedzialny biznes? Nie, to nie jest odpowiedzialny biznes. Gastronomia. Są restauracje. Gesslerowa nie pozwoliłaby sobie na to, żeby dawać na wynos. Wiesz, to jej nie będzie w tym biznesie. Skoro stać ją na taki ekskluzywny szał, to stać ją na to, aby przez rok opłacać swoich pracowników. Albo nie opłacać, a wyrzucić. I nie płacić czynszu w kamienicy. A są ludzie, którzy mają ładne, fajne restauracje. Nigdy nie mieli opcji na wynos, a potrafili to zmienić, kucharze przychodzą. Sporo jest takich rzeczy. Tak, jak uważałem, że wsparcie dla działalności jest złe, tak uważam, że my sobie damy radę. Tym bardziej my, Polacy. My sobie mega damy radę.

**A kojarzysz, co będzie w trzecim etapie?**

Pewnie wszystko przepuszczą.

**Nie, <czyta>.**

Kiedy to planują?

**Nie wiem, tu nie ma dat, jest tylko kolejność.**

Boję się, że przed 10.05. postanowią otworzyć galerie. Teraz prawda jest taka, że rządzący, którzy mają nad nami dużą władzę, mogą bardzo mocno wykorzystywać stan koronawirusa. Jakiś mądry ekonomista pracujący dla rządu obliczy, że potrzymamy galerie zamknięte jeszcze z miesiąc. Ta reszta zbuduje się finansowo, te małe zakłady. To wszystko da się odbudować, to jest gospodarka. Jeśli chodzi o koronawirusa, to jest złe, to odpuszczanie bez konkretnej wiedzy na temat możliwości ratowania się  przed zakażeniem. U nas tak szybko reagowali, wszystko zamknęli, co mnie cieszyło. A teraz z niewiadomych tak naprawdę przyczyn - bo nie jest powiedziane do końca, z jakiego powodu oni są już na tyle pewni, żeby to wszystko otwierać.

**Myślisz, że naukowcy naprawdę nie wiedzą nic o tym koronawirusie?**

Myślę, że dużo wiedzą i dlatego na przykład osoby rządzące krajami, świetnie to wykorzystują. Wyobraź sobie, że teraz takie rządy w każdym kraju mogą sobie kreować własną gospodarkę w każdym kraju na nowo. Mogą udawać, że nie chcą zagranicznych produktów. My mamy z Ukrainą umowę na 30 lat, na zboże. Oni mogą teraz powiedzieć, że ze względu na koronawirusa my jeszcze potrzymamy zamknięte granice przez pół roku. I może będą brali warzywa czy owoce od polskich rolników. Można teraz bardzo mocno wykreować swoją gospodarkę. Nie mówię, że tylko w naszym kraju. Każdy mądrzejszy kraj będzie starał się to zrobić.

**No dobra, w trzecim etapie jest otwarcie fryzjerów i kosmetyczek.**

No właśnie, dobrze, że mi powiedziałaś, bo dla mnie męczarnią jest, że nie mogę iść do fryzjera.

**A pójdziesz, jak otworzą?**

Ja akurat pójdę. Ale nie ważne, co otwierają, ze względu na ich brak wiedzy, uważam, że to jest złe. Ale jeśli to robią, to chciałbym i wierzę mocno w mojej głowie, że zrobią to na tyle mądrze, żebyśmy skorzystali na tym gospodarczo. Kiedyś i tak będą musieli to zrobić.

**Skupmy się na tych fryzjerach i kosmetyczkach. Myślisz, że ważniejsze jest, aby to otworzyć, bo ludzie muszą się ostrzyc, czy żeby ruszyć gospodarczo?**

Ja myślę, że żeby gospodarczo znowu ruszyć.

**Masz pojęcie, jak duża jest to w skali kraju gałąź gospodarki?**

Nie mam świadomości, ale większość tego typu usług w Polsce to są usługi i małe działalności gospodarcze. Jest tam zatrudnionych niewiele osób, najczęściej wynajmują też miejsca swojego wykonywania działalności. Oni muszą płacić czynsze. Mieli te trzy miesiące, które dał rząd, że nie trzeba było płacić składek, więc pewnie z tego skorzystali. Więc teraz dlatego to ich powoli uruchamiamy, żeby jednak było na te opłaty czynszu.

**No, ale za czynsz musieli płacić.**

Tak i nie.

**No tak, musieli.**

W Gdańsku główny deweloper przy deptaku, który ma tam prawie wszystkie lokale, napisał, że „słuchajcie, pomagamy sobie, do odwołania nie płacicie mi czynszu”. Wyobraź sobie, że cały deptak w Gdańsku - to są kolosalne pieniądze dla dewelopera. A on napisał do wszystkich maila, że odpuszcza.

**To Gdańsk. A reszta?**

Niektórzy wynajmują mieszkania. Pierwszy pomysł, który przychodzi do głowy, to napisać do właściciela o obniżenie czynszu. On może oczywiście nie obniżyć wynajmu. Niektórzy w ogóle nie zapytają, jak ja. Ale są ludzie, którzy próbują w ten sposób negocjować. Każdy musi próbować. Te małe działalności są najważniejsze dla naszego kraju, bo prawie wszystko, co zarobią, oddają podatkowo do naszego kraju. Im większy moloch, tym więcej pieniędzy ucieka poza granice naszego kraju. Więc jeśli mają z powrotem otwierać tę gospodarkę, żeby to zadziałało i nie było wielu strat, na samym końcu uważam, że powinni otworzyć galerie handlowe.

**Ale w galeriach są też polskie sklepy.**

Jakie? Nie wiem, czy jest jakiś polski sklep w markecie.

**Reserved jest polski.**

Polski, ale ma opodatkowanie w trzech różnych krajach, nie oddają u nas całego podatku.

**Wittchen jest polski.**

Tylko pracowników ma zatrudnionych na oddziale matka, nie?

**No nie.**

A gdzie oddaje składki?

**W Polsce, jest spod Poznania. Tu produkuje, tu ma fabrykę.**

A co z tego, że ma tu fabrykę? Jak masz 100 tys. obrotu miesięcznie, to miałabyś markę, firmę zarejestrowaną w Polsce?

**No nie wiem.**

Nie.

**Uważasz, że wszyscy tak kombinują?**

To nie jest kombinowanie. To jest rynek. Ze 100 tys. zł zarobionych w Polsce musisz oddać - uśredniam - 20 + 20. To jest 40% ze 100 tys. Jeśli zrobisz w Polsce córkę, a matkę, no nie wiem, boom, w Wielkiej Brytanii, to przy 100 tys., oddajesz 6%. To każdy rozsądny, prowadzący tu biznes, ucieka stąd. Dlatego uważam, że molochy powinny być najdłużej trzymane, bo to małe działalności tworzą nasz biznes, wartość rynku gospodarczego.

**A co z ludźmi zatrudnionymi w galeriach?**

Załóżmy, że jest jakaś pani, która pracuje w Biedronce i utrzymuje męża i dziecko. I ona będzie teraz pracowała na pół etatu, albo w ogóle. To są takie tragedie.

**Jaka to jest skala, takich ludzi, którzy mają zmniejszone dochody, lub je tracili?**

Ja myślę, że to około 30-40% ludzi w Polsce.

**To jest dobre dla gospodarki?**

Nie, ale muszą to jakoś uruchomić. Jeśli nie będziemy konsekwentnie i odpowiedzialnie uruchamiać tej gospodarki, to nie będziemy zaraz mieli pieniędzy na emerytury, 500+, wsparcie dla przedsiębiorstw, które dostały i wykorzystały to wsparcie, jak 3 miesiące niepłacenia ZUSu. To są ogromne pieniądze, na to musimy skądś mieć.

**One mogą nie płacić teraz ZUSu?**

Tak.

**A to nie jest tyko przesunięte, odsunięte w czasie?**

Ja się spotykam z ludźmi, którzy już korzystają.

**Gdybyś Ty otwierał gospodarkę, jak byś to zrobił?**

Od najmniejszych, od tych działalności, to oni tworzą nasz rynek. Fryzjerzy, kosmetyczki, małe sklepy, te osiedlowe - gdzie jest właściciel i 2-3 osoby. Od nich bym zaczął.

**A co potem?**

Niestety, ale u mnie najbardziej po dupie dostałaby kultura.

**Dlaczego?**

Bo to najmniejszy przyrost gotówki do budżetu państwa.

**Myślisz, że powinno się też brać pod uwagę aspekt zdrowia psychicznego ludzi, oprócz aspektu gospodarczego?**

A nie uważasz, że otwieranie punktów od najmniejszych do największych, to też jest rozsądne podejście z psychologicznego punktu, dla ludzi? Uważam, że to też jest dobre. Otworzysz coś dużego i teraz - pójdziesz, czy nie pójdziesz? Ja podchodzę do tego normalnie. Ja nie pójdę, nie dlatego, że jest koronawirus, tylko dlatego, że po prostu nie lubię tłumów. Ale wczoraj rozmawiałem z człowiekiem, który nas serwisował, wspierał stację benzynową. I on powiedział Ewelince, że on jest przerażony. Facet 30 parę lat. Od paru tygodni nie wychodzi z domu. Byłem w szoku. My tu pracujemy trzy razy więcej, w ogóle nie zwracamy uwagi, a tu facet taki mówi nam, że on się nie rusza z domu. Zanim on według mnie będzie psychicznie przygotowany, żeby iść do jakiegoś motłochu typu galeria, do kina na film, to uuu, jeszcze minie dużo czasu od tego, kiedy to wszystko otworzą.

**No dobra, ale jest dużo ludzi, którzy się nie boją i cierpią na tym, że nie mogą decydować o sobie.**

No.

**I dla ich zdrowia psychicznego nie powinno się pootwierać miejsc, aby mogli spędzić czas, gdzie lubią?**

Wydaje mi się, że to jest błędne myślenie.

**A otwieranie w trzecim etapie przedszkoli i klas 1-3. Co sądzisz?**

W perspektywie prywatnej - jest to błędem. Natomiast jest to wymuszone i jednoznaczne z tym, że chcą otwierać te małe lokale usługowe. Skoro ludzie będą już mogli wrócić do pracy, mimo wszystko będą musieli komuś te dzieciaki zostawić. To jest jakby automatyczne.

**Ale mogliby siedzieć na tym zasiłku?**

Na którym zasiłku?

**Jest teraz taki specjalny zasiłek.**

Myślisz, że on będzie trwał rok? Że to pociągniemy, jak nie otworzymy tych małych lokali?

**No nie wiem.**

Na szczęście ten - jak słyszymy często - zły rząd, był w stanie pomóc. Natomiast państwo nie jest w stanie utrzymać 30 milionowego społeczeństwa na zasiłkach. Musi to być jednoznaczne. Skoro pani w Zarze chce już iść do pracy, to dobra.

**Myślisz, że ona chce wrócić? Siedzi teraz w domu, ma 80% zasiłek, nie musi chodzić do pracy. Myślisz, że ona chce wrócić do pracy?**

To społeczeństwo jest podzielone. Mamy część, która już nie może się doczekać, aż zacznie normalnie funkcjonować, bo to normalne funkcjonowanie dla niego to pójście do pracy, spotkanie się z kimś. A jest towarzystwo, które wolałoby brać zasiłek. To tak, jak przy 500+. Są tacy, którzy dostają 2000 zł za czwórkę dzieci i nie chcą pracować, i tacy, którzy biorą, ale sobie pracują. Słuchaj, różni są ludzie. Ja, gdybym miał wybierać, to chyba nie wytrzymałbym dwóch miesięcy. Już wolałbym nie brać tego zasiłku, a funkcjonować normalnie, tak, jak to wygląda w mojej głowie. To jest każdego osobista sprawa. Natomiast zasiłek nie będzie cały czas, bo on był stricte na daną sytuację.

**Ale dalej jest koronawirus.**

Dlatego nie jestem do końca przekonany, czy odpuszczanie tego jest dobre. Natomiast jeśli odpuszczamy, to rozumiem tę procedurę. Jeśli otworzymy mały lokal, ale żłobki nie, to zadzwoni taka dziewczyna do szefa i powie, wie pan co, ja bym przyszła do tej pracy, ale jeszcze są żłobki zamknięte. Dlatego to musi iść jakoś jedno w drugim, skoro już idzie.

**A czy Ty wiesz, jak Szwecja podeszła do tematu pandemii?**

Nie wiem. Kiedyś czytałem z zainteresowaniem, jak jest w innych krajach. Teraz, nic nie wiem.

**<czyta>  Co sądzisz?**

Fajnie brzmi, bo jest mało obostrzeń. Tylko że, musimy brać pod uwagę, że Polska to jakieś 38-40 milionów, a Szwecja to 10 milionów mieszkańców. Trzeba by brać też chyba pod uwagę mieszkańca na metr kwadratowy państwa. Mówi się, że w USA jest taka tragedia. Tylko wiesz, Texas jest większy od naszego kontynentu. Patrzmy na taką Szwecję. Kraj skandynawski, który jest ogólnie odcięty od rzeczywistości - wystarczy, że zamkną swoje doki. Dają sobie świetnie radę, jako trzy kraje skandynawskie.

**Ale oni nie zamknęli granic.**

Ze Szwecji nie mogą wrócić. A nie, to ze Szwajcarii, przepraszam. Rozumiem ich podejście niezafiksowania się - to jest fajne. Natomiast też są pewnie złożone cyfry i liczby, które pozwalają im to robić. Mentalność też jest inna, to kraj skandynawski.

**No dobra, a Nowa Zelandia. Oni dali ludziom 48 h na przygotowanie się, ogłosili, że będzie lock down, po czym zamknęli wszystko na 4 tygodnie. Wszystko. Zostały tylko wodociągi i elektrownie, żeby był prąd. Nie można było wychodzić, iść do sklepu. Co o tym sądzisz?**

Z punktu widzenia mojego podejścia tyrana, ciekawe jak im to wyszło, ale jeśli to by im wyszło, to są mega krajem, jeśli chodzi o...

**Jeśli u nas by to zrobili, Ty też musiałbyś zamknąć swoją stację na pięć tygodni.**

A co się z tym wiąże?

**Z czym?**

Ty mi rzucasz hasła, w tym kraju zrobili tak, w tamtym tak. Co się z tym wiąże? W moim kraju rząd dał mi trzy miesiące zwolnienia z ZUSu. Co w takim kraju, gdzie oni to zamknęli? Może rząd dał im po 10 tysięcy co miesiąc?

**Nie wiem.**

No właśnie, ja też tego nie wiem, to jest zrzucanie...

**Chodzi mi o to, co uważasz za lepsze, ostre działania, czy takie bardzo łagodne? Podejście którego kraju jest Ci bliższe?**

Nie mam zdania. Z prostej przyczyny. Gdyby się okazało, że to była tylko nakręcanka i taka Szwecja zrobiła bardzo mało obostrzeń i wszystko jest super, gospodarka działa, bo zrobili mało - super. Zajebista jest Szwecja, wszyscy tak mogli zrobić. Ale gdyby się okazało nagle, że ten koronawirus wybił tam połowę osób, to by wszyscy mówili, boże, jacy idioci, mogli zrobić tak samo, jak my. Na przykład. I w drugą stronę. Nie jesteśmy w stanie tego określić, nie mam zdania.

**A jakich danych być potrzebował, żeby móc powiedzieć, który system był lepszy?**

Pierwsze dane to są te o ilości osób na metr kwadratowy i śmiertelność, zarażenia, zakażenia. Druga sprawa to dane gospodarcze. To razem mówi wszystko.

**Temat dbania o siebie. Widzisz jakieś zmiany u siebie?**

Tak, mam wszystko gdzieś.

**No tak, ostatnio powiedziałeś mi nawet, że chodzisz w podartych dresach.**

No jest. Olewa się większość rzeczy. Chociaż ostatnio z Ewelinką - ona kupiła jakiś peeling na nogi i mnie namówiła. Siedzieliśmy sobie w takich skarpetkach na nogach i sobie rozmawialiśmy.

**I co? Pierwszy raz to robiłeś?**

I dupa z tym peelingiem za przeproszeniem. Ogólnie peeling w skarpetkach tak, robiłem pierwszy raz, ale peeling ogólnie nie, nie pierwszy raz. Był taki czas, że może częściej chodziłem na paluszki do kosmetyczki niż niejedna kobieta, więc nie nie nie, spokojnie.

**Teraz nie chodzisz. Brakuje Ci?**

Ja już nie chodziłem od kilku lat. Więc na spokojnie.

**Jak Ewelina powiedziała Ci, że masz podarte dresy, kupiłeś w końcu coś?**

Ja nie, Ewelina mi kupiła.

**Dlaczego jej zależy, żebyś nie chodził w tych podartych?**

Bo nie lubi słuchać, jak narzekam. Chodzę, bo jestem facet. Ale narzekam jak baba.

**Co jeszcze, oprócz peelingu, zrobiłeś sobie z nowości kosmetycznych?**

Nic. Stwierdziliśmy, że jest beznadziejny i że może będziemy robić jak Lewandowscy - ubierać te skarpetki na noc i spać w nich – może wtedy będzie lepszy.

**Myślałeś o tym, żeby Ewelina Cię ostrzygła?**

Nie.

**Dlaczego?**

Takiego ryzyka nie podejmuję. Mi nie przeszkadza to, jak one rosną. Ja mogę mieć długie, krótkie. Mi też mogą rosnąć, uwierz mi. Natomiast poszedłbym. Po prostu sam dla siebie. Ja uwielbiam u fryzjera zasypiać i tam jest mi dobrze.

**Golisz się teraz?**

Jeden pierun. Ja się golę, jak chcę. Ja wiecznie wyglądam jak żul. Mało kiedy golę się do zera.

**A Ewelinie brakuje jakichś rzeczy? Kosmetyczki, takich rzeczy?**

Ewelinka daje sobie radę na telefon. Przyjeżdża do nas taka pani i...

**Myślisz, że to bezpieczne?**

No nie, ostatnio o to walczyłem, to jedną odpuściła. Ale ostatnio powiedziała już, że nie daje rady i musi pani przyjechać. To przyjechała.

**Ale oficjalnie salony są zamknięte.**

Oficjalnie. Ale wyobrażasz sobie, że wszystkie kobiety nie korzystają z tego?

**Myślisz, że to podziemie jest duże?**

Yhm.

**To jakaś pani fryzjerka nie mogłaby przyjechać i obciąć Ci włosów?**

Mogłaby, ale ja nie jestem na tyle sfokusowany, że to musi być. Natomiast poszedłbym.

**Ewelina miała paznokcie. Coś jeszcze?**

<Misiek, jesteś tu?

No.

Jak był koronawirus, sprzątali nam dom?

Nie.>

A, no to nie. To tylko to.

**Normalnie macie jeszcze panią sprzątającą?**

Różnie. Kilka mamy tu, a jedną tam. Mieliśmy taki plan, żeby też panią sprzątaczkę wykorzystać w Warszawie, że jest koronawirus, dać jej klucze i żeby nam na błysk zrobiła dom. Ale jakoś to umarło. Świetny pomysł na wykorzystanie to był.

**Powiedz mi jeszcze o tych paznokciach. Bo jednak nie wolno.**

Z tym to była awantura. Długa. Z Markiem utrzymywaliśmy, że nie, ale no dobra, już. Każdy ma swoje potrzeby i ok, zrozumieliśmy. Markowi kazałem się spytać pani na wejściu, czy nie ma koronawirusa, żeby mogła wejść do domu. Chyba nie spytał, bo się wstydził i tyle. No dobra.

**Ja tak serio pytam, uważasz, że to jest w porządku?**

Absolutnie, no nie! Nie zgadzam się z tym, że to w porządku. Jak się dowiedziałem, to nie, wyjazd, nie ma takiej opcji. Później odpuściłem już. Bo było oj proszę, no muszę już, muszę. No to odpuściliśmy faktycznie. De facto pani przyjechała w masce. W naszym przypadku byłoby to zresztą hipokryzją - na naszej stacji przewraca się mega wiele osób. Kobiecie zresztą ostatecznie i tak nie powinno się odmawiać, trzeba odpuścić.

**Ale nie wiecie, gdzie ta pani była wcześniej.**

Nie wiemy też, kto jest na stacji benzynowej, kto na nią przyjeżdża. Po trzech tygodniach u nas ktoś się przyznał, że miał kontakt z osobą, u której stwierdzono koronawirusa. Najgorsze jest to, że z tego, co przez ten czas można było wywnioskować, to ci ludzie, którzy naprawdę mogli być zarażeni, to oni bali się mówić innym ludziom. Nie wiem, może bali się odrzucenia? Nie wiem, o co chodzi. To były ze 3-4 przypadki. Jak już się dowiedziałem o osobach z rodziny i tym, że one się przyznają po 3 tygodniach do takich rzeczy, a jeszcze w międzyczasie potrafią przyjeżdżać z dzieciakami do innej rodziny - to jest mega nieodpowiedzialne.

**Myślisz, że z gospodarczego punktu widzenia, to lepiej, że ta pani robi to na czarno, niż otwierając jakiś mały zakładzik?**

Myślę, że ta pani ogólnie robi to na czarno. To nie jest osoba, której zależy na naszej gospodarce. Akurat, kiedy mówimy o osobach, które jeżdżą po domach. One po to to robią, żeby uniknąć opłat.

**Ale większość z tych dziewczyn, które kiedyś miały swoje lokaliki, teraz jeździ po domach, przeszły do szarej strefy. Kiedyś, pracując w salonach, odprowadzały podatki.**

My nie jesteśmy narodem jak Szwajcaria, Norwegia, czy Nowa Zelandia. My jesteśmy Polakami. Jesteśmy z PRLu nauczeni kombinowania i głupot. I to jest właśnie nasze, jeszcze genetycznie PRLowskie wynaturzenie, że jak nam nie dali, to będziemy robić tak. Są tego plusy, minusy, ale to jest właśnie taki naród. I u nas się tego nie wypleni. Może dzieci Twoich dzieci - może ich generacja będzie już normalna, że coś się robi tak, albo tak.
