# Supplementary material for: The challenges arising from the COVID-19 pandemic and the way people deal with them. A qualitative longitudinal study
Source: PLoS One. 2021 Oct 11;16(10):e0258133. doi: 10.1371/journal.pone.0258133 (PMC8504766; doi:10.1371/journal.pone.0258133)
Supplement: S1 Dataset — (ZIP) [file pone.0258133.s003.zip › Transcriptions/stage 3/20.3_F_25_couple, with child.docx]

**20.3._F_25_couple with child**

**Co się u ciebie działo przez ostatnie dwa tygodnie?**

To chyba wszystko bez zmian. Wszystko robiłam to, co robiłam wcześniej. No święta były. Święta spędziliśmy w rodzinnym gronie w domu, tak jak mówiłam. Nigdzie nie jechaliśmy oprócz do nas na działkę. I tyle, chyba nic. Babcię badają ciągle na dializach. Już chyba 3 testy jej robili i wszystko jest ok. Więc u nas nikt nie choruje, nawet z dalszej rodziny, ze znajomych. Więc złych wiadomości nie mam.

**Święta w rodzinnym gronie, czyli tak, jak mieszkacie razem - rodzice, babcia i wy?**

Tak. Z domownikami.

**Jak te święta wyglądały w porównaniu do świąt, które spędzałaś dotychczas?**

No słabo. Zawsze spędzaliśmy bardzo aktywnie święta. Mamy sporą rodzinę, więc wszędzie jeździliśmy przez te 2 dni plus jeszcze spotykaliśmy się ze znajomymi, a tutaj nie było takiej możliwości. Ja raczej duchowo tych świąt nie przeżywałam. Nie wiem, dlaczego, ale nie przeżywałam ich duchowo. Inne są te święta bożonarodzeniowe, a te takie - no były, bo były. Nawet nic tam za bardzo się nie zmieniło z tego względu, że nie była to forma odpoczynku, bo odpoczywamy już bardzo długo. Więc dzień jak co dzień. Tylko tyle, że wiadomo, była święconka, były potrawy, był barszczyk, itd. Ale raczej odbyły się i tyle.

**Z koszyczkiem do kościoła nie poszliście?**

Nie.

**A pojawiły się jakieś nowe rzeczy?**

Tak. Będę robiła córce pokój z nudów po prostu. Znaczy z nudów, nigdy nie było się czasu za to wziąć po prostu. Mamy taki jeden wolny pokój, który jest niewykorzystany, więc jestem w trakcie remontu tego pokoju. Czyli zamawianie mebli, czekanie na paczki.

**Dwa tygodnie temu wspominałaś o tym. Mieliście już wtedy jakieś plany?**

Powstał plan niedawno. Jak jest kwarantanna, jak jest korona wirus, to rozmawiałam z Michałem:*a może zrobimy pokój*. I robimy pokój. Tam jakieś meble nam dochodzą, czekamy jeszcze jak wszystko przyjdzie, to będziemy malować. Byliśmy w Castoramie np. W tej Castoramie też tragedia, w tych maskach się nie da chodzić. W ogóle byłam w sytuacji, gdzie oglądałam dywany i 2 pracownice ze sobą rozmawiały i mówią, że już pierdzielą, że zdejmują te maski, bo już nie mogą w nich wytrzymać. Ale wierzę, bo ja byłam tam pół godziny czy 40 min. i już byłam bardzo zmęczona tą maską, w takim sensie, że naprawdę brakuje powietrza i nie ma takiego normalnego przepływu. Współczuje tym pracownikom, którzy są tam 8 godzin.

**Czy według ciebie jest różnica pomiędzy kwarantanną a izolacją?**

Ja tylko teraz tak powiedziałam, bo nie mamy kwarantanny, tylko to właśnie ta izolacja. Jest różnica. No kwarantanna - tak mi się wydaję, nie wiem, ale to jest jednak jakoś nałożone odgórnie. A izolacja jest dobrowolną czynnością.

**Subiektywnie, w codziennym użyciu, jak mówisz?**

Raczej nie używam izolacji, raczej kwarantanna. Chociaż mówię, używam to dość potocznie. Chociaż różnicę od razu bym podała i wiem, że niepoprawnie tego używam. Ale raczej wszędzie trąbią kwarantanna, kwarantanna, no to tak się mówi potocznie. Ale wiem, że to, co robimy, to nie jest kwarantanna, tylko izolacja.

**Pojawiły się jakieś nowe rzeczy, które są dużym wyzwaniem?**

Wyzwaniem? Chyba nie. Bo kolejki tak, jak są, tak były. W o ogóle otwierają już te sklepy. Pepco jest otwarte u nas. Byłam w szoku, jak się dowiedziałam, bo siostra napisała, czy chcę coś z Pepco. Okazało się, że jest to otwarte, tylko tam nie ma żadnej żywności. Wiadomo, co jest w Pepco - jakieś głupoty do domu czy ubranka. Ale jest otwarte. Nie wiem, dlaczego, nie mam bladego pojęcia. A co się pojawiło? teraz bardzo dużo osób jest na ulicach. Jak wracaliśmy z tej Castoramy, gdzie byliśmy w mieście i jest tam akurat taka 3 km prosta droga, gdzie obok jest chodnik, dla rowerzystów, itd., to po prostu wysyp ludzi. Ludzie znowu zaczęli biegać, spacerować, itd. Może te 2-3 metry były odległości, ale chodzi mi o sam fakt, że na początku nie było nikogo. Naprawdę nie było nikogo. W ogóle umarłe miasto. A teraz tak już chyba ludzie dali sobie trochę na luz. Z resztą też się nie dziwie, bo te już długi czas. No mówię, ja mam możliwość tutaj trochę inaczej odpocząć. A jednak ludzie w mieście, którzy mieszkają w blokach, to współczuje im bardzo. I dlatego się nie dziwię, że jeżeli mają możliwość wyjść do tych parków i na ulicę, to wychodzą. Ale naprawdę jest bardzo dużo ludzi.

**Może są jakieś rzeczy, z których zrezygnowałaś albo które ograniczyłaś?**

Nie. Chyba nie. Nic mi nie przychodzi do głowy. Wszystko jest tak, jak było. Raczej nic się u nas nie zmieniło, wszystko jest tak samo. Nadal mąż jeździ do pracy, ja siedzę w domu z dzieckiem. Także nic się nie zmieniło.

**W ciągu tych ostatnich 2 tygodni nie pojawiło się nic, co zaczęłoby jakoś bardziej doskwierać?**

Raczej nie. Jedyne co, to trochę śmieszy mnie fakt tych masek. Nie w takim sensie, że walczę z nimi, że nie chcę ich nosić, to absolutnie. Pamiętam, jak na początku całego tego zamieszania, ludzie wykupowali te maski, a rząd mówił, że absolutnie, to jest w ogóle do niczego, w niczym to nie pomoże. Poblokowali przecież wszędzie na Facebookach, itd. Że to jest do dupy, to nas nie ochroni, itd. A teraz jest nakaz noszenia ich *[śmiech]*. Nie rozumiem, o co w tym chodzi i dlaczego, tak to się stało. Ale śmiesznie. Kupili z Chin maseczki i teraz już można chodzić i sprzedawać.

**Jak postrzegasz takie działania rządu?**

No beznadziejnie to postrzegam, ale cóż, wolę ją nosić niż dostać mandat. Pamiętam, jak jeszcze na początku to się pojawiało, to w wiadomościach huczało, że ludzie głupieją, bo wykupują maseczki, bo wykupują żele. A teraz co? Na każdym wejściu musi stać żel, żeby umyć ręce, muszą być rękawiczki założone, gdzie rząd jakby potępiał takie zachowania, jak ludzie na początku robili zapasy. I absolutnie maseczka nam w niczym nie pomoże - to nie rozumiem, po co jest nakaz tego noszenia w miejscach publicznych. No ale wszędzie jest miejsce publiczne - park, droga, itd. Więc trochę nie rozumiem i beznadziejnie no.

**Jakie emocje w kontekście tej niekonsekwencji rządu odczuwasz?**

Mówię, tak jak ja jestem poza tą całą polityką, więc ja tam za bardzo się tym nie przejmuję, to jedyne co, to mnie śmieszy ten fakt i jest żenujący. Bo gdyby włączyć teraz im wiadomości sprzed miesiąca, no to... I włączyć im wywiady z ludźmi, którzy mówili, że to jest do niczego i że to my jesteśmy głupi, bo to kupujemy, a to w żaden sposób nie zabezpieczy nas od wirusa. A teraz nagle mówią, że jednak nas zabezpieczy. To nie jest jakieś tam małe uchybienie, tylko po prostu zaprzeczanie sobie, tego, co mówili miesiąc temu. Ale mówię, ja raczej nie interesuję się polityką, nie interesuję się tym rządem, żyję sobie po swojemu. Trzeba teraz nosić maski, to je noszę. Od początku uważałam, że wiadomo w 100% nas nie zabezpieczą, ale może w jakiś tam procentach nas uchronią, jak wszystko - jak rękawiczki, żele, itd. Ale teraz, jak się pójdzie do sklepu, to jest żel do dezynfekowania, rękawiczki, trzeba założyć maski, a na początku to było be i fuj i niepotrzebne. Gdzie w innych krajach się chodziło w maskach... No nieważne, tak było.

**To zażenowanie jest dosyć duże czy jest sobie i nie przeszkadza?**

Mi w codziennym życiu nie przeszkadza, bo ja się tym nie interesuję w dużym stopniu. Jak w telewizji usłyszałam, że teraz będzie ten nakaz noszenia maseczek, to jedynie to zażenowanie i mnie to śmieszyło, że jeszcze miesiąc temu mówili po prostu co innego. Dlatego mam kolejny dowód na to, że nie trzeba się słuchać innych, tylko robić, jak się uważa, bo tam, gdzie jest kasa, tam jest zdanie, tam jest racja.

**Robić, jak się uważa, ale jednak zakładasz maseczkę, bo nie chcesz dostać mandatu, tak?**

Tak. Znaczy ja od początku myślałam, że to jest jakieś zabezpieczenie i ludzie powinni chodzić w maseczkach z tego względu, że, jeżeli ja będę miała maseczkę, a ktoś nie będzie jej miał, to trochę głupio, nie? W takim sensie, że jest wtedy jakaś pół na pół szansa. A jeżeli ja będę miała maseczkę i osoba, która będzie koło mnie też będzie miała maseczkę, to jest większa szansa zabezpieczenia. Tylko, jeżeli rząd na początku mówił, że to jest bez sensu, no więc ludzie jedni mieli, drudzy nie mieli. Więc ja ogólnie jestem za tym, żeby w miejscach publicznych nosić maseczki. Poza tym, mieliśmy przykład z Chin, gdzie tam, gdzie ten wirus już umierał, to ludzie na ulicach mieli maseczki - wszyscy. Nawet, jak pokazywali - no były filmy, nie filmy i na YouTube Polacy, którzy pokazywali to. A u nas nie, maseczek nie wolno. Więc dla mnie to jest po prostu beznadziejne i tyle. Chociaż ja jestem za tym, żeby nosić te maseczki, tylko śmieję się z ich decyzji i z tego, co oni mówią.

**Nosiłaś wcześniej maseczkę, jak nie było nakazu?**

Nie nosiłam. Jak byłam w sklepie, to rękawiczki, itd., ale maseczek nie nosiłam. Było to bez sensu, bo byłabym wtedy w sklepie jedyną osobą, która miałaby maseczkę. Bo nawet obsługa nie miała.

**To jak to jest, według ciebie ta maseczka faktycznie kogoś chroni?**

Tak, no na pewno w jakimś stopniu chroni, tylko mówię, że jeżeliby wszyscy nosili, tak, jak jest teraz. Że ja wchodzę do sklepu i nie ma osoby, która nie ma maseczki. A jak u nas, tutaj na wsi w tych sklepach, to rękawiczki miała 1 osoba na 10. Nawet czasem obsługa nie miała tych rękawiczek. A maseczek, to nawet, jak osoba miała maseczkę, to ludzie inaczej się na nią patrzyli. Na początku tej sytuacji, nie teraz. Bo pewnie słyszeli w wiadomościach - no głupia, po co nosi, i tak ją to nie chroni. Więc ja bym była jedyną osobą, która by miała tą maseczkę w sklepie. Więc bez sensu.

**A poza sklepem, jak zdarza ci się wyjść z domu, to nosisz maseczkę?**

Raczej nie zdarza mi się nigdzie tutaj dalej wychodzić, bo ja nie chodzę do parku, nie chodzę do miejsc publicznych, tak, jakbym żyła w mieście, bo żyję na wsi. To u mnie na podwórku nie będę nosiła maseczki. A nie byliśmy w ostatnim czasie. I na stację benzynową, jak wchodzę, to też oczywiście ją zakładam. Ale nie bardzo mam, gdzie indziej ją zakładać.

**A zdarzyło ci się nie mieć maseczki od wprowadzenia nakazu?**

Nie, nie zdarzyło mi się, bo mama mnie pilnuje, jak jadę do sklepu: *tylko pamiętaj wziąć maseczkę*. Ale ja byłam w sklepie 2-3 razy. Bo my się wymieniamy - raz jeździ mama, raz mąż. To nie jest tak, że jestem sama z zakupami, że przez 2 tygodnie tylko ja robię zakupy.

**Widziałaś może, żeby ktoś nie miał maseczki?**

Chyba nie. Nie widziałam. Jak byłam w sklepie, to wszyscy mieli. Na stacji też. więc raczej mają. I w Castoramie też wszyscy mieli. Jedynie co, to parę pań nie miało, ale miały takie bandanki, które zakrywały nos i buzię. I te panie, które zdjęły, bo powiedziały, że nie wytrzymują i nie dają rady w tej maseczce. To z obsługi panie.

**Jaka była reakcja otoczenia na to, że te panie zdjęły maseczki?**

Nie zauważyłam, bo w Castoramie jest tak, że w jednej alejce mogą być tylko 2 osoby. Ja byłam w alejce z dywanami i te panie układały, więc obok nie było innych osób, które mogłyby tam w jakiś sposób zareagować, więc się nie rozglądałam za bardzo. Tylko za plecami słyszałam ich rozmowę. Jak się odwróciłam, to już miały zdjęte te maseczki. Nie widziałam innych osób tam, które mogłyby zareagować, więc nie jestem w stanie powiedzieć. Ale, jak wracaliśmy z tej Castoramy i na długim chodniku, to nowe osoby, które biegały, to miały założoną maseczkę. Im bardzo współczuję, bo nie wiem, jak można w niej biegać. Ale miały założone. Na rowerze też wszyscy mieli.

**Obrazki. Który z nich najlepiej oddaje twoje emocje teraz i w ciągu dwóch tygodni?**

Chyba znów wybiorę to, co ostatnio wybrałam. 6 i 13.

**6**

Wybrałam podobnie, jak 2 tygodnie temu z tego względu, że bardzo dużo czasu spędzamy na świeżym powietrzu i na działce. A krajobraz jest bardzo podobny. Mamy fajną pogodę. Tym bardziej, że ja mam małe dziecko. Co innego, jak ktoś jest sam, to może sobie ćwiczyć w domu, oglądać różne seriale. Ja niestety nie mogę. A w domu ciężko jest przesiedzieć cały dzień i z dzieckiem się bawić, więc spędzamy czas na dworzu. Na dworzu mamy bardzo dużo zajęć. Moja córka ma i rowerek, i skuterek, i samochodzik. Wczoraj jej zamontowaliśmy piaskownicę na podwórku, bo ma jedną na działce i się strasznie lubi bawić. Więc spędzamy czas na podwórku. Mój mąż ciągle myje samochody, dywany pierze. Ale wszystko robimy na dworzu, żeby wyjść z tego domu, bo już wariacji można by było dostać.

**Powiedziałaś, że nie możesz robić pewnych rzeczy. Mogłabyś to rozwinąć?**

W sensie, jak był boom na Netflixa, oglądanie seriali, ja nie mam na to czasu. Z tego względu, że moja córka idzie spać dopiero ok. 23. A o 23 to ja już marzę o tym, żeby się położyć spać, a nie coś oglądać. Mój mąż też chodzi wcześnie spać, bo wstaje w nocy do pracy. Więc ja nie mam, kiedy obejrzeć po prostu tego serialu. Tak, jak osoby ćwiczą, ja też nie mam, kiedy. Bo córka jest ciągle koło mnie i ona jest taka, że sama się nie potrafi zabawić w ogóle. Więc wszystko trzeba zrobić z nią niestety. Jak lalkami, to z nią, jak w domku, to z nią i tak ciągle.

**Jakie emocje powiązałabyś z tym obrazkiem nr 6?**

Ja w ogóle wrzuciłam na luz ostatnio bardzo, bardzo. Jakoś tak, dla mnie jakby tego koronawirusa już nie było i strasznie się przyzwyczaiłam do tego, jak jest teraz. Teraz to sobie nawet nie wyobrażam, jak wrócę do szkoły i do jakichś zajęć, gdzie trzeba wstać na godzinę rano, wszystko jest uporządkowane. I naprawdę tak mi zaczyna być dobrze trochę *[śmiech].*Oprócz tego, że tam nie można spotkać się ze znajomymi, odwiedzić rodzinę czy coś kulturalnie zrobić. No kulturalnie, wyjść do kina, kino nie jest bardzo kulturalne, ale nie wiem, no przyzwyczaiłam się do tego, że tak jest. Wydaje mi się, że będziemy się zachowywać jak jakaś dzicz wypuszczona, ja nie wiem. Ale mi jest tak dobrze, naprawdę. Jest spokojnie, wszystko harmonijnie, nikomu się nie spieszy. Zaczęliśmy bardziej chyba doceniać - wszyscy, chociaż ja kiedyś dużo też spędzałam czasu na świeżym powietrzu, ale teraz się docenia ognisko, teraz się docenia, jak smakuje kiełbaska z grilla. A kiedyś to było takie normalne i ludzie woleli wyjść do klubu niż w lato posiedzieć przy ognisku. I ja mam tak samo, że zaczęło się doceniać bardzo przyrodę i to, jakie się ma warunki. Tym bardziej bezpłatnie. Nie za żadne pieniądze. Bo wydaje się ich dużo, jeśli chcemy skorzystać z jakiejś rozrywki. A tu mamy rozrywkę wkoło siebie i tego nikt nie doceniał wcześniej.

**13**

Bardzo podobny, bo tez przedstawia przyrodę. Ale tu jeszcze mi się skojarzyło z sadzeniem, bo tata teraz dużo rzeczy sadzi: maliny, truskawki, coś tam cuduje na tych działkach. Tak jakby wszystko budzi się do życia i zawsze o tej porze to robił. Jedyna zła wiadomość to taka, że lis zadusił tacie wczoraj 30 kur i dzisiaj jest po prostu załamany. Ale bardzo dużo czasu spędzam tam u nas na działce. I tata tam sobie traktorem jeździ. On jak miał kryzys wieku średniego, to tak postanowił dużo rzeczy takich dziwnych robić. Ale no fajnie. Moja córka się cieszy, bo mamy króliki, kury, gęsi i nawet świnki tata sobie kupił, domek dla nich zrobił. Więc fajnie. Jakoś w ogóle już bardzo mało rozmawiamy o tym korona wirusie. Poza tym, wydaje mi się, że już niebawem będzie koniec. Jeszcze miesiąc, może półtora i znowu wszystko wróci do jakiejś normy. Chociaż wydaje mi się, że teraz jest fajnie. A tak, jak było wcześniej, to trochę my jako ludzie się zagubiliśmy. I spędziliśmy za czymś, co w ogóle nam nie jest potrzebne do szczęścia.

**Te rzeczy, które zaczął robić twój tata, pojawiły się przed epidemią?**

Tak, bo to tak ze 3-4 lata temu. Tam miał spokój, ciszę. Ja wiadomo, pomagałam mu, ale tam się zbytnio nie angażowałam. A teraz tak się człowiek cieszy, że trzy jajeczka od kury zebrał.

**Teraz jesteś bardziej zaangażowana w to, co robi twój tata?**

Tak. Non stop jesteśmy tam i na działce i tutaj i jesteśmy ze sobą. Wcześniej tata jechał na działkę, a ja jechałam do znajomych. Bo wiadomo. Mama też robiła sobie coś innego. A teraz spędzamy czas wszyscy razem, wspólnie. I myślę, że i więzi w jakiś sposób się bardziej zawiązały. Chociaż my mieliśmy bardzo dobre relacje. Ale dużo rzeczy robimy po prostu wspólnie, dużo się rozmawia, itd. I nie mam tak, jak są teraz memy i śmieszne filmiki, ile ludzi będzie się rozwodzić. To mam tak, że tak, jak zawsze mi brakowało tego czasu z mężem, bo byliśmy zaganiani, itd. Tym bardziej, że bardzo szybko mieliśmy dziecko po ślubie, więc nie zdążyliśmy się sobą nacieszyć, tak teraz jesteśmy non stop ze sobą. Możemy normalnie porozmawiać, możemy coś porobić. Wcześniej było tak, że np. mój mąż zabierał córkę na spacer, bo ja w tym czasie musiałam jakieś zajęcia przeprowadzić. A teraz razem możemy iść na spacer, razem samochód umyć, razem coś zaplanować. A wtedy było takie wymienianie się. Teraz możemy wyjść na podwórko i się bawić wspólnie, a wcześniej było na zmianę, bo każdy miał pracę. Więc trochę boję się, że, jak się to wszystko skończy, to ludzie znowu... Na pewno zapomną o tym, co się działo i znowu będzie pogoń tylko i wyłącznie za pieniądzem, za pracą i tyle.

**Czyli wydaje ci się, że niekoniecznie to, co jest teraz w sferze relacji, się utrzyma?**

Myślę, że nie. Już nieraz były już takie... Znaczy może nie jakieś epidemie. Ale czasami dzieją się złe rzeczy też w rodzinie. Jest jakaś sytuacja i mija rok dwa i daje to nauczkę tylko na krótki okres. A potem się wraca do wszystkiego. Niestety świat też to wszystko napędza. Nie możemy... Tak, jak ja bym chciała sobie żyć, tak jak teraz żyję za dwa miesiące, to mi na to nie pozwoli szkoła, praca. Nie będę miała żadnych dochodów, itd. To nie mogę ja jedyna, sama odstawać od wszystkiego, bo to nie jest możliwe w tym świecie. A to świat sam siebie napędza. I to musieliby wszyscy ludzie zrezygnować z takiego pędu. A nie zrezygnują. Bo światem rządzi pieniądz i nie ma takiej możliwości. Teraz fabryki nie dadzą znowu weekendów wolnych, wszystkiego wolnego, żeby ludzie mogli spędzić czas z rodziną. Tylko już się dzieją żłobki, powroty do żłobków. No po co do żłobków? Żeby matki mogły iść do pracy. Bo dziecko z podstawówki można w domu zostawić, a teki dzieci roczne, dwuletnie, to wiadomo, że matki muszą wrócić. I rząd robi wszystko, żeby matki wróciły do pracy. Bo zależy na tym, żeby były pieniądze. Więc nie będzie się dało żyć tak, jak teraz. Chociaż przez jakiś czas, nie wiem, jaki, ludzie będą o tym pamiętać na pewno i zaczną doceniać różne rzeczy. Ale to szybko minie.

**Myślisz, że u was w kwestii relacji też to się zmieni?**

Wydaje mi się, że my nie będziemy mieć możliwości spędzać razem tyle czasu, co teraz. To właśnie ten koronawirus, który jest przekleństwem, dał nam taką możliwość. A niestety, jak ja wrócę do swoich obowiązków, gdzie znowu będę musiała prowadzić zajęcia, jeździć na swoje zajęcia, iść na praktyki, itd., to znowu nie będziemy mieć tego czasu. Nie mogę z tego zrezygnować. No, niby mogę, tylko wtedy bym nie zarabiała.

**Jakie emocje odczuwasz w tym kontekście, jak myślisz o tym, co będzie, kiedy to się skończy?**

Wiadomo, że z jednej strony chce się, żeby się to skończyło. Żeby można... Najbardziej mi przeszkadza to, że nie można się spotykać z ludźmi. Bo już prawdę, ta galeria itd. już się nauczyłam przez ten Internet zamawiać, odsyłać, kurierzy przyjeżdżają, odjeżdżają, itd. Ludzie daliby sobie radę. Spożywczaki są otwarte, spoko. To kino, gdzie uwielbiałam chodzić do kina i musiałam nawet raz na dwa tygodnie iść, to teraz już 2 miesiące i nie jestem w kinie - i można. Można się od wielu rzeczy odzwyczaić. Ale jednak tego kontaktu z ludźmi mi bardzo brakuje i tego się nie mogę doczekać. A jakiś innych rzeczy, to... O i wyjazdów, np. Żeby wsiąść w samochód i sobie pojechać gdzieś. Tak, jak mieliśmy wakacje zaplanowane, weekendy, bo my często też wyjeżdżamy. No nie możemy. Więc tego mi brakuje. Ale nie tam, że tego kina, galerii, itd. To mogłoby ich nie być.

**Powiedziałaś, że pojawił się spokój, harmonia, radość z małych rzeczy. Czy coś jeszcze się pojawiło? Gdybyś mogła wymyślić inny obrazek, to coś byś zmieniła?**

Nie. Trudne pytanie zadałaś. Ja wiem, jak na początku było całej tej sytuacji, to każdy czuł się naprawdę okropnie. Bo jak, teraz nie mogę, tego, tamtego. Ale z biegiem czasu już się do tego przyzwyczailiśmy. I jak ja patrzę na to, co było wcześniej, a jak jest teraz, to teraz jest o wiele lepiej. Oprócz tych spotkań z ludźmi, bo tego mi brakuje. I wyjazdów. Ale te wszystkie centra handlowe, itd., te kina... No, może jeszcze tych restauracji. Ale teraz jest bardzo spokojnie i bardzo fajnie. Wstaje się o której się chce. Tylko jeszcze całe szczęście, że mój mąż pracuje. Bo gdyby mój mąż nie mógł pracować, to pewnie bym była załamana. Ale mój mąż może normalnie pracować. Bardzo dużo czasu spędzam z dzieckiem, gdzie wcześniej na zmianę się opiekowaliśmy Helenką. Moja mama też się nią sporo zajmowała. Więc te pewne chwile i momenty uciekały. A teraz jest wszystko po prostu na bieżąco. Wszystko, co powie nowego, to się cieszę, że jestem cały czas przy jej rozwoju. Gdzie i tak dużo czasu z nią spędzałam. Ale było tak, że a to mama mi coś powiedziała, że Helenka coś zrobiła, a to Michał. Nie wiem no, jest fajnie. Mamy dużo czasu, żeby spędzać ze sobą i jest ok. Tylko wiem, że to jest na chwilę. Że zaraz, jak to wszystko wróci do normy za chwilę, to znowu tego czasu nie będzie. Ale naprawdę, tak jak widzę po mojej mamie, to wcześniej siedziała i oglądała te wiadomości. A teraz, o, siedzi na podwórku opala się *[śmiech].*

**Czyli ani ty, ani twoi bliscy nie czujecie dużego zagrożenia w związku z sytuacją?**

Teraz nie, teraz już naprawdę nie. Na początku był ogromny strach. Ale teraz, już po tych świętach, to każdy żyje normalnie. Nawet nie widzę, żeby jacyś smutni chodzili. Tata się jeszcze cieszy, bo - to też jest dobre - bo wyczytał, że to COVID-19 od tyłu to jest coś tam czytane i to jest nazwa psalmu jakiegoś tam. I ten psalm, mówi przeczytaj sobie Anita. Bo w tym psalmie pisze właśnie o tej całej epidemii i zarazie. I mówi, że tam jest napisane, że ci, co wierzą i ufają, to im nic nie będzie. I tata mój jest takiego zdania, że jemu nic się nie stanie *[śmiech]. I że co ma być, to będzie.*Jak ma się zarazić, to się zarazi w sklepie, itd. Mój tata, to w ogóle jest taki. Mówiłam ci, jest bardzo wierzący, więc on ufa, co tam się dzieje na górze, to tak ma być. On raczej nie przejmuje się takimi ziemskimi rzeczami, bo uważa, że wszystko jest w jakiś sposób dla nas zaplanowane i to, co da Pan Bóg, to tak będzie.

**Ta informacja go uspokoiła?**

Tata od początku się tym nie przejmował. A teraz jeszcze, tylko zobacz sobie Anita, zobacz sobie. A ja w końcu nie zobaczyłam. W każdym razie on jest bardzo spokojny i uważa, że nic mu się nie stanie na pewno. A jak mu się stanie, to widocznie tak miało być. I nie będzie się przejmował takimi rzeczami. Chociaż, jakbyś zobaczyła, jak wygląda mój tata, to byś w ogóle nie powiedziała, że to jest taki człowiek. Wygląda bardzo groźnie *[śmiech].*

**O jakich zmianach w obowiązujących ograniczeniach słyszałaś?**

To, że można wyjść do parków, na spacer i chyba liczba osób w sklepie. I coś słyszałam o kościołach, że 15 m2 na jedną osobę. Bo mama się mnie pytała, ile to jest 15 m2 i musiałam jej powiedzieć, ile to jest. Ale moi rodzice jeszcze nie chodzą do kościoła, jeszcze nie byli. Więc o tym słyszałam. Wiem, że są te stopnie, które mają wprowadzać. To w pierwszej kolejności te żłobki, a potem na samym końcu jakieś restauracje i cała reszta.

**Porozmawiajmy najpierw o zmianach w kościele - co o tym sądzisz?**

Hmm... Nie wiem w ogóle, jak ludzie będą w tym kościele wyznaczać to 15 m2. Bo tak, jak moja mama, która jest inteligentną kobietą się mnie pytała, ile to jest.

**To jest do zrobienia, żeby to samodzielnie określić?**

Znaczy może ograniczenie, to dobrze, żeby powoli wracać do normalności. Ale dla mnie 2 metry w kolejce jest ok, bo ludzie na oko będą mniej więcej wiedzieli, ile to jest. Ale jak podali 15 m2 na jedną osobę, to ciężko chyba. Ja wiem, jak to mniej więcej wygląda, ale ludzie sobie chyba nie zdają sprawy, że to jest 5m na 3m i to jest tylko twój prostokąt i nikt nie może wejść w ten prostokąt. A to jest dużo. To nie jest metr na metr czy 2 na metr, jak mogą sobie w głowie przypomnieć, jak to było w kolejkach. 15m2 to jest duży obszar, więc wątpię, że babcie będą stały w takim prostokącie, a nie usiądą obok siebie w ławkach. A raczej w kościele nie ma ochroniarzy, którzy będą je ustawiać.

**Czyli myślisz, że to może być ciężkie do przełożenia w praktyce?**

W praktyce na pewno. Bo moja mama, która ma 45 lat miała problem z 15m2. A tym bardziej babcie, gdzie zazwyczaj starsi ludzie chodzą do kościoła. Więc takiej babci się nie wytłumaczy, że ona tam nie może usiąść.

**Co sądzisz o możliwości przemieszczania się w celach rekreacyjnych?**

Że fajnie, że mogą jeździć na tych rowerach, bo ja wiem, że niektórzy mogą mieć bzika. Jak moja koleżanka, która dzień w dzień biegała. Potem nie mogła już biegać. No fajnie, niech ludzie coś robią, bo to można zwariować w tym domu. I wierzę, że ludzie powinni wychodzić. Tym bardziej, że siedząc w domu osłabiamy swoją odporność - tak na logikę. Więc niech ci ludzie wyjdą z tego domu, zaczerpną świeżego powietrza i niech coś robią. Czasami ludzie też mają tak, że jak pobiegają albo pojeżdżą na rowerze, to dają upust emocjom. Wiesz o co mi chodzi? Chociaż podobno, czytałam badanie, że sport nie tłumi agresji, ale ją aktywuje. Fajnie, niech wychodzą, tylko ja nie wiem, jak oni jeżdżą z tymi maseczkami. Zależy, jaki jest przepływ powietrza. Ja mam takie 3-warstwowe. Ale ciężko. Ale już bym wolała jechać z maseczką niż w ogóle jej nie mieć. Chociaż na rowerze, jak jadą obok siebie i ktoś kaszlnie, kichnie... Ale jak jedzie 1 rowerzysta, to jak on ma zarazić inne osoby?

**To potrzebna jest ta maseczka czy nie?**

Chodzi o to, że jak u nas na wsi jedzie jeden rowerzysta, to on nie ma styczności z innymi ludźmi. Ale jak zobaczyłam tą sytuację w Radomiu, gdzie po prostu biegają, jeżdżą na rowerze, na rolkach jeden za drugim, to jednak jest ta styczność z ludźmi duża, w miastach jest duża, więc chyba trzeba. Bo tam jest większe zagęszczenie ludzi. Bo na wsi, to jedzie jeden rowerzysta i za pół godziny drugi. A jak tam zobaczyłam, to byłam w szoku - już dawno tyle ludzi na zewnątrz nie widziałam.

**A nowe zasady w handlu - co o nich sądzisz?**

Słyszałam tylko tyle, że może być więcej osób. Tak samo myślałam, że w tej Castoramie się naczekamy w kolejce, jak wszędzie. A tam od razu mogliśmy wejść, więc oni chyba tego nie monitorują, ile osób jest w sklepie. Bo nie wiem, jak mogliby to monitorować, jak ludzie wchodzili, wychodzili i ja też po prostu weszłam. Nikt nas tam nie policzył.

**A powinien?**

No chyba. No byłam w szoku, jak tutaj wszędzie muszę wejść, to muszę czekać 40 minut, żeby wejść do sklepu spożywczego, przed Biedronką, itd. A tu do Castoramy sobie po prostu weszłam. I nie było osoby, która by w jakiś sposób liczyła ludzi. Bo u nas to zostawiali np. 1 wózek czy 2 i mogłaś wejść tylko z wózkiem. Później, jak osoba wychodziła, zostawiała ci wózek, wtedy ty wchodziłaś. I tak sobie radzili. Tylko zerkali. Jak ktoś nie miał wózka to wypraszali. A w tej Castoramie po prostu weszliśmy sobie. Pełno ludzi na parkingu w środku tak samo. Tylko tyle, że jak chcieliśmy do żyrandolu poprosić panią, to powiedziała, że nie może podejść, bo mogą być tylko 2 osoby w alejce i dopiero wtedy ona może podejść.

**Z czym wiązał się ten szok? To było poczucie zagrożenia?**

Nie, raczej takie... Nie no, ja też nie jestem jakąś panikarą. Nie mam tak, że *o boże, teraz to wychodzę.*Ale no po prostu dziwne, bo pierwszy raz nie czekałam w kolejce już od długiego czasu, tylko po prostu sobie weszliśmy i nie było tam żadnego zabezpieczenia.

**To dobrze, że nie czekałaś w tej kolejce?**

Nie no, dla mnie to dobrze, że nie czekałam. Ale nie zastanawiałam się nad tym. Teraz ci mówię, bo dopiero teraz mi się o tym przypomniało. Nawet się nad tym nie zastanowiłam. Tylko po prostu dziwne, bo mówiłam: *zobacz Michał, nawet nie ma kolejki?*A Michał mówi, że to musi być wejście jakieś z drugiej strony. Ale patrzymy, tu otwierają się drzwi, możemy wejść. To poszliśmy i zajęliśmy się innymi rzeczami, a nie rozmyślaniem nad tym, czy tak powinno być.

**A to, że osoby pow. 13 r.ż. mogą przemieszczać się bez opiekuna - to dobrze?**

Hmm... Nie wiem, czy tak powinno być. Nie mam 13 latki w rodzinie. Chociaż mój Michał ma, która się bardzo ucieszyła. Tylko powiedz mi, gdzie ona może pójść?

**Na spacer z psem np.?**

To jak wyjdzie sobie na spacer z psem, to spoko. Ale 13-latka nie robi zakupów. Wiadomo, że czasem mama wyślę ją po coś do sklepu, ale ona nie ma potrzeby wchodzenia do tego sklepu, bo mama zrobi te zakupy. Rozumiem 18-latkowie, ale to już dorośli ludzie, to nie ma o czym mówić. Wiadomo, że próbują... Teraz będzie coraz więcej jakichś upustów po to, żeby wrócić do normalności, tak, jak było wcześniej. Więc ja nie zastanawiam się nad tym, czy to jest dobre czy nie, tylko po prostu dają więcej luzu. Ale nastolatka też się raczej nie spotka... Jak jest ogólny zakaz spotykania się, to mama jej nie wyśle raczej do koleżanki - *dobrze, idź sobie córciu sama do koleżanki*. Bo się nie chodzi do koleżanki. Jedyne, gdzie teraz może pójść, to do parku na spacer albo do sklepu. A nie pójdzie 13-latka do sklepu.

**Przestrzegasz tych wszystkich ograniczeń?**

Raczej wszystkiego przestrzegam. Czego nie przestrzegam? Nie no, raczej przestrzegam. Pewnie, jak bym mieszkała w Radomiu, to bym po kryjomu jeździła gdzieś tam do lasu. Bo bym na pewno nie wytrzymała w domu, ale to dlatego, że od dziecka mieszkałam na wsi. Więc, jak ja studiowałam w Warszawie, to dla mnie była katorga mieszkać w mieszkaniu. A wierzę, że ludzie, którzy mieszkają w mieszkaniu, też niekoniecznie odnajdą się na wsi, bo są przyzwyczajeni do innego trybu życia. Więc ja nie mówię, co jest lepsze, gorsze, tylko, że ja jestem przyzwyczajona tak. Więc gdybym ja teraz była w Radomiu, to bym oszalała. Tym bardziej z dzieckiem. Nie wyobrażam sobie, że nie mogę wyjść z dzieckiem na dwór, gdzie to jest taki jedyny ratunek. Bo całą zimę się czeka na to, żeby wyjść wreszcie na dwór, bo w domu niestety - zobaczysz, jak będziesz miała swoje dziecko, można naprawdę... *[śmiech]*

**Wtedy byś wychodziła pomimo zakazu?**

Na pewno, na 100%. W życiu bym nie wytrzymała. Na milion procent bym wychodziła, to już teraz mogę powiedzieć. Ale, że mam taką możliwość, jaką mam, nie łamię zakazów.

**Przestrzegasz ich, bo żaden z nich ci na tyle nie przeszkadza, żeby go nie przestrzegać?**

Tak. Właśnie o to chodzi, że mi nie przeszkadzają, więc ich przestrzegam. Ale gdybym miała taką sytuację, że nie mogłabym wyjść z tego domu, to na pewno bym wyszła. Na 100%.

**Co słyszałaś o etapach łagodzenia restrykcji?**

Widziałam piąte przez dziesiąte, że są te etapy. To, co teraz wprowadzają, czyli wyjście do parków, itd., ale mają być zasłonięte usta. Potem zobaczyłam, że mają otwierać żłobki. To od razu lampka w głowie, że po co żłobki. Przecież zazwyczaj małe dziecko jest przy matce. To po to, żeby matki miały, jak wrócić do pracy, bo nie ma kto się dziećmi zajmować. I potem zobaczyłam, że te restauracje mają być pootwierane. A jeszcze był jakiś etap, że galerie tylko z dziełami sztuki. Ja nie wiem, to prawda jest? Dobrze usłyszałam?

**Tak, mają być otwierane galerie sztuki w drugim etapie, więc dosyć szybko. Razem z bibliotekami i muzeami.**

Właśnie to słyszałam, że mają być otwarte biblioteki i galerie z dziełami sztuki. Mówię, no na pewno... Chociaż nie wiem, może są tacy ludzie, którzy lubią chodzić do galerii i im tęskno do takich rzeczy. Ale akurat ja nie jestem taka galeriowa i kulturalna *[śmiech].*

**Jak oceniasz te plany luzowania ograniczeń?**

Nie oceniam ich za bardzo, bo się w nie nie zagłębiałam i nie patrzyłam dokładnie. Tylko piąte przez dziesiąte słyszałam, więc nie chcę mówić tutaj głupot. Zwróciłam tylko uwagę na te żłobki, że pierwsze będą żłobki. Chociaż w ogóle teraz, nie wyobrażam sobie, żeby dzieci poszły do szkoły, żebyśmy my wróciły na studia, żeby normalnie iść do pracy, bo jest na to za wcześnie. Jak dla mnie.

**A co jest taką granicą, żeby można było luzować ograniczenia?**

Np. gdzie dziennie zachorowań będzie kilkadziesiąt załóżmy, a nie kilkaset. Gdzie dziennie będą odnotowywać spadek zachorowań. A na razie, to nie wiem. Chyba ostatnio było nawet jakoś dużo, ok. 400 czy 500. Nie interesuję się tym za bardzo, więc nie wiem. Ale dużo jest tych zachorowań. Jeżeli będzie tak, że będzie spadać codziennie i dojdzie do 30-40 osób, to można coś tam luzować. Bo wydaje mi się, że jak teraz znowu ludzie wyjdą wszyscy do pracy, itd., tym bardziej też dzieci do żłobków, do szkoły, to żeby nie było tego samego.

**Które ograniczenia powinny zostać na dłużej, a które znoszone w pierwszej kolejności?**

W pierwszej kolejności, wydaje mi się, że spotkania z innymi osobami, takie prywatne. Bo skoro możemy iść do sklepu, tak jak np. ja do Castoramy, gdzie jest pełno, ale to pełno ludzi, to dlaczego ja np. nie mogę się spotkać z kimś na grillu? I będziemy też wtedy nosić maseczki. Znaczy nie można... Jest ta izolacja, ale to nie jest... Są tam jakieś mandaty itd., bo siostra mi opowiadała, że nie można chodzić w więcej niż 2 osoby, itd. Więc dla mnie to się trochę mija z celem, bo idę do Castoramy i jest tam załóżmy 150 osób, a ja nie mogę nadal swobodnie, bez potępienia spotkać się ze znajomymi. Gdzie równie dobrze możemy sobie siedzieć na podwórku, gdzie jest mniejsze zagrożenie wydaje mi się, niż w zamkniętych pomieszczeniach. I chyba to jako pierwsze. Żeby można było się spotykać z tymi rodzinami. Oczywiście, chodzi mi z osobami - nie znowu w kinie, gdzie jest 100 osób, których nie znam i nie wiem, co robiły dotychczas. Ale z takimi osobami zaprzyjaźnionymi i z rodziną. Chyba to jest dla mnie najważniejsze. A nie miejsca publiczne, gdzie każdy może pójść i jeden drugiego nie zna.

**Co najdłużej powinno zostać zamknięte?**

Na pewno te centra handlowe. Tym bardziej, że w centrach handlowych jest bardzo dużo osób bezdomnych, gdzie raczej oni nie mieli wykonywanych testów i raczej mało zwracają uwagę na swoje zdrowie. Nie wiedzą czy są chorzy czy nie są. Tym bardziej, że często są pod wpływem alkoholu i tak, jak alkoholicy, piją non stop, więc raczej mało uwagi zwracają na to, czy są zakażeni czy nie, dopóki nie umrą. Taka jest prawda. Więc myślę, że takie miejsca publiczne, gdzie każdy może wejść bez większej weryfikacji. Ale myślę, że takie spotkania u siebie na podwórku ze znajomymi czy najbliższą rodziną, to powinny już zaczynać być traktowane normalnie.

**Do tego zaliczyłabyś też kina albo szkoły i żłobki?**

Tak, też bym zaliczyła. Nie wiem, dlaczego... To znaczy wiem, dlaczego otwierają te żłobki. Żeby matki wróciły do pracy. Bo ja dziecko nie będzie chodziło 13-letnie, to może zostać w domu i nikt nie robi z tego awantury. Więc myślę, że szkoły w ogóle nie powinny... Tylko nie wiem, jak oni by to zorganizowali. Tak jak u nas liczy załóżmy 400 dzieci. I teraz dzieci z 400 innych domów mają iść do szkoły. Ja nie interesuję się tym, więc nie wiem. Mogę powiedzieć, jak ja uważam jako szary człowiek. A nie doktor, który pewnie wie ode mnie lepiej. Albo jakiś, który robi statystyki i wszystko bada.

**Nas interesuje twój punkt widzenia.**

Nie chcę mówić głupot. Ale ja bym najpierw wróciła te kontakty najbliższe, a nie, że oni teraz żłobki otwierają, a ja nadal nie będę mogła z koleżanką się spotkać, bo sąsiad mnie zgłosi, że ona tutaj nie powinna być.

**Czy te miejsca po otwarciu powinny funkcjonować na nowych zasadach czy tak samo jak przed epidemią?**

Wszystko zależy od tego czasu jak to będzie. Jeżeli teraz by otworzyli basen, to bym w życiu na niego nie poszła. I myślę, że mało byłoby osób, które by na niego poszły. Jeżeli ktoś idzie na zakupy do Biedronki, to myślę, że nie będzie miał problemu z pójściem do galerii handlowej, bo to jest mniej więcej to samo. Już pójście do kina trochę nie, bo to już jednak siedzi się bardzo blisko tych osób, więc mogą się ludzie obawiać. Ale myślę, że jest mała różnica między spożywczakiem, a galerią, gdzie się ogląda ubrania, bo to mniej więcej takie same zasady. Jakie było pytanie?

**Czy te miejsca po otwarciu powinny funkcjonować na nowych zasadach czy tak samo jak przed epidemią?**

Właśnie chodzi o to, że ta epidemia ciągle nie minęła i dopóki nie będzie nowych przypadków zachorowań, a wiadomo, że jeszcze przez jakiś czas będą, to wydaje mi się, że te maseczki, rękawiczki, itd. powinny być miejscach publicznych.

**Czyli te zasady powinny być troszkę zmienione?**

Tak. Jeżeli ta epidemia minie i przez miesiąc czy tam ileś nie będzie nowych przypadków zachorowań, no to niech wszystko wróci do normy. Chociaż to częste mycie rąk, itd. powinno zostać jako nawyk u ludzi. Gdzie wchodzę z jakiegoś miejsca i myję ręce - to powinno zostać, a myślę, że mało osób tak robiło. A jednak dużo bakterii przenosimy przez dłonie. Nie tylko tego koronawirusa, ale milion innych bakterii i zarazków.

**Słyszałaś, w jakiś sposób Szwecja podeszła do pandemii?**

Tak, coś czytałam, jakiś artykuł. Że bardzo luźno. Nadal wszystko jest tak jak było.

**Praktycznie tak. *[Wyjaśnienie sposobu radzenia sobie przez Szwecję.]***

**Co o tym sądzisz?**

To niech poczekają... To skończą tak, jak Włosi. Przecież był klasyczny przykład Włochów, co Włochy zrobiły, gdzie oni raczej zaczęli wprowadzać te zakazy, to już było za późno. Niech czekają. Mają przecież przykład na innych państwach. Dla mnie to jest głupota. Co mogę więcej powiedzieć? Głupota. Tak, jak mówisz. Zamknęli tylko uczelnie, bo tam się uczą raczej studenci i nie pracują. Ale żłobki i podstawowe szkoły zostały nadal otwarte z tego względu, żeby rodzice mogli pracować. Wydaje mi się, że martwią się o gospodarkę, żeby nie było tak, jak u nas albo w wielu innych państwach, że jednak te skutki będą dość spore. Ale wydaje mi się, że to nie jest jednak zamknięte państwo na rok, tylko mamy teraz 1,5 miesiąca, więc jak będzie jeszcze miesiąc, to myślę, że aż takich tragicznych skutków nie będzie. To nie jest powódź, która wszystko zniszczyła. Tylko z powrotem każdy wróci do pracy i te pieniądze będą zarabiane i tak samo będą trafiały do skarbu państwa. Te skutki nie będą tak ogromne i jakoś sobie z tym poradzimy. To tak, jak być bezrobotnym przez 2 miesiące dla nas. Wydaje mi się, że każdy dałby radę być bezrobotny przez dwa miesiące. A tak, to głupota. Zobaczymy, okaże się, nie wiem.

**Nie chciałabyś, żeby w Polsce było takie rozwiązanie?**

Nie, bo wydaje mi się, z tego, co tak obserwuję, że te zakazy były wprowadzone dość szybko, a nawet bardzo szybko w porównaniu do innych krajów, to jednak ta liczba zachorowań nie jest taka ogromna. Teraz w najgorszym przypadku było ok. 500 zachorowań dziennie, a u niektórych jest kilka tysięcy, czy kilkanaście tysięcy dziennie. Być może to też się wiąże z wielkością państwa, ale ten przyrost jednak jest dość mały w mojej opinii. W porównaniu do Włoch. Tak samo liczba zgonów i całej reszty. Poza tym, można to też w ten sposób odwlec to w czasie i jakoś się przygotować - kupić sprzęty, zabezpieczyć się w jakiś sposób.

**A jak oceniasz pozostawanie w sferze rekomendacji zamiast zakazów?**

Sądzę to, że te rekomendacje będą do dupy. Z tego względu, że ja by sobie z takich rekomendacji nic nie zrobiła. Jakby ktoś mi powiedział: *wiesz co, lepiej, żebyś się nie widywała ze znajomymi,*to ja bym i tak się widywała. Może bym bardziej uważała, ale i tak bym to robiła.

Myślę, że gdyby to było tylko rekomendowane, to nadal np. kosmetyczki, fryzjerki, itd. by przyjmowały. Tak uważam. Dopóki nie została nałożona kara pieniężna na to, ok. 5 tys zł grozi, więc one boją się tylko i wyłącznie kary, a może nie tak bardzo tego, że ktoś może przyjść i je zarazić. I tak, jak słyszę, jak inni rozmawiają, to dla nich najbardziej bolesna byłaby ta kara pieniężna. Boją się, bo jakby odjechały samochodem i policja by ich zatrzymała, to co by powiedziały. Boją się kary, a nie tego, o co naprawdę chodzi, czyli to jest jakby robione dla naszego dobra, a nie żeby nałożyć nam karę. Zawsze ludzie boją się kary. To tak, jak dziecko zazwyczaj, jak rodzice stosują złe metody wychowawcze, to boją się kary, a nie tego, że nie może wkładać tam paluszka, bo się oparzy i będzie bolało. Tylko boi tego, że mama na niego nakrzyczy, czyli kary. I wydaje mi się, że w tym przypadku byłoby tak samo.

**Czyli jednak niestety, ale w Polsce potrzebujemy zakazów?**

Tak. Ja tak uważam, że są te zakazy, może niekoniecznie dla wszystkich wygodne, ale one być powinny. Ale jestem bardzo ciekawa sytuacji tej Szwecji, co tam się wydarzy. Czy oni takim podejściem uchronią się w jakiś sposób czy będzie tak samo, jak w państwach, gdzie są nałożone zakazy, czy będzie bardzo słabo i rozwinie się gorzej. Bo to tylko czas może pokazać, jak to będzie wyglądać. Pożyjemy, zobaczymy.

**Jak wygląda twoje dbanie o siebie? Co się zmieniło?**

No to tak, nogi mam nieogolone. I paznokcie mam do połowy pomalowane. Tak to wygląda. Nie dbam o siebie, bo nie ma tak jakby potrzeby. Mąż mnie zna od stóp do głów, więc raczej nie muszę się przed nim stroić. Chociaż wiadomo, zawsze, jak gdzieś wychodzimy, to staram się chociaż raz w tygodniu ładnie wyglądać. Ale nie mówię teraz, bo teraz nie wychodzimy, tylko ogólnie. Wiadomo, że facet też musi trochę oko nacieszyć, więc nie chcę też codziennie wyglądać domowo i w dresach. Ale teraz chodzę w dresach non stop. Nie chce mi się golić nóg, no bo mi się nie chce, bo kto mnie zobaczy. Ewentualnie tam trochę się ogarniam, jak idę do sklepu. Ale takie rzeczy... Ja sobie woskuję nogi. teraz bym musiała je ogolić, czego się boje i czekam aż kosmetyczki wrócą do pracy. To jest głupie, ale to się wiąże z tym, że jak ja ogolę, to potem będę miała twardy zarost i będę musiała znowu czekać, żeby je wydepilować kolejny miesiąc. Więc tak to wygląda. No nie dbam o siebie. Bo zazwyczaj jest tak, że się dba, żeby jak się wychodzi, to jak ktoś inny cię postrzega, a sama dla siebie raczej... Mi to nie przeszkadza.

**Zrezygnowałaś z używania jakichś kosmetyków?**

Nie. Kosmetyków używam. Znaczy zrezygnowałam z fluidów i całej ruszy, chociaż ja i tak się bardzo mało maluje. Ale jakiś fluid mam. Tylko tego nie używam, ale jeżeli chodzi o takie balsamy, kremy, itd. to używam. Nawet więcej niż wcześniej, bo mam na to czas. Jakieś olejki do buzi. Chodzi mi bardziej takie pielęgnacyjne rzeczy, to używam częściej, bo mam na to czas, żeby się wykąpać i coś zrobić. Ale jeżeli chodzi o malowanie, to już w ogóle się nie maluję. Gdzie wcześniej mi się zdarzało.

**Teraz używasz więcej kosmetyków do pielęgnacji?**

Tak. Zawsze je miałam, nie, że zamówiłam je sobie specjalnie. Ale zawsze kąpałam się na szybko, bo trzeba dziecko położyć, a teraz mam na to dużo czasu, więc jakieś olejki do buzi, kremy na noc, jakieś balsamy, to używam.

**Z czego wynika to, że masz paznokcie pomalowane do połowy?**

Z tym, że sama sobie nie umiem... *[śmiech]*Może przesadziłam, że do połowy, ale już są takie nieładne. Zawsze robiłam sobie hybrydę. Ja nie umiem sobie sama malować paznokci, więc u rąk i stóp mam hybrydę. A, że nie ma mi, kto tego zrobić, bo kosmetyczek nie ma... A ja sama sobie nie zrobię.

**Nie ściągasz tego sama, tylko czekasz aż zrośnie?**

Boże, jak to brzmi, masakra. *[śmiech]*Chodzi o to, że z rąk sobie zdjęłam sama. Mam gładkie paznokcie niepomalowane. Ale u nóg mi to mniej przeszkadza, bo mnie nikt nie widzi, więc mam takie z odrostami. I czekam, bo może jeszcze 2 tygodnie, może ileś, to kosmetyczki zaczną przyjmować i sobie zrobię. Bo ja mam ogólnie bardzo słabe paznokcie i tak, jak na rękach teraz nie mam, to mi się strasznie łamią, rozdwajają, itd. Ale u rąk zdjęłam całkowicie, a u nóg mam.

**Brakuje ci wizyt u kosmetyczki, u fryzjera?**

Nie za bardzo mi brakuje, bo nie mam... Brakowałoby mi, jakbym teraz musiała wyjść na miasto. To by mi brakowało, bo jak ja wyglądam. Musiałabym to sobie zrobić sama, a ja jestem anty dziewczyńska i ja nie umiem takich rzeczy robić. Więc by mi brakowało. A na chwilę obecną nie brakuje, bo nie mam potrzeby wyglądać super.

**Na ile czujesz, że to jest już czas na takie wizyty?**

Chciałabym się ogarnąć, tylko, że nie czuję ciśnienia i potrzeby. Co innego, jakbym teraz miała wyjść w krótkich spodenkach na miasto, gdzie są ludzie i każdy by widział, że mam nieogolone nogi, to bym się krępowała i musiała już iść teraz, zaraz. Bo raczej dbam o swój wygląd na porządku dziennym. A teraz nie mam potrzeby, więc nie mam ciśnienia.

**Dlaczego nie widzisz potrzeby?**

Nie to, że mi się nie chce. Ja sama nie potrafię, więc mi ktoś to musi zrobić. Skoro nie ma możliwości, to nie robię. Gdybym miała iść teraz nie wiem, założyć klapki, żeby ktoś widział moje paznokcie, to bym się bardzo źle czuła i musiała zrobić to już. A że nikt mi tego nie widzi, tylko ja, więc nie mam ciśnienia i nie muszę teraz, zaraz.

**Kupujesz teraz nowe kosmetyki?**

Nie, nie kupuję. Mam dużo. Ja mam sporo kosmetyków, chociaż jestem anty dziewczyńską i zawsze ktoś mi musi powiedzieć co kupić i czego używać, bo ja się nie znam. Jak mi koś powie, że to jest dobre, to ja to kupię, ale użyję załóżmy maseczki przez 2 dni, a potem już nie mam na to czasu. Dopiero teraz sobie tego wszystkiego... Ale to są rzeczy do pielęgnacji, to teraz mogę tego używać.

**Dlaczego postanowiłaś teraz wprowadzić te rytuały pielęgnacyjne?**

Bo nigdy nie miałam na to czasu wcześniej. Trochę pieniędzy wydałam na te kosmetyki i zawsze mąż się na mnie drze: *a, bo nie używasz*. Ja bardzo lubię ładnie pachnieć, itd. Ja lubię takie rzeczy. Nie lubię się malować, ale takie rzeczy do pielęgnacji, nawet stóp, lubię olejki, nie olejki. A nigdy nie miałam na to czasu. Więc teraz wykąpię się i mówię, no, mam czas, nie muszę nic zrobić, na tym laptopie siedzieć, itd. więc sobie po prostu to robię. To tylko się wiąże z tym czasem, że kiedyś nie miałam, a teraz mam.

**Teraz się w ogóle nie malujesz?**

Teraz w ogóle. Ja tak w ogóle się bardzo rzadko maluję. Ja raczej tylko na wyjścia. Ale np. do szkoły fluid i rzęsy nakładałam. Ja żadnych innych kosmetyków nie używałam. Tylko, jak szłam na jakąś imprezę, to szłam do kosmetyczki i kosmetyczka mnie maluje. I nigdy nie noszę pełnego makijażu, tylko lekki fluid i rzęsy. A teraz, to już w ogóle się nie maluje.

**Przed epidemią chodziłaś na basen, siłownię?**

Na basen jeździliśmy rekreacyjnie z córką. To całkowicie inne życie przed córką i teraz. Przed córką chodziłam na siłownię, itd. Na basen też raz w tygodniu i tak aktywnie spędzałam czas. Ale odkąd mam córkę, to brakuje mi na to czasu, bo już w ogóle by mnie w domu nie było. Więc z czegoś trzeba było zrezygnować. I zrezygnowałam z jakiś tam przyjemności. Do pracy muszę chodzić, do szkoły muszę chodzić - to jest nieuniknione. Ale z takich rzeczy dodatkowych, jak siłownia, która zajmowała mi 3-4 dni w tygodniu, po 2-3 godziny, to mogłam zrezygnować i zrezygnowałam. A na basen nie jeżdżę dla sportu, tylko jeżdżę dla rekreacji z dzieckiem. Całkowicie inne życie.

**Brakuje ci tych rekreacyjnych wyjazdów z córką na basen?**

Na razie jeszcze nie, z tego względu, że jeszcze nie jest tak ciepło. A zawsze chce się wody, jak jest ciepło. Na razie tych basenów raczej mi nie brakuje. Ja byłam na wyjeździe - 29 lutego wyjechaliśmy. Byliśmy w górach i tam na basenie codziennie byliśmy. Wróciliśmy i zaraz się zaczęło wszystko z koronawirusem. Więc tam się zrelaksowaliśmy dostatecznie.

**Byliście w Polsce na wyjeździe?**

W Polsce, w Białce Tatrzańskiej.

**Brakuje ci chodzenia do sklepów stacjonarnych?**

Brakuje mi tego, żebym mogła przymierzyć i zobaczyć, czy coś jest wygodne. I to jest najgorsze, bo tak, jak mówiłam, ja lubię rzeczy ładne, ale muszą być też wygodne. Bo jak nie będą wygodne, to ja tego nie założę. I to mnie denerwuje, że czasem coś przyjdzie i jest niewygodne. Muszę to odsyłać i czekać kolejny czas, zanim przyjdzie coś znowu.

**Internet nie bardzo jest w stanie zastąpić te zakupy?**

Nie bardzo, ale się przyzwyczaiłam. Tak samo się przyzwyczaiłam, jak do tych kolejek do sklepu. Wczoraj był problem, bo trzeba było mojej mamie zamówić spodnie, a moja mama jest wygodnicka i musi mieć materiał taki i to, i szukaliśmy jej 4 godziny jednych spodni. Pogoda się zmienia i chciała coś lekkiego. Ale mojej mamie dogodzić, to...

**Jaką funkcję mają te zakupy ubraniowe?**

Po pierwsze jest przymus, bo zmienia się pora roku. U mojej córki to ja muszę - oczywiście nie tyle. Ale musiałam jej zmienić wszystko, bo ona wyrasta z rzeczy. Ja też dużo rzeczy nie miałam. Nawet głupich dresów nie miałam. Kiedyś zrobiłam sobie zapas na wyprzedaży letnich rzeczy, to ich mam pod dostatkiem. Ale takich na teraz nie miałam. Więc nie kupuję tego dużo, ale tam 2 pary spodni, które są potrzebne. Oczywiście jest przyjemność duża. Bo mówię, chyba każda kobieta się cieszy, jak przyjdzie coś nowego. Może facet na to nie zwraca uwagi. Ale dziewczyny się cieszą, to chyba już tak w naszej naturze - fajnie, jak paczka przychodzi. Zbędnych rzeczy nie kupuję. Może więcej niż powinnam, ale to nie są rzeczy zbędne, których nie założę. Różnie. Jedna osoba może chodzić w jednych spodniach tydzień i to jest dla niej normalne, a inna musi codziennie coś innego nosić.

**Kupujesz więcej niż przed epidemią?**

Nie. Ja kupowałam w galeriach też sporo, w sklepach stacjonarnych. Nie, żebym była jakąś zakupoholiczką, ale córce kupuję wszystko, co mi się podoba. Nie patrzę, czy ma już 5 par spodni czy 8, czy ma już taki kolor legginsów. Ale jak mi się podoba, to kupuję. To są trochę takie niepotrzebne rzeczy, bo wystarczyło by jej mniej. Ale to kupowałam wcześniej też, przed epidemią. Teraz jest dużo tego kuriera dla mnie, bo wcześniej wszystko kupowałam stacjonarnie, bardzo rzadko przez Internet. Tylko jakieś sprawdzone firmy, gdzie znałam rozmiar. Po prostu dużo jest paczek, ale tyle samo bym kupowała w sklepie normalnym.

**Z punktu widzenia konsumenta czego ci najbardziej brakuje?**

Tak, to restauracje chyba najbardziej. Razem z kinem.

**Coś jeszcze?**

Nie, nie. Na dyskoteki się już odzwyczaiłam chodzić, więc mi tak bardzo nie brakuje, bo chodzimy raz w miesiącu albo i nie. Rzadko chodzimy już do klubów. Chyba nie. Ja nie jestem taka, że chodzę do galerii sztuki - nie chodzę, nie oglądam. Nie mam na to zajawki. Na koncerty bardzo lubię chodzić, ale to też rzadko. Raz na 2 miesiące, jak się trafi koncert, to jest super. A do kina i restauracji chodziłam regularnie, więc tego mi najbardziej brakuje, z czego najwięcej korzystałam.

**Dlaczego było ważne, żeby iść do kina, restauracji?**

Bo ja bardzo lubię wychodzić i bardzo lubię jeść *[śmiech].*To są dwie podstawowe rzeczy. Ja uwielbiam wychodzić z domu. Ja nie umiem być sama ze sobą, siedzieć w pokoju, itd. Uwielbiam ludzi, uwielbiam wychodzić.

**Zawsze ze znajomymi?**

Tak. Albo z mężem. Ze znajomymi, z mężem, nawet z mamą. Naprawdę, bardzo lubimy wychodzić z domu. Nie jesteśmy typem domownika, który potrafi i lubi spędzać czas tylko i wyłącznie w domu. Ja zawsze lubiła, jak coś się dzieje. Jak chciałam coś zjeść, to ja potrafiłam jechać do McDonalda i zjeść w samochodzie, bo byłam nieubrana, ale nie wyobrażałam sobie zamówić tego McDonalda do domu, bo to już nie taka przyjemność. Nie wiem, dlaczego tak mam, ale tak mam i tyle. Wolę tysiąc razy obejrzeć film w kinie niż w domu, bo to całkowicie inny klimat. To się wydaje nienormalne, bo można obejrzeć ten sam film. Ale dla mnie to jest różnica. Kupić sobie popcorn i w ogóle. Tak fajnie.
