# Supplementary material for: The challenges arising from the COVID-19 pandemic and the way people deal with them. A qualitative longitudinal study
Source: PLoS One. 2021 Oct 11;16(10):e0258133. doi: 10.1371/journal.pone.0258133 (PMC8504766; doi:10.1371/journal.pone.0258133)
Supplement: S1 Dataset — (ZIP) [file pone.0258133.s003.zip › Transcriptions/stage 2/1.2_F_25_single.docx]

**1.2_F_25_single**

**Obrazki. Który z tych obrazków najlepiej oddaje twoje emocje w tej chwili?**

Chyba 13 najbardziej teraz pasuje. To jest kwestia tego słońca na obrazku, bo mamy fajną pogodę i to zachęca do wychodzenia. Poza tym dzisiaj jest mój pierwszy dzień po kwarantannie. Byłam już w sklepie, także trochę wolności i w końcu poczucia, że... Wiadomo, że nie ma za bardzo, gdzie iść, ale jak coś się nadarzy, to będę mogła, bo nikt już mnie nie przyjedzie sprawdzić. Także chyba ta wolność właśnie i słońce najbardziej.

**A z jakimi emocjami ci się to kojarzy?**

Radości w końcu, że mogę wyjść. Nie wiem, takiej trochę też nadziei, że to siedzenie w domu będzie trochę bardziej urozmaicone, bo mogę wyjść pobiegać, mogę wyjść z psem na dłuższy spacer. Czy właśnie na wycieczkę do sklepu, co też jest urozmaiceniem. Więc właśnie tak głównie radość i trochę nadziei na rozrywkę.

**Co wpływa na te emocje poza zakończeniem okresu kwarantanny?**

Trochę ten tydzień był lepszy niż poprzedni. Bo parę rzeczy poszło do przodu, bo miałam dwie rozmowy o pracę, więc to też było motywujące, że coś tam się dzieje i jakoś tą pracę może zdalnie, ale uda się znaleźć. Ale nawet całkiem fajną, bo to były takie opcje pracy, które by mnie interesowały. Dostałam też maila z zaproszeniem na staż. To nic pewnego, ale zawsze jest to konkretna rzecz do robienia, nie tylko, żeby teraz zająć czas, ale to jest rekrutacja na lato, na lipiec, gdzie myślę, że szanse są takie, że wrócimy już do lipca do normalności na pewno, że będę mogła na ten staż do biura jeździć. Planowanie dalsze, ale bliższe niż w stylu "za rok" jest motywujące do tego, żeby coś tam robić. Już patrzyłam trochę na mieszkania. Jak tylko będzie możliwość umówić się na oglądanie czy podpisanie umów, to pojadę zobaczyć to mieszkanie i coś szybko sobie wynajmę. Więc to jest takie napędzające i dające troszeczkę motywacji i nadziei na to, że ta sytuacja będzie mogła się niedługo zmienić. Jakoś sobie ją przeorganizuję, bo, jak mówiłam, miałam mieszkać w Londynie, więc to spora zmiana. Gdyby nie było obecnej sytuacji to, poza tym, że nie byłabym tu, to i tak bym aplikowała, szukała pracy i miała nadzieję, że coś dostanę. To daję też poczucie, że nie wszystko się totalnie pozamykało i zmieniło. To ułamek normalności, bo i tak, takie rzeczy, jak rekrutacje i tak normalnie się dzieją, więc to pokazuje, że nie jesteśmy jeszcze w totalnym odcięciu, na zasadzie, że nas zamykają, wszystko się zamyka i jest jakaś hibernacja życia. To by było już bardzo dla wszystkich stresujące. A takie scenariusze też niektórzy mają podobno.

**Czy fakt, że podejmujesz kroki, żeby znaleźć pracę, daje ci poczucie kontroli nad tą sytuacją?**

W pewnym ułamku, jakie to daje, to tak. Poczucie kontroli, kiedy się szuka pracy może jest ogólnie małe. Robię, co mogę, ale to nie zależy od ciebie, czy ci odpowiedzą, czy cię będą chcieli, itd. Tylko w normalnej sytuacji rynkowej się ma przeświadczenie, że gdzieś się na pewno odezwą, bo wszyscy szukają. To jest kwestia tylko bycia lepszym od innych kandydatów, których jest stosunkowo normalna ilość. A w obecnej sytuacji jest to o tyle niepewne, że sporo ludzi potraciło pracę i może być większa ilość ludzi, przeciwko którym się konkuruje w rekrutacjach. Plus nie wiadomo, który biznes będzie stopował rekrutacje. Teraz dostaję maile z rekrutacji ze stycznia z Londynu, że zawieszają rekrutacje ze względu na epidemię. Więc jakiś ułamek kontroli jest, ale mniejszy niż normalnie, bo jest świadomość tego, że nie będą odpowiadać albo będą zawieszać rekrutację. W jednej pracy można zacząć dopiero w biurze, nie zatrudniają nowych pracowników zdalnie. Więc jest też czekanie z mojej strony, kiedy to będzie. I to daje poczucie niepewności. Ale jest to jedna z nielicznych rzeczy, które w tym momencie mogę zrobić, która daje poczucie, że coś się posunie do przodu.

**Czy twoje działania były nakierowane na to, żebyś zaczęła odczuwać radość i nadzieję?**

Szukanie pracy nie jest tylko ukierunkowane na odczucie radości i nadziei, że jakoś to będzie, tylko ja tak mam, że jak jest jakaś sytuacja, którą można poprawić. W tym momencie zarabianie jest esencjonalne, potrzebne, żebym mogła mieć pieniądze i nie musiała siedzieć u rodziców w domu. Więc ja mam tak, że jak jest coś do zrobienia, to nie lubię siedzieć i czekać aż się samo stanie, tylko muszę mieć poczucie, że zrobiłam, co mogłam, żeby sytuację poprawić. A reszta nie zależy ode mnie, więc akceptuje co tam się dzieje. Ale ja mogę siedzieć i wysyłać CV i czekać aż ktoś się odezwie. Mam mnóstwo czasu na to, więc mogę wysyłać nawet tam, gdzie jest nikła szansa. To jest bardziej kwestia tego, że ja muszę tak sobie trochę sumienie uspokoić, a trochę mam taką osobowość, że lubię działać, jak jest coś do zrobienia. Nie lubię usiąść i nic nie robić zbyt długo, kiedy wiem, że coś jest do zrobienia. Przy okazji to szukanie pracy daję mi radość i poprawę samopoczucia, ale nie poprawa samopoczucia była celem, tylko to, że lubię działać, kiedy trzeba.

**Jak jest u ciebie teraz z odczuwaniem niepokoju?**

To, że odezwali się w sprawie pracy, jest poprawieniem tego stanu, jeżeli chodzi o moją sytuację na rynku pracy. To oznacza, że coś tam prędzej czy później się znajdzie. Ja też z racji pogłębionej duchowości mam takie podejście, że pewne rzeczy dzieją się w odpowiednim czasie i mają dla nas odpowiednie znaczenie. I jak nie jedna praca, to może ta druga okaże się lepsza. Nie mam ciśnienia ze względów bezpieczeństwa czy też poziomu tego życia w domu, jedyne co, to, że wolałabym nie mieszkać z rodzicami, aczkolwiek dopóki nie mogę wychodzić na imprezy, spotykać się ze znajomymi, to i tak niewielkie to ma znaczenie. Bo dopiero, jak będę mogła wrócić do życia społecznego, jak będę chciała się widzieć z ludźmi i chodzić na imprezy, to dopiero zacznie mi doskwierać mocno to mieszkanie pod Warszawą z rodzicami. Na razie jest to tylko kwestia ewentualnych sprzeczek z nimi, ale też nie ma za bardzo o co, bo się nic takiego nie dzieje. Więc gdzieś niepokój o rynek pracy i sytuację na rynku pracy się uspokoił. Ten niepokój jest bardziej w kwestii jeszcze innych nieprzewidzianych przez nas wypadków. Nieprzewidziana jest długość kwarantanny i hibernacji, zamknięcia całej gospodarki. Im dłużej to będzie zamknięte, tym gorsza będzie sytuacja finansowa taka, żeby rząd był w stanie pomóc. Coraz mniej jest możliwości, a też nie jest tak, że rząd ma worek bez dna z pieniędzmi. Więc to jest bardziej niepokój o to, co się będzie działo gospodarczo i ekonomicznie w kraju, bo to dotknie nas wszystkich - od firm po pracowników, bezrobotnych, każdego.

**Ten niepokój mocno ci doskwiera na co dzień?**

Mocno nie, bo myślę, że zmieniałoby się dla nas mocno, gdybyśmy zaczęli odczuwać to w stylu, że rzeczywiście przestałaby być wystarczająca ilość jedzenia, gdybyśmy mieli wszystkie półki puste, a nie tylko te z papierem toaletowym i drożdżami. Zaczynają się dziać takie dziwne rzeczy, jak niektóre zachowania ludzi, którzy zaczęli być przewrażliwieni na punkcie tego, że ktoś nie ma rękawiczek albo że ktoś kaszlnie, kichnie.

**Podejmujesz jakieś działania, żeby zniwelować uczucie niepokoju?**

Tak, w tym moim ekonomicznym poziomie to szukanie pracy, bo to kwestia mojej bliższej i dalszej przyszłości. Zmieniły mi się plany na najbliższe półtora roku, ale stwierdziłam, że to nawet może lepiej i przegadywałam z terapeutą tę kwestię. Teraz będziemy układać mi plan celów na najbliższy czas. Ale po przemyśleniu tych studiów doszłam do wniosku, że może powinnam jednak inny kierunek wybrać, także to się trochę zmieniło. A jeśli chodzi o ten niepokój, to tak, szukanie pracy i takie myślenie, co zrobię, jak tylko już będzie można, czyli praca i wyprowadzenie się, jak tylko już będzie można się ruszać. Dla mnie też istotna jest kwestia zaplanowania jakiegoś wyjazdu, bo to, co mnie męczy bardzo, to jest siedzenie w jednym miejscu. Od dwóch lat byłam w Warszawie bez zbędnego podróżowania, bo oszczędzałam na studia w Anglii. Teraz te studia się odsuwają w czasie i wiem, że muszę jakiś wyjazd sobie zaplanować, bo czuję się źle bez możliwości wyjazdów. Tylko teraz dochodzi pytanie, kiedy będziemy mogli latać, gdzie będziemy mogli latać i jak będzie w ogóle sytuacja linii lotniczych.

**Co się zmieniło przez ostatni tydzień u ciebie?**

Przez resztę tygodnia już *binge watchingowałam* Dom z Papieru, bo wyszedł nowy sezon w piątek i wczoraj skończyłam. Wmawiałam sobie, że to jest nauka hiszpańskiego, bo oglądałam z napisami i to była trochę nauka, bo mi to w ten sposób wchodzi do głowy. Także nie, chyba nie zmieniło się za bardzo, wciąż dość leniwie. Z tym że dzieliłam ten czas na szukanie pracy, wysyłanie maili. Nie zabrałam się do sprzątania - to jest jedyna rzecz, do której się nie zebrałam, ale to mam plan, żeby zrobić to dzisiaj. To też, że jest ładna pogoda, można wyjść na dwór więcej, to dodaje trochę energii. Ale wciąż za wiele się nie zmieniło. Nawet coś upiekłam jednego dnia. Może więcej razy zmotywowałam się do zrobienia czegoś innego niż tylko siedzenie przez komputerem. Wychodziłam z psem do ogrodu co najmniej raz dziennie. Do dzisiaj, kiedy mogłam wyjść do sklepu, potem będę mogła wyjść na dłuższy spacer z psem, iść pobiegać, to nie, nie za wiele się zmieniło.

**Te zmiany mogą nastąpić teraz, bo kwarantanna się skończyła?**

Tak. Od dzisiaj mogę trochę więcej się przemieszczać.

**A z czego wynikało to, że spędzałaś mniej czasu przed komputerem?**

Trochę ze zmęczenia tym siedzeniem. Były dni, kiedy za mocno oglądałam wieczorem serial, bo się tak wciągnęłam, to rozbolała mnie głowa. Rozregulowałam sobie przez te ostatnie dwa tygodnie mój normalny tryb spania, gdzie zazwyczaj ok. 22/23 to już lubię usnąć, a wstawałam ok. 8:00, a teraz to się przesunęło. W ostatnim tygodniu zmuszałam się do wstawania ok. 8:00, ale wciąż to nie jest mój normalny tryb funkcjonowania, tylko przesunięty o 2-3 godziny.

**Wspomniałaś o wypiekach. Wiele osób teraz piecze i...**

Brakuje drożdży w sklepach. Półka z proszkiem do pieczenia też była pusta.

**Skąd się u ciebie wzięło to, że miałaś ochotę coś upiec?**

Jeżeli chodzi o gotowanie, to ja w ogóle wolę pieczenie ciast i ciasteczek - zawsze tak miałam. W podstawówce, gimnazjum piekłam coś co najmniej raz na tydzień. Mój młodszy się śmiał, że jestem "cukiernicą" w domu. Raz, że ja lubię jeść też słodkie i to był sposób na zrobienie zdrowszego słodkiego niż rodzice mi kupią w sklepie. Ale mi też sprawiało przyjemność samo pieczenie.

**A są jakieś czynności, z których zrezygnowałaś przez ostatni tydzień?**

Hmm... Chyba nie.

**A coś ci zaczęło mocniej przeszkadzać?**

Przeszkadza mi to, że nie mogę się zmotywować i ruszyć do ćwiczeń, bo tego mi się w ogóle nie udało zrobić. Zaczęłam czuć, że jestem coraz mniej rozciągnięta, bo wcześniej robiłam jogę regularnie. Więc chcę wrócić teraz do jogi, może do samego rozciągania, szczególnie, jak zacznę teraz biegać. Przez ostatnie dni mieliśmy wybrakowany wybór jedzenia, nie mieliśmy nic świeżego. W ogóle śmieszna sytuacja, bo w tym tygodniu rząd zdecydował, że kwarantanna będzie dotyczyć wszystkich domowników, jeśli ktoś wraca z zagranicy. Ale to miało nie działać wstecz. I do środy sprawdzali tylko mnie, a w czwartek już policja spytała o resztę domowników, tata musiał podać nazwiska i w sobotę i wczoraj pytali o wszystkich nas. Więc od czwartku rodzice tez nie mogli wychodzić z domu. Nie była to tragedia, bo wiedzieliśmy, że będzie to tylko do niedzieli. Zakupy nie były jakoś bardzo potrzebne, trzeba było zjadać to, co jest w domu. Więc kombinowałyśmy z mamą. Mój brat, który studiuje w Warszawie, chciał przyjechać w sobotę i tata chciał pojechać po niego samochodem w sobotę, ale stwierdziliśmy, że skoro tata już jest w tej bazie, to mógłby dostać ten mandat 30 tys. zł. W ogóle wczoraj sprawdzili nas podwójnie. Ja i tak nie wychodziłam, bo nie mam gdzie. Ja wylądowałam po powrocie z Londynu o 20:40 i teoretycznie ta kwarantanna kończyła się wczoraj o 20:40, ale wiadomo, że oni nie liczą godzinami, tylko już do końca dnia. I przy porannej kontroli tata się dopytywał policjanta, czy o 21:00 to już będzie koniec kwarantanny, bo chciał wczoraj jechać po brata. I później sprawdzili nas tego dnia drugi raz, myślę, że przez to, co tata powiedział. Oczywiście wiadomo, że nie jest to jakoś narażające ludzi, bo tata wsiadłby w samochód, pojechał po brata, przywiózł go i co to jest za różnica, czy zrobiłby to wczoraj czy dzisiaj - żadna. Więc nie czuliśmy, że to jest stawiające kogokolwiek na jakieś narażanie. Ale faktem jest, że gdyby przyjechali akurat, kiedy tata by pojechał, to mogliby ten mandat wystawić i byłoby ciężko z odwoływaniem się od niego.

**Jaki jest twój stosunek do nowych ograniczeń wprowadzonych przez rząd?**

Tak, o tyle, że nigdzie się nie przemieszczamy, nie jeździmy komunikacją. Jedyne co, to na zakupy. Ja rozumiem to w miastach z tymi lasami i parkami - to były miejsca, gdzie ludzie mogli się gromadzić i trzeba było to kontrolować. Ale jest to o tyle przesadne, szczególnie jeśli chodzi o las. Bo park rozumiem, parków jest więcej też w mieście i podobno matki z dziećmi na spacerkach się zbierały. Teraz idzie ciepła pogoda, więc tym bardziej by to robiły. Ale zabronienie ludziom chodzenia do lasu, kiedy mieszkają na wsi i u nas tutaj na osiedlu jest dużo mniejsze zagęszczenie ludzi na teren niż w miastach. Więc ja, wychodząc na spacer, spotykam może jedną osobę, psy się powąchają i tyle albo wcale, jak im nie pozwolimy. Ja nie spotykam tu nikogo. Jedyne miejsce, gdzie spotykam, to jest sklep, ale wiadomo, to jest pomieszczenie, do którego wchodzimy. U nas naprawdę, wychodząc biegać, w ogóle nie mam z nikim kontaktu, jak wychodzę sama, więc dlaczego miałabym nie móc pobiegać w lesie? My tu mamy taki mały lasek, ja nie wiem czy to jest las państwowy, ale już chorym wydaje mi się, żebym nie mogła tam iść pobiegać. Widziałam już jakieś petycje dużo osób podpisuje z tym wchodzeniem do lasu, no bo zgromadzenia, zgromadzeniami - to można karać, ale nie można zabronić iść matce z dzieckiem do lasu na spacer, bo ludzie potrzebują kontaktu z naturą, jak już nie mogą nic innego robić. Szczególnie, że teraz jest lepsza pogoda... Szczególnie współczuję tym w blokach bez balkonu, bo im zostaje tylko otworzenie okna.

**Planujesz iść pobiegać do tego lasu mimo tych ograniczeń?**

Tak. Bo on jest tak mały, że ja zdążę przebiec zanim mnie dogonią czy zobaczą *[śmiech]*, więc już będę poza tym lasem. To jest taki mały wycinek lasku tuż przy domach, a dalej jest łąka już. Poza tym, u nas na osiedlu policja przyjeżdża skontrolować ludzi. Wracając ze sklepu widziałam, że policja była w nieoznaczonym samochodzie i oni patrolowali okolicę, jakby ktoś się zgromadził na chodniku.

**Znać jakieś osoby, które nie przestrzegają niektórych ograniczeń?**

Nie. To znaczy mam jednego znajomego, który miał kwarantannę też po powrocie z Londynu. On siedział w domu, ale wyszedł sobie na spacer w sobotę i policjanci go zatrzymali na kontrolę i pytali, co robi, dokąd idzie. Mam przyjaciółkę, która poszła pobiegać. Ona twierdzi, że nie można biegać, tak mi mówiła. Ale skonsultowałam się z kolegą, że na samo bieganie nie ma zakazu, tak samo jak spacer. Tylko ona chyba biegała po prostu w miejscu, który był uznany za park czy skwer. Ale jest osobą o dużym talencie aktorskim i potrafi wymyślać różnego rodzaju wymówki, więc pomachała im [policjantom] Xanaxem, twierdząc, że ma depresję i jakby siedziała w domu, to się zabiję. Więc to było mocno przekoloryzowane. Ale biegała tylko, więc nie do końca jestem przekonana, czy złamała przepisy.

**Twoim zdaniem to, że ona poszła do parku pobiegać, jest w porządku?**

Nie wiem, czy to był park, czy co to było. Jeżeli to był park, to to jest bez sensu narażanie się, bo można wybrać po prostu inne miejsce do biegania. Ale jeżeli poszła biegać sama i nie jest z nikim, nie jest to miejsce w stylu zamknięte pomieszczenie, to nie rozumiem, czemu nie mogłaby tego zrobić. To jest jedyna możliwość wydzielenia trochę energii na zewnątrz. Ona jest, podobnie jak ja, mocno ekstrawertyczna, więc my już jak obie rozmawiamy, to wariujemy od tego siedzenia wewnątrz, więc ja ją doskonale rozumiem. To doprowadzi do tego, że po miesiącu takiego zamknięcia więcej ludzi będzie w psychiatrykach niż w szpitalach. Ludzie będą bez pracy, zestresowani, że nie mają za co jeść i jeszcze do tego, to, że byli zamknięci... Oczywiście jest to żart i koloryzowanie, ale ludzie nie będą w dobrym stanie psychicznym po takim miesiącu wewnątrz. Więc o zdrowie mentalne też trzeba dbać.

**Czyli, nawet jeśli poszła pobiegać do parku, to twoim zdaniem nie jest to szkodliwe, skoro nie minęła tam innych ludzi?**

No nie jest to szkodliwe, ale rozumiem, czemu zamknęli parki. Bo to były miejsca, w których matki z dziećmi szły na spacer, dzieci się bawią i tam się spaceruje raczej powoli niż szybko. Więc jeżeli się zebrały trzy matki, no to już się robiło z tego jakieś zgromadzeni i było to bardziej niebezpieczne.

**A z twoich obserwacji, spotkałaś się z tym, że ktoś nie stosuje się do ograniczeń?**

Nie, w okolicy to byłam dzisiaj w sklepie i to mnie jedna pani strofowała, bo nie zauważyłam rękawiczek. Rodzice mi powiedzieli, że to jest obowiązek teraz, że nie wpuszczają do sklepów bez rękawiczek, a pani na wejściu mnie nie poinstruowała, więc wzięłam wózek i weszłam do środka. My nie mamy rękawiczek w domu, a podejrzewam, że nie można ich nigdzie kupić, plus jest straszny problem z przesyłkami - ja czekam już na jedną przesyłkę od dwóch tygodni, tata na coś czeka, paczkomaty są pozatykane, więc no jest problem z przesyłkami, strasznie długo idą. Więc nie wiedziałam z tymi rękawiczkami. Ja się nie stresuję. Też na wejściu do sklepu jest żel antybakteryjny. Ale po 15 minutach mojego bycia w sklepie, jak już zdążyłam kupić wędlinę dla babci, jak już przeszłam parę alejek, to jedna pani zwróciła uwagę pani na dziale mięsnym, że ja nie mam rękawiczek i nikt nie zwrócił mi uwagi. Bo faktem jest, że nikt nie zwrócił mi uwagi. Więc wyszłam, zdezynfekowałam ręce i założyłam te duże foliowe rękawiczki. Ale ani obsługa mnie nie strofowała, tylko pani kupująca. W ogóle, ja szczerze mówiąc wątpię, żeby to za wiele dawało. Większym problemem w takich sklepach jest nasze oddychanie w nich. Bo nie dotknęłam, nawet myślałam o tym przez cały czas bycia w sklepie, nie dotknęłam ani oka, ani nosa, ani ust od wyjścia z domu.

**A miałaś maseczkę?**

Nie. Nie mamy tych jednorazowych... A nie, przepraszam, kłamię. Dostaliśmy od policji, a właściwie od gminy taką kopertę z maseczkami. Tam dostaliśmy chyba ze 3 maseczki. To mamy te 3 jednorazowe. Ja mam taką wielorazową, którą przywiozłam z Nepalu i ona chroni przed pyłem, tylko muszę ją wyprać, bo już miałam ją wiele razy i to nie ma sensu, jak się jej nie wypierze w wysokiej temperaturze.

**Planujesz ją wyprać i jej używać?**

No, może wychodząc do sklepu... Aczkolwiek, no ja mam podejście do tego takie, jak mówią, czyli, że to niewiele daje. Bo ci, co się zajmują wirusami, mówią, że te, które dają, to są te, które szczelnie zamykają nam ten obszar ust. Są takie, które są szczelne i mają filtr. Te maseczki nauszne mają dziury przy policzkach, mają dziury przy nosie i to jest niewystarczające według specjalistów. Podobno te wirusy akurat mają tak małe cząsteczki, że i tak się mogą wydostać. Pewnie mniej się wydostają niż bardziej, ale... Nie wiem, może jak będę szła do sklepu czy miejsca, gdzie może być więcej ludzi, to tak, ale mam taką świadomość, że to też nie jest stuprocentowe zabezpieczenie. To samo z tymi rękawiczkami. Widziałam w sklepie ludzi w rękawiczkach, a bez maseczki. Byli i w maseczce i rękawiczkach, ale byli też tacy w samych rękawiczkach. Więc z tymi rękawiczkami, to rzeczywiście, zdejmą, wyrzucą, nie wyniosą ze sklepu tego wszystkiego, co dotykali na rękach. Ale to bardziej chodzi o to, co unosi się w powietrzu w pomieszczeniu i czy my to wdychamy czy nie.

**Czy czujesz się zagrożona obecną sytuacją?**

Ekonomicznie trochę tak. Pomijając to, że miałam zarabiać w Londynie, a nie tu, a będę zarabiać tu. Poza kwestią tego, jak to będzie wyglądało z zarobkami, to nie. Poczucia zdrowotnego zagrożenia nie mam.

**A jeśli chodzi o twoich bliskich, to widzisz u nich jakieś zmiany emocjonalne?**

Nie, dlatego, że dla taty zmieniło się tylko to, że nie może iść na basen i chodzenie do sklepów, urzędów jest trochę bardziej ograniczone. Ale tata już nie pracuje od dłuższego czasu i on tylko narzeka, że nie może iść na basen, ale to są inne aktywności. Tata pracował w ogrodzie, zajmował się domem i ogrodowo-budowlanymi rzeczami i to się nie zmienia, on dalej to robi. Więc dla niego się nic nie zmieniło. A mama, to jakby miała wcześniejsze wakacje niż lato. Ale jej to pasuje, że nie musi jeździć do pracy, bo ona jest w kontakcie z uczniami mailowo. Aczkolwiek jej jest ciężko prowadzić zdalne lekcje, a jeśli chodzi o kwestie wideo, to jej szkoła tego nie ma. Mama jest nauczycielem języka migowego, więc teoretycznie mamy technologię, które umożliwiłyby dzieciakom bycie z nią w kontakcie i rozmawianie, ale szkoła i dyrektor tego nie zorganizowali. Więc ona zajmuje się domem, ma czas ogarnąć różne rzeczy. Pensja marcowa będzie mniejsza, nie wiem jak z kwietniową, ale te dwie niższe pensje, to nie jest aż tak ważne dla naszego całego budżetu. Jedyne o co się martwię, to, żebyśmy nie przynieśli jakoś tego do domu, bo jeżeli ktoś miałby mieć jakieś objawy i się zarazić i chorować, to jest to moja babcia. Ale ja bardziej o tym myślę. Jak z nią przebywam, to się do niej nie przytulam i ograniczam bliski kontakt, żeby niczego nie przenieść. Jest jakaś szansa, bo może w małym stopniu, ale korzystamy jednak z tych samych przestrzeni w domu.

**Jak twoi rodzice radzą sobie w tej sytuacji?**

Oni mają swoje różne teorie spiskowe i coraz to nowsze wymyślają albo sobie przedyskutowują. Oni od dłuższego czasu powtarzają, że coś się musi systemowo i kwestii tej bańki finansowej pozmieniać. Plus są też, tata w szczególności, przeciwnikami tej sieci 5G, więc im się idealnie wszystko składa. Więc oni sobie tak to widzą. Ale oni wychowywali nas bardziej w takim duchu pozytywnego myślenia, też na zasadzie, że jakoś to będzie i oni się nie przejmują, nie jest to dla nich przerażające ani nic takiego. Im to nie doskwiera. Bratu też i on może i studiować i pracować, a jego branża IT wręcz będzie rozkwitać w tej sytuacji. Nie słyszałam jeszcze od znajomych, żeby ktoś tracił pracę. Jakoś każdy sobie radzi. Znam parę osób, które nie może pracować. Nie wiem o nikim znajomym, kto byłby zarażony i dotknięty samym przetestowaniem i pozytywny wynikiem wirusa.

**Jakie dziwne zachowania w radzeniu sobie z tą sytuacją zaobserwowałaś u ludzi?**

Bardziej mi chodzi o takie przewrażliwienie, że nie wiadomo w co wierzyć, więc dla niektórych to jest duże zagrożenie. Ja osobiście uważam, że ten wirus może nie być aż tak dużym zagrożeniem, jak to się rysuje. Przy tym, jakie są statystyki, jak statystyki są nieprawdziwe, jak nieprawdziwa jest umieralność w porównaniu do innych wirusów itd. Jest on mocno zarażany, to fakt, ale żeby tak naprawdę go powstrzymać, to trzeba by było zahibernować ludzi na dwa tygodnie do miesiąca. A jest to zwyczajnie nierealne. Ja jestem w sytuacji, w której nie pracuje, więc nie jeżdżę do tej pracy, nie narażam się codziennie. Ale nie muszę, bo nie mam tej pracy, a jak znajdę pracę, to raczej zdalną.

**Po czym poznajesz, że ludzie są przewrażliwieni?**

Jak ta pani kupująca, która zwraca mi uwagę, a nie obsługa w sklepie o te rękawiczki. Gdzieś tam słyszałam, że jakąś młodą dziewczynę babcia skrzyczała za kichnięcie w autobusie. Teraz ktoś kichnie, kaszlnie, to będzie na niego krzyk. Też obserwowałam sytuację z Londynu, gdzie oni nie mają jeszcze zakazu a propos parków i przy tej dobrej pogodzie jacyś ludzie wczoraj opalali się na skrawku trawy i zostali skrzyczani przez innych, żeby z tej trawy zeszli. No bo im więcej się będzie ludzi zbierało na trawie, tym bardziej wszystkie obszary zielone zaczną być strefami, gdzie jest zagrożenie, więc rząd zacznie zamykać parki i nie pozwalać ludziom w ogóle wychodzić. Więc ci, co tego nie robią, boją się, że ci, co ignorują zasady i siedzą w parkach, sprawią, że dla wszystkich zasady się zaostrzą. Z drugiej strony, jeżeli by chcieć wyeliminować kompletnie zagrożenie, to trzeba by było zahibernować ludzi, a nie można. Są tacy, którzy wciąż pracują, jeżdżą do pracy i muszą to robić, bo nie będą mieli za co żyć. Trochę mnie irytuje, jak większość influencerów online nawołuje do #zostańwdomu. Ja mam ten przywilej, że nie pracuję, siedzę w domu, niewiele się dla mnie zmienia i mam ten komfort. Finansowo nie jest to dla mnie idealna sytuacja, ale też nie umieram, dzięki temu, że mam oszczędności. Ale jest masa ludzi, którzy tak nie mają. Gdybym nie miała tych oszczędności, które dają mi komfort tego, że mam za co jeść teraz i jak już się wszystko skończy, to mogę wystartować do znalezienie mieszkania, itd., to bym już się tak dobrze nie czuła i miałabym w nosie to, że ktoś mi mówi: "siedź w domu, bo umrzesz". Jeżeli mam umrzeć z głodu za chwilę albo wpaść w inne problemy finansowe, czy nie mieć za co zapłacić za prąd i gaz. Stawiając się w sytuacji tych ludzi, którzy muszą iść do pracy, żeby mieć za co jeść za dwa tygodnie czy za miesiąc, to gadanie celebryty, który ma pewnie większe oszczędności, ma mnóstwo możliwości zarabiania, bo zarabia online, ale ktoś, kto pracuje w sklepie, piekarni - tych miejscach, które nas zaopatrują w esencjonalne rzeczy, może nie mieć tego szczęścia, ale robi coś, co jest potrzebne. Więc takie gadanie niektórych "nie bądź egoistą, siedź w domu", w momencie, kiedy, to bycie egoistą to jest pójście do pracy, żeby mieć za co jeść, to mnie, gdybym była w tej sytuacji, mocno by wkurzało. Siedzę w tym domu, robię to, co wszyscy, ale doskonale mogę sobie zdać sprawę, dla których siedzenie w domu jest końcem wielu rzeczy i dużym problemem. Już pomijając kwestię psychicznego samopoczucia. I takich ludzi pewnie jest sporo, bo ilu ludzi ma poduszki finansowe w tym kraju? Już pomijam przedsiębiorstwa, firmy i inne takie miejsca.

**Czy widziałaś jeszcze jakieś inne dziwne zachowania?**

Ja nie widziałam, ale mam przyjaciela zakonnika i on mieszka w domu zakonnym i mają w kościele msze. Jego pracą jest stanie przed kościołem i odsyłanie kogoś, kto przyszedł ponad limit. To jest dla mnie śmieszne, że zamknęli parki i lasy, ale msze wciąż mogą się odbywać, co prawda do 5 osób, ale wciąż. Jest ktoś w tej kaplicy, kto widzi się z tymi 5 osobami razy ileś codziennie. I ten mój przyjaciel opowiadał mi, że dziś po zaostrzeniu przepisów, przyszło 17 osób na mszę, kiedy może być 15. I to są te starsze osoby, które są najbardziej zagrożone. Ale to są ludzie, głównie na emeryturze, może nie mają zajęcia i to są ludzie, którzy chodzili na te msze dzień w dzień o 7:30 i taki był ich rytm. Więc, jak im się to nagle wybija, to jest mocno problematyczne. A ci ludzie, z którymi on rozmawia i musi ich odesłać, bardzo często odchodzą bardzo obrażeni i nie mogą uwierzyć w to, że mszy nie ma, no bo jak to, nawet w czasie wojny czy stanie wojennym, mimo wszystko msze się odbywały. Ja wiem, że starsi ludzie nie zawsze mają dostęp do technologii, żeby te msze obejrzeć. Normalnie na codzienne msze poranne przychodzi ok. 40 osób, więc jak teraz 17 przychodzi, to jest połowa. Więc jak oni są odsyłani po 5 osób do kaplicy albo do salki, gdzie ksiądz normalnie też odprawia mszę, to oni są obrażeni i nie chcą, bo dla nich konieczne jest wejście do kościoła. Ostatnio dyskutował z panią nt. przyjmowania komunii - ksiądz daje komunię do ręki, żeby ludzie sami ją wzięli. I ta kobieta mówiła, że dla niej to nie do przeżycia, żeby nie mogła wziąć komunii od księdza, bo tak ją nauczono. I po dyskusji ona sama doszła do wniosku, że to jest irracjonalne, że nie jest w stanie tego zaakceptować. Ale są ludzie, którzy mają swoje rytuały, że to jest dla nich trudne do zaakceptowania. Plus dochodzi do tego wierzenie na zasadzie, że jak idę do pracy, to się mogę zarazić, ale jak idę na mszę, to pan Jezus chroni tarczą i to jest dla mnie straszne. To są opowieści znajomych, ja nie widziałam na własne oczy.

**Jak najczęściej robisz w obecnej sytuacji zakupy - przez Internet czy na miejscu?**

Ciężko stwierdzić, bo ostatnie dwa tygodnie mogłam robić je tylko online. Dziś był pierwszy dzień, kiedy poszłam do sklepu. Zrobiłam zakupy dla babci, dla siebie i wydałam mnóstwo pieniędzy, gdzie normalnie bym tyle nie wydała na siebie. A też nie kupiłam takich esencjonalnych rzeczy do jedzenia. Kupiłam chleb, coś do chleba, kilka warzyw. Ale ponieważ jak ja siedziałam w domu, to rodzice chodzili na zakupy i miałam przymusową dietę od słodyczy. Mama nie kupowała słodyczy, bo niezdrowe. Więc ja dziś zaopatrzyłam się w słodycze i też już w słodycze na czas Wielkanocy, takie wielkanocne czekoladki. Wymyśliłam kolejne pieczenie, więc też kupiłam trochę składników do pieczenia w tym momencie. Więc można powiedzieć, że moje zakupy stoją teraz pod znakiem słodyczy albo takich rzeczy, których rodzice na pewno nie kupią.

**Co w ostatnim czasie zamawiałaś przez Internet?**

Głównie były to ciuchy. Zamówiłam jedne buty, ale już je zwróciłam. Zamówiłam dwie sukienki i torebkę. Jedna sukienka miała nieodpowiedni rozmiar, więc dziś ją odesłałam. Mamie kupiłam filmy, które są jej potrzebne do pracy. Niewiele zamawiałam już dla siebie w tym tygodniu. Od naszej rozmowy nic dla siebie nie zamówiłam. Czekam wciąż na przesyłkę z olejkami eterycznymi. Myślę o tym, żeby dokupić suplementy diety, bo mi się pokończyły, takie jak cynk, itp. Ale trochę mam takie, że nie chcę zamawiać przez Internet, bo zastanawiam się, ile ta przesyłka będzie trwała. Na coś chyba jeszcze czekam, ale nie wiem na co... No właśnie, są rzeczy, o których zapomniałam, że na nie czekam, bo tak długo idą.

**Gdyby w tym momencie nie było epidemii, to kupiłabyś te sukienki online czy raczej stacjonarnie?**

Pewnie do stacjonarnego sklepu. Myślę, że bym ją przymierzyła, bo nie lubię tego procesu oddawania. Sam fakt, że coś kupię, a później muszę czekać na zwrot, blokuje mi te środki na jakiś czas. Więc jak się ma dużo środków, to to nie przeszkadza, ale jak się ma ograniczoną ilość... A ja oszczędności mam na drugim koncie i nie chcę ich ruszać. W przypadku ciuchów, szczególnie spodni nie kupiłabym przez Internet. Dużo butów zamawiam przez Internet tak czy siak, bo zamawiam je w sklepach stricte internetowych, bo rozmiary buta znam i wiem, że łatwiej jest utrafić, ale sukienkę pewnie poszłabym przymierzyć. Kupiłam dwie sukienki i jedna okazała się za duża. Są tak różne czasami rozmiarówki, więc nie wiadomo jak utrafić czasami.

**Obecnie kupujesz przez Internet więcej niż przed epidemią czy mniej?**

Myślę, że tyle samo. Trochę mam tak, że dopóki nie zarabiam, to wiem, że jak zacznę tak kupować, to przejem pieniądze, które są na najbliższy czas. Gdybym zaczynała staż w lato, to on nie będzie płatny... Jak będzie płatny połowę tego, co zarabiałam przed wyjazdem do Londynu, to i tak byłoby dobrze. Gdzieś mam w głowie, że te pieniądze są potrzebne i wolałabym wydać mniej oszczędności niż więcej na sytuację niekontrolowaną, więc to było mocno na poprawę nastroju z tymi sukienkami i torebką. To był dość spory wydatek na raz. Ale powiedzmy, że takie jedno szaleństwo mi wystarczy i potem się hamuje z szaleńczymi wydatkami na ciuchy czy coś, no bo, pewnie mogłabym niektóre rzeczy znaleźć taniej, gdybym poszła do większej ilości sklepów albo iść do lumpeksu niż zawsze kupować w necie. Są na pewno tańsze opcje. Myślę, że normalnie nie kupiłabym tych trzech rzeczy na raz, tylko tę jedną sukienkę.

**To były takie produkty, których nie kupiłabyś w normalnej sytuacji?**

Nie, to była sukienka w normalnej cenie. Może z torebką było szaleństwo, bo była droższa niż reszta rzeczy. Ale z torebką było tak, że od dłuższego czasu o takiej myślałam i to było coś, co się wpasowało w mój plan zakupowy. Pewnie bym je kupiła, ale może wahałabym się bardziej na zasadzie, czy jest mi to potrzebne i cen wydatek jest ok. Ja zakładam sobie zawsze w budżecie miesięcznym jakąś sumę na ciuchy i sumę wolnych wydatków na zasadzie, że zachce mi się coś kupić, może nie esencjonalnego, to żebym na jedną taką rzecz w miesiącu mogła sobie pozwolić. W tym miesiącu to był taki wydatek, a że nie wydaje na nic innego, dostałam zwrot za mieszkanie w Londynie, no to...

**Czy te zakupy miały jeszcze jakąś funkcję dla ciebie, poza poprawą nastroju?**

No taką, że przyda mi się więcej sukienek na lato.

**Jakie są twoim zdaniem wady i zalety zakupów przez Internet?**

Jedynie co, to niemożność przymierzenia i tego, że te środki są zablokowane. Chyba Zalando ma taką usługę, że mogę zamówić kilka rozmiarów i dopiero, jak wybiorę jeden rozmiar, to za niego płacę, a resztę odsyłam. I to jest fajne. Zalety to to, że nie muszę się nigdzie ruszać. Wybór jest duży bez łażenia po sklepie, można przefiltrować, jest to mniej męczące. Jak się zna rozmiarówkę danego sklepu, to też można kupić bez przymierzenia. Wadą jest to, że koszt przesyłki czasami zwiększa kwotę. I jak chcę coś na już i chcę pominąć koszt przesyłki, to czasem wybieram w punkcie odbioru.

**Jak wyglądają obecnie twoje zwyczaje żywieniowe?**

Ono nie zmieniło się bardzo od takiego żywienia weekendowego. Na moje żywienie bardzo wpływa to, czy wychodzę i jestem w pracy. W pracy to albo miała obiady w pracowej stołówce albo coś sobie zrobiłam. Generalnie nigdy nie miałam jakiś genialnych nawyków żywieniowych i odpowiednie odżywanie się to jest u mnie problem, bo ja np. zajadam stres, nudę. Słodycze są istotnym poprawiaczem humoru.

**Podczas kwarantanny też to obserwowałaś?**

Tak. Najbardziej to ja to widziałam, jak byłam w Londynie, bo wtedy się najbardziej stresowałam. W Londynie wychodziłam raz dziennie tylko po to, żeby kupić jakieś ciastka. Po powrocie byłam uzależniona od tego, co kupi mama, więc tego było mniej, z czasem w ogóle. Ale to np. upiekłam bułki - bułki były po to, żebym mogła coś słodkiego sobie zjeść. Ja mam tak, że lubię cieszyć się na myśl, że za chwilę mam jakiś dobry obiad. Budzę się rano. Z rana jestem w stanie nie jeść tak do 13:00, jeżeli nic nie robię, bo lubię to uczucie głodu z rana. Ostatnie dni, ponieważ wcześniej jadłam dość dużo, to miałam takie dni, że byłam przejedzona i potrzebowałam tego uczucia pustego żołądka. Ostatnie dni, to jadłam jeden albo dwa mniejsze posiłki dziennie. Ale dziś mogłam wyjść do sklepu, więc zaopatrzenie słodyczowe jest. Więc ono się nie zmieniło od żywienia weekendowego, kiedy byłam w domu. Jest to co innego niż kiedy mam dzień w pracy.

**Jak wygląda u was przygotowywanie posiłków?**

Różnie. Śniadania raczej robię sobie sama zazwyczaj.

**Jesz też sama?**

Tak, bo my mamy wszyscy inne pory jedzenia. Nie jemy teraz śniadań razem, bo każdy ma inny swój tryb. Czasem jemy obiad razem, ale też różnie, bo czasem jak mama zrobi i jesteśmy godne, to zjemy, a tata robi w tym czasie coś innego i zje później. Mama nie je ze mną codziennie. Zdarzają się sytuacje, że robi coś innego i nie je, ale robi obiady, więc gotuje codziennie. Wczoraj akurat zrobiła mi obiad. Więc raczej obiady są zrobione przez mamę, czasem jemy je razem, czasem, kto kiedy jest głodny. Kolacji się u nas raczej nie jada. Ja też kolacji zazwyczaj nie jem, to się zdarza rzadko.

**Zazwyczaj jesz dwa posiłki dziennie?**

Jeszcze taki podwieczorek, który nie jest kolacją, ale takim wcześniejszym posiłkiem.

**I te podwieczorki zazwyczaj każdy przygotowuje sam?**

Tak. Nie mamy rytuału wspólnych kolacji. Nigdy nie mieliśmy.

**To się nie zmieniło?**

Tak, to się nie zmieniło. Raz na jakiś czas zjemy razem obiad, teraz kiedy spędzamy czas w domu. Kiedy ja i brat nie mieszkaliśmy z rodzicami, to w dni, kiedy przyjeżdżałam do domu, z racji tego, ze było to niezwykłe, że jesteśmy wszyscy razem, to się jadło wspólne śniadanie. Ale to jest w tych momentach. Teraz, kiedy jesteśmy wszyscy w domu, to nie robimy wspólnych śniadań, bo każdy ma swój tryb.

**A zamawiasz jedzenie z dostawą? Przez Internet lub telefon.**

To jest to nieszczęście, że tutaj na naszym zadupiu ciężko z tym. Uber Eats tu nie dojeżdża, Pyszne.pl ma ograniczoną ilość restauracji. Zamówiłam sobie pizzę w sobotę. Jakoś w sobotę naszła mnie straszna chęć na pizzę. Tylko że to u nas jest koszt 50 zł za jedną pizzę, bo musiałam wybrać zamówienie, które przekroczy minimalny koszt. Jeszcze akurat rodzice coś innego mieli na obiad, więc jadłam ją sama na raty.

**Czyli tą przeszkodą są problemy z dostawą?**

Tak. To znaczy dostawy są, bo to była pizzeria u nas w miejscowości [Nadarzyn], ale jest mniejszy wybór. Ja na Pyszne.pl mam tu chyba 4 restauracje i dowożą, wszystko spoko, ale minimalny koszt zamówienia plus dostawa, to z tego się robi, no, "raz w tygodniu zrobię sobie imprezę". Może teraz, jak brat przyjedzie, to go raz w tygodniu namówię na taką pizzę na pół, to wtedy to jest trochę mniejszy koszt.

**Zamawianie jedzenia dla ciebie w tym momencie to sposób na świętowanie czegoś, np. weekendu?**

No, coś w tym stylu *[śmiech].* Nie codziennie, bo nie ma takich opcji. Gdybym była w mieście, to byłoby tych opcji więcej na różne kuchnie.

**Co myślisz o zamawianiu jedzenia z dostawą? Czy to jest bezpieczne?**

Ja myślę, że nic nie jest bezpieczne, jakby się tak uprzeć. Więc, mówię, żebyśmy się mogli uchronić całkiem od tego wirusa, to trzeba by było się zahibernować.

**Gdzie jest tutaj to ewentualne niebezpieczeństwo?**

Albo na pudełku coś będzie. Nie wiem, czy oni ustalili, jaka jest żywotność wirusa na różnych powierzchniach. Ktoś tam twierdził, że na tym plastiku, to się utrzymuje od kilku godzin do kilku dni nawet. To by było przerażającą diagnozą, bo taka plastikowa powierzchnia, jeśli ktoś jej nie przetrze, to się przez kilka dni utrzymuje wirus. Teoretycznie jest dostawa bezkontaktowa, że mogą to odłożyć i pojechać. Ale u nas nie mogą położyć pod drzwiami, bo jest furtka. Ja musiałam wyjść i pani mi to podała przez furtkę. Byłyśmy na odległość mojej i jej ręki, więc to może były ze 2 metry. Nie wiem, no ja tu nie widzę jakiegoś wielkiego zagrożenia i dla mnie to już jest przewrażliwienie. Poza tym, jeżeli ktoś z nas ma się zarazić, to się tego uniknie chodzeniem do sklepu, wchodzeniem do miejsc, gdzie są inni ludzie w postaci obsługi, która przez cały dzień ma kontakt z mnóstwem ludzi i są potencjalnymi nosicielami, myślę najlepszymi miejscami, gdzie ten wirus może być przeniesiony.

**Uważasz, że nie mamy wpływu na to, czy się zarazimy?**

Możemy próbować zmniejszyć prawdopodobieństwo. Myślę, że maseczki... Nie wiem, co mam myśleć o maseczkach. Na logikę, to się trochę wydaje dawać poczucie bezpieczeństwa. Ale skoro specjaliści twierdzą, że to nie jest w stu procentach bezpieczne, to nie wiem. Może rękawiczki tak. Ale najgorsze jest to wdychanie i oddychanie w pomieszczeniach. Więc najłatwiej nie wchodzić do pomieszczeń. Tylko jest to zwyczajnie niemożliwe, bo w którymś momencie, musimy pójść do sklepu. Największy problem z tym wirusem to to, że jest to niewidzialna mała cząsteczka w powietrzu. Więc dostanie się do naszych ust, oczu czy nosa... No, ok, może maseczka mogłaby pomóc, ale nie w 100%, więc jest jakaś szansa zawsze, że nie unikniemy tego pomimo różnych działań. Nie wszyscy noszą maseczki. Ja widziałam panie ekspedientki, które miały maseczki i takie, które nie mają maseczek. Przyjechał pan dostawca do sklepu bez maseczki i coś tam podawał. Więc jest tyle ludzi, których trzeba byłoby pilnować, bo nie każdy się pilnuje. I nic dziwnego, bo jest to trochę paranoiczne już, gdybyśmy się mieli tak zabezpieczać. Gdyby każdy miał chodzić w kombinezonie, to byłaby to paranoja już. Nie mówiąc o tym, że nie ma tylu kombinezonów. Nie da się zahibernować życia, ludzie muszą pracować, jeździć autobusami. Jakiś kierowca autobusu się żalił, że on daje komunikat, żeby ludzie wysiedli, bo jest ich za dużo, ale oni się nie słuchają. Z jednej strony można pomyśleć, co za egoiści, nie myślą o bezpieczeństwie innych ludzi. Ale z drugiej strony, jest mniejsza ilość autobusów, są rozkłady świąteczne i ludzie muszą dojechać do pracy. Jakbym miała się bać o to, czy wsiądę czy nie wsiądę do autobusu, żeby do pracy dojechać i wychodziła do tej pracy 3 godziny wcześniej niż muszę, to był żyła w dużym stresie.

**Obecnie płacisz częściej kartą niż gotówką?**

Ja zawsze płaciłam kartą. To znaczy kartą, telefonem tak naprawdę. Teraz jest plus ten, że mam iPhone z rozpoznawaniem twarzy, więc tylko muszę nakierować go na twarz. Ale wcześniej miałam ten z kciukiem. Więc, kiedy płaciłam telefonem, to musiałam przyłożyć kciuk. Więc musiałabym zdejmować rękawiczkę, dotykać tego telefonu wtedy, żeby zapłacić tym telefonem. Teraz nie muszę, ale tak sobie tylko pomyślałam o tym wcześniejszym modelu. Teraz nie muszę dotykać terminala, wyciągać karty z portfela. Telefonem też nie muszę niczego dotykać, wystarczy, że go przybliżę. Co było zabawne, widziałam terminal owinięty folią jednorazową i podejrzewam, że ją zmieniają albo przemywają folię płynem dezynfekującym. To było zabawne, bo sygnał nie jest zaburzony, ale no, było to śmieszne.

**Uważasz, że ta folia była realnym zabezpieczeniem?**

Mało kto dotyka terminala, może poza obsługą. Jest to dla nich ułatwienie, bo oni muszą dezynfekować wszystko. Jest to powierzchnia, której dużo kart może dotykać, więc ułatwia im to przemycie płynem. Bo stała na wejściu osoba, która każdy oddany po zakupach wózek, przemywali tę rączkę. No i przemycie rączki wózka to nie jest głupi pomysł. Jest taki mem, że dobrym skutkiem epidemii jest to, że w Biedronce umyli koszyki po raz pierwszy. Bo tak normalnie, to chyba jest w ogóle niemyte. Dezynfekcja koszyków nie jest negatywna.

**Twoim zdaniem w obecnej sytuacji to jest bezpieczne, żeby płacić gotówką?**

Pieniądze są tak brudne. Zawsze się upomina dzieci, żeby nie wkładały pieniędzy do buzi. Bo na pieniądzach, poza wirusami mówię, jest najwięcej bakterii z tych przedmiotów, których używamy. Nie wiem, to jest tak samo, jak na wszystkim innym, czego dotykają ludzie. Ekspedient ma rękawiczki i jeżeli się pilnuje, żeby tą rękawiczką nie podrapać się po twarzy, oku czy niczym innym, to dobrze. Ale jeżeli nie, to wszystkie produkty, które przeszły przez jego ręce, na które ludzie nachuchali, nakichali, itd, to sam się może zarazić, dotykając tymi rękawiczkami i przenieś to dalej. Więc to jest paradoks tych rękawiczek. Bo rękawiczki chronią powierzchnię naszej skóry, ale to nie przez skórę się zarażamy, tylko zależy, czy się dotkniemy twarzy. Jeśli ja wchodzę do sklepu, to ma to o tyle sens, że mam ręce czyste, wszystko w sklepie dotykałam rękawiczkami, więc ja tam nic nie rozniosłam i później, zdejmując rękawiczki, wyrzucając je, ze sklepu nic nie przyniosłam na siebie, jeżeli nie dotykałam twarzy. Więc tu jest dużo takich, ale a propos tego. Nie wiem, czy pieniędzmi bym się stresowała. Myślę, że tak samo, jak wszystkim innym, czyli samym faktem bycia w sklepie, to nie.

**Jak teraz będziesz planować zakupy spożywcze? Jak często będziesz chodzić do sklepu?**

Myślę, że maksymalnie raz na dwa dni. Podejrzewam, że największą motywacją będzie wyjście po słodycze, jak już będę miała duże parcie. Im bliżej będzie okresu, tym większe będzie parcie. Jedno, to że rodzice będą robili zakupy generalne, a ja myślę, że minimum raz na tydzień, a maksimum raz na dwa dni, chociaż to też pewnie nie ciągle.

**Opowiedz mi o swojej dzisiejszej wizycie w sklepie.**

Rodzicom zdarzało się czekać w kolejce, więc ja, wiedząc, że 8:15 otwierają, a 10-12 jest dla seniora, stwierdziłam, że pojadę od razu, bo więcej jest świeżych rzeczy, tak jak chociażby chleb. Więc byłam 8:15 pod sklepem i nie było kolejki, weszłam bez czekania. Nie było też tłumu ludzi w środku, więc pewnie się rozkręcą później ludzie. Brat wysyłał zdjęcia kolejki do Biedronki na Mokotowie, gdzie spędził 20 minut w samej kolejce. Tylko to jest kwestia zagęszczenia ludzi. Wiadomo, że na Mokotowie jest tych ludzi na tyle dużo, że do Biedronki czeka ich więcej. Nasz największy supermarket jest sklepem pomiędzy normalnym cenowo sklepem, a jakąś Almą, czyli są tam też jakieś lepsze produkty. W tym sklepie są też normalne ceny, ale to nie jest sklep, do którego ludzie idą, żeby kupić taniej. Więc myślę, że w Lidlach i Biedronkach kolejki są większe z tego powodu też. Nie zauważyłam płynu do dezynfekcji, bo był na zewnątrz i rękawiczek też nie. A pani, która mnie wpuszczała i dawała mi wózek, nie powiedziała mi ani o żelu, ani o rękawiczkach, więc ja przez dobre 15 minut chodziłam bez nich. O maseczce zapomniałam, że mamy te jednorazowe w domu. W ogóle zapomniałam o całej kwestii bezpieczeństwa, chyba z ekscytacji pójścia do tego sklepu. Rękawiczki założyłam później i zdezynfekowałam ręce. W sklepie wszystko jest. Pusta była tylko półka z proszkiem do pieczenia.

**Miałaś listę?**

Tak. To znaczy swojej nie, bo ja wiedziałam, co chcę, ale miałam listę od babci z rzeczami dla niej. Mama poprosiła mnie też o kilka rzeczy. Ja głównie kupiłam składniki do tych ciastek, chleb, coś do chleba, warzywa.

**A wcześniej robiłaś zakupy z listą?**

Jak idę sobie tak, że mam ochotę coś kupić, to bez listy. A jak miałam konkretny przepis to z listą, żeby kupić wszystko z tego przepisu. Ale z tymi ciastkami, to udało mi się zapamiętać.

**Jak się czułaś podczas tej wizyty w sklepie? Czułaś jakieś zagrożenie?**

Nie. Poza tym, że obsługa chodziła w maseczkach, rękawiczkach. Maseczek nie mieli wszyscy. Jedna pani miała tą plastikową osłonę na twarzy, więc w różnym stopniu byli pozabezpieczani.

**Robiłaś te zakupy szybko, żeby jak najmniej spędzić czasu w sklepie?**

Nie. Chodziłam, oglądałam wszystkie alejki, przypominałam sobie, myślałam, czy coś jeszcze potrzebuje.

**A jak zachowywali się inni?**

Na pewno mieli bardziej wypełnione koszyki. Ludzie robili naprawdę duże zakupy albo już szykowali się na świąteczne zakupy. Ponieważ było teraz mało ludzi, to myślę, że ludzie są świadomi, że im bliżej świąt, tym większe kolejki w sklepach, bo normalnie przed świętami jest dużo więcej ludzi. A z racji okrojonych godzin i osób w środku... Ja w ogóle nie jestem pewna, czy oni liczyli, ile nas było. Nie było tłumu, ale nie wiem, czy nas było 10. No nie wiem, może nas było 10, może nie więcej. Jest 3 główne kasy i czwarta przy alkoholach. Ale otwarta była jedna i drugą otworzyły panie na prośbę jednej klientki z ogromnym koszykiem. Ja nie miałam tak dużych zakupów.

**Jak planujesz spędzić Wielkanoc?**

Bez zmian, w domu. Gdybym normalnie była poza domem, to bym przyjeżdżała na święta do domu. Brat przyjeżdża i zostaje na cały tydzień i na święta.

**Brat przyjeżdża specjalnie na świata?**

To jest chyba kwestia tego, że on i tak siedzi w Warszawie sam w mieszkaniu i chciał już tydzień temu przyjechać. A mamy ogród, więc przy tej dobrej pogodzie, która będzie w tym tygodniu, może wyjść, zająć się psem. Pracować sobie może, nie musi wracać do Warszawy. Przyjemniej jest tutaj u nas.

**Planujecie obchodzić wasze zwyczaje Wielkanocne tak jak zawsze czy inaczej?**

Myślę, że tak. Nie wiem, co mama planuje gotować, coś już wymyśla. Ja nie jestem fanką tego, żeby na święta było więcej jedzenia, bo ciągle trzeba jeść i jesteśmy przejedzeni. Myślę, że to niepotrzebne, bo ciężej mi nie jeść, jeśli mam tyle dobrych rzeczy przed nosem. Podejrzewam, że zrobimy śniadanie wielkanocne w poniedziałek albo w niedzielę i poniedziałek - nie pamiętam, jak to robimy szczerze mówiąc. To jest teoretycznie święto ważniejsze w kościele, ale biorąc pod uwagę tradycje, to ważniejsza jest Wigilia.

**Jak wyglądają przygotowania?**

Sprzątamy więcej w domu i mama powtarza, żebym umyła okna. Porządki ewentualnie w domu i gotowanie, ale to mama z babcią się zabiorą za to w sobotę i tyle.

**Planujecie większe zakupy na Wielkanoc?**

Rodzice idą na zakupy jutro i pewnie już pod kątem Wielkanocy. Normalnie pewnie zrobiliby je bliżej weekendu. Normalnie pewnie byłyby już tłumy w sklepach w czwartek/piątek.

**Co planują kupić? Coś innego niż zazwyczaj?**

Poza jajkami, to chyba nic. Nie sprawdzałam, jak wygląda święcenie koszyczków w tym roku.

**Święconka to jest dla was ważny element świąt?**

Rodzice nie chodzą do kościoła regularnie już nie wiem od ilu lat. Ja jestem protestantką i jak miałam 13 lat to przestałam chodzić do kościoła katolickiego, a w wieku 15 lat przeszłam na protestantyzm, a brat zaraz za mną. Dla mnie te tradycje kościelne przestały mieć znaczenie. Ale mama chodziła z babcią poświęcić koszyczek do kościoła. To jest takie mocno tradycyjne bardziej niż religijne. Ale dla nas to by było do przeżycia, jeżeli tego nie będzie. Mama coś się śmiała, że sami będziemy święcić.

**Twoja rodzina planuje pójść do kościoła?**

Nie, to jest ostatnia rzecz, którą byśmy planowali. Te zgromadzenia, są ważne dla ludzi, rozumiem, dlaczego, ale w przypadku, kiedy inne zgromadzenia są nielegalne, to jest to trochę głupim pomysłem.

**Planujecie kupować kwiaty, dekoracje?**

Nie. Dla taty to byłby zbędny wydatek. A mama zebrała bazie z ogrodu. Podejrzewam, że jak jutro będą w sklepie, to może kupi po prostu jakieś, żeby były świeże. Czasami coś z ogrodu też się przynosi. Więc za specjalnie innych dekoracji nie.

**A ubrania na święta?**

Nie. Każdy z nas ma coś bardziej odświętnego, więc my zakładamy to, co jest. Ja nie mam tendencji do kupowania ubrań świątecznych, kiedy mam siedzieć w domu. Bo kiedy jestem z moją rodziną, to mi to nie jest potrzebne, bo oni na to nie zwracają uwagi, więc bez sensu wydawać pieniądze. Gdyby było to jakieś wyjście, to tak, ale w domu nie, nieszczególnie.
