# Supplementary material for: The challenges arising from the COVID-19 pandemic and the way people deal with them. A qualitative longitudinal study
Source: PLoS One. 2021 Oct 11;16(10):e0258133. doi: 10.1371/journal.pone.0258133 (PMC8504766; doi:10.1371/journal.pone.0258133)
Supplement: S1 Dataset — (ZIP) [file pone.0258133.s003.zip › Transcriptions/stage 2/7.2_M_28_couple, no children.docx]

**7.2_M_28_couple, no children**

**Co się zmieniło od kiedy się widzieliśmy?**

Co się zmieniło...Nadszedł weekend i urządziliśmy sobie małą imprezę przez Skype. Nie podejrzewałam, że może tak wyglądać, jak wyglądała, tzn. było bardzo miło, trwała 3-4 godz., jakiś tam delikatny alkohol i nawet delikatne tańce pod koniec, po spożyciu. To jest taka nowość, bo w sumie wcześniej tego nie robiliśmy. Wiedziałem, że niektórzy to robią, niektórzy się podśmiewają z tego, ale uważam, że nic w tym nie ma chyba złego ani głupiego. Co potem? Początek tygodnia to był taki okres zakupów online i odbierania paczek, które przychodziły do mieszkania tak 5-6 razy. Od jakichś tam głupich rzeczy jak kapsułki do ekspresu do kawy po zamówienie rowerka stacjonarnego do domu, żeby się trochę poruszać, bo trochę tego ruchu brakuje i w sumie to coraz bardziej doskwiera. No i dzisiejszy dzień z kolejnym rozwojem sytuacji ze strony rządu. I tak w skrócie te kolejne kroki od rządu sprawiły, że też jakby...Wiedziałem, że to się pewnie przedłuży - ten okres zamknięcia szkół i wszystkich lokali, gdzie mogą zbierać się ludzie, ale jakoś tak automatycznie zadzwoniłem od razu do rodziców, czy aby mają te maseczki i rękawiczki, które teraz trzeba mieć obowiązkowo. Sam zamówiłem rękawiczki, bo w sumie mieliśmy tylko kilka z takim jeszcze w miarę oczekiwaniem, kiedy to się może zakończyć, ale nie wygląda, że to się zakończy tak szybko, więc lepiej mieć na zapas.

**Zdjęcia emocje**

**4 i 6**

**Który z nich dobrze oddaje emocje, które przeżywałeś?**

W zeszłym tygodniu wybrałem 4 i też 4 wybiorę dzisiaj. Wybrałbym pewnie 6...Na tych bym się zatrzymał.

**4**

Widzę kolejny okres takich innych relacji, które występują nawet w rozmowach z przyjaciółmi. Więcej rzeczy się np. u nich działo. U mnie akurat zawodowo czy prywatnie nie. Jakieś problemy w pracy, jakieś nowe kroki od ich pracodawców, cięcie pensji czy coś takiego i znacznie więcej razy padało jakieś sformułowanie w stylu: no nic, musimy się wspierać, musimy czekać, aż to się rozwiąże, najważniejsze, że się wspieramy. Jakieś takie wyrazy wsparcie i pomocy z różnych stron. Z drugiej strony właśnie ta pseudo impreza, czyli też nowość dla mnie, ale w sumie wypadło to niewiele gorzej niż spotkanie z tymi przyjaciółmi. W sensie, że nie czułem żadnej różnicy, bardzo dobrze spędziłem czas wtedy. Czułem różnicę oczywiście, bo byliśmy w innych miejscach, ale jakby na takim stopniu rozmowy, śmiechu, spędzenia czasu. Było super. I trzecia rzecz to taki odruch, który dzisiaj miałem, jak minister powiedział, że trzeba już obowiązkowo zakrywać twarze. Uświadomiłem sobie, kurde, nie rozmawiałem w sumie z moimi rodzicami, czy oni mają np. maseczki i od razu automatycznie 5 min. później do nich zadzwoniłem czy niczego nie potrzebują.

Jak pomyślałeś, że rodzice, że musisz zadzwonić, zapytać, to jakie emocje ci towarzyszyły wtedy?

Taka obawa, taka chęć pomocy, zatroszczenie się o rzeczy, o które oni może nie mają np. możliwości się zatroszczyć, bo mieszkają w mniejszym mieście i mają tam może mniej możliwy dostęp do różnych rzeczy. Troska - tak bym to chyba ujął.

**6**

**Co tam jest, co ten obrazek przedstawia dla ciebie?**

To jest taki symboliczny obraz tego, że mam wrażenie, że sytuacja powoli może zacznie zmieniać się na plus, tzn. np. po takich sygnałach z Austrii czy z Norwegii, gdzie ten procent nowych zachorowań jakby zmniejsza się i w Norwegii to już jest chyba nawet poniżej 1%. Taki sygnał, impuls, że może za jakiś czas w końcu u nas też to zacznie się powoli normować i że może to jest jakoś do przebrnięcia w miarę bezboleśnie, przynajmniej dla mnie i dla tych osób, o które się najbardziej obawiam i troszczę. No i też z drugiej strony słońce, bo pogoda też daje się we znaki w taki sposób, że coraz jakby większa niecierpliwość się we mnie pojawia, że to jest taka nadzieja, że niedługo może w miarę sobie jakoś z tym poradzimy.

**Ta niecierpliwość? O co tu chodziło?**

No taka, że kiedy znowu będziemy mogli wyjść z domu na zewnątrz, wyjść do natury, trochę skorzystać z tej pogody i poczuć się inaczej niż po tych zimniejszych miesiącach i po tym pozostawaniu w domu.

**Czujesz tę niecierpliwość w różnych momentach dnia, że już chciałbyś wyjść?**

Są takie impulsy, to jest bardzo nieregularne. Są dni, kiedy w ogóle tego nie zauważam, tzn. dzień mija i już. Jakby trochę zapominam o obecnej sytuacji. A le był też taki jeden dzień, to był chyba poniedziałek, gdzie już byłem bardzo jakby zdenerwowany, źle się czułem, tak to na mnie działało, że dostałem jakiegoś bólu głowy, bo...Nie wiem, czy sam sobie wkręciłem to, że potrzebuję powietrza, może to było jakieś małe przesilenie czy po prostu taki moment, że myślałem sobie, że mam dość.

**Co się zdarzyło w ten poniedziałek, że akurat wtedy był kryzys?**

Tak naprawdę nie wiem. Jakoś tak po prostu mnie naszło, że to był taki kiepski dzień dla mnie przez jakieś kilka godzin. Nic konkretnego nie wpłynęło na to, że coś się wydarzyło i tak się poczułem. Po prostu może to narastało we mnie przez jakiś dłuższy czas i jakoś wyszło teraz.

**Jak sobie z tym poradziłeś?**

Tych kilka godzin było trochę męczących i jakoś nie umiałem sobie znaleźć miejsca w domu, ale też wsparcie i pomoc Dominiki, to że porozmawialiśmy sobie potem. Złapałem się na tym, że mogłem być nieprzyjemny w ogóle dla niej i z kilkoma osobami też rozmawiałem w ciągu dnia, że powinienem się ogarnąć po prostu. To nie jest moja wina i to nie jest niczyja wina spośród tych osób i nie powinienem im dawać jakichś takich sygnałów czy stawać się nieprzyjemny. Chociaż nie byłem, nie kłóciłem się, nie krzyczałem czy coś, nie mówiłem jakichś niemiłych słów, ale po prostu stwierdziłem, że jest tak jak jest, tak na razie musi być i pora się ogarnąć.

**Jak byś to nazwał? To był zły humor czy...?**

Tak, to był zły humor, bo też miałem jakiś problem ze snem akurat tej nocy. Wstałem o innej porze niż zazwyczaj, bo dużo wcześniej. Zazwyczaj wstaję ok 8, a wstałem o 6.30 i też samo to mnie od razu wyprowadziło równowagi. Nie dość, że mamy ograniczenia, to jeszcze mam problem ze snem nie wiadomo dlaczego, bo nawet nie mam kiedy się zmęczyć w ciągu dnia, skoro siedzę w domu. I tak to się nałożyło może.

Czy był jeszcze jakiś moment w poprzednim tygodniu, że jakieś emocje cię nachodziły, poza tym fatalnym poniedziałkiem?

Był też taki dzień, nie pamiętam który. To był wtorek chyba, czyli kolejny dzień po tym, kiedy akurat np. Dominika miała gorszy dzień, tzn. gorszą pierwszą połowę dnia. Wydaje mi się, że troszkę zamieniliśmy się rolami i to ja akurat byłem tą stroną uspokajającą i starałem się jakoś tak wspierać ją w tym jakimś tam trudnym czasie. Inne emocje? Taki wyraz mały smutki, że u mnie święta w domu rodzinnym przeżywa się tak bardzo klasycznie, tzn. jest spotkanie z rodziną, oczywiście jedzenie, jakieś wspólne spędzanie czasu, a w tym roku tego nie będzie, więc też odczułem taki brak i tęsknotę do tego.

**W zeszłym tygodniu było w tobie dość dużo lęku. Jak jest teraz?**

Wspominałem nawet o tym Dominice w tym tygodniu, że to jakoś zaczyna wyglądać tak, że jakby się przyzwyczajam. Wiem, że muszę siedzieć w domu i ograniczać ryzyka z każdej strony oczywiście, ale mam troszkę mniejszy lęk, że się zarażę, ale też nie w taki sposób, że staję się mniej odpowiedzialny. Jakoś to we mnie jest teraz, że ok., jest jak jest, ale mam mniejszą obawę, że to może jakoś zagrażać.

To jest takie pogodzenie się trochę z sytuacją i dostosowanie się?

Tak właśnie mi się wydaje, że to jest takie już...Trochę starałem się do tego przywyknąć.

**Powiedziałeś, że zaczyna brakować ci ruchu. Pojawiło się coś jeszcze, co zaczęło przeszkadzać?**

Myślę, że tak można to nazwać, jeśli chodzi o rzeczy, które można robić w domu. Nachodzi już pewne takie znużenie, że jakby mam trochę ograniczone możliwości, bo jednak nie da się wymyślić wielu różnych rzeczy, żeby je robić, żeby jakoś spędzić czas. Oglądanie filmów, słuchanie muzyki, pracowanie, rozmawianie, przebywanie razem...To jednak jest pewne ograniczenie i bardzo fajnie byłoby to zmienić.

**Myślałeś, żeby sobie jakieś nowe aktywności powprowadzać?**

No właśnie ten rowerek i wcześniej jeszcze zamówiliśmy takie gumy do treningów domowych, które zastępują jakieś maszyny czy przyrządy z siłowni. To jest i z tego się też cieszę. Z innych rzeczy to...W sumie częściej się odzywam sam do różnych osób, w sensie częściej nawiązuję rozmowę. Wtorek czy środa był takim dniem, że zadzwoniłem do swojego brata, z którym nie rozmawiałem ze 2 tyg. i stwierdziłem, że do niego zadzwonię, chociaż to nie jest jakby...To nie jest normalne w naszych relacjach. To było coś takiego innego. Mamy takie relacje z moim bratem, że są po prostu okresy, gdzie nadrabiamy zaległości co u nas wzajemnie się dzieje, a nie na zasadzie, że dzwonimy do siebie co tydzień czy co 2 tyg. To raczej jest raz w miesiącu, ale obaj nie odczuwamy z tego powodu jakiegoś braku. taki jest po prostu sposób naszej relacji. Co jeszcze...W sumie chyba to tyle.

**Próbowałeś korzystać z jakichś ofert online, które się pojawiają w ilości dużej?**

Zastanawiałem się nad tym. Zastanawiałem się nad jakimś językiem, tylko nie mam takiego przekonania teraz, że to jest właściwa metoda nauki języka. Może powinienem spróbować a nie jakoś wątpić. Z innych online rzeczy, to np. Dominika korzysta z jogi. Pytała, czy nie chcę z nią poćwiczyć, ale ja akurat do jogi nie za bardzo jestem...Nie mam przekonania też do jogi. Bardzo chciałbym to ćwiczyć, ale po prostu nie jestem wystarczająco jakoś rozciągnięty. No jakoś tak nie korzystam z takich rzeczy.

J**ak usłyszałeś, że będą te nowe obostrzenia, że maseczki, to co sobie pomyślałeś?**

Z jednej strony nie byłem zaskoczony. Podejrzewałem, zakładałem, że to się przedłuży. Z drugiej strony np. to, że matury czy egzaminy dla 8-klasistów będą przełożone wg planu na połowę czerwca, dało mi jednak taką małą nadzieję, że to może już być taki w miarę realny czas, kiedy to może nastąpić i to też się wiąże z moją sytuacją zawodową, czyli festiwalem, nad którym pracujemy przez cały rok. On jest zaplanowany na koniec lipca co roku i cały czas nie mamy pewności, czy to faktycznie wyjdzie tak jak powinno wyjść, bo jednak wydarzenie na 20-25 tys. ludzi to jest jednak duże wyzwanie w tych czasach. Nie wiem, czy to będzie możliwe i cały czas tak balansujemy, że to może być już taki czas, że sobie z tym poradzimy i po prostu trzeba będzie wprowadzić jakieś dodatkowe środki ostrożności, ale jednak samo wydarzenie się odbędzie. Z drugiej strony to jest w sumie niedługo, więc jak usłyszałem, że te matury, egzaminy może odbędą się w połowie czerwca, to też mi daje takie pozytywne odczucia i nadzieję, że i z naszym festiwalem będzie ok. I właśnie samo to, że pomyślałem o moich rodzicach od razu. Tak to u mnie przebiegło.

**Pojawiły się takie rzeczy w tym tygodniu, że poczułeś się jakoś bardziej zagrożony, że pojawił się lęk?**

Zastanawiałem się nad tym, bo niedawno pojawił się taki news, który później widziałem, że pojawiał się w mediach ogólnopolskich dużych, np. na Onecie. W pobliżu nas, w szkole pożarniczej, która jest na Żoliborzu, czyli 700 m od nas, że tam była jedna czy kilka osób zarażonych i na takich nawet grupkach FB mieszkańców pojawiły się sygnały, że pewnie uczniowie tej szkoły roznieśli po okolicznych sklepach, miejscach. Były takie głosy, że nie uważali, że to ich wina, z czym się kompletnie nie zgadzam. Nie podzielam takich opinii i takiej paniki, ale na początku to był taki pierwszy odruch bez zastanowienia, taki emocjonalny, że kurde, może byłem w tych miejscach, gdzie były te osoby zarażone. Po chwili pomyślałem sobie, że przecież mogłem być gdziekolwiek i gdziekolwiek mógłbym być zarażony, że to nie ma żadnej różnicy. Warszawa to jest duże miasto, mnóstwo osób i można zostać zarażonym wszędzie. To mi się pojawiło, to był taki ułamek sekundy, na gorąco, który bardzo szybko minął. To był też taki impuls uświadamiający być może, że jak przekonamy się o tym, że to jest blisko nas, to może zadziałać tak, że bardziej się boję, że bardziej się boimy. Tak naprawdę to, że jest blisko nas oznacza, że jest wszędzie i to może się wydarzyć gdziekolwiek, więc teraz takie mam podejście do sytuacji.

**Myślisz, że ludzie się stosują do obostrzeń i dostosują się do tych nowych?**

Mam nadzieję, że tak, bo dzisiaj nawet wychodząc na zakupy minąłem może 15-20 osób na ulicy, w sklepach i jedna czy dwie osoby tylko nie miały tej maseczki czy rękawiczek. Obserwując to byłem pozytywnie zaskoczony, że tak jest, ale z drugiej strony nie wiem, co się może wydarzyć w Wielkanoc. Mam taką obawę, że część osób stwierdzi, czy nie wytrzyma po prostu i pojedzie do rodzin, spotka się z ludźmi i nie wiadomo, jaki to później będzie miało efekt. Ze względu na okoliczności ta odpowiedzialność zejdzie trochę na bok.

**To pogadajmy może o tej Wielkanocy. Jak planujesz spędzić Wielkanoc, jakie masz przemyślenia na temat nadchodzących świąt?**

Wydaje mi się, że to jest mocno rodzinny czas i znaczna część Polaków spędza zwykle Wielkanoc spotykając się ze sobą. W takiej swojej perspektywie, choć wiem, że ja się nie pokuszę o to, żeby jechać i spotkać się z kimkolwiek, to mam taką obawę, że jednak znajdą się takie osoby - że to jest jednak Wielkanoc, święto, które jest raz w roku, taki wyjątkowy czas. Dla niektórych osób na pewno także pod względem religijnym, ale dla innych po prostu pod względem rodzinnym. Nie wiem, jak zachowają się ludzie. Dla mnie np. ważniejsze jest Boże Narodzenie i nawet nie wiem, jak wtedy ja bym się zachował. Wielkanoc jestem w stanie przeżyć w taki jeszcze w miarę bezbolesny sposób, ale z Bożym Narodzeniem pewnie miałbym większy dylemat, choć nie sądzę, żebym też złamał to postanowienie. Raczej jestem tego pewien. Mam nadzieję, że ludzie jednak zachowają ostrożność.

**Znasz kogoś, kto może się złamać?**

Zastanawiam się nad tym co zrobi mój brat ze swoją rodziną, bo nie byłem w sumie przekonany - wcześniej z nim o tym nie rozmawiałem, czy zostaje u siebie w domu, czy będą się poruszać do naszych rodziców albo do rodziców żony mojego brata.  Okazało się błędem, że tak myślałem, bo po rozmowie z nim wiem, że w ogóle nie było o tym mowy. Z drugiej strony moja przyjaciółka, która mówiła, że na pewno nie spotka się z rodzicami, pojechała do domu. Tylko tam wystąpiły inne względy, bo jej mama znalazła się w szpitalu, jakieś poważne względy zdrowotne, więc pojechała z zastrzeżeniem tego, że ani ona nie wychodziła z domu przez ostatnie 3 tyg., ani rodzice praktycznie też się nie poruszają. Jestem w stanie jakoś praktycznie to zrozumieć. Ale z tego co słyszę od innych osób, to raczej wszyscy zostają sami nawet w domach nie ryzykując.

A co twoi rodzice na to, że nie przyjedziesz?

Nie było nawet takiej namowy od nich, że może jednak. Była rozmowa, jak ja i Dominika spędzamy święta. Nie było mowy o tym, że się spotkamy. Taka oczywista rzecz, że musimy spędzić te święta inaczej niż zazwyczaj po prostu. Dla wszystkich to było oczywiste.

**Jak wyglądają wasze przygotowania do Wielkanocy?**

Wyjeżdżamy na wieś. Do domku na wsi, który należy do babci Dominiki. Mam tylko taką obawę, że może nas zatrzymać policja po drodze i robić jakieś problemy, ale wyjeżdżamy tam sami, będziemy tam sami, bo tam nikt nie mieszka na tej działce, więc tylko wychodzimy z naszego domu, wsiadamy do samochodu i jedziemy tam. Nie stwarzamy jakiegoś zagrożenia dla innych. No i pewnie jakieś jedzeniowe sprawy. Zamówiliśmy sobie jakieś tam jedzenie, sami też będziemy coś przygotowywać...Z lekkim zachowaniem ducha wielkanocnego w postaci potraw, a z drugiej strony ten wyjazd na wieś, żeby może trochę skorzystać ze słońca i ładnej pogody, trochę się oderwać i wyrwać się z domu.

Normalnie jakie jeszcze elementy wielkanocne były ważne, poza jedzeniem?

Ze strony moich rodziców na pewno ta religijna część, że to jednak było już praktycznie od czwartku, że codziennie do kościoła, bo odpowiednia msza czy coś. W niedzielę czy poniedziałek też w sumie chodziłem z nimi do kościoła. Jeździliśmy do kogoś, ktoś przyjeżdżał do nas z rodziny, mój brat przyjeżdżał, więc spotykaliśmy się w większym gronie. I to tyle w sumie chyba. I na pewno, jeśli chodzi o samo jedzenie, to były takie ilości...Takie typowe świąteczne obżarstwo, którego w tym roku pewnie nie będzie i dobrze.

**Jak rodzice sobie radzą z tym, że nie idą do kościoła w tym roku?**

Właściwie nie rozmawialiśmy o tym. Wiem, jak to wygląda w zwykłą niedzielę. Oni zostają w domu i oglądają mszę w tv czy słuchają tego w radiu, jak gdyby godząc się z tym bez żadnego zastanawiania się, czy może jednak dzisiaj warto by było pójść. Nie, nie ma czegoś takiego u nich.

**A święconka w sobotę?**

O kurczę...To jest bardzo dobre pytanie. Zapomniałem o tym. Nawet nie wiem, jak to jest teraz...Rozumiem, że nie ma tego. Nie zainteresowałem się tym, szczerze mówiąc i nawet ich nie zapytałem, jak to wygląda. Nie wiem, czy jest to święcenie w kościołach, czy nie.

**Rozumiem, że dla ciebie ta święconka nie jest ważna?**

No nie. Raczej to się pokrywało z tym, że ja zawsze te święta spędzałem z nimi i od mojego urodzenia tak to zawsze wyglądało. Teraz jest taka sytuacja, że nie wiem jak rodzice, a my nie będziemy przygotowywać tej święconki.

**Co sobie zamówiliście do jedzenia na Wielkanoc?**

Zamówiliśmy wegańskie jedzenie przede wszystkim, bo przez to, że Dominika jest weganką, ja praktycznie prawie w ogóle nie jem mięsa. Na pewno nie jem mięsa w domu. Zdarza mi się zjeść mięso u rodziców Dominiki albo u mnie w domu, co jest bardzo rzadkie i też zupełnie się do tego przekonałem i robię to w pełni świadomie, a nie dlatego, że muszę. Zamówiliśmy żurek wegański, pasty czy pasztety, mazurka wegańskiego - też, żeby wspomóc naszą lokalną wegańską restaurację, do której często chodziliśmy kiedyś. I sami też coś sobie przygotujemy.

**Co przygotujecie?**

Nie wiem jeszcze. Może jakieś bułeczki, jakieś dodatkowe pasty, pewnie jakiś obiad. Nie zaplanowaliśmy tego jeszcze tak konkretnie. Jest kwestia tego wyjazdu i tego, że tam już raczej nie będziemy mieli dostępu do sklepu, więc wszystko będziemy musieli zabrać stąd w sobotę.

**Masz takie produkty, bez których nie wyobrażasz sobie świąt? Taka przyjemność, że muszę je mieć?**

Właściwie chyba nie, bo byłem przyzwyczajony klasycznie, że to jest jedzenie jajek. Ok., jest żurek, ale on też u mnie w domu wcale tak często nie występował. W sumie nie, nie mam takich rzeczy. Fajnie jest po prostu zjeść coś fajnego innego niż w ciągu roku.

**Dominika jest weganką, tzn., że ty jajek sobie nie zrobisz?**

Myślę, że nie. Jajka jem dosyć rzadko już teraz i nie myślę w ten sposób, że jak Wielkanoc, to muszę zjeść to jajko. Jak będę miał ochotę, to sobie zrobię. Po prostu nie planuję tego. Jak sobie w sobotę pomyślę, że może skoro jutro jest Niedziela Wielkanocna, to może zjem sobie jajko, to sobie zjem jajko.

Zakupy już zrobione czy jeszcze nie?

Jutro pewnie będziemy robić te zakupy.

**Macie listę, czy robicie bez listy?**

Jeszcze nie mamy, ale będziemy mieć. Już teraz bez listy jest nam ciężko. Staramy się ograniczać czas przebywania w sklepach, więc lista to jak najbardziej ułatwia. Ta lista się na pewno pojawi.

**Przejdźmy do robienia zakupów. Co dzisiaj kupowałeś?**

Dzisiaj zakupy były przy okazji, bo wyszedłem odebrać paczki z paczkomatu. Zaszedłem tylko do sklepu po drodze i to były takie nieistotne sprawy. Gdybym nie wychodził po te paczki, to na pewno nie wybrałbym się po to. Odbierałem paczkę, która była w kiosku, kiosk był chwilowo zamknięty, musiałem poczekać 15 minut nie wracając do domu, więc zaszedłem do sklepu. Normalnie zakupy wyglądają tak, że sporządzam listę, Dominika czasami ją uzupełnia, chociaż zazwyczaj to ja zajmuję się tym, czego nam brakuje w domu, jeśli chodzi o jedzenie, chemię czy jakieś inne rzeczy. No i zakładam rękawiczki, zakładam maseczkę, biorę ze sobą torby i zazwyczaj wracam obładowany. Chodzę na piechotę i tylko tutaj pod domem.

**Od naszego ostatniego spotkania w czwartek, ile razy robiłeś zakupy?**

Takie większe, faktycznie potrzebne to były 2 razy i ten dodatkowy raz dzisiaj raczej przy okazji i dla zabicia czasu. Nie planowałem tego, bo miałem tylko zejść do okolicznego kiosku. Byłem o 13.45 i była kartka, że przerwa do 14. Nie opłacało mi się wracać do domu, więc nie wiem, czy nazwałbym to zakupami.

**Teraz bardziej planujesz zakupy niż wcześniej?**

Wcześniej też starałem się zawsze robić listę, ale też czasami lubiłem sobie pochodzić albo przechodząc obok jakiejś półki się zatrzymać, że o kurczę, może to by mi się przydało albo nam przydało. Jakoś tak nie przejmowałem się czasem spędzany, w sklepie, a w tej sytuacji teraz jest tak, że staram się w tym sklepie przebywać jak najkrócej, jak najmniej osób minąć i jak najszybciej wrócić.

**Dlatego, że się nie czujesz bezpiecznie? Masz maseczkę, rękawiczki, ale nadal masz wrażenie, że ten wirus gdzieś...?**

No ta, w sumie tak. Przechodząc obok kogoś staram się zachować dystans. Nawet, jak niektóre osoby się tym w ogóle nie przejmują, co uważam, że jest trochę nieodpowiedzialne i wchodzą w kogoś innego nie mając maseczek i rękawiczek. To nie jest jakaś diametralna zmiana, że przygotowuję listę zakupów, ale ta lista jest pewnie bardziej skrupulatna i nie ma oglądania w sklepie.

**Teraz są limity osób w sklepie. Zauważyłeś, że teraz ludzie się jakoś inaczej zachowują, pilnują tego?**

Raczej tak, ale też nie wszyscy. Nawet jak są wywieszone kartki z konkretną informacją, że o tym, ile osób może przebywać w sklepie i o tym, ile osób może jeszcze wejść do sklepu decyduje liczba wystawionych koszyków. Jednak widzę, że niektóre osoby nie zwracają na to uwagi. Nie czytają tych kartek, nie biorą koszyków, chociaż jest taka prośba, żeby brać, żeby zaznaczyć dla pracowników sklepu, ile tych osób fizycznie jest w tym miejscu...Jednak czasami to nie działa, ale raczej większość tak. Carrefour, który mamy pod blokiem to nie jest mały CF ani duży CF, to jest taki powiedzmy średni, gdzie miejsca nie jest jakoś super dużo pomiędzy półkami. To przejście pomiędzy alejkami nie jest czasami bezpieczne, jeżeli oczywiście jest tam ktoś inny.

**Ludzie czekają pod sklepem, żeby wejść?**

Akurat w tym sklepie czekają, bo pilnuje tego ochroniarz, ale np. mamy taki sklep trochę bardziej eko i też bezglutenowe rzeczy i wegańskie, to tam czasami jednak tak to nie wygląda. Mamy sklep alkoholowy, w którym byłem właśnie przed tą imprezą i tam to już kompletnie...Niektórzy po prostu wchodzą sobie, ci sprzedawcy zwracają uwagę i te osoby są bardzo zaskoczone tym, że nie mogą sobie wejść bez koszyka, bo wchodzą po 1 piwo, czy jedno wino. Jakby nie rozumieją sensu tej sytuacji. Ale zazwyczaj czekają, ja na pewno czekam i widzę, że jest jednak takie poczucie odpowiedzialności, bo nawet jak ktoś podchodzi i nie jest pewny, czy to jest kolejka, to pyta i jednak ustawia się gdzieś 1.5-2 m dalej.

**Co myślisz o tych, co wchodzą bez koszyka, próbują wejść, bo tylko po jedną rzecz, itp.?**

Właśnie mam z tym problem, bo czasami staram się te osoby zrozumieć. Staram się nie oceniać negatywnie tych osób i nie myśleć, że co za nieodpowiedzialni ludzie, co oni w ogóle robią? Staram się pomyśleć, że może ktoś się zamyślił, ktoś się zagadał, może nie zauważył tych innych osób, które są w sklepie, ale jednak dochodzę do wniosku, że jednak w obecnej sytuacji, która nie trwa od wczoraj, tylko trwa od dłuższego czasu...  Przekaz we wszystkich mediach i w mediach społecznościowych...Ciężko nie dowiedzieć się, jak się powinno zachować w takich sytuacjach, więc jednak trochę się dziwię. Tym bardziej, kiedy drzwi do tego sklepu mają 1.5-2 m, ta kartka zajmuje połowę tych drzwi, to zastanawiam się, jak oni tego mogą nie zauważyć?

**Myślisz, że oni robią to specjalnie? Olewają specjalnie te zakazy?**

Może tak być. Już będąc dalej od tego sklepu widziałem parę 25-30latków, którzy przyjechali na rowerach do tego sklepu, nie mieli ani maseczek, ani rękawiczek i też weszli do tego sklepu bez koszyka, nie czytając tej kartki i jakby nie sprawiali wrażenia przejętych całą tą sytuacją. To dało jakiś taki obraz, że mogli to faktycznie olać.

**A inni ludzie jak reagują na tych nieprzestrzegających zasad?**

Akurat tam poza nimi tylko ja byłem w sklepie i jak wchodzili, to chyba sprzedawcy byli zajęci czymś innym. Kiedy zauważyli, że oni nie mają rękawiczek i nie wzięli tego koszyka, to oni już byli przy kasie, więc odniosłem wrażenie, że nie zwracali im uwagi, bo już jest po fakcie, że już tylko zapłacą za te rzeczy i wyjdą sobie. Jakiś czas temu byłem w jeszcze innym sklepie, też Carrefourze, który jest troszeczkę dalej i tam widziałem taki przypadek, że sprzedawcy prawie wyprosili jedną osobę ze sklepu krzycząc, że kartki trzeba czytać, co to ma być. Reakcja byłą dosyć stanowcza i niemiła.

**Miałeś szansę zaobserwować te godziny dla seniorów i jak to działa? Słyszałeś w ogóle o tym?**

Tak i dlatego w ogóle nie wychodzę między 10 a 12, bo wiem, że to są godziny dla seniorów. I fajnie, i dobrze, że mają taką możliwość, ale nie wiem jak to fizycznie wygląda w środku. Czy inni próbują wtedy też się dostać, czy nie.

**Jak teraz płacisz w sklepie?**

Płacę telefonem. Zazwyczaj płaciłem kartą a teraz jakby łatwiej jest mi telefonem. Może dlatego, że biorę ze sobą mniej rzeczy? Nie muszę brać portfela. Akurat nigdy nie miałem żadnej awarii czy jakiegoś problemu z tym płaceniem przez telefon i tym bardziej że listę zakupów też często miewam w telefonie zapisaną, więc zerkam na tę listę i nawet później nie chowam tego telefonu, tylko od razu płacę za jego pomocą.

**Przerzuciłeś się z karty na telefon i to wynika z sytuacji koronawirusowej?**

Trochę tak, a trochę z takich praktycznych względów. Nie noszę już ze sobą gotówki, tzn. teraz. Wcześniej zazwyczaj starałem się mieć jakąś gotówkę przy sobie. Zazwyczaj płaciłem kartą, a teraz noszę tylko telefon. Jakoś zaufałem temu telefonowi. Może jak kiedyś będę miał jakiś problem, to się to zmieni, ale to mi się właśnie zbiega z tą listą, bo i tak korzystam z telefonu w czasie robienia zakupów.

**Kurier was odwiedzał w tym tygodniu wiele razy, paczkomat też odwiedzałeś. Co zamawialiście poza rowerem i kapsułkami?**

Zamówiliśmy gumy do ćwiczeń, Dominika chyba 2 razy miała kosmetyki, czy 3 razy nawet. Co jeszcze...Dzisiaj też coś z paczkomatu odbierałem dla niej. I chyba o czymś zapomniałem...A, i dzisiaj zamówiłem też rękawiczki i jeszcze coś...kurczę...

**Czy to były rzeczy, które normalnie byście zamówili też w sklepie internetowym, czy kupilibyście stacjonarnie?**

Nie mam pewności. Rower może zamówilibyśmy online, ale odebralibyśmy go np. w sklepie. Nie wiem, jak z tymi kosmetykami Dominiki, czy one są dostępne w sklepach. Gumy pewnie też byłyby dostępne w sportowych sklepach, więc chyba to są zamówienia ze względu na sytuację bardziej.

**A kapsułki zwykle kupujecie online?**

Nie, pierwszy raz. Normalnie kupuję w jakimś punkcie Nespresso. W Arkadii zazwyczaj.

**Myślisz, że zostaniesz już z kapsułkami zamawianymi online?**

Myślę, że nie. Ze względu na to, że te paczki jednak wliczając koszty dostawy tych 5-6 paczek, to wychodzi ok. 50 zł. Zdarza nam się zamawiać różne rzeczy online, bo czasami nie mamy czasu lub nie są dostępne i to jest ok, ale nie w takich ilościach. Czasami po prostu jesteśmy w Arkadii i to nie jest problem, żeby zajść na 5 min i te kapsułki wziąć. Koszty dostawy czasami są jednak...Nawet mimo tego, że to jest paczkomat, to w przypadku kawy chyba płaciłem za to 9 zł. Zliczając to wszystko i jeszcze kolejne paczki, które pewnie będą to są już jakieś środki, które można wykorzystać zupełnie inaczej.

**Zdarzyło się wam zamówić jakieś jedzenie przez internet w ostatnim czasie?**

Tak, to nam się zdarza i korzystamy z takich rzeczy. Nie tylko w sumie teraz, bo są takie sytuacje, kiedy nie mamy czasu czegoś zrobić albo mamy na coś specjalnego ochotę, ale to jest raz na tydzień, półtora, może 2 tygodnie.

**A w ciągu ostatnich 2 tyg.?**

3-4 razy myślę, że tak. Pizza, indyjskie, raz zamówiliśmy sobie obiad z takiej naszej lokalnej knajpki, też, żeby trochę im pomóc. I chyba tyle.

**Myślisz, że to jest bezpieczne w kontekście wirusa? Jak jest z tym dostawcą jedzenia?**

Teraz dostawa jest głownie bezdotykowa, tzn. zostawiali nam pod drzwiami to jedzenie i odchodzili, żeby nie było bezpośredniego kontaktu, ale z drugiej strony coś nam zostawiają i nie wiadomo, czy ten wirus nie został na tym opakowaniu i dlatego jak odbieramy takie jedzenie to od razu myjemy ręce i się odkażamy, i staramy się to już jeść w takich bezpiecznych warunkach.

**Trzeba przełożyć to jedzenie? Jak to technicznie robicie?**

Tego indyjskiego akurat nie przekładaliśmy. Nie wiem, czy jest w ogóle możliwość, żeby to jedzenie samo odkazić.

**A opakowanie? Pudełko od pizzy np.?**

Pizzę zazwyczaj jemy na talerzach a z tym indyjskim, to w sumie nie. Ten obiad, który jedliśmy to też przełożyliśmy na talerze. Po odbiorze zawsze staram się myć ręce, ale fakt faktem to faktycznie może gdzieś zostać. To jest możliwe i pewnie mamy to z tyłu głowy.

**Czemu teraz zamawialiście częściej?**

Ta pizza to był akurat ten dzień, kiedy miałem zły nastrój i pomyślałem, że zjem sobie pizzę, bo mam na to ochotę i może sobie poprawię ten nastrój. Ten obiad to z tego powodu, że trochę nie mieliśmy czasu, żeby gotować akurat tego dnia, bo Dominika ma dużo pracy, ja też miałem. Indyjskie z kolei to z tych samych powodów co pizza, bo akurat Dominika jest wielką fanką takiego jedzenia, więc to było dla przyjemności. Raczej dla przyjemności w sumie zamawialiśmy.

**Zdarzyło ci się zamówić dostawę zakupów spożywczych?**

Miałem taką myśl jakieś 2 tyg. temu i powiedziałem o tym Dominice. Powiedziała, że chyba raczej teraz to nie wyjdzie, bo są dosyć długie dostawy, bo pewnie sporo osób chce w taki sposób sobie poradzić i w sumie nawet nie sprawdzałem tego, ile czasu takie dostawy mogą wynieść Nigdy tego nie robiłem, ale kiedyś zainteresowałem się tym i zazwyczaj było tak, że dostawa w 1-2 dni. Teraz obstawiam, że to może być powiedzmy tydzień, więc zrezygnowałem. A z drugiej strony to pójście na zakupy to możliwość wyjścia z domu.

**Nie chcesz tego oddać tak do końca?**

Tak. gdyby przywieźli te zakupy, to już nie miałbym wymówki, tak, dokładnie.

**Masz jeszcze jakieś plany zakupowe, o których wiesz na pewno, że będą online ze względu na koronawirusa?**

A, Dominika jeszcze kupiła książki. O tym zapomniałem. Właściwie nie wiem, bo z takich rzeczy przydatnych w domu, to raczej już wszystko mamy. Elektronika, sprzęt do ćwiczeń, to też już zostało załatwione. W sumie nie spodziewam się. Nie planowałem kupować kolejnych książek czy płyt, czy czegoś takiego.

**Jak z zakupami radzą sobie twoi rodzice. Oni nie mieszkają w Warszawie?**

Oni wychodzą sami po prostu, ale z tego co mi mówią, to raczej 2 x w tyg. To jest rzadziej niż zwykle, bo oni lubili sobie chodzić codziennie lub prawie codziennie. A to kupić tylko pieczywo, a to tylko coś tam, więc dla nich to jest zupełnie inna sytuacja.

**Masz okazję obserwować wiele starszych osób na Żoliborzu. Dużo chodzą?**

Tak, mnóstwo. Właściwie, kiedy tylko spojrzę przez okno, to najczęściej widzę jakąś starszą osobę, która stoi albo w kolejce do apteki, albo w tym Carrefourze. Zazwyczaj ktoś jest. Ale też na ulicy, jak sami wychodziliśmy, to też nam się zdarzyło widzieć. Jest takich osób dużo.

**Bardziej się rzucają w oczy niż normalnie? Proporcjonalnie jest ich więcej?**

Chyba nie, tylko bardziej zwracamy na nie uwagę ze względu na to, że są w tej grupie ryzyka, i też dlatego, że kurczę, mogliby liczyć na jakąś pomoc. My też spotkaliśmy taką osobę 2 razy i pytaliśmy się, czy pomóc, czy coś. Raczej była taka reakcję, że nie, że dziękuję, radzę sobie. Nie wiem, czy oni po prostu muszą wychodzić, czy chcą wychodzić, bo to dla nich też jest forma wyjścia z domu?

**Zdarzyło wam się zamówić jedzenie w knajpie i pójść je odebrać?**

Nie zdarzyło nam się jeszcze. Jeszcze, bo mamy taki plan, żeby jutro zrobić sobie jedzenie izraelskie, a takich rzeczy jak kiszone buraki czy kiszone marchewki nie jesteśmy w stanie zrobić w ciągu 1-2 dni. Powstał taki pomysł, żebyśmy zamówili to sobie w izraelskiej knajpie i podeszli czy podjechali to odebrać. Mamy Tel Aviv na Żoliborzu i zazwyczaj tam chodziliśmy po prostu.

**Skąd taki pomysł, żeby izraelskie jedzenie?**

Bo dużo gotuję ostatnio i co tydzień w piątek mieliśmy ostatnio taką kolację, gdzie robię takie rzeczy z innych kuchni. Raz była kuchnia meksykańska, to zrobiłem tacos z różnymi rzeczami, tydzień temu była azjatycka i były takie bułeczki na parze, tempura i tego typu rzeczy i w simie szukaliśmy pomysłu, co można zrobić teraz. Padło akurat na Izrael. kiszonek nie jesteśmy w stanie zrobić, a Dominika powiedziała, że bez kiszonek to ciężko.

**Wy na co dzień też tak jadacie i ty też tak gotujesz?**

Nie. Zdarza nam się różne rzeczy gotować, ale tych akurat nie gotowaliśmy wcześniej. W sumie ja się chyba tak dosyć poświęciłem temu kucharzeniu ostatnio. Dla mnie to też jest forma spędzania czasu. W sumie o tym ci nie wspominałem, że są takie 2-3 wieczory w tygodniu, kiedy lubię gotować, żeby to mi zajęło 2 godz. Z jednej strony może być z tego coś pożytecznego i może z tego wyjść coś dobrego, z drugiej mam jakieś spędzenie czasu, może się czegoś nauczę, trochę czegoś popróbuję. To jest taka nowa aktywność. Ja często gotuję, ale w taki trochę inny sposób. Gotowałem obiady, czasem zdarzało mi się kolację, a tutaj akurat np. ostatnio zrobiliśmy chałkę, chleb. Wiem, że dużo osób teraz takie rzeczy robi i stwierdziliśmy, że spróbujemy i akurat udało mi się to zrobić. Takich rzeczy w ogóle nigdy nie robiłem.

**To znaczy, że również zmieniły się wasze zwyczaje żywieniowe?**

Troszkę może się zmieniły. Nie powiem, że kuchnia meksykańska czy azjatycka to są dla nas obce rzeczy, ale w domu zazwyczaj tego nie gotowaliśmy. Tego typu akcje to zazwyczaj było pójście raz na jakiś czas do jakiejś fajnej restauracji i tego typu spędzenie czasu. Robimy to samo praktycznie, tylko gotując w domu i siedząc w domu. Czy to nam zostanie? Nie wiem, ale właściwie nie miałbym nic przeciwko, że czasami zamiast pójść do restauracji sami coś ugotujemy i w taki sposób spędzimy czas dla rozrywki. Dla mnie ok. To jest trochę rozrywka, trochę uczenie się nowych rzeczy. 2 w 1.

**Śniadania też macie inne niż do tej pory?**

Nie, chyba nie. Zazwyczaj Dominika się specjalizowała w robieniu śniadań - jakichś specjalnych past czy jakichś innych rzeczy niż zazwyczaj robimy. Ja zazwyczaj byłem od robienia takich rzeczy na obiad/ kolację i to chyba nam zostało. Faktycznie więcej gotuję. Sprawia mi to przyjemność po prostu.

**Rozumiem, że jest w planach też sport?**

Zacząłem już z gumami i też byłem pozytywnie zaskoczony, że to daje jakiś fajny efekt, że czuję faktycznie te ćwiczenia. Bardzo wyczekiwaną rzeczą był ten rower, bo nie możemy chodzić na siłownię. Ja nie byłem takim sportowcem, że spędzałem godziny na siłowni 5 x w tyg., tylko to było takie raczej zdrowotne, sportowe chodzenie od kilku miesięcy 2-3 x w tyg. Trening nie jakiś specjalistyczny tylko taki z chęci poruszania się, trochę poprawienie sylwetki, itd. A mimo wszystko tego zaczęło nam obojgu braknąć mocno. Ja może pokusiłbym się, żeby zamówić jakąś sztangę, obciążenie, ale niestety nic chyba z tego nie wyjdzie, bo nie mamy miejsca, żeby to trzymać w wygodny sposób. Siłownię mamy po drugiej stronie ulicy tylko nie mamy pewności, czy ona przeżyje obecny okres.

**Czy jeszcze coś się pojawiło w twoim rytmie dnia co pomaga ci w obecnej sytuacji fajniej spędzić czas?**

Zdarza nam się też pograć w planszówki. Trzy tyg. temu chyba zamówiliśmy sobie jedną czy dwie nowe planszówki, żeby mieć jakąś nową fajną grę. Gramy we dwójkę. Co jeszcze? Ostatnio czytaliśmy książkę na głos sobie nawzajem. To też jest jakiś taki sposób. To chyba tyle.

**Czy zauważyłeś jakieś dziwne zachowania związane z epidemią? Np. moja koleżanka ma w łazience pudełko i tam wrzuca rzeczy, które przynoszą do domu i to odbywa kwarantannę. Może ciebie też coś zaskoczyło?**

Ciężko powiedzieć...Chyba nie. Tak się zastanawiam...Jest jedna taka rzecz i nie wiem, czy to można na tej samej półce ustawić, ale mamy taką koleżankę, która założyła sobie profil na Instagramie, na którym gotuje, ale robi takie rzeczy w stylu: ugotowałam dziś owsiankę. Pokazuje, że ugotowała owsiankę i rozpisuje przepis, że do owsianki są potrzebne np. owoce i płatki owsiane - takie zupełnie oczywiste rzeczy. I ja np. tego nie rozumiem.

**A to jest związane z koronawirusem? To jej zachowanie?**

Nie mam pojęcia w sumie, ale dodaje to codziennie albo 2 x dziennie, a z tego co wiem, to ona za bardzo nie gotowała wcześniej. Nie wiem, czy to jest dziwne do końca.

**A rzeczy, które ludzie umieszczają w social mediach? Zdarzyło ci się zobaczyć coś związanego z koronawirusem, co wydało ci się dziwne, że ktoś to wstawił?**

Staram się przeglądać. Może taka rzecz, że zauważyłem, że niektórzy traktują ten czas jako jakiś challenge. Mój szef ostatnio wrzucił post, że w 2 tyg. zamierza schudnąć 20 kg, co jest dość odważne. Też widziałem, że ktoś pisał, że w całym tym czasie nie pije alkoholu. Zastanawiam się, dlaczego. Ja do tego tak nie podchodzę, że to jest taki czas, w którym ja muszę sobie czy komuś coś udowodnić. Nie oceniam tego, że to jest złe. Może ktoś tego potrzebuje, może to jest w sumie taka pozytywna korzyść tego czasu, którą ktoś wyszukuje.

**Nie masz wyzwania nauczyć się gotowania 100 nowych potraw?**

Nie. Podchodzę do tego tak, że jak mam na coś ochotę, to chcę to zrobić po prostu. Nie na zasadzie, że do końca kwarantanny muszę się nauczyć włoskiego czy liczyć do 20 po włosku albo tych 100 przepisów, przeczytać 80 książek. Ja w tym czasie potrzebuję możliwie jak największej swobody nawet w tym życiu w domu, skoro nie mogę mieć swobody w wychodzeniu.
