# Supplementary material for: The challenges arising from the COVID-19 pandemic and the way people deal with them. A qualitative longitudinal study
Source: PLoS One. 2021 Oct 11;16(10):e0258133. doi: 10.1371/journal.pone.0258133 (PMC8504766; doi:10.1371/journal.pone.0258133)
Supplement: S1 Dataset — (ZIP) [file pone.0258133.s003.zip › Transcriptions/stage 2/20.2_F_25_couple, with child.docx]

**20.2_F_25_couple with child**

**Emocje od ostatniego spotkania**

Działo się bardzo dużo, jestem przeokropnie zła. Mój mąż, będąc w czwartek (2 kwietnia) na poczcie i chcąc nadać przesyłkę, usłyszał od pani w okienku, że nie zostanie obsłużony, bo on do 9 kwietnia jest na kwarantannie. Bardzo się z dziwił, bo nie mieliśmy żadnej informacji na ten temat, a wedle wypowiedzi tej pani, jego kwarantanna powinna trwać już mniej więcej od tygodnia. Byliśmy w szoku. Ja i mama zadzwoniłyśmy na policję, która nic na ten temat nie wiedziała oraz do sanepidu, który w ogóle nie odbiera. Próbowaliśmy uzyskać jakiekolwiek informacje. Domyśliliśmy się, że sytuacja może być związana z babcią, która regularnie bywa w szpitalu, ze względu na dializy, którym musi się poddawać. Zadzwoniliśmy więc tam. Lekarz babci zaprzeczył jej rzekomej kwarantannie - powiedział, że miałby to w systemie, a nie widzi takiej informacji, więc wszystko jest w porządku. W dalszym ciągu nie wyjaśniło to jednak sytuacji na poczcie. Nikt nic nie wiedział, ale pani na poczcie utrzymywała, że mamy kwarantannę. W poniedziałek informacja się potwierdziła. Wtedy to listonosz przyniósł zawiadomienie z dializ, że z racji tego, że babcia mogła mieć w szpitalu kontakt z osobą zarażoną, została poddana kwarantannie do 9 kwietnia. Sytuacja jest o tyle absurdalna, że informacja przyszła trzy dni przed końcem tego okresu. Podjęliśmy więc kolejną próbę - nieudaną zresztą - kontaktu z sanepidem oraz - również chybioną - z dializami. Do naszego domu przyjechała za to policja, aby sprawdzić przestrzeganie kwarantanny przez babcię. Jednak, jak wspominałam ci ostatnio, babcia z rodzicami już jakiś czas temu przeprowadzili się na działkę, ponieważ obawialiśmy się o zdrowie naszej córeczki - o jej ewentualny kontakt z babcią, która mogłaby mieć w szpitalu styczność z osobą zarażoną. Jednak tamta decyzja o tymczasowej przeprowadzce była wtedy tylko i wyłącznie naszym, rodzinnym postanowieniem. Babcia akurat i tak nie wychodziła z domu, a testy, które zrobiono jej w międzyczasie, miały negatywny wynik. Ale gdyby kwarantanna dotyczyła osoby, która mogłaby mieć wynik pozytywny i wychodziłaby z domu? Lub na przykład mojej mamy, która opiekuje się babcią i normalnie robiła zakupy? My chodziliśmy na pocztę. Co by się stało, gdyby to na nas była nałożona kwarantanna? W przypadku kontroli dostalibyśmy karę, pomimo tego, że nikt nas nie poinformował. Poza tym, jest jakieś zagrożenie, a oni informują o tym na trzy dni przed końcem? To jest nie do pomyślenia. Według mnie, mają tam niezły burdel i tyle.

**Obrazki**

Wybieram 6. Widzę tu naturę, las, piękne słońce i zieleń. Ostatnio dużo czasu spędziliśmy na działce, na odludziu, w dodatku pogoda była bardzo ładna. Było miło, fajnie, sympatycznie, bardzo spokojnie - w ogóle nie myśleliśmy o tym, co dzieje się na świecie. Było tak, jak dawniej. Dość normalnie, jak gdyby nic się nie działo. Jeździmy tam dosyć często, choć teraz, kiedy babcia z rodzicami się tam przeprowadzili, poczekaliśmy na wynik testu babci. Kiedy okazało się, że jest zdrowa, postanowiliśmy tam pojechać.

**Emocje wśród bliskich**

Oni bardzo się cieszą - dziś jest ostatni dzień kwarantanny, więc wracają do domu. Mama już dziś wróciła, babcia przyjedzie dopiero wieczorem, ponieważ może mieć jeszcze kontrolę na działce - powinna tam zostać do końca dzisiejszego dnia. Mama bardzo się cieszy, babcia jest stęskniona, zwłaszcza za wnuczką, ale też cieszy się, że już dziś się zobaczymy. Ważne też w tym wszystkim jest to, że zbliżają się święta i spędzimy je razem.

**Zachowania**

Wciąż nie oglądamy telewizji. Ja czytam też mniej wiadomości - sprawdzam tylko wieczorem, ile jest nowych zachorowań - głównie statystyki. Jest u nas dużo spokojniej i mniej przejmuję się całą sytuacją, która jest na zewnątrz. Mam też większą nadzieję, że to musi się skończyć i będzie to już niebawem. Praktycznie miesiąc jesteśmy już w domu, wiec skoro tyle wytrzymaliśmy, wytrzymamy kolejne tyle. I mam nadzieję, że za miesiąc będzie już wszystko dobrze. Nie czuję się zagrożona, bo nie bardzo mam gdzie tego zagrożenia dotknąć. Mam na myśli to, że wychodząc z domu zabezpieczam się, wracam - myję ręce; nie miałam styczności z innymi osobami. Gdybym udzielała się społecznie, mogłabym się bać, ale z racji tego, że raczej jesteśmy tu sami - raczej się nie boję. Jeśli chodzi o bliskich, nawet nie rozmawiałam z nimi za bardzo na takie tematy - poruszaliśmy raczej sprawy organizacyjne i to, że będąc na działce, tęsknią.

**Czy pojawiło się coś, co doskwiera bardziej, niż w poprzednim tygodniu?**

Chyba nie, choć wszędzie są coraz większe kolejki. Dotychczas w jednym z większych sklepów w okolicy - Stokrotce, ludzie chodzili normalnie. Było tam sporo miejsca, więc nie trzeba było zwracać szczególnej uwagi na zachowanie odległości, itd. Teraz wchodzą tylko dwie osoby. Są dwa koszyki i tylko osoba mająca jeden z nich może wejść, przekazując go przy wyjściu następnej. To chyba jedyna możliwość, aby jakoś kontrolować liczbę klientów, bez angażowania personelu sklepu. Trzeba więc poczekać mniej więcej godzinę, zanim będzie można wejść - wcześniej zakupy przebiegały dużo sprawniej. W mniejszych sklepikach, jeszcze przed wprowadzeniem tych zaleceń dotyczących limitu klientów, wchodziło się pojedynczo. Więc tam nic się nie zmieniło. Ludzie w kolejkach pytali tylko, kto jest ostatni. Nie widziałam, żeby ktoś wyrażał swoje niezadowolenie - wydaje się, że przyjmują po prostu to, że tak powinno być i jest.

**Jak odnosisz się do zasad, które zostały ostatnio wprowadzone?**

Nawet nie bardzo wiem, jakie zasady zostały w tym tygodniu wprowadzone. Te, o których wiem, nie przeszkadzają mi i ich przestrzegam. Przyjechała mama, więc jest włączone TVP Info - usłyszałam coś, że od 16 kwietnia będzie można wychodzić w miejsca publiczne tylko w maskach. Uważam, że to bardzo dobrze - zawsze to jakieś zabezpieczenie. A tak, to jedna osoba założy, a inna już nie. Z tego, co widzę, ludzie dotykają owoców bez rękawiczek. Ktoś inny później je kupi - wiadomo, że powinien je umyć, ale to i tak może zwiększać prawdopodobieństwo zarażenia. Jeśli wszyscy będą przestrzegać zasad, bo będą musieli, będzie lepiej.

**A co sądzisz o zakazie korzystania z terenów zielonych?**

Wierzę, że inne osoby mogą mieć z tym problem. Ja akurat mieszkam na wsi, jestem otoczona zielenią, więc nie muszę iść do parku, żeby pospacerować. Mieszkam w domku z ogrodem. Mam też trzy hektary pola na działce i otaczający je las, więc nie odczuwam potrzeby wyjścia do parku. Rozumiem jednak, że jeśli ktoś mieszka w bloku, taki park jest dla niego jedynym miejscem, gdzie może się przespacerować i odetchnąć czystym powietrzem.

**Jak postrzegasz osoby, które nie stosują się do tych zasad?**

Nie zastanawiałam się nad tym za bardzo, ale w sumie to bardzo im współczuję. Uważam, że wyjście na spacer to nie jest nic złego. Tak samo, jeśli ktoś sam wychodzi na rower i nie dotyka niczego wokół. Rozumiem, że niektóre miejsca robią się przez to zatłoczone, bywa tak, że są urządzane ogniska. Ostatnio też było głośno o przypadku, kiedy to policjanci zrobili sobie grilla nad jeziorem. Tego nie pochwalam. Ale jeśli idą sobie trzy osoby, a kilka metrów za nimi kolejne trzy, to nie uważam, że to coś złego. Z drugiej strony, jeśli zostały nałożone takie, a nie inne sankcje, powinniśmy się jakoś dostosować. Ten miesiąc to w skali życia nie jest bardzo długi czas. Chociaż nie wiem. Jeśli ludzie nie przestrzegają zasad, to ich nie przestrzegają - nie uważam, że są w takim przypadku jakimiś zwyrodnialcami.

**Co dla Ciebie jest najważniejsze w kwestii tych zasad?**

Ludzie nadal chodzą do pracy. Sklepy meblowe, czy supermarkety są otwarte. Nie jesteśmy w stanie ograniczyć wszystkich kontaktów. Nawet nasza rodzina ma ograniczone możliwości rozdzielenia się i odbywania izolacji, zwłaszcza z dala od siebie. Mój tato ma serwis opon, który wciąż jest otwarty - ludzie w nim zachowują oczywiście odpowiednią odległość. Ale gdyby tato chciał zrezygnować z wszelkich kontaktów, to chyba by zbankrutował. Tak samo mój mąż, który rozwozi mięso. Od miesiąca ja siedzę w domu, jednak on pracuje. Zachowuje odpowiednie środki bezpieczeństwa - płatności odbywają się teraz przelewem, zamiast gotówką przy odbiorze. On jedynie zostawia towar i odjeżdża. Nie jesteśmy jednak w stanie restrykcyjnie przestrzegać izolacji. Musielibyśmy zamknąć się w osobnych pokojach i nie wychodzić przez dwa tygodnie. A trzeba też jakoś funkcjonować. Niedługo to się skończy i co potem? Gdyby pozamykali wszystkie firmy, nie było wypłat, niczego. Jak mielibyśmy później żyć i z czego się ponieść? Dlatego coś trzeba robić. Najważniejsze jest, aby ograniczyć bliskie kontakty z ludźmi, co do których nie wiemy, z kim się spotykali w ciągu ostatniego tygodnia, czy dwóch. Ale jeśli wiem, że ktoś był w domu od miesiąca, nie widzę niczego złego w spotkaniu się z taką osobą. Nie zaryzykowałabym jednak styczności z osobą z ogłoszenia.

**Czy pojawiły się jakieś nowe wyzwania?**

Nie, wyzwania nie. Mam jednak bardzo dużo pracy na uczelni. Miałam też od 1 kwietnia mieć praktyki w więzieniu. Przez zaistniałą sytuację, nie mogłam ich rozpocząć, ale kończę już licencjat i powinnam wyrobić 60 godzin praktyk. Wczoraj pani doktor, opiekun naszych praktyk, zarządziła, że nie będzie wyznaczonego innego terminu, ale w ramach tego, mamy pomagać ludziom - robić zakupy starszym osobom, być wolontariuszami. To mam siedzieć w domu, czy się "wolontariuszować"? Coś jest chyba nie tak. Mamy zajęcia przez internet, żeby zachować bezpieczeństwo, a nagle, w ramach praktyk, mam codziennie wychodzić i robić ludziom zakupy. Sama staram się to ograniczać, być w sklepie raz na trzy dni, a teraz powinnam zbierać paragony, nagrywać rozmowy z osobami, którym codziennie pomagam? Uważam, że ja najbardziej pomogę ludziom, kiedy będę siedziała w domu. Są na tym świecie, w kraju oraz w naszym mieście wolontariusze, którzy się tego podjęli i wykonują to od miesiąca. Jestem też przekonana, że oni nie mieszkają z siedmioosobową rodziną i małym dzieckiem. Skoro to robią, to myślę, że są odizolowani tak, jak lekarze i pielęgniarki. Nie chodzi o to, że jestem mało pomocna, ale w tym momencie trzeba wybrać dla siebie lepsze dobro. A dla mnie lepszym dobrem jest to, żeby uchronić moją babcię, czy córkę przed niebezpieczeństwem. Jeśli będę chodziła codziennie do supermarketów i robiła zakupy, to to niebezpieczeństwo niestety zwiększę. Ona wymyśliła jakieś cyrki i każdy jest teraz w kropce, nie wie, co z tym zrobić. Ale czekamy jeszcze na zatwierdzenie tej decyzji przez rektora. Cała grupa podchodzi do tej sprawy podobnie, jak ja. Jesteśmy zdania, że praktyki powinny odbyć się w lecie, bo pewnie obrony i pozostałe kwestie i tak się wydłużą. A teraz, lepszym rozwiązaniem będzie, jeśli zostaniemy w domu.

**Zauważyłaś w swoim otoczeniu lub u innych ludzi jakieś dziwne zachowania?**

Nie, nie przypominam sobie niczego takiego, co wydałoby mi się dziwne w czyimś zachowaniu.

**Zakupy przez internet**

Od naszego ostatniego spotkania nic się nie zmieniło. W sieci kupuję głównie ubrania. Przed epidemią też zdarzało mi się robić zakupy przez internet. Teraz jest jednak więcej czasu, kuszą promocje - są teraz bardzo duże, nawet po 30% - to jest bardzo dużo. Kiedyś, jeśli chciałam coś kupić, szłam do galerii. Oglądałam rzeczy i kupowałam jedną wybraną. Teraz zamawiam hurtowo - po pierwsze ze względu na dużą ilość czasu - mogę sobie przeglądać, oglądać. W dodatku został wydłużony czas na zwrot - jest na to chyba 90 dni. Po drugie mam świadomość, że nie mam dostępu do normalnych galerii i normalnych sklepów, wydaje mi się, że pozostał tylko internet. I trzeba kupować, trzeba zmieniać odzież, bo robi się ciepło. Moja córka nie miała ani jednej wiosennej rzeczy, bo ubranka z zeszłego roku są już za małe. Zamawiałam głównie dla córki, ale dla siebie też, nie powiem, że nie. Z nadmiaru czasu stwierdziliśmy też, że zrobimy córce dodatkowy pokój, więc teraz doszło jeszcze oglądanie mebli i całej aranżacji - znów mamy zajęcie.

**Jak postrzegasz robienie zakupów?**

Nie kupuję tylko najpotrzebniejszych rzeczy. Lubię zakupy i lubię też robić prezenty. Nie jestem zakupoholiczką, broń boże! Ale jeśli jest więcej pieniędzy i mogę je wydać dla kogoś, cieszę się z tego. Więc kiedy teraz przychodzą te paczki, ja naprawdę się cieszę, sprawia mi to przyjemność. To jest zawsze coś innego niż siedzenie w domu i nie robienie normalnych zakupów. Jeśli mam świadomość, że nie mogę tych rzeczy kupić stacjonarnie, cieszę się, że w ogóle mogę je kupić. Nawet przebieranie się, oglądanie w domu tego wszystkiego, co przywiezie kurier - to jest fajne.

**A jeśli o zakupy spożywcze, zdarza Ci się robić je online?**

Nigdy z czegoś takiego nie korzystałam. Wiem, że w Warszawie jest taka opcja - moja siostra, która tam mieszka, często robiła zakupy w ten sposób. U nas nigdy się tego nie praktykowało, więc ja nawet nie szukałam aplikacji, informacji, czy u nas coś takiego istnieje. Chociaż to wydaje mi się super, bo można kontrolować to, co się kupuje, ceny. Na pewno to jest lepsze. Tutaj, kiedy wchodzę do sklepu, robię to w celu kupna czterech rzeczy, a wychodzę z całym koszykiem. Przechodząc między regałami, wszystko kusi. A najgorzej, kiedy pójdę do sklepu głodna, wtedy to już w ogóle - wszystko kupuję. Przez internet na pewno byłoby to bardziej racjonalne, ale tak, jak mówiłam - mieszkam na wsi, robię zakupy w małych sklepikach i nawet nie myślałam, żeby coś takiego sprawdzić, bo nawet nigdy nie przyszłoby mi do głowy, żebym tak mogła, robić zakupy w ten sposób.

**Co jest dla Ciebie wadą w zakupach online?**

To, że nie mogę przymierzyć, zobaczyć, czy jest wygodne. Ja muszę mieć bardzo wygodne rzeczy - nienawidzę mieć niewygodnych. I to jest w internecie denerwujące - kiedy na zdjęciu coś wygląda ładnie, później przychodzi - nadal jest ładne, a później kiedy to zakładam, okazuje się, że jest beznadziejne. Kiedy jestem w sklepie, mam możliwość przymierzenia i od razu zdecydowania, czy chcę coś kupić. A tak, umyślę sobie w głowie, że bluzka będzie pasowała do spodni, a nagle okazuje się, że spodnie są beznadziejne. I co teraz? Ostatnio kupiłam tak buty - wszystko wyglądało dobrze, a teraz, kiedy w nich chodzę, okazuje się, że są niewygodne i mnie drażnią, bo już sam fakt ich wkładania jest trudny. Ale nie zamawiałam już drugich, bo nie mam pewności, że historia się nie powtórzy. Po prostu poczekam, aż będę mogła pójść do sklepu, poprzymierzać sobie i wtedy jakieś wybrać. A na razie męczę się w tych. Jeśli otworzą sklepy, na 100 % znów zacznę robić w nich zakupy. Wiadomo, nie zawsze wszystkie rzeczy są dostępne. Czasami brakuje na przykład jakiegoś rozmiaru. Ale już przymierzyłam tę rzecz i wiem, że będzie fajna, więc zamówię ją sobie przez internet. Tak samo ubrania dla córki - na tę chwilę ubieram ją, jak chcę, bo ona nie powie mi jeszcze, ze coś jej się nie podoba. Kupuję jej leginsy i dresiki, zamiast jakichś sztywnych spodni. Takie ubrania też łatwiej kupić bez mierzenia. Kiedy potrzebuję bardziej eleganckich ubrań, trudniej dobrać mi je w internecie, muszę je mierzyć. Bardziej niż na wygląd, zwracam uwagę na wygodę. Jeśli nie wiem, czy coś będzie wygodne, trudniej mi to kupić.

**Jak postrzegasz jedzenie?**

Uwielbiam jeść, to jest mega przyjemność, chyba jedna z największych w życiu. Poza spaniem. Bardzo lubię jeść i lubię też rzeczy niezdrowe - chipsy, fast foody, uwielbiam wszystkie hamburgery i pizze, dużo ziemniaków. Wszystko smażone - absolutnie jakieś duszone, czy coś. W ogóle nie jem zdrowo - to nie ma się czym chwalić, ale jakieś sałatki i inne rzeczy odpadają - jestem bardzo mięsożerna. Ale to ktoś mi kiedyś powiedział, że to może być związane z moją grupą krwi. Ja mam 0 Rh+, więc jem dużo mięsa. Jem bardzo mało warzyw. Bardzo chciałabym je lubić, ale jem je na siłę. Jem, bo jem. Bo jestem dorosła i wiem, że trzeba takie rzeczy jeść - zjeść tego ogórka, czy pomidora, albo czasami zupę brokułową, ale nie sprawia mi to przyjemności. Jeśli ktoś idzie i zamawia sobie sałatkę, dla mnie to jest masakra. Może zjeść tyle dobrych rzeczy, a je sałatkę?

**Czy Twoje nawyki żywieniowe uległy zmianie podczas epidemii?**

Mam więcej czasu, wiec więcej jem. Człowiek się nudzi, więc ma takie...jak chyba każdy, że chciałby mieć coś pod ręką. Jeśli jestem zajęta, mogę nie jeść cały dzień. Zdarzało mi się jeść w takie dni dopiero po południu i nie przeszkadzało mi to. Zawsze miałam duży apetyt, a teraz jem więcej. Te same rzeczy, co zazwyczaj, jednak zdecydowanie w większej ilości. Nawet wieczorami, tak raz w tygodniu, ganiam męża po Mc Donald'sy.

**Czy zmienił się u Was podczas epidemii sposób przygotowania i uczestniczenia w posiłkach?**

Nie. U nas zawsze jest problem z posiłkami, ponieważ jemy "na raty" - każdy o innej porze. Tato, mąż i brat pracują i przychodzą w różnych godzinach, więc nie ma jednej pory obiadowej, jak w niektórych domach. Uważam, że to byłoby bardzo dobre, ale u nas fizycznie nie da się tego zrobić, choć mama walczy o to przez całe życie. Moglibyśmy ewentualnie spotkać się na kolacji o 20. Wtedy MOŻE wszyscy byliby w domu - jest po prostu za dużo osób i każdy ma inny tryb życia, pracy - zdarza się, że pracują do nocy, a czasami o 13 leżą w domu. Niestety nie da się u nas zjeść posiłku o jednej godzinie i to nie zmieniło się w czasie epidemii, bo nikt u nas nie stracił pracy - każdy pracuje normalnie. Ostatnio nie było rodziców. Z mężem jestem jakoś w stanie się zgrać, tym bardziej, że teraz siedzę w domu, a mąż wraca o 9 rano. To faktycznie, jedliśmy wtedy razem obiad o takiej porze, o jakiej go akurat zrobiłam.

**Zamawiacie teraz jedzenie na dowóz?**

Knajpy wokół nas są zamknięte. To prywatne restauracje i tam, gdzie kiedyś była możliwa opcja dowozu, pracownice bały się pracować, więc lokal zamknięto. Najbliższe restauracje-sieciówki są 15 km od nas, w Radomiu. Myślałam, że nie dowożą, bo tak było zazwyczaj, ale ostatnio brat zamówił pizzę. Ale nie zagłębiałam się w to, kto dowozi, nie miałam takiej potrzeby. Nigdy też nie używałam tych aplikacji typu Pyszne.pl, bo nie lubię jedzenia na dowóz. Wolałam zawsze wyjść do restauracji, Mc Donald'sa. To dla mnie przyjemność, kiedy mogę spotykać się przy jedzeniu. Lubię jeść, ale też umawiać się na jedzenie, dlatego rzadko zamawiam na dowóz. A teraz się denerwuję, bo kiedy mąż przywozi mi Mc Donald's, frytki są już zimne, niedobre. Choć on kiedyś też często mówił mi, że beznadziejnie robię, bo jechaliśmy do Mc Donald'sa, a mi nie chciało się wychodzić z samochodu, więc jedliśmy w środku. On twierdził, że równie dobrze mógł mi w takim przypadku przywieźć to jedzenie. No ale ja naprawdę nie lubię dowożonych jedzeń.

**Rozumiem więc, że nie zamawialiście wcale?**

Czasami, bardzo rzadko, zamawialiśmy właśnie z tej restauracji - "Chińskie jadło", gdzie mają też pizzę - to jakieś 3 km od nas, więc przywiezione jedzenie zawsze było ciepłe. Ale i tak wolę jechać tam i zjeść na miejscu, niż zwozić to do domu w tych kartonach, plastikach, takich tam. Mamy dużą rodzinę, więc czasami ciężko jest się wybrać razem, zwłaszcza z małym dzieckiem. Pilnowanie i zabawianie jej podczas jedzenia kosztuje nas dużo nerwów, biegania za nią. Musimy jeść turami, zamiast faktycznie siedzieć rodzinnie, razem uczestniczyć w posiłku. Zazwyczaj jeździmy więc osobno. Ale czasami jest niedziela i się nudzimy, wtedy postanawiamy, że pojedziemy albo zadzwonimy po pizzę – to zależy od tego, czy akurat pracuje u nich jakiś dostawca - i zjemy ją w domu.

**Myślisz, że zamawianie jedzenia jest teraz bezpieczne?**

Nie zastanawiałam się nad tym, bo ciężko stwierdzić, czy cokolwiek jest teraz bezpieczne. Zawsze jest to ryzyko. Ze dwa tygodnie temu byliśmy w Mc Donaldzie. Osoba, która przyjmowała pieniądze miała rękawiczki, ale już ta, która wydawała jedzenie - nie. To był szok, choć wzięliśmy te papierowe pudełka i po prostu zjedliśmy, co było w środku. Nie myślałam o tym w kategoriach, że to niedopuszczalne, boję się i wyrzucam. Po prostu zdziwiłam się tą niekonsekwencją. Nie będziemy wiedzieć, kto i w jaki sposób przygotowuje i pakuje jedzenie w sieciówkach. Może inaczej byłoby w przypadku prywatnych restauracji, ale te są już wokół nas zamknięte, jak mówiłam wcześniej. Wtedy znając tych ludzi, widząc ich, ma się większe zaufanie. Wierzę, że właściciele takich placówek dbają o to, żeby standardy były spełnione, bo gdyby ktoś u nich zachorował, musieliby zamknąć firmę. Każdy też dba o siebie - nikt nie chce być chory, więc pewnie każdy się jakoś zmobilizował i zachowuje to bezpieczeństwo, ale czy w 100%? W 100% na pewno nigdy nie. Sam fakt ściągania rękawiczek - nie wiem, czy ktoś się trudzi 30 sekund, żeby je odpowiednio zdjąć, czy po prostu ściąga, jak ściąga.

**Płatności**

Staram się więcej używać karty, ale kiedyś nie używałam jej wcale. Obecna sytuacja wiąże się z tym, że muszę najpierw wpłacić pieniądze we wpłatomacie, a dopiero później płacić bezgotówkowo. Mój mąż też bardzo mało korzystał z karty. On jest w ogóle dziwny, nie chce, aby ktoś wiedział gdzie i jakich transakcji dokonywał. Uważa, że nawet to, że zrobił zakupy w jakimś sklepie, dostarcza o nim informacji. Teraz staramy się robić te przelewy kartą, jeśli o to proszą. Rozumiem też pracowników kas. To jest teraz jakieś utrudnienie - zawsze używałam gotówki - ale jakoś się przestawiłam, nie robi to dla mnie większego problemu. Choć pewnie jeśli tylko będę mogła, znów wrócę do gotówki - używałam jej tyle czasu. To może jest głupie, bo teoretycznie to jest to samo, ale jeśli płacę gotówką, wiem, ile mam, i tak dalej. A tą kartą to... Miałam kiedyś takie sytuacje, że nie wczytało mi jej, albo pobrało dwa razy za to samo i jeszcze zablokowało środki w przelewach oczekujących. I ani nie mogłam zapłacić za zakupy, ani tych pieniędzy wypłacić - jakieś takie cuda. A tu jak mam gotówkę, to wiem, że mam i mogę zapłacić bez względu na to, co stanie się w systemie.

**Zwracasz uwagę na to, czy dotykasz terminala?**

Ostatnio robiłam raczej mniejsze zakupy, więc płaciłam bezdotykowo. Nie przypominam sobie, żebym ostatnio wpisywała PIN. Zresztą, nawet, gdybym musiała, i tak zawsze w sklepie mam na sobie rękawiczki - nie przeszkadza mi to, gdybym miała dotknąć terminala.

**Jak często robisz teraz zakupy?**

Względem zeszłego tygodnia - nie wiem. Tak samo chyba. Wcześniej co dwa, teraz robiliśmy tak co trzy dni. Ale to dlatego, że rodziców i babci nie było w domu. Dziś mama już prosiła mnie, żebym pojechała do Radomia, aby zrobić jakieś większe zakupy przed świętami. Będę tam chyba pierwszy raz od miesiąca. A tak, zaglądam tylko do lodówki, nie robię list zakupów. Chodzę do małych sklepików po kilka rzeczy, choć często kończy się na tym, że kupuję dużo więcej, niż planowałam. Dlatego spożywcze zakupy internetowe mogłyby być lepsze - nie wydawałabym niepotrzebnie pieniędzy.

**Zamierzasz przygotować się jakoś do tych przedświątecznych zakupów?**

Tak, zawsze przed świętami i innymi ważnymi wydarzeniami jest lista, ponieważ to duże zakupy. Są na niej wypisane potrawy, które będą przygotowane, a do każdej z nich produkty, jakie będą potrzebne. Mama już teraz robi tę listę. Ale to jest wyjątek - poza takimi sytuacjami, nigdy nie robimy list. Ewentualnie jak wracam ze szkoły, mama napisze mi SMS, że czegoś potrzebuje. Ja wtedy wchodzę po to, o co ona mnie prosi, a kupuję jeszcze 10 innych rzeczy.

**Czy zmieni się coś, jeśli chodzi o środki ostrożności, jakie planujesz podjąć?**

Dotychczas zakładałam jedynie rękawiczki, maski nie, bo nie miałam kontaktu z tymi ludźmi. Dzisiaj już naszykowałam sobie też maseczkę, z racji tego, że jak trąbią o tym w telewizji, to trzeba założyć. Ale u nas nie było możliwości spotkania się z inną osobą, bo i tak do sklepu wchodziło się pojedynczo. Poza tym, ci prywaciarze pozakładali sobie takie szyby. Firmy bardzo dobrze zabezpieczyły się same. Teraz nawet przed wejściem na Orlen stoi płyn dezynfekujący, papier i rękawiczki. Więc najpierw myje się ręce, później zakłada rękawiczki i dopiero wtedy wchodzi na stację.

**Jak zamierzacie spędzić święta?**

Zostajemy w domu. Dzwoniłam do teściów, ale tam jest taka sytuacja, wspominałam Ci o tym w zeszłym tygodniu, że siostra mojego męża wróciła dopiero ze szpitala w Warszawie, gdzie zostało jej nowo narodzone dziecko. Ze względu na nią, oni z nikim się nie widują, ponieważ ona ma teraz obniżoną odporność. Dlatego teściowie nie robią świąt, choć myślałam, że może uda nam się do nich pojechać, zwłaszcza, że córeczka bardzo za nimi tęskni. Oni nigdzie nie wychodzą, więc mogłabym się z nimi spotkać, ale nie robią świąt. Mówią, że zobaczymy się, kiedy to wszystko się skończy.

**Na ile te święta będą inne?**

My mamy bardzo dużą rodzinę, spędzamy święta w bardzo dużym gronie, więc te na pewno będą inne. Zawsze widywaliśmy się z ciotecznym rodzeństwem i znajomymi - jeździliśmy od domu do domu. Teraz będziemy tylko z domownikami. Choć nie wiem, czy przygotowania będą inne. Mama już cuduje bardzo dużo potraw, chociaż mówię jej, że przecież sami tego nie zjemy. Ale pewnie zrobimy to, co zazwyczaj. Przygotowania duchowe może będą mniejsze, z racji tego, że teraz nie chodzimy także do kościoła. Moi rodzice są bardzo religijni. Nie poświęcimy koszyczka, ale rodzice jakoś tego nie przeżywają za bardzo. Radzą sobie sami - dużo się modlą - wspólnie, wieczorem.

Mama była w kościele raz od wprowadzenia stanu wyjątkowego. W pierwszą niedzielę od ogłoszenia tej informacji, co miało miejsce chyba w czwartek. Mówiła, że było w nim bardzo mało osób, dosłownie jakieś 10, gdzie zazwyczaj jest 150. Później przez cały tydzień trąbili o tym w telewizji, więc ona stwierdziła, że odpuszcza sobie i nie będzie w ogóle chodziła. I od tamtej pory nie chodzą z tatem do kościoła.

**Czy brak święconki jest problemem?**

Nie wiem, jak dla moich rodziców, nie rozmawiałam z nimi na ten temat, ale ja nigdy nie czułam tych świąt. Pomimo tego, że dla Chrześcijanina powinny one być ważniejsze. Dla mnie one były, bo fajnie, bo widzimy się z rodziną, ale jakoś emocjonalnie ich nie przeżywam. Naprawdę, bardziej te bożonarodzeniowe. Chociaż wiem, że to te powinny być ważniejsze i one są najważniejsze dla naszego Kościoła. Więc lubię tradycje z nimi związane, ale nie przeżywam tego, że nie pójdę z koszyczkiem - bo odkąd jesteśmy "duzi", mama go szykuje i to nas wygania z nim do kościoła. Mi to nie będzie robiło różnicy. Ale na pewno przygotujemy koszyczek, tata nawet upiekł go w tym roku z chleba, bo dostał od znajomych zakwas. Dotychczas koszyk był wiklinowy. Więc na 100% będzie koszyczek, barszcz biały - mama kupiła kiełbasę.

**Jakie produkty masz zamiar kupić przed świętami?**

Kiedyś zamawialiśmy na święta cateringi, ale teraz, skoro będziemy świętować w mniejszym gronie, to bez sensu - robimy wszystko sami. Nie będzie świętowania jak dotychczas, będzie to raczej jak normalny dzień. Nasze święta zawsze są bezalkoholowe, więc nie kupujemy żadnych trunków. Mama robiła listę i jeszcze nie wiem, co na niej będzie. Mąż ma jutro kupić jajka. A reszta to rzeczy, które będziemy chcieli sobie przez te parę dni jeść. Dla przyjemności zamierzam na pewno kupić chipsy. Zobaczę, co jest na półkach i coś sobie wybiorę. Nie myślę o tym z wyprzedzeniem, choć często to są produkty, których raczej nie dostanę w sklepikach w Jedlińsku. Na przykład bagietki czosnkowe do piekarnika - często je kupuję. Często też tatara, bo na przykład jest w Biedronce i jest bardzo dobry. Dużo jest takich rzeczy - łosoś, pasty rybne. Na co dzień się tego nie je, bo robimy normalne polskie obiady. Bardzo lubię też tortellini z szynką parmeńską - u nas tego nie ma. To nie tyle z okazji świąt, co dla przyjemności tak w ogóle, bo te święta to będą dla mnie normalne dni. Więc to będą takie normalne, nie świąteczne rzeczy, które nam będą smakować. Nie będziemy piec mazurków i takich tam. Kupię to, czego nie mogłam kupić tutaj, bo trzeba po to pojechać np. do Biedronki, w której nie byłam miesiąc.
