# Supplementary material for: The challenges arising from the COVID-19 pandemic and the way people deal with them. A qualitative longitudinal study
Source: PLoS One. 2021 Oct 11;16(10):e0258133. doi: 10.1371/journal.pone.0258133 (PMC8504766; doi:10.1371/journal.pone.0258133)
Supplement: S1 Dataset — (ZIP) [file pone.0258133.s003.zip › Transcriptions/stage 2/17.2_F_35_single, with child.docx]

**17.2_F_35_single with child**

**Jakie emocje towarzyszyły ci od czasu naszej ostatniej rozmowy?**

To była huśtawka po prostu. Od złości, że nas zamknęli, że nie można iść do pracy, przez to, że moje dziecko ma 9 lat i ja nie dostanę opieki, więc tak naprawdę państwo mi nic nie da, po to, że w sumie fajnie, bo sobie siedzę w domu z dzieckiem i na spokojnie sobie wszystko robimy. Teraz jest tak, że dobra, przeczekajmy, nie mamy na to wpływu i jakoś musimy to przeżyć.

**Jakie wydarzenia wpływały na to, że odczuwałaś takie emocje?**

Ten moment, kiedy mi zabronili iść do pracy i salony kosmetyczne zamknęli to był tak jakby gwóźdź do trumny. Ja byłam naprawdę wściekła, już nie wiedziałam co mam robić. Niby ta pomoc jest od państwa, ale wcale nie ma i dlaczego moje dziecko ma 9 lat a nie 8? bo jakbym miała 8-latka, to bym dostała tę opiekę, a ja mam 9-latka i co ja mam zrobić? Potem to kwestia uspokojenie się, przespania się z tym 2 dni i mówię, że wszystkim nie pomogą, nikt nie ma na to większego wpływu, trzeba to jakoś przeczekać. Potem śmiałam się z dziewczynami z pracy, że dobrze, bo konkurencja trochę upadnie i może nie będzie tak źle, zostaną tylko najmocniejsi. Zaczęłyśmy się śmiać, bo trzeba się jakoś ratować. No nic, zobaczymy.

**Emocje - zdjęcia**

**15**

To jest jakaś ewidentna burza, na dodatek jest fala. Te moje emocje były od Sasa do lasa i w pewnym momencie sama już nie wiedziałam co mam na ten temat myśleć i mniej więcej tak wyglądało. Przemawia do mnie, że jest tam jakieś słońce i zawsze po tym deszczu wyjdzie słońce, więc jest nadzieja, że to wszystko się w końcu skończy.

**Czyli pojawiła się nadzieja. Coś jeszcze?**

Na razie chyba taki spokój, przeczekanie. Jeszcze kilka dni temu rzeczywiście na coś tam czekałam, a teraz to jest kwestia, że miesiąc i może się uspokoi.

**Powiedziałaś, że czułaś złość. Co robisz, żeby się uspokoić? Co pomaga ci radzić sobie z takimi emocjami?**

Dwie rzeczy robię - idę spać i przestaję rozmawiać z ludźmi na temat koronawirusa. ten natłok informacji dobija, bo gdzie się człowiek nie obejrzy, jest co innego powiedziane, inaczej zinterpretowane i po prostu już w pewnym momencie nie chciałam tego słuchać. Ewentualnie przeczytałam wiadomości i poszłam spać. I absolutnie się w ogóle wyłączyłam na 2 dni z życia. I przeszło.

**Żartowałaś z koleżankami z pracy. Jesteście w kontakcie cały czas, jakoś się wspieracie?**

Cały czas jesteśmy w kontakcie, non stop. Jakby grupowo miałyśmy te wahania nastroju, że najpierw byłyśmy złe, potem, że jakoś to będzie, że przecież inni mają gorzej, nie ma tragedii, mamy za co żyć. Cały czas sobie tłumaczyłyśmy, że to nie jest sytuacja...Że nam się nie spalił salon i trzeba się odbudować. My wszystko mamy i zaraz nam pozwolą go otworzyć i to jest kwestia miesiąca, no może dwóch i wszystko zacznie wracać do normy, i zaczniemy na nowo pracować. Tak się trochę miło podbudowałyśmy, że gdzieś nam może konkurencja upadnie, takie małe salony. [śmiech]

**Coś się zmieniło w twoich zachowaniach w ostatnim tygodniu?**

Nie, u mnie jest cały czas to samo, ale ja i cała moja rodzina respektujemy te wszystkie zasady niewychodzenia, niespacerowania, nie łażenia po lasach. Raz byliśmy dosłownie. Wychodzę na zakupy tylko ja - raz w tygodniu, góra dwa. My w sumie od momentu zamknięcia szkół wszyscy w domu żyjemy pod dyktando koronawirusa.

**Czy to jest trudne/ łatwe?**

Nie jest to jakieś mega trudne, da się z tym pogodzić, tylko nas też jest sporo w domu, bo my jesteśmy całą rodziną + są moi rodzice, więc my nie jesteśmy tylko we trójkę. Są też rodzice, więc mamy tych bliskich obok siebie. 7 domów dalej mieszka moja siostra, do której mogę codziennie pomachać i pogadamy przez płot. Ze znajomymi? Wiadomo, że brakuje nam jakichś wyjść, żeby polecieć gdzieś na spacer, na lody, natomiast nie odszukujmy się, że w trybie pracy, jaki ja miałam, to nie było częste. Ja pracowałam do późnych godzin. Nie jest mi z tym jakoś bardzo ciężko. Z tego wszystkiego, to najbardziej mi szkoda syna, bo on bidny musi siedzieć w domu i tu on traci te kontakty z kolegami. Myślę, że on najbardziej odczuwa to z nas wszystkich.

**Na ile czujesz się zagrożona obecną sytuacją?**

Czuję niestabilność finansową przede wszystkim. Tym się czuję zagrożona, bo ja nie wiem, czy nie trzeba się będzie przebranżowić, bo to nigdy nic nie wiadomo. Nie boję się jakoś, że się zarażę, chociaż w Radomiu jest Armagedon w szpitalu. Myślę, że zachowując te wszystkie środki ostrożności to nie ma szans...Niby są szanse, ale tak jakby 1 do nie wiem ilu, żebym się miała bać tego koronawirusa. To chyba nie. To chyba tylko te kwestie finansowe będą gdzieś z tyłu głowy jeszcze miesiącami.

**Jak reagują na to wszystko twoi bliscy?**

My w domu siedzimy wszyscy, moi rodzice siedzą, moja siostra siedzi i szyje maski na potęgę dla szpitali, szwagier wychodzi na zakupy. Wszyscy się zamknęli. Śmieję się, że mamy 2 obozy. Mamy obóz racjonalny, który mówi ok, no jest wirus i mamy obóz, który mówi, że Chiny wytworzyły wirusa, żeby nas zabić. I tu mamy zgrzyty w rodzinie. Ja jestem ten obóz racjonalny i twierdzę, że po prostu jest wirus, który mógł zmutować i zabił wielu ludzi, ale to bardziej też z tego względu, że zbyt wielu ludzi się zaraziło naraz. Moja siostra uważa, że to Chińczyki chcieli nas zlikwidować, bo jest nas za dużo i wypuścili wirusa. To jest jej teoria, że wszyscy są chorzy tylko nikt o tym nie mówi, nie robią badań. W ogóle wszystko tajne i poufne.

**Siostra szyje maski. To jest związane z jakimiś emocjami czy jest to racjonalne działanie? jak to postrzegasz?**

Ona generalnie szyje - ma swoją firmę i szyje różne takie rzeczy dla dzieciaków, itd. jak zaczął się ten koronawirus, ale to było jeszcze daleko od Europy, to ja mówiłam do niej: zacznij szyć maski. I ona się z tego śmiała, natomiast w momencie, gdy zaczął się kryzys u nas i rzeczywiście tych masek nie było, i kiedy szpital już nie miał masek, ona zaczęła szyć z 2 dziewczynami z Radomia na zasadzie, że uszyjemy i wam damy. Top poszło już na taką skalę, że one od końca marca do teraz uszyły już 10000 masek dla szpitala. One są tak wkręcone, żeby pomagać tym ludziom. Naprawdę ona po nocach siedzi i szyje maski, i robi to charytatywnie. Absolutnie nie bierze za to żadnych pieniędzy. Mają jedynie jakąś zrzutkę gdzieś założoną, żeby na materiał ewentualnie mieć i tyle. Bardzo emocjonalnie do tego podeszła, bardzo. Wydaje mi się, że dla niej to jest kwestia tego, żeby lekarze i pielęgniarki się nie pozarażali. Żeby ci, którzy są na pierwszej linii frontu i mogą nam pomóc w razie W, to żeby oni byli, bo jak ich zabraknie, to wtedy będzie Armagedon. Ona się o nich troszczy nie dlatego, żeby w razie gdybym zachorowała, to żebyście mi pomogli, tylko, żeby oni w ogóle mogli pomagać. Ona poświęca swój czas, w którym mogłaby zarabiać pieniądze, żeby szyć maski. Mogłaby uszyć 100 czy ileś i mieć to w nosie, a ona siedzi kolejny dzień i szyje maski. Jeździ, rozwozi i dostarcza, przerzuca przez płot. Ubaw nie z tej ziemi z tego, bo bardzo się wkręciła, bardzo.

**Gdyby nie szyła masek, to miałaby inne zamówienia?**

Myślę, że tak. bo ona wysyła i nie ma kontaktu z klientami. Coś tam by na pewno robiła, bo i tak robi coś w międzyczasie. Pewnie mniej byłoby tej pracy, natomiast ani razu nie wspomniała, że porzuca swoją pracę na rzecz szycia masek. Łączy jedno z drugim a nie szyje, bo nie ma co robić. Najciekawsze jest to, że to nie jest ona jedna. One zaczęły od 3, a w tej chwili ich jest chyba 40 i to są dziewczyny, które miały gdzieś starą maszynę w domu i ok., mam maszynę, pokażcie mi jak to robić. Siedzą i szyją, i większość jest nawet incognito - nie wiadomo, kto to jest.

**Jak one stworzyły taką społeczność?**

Na FB oczywiście. Gdzieś się pojawiła grupa Widzialna Ręka, że będą szyć maski i jedna drugiej, druga trzeciej, potem założyły swoją grupę, żeby tam się spotykać i inne osoby zaczęły dołączać. Na FB bywają też lekarze, pielęgniarki i zaczęli prosić o te maski sami. Impreza taj jakby się sama kręciła.

**Są jeszcze jakieś działania, które uważacie, że mogą wpłynąć na sytuację czy to jest raczej kwestia przeczekania?**

Jedyne, co wg mnie ja mogę zrobić, to siedzieć w domu i nie wypuszczać dziadków z domu. Nic innego nie przychodzi mi do głowy.

**W jaki sposób to pomoże ci poradzić sobie z tą sytuacją?**

Mamy duże prawdopodobieństwo, że się nie zarazimy, bo ja bardzo bym nie chciała, żeby ta choroba...Już i tak jest to tak nakręcone i jest to tak blisko nas...Bardzo bym nie chciała, żeby to dotknęło moich najbliższych, bo nie wiem co, jak by to zadziało się. Siedzenie w domu ogranicza do minimum zarażenie siebie bądź ich. Nie ma innej możliwości w tej chwili i wydaje mi się, że jakby każdy wziął to sobie do serca i rzeczywiście wychodził tylko ten, co musi, to w miarę byśmy to ogarnęli - tego wirusa. Zresztą nie jest źle teraz.

**Mówiłaś, że przestrzegasz zasad, które zostały wprowadzone. Znasz osoby, które tego nie robią?**

W moim bliskim otoczeniu nie. Jeżeli ktoś musi się przemieszczać, to ma ku temu powód, musi jechać do pracy. Nie znam nikogo, kto by wychodził na spacery. Wszyscy siedzą grzecznie w domach.

**Co sądzisz o nowych zasadach, które wprowadzono?**

Trochę frustrujące jest, że ja nie wiem, o której iść do sklepu, żeby nie stać w kolejce. Ok., rozumiem, bo w tych sklepach rzeczywiście mógł być Armagedon, chociaż ja tego nie odczułam. Jak się poszło w dobrej godzinie, to jakoś było porządku, a teraz rzeczywiście nie wiem, kiedy iść, bo zawsze będzie dużo ludzi. I tak się teraz 2 dni cykam, już mam prawie pustą lodówkę. Jedyne, co jest dla mnie nie do pojęcia, to że osoby sobie bliskie mają chodzić od siebie 2 metry, czyli ja z moim synem mogę iść za rękę, a mój partner ma być ode mnie 2 m, ale mieszkamy w jednym domu. To jest w ogóle jakiś kabaret. I rzeczywiście ostatnio szliśmy do sklepu i obawiałam się, że wystawią nam mandat. Generalnie nie ma co się buntować przeciw takim zasadom, bo ja i tak ich nie zmienię. Nie odsunęliśmy się wtedy od siebie na 2 m, ale mieliśmy ubaw z tego, bo bez sensu - jechaliśmy w jednej windzie, w jednym samochodzie, mieszkamy w jednym domu a potem mamy iść 2 m. Tłumaczyli gdzieś w wiadomościach, że prawidłowo jest, jeśli idzie mama, tata i dziecko. Nie wychodzimy razem i jadę sama na zakupy.

**A inne zasady, które zostały wprowadzone. Czy ludzie ich przestrzegają?**

Różnie to bywa. Najbardziej widziałam tę aferę, że lasy są zamknięte, chociaż to zamknęło trochę ludziom furtkę na wychodzenie z domu, bo to było na zasadzie, że ach, to ja sobie pójdę do lasu. I takich ludzi była setka i nagle ta setka spotkała się w tym lesie. Trochę jest irracjonalne, że jak się jedzie na rowerze...Policja stała na ścieżce, zatrzymywała rowerzystę, który jechał sam i cofała go do domu. A jak nie, to mandat. Ale on jechał sam...Nie da się zrobić tak, żeby wszystkim dogodzić. Ja myślę, że powinniśmy wszyscy przesiedzieć te 2 tyg. w domu. Ci, którzy wychodzą uprawiać sport może nie robią jakiejś tragicznej rzeczy i to nie jest najstraszniejsza rzecz, ale mogliby przesiedzieć 2 tyg. bez treningu czy bez jazdy na rowerze. Nic by się nikomu nie stało, a nigdy nie wiesz. Po co ryzykować? To tylko 2 tyg. Co to są 2 tyg. w skali całego życia? Czy nawet miesiąc? Mogliby się przemęczyć, bo wiesz, jak to jest - jeżeli ktoś wyjdzie, to ktoś inny powie, że w sumie to on też pójdzie. I takich osób zaraz znowu będzie setka. I wtedy to, co powinno być zrobione nie zostanie zrobione. Te zasady jednak działają. Nawet te przed obostrzeniami, bo gdyby nie one, to mielibyśmy teraz 10000 chorych. jak działa to czemu tego nie robić?

**Co się zmieniło w ostatnim tygodniu, jakie działania podejmowałaś, żeby radzić sobie w obecnej sytuacji?**

Przede wszystkim zmieniło się, że już nie mogę iść do pracy, a druga to zakupy. One były jakoś tam ograniczone, natomiast teraz to jest wieczne stanie w kolejce. Nic więcej się nie zmieniło. Żyjemy tak samo od miesiąca.

Widzisz jakieś nowe wyzwania?

Zakupy. Ostatnio byłam o 21.30 w Lidlu i stałam w kolejce, więc zakupy są dla mnie wyzwaniem naprawdę. I od 2 dni siedzę, bo trzeba zrobić zakupy na święta, moja mama mnie pyta, kiedy pojadę, a ja chodzę w kółko i się zastanawiam, o której pojechać, żeby nie zmarnować tam 2 godz.  na staniu w kolejce. Zakupy teraz to jest jakieś szaleństwo.

**Czy zaobserwowałaś jakieś dziwne zachowania ludzi w najbliższym otoczeniu?**

Nie, wszyscy się chyba zachowują najbardziej naturalnie jak się da na taki moment w życiu, jaki mamy.

**A w dalszym otoczeniu?**

tak. Ludzie bardzo się boją kontaktów między sobą. Wczoraj pojechałam do sklepu kosmetycznego i pan, który mnie obsługiwał...Nie mówię już o tym, że nie miał pojęcia do czego służy maska, miał ją źle założoną i dotykał jej cały czas rękami. Miałam tylko ochotę mu powiedzieć, żeby ją zdjął, bo ona mu nić nie da. I na każdy mój krok, prób kroku zbliżenia się do niego on robił taki unik, jak ja bym miała go od razu zarazić. Aż tak się źle czułam w tym sklepie, że wzięłam, kupiłam, wyszłam i powiedziałam, że nigdy więcej nie wejdę do tego sklepu. To było tak okropne uczucie, ale o k r o p n e. I zauważyłam, że wszędzie ludzie patrzą, czy masz rękawiczki, pomimo, że ich używają absolutnie nie mając pojęcia, jak je nosić, bo mają te same, wyciągają z kieszeni, potem je chowają do kieszeni...Ludzie zaczynają się bać ludzi. To jakiś koszmar będzie. Jestem pewna, że nasza mentalność się zmieni na lata, że my będziemy tak nieufni wobec siebie, że nawet nie wiemy, że możemy być tak nieufni. Jeżeli ja teraz idę do sklepu i nie kaszlę, nie kicham, nie prycham, nie mam zaczerwienionego nosa, nie próbuję się przytulać do sprzedawcy, tylko stoję normalnie w odległości nawet 2 m i on automatycznie robi przede mną taki unik do tyłu...Jeżeli to się zaczyna teraz dziać, to za chwilę będzie jeszcze gorzej. Jeżeli ta choroba dotknie kogoś z bliskiego otoczenia, to ci ludzie już będą mieli w ogóle wariactwo. Strasznie będzie.

**Zdarza ci się robić zakupy przez internet?**

Rzadko. Ubrania tak, ale okazuje się, że wcale nie potrzebujemy tyle ubrań. Ta dam! Niesamowite to jest. Spożywcze jednak wolę kupić w sklepie. Idę z listą i kupuję to, co jest na liście, ale już się przyzwyczaiłam i nie potrafię się przestawić na internetowe.

**Próbowałaś?**

Raz miałam podejście, ale mi w ogóle nie wyszło, więc nigdy nie zamówiłam. Grzebałam po tym sklepie, bo u nas w L'eclercu można tak kupować i nie wiem, czy to była kwestia tego, że jestem przyzwyczajona do Lidla i tam nie było tych produktów, które chcę. W sensie, że były, ale nie dokładnie te. Ta ilość tych produktów, które trzeba było przegrzebać też mnie przytłoczyła. Rzuciłam to w kąt i już nigdy się tym nie zajęłam.

**Zamawianie produktów przez telefon. Słyszałaś o tym?**

Nie

**Byłabyś chętna spróbować?**

Nie mam pojęcia.

**Czy teraz kupujesz przez internet częściej/ rzadziej?**

Ja nic nie kupuję. Ja będę miała tyle oszczędności, że nie będę wiedziała co z tym zrobić. Nagle się okazało, że ja wcale nie potrzebuję tej całej szafy ubrań. To jest coś, czego nie jestem w stanie ogarnąć, bo ani nie kupuję kosmetyków, ani ciuchów, ani młodemu nic nie kupuję. W ogóle nie kupuję nic oprócz jedzenia. Nagle okazało się, że mam całą szafę ubrań i żadna nowa sukienka nie jest mi potrzebna. Może to jest też to, że częstotliwość wyjścia z domu jest dużo rzadsza, więc zazwyczaj w domu jest dres. Może też to, że w najbliższej przyszłości nie mam zaplanowanych żadnych imprez, żadnych urodzin, żadnych wyjść. Więc po co mi nowe? Bo zazwyczaj to właśnie przed imprezką, urodzinami trzeba kupić. Totalnie nic nie muszę i nawet się śmieję, że jak młody znosi, zniszczy te ubrania, które ma, to dopiero się kupi, a nie, że kolejna para spodni.

**Wcześniej często zamawiałaś?**

Ubrania to ja 99% zamawiam przez internet, bo ja nienawidzę chodzić po GH. Dla mnie spędzenie wieczoru w GH to jest dramat. Nienawidzę i idę tylko jak muszę, więc internet jest dla mnie wygodny. GH to szkoda czasu, nie lubię przymierzać, nie lubię tam łazić, a przed komputerem to siądę w wolnej chwili, przejrzę, zobaczę...W ogóle mnie nie kręci chodzenie po sklepach. Dla syna też zawsze zamawiam ubrania przez internet. Jak zamykali szkoły...Od tamtej pory mam jeszcze 2 sukienki w koszyku internetowym w Zarze, ale sobie myślę, że po co je kupować, skoro ja ich nigdzie nie założę? I one tak leżą. I jednak sytuacja finansowa jest na tyle niestabilna, że ja nie wiem do końca, czy ja mogę sobie na to pozwolić i czy te pieniądze, które ja wydam na sukienki, które mi nie są potrzebne, nie lepiej zatrzymać na czarną godzinę. Nie zamawiam, nie kupuję. Na pewno od momentu, kiedy ten kryzys się pojawił, to nic nie kupiłam. Kupuję rzeczy niezbędne, czyli jedzenie.

**Jaką funkcję pełni u ciebie jedzenie?**

Chodząc do pracy to była kwestia, że trzeba zjeść, a jak się zapomniało, to się zjadało wieczorem. Starałam się pilnować godzin jedzenia, żeby to jakoś funkcjonowało, ale zazwyczaj było to bardzo monotonne, w kółko to samo, bo niby nic innego nie można ugotować. A od czasu, kiedy siedzę w domu to jestem Magdą Gessler własnego domu. To naprawdę jest zaskakujące, że ja potrafię gotować. Nie wiedziałam. No i się okazuje, że żeby gotować smacznie i zdrowo nie potrzeba milionów, bo można ugotować z tego, co się ma w lodówce i można ugotować tyle fajnych potraw, które się gotuje w pół godziny. I jemy wszyscy razem przy stole jak biali ludzie, normalni. To się bardzo zmieniło, bo razem jadaliśmy tylko w niedzielę i czasem w sobotę, bo każdy przychodził do domu o innej godzinie, ja pracowałam do późna i wpadałam na kolację. Nie zdarzało nam się razem jeść, może śniadanie jeszcze czasami, o ile ja jadłam to śniadanie. Coś w tym jest, jak mówią, że wspólne posiłki scalają rodzinę. Fajnie tak.

**Mówiłaś, że pieczesz sama chleb teraz. A czy pojawiają się jakieś nowe produkty, może z czegoś zrezygnowałaś?**

Myślę, że nowe nie, ale bardziej przykładam wagę do tego, co jemy. Jemy więcej warzyw, jemy zdrowiej, pijemy wodę. Zdecydowanie jest to bardziej przemyślanie, już nie na tej zasadzie, że otwórzmy lodówkę i zjedzmy co jest, tylko żeby to było wartościowe, żeby to nie było tylko, żeby się najeść i zaspokoić głód, ale żeby to miało jakieś jeszcze wartości odżywcze.

**Z czego wynika ta zmiana?**

Bo mam czas. Teraz wstaję rano, ogarnę coś tam...W sumie jak codziennie ogarnę dom, to on jest ogarnięty cały czas i potem mam czas w ciągu dnia, żeby zrobić ten obiad. W normalnym trybie życia, wracając wieczorem, to modliłam się, żeby ugotować cokolwiek, albo gotowała moja mama, żeby tylko był obiad na następny dzień.  W tej chwili jemy o 15 i to można gotować świeżo, zdrowo. Mam czas.

**Sprawia ci to przyjemność?**

Tak. Ja całe życie myślałam, że ja nie lubię gotować, że gotowanie to jest w ogóle coś za co ja w ogóle nie powinnam się zabierać. Okazało się, że wcale tak nie jest, tylko kwestia czasu. Wydaje mi się, że człowiek wpadł w wir pracy, była praca, praca, praca, a gdzieś pomiędzy pracą może wpadnę do domu i coś zjem, może coś posprzątam i trochę wychowam dziecko. Praca to było...Focus na pracę. Nie jest to dobre.

**Zdarzało się wam zamawiać jedzenie z dowozem? Teraz się zdarza?**

Teraz w ogóle nie, natomiast kiedyś bardzo, bardzo często. Szczególnie w jakieś weekendy. Dużo zamawialiśmy. Pizza była standardowo, sushi zamawialiśmy i indyjskie jedzenie, czasami chińczyka. Raczej zamawialiśmy to, czego nie potrafiliśmy ugotować w domu.

**Dlaczego teraz nie zamawiacie?**

Bo możemy ugotować. Nagle się okazuje, że mamy świetne sprzęty do gotowania, które stały nieużywane i wystarczy ściągnąć przepisy, których jest miliony w internecie i po prostu zacząć eksperymentować. Wszystko się da ugotować. Jak teraz nam się coś chce - pizzę czy cokolwiek, to ok, to chodź zrobimy, Nie ma problemu.

**Gdyby wam się nie chciało gotować, to miałabyś jakieś obiekcje, żeby zamówić jedzenie z dowozem teraz?**

Pewnie bym się trochę biła z myślami, czy w tej chwili wydawać...Na prawdę. Myślałam, że tylko ja tak mam, ale okazuje się, że moje koleżanki też. W momencie, kiedy jest na tyle niestabilna sytuacja gospodarcza i na rynku pracy, to lepiej ugotować te ziemniaki z kotletem niż zamawiać obiad za 2 x więcej. To by mnie pewnie zastanowiło i pewnie bym ugotowała, znając siebie.

**Czy zmieniło się coś w twoich sposobach płatności?**

Sugerują wszyscy, żeby używać karty, natomiast ja więcej obracam gotówką, więc na tyle, na ile mogę to ja używam karty, natomiast ja mam gotówkę i nadal płacę, jak płaciłam, czyli pół na pół generalnie. To co mogę to płacę kartą, ale gotówkę normalnie mam w obrocie. mój sposób myślenia o tym się nie zmienił, ale mam gdzieś z tyłu łowy, że jak płacę gotówką, to wszyscy mnie zabijają wzrokiem. I ludzie w kolejce, i kasjerzy. Nigdy mi absolutnie nikt nic nie powiedział, ale to z tyłu głowy...Sama wiem, że powinnam płacić kartą, więc gdzie mogę, to płacę tą kartą.

**Jakieś inne sposoby płatności?**

Tylko karta albo gotówka. Nie używam blika, bo mam kartę. nie mam tej płatności w telefonie i jakoś sceptycznie do tego podchodzę. Karta to karta. Wiem, że mogą mi ja ukraść, ale jakoś tak...

**Nie korzystasz z innych metod, bo czujesz, że to jest jakieś ryzyko?**

Trochę tak, bo na tym telefonie mam wszystko. Nie chciałabym, żeby ten telefon mi ktoś ukradł. Jak jeszcze miałabym tam płatność telefonem to już w ogóle. W dobie tego, że wszędzie wszystko jest, wszyscy wszystko o nas wiedzą, to trochę zwiększa się moje bezpieczeństwo jak nie używam tego telefonu do płatności. Nigdy nie miałam potrzeby innych metod i nawet się nad tym nie zastanawiałam.

**Czy zmieniły się twoje zwyczaje w kwestii zakupów?**

Nic się nie zmieniło, jest dokładnie tak samo. Biorę listę i robię zakupy. Pełen wózek, bo robię dla całego domu, prawie te same produkty. Mogłabym listy nie wyrzucać i jeździć z tą samą.

**Czy poza listą jeszcze jakoś przygotowujesz się do zakupów?**

Teraz nie wiem, w jakich godzinach jechać, ale jeszcze tydzień temu starałam się wybrać taką godzinę, żeby ominąć te największe tłumy. Jeździłam 10-11. Teraz nie ma takiej godziny, więc przygotowuję się na dzisiejszy wypad do sklepu po południu i nie wiem, którą godzinę wybrać. Od 10 do 12 kupują emeryci, koło 15 zaczynają ludzie z pracy wychodzić i zawsze były w tych godzinach kolejki. Może jak ok 19 pojadę, to już będzie spokojniej, chociaż ostatnio o 21.30 stałam w kolejce, więc...Nie wiem.

**Byłaś już w sklepie od momentu wprowadzenia limitu klientów?**

Byłam w piątek i to był 1 dzień, kiedy Lidle były czynne do 24. Pojechałam o tej 21.30 i takich jak ja było dużo. Było dosyć pusto na półkach i wtedy byli ludzie, którzy absolutnie nie trzymali się zasad tego odstępu między sobą, pchali się na siebie. Ja się już zaczęłam denerwować, ekspedientka zwróciła uwagę, bo wykładając zakupy już czułam oddech następnej osoby na plecach i to byli młodzi ludzie. Naprawdę zrobił się tłok i dlatego muszę wybrać inną godzinę. Ja nie lubię zakupów w sklepach, marketach i łażenia, a w tych czasach, kiedy jest ten wirus, to już w ogóle nie ma co ryzykować i pchać się tam, gdzie jest dużo ludzi.

**Czy w kolejce przed sklepem jakoś inaczej to wyglądało?**

Ja akurat weszłam, jak kolejki przed jeszcze nie było, ale przed jest fajnie, bo ludzie naprawdę stoją w takich 2 m odstępach od siebie. Bardzo się pilnują, bardzo grzecznie wchodzą.

**Teraz robisz niezbędne zakupy. Wcześniej zdarzało ci się robić je dla przyjemności?**

Tak, często wpadałam do sklepu i że dzisiaj może akurat zjemy to i to kupię, a potem leżało przez 3 dni, nie zdążyłam ugotować i się wyrzucało. Teraz są to bardzo przemyślane zakupy, takie racjonalne.

**Jakie produkty kupowałaś dla przyjemności?**

Bardzo różnie. Jeżeli chodzi o jedzenie, to czasem pomysł, że ugotujemy jakąś dziwną potrawę, której nigdy nie gotuję. Jeśli chodzi o kosmetyki i ubrania, to jeszcze jakiś czas temu bym myślała, że to wszystko jest potrzebne. Ten miesiąc w domu trochę mnie zmusił do refleksji, że większość tych ciuchów czy tych kosmetyków było kupowanych tylko dla przyjemności. Ja nie mówię o mydła i paście do zębów, tylko 3-cia odżywka do włosów czy kolejny podkład. Nie z potrzeby, tylko kupię, bo kupię, bo ktoś polecił. nie były to rzeczy niezbędne. teraz kupuje rzeczy niezbędne.

**Co planujesz w związku ze świętami?**

To będą takie święta nie święta. Niby święta, ale niczym się nie będzie różnić niedziela od ostatnich dni. Ja mieszkam z rodzicami, więc jestem w trochę lepszej sytuacji, bo będziemy z nimi świętować, czyli zjemy razem obiad i śniadanie wielkanocne, natomiast nawet dzisiaj rozmawiałam z mamą, że tak naprawdę nie ma co gotować, nie ma co piec, bo nie ma tego kto zjeść. Nagle nie ma co robić 3 ciast, gdzie nas jest 5. Kto to zje? U nas święta zawsze były bardzo rodzinne i bardzo liczna z nas rodzina, więc śmialiśmy się zawsze, że powinniśmy mieć jeszcze jeden dzień wolny, żeby odpocząć po świętach. Teraz tak nie będzie i trochę nam przykro, a najbardziej z tego powodu, że ja mam babcię, która ma 89 lat. I zawsze jak idziesz na te święta, to sobie myślisz, że trzeba iść, bo nigdy nie wiadomo, co to będzie, a tu nie możemy iść, bo nie możemy jej narażać. I, kurczę, święta bez babci? Dziwne. Jakoś trzeba to przeżyć. Nie wynurzamy się z domu. Niby się tak bardzo nie boimy tego wirusa, ale nie będziemy się mu nadstawiać. Siostra też siedzi u siebie, nie ruszamy się z domu i nie ryzykujemy. Stwierdziliśmy, że jak teraz przeczekamy, to się spotkamy za 2 tyg. To nie ten czas.

**A kwestia kościoła?**

Nie byliśmy już z palmą i to było takie dziwne. Teraz nie pójdziemy ze święconką. No stracą te święta. Dla mnie to nie będą święta. To będzie kolejna niedziela. Absolutnie nie planujemy brać udziału w jakichś nabożeństwach.

**Co świątecznego planujecie zrobić?**

Na pewno ugotujemy żurek, śmiejemy się, że się ubierzemy odświętnie, usiądziemy wspólnie do stołu i tyle. W poniedziałek polejemy młodego wodą, żeby miał i tyle. Rozmawiałyśmy z mamą, czy pieczemy jakieś mięsa, stwierdziłyśmy, że może jedno, ale też się zastanawiamy, bo nie będzie miał kto tego zjeść. Zrobimy jakiś bardzie odświętny obiad, ale myślę, że nie będzie jakiś typowo wielkanocny. Jakieś ciasto zrobimy, jakiś sernik.

**Planujesz kupić teraz coś dla przyjemności?**

Może Colę kupimy dla młodego, a tak to chyba nie. Jakoś w ogóle nie czuję tych świąt. Coli nie ma u nas w domu na co dzień, ona jest tylko od święta. Cola i chipsy. Na Boże Narodzenie też jest.

**Co jest ważne dla ciebie w święta?**

Najważniejsze są te spotkania rodzinne. Jest okazja, żeby się spotkać i tak naprawdę nie ma wymówek. Raczej się nikt nie wykręci, że jest w pracy i u nas te 2 x do roku i jeszcze w babci urodziny są takie święte święta, gdzie się wszyscy spotykaliśmy. Przez kilka miesięcy każdy żył własnym życiem, a święta, co by się nie działo, to się jechało do babci i tam się spędzało święta. 35 lat rok w rok tak samo i nigdy w życiu nie było inaczej, że np. święta były przeniesione do mojej mamy czy do jakiejś cioci. Zawsze były u babci i nikt nawet nikogo nie zapraszał, bo to była zawsze konkretna godzina. No i co? Po 35 latach nie? Dlatego może ja nie umiem obchodzić inaczej świąt? Nie będą to dla mnie święta.

**Na ile ważna jest święconka i palma?**

Dla mnie jest to bardziej tradycja niż obrządek kościelny. Zawsze się chodziło z palmą i święconkiem. Zawsze u nas było tak, że to dzieci z tym chodzą. Ostatnio młody zaczął chodzić, więc to ja musiałam z nim chodzić. I znowu ta tradycja, która jest u nas zachowywana od lat...Ktoś mi ją zabrał.

**Jak się z tym czujesz?**

Źle mi z tym. Tak jakby w ogóle to nie były święta tylko kolejny tydzień kwarantanny i siedzenia w domu. Nie ma świąt, zabrali święta. Mam z mamą mały zgrzyt, bo ona, że jak to nie ma świąt? Dla mnie nie ma. Nie byłam z palmą, nie ma świąt. Dla mamy nadal są i ona nadal chce je celebrować
