# Supplementary material for: The challenges arising from the COVID-19 pandemic and the way people deal with them. A qualitative longitudinal study
Source: PLoS One. 2021 Oct 11;16(10):e0258133. doi: 10.1371/journal.pone.0258133 (PMC8504766; doi:10.1371/journal.pone.0258133)
Supplement: S1 Dataset — (ZIP) [file pone.0258133.s003.zip › Transcriptions/stage 2/18.2_F_48_couple, with children.docx]

**18.2_F_48_couple with children**

**Obrazki. Który z obrazków najlepiej oddaje Twoje emocje w tej chwili?**

12 i 14.

**12**

Tutaj widzę właściwie taki ciężar, takie obciążenie. Przytłaczające takie duże obciążenie. Wynikające z całej sytuacji, z natłoku różnych zdarzeń, różnych sytuacji. Wiadomości plus zachowania ludzi przytłaczające, ciężkie.

**Jakie to są zachowania?**

Chaos wśród ludzi, niedbałość i to mnie przygniata.

**Jak odczuwasz ten ciężar?**

Pozbawia mnie takiej energii, tego, co miałam. Takiego entuzjazmu, tego, że idę do przodu. Te zachowania mnie stopują, pozbawiają energii. To przytłacza. Jakbym osadzała się w miejscu, nie szła do przodu.

**Masz jakieś sposoby na minimalizowanie tego stanu?**

Branie tego z humorem. Rozładowywania napięcia poprzez żarty. Przekłucie tego na jakieś... Tak, żeby to wziąć na wesoło. Nawet, jak widzę, że jest jakaś trudna sytuacja, to, żeby z tego coś takiego... Przekuć to na żart, z humorem.

**Udaje ci się?**

Tak, raczej tak.

**Czy ten ciężar odczuwasz w ciele fizycznie?**

Tak, to jest takie... Ja wiem, taki odpływ energii. Czuję taką ociężałość wtedy, jak te kamienie. Co prawda one są małe i takie nawet ładne, ale ja bardziej się czuje, jak taki głaz.

**Zwalone skały byłyby bardziej adekwatne?**

Tak.

**Jak przekuwasz to w żart, to to wymaga od ciebie wysiłku czy samo wychodzi?**

Nie, nie. Dużego wysiłku nie. Ja tak sobie ogólnie radzę ze stresem, że bardziej przekuć to w żart, w jakiś dowcip, dopasować do sytuacji niż w jakąś złość czy smutek. To może potem przychodzić, jak już sobie nie daję rady humorem.

**Co bardziej?**

Bardziej złość.

**Na co to jest złość?**

To bardzo różnie. To też zależy od sytuacji czy też rodzaju zdarzenia. Ale bardziej na sytuację, na osobę i na końcu na siebie.

**14**

Tutaj to jest takie jakby ściśnięcie w emocjach. Nie wiem, jak nazwać tą emocję. To jest takie uwikłanie, takie splątanie. Bardziej to mnie ogranicza... to nie jest emocja. Te kamienie to mnie z góry obciążają, a tutaj trochę bezradność, bardziej uwikłanie w sytuację.

**To jest bardziej wewnętrzne a tamto bardziej z powodu sytuacji?**

Tak.

**Które z nich towarzyszy bardziej stale?**

Chyba to przy...*[problem techniczny - przerwa]*Bardziej te kamienie. To mnie obciąża.

**A ten supeł, czy masz sobie sposób, żeby sobie z tym radzić?**

Radzę sobie bardzo praktycznie, zajmując się czymś. Bo te kamienie to są bardziej takie, jak jestem w pracy, wśród ludzi. Natomiast ten supeł, węzeł, to jest jak zostaję sama i jest więcej wiadomości takich, co się dzieje. I wtedy, to mnie tak ogranicza. Poza tym... Ostatnio, jak czułam się uwikłana w domu co prawda tylko przez chwilę, bo ja wychodzi do pracy, to aż chciało mi się wyjść na dwór, więc jakiś taki krótki spacer poczyniłam, żeby poczuć taką wolność.

**Pomogło?**

Pomogło, tak. Po prostu miałam wrażenie, że oddycham podwójnie. Krótki czas, krótki odcinek, znane miejsce, otoczenie, a tyle dało.

**Jeszcze znalazłaś jakiś sposób?**

Zajęcie się czymś innym, czyli obejrzenie filmu, z rodziną przebywanie, rozmowy zupełnie o czymś innym, nie o danej sytuacji i wtedy następuje odciągnięcie uwagi i myśli.

**Czy jak się pojawiają takie emocje, to szukasz od razu sposobu, żeby je zminimalizować, czy masz chęć, żeby się temu poddać?**

Nie *[śmiech]*. Poddać to nie.

**Czyli raczej cię to motywuje, żeby temu przeciwdziałać?**

Tak. W zasadzie to jest odruch obronny. Wiem, że jakbym się poddała to... Staram się mieć nad tym kontrolę, żeby się tak nie ponieść całkiem tym emocjom i nie wkręcić się w tą sytuację.

**Mam wrażenie, że to się zmieniło od zeszłego tygodnia. Co się zmieniło?**

Jest ciężej pod tym względem, że też jakby jest coraz mniej osób takich dzielnych wokół i radzących sobie. I jak widzę takie przygnębienie wśród ludzi i jakieś takie dylematy, to też widzę, że wokół się ten świat zmienia i ja też się zmieniam.

**Jak wygląda ta zmiana u ludzi?**

Oj, bardzo różnie. Bo jedni mówią wprost o tym, że jest im ciężko w domu, co tam się dzieje, itd. Nie wiem, jakaś staruszka nagle rozpłakała się, że tak jest jej szkoda tych młodych. I taka sytuacja, że jak widzę kobietę, że ojej, ja już mam tyle lat, no jakoś sobie radzę, ale tak mi jest szkoda tych młodych. I wtedy tak się robi ciężko z tym. I to się udziela.

**I sama zaczynasz myśleć, że ci szkoda tych młodych?**

Nie. Natomiast szkoda mi bardziej tej osoby, która to odczuwa, jest jej smutno i stara się jakoś pomóc, ale nie ma, jak pomóc. To w ten sposób bardziej. Bardziej jest tak, że ludzie są nerwowi, są już tak troszeczkę rozdrażnieni. Więc tak trzeba delikatnie. Trudno z tym humorem i postawą... Jakoś staram się rozładowywać, ale jest już trudniej. Jest ciężej w tym funkcjonować, nawet z takim zadaniowym podejściem, poczuciem humoru, jest ciężej się przebić, bo taki się trochę murek robi. Może jeszcze nie mur, ale murek.

**A ty zaobserwowałaś u siebie nowe zachowania?**

Na pewno więcej śpię. To znaczy śpię... W sensie szybciej się męczę i tak do tego spania mnie ciągnie. Tak jakby to było takie lekarstwo, że jak zasnę, to odpocznę i ta regeneracja będzie i tak świadomie do tego wyspania się...

**Udaje ci się wyspać?**

Znaczy, udaje, bo jak już śpię, to śpię. Natomiast długości nie wydłużyłam za bardzo. Ale no tak jest inaczej.

**Coś jeszcze nowego?**

Jestem, no może nie spokojniejsza, ale ta moja charyzma to się wyciszyło. Nie mam takiej twardej postawy, ale mam taką, myślę, że rozsądną dalej i spokojną, ale nie już twardą.

**Można to nazwać przygaszeniem?**

Tak, o, bardzo dobre słowo.

**To ci przeszkadza czy pomaga?**

Jest to coś nowego. I na razie się tego uczę i obserwuję, jak to jest.

**Chciałabyś, żeby tak zostało?**

Trudno mi powiedzieć, bo nie jest to coś, co mi aż bardzo źle robi. Bo to jest w kierunku, no wygaszenia, ale też spokoju, bez lęku i jakiegoś napięcia. Na takim równym poziomie. Bardziej z pogodzeniem się z sytuacją i takim tu i teraz.

**Jakaś jeszcze zmiana?**

Jest we mnie więcej złości. Ale złości no to chyba jak u większości, jak słucham o wyborach. No to rzeczywiście, tutaj z tego wyciszenia, to jest takie wkurzenie, co by gorzej tego nie nazwać.

**Coś jeszcze cię złości?**

Złości mnie np. takie... Jak obserwuję, że jest grupa ludzi, która dostosowuje się do tych zaleceń, przestrzega różnych zasad. A złości mnie takie podejście, że nagle tu chodzą jakieś grupy, że "ojej, co to będzie, przecież to nic takiego". I to mnie złości, że, no jest to takie... No mówię, z jednej strony jest grupa, która się dostosowuje i to jest fajne i gdybym ja nie pracowała tu, gdzie pracuję, to bym się dostosowywała. A są ludzie, którzy traktują to lekko, jakby to był wydłużony urlop, czy jakaś sytuacja wirtualna.

**Po czym to poznajesz?**

Jak ktoś przychodzi czwarty raz do sklepu po jakieś mało istotnie rzeczy, to nie, nie. Albo mamy ewidentnie zakaz dotykania towaru, mamy zabezpieczone rękawiczki, płyny i ktoś z tego nie korzysta, wchodzi, jak gdyby nigdy nic. A ktoś stoi i pilnuje tego, że zdobył maseczkę i korzysta z tego, to tu nie ma mojej zgody. Poza tym, że ktoś siedzi 2 tygodnie w domu i co 2 tygodnie idzie na te zakupy, takie bardzo podstawowe, a druga osoba... No nie ma tego efektu, bo ktoś jest poszkodowany. No bo się stara i tak naprawdę ktoś, kto spotyka się z osobą, która to lekceważy, to ten jego wysiłek, to siedzenie w domu, może iść na marne. To mi bardzo przeszkadza, to mnie złości.

**Jeszcze jakieś zachowania ludzi cię złoszczą?**

*[Śmiech]*Ta cała sytuacja z godzinami dla seniora. To jest całe zamieszanie i chaos z tym. Tez takie niezrozumienie... Ale to też wynika z tego, że to się dzieje szybko, jest takie niedopracowane, niedopowiedziane. Ja w pierwszym takim, no nie wnikałam bardzo na początku, zrozumiałam, że to są te dwie godziny dla seniorów, że oni tylko wtedy wychodzą. A to się okazało, że oni mogą wychodzić cały dzień, a te 2 godziny to są jeszcze takie ekstra uprzywilejowane. A też mam wrażenie, że ci seniorzy odebrali to, jako taki wręcz, że wy macie iść w ciągu tych dwóch godzin - że to jest taki przywilej, takie ekstra. I oni się dostosowali do tego, że czy potrzebuję czy nie, to wyszedł przekaz 2 godziny seniora i ja idę wtedy. W pierwszy dzień, to mnie strasznie rozbawił widok [*śmiech*]. Wybiła godzina 10 i pojawili się sami emeryci, ale tak tłumnie. Przychodzą cały czas, bo okolica jest taka, że tych osób starszych sporo mieszka, ale to był taki wysyp starszyzny. No wyglądało to komicznie, bo jak w pewnym momencie zaczął wchodzić balkonik, kula, to mnie to rozbawiło. Nie wiedziałam o co chodzi, bo to taka zupełnie nowa sytuacja. Była też taka sytuacja, że była za 10 dwunasta i pani chciała ewidentnie wyrzucić pana ze sklepu, bo jej się to należy. Więc jakaś taka sprzeczka jeden na drugiego, itd.

**Osoby młodsze nie mogą wejść do sklepu w czasie godzin dla seniora?**

To znaczy, to jest też bardzo rozbieżne. Ja nie czytałam tej ustawy, bo naprawdę to mnie... Ale jak widzę, jak to jest w sieciowych sklepach, to nie mogą być osoby poniżej 65 roku życia. Ale koleżanka mi mówiła, że jest dopisek, że jeśli nie ma seniora w sklepie, to mogę obsłużyć osobę młodszą. Natomiast od strony mojej pracy to jest też bardzo niekomfortowe, bo jak mam sprawdzić, czy ktoś ma lat 62, 65 czy 68. Bo pani 60-letnia może nieraz wyglądać na staruszkę, a starsza pani na lat 60. Więc nie mam takiego narzędzia i prawa, żeby ich pytać o dowód, o wiek. No tutaj jakieś straszenie karami. Więc też to jest bez sensu. Ja stwierdziłam, no fajnie, tylko to mi blokuje cały przepływ i efekt był taki, że w sobotę po 12 młodzi ludzie, którzy się dostosowali do tego, musieli stać w kolejce i wydłużył nam się czas pracy. A przez te dwie godziny było luźniej. Więc to zupełnie rozbija... Ale izba handlowa zaprotestowała, bo rzeczywiście, bardzo to w mniejszych sklepach - bo ja nie rozszerzę, jak Biedronka na 24h, to bardzo wprowadza chaos. I nie wiem czemu to służy, niczemu. Takie sytuacje mnie złoszczą. Albo zapewnienie rękawiczek dla klientów - no fajnie, tylko ich nie ma albo są za jakieś pieniądze... No ja sobie radzę. Udało mi się, tylko złości mnie to, że ktoś coś wymyśla, ja bardzo bym się chciała z tego wywiązać, bo uważam, że w porządku, natomiast nie mogę, fizycznie nie mogę, choćbym nie wiem, jak chciała. I to jest właśnie takie, to bardzo rozbija. Takie przyziemne sprawy i to mnie złości. I jak patrzyłam, jak w Austrii sobie radzili, że rząd w sklepach rozdawał maseczki i rękawiczki dla wszystkich, bez wyjątku, to zupełnie jakbym była w innym świecie. No to mi się zrobiło smutno, że znowu jesteśmy na końcu tej Europy.

**Na ile się obecnie czujesz zagrożona sytuacją?**

Właśnie obawiam się, że ten brak dostępu do testów, że ich jest mało i nawet, jak ktoś przychodzi, to nie wiem, czy jest nosicielem, czy nie. Że ta mała ilość testów - tego się boję. I tego dostępu, że słyszę, że ci lekarze są bardzo narażeni, nie mają ochrony - to są moje największe obawy.

**Podjęłaś jakieś nowe środki ostrożności w tym tygodniu?**

Dołożone płyny do dezynfekcji dla klientów i przypominamy klientom, żeby skorzystali, jak wchodzą i zdecydowanie przypominam, że proszę skorzystać, założyć, nie dotykać towaru i większość się stosuje. Jak musiałam pozałatwiać jakieś sprawy, to tak się zastanowiłam, bo ja założyłam tą maseczkę, bo jej używam, ale nie wiem, czy to masz aż taki sens duży, bo skoro jest w sklepie i nie używam jej... Ale mamy taką ochronną pleksi i to daje takie poczucie bezpieczeństwa.

**Co się stało, że zaczęłaś używać maseczki?**

Chyba te wiadomości, że gdzieś tam w Chinach kazano. W tej Austrii. Zobaczyłam, że u nich nie postępuje tak, a większość nosi te maseczki. Więc, jakby na zasadzie, że nie zaszkodzi, a może pomoże.

**Jak z oglądaniem wiadomości?**

Bardzo delikatnie. Jeszcze bardziej staram się ograniczać. Nawet nie wiem, ile było... W ostatnie dwa dni, to zupełnie. tez mi to właściwie nic nie da, to nie wniesie nic więcej w moje życie codzienne, bo nie mam na to wpływu.

**Masz wrażenie, że to jest poza tobą, że sama nic nie możesz?**

Nie, nie. Tylko jakby, jestem świadoma zagrożenia, że jest to coś, co może spotkać każdego z nas. To, co mogę, robię, ale no cóż mogę więcej.

**Co myślisz o zakazie pójścia do lasu?**

To jest takie... Nie do końca się z tym zgadzam, bo to jest traktowanie wszystkiego wybiórczo. Na zasadzie, że zamknięta jest galeria, nie mogę kupić butów, ale mogę iść do sklepu budowlanego kupić sobie tapetę. Więc ja nie widzę różnicy, czy spotkam się z ludźmi w sklepie budowlanym, czy w galerii handlowej. Chodzenie do lasu to też kwestia, jak ludzie się zachowują w tym lesie. Na Helu, jak było głośno, że masowo ludzie wyruszyli. To jest takie traktowanie, jakby to był piknik, dłuższy weekend, może wakacje. I tam nie widzę powagi tych ludzi. Ale z drugiej strony ten mój krótki spacer, gdzie mam ogródek i mogłabym sobie siedzieć w ogródku, to dla ludzi w blokach, to uważam, że jakiś dar z niebios wyjście do lasu. Nijak się to ma... Zezwolenie na prowadzenie sklepów całodobowych, ale jak ktoś jedzie na myjnię, zakłada rękawiczki i jest sam ze swoim samochodem, to jest w ogóle dla mnie bez sensu.

**Którego ograniczenia tobie najtrudniej przestrzegać?**

Nie mam takich trudności. Bo ten mój krótki spacer... Chociaż, jak wyszłam na ten spacer, to się czułam trochę, jakbym coś ukradła. Miałam dyskomfort, że robię coś nie tak. Nie było nikogo wokół, tylko mój mąż, ale taki był dyskomfort, że to było takie wykradzione.

**Korciło cię, żeby jeszcze jakiś zakaz złamać?**

Nie. Jak patrzę, jak ludzie spotykają się, jakieś grupki - np. ostatnio widziałam 4 panów pijących alkohol gdzieś w bramie, to mam ochotę zrobić takie mocne cięcie i zamknąć wszystko. Na zasadzie, tak jak my, zamykamy nasz sklep jako źródło dochodu, to żeby to było konsekwentne i żeby wszyscy się stosowali. Ale to jest nierealne *[śmiech].*

**W twojej okolicy ludzie stosują się do zasad?**

W mojej okolicy stosują się. Nie spotkałam się wśród znajomych, czy nawet teraz święta nadchodzą. W mojej okolicy każdy chce tego przestrzegać. Chociaż koleżanka, która się opiekuje ciocią i mamą, mówi, że one jej się po prostu wymykają, że nie chcą słuchać. Mówi, że jest przerażona, ale nie może inaczej. Z tymi starszymi osobami trochę mają problem znajomi.

**Jak sobie twoi bliscy radzą z sytuacją?**

Mąż jest bardzo podobnie, jak ja, taki zmęczony. On więcej słucha wiadomości, więc jego poziom zdenerwowania jest większy od mojego, bo on dzielnie siada, czyta i ogląda.

**Poziom obaw też jest większy?**

Chyba nie. Chociaż może tego nie dawać poznać, bo to jednak męska natura jest trochę inna. Syn chyba sobie dobrze radzi, może tak... Spotkał się z kolegą i się śmiał, że mieli miarkę, żeby zmierzyć odległość. Ale to chyba bardziej w formie żartu. Ale on nie narzeka, chociaż stracił apetyt. Większość klientów, itd. mówi, że młodzież siedząca w domu cały czas je, to on ma odwrotnie.

**A myślisz, że czemu?**

Ja się go pytałam i mówił, że jak tak nie chodzi, nie spotyka się, tak mu się po prostu nie chce.

**Raczej przez brak aktywności fizycznej a nie stan psychiczny?**

Tak, zdecydowanie. Bo on raczej jest pogodny, zadowolony z tego, że nie musi chodzić na uczelnię.

**A jakieś twoje, nowe zachowania, któryś byś się nie spodziewała?**

Jeszcze nie. Jeszcze nic się nie zmieniło. Ale mówię jeszcze, bo myślę, że to jest sytuacja rozwojowa. Ale nie. Na razie jest wszystko takie...

**A u kogoś ze znajomych coś, co cię zdziwiło?**

Na razie nie. Ale np. dzwonią do mnie koleżanki, które... Fakt, że my się więcej spotykałyśmy niż rozmawiałyśmy, bo ja się wolę spotkać niż siedzieć na telefonie. Ale zdarzyło się, że koleżanki dzwoniły, które nie podejrzewałabym, że zadzwonią na długą rozmowę. To była taka różnica, że nie ja pierwsza, że ta rozmowa i że ta rozmowa będzie tyle trwała. Że była potrzeba dłuższej rozmowy.

**A jakieś sposoby na radzenie sobie z emocjami?**

Dwie koleżanki zaczęły robić remont w domu. Dwie koleżanki zajęły się wolontariatem - jedna na infolinii w centrum medycznym. A druga, co prawda ona jest terapeutą, ale gdzieś tam sobie odnalazła w centrum kryzysowym, czy coś takiego. Ale to ewidentnie, żeby nie siedzieć w domu, ale nie z taką potrzebą zarobkową, tylko, żeby jakoś zająć czas.

**Widzisz wzrost działań na rzecz innych ludzi?**

Tak. W takiej naszej małej społeczności widzę, że coraz więcej osób robi zakupy dla sąsiadów. Że to się rozszerza, że nie tylko rodzina, tylko się rozszerza, że może koleżanka, może sąsiadka. Taka pomoc sąsiedzka to bardziej jest widoczna.

**Nadal jeździsz do rodziców?**

Tak.

**A jak oni w tej sytuacji?**

Oni się dobrze odnajdują. Mama się chyba cieszy, bo ten kontakt się zrobił częstszy. Ona dzwoni do mnie częściej, jak czegoś potrzebuje. Moja mam bardzo szuka zainteresowania i pyta, co robisz, itd. i mi to przeszkadzało i pytałam, co chcesz konkretnie, bo ja jestem w pracy i nie mogę. Teraz ja mam większe przyzwolenie na to, że ona dzwoni i mi to nie przeszkadza. To nie są długie rozmowy, ale na zasadzie, że mamy temat. Mówi konkretnie co potrzebuje, albo jak mi minął dzień, ale mi też jest łatwiej, bo dzwoni z konkretami.

**Nadal jesteś taka zajęta?**

Tak.

**I nadal to pomaga?**

Tak, zdecydowanie. Ostatnio też mówię, no nie... No ciężko by mi było.

**Czy coś się zmieniło w tym, jak robisz zakupy?**

Nie. Może nawet trochę mniej niż wcześniej, bo mniej mam czasu na gotowanie. Wykorzystuje to, co mam w domu. Ostatnio nawet mi się podobało, bo jakiś fragment wywiadu z Makłowiczem słuchałam i on mówił, że nie dokupił nawet paczki makaronu, bo on ma swoją podręczną spiżarnię. I ja stwierdziłam, że tak, no ja właściwie na tej samej zasadzie, bo ja też gotuję. I on pokazał przepisy, co można zrobić z tego, co mamy w domu. Nic nie dokupuję więcej, co bym kupowała wcześniej.

**Planujesz bardziej zakupy?**

Na pewno są bardziej przemyślane i jak już kupuję, to myślę, co mam kupić, żeby nie rozciągać tego. Może minimalnie, też nie jakoś bardziej. Bo ja nie lubię robić takich zakupów, że ciągle biegać na zakupy, więc ja sobie zawsze robię taki plan, że to, co potrzebuję na tydzień, to z reguły tak kupuję.

**Byłaś już na zakupach po nowych obostrzeniach?**

Tak.

**Jak to odebrałaś?**

Jakby z takim, może nie z pokorą, ale tak jest. Nie zrobiło to na mnie jakiejś... Więc chyba wprowadziło większy porządek. Bo i tak przestrzeń, którą ludzie zachowują między sobą - mam wrażenie, że to jest trochę spokojniejsze. Jak byłam w Lidlu, to tak w cudzysłowie na pocieszenie, to nie zauważyłam linii, gdzie mam stanąć i kasjerka mi pokazała, że powinnam stanąć dalej. Ale już pani za mną z taką agresją do tej kasjerki się odnosiła, że ona panu pozwoliła, a jej już nie pozwoliła no a pan miał jakieś 2 rzeczy - ja odchodziłam, a on zaraz za mną podszedł. No i dostało się pani kasjerce, że nie jest konsekwentna.

**Czyli widać było to rozdrażnienie?**

Tak, widzę to rozróżnienie w różnych miejscach. Czy to jest mały sklep, czy duży, poziom jest ten sam. Może nawet w tych większych sklepach, może trochę większy, bo tu mamy chyba trochę bardziej wyselekcjonowanych klientów, bo sporo osób się zna.

**Pojawiła się jakaś strategia robienia zakupów?**

Bardzo się to nie zmieniło, dlatego, że ja wcześniej też najczęściej robiłam zakupy w ciągu dnia - godz. 12/13. Jak pracowałam w salonie kosmetycznym, to jest taka pora, że jest najmniej klientek, więc miałam ten komfort, że mogłam wyjść i zrobić zakupy. Więc ja tak funkcjonowałam i w tej chwili też tak funkcjonuję i to jest też taka pora, gdzie jest najmniej klientów. I tak mi jest najwygodniej, bo to są takie godziny albo obiadowe - no nie wiem, od czego to zależy w sklepie. I ja robię zawsze tak zakupy, że z zapasem. Nie biegnę po jedną rzecz - to bardzo rzadko.

**Kupujesz inne marki niż wcześniej?**

Nie. Bo nawet skończyła mi się chemia i pojechałam do sklepu, w którym zawsze kupuję i dokupiłam rzeczy, po które normalnie bym poszła do Rossmana, ale stwierdziłam, że skoro tam były, to już nie będę biegała i traciła czasu. Byłam szczęśliwa, że po prostu w jednym miejscu zrobiłam hurt. Ale ja tak kupuję zawsze.

**Przed tą sytuacją poszłabyś oddzielnie do Rossmana?**

Nie, chyba aż tak nie.

**Zauważyłaś u ludzi, którzy kupują u ciebie w sklepie, że kupują inaczej?**

To, że jest ilość inna to jest jedna sprawa. Ale np. zaobserwowałam, że dużo więcej sprzedaje się słodyczy. Zdecydowanie - ciastka, ciasteczka, ciasta. W ogóle wszystko zdecydowanie większa ilość się sprzedaje. Takie chrupkie przekąski - paluszki, chipsy, tego typu rzeczy. To przeważa. Owoce u nas zawsze górowały, teraz też trochę więcej się tego sprzedaje, ale to jest tai wyrównany poziom. Bo nawet klienci mówią: dobrze, to te ciasteczka zamienimy na jabłka. Myślą trochę nad tym. Ale zdecydowanie górują słodycze.

**To są zakupy, żeby zrobić sobie przyjemność?**

Tak. To się bardzo zmieniło od tego początku. Bo początek to były takie podstawowe rzeczy, ten ryż, makaron, ziemniaki, warzywa, mrożonki. Natomiast teraz to są bardziej przyjemności.

**Zauważyłaś różnicę w kupowaniu marek premium?**

U nas jest specyficzny klient, który wybiera marki, nie dyskontowe, czy też wyprodukowano dla, tylko to jest taka grupa klientów, która przyjeżdża i musi mieć wyselekcjonowane marki, że nie majonez jakikolwiek, tylko muszą być konkretne marki. Natomiast zmieniło się o tyle, że osoby, które w normalnej sytuacji zwracałyby uwagę na cenę, to biorą, co jest. Trochę to też wynika z tego, że klienci sami mówią, że jak mają stać w Biedronce w kolejce ileś czasu, to szkoda im tego czasu i wolą zapłacić drożej, chociaż to nie zawsze jest drożej. Albo boją się, że nie chcą być w dużej powierzchni, w dużym skupisku. Wolą przyjść do małego sklepu, gdzie widzą, że my pilnujemy, żeby były dwie osoby, że my podajemy towar. Więc już nie patrzą na cenę, tylko że kupują coś lepszego niż zwykle, bo poczucie bezpieczeństwa jest ważniejsze.

**Zauważyłaś zmiany w sposobach płacenia?**

Bardzo się zmieniło, większość przeszła na kartę. Już nawet te wszystkie babcie, staruszki mówią, że *"córka mi kazała płacić kartą*". Więc już się wyedukowały. Trzeba było też więcej czasu poświęcić, żeby nauczyć, pokazać, bo pierwszy raz pin wprowadzały, czy w ogóle pierwszy raz posługiwały się kartą. To się bardzo zmieniło.

**Masz wrażenie, że u tych osób został te nowe sposoby płacenia?**

Myślę, że częściowo tęsknią. Że to jest takie nowe i ok, w tym momencie dostosujemy się, ale myślę, że nie wszyscy.

**A u ciebie coś się zmieniło?**

Tak, zaczęłam częściej korzystać z karty. Ja od niedawna jestem posiadaczem swojej karty i zbiegło się to z tą sytuacją, bo przekornie, trochę się ze mnie śmiali, że jestem jak Kaczyński, że nie mam swojego konta i karty *[śmiech].*Po prostu mam 2 firmowe, gdzie muszę dokonywać przelewów i płatności z karty, więc stwierdziłam, że posiadanie kolejnego konta i płacenie za nie nie jest mi do niczego potrzebne. Ale jak mi się zdarzyło, że stałam w kolejce tylko dlatego, że płaciłam gotówką, nie miałam karty, a to by skróciło mój czas, to mnie to skusiło. Albo jak kupowałam przez Internet i korzystałam z męża konta, ale jego nie było i to mnie ograniczało, więc stwierdziłam, że założę. I więcej zaczęłam korzystać. Pani w aptece mnie poprosiła, czy mogę zapłacić kartą, a chciałam gotówką. Ale powiedziałam, że dobrze, jeżeli wolicie kartą, to tak. Więc więcej tą kartą korzystam.

**Myślisz, że ten nawyk ci zostanie?**

Myślę, że mi tak do końca nie zostanie, bo ja jestem starej daty i lubię, że jak mam tyle, to widzę, dotykam i ja wiem. Natomiast to jest tak rytualne i tak lekko się wydaje, że nie do końca jest to moje.

**Masz większe poczucie kontroli nad wydawanymi pieniędzmi, jak masz je fizycznie?**

Tak.

**Czy coś się zmieniło w zakupach online?**

Ja osobiście nie. Ale np. mąż zamówił więcej rzeczy, których normalnie by nie kupował przez Internet i korzystamy z tego. Bo raz, że nie mamy czasu, a dwa, że jest wygodniej, bo wieczorem można sobie przejrzeć, zobaczyć.

**Co zamówiliście w ciągu ostatnich 2 tygodni?**

Rękawiczki do sklepu, maseczki. Mąż zamawiał bieliznę. I prezent dla syna. Co prawda ten prezent byłby kupiony i tak przez Internet, bo jest najwygodniej.

**A bielizna też byłaby kupiona przez Internet?**

Myślę, że gdyby była taka możliwość, żeby iść do sklepu, to bardziej do sklepu. Ale tutaj mąż się bardziej zmobilizował, żeby przez Internet kupić.

**Coś jeszcze chcieliście kupić?**

Ja chciałam zamówić buty, ale stwierdziłam, że skoro dostałam SMS od zaprzyjaźnionego sklepu lokalnego, że pracują i zapraszają, to zdecydowanie wybrałam tę opcję, że pojechałam do sklepu. Tak na zasadzie wspieramy lokalne firmy - dziewczyny pracują i się ratują. Ale to w ogóle było wielkie przedsięwzięcie, bo wybrałam takie buty, jakie ma koleżanka i wiem, że są dobre, bo ja muszę dotknąć, zobaczyć, przymierzyć. Jestem też leniwa na zasadzie, no może nie leniwa, ale odesłać coś. Zanim ja bym się zebrała, zanim wysłała, minąłby tydzień. Nie wiem, czy bym się wyrobiła w tym czasie, kiedy trzeba odesłać.

**To były buty, których potrzebowałaś? Czy dla przyjemności?**

Tak. Nie poprawa humoru, tylko zdecydowanie. Sezon letni i praca inna.

**Kupiłaś ostatnio jakieś rzeczy na poprawę humoru?**

Nie. I nawet jakoś tak nie mam.

**Jak jest z dostawą zamawianych rzeczy przez Internet?**

Ja jeśli zamawiam, to kurierem. Natomiast mąż się przekonał do paczkomatów i z nich korzysta. Przekonał się wcześniej już.

**Epidemia miała jakiś wpływ na opcje dostawy u was?**

Teraz korzysta z paczkomaty, ale chyba bardziej z tego względu, że... No wybrał paczkomat, nie kuriera. Ale to chyba bardziej, że będzie mógł sobie podjechać w każdej chwili, że będzie wygodniej, żeby nikt nam w domu nie przeszkadzał telefonem, że o tej godzinie musi być ktoś w domu. Odbierze, kiedy będzie mu pasować.

**Macie jakiś system odbioru tych paczek? Dezynfekcja, itp.**

Nie. Nie zanoszę kartonu do kuchni i nie stawiam na stole, tylko rozpakowuję na podłodze najpierw. Ale to zawsze tak robiłam. Czy to jest taki wirus, czy taka bakteria - to, co z zewnątrz do domu, to z ostrożnością zawsze.

**Czy coś się zmieniło w kwestii żywienia?**

Czy coś się zmieniło... *[śmiech].*Mniej gotuję, bo mam mniej czasu i mniej energii na wymyślanie. Owszem zdrowo i to, co zawsze, że te warzywa u nas zawsze królowały. Natomiast mniej twórcza jestem, ale to wynika z braku czasu, siły, itd. Weekendy przejął syn i to, co się zmieniło, to, że mamy kolejny weekend z pizzą. I to jest fajne, bo on wtedy serwuje.

**Ale on robi czy zamawia?**

Robi. Nie zamawiamy jedzenia od czasu...

**Przedtem zamawialiście?**

Ja bardzo mało, ale syn tak. Jakaś pizza, kebab. To jest taki wiek poszukujący. Ja gotuję, więc bardziej wyjścia niż zamawianie. Syn, mąż zamawiali. Natomiast teraz to się skończyło.

**Dlaczego?**

Syn się boi. To zdecydowanie, bo on stwierdził, że marzy mu się kebab, zjadłby go, ale się zastanawia, że skoro ludzie mniej kupują, to to może być nieświeże. On robi świetną pizzę. Ja nie jem takiej zamawianej, bo się zawsze po niej źle czuję. Natomiast bardzo lubię, jak robi sam. Ja gotuję i wolę domowe jedzenie, a on się realizuje, bo rzeczywiście robi dobrą pizzę. No i mamy frajdę z tego.

**A mąż co zamawiał?**

On bardzo mało. Tą pizzę z synem. Ale to właśnie było takie lenistwo, a teraz jest wymuszone, że trzeba zrobić samemu.

**A ty byś obawiała jedzenia zamówionego z zewnątrz?**

Ja tak, ale jak nigdy nie byłam fanką jedzenia zamawianego. Jeśli już, to wolę wyjść do restauracji. Sporadycznie, naprawdę bardzo rzadko. Nigdy nie byłam przekonana do końca.

**Zmieniła się forma posiłków - jecie wszyscy razem?**

Teściowa chętnie obiady gotuje i w tym tygodniu troszkę ulegliśmy, że ona gotuje te obiady. Nie do końca, bo to jest inna kuchnia. Ja też lżej, mniej gotuję. Ja wiem, że ona chce nam dogodzić, że zupa, drugie danie, kisiel - za dużo naraz. Ale jemy razem, bo ona dostosowuje, kiedy możemy się pojawić na obiedzie. Śniadanie każdy je oddzielnie. Wieczorami staramy się razem spotykać i jeść wspólnie. Teraz wieczorami jemy częściej razem niż kiedyś, bo wcześniej ja wieczorami często pracowałam. A teraz kończymy z mężem o tej samej porze, więc automatycznie te wieczory są wspólne i to jest takie na plus.

**Wtedy jecie też we 4 z teściową?**

Nie. Z teściową jest tylko obiad.

**A zmieniły się rzeczy, które jecie?**

Właśnie z tym glutenem mi się trochę umknęło. Bo ja jestem tarczycowa i gluten, i nabiał mi szkodzą. Ale to na zasadzie, że sama bym sobie nie zrobiła, ale jak idę do teściowej i jest makaron pszenny, to go zjem.

**Masz z tym problem czy traktujesz jako dyspensę?**

Niby robię sobie dyspensę i robię to w szczytnym celu, ale nie do końca się z tym dobrze czuje.

**Sama byś sobie tego nie zrobiła?**

Nie.

**Czy u was w domu pojawiły się przyjemności, jak np. słodycze?**

Ja z tym walczę, ale czy się pojawiły? Mąż przyniósł jakieś cukiereczki, których był zakaz. Więc przyniósł sobie. My nadrabiamy orzechami, ale orzechami zawsze zastępowałam, żeby cukiereczków nie było. Ale troszeczkę się to rozmywa. To syn przyniósł jakieś chipsy, właściwie ich nie ruszał, one tak leżały. Ale jak oglądaliśmy jakiś film, to stwierdziliśmy, no może otworzymy tę paczkę chipsów, ale to jest bardziej na zasadzie próbowania - skusić się czy nie. Może na zasadzie, że wynagrodzimy sobie to siedzenie, może nie, ale nie popłynęliśmy jeszcze z tym.

**Ale pojawiły się takie rzeczy w domu, że jest po co sięgnąć?**

Może nie aż tak, bo pilnuję. Nie, nie, takich zapasów nie ma u mnie.

**A sam styl odżywiania się zmienił?**

Nie. Nie mam na to czasu. A poza tym ja uważam, że taka zbilansowana dieta jest potrzebna, żadnych skrajności. Brakuje mi urozmaicenia trochę, ale to wynika z braku czasu. Brakuje mi też takiej - bo ja starałam się jeść systematycznie, żeby nie robić za dużych przerw z zalecenia lekarza. I raz, że mam trochę kaca moralnego, że mi się to rozmywa i źle się z tym czuje tak psychicznie. Ale też czuję, że mój organizm trochę daje znać, że to nie jest tak.

**To wynika ze zmiany twojego trybu życia w tej chwili?**

Tak. Ja nie mam tego czasu w tej chwili. Staram się, że mam jakąś paprykę, którą pokroję, umyję wcześniej. Ale tak, jest problem, bo ja muszę rozebrać się z tych rękawiczek i więcej czasu to zajmuje, dłużej te ręce muszę być i to wszystko zajmuje dłużej niż zwykle.

**Jeszcze coś nowego się pojawiło?**

Nie. Panowie robią sobie, degustują piwa. No tak, to się zmieniło, bo mój mąż nie pije innego alkoholu niż piwo, a piwa to tylko określone sobie wybiera. I w te soboty, jak już mamy taki spokój i mamy pizzę i film, to syn - już wcześniej kupili jakieś gatunki piwa, i piją piwo na pół i sobie smakują, które piwo jest lepsze. Mają taki męski wieczór na taką degustację.

**A jak kupują te piwa?**

Kupuje je syn w lokalnym sklepie.

**Jakie są wasze plany na Wielkanoc?**

Zaczęłam się zastanawiać nad tą Wielkanocą. Plany są takie, że każdy spędza u siebie. Nie spotykamy się z rodzicami, bo nie i mama sama wyszła z taką inicjatywą, żebym nic nie szykowała, bo zawsze ja szykowałam święta. Więc mama od razu powiedziała: My nie przyjeżdżamy, więc nic nie szykuj. Ja powiedziałam, że no tak, przecież się nie spotykamy, więc bez sensu byłoby w święta się spotykać. Więc u mamy już to przeszło, co w pierwszym tygodniu, że ona pojedzie po receptę do przychodni. Teraz już sobie to ułożyła. Córka też nie przyjedzie, ona ma jechać na działkę. Tak naprawdę my jesteśmy największym zagrożeniem dla naszych najbliższych. Teściowa mieszka z nami w jednym domu, więc będziemy razem. Konsekwentnie zostaniemy w domu i nastawiamy się na odpoczynek.

**Przygotowujesz święta?**

Właśnie się nad tym zastanawiałam i to jest dla mnie takie nowe, że nie wiem, jak to będzie Trochę tak stwierdziłam, że... Trochę taki lęk, taka obawa - tutaj miałam tak aż mnie za gardło ścisnęło. Bo jak to nie będzie rodziców. Córka na Wielkanoc w poprzednim roku nie była, ale to było naturalne, że ma swoje plany i coś zorganizowała. Z tym nie miałam problemu. Natomiast tutaj ta myśl, że nie będzie rodziców, to tak mi jest jakoś nie wiem jak. A z drugiej strony nie wyobrażam sobie, żeby po prostu wstać i żeby było bez wspólnego śniadania. Nawet z mężem, z synem.

**Mieliście rytuały świąteczne?**

Tak. Nawet szykowanie tego koszyczka. Dzieci, jak były młodsze, to z babcią zawsze szykowały te jajka i były ich niezliczone ilości. To minęło, ale też jakieś ciasto wielkanocne, co się kojarzy ze świętami. No zupełnie nie mam na to pomysłu.

**I będziesz robiła takie rzeczy, jak mazurki?**

Ja myślę, że zostanę przy tej tradycji takiej. No, może mazurki nie... Chociaż nie no, jeden zrobię, taki nasz ulubiony. To jest tak, jak dzieci były małe, to robiłam pierniczki, których nie lubiłam robić. I robiłam dlatego, że to wypada, że wszyscy robią i to dłubanie, ten bałagan... Ale robiłam to. Robiłam, bo dzieciaki się bawiły, no i fajnie, robiliśmy te pierniczki, więc jak urosły i nie były zainteresowane, to ja odetchnęłam, że uff. Po czym ze trzy lata temu córka stwierdziła, że oni będą robić te pierniczki, że jak to bez pierniczków. No i tutaj, nawet jeśli to się robi nie do końca z takim, że "wow, fajna zabawa", to gdzie tam to zostaje. To zaprocentowało i to, jak oni robią sami teraz te pierniczki - ja już nie muszę w tym na szczęście uczestniczyć. To to jest fajne, że coś dawało to robienie pierniczków, które nie zawsze były zjedzone, bo czasem były twarde jak głaz. Ale to takie minimum, żeby było na święta.

**Mówisz, że zrobisz 1, a zwykle robiłabyś ile mazurków?**

Nie, mazurków za dużo bym nie robiła. Pewnie zrobię szarlotkę, którą wszyscy lubią i to jest ciasto podstawowe na święta i dla wszystkich. Ale znowu też będzie nas mało, więc bez sensu robić tego, nie wiadomo ile, żeby wyjadać. U mnie na święta nie ma takiego przepychu, żeby to zostało i później jeść to tydzień.

**A są takie rzeczy, z których zrezygnujesz?**

Jedyną rzeczą jest to, że się nie spotkamy z dalszą rodziną - tego nie będzie.

**A sprzątanie, dekoracje?**

Kupiłam sobie nawet taki stroik. Przejeżdżałam obok kwiaciarni i kupiłam stroik, bo stwierdziłam, a dlaczego ma nie być takiego stroika, tego zajączka, więc tak sobie kupiłam, tak, żeby był. Właśnie się zastanawiam, może będzie ten koszyczek i będzie stał. Ja mam zawsze świeże kwiaty, bo jest wiosna, więc żonkile, tulipany muszę mieć co tydzień świeże, więc one będą. Tak dużo się chyba nie zmieni... Znaczy dużo - no bardzo dużo, bo nie będziemy razem. Ta cała otoczka chyba się nie zmieni.

**A kwestia święconki i kościoła? Czy to jest ważne?**

Ja to jestem taka przekorna, bo mój mąż się śmieje "ty to nie jesteś taka katoliczka prawdziwa" *[śmiech].*Ja nie mam z tym problemu, że nie pójdę do kościoła z tą święconką. Nie mam też problemu z tym, że nie pójdę do kościoła w święta. Ja z wiary korzystam... No jak to zabrzmiało - korzystam. No korzystam z wiary, ale to nie musi być takie celebrowane, że ja muszę i to musi być ta msza. Może trochę wybiórczo, może nie tak. Ale mnie przekonuje papież. Jego słowa, jak on już dużo wcześniej, że nie będzie tych obchodów, że kościół przychodzi do nas, a nie my do kościoła - no to jest tak moje, że ja w ogóle nie mam z tym problemu. Mnie wręcz, co też moja koleżanka tak przeżywała, że "ojejku, no jak to święta bez koszyczka, bez kościoła" - ale ona chodzi dwa razy w roku do kościoła. Tutaj jest mój zgrzyt, że niedziela palmowa, której nie było... Ja mam takie etapy, że chodzę co niedziela do kościoła z takiej potrzeby, ale jak nie mam potrzeby, to nie idę i nie widzę z tym problemu. Jak chodzę co tydzień, nagle jest niedziela palmowa i wielki tłum, to tak mnie to... A gdzie byliście wcześniej? Teraz nagle idą, bo wypada, bo fajna palemka, bo ten koszyczek. Więc z tym nie mam problemu.

**W tym roku też będziesz miała koszyczek, chociaż niepoświęcony?**

Tak.

**A palemka?**

Też zawsze miałam, a w tym roku nie miałam,

**Pomyślałaś o tym w kategoriach, że ojej, nie ma palemki?**

Nie. Jest taki czas, jest inaczej i po prostu nie ma.

**Czy są w tobie obawy, że ludzie nie będą przestrzegać ograniczeń i się rozjadą na święta?**

Tak, myślę, że tak. Tu są moje obawy. Znaczy myślę, że to się bardzo podzieli. Tak jak ze wszystkim, że część się dostosuje. Tak jak słyszę, że ludzie się nie spotykają, nawet z najbliższą się nie spotykają. Jest też część, że nawet patrząc po zakupach, jakie robią, to nie są zakupy dla 2-3 osobowej rodziny. U nas te święta są zakorzenione jako rodzinne, że trzeba się spotkać, bo jak to się nie spotkać. Tak jak to, że trzeba iść do kościoła, nawet, jak się nie chodziło cały rok, bo w święta to trzeba. Potem narzekają, ale to się trzeba spotkać. Jest to zakorzenione. Ja pamiętam, jak którejś Wielkanocy nie wyjeżdżaliśmy wcześniej na urlop i mąż mówi, że nie mamy czasu wyjechać, to może na Wielkanoc ze znajomymi wyjedźmy. Ja nie mam rodzeństwa, więc rodzice sami. Mąż mówi, że pojedziemy ze znajomymi, dzieciaki na nartach może pojeżdżą. Ja pamiętam, ze ja byłam przerażona, jak ja to powiem rodzicom, że mnie nie będzie na święta. Miałam wyobrażenie, że to trzeba, że moja mama jest przeszczęśliwa, że te święta organizuje. To było przekomiczne, bo dorosła osoba, dzieci, mąż, oddzielna rodzina i ja w końcu musiałam się z tym zmierzyć. I powiedziałam w końcu: "mamo, wyjeżdżamy na Wielkanoc, nas nie będzie". A mama: "ojej, jak dobrze, nie będę w tych garach siedziała, pojadę to tu, to tam". A ja miałam takie wyobrażenie, że te święta to trzeba z rodziną. Teraz wiem, że nie trzeba. Ale myślę, że sporo osób sobie nie wyobraża, że można inaczej.

**Wyjazd na działkę to nie jest złamanie zakazu?**

To jest prywatna, więc można.

**A gdyby nie wyjeżdżała, to przyjeżdżałaby do was?**

Nie, nie widujemy się w tej chwili.

**Jest jeszcze coś, co w tym tygodniu cię uderzyło i chciałabyś coś dodać?**

Myślę, że ten kocioł z wyborami. To jest dla mnie coś, co jest tak abstrakcyjne i oderwane od rzeczywistości, jaka jest tu i teraz i od problemów, z jakimi ludzie się zmagają, z życiem codziennym. Ten wirus już stał się bardziej realny, namacalny, a te wybory to dla mnie taki *science fiction*, że nie pasuje do rzeczywistości.

**Taki opar absurdu?**

Tak. Aż ciężko uwierzyć, że to się dzieje.

**Wzięłabyś udział w takich wyborach, gdyby były?**

Nie. To w ogóle nie.

**Coś jeszcze?**

Na pewno to, że się zmniejsza ilość w moim otoczeniu ludzi. Że mniej samochodów, mniej chodzących, spacerujących. Jest różnica, to widzę. I jeszcze nie dokończyłam taki artykuł o targu w Chinach, tam skąd się miała wziąć epidemia i jeszcze muszę to przeanalizować i dokończyć. Ale to mi dało do myślenia, inne spojrzenie.

**To była nowa informacja?**

To, że stamtąd to się wzięło, to nie. Ale to, jak się odbywają te targi. Epidemiolodzy, biolodzy od dawna przewidywali, że coś wybuchnie. Musiałabym sobie podzielić ten artykuł, bo... No to było tak przerażające, że musiałam podzielić. Ale ma to sens, jak miesza się to wszystko. Azja jest mi daleka i ja zawsze myślałam, że to, że oni tam jedzą różne zwierzęta, wynika z głodu i braku wyboru. A okazuje się, że to jest fanaberia, dla koneserów. I to jak się to odbywa, to rzeczywiście, te wirusy, bakterie, to się przenosi. To nie trzeba być naukowcem, żeby to zobaczyć.

**Czy myślisz o tym w kategoriach, że to kara od natury?**

Ja bym bardziej powiedziała, że zemsta, może bardziej krzyk natury. Nie lubię rozdzielać wina - kara.

**Masz potrzebę poszukiwania przyczyny?**

Przemknęła mi taka refleksja, że może po prostu było nam już za dobrze, nam jako ludziom. Że przestaliśmy doceniać drobne rzeczy i że gdzieś to... No bo, jak przeczytałam, że to milionerzy chcieli zjeść żółwia, bo tak chcą, to nie jest naturalne. Jestem w stanie zrozumieć, że ludzie w łagrach zjadali drugiego człowieka z głodu - to jest instynkt, walka o siebie. Ale jeśli to jest bardzo komercyjne, takie dla swojego, bo już nie wiem, co mam zrobić, to nie. Za daleko to poszło. W artykule to było bardziej pod kątem warunków, jak to się odbywa. Oni przewidywali, że te choroby odzwierzęce mogą mieć tam źródło.
