# Supplementary material for: The challenges arising from the COVID-19 pandemic and the way people deal with them. A qualitative longitudinal study
Source: PLoS One. 2021 Oct 11;16(10):e0258133. doi: 10.1371/journal.pone.0258133 (PMC8504766; doi:10.1371/journal.pone.0258133)
Supplement: S1 Dataset — (ZIP) [file pone.0258133.s003.zip › Transcriptions/stage 2/16.2_F_36_couple, with children.docx]

**16.2_F_36_couple with children**

**Zdjęcia - emocje**

**16, 14, 1**

**16**

Zaczyna się już palić. U nas się jeszcze nie pali, ale już wokół zaczyna się palić. Myślę bardziej o sytuacji gospodarczej kraju, że już zaczyna być coraz gorzej. Już słyszymy, że ludzie zaczynają tracić pracę. Tu, gdzie ja stoję i robię to zdjęcie, to jeszcze jestem bezpieczna, ale już widzę, że drzewa dookoła się palą.

**Jakie to są emocje, uczucie? Co czujesz, jak o tym opowiadasz?**

Taki niepokój, że nadciąga żywioł, katastrofa jakaś i że trzeba się przed tym zdystansować, uciec jakoś.

**Jak doznajesz takiego uczucia, to co ono z tobą robi? Co ono robi z tobą, z twoim ciałem?**

Ja bardziej myślę o tym, nie odczuwam tego jakoś bezpośrednio?

**Masz jakieś sposoby na minimalizowanie tego niepokoju?**

Niekoniecznie, chyba nie umiem na to odpowiedzieć. To jest niepokój związany z analizą sytuacji niż uczucie.

**14**

Ja tu widzę węzeł gordyjski, który jest niemożliwy do rozwiązania. Nie wiadomo jak rozwiązać tę sytuację, w której jesteśmy. Właściwie nie możemy nic zrobić. My, jako ja, jedna osoba i trochę jesteśmy w takim punkcie, że jesteśmy związani, nie wiadomo jak to rozplatać i nie wiadomo, czy ktokolwiek wie, jak rozplątać tę sytuację, jak powrócić do normalności, jaki jest plan. Wydawałoby się, że trzeba by to wszystko przeciąć i zacząć od nowa wszystko ustawiać. Tak bym chciała, żeby było, ale czy to jest możliwe...

**Jakie to są emocje, uczucia?**

Taka bezradność. Wobec tak skomplikowanej sytuacji, że nie wiem, czy ona w ogóle da się rozwiązać. Bezradność, nadmiar komplikacji.

**1**

To jest taka tęsknota za tym, żeby tak było, żeby był korek znowu na mieście. Chciałabym postać w korku. To jest sentyment, zresztą widać, że jest to jakiś kraj dalekowschodni, więc to też jakiś sentyment za daleką podróżą.

**Które z tych emocji teraz dominują?**

Chyba na razie ta 16, niepokój. Na razie jest taka sytuacja, że mam wrażenie, że coś się zaczyna dziać niedobrego. Jeszcze bardziej i coraz bardziej się zbliża do drzwi.

**Co zdarzyło się w ostatnim tygodniu?**

Może nie tak bezpośrednio nas dotykają jeszcze takie rzeczy, ale mamy już głosy od znajomych, że ludzie tracą pracę po prostu. Mój mąż też trochę w usługach pracuje i już mu niektórzy porezygnowali, wynajmujemy mieszkanie i też nam już dziewczyna zrezygnowała z wynajmu, więc też nas to dotyka w takim sensie, że trochę będziemy w plecy, jeśli chodzi o pieniądze. Tak przybliża się...A jakby nie widać końca, nie widać pomysłu i planu jakiegoś. Ja przynajmniej nigdzie nie spotkałam się jeszcze, żeby ktoś jakiś plan podał, jak mamy z tego wszystkiego wychodzić.

**Mówiłaś, że czujesz się bezradna. Masz też poczucie bezradności dookoła?**

Tak. Nie da się realnie...My nie mamy jak - ja, jako ja, my, jako rodzina, w ogóle wpłynąć na tę sytuację, bo to w ogóle nie jest w naszych kompetencjach zarządzić koniec epidemii albo zwalczyć wirusa. Czekamy na decyzję innych ludzi a propos tego, jak będzie wyglądać nasze życie. No i jest taka bezradność.

Czy twoje zachowanie, działania jakoś się zmieniły w ostatnim tygodniu?

Nie, chyba nie. Cały czas robię to, co robiłam. Nic takiego sobie nie przypominam.

**Czy szukasz jakichś sposobów redukcji negatywnych emocji?**

Wczoraj strasznie się zdenerwowałam jak przeczytałam wiadomości. Strasznie mnie zdenerwowało to, co się działo w sejmie, jakieś głupoty powymyślane. Zaczęłam się denerwować i poszłam do męża, żeby mu to pokazać i spytałam, dlaczego jego to nie denerwuje. Powiedział, że też go to denerwuje, ale że to nie jest najważniejsze. Bardzo mnie tym uspokoił, że to nie jest teraz najważniejsze, że nie mamy na to wpływu i że po co się tym denerwować, złościć. Mamy tutaj życie, które się toczy cały czas. Trzeba się skupić na tym, że nasze dzieci dzisiaj coś fajnego zrobiły, że czegoś się nauczyły, coś przeżyły, gdzieś urosły. Tym trzeba się zająć, bo na to mamy wpływ a na tamto wpływu nie mamy, więc nie ma co tracić takiej energii na to. Mnie to bardzo uspokoiło, bo faktycznie miał rację.

**Zastanawiałaś się, na co masz wpływ a na co nie masz?**

My tak żyjemy od dosyć dawna, że na pewne rzeczy nie mamy wpływu i musimy się z tym pogodzić. Czasami, jak tyle rzeczy się dzieje wokół nas, na świecie, to zapominamy o tym i mój maż mi o tym przypomniał wczoraj. Są rzeczy ważne i ważniejsze i nie ma się co denerwować. Mam wpływ na moje najbliższe otoczenie, na ludzi, których znam, na moją rodzinę, przyjaciół. Jakiś pośredni niby wpływ na te rządy mam, ale on jest tak mały, że jakby pomyśleć o tym na serio, to jest aż śmieszne. że mam aż tak mały wpływ. Są rzeczy. Są rzeczy, na które kompletnie nie mam wpływu i np. zdaję sobie z tego sprawę za każdym razem jak wsiadam do samochodu. Mam wpływ na to, jak szybko będę jechać, ale już nie na to, że spotkam pijanego kierowcę i on we mnie walnie.

**Ta sytuacja z koronawirusem to jest takie spotkanie z pijanym kierowcą?**

Tak, właściwie tak, bo mogę się tam jakoś zabezpieczać, żeby ten wirus się do nas nie dostał do domu, ale tak do końca to nie wiem, czy się da odciąć kompletnie. Nie znam się na tym. Myślę, że do końca nie da się od tego odciąć i trzeba się pogodzić z tym, że nas też może to spotkać. Muszę wyjść do sklepu, mogę kogoś potkać...A jak nie ja, to moi rodzice, rodzina, znajomi.

**Mówiłaś, że trzeba wiedzieć, co jest ważne, co nie. Czy zastanawiasz się nad tym teraz?**

Nie, bo ja mam hierarchię ważności ustaloną już dosyć dawno i to na nią nie wpływa.

A to uczucie złości, to jest coś, co pojawia ci się teraz rzadziej, częściej, tak samo?

Pojawiało mi się. Czytam te różne wiadomości i denerwuję się czasami. Teraz wydaje mi się, że jest natłok tego - tych decyzji różnych i tych spraw. Ja przeżywam bardzo jak wydaje mi się, że ktoś gada głupoty. Mój mąż spokojniej jakoś do tego podchodzi. Moi rodzice np. teraz za dużo oglądają tv, bo nie ma teraz sportu, który lubią oglądać. Włączyli jakieś obrady sejmu i moja mama też była taka zdenerwowana. Powiedziałam jej, żeby to wyłączyła, żeby lepiej jakąś książkę sobie przeczytała, bo to bez sensu to oglądać.

**Ostatnie obostrzenia - czy one coś zmieniły w twoim funkcjonowaniu, zachowaniu?**

Oprócz tego, ze czuję się dyskryminowana ze względu na wiek...To była moja pora robienia zakupów. Następnego dnia mąż o 9.30 radośnie mi powiedział, że mogę jechać na zakupy, bo jemu coś odwołano. [śmiech] Od tamtej pory byłam tylko raz na zakupach, zrobiłam większe zakupy, żeby nie musieć jechać po raz drugi, bo...Oprócz tych godzin dla seniorów wprowadzili też jakieś ograniczenia liczby osób w sklepie. Chyba 3 osoby na kasę. Jechałam samochodem i pod każdym sklepem były kolejki, a jak dojechałam do tego, co chciałam, to była gigantyczna kolejka. Pojechałam do Carrefoura. Pojechałam tam, bo dla mojego syna chciałam kupić papier kolorowy, krepinę, itd., żeby jakieś prace ręczne...Palemkę zrobiliśmy na niedzielę palmową. Takich rzeczy w Lidlu czy Biedronce nie ma. jak zobaczyłam tę kolejkę, to byłam załamana, bo mąż mi dał tylko 2 godz. na zakupy. Jak zobaczyłam tę kolejkę, to chciałam się od razu zawinąć i wracać do domu. Ale stwierdziłam, że stanę i najwyżej po godzinie zrezygnuję i wrócę do domu i pojadę w nocy o 12 gdzieś na zakupy. Na szczęście szybko poszło dosyć, więc się wyrobiłam.

**Myślisz o tym, żeby zacząć jeździć nocą do sklepu?**

Nie wiem jeszcze. Zobaczę, bo muszę jutro jechać. Pojadę wcześnie rano, bo potem nie mogę, potem już musiałabym dopiero wieczorem...W sumie wolę chyba rano niż wieczorem...Zobaczę. Ograniczę się już do raz w tyg. teraz i mam nadzieję...Żeby te owoce chociaż były świeże jak pojadę. Przed świętami na pewno pójdę raz i potem dopiero po świętach.

**Ale dlatego, że są te obostrzenia, czy z jakiegoś innego powodu chcesz rzadziej robić zakupy?**

Po prostu nie znoszę tych kolejek. Jak widzę kolejkę, to od razu mam ochotę uciec. Wyzwala to we mnie...Mimo, że ja nie pamiętam tego dokładnie. Nie robiłam zakupów mając 4 lata, ale pamiętam te kolejki i pamiętam to stanie w tych kolejkach. Od razu mi się to kojarzy ze staniem w tych długaśnych kolejkach, w których nic się nie działo, dochodziło się do lady i tam nic nie było w tych sklepach. Pamiętam to naprawdę. [śmiech] Kojarzy mi się to z biedą, z tym, że nic nie ma i że nie można wejść do sklepu i nic kupić normalnie. Mam alergię na te kolejki i strasznie nie lubię. To mnie złości. Strasznie mnie złoszczą kolejki.

**A te inne obostrzenia, które weszły tydzień temu?**

No właśnie jak czytam, to nie do końca wiadomo co wolno, a czego nie wolno. Gdzieś czytam, że nie wolno myć samochodów na myjni, bo mandaty...Ja już z dziećmi nie wychodzę poza osiedle, bo jeszcze jakiegoś kretyna policjanta spotkam. Oni chyba sobie sami decydują, co wolno, czego nie. Nie chce mi się z nimi użerać, więc w zeszłym tygodniu tylko trochę po osiedlu pochodziliśmy, a teraz jest cieplutko, mamy duży balkon, więc sobie siedzimy na balkonie. Przynajmniej dobre to, że mamy ten balkon. Myśmy do lasu i tak w zasadzie nie jeździli i całe nasze życie się kręci w Warszawie, więc nie mamy za bardzo gdzie jeździć poza miasto.

**Co jest teraz dla ciebie największym wyzwaniem?**

Ta cała izolacja mnie wkurza. Nie wiem, na ile jest zabronione spotykać się w rodzinie, ale jednak wszyscy tego przestrzegają. To mnie najbardziej denerwuje. Już chyba nawet zapadła taka decyzja przez aklamację, że święta też spędzamy każdy w swoim domu.

**Znasz osoby, które nie przestrzegają ograniczeń?**

Chyba nie. Wśród znajomych to raczej wszyscy są tylko w swoim gronie rodzinnym. Mam takie sygnały, od niektórych znajomych, że ich rodzice, dziadkowie domagają się tego, żeby się z nimi spotykać, a oni, ci młodzi nie chcą, bo boją się o tych starszych. To mówiła koleżanka, która jest dziennikarzem, więc ona chyba jeszcze chodzi do pracy. Ona mówi, że ona nie pojedzie do swojej mamy i babci, bo się boi i niestety nie ma zrozumienia po drugiej stronie. To ją boli, bo jej mama uważa, że to jest wymówka, że epidemia jest wymówką, żeby nie przyjechać. Dla tej koleżanki to jest bardzo przykre. Ona czuje się niezrozumiana przez swoją rodzinę. To samo mówił kolega, którego rodzice też nalegają, żeby się spotkać na święta. I to jeszcze jego mama jest lekarzem i to ona nalega, żeby os się z nimi spotkał. I jeszcze z babcią. On też mówi, że jest w takiej sytuacji, że po prostu nie wie, co on ma zrobić. Ja też nie umiem mu powiedzieć, co powinien zrobić. On z jednej strony wie, że to jest zagrożenie, ale jednocześnie ufa swojej mamie, która jest lekarzem.

**A gdyby twoi rodzice tak się zachowywali?**

Nie wiem. Na szczęście tacy nie są. Ja starałabym się wytłumaczyć mojej mamie. Naprawdę nie potrafię hipotetycznie odpowiedzieć na to pytanie. Gdyby to mi się przytrafiło to szukałabym rozwiązania. Na pewno moim znajomym nie powiedziałabym co mają robić, czego nie. To jest ich decyzja. To też nie jest tak, że takie spotkanie na pewno kogoś zarazi, że tek ktoś umrze, że to jest katastrofa, itd. Rozumiem, że to trzeba ograniczyć, żeby się nie rozprzestrzeniało, ale też ja nie potępiam takich ludzi.

**Są takie zakazy, obostrzenia, których łamanie uważasz za niedopuszczalne?**

Na pewno bym potępiała nieprzestrzeganie tych zasad w sklepach, jak jest kolejka i ktoś się próbuje wbić, żeby nie stać w tej kolejce, żeby to ominąć czy oszukać w tej kwestii. To by mnie zdenerwowało najbardziej.

**Zaobserwowałaś u siebie jakieś nowe zachowania? Czy robisz jakieś rzeczy, których wcześniej nie robiłaś?**

Nie chyba nie. U znajomych też nie.

**Masz potrzebę poszukiwania dla siebie czegoś nowego?**

Nie mam czasu. Mój dzień się jakoś diametralnie nie zmienił. Oprócz tego, że nie wychodzimy, to codziennie tak samo trzeba zrobić obiad, nakarmić, przewinąć, ubrać, uprać, przeczytać książkę, pomóc zrobić jakieś domowe przedszkole, zagrać.

**Pojawił ci się silniejszy niepokój o sprawy gospodarcze. Czy jakiś jeszcze się nasilił albo pojawiły się nowe obawy?**

Myślę, że nasze państwo coraz gorzej sobie radzi z tym wszystkim. Jeśli chodzi o sytuację światową, to się zaczyna robić coraz ciekawiej, bo już dochodzą głosy o tym przetasowaniu światowym, które może nastąpić. To są te ciekawe czasy, w których się nie powinno żyć. "Obyś nie żył w ciekawych czasach" Ciekawa jest ta obserwacja, ale zobaczymy jaki to będzie miało wpływ na nasze życie - czy pozytywny, czy negatywny, bo to trudno oceniać.

**Środki ostrożności - jak jest teraz? Coś się zmieniło?**

Kazali nosić rękawiczki do sklepów, więc zakładam te rękawiczki. Każą nosić to noszę. Maseczki jeszcze nie. Z tymi maseczkami to też różne są...Chronią/ nie chronią? Powinni chyba nosić ci, co są chorzy chyba. Jest jakiś zamęt, ale...Myślę, że one chyba są dobre i powinno się je nosić, ale póki co jest jak jest.

**Zakupy przez internet - czy coś się u ciebie tu zmieniło w ostatnim czasie?**

No właśnie ja teraz nie robię zakupów przez internet. Zazwyczaj przez internet ja kupuję takie rzeczy ekstra, takie, które nie są pierwszej potrzeby - ekstra herbatę, której nie dostanę w sklepie, kawę, filtry do wody. Dla dzieci też kupuję trochę takich pierdułek, ale to kupuję na Aliexpress - zdrapywanki, wycinanki, naklejki. Raz na jakiś czas robię takie zakupy, ale ostatnio nie, bo jeszcze nie przyszły do mnie poprzednie i nie wiem czy teraz będą jakieś dostawy. Przez ostatnie 2 tyg. nic nie kupiłam przez internet. Kawę już kupuję w sklepie, bo nie bawimy się już w ekstra kawę i herbatę.

**Dlaczego?**

To nie jest na to czas i stwierdziliśmy, że musimy trochę pooszczędzać. Nie ma co szaleć teraz i trzeba się trochę uspokoić.

**Kwestia dostawy ma dla ciebie znaczenie? Kurier/ paczkomat?**

Zwykle był paczkomat u nas, ale teraz już bym chyba nie zamawiała do paczkomatu. To było kiedyś wygodne, bo zawsze jakoś było po drodze. A jak nie wychodzę, to musiałaby, się specjalnie do tego paczkomatu wybierać. Nie wiem jakie są ceny i co się bardziej opłaca. Nie patrzyłam nawet na to, bo stwierdziłam, że nie będę na razie nic kupować. To nie jest tak, że mam jakieś stałe rzeczy, które zawsze kupuję i raczej to jest zbytek.

**Rozważałaś opcję zakupów spożywczych przez internet?**

Rozważałam, ale z tego, co piszą ludzie na portalach, to terminy są na za miesiąc, bo się nie wyrabiają, a ja potrzebuję teraz. Zresztą troszeczkę nie mam zaufania do kupowania warzyw i owoców w ten sposób, bo ja lubię sobie wybrać i boję się, że ki przyjadą nie takie jak ja chcę. Jak wybieram jabłka w sklepie, to ja muszę je powąchać. Teraz oczywiście tego nie robię, bo nie chcę, żeby na mnie patrzyli ludzie źle. Raz chyba kupiłam spożywcze przez internet, ale nie byłam z tego zadowolona. Z warzyw i owoców szczególnie. Oni pakują, co im tam leci pod rękę.

**Czy jakoś zmieniły się ostatnio wasze zwyczaje jedzeniowe w domu?**

Nie, zupełnie nie. Ja staram się kupować takie rzeczy, które kupowałam normalnie i robić to, co normalnie jemy. Teraz w ogóle mamy z mężem taki czas, że jest post i ja w czasie postu nie jem słodyczy i nie pijemy alkoholu w ogóle. Teraz jeszcze nie kupuję niczego do jedzenia dla samej przyjemności, ale za tydzień, jak się spotkamy, to może będzie inaczej. Dałam się namówić mojemu synowi na czekoladki, bo strasznie chciał. Kupiłam mu ptasie mleczko i dałam na deser. Bardzo się ucieszył. Ale dzieciom postu nie robimy. Po prostu to ptasie mleczko to było coś ekstra.

**Czy okres postu odbieracie w tym roku tak samo jak zwykle?**

Ostatnio mąż miał taki moment, że mówił " już kup jakieś piwo, jakieś wino, już się napijmy", ale ja powiedziałam, że tym bardziej ze względu na trudne czasy musimy wytrzymać post.

**A zamawianie jedzenia do domu?**

Zamawiamy jedzenie. Generalnie wolimy chodzić do restauracji niż zamawiać, bo zawsze fajniej jest gdzieś wyjść. Nawet nasze dzieci już są takie, że można z nimi wyjść i nie ma problemu. Zamawiamy takie rzeczy obiadowe albo na kolację.

**Jak to było w czasie ostatnich 2 tyg.?**

Akurat właśnie dzisiaj zamówiliśmy. Zamawiamy tak raz w tygodniu powiedzmy. Zazwyczaj z okazji niedzieli, bo w niedzielę zazwyczaj robimy sobie wieczorem randkę taką domową. Czasem...Dzisiaj akurat odkryłam nową knajpę, która dostarcza i stwierdziliśmy, że dobra, zamawiamy już, bo nic nie ma i musiałabym coś odmrozić, żeby zrobić. Lubimy sobie zamawiać takie dania chińskie, tajskie, bo lubimy taką kuchnię a nie do końca ja umiem coś takiego przygotować. Dzisiaj zamówiliśmy chińszczyznę od takiego taniego Chińczyka i to był nie najlepszy pomysł, ale przynajmniej nie było drogo.

**Czy jest takie zamawianie dla ciebie?**

Dla mnie zawsze każde zamawianie jedzenia to jest święto, bo ja nie muszę robić.  My mamy takie okresy, że czasem częściej, czasem rzadziej zamawiamy.

**A teraz?**

Na początku mieliśmy takie założenie, że już nie zamawiamy, bo trzeba oszczędzać, a potem stwierdziliśmy, że jednak raz na jakiś czas nam się należy, że w sumie trochę do tego przywykliśmy, że raz na jakiś czas jemy coś innego niż to domowe jedzenie.

**Robicie coś innego niż dawniej, jak zamawiacie jedzenie?**

Słyszałam, żeby zamawiać ciepłe a nie sushi, bo na zimnym ten wirus może się bardziej...Więc nie zamawiam już sushi. Wcześniej zamawiałam, bo lubię.

**Coś robisz jeszcze z tym jedzeniem, jak przywiozą, np. podgrzewasz?**

Nie, bo zazwyczaj przychodzi ciepłe. Ci panowie stawiają to na wycieraczce, sami stają trochę dalej. I jeszcze zależy, czy trzeba zapłacić czy nie. Dzisiaj był pan w masce, w rękawiczkach.

**Co ze sposobami płatności?**

Staramy się zamawiać bezpośrednio w restauracji, bo słyszałam, że Pyszne.pl ma dużą prowizję i sama zauważyłam, że na Pyszne czasem dostawa jest płatna a bezpośrednio nie jest. Staram się zawsze wchodzić na stronę restauracji i zamawiać bezpośrednio przez telefon. Przez portal się płaci od razu kartą, a tak jak dzisiaj to chyba płaciliśmy gotówką.

**A jak płacisz w sklepach?**

Teraz kartą. Przedtem raczej gotówką. To są tylko takie względy praktyczno-techniczne, że wcześniej płaciłam gotówką a teraz kartą. Oszczędzam gotówkę można powiedzieć.

**Płacisz kartą kredytową?**

Zwykłą płatniczą.

**Więc i tak ci gotówka schodzi z konta?**

Z konta tak, ale nie z ręki. Uważam, że trzeba dywersyfikować i mieć jakąś rezerwę zawsze.

**Lepiej się czujesz płacąc gotówką czy kartą?**

Właściwie to jest mi wszystko jedno. Nie płacę telefonem, Blikiem. Mam wrażenie, że ktoś mi telefon ukradnie, bo to jest taka rzecz, której ja ciągle szukam, zostawiam go w dziwnych miejscach czasami. Nie znam się na tym i nie wiem, jakie tam są zabezpieczenia i mam wrażenie, że jak mi ktoś ukradnie telefon albo go zgubię, to mi wyczyszczą konto.

**Jakoś bardziej teraz planujesz zakupy niż jeszcze tydzień, dwa temu?**

No właśnie teraz muszę bardziej zaplanować ze względu na święta. Chcę zrobić jakieś takie bardziej świąteczne rzeczy, których nie robię na co dzień, więc muszę sobie przygotować listę, bo nie znam na pamięć tych przepisów.

**Co planujesz na święta?**

Za dużo nie planuję, bo jak będzie nas tylko 4...Chciałabym zrobić sernik, więc muszę wcześniej sprawdzić przepis, bo nie chcę potem w sklepie dłubać w telefonie. Zrobię żurek, więc muszę kupić białą kiełbasę, jajka. Jeszcze trzeba te jajka zabarwić jakoś, żeby mieć jakąś frajdę. Reszta to chyba tak jak normalnie. Co my zjemy w tą czwórkę? Zawsze się dzieliliśmy, co kto robi a teraz...No nie wiem.

**Zrezygnowałaś z robienia czegoś, co zwykle robiłaś?**

Na pewno nie będę robić mazurka.

**Będziecie malować jajka. A jakieś ozdoby świąteczne planujesz w tym roku?**

U nas nie ma tradycji, żeby jakieś prezenty dzieciom dawać. Nie mam takich stroików na Wielkanoc. Zawsze jakieś kwiaty kupowałam - żonkile, bukszpan. Na Wielkanoc nie mam takich ozdób, a mój mąż mi zabrania kupować, bo to bez sensu wydawać pieniądze na takie rzeczy. On nie lubi jak jest tak bez sensu porozwieszane, takie pierdułki po domu.

**Planujesz z okazji świąt kupić coś ekstra?**

Nie. Ja kupuję takie rzeczy jak zawsze. Nie planuję nic specjalnego, oprócz sernika, który jest wyborny.

**Palemka, święconka, wyjście do kościoła?**

Palemkę mamy, zrobiliśmy ją i byliśmy na naszym kanapowym kościele. Siedzieliśmy na kanapie, Julian nawet dzielnie trzymał tę palemkę. Tłumaczyłam mu, że normalnie byśmy poszli do kościoła. Akurat nie było święcenia tych palemek, żeby tej wody nie lać. Kościoła w święta nie będzie. Generalnie jest mi przykro, że nie będzie takich rzeczy, ale jednocześnie uważam, że jest to jedynie część tradycji a nie jakiś dogmat wiary. Post jest rezygnacją z czegoś, jest po to, żeby mnie do czegoś zmusić. To jest takie działanie do wewnątrz, natomiast bez święconki będzie inaczej, ale nie jest tak, że przez to świąt nie będzie, że one się nie liczą. Spędzimy je w domu, zrobimy koszyczek z jajkami, pomalujemy jajka, postawimy to na stole. No tak, nie pójdziemy do kościoła tego dnia, ale są te wszystkie transmisje w tv. My z dziećmi i tak nie poszlibyśmy na Liturgię Wielkiego Piątku, bo zawsze tam były tłumy dzikie. W zeszłym roku byliśmy na Drodze Krzyżowej. W sobotę jest wszystko w nocy, więc i tak byśmy nie poszli, więc właściwie tylko ta święconka i to, że do kościoła w niedzielę nie pójdziemy.

**A to, że nie idzie się do spowiedzi, do komunii? Masz poczucie dyskomfortu z tego powodu, jakiejś straty?**

Dla mnie to są jakieś zewnętrzne rzeczy. Uważam, że bez tego można przeżyć. Te rzeczy nie są konieczne. Ważniejsze jest to, co ja mam w środku a nie to, co robię na zewnątrz. W ogóle mnie nie ogranicza ten kult zewnętrzny w mojej relacji z Panem Bogiem. On mi pomaga w jakiś tam sposób, ale to nie jest tak, że to jest rzecz konieczna dla mnie. Uważam, że na tym polega całe chrześcijaństwo - że to ja mam relację z Panem Bogiem. Codziennie przez cały dzień. pójście do kościoła jest ważne, ale nie jest najważniejsze.

Myślę, że już się ludzi oswoili z myślą, że w tym roku tak będzie, choć niektórzy na pewno mają z tym kłopot. Ci biskupi zaczęli wyjaśniać różne rzeczy, niektórzy księża też.

**Obawiasz się tego, że ludzie zaczną się jednak gromadzić w kościołach?**

Nie, raczej nie, bo wydaje mi się, że te 5 osób zostało, a wprowadzili to ograniczenie 50, jeśli chodzi o koncelebrę, żeby nie było tak, że nagle mamy 100 ministrantów.  %0 jest ze względu na to, że są różne zgromadzenia zakonne, itd.  Dobrze, że to ograniczenie się stało na długo przed świętami i już ludzie się z tym oswoili. Jest bardzo dużo internetowych transmisji mszy, rekolekcji, te wszystkie liturgie też mają być transmitowane. Ten przykład z Watykanu też poszedł, że Papież też sam będzie spędzał święta.
