# Supplementary material for: The challenges arising from the COVID-19 pandemic and the way people deal with them. A qualitative longitudinal study
Source: PLoS One. 2021 Oct 11;16(10):e0258133. doi: 10.1371/journal.pone.0258133 (PMC8504766; doi:10.1371/journal.pone.0258133)
Supplement: S1 Dataset — (ZIP) [file pone.0258133.s003.zip › Transcriptions/stage 2/11.2_M_35_couple, with child.docx]

**11.2_M_35_couple with child**

**Co się u Ciebie zmieniło od momentu, kiedy się widzieliśmy?**

Nic się u mnie nie zmieniło. Jestem zmęczony tym koronawirusem.

**Czym najbardziej?**

Sytuacją całą, tą samą co zawsze. Nie można wyjść, nie można się spotkać z ludźmi, nie można iść na spacer. I tak wychodzimy, ale w miarę krótko i w miarę rozsądnie.

**Jesteście na wsi?**

Tak, dzisiaj przyjechaliśmy. W sobotę pojechaliśmy, dziś wróciliśmy (wtorek).

**Na święta pojechaliście?**

Można tak powiedzieć, o ile w ogóle to były święta.

**Opowiedz mi o twoim zmęczeniu.**

Nie mamy możliwości poruszania się, ruchu. Pewnie w ten weekend byśmy gdzieś wyjechali dalej. Już kombinowałem, że jak nas zatrzymają, gdzieś na trasie, to będę mówił, że jedziemy do wujka do Świnoujścia, który jest sam, jest starszy i jedziemy mu pomóc. Ale jakoś nigdzie nie pojechaliśmy. No męczące to jest ogólnie. Brak możliwości w naturalny sposób poruszania się czy to autem, czy pieszo, w moim przypadku jest... irytujący? Nie wiem.

**Wyglądasz na zmęczonego sytuacją.**

Zmęczony jest ogólnie po dwóch leniwych dniach leżenia w salonie, grania z dzieciakiem i oglądania filmów, bo nic więcej się nie robiło. To nie jest w naszym przypadku codzienność albo życie, które nam odpowiada. Siedzenie w zamknięciu raczej nie.

**Co jeszcze ci przeszkadza?**

Chodzenie w rękawiczkach. A zaraz będzie chodzenie w maskach.

**Co było najbardziej wkurzające w poprzednim tygodniu?**

Dla mnie i dla nas to ogólnie... Nie wiem, my tak bardzo nie widzimy różnicy, bo my zajmujemy się cały czas lokalnie stacją i mamy te same problemy, które mieliśmy i albo troszkę więcej albo trochę mniej. My nie jesteśmy zmęczeni sobą, nie mamy problemu znaleźć sobie jakieś tam dzieciakowi zajęcia. Zresztą jak ma lekcje, to go nie ma. My ogólnie nie mamy takiego problemu, że coś jest męczące albo, że bardzo bardzo się różni nasze życie od tego, co było. Ale de facto nam nie odpowiada na pewno siedzenie w domu, bierne spędzanie czasu.

**Ostatnio byłeś pozytywniejszy. Co się zmieniło?**

Już pewnie w te święta z dwudniową czy trzydniową przerwą, to byśmy gdzieś wyjechali. No wiesz, hotele zamknięte, no to tak trochę jest... Świąt w ogóle nie było, nie odczuwało się. My nie odczuliśmy świąt. Nie byliśmy ani w kościele, ani ze święconką ani nic z tym związane. Jedyne, co było wspólnego ze świętami, to obiad. Coś tam jest zabierane tego normalnego życia. Pytałem się ludzi, czy byli w kościele, to są tacy, którzy codziennie chodzili do kościoła. Ja nie czułem tych świąt. To były dla mnie normalne dni wolne. A jak wolne, to obiecałem małemu, ze względu na to, że w tygodniu nie miałem czasu, że pogram z nim. Więc siedziałem pół dnia na graniu, pół dnia na oglądaniu filmu. Więc tak trochę kiepsko.

**To wynikało z tej sytuacji?**

Tak, tylko i wyłącznie. Na pewno byśmy nie siedzieli w domu. To raczej nie my. Ukradkiem gdzieś tam z tyłu poszliśmy sobie na spacer, tylnym wyjściem z osiedla. Ale to 10 minut spaceru i tyle. Dla mnie to jest męczące.

**A czemu poszliście ukradkiem?**

Nie wiem. My jak przyjechaliśmy do Warszawy, to pierwsze, co usłyszeliśmy rano przez głośniki, to dla mnie był szok, jak okno otworzyliśmy. I koło naszego osiedla faktycznie się te służby kręciły. A Marek, jak się dowiedział, że to jest 3 tys. zł mandatu, to się bał, że będzie musiał dać z kieszonkowego.

**Ale i tak go wzięliście na spacer?**

Tak. Poszliśmy do Żabki i z powrotem.

**To spacer do sklepu?**

Na lody. O, właśnie, poszliśmy na lody.

**Co najbardziej wpływa na to, że czujesz, że normalne życie jest ci zabierane?**

W naszym przypadku głównym elementem jest to siedzenie w domu. Narzucenie siedzenia w domu. Innych chyba nie odczuwam. Dla nas to wszystko, bo my sobie jedziemy autem z tej wsi do Warszawy i u nas niewiele trzeba, żebyśmy zamiast tych dwóch dni w domu, pojechali gdzieś dalej. Zazwyczaj te wyjazdy są mocno spontaniczne, 2-3 dni, bo dłużej niestety nie możemy. I mamy tak, że jedziemy i jest myśl: "gdzie jedziemy? nad morze czy w góry?" a tu dupa, za przeproszeniem. Ja z drugiej strony bardzo lubię... Może nie jestem takim uczęszczającym cyklicznie chrześcijaninem, natomiast święta są taką rzeczą dość dla mnie istotną. A tu mi tych świąt brakowało.

**Obrazki. Który z obrazków najlepiej oddaje twoje emocje w ostatnich dniach?**

Cały tydzień miałem bardzo ciężki i bardzo intensywny, więc ja bym dał 3, bo skojarzyło mi się z bieżnią. Bo cały tydzień zapierniczałem. Nie patrząc na pozostałe uczucia, bo na nie nie miałem czasu. Ta 3 jest taka odpowiednia, bo schodziło mi ze wszystkim do później nocy.

**A dzisiejsze emocje?**

Dzisiaj się mogę czuć kiepsko, bo jestem zmęczony leżeniem. Poza tym, jak nie ma słońca, to ja inaczej funkcjonuję. Jest wietrznie, ja nie lubię zimna. Dla mnie, jak jest zimno, to może nie być dnia. Może to będzie 7. 7 może się z jakąś dziwną drogą kojarzyć. 11 to smutno, nie wiem... Nie czuję się smutno, ale nie jest to najlepszy dzień.

**Który z tych obrazków najbardziej pokazuje twoje wkurzenie?**

Tylko 16. Akurat rzeczy przy 15 to uwielbiam oglądać. Tak, bo ciężko mnie wyprowadzić z równowagi, więc 9 odpada. Raczej 16, bo gotuje się we mnie i lepiej do mnie nie podchodzić.

**Jak sobie radzisz z tym, że się w tobie gotuje?**

Idę do lodówki, biorę 3 kostki lodu, Martini i tyle. Nie mam nic lepszego.

**Ile takich kuracji zastosowałeś w zeszłym tygodniu?**

Nie wiem, 6 na 7 dni, nie wiem. Myślę, że z 5 dni to na spokojnie. Martini jest więcej wieczorem niż go nie ma.

**Jak w tej wściekłości twojej odnajduje się Ewelina i Marek?**

Ja zawsze się zachowuję tak samo. Ja nie jestem osobą, która krzyczy, nie z tych trzęsących się. Ale czasami jest moment, że mówią do siebie, no to jest chwila, w której lepiej się do Oskara nie odzywać.

**Jak oni się odnajdują w tej sytuacji, że trzeba nie wychodzić?**

Mamy to szczęście, że nie jesteśmy zmęczeni sobą. Więc nam nie robi to wielkiej różnicy. I Marek się odnajduje, bo zaraz znajdziemy mu coś do zabawy, i Ewelina się odnajduje. Więc wspólnie sobie dajemy radę. Nie ma u nas takiej, że cała 3 chodzi jak tykająca bomba.

**Ewelina się tak nie wkurza jak ty?**

Wydaje mi się, że podobnie. Nam się ani nie zmniejszyło, ani nie zwiększyło obowiązków, ani nie zmieniły się diametralnie. Jedyne, co doszło Ewelince, chociaż ja obiecałem wychowawczyni, że będę to robił, to jest siedzenie z dzieckiem podczas odrabiania lekcji.

**Dlaczego ty nie siedzisz z Markiem nad lekcjami?**

Wykorzystuję ten czas [że jesteśmy razem za wsi] i zajmuję się stacją. Bo to Ewelinka [zazwyczaj] przyjeżdża i zajmuje się stacją, a ja siedzę w Warszawie. Ale jak już jesteśmy razem, to jest tyle rzeczy do zrobienia, że się siedzi i robi.

**Łatwiejsze jest dla ciebie zajmowanie się stacją czy Markiem?**

Hmm... Nie widzę problemu ani tu, ani tu, natomiast Marek by nie chciał. Bo jak Marek ma problem albo mu się nie chce odrabiać lekcji, to Ewelinka mówi, że zaraz Oskarek przyjdzie.

**Czyli jesteś takim postrachem?**

Nie, ja jestem konsekwentnym człowiekiem i jak mi pisze brzydko zdanie, to powtarza, aż napisze ładnie. I tyle. Taki terrorysta.

**Czyli postrach?**

Muszę się Marka zapytać, ale może postrach, nie wiem. Chcę go po prostu nauczyć, że jak cokolwiek robi i się podejmuje, to żeby to robił dobrze.

**Jeszcze jakieś ograniczenia cię denerwują? Np. limit osób w sklepie?**

Mnie to nie dotyczy. Ja to widzę, ale tylko widzę z auta. Nie tyczy mnie to. Ja nie mam z tym problemu. Tak jak byliśmy na spacerze w sklepie, to przed tą Żabką czekaliśmy aż wyjdą ludzie, ale u mnie to jest na tyle sporadyczne, że mnie to nie frustruje. To był pierwszy raz, kiedy stałem w kolejce od koronawirusa. To do mnie na całe szczęście nie dotarło.

**Jak widzisz z samochodu tych ludzi, którzy stoją, to co myślisz?**

To jest kiepskie, bo myślę też o klientach, którzy stoją na stacji. Więc ja patrzę na to dwojako: od tego, co nam nakazują i od strony klienta. To jest trudne, nie ma co się kłamać. Tym bardziej, że może jakby było słoneczko, było przyjemnie. U nas jest o tyle dobrze, że ludzie w samochodach czekają. A np. pod Biedronką widziałem, że kolejka bym długa. To jest na pewno trudne, męczące i poniekąd uwłaczające.

**Uwłaczające dla ludzi, którzy muszą stać?**

Tak mi się wydaje.

**Myślisz, że oni też tak o tym myślą?**

Nie wiem. Ja bym to pieprznął i bym nie stał. Ja tego nie rozumiem.

**Pytałeś co ludzie na stacji sądzą o tym, że muszą czekać?**

97% jak najbardziej to rozumie i nie narzeka. 2% to są osoby, które może jednak, bo jest zimno, nie rozumieją całego, skąd to się wzięło. Spotkałem ze dwie osoby, które narzekały i były to osoby starsze, bo im najbardziej będzie doskwierało stanie na wietrze. Młodemu też to będzie doskwierało, ale on albo tego nie powie albo ma dłuższą wytrzymałość. Chociaż różnie to bywa. Większość osób to rozumie. Ja się zastanawiałem, co będzie, jak będą godziny tylko dla osób starszych. Uważałem, że to był dobry pomysł, ale tutaj ludzie w innym wieku mogą mieć powody do złości, że wyłączają ich z tego. Ale to nie był głupi pomysł moim zdaniem.

**Ci ludzie mają prawo do tej złości, że są wyłączeni?**

Ogólnie mamy do wszystkiego prawo. Każdy ma prawo do swoich nerwów i swojej frustracji. Ja uważam, że to jest naturalne. Słuchaj, każdemu jest teraz trudno. Jeden to przechodzi mniej, drugi bardziej. Tak, jak są różni ludzie, tak w innej skali każdy to odbiera. I jest laba. Myślę, że ta laba wpłynie na nas jakoś mentalnie i to będzie trwało. Ale może się okazać, że za jakiś rok, pomimo, że nie będzie już wirusa w takich ilościach, tylko w takich ilościach jak grypa, do okiełznania lekami, to nie będziemy patrzeć na Azjatów, chodzących w maskach normalnie, że to nie jest coś nienormalnego. To będzie naturalne. Kto będzie chciał, założy dla własnego bezpieczeństwa. Może to pozytywnie na nas wpłynie.

**Byli tacy, co codziennie chodzili do kościoła - co o nich myślisz?**

Będę szczery. Pytałem celowo ludzi, którzy pracują na stacji, żeby im zamknąć usta, gdyby chcieli krótsze godziny pracy czy coś. Pytałem mojej mamy, która jest mega katolicka i dla niej są duże święta rodzinne związane z kościołem. Wolałbym, żeby nie poszła niż miała iść. Ale celowo się zapytałem pracowników, bo nie lubię hipokryzji. Albo się czegoś trzymamy od A do Z albo nie trzymajmy się. Dla mnie święta są rzeczą ważną i z chęcią bym poszedł z rodzinką do kościoła. Ale nie poszedłem naturalnie, mając świadomość, że może być tam skupisko osób i starszych i w ogóle skupisko osób. Mi nie przeszło przez myśl, żeby iść do kościoła. A co uważam o ludziach, którzy chodzą? Ja jestem wierzącą osobą i uważam, że gdyby poszli sami na łąkę i się pomodlili to jeszcze większą by zrobili dobroć sobie i temu, do kogo się modlą. Nie pytam, do kogo się modlą. Zrobiliby sobie, swojej rodzinie dużo lepiej. Uważam, że chodzenie do kościoła w tym okresie jest tak samo głupim uczynkiem, jak chodzenie na imprezy. Na równo to traktujemy. Pomimo tego, że uważam się za osobę wierzącą.

**A o co chodzi z tymi pracownikami?**

Ja wpadam i pytam o różne rzeczy wyrywkowo, które później zlepiam w całość i później ma obraz danej osoby. A z pracownikami powinno się to robić często. Robię to celowo, bo później sobie to szufladkuję i mam w swojej bazie danych w głowie, a tą bazę danych wykorzystuję do różnych celów. Jest osoba, która jest najbardziej przestraszona z pracowników i widać było ten lęk. Nawet ze względu na tą osobę uważam, że podjęliśmy trochę za szybko decyzję z pewnymi sprawami organizacyjnymi na stacji. Po czym tę samą osobę pytam później, czy była w kościele i ona mi mówi, że była w sobotę, niedzielę i poniedziałek. Więc ja muszę mieć to w głowie, że jak będzie się bała, to muszę to zrównoważyć. Ja jestem człowiekiem i jak widzę, jak ktoś się lęka, to reaguję jak człowiek do człowieka. Tak zareagowałem wtedy i teraz jestem w stanie powiedzieć, nie wiedząc, że ta osoba ma do końca świadomość tego. Albo jakiś wpływ społeczny ją ściska w głowie, że tu się nie może odnaleźć i jest zlęknięta. Ale potrafi wziąć do kościoła całą rodzinę i tu nie ma takich negatywnych myśli. Ja zbieram takie dane. To pomaga w podejmowaniu decyzji, zarządzaniu biznesem. Była tak sytuacja, co już mówiłem - nie u nas akurat, że pracownica wyleciała ze sklepu z piskiem, rozebrała się z tych ubrań roboczych, bo nie wytrzymała jakiejś presji. Też trzeba brać pod uwagę, że oni cały czas mają kontakt z ludźmi, ktoś kaszle, siedzi, ktoś im zabiera, wymienia produkt. Różne zachowania mogą wystąpić. Ale kiedy spokojnie chcemy coś wprowadzić, a ta osoba naciska, to mogę być typowo osobą... Biznesowo podejść do tego. I mogę powiedzieć: przestań lamentować, bo tu jesteś przestraszona, a tam chodzisz do kościoła.

**Czy to chodzi o to, że chcesz ich edukować, tłumaczyć sytuację?**

Miałem z nimi kilka rozmów, w których to ja jestem osobą najmniej zdenerwowaną sytuacją. Ja jestem dobry gracz, więc wszystko przyjmuję, przytakuję i na spokojnie mogę wypowiadać się bez żadnej nerwowości, podtekstów, uszczypliwości. I jestem w stanie z nimi pogadać... Miałem kilka razy sposobność rozmawiać, że kilka razy ich uspokajałem. W moim mniemaniu, uważam, że dosyć specyficznie myślą i fajnie, jak ktoś spoza im powie, że może tak, czy może siak. Że może ten koronawirus to nie jest koniec świata. Wszystko powinno się z dystansem przyjmować. Miałem kilka rozmów, w których ich uspokajałem i na pewno - może nie edukowałem, bo nie uważam się za taką osobę, która mogłaby edukować, ale żeby inaczej spojrzeli na wszystko dookoła.

**Zaobserwowałeś jakieś dziwne zachowania wśród ludzi?**

Lubię obserwować ludzi i pewnie każdą osobę, którą spotykam, lubię chwile popatrzeć na nich i poobserwować i każdy inaczej się zachowuje. Natomiast ja nie oceniam tego w ten sposób dziwnie - niedziwnie. Każdy ma swój sposób bycia. Widać różnice po osobach, które były rozrywkowe, gadatliwe, a teraz zupełnie wyalienowani, jakby sobie nie dawali rady z sytuacją albo bardzo się nią przejmowali. I sporo jest takich osób. I to są osoby na różnych szczeblach zawodowych, różnorodni intelektualnie. Ale jest dużo takich. Są osoby, które przeszły ze skrajności w skrajność. Ich obecne zachowanie jest przynajmniej o 120 stopni inne niż było.

**Jakiś przykład?**

Tą osobę, o której ci powiedziałem, która z daleka jest osobą, potrafiącą rozmawiać z ludźmi i na tyle się zbliżyć do innych, że po chwili może poklepać po ramieniu kogoś, z kim nawiązała dobre relacje. A teraz ludzie są dla niej, nie wiem... Każdy człowiek to jedna wielka bakteria. I to nie jest odległość 2 metry, tylko wchodzi, patrzy, ile jest osób w sklepie i jak jest 3 to nie wejdzie. Bo uważa, że 2 to za dużo. Mam przykład mojego szwagra, który ogólnie jest taki inteligentny, twarz facet. Jest sędzią, więc z natury silny człowiek o silnym usposobieniu. Kilka dni temu się przeziębił i faktycznie przeziębił, bo miał robione badania. Siostra też jest sędzią, więc jak się dowiedzieli, że on jest przeziębiony to ją na zwolnienie, a oni wszyscy zostali przebadani. To powiem ci, że on już prawie się żegnał z nią, że taki stan. Może to jest zabawne - nie chciałbym być w jego sytuacji ani w mojej siostry. Ale nie uwierzyłbym, że on, twardy facet już się żegnał, chciał przechodzić na tamten świat.

**On robi teraz jakieś nieadekwatne rzeczy do sytuacji?**

Nie znam na bieżąco czy ma jakieś odchyły. Na pewno obydwoje siedzą w domu, nie wychodzą. W zeszłym tygodniu jeszcze wychodzili na spacery, tak w tym tygodniu nawet Paweł okna nie chce wietrzyć. Słyszałem przez telefon, że nawet okien nie otwierają.

**A jak robią zakupy?**

Nie wiem, nie mam pojęcia. Uważam, że to jest dobry moment na robienie zakupów online. Ja, jakbym miał stać w kolejce, to bym zamawiał wszystko online. Aczkolwiek powiem ci, że byliśmy w Warszawie i pierwsze co, to fast food. I pierwszy raz miałem kłopot z odebraniem żywności przy drzwiach.

**Dlaczego?**

Otworzyłem te drzwi. Jestem przyzwyczajony, że zazwyczaj robią to cudzoziemcy. Otworzyłem te drzwi, spojrzałem, mówię: Boże, jak ja teraz się z nim wymienię tym jedzeniem. Do tego stopnia, że zamknąłem te drzwi, rozerwałem reklamówkę, wyciągnąłem zafoliowane posiłki i zostawiłem je przy drzwiach, a reklamówki się od razu pozbyłem. I myłem ręce. To faktycznie, powiem ci, że tu u mnie zmiana jest *[śmiech]*.

**Spodziewałeś się, że tak zareagujesz?**

Nie.

**Później powtórzyłeś zakup czy już nie?**

Nie no, powtórzyłem.

**Udało się później już to opanować?**

Tak. Powiem ci, że sam się zdziwiłem. Do momentu, jak otworzyłem drzwi. W momencie jak otworzyłem drzwi nagle konsternacja i jak to zrobić. Spojrzałem, że ma rękawiczki i pomyślałem, ciekawe, kiedy zmieniał te rękawiczki.

**Myślałeś o tym, żeby założyć rękawiczki podczas brania jedzenia?**

Myślałem, ale przyjąłem cały posiłek do rąk i tyle. Natomiast ten dostawca wiecznie ma problem z pin-padem, więc i tak wziąłem do okna ten terminal, żeby płatność przeszła i już machnąłem ręką. Nici z tego, może przeżyjemy.

**A dlaczego zamówiliście fast food?**

Bo jesteśmy nałogowymi osobami jedzącymi na telefon.

**Jesz przez telefon tylko jak jesteś w Warszawie?**

Tak. Aczkolwiek, teraz będąc przez te dwa dni, wczoraj albo dziś rano Ewelinka mnie poinformowała, że nic nie zamówiliśmy przez telefon. Więc grubo. Niedziela i poniedziałek nic.

**Zamówiliście z tej samej, co zazwyczaj?**

Tak. Mamy swoich ulubionych 4 czy 5 knajp. Jak się codziennie jada, to z jednej to nie bardzo.

**Czego się bałeś przy odbiorze jedzenia?**

Nie wiem. Wszędzie. Ja akurat wtedy brałem kebaba. A jak widzę, jak oni tego kebaba robią, to on był wszędzie.

**Ale to nie przeszkodziło w zamówieniu?**

Nałogowemu człowiekowi wiele rzeczy nie przeszkadza.

**Jak jedliście przez ostatnie 2 tygodnie?**

Na wsi sobie normalnie gotujemy. Przed świętami Ewelinka i jej mama przygotowała na tyle jedzenia i mieliśmy tyle rzeczy, a resztę sobie przygotowaliśmy na miejscu. To też były święta, więc trochę normalniejsze posiłki, nie *fast foodowe*, czas żeby co innego zjeść.

**Jakie to są normalne posiłki?**

Dla mnie normalny posiłek, to jest z miejsca mojego pochodzenia. Czyli posiłki polskie jadam w święta. I niedziela mi się kojarzy z polskim posiłkiem. Jak byłem dzieckiem, to zawsze miałem polski posiłek w niedzielę: rosół, kotlet, ziemniaki, jakiś kurczak. Normalny posiłek to jest polski posiłek. Wszystko inne zamawiamy telefonicznie i nam przychodzi.

**Co dziś jadłeś od rana?**

Dwie kawy, paczkę papierosów, żurek pyszny z dokładką.

**Jadasz śniadania?**

Zależy co pierwsze czy kawa, czy śniadanie. Jadam. Natomiast, jak jestem na coś sfokusowany, jak mam jakieś zajęcie, to ciężko mnie zwabić do kuchni.

**Jak się zmieniło wasze jedzenie?**

Jemy nieco, może nie zdrowiej, ale naturalniej. Przygotowujemy własne posiłki i nie są bardzo wymyślne, bo się na wymyślne nie chce czasu spędzać, bo szkoda. Jemy normalnie i w miarę wiemy, co jemy.

**Daj mi przykłady normalnych posiłków.**

Jemy ostatnio dużo zup, a nie jedliśmy. To jest pozytywne, bo nie jadaliśmy tak dużo zup. Ale żurki, pomidorówki. Rosół u nas jest zawsze, bo musi być, ale nie każdy nazywa rosół zupą. Zup doszło sporo w menu. Mały zawsze je to samo - u niego nie ma różnicy. Normalne jedzenie? Polskie jakieś, ziemniaki. My też różnie jemy, bo ja zjem rybę, oni już nie zjedzą. U nas 3 osoby przy stole i 3 osoby jedzą co innego.

**Jak jeszcze można określić to, jak jecie teraz?**

Na pewno moi jedzą zdrowiej. Ja też w sumie, bo jak te zupy jadam to jest zdrowsze.

**Jak na co dzień jecie gotowe posiłki, to one są mniej zdrowe?**

Na pewno zupy byśmy nie zamówili w knajpie, żeby nam przywieźli, tylko na miejscu. Więc zupy nam odchodzą, a z tego, co wiem, są zdrowe dla żołądka. mamy ten komfort, że kupujemy wędlinę robioną rzemieślniczo, a nie masowo. Też często jadamy dziczyznę. Więc jeśli my przygotowujemy posiłki, to jemy zdrowiej, bo mamy możliwość doboru ziemniaków, które wiemy, jakiego są pochodzenia. Warzywa tak samo wiemy, jakiego są pochodzenia, jak już się decydujemy na robienie przez siebie samych. Ja ogólnie nie jem zdrowo. Moja jajecznica to jajka w tłuszczu.

**Zamawiasz coś przez Internet?**

Nienawidzę zamawiać przez Internet. Bo jestem zwykłym facetem - muszę dotknąć, zobaczyć i kupię.

**Ale teraz trudno jest dotknąć, zobaczyć.**

Powiem ci, że ubrania mam coraz gorsze. Już zwróciła mi Ewelinka uwagę, że mi kupi przez Internet dresy po domu. A ja nie mam z tym problemu, że są zniszczone. I tak nie wychodzimy, i tak siedzę sam w zamknięciu. Jak otworzą galerię, to znaczy, że koronawirus minął, więc wtedy będziemy mogli chodzić po ciuchach. Ewelinka kupiła mi coś przez Internet kiedyś. Ja nigdy bym nie kupił.

**A dla Marka kupujesz przez Internet?**

Karty Pokemon.

**A Ewelina kupuje przez Internet?**

Tak.

**A co kupuje?**

Nie widziałem AGD, żeby kupowała przez Internet, a tak to chyba wszystko. Nie ma wpływu koronawirus - wszystko potrafi kupić przez Internet.

**Teraz dostajecie więcej paczek?**

Dobre pytanie, zastanawiam się. Ostatnia paczka przyszła dzisiaj, a przedostatnia? W zeszłym tygodniu, w czwartek chyba. Może faktycznie jest więcej. Nie zastanawiałem się nad tym, ale może faktycznie. Patrząc na ostatnie 2 tygodnie, to tak.

**A tobie nie przyszło do głowy, żeby coś kupić?**

Nie, bo ja pojechałem do sklepu. Kupowałem narzędzia.

**Jaki to był sklep?**

Ja jestem biednym człowiekiem. Nie wydaję na rzeczy nieprofesjonalne. Byłem w sklepie profesjonalnym. I tam się obkupiłem.

**W jaki sposób ludzie teraz płacą [u niego na stacji]?**

Zwiększyło się kartą. O dziwo, bo tu jest rejon, gdzie ludzie nie lubią płacić kartą. Ludzie chyba na wsi, mając gotówkę, odczuwają większą wartość pieniądza. I tu bardzo długo nie było kart, bo nie było takiej potrzeby. Teraz już jest częściej.

**Umiesz oszacować proporcje?**

Zwiększyło się tak o 1/5 kartą. To jest sporo, bo tutaj są ludzie, którzy zajeżdżają samochodem i nie są stąd - ta część płaciła kartą. A teraz się to zmienia na ludzi, którzy tu mieszkają. To jest de facto dużo procentowo.

**Myślisz, że oni to robią ze względów bezpieczeństwa?**

Myślę, że to bezpieczeństwo jest na drugim, trzecim miejscu. Wszędzie jest łatwiej płacić kartą. Dużo osób posiadało kartą i z niej nie korzystało, bo nie chcieli. Potrafili sobie wypłacać pieniądze przed zakupami, żeby zrobić zakupy gotówką. To trochę im ułatwia, żeby płacić teraz kartą. Samo spędzenie czasu jest krótsze, nie musisz walczyć z drobnymi. Fajnie, ludzie się do tego przekonują. Ja bym wolał, żeby 100% płaciło kartą.

**Oni płacą zbliżeniowo?**

Ciężko mi rozgraniczyć. Zdarza się tak i tak. Sama kwota 100 zł to duża kwota bez PIN. Więc można zrobić tych zakupów. Nie mówimy o dużych zakupach na weekend, tylko o bieżących.

**Uważasz, że ludzie teraz inaczej kupują?**

Te koszyki zmieniły się tylko w momencie tego boomu. żywność długoterminowa, papier toaletowy, takie rzeczy. Natomiast teraz to wraca do normy. Jedyne, czego do tej pory nie włączyliśmy, to nie włączyliśmy posiłków fast food na stacji typu hot-dog. Był duży spadek i nie chcemy próbować na razie. Dzisiaj rozmawiałem z człowiekiem, który zajmuje się lodami gałkowymi na mazowieckie i oni mają porażkę w tym roku. Więc widzę, że tego typu produkty mają spadek. Ale u nas nie jest tego aż tak dużo, że bym widział zmianę koszyków. My na razie tego nie wprowadzamy, włączyliśmy, bo nam spadło strasznie i nie było sensu tego trzymać. A ogólnie koszyki wróciły chyba do normy wszędzie.

**A ludzie się inaczej zachowują w sklepie?**

Tego już mi program nie patrzy. Nie mam czasu na tyle być w sklepie. Dla komfortu kupujących i własnego, nie dodaje kolejnej osoby na sklepie przez swoją osobę.

**Masz poczucie, że w okolicy ludzie źle odbierają te limity?**

Spotkałem chyba jedną osobę, która się skarżyła, ale to osoba, która ogólnie się skarży, że świat tak wygląda, więc jej bym nie brał pod uwagę. Zauważyłem jedno. Ludzie teraz często podjeżdżają, widzą kolejkę i odjeżdżają. Nie chce im się stać. Załatwi to może w innym miejscu. Dzisiaj nawet zauważyłem, że potrafią podjechać i odjechać. Akurat ta osoba wróciła po jakimś czasie. Ale pewnie jest teraz tak w większości miejsc, że sprawdzają, jaka jest kolejka i odjeżdżają. Albo wreszcie się może nauczą korzystać z Google, że tam można sobie sprawdzić, jakie są kolejki w sklepach. Ja nigdy nie sprawdzałem na stacji czy u nas pokazuje, ale w Warszawie sprawdzam. I przed koronawirusem sprawdzałem też. Jednocześnie to idzie z lękiem, strachem, że koronawirus nas pozabija, a nie że wszyscy ogłupieli i dajcie spokój. Na wsi chyba lęk przed wirusem wypiera nieodpowiedzialne rzeczy. Aczkolwiek trafiają się ludzie, którzy wchodzą - różnie jest.

**Dlaczego pojechaliście na Wielkanoc do Warszawy?**

Bo nam jest tam lepiej.

**Ostatnio mówiłeś, że wsi macie ogród, świeże powietrze...**

Tak, tylko cały czas jest świadomość, że jesteśmy cały czas w motłochu swojej pracy. Za siatką mamy stację. Więc są telefony, różne takie rzeczy. Lepiej mi się tam śpi. Młody wie, że tutaj nie mam dla niego czasu, a tam znajdę czas.

**Jak wyglądały wasze 3 dni w Warszawie?**

W sobotę skończyłem tutaj o 22:00. Tak naprawdę tylko sen. W niedzielę spaliśmy do oporu. Ewelinka jeszcze chyba dłużej nawet. Ja z Markiem grałem i oglądaliśmy filmy. Jak my graliśmy, to Ewelinka robiła jedzenie, a jak nie graliśmy, to oglądaliśmy filmy.

**Jedliście coś świątecznego?**

Były świąteczne elementy z jajeczkiem. Zupka świąteczna. Ale nawet dom był nieubrany świątecznie. Ja to lubię na przykład. Lany poniedziałek - mówię po południu do Marka, że może bym cię oblał wodą, jest lany poniedziałek. A on: tak? lany poniedziałek jest? Totalna porażka. Tego się nie czuje. Tak to by się wyszło na ulicę. Może poszłoby się coś kupić do galerii. Może w tych galeriach już są jakieś pisaneczki, pioseneczki, itd. Pewnie gdzieś by się widziało palmy w tamtym tygodniu, to już by się człowiek powoli nakręcał. A teraz nic. Jedyne, co nas zszokowało, to na naszym osiedlu... Mamy takie dość specyficzne osiedle, gdzie nikt nikogo nie znał. Każdy ma swoje życie. Czasem się zdarza, że ktoś powie dzień dobry. A my w szoku - w niedzielę wychodzimy, a tam wszyscy dzień dobry, sąsiedzi na dole się znają, wspólne picie alkoholu, rozmowy do godziny 12:00. Oczy wielkie, ludzie dostają psikusa i wychodzą do siebie. W takim sensie, że każdy na swoim balkoniku oczywiście, ale okno w okno ludzie ze sobą gadają. Jakiś sąsiad z dołu wyniósł jakiś bimber, zaczął polewać, wszyscy z nim gadają - oczy wielkie. Na osiedlu, na którym tylko widać dzieci, bawiące się na telefonach, nic więcej. Nagle - o, kurde. Więc fajnie.

**Co jeszcze świątecznego przygotowaliście?**

Nic. Nie zrobiliśmy jakiegoś specjalnego obiadu sobie. Tylko następnego dnia dopiero zjadłem sałatkę. Więc wszystko strasznie rozmyte. W naszym przypadku nie było czuć świąt.

**Myśleliście, żeby coś sobie kupić dla przyjemności?**

Nie było takich pomysłów. My dla przyjemności wyjeżdżamy.

**Ale nie można wyjechać. Coś innego na poprawę humoru?**

Może to płytko zabrzmi, ale my sobie poprawiamy sami humor. Nie mamy takich planów nigdy.

**A czego najbardziej ci zabrakło w tych świętach?**

Nie wiem, ja się nie wczułem, więc nie zrobiliśmy klimatu. Brakowało mi tego klimatu. Bo to trwa tylko 2 dni, ale te 2 dni może trwać dużo odpoczynku, siły, nakręcenia się. Ja lubię się wczuwać w święta. Galerie to jedno, ale nie czuliśmy w domu świąt. Żeby poczuć święta, to wychodzi się zobaczyć lampki. Jak brakuje mi śniegu, to już coś nie gra. Jak święta, to Kevin sam w domu. Nie mówię o filmie, tylko o śniegu i świecących lampkach.

**Jakie są ważne elementy Wielkanocy?**

Nie było rzeżuchy, bazi, zajączka. Nie zrobiliśmy tego. Nic. Wyłączyliśmy te święta.

**Czy na święta się spotykacie z rodziną?**

Tak.

**Brakowało ci tego?**

Ja nie mam z tym problemu czy mi brakowało czy nie. Nie wiem, jak Ewelinka. Ale też chyba nie brakowało. To chodzi o taki klimat w swoim domu. My bardzo często na święta wyjeżdżamy, więc nie spotykamy się z rodziną. Od jakiegoś czasu, to wszystkie święta jesteśmy gdzieś.

**Dlaczego tej rzeżuchy nie wysialiście?**

My tego nie czuliśmy i tego nie zrobiliśmy. Ja przybieram dom na święta zazwyczaj. Gdzieś to mi umknęło całkowicie. Nie było tego.

**Nie rozumiem, dlaczego ci to umknęło?**

Nie wiem. Ciężko mi jest odpowiedzieć. Zapracowanym jest się naturalnie, natomiast... No nie idziesz ulicą, nie widzisz za witrażem jakichś dekoracji - tego się nie widzi. Nie wczułem się. U mnie nie jest tak, że jest data w kalendarzu i są święta. U mnie jest proces, w który ja się wkręcam. Do tego stopnia, że u nas nic nie ma, a nagle jedzie Marek z Ewelinką, przyjeżdżają i jest cały dom ubrany. Więc jakiś proces, w którym ja się przygotowuję.

**Co jest zapalnikiem procesu?**

To może być cokolwiek. Niedzielna msza, spacer sobotni - coś, że to jest już ten moment. Tu nie było tego zapalnika. Po prostu od rana do wieczora pracowaliśmy codziennie. Nagle weekend i jedźmy stąd. Wysyłali mi ludzie życzenia, a ja nie składałem, tylko pisałem ok, dzięki. Dla mnie święta to nie jest ta data, tylko jakiś proces w głowie i w otoczeniu. Nie pykło.

**I nie dało się pyknąć?**

U mnie się tak nie da.

**Marek odczuł, że są święta?**

Dla niego to był weekend, po prostu, nic więcej. W poniedziałek po południu, spytał się, o Boże, to jest lany poniedziałek?

**Nie zrobiło ci się przykro, że się nie zorientował, że były święta?**

Nie. Nie pomyślałem o tym, że ja coś zdupczyłem, tylko mi się przykro zrobiło, że to jest taki czas. Przeszły te święta obok nas. Nie wiem, co jest powodem, ale przeszły. Dobrze, że z tobą pogadałem, to dało mi to do myślenia.
