# Supplementary material for: The challenges arising from the COVID-19 pandemic and the way people deal with them. A qualitative longitudinal study
Source: PLoS One. 2021 Oct 11;16(10):e0258133. doi: 10.1371/journal.pone.0258133 (PMC8504766; doi:10.1371/journal.pone.0258133)
Supplement: S1 Dataset — (ZIP) [file pone.0258133.s003.zip › Transcriptions/stage 2/3.2_F_54_single.docx]

**3.2_F_54_single**

**Coś się zmieniło od ostatniego tygodnia?**

Zmieniło się to, że przyjechała do mnie córka.

**Miała w piątek przyjechać.**

Tak jak mówię, tak się stało właśnie. A miała przyjechać i pracować tutaj online i zostać już ze mną. A prawie wszystko się udało oprócz tego, że ją zwolnili z pracy jednak. To znaczy, no, firma zawiesiła, bo to jest centrum targowe PTAK. Więc oni zawiesili po prostu działalność i chyba wszystkich pracowników na umowach cywilno-prawnych, po prostu te umowy zostały rozwiązane przedwcześnie. A reszta pracowników została, nie wiem, zawieszona, zwolniona, cokolwiek. Wszystko jedno. W każdym razie moim zdaniem, no oprócz tego, że oczywiście jej jest przykro, bo to nie jest miła sytuacja, to powiedziała, że z naszego punktu widzenia jest super po prostu. Obecnie z naszego punktu widzenia. My jesteśmy razem, wyrąbista pogoda, mamy, wie pani, hektar obejścia, który wymaga jakichś prac. No i jesteśmy dwie i obie to lubimy. Po prostu… A jeszcze na dodatek… No, więc tak się zmieniło, jest bosko, no.

**Emocje – zdjęcia.**

Ale mówimy o w ogóle moich emocjach czy emocjach związanych z sytuacją obecną?

**Myślę, że możemy pogadać o obu, bo myślę, że one się zazębiają trochę.**

Ja myślę, że one się w ogóle nie zazębiają akurat (śmiech). One są po prostu tak różne w tej chwili. One się jakby połączyły w tym sensie, że obie wynika z tego, że panuje pandemia. Ale jakby osobiście to się zmieniło w tym sensie, że ja jestem po prostu w lepszej kondycji. I jak mam dziecko przy sobie jedno, to mam super.

**To emocje związane z tym, że Julka jest z panią, to który to byłby obrazek?**

Myślę, że ten las taki z tym słońcem.

**6. A emocje związane z sytuacją, z pandemią, z tym, że jest ten koronawirus dalej.**

Jest tak i jest dziura w gospodarce i jest dupa wołowa ogólnie rzecz biorąc.

**To który obrazek najlepiej te emocje pokazuje, to, co pani czuje?**

To może ta 8.

**Zacznijmy od 6. Ona jest pozytywna?**

Tak, zdecydowanie jest pozytywna.

**Co to byłaby za emocja, co to byłyby za uczucia?**

Wie pani co, to jest tak. Jest takie większe poczucie bezpieczeństwa na pewno. Bo jesteśmy razem i się nie muszę martwić o córkę w takiej chwili. Bo jest ze mną i jakby we dwie zawsze nam się, znaczy mnie się lepiej funkcjonuje, myślę też, jak jesteśmy razem. No i co, no wie pani, jak mi już odeszło pół niepokoju, to myślę, że tak lepiej.

**No właśnie, bo w zeszłym tygodniu było tak, że ten lęk się składał z dwóch aspektów. Tego lęku o dzieci i trochę aspektu finansowego. Ale ten lęk o dzieci był taki przeważający. Teraz odszedł lęk, bo Julka jest z panią, więc ten jeden odpadł.**

No wie pani, Julka jest ze mną, odpadł może nie całkiem, bo jeszcze drugie dziecko mam. Ale tamto dziecko nie martwię się, bo są zaopiekowani jakby sobą. A Juleczka sama z chorym kotem. Teraz mam cały komplet tutaj. Znaczy Julkę z chorym kotem, w tym sensie.

**Mamy jeszcze tą 8, która bardziej odnosi się do sytuacji koronawirusowej. Tu jakie są emocje? Jeśli odszedł lęk o córkę, to co zostało?**

No wie pani, zostało jakby, niepewność taka tego, co się stanie, niepewność o taką najbliższą przyszłość, w sensie takim bardzo namacalnym, czyli ekonomicznym. Tak naprawdę najbardziej ekonomicznym. Bo tutaj, na tej wsi mamy taki, jesteśmy tak daleko tak naprawdę od sytuacji bieżącej, że no na pewno nie martwię się o jakieś osoby, o znajomych. Bo nic nie wiem o tym, żeby chorowały. A tak naprawdę to tutaj jak się nie chce, to się nie ma kontaktu z rzeczywistością, taką otaczającą. Tak że no jest taki strach o to, co będzie. Trochę mnie teraz…. Znaczy, no nawet trudno, że denerwują powiedzieć, trochę mnie wkurza. Trochę mnie wkurza a trochę mnie smuci tak naprawdę to, co się dzieje w polityce, tak? Ale to nie jest dla mnie jakieś zaskoczenie, bo ja jestem za duża na to, żeby to mnie zaskakiwało. Chociaż to, że ci ludzie po prostu wszyscy walczą z pandemią, a my kombinujemy jak tu koniecznie wybrać pana Dudę drugi raz na następną kadencję, to mi się rzygać chce po prostu, wie pani. Zwyczajnie i po prostu.

**To zaraz do tego wrócimy. Ale chciałam o tej niepewności trochę pogadać. Pani mówi, niepewność tego, co się stanie w tej najbliższej przyszłości ekonomicznej. A jak sobie radzić z tymi emocjami? Co pani robi, żeby to nie było tak, że to cały czas gdzieś się telepie z tyłu głowy?**

Wie pani co, ja mam, powiedziałabym dość… Myślę, że ja nic nie robię. Ja mam po prostu dobrą sytuację. Jestem pewnie w lepszej sytuacji niż bardzo wiele osób. To znaczy ja jestem co prawda zależna finansowo od mojego męża, który mi płaci alimenty. Ale po pierwsze on jest bardzo sprawny biznesowo. Jakby mnie to nie dotyczy, ja nie straciłam źródła dochodu. Ale oczywiście mam z tyłu głowy to, że ponieważ u niego sytuacja na pewno jest dużo gorsza, więc ja mogę, nie wiem, nie mieć takiej sytuacji za miesiąc czy za 2, jak mam teraz. Niemniej jednak mnie w dalszym ciągu stać na wszystko, nie wali mi się na głowę żadna firma na przykład. Nie wiem, nie miałam restauracji, baru ani nic takiego, co spowodowało, że przestałam mieć źródło utrzymania. Więc myślę, że to nie jest jakaś żadna moja zasługa, że ja sobie z tym radzę. No, może też nie mam takiej skłonności do zamartwiania się po prostu wszystkim, co się może ewentualnie kiedyś w życiu albo mnie, albo komuś z mojej rodziny stać. Nie jestem tak skonstruowana. Nie powiem, żebym była jakaś beztroska szczególnie. Ale też nie mam jakiegoś takiego, nie wiem, jak to się nazywa w ogóle, ale nie jestem, pod tym względem staram się jakby patrzeć do przodu i ewentualnie zmieniać to, na co mam wpływ. Chociaż rzeczywiście łatwo się mówi, jak się ma za co pójść do sklepu i nie ma problemu. Wiem, że taka postawa mogłaby się zmienić szybko, gdyby się okazało, że po prostu nie mam za co. I wcale bym pewnie nie była taka radosna i pogodna, jak jestem.

**A możemy też chwilę porozmawiać o tym, jak to jest z emocjami u Julki? Jak ona w tej sytuacji się odnalazła?**

Tak, znaczy mówimy o tej pracy?

**O pracy z jednej strony, ale z drugiej strony o tym, że zmienia środowisko, tak? Bo ona jednak z Warszawy przyjechała na czas pewnie dłuższy niż te kilka dni.**

Tak, zdecydowanie. Mam nadzieję, że to jest kwestia miesiąca albo więcej. Bo to się szybko nie zmieni. A ją nic nie ciągnie z powrotem, w sensie jakichś zobowiązań. Ja myślę, że tak, na pewno po pierwszym, takim bardzo nieprzyjemnym momencie, jak nas zwalniają z pracy, na który zresztą reaguje dość zawsze chyba bardzo adekwatnie, czyli była wkurzona po prostu maksymalnie na to, co się stało, jak to się stało itd., to… Myślę, że podeszła tak, wie pani, w sumie mówi, to dobrze, że i tak do ciebie jadę, to się tak złożyło, że ona i tak miała przyjechać. Więc to nie jest jakaś zmiana planów czy cokolwiek. Ale jak przyjechała tutaj, to dla niej to jest, myślę, ogromna zmiana. Po pierwsze dobrze się czuje psychicznie. Wychodzi, świeci słońce, ćwierkają ptaszki. Ona była miesiąc w zasadzie zamknięta w czterech ścianach z kotem. Nawet w pewnym momencie zarządziła sobie własną taką kwarantannę, bo miała podejrzenia, że jej koleżanka jest chora. Więc nawet ktoś tam jej robił zakupy, zostawiał jej na klatce schodowej. Mieszkając sama w domu, na pięćdziesięciu paru metrach, no kot jest, ale ja myślę, że można oszaleć po prostu. Ona sobie oczywiście bardzo dobrze radziła, znaczy młodzież. W sensie takim, że spotykali się na Skypie, grali w jakieś gry, na jakichś platformach wspólnie i coś. No to jest wszystko super, ale takiego kontaktu fizycznego po prostu jest brak, takiego spojrzenia, dotknięcia. Więc myślę, że ona tutaj odetchnęła. Przytulamy się bez przerwy prawie. Robimy rzeczy razem, chociaż nie musimy robić tej samej rzeczy. Robimy je na dużej przestrzeni, widzimy się. Nie wiem, ona gotuje coś, ja coś robię, jakieś jajka wielkanocne komuś. No, krótko mówiąc, spędzamy super czas. Mamy kartkę z rzeczami do zrobienia dłuższą na pewno niż miesiąc, więc nie ma problemu. A na razie jest super. Ja nie wątpię, że kiedyś przyjdzie taki moment, ponieważ obie mieszkamy jednak same, że możemy mieć jakieś zwarcie. Albo możemy mieć się po prostu zwyczajnie dosyć. Ale to nie jest 52 metry. 50 metrów to tu jest mieszkania, ale mamy tu hektar miejsca, prywatny las, po którym jak rozumiem, nawet pan Kaczyński nie może mi zabronić chodzić. W której nie ma nikogo oprócz mnie i saren. Więc niech spadają. Na pewno się tu nie zgotujemy w czterech ścianach. I też, wie pani co, też mnie, prawda w sensie takim, że nie musi się ona zamartwiać w takim sensie, że gdzieś poszłam czy coś. Bo chociaż myślę, że ona się bardziej martwi, bo teraz widzi, że ja gdzieś idę, na jakieś zakupy na przykład. A wtedy tylko słyszała, że idę (śmiech).

**A teraz wszystkie wyjścia widzi.**

Tak, wszystkie wyjścia, no właśnie, wszystkie, tak. Ale naprawdę myślę, że tutaj, nawet ona mówi, już prawie nie oglądam wiadomości, dosłownie włączam na 10 minut jak jej nie ma, bo ona mówi: mamo, ja już tego nie mogę oglądać. Więc w ogóle nie oglądamy. A jak w ogóle nie oglądamy tych wiadomości, to tutaj te informacje w ogóle nie występują jakby. Więc oprócz, jak się wychodzi do sklepu, no to tak, bo ma pani ten porządek jakiś tam funkcjonowania w tych kolejkach. Ale gdyby nie to, to jak byśmy tak tu były na wsi tylko, to byśmy nie wiedziały, czy jest wirus, czy się zwiększa czy się zmniejsza, czy cokolwiek. Na pewno jest dużo zdrowiem. I dla psychiki. I szansa, oczywiście poza wszystkim, szansa zarażenia jest na pewno dużo mniejsza. Ale dla psychiki to jest świetne po prostu.

**A czy jeszcze coś się nowego pojawiło w ostatnim tygodniu, poza tym, że przyjechała córka, poza tym, że spędzacie dużo czasu razem?**

(śmiech) Dużo nowych rzeczy w ogóle nie ma tak naprawdę teraz. Bo człowiek jest taki trochę wyizolowany, to jest odłączony. Ja miałam to szczęście, że przyjechała Julka, w związku z powyższym dla mnie się coś nowego zdarzyło. Ale tak generalnie to ludzie się nudzą trochę w tych domach.

**No właśnie, jak tu jest w pani okolicy. Bo pani jest takim dobrym źródłem wiedzy o tym, co się dzieje poza dużym miastem.**

Wie pani co, tutaj jakby… Jak by to pani powiedzieć. Ci, co siedzą w domach, czyli ci rolnicy, nazwijmy ich, tak? To im się dużo nie różni. Oprócz tego, że tam sobie smarkają i jęczą w sprawie tych zakupów. Bo tak, no to pojechali, to mogli pojechać i kupić, kiedy chcieli. I wracali do domu. A teraz to jest troszkę nie tak. Bo rano, no to rano, a potem między 10 a 12 to już teraz tylko seniorzy, więc nie można wejść do sklepu. A potem to jest kolejka duża. A potem to jest zaraz przerwa techniczna. Więc są nieszczęśliwi. Ale się przystosują do tej sytuacji. Bo to nie jest takie… Wie pani, oni nawet, jak są wkurzeni, to nie jest tak, że nie wiem, on będzie wkurzony 2 dni z tego powodu. Nie. Poszedł, nie kupił, dobra, no. Tak tutaj taka… No jest jak jest, bym powiedziała, jest bardziej zgodne jakby z ich… Nie wiem, oni nie wojują. Jest jak jest i trzeba się do tego przyzwyczaić. Tam sobie coś posmęcą pod nosem, ale nie wejdą do takiego sklepu, jak nie można na przykład. Nie będą się starali tam jakoś przemknąć czy coś. Jest jak jest. I bardzo się nie denerwują tą sytuacją. A ci, co pracują, no to oni bardziej. Bo gdzieś jeżdżą, do tego Płocka albo gdzie indziej, to może oni się bardziej wtedy tym, dla nich to jest jakaś różnica. Ale ja tutaj nie mam dużo takich osób. Więc nie mogę powiedzieć. U sąsiadów też jakieś na przykład remonty teraz są wykonywane (przerwa techniczna spowodowana pojawieniem się kota). No, nie wiem, o czym rozmawiałyśmy

**O tym, co się dzieje w okolicy, że remonty się pojawiły. To wynika z tego, że oni nie wychodzą do pracy?**

Nie, ale pojawiły się takie remonty, wie pani, że jak oni są w domu… Pani nie wiem, tutaj remonty to się przeprowadza we własnym zakresie. Więc panowie, jak są w domu teraz, niektórzy, bo ci co zawsze są w domu i piją, to dalej robią to samo i to się w ogóle nie zmienia. Natomiast ci, którzy pracowali i na przykład zjechali teraz i zostali w domu, to na przykład u mojej jednej sąsiadki widzę, że jest remont łazienki. Więc tam jakieś kafelki, coś tam. Z pomocą jakiegoś kolegi, co przywiózł tam teścia. Czyli w ścisłym takim gronie rodzinnym no coś tam robią sobie takiego, na co wcześniej nie było czasu po prostu. Czy tam było zaplanowane na przykład na lato albo na kiedyś, to teraz jest wykonywane. Na przykład remont u jednej mojej sąsiadki. Druga moja sąsiadka, która ma dziecko w wieku szkolnym po prostu mówi, że wzięła zwolnienie, nie pracuje, jest na zwolnieniu, bo poza wszystkim jest też chora. To jest po prostu poruszona rolą, którą obecnie pełni, czyli rolą nauczyciela. Mówi, że bardzo im się poprawiły oceny. Ponieważ ona wykonuje część prac osobiście. I to ją bardzo cieszy. Wie pani co, ja uważam, że to jest świetne. Ponieważ dla mnie ten system oświaty jest tak absolutnie abstrakcyjnie niedopasowany do tego, co się dzieje na wsiach, że ja zawsze uważałam, że moja recepta, jak ktoś się do mnie zwraca o pomoc dziecku w lekcjach czy w czymś tam, to ja zawsze mówię, że to jest absolutnie absurdalne, czego oni muszą się uczyć tutaj. Oczywiście tego samego, co w mieście. Ale to jest, no to jest absurd. To, że oni się uczą takich rzeczy i sposób, no to szkoda gadać w ogóle. To jest absolutnie nieadekwatne do ich możliwości. Do tego, co im się ewentualnie kiedykolwiek w życiu mogłoby przydać. No więc, ja mówię do niej, żeby się cieszyła ,że czyta o Mieszku I, bo na pewno nie pamięta, jak czytała sama o tym Mieszku 40 lat temu. A teraz przynajmniej czyta to z przyjemnością. I dzwoni do mnie, żeby mnie poinformować, że ten Mieszko to był straszny kobieciarz i ile lasek on miał oprócz tej Dąbrówki itd. No mówię, widzisz, historia jest ciekawa, tylko nie w wieku 10 lat po prostu. No i takie mamy tutaj spostrzeżenia. Tak sobie tutaj żyjemy, generalnie w miarę spokojnie.

**A myśli pani, że u pani sąsiadów tych najbliższych jest w ogóle jakikolwiek lęk związany z tą sytuacją?**

U moich najbliższych sąsiadów… No nie wiem, ostatnio nie rozmawiałam z taką najbliższą, najstarszą sąsiadką, już ze 4 dni z nią nie rozmawiałam albo 5. Miała taki moment, że była taka poruszona, bo coś odsłuchała, z nudów jakieś wiadomości w telewizji. To tam było rzeczywiście… Trudno mi powiedzieć. Myślę, że nie za bardzo. No, są poddani tym restrykcjom, no więc wiedzą, że coś się dzieje. Ale cały czas, jak rozmawiamy, to mówią, że a jak to się już uspokoi, no to oni coś tam zrobią. Czyli może się tym nie przejmują, ale wiedzą, że coś się dzieje. Bo są rzeczy, których nie można robić. Albo też mam sąsiadkę jedną, która ma jakieś tam komunalne mieszkanie w Gdańsku, bo kiedyś ją tam los rzucił. No to mi opowiada, że jak ona już tutaj, bo tam jej lokator jakiś mieszkał w tej kawalerce, no ale w tej chwili się wyprowadził. No to jak to się już wszystko skończy, to ona pojedzie do tego Gdańska i tam coś załatwi. Więc jak to się już wszystko skończy czy uspokoi. Więc jest to coś, ale moim zdaniem to coś jakby ich nie dotyczy. I myślę, że mają dużo racji w tym, że ich to nie dotyczy. Że to nie jest zupełnie bezpodstawne. Bo tutaj, w Płocku mamy 4 przypadki koronawirusa. Czy też mieliśmy ze 3 dni temu. Bo teraz to naprawdę muszę pani powiedzieć, że od kiedy jest Julka, to nawet, jak by mnie pani przepytała, ile jest osób zakażonych itd., no to chyba wczoraj widziałam, że 111 zgonów. Ale tylko dlatego to pamiętam, że mam pamięć do cyferek. Chyba. Bo dzisiaj na przykład to się nie skaziłam telewizją rano. Bo co chwila się wyłączał prąd, więc myślę sobie, a, dobra, nie będę tego oglądać. A Płock to jest największa koło nas aglomeracja miejska. Więc jeżeli tam mamy 4 przypadku. No plus tam oczywiście ileś ludzi siedzi w domach z temperaturą i z czymś, ale gdzie oni w ogóle nie mają żadnej szansy nawet na to, żeby się dowiedzieć, czy są chorzy. Może za pół roku będą mieli szansę się dowiedzieć, czyli byli, jeśli w ogóle ich to będzie interesować na przykład. Albo jak zostaną przeprowadzone jakieś, nie wiem, narodowe testy czy cokolwiek. Natomiast tak, to rzeczywiście. Jakaś pani wczoraj w sklepie była, ten wózek odprowadzałam, wózeczek do sklepu. I ona wystartowała do mnie po ten wózek z gołymi rękami. Ja mówię, może pani chociaż by jakieś rękawiczki założyła. A ona mówi: a, to w sklepie będą. Ja mówię: no będą w sklepie, ale jak pani dotknie teraz już tą poręcz tego wózka bez tych rękawiczek, to w zasadzie może pani ich nie zakładać, nie? A, ona się tego nie boi, bo u nas tego nie ma. Nie, tutaj tego nie ma.

**To fajne podejście, powiedziałabym, tutaj tego nie ma (śmiech).**

Ja jej mówię: no wie pani co, jeszcze nie ma. Do czasu, aż się pojawi jakiś przypadek. To mówię szkoda nie… Ale dobra, no co, dorosła osoba. Ale młoda dziewczyna. Nie nastolatka, w żadnym wypadku, bo teraz nie ma w ogóle dzieci samodzielnych. No, ale… Ale faktem jest, że jakby trudno im w to uwierzyć. Bo to się tak trochę dzieje, jak u nas na początku moim zdaniem. Czyli to jest tam gdzieś. W takich Chinach albo we Włoszech. To dla nich to są, z punktu widzenia tutejszej ludności, to to jest… Ja pani podam przykład, moja sąsiadka mówi: a, bo mój syn to wyjechał do Australii. Ten syn jest kierowcą TIR-a. Ja mówię, to ja jej zrobię zakupy. A ja mówię Lucyna, do Australii to on chyba nie pojechał tym TIR-em, może do Austrii? A, no może, ale ona się na tym nie zna, mi mówi (śmiech). Więc rozumie pani? Więc czy to są Włochy, czy to są Chiny, czy to jest na przykład… No, może Czechy, Niemcy, to tam niektórzy jeżdżą do pracy, to oni wiedzą. Ale to jest zasadniczo tak samo daleko. W sensie psychicznym. Po prostu. Oni nie mają żadnego kontaktu z tym miejscem. Ani wyobrażenia, ani nigdy tam nie byli, ani nigdy tam nie pojadą, ani nie mają takich marzeń, żeby w ogóle tam pojechać. Więc co? No nie ma tego tutaj. Takie mają do tego podejście. No, do pierwszego przypadku oczywiście to się stanie. I wtedy będzie katastrofa. Bo widzę, że nie potrafią. Używanie rękawiczek, chodzenie w tych rękawiczkach cały dzień, w tych samych. Jak ona idzie do sklepu w tych rękawiczkach, potem wychodzi, dotyka samochodu, kierownicy, wysiada… To co tu dużo mówić.

**Czyli nie spełniają swojej funkcji te rękawiczki?**

W większości przypadków nie. Jak ja patrzę, jak ci ludzie… No jedną panią widziałam, która wyszła ze sklepu, otworzyła sobie bagażnik samochodu za pomocą pilota. Wsadziła tam torbę z zakupami, po czym zdjęła rękawiczki. I to w prawidłowy sposób, czyli nie dotykając, tylko środkową część, zdjęła je do góry nogami, podeszła do kosza na śmieci i je wyrzuciła. Sztuk jeden. A wszyscy chodzą w rękawiczkach. Ale wie pani, kupili sobie za złotówkę jedną parę, dopóki się nie podrze, będą w niej chodzić.

**A pojawiają się osoby w maseczkach?**

Widziałam wczoraj w sklepie 2 osoby w maseczkach. Ale nawet już panie, chyba ktoś stwierdził, że to jest po prostu jakiś absurdalny pomysł, żeby te ekspedientki na przykład chodziły… Taka pani w dziale mięsnym stoi za tą ladą mięsną, sprzedaje w maseczce. Może nie zaraz schabu karkowego, tak? Ale sama się udusi w tej maseczce oczywiście. Więc już widzę, że te panie sklepowe w maseczkach chodzą tylko te, które chodzą po sklepie. A ta pani, która stoi za ladą na przykład w mięsnym już szczęśliwie ktoś jej pozwolił zdjąć tą maseczkę. Bo wie pani, nie można wylać dziecka z kąpielą. Powoli może zaczynamy coś tam myśleć. Chociaż jak taki rząd wprowadził obostrzenia, no to po co tu myśleć? Jak naślą pani zaraz kogoś, jakiś uprzejmy sąsiad zazdrosny przyśle kogoś tam na jakąś kontrolę na przykład. No, to jest jedyna rzecz, której oni się boją. Oni się bardziej boją sąsiada, który powie, że coś tam, niż wirusa. Bo ten sąsiad jest na pewno bardziej realny niż ten wirus dla nich tutaj.

**Powiedziała pani jeszcze coś takiego, o tych zakazach, które wprowadzili, że jest ten zakaz wejścia do lasu. Pani ma swój las, więc tutaj nie mogą zakazać. A co pani sądzi w ogóle o tym zakazie wejścia do lasów?**

Myślę, że to jest też absurdalne. To znaczy zakaz wejścia do lasu wydaje mi się mało sensowny. Chociaż rozumiem, że chodzi o to, żeby w obliczu pogody, która w tej chwili się dzieje, myślę, że… No nie wiem można się spotkać na przykład na parkingu przed lasem, czy na jakimś wejściu do lasu. Gdzie nagle podjedzie pierdyliard samochodów. Bo w samym lesie, umówmy się, nawet jak jest pora grzybobrania czy coś, no to nie ma tylu ludzi, żeby się o nich potykać. Te lasy są jednak duże. Jak idziemy na spacer w parku, owszem. Ale w lesie, no myślę, że… Chociaż z drugiej strony, jak nie wprowadzimy obostrzeń… To jest tak, że trudno jest postawić chyba granicę. Jak zawsze. Gdzieś ona musi przebiegać. I zawsze te przypadki blisko tej granicy są kontrowersyjne. Tak jak nie wiem, człowiek jest pełnoletni, ma 18 lat. A jak ma 18 i jeden dzień, to jest, a jak ma 18 bez jednego dnia, to nie jest. No tak to jest po prostu. Wie pani, znowu, mnie jest wygodnie o tym mówić, bo ja mieszkam w miejscu, gdzie nie ma wirusa na razie, jestem na odludziu i mam własny las, więc w sumie chrzanię tego pana. Ale z drugiej strony, jeśli myśliwy może wejść do lasu i strzelać, to ci ludzie tutaj nie bardzo rozumieją, dlaczego on może, a oni nie mogą. No to teraz im trzeba wyjaśnić, że oni nie mogą, bo w tych samochodach, co oni podjadą pod ten las… A oni nie będą jeździć żadnymi samochodami. Moim zdaniem tak naprawdę powinny być inne zasady, czy też być jakoś modyfikowane, jeśli to jest w ogóle możliwe, dla miast, dla mieszkańców miast i mieszkańców wsi. Bo jednak to życie jest po prostu absolutnie inne. Oni tu w ogóle nie jeżdżą samochodami prawie, jeżdżą na rowerach. Jak by ta pani sobie pojechała do lasu na rowerze, no to nikomu nic się nie stanie. Bo ona na ten rower, nawet jak by chciała, to nikogo więcej nie weźmie, prawda? A z drugiej strony ja rozumiem, że trzeba wprowadzić jakieś zasady. No i trzeba je wprowadzić. Może nie należy się rozdrabniać na to, czy ktoś mieszka w dupie wołowej czy Warszawie. Chociaż myślę, że tu akurat jest zasadnicza różnica. Ale nie wiem, na przykład zawsze się staram przemyśleć, co ja bym zrobiła, jak ja bym była na miejscu takiego pana ministra zdrowia. No to naprawdę on ma przerąbane. I zresztą jako jedyny wzbudza we mnie litość w tym całym zespole rządzącym. Bo widać, że on się coraz gorzej czuje już po prostu, oczy sine i… Ale czy on ma się zajmować tym, żeby mieszkańcom wsi było jakoś szczególnie dobrze? Czy, no po prostu, wprowadza jakieś obostrzenia i ma na pewno więcej problemów na głowie. Niewątpliwie, gdybyśmy z tego wyciągnęli takie wnioski, że byśmy wprowadzili czy przygotowali pewne mechanizmy czy rozporządzenia, które… Bo teraz to jest taka, wie pani, na żywym organizmie próbujemy. Na żywym organizmie państwowym, w określonych okolicznościach ekonomicznych i powiedzmy rozwoju cywilizacyjnego. Wiadomo, że przygotowane procedury 30 lat temu w tej chwili będą absolutnie bez sensu. Teraz robimy jakieś próby. No, a jak byśmy to potem ubrali w jakiś, w jakąś, nie wiem, zestaw zachowań, bloki jakichś zachowań, które by można było wprowadzać wtedy, w zależności od sytuacji, czy to jest, nie wiem, wirus czy skażenie jakieś powietrza czy czegoś, to być może bylibyśmy bardziej przygotowani. Ale skąd mamy być tak naprawdę? Myślę, że nie ma jakichś zupełnie idiotycznych rozporządzeń. Nie robią czegoś, co jest zupełnie bez sensu. Z punktu widzenia państwa oczywiście. Chociaż myślę, że z punktu widzenia poszczególnych obywateli czy grup obywateli być może można by to było zrobić… Ja wiem, może sensowniej po prostu. Chociaż wie pani, no nikt tu nie cierpi jakoś strasznie. Bo znowu, tak naprawdę to miastowi najbardziej cierpicie z tego powodu, że nie możecie pójść do lasu. Bo ja do tego lasu mogłam pójść, mogę pójść i nawet, jak by mi zakazali, to oni tego państwowego lasu, co go tu mam po drugiej stronie drogi też nie pilnują. I nie będą tego robić. Więc być może dobrze, że jest zakaz wejścia do lasu, bo połowa Warszawy wyjechałaby w weekend do lasu.

**Pewnie tak, patrząc na to, co działo się, jak jeszcze były otwarte lasy wokół Warszawy.**

Bulwary, parki. Ale co tu się dziwić. Siedzimy w domach tą zimę, czy w tygodniu jeździcie do pracy. I ten ciągły pośpiech i ciągle… I nagle po pierwsze zamykają wszystkich w domach, dostają wszyscy, czy część, czy znakomita pewnie większość świra po prostu. I te wszystkie sielankowe w telewizji spoty, zostań w domu i te dzieci leżące na stołach i coś tam, i w ogóle w takiej sielance. To umówmy się, że to się dzieje w 1% domów pewnie, tak? A w reszcie jest gorzej. A w części jest fatalnie po prostu. Więc jak ci ludzie by chcieli troszkę tego stresu sobie ująć i pójść do lasu, no to nie byłoby nic w tym niezrozumiałego. No więc może i dobrze, że tak nie jest. Bo ten zakaz wejścia do lasu, no tak jak mówię, to tak naprawdę dotyczy was, a nie nas.

**A czy jest takie ograniczenie, obostrzenie z tych rządowych, które, jeszcze sobie pani myśli, że jest takie niezrozumiałe, dziwne, albo niepotrzebne, albo mało znaczące w tej sytuacji, którą mamy?**

Myślę, że tym seniorom, to powinni ich wcześniej wpuszczać do tych sklepów. Bo seniorzy, z mojego doświadczenia wynika z pracy z seniorami na przykład w Płocku, że oni w ogóle prowadzą taki tryb życia trochę inny, oni wcześniej wstają. Oczywiście, pewnie są jakieś wyjątki. Mam jednego seniora, który nie lubi wcześnie wstawać. Ale oni wcześniej wstają i woleliby rano sobie pójść do tego sklepu. Oczywiście, oni mogą iść cały czas. Bo to nie jest tak, że ci seniorzy nie mogą chodzić. Ale te 2 godziny dla nich, myślę, że jak by było od 8 do 10, to by było sensownie. Bo oni po prostu potrzebują, rano wstają, by sobie to kupili, a teraz tak… Wie pani, no niby mogą wcześniej pójść, no ale są dla nich te godziny. Ale tak, żeby było coś absurdalnego, jakiś zakaz albo nakaz, to nie wydaje mi się.

**A przestrzegane są te godziny dla seniorów w sklepach okolicznych?**

Tutaj tak. Ja wczoraj wyjechałam po siatkę, bo ja też wykonuję, w związku z tym, że jest Julka, to zamierzamy zrobić wybieg dla psa jednego, którego wzięłam w październiku, który już mi zjadł wszystko, co było do zjedzenia, wszystkie drzewa, wszystkie kwiaty. Wszystko, co było takie w miarę, oprócz drzew takich wielkich, to wszystko… Jak to mówimy, pies patologiczny (śmiech). No może coś w tym jest. No młody po prostu i ogromny jest. I tylko po prostu jest straszny szkodnik. I robimy mu wybieg taki. Bo nie mogę na niego patrzeć, musiałam go uwiązać na łańcuch. Na co po prostu, jak go widzę na tym łańcuchu, to mi serce krwawi. I robimy dla niego wybieg. Pojechałam po siatkę. I byłam o wpół do 12 już po całej akcji z siatką, siatka w bagażniku, mówię dobra, to podjadę do sklepu, zrobię zakupy. No i chciałam wejść do sklepu. I myślę sobie dobra, w tym supermarkecie to mnie na pewno nie wpuszczą. Ale to pójdę do takiego małego sklepu, to tam na pewno zrobię zakupy. Wkładam głowę, a pani mówi: zapraszamy od 12, bo teraz tam coś tam.

**A byli ludzie w sklepie?**

Tak. Byli. Tam ze 3 osoby.

**Pytam, bo słyszałam o przypadkach, kiedy w sklepie nie było ludzi, a i tak nie chcieli obsłużyć nie seniorów. Dlatego pytam, czy w ogóle byli ci seniorzy w tym sklepie.**

Byli. Nie, no to ja uważam, że jeżeli już mamy takie godziny dla seniorów, no to dobrze, że nie wpuszczają innych ludzi do sklepu. PO prostu. Bo jak wejdzie kto inny, a potem wejdzie ten senior, no to co zrobić z tamtą osoba, która już robi te zakupy? Wyprosić ją czy coś? No nie bardzo. Tak jest, dobra, tak wymyślili, to się do tego stosujmy. Ja myślę, że to siedzi (niezrozumiałe) to są po prostu osoby żądne władzy. I na tym polega z mojego punktu widzenia ich niekompetencja. Czy to, że nie są na właściwym miejscu po prostu dla siebie. Natomiast, no jakiś tam iloraz inteligencji mają. Jak coś wymyślają, a najczęściej nie wymyślają, tylko kopiują jakieś rozwiązania, na szczęście może, ale jakieś rozwiązania innych krajów, no to nie mogą nam zrobić jakiejś strasznej krzywdy, tak? Może coś być bardziej lub mniej dla nas upierdliwe. Ale starajmy się to zrozumieć. Myślę, że to ma sens. Skoro już ci seniorzy muszą wyjść albo muszą, w sensie, że nie mają co jeść, albo muszą, bo po prostu się uduszą. To dajmy im tą możliwość, żebyśmy nie chodzili po tych sklepach. Wie pani, młodzi ludzie, którzy robią te zakupy szybciej, 17 razy koło takiego seniora przejdą. Ja sama na przykład jak czasami jestem w sklepie, to wykonuję usługę pod tytułem zdjęcie z wyższej półki dla takiej osoby, jak widzę, że ona stoi i się patrzy gdzieś i nie wie, jak sobie poradzić, żeby ten towar z tej górnej półki w tym koszyku umieścić. Albo czytam im te takie ulotki, co są, co tam jest w tym, czy czego tam nie ma. Więc no mamy kontakt z tymi ludźmi, no nie oszukujmy się. Nawet, jak się przemykamy po tych sklepach. Ja myślę, że trzeba tutaj sobie cały czas przełożyć tą sytuację na miasto. Że to naprawdę ma sens. No oczywiście, że czasem się zdarzy, że jest pusty sklep i coś. No i dobra, nic się z tego powodu nie stanie, że będzie pusty przez 15 czy przez 5 minut. Jakiś senior może przyjdzie.

**A jak wygląda teraz wasz dzień, od kiedy Julka jest w domu? Jak spędzacie?**

Tak wygląda, że Julka pierw śpi. Bo na przykład nie wstała. Ja w tym czasie, no różnie, jakieś śniadanie robię albo coś pozmywam, napalę w piecu, nakarmię zwierzęta. Wyjdę sobie z kawą na taras. Zajebiście jest teraz, na przykład takie słońce świeci cudne. Tak że po prostu mam jak w raju, bo mam ten teras, stół na tarasie z widokiem na las. No w ogóle co tu dużo mówić. A potem, ja Julka wstaje, no to tam ustalamy sobie, co będziemy robić. I robimy. Wczoraj na przykład uruchomiłyśmy karchera i Julka ogarnęła taras. Na którym zwykle zimą stoją budy i coś tam, więc jest straszny syf, bo słoma z tych bud się wszędzie wala. I mam takie drzewo obudowane tym tarasem, więc na dole są kamyczki.

**Czyli wczoraj były porządki.**

Plany mamy różne. Mamy mnóstwo rzeczy do zrobienia. Więc tylko po prostu ustalamy, na co mamy ochotę. Co mamy ochotę zrobić tak naprawdę. I to jest z dużej puli, bym powiedziała. No, którą oczywiście w jakimś tam trybie trzeba zrobić. Julka jest na szczęście taką osobą, która lubi kończyć rzeczy. Nie jest to taki typ, że zacznie tysiąc i żadnej nie skończy. Wręcz przeciwnie, lubi kończyć. Czyli wszystko to, co tam… Nawet jak jej się nudzi już w trakcie, ja to po prostu zostawiam. I czekam na inny dzień, że mi się zachce dokończyć. A ona kończy, bo to jest takie zwieńczenie i jej się lepiej w tym funkcjonuje. Więc to jest po prostu cudne. A wczoraj właśnie zrobiła ten taras, wczoraj zaczęłyśmy grodzić, bo tutaj taki mój znajomy porobił nam słupki, pospawał. Kupiłyśmy siatkę i zaczęliśmy grodzić, zabetonowaliśmy słupki. I będzie cudak miał taki wybieg. No, taki kojec. Taki kojec 20 metrów na 4, więc powiedziałabym taki nawet nie najmniejszy. Ale będzie przynajmniej można coś tu zrobić. Na przykład nie mogę wysiać nasionek marchewki i pietruszki oraz innych, ponieważ on mi to przekopie za chwile to pole, ten pies. Więc muszę poczekać na kojec dla psa. I takie tam. Tak że ustalamy, co robimy. Robimy, w międzyczasie jemy, kiedy jesteśmy głodne. Naprawdę możecie nam zazdrościć.

**A zwyczaje żywieniowe wam się zmieniły, to jak jecie w ciągu dnia? Czy to jest tak jak było u pani?**

Nie chyba. Nie zmieniły się specjalnie. To znaczy Julka może tak, może ona je ciut więcej, bo tu jest na świeżym powietrzu, to nawet jest głodna. Ale chyba nie za bardzo. Może więcej tego jedzenia jest w tej lodówce. No, przede wszystkim, jak ona jest, no to jest zawsze jedna osoba więcej, to jest różnica. Zwykle, jak ja robiłam zakupy, to więcej jedzenia zawsze kupowałam dla zwierząt niż dla siebie. Teraz myślę, że jest mniej więcej po równo (śmiech). Więc na pewno jest więcej jedzenia i w tej lodówce, jak ja jestem sama czy z tym znajomym, no to tam dużo nie trzeba w tej lodówce. A jak jest Julka, no to już bym chciała, że tam jak zajrzy do tej lodówki, to żeby mogła sobie coś wybrać, a nie zjeść tylko to, co jest. Więc trochę jest może więcej tego jedzenia. Ale nie jakoś… Znaczy zawsze jest więcej, jak są dzieci. Zawsze jest więcej słodyczy w szafce, jak są dzieci. Zawsze się trochę bardziej na bogato to robi.

**Ale dzieci są dorosłe, to dla nich też słodycze?**

Tak, oczywiście! Wie pani, jak one się cieszą? Naprawdę. Mój 30-letni syn, jak dostaje jajko z niespodzianką na Wielkanoc, takie z króliczkiem, Julka też, to jest absolutnie bardzo fajnie. To nie przechodzi.

**A jakie takie produkty specjalnie, których na co dzień pani nie kupuje, ale się pojawiły ze względu na to, że Julka przyjechała? Co pani kupiła takiego innego do lodówki?**

Kupiłam na pewno Pepsi, zero czy tam jakąś inną, co to ona pije. Ja nie pijam na przykład Pepsi czy tam Coli w takich… Ja robię kompot, bo mam tutaj 100 drzew owocowych, więc kompoty. No nie wiem. Jest jakiś, nazwijmy go, wybór wędlin. Więc jest na przykład jakaś kiełbasa taka cienka i jakieś, jak to się tutaj mówi, plasterki. Kup mi coś do chleba. Ja mówię, ale co? Plasterki. Tak mówi moja sąsiadka do mnie. Więc nie dostaje pani informacji, jaką wędlinę, tylko że wędlinę jakąś. No jakieś tam plasterki. Ja ostatnio nie kupuję plasterków, bo ja sama w zasadzie nie jem. Ale Julka lubi bułę z plasterkami i na to ser żółty i coś. Danonki na przykład kupuję mojej córce. 26 lat skończyła 2 dni temu, 6 kwietnia. Więc tak, co tam jeszcze w tej lodówce takiego jest innego. No jakieś tam mięcho kupiłam, kawałek. Ale to już wcześniej kupiłam, wczoraj nie kupowałam. Nie, głównie to chyba właśnie jakaś kiełba, serki, jakieś tam takie Fantazje, takie wie pani, te serki w tym kubeczku, co tam się wrzuca jakieś groszki do czegoś. No czekolada. No to takie historie zaistniały dodatkowo.

**A czy w związku z tym pani sobie też coś dla siebie takiego specjalnego kupiła? Czy wszystko jest z myślą o Julce?**

Nie, no kupiłam resztę rzeczy, którą kupuję zwykle.

**No tak, ale coś takiego specjalnego, czego zazwyczaj nie ma.**

Nie, chyba nie.

**Coś dla własnej przyjemności, inaczej niż na co dzień?**

Nie, chyba nie, nic takiego specjalnego. Ja sobie czasem, wie pani, ja sobie robię przyjemności jak… (respondentka odbiera telefon). Ja nie kupuję nic takiego szczególnego. Bo na co dzień, jak mam na coś ochotę, to to sobie po prostu kupuję. Też nie mam jakichś takich żywieniowych ekstrawagancji, bym powiedziała. Czasem mam taki ciąg. No to jak mam jakieś ciągi, takie dwutygodniowe, takie ciążowe bym powiedziała bardziej, np. na marchewkę z groszkiem, to sobie ją robię po prostu (śmiech). Mam swoją marchewkę, swój groszek konserwowy w słoikach, więc…

**A gotujecie cały czas? Czy coś wam się zdarza gotowego kupić z takich gotowych dań?**

Nie, nie kupujemy gotowych dań.

**W ogóle?**

Nie kupujemy gotowych dań w ogóle, bo po pierwsze ja lubię gotować. To znaczy lubię gotować nie dla samego gotowania, tylko jak mam odbiorcę. Jak jestem sama, to często nie gotuję. Ale zasadniczo tak, lubię gotować i dobrze gotuję. Zwykle jestem skromniejsza, ale co będę owijać w bawełnę, dobrze gotuję (śmiech). Więc w zasadzie nie kupuję żadnych gotowych dań w ogóle. Nie wiem, może mam jakieś jedno w lodówce. Znaczy ja nie wiem, czy ono jest gotowe. Bo ja je kupiłam, ale bardzo mi się spodobało pudełko takie, mrożonka taka. Tam jakieś krewetki w czymś tam. I myślę sobie, dobra, to kupię i kiedyś zjem. I to po prostu leży w lodówce. Ale może teraz, jak jest Julka, to jest szansa, że to zjemy. Bo ja sobie myślę, że o, na jedną osobę, to mi się nie chce tego wyciągać, dobra, to niech leży. I tak leży. Jak będzie Julka, to może nam się uda. Bo ona lubi kończyć i lubi też na przykład jakby jeść to, co jeść. W sensie takim wyjeść. Ja raczej więcej kupuję niż zużywam. No jak prowadziłam dom na 4 osoby i 3 zwierzęta i jeszcze koleżanki córki, które przychodziły na zupę, bo ich mamy zup nie gotowały, to ja mam w ogóle kłopot, żeby ugotować mało. Więc jak już ugotuję, to jest zwykle dużo. Więc tu zamrożę, tu odłożę. I potem to wszystko tak… Tak to woziłam dzieciom do Warszawy.

**A teraz leży.**

A teraz leży.

**A w ogóle u was w okolicy na przykład można zamówić coś do jedzenia? Czy do Płocka trzeba by pojechać?**

Nie. Mamy w Staroźrebach jedną pizzerię. I tam podobno – podobno – można zamówić coś do jedzenia nawet innego niż pizza. Ale tak jakoś, no nie wiem, co tam jeszcze, na pewno jakiś kebab, czyli takie cokolwiek. Nie wiem, może jakiś kotlet jeszcze. Tego nie wiem, ale podobno coś można zamówić. Niemniej jednak ja tam w ogóle nie występuję oraz nie kupuję takich rzeczy. A tak, to by trzeba było do Płocka pojechać. Ale też nie wiem za to, gdzie by w tym Płocku, no, ale pewno tam coś można było… Tam jest KFC, McDonald ’s jest, są takie miejsca.

**A jak Julka w Warszawie mieszka, to ona zamawia jedzenie takie na wynos? Takie, że właśnie przywożą.**

Pizzę na pewno zamawia raz na jakiś czas. Ale z rzadka zasadniczo. Ale tak, ona lubi pizzę i czasem zamawia.

**Pewnie w okolicy ludzie też raczej nie zamawiają, skoro jest tylko ta jedna pizzeria?**

Na pewno nie zamawiają nawet.

**A to jest tak, że ta pizzeria się cieszy popularnością, czy raczej nie bardzo?**

No więc powiem pani, że akurat ta pizzeria… Teraz nie, bo teraz jest zamknięte, nie można w środku jeść. Ale ona w ogóle jest otwarta, drzwi są otwarte, może pani wejść, zamówić na wynos. Więc trudno mi powiedzieć, jak to teraz wygląda, ale wcześniej to zawsze tam ze 2 czy 3 osoby przy stolikach widziałam. I kiedyś na przykład, ja tam byłam raz z Julką, jesienią, jak wymieniałyśmy w jej samochodzie opony. Bo zakład oponiarski jest obok. No i coś trzeba było ze sobą zrobić, to poszłyśmy się tam kawy napić, do tej pani. Za 6 zł chyba dwie kawy, czy… Więc w sumie tak, po pierwsze na pewno jest tanio. Po drugie, czy tam ktoś z tego korzysta, no, są osoby, które tam korzystają. Głównie młodzi ludzie. Przychodzą, kupują sobie ten taki na wynos kebab albo coś. Jeśli tam kogoś widziałam, to młodych ludzi. Jest też druga pizzeria u nas, która obecnie jest zamknięta. Myślę, że na okres zimowo-jesienno-wiosenny. Ale nie mogę powiedzieć, czy ona się otworzy, ponieważ to jest własność pana wójta, który został niedawno wybrany, rok temu w wyborach. Więc być może on ją zamknął, bo teraz nie ma, jakby jak tego ogarnąć. Trudno powiedzieć, bo jego żona ma w tej chwili małe dziecko. Ale w tej drugiej pizzerii, to nie wiem, może raz widziałam jedną osobę. Więc myślę, że jedna pizzeria na takie Staroźreby.

**Wystarczy.**

Myślę, że zdecydowanie wystarczy. I dwie się po prostu nie utrzymają, bo nie będą miały z czego. Bo nawet ci młodzi ludzie, którzy tam bywają, no to skąd oni mają kasę, tak? No znikąd tak naprawdę, nie mają jej. Tam gdzieś od rodziców albo może tam coś sprzedadzą albo coś zakombinują. Tak że mamy jedną pizzerię, była druga pizzeria, która została przez tą pizzerię wykończona od strony takie powiedzmy mało uczciwej konkurencji, która się przeniosła do innego takiego małego miasteczka. Wieś gminna, Radzanowo. I tam też byłam raz, to były 2 stoliki zajęte. No i pan jeździł, wyjeżdżał z pizzą i zawoził. Bo tutaj to chyba nie wożą, tylko trzeba przyjść po nią. A tam wychodzi pan z tej pizzerii i tam ze 3 pizze wywiózł w ciągu dnia. Ale to w zasadzie tutaj, myślę, że ludzie dorośli, tacy nie wiem, 40 plus to nie korzystają z tego w ogóle. To jest przede wszystkim dużo droższe. Tak naprawdę, żeby nakarmić rodzinę, no to…

**A jak pani była teraz w tym sklepie robić zakupy. Czy obecna sytuacja, to że mamy koronawirusa, wpływa w jakikolwiek sposób na to, jak pani płaci, czy to jest gotówka, karta, telefon?**

Na to, jak ja płacę nie wpływa, bo ja zawsze płacę kartą.

**A myśli pani, że ludzie więcej płacą kartą w okolicy?**

Myślę, że nie. Ponieważ oni nie mają kart.

**Nie używają na co dzień.**

Nie używają ich w ogóle na co dzień. Jeżeli mają kartę do konta, bo zostali jakby zmuszeni przez sytuację, żeby takie konto założyć, bo oni jakieś chyba, taką namiastkę konta to mają wszyscy w Banku Spółdzielczym. Ci, którzy nie mają gdzie indziej. Bo ten Bank Spółdzielczy jakby otrzymuje w tej chwili emerytury. I te emerytury są wydawane nie na poczcie, tylko w banku. No więc część osób sobie po prostu pozakładała konta. Kart w ogóle nie brali, bo nie wiedzieli, do czego one służą. Niektórzy, jak wzięli karty, no to one im służą wyłącznie do wypłaty pieniędzy w bankomacie. Pomimo tego, że w większości sklepów można płacić kartą. Chociaż w dalszym ciągu nie we wszystkich. No to mają te karty. Ale w ogóle nie korzystają w sensie płatniczym, tylko wyciągają pieniądze z bankomatu. Najczęściej zresztą nie sami, bo nie wiedzą, jak to zrobić i się boją w ogóle tego bankomatu. Że on im zabierze tą kartę albo nie da tych pieniędzy albo coś. Nie wiedzą w ogóle, jak to działa. No i rodziny wypłacają. Czy na przykład jakiś młody człowiek ma tą kartę tej babci i wypłaca tej babci pieniądze z bankomatu. No to jest takie..., specyficzne.

**Bo w Warszawie dużo się mówi, żeby w sklepach płacić kartami, ze względu na to, że na tych pieniądzach ten wirus itd. Czy to dotarło na prowincję, taki przekaz, że karta jest po prostu bezpieczniejsza, czy oni machnęli na to ręką?**

Może jest bezpieczniejsza, ale trzeba by było teraz pójść do banku, wystąpić o tą kartę pewnie. Poza tym, tak jak pani mówię, oni się w ogóle nie posługują tym. Więc… Chociaż sama karta, coś takiego jak karta to w ogóle funkcjonuje tutaj, bardziej w sensie kart takich lojalnościowych. Karta, nie wiem, Moja Biedronka czy Delikarta, bo my tu Biedronki nie mamy żadnej, ale mamy ten sklep, który jest, to ma jakąś tam kartę lojalnościową. Więc można powiedzieć, że w tej chwili ci ludzie wiedzą, co to jest karta, może nie karta płatnicza i może do końca tego nie rozumieją. Ale w ogóle coś takiego jak karta już jakoś zafunkcjonowało w świadomości ludzkiej. Ale to, że pieniądze są bardziej niebezpieczne niż karta, to myślę, że nie. Zwłaszcza, że moim zdaniem, na przykład, jeżeli musimy wystukać ten pin na tym padzie, no to co za różnica, czy ja dotykam pieniądze, może nawet pieniądze są bezpieczniejsze. Bo jak by wszyscy stukali tym palcem po tych klawiszach, no to co? No to i tak zbieramy te wirusy. Przecież one nie są w żaden sposób czyszczone, tak? Bo nie może pani tego pada zapsikać płynem dezynfekcyjnym, bo to tak jak klawisze komputera, zaraz przestanie działać. Ja to nie wiem, czy to jest rzeczywiście takie dużo bezpieczniejsze. Myślę, że moich pieniędzy dotykam ja i pani kasjerka. Oczywiście, ktoś tam jeszcze miał je w ręku. Jak byśmy wszyscy macali ten pad, zważywszy na to, że tych rękawiczek nie zmieniają, to nie wiem, czy to byłoby takie… Nie, ale tutaj karta w zasadzie, no oczywiście część ludzi młodych… Jak ktoś dostaje pieniądze na konto w tej chwili, wypłatę, ma te pieniądze na koncie i ma kartę do tego konta, no to z tej karty czasem korzysta. Ale starsi ludzie absolutnie nie, w ogóle nie ma mowy.

**Czy pani się zdarza kupować przez internet coś?**

Zdarza mi się. Rzadko.

**A zdarzyło się ostatnio? A co się zdarza?**

Ostatnio tak, książki na przykład kupuję przez internet. Bo jest dużo bezpieczniej niż jak pójdę do księgarni. Dużo mniejsze zakupy robię przez internet niż w księgarni.

**Czyli bezpieczniej dla portfela.**

Tak. Jeśli chodzi o księgarnię. Są takie rzeczy, które jest mi wygodniej. Bo zasadniczo to jest tak, że niby się kupuje… Jak mam jedną, konkretną rzecz, którą bym chciała kupić, na przykład mam jakieś zamówienie albo dla dziecka chcę coś kupić. Albo tu mój znajomy mnie pyta, czy ja bym mu nie mogła kupić coś tam do samochodu, jakąś część. Ja mówię, jak mi dasz wystarczająco dużo danych, żebym ja mogła kupić na pewno tą część, a nie jakąś inną, to proszę bardzo. Albo jak ktoś przychodzi tu w takiej sprawie, że chce kupić żelazko. Ja mówię, proszę bardzo. Ostatnio kupowałam telewizor dla sąsiadki. Bo jej się zepsuł telewizor. No więc takie rzeczy, jak mam jakąś jedną, konkretną rzecz, co mam ją kupić, to chętnie. Natomiast w ogóle żadnych takich akcji, żeby na przykład usiąść sobie i sobie coś przejrzeć, co ja bym sobie mogła kupić. Albo, że na przykład kupiłabym sobie sukienkę, abstrahując od głupoty tego pomysłu, no i że na przykład siedzę i godzinę albo dwie, albo pięć przeglądam te sukienki. W ogóle odpada taka sytuacja. Więc, no kupuję. Ale na pewno to nie jest jakby jakoś istotnie więcej. Na przykład dużo kupuję przez internet przed świętami, przed gwiazdką. Bo jak mam jakieś zamówienia, czy mój brat mi powie, bo on ma trójkę dzieci, to co tam dla tych dzieci i dla niego, jakieś rękawiczki czy coś, no to jak nie mam innych pomysłów… Bo ja staram się większość prezentów robić w ogóle sama. Ale jak nie mam innych pomysłów, czy jakieś dziecko chce, nie wiem, Lego czy zestaw jakiś, proszę bardzo, to tak, chętnie. Bo to na pewno jest dużo szybciej. Ale wszystko to, co ja bym miała na przykład… Co ja ostatnio… Coś ja takiego chciałam sobie kupić i zaczęłam przeglądać ten internet. I nawet coś sobie kupiłam, cholera, tylko ja nie pamiętam, co to było. Coś sobie chciałam kupić i zaczęłam przeglądać internet na okoliczność… Jakiś był sprzęt gospodarstwa domowego. W każdym razie (niezrozumiałe) coś mnie zainteresuje, zobaczyłam, myślę sobie a, to może ja sobie to kupię. I w końcu jak się zatrzymałam w pewnym momencie, myślę sobie, zamiast kupić sobie coś za 50 zł, co miałam kupić, to mam w tym koszyku za 500. Bo jeszcze sobie wymyśliłam, że mi się zepsuła wyciskarka, to jak ja sobie ją kupię, to wyciskarki samej to nie, to może sobie kupię maszynkę do mięsa, co ma takie wymienne nożyki, to będę mogła sobie robić… No i tak… Tarkę, co będzie miała w sobie tarkę, bo mi się zepsuła tarka. No dobrze, to będzie miała tarkę. Ale potem nie, tu widzę, że jedne mają tarkę do ziemniaków na placki ziemniaczane, a inne nie mają. A, to ja teraz poszukam takiej, co ma tą tarkę, prawda? A jak w życiu nie robię placków ziemniaczanych. Ponieważ nie lubię placków ziemniaczanych (śmiech). I nie dość, że spędziłam przy tym ze dwie godziny, to jeszcze nie kupiłam nic, bo na szczęście dotarło do mnie, kupię sobie to i co ja z tym zrobię? Wsadzę do szafki, bo i tak w życiu tych placków nie zrobię. Więc mówię do siebie, zrób na zwykłej tarce, zetrzyj, zrób 5. I wtedy pozwolę ci kupić sobie elektryczną maszynkę do mięsa z tarką do ziemniaków. I tak to się na szczęście skończyło. Mnie to jest szkoda czasu. Bo to strasznie… Znaczy ja pracuję sprawnie na komputerze, dużo na nim robiłam, w różnych programach, kiedy pracowałam, więc nie mam kłopotów z obsługą różnych programów, nawet bardziej skomplikowanych. Ale to poszukiwanie i ten, jakby specyfika ułożenia tych rzeczy, czy… Nie wiem, jak to nazwać, ale specyfika tych zakupów internetowych jest taka, że ja mam potem poczucie straconego czasu po prostu straszne. I to mnie dobija. I tracę humor. Jak chcę coś kupić konkretnego, to to jest bardzo przydatne narzędzie. Tak jak na przykład kiedyś jeździłam często do Hiszpanii, bo to jest kraj mojego ojca i często tam bywałam. I na przykład uwielbiam jakiś płyn do kąpieli, który zawsze sobie w tej Hiszpanii kupowałam, to teraz jak się okazuje, że z powodów osobistych nie mogę tam jeździć czy finansowych, to w dalszym ciągu mogę sobie to wbić w komputer i z jakiejś tam drogerii przywożą mi ten płyn, pan kurier mi przywozi na wieś. I to jest na przykład super.

**A czy w ciągu ostatnich 2-3 tygodni pojawił się jakiś zakup przez internet, który był przez internet ze względu na koronawirusa, a tak, to by się odbył w zwykłym sklepie?**

No nie właśnie. Mieliśmy robić takie zakupy urodzinowe. Ale moje dzieci stwierdziły, że ponieważ się nie będziemy widzieć na te urodziny i w ogóle, może Julka przyjedzie, a może nie przyjedzie, jeszcze wtedy nie było wiadomo, to sobie, kiedy indziej zrobimy. Bo tak, to by były. Bo tak, to bym zakupiła i Julce, i Hubertowi. Ale w związku z tym, że nie ma tych urodzin takich oficjalnie, nie robimy sobie na razie prezentów i czekamy, no to chyba nie. Oprócz telewizora dla sąsiadki, po który być może bym pojechała i wzięła jej w sklepie po prostu w Płocku, będąc przy okazji, no to teraz kupiłam przez internet. Ale tak, to myślę, że nie. Można powiedzieć, że jeśli o mnie chodzi, to nie.

**Ale ten telewizor, to rozumiem (niezrozumiałe), pani po prostu nie jeździ do Płocka. Że to jest jakby przyczyna…**

No tak, no teraz kupiłam, dlatego przez internet… No tak, no chociaż wie pani, miałam zapotrzebowanie na konkretny telewizor. I tak bym nie zabrała ze sobą tej sąsiadki. Miałam zapotrzebowanie na konkretny, który wtedy przyjeżdża do domu. I jeszcze na dodatek ja to mogę tak załatwić, że oni płacą przy odbiorze. Więc ja jakby nie pośredniczę pieniężnie. No, to może nawet i też bym tak wybrała. Więc myślę, że można powiedzieć, że ja nie wykonuję takich zakupów.

**To jeszcze chciałam zapytać o plany zakupowe na najbliższe dni, jak to z tymi zakupami będzie. Będzie więcej wizyt w sklepie, tyle samo? Jak pani myśli?**

Ja chyba jestem zbyt dobrze zorganizowana, Julka zresztą też. Więc myślę, że to będzie raczej więcej w czasie jednych zakupów niż więcej wyjść na zakupy. Tak że tutaj…

**Ale to chodzi o to, żeby jednak ograniczyć te wizyty wśród ludzi, czy z innych powodów?**

No tak, no zdecydowanie. Bo tak jak Julka przyjeżdżała zwykle, to robiłyśmy jakoś więcej tych zakupów, prawda? To znaczy częściej może jeździłyśmy, jeździłyśmy na przykład do Płocka, żeby zrobić jakieś zakupy, żeby do galerii. Żeby ona mogła, wie pani, tu jest dużo mniej osób w tych galeriach i te zakupy są po prostu przyjemniejsze, jak może pani przejść spokojnie. Tam ten Płock jest malutki w ogóle, więc jest na pewno wygodniej to robić. Teraz na pewno się nigdzie w takich sprawach nie wybieramy. Ale ja zawsze funkcjonowałam na zasadzie karteczkowej. Miałam karteczkę, na której się zapisuje wszystko, co trzeba kupić. I w związku z powyższym, jak się tej karteczki oczywiście nie zapomni do sklepu, to w zasadzie można powiedzieć, że się udaje zakupy wykonać… My nie mamy na tych karteczkach… No mam na przykład na karteczce kacze nogi, które przepisuję z dnia na dzień. Bo mogłabym może pojechać do Płocka. No może pojedziemy jutro albo wieczorem czy w nocy do Płocka, żeby zrobić jakieś zakupy takie świąteczne. Albo pewnie nie pojedziemy.

**Jakie macie plany świąteczne? Czy Hubert przyjeżdża?**

Nie, Hubert nie przyjeżdża z Magdą, bo oni będą albo sami albo pojadą do rodziców Magdy. Więc, żeby już nie mnożyć tych kontaktów, ponieważ my jesteśmy w ogóle ateistami. I te święta, no my spędzamy je zwykle, bo moja część rodziny, jakby z mojej strony jest niewierząca, moje dzieci są również niewierzące, nieochrzczone itd. Ale druga część rodziny, babcie, dziadkowie ze strony mojego męża są wierzący, więc święta spędzaliśmy… Teść święcił, póki żył ten koszyczek czy cokolwiek. No i dzieliliśmy się jajkiem, mieliśmy potrawy, było bardzo sympatycznie, rodzinnie. Wszyscy mnie pytali, no jak to, jesteś taka niewierząca i ty święta? Ja mówię normalnie, święta są fajne, jest rodzina, wszyscy się spotykają, jest zajebiście. Tylko po prostu no to, że jesteśmy niewierzący, to w ogóle nie przeszkadza w świętowaniu. Przyjeżdżał mój brat z dziećmi, po prostu ful, 15 osób. A teraz, tylko będziemy we 3 osoby. To znaczy ja, Julka i mój znajomy tutaj stąd, który też jest samotny. I nie zamierzamy robić żadnych, wie pani, tutaj stołów. Sprzątniemy ze stołu ewentualnie jakieś zalegające na nim różne historie (śmiech). Zrobimy może jajka faszerowane, bo Julka bardzo lubi. A tak poza tym, to nie zamierzamy wykonywać takich wystawnych, że tutaj postawić na stole 5 dań i potem je tylko przekładać znowu do lodówki, a potem z lodówki do stołu itd. Zrobimy jakieś ciasto. No, na pewno, jajek faszerowanych nie robimy na co dzień. Chociaż może szkoda. No to teraz zrobimy. Zrobimy jakieś… Nic specjalnego. Chociaż ja mam takie, chciałabym na przykład upiec nogi kacze. Czasem je piekłam jakby w innym terminie, to nie jest tak, że one są zarezerwowane na święta. Ale tak sobie pomyślałam, że bym kupiła. Ale też, jak ich nie kupię, to zrobię, nie wiem, wszystko jedno co. Coś zrobimy na pewno do jedzenia, coś, co nam będzie smakowało. I na pewno tu nie będziemy siedzieć przy stole. Nie będzie się, te dni moim zdaniem nie będą się różniły od innych dni, które tutaj spędzamy razem w tej chwili. Wszyscy jakby w takim trybie niepracującym. Bo ja tak nie jeżdżę i nie uczę dzieci, ani nie jeżdżę do seniorów, ani nie mam z nimi zajęć. Staś też w zasadzie nie jeździ teraz do ludzi i tam nie naprawia sprzętów. Bo też się stara jakoś ograniczać. Julka nie pracuje. No więc pracujemy sobie tutaj u nas, cały czas coś robimy.

**Czyli te faszerowane jajka, bo lubicie, będą takie trochę inne, ale jakby niedużo. Czy coś jeszcze planujecie z takich rzeczy niecodziennych, żeby zjeść, poza jajkami i kaczką?**

No nie wiem. Zrobimy może ciasto orzechowe, Julka lubi bardzo. Znaczy wybierzemy sobie z tych świąt po prostu takie rzeczy, które my lubimy w szczególności. Zrobimy ciasto orzechowe, to jest taki, u nas zawsze w domu robione zamiast mazurka. Bo mazurka nie wszyscy lubią, dzieci nie lubią takich rzeczy w cieście, więc taki… Taki, nazwijmy go, mazurek orzechowy. Jajka faszerowane. I może upieczemy kaczkę. A może nie. Bo jak jej nie będzie, to jej nie urodzimy. A na pewno nie będziemy zabijać tu żadnej kaczki w tej sprawie szczególnej. No i tak. Bez napinki.

**A myśli pani, że będzie pani brakowało tej rodziny, tego, że nie przyjedzie te kilkanaście osób, nie spotkacie się?**

O, na pewno. Na pewno będzie mi brakowało syna z Magdą. Bo z bratem jestem w kontakcie, jesteśmy wszyscy rozsądni, no byłoby miło, gdyby byli. Ale jak ich nie będzie, to nie będzie pierwszy raz, kiedy ich nie ma. Bo oni mają te dzieci młodsze niż moje, więc czasem a to któreś dziecko chorowało czy coś. No to Wielkanoc się zdarzało, że nie zawsze byli w Wielkanoc. W święta Bożego Narodzenia zawsze, a… No znajomi nie przyjadą. Więc z jednej strony będzie mi brakowało, tak, oczywiście. Ale z drugiej strony, wie pani, jak by to powiedzieć… No, ja się dobrze czuję z moimi najbliższymi też. Więc to nie jest tak, że… Brakuje mi znajomych. Zagrałabym w brydża, posiedziałabym chętnie, zrobiłabym grilla na 10 osób, byłoby fajnie. Ale jak tego nie ma, też jest fajnie. To nie jest tak, że ja tu ubolewam i że to jest jakaś strata. Jeszcze będziemy się spotykać w kupie, nie ma problemu. Jest wyjątkowa sytuacja, no i się nie spotkamy.

**A czy w sklepach widać przygotowania do Wielkanocy tych ludzi z okolicy? Że oni kupują więcej, szykują się na…**

Kupują mięsa więcej chyba. No tak, jak byłam wczoraj, to pani kupowała jakieś, nie wiem, 2 kilo kiełbasy białej, jakiś schab taki. No kupują. Myślę, że kupują więcej. Ale myślę też, że chodzą rzadziej do tych sklepów. Bo normalnie przed tymi sklepami zawsze było dużo samochodów. I po tym można stwierdzić. No teraz jest mniej koszyków, więc tych koszyków nigdy nie ma. Ale ludzi jest zdecydowanie mniej w tych sklepach. Stąd myślę, że robią po prostu większe zakupy. Bo tak, to by musieli codziennie tam dmuchać. I codziennie stać przed tym sklepem w kolejce. No coś tam na pewno robią, jakieś większe zakupy, ale żeby jakoś… Też nie obserwuję tego bardzo, bo rzadko tam jestem.

**A myśli pani, że ludzie pójdą, w okolicy, bo pani nie chodzi do kościoła. Ale myśli pani, że ludzie pójdą na święta czy ze święconką, czy w niedzielę wielkanocną?**

Nie mam bladego pojęcia, mówiąc szczerze. Nie pytałam ich o to, oni też… Nie wiem. Myślę, że część takich bardzo kościelnych pójdzie. Ale tutaj ta wiara nie jest jakaś taka… Tu praktykujących osób to nie jest dużo. Takich, co chodzą w niedzielę do kościoła. Może w samych tych Staroźrebach, czyli w tej gminnej miejscowości, gdzie stoi ten kościół, to może tak. Ale żebym tutaj z tej okolicy, to ja nie wiem, czy tu ktoś chodzi na przykład w niedzielę do kościoła. A w szczególności trudno mi powiedzieć, czy będą sobie coś święcić czy nie, bo ja trochę nie jestem na bieżąco w tym zakresie.

**Czy jest coś, czego jeszcze będzie pani brakować?**

Tak.

**Coś będzie inaczej?**

Nie. To znaczy ja mam takie kiepskie poczucie tych świąt. Kiepskie, w sensie, że nie czuję tych świąt. Bo z mojej perspektywy te święta, to nie jest religijne przeżycie czy duchowe, tylko to jest przeżycie czasu z rodziną. Więc tego mi na pewno będzie brakować, czyli większej grupy tutaj moich najbliższych. Ale jeśli chodzi o duchową sprawę, no nie ma takiego, tego czegoś, tego napięcia świątecznego jakiegoś tam. Bo nie ma. Ale i tak będzie fajnie.

**A fakt, że mniej będziecie gotować? Bo jak przyjeżdża więcej osób, to siłą rzeczy się robi po prostu więcej jedzenia. Czy myśli pani, że to będzie lepiej czy gorzej, że tak mniej tego gotowania będzie?**

Ja wiem? Po prostu będzie inaczej, po prostu będzie mniej gotowania. Ja nie mam kłopotów z ugotowaniem dla większej ilości osób. Chociaż oczywiście no to jest jakieś tam wydarzenie. Że trzeba ileś tam tych potraw i coś tam. Jakoś to zgrać, przechować w lodówce itd. A teraz nie będzie takiego kłopotu. Ale nie jest to, ani ugotować ani nie ugotować, nie jest to jakiś… Trudno powiedzieć. Inaczej będzie. Nie będzie lepiej ani gorzej.

**Czy są jeszcze jakieś rzeczy, których nie poruszyłyśmy w kontekście obecnej sytuacji?**

Na razie chyba nic takiego. Wyczerpałyśmy temat.

**Dziękuję.**
