# Supplementary material for: The challenges arising from the COVID-19 pandemic and the way people deal with them. A qualitative longitudinal study
Source: PLoS One. 2021 Oct 11;16(10):e0258133. doi: 10.1371/journal.pone.0258133 (PMC8504766; doi:10.1371/journal.pone.0258133)
Supplement: S1 Dataset — (ZIP) [file pone.0258133.s003.zip › Transcriptions/stage 2/9.2_F_25_couple, no children.docx]

**9.2_F_25_couple no children**

**Emocje – zdjęcia.**

**6**

Myślę, że z takich prozaicznych powodów. Chyba chodzi o to, że po prostu się zrobiła ładna pogoda. I to było najweselsze zdjęcie, ze słońcem, z całej tej puli. Więc tak mi się skojarzyło. No i trochę też chce się wyjść do takiego lasu, jak już tak człowiek długo posiedzi w domu.

**Czyli pogoda sprawia, że się czujesz…**

Dobrze.

**Znaczy dobrze w sensie psychicznym.**

Tak.

**A czy jest coś takiego, co mimo tego, że generalnie czujesz się dobrze przez pogodę, czy jest coś, co się martwi, czy coś cię stresuje teraz?**

Ogólnie w pracy jest bardzo ciężko. Tak jak mówiłam, pracuję dla firmy medycznej. W sensie księgujemy im faktury. Więc ich obroty się bardzo zwiększyły teraz. I jest po prostu bardzo stresująco, cały czas jakieś nowe wymagania. Tak że dużo stresu, ale chociaż ta pogoda mnie pociesza.

**Ale to jest tak, że ta sytuacja w pracy, ona się zmieniła właśnie ze względy na koronawirusa też, tak?**

Tak. Po pierwsze też jest trudniej, bo ja pracuję w tym miejscu od września. Czyli nie jakoś bardzo długo. I w biurze miałam ten komfort, że wystarczyło zapytać się koleżanek. A teraz wszystko trzeba wyjaśniać sobie na Skypie. I to jest po prostu skomplikowane, bardzo pracochłonne, bardzo męczące. Zostałyśmy, bo z koleżanką byłam wspólnie przyjmowana we wrześniu, zostałyśmy wrzucone nagle w ten wir wszystkiego. I tak naprawdę nie mamy nawet do czego się odnieść. Właśnie przez tego koronawirusa. Bo jesteśmy same w domach.

**Czyli masz więcej pracy niż wcześniej.**

Bardzo, tak.

**To chyba taka dosyć nietypowa sytuacja, bo tak jak obserwuję, ludzie raczej siedzą i nic nie robią, a ty masz więcej pracy.**

Tak. Właśnie mąż od jutra przechodzi na postojowe. A u mnie rzeczywiście te obowiązku eskalują, zwiększają się cały czas.

**Bo ostatnio jak rozmawiałyśmy, to mówiłaś, że twój mąż ma obniżoną pensję.**

Tak, tak. Bo to jeszcze nie było pewne. To znaczy na pewno wszyscy, cała firma przeszła na 80% etatu. Ale w tej chwili już podjęto decyzję, że osoby nowozatrudnione są właśnie zostawione na tym postojowym. To jest 64% etatu. Natomiast są już informacje, że… Najtrudniejszy jest ten okres adaptacyjny, kiedy firmy musiały się dostosować do tego, jakie są wymagania związane z pandemią. Natomiast wiem, że jego firma już chyba od czerwca opracowała jakieś tam plany i mają od nowa ruszyć.

**To znaczy, że od czerwca normalnie zaczną pracować tak jak wcześniej?**

Tak, na to wygląda. Bo mąż jest inżynierem. I jakby cała ich praca stanęła, bo stanęła produkcja. I oni nie mogą niczym się w tej chwili zajmować, bo nie mają jak przeprowadzić prób itd. No i się okazało, że od czerwca ma wrócić produkcja, jakieś wdrożyli nowe rozwiązania. I że mają zacząć już normalnie funkcjonować.

**A czy to obniżenie wypłaty twojego męża to jest coś, co obciąży bardzo wasz budżet domowy?**

Może jakoś nie bardzo. To znaczy jest to duże obniżenie, bo to jest połowa pensji tak naprawdę. Ale nie ma tragedii.

**Czyli ty o swoją pracę się nie martwisz, tak powiedziałaś tydzień temu. Tutaj nic się nie zmieniło?**

Przez to, że zadałaś mi te pytania, zaczęłam się nad tym zastanawiać. I zaczęłam się trochę martwić. Ja też pracuję z językiem niemieckim. I przyznaję, że no ten mój niemiecki to nie jest jakieś tam C2 czy C1. Natomiast wydaje mi się, że całkiem nieźle mi idzie. Ale zaczęłam się zastanawiać, bo nawet teraz, jak bym chciała iść na jakiś kurs czy tam sobie coś popowtarzać, no to mam pewne ograniczenia tak naprawdę. Bo ja nie przepadam za taką metodą uczenia się, że się na Skypie spotyka z wykładowcą, tylko raczej potrzebuję się spotkać z jakąś osobą i żeby mi coś po prostu wytłumaczyła. No i nagle widzę, że nie mam możliwości. Że nawet, jak bym miała czas, to tak nie do końca jest jak.

**Powiedziałaś, że zaczęłaś się martwić, jak zaczęłam zadawać o to pytania. Rozumiem, że to jest takie martwienie się o brak możliwości rozwoju w tej pracy?**

To po pierwsze. A po drugie zaczęłam się zastanawiać, bo jestem mocno przeciążona ostatnio. I tak planowałam… Myślałam, że jak by co, jak mi się nie spodoba w tej pracy, no to, że zostanę powiedzmy do września, bo wtedy planowaliśmy wziąć kredyt, więc żeby mieć umowę na czas nieokreślony. No i wtedy będę szukała spokojnie czegoś innego. Ale teraz zaczęłam się zastanawiać. Bo nagle wszystkich ludzi zwalniają. Tak jak mówiłaś, ta sytuacja na rynku robi się trudna. I zaczęłam się zastanawiać, czy mam się jeszcze trochę pomęczyć. Czy w ogóle jest jakaś… Gdybym teraz powiedzmy zdecydowała się odejść albo nie wiem, coś by się rozsypało, nagle by się okazało, że… Jako że jesteśmy najmłodsze z koleżanką w zespole, to nie wiem, ze względu na jakieś cięcia tam finansowe chcą nas zwolnić, to zastanawiałam się, jaka jest realna szansa, że ja znajdę tą pracę, w jakim czasie.

**I jak myślisz, jaka jest realna szansa, że znajdziesz pracę?**

Przyznaję, że nie jest trudno znaleźć pracę w księgowości. I ogólnie wszyscy się śmieją, że w księgowości pracują ludzie tacy jak ja. Czyli po filologiach polskich, po geologiach. Koleżanka jest po biologii. Tak że ogólnie wystarczy chyba tylko chęć do nauki. I ja tą nową pracę znalazłam w przeciągu miesiąca. Ale to już tak, że mi zaproponowali, że mi się wszystko podobało. I OK, przyjęłam. Tak że byłam na kilku rozmowach. I tak jak patrzę na męża, który bardzo długo szukał pracy, to wydaje mi się, że to dość dobry wynik. I z jednej strony ten fach, jest ta łatwość w znajdowaniu pracy. Nawet teraz na Linkedinie cały czas dostaję jakieś tam informacje. Ale teraz, jak to będzie wyglądało realnie? Jeżeli ja powiem komuś z rekruterów, rzeczywiście chciałabym podjąć się tej pracy, czy tam chciałabym na tą pozycję się zgłosić. To jak to będzie wyglądało naprawdę? Jak oni przeprowadzą rozmowę? Przez Skype, ale co potem? I czy oni rzeczywiście kogoś potrzebują, czy wrzucili ogłoszenie jakiś czas temu i ten HR tam sobie te follow upy wysyłał, wysyłał, bo to jest ich obowiązek. Jakby nie wiem, jak wygląda ten rynek w tej chwili.

**Ale póki co nie myślisz, tak zanim ja nie wywołałam dopytywaniem.**

Nie, nie. Nie zastanawiałam się nad tym.

**Czy w tym tygodniu, od kiedy ostatni raz rozmawiałyśmy, coś się w waszym życiu zmieniło? Coś robisz, czego nie robiłaś wcześniej albo coś przestałaś robić?**

Nie. Nie.

**Czy coś się pojawiło nowego, co ci bardziej przeszkadza niż wcześniej?**

Nie zmieniło się nic tak naprawdę. Cały czas taka, powiedziałabym, może nie nuda, ale stagnacja (śmiech).

**Pamiętam, że jak mówiłaś tydzień temu o tej stagnacji, to zrozumiałam to tak, że to jest po prostu taka sytuacja, że wstrzymujesz różne plany.**

Tak.

**Tak jak mówiłaś o tej zmianie miejsca zamieszkania. Czyli ta stagnacja teraz to nadal jest takie wstrzymanie się w różnych działaniach?**

Tak. Też, jeżeli chodzi o zmiany, no to jeszcze w tamtym tygodniu, jeszcze tak byłam trochę taka podbudowana, bo tak myślałam sobie, a może jednak pojadę do tego domu na święta, długo nie widziałam rodziców. No, ale teraz już w tym tygodniu zdecydowaliśmy, że na pewno nie pojedziemy. Więc tyle się zmieniło, że jest mi przykro. A po drugie zaczęłam się zastanawiać, co mam zrobić na te święta. I myślałam, a może kuzynkę zaproszę, a może kolegę zaproszę. A mąż mówi, no jak będziesz zapraszała, skoro jest koronawirus i to jest niewskazane. No i tak smutno, takie święta we dwójkę.

**Czy może kojarzysz jakieś nowe ograniczenia, które zostały wprowadzone w tym tygodniu w Polsce?**

Na pewno te dzisiejsze. Czyli od 16 kwietnia chyba mają wejść te rozporządzenia, że trzeba wszędzie zakrywać noc i usta. I nosić maseczki. Maseczek w ogóle nie mamy. Nie wiem, cudem mi się udało, zamawiałam mężowi prezent na zajączka i przypadkiem dorzucili mi do tego prezentu maseczkę (śmiech). Tak że to jest jedyna maseczka, jaką mam. No to to. I jakieś tam jeszcze były zaostrzenia, coś mówili o szkołach, o maturach. To tak naprawdę pozostały chyba te same, które były wcześniej, mamy je kontynuować i starać się nie wychodzić z domu. Podobno Biedronka zaczęła dowozić rzeczy do domu.

**A czy te ograniczenia, które zostały teraz wprowadzone, co myślisz o tych ograniczeniach?**

Myślę, że mogły być wprowadzone wcześniej. Bo wiadomo, że to się… Wszyscy wiedzieli, że wirus przenosi się drogą kropelkową. Więc nie wiem, co jest logiczniejsze, zakrywać twarz szalikiem czy wkładać rękawiczki (śmiech). Wydaje mi się, że każdy ma możliwość, ma jakąś chustę w domu. I byłoby to bezpieczniejsze. No, ale wydaje mi się, że za późno wydali ten komunikat. Ale dobrze, że w ogóle.

**W tym tygodniu wychodziłaś z domu?**

Wychodziłam. Mam pielgrzymki do paczkomatu. Do paczkomatu, no to przyznaję, że byłam kilkukrotnie w tym tygodniu. Ale mamy bardzo blisko ten paczkomat. I biorę rękawiczki, wszystkich omijam po drodze. Tak że powiedziałabym, że to jest taki spacer, żeby nie zwariować. I byłam też na zakupach w Rossmannie.

**Co dla ciebie teraz, w tej sytuacji jest takim największym wyzwaniem?**

W tym tygodniu bardzo zaczęłam odczuwać, nad tym się zastanawiałam też… Dlatego, że strasznie przeżywam każdą trudną sytuację, która jest w pracy. A wiem, że w biurze bym tego nie przeżywała. I że miałabym to tak, ech, OK, to pogadajcie sobie. Ale tutaj strasznie mnie wszystko rozkleja. Ktoś do mnie napisze coś niemiłego, jakiś szef mówi, że coś ma być zrobione, a nie miało być. No wiadomo, takie codzienne sytuacje. I od razu chce mi się po prostu płakać. I ja jestem pewna, że to chodzi o to, że cały czas siedzę w domu. I nie mam żadnego innego środowiska. I tak naprawdę nie mam żadnej odskoczni od tego trybu życia. I to jest po prostu tak strasznie męczące, że człowiek zaczyna wszystko bardzo personalnie przyjmować. Bo tak naprawdę całe życie moje teraz się kręci wokół mnie i mojego męża. I to jest bardzo dziwne. Bo tak zazwyczaj to są jeszcze znajomi, jest jakaś perspektywa, że się do tych rodziców pojedzie. Są inni ludzie, z którymi się cały czas człowiek styka na ulicy czy w pracy. I większość tej empatii przekazuje powiedzmy czy jakichś uczuć na tę drugą osobę. A tutaj jesteśmy we dwójkę i po prostu wszystkie sprawy narastają. Nie ma odskoczni i to jest bardzo trudne.

**Czyli zwykle, jak takie sytuacje miały miejsce w pracy, to co kiedyś robiłaś?**

Zależy od dnia. Wiadomo, że czasem się chciało płakać. Ale raczej po prostu wychodziłam, szłam po kawę. I to już było coś innego. Albo nawet, moja koleżanka miała taką metodę, że jak widziała, że coś się dzieje, albo ja widziałam, że z nią już jest niedobrze, to po prostu wychodziłyśmy z naszego piętra i jechałyśmy kilka pięter wyżej. Żeby zupełnie się oderwać od biura. I sobie w innym biurze gdzieś tam piłyśmy tą kawę, jadłyśmy jabłuszko. I to było takie coś zupełnie innego. A teraz ktoś na mnie naskoczy i co ja mam zrobić? Do łazienki pójść?

**I co robisz, jakoś starasz się sobie z tym radzić?**

Też nie za bardzo mam możliwość. Dlatego że jesteśmy, mamy obsługiwać telefon. I kazali nam zainstalować aplikację, to się nazywa awaja. Ogólnie okropnie to działa, cały czas się zwiesza. Ludzie dzwonią, ja nie mogę odebrać. Potem ci ludzie się denerwują, że nieodebrane. I tak naprawdę nawet nie mamy możliwości odejść za bardzo. Bo ktoś może dzwonić. Też sprawa jest taka, że nie mogę mieć, nie słyszę w ogóle telefony, bo muszę mieć włączoną tą awaję na słuchawkach. Bo inaczej nie mogę się dogadać z dostawcą. Bo komputer nie wyłapuje w ogóle dźwięków. Więc jak chcę iść do toalety, to muszę pisać koleżance, czy ona jest na telefonie. I co najmniej ileś osób musi być na telefonie. Wcześniej w biurze było tak, że po prostu mówiłam do tej swojej Mirki: dobra, Mirka, wychodzimy. Wystarczyło powiedzieć jednej osobie – jesteś na telefonie? I ktoś mówił, jest. I mogłyśmy sobie iść. A teraz nagle się okazało, że są jakieś nowe zasady i wszyscy mają być na tym telefonie. Jedna osoba tylko może zejść. Więc na 3 osoby jest trudno.

**Czyli co wtedy robicie, jak jedna osoba chce odejść?**

No cały czas jesteśmy, mamy jakąś grupową konwersację, w 3 osoby i musimy się umawiać. I pisać, idę do toalety. Albo idę na obiad, wracam za tyle i tyle. No i trzeba się dogadywać. Bardzo niewygodne.

**Czyli jeśli w trakcie takiego dnia pracy coś się takiego wydarzy, co sprawia, że jest ci przykro czy chce ci się płakać czy coś takiego…**

Nie mam gdzie uciec (śmiech).

**To nie masz możliwości zareagowania na to jakby w trakcie pracy.**

Tak. Znaczy mogę płakać, tyle mi zostało.

**Ale zdarzyło ci się popłakać w trakcie pracy przez to?**

Mi raczej płacz przychodzi bardzo łatwo. Ogólnie rozumiem, że są ludzie, którzy nie płaczą w ogóle. Ale na przykład, nie wiem, złoszczą się i, nie wiem, boksują sobie albo coś ćwiczą czy coś. Ja, najprościej mi się jest popłakać. I ja potem mam naprawdę już do wszystkiego… Już mi jest wszystko jedno. Tylko to jest najgorsza moja wada. Bo tak naprawdę najbardziej nieodpowiedni sposób reagowania na emocje. A tutaj mam ten komfort, że jak się coś stanie i uronię łezkę, nikt się nie będzie śmiał, nikt mi nic nie powie. A w pracy nie można, nie da się.

**Czyli też masz trochę możliwość wyrażania tych emocji w trochę inny sposób niż zwykle.**

To tak. Też bardzo dobrze, jak się zdenerwuję, to zazwyczaj zaczynam gadać do męża: ja nie mogę, oni są jacyś nienormalni! Tak sobie pogadam, pogadam, opowiadam historię, to co się wydarzyło. I mi przechodzi.

**Czyli jak skończysz ten cały dzień pracy, to co zwykle wtedy robisz?**

Czytam, oglądam serial.

**Bo jak o tym opowiadasz, to brzmi strasznie stresująco, że musisz być cały czas przy tym telefonie, nie możesz sobie zrobić przerwy wtedy, kiedy chcesz.**

No nie bardzo. I jeszcze najgorsze, jak coś by się stało i nie daj boże nie odbierzemy telefonu, to przychodzi raport. I zaraz są spytki, dlaczego telefon nie został odebrany. I każdy telefon musi być zalogowany. Więc czasem dzwoni ktoś, gadają po tym niemiecku bardzo szybko albo z akcentem, ja ich nie rozumiem w ogóle. I ja mam wiedzieć, kto do mnie dzwonił. Ja muszę to w specjalnym narzędziu zalogować, bo inaczej wyciągają konsekwencje. Jakoś to liczą tam. Tak że jest stresująco pod tym względem.

**Powiedziałaś, że jak kończysz pracę, to sobie oglądasz film i czytasz.**

Tak, tak.

**I to pomaga ci to napięcie zniwelować?**

Myślę, że bardzo. Szczególnie czytanie.

**A widzisz, jak sobie inni ludzie dookoła ciebie radzą z tą sytuacją? Mówię o koleżankach z pracy, ale nie tylko.**

Jesteśmy tak poddenerwowane, że jak rozmawiałyśmy na Skypie o Wielkanocy, to wszystkie trzy płakałyśmy (śmiech).

**Ale one też przez pracę?**

No, ogólnie. Wiadomo, że stresy, też dostajemy dziwne, dodatkowe zadania, które w ogóle nie wiążą się z naszym krajem. To też jest irytujące, bo przez to zaniedbujemy pracę na naszej stronie. No i one też, tak samo załamane, że nie mogą do domu jechać. Bo wszystkim się bardzo dobrze… Może nawet nie chodzi o święta, ale wszystkie potrzebujemy odpoczynku. I bardzo dawno nie było już żadnej takiej przerwy. Ja na przykład miałam wzięty dodatkowy dzień wolny we wtorek, bo liczyłam z mężem, że trzeba będzie przebić się przez korki, jak będziemy wracali po Wielkanocy. I mimo, że jestem w domu, to i tak sobie zostawiłam ten dzień wolny. Bo po prostu potrzebuję, przez te 4 dni posiedzieć i żeby nikt mi nie truł i żebym się nie denerwowała. I w końcu wrócić do tej mojej idealnej równowagi.

**Ale ta sytuacja, o której opowiadasz teraz, takiego dużego stresu w pracy, ona trwa od samego początku, jak przeszłaś na home-office?**

Tak. Tak. Znaczy, no jeszcze tam powiedzmy pierwsze 3 dni, no to jeszcze super, super. Ale człowiek po pierwsze dziczeje. A po drugie, naprawdę trudno się… Ja wiem, że się powtarzam o tym zdystansowaniu. Ale trudno się zdystansować i zrozumieć, że ktoś nie mówi mi czegoś złośliwie, tylko po prostu nie wiem, mam zmienić coś w swoim działaniu albo coś inaczej zrobić. Trudno się oderwać.

**Trudno się oderwać, bo tak jak powiedziałaś, nie możesz gdzieś wyjść czy się spotkać ze znajomymi.**

Tak.

**Ale to powiedzmy czytanie książek po pracy pozwala trochę jednak się…**

No jednak wkracza się wtedy w inny świat. I nie myśli się o tym swoim. Więc tak, uważam, że to pomaga. Tak samo seriale. To są jakieś tam opowieści o innych ludziach. I nie wiem, czy pamiętasz, ale mówiłam ci, że jak się stresuję, to czasem tak sobie wyobrażam, że jestem tak mała i nieznacząca, że te problemy są tak małe i nieznaczące. I jak siedzę przy komputerze i coś mnie denerwuje, to trudno mi się jest w ten sposób, w każdym razie w domu, zdystansować. Natomiast, jak już jestem po pracy i siedzę, oglądam te filmy o innych ludziach, którzy mają jeszcze gorsze problemy, myślę sobie – i po co ja się martwiłam? Jutro na luziku podejdę do tej pracy. No i co? Idę do tego biurka, siadam. I znowu coś ode mnie chcą, i znowu mnie denerwują (śmiech).

**Ale w weekendy nie pracujesz?**

Nie, nie, nie. No, ale to… Wypadałoby posprzątać w domu, no to prawie pół soboty już zajęte.

**Czyli nie starcza ci ta sobota i niedziela, żeby się tak jakby oderwać od tego?**

Nie, nie, nie. Zupełnie.

**Czyli jeśli chodzi o twoje koleżanki z pracy, to one też tak samo reagują na tę sytuację w pracy jak ty. A jakieś jeszcze inne osoby w twoim otoczeniu, czy widzisz, jak sobie teraz radzą, jakie działania podejmują?**

Koleżanki, zakładam, że się mniej stresują. Też zauważyłam, że zależy od dnia każdej. Na pewno się denerwują tą sytuacją świąteczną. I na pewno denerwuje je to, jak wygląda teraz praca, w ten sposób. A inne osoby, no powiem szczerze, że tak jak sobie myślę, to bardzo dużo osób jest poirytowanych. Bo projekty stanęły, nie wiadomo, co zrobić. Wszyscy są w impasie ani w tą ani we w tą. Tutaj ostatnio rozmawiałam z kolegą, jego firmę mieli przenosić, zmieniać lokację. No i ta sama sytuacja, nie bardzo wiedzą, co mają robić. Tutaj jakiś szef jest, jakiegoś tam nie ma, nikt nie jest do końca decyzyjny, a decyzje są wycofywane cały czas. I takie trwanie w zawieszeniu, bardzo nie lubię takich rzeczy. Bo lubię mieć pewniki zawsze. A nie takie ni w tą ni we w tą.

**A czy obserwujesz, jakie działania oni podejmują, żeby sobie radzić teraz?**

Tak sobie teraz myślę i wydaje mi się, że wszyscy po prostu czekają. I raczej pocieszają się myślą, że wcześniej czy później ta sytuacja się rozwiąże. Ale swoje trzeba przecierpieć i przeczekać.

**Czyli tak jak ty trochę.**

Chyba tak.

**A słyszałaś o jakichś takich zachowaniach wśród twoich znajomych i rodziny, które są dziwne? Są związane z epidemią i są dziwne dla ciebie.**

Na pewno przypomniało mi się jeden, to koleżanka mi opowiadała. To jeszcze było przed tym wielkim wybuchem, przed tymi home office’ami. Ale opowiadała, że jej teściowie są tak strasznie przerażeni, że ona poszła do łazienki i chciała umyć ręce. I naciska ten nie dyfuzor, tylko jak to się nazywa? No, naciska ten żel, a tam jej się normalnie woda leje na ręce. No i ona tak nalała sobie tę wodę na rękę, rozsmarowała, chciała spłukać, pochyliła głowę – alkohol. Mówi normalnie, że wlali tam alkohol. I że właśnie odkażali wszystkie klamki. I wtedy ją to bardzo śmieszyło. Natomiast na pewno przesadzali mocno. Jeżeli chodzi o jakieś dziwne jeszcze zachowania… Nie, to chyba nie słyszałam. Oprócz tego, to było bardzo zabawne. Raczej znam ludzi, którzy tak racjonalnie do tego podchodzą.

**Racjonalnie teraz podchodzić do tego, to znaczy jakie to są zachowania?**

Zachowywać wszelką ostrożność, natomiast sprawiać, żeby te zasady no nie stały się właśnie takie, może nie nienormalne, bo to jest złe słowo, ale żeby nie stały się manią, o to chodzi. Nie dać się zwariować.

**To chciałam z tobą porozmawiać o zakupach. Jak teraz wyglądają twoje zakupy?**

Ja bardzo dużo wcześniej już kupowałam przez internet. Bo nie lubię chodzić, znaczy lubię chodzić po sklepach, ale ogólnie nie lubię grzebać. Na przykład są kobiety, które bardzo lubią iść na przecenę i wygrzebywać jakieś tam ciuszki. A mnie to denerwuje. Ja albo wchodzę do sklepu i widzę, że coś mi się podoba. Albo mi się nie podoba, to nie kupuję. No i internet jest super rozwiązaniem, bo wybieram dokładnie to, co chcę. I nie muszę chodzić na kompromisy. Więc tutaj się nic nie zmieniło. Oprócz tego, że pozwalam sobie zamawiać więcej książek. Chociaż mam też zasadę, zamawiam tylko te książki, które wiem, że przeczytam więcej niż 1 raz. Bo takie, które przeczytam tylko 1 raz, to ściągam na Kindle ‘a. Więc ostatnio właśnie przyszło kolejne duże zamówienie. Widzę różnicę, bo wcześniej zamawiałabym te wszystkie rzeczy jak najtaniej, czyli Pocztą Polską. A teraz zamawiam wszystko do paczkomatu. Żeby po prostu zmniejszyć to chodzenie. Nawet gazetę zamówiłam przez kuriera. Bo okazało się, że Przekrój wysyłają. Więc Przekrój mi też przyszedł. Przyjechał pan, zadzwonił do domofonu, powiedział, żebym zeszła. Ja zeszłam, on na ziemi przed blokiem zostawił ten Przekrój w kartoniku. I patrzył, jak odbieram (śmiech). Jeżeli chodzi o inne sprawy, no to takie rzeczy jak pieczywo i produkty spożywcze, to myśleliśmy, żeby je zamawiać, ale problem jest taki, że teraz oczekiwanie na zamówienie to jest bardzo długi czas. Więc raz w tygodniu staramy się jeździć na takie duże zakupy. Żywimy się głównie chlebem tostowym. Bo się nie psuje (śmiech). A jeżeli chodzi o Rossmanna, to też chciałam zamówić z Rossmanna rzeczy, ale bardzo długo trwało oczekiwanie. A ja potrzebowałam płynu do naczyń i Domestosu teraz. Więc po prostu poszłam. Tylko wiem, że oni też pakują zamówione produkty i można odebrać na miejscu. No, ale nie widzę sensu w tym. Jak już się wychodzi z domu, to już chyba lepiej sobie wybrać to, co się chce. Tak mi się wydaje.

**A z tym Rossmannem to wcześniej zamawiałaś produkty z dostawą do domu?**

Nie, nie, zawsze kupowałam… Tak że tym razem zrobiłam tak samo. Zresztą moja mama powiedziała, że była w Rossmannie. Więc stwierdziłam, jak już mama była w Rossmannie, no to może też się przejdę.

**Czyli w tym tygodniu, te książki, które zamówiłaś, to zamówiłaś je w tym tygodniu, czy przyszły w tym tygodniu?**

Zamówiłam i przyszły w tym tygodniu.

**A gdzie zamawiasz książki?**

Kiedyś zamawiałam na Chodniku Literackim. Ale teraz widzę, że mają duże braki. I ostatnio zorientowałam się, że na stronie Znaku jest bardzo dużo książek. Takich, na które trudno trafić. Więc zamówiłam sobie właśnie ze Znaku. A od męża poprosiłam na zajączka z wydawnictwa Czarnego. Coś miał mi wybrać z listy. Nie wiem, co wybrał, więc zobaczymy.

**Czyli nie ma czegoś takiego, że wcześniej kupowałaś to stacjonarnie a teraz zaczęłaś to kupować przez internet.**

Nie. A może są. Apteka. Apteka. Zamówiłam Collaflex na stawy (śmiech). Jakąś tam polopirynę. I stwierdziłam, że to bezpieczniejsze. Dlatego, że nie potrzebowałam tych rzeczy na już. I byłam pewna, że pomimo, że tam było napisane, że 10 dni roboczych, ja byłam pewna, że mi przyślą wcześniej. Oczywiście przysłali po 14 dniach roboczych, więc naczekałam się jak głupia. Ale rzeczywiście wcześniej normalnie bym poszła do apteki, nie zamawiałabym. Tak że ta jedna rzecz.

**A myślisz, że to, że teraz masz już jakieś takie doświadczenie z tego zamawiania lekarstw online, czy jak już cała sytuacja z pandemią się skończy, czy myślisz, że mogłabyś też takie lekarstwa zamawiać przez internet?**

Jak najbardziej. Znaczy, nie wiem, jak to działa z lekarstwami na receptę. Ale w sumie takich nie zamawiam. No, jak bym miała możliwość, to jak najbardziej. Myślę, że jest to wygodniejsze. No i tańsze tak naprawdę. Bo apteki bardzo często mają wysoką marżę. Na przykład ta apteka Gemini, powiedziałabym, że ma ceny konkurencyjne.

**Czyli te rzeczy, które zamawiałaś w ciągu kilku dni, to jeśli to były rzeczy, które były ci jakoś niezbędne, potrzebne na już, to wtedy szłaś do sklepu. A jeśli to coś, co nie jest niezbędne na teraz, to zamawiałaś to przez internet.**

Tak.

**Czy ty te zakupy książkowe traktujesz jako takie zakupy dla przyjemności, czy jako takie, które są ci potrzebne? Jakie masz podejście do tych zakupów?**

Folguję sobie (śmiech). Ogólnie… Może nie, że mam problem, ale bardzo, bardzo, zawsze lubiłam kupować książki. I przyznaję, że jak już jestem dorosła, to potrafię rozróżnić książkę, która powinna być w moim księgozbiorze i mam nadzieję, że moje dzieci będą ją czytać. Od takiej, którą przeczytam tylko raz. Więc powiedziałabym, że robię teraz świadome zakupy. No i wynagradzam sobie to, że nie wychodzę z domu, że trudno o jakąkolwiek inną rozrywkę. Należy mi się za te stresy w pracy (śmiech).

**Czyli teraz książek kupujesz więcej niż przedtem.**

Tak. No naprawdę, wcześniej starałam się bardzo, bardzo ograniczać. I zazwyczaj zamawiałam sobie książki jakieś tam określone, a to na urodziny, a to pod choinkę. No już abstrahując od tego, że nigdy nikt nie realizował mojej listy (śmiech). Moja ciocia raz, 3 lata temu kupiła mi książkę z listy. Bo u nas przed świętami robi się listy w rodzinie. A teraz po prostu patrzę, chcę, zawsze chciałam. I myślę: dlaczego ja tego wcześniej nie zrobiłam, przecież ta książka była w moim koszyku już od roku.

**Ale robisz te zakupy książkowe tak, że masz swoją listę książek, które chcesz przeczytać i potem je wyszukujesz? Czy w jaki sposób wyszukujesz te książki?**

Wchodzę na stronę i po kolei przeglądam każdą kategorię. I wtedy mi się przypomina, jaką książkę miałam kupić i chciałam mieć. No i plus te książki, które od dłuższego czasu leżą w koszyku, ale czas sobie myślałam, no nie, odpuszczę sobie, są ważniejsze rzeczy. No a teraz…

**W jakim koszyku?**

Że mam konto na stronie i po prostu do koszyka wkładam. One tam sobie wiszą, wiszą, dopóki ich nie kupię. Albo dopóki nie znikną. O właśnie, to było najtrudniejsze, co chciałam zrobić. Bo próbowałam zrobić zakupy na Rossmannie przez internet. Ale co wrzucałam rzeczy do koszyka, to po 5 minutach wchodziłam do koszyka i już jej nie było. Tak szybko tam rozchodziły się rzeczy. I to takie rzeczy typu kapsułki do prania. Albo Domestos. Wrzuciłam Domestos żółty i wyskoczyło mi po 10 minutach, że go już nie ma. Zanim ja zdążyłam skończyć zakupy. A Coccolino to już o w ogóle.

**Z tymi zakupami w Rossmannie było tak, że pododawałaś sobie te produkty do koszyka, jakby chciałaś to zamówić online.**

Tak, chciałam.

**W którym momencie stwierdziłaś, że jednak nie?**

Jak mi któryś raz te rzeczy z koszyka uciekły. A i tak miałam wątpliwość, bo było kilka rzeczy, które rzeczywiście no… Tak jak ten płyn do mycia naczyń, naprawdę już nie było płynu do mycia naczyń. Ale stwierdziłam dobra, rozcieńczę wodą, może jakoś przejedziemy te kilka dni. I też miałam tą głupią nadzieję, jakie tam 10 dni roboczych, może szybciej przyjdzie. Ale zdenerwowałam się rzeczywiście, jak się okazało, że wszystko się z tego koszyka rozchodzi, że te zakupy w ogóle nie mają sensu tak naprawdę.

**Czyli po prostu, jak zobaczyłaś, że robisz te zakupy, te rzeczy ci wypadają i…**

I ja musze cały czas zastępować je nowymi. A kilka rzeczy, wchodziłam do koszyka i widziałam, że jest komunikat, ten produkt jest już niedostępny. Ale kilka razy tego komunikatu nie zauważyłam, więc dodawałam do koszyka. I później nie wiedziałam, co z niego uciekło, co ja miałam kupić, a co nie. A przeglądałam Rossmanna zakładkę po zakładce, żeby o niczym nie zapomnieć. Tak że to było bardzo irytujące.

**Ale to myślisz, że to jest tak teraz przez tę sytuację, którą mamy?**

Na pewno. Na pewno, bo… Znaczy pewna nie mogę być, ale tak mi się wydaje. Dlatego że no ile osób robi sobie jakieś tam większe zakupy? Myślę o głupim Coccolino. Jak to jest, ile oni mieli sztuk tego Coccolino, skoro wszystkie zapachy się wykorzystały? Weszłam, były wszystkie. Ale zanim doszłam do zakładki z płynami do płukania, no to wrzuciłam do koszyka i po chwili wypadł. Tak że… No, wydaje mi się, że ludzie właśnie zamawiają takie rzeczy przez internet, bo to nie jest produkt pierwszej potrzeby. Oni nie muszą, ograniczają wyjścia do sklepów. I stwierdzają: o, super, Rossmann dostarczy, to sobie zamówimy w Rossmannie. Nie potrzebujemy niczego na cito, więc…

**Jak zrezygnowałaś z tych zakupów w Rossmannie, zdecydowałaś się do niego pójść stacjonarnie. A nie miałaś jakieś takiej myśli, żeby to zamówić online, tylko w jakimś innym sklepie?**

Próbowałam. I nawet przypadkiem, ja nie wiem, jak to zrobiłam, ogólnie mam ostatnio papkę z mózgu, już jestem po prostu tak… Byłam pewna, że loguję się na Hebe. Założyłam konto, wszystko w porządku. Patrzę, dostaję e-maila rejestracyjnego, a sklep nazywa się Hefe (śmiech). I założyłam sobie konto na jakiejś drogerii Hefe czy czymś takim. Próbowałam usunąć to konto, bo co ja tam będę kupowała? Ale nie wiem jak, nie wiem, chyba muszę im wysłać jakiegoś, muszę poszukać. I stwierdziłam, no dobra, jak już założyłam sobie konto na jakiejś stronie, której w ogóle nie znam. Nawet jej nie sprawdziłam, tylko po prostu założyłam tam konto, no to były tam produkty takie drogeryjne. No i myślę, no nic, no to mogę sobie spróbować zamówić stąd. Szczególnie, że był wielki napis, że nie zmienił się czas przygotowania wysyłki. No i próbuję szukać i co? Domestos- nie ma, Fairy – nie ma. No to co ja tam kupię? Więc nie było tych rzeczy. I nie wiem, czy to dlatego, że to jakiś mały sklep i wszyscy ludzie zaczęli nagle tam kupować Domestosy. Czy po prostu ja zalogowałam się na jakimś, nie wiem, nie wiem.

**Czyli w Hefe nie kupiłaś tych rzeczy. A wróciłaś w końcu na tę stronę Hebe?**

Tak, tylko znowuż w Hebe był bardzo mały wybór. I z tego, co pamiętam, wydaje mi się, że był jakiś produkt, który bardzo, bardzo chciałam i go tam nie było. Wydaje mi się, że to było to Coccolino. Naprawdę, ja mam taki sentyment do Coccolino, że musi być Coccolino. I chyba nie było. Ale stwierdziłam…

**Ale masz swój jakiś taki ulubiony zapach?**

Niebieski. Niebieski Coccolino. Intense nie jest na moją kieszeń (śmiech).

**To jest tak, że one się różnią zapachami?**

Tak. Tak. Bo są takie duże, tańsze i one są jakby zwykłe. I są Intense, które pachną bardzo mocno. I ten zapach utrzymuje się dłużej. I one są mniejsze i droższe. Ale ja nie kupuję Intense, bo moją wielką wadą jest to, że leję bardzo dużo płynu. No i jest to nieekonomiczne po prostu w moim gospodarstwie domowym, żebym miała Intense.

**Czyli głównym takim produktem, bez którego by się te zakupy nie odbyły, to właśnie był ten płyn do płukania.**

Płyn do płukania, tak.

**No dobrze, to poszłaś do Rossmanna na zakupy. Czy jakoś się przygotowywałaś?**

Tak. Nie wiem, dlaczego, ale pomyślałam, że dobrym pomysłem będzie związanie włosów, żeby mi się nigdzie tam nie pałętały. Oczywiście wybrałam sobie kilka par rękawiczek.

**A o co chodzi z tymi włosami?**

Jakoś tak wydaje mi się, że jak są rozpuszczone, to cały czas człowiek poprawia, coś majstruje przy twarzy. I stwierdziłam, że skoro już teraz są takie zaostrzenia higieniczne, no to byłoby raczej nierozsądnym iść na zakupy, majtać sobie tutaj najpierw na koszyczku rączką, na koszyczku, który dotykało kilka osób, a potem gdzieś przy włosach. Tak że miałam rękawiczki i związane włosy.

**I miałaś jakąś listę zrobioną?**

Tak, miałam listę. Jako że cały czas mi wypadały na stronie Rossmanna rzeczy z koszyka, to w końcu wszystkie zaczęłam zapisywać. Jak już się zorientowałam, że te zakupy nie mają sensu, no to przynajmniej miałam listę.

**Jak już byłaś w sklepie, czy to już było, bo ja nie wiem, jakie są teraz obostrzenia, ile osób może być w sklepie?**

5. Znaczy 5 w Rossmannie. I akurat trafiło mi się. A, jeszcze jedno przygotowanie – nie ubrałam butów na obcasie. Ubrałam adidasy. Jak bym stała bardzo długo w kolejce.

**Byłaś przygotowana na to.**

Tak. Ostatnio, w tamtą sobotę, mąż stał do Aldiego godzinę. I kolejka zawijała się aż dookoła dyskontu. Tak że ja włożyłam adidasy. Ale miałam szczęście, bo w sklepie było 6 osób. Bo ja najpierw, przy sklepie był stoliczek z płynem do dezynfekcji. No więc zdezynfekowałam ręce, włożyłam rękawiczkę jedną, żeby nie marnować. Stwierdziłam, że to jest rękawiczka na koszyczek. I weszłam do sklepu. I pani mi mówi, że jest za dużo osób i muszę poczekać. I dosłownie 3 minuty czekałam. I dwie osoby wyszły. I pani zawołała: można już wejść. No więc weszłam i zrobiłam zakupy. I w ogóle okazało się, że jak wychodziłam, to już była bardzo duża kolejka, także satysfakcja. Ale niestety odruchy były silniejsze na tych zakupach ode mnie. Czyli tak, weszłam do sklepu, minęła minuta, przełożyłam koszyk z ręki z rękawiczką do drugiej ręki. Po czym się zorientowałam, przecież ja mam tę rękawiczkę po to, żeby trzymać w tej ręce koszyk. I znowu przełożyłam. Po czym pomyślałam, no i co teraz z tą ręką? Będę dotykała produktu, a potem będę… I nie wiedziałam już jak zareagować. Po czym tak się tym zafrasowałam, że myślę, co ja teraz mam zrobić? A na koniec wyszłam ze sklepu i stwierdziłam: teraz dezynfekuję ręce jeszcze raz. No i odruch – ręką naciska. Ludzie patrzą na mnie, jak ja mogę ręką naciska. Ja tak: łokciem, łokciem (śmiech).

**Czyli bardzo dużo to wymaga takiego myślenia cały czas o tym wszystkim, co robisz.**

Skupienia, tak, tak. Bo nawet tak zastanawiałam się, czy jest sens mieć rękawiczki na takich zakupach. Skoro ja tymi rękawiczkami dotykam produktów, które potem będę bez rękawiczek dotykać w domu. No to… No nie wiem, nie wiem, jak to zorganizować. Przecież nie będę ich płukała.

**A widziałaś jak się inni ludzie zachowywali w tym sklepie?**

Całkiem normalnie. Nie mieli list. Ogólnie było dużo kobiet, które po prostu wybierały sobie tusze do rzęs. Albo stały przy kolorówce. Ja myślałam, że będą głownie w takich celach jak ja, takich tutaj zdroworozsądkowych, Domestos, płyn do prania. Ale nie, panie tylko przyszły po tusz do rzęs. I widziałam kilka osób, które wychodziły bez niczego. Czyli przyszły, nie znalazły czegoś i wyszły. Albo przyszły pooglądać kosmetyki.

**I co myślisz o takich zakupach teraz?**

Znaczy ja rozumiem, że jeżeli jakaś pani musi pracować z biura, no to jak jej się skończyła ta maskara, no to oczywiście musi ją gdzieś kupić. Ale naprawdę widziałam, może nie kilka osób, bo była nas tylko piątka, ale widziałam, jako że te osoby cały czas rotowały i zmieniały się, no to widziałam 3 panie, które po prostu wybierały sobie takie… rzeczy, które nie są niezbędne. Ja bym takie zakupy zrobiła przez internet. W sumie też kosmetyki kupuję przez internet.

**A teraz takie zakupy kosmetyczne robiłaś od tego czasu, kiedy się zaczęła…**

Jeszcze nie. Ogólnie raz na 3 miesiące robię takie ogromne zakupy. I kupuję parę podkładów, parę pudrów. Takie, które są dla mnie najlepsze. I jakieś tam rzeczy, których też nie muszę kupować, ale je kupuję (śmiech). Tak że robię to raz na 3 miesiące, bo akurat na tyle potem mi starczają. Tak że wypada, że za miesiąc będę robiła takie duże zakupy. Zresztą może nawet później, bo teraz w domu nie używam. Raczej tylko kremu i ewentualnie pudru ryżowego.

**Czyli się to zmieniło trochę.**

No tak. Nie maluję się w domu. Więc rzeczywiście nie wykorzystuję tak tych kosmetyków, takich do twarzy przynajmniej.

**Jeszcze chciałam do Rossmanna na chwilę wrócić. Jak płaciłaś za te zakupy?**

Kartą. I właśnie nie byłam też pewna. Bo podeszłam do kasy i byłam, nie wiedziałam, jak się zachować, czy mogę kasować w kasie samoobsługowej, czy oni mają jakieś zalecenia, że… Chociaż to by było bezsensowne, bo to by właśnie jakby przeczyło tym zaleceniom, gdyby panie sprzedawczynie kasowały produkty. Tak że tak stałam przy tych kasach, nie bardzo wiedziałam, co zrobić. Ale pani nie było, więc zaczęłam kasować sama produkty. Po chwili podeszła do drugiej kasy samoobsługowej druga pani, też nie wiedziała co zrobić, więc też zaczęła kasować. No i płaciłam kartą. I to też ciekawe, że jakoś nie zwróciłam uwagi na informację, że zwiększono możliwości na płatność zbliżeniową. Jak już byłam w tej galerii handlowej, bo byłam w Rossmannie, który ogólnie jest w takiej galerii, no jak wychodziłam, to poszłam do apteki. Bo tak długo czekałam na zamówienie z tej internetowej apteki, że w ciągu tych 14 dni roboczych kilka rzeczy musiałam znowu kupić. I właśnie w tej aptece było tak, że wyszło mi chyba siedemdziesiąt coś złotych. I zastanawiałam się… Znaczy byłam na siebie zła, bo wiedziałam, że muszę wyjąć rękawiczkę i wpisać PIN. A pani właśnie powiedziała, że jest teraz zwiększony limit i za stówkę można płacić zbliżeniowo bez PIN-u.

**A normalnie za zakupy też płaciłaś kartą?**

Zawsze płacę kartą. Ja ogólnie się trochę brzydzę gotówki. Bo ludzie dotykali, ja też w tramwaju nigdy nie chwytam się tych rurek, nie siadam na tych krzesełkach. Zawsze sobie stoję z tyłu i się jakoś tak…

**Czyli w tej galerii byłaś w Rossmannie i w aptece. Jeszcze gdzieś poszłaś?**

Nie.

**Powiedziałaś, że planowałaś te zakupy w takim sensie, że przygotowałaś sobie tą listę, związałaś włosy, wzięłaś jedną rękawiczkę. Ale chodzi mi o to, czy planowałaś, kiedy tam pójdziesz, o jakiej godzinie, jakiego dnia?**

Raczej jakiego dnia wynikało z tego, że poprzedniego dnia nie udało mi się zamówienie na stronie Rossmanna, a nie mieliśmy już płynu do mycia naczyń. I poszłam po prostu po pracy. I miałam nadzieję właśnie, że mało ludzi będzie o tej godzinie. I się nie myliłam. Bo rozmawiałam z koleżankami. I podzieliły się swoim doświadczeniem, które wygląda tak, że wstawały o 5 rano albo o 6, żeby jechać na zakupy z samego rana, jak tylko otworzą market. I kończyło się to tak, że stały w okropnych kolejkach, bo wszyscy ludzie myśleli tak samo. Tak że zakupy rano o 7 chyba nie bardzo mają sens. A nawet, mieszkamy naprzeciwko, nie wiem, taki pasaż handlowy, takiego pasażu handlowego. I w tym pasażu, znaczy patrzę przez okno, jak pracuję, to patrzę cały czas przez okno. I rzeczywiście ludzie się kręcą cały czas od ósmej, czyli tak jak on jest otwarty, do powiedzmy 11. I tam jest wtedy największy ruch. A potem nie ma prawie nikogo.

**Po pracy to o której mniej więcej?**

Byłam tam około 18.

**A do tego pasażu naprzeciwko też chodzisz teraz?**

Byłam w tym tygodniu. Wiem, że pytałaś o to, ale teraz mi się przypomniało. Jak szłam do paczkomatu po te książki, to stwierdziłam, że jak już idę, to kupię mężowi bułki na śniadanie Bo wygląda tak, że to jest zbiór takich malutkich sklepików. I ogólnie wszystkie sklepiki, typu jakieś tam sklepiki, takie niespożywcze się pozamykały. Więc zostały same piekarnie Julki, o takie o. I mięsny. I rano, przed odebraniem tej paczki, poszłam po te bułki. A jeszcze mi się przypomniało, że jest tam sklep monopolowy. Nie, nie, nie kupowałam nic dla siebie (śmiech). Jak już stwierdziłam, że jak już weszłam do tej Areny, bo tak to się nazywa, no to może kupię jakiś likier dla rodziców i im wyślę. Na święta. I tak właśnie zrobiłam. Tak że poszłam odebrać paczkę i poszłam też po bułki, po alkohol. Ale planowałam wszystko tak, żeby zamknąć to w tym jednym wyjściu. Tak że musiałam iść do paczkomatu i specjalnie czekałam do następnego dnia, żeby od razu kupić te bułki i alkohol. Ale nie dla mnie.

**A dlaczego do następnego dnia czekałaś?**

Bo dostałam powiadomienie tam powiedzmy o 17 we wtorek. A poszłam w środę rano, bo stwierdziłam, że od razu będę mogła bułki kupić. I likier.

**A te zakupy w tym sklepie konkretnym, to tam jakoś też się zabezpieczałaś?**

Tak. Zdarzyło mi się już kilkakrotnie, może niewiele razy, ale czasem jest tak, że potrzebujemy czegoś do domu naprawdę na już. Zresztą tam jest dobry sklep wędliniarski, więc opłaca się na przykład kupić więcej wędliny i potem ją zamrozić. Bo to nie jest taka delikatesowa, pakowana próżniowo, tylko taka normalna. Ja akurat za mięsem nie przepadam, ale tutaj mówię o mięsożernej części rodziny. Zresztą mają tam jajka z wolnego wybiegu. W każdym razie to są takie malusie sklepiki i zazwyczaj ci przedsiębiorcy są też właścicielami. I to są takie stoiska. I w ogóle to prześmiesznie wygląda, bo teraz te wszystkie stoiska są otoczone taką pleksą. Ci ludzie powiesili sobie pleksi, bo przyklejali. I są tylko takie małe okienka powycinane. NA pieniądze, na czytnik kart. I ewentualnie taka dziurka, żeby podać produkt. Tak że oni też są zabezpieczeni. I przez wejściem trzeba oczywiście odkazić ręce. W ogóle bardzo kręcą się w tej chwili tam ochroniarze. Myślę, że to jest ze względu też na to, że dużo sklepów z odzieżą zostało tam pozamykanych, więc pewnie pilnują tego dobytku tam. Ale oczywiście szłam w rękawiczkach, dwóch już.

**Jeszcze powiedz mi o tych wycieczkach do paczkomatu. Nie wiem, czy ty traktujesz to jako wycieczki?**

Tak. To jest zawsze jakaś odskocznia. No i fajnie się przejść, troszeczkę pooddychać świeżym powietrzem, tak że jest przyjemnie. Miałam taką śmieszną historię, to w tym tygodniu. Nadawałam paczkę do siostry męża, zapomniałam, jak się nazywa to połączenie? Szwagierka? W każdym razie nadawałam do niej paczkę. No i poszłam do tego paczkomatu i było już koło 18. Przyznaje, że pierwszy raz nadawałam paczkę paczkomatem i nie wiedziałam, jak to zrobić. I poszłam w dwóch rękawiczkach, stoję z tą paczką. Ale uprzedził mnie pan, ojciec rodziny. Maseczka, rękawiczki, cały okutany, tylko oczy było widać. I wielka torba z pampersami. I pan stanął o odbierał paczkę. No i stanęłam za nim kilka metrów. No i on już odchodził… Zaczepiłam go. Przepraszam, czy wie pani może, jak mam wskanował ten kod QR, bo pierwszy raz nadaję paczkę paczkomatem. Facet się tak przestraszył, że ja do niego mówię. I tak, odszedł kilka kroków, popatrzył na mnie. Nie wiem, może patrzył, czy nie jestem chora. I mówi, ma pani tam takie oczko, to chyba tam. I tak odsuwał się, jak mówił. I ja tak: dziękuję bardzo. On się tak szybko odwrócił i zaczął bardzo, bardzo szybko iść. I opowiadałam potem mojej kuzynce, z którą rozmawiałam przez telefon, która jest matką trójki dzieci. I ona mówi: no co ty się dziwisz. Nie dość, że żona go wygoniła na zakupy, jak ma jeszcze małe dziecko w domu, no to się stresuje. Ale był tak przerażony tym, że ja do niego mówię (śmiech).

**Czyli ty nie masz takiego strachu przed ludźmi, których spotykasz gdzieś na ulicy?**

Nie, nie, raczej nie. Ja bym się bardziej bała, że ja będę wektorem. Czyli że ja komuś przekażę. A nie, że ktoś mnie zarazi.

**I te paczki, które wtedy nadawałaś, to rozumiem, że takie wielkanocne.**

Tak.

**Normalnie takie paczki wysyłałaś Pocztą Polską, tak?**

No tak, zazwyczaj wybierałam ten najprostszy, najtańszy sposób dostawy. Ale teraz te paczkomaty naprawdę okazują się też bardzo higieniczne. Mam zawsze jedną rękawiczkę, którą biorę do paczkomatu. W sensie świeżą za każdym razem. I po każdym wpisaniu kodu ją po prostu wyrzucam, jak już odbiorę paczkę i wiem, że mam czyste łapki. Ale to z tym kodem QR było bardzo wygodne też. Wystarczyło sczytać, to się w sumie wszystko samo zrobiło. Nic nie trzeba było wpisywać, albo nie pamiętam, że trzeba było.

**A z tymi paczkami to myślisz, że mogłabyś się przerzucić w przyszłości też na nadawanie przez paczkomat?**

Myślę, że teraz będę tylko tak robiła. Właśnie dzisiaj chcę wysłać likier rodzicom. Ale to też chcę połączyć z wyjściem, bo wiem, że do paczkomatu przyszła jakaś książka z wydawnictwa Czarnego, którą mam dostać na Zajączka. Więc mąż czeka z odebraniem tej paczki, dopóki ja nie skończę tej swojej, żeby już wszystko załatwić za jednym razem.

**Czyli tak planujecie wszystko strategicznie, żeby nie chodzić za dużo.**

Tak jest.

**Zakupy spożywcze powiedziałaś, że raz w tygodniu robicie teraz?**

Tak.

**I jeździcie razem, czy tylko ty, czy twój mąż?**

Wcześniej jeździliśmy razem. Dlatego że ja nie lubię, jak mąż robi sam zakupy, bo on kupuje tylko rzeczy z listy. A wiadomo, że ja oczekuję jakiegoś smakołyku z tych zakupów, a tutaj nigdy nie ma, jak nie wpiszę na listę (śmiech). Tak że zawsze jeździliśmy razem. I jak zaostrzyła się sytuacja, też jeździliśmy razem. No, ale teraz nie wpuszczają już chyba w dwójkę. Więc od dwóch dni robię listę na Wielkanoc, myślę bardzo ciężko nad tym… Bo trzeba mu napisać dokładnie. Na przykład napiszę mu owoce, to on nie wie, co ma kupić. Bo muszę napisać 4 jabłka, 5 bananów. Także rozpisuję wszystko, warzywa, owoce, żeby wiedział co kupić.

**Dlaczego w takim razie on jeździ a nie ty?**

Dlatego, że ja mam prawo jazdy, ale ostatni raz siedziałam za kierownicą 8 lat temu. I ogólnie miałam robić taki kurs powtarzający. W sensie chciałam sobie wykupić jazdy i zacząć jeździć autem. Bo bardzo mnie denerwowało to, że jestem niesamodzielna. Bo dobrze jest wsiąść w auto, pojechać po wodę i kogo nie prosić o nic. Ale niestety ze względu na okoliczności, najpierw czekałam, aż zrobi się troszeczkę cieplej. A jak się zrobiło troszeczkę cieplej, to przygnało koronawirusa. I czekam.

**Czyli teraz te smakołyki wpisujesz po prostu na listę?**

No próbuję wymyślać, co tam będę chciała. Ale wiadomo, że fajnie jest pójść do sklepu i patrzeć: promocja na Milkę! Albo są sklepy, w których są takie szczególnie produkty, które warto kupić, ale o których się normalnie nie pamięta.

**A twój mąż jakoś te zakupy planuje, jeśli chodzi o godzinę, w której jeździ?**

Na pewno jak był w tamtym tygodniu na zakupach… Bo to też jest problem, bo jak ja chcę iść na zakupy, no to… Strasznie się o to kłócimy zawsze. Ale mąż się bardzo denerwuje, jak noszę za ciężkie rzeczy. A ja jestem przyzwyczajona. Od zawsze robiłam duże zakupy, jestem przyzwyczajona, że się robi duże zakupy i że trzeba je nosić. Teraz jest problem, bo jak już robimy na cały tydzień, to one zazwyczaj są bardzo ciężkie. Więc w tym tygodniu poszedł sam. Stał tam godzinę w kolejce do Aldiego. Ale poszedł w takich godzinach, żeby po pierwsze ominąć tą godzinę dla seniorów. I po drugie mieliśmy nadzieję, że ludzie po południu będą gotowali obiady. No, okazało się, że stali w kolejce do Aldiego akurat (śmiech).

**Czyli teraz będziecie planować w jakiejś innej godzinie?**

Myślę, że tak. Ja tak chciałam zaproponować, robię właśnie, kończę tą listę, którą zaczęłam i myślę, że może jutro, właśnie też tak około 18-tej. Chociaż… Mam też nadzieję, że jako że ta niedziela była handlowa, mam też nadzieję, że ludzie się trochę poobkupywali, że będzie mniejsze zamieszanie.

**Czy u was się zmieniło coś pod względem tego, co jecie?**

Tak. Wcześniej nie gotowaliśmy w ogóle. Mąż jadł obiady w pracy. Ja też jadłam obiady w pracy. I mąż ma jakąś tam swoją kantynę, natomiast u nas jest tak, że mamy za darmo kanapki, jakieś tam różne rzeczy. Więc zazwyczaj sobie po prostu do tego dokupywałam jakąś zupę itd. Raczej w ten sposób było. A teraz gotujemy. I mąż sobie gotuje sam, bo ja raczej nie przepadam za takimi rzeczami, które on lubi. Największym hitem jest tak zwany przysmak dresa (śmiech). Otóż, jak wygląda przysmak dresa, ja bym tego nie zjadła. Gotuje się makaron, muszą być świderki. Do tego musi być parówka. Parówkę należy podsmażyć na oleju, ma być tłusto. Potem tą podsmażoną parówkę podsmażamy z tym makaronem i z serem. I to wszystko zalewa się ketchupem. I mój mąż je takie rzeczy (śmiech). Przysmak dresa.

**Ale to teraz przysmak dresa częściej gości w waszej kuchni niż częściej?**

No właśnie wcześniej mąż nie miał kiedy sobie robić tego przysmaku dresa. A teraz ma czas. Ale nie, no przyznaję, też sobie tam jakieś kotlety robi itd. Ale on sobie gotuje. Ja natomiast gotuję sobie zupę na 3 dni. I zazwyczaj jem do tego tosty. Bo uważam, że lepiej zjeść mały obiad i duży deser (śmiech).

**Ale powiedziałabyś, że to jecie teraz jest mniej zdrowe od tego, co wcześniej?**

Nie. Wydaje mi się, że nie. Znaczy pod jednym względem, jem teraz czekoladę, a nie powinnam. I chodzę cały czas z wysypką. Więc jest jeden minus. W ogóle przed tą rozmową mi się przypomniało, że przecież jestem niepomalowana. Więc tak troszeczkę zakryłam sobie tutaj i mam nadzieję, że nie widać. Ale naprawdę strasznie mnie wysypało.

**Niestety nie widać za bardzo, czy ktoś jest pomalowany czy nie.**

Ale ja się lepiej czuję. Ja się lepiej czuję.

**Czyli więcej gotujecie. Znaczy w ogóle gotujecie w domu, bo wcześniej raczej nie bardzo.**

Znaczy ogólnie lubimy gotować. Ale to jest też tak, że mamy małą kuchnię, jest po prostu trudno. I tak zazwyczaj było na każdym naszym mieszkaniu. A jeszcze jak się mieszkało ze współlokatorami, to też było utrudnione.

**Ale gotujecie te posiłki razem czy oddzielnie?**

Nie, nie, oddzielnie. Też mąż pracuje, pracował w innych godzinach. I też o innych godzinach jesteśmy głodni. W weekendy jemy razem. No, ale teraz osobno, jak któreś jest po prostu wygłodniałe już. No i tak jak mówiłam, ja też mam inne preferencje żywieniowe.

**Czyli w weekendy teraz też już nie jecie razem?**

Nie, w weekendy właśnie jemy razem.

**A zamawiacie coś do jedzenia przez internet czy przez telefon?**

Tak. Ogólnie to był zawsze nasz zwyczaj, że w sobotę jest zamawiane. Bo tak naprawdę na tych obiadach w pracy raczej oszczędzamy, myślę. No, bo… Myślę, że tak, dlatego że kupuje się już gotowy produkt, to na pewno. Dlatego w sobotę sobie zawsze pozwalaliśmy na zamówienie czegoś. I teraz trochę nam się przesunął ten tydzień. Bo w tamtym tygodniu w tygodniu zamówiliśmy pizzę. Więc ja zakładam, że jutro może być ten dzień z zamawianiem. Ale zamawiamy. Jest teraz taka możliwość wyboru bezdotykowej w ogóle dostawy, czyli zostawiają na progu i pukają. Chociaż nie widzę w tym sensu, bo i tak dotyka ten karton pan. Ja też go dotknę. No, ale…

**Czyli to zamawianie to jest dla was taki zwyczaj.**

Raz w tygodniu, tak.

**A teraz się to przesunęło czy po prostu jest częściej?**

Przesunęło się na inny dzień jakiś tam.

**I to jest tak, że macie coś takiego, co… Chodzi mi o to, czy to jest jakieś takie inne jedzenie od tego, co jecie zazwyczaj? Czy to jest taka większa przyjemność to jedzenie na wynos?**

Teraz, w czasie tego koronawirusa myślę, że tak. Dlatego też w tamtym tygodniu chyba nie doczekaliśmy do tej soboty i zamówiliśmy wcześniej. Bo… No to jest zawsze coś innego. A tak jak na przykład patrzę na tego mojego męża. Kupił sobie kilogram mielonego. I zrobił sobie 4 mielone. I on codziennie je te mielone. No to zbrzydłoby w końcu. Albo ja przez 3 dni ta sama zupa. Jeszcze jak kupowałam zupy z Biedronki, to codziennie była inna. A tutaj cały czas to samo. Więc rzeczywiście jest przyjemniej.

**A skąd zamawiacie zwykle?**

Jest taka pizzeria we Wrocławiu, nazywa się Mania Smaku. I zazwyczaj zamawiamy właśnie z niej pizzę. Chociaż ostatnio mieliśmy ochotę na taką tłustą, studencką pizzę, tak że Pizza Station, sieciówka wjechała.

**Wydaje mi się, że byłam 2 czy 3 razy tylko we Wrocławiu, ale kojarzę te pizzerie. Nie wiem, czy ona jest aż tak sławna.**

Jest znana. I ogólnie Wrocław pizzą stoi ostatnio. Najlepsza pizza we Wrocławiu to jest w Niezłym Dymie. Oni mieli food truck. Ale no niestety nie dowożą. Jest jeszcze Si, też świetna.

**A zamawiacie bezpośrednio z tej pizzerii czy przez jakiś serwis do zamawiania?**

Z pizzerii bezpośrednio.

**I powiedziałaś, że ta opcja tej dostawy bezkontaktowej…**

To jest chyba, bo oni i tak realizują to zamówienie przez jakiś portal ostatecznie. Bo wiem, że pan był z Pyszne ostatnio. A zamawiałam przez stronę. Nie wiem, jak to tam działa.

**Czyli nie masz jakichś obaw przy zamawianiu tego jedzenia?**

Nie. Chociaż przyznaję, 2 tygodnie temu chyba zamawiałam pizzę w sobotę jeszcze. I przyszedł pan. Ale pan, ja nie wiem, czy on był tak skacowany. Taki, powiedziałabym po trzydziestce. Czy on był tak skacowany, czy on był tak zmęczony, ale wyglądał na strasznie chorego. I mieszkamy na piątym piętrze bez windy (śmiech). I pan, jak do nas dotarł, ja jak zamknęłam za nim drzwi, to patrzyłam, czy on ze schodów nie zleciał. Bo był tak wykończony. I zastanawiałam się, czy on jest chory, czy on jest właśnie jakiś wczorajszy. Ale dotarł do samego dołu, więc… Znaczy nie znalazłam go na klatce (śmiech). Ale zaczęłam właśnie o tym myśleć, czy oni są zdrowi. Ale z drugiej strony, no co, na karton mi nakaszle? Nie wydaje mi się, żeby to było niebezpieczne.

**Mówiłaś trochę o tym, że są takie produkty, których potrzebujesz na teraz, na już. I po nie jesteś gotowa wyjść na zakupy bez takiego większego planowania. Ale są też takie, które gdzieś tak odkładasz. I chciałabym zrozumieć, które to są te produkty, że możesz wyjść tylko po to, żeby to coś kupić.**

Zastanawiam się, co to mogłoby być. Myślę, że mógłby być to jakiś ewentualnie lek. Gdyby mi jakiegoś zabrakło, bo apteczkę mam dobrze zaopatrzoną. Ale myślę, że taki podstawowy produkt gospodarstwa domowego, na przykład ten płyn do mycia naczyń. Niby taka głupia rzecz, ale tak w rzeczywistości to jak mam inaczej umyć naczynia bez tego płynu? Albo taki Domestos. Tak że wydaje mi się, że podstawowe właśnie produkty używane w gospodarstwie domowym. I na pewno mleko. I pieczywo. Myślę, że to są takie…

**Jak ty planujesz spędzić Wielkanoc w końcu?**

Raczej, znaczy zastanawiałam się, co zrobić do jedzenia. Tylko jakoś wyżyn nie osiągnę. Ogólnie bardzo lubię piec, ale mamy w mieszkaniu kuchenkę gazową Ewa. Nie wiem, czy kojarzysz. To jest stara kuchenka gazowa. Ogólnie, jak się zapali zapałkę tam w dziurce. A mieszkanie jest w porządku, nie wiem, co oni z tą Ewą zrobili, że ona tam jest. Ale jest. I zapala się zapałką tam na dole. Najśmieszniejsze jest to, że jak to się pali, to nie ma żadnego odgranicznika. Po prostu patrzy się do kuchenki i tam się pali ogień. I pod tym dnem takim kuchenki widać normalnie ogień w środku. Ja się bardzo boję tej kuchenki. A po drugie jest wielki z nią problem, bo ona mi psuje każde ciasto. Żadne tam nie wychodzi, bo wszystkie się palą. Dlatego, że ona nie ma miarki z temperaturą. Ma ośmiostopniową skalę. I nie wiadomo jaki numer to jaka temperatura. Więc spaliłam już na wiór kilka razy tartę. Bo według naszych obliczeń, jakiś tam numer powinien być 180 stopniami, a okazał się 230. Jedyne, co wychodzi z niej to ciasto drożdżowe, bo drożdże lubią ciepło. Więc zakładam, że spróbuję upiec jakieś ciasto drożdżowe. Ale nie wiem, jak to wyjdzie w tej kuchence. Też nie widzę sensu, żeby robić dużo rzeczy, bo jesteśmy tylko we dwójkę. I nie chcę, żeby później się marnowało. A mąż też jest wybredny. On sernika bez rodzynek to be, a ja znowuż jestem bezrodzynkowa. Tak że myślę, że spróbuję po prostu na małej blaszce upiec jakieś ciasto drożdżowe, może jakieś ciasteczka ewentualnie. No i co? No raczej jajka, może jakiś gyros zrobię. Na pewno nie będę przesadzać. Tylko tam symbolicznie. I myślę, że postaram się kupić jakiś kawałek kiełbasy białej, jakieś pieczywo. Znaczy u mnie w domu też się raczej, raczej nie ma przepychu, jest tak normalnie, żeby można było to zjeść. Tylko tyle, że w Wielkanoc mama piecze mięsa. No i u nas ogólnie jesteśmy bardzo słodyczowi wszyscy. Więc w Wielkanoc jest dużo słodkiego. Więc mama rurki robi, takie dobre rzeczy. Ale, no cóż, rurek tutaj nie upiekę.

**Czyli rozumiem, że siedzicie po prostu sami?**

W domu, tak.

**A nie pamiętam dokładnie, czy tydzień temu mówiłaś, że jeszcze rozważaliście to, żeby pojechać gdzieś?**

Tak. Właśnie jeszcze w tamtym tygodniu myślałam, że pojadę do domu. Jeszcze tata mi wysłał w ogóle w poniedziałek… Nie, w niedzielę, bo była niedziela palmowa. I moi rodzice sobie zrobili żarty. I tata ścinał wierzbę. I wierzba ma takie długie witki. I ten ścięty krzak tej wierzby powiązali i kokardą obwiązali, no i że ha, ha, ha, taka ich palma w tym roku. Ale mój tata tak dla żartów napisał, że coś tam, coś tam, do Karpacza zapraszamy. Ogólnie taki wierszyk był. Ja rozumiem, że on żartował, ale przykro mi się zrobiło, bo wiedziałam, że… Jakby wtedy już się zorientowałam, że nie ma co jechać. No przykro mi tak było, szczególnie, że ja chciałam i widziałam też, że rodzice są rozczarowani. No, ale…

**Czyli twoi rodzice chcieli, żebyś przyjechała mimo wszystko?**

No, ja rozumiem, że chcieli. Ale tak mi się wydaje, szczególnie po tym wierszyku, bo to niby taki ha, ha, ha, żarcik. Ale wydaje mi się, że chciał mi zakomunikować, że ma nadzieję, że przyjadę. Ale potem, jak rozmawiałam z mamą przez telefon, no to jakby rozmawiałyśmy z tej perspektywy, że to oczywiste, że spędzamy osobno.

**Czyli nie musiałaś jakoś tłumaczyć tej decyzji.**

No nie. Też moja mama, ja się boję, że zarażę rodziców. No już nie są pierwszej młodości jednak. Mama z kolei się martwi, że nas pozaraża, bo jest pielęgniarką, więc tak naprawdę nie wie, z czym ona tam w tym szpitalu swoim jest.

**A jak sobie radzisz z tym, że właśnie była niedziela palmowa, teraz będzie niedziela. Nie wiem, czy właśnie będziesz szła do kościoła czy nie?**

Wydaje mi się, że to bardzo nierozważne. Chociaż rozmawiałam z moim wujkiem. Wujek ma z sześćdziesiąt coś lat. Wyobraź sobie takiego starego kawalera, który ma wąsa takiego. Ubiera swetry takie, jeździ na rowerze i zawsze ma takie czarne spodnie, takie trochę w kant. I pantofle do tego. To jest mój wujek, stary kawaler, który jąka się i karmi kury. I ogólnie, no to stereotyp. I wczoraj wujek do mnie dzwoni i się pyta: no Hania, bo jemu się wydaje, że ja mam na imię Hania, ja już go nie wyprowadzam z błędu od wielu lat, no Hania, czy ty jedziesz do domu na święta? Ja mówię, że nie jadę wujku, bo tutaj pandemia itd. A do kościoła idziesz? Bo mieszka na wsi, więc on cały czas do kościoła chodzi. Ja mówię, no wiesz wujku, to niebezpieczne. Pewnie będę oglądała transmisję. A wujek: ha, ja chodzę. Mówię, o wujku, no to chyba niezbyt zdrowe. A on mówi, e, 8 osób w kościele i same stare baby (śmiech). Tak że na wsi się nie przejmują. Więc może będę oglądała transmisję. Tylko, to też jest tak, że… Ja po prostu bardzo lubię te święta. To nie jest tak, że jestem jakąś dewotką, chociaż jestem wierząca mocno. Ale bardzo lubię samą otoczkę, jakby ceremoniał. I nie wiem, jak to będzie wyglądało po prostu. Bo na przykład w niedzielę palmową stwierdziłam, ach, obejrzę sobie mszę z Jasnej Góry. I szczerze mówiąc nie polecam (śmiech).

**A czemu?**

Ogólnie Jasna Góra to była zawsze taka dla mnie wydumana. To po pierwsze. Bo wiadomo, tutaj obrona przed wrogimi wojskami, cud itd. W serduszku Polski bije Jasna Góra. Ale tak naprawdę no to mam wrażenie, że jest trochę niedofinansowana. I rzucają raczej tam, chyba nie ma tam żadnych młodych kapłanów. I szczerze mówiąc, ja bym chciała jakieś takie, no usłyszeć coś z korzyścią dla umysłu może. A oni tak wszystko robią to mechanicznie. I naprawdę nie ma w tym żadnej głębi. Ale wiem, że we Wrocławiu jest… Bo ogólnie we Wrocławiu też jest trudno z kościołami. Ogólnie albo są kazania politycznie, tak że nie słucham. Albo mówią bzdury. Albo mówią coś w stylu: przykładam się do tego, żeby moje kazanie miało sedno, po czym kazanie nie ma sedna (śmiech). I jest jeden, jedyny kościół tutaj, który jest powiedzmy normalny. I to są dominikanie. I wiem, że dominikanie mają streamingować mszę. Więc zamierzam to obejrzeć i zobaczyć, co tam się będzie działo. Bo wydaje mi się, że to będzie najrozsądniejsze. Ale powiem szczerze, nie jest łatwo obejrzeć cokolwiek normalnego teraz, jak człowiek chce. Nie jest łatwo.

**Czyli będziesz próbowała to oglądać. A jeśli chodzi o święconkę?**

Na pewno nie będziemy nigdzie wychodzili. Wiadomo, że w tych wszystkich ceremoniałach tkwi wielka metafora tak naprawdę. I na pewno odłożę jakieś rzeczy, tam ze 2 jajka, jakieś pieczywo na bok w sobotę. Tak, żebyśmy mogli je tak bardziej podniośle zjeść w niedzielę. Natomiast wydaje mi się, że tutaj jest jedynym rozwiązaniem, chyba można sobie błogosławić w domu, dlaczego by nie. Więc wydaje mi się, że jest to jakieś rozwiązanie. Zresztą tak jak mówię, to jest tylko jakaś metafora. I przecież błogosławieństwo nie spadnie na to jedzenie nagle.

**Mam takie wrażenie, że mimo, że jesteś wierząca, to dla ciebie w tej całej Wielkanocy najważniejsze jest to, że to jest jakiś ważny czas. I te przygotowania do tego. I że bardziej z tego powodu jest ci przykro, że tego nie ma niż z powodu tego, że nie możesz iść do kościoła na przykład.**

Oczywiście, tak.

**Czy teraz jest tak, że czujesz się przez to jeszcze gorzej, czy już trochę lepiej czy tak samo?**

Chyba lepiej. Na pewno to nie będą święta takie, jak zawsze. I będą miały zupełnie inny wymiar. Ale może jest to jakieś miejsce na refleksję tutaj, taką inną niż zwykle. Zresztą też tak naprawdę to jesteśmy młodym małżeństwem i to jest nasza pierwsza Wielkanoc. I będziemy we dwójkę. Może jakieś nowe zwyczaje wprowadzimy. Może zamówię pizzę na Wielkanoc. Dlaczego nie (śmiech).

**To jeszcze bym chciała się dowiedzieć, co macie na tej liście na Wielkanoc?**

Była rzeżucha, ale udało mi się kupić ją w Rossmannie. I barwniki do jajek też. Pamiętam, u mnie w domu największa awantura była zawsze o barwniki do jajek. Bo mama chowała je, wyciągała je raz w roku. I nikt nie wiedział, gdzie ona je schowała potem znowu. I pamiętam jedne święta, ja nie wiem, ile ja mogłam mieć lat, 6 chyba. Strasznie mama nakrzyczała na tatę, że to on pewnie gdzieś schował te barwniki. Bo moja mama jest taką nerwuską. I mama tak była zła, że tata mówi do mnie, bo byłam dzieckiem: pójdziemy na spacer. I wyszliśmy z domu. I wyobraź sobie, że na chodniku znaleźliśmy barwniki do jajek. Ktoś zgubił te barwniki i myśmy je wzięli. I te barwniki, bo to bardzo mało się tego wykorzystuje. A na 3 osoby, no to ile tych jajek zabarwisz? Z 5? No teraz 4 z Kubą. No to te barwniki do dziś mamy. To 6 lat. A teraz mam 26. 20 lat barwniki (śmiech). Tak że są barwniki, jest rzeżucha. Na pewno kupię szczypiorek, na pewno kupię jajka. Ale jakieś takie lepsze, nie marketowe, tylko z Areny, takie od kurki. Wędlinka, ale też jakąś lepszą. Pasztet, ale nie taki pasztet Profi z kurką, tylko jakiś taki lepszy. Chcę zrobić coś z tym ciastem drożdżowym, więc mam tam drożdże. I chcemy zrobić też takie już przy okazji normalne zakupy, więc mleko, woda.

**A dopisałaś na tą listę jakieś takie smakołyki wielkanocne dla siebie?**

Tak naprawdę do smakołyków jeszcze nie doszłam. Na razie próbuję sobie wyobrazić, co bym kupiła w Biedronce. A Biedronka, o której myślę, zaczyna się od warzywnego. Więc muszę się namyślić dobrze. Mam nadzieję, że jak coś zobaczy dobrego, to będzie dzwonił.

**Jeśli chodzi o Wielkanoc, o takie rzeczy, żeby sobie sprawić przyjemność, to z jakiej to są kategorii rzeczy?**

Mi się lody kojarzą z Wielkanocą. Ja jem całe życie lody, więc… W sensie jesień, zima, mi wszystko jedno. Ale może pozwolę sobie na Manhattany z okazji Wielkanocy. Z takich smakołyków… No szczerze, to raczej nie kojarzą mi się rzeczy przetworzone. Tak że czekolada mało. Nawet Kinder Niespodzianka, która tak zgubnie wpłynęła na moją twarz, bo dostałam od siostry Kuby z paczkomatu Kinder Niespodziankę. Najbardziej kojarzą mi się takie właśnie zrobione. Więc może jakiś sernik zrobię ewentualnie.

**Dziękuję bardzo.**
