# Supplementary material for: The challenges arising from the COVID-19 pandemic and the way people deal with them. A qualitative longitudinal study
Source: PLoS One. 2021 Oct 11;16(10):e0258133. doi: 10.1371/journal.pone.0258133 (PMC8504766; doi:10.1371/journal.pone.0258133)
Supplement: S1 Dataset — (ZIP) [file pone.0258133.s003.zip › Transcriptions/stage 2/10.2_F_55_couple, no children.docx]

**10.2_F_55_couple no children**

**Zdjęcia - emocje**

W zeszłym tygodniu wybrałam **8** i to się niewiele zmieniło w tej chwili, bo nadal nie wiadomo, co będzie. W tym momencie nic innego mi tu nie przychodzi, bo ani ta 1, ani 2 - to w ogóle jest dla mnie jakieś dziwne; 3- nie rozumiem, o co chodzi; 4 - bądźmy razem, łączmy się. Pamiętam, że jak byłam dzieckiem, to w ten sposób robiłyśmy siodełko dla koleżanek. 5 - w ogóle nie wiem, co tu jest; 6 to jest ewidentne światło, słońce w lesie, więc to absolutnie nie kojarzy mi się z obecną sytuacją; 7 to jest jakby droga donikąd, natomiast najbardziej nadal to 8. Między **7** a **8**, bo nadal nie wiadomo, dokąd to wszystko zmierza i jak to się zakończy.

Jakie są w pani emocje, co pani czuje w związku z obecną sytuacją?

Po prostu jest to sytuacja nowa dla wszystkich na całym świecie i tak naprawdę nikt nie wie, jak to się zakończy, w jakim kierunku...Już pomijając samą historię koronawirusa i choroby, ile to będzie trwało, jak długo, czy wróci. Nadal nie wiadomo co będzie z ludźmi, z gospodarką, czy będą mieli za co żyć wszyscy. Wielka, wielka, wielka niewiadoma.

**Czy intensywność tego uczucia jakoś się zmieniła?**

Nie jest bardziej nasilone, ponieważ powiem szczerze, że się tak troszeczkę od tego jakby odcięłam. Nie oglądam tych wiadomości, staram się nie oglądać, bo czasami coś tam oczywiście się przemyci. Zajęłam się porządkami w domu. Wykorzystuję ten czas, żeby tutaj porobić takie zaległe rzeczy po zimie albo różne takie, co nigdy na nie nie ma czasu. Oprócz tego wieczorem w czwartek jeszcze stłukłam sobie strasznie 2 palce i ledwo chodzę, więc dodatkowo jestem taka troszeczkę uziemiona, pomijając, że staram się już prawie w ogóle nie wychodzić. Tyle, co muszę po te zakupy i z psem. Nie powiem, że jestem spokojniejsza, jeśli chodzi o sytuację, tylko po prostu nie jestem taka targana, bo tak staram się tego nie...A nie, jeszcze raz było coś takiego w poniedziałek. Napracowałam się mocno schylając gdzieś tam pod łóżkiem i musiałam sobie coś naruszyć w kręgosłupie, w kręgach. W każdym razie po jakimś czasie takiej straszliwej niemocy, której dostałam...Takiej, że naprawdę nie byłam w stanie ani stać, ani w ogóle nic podnieść...I to się przerodziło w taki jakiś totalny atak paniki. Takiej naprawdę...Oczywiście natychmiast zaczęłam czytać, że jednym z objawów jest totalne zmęczenie i w ogóle...Wpadłam w taki prawie szał - płakałam i w ogóle nie wiedziałam...Ale oczywiście wrócił mąż, powiedział, żebym się kopnęła w głowę i że to na pewno od kręgosłupa. I faktycznie za jakaś godzinę to zmęczenie tak ustąpiło, natomiast wszedł mi taki typowy mój lędźwiowo-krzyżowy ból pleców, że musiałam leżeć z nogami w takiej krzesełkowej pozycji, żeby to mi się wszystko poustawiało. Nie mam żadnych innych objawów, a wpadłam w panikę do siostry, że mam na pewno nietypowe objawy koronawirusa i co to będzie, co to będzie. Oczywiście potem to wszystko się jakoś uspokoiło. To był jeden moment, że wpadłam w taką histerię, ale to było w poniedziałek i potem już nie.

**Powiedziała pani o tej niepewności co będzie. Czy nadal jest w pani ten lęk przed zachorowaniem, o którym mówiła pani w zeszłym tygodniu?**

Nie, w tej chwili jakoś nie. Był totalny atak tego lęku i paniki w ten poniedziałek, że ja już jestem rzeczywiście chora, ale to się uspokoiło i w tej chwili np. nie odczuwam takiego lęku. Był tutaj też syn...A, bo on miał przyjechać w poniedziałek, coś tam musiałam mu podpisać i też w ogóle zrobiłam aferę, że absolutnie, że on nie może w ogóle wchodzić do mieszkania, że tylko mąż mu poda i takie tam rzeczy. I nie, teraz jakoś nie ma...Rozmawiałam też w międzyczasie - mam 2 bliskie znajome, które są lekarzami. Jedna jest internistką, więc też tak...Może nie tyle zbagatelizowała, bo to nie o to chodzi, że te maseczki, które nosimy...Że generalnie tak czy owak ten wirus jest w formie sprayu w powietrzu, więc wystarczy, że niech rzeczywiście ktoś tam kichnie w pobliżu, to jeśli nie mamy zasłoniętych oczu, to tak czy siak wszystko się dostanie. Ja akurat mam te maseczki i noszę, ale...Z kolei ta druga powiedziała, że nie jest tak źle, pracuje zresztą w jednym z tych jednoimiennych szpitali i że jeśli sytuacja będzie tak wyglądała, jak jest w tej chwili, to absolutnie nie przypomina to sytuacji, jaka była we Włoszech i że będziemy mogli uznawać się za szczęściarzy. To było takie budujące, bo ja się o nią tak troszeczkę martwiłam, bo wiem, że ona jest specjalistą chorób zakaźnych i bałam się, że ją tam w ogóle uziemią, ale nie.

**Robiła pani coś konkretnego, gdy był ten atak paniki, żeby to się uspokoiło, czy ten stan sam minął?**

Nie, nie robiłam. Najpierw się po prostu tak położyłam i leżałam, bo to się stało tak po południu 16-17-18, a potem chyba włączyłam jakiś film, wzięłam gorący prysznic szczególnie na te plecy no i tak zaczęłam planować, co ja zacznę robić od jutra, czyli od wtorku.

**Ma pani jakieś sposoby radzenia sobie z emocjami generalnie, w tej sytuacji? Mówiła pani, że trochę odcięła się od informacji, rozmawiała ze znajomymi. Czy to pomogło jakoś lepiej się poczuć w tej sytuacji?**

Chyba tak, ale generalnie na pewno przeważyło tutaj to, że ja tego nie oglądam. Po prostu celowo nie chcę. Oczywiście wiadomo, że z dnia na dzień te liczby są coraz wyższe i więcej jest i zgonów, i tych zachorowań w ogóle, więc celowo się od tego jakby odcięłam.

Co spowodowało, że podjęła pani decyzję, żeby się odciąć?

To bardziej było tak naturalnie, że po prostu bardzo byłam zajęta i potem tak już...To było potem nawet na zasadzie takiego odruchu, jak włączałam tv i jak tylko słyszałam, że coś, to natychmiast to przełączałam. Nie, nie, nie, dziękuję, nie chcę.

**Jeszcze jakieś sposoby radzenia sobie z tą sytuacją się pojawiły?**

Nie.

**A czy coś zmieniło się w pani życiu codziennym w ciągu ostatniego tygodnia?**

Nie. No, oprócz tego, że tę nogę mam stłuczoną. Musiałam też zamknąć katalog, ponieważ jestem liderem Avonu i my mamy ten system trzytygodniowy i teraz w środę był koniec, aczkolwiek powiem szczerze, że nawet nie miałam ochoty dzwonić do tych wszystkich swoich ambasadorek i zawracać im głowę w tym momencie zamówieniami. Stwierdziłam, że każdy ma w tym momencie co innego na głowie. Na pewno to się przełożyło też na moje zarobki w tym momencie, bo są dużo, dużo, dużo mniejsze, ale stwierdziłam, że to nie jest czas, żeby teraz akurat...Pracę odpuściłam zdecydowanie. poza tym też postanowiłam i bardziej zajęłam się tym domem. To jest dla mnie teraz może nie tyle priorytet, bo bez tego można żyć, ale jak ja już zacznę coś robić, jak coś zaplanuję, to ja to już muszę skończyć. Nie potrafię tego odłożyć i tak naprawdę w tych porządkach to nie samo sprzątanie zajmuje mi najwięcej czasu tylko uporządkowanie wszystkiego, żeby można było sprzątać. Mam taką zaprzyjaźnioną panią, która do nas przychodzi od wielu lat, ale teraz w tym okresie my się rozstałyśmy. Powiedziałam, żeby nie przychodziła ze względu i na nią, i na siebie, więc strasznie mi jest ciężko z tym sprzątaniem i z tymi porządkami. Zajęło mi to bardzo dużo czasu i nadal jeszcze nie skończone, oczywiście. Mam nadzieję, że dzisiaj może, ale śmiałam się z siostrą, że ja jeszcze w święta będę sprzątać.

**Czy jeszcze jakieś nowe czynności, poza porządkami się pojawiły?**

Obiecywałam sobie, że w tym roku już absolutnie nie zrobię sobie żadnych kwiatów na balkonie, bo potem to tylko śmieci, brudzi i oczywiście zupełnie nie zrozumiale dla mnie zajęłam się też kwiatkami i zrobiłam sobie ten balkon z kwiatkami, więc i ziemia, i kwiatki. Niedużo, ale jest i cały dzień na pewno mi to zajęło.

**Czy te porządki, to zajmowanie się kwiatami to są czynności, które poprawiają pani nastrój?**

Nie tyle poprawiają, co jak już sobie postanowię, to zrobię. I jest. Jestem zadowolona, że to zrobiłam.

**Pojawiły się jakieś nowe rzeczy, które zaczęły pani przeszkadzać?**

Nie.

**A pani poczucie zagrożenia obecną sytuacją, Coś się zmieniło?**

Hmm...Nie tyle, że się zmieniło, ale ja jestem finansowo uzależniona od męża ewidentnie. Moja działalność, którą prowadzę w Avonie nie jest na potrzeby życia, bo to by nie wystarczyło. Takim oparciem dla mnie jest mąż i na nim polegam. Na razie, z tego co mi powiedział, na 2-3 miesiące mamy za co żyć, ale co będzie dalej, jeśli to się będzie wszystko tak...On też nie wie. Jego firma też stoi zamknięta i jak to zacznie się odbudowywać, to nikt tego nie wie. Jest to okropne. To jest coś nowego i to jest bardzo...

**Obserwuje pani jakieś nowe sposoby radzenia sobie przez osoby w pani otoczeniu?**

Nie, ponieważ prawie z nikim nie mam kontaktu. Telefonicznie mam jedynie z siostrą. Ona jest już osobą na emeryturze, więc wiadomo, że ma emeryturę, pomaga dzieciom, ale oczywiście w tej sytuacji jest cały czas w domu, nigdzie nie wychodzi, nigdzie się nie przemieszcza. Oczywiście boimy się, co będzie dalej, ale wspólnym naszym tematem głównym jest nasz tata, który nie radzi sobie trochę z tą sytuacją, bo nie rozumie, dlaczego my nie przychodzimy. On ma opiekunkę, która z nim mieszka, ale zawsze siostra była tam bardziej aktywna, bo ja pracowałam i częściej mnie nie było, nie miałam czasu. Siostra tam częściej przyjeżdżała, żeby pobyć. Ja jestem od ciężkiej roboty - zakupów, przywożenia, bo jeżdżę samochodem, siostra nie. Teraz żadna z nas nie przychodzi. Ja, jeśli nawet te zakupy przywożę, to wstawiam po prostu przez drzwi i wychodzę. Widzę, że na niego to troszeczkę tak źle wpływa, ale nie mamy wyjścia i robimy to po to, żeby właśnie uchronić wszystkich, szczególnie, że ta osoba, która się nim opiekuje jest z Ukrainy i co będzie, jeśli nie daj Boże jej się coś stanie. Oczywiście kupiłyśmy jej ubezpieczenie zdrowotne, ale nie wyobrażam sobie, żeby tam się coś zadziało, bo nie wiem, co my wtedy zrobimy.

**Czy widzi pani jakieś zmiany u osób z pani otoczenia, jeśli chodzi o nastrój?**

U moich synów w tej chwili nie ma jakichś dramatycznych nastrojów. Młodszy syn pracuje zdalnie z domu i jest bardzo zapracowany. Mieszka z dziewczyną i z kolegą, i z tego, co widzę to mają się świetnie. Właśnie coś tam sobie postanawiają ugotować wspólnie na święta, mają dzwonić zapytać, jak się robi to, jak to. I to samo jest u drugiego syna. Jego partnerka też do mnie dzwoniła, że mamo, jak się robi te śledzie, jak się robi tamto, więc jakoś sobie szykują te święta. Też sami. Jedno jest takie pewne, że wszyscy starają się utrzymywać tę izolację i nawet jeśli coś się dzieje, to tak jak mówiłam - syn wszedł, zostawił papiery, ja podpisałam i wyszedł. Nie było ani podania ręki, ani nic.  Przedtem nie widzieliśmy się od połowy marca.

**W zeszły tygodniu wprowadzono nowe ograniczenia. Co pani o nich sądzi?**

Uważam, że dobrze, że powinny być takie ograniczenia.

Przestrzega ich pani?

Tak. Do sklepu nie wejdę w tym czasie, bo mnie nie wpuszczą, ale nie wybieram się również, bo wiem, że to są godziny dla...Poza tym w ogóle staram się raz w tyg. robić takie zakupy większe i jeśli czegoś mi brakuje, to staram się iść do takiego sklepu blisko mnie, gdzie wiem, że jak wejdę, to nikogo prawie nie ma. Jak najbardziej tak.

**Zna pani osoby, które nie przestrzegają jakichś ograniczeń?**

Nie.

**Obserwuje pani takie osoby gdzieś w otoczeniu, na ulicy?**

Na pewno już troszeczkę mniej widzę takich spacerujących ludzi, jak wtedy mówiłam, że uważają, że są na wakacjach. Ja prawie nie wychodzę, więc nawet...Dzisiaj będę musiała rzeczywiście wyjść i też się zastanawiam, ile będę stała w tych kolejkach, ale nie mam wyjścia, bo muszę zaopatrzyć mojego tatę, teściową - tutaj troszeczkę jestem wściekła, ale to już inna historia i dla nas zrobić zakupy, bo jednak w miarę jakoś...Nie spotykamy się wszyscy razem, nie zamierzam uskuteczniać takiego pieczenia i wypieków, co to zawsze są co roku, ale coś kupić muszę.

**To, że jest mniej spacerujących ludzi wynika wg pani z tych ograniczeń?**

Mam nadzieję, że ta świadomość jednak do ludzi dochodzi, że to jest jedne wyjście, żeby po prostu nie wychodzić.

**Co obecnie jest dla pani największym wyzwaniem w codzienności?**

Posprzątanie domu, z którym sobie nie radzę, bo nie mam siły. Jakie przyziemne rzeczy. Jak mówiłam, może się tak celowo troszeczkę odcięłam od tych spraw bardzo ważnych, ale właśnie po to, żebym mogła się tak tu tym zająć, żebym tę głowę miała taką luźną, żeby to była namiastka, tak jakby rzeczywiście były święta i nic się nie zmieniło. Tyle tylko, że nie będziemy ich spędzać razem. Taka namiastka normalności.

**Czy zaobserwowała albo słyszała pani o jakichś dziwnych sposobach radzenia sobie z tą sytuacją? Które pani wydały się dziwne?**

Nie.

**Jak u pani będą w tym roku wyglądały święta?**

Dzisiaj jeszcze, jak tylko skończymy, to będzie ta pora, kiedy będę mogła pojechać na zakupy, więc przede wszystkim muszę zrobić te zakupy dla taty, dla teściowej, dla nas. I jak wrócę, to planuję dokończyć to sprzątanie. Jutro zamierzam coś pogotować, może coś poczytam. Na pewno będę dzwoniła do dzieci. Teraz rozmawiamy sobie przez video, messengery i w ogóle. Zamierzam trochę tak odpocząć i zająć się sobą.

**W jaki sposób?**

Np. dzisiaj zamierzam sobie sama obciąć włosy i jest to mój „a must” na dzisiaj. Zamierzam doprowadzić do porządku swoje ręce, stopy, jakieś tam maseczki. Takie domowe SPA. Jeszcze mnie czeka dzisiaj i jutro moja zawodowa część, bo muszę też okleić wszystkie swoje katalogi, które mam i też poroznosić, porozkładać tam, gdzie to zawsze robię. Przypinam je do tych tablic na klatkach. Ja zawsze to robiłam w koszulkach, bo to była jedyna możliwość, żeby je przypiąć przez tę dziurkę, a teraz to w ogóle w tych koszulkach, bo każdy może wziąć, odczekać, wyjąć. Jutro to planuję zrobić.

**Mówiła pani o gotowaniu. Jakie to będą potrawy?**

Na pewno tradycyjne potrawy się znajdą, typu barszcz biały czy żurek, ale to też na zasadzie czyszczenia szafek w kuchni i nie będą to jakieś wymyślne, tylko zupki z proszku polecam w tym roku. Sałatkę jarzynową, sos tatarski zrobię, bo zawsze to lubimy. Nie zamierzam piec w tym roku jakichś mięs, schabów, karkówek, jedynie może upiekę białą kiełbaskę, bo też to lubimy. Nie będę robiła żadnych mazurków ani ciast, nic z tych rzeczy. Po prostu zamierzam sobie kupić jakiś kawałeczek ciasta gdzieś, nawet w Biedronce, bo wszystko mi jedno, ale jak będę chciała coś słodkiego, to żeby było na wszelki wypadek. Chodzą za mną jajka faszerowane, które zawsze są na święta i może sobie zrobię, ale nie wiem, bo może mi się nie będzie chciało. Ostatnio też oglądając tv wpadłam na przepis, który mi cały czas siedzi w głowie i nie wiem, czy mam się za to zabierać, czy nie. To jest coś zupełnie nowego, czego nigdy nie robiłam i nie wiem, może się zdecyduję, żeby to zrobić. Taki pasztet z kurcząt, ale gotowany w takiej folii spożywczej w wodzie. Nie wiem. Zamierzam sobie zakupy zrobić na to, ale czy to zrobię, to nie wiem.

**Wcześniej, przed epidemią pewne rzeczy musiała pani zrobić?**

Zawsze robiłam. Ja co roku sobie mówię, że ja nie muszę tego robić i w ogóle, ale też dzieci proszą, chcą czy coś i efekt jest tego taki zawsze, że ja jestem wykończona, zapracowana w tej kuchni i w tym roku mąż mówi, że wreszcie mamy możliwość, że nie muszę się ja napracować, że spędźmy to tak w ten sposób. To jest pozytywna strona tej sytuacji, że jest mniej pracy i mniej presji.

**Jak planuje pani zrobić zakupy wielkanocne?**

Dzisiaj. Ja zawsze robię albo w Lidlu, albo w Biedronce. Mam jedno i drugie blisko. Jedne rzeczy lubię z Biedronki, jedne z Lidla, ale ponieważ...Zacznę od Lidla i jeśli uda mi się zrobić wszystkie tam, to już do Biedronki nawet nie pojadę. Jeśli nie, to wtedy wracając jeszcze podjadę do Biedronki.

**Czym te zakupy będą się różnić od tych, które pani robi zazwyczaj w czasie epidemii?**

Niewiele się będą różnić. Tu jest lista. to wszystko pogrupowane to są moje listy, tutaj z kolei jest babcia Elżbieta, dziadek. Niewiele się będą różnić, oprócz tego, że doszły jakieś produkty, które są konkretnie potrzebne czy na sałatkę jarzynową, czy tam na coś.

**Zazwyczaj robi pani zakupy z listą?**

Zawsze. Moja mama się śmiała ze swojej koleżanki, która zapisywała na kartce, że musi kupić mleko, masło, chleb i mówiła, że ona jakaś głupia, że to wszystko sobie piszę. A ja się śmiałam, że ja też zawsze robię listę, bez względu na to, co mam kupić, bo jeśli na tej liście nie wpisze sobie tego masła, to ja go nie kupię po prostu. Zajmę się czymś i mogę zapomnieć. Lubię zawsze mieć listę i do tego stopnia, że lubię tę listę przepisywać co chwila, gdzieś coś zmieniać. Tutaj nawet to jest pogrupowane, bo tu jest część warzywno-owocowa, tutaj jakieś kuchenne rzezy, tu tzw. suche. Oczywiście uwielbiam w sklepie odhaczać to wszystko. Teraz może, bo też proszą, żeby się nie zastanawiać długo, więc będę się starała też szybko. Potem będę musiała te zakupy zawieźć do taty i do teściowej. Zwykle jak są święta, to uwielbiam pojechać sobie na giełdę, kupić sobie swoje ulubione kwiatki...Teraz nie pojadę na pewno. Wiem, że giełda jest otwarta, ale kupienie w tym momencie akurat dla mnie kwiatków nie jest tą potrzebą największą. Jeżeli kwiatki będą, to ja je kupię w Biedronce i nie będę jechała specjalnie po to.

**A właśnie, te produkty dla dekoracji, dla przyjemności na święta. Takie też pani planuje?**

Kwiaty już stoją, bazie też są, tylko nie ma jeszcze tych dekoracji zrobionych. Mam w tej chwili posprzątaną połowę mieszkania i została mi ta część salonu z kuchnią i tutaj te ewentualne dekoracje, ale takie bardzo skromne w tym roku. Zdecydowanie skromniejsze niż zwykle.

**Dlaczego skromniejsze?**

Po to, żeby potem tego nie chować, nie...Tak z wygody. Coś tam sobie już wyjęłam - kurkę, baranka, coś stoi, te kwiaty ustawię i tyle.

**A takie produkty jak ubrania na święta?**

Nie, w tym roku nic sobie nie kupiłam, a wręcz te porządki zaczęłam robić od maksymalnych porządków w szafach. Mam naszykowane 3 wielkie worki ubrań, które czekają, że może ktoś coś chce z rodziny, jak się już wszystko uspokoi, albo oddam opiekunce ojca, bo zawsze też wywozi na Ukrainę i tam się przydają. Nic sobie nowego nie kupiłam i nie zamierzam.

**A takie produkty trochę lepsze niż na co dzień, bardziej premium. Będą jakieś takie z okazji świąt?**

Cały czas chodzi za mną ten pasztet w folii. On jest z jakimiś pistacjami, z czymś tam. Ale nie wiem, czy będę to robić, bo potem niby tylko to, tylko to i cały dzień jestem zajęta, zmęczona. Obiecałam sobie, że nie chcę teraz tego czasu w ten sposób spędzić. Co prawda generalnie mamy teraz dużo wolnego, ale...Nie wiem. Z drugiej strony to mogłoby być fajne - taka nowa rzecz. Ja to kupię, bo to musi być mięso z kurczaka i z piersi, i z udek, koniak, bo to ma się w nim marynować i te pistacje, śmietana kremówka. To taka terrina francuska wyjdzie. To trochę tak, żeby sobie dogodzić jakimś lepszym produktem. Coś innego. Przeważnie na każde święta staram się zrobić jakąś nowość oprócz tego, że to, co zawsze. Zobaczymy, dowie się pani za tydzień.

**Czy aspekt religijny tych świąt jest dla pani ważny?**

Nie. Jest oczywiście ważny, jeśli chodzi o taką tradycję, natomiast ja nie jestem osobą wierzącą, nie chodzę do kościoła i tutaj...No nie. Nie mówię, że ja w nic nie wierzę, bo każdy w coś tam wierzy, ale ja nie lubię kościoła, więc...

**A święconka? Bo to też jest taki element...?**

Już nie. Jeszcze...To takie trochę jest dziwne. Moi synowie byli chrzczeni, byli u komunii, do bierzmowania już nie przystąpili. Jako dzieci i jeszcze żyła wtedy moja mama, więc to tak się bardziej i tradycyjnie, i bardziej też kościołowo obchodziło. Był taki rytuał chodzenia ze święconką, ale teraz tego już nie robimy. Ani oni, ani ja.

**Czy robi pani zakupy przez internet?**

Dla siebie nie robię, ale z racji mojej działalności avonowskiej składam zamówienia przez internet. Teraz nawet nie ma innej możliwości. Klientki składają mi zamówienia, ja muszę to zamówić i później rozdać. Oczywiście zdecydowanie mniej jest w tej chwili, ale w zeszłym tygodniu w piątek, to chyba byłam u 4-5 klientek jednak z zamówieniami. Coś się jednak działo.

**Czy zauważyła pani jakąś zmianę w tym co klientki zamawiają?**

Zauważyłam, że zamawiają dużo mniej.

**A czy zamawiają bardziej jakieś produkty dla przyjemności, dla pielęgnacji?**

Nie, to się nie zmieniło. Zmieniło się tylko to, że jest mniej.

**Wracając do pani zakupów przez internet. Pani nie kupuje dla siebie przez internet w ogóle?**

Wiele razy kupowałam. W zeszłym tygodniu kupiłam przez internet maseczki z tymi filtrami 2FPP. Ostatnio to jest mój jedyny zakup. Mąż kupił mikrofalówkę, która nam się zepsuła i akurat wczoraj została dostarczona. Więcej chyba nic. Nie mam potrzeby kupowania przez internet. Kiedyś spożywcze zakupy usiłowałam robić przez internet - właśnie z dostawą, żeby...I byłam totalnie niezadowolona z tego, bo nie takie warzywa, nie takie coś tam, więc to akurat...Poza tym ja lubię robić zakupy, lubię wejść do sklepu i zrobić te zakupy, natomiast oczywiście, jeśli czegoś poszukuję i nie mogę tego kupić, to wtedy wiadomo, że szukam w internecie. Tak, jak ostatnio z tą kamerką. Nadal to jest jeszcze ta kamerka pożyczona przez moją nadrzędną liderkę, ale starałam się sama zakupić. Ciężko było chociażby z tego powodu, że ja sobie jakiś limit cenowy wyznaczyłam. Wszystkie, po pierwsze są z Chin i teraz czeka się po 3 tygodnie, więc nie kupiłam tego przez internet. Czekam i zobaczymy, co będzie dalej. Jeśli czegoś nie mogę kupić normalnie, to ja kupuję przez internet, jak najbardziej, tylko w ostatnim czasie nie było takiej potrzeby, żeby coś. Ale to nie ma związku z epidemią.

**Chodzi pani teraz do sklepu raz na tydzień. To jest rzadziej niż przed epidemią?**

Tak. Rzadziej, żeby nie być w tym skupisku ludzi, żeby jednak jak najbardziej się izolować. To są względy bezpieczeństwa.

**Była pani w sklepie już po tych ostatnich obostrzeniach?**

Tak. Stała osoba, która nie wpuszczała, jeśli było za dużo osób i jak już ktoś wychodził, to zapraszała następną osobę. Grzecznie mówiła, że jest limit i trzeba poczekać, bo ustawiała się duża kolejka. Generalnie to i tak bardzo szybko przebiegało, bo mimo tego, że ta kolejka była długa, to te zakupy zajęły mi mniej więcej tyle samo czasu, co tydzień wcześniej. Przy wejściu są rękawiczki i każdy je zakłada, jest też spray jakiś i tyle.

**Inni klienci robili zakupy szybko?**

Nie obserwowałam. Ja starałam się szybko, żeby jak najkrócej być w sklepie.

**Jakieś szczególne zachowania obsługi sklepu?**

Teraz może nie, ale wcześniej, jak były te pierwsze ograniczenia, że 1.5 m, że coś, to był taki młody chłopak siedzący w kasie, który bardzo krzyczał na wszystkich, żeby nie podchodzić, żeby zachować odstęp. Bardzo tego przestrzegał. Wszyscy się uśmiechali trochę, bo on to robił w taki sposób bardzo...Ale wszyscy się dostosowywali, nikt się nie sprzeciwiał.

**Jak się pani czuła w czasie tej wizyty w sklepie. Były jakieś emocje negatywne, jakiś lęk?**

Nie, tylko starałam się, jeśli stałam przy jakiejś ladzie i wyjmowałam coś i ktoś stawał koło mnie, to byłam niezadowolona, bo przecież ma zachować ten odstęp i dlaczego podchodzi w tym momencie? Ja, jeśli chciałam się udać po coś i ktoś tam stał, to czekałam, aż ta osoba odejdzie, żeby jednak się tak nie zbliżać.

**Była pani czujna na to, co robią inni ludzie?**

Tak.

**Kupuje pani dużo więcej, jeżeli chodzi o jednorazowe zakupy, bo robi je pani rzadziej?**

Robię rzadziej, ale teraz się generalnie wszystko zmieniło, bo od tych kilku tygodni my jesteśmy sami z mężem. Wtedy tutaj przez 4 dni w tygodniu był mój syn z partnerką, z 2 dzieci. Wtedy siłą rzeczy te zakupy były większe, bo Kasia też oczywiście po zakupy wychodziła, ale w większości to ja je robiłam. Teraz są mniejsze. Zapasów nie zwiększamy, wręcz odwrotnie, zaczęłam...To było z tymi porządkami też związane, ponieważ całe łóżko miałam w tych zapasach a musiałam pod to łóżko też różne rzeczy pochować. Te zapasy syna nadal tam są gdzieś poukładane, natomiast to, co było kupione dla nas, to ja to już rozdzieliłam do szafek i już z tego korzystam. Nie ze wszystkiego, bo tutaj widzę, że część rzeczy jest takich, których rzeczywiście nigdy nie zjemy - jakichś pulpetów w sosie pomidorowym, gołąbków w słoikach. Nikt tego nie je. Nie wiem, po co to było kupione, ale to nie ja kupowałam.

**Mówiła pani poprzednio, że widok tych zapasów wzbudzał w pani pewną panikę, że tego jest tak dużo?**

Nie, paniki nie wzbudza, ponieważ teraz widzę, że w sklepach wszystko znowu jest. Był moment, że nie było ryżu, nie było czegoś. Teraz wszystko wróciło, więc tu jakoś się uspokoiłam. Natomiast mój syn o tych zapasach wręcz zapomniał.

**Jak pani płaci za zakupy?**

Przeważnie kartą, aczkolwiek ostatnio właśnie jak kupowałam kwiatki dla siebie w kwiaciarni obok, to gotówką, bo ta pani nie przyjmuje płatności kartą. Teraz bardziej staram się karty używać niż przed epidemią z tego względu, żeby nie obracać tak bardzo tymi pieniędzmi.

**Kartą płaci pani zbliżeniowo?**

Tak, ale i tak trzeba PIN wpisać.

**Czyli dotknąć terminala też trzeba.**

Tak.

**Obracanie gotówką jest niebezpieczne, powinniśmy tego unikać?**

Nie mam na ten temat zdania. Nie wiem generalnie jak się mają te wszystkie rękawiczki też do tego. Wszystko chodzi o to, żeby potem nie dotknąć twarzy, ust, oczu, nosa. Wiem, że kiedyś na to nie zwracałam uwagi, a teraz bez względu na wszytko staram się tego nie robić, czy mam rękawiczki, czy nie. Zaczęłam zwracać na to uwagę. Coraz częściej w danym momencie tu swędzi mnie oko, tu bym się chciała podrapać, ale nie mogę.

**Ta gotówka może być źródłem dodatkowych zarazków?**

Wg mnie tak naprawdę nie, bo gotówka jest w portfelu zamknięta, na tej samej zasadzie wyjmujemy kartę czy gotówkę, nikt w to nie pluje.

**Ale jednak zdecydowała się pani częściej używać karty? Dlatego, że były takie komunikaty?**

Tak. Też ze względu na to, że ostatnio po prostu mniej miałam gotówki.

**Czy zmieniły się pani zwyczaje żywieniowe, w stosunku do tego co było przed epidemią?**

Nie zmieniły się generalnie, aczkolwiek ja obiecuję sobie już od bardzo dawna, że w ogóle zmienię soje zwyczaje żywieniowe, chociażby z tego względu, że chcę przestać jeść mięso. Stwierdziłam, że nie będę teraz robić jakichś drastycznych zmian przed tymi świętami. Część rzeczy tak tradycyjnie jemy na święta, one się teraz powtórzą, dlatego stwierdziłam, że nie będę tego teraz tak zmieniała. Ja w ciągu ostatnich kilku lat staram się odchudzić i w związku z tym zmienić swoje nawyki, ale jest mi bardzo ciężko.

**Pojawiły się jakieś nowe produkty, nowe dania?**

Ponieważ teraz jestem w domu, to zaczęłam po prostu codziennie szykować obiad dla męża. Prawie codziennie jest inny, świeżo przygotowany, czego wcześniej nie robiłam. Nie wiem z czego to wynika, bo czasami jestem bardzo teraz zapracowana w domu. Kiedy indziej bym powiedziała, że nie ma mowy, jestem zajęta, żadnego obiadu nie zrobię. Teraz jednak mam to jakoś usystematyzowane i zawsze zrobię.

**Określiłaby pani te dania teraz, jako zdrowsze/ mniej zdrowe/ nic się nie zmieniło?**

Generalnie, ja już od dłuższego czasu staram się takie zdrowsze. Jeśli mięso, to głównie jadamy drób, wieprzowiny ostatnio bardzo mało, czasami trochę wołowiny, ale to mój mąż nie lubi, bo mu w zęby wchodzi. Dużo warzyw duszonych. Ja lubię robić takie potrawy, może nie jednogarnkowe, ale takie, jak np. wczoraj - na szybko jakieś fileciki z kurczaka i do tego i cukinia, i pomidory, i zalane to też pomidorami, i to się wszystko tam poddusiło.

**Pani przygotowuje posiłki w domu?**

Tak.

**Wszystkie?**

Nie, tylko obiad. Ja nie jadam śniadań, chociażby dlatego, że (bardzo zły nawyk) podjadam wieczorem, więc jak zjem wieczorem o 11, to rano nie jestem głodna. Mój mąż od kilku lat je codziennie to samo na śniadanie, na co ja nie mogę patrzeć, więc staram się nawet mu nie towarzyszyć, jak on to je. Je płatki owsiane górskie i białka jajek. Z reguły u nas jest taka obiadokolacja, bo my jemy przed 19. Mąż wraca 18.30, zjadamy i włączamy Fakty. Wieczorem, jeśli ktoś chce coś dojeść, to już sam. Teraz będzie może troszkę inaczej w te święta i zjemy razem jakieś śniadanie, na które on niekoniecznie będzie jadł te płatki.

**Obiadokolację zazwyczaj jadacie wspólnie?**

Tak.

**To jest ważne dla pani, żeby jeść wspólnie posiłek?**

Nie. Ostatnio np. w sobotę...W sobotę z reguły mąż wraca wcześniej, ok 16, ale że ja byłam głodna, to zadzwoniłam do niego, że ja wcześniej zjem sama, potem odpocznę a on przyjdzie, to już sam zje. Nie jest to takie, że...Aczkolwiek teraz częściej jadamy razem niż wcześniej. Wcześniej, to w ogóle każde oddzielnie, ale to też wynikało z tego, że trudno na było pojawić się w tym samym czasie w domu. Do końca zeszłego roku, jak ja pracowałam w Złotych Tarasach w Avon Studio, to często miałam dyżury takie, że w ogóle się mijaliśmy. Wracałam np. o 11 w nocy a szłam na 16.

**Zamawiają państwo czasem posiłki z dostawą do domu?**

Czasami tak, ale w ostatnim czasie akurat nie. Właśnie wcześniej, jak ja pracowałam jeszcze w tych Złotych Tarasach i przyszła sobota, i byłam taka zmęczona, i w ogóle, to coś tam sobie zamawialiśmy. Teraz tego nie robimy, bo ja robię posiłki.

**W obecnej sytuacji zamawianie jedzenia z dostawą jest bezpieczne?**

Myślę, że tak. Aż się zastanowię nad tym...Skoro chodzimy i robimy zakupy w sklepach, to równie dobrze możemy zamówić jedzenie do domu. Raczej nie zdarzyło się, żeby zarazić się przez żywność. Tak, jak najbardziej.
