# Supplementary material for: The challenges arising from the COVID-19 pandemic and the way people deal with them. A qualitative longitudinal study
Source: PLoS One. 2021 Oct 11;16(10):e0258133. doi: 10.1371/journal.pone.0258133 (PMC8504766; doi:10.1371/journal.pone.0258133)
Supplement: S1 Dataset — (ZIP) [file pone.0258133.s003.zip › Transcriptions/stage 2/4.2_M_32_couple, no children.docx]

**4.2_M_32_couple no children**

**Obrazki**

12 i 15, jak ostatnio. Nadal jest trochę spokoju wewnętrznego związanego z tym, że taka postawa życiowa, takiego Zen, czy stoicyzmu, jest dobra, zwłaszcza w takich trudnych momentach. Ta 15 to dlatego, że nie ma obrazka, który przedstawiałby eksplozję, chęć wyrwania się. Rozbijająca się fala coś nakrywa, przykrywa, ale nie przelewa się. A nam ulewa się już siedzenie w domu - to by pasowało. W związku z tym siedzeniem jest coraz więcej frustracji, narasta we mnie poczucie, że to do niczego nie prowadzi - ta izolacja, ograniczenie kontaktów, zaostrzenia, których zasadniczo jestem zwolennikiem, bo to są potrzebne kroki. Ludzie zawieszają działalności, kotłują się w domach na małej przestrzeni. Kupujemy czas dość drogo - ekonomicznie, społecznie, zdrowotnie - żeby coś mogło się wydarzyć. A w moim odczuciu to się nie wydarza. Nie mam dostępu do wszystkich danych, nie chciałbym ogłaszać tutaj jakiegoś definitywnego sądu. Jednak według mnie, państwo słabo sobie z tym radzi, nie widzę na razie żadnego planu, pomysłu. Nie zrodziło się nic z zarządzania kryzysowego. To nie jakby człowiek żył z jakimś planem, tylko każdego dnia człowiek wstanie i zobaczy, co to tam będzie, co się wydarzy. Może pójdzie w lewo, może w prawo i tak trochę nie wiadomo, co go czeka. To dla nas jest w tym momencie kłopot. Nie ma zarysowanych scenariuszy, nie wiadomo kiedy to się skończy, co będzie się działo następnie. Na razie wiemy tylko, że zachorowań ma być mniej, dlatego siedzimy wszyscy w domach... No i właśnie - co dalej? Co, kiedy zachorowań będzie więcej, co kiedy mniej? Co zaczniemy zmieniać, kiedy, w jakiej kolejności? Co z naszymi możliwościami wykonywania testów, czy możemy coś zmienić w tej kwestii i do jakiego stopnia? Czytałem i rozmawiam ze znajomymi na temat tego, jak to wygląda we Francji i w Niemczech. Wiem, że Francuzi na początku mocno zareagowali, ponieważ ludzie nie stosowali się do zakazów - społeczeństwo podeszło do nich dość frywolnie. Mieli więc policję na ulicach, która pilnowała, aby ludzie pozostawali w domach. Wiem jednak, że teraz mają naszkicowane plany, możliwe scenariusze rozwoju sytuacji, kroki, jakie państwo chce podjąć. U nas w ogóle tego nie widzę. Rodzi się we mnie trochę poczucie bezsensu. Dla mnie byłoby nawet całkiem spoko, gdyby państwo nie ucięło mi, w moim odczuciu bezmyślnie, możliwości spacerowania po terenach zielonych - terenach nadwiślańskich - nie mówię tu o bulwarach, tylko miejscach bardziej wyludnionych, łąkach, polach, lesie. Tę decyzję krytykuje zresztą część lekarzy i epidemiologów - możliwość wychodzenia na spacer jest bardzo ważna dla zdrowia fizycznego i psychicznego. Wiem jednak, że mi i żonie udaje się dosyć niedużym kosztem fizycznym i psychicznym, dostosować do zaleceń. Przeszliśmy na home office, mamy średniej wielkości mieszkanie, w którym udaje nam się nie potykać o siebie, nie mamy dzieci, a nasze dochody nie spadły - nie cierpimy jakoś bardzo. Zmieniliśmy tylko sposób robienia zakupów, o czym już mówiłem. Nie spotykamy się też z rodziną i znajomymi, co jest naszą największą bolączką. Moja subiektywna ocena jest taka, że państwo robi niewiele. Z tego, co ostatnio opowiadałem, szpitale mają niedobory i nie widzę jakichś gwałtownych ruchów, żeby to się zmieniało. Oczywiście, część rzeczy na pewno dosyłają, zamawiają - natomiast wydaje mi się, że to wciąż za mało w stosunku do potrzeb. Jest wiele doniesień na temat tego, że szpitale są miejscami, gdzie wirus rozprzestrzenia się niesamowicie szybko i nie potrafimy mieć tego pod kontrolą, on się wymyka. Przez źle podjęte interwencje, lekarze i pielęgniarki zarażają w kluczowych dla nas miejscach. Czy to zła organizacja pracy, czy niedostatek środków ochrony osobistej - nie wiem - jednak wygląda na to, że to w sumie działa źle. Wydaje mi się, że przyczyną są nie tylko długofalowe działania rządu przez kilka lub nawet kilkanaście ostatnich lat różnych zaniedbań, kiedy to różne rzeczy zostały w kraju rozmontowane. To też przygotowanie nas w ciągu ostatnich kilku miesięcy, w lutym, marcu - kiedy już wiadomo było, że coś się będzie dziać. Działania nie zostały podjęte, a teraz, w działaniach bieżących, też nie widzę szczególnych zmian. Wydaje się, że rządzący zajmują się w tej chwili głównie zmianami w prawie wyborczym. Wiem, że to wszystko jest skomplikowane prawnie, jednak nie zmienia faktu, że to brzmi jak absurd.

**Czy ta frustracja wpływa na Twoje codzienne funkcjonowanie? Jak sobie radzisz?**

Raczej nie, nie. Choć jeśli ona się kumuluje, może dostanę 10 lat wcześniej zawału. Ale w tej chwili - co mogę zrobić? Musiałbym przejść jakieś załamanie nerwowe, a od tego jestem daleko. Choć jest mi po prostu przykro, bo mam poczucie, że ludzie będą niepotrzebnie umierać. Później część będzie cierpiała ubóstwo, niedostatek. W dodatku nie wiadomo, czy nie będziemy za chwilę żyć w jakimś dziwnym kraju, w którym dzieją się dziwne rzeczy. Więc trochę mi jest szkoda. To jest takie smutne i nie napawa optymizmem, ale nic z tego nie wynika, prócz gorszego samopoczucia. Mam też trochę poczucie bezradności - próbowałem znaleźć sposób, w jaki mógłbym wpłynąć na sytuację - niektórzy na przykład szyją maseczki. Nie znalazłem jednak niczego takiego, co mógłbym zrobić. Myślałem, aby dołączyć do jakiejś akcji, ale nie bardzo wiem jak, bo przecież nie wolno wychodzić z domu, a ja nie jestem w tym momencie nigdzie zatrudniony. Jest mi więc smutno, ale nie biorę tego też bardzo do siebie. To, co mogę zrobić, to ewentualnie zadbać o siebie, jakoś się przygotować - pomyśleć na przykład, czy ze względu na ewentualną hiperinflację, nie powinienem ulokować swoich oszczędności w innej walucie. Mógłbym ograniczyć ilość negatywnych informacji, które do mnie dopływają, ale trochę nie chcę tego robić. Mam wrażenie, że daje mi to poczucie jakiejś minimalnej kontroli, przynajmniej wiem, co się dzieje. Mogę reagować - nie wiem jak i na co, ale zawsze. Wydaje mi się, że jestem w stanie znieść te negatywne informacje i nie chcę się zupełne od nich odcinać. Zastanawiam się też, jak wyglądałaby epidemia bez takiej technologii, jaką dysponujemy - myślę, że mogłoby dochodzić do lokalnych wybuchów paniki, ze względu na brak dostępu do informacji, który jednak chyba trochę uspokaja. Nie sprawdzam oczywiście tych informacji cały czas, jednak w miarę regularnie. W poniedziałek zająłem się robieniem gigantycznych zakupów dla siebie i rodziny, wydałem prawie 1000 zł. Zanim wszystko kupiłem, podzieliłem i porozwoziłem, minęło sporo czasu. W niedzielę cały dzień majsterkowałem, bo zepsuło nam się trochę AGD. Te czynności faktycznie były relaksujące, pozwoliły zapomnieć trochę o całej sytuacji. Czasami też oglądanie filmów pomaga, kiedy człowiek zanurzy się w jakiś inny świat. Nie są to jednak celowe działania, aby radzić sobie z sytuacją i o niej nie myśleć. Taką funkcję spełniały dotychczas spacery, ale teraz ta możliwość została trochę ukrócona. Czuję niepokój przed tym, że gdybym chciał skorzystać z terenów zielonych, mogłyby mnie spotkać z tego tytułu jakieś reperkusje. To niesympatyczne uczucie.

**Przestrzegasz zakazu korzystania z terenów zielonych?**

Tak, mam potrzebę praworządności i nie dawania złego przykładu, więc trochę nie chcę łamać pewnych przepisów. Choć jeśli czuję, że coś jest niesprawiedliwe, ciężko się podporządkować. Czuję, że ten przepis to ograniczenie mojej wolności, swobody. Z drugiej strony kary są bardzo wysokie, więc nie chciałbym dostać mandatu - to mnie trochę zniechęca. Nie wiem, jak będę sobie radził. Na razie wychodzimy z żoną na spacer po osiedlu, co chyba też jest trochę nielegalne. Przyjęte ostatnio przepisy są bardzo płynne. Wychodzić można w celu realizacji podstawowych potrzeb życiowych, choć nikt do końca nie wie, czym one są. Mam tylko nadzieję, że chodząc po osiedlu nie zostaniemy przez nikogo pociągnięci z tego powodu do odpowiedzialności. Czuję się jednak pokrzywdzony, uważam, że to niesprawiedliwe i nieuzasadnione. Jadąc do lasu, nie stwarzam żadnego zagrożenia, przynajmniej większego niż kiedy pójdę do sklepu, na spacer z psem i tak dalej. Wręcz odwrotnie - jeśli będę spacerował z psem po osiedlu, mam styczność z większą liczbą ludzi. Wyjeżdżając do lasu sprawiam, że w mieście jest mniej osób, więc nie stwarzam zagrożenia, a tym samym mam mniejszą styczność z ludźmi. Z mojego punktu widzenia jestem rozgoryczony, uważam, że to bez sensu i wylano dziecko z kąpielą. Rozumiem zamysł, chciano dobrze, zakaz miał służyć temu, by się nie grupowano. Jednak takie zasady przynoszą odwrotny skutek - to trochę jak prohibicja, która miała spowodować, że ludzie będą pić mniej alkoholu i okazało się, że pili tyle samo, a wzrosła przestępczość zorganizowana. Myślę, że ludzie nadal będą wychodzić, może nieco mniej będą się grupować, jednak zakaz obciąży niepotrzebnie ich psychikę. Mam takie osoby wśród znajomych, które ta sytuacja zaczyna już bardzo męczyć. I one nie mogą teraz choćby pojechać na rower, co mogłoby pomóc. Wiem, że ich to dużo kosztuje psychicznie, że to dla nich jest trudne; a dla mnie to smutne. Rozumiem intencje, ale uważam, że ten zakaz jest błędny. I potrafię znaleźć inne tego typu przykłady. A jeśli mamy bardzo zaostrzone różne zasady, a nie widać szerszego planu, koncepcji, to to obniża morale - myślę, że nie tylko moje. Według mnie, jest to przejaw bezsilności państwa. Mamy restrykcje, lecz nie mamy planu. Biorąc pod uwagę statystyki, będziemy siedzieć w domach jeszcze ze dwa lata. Aby uzyskać odporność populacyjną, kiedy to zachorować musiałoby ok. 60% społeczeństwa, w obecnym tempie rozwoju sytuacji, epidemia w Polsce skończy się mniej więcej za 20 lat. Biorąc pod uwagę bardziej optymistyczny wariant, że chorych jest dużo więcej, to nadal minimum dwa lata.

**Co według Ciebie należałoby zrobić?**

Nie jestem ekspertem. Gdyby ode mnie zależały decyzje, zależałoby mi na tym, aby znaleźć ludzi, którzy się na tym znają - statystyków, epidemiologów. Gdyby jedna osoba miała być tą zarządzającą, nie chciałbym być w jej skórze. To by oznaczało przejęcie odpowiedzialności za cały dotychczasowy bałagan. A rozwiązanie sytuacji nie jest proste, jest w niej wiele sprzeczności. Choć może dałoby się na przykład do pomocy zaangażować jakoś wojsko? Nie rozumiem, dlaczego na terenie Warszawy, która jest dużym ośrodkiem i ma zarówno duże potrzeby, jak i duże możliwości udzielania pomocy, nie rozstawiono jeszcze szpitali polowych? Jest na to przestrzeń choćby w centrach wystawienniczych, typu Expo, czy na Polu mokotowskim. Utworzenie takich jednostek mogłoby ułatwić przyjmowanie i leczenie osób zarażonych w izolacji. Może można by wprowadzić ten stan nadzwyczajny, klęski żywiołowej lub jakiejś innej - choć to trzeba by popytać prawników. Może można by w ten sposób za jakąś sensowną rekompensatą – choć nie wiem, w jaki sposób ustaloną – wykorzystać niefunkcjonujące obecnie hotele w centrum miasta - przerobić je na jakieś szpitale, centra organizacyjne. Chciałbym, żeby organizacyjnie było to wszystko lepiej rozwiązane. Nieprawdopodobne wydaje mi się też, że jako kraj - środkowowschodni, ale jednak - europejski, nie byli w stanie zlecić i wyprodukować jakiegoś płynu do dezynfekcji, albo maseczek. Teraz nie jest czas na liberalizm, a interwencjonizm. Dlatego nie rozumiem, dlaczego nie możemy zlecić szwalniom milionów maseczek na np. za miesiąc. Chyba są jeszcze w tym kraju zakłady, które coś szyją. Gospodarczo - tarcze antykryzysowe - one są niesamowicie skomplikowane, są liczne wnioski, komisje, a profity niewielkie. Te rzeczy powinny być automatyczne - nie wiem, jakie to są koszty. Możliwe, że na tyle duże, że taka biurokracja ma za zadanie utrudnić pozyskanie środków z państwa, choć na sztandarach wpisujemy sobie, że wspieramy przedsiębiorców. Jednak jeśli te pieniądze są, to powinno być automatyczne, niewymagające działań ze strony wnioskodawcy.

**W ostatnim tygodniu weszły jeszcze jakieś obostrzenia?**

Wydaje mi się, że nie i że w ostatnim czasie kwestie związane z wyborami są ważniejsze od walczenia z epidemią. Zamroziliśmy wszystko i zamiast się tym zająć, zajmujemy się czymś innym - takie mam wrażenie.

**Czy zacząłeś robić coś, czego dotychczas nie robiłeś?**

Trochę w ramach wygłupów, zaczęliśmy dziś robić jakieś przysiady, pompki, pajacyki. Powtarzamy sobie, że skoro nie wolno wychodzić, powinniśmy zacząć ćwiczyć w domu, żeby zupełnie nie zesztywnieć. Ale na razie to wciąż pozostaje w sferze deklaracji i gadania o tym. Może przyjdzie taki dzień, kiedy zaczniemy działać, choć on na razie nie nastąpił.

**Czy słyszałeś o nietypowych zachowaniach podczas epidemii, wśród swoich znajomych?**

Nietypowe jest dla nas to przejście do online-u i na przykład granie tam w gry planszowe. Zdarzało nam się już kilka posiadówek na Messengerze, takich wirtualnych spotkań na piwo. Jest to dziwny substytut spotkania, ale możliwy. Z rodziną też kontaktowaliśmy się w ten sposób już kilka razy. Jesteśmy stale w kontakcie poprzez grupę na Messengerze. No, poza moją babcią, której wciąż nie udało się scyfryzować. Wcześniej ta grupa służyła nam tylko do kwestii organizacyjnych - zapraszania się na obiad, szybkiego, jednorazowego informowania o czymś wszystkich członków rodziny na raz. Teraz wymieniamy się tym, co u kogo słychać. Moja siostra wrzuca tam na przykład filmy ze swoimi synami, przesyłamy sobie przepisy, zdjęcia, tego typu rzeczy. Wszyscy mieszkamy w Warszawie, więc przed epidemią nie było problemu z tym, żeby się spotkać. Teraz, nasze życie społeczne, ze względu na okoliczności, przeniosło się do sieci.

**Masz wrażenie, że te relacje są teraz bardziej intensywne?**

Nie, są trochę inne. Ani mniej, ani bardziej intensywne, inne. Właśnie dziwne. Wcześniej rozmawialiśmy w realu, a teraz robimy to w sieci. Wydaje mi się nienaturalne, żeby składać życzenia, czy rozmawiać o jakichś głębszych przeżyciach przez internet. Do tego dotychczas służyły spotkania i to one są bardziej naturalne.

**Jak robisz zakupy?**

Raz na tydzień lub nawet rzadziej, raz na 10 dni, jeżdżę do Biedronki. Robię też wtedy zakupy dla rodziny - mam regularnie trzy listy zakupów. Z naszego domu, od szwagierki i teściowej. Czasami robię też zakupy dla rodziców i babci. Babcia niechętnie mówi, czego jej potrzeba - staram się to na niej trochę wymusić. Inaczej ona sama pójdzie do sklepu, będzie chodzić po kilka rzeczy, stać w tych kolejkach. Mieszkamy z żoną w małym bloku, z widzenia znamy wszystkich sąsiadów. Mamy jedną starszą, schorowaną sąsiadkę. Dotychczas nie łączyła nas z nią żadna relacja, poza tym, że mówiliśmy sobie dzień dobry. Teraz, tak raz w tygodniu, do niej zachodzę, pytam, czy niczego jej nie potrzeba. Wtedy zdarza mi się jeszcze kupić kilka rzeczy dla niej. Osoba, która dotychczas jej pomagała, jest lekarzem, więc teraz nie bardzo ma czas, aby regularnie ją odwiedzać. Dlatego do niej biegamy, choć ona deklaruje, że nie chce pomocy. To jest w sumie rzecz, która w ostatnim czasie się u nas zmieniła - mam wrażenie, że Polacy są dosyć anonimowi w swoich blokach, nie budują relacji z sąsiadami. Teraz, w trudnych czasach, uruchomił się w ludziach jakiś rodzaj solidarności. Nie jakiejś mocnej, skrajnej. Ale jest choćby to pytanie, co słychać, czy wszystko w porządku, trochę śmieszkowania i też właśnie rzeczy praktyczne, jak robienie zakupów, zdarzają się.

**Czy byłeś w sklepie od momentu wprowadzenia limitu klientów?**

Tak, wczoraj. Miałem jechać w poniedziałek rano, ale zanim zdążyłem się wybrać, minęło trochę czasu. Nie chciałem trafić na przerwę, ani godziny zakupów dla seniorów, więc ostatecznie pojechałem na te zakupy o 14. Miałem listy, o których wcześniej wspominałem. Słyszałem też, że w Galerii Młociny, która jest ogólnie mało popularnym miejscem, jest otwarta Biedronka i są w niej wszystkie produkty. Nie jest to mój najbliższy sklep, to czwarta z kolei Biedronka. Kiedy przyszedłem, nie było żadnej kolejki. W sklepie, oprócz mnie, było może 5-7 osób. Robiłem zakupy długo, bo miałem na liście 112 pozycji. Dla jednej osoby było to pewne wyzwanie logistyczne. Około 17 troszkę się w tej galerii zagęściło. Podejrzewam, że przez czas, kiedy sporo osób kończy pracę. Ale to i tak nie było bardzo dużo ludzi - troszkę mniej, niż w mojej osiedlowej Biedronce przed epidemią. To nie był tłum, z tym, że te 1,5 m odstępu od ludzi było już nie do zrobienia. Myślę, że gdybym pojechał o tej 6, czy 7 rano, jak wcześniej planowałem, nie byłoby z tym problemu. Teraz ma być tak, co bardzo mnie cieszy, że Biedronki mają być całodobowe. Gdyby nie to, że wczoraj zrobiłem już zakupy, pewnie pojechałbym np. o 23, albo o 2 w nocy. Po to, aby unikać niepotrzebnego kontaktu i zminimalizować ryzyko zarażenia, zwłaszcza, że pracując zdalnie i nie przemieszczając się w duże skupiska mamy jednak spore szanse pozostania zdrowymi. Te zakupy są teraz najbardziej newralgiczną sytuacją, więc miałbym wtedy poczucie, że robiąc je o takich porach, minimalizuję ryzyko. Wcześniej nie miałem maseczek i rękawiczek, bo nie mogłem ich kupić, ale po ostatniej wymianie zakupów dostałem kilka maseczek z jakimś lepszym filtrem FFP od szwagierki, która dostaje je jako przydział ze swojej korporacji. Ona wychodzi tylko z psem, więc nie jest w stanie ich wszystkich zużyć. To nie są co prawda maseczki jak do sprzątania Czarnobyla, ale są zdecydowanie dobre. Na pewno lepsze od - też zresztą nie tak złych - maseczek szmacianych.

**Jak zachowywali się inni ludzie w sklepie?**

Większość normalnie. Choć trochę dziwi mnie zachowanie pracowników - miałem wrażenie, że oni się za bardzo nie przejmowali. Nie wyglądało to tak, jakby oni się jakoś bardzo chowali za tymi pleksi, czy bardzo pilnowali, żeby ludzie mieli rękawiczki. Nikt z nich nie miał też maseczki. Może to pewien rodzaj wyparcia, skoro nie mogą z tym nic zrobić? A może się tym nie przejmują, bo uważają to za banialuki. Nie wiem. Ale jeśli chodzi o klientów, ponad połowa była w maseczkach, wszyscy w rękawiczkach, bo teraz jest już przepis. Choć trochę nie rozumiem tych rękawiczek. Wydają mi się one dużo mniej istotne, niż maseczki, ale nie wiem.

**Jak płaciłeś za te zakupy?**

Kartą, zbliżeniowo, jak zwykle. Zdarza mi się płacić gotówką, choć teraz nie robię tego w ogóle. Nie jest to w moim przypadku jakaś diametralna zmiana.

**Kupiłeś coś ostatnio przez internet?**

Moja żona kupiła. My w ogóle mamy taki podział, że to ona głównie kupuje przez internet. Nie lubi chodzić po galeriach, robi to rzadko. Od dawna kupujemy wiele rzeczy w sieci, ale nie ze wszystkim się da, niektóre rzeczy dobrze przymierzyć, obejrzeć, jak buty. Ale je też zdarzyło się nam kupować online. Wtedy bierzemy w trzech rozmiarach takie, które nam się podobają i dwie pary odsyłamy. Ostatnio mieliśmy zrobić zakupy w aptece. Kupić takie leki, które nie wymagały recept. Pierwszy raz zamówiliśmy je do paczkomatu. Złożyliśmy też po raz pierwszy zamówienie online na kilka rzeczy ze sklepów specjalistycznych. Trochę zaczyna brakować mi marketów budowlanych. Nie wiem nawet, czy one są otwarte - chyba nie. Ale chciałbym uniknąć jeżdżenia tam, a nie jestem w stanie w ciemno nabyć tych rzeczy online - wolałbym je pooglądać na półce, zobaczyć - na przykład elektronikę. Poprzepalały mi się ostatnio przewody w odkurzaczu i musiałem je wymienić. Jedną z możliwości byłoby pojechać na giełdę z elektroniką, koło GUSu, ale nie chciałoby mi się tam wybierać specjalnie po te dwa małe elementy i ryzykować zarażeniem. Kombinowałem więc z lutowaniem tego, co miałem w domu. Tuż przed wybuchem koronawirusa chcieliśmy wymienić materac, bo obecny jest już bardzo stary. Zastanawiamy się teraz, czy go kupić, czy nie, czy nas w ogóle stać, bo może trzeba oszczędzać. Plan był taki, żeby wziąć go z małej firmy. Teraz nie wiem nawet, czy oni działają - będę do nich dzwonił w tym tygodniu, czy oni w ogóle nie upadli. Siedzimy teraz w domu, więc aż się prosi, aby z okazji wiosennych porządków zrobić w domu niektóre rzeczy. Zwykle miałem taki zwyczaj konsumpcyjny, że jechałem sobie do Leroy lub do jednego takiego znanego mi sklepiku z elektroniką i kupowałem rzeczy, które były mi potrzebne. Mogłem je obejrzeć, porównać ceny i porozmawiać ze sprzedawcami, którzy są w stanie mi doradzić. Ale to jest mój model zakupowy. Mojej żonie pewnie to nie przeszkadza. Ona najpierw czyta poradniki w sieci, szuka na różnych stronach, porównuje oferty i zamawia online. Do mnie przemawia inna opcja i to mnie trochę blokuje. Niedługo będę potrzebował kupić farbę do metalu, więc pewnie będę musiał trochę poczytać i wybrać coś przez internet.

**Czy masz poczucie, że jak pandemia się skończy, będziesz więcej rzeczy kupował online?**

Jeśli chodzi o leki, już od dawna myślałem o tym, że powinniśmy to robić online. Nie kupujemy ich moim zdaniem w dużej ilości, w porównaniu do innych Polaków. Część tych leków jest jednak na receptę, więc nie można ich zamówić przez internet. Nawet pomimo wprowadzenia elektronicznych recept, niektóre z nich trzeba realizować osobiście w stacjonarnej aptece. Ale gdyby można było wszystko kupić w jednym miejscu, bez wychodzenia z domu, chętniej robiłbym takie zakupy w sieci. Jednak jeśli chodzi o markety, pewnie nie zmienię moich zwyczajów konsumpcyjnych. Gdybym czuł się profesjonalistą, wiedział, co wybrać, wtedy pewnie przeklikiwałbym szybko przez jakąś stronę internetową i po prostu to zamawiał. W związku z tym, że się nie znam, lubię pójść, poczytać etykiety, popatrzeć na produkty, skonsultować ze sprzedawcami, którzy mają jakieś tam pojęcie i dopiero wtedy dokonać wyboru. To jest dużo przyjemniejsze, niż siedzenie nad komputerem.

**Czy zmieniło się coś, jeśli chodzi o to, co Ty i Twoja żona teraz jecie?**

Odrobinę tak. Zawsze staraliśmy się robić jedzenie w domu i nie kupować go w pracy. To musiały być jednak rzeczy, które nadal będą smaczne po odgrzaniu w mikrofali oraz łatwo się je przenosi. Rzadko nosiliśmy na przykład kotleta z ziemniakami i surówką, bo to stałoby się jedną paćką albo wymagałoby od nas niesienia tego w trzech osobnych pojemnikach. Zupa odpadała, bo się rozlewa. W związku z tym, preferowaliśmy na przykład ryż lub makaron z sosem. Teraz mamy większe możliwości, jemy mniej makaronów, dań jednogarnkowych. Częściej robimy sobie potrawy bardziej tradycyjne, czyli takie, gdzie jest jakiś zapychacz - na przykład te ziemniaki. Do tego jajko, czy kotlet - w naszym przypadku raczej bezmięsny, ale to się nie zmieniło, preferujemy kuchnię wegetariańską. I jakaś surówka. Na śniadania jemy mniej owsianki. Oboje jesteśmy w home office, więc częściej jemy teraz jakieś przyjemne kanapki z herbatą, jajko na miękko - jest na to więcej czasu. Piję też więcej alkoholu - siedzę wieczorem w domu, mało jeżdżę samochodem - wieczorami chętniej odpalam sobie piwo. Wcześniej nie piłem go tak często, teraz robię to mniej więcej co drugi dzień. Jeśli chodzi o pozostałe aspekty, raczej nic się nie zmieniło.

**Sposób przygotowania posiłków**

Wcześniej częściej jadaliśmy osobno, ponieważ byliśmy w pracy. Razem, ale nie wspólnie jedliśmy śniadanie. Mam tu na myśli, że nie było tej celebracji, koncentracji na posiłku. Każdy robił w międzyczasie dużo innych rzeczy związanych z porannymi przygotowaniami - prysznic, spacer z psem. Obiad był raczej osobno i obiadokolacja raczej razem, chociaż to zależało akurat od naszego planu dnia, ewentualnych zajęć dodatkowych, czy innych obowiązków. Ale w przypadkach, kiedy żona wracała później, starałem się naszykować dla niej coś do jedzenia, żeby choć na chwilę usiąść razem. Teraz śniadania są bardziej celebrowane, zazwyczaj mamy na to czas. Chyba, że akurat żonie wypadnie w tym czasie telekonferencja - wtedy nie.

**Zamawiacie coś?**

Teraz nie. Myślałem, że będziemy to robić, ale jest chyba tyle czasu wolnego na zrobienie jedzenia, że nie. Ewentualnie może zrobimy to dla przyjemności, jednak nie ma takiej konieczności. Kiedyś bywało tak, że byliśmy zmęczeni lub czegoś brakowało. Zamawialiśmy jakiś obiad raz na tydzień/dwa, ot tak, że był - byliśmy głodni, chcieliśmy to na już, żeby ktoś przywiózł i tyle. Teraz jest mniej zamawiania. Ale troszkę mniej, bo nigdy nie było go dużo. To są też rzeczy, których nie bardzo jesteśmy w stanie ugotować samodzielnie. Pizzę, chińczyka czy falafel potrafimy zrobić w domu – zastanawiam się za to nad zamówieniem czegoś z hinduskiej restauracji w okolicy, oni gotują dobre rzeczy, a my nie odtwarzamy tego smaku.

**Uważasz, że zamawianie jedzenia jest teraz bezpieczne?**

Nie chciałbym odpowiadać na to pytanie 0/1. Są rzeczy, które są w 100% niebezpieczne - jeśli umówisz się z kimś, kto ma stwierdzonego koronawirusa i kaszlnie Ci w twarz, to to takie jest. Choć może też nie do końca w 100%, bo możesz przejść to bezobjawowo. W każdym razie to jest niebezpieczne. Jeśli chodzi o rzeczy bezpieczne - chyba nie ma takich w pełni bezpiecznych. Ewentualnie izolacja w domku, w Bieszczadach. Zamawianie jedzenia to dla mnie (nie)bezpieczeństwo tego samego stopnia, co zakupy spożywcze, czy minięcie się na klatce schodowej z sąsiadem. To nie jest coś bardzo niebezpiecznego, a raczej ryzyko, którego nie unikniemy. Możemy je tylko minimalizować, ale ono zawsze jakieś będzie. Chodzi głównie o kontakt z człowiekiem - bardzo nieduży. Mam na myśli na przykład to, że dostawca może być chory. Może przenieść wirusa na powierzchnię, z której na przykład trafi on nam do oka. Powyżej 60 stopni chyba koronawirus ginie, więc drogą pokarmową raczej nie można się zarazić. Uważam, że to ryzyko jest na tyle nieduże, że raz na jakiś czas można sobie na nie pozwolić.

**Wielkanoc**

Ona jest dla mnie ważna z powodów społecznych i tradycyjnych. Jeśli chodzi o kwestię religijną - też właściwie jest ważna. To mi się przez lata zmieniło - ocena tego święta jako religijnego przeżycia. Moja rodzina nie jest mocno religijna, większość osób jest nawet niewierząca, a z niektórymi trochę nie wiadomo. Dlatego Niedzielę Palmową i Wielki Tydzień obchodziłem z żoną trochę na osobności. Uczestniczyliśmy w tych wydarzeniach duchowych sami, natomiast w Niedzielę Wielkanocną wskakiwała ta część społeczna świąt. To jest teraz trudne, zazwyczaj spędzaliśmy je w dużym gronie, około 12 osobowym. Odwiedzaliśmy się też wzajemnie, jeździliśmy do ciotek. Teraz na pewno nigdzie nie pojedziemy. Z moimi rodzicami umówiliśmy się już, że się nie spotkamy. Oni spędzają święta z moją młodszą siostrą, która z nimi mieszka i z babcią, bo tata i tak się z nią ciągle kontaktuje. Na wszelki wypadek my już do nich nie dołączymy. W rodzinie mojej żony jest kłopot. Ona, jej mama i siostry chcą się spotkać tylko online. Ja jestem przeciwny. Wydaje mi się, że oczywiście, niesie to ze sobą pewne ryzyko, ale wszyscy jesteśmy dość mocno zaizolowani od dłuższego czasu. Wydaje mi się, że jeśli do soboty nie będziemy mieć żadnych objawów grypopodobnych - co oczywiście nie musi świadczyć o tym, że nie jesteśmy cichymi nosicielami - to może byśmy się jednak spotkali, bo już bez przesady. Ale dyskusja trwa i zobaczymy, choć wydaje mi się, że to jest ryzyko, które jestem w stanie zaakceptować. Gdybyśmy chodzili do pracy, spotykali się z ludźmi na co dzień, ryzyko byłoby większe i stwierdziłbym, że nie powinniśmy się spotykać. Ale że we wszystkich trzech domach jesteśmy mocno odizolowani, uważam, że niebezpieczeństwo związane ze spotkaniem jest małe. Dla mnie byłoby bardzo dziwne, gdybyśmy się nie spotkali. To byłyby strasznie dziwne święta. A już samo to, że nigdzie później nie pojedziemy, w żadne odwiedziny - jest dziwne. Nie zamierzam jednak iść do kościoła. Ostatnio było mi tam mniej po drodze, ale nawet gdyby było zupełnie na odwrót, ze względów epidemiologicznych, byłbym w stanie to całkowicie odłożyć. Zawsze uważałem, że to, co dzieje się w kościele, to raczej rzeczy symboliczne, że to jakieś uzewnętrznienie spraw wewnętrznych. Te obrzędy, rytuały są ważne, bo inaczej nie czuje się tej całej odmiany, wyjątkowości. To tak, jak tort podczas urodzin - podkreśla odmienność, wyjątkowość tego dnia. One nie są jednak najważniejsze. Jestem w stanie całkowicie zrozumieć msze na odległość, modlitwę w domu, nawet tzw. komunię duchową. Pewne tradycje są potrzebne, jednak nie są istotą. Dlatego jestem w stanie z tej otoczki zrezygnować - to nie tak, że święta bez koszyczka, czy pójścia do kościoła się nie odbędą. Będzie mi tego brakować, jest to jakieś zubożenie tych świąt, ale nie niszczy ich istoty.

**Przygotowujecie się jakoś do Wielkanocy?**

Tak, musieliśmy podczas układania listy zakupów wziąć pod uwagę potrawy, które chcielibyśmy zrobić z tej okazji. Kupiłem barwniki do jajek, składniki na ciasto, wanilię, jakieś bakalie, pistacje - to taka rzecz do jedzenia bardziej odświętna, na co dzień staram się ich nie kupować, bo uważam, że są bardzo drogie. Jako już zamożne społeczeństwo jesteśmy w stanie jeść na co dzień rzeczy, które kiedyś pojawiały się na stołach tylko podczas szczególnych okazji, jak kiełbasa. Uważam, że ten rarytas od święta jest potrzebny i dla mnie są nim pistacje. Chciałem kupić chrzan, którego nie było. Wziąłem rzeżuchę, żurek w butelce, produkty na sałatkę warzywną. Kupiłem baranka, ale to tak dla jaj. Żona pytała, czy będziemy robić koszyczek - ja nie jestem ich fanem. No, ale kupiłem już tego baranka, jakiegoś takiego czekoladowego, z Milki. Ale w porównaniu do corocznych przygotowań - nie są one ani dużo większe, ani dużo mniejsze. Parę lat temu Wielki Post był dla mnie większym wydarzeniem, zwracałem większą uwagę na kalendarz liturgiczny, byłem z nim na bieżąco. Teraz jestem mniej, więc podejrzewam, że i tak zaczęlibyśmy te przygotowania tak na tydzień przed Wielkanocą.

**Czy w trakcie pandemii zdarzyło Ci się kupić jakieś rzeczy dla przyjemności, jak te pistacje?**

Trochę tak, trochę nie. Wspominałem Ci, że piję teraz więcej piwa. Jeszcze zanim zostaliśmy zamknięci w domach, była promocja w Biedronce. Ja nie mam wyszukanego gustu, jeśli chodzi o ten trunek, lubię takie zwyczajne. Znalazłem Kasztelany w dobrej cenie, kupiłem ich wtedy 16. Pomyślałem sobie, że mam szczęście, że jest promka, bo jak już będziemy siedzieć w domach i się nudzić, to ja sobie wieczorem tak usiądę, odpalę piwo, obejrzymy sobie film lub serial, do tego coś do pochrupania - i tak w przyjemny sposób spędzimy nieprzyjemny okres epidemii. To było z takim zamysłem, że będzie to pewien rodzaj odskoczni, przyjemności.

**Zdarza Ci się wyskoczyć do sklepu po jedną lub kilka rzeczy?**

Zazwyczaj tak robiłem, teraz nie. Dlatego poza brakiem chrzanu, cierpimy teraz niedostatek dobrej herbaty. O ile na wielu rzeczach oszczędzamy i staramy się być zdyscyplinowani, jeśli chodzi o zakupy, tak herbata jest tym produktem, do którego przyzwyczaiła mnie żona i na który wydajemy więcej. Zazwyczaj kupowaliśmy ją w osiedlowym Samie, wielkości Biedronki. Ostatnio po prostu, podczas tych dużych zakupów, kupiłem Lipton, żeby mieć jakąkolwiek herbatę. Żona była niepocieszona. Do Sama chodziłem też po kilka innych rzeczy, których normalnie nie mogę dostać w Biedronce, jak ajwar. Teraz są tam strasznie duże kolejki, dziś rano przed sklepem stało chyba 40 osób. Dlatego dobrą herbatę wypijemy w lipcu. Święta, ze względu na chrzan, może też przełożymy, trudno. :) Nie są to takie potrzeby, dla których chciałbym narażać się na wirusa i te tłumy. Może uda nam się zamówić to przez internet, ale nie chcę też zamawiać sześciu paczek z pojedynczymi produktami. Gdyby udało się to zamówić w jednym miejscu, poczekalibyśmy nawet dwa tygodnie, żeby to do nas przyszło. Ale jak mam zamawiać jedną rzecz i drugie tyle dopłacać za przesyłkę, na chwilę obecną wydaje mi się to bez sensu - nie są to rzeczy niezbędne dla naszej egzystencji, albo mamy na razie jakieś ich substytuty. Choć może jak nam się takich produktów nazbiera, postanowimy je zamówić lub pojechać po nie do jakiegoś innego sklepu.
