# Supplementary material for: The challenges arising from the COVID-19 pandemic and the way people deal with them. A qualitative longitudinal study
Source: PLoS One. 2021 Oct 11;16(10):e0258133. doi: 10.1371/journal.pone.0258133 (PMC8504766; doi:10.1371/journal.pone.0258133)
Supplement: S1 Dataset — (ZIP) [file pone.0258133.s003.zip › Transcriptions/stage 2/12.2_M_33_couple, with children.docx]

**12.2_M_33_couple with children**

**Jakie emocje Ci towarzyszyły w tym tygodniu? Ogólnie**

Ogólnie same emocje miałem takie pozytywne. Nie wiem, dlaczego. Jakoś nie wnikałem za bardzo w sytuację taką co się dzieje na zewnątrz. I wydaje mi się, że tutaj dużą rolę odegrała pogoda. Można sobie wyjść na zewnątrz. Jest ciepło. Fajnie, przyjemnie. Także nie miałem większych sytuacji stresowych, które bym zapamiętał. Wręcz przeciwnie. Dużo fajnych chwil spędziłem z rodziną w tym czasie. I tak jak mówię na przestrzeni tygodnia z oderwaniu od tej sytuacji, która się wokół dzieje, emocje miałem takie dosyć pozytywne.

**Obrazki.**

13. Rzuciła się w oczy. Skojarzyła mi się najbardziej. Ona odzwierciedla dokładnie to, co przeżywałem w tym tygodniu. Kojarzy mi się z naturą, dużo czasu spędzałem na zewnątrz. Z rodziną. Bawiłem się  z psami. Już planowałem koszenie trawy. Sprawdzałem, czy sadzonki winogron ruszyły. I bardziej tak spędzałem czas - ten obrazek może to odzwierciedlać. Słońce, dużo pomogło. I plusowe temperatury. Mentalnie wychodzę z zimy.

**Gdybyś miał nazwać konkretne emocje, to co by to było?**

Emocje tak? Radość. Zadowolenie z planów, które mi się gdzieś w głowie rodzą. Takich przyszłościowych. Bardziej tutaj mam na myśli właśnie wszystko co się znajduje na posesji. Też takie szczęście czyste. Jak widzę dzieci, które skaczą na trampolinie. I  taka wesołość. One tak bardzo nie czują tego jakiegoś zagrożenia, dla nich to jest taka sytuacja - może już stała się taka naturalna? Bo dzieci chyba ogólnie tak mają, że szybko się dostosowują do nowej sytuacji. Chyba bym tak opisał te emocje. Bardziej w kierunku pozytywnym. Szczęście, zadowolenie, planowanie. Zadowolenie z planów, które rodziły się w głowie. Jakieś takie myśli pozytywne o przyszłości. Dużo w tym tygodniu mogłem sobie powyobrażać. A z tym jest związana np. o jakieś zakupy, typu tablica z koszem do powieszenia, żeby pograć w kosza z dzieciakami. Albo zestaw leżaków na trawkę. Super sprawa. W tym kierunku moje myśli dążyły.

**Przejdźmy może do doświadczeń. [...] Co jeszcze zmieniło się w ostatnim tygodniu jeśli chodzi o zachowania? Czy coś doszło, czy czegoś ubyło?**

Nie myślę, że tak ogólnie nie ma dużej różnicy. Wszyscy jesteśmy ograniczeni, tak jak rozmawialiśmy ostatnio, jeśli chodzi o to wychodzenie na zewnątrz. Rytuały zostały mniej więcej te same. Skupiamy się na pracy. Popołudniem skupiam się, żeby jak najefektywniej się zrelaksować. Z dziećmi, z rodziną. Teraz korzystając  z tego, że można wyjść na zewnątrz, połapać trochę tego słońca. Za dużo u mnie się nie zmieniło. Tych zmian, to one się tyczą bardziej tego, że bardziej myślę o przyszłości i o tym, co mam do zrobienia. Taką checklistę sobie w głowie układam - tego co się będzie u mnie działo na posesji, co mam zaplanowane w najbliższym czasie. Czekamy na kuriera z koszem. Z dzieciakami miałem wyprawę w sobotę - pojechaliśmy sobie ciągnikiem po piasek, żeby piaskownicę zrobić. Już się bawią tam. Być może to mnie tak... Wyjazd, las, pola, ciągnikiem - to mnie tak nastawiło fajnie, pozytywnie.  I być może dlatego ten obrazek mi się tak skojarzył.

**Czy te wyjazdy z dzieciakami zawsze?**

Czasem sam. Ale teraz jak już dzieci mam w takim wieku, że ich to bardzo interesuje, i widzę też po nich, że chcą tego kontaktu ze mną - bo jednak cały dzień siedzę w tym pokoju i tylko od czasu do czasu wyjdę i nie raz pokrzyczeć, że za głośno, albo że coś niedobrego robią - to one szukają tego kontaktu i mają frajdę taką jak pojedziemy na wyprawę. Ja pamiętam swoje dzieciństwo i wiem, że to jest straszna frajda coś robić z tatą. I coś innego to jest. Ja się staram zawsze dzieciom zapewniać takie - jak to powiedzieć - męskie rozrywki, tak zapraszam jak pracuję przy drzewie to też ich angażuję, że sobie zbierają na taczkę. I dla mnie to jest praca wykonana, którą one zrobią, a dla nich to jest 100% zabawa. I widzę, że to się im podoba. I też ich tego próbuję nauczyć, że są obowiązki na posesji. Im bardziej będą starsze, tym bardziej będą pomagać. Im to sprawia przyjemność. Daję im pokierować ciągnikiem i jest fajnie.

**Ostatnio opowiadałeś o pracy, a teraz pojawił się ten ciągnik. Nie bardzo wiem, jak ja mam to łączyć - czy uprawiacie ziemię?**

U nas to jest tak, że dawniej dziadkowie prowadzili gospodarstwo, później przez chwilę teść. Natomiast wszystkie te budynki, zabudowania, maszyny rolnicze - ciągnik jest na miejscu - my już nie uprawiamy ziemi, nie mamy zwierząt hodowlanych. Ale maszyny zostały i korzystamy z tego. Mamy też swoje pole, ale nie uprawiamy. Możemy pojechać po piach, bo niedaleko jest betoniarnia. Ale lepiej jest zabrać dzieci, pojechać gdzieś do lasu czy na pole - dzieci na polu zbierały kamienie i też miały frajdę. Coś miały innego niż mają na co dzień. I jedną stronę córka kierowała pod moją kontrolą, a w drugą stronę syn. Teraz tylko czekają kiedy ich tylko zaproszę do "pracy".

Stąd też to planowanie na posesji, bo to jest dosyć duże. Jest część terenu, gdzie jeszcze ręki nie dołożyłem - jakieś krzaki są. Od frontu już zrobiłem dla dzieciaków prawie 100 m2 trawnika, jakieś domki, trampolina. Ale jeszcze jest dużo do zrobienia i można sobie planować na kilka lat do przodu.

**Kiedy to wszystko zrobiłeś? To świeża sprawa?**

Trawnik w zeszłym roku. Chyba w czerwcu już fajna trawka była. Wtedy też zbudowaliśmy domek, w którym dzieciaki się bawią. Trampolinę mieliśmy wcześniej. Ale musiałem zburzyć płot, który dzielił posesję, żeby powiększyć tą przestrzeń dla dzieciaków.

Ale jest jeszcze dużo do zrobienia.

Zauważyłem w tym tygodniu, że dzieciaki w drugiej części, tej oddalonej od drogi, w ogóle nie przebywały, nie chodziły, albo tylko ze mną. Albo po tej sobocie się otworzyły i częściej ich widzę jak idą za stodołę i po chaszczach się szlajają. Wczoraj córka przybiegła, że bratu noga gdzieś utknęła. To pobiegłem pomóc. I od razu mi się przypomniało moje dzieciństwo, że się tak chodziło po wertepach. To jest odkrywcze dla nich, ja im nie zabraniam.

**Czy jest coś co Cię denerwuje?**

Chyba mnie denerwuje jak się kłócą dzieciaki. Nie wiem, czy dzieci się zirytowały przez tak długi czas, że są tutaj w domu i nie opuszczają w ogóle tego podwórka. Ale zaczęły się dziwne odzywki do rodziców - nad tym już zapanowałem. Odreagowują. Nie wiem jak to nazwać - trochę się stawiają. Natomiast już się dogadaliśmy. Ale potrafią się nie raz tak pokłócić, że się pobić i w ogóle. Ale to chyba też jest normalne.

**Co cię w tych ich kłótniach najbardziej denerwuje?**

Takie zachowanie dziecinne najbardziej. Ja bym je chciał uczyć, że można się czymś podzielić, wyznaczyć, jak będą korzystali z czegoś (siostra tyle razy, brat tyle razy). Przy oglądaniu telewizji chociażby.  Ja im cały czas próbuję wytłumaczyć, że bicie, płacz i wszystko co za tym idzie nie jest rozwiązaniem. Że to tylko pogarsza sytuację. Bo ja przychodzę, mówię "nie potraficie się dogadać, to nie będziecie oglądać, znajdźcie sobie zajęcie u siebie w pokojach". Albo jak o zabawkę się kłócą, to zabawka nie będzie ani dla jednej osoby, ani dla drugiej. I próbuje ich tego nauczyć, że to nie będzie z korzyścią dla nikogo. Nie wiem, czy widzę zalążek tego, że bardziej się dogadują, ale chyba tak. Natomiast chcę ich nauczyć, że jak się sami dogadają, to będą mieli tą korzyść.

**A czy myślisz, że te kłótnie są powiązane z tym, że musza siedzieć w domu i z sytuacją z koronawirusem?**

Niekoniecznie bezpośrednio, natomiast na pewno mają wpływ z tego względu, że wcześniej tak długo ze sobą nie przebywali w ciągu dnia, bo oni są w różnych klasach, mają różne zajęcia po szkole. Wieczorami zostawał czas na krótką zabawę, kolację i potem już spać. A teraz spędzają z sobą praktycznie 24 godziny na dobę, bo od rana razem do momentu aż pójdą spać. Z jednej strony to fajnie, bo mogą się zająć sobą i naprawdę fajnie się bawią. W 80% czasu to jest super zabawa między nimi i cieszę się, że te 1,5 roku różnicy znika między nimi. Natomiast to też jest tak, że jak spędzają ze sobą tyle czasu, to jakieś konflikty wypływają. I więcej jest tych konfliktów, bo i więcej czasu. Na pewno ta sytuacja ma wpływ.

**A czy ta sytuacja ma w jakiś sposób wpływ na Ciebie? Czy pojawiło się coś co zaczyna Ci bardziej doskwierać niż wcześniej?**

Chyba tak naprawdę już myślę o świętach. Spędzonych w takim gronie rodzinnym ściśle. A nie całą rodziną. Do nas na święta wielkanocne, czy na boże narodzenie to do Wigilii siada 25 osób. Na Wielkanoc tyle samo jest przy stole. Wielki stół, wynoszenie kanapy. Kombinowanie jak stoły. Ludzie przyjeżdżają, nocują. Chyba to mi doskwiera w tym momencie. Bo będą też święta dziwne. Będziemy mieli niby wszystko naszykowane. Być może ktoś z najbliższej rodziny odwiedzi, ale to dosłownie na chwilę i to z zachowaniem różnych dystansów, maseczki, itd. Ale to nie będzie w takiej formie jak zawsze. To jest taka myśl, która mnie przygnębiła. Nawet miałem jeszcze taką nadzieję, że jednak ta najbliższa rodzina, z którą ten kontakt jest - taki nawet codziennie, co dwa dni. Żona jeździ do pracy do rodziców. Myślałem, że coś się uda zrobić, żeby przynajmniej  w okrojonym gronie ale żeby te święta były inne. Natomiast chyba do mnie dzisiaj dotarło, że chyba tak nie będzie możliwe. Żeby więcej osób do obiadu przy stole było. Żeby pogościć się po polsku w większej liczbie osób. To mnie przygnębiło.

**To dotarło do Ciebie dzisiaj ponieważ... Coś usłyszałeś? Zobaczyłeś? Co miało na to wpływ?**

Usłyszałem, że takie decyzje zapadły. Bo do końca te decyzje były przesuwane i gdzieś brałem pod uwagę taką sytuację. Natomiast to nie było pewne.

Ja w tej kwestii nie decyduję, bardziej chodziło o decyzje teściów - bo to chodziło, czy oni przyjadą do swoich rodziców. Natomiast miałem nadzieję, że ta bliska relacja sprawi, ze przyjadą. Na ten jeden dzień, posiedzą. Ale chyba raczej nie  - tak już dzisiaj usłyszałem, bo to jest duże ryzyko ze względu na dziadków. Szczególnie, że teściowie nie mają czegoś takiego, że są na kwarantannie u siebie zamknięci i ograniczony mają kontakt. Tylko prowadzą biznes i z różnymi osobami się widzą.

Cała rodzina patrzy w kierunku dziadków - tu jest największa obawa. Bo dziadkowie są wiekowi. 87 i 85 lat. I każdy się boi. I żeby jak najbardziej ograniczyć kontakt.

Miałem ciekawą sytuację w tym tygodniu, siedziałem w domu i widzę idzie sąsiad z reklamówką jajek chyba. Dla dziadków. Musiałem wyskoczyć przez furtkę i go zatrzymać. Powiedzieć, że bardzo dziękujemy, przekażę dziadkom ale spotkać się nie da rady, nie mogę cię wpuścić. Był zdziwiony. Tacy najbliżsi sąsiedzi to wiedzą, a on z dalej przyszedł. No niestety. Taka sytuacja. I póki nie minie ta kwarantanna powiedzmy całego narodu i ta sytuacja ogólnie to będziemy musieli tego pilnować.

**Jak planujecie przygotować się do Świąt? Czy to będą szerokie przygotowania czy w związku z tym, że grono będzie mniejsze to przygotowania też mniejsze?**

Oczywiście na mniejsza skalę. Normalnie to 25 osób do stołu siadało, a teraz będzie 7 osób z dziećmi licząc. Więc nie będzie wynoszenia (kanap?), przestawiania w salonie. Dom wiadomo będzie wysprzątany, jak zawsze. Obiad będzie uroczysty, usiądziemy z dziadkami przy obiedzie. Natomiast całej tej otoczki pozostałej czyli pełnego domu, harmidru, 9 dzieci, które tu biegały - to jest coś się kojarzy ze świętami i chociaż jest mega głośno, i mega roboty przy tym, to zawsze sprawia przyjemność [całą opowieść o świętach słychać smutek w głosie, taki żal].

**Jak to u Was jest, co jest w świętach najważniejsze. Pewnie te spotkania z bliskimi. Czy w świętach jest coś jeszcze co jest dla Ciebie istotne?**

Wiadomo, jak dla każdego katolika, to te wszystkie aspekty religijne. Staramy się z dziećmi uczestniczyć w niedzielę w mszy świętej - teraz przez transmisję telewizyjną. Ale zawsze jeździliśmy do kościoła. Zawsze święcenie święconki - dzieci zawsze jeździły i to też ma ogromną wartość. Są dwie takie wartości, które w Świętach na pierwszym miejscu były stawiane czyli spotkania rodzinne plus te wydarzenia religijne. Chyba powinienem mówić w odwrotnej kolejności, nie? Ale może te święta bożego narodzenia się bardziej czuje, atmosferę świąteczną - choinka, pasterka, itd. Wielkanocne pewnie są ważniejsze z punktu widzenia wiary, natomiast miałem zawsze problem, żeby dostosować się do tych świąt, przestawić się - ten wielki post. wtedy chyba za dużo się dzieje na zewnątrz. Łatwiej mi chyba taki rachunek w adwent zrobić, wyciszyć się, niż przed Wielkanocą. Kojarzą mi się spowiedzi na ostatnią chwilę.

**Teraz nabożeństwa w telewizji. Jak to będzie podczas Świąt? Planujecie pójść do Kościoła, czy raczej też z domu?**

Zdecydowanie w domu. Śledzimy na bieżąco to co jest przekazywane przez media i zarządzenia biskupów. Myślałem o tym, żeby się umówić ze znajomym księdzem na spowiedź, ale są zalecenia, że można to zrobić indywidualnie, wewnętrznie pogodzić się z bogiem. Później przyjąć komunię w sposób symboliczny, nawet uczestnicząc w mszy przed telewizorem. Chyba w tej sytuacji kościół też dosyć elastycznie do tego podszedł. I bardzo fajnie ostatnio było na mszy powiedziane - to była transmisja ze świątyni opatrzności bożej - że bóg może wszystko i to nie jest dla niego problemem, żebyśmy my mogli uczestniczyć w tej mszy chociaż jesteśmy zdalnie. I to jest budujące. W ten sposób sobie to zorganizujemy. Na pewno będziemy oglądać transmisję, uczestniczyć we mszy, bo to nie jest tylko oglądanie transmisji tylko uczestniczenie we mszy świętej. Z całą rodziną.

**Wspomniałeś o święconce. Wiele osób podkreśla też te tradycje święceni palmy. Czy to dla Ciebie jest istotne?**

Tak. W niedzielę palmową też byliśmy na transmisji, podczas transmisji trzymaliśmy palmę, dzieci też trzymały. Podtrzymujemy te tradycje na ile możemy, na ile jesteśmy w tym momencie w stanie. Nie będziemy ryzykować, szczególnie, że były sytuacje u nas w okolicy, że ludzie przyszli do tego kościoła, przyjechała policja i ludzie musieli się rozejść. Na kolejną mszę ksiądz był na tyle poinformowany, może nie tyle poinformowany, tylko dostał ostrzeżenie, że on jest też za tych ludzi odpowiedzialny, no i sam namawiał do tego, żeby skorzystać z transmisji telewizyjnej. My z tego po prostu korzystamy. Na szczęście mamy taką możliwość.

**A jak ze święconką?**

Tutaj z tego co słyszałem zalecenie jest takie, żeby poświęcić wodą święconą, którą mamy w domu, przez najstarszego członka rodziny. Coś w ten deseń. I taki jest plan. Żeby przygotować święconkę, poświęcić ją w domu, pomodlić się i w ten sposób poradzić sobie z tym, żeby zachować tradycję.

**Przejdźmy może do tych zasad, które ostatnio zostały wprowadzone**. **Czy stosujesz się do nich?**

Nie do końca się stosuję, w sensie takim, że - np. to jest kwestia wymiany opon w aucie. Nawaliło też nam auto, a my potrzebujemy dwóch samochodów. Nie widzę takiej potrzeby, żeby czekać z wymianą opon do lipca. Nawet mi się gdzieś artykuł przewinął dzisiaj że policja potrafi mandaty za to wlepiać, że nie jest to rzecz która jest pierwszej potrzeby, itd. Natomiast jeśli są zachowane te wszystkie bezpiecznościowe sprawy - ochrona siebie i kogoś. Aż tak bardzo nie podchodzę restrykcyjnie do tego. Jak mam potrzebę, żeby wymienić sobie opony, to jadę, zostawiam u mechanika samochód, wracam potem jadę odebrać. Wszystko w rękawiczkach, jeśli jest taka potrzeba to maseczka, płyn do dezynfekcji. I mam to zrobione. Aż tak restrykcyjnie się nie trzymam tego co jest zalecone.

Byłem ostatnio w sklepie na zakupach, na które musiałem pojechać, ale wróciłem sobie dłuższą drogą, bo mi się fajnie jechało. To też jest takie... No, nikomu nie zagrażam.

**Czyli rozumiem, że ważniejsze dla Ciebie jest to, aby to co robisz robić z głową, nie zagrażać sobie ani innym, niż stosować do wszystkich zaleceń.**

Oczywiście, że tak. Myślę, że każdy ma swój rozum i trzeba to wziąć na logikę. Jeśli coś jest zrobione dobrze, to nie ma powodu, żeby sobie coś zarzucać. Być może te restrykcje się pojawiły przez to, że są tacy ludzie, którzy nie biorą sobie tego poważnie. Na logikę tego nie biorą. Nie podchodzą poważnie do sprawy. I przez to nakładane są restrykcje na wszystkich.

**A co myślisz o tych zaleceniach, które zostały wprowadzone w ostatnim tygodniu?**

To były chyba restrykcje związane z ilością osób w sklepie. Ten zakaz osób poniżej 18 roku życia bez osoby dorosłej na ulicach - to mi się wydaje, że to jest idealny przykład tego do czego się odnosiłem wcześniej. Czyli to, że te młode osoby 16-17 lat nie brały sobie na serio tych wszystkich obostrzeń i zakazów i to nagminnie łamały. I dlatego ten zakaz powstał dla ogółu. Ale na moją logikę to dziwne, żeby ktoś kto ma 17 lat musiał z osobą dorosłą się przemieszczać na ulicach. Ciężko mi sobie to wyobrazić. Natomiast domyślam się skąd się to wzięło.

**A jak myślisz, jakie są przyczyny, że ludzie nie przestrzegają tych zaleceń?**

Myślę, że jest to spowodowane - nie wiem, ciężko powiedzieć. Ponieważ to chyba dużo zależy... Z tego co słyszałem słabo przestrzegają osoby starsze i osoby bardzo młode. Nie można powiedzieć jednoznacznie, bo każda pewnie grupa wiekowa ma swoje powody. Młodzi to ciężko mają usiedzieć w domu, gdzie 80% czasu spędzają na zewnątrz. Nie raz jest to tak, że ktoś z bloku wychodzi, z mieszkania 40 metrów i tak naprawdę żyje na zewnątrz, a wraca tylko do domu spać, posiłki, krótka rozmowa z rodzicami i dalej na zewnątrz. Młode osoby ciężko mają się przestawić. Starsze znowu mogą sobie tłumaczyć, że "tyle lat przeżyłem, to mnie nic nie grozi". Często też wstydzą się poprosić o pomoc. Np  ze zorganizowaniem jakiś produktów spożywczych, czy czegokolwiek. Stwierdzają, że sami sobie poradzą. Może w ten sposób.

**Jeżeli chodzi o działania, które podejmujesz teraz w porównaniu do zeszłego tygodnia. Czy coś się zmieniło?**

Raczej nie. Trzymam jeden standard.

**A jeżeli chodzi o wyzwania, które pojawiły się na Twojej drodze?**

Ogólnie cały czas się pojawiają wyzwania jeśli chodzi o sprawy związane z pracą. To jeśli chodzi o wyzwania starczy, nie szukam nowych wrażeń. Natomiast jeśli chodzi o część prywatną, to nie można mówić o jakiś wyzwaniach. Bo tak naprawdę jak się ma 3 dzieci to cały czas są jakieś wyzwania. Trudne tematy do wytłumaczenia. Chociażby ta sytuację trzeba im wytłumaczyć, i to też jest dla mnie wyzwanie, bo widzę po sobie, ze nie jestem idealnym rodzicem i czasami rzucę im karę, zabronię czegoś. Np rzucę, że karą jest nieoglądanie telewizji, a potem sobie myślę, że trzeba teraz wytrzymać w tych postanowieniach. Takie wyzwania życia codziennego.

**Czy te wyzwania w pracy są związane z koronawirusem?**

Są tez takie. Chociażby to, ze część rzeczy musimy zrobić u nas na symulatorach, w laboratorium na miejscu. Długo była przeciągana decyzja w sprawie tego, czy będą odbiory z klientem w Puławach. Dla mnie to już było wyzwanie, bo po pierwsze nie ma bazy noclegowej, pod drugie trzeba by zapewnić bezpieczeństwo. Odstępy miedzy ludźmi. Rozstawić sprzęt tak, żeby jak najmniejszy kontakt był pomiędzy osobami. Kolejnym wyzwaniem było, żeby zorganizować jakieś zdalne odbiory, tzn. postawić kilka komputerów w siedzibie PKP i miałoby to zdalny dostęp do laboratorium naszego w Warszawie. Bardzo dużo tych wyzwań w pracy krąży wokół tego koronawirusa.

Kolejnym wyzwaniem, o którym już myślę, to jest to że jak skończy się to wszystko to coś co było rozłożone w czasie na kilka miesięcy - marzec, kwiecień, maj, będziemy musieli zrealizować w półtora miesiąca. I po takim długim spędzeniu czasu w domu, nagle będzie tak, że tydzień będę w domu, a dwa tygodnie na wyjazdach. Na odbiorach - Poznań, Warszawa, czy cokolwiek. Też już o tym myślę.

**O Twoich bliskich. Czy na tej płaszczyźnie zauważyłeś jakieś zmiany? Czy o czymś teraz więcej opowiadają?**

Ja widzę ogólnie,   że nie raz udziela się żonie ten pesymizm trochę. Kiedy pojedzie się spotkać z rodzicami, bo oni też mają lepsze i gorsze dni. I nie raz potrafią ją tym zarazić. Że przyjedzie zdołowana. To widać, że ludzi to odczuwają. I pytanie tylko na ile pokazują nam prawdziwe emocje. Bo jak ja z sąsiadami rozmawiam, to nikt nie ma obaw, każdy robi swoje, jest super... Pytanie też na ile oni okazują to prawdziwe. Nieraz jest tak, że jak ktoś się zaczyna przejmować, to to przesiąka do mojego najbliższego otoczenia.

**A czy zauważyłeś u ludzi jakieś zachowania, które według Ciebie wydają się dziwne?**

Dziwne jest to dla mnie, że ludzie się tak tłumaczą. Jak rozmawiam  np. z bratem, to ja sobie doskonale zdaję sprawę, że on ma taką pracę gdzie musi się spotykać z wieloma osobami i mam wrażenie, że on jak rozmawia ze mną to trochę mi się tłumaczy. A dlaczego on jeździ po hurtowniach, a dlaczego to, a tamto... Ja go nie oceniam. Ale mam wrażenie, że ludzie mają takie dylematy - na szczęście ja takiego nie mam - na ile muszą sobie sami te restrykcje wprowadzić. Co już jest granicą, gdzie trzeba tego koronawirusa odłożyć na bok i robić dalej swoje, a co już musi się zmienić. I moim zdaniem w momencie kiedy biją się z myślami trochę, to próbują się wytłumaczyć przed kimś. To nie tylko mój brat. Już tydzień temu jak rozmawiałem z sąsiadem, to on tak jakby mi się trochę tłumaczył. A ja go nie osądzam. Każdy ma swój rozum. Ale też każdy ma chyba takie odczucia swoje, że ciężko jest postawić tą granicę. U mnie jest łatwiej zdecydowanie. Chociaż też przez chwilę miałem stres, bo jak mi powiedzieli o tych Puławach, to już miałem zgryzotę, bo mówię sobie, że albo będę musiał wysłać jakiegoś człowieka ode mnie z zespołu i powiedzieć mu "jedź do 5-6 obcych osób, dojeżdżaj 100km w jedną stronę bo nie ma gdzie cię przenocować" albo myślałem, że ja wezmę to na siebie i będę tam jeździł. I też miałem takie myśli, czy to jest warto? Czy ze względu na dziadków powinieniem? Gdzie tą granicę postawić? Myślę, że każdy sobie zadaje to pytanie. Pewnie jakby się te odbiory odbywały i musiałbym jeździć, to bym się tłumaczył przed żoną, że nie mogłem nikogo wysłać, bo to jest moja odpowiedzialność. I ja nie mogę siedzieć w domu, a ktoś będzie się narażał. Później byłby problem tego typu, gdzie  ja bym sobie postawił granicę, czy ja bym w ogóle nie wchodził do dziadków - chociaż to bez sensu, bo jakbym coś złapał to bym przekazał żonie, żona dzieciom, dzieci dalej, itd. Bo można powiedzieć, że jak jedna osoba zachoruje, to pewnie już wszyscy. Czy ja powinienen się wtedy izolować, czy  na przykład po takich odbiorach które by trwały tydzień czy nie pojechać do mieszkania rodziców na dwa tygodnie. Takie wyznaczanie swoich granic. Myślę, że bez względu na to gdzie tą granicę postawimy, to nigdy nie będzie tak naprawdę dobrze, chyba że pójdziemy już naprawdę po bandzie.

**To tłumaczenie z jednej strony jest dziwne, ale z drugiej czujesz, że gdybyś był w podobnej sytuacji  to możliwe, że zachowałbyś się podobnie?**

Tak, bo sobie tak myślałem skąd to się bierze. I do takiego wniosku doszedłem. I nie tyle to było dziwne, tylko było inne. Bo żebym ja mógł go dobrze ocenić, to bym musiał dokładnie wiedzieć, co on robi.  Wszystkie wiedzieć, jakie te relacje z ludźmi. Czy się wita. Ja tego nie wiem tak naprawdę.

**Czy kupujesz rzeczy przez Internet? Albo zamawiasz rzeczy przez telefon? Czy są sytuacje, kiedy zamawiasz inaczej niż będąc bezpośrednio w sklepie.**

No nie. Raczej nie. Natomiast wiadomo, że ograniczam wyjazdy do sklepów do minimum. Spożywczych produktów nie zamawiam przez Internet. Być może teraz jak siedzimy w domu, to więcej ogólnie zamawiamy rzeczy przez Internet. Nie wiem, czy to jest forma jakaś odstresowania się. Ale sporo zaczęliśmy zamawiać. Ale to też są takie rzeczy, które widzimy, że jest potrzeba. No może nie byłoby takiej potrzeby, np. drukarkę, tablica do kosza, leżaki. Jak tak teraz na to patrzę, to te zakupy wymusiła poniekąd ta sytuacja która jest teraz. W niedzielę była pogoda, wyszliśmy na zewnątrz i była kłótnia o te leżaki, co mieliśmy wcześniej. To potrzeba się zrodziła. Drukarki w domu nie mieliśmy potrzeby, bo drukowałem  w pracy wszystko i żona też, a od momentu kiedy jesteśmy w domu i dzieciaki non stop potrzebują jakiś materiałów, to musieliśmy zamówić.

Skrzynka na listy zamówiona i założona, żeby listonosz nie musiał przychodzić. Tak sobie teraz uświadamiam, że zakupy robiliśmy potrzebne w danym momencie. Niż takie ubraniowe na przykład. Bo o tym się nie myśli teraz.

A spożywkę zawsze kupowałem i będę w sklepie, dopóki mogę. Jak skończymy, to też będę jechał na zakupy. Nie planowałem, żeby ktoś mi przywoził - raczej założę sobie tą maskę i rękawiczki i pojadę.

**A wcześniej jakiego typu produkty kupowałeś przez Internet?**

Moje zakupy na Allegro, czy innych sklepach ograniczały się do zakupów bardzo specyficznych, np. części do samochodów, elektronika bo jest tańsza przez Internet. Nie miałem tak, że sobie siadałem i szukałem. Jeśli chodzi o zakupy odzieży to zdecydowanie wolę iść do sklepu, robię strzał raz na jakiś czas się obkupię.

**Myślisz, że to jak teraz kupujesz wpłynie na to, jak będziesz kupował po epidemii? Czy może coś się zmieni?**

Sam jestem ciekaw czy coś się zmieni. Bo pytanie, kiedy będzie po epidemii. Bo mogą być zniesione restrykcje, może być mała liczba zachorowań i będą tylko ludzie zdrowieli i wskaźniki będą szły w dół, ale pytanie ile jeszcze zostanie takiego  dystansu, do zgromadzeń większych. Kiedy na przykład zdecyduję się jechać na giełdę, gdzie człowiek obok człowieka przechodzi ramię w ramię. Tak bez myśli o koronawirusie. Czy to będzie tak, że wskaźniki zaczną iść w dół np. z początkiem czerwca, w lipcu niech teoretycznie będą wyleczeni, nie będzie nowych przypadków, to czy to wszystko wróci do normy jeśli chodzi o restrykcje. Ale pytanie kiedy to w głowie tak naprawdę wróci.

My też mamy wakacje na wrzesień, więc myślę też w tym kontekście, czy będziemy chcieli jechać, czy nie. Jestem sam ciekaw jaką decyzję podejmę w sierpniu.

**Ograniczenie kontaktu, niższe ceny. Czy są jeszcze jakieś zalety zakupów przez Internet?**

Taka szybka możliwość zwrotów. Mogę przetestować produkt przez 2 tygodnie i zwrócić go bez poddania przyczyny. I z tego korzystam. Biorę pod uwagę to że wydam pieniądze na kuriera w drugą stronę, ale przynajmniej nie będę musiał się tłumaczyć w sklepie, bo to jest zupełnie inna rozmowa wtedy.

**Tych zakupów przez Internet teraz jest więcej?**

Myślę, ze troszkę tak. Bo teraz mamy trochę inne potrzeby. Dostosowane do tej sytuacji. Plus to, że te sklepy są pozamykane. Drukarkę mógłbym pojechać do sklepu obejrzeć, ale to też zaleta, że można sobie wieczorem usiąść, poszukać, poczytać opinie. A u mnie ciężko czasami ten czas znaleźć, żeby się gdzieś udać do sklepu. A tu mam 14 dni na zwrot.

**Do tematów około jedzeniowych. Jaką funkcję dla Ciebie pełni jedzenie?**

Dobre pytanie. Ogólnie jeszcze pół roku temu to się życie kręciło wokół jedzenia. Dużą ma wartość dla mnie jedzenie, nie traktuję go tylko jako zdobycie potrzebnych do życia węglowodanów itd., energii. Bardzo poważnie traktuję jedzenie. Nie wspomniałem o moim zainteresowaniu, ale bardzo lubię gotować. Dużo gotuję, mnóstwo czasu spędzam w kuchni. Ogromną wartość ma u mnie jedzenie i wytwarzanie jedzenia. Lubię zrobić coś dobrego i żeby ludziom smakowało.

**Zawsze tak było, że lubiłeś gotować, czy to się zmieniło?**

Jeszcze pół roku temu i kilka lat wstecz, nie miałem czasu na gotowanie. Przynajmniej tak mi się wydawało. Dużo rzeczy kupowałem, zamawiałem. Lunche w pracy. Kebab. Pizza. Takie śmieciowe jedzenie. Na kolacje jak nie parówka, to jeszcze coś innego. I od momentu, kiedy zmieniłem nawyki żywieniowe, to też mi się zmieniły nawyki po powrocie z pracy. Jak chcę jeść coś innego, to trzeba to ugotować. Zawsze lubiłem gotować, a teraz mam okazję się rozwinąć. Lubię spędzać czas w kuchni. Jak ugotuję obiad czy kolację zrobię, skończę gotować i mam blaty czyste i poukładane na swoje miejsce wszystko to mnie mega odstresowuje. Gotowanie i organizacja pracy w kuchni to relaks po pracy. A rodzina i żona korzysta. Ja mogę w kuchni siedzieć godzinami.

**A jeśli chodzi o ostatni czas, to czy Twoja dieta się jakoś zmieniła?**

Troszkę się zmieniła, bo ogólnie trzymałem dietę - może nie ścisłą, ale jak jeździłem do pracy to miałem schemat, gdzie zabierałem jedzenie na cały dzień. Śniadanie, drugie śniadanie, obiad, podwieczorek. I do domu tylko na kolacje wracałem. A teraz jak się w domu siedzi to żona jak zrobi ciasto, to ciężko sobie odmówić. Ale też taki pozytyw jest z tego, że więcej czasu jest na gotowanie. Ale można sobie powymyślać, próbować nowych rzeczy. Ogólnie dużo nowości zacząłem wprowadzać. To co mieliśmy wcześniej, taki schemat wypracowany - takie zdrowe i w ogóle, to teraz  odjechałem i zacząłem robić inne rzeczy. Zrobiłem devolaye, zrazy zrobiłem. Chińskie dania. Krewetki różne. Mam trochę więcej czasu i trochę pewniej się poczułem w kuchni.

**Czy to znaczy, że na zakupach też wybierasz jakieś nowe produkty?**

Jak układam sobie listę zakupów, to wpisuję. W samym sklepie to już nie. Wcześniej świat się kręcił wokół kurczaka, a teraz jakieś kaczki, wołowina, hulasz, placki po węgiersku. Rosołki z królika. Takie dla mnie wyższy poziom. A to że jestem w domu cały czas, to tylko pomaga mi się rozwinąć.

**Jak byłeś w pracy, to zdarzało Ci się zamawiać jedzenie do pracy.**

To był taki czas, że codziennie pizza, kebab albo chińskie. Taki trójkąt bermudzki. Ale już się teraz koledzy przyzwyczaili, że już nie wychodzę z nimi. Nie zabierałem obiadów w ogóle do pracy. Czasami od pana kanapki kupowałem.

**A zdarzało Wam się zamawiać jedzenie do domu?**

Wtedy się zdarzało i teraz też nam się zdarza. Bo lubimy z żoną raz na jakiś czas sobie zamówić czy chińskie danie, czy cokolwiek. Ale to bardziej przy weekendzie, jak chcemy sobie podkręcić końcówkę tygodnia, to sobie mówimy, że  w piątek np. jadę po burgery. Albo po chińskie i robimy sobie wieczorek z filmem. Bo jednak to jedzenie jest ważne moim zdaniem. I też wyjazd do restauracji raz na jakiś czas jest ważny.

To jest dla nas ważny aspekt życia.

**A dlaczego to ważny aspekt?**

Myślę, że to zbliża ludzi, takie wspólne wyjście, urozmaica tydzień. Nie sprawia frajdy to, co się dzieje cały czas, tak jak w moim przypadku te kebaby, lunche. Sprawia frajdę to co się dzieje raz na jakiś czas. I warto to sobie dzielić z rodziną. Więc do restauracji jest fajnie wyjść. I jeszcze się zje coś smacznego. To przyjemnie spędzony czas.

**A teraz w czasie epidemii zdarza się Wam zamawiać do domu?**

W ogóle nie zamawiamy teraz. Raz tylko mieliśmy sytuację taką, że zrobiliśmy sobie wycieczkę całą rodziną żeby zaprowadzić samochód do mechanika. No i na powrocie do KFC zajechaliśmy. Jest wycieczka, więc robimy sobie odstępstwo od normy. Każdy zadowolony.

**Czemu teraz wcale nie zamawiacie?**

Bo gotuję. No i teraz też nie chcemy za bardzo podnosić tego ryzyka. Do nas też raczej się nie dowiezie, trzeba samemu jechać odebrać. To jest poza zasięgiem tych dostarczycieli jedzenia. Nigdy nie było tak, że coś zamawiałem i ktoś mi przywoził pod bramę. Zawsze musiałem się samemu ruszyć. Więc teraz jak tylko byliśmy po trasie, to zrobiliśmy sobie wyjątek - podjechaliśmy do okienka i zamówiliśmy jedzenie. Ale tak specjalnie to nie wyjeżdżam. Być może to jest spowodowane tym zmniejszaniem ryzyka.

**Ale to ryzyko z czym jest związane?**

Z kontaktem. Bo wiadomo ktoś przygotowuje to jedzenie też. Nie wiemy kompletnie nic o tej osobie, która to przygotowuje i jakiś strach jest. Jak zamówiliśmy to jedzenie w KFC, to też taka nieciekawa myśl się przemknęła przez głowę, że to też jest jakieś ryzyko. Ale podjęliśmy z żoną to ryzyko, bo stwierdziliśmy, że takie restauracje też mają jakieś zasady higieny, bezpieczeństwa. Ale płacenie już się odbywało przez gumową rękawiczkę. Cała obsługa tego terminala. Ale była przez chwilę myśl, że może niepotrzebne ryzyko.

**A jeżeli chodzi o płacenie. Coś się u Ciebie zmieniło?**

Gotówki używam tylko w jedną stronę - jak ja ją wydaję. Albo jak trafię w sklepie, że  mam 50 z groszami, to wyjmuję z portfela. Jeśli ktoś ma mi wydać złotówkę, to mówię, że bez reszty. Nie chcę obracać pieniędzmi, bo tam się dużo potrafi osadzić. Większość transakcji jest więc realizowana przez kartę. I zdecydowanie wolę przez rękawiczkę wpisać pin na terminalu niż nosić w kieszeni pieniądze, które mi ktoś dał.

**A wcześniej zwracałeś uwagę jak płacisz?**

Ciężko powiedzieć. To chyba zależało od sytuacji za co się płaciło. Ja wychodziłem na miasto wieczorem z kolegami, to wolałem mieć kartę. Żeby mieć jeden środek płatniczy. Niż mieć pełną kieszeń drobnych. W innej sytuacji wolę gotówkę. Teraz przez tą sytuację z koronawirusem zdecydowanie wolę kartę.

**A czy poza kartą jakieś inne bezgotówkowe metody?**

Yyyy. Chyba tylko takiej "później ci oddam".

I też jak teraz się rozliczam z rodziną i np. coś kupuję dla siostry to już się nie rozliczamy gotówkowo tylko przelewem.

**Zakupy. Czy coś się zmieniło jeśli chodzi o częstotliwość zakupów?**

Nie. Jedyne co się zmieniło, to tyle że są święta i robię w innych miejscach zakupy. Nie jadę do Lidla, bo stwierdziłem że będzie dużo osób, duża kolejka przed, produktów będą wyprzedane. Więc robię w lokalnym sklepie zakupy na święta. Pewnie za tydzień wszystko wróci do normy. A teraz są te kolejki przed sklepem, bo wpuszczają po kilka osób. Dużo czasu się traci, więc szybciej mogę to zrobić lokalnie.

**A czy byłeś w sklepie od momentu wprowadzenia limitu klientów? Opowiedz o tych zakupach**

Byłem w lokalnym sklepie i rzeczywiście jak ktoś chce wejść to sprzedawca reaguje, że musi poczekać. Ktoś wychodzi, ktoś wchodzi. W większym sklepie już ostatnio, tydzień temu zauważyłem kolejkę jak wychodziłem. I ta kolejka była dosyć długa, więc stwierdziłem, że słabo byłoby stać w takiej długiej kolejce. I to pomogło podjąć decyzję w tym tygodniu. Ale jest ogólnie mniej osób w sklepie, i to widać.

**Co planujesz kupić na tych zakupach przedświątecznych?**

Standardowy pakiet świąteczny. Na obiad. Produkty na sałatki. Warzywa. Owoce. Napoje. Alkohole. Chociaż nie będzie za bardzo jak w tym roku posiedzieć przy alkoholach. Ale takie standardowe zakupy, tylko w mniejszej ilości.

**A czy planujesz kupić coś typowo dla przyjemności?**

Jakieś dobre piwo. O przyjemności żony - wino bezalkoholowe, albo piwo dla niej. Coś chrupkiego na wieczór, bo jak są święta to sobie można pozwolić. I takie produkty związane ze świętami, bo dla mnie od soboty popołudnia do poniedziałku popołudnia to dla mnie jest święto i nie muszę myśleć, żeby pilnować kalorii, ćwiczyć, itd. Mogę zrobić to na co czekam. Żeby tak sobie poluzować trochę. Może chipsy serowe.

Piwo bym chciał takie, nie które kupuję przeważnie. Lubię dobre piwo, ale szkoda mi wydać na butelkę piwa, szczególnie jak kupuję 4 sztuki. Zależy od tego, czy idę na ilość, czy idę na jakość to kupuję różne piwa. Przy normalnym weekendzie to kupuję tańsze: Tyskie, żywiec, Lecha. Bo wtedy kupię 20, bo ktoś może przyjedzie. A jak wiem, że te święta szykuję się jakie się szykują, to sobie kupię takie żeby trochę odróżnić. Wyróżnić ten dzień świąteczny. To może sobie kupię fajne piwo pszeniczne. Nawet za 5 zł. Coś z Kormorana (?) Może.

**A wino dla żony też z taką zasadą?**

Wiem, co ona lubi. Ale że ona teraz jeszcze bezalkoholowe musi, to patrzę co jest. Próbuję zapolować na te produkty dla niej.
