# Supplementary material for: The challenges arising from the COVID-19 pandemic and the way people deal with them. A qualitative longitudinal study
Source: PLoS One. 2021 Oct 11;16(10):e0258133. doi: 10.1371/journal.pone.0258133 (PMC8504766; doi:10.1371/journal.pone.0258133)
Supplement: S1 Dataset — (ZIP) [file pone.0258133.s003.zip › Transcriptions/stage 2/15.2_M_43_couple, with children.docx]

**15.2_M_43_couple with children**

**Obrazki. Który z obrazków najlepiej oddaje Pana emocje w obecnej chwili?**

Wie Pani co, ja bym pozostał w zasadzie przy tym samym zestawie, rozszerzonym jedynie o 13, której chyba ostatnio nie było. Czyli mamy 3,4,6,12 i 13.

**O obrazkach 3,4,6,12 opowiadał Pan poprzednio. Chciałby Pan coś dodać?**

Nie, podtrzymuję. 3 to jest trochę bycie w blokach startowych i odliczanie do czegoś, co nie nadeszło - mówię tutaj o szczycie pandemicznym z punktu widzenia mojego zawodu. 4 to w dalszym ciągu współpraca i jedność, która była. 6 to podążanie ku konkretnemu celowi, gdzie gdzieś tam jest widoczne słońce. 12 to wspomnienia bałwana i pewnego rodzaju rodzinność, wspólnota, spędzanie sporej części wolnego czasu razem. A 13, to powiedziałbym, że wymowa jest taka, jak w 6, czyli źródło światła, nadziei, w kierunku którego się idzie.

**Z jakimi emocjami wiąże się dla Pana 13?**

Radość, nadzieja, optymizm. Taka lekkość, ale to może bardziej fizjologiczna. Coś przyjemnego, powiązanego z ciepłem, z nadchodzącą wiosną. Takie skojarzenia.

**Skąd te emocje się biorą?**

Ja bym powiedział, że to raczej predyspozycje indywidualne. Od zeszłego tygodnia niewiele się zmieniło. Pewnie w zeszłym tygodniu też mógłbym dołączyć ten sam obrazek. Nie nastąpiła żadna istotna zmienność.

**Pana działania są nakierowane na to, żeby odczuwać pozytywne emocje?**

Nie, nie robię niczego szczególnego. Wypełniam swoje obowiązki zawodowe, rodzinne. Robiłem, to co robiłem do tej pory. Nie wydarzyło się nic istotnego, co by wpłynęło w sposób pozytywny na mój nastrój. Utrzymujemy *status quo*.

**Czy w pana życiu codziennym nastąpiły jakieś zmiany?**

Jest trochę więcej obowiązków zawodowych, w związku z poszerzeniem ich. Miałem okazję pracować zdalnie w sobotę i w niedzielę nad programami pomocowymi dla instytucji w województwie Wielkopolskim. To były głównie telefony, ale jednak pracowałem. Więc trochę więcej obowiązków zawodowych. Poza tym, nic więcej.

**Czy to, że ma pan więcej obowiązków zawodowych skutkuje zmniejszeniem czasu spędzanego z rodziną?**

Nie, w żaden sposób się to nie ograniczyło. Po prostu co jakiś czas muszę odebrać telefon, podjąć jakąś decyzję albo wydać dyspozycję. Albo wysłuchać kogoś w jakiejś sprawie i to wszystko. Natomiast nie rujnuje to mojego życia w żaden sposób. Jest tego więcej, zakres odpowiedzialności zrobił się większy.

**Jest coś, z czego pan zrezygnował?**

Zacząłem unikać Whisky i pije tylko i wyłącznie wino. Bo już mam po dziurki w nosie Whisky. I tak samo jem zdecydowanie mniej posiłków mięsnych, bo już mam po dziurki w nosie mięsa, więc zdecydowanie więcej warzyw w tym tygodniu.

**Coś zaczęło panu szczególnie doskwierać?**

Nie, utrzymuje się na tym samym poziomie. *Status quo* względem zeszłego tygodnia.

**Jak wygląda pana obecne poczucie zagrożenia? Czy coś się zmieniło?**

Też nic się w tym zakresie nie zmieniło. Nie odczuwam jakiegokolwiek większego poczucia zagrożenia. Raczej określiłbym to na skali 2 na 10, gdzie 1 oznacza brak jakiegokolwiek poczucia zagrożenia.

**Jak pan się czuje z nowymi ograniczeniami wprowadzonymi przez rząd w ostatnim tygodniu?**

Chyba też podtrzymam swoje zdanie z zeszłego tygodnia. Zakładam, że one mogłyby być jeszcze bardziej restrykcyjne. Bo wydaje mi się, że jak w zeszłym tygodniu rozmawialiśmy, to one już były, jeśli dobrze pamiętam. Więc tutaj się niewiele zmienia - jest to pójście w dobrym kierunku. Ale też oczywiście, niezależnie od obostrzeń administracyjnych, wiadomo, że przede wszystkim my jesteśmy za to odpowiedzialni.

**Czy pan przestrzega tych ograniczeń?**

Tak, przestrzegam tych ograniczeń.

**Zna pan jakichś ludzi, którzy tego nie robią?**

Nie, w moim otoczeniu bezpośrednim nie znam takich osób. Natomiast widzę, że skala jest całkiem spora, ponieważ przez moją instytucję, w której pracuję, przechodzi cały szereg pytań na ten temat. Ludzie dzwonią na nr 112 i pytają się: "czy ja mogę iść na ryby", "czy mogę odwiedzić grób ojca/męża na cmentarzu". Także zainteresowanie przemieszczaniem się jest, ludzie mają wątpliwości i szukają potwierdzenia bądź zanegowania możliwości korzystania z nich. Ale w moim najbliższym otoczeniu ja nie widzę, żeby ktokolwiek łamał te obostrzenia, które są narzucone.

**Jak pan ocenia to, że ktoś łamie ograniczenia?**

Hmm... No właśnie, jak do tego teraz podejść? Powiem tak, że bardziej bym podchodził do tego indywidualnie, jednostkowo. Bo faktycznie te obostrzenia są dość ogólnikowe i teraz nie wiem, czy ktoś, kto wychodzi przed dom na swojej posesji, to łamie te ograniczenia czy nie. To, co nam doskwiera, to jest bardzo brak bardzo jasnych i klarownych wytycznych. Teraz jest taka informacja, że mamy się nie kontaktować i nie wychodzić z domów, czyli w domu mogę być, ale już np. wsiąść w samochód i pojechać o 5:00 nad ranem nad rzekę, gdzie będę zupełnie sam, połowic ryby przez godzinę, wsiąść do samochodu i wrócić o 6:30 już teoretycznie nie mogę. Kiedy w zasadzie jestem bardziej sam na tych rybach, aniżeli w domu. Więc w ujęciu jednostkowym ja rozumiem, że ludzie się o to pytają. I przede wszystkim nie mogę ani zanegować czyjegoś działania ani go pochwalić. Każdy z nas jest odpowiedzialny za siebie. Mi nie przeszkadza brak odpowiedzialności innych osób. Nie oceniam negatywnie ludzi, którzy chcą się wyrwać na cmentarz czy chcą wyjechać na własną działkę rekreacyjną i tam spędzić czas, bo może, jakbyśmy zaczęli szukać, to byłoby to nawet dopuszczalne względem obostrzeń, które są. Bo jeśli mam być w swojej posiadłości, to mogę być i na działce, i w mieszkaniu, a jeśli mam dwa różne mieszkania, to w którym z nich mam być? Natomiast, oczywiście nie jestem skłonny zaakceptować już takiego ewidentnego łamania tych zasad, które są, czyli np. organizacja chrzcin w restauracji, co ostatnio miało miejsce w województwie wielkopolskim. No to to już jest, powiedzmy sobie, wręcz samobójstwo nie wprost pod względem zdrowotnym.

**Dlaczego pan przestrzega ograniczeń?**

Pytanie o motywację. Jest to motywacja zarówno też wewnętrzna, ponieważ chcę żyć w zgodzie z samym sobą i też z rodziną - skoro wszyscy przestrzegamy, dlaczego ja miałbym narażać swoją rodzinę. Ja to rozumiem i w tym czuję też swoją wewnętrzną wolę do przestrzegania. Z drugiej strony, jestem odpowiedzialny za współpracowników. Im mniej mam kontaktów zewnętrznych, tym wszyscy w firmie będziemy zdrowsi. Poza tym, jestem w korpusie służby cywilnej, w związku z tym, składając ślubowanie, ślubowałem również przestrzeganie norm i zasad prawnych i społecznych, które obowiązują. Są to też normy etyczne. Ale powiedziałbym, że wiadomo, z normami etycznymi narzucanymi przez instytucje może bywać różnie. W głównej mierze jest to moja własna motywacja do pozostawania w obrębie tych uregulowań, które są.

**Wspomniał pan o odpowiedzialności. Czy czuje się pan odpowiedzialny za to, żeby nie stwarzać zagrożenia w kontaktach z bliskimi?**

No tak, to też powiedziałem, kiedy była mowa o motywacji takiej bardziej wewnętrznej. Wiadomo, że o swoich się dba bardziej niż o innych - my vs. oni. Więc to wewnętrzna motywacja, moja własna wola jest bardziej w kierunku rodziny, natomiast przyzwoitość zawodowa bardziej w kierunku współpracowników.

**Na ile w tej motywacji do przestrzegania ograniczeń pojawia się myśl o zagrożeniu wirusem?**

Nie pojawia się w zasadzie. Nie ma czegoś takiego. Nie jest to podyktowane w żaden sposób poczuciem zagrożenia moim własnym, czy gdzieś tam obiektywnie postrzeganym.

**Czy obserwuje pan u swoich bliskich zmiany w zachowaniu, w sposobach radzenia sobie z sytuacją?**

Zauważam jedną rzecz względem zeszłego tygodnia, że mój ponad 6-letni syn wchodzi w fazę już kryzysową, która wynika z odosobnienia i braku kontaktu z rówieśnikami, których miał cały czas całkiem sporo. Rozmowy przez video konferencje nie są już fajne i atrakcyjne, bo nie są efektem nowości, który był wcześniej. Więc widzę, że teraz częściej traci dobry nastrój, częściej bywa płaczliwy, marudny. Kontestuje nasze propozycje rodzicielskie odnośnie zabawy, czasami nawet pewne sugestie odnośnie zachowań. Potrafi czasami się zbuntować, popłakać się, pójść do pokoju, trzasnąć drzwiami, spędzić tę chwilę czasu w samotności. Więc to jest taka zmiana negatywna względem zeszłego tygodnia, którą zauważam w rodzinie.

**Co państwo robią, żeby pomóc synowi przetrwać ten czas?**

Możliwości są dwie. Obydwoje z małżonką funkcjonujemy trochę inaczej. Moja małżonka stara się spacyfikować syna za pomocą nakazów, zakazów, gróźb i przyjmowania bardziej dyrektywnej, rodzicielskiej postawy względem syna. Ja mu trochę bardziej przyzwalam na to, żeby on pobył jednak sam, żeby, chcąc się od nas odciąć, sam wyregulował swoje emocje i znalazł zajęcie. Obydwie strategie działają. Da się spacyfikować go jeszcze, ale czasami można mu odpuścić i on sam się wyreguluje w tym zakresie. W zależności od tego, kto jest bardziej pod ręką i kto się nim zajmuje, taka strategia wchodzi w życie i obydwie są okej, w znaczeniu, że działają na niego.

**A zauważył pan nowe sposoby radzenia sobie w obecnej sytuacji w pana otoczeniu?**

W zasadzie, to nie. Jedynie mój ojciec zakupił sobie coś w rodzaju żyroskopu, montowanego do łóżka szpitalnego, po to, żeby nie musiał montować telefonu w rękach podczas rozmów na video Messenger. Jest bardzo zadowolony z gadżetu, który kupił przez Internet. Tylko tyle, że może sobie uprościł życie, ale też pewnie z nudów szukał, z nudów znalazł i z nudów pieniądze wydał. Może trochę taka strategia unikowa, żeby nie myśleć, to przeszukuje różne portale, nowości, żeby nie myśleć o tym, co się dzieje. Ale to jest moje założenie, niezweryfikowane.

**Zauważył pan jakieś zmiany w emocjach ludzi?**

Była taka fala wątpliwości i pytań po wprowadzeniu drugiej tury obostrzeń, bo ludzie nie wiedzieli, co mają ze sobą zrobić, co wolno, a co nie. I tak w zasadzie jeszcze w poniedziałek takie telefony do 112 trafiały. Na ten moment widzę, że to się uspokoiło. Ci, którzy nie wiedzieli, jak się mają zachować, już się dowiedzieli. Ci, którzy mieli wątpliwości wydzwonili do nas wiele razy i na ten moment chyba taka sytuacja informacyjna się ustabilizowała. Czy są jakiekolwiek inne nastroje społeczne o charakterze negatywnym - nie wiem. Widziałem, że dzisiaj pojawiła się propozycja kolejnej tarczy…

*[przerwana rozmowa - problemy techniczne].*

Mogę jedynie zakładać, że część przedsiębiorców będzie zadowolona i zabezpieczona poprzez tę tarczę, natomiast nie wiem, jakie są nastroje społeczne w tym zakresie. Obserwuje jedynie doniesienia medialne, jakieś fakty, które się pojawiają. Może bardziej wyłapuje punkty zapalne, które mogą stanowić zagrożenie dla poczucia stabilności może społecznej, ale to są tylko i wyłącznie moje mniemania. Oprócz funkcjonowania gospodarczego, mamy teraz wątpliwości odnośnie funkcjonowania matur, jak one miałyby przebiegać, więc to też może być jakiś punkt zapalny: egzaminy 8-klasistów, nabory do szkół. Natomiast ja nie dostrzegam, oprócz tego, że mogą być to punkty zapalne, nie dostrzegam negatywów, ale też nie jestem bacznym obserwatorem społeczeństwa na ten moment. Nie mamy kontaktu jednak z szerszymi grupami społecznymi. Ja też nie czytuje żadnych opinii w mediach społecznościowych, więc nie wiem, ale zakładam, że mogą to być punkty krytyczne. To samo wybory prezydenckie pewnie dzielą jednych i drugich i to też może być taki punkt, gdzie część może czuć się zadowolona, a część może czuć się zagrożona taką organizacją.

**Jak obecną sytuację postrzegają pana najbliżsi?**

Jeśli chodzi o dalszą rodzinę, to z perspektywy rodziców żony, to oni są tak samo wycofani, jak byli wycofani. Po prostu starają się auto izolować na tyle, na ile jest to możliwe i robią to bardzo skutecznie i szczelnie. Natomiast my rozmawiamy o rzeczach raczej codziennych. Staramy się pogadać o dzieciakach, o tym, co się dzieje, natomiast nie prowadzimy dyskusji na tematy ogólnospołeczne, więc nie wiem, jaka *de facto* jest ocena przez nich tej sytuacji. To samo dotyczy moich rodziców. Raczej koncentrujemy się na pozytywach, nie prowadzimy debat politycznych ani takich społeczno-gospodarczych. Wobec takiej niejasnej przyszłości nie myślimy o tym, co będzie za dwa miesiące, raczej niczego nie planujemy, natomiast staramy się koncentrować na pracach tu i teraz. Takie codzienne funkcjonowanie.

**Zaobserwował pan jakieś dziwne zachowania, sposoby radzenia sobie z tą sytuacją?**

Chyba znowu odniosę się do doświadczeń zawodowych. Wzrasta poziom donosicielstwa. Okej, to może jest za duże słowo. Ja domyślam się, że ludzie są po prostu sfrustrowani i też wyposzczeni, jeśli chodzi o napływ bodźców. Np. pojawiają się takie informacje: proszę przesłać tutaj policję, bo tutaj w piaskownicy siedzą rodzice z dwójką małych dzieci, a przecież niewolno i prawdopodobnie pan pije piwo. I odpowiedź pracownika jest taka, że rodzice mają prawo się zajmować dziećmi i nie muszą zachowywać dwumetrowego odstępu, tym bardziej, że jeśli to są małe dzieci, to wymagają takiej opieki, jak zawsze. Tym bardziej, że zamknięte są większe obiekty typu place zabaw. Ale jeśli gdzieś przed domem jest piaskownica i siedzą w niej rodzice, to nie jest to zabronione. I wtedy np. operator mówi, że jeśli pani ma pewność, że pan pije alkohol w tej piaskownicy, to wyślemy patrol. I ta osoba mówi: nie, nie, to chyba jednak nie jest wcale alkohol. Jest to jakiś inny napój. Albo ktoś dzwoni, że po ulicy prawdopodobnie 15-latka i ma 15 lat, nie więcej i że idzie bez opieki rodzica. Albo że kogoś zdaniem w Inter Marche jest więcej ludzi niż powinno być, ponieważ są 3 kasy, a on przeliczył, że tam jest więcej niż 9 osób. takie doniesienia się pojawiają. Natomiast, ja nie mam pewności czy to wynika z braku poczucia bezpieczeństwa czy po prostu z faktu jakiejś formy frustracji i szukania zastępczych obiektów agresji. Więc to są takie nietypowe zachowania, których wcześniej nie obserwowaliśmy. Ale to są obserwacje nie wprost. Widzę to w pracy po profilach rozmów i osób dzwoniących, natomiast w zewnętrznym świecie ja takich dziwnych zachowań nie widzę. Nie mam też wielkiego kontaktu z otoczeniem zewnętrznym, bo przemieszczam się samochodem do pracy, a mieszkam w takiej okolicy, gdzie też niewiele widzę, bo ulice mam opustoszałą i zbyt wiele ludzi nie wychodzi. Więc dziwne zachowania tylko z tych rozmów na 112.

**Jaki pan ma stosunek do tego typu zachowań?**

Biorąc pod uwagę moje wykształcenie i doświadczenie zawodowe w tej materii, akceptuje je w pełni, ponieważ wiem, że ludzie w mocnej fazie stresu mogą bardzo różnie reagować, od dysocjacji po konwersję. Więc przyzwalam na nie w takim znaczeniu społecznym. Rozumiem, że ludzie w ten sposób mogą tracić kontrolę nad swoimi własnymi zachowaniami.

**Co jest dla pana największym wyzwaniem obecnie?**

W dalszym ciągu, tak jak to było mniej więcej w zeszłym tygodniu, realizacja obowiązków dydaktycznych, rodzicielskich i wychowawczych względem dziecka, które nie ma opieki przedszkolnej w zerówce.

**Czy ma pan poczucie wpływu na obecną sytuację?**

Mam wpływ na swoje decyzje, zachowania i swoje najbliższe otoczenie. Mogę też swoimi zachowaniami i działaniem w minimalnym stopniu obniżać ryzyko szerzenia epidemii. Natomiast w kontekście samego zjawiska przyrodniczo-biologicznego, to nie mam tutaj żadnej kontroli. Siłą rzeczy. Mogę zarządzać sobą, swoją rodziną, swoimi bliskimi, żeby minimalizować ryzyko zakażenia, ale ono tak czy inaczej gdzieś tam się będzie rozprzestrzeniało, patrząc na wzorce z Chin, Stanów czy Europy. W skali kraju nie mam absolutnie żadnej możliwości kontrolowania ani wpływania na to. Natomiast w skali mikro jak najbardziej tak.

**Czy kupuje pan przez Internet?**

Zawsze kupowałem całkiem sporo przez Internet. Zakupy spożywcze robiłem raz na dwa tygodnie. Ale wydaje mi się, że tych zakupów online jest więcej. Zdecydowanie więcej. Powiedziałbym, że jest tego dwukrotnie więcej niż było przed okresem obostrzeń. Ale to też wynika z tego, że więcej rzeczy w domu robię: jakieś modyfikacje, porządki, remonty, w związku z czym, potrzebuję do tego więcej rzeczy. Typu w niedzielę naprawiałem instalację hydrauliczną, modyfikowałem pierwszy raz w życiu coś w kuchni, to musiałem mieć jakąś wylewkę, kran, adaptery do przewodów wodnych i to kupiłem przez Internet. Takich rzeczy jest więcej, w związku z czym też więcej kupuję. Skończyły mi się teraz kremy do twarzy, to też musiałem je zamówić. W sklepie, w którym będę robił zakupy spożywcze dzisiaj w pracy, nie kupię sobie kremu, który stosuje od lat. Więcej rzeczy kupuję, bo więcej rzeczy robię, a też zaczynają mi się kończyć zapasy, które miałem z kiedyś.

**Dlaczego tych zakupów jest obecnie więcej?**

Kończą się zapasy, to jest jeden powód. Druga sprawa, że plany modernizacji domu, które były rozciągnięte na dłuższy czas i te inwestycje też były rozciągnięte na dłuższy czas, one się nagle skumulowały w jednym momencie. Po prostu więcej spędzam czasu w domu, więcej rzeczy w domu robię, więc też więcej kupuję.

**Co kupił pan przez ostatnie dwa tygodnie przez Internet?**

Zakupiłem 1 zestaw klocków LEGO Ninjago dla syna, ponieważ idzie Wielkanoc, którą będziemy świętować w wewnętrznym gronie. Kupiłem 2 przewody zasilające do iPhone, ponieważ jeden mi się przerwał i nie miałem czym ładować, a w zestawie były 2, więc kupiłem 2. Później farbę Tikkurilla do ścian, którą kupiłem do pomalowania pokoju. Później kupowałem jeszcze silikon do uszczelniania podłóg. Kremy ZIAJA seria YEGO do twarzy.  Zamawiałem małżonce farby do włosów i hennę, ponieważ chce zrobić prace nad swoim wyglądem samodzielnie. Czy coś jeszcze kupiłem w ostatnim czasie? Hmm... Nie przypomnę sobie, ale jakieś drobiazgi jeszcze pewnie były.

**Gdyby nie epidemia, to zakupiłby pan te rzeczy stacjonarnie czy też przez Internet?**

Pewnie stacjonarnie bym kupił rzeczy typu farba. Bo to trzeba jednak pójść, zobaczyć próbki - jest zdecydowanie prościej. Tutaj musiałem polegać tylko na tym, co jest na ekranie. Takie rzeczy raczej stacjonarnie. Kosmetyki tak samo.

**Pojawiły się jakieś zakupy typowo przyjemnościowe?**

A widzi pani, tak, pojawiły się takie zakupy. Kupiłem sobie 3 koszulki polskiego zespołu Mgła pana Mikołaja Zientary, którego sobie bardzo cenię. Tak, to była forma przyjemności.

**Dlaczego akurat teraz kupił pan te koszulki?**

Bo idzie lato i się zrobiło ładnie na dworze.

**Klocki LEGO to prezent dla syna czy też sposób spędzania wspólnie czasu z synem?**

Jest to prezent dla syna z okazji Wielkanocy, ale też pewnie będziemy wspólnie spędzać czas, budując zestaw.

**Jak wyglądają obecnie pana zwyczaje żywieniowe?**

To, co się zmieniło od zeszłego tygodnia, to niechęć do jedzenia śniadań. Pewnie też chodzi o samą objętość posiłków. Zauważam jednak, że jemy zdecydowanie więcej, a ruchu jest mniej albo ma on inny charakter, jak sprzątanie czy remontowanie. W związku z tym nie jadam śniadań. Jadam pierwszy posiłek ok. godziny 11/12, kiedy zaczynam odczuwać głód. Zdecydowanie wysyciłem się produktami mięsnymi, bo miałem jakiś tam zapas indyków, kurczaków, w związku z tym zjadłem tego, ile mogłem zjeść, ale na ten moment już nie mogę patrzeć na drób. Zdecydowanie więcej potraw jarskich, warzywnych, przyswajam na ten moment. I też, tak jak powiedziałem, w okresie fascynacji i zdegustowaniu wszystkich gatunków Whisky, które sobie kolekcjonowałem, już nie mogę patrzeć na Whisky. Zaniosłem do gabinetu, schowałem i niech czekają na lepsze czasy. Gdybym miał się napić, to napiłbym się chętniej wina czy piwa bezalkoholowego, jeśli mówimy o formie przyjemności, czy po alkohole generalnie. Żadnych mocnych alkoholi, bo już to jest wręcz bodziec awersyjny.

**Mógłby pan o tym opowiedzieć z perspektywy zmian nie w ostatnim tygodniu, a porównać stan sprzed epidemii do stanu w jej trakcie?**

Przed epidemią starałem się jeść 5 niewielkich posiłków dziennie, które sobie szykowałem, które zabierałem w różnych formach albo jadałem je w domu, a teraz faktycznie nastąpiło takie przejście do kuchni staropolsko-rubasznej, więc wszystko dużo, niekoniecznie bardzo fit, tylko jakieś tradycyjne polskie dania typu Lasagne albo schabowy. W zasadzie nawet trudno mi to jest wyjaśnić. Może w ten sposób, że posypało się część schematów życiowych, dotyczących codziennego funkcjonowania, to żywieniowy też się troszeczkę zmienił.

**A jak wygląda przygotowywanie posiłków u państwa w domu?**

Ja w dalszym ciągu jestem główną osobą odpowiedzialną za przygotowywanie posiłków, ponieważ moja małżonka nie ma talentu w tym kierunku i od zeszłego tygodnia jej talent się nie poprawił.

**Przed epidemią też głównie pan gotował?**

Tak, to się nie zmieniło.

**Państwo spożywają razem posiłki?**

Tak, te które jesteśmy w stanie, jemy razem. Też jesteśmy aktywni zawodowo i często jest tak, że trzeciej osoby z naszej trójki nie ma w domu.

**Przed epidemią też tak to wyglądało?**

Tak. Jak tylko było to możliwe, jadaliśmy razem.

**Dlaczego to jest ważne, żeby jeść razem?**

To jest bardzo trudne pytanie. Szczerze mówiąc, nie wiem. Jakby się mnie pani spytała, dlaczego jadaliśmy razem, to też bym odpowiedział, że nie wiem. Może po prostu jakaś forma tradycji rodzinnej. Przyzwyczajenie.

**Zamawiają państwo jedzenie z dostawą?**

Jednokrotnie nam się to zdarzyło. To był 27 marca. Zamówiliśmy pizzę, to były urodziny mojej żony. Tak w formie imprezy dwu i półosobowej.

**A przed epidemią zamawiali państwo jedzenie?**

Zdarzało się to, ale też przy okazji spotkań towarzyskich. Też oczywiście w szerszym gronie. Gdzieś tam znajomi, rodzina, czy szwagry. Jak szwagry, to wiadomo, że musi być pizza.

**To była forma świętowania?**

Tak, dokładnie. Poza tym zamawianie *fast foodów* to też jest jakaś tam kaloryczność. Nie do końca pożądana.

**Co pan sądzi o zamawianiu jedzenia z dostawą w obecnej sytuacji? Jest to bezpieczne?**

Hmm... No tak, jest to bezpieczne, jeśli zachowamy zasady bezpieczeństwa, które nas obowiązują. Wiadomo, zawsze jest jakieś ryzyko, że ktoś chory będzie szykował te posiłki, ale jest ono jednak stosunkowo niewielkie. Eksperci mówią, że raczej drogą pokarmową nie albo prawdopodobieństwo jest bardzo niewielkie, więc nie neguję dostarczania jedzenia przez firmy zewnętrzne, żadnych form cateringu. Po prostu z tego nie korzystam, ale nie mam absolutnie żadnych obaw z tym powiązanych. Rozumiem, że ludzie chcą tak robić. Czerpią z tego jakąś przyjemność. I rozumiem, że firmy, które oferują takie usługi, chcą generować zyski, więc jestem w pełni za.

**Czyli nie widzi pan zagrożenia, a to, że nie zamawia pan często wynika z własnych zwyczajów a nie obecnej sytuacji?**

Tak, dokładnie.

**Czy zmieniły się stosowane przez pana sposoby płatności?**

Bardzo rzadko miałem okazję nosić przy sobie gotówkę przed okresem epidemii i to się na ten moment nie zmieniło. Może jedynie tyle, że mam tej gotówki nieco więcej w domu, aczkolwiek nie wiem po co. Na wszelki zaś, jakby wszystko padło. Ale płaciłem zawsze za pomocą karty, ewentualnie płatności online typu BLIK i to się nie zmieniło w żaden sposób.

**A uważa pan, że obecnie bezpiecznie jest korzystanie z gotówki?**

Jest równie bezpieczne, jak używanie jej przed. Prawdopodobieństwo, że ktoś na mnie napadnie i ukradnie mi 20 tys. zł w gotówce jest równie niewielkie, jak było przed. Natomiast jeśli chodzi o wymianę gotówki z rąk do rąk, to banknoty są nośnikiem i zawsze były. Tutaj też nie za wiele się zmieniło. I tak zawsze po dotykaniu pieniędzy trzeba było dezynfekować się czy umyć ręce. Teraz może bardziej trzeba na to zwracać uwagę, ale te banknoty nie są ani brudniejsze, ani czystsze aniżeli były kiedyś.

**Czy obecnie robi pan rzadziej zakupy spożywcze niż raz na dwa tygodnie, jak pan wspomniał, że miał w zwyczaju wcześniej?**

Na ten moment dzisiaj po pracy jadę na większe zakupy, mam już listę i będę musiał je zrobić. Ale to tak wychodzi ok. 3 tygodni od ostatnich większych zakupów. Więc powiedzmy sobie, że nominalnie rzecz ujmując, o tydzień dłużej te zakupy zostały przeciągnięte. Normalnie zapasy starczają na 2 tygodnie, teraz na 3 wystarczyły, ale to dlatego, że mieliśmy trochę więcej rzeczy kupionych.

**Kupienie większej ilości rzeczy było celowe, żeby rzadziej chodzić do sklepu?**

Nie wiedzieliśmy, w którym kierunku pójdą obostrzenia, że może będą jakieś ograniczenia w funkcjonowaniu sklepów. Ale to było tak raczej na wszelki zaś, a nie było ku temu żadnych konkretnych przesłanek. Nawet nie stanowiło to żadnego zagrożenia dla nas, ale stwierdziliśmy, że można kupić więcej, że nie stanowi to żadnego zapasu. Więc część tych zapasów nadal jest, ale zostały już takie monotematyczne, jakieś zaprawy w słoikach z zaprzyjaźnionej spółdzielni rolniczej, ale jedzenie tych samych szparagów ze słoika po 3 tygodniach zaczyna wychodzić bokiem. Trzeba to jakoś zmodyfikować i dzisiaj jest ten czas, żeby dokonać zakupów produktów podstawowych, typu ser żółty, mleko. Bo to się po prostu skoczyło.

**To, że państwo kupili więcej rzeczy, było podyktowane niepewnością, w którą stronę pójdą ograniczenia?**

Tak, dokładnie.

**Nie była to kwestia zminimalizowania zagrożenia zarażeniem?**

Nie, tylko kwesta decyzji administracyjnych. Ewentualnych ograniczeń rządu.

**Czy przed epidemią też stosował pan listę na zakupy?**

To zależy od liczby zakupów. Do 15 pozycji jestem w stanie zapamiętać, a powyżej tej liczby już nie. A teraz mam więcej niż 15, więc musiałem mieć listę.

**Czy był pan w sklepie od naszej ostatniej rozmowy?**

Byłem w Żabce, ponieważ Żabka jest punktem, gdzie można odebrać paczkę. Kupiłem kwasek cytrynowy przy okazji.

**Czy podczas tej wizyty zaobserwował pan jakieś zmiany w sklepie?**

W Żabce były już wcześniej ograniczenia co do liczby osób. A teraz pojawiły się rękawiczki i płyn do dezynfekcji. Są dostępne przy wyjściu. Pani sprzedająca była ubrana w rękawiczki lepszej jakości niż te, podawane klientom. Miała kasę i dostęp przez ladę przesłonięty kawałkiem szyby pleksi i sama dysponowała płynem do dezynfekcji rąk

**Stał pan w kolejce?**

Nie. Żabka, do której uczęszczam, nie jest uczęszczaną Żabką.

**Miał pan okazję obserwować innych klientów?**

Nie, byłem sam. Aczkolwiek, przejeżdżając przez miasto, widzę, że zdarzają się kolejki przy sklepach typu Lidl czy Biedronka. Ludzie stoją z koszykami w odległościach od siebie czasem większych niż 2 metry. I te kolejki ze 2 razy udało mi się widzieć.

**Czy podczas zakupów sprzed 3 tygodni sprzedawcy w sklepie byli specjalnie zabezpieczeni?**

Tak, pamiętam, ze szyba pleksi już była zainstalowana. I były naklejki przy kasach, które zaznaczały obszar, w którym może się znajdować klient, żeby zachować 2 metry. I ludzie też w odstępach 2-metrowych od siebie stali.

**A inni klienci nosili maseczki, rękawiczki?**

Tak, widziałem i to był pierwszy moment, kiedy widziałem maseczki u kogokolwiek. Były to pojedyncze osoby, ale tak mi się wydaje, że 2 osoby były w maseczkach, kolejne 2 w bandanach na twarzy.

**Planuje pan używać maseczki i rękawiczek podczas wizyty w sklepie?**

Tak, skoro jest to obowiązkowe, to tak. Znaczy tutaj mówię bardziej o rękawiczkach. W maseczce byłem dzisiaj w szpitalu, ponieważ mam kontrakt ze szpitalem i musiałem dowieźć dokumenty, to ze względu na szacunek do ludzi, z którymi pracuję, a jednocześnie też swoje własne bezpieczeństwo, założyłem po raz pierwszy maseczkę na krótką, 15-minutową wizytę w szpitalu.

**Planuje pan dziś w sklepie używać maseczki?**

Tak, założę maseczkę.

**Uważa pan, że to skuteczna forma ochrony przed wirusem?**

Zwiększają w niewielkim zakresie bezpieczeństwo, natomiast ja jestem człowiekiem aktywnym zawodowo, dzisiaj byłem w szpitalu, pracuję w grupie 70 osób w numerze 112 i de facto sam nie wiem, czy ktoś z nas nie przyniósł tego, więc staram się zabezpieczyć innych, którzy są w moim otoczeniu. Być może mi się uda kichnąć w Lidlu. Ne chciałbym kichać na kiełbasę myśliwską albo twarz emerytki. To działa dwustronnie. Ja sam nie będę generował materiału biologicznego na zewnątrz, a może jak ktoś kichnie z metra mi w twarz, to ta maseczka będzie mogła mi pomóc, mimo, że to podstawowy model, bez filtra HEPA. Fizyczne zabezpieczenie przed kontaktem z materiałem biologicznym, jak ktoś mnie dotknie albo ja dotknę ust czy nosa albo ktoś kichnie - tylko takie fizyczne zabezpieczenie. Mógłbym mieć cokolwiek na twarzy dzisiaj i poziom zabezpieczenia byłby ten sam. Mam podstawową maseczkę i ona nie filtruje powietrza.

**Jak planuje pan spędzić Wielkanoc?**

Wydaje mi się, że w sposób bardzo zbliżony do wszystkich ostatnich dni. Pewnie uszanujemy jakąś tradycję Wielkanocną. Pewnie dziecko będzie miało jakieś prezenty, będzie szukało w ogródku z koszyczkiem słodyczy, pewnie też klocków LEGO. Pewnie przygotujemy potrawy charakterystyczne dla tego okresu. Może niekoniecznie będę w dresie, tylko jakoś bardziej przyzwoicie niż na co dzień się ubiorę. Może założę jakąś marynarkę, koszulę. W jakimś podstawowym zakresie małżonka będzie pewnie też kontynuowała obrządek religijny, który jest w tym okresie ważny. Więc nie wiem, czy będzie uczestniczyła w Triduum Paschalnym online. Jeszcze o tym nie rozmawialiśmy. Natomiast ustaliliśmy, że będzie święcenie pokarmu w wersji dozwolonej. Jeden z moich bliskich kolegów jest szafarzem i poproszę go o poświęcenie pokarmu w wersji online. czyli zadzwonimy do niego i jakąś tradycję podtrzymamy. A jeżeli nie, to sam się zapoznam z instrukcją jako człowiek głęboko wierzący i zupełnie niepraktykujący po prostu sam poświęcę ten pokarm w sposób, który będzie wskazany przez Kościół Katolicki.

**Święconka jest ważnym elementem świąt?**

Z mojej perspektywy nie, ale jest atrakcją dla dziecka.

**Zamierza pan iść do kościoła z okazji tych świąt?**

Nie. Nawet przed okresem epidemii starałem się tego unikać, jak mogłem.

**Jak wyglądają państwa przygotowania do Wielkanocy?**

Jeszcze jest szmat czasu. Nie ma sensu niczego robić na razie. Przygotowania zajmą pewnie nie więcej niż 1 dzień.

**To będą głównie potrawy czy też porządki?**

Porządki robimy od 3 tygodni i nie ma sensu bardziej.

**Zakupy Wielkanocne planuje pan także dziś zrobić?**

Trudno mi ocenić, czym są zakupy wielkanocne. No tak, może kupię potrawy typu czekoladowe baranki i zajączki do poszukiwań, klocki LEGO są już zakupione. Pewnie kupię białą kiełbasę. I to będzie tyle przygotowań. Resztę rzeczy, to są rzeczy codziennego użytku. Sałatkę warzywną można zrobić z tego, co się je na co dzień. Chyba tym wyznacznikiem kulinarnym Wielkanocy jest biała kiełbasa.

**A jakieś produkty dla własnej przyjemności?**

Nic ponad to, co mamy teraz, ponieważ już od 3 tygodni sprawiamy sobie przyjemności. Więc nie będzie niczego specjalnego. Ten czas jest formą przyjemności sam w sobie. Spędzanie czasu razem czy jedzenie schabowego, którego się nie jadło od dawna. Więc nie będzie niczego specjalnego na święta.

**A dekoracje, kwiaty?**

Ja myślę, że to będą prace plastyczne syna. Ja jestem przeciwnikiem zagracania domu. I nie będę miał takich rzeczy, glinianych zajączków za 3 zł kupionych tylko po to, żeby je gdzieś tam postawić. Będzie święconka. I będziemy mieli jajka w różnych formach kolorystycznych i to będzie taka forma tradycyjna. Pisanki w ramach terapii zajęciowej z synem zrobimy sami.
