# Supplementary material for: The challenges arising from the COVID-19 pandemic and the way people deal with them. A qualitative longitudinal study
Source: PLoS One. 2021 Oct 11;16(10):e0258133. doi: 10.1371/journal.pone.0258133 (PMC8504766; doi:10.1371/journal.pone.0258133)
Supplement: S1 Dataset — (ZIP) [file pone.0258133.s003.zip › Transcriptions/stage 2/13.2_M_46_couple, with children.docx]

**13.2_M_46_couple with children**

**Obrazki. Który z obrazków najlepiej oddaje Pana emocje w obecnej chwili?**

Nic się nie zmienia dla mnie. 4,7,1 i zostaje to samo. Dalej tkwimy w miejscu, w niewiadomej, jeśli chodzi o epidemię. Patrząc z punktu widzenia medycznego niewiele wiemy, jak sobie poradzić na razie z epidemią, z chorobą. Zwykłe zasady stosowane w przypadku epidemii, chora zakaźnych i nic więcej. Nie mamy specyficznych leków na to, nie ma szczepionki i pojawiają się różne ciekawe doniesienia, nie wiadomo, które są prawdziwe, a które nie. Czytałem gdzieś w sieci, że tygrysica zachorowała, więc różne śmieszne rzeczy. One są trochę straszne, trochę śmieszne, więc tkwimy dalej w tym samym punkcie, który był. Tkwimy w tym samym punkcie nie tylko z punktu widzenia medycznego, ale patrząc też na to, co robi władza. Dalej stoimy w tym samym miejscu, dalej się panowie szturchają o wybory zamiast się zainteresować tym, że np. pielęgniarki na 12-godzinną zmianę mają jedną maskę na całą zmianę. To są żarty, a panowie ustalają nowe zasady głosowania w wyborach prezydenckich, które większość ludzi inteligentnych uznaje za niepoważne w tej chwili. Drugi obrazek - 4 - my się staramy coś zrobić, ale na dole. Nie ci, co rządzą, tylko ci, co mają styczność z pacjentami, z bliskimi. U mnie się nic nie zmieniło właściwie, jeśli chodzi o emocje nastroje i 7 zdjęcie to samo - pustka, szaro-buro. Gdyby miało coś jeszcze do tego dojść, to dołączyłbym jeszcze 16, bo dziś jestem wściekły z powodu idiotyzmów i żenującego poziomu zarządzania szpitalem. I to byłoby więcej niż ten pożar w lesie, tylko chyba jakiś wybuch bomby atomowej, coś takiego.

**Te emocje dotyczą decyzji wewnątrz szpitala?**

Wewnątrz szpitala, ale dość szczególnej sytuacji, która jest związana z epidemią. Pojawia się mnóstwo różnych rozporządzeń w szpitalu, dotyczących funkcjonowania naszej pracy, itd., natomiast brakuje rozporządzeń podstawowych. Tak jakby wpuszczało się do szpitala wszystkich ludzi, nie czuwając nad tym, czy ktoś może być zarażony, czy też nie, bo to jest sobota, niedziela, święto, nikt nad tym nie panuje. W zwykły dzień próbują zebrać wywiad epidemiologiczny i ewentualnie zareagować, a w sobotę, niedzielę i święta, nikogo to nie interesuje. Także u mnie jest pożar, ale po bombie atomowej - tam powinny być zgliszcza. To są bzdury, idiotyzmy, które funkcjonują na każdym kroku.

**Czy podejmuje pan jakieś działania, żeby poradzić sobie z tą złością?**

Oczywiście. Idę do ogrodu poczyścić meble ogrodowe, które wymagają renowacji, czyszczenia, itd., albo wsiadam na rower - trenażer, i mam godzinę intensywnego wysiłku. To jest częściowo moje hobby, częściowo konieczność, ale tak naprawdę rzeczy, które robię z przyjemnością.

**Mówiąc o tych emocjach odnosił się pan głównie do perspektywy pana zawodu. Czy z perspektywy osobistej pojawiły się jakieś nowe emocje?**

Nic się nie zmieniło. Ja uważam, że powinniśmy kontynuować jakiś kierunek naszych zachowań w stosunku do najbliższych i nie planuję na razie tego zmieniać, dopóki sytuacja się nie zmieni, i to wszystko. Może bardziej tęsknię za bliskimi, z którymi się normalnie widywałem częściej - za moimi rodzicami. Ale generalnie, uważam, że tak jak jest, tak musi być i dla mnie to jest najlepsze rozwiązanie. Będą inne sposoby na to, żeby leczyć chorobę, zaszczepić się, to będziemy mogli wtedy rozmawiać o innych pomysłach na funkcjonowanie, a za tym się pojawią emocje, a na razie jest wszystko zamrożone. To dotyczy moich najbliższych. Bo oczywiście moje emocje, dotyczące sytuacji w Polsce się pozmieniały.

**A co się pozmieniało w tej kwestii?**

Ktokolwiek ma odrobinę chociaż uwagi skierowanej na sytuację polityczną, to widzi, co się dzieje. To, co powiedziałem przy poprzedniej rozmowie, że nasze władze zamiast się zająć porządnie tematem epidemii, to partia rządząca zajmuje się tematem wyborów. Te ostatnie ruchy różnego rodzaju na arenie politycznej budzą we mnie wściekłość. Ta arogancja władzy, która daje ludziom do zrozumienia, że oni są niczym, że są potrzebni jako elektorat. I na tym się kończy zainteresowanie władzy społeczeństwem. Sączyć jad, załatwić swoje, zebrać pieniążki zrobić przy tym więcej interesów, a lud niech się odczepi. To jest niepoważne. Mądrzy ludzie mówią, co należałoby zrobić, a aroganccy przedstawiciele władzy tego nie słuchają. I to jest przykre, to budzi we mnie wściekłość. I to się zmieniło bardzo od tamtego tygodnia, bo się bardzo zmieniło na poziomie decyzji politycznych. Dalej nikt nie mówi nic sensownego i konkretnego dla ludzi, którzy tracą źródło dochodu i zaraz będą na bruku. Nasze państwo w ogóle nie jest na to przygotowane. Z resztą, patrząc na to, co się dzieje na świecie, chyba żadne państwo nie jest przygotowane. Nawet te bogate są zaskoczone i nie jest tak różowo, jak mogłoby się wydawać. Epidemia się zapowiada na dłużej niż ludzie oczekiwali i z większą burzliwością to wszystko przebiega. To jest dla mnie przykre i to budzi we mnie wściekłość, takie zachowanie władzy, też na poziomie regionalnym. Akurat dzisiaj jest taki dzień... Trafiła pani w sam wybuch wulkanu. Tornado przeszło.

**Epidemia bezpośrednia wpływa na sytuację w pana szpitalu?**

Jak najbardziej. W związku z pandemią i trudną sytuacją w kraju, w związku z rozporządzeniem ministra, wprowadzamy następujące zmiany... Nic dyrekcji nie ogranicza, bo wszystkie decyzje są argumentowane: "w obliczu epidemii", "w związku z rozporządzeniem ministra" i koniec. Różne głupie rzeczy są podejmowane, za które ludzie powinni odpowiedzieć przed sądem, bo to stwarza zagrożenie dla pacjentów. Już nie mówię o różnych zagrożeniach dla personelu, związanych z tymi decyzjami. Bardzo łatwo wykorzystać epidemię do usprawiedliwienia niekorzystnych działań.

**Czuje się pan osobiście zagrożony obecną sytuacją? Czy coś się zmieniło w tej kwestii?**

Nie, jest tak, jak było. Mam obawy o sprawy materialne. O sprawy medyczne, to nie muszę mówić, bo to wiadomo... Czuję się odpowiedzialny za to, że jestem najbardziej prawdopodobnym ogniwem, łączącym moją rodzinę i moich bliskich z ludźmi chorymi, z nosicielami wirusa. To zostaje tak, jak było. To co możemy robić, to robimy w domu i wśród najbliższych. Ale obecna sytuacja nie pozwala zmienić podejścia i nie zmienia się nic ani na lepsze, ani na gorsze.

**Co Państwo robią w kwestii wprowadzanych ograniczeń?**

Rzeczywiście nie wychodzimy z domu. Ograniczamy się do minimum z zakupami. Zakupy robimy raz na kilka dni większe od razu. Myśmy przestali chodzić na rower, spacerować. Mamy wystarczająco dużo przestrzeni, można chodzić wokół domu. Ludzie, którzy mieszkają w bloku, nie mają takich możliwości i muszą wychodzić na spacer pomiędzy innych. My mamy taką możliwość, żeby ograniczyć, więc szanujemy to. Ani ja, ani żona nie wychodzimy. Tak jest i już. Skupiamy się na tym, co mamy do zrobienia w domu. Ja w pracy stosuję zalecone środki zabezpieczeń. Mamy w domu preparaty do odkażania i mamy w razie potrzeby i rękawiczki i maseczki. To, co jest ogólnie zalecane, to mamy. I tak naprawdę chodzi o profilaktykę, o prewencję, bo nic innego zrobić nie można i to ograniczenie kontaktu to jest najsensowniejszy sposób. Bo nic innego nie daje aż tyle gwarancji, co odizolowanie.

**Czy zmieniło się coś w kwestii spędzania czasu, codziennych czynności?**

Nie. Mam trenażer w piwnicy i z niego korzystam. Poza tym prace w ogrodzie. Jest akurat wiosna, ładna pogoda i można z tego skorzystać. Jeśli ktoś lubi takie rzeczy i ma na tyle sprawności w rękach, to część z moich kolegów i ja też. Mnie się to podoba i ja część rzeczy drobnych koło domu mogę zrobić sam.

**Pojawiło się coś, co zaczęło panu mocniej doskwierać?**

Nie. Kwestia niemożności spotykania się ze znajomymi, to jest na stałym poziomie. Ja i tak mam troszkę inną sytuację, bo ja się musze spotykać ze znajomymi w pracy. Co drugi dzień chodzę do pracy, więc widzę kolegów. Moja żona np. pracuje tylko online w tej chwili, więc ona widzi ludzi tylko w komputerze.

**U Pana kontakty w pracy pozwalają wypełnić częściowo te potrzebę?**

Jest to taka sytuacja, która różni mnie w postrzeganiu tych uciążliwości od osób, które nie wychodzą z domu i nie widują się ze znajomymi z pracy, ze znajomymi poza pracą też nie. Myślę, że z tego powodu mam łatwiej.

**Czy w pana otoczeniu zaobserwował pan u ludzi jakieś zmiany?**

Oczywiście. Nasila się wściekłość u ludzi, którzy uprawiają aktywnie sporty - rowery, bieganie. I część z nich próbuje sobie usprawiedliwiać, że może można by było pojechać na rower, bo to i to albo pójść z kijkami albo coś tam innego zrobić. Oczywiście irracjonalne podejście i jeżeli się zastanowią dobrze nad tym, co mówią, to są to próby usprawiedliwienia siebie w łamaniu ogólnie panujących zasad, a nie ma to nic wspólnego z ich zdrowiem, czy rzeczywistymi potrzebami. Oczywiście, uprawianie sportu dla części z nas jest bardzo ważne, jest nieodzowną częścią funkcjonowania - ja to rozumiem i dla mnie też jest. Ale, że tak powiem, potrafię to odsunąć na później, bo tego wymaga sytuacja. A część ludzi coraz bardziej jest wściekła i szuka sobie coraz to nowych furtek, żeby spróbować to usprawiedliwić.

**Czy pana zdaniem ci, którzy teraz usprawiedliwiają wyjście pobiegać, mogą za chwilę złamać też inne ograniczenia?**

Nie wiem. To zależy od ludzi. Każdy z nas indywidualnie traktuje tę sytuację i indywidualnie traktuje zakazy. Niektórzy uważają, że jeżeli coś jest w ich pojęciu nieracjonalne albo nie do końca zgodne z prawem. A właściwie, jeśli można podważyć prawne regulacje takich decyzji, to ci ludzie starają się usprawiedliwiać to, że złamali te zakazy, czy też zalecenia. Więc to jest tak: "A mnie się wydaję, że gdyby było można wpuścić więcej osób do sklepu, to nic by się nie stało". Więc to takie usprawiedliwianie siebie i tłumaczenie wszystkim, że ja mam rację, a nie ma podstaw prawnych, żeby zakazać ludziom wychodzić, bo nie ma stanu wyjątkowego, itd. Wczoraj miałem rozmowę z naszymi pielęgniarkami na ten temat. One są w dużo gorszej sytuacji niż lekarze, bo z tymi pacjentami przebywają stale. Więc się boją, pytają i rozmawiamy na ten temat. I też mówiliśmy o tym, że ludzie się usprawiedliwiają, żeby szukać wymówek na różne rzeczy.

**Uważa pan, że to usprawiedliwianie jest odpowiedzialne?**

Oczywiście, że nie. Bo należy zrozumieć istotę problemu. I nie przejmować się tym, czy to jest w 100% zgodne z prawem, tylko czy to ma sens i uzasadnienie merytoryczne. Jeśli tak, to nie czepiajmy się tego, że ktoś nie ogłosił stanu wyjątkowego. Moim zdaniem powinien być ogłoszony z zupełnie innych przyczyn. Ale jeśli ktoś z mądrych ludzi powiedział o tym, że powinniśmy unikać kontaktu z innymi osobami, bo to zmniejszy ryzyko zakażenia się i intensywność epidemii u nas, to z tego czerpmy wskazówki. Z idei, a nie próbujmy łamać zalecenia, bo przepis jest nie do końca zgodny z Konstytucją. Jest szereg rzeczy, które przeszły, a są niezgodne z Konstytucją i były łamaniem prawa obowiązującego, to ludzie na to nie zwrócili uwagi. Zwracają uwagę na rzeczy, które są dla nich niewygodne, nieprzyjemne, burzą dotychczasowy porządek, więc ludzie zaczynają szukać furtek, żeby to ominąć.

**Czyli tych ograniczeń, nawet jeśli są niezgodne z Konstytucją, powinniśmy przestrzegać?**

Nie mówię, że same zasady są niezgodne z Konstytucją, tylko nie ma podstaw prawnych, żeby coś takiego sankcjonować, żeby karać ludzi, że idą na spacer do lasu czy siedzą nad rzeką. Nie mówię, że mają to być rzeczy niezgodne z Konstytucją. Tylko ktoś, kto nie stosuje się do zaleceń, pójdzie biegać, a później będzie się kłócił, że nie ma podstaw prawnych do tego, że Konstytucja mówi co innego, itd. Chodzi mi o istotę tych zaleceń. My powinniśmy patrzeć na to, jakie są korzyści z tych procedur, na tym się skupić i to uszanować. I jednak nie wychodzić. A jak ktoś nam zwróci uwagę, to uszanować to, a nie wykłócać się. Chodzi mi o odpowiedzialność ludzi. Natomiast zdaję sobie sprawę z tego, że społeczeństwo, składa się z jednostek, które zawsze będą nastawione źle do jakichkolwiek regulacji. Z ludzi, którzy zawsze będą chcieli prowadzić sobie życie po swojemu i pewne łagodne formy przekazu, informacje w mediach, list skierowany indywidualnie do każdego, nie zrobi tyle dobrego, co zalecenia, które są w jakiś sposób egzekwowane w formie kar za łamanie zaleceń. Tacy jesteśmy. A dobro 5% subiektywne nie może być podstawą do tego, żeby stwarzać ryzyko reszty społeczeństwa. Mamy przykład z Włoch, co tam się dzieje w tej chwili. I mimo tej sytuacji zdjęcia z ulic dużych miast, wcale nie pokazują nam, że Włosi wyciągają z tego wnioski.

**Co jest dla pana największym wyzwaniem w obecnej sytuacji?**

Tak prywatnie, to wyzwaniem jest odpowiadanie na stałe pytania ludzi do mnie kierowane. Ja jestem chirurgiem i mogę rozmawiać na temat chirurgii. A na temat chorób zakaźnych, zwłaszcza epidemii, nie chcę rozmawiać. To nie jest moja branża i dla mnie jest to wyzwanie, kiedy co chwila mnie ktoś pyta, co sądzę o maseczkach itd. Tego typu pytania są największym wyzwaniem. Ja mam jakieś swoje zdanie na ten temat i ono nie musi się pokrywać z oczekiwaniami ludzi. Najczęściej się nie pokrywa. Ludzie chcą usłyszeć, że wszyscy powinniśmy nosić maseczki i to co jest, to jest źle, bo się pozwala ludziom chodzić bez. Wszyscy w maseczkach, wszyscy w rękawiczkach. Ale ludzie nie wiedzą, jak tego używać. Co z tego, że się każe ludziom nosić maseczki, jak będą kichać w nie przez 12 godzin, nosić to w samochodzie i tak naprawdę będzie to robiło więcej szkody niż pożytku. Więc tłumaczenie ludziom, że to nie jest dobry pomysł, że maski powinni nosić ci, którzy są źródłem infekcji. Oczywiście są tacy, którzy chodzą pośród nas, a nie mają objawów i ok, oni też powinni. Ale to w takim razie zróbmy tak, niech wszyscy noszą, dajmy ludziom maseczki, dajmy ludziom rękawiczki - nawet możliwość kupienia, a nie tak, że maska jest droższa 30 razy niż była, bo to żarty są. I to jest dla mnie największe wyzwanie - odpowiadanie na pytania, kiedy nie mam takiej odpowiedzi, jakiej chciałbym udzielić, czyli popartej dowodami naukowymi.

**Widzi pan u ludzi w swoim otoczeniu nowe sposoby radzenia sobie z sytuacją?**

Tak. Ludzie zaczęli jeździć do pracy na rowerze, bo nie mogą inaczej, a mają prawo jeździć na rowerze do pracy.

**Jak pan ocenia to zachowanie?**

Myślę, że to jest w porządku. Bo w mojej opinii jazda na rowerze nie jest czynnikiem, który sprzyja kontaktom z innymi osobami. Jeśli robimy to w odpowiedni sposób, to ja się w ogóle z nikim nie muszę kontaktować. Fakt, że się przemieszczam, raczej sprzyja, zwłaszcza w miejscach, gdzie nie ma ruchu pieszego, gdzie ludzie nie chodzą, to sprzyja izolacji, a nie pogarsza. Natomiast, szanując przepisy, nie chodzi na ten rower. Ale jest pewnego rodzaju furtka, która pozwala ludziom na to, żeby skorzystać z tego roweru, nie łamiąc przepisów. Może to być w sprzeczności z tym, co powiedziałem wcześniej, że ludzie sobie próbują usprawiedliwiać. Można różnie do tego podchodzić. Ja mam dojazd do pracy taki, że nie spotkam nikogo. Wiadomo, że na ścieżce rowerowej pieszych nie ma. Rowerzysta z rowerzystą mija się w jakiejś tam odległości i nie jest to czynnik ryzyka z punktu widzenia epidemiologii. Oczywiście zawsze się może zdarzyć, że wjedziemy w chmurę kropelek, bo ktoś przed nami zakaszlał, ale jest to o wiele mniejsze ryzyko niż w codziennym życiu, w sklepie.

**Zaobserwował pan jakieś dziwne zachowania w otoczeniu?**

Tak. Dziwne jest dla mnie to, że ktoś jedzie w samochodzie sam jeden i ma ubraną maseczkę - to już kompletny idiotyzm. To już nawet nie jest dziwne. To jest głupie, irracjonalne i świadczy o tym, że ktoś kompletnie nie ma pojęcia o tym, co robi. Słyszał coś, ale nie wie, co robi.

**Myśli pan, że ta osoba uważa, że się chroni, nosząc maseczkę?**

Tak. Myśli, że się chroni przed infekcją, przed wirusem, natomiast nie ma świadomości, że to nie jest absolutnie sposób uchronienia się przed infekcją.

**Jeszcze jakieś dziwne zachowania?**

Czasem nerwowe ruchy u niektórych osób. W szpitalu u nas widzimy, że niektórzy się denerwują, na przykład, że przyjmujemy pacjentów. Część pielęgniarek ma pretensje o to, że dalej przyjmujemy pacjentów, chociaż jest epidemia, a powinniśmy przestać, żeby się nie zakazić. To jest dla mnie też irracjonalne, bo pracujemy nie po to, żeby przychodzić do pracy, tylko po to, żeby leczyć ludzi, którzy tego wymagają. My nie operujemy w tej chwili osób, które tego nie wymagają. Więc tych zachowań też nie bardzo rozumiem. I one są dla mnie irracjonalne. Bo skoro ktoś nie chce pracować, to niech weźmie urlop, cokolwiek, ale niech nie ma pretensji o to, że wykonujemy swój zawód. Przecież obok infekcji wirusem pacjenci dalej chorują na te choroby, na które chorowali. Więc to jest dla mnie niezrozumiałe zachowanie wśród pielęgniarek. Nie spotkałem się na razie z takim zachowaniem wśród lekarzy. Jeszcze nie. Może akurat trafię, ale na razie nie. Ale kilka pielęgniarek w takiej dziwnej sytuacji zastałem, jak się denerwowały, no bo "myśmy powinni przestać, bo się zarazimy". To jaki ma sens chodzenie do pracy w ogóle w takim razie. Przestańmy w ogóle chodzić, zamknijmy oddział i niech sobie ludzie robią, co chcą. Pytanie, co będzie, jak osoba z najbliższego grona tych pań, które się denerwują, trafi do szpitala z potrzebą pomocy. A my nie będziemy mogli udzielić tej pomocy, ponieważ nie będzie nikogo, bo wszyscy się wystraszyli wirusa.

**Z czego wynika zachowanie tych pielęgniarek?**

Z indywidualnych cech charakteru i może poziomu inteligencji. Społecznej świadomości, że to, co robimy - przynajmniej w moim odczuciu, w sferze zawodowej, nie może być postrzegane tylko przez pryzmat własnych korzyści, zagrożeń dla nas, ale funkcjonujemy w jakimś konkretnym celu. Więc albo się wpasujemy w sytuacje. A cały świat dąży do tego, żeby dopasować obecne zachowania do trudnej sytuacji epidemiologicznej i spróbować ograniczyć koszty tych zachowań. A to, żeby wypracować odpowiedni model, to trzeba próbować. A nie stwierdzić, że chowamy się do piwnicy, nie wychodzimy i koniec. Bo też nie ma to sensu.

**Jak pan ocenia najnowsze ograniczenia wprowadzone przez rząd? Limity osób w sklepie, godziny dla seniora, itd.**

Dobrze. Podoba mi się reakcja sklepów na to, co się dzieje wokół nas. To, że oni dopasowali godziny pracy, bo wiemy o tym, że w niektórych sklepach można robić zakupy całodobowo, chociaż wcześniej tego nie było. Personel jest dobrze nastawiony. Nigdy nie zauważyłem, od momentu poważnego traktowania epidemii w naszym kraju, niechęci i dziwnych zachowań ze strony pracowników sklepów. To są tak naprawdę ludzie najbardziej narażeni na infekcje. Oni się stykają z największą ilością osób. Więc zareagowali bardzo dobrze. A te wszystkie regulacje, które weszły, uważam, że są z korzyścią. One oczywiście stwarzają mnóstwo sytuacji wątpliwych, czy wręcz niezgodnych z prawem. Wydaje się, że są niemożliwe do rozwiązania, jak np. to, że mamy nieletnich rodziców, którzy nie mogą pójść sami do sklepu - i oni muszą sobie jakoś radzić. To są różne rzeczy trudne do rozwiązania na już, ale pewnie szybko się uda z tym uporać. Mam nadzieję. Natomiast generalnie te obostrzenia uważam za dobre. I czepianie się ludzi, że mogłoby być inaczej, bo powinno być w dużym sklepie więcej ludzi, itd., to jest chyba niepotrzebnie zupełnie.

**Zdarzyło się panu coś kupić przez Internet podczas epidemii?**

Oczywiście. Różne, bardzo różne rzeczy. Elementy wyposażenia samochodu, grę dla syna, elementy do ogrodu, kosmetyki.

**Czy te produkty w innej sytuacji kupiłby pan stacjonarnie?**

Nie. Większość rzeczy kupuję przez Internet i tu się nic nie zmieniło.

**Obecnie kupuje pan przez Internet tyle samo, co przed epidemią?**

Tak, tyle samo.

**Jakie są pana zdaniem zalety i wady zakupów przez Internet?**

To zawsze tak samo było dla mnie. Podstawową wadą jest to, że nie mogę ocenić jakości produktu w taki bardzo podstawowy sposób. Czyli wziąć do ręki, pooglądać i zobaczyć, jak jest wykonane. Na zdjęciach wszystko wygląda dobrze, a czasami przychodzi coś zupełnie różnego od tego, co na zdjęciu. Ostatnio kupiłem czajnik do pracy, który miał być czerwony, dobrej firmy, przyszedł brązowy. Może drobiazg, może nie. Akurat w tej sytuacji nie miało to większego znaczenia, ale jakby ktoś chciał sobie dopasować ten czajnik, to musiałby odsyłać. Tego nie lubię w Internecie. Nienawidzę odsyłania produktów. To jest dla mnie podstawowa wada zakupów przez Internet, że nie mogę ocenić organoleptycznie. A reszta, to same zalety. W momencie, jak się pojawiły paczkomaty, to już w ogóle.

**U pana w okolicy nie ma problemów z przepełnieniem paczkomatów?**

Nie. Ja miałem dwa razy taką sytuację, ale to przed epidemią. A korzystam z paczkomatów od kiedy się pojawiły.

**Produkty, które pan kupił - elementy do samochodu i ogrodu, przydają się podczas spędzania wolnego czasu czy miały inne funkcje?**

Różnie. Elementy bagażnika są potrzebne najczęściej, żeby pojechać na urlop, wpiąć narty, itd., więc to tego typu. Do ogrodu, to wiadomo, musi być coś, jeśli mam wyczyścić meble czy użyć systemu nawadniania.

**To były raczej produkty użytkowe?**

Użytkowe, zdecydowanie tak.

**A zdarzyły się panu zakupy czysto dla przyjemności?**

*[Śmiech]*Czysto dla przyjemności zakupiłem sobie licencję na oprogramowanie do trenażera.

**I to się wiąże z pana hobby?**

Tak. To jak najbardziej dla przyjemności, bo akurat ten program mi się spodobał najbardziej i powiedzmy, że była to kwestia wyboru, nie przymusu, bo ten program jest obsługiwany przez trenażer, tylko chciałem akurat ten, więc go kupiłem.

**A jeśli chodzi o grę dla syna, to gra, w którą mogą państwo grać razem?**

Tak. Gra komputerowa, ale z trybem takim, że jednocześnie gra dwóch graczy. Czasami gram z synem. *[Śmiech]*Ale to jest katastrofa, bo te gry zupełnie odbiegają od moich wyobrażeń o grach. To jest coś dla mnie dziwnego. Ja jako nastolatek grywałem w gry, natomiast te obecne gry, kierowane stricte do dzieci, znane i lubiane, są bardzo ograniczone w sensie przekazu. Tylko strzelanie, rozwalanie, chociaż są dla dzieci.

**Przed epidemią też pan grał z synem w tego typu gry?**

Tak. To się nie zmieniło.

**A jeśli chodzi o kosmetyki, to były to kosmetyki, których zawsze pan używał czy coś nowego?**

Nie, to co zwykle.

**Można więc powiedzieć, że pana zwyczaje zakupów przez Internet, nie zmieniły się w stosunku do sytuacji sprzed epidemii?**

Tak, nie zmieniły się. W przypadku oprogramowania tylko epidemia przyspieszyła moją decyzję.

**Czy zmieniły się pana zwyczaje żywieniowe?**

Oczywiście, że tak. Jeżeli jestem co drugi dzień w domu, mogę zrobić śniadanie w domu i wygląda to zupełnie inaczej. Mogę zjeść z żoną śniadanie wspólnie. Więc myślę, że te zwyczaje już się bardzo różnią w stosunku do tego, co było przed epidemią, bo przynajmniej co drugi dzień możemy zjeść wspólnie śniadanie. A wcześniej tego nie było poza dniami wolnymi. To na pewno. Jak jest więcej czasu na zrobienie jedzenia, to można zrobić coś bardziej wymyślnego. Zamiast jeść kanapki, można jeść sałatkę i trochę więcej czasu poświęcić na zrobienie tego. To, że można zjeść z bliskimi śniadanie to jest duża zmiana dla mnie.

**Wcześniej jak wyglądały pana śniadania?**

Jadłem w pracy. Bo zjedzenie śniadania o 6:00 to dla mnie nie jest... Nie radziłem sobie z tym.

**A te posiłki na śniadania w domu są bardziej wymyślne, czy robione na szybko?**

Nie, to jest na spokojnie właśnie. To jest cała przyjemność z tego, że można spokojnie to śniadanie zjeść bez pośpiechu niepotrzebnego.

**Czy zmieniły się w jakiś sposób pana zasady żywieniowe?**

Nie.

**Jak wygląda obecnie przygotowywanie posiłków?**

Wspólnie nie gotujemy. Zazwyczaj jedna osoba gotuje, a druga korzysta z tego czasu, bo to bez sensu. Dwie osoby na raz w kuchni to jest straszny galimatias *[śmiech]*. Każdy ma swoje zasady przygotowywania posiłków i ja nie przeszkadzam żonie, żona mnie i myślę, że to jest dobre rozwiązanie. Ale to, że się możemy podzielić, że raz to robię ja, a raz żona, to jest w porządku. Częściej robi żona oczywiście, ale ja mam swoje potrawy, które ja tylko przygotowuję, więc trochę się dzielimy.

**Jedzą państwo teraz wszystkie posiłki wspólnie?**

Większość tak. Syn z racji wieku je częściej i ostatni posiłek zjada później niż wszyscy. I tu się różnimy czasami, że np. ja zjem kolację, a żona nie zje. Reszta posiłków udaje się jeść. Ja nie mam dyżurów, nie jestem w pracy. Teraz pracuje po 12 godzin co drugi dzień, to w taki dzień nie mam szans, żeby zjeść z rodziną.

**To jest dla pana ważne, żeby jeść posiłki razem?**

Oczywiście, że tak. Dla mnie tak.

**Dlaczego?**

Bo to scala rodzinę.

**Zamawia pan jedzenie przez Internet/ z dostawą?**

Tak.

**To się zmieniło przez epidemię?**

Tak. Myślę, że częściej trochę zamawiam niektóre rzeczy i żona zamawia teraz produktu z takiej naszej lokalnej piekarni. Tam oprócz chleba są też inne wyroby typu racuchy, proziaki, można zamówić zupę w domowy sposób przygotowaną, z resztą wyborną. Troszkę jedzenia więcej kupujemy na zasadzie takiej, żeby pomóc ludziom, którzy prowadzą ten swój biznes, żeby im się to nie zapadło. Bo robią naprawdę świetne rzeczy i trzeba o to dbać, więc trochę zamawiamy.

**Wsparcie lokalnego biznesu to jeden z powodów, dla których państwo zamawiają jedzenie?**

Tak, oczywiście.

**I to jest też kryterium wyboru?**

Zdecydowanie jesteśmy anty fast-foodom, zwłaszcza sieciowym. Ja właściwie nie korzystam z takich miejsc i żona też. Czasami synowi się udaje namówić na jakiś wypad, ale to tak na zasadzie atrakcji sezonowej raz na jakiś czas.

**Są jeszcze jakieś powody częstszego zamawiania jedzenia?**

Chyba tylko fakt, że w ten sposób możemy komuś pomóc, troszkę nas zachęcił, żeby częściej to robić.

**Uważa pan, że zamawianie jedzenie z dostawą w obecnej sytuacji jest bezpieczne?**

Myślę, że jest tak samo bezpieczne, jak chodzenie do sklepu.

**Czy zmieniły się używane przez pana sposoby płatności?**

Raczej nie. Ja w większości używam bezgotówkowych płatności. Robiąc zakupy przez Internet, korzystam z bankowości Internetowej, wszelkie rachunki, a w sklepie w większości płacę kartą. Odkąd się pojawiły opcje płatności zbliżeniowych - nie korzystam z telefonu - natomiast karta do płacenia zbliżeniową jak najbardziej. I uważam to za niezwykle wygodne. I akurat w tej sytuacji, która jest w tej chwili, też może... Nie wiem, to trzeba byłoby zobaczyć jakieś naukowe opracowania, jaki to ma wpływ na przenoszenie patogenów, ale myślę, że całkiem spory. Pieniądze wiadomo, że są brudne. *[uśmiech]*W różny sposób można to dopasować.

**Jak wyglądają pana zwyczaje zakupów spożywczych obecnie?**

Robię zakupy raz na 3-4 dni do sklepu.

**Ta mniejsza częstotliwość chodzenia do sklepu jest w odpowiedzi na zagrożenie zarażeniem?**

Tak. Robimy większe zakupy, rzadziej. Wcześniej nie planowaliśmy, tylko robiliśmy na bieżąco, wracając z pracy, czy przy okazji kupowaliśmy coś, co nam będzie potrzebne na następny dzień.

**Czy od początku epidemii chodził pan rzadziej do sklepu, czy to się zmieniło pod wpływem wprowadzanych ograniczeń?**

W momencie jak to zagrożenie się stało realne u nas, czyli pojawiły się informacje, że są zachorowania u nas, to myśmy zaczęli ograniczać. A już na pewno od momentu, jak zdecydowaliśmy się, żeby nie kontaktować się z rodzicami, tak, żeby się spotkać i pogadać, tylko na zasadzie zrobienia im zakupów i podania przez drzwi, to chyba od tego momentu zdecydowaliśmy, żeby zrobić zakupy rzadziej, ale większe. To ewoluowało, to nie była decyzja, że od dzisiaj tak zrobimy, bo tak, bo wyszły przepisy, zalecenia. To był proces.

**Teraz chodzą państwo na zakupy z listą?**

Tak. Można zrobić rzadziej. A ja ostatnie zakupy zrobiłem w 20 minut. Trzeba mieć jeszcze do tego rozeznanie, gdzie co jest w sklepie.

**Mógłby pan opowiedzieć o swojej ostatniej wizycie w sklepie, już po wprowadzeniu najnowszych ograniczeń?**

W tej chwili w każdym sklepie, do którego wchodzę, to przy wejściu albo jest pracownik sklepu, który daje ludziom odkażalnik i wiszą rękawiczki lub leżą w pudełku, które trzeba ubrać. Wchodzi się do sklepu, jest preparat do odkażania, są rękawiczki, najczęściej foliowe.

**A inni klienci? Nosili maseczki?**

Część ludzi nosi, część nie.

**Pan nie nosił?**

Nie. Noszę w sytuacjach bezpośredniego narażenia. W sytuacjach, kiedy jestem w szczególny sposób narażony na możliwość kontaktu z wirusem. Przyjeżdża pacjent nieprzytomny, nic nie wiemy, nie można zebrać wywiadu, to wiadomo, że nie będzie nikt ryzykował zarażenia, tylko się ubieramy, tak jak w zaleceniach.

**A osoby pracujące w sklepie były jakoś zabezpieczone?**

Widać, że obsługa pracuje w maseczkach i rękawiczkach. Nie przypominam sobie, żeby ktoś pracował w okularach ochronnych, goglach. Część osób ma maseczki chirirgiczne, część ma uszyte, część ma z filtrami profesjonalnymi. Patrząc na to z punktu widzenia medycznego, ludzie w sklepie to osoby, które spotykają się z bardzo dużą ilością ludzi na co dzień, więc potencjalnie mają szansę na zarażenie się. A tym samym są jednym z bardziej prawdopodobnych źródeł infekcji dla innych. To, że panie w kasie, czy te, które gdzieś chodzą przy półkach, to jest moim zdaniem uzasadnione.

**Były kolejki przed sklepem, że trzeba było stać w odstępach?**

Czasami tak. Przed sklepem, przed pocztą.

**Patrząc na zachowanie innych klientów, zauważył pan, że robią zakupy szybciej?**

Ostatnio jest tak mało ludzi w sklepie, że nie zwróciłem uwagi, jak się ludzie zachowują, bo mało kogo widać wokół.

**Teraz było mniej ludzi w sklepie?**

Zdecydowanie. Dużo, dużo mniej ludzi. A ponieważ ja się spieszę, więc nie zwracam uwagi na to, co się dzieje wokół mnie. W kasie już nerwowości nie widać. Myślę, że wszyscy ludzie starają się szybko robić zakupy. Tak, jak ja to robię. Pewnie, może nie jest to z nerwowością, ale szybciej niż zwykle.

**Dlaczego szybciej? Żeby jak najkrócej być w sklepie?**

Tak. Większość ludzi się boi infekcji, więc robi zakupy szybciej.

**W jakim sklepie robił pan zakupy?**

Lokalna sieć, ale dosyć duże sklepy. I w sklepie z materiałami budowlanymi sieć dużych sklepów.

**W obu typach sklepów widział pan podobne zachowania?**

Tak. Niedużo ludzi i mam wrażenie, że ludzie dość sprawnie się przemieszczają.

**Jak planuje pan spędzić Wielkanoc?**

W domu. Nie przekraczając posesji. Z żoną i synem. Zdecydowaliśmy się na to, żeby nie widzieć się dalej z rodzicami, pomimo świąt. Jeśli to ma być konsekwentne zachowanie, to te święta nie mają tutaj znaczenia. Potraktujemy to w sposób bardziej naukowy i pragmatycznie podejdziemy do sprawy, a nie z emocjami, że święta to już sobie robimy dyspensę od reguł.

**Przygotowują się państwo do tych świąt, tak jak zwykle?**

Porządki nie. Potrawy trzeba zrobić, żeby przypomnieć sobie, jak co roku, nasze świąteczne zwyczaje i tradycje, ale to nie jest na pewno nic tak rozbudowanego, jak było to wcześniej przed epidemią. Tradycyjne potrawy, ale w zdecydowanie mniejszej ilości. Zwykle śniadanie spędzaliśmy z rodzicami, przyjeżdżali do nas, a teraz tego nie będzie, więc trochę skromniej.

**Planują państwo specjalne zakupy na Wielkanoc?**

Może trochę. Niektórych produktów nie używa się na co dzień, więc może trochę więcej.

**A dekoracje, kwiaty?**

Nie sądzę, żeby można było się obyć bez dekoracji *[śmiech].*

**To ważny element, który się pojawi też w tym roku?**

Tak, oczywiście.

**A planują państwo kupić jeszcze jakieś przyjemnościowe produkty?**

Słodycze świąteczne to takie połączenie dekoracji z przyjemnością myślę.

**To ma za zadanie stworzyć atmosferę?**

Tak, mamy 10-lata w domu, więc on poznaje świat przez różne rzeczy, które go otaczają, nasze zachowania, tradycje, itd., więc to chyba ważne, żeby nie rezygnować z takich rzeczy.

**To będzie namiastka normalności?**

Tak. Poza tym, że najważniejszym elementem świąt, tradycyjnym u nas, czyli spotkaniem z rodziną, które dla mnie jest t taka podstawowa część tych obu świąt - i Bożego Narodzenia i Wielkiejnocy, to jest spotkanie z najbliższymi. Takie bardziej odświętne, wszyscy razem, nie teściowa w poniedziałek, rodzice w czwartek, tylko wszyscy razem siadamy przy śniadaniu, żeby spędzić ten czas wspólnie. Tego nie będzie, więc trzeba, żeby zostały inne, powierzchowności.

**Planują się państwo spotkać z rodziną online?**

Na pewno.

**Czy jest to dla państwa ważne, żeby iść do kościoła?**

Nie tak, jak kiedyś to było. W tej chwili już nie jest to ważne.

**Kiedyś w znaczeniu przed epidemią, czy ogólnie, wcześniej?**

Kiedyś w życiu. Obecna sytuacja nie a z tym związku.

**A kultywują państwo tradycję święconki?**

Tak.

**I jak w tym roku mają państwo zamiar to zrobić?**

Nie będzie. Jak mamy być konsekwentni, to nie ma wyjątków. Ponieważ syn pamięta doskonale, co było w ubiegłym roku, a mam nadzieję, że w przyszłym będzie normalnie, to uważam, że nic złego się nie stanie, jeśli w tym roku nie pójdzie święcić.
