# Supplementary material for: The challenges arising from the COVID-19 pandemic and the way people deal with them. A qualitative longitudinal study
Source: PLoS One. 2021 Oct 11;16(10):e0258133. doi: 10.1371/journal.pone.0258133 (PMC8504766; doi:10.1371/journal.pone.0258133)
Supplement: S1 Dataset — (ZIP) [file pone.0258133.s003.zip › Transcriptions/stage 2/19.2_F_39_couple, with children.docx]

**19.2_F_39_couple with children**

**Wróćmy do obrazków sprzed tygodnia. Który z nich oddaje Twoje aktualne samopoczucie?**

Myślę, że 6. Może pogoda ma wpływ na to. Raczej dobrze się czuję, słońce dodaje energii, a wczoraj i dzisiaj byliśmy na powietrzu.

**Tydzień temu wybrałaś obrazek z drogą, mniej pozytywny. Co się zmieniło przez ten czas?**

Myślę, że najbardziej słońce i to, że jesteśmy razem z rodziną. Popołudnie możemy spędzać na powietrzu, to czas z rodziną, to jest ważne. Mamy spore podwórko i głównie tam wychodzimy. Doładowujemy energię słońcem, bawimy się z psem, wykonujemy jakieś małe prace ogrodowe. To daje spokój.

**Czy przekłada się to na całą sytuację związaną z koronawirusem?**

Na razie jestem w zawieszeniu, nie ma reakcji bardzo pozytywnych lub negatywnych. Jest neutralnie. Zawieszenie to praca, kontakt z ludźmi, wychodzenie - swoboda jest zawieszona. Też nie wiadomo, jak będzie ze zdrowiem. Przestrzegamy kwarantanny i dbamy o siebie, nie jesteśmy bardzo narażeni na czynniki zewnętrzne, więc wiele zależy od nas. O to jestem spokojna. Mąż robi zakupy, więc on jest najbardziej narażony, ale dbamy o higienę i używamy maseczek.

**Mówiłaś, że masz trochę mniej pracy jako księgowa. Czy to się zmieniło w tym tygodniu?**

Przez to, że pracujemy z domu i mamy przeciążone łącza, zmieniło się tempo mojej pracy. Nie da rady przetworzyć wszystkiego w szybszym tempie, więc tej pracy mi nie ubywa, dłużej wszystko trwa zanim systemy załapią i to się przemieli. Jeśli chodzi o pracę, to są trochę zmiany. Zadzwoniła przełożona i powiedziała, że wysyła ludzi na urlopy i tej pracy będzie mniej. Ja jako jedyna mogę iść na opiekę nad dziećmi, więc chcą, żebym z tego skorzystała, żebym popracowała jeszcze do końca tego i przyszłego tygodnia. Ona mnie wtedy zastąpi, bo pewnie chce się chronić żeby pewnie nie iść na urlop, a ja pójdę na opiekę. Musiałam wysłać harmonogram pracy, która mi jeszcze została. To jest gdzieś wyżej raportowane i oni podejmują decyzję co dalej z pracownikiem. Ja zdawałam sobie sprawę, że będę musiała iść na opiekę prędzej czy później. Płaci za to ZUS, więc to dla firmy odciążenie, tyle że 80% mojego wynagrodzenia. W jakimś sensie jestem trochę zabezpieczona. Dziś mieliśmy wideorozmowę z prezesem, powiedział, że obcinają nam wynagrodzenie o 10%. Wiemy więcej, to jest lepsza opcja niż zwolnienia. Słyszałam, że takie firmy, które podlegają pod spółkę-matkę, na przykład w Irlandii zwolniono 500 pracowników z uwagą, że przyjmą ich ponownie po poprawie sytuacji. We Francji było zmniejszone wynagrodzenie o 20% pracownikom i starają się o jakąś subwencję żeby to zniwelować. U nas zdecydowano, że skoro wszędzie są zmiany, to u nas też muszą ratować firmę. Przetrwały tylko dwa największe kontrakty i zdecydowali, że zmniejszają o 10% solidarnie wszystkim. To nie nastraja mnie jakoś negatywnie, zdawałam sobie z tego sprawę.

**Czy na tę opiekę jest jakieś ograniczenie czasowe?**

Tak, chyba 60 dni. Wyliczyłam, że jakbym miała to brać ciągiem, to starczyłoby do dwudziestego któregoś czerwca. Zobaczymy. Na razie wysłałam harmonogram i oni podejmą decyzję, od kiedy miałabym iść na tę opiekę. Na razie pracuję.

**To duża zmiana dla ciebie?**

Wiadomo, że to przykre, jak obcinają pieniądze. Każdy jakoś sobie wynegocjował warunki. Pracuję tu, w tym roku minie mi dwa lata, jak zmieniałam pracę, to na lepsze warunki niż poprzednio. Jakiś czas temu dostałam podwyżkę, u nas co roku są rozmowy i coś tam się zawsze wynegocjuje. W tym roku w marcu zaczęły się rozmowy roczne i ciach, trzeba było przejść na pracę zdalną. Z podwyżek nici i jeszcze obniżenie. To przykre. Słyszałam, że 500+ też mają zatrzymać. Może ceny usług będą wzrastały; jeśli chodzi o artykuły spożywcze, to już to trochę widać.

**Czy utrata 500+ mocno nadwyręży wasz domowy budżet?**

Myślę, że może nie mocno, ale jednak to spory zastrzyk. Jesteśmy trochę odcięci od świata i nie korzystamy z tych atrakcji co zwykle - nie wyjedziemy za granicę, nie mamy rozrywek, więc nie ubędzie tak bardzo. Można się zorganizować, aby przeżyć bez atrakcji.

**Czyli w normalnej sytuacji zmniejszenie budżetu 500+ byłoby dotkliwe, ale przez siedzenie w domu nie będzie to obciążające?**

Tak, w innej sytuacji musielibyśmy sobie czegoś odmówić. Trzeba by było wybierać rzeczy bardziej ważne.

**Obawiasz się zupełnej utraty pracy?**

Patrząc racjonalnie, jest jakaś wersja dla wszystkich - te urlopy i powroty po nich, to jest wersja oficjalna. Ale i tak nie wiemy, co będzie za jakiś czas. Jeśli chcą kogoś zwolnić, to i tak go zwolnią. Jak będzie trzeba to człowiek sobie pracę znajdzie, a w księgowości zawsze jest trochę ofert. Wiadomo, że to dla mnie dogodne miejsce pracy, bo to miejscowość mojego zamieszkania, nie tracę czasu na dojazdy. Finansowo też trochę poprawiłam, to dwa plusy. Jestem dobrym pracownikiem i chyba to widzą, ale jaką podejmą decyzję , taka będzie.

**Jak o tym myślisz, że teraz będziesz w domu i nie będziesz pracowała w ogóle?**

Może bardziej się zaangażuję w naukę młodszej córki. Teraz nie naciskam, jak jej się nie chce to leci na podwórko. Jak wraca to jej odpuszczam. Czasami materiały od wychowawczyni drukujemy innego dnia i to się zbiera. W tej sytuacji po prostu bardziej poćwiczymy czytanie, a widzę, że też niechętnie do tego podchodzi i ma trudności ze złożeniem sylab. W tą stronę może pójdę. Może też bardziej sobie poczytam, tak dla siebie. Może poprzebywam więcej z dziećmi na podwórku.

**Czyli nie obawiasz się tego, że twój dzień będzie wyglądał inaczej?**

No taki mam plan. Może będzie tak, że pierwszy tydzień wykorzystam, a potem zacznę się nudzić. Nie wiadomo.

**Czy zaczęłaś się rozglądać za inną pracą?**

Nie, na razie jakieś zabezpieczenie jest. Może dlatego się tak nie załamuję, bo widzę, że większość ludzi jest w takiej sytuacji. To jest ogólnoświatowa sytuacja i nie jestem wyjątkiem. Ludzie są zwalniani, w zawieszeniu, ja nie jestem w sumie w takiej złej sytuacji.

**Czy w twoim bliższym otoczeniu jest ktoś w złej sytuacji?**

Może nie blisko, ale pocztąą pantoflową dowiedziałam się, że u szwagra w pracy zwolniono 28 osób, a docelowo mają zwolnić jeszcze 90. To już słabe.

**Czy w tym tygodniu coś się zmieniło w twoim życiu? Pytam o codzienną rutynę.**

Chyba nie. Próbuję czytać, ale nie mam na to czasu. Oszczędza się na dojazdach do pracy, ale i tak cały czas coś się robi, takie stałe zajęcia. I tak obiad o szesnastej, zmywam potem naczynia. Tylko tyle, że pogoda się poprawiła i wychodzimy na powietrze. Dzisiaj, przed tą rozmową, zebraliśmy się wszyscy na podwórku - siostra, mama, szwagier, dzieci. Posiedzieliśmy, dzieci grały w piłkę. To może to. Można też porozmawiać z sąsiadką, która też wyszła do ogródka.

**Czy słyszałaś o jakichś nowych obostrzeniach?**

Wiem, że liczba osób jest ograniczona jak się wchodzi do sklepów, na jedną kasę trzy osoby bodajże. Jakoś tak. Pilnują tego dobrze. Są odstępy, dezynfekowane ręce przez ochroniarzy, obowiązkowe rękawiczki. Ale to tyle co usłyszę i mąż mi powie. Zamówiłam do paczkomatu książkę i jakieś drobiazgi, miałam nadzieję, że wyrwę się rowerem do paczkomatu. Ale mąż jechał po zakupy i sam mi te paczki odebrał, znowu nie udało się wyrwać do świata.

**Czemu akurat mąż robi zakupy?**

Może on jest ostrożniejszy; uważa, że jak jedna osoba wyjdzie, to zagrożenie się zmniejszy. On i tak zawsze odpowiadał za jakieś większe zakupy, ja raczej za drobiazgi. Teraz stara się raz w tygodniu zrobić zakupy. Ja w tym tygodniu raz poszłam rano po pieczywo do sklepiku osiedlowego, czułam się z tym źle. To już jest trochę obcowanie. Założyłam rękawiczki, weszłam do sklepu po chleb, jakiś facet wchodzi i na mnie napiera. Nakrzyczałam na niego, żeby zachował dystans. Później pani przy kasie chce wszystko w ręce brać. Denerwujące, jedni się stosują, inni nadal nie.

**Co myślisz o tych wszystkich ograniczeniach, przepisach?**

Jeśli chodzi o naukowe podejście to nie mam pojęcia, ale skoro narzucili, to chyba jakiś sens to ma. Te rękawiczki chyba najbardziej, dystans też na plus, dezynfekowanie, spryskiwanie. Zastanawiałam się nad obowiązkiem noszenia maseczek. Na pewno jakieś podstawy i sens to ma, żebyśmy wszyscy solidarnie tego przestrzegali. Jak wyszłam do sklepu założyłam maseczkę i rękawiczki, ale nie jest to wszechobecne wiec głupio się czułam z ta maseczką.

**Znasz kogoś, kto nie przestrzega tych zakazów?**

Tak, ta znajoma, o której wspominałam w zeszłym tygodniu. W niedzielę przyjechał do niej kolega i się bawili. Na święta jej chłopak się zastanawia, czy nie jechać do byłej partnerki i dzieci, ona też ma dziecko z byłym partnerem gdzieś na wsi i dziadek tego chłopca ma raka. Dziecko jest tam od początku kwarantanny. Ona nie jeździ, bo to ryzyko, ale też się zastanawia czy nie jechać. To nieodpowiedzialne. Ona twierdzi, że nie stosuje środków zaradczych i nic jej nie jest. Jak mieli zamykać salony kosmetyczne, to jeszcze szybko pobiegła paznokcie sobie zrobić.

**Jak myślisz, dlaczego ona zachowuje się w ten sposób?**

Nie wiem, może brak wiedzy? Może nie ma autorytetu, który mógłby ją przekonać? Upominaliśmy ją, że może zaszkodzić innym. My chyba nie jesteśmy dla niej takim autorytetem.

**Kto jest autorytetem dla ciebie?**

Ja liczę się ze zdaniem męża, to taki mądry facet i on ma wpływ na mnie.

**Czy słyszałaś jeszcze o jakichś dziwnych zachowaniach związanych z koronawirusem?**

Teść opowiadał, że był w sklepie i ktoś kaszlał ostentacyjnie. Jak zwrócili mu uwagę, to jeszcze złośliwie kaszlał zanim wyszedł.

**Czy decyzja o zamknięciu salonów kosmetycznych jest twoim zdaniem słuszna?**

Tak, tam nie da się odległości zachować, tam robi się wszystko przy skórze człowieka, przy twarzy. Dostałam telefon od fryzjerki bo chyba akurat dzisiaj miałam iść na wizytę. Powiedziała, że gdybym była w potrzebie, to ona zrobi mieszankę, a ja ją odbiorę w zakładzie i sama sobie nałożę. Super opcja, jak będzie się pogarszał mój stan psychiczny, to jest to dobra opcja, a oni coś zarobią zawsze. No i utrzymują klientów, a niektóre panie są bardzo przewrażliwione na punkcie swojego wyglądu. Jeszcze poczekam i sobie poprawię humor farbowaniem. Za tydzień mieliśmy mieć chrzciny i dlatego zapisałam się na tą farbę. To było tydzień temu, zadzwonił do nas ten brat cioteczny, ojciec chłopca, i powiedział, że bali się jak to będzie i wzięli po prostu pierwsze lepsze dwie osoby z rodziny i poszli do kościoła szybko ochrzcić dziecko poza mszą. Wiadomo, że nikt nie będzie miał żadnych pretensji o to. Córka nie jest w wieku komunijnym, ale mamy mieć wesele jakieś 23 maja i zastanawiamy się, jak to będzie. Wesele to większe przedsięwzięcie. Jeszcze nie jest odwołane, ale do 23 kwietnia trzeba potwierdzić obecność. Nie wiem czy pojedziemy, jeszcze zrobimy wywiad w rodzinie. Może premier ogłosi co dalej. Słyszałam, że niektórzy ludzie odwołują wesela i przekładają na później.

**Gdyby chrzciny odbyły się tydzień po świętach to byście pojechali?**

Gdyby nie było tego chrztu tylko sama impreza to nie wiem, trudno powiedzieć. Ale ta rodzina jest racjonalnie podchodząca więc nie naraziliby nikogo.

**Czy widzisz jakieś sytuacje, które usprawiedliwiają spotkanie się?**

To nie jest sytuacja do usprawiedliwienia. Nawet te święta, my zawsze spotykamy się w większych gronach, ale każdy jednak został w domu. My zawsze jeździliśmy do babci, tam jest druga córka mojej babci i ona ma cztery córki i wszyscy też tam zjeżdżali. Ale teraz wszyscy zostają w domu. Wujek gotuje barszcz, pieką mięsa, każdy ma przyjechać poodbierać, ale wszyscy zostają u siebie. To usprawiedliwione, nikt się nie spotyka.

**Czy masz jakieś sposoby na poprawę humoru w tej sytuacji?**

Jak mam sprzeczkę z mężem albo coś mnie zdenerwuje czy ogólnie mam dosyć wszystkiego, to najlepiej jest poćwiczyć godzinę i wszystkie złe myśli odpływają. Obrażenie znika. Albo zrobię sobie kawy i posiedzę na słońcu na balkonie, resetujesz sobie głowę. Ćwiczenia stosowałam też wcześniej, później jeździłam też dużo na rowerze, jak wiosna się zaczynała, to tak na plus dla mnie było. W ogóle lubię powietrze i przyrodę. Teraz to, że nie chodzę i nie spacerujemy, to działa na minus, muszę coś innego robić. Moja córka robi jakieś ćwiczenia pływackie, jakąś metodę Jacobsona, 15 minut odprężenia ciała. Ona mówi, że tak się świetnie czuje, więc ostatnio dwa dni zrobiłyśmy i męża zawołaliśmy. On wiadomo, że nie jest sportowiec, bo jest taki grubszy. Leżenie i napinanie palców u stóp to taki wysiłek fizyczny dla każdego. I kolejne etapy. Sama ćwiczę mięśnie brzucha, pośladki, uda, ogólnie ciało. Z hantlami, skaczę na małej trampolinie. Ćwiczę to sama. Dzieciom ogródek pozwala się wybiegać, szczególnie młodszej córce. Ona robi się wieczorami taka rozbrykana. Oglądamy sobie "M jak miłość", ona zaczyna skakać po tej córce, ona chyba tak to rozładowuje. To chyba przez kwarantannę. Ale to mnie bardziej śmieszy niż niepokoi.

**Zakupy robisz przez internet czy w sklepach?**

Spożywcze to mąż, a takie dla przyjemności to przez internet - książka, kosmetyk. Wcześniej robiłam zakupy internetowe tylko czasem, ale były nieudane. Czasami się powstrzymałam, bo pewnie będę znowu zwracać. Nie zawsze jest czas na sklepy i wtedy czasami trzeba było zamówić przez internet. Teraz robię to dlatego, że nie można stacjonarnie pójść sobie pochodzić. W tym tygodniu zamawiałam książki i świece z wosku pszczelego. Pisanki będziemy robić i chcemy je ozdobić woskiem. Książki kupowałam przez internet też wcześniej, tak taniej było. Teraz nie zamawiałam w Empiku, patrzę na to, żeby było taniej i odbiór w paczkomacie. Paczkomaty są dobre, bo mogę się przejechać i nie ma kontaktu z kurierem. Ale ostatnio jak siostra zamówiła, to kurier podjechał pod ogrodzenie i przerzucił przez płot na prośbę szwagra. Wosk kupiłabym raczej stacjonarnie gdyby nie ta sytuacja, przez internet kupiłabym go tylko gdyby nie było takiej możliwości stacjonarnie.

**Czy masz wrażenie, że przyzwyczajenia zakupowe, które pojawiają się teraz, zachowasz na czasy po epidemii?**

No takie pewniaki pewnie tak, jak książki czy kosmetyki takie. Można poczytać opinie albo ponowić zakup. Ale ubrania, buty czy rzeczy dla dzieci wolę stacjonarnie, bo można przymierzyć, nie bawię się w zwroty przez internet. Kosmetyki do włosów zamawiałam przez internet, ale inne, na przykład do twarzy, wolałam stacjonarnie żeby je obejrzeć.

**Czyli są produkty, które wolisz dotknąć osobiście, ale są też takie, które wolisz zamawiać?**

Tak, bo wiem, że nie będę ich zwracać.

**A ubrania, które zamówiłaś ostatnio przez internet?**

Już przyszły i są ok. Spodnie trochę na wyrost, ale mogą być. Kurteczka taka na teraz. Jeszcze zobaczę, czy będę ją zwracać, teraz powydłużali zwroty do 90 dni. To też mnie zachęciło do zakupów przez internet. Łatwiej jest zwrócić. Sam proces zwrotu chyba przebiega tak jak kiedyś. Oddaję w jakimś punkcie albo zamawiam kuriera po odbiór towaru. Nie wiem czy można przez paczkomat, to zależy od sklepu. Czasami można.

**Jeśli chodzi o lekarstwa, robiliście jakieś zakupy w aptece?**

Jakiś czas temu jakieś tabletki na gardło, ale to przez internet do apteki i tam odbiór. Mają tak duże marże stacjonarnie, że to aż nieuczciwe. Jak fizycznie poproszę o ten sam lek w aptece, to jest on droższy niż odbiór leku zamówionego przez internet. To jest chyba najdroższa apteka u nas, zawsze była z tego znana. Wcześniej też kupowałam tam po wcześniejszym zamówieniu przez internet.

**Widziałaś gdzieś jeszcze wzrost cen?**

Nie, raczej nie. To chyba tu najbardziej.

**Zamawiacie do domu jedzenie z dowozem?**

Przed kwarantanną czasami pizzę. Fast-foody kupowaliśmy na miejscu. Teraz nie zamawiamy jedzenia z dowozem, nie chcemy mieć tego kontaktu i sami wymyślamy dania, dużo gotujemy. Nie chcemy jeść ciągle tego samego, żeby się nie nudziło.

**Czy jecie teraz więcej?**

Tak, jak lodówka jest po drodze to często się do niej zagląda. Co chwilę się podchodzi. W pracy tego nie ma. Gotujemy razem i razem jemy posiłki, ale tak było u nas zawsze. Teść zawsze mówił, że w domu musi być stół i rodzina musi raz w tygodniu usiąść razem. I już się tak przyzwyczailiśmy. Nawyk. Teraz na pewno częściej jemy razem posiłki bo jesteśmy razem non-stop. Nawet córki sobie wymyśliły z nudów, że zrobią ciastka. Z nudów każdy lubi coś sobie zrobić i zrobi dla wszystkich. Albo też pieczywa nie kupujemy codziennie, robi się większe zakupy, ale zjadamy je. Jak jakaś bułka sczerstwieje to wkładamy do piekarnika. Staramy się wykorzystać wszystko, żeby nie wywalać. Mała też się garnie, żeby coś samemu robić.

**Czy są jakieś potrawy, których jecie więcej lub mniej?**

Może makarony bo mamy ich bardzo dużo. Jest czas na zrobienie rzeczy czasochłonnych, na przykład te pierogi. Wiadomo, że kłótnia zejdzie, ktoś wałkuje. Ostatnio dużo tych pierogów jemy. Kiedyś nie było na to czasu, to musiał być specjalny dzień żeby wszyscy się zaangażowali.

**Twoje jedzenie jest teraz zdrowsze lub mniej zdrowe, niż wcześniej?**

Nie wiem, nie jest niezdrowe na pewno. Jest sycące bo konkretne. Myślę, że takie bardzo domowe, tradycyjne różne rzeczy. Wcześniej po powrocie z pracy robiliśmy jedzenie raczej na szybko. Nie zawsze to było super jakieś, w weekendy fajniejsze obiady dwudaniowe. Teraz mamy bardziej różnorodnie, zawsze w lodówce jest coś do podgrzania. Teraz jest taki większy wybór i tak jakoś więcej rzeczy obiadowych mamy. Nie planujemy posiłków z wyprzedzeniem, ale czasami o tym rozmawiamy.

**Opowiedz mi o swoich zakupach robionych stacjonarnie, a także o zakupach męża.**

Zakupy mąż robi raz w tygodniu. Robimy listę, wymyślamy sobie co kupić. Teraz będziemy świętować święta i już w sumie wszystko mamy. Przed epidemią też robiliśmy listę na zakupy. Wcześniej częściej chodziliśmy na zakupy, wtedy łatwiej się na coś skusić, kupić więcej. Teraz nie jemy słodyczy, a wcześniej zawsze ktoś na coś namówił. Teraz bardziej w kierunku zdrowego. Teraz siedzimy w domu i mniej się ruszamy, więc nie ma sensu. Wcześniej starsza miała basen, szczupła jest to niech tam sobie coś słodkiego zje. Młodsza chodziła na gimnastykę artystyczną i basen więc też. Teraz nie ma potrzeby tego jeść bo jesteśmy cały czas na miejscu.

**Jesz coś czasem dla przyjemności?**

Lubię wafelki w czekoladzie, czasami chińszczyznę zamawialiśmy. Teraz nie zamawiamy takich rzeczy. Jak byłam po pieczywo to kupiłam po jednym wafelku dla każdego, ale na tym koniec.

**Planujecie zakupy bardziej, niż wcześniej? Jak one wyglądają?**

Nie, wszystko mamy więc większej ilości rzeczy nie trzeba kupować. Trochę uzupełniamy, jak coś się kończy. Mąż najczęściej jeździ do Lidla, ale jak wprowadzili ostatnio obostrzenia to nie wiedział czy jechać wieczorem, czy na przykład z samego rana następnego dnia. Zdecydował, że pojedzie wieczorem i to był zły pomysł. Towar wystawiają rano i nie było w czym wybierać. Warzywa zaczęły się prędko marszczyć, może były trzymane w chłodni. Zastanawiał się, kiedy pojechać, żeby tłumu nie było, żeby nie stać przed wejściem. Pojechał koło 23, a Lidl jest do 24. Był piąty w kolejce. Wszedł po 23, zrobił szybko zakupy. Mówił, że ludzie najpierw rzucili się do pieczywa. Pytał obsługę,do której można wejść i powiedzieli, że do północy, ale to dla nich nie fajne, bo pracownicy muszą siedzieć do ostatniego klienta. Teraz wpuszczają 3 osoby na jedną kasę. Jak jedna wyjdzie, to jedna wchodzi. Nie można pójść jako mąż z żoną, bo zwracają uwagę i nie wpuszczą inaczej niż osobno. Chyba chcieli ograniczyć to, żeby rodzinami tak nie jeździć. Teraz zakupy zajmują mu więcej czasu.

**Powiedz mi teraz o swojej wizycie w sklepie.**

Byłam wczoraj rano. Miałam przygotowaną kartę, żeby zapłacić bezdotykowo, rękawiczki, maseczka, torba. Kupiłam słodycze, pieczywo, starałam się, żeby też pani przy kasie tego nie dotykała. Starałam się uważać, ale ten facet na mnie wszedł. Nie czuję się bezpiecznie na zakupach, w jakąś paranoję wpadam, lęk się robi na tych zakupach. Chcesz uciekać od ludzi jak przed trędowatym. Jak widzisz ludzi stosujących się do zasad to się nie boisz, co innego jak ktoś nic sobie z tego nie robi. Sprzedawczyni miała rękawiczki, mają też ekrany z pleksi, że nie mają kontaktu z oddechem. W tym sklepie limitu wejść nikt nie pilnował. Była wywieszka w drzwiach, ale w środku jeden pracownik. Ktoś czekał przed drzwiami, ale w sklepie były trzy osoby.

**Czy są sytuacje, kiedy przezwyciężasz ten lęk?**

Tak, jak to pieczywo albo leki gdy coś się dzieje. Wtedy to jest bardzo ważna sprawa i można przezwyciężyć lęk.

**Zazwyczaj płacisz kartą czy gotówką za zakupy?**

Kartą, gotówki praktycznie nie noszę ze sobą. To bezpieczniejsze bo bezdotykowe, i tak robiłam to wcześniej dla wygody.

**Jak planujecie wielkanoc?**

Jutro jest wielki czwartek więc obejrzymy jakieś nabożeństwo w TV, w wielki piątek pewnie też, w sobotę też, a w niedzielę wiadomo - jakieś śniadanie. W sobotę chcemy poświęcić pokarm, zrobi to głowa rodziny. Mamy już kupione rzeczy. Pewnie upieczemy mięso, ciasto. Umówiliśmy się, żeby nie chodzić po domach, może wyjdziemy na kawę na powietrze. Nikt do nas nie przychodzi, to ustalone już w całej rodzinie. Nie idziemy do kościoła, tylko dom i telewizja. Tam to chyba zagrożenie większe. Słyszałam, że do 50 osób może być w kościele, ale nie potwierdzałam tego. Tam i tak zagrożenie jest chyba największe. Nie wiadomo jak kto usiądzie. Ludzie siedzą tam długo, godzinę. Nie wyobrażam sobie, żeby iść.

**Czy jest ci przykro z tego powodu?**

Na pewno inaczej, jak się pójdzie to odczuwa się klimat tych świąt, refleksje. W domu są rozpraszacze. Łatwiej jest o skupienie w kościele. Poświęcić pokarm można w domu, są takie przewodniki. Oglądaliśmy wypowiedź księdza o Oazie Krakowskiej, która ma przewodnik w internecie, jak spędzić w domu czas na wzór nabożeństw. Poczytam sobie, zobaczymy.

**Przygotowujecie się już do wielkanocy?**

Jakieś ogólne sprzątanie zrobione, zakupy zrobione. Jakieś gotowanie to pewnie w piątek. Teraz nic nas nie goni, bo się nie wyjeżdża. Można spokojnie przygotowywać. Gotujemy wszystko tak, jak tradycyjnie.

**Ostatnie zakupy męża uwzględniały jakieś wielkanocne produkty?**

Tak, te które można dłużej trzymać. Te, które trzeba na świeżo, to dzisiaj rano pojechał mięso dokupić. Na święta kupił jeszcze składniki do ciasta, coś do picia, jakieś bakalie, teraz to mięso, jakieś warzywa, tam jajka, kiełbasę białą, żur, takie rzeczy na tej zasadzie. Kupiliśmy kaczkę bo wszyscy lubimy, dla przyjemności. Będą jabłka pieczone. Dobre ciasto będziemy robić. Kupił dzieciom jakieś sikawki na lany poniedziałek. Kupił pistoleciki, ale są takie, że jeden już pękł. Lepiej leje się butelką z dziurką. Dekoracje wyciągnęłam te, co miałam. Planowałam kupić jakąś figurkę zająca czy kurczaka, ale nie ma już czasu i przez internet nie wiadomo, jak to dokładnie wygląda. Takie jakieś akcenty. Dekoracje wystawiłam tydzień temu, półtora może.

**Jak w ogóle czujesz się z tymi świętami?**

Myślę, że bardzo domowe będą. Może nie takie uroczyste. Jak się gdzieś jedzie, to każdy się wystroi. Teraz będzie atmosfera raczej zbliżona do tego, co mamy zazwyczaj. Może postaramy się ubrać jakoś ładniej, nie w dresach.

**Co jeszcze zrobicie, żeby święta były świętami?**

Dobre jedzonko. Zazwyczaj jemy w kuchni, po prostu przeniesiemy się do salonu i to będzie takie biesiadowanie w małym gronie. Tak żeby odczuć to. Może jakieś fajne filmy pooglądamy.

**Czy dla córek to ważne?**

Myślę, że tak. Najbardziej chyba lubią spotkania z rodziną. Teraz mówią, że będzie smutno, nudno. Mamy dużo dzieci w rodzinie, może lubią się spotkać.

**Zastanawia mnie, czy młodsza córka rozumie tę sytuację.**

No nie wiem, czy tak do końca rozumie. Dla nich to jednak wolne od szkoły, podwórko mają. Może w bloku byłoby inaczej. A tak to trochę wakacje, dobrze się bawi na powietrzu. Dzisiaj też już się wygłupia, jeździ na rowerku. Z tym kuzynem, synem mojej siostry, też się pointegrują.

**Nie masz obaw o zarażenie z tym kuzynem?**

Na początku pilnowałam, żeby zachowywali odstęp, ale to chyba można zwariować. On niby nigdzie nie chodzi, jego mama pracuje parę dni w tygodniu, ale zachowuje higienę. Moja mama zrezygnowała na miesiąc z pracy, więc nie ma kontaktu z obcymi. Też się dezynfekują. Jakoś trzeba żyć, nie można się zamknąć totalnie. Pilnuję, żeby myła ręce.

**Tydzień temu byłaś smutna, że nie ma klimatu wielkanocnego.**

Tak, ale teraz widzę, że wszyscy się przystosowali. U większości tak będzie więc nie jestem sama w tym wszystkim. Każdy to rozumie i wiele osób ma takie podejście.

**Twoi znajomi tak samo zostają w domu?**

Tak, wszyscy tak zostają, nie przemieszczają się.
