# Supplementary material for: The challenges arising from the COVID-19 pandemic and the way people deal with them. A qualitative longitudinal study
Source: PLoS One. 2021 Oct 11;16(10):e0258133. doi: 10.1371/journal.pone.0258133 (PMC8504766; doi:10.1371/journal.pone.0258133)
Supplement: S1 Dataset — (ZIP) [file pone.0258133.s003.zip › Transcriptions/stage 2/5.2_M_39_single.docx]

**5.2_M_39_single**

**Zdjęcia - emocje**

**11 i 2**

6 i 13 to takie, które pokazują to, co chciałbym, żeby do mnie wróciło, czyli natura. Do obecnej sytuacji pasują 11 i 2, bo człowiek jest taki uwięziony. Niby na dworze a jednak coś stopuje.

**11**

**Jakie to są emocje?**

Tak, jak mówiłem poprzednio. Człowiek widzi, że się już na dworze robi coraz fajniej, bo idzie wiosna, a jednocześnie nie może wyjść z domu.

**Jak odczuwasz tę emocję, co stan związany z tym uczuciem tobie robi?**

Dobrym słowem chyba to będzie samotność, że człowiek jest sam z tym wszystkim i większość chyba tak przechodzi.

**Ty się czujesz samotny i odizolowany w tej chwili?**

Trochę tak. I dlatego właśnie przyjąłem tę propozycję, żebyśmy się spotykali. To wszystko wpływa na ogólny stan psychiczny - to jest nerwowość z bezradnością. Nerwowość jest może też spowodowana bezsennością. Wcześniej byłem poddenerwowany, że może jestem chory, teraz jestem poddenerwowany, że jestem w zamknięciu. Chciałbym móc oddechu trochę złapać, życia jakiegoś.

**To podenerwowanie to jest irytacja, złość czy może coś innego?**

Nie, bezsilność jakaś taka. Chciałbym już tam być za tą szybą.

**2**

Człowiek jest jakiś przyblokowany. Niby może, ale nie może. Chciałbym wrócić do ludzi, do natury.

**Jakie to jest uczucie, jaka emocja. Tam było podenerwowanie, bezsilność, a tu?**

No, że człowiek już chciałby się oderwać od tej gumy i pójść do przodu, do swoich spraw, zajęć, przyjemności, które robi człowiek poza domem, do wyjścia na dwór, do spacerów.

To jest zatrzymanie, zblokowanie, stanie w miejscu.

**Są emocje, uczucia, które ci się nasilają, a może takie, które bledną? Coś się zmieniło w ciągu ostatniego tygodnia?**

Wróciłem do pracy w sobotę i człowieka trochę traktowali jak trędowatego, bo jako jedyny byłem na chorobowym w tym najgorszym okresie. Głupie teksty, docinki i irytacja była od razu z tym związana. Poza moimi plecami, jak mnie nie było, mnóstwo o mnie rozmawiali. Ktoś mi o tym powiedział, ale i tak dało się to wyczuć. No i oprócz tego, że nie mogłem spać, to pobudka o 4.55 do pracy, kontrola w pracy - temperatura, itd. i dopiero można wejść na zakład. Wiadomo, wszyscy gadają o jednym. Już wolałem być w domu, bo po pierwsze nie chce mi się o tym rozmawiać, bo już tego jest tak dużo...Już bardziej teraz wolę przeczytać pozytywne rzeczy niż słuchać tego pieprzenia w kółko o tym samym.

**Na czym polegało to traktowanie jak trędowatego?**

"No i co? Zdrowy?" Takie teksty w stylu "I co? Co ci było? A może ty jesteś chory? A czemu tak długo?" Kolega mi przekazał, że rozmowy były poza mną, że pewnie siedzi w domu i przeciąga, bo nie wie czy nie jest chory na koronawirusa. Przez chwilę człowiek jest tym zirytowany a potem już macha ręką. Zawsze jest tak, że wszędzie człowieka skomentują. W pracy nie ma kolegów. Z brygadą, z moimi ludźmi rozmawiałem raz przez telefon jak byłem na zwolnieniu. Miałem ochotę wrócić do pracy chociaż na dzień, dwa, żeby trochę się odciąć, ale jak już stamtąd wróciłem to już nie mam ochoty. Wolałbym w domu zostać.

**Spodziewałeś się czegoś innego po tym powrocie do pracy?**

Myślałem, że lżej to jakoś przyjmę. Człowiek się zaraz irytuje, jeżeli słyszy te komentarze. Myślałem, że mniej to mnie będzie irytować, że w mniejszym stopniu będą takie rozmowy.

**Próbuję zrozumieć, co cię tak irytowało?**

Jakaś taka ludzka nieufność i takie...Ja na to mówię, że niektórzy lubią po prostu innym psuć krew. Aż się krew buzuje...Podkurzyć kogoś takimi głupimi historiami.

**Miałeś wrażenie, że ludzie się ciebie boją?**

Nie...Wiadomo nikt się nie wita po staremu żółwikiem, jest mnóstwo płyny dezynfekującego u nas w pracy, bo my go produkujemy. To jest jeden z plusów, że mam taki płyn.

**A czy sama zmiana funkcjonowania dała ci coś pozytywnego?**

Myślałem, że tak będzie, ale albo to były uczucia neutralne, albo irytacja przez te docinki i gadki. Wróciłem do domu wczoraj, bo miałem dwa dni pracy, dzisiaj mam wolne a jutro idę na nocną zmianę...Wczoraj się wkurzyłem, bo nadal mnie to gardło boli, z rana muszę kasłać...Wczoraj, kiedy byłem u rodziców na obiedzie po pracy, kolega do mnie napisał czy jakiegoś drinka online nie wypijemy? Powiedziałem, że nie wiem, bo mnie to gardło boli, ale taki byłem poirytowany tym wszystkim, zmęczony tą pracą, że poszedłem do Biedronki, kupiłem sobie 3 wina, bo nie będę chodził kilka razy po jedną butelkę. Niech będzie już w domu sobie stało. I wczoraj na face time sobie siedzieliśmy, i wypiłem sam 1.5 butelki wina. I to mi było tak potrzebne...Po prostu ze mnie to wszystko zeszło i usnąłem po raz pierwszy od 3 tygodni takim ciężkim snem.

**Miałeś poczucie ulgi?**

Tak, bardzo. Ja nie mam często takich ciągotek i nie zapijam wszystkich smutków, ale tutaj potrzebowałem wyluzowania się po prostu. Wysączyłem te 1.5 butelki wina i jak zgasiłem światło, to było mi już tak wszytko jedno...Chcę usnąć i padłem od razu. Spałem z 7 godzin. Na pewno więcej niż ostatnio.

**To była pierwsza impreza online, w której uczestniczyłeś?**

Tak.

**Poszedłeś do rodziców?**

Tak, przez płot...Mama wkurzyła się na mnie, że nie chcę wejść. Zdenerwowana była, że nie chcę wejść. Powiedziałem, że się cały czas dziwnie czuję, że jak oddycham to mi jest różnie, że czasem muszę kasłać, a mama mówi, że jestem przewrażliwiony i żebym wchodził. No i wszedłem nie witając się z nimi za specjalnie, zjadłem i wyszedłem.

**Masz niepokój, że możesz im coś "sprzedać"?**

Tak. Jakbym był zdrowy...Nie chcę się z nimi widzieć na razie. Wiem, że są w grupie ryzyka.

**Pojawiły się jakieś nowe rzeczy, które ci przeszkadzają?**

Blokowanie życia codziennego. W tym momencie to chyba jest jakieś fatum. Zaczął mi kibel przeciekać. Nie mogę iść do OBI, bo nie chcę tam wchodzić. Odkurzacz mi się przyblokował, więc do wymiany jest worek, którego nie mam. To tak samo wiąże się z pójściem do jakiegoś sklepu. Na razie to przeciągam, żeby jak najrzadziej wchodzić do tych sklepów. Nie wiem, co jeszcze mi się popsuje w domu. Taka irytacja. Wczoraj tylko na szybko do Biedronki wszedłem, kupiłem 3 wina i wyszedłem.

**Na ile się czujesz teraz zagrożony sytuacją?**

Teraz ze 20, trochę jakby zeszło...Nie wiem...Po jedzeniu mi się zrobiło gorąco, to się człowiek zdenerwował trochę, że może znowu temperatura. Aż zmierzyłem temperaturę. Bardziej się nerwowy zrobiłem, ale coraz bardziej obojętnieje mi to wszystko. Zobojętniało mi trochę. Obawa o moje zdrowie jest na podobnym poziomie, co tydzień temu, natomiast sam koronawirus w kraju i na świecie mi spowszedniał. Teraz już mam to w dupie i zaczynam myśleć, że to jest za mocno wyolbrzymione. Już przechodzę w stan krytyki państw, że dały się wciągnąć w taki medialny kocioł, który został nakręcony, napędzony przez wszystkich. Coraz bardziej się skłaniam ku teorii chińskiej i jak oni na tym wszystkim wyjdą. Tyle profitów będą mieli z tej epidemii, że to będzie woda na młyn dla ich gospodarki. Byli potęgą a teraz to już w ogóle wszystkich wyprzedzą.

**Pojawiły się jakieś nowe zagrożenia?**

Obawiam się upadku gospodarki. Nasz kraj nie chce wprowadzić stanu wyjątkowego po pierwsze z powodu wyborów, a poza tym, kurwa, nie mają na to pieniędzy. Są spłukani, więc nie mogą i nie chcą wprowadzać i dlatego wprowadzają takie a'la...To jest wszystko niezgodne z konstytucją. Teraz jakby mnie ktoś zatrzymał i jakiś mandat chciał mi dać, to ja żadnego mandatu nigdy nie przyjmę.

**Pojawiły się nowe ograniczenia. Jak to odbierasz?**

Władza sobie robi co chce. Nie ma pieniędzy, to jeszcze będzie chciała przytrzymać ludzi karami finansowymi, co jest niezgodne z konstytucją.

**A co myślisz o sensowności ograniczeń, które wprowadzono?**

Niektórzy ludzie są naprawdę tak tępi, że niektórych może tylko kara finansowa powstrzymać, jeśli już mamy być w kwarantannie.

**A te nowe ograniczenia - limit osób w sklepie, zakaz wstępu do lasu?**

W piątek wprowadzili a ja w piątek pojechałem do lasu. Stwierdziłem, że w dupie to mam. Jutro zaczynam pracę, siedziałem tyle czasu w domu, ja muszę oddech złapać. To nie jest tak, że ja nie muszę przestrzegać ograniczeń. Ja chcę się przyłożyć też do tego, żeby zatrzymać koronawirusa. Nie postrzegam tego tak, że ktoś mnie w czymś ogranicza, ale jak oni coś ogłaszają, to ja mam to w dupie.

**Których ograniczeń przestrzegasz?**

Ja w ogóle nie nazwałbym tego przestrzeganiem.

**A jak?**

Te, które są dla mnie sensowne, to sam sobie je wprowadziłem a nie to, że państwo mi e jakiś sposób coś narzuca. To jest zdroworozsądkowość. Nie zbliżam się do ludzi, żeby ktoś czuł się bezpiecznie, żebym ja czuł się bezpiecznie. Wchodzę do sklepu i zakładam komin, już teraz mam rękawiczki więc wchodzę w rękawiczkach, mam przy sobie żel, omijam ludzi, oni mnie. Do lasu pojechałem - byli tam starsi ludzie, byli też młodzi, którzy biegają. Dla mnie to jest jeden z najgłupszych zakazów. Jeśli nie pozwalają chodzić po mieście...A w lesie są ci sami ludzie, którzy są ze sobą w domu. To jest w ogóle jakaś głupota. To jest jedyne miejsce, gdzie pomaga nam to nie wpaść w nerwicę i w depresję, bo z tego co wiem, po pierwsze, 20 min na łonie natury zmniejsza poziom stresu. Świeże powietrze to jest dwa. Samo obcowanie z naturą jest czymś pozytywnym. Czemu ludziom to odbierać? To jest wpływanie na ich jeszcze gorszy stan psychiczny. Rozumiem po mieście, po sklepach, nie gromadzą się, nie ma imprez. Kto jest w tym lesie? To są pary, to jest ojciec z żoną i dwójką dzieci, to jest człowiek, który uprawia bieganie albo chce trzymać kondycję. Każdy jest w tym samym gronie, w którym jest w domu, nie rozmawia, mija...Spotkałem znajomych w odległości 3 m sobie pogadaliście.

Co dało ci to wyjście do lasu. Mówiliśmy o ty, co dało ci poprzednio, a co teraz?

Taki głęboki oddech. Ja to odczuwam, że ze mnie schodzi to. Tak samo, jak się wczoraj lekko upiłem. To jest takie samo uczucie. I jak człowiek przed snem zrobi sobie taki głęboki wydech. To jest to samo.

**Na jak długo to starcza?**

Nie wiem. Na drugi dzień poszedłem do pracy i od razu mnie wkurwili. [śmiech] Nie mogę się wypowiedzieć. Musiałbym pójść i mieć potem tydzień wolnego.

**Co myślisz o łamaniu zasady spotykania się?**

Ludzie nie wytrzymują, pękają. Niektórzy nie do końca wierzą w to, uważają, że to jest nakręcone i chcą się wyluzować.

**A ty?**

Ja to rozumiem, ale nie chciałbym w tej chwili nikogo narażać ani też na drugi dzień myśleć, czy ten ktoś był zdrowy czy chory. Nie popieram tego w tej chwili. Jak ktoś robi coś takiego nagminnie, to jest totalnym idiotą.

**Gdyby twoi sąsiedzi urządzili imprezę, to co byś zrobił?**

Nic. W życiu nie donosiłbym służbom. Pomyślałbym sobie, że to jest ich wybór. Znam takich, którzy w ogóle siedzą w domu 2-3 tyg. i nigdzie nie wychodzą. Narobili tyle zapasów, że w ogóle nigdzie nie wychodzą. W jakiś sposób to też ma sens. To też jest nakręcane, że ludzie zdrowi też mogą przenosić chorobę...Każdy ma swój wybór, ja bym się nie zdecydował. Ale też nie będę w tym taki święty, bo jakbym chciał, żeby przyszła do mnie koleżanka, to...No chciałbym, bo mi sprawi to przyjemność i jej też.

**Nie było jej od naszej ostatniej rozmowy?**

Nie. Nie dlatego, że są obostrzenia. Ona pracuje od pon. do piątku, a ja od soboty zacząłem. Jakieś zakusy do tego spotkania zrobiłem, no ale nie przyszła. To nie chodzi o obawy przed koronawirusem tylko bardziej o nasze relacje w tym momencie.

**A zauważyłeś u siebie jakieś nowe zachowania, nowe środki ostrożności? A może z jakichś zachowań zrezygnowałeś?**

Nie, wszystko jest tak samo, chociaż dołożyłem płyn, który mam z pracy i rękawiczki. To minimum spełniam. Człowiek, jakby się nie zabezpieczał, to nie wie, czy na makaronie, kurwa, czy na jakiejś reklamówce nie wniesie czegoś. Jak coś ma się wydarzyć to się wydarzy. Jedynie co można, to jakieś środki zapobiegawcze, które już mam.

**Biorąc pod uwagę wszystkie ograniczenia, które teraz mamy, co jest dla ciebie największym wyzwaniem?**

Właściwie wszystkie. Moje granie i występy to akurat dobrze, że jest pauza, bo mogę odpocząć. Minus, że nie zarabiam dodatkowo. Chciałem odpocząć, ale też tęsknię za tym graniem. Ładna pogoda, jakieś urlopy poplanowane, wiosna...Chętnie bym gdzieś pojechał. Podróży mi brakuje i natury. Potrzebę natury chyba jeszcze bardziej wszyscy odczujemy w przyszłym tygodniu.

Jak radzą sobie osoby z twojego otoczenia?

Myślę, że podobnie ma każdy.

**Zauważyłeś u ludzi jakieś nowe zachowania, działania?**

Tak, każdy sobie na swój sposób radzi z zabiciem czasu. U niektórych jest to nazywane nudą. Niektórzy się bardziej wzięli za te rzeczy, które odkładali, niektórzy zaczęli nowe rzeczy robić.

**Co na przykład?**

Joga zaczęła się robić modna, jakieś ćwiczenia rozciągające.

**A ty zacząłeś robić coś nowego?**

Ja nawet na jednych zajęciach jogi online byłem. [śmiech] I myślę, że będę wracał do tego, żeby się może lepiej poczuć.

**Co ci dały te zajęcia?**

Było wydarzenie - ktoś udostępnił na FB, że będą zajęcia przez 2 tyg. codziennie i ktoś tam uczy.

**Z jakiego powodu wziąłeś w tym udział?**

Myślę, że joga jest wielopoziomową sprawą. Ja po prostu muszę się bardziej zmotywować, żeby to pociągnąć, ale z tego co ludzie mówią, po samych zajęciach i z tego co czytałem, to człowiek po niej ma lepszą kondycję dla ciała, ale i dla ducha, bo uspokaja bardzo joga. Chciałbym mieć lepszą kondycję fizyczną i psychiczną. Nadal układam puzzle i robię muzykę. Będę się też przygotowywać do grania na żywo - taki stream. Kupiłem sobie kamerę, więc może w czwartek albo w piątek. Raczej to samo robię, bo filmy, puzzle i tyle.

**Czy zauważyłeś jakieś nietypowe, dziwne zachowania innych ludzi?**

Mnóstwo ludzi sobie jaja robi, wiadomo, bo tak im się nudzi w domu. Matki z córkami robią sobie W-F domowy, mnóstwo ludzi gra w gry, nakupowali jakieś gry komputerowe, grają w planszowe gry. Takie szukanie zajęcia też jest kreatywne. Nawet to, co ty robisz.

**Tę kamerę kupiłeś przez internet, jak pamiętam. Czy sytuacja, którą mamy jakoś wpłynęła na twoje podejście do zakupów internet vs stacjonarne?**

Przed zakupem kamery zapytałem się na FB ludzi co myślą o zakupie internetowym - czy wybrać opcję kurier, czy paczkomat. I tu, i tu kurier przynosi, ale jednak w paczkomacie poleży sobie trochę. I wybrałem paczkomat. Przywiozłem tę paczkę, obsiukałem całą...

**A jak przyniosłeś wino z Biedronki to też całe obsiukałeś?**

Kurde, nie! Nie mam pojęcia, dlaczego tak z tą paczką...Jakoś zrobiłem to z automatu po tym, co mi na FB radzili i ktoś napisał, że nie ma problemu, bo bierzesz paczkę, obsiukasz w domu i już?

**A przed koronawirusem jak było z kupowaniem przez internet?**

Paczkomat, żeby odbierać wtedy, kiedy ja chcę. Kupuję płyty winylowe i średnio raz w miesiącu przychodzi ta paczka. Ilościowo zamówień robię tak samo jak kiedyś. Sporo ludzi teraz więcej kupuje, bo siedzą w domu i chcą coś robić. U mnie ilość zakupów się nie zmieniła i myślę, że to jest też zły czas na wydawanie pieniędzy na jakieś bzdury. Zawsze czasami człowiek sobie zrobi przyjemność kupieniem jakiejś bzdury, ale nie. Nie, tym bardziej, że nie mam dodatkowych funduszy z grania, więc ten budżet jest trochę mniejszy. Ale też mniej się wydaje, bo siedzi się w domu.

**Jak kupujesz jedzenie?**

Szybkie zakupy w Biedronce. Nigdzie więcej nie chodzę w tym momencie.

**A zakupy spożywcze przez internet/ telefon?**

Nie. Wolę sam na coś spojrzeć i wiedzieć czy będę to chciał. Czasami człowiek idzie do sklepu po coś, ale zmieni po drodze zdanie. Nie chcę, żeby były dodatkowe zakupy, bo potem się wyrzuci to jedzenie.

**Czy zaobserwowałeś, że coś się zmieniło w twoich zwyczajach, preferencjach żywieniowych?**

Na pewno w mojej diecie na porządku dziennym jest cebula, czosnek, imbir, papryka, cytryna, miód, sok malinowy. To są te rzeczy, które królują teraz.

**Od kiedy?**

Od początku. Jak tylko się w Polsce pojawiło? Spodziewam się, że po tych rzeczach odporność będzie trochę większa. Ona nie jest od razu, więc buduje się ją.

**Coś jeszcze się zmieniło? Liczba posiłków, to na co masz ochotę, itd.?**

Chyba nie. Sporo jedzenia dostawałem też od rodziców, więc dosyć zróżnicowane było to jedzenie. Z rana często jadam jajka w pomidorach z czosnkiem, z cebulą, na ostro. Robię sobie szakszukę. Raczej staram się jeść się jeść na ostro. Obiad to wiadomo - jakieś mięso, warzywa, ziemniaki, a wieczorem to już jest bardzo różnie.

**Pojawia ci się czasem taka chęć zrobienia sobie przyjemności jedzeniem?**

Zrobiłem sobie chyba największą przyjemność - w sobotę po pracy pojechałem sobie do pizzerii po pizzę. Zamówiłem przez telefon, pojechałem, zapłaciłem szybko kartą i odebrałem.

**Dlaczego nie zamówiłeś do domu?**

Bo wracałem z pracy i było mi po drodze.

**Często zamawiasz jedzenie do domu?**

Jak siedzę w domu, to tak. W ogóle jadanie na mieście - pizza, burger to jest taki standard. Średnio raz na 2 tyg. pizzę zjem na mieście czy do domu. Ostatnio zamawiałem jedzenie od mojego przyjaciela, który ma klub, w którym grywam. Klub jest niestety zamknięty i nie zarabiam, ale jest tam też restauracja, która też nie zarabia - jedynie na dowozach i chcę go podratować, bo jest tam straszny stan. Domyślam się, że jest bardzo źle. Wziąłem się za tzw...Chcę mu pomóc i załatwiłem sprawę tak, że uszyjemy 100 sztuk maseczek materiałowych i cały zysk ze sprzedaży tych maseczek będzie dla niego. Dam mu w kopercie na przeżycie. Ze 3 razy zamówiłem tam jedzenie, żeby mu się dołożyć do życia. Po pierwsze to było ze względu na niego, ale po drugie mogłem zróżnicować to, co ja robię w domu, bo nie umiem robić leniwych...Chętnie wziąłem, jakaś zupa czy coś...

Wczoraj ten alkohol był też taką przyjemnością, którą sobie zrobiłem. Myślę, że każdy się teraz z drobnych rzeczy cieszy. Wszystkie przyjemności są teraz bardziej doceniane, nie? Ludzie teraz będą doceniali takie małe rzeczy.

**Zamawiasz teraz częściej jedzenie do domu?**

Nie.

**Takie zamawianie jedzenia to jest bezpieczne rozwiązanie?**

Akurat od przyjaciół zamawiam. Oni wiedzą, że muszą bardzo uważać. Robią to sami, pracownicy są na zwolnieniach. Jak człowiek ma się zarazić, to czy to będzie sklep, czy zamówienie jedzenia...2 razy mi przywieźli, jak byłem chory, na wycieraczkę, raz pojechałem sam, bo była ładna pogoda.

**A jak zamawiasz pizzę z domu, to raczej sam ją odbierasz czy ci ją przywożą?**

Raczej przywożą.

**I to też jest bezpieczne?**

No...Ostatnio potrzeba była bardzo silna. W ogóle im mniej kontaktu tym lepiej, ale człowiek nie może sobie wszystkiego odmówić, bo to już jest szaleństwo

**Sposób płacenia. Czy tu coś się zmieniło?**

Wolę płacić kartą. Przedtem wolałem płacić telefonem. Teraz kartą, bo do telefonu trzeba odcisk palca a w rękawiczce się nie da. Przesłanie pieniędzy telefonem się nie uda bez odcisku palca. Jak będę mógł, to wrócę do płacenia telefonem, bo tam nie trzeba wpisywać pinu. Jak przez pyszne.pl to od razu pobierają z konta. Tylko ta karta to jest zmiana, reszta tak samo.

**Byłeś w Biedronce po ostatnim zaostrzeniu przepisów?**

Tak, wieczorem ok 23, bo wtedy nie ma ludzi i było wszystko co chciałem kupić.

**Czy planujesz teraz zakupy bardziej niż przedtem?**

Tak, żeby się nie marnowało to jedzenie. Kupuję sporo tego jedzenia, żeby gotować w domu i muszę planować, żeby nie kupić za dużo i potem tego nie wyrzucać do śmieci. Robię sobie listę w notatniku w telefonie.

**Zauważyłeś jakieś zmiany przy okazji ostatnich zakupów?**

Są te kreski narysowane, żeby zachować odległość, ludzie są w maseczkach, w rękawiczkach, kasy są obstawione pleksi. Już mi to spowszedniało.

**A czujesz się bezpieczniej teraz?**

Myślę, że oni się czują bezpieczniej.

**Zbliża się Wielkanoc. jak zamierzasz spędzić święta?**

U rodziców, ale wtedy też pracuję, więc będą takie łączone. Nie robię żadnych przygotowań. Zwykle spędzam święta u rodziców, więc nigdy nie robiłem żadnych przygotowań.

**Święconka, pójście do kościoła. Czy to jest dla ciebie ważne?**

Już nie. Kiedyś bardziej, jak byłem młodszy. Ja nie pójdę. Nie wiem, jaką decyzję podejmą decyzję, jeśli o to chodzi. Zwykle ja z bratem chodziłem ze święconką. Jako najmłodsi w domu, wiadomo. Nie rozmawialiśmy, jak to będzie w tym roku.

**Jak było z niedzielą palmową?**

Ja nie byłem, tata też na pewno nie i myślę, że mama też nie. Myślę, że przeżyją to, że nie pójdą do kościoła w tym roku.

**Jak sądzisz, ludzie będą się przemieszczać w te święta?**

Obawiam się, że niestety tak.

**Masz jakieś jeszcze przemyślenia z ostatniego tygodnia, o których nie rozmawialiśmy?**

Chyba ta samotność jest taka coraz bardziej...Przybija.

**Obmyślasz jakieś rozwiązania, żeby ją minimalizować?**

Gdyby to życie prywatne się poprawiło, ale nie chcę, żeby tu cokolwiek poszło na siłę, bo jest to związane z emocjami, które są wymuszone przez otoczenie w tym momencie. Chcę, żeby decyzje były podejmowane w normalnych, naturalnych warunkach. To jest na zasadzie, jak bycie na bezludnej wyspie - przybija ktoś przypadkiem do brzegu i...Jest taki film, który doskonale opisywał te moje myśli - z Jennifer Lawrence. Film się nazywa Pasażerowie. On się wybudza za wcześnie, potem ją wybudza. Ona o tym nie wie na początku, ale może na co dzień np. w ogóle by nie zwróciła na niego uwagi. Są na siebie skazani, rodzi się jakieś uczucie...W tym momencie jest tak, że ludzie się nie mogą spotykać, randkować, poznawać z obcymi ludźmi. Jest jakaś osoba i specjalnie innych możliwości nie mamy. Uczucia wymuszone nie wiadomo, czy naprawdę takie są, jak nam się wydaje w nienormalnej sytuacji. Można coś nadinterpretować. ja znam siebie trochę i nie chcę w nic wejść głębiej, bo nie wiem, czy to jest dlatego, że ja czuję się samotny, czy ja rzeczywiście za kimś tęsknię.
