# Supplementary material for: The challenges arising from the COVID-19 pandemic and the way people deal with them. A qualitative longitudinal study
Source: PLoS One. 2021 Oct 11;16(10):e0258133. doi: 10.1371/journal.pone.0258133 (PMC8504766; doi:10.1371/journal.pone.0258133)
Supplement: S1 Dataset — (ZIP) [file pone.0258133.s003.zip › Transcriptions/stage 2/6.2_M_24_couple, no children.docx]

**6.2_M_24_couple_no children**

**Emocje - zdjęcia**

**13**

Dlatego, że świeci słońce, jest ładna pogoda.

**To sprawia, że czujesz się lepiej?**

Tak, trochę lepiej. Z drugiej strony też powoduje to, że jeszcze bardziej chciałoby się wyjść z domu, ale jakoś nastraja też optymistycznie

**W tamtym tygodniu wybrałeś 10. Nie masz już takiego poczucia...?**

Dalej jest, ale oprócz tego teraz wybrałem jeszcze 13.

**Czy w ogóle wychodzisz teraz z domu?**

Wczoraj byłem na trochę dalszej wyprawie do Rossmanna i tak się właśnie czułem, jakbym szedł na jakąś wyprawę, bo ten Rossmann jest mniej więcej 2 km ode mnie. Zajęło mi to jakąś godzinę, bo jeszcze tam trzeba było czekać pod tym Rossmannem w kolejce. I byłem też w sklepie, żeby zrobić jakieś zakupy na śniadanie wielkanocne. W zasadzie tyle i jeżeli wychodzę, to do sklepu.

**Czy w tym tygodniu coś się zmieniło w twoich zachowaniach?**

Nie, nic się nie zmieniło.

**Słyszałeś o nowych ograniczeniach, które weszły w życie?**

Tak, w przyszłym tygodniu każdy, kto będzie w przestrzeni publicznej ma albo nosić maseczkę, albo jakąś chustkę zakrywającą twarz. O tym słyszałem. Nie wiem, czy to jest dobry pomysł. Myślę, że jeżeli chodzi o osoby, które faktycznie pracują w sklepach, bankach i miejscach, które są otwarte, to jest to uzasadnione działanie, bo potencjalnie mogą się łatwiej zarazić niż zwykłe osoby i one powinny takie maseczki nosić. Jeśli chodzi o osoby zdrowe, to w zasadzie uważam, że noszenie maseczek może nawet sprzyjać zarażeniu się, bo wiadomo - jak mam maseczkę to pod nią mam środowisko sprzyjające rozwojowi bakterii i nawet może to sprzyjać zarażeniu się inną chorobą. Z tego, co słyszałem na kanałach informacyjnych, to lepiej by było nie nosić maseczek przez osoby zdrowe, ale tak naprawdę może być taka sytuacja, że osoba nie wie, że jest chora. Jeżeli to ma trochę ułatwić poruszanie się większej liczbie osób, to czemu nie?

**Będziesz się stosował do tych zasad?**

Tak, myślę, że tak. Oczywiście będę się starał nadal siedzieć jak najwięcej w domu, dopóki to wszystko nie przycichnie, ale jeżeli będę miał potrzebę wyjścia gdzieś na zakupy czy udać się na jakąś większą wyprawę do drogerii, to będę się stosował. Jak najbardziej.

**Widzisz/ znasz osoby, które tych zasad nie przestrzegają?**

W otoczeniu najbliższym nie, ale na FB, na różnych grupach...Ja akurat nie śledzę bezpośrednio tych grup, ale czasem ktoś mi coś podsyła. Ludzie w ogóle negują, że jest jakakolwiek pandemia. Wczoraj dostałem screen komentarza, gdzie właśnie pani się wypowiadała, że to jest zbędna panika, że nie ma żadnej pandemii.

**Dlaczego ci ludzie tak myślą, jak sądzisz?**

Nie mam pojęcia, szczerze mówiąc. Nie wiem, jakie dowody musieliby dostać, żeby zmienili swoje przekonanie, że faktycznie jednak coś się dzieje. Wydaje mi się, że to, że ludzie zwyczajnie chorują i to raczej nie jest wielka tajemnica, że ludzie leżą w szpitalach, szpitale są przepełnione...Nie wiem jakich jeszcze dowodów trzeba dostarczyć.

**Czy obserwujesz w swoim otoczeniu jakieś sposoby radzenia sobie z tą sytuacją, które są dziwne?**

Tak. W zasadzie kolejki przed sklepami. Stałem wczoraj 20-25 minut w kolejce przed drogerią i te kolejki to jest taki wężyk, osoby stoją w odległości mniej więcej 3 m. Przed Auchan, gdzie chodzę na zakupy też się takie kolejki robią, ale jest troszkę mniejszy odstęp. Kolejka zwalnia się w ten sposób, że jak jedna osoba wychodzi ze sklepu, to za nią wchodzi ta z kolejki. To uważam za jeden z takich sposobów na radzenie sobie z tym wszystkim i ograniczenie ilości osób w sklepie, zmniejszenie kontaktu.

**Na ile obecnie czujesz się zagrożony tą sytuacją?**

Myślę, że nic się nie zmieniło od zeszłego tygodnia. Niepokój na poziomie 50% dalej.

**Czy masz jakieś działania, sposoby radzenia sobie z tą sytuacją?**

Wydaje mi się, że nic nowego się nie pojawiło.

**A robisz coś dla siebie, co po prostu sprawia ci przyjemność?**

Cóż...W sumie to nie bardzo. Gram na gitarze dalej, czasami wypiję sobie piwo - wiadomo, to też sprawia mi przyjemność. Co jeszcze...Oglądnę film i to tyle.

**W jaki sposób teraz robisz zakupy. W sklepach, przez internet?**

W sklepach. Chodzę do sklepu z listą i staram się spędzać jak najmniej czasu w sklepie. Internetowe zakupy też robię, ale nie są to zakupy spożywcze, tylko rzeczy typu żwirek dla kota czy książka. Takie nie do jedzenia.

**Czy wcześniej, przed epidemią też robiłeś zakupy online?**

Tak i nawet spożywcze też się zdarzały, ale dość dawno temu.

**Żwirek też dawniej kupowałeś przez internet?**

Tak, głównie przez internet, dlatego, że uważam, że stosunek jakości do ceny tego co mogę znaleźć w internecie jest najlepszy. Zazwyczaj w sklepie zoologicznym, który miałem obok siebie pod wcześniejszym adresem, to tam nie mogłem znaleźć takiego żwirku, który mi odpowiadał, a z Allegro zamówiłem kiedyś taki, który kosztował 80 zł, objętość miał 40 l i był świetny, bo nie wydzielał żadnego zapachu - bardzo neutralny. Jak już raz taki znalazłem, to zacząłem cały czas go kupować.

**Jaką wybrałeś formę dostawy?**

Kurierem.

**Nie miałeś tu żadnych obaw związanych z epidemią?**

Widzę, że kurierzy noszą rękawiczki cały czas, chociaż o nie jest może do końca wyznacznik czy to jest bezpieczne, bo on mógł np. nie zmieniać tych rękawiczek. Ja stosuję zasadę ograniczonego zaufania i jak dostaję coś takiego, to zazwyczaj sam staram się to jakoś wytrzeć albo umyć, zdezynfekować i tak samo jest w miarę możliwości z zakupami spożywczymi. Jak przyniosę tylko ze sklepu, to staram się albo pod bieżącą wodą gorącą, albo wytrzeć czymś, co jest nasączone alkoholem. mam ściereczki do okularów- one mają zawartość alkoholu.

**Widzisz, że kurierzy noszą rękawiczki, ale nie do końca ufasz tym zabezpieczeniom?**

Też nie do końca. Nie wiek, jak to określić. Ufam, bo ja sam też noszę rękawiczki, tylko może bardziej ludziom nie ufam, ale wolę, żeby mieli te rękawiczki niż żeby ich nie mieli.

**Nie myślałeś o dostawie do paczkomatu?**

Myślałem, jak najbardziej, tylko żwirek miał objętość 40 l, więc jest dosyć ciężki. Raz już tak zamówiłem do paczkomatu i stwierdziłem, że następnym razem zamówię kurierem. Ostatnio tez zamówiłem książkę i przyszła mi do paczkomatu, który znajduje się jakieś 5 min drogi ode mnie z domu.

**Czemu ten paczkomat wybierasz?**

Zamawiając książkę miałem też możliwość dostawy kurierem, ale wybrałem paczkomat ze względu na zmniejszenie kontaktu między mną a kurierem. Jeżeli mogłem to zrobić, to zrobiłem. Poza tym dzięki temu wyszedłem też z domu.

**Taka kolejna wycieczka?**

Tak, powiedzmy, ale w jakimś celu. Nie jest tak, że wychodzę z domu bez celu, chociaż zawsze mogę mieć cel, że wychodzę, żeby sobie pochodzić, ale chyba rozumiesz, o co mi chodzi.

**Wcześniej zamawiałeś książki przez internet?**

Zdarzyło mi się, ale szczerze mówiąc nie zamówiłem wiele książek w życiu. Tę akurat zamówiłem, bo była promocja. Do 7.04. dostawa była za darmo, a książka jest pana, o którym wspominałem w zeszłym tygodniu - on prowadzi ten swój kanał na you tube. Książka jest bardzo ładnie wydana, ale jeszcze nie miałem okazji zacząć czytać, bo czytam coś innego. Jeżeli zamawiałem książki poprzednio, to zamawiałem je z Allegro i to była książka potrzebna mi na studia. Tam akurat mogłem ją znaleźć w dobrej cenie - używana. Przeważnie chodziłem albo do księgarni, albo do jakiejś oficyny wydawniczej swojej uczelni, albo pożyczałem z biblioteki.

**Ta zamówiona teraz, gdzie ją zamówiłeś?**

Strona nazywa się genyaltenberg.pl i to jest jedyna strona, na której można tę książkę dostać. To jest prawdopodobnie życzenie autora. Sam pan Dawid Myśliwiec mówił, że tylko z tej strony można ją zamówić i właśnie do 7.04.

**Coś jeszcze zamawiałeś w ciągu ostatnich 2 tygodni?**

Karmy dla kota nie zamawiałem, ale muszę to zrobić. Nie, nic więcej nie zamawiałem. Jeżeli to się liczy, to pizza.

**To jedzenie dla kota jak zazwyczaj kupujesz?**

Często kupuję rossmannową karmę, bo ona chyba jakościowo jest niezła i też na taką jedną trafiłem. Winston się nazywa w opakowaniu za 8 zł i ona ma chyba ponad 50% jakiegoś mięsa. Raz na jakiś czas zamawiam w internecie karmę Applaws. Też ma całkiem niezłe opinie i chyba niezły skład i jest całkiem przystępna cenowo. Też nieregularnie to jest, bo raz się zdarzało 1x na 3 miesiące, raz 1 x na 4 miesiące tę karmę zamówić.

**Gdzie ją zamierzasz kupić?**

Myślę, że też na Allegro, a jeżeli jej nie będzie, to poszukam gdzieś indziej.

**Powiedz o zamawianiu jedzenia. W jaki sposób zamawiasz?**

Korzystam z pizza.pl albo pizzaportal.pl i tam jest opcja, że wybiera się jedzenie, dodaje się do koszyka sosy, robi się przelew przez stronę albo blika, potem przychodzi potwierdzenie, czas dostawy i przyjeżdża dostawca. Zazwyczaj zostawia to jedzenie przed drzwiami i dzwoni. Czasami też robię tak, że zaglądam na pyszne.pl, szukam sobie restauracji dostępnych, bo wiem, że pyszne.pl nalicza sobie czasami prowizję, więc czasami taniej jest zamówić bezpośrednio ze strony restauracji. Szukam sobie takiej restauracji i ewentualnie wchodzę na jej stronę.

**Czy ta forma dostawy jest dla ciebie bezpieczna?**

Pewności nie mam właśnie. Oczywiście mógłbym wchodzić na stronę każdej restauracji i się doszukiwać, czy oni trzymają się w obecnych czasach jakichś standardów i czy pakowanie tego jedzenia przebiega bezpiecznie, ale ufam, że raczej tak jest i że każda restauracja powinna sama o to zadbać, bo konkurencja nie śpi. Poza tym chyba nikt nie chce żadnych problemów związanych z tym, gdyby coś się komuś stało z klientów. Raczej ufam, ale też, jeżeli dostaję takie jedzenie pod drzwi, to zazwyczaj staram się przetrzeć jakąś ścierką wierzch takiego pudełka. Nie wiem, czy to w jakikolwiek sposób coś daje, czy to jest wyłącznie moje uspokojenie.

**Jak często zamawiasz teraz?**

Mniej więcej raz na tydzień, zdarzało się 2 razy, ale ostatnio dostałem paczkę jedzenia z domu, więc ostatni raz zamawiałem pizzę chyba ponad tydzień temu.

**Przed epidemią też zamawiałeś jedzenie przez internet?**

Tak.

**Opowiedz o ostatniej sytuacji, kiedy zamawiałeś. Kiedy, o jakiej godzinie, z czego to wynikało?**

Sprawdzam...Chyba 6.04...To było 3.04., czyli równo tydzień temu i to były burgery akurat.

To było o 16.38 i powód był taki, że jedzenie, które mamy akurat w tej chwili możemy chyba zostawić sobie na jutro, nie będziemy musieli iść do sklepu i dzisiaj zjemy sobie burgery. Trochę widzimisię, trochę ochota na burgera a trochę też chcieliśmy odłożyć to wyjście do sklepu. Poza tym chyba też nie chciało mi się przygotowywać tego jedzenia i wolałem dostać coś gotowego.

**Czy to jak teraz jecie jakoś się zmieniło?**

W zasadzie tak, bo o takich bardziej ustabilizowanych porach. Wcześniej zdarzało się często, że obiad jedliśmy o 18, a teraz zazwyczaj to jest 16.30-17. Zazwyczaj już mamy czas na śniadanie, a bywało tak, że albo jadłem w pracy, albo zabierałem ze sobą, ale w zasadzie nie jadaliśmy go wspólnie z dziewczyną. Teraz zazwyczaj jest na to czas.

**Zmieniło się też to, co jecie?**

Nie wydaje mi się, żeby jakoś diametralnie. Chociaż tak...Jemy więcej słodyczy, to na pewno, ale jeśli chodzi o pozostałe jedzenie, to jem to, na co mam ochotę i w umiarkowanych ilościach.

**Rozumiem, że na co dzień nie jedliście za bardzo słodyczy?**

Zdarzało się też. ja bardzo lubię słodycze, więc zdarzało się czasem kupić, ale teraz jest zdecydowanie częściej i jeżeli chodzę do sklepu, to zazwyczaj biorę coś też więcej, na zapas. Często też jakiś napój dodatkowy, żeby stał w lodówce, jakieś chipsy na wieczór, a tego praktycznie się nie zdarzało kupować wcześniej.

**Jak myślisz, dlaczego tego teraz jest więcej?**

Dlatego, że i tak siedzę w domu, jest dużo seansów filmowych i po prostu lubię do seansu coś podjadać. Nie mam też jakichś wyrzutów sumienia z tego powodu, bo wiem, że pewnie dużo ludzi tak robi i usprawiedliwiam się też tym, że jeżeli się to wszystko skończy i wróci w miarę do normy, to wtedy z kopyta ruszę na siłownię albo na basen i to spalę. Mam już motywację do tego.

**Poćwiczenie i powrót do formy będzie dopiero po epidemii?**

Tak, myślę, że tak. W domu, póki co jeszcze nic nie ćwiczyłem, ale gdzieś na dniach planowałem, żeby chociaż jakieś pompki, jakieś rozgrzewki, bo już trochę zaczyna mi brakować ruchu. Jeszcze tego nie zrobiłem i nie spieszy mi się jakoś bardzo.

**Opowiedz o wyprawie do Rossmanna. Jakoś się przygotowywałeś do tego?**

Tak. Zrobiłem listę, której i tak zapomniałem, ale pamiętałem, co miałem kupić, bo miałem niewiele do kupienia. Musiałem kupić karmę, żyletki do maszynki do golenia, tampony dla mojej dziewczyny. Chciałem jeszcze kupić rękawiczki ochronne, ale nie było w Rossmannie. pytałem też w aptece i też nie było, ale to spontanicznie bardziej. Coś jeszcze chciałem kupić w Rossmannie, ale nie mogę teraz tej listy znaleźć.

**Jeszcze w jakiś sposób się przygotowywałeś do zakupów?**

Wziąłem sobie 2 dodatkowe torby materiałowe, bo myśl była taka, żeby iść do Rossmanna i potem od razu na zakupy, ale ostatecznie tak nie wyszło. Wracając z Rossmanna i tak miałem po drodze dom, więc wolałem sobie zostawić zakupy i dopiero pójść na kolejne. Koniec końców wróciłem, posiedziałem trochę w domu, zjadłem obiad i wtedy poszedłem na zakupy.

**Brałeś jakieś maseczki, rękawiczki, żele?**

Rękawiczki tak. Od siebie z domu, nitrylowe, ale to głównie z myślą o komunikacji miejskiej i o tym, że wejdę do sklepu i będę czegoś dotykał. Musiałem pojechać tramwajem.

**Jak się czujesz teraz z jeżdżeniem tramwajami?**

Dość...Ja mieszkam na Białołęce i pętla, którą mam obok siebie to jest pętla, czy li startuje stamtąd. Jak wsiadłem, to ten tramwaj jeszcze trochę stał, więc miałem czas się rozejrzeć po tym tramwaju, czy jest mało osób i żeby znaleźć sobie miejsce. Było niewiele osób, więc było ok. Wracając, było dość sporo osób w tym tramwaju i na prędce chyba nie naliczyłem 14, więc wszedłem. Tramwaj zajechał i dużo bardzo osób wyszło z tego tramwaju, więc było więcej niż 14, ale te osoby, które już zostały i te, które weszły razem dały chyba 12-13 osób. Na tramwajach są takie karteczki, że max.14 osób. Nie wiem, jak to egzekwować prawdę mówiąc.

**Te karteczki sprawiają, że czujesz się bezpieczniej jadąc tramwajem?**

Tak, bo to jest jakiś sygnał ostrzegawczy do ludzi, że może zastanów się jednak i jak widzisz, że jest przepełniony tramwaj, to może nie wchodź, zaraz przyjedzie następny. ja akurat nie widziałem, żeby był bardzo przepełniony, było chyba mniej niż 14 osób i to wychodziło tak ze 2 m odstępu od innego człowieka. Chyba było w porządku, czułem się w miarę bezpiecznie, ale była jedna sytuacja, które wywołała we mnie jakby niepokój. Jakaś pani rozmawiała przez telefon i stanęła bardzo blisko innej pani. Naprawdę blisko. I dosłownie w tej samej chwili zobaczyłem komunikat na ekranie, żeby nie rozmawiać przez telefon, nie zbliżać się do innych pasażerów. To była taka synchronizacja. Troszkę krzywo się na to popatrzyłem i zdałem też sobie sprawę, jak ta cała sytuacja zmieniła postrzeganie nasze rzeczywistości, innych ludzi, transportu i tego jak na co dzień żyjemy. Człowiek idąc ulicą wcześniej, przed epidemią, czuł się całkiem swobodnie, a teraz staram się zachować dystans od innych ludzi, omijam ich szerokim łukiem, jeżeli wchodzę do tramwaju, to staram się znaleźć ustronne miejsce. No i też nie wiem, na ile w tym wszystkim jest prawdy, na ile to jest podyktowane tym, że ktoś zaleca, żeby tak było, a na ile to jest prawda. Staram się stosować do zaleceń.

**Jak same zakupy w Rossmannie przebiegały?**

Starałem się je zrobić jak najszybciej, może trochę nerwowo, ale myślę, że całkiem niepotrzebnie, bo wyczekałem swoje w tej kolejce. Wiedziałem, że czekają tam inni ludzie, że zbiera się ich sporo przed tym Rossmannem. To było wczoraj, więc już po tych dodatkowych ograniczeniach, które wprowadzono.

[moderator kaszle, idzie napić się wody]

**Kasłanie jest teraz bardzo niemile widziane, prawda?**

No właśnie i to jest chociażby jedna z tych rzeczy, która zmienia nasze postrzeganie obecnej rzeczywistości. Wcześniej takie pokasływanie przez kogoś na ulicy nie było niczym szczególnym, a teraz coś się zapala w głowie, prawda? Zwracam też uwagę na takie rzeczy, jak to wszystko się zmienia i moje procesy myślowe też się trochę zmieniają w ten sposób, ale też nie chcę popadać w jakąś paranoję.

**Czyli te zakupy były...?**

Troszkę chaotyczne, nerwowe, na szybko. Raz, że też nie miałem tej listy, więc starałem się sobie przypomnieć, czy to aby na pewno wszystko. Do tego starałem się sobie przypomnieć, czy może coś jeszcze trzeba kupić, może jakiś płyn do mycia naczyń, a może to, może tamto? To też trochę taką nerwówkę wprowadzało. Do tego nie znałem też rozkładu tego Rossmanna - byłem tam 1-szy raz, a on był dość spory, więc też szukałem trochę tych rzeczy. Koniec końców udało się wszystko kupić.

**Jak płaciłeś za zakupy?**

Kartą. Cały czas płacę kartą teraz. Wcześniej też przeważnie używałem karty, gotówki bardzo rzadko. Zdarzyło mi się zgubić portfel, a w nim była spora suma gotówki. Miałem po prostu pecha, bo nie zdążyłem jej wpłacić. Generalnie od kiedy założyłem sobie konto w banku i dostałem kartę - nie kredytową, nie debetową, tylko płatniczą, to staram się jej używać, bo jest wygodna bardzo. Do tego jeszcze jak weszły płatności telefonem, to nie od razu, ale telefonem płacę mniej więcej od jesieni zeszłego roku.

**Czyli teraz ten sposób się nie zmienił?**

Dokładnie.

**Obserwowałeś, jak się zachowywali inni klienci w Rossmannie?**

Nie. Raczej nie obserwowałem i nie zauważyłem też, żeby ktoś faktycznie chodził, podziwiał coś, szukał czegoś, zastanawiał się. Raczej każdy był konkretnie zorientowany na to, co mu jest potrzebne i co miał do kupienia, to kupował i wychodził.

**A obsługa?**

Była zajęta. z tego, co pamiętam 2 ekspedientki były dosyć ruchome między kasą a półkami, miały też rękawiczki, ale jedna nie miała maseczki, druga chyba też nie miała maseczki, ale ochroniarz, który też siedział przy wejściu miał i maseczkę, i rękawiczki.

**Obsługa powinna mieć i maseczki, i rękawiczki?**

Być może. Raczej powinni mieć, ale może coś się zdarzyło i musieli na chwilę zdjąć, Też nie wnikałem w to, szczerze mówiąc i dopiero teraz o tym pomyślałem, że nie miały tych maseczek.

**Jak się czułeś robiąc te zakupy?**

Czułem, że jest inaczej, zdecydowanie. Nie znam czasów moich rodziców i poprzedniego pokolenia, kiedy faktycznie były kolejki do sklepów, ale takie skojarzenie mi się nasunęło. Już pierwszy raz te kolejki zobaczyłem przed Auchan 1.5-2 tyg. temu; wczoraj jadąc do Rossmanna widziałem kolejki przed sklepem mięsnym, do którego też miałem wejść, ale zrezygnowałem, bo `kolejka tam była chyba na pół godz. stania; no i przed tym Rossmannem też były kolejki, więc w ten sposób jakby to postrzegam, ze jakby powrót do tamtych czasów pod tym względem. Nie mamy jedzenia na kartki, ale kolejki są i produktów też czasami nie ma aż tak dużo. Jak wczoraj byłem w Auchan, to wymiecione praktycznie wszystkie warzywa, z mięsem nie było tak źle, ale mąki w ogóle nie było w sklepie.

**Jak ty się czujesz, jak widzisz, że nie ma tych produktów w sklepie?**

Ja się trochę irytuję, wiadomo. Po to przyszedłem do sklepu, żeby to kupić i nastawiam się, że to będzie. Irytuję się tym bardziej, że znowu będę musiał przyjść do tego sklepu, dajmy na to, tylko po tę jedną rzecz i też się trochę narazić przez to, stracić czas. Wolałbym, żeby to było.

**Kiedy ostatni raz byłeś na zakupach w Auchan?**

Wczoraj. Zrobiłem sobie listę i tym razem już ją wziąłem ze sobą. I wziąłem 2 torby materiałowe, z których jedna się przydała, plecak, rękawiczki ochronne, okulary. Zazwyczaj noszę okulary na co dzień. Zamysłem ich było stosowanie ich tylko do komputera, ale okulista powiedział mi, że mam astygmatyzm i dobrze by było nosić je cały czas. Często jest tak, że nie chodzę, ale zazwyczaj się staram w nich chodzić. Założyłem też okulary. Myślałem, że może nie będę ich brał, ale wziąłem pod tym kątem, że to też jest jakaś bariera ochronna.

**Planowałeś, którego dnia pójdziesz na te zakupy i jakąś konkretną godzinę?**

Tak. To były zakupy robione głównie pod śniadanie wielkanocne. Mamy z dziewczyną listę kilku takich rzeczy, które chcielibyśmy sobie zrobić na to śniadanie - niekoniecznie związane z tradycją. Zakupy chciałem zrobić po tym momencie, jak dostanę tę paczkę od rodziców z jedzeniem, żeby zobaczyć, czego brakuje nam na święta. Paczkę dostałem w środę, więc wiedziałem, że na zakupy pójdę albo w czwartek, albo w piątek. Wczoraj już poszedłem po część produktów, żeby nie obładowywać się na raz i po część albo pójdę dzisiaj, albo pójdzie moja dziewczyna, albo jutro rano.

**Sam sposób robienia zakupów w tym sklepie?**

Jeżeli chodzi o moją listę, to nie zgrupowałem jej pod tym kątem, że warzywa razem, owoce razem, jakieś słodycze razem, tylko to, co mi przychodziło do głowy, to spisywałem. W takiej też kolejności, patrząc na listę starałem się udawać w te działy. Jeżeli widziałem, że pozycja 1 na liście i pozycja 4 są w miarę blisko siebie, to starałem się iść tam najpierw.  Raczej w zorganizowany sposób starałem się zrobić te zakupy.

**A ludzie, którzy byli w tym sklepie?**

Też myślę, że w ten sposób, skoro widziałem ludzi z listą. Każdy stosował się do tego, że trzeba skorzystać z płynu do dezynfekcji rąk, każdy stał w tej kolejce i nikt nie próbował się do niej wbić. Myślę, że wszystko przebiegało w sposób zorganizowany i tyle.

Jakie tam zasady obowiązują teraz?

Jest stoisko z płynem do dezynfekcji zaraz naprzeciwko wejścia, są też rękawiczki foliowe - ja akurat miałem swoje i sporo osób przychodzi już w swoich. Rękawiczki staram się zakładać bezpośrednio przed wejściem do sklepu, a nie wychodząc z domu. Nie wiem już właśnie, czy myślenie o takich rzeczach to nie jest już troszkę paranoja, że zaraz przed wyjściem z domu, bo dotknę klamki w klatce i czy powinienem w ten sposób funkcjonować. No ale powiedzmy, że ja zakładam na razie rękawiczki przed wejściem do sklepu. Jeżeli chodzi o kolejki to nie wiem, czy to jest jakieś obostrzenie, czy ludzie sami z siebie to zaczęli robić, bo ja nie słyszałem o tym wcześniej, żeby jakaś konkretna ilość ludzi w sklepie miała być, tylko raz przyszedłem pod Auchan i widzę, że jest kolejka, ok., to ja też się dołączę. To wydaje się dosyć logiczne, żeby było jak najmniej ludzi w sklepie i żeby się zamieniały - jedna wchodzi, druga wychodzi. Jeżeli o to chodzi, to nie wiem, czy to jest obostrzenie, ale zakładam, że jakieś jest, żeby po prostu było jak najmniej ludzi w tym sklepie. Chyba widziałem też jakąś informację, żeby jak najmniej czasu spędzić, ale może wymyślam już teraz. Sporo karteczek wisi na drzwiach tego sklepu, ale nie przypatrywałem się. Komunikaty jakieś są też w tym Auchan porozwieszane. Pamiętam, że zwróciłem na nie uwagę jak wszystko się zaczynało, ale wtedy jeszcze nikt nie miał maseczek, nie było tych plastikowych ekranów, czyli to wszystko się dopiero zaczynało, ale było już słychać o koronawirusie. Pamiętam, że były komunikaty, że w związku z pandemią COVID 19 tutaj jest nr alarmowy i gdybyś wrócił z jakichś krajów, w których ta choroba jest, to zgłoś się pod ten numer. Także bardziej takie informacyjne rzeczy. Tęż instrukcja, jak myć ręce tam była rozwieszona, Jeśli chodzi o obecne obostrzenia to nie przypatrywałem się aż tak bardzo.

**Te kolejki do Auchan są dla ciebie zauważalne i przeszkadzają ci?**

Są zauważalne, pewnie, że tak. Czy mi przeszkadzają? Właściwie to nie bardzo. Jeżeli faktycznie miałbym spędzić 40 min w kolejce przed sklepem, to by mi to trochę przeszkadzało, ale jeżeli kolejka jest na 10 min czekania, to jeszcze nie jest tak źle, nie denerwuję się z tego powodu, bo każdy musi w niej stać i po prostu tak to dzisiaj wygląda.

**Zdarza ci się też robić takie mniejsze wypady na zakupy?**

Zdarza się bardzo sporadycznie. Mam Żabkę naprzeciwko i zdarzało się pójść do Żabki po takie dosłownie marginalne rzeczy, ale to może raz na 2 tyg. tak wyjdę? Raczej staram się właśnie to co trzeba kupić raz i nie wychodzić niepotrzebnie.

**Dostałeś paczkę od rodziców. To się pojawiło teraz, czy wcześniej też rodzice ci przysyłali takie paczki żywnościowe?**

Zdarzało się, że mi przysyłali, szczególnie jak jeszcze byłem na studiach. Czasami miałem ciężki tydzień, zaliczenia albo dużo nauki. Moja dziewczyna chodziła do pracy, więc czasami nie było jak sobie tego jedzenia zrobić albo nawet pójść na większe zakupy, więc prosiłem o taką paczkę i zazwyczaj było tak, że rodzice mi przysyłali taką paczkę w niedzielę pociągiem z Przemyśla przesyłką konduktorską. Ja ją sobie odbierałem na Centralnym, bo wtedy jeszcze mieszkałem na Tamce. Taką paczkę dostawałem nieregularnie, ale zdarzało się powiedzmy co 2 miesiące, potem po 3 tyg. Nieregularnie, ale zdarzało się. A teraz w czasie pandemii dostałem już 2-gą taką paczkę - w tę środę i 2 tyg. wcześniej.

**Co teraz było w tej paczce?**

Gołąbki, pierogi. Gołąbki zamroziłem, pierogi zjedliśmy praktycznie od razu. Nie lubię mrożonych pierogów, więc wolałem je na świeżo. Do tego były jeszcze 2 świeże chleby żytnie. bo mamy w Przemyślu taka panią, która ma piece do chleba i to jest jej pasja - wypieka bardzo dobre pełnowartościowe chleby. W zasadzie mogą być świeże nawet 1.5 tyg. Dostaliśmy też trochę słodyczy, jakieś pierniki, ciasto czekoladowe, czekoladki Lindt, kilka opakowań sera żółtego, schab upieczony przez moją mamę, chrzan, który moja babcia zrobiła. Nie mogę sobie przypomnieć, czy coś jeszcze.

Czy wy macie już zaplanowane zakupy wielkanocne?

Mamy zrobioną listę, jeśli chodzi o te potrawy na Wielkanoc, ale tylko część produktów została spisana na listę i już zakupiona, a jeszcze resztę trzeba sporządzić i pójść dzisiaj albo jutro.

**Co planujecie przygotować?**

Żurek, sałatka jarzynowa, sałatka warstwowa, roladki schabowe, sernik na zimno, batony Bounty - to ja znalazłem kiedyś taki przepis i teraz sobie o nim przypomniałem. Stwierdziłem, że możemy to teraz zrobić, bo jest jakaś okazja. Jajka faszerowane i ciasto francuskie z parówkami.

**Bardzo różnorodna kuchnia?**

Tak i stwierdziliśmy, że nakupujemy tych składników i po prostu nie, że wszystko zrobimy naraz, albo nawet wszystko możemy zrobić naraz, ale w małych porcjach. Różnorodnie, ale niewiele.

**Będziecie razem przygotowywać te potrawy czy ktoś u was gotuje bardziej?**

Myślę, że razem.

**Jakie macie plany na święta oprócz jedzenia?**

W zasadzie nie wiem czy mogę o nich mówić. Może następnym razem - skonsultuje to z dziewczyną.

**Ale zostajecie w domu?**

Tak, jak najbardziej. Nie planujemy nigdzie jechać i zresztą to już dawno było wiadomo, że do domu nie będziemy zjeżdżać, chociaż moja mama próbowała mnie do tego przekonać. Już wtedy wiedziałem, że bardziej odpowiedzialnie będzie zostać w domu, bo gdybyśmy pojechali na święta i nagle by się okazało, że nie możemy wrócić do Warszawy a mamy 2 koty, to tak średnio, żeby je u kogoś zostawiać na tak długo. Zresztą nie wiem co byśmy zrobili - czy poprosilibyśmy kogoś, żeby tu przychodził do kotów, czy koty byśmy u kogoś zostawili. Łatwiej będzie pojechać do domu jak to się wszystko skończy. Generalnie planowaliśmy po prostu siedzieć w domu i jeść dobre rzeczy

**Jakie argumenty miała twoja mama, żebyś przyjechał?**

Takie, że się dawno nie widzieliśmy i w zasadzie tyle. Powiem wprost, że to było trochę krótkowzroczne, ale moja mama czasami takie pomysły dziwne ma.

**Jakie miałeś argumenty, że jednak nie przyjedziesz?**

Chociażby takie jak powiedziałem, że coś może się nagle w prawie zmienić i zamkną miasta, i nie będzie można w ogóle się przemieszczać. Powiedziałem, że ja nie wiem czy ja jestem zdrowy, czy nie, bo ja mogę być chory, tylko przechodzić to bezobjawowo i zaraz by się zaraziła i tata, brat, a może jeszcze babcia przy okazji. Do Przemyśla jest 7 godz. jazdy pociągiem, więc ta podróż jest zawsze dość męcząca dla mnie i wiedziałem, że teraz jazda pociągiem to teraz też tak w ogóle nie byłby zbyt dobry pomysł. Ponadto słyszałem, że w Przemyślu jakiś czas temu na dworcu był jakiś problem - jakieś duże zgromadzenie Ukraińców, którzy wracali do siebie do domu i oni też wracali gdzieś z Włoch, itd., gdzie była już ta epidemia. Uciekali przed nią, więc to też nie byłoby bezpieczne i chciałbym uniknąć takich miejsc. Tutaj mój tata wyszedł z propozycją, że mógłby po nas przyjechać do Warszawy, ale stwierdziłem, że lepiej po prostu będzie pojechać jak to wszystko się uspokoi i spotkać się jak już będzie bezpiecznie. Mam nadzieję, że jej wystarczyły te argumenty.

**Jak się czujesz z tym, że spędzisz święta w taki sposób?**

Jest dość dziwnie. To są też moje pierwsze święta z moją dziewczyną, więc w sumie też cieszę się na swój sposób. Jest fajnie, ale jest inaczej zupełnie, np. z rodziną spotkamy się na takiej wideokonferencji, jak z tobą - ja, wujek, mama, tata, mój brat. Moja babcia nie, bo nie umie korzystać z komputera i takich rzeczy. Śniadanie będzie w ten sposób i jest to coś nowego. Jest inaczej, trochę dziwnie, ale nie jakoś super dziwnie. Ja, szczerze mówiąc nie jestem religijnym człowiekiem za bardzo. Nie przykładam do tego wielkiej wagi i tak samo do tego, że dzisiaj jest Wielki Post. Mógłbym zjeść mięso i nie zrobiłoby mi to różnicy, ale wiem, że to jest jakaś tradycja. Szczególnie moja babcia jest bardzo wierząca i zawsze mi coś mówi na ten temat, Ja się czasem denerwuję, bo dlaczego ktoś ma mi coś narzucać co mam myśleć i jak mam sądzić. Przymykam na to oko, bo wiem, że babcia to jest babcia, ma swoje lata i też bardzo dużo dobrego kiedyś i cały czas dla mnie robi. Tak samo babcia mnie pytała, czy mam jakieś palmy, pisanki na Niedzielę Wielkanocną. Mówiłem, że mam, ale oczywiście nie mam. Jeśli chodzi o święta, to ja mam podejście, że to jest czas nie jakiś super duchowy, tylko to jest czas na spotkanie się z rodziną, na spędzenie czasy razem i tyle. I to jest w sumie pozytywne też, więc dlaczego nie. U mnie wewnętrznie zawsze święta były pozbawione tego czegoś religijnego. Jak zjeżdżałem do domu, to mój kontakt z duchowością polegał na tym, że szedłem, bo musiałem do kościoła z koszyczkiem z moim bratem, bo zazwyczaj tata był w pracy, bo jest lekarzem, a mama ma chyba jeszcze mniej wspólnego z kościołem niż ja, więc zazwyczaj padało na mnie, że chodziłem z bratem z koszyczkiem. Na Boże Narodzenie zawsze wygląda to w ten sposób, że w sumie czyta się u nas Biblię i tylko je się kolację wigilijną i w zasadzie tyle. Nie ma jakiegoś celebrowania, kolędowanie, itd.
