# Supplementary material for: The challenges arising from the COVID-19 pandemic and the way people deal with them. A qualitative longitudinal study
Source: PLoS One. 2021 Oct 11;16(10):e0258133. doi: 10.1371/journal.pone.0258133 (PMC8504766; doi:10.1371/journal.pone.0258133)
Supplement: S1 Dataset — (ZIP) [file pone.0258133.s003.zip › Transcriptions/stage 2/14.2_M_55_couple, with children.docx]

**14.2_M_55_couple with children**

**Zdjęcia**

Na pewno 16. I 1.

16 - widzę tu pożar, czyli siłę niszczącą. Uważam, że to, co dzieje się teraz na całym świecie, może doprowadzić do zniszczenia układów - głównie gospodarczych, ekonomicznych, ale też społecznych, które w tej chwili mamy. Ja już odczuwam to na mikro poziomie, jeśli chodzi o Polskę i firmę, którą prowadzimy. Coraz więcej klientów odmawia płacenia. Uważam, że dojdzie w kraju do sytuacji, w której będzie można nie wywiązywać się z własnych zobowiązań ze względu na koronawirusa. Nie chodzi w tym przypadku o faktyczne problemy z nim związane, lecz o to, że moralność ludzi upada - zaczynają używać pandemii jako przyzwolenia na niedotrzymanie obietnic, są zdania, że mają prawo nie płacić. Czuję w związku z tym niepewność i zaczynam odczuwać strach o to, jak będzie wyglądać przyszłość. Ten niepokój jest w dodatku podsycany przez rządzących, którzy wprowadzają przepisy sprzyjające takim zachowaniom - to demoralizuje. Nie wiem, jak radzić sobie z tymi emocjami - nie zajmowałem się tym jeszcze, ponieważ obecnie ważniejsza jest dla mnie *damage control* naszej firmy. Działam, a uczucia odkładam na dalszy plan.

Jeśli chodzi o obrazek 1. - mam skojarzenie z totalnym chaosem - przepychanką, korkiem. To absolutnie nie odzwierciedla obecnej sytuacji drogowej, która jest zupełnie inna, ale symbolicznie oznacza zamieszanie i brak możliwości poruszania się do przodu. Wynika to ze stanu prawnego, który powstał obecnie w Polsce. Przepisów napisanych na kolanie, szybko i niedokładnie. Zamiast uspokoić, usystematyzować sytuację, wprowadziły dużo więcej bałaganu i niepewności. Kiedy o tym myślę, czuję wściekłość - nie wiem, jak można być takim idiotą, wprowadzać przepisy niezgodne z jakimkolwiek porządkiem prawnym. Kiedy klienci przestają płacić, próbuję w związku z tym zmniejszyć koszty własnej działalności, renegocjować umowy najmu. Ale wtedy też jestem odbierany jako ten, który próbuje znaleźć luki, a takie zachowania to według mnie totalna demoralizacja. Ten łańcuch powiązań obecnych w biznesie zaczyna się rozpadać i będzie to miało ogromne negatywne konsekwencje dla gospodarki. W obliczu tej wściekłości wino (Grüner Veltliner, Primitivo, Chianti) jest świetnym sposobem :) - niewątpliwie uspokaja. Czuję też złość, kiedy nie mogę pójść do lasu pobiegać. To nie tyle pomaga, co nie przeszkadza. Choć wolałbym to robić w sposób bezstresowy. Oczywiście nadal biegam - mieszkam w lesie i mam gdzieś te przepisy, nie widzę powodu, dla którego nie mógłbym pobiegać koło domu. Ale łapię się na tym, że kiedy idę to robić, wyłączam lokalizację i sieci komórkowe - zaczynam się bać, że mnie namierzą. Nie robiłem tego poprzednio, a to zaczyna robić się teraz jakąś obsesją. Nie wiem już, czy jesteśmy śledzeni, czy nie.

**Jakie jeszcze zachowania się u Ciebie pojawiły?**

Może też stałem się bardziej brutalny w moich zachowaniach biznesowych. Skoro wszyscy wokół nas się tak zachowują, stałem się wobec nich identyczny. Dlaczego miałbym być tym jedynym naiwnym, który wszystko będzie robił prawidłowo? Przestałem też zupełnie jeździć na rowerze. Boję się dyskutować z jakimś stójkowym na temat tego, czy ja mogę na tym rowerze jechać, czy nie. Zawsze mogę powiedzieć, że jadę do pracy. Ale jak zacznie się w to zagłębiać, nie wiem, czy potrafię kłamać.

**Co Ci w tej chwili najbardziej przeszkadza?**

Bardzo przeszkadza mi sama świadomość, że nie mogę sobie w każdej chwili wsiąść w samochód, czy samolot i pojechać, dokąd chcę. To nie jest coś, co muszę robić na co dzień, jednak wolność przemieszczania się i podróżowania jest dla mnie bardzo istotna. Podejrzewam, że takie zakazy pozostaną u nas na dużo dłużej i tych ograniczeń bardzo się boję. Druga rzecz, której się boję to to, że ten niejako wspomagany przez rządzących upadek moralności w biznesie, przerzuci się na naszą firmę i w pewnym stopniu zostaniemy pozbawieni źródeł utrzymania.

**Jakie zagrożenia czują bliscy?**

Moja żona przejmuje się ciągle sytuacją, która ma miejsce u nas w firmie. A my już i tak w znacznym stopniu pozbawiliśmy się dochodu, obniżając czesne w przedszkolu o połowę. Wiemy, że będziemy stratni i będziemy przez jakiś czas dopłacać do biznesu. Mamy jeszcze pewną poduszkę finansową, jednak nie wiemy, na jak długo ona wystarczy. Poza tym wiemy, że nie ma takiej niegodziwości, do której nie mogliby posunąć się rządzący, gdy zabraknie im pieniędzy. Nie jestem pewien, czy w pewnym momencie nie postanowią skonfiskować bankowych depozytów. I tego obawiam się bardziej, niż tydzień temu - jest to rezultat spadku moralności, który obserwuję. Kiedy słyszę, że rządzący twierdzą, że można nie płacić czynszu należnego innemu podmiotowi, w pewnym momencie na tej samej zasadzie mogą powiedzieć, że skoro masz pieniądze, to ci je zabierzemy.

O ile tydzień temu nie bałem się jeszcze zachorowania, tak teraz zaczynam się obawiać tego, że w takim przypadku mógłbym zostać umieszczony w jakimś bydlęcym szpitalu, w którym będę musiał jakiś czas przebywać. Przymusowo zostanę wyłączony z mojego środowiska i nie będę mógł na przykład pracować, a to na właśnie moich działaniach opiera się cała firma. Nie obserwuję takiego wzrostu obaw w moim otoczeniu, ale dochodzi do tego, że boję się na przykład iść do sklepu.

**A kiedy ostatnio byłeś w sklepie?**

Tydzień temu.

**Czy jakieś obawy odeszły?**

Nie.

**Jak odnosisz się do ostatnio wprowadzonych ograniczeń?**

Uważam, że są totalnie bez sensu. Rozumiem, dlaczego je wprowadzono. Rozumiem, że jest całe mnóstwo idiotów w naszym kraju, których jeśli nie weźmie się za pysk, to będą zachowywać się nieodpowiedzialnie i nieracjonalnie. Rozumiem też, dlaczego zostało wprowadzone ograniczenie wejścia do lasu - chodziło o ograniczenie możliwości organizowania pikników, spotkań. Ale uważam też, że to trochę tak, jakby zakazać wszystkim poruszania się samochodami, ponieważ ktoś może jechać za szybko. To niech w takim przypadku rząd zajmie się kontrolowaniem tego, żeby nie było nielegalnych zgromadzeń w lesie, a nie zakazywaniem wszystkim uprawiania sportu w lesie.

**Wtedy akceptowałbyś drony, które latają i wyszukują grupy?**

Tak, oczywiście, że tak. Niech sobie latają. Tylko nie jeśli biegam samotnie po alejce, a ten las wokół zawsze taki był, że nikogo wokół tu nie było. To mi przeszkadza.

**Czy widzisz jakieś inne mało sensowne zakazy?**

Jeśli chodzi o pozostałe zakazy, raczej nie mam problemu z ich przestrzeganiem. Inne zakazy wydają mi się sensowne. Choć jeszcze ten, dotyczący zakazu samodzielnego wychodzenia z domu osób poniżej 18 roku życia również wydaje się absurdalny. Przykładem może być córka znajomych, która nie może wyjść z psem, ponieważ 18 lat ukończy za dwa miesiące. Z jeszcze innej strony uważam, że jeżeli wprowadza się jakieś zasady, powinny one mieć właściwą podstawę prawną. W tym momencie tak nie jest. Wszelkie zmiany, ograniczenia swobód, nie mogą być wprowadzane poprzez rozporządzenie - jak dzieje się to obecnie, lecz poprzez ustawę.

**Znasz osoby, które nie stosują się do tych przepisów?**

Tak, sam ich nie zawsze przestrzegam. Spotykam też znajomych, czy po prostu widzę innych ludzi, którzy korzystają z terenów zielonych, czy nie zachowują dwóch metrów odstępu pomiędzy sobą. Uważam, że akurat ten zakaz dotyczący utrzymywania odpowiedniej odległości jest rozsądny, a ci ludzie, którzy go nie respektują – nieodpowiedzialni. To mnie złości. Uważam też, że są miejsca, w których brakuje odpowiednich regulacji. Od 12 kwietnia w kościołach będzie mogło znów zebrać się 50 osób. Mam nadzieję, że to zostanie zmienione. Wkurza mnie też niepewność związana z obecnym stanem prawnym, zgodnie z którym mamy we wtorek otworzyć przedszkole. To wciąż nie zostało zmienione. To jest totalny absurd, jednak planujemy to zrobić, ponieważ taki jest stan prawny na dzień dzisiejszy. Planujemy zakupy spożywcze przed świętami, aby kuchnia mogła ruszyć ze śniadaniem od wtorku - nie wiemy, jaka będzie decyzja rządu w tej sprawie i musimy być przygotowani na to, że rodzice mogą chcieć przyprowadzić dzieci. Możliwe, że dla rządzących to jest oczywiste, jednak na chwilę obecną nie ma oficjalnej decyzji w tej sprawie. To mnie strasznie denerwuje, wywołuje wściekłość. Tym bardziej, że wcale nie jest aż tak nieprawdopodobne to, że nie przywrócą działania szkół. Bo jeśli im bardziej zależy na wyborach, niż zdrowiu ludzi, a coraz częściej mam takie wrażenie, to oni to mogą zrobić.

**Czy znalazłeś jakieś sposoby, które pomagają Ci radzić sobie w tej sytuacji?**

Nie, choć szczerze - nie szukałem. Nie mam czasu. Zapracowanie w pewnym stopniu pomaga - kiedy muszę się na czymś skupić. Tylko też ciężko odciąć się od całego otoczenia. Są telefony, maile, przychodzą rodzice i mówią, że jesteśmy wstrętnymi kapitalistami i nie obniżamy czesnego w szkole. Pomimo tego, że szkoła realizuje program. Ale rodzice twierdzą, że z powodu koronawirusa, ich nie stać, poza tym muszą teraz spędzać dużo więcej czasu z dziećmi, pomagać im w nauce szkolnej. Dla mnie to jest absurdalne, czy oni dopiero teraz zauważyli, że mają dziecko?

**Tłumaczysz im?**

Nie tłumaczę, uważam, że to byłaby bezczelność z mojej strony. Pomimo tego, że rodzice absolutnie nie ograniczają się w argumentach wobec nas, staram się nie pouczać innych. Wiem, że to tylko wywołuje negatywna reakcję. Próbuję racjonalnie i rzeczowo - na tym polega większość mojego dnia, na pisaniu pism.

**Czy pojawiły się u Ciebie lub osób w Twoim otoczeniu nietypowe zachowania?**

U mnie nie. Zauważyłem jednak, że mój dwudziestoletni syn, sam z siebie, zaczął odkurzać cały dom. Sam kupił sobie odkurzacz - mieszkając z nami. Zamówił go online - fajny, drogi, jakiś hi-tech. Możliwe, że ten odkurzacz pojawił się ze względu na mieszkanie, które dla niego remontujemy w centrum Warszawy, ale zanim będzie tam mógł zamieszkać, przecież minie jeszcze dużo czasu. Syn wcześniej absolutnie nie zajmował się sprzątaniem, ciężko było go zmusić do tego typu czynności. Nie wiem, co mu to daje, może to jakaś odskocznia, ciężko powiedzieć. Ale siedzi teraz w domu, ze względu na sytuację związaną z koronawirusem; nie pracuje – wcześniej pracował na lotnisku, pewnie już też dostaje hopla. Od jakiegoś czasu gra też w gry komputerowe.

**Czy w ciągu ostatnich dwóch tygodni zrobiłeś jakieś zakupy przez internet, których nie planowałeś?**

Nie było to nic nietypowego, choć te zakupy były spontaniczne. W ciągu ostatniego tygodnia kupiłem dużo kawy. Kupiłem też wino u Kondrata - 10 butelek. Jakieś inne, niż dotychczas - trochę lepsza półka. Pomyślałem sobie, dlaczego miałbym nie spróbować jakiegoś lepszego wina. Dostałem wtedy mailing od niego i to skłoniło mnie do zakupów. Kupuję zazwyczaj 50/50 jeśli chodzi o proporcję zakupów online do tych stacjonarnych. Nie myślałem, żeby kupować teraz więcej, z niczego też na razie nie rezygnowałem. Kiedy zamawiam w sieci, preferuję dostawy do paczkomatów i to się u mnie nie zmieniło. Codziennych zakupów spożywczych nie robię przez internet. Miałem taki moment, że chciałem spróbować, ale zauważyłem, że są bardzo odległe terminy, np. tygodniowe. Więc stwierdziłem, że to bez sensu.

**Czy zmieniło się coś w Waszych zwyczajach żywieniowych?**

Wcześniej jadało się więcej na mieście - teraz w zasadzie tylko w domu. Jeśli chodzi o to, co jemy, nic się nie zmieniło poza tym, że piekę więcej chleba, bo teraz więcej go jemy.

**Jak to jest, jeśli chodzi o zamawianie jedzenia z dowozem?**

W ciągu ostatnich dwóch tygodni zdarzyło nam się zamówić dwa razy sushi. Normalnie zamawiamy je bardzo rzadko - w ogóle bardzo rzadko zamawialiśmy jedzenie restauracyjne do domu. Wcześniej po prostu jadaliśmy je na mieście. Teraz zamawialiśmy już dwa razy i zastanawiam się, czy nie robić tego częściej. Nie odbieram tego jako jakiejś namiastki "normalności". Po prostu lubię zjeść co jakiś czas coś innego i nie chcę sobie tego odmawiać. Można zamówić wiele rzeczy, jedynym minusem jest długi czas oczekiwania - logistyka jest jeszcze dość kiepska, restauracje nie mają wystarczającej liczby kurierów. Ale ostatnio, jak zamawiałem sushi, wolałem sam pojechać i je odebrać, niż czekać na dostawę. Musiałbym czekać trzy godziny, a kiedy zaproponowałem, że przyjadę sam, była to godzina oczekiwania.

**Czy masz jakieś obawy związane z dostawą jedzenia?**

Nie. Jedyne, co zmieniłem, to to, że teraz płacę od razu przy zamówieniu online, żeby nie przekazywać żadnych pieniędzy. Kiedyś pewnie zapłaciłbym przy odbiorze. Teraz staram się unikać gotówki. Wcześniej też starałem się płacić kartą, nie przepadałem za gotówką, jednak teraz płacę już tylko kartą. Telefonem nie płaciłem, nie umiem.

**Jakie podejmujesz środki ostrożności, wychodząc z domu?**

Idąc na zakupy, zacząłem oprócz rękawiczek zakładać maseczkę - nie tyle ze strachu, co po to, żeby nie stresować innych. Chociaż ona mi strasznie przeszkadza, bo parują mi przez to okulary. Ale to robiłem już tydzień temu.

**Jak często robicie teraz zakupy?**

Mam wrażenie, że żona też boi się trochę bardziej. Wcześniej robiliśmy zakupy na 2-3 dni. Teraz staramy się raz na tydzień. Tylko ostatnio żona wybrała się na zakupy bez listy. Zapomniała o wielu rzeczach i niedługo trzeba będzie, wcześniej niż mogłoby być to konieczne, wybrać się do sklepu, aby je kupić, przez co jestem wściekły.

**Jak wyglądają przygotowania do zakupów?**

Po prostu jest w domu lista i domownicy wpisują na nią, co trzeba kupić. Kiedy lista się zapełni to znak, że trzeba jechać na zakupy. Kiedyś to było bardziej spontaniczne. Obecnie jest to wyprawa. Na miejscu są kolejki w których trzeba odstać swoje, ze względu na limit klientów. Później trzeba zrobić zakupy w miarę sprawnie, ponieważ jest presja, aby zrobić miejsce dla innych, którzy oczekują na zewnątrz. Z całą tą sytuacją czuję się źle. Nie lubię czekać, stać w kolejkach, uważam, że to jest takie upodlenie człowieka, to mi się kojarzy z PRLem. Uważam, że formuła, którą wprowadzono - 3 x ilość kas - jest bez sensu. Ona powinna być w stosunku do powierzchni sklepu. Cieszy mnie jednak, że wydłużono czas pracy niektórych sklepów i można pójść na zakupy około godziny 23, kiedy nie ma nikogo, a produkty i tak są dostępne. Nie zdarzyło mi się jeszcze stać w tej kolejce przed sklepem, jednak kiedy to nastąpi, pewnie będę słuchał książki, czy coś czytał w telefonie. Absolutnie nie mam ochoty na żadną interakcję z kolejkowiczami i tak było zawsze. Ja w ogóle jestem aspołeczny, więc nakaz ograniczenia kontaktów społecznych nie dotyka mnie w dużym stopniu.

**Czy zauważyłeś zmiany w zachowaniu ludzi w sklepie?**

Zdecydowanie. Noszą rękawiczki, maseczki, trzymają odległość, raczej też ze sobą nie rozmawiają. Kiedy zdarzyło mi się podejść zbyt blisko, od razu spotkałem się z negatywną reakcją. Ludzie się obawiają innych. Nastąpiła obawa przed fizycznym kontaktem z drugim człowiekiem.

**Czy planujecie przygotowania do Wielkanocy?**

Nie przygotowujemy się, nigdy też nie chodziliśmy do kościoła. Dotychczas żadne święta nie miały dla nas większego znaczenia poza tym, że były raczej kłopotliwe. Szczerze mówiąc, nawet się teraz cieszę, że nie muszę nigdzie jechać i niczego specjalnego robić w domu, żeby zaprosić rodzinę na tę Wielkanoc. Wcześniej potrawy świąteczne były, ze względu właśnie na gości. To był ogromny stres związany z przygotowaniami. Więc obecnie dla mnie to jest raczej ulga i święty spokój, niż jakiś brak.

**A czy pojawiła się jakaś presja ze strony rodziny, aby jednak się spotkać?**

Prawnie i formalnie mamy jak najbardziej możliwość spotkania się z rodziną. Dzwoniła nawet teściowa, aby ustalić coś w tej kwestii. Jednak ja powiedziałem, że skoro się nie spotykamy, to nie spotykajmy się z nikim. Nie wiem, z kim widziała się teściowa i tak samo nie wiem, czy nie istniałoby ryzyko, że ja ją zarażę. Tym bardziej, że jest w podeszłym wieku. Stwierdziłem, że jeśli się nie spotkamy, nawet na święta, będę się z tym lepiej czuł. Pewnie zjemy jakieś śniadanie z żoną i synem, ale bez specjalnego gotowania, pieczenia.

**Refleksje**

Oglądając wiadomości, obserwując, co dzieje się na świecie, przygnębienie u mnie narasta. Myślałem, żeby odciąć się od informacji, jednak to mi się nie udało. Nie mam tego komfortu, aby zamknąć się w zupełnej izolacji i czekać. Mam biznes. Muszę monitorować choćby to, czy otworzą szkoły. To dla mnie życiowo istotne - ja muszę podejmować decyzje. Widzę też, że w Chinach, gdzie sytuacja zaczynała wyglądać lepiej, następuje nawrót. Podejrzewam, że w tej sytuacji rządy nie będą chętne do szybkiego cofania restrykcji. Nie wydaje mi się, żeby powrót do normalności miał szybko nastąpić. Wcześniej myślałem, że może jesienią, od września. Teraz myślę, że jeśli to nastąpi, to prędzej za rok. Podejrzewam, że stan ograniczeń będzie trwał nie dwa miesiące, a rok. Do czasu, aż zostanie wprowadzona szczepionka. Gdyby już była, zaszczepiłbym się.
