# Supplementary material for: The challenges arising from the COVID-19 pandemic and the way people deal with them. A qualitative longitudinal study
Source: PLoS One. 2021 Oct 11;16(10):e0258133. doi: 10.1371/journal.pone.0258133 (PMC8504766; doi:10.1371/journal.pone.0258133)
Supplement: S1 Dataset — (ZIP) [file pone.0258133.s003.zip › Transcriptions/stage 2/2.2_F_27_single.docx]

**2.2_F_27_single**

**Emocje – zdjęcia.**

**11, 6**

Najbardziej 11, ale 6 też ewentualnie.

**Co dla ciebie przedstawia ten obrazek (11)?**

Że generalnie teraz jest słabo, ale trochę w sumie lepiej, gdzieś jest lepiej.

**A na czym polega to, że teraz jest słabo?**

Chyba to jest kwestia tych kropli, które w sumie mogą znaczyć, że jeszcze trochę tutaj parę gdzieś w oddali jest… tęcza. No, generalnie taka pogoda troszeczkę po deszczu. I takie zamieszanie, jakby ciągle… nie jest superpozytywnie, ale widać, że gdzieś tam jest dobrze.

**A jak ty się czujesz w tym, że jest słabo? Co przede wszystkim czujesz?**

Szczerze mówiąc to ja się nie czuję, że jest słabo, ale generalnie wiem, że jest, no nie poprawiła się sytuacja, odkąd ostatni raz rozmawiałyśmy. Mam wrażenie, że już jakoś tak i ja przywykłam do tej sytuacji i ludzie przywykli do tej sytuacji. No, a to, że jakby już w niektórych państwach, w Chinach już otwierają te miasta, w których to się zaczęło, no to jest jakby już jakąś nadzieją.

**Czyli ta tęcza tutaj to jest nadzieja, że tak już gdzieś widać?**

Tak, coś takiego. Albo coś takiego, że po prostu… może nawet nie nadzieja, ale po prostu, że już się poprawia. Jakby nie chodzi o to, że nadzieja, tylko po prostu, że to jest taki kolejny etap. Jakby, no, nigdy nie będzie tak, że będzie padało do końca życia, kiedyś w końcu zacznie świecić słońce. Chociaż nie wiem, może będzie. Ale raczej, generalnie tak jest, że zawsze po deszczu jest, przestaje padać deszcz. No i właśnie to też może jest coś takiego, że potwierdzenie też tego, że ta sytuacja nie będzie trwała zawsze, tylko już może się gdzieś kończyć. Co oznacza, że kiedyś może się też skończyć w Polsce.

**A jak myślisz o swoich emocjach, bo rozumiem tą racjonalną część z jednej strony. Z drugiej strony są już państwa, gdzie to powoli idzie ku lepszemu, to co ty czujesz w tym momencie?**

Szczerze mówiąc nic konkretnego. Że to jest po prostu naturalna kolej rzeczy i trzeba swoje odczekać, trzeba się przyzwyczaić i trzeba się nauczyć żyć w tym środowisku, w takich warunkach. A wiedząc, że tam jest lepiej, to po prostu jest… Może powiedziałabym, że nadzieja. Ale nie wiem, czy czuję, że to jest nadzieja. Tylko raczej po prostu coś takiego, że potwierdzenie albo pewność tego, że też będzie tak tutaj.

**A co jest na 6 dla ciebie?**

Na podobnej zasadzie. Czyli też, tutaj jest więcej słońca, czyli jakby więcej jest tej pewności powiedzmy, że wszystko się unormuje. Ale to też nie jest pełne słońce, bo jest troszeczkę przykryte drzewami, może na jakiejś polanie dalej jest na przykład słońce, w którym się można opalać albo… Tak to widzę.

**Odniosłam takie wrażenie, że to, co ty czujesz to jakiś taki spokój większy niż w zeszłym tygodniu?**

Tak. Wydaje mi się, że to jest po prostu kolejny etap mojego bycia w tej sytuacji, że po prostu przywykłam. Totalnie się zaaklimatyzowałam, mogłabym tak żyć, uważam, że długo. W ogóle też… Rozmawiałam z ludźmi, czy coś takiego. I ludzie mówią, że się źle czują, że ktoś tam płakał, że coś tam. I ja mówię, no to pewnie dlatego, że się nie widzisz z ludźmi, że jakoś dziwnie jest wszystko. No i to jest zrozumiałe. Ale odkąd, to już jest ponad 20 dni, kiedy powinno się siedzieć w domu, nie miałam ani jednego takiego załamania, że jakoś się specjalnie martwiła, czy że płakałam, czy coś takiego. Zupełnie czegoś takiego nie miałam. Po prostu nowa to była sytuacja, w której się czułam dziwnie. A teraz już się czuję powiedzmy OK, mogę podążać tymi ścieżkami, którymi trzeba podążać.

**W zeszłym tygodniu mówiłaś, że czujesz się trochę jak w grze komputerowej. Że takie nierealne. Dalej masz takie odczucie?**

Tak. Wciąż mam takie odczucie. Ale przez to, że już tydzień, odkąd to powiedziałam, tydzień więcej, to już wydaje mi się, że po prostu tak musi być. Chociaż… Ciągle to wygląda nienaturalnie oczywiście, ale jest to już rzeczą, którą po prostu można przełknąć. (niezrozumiałe) że więcej osób wychodzi jednak na ulice. Albo może po prostu zachowują się na tyle pewnie i na tyle normalnie, że nie zwracam na to aż takiej uwagi.

**Mówisz, że to nie jest spokój, bo to nie jest dobre. Czy to jest obojętność, wobec tego, co się dzieje? Czy masz takie poczucie, że już jest ci wszystko jedno?**

Nie, absolutnie nie.

**A jak można by nazwać ten stan, w którym jesteś? To jest pozytywny czy negatywny stan?**

No obojętność właśnie… Obojętność jest obojętna. Ale w tym kontekście, jak ty powiedziałaś, no to raczej obojętność jest negatywna. Czyli już mam dość, już mnie to nie interesuje, już mnie nic nie obchodzi. Ale nie, to po prostu taka całkowita akceptacja raczej.

**Jak jeszcze mogłabyś opisać swoje uczucia teraz? Akceptacja, to jest przywyknięcie tak technicznie?**

Tak. Przystosowanie się. No, coś takiego.

**A masz jakieś takie ewidentnie negatywne emocje, związane z tym, co się teraz dzieje, w tym momencie?**

Nie.

**Czy jest jakaś taka, która się pojawia u ciebie teraz, taka ewidentnie negatywna, w która się źle czujesz?**

Nie. Nie, naprawdę nie. Ja się po prostu czuję tak, no nie wiem, można by to porównać, jak się szło do pierwszej klasy, pierwszy raz do szkoły. I na początku było dziwnie, nie znało się nikogo, panie, trzeba było siedzieć w ławce i robić coś, co ktoś ci każe przez cały dzień. A potem, nie wiem, ciężko mi powiedzieć, jak było w pierwszej klasie (niezrozumiałe) czy coś takiego. Ale wydaje mi się, że już się wszystko unormowało, wszystkich znałam. I przywykłam do tego, że trzeba chodzić do tej szkoły, uczyć się, robić to, co ci każą. I rozmawiać z ludźmi, z którymi się nie chce i robić to, co się nie chce. I wydaje mi się, że to jest na takiej samej zasadzie, że na początku to było coś nowego, nie wiedziałam, jak się zachowywać i co mam robić. A teraz już wiem. I po prostu to robię.

**A co się w ogóle u ciebie zmieniło w ciągu tego czasu, kiedy rozmawiałyśmy, od środy zeszłej?**

W życiu… Chyba nic.

**Dalej chodzisz do pracy, czy udało ci się zdalnie pracować?**

Nie, nie udało mi się zdalnie, ale wiem, że już mi się udaje kończyć, żeby można było pracować zdalnie. Nie wydaje mi się, żeby coś się zmieniło jakoś wyjątkowo, byłam 2 razy w sklepie. Raz wybraliśmy się w niedzielę, bo celebrujemy każdą niedzielę handlową, nawet w takich warunkach. Dla tych ludzi, którzy pracują, żeby wiedziały, że naprawdę jest to dla mnie ważne, że te sklepy są otwarte w niedzielę. Więc byliśmy w niedzielę w Lidlu. Ale właściwie to też… OK, były takie (niezrozumiałe), czyli kolejka na 20 metrów, bo ludzie stoją tak daleko od siebie, trzeba umyć ręce, założyć rękawiczki. Ale to też nie było… Było to dziwne, ale nie było to coś, co by mnie w jakiś sposób denerwowało albo jakoś przestraszyło. Nie. PO prostu tak było, ustawiliśmy się w kolejce, weszliśmy, zrobiliśmy zakupy, wróciliśmy do domu. Trochę przygoda w sumie. Nawet można z tego czerpać trochę korzyści takich, że się robi coś innego niż zawsze i to jakoś jest urozmaicenie może.

**Trochę jak przygoda.**

Trochę tak.

**Mi się przygoda kojarzy z czymś fajnym.**

Tak, no troszeczkę tak. Znaczy wiesz, jeżeli się siedzi w domu przez tyle czasu albo się wychodzi tylko do pracy, to przygoda pójścia do sklepu i popatrzenia na innych ludzi może być czymś fajnym generalnie. No więc było to troszeczkę pozytywne, jeśli chodzi o to wyjście z domu. I to nie sama, nie do pracy i nie załatwiać rzeczy, tylko po prostu sobie troszeczkę dla przyjemności iść do sklepu. Ale tak, nie uważam, żeby się coś zmieniło w moim życiu.

**Na razie te wszystkie zmiany są takie, że albo się przystosowałaś albo że pojawia się coś takiego, co można odczuć pozytywnie, jak to wyjście do sklepu. A czy pojawiło się coś, co zaczęło ci przeszkadzać?**

Nie. Mam ochotę powiedzieć, że na przykład jest ciepło i się ma ochotę wyjść na dwór, ale coś takiego mogłabym powiedzieć, gdybym musiała ci odpowiedzieć coś, to bym wymyśliła to. Ale przez to może, że wychodzę trochę do pracy, to spędzam czas na tym świeżym powietrzu i nie brakuje mi tego aż tak. Więc nie, niczego mi nie brakuje. Właściwie no, jak słyszysz już zaczęłam odczuwać pozytywy w ogóle generalnie tej sytuacji.

**A zdziwiona jesteś tym, że odczuwasz pozytywy?**

Nie. Dlatego, że nauczyłam się o sobie tego, że jak już się przyzwyczaję do jakiejś sytuacji, to potem bardzo szybko sobie zbieram jakieś pozytywy, żeby łatwiej mi było sobie żyć. Więc nie dziwi mnie to. Ale może to dziwić innych albo może być jakieś takie…

**A dlaczego to może dziwić innych?**

Bo jak widzisz, zupełnie, jeszcze bardziej nie martwię się o to, że mogę zachorować, że ktoś inny może zachorować. Mimo, że ludzie chorują i ta epidemia rośnie, a nie maleje, przynajmniej w Polsce. Raczej staram się patrzeć na to z perspektywy mojej osoby, a nie z perspektywy całego społeczeństwa.

**A dalej śledzisz te wszystkie liczby?**

Śledzę te liczby, ale już właśnie, to sobie uświadomiłam, że troszeczkę mniej. Dlatego, że jeżeli dziennie choruje tam 250 osób, to one są dużo bardziej anonimowe mimo wszystko, niż na przykład 5, które zachorowało jednego dnia. (niezrozumiałe) imion i nazwisk, ale oczywiście, jeżeli to rośnie w tak szybkim tempie, to troszeczkę mniej się na to zwraca uwagę. I też przestałam troszeczkę może na to zwracać uwagę i zaczęłam zwracać uwagę na po prostu siebie.

**Czyli poszliście 2 razy do sklepu, czyli to nie jest tak, że jeszcze bardziej ograniczacie te wyjścia. A w związku z tym, że wychodzisz codziennie masz takie poczucie, że cokolwiek ograniczałaś bardziej w tym tygodniu? Jakieś zachowania, które myślisz sobie, że są bardziej niebezpieczne albo nierekomendowane? I związku z tym sobie myślisz, a, nie wiem, to nie pójdę do sklepu, pójdę raz mniej albo nie zrobię czegoś. Miałaś takie momenty?**

No właśnie rozdzieliłabym na te nierekomendowane i te niebezpieczne. Bo żadne mi się nie wydają niebezpieczne. Wydaje mi się, że niebezpieczna mogę być bardziej ja dla starszych ludzi niż jacykolwiek ludzie dla mnie. Bo chodzę w tej maseczce, w tych rękawiczkach. Wiem, że można się zarazić, oczywiście, że tak, nawet uważając. Ale… Żadna sytuacja mi się nie wydaje niebezpieczna, szczególnie do sklepu. No, chyba że na przykład pójście do szpitala, gdzie faktycznie są tam chorzy. Ale to jest sytuacja, której nie zamierzam w ogóle robić. I czy ograniczyłam te sytuacje, których nie powinno się robić? Znaczy nie, wydaje mi się, że ja przestrzegam tych zasad. To, że pójdę 2 razy w tygodniu do sklepu, to akurat… No nie wiem, mogłabym pójść raz faktycznie.

**Chodzi mi o to, czy czułaś, że to niewłaściwie, że poszłaś dwa właśnie zamiast raz. Czy miałaś takie poczucie, że można to było upchnąć na jednych zakupach, czy myślisz sobie OK, to jest coś jeszcze akceptowalne, dozwolone, niezagrażające innym?**

Ani jedno, ani drugie. Po prostu za pierwszym razem nie mogłam wszystkiego donieść, nie mogłam kupić za dużo rzeczy, bo poszłam sama. Za drugim razem pojechaliśmy taksówką we dwójkę, więc kupiliśmy bardzo dużo rzeczy. I to dlatego. I teraz możliwe, że nie pójdziemy przez długi czas do sklepu, bo po prostu mamy bardzo dużo rzeczy. Ale nie uważam, żeby to było w jakikolwiek sposób niestety… No właśnie niestety, bo wiem, że może jeżeli bym się troszeczkę bardziej bała, troszeczkę bardziej bym może uważała, albo może… No właśnie nie wiem. Nie, wydaje mi się, że to jest w porządku.

**Powiedziałaś, że w ogóle ci nie wzrósł ten lęk o to, że się zarazisz, że będziesz chora. Tutaj ci się nie pojawiło się takie. Bo w zeszłym tygodniu o tym mówiłyśmy, że właściwie ty się o tego nie boisz. Pamiętam, że bałaś się różnych innych rzeczy. Ten lęk, o którym mówiłyśmy, on się składał u ciebie bardziej z lęku o pracę, o finanse, o to, jak firmy będą funkcjonować, jak twoi znajomi finansowo się odnajdą. A w ogóle się nie pojawił lęk o chorobę. I nadal go nie ma, tak?**

Nie. A nawet te lęki, o których mówisz teraz troszeczkę się zmniejszyły. Dlatego, że widzę rozwiązania na tą sytuację. I jakby to też jest troszeczkę może ta tęcza, która była na tym obrazku. Jakoś tam ludzie sobie radzą.

**A podasz mi przykład tego, jak ludzie sobie radzą? W sensie taki, który pokazuje, że ten twój lęk może się zmniejszać dzięki temu?**

Nie (śmiech). Nie wiem.

**W zeszłym tygodniu dość dużo rozmawiałyśmy o tym, że znajomi mają mniejszą kasę za pracę, obcinają pensje itd. Jak ludzie sobie z takimi rzeczami radzą?**

No nie wiem, na przykład w mojej firmie rozwiązaniem na tę sytuację jest… No generalnie pracuję w firmie, która jest łącznikiem między influencerami a firmami. No i jesteśmy pośrednikiem, żeby reklamować na Instagramie i na różnych mediach społecznościowych produkty. No i odpowiedzią naszej firmy była po prostu kampania, związana właśnie z tym wirusem. Czy jakieś dodatkowe narzędzia powstały, właśnie takie zdalnie. Więc pokazuje to, że z każdej sytuacji ludzie są w stanie wyjść. Albo jakieś takie, to już chyba było wcześniej, ale chyba o tym myślałam, że można kupić bony na kawę, żeby teraz zapłacić kawiarni, a odebrać ją, kiedy indziej. Albo widziała, że w Starbucksie na Nowym Świecie zrobili coś takiego, że można… Otworzyli tego Starbucksa po prostu. W sensie są otwarte drzwi, jest tam pleksi, nie przyglądałam się, bo byłam po drugiej stronie ulicy. Ale jakby znaleźli rozwiązanie na to. Kolejnym rozwiązaniem jest to, że mimo że mało osób może wchodzić do sklepu, no to wydłużyli godziny otwarcia. Nie wiem, czy to jest dobre dla ludzi, którzy tam pracują, bo siedzieć 24 godziny… Znaczy wiem, że to nie jest 24 godziny, ale jakoś się wydłuża ta zmiana. Jakby pokazuje to, że z każdej sytuacji jest wyjście. Czyli jak mało osób może wejść do sklepu, no to w takim razie wydłużymy godziny otwarcia. Może też wtedy te osoby, które tam pracują, się odbiją jakoś finansowo, jeżeli będą musiały pracować więcej czy w nocy.

**A jak sobie radzi Filip? Bo mówiłaś w zeszłym tygodniu, że on tak trochę gorzej niż ty tą sytuację znosi?**

Chyba też coraz lepiej. Chyba też powoli przywykł. A może nawet, jeśli nie przywykł do końca, tak jak ja, to wydaje mi się, że przez to, że dużo czasu spędzamy ze sobą i to więcej niż w zeszłym tygodniu, jakoś tak też jest spokojniejszy.

**A czym ten spokój u niego się objawia?**

Mniej pije?

**A rodzice? Mówiłaś, że rodzice, no nie jeździsz do nich. Byłaś u nich w międzyczasie?**

Nie. Ja do nich nigdy nie jeżdżę, więc… Mimo, że to jest 10 minut drogi samochodem. Nie, nawet się nie widziałam. Coś tam z nimi pisałam, ale… No właśnie! Nawet w tym tygodniu się nie spytali, jak się czuję ani ja się ich nie spytałam. Po prostu rozmawiamy normalnie o jakichś tam rzeczach bieżących.

**A ze znajomymi utrzymujesz w tym tygodniu ostatnim, jakoś miałaś więcej kontaktów? Albo wyciszają się te kontakty, jak to u ciebie wygląda?**

Chyba się właśnie unormowały. Tak jak w zeszłym tygodniu, nie wiem, czy o tym mówiłam, wydaje mi się, że tak. Ale na pewno o tym kiedyś pomyślałam, że właśnie jakoś ludzie chcą ze sobą rozmawiać, na tym Zoomie dzwonić do siebie, jakieś konferencje, jakieś imprezy. To tak mam wrażenie, że teraz to już wróciło do takiej normalności. Czyli się rozmawia z tymi, co się normalnie rozmawia. A z tymi, co się nie rozmawia, to się raczej nie rozmawia.

**A wcześniej było tak, że tak było aż za dużo tych kontaktów? Za dużo tego inicjowania kontaktów z różnych stron?**

Mi osobiście nie. Nie wiem, czy ktoś jakoś bardziej utrzymywał kontakt niż wcześniej. Ale widziałam, że ludzie tam rozmawiają ze sobą. Też zazwyczaj… Nie wiem, czy nie rozmawiają, ale mam wrażenie, że są dalszymi znajomymi niż… No nie muszą się naprawdę spotkać na tym Zoomie, to akurat… Ale to jest gdybanie, nie wiem tak naprawdę. Może się okazać, że tak to nie jest. Ja mam takie wrażenie.

**Powiedziałaś: kontakty towarzyskie wracają do normy. Co jest dla ciebie taką normą kontaktów towarzyskich?**

Nie ma takiej sztucznej potrzeby wracania do jakichś znajomych, z którymi się nie rozmawia przez cały czas. W sensie, no mam znajomych, z którymi rozmawiam codziennie, no i faktycznie rozmawiam z nimi codziennie. I zawsze mam coś do powiedzenia. Znaczy nie chodzi o to, że przez cały czas. Ale w ciągu dnia zdarzają się takie rzeczy, o których chciałabym powiedzieć komuś, więc po prostu im to mówię. Więc to po prostu oznacza, że rozmawiamy codziennie. Tam się w jakiś sposób kontaktujemy. Ale na przykład nie ma już tak, że rozmawiamy na przykład z ludźmi z pracy i nie o pracy.

**A to było w zeszłym tygodniu.**

Tak, to robiliśmy. A w tym tygodniu mniej. Wydaje mi się, że mamy więcej pracy. Po prostu już tego nie ma. No, przywykliśmy, czy…

**Myślisz, że to wynika z tego, że ludzie już przywykli do tej sytuacji?**

Nie wiem właściwie. Bo 2 dziewczyny, które są na tej grupie, z którymi mogłabym rozmawiać, rozmawiam z nimi tak czy inaczej prywatnie codziennie. A z jednym chłopakiem rozmawiam codziennie, ale o pracy… Znaczy rozmawiamy po prostu służbowo. Ale w międzyczasie rozmawiamy troszeczkę o jakichś tam innych rzeczach. I jest jeszcze jedna dziewczyna, z którą nie rozmawiam, ale nie rozmawiałam z nią nigdy poza pracą. Nie wiem. Może po prostu już wszyscy żyją takim swoim życiem domowym.

**A u tych swoich znajomych obserwujesz jakieś zmiany, jakieś inne emocje się u nich pojawiają? Oni jakoś przezywają tą sytuację jakoś inaczej? Czy już właśnie przestają, tak jak ty, jakby się koncentrować na tym, co się dzieje?**

Mam wrażenie, że przestają się koncentrować. Moja szefowa sobie właśnie teraz pojechała do rodziców do domu, na Włochy i tam sobie właśnie zrobiła biuro w ogrodzie. Więc jakby to też jest kolejny przykład na to, że ludzie sobie radzą z każdą sytuacją. Albo przynajmniej starają się czy znajdują jakieś inne rozwiązania. I się jakoś tam powoli przystosowują.

**A znasz jakieś osoby, które się nie dały rady oswoić z tą sytuacją, jeszcze cały czas przeżywają ją w taki negatywny sposób?**

Nie. Ale tak naprawdę to od rozmowy z tobą rozmawiałam może z siedmioma osobami prywatnie. Z pracy może kolejne, ale nie rozmawiam z nimi w ogóle, więc to nie ma sensu. No z siedmioma osobami może rozmawiałam o jakichś prywatnych rzeczach. Więc z tych osób żadna, raczej nie wydaje mi się, żeby przejawiała objawy strachu.

**Od czwartku chyba wprowadzano nowe zasady korzystania ze sklepów, te godziny dla seniorów, liczba osób na kasę itd. Jak ty się w ogóle czujesz z tym, że są nowe ograniczenia? Bo tydzień prawie z nimi żyjemy.**

Tak jak mówię, chyba nie… Znaczy zauważyłam, że są, ale nie zauważyłam, żeby mi zaczęły przeszkadzać. No fakt, troszeczkę trzeba dłużej postać w kolejce na przykład, bo mniej osób jest wpuszczanych do sklepu. Ale właśnie tez wydaje mi się, że jakoś to jest, każdy wie, że trzeba zrobić te zakupy szybciej, że jakoś to idzie sprawnie. Ten pomysł, że tylko starsze osoby mogą być rano w sklepie, to w ogóle jest super pomysł.

**A dlaczego myślisz, że to super?**

Bo jest mniejsze podobieństwo, że jakieś młodsze osoby zarażą starsze.

**A myślisz, że ci starsi wykorzystują te godziny? Starają się ograniczać do dwóch godzin, żeby mieć sklep tylko dla siebie?**

Wydaje mi się, że część tak, część nie.

**A jak myślisz, od czego to zależy?**

Od świadomości tych ludzi.

**Ci, którzy są bardziej świadomi, chodzą w tych tam 10—12?**

Tak. Nie wiem, czy sami oni są bardziej świadomi, czy może mają bliskie osoby, jakieś takie młodsze, które ich uświadamiają. Ale na przykład spotkałam, byłam jeszcze więcej razy w sklepie, oczywiście, niż to, co powiedziałam, bo jeszcze byłam raz na dole po pomidory. Znaczy to jest sklep na dole, to zupełnie nie traktuję tego jak duży sklep, w którym byłam zrobić większe zakupy. To tam byłam przed 10 chwilkę, to była starsza pani, która czekała przed wejściem do 10, żeby móc do niego wejść.

**A jak myślisz, dlaczego czekała? Było tyle ludzi w sklepie?**

Nie, byłam tylko ja. Ciężko powiedzieć właściwie. Nie wiem, może po prostu chciała przestrzegać zasad. Albo chciała właśnie brać udział w tej akcji, żeby pokazać, że jest starsza, że będzie chodziła na zakupy później, żeby pokazać, że jest świadoma i że interesuje ją zdrowie swoje i innych.

**No tak, ale mówisz, że poza tobą nikogo nie było w sklepie, to nie byłoby dużym zagrożeniem dla niej zrobić te zakupy, co?**

Nie, absolutnie.

**No właśnie.**

Przecież to powietrze się tak bardzo nie wymieszało od tego, gdy ja weszłam. Nie wiem. Nie wiem, naprawdę ciężko mi powiedzieć. Naprawdę, to była dziwna sytuacja. Dlatego o niej wspominam, bo aż zwróciłam na nią uwagę.

**A jak pojechaliście do tego Lidla w niedzielę i była ta kolejka, w której musiałaś stanąć, to jak się czuliście z Filipem? Rozmawialiście w ogóle o tej sytuacji, że taka…**

Patrzyliśmy na siebie, że dziwnie. Bo w ogóle Filip mi powiedział, co nie wiem, czy jest prawdą, ale posłuchaliśmy się, czyli tego, że 2 metry od siebie trzeba iść, nawet jak się mieszka w jednym domu. No i tak szliśmy. To było dziwne, ale to już było troszeczkę przerysowane. Szczególnie, że w domu byliśmy dużo bliżej niż 2 metry od siebie. Więc… Było to dla nas dziwne. Dziwne było w ogóle wszystko. Też a propos tych starszych ludzi w sklepach, to była właśnie starsza pani, która nie była absolutnie świadoma. Bo nie dość tego, że przyszła wtedy, kiedy było bardzo dużo ludzi, to jeszcze stała bardzo blisko i nas i takiej osoby, która stała za nami. Więc chyba… Ale denerwowało nas to, że ta pani tak blisko podchodzi. I to nawet nie dlatego, że się boimy o nią, że się boimy o nas, tylko właśnie dlatego, że ktoś nie przestrzega zasad.

**Ale to było takie, że byliście źli na nią? Że ktoś za tobą przestrzega i przychodzi ktoś, to jakby wyłamuje się?**

Tak, można powiedzieć, że trochę byłam zła. Wydaje mi się, że Filip też i potem jeszcze o tym rozmawialiśmy. Nawet chyba 2 razy o tym rozmawialiśmy. Znaczy nie była to jakaś długa rozmowa, ale wspomniane było o tej pani. Więc obydwoje na to zwróciliśmy uwagę.

**Widziałaś jeszcze jakieś zachowania ludzi, o których myślałaś, że to nie jest fajne?**

Nie, ale słyszałam, że ludzie widzieli. Że na przykład ludzie jacyś się grupują… Znaczy grupują, to okropnie brzmi (śmiech). Znaczy wiem, że nie można tego robić, ale brzmi to tak, jakby naprawdę to było coś jak w Korei Północnej. Ale właśnie słyszałam, że jacyś ludzie tam sobie stoją, jakaś młodzież pije coś. Nie wiem. Coś takiego słyszałam, ale nie byłam świadkiem.

**Byłaś świadkiem jeszcze jakichś zachowań, które wydają ci się niewłaściwe na obecny moment?**

Nie, chyba nie. Nie, nic takiego sobie nie przypominam.

**A obserwujesz na przykład ludzi, którzy chodzą na spacery, widujesz takich?**

Właśnie nie. Właśnie nie widuję takich ludzi. Dzisiaj może trochę widziałam więcej z samochodu. Że spacerowali, ale w sumie to byli ludzie z psem w sumie. Więc nie wiem, może ten spacer z psem mógłby być krótszy. Ale wydaje mi się, że też ludziom troszeczkę odpuszcza stres może i… I chcą chodzić więcej.

**A co sobie myślisz na przykład o rodzicach, którzy wychodzą z dziećmi na spacer? Bo są tacy.**

Tak. Nie wiem. Jestem pewna, że też bym wyszła, gdybym miała dziecko. Zresztą, jeżeli to jest spacer, gdzie nie dotyka się do żadnych publicznych części w sumie… Tylko, że właśnie, dziecko nie będzie dotykało rzeczy, które są publicznie dostępne. Ale no, nie, wydaje mi się, że to nie jest złe, jeżeli to będzie krótki spacer. I to naprawdę będzie spacer, a nie na przykład na plac zabaw. Znaczy to jest zamknięte, ale no można to jakoś może obejść czy coś, nie wiem.

**Mówisz, że jak byś miała dziecko, też byś wyszła.**

Myślę, że tak.

**A czemu?**

Nie wiem, bo chciałabym, żeby spędziło trochę czasu na dworze. No, zależy też, gdzie bym mieszkała, czy byłoby na przykład miejsce, żeby to dziecko się wybiegało czy nie.

**Ty nie jeździsz autobusami, prawda? Ty raczej taksówkami się poruszasz?**

Tak, tak, tak, w pracy mam to zapewnione. Ale jechałam metrem raz.

**I jak się czułaś w komunikacji publicznej?**

Trochę jak w grze, ale nie było to tak uderzające, jak… Wszystko jedno, tylko było prostu mało ludzi.

**A sprawdziłaś, ile jest tam osób przed wejściem?**

Nie, bo ja wiedziałam, że nie muszę ich liczyć, bo było dużo mniej niż 20.

**(niezrozumiałe). Bo ja na przykład nie wiem, ile powinno być ludzi w metrze.**

Bardzo lubię czytać te naklejki, które są na środkach komunikacji miejskiej. I wiem, że w tramwajach może być 17, w różnych autobusach 20, 21, 22. W metrze może być 20.

**Na wagon, tak?**

Tak. Ale po prostu zwracam na to uwagę, bo sobie to czytam i zapamiętuję. Zaczęłam czytać tak z ciekawości, a nie dlatego, żebym naprawdę liczyła.

**A myślisz, że gdyby było więcej, to byś przeliczyła tych ludzi w wagonie przed wejściem?**

Przed wejściem nie, ale może jak bym była w środku, to bym się zastanowiła, ile jest. To bym przeliczyła, czy… Ale nie wiem, czy bym wysiadła, raczej nie. Tylko bym zwróciła sobie uwagę na to, że jest za dużo.

**I co, dalej byś jechała?**

Myślę, że tak.

**Jak by było 25?**

Myślę, że tak.

**A czemu? To byłoby złamanie zasady.**

Ale nie miałabym pewności, że ja wsiadłam dwudziesta pierwsza.

**A pojawiło się u ciebie jakiekolwiek zachowanie, które sprawia, że jakoś łatwiej ci się odnaleźć w tej sytuacji, którą mamy?**

Na pewno pogoda jest sytuacją, która mi się pozwala odnaleźć w sytuacji lepiej.

**Tak? To, że jest ładnie?**

Tak, ja po prostu lubię, jak jest ładnie.

**A nie kusi cię wtedy, żeby wyjść.**

No, ale właśnie, wyjść i co mam robić? Jakby nie mam pomysłu, co mogłabym robić. Mogłabym iść na spacer, faktycznie, czy coś takiego. No nie wiem, nie miałam też za bardzo czasu, bo… Wydaje mi się, że jak jesteśmy we dwójkę w domu i nie ma pracy, to zwracamy uwagę na to, żeby jednak siedzieć w tym domu. Znaczy pomijając tego Lidla, ale się umówiliśmy na niego naprawdę dawno i to było zaplanowane, to jednak siedzimy w domu mimo wszystko.

**A co robicie? Jak spędzacie czas teraz? Bo mówiłaś, że trochę więcej z Filipem spędzacie czasu.**

Gotujemy. I… niestety oglądamy Eurowizję.

**Dlaczego niestety?**

Filip już to oglądał kiedyś. Ja tego nigdy nie oglądałam i nie interesowało mnie to za bardzo. Ale on uznał, że obejrzy sobie podczas kwarantanny wszystkie Eurowizje od któregoś tam roku. Nudzi mnie to, ale… Znaczy i tak znalazłam sobie, to jest to, doskonały przykład na to, o czym mówiłam wcześniej, czyli po prostu coś mi się nie podoba albo coś nowego. Ale znajdę sobie jakiś sposób, żeby się z tego cieszyć. Więc robimy sobie tak, że oceniamy każdy występ według naszej skali, od 1 do 10, potem według punktów Eurowizji i potem wybieramy zwycięzcę, który by wygrał, gdybyśmy my byli jurorami. Mimo, że te występy mnie nudzą i cały ten program też, no to jednak znalazłam coś pozytywnego. I sobie mogę tak spędzać czas. Ale to jest naprawdę strasznie długie, to trwa 3 godziny.

**No dobra, a gdybyś miała sobie wymyślić, powiedzmy, że nie ma tej Eurowizji, to co ty byś chciała robić w tym czasie? Na co byś miała ochotę?**

Nie wiem. Próbowałam grać w Simsy, znaczy już dawno, zanim w ogóle zaczęłyśmy rozmawiać ze sobą, ale… Nie wiem jako nie potrzebuję, umiem sobie organizować zajęcie, za niczym nie tęsknię. Raczej mam co robić, jeżeli nie ma co robić generalnie. I to nie są żadne takie nowe rzeczy, typu sprzątanie w szafie czy sprzątanie w kuchni, jakieś tam patrzenie na te rzeczy. Nie, tylko po prostu jakieś takie normalne rzeczy, które bym robiła, gdyby pewnie można było wychodzić z domu, ale bym nie chciała.

**A myślisz sobie, że bardziej lub mniej niż w zeszłym tygodniu brakuje ci wyjścia do knajpy?**

Może mniej. Nie, nie umiem powiedzieć, że w ogóle mi nie brakuje. Ale ani razu nie miałam tak, że pomyślałam sobie, że chciałabym wyjść gdzieś.

**A miałaś taką rzecz, o której pomyślałaś, kurczę, no zrobiłabym, ale nie mogę.**

Nie.

**No dobra, to knajpa tak troszeczkę. Ale nie jakoś tak, że aż…**

No nie. Jakby ktoś zadzwonił, czy bym gdzieś z nim wyszła, to bym pewnie z nim wyszła. Ale to nie jest tak, że ja bym na to… Nie wiem, mogłabym nawet na to wpaść. Ale to też nie jest rzecz, za którą jakoś wyjątkowo tęsknię. Oczywiście – będzie można, no to będę chodziła. Ale jak nie można, to nie można.

**A czy jest cokolwiek za czym tak wyjątkowo tęsknisz? Czy ty się tak dobrze przystosowałaś do tej sytuacji, że już nie tęsknisz?**

Trochę tęsknię za chodzeniem do biura, gdzie są wszyscy ludzie. I troszeczkę za taką, no jednak rutyną. Bo jak się pracuje zdalnie, znaczy, jak ja pracuję zdalnie, to właściwie trochę pracuję zdalnie, a trochę jeżdżę do biura. I nie wiem, czy będę w biurze tego dnia, czy nie. Czy będę musiała coś załatwić, czy będę musiała tam być, czy nie będę musiała. Więc troszeczkę za rutyną. I tyle chyba.

**Mówiłaś w zeszłym tygodniu, że u ciebie te wyjścia to knajpy to bardziej takie jakby… Ważniejsze jest to, z kim idziesz się spotkać. To myślisz sobie, że takich spotkań face to face to brakuje ci? Czy sobie myślisz, OK, jakoś tam będzie.**

To nie jest do końca tak, że ja się nie widuję z tymi ludźmi. Bo jednak w pracy widuję się z koleżanką z pracy. I właśnie to jest moja najbliższa koleżanka. I nie widujemy się codziennie, faktycznie, ale zdarza się, że się widzimy w biurze. Widziałyśmy się na pewno w tym tygodniu 2 albo 3 razy. No i to jakby połowa może normalnego dnia pracy. Więc, no jakiś tam kontakt miałam z kimś innymi niż z Filipem. I no właśnie nie jestem taką typową osobą, która generalnie w tej sytuacji, bo ja ani nie siedzę w domu, też się widzę z ludźmi. I to jakby nie jest moje widzimisię, tylko muszę się z tymi ludźmi spotkać, bo jednak to są jakieś służbowe sprawy. Widzę też jakichś innych ludzi z działu IT, którzy coś tam podłączają w biurze. Więc to też jest takie troszeczkę, cząstka tego normalnego życia, że są. Osoby, z którymi normalnie nie rozmawiam w pracy nawet teraz z nimi nie rozmawiam, ale przez to, że są, jest tak normalniej. Więc widuję się z ludźmi, ale z takimi innymi znajomymi, z którymi bym się może spotkała… Nie wiem, rozmawiałam na Facetimie jeszcze z jedną koleżanką, którą… Ale to też tak na szybko, po prostu zadzwoniłam do niej na chwilę i rozmawiałyśmy 20 minut. Ale to też nie było nic takiego, że co u ciebie, tylko po prostu jakieś bieżące sprawy.

**A gdybyś miała wybór, bo to cię w zeszłym tygodniu nie zapytałam właściwie i mogła zdecydować się tylko na pracę zdalną, w sensie, twoje aktywności zawodowe by pozwalały na to, żebyś pracowała tylko i wyłącznie w domu, to byś się na to zdecydowała?**

Nie.

**A czemu?**

Ja bym mogła prędzej mieszkać w pracy niż pracować stąd (śmiech). Ja potrzebuję cały czas widzieć jakichś ludzi. (niezrozumiałe) się nie potwierdza. Mogłabym się przyzwyczaić do niewidzenia tych ludzi i byłoby to OK. No, ale teraz ja wiem, że kiedyś będę na nowo widziała tych ludzi normalnie. Wiem, że to jest okres przejściowy tylko. I że jak to się skończy, to normalnie wszystko wróci do normy i będę widziała tych ludzi. Ale wiedząc, że nigdy już tak nie będzie, czy to nie wiem, na chwilę obecną nie będzie tak, to nie, nie chciałabym.

**A wyobrażasz sobie taką sytuację, nie wiem, na Cyprze chyba tak jest, nie wiem jak we Francji, że tam w ogóle wszystko jakby na 2 tygodnie zamknęli. I nie ma, siedzimy w domu i nie wychodzimy, tylko do sklepu. I to trzeba mieć pozwolenie, zgłaszać itd. Wyobrażasz sobie siebie w takiej sytuacji?**

No jak trzeba będzie, to tak… Znaczy no ja mam świadomość tego, że po prostu to nie ja decyduję i nie ja ustalam te zasady. I też nie ja roznoszę tego wirusa, chociaż może chodząc, roznoszę. Ale generalnie chodzi o to, że… Jakby nie mam żadnej władzy nad tym. Więc jedyne, co mogę zrobić, to mogę po prostu zobaczyć się w tej sytuacji i być w niej. Na pewno bym nie łamała w żaden sposób prawa czy tych ustalonych porządków. Wtedy mogłoby być mi ciężko, oczywiście.

**Zastanawiam się, czy obserwujesz takie dziwne dla ciebie zachowania wśród osób twoich znajomych? Albo słyszałaś o jakichś takich dziwnych zachowaniach, które ludzie teraz mają? Tak z twojej perspektywy.**

Chyba nie. Wydaje mi się, że już wszystko tak wraca… No, nie wraca do normalności może, ale właściwie tak się staje normalnością może. Nie spotkałam się z czymś dziwnym, co by mnie zdziwiło wśród moich znajomych.

**A myślisz, że to dobrze, że to jakby staje się taką normalnością, ta sytuacja, którą teraz mamy?**

I tak i nie. Bo tak jak mówiłam w zeszłym tygodniu, jednak ludzie powinni się bać, żeby uważać i żeby ten wirus nie rozprzestrzeniał się. Ale z drugiej strony dla dobra psychicznego może tych ludzi lepiej, żeby traktowali tą sytuację jako normalną?

**Następny temat taki duży, który mamy dzisiaj, to jest robienie zakupów. Najpierw chciałabym pogadać w ogóle z tobą o kupowaniu przez internet. Czy ty w ogóle w tej chwili kupujesz jakieś rzeczy przez internet z dostawą? Zamawiasz coś? Chodzi mi o wszelkie rzeczy, które można kupić przez internet. W ciągu ostatniego tygodnia, dwóch, zdarzyło ci się coś kupować?**

Jedzenie tak. Jedzenie, ale w sensie gotowe.

**A co zamawiałaś, co zamawiacie?**

Ramen. Pizzę chyba też zamówiliśmy. I coś może.

**A to w pracy czy w domu?**

W pracy. W domu nie zamawialiśmy, Filip dzisiaj zamawiał McDonalda.

**A w pracy to jest tak, że zazwyczaj zamawiacie? To jest taki element waszej codzienności pracowej?**

Tak, tak, tak, raczej tak.

**A teraz jest tego zamawiania więcej, mniej?**

Chyba tyle samo. Jesteśmy we dwie i właściwie cały czas zamawiamy, więc… No, raczej tyle samo. Nie, no właściwie to więcej, bo na pewno się zdarzyła sytuacja, w której nie zamówiłyśmy. A odkąd teraz pracujemy w tym nowym biurze, to chyba za każdym razem zamówiłyśmy. Więc częściej, ale przez to, że to jest krótszy okres może… Możemy to rozłożyć wszystko, odkąd się znamy i porównać do tego czasu, że wyszłoby tyle samo.

**A coś jeszcze przez internet poza takim gotowym jedzeniem zdarza ci się kupować?**

W życiu generalnie tak. A w tej sytuacji chyba jeszcze nie.

**A co ci się generalnie zdarza kupować przez internet?**

Ubrania wszystkie. No tylko ubrania.

**A spożywkę zdarza ci się?**

Zdarzało nam się kiedyś to robić. Ale jednak ja wolę pochodzić po sklepie i popatrzeć.

**A kosmetyki zdarza ci się przez internet kupować?**

Raczej nie.

**A jakieś książki albo elektronikę jakąś, takie rzeczy? To ci się zdarza czy raczej wchodzisz do sklepów?**

Zastanawiam się, jak często kupuję takie rzeczy. Książki faktycznie zdarza mi się czasem zamówić. Właściwie to nawet wszystkie, jakie kupuję, to zamawiam, ale nie jest to jakoś często. I ewentualnie kupuję jakieś takie zdrowe rzeczy albo takie (niezrozumiałe), których po prostu, nie wiem, gdzie są takie sklepy, jakieś bio czy coś i nie chce mi się do nich jeździć. Takie rzeczy.

**A w czasie tego okresu kwarantannowego, nie miałaś takiej potrzeby, żeby sobie coś bio kupić?**

Miałam potrzebę i nie było w tym sklepie internetowym tej rzeczy, którą chciałaś kupić, więc jej po prostu nie kupiłam.

**I nie miałaś tak, że sobie myślałaś, że chcę, więc poszukam w innym.**

Miałam, miałam nawet. Ale nie znalazłam dokładnie tego, co chciałam. I nie wiedziałam też, czy to, co znalazłam będzie takie samo jak to, co miałam. I w sumie, po prostu, bo kiedyś to się skończy, kiedyś pojawi się ten produkt w sklepie, to sobie kupię na nowo.

**Czyli to nie jest tak, że ty jak czegoś chcesz, to masz taką potrzebę, żeby jednak zrealizować to szybko? Jesteś w stanie tak czekać na różne rzeczy?**

Wiesz co, to nie jest rzecz, którą bardzo chcę mieć, bo to jest po prostu… To jest taki proszek z gorzkiego melona, ekstrakt z gorzkiego melona. Który wsypuję sobie łyżeczkę do wody. I wypijam to rano i wieczorem. I to jest po prostu gorzkie. I smakuje jak gorzka herbata, ale bez smaku herbaty. Piję to, żeby pobudzić metabolizm. I generalnie jest to dość zdrowe, żeby to pić, w sensie jak suplement mniej więcej. No, nie jest to smaczne, zależy mi na tym, żeby to pić. Ale nie jest to rzecz, którą chcę, tylko rzecz, którą może powinnam pić. Więc jeżeli jej nie mam, to jest troszeczkę inaczej.

**Czyli z jednej strony powinnaś, ale jak nie ma, no to trudno.**

No, to jest troszeczkę taki dzień dziecka.

**Czyli odpuszczasz sobie wtedy ten element takiego dbania o ten swój metabolizm i o siebie.**

Tak. I mam też (niezrozumiałe). Ale to też jest, wydaje mi się… właśnie nie pamiętam, ile to kosztuje, ale w mojej głowie, mam takie wrażenie, że to jest trochę drogie, więc… Może trochę szkoda mi pieniędzy teraz na to, jak była świadomość, że może troszeczkę być… Że na przykład mogą mi się skończyć pieniądze. No to może nie powinnam tego kupować, może powinnam się skupić, nie wiem, na jedzeniu czy na czymś takim, bez czego na pewno nie mogę żyć. A to nie jest rzecz, bez której nie mogę żyć.

**A masz jeszcze jakieś takie produkty w ogóle, które kupujesz przez internet i tylko przez internet? Ten proszek z melona, coś jeszcze masz takiego, co zawsze przez internet?**

No ubrania chyba tylko, buty. I tyle.

**A z ubraniami, dlaczego przez internet ubrania?**

Bo jest bardzo dużo ludzi w sklepach. I nie lubię jeździć w ogóle do sklepów za bardzo. W sensie centrów handlowych. Jest mi tam gorąco, nie chce mi się nosić kurtki. No nie wiem, wygodniej mi się po prostu robi zakupy w internecie.

**Czyli wygoda.**

Tak.

**A w związku z tym przez ostatni miesiąc żadnych ciuchów nie kupiłaś?**

Ja generalnie kupuję bardzo mało ubrań, więc… Nie, chciałam sobie kupić nawet ubrania, ale pomyślałam sobie, że muszę oszczędzać, więc nie kupiłam po prostu.

**A są jeszcze jakieś inne rzeczy, których teraz nie kupujesz z taką myślą, że trochę za drogie albo że może niekoniecznie potrzebne, może warto oszczędzić więcej.**

No generalnie teraz kupuję po prostu tylko takie spożywcze rzeczy. Ale też nie jestem pewna, czy to jest przez tego wirusa, czy po prostu przez to, że nic mi nie przyszło do głowy, nie miałam czasu ani ochoty o tym pomyśleć, czy coś potrzebuję.

**A kupiłaś sobie w ostatnim czasie, nawet będąc w sklepach stacjonarnych, coś tak dla przyjemności, nawet jedzeniowego? Żeby sprawić sobie przyjemność.**

Nie, ale byłam na paznokciach. To mi się przypomniało, co robiłam, odkąd nie widziałyśmy się. Mimo, że nie można.

**Mimo, że nie można? Ale byłaś w salonie?**

Wiesz co, taka dziewczyna robi w domu. I to nie jest jej dom, tylko wynajęła mieszkanie, żeby robić paznokcie. I wtedy chyba jeszcze można było tak naprawdę. Nie było tak otwarcie powiedziane, że nie można, tak jak teraz. Bo to było w sobotę, jak byłam. Ale to też jest troszeczkę… No, każdą sytuację można sobie tak usprawiedliwić i właściwie powiedzieć, że wszystko jest w porządku. Ale ona jest za długo moją koleżanką, trochę jak bym się spotkała z koleżanką w tym domu po prostu. I ona z tego co mówiła, to też, ona w ogóle była w megaprofesjonalnej maseczce i myła ręce 5 razy podczas tego, jak robiła mi paznokcie i jeszcze kazała 5 razy ręce myć mi. I też ona bardzo ograniczyła listę klientek do właściwie tylko takich, powiedziała, że nawiedzonych. Czyli takich, co wie, że na pewno wychodzą z domu tylko na paznokcie i tylko w tej maseczce i jakoś tak może być. I do jakichś bliskich, zaufanych.

**To pogadajmy trochę o tym, jak jecie. Powiedziałaś, że w wolnym czasie gotujecie. W ogóle gotujecie więcej niż wcześniej?**

Gotujemy. Po prostu gotujemy.

**A co zaczęliście gotować?**

To, co ja wymyślę właściwie.

**To opowiedz mi, co tam powymyślałaś.**

Robiliśmy placki ziemniaczane. Frytki z batatów też robiliśmy. Znaczy, po prostu chodziliśmy po tym Lidlu i patrzyliśmy sobie na produkty. I patrząc na nie, wymyślaliśmy jakieś rzeczy. Jak Filip zobaczył bataty, spytał się, czy możemy zrobić frytki z batatów. No jasne, możemy zrobić. Robiłam też boczniaki w panierce, mizerię zrobiliśmy, buraczki zasmażane. Marchewkę z groszkiem.

**Coś jeszcze takiego, co nie robicie na co dzień się pojawiło?**

Filip chyba zrobił kotlety jajeczne, ale nie wiem, nie jadłam tego.

**A to wy na co dzień w ogóle nie gotujecie, tak w normalne dni?**

Raz na tydzień. Nie, raz w tygodniu roboczym i raz w weekend. Czasami 2 razy w weekend.

**A teraz gotujecie, bo macie czas, czy wam się nudzi?**

Mamy czas. Tak jak już mamy świadomość, że codziennie puszczamy tą zmywarkę, to możemy dołożyć tych garnków. Bo niestety zmywarka teraz chodzi codziennie. I śmieci też wyrzucamy codziennie.

**I to się zmieniło w porównaniu z tym, co było przed?**

Tak.

**A czy to, że więcej gotujecie sprawiło, że twój styl jedzenia się zmienił, takie zwyczaje żywieniowe wam się zmieniły?**

Wydaje mi się, że jem bardziej niezdrowo. Wydaje mi się, że mniej zwracam uwagę na to, co jest zdrowe, albo… jakieś takie powiedzmy fit, a dużo bardziej jem to, na co po prostu mam ochotę. Bez większych wyrzutów sumienia.

**A bardziej niezdrowo, to co zjadłaś takiego, co myślisz, że nie zjadłabyś tego normalnie, a teraz po prostu pozwoliłaś sobie?**

Jem puree ziemniaczane z ogromną ilością masła i śmietany od trzech dni i nie mam żadnych wyrzutów sumienia. Tak samo, zaczęłam jeść w ogóle masło, ja nie jadłam nigdy wcześniej masła. A teraz jem chleb z masłem na przykład.

**Na nic ci się nie pojawiły wyrzuty sumienia?**

Nie.

**A to jest tak, że tylko sobie na ten ciepły posiłek pozwalasz na więcej, czy to jest tak, że każdy posiłek jest inny niż był przed epidemią? Nie wiem, śniadanie, w ogóle jesz śniadania?**

Tak, jem śniadania. No właśnie przez to, że jest więcej czasu na przygotowanie, to codziennie można zjeść coś nawet fajnego na śniadanie. Zawsze się staram, żeby to było fajne śniadanie. A teraz jest fajne-smaczne, a nie fajne takie, które jest zdrowe.

**A daj mi przykład takiego, które jadłaś wcześniej. Takiego, które było fajne i zdrowe.**

Przede wszystkim jadłam chleb bez jakiejkolwiek pszenicy. Po prostu chlebek z ziaren. A awokado najczęściej. Potem się dowiedziałam, że nie powinnam za bardzo awokado jeść. No, ale zjadłam z tym awokado, generalnie rukolą, może cebulą, z pomidorem. A teraz już nie ma w tym domu czegoś takiego jak chleb bez pszenicy. Jest niestety tylko niezdrowe. W sensie nie jest to po prostu tak zdrowe, jak było wcześniej.

**No dobra, to co dzisiaj na śniadanie jadłaś, pamiętasz?**

Dzisiaj jadłam hot-dogi.

**Hot-dogi jadłaś na śniadanie?**

Tak. Znaczy w dalszym ciągu są to hot-dogi bez mięsa i mam nadzieję, że przez to nie zacznę jeść mięsa. Ale tak, hot-dogi…

**Masz nadzieję, że nie zaczniesz jeść mięsa. Trochę się boisz, że cię skusi?**

Tak. Nie wiem, czy to jest kwestia tej… Nie wydaje mi się, żeby to była kwestia koronawirusa, tylko po prostu już od dłuższego czasu mam troszeczkę ochotę. A że teraz sobie pozwalam na więcej, to może sobie pozwolę. Znaczy na razie wiem, że nie, ale może mi przyjdzie taki pomysł do głowy.

**A na przykład jecie razem też?**

Tak.

**Chodzi mi o to, że razem, w tym samym momencie, to samo.**

Tak, tak, tak, tak.

**A wcześniej jedliście razem?**

Nie. Czasami, nie zawsze, ale razem tak częściej chyba niż wcześniej.

**Ale to częściej, bo?**

Nie wiem, mam tylko odpowiedź, dlaczego moglibyśmy na myśl taka. A czemu częściej? Nie wiem, już chyba po prostu… Przede wszystkim to jest tak, że ja w swoim pokoju nie spędzam w ogóle już teraz czasu, tylko cały czas spędzamy w jednym pokoju. I nawet żartujemy, oczywiście to się nie stanie, żartujemy, że moglibyśmy totalnie wynająć ten pokój komuś, bo i tak nikt w nim nie spędza czasu. I właśnie dlatego, że spędzamy czas ze sobą, to w tym samym czasie też możemy zjeść.

**A skoro tyle gotujecie, to nie rozważałaś na przykład tego, żeby sobie ugotować i wziąć do pracy? Na przykład gotować wieczorem i sobie zapakować i coś wziąć ze sobą do jedzenia i nie zamawiać?**

Tak. Ale zamówienie jedzenia w pracy to też jest rytuał i to jest rzecz, którą robimy razem. Razem wybieramy, razem odpuszczamy, jeżeli czegoś nie chce ta druga osoba. Więc to jest raczej też taka forma czegoś fajnego. A nie konieczność zjedzenia. Jasne, że mogłybyśmy sobie robić jedzenie. Ale to raczej chodzi o sam rytuał.

**A jak się pojawił ten Lidl, w którym byliście i gdzieś jeszcze byliście razem na zakupach?**

Nie, byłam sama w Biedronce dzień wcześniej.

**No to jak wyglądała taka lista zakupów takich biedronkowych?**

Mieliśmy sobie zrobić listę zakupów. I nawet dzień wcześniej czy 2 dni przed tym Lidlem sobie tak usiedliśmy i postanowiliśmy, że chcielibyśmy zrobić listę rzeczy, które będziemy jeść przez cały tydzień. I kupić produkty tylko do tych rzeczy. I jeszcze chcieliśmy przejrzeć szafki kuchenne, żeby kupić tylko rzeczy, które są potrzebne. Ale nie wyszło to w ogóle.

**Ale to w ogóle bez listy pojechaliście?**

Tak, tak, tak. My nigdy nie robimy listy. Filip sobie robi ewentualnie czasem listę, to są takie rzeczy, które mu brakują. Ale to nie są spożywcze rzeczy, tylko na przykład chusteczki albo coś takiego, Pronto. Ja nigdy nie robię listy, bo to nie jest nigdy tak, że ja coś potrzebuję. Chcę kupić to, na co mam ochotę.

**No dobra, ale powiedzmy, że wiesz, że będziesz jechała w niedzielę do tego Lidla, bo już zaplanowaliście, że będziecie jechać. I w piątek przychodzi ci na myśl, że zjadłabyś coś, to nie wpisujesz tego na przykład na listę, z myślą o tym, żeby pamiętać w niedzielę?**

Ja pamiętam.

**No dobra, to poszłaś do Biedronki w piątek, w sobotę…**

My też bardzo uważnie chodzimy po sklepach. I oglądamy każdy produkt. Więc jakby nic nam nie umknie, bo wszystko nam wpada w oczy. Więc wiemy, czy to potrzebujemy czy nie.

**To poopowiadaj mi trochę, co w tym Lidlu kupiliście. Pamiętasz, co tam było?**

Filip kupił bardzo dużo mrożonek. Tłumaczył mi dzisiaj, dlaczego to kupił, powiedział, że na wszelki wypadek.

**To takie zapasy.**

No właśnie nie wiem, czy nuggetsy można nazwać zapasami. Ale tak powiedział. Kupiliśmy dużo warzyw. Warzyw i nabiału. Śmietanę, jogurt. Pieczywo kupiliśmy też. Filip kupił dużo alkoholu, ja kupiłam piwo bez alkoholu, picie. Bo niestety rzecz, którą nie mogę się powstrzymać, to są po prostu słodkie picia, które muszę pić zamiast wody.

**Czyli cola, tego typu rzeczy?**

Nie, niegazowane.

**Co to jest słodkie picie?**

Icetea, coś takiego. I nie wiem, kupiliśmy bardzo dużo rzeczy, więc dziwne, że mi nic nie przychodzi na myśl. Były to puszki, ale było tylko po jednej puszce kukurydzy i fasoli, bo to po prostu zawsze musi być jedna w domu, no i akurat nie było. A tak to warzywa, nabiał… I pieczywo.

**Kupiliście coś, czego na co dzień nie kupujecie, ale pomyślałaś sobie, że należy mi się taka przyjemność w tym trudnym czasie. I kupiłaś sobie coś z jedzenia takiego super?**

Taką rzeczą jest mrożona pizza, ale kupujemy ją zawsze, jak się wybieramy na większe zakupy.

**A kupiłaś więcej w związku z tym?**

Czy więcej kupiłam?

**No, tej pizzy.**

Nie. Filip kupił więcej, ja kupiłam tylko jedną. Ale możliwe, że Filip też częściej kupuje niż ja. Bo ja to kupuję raz na 2 miesiące może.

**Mówisz, że jak jesteście w sklepie, to bardzo dokładnie oglądacie, co tam jest i dzięki temu kupujecie wszystko to, co jest potrzebne. I nadal taki rytuał zachowaliście?**

Ja, szczerze mówiąc, czułam, że już chciałabym wyjść z tego sklepu. Bardzo mnie to zmęczyło i miałam świadomość tego, że powinnam być w tym sklepie krócej. Dlatego, że ludzie czekają, że trochę nie chcę tam być. Ale Filip bardzo długo robił zakupy.

**A byłaś na niego zła za to, że tak długo tam jesteście?**

Zirytowana.

**I powiedziałaś mu to?**

Nie. Nie, też nie chciałam, żeby się czuł w jakikolwiek sposób zestresowany w tym sklepie albo zdenerwowany. A ja nie bałam się o swoje życie czy coś takiego, tylko już po prostu nie chciałam tam być. Ale to nie była kwestia chyba… Nie wiem, czego to była kwestia, po prostu już nie chciałam tam być. Ale poradziłam sobie jakoś.

**A jeszcze chciałam cię zapytać o plany takie zakupowe na najbliższe dni. Jak myślisz, kiedy następne zakupy?**

Wydaje mi się, że do niedzieli raczej nie.

**Czyli do Wielkanocy?**

Tak, myślę, że tak.

**A te zakupy w Biedronce dzień wcześniej? Chciałaś coś konkretnego kupić, coś ci brakowało?**

Brakowało mi chyba wszystkiego, ale to było za długo do czekania do Lidla, a w tym Carrefourze na dole nic nie ma. Nie mam żadnego innego sklepu w okolicy, więc poszłam do Biedronki. Też dlatego, bo to był dzień, kiedy cały dzień pracowałam zdalnie i właściwie zrobiłam wszystko szybko. Dlatego, że wstałam jakoś też dużo wcześniej, zaczęłam robić te rzeczy wcześniej. Więc miałam chwilę, żeby… Miałam tak, że po prostu nic nie musiałam zrobić albo mogłam to zrobić później, więc po prostu uznałam, że się przejdę. I kupiłam jakieś rzeczy, ale wykorzystałam je od razu na obiad, czy na… Nie pamiętam właśnie co… Dużo wtedy zrobiłam strasznie do jedzenia, ale nie pamiętam… Aha, ugotowałam zupę i zrobiłam sałatkę ziemniaczaną. (niezrozumiałe) tylko śmietanę i pomidory i włoszczyznę. I jakieś rzeczy na sałatkę, czyli ziemniaki, majonez.

**A jakie masz wrażenie, jak sprzedawcy w sklepie się zachowują w obecnej sytuacji?**

Przepraszam, bardzo mi przykro, nie zwróciłam uwagi. Ale właśnie! Może dlatego nie zwróciłam uwagi, dlatego że… Bo inaczej zazwyczaj właściwie zwracam uwagę, rozmawiam ze sprzedawcami bardzo dużo albo żartuję albo coś takiego. A nie wydaje mi się, żebym miała w ogóle jakąkolwiek interakcję między ludźmi.

**A myślisz, że to wynikało z ciebie czy z nich, ten brak interakcji?**

Obydwu, wydaje mi się. Oni może byli zmęczeni. Zresztą, zawsze jak stoję przy kasie, to mam taką refleksję, że tak bardzo dużo ci ludzie na tej kasie, którzy siedzą, mijają ludzi. I mogą się zwyczajnie bać albo po prostu być narażonym na wirusa.

**A czy obecna sytuacja w jakikolwiek sposób wpływa na to, jakich ty rodzajów płatności w sklepie używasz?**

Nie, zawsze płaciłam telefonem i cały czas to robię.

**To pogadajmy chwilę o Wielkanocy, bo się zdziwiłaś, że to już.**

Tak, zdziwiłam się.

**Masz jakieś plany na Wielkanoc?**

Wydaje mi się, że właśnie nie mam planów. Pytałam się mamy, czy coś robimy, a ona powiedziała, że niestety chyba właśnie nic nie robimy. Więc wydaje mi się, że nic nie zrobię.

**A czego byś się spodziewała, pytając mamę.**

A, zawsze idziemy do babci w sobotę. W sobotę idziemy zamiast w niedzielę, dlatego że w poniedziałek chcemy odpoczywać. Więc odkąd chyba się urodziłam pewnie, jest tak, że w sobotę najpierw mama idzie tam z koszykiem, a potem idziemy do babci. Później, w niedzielę idziemy do drugiej babci. I to koniec. Potem właśnie, żeby sobie w poniedziałek móc normalnie nic nie robić. I nie siedzieć przy tym stole. Znaczy albo ze znajomymi się rodzice spotykali czy coś takiego. A teraz wydaje mi się, że po prostu nic nie będziemy robić. W sensie oni będą siedzieli w domu, ja będę siedziała w domu i babcia.

**A jak się czujesz z tym, że nic nie będziecie razem robić?**

Cieszę się, dlatego że ja nie lubię w ogóle takich rzeczy robić z rodziną. Ale jest mi troszeczkę przykro, bo może mogłabym jakoś wesprzeć babcię, czy coś z nimi porobić. Jakoś wydaje mi się, że jak teraz nie mogła wychodzić, to może być dla nich troszeczkę gorzej. Dlatego chciałabym mieć obowiązek pojechania tam, bo wiem, że byłoby jej na pewno miło, jak bym tam pojechała. A jak mam przyzwolenie, żeby nie jechać, no to raczej nie pojadę. I wiem, że jej będzie przykro.

**A jest zakaz, żeby pojechać?**

Nie, ona nawet tego nie organizuje.

**A tak, żebyś sama się wybrała do babci w odwiedziny chociaż zobaczyć babcię, myślałaś o tym, czy nie?**

Ja o tym myślę raz na 3 miesiące jak muszę to zrobić. Bo sobie tak uświadamiam, że muszę. No muszę, a tymczasem po prostu, zwyczajnie mi się niestety nie chce.

**A z rodzicami się zamierzasz spotkać?**

Podczas tej kwarantanny? Nie, raczej nie.

**A będziesz jakoś specjalnie się szykować do Wielkanocy? Myślisz o tym, że jest Wielkanoc, czy po prostu będzie to taki weekend przedłużony o 1 dzień?**

Nie wiem. Właśnie dlatego, że Filip akurat będzie się spotykał z rodzicami. Nie wiem zupełnie, może właśnie o tym porozmawiam z mamą. Dlatego, że nie wiem, może na przykład pojadę po prostu do domu i będzie siedziała z rodzicami. Ale tak właściwie, skoro jest jedna babcia, to ta babcia też mogłaby do nas przyjść, albo my też moglibyśmy iść do babci. Więc nie wiem, ale na pewno poddam się temu, co ktoś z nich zadecyduje.

**Czyli pewnie mama albo tata.**

Tata na pewno nie, ale wydaje mi się, że babcia może zadecydować, czy na przykład ma siły coś przygotowywać, albo czy ma ochotę wychodzić albo czy ma się ochotę spotykać z nami, którzy jednak wychodzą z domu częściej troszeczkę niż ona.

**A zakładając, że nie wiem, babcia nie będzie ryzykować, to jakoś przygotujesz się do Wielkanocy, czy po prostu potraktujesz to jako weekend?**

Potraktuję to jako weekend.

**Czyli żadnych przygotowań.**

Żadnych. Chcieliśmy kupić bazie, ale nie było.

**A jeżeli będziecie jechać do babci, to będą jakieś przygotowania, czy po prostu pojedziesz?**

Po prostu pojadę.

**Bazie chciałaś kupić?**

Filip chciał kupić bazie, bo on lubi takie tematyczne ozdoby w domu.

**A macie jakieś inne, poza baziami, jakby ozdoby?**

Nie. Wydaje mi się, że zapomnieliśmy w ogóle o tych świętach w tym roku. Bo w zeszłym roku faktycznie, coś tam nakupował, jakieś kurczaki czy coś. I to stało. A teraz nie.

**A ty w ogóle nie jesteś taką osobą, którą tak jakoś celebruje święta? Te czy Boże Narodzenie?**

Raczej nie.

**To powiedzmy, że Filip pojedzie do rodziców, babcia powie, że nie idziecie, to jak wyobrażasz sobie tą sobotę, niedzielę, poniedziałek? Bo dla ciebie zawsze święta już były w sobotę.**

No tak, ale dla Filipa już nie są w sobotę, więc w sobotę Filip pewnie będzie w domu, więc będzie to normalna sobota. A w niedzielę, nie wiem, może tak naprawdę się spotkam, kogoś zaproszę tutaj, żeby przyszedł, co się troszeczkę mija z celem. Nie wiem, zupełnie nie wiem. Może po prostu odpocznę i nie będę robiła nic, sama.

**A myślisz, że takie 2 dni, niedziela, poniedziałek sama to jest coś, co w tym momencie jest fajne? Jakby cieszysz się na to?**

Wydaje mi się, że to nigdy nie jest fajne, nie lubię być 2 dni sama. Ale w sumie już dawno nie byłam sama. Więc może przynajmniej 1 dzień będzie fajny. A drugi może być trochę ciężki, ale wtedy może znajdę sobie jakieś inne zajęcie. Albo pojadę do tego nieszczęsnego domu.

**Ale bardzo chyba nie chcesz, co?**

Nie, za bardzo tam nie lubię, bo już nie mam już tam swojego miejsca. Mój brat się przeprowadził do mojego pokoju, bo mu się bardziej podoba i… Jakby już po prostu się nie czuję za bardzo jak u siebie, tylko jak gość.

**A nie myślisz o tym na przykład, żeby skoro Filipa nie będzie, żeby to rodzice ciebie odwiedzili?**

Nie wiem, jak by chcieli, to mogą przyjechać, ale nie będę ich zapraszać (śmiech).

**Czyli tak, zero przygotowań do Wielkanocy u ciebie, tak naprawdę. A to, jak spędzisz, to zależy od tego trochę, co rodzice zdecydują.**

Tak, raczej tak. Tak.

**A masz jakiekolwiek pomysły na dalsze spędzanie takiego czasu w tej kwarantannie? Poza gotowaniem i oglądaniem Eurowizji? Ile wam jeszcze tych lat zostało do obejrzenia?**

(westchnięcie) Sześć.

**Wiesz, jaką miałaś minę teraz (śmiech)?**

One są strasznie długie.

**Ja nie wiem, bo nigdy nie oglądałam, więc trudno mi sobie wyobrazić.**

Ja też nie oglądałam. A są jeszcze półfinały. Są 2 półfinały i 1 finał. Więc jeszcze naprawdę sporo oglądania.

**To jeszcze trochę przed wami, macie czym wypełnić ten czas.**

Tak. Tak. Dzisiaj chyba nie będziemy oglądać, bo spytałam, czy możemy dzisiaj nie oglądać.

**I co będziecie dzisiaj robić?**

Nie wiem.

**A to jest tak, że miałabyś ochotę pobyć już trochę sama. W sensie to, że gotujecie z Filipem albo oglądacie razem tą telewizję, to myślisz sobie: pobyłabym wieczór sama?**

Nie, bo ja wiem, że mam taką możliwość. W sensie… Akurat bardzo ładnie nauczyliśmy się mówić sobie, jeżeli chcemy spędzać czas sami, że chcemy dzisiaj być sami. I wiem, że mam taką możliwość, jak bym chciała, to bym spędziła ten czas sama. Ale nie mam ochoty tego robić jeszcze. Znaczy ja po prostu bardzo rzadko mam ochotę spędzać czas sama, w ogóle Filip częściej. Ale Filip też nie ma ochoty.

**Czy masz jakieś jeszcze refleksje związane z naszą rozmową?**

Nie, właśnie jak myślałam o tym, że będziemy rozmawiać, to właśnie chciałam zwrócić uwagę na to, że się czuję dużo spokojniej i dużo tak normalniej niż wcześniej.

**To załapałam, tylko cały czas nie do końca rozumiem, z czego wynika to, że ty się czujesz tak dużo spokojniej i normalniej? Bo sytuacja się, przynajmniej tak mi się wydaje, że nie idzie jakby…**

Nie, gdzieś tam idzie. Nie, no po prostu… Nawet nie chodzi o to, że idzie, tylko po prostu wiem, że kiedyś zacznie iść. Zresztą też troszeczkę mniej oglądam w sumie wiadomości. (niezrozumiałe) ale to nie jest tak, że codziennie oglądam albo o tym słyszę.

**Ale tak celowo ograniczyłaś?**

Nie, chyba przypadkowo.

**Dziękuję bardzo.**
