# Supplementary material for: The challenges arising from the COVID-19 pandemic and the way people deal with them. A qualitative longitudinal study
Source: PLoS One. 2021 Oct 11;16(10):e0258133. doi: 10.1371/journal.pone.0258133 (PMC8504766; doi:10.1371/journal.pone.0258133)
Supplement: S1 Dataset — (ZIP) [file pone.0258133.s003.zip › Transcriptions/stage 5/5.5_M_39_single.docx]

**5.5_M_39_single**

**Co się działo u ciebie przez ostatni miesiąc?**

Leki, które przyjmuje działają i czuje się już dobrze. Przy okazji zacząłem chodzić do mongolskich lekarzy, którzy postawili mi bańki, zrobili akupunkturę, nastawiali mi kręgosłup, itd. I ja przewiałem te bańki. Tak mnie połamało, że ja nie miałem siły leżeć nawet.

**Skąd pomysł, żeby iść do mongolskich lekarzy?**

Bo ja już nie mam pomysłu na swoje dolegliwości. Wiecznie w ogóle mam zawroty głowy od siedzenia, więc nie miałem już na to pomysłu, a ich sporo ludzi chwali. Ona jest lekarzem w ogóle - ta laska, która tam przyjmuje i ona łączy konwencjonalne z niekonwencjonalnym. Jak wychodzę od nich, a dopiero zaczęliśmy i to jest początek, ale jak wychodzę to się czuję lepiej. Kwestia tego, żeby na stałe tak zostało i żeby to nie było aż tak uciążliwe, jak jest teraz. Zajechałem siebie, zajechałem kręgosłup i stwierdziłem, że się biorę za siebie ze wszystkich stron, bo to jest ostatni gwizdek. 40-tka pyknęła, wróciłem do sportów i dźwigam się. Nie wiem, jak długo tam będę chodził. Myślę, że raz na jakiś czas ja się będę musiał tam pojawić i tak to już zostanie. Mam pospinany tak cały kręgosłup i nastawiają mi kręgi, bo mi wyskakują, i...Wg niej to się nazywa fachowo nerwica wegetatywna. Dwutorowo idę i każda strona o tej drugiej stronie mówi, że to jest dobrze, że w dobrą stronę. To mnie uspokaja, nie wprowadza w stany nerwowe, więc nie pobudzam tych stresów, które powodują napięcie mięśni, więc zostają stany zwyrodnieniowe i przemęczenie kręgosłupa, a już nie do końca czynnikiem jest stres.

**Mieszkasz teraz u siebie, u rodziców?**

U siebie. Dobrze się stało, że wróciłem do siebie, bo człowiek zaczął powoli się zbierać. Dużo na tle kariery muzycznej się rozpędziło znowu, bo tam gdzieś za granicą wychodzą moje wydawnictwa, więc to też miło i jakoś szukam zawsze dobrych impulsów i w ogóle impulsów do działania, więc człowiek zaczął wychodzić z domu, za sporty się wziąłem, spacery, deskorolka, rower, zacząłem grać w tenisa po kilku latach przerwy. Najbliższe grono znajomych bardzo mi pomogło w tym wszystkim. Nieliczni są uświadomieni, o co chodzi w moim życiu w tym momencie, że bardzo potrzebowałem pomocy, przyjaźni, towarzystwa i samych dobrych emocji.

**Pracujesz teraz czy jesteś jeszcze na zwolnieniu?**

Po nocnej zmianie idę dzisiaj na nocną zmianę. Wróciłem wczoraj do pracy. Tam panują wewnątrzfirmowe zasady i na wejściu mierzą ci temperaturę i w drodze do swojego budynku trzeba być w maseczce, ale to tylko przechodząc przez bramę zakładu ludzie to stosują i ewentualnie w autobusach wewnętrznych, bo Orlen jest tak ogromny, że się dojeżdża autobusami wewnętrznymi. Tylko wtedy ludzie stosują, a tak to...

Były jakieś przełomowe dla ciebie momenty w czasie tego miesiąca?

Chyba tylko to, że zgrało się to, że zapragnąłem wrócić do tenisa i była taka możliwość, że można wejść pod kryty balon i nie ma przeciwskazań. No to, że te sporty, które ja uprawiam można bez problemu. Nie ma obostrzeń i nie muszę o tym myśleć.

**Czy twoje życie wróciło już do takiej normy sprzed epidemii, czy jeszcze coś zostało inne niż wcześniej? Coś przeszkadza?**

No kluby, nie? Wesela można robić na 150 osób, a kluby zamknięte. To jest w ogóle jakaś paranoja. Albo tak, albo tak. To są debile, zresztą znasz moje poglądy...W UK mam kolegę, z którym wczoraj rozmawiałem i on mówi, że u nich jeszcze totalny jest ten lockdown. Teraz jeszcze tam wprowadzili kwarantannę dodatkową. W Irlandii jest tak samo jeszcze i jest przechlapane. Dopiero w lipcu coś tam ma ruszyć. Co kraj, to obyczaj. Słowacy otwierali granice, ale nie dla nas. To jest bzdura totalna to wszystko.

**Poza klubami, jak patrzysz na swoje życie, to jeszcze czujesz tę pandemię?**

Czuje się, bo ten temat, nie ma siły, ale jednak się przewija w rozmowach z przyjaciółmi. Jak nie w ten, to w inny sposób. Z tymi, z którymi rozmawiałem w trakcie, to mam to przegadane, z kim nie gadałem, to rozmawiamy na nowo. Pytamy się jak tam było w tej pandemii, jak przeżyliśmy, jak przetrwaliśmy, jak finansowo. Ostatnio byli u mnie znajomi a z Wrocławia i mówią, że tam jest tak, jakby w ogóle nie było jakiejkolwiek pandemii. Open'erowe miejsce, gdzie jest muzyka, to są balety i na kilkaset osób, więc...

**A ty sam jeszcze się czegoś obawiasz?**

Zero. Został 1% takiego wiesz...Jak chodzę do supermarketu, to jednak zakładam. Byłem wczoraj w Auchan i jednak komunikaty cały czas w tle, że niezbędne są maseczki, że proszę nosić. Znajomy mi mówił, że ochroniarz mu zwrócił uwagę, żeby założył maseczkę. Kurde, ja myślę, że to w ogóle już tak zostanie, nie? Nasze państwo będzie akurat manipulowało pod siebie, ale totalnie idealna sytuacja im się trafiła, żeby manipulować pod siebie, czyli to, co jest dobrze im, to będzie ok., a to, żeby się ludzie nie zbierali, żeby protestów jakichś nie było, to będą tak manipulować kurde. Twój ból jest większy niż mój, nie?

**Emocje- zdjęcia**

13 - doszła taka wolność natury, że można już

6 - tak samo

9 - doszła, bo jest to spowodowane tym, że koronawirus już człowiekowi w głowie siada, ale sytuacja na świecie i susze, i klęski żywiołowe będą teraz myślę, że też tematem bardzo ważnym. No niestety, ale ta planeta umiera.

4 - z pewnymi ludźmi, z którymi była więź przed pandemią jest dzisiaj jeszcze większa. Nie tylko z moich wniosków, ale i z wniosków moich przyjaciół wynika, że z tymi, z którymi byliśmy blisko, przyjaźniliśmy się, to jesteśmy jeszcze bliżej, bo z nimi najczęstszy kontakt mieliśmy, wręcz bez przerwy.

1 - do tego jeszcze daleko, jeszcze nie ma aż tak, nie ma jeszcze takiego ruchu, jeszcze ludzie niektórzy podchodzą sceptycznie. tak, jak mówiłem o przesuwaniu się tego od takiego myślenia i przejmowania się koronawirusem do takiego myślenia, żeby mieć to totalnie w dupie. To widać. ja byłem na mieście u mnie w Płocku i jest tak, że jest mnóstwo ludzi na mieście, ale jednak nadal to widać na mieście, że bardzo słabo jeszcze się to kręci. Jest takie jeszcze trochę zamknięcie się.

**Jakie to są uczucia, które odpowiadają tym zdjęciom?**

13 i 6 - totalna wolność w tym momencie związana z łonem natury, że w końcu mamy do niej bezpośredni dostęp. Nic nas już nie ogranicza, jeśli chodzi o naturę i przyrodę.

9 - owszem, jest 6 i 13, ale pamiętajmy, że są też inne problemy życia, o których na chwilę zapomnieliśmy, a za chwilę to wróci. Klęski nieurodzaju, susze. Dobra, z koronawirusem ludzie potencjalnie sobie poradzili, przynajmniej w głowach, ale teraz trzeba pamiętać, że jest mnóstwo innych spraw ważnych. Tak, jak jest piramida Maslowe'a, to myślę, że najpierw człowiek walczy, żeby w ogóle przeżyć, a potem, jak już ma to wszystko, to sięga po jakieś wrażenia wyższe, po jakieś wyższe doznania. Dzisiaj myślimy o tym, co jeść, bo sklepy zamknięte, bo własne interesy pozamykane i człowiek myślał, jak mieć pieniądze na przeżycie, a dzisiaj, kiedy to wróciło, myśli o tym, żeby to odbudować i mieć czas w końcu na te przyjemności, nie?

1 - chyba nikt nie tęskni za takim obrazkiem, natomiast on świadczy o pogoni za wszystkim. Sporo ludzi zwolniło dzisiaj i doszło to, że jeszcze nie wszystkie gałęzie gospodarcze wróciły i to ma wpływ na ten widok nr 1.

**Co jeszcze nie jest takie same?**

Myślę, że wszystkie te branże eventowe. Nie ma tylu ludzi na mieście, co dawniej. Ludzie, którzy mają biznesy mówią, że to nie jest jeszcze to, że w tym miesiącu to w ogóle byli pierwszy raz na zero. Są do tyłu, nie?

**Byłeś już w jakiejś restauracji/ kawiarni?**

Normalnie chodzę. W barze mlecznym, do którego zaglądam, co 2 stolik jest zajęty, czyli zdezynfekowany z tabliczkami, a ludzi to prawie nie ma w ogóle. Tragedia jest. Tam zawsze przynajmniej 2/3 stolików było zajęte, a teraz jest połowa dostępnych i siedzą 3 osoby, w tym ja. Naprawdę będzie bardzo źle. Rynek pracodawcy będzie teraz wytyczał reguły gry. Ci znajomi z Wrocławia opowiadali, że ich znajomy, który ma sklep spożywczy, to pa pandemii i w trakcie, to ci pracownicy to odmiana o 360 stopni - mili, proszę, dziękuję. Martwią się o swój stołek, nie? Dbają bardzo.

**Kina, siłownie - poszedłbyś?**

Do kina na pewno. Pewnie zrobią co 2 fotel albo 2 puste, 2 zajęte czy coś...

**Co o tym myślisz, to jest bezpieczne?**

Mam jeden wniosek w ogóle - wyższe środki ostrożności dla starszych. Jeśli wśród nich jest jakaś umieralność i jakieś choroby współistniejące, to żeby wszystko to chodziło, to część z nich po prostu bardziej izolować albo niech sami się izolują i tyle, nie?

**A jak to można zrobić?**

Nie wiem...Starzy ludzie, powyżej powiedzmy 60 r.ż. niech mają w sklepie te swoje godziny i tyle.

**Tak na stałe?**

Nie wiem. Słyszy się, że to wraca, potem się słyszy, że w ogóle ci bezobjawowi nie zarażają, więc myślę, że i ja, i ty, jak byśmy mieli zachorować, to byśmy już zachorowali. Ja już może przeszedłem, nie wiem i nie chcę wiedzieć. Znowu bym się zapętlił w jakichś myślach a w tym momencie jest mi to totalnie zbędne i niepotrzebne. Ja w tym momencie o samych pozytywnych rzeczach chcę myśleć, a nie się czymś zamartwiać. Zdrowie psychiczne ma mi wrócić na 100%, więc jeszcze się zamartwiać koronawirusem, który na 99,9 nie zabije mnie, a pewnie na 0,99 jest prawdopodobieństwo, że ja się zarażę jakoś i cokolwiek się wydarzy.

**Należy chronić starszych na wszelki wypadek i jeszcze kogoś?**

Nie.

**Jedyne miejsce, gdzie teraz zakładasz maskę to jest sklep?**

Tak. Ja zakładam, dezynfekuję ręce na wejściu i na wyjściu, i to mi zostało, natomiast wchodząc do domu już tylko myję ręce.

**Robisz to dlatego, że jest taki nakaz, czy masz taką potrzebę? Dla siebie/ dla innych?**

Myślę, że jedno i drugie trochę i to jest jedyna rzecz, która w głowie gdzieś tam zostaje. No dobra, nic mi się nie przytrafi, ale żeby mieć święty spokój...W tych sklepach maseczka, to żeby inni się czuli bezpieczniej. Dezynfekcja rąk też jest raczej dla ludzi, bo człowiek dotyka towarów. Bardziej wychodząc i dezynfekując robię to dla siebie.

**Korzystasz czasem z komunikacji miejskiej?**

Tylko z tej w pracy, jeżeli już. Powinno się zakładać w niej maski, bo taki jest przykaz. Powiedzmy, że 8 na 10 ma je na sobie, żeby nikt się nie przypieprzył, a tak, to mają to gdzieś.

**Co myślisz o tym, że ludzie tego nie przestrzegają?**

Zmęczyli się i widzą, że to jest, kurna, niegroźne, i zostaliśmy zapędzeni w jakiś kozi róg, zrobili z nas debili.

**Spotykasz się już z większą liczbą ludzi na raz?**

Tych momentów nie ma za dużo, bo ja też za dużo nie wychodzę. Ostatnio była też straszna pogoda. Ale tak, nie mam nic przeciwko temu, żeby się spotkać w gronie wieloosobowym.

**A na wesele na 150 osób byś poszedł?**

Nie lubię wesel, ale bym poszedł. Nie miałbym obaw.

**Pewnie dotarły do Ciebie wiadomości ze Śląska...**

No dotarły. No i co? Ja to traktuję po prostu jak grypę. Ilu z tych górników umrze? Pewnie nikt. Czy ktoś zapadnie na ciężką chorobę? Może 1 kurde na 100? Może. Nasz rząd to są debile i hipokryci. Minister Zdrowia mówi, że maseczki nam w niczym nie pomagają, potem je każe nosić, a później w wywiadzie na pytanie, dlaczego nie ma maseczki, odpowiada "bo jestem bezobjawowy". No żesz kurwa! Żenada.

**APLIKACJE**

**Słyszałeś o takich, które powstały przy okazji pandemii?**

Nie słyszałem o żadnych.

**Kategoria 1**

**Aplikacje analizujące dane osobiste (bieżące informacje o stanie zdrowia, historię przemieszczania się i kontaktów z innymi ludźmi) w celu monitorowania rozprzestrzeniania się koronawirusa.**

To taka, co tam Bill Gates coś tam proponował? Jeśli będą to chcieli wprowadzić na siłę, sam idę na proces. Nie ma szans. Inwigilacja. W życiu bym tego nie założył i mało tego, bo jest przykład tej pielęgniarki, która zajmowała się chorymi i później naznaczona była w miejscowości, w której mieszkała, i mąż musiał zakupy robić. Bardzo niebezpieczne. Oglądałaś serial "Czarne Lustro"? Tam jest jeden z odcinków, jak nie więcej, gdzie właśnie w ten sposób było. Ten futurystyczny świat za chwilę w większości przypadków może się pojawić albo już się pojawia. W takich Chinach to już jest wprowadzane. Kategoryczne nie ode mnie.

**Aplikacje, które na podstawie danych lokalizacyjnych monitorują przestrzeganie kwarantanny domowej.**

To jest też niebezpieczne, to jest już na skraju...I tak jesteśmy monitorowani non stop przez smartfony, przez samo to, że je mamy, czy przez smartwatche.

**Instalując sobie jakąś aplikację sprawdzasz, do czego ma dostęp i od tego zależy, czy ją sobie zainstalujesz?**

Aplikacja wiem, że i tak, i tak mnie śledzi, i nie mam na to wpływu od dnia, w którym dostałem swoją pierwszą komórkę. To są bezcenne dane, które są handlowane, które są kradzione przez odpowiednie organy, pewnie służby, CIA korzystało z FB od samego początku, czy tam FBI. To jest oręż w rękach państwa do lepszego manipulowania, śledzenia i dla mnie to wszystko jest ograniczenie wolności, natomiast zamykanie tak ludzi na tę kwarantannę, ze śledzeniem jakimś? Kurwa, ludzie jednak powinni chyba mieć swój rozum, nie?

**Aplikacje oparte na automatycznej lokalizacji użytkowników, informujące ich, że znajdowali się w miejscach zagrażających zarażeniem się koronawirusem.**

To jest bez sensu, bo chyba każde miejsce jest takim miejscem.

**Monitoring z automatycznym systemem rozpoznawania twarzy, w celu szybkiej identyfikacji osób nieprzestrzegających zaleceń władz.**

Telefony mają już system rozpoznawania twarzy, ale jeśli to miałoby być przez jakiekolwiek organy, to jestem przeciwny. To jest nadal inwigilacja.

**Ty używasz systemu rozpoznawania twarzy w telefonie?**

Nie mam jeszcze takiego najnowszego. Używam odcisku palca.

**I tu nie masz obaw, że twój odcisk gdzieś krąży?**

[śmiech] Pewnie od dnia, kiedy przycisnąłem pierwszy raz. Pewnie gdzieś tam jest. Trzeba by było w ogóle przestać używać komórki, przestać używać internetu, odciąć się całkiem. Wiadomo, trzeba gdzieś wypośrodkować.

**Jeżeli to jest użyteczne dla ciebie, to jesteś w stanie zaakceptować też tę drugą stronę tego, ale nie chcesz, żeby jeszcze dokładać jakieś aplikacje, które tym bardziej będą cię śledziły?**

Tak.

**Kategoria 2**

**Drony dostarczające produkty medyczne i inne towary osobom potrzebującym.**

To by się pewnie znalazło w jednym z odcinków "Black Mirror". To się wydarzy. Jeśli ktoś się godzi na takie dostarczanie...Jeśli nie mógłbym wychodzić, bo byłbym bardzo chory, to jasne, że bym się zgodził na to.

**Są jakieś niebezpieczeństwa tego rozwiązania?**

Przekazywanie bezpośrednio osobom postronnym swojej lokalizacji. Trzeba się pilnować z tym, gdzie to wszystko trafia. Sam adres mailowy - wszędzie zaznaczam, że nie chcę żadnych newsletterów, czy spamu jakiegoś. Nie chcę być targetowany.

**Aplikacje, w których użytkownicy mogliby informować się o tym, czego potrzebują i dzięki temu pomagać sobie nawzajem.**

To jest ok. Oczywiście, jak ktoś by posiadał te dane, to jest też targetowanie. Musiałbym to zobaczyć, przeczekać początek tego wszystkiego i zagrożenia same by się ujawniły.

**To jest w ogóle potrzebne?**

Nie wiem. Musiałoby być i dopiero wtedy bym się zastanowił. Nie odrzucam tego, ale mam za mało danych.

**Aplikacje sztucznej inteligencji, decydujące na podstawie zebranych danych, gdzie skierować największe środki i wysiłki do walki z pandemią.**

To chyba jest po stronie rządu a nie po mojej. W końcu jakaś inteligencja. Jak ma to usprawnić działania rządu i gospodarkę, to ok.

**ProteGo Safe**

Broń Boże nigdy nie będę nic od rządu pobierał.

**Gdybyś żył w innym kraju z innym rządem, to byłoby inaczej?**

No.

**Jakie jest twoje wrażenie po przeczytaniu?**

To będzie na zasadzie, że jak chcesz sobie pomóc, to wejdź sobie i zainstaluj. Będą tak odsyłać i będą zmuszać pewnie w ten sposób ludzi do takiej formy. To jest jasne. To nie będzie zmuszanie bezpośrednie. To jest na zasadzie, że zadzwoni człowiek, będzie szukał pomocy, to powiedzą, że dobrze, ale my chcemy znać pana/ pani historię, co było, jaki stan zdrowia. Ja na to powiem, że ja opowiem, a oni, że nie, nie, proszę pana. Trzeba prowadzić ProteGo Safe, która jest udostępniona dla wszystkich za darmo i proszę tam wypełniać. Na takiej zasadzie, że chcesz pomocy, to uzupełniaj. Nie, nie, nie, nie. To jest wszystko manipulacja, kurde. Jestem na totalne nie.

**Widziałbyś tu jakiekolwiek korzyści?**

Pozornie tylko, ale nie, nie, nie. Nie ufam temu. Niby człowiek będzie sobie chciał pomóc, będzie prowadził coś tam, będzie miał do czegoś dostęp...Myślę, że strona gov z zakładką postępowania i tyle, a nie logowanie się i podawanie swoich wszystkich danych.

**To mogłoby być wykorzystane przeciwko tobie?**

Oczywiście, wszystko. Pod pretekstem zagrożeń i bezpieczeństwa jest targetowanie, inwigilacja. Już jest, a będzie jeszcze bardziej na porządku dziennym. To są drogi po nitce do państwa totalitarnego, a jak widzimy na świecie, robi się ich coraz więcej, bo wszędzie jest źle, jest większy kryzys i wszędzie trzeba ludność trzymać za mordę. Zaczyna być to coraz bardziej widać, coraz bardziej państwa zaczynają się robić prawicowe, czasami skrajnie, pod pretekstem bezpieczeństwa, zdrowia, ochrony, zagrożeń. Najłatwiej manipulować społeczeństwem, które mówi "nie mamy pracy, dajcie nam pracę". I wtedy rząd mówi "nie wpuszczajmy obcokrajowców, bo zabierają przecież wam pracę". To ludzi nastraja do zachowań rasistowskich i tego typu rzeczy prawicowych, które potem pod przykrywką bycia patriotą...I można w ten sposób ludźmi manipulować.

**Czy komuś taka aplikacja mogłaby być przydatna? Może jakiejś grupie ludzi?**

Starym ludziom - proszę bardzo, chociaż oni mieliby trudniejszy dostęp, więc nie, przepraszam, źle. Oni nie mają dostępu do sieci albo w małym stopniu. Gdyby mieli i umieli się tym posługiwać, to jest na zasadzie, że jak się dostaje jakiejś zapaści, zawału...Wtedy może będzie szybszy dostęp do pomocy.

**Kwarantanna Domowa**

Czyli to już wprowadzają?

**To już jest.**

[śmiech] I teraz dobrze wiedzą wszystko. Przecież na kwarantannie zawsze będą jacyś ludzie. W ten sposób cały czas będą mieli kontrolę. "Pomóż sobie, pomóż innym" - kurde, dobrze tu manipulują. Czysta manipulacja.

**Co ty na to, że ta aplikacja jest obowiązkowa?**

Ale to jest na każdym szczeblu prowadzone przez ten rząd. W ten sposób trzymanie ludzi za mordę. Jest taki serial "Opowieści Podręcznej" i tam było, że terror i tyrania nie są wprowadzane z dnia na dzień i totalitaryzm. To jest jak siedzenie w garnku z gorącą wodą i podgrzewanie. Od razu się nie ugotujemy. To są właśnie te sposoby.

**Ale jednak jak ktoś jest w kwarantannie, to jest to dobry pomysł, żeby jej nie łamał?**

Nie wiem, dla mnie to jest ewentualnie dla ludzi chorych. Jak ktoś jest na kwarantannie, to nie znaczy, że są chorzy. Jak ja bym był na kwarantannie, to co ja mogę w tym momencie...No kurde, trzeba obalać rządy, a nie kurde...

**Gdzie tu są mniejsze/ większe zagrożenia?**

Nie wiem, zobaczymy jakie to będzie miało konsekwencje.

**A jakich konsekwencji się spodziewasz?**

Każda kula śnieżna jest tylko pigułą na początku. Nie widzę nic dobrego w tej aplikacji. Jest chyba jedyny plus, jeśli naprawdę miałem kontakt z osobą chorą, to chyba obciachem by było, żeby mnie policja sprawdzała codziennie. Przychodziła i sprawdzała, czy jestem, kurwa, na kwarantannie. Bo wiesz, jak potem by było? Jak z tą lekarką. To tak cała klatka by na mnie potem reagowała. Mój kolega z Łodzi mówił, że w klatce na II piętrze 3 studenciaków było na kwarantannie i opowiadał mi, że tam na II piętrze są zarażeni. Teraz pewnie będzie ich mijał szerokim łukiem.

**Ta aplikacja już jest. Co o tym myślisz?**

To nawet nie wiedziałem, bo nie chcę nawet o tym wiedzieć. Nie chcę nic wiedzieć na temat koronawirusa, bo ja mam większe w tym momencie problemy niż ten cały koronawirus.

**Gdyby było tak, że to jest tylko dla osób łamiących zasady kwarantanny, to ok?**

Ja nie wiem. Świat zwariował. Dzisiaj nie jestem w stanie jednoznacznie powiedzieć, co jest do końca dobre, co jest do końca złe, natomiast, jeśli ktoś nie choruje...Jeśli się okaże, że osoby, które miały kontakt i zostały zarażone, ale chorują bezobjawowo i oni nie zarażają, to kurwa, co z tymi co podostawali po 20000 kary? To jest paranoja. O co tu chodzi? Bo dzisiaj gówno wiemy, ale oni pod przykrywką bezpieczeństwa robią co chcą i dlatego ja im nie ufam. Ten rząd tak działa. Rządy w innych państwach albo to też wykorzystały, albo nie chciały zniewolić narodu. Na Białorusi i tak są zniewoleni na co dzień, więc tam czy dołożą obostrzenie, to jeden grzyb.

**Gdybyś miał do wyboru - aplikacja czy opaska na nogę, jak w areszcie domowym?**

Żadnych opasek, żadnych aplikacji, jestem na nie.

**Twoja przyszłość w kontekście pandemii. Co zaprząta twoją uwagę, czego się obawiasz?**

Że życie klubowe nie wróci. Myślę poważnie już nad tym, żeby życie zmienić pod kątem ustatkowania się, ale na moich zasadach, a nie, że się wszystko pokończyło i czas się ustabilizować. Teraz dla mnie poznanie kogoś jest ekstremalnie trudne i nawet często tych ludzi nie widać, bo mają maseczki. Zniesienie zakazu noszenia maseczek - niech idzie ku temu, bo ludzie zdziczeją. Niektórzy się jeszcze nie witają, niektórzy trzymają dystans?

**Ty podajesz rękę, witasz się normalnie?**

Ja już normalnie wyciągam rękę, z niektórymi znajomymi ściskam się na przywitanie, niektórzy widzę, że jeszcze trzymają ten dystans. We mnie oporu przed tym nie ma prawie wcale.

**Twoje przewidywania na temat sytuacji gospodarczej, ekonomicznej w kraju, na świecie?**

No będzie źle, gówno się rozlało. Ci, co odłożyli, będą w stanie się dźwignąć. Ci, co nie odłożyli i jeszcze byli w czarnej d, to będą mieli naprawdę źle. Są tacy, którzy zarobili w tym czasie i to duże pieniądze, np. na handlu z Chińczykami. Apteki nie miały spadku, spożywcze chyba też nie miały najgorzej, bo ludzie musieli kupować, branża lotnicza to dramat, branża eventowa to samo. Branża turystyczna to w ogóle dopiero to rusza, ale za chwilę wejdzie jesień i znowu wejdą jakieś obostrzenia. Tak będzie. Ja wiem, co zrobią. Znowu powprowadzają jakieś obostrzenia, ale jakie, co oni wymyślą, t już...

**A zmiany społeczne?**

No tak. Ostatnio rozmawiałem z kolegą na temat uprzedzeń, rasizmu, ksenofobii i takich rzeczy. Ludzie, którzy nie są dojrzali, są tępi i manipulowani przez państwo, to z następnymi pokoleniami będzie jeszcze gorzej. Przyspieszy to, bo wg mnie dziś młodzież ma problemy z komunikacją. Ja i ty wychowaliśmy się na podwórku, widzieliśmy emocje ludzi, uczyliśmy się ich bezpośrednio, a dzisiaj dzieci nie widzą tych emocji i dlatego mamy dzieci, które chodzą do psychologów, bo chcą sobie coś zrobić, bo jakieś dziecko dziecku zrobiło zdjęcie i je szantażuje, wieszają się takie dzieci albo w ogóle cuda się dzieją. Dzieci będą miały jeszcze gorszy dostęp do nauki ludzkich uczuć.

**Masz wrażenie, że to ma związek z pandemią?**

Ja widzę to tak, że ta izolacja jednak była. Dzieciak siedział w domu na zajęciach albo wolał grać na komputerze, albo kuł, bo matka z ojcem kazali się uczyć. Dziecko trzeba było wypychać na dwór, bo wolało komputer, a dzisiaj izolacja przez to, że już rodzice nie chcą, żeby dzieci wychodziły, po drugie maseczki...To też jest brak tej nauki mimiki twarzy, czyli tych emocji dobrych/ złych. To też wpłynie na to, że te dzieci będą miały problem z wyrażaniem swoich uczuć, z odczytywaniem uczuć innych. Ja już tu tak głęboko socjologicznie podszedłem do sprawy.

**A czy jakieś korzystne sprawy też mogą wyniknąć z tego doświadczenia pandemii?**

Ludzie się pozbliżali jeszcze bardziej do siebie przez izolację, przez brak kontaktów, przez złą sytuację finansową. Ci, co chcą pomóc, naprawdę pomagają, bo wiedzą, że równie dobrze mogą być w takiej samej sytuacji jutro, nie? Ogólnie Polak Polakowi wilkiem jest i to od zawsze wiadomo, natomiast myślę, że wśród przyjaciół, którzy są prawdziwymi przyjaciółmi, jeszcze bardziej się to zacieśniło.

**Gdyby była 2 fala, to czego się spodziewasz? Że co się stanie?**

Obowiązek maseczek. Myślę, że lasów nie pozamykają, bo to jest bezsens, ale myślę, że wrócą maseczki. Co więcej, to nie mam pojęcia.

**Dobrze, że poluzowano tak jak poluzowano, czy jednak pewne ograniczenia powinny były zostać na dłużej/ na zawsze?**

Myślę, że trzeba bardziej rozwiązać temat starszych ludzi. To jest jedyna taka logiczna rzecz, którą z tego wyciągam. faktycznie ten wirus jest bardziej zaraźliwy niż zwykła grypa, ale nie jest aż tak śmiertelny. Tak straszą, ale po statystykach widać, kto to ewentualnie umiera. Jeśli tak, to pomóc tym starszym.

**Mówić im, że mają nie wychodzić?**

No nie, nie aż tak. Ci ludzie i tak prawie nie wychodzą, nazwijmy rzecz po imieniu. Na siłowniach nie widzę dziadków. Gdzie są starsi ludzie? W kościołach, w sklepach, na bazarach...Są jeszcze jakieś miejsca? Zostawić jakiś nakaz, bo oni raczej już nie będą szukali drugiej miłości w wieku 70 lat. A jak tak, to niech jadą do Ciechocinka. Sanatoria niech sobie działają.

**Mówiłeś, że u cienie w pracy mierzą wszystkim temperaturę. Czy takich miejsc powinno być więcej?**

To jest strategiczny zakład i ja to rozumiem. Wolę, że mierzą temperaturę niż mieliby mnie skoszarować w pracy.

**A inne miejsca - urzędy, sklepy, galerie?**

Bardzo możliwe, że tak będzie. To jest Czarne Lustro, które jest bardzo bliskie. Tutaj mógłbym się na to zgodzić. To jest to, co moja maseczka, jak wchodzę do supermarketu. Ja mogę kogoś zarazić. Nie wiem, czy z wysoką temperaturą ktoś chodzi na zakupy...No raczej nie chyba. Nie da się nie zauważyć, że się ma 38. To nie jest jakiś atak na prywatność. Maseczki w sklepach też na razie mogą zostać.

**Czy jakieś poluzowanie uważasz za bezsensowne i szkoda, że coś poluzowano?**

No nie, ale ja nie jestem zwolennikiem całej tej pandemii...

**Czy jakoś powinniśmy się przygotować do ewentualności 2 fali?**

No nie wiem...Jeśli faktycznie zachorowalność będzie, wirus się jakoś zmutuje, to żeby system zdrowia się nie załamał. Zaopatrzyć szpitale, jakieś dofinansowania na szpitale, na większą liczbę etatów w szpitalach. No, żeby tak odciążyć te szpitale. Chorować chorujemy i chorować będziemy, umierać umieraliśmy i nadal będziemy umierać.

**Twoi rodzice, ty, powinniście się jakoś przygotować?**

Ja myślę, że przygotować się możemy na to, żeby lepiej spędzać czas w domu. kupić sobie planszówki w lato, bo nie będzie może aż takiego zapotrzebowania, kamerę już kupiłem, może studio sobie lepiej zaopatrzyć, żeby muzykę jeszcze fajniej robić. takie rzeczy, co ludzie lubią robić w domu albo co by chcieli robić w domu. Nauczyć się czegoś w domu, gitarę niech ktoś sobie kupi...Takie rzeczy, żeby nie zwariować. Maski i rękawiczki i tak będę miał z pracy zapewnione. Ludzie jeszcze pewnie kupią rzeczy, które im zapewnią kondycję - kupią sobie do domu bieżnię, rowerki.

**Czy myślisz, że w jakieś sprawie związanej z pandemią nasz rząd dobrze zadziałał?**

[śmiech] To jest dobre pytanie...Nie wiem. Znasz moje poglądy...Wiele rzeczy zrobili pod publikę. Tak działają rządy, żeby uzyskać poparcie elektoratu. Na pewno się zdyskredytowali tymi wyborami, które się miały odbyć, ta afera z Szumowskim, wotum nieufności, które sobie sami przyklepują. Nie wiem.

**Punkty przełomowe czasu pandemii dla ciebie?**

Najbardziej to chyba zamknięcie klubów. Brakuje mi. Powoli mi zaczyna brakować. Na początku odpoczywałem, a teraz już miałbym ochotę pograć. Zapadło mi w pamięć to przechodzenie niektórych z totalnej paniki do totalnie mieć wszystko gdzieś. Tęsknię za przyjaciółmi, którzy są gdzieś daleko, czekam na swojego kumpla, który ma z Irlandii przylecieć, ale chyba nawet nie ma lotów uruchomionych na razie. To zamknięcie granic mi się zapamięta.

**Coś jeszcze?**

Na pewno to zacieśnienie przyjaźni. Na pewno część ludzi przewartościowała pewne rzeczy, że dziś one są, a jutro już ich nie ma i będą już tak podchodzili. No w ciekawych czasach żyjemy i tak się jeszcze nie zdarzyło. To ludzie będą brali pod uwagę, że w życiu nie jest tylko pewna śmierć i podatki, może nie będą ludzie już tak szaleli finansowo, może będą oszczędzali bardziej.

**Tobie się coś przewartościowało?**

Nie wiem. Może jak będą jakieś ruchy finansowe, to będę się jeszcze dłużej zastanawiał. Na razie nie robię żadnych, więc nie myślę w tych kategoriach.

**Myślisz, że otworzą te kluby?**

Nie wiem. W Niemczech jest dofinansowanie do kultury, u nas nie ma. Streaming, który robiłem teraz nie ma sensu, bo ludzie chcą wyjść na zewnątrz i nikt tego nie ogląda już. Jesień/ zima i wrócę z tym i może będzie można w końcu zrobić coś w plenerze.
