# Supplementary material for: The challenges arising from the COVID-19 pandemic and the way people deal with them. A qualitative longitudinal study
Source: PLoS One. 2021 Oct 11;16(10):e0258133. doi: 10.1371/journal.pone.0258133 (PMC8504766; doi:10.1371/journal.pone.0258133)
Supplement: S1 Dataset — (ZIP) [file pone.0258133.s003.zip › Transcriptions/stage 5/7.5_M_28_couple, no children.docx]

**7.5_M_28_couple no children**

**Co tam u Ciebie przez ostatni miesiąc?**

Na szczęście włosy mi trochę odrosły. Muszę zerknąć w kalendarz. Chwilę po majówce zmarła moja babcia, u której byłem ostatnio i o której ci opowiadałem. To był taki główny punkt zdarzeń ostatnich. Nie byłem na pogrzebie. Widziałem się z nią zanim zmarła i jeszcze wtedy obowiązywały mocniejsze restrykcje, więc oddałem swoje miejsce komu innemu, żeby mógł przyjechać na pogrzeb. Co było później? Byłem służbowo we Wrocławiu, obserwowałem, jak to wszystko tam wygląda i teraz jesteśmy 2 raz na wsi. Byliśmy już raz na Podlasiu i teraz też jesteśmy na Podlasiu, ale w innej części. I właściwie tyle w skrócie.

**Skąd pomysł na wieś?**

Żeby uciec z miasta w czasie pandemicznym, a z drugiej strony, to zawsze natura była bliższa nam do ucieczki. W czasie restrykcyjnym udawało nam się uciec do lasu, ale jeszcze nie był możliwy najem krótkoterminowy i dlatego teraz tak sobie to odbijamy. I też nieudaną podróż do Włoch wielkanocną. Taka wieś, taka ucieczka lekka.

**Najbardziej od czego uciekasz?**

Ojej...Wydaje mi się, że przede wszystkim chcę zostawić zgiełk i ruch, a w tym czasie teraz bardzo mi się podoba, że na wsi to wygląda zupełnie inaczej. Praktycznie czasami cała ta sytuacja nie jest odczuwalna, dopóki nie zobaczy się po jakimś dłuższym czasie jakiejś jednej osoby, która idzie w maseczce albo nie idzie w maseczce i też sobie przypominamy, jak to wygląda w Warszawie. Inny rytm tutaj panuje całkowicie, inne podejście. Byłbym skłonny zapomnieć o całym koronawirusie tutaj.

**Ludzie nie przestrzegają tych obostrzeń?**

Trochę tak. Chyba wydaje im się, że nie muszą i obserwując, jak ktoś idzie po chodniku raz na godzinę, to faktycznie można tak myśleć. Czasami w ogóle infrastruktura nie stwarza takich potrzeb, bo wszyscy mieszkają w oddzielnych domach, wszyscy są pochłonięci jakąś pracą przy swoich domach czy gdzieś na polach. Takiej konieczności noszenia maseczki to nawet chyba nie ma właściwie.

**A dziś, jak byliście na zakupach, to mieli maseczki?**

Częściowo tak, a częściowo faktycznie nie mieli. Byliśmy w Augustowie w Lidlu i niektórzy tak sobie wchodzili bez maseczki. Może wszyscy mieli astmę.

**Jak ty się z tym czułeś, że nie mieli?**

Dziwnie, bo jak wyszliśmy ze sklepu, to obejrzałem się w kierunku tych osób, które nie miały maseczki, lekko zdziwiony. A one nie były zdziwione jakoś tak, nie wyglądały na zakłopotane, że zapomniały czegoś. Tak wyglądało, że to jest norma dla nich. Dla mnie to jest irytujące i to jest taka irytacja, że kurczę, dlaczego? Są jakieś wytyczne, każdy ma swój rozum też i każdy powinien myśleć o własnym bezpieczeństwie czy jakimś ryzyku wobec innych, a tutaj tego nie ma. To jest takie zdziwienie połączone z irytacją.

**Na ile jesteście tam teraz?**

Przyjechaliśmy dzisiaj i zostajemy do niedzieli. Poprzedni wyjazd to też były 4 dni.

**Planujecie wyjazd na długi weekend?**

Planujemy, ale dopiero w sobotę, czyli właściwie nie w długi weekend. Planujemy jechać na Mazury, bo rodzice Dominiki mają działkę ma Mazurach nad jeziorem i tam się umówiliśmy ze znajomymi, ale to raczej tylko 1 dzień, 1 noc i w niedzielę powrót.

**Opowiedz o wyjeździe do Wrocławia.**

To był 2 tydzień mają. Pojechaliśmy samochodem z moim szefem, jechaliśmy tam służbowo oglądać tereny, które będziemy adaptować pod nowe wydarzenie. To był jeszcze zupełnie inny okres, ale też miałem wrażenie, że tam ludzie troszeczkę inaczej reagowali niż w Warszawie, a może to było jakieś złudzenie albo w takim miejscu akurat byłam, ale ludzie w centrum miasta nie poruszali się w maseczkach w 100%. Widać było, że niektórzy tak...Nie chcę powiedzieć, że wyglądali, jakby się nic nie działo, bo nie wiem, co mieli w głowach, ale nie mieli ani rękawiczek, ani maseczek. Na pewno wtedy jeszcze były dużo mocniejsze restrykcje.

**Jak się czułeś z tym, że musiałeś się ruszyć z domu?**

Świetnie. To była dosyć odległa podróż, byłem we Wrocławiu może 3-4 razy w życiu i sama opcja wyjazdy była dobra. Bardzo mnie to ucieszyło, że się wyrwę z domu. Myśl, że wyjdę z tego otoczenia, w którym jestem cały czas. Zmiana otoczenia, zmiana widoków. środowiska. Miałem nadzieję, że zobaczę cokolwiek innego.

**Miałeś jakieś obawy związane z wirusem, jak tam jechałeś?**

W pierwszej chwili to tak. Nawet samo to, że będę jechał z szefem w samochodzie w odległości bliższej niż 2 m, raczej pół metra. Tym bardziej, że wiem, że mój szef miał kilka spotkań. On też siedział w domu i przestrzegał zasad, ale wiem, że ok tydzień przedtem miał spotkanie, na którym musiał być i na nim był. Z kilkoma osobami. Miałem takie obawy i myśli, czy nic nam się nie wydarzy, czy nic nie przyniósł, ty bardziej, że był w biurowcu, gdzie pracowali ludzie. Nic się nie stało. We Wrocławiu poruszaliśmy się już na wolnym powietrzu, więc takich obaw nie miałem i nie przebywaliśmy z innymi osobami w bliskiej odległości. Chciałem zaobserwować, jak to tam wygląda.

**A jak sobie radziliście z jedzeniem?**

W jedną stronę jadąc, zajechaliśmy na stację po kawę i to było nasze śniadanie, a w drodze powrotnej po kilku godzinach wzięliśmy sobie jedzenie na wynos z restauracji. Nie próbowaliśmy kombinować. Nie zostawaliśmy tam na noc i też pewnie z tego względu uniknięcia ryzyka. Normalnie pewnie pojechalibyśmy tam na 2-3 dni i byłoby co robić, ale tu nie było takiej możliwości. Sami ustaliliśmy, że lepiej nie, że lepiej się przemęczyć. Wyjechaliśmy o 7 rano i wróciliśmy o 20.

**A jak maj w pracy? Dużo, mało?**

W pracy jest tak, że w sumie nie narzekam. Ostatnie kilka dni miałem luźniejszych, ale to głównie dlatego, że czekam na różne procedury, dzięki którym będę mógł pójść dalej z festiwalem. Po majówce wiele się nie zmieniło. Cały czas jest praca przy jakichś tam projektach sponsorskich, festiwalowych. Ogłaszaliśmy jakiś dodatkowy dzień festiwalu w przyszłym roku, więc tej pracy mi nie brakowało. Może nie aż tyle, jak w momencie odwoływania, bo nie pracowałem np. w weekendy, mniej wieczorów było zajętych. Trochę mniej, ale też nie tak, że nie miałem nic do roboty.

**Czy jest coś, co wróciło do stanu sprzed epidemii w twoim życiu?**

Zaczęliśmy się już spotykać ze znajomymi w małych gronach, wróciło to, że już nie czuję takiego uwięzienia w domu albo nie czuję też tego, że jak wychodzę z domu to muszę być szczelnie opatulony w maseczkę i mieć rękawiczki koniecznie, co też było męczące czasami. Mogę wyjść z domu nie mając maseczki i pójść się przespacerować. Mam też możliwość umówić się z kimś w weekend. Teraz wracają kina w końcu i też myślę, że skorzystamy z tego kina. Mniej strachu chyba jest we mnie. Może właśnie przez to, że są poluzowania, że pewne rzeczy można robić, nie ma takiego rygoru i czuję się tak inaczej.

**To mniej strachu wynika z tego, znoszą obostrzenie, że więcej już możemy? To jest główny powód?**

Trochę tak, ale myślę, że też trochę dlatego, że trochę czasu minęło o może jest we mnie takie uczucie, że mogłem się już zarazić, a się nie zaraziłem i teraz troszkę mniej się boję. Ja wiem, że to jest złe, ale taki mam trochę sposób myślenia, że...Staram się nadal uważać. Ostatnio się złapałem na tym, że te poluzowania, a nadal trzeba się dezynfekować, nadal trzeba przestrzegać tych zasad, ale wróciło takie poczucie wolności.

**Gdzie nosisz maseczkę?**

We wszystkich miejscach publicznych, na pewno w sklepach, w kinach trzeba będzie mieć maseczkę, co może być męczące, ale tak musi być. Nawet stojąc w kolejce do sklepu, mimo, że to jest na ulicy, to np. wczoraj, czekając w kolejce do piekarni, założyłem maseczkę mimo wszystko. Wszystkie publiczne miejsca, gdzie jestem wewnątrz z innymi ludźmi. Na razie nie byłem w miejscach plenerowych, gdzie było dużo ludzi.

**Czy jeszcze coś wraca do normalności?**

Sam nie wiem. Ogólnie bym powiedział, że wróciło przebywanie jakkolwiek na powietrzu. Mogę wyjść z domu, kiedy chcę i to bardzo dobrze na mnie wpłynęło i dużo mi w głowie zmieniło.

**Kto zainicjował pierwsze spotkanie ze znajomymi?**

Ja. Sprawdzę, kiedy to było...2.5 tyg. temu. Stało się to, że potrzeba...Zmieniło się chyba to, że już nie wytrzymałem. Tak bym to nazwał. Oczywiście z zachowaniem ostrożności i w małym gronie, itd., ale już po prostu nie wytrzymałem. Zrobienie czegoś innego niż siedzenie w zamknięciu, wyjście do ludzi, wyjście na powietrze. Wszystko razem. Spotkaliśmy się na zewnątrz. Pierwsze spotkanie było na powietrzu i właściwie drugie, które było tydzień później, też.

**Kiedy przyjdzie czas na twoje spotkania w domu?**

Hmm...Myślę, że niedługo pewnie, raczej. Raczej nie w dużym gronie też na początku, ale to pewnie wróci w ciągu kilku tygodni na pewno.

**Na ile przeszkadza ci, że np. nosisz cały czas maseczkę do sklepu?**

Wydaje mi się, że prawie w ogóle mi to nie przeszkadza. Przeszkadzało mi bardziej na ulicy, kiedy nie było obok nikogo, bo męczy mnie to gorzej mi się oddycha i jest mi niewygodnie. W takich zamkniętych przestrzeniach to raczej mi nie przeszkadza, bo mogę założyć tę maseczkę, a później mogę ją po prostu zdjąć, Po prostu to jest konieczne i jestem z tym ok całkowicie.

**Emocje - zdjęcia**

Wrocław - z jednej strony powiedziałbym, że 2, a z innej 6. Te pozostałe rzeczy to 13 i 6.

2 - z jednej strony bardzo się ucieszyłem, że tam jadę, ale już przebywając tam, po jakimś czasie straciłem to wrażenie świeżości, że jestem w innym miejscu, bo to po prostu było duże miasto, czyli takie wejście w gumę, że niby gdzieś jadę, gdzie ma być inaczej, ale tak naprawdę było tak samo i trzeba było robić to samo - chodzić w maseczce, nie można było iść do restauracji i dalej było bardzo dużo ludzi. Oczekiwałem chyba czegoś innego i myślałem, że da mi to trochę więcej, pozwoli mi troszkę lepiej odpocząć albo poczuć się inaczej, a właściwie było podobnie.

**Jakie to były uczucia?**

Może smutny to nie, ale po powrocie stwierdziłem, że wyglądałoby tak samo, gdybym pół dnia jeździł z moim szefem po Warszawie.

6 - bo spędziłem pół dnia w pięknym parku przy ładnej pogodzie i to było takim plusem, ale z drugiej strony, tak samo by, się poczuł będąc w parku w Warszawie. Myślałem, że może się odetnę trochę w tym Wrocławiu.

6 - Podlasie. To taki promyk nadziei powrotu do normalności.

13 - jesteśmy w takich miejscach na Podlasiu, gdzie jest naprawdę dużo natury, tzn. w małych wioskach i bardzo dużo po tej części przyrodniczej chodzimy. Sam nawet układam tak rękę i dotykam tych roślin, i cieszę się z tego, że mam możliwość, żeby to zrobić.

**Jak byś nazwał te emocje?**

Jednym słowem powiedziałbym - ulga. Ulga, że mogę zrobić coś innego, że świat może wyglądać inaczej i że po prostu odpoczywam.

**Jakieś obserwacje otoczenia? Radzenie sobie w tej sytuacji?**

Wydaje mi się, że już większość osób czuje takie rozluźnienie, że mniej rozmawia się o zagrożeniach, o jakichś złych rzeczach, o tym, że dalej jest ileś tam zachorowań i zgonów. Już nie pamiętam, kiedy o tym z kimś rozmawiałem. Prawdopodobnie po prostu z Dominiką w domu całkowicie przypadkowo. I to też na zasadzie: "Wiesz. ile jest teraz zachorowań? Nie, raczej nie. A ty? Nie." Nie sprawdzam, całkowicie nie. Mówiłem kiedyś, że sprawdzam codziennie, a teraz już całkowicie nie. Czasami siłą rzeczy gdzieś ta informacja do mnie dotrze, bo otworzę Onet i spojrzę na pasek, ale sam specjalnie tego nie sprawdzam. Nawet nie patrzę czasami w tę stronę, bo wiem, że na górze jest pasek i od razu przesuwam w dół i szukam informacji, która mnie interesuje w sporcie, kulturze czy w innych. W maju o tyle jeszcze obserwowałem, chociaż nie było to 100% mojej uwagi, ale co w kolejnych etapach się odmraża. Nie przywiązywałem się do tego i nie wyczekiwałem, że teraz nagle jest coś i coś. To następowało i już. To była też taka ciekawość i doinformowanie, że teraz jest tak a nie tak. Na pewno maj był bardziej niespokojny, jeszcze to było bardziej intensywne, jeszcze było więcej restrykcji, jeszcze troszkę inaczej to wszytko wyglądało. Teraz ten czerwiec to już czuć taką wolność. Większą na pewno niż była.

**Zdarzyło ci się powtórzyć wypad do GH?**

Nie, na szczęście nie.

**A byliście już w knajpie?**

Byliśmy, ale w trakcie tego pierwszego wyjazdu na Podlasie. W Warszawie nie byliśmy jeszcze. Mieliśmy taki plan, ale jakoś tak czasowo nam się nie zgrało. Na Podlasiu wyglądało to tak, że nie nosi się maseczek...Poczekaj, żebym ci nie skłamał...Czy byliśmy w Warszawie też?

**Ok., jak było na Podlasiu?**

Co 2 stolik był wolny, zanim usiedliśmy, to była tam karteczka "zdezynfekowane", co było znakiem, że już możemy tam usiąść. Obsługiwano nas tam bez maseczki na pewno i właściwie tyle. Było znacznie mniej osób, więcej przestrzeni. Nie wydaje mi się, że czułem obawy, że używam sztućców albo siedzę tam, gdzie siedziały inne osoby.

**Zamknięta przestrzeń, a ty nie masz maseczki? Jak z tym się czułeś?**

Tam akurat faktycznie i obsługa, i inni, którzy siedzieli przy stoliku 3-4 m dalej...Naprawdę, tam było mnóstwo przestrzeni, tym bardziej, że w tej sali stoliki są dosyć duże i siedzieliśmy w przeciwległych rogach. A, byliśmy w Warszawie. Byliśmy też w Jaskółce na Żoliborzu, ale tam właściwie wyglądało to dosyć podobnie. Siedzieliśmy na zewnątrz i tam też przestrzeń między stolikami jest, a obsługa już ma maseczki i żeby wejść do toalety do środka to też trzeba założyć maseczkę. Też była karteczka, że zdezynfekowano, była przestrzeń, więc dosyć pewnie się czuliśmy.

**Masz takie wrażenie, że to już jest bezpiecznie tak pójść do knajpy?**

Wydaje mi się, że jeszcze jednak nie do końca. Na razie nie wracam do częstotliwości chodzenia do knajp czy do miejsc sprzed pandemii, czy w ogóle z okresu, który był rok temu o tej porze, bo też było ciepło, też było miło i wychodziliśmy dosyć często. No raczej nie, to jest raczej sporadyczne jeszcze. Jeszce z taką lekką niepewnością, przynajmniej u mnie.

**Opowiedz o tym planowanym kinie?**

Właśnie dzisiaj nawet słuchaliśmy audycji w Tok FM z szefem sieci Helios o tym, że teoretycznie można zająć 50% miejsc w sali kinowej, ale fizycznie wychodzi na to, że jeszcze mniej, bo można siedzieć w rzędach naprzemiennie i co 2 miejsce. Wiem, że na pewno trzeba siedzieć w maseczce przez cały film i są też dodatkowe obostrzenia w łazienkach, że muszą być częściej dezynfekowane i sprzątane, że są żele. Podobno jest taki pomysł, że wszystkie osoby, które są na filmie, muszą dostawić do siebie dane kontaktowe, żeby w razie czego znaleźć osobę, która była nosicielem wirusa. Przez te maseczki, to zastanawiałem się dzisiaj, bo kina dużo też zarabiają na napojach, pop cornie, itd. Jeśli trzeba siedzieć w maseczce, to czy w ogóle te bary będą otwarte? A jeśli tak, to na jakich zasadach można jeść w sali? Tak naprawdę nie wiem.

**Myślisz, że ludzie będą przestrzegać siedzenia w maseczkach?**

Sam jestem ciekaw, ale wydaje mi się, że jeśli to będzie sprawdzane - np. 3 x w trakcie filmu obsługa przejdzie się po sali, to jednak niektórzy przynajmniej będą tego przestrzegać, mam nadzieję. Może być tak, że zdejmą, a jak zobaczą, że wchodzi obsługa, to założą, ale też jest monitoring na salach. Mam nadzieję, że tak, że osoby, które w ogóle odważą się pójść do kina...To jednak jest mocno zamknięta przestrzeń i to jest dopiero kolejny etap odmrożenia. Na niektórych to też działa, że skoro to tak późno zostaje odmrożone. tzn., że jest większe ryzyko.

**A może pójdą tylko ci, którzy się nie boją koronawirusa?**

Hmm...Być może tak, ale mam nadzieję, że to będzie tylko jednorazowe wyjście, bo ktoś wywali ich z sali albo zwróci im dość mocno uwagę i stwierdzą, że nie ma sensu próbować, bo się nie da obejść przepisów

**A do fryzjera się wybierasz?**

Wybieram się pod koniec czerwca. Taki mam plan. Trochę nieprzypadkowo, bo mam urodziny 5 lipca i przed tymi urodzinami chciałem pójść i spotkać się ze znajomymi. Zastanawiałem się, czy pójść już teraz i jakoś sobie wykierunkować tę dziwną fryzurę, którą mam i z którą nie wiem, jak się w ogóle obchodzić, ale nie, stwierdziłem, że właśnie jeszcze poczekam. Słyszałem, że ostatnio w jednym zakładzie podobno zakaziło się kilkadziesiąt osób i jeszcze mam obawy. Po prostu nie wiem, jak to wygląda. Może to też strach z niewiedzy?

**Jak sobie wyobrażasz taką wizytę?**

Wydaje mi się, że muszą mieć maseczkę i rękawiczki na pewno. Czy nie?

**Ty też będziesz musiał je mieć.**

Aha...O, tego nie wiedziałem...Wydaje mi się, że to jest ok, że to jest do zniesienia, żeby...Przynajmniej w moim przypadku, bo to będzie szybka sprawa. Nie mam nic do tego. To nie jest powód, dla którego nie poszedłbym do fryzjera.

**Planujesz wrócić na siłownię?**

Maja się otworzyć chyba od soboty 6-go. Sam nie wiem...Ja chodziłem na siłownię, która jest dosyć mała i bywały godziny, gdzie nie było nikogo, albo była jedna osoba poza mną, ale są też godziny, że jest tam 30 osób, a naprawdę jest mała. Czułbym się dosyć mocno zagrożony w takiej sytuacji, nie czułbym się dobrze i dlatego wydaje mi się, że jeszcze poczekamy z tą siłownią, albo będziemy wybierać takie godziny, które wiemy, że są mniej oblegane. Siłownia jest 24 h czynna. Rozumiem wszystkie osoby, które pójdą na tę siłownię. Rozumiem takie powody, że mają faktycznie taki tryb życia, że im tego brakuje, że robiły to przez długi czas wcześniej, że potrzebują ruchu i aktywności i że to dla nich pewnie dobrze. Natomiast uważam, że to nie jest najbezpieczniejsze miejsce, bo mimo wszystko ćwiczy się na tych samych maszynach, korzysta się z tych samych urządzeń, wspólna szatnia, wspólna łazienka. Wydaje mi się, że kilka zagrożeń tam jest co w co najmniej kilku miejscach i ja chyba jeszcze poczekam z tą siłownią. Nie wrócę w czerwcu, nie wiem, co będzie w lipcu.

**Zastanawiam się, czego jeszcze mogłoby ci brakować...**

Chodziłem też do klubów grać, jako DJ, ale tych klubów nie ma. Kluby będą otwierać na samym końcu.

**Masz pomysł, kiedy?**

Nie wiem. Teraz troszkę ratuje je sytuacja taka, że są dozwolone zgromadzenia do 150 osób, a część tych miejsc` imprezowych ma jakieś ogródki, jakieś patio czy jakąś taką otwartą przestrzeń. Z tego co widzę, to nie ma, póki co tańczenia i muzyki, tylko raczej te miejsca działają na zasadzie barów.

**Jak jest teraz z zakupami?**

Dalej rzadziej i z takim przygotowaniem raczej do tych zakupów. 2-3 tyg. temu byliśmy w Lidlu i zrobiliśmy zakupy na 2 tygodnie. Teraz byliśmy w Lidlu i mamy plan jechać do Lidla, ale po to, żeby zrobić sobie duże zakupy na 1-1.5 tygodnia, żeby nie chodzić, nie dokupywać.

**Myślisz, że taki sposób już z tobą zostanie?**

To się głównie wiązało z tą wolnością. Kiedyś siedziałem sobie wieczorem i o kurczę, tego nie mam w domu, albo chcę coś zjeść i ok., idę i za 5 minut wracam. W czasie pandemii się tego pozbyłem, bo jednak sam proces, czyli założenie maseczki, chodzenie w rękawiczkach, które są strasznie niewygodne dla mnie. Już to mnie zniechęcało, nie mówiąc już o zagrożeniach. Myślę, że trochę wróci to, że jeśli któregoś dnia pomyślę sobie, że chce mi się czegoś, to będę miał więcej odwagi, żeby pójść do sklepu niż w czasie pandemii, ale myślę, że i tak będę tego unikać sposobem większych zakupów jednak.

**APLIKACJE**

**Spotkałeś się z takimi na czas pandemii?**

Słyszałem tylko o tej do meldowania się na kwarantannie. Nie wiem, czy faktycznie tak to wyglądało w Polsce, ale osoby przebywające na kwarantannie muszą meldować się w aplikacji i jak tego nie zrobią, to przychodzi do nich SM lub policja i muszą pomachać przez okno udowadniając, że są w domu. Nie wiem, czy miało się zrobić sobie zdjęcie w domu, czy był jakiś lokalizator? Tylko tyle słyszałem.

**Kategoria 1**

**Aplikacje analizujące dane osobowe...**

Pierwsze wrażenie jest takie dosyć negatywne, bo to jest pewien sposób inwigilacji i gdzie później te dane trafią? Do kogo i po co, w jakim celu? Mam takie poczucie, że to niebezpieczne dla nas. Zamysł pewnie byłby taki, żeby to pomogło, żeby to miało dobry skutek, ale nie wiem, czy potencjalnych zagrożeń nie jest więcej niż plusów.

**Jaki mógłby być plus?**

Taki, że jakby faktycznie wszyscy rzetelnie raportowali o tym wszystkim, to faktycznie może dałoby się nad tym jakoś zapanować, kontrolować, zdobyć informacje, dojść do tego, kto zaraził.

**Aplikacje, które monitorują kwarantannę domową.**

Tutaj akurat...To jest nadal sposób śledzenia nas wszystkich, natomiast udostępniając lokalizację w telefonie, korzystając z map, itd., to często się to robi. Wydaje mi się, że nie zastanawiamy się wyznaczając trasę do innego punktu, że ktoś może też to wiedzieć. To wydaje mi się mniej niebezpieczne i mi osobiście by to mniej przeszkadzało. Mogę udostępnić lokalizację, że jestem w domu, ale nikt nie wie co robię, nie zna mojej historii choroby, moich danych osobistych i bardziej osobistych spraw.

**Lokalizacja użytkowników i informowanie o znajdowaniu się w miejscu narażenia na zakażenie**

To może być ok, bo mogę być nieświadomy, że w tym miejscu akurat było jakieś ognisko koronawirusa, albo były osoby, które go miały, tylko dostaję taką informację i co dalej? To jest na zasadzie ostrzeżenia i teraz zrób coś z tym, zgłoś się do odpowiednich służb? To mnie informuje, ale do niczego mnie to nie obliguje. To może być jakaś wytyczna, wskazówka, spoko, fajnie, natomiast nie mamy pewności, że wszyscy, którzy dostaliby takie powiadomienie zrobiliby coś z tym.

**Tutaj musisz włączyć lokalizację, żeby aplikacja wiedziała, gdzie byłeś przez cały dzień. To jest warte tej informacji?**

Kurczę...Wydaje mi się, że to nie jest warte, ponieważ ktoś widzi każdy mój ruch, a raczej jesteśmy świadomi tego, że w miejscach ruchliwych, publicznych jest możliwość zarażenia. Uważam, że trzeba tak czy tak uważać.

**Monitoring z rozpoznawaniem twarzy...**

Oj, to bardzo niebezpieczne mi się wydaje. Od razu pojawia mi się takie myślenie futurystyczne, science fiction. Za chwilę, skoro już mamy taki system do twarzy, to za chwilę będzie też wykorzystywany np. też do tego, żeby...Będę sprawdzany, czy np. korzystam` z takich produktów albo, czy spotykam się z tą osobą i w ogóle wszystko. To jest niebezpieczne.

**Drony**

Np. przez okno? Wydaje mi się, że jeśli byłyby takie drony, były niezawodne i mogły pomóc w taki sposób, to czemu nie. Żeby dostarczyć pizzę czy jedzenie, jak ktoś był na kwarantannie, to ktoś bliski musiał dostarczać, to czemu nie mogą tego robić drony?

**To jest potrzebne?**

Sam nie wiem. Uważam, że to jest trochę nieprawdopodobne - 10 dronów pomiędzy oknami...Wydaje mi się, że jeśli pomogłoby to starszym osobom, które są w grupie ryzyka, które musiały same sobie radzić, nie mają wsparcia, to tak. Nie widzę nic złego.

**Aplikacje informujące wzajemnie uczestników.**

Wydaje mi się, że to jest ok. Na pewno na FB funkcjonowała grupa/ ruch Niewidzialna Ręka i właściwie polegało to na tym samym, ale aplikacja mogłaby to usprawnić, np. żeby wiedzieć, że dane osoby mieszkają w danej dzielnicy. W taki sposób logistyczny mogłoby to pomóc i usprawnić.

**Aplikacje sztucznej inteligencji.**

Myśląc o scenariuszach futurystycznych, że maszyny zastąpią człowieka, to wydaje mi się, że nie mam takiego strachu, że maszyny opanują świat czy coś takiego. Raczej zastanawiam się, czy maszyny nie myliłyby się pod takim względem, że akurat ta sytuacja, którą mamy była często ruchliwa dosyć. Nagle w Polsce wybuchło wielkie ognisko na Śląsku...Też zastanawiam się czy te dane będą wystarczająco zabezpieczone, chociaż to już w dalszej kategorii.

**Kwarantanna domowa**

Uważam, że to jest trochę dziwnie zakomunikowane. Samo sformułowanie, że dostajemy zadanie jest to komunikacja jak do dzieci, żeby je zachęcić. Wyzwanie. To jest raczej raportowanie niż wykonywanie zadań. Może ktoś się obawiał, że raportowanie będzie źle brzmieć albo zniechęcać. Nie wiem, czy można nazwać zadaniem zrobienie sobie zdjęcia. Wydaje się być...To, że 20 minut i że te zadania przychodzą w różnych momentach jest dobre, bo nie daje możliwości tego, żeby to obejść. Rozpoznawanie twarzy, geolokalizacja...Ok., to jest jakiś środek dodatkowy. To, że można się z kimś skontaktować w razie potrzeby...Sam nie wiem.

**Dobrze, że jest obowiązkowa?**

W ogóle nie wiem, jakie to ma podstawy prawne i nad tym się zastanawiam.  Czy można kogoś zmusić, żeby miał aplikację w swoim telefonie? Przede wszystkim trzeba mieć telefon odpowiedni, żeby mieć aplikację w ogóle. Nie wiem, czy o tym akurat pomyślano. To jest obowiązkowe, ale dlaczego i co mówi o tym prawo? Co, jeśli zrobię sobie zdjęcie wcześniej i wyślę im to samo zdjęcie? Czy tam jest osoba, która to weryfikuje, czy automat, który porównuje twarze? Smsy o różnych porach, ale nie zawsze wiadomo, czy jest dzień czy noc...

**Ale tam jest jeszcze geolokalizacja.**

No tak, ale zawsze może to zrobić moja mama, przez mój telefon, jak nacykam sobie wcześniej zdjęć. Trzeba by zaufać ludziom, że to robią.

**Jak ty byś był na kwarantannie, pobrałbyś to?**

Tak, nie widziałbym problemu.

**ProteGO Safe**

Na plus to uważam, że regularne zapisywanie informacji o tym jak się czujesz i w jakiej jesteś kondycji. Niekoniecznie może do tego potrzeba aplikacji, ale skoro może pomóc, to czemu nie? Test oceny ryzyka też może być ok. Rekomendacja zachowań - wydaje mi się, że te rekomendacje są raczej bezpieczne. Raczej kierują w dobrą stronę. Pomocne dla lekarzy - ok. Raczej zaciekawił mnie akapit 2 (moduł Bluetooth). Czyli mając smartfony i przebywając w danym miejscu, będzie tam taka technologia, która namierzy wszystkie urządzenia innych osób, które tam były, moje też i będzie mi mówić...To może być takie trochę...

**Co ona ci będzie mówić?**

Zastanawiam się jak to wygląda. Czy później dostaję informację o tym, że dzisiaj w takim i takim miejscu przebywały osoby, które są zakażone koronawirusem?

**Jak są zakażone, to chyba nie powinny chodzić?**

No właśnie...Mam wrażenie, że to jest aplikacja, która jest raczej zbiorem informacji, które mają pomóc, a nie czymś, co ma powodować, że czujemy się bezpieczniej albo lepiej.

**W czym te informacje mogą pomóc?**

Dziennik zdrowia, jak ktoś sprawdza czy ma jakieś objawy i zapisuje sobie przez kilka dni. Jeśli ma kilka dni objawy, to jest to impuls, żeby pójść się przebadać albo coś zrobić z tym dalej. Ankieta stworzona przez lekarzy jest ok., bo może niektóre osoby nie są świadome różnych objawów, że świadczą o czymś. Jeśli to ma pomóc temu, że w razie czego można poszukać pomocy, to myślę, że ok.

**Jak sobie wyobrażasz test oceny ryzyka?**

Jeśli znajdziemy w nim takie pytania, jak: "Czy przebywałaś w miejscu, gdzie przebywało dużo osób", to wydaje mi się, że to bez sensu. Taki test powinien być bardziej konkretny, dawać nam coś, czego może nie wiemy albo...Nie wiem. Na pewno miałbym z tym problem, że pojawia się safe w nazwie, bo niektórzy mogą to potraktować, że ta aplikacja da poczucie bezpieczeństwa albo pomoże w tym bezpieczeństwie. Jakoś pośrednio tak, ale nie bezpośrednio.

**Gdyby tam było pytanie na co chorujesz, to chciałbyś się tym dzielić w takiej ankiecie?**

W ogóle mam wrażenie, że z aplikacjami jest takie ryzyko, że one nie dają bezpieczeństwa naszych danych osobistych i nie wiadomo, jak one będą wykorzystane. Powiedzmy, że w takiej ankiecie ujawnimy informację, o tym, że mamy jakieś schorzenie albo bierzemy jakieś leki, a później będziemy dostawać reklamy i powiadomienia targetowane do nas, bo jesteśmy takimi a nie innymi pacjentami. Tego bym się też obawiał.

**Zainstalowałbyś ją sobie?**

Wydaje mi się, że chyba nie. Odpycha mnie to ujawnianie danych. To jest niby aplikacja, która ma służyć i pomagać, natomiast nadal nie wiem, komu daje te dane, nie wiem, po co one są i kto mi pomoże.

**W czym ci może pomóc?**

Może mi chyba pomóc w samokontroli i w jakimś uzupełnieniu informacji.

**Jeszcze masz jakieś przemyślenia? A ostatnia kropka?**

Czy to miałaby być aplikacja, którą rekomenduje MZ w Polsce? Albo MZ ją wykonało? Czy to jest aplikacja, którą po prostu można znaleźć w sklepie? Jest napisane, że narzędzie pomocne dla lekarzy, ale jakich lekarzy i skąd? Mam wrażenie, że jest tu dużo niewiadomych i z tym mam problem. Nie wzbudza to mojego zaufania. Pierwszą rekomendowało ministerstwo i dzięki temu zrobiłbym to. Po prostu. Bo tak może powinienem zrobić, bo może to ułatwi pracę policji, która nie musi do mnie przychodzić. Tutaj trochę mnie to zniechęca i nie czuję takiej potrzeby.

**Potrzebowałbyś aktualnych informacji od aplikacji?**

Wydaje mi się, że nie.

**Jak będzie za miesiąc, dwa, rok?**

Myślę, że jest taka odwilż wakacyjna teraz, ale nie wiadomo, jak będzie za miesiąc, co będzie po wyborach, po rożnych zgromadzeniach do 150 osób. Mówi się o tym, że będzie kolejne ognisko, że to do nas wróci. Z drugiej strony ma być szczepionka. Nie wiem jeszcze, jak droga, jak dystrybuowana, jak skuteczna. I powiedziałbym, że teraz faktycznie czuję się lepiej, czuję te poluzowania, czuję więcej wolności, ale nie uważam tego za coś stałego i że to już się nie zmieni. Tym bardziej chcę korzystać z tego co jest teraz, bo potem znowu może być inaczej. To się też spina z moją sytuacją zawodową, z tym co będzie za rok...Z sytuacją zawodową innych osób, bo nie mam takiego komfortu, żeby nie pracować przez rok albo nie mam takiej tarczy, dzięki której...Mam przyjaciela, który pracuje w Szwajcarii i 80% jego pensji funduje rząd. U nas niestety nie ma czegoś takiego, więc czuję się niepewnie. Czuję niepokój.

**A zdrowotnie? Myślisz, że to wróci i będzie łatwiej się zarazić?**

Mam taką obawę, że cały czas może wystarczyć moment nieuwagi. Im więcej poluzowań, im bardziej swobodnie się czujemy, tym łatwiej o taki moment. Jak to znowu do nas wróci, to też to może być nagłe. Ta sytuacja się może zmienić bardzo szybko. Myślę też o tym, że minęło za mało czasu, żeby ocenić, jakie skutki będzie miał koronawirus dla tych osób, które nawet przejdą go mniej czy bardziej problemowo. Interesowałem się tym akurat i czytałem, że zostawia jakieś tam skutki w płucach, w organizmie, natomiast mamy to dopiero od 3 miesięcy, więc trzeba będzie pewnie długich badań i po krótkim czasie, i po długim czasie, żeby się tego dowiedzieć.

**Myślisz, że u nas jest już tendencja w dół?**

Sam nie wiem. Mam wrażenie, że trochę tak, natomiast cały czas nie mam takiej pewności, że wykonuje się tyle testów, ile się powinno wykonywać. Gdybym miał podejrzenie u siebie, to sam nie wiem, czy udałoby mi się przebadać. Nie mam takiej pewności. Nie czuję się bezpiecznie i że jak coś się wydarzy, to teraz wiem, jak się zachować. Wcale nie.

**Myślisz o tym, jakie będą skutki ekonomiczne, gospodarcze w kraju?**

Mocno o tym myślę. Zaczynając od mojej branży imprez masowych i kultury, ciężko jest o tym myśleć, kiedy nie wiemy, co się wydarzy, bo ciężko jest znaleźć inną branżę. Uważam, że to jest branża bardzo specyficzna. Artyści czasami robią to przez całe życie, co mają zrobić i to nie będzie tak, że po prostu jak tutaj stracimy pracę, to za chwilę sobie znajdziemy nową w jakiejś stabilnej branży. Wydaje mi się, że nie do końca tak jest, że tych miejsc pracy po prostu zabraknie. Jest realne zagrożenie, że stracimy dochody i nie wiadomo co wtedy. A jeśli chodzi o gospodarkę, to w drugiej kolejności, tzn. myślę bardziej o gospodarce względem pojedynczych osób, których to dotyka a nie na zasadzie PKB czy czegoś tam. To w drugiej kolejności raczej, że to osłabi nasze państwo czy coś takiego.

**A sytuacja społeczna się zmieni?**

Tu jest największa niewiadoma dla mnie. Cały czas i w jedną i w drugą stronę różne zjawiska mnie zaskakują. Nadal widzę osoby, które mimo tego, że już możemy sobie chodzić bez maseczek po ulicy, nadal to robią. Nadal konsekwentnie w maseczkach czy w przyłbicach. Uważam, że to jest super. Z drugie strony są osoby, które jakby nic sobie z tego nie robią, czy są w takiej przestrzeni, czy w innej, czy są jakieś restrykcje, jakieś środki. Zupełnie nie, więc ciężko mi jest przewidywać. Wolę założyć najgorszy scenariusz niż ten najlepszy i dlatego właśnie mocno się obawiam tego, że to do nas wróci przez nieuwagę pewnych osób i nieprzestrzeganie zasad różnych.

**Myślisz, że zmienią się relacje pomiędzy ludźmi?**

Hmm...Wydaje mi się, że nie. Wydaje mi się, że może być taki chwilowy zryw, że np. ok., teraz możemy wychodzić, spotkać się ze znajomymi i to będzie pewnie chwilę trwało, ale to wróci do takiej normalności, czyli stanu sprzed wirusa.

**Czy jakieś ograniczenia powinny z nami trochę dłużej zostać?**

Ja np. nie mam nic do maseczek w sklepie. Jestem w stanie wytrzymać 15-30, czy 45 minut w takim celu, żeby nie ryzykować też zdrowiem innych, bo mam wrażenie, że tego czasem brakuje ludziom - że myślą o sobie, a nie myślą o tym, że mogą zarazić kogoś innego zdejmując maseczkę. Brakuje mi takiego altruizmu wśród ludzi. Uważam, że takie obostrzenia powinny zostać dłużej dlatego, że może być najgorszy scenariusz a nie ten najlepszy. Maseczki w miejscach publicznych jak najbardziej mogłyby zostać.  Nie mam nic do tego. Też wydaje mi się, że limity osób na danej powierzchni. To też jest ok, czemu nie? Mam dylemat i uważam, że nie jestem obiektywny, jeśli chodzi o te zgromadzenia. Uważam, mimo tego, że w moim interesie jest, żeby były te zgromadzenia coraz większe, to uważam, że nie powinno ich być.

**Są jakieś grupy, które powinniśmy szczególnie chronić?**

Myślę, że tak. Nadal nie wiem, jak to wygląda w przedszkolach, tzn. dezynfekowanie zabawek po każdej zabawie każdego dziecka. To wszystko brzmiało...

**Jest reżim sanitarny.**

Jest? Ok., to super, bo byłem sceptyczny. No to świetnie.

**Jak wyobrażasz sobie taki reżim sanitarny?**

Myślę takim idealnym scenariuszem, czyli faktycznie dezynfekowanie po każdym dziecku, uważanie, co robi, itd. Uważam, że nad dzieckiem troszkę nie da się zapanować aż tak, żeby wszystko sprawdzić, żeby sprawdzić wszystkie scenariusze i być w 100% pewnym, że nie dotykało czegoś, kogoś. Nie wiem.

**Myślisz, że to jest dobre dla tych dzieci?**

A, pod tym względem...No właśnie. Wprowadzanie takiego reżimu też może być niebezpieczne dla psychiki dziecka. No nie wiem...To jest trudny temat. Nie wiem jeszcze jak z egzaminami maturalnymi...

**Są.**

Ale stacjonarnie normalnie?

**W szkole. Ruszają w poniedziałek.**

Aha, ale...Bo ja nie wiem czy w maseczkach, rękawiczkach? W rękawiczkach to chyba nie...Wydaje mi się, że matura czy egzamin to jest duży stres i jeszcze siedzieć w maseczce to musi być niełatwe. We wszystkich tych kwestiach bardziej myślę o tej jednostce niż o ogóle.

**Od września te reżimy powinny być utrzymane i ta nauka zdalna?**

Jeżeli sytuacja będzie wyglądać podobnie, to chyba tak. Nie wiem wtedy co z rodzicami, jak znajdą opiekę, czy sami będą musieli rezygnować ze swojej pracy. To jest bardzo trudne. Nie wiem. Nie wiem też w ogóle, jak to oddziałuje na dzieci, jaki jest % zachorowań w ogóle u dzieci, czy przenoszenia tego wirusa.

**A starsi?**

Starsi na pewno też. Mogłyby być nadal i jeszcze przez długi czas te 2 godz. dla nich od 10 do 12. Nie mam nic do tego. Jeśli to ma komuś pomóc, a nie zaszkodzić, to czemu nie?

**A gdyby była utrzymana izolacja dla osób starszych?**

To jest takie trochę dla nich niebezpieczne, bo wydaje mi się, że osoby starsze, często schorowane, to dla nich samo wyjście z domu, obcowanie z jakimiś ludźmi czy rozmowa to jest jak lekarstwo czasami. Czują się wtedy dużo lepiej, więc jak mówimy o takim zamknięciu całkowitym nie zakładając, że do takiej osoby musi ktoś przyjść czy wyjść z nią na spacer, to ciężko. Wydaje mi się, że niektórzy woleliby zaryzykować i wyjść niż po prostu siedzieć do końca życia samemu.

**Mierzenie temperatury w różnych miejscach?**

Dla mnie to nie jest problematyczne, tylko...Akurat Dominika była przedwczoraj w bibliotece UW i tam też było mierzenie temperatury i ok, tylko co się stanie, jak ta temperatura będzie wysoka? Co się wtedy dzieje? Czy po prostu dostaję informację, że nie mogę wejść, bo mam wysoką temperaturę, czy jest wzywane pogotowie, czy Sanepid, czy są jakieś kroki. Samo zmierzenie temperatury wydaje mi się sprawą chwilową, która może pomóc a nie zaszkodzić. Co dalej się z tym zadzieje? Czy ten człowiek sobie pójdzie i zarazi kolejnych 50 osób w innym miejscu? Wtedy nic z tego nie ma.

**Najważniejsze dla ciebie wydarzenia w trakcie?**

Myślę, że...To, że trzeba było zostać w domu bez wychodzenia bez potrzeby, bo to fizycznie sprawiło, że przestałem spotykać się z ludźmi, nie wychodziłem faktycznie z domu, poza koniecznymi wyjściami do sklepu. Zamknięcie szkół też było takim momentem, mimo, że nie zmieniło mojego życia bezpośrednio, natomiast w Polsce było to rzadkie zjawisko. Są miejsca na świecie, gdzie spadnie trochę śniegu i są zamykane szkoły i to się zdarza, i oni są do tego przyzwyczajeni. Ja nie przypominam sobie innej sytuacji, kiedy zamknięto szkoły. Myślę, że świadomość tego, ilu ludzi zginęło we Włoszech. Włochy chyba zrobiły największe wrażenie na mnie. Z jednej strony ze względu na skalę, jaka tam była, z drugiej, bo bardzo lubię Włochy i jeżdżę tam, i planowałem tam jeździć bardzo często, więc też z takiego sentymentu. No i tyle zgonów w Polsce jednak, czyli takie uświadomienie sobie, że ten wirus serio zabija.

**Masz jeszcze jakieś refleksje?**

Że dla mnie to jest nadal szok, że to się wydarzyło, że nagle pojawiło się coś, co sparaliżowało cały świat i to w takim tempie, że nikt się tego nie spodziewał. Myśląc o tych samych miesiącach, ale rok temu, chciało się zupełnie co innego. Zupełnie inny świat i to jest dla mnie taki scenariusz filmowy trochę, ale niestety prawdziwy.
