# Supplementary material for: The challenges arising from the COVID-19 pandemic and the way people deal with them. A qualitative longitudinal study
Source: PLoS One. 2021 Oct 11;16(10):e0258133. doi: 10.1371/journal.pone.0258133 (PMC8504766; doi:10.1371/journal.pone.0258133)
Supplement: S1 Dataset — (ZIP) [file pone.0258133.s003.zip › Transcriptions/stage 5/12.5_M_33_couple, with children.docx]

**12.5_M_33_couple with children**

**Co się u ciebie działo przez ostatni miesiąc?**

Ciężko wszystko przytoczyć, ale ogólnie bardzo dużo się pozmieniało. Wróciłem na pełne obroty, jeśli chodzi o pracę, dom, przyjemności. Wszystko wróciło na pełne obroty i historia z COVID została z tyłu gdzieś daleko.

**Były jakieś ważne momenty?**

Chyba tak. Ogólnie pojawiła się u mnie w firmie możliwość zrobienia testów serologicznych i skorzystałem z takiej możliwości. I chyba troszeczkę myślałem o tym, jaki będzie wynik. To mnie skłoniło myślami, żeby pomyśleć o tej całej sytuacji trochę bardziej.

**Masz już wyniki?**

Tak, mam. Ujemne, także nie chorowałem, nie miałem styczności. Liczyłem po cichu, że jednak będą plusowe. Ale w sumie, tak jak w tym naszym badaniu, że nie ma wyniku złego lub dobrego. Chyba to był bardziej istotny moment, który by kierował moje myśli w kierunku całej sytuacji, wirusa, COVID itd.

**Jak wygląda teraz twoja codzienność?**

Niemalże wróciła do tego trybu z początku marca. Jeżdżę do pracy, bo mamy bardzo dużo praca. Wracam i dzieci, dom - wszystko kwitnie, rośnie na podwórku, więc i na podwórku mam dużo pracy. Także tak naprawdę intensywnie bardzo. Po tym chwilowym przestoju, zwolnieniu tempa, teraz wraca.

**Co jeszcze pozostało z tych zachowań związanych z kwarantanną?**

Tzn. chyba takim standardem się stał zapach środka dezynfekującego wszędzie, gdzie się nie wejdzie. Jeśli chodzi o jakieś zachowania międzyludzkie, to zauważam, że trochę wróciło takie witanie się. Nieraz takie krępujące sytuacje, nie wiadomo, jak podać rękę i to później dziwnie wychodzi. Ale gdzieś to się zaciera w taką normę.

**Co wpływa na to, że pewne obszary wracają do stanu sprzed epidemii?**

Chyba każdy z nas na to wpływa i tym samym zaraża myśleniem otoczenie, otoczenie to podchwytuje i idzie dalej to falowo. To jest tak, jakby po prostu w pewnym momencie wszyscy ludzie, których znam, pokonali te bariery, granice i wszystko zaczęło wracać do normy.

**Był jakiś przełomowy moment, kiedy nagle wszyscy stwierdzili, że wracamy do normalności?**

Moim przełomowym momentem to była majówka. To było takie fajne wyrwanie się z klatki.

**A dla otoczenia też był taki moment?**

Myślę, że po majówce. Ja zauważyłem, jeżdżąc do pracy, że więcej ludzi zaczęło się w biurach pojawiać. Jak pierwszy raz pojechałem, to było mało ludzi, cicho. A jak dziś pojechałem, to było mnóstwo ludzi. Został zmieniony tryb, tzn. tydzień zdalny, tydzień w biurze. Natomiast ja przez ostatnie 2 tygodnie codziennie byłem w biurze ze względu na obowiązki. Ludzie się zaczęli umawiać na jakieś wyjścia, na piwo, nad Wisłą posiedzieć. Takie przełamanie dosyć duże poszło, jeśli chodzi o tą sytuację i o podejście ludzi. Wydaję mi się, że tak w połowie maja było już to duże rozluźnienie. My się zdecydowaliśmy na jakąś imprezę w domu, rozszerzając ją o rodzinę, ale z którą był dalszy kontakt. Te bariery gdzieś tam zniknęły.

**Te zmiany w pracy są związane z COVID czy już na stałe wprowadzone?**

Na razie jest to jeszcze jako przeciwdziałanie w czasie wirusa. Natomiast już widzę, że głosy się pojawiają w firmie... Z resztą ostatnio wypełniałem ankietę na prośbę przełożonego, jak oceniam pracę zespołu, czy taki tryb będzie fajnie wprowadzić na stałe. Bardziej się wypowiadałem, jak wyglądała komunikacja, efektywność zespołu, itd. Myślę, że jak najbardziej. Będziemy wszyscy dążyć do tego, bo i mój przełożony jest tego zdania, że w naszej pracy jest to możliwe i sprawdziło się w tej próbie teraz podczas tej sytuacji.

**Czy jest coś, co nadal przeszkadza?**

Tak. Fajnie by było, jakby już... Nie wiem, czy tak przeszkadza, bo to się już zaciera. Np. noszenie tej maski w sklepach to mi trochę przeszkadza. Maska mi zaczęła przeszkadzać w kościele czy gdziekolwiek. Brakuje mi jeszcze trochę tego otwarcia dla dzieci np. placów zabaw, ośrodków rekreacji.

**Dlaczego maska zaczęła przeszkadzać?**

Nie wiem, może się cieplej zrobiło i więcej przebywam poza domem. Mniej mi przeszkadzała, jak większość czasu byłem w domu. Jak wyjeżdżałem na chwilę do sklepu czy coś załatwić, to bardziej mnie interesowało otoczenie niż ta maska. A teraz jest taka zwykła codzienność, gdzie noszę tą maskę w kieszeni i jak trzeba, to wyjmę, ale po prostu przeszkadza.

**Jak się czułeś w ciągu ostatniego miesiąca w tych ważnych momentach?**

Pierwszy ważny moment to był powrót do pracy biurowej. Czułem taką radość wręcz z tego, że to wraca namacalnie do normalności. Mogłem się spotkać ze znajomymi z pracy i to było bardzo pozytywne. Następnie miałem kilka wyjazdów gdzieś w teren kolejowy. Zawsze mi to sprawiało przyjemność i teraz tym bardziej. Też z rodziną planujemy już gdzieś się wybrać, jakoś zaplanować wakacje. To są takie pozytywne momenty i bardziej takie odetchnięcie, że to wraca do normy. Natomiast, śledząc relacje prasowe widzę, że te ogniska są, zachorowania są. Tylko mi się wydaje, że jeśli ktoś osobiście tego nie zauważył - bo ta pierwsza reakcja była bardzo zachowawcza i to pewnie widać na tych pierwszych rozmowach u ciebie, jak do nich wrócisz. Natomiast człowiek też obserwuje i analizuje to, co się dzieje wokół. Ja nie zauważyłem, żeby ktoś z najbliższego czy dalszego otoczenia, zachorował, ciężko to przeżył, zmarł, itd. To daje do myślenia. Przestałem troszkę myśleć o tej sytuacji i mój wzrok się skupił na takich osobistych celach czy to w pracy czy w domu.

**Znalazłeś jakieś obrazki, które oddają twoje emocje?**

Nie szukałem, bo nie miałem czasu za bardzo, ale dosłownie przez chwilkę przed naszą rozmową pomyślałem o tym i wszystko - to może zboczenie zawodowe - mi się kojarzy z pędzącym pociągiem. Gdzie już to życie nabiera tempa, powoli się rozpędza i mało jest rzeczy, które potrafią lokomotywę zatrzymać [śmiech].

**Kiedy ta lokomotywa ruszyła?**

Myślę, że to jest połowa maja, ok. 15 maja. Wtedy zaczęła powoli ruszać, a teraz jest już nieźle rozpędzona. Do jakichś 60 km/h.

**Czyli będzie rozpędzać się jeszcze dalej?**

Tak, tak, jak najbardziej.

**Co przeszkadza w dalszym rozpędzeniu się?**

Mało jest takich rzeczy, które przeszkadzają, bo te, które doskwierają, bardziej dotyczą takiej rekreacji, wypoczynku, itd. U nas idzie pełną parą i nie widzę na razie przeszkód, które można by było napotkać związane z wirusem. Chyba takie obawy tylko, że gdzieś znowu się pojawią jakieś liczne przypadki i będzie nawrót tej sytuacji z marca, kwietnia. Analitycy mówią, że to może nastąpić na jesieni, w okolicach września, października. Chyba tego się trochę obawiam, bo to znowu zaburzy rytm.

**Obrazki. Które z nich oddają twoje emocje z ostatniego miesiąca?**

1 nie wybiorę, chociaż korki do Warszawy już się zaczynają pojawiać. Ale chyba 15 ogólnie. Chyba to odzwierciedla - taka burza, siła, moc, która gdzieś drzemie, ale została uwolniona. Chyba z tym mi się najbardziej kojarzy. Chociaż to jest dziwne, bo tak, jakbym pierwszy raz widział ten obrazek. Jakbym go pierwszy raz zauważył. Był on wcześniej?

**Tak.**

Tak jakbym go wcześniej nie widział, nie brał pod uwagę. Teraz jak najbardziej ten oddaje to, co czuję. Taką ekspresję, moc. Jest gdzieś siła, która zostaje uwolniona.

**Czujesz się zagrożony tą sytuacją jeszcze?**

Nie. W ogóle zmieniło się moje nastawienia od neutralności wręcz do... Nie boję się tego i wręcz przeciwnie - wyzywająco patrzę na tego wirusa i mimo, że może mi zaszkodzić zdrowotnie bardzo, jakoś rzuciłbym mu wyzwanie. Ja też nie lubię tak mówić, bo to trochę takie głupie gadanie - "rzucać wyzwanie wirusowi". Natomiast nie boję się go i jestem takim chojrakiem trochę w tym momencie. Nawet, jakbym zachorował, to mam taką świadomość wewnętrzną, że musiałbym sobie z tym poradzić, musiałbym to przechorować i miałbym z głowy wręcz.

**Jakie emocje czułeś, kiedy pojawiła się możliwość testów? I jak otrzymałeś wynik.**

Pracowałem z klientem bezpośrednio. Oczywiście też takie rozluźnienie - ja byłem przygotowany: rękawiczki, maski, itd., a klient luźniej do tego podszedł i ja też zacząłem po pewnym czasie. Wyczytałem w mailu, że pojawiła się taka możliwość, ale była skierowana do osób, które przez cały ten okres pracowały w biurze, bo musiały, lub pracowały z klientem. Natomiast pojawiła się taka szansa i od razu napisałem zgłoszenie, z zaznaczeniem tego, że pracowałem większość czasu w domu, natomiast jeśli gdyby zostałoby tych testów, to chciałbym się znaleźć na liście rezerwowej. Już na drugi dzień dostałem informację, że jak najbardziej jest możliwość. I wszystko się działo bardzo szybko. We wtorek zobaczyłem maila, w środę dostałem informację, że mogę, w czwartek były badania i z tego, co się później dowiedziałem, w piątek już były wyniki. Natomiast wyniki odebrałem dopiero we wtorek, także cały weekend sobie o tym myślałem. Podszedłem o tego w ten sposób, że nie wiedziałem czy przechorowałem czy nie i bardziej z ciekawości. Rodzina też była ciekawa, bo jak ja bym miał, to pewnie oni też. Ale to było na zasadzie takiej, że oszacowałem te szanse, że miałem na jakieś 20% maksymalnie. Bo jednak widziałem się z dziadkami przez cały ten okres i jeśli ja bym chorował nawet bezobjawowo, to pewnie oni mieliby gorzej. I powiedzmy, że chociaż jedna osoba miałaby jakieś objawy w tym otoczeniu, w którym się obracałem. Więc podchodziłem z taką dużą rezerwą. I trochę się tliła we mnie nadzieja, że może jednak pozytywny i byłoby z głowy. I bym wiedział, że jestem na tyle odporny, że nawet tego nie poczułem. Jak odebrałem testy we wtorek to tak otworzyłem i stwierdziłem: e, negatywne. Przewaga, bo te 80% to jednak negatywnie. Z tego, co słyszałem, to wszyscy, z którymi rozmawiałem mieli wynik negatywny. To też daje do myślenia, że kurczę, nie spotkałem osoby... O jakiejś tylko się słyszało, że ktoś zachorował, ale takiej bezpośrednio z bliskich znajomych, nie miałem tego przypadku.

**Jak osoby z twojego otoczenia odczuwają tę sytuację? Jakieś nowe emocje?**

Tak. Zauważyłem chęć spożycia alkoholu na otwartym terenie, taką wzmożoną, bo już kilka osób na ten piątek rzucało propozycje. Poza tym, zauważyłem, że nagle ci wszyscy znajomi nasi z żoną... Można by było chodzić na imprezy co weekend i w sumie tak się dzieje. Każdy jakoś otworzył się bardzo i wszyscy mają ochotę, żeby się spotkać, porozmawiać, napić się piwa, pośmiać się, pograć w gry. I to widać. W pracy widać, w znajomych, w rodzinie. Gdzieś jest takie parcie na to, żeby się spotykać i spędzać razem czas.

**Myślisz, że ludziom brakowało tego i jest bardziej teraz niż byłoby normalnie?**

Myślę, że jest taki troszeczkę peak do góry, że jednak spotykamy się. Przykładowo w piątek mamy spotkanie z jedną parą znajomych, w sobotę z drugą parą znajomych. Niedziela jest jeszcze nieobstawiona, ale coś mi się wydaje, że ktoś przyjedzie do nas. I tak weekendy zaczynają wyglądać od jakichś 2 tygodni, że z kimś się spotykamy.

**Jak wyglądają zakupy teraz?**

Utrzymujemy tryb, że 1 w tygodniu są zakupy. Natomiast wybrałem się ostatnio do Centrum Janki i zauważyłem, że jest bardzo dużo ludzi. Po prostu galerie tętnią życiem.

**Po co wybrałeś się do galerii?**

Musiałem zrobić zakup konkretnej rzeczy. Córka poprosiła o Twistera na Dzień Dziecka. To było w poniedziałek, więc wstąpiłem. Przy okazji odwiedziłem kilka sklepów. Nic nie kupiłem, bardziej się rozejrzeć. Natomiast zauważyłem, że jest bardzo dużo ludzi, parking pełen. To w sumie pierwsza wizyta w jakiejkolwiek galerii od lutego.

**Były jakieś zachowania, które szczególnie zwróciły twoją uwagę?**

Ja już bym się wbił do Auchan bez rękawiczek, ale jednak ktoś mnie zatrzymał i poprosił, żebym założył rękawiczki [śmiech].

**Tam na wejściu stały rękawiczki?**

Tak, stały rękawiczki. Już rękawiczek w kieszeni nie noszę.

**Maseczkę nosisz w tych sklepach?**

Tak. Miałem sytuację, że się wybierałem do sklepu i się wracałem po maseczkę do samochodu. Bo może być tak, że ktoś by mnie nie obsłuży, wyprosił albo by się na mnie dziwnie patrzył.

**Otwarto ostatnio restauracje i kawiarnie. Korzystałeś już?**

Nie, jeszcze nie wybraliśmy się na posiłek do restauracji, ale wybieramy się w piątek ze znajomymi. Umówiliśmy się w lokalu z ogródkiem, z placem dla dzieci na zewnątrz. Więc będę miał okazję w piątek, a do tej pory jeszcze nie. Bardziej zamawiamy jakieś jedzenie na wynos.

**Do pracy czy do domu?**

Do domu jeśli już. W pracy już nie zamawiam. Przywożę swoje. Dalej noszę z domu. Gotuję. Jeszcze dzisiaj mam ugotować obiad. Jeszcze wieczorne atrakcje w kuchni.

**Co sądzisz o tym, że otworzyli restauracje, kawiarnie?**

Trochę mi się zmieniło myślenie i myślę, że ok. Był ten moment, kiedy trzeba było zamknąć, żeby ludzie rzeczywiście zostali w domach. I pewnie dlatego nie widzę takich osób w moim najbliższym otoczeniu, którzy chorują czy wręcz umierają właśnie przez to, że taka kwarantanna się odbyła. Natomiast, jeśli ten wirus jest i gdzieś krąży, to musielibyśmy siedzieć rok czasu w domu, żeby czuć się bezpiecznie. Myślę, że to jest ten moment, żeby ci, którzy prowadzą biznesy restauracyjne mogli zacząć zarabiać. I ludziom też to jest potrzebne, żeby się spotkać, usiąść sobie nawet w tym ogródku piwnym, porozmawiać ze sobą i poczuć trochę takiej normalności.

**Myślisz, że to bezpieczne?**

Ja wiem... Powiem ci, że dla mnie w tym momencie troszkę się pozmieniały kryteria, co jest bezpieczne, a co nie. Ciężko to powiedzieć. Jest ryzyko na pewno i myślę, że - przynajmniej ja - akceptuję to ryzyko. Nie będę myślał o tym, czy się zarażę czy nie, jak pójdę do restauracji, dlatego, że już przestałem o tym myśleć w biurze, w pracy. Wychodzę, łapię za klamki, chodzę do sklepów, korzystam z bankomatów. Nie robię tego w rękawiczkach. Co jakiś czas zdezynfekuję ręce. Ale mniej działań podejmuję prewencyjnych. I mi się wydaję, że to ryzyko będzie i jest, ale już tak bardzo się o tym nie myśli. Ja mówię o sobie, ale jeszcze teraz nie wiem, jak z dziećmi. Nie powiedziałbym, czy to jest bezpieczne czy niebezpieczne - nie da się powiedzieć. Bardziej jest to poziom ryzyka, który jest albo akceptowalny albo nie, w zależności od sytuacji.

**Ostatnio zostali otwarci fryzjerzy, kosmetyczki, kina, siłownie - co o tym sądzisz? Dlaczego są otwierane?**

Bo ludzie tego potrzebują do życia i to też jest takie akceptowalne ryzyko przez wszystkich - przez korzystających, przez rząd. I na pewno na korzyść dla osób, które prowadzą takie biznesy. Jednak o nich też trzeba pomyśleć. I taki fryzjer już miał długą przerwę w interesie i wydaje mi się, że to jest dobry moment, żeby też mógł zacząć zarabiać.

**Czy byłeś w takich miejscach?**

Byłem, ale jeszcze przed zniesieniem tego [śmiech].

**Podziemie?**

Dokładnie i dosłownie fryzjer w piwnicy.

**Jak to wyglądało?**

Nie. Pani fryzjerka się zapytała: zakładać maskę? I nie wiedziałem, w którą stronę ona pyta - czy żeby zwiększyć bezpieczeństwo dla mnie czy dla siebie. To ja jej odpowiedziałem, że ja się nie czuję chory [śmiech]. Aczkolwiek śmieszna sytuacja, bo znalazłem takie dojście i pojechałem do kogoś na posesję. Zeszliśmy poziom niżej do takiej sutereny gdzieś, a'la garaż, mały pokoik. Wchodzę, a tam salon kosmetyczny.

**Nie założyła tej maski?**

Nie. Tak mnie krótko ostrzygła, że do dzisiaj jeszcze nie potrzebuję.

**Słyszałeś coś o aplikacjach stworzonych na potrzeby pandemii?**

Słyszałem, że Chińczycy znaleźli prosty sposób, żeby inwigilować społeczeństwo, bo teoretycznie ma to służyć temu, że jeśli osoba zarażona była w pobliżu kogoś, to ta aplikacja pozwalała śledzić, kto był w pobliżu, itd. Nie wiem czy to prawda, bo cały ten COVID jest owiany teoriami spiskowymi. Natomiast, jak mnie ktoś mówi, że uważaj, nie wpisuj czegoś do sieci albo nie kupuj Huiwei, to ja się trochę z tego śmieję, bo nie uważam się za taką personę, którą warto by było śledzić, szpiegować [śmiech]. Pochlebiłbym sobie, jakbym się tak bardzo chował ze wszystkim. Mam takie podejście dosyć luzackie w tym temacie. Przypomniałem sobie mema, jak stał sobie taki żołnierz chiński z łezką w oku i było podpisane: "Kiedy zrywasz ze swoją dziewczyną, z którą bardzo długo byłeś i twój Chińczyk na podsłuchu się wzruszył".

**Opis dwóch kategorii aplikacji. Co dają takie rozwiązania?**

Ciekawe pytania zadajesz. Wydaje mi się, że nie unikniemy tego typu rozwiązań w tych czasach. Komuś na pewno coś dają, niekoniecznie zgodnie z pierwszym zamysłem pomocy. Coś na pewno komuś dają. Ja jestem świadom, że nie powstrzymamy tego w ogóle. Są systemy w Chinach, gdzie rzeczywiście sczytywane są twarze i nie trzeba kupować biletu na komunikację. Ja byłem w szoku, że ja mogę sobie wejść na stronę Google i zobaczyć, gdzie byłem 2 miesiące temu, linia po linii, jak się przemieszczałem. Natomiast, jakby ktoś dał mi taką władzę, żeby to zatrzymać, to chyba bym tego nie zrobił, bo być może... Ja się czuję neutralny. Czuję się osobą, która, jak Chińczyk chce siedzieć i mnie szpiegować, to proszę bardzo, mnie to nie przeszkadza. Nie mam w zamyśle napadać na banki, robić oszustw gospodarczych. Mam w zamyśle normalnie żyć. Natomiast, jeśli komuś miałyby pomóc te aplikacje, to jak najbardziej, bo są osoby, które nie mogą na kogoś liczyć bliskiego, chociaż mają bliskie osoby. Spotkałem się z takim przypadkiem, gdzie starsza osoba potrzebowała jakiejś pomocy. Odezwała się do mnie, gdzie mnie trochę znała, a wiem, że miał osoby, które mogłyby mu pomóc. I być może takie aplikacje mogłyby pomóc takim osobom. Ale to jest tak naprawdę już bardziej zaawansowane, jak sztuczna inteligencja. Bo drony to już są, roznoszą produkty. Dane lokalizacyjne też. Co mnie jeszcze tam zaciekawiło? Informacje o stanie zdrowia. Też są. Zwykły smart watch już mierzy tętno, itd. i alarmuje, daje alerty. Także to powoli w nas wnika, przenika. Jedna rzecz mi się nie podobała. Coś ze sztuczną inteligencją?

**Powtórzenie opisu.**

Tutaj już mi pachnie trochę Pirxem i nie wydaje mi się, żeby to był dobry pomysł. Komputer się będzie opierał na czystej statystyce, a jednak czynnik ludzki jest najważniejszy. Obym nigdy nie dożył takich czasów, że sztuczna inteligencja będzie decydowała o losach wielu ludzi. Komputery, które będą wspomagać człowieka, jak najbardziej, ale nie decydować.

**Widzisz różnice pomiędzy tymi 2 kategoriami?**

Na pierwszy rzut oka, ta pierwsza zdaje się być trochę bardziej kategorią, która zbiera dużo informacji i je przetwarza. I te informacje mogą być przetworzone w celu dobrym lub złym. Natomiast ta druga kategoria mi się skojarzyła - drony, aplikacja informująca o potrzebie pomocy - to pewnie te dwie mogłyby pomóc. Oprócz tej sztucznej inteligencji. I one są takiej bardziej dla ludzi, a nie dla statystyk.

**Twoje obawy budzi podejmowanie decyzji przez sztuczną inteligencję?**

Tak, to chyba najbardziej.

**Prezentacja aplikacji ProteGO Safe. Co myślisz o tej aplikacji?**

Jest to jakiś wstępny wywiad o stanie zdrowia, o chorobach, jakie się leki ma, jakie się przyjmuje. Taki wstępny kwestionariusz, który już skieruje osobę badaną w jakąś grupę ludzi, jakiegoś ryzyka być może - taką, żeby ktoś był świadomy. Ale tutaj było też napisane, że pobiera informacje o miejscach, gdzie się przebywa. Wystarczyłoby, że ktoś w tej aplikacji zmieni status na "COVID", że zachorował i po jakimś czasie wyciągnięte by zostały wszystkie te kontakty, które miał po drodze, z kim się mijał na ulicy. Żeby to zadziałało, to dużo osób by musiało mieć tą aplikację. Ale nie wiem, szczerze mówiąc... Ja bym chyba nie zainstalował.

**Coś ci się podoba w tym opisie?**

Ogólnie to wygląda bardzo dobrze. Jak się to czyta z takim podejściem, że to jest dla nas, dla naszego zdrowia. Natomiast, uważam, że nie wiem komu by to mogło być potrzebne. Nie jest chyba czas na to, żeby to było efektywne.

**Co budzi twoje obawy?**

Połączenie się Bluetooth z innymi urządzeniami. Bluetoothem można dużo rzeczy przesłać - jakieś zdjęcia, treści. Nie tylko informacje z samej aplikacji. Chociaż ja mam Bluetooth non stop włączony. Nie wiem...

**Widzisz w tym potencjalne zagrożenie dla swojej prywatności, bezpieczeństwa?**

Tak. Powiedziałem, że nie przeszkadzałby mi ten Chińczyk, który by mnie obserwował. Ale ja bym chyba tego nie zainstalował. Ciężko to wytłumaczyć. To już zakrawa takimi wizjami przyszłości, że zaraz wszyscy będą zachipowani i takie ograniczenia zostaną wprowadzone. Bo to jest taki pierwszy krok, żeby zachęcić ludzi, żeby wszyscy mieli to samo. Bo rzeczywiście, żeby to było efektywne, to musieliby mieć wszyscy.

**Wiesz, że Facebook na tej zasadzie podpowiada ci znajomych?**

Tak. Zdaję sobie sprawę, że jak tylko czytasz ciasteczka, to już nas otacza. Nieraz są wnerwiające te ciasteczka, jak sobie raz zobaczę np. opony, to później przez tydzień oglądam różne opony. Ale przed tym się chyba nie ucieknie. To jest kwestia wyboru. Mogę też odinstalować Facebooka czy kupić sobie Nokię 3310 i żyć bez smartfona. Ta aplikacja jest dla mnie podejrzana.

**Uważasz, że rząd powinien tworzyć takie aplikacje?**

Hmm... Myślę, że tak. Z tego względu, że to wszystko globalnie idzie w jakimś kierunku. To idzie w takim kierunku, gdzie wszędzie nas będą otaczać takie aplikacje. Nie wiem czy jest to dobre czy złe. Dla mnie raczej złe, bo coraz więcej jest bodźców, które nas otaczają. Chociażby te ciasteczka, jak z tymi oponami, to jest już wnerwiające. Natomiast rząd będzie gonił za tym postępem. Nie zostanie z tyłu za Chińczykami zbyt daleko i za Amerykanami. Chociaż pewnie jesteśmy daleko, ale będzie to wprowadzał.

**Prezentacja aplikacji Kwarantanna Domowa.**

To był chyba nakaz korzystania z tej aplikacji?

**Tak. Dalszy opis. Co sądzisz o tej aplikacji?**

Jestem zaskoczony, bo wiedziałem, że jest jakaś aplikacja, ale nie wiedziałem, że działa na takich zasadach. Ogólnie nie podobają mi się - ani jedna aplikacja ani druga. Wydaje mi się, że ludzie są myślący. Kto ma narażać kogoś, to i tak, mając tą aplikację nawet, będzie narażał. Wyskoczy po piwo, do sklepu na chwilę. Wydaje mi się to przesadą tak szczerze mówiąc.

**Czemu służy ta aplikacja?**

Największy plus tej aplikacji to jest kontakt z lokalnymi ośrodkami pomocy społecznej, gdzie można napisać, że potrzebne są artykuły spożywcze, leki, itd. Natomiast takie sprawdzanie i robienie sobie selfie, naprawdę?

**Tak to działa.**

To powiem, że jestem zaskoczony. Nie podoba mi się ta aplikacja. Wydaje mi się, że jakieś służby mogą sprawdzić, czy ktoś jest w domu. Z jednej strony jest to sprytne, żeby kontrolować czy ktoś rzeczywiście jest na tej kwarantannie. Ale z drugiej... Nie wiem, czy ta pierwsza, czy ta druga... Te aplikacje same w sobie są podejrzane. Mogą dawać bardzo dużo informacji komuś, kto może je jakoś wykorzystać. Nawet robiąc sobie selfie, to przecież można coś w tle sfotografować. Różne takie informacje dodatkowe można komuś przekazać.

**Gdybyś miał wybór, to pobrałbyś tę aplikację na swój telefon?**

Nie. Jeśli miałbym wybór, to nie. Jakbym miał być na kwarantannie, to po prostu bym był. To jest kwestia odpowiedzialności. Tutaj trochę robią z ludzi, no nie wiem, małpy. Bo to jest tak, jakby każdy chciał od razu uciekać, gdzieś wychodzić, coś kombinować, wiedząc, że mogą być chorzy.

**Czy gdybyś był na kwarantannie to pobrałbyś tę aplikację, czy starałbyś się jakoś to obejść?**

Nie szukałbym sposobu. Pewnie jakbym był na kwarantannie, to po prostu bym to pobrał i robił sobie selfie.

**Ale nie podoba ci się to?**

Nie podoba mi się ogólnie. Bo to takie... Jak ja bym miał 2 tygodnie być na kwarantannie, to bym po prostu był. Kwestia własnego rozumu.

**Czy uważasz, że rząd powinien tworzyć takie aplikacje?**

Myślę, że tak. Ale na tej zasadzie tej pierwszej aplikacji, że jeśli chcesz, to sobie to pobierz. Bo być może ci się to przyda. Bo być może ktoś bierze jakieś leki, gdzie rzeczywiście choruje na bardzo rzadką chorobę i z tej aplikacji będzie można uzyskać takie informacje. Natomiast wszystko powinno być robione na zasadzie dobrowolności. Ktoś chce, to sobie pobiera, ktoś nie, to nie. Ktoś więcej poczyta, porozmawia ze znajomymi - może ktoś będzie miał więcej informacji na temat tej aplikacji. Na tej zasadzie. Nikt nie lubi, jak coś się komuś narzuca. Bo nawet z tym Facebookiem - ja mam go zainstalowanego, chociaż go nie aktualizuję już od kilku lat, nic tam nowego nie wrzucam. Czasem mam chęć, a później znowu pół roku nic z tym nie robię. Bardziej mi służy do komunikacji, czyli przez Messengera się z kimś komunikować, coś obejrzeć. Bardziej treści, które mam polubione sobie np. pociągi pooglądam, itd. Natomiast w każdej chwili mogę odinstalować, itd. Moim zdaniem trochę przesada, że jest to obowiązkowe i trzeba. I trzeba sobie robić kilka razy dziennie selfie. To jest dla mnie trochę śmieszne wręcz.

**Byłoby to akceptowalne, gdyby pozostawić ludziom wybór?**

Chyba tak. Aczkolwiek nie wiem, czy wtedy ktokolwiek by się zdecydował.

**Myślisz o przyszłości po pandemii?**

Ja myślę o tym, że to jest chwilowe. Tzn., że nie będziemy mieli jakichś problemów w przyszłości takiej do 5 lat. Że w końcu pojawi się szczepionka. Dużo osób mówi, że nie będzie się szczepić. Ja np. nie należę do tego ruchu i pewnie, jak się pojawi, to będzie ten wirus gdzieś tam krążył, ale być może tak, jak grypa. Nie widzę jakiś strasznych konsekwencji tego, że zmieni się bardzo życie. Na pewno zostaną jakieś nawyki w głowie typu, żeby w domu maseczki trzymać, jakieś środki dezynfekujące. Natomiast, mnie się wydaje, że to wróci do normy - koncerty, tłumy ludzi w jednym miejscu. I tak to widzę. Że będzie to taki epizod, o którym będzie się opowiadało wnukom [śmiech].

**Co najbardziej zaprząta twoją uwagę?**

Takie rzeczy, o których normalnie bym myślał, nawet, jakby nie było pandemii. Gdzieś widzę jakąś przyszłość za 5 lat. Mam pewne plany, które realizuję. Myślę o tych planach, itd. Myślę o tym, co się będzie działo w firmie. Bądź, co bądź pracuję już w tej firmie 9 rok. Za 5 lat, to już będzie 14 lat w jednej firmie. Zastanawiam się czy będę pracował, czy zrezygnuję. Raczej nie zrezygnuję. Ciężko powiedzieć. Ale kompletnie nie myślę o tym, że to będzie coś związanego z pandemią. Chociaż z drugiej strony miewam takie myśli... Z resztą widzi się takie treści w Internecie, że to nie pierwszy taki wirus, jaki nas dopadnie. Że te lodowce topnieją, itd. To jest życie, różnie może być. Może pojutrze wybuchnąć wulkan pod Yellowstone [śmiech]. To jest takie... Odkładając te rzeczy, typu pandemie, katastrofy, to planuję normalne życie z dziećmi, które będą dorastać, z jakimiś inwestycjami, które będą rosnąć, o ogórdku, itd. Ja nie mam jakichś oszałamiających planów na swoje życie, że za 10 lat, to rzucę wszystko i pojadę w podróż dookoła świata. Po prostu będę zadowolony, jak będę mógł wychować dzieci i później się cieszyć taką jeszcze nie starością, ale większą swobodą. Zawsze żonie powtarzam: spokojnie, zobaczysz, za 15 lat, to sobie tak będziemy żyć. I na wycieczki jeździć, i na działeczce siedzieć i pływać łodzią po jeziorze w zacisznych miejscach. Do tego dążę. Zobaczymy, jak to się wszystko potoczy. Ale na razie idzie w tym kierunku. I bardziej takie rzeczy, jak pandemia, to jakiś epizod, który nawet, jak się pojawi, to się przeżyje. I będzie tak, jak z COVID, że różne emocje będą targać, ale koniec końców, później się będzie o tym opowiadać.

**Masz jakieś przemyślenia co do sytuacji gospodarczej?**

Już się zmieniła troszeczkę. Widać taki ruch, jeśli chodzi o pracowników. Dużo osób wróciło do Polski. Ciekawe, czy to nie wyjdzie na plus. Przed pandemią brakowało pracowników fizycznych i musieliśmy się posiłkować w wielu przypadkach ludźmi zza wschodniej granicy. Natomiast mały przewrót jest. Może to wyjść na plus, kto wie. Ktoś na pewno też zarobi na tym kryzysie. Coś w stylu, że nie można zmarnować dobrego kryzysu, bo zawsze ktoś na tym zarobi. Nie wiem, czy akurat na maseczkach, czy jakichś innych, ale jakoś bym nie widział trzęsienia ziemi, jeśli chodzi o gospodarkę. Dzisiaj słyszałem, że są plany wybudowania 1800 km nowych linii kolejowych w Polsce, także się nie boję. Będzie nad czym pracować.

**A sytuacja społeczna. Są jakieś grupy, których szczególnie mogą dotknąć zmiany?**

Tak. Najbardziej chyba ucierpią mali przedsiębiorcy, takie zakłady fryzjerskie, osoby, którzy zatrudniają niedużo osób. Np. moi rodzice mają sklep i to jest tak, że jakiś kokosów na tym nie ma. Takie przedsiębiorstwa, które są bardzo wrażliwe na to, co się dzieje na rynku, to mogą ucierpieć. Mogą być zwolnienia. Słyszy się w radiu, że ludzie dzwonią, pytają różnych specjalistów, że dostają połowę pensji, itd. To może uderzyć w takich przedsiębiorców. I teraz kopalnie - to może uderzyć w kopalnie, gdzie ludzie pracują razem i tam, gdzie pojawiło się ognisko, to każdy przestój kosztuje. W takich przedsiębiorców najbardziej.

**Któreś z ograniczeń powinny zostać utrzymane na dłużej?**

Chyba tak dla pewności, to takie typowo duże imprezy masowe. Chociażby mecze, koncerty. Chyba tego typu rzeczy, bo to jest wtedy, kiedy mamy największą masę różnych ludzi, która spotka się w jednym miejscu. To powinno być gdzieś przedłużone chociażby do Nowego Roku. Z tego względu, że jeśli ktoś prognozuje, że na jesień będzie druga fala zachorowań, to warto z tym się wstrzymać.

**Któreś z rozwiązań, które wprowadziłeś w trakcie pandemii, planujesz utrzymać dłużej?**

Może zachowań. Ta dezynfekcja rąk już takim standardem się zrobi. Te żele już kady ma pod ręką. Te rękawiczki może nie, bo są one uciążliwe w noszeniu. Częste mycie rąk na pewno. Bardzo częste. Specjalnie się chodzi, myje ręce. Bardziej zwracanie uwagi na to dzieciom, żeby np. nie lizały poręczy, czy tego typu rzeczy. I chyba w ten sposób. Bardziej tak dbać o higienę. Mieć świadomość, że bakterie, wirusy gdzieś tam są i mogą zrobić jakąś krzywdę. Natomiast ja już widzę, że normalnie się witam z ludźmi, czyli to już u mnie wraca w tym akurat aspekcie. Sam chętnie wypatruję, kiedy basen otworzą. Już niedługo mają być otwarte?

**Większość basenów od 8.06.**

To jest bardziej pod kątem obserwacji, czy te zachorowania... Bo one chyba jakoś bardzo nie słabną? Nie śledzę mediów. W tym momencie jest jeszcze jakiś dystans, taka rezerwa. Na mniejszym poziomie niż wcześniej, ale jest. Tak mówię o basenie, ale nie wiem czy bym się wybrał teraz. Ja bardziej planowałem gdzieś na jesieni. Na razie i tak nie mam czasu. Na jesieni będziemy obserwować, jak to będzie wyglądało.

**Dlaczego teraz byś się nie wybrał? Ze względu na to, że nie masz czasu, czy że ryzyko jest zbyt duże?**

To drugie chyba. Nie czekałbym na otwarcie od razu, tylko jeszcze bym poobserwował. Poczekałbym jakieś 2-3 tygodnie, jak ruszą te baseny, czy nie pójdzie jakaś druga fala. Tak bardziej z rezerwą. Także, jak otworzą od czerwca, to może od lipca bym się wybrał. Taki jeszcze miesiąc dodatkowy dla obserwacji.

**Są jakieś grupy, które powinny być chronione szczególnie?**

Chyba osoby starsze. Mnie się wydaje, że takie zasady sanitarne w domach opieki społecznej, w hospicjach, w szpitalach, powinny być na bardzo wysokim poziomie. Bo tam są osoby chore, starsze i tak naprawdę one są najbardziej narażone na konsekwencje zarażenia.

**Jeszcze w jakiś sposób można by było chronić tę grupę?**

Nie mam pomysłu w tym momencie. Nie wiem, czy te godziny dla seniorów to jest dobre rozwiązanie. Jak wszystko wróci do normy, to dlaczego mieliby nie wpuszczać ludzi do sklepów w tym samym czasie. Wydaje mi się, że w takich ośrodkach to musi zostać na wysokim poziomie, bo tam największe żniwo zbiera ten wirus.

**A izolowanie starszych w ich domach?**

Właśnie co znaczy izolowanie. Bo człowiek starszy też potrzebuje spotkać się z ludźmi, wyjść na zewnątrz. Nie widziałbym takiego rozwiązania, żeby ich izolować.

**Raczej nie powinniśmy ograniczać ich wolności?**

Tak. To jest trochę czarny humor, że po prostu, może ktoś będzie żył dłużej, ale co to za życie. U mnie dziadkowie i tak nie wychodzą. Są w tak sędziwym wieku, że mają tutaj swój własny świat w domu, gdzieś tam na podwórko [wyjdą]. Ale wiem, że dużo jest takich starszych osób, które gdzieś szaleją po mieście z torbami czy wózeczkami. Na pewno bym nie kazał takim osobom siedzieć w domu. Prędzej wpadną w jakąś chorobę siedząc w domu - przynajmniej moim zdaniem, może nawet jakąś psychiczną - niż tak jakby sobie utrzymywali normalny tryb życia.

**Są jakieś zachowania, rozwiązania, które powinny zostać z nami na zawsze?**

Myślę, że tak. Płyny dezynfekujące w sklepach, w firmach. Mogą to być też większe dezynfekcje, typu kościół, sklepy. To są bakterie, wirusy. Powinniśmy być świadomi, że tak naprawdę nie tylko 1 COVID jest na świecie, który zagraża i szybko się przenosi. Za chwilę może to być coś innego i myślę, że takie prezencyjne działania powinny stać się standardem. Powinien być zwiększony budżet na tego typu rzeczy, żeby być przygotowanym na taką sytuację. Na to, żeby przystosować szpitale. Niech to będzie na zasadzie straży pożarnej, że są duże oddziały przystosowane do tego, żeby bardzo szybko stać się jednoimiennymi oddziałami. Gdzieś powinny się znaleźć na to pieniądze. Myślę, że taki COVID to będzie troszkę na orzeźwienie, czy też na spojrzenie na to wszystko z innej strony. Taki sygnał, żeby pomyśleć o tego typu zagrożeniach.

**Co sądzisz o nowych rozwiązaniach w miejscach nowo otwartych? Kina, przedszkola, obiekty sportowe.**

Tak. Moje dzieci już od tego tygodnia są w przedszkolach. Wysłaliśmy tydzień temu: idźcie powolutku [śmiech]. Też krążył taki mem. Już są w tym tygodniu w przedszkolu. Zależy, jakie to są ograniczenia. Jak np. w kinie, jak siadamy co drugie krzesełko. Na stałe nie wydaje mi się, że będzie to konieczne. Na ten okres przejściowy ok. Po tym, gdzie zwalniamy obostrzenia, do momentu, kiedy nie będziemy mieli zachorowań to jak najbardziej trzeba to wprowadzić i trochę może to pomóc. Czy też dezynfekcje, odstępy, odstępy w kolejkach. Nikomu nie przeszkodzi stanąć w kolejce 2 metry od kogoś. Można takie zachowania jak najbardziej utrzymać. Natomiast docelowo myślę, że to wróci do normy takiej, jak przed COVID.

**Jak się odnosisz do mierzenia temperatury np. w przychodniach, na lotniskach?**

Mi też mierzą temperaturę 3 razy dziennie jak jestem w pracy. Dla mnie to też jest takie śmieszne. Bo 1 osoba chodzi w budynku, gdzie w firmie jest 300 osób i mierzy temperaturę. Ma mierzyć temperaturę 2 razy dziennie każdej osobie. Rozmawiałem z taką osobą i ona, jak za drugim razem mi mierzyła temperaturę, to była już padnięta.

**Zatrudnili nową osobę do tego?**

Nie. Jest taka pracownica. Nie wiem czy troszkę niepełnosprawna. I tak pomaga np. kawę robić, coś przygotowywać. I ona biega tak z tym termometrem. Aż mi się jej szkoda robi, bo naprawdę. Od pokoju do pokoju, na korytarzach i to trochę trwa, tak trzeba trzymać tą rękę... Dla mnie to jest bez sensu. Bo każdy czuje czy ma temperaturę. Nie wydaje mi się, żeby ktoś, źle się czując, specjalnie narażał innych i przychodził do pracy. Moim zdaniem to jest na wyrost. Każdy chyba czuje, że jeśli jest rozpalony... Z resztą można się samemu zmierzyć. Mógłby być gdzieś 1 termometr, gdzie jakby ktoś miał wątpliwości, to by sobie zmierzył temperaturę.

**Myślisz, że takie rozwiązania są skuteczne?**

Jeśli chodzi o mierzenie temperatury, to nie.

**A pozostałe?**

Zależy. Ta dezynfekcja na pewno nie zaszkodzi, a może pomóc. Takie mierzenie temperatury, to mały efekt tego widzę. Co innego jest sobie przechodzić, jest płyn, zrobić cyk i iść dalej, niż jakby ktoś miał biegać z tym termometrem i mierzyć. Mało zyskujemy, a duże energii to wkładamy. Jeśli chodzi o żele, płyny dezynfekujące, to w drugą stronę. Mało wkładamy, bo sobie psikniemy czy cokolwiek, a możemy dużo zyskać. Bo wszystkiego dotykamy rękoma. Część można byłoby zostawić, są sensowne, a część nie. Tak samo, jak w maseczkach. Zdjęli obostrzenie i na wolnym powietrzu można chodzić bez maski. I widzę, że niektórzy chodzą jeszcze, bo chcą. Natomiast takie uprawianie sportu, to było takie na wyrost bardzo.

**Dlaczego takie mierzenie temperatury zostało wprowadzone?**

Ja się uśmiecham pod nosem zawsze. Dla mnie to jest takie... Ja mam takie odczucia, że jest to na wyrost. Może, żeby pokazać, że taka kontrola, że się tak pilnuje, itd. Nie wiem, czy ma to jakiś czynnik psychologiczny. Każdy mierzy ludzi własną miarą. Mi się wydaje, że to jest trochę ośmieszanie społeczeństwa. Tak samo, jak mamy aplikacje, gdzie trzeba sobie robić selfie 2 razy dziennie, czy że ktoś mi mierzy temperaturę to jest dla mnie na wyrost. Każdy jest myślący i ma jakąś świadomość. Pracujemy w poważnej firmie. Wydaje mi się to na wyrost zdecydowanie.

**A przed koronawirusem zdarzało się, że ktoś z objawami chorobowymi przychodził do pracy?**

Raczej nie. Jeśli już przyszedł, to szybko wychodził, jeśli się zorientował, że ma temperaturę. To jest taki biurowy temat tabu trochę. Nie da się przyjść z temperaturą i narzekać "jaki jestem chory". Zaraz ludzie dadzą do zrozumienia, że nie chcą pracować w otoczeniu kogoś, kto jest chory - idź do domu.

**Nie było społecznego przyzwolenia, żeby przychodzić chorym do pracy?**

Nie. U nas w firmie jest taka normalność, że ktoś kicha, to ludzie wręcz mówią: pozarażasz nas, idź do domu. Bezpośrednio. Miałem kolegę, gdzie 2 razy kichnąłem, siedział na przeciwko mnie przy biurku, to zasłonił się kartonami [śmiech]. Zrobił sobie murek. To było dawno, z 7 lat temu. Ale on to wyjątkowo, większość życia spędza na L4. Specyficzny przypadek. Ale ogólnie nie ma takiego przyzwolenia. Pamiętam, że jechałem kiedyś do pracy i czułem się źle. Ale to był poniedziałek rano i przeważnie się w poniedziałki rano czuję źle. Sądziłem, że to jest choroba poweekendowa jeszcze. Ale usiadłem i jak mnie stawy zaczęły boleć, to od razu do EnelMedu, szybko dostałem diagnozę. I jak wróciłem do domu to już miałem prawie 40 stopni gorączki. Taka grypa porządna. Ale to bardzo szybko się zwinąłem. Jakby ludzie wiedzieli, to by mnie zlinczowali.

**Co sądzisz o prognozach 2 fali zachorowań?**

Myślę, że jest to prawdopodobne z tego względu, że może się to wymieszać wszystko razem z takimi zachorowaniami na przejściu z jesieni w zimę czy też z lata w jesień. Takimi zwykłymi przeziębieniami, grypą, itd. O ile teraz było tak, że jak teraz ktoś miał temperaturę, to już podejrzewa to [COVID]. Może tak być na jesieni też. Dlatego mam podejście, że trzeba jak najszybciej korzystać z tych ulg, które się pojawiły. Korzystać z życia. Zrobić to, tamto, wyjechać, pozałatwiać, bo później może być znowu problem, żeby się gdzieś ruszyć.

**To jest coś, czego się najbardziej obawiasz?**

Raczej tego. Wiadomo, że zdrowie jest najważniejsze. Natomiast, jeśli coś przeżyliśmy, zaobserwowaliśmy, to większość poglądów opieramy na tym, co było w marcu, kwietniu. Dużo było kroków, żeby unikać kontaktu z ludźmi, unikać sposobności zarażenia. Wiadomo, nic się nie wydarzyło, to pewnie człowiek będzie miał takie podejście, bazując, na doświadczeniu, inne.

**Myślałeś, żeby przygotować się do tej 2 fali?**

Jeszcze nie. Na razie, to wychodzę z tej 1 fali dopiero. Na pewno będę musiał pomyśleć. Przeanalizować, co się działo u nas w domu przez te 2 miesiące. Czy może dzieci nie potrzebują jakiejś przestrzeni dodatkowej. Córka już będzie w szkole, więc będę musiał pomyśleć, czy nie zapewnić jej komputera dodatkowego, z którego będzie mogła ona korzystać na jakieś zajęcia online. Będę musiał pomyśleć, jeśli coś takiego będzie wisiało w powietrzu. Ale każdy już będzie wiedział, jak się zachować mniej więcej z tego doświadczenia, które się miało.

**Jak rząd powinien zareagować, kiedy będzie 2 fala?**

Myślę, że powinien wprowadzić znowu ograniczenia. Powinny zostać zamknięte szkoły, przedszkola, żłobki. Powinno znowu wisieć hasło "zostańcie w domu" gdzieś w tle. Dodatkowo wzmożone dezynfekcje, środki komunikacji publicznej dezynfekowane. Dużo rzeczy można było podpatrzeć teraz, co się działo. Mi się wydaje, że to spełniło jakąś swoją rolę. Możemy teraz spróbować wrócić do normalności. Natomiast na pewno bym nie wprowadził zakazu przemieszczania się dla osób pon. 18 r.ż., nakazu noszenia maseczek na otwartych przestrzeniach. Nie pozamykałbym barów, restauracji całkowicie, tzn. bez możliwości zamówienia jedzenia na wynos. Bo tak było przez chwilę, że wszystko było pozamykane, gastronomia. Dużo takich nawyków pewnie zostanie z wiosny, jeśli chodzi o jakieś stacje benzynowe, odstępy - to się pewnie nie zmieni przez dłuższy czas. Wiadomo, niektóre pomysły były trafione, niektóre nie.

**Jakie były dla ciebie najważniejsze wydarzenia w trakcie trwania pandemii?**

Pierwsze wydarzenie to była informacja o tym, że gdzieś w okolicy ktoś zachorował i że jest to w naszej gminie - takie znaczące. Drugie wydarzenie to był najwyższy stan obostrzeń, tzn. kiedy te obostrzenia rosły z wystąpienia na wystąpienie, że zamykamy to, tamto, itd. To był taki moment, kiedy napięcie rosło. Kolejny moment był taki, gdzie napięcie sięgnęło zenitu. Jak rozmawialiśmy, to pamiętam, że musiałem kogoś przegonić spod domu, bo do dziadków się wybierał w odwiedziny. Brama zamknięta, wszyscy w domu siedzą, nie widzimy się ze swoją najbliższą rodziną - w czasie świąt, po świętach. I później to już tak typowo odpuszczamy. Majówka zdecydowanie. Wyjazd z domu, porozmawianie trochę w innym środowisku z ludźmi. I ostatni etap jest teraz, kiedy ja czuję, że wychodzę z tego trybu COVID. Trochę więcej zacząłem o tym myśleć, jak z Tobą rozmawiam. Ale jeszcze 2 godziny temu totalnie nie myślałem o COVID. Takie rzeczy typu noszenie maseczki w kieszeniach, dezynfekcja rąk, to nie myślę. Już nie wczuwam się, że dezynfekuję ręce, z tego względu, że może być wirus albo czegoś dotknąłem. Już to robię automatycznie.

**Pierwszy chory w okolicy - dlaczego przełomowe?**

Dlatego, że to był sygnał dla mnie, że to nie jest błahostka, wymysł. Dotarło to. Nie sądziłem, że dotrze to do nas tak szybko. Gdzie jedna z pierwszych osób w mazowieckim to był właśnie ten kierowca, który potem pozarażał jeszcze inne osoby. To było w mojej gminie. Dosłownie 2 km od miejsca, gdzie on przejeżdżał z tymi ludźmi. I to było takie, że o, szybko. Może jakbym mieszkał w innej gminie, to było szybko. Już była rozmowa z sąsiadami, że jeździła z nim taka dziewczyna, która mieszka 3 domy dalej. I ona jest na kwarantannie, ale wszyscy domownicy normalnie funkcjonują. To było takie nie do pomyślenia dla mnie. Później dopiero się zmieniło, że jeśli 1 osoba jest na kwarantannie, to wszyscy domownicy są. Natomiast takie zdziwienie, że to się dosyć szybko pojawiło w okolicy.

**Kiedy rosły obostrzenia - dlaczego przełomowe?**

To już była taka nerwówka. Nie spotykamy się z najbliższą rodziną. Rękawiczki, maska, żele, kontakty tylko w najpotrzebniejszych sprawach. Zakupy szybko zrobić, wrócić. Dezynfekcja, odstępy od siebie. Praca zdalna. To wszystko robiło swoje, że odizolowaliśmy się totalnie.

**Najgłębsza izolacja - dlaczego?**

Takie były zalecenia. Rozsądek podpowiadał, że nie ma co kusić losu i się dostosowaliśmy. Kolejny etap to był, kiedy obostrzenia zaczęły się powoli - chociaż nie wiem, czy były już zdejmowane, ale chyba wtedy się pojawiło jedzenie na wynos. Przełomowym momentem była majówka, wyjazd. Gdzie pojechaliśmy w zupełnie inny region Polski, pospacerowaliśmy po plaży, porozmawialiśmy z tamtejszymi ludźmi, którzy trochę inaczej podchodzili do sprawy, że jaki COVID, itd. Od tamtego momentu, tak się zaczęliśmy rozluźniać troszeczkę. Wróciliśmy, to się zobaczyliśmy z rodziną. Gdzieś tam zaczęliśmy się otwierać.

**Wyjście z trybu COVID - jakiś jeden punkt czy cały czas po majówce?**

To jest jakieś 2 tygodnie temu, gdzie zacząłem normalnie pracować. Zacząłem jeździć do klienta, do pracy, do biura, spotykać się z ludźmi. Takie początki w maju, to chodziłem w korytarzu w masce. Teraz to wróciło do normalności. Te spotkania towarzyskie, to wszystko już ruszyło. Nie mam czasu już o tym myśleć, że na siebie uważać, itd.

**Co było przyczyną tego, że postanowiłeś skorzystać z podziemia fryzjerskiego?**

Że tak powiem, miałem już za dużo na głowie [śmiech]. Musiałem skorzystać. Pojawiła się okazja.

**Szukałeś tej okazji?**

Żona mi podsunęła, że usłyszała, że ktoś świadczy takie usługi. Mówię spoko, to korzystam.

**Wcześniej mówiłeś, że jakaś maszynka w domu poszła w ruch.**

Tak. Przez ten okres strzygłem się 2 razy. 1 żona mnie ostrzygła w tym największym momencie izolacji. I zadowolony ogólnie byłem. A później to już rzeczywiście miałem dużo na głowie i był już taki czas.

**Skoro byłeś zadowolony, to dlaczego postanowiłeś iść do podziemia?**

Nie wiem, czy może też z ciekawości, jak to będzie wyglądało. Czy też... Żona mnie strzygła w niedzielę. Niedziele to są takie, że dużo czasu, itd. Nie mamy tak za bardzo czasu. Po nocach chyba musiałaby mnie strzyc gdzieś w domu. Ale to jest wygodniej jechać do fryzjera. To jest wygodne. Pod tym względem, że w domu, to jeszcze, że trzeba to posprzątać, a nie wiadomo, gdzie, zaraz włosy, najlepiej to na zewnątrz. W dzień ja pracowałem, żona z dziećmi, itd. Wygodniej mi pojechać do fryzjera. I będę jeździł. Wróci do normalności, a maszynka zostanie na w razie czego. Na jesień [śmiech].

**Chciałbyś coś dodać?**

Tylko, że pytania o te aplikacje mi sprawiały problemy. To były takie tematy, pytania dające do myślenia. I na pewno mi to zostanie na dłużej, bo jest to taki większy trochę temat. Na pewno nie pod kątem COVID, tylko ogólnie, w jakim kierunku zmierzamy. Czytałem jakąś książkę ostatnio Dana Browna, chyba "Początek". I tam był przedstawiony przekrój ludzkości, że człowiek ewoluował, przechodził w inne formy, że to trwało tyle. Nawet nie mówię, że od Homo Sapiens, tylko dużo wcześniej. Tam było pokazane, że bardzo szybko ludzkość wyprze inna forma. I tam było pokazane obrazowo, że ludzkość zostanie zastąpiona przez inną formę bardzo szybko. Przykład był, że coraz bardziej się otaczamy elektroniką. Korzystamy z implantów, chipów, technologii, itd. I że to zmierza w takim kierunku, gdzie tego się raczej nie zatrzyma. To daje do myślenia, czy warto z tym walczyć, czy nie, czy lepiej się poddać, czy zaufać komuś, jak np. patrząc na te aplikacje rządowe. Nie wiadomo tak naprawdę. To jest dylemat. Ciekawy temat, który będę dalej zgłębiał.
