# Supplementary material for: The challenges arising from the COVID-19 pandemic and the way people deal with them. A qualitative longitudinal study
Source: PLoS One. 2021 Oct 11;16(10):e0258133. doi: 10.1371/journal.pone.0258133 (PMC8504766; doi:10.1371/journal.pone.0258133)
Supplement: S1 Dataset — (ZIP) [file pone.0258133.s003.zip › Transcriptions/stage 5/15.5_M_43_couple, with children.docx]

**15.5_M_43_couple with children**

**Co się u pana działo przez ostatni miesiąc?**

Zaczął się wielki powrót do rzeczywistości. Takiej, którą chyba znałem wcześniej, mimo obostrzeń i w dalszym ciągu 200-300 osób zakażonych miesięcznie, zakończył się ten radosny okres nietypowego rozwoju i powróciliśmy do normalnego funkcjonowania już. Zawodowo do pracy w szpitalu, zakończyłem zajęcia dydaktyczne przewidziane na ten rok, szykujemy się do obrony prac magisterskich, żona wróciła do pracy do sądu, dziecko wróciło do przedszkola od poniedziałku. Normalność.

**Syn wrócił do przedszkola na takich zasadach jak wcześniej?**

Przedszkole jest otwarte w regularnych godzinach, ale wdrażamy syna w nieci mniejszym wymiarze czasowym i będziemy ten czas wydłużać. Wczoraj był 9 -12, przedwczoraj do 14, dzisiaj nawet nie wiem.

**Czy były jakieś ważne momenty/ zmiany w ciągu ostatniego miesiąca?**

Ta normalność nastąpiła jakby nagle i ostatnie 2 tyg. już były takie stricte robocze. Tylko tyle, że musieliśmy się nastawić na powrót do dawnej rzeczywistości i wdrożyć to.

**Czy powrót do pracy to był ważny moment?**

Nie, nie powiedziałbym. Po prostu to się zmieniło i trzeba było wrócić do pracy, ale też chyba powróciłem do tej pracy z jakimś takim nastawieniem bardziej negatywnym niż optymistycznym. To jest powrót do normalnego funkcjonowania, czyli zakończył się okres wakacyjny, remontowy, wypoczynku, spania, picia wina i trzeba było wrócić do roboty. I nie zapowiada się, żeby był urlop kolejny w trakcie wakacji, bo z racji tej przerwy, to projekt naukowy będzie trwał do końca sierpnia. Nie wyjeżdżamy nigdzie. Trudno być w pełni radosnym.

**Jak z remontem?**

Ekipa weszła teraz w trakcie naszej przerwy, wyburzyli cały salon, ścianę zewnętrzną i wstawili piękne duże okno na całą ścianę. Przetrwaliśmy remont, wielkie sprzątanie i teraz tylko jakieś drobne poprawki tynku i malowanie. Jest ok. Udało się to zamknąć.

Nie miał pan obaw, że obcy ludzie wchodzą do domu w trakcie epidemii?

Tak, oczywiście. To nie były obawy, ale wiedziałem, że ustalamy pewne zasady, że przynajmniej panowie, dopóki nie wyburzą ściany i nie zapewnią świeżego powietrza, to pracują na tyle, na ile się da w maseczkach, natomiast rodzina po prostu została odseparowana na górze. Nie dało się funkcjonować na parterze, więc wszyscy byli odizolowani na piętrze. Jedynie ja krążyłem. Ja się jakoś bezpośrednio nie zabezpieczałem, a panowie na początku pracowali w maseczkach. Jak już wywalili ścianę, to zrobił się przewiew. Oni byli gotowi do pracy w maseczkach i oni już przyjechali z tymi maseczkami. Jakbym powiedział, że nie trzeba, to pewnie by nie mieli, ale nie zdziwiło ich to i byli na to przygotowani. To taki quasi środek bezpieczeństwa, ale dopełniliśmy procedur formalnych, prawnych.

**To bardziej było ze względu na procedury?**

Tak, chyba tak. Oni się mogli ewentualnie mnie bać, bo ja mam pewnie więcej kontaktów codziennych w szpitalu i w urzędzie.

**Jak wyglądają teraz kontakty z ludźmi?**

Spotkań towarzyskich jeszcze nie mieliśmy okazji odbywać poza domem. Fakt, że mieliśmy spotkanie takie, że dzieci bawiły się razem w piaskownicy na terenie mojej posiadłości i rodzeństwo mojej żony też przyszło, ale nie miało to formy jakiejś zorganizowanej imprezy. Po prostu posiedzieliśmy w ogrodzie. Na życie towarzyskie zewnętrzne, na chodzenie do knajp po prostu nie było czasu. Tutaj już nie mam żadnych ograniczeń, chociaż nie, nie chce mi się. Nigdzie bym nie poszedł.

**Jak była rodzina to były jakieś maseczki, czy już było luźno?**

Na początku tak, mieliśmy maseczki, ale finalnie te maseczki poszły gdzieś tam w kąt. Na otwartej przestrzenie to tak...

**Obserwuje pan zmiany w otoczeniu?**

Tak. Zarówno moi teściowie, jak i moja mama wrócili do pracy do szpitali. Teść długo się opierał, ale dostał polecenie służbowe powrotu i nie miał wyboru. Musiał jechać do pracy i koniec.

**Widzi pan jeszcze jakieś zmiany?**

Na pewno bez problemów można zrobić zakupy. Mniej osób chyba robi zakupy, ale może dlatego, że już je porobili. Taka normalna obecność ludzi w sklepach, żadnego odliczania ludzi. Oczywiście w maseczkach, ale chyba zrobiło się całkowicie normalnie.

**Jest coś, co nadal przeszkadza?**

Nie, zupełnie nic. Samo ruszenie z powrotem do życia odbyło się bez problemu, ale teraz zarówno kontrahenci jak i pracodawcy chcą nadrobić stracony czas, więc teraz nagle dostaję 3 maile i wszyscy się pytają, kiedy zrobimy cykl zaległych szkoleń i wszyscy nagle chcą na jutro, na pojutrze. Zrobiło się tego całkiem sporo i jest to o tyle obciążające, że teraz trzeba szybko nadgonić to, co się straciło w okresie pandemicznym. Robi się tej pracy całkiem sporo. Byłem do tego przyzwyczajony, ale stresujące jest to, co było też przed okresem pandemii, że jeśli ja funkcjonuję w 4 różnych rzeczywistościach zawodowych, to muszę sam zsynchronizować swój czas aktywności, co czasami jest trudne, bo oczekiwania pracodawców i kontrahentów są dość sztywne. Pojawiają się trudności w synchronizacji kalendarza działań, ale to było już wcześniej, tylko teraz może jest tego po prostu więcej, bo wszyscy chcą nadgonić.

**Emocje - zdjęcia.**

1 - czułem się bardziej przytłoczony natłokiem, który się objawił, więc 1 jest idealna. Dużo spraw na głowie, które trzeba uporządkować nagle w tempie ekspresowym.

Czy coś jeszcze? Musiałbym bardzo na siłę szukać. Poprzednio była i 6, i 13, natomiast ten [niezrozumiałe] już nadszedł. Te 2 zdjęcia straciły już swoją ważność. Nie powiedziałbym, że jestem w nastroju burzowym, bo od tych korków i natłoku spraw do kryzysu burzowego to jeszcze jest daleko.

16 - może to. Pożary, które trzeba teraz gasić. Nie są to kryzysy zawodowe, tylko zadania do zrealizowania. Dużo, spadły nagle, niespodziewanie i gdzie człowiek zagasi w jednym kącie, to w drugim zagajniki zaczyna się palić od nowa.

**Myśl, że trzeba wrócić do normalności. To też powodowało emocje?**

Nie, pierwotnie byłem nastawiony neutralnie, natomiast teraz po tych 2 tygodniach już się czuję zmęczony tym okresem. Teraz jest na pewno więcej negatywnych emocji niż było w trakcie okresu całej pandemii.

Jakie to są emocje, poza przytłoczeniem nadmiarem obowiązków?

na pewno jest irytacja liczbą rzeczy do uporządkowania, złość, która nie jest ukierunkowana na nic konkretnego ani na nikogo konkretnego. Po prostu złość na tę sytuację, że się pojawiła i że jest. To było brutalne uderzenie obuchem w łeb.

**Ma pan jakieś sposoby na radzenie sobie z tymi emocjami?**

Uporządkowanie wszystkiego i zrealizowanie wszystkich zobowiązań.

Czuje się pan czymś zagrożony w związku z pandemią?

Pośrednio tak. Zmianami w MNiSW i zmianą ministra nauki, bo teraz po raz kolejny zmienili nam zasady funkcjonowania uczelni. Dostaliśmy do wglądu nowe kryteria oceny, więc martwi mnie habilitacja, której pewnie nie zrobię, bo muszę ją zaczynać znowu od początku. To jest pośrednio związane z pandemią, bo też kryzys rządowy, wyborczy, odejście Gowina wiąże się z pandemią, a to pociągnęło za sobą kolejne konsekwencje uderzające też bezpośrednio również we mnie. Niepokój powiązany z okresem pandemicznym. Nie wprost, ale jednak powiązany. Gdyby nie pandemia, Gowin pewnie nadal by był ministrem i nadal byśmy tkwili w tym, co mieliśmy, a tak mamy zmiany.

**A w kwestii zagrożenia chorobą?**

Nie, nie, zupełnie nie.

Jak wyglądają emocje u osób z pana otoczenia?

Jakoś zmian nie dostrzegam za bardzo. Najbliższa rodzina - rodzice, teściowie, musieli też powrócić do rzeczywistości, ale nie jestem w stanie powiedzieć, czy są w lepszej kondycji psychicznej, czy też nie. Trochę zatraciłem kontakt z osobami, z którymi pracowałem, ale kolegowaliśmy się też prywatnie. Nawet nie wiem, co się u nich teraz dzieje, więc to też jest jakaś forma zmiany, że straciliśmy sobą zainteresowanie, po pierwotnej fascynacji możliwościami komunikatorów video, pogadania przez What's App. nagle jakoś straciliśmy zainteresowanie i nie potrafię wyjaśnić, dlaczego to tak wygląda. Wydaje mi się, że to jest chwilowe i że jak powrócimy do jakiejś normalności uczelniano - biurowej i będziemy mieli więcej kontaktów osobistych już, to się unormalizuje znowu.

**Patrząc na ludzi, widzi pan jakieś zmiany w ich zachowaniu?**

Widzę przede wszystkim ich twarze, bo już nie mają maseczek i ta komunikacja niewerbalna jest bardziej spójna. Tak, poza tym chyba nie widzę żadnych różnic. Ludzie przemieszczają się nadal...Może coraz więcej korków, co też jest oznaką jakiejś normalności, bo po prostu powróciły korki w pełnej formie. No i dzieci nie chodzą do szkół w większości przypadków. Poza tym trudno mi powiedzieć.

Jak nie ma nakazu, to nosi pan maseczki też w przestrzeni otwartej?

Zdarza mi się nosić przy przejściu skądś dokądś z czystego lenistwa - pomiędzy urzędami albo sklepami, bo nie chce mi` się ich ściągać. Do sklepu wchodzę w ekwipunku.

**Jak wyglądają teraz zakupy?**

W ostatnim czasie nie miałem większej możliwości robienia zakupów. Żona ostatnio robiła. Spadło moje zainteresowanie Castoramą online, bo już zakończyłem remont, zakupy firmowe czy ogólne online też nie, więc ostatnio szczęśliwie nie wydałem zbyt wiele. To wynika z braku czasu. Byłem raz w GH w sobotę. Spędziłem tam 7 min, ponieważ wbiegłem tylko do Auchan, żeby kupić ścisk stolarski, bo klej mi stygł. Gdzie indziej nie miałem możliwości zakupu takiego urządzenia. Było dużo ludzi i powiedziałbym, że taki normalny tryb weekendowy. Ludzi było bardzo, bardzo dużo. Parkowanie jest co 2 miejsca i taka była różnica. Taka zasada została wprowadzona i to jest dobre rozwiązanie, bo skończy się obijanie drzwi.

**Były jeszcze jakieś dodatkowe zabezpieczenia?**

Tak, był dystrybutor płynu do rąk i rękawice foliowe.

**Czy myśli pan, że teraz już będą u pana zwyczaje zakupowe jak przed epidemią, czy coś się zmieni?**

Ja nigdy nie przepadałem za zakupami. Nie jestem pasjonatem krążenia po galeriach. To się nie zmieniło i raczej się nie zmieni, zakupy zawsze robiliśmy raz na 2 tygodnie, takie większe. Słyszałem natomiast wypowiedź jednego z dyrektorów CH, że zmieniają się nawyki ludzi i że teraz jest więcej ludzi, którzy przychodzą na krótko i z konkretną listą zakupów. Zakupy i krążenie po galeriach przestało być formą rozrywki i ludzie kupują to, czego potrzebują. Nadmierny konsumpcjonizm to nie jest dobre rozwiązanie, więc z dobrodziejstwem dla nich. Może ludzie znaleźli inne sposoby na zagospodarowanie wolnego czasu. U mnie pewnie będzie tak samo jak przed epidemią.

**A zakupy online?**

Już nie będę kupował farby i gipsu z dowozem za 50 zł, bo to nie bilansuje się ekonomicznie. To, co wcześniej kupowałem stacjonarnie zostanie stacjonarne i tak samo jest z online.

**Otworzono restauracje. Miał pan okazję być?**

Byłem raz na zasadzie przypadku w McD, ale to tylko z racji tego, że umówiłem się na odbiór zwierzęcia z osobą na parkingu i ta osoba zakomunikowała mi, że będzie pół godz. później. Byłem z synem, to stwierdziłem, że trudno i pójdziemy do kapitalisty na frytki.

**Pojawiło się zwierzątko w domu?**

Tak. To było dawno obiecane. Pies - zakup na Dzień Dziecka. To było zaplanowane już na jesieni zeszłego roku na zasadzie kontraktu z synem.

**Wszedł pan do środka do McD?**

Tak, nie chcieliśmy stać w kolejce do McDrive. Syn został na zewnątrz, ja wszedłem i skorzystałem z urządzenia do zamawiania, pobrałem numerek, ponieważ nie odbiera się towaru w środku i nie czeka się w środku. Usiedliśmy na zewnątrz i pracownik nam te frytki wyniósł do stolika z numerkiem zamówienia. Nie wolno było zjeść w środku. Zakładam, że to może być inwencja własna właścicieli, bo ten McD jest bardzo mały. Ja nawet nie wiem, czy można było zamówić bezpośrednio a nie przez maszynę. Z 3 maszyn były czynne 2, a środkowa była wyłączona, żeby były większe odległości między ludźmi, w związku z czym wszyscy tłoczyli się przy 2 pozostałych.

**To było bezpieczne? Miał pan jakieś obawy?**

Myślę, że to raczej ja jestem większym zagrożeniem dla ludzi niż oni dla mnie, ale zarażenia absolutnie się nie obawiałem. Nie mamy pewności, czy nie przeszliśmy już tego bezobjawowo. Dla mnie to był temat nieistniejący w momencie, kiedy otwierałem świeżo zdezynfekowane drzwi do McD. Zabezpieczenia były przestrzegane, cały personel ubrany i zabezpieczony. Sam byłem świadkiem dezynfekcji drzwi i klamek, niemożność odebrania jedzenia w środku. Było to zauważalne bardziej niż bardzo.

**Co pan sądzi o tym, że otwarto kawiarnie i restauracje?**

Chyba na siłę będę szukał jakichś refleksji i przemyśleń. Jest mi to temat zupełnie obojętny. Nie wybieram się do restauracji. Bardziej jest istotne, żeby ci przedsiębiorcy wreszcie ruszyli do przodu i może to jest zbawienne dla podtrzymania statusu finansowego tych ludzi. Pod tym względem jest to ok.

**A z perspektywy epidemii to jest jakieś zagrożenie?**

To uwolnienie społeczne może doprowadzić do utrzymania się całkiem wysokiej liczby zakażeń, ale wiemy, że w większości nam to nie grozi, szczególnie młodym osobom, że na ten moment ta liczba jest stała mniej więcej dziennie, że opieka medyczna jest jako tako wydajna, więc wydaje mi się, że ludzie po prostu przestali się obawiać.

**Fryzjerzy i inne punkty?**

Fryzjer tak, zdecydowanie. Fryzjer, z którego korzystam od lat sam się skontaktował w momencie otwarcia i zaproponował 1-szy wolny termin za 3 tygodnie, z którego skorzystałem. Zabezpieczenia były - rękawiczki, w których pracował fryzjer, maseczki, z których można było skorzystać, tuż przed moją wizytą dezynfekcja i rzeczywiście klienci umawiani co godzinę, tylko 2 pracowników. Czułem się bezpiecznie, natomiast chyba nie czułem się bezpiecznie z tego tytułu, że posługujemy się artefaktami w postaci maseczek i płynów, tylko dlatego, że prawdopodobieństwo, że coś się przydarzy jest praktycznie bliskie zera. A jak coś się wydarzy, to nie będzie stanowiło zagrożenia dla życia ani zdrowia.

**Jak pan myśli, dlaczego takie miejsca zostały otwarte?**

To też trochę powód ekonomiczny, ale też są to takie usługi, z których korzysta się dosyć często i wydaje mi się, że dużo ludzi już potrzebowało tych usług. I szukając pokrętnych wytłumaczeń, to, skoro posłowie partii opozycyjnych i rządzących byli ostrzyżeni, a nie mogli być ostrzyżeni i to niekoniecznie żony strzygły, to trzeba było może trochę uwiarygodnić te zmiany w owłosieniu u ministrów i posłów. To tak w formie żartu.

**Mają być otwarte siłownie, kina?**

Ubolewam, że pewnie nie będę miał czasu skorzystać z żadnej formy aktywności fizycznej. Jeśli jest w knajpie 200 osób i są 3 na siłowni, to potencjalne ryzyko jest takie samo lub nawet mniejsze. Liczba zachorowań pewnie się będzie utrzymywała albo będzie minimalnie większa lub mniejsza. Nie będzie być może wyciszenia tej pandemii na okres letni tylko ona będzie się cały czas utrzymywała na tym samym poziomie, natomiast przywykliśmy już do tego i chyba nie stanowi to aż tak dużego zagrożenia.

**APLIKACJE**

Słyszałem o aplikacji kwarantannowej, która jest uciążliwa nawet dla ludzi już po kwarantannie, ponieważ dane nie są usuwane z baz danych i jakieś osoby w dalszym ciągu dostają powiadomienia, chociaż nie są już w kwarantannie. Słyszałem też o jakichś aplikacjach w programie popularno- naukowym. Chyba chodziło o Tajwan, Hong Kong albo Singapur, gdzie można było wprowadzić dane, że jest się zakażonym albo po zakażeniu i w tym momencie, kiedy inne telefony z tą aplikacją zbliżały się do tego telefonu, to na tamtych telefonów wyświetlała się informacja ostrzegająca.

**Kategoria 1**

Kojarzy mi się to ze spuścizną orwellowską i powiedziałbym, że to już jest za dużo. Mówimy tu o takiej inwigilacyjnej kontroli wszystkich i wszystkiego. W sensie ideologicznym powinienem wykazać oburzenie na taki pomysł, ale rozumiem, że w sensie zdrowotnym może to mieć jakieś krótkotrwałe pozytywne oddziaływanie, żeby tylko nie stanowiło pewnego wyłomu w murze, że w ten sposób będziemy mogli również po okresie pandemicznym w jakimś zakresie być monitorowani i nadzorowani. To jest jednak uderzenie w wolność większe aniżeli obostrzenia, które były narzucone przez rząd, ponieważ już w jakimś zakresie zautomatyzowane i trudniej kontrolowalne. To może mieć sens, jeśli ktoś ma jakieś obawy i faktycznie korzysta z takiej aplikacji, i może być taki system ostrzeżenia. Jeżeli komuś to jest potrzebne, to krótkofalowo może to być całkiem niezłe.

**Dobrze, że takie rozwiązania są tworzone?**

Może stanowią dopełnienie innych mechanizmów nadzoru, jak chociażby nadzór policyjny przy kwarantannie, ale ich użyteczność określiłbym raczej jako niską. Jak ktoś się boi, to ok, ale ja osobiście nie przykładałbym do tego zbyt wielkiej uwagi. Jeśli miałbym to pobrać, to kierowałaby mną bardziej ciekawość poznawcza i wypróbowałbym ją przez godzinę czy dzień potem bym ją odinstalował. Zobaczyłbym, jak to wygląda od kuchni, ale nie dla zabezpieczenia się.

Kategoria 2

Nie można zdjąć obowiązku z władz państwowych i zastąpić go sztuczną inteligencją, bo one są odpowiedzialne tak czy inaczej za decyzje. Wydaje mi się, że czy to sztuczna inteligencja, czy Minister Szumowski, to wyniki byłyby bardzo podobne i te środki zostały zadysponowane w sposób w miarę właściwy. Po fascynacji Big Data i próbie podjęcia jakichś działań 2-3 lata temu, stwierdziłem, że to też jest ślepy zaułek i jestem raczej przeciwnikiem przetwarzania dużych zestawów danych potencjalnie ze sobą niepowiązanych. To jest jednak też uderzenie w jakieś tam struktury wolności.

same drony to całkiem niezły pomysł, chociażby ze względów ekonomicznych. Dron jest tańszy niż kurier i listonosz, więc ok. Dostarczanie medykamentów, to nie wiem na jakiej zasadzie, bo jeśli to Apap, to bez sensu chyba, a jeśli jakieś szczepionki to tak czy tak są potrzebni pracownicy medyczni, żeby zaaplikować, więc nie bardzo wiem, dlaczego medykamenty akurat, ale jakaś forma narzędzi działania logistycznego, jak najbardziej ok. Myślę, że niezależnie od pandemii.

**Czy ta kategoria budzi jakieś obawy?**

Wymiana potrzeb i możliwości ich realizacji - takie narzędzia też są i chyba tworzenie dedykowanej aplikacji...Nie wiem, czy jest potrzebne. Przychodzi mi na myśl, że to jest produkt dla generacji młodszych ludzi, do których ja już nie należę i to jest poszukiwanie czegoś, co jest ciekawe, nowatorskie i takie wow, i wszyscy teraz zamiast w tik toka, będą wrzucali informacje w taką aplikację, i będą się strasznie jarali, że z tego korzystają. Czy to jest pragmatyczne? Nie wiem, bo mogę napisać do szwagra na What's App, żeby mi przywiózł skrzynkę piwa i pewnie przywiezie. Nie widzę pragmatycznego sensu tego typu narzędzi. To jest nadprodukcja aplikacji i trochę rozdmuchiwanie potrzeb, bo te potrzeby można spokojnie zaspokoić z wykorzystaniem istniejących już narzędzi. Nie widzę tu żadnego zagrożenia, tylko po prostu jest to straszna wydumka.

**Kwarantanna domowa**

Jest to zaczerpnięte z istniejących wcześniej rozwiązań w systemie kar wolnościowych chociażby we Włoszech, gdzie ludzie są w aresztach domowych i muszą zrobić sobie zdjęcie na tle telewizora, który wyświetla aktualny program tv z godziną, czyli najlepiej na tle programu informacyjnego. Od strony technicznej nie jest to niczym zaskakującym i niczym nowym.

**Czemu ta aplikacja ma służyć?**

Ma wspierać służby w nadzorze nad osobami w kwarantannie i jednocześnie pomagać w zabezpieczaniu innych obywateli, którzy mogliby być zarażeni przez te osoby. Wspiera procedury nadzoru. Tam są jakieś wartości dodane w sensie kontaktu z pracownikiem socjalnym, ale nie wiem czy to by rzeczywiście działało.

**Jakie informacje o osobie zbiera ta aplikacja?**

Na pewno zwielokrotniony wizerunek, być może z tego da się wykorzystać jakieś informacje na temat, jak ta osoba mieszka, jak ma urządzone mieszkanie, jakie sprzęty się znajdują, pewnie da się ściągnąć dane na temat systemu operacyjnego urządzenia elektronicznego. Tak na dobrą sprawę, jeżeli jest odpowiedni kod, to da się ściągnąć wszystkie dane ze smartfona, łącznie z przeglądanymi aplikacjami, stronami internetowymi - przejrzenie całej zawartości smartfona. Pewnie jest też możliwa weryfikacja czasu reakcji. Jeśli te informacje przekazywane by były szybko, to znaczy, że ta osoba ma większy kontakt ze swoim smartfonem, jeśli później, to wiadomo, że smartfon leży w kącie i może nie stanowi jakiegoś obiektu zainteresowania danej osoby.

**To budzi jakieś pana obawy?**

Raczej nie. Ja nie sądzę, żeby takie rzeczy się działy. To by była za duża afera, gdyby to było możliwe, ale to, że będzie kilka zdjęć, smsy i geolokalizacja, to też nie jest niczym nowym. De facto niewiele da się z tego wykorzystać. Na pewno żadnego profilowania nie da się zrobić, bo i tak człowiek jest cały czas w jednym miejscu, nie da się wychwycić, czy był w sklepie, czy był w restauracji, jeśli okolica jest ta sama. Nie wzbudza to jakichś moich większych obaw.

**A jest coś, co się panu podoba?**

Jest dla mnie całkowicie neutralna, a sam system nie jest niczym nowym i chyba jest skuteczny.

**Pobrałby pan tę aplikację dobrowolnie?**

Myślałem o tym z czystej ciekawości i nawet nie wiem, czy da się ją ściągnąć bez tego smsa z linkiem aktywacyjnym. Ale też już na samym początku były jakieś informacje, że nawet po odinstalowaniu tej aplikacji, wciąż przychodziły jakieś smsy o dziwnej treści, więc instalowanie jej bez wyraźnej potrzeby nie byłoby chyba rozsądne.

**Jakie korzyści może mieć użytkownik?**

Żadnych. To jest element kary formacyjnej i ma do tego dodatkowe narzędzie nadzoru, więc gdzie te korzyści?

**Ta aplikacja powinna być obowiązkowa dla osób na kwarantannie?**

Jest to jakieś ograniczenie wolności i swobód, ale zakładam, że tym osobom w kwarantannie mogła być ona wysyłana i powinni oni ją sobie instalować.

Czy rząd powinien tworzyć takie rozwiązania?

Tak, bo takie rozwiązanie działa też na sferę motywacyjną, psychiczną, że jeśli bym jej nie miał, to oceniłbym, że prawdopodobieństwo, że odwiedzi mnie patrol policji i nie znajdzie mnie w domu jest znikome. Jeśli mogę się spodziewać jakiegoś komunikatu i reakcji na ten komunikat ze strony aplikacji, to wydaje mi się, że część osób pozostawała w tej kwarantannie z racji przewidywalności tego systemu. Dodatkowy czynnik motywacyjny do pozostania w domu.

**ProteGo Safe**

Nie wnosi ona niczego nowego z jakiejś wiedzy ogólnej. To jest aplikacja dla pokolenia, które będzie chciało z niej korzystać. Z praktycznego punktu widzenia ona nie wnosi nic nowego. Będąc człowiekiem świadomym i samoświadomym, dobrze wiem, w jakim stanie aktualnie jestem i jak się czułem wczoraj. Faktycznie pojawia się tu informacja na temat modułu Bluetooth. O tym rozwiązaniu słyszałem już wcześniej z krajów azjatyckich. Ankieta stworzona przez lekarzy - jak to zwykle bywa w przypadku takich narzędzi błąd pomiaru jest przeogromny. To jest tylko takie bardzo ogólne narzędzie przesiewowe, które absolutnie nic nie da, nic nie zmieni, nie pomoże mi być zdrowszym ani nie pomoże lekarzowi, który będzie się ze mną kontaktował, ponieważ pewnie zupełnie zleje taką aplikację i będzie opierał się na mojej autoanamnezie i na tym, co sam zaobserwuje. Nie bardzo rozumiem istotę tworzenia takiego narzędzia. Sens rozumiem jest taki, że będzie grupa odbiorców z pokolenia dzisiejszych 20-latków, gdzie dla nich zamykanie wielu sfer własnego życia w aplikacji jest rzeczą zupełnie naturalną i normalną. Dla nich może to być zrozumiałe, czytelne narzędzie, które wg nich coś może im faktycznie dać. Możliwość rozszerzania jej o kolejne użyteczności jest też zrozumiała.

**Jakie dane pobiera ta aplikacja?**

Ona już będzie zbierała większą ilość informacji na temat przemieszczania się, będzie mogła zbierać informacje na temat urządzeń w jakimś podstawowym zakresie. Będzie możliwość tworzenia siatki innych urządzeń, siatki miejsc, mijanych osób poprzez kontakty tych innych osób z kolejnymi osobami. Dałoby się stworzyć faktycznie taką sieć relacji, które użytkownik posiada. Czy to jest bezpieczne i zdroworozsądkowe, dobre dla samego użytkownika? Nie wiem. Daje pewnie duży wgląd i tutaj profilowanie i segmentacja takiego użytkownika będzie możliwa. Nie wiem, jakie jest założenie pierwotne twórców tej aplikacji, na ile oni będą przetwarzali te dane, na ile będą je wykorzystywali w taki sposób anonimowy, ale starając się sprofilować użytkowników do różnych celów. Tego nie wiem, ale ta aplikacja daje na pewno dużo szersze możliwości aniżeli aplikacja kwarantannowa. Już z założenia. Nie wiem, jaki możliwości ta aplikacja jeszcze da w kontekście poznawania samego użytkownika, bo nie znam założeń twórców, ale możliwości są bardzo, bardzo szerokie.

**To budziłoby obawy przed pobraniem tej aplikacji?**

Z ciekawości może bym ją pobrał i zobaczył, jak wyglądają poszczególne funkcjonalności, ale na co dzień zwyczajnie nie chciałoby mi się z niej korzystać. Po 2 dniach poszłaby w odstawkę zwyczajnie, bo nie widziałbym sensu korzystania z niej.

jak by pan ją na chwilę pobrał, to nie miałby pan takich obaw, że te dane gdzieś tam zostaną i nadal będą przetwarzane?

Tak, ale to byłby jakiś wyrywek tego, co zaprezentowałem swoimi zachowaniami i wpisami i takie krótkie testowanie, wydaje mi się, że dałoby jakiś podstawowy obraz mnie samego, ale też nie byłbym chyba łakomym kąskiem dla twórców tej aplikacji i może mój profil zostałby zwyczajnie skreślony.

**Czy tego typu aplikacje powinny być tworzone przez rząd?**

Rozważam sferę etyczną i też poziom` zaufania do twórców aplikacji w sensie komercyjnym.  Już chyba bardziej bym zaufał twórcom, którzy to robią dla pieniędzy niż sferze rządowej, która pewnie nie potrafiłaby tym dobrze zarządzać i oni byliby pewnie najbardziej zainteresowani tym, czy ja, aby na pewno głosuję na X a nie na Y, czy byłem na wyborach. Tutaj mamy znowu Orwell'a w pełnym zakresie. Gdyby twórca był komercyjny, to pełnego zaufania też nigdy bym nie miał, bo wiedziałbym, że z tych danych da się wyciągnąć bardzo dużo. Do bazy danych i tak mogliby zastukać smutni panowie z różnych służb i powiedzieć, że potrzebują dane i proszę nie marudzić, proszę o udostępnienie. To nie jest tak, że takie dane będą bezpieczne.

**Przyszłość**

Myślę o rzeczach, które muszę zrealizować w najbliższym czasie. Nie dzielę czasu na teraz i po pandemii, zupełnie nie.

**Zastanawiał się pan, co się może zmienić na poziomie Polski, jako kraju?**

Na pewno może się zmienić profil zatrudnienia, tzn., że część ludzi pozmienia pracę. Może z korzyścią dla samych siebie. Mogą być chwilowo niższe uposażenia, bo pracodawca zawsze stara się skorzystać na takich okazjach, czyli urealnienie płac. Na pewno koszty funkcjonowania firm powrócą do starych poziomów, paliwo będzie chyba trochę tańsze, ale nie będzie już za 3.38 i ten czas już minął pewnie bezpowrotnie. Mogą się zmienić chyba ceny nieruchomości, ale w niewielkim zakresie. Wzrosną ceny nieruchomości rekreacyjnych, bo trudniej będzie wyjechać do hotelu, będzie więcej obostrzeń i więcej można zarobić na sprzedaży apartamentów, działek rekreacyjnych. to już jest zauważalne i wypoczynek indywidualny będzie chyba bardziej popularny. Jeśli można na tym zarobić, to też nie widzę w tym niczego złego. Cieszę się, że mam taką lokalizację kupioną wcześniej i jej wartość pewnie wzrosła. Może zmienić się też struktura wydatków domowych. Będzie mniej zbytków, bo ludzie teraz bardziej będą myśleli o tym, że jednak nie są w czasach 100% stabilnych, że zawsze mogą stracić pracę, zawsze będą mogli być zamknięci kolejnym dekretem głów państwa. Wydaje mi się, że nie będzie jakiegoś wielkiego kryzysu gospodarczego. Owszem, problemy mogą mieć firmy, które zapewniają rozrywkę - imprezy masowe, być może kluby sportowe, być może gwiazdy muzyki i organizatorzy dużych eventów, bo to się może faktycznie zmienić. Być może będziemy mniej skoncentrowani na życiowych zbytkach, a będziemy się koncentrować na rzeczach istotnych życiowo.

**Ta zmiana będzie spowodowana sytuacją ekonomiczną czy przewartościowaniem u ludzi?**

Przewartościowaniem bardziej. Myślę, że będziemy bardziej ostrożni w tych zakresach, może sport i sztuka przeniosą się bardziej do sfery online, czyli będzie obraz i dźwięk, ale nie będzie obecności, nie będzie przeżywanych emocji na miejscu. Na pewno też ten czas unormuje trochę sferę próżności, czyli sportu, rozrywki, celebryctwa. Powrócą takie pierwotne wartości. Trudniej może będzie zarobić na niczym - na umiejętności łapania piłki, nie byciu wcale wirtuozem ani genialnym wokalistą, czy umiejętności prezentowania braku jakiejkolwiek umiejętności na YT za duże pieniądze.

**Z perspektywy całego świata, na poziomie globalnym, jakichś zmian pan oczekuje?**

Nie oczekuję żadnych zmian, ale one się po prostu wydarzą. Ograniczenia komunikacyjno-transportowe. Przez pierwsze miesiące, może lata, będziemy mniej latać, mniej się przemieszczać, może poza jakimiś celami biznesowymi, chociaż to pewnie może być realizowane online. Turystycznie na pewno mniej.

**Jest coś, czego się pan obawia?**

Nie obawiam się niczego, bo te potencjalne zmiany nie dotykają sfery moich zainteresowań ani możliwości też. Muszę się chyba raczej pogodzić z tym, że nie będziemy raczej wylatywać na wakacje w takiej formie, jaką znaliśmy, być może wakacje będą również droższe. Jak będą droższe to trudno, ale jeśli będą bezpieczne, to myślę, że będziemy z nich korzystać. To jest jednak melodia przyszłości. Na pewno nie w najbliższe wakacje.

**Czy zmieni się sytuacja społeczna?**

Na pewno jakieś sfery wpływów politycznych, bo ta sytuacja wahnęła całym systemem politycznym Polski. Ci, którzy byli pewni swego może wcale tak pewni już nie są. Ci, którzy byli zaangażowani biznesowo w sferę rozrywki mogą mieć problemy, bo tej rozrywki nam znanej pewnie nie będzie przez jakiś czas albo i w ogóle, bo być może nie będzie już też chętnych odbiorców. Patrząc na margines społeczny, to oni się nie zmienią. Zawsze korzystali z pomocy, nadal korzystają, zawsze pili, nadal piją, zawsze urządzali burdy i urządzają je nadal. Nasza klientela w tym zakresie się nie zmieniła i u nich się raczej nic nie zmieni. Wydaje mi się, że uczniowie i studenci będą mieli więcej zajęć online, bo to okazało się wygodne i dużo tańsze, przynajmniej na poziomie mojej uczelni.

**A ograniczenia, czy te, które teraz są powinny zostać na dłużej?**

Całkowicie nie mam zdania na ten temat. Możemy sobie jedynie a priori założyć, że jak je zdejmiemy, to będzie więcej zakażeń, ale być może to będzie poziom zakażeń akceptowalny w dalszym ciągu. Z drugiej strony, tyle rzeczy już zostało poluzowanych i możliwości adaptacyjne społeczeństwa są na tyle duże, że nawet jak siłownie są zamknięte to można poćwiczyć gdziekolwiek. Nie myślałem o tym, nie mam żadnych przemyśleń osobistych z tego obszaru. Nie było i to do niczego potrzebne.

**Czy niektóre ograniczenia/ zmiany powinny zostać na zawsze?**

Chyba większa dbałość i higienę to jest dobra rzecz ogólnie, większa dbałość o czystość. Zobaczyliśmy też, że ranga polityków i polityki jest dużo mniejsza niż oni myśleli i niż my sami myśleliśmy. Taki jakiś głos rozsądku i też mniejszego zainteresowania tą sferą to jest dobra rzecz. Ludzie może przestali się tym interesować tak bardzo, może stali się bardziej świadomi tego, w jaki sposób nami się zarządzą. Okazało się, że politycy mogą mówić bardzo różne rzeczy, a świat i tak podąża sam, nie musimy ich słuchać. I tak żyjemy, i tak żyjemy całkiem dobrze. Taka świadomość społeczna mogłaby zostać - żebyśmy byli bardziej świadomi tego, w jaki sposób nami się zarządza, Nie jestem zwolennikiem e-learningu, bo to bardziej utrudnia życie niż ułatwia. Pewnie, jak bym był wojującym ateistą, to powiedziałbym, że ograniczenie liczby wiernych do 15 w kościele, ale nie jest to moja sfera zainteresowań. Większa refleksyjność odnośnie wydatkowania środków finansowych, domowych - to jest dobra rzecz. Pilnowanie siebie, oszczędzanie, niewydawanie na bzdury w CH, w galeriach i może znalezienie alternatyw dla prymitywnej rozrywki, jaką jest zwiedzanie GH.

**Uważa` pan, że któreś grupy powinny być szczególnie chronione? Powinna być taka strategia?**

Tak. Jest top dosyć dobre rozwiązanie. W zasadzie można było sugerować izolowanie tylko osób, które są w grupach ryzyka. Można by było bardziej wspierać osoby starsze i zagrożone, zapewnić im taką pomoc nie partyzancką, jak było teraz, tylko taką bardziej usystematyzowaną, że są robione zakupy, że takie osoby mają pierwszeństwo w opiece medycznej, że być może powinien być niezarażony opiekun dedykowany jakiejś grupie osób starszych, który nawet może przeszkoliłby ich w zakresie telemedycznym, jakieś aplikacje, e-recepty, itd. Ta grupa ryzyka, która najbardziej ucierpiała teraz, czyli osoby starsze to mogłyby być grupy, co do których powinien być bardzo konkretny plan wspierania, ale takiego nie odciążającego wspierania, czyli nie zamykania ich w jakichś ośrodkach zamkniętych, tylko jakiejś usystematyzowanej opieki gotowej, środków finansowych na tę opiekę, środków kadrowych. Wsparcie a nie zakazy i przymusowa izolacja.

**Pomiar temperatury, większe odstępy między stolikami. Co pan myśli o takich rozwiązaniach?**

W ograniczonych miejscach mogłoby to być sensowne. Tam, gzie są zamknięte przestrzenie, gdzie jest ryzyko pojawienia się większej liczby osób. Może tak faktycznie być. Sam mam mierzoną temperaturę przy każdym wejściu do szpitala. Tam, gdzie są ci, którzy wymagają większego zabezpieczenia, jak np. pacjenci w szpitalach. Jestem za takim przesiewem.

**To jest skuteczne?**

Myślę, że tak, ale odwołujące się raczej do dobrej woli osoby, która będzie miała podwyższoną temperaturę. Nie możemy zabronić takiej osobie wejścia gdziekolwiek. Możemy ją jedynie poprosić, poinformować o tym, zasugerować, żeby nie wchodziła, żeby oddaliła się z tego miejsca i zainteresowała się przede wszystkim własnym stanem zdrowia.

**II fala. Co pan o tym myśli?**

Wydaje mi się, że nie będzie jako takiej wyrazistej II fali, ale utrzyma się wysoki poziom zachorowalności, który jest teraz na dłuższy okres czasu. Myślę, że powinno być mniej partyzanckich działań rządu, mniej działań wizerunkowych, jak nietrafne inwestycje. Działanie z większą dozą rozsądku. Jeśli chodzi o samą skuteczność działań, to nie mam żadnych zastrzeżeń i to mogłoby być podtrzymane, ale może niekoniecznie kupujmy chińskie maseczki, tylko pomyślmy nad prawdziwym zabezpieczeniem. Może zacznijmy produkować takie w Polsce?

**Pan się planuje jakoś przygotować na to?**

Nie mam pojęcia. Może wstawię sobie sprzęt siłowniowy do piwnicy. do gabinetu? Nie, nie myślałem jeszcze w ogóle o tym.

**Ten trend odmrażania powinien się utrzymać?**

Tak chyba powinno być i tak chyba będzie, bo okazuje się, że nie taki diabeł straszny, a nawet jak jest dużo zachorowań...Umrą pewnie osoby, których i tak nie da się uratować z racji poważnego stanu a nie z racji braku miejsc w szpitalach czy z braku sprzętu. Te ofiary powszednieją, ta śmierć powszednieje. Wydaje mi się, że nie będzie jakichś specyficznych działań, które będą przywracały ten okres kwarantannowo-izolacyjny.

**Jakie dla pana były najważniejsze momenty tej sytuacji?**

Na pewno zamknięcie przedszkola i to w takim sensie negatywnym. W sensie pozytywnym to było zawieszenie zajęć dydaktycznych, które było zaskakujące i dało sporą część czasu wolnego. Później wdrożenie e-learningu, czyli zupełna zmiana stylu pracy dydaktycznej, odkrycie w sobie pokładów malarza, tynkarza, murarza, specjalisty od wszystkich spraw technicznych, wymiana okna do zapamiętania na długo i dobrze, zakup psa, chociaż nie był powiązany bezpośrednio z tym. I może jeszcze wyjazd nad morze w czasie tej największej kwarantanny, ale wyjazd do własnego mieszkania. To była ta pusta plaża, gdzie było kilkadziesiąt osób zamiast kilku tysięcy. Jeszcze pozamykane sklepy i restauracje nadmorskie. No i teraz już powrót do aktywności.

**A z perspektywy kraju?**

Zatrzymanie rozgrywek ligowych, życie stało się nudniejsze. Na pewno na długo zapamiętam chaos wyborczy i przepychanki wąskiej grupy interesariuszy, którzy chcieli albo nie chcieli wyborów, bez patrzenia, jak to jest odbierane społecznie.  Nie wiem, czy coś jeszcze. Wiele z tych rzeczy spowszedniało i one straciły swoją siłę rażenia - kolejne statystyki koronawirusowe. Takie rzeczy, które uderzały w podstawowe nawyki i przyzwyczajenia. To najbardziej zapamiętujemy.
