# Supplementary material for: The challenges arising from the COVID-19 pandemic and the way people deal with them. A qualitative longitudinal study
Source: PLoS One. 2021 Oct 11;16(10):e0258133. doi: 10.1371/journal.pone.0258133 (PMC8504766; doi:10.1371/journal.pone.0258133)
Supplement: S1 Dataset — (ZIP) [file pone.0258133.s003.zip › Transcriptions/stage 5/13.5_M_46_couple, with children.docx]

**13.5_M_46_couple with children**

**Co działo się przez ostatni miesiąc?**

U mnie w domu zmieniło się to, że zaczęliśmy częściej go opuszczać, wyjeżdżać na wycieczki rowerowe. W pracy zmienił mi się sposób funkcjonowania, bo wróciliśmy do normalnych zasad, tj. godziny 7-15 codziennie, a nie co drugi dzień 12 h. Poradnie wróciły do normalnej pracy. Ja przeniosłem prywatny gabinet w inne miejsce, on już też jest otwarty, więc pracy jest więcej. Pierwszy raz byłem w nim w zeszłym tygodniu.

**Ważne momenty**

To nie są momenty, jakichś ważnych zmian nie było, to są raczej konsekwencje sytuacji, która nas otacza i właściwie tyle. Poza przenosinami gabinetu, które są uciążliwe - to jak przeprowadzka w mieszkaniu, gdzie trzeba przenieść rzeczy, posprzątać, jest tego dużo - to nic.

**Jak z synem?**

On nadal nie wrócił do szkoły. Pracuje z komputerem i lekcje są prowadzone przez internet. Ponieważ wróciłem do pracy i nie mogę poświęcać mu czasu na pomoc w nauce, tylko i wyłącznie dlatego, że żona jest w domu, udaje nam się to zrobić. Inaczej trzeba by było stosować jakieś wymyślne rzeczy, żeby to zadziałało. Żona pracuje teraz w domu, dzięki temu to jakoś działa. Gdyby było inaczej, pewnie byśmy się jakoś dzielili, ja bym wziął kilka dni wolego, tak by to trzeba było zrobić.

**Ma pan wrażenie, że życie wróciło do normalności sprzed pandemii?**

Nie. Szkoła nie wróciła i szkolnictwo wyższe. Moja żona nadal pracuje w domu, nie wyjeżdża. Z jednej strony pewnie jest jej to na rękę, a z drugiej pewnie nie, bo pewnie chciałaby już normalnie funkcjonować, a nie tylko siedzieć w domu i przed kamerą tyko się pokazywać. Nie jest to najlepszy sposób funkcjonowania. A też fakt, że syn uczy się w domu - to też nie jest normalne, bo ja nie potrafię zapewnić mu tego, co zapewniają mu nauczyciele. Z różnych względów. I to nie jest normalne. Także nie wróciło do normalności. Do kina też nie chodzimy, gdybyśmy chcieli pojechać na wakacje, to też nie pojedziemy. Do normalności jest jeszcze daleko.

**A czy są jakieś obszary, które wróciły do normalności? Wspomniał pan, że godziny pracy wróciły do tego stanu sprzed.**

Tak, praca jest tak samo uciążliwa, jak była przed epidemią. Jest jej tak samo dużo, albo nawet więcej. No i tyle. Do normalności wróciły może zakupy. Można pójść do sklepu i kupić coś w normalnym sklepie, nie tylko przez internet. Nie mam takiej dużej potrzeby - poza zakupami spożywczymi oczywiście - robienia często innych zakupów, więc to nie jest nic takiego wielkiego. Natomiast - można. To się zmieniło na plus.

**Jak wyglądają spotkania ze znajomymi/ bliskimi? Pojawiły się już jakieś?**

Tak. Z najbliższymi. Umawiamy się np. na rower, obiad w domu. To było raz czy dwa, ale zdarza się.

**Raczej na zewnątrz czy w domu?**

Zdecydowanie częściej na zewnątrz.

**To ze względów bezpieczeństwa?**

Tak. Choć będąc konsekwentnym, nie należałoby się w ogóle spotykać. Bezpieczeństwa, ale chyba też jakiś tam indywidualny rozkład zajęć. Więc może udało nam się ze dwa razy widzieć ze znajomymi w tym czasie.

**Syn już spotykał się z kolegami?**

Tak. Z rzeczy, które jeszcze trochę zbliżyły się do sytuacji sprzed epidemii, to to, że syn wrócił do treningów tenisa. Bo on przez dłuższy czas nie uprawiał tego sportu - my, ani nikt z naszych sąsiadów czy kolegów nie ma kortu tenisowego, żeby można było skorzystać. A obiekty sportowe były zamknięte. Teraz, jak wróciło to do funkcjonowania - w troszkę zwariowanych warunkach, ale jednak - Mikołaj zaczął jeździć na treningi.

**Na tych treningach są jakieś zabezpieczenia/ środki ostrożności?**

Są środki ostrożności. Ja nie byłem w Rzeszowie. W Przemyślu, czyli miejscu, gdzie mieszkamy, te treningi są na otwartym powietrzu, więc jakichś szczególnych zabezpieczeń nie ma. Kwestia tylko tego, że nie ma dużej ilości osób na korcie równocześnie. Przychodzą sami zainteresowani tylko. Natomiast w Rzeszowie, gdzie Mikołaj jeździ, jest hala - tam jest specjalnie zatrudniona pani, żeby odkażać poręcze, uchwyty, klamki i pilnować, żeby nikt nie wchodził - poza użytkującymi obiekt. Czyli rodzice nie wchodzą, mogą patrzeć przez okno, ale nie wchodzą na teren obiektu.

**Co pan sądzi o tych zabezpieczeniach stosowanych na kortach?**

<śmiech> To wszystko są jakieś pozory. Dają ludziom takie poczucie, że coś jest robione. Ale jakby się z naukowego punktu widzenia temu przyjrzeć, to nic to nie daje. Natomiast głowa spokojna, takie placebo, które troszkę uspokaja nastroje społeczne.

**Jak to jest w przypadku tych spotkań syna z kolegami? Dbają państwo o zachowanie pewnych zasad?**

Chłopcy biegają po podwórku, więc raz jest dystans, a raz nie. Bawią się na zewnątrz. Jak się bawią w domu - jest dwóch kolegów. Jeden to ten, z którym gra w tenisa, a z drugim zna się od niemowlęctwa. Z nimi czasami spotyka się też w domu. Więc nie stosujemy specjalnych zabezpieczeń. To nie ma sensu.

**Dlaczego państwo zdecydowali się te spotkania umożliwić?**

Syn tego potrzebował. Dziecko jest izolowane od kolegów - poza komputerem - od kilku miesięcy. On inaczej postrzega świat. My możemy sobie różne rzeczy wytłumaczyć, jemu jest trudniej. Gdzieś musi wytracić swoją energię. Zupełnie inaczej funkcjonuje, jak spotka się z kolegą, niż jak częściowo te same rzeczy będzie próbował zrobić z rodzicami.

**Jakieś spotkania z rodziną się u państwa pojawiły?**

Tak i też bardzo krótkie. Nie robimy ogniska, grilla, jakiś większych spotkań, obiadów. Widujemy się z rodzicami. Nie ma takiego ścisłego reżimu, jak kiedyś. Troszkę luźniej jest, ale to jeszcze nie wróciło do wcześniejszego stanu.

**A czy u osób w pana otoczeniu to wróciło do poprzedniego stanu?**

Wygląda prawdopodobnie tak, jak u mnie. Z tymi ludźmi, z którymi rozmawiałem, doszliśmy do wniosku, że to wygląda podobnie. Nie ma pełnej swobody w zachowaniu, ale też nie ma bardzo ścisłego rygoru. Nie wyjeżdżamy z miejsca zamieszkania - poza dłuższymi wycieczkami rowerowymi. Zdecydowaliśmy się na nadchodzący długi weekend pojechać z żoną na takie spotkanie ze znajomymi, ale nie jednodniowe, tylko już z noclegiem. Też na świeżym powietrzu, rowery i aktywność fizyczna.

**Jak się panu wydaje, o spowodowało rozluźnienie rygoru - nie tylko u pana, ale i w otoczeniu?**

Tak szczerze, to po pierwsze przyzwyczajenie - habituacja. Mamy stały bodziec, aż w końcu nasze receptory - porównując nasz organizm do receptorów - przyzwyczajają się do bodźca na tyle, że on przestaje być tak bardzo drażliwy. Przestajemy czuć zagrożenie w takim stopniu, w jakim to było na początku. Poza tym bodźce w postaci przywracania funkcjonowania niektórych dziedzin naszego codziennego życia - zakupy, galerie handlowe, obiekty sportowe, itd. To powoduje, że ludzie mają wrażenie, że jest coraz bezpieczniej. Jak widać w mediach, na przykładzie tego, co dzieje się na Śląsku, wcale bezpiecznie nie jest. No i tyle. I wraca ta sama kwestia - gdyby sprawdzić testami, ile ludzi zakażonych przybyło, dopiero mogłoby się okazać, jaka to była skala. Może niepotrzebnie się już martwimy, może należałoby zrobić większej ilości populacji testy i przekonać się, że już przebyła zakażenie - bezobjawowe - i nie trzeba się aż tak bardzo martwić, bo mamy temat za sobą. Więc jest, jak jest. Poza tym, uważam, że jesteśmy zmęczeni tą całą procedurą, izolacją. Wszyscy chcieliby normalności, więc jakoś podświadomie próbują wracać do tego, co było i nie czują tak bardzo zagrożenia, a chęć powrotu do normalności jest coraz silniejsza. Takie mam wrażenie, a jak jest faktycznie? Może pani badania to pokażą.

**Pan odnosi to też do siebie? To, że z jednej strony jest jest zmęczenie izolacją, a z drugiej chęć powrotu do normalności?**

Tak, mówię to na własnym przykładzie. Obserwuję też otoczenie. Może, gdybym mieszkał na Śląsku, myślałbym inaczej. Ale mieszkam na Podkarpaciu i wiem, że tu się robi bardzo mało testów. Szpitale wróciły do normalnego funkcjonowania, nie mamy już szpitali jednoimiennych. Ponieważ nie widzę wśród mojego otoczenia - rodziny, otoczenia, pacjentów - nie widzę przypadków choroby, mi się ta wrażliwość i czujność na problem zmniejsza. Gdybym widział wokół chorych, pewnie funkcjonowałbym inaczej. A ponieważ tego nie ma, traktujemy temat w sposób taki, jakby był coraz mniej ważny. Zdaję sobie sprawę, że naukowo jest inaczej, ale z mojej strony widzę to w ten sposób. Jakieś takie rozluźnienie w społeczeństwie myślę, że z tego powodu wynika. U mnie wynikałoby z tych powodów.

**Czy jest coś, co panu nadal przeszkadza?**

To samo - fakt szkoły, to, że moje dziecko uczy się w domu. Taka nauka nie jest pełnowartościowa - niezależnie od tego, jak wiele serca poświęca w tą naukę moja żona i ja. Moja żona bardzo dużo, robi to perfekcyjnie i jest świetna, ale uważam, że to nie jest to samo. Syn nie ma kontaktu z rówieśnikami, nie ma obowiązku od do. Moje dziecko funkcjonuje inaczej, męczy, irytuje go dużo krótszy okres, który musi poświęcić na naukę - dużo krótszy, niż poświęcałby w szkole stacjonarnej. Musi zrobić kilka zadań, przed komputerem zajmuje mu to mało czasu, a on i tak jest zdenerwowany i poirytowany. Więc myślę, że to jedna z takich rzeczy, które najbardziej dokuczają. To, że syn nie funkcjonuje w tym normalnie. Żona mu to świetnie organizuje, ja nie potrafię tego tak zrobić, nie mam czasu, nie poświęcam tyle czasu, co ona, na organizowanie. Łatwiej przychodzi mi z nim usiąść i zrobić zadanie, niż zorganizować mu tą naukę tak, jak robi to moja żona.

**Znaleźli państwo jakieś nowe sposoby, aby pomóc w tej sytuacji synowi?**

Mogę mówić za siebie. Ja nie znalazłem.

**To, że syn wrócił na tenisa i zaczął spotykać się z kolegami, w jakiś sposób mu pomaga?**

Tak, bardzo cieszył się po ostatnich treningach.

**Emocje - jak się pan czuł przez ostatni miesiąc w związku z sytuacją pandemii?**

W związku z sytuacją pandemii, nic się u mnie nie zmieniło. Czuję się bezpieczniejszy, spokojniejszy o najbliższych. Czuję, że na Podkarpaciu nie ma tak dużego zagrożenia jak w takich aglomeracjach, jak Śląsk, czy Warszawa i okolice. Duże miasta funkcjonują inaczej, małe miasteczka trochę inaczej.

**Obrazki - emocje w ciągu ostatniego miesiąca**

1. zostaje, ale zmieniamy 7. na 15. i 16. 1. - jak mój syn pójdzie do szkoły, a żona wróci do pracy i wszyscy zafunkcjonują trochę normalniej - oprócz mnie, bo w sensie zawodowym ja już funkcjonuję - to wtedy, jak moja rodzina wróci do normalnego funkcjonowania, to ten obrazek się zmieni wtedy. 15. - parę jakichś indywidualnych sytuacji miałem w pracy. To dotyczyło układów zawodowych. I ten 15. i 16. obrazek to tak oddaje. Uderzyła fala, był taki jeden moment, który mocno mnie dotknął, a później się po tym pali. Fala przechodzi, a ogień trwa.

**Pamiętam, że wspominał pan o niewiadomej w sytuacji z ordynatorem - jak to się rozwiązało?**

Ordynator i zespół został. Ta fala to decyzja, która miała związek z rozwiązaniem konfliktu. Ponieważ nie zostało to rozwiązane w sposób mądry - wg mnie w ogóle nie zostało rozwiązane - to konsekwencją tego jest 16 obrazek.

**Jak się pan w związku z tym czuje z nierozwiązaniem konfliktu, z tym pożarem?**

Chcę być obojętny. Zostały mi odcięte możliwości wpływania na rozwój tej sytuacji, więc się odcinam. Zostałem kompletnie zaskoczony pewnymi decyzjami mojego szefa. Konsekwencja jego zachowania jest taka, że ja próbuję się od tego odciąć. Emocje są dalej, natomiast ja przynajmniej - w danej chwili - nie jestem tak bardzo wzburzony. Gdybym codziennie reagował na te elementy układanki, codziennie byłbym bardzo zdenerwowany, a na to nie mam ochoty. Więc generalizując mój stan, tak to wygląda. Choć jest to związane z wybranym elementem mojego życia, choć bardzo ważnym.

**Wcześniej pojawiała się złość na decyzje polityczne odejmowane w kraju. Czy to nadal jest, czy się zmieniło?**

Oczywiście. To już są różne dziwne rzeczy. Nie mam nic do dodania do tego, co było poprzednio. Nadal tkwimy w tym samym miejscu, nie ma opcji w tym momencie do zmiany mojego podejścia. Nie wiem, czy rozmawiałem już z panią po zniszczeniu Trójki?

**Przed**

Moje ulubione radio. Słuchałem go od 6 r.ż. Ja chyba pani wspominałem w którejś z rozmów, że odchodzą redaktorzy z tej rozgłośni. Których ja bardzo lubiłem i dla których włączałem czasami radio, żeby ich posłuchać - nie tylko muzyki, bo ją mam w różnych serwisach streamingowych. Warto było ich posłuchać, opinii, zdania. Czasami tego, jak prezentują nowości lub przypominali stare rzeczy. Radio się rozleciało prawie całkiem w takim wymiarze, w jakim je lubiłem i dla jakich wartości go słuchałem. I szkoda, a to jest tylko i wyłącznie następstwo jakichś tam politycznych układów i próba podlizania się politykom. Żenujące.

**Od tej sytuacji politycznej też stara się pan odcinać, dystansować?**

Nie komentuję tego w mediach społecznościowych - bo to nie ma sensu moim zdaniem. Nie mogę się całkiem odciąć. To nie jest tak, że ja zapominam i myślę sobie, a, nie będę się tym denerwował, bo to nie ma związku. Jest to niezależne ode mnie. Widząc kolejne informacje o aferach, o zaprzepaszczaniu dziedzictw kultury naszego kraju - bo za to uważałem tę rozgłośnię. Niszczy się rzeczy wartościowe, rozpuszcza pieniądze publiczne. Jak się zastanowię nad tym, że regularnie chodząc do pracy, płacąc podatki, ubezpieczenie - utrzymuję całe mnóstwo osób, które nie pracują i nie mają nawet zamiaru pracować. Nie mówię o przypadkach, kiedy niektórzy nie mają takiej możliwości, bo mają w tym momencie zakaz. To jestem w stanie jakoś znieść. Ale ci, którzy nie mają zamiaru pracować i dostają pieniądze, bo państwo uważa, że trzeba wszystkim pomóc - niezależnie od podejścia tych ludzi do codzienności - to mnie denerwuje, ciężko mi przejść nad tym do obojętności. Ja ciężko pracuję, poświęcam swoje życie rodzinne - taki mam zawód, że on najbardziej uderza w rodzinę, a to mi przeszkadza. I kolejna decyzja naszych polityków, żeby znowu jakieś pieniądze zapłacić, co ma na celu tylko jedno - utrzymanie elektoratu. To jest dla mnie nie do przyjęcia i nie mogę przejść nad tym tak zupełnie bez emocji, tyle.

**Czy są jeszcze jakieś emocje wokół sytuacji epidemii? Jakaś irytacja?**

Nie, nie irytuję się z powodu obostrzeń, zakazów. Nie raz podkreślałem, że jedynym sensownym środkiem zapobieżenia rozprzestrzeniania się epidemii jest izolacja. Ja dobrze o tym wiem, zdaję sobie z tego sprawę. Dowodów na to było wiele, leków na to nie mamy. Czym innym się chronić? Różne grupy ludzi różnie reagują na kontakt z patogenem, więc jest inny przebieg choroby. W związku z tym, nie wiemy jeszcze, jak radzić sobie z zakażeniami. Może za paręnaście miesięcy będziemy wiedzieli, że Włosi muszą uważać bardziej, Japończycy mniej, a Peruwiańczyków problem w ogóle nie dotyczy. No nie wiem, zobaczymy. Nie denerwuje mnie izolacja, a to, że przy okazji pandemii są forsowane pewne pomysły i zmiany w prawie, przepisach, funkcjonowaniu firm - jak w moim szpitalu, które bez epidemii trudno by było wprowadzić, a teraz się to tłumaczy potrzebą ochrony populacji, ochrony społeczeństwa przed zakażeniem. Tym tłumaczy się różne głupie rzeczy.

**Wspomniał pan, że jest pan spokojniejszy o najbliższych. A czy jakieś poczucie zagrożenia się utrzymuje?**

Cały czas. Dla mnie cały czas, bo chodzę do pracy i chodzę do pracy w szpitalu. Gdybym chodził do pracy w sklepie, byłoby to pewnie jeszcze większe. I we wszystkich miejscach, gdzie spotyka się dużą ilość ludzi i spotyka się z ludźmi. Te miejsca są dla mnie możliwym ogniskiem zakażenia i bałbym się jeszcze bardziej. Ponieważ pracuję w szpitalu na oddziale, nie mam tak dużego kontaktu z dużą liczbą osób, jak w sklepie na przykład. Ale też jest, trafiają pacjenci w różnym stanie, część z nich może być zainfekowana, więc dalej jakiś poziom strachu jest.

**Jak sobie pan radzi z różnymi emocjami? Wcześniej mówił pan, że to hobby pomaga przewietrzyć głowę.**

Tak jest cały czas. Aktywność fizyczna, praca wokół domu. Aktywność fizyczna z większym wysiłkiem bardzo pomaga człowiekowi uzyskać spokój ducha. Oczywiście, praca z wyboru. Nie niewolnicza i katorżnicza - to co innego.

**Czy kiedy obserwuje pan otoczenie, zauważa pan zmieniające się emocje?**

Wśród kolegów z pracy jest tak, jak było. U niektórych osób, które w bardzo hipochondryczny sposób traktowały początek epidemii i z bardzo dużym strachem oceniali sytuację - niektórzy się bardzo uspokoili, a część wykazuje wręcz zachowania w drugą stronę. U tych drugich kiedyś był strach przed zakażeniem, poczucie, że choroba jest bardzo groźna, nieunikniona i prawie że zabija wszystkich. W tej chwili prawie się nie boją, pomimo, że na początku byli oburzeni, że nie ma od początku nakazu noszenia maseczek, że środki do dezynfekcji były na początku niedostępne, później drogie. W tej chwili nie mówią już nic o maseczkach, o odkażaniu. Mam takich paru znajomych, którzy prawie w obsesyjny sposób traktowali odkażanie rąk. Wszędzie mieli porozmieszczane pojemniki do odkażania rąk - w samochodzie, tuż przy wejściu do domu. To się uspokoiło, już tego nie robią. I nie mają już poczucia, że choroba ich zabije. Zaczynają wręcz w drugą stronę - szukać spiskowych teorii, które by wyjaśniały obecną sytuację. I czasami wysuwają wręcz absurdalne wnioski. Wcześniej wypytywali mnie o medyczne rzeczy, teraz słyszę od nich teorie spiskowe dotyczące obecnej sytuacji.

**Co pan sądzi o tym, że niektórzy uciekają w takie teorie?**

Potrzebują wyjaśnienia. Myślę, że część ludzi źle funkcjonuje, jeśli musi przyjąć sytuację bez wyjaśnienia. Nie mają świadomości, jaka była przyczyna i oni źle to znoszą. Żona by powiedziała, że mają potrzebę domknięcia. Mam wrażenie, oni rozmawiając wcześniej, jak ja próbowałem jakoś tak... Może nigdy nie bagatelizowałem. Mam wrażenie, że na początku mogłem nie doceniać krótkotrwałych skutków epidemii. Wydawało mi się, że ten przebieg nie będzie aż tak gwałtowny jak w Chinach, czy we Włoszech. Tam rzeczywiście na niedużym obszarze wyglądało to dramatycznie. Ale jak się zastanowić, w ogólnej ocenie ilości ofiar spowodowanych chorobą, nie jest to tak strasznie groźna infekcja, jak to było z grypą Hiszpanką kiedyś. Tam było dużo więcej ofiar. Ten wirus schematem zbliża się raczej do zwykłej grypy, na którą chorujemy. A rzadko chorujemy na grypę - pomimo, że ludzie mówią, że mieli grypę, to często to nie była grypa. Powikłania przy grypie są groźne, część ludzi cierpi i część umiera. My sobie z tego nie zdajemy sprawy i przechodzimy nad tym do porządku dziennego. Ja na początku miałem taki pogląd i ocenę sytuacji. Później mi się to trochę zmieniło patrząc na to, co dzieje się w Chinach i we Włoszech. Ale zobaczymy za kilka-kilkanaście miesięcy, jak to będzie wyglądało. Mam wrażenie, że ludzie, którzy wcześniej wypytywali mnie o tą całą sytuację ze strachem i ja próbowałem to jakoś łagodzić, teraz oni przeszli na drugą stronę. Moje podejście, które wynika z medycznego postrzegania świata jest podobne, jak było. To się nie zmieniło, choć nie wiem, jak teraz ci ludzie na mnie patrzą. Czy pamiętają to, co było trzy miesiące temu, czy już zapomnieli. Ich nerwowość, jakieś tam dopytywanie.

**Zakupy - jak wyglądają zakupy spożywcze? Czy coś się zmieniło?**

Niewiele. Nie ma rękawiczek w sklepach - kiedyś wisiały jakieś foliowe rękawiczki, teraz w niektórych sklepach nie ma ich w ogóle. Jest pojemnik z płynem do odkażania rąk. Jest trochę luźniej - nadal nie ma jakiegoś strasznego tłoku, mam wrażenie, że ludzie robią duże zakupy, żeby nie robić ich zbyt często. Moje podejście się nie zmieniło. Nie chodzę zbyt często, robimy te zakupy starając się też zrobić tak trochę na zapas, żeby często do sklepu nie chodzić po drobiazgi. Większość rzeczy, które można zrobić przez internet, zamawiamy online - sprzęt AGD, komputerowy, kosmetyki. Te rzeczy nadal funkcjonują w formie zakupów internetowych. Przed epidemią kupowaliśmy podobnie. Zakupy przez internet są dla nie najwygodniejszą formą. Kiedy mogę odebrać przesyłkę z Paczkomatu, to w ogóle jest super sprawa - nie muszę się przejmować oczekiwaniem na kuriera, czy odbiorem gdzieś na poczcie. Tak mi było zawsze najwygodniej. Robię zakupy wtedy, kiedy mam czas, mogę siąść w środku nocy przed komputerem i kupić, co mi jest potrzebne. Więc jest to jest dla mnie najwygodniejsza forma I kupuję przedmiot ten, który chcę, a nie ten, który właśnie jest dostępny na półkach i muszę się domyślać lub rozmawiać ze sprzedawcą, co jeszcze mają w asortymencie. W internecie szukam konkretnej pozycji i kupuję w tym sklepie, w którym jest.

**Państwo robili zakupy z listą?**

Tak, bez niej zapomniałbym połowy rzeczy, więc ona jest niezbędna. Przyspiesza zakupy. Kiedy robiłem zakupy w takim jednym supermarkecie i nauczyłem się w końcu, jak są rozmieszczone produkty, lista pozwoliła mi robić zakupy o połowę szybciej. Układałem wtedy listę strefami w sklepie.

**Są już otwarte galerie handlowe. Był już pan w galerii?**

Raz lub dwa. Było to właściwie wyjście ze schodów do sklepu, który był tuż przy nich. Później wyszedłem, bo nie było mi nic potrzebne. Nie chodzę po galeriach w celach rozrywkowych. Jest to dla mnie uciążliwe, a nie przyjemne.

To był sklep z...?

Empik. Był mi potrzebny prezent, a wiedziałem, e tam go znajdę.

**Ostatnio otworzono też restauracje i kawiarnie. Co pan o tym sądzi?**

Odcinając się od medycyny, powiedziałbym, że nareszcie, bo trochę ludzi odetchnie - zarówno właścicieli lokali, jak i klientów. Z punktu widzenia medycznego, zwiększa to ryzyko infekcji. Nie chcę się wypowiadać więcej na ten temat. Moim zdaniem, dłuższe utrzymywanie sytuacji, jaka miała miejsce, tj. zamknięcia lokali gastronomicznych, myślę, że obiektów sportowych też, myślę, że doprowadziłaby do finansowej ruiny. Poniesione koszty chyba nie byłyby adekwatne do korzyści. Ludzie się zarażą wirusem również w kościele, poczekalni, na dworcu i w innych takich miejscach. Na bazarze z warzywami - tam jest kompletny chaos, jeśli chodzi o zachowanie jakichś środków - i odległości, i aseptyki - to nie funkcjonuje. Myślę, że trudniej zarazić się wirusem w restauracji, niż robiąc zakupy na bazarze warzywnym.

**Był pan już w restauracji lub kawiarni?**

Nie.

**Z jakiegoś konkretnego powodu?**

Chyba nie mieliśmy jeszcze takiego pomysłu. Pożytkujemy wolny czas na inne rzeczy, a na razie jeszcze się nie umawialiśmy, ale to pewnie niebawem nastąpi.

**Teatry, kina, obiekty sportowe traktuje pan podobnie w kwestii bezpieczeństwa?**

Tak

**Te zabezpieczenia na kortach, o których pan wspominał, są przez pana traktowane jako czynności, które raczej uspokajają ludzi, niż przekładają się na faktyczne bezpieczeństwo?**

Nie wiem, nie potrafię powiedzieć. To trzeba by robić badania, żeby móc udzielić na takie pytanie rzetelnej odpowiedzi. Ja mogę powiedzieć tylko, że takie mam wrażenie, tak mi się wydaje. Ale takie badanie trzeba by precyzyjnie skonstruować, co myślę, że jest w obecnych warunkach trudne do wykonania.

**Ma pan jakieś obawy związane z tym, że syn korzystał z tych obiektów sportowych?**

Nie.

**Rozwiązania technologiczne. Czy słyszał coś pan na ten temat? O jakichś aplikacjach stworzonych na potrzeby sytuacji?**

Ja niczego takiego nie używam. Gdyby pani mi opowiedziała o czymś takim, przeczytała, być może bym sobie przypomniał, ale teraz nic nie przychodzi mi do głowy.

**<opisy rozwiązań pierwszej kategorii>**

O, słyszałem o aplikacji służącej do monitorowania kwarantanny domowej.

**Ok, to do tego wrócimy. <czyta do końca opisy z pierwszej kategorii> Czy uważa pan, że takie rozwiązania są potrzebne?**

To zależy komu.

**Komu mogłyby być potrzebne?**

<śmiech> Nie chcę być postrzegany jak <śmiech> ktoś, kto... A, właściwie to wszystko mi jedno. Uważam, że rząd wykorzystuje - zresztą nie tylko ten, jakikolwiek - rządy mogą wykorzystywać takie aplikacje po to, żeby mieć troszkę większą kontrolę nad społeczeństwem. Służby specjalne, wywiad, różnego rodzaju agencje, których my nie znamy, a nawet nie wiemy, że takie istnieją, funkcjonują. Różnego rodzaju służby, które dbają o nasze bezpieczeństwo, ale też kreują funkcjonowanie naszej rzeczywistości.

**Czy te aplikacje mogą dawać coś ludziom, którzy je pobierają?**

Ja osobiście nie chciałbym być obiektem monitorowania przez takie aplikacje. Nie sądzę, żeby to dawało mi cokolwiek. Denerwuje mnie pojawianie się reklam spersonalizowanych. Jak przeglądam coś w sieci, a później przez najbliższe trzy miesiące widzę buty, albo rakiety do tenisa, albo wzmacniacz, gitarę, cokolwiek, to mi się robi od tego niedobrze. Nie lubię czegoś takiego, nie podoba mi się to. Dla mnie tego typu rzeczy nie dają nic tej osobie, która jest monitorowana. Oczywiście, możemy mówić, że mamy guzik, i jak jesteśmy monitorowani i wciśniemy ten guzik, to nam pomoc przyjedzie. Można w to jeszcze wiele innych rzeczy włączyć i ktoś będzie tłumaczył tego przydatność, ale ja jestem specyficzny, jeśli chodzi o aktywność w sieci i jestem na bakier z takimi pomysłami. Z własnego wyboru. Nie lubię się chwalić, nie publikuję treści sam z siebie. Oczywiście, w rozmowach ze znajomymi tak, ale na forum, w mediach społecznościowych - nie. Prywatnie tak, wymieniam się różnymi informacjami. Wiem, że one mogą krążyć w sieci, natomiast nie jest to potrzeba pokazywania się i tego, co mnie otacza publicznie, aby każdy, kto chce, mógł to oglądać. Tego typu aplikacje uważam, że wchodzą w taką strefę prywatności, którą ja nie chcę się dzielić. Nie chcę wiedzieć, o tym, że ktoś mnie śledzi i wie, gdzie ja się aktualnie znajduję. Oczywiście może to zrobić, bo chodzę z telefonem. I niektóre służby mogą to zrobić, jeśli będą chciały. Ale ciągle są to jeszcze rzeczy mało popularne, nie stosowane rutynowo. Jak zaczniemy instalować tego typu aplikacje, jak ta dot. kwarantanny, a tym gorzej, jeśli będzie ona obowiązkowa, to okaże się, że wszyscy jesteśmy monitorowani urzędu i okazałoby się, że dane, lokalizacja każdej osoby są zapisywane, monitorowane. A tak zawsze jest furtka, że może nie jestem obiektem obserwacji różnych służb. Nie robię nic złego, ale tym bardziej nie chciałbym być obserwowany.

**<opisy drugiej kategorii aplikacji> Co pan sądzi?**

Makabra. Z mojego punktu widzenia - nie jesteśmy w stanie obyć się bez AI, natomiast powierzanie jej takich zadań, jak zarządzanie środkami w czasie epidemii - biorę pod uwagę wszelkiego rodzaju środki: materiały medyczne, pieniądze, żywność, siły medyczne, itd. - to dla mnie jest zbyt daleko posunięte. Sztuczna inteligencja - takie jest moje wrażenie - wszystkie nowe technologie są wprowadzane, jak wojsko jest już przekonane, że używanie tego w życiu codziennym nie będzie stanowiło zagrożenia dla tajemnic wojskowych. Wtedy wypuszcza to do codziennego funkcjonowania. Więc skoro my mamy AI na takim poziomie, to nie wiem co jest w wojsku. I może jest tak dobry poziom zaawansowania tych technik, który pozwala czuć się bezpiecznie. Jednak dla mnie to jest zawsze furtka do tego, żeby się nam <śmiech> maszyny zbuntowały. Takich filmów SF mamy całe mnóstwo, ale filmów o epidemiach też było kilka. Ciągle traktowaliśmy to jako film sensacyjny, thriller, a tu mamy rzeczywistość. A problemy z buntem maszyn - różnie wyrażonym i sztuczną inteligencją - też będziemy mieli niedługo. Coraz więcej urządzeń jest bardzo autonomicznych. To powoduje, że człowiek robi się coraz mniej czujny, przestaje myśleć analitycznie i tak szczegółowo, jak przed erą maszyn. To, nad czym kiedyś myślał trochę więcej, zostawia sztucznej inteligencji i to się stanie kiedyś kolejnym problemem. Awaria systemu zablokuje nam elektrownię - nie chodzi mi o jakieś nie wiadomo jakie scenariusze, a o takie proste rzeczy, typu urządzeń w elektrowni, elektrociepłowni, sieci telekomunikacyjnej. Okaże się, że jest jakiś drobny błąd w systemie, którego skutki będziemy naprawiać przez wiele tygodni, bo coś się rozleci. Także ja mam podejście takie ostrożne. Zdaję sobie sprawę, że rozwoju techniki powstrzymywać nie wolno, to jest dobre, że on jest. Tylko zbyt pochopne ufanie nowym, nie sprawdzonym do końca technologiom, chyba nie jest dobre. Mnie się tego typu aplikacje nie podobają. Myślę, że jest wystarczająco dużo możliwości komunikowania się z innymi ludźmi, obwieszczania o swoich potrzebach przy pomocy dostępnych środków, aplikacji, możliwości, że tworzenie nie wiadomo jakich, specjalnie przeznaczonych do użytku w czasie epidemii rozwiązań, jest chyba tylko sposobem na zaistnienie na rynku. To jest sztuczne kreowanie potrzeb u ludzi, chęć sprzedania produktu, zaistnienia na rynku, wykorzystania sytuacji. Większość sklepów ma swoje strony internetowe i dzięki tym narzędziom całkiem normalnie funkcjonuję - czy to w czasie epidemii, czy nie. Robienie tego specjalnie od nowa, tworzenie aplikacji czy zakładki w aplikacji na potrzeby pandemii, uważam za niepotrzebne. Media społecznościowe, z których korzystałem, działają dalej. Jeśli chcę z kimś porozmawiać, korzystam z nich lub innych komunikatorów, nie ma problemu. Może na jakimś bardzo zaawansowanym poziomie to ma sens, ale dla przeciętnego obywatela - nie jest mi to potrzebne. Więc uważam, że tworzenie takich aplikacji nie koniecznie jest bardzo potrzebne. Drony... ok. Znowu będę mówił, że będziemy inwigilowani. Bo dron, który będzie dostarczał nam jedzenie, zrobi nam przy okazji kilka zdjęć i nagra film z miejsca, do którego się dostał. Z jednej strony są super, do różnych celów, proszę bardzo, można to zrobić tak, żeby ta technologia faktycznie ludziom służyła - i pewnie będzie. I pojazdy bezzałogowe też będą i będą ludziom dostarczać żywność, wydawać paczki i robić inne rzeczy.

**Aplikacja "Kwarantanna domowa" - co pan sądzi?**

Myślę, że nic ponad to, co powiedziałem. Ona oczywiście może być przydatna, aczkolwiek jest to taka inwigilacja, że coś strasznego. Telefon naładowany... I pani i ja dobrze wiemy, że telefon to świetny sposób na to, aby nagrać rozmowy, użyć telefonu do przekazania do tego, co się akurat dzieje, gdzie on leży. Służby potrafią to zrobić.

**A jakie informacje o osobie ta aplikacja pobiera?**

Wygląd, zdjęcie, miejsce w którym znajduje się osoba. Poza tym, jeśli aplikacja porównuje zdjęcia, będzie widziała, co dzieje się w domu. Dopasuje wygląd wnętrza do poprzednich zdjęć, sprawdzi, czy to jest tak, czy nie. A to jest furtka też do używania aparatu, do tego, żeby zobaczyć, co jest w domu. No i tyle.

**Czy jest w tym opisie/ aplikacji coś, co się panu podoba?**

Oczywiście. Idea aplikacji jest fajna. Gdyby nie było mojej obawy przed inwigilacją, to jest w porządku. Ktoś ma telefon, używa go w taki sposób, żeby ułatwić innym funkcjonowanie - tu jest informacja o tym, że policja dalej może zajrzeć. Jakiekolwiek inne służby, które miałyby kontrolować, mogą sprawdzić, czy ta osoba nie oszukuje, czy tym telefonem nie próbuje oszukać jakiegoś organu nadzorującego. Idea dobra, ułatwia zwłaszcza tym nadzorującym. Powoduje, że oni są mniej narażeni na możliwość infekcji od osoby objętej kwarantanną. To jest na plus. Każdy ma w tej chwili telefon, więc jest łatwość nadzorowania takiej osoby, łatwość sprawdzenia - niesamowicie duża.

**Czy rząd powinien tworzyć takie aplikacje?**

Powiedziałbym, że tak, ale dać ludziom wybór, czy chcą z nich korzystać, czy nie. To, o czym ja powiedziałem, nie musi być dla niektórych problemem. Ktoś się tym nie przejmuje, że jest pod ciągłym nadzorem. Ja tego nie lubię, nie chcę, wolałbym rzeczywiście, żeby ktoś raz na jakiś czas zapukał, ja bym się pokazał przez okno. Czy zadzwonił do mnie, nie musząc wchodzić na teren posesji, żebym ja pokazał się w oknie, stanął przed domem. Cokolwiek. Można tak to zrobić. Ale jak ktoś chce używać, niech używa. Ja bym nie używał.

**Ona jest teraz obowiązkowa. Czy gdyby był pan na kwarantannie, szukałby pan sposobów, żeby jej nie pobrać?**

Nie. Szanuję prawo, stosuję się do ogólnie panujących zasad - to nie tylko kwestia prawa pisanego, ale też zasady międzyludzkie. I tyle. Więc skoro taki byłby obowiązek, pewnie bym to zrobił, ale z wyboru bym tego nie zainstalował. A, jeszcze jedna rzecz mi przyszła do głowy. Jak rozmawialiśmy na temat tej aplikacji - ja nie wiem, czy odinstalowując tę aplikację po kwarantannie, nie mam pewności, że specjalista informatyk nie zostawił tam jakiejś części, która nadal będzie prowadzić inwigilację. Części oprogramowania, która być może nie będzie widoczna, a zintegruje się z systemem telefonu, komputera czy tabletu i dalej będzie śledzić, i dostarczać dane. Może nie aż w tak ogromnym zakresie jak tutaj, ale różne inne, które nie byłyby standardowo udostępniane i przesyłane do jakichś serwerów, żeby to magazynować i zapisywać. I powiem, że spora część moich znajomych ze szpitala, kolegów lekarzy ma takie właśnie podejście do tego.

Z własnego wyboru. Nie lubię się chwalić, nie publikuję treści sam z siebie. Oczywiście, w rozmowach ze znajomymi tak, ale na forum, w mediach społecznościowych - nie. Prywatnie tak, wymieniam się różnymi informacjami. Wiem, że one mogą krążyć w sieci, natomiast nie jest to potrzeba pokazywania się i tego, co mnie otacza publicznie, aby każdy, kto chce, mógł to oglądać. Tego typu aplikacje uważam, że wchodzą w taką strefę prywatności, którą ja nie chcę się dzielić. Nie chcę wiedzieć, o tym, ze ktoś mnie śledzi i wie, gdzie ja się aktualnie znajduję. Oczywiście może to zrobić, bo chodzę z telefonem. I niektóre służby mogą to zrobić, jeśli będą chciały. Ale ciągle są to jeszcze rzeczy mało popularne, niestosowane rutynowo. Jak zaczniemy instalować tego typu aplikacje, jak ta dot. kwarantanny, a tym gorzej, jeśli będzie ona obowiązkowa, to okaże się, że wszyscy jesteśmy monitorowani z urzędu i okazałoby się, że dane, lokalizacja każdej osoby są zapisywane, monitorowane. A tak zawsze jest furtka, że może nie jestem obiektem obserwacji różnych służb. Nie robię nic złego, ale tym bardziej nie chciałbym być obserwowany.

**ProteGO Safe <prezentacja> Co pan o tym sądzi?**

Ta jest dla mnie dużo bardziej akceptowalna. Po pierwsze dlatego, że jest narzędziem dobrowolnym - mogę ją sobie zainstalować albo nie. To, co mi się nie podoba, to ten fragment o wykorzystaniu modułu bluetooth, aby łączyć się z innymi urządzeniami, byłby chaos niesamowity.

**Czym spowodowany?**

Łączeniem się ze wszystkimi urządzeniami. Każdy w tej chwili ma telefon w kieszeni, torebce, torbie. To, co by się działo, ja sobie tego nie wyobrażam, gdyby aplikacja musiała się łączyć. Powiedzmy, stoimy w kolejce w sklepie i co ja mam zrobić? Uciekać z tej kolejki? Aplikacja mi powie, że w tej kolejce... Ja nie wiem, jak to miałoby wyglądać. W tej chwili widzimy urządzenie, z którym jesteśmy połączeni, powiązani, jak by to miało wyglądać, że aplikacja by nas ostrzegała, że w promieniu trzech metrów znajduje się osoba potencjalnie zakażona wirusem? Łączenie się urządzeń bluetooth zajmuje kilka sekund. Stoję w kolejce w sklepie i co? Ze wszystkimi urządzeniami się połączę? Nawet telefon nie ma takiej możliwości, żeby łączyć się z więcej niż dwoma na raz. Więc jak to? Które sobie wybierze? Którego kolejkowicza telefon sobie wybierze? Tego najbliżej? Czy będzie szukał tego z wirusami? Żeby go znaleźć, musiałby się najpierw połączyć, więc jak to będzie wyglądało? Dla mnie z technicznego punktu widzenia - coś takiego jest nie do zaakceptowania. Reszta ok, sam korzystam z aplikacji przypisanej do samochodu, gdzie można szybko przejrzeć instrukcję - nie muszę wozić książki. Mogę sprawdzić tam stan samochodu, przerzucić w aplikacji lokalizacje do nawigacji samochodowej i skorzystać z wielu innych ułatwień, które poprawiają mi komfort życia. Ale ten fragment o łączeniu się i ostrzeganiu przed innymi, mogącymi być potencjalnym zagrożeniem - to dziwne. Dziwne i dla mnie nie do zaakceptowania.

**A z medycznego punktu widzenia. Jak pan to ocenia i to, że może to być pomocne dla lekarzy?**

Testy oceny ryzyka są stosowane w medycynie od dawna. My ich używamy i to jest na co dzień pomocne narzędzie.

**Gdyby trafił do pana pacjent z wypełnionym testem, czy skorzystałby pan z takich informacji? Traktowałby pan je jako wiarygodne?**

Nie wiem, na pewno bym to zweryfikował. Aplikacja to tylko narzędzie. Jako lekarz leczę pacjenta, a nie wyniki. Tak należałoby potraktować ankietę z aplikacji. I tyle. Na pewno nie byłoby to jedyne narzędzie oceny zdrowia pacjenta i oceny ryzyka - czy nosicielstwa, czy zakażenia. To może być pomocne, ale na pewno nie jedyne i nie na pierwszym miejscu. Trochę z przekorą to powiem - myślę, że 80% pacjentów ma problem ze sformułowaniem zdania podrzędnie złożonego i zawarcia logicznej treści w taki sposób, aby ją przekazać drugiej osobie. Zacząć i skończyć wypowiedź. Niech to będzie prosta wypowiedź - kilka zdań. To wygląda makabrycznie. Więc też można się domyślać, że używanie tej aplikacji, jej wyniki, nie zawsze, nie u wszystkich byłyby wiarygodne. Interpretacja rzeczywistości przez niektóre osoby jest zaskakująca.

**Jak się panu wydaje, czemu służy ta aplikacja?**

Pomocy. Pomocy ludziom, ułatwieniu uzyskania ważnych informacji, dostępu do nich, w jednym miejscu. To jest wg mnie bardzo mądra idea. Kiedy czegoś szukamy, jeżeli szukam informacji z mojej branży, jestem w stanie szukać ich w sposób przemyślany i celować w konkretne stron internetowe, artykuły. Ale jeśli szukam czegoś spoza swojej branży, tematu, na którym się nie znam i próbuję szukać wyjaśnień, tłumaczenia, rozwiązania problemów, to mam problem z tym, żeby wybrać właściwe informacje. Tego typu aplikacja z założenia powinna nam udostępniać właściwe informacje, sprawdzone, dobre. Jeśli się pod tym Ministerstwo Zdrowia i Inspektorat Sanitarny podpisuje, to należy uważać, że te informacje zawarte w aplikacji są sprawdzone, naukowo udowodnione i rzeczywiście pacjentom pomogą. I te osoby, które mają problem z wyszukaniem odpowiednich aplikacji, najprawdopodobniej w takiej aplikacji najłatwiej by je znalazły.

**Czy ta aplikacja budzi jakieś pana obawy? Poza tym, że technologicznie jest to nie do zrobienia?**

Obawy nie obawy - tak, jak mówiłem, to, co pacjent mówi, i tak weryfikuję jeszcze sam. I to na kilka sposobów. Więc nawet, jeśli ta ankieta byłaby wypełniona niewłaściwie, dla mnie, jako dla lekarza, nie byłby to problem. I tak musiałbym od nowa zrobić swoją. Natomiast dla użytkownika programu nie budzi obaw.

**Jakie informacje o użytkowniku pobiera aplikacja?**

Na podstawie tego, co pani zaprezentowała, wydaje mi się, że jest to tylko ocena stanu zdrowia na podstawie ankiety, a nie zaangażowania innych środków - zdjęć, pomiaru tętna.

**Przekazanie takich danych nie budzi pana obaw, bo jest to inaczej traktowane niż dane w innych aplikacjach?**

Tak.

**Czy rząd powinien tworzyć rozwiązania tego typu, tworzyć takie aplikacje?**

Myślę, że tak. Takie pomocowe aplikacje, jak najbardziej.

**Myśli pan o przyszłości o pandemii?**

Oczywiście.

**Co najbardziej zaprząta pana głowę w tych myślach?**

Na czym się skupiam? Chciałbym pojechać na wakacje. Jeśli chodzi o mnie i moją rodzinę, doszliśmy z żoną do wniosku, że w tym roku chyba się nam to nie uda, uskutecznić tej naszej corocznej aktywności. Przeznaczymy czas i środki na co innego. Boję się też, jak będzie wyglądała nasza gospodarka, o czym nie raz mówiłem. I państwo - cóż nasz rząd jeszcze nawywija, żeby utrzymać władzę. Co oni jeszcze zrobią, wymyślą, zepsują, aby utrzymać się u władzy, a konsekwencje tego będziemy później naprawiać przez dziesiątki lat.

**Te konsekwencje decyzji politycznych budzą pana obawy?**

Tak.

**W związku z przyszłością pana i pana rodziny, ma pan jakieś obawy?**

Raczej nie. Uważam, że szkoły wrócą wcześniej lub później do funkcjonowania. Tu problem raczej nie będzie. To też wiąże się z aktywnością zawodową mojej żony - taką "stacjonarną", czyli na uniwersytecie, a nie internetowo. Kiedy szkoły podstawowe wrócą, wrócą też uczelnie. Uniwersytety są autonomiczne w decyzjach, więc niektóre z nich mogą też zachować się troszkę inaczej niż dyktuje trend ogólny. Mojej zawodowej przyszłości też się nie obawiam. Jestem przekonany, że pacjentów nie zabraknie. Pacjentów zawsze będzie tyle samo, ludzie zawsze będą chorować, cierpieć, pacjentów będzie tyle samo. Specjalność też mam taką, że... Struktura zachorowalności na choroby układu krążenia w Polsce jest taka, że nie muszę się obawiać o pracę. Nie tylko w Polsce zresztą. I prognozy na przyszłość wcale nie są optymistyczne Dalej nie znamy sposobu na leczenie miażdżycy, więc właściwie to jedno już załatwia temat mojej przyszłości. Jeśli chodzi o mój najbliższy świat, który mnie otacza - nie wiem, co będzie z oddziałem, natomiast jeśli chodzi o gabinet - tydzień temu zacząłem przyjmować. Pacjenci są, zapisują się, więc myślę, że to będzie dalej funkcjonowało. Kupiliśmy nowy aparat USG do gabinetu w związku z przenosinami, więc robimy też nowe inwestycje i mamy nadzieję, że będą uzasadnione i w jakiś sposób będziemy mogli je finansować. Więc tyle mogę powiedzieć w temacie przyszłości. Uważam, że należy patrzeć optymistycznie. Ale to z mojego punktu widzenia. Dla ludzi, którzy potracili środki, sposoby na uzyskanie przychodu - potracili hostele, hotele, motele i muszą jakoś sobie z tym radzić, wcale nie jest łatwo. Małe firmy, które musiały wstrzymać produkcję, bo nie było zbytu, nikt tego nie chciał brać. To jest też problem i ci ludzie na pewno też inaczej patrzą w przyszłość. Dla nich to jest wyzwanie teraz.

**Patrząc na sytuację gospodarczą, jak pana zdaniem może ona się teraz zmienić?**

Nie jestem osobą, która zna się na biznesie, finansach, funkcjonowaniu gospodarki. To jest dla mnie obcy temat. Uważam, że większe znaczenie ma to, co państwo zrobiło z funkcjonowaniem gospodarki wcześniej, przed epidemią. Różne zapisy w prawie, rozdawnictwo środków publicznych - niezwiązane z epidemią - ma większe znaczenie, niż ta epidemia. To są ruchy, które trwają od kilku lat i prowadzą do pogarszania się stanu gospodarki. Ostatnio widziałem pomysł powołania jakiegoś konsorcjum sklepów państwowych, w których ceny byłyby ustalane państwowo. To krok jeszcze i będziemy mieli wprowadzoną reglamentację, kartki. Różnego rodzaju produkty, będziemy mieli sklepy Społem i to już chyba kiedyś było i do niczego dobrego nie prowadzi. To pomysły żywcem wzięte sprzed 40 cz 50 lat - przykre. Przykre jest, że po krótkim okresie - tak mi się wydawało - funkcjonowania wolnego handlu, mądrego podejścia do gospodarki, rynku, znowu ktoś zaczyna wprowadzać mechanizmy, które się nie sprawdziły. To jest bardzo świeże. Mamy też przykłady zagraniczne, które pokazują, że takie zachowania nie prowadzą do niczego dobrego. To bardziej budzi moją obawę o przyszłość gospodarki, kraju. Zmiana prawa, konstytucji, takie kształtowanie prawa, które pozwala politykom będącym u władzy wpływać na wszystko. A ludzie przestaną mieć jakiekolwiek mechanizmy obrony i zabezpieczenie przed niekorzystnymi decyzjami rządzących. Bo jak prezydent czy premier powie nam, że tak mamy robić, to tak mamy robić. Sądy uzależnione od polityki, oddalą nasz sprzeciw i sprawy nie będą rozpatrywane. Zresztą, widać to na co dzień już w tej chwili.

**Ma pan jakieś przemyślenia, jak może zmienić się sytuacja społeczna? Czy ta sytuacja może dotknąć jakieś konkretne grupy ludzi?**

To znowu będzie wiązało się z gospodarką. Przedsiębiorcy. Drobni przedsiębiorcy, którzy stracą źródło dochodu, będą w trudnej sytuacji codziennej. Jest całkiem spore grono ludzi, którzy dobrze sobie radzą z dnia na dzień. To nie chodzi o nie wiadomo jakie biznesy i pieniądze, ale starcza im na to, żeby normalnie funkcjonować. Ta sytuacja ostatnio, która doprowadziła część z tych osób do bankructwa, spowoduje, że oni będą mieli problem. To przełoży się na ich funkcjonowanie w społeczeństwie. Też na obowiązki państwa wobec tych osób. Myślę, że sporo się zmieni.

**Czy któreś z ograniczeń powinny zostać utrzymane na dłużej/ na zawsze?**

Na zawsze? Niee, na zawsze nic. Nic na zawsze, o. <śmiech> Na dłużej... Ci pacjenci, którzy trafiają do nas do szpitala, wypełniają ankietę. Taką najprostszą, epidemiologiczną. Czy nie mieli kontaktu z osobą chorą. Tego typu rzeczy powinny zostać. Tak, jak wypełniamy przy przyjęciu do szpitala inne ankiety, ta ankieta powinna być dołączona do standardu, nie powinno się z niej rezygnować.

**Pan planuje utrzymać jakieś zachowania na dłużej? Na przykład noszenie maseczek?**

Absolutnie. Ja noszę maseczki po 6-8 godzin w ciągu dnia, stojąc przy stole operacyjnym. Mam po dziurki w nosie maseczek. Czasem wsiadam do samochodu i zapominam jej ściągnąć, co mnie strasznie denerwuje, bo po 300, czy 500 metrach jazdy samochodem orientuję się, że mam na twarzy maseczkę. Ona jest dla mnie tak niezauważalna z racji tego, że codziennie ją zakładam. Więc denerwuję się, kiedy zapomną o jej zdjęciu, bo jazda samochodem w maseczce to da mnie idiotyzm. Nie planuję utrzymywać żadnych z tych środków. Ręce odkażam spirytusem kilkadziesiąt razy dziennie w pracy. Nie potrzebuję robić tego jeszcze poza pracą. Mam tego po dziurki w nosie. Mam kłopot z utrzymaniem dobrej kondycji rąk przez te środki, a że w szpitalu ciągle są nowe, bo może ten będzie tańszy, bo ktoś inny wygrał przetarg, to też nie można zamówić jednego, tego, który nam najbardziej będzie odpowiadał. Z punktu widzenia naukowego to cały ten bałagan z rękawiczkami w sklepach, to idiotyzm. On w ogóle nie działa. Personel medyczny, który pracuje w rękawiczkach wie, jak je ściągać, ale reszta społeczeństwa nie ma na ten temat zielonego pojęcia. Ściąga te rękawiczki albo wsiada w nich do samochodu - bo widać ludzi jeżdżących w ten sposób. Co robić z tymi zakupami? Wyciąga je pani z koszyka i ma pani je w domu odkażać spirytusem? Każde opakowanie? Worek z mąką? To jest chaos, bałagan.

**Zabezpieczenia w kinach, restauracjach, lotniskach. Co pan sądzi o tego typu rozwiązaniach?**

Bardzo proszę, temperaturę niech mierzą. Jest to sposób stosowany w krajach azjatyckich, gdzie funkcjonują różnego rodzaju inne choroby, infekcje i tam się szuka żółtej febry, innych chorób. Tam kamery termowizyjne są od dawna, to nie jest nic nowego, one są i bardzo dobrze, że są.

**Czyli to jest skuteczna forma zabezpieczenia?**

Raczej bym powiedział, że to jakaś forma zmniejszenia ryzyka, czy zwiększenia prawdopodobieństwa wyłapania osoby zakażonej. Bo gorączkę można mieć z różnych powodów. Gorączkę może nie, ale podwyższoną temperaturę tak. Kwestia czułości urządzeń - to są skanery, urządzenia, które działają w sposób przesiewowy, więc tak naprawdę ta podejrzana osoba jest badana dokładnie dopiero później. Ale uważam, że ten screening, ankiety - można by zostawić. Nie wiadomo, czy wszyscy w ankietach piszą prawdę, ale to akurat jest do sprawdzenia. Kupując bilet, podajemy swoje dane, a one krążą wszędzie. Służby zaangażowane do monitoringu ruchu pasażerskiego - myślę, że stworzą aplikacje, które bardzo ładnie połączą używanie karty kredytowej, logowanie telefonu, zakup biletów lotniczych. Te dane wrzucone w jeden program, kiedy kupimy bilet i za dwa miesiące znajdziemy się na lotnisku, spowodują, że w jednym miejscu będą wszystkie informacje o nas - łącznie z zakupami w kinie, sklepie. Aplikacja - to, o czym rozmawialiśmy przy AI - tak przetworzy dane, że osoba przy odprawie powie, "oj, to pana zaprosimy na bok, bo w ciągu ostatnich miesięcy był pan w Azji, Ameryce pd, Włoszech, Wuhan i jeszcze gdzieś indziej. Pana akurat będziemy musieli przebadać dokładniej". I tyle. Nie będzie do tego potrzebna nasza ankieta, tylko imię, nazwisko, nr paszportu i karta kredytowa.

**Pana zdaniem któreś grupy powinny być chronione szczególnie w kontekście epidemii?**

Wiemy, że osoby w podeszłym wieku i obciążone różnymi chorobami, mogą być bardziej podatne na infekcje i choroba będzie miała u nich cięższy przebieg. Więc można zastanowić się nad tym, czy w jakiś sposób tych ludzi starszych lub mocno schorowanych nie chronić przed kontaktem z otoczeniem. Ale w jaki sposób, żeby nie odebrać tym ludziom godności i nie zabrać im jeszcze - przepraszam, że tak powiem, może źle to zabrzmi, ale - resztek przyjemności z życia? Bo jeśli ktoś ma 80 lat i 10 chorób, które go tam gnębią i odbierają przyjemność z życia, to jeśli zabronimy mu wychodzić na spacer, a on ma swój rytuał i raz dziennie idzie sobie posiedzieć na ławce nad rzeką, a my mu powiemy, że to nie jest dobre miejsce, bo tu za dużo ludzi chodzi i niech pan sobie siądzie na balkonie, to jeszcze dołożymy 11 zgryzotę temu człowiekowi, oprócz 10, które miał do tej pory. Więc nie wiem, jak do tego podejść. Myślę, że psychologia jako nauka, będzie grała tu dużą rolę. Żeby stworzyć sobie taki sposób na radzenie sobie z problemem, żeby ludzi dodatkowo nie pognębiać.

**Słyszał pan o tym, że mówi się o drugiej fali zachorowań.**

Tak

**Co pan o tym myśli?**

Pewnie, że nadejdzie. Będziemy mieli cykliczne zachorowania tak, jak to jest z innymi infekcjami wirusowymi, które pojawiają się w społecznościach w okresie, kiedy populacja ma obniżoną odporność. Tak było i jest.

**Czy rząd powinien wtedy znów wprowadzić lockdown?**

Czy nasze państwo jest stać na to? Robienie niepotrzebnych dużych ruchów będzie pomyłką. Jednak jeśli będzie taka potrzeba, to tak. Zobaczymy, jak będzie wyglądała druga fala. Spodziewamy się tego, że część społeczeństwa przechorowała infekcję, część przeszła bezobjawowo, nabyła odporność. Tak naprawdę nie mamy jeszcze doświadczenia z tym, jak kształtuje się odporność po zachorowaniu, czy to jest stała, czy czasowa odporność, u jakiego odsetka populacji, która miała kontakt z patogenem, odporność się wykształci. To jest wielka niewiadoma, więc myślę, że to jest taki poligon. Dlatego też jestem zdania, że druga fala będzie trudna. Ale zblokowanie znowu państwa może być nie do osiągnięcia w taki bezbolesny sposób. To znów może być coś potężnego i spowoduje jakąś gospodarczą zapaść.

**Pan planuje się jakoś przygotować?**

Oprócz zdrowego trybu życia i normalnego funkcjonowania - nic więcej. Myślę, że odpowiedź na infekcję to jest sprawa naszej odporności. Nie wiemy, jak każdy z nas zareaguje. Część z nas nawet nie wie, że ma choroby współistniejące. Młodzi ludzie chorują, nie wiedząc zupełnie o tym. My się przekonujemy o tym dopiero w takich sytuacjach. Że ktoś chorował, a nie wiedział o tym, nie leczył się. Ja postaram się żyć normalnie, nic nie zmieniać, uważam, że to będzie nieskuteczne. Jakieś próby izolowania się. Ja chodzę do pracy w miejsce, gdzie nie mam szans izolować się od ludzi. Noszenie maseczki przed niczym mnie nie zabezpieczy. Jest ono uzasadnione w trakcie zabiegu chirurgicznego. I chroni przed bakteriami, a nie wirusami. Bakterie są większe, niż wirusy. Mogę powiedzieć tak, jak już powiedział niejeden z naukowców - że wszyscy przechorujemy, albo prawie wszyscy. Nieliczni przechorują w sposób ciężki. Część będzie miała takie objawy, których nawet nie skojarzymy z infekcją. Mam przykład pośród moich znajomych - jedna z moich koleżanek, która przebyła infekcję, miała napady migreny. Chyba dwa. I to było wszystko. Jest lekarzem, pracuje, miała zrobiony test i wyszedł pozytywny. Kwarantanna 14 dni, a nie miała żadnych innych objawów. Jej komentarz był taki, że dostała nieplanowany urlop, który spożytkowała, bo ani nie była osłabiona, ani nie miała żadnych innych objawów. Więc tak to może wyglądać, że będą nas boleć kolana przez trzy dni, później to ustąpi, a po badaniach okaże się, że przeszliśmy infekcję koronawirusa.

**Ważne momenty**

**Jakie istotne momenty, jakie pan zapamiętał? Z perspektywy osobistej.**

Zakaz przemieszczania się. Nie było bardzo skrupulatnego, ale było ograniczenie przemieszczania. Nasz nieudany wyjazd zimowy w Bieszczady - to był dla mnie start epidemii w takim osobistym odczuciu. Później były zmiany w pracy, tuż za nimi zamknięcie szkół, ale też uczelni wyższych. Żona wtedy pojechała i wróciła właściwie po jednym dniu, bo zamknięto uniwersytet. Później były zmiany w mojej pracy, związane z zachowaniami dyrekcji. Teraz wiadomo - ważny powrót mojej pracy do funkcjonowania w takim wymiarze, jak było to przed epidemią. I możliwość - wreszcie, o to jeszcze było przed moją pracą - uprawiania sportów. To jest ważne. I teraz przywrócenie działania obiektów sportowych, więc aktywności już nie tylko na zewnątrz. Mój syn może teraz pojechać na tenisa - akurat te ciekawsze treningi ma w hali. Ważne też, że my w końcu mogliśmy zacząć jeździć na rowerze. I nie jest to przejaw jakiegoś cwaniactwa, czy robienia na złość władzy, a normalna aktywność, akceptowana przez obecne przepisy prawne. I chyba tyle przychodzi mi do głowy. To bardzo ważne, bo w końcu zaczęliśmy całą rodziną jeździć na rowerze, jak chcieliśmy wcześniej.

**Z perspektywy kraju**

Jest ich całe mnóstwo, tylko jak je uszeregować. Myślę, że najbardziej dotkliwe to było zamknięcie obiektów użyteczności publicznej - restauracji, barów, kawiarni, obiektów sportowych. Tych obiektów, które wiążą się z aktywnością poza zawodową. Na terenie całego kraju, bo nigdzie nie można było z tego korzystać. Zmiany, które się też przekładają na kondycję firm, przedsiębiorstw. Ludzie potracili hotele, hostele. Bałagan wyborczy - coś strasznego. Podejrzewam, że gdybyśmy nie mieli epidemii, mielibyśmy normalną kampanię wyborczą. Jakie by były tego skutki - nie wnikam. Natomiast to, co obóz rządzący wyprawiał w związku z planowanymi wyborami, to jest karygodne. Oni zmarnowali mnóstwo pieniędzy na wybory, które nie doszły do skutku. Pieniędzy, które przydałyby się w innym miejscu. Jakimkolwiek, nie koniecznie związanym z epidemią. Organizacja wyborów była łamaniem prawa. Prawa, które wszyscy znamy, oczywiście nie szczegółowo, ale ja też wiem, że wybory mają być powszechne, ogólnodostępne, itd. Te prawa nie były zapewnione ludziom. Było na poczekaniu wymyślanie jakichś dziwnych mechanizmów, które miały pomóc utrzymać władzę obecnej ekipie. Na szczęście to nie wypaliło, natomiast nikt nie wyciąga teraz odpowiedzialności za całe to zło i decyzje, które były łamaniem prawa. Powinny być osoby pociągnięte do odpowiedzialności, a nikt nie odpowie za marnowanie pieniędzy społecznych. Ważny moment to też powrót do funkcjonowania galerii handlowych i dużych sklepów. Może ja z tego tak nie korzystałem, ale jest to uciążliwość. Czasem było mi to potrzebne. Żeby obejrzeć jakiś produkt w sklepie, nie tylko w internecie. Obiekty sportowe - to pewnie też ruszy, choć na razie tego nie widać. A, zapomniałem powiedzieć o jednej rzeczy. Dostałem pożyczkę dla mikroprzedsiębiorców. To był bardzo krótki proces. Zgodnie z przepisami ona mi się należała, ale nie wszystko, co się należy, się dostaje. A tutaj - dostałem po kilkunastu dniach. Nie są to duże sumy, ale dlaczegóż by z tego nie skorzystać? Akurat uważam, że moja praktyka ucierpiała na tym dość poważnie. Myślę, że 40% dochodu mniej to całkiem dużo, choć niektórzy tracili więcej. O tym nie powiedziałem, to mi umknęło. Cieszę się, że mam te pieniądze na koncie, tym jestem pozytywnie zaskoczony.
