# Supplementary material for: The challenges arising from the COVID-19 pandemic and the way people deal with them. A qualitative longitudinal study
Source: PLoS One. 2021 Oct 11;16(10):e0258133. doi: 10.1371/journal.pone.0258133 (PMC8504766; doi:10.1371/journal.pone.0258133)
Supplement: S1 Dataset — (ZIP) [file pone.0258133.s003.zip › Transcriptions/stage 5/2.5_F_27_single.docx]

**2.5_F_27_single**

**Jak ci minął ten ostatni miesiąc?**

Tak jakby nigdy nie było koronawirusa. Ale nie, właśnie chciałam ci się pochwalić, że usłyszałam o osobie, która się naprawdę bała. Nie wiedziałam, że takie istnieją, nie zdawałam sobie z tego sprawy. Ale dowiedziałam się o takiej osobie, która się naprawdę bała. I ona się do tego stopnia bała, że jeżeli wychodziła z domu, to myła się cała przedtem, zakładała nowe ubrania, wychodziła do sklepu, ale też raz na 2 tygodnie. Oblewała się płynem takim antybakteryjnym, potem wracała, wrzucała ubrania prosto do prania. Przebierała się, myła się. I jedzenie zamawiała sobie przez Ubera albo przez jakiegoś tam Volta, ale raz na 2-3 dni.

**I co sobie pomyślałaś wtedy?**

To jest niesamowite. Nie umiałabym tak żyć w ogóle. Już abstrahując od wszystkiego, nie wiem, jak strach mógłby wpłynąć na mnie aż tak, żebym w taki sposób się zachowywała. Nie umiałabym tak żyć. Zdziwiłam się niesamowicie.

**Ale zdziwiłaś się, bo w ogóle takie zachowanie jest dziwne, czy u tej osobie było dziwne?**

Nie, nie znam tej osoby, to jest osoba z pracy. Wiem kim jest, oczywiście rozmawiałam z nią kilka razy. Ale generalnie dziwne dla osób, dla ludzi w ogóle.

**Że można tak reagować.**

Tak. I to było takie pierwsze zderzenie.

**A u ciebie było tak, jakby nie było koronawirusa?**

To znaczy no wiesz, to był miesiąc, więc na początku jeszcze było to chodzenie w maseczce i jakieś takie uważniejsze przestrzeganie zasad. No takich społecznie ustalonych, nie jakichś wewnętrznych moich. Ale z każdym zdjęciem tych obostrzeń coraz mniej w ogóle o tym myślałam. Mogę ci powiedzieć, że w tym momencie nie mam pojęcia, co się dzieje w koronawirusem w Polsce. Nie wiem, po prostu nie wiem, nie widzę tego, nie interesuję się tym tematem za bardzo. No ja już chodziłam do biura jak rozmawiałyśmy ostatnio razem. I teraz chodzę tak samo. Ale nie dlatego, że musimy, nie musimy. Tylko dlatego, że nie chce mi się już siedzieć w domu trochę. No zresztą taka rutyna wstawanie rano i chodzenie do biura dobrze mi robi. Zresztą, jak siedzimy we dwie w biurze, bo można do nas przychodzić, w biurze może być nas 25 osób, a jesteśmy we dwie na piętrze. I dodatkowe dwie są zawsze chyba też na innym piętrze. Ale inne osoby się pojawiają tylko jak muszą, albo jak chcą się skupić, a coś im przeszkadza. Ale maksymalnie w biurze było 7 osób może. Więc jeśli chodzi o taką normalność, to w moim życiu jest. Ale jeśli chodzi o resztę społeczeństwa, to widzę, że nie do końca.

**A kiedy w twoim odczuciu pojawiła się taka już twoja normalność?**

Myślę, że wtedy, kiedy otwarto restauracje. I też chyba rozmawiałyśmy o tym, czy pójdę, czy pierwsze co będę wyczekiwać. I chyba nie aż tak ja wyczekiwałam, jak moi znajomi z pracy, którzy mówili: dobrze, to idziemy do Dzika koniecznie, do Dzika, bo już można. I poszliśmy do tego Dzika. Ale to pierwsze wyjście do Dzika, bo byłam w Dziku 2 razy od tamtej pory. Pierwsze wyjście do Dzika było takie bardzo zgodne z zasadami, dlatego, że… Chyba nikt, nie, trochę osób tańczyło, ale to było tak bardzo… I to było wszystko w ogródku, nie było tej części wewnątrz otwartej. Tam trochę osób tańczyło, ale my nie tańczyliśmy, tylko było bardziej jedzenie, rozmawianie i picie alkoholu niż jakaś tam zabawa typowo jak w Dziku. Ale byłam w Dziku w sobotę. I było to tak, jakby koronawirus nigdy nie istniał i w ogóle nikt nie wiedział, kto to jest. Wydaje mi się, że ktoś po prostu w pewnym momencie przestał kontrolować ilość wchodzących osób. I tych osób było bardzo dużo. Bardzo dużo. Ale czy się bałam, czy coś takiego, nie, absolutnie nie. Tylko po prostu sobie pomyślałam, że fajnie.

**Że fajnie, że w końcu.**

Tak.

**Mówisz, że jak obserwujesz, to ty już wróciłaś do normalności, ale ludzie jeszcze nie do końca. Czego brakuje, żeby ludzie, tak jak ty ich obserwujesz, to czego ci brakuje w ich zachowaniu?**

Wiesz co, tak właściwie powiedziałam, że ludzie inni, ale nie wiem, czy ludzie inni. Wiem, że ludzie w mojej pracy, którzy nie przychodzą do tej pracy. Nie mogę zrozumieć tego, czemu. Bo zwalałabym to na lenistwo, w sensie no może wygodniej jest komuś pracować z domu, jak nie musi, to tego nie robi. Ale słyszę, że ludzie mówią, że na przykład widzieli w biurze, że ktoś był, a ta osoba często wychodzi sobie, gdzieś tam sobie na Instagramie widzą, że ta osoba gdzieś wychodzi, więc oni nie chcą przychodzić do biura, bo się boją, że spotkają tę osobę i ta osoba mogła się zarazić. Więc to nie jest do końca lenistwo, tylko to też są jakieś obawy.

**Czyli oni mają potrzebę takiej samoizolacji jeszcze?**

Właśnie tak. I wydaje mi się, że będzie w 100% dla mnie normalnie, jeżeli ci ludzie po prostu zaczną się pojawiać w biurze. I nie mówię o 5 dodatkowych osobach, tylko normalnie, przynajmniej 20 osób przecież mogłoby przyjść do tego biura.

**Żeby ten limit osiągnąć, co?**

Tak, no coś takiego. Żeby było widać, że to biuro to nie jesteśmy tylko we dwie i możemy, które sobie siedzimy i możemy na głos rozmawiać o wszystkim, tylko jeszcze jacyś ludzie pracujący.

**Czyli pracowo jeszcze ty już wracasz do normalności, koleżanki, koledzy jeszcze nie. Jakie jeszcze obszary, takie masz poczucie, że twoje wróciły do normalności?**

No to właśnie wychodzenie sobie gdzieś po prostu, żeby sobie posiedzieć w miejscu publicznym, a nie w domu?

**Gdzie byłaś? Jakie takie fajne sobie wyjścia zrobiłaś, że myślisz sobie, brakowało mi tego i to było fajne.**

Nie myślałam, że mi tego brakowało, bo właściwie nigdy wcześniej tego nie robiłam, o czym teraz powiem. Ale byłam w takiej… Magda z mojej pracy chodzi do takiej restauracji, która jest przy jej domu. I przez to, że jest przy jej domu, to ona tam często chodzi i się poznała z tymi właścicielami, którzy są właściwie w naszym wieku. I oni tak sobie we dwójkę to prowadzą bardzo luźno. I ona tam chodziła dość często. I kiedyś po pracy się zapytała, jak już otworzyli, czy ja nie chcę iść z nią. No i poszłam i byłam tam przez ten miesiąc może z 4-5 razy, nawet może więcej. Też oni robią co niedzielę odkąd można, takiego grilla. Gdzie wystawiają po prostu grilla na ten ogródek. I na tym grillu było ze 30-40 osób. I dzieci normalnie biegały, nikt nie miał maseczki. Znaczy to był ogródek, więc nie trzeba było. Ale też było bardzo normalnie. Więc tak, takie rzeczy robiłam. I byłam tam też nie na tym grillu, ale… I chyba z restauracji to byłam tylko tam, no i byłam też w tym Dziku.

**A zakupy?**

Nie byłam w centrum handlowym, w dalszym ciągu nie udało mi się tam pojawić, nie miałam takiej potrzeby. Niestety już przestaliśmy się wybierać na wycieczki do sklepów.

**Czemu?**

Bo wiem, że nie trzeba stać w kolejce ani nigdzie indziej do sklepów bliżej. No nie wiem, nie chce nam się robić jakichś zapasów. Trochę też się nie zgrywaliśmy, dużo czasu też spędzaliśmy osobno, a samej mi się nie chciało. Nie wiem, chętnie bym się wybrała, ale jakoś nie mam potrzeby.

**Czyli wróciłaś do swojego stylu zakupów, blisko, w Żabce, w Biedronce?**

Tak, wróciłam, ale raczej zostało mi to narzucone. Myślę, że jakby Filip chętnie chciał tam pojechać, to bym jechała z nim.

**Czyli nie masz towarzysza do tych wyjazdów swoich?**

Myślę, że tak.

**A miałaś jakieś plany, co jeszcze chcesz odwiedzić sklepowo, gdzie byś jeszcze pojechała?**

Nie, no ja lubiłam po prostu jeździć do tych sklepów, chętnie bym pojechała do każdego, w którym byłam już kiedyś.

**Co jeszcze, masz takie poczucie, że jest normalnie? Już możesz sobie wyjść, kiedy masz ochotę.**

Widziałam się z mamą. Niestety już teraz nie dała się nabrać na to, że nie można się spotykać. I chciała, żebym się spotkała z całą rodziną.

**No i co?**

Ale powiedziałam, że nie można. Nie, no musimy się umówić, ale jeszcze nie teraz.

**Czyli na razie tylko z mamą?**

Tak.

**A dlaczego tak bardzo mama nalega, żebym się spotkała z całą rodziną?**

Bo lubi spędzać ze mną czas i wie, że oni lubią ze mną. Ja też całkiem z nimi lubię, ale jakoś… No jak mam do wyboru robić coś innego niż spotkać się z moim bratem i moim tatą, to wolę robić coś innego.

**A jak się spotykacie, to jecie obiad, gadacie, jak wyglądają te rodzinne spotkania?**

Nie wiem, no to one nie są częste, właśnie są za każdym razem inne. Chyba mi najbardziej przeszkadza po prostu dojechanie tam.

**A to daleko?**

Nie (śmiech). Nie, właśnie nie, dlatego to jest jeszcze gorsze. Nie, powinnam się z nimi spotkać, ale jakoś tak... Ale zdarzało mi się z nimi rozmawiać na Facetime, więc…

**Coś jeszcze z takich normalnych rzeczy, w sensie takiej codzienności już wróciło do normy?**

No, tak jak mówię, wydaje mi się, że wszystko. Wstaję rano, idę do pracy. Ale też mam poczucie takie, że trochę takich wakacji. Dlatego, że nie śpieszę się, żeby był na 9. Prawdopodobnie nigdy nie musiałam się śpieszyć, ale miałam poczucie, że muszę. A teraz jak dzisiaj weszłam za dziesięć 9, to nie wiem, nikt nie zauważył, być może dlatego, że byłam jedyna w biurze wtedy jeszcze. Ale właśnie i tak jest troszeczkę bardziej tak na luzie. Nie muszę się zbierać szybko, żeby wstać, nie muszę się śpieszyć. I to jest fajne. Ale też sobie zdaję sprawę z tego, że przez ten czas sobie pozwalałam dużo więcej niż pozwalam sobie w normalnym życiu. I chciałabym trochę przestać. W sensie bardziej uważać na to, co jem, mniej wydawać pieniądze na głupoty. I jakoś tak się bardziej zdyscyplinować.

**Dobra. Czyli największe grzechy okresu koronawirusowego twoje. Jedzenie byłoby w takich grzechach?**

Tak.

**Czego najbardziej żałujesz?**

Nie, no absolutnie niczego nie żałuję, bo nie zrobiłam nic, czego mogłabym żałować.

**To co chciałabyś zmienić, do czego chcesz wrócić?**

Jadać bardziej regularnie i zwracać uwagę na to, co jem. Nie wiem, dlaczego nie zwracałam wcześniej uwagi na to. Znaczy po prostu myślałam, że wakacje są, więc można jeść codziennie pizzę albo zamówić sobie to, na co się ma ochotę. Jakoś tak dawałam sobie przyzwolenie na to, a teraz już chciałabym jednak… Znaczy nie chciałabym, bo przyjemnie jest tak, ale wiem, że już powinnam.

**Czyli wiesz, że już powinnaś do tego swojego stylu jedzenia, który był przed. On był dla ciebie lepszy.**

Tak.

**Co jeszcze?**

No też wydaje mi się, że mogłabym jakoś tak bardziej kontrolować swoje wydatki. I to nie jest tak, że kupowałam coś specjalnego, chociaż kupiłam ten dywan, o którym ci mówiłam. Ale zapłaciłam za niego 70 zł, więc to był super zakup. Ale to nie chodzi o konkretne rzeczy, tylko na przykład kupowałam sobie wszystko, na co miałam ochotę w Żabce, dlatego że… Nie wiem, ale miałam poczucie, że… Nie wiem, nie wiem, jakie miałam poczucie, po prostu chciałam sobie sprawić jakąś przyjemność i kupowałam rzeczy, których nie potrzebuję i nawet nie mam jakiejś wyjątkowej ochoty. Tylko po prostu jest w sklepie, więc sobie spróbuję.

**Jakieś jeszcze rzeczy, które myślisz sobie, że tak z perspektywy czasu to już trzeba je zakończyć?**

Wydaje mi się, że więcej palę niż paliłam. I postanowiłam sobie, że będę paliła, dopiero pierwszego papierosa mogę zapalić o 17-tej, jak wrócimy już do biura. Tylko, że właśnie nie wiem, kiedy jeszcze wrócimy do biura, bo ja wróciłam, ale nikt inny nie wrócił, więc…

**Ale nie ma biura.**

Tak, no właśnie. Dlatego jeszcze nie wiem. Nie wiem, czy to już.

**Czyli nadal palisz od rana?**

Nie, no to nie jest od rana, to jest od dwunastej. Coś takiego. Ale palę więcej na pewno niż paliłam przed koronawirusem. Czy to jest związane z tym, nie mam pojęcia. Ale wydaje mi się, że po prostu sobie na dużo więcej pozwalałam, dlatego że…. Było coś beznadziejnego w życiu, więc jakoś sobie chciałam poprawić nastrój. Albo na przykład nie uważałam, że trzeba oszczędzać, bo i tak mam mało pieniędzy, więc mogę je wydawać na co chcę. Nie mało, ale mam mniej pieniędzy, więc mogę sobie… Że to nie jest okres na oszczędzanie, tylko to jest okres na wydawanie.

**Jedzenie, kontrola wydatków, palenie fajek, co jeszcze? Jest jeszcze coś?**

Nie. Myślę, że nie.

**A weź mi opowiedz o tym pierwszy wyjściu do Dzika, jak to było?**

Chyba się dogadali… Jakoś, jak tylko powstało wydarzenie na Facebooku, że się idzie do Dzika, to można iść do Dzika. To chyba Patrycja zadzwoniła, zrobiła rezerwację. I po prostu tam poszliśmy.

**A było inaczej niż wcześniej?**

Niż wcześniej w Dziku?

**Tak.**

No jasne, bo teraz to było pójście na obiad do Dzika, co nie było nigdy wcześniej.

**Czyli takie celebrowanie w ogóle tego powrotu.**

Nie, po prostu nie (niezrozumiałe), nie było nawet warunków do tego, żeby (niezrozumiałe), tylko było tak bardziej stolikowo. Siedząca to była impreza. I wydarzenie było napisane, że to w ogóle w takim będzie raczej klimacie. Nie spodziewaliśmy się niczego innego. Tam troszkę tańczyli, bo był tam DJ, to byliśmy zdziwieni.

**Czyli normalnie nie chodziliście na obiad do Dzika, teraz poszliście sobie zjeść.**

Normalnie to chodziliśmy do Dzika o 23 i wychodziliśmy o 5. A teraz się umówiliśmy o 19 (niezrozumiałe).

**No i co?**

No i też nie było żadnego befora przez tym Dzikiem, tylko po prostu każdy sobie z domy wyszedł i pojechał taksówką grzecznie do Dzika. Zjedliśmy tam, wypiliśmy piwko no i poszliśmy do domu.

**Czyli takie zupełnie inne wyjście niż normalnie.**

Tak. Zupełnie inne niż normalnie. Ale przez to, że byliśmy tam i zobaczyliśmy się razem po jakimś tam dłuższym czasie nie w biurze, no to było miło.

**A jakieś jeszcze wyjścia? Gdzie jeszcze była poza Dzikiem i tą knajpą tych znajomych?**

Byłam po Dziku jeszcze na piwie na plaży. Na plaży Żoliborz. I tam też nie było koronawirusa już w ogóle. Mimo, że to było dzień po Dziku, w którym jeszcze troszeczkę tam koronawirus panował. W sensie mówię tak mentalnie. Mimo, że też, no już… Na tej plaży w ogóle nie było żadnego koronawirusa.

**A na czym polegało to panowanie koronawirusa w Dziku?**

Że na przykład ludzie ostrożnie do siebie pochodzą albo chodzą w maseczce. Albo nie wiem, nie piją picia z jednej szklanki albo piwa z jednej butelki. Albo nie robią grilla na plaży, bo nie można tego robić (śmiech). Bo w tym Dziku to jeszcze były jakieś tam zasady, zachowania ustalone przez kogoś. Ale na tej plaży nikt nie pilnował, więc nie było żadnej zasady. Wyglądało to tak, jak bycie na plaży każdego innego dnia.

**Gdzieś jeszcze byłaś? A byłaś gdzieś w takim zamkniętym miejscu?**

Nie, ale ja nie chodziłam nigdy w takie miejsca chybam, więc nie czułam też takiej potrzeby. Nie. Ale zastanawiam się, co robiłam w jakieś pozostałe weekendy. Bo to był jeden, który ci opowiedziałam, w inny był Dzik. Nie, może siedziałam w domu, ale na pewno nie byłam… Zapamiętałabym, jeżeli byłabym w jakimś lokalu.

**Zakupy to powiedziałaś, że trochę wracasz do tego, jak to było…**

Tak, ale ubolewam nad tym. Wydaje mi się, że to był rozsądny w ogóle sposób robienia zakupów, planowanie mniej więcej, co się będzie jadło przez cały tydzień i robienia zakupów pod to.

**OK, czyli to, żebyście tak chodzili po tym sklepie i wybierali, myśląc to zjemy jutro, to zjemy pojutrze to było mądrzejsze niż to, jak teraz działacie?**

Tak.

**A jak byś teraz określiła swój styl zakupów?**

Nie robię żadnych zakupów (śmiech), właśnie chyba to mi najbardziej przeszkadza. Byłam na zakupach w sobotę rano. I kupiłam tylko rzeczy, które chciałam zjeść… Nie, chciałam zjeść coś na śniadanie, ale przypomniało mi się, że mam to w domu i nie kupiłam w końcu nic specjalnego. I nie jadłam w domu w sumie w sobotę obiadu ani nie jadłam go też w niedzielę. I do dzisiaj od soboty nie jadłam w domu obiadu.

**To co jadasz teraz? Wróciłaś totalnie do zamawiania?**

W sobotę byłam w tej knajpie, o której rozmawiałyśmy, jadłam tam. W niedzielę byłam u Magdy i zamówiłyśmy. I w poniedziałek i wtorek też.

**Czyli wróciłaś do swojego stylu zamawiania jedzenia w pracy.**

Tak, ale niechętnie, bo właśnie już teraz mam taką refleksję, z Filipem o tym rozmawialiśmy, że jak byliśmy dziećmi i rodzice zamawiali jedzenie, znaczy no dziećmi… Że to było jakieś święto, takie zamawianie jedzenia i się naprawdę czekało albo można było na przykład dyskutować, na co się ma ochotę. A teraz to jest jakiś taki przykry obowiązek, że po prostu trzeba coś zjeść. I na przykład, jeżeli pracujemy z domu, to nie jesteśmy w stanie wyjść, żeby zrobić zakupy, więc trzeba było o tym pomyśleć wcześniej. A jeżeli o tym wcześniej nie pomyśleliśmy, to nie ma. I trzeba zdecydować spośród rzeczy, na które może się mieć ochotę. Co w ogóle dla mnie 10 lat temu było nie do pomyślenia, że patrzę na te rzeczy i nie mam na nic ochoty. A teraz właśnie na nic nie mam ochoty i to jest takie jedzenie, bo się musi jeść. I strasznie mi jest z tego powodu przykro, że to tak spowszechniało. Bo kiedyś to był fajny sposób celebrowania różnych rzeczy.

**A teraz już nie ma takiego sposobu, już nie możecie uczcić jedzeniem, zamawianiem jedzenia.**

No teraz z Filipem, jak coś chcemy uczcić, to sobie coś robimy do jedzenia.

**A myślisz o tym? Bo już nie pamiętam, na którym spotkaniu, chyba na trzecim mówiłaś, że może będziesz gotować do pracy. Był taki moment, że powiedziałaś, że może trzeba będzie gotować. Przeszło ci to przez myśl znowu?**

Wiem, że będę musiała to robić. Umówiłyśmy się nawet z Magdą. Tylko Magda się teraz przeprowadza i ona jeszcze nie mieszka w swoim mieszkaniu. Nie wiem, czego to jest wyznacznik tak naprawdę, nie pytałam się, jej, ale może gotować tak samo w tym mieszkaniu, jak w tym co ma. Ale powiedziała, że jak się przeprowadzi, to wtedy będzie gotować. I ja wtedy powiedziałam, że mogę gotować. A ona się przeprowadza 1 lipca, więc mogę poczekać.

**I jaki wtedy jest plan?**

No, że będę gotować. Nie, tak naprawdę to myślę o tym. I sobie tak czasem chodzę po tej kuchni i sobie planuję już teraz, co ja będę jadła w te dni, bo nie mam żadnego pomysłu.

**To jeszcze masz dużo czasu do 1 lipca, żeby wymyślić, co będziesz jadła.**

No tak, ale mam też całe życie do gotowania tych rzeczy, więc chyba jeszcze sporo muszę wymyślić.

**Czy jeszcze coś, jakieś miejsca, gdzie się pojawiłaś, bo wcześniej nie chodziłaś?**

Nie, nie było takich miejsc. Ale też mogę powiedzieć, nie wiem, na ile to jest związane. Bo teraz trzeba chodzić w maseczce w sklepach, w metrze. I do miejsc, które mam pod domem nie chodzę w maseczce, na przykład do Żabki. I zdarzyła mi się taka sytuacja, że, ja zawsze noszę tą maseczkę w kieszeni na wszelki wypadek. Tylko że tak jak sobie myślę, że to uważam, że jest to głupie, nosić maseczkę, którą noszę w kieszeni, dlatego dotykam rękami tyle rzeczy, nawet przez przypadek, że jeżeli wezmę tę maseczkę i zacznę ją dotykać, a potem ją wezmę do twarzy, to nie ma to najmniejszego sensu. W kieszeni mam też inne rzeczy, które też mogą być brudne. Więc już nawet nie chodzi o tego koronawirusa, tylko generalnie brud, którzy przykładam do twarzy, nie chcę tego robić. Dlatego staram się tego nie robić, chyba, że ktoś mi zwróci uwagę. I właśnie weszłam do Żabki, w której znam tego pana, codziennie go prawie widzę. I on mówi: a maseczka? Ja mówię, już wyciągam. Nie, no żartowałem przecież, co pani (śmiech). I tak sobie pomyślałam, że to w ogóle nie jest śmieszne, tylko jakieś zasady, których trzeba przestrzegać.

**Myślisz, że dobrze, że on tak jakby… Że on pozwala na chodzenie bez tych maseczek?**

No nie wiem, wyciągnęliśmy z tego wniosek taki, że jeżeli którekolwiek z nas by miało koronawirusa albo jego pracownicy albo ja albo Filip, gdybyśmy i tak mieli, bo widywaliśmy się podczas kwarantanny tej takiej zamkniętej sporo. Ale czy dobrze, że się śmiejemy? To jest jego sklep, więc on się powinien raczej przejmować tym.

**Na paznokcie chodziłaś w trakcie całości. A poszłaś w końcu do fryzjera?**

Nie. Już przestałam mieć ochotę.

**Już odeszło.**

Tak.

**Bo mówiłaś w pewnym momencie, że tych fryzjerów, fryzjerów. I żadnej zmiany na głowie.**

Nie, żadnej zmiany na głowie. Ale na paznokciach byłam parę razy od tamtej pory.

**OK, bo to pamiętam, że chodzisz regularnie, zapisujesz się od razu. A właśnie, jak wasze ćwiczenia?**

Filip ćwiczy do teraz, ja nie. To znaczy zmuszałam się przez pierwszy tydzień chyba nawet. Ale potem sobie uświadomiłam, że mi to sprawia straszną nieprzyjemność. I wstaję rano i myślę o tym, że ja muszę ćwiczyć. Bo kolejnego dnia mieliśmy wstać o 7 chyba rano i robić te ćwiczenia. I wstaliśmy i robiliśmy te ćwiczenia. I ja przez cały dzień myślałam, że to jest beznadziejne, że w ogóle nie chciałam tego robić i się musiałam zmuszać. I przez parę dni kolejnych też to robiłam. I sobie wstawałam rano i sobie myślałam, że tego nienawidzę. Więc w pewnym momencie po prostu uznałam, że skoro to mi sprawia taką nieprzyjemność, to ja nie chcę tego robić. I jeżeli ma mnie to stresować albo sobie myśleć w pracy, o Jezu, jeszcze muszę wrócić do domu i ćwiczyć, to po prostu uznałam, że tego nie będę robić.

**A na przykład kino? Tęsknisz za kinem, chodziłaś do kina wcześniej?**

Nie, nie chodziłam do kina i nie tęsknię za kinem. Filip chyba dość często chodził do kina. Ale on też jeszcze nie był w kinie. Nawet nie jestem pewna, czy można chodzić do kina szczerze mówiąc.

**No coś tam otworzyli, ale nie wiem, na jakich zasadach.**

Więc to akurat nie, absolutnie nie. No nie.

**Emocje – zdjęcia. Jest tu taki, który pasuje do tego, jak się czujesz teraz?**

Nie… Ja jeszcze mogę szybko powiedzieć, to nie jest temat obrazków, ale wybieram chyba 1 bo…

**To powiedz, co chciałaś szybko powiedzieć.**

Bo mówiłam ci, że się boje, nawet nie tyle powrotu do życia, że się będę mogła jakoś zarazić, tylko bałam się tego, że nie będę umiała z ludźmi w normalny sposób rozmawiać. Albo że będzie jakoś dziwnie po takiej rozłące długie. Albo że trzeba będzie zachowywać jakieś tam zasady. To bezpodstawne były moje obawy, bo w ogóle całkowicie jest normalnie. I może to jest kwestia ludzi, z którymi się spotkałam, ale zupełnie normalnie, więc cieszę się, że tak wyszło. A obrazek wybieram 1.

**Czyli co to jest, jak byś to opisała?**

Jakiś taki normalny dzień w jakimś tam mieście.

**Czyli to jest normalność.**

Tak. Teraz często jeżdżę sobie metrem do pracy. I w tym metrze już jest dużo ludzi. Już nie jest tak, jak było 3 tygodnie temu. Tylko zdarza się, że muszę się rozglądać za tym, żeby gdzieś usiąść.

**A czemu się zdecydowałaś na metro? Bo wcześniej jeździłaś taksówką.**

No, ale ile można wydawać pieniądze na taksówki? O ile to jest za służbowe pieniądze, to mogę sobie jeździć, ale jeżeli… Nawet pewnie ja bym swoich pieniędzy nie oszczędzała aż tak, ale przez to, że Magda kupiła mieszkanie i teraz je remontuje i to ona wprowadza jakąś taką atmosferę oszczędzania pieniędzy. Więc jeżeli ona wraca pieszo do domu, to ja mogę wrócić metrem. Jak byśmy wracały razem, no to OK, ale sporo… Poza tym spoko jest tym metrem, naprawdę. Ile się trzeba wkurzać, żeby ten pan z Ubera przyjechał, czekać. A my do metra mamy, okazuje się bardzo blisko. I z metra też blisko do pracy. Wiec zajmuje mi to 14 minut może dojechanie tym metrem.

**Czyli właściwie czasowo prawie tyle samo?**

Tak.

**Powiedziałaś, że jest ta jedna osoba, która się strasznie bała i to cię bardzo zaskoczyło. A jak inni? A jak Filip się odnajduje? Ma jeszcze jakieś wyzwania?**

No wyzwanie Moniki Kołakowskiej trzydziestodniowe, które sobie ćwiczy. Wyzwanie ma też takie, że awansował, więc… Awansował, a właściwie jego teamleaderka jest w ciąży i odchodzi już właśnie. Więc on jest temleaderem, więc wyzwanie ma takie, że ma dużo pracy strasznie. I to jest jego wyzwaniem. I był teraz tydzień nad morzem.

**A rodzice wrócili już do normalnego funkcjonowania?**

Nie, moja mama nie wróciła, ale mój tata wrócił. A mój brat nie wrócił.

**A z czego to wynika, że twoja mama nie wróciła?**

Bo w mojej mamy pracy nie pozwolili jeszcze wrócić.

**Czyli to nie jest jej decyzja.**

Nie, ale rozmawiałam z nią o tym, że nawet, jeżeli… bo może ktoś może wrócić. Ale powiedziała, że ona i tak by nie chciała wrócić. Tylko i wyłącznie przez wygodę.

**Bo jest jej łatwiej w domu.**

Tak. Ona już szykuje sobie na działce stanowisko i zamierza tam pracować całe wakacje.

**Czyli wygoda bierze górę.**

Myślę, że tak.

**A poza tą osobą, o której mi opowiadałaś, znaczy jeszcze kogoś, kto sobie w tym momencie gorzej radzi? W sensie ma takie duże wątpliwości albo duże obawy?**

Nie. Ale ja nie znam takiej osoby, która miała wcześniej.

**A jak tam ta twoja Basia, szefowa?**

Basia szefowa, wyhaczyłam, że ona zaczęła wychodzić z domu. I mimo, że nie przychodzi do biura, to na przykład wychodziła na terapię. To też jest jej koleżanka, więc trochę inaczej, ale do niej do domu, więc była tam, zauważyłam to. Ale nie przychodzi do biura. Ale też robiła urodziny na 50 osób.

**I co sobie myślisz o niej, o jakby takim jej podejściu?**

Znaczy ja wiem, dlaczego ona nie przychodzi do biura i doskonale to rozumiem. Ona mi powiedziała, że będzie przychodziła do biura, kiedy będzie miała się z kim spotkać w tym biurze. Ale jeżeli ona ma siedzieć tam ze mną, no to również dobrze może pracować z domu. Bo większość jej czasu w pracy, ona się spotyka z ludźmi i z nimi rozmawia. Albo z jakimiś ludźmi od nas z biura albo z biurami w innych krajach na Zoomie. Ale to jest tylko tyle. A jeżeli ona ma się z nimi spotykać na Zoomie w biurze, to może też siedzieć w domu po prostu. Ona jest gotowa wrócić do biura w każdym momencie.

**A gdyby ona zaczęła przychodzić do biura, to ludzie nie mieliby takiego poczucia, że już pora wracać?**

Może to jest właśnie takie koło, które się kręci.

**Bo rozumiem, że w waszym biurze ona jest główna tam. I tak się zastanawiam, czy jeżeli ona nie przychodzi, to inni sobie myślą, że to jeszcze nie czas.**

Nie do końca, bo nikt nie wie, czy ona przychodzi czy nie. Bo to nawet nie jest tak, że ktoś się nas pyta, a w ogóle ktoś przychodzi do tego biura? Nie, po prostu nie przychodzą. I była jakiś czas temu dziewczyna w biurze, nie pamiętam po co, ale po coś tam przyszła, coś tam popracowała. I ona tak przychodzi do mnie i mówi: wiesz co, fajnie tutaj jest w sumie. Myślę, że tak raz na tydzień to mogłabym tu przychodzić popracować. I strasznie mnie to zdziwiło, raz na tydzień? Przecież mogłaby codziennie przychodzić. Raz na tydzień by mogła sobie, fajnie.

**A to nowe biuro, które tam przeprowadzałaś?**

Tak, tak, tak.

**Coś jeszcze z takiego otoczenia, masz obserwacje, jak to wygląda?**

Przepraszam, byłam w Ikei. Zapomniałam o tym, bo byłam z pracy w Ikei. Bo Basia właśnie organizowała te urodziny. I to były urodziny dużo niżej budżetowe niż jej każde urodziny. Ale trzeba było strasznie dużo rzeczy kupić. I właśnie pojechałam kupić te rzeczy. Bo z Basią to jest tak, że wszystko z dnia na dzień. Ale to i tak było z tygodniowym wyprzedzeniem, ale to i tak było mało czasu na ogarnięcie tego wszystkiego. I pojechałam do Ikei, bo dostawa była na za jakoś strasznie długo. I pojechałam do Ikei. I w Ikei też za dużo ludzi nie myślało o koronawirusie. Trzeba było chodzić w maseczce, faktycznie tego ochroniarz bardzo pilnował. Miałam w ręku picie i pan mi powiedział, że nie mogę przez (niezrozumiałe) w tej Ikei się napić. Ale było bardzo dużo ludzi i w ogóle tak dość klasycznie.

**Czyli jak w Ikei.**

Tak.

**Pogadajmy może trochę o tych obostrzeniach. Co sądzisz, że te maseczki tak znieśli w połowie? W sensie na zewnątrz znieśli, a w środku zostały?**

Szczerze mówiąc bardzo (urwane), dlatego że w pewnym momencie zaczęły (urwane). Były momenty, w którym po prostu miałam odruch wymiotny (urwane) bałam się, że albo że będę musiała coś tej policji tłumaczyć (urwane).

**Czyi zaczęły te maseczki ci bardzo przeszkadzać, zaczęłaś się dusić.**

Tak, bardzo. Może, nie wiem, nie jestem w stanie ci powiedzieć, czy się zawsze dusiłam czy nie. Ale był już taki moment, w którym bardzo nie mogłam znieść noszenia maseczki. Bardzo. Więc cieszę się, że już jej nie muszę nosić. No, ale tak jak już mówiłam o tych maseczkach, które trzeba nosić ze sobą, żeby zakładać w różnych miejscach, jak się wchodzi do nich, no to uważam, że jest głupie po prostu. Bo nie wiem w jaki sposób bym miała zabezpieczać tą maseczkę w torebce czy coś, żebym nie brzydziła się jej założyć.

**OK, czyli żeby ona była higieniczna.**

Tak. Bo to jest coś, jak bym wkładała rzeczy, które mam w kieszeni do twarzy sobie. No nie wiem, oczywiście mogłabym sobie brać strunowy woreczek i to przekładać. Ale właśnie też zauważyłam, że się wytworzyła taka fajna kultura ludzi, czy można być bez maseczki. Na przykład bardzo często w taksówce, wsiada: dzień dobry, nie będzie panu przeszkadzało, że będę bez maseczki? Nie, a panu, że ja nie? Tak, nie będzie przeszkadzało. No i jedziemy zadowoleni. I tak właściwie to jest strasznie głupie (śmiech). No, ale zauważyłam, że takie pytania bardzo często się pojawiają.

**W taksówce, gdzie jeszcze jest taka kultura pytania, czy mogę bez maseczki?**

No właśnie w tej Żabce u nas pod domem się zdarza. A w innych to po prostu wchodzę bez maseczki i czekam, aż ktoś mi zwróci uwagę.

**A myślisz, że to dobrze tak z punktu widzenia chorobowego?**

Myślałam o tym, bo wiedziałam, że będziemy rozmawiać, więc tak sobie wzięłam ten temat pod lupę. I wiesz co, wydaje mi się, że nie jest tak, że jakoś dużo więcej osób choruje teraz. Więc no chyba nie tak źle.

**A wiesz, ile mamy teraz zachorowań?**

Nie. Wiem, że jakiś czas temu było około 200 osób chorych na dzień nowych. Bo ktoś się tam spytał, więc sprawdziłam. A, ktoś z biura w Singapurze się spytał nas, ile mamy zachorowań i sprawdziłam wtedy.

**Wiesz co, bo chyba wczoraj padł rekord dzienny.**

Tak? Czyli jednak nie mam racji.

**Tak mi się wydaje. Wydaje mi się, że wczoraj było 600 osób.**

I to jest najwięcej od początku koronawirusa?

**Dziennie, tak.**

Ale czy to nie było związane z tym, bo jednak coś tam wiem, że w jakiejś kopalni ktoś miał koronawirusa i przez to wszyscy mają?

**Zaraz ci przeczytam.**

Bo wiem, że na pewno brat mojej koleżanki ma koronawirusa, bo on jest właśnie górnikiem.

**No, wczoraj było 599 nowych zakażeń.**

Czyli nie spada liczba zakażonych.

**W niedzielę 575, w sobotę 576. W piątek 362, w czwartek 361.**

Czyli moja teoria całkowicie została zdmuchnięta (śmiech). Mogłam najpierw sprawdzić a potem wymyślać.

**Ale dla mnie to jest fascynujące, że przez obserwację miałaś podstawę, że na pewno jest lepiej.**

Nawet wiesz co, nie przez obserwację tylko przez to że nie słyszałam jakichś takich, nigdzie nie przeczytałam, że to jest rekordowa… Wydaje mi się, że powinno to być w jakiś sposób nagłośnione, żeby jednak ktoś zauważył, że coś jest nie tak. Ale wyszłam z założenia, że jeżeli nie czytałam takiej informacji, nic nie wpadło mi w oczy, nawet nie to, że szukałam, nie wpadło mi w oczy, to znaczy, że jest lepiej. No to czy to jest dobrze w takim razie, nie wiem.

**Ale myślisz, że powinniśmy jako społeczeństwo coś z tym zrobić, na przykład wrócić do tych różnych obostrzeń?**

My jako społeczeństwo czy władza?

**No nie wiem. Możesz na przykład utrzymać ten, w sensie możesz sama z siebie nosić maseczkę częściej.**

Ale przecież nikt nie będzie tego robił. Ja na przykład się trzymałam tych zasad, ale teraz nie ma opcji, że ja sama z własnej woli będę chodziła w maseczce na ulicy.

**Czyli miałoby to być nakazane znowu? Żebyś pomyślała, dobra, żeby nie dostać mandatu, będę chodzić w maseczce.**

Tak. Trochę mnie przestraszyłaś z tymi liczbami. Bo zupełnie czego innego się spodziewałam po prostu.

**A czy kojarzysz jeszcze takie obostrzenia, które z nami zostały? Coś jeszcze wiesz, że jest ograniczone, zamknięte, nie można?**

Nie wiedziałam, czy to kino, ale powiedziałaś, że tak.

**Myślisz, że kina powinny zostać dłużej zamknięte?**

Myślałam o tym też pod tym względem, że bardzo ciężko jest wytrzymać w maseczce. Bo rozumiem, że tam trzeba być w maseczce, tak jak w sklepie. Bardzo ciężko jest wytrzymać w maseczce tyle, ile trwa film. I wydaje mi się, że po prostu nikt tego nie będzie robił. Ale też widziałam, że baseny są otwarte, a to jest dla mnie szokujące. Nie rozumiem, na jakiej zasadzie to… I tam może być 1 osoba na torze co drugi tor, coś takiego. Ale no woda się miesza, więc nie rozumiem… Rozmawiałam o tym z Filipem, a Filip powiedział, że przecież nikt nie pluje do wody. Ale przecież to można niechcący napluć do wody. Albo wpadnie ta woda, wypłynie nosem. To jest strasznie dziwne dla mnie.

**A myślisz, że woda w basenie jest zwykle brudna? W sensie, że tam łatwo się zarazić różnymi rzeczami?**

No nie wiem, tak by mogło się wydawać.

**Siłownie jeszcze z takich rzeczy, które właśnie nie wiem, czy są otwarte czy zaraz mają otworzyć.**

Wiem, że na pewno solaria są otwarte, ale jeszcze nie byłam. A na solarium akurat bardzo czekałam. Ja zapomniałam o tym, nawet tobie nie mówiłam o tym, że to jest moje marzenie, iść na solarium. Bo zupełnie zapomniałam o tym. Ale przypomniało mi się, że jestem strasznie blada i muszę iść na solarium. I zanim ogłosili, że ja muszę iść na solarium, to ja sobie już wyhaczyłam takie solarium, które jest otwarte, już pani powiedziała, że w poniedziałek. A oni to zdjęli w jakiś im inny dzień. W każdym razie to było wcześniej. Więc już planowałam, żeby tam się wybrać. Ale jakoś zapomniałam. Potem się umówiłam z Magdą, że pójdziemy razem. I w końcu nie poszłyśmy i tak... Liczyłam, że na to, że pójdziemy do tego solarium, ale się okazało, że da się wytrzymać bez jakiś czas.

**Jeszcze jakieś miejsca, które myślisz, że są takie bardziej niebezpieczne?**

No właśnie nie wiem, czy te miejsca są w ogóle niebezpieczne. Bo tak samo się mijam z tymi ludźmi i mogę na nich nakichać w sklepach i w jakichś innych miejscach. A w sumie na przykład u fryzjera to jest mniej osób niż w sklepie teoretycznie. Więc nie wiem, czy te wszystkie miejsca, no nie wiem, na przykład szkoły czy jakieś takie zgrupowania to może faktycznie, ale… Takie usługowe punkty, gdzie jest stricte jedna osoba obsługująca i 1 klient? Ciężko powiedzieć, ale wydaje mi się, że tam jest mniej osób niż na przykład w szkole.

**A szkoły są zamknięte.**

Ale były matury.

**No matury są, tak, ale w szkole nie ma lekcji.**

Ale przedszkola są otwarte. Klasy I-III?

**Wiesz co, w klasach I-III jest tylko opieka, nie ma lekcji. Można oddać tylko na świetlicę dziecko.**

Ale to, że nie ma lekcji albo są, to nie znaczy, że te osoby nie są w szkole i można się zarazić albo nie.

**Myślisz, że są dzieci w szkole? Wiem, że to w ogóle cię nie dotyczy…**

Myślałam, że są, skoro można prowadzić dzieci do szkoły.

**No dobra, to szkoły, przedszkola. No przedszkola są otwarte w tak zwanym reżimie sanitarnym.**

No właśnie ja rozmawiałam z takim dzieckiem ostatnio i mówił, że jest 10 osób tylko, 10 dzieci.

**A co sądzisz o tym, że te przedszkola otworzyli?**

Słyszałam, że w jednym przedszkolu wszyscy byli zarażeni. Ale to są jakieś takie rzeczy, które ja gdzieś przeczytałam, wpadło mi w oko, a nie to, że się tym zainteresowałam. No nic nie sądzę. No wydaje mi się, że już trzeba… Znaczy właśnie wszystko, co myślałam, bo myślałam źle, bo się okazało, że dzisiaj czy wczoraj padł rekord zarażonych osób. Ale powiem ci, co myślałam po prostu i tyle, nie będę się wycofywać. Myślałam, że po prostu to powoli jakoś musi ruszyć do przodu, odmrażać się powoli. I podejmujemy te kroki. I ktoś, kto się na tym zna, podejmuje te decyzje. I to wszystko prowadzi ku temu, żeby tego koronawirusa już… Żeby on się tak nie rozpowszechniał szybko jak do tej pory. A teraz trochę inaczej wygląda wszystko.

**Trochę ci rozbiło myślenie.**

Tak.

**A w kontekście tego, że wiesz, że mamy teraz ten nowy wzrost, to myślisz sobie, że jakieś działania rząd powinien podjąć? Czy to powinno po prostu tak się toczyć, jak się toczy?**

Nie, no nie powinno się toczyć, jak się toczy, skoro przez 2 dni zachorowało 1000 osób. I nie wiemy, jak jest dzisiaj. Tylko, że też właśnie nie wiem, bo coś tam słyszałam, że w jakiejś kopalni. Więc jeżeli te 500 osób jest z jednej kopalni, w dalszym ciągu to nie jest coś takiego, że… powinniśmy przestać wychodzić na przykład albo przestać się nie pilnować, zacząć się pilnować znowu. Tylko po prostu to oznacza, że w danym miejscu było jakieś skupisko. Więc no nie wiem. A jeżeli zakładamy, że po prostu ludzie chorują w różnych częściach Polski, no to w takim razie coś trzeba zrobić z tym.

**Chciałam z tobą porozmawiać o różnych aplikacjach związanych z pandemią. Słyszałaś o jakichś aplikacjach związanych z koronawirusem?**

Słyszałam o takiej aplikacji, że jeżeli ktoś ma kwarantannę, to może sobie zainstalować tą aplikację i wysyłać zdjęcie, że jest w domu. Słyszałam też o takiej aplikacji, która jest na Białorusi. To jest porównanie do Polski, bo ludzie przekraczający granicę z Polski na Białoruś, oni dostają taką aplikację, muszą ją ściągnąć. I policja im mierzy czas, w jakim powinni dojechać do miejsca docelowego, żeby nie jeździć nigdzie po Białorusi. A w Polsce nie ma czegoś takiego. Tylko po prostu… Opowiadał mi to pan z Ubera generalnie. I on powiedział, że jak on wracał z Białorusi do Polski, to przed 14-dniową kwarantanną pojechał sobie jeszcze do Ikei i Leroy, Carrefour, Auchan, jakieś 5 jeszcze innych sklepów wymienił. Naprawdę byłam bardzo zdziwiona, o czym on mówi w ogóle. No i on sobie tam robił zakupy, żeby siedzieć na balkonie przez 14 dni. I tak sobie pomyślałam, że właściwie po co on siedział te 14 dni w domu, skoro przez 4 godziny mógł zarazić więcej osób, niż jak by mógł wychodzić. Ale o takich dwóch aplikacjach słyszałam.

**Pokażę ci pomysły a potem konkretne screeny ze stron. Pomysł pierwszy aplikacja analizująca dane osobiste, bieżące informacje o stanie zdrowia, historie przemieszczania się i kontaktów z innymi ludźmi, w celu monitorowania rozprzestrzeniania się koronawirusa.**

Na jakiej podstawie są te bieżące informacje o stanie zdrowia?

**No a jak ci się wydaje?**

No wydaje mi się, że trzeba je wpisywać.

**No i co sądzisz o takiej aplikacji, gdzie trzeba wpisywać informacje o swoim stanie zdrowia?**

No w porządku, ale czy to nie było coś takiego, że ludzie kłamali o swoich objawach i ukrywali to, że mają koronawirusa na początku, bo się wstydzili? I tak chodzili do pracy i przez to ten koronawirus się rozprzestrzeniał?

**Czyli jak trzeba by wpisywać, jaki jest twój stan zdrowia, to część ludzi będzie kłamać?**

Tak. A druga rzecz jest taka, że trzeba… No nie wiem, na przykład temperaturę, to trzeba mieć termometr. Ja na przykład czegoś takiego nie mam w domu, więc nie zmierzyłabym sobie po prostu. Jeżeli trzeba by wpisać, to prawdopodobnie wymyśliłabym. Bo nie wiem… Nie wiem, uważam, że to też dużo czasu by zajmowało. Nie wiem jak bardzo ludzie by to chcieli zrobić.

**A historia przemieszczania?**

No właśnie, jeśli tu jest napisane, analizujący to przemieszczenia, to pomyślałam sobie, że to byłby jakiś GPS. Ale nie wiem, czy ludzie by chcieli, żeby ktoś jawnie śledził, gdzie oni chodzą. Bo o ile zdaję sobie sprawę z tego, że te aplikacje generalnie monitorują, gdzie chodzimy i jacyś ludzie mają do tego dostęp. Nie wiem, czy świadomie by na to… Znaczy, kupując telefon, świadomie na to wyrażają zgodę. Ale czy świadomie… Nie wiem, wydaje mi się, że mogłyby powstać jakieś takie teorie spiskowe, że na przykład rząd chce śledzić wszystkich i każe ściągnąć taką aplikację, żeby zbierać takie informacje. Jeżeli ja bym miała wypisywać, gdzie ja byłam, to absolutnie nie chciałoby mi się tego robić, jak już trzeba by.

**Czyli myślisz, że to jest coś, co budziłoby raczej sprzeciw?**

Ta historia przemieszczania się?

**No w ogóle taka aplikacja, która by takie rzeczy zbierała i analizowała.**

No myślę, że tak. Skoro ludzie uważali, że… Jakby nakłaniają do płacenia kartą zamiast gotówką, żeby monitorować wydatki, no to myślę, że tak samo aplikacja, która zbiera historię przemieszczania się i kontakty z innymi ludźmi, mogłaby podobne wnioski nasuwać.

**A myślisz, że tutaj jest jakaś wartość? W sensie, że na przykład monitorowanie tego rozprzestrzeniania się koronawirusa byłoby możliwe dzięki tej aplikacji. Myślisz, że to jest coś, na co warto się zgodzić w związku z tym?**

Ale ja, czy generalnie?

**Zacznijmy od ciebie. Myślisz, że ty byś sobie pomyślała, dobra, żeby ta pandemia się nie rozprzestrzeniała, to ja sobie tą aplikację zainstaluję.**

No tak jak mówię, jak by trzeba było ściągnąć, no to bym pewnie ją ściągnęła. Ale z własnej woli bym na pewno tego nie zrobiła.

**A ludzie?**

Ludzie wydaje mi się, że też by tego nie zrobili. W ogóle ludzie bardzo pilnują takich swoich danych.

**To następna. Aplikacje, które na podstawie danych lokalizacyjnych monitorują przestrzeganie kwarantanny domowej. To jest to chyba, o czym mówiłaś.**

Tak. Nie wiem, czy to jest to, bo tam mi się wydaje, że trzeba było wysłać zdjęcie. Nie wiem, coś o tym słyszałam i czytałam. Tak, w porządku jest ta aplikacja. Uważam, że powinna taka być. Dlatego, że akurat w tym momencie ingerowanie w czyjeś życie, znaczy taką prywatność, gdzie są w danym miejscu jest potrzebne, bo takie są zasady, że mają nie wychodzić z domu. A nie wyobrażam sobie, w jaki inny sposób można byłoby to sprawdzać. Bo wiem, że ludzie i tak wychodzili z domu jacyś tam, podczas kwarantanny. Zresztą sam fakt, że podnieśli ten mandat z 5 tysięcy na 30, no to o czymś świadczy, że ktoś tam nie przestrzegał… Na ile ta kwarantanna jest słuszna nie wiem, ale ta aplikacja wydaje mi się być w porządku.

**Aplikacja oparta na automatycznej lokalizacji użytkowników i informujące ich, że znajdowali się w miejscach zagrażających zarażeniem się koronawirusem.**

Bardzo fajne.

**Czemu?**

Bo chyba też taką obawą koronawirusa było to, że nie wiadomo, w jakim miejscu się można zarazić, nie wiadomo, gdzie ktoś wcześniej był. Nie wiem, czy to by nie siało paniki. Ale jeżeli dowiedziałabym się, że na przykład wchodzę… Tak sobie to wyobraziłam, że dostałabym dźwięk SMS-a, że tutaj był kiedyś ktoś z koronawirusem czy coś takiego, no to mogłabym na przykład w tym miejscu na siebie uważać.

**A skąd oni by wiedzieli, że tam ktoś był z koronawirusem?**

Nie wiem, nie jestem informatykiem, ale taką sobie wyobraziłam fajną aplikację. No nie wiem, na pewno byłoby to już przestarzałe, ale jeżeli ktoś by miał koronawirusa i spisywał sobie miejsca w których był, ale przez jaki czas? Nie wiem, fajnie ta aplikacja brzmi, ale nie wiem, na jakiej zasadzie miałaby działać.

**A co jest w niej takiego zachęcającego? To, że ty byś mogła wtedy też uważać bardziej?**

Tak.

**A jest coś, co tutaj ci się nie podoba w tym pomyśle?**

Nie, bo wszystkie się opierają na lokalizowaniu ludzi. Ale ta ma taki bardziej natychmiastowy skutek tego, jakby… Są jakieś plusy tego, że się udziela tej lokalizacji. W sensie ja mówię ci, gdzie jestem, a ty mi mówisz, czy jest tam koronawirus czy nie. A jeśli ja po prostu pokazuję swoją lokalizację w celu jakichś badań? No nie wiem, nie umiem tego wyjaśnić, ale wydaje mi się ta fajniejsza aplikacja niż ta pierwsza.

**Czyli ty być mogła dzięki temu się lepiej chronić?**

Tak. Chociaż prawdopodobnie, uważam, że ta aplikacja pierwsza, mogłaby przynieść jakieś większe rezultaty, bo wtedy ludzie by się, nie wiem, badali bardziej ogół.

**Następna. Monitoring z automatycznym systemem rozpoznawania twarzy, w celu szybkiej identyfikacji osób nieprzestrzegających zaleceń władz.**

Nie rozumiem. Chodzi o kamery na ulicach czy chodzi o monitoring z telefonu?

**Chyba na ulicach.**

Bo skoro mówimy o aplikacjach, to ja tak sobie wyobraziłam, że telefon patrzy, czy mam maseczkę czy nie. Nie, no monitoring na ulicach, tak?

**Nie wiem, może jeden i drugi, nie wiem. Ty sobie wyobraziłaś, że idziesz z telefonem i telefon patrzy, czy ty masz maseczkę.**

Nie, wydało mi się to absurdalne, dlatego pomyślałam od razu o kamerze na ulicy.

**To jest pewnie jakaś taka aplikacja, która jest w kamerze ulicznej albo w kamerze sklepowej na przykład.**

Nie, no w ogóle nie wierzę, że coś takiego miałoby prawo istnieć. Znaczy wierzę w technologię, więc może ktoś by mógł coś takiego zrobić. Ale było tyle innych rzeczy, gdzie ludzie nie używali akurat tego, więc nie wiem, dlaczego do zatrzymania koronawirusa by mieli coś takiego stworzyć.

**Ale to jest w ogóle pomysł, który ma szansę być zaaprobowany przez ludzi?**

Wydaje mi się, że nie może nie być, ale tak naprawdę nie rozumiem, dlaczego. Bo teoretycznie i tak wszędzie są kamery i monitoring jest. I tak jesteśmy nagrywani. A jeżeli to miałoby być w jakimś dobrym celu, chociaż w zasadzie monitoring jest po to. Ale ta identyfikacja twarzy też bardzo jakoś tak… Nie wiem, jak by to miało działać. Równie dobrze mogłoby coś takiego być w sklepach przecież i wtedy by nie było żadnych złodziei, bo… Znaczy byliby złodzieje, ale byliby szybko łapani, bo ktoś by ich identyfikował od razu.

**A na przykład do tego, żeby identyfikować ludzi, którzy nie chodzą w maseczce w sklepie?**

No to chyba coś takiego jest. Na przykład ochroniarze oglądają i każą założyć maseczkę.

**Czyli taki pan aplikacja?**

No chyba tak. Nie wiem, czy warto poświęcać pieniądze czy jakiś czas na coś takiego.

**Następne. Drony dostarczające produkty medyczne i inne towary osobom potrzebującym.**

Nie wiem, co mogłoby być w tym złego, może być.

**A co jest w tym fajnego?**

Że żadne osoby nie muszą dostarczać osobom potrzebującym jakichś towarów.

**To jest potrzebne rozwiązanie?**

Jeżeli są jakieś wolne drony, no to tak.

**A chciałabyś, jeżeli na przykład musiałabyś być na kwarantannie, to chciałabyś, żeby dostarczał ci ktoś rzeczy czy na przykład dron pod drzwi?**

Nie miałabym żadnego zdania. Na pewno z dronem byłoby fajniej, ale rezultat byłby ten sam. Bo nie widziałabym tej osoby przecież, jak by zostawiała mi to pod drzwiami. Więc to nie jest żadne spotkanie z człowiekiem. Fajne, fajne zastosowanie technologii.

**Aplikacje, których użytkownicy mogliby informować się nawzajem, czego potrzebują i sobie pomagać.**

Ta aplikacja chyba jest, to znaczy wszystkie są. O tej słyszałam aplikacji, że coś takiego jest. A (niezrozumiałe) było coś takiego, jak wieszanie kartek, jeżeli ktoś potrzebuje pomocy, to mogę pomóc. I fajna jest ta aplikacja, ale szczerze mówiąc nie korzystałabym z niej jako żadna strona.

**Aplikacja sztucznej inteligencji, decydująca na podstawie zebranych danych, gdzie skierować największe środki i wysiłki do walki z pandemią.**

No dobrze. Nie wiem, co mam na ten temat powiedzieć, myślałam, że coś takiego w ogóle jest. Chyba, że to ludzie decydują, a nie sztuczna inteligencja.

**A jak myślisz, jak to teraz jest? Myślisz, że to jest tak, że człowiek mówi, to teraz tam? Czy to jest jakiś komputer, który wylicza i mówi dobra, tu jest najwięcej potrzebne?**

Wydaje mi się, że to jest połączenie obydwu, i człowieka, i komputera. No człowiek ręcznie tego nie liczy. Ani, nie wiem, jakiegoś wzrostu ani jakichś takich tendencji. Może to policzyć ręcznie, ale pewnie też to jest liczone na komputerze, to jest kombinacja komputera i człowieka. Jeżeli to chodzi o na przykład w pełni zaufanie komputerowi, to nie wiem.

**To nie do końca.**

Nie wiem, co ja bym na ten temat myślała. Dlatego, że tak jak mówię, teraz nie wiem, jak to działa, może tak naprawdę komputery tylko wyliczają, a człowiek to tylko… Nie, no wydaje mi się, że i tak człowiek musi to kontrolować w jakiś sposób, gdzie się wysyła te rzeczy. Po prostu.

**Pokażę ci teraz opis „Kwarantanny Domowej” (prezentacja aplikacji). Co sądzisz?**

Bardzo fajny pomysł.

**Co jest najfajniejszego w tym pomyśle?**

Wykorzystanie technologii do kontroli, znaczy no właśnie to brzmi okropnie, ale no jakby… Jakby wykorzystanie tego, że używamy cały czas telefonu do jednak jakiejś dobrej rzeczy. Czyli do kontrolowania, czy ludzie, którzy powinni siedzieć w domu, naprawdę w tym domu siedzą.

**Bo ty masz takie poczucie, że nie siedzieli i dobrze by było, żeby mieć większą kontrolę nad tymi, którzy mają na tej kwarantannie być.**

Nawet nie mam poczucia, że nie byli w domu. Nie wiem, czy byli. Ale bardzo dużo osób było na tej kwarantannie. W sensie miało taką obowiązkową kwarantannę. I wydaje mi się, że jest bardzo ciężko sprawdzić te wszystkie osoby. Bo na pewno jest mniej policjantów niż tych ludzi, są porozsiewani. Wydaje mi się, że to jest bardzo ciężka praca, chodzić i sprawdzać te osoby generalnie. I też może trochę niebezpieczna, ryzykowna. I rozumiem, że jakby nie zastępują tych wizyt policji, jasne. Ale fajny sposób… W sensie jakby na przykład można oszukać policję, żeby wyjść z domu potem jak policja była. A tutaj zawsze jest świadomość tego, że można dostać tego SMS-a, który sprawdzi, gdzie jesteś. Więc uważam, że super.

**A widzisz jakieś zagrożenia, coś, co jest niefajne w tej aplikacji, w tym pomyśle?**

No może być takie właśnie bardzo ingerowanie do ludzkiego życia. Tylko, że nie wiem do końca, czy trzeba się tym przejmować podczas pandemii. Nie wiem, czy to nie jest tak, że… Nie wiem, wydaje mi się, że jeżeli ktoś musi siedzieć w domu, to musi. Ale ciężko jest uwierzyć, że ludzie będą siedzieli w domach. Więc fajnie, że jest taki system. Zresztą jest XXI wiek, 2020 rok, więc fajnie, że wykorzystujemy aplikacje w takim celu. A nie jakimś innym. A podejrzewam, że ściągając jakieś inne aplikacje ze sklepu w telefonie, zgadzamy się na lokalizację i jakieś inne rzeczy, które są w tej aplikacji.

**To droga aplikacja ProteGO Safe (prezentacja aplikacji). Co sądzisz?**

Nie twierdzę, że jest niepotrzebna, fajny pomysł. No… fajny pomysł.

**A co jest w tym fajnego? Co ci się podoba?**

Znaczy bardzo podobało mi się to zdanie, że to jest pomoc dla lekarzy, jeżeli coś tam się wydarzy, to będą mieli już jakiś podgląd na twoją historię niemedyczną, tylko historię tam życia, jak się czułeś przez ostatni czas. Nie ma na przykład gwarancji, że ktoś zapomniał, jak się czuł czy coś. Bo jeżeli ktoś to spisywał, to ma to w telefonie i będzie mógł pokazać.

**A dla ciebie? To jest aplikacja, którą ty mogłabyś mieć i wypełniać?**

Nie będzie mi się chciało tego robić. To jest na takiej samej zasadzie jak aplikacje do kontrolowania okresu. Jeżeli ja wpiszę, że się zaczął i skończył, to już jest dla mnie naprawdę bardzo dużo. A tam jeszcze się wpisuje, czy tam się brzuch bolał czy nie. I jak się czułaś. Generalnie to na pewno ginekologowi to by bardzo pomogło czy tam endokrynologowi. Ale po prostu nie robię tego, bo nie chce mi się, nie mam na to ochoty, zapominam.

**I tu myślisz, że też byś nie robiła tego?**

Mogłabym ją zainstalować i sobie popatrzeć. Ale, żeby robić to codziennie, to na pewno bym tego robiła. Mogę spróbować (śmiech). Ale to nie jest tak, że od razu zakładam, że nie chce mi się tego robić. Tylko po prostu wydaje mi się, że…

**A ten punkt drugi, druga kropka?**

To jest właśnie to, nad czym się zastanawiałyśmy, jak to może informować o spotkaniach z chorymi. Nie wiem, no jakby nie rozumiem tego. Wiem, co jest napisane i rozumiem, jaki jest tego sens, ale ciężko mi jest zrozumieć, że coś takiego jakby działało. Trzeba by było mieć włączony bluetooth przez cały czas. Nie wiem. Dla mnie coś takiego jest naprawdę niepojęte.

**No to bluetooth jedno, ale byś musiała wpisywać, że jesteś chora.**

No tak. I jeszcze bym miała świadomość tego, że wiem, że jestem chora i to napisałam w aplikacji. I mam włączony bluetooth. I teraz ludzie, którzy mając aplikację i będą obok mnie przechodzić, będą się dowiadywać o tym, że jestem chora. Nie wiem, czy bym tak chciała.

**Nie wiesz, czy byś chciała, żeby wszyscy wiedzieli, że jesteś chora?**

Nie. Znaczy w teorii powinni, żeby uważać i żeby się wirus nie rozprzestrzeniał. Nie wiem, czy to nie jest jakiś taki ostracyzm. Zresztą z drugiej strony, jak już jestem chora, no to chyba nie chodzę po ulicy, tylko jestem w domu w najgorszym wypadku.

**Czyli jakby nie tyle trzeba wpisać w aplikacji, że człowiek jest chory, tylko trzeba po prostu nie wychodzić?**

No tak. No tak, no wydaje mi się, że w ogóle takim pierwszym (niezrozumiałe) jak się czuje, że jest się trochę chorym, to po prostu nie wychodzić, żeby zarażać jak najmniej osób. Bo teoretycznie może to być koronawirus. Nawet, jeżeli nie, to bezpieczniej posiedzieć, nie wiem, 5 dni w domu i się nie spotykać z nikim. Ale jeżeli na przykład, że ma takie poczucie, że czuje się słabo, ale wpisałem w aplikację, więc mogę już robić, co mam ochotę… Nie wiem, strasznie ciężko mi jest zrozumieć te aplikacje, na jakiej zasadzie one miałyby działać. I też ciężko mi zrozumieć, że naprawdę ludzie by wpisywali do tej aplikacji coś. Pewnie tacy ludzie są, którzy by robili to systematycznie… Nie wiem.

**A ty nie. Ktoś by może robił.**

Tak.

**Ty w ogóle myślisz o przyszłości? Będzie ten koronawirus długo z nami, nie będzie? Myślisz w ogóle sobie o tym? Czy myślisz sobie, jak cię zapytam.**

Myślę o tym, że możesz mnie zapytać, więc myślę. No tak, ale generalnie wszystkie myśli, które są związane z koronawirusem to są takie, że wiem, że się umówiłyśmy, więc muszę mieć jakieś przemyślenia. I wtedy sobie o tym myślę. A co do przyszłości koronawirusa, to podejrzewam, że jeszcze może wrócić koronawirus. Ale nie są to moje przemyślenia, tylko rzeczy, które gdzieś tam przeczytałam albo usłyszałam. Więc wierzę w to.

**Wierzysz w to, że wróci? Jak powinniśmy się wtedy zachować jako kraj, jak rząd się powinien zachować, ludzie?**

No właśnie przez to, że naprawdę byłam przekonana, że ten koronawirus się kończy… A nawet nie tyle kończy, tylko jest po prostu tak samo, jak było miesiąc temu. Można więcej rzeczy robić, a choruje tyle samo osób. Z takiego wychodziłam założenia. Że po prostu nie zmienia się nic, że nie jest gorzej. A jak się dowiedziałam teraz, że jest gorzej, to nie wiem, bo chciałam powiedzieć, że można podjąć takie same kroki. Ale jak widać nie przynoszą rezultatów. A może przynosiły na początku, ale przez to, że zostały zniesione, nie przynoszą. No nie mam pomysłu, co można by zrobić innego niż to, co było zrobione.

**A teraz myślisz albo czujesz albo widzisz jakieś skutki, że były te obostrzenia, te zamknięcia?**

Ale że zostało zniesione czy że były?

**Na przykład, że był ten lockdown i jakoś tam gospodarka gorzej działa albo ludzie, nie wiem, nie mają pieniędzy czy coś takiego.**

A to takie skutki. No to widzę, mówiłam o nich od samego początku i w dalszym ciągu te skutki są. Ale w ogóle jeszcze tego nie powiedziałam, bo zapomniałam o tym, ale jest dużo takich sytuacji, w których te obostrzenia są oszukiwane w taki dość jawny sposób. Na przykład kluby są zmieniane w kawiarnie albo w jakieś galerie sztuki czy coś takiego. Że można było jednak je otworzyć, mimo że nie można. Zdaję sobie sprawę z tego, że ci ludzie w jakiś sposób muszą zarobić pieniądze i nie spodziewali się, że takie ogromne straty cokolwiek im przyniesie. Nawet, jeżeli mieli jakieś oszczędności i zapewnili sobie coś na czarną godzinę, to nie spodziewali się, że tak ogromne prawdopodobnie. No a skutki, tak, są jakieś skutki. Wydaje mi się, że pewnie są też droższe produkty różnego rodzaju.

**Ale jakie na przykład? Jakiego typu te produkty mogą być droższe?**

Spożywcze, nie, no wydaje mi się, że wszystkie. Nie zauważyłam żadnego wzrostu cen, ale prawdopodobnie wszystkie.

**Obawiałaś się wcześniej, że nie będziesz się umiała normalnie zachowywać, że będzie to takie nienaturalne. Nie ma tego. A myślisz, że będą osoby, które długo się będą tak inaczej zachowywać? Czy to będzie widoczne?**

Tak, będzie to na pewno widoczne. Dlatego, że ja widzę, że są ludzie, którzy no może nie bezpośrednio ze mną, ale widzę, że ludziom generalnie odbija troszeczkę od niewychodzenia z domu. I mam wrażenie, że nie wychodzą z domu nie dlatego, że się boją, tylko dlatego, że już przywykli po prostu do tych wakacji, o których ja mówiłam, że są. I to nie były absolutnie osoby, które się bały. Tylko po prostu to były osoby, które normalnie w miarę funkcjonowały, nie wychodziły z domu. A teraz spodobało im się to trochę. I teraz widzę, że trochę dziczeją i się zachowują w inny sposób. Jakoś tak, że trochę się nie możemy dogadać. I nie wydaje mi się, żeby to była moja zasługa, że nie możemy się odnaleźć.

**Czyli co, że im się już nie chce?**

Trochę tak.

**Rozleniwili się?**

Tak.

**Tak społecznie się rozleniwili, nie chce im się spotkać, nie chce im się wyjść.**

Tak, ale z drugiej strony łakną strasznie kontaktu i uwagi. Której nie dostawali przez ten cały czas, bo nie wychodzili, więc jakby mieli ograniczoną liczbę, ilość osób.

**Myślisz, że jakieś ograniczenia powinny zostać dłużej?**

Szczerze mówiąc niechętnie, ale uważam, że można by jeszcze trochę… Ale właśnie nie wiem, bo wszystkie te obostrzenia mają za sobą skutki gospodarcze. I tak naprawdę ciężko jest mi powiedzieć, co jest gorsze. To znaczy nie to, że mam do wyboru życie ludzkie albo gospodarkę. Tylko raczej… No też nie wiem, bo na przykład pomyślałam, że może faktycznie jeszcze nie powinny móc się otwierać restauracje. Ale z drugiej strony ci ludzie w jakiś sposób muszą zarabiać pieniądze i ta gospodarka musi iść do przodu. Więc nie jestem pewna, nie wiem, ile osób się zaraziło w restauracji. Jeśli chodzi o kluby, tak, raczej nie powinny być otwarte. To, jak byłam w Dziku w zeszły weekend pokazuje, że można to obejść. Czy był tam ktoś chory, czy się zaraził, nie wiem. Ciężko mi jest odpowiedzieć na to pytanie, nie wiem.

**A czy masz poczucie, że jakieś grupy się powinno szczególnie chronić? Dzieci albo osoby starsze. Myśląc o tym, że będzie ta druga fala i jakoś można się przygotować do tego.**

Tak, to przede wszystkim osoby starsze należy chronić. Ale też wydaje mi się, mam takie poczucie, ale nie jest niczym poparte, że starsze osoby, te które znam, albo które widziałam, o których słyszałam, dość dobrze sobie radziły w tym okresie. I nie tylko same, ale młodzi ludzie bardzo pomagali albo w jakiś sposób wspierały jak mogły. I wydaje mi się, że jesteśmy całkiem nieźle do tego przygotowani. I też wydaje mi się, że dużo starszych osób, ale to wymyślam, mogą się nauczyć na przykład dużo bardziej technologii albo obsługiwania Skype czy coś. Tak po prostu dla siebie, bo nie mogłyby się spotykać z wnuczkami. Ja na przykład rozmawiałam z moją babcią na Facetime. Nie jestem w stanie stwierdzić, czy miała to przed koronawirusem czy nie. Ale bez problemu z nią rozmawiałam i nie było to dla niej żadnym problemem. I nie odpowiadam w ogóle na pytanie, ale uważam, że to jest coś, co starsi mogli wyciągnąć z koronawirusa, to właśnie to. Ale tak, uważam, że trzeba ich chronić. Więc może, jeżeli będzie druga fala na przykład, to nie będą aż tak przestraszone czy coś. Bo będą wiedziały, że oprócz tego, że umieją korzystać z telefonu, to też mają wsparcie, bo za pierwszym razem tak było.

**Teraz jak przeglądam internet, media, to widziałam wypowiedzi, że drugiego lockdownu nie da się zrobić ze względów gospodarczych, trzeba będzie wymyślić coś innego. Czy myślisz, że taki model, żeby osoby starsze nie wychodziły z domu, po prostu, w sensie, bo inni trochę muszą, bo muszą pracować, a osoby starsze żeby nie wychodziły z domu.**

Ale wiesz co, tak naprawdę to się okazuje, że dużo więcej osób nie musi wychodzić z domu niż by się wydawało. Bo dużo firm może pracować całkowicie zdalnie albo w 80% zdalnie. I na przykład moja firma radzi sobie dość dobrze, pracując zdalnie. I Filipa. I niektórych ludzi, których znam. Więc może takiego całkowitego się nie da, ale trochę się da. Może całkowitego nie, ale dużo osób może nie wychodzić z domu.

**Bo ten całkowity to miałam na myśli, że znowu zamkną knajpy, fryzjerów, sklepy wszystkie poza spożywczymi, produkcję w fabrykach niektórych.**

Ale z drugiej strony sobie myślę, że jeżeli ludzie mogliby wychodzić do knajpy, to czemu mają nie wychodzić do pracy? W sensie do budynku firmy, o to mi chodzi. Osoby starsze żeby nie wychodziły z domu, mogą nie wychodzić, ale czy to w jakiś sposób zaprzestanie rozprzestrzenianiu się koronawirusa? To nie jest tak, że tylko starsze osoby przenoszą tego koronawirusa. Właśnie to chyba bardziej młode, a starsze bardziej są podatne na to.

**No tak, gorzej przechodzą. Więc żeby ich chronić przed tym gorszym przechodzeniem, żeby nie miały kontaktu. Myślisz, że to jest OK czy nie bardzo?**

Nie, bo to brzmi niedobrze, żeby… Zmuszanie kogoś do siedzenia w domu jest w ogóle okropne. Jeżeli to jest ktoś starszy, kto mieszka sam, no to może się wydawać bardzo złe. Ale z drugiej strony nie mam innego pomysłu, jak można by chronić te osoby.

**Masz jeszcze jakieś przemyślenia związane z koronawirusem?**

Tak. Jak mówiłaś o tym, kogo jeszcze trzeba chronić. Powiedziałam osoby starsze, ale uważam też, że dzieci. I nie dlatego, że w jakiś sposób słabo przechodzą koronawirusa, tylko właśnie dlatego, że go bardzo łatwo mogą przenieść. I to jest bardzo niepokojące, bo dzieci bardzo dużo rzeczy lubią sobie wkładać do buzi i dotykać siebie, a potem rodziców. I też sobie nie wyobrażam takiej sytuacji, że na przykład rodzice z racji tego, że jest koronawirus, to mnie przytulają swoje dziecko albo mniej dotykają, bo to dziecko było w przedszkolu i mogło dotknąć kogoś, kto miał koronawirusa. Więc wydaje mi się, że na dzieci też trzeba zwrócić dużą uwagę.

**A w jaki sposób te dzieci można tak bardziej ochronić, jak ci się wydaje?**

No właściwie to chyba nie posyłać do przedszkola. Ja rozumiem, że nie każdy może i nie każdy ma taką możliwość. Ale z drugiej strony, jak by na przykład… Nie, no nie wiem. Bo wpadłam na pomysł, żeby dzieci zamknąć z dziadkami. Ale to raczej nie przejdzie. Nie, no najpierw trzeba byłoby dzieciom zrobić kwarantannę. Oczywiście, że wszyscy dziadkowie, którzy mogą, to nie jest tak, że (niezrozumiałe), tylko jak ktoś jest taki bardziej żywotny. Ludzie, no nie wiem, 50 do 60 lat?

**Ale oni pracują.**

Ale właśnie oni by mieli przymusową pracę w domu. No nie wiem, to jest pomysł, który wymyśliłam w tej sekundzie i pewnie nie ma racji bytu generalnie. Ale oprócz tego, to jeżeli wzajemnie by siebie chronili?

**Czyli myślisz, że jakimś rozwiązaniem byłoby jednak ograniczenie chodzenia dzieci do przedszkola, po prostu, żeby się nie spotykały?**

Tak. Dzieci, pracowałam przez półtora roku w przedszkolu, więc dokładnie wiem, w jaki sposób dzieci przenoszą na siebie ślinę, wszy i wszystko inne możliwe. Tak że koronawirusa zapewne też.

**A myślisz tak samo ze szkołami, podstawówkami?**

No dzieci z podstawówki to są też małe dzieci. Mimo, że mi się wydawało, że byłam dorosła, to naprawdę te dzieci są malutkie. I nie jest tak, że nie starają się czy coś, tylko po prostu nie myślą o konsekwencjach i sobie… Nie wiem, dadzą cukierka z buzi spróbować, albo się napiją z jednego picia.

**Tak jak wy robicie ze znajomymi na piwie?**

Tak, dokładnie tak.

**A gdybyś tak miała, podsumowując spotkanie, cofnąć się do początku pandemii w Polsce czy na świecie i pomyśleć o takich przełomowych, ważnych wydarzeniach, to co to by to było?**

Jak się dowiedziałam w ogóle o tej pandemii i trochę nie do końca wiedziałam w ogóle, co się dzieje, że coś tam jest, ale daleko. Ale ludzi to ciekawiło, więc ja też się zainteresowałam. Kolejny, jak ten koronawirus był coraz bardziej w Europie, ale nie w Polsce. Ale już wtedy było oczywistym, że wcześniej czy później w Polsce się pojawi. Potem chyba zamknięcie prac i szkół. To był chyba ten sam tydzień, podobne dni. Nie, no jeszcze w międzyczasie ta pierwsza osoba chora na koronawirusa. Zamknięcie szkół... Ale chciałabym powiedzieć, że na przykład pierwsza osoba, która umarła na koronawirusa, ale absolutnie nie pamiętam tej sytuacji w ogóle. Nie wiem, czy to była kobieta czy mężczyzna, ile miał lat ani nic. Więc mogłabym chcieć to powiedzieć, ale nie. Potem przez długi czas wydaje mi się, że zupełnie… Nie, no potem nakaz chodzenia w maseczkach. I potem, wydaje mi się, że długo, długo nic do momentu, kiedy była ta konferencja, gdzie ogłoszono 4 plany wyjścia z tego. Aha, nie mam nic więcej do dodania pomiędzy tym, oprócz tego, że nie trzeba chodzić w maseczce po ulicy.

**I to jest też ważny moment dla ciebie.**

No i otwarcie restauracji też. Ja nawet nie wiem, czy bym zwróciła uwagę na te maseczki, gdyby nie to, że kiedyś ty się mnie spytałaś, kiedy będę uważała, że jest koniec, a ja wtedy powiedziałam, że jak nie będzie trzeba nosić maseczki. I teraz nie jest tak do końca koniec. Znaczy widzę, że nie jest. Ale nie trzeba chodzić w maseczce.

**To po co maseczka? To, że trzeba było ją nosić, było ci z tym bardzo źle. To takie największe rzeczy, które ci przeszkadzały? W sensie taką było trudnością, dyskomfortem poza maseczką?**

Na pewno obniżenie wypłaty. Znaczy no niefajne, no po prostu był taki skutek pandemii, ale no to mogło być niefajne na pewno. Nie myślałam, że to jest niefajne, ale jest fajne, jak można czyli chodzenie do restauracji. I absolutnie za tym nie tęskniłam, ale po prostu fajnie sobie posiedzieć przy jakimś stoliku, który nie jest w domu. I żeby ktoś ci przyniósł jedzenie, mimo że to w dalszym ciągu jest jedzenie zamówione a nie zrobione. I tyle z niefajnych rzeczy. Generalnie jak już mówiłam, mi się całkiem podobało.

**Właśnie, chciałam cię zapytać o największe plusy pandemii.**

Wrażenie wakacji. I jakby mimo, że na początku mówiłam, że to przeszkadza mi już teraz i przeszkadza, ale fajne było takie zluzowanie. I to nie były wyjazdowe wakacje, ale to były po prostu takie wakacje od normalnego życia, które prawdopodobnie już od teraz będziemy… No nie od teraz, ale może tam od przyszłego roku, będziemy już prowadzić do końca życia. Czyli wstawanie rano, chodzenie do pracy, jakaś taka rutyna. Na którą sobie nie można było pozwolić podczas pandemii, bo nigdy nie wiadomo było, co się wydarzy. I też takie fajne było to, że można było sobie wszystko usprawiedliwić tym koronawirusem. I złe samopoczucie i to, jak się było dla kogoś niemiłym. I potem się mówiło: sorry, ale tak przezywam to, że jest pandemia. No wydaje mi się, że to było w porządku.

**(niezrozumiałe)**

Naprawdę mam takie odczucie, że bardzo… No nie tyle przyjemne, bo ludzie umierali i byli chorzy. Ale dla mnie osobiście był to też taki ciekawy okres odpoczęcia od jakichś nawet takich swoich zasad, które sobie ustaliłam kiedykolwiek. I chciałabym się ich trzymać generalnie, ale miałam takie przyzwolenie, żeby ich się nie trzymać.

**Masz jeszcze jakieś przemyślenia?**

Nie, strasznie mi przykro, że to jest koniec. I nawet sobie zaczęłam szukać terapeuty, żeby móc z nim porozmawiać po prostu.

**Dziękuję.**
