# Supplementary material for: The challenges arising from the COVID-19 pandemic and the way people deal with them. A qualitative longitudinal study
Source: PLoS One. 2021 Oct 11;16(10):e0258133. doi: 10.1371/journal.pone.0258133 (PMC8504766; doi:10.1371/journal.pone.0258133)
Supplement: S1 Dataset — (ZIP) [file pone.0258133.s003.zip › Transcriptions/stage 5/9.5_F_25_couple, no children.docx]

**9.5_F_25_couple no children**

**Co się u ciebie działo przez ostatni miesiąc?**

Nie jestem pewna czy dużo, natomiast pojechałam do domu. Miałam urodziny. Były już pierwsze imprezy rodzinne i takie spotkania ze znajomymi jakieś wypady do lasu. I to chyba tyle.

**Czy ten stan już wrócił o tego, jak było przed pandemią?**

Może nie do końca, dlatego, że wciąż pracuję z domu. Natomiast z domu będę pracować do sierpnia, bo mamy remonty i się wszystko przedłużyło. I tak naprawdę to jest jeden taki nienaturalny stan, który pozostał. Poza tym tak samo, jak przed koronawirusem.

**Pamiętasz jakieś przełomowe momenty w ostatnim miesiącu?**

Myślę, że to właśnie ten wyjazd do domu. Jechałam pociągiem. Tak się złożyło, że miał po mnie przyjechać tata, ale okazało się, że coś jest z kołami i nie może wyjechać na autostradę. Miał mnie zawieźć mąż, ale okazało się, że podpisali źle umowę na ubezpieczenie i jest nieważna, bo pomylili dowody rejestracyjne. Więc z musu pojechałam tym pociągiem. Trochę się obawiałam, ale okazało się, że pociąg wyglądał jak zawsze. Było bardzo dużo ludzi, bo jechali też do Szklarskiej Poręby i do Karpacza. Usiadła obok mnie taka rodzinka z dziećmi. Nie wiem, jak to jest, bo tyle pustych miejsc, a oni usiedli obok mnie w tej czwórce. Oczywiście dorośli mieli maseczki, ale dzieci nie. Tak całkiem normalnie się zrobiło. Byłam prawie 2 tygodnie w domu i oprócz tego, że pracowałam z domu, to wszystko nabrało jakiegoś takiego naturalnego stanu. Tutaj mieszkamy w bloku, więc każde wyjście wiąże się z jakimś przygotowaniem. Tam byłam u siebie, mogłam wyjść na balkon, miałam dużo podwórka. Na zakupy chodził tata, więc nawet nie musiałam maseczki wkładać i kompletnie zapomniałam, że coś jest nie tak. Jeszcze dodatkowo mój tata miał 60 urodziny, więc rodzina stwierdziła, że zrobi mu niespodziankę. I przyjechali, więc mieliśmy małą imprezę rodzinną. Mój mąż mówi, że to bardzo nieodpowiedzialne, ale uważam, że jako że zjechali się sami dorośli po 50 to jest ich decyzja i ich wybór. Były też urodziny kuzyna, byliśmy na cieście. Tak zupełnie się już zwyczajnie zrobiło.

**Po powrocie do mieszkania nadal było zwyczajnie?**

Trochę nie. Może ze względu na to, że jakoś w domu to było naturalniejsze. Bo ja nie bałam się nigdy o siebie, tylko o innych ludzi i w domu bałam się o tych bliskich. A tutaj się okazuje, że... Moja mama jest pielęgniarką, więc zakładam, że ma rozsądne podejście do tych spraw. Widziałam, że rodzice dbają o higienę i przestrzegają podstawowych zasad, natomiast nie panikują. I to mnie bardzo uspokoiło, bo tak naprawdę to oni są w grupie ryzyka. A oni się wydawali jakby... Może nie przyzwyczajeni, ale bardzo normalnie traktowali tą sytuację. To mnie bardzo uspokoiło. Bardzo się zrelaksowałam w domu. I teraz tu we Wrocławiu jest większe zagęszczenie ludzi, więc bardziej widoczne jest, że coś się dzieje, bo ludzie chodzą jeszcze w maseczkach, mimo że już nie trzeba. Wczoraj też widziałam się z kolegą, więc pierwszy raz wyszłam do centrum od tego czasu i ten dzień nie różnił się niczym od tych innych dni przed pandemią. Było dużo ludzi, wszyscy chodzili bez maseczek.

**Jak się czujesz, jak jest dużo ludzi i są bez maseczek?**

Ja przestrzegam tych podstawowych zasad. Te stoliki też są porozkładane w odpowiednich odległościach. Natomiast prawda jest taka, że jakbym się miała zarazić, to równie dobrze mogłabym się zarazić od kogoś w Biedronce, kto ma maseczkę. Mam nadzieję, że ludzie są na tyle rozsądni, że jak czują się źle, to starają się nie wychodzić z domu albo noszą maseczki. Nie czułam jakiegoś niebezpieczeństwa. Z resztą ja zakładam, że już przechorowałam tego koronawirusa, więc czuję się z tym bardzo dobrze.

**Spotkania z ludźmi już wyglądają jak przed pandemią?**

Powiedzmy. Widziałam, że tata się cieszył z niespodzianki, ale był trochę zdystansowany ze względu na to, że chyba przeraziła go myśl, że to jest nieodpowiedzialne, ale już nie miał niczego do powiedzenia. Najbardziej w domu rodzinnym się martwiliśmy o babcię, a babcia jest po prostu pierwszy wyjadacz. Ona pierwsza wyjdzie na zakupy, choćby się ją pilnowało, prosiło. Więc, co ja będę zakazywała jej, że ona chce sobie kości rozprostować? Ona jest pierwszym pershingiem, który wychodzi na zakupy, wszystkie sklepy przechodzi. Powiem szczerze, że głównie na babcię krzyczymy, bo nosi maseczkę, ale stwierdziła, że rękawiczek już nie musi na zakupach. Jak widzę z tej perspektywy, że osoby, o które najbardziej się martwiłam są trochę zdystansowane, nie powiem, że niefrasobliwe [śmiech]. Zakładam, że babcia najszybciej by się nabawiła tego koronawirusa w sklepie, do którego ona musi koniecznie pójść, choć naprawdę nie musi, niż gdyby złapała od nas.

**Uważasz, że takie starsze osoby powinny być w jakiś specjalny sposób chronione?**

Myślę, że tak, bo są najdelikatniejsze i najbardziej podatne na jakiekolwiek zachorowania. My już wcześniej mówiliśmy babci przy tych epidemiach grypy, żeby nie wychodziła, że jej zrobimy zakupy. Ale z drugiej strony, rozumiem, że ona ma 80 lat i to też nie jest dużo, a jest sprawna i myśląca, więc nie możemy jej zabronić wychodzić z domu, pogadać z jakimiś ludźmi w sklepie, bo rozumiem, że to wpływa dobrze na jej zdrowie psychiczne.

**Powinno się jakoś zamykać te osoby?**

Ja myślę, że bardzo w porządku były te godziny dla seniorów i uważam, że byłoby dobrze, gdyby one do czasu wygaśnięcia tej pandemii one funkcjonowały. Może nie w tak szerokim zakresie, natomiast gdyby była jakaś godzina dedykowana dla seniorów, że oni mają pierwszeństwo w sklepach, nie muszą stać w kolejkach i nawdychać się oparów oddechowych, to myślę, że to byłoby dobre rozwiązanie. Bo na początku pandemii śmiać mi się chciało, bo najbardziej się wszyscy martwili o seniorów, a oni najmniej przestrzegali wszystkich zasad. Natomiast teraz widzę, że starsze osoby wciąż noszą maseczki i to jest profilaktyka. Chyba jeszcze, gdyby się tak dodatkowo chroniły. Np. pan w sklepie powiedział babci, że ona nie musi nosić rękawiczek, więc ona stwierdziła, że jak nie musi, to nie będzie.

**Teraz we Wrocławiu już się spotykasz w większych grupach?**

Zaraz jak wróciłam z Karpacza, to zdecydowaliśmy się na organizację, może nie imprezy, ale spotkania. Ale u nas w domu, nie wychodziliśmy nigdzie. To jest ogólnie doroczna impreza, która się odbywa raz w roku it o było spotkanie nr 5. Wszystkich uprzedziłam, że rozumiem, jeśli nie przyjdą, bo obawiają się spotkania z większą grupą. nie było nas jakoś bardzo dużo - może z 7, razem z nami 9. Także to było pierwsze spotkanie. Tak naprawdę ja nie żałuję, bo wszyscy jesteśmy odpowiedzialni i mam nadzieję, że unikamy potencjalnych źródeł. Np. mój mąż, którzy chciałby pojechać na Dzień Ojca do domu, a jego dziadek ma nowotwór płuc i oni się bardzo pilnują, to wiem, że tak sobie obliczył to spotkanie ze znajomymi, żeby przeczekać ok 3 tygodnie i za 3 tygodnie zamierza pojechać do domu. Żeby w razie czego odczekać. Więc tak staramy się planować jakieś rzeczy, żeby on mógł w razie czego. Miałam jeszcze taką propozycję, bo moi rodzice wyjechali w góry. W tym roku urlop w Polsce i stwierdzili, że góry będą najlepsze. Myśleli, że ja przyjadę ze znajomymi. W sumie ja pracuję, ale jak już wiedzą, że mogę pracować z domu, to znajomi sobie gdzieś pójdą, a chociaż wieczór spędzimy. I miałam nadzieję, że pojedziemy z mężem i ze znajomymi - z 2 przyjaciół. I okazało się, że ten wyjazd nie zgrywa się w tym czasie jego kwarantanny. Gdyby pojechał, to byłby już bardziej narażony. I ja go rozumiem, że gdyby coś się stało dziadkowi, to brałby to na siebie. Odpadła ta opcja.

**Ona ma takie ustalenia, a ty też próbujesz coś takiego robić?**

Nie mam takiej sytuacji jak on. Po prostu unikam spotkania z teściami. Tylko zawiozłyśmy im ostatnio ciasto z mamą, ale to normalnie na 2 metry im podawaliśmy. Chociaż zaczęłam sobie myśleć, że ja też mogę być takim wektorem, więc może rzeczywiście ograniczę takie spotkania do minimum, żeby nie stresować męża w razie czego.

**Staracie się unikać takich sytuacji, w których moglibyście się zarazić i przekazać dalej?**

Od pewnego momentu, bo po przyjeździe z Karpacza spotkaliśmy się ze znajomymi, z bardzo dużą grupą osób, więc przez kilka dni narażaliśmy się bardzo dużo. I byliśmy też u siostry Kuby, także wykorzystaliśmy jakiś swój limit, a teraz będziemy się kulturalnie przez dłuższy czas kontrolowali.

**W tym kontrolowaniu chodzi o to, żeby się nie spotykać z większą liczbą znajomych?**

Tak.

**Jeszcze coś teraz robicie, żeby nie dopuścić do zakażenia?**

Tak naprawdę cały czas to samo, czyli myjemy ręce, dezynfekujemy w miejscach publicznych, np. w galerii handlowej. Wiadomo, w galerii handlowej w maseczkach. Natomiast, jak ja wczoraj byłam na mieście, to tak naprawdę miałam tylko maseczkę 2 razy w tramwaju.

**W tramwaju trzeba?**

Tak. To jest w ogóle bardzo śmieszne, bo trzeba mieć w tramwaju i kiedy się wchodzi do kawiarni, restauracji, ale jak się usiądzie przy stoliku to nie trzeba.

**Potrafisz jakoś to wytłumaczyć?**

Ja rozumiem, że tutaj chodziło chyba o jakieś zamknięte miejsca publiczne. Jednak tramwaj jest zamkniętym miejscem publicznym, bo w przestrzeni publicznej, na podwórku, można bez maseczek. Rozumiem, że jak wchodzę do restauracji, to wchodzę do zamkniętej przestrzeni publicznej, ale jak już zajmę swój stolik, to już mam wydzieloną strefę co 2 metry, więc wtedy już można zdejmować. Tak nawet było napisane.

**A to są 2 metry odległości pomiędzy stolikami?**

Tak. Pomiędzy krzesłami jakby. Wszędzie chyba jest taki obowiązek, bo byliśmy na kawie w weekend i ja byłam wczoraj i na stolikach są informacje, że stoliki były dezynfekowane.

**Tobie to przeszkadza, że są takie nowe zasady?**

Wydaje mi się, że to normalne, że przecierają stoliki po każdym kliencie. Przyznaję, że jak wczoraj weszłam do kawiarni, to jako że wszyscy ludzie wchodzili z ulicy, czyli z otwartej przestrzeni, to ja widziałam może ze 2 osoby, które miały maseczki i przy stoliku zdjęły. Także to tak nie działa chyba.

**Jest coś, co ci jeszcze przeszkadza w tej sytuacji?**

Chyba nie.

**Jak widzisz powrót do pracy?**

Bardzo dobrze mi się pracuje w domu. No zobaczymy, będzie mi bardzo trudno wrócić do biura na pewno. Tak oficjalnie, to wracamy do biura w lipcu, ale przez jakiś remont przetrzymują nas w domu. Ostatnio rozmawiałam z tatą, jakie oni na nas oszczędzają na nas pieniądze teraz. Nie ciągniemy prądu, nie pijemy ich kawy i herbaty. Dostarczali nam kanapki czy owoce, to też nie muszą za to płacić. Nie trzeba sprzątać, robić składek na urodziny. Nagle się okazuje, że dla firm jest to opłacalne. Jestem ciekawa, jaki będzie odzew po tej sprawie. Może będą chętniej dawali home office.

**Nie jesteś zadowolona, że wracasz?**

Nie.

**Mają być u was jakieś specjalne zasady?**

To już mówili od dłuższego czasu i dlatego tak długo jesteśmy na home office, bo w tym biurze nie są w stanie nam zagwarantować tych 2 m odstępów i oddzielających ścianek. Mówiło się, że wróci połowa ludzi, tak, żeby jedno biurko pomiędzy było wolne. Ale w praktyce bardziej im się opłaca trzymać nas w domu. Wiadomo, że też problemy, bo rozumiem, że trudniej im kontrolować nas. Ale z drugiej strony wydaję mi się, że to jest opłacalne.

**Ty wracasz normalnie, jak wcześniej, że na co dzień do biura?**

No tak myślę, niestety [śmiech]. Kto wie, może gdzieś uda się ugrać potem, ale myślę, że raczej będą niechętni. Takie pojedyncze dni ok, może częściej - bo tak to miałam raz w miesiącu. To może zwiększą tą liczbę, natomiast raczej się nie zgodzą na całkowite pozostanie.

**Ty byś mogła zostać?**

Mogłabym. Jedyne, co działałoby niekorzystnie, to jednak dobrze mieć kontakty w biurze. Są koleżanki. Jednak nie chciałabym być poza zespołem, jak już w nim jestem. Byłoby mi przykro.

**Obrazki. Emocje w odniesieniu do różnych momentów.**

Ja miałam bardzo duży przeskok. W domu było tak miło, ciepło, ładnie, zielono. W urodziny mój tata wstał o 5 rano, żeby pójść mi zebrać konwalie. To było takie miłe. Tak wróciłam do Wrocławia i tak... Kto mi tu nazbiera konwalii?

**Obrazki.**

W domu czułam się na 5. Wszechświat, galaktyka. Po pierwsze, we Wrocławiu w ogóle nie widać nieba. Zawsze widać tylko kawałeczek i trudno zobaczyć coś więcej. A w domu pierwszy raz od czasów zimowych widziałam takie w całości rozgwieżdżone niebo. To mi się kojarzy ze spokojem, z głębią, z czymś duchowym. I tak było w domu. A tu we Wrocławiu kojarzy mi się 6. Też jest spokojnie, malowniczo w życiu. Jakbym miała przyrównać życie w domu, to wczesny poranek. A życie we Wrocławiu to popołudnie cały czas. Ja bardzo lubię popołudnia, więc...

**Czyli u siebie w mieszkaniu też odczuwasz spokój?**

Tak. Ale trochę mi tęskno do zieleni.

**Teraz czujesz jakieś negatywne emocje w związku z sytuacją?**

Z koronawirusem nie. Z czymkolwiek tak, bo w domu miałam taką... Straciłam dofinansowanie, związane z tym, że nie skończyłam 26 lat. I to był naprawdę bardzo duży dodatek i kiedy zatrudniałam się w tej nowej firmie we wrześniu, to zgodziłam się na nieco mniejszą pensję niż w poprzedniej firmie, bo wiedziałam, że dostaję dodatek. Byłam pewna, że do maja sobie wszystko ureguluję. Moja przełożona odchodziła i poprosiłam ją o podwyżkę. Okazało się, że polityka firmy jest taka, że nie dostaje się podwyżek po mniej niż roku pracy. Jeżeli zacznę się starać we wrześniu, to i tak mi odmówią, bo w pierwszej kolejności podwyżkę dostaną osoby, które prosiły wcześniej ode mnie. Bardzo mnie ta sytuacja przytłoczyła. Dodatkowo jedna z członkiń zespołu strasznie się wyżywa na mnie. Może na wszystkich, ale ja za bardzo to biorę do siebie. Jeszcze ostatnio cały czas robię babole głupie, więc się dołuję. Jeśli chodzi o sferę pracy, to to mnie teraz stresuje.

**To wyżywanie się jest przez Internet?**

Jest nieuprzejma. Czyta się jej wiadomości i widać, że jest zdenerwowana, ironiczna i to, co pisze to jest sarkazm. Może gdybym tylko ja tak myślała, ale wszyscy to wiedzą, tylko niektórzy nie biorą do siebie, a mi jest przykro, bo ją bardzo lubię i to nieprzyjemne, że sobie co jakiś czas sobie znajduje jakąś ofiarę. To tak na zmianę, że raz ja, a raz koleżanka. Teraz ja mam miesiąc ofiary i jest ciężko.

**Planujesz coś zrobić z tą pracową sytuacją?**

Tak, dlatego, że ta poprzednia przełożona odeszła i mąż powiedział, żebym spróbowała jeszcze raz z nową przełożoną. Tak myślę, żeby spróbować przed urlopem. Dziś ustaliłam urlop na początek lipca i spróbuję wtedy. Najwyżej, jak dostanę kosza, to naładuję akumulatorki na urlopie. Ta koleżanka, którą lubię, ale jest niemiła, będzie się prawdopodobnie zwalniać i może to być argument, żeby dali mi podwyżkę wcześniej.

**Nie myślisz o tym, żeby zmieniać pracę?**

Myślę, ale dostałam obietnicę, że dostanę we wrześniu umowę na czas nieokreślony i to nam pomoże przy kredycie, więc do wtedy chcę dotrwać. Stwierdziłam, poza tym, że muszę swój niemiecki poprawić, więc jak wyniosę go do odpowiedniego poziomu, to wtedy będę szukała czegoś innego.

**Co planujesz na urlop?**

To było tak, że musieliśmy się określić. Oczywiście osoby starsze stażem sobie wzięły najlepsze terminy. Nie bardzo wiedziałam, co powie mój mąż, ale wrócił już z postojowego do pracy i tam go poprosili, żeby wybrał urlop. I okazało się, że 6-17 lipca nikt u mnie nie ma urlopów i on też mógłby iść. Trochę mąż niezadowolony, bo lipiec deszczowy, ale zaklepaliśmy ten termin. Ja myślałam o tym, żeby na Mazury się wybrać, bo nie byłam nigdy. Albo w Bieszczady.

**Czyli w Polsce?**

Tak.

**Myśleliście o wyjazdach zagranicznych w tym roku?**

Nie. Przed koronawirusem oczywiście, ale teraz nie. Gdyby sytuacja była klarowniejsza... Teraz zastanawiam się, czy ja będę w stanie zorganizować te wakacje na miesiąc przed. Ale zakładam, że będę i jakoś to ogarnę. Natomiast, gdybym wiedziała, że nie zastanie mnie w innym kraju coś nieprzyjemnego, a wolałabym w tym roku nie ryzykować z wypadami do Włoch, Hiszpanii, to bardzo chętnie pojechałabym dość niedaleko, bo np. na Bawarię. Ale tu jest problem, bo jakby pojawiła się jakaś druga fala, to istnieje możliwość, że utkniemy w tych Niemczech i jest to nieprzyjemne. Nie wiem, jak miałabym to zrobić.

**Kiedy takie wyjazdy dla ciebie będą bezpieczne?**

Na pewno pojawi się jakaś druga fala. Myślę, że jak już ta 2 fala ucichnie. Ja dopiero pod uwagę biorę kolejne wakacje Natomiast myślę, że będzie można sobie robić jakieś *city break* w listopadzie, grudniu.

**Jak widzisz emocje w otoczeniu u innych?**

Mam wrażenie, że podejście jest podobne, jak u mnie. Może nawet ja się za bardzo przejmuję, bo mamy znajomych, gdzie kolega jest fizjoterapeutą, czyli jeździ po tych wszystkich babciach, itd., a mimo to, co weekend jeździ do domu. A nie musi, jeździ z nudów. Ja bym starała się może to ograniczyć, wiedząc, że mam cały czas kontakt z różnymi ludźmi. Także powiedziałabym, że zachowują się zupełnie jak przed pandemią. Tyle, że jak wszyscy do nas przyszli, to pierwsze co, to myjemy ręce. I nie pijemy ze swoich kubeczków.

**Myślisz, że to zostanie na dłużej? Jak mycie rąk np.**

Myślę, że może zostać, bo to już taki odruch się zrobił.

**Jak u was teraz wygląda planowanie zakupów?**

Teraz jest tak, że mąż jest na 80% etatu, czyli ma piątki wolne. Ze względów praktycznych, żeby nie stać w kolejce po południu czy w sobotę rano, chodzi na zakupy w piątek rano. Więc ta lista pozostała. Jako że teraz oboje pracujemy w domu i po pracy nam się nie chce wychodzić na zakupy, to ta lista jest dobrym rozwiązaniem, żeby zapewnić jedzenie na cały tydzień.

**Przed pandemią też była lista?**

Nie. Bardzo rzadko. Ja zazwyczaj robiłam kilka małych zakupów i to było nieoszczędne mocno.

**A teraz według ciebie jesteśmy w trakcie czy po pandemii?**

Ja się czuję tak, jakbyśmy byli już po. Natomiast wiem, że tak realnie jesteśmy w trakcie. Mnie spokój już ogarnął.

**Te nowe rzeczy związane z zakupami zostaną na dłużej?**

Mam nadzieję, że tak.

**A jest coś, że wróciło z zakupami do stylu sprzed epidemii?**

Już się swobodniej chodzi na zakupy. Jak organizowałam to spotkanie, to mąż poszedł i do Biedronki, Areny, po piwo, czyli przeszedł kilka sklepów, a wcześniej poszedłby do jednego.

**Zauważyłaś, że dzięki takim zakupom jak podczas pandemii, bardziej oszczędzacie?**

Tak. Dlatego, że jak robiłam kilka zakupów, kilka małych rzeczy, to one bardzo szybko znikały i cały czas musiałam dokupować coś nowego. To trochę dziwne, bo w sumie teraz kupujemy tyle samo. Może chodzi o to, że jakieś bzdurki dokupowałam przy mniejszych zakupach, których nie potrzebowaliśmy. A teraz robimy jedne duże, to wiadomo, że jak człowiek patrzy, że ma dużo rzeczy w koszyku, to raczej się ogranicza.

**Opowiedz o tej wizycie w galerii.**

Jeszcze przed wyjazdem do domu poszłam pierwszy raz do galerii. To była totalnie nieudana wyprawa dlatego, że wtedy znosili te obostrzenia dopiero i trzeba było... Teraz też trzeba nosić rękawiczki niby, ale inaczej to hula. Jak wtedy poszłam, to na palcach 2 dłoni można było policzyć ilość ludzi w galerii. Było mało ludzi, ja miałam grubą maseczkę i myślałam, że zemdleję z tymi zakupami. A musiałam iść kupić sobie skarpetki. Dostałam kartę podarunkową od teściowej. Nie wiedziałam, do kiedy jest ważna karta. Poszłam do tego Medicine, wybrałam te skarpetki, napociłam się jak głupia - po prostu spływałam potem. Jeszcze stwierdziłam, że pójdę na zakupki malutkie i się niedobrze skończyło w tym Carrefour. Wracałam do domu, pot się ze mnie lał, zrobiło się ciepło, a ja byłam w jakiejś grubej kurtce. Nie wiedziałam, co, gdzie dać. Jeszcze dodatkowo zrobiłam sobie koka i ten kok mi się rozwalił z tego potu i wyglądałam, jakbym wyszła ze śmietnika. Ja się dziwiłam, dlaczego ochroniarze się na mnie patrzą. Także to wyjście to była katastrofa. Ale potem zaczęli znosić kolejne obostrzenia i w sobotę byliśmy z mężem najpierw na kawie w tej samej galerii. I dostaliśmy na Dzień Dziecka od teściowej kolejne karty do Medicine i chcieliśmy coś kupić, ale nie było nic fajnego. Więc ostatecznie przeszliśmy ten Medicine, poszliśmy do Rossmana i wyszliśmy. Ale ludzi było już bardzo dużo. Bardzo mało ludzi w rękawiczkach, wszyscy w maseczkach. Także jak normalne wyjście. Oprócz tego, że wszędzie płyny odkażające.

**Jak teraz w galeriach jest z ograniczeniami?**

W Medicine, bo mąż przymierzał, że wpuszczają pojedynczo do przebieralni i po każdym kliencie przebieralnia jest dezynfekowana, blokowana na jakiś czas. Wpuszczają do co drugiej, żeby zachować odległość. Tą, z której wyszedł klient dezynfekują i zamykają. Np. parownicami zasłonki czyszczą i te mierzone rzeczy też.

**Co o tym myślisz?**

Myślę, że to właściwe, tylko czy to tak realnie... Ja rozumiem, że ta para jest gorąca, ale też nie może być za bardzo. Myślę, że to taka pro forma. Natomiast człowiek czuje się bezpieczniej, jak widzi się, że higiena jest zachowana.

**Maseczki trzeba nosić?**

Tak.

**Byłaś w restauracji?**

W sobotę w galerii byliśmy w kawiarni i ja wczoraj też byłam w kawiarni.

**Fryzjerzy, kosmetyczki - byłaś już?**

Oczywiście. Paznokcie zrobiłam. Miałam obgryzione, więc nie widać w ogóle, ale czuje się lepiej. W ogóle, dlaczego wróciłam po 2 tygodniach do Wrocławia? Bo miałam od 3 tygodni klepnięte paznokcie, więc dlatego wróciłam. W salonie było tak samo po tym względem, że te panie zawsze mają maseczki, jak piłują paznokcie. Ale od pani oddzielała mnie ścianka z pleksi. Także paznokcie wkładało się w dziurkę i ona robiła to, co umie, bo ja nie umiem [śmiech].

**Ty myślisz, że te ścianki, itd. rzeczywiście przed czymś chronią?**

Myślę, że tak, bo jednak, kiedy podawałam dłoń przez szybkę, to ona mi ręce odkaziła, ale za każdym razem tak robią. Zastanawiałyśmy się z koleżankami z pracy czy to było sensowne, żeby zamykać takie salony, bo takie salony kosmetyczne dbają cały czas o higienę. Nie dość, że stoliki i sprzęt są odkażane, to też lampy są czyszczone. Wydaje mi się, że w dobrym salonie cały czas się dba o higienę. Problemem była tylko ta odległość, ale to można było rozwiązać obowiązkową ścianką z pleksi. Nie wiem, jak obejść wtedy zabiegi na twarz, ale zakładam, że dało się coś wymyślić - może te przesłony na twarz. Tutaj niepotrzebnie zatrzymywali przedsiębiorców.

**W salonie ty nie musisz mieć maseczki?**

Miałam. Nie pamiętam, czy klientki wszystkie miały, ale chyba tak. Nie wiem, czy są obowiązkowe.

**Jeszcze z jakichś usług korzystałaś?**

Nie.

**Niedługo mają być otwierane kina, siłownie, teatry. Planujesz się wybrać na siłownię?**

Już się umawiałam z koleżanką od dawna, że zaczniemy na stepy chodzić. I już dostałam wiadomość... Tylko ja nie wiem czy to siłownie, czy miejsca aktywności takie otwarte. Chodziło bardziej o boiska, korty. Wiem, bo odblokowali mi Multisporta, który był zamrożony na czas pandemii i stąd wiem. I o kinach i teatrach też słyszałam, tylko nie wiem, w której fali - czy teraz, czy jakaś kolejna będzie.

**Czyli siłownie mają być nadal zamknięte?**

Nie jestem pewna. Zrozumiałam z tego maila tyle, że... Oglądałam na bieżąco te informacje o kolejnych zniesionych zaostrzeniach, ale nie pamiętam teraz. Wydaje mi się, że chodziło tylko o takie otwarte.

**Śledzisz nadal informacji?**

Ja ogólnie bardzo mało się w to angażowałam od początku. Najważniejsze fakty raczej mnie nie ominą i też nie chcę być taką ignorantką. Natomiast staram się sortować te wiadomości. Nie wczytywać się mocno w tematy, nie dywagować, tylko śledzić, co się dzieje najważniejszego. Czyli wiem, jakie są etapy, słuchałam, wiem jaka jest liczba zachorowań w Polsce, mniej więcej wiem, co się dzieje w innych państwach, natomiast nie zagłębiam się mocno w te tematy.

**Czyli śledzisz liczbę zakażonych?**

Tak i trochę mnie to przeraża.

**Ile jest teraz?**

Nie pamiętam dokładnej liczby. Zapamiętałam tylko odczucie, że jak ostatni raz to widziałam ze 3 dni temu, to zaskoczyło mnie, że to tak duża liczba.

**Szukałaś informacji, ile jest zakażonych?**

Nie. Gdzieś mi wpadło.

**Technologia. Słyszałaś o jakichś aplikacjach na czas pandemii?**

Czytałam ostatnio o jakimś pomyśle na aplikację. Nie wiem, czy taka istnieje. Nie pamiętam, co to był za pomysł. Pamiętam tylko, że związana z koronawirusem. Wiem na pewno, że dużo galerii handlowych na stronach podaje, że mają jakieś systemy liczenia ludzi na m2. Nie wiem, co to za system. Zakładam, że jak ktoś wchodzi, to liczą, ile osób... Ale nie wiem, w ogóle się tym nie interesowałam. Ja ostatnio w ogóle usunęłam Facebooka, więc... Tzn. z telefonu, nie, że w ogóle.

**Tak robisz raz na jakiś czas?**

Tak.

**Opis pierwszej kategorii - pomysł 1.**

Myślę, że to aplikacja japońska. Skojarzyło mi się, nie wiem, dlaczego. Oni mieli tam bardzo zakrojoną technologicznie kontrolę. Uważam, że jeżeli mielibyśmy złą sytuację. Jest to jednak aplikacja śledząca. W wyjątkowych okolicznościach mogłaby zostać użyta z korzyścią, natomiast niepokoi mnie mocno fakt, że ma dostęp do takich informacji, dotyczących zdrowia. Rozumiem, że podczas stanu wyjątkowego byłaby zalecana, natomiast pytanie, jakie dane zebrała i co z nimi będzie robiła.

**Co to by było - ta wyjątkowa sytuacja?**

Mówię stan wyjątkowy, czyli mamy pandemię i państwo wprowadza stan wyjątkowy.

**Mówiłaś wcześniej, że powinien być wprowadzony taki stan?**

Tak.

**I w takiej sytuacji można takie aplikacje wprowadzić?**

Myślę, że tak. Jest to trochę zamach na naszą personalną stronę, natomiast są sytuacje, w których jestem w stanie to zrozumieć. Dla dobra ogółu.

**Jak podchodzisz do prywatności w Internecie?**

Nigdy nie ustawiam jakiegoś namierzania w telefonie - nie lubię takich rzeczy, denerwują mnie. Zawsze mam potem problem z nawigacją na Google Maps, bo mi nie może złapać, jak już włączę. Jestem mocno zdystansowana, boję się. Uważam, że te dane mogą łatwo wypłynąć. Staram się danych krytycznych nie podawać. Mam aplikację bankową, ale nie aktywowałam wszystkich funkcji - mogę tylko sprawdzać stan konta i nic więcej. Bo jednak uważam, że taka dobra stara zdrapka jest bezpieczna. Rozumiem, że te weryfikacje dwupoziomowe też są bezpieczne i trzeba mieć też bezpieczne hasło. Ale mimo wszystko przezorny zawsze ubezpieczony. Z resztą, nie mam teraz Facebooka, nie tylko dlatego, że robię sobie przerwę, tylko ściągnęłam wirusa na telefon. Zaczęliśmy oglądać serial "Co robimy w ukryciu". Bardzo spodobał mi się motyw przewodni i stwierdziłam, że ściągnę to sobie na dzwonek. Zamiast zrobić to na komputerze, zrobiłam to przez telefon na nie wiem, jakiej stronie. I zdziwiło mnie, że coś się ściągnęło i nie wiadomo, gdzie. Nie mogliśmy znaleźć tej strony potem i doszliśmy do wniosku, że mogłam ściągnąć wirusa. I stwierdziłam, że już mi wszystko jedno i usunęłam wszystko z telefonu. Nawet, jak byłam w Rossmanie, to nie miałam aplikacji ściągniętej.

**Opis pierwszej kategorii - pomysł 2.**

Uważam, że potrzebna i jak najbardziej tak. Kwarantanna to jest coś z góry narzuconego i to nie jest coś, co robimy, bo chcemy albo możemy odmówić. Tylko uważam, że skoro jesteśmy potencjalnym zagrożeniem, to jesteśmy zobligowani wręcz do tego, żeby przestrzegać pewnych zaleceń i zgodzić się na uchylenie rąbka prywatności. Jak najbardziej się zgadzam. Szczególnie, że ludzie nie przestrzegali kwarantanny, wychodzili i zarażali innych. Słyszałam historie od kolegi policjanta, że sprawdzali kogoś na kwarantannie i najpierw dzwonili, że będą jechać. Dzwonili do członka rodziny, który nie jest zarażony i ten członek zaczął mówić po arabsku, więc oni się zdziwili. Podjechali i się okazało, że rodzina sobie zorganizowała kilkudniową libację, więc byli tak pijani, że nie dało się ich zrozumieć. To mi dało do myślenia, bo jak już się upijają, to może mieliby ochotę wyjść. Z tego co wiem, próbowali uciekać przez okno.

**Jak te aplikacje miałyby działać?**

Musiałoby to być trochę jak opaski policyjne, jak się wychodzi na warunkowym. Telefon mogę komuś przekazać, a opaski byłyby dobrym rozwiązaniem. Czyli 2 tygodnie siedzę w domu, opaska mnie namierza, żadna policja nie musi przyjeżdżać. Ewentualnie tak warunkowo, żeby sprawdzić, że nie zdjęłam jej w nielegalny sposób. Myślę, że oszczędność czasu i pieniędzy to byłaby na pewno, tylko teraz pytanie, ile taka opaska kosztuje. Myślę, że nie musi być jakoś specjalnie droga - teraz wszystko ma GPS.

**Masz jakieś obawy, związane z taką aplikacją?**

Zawsze można prawa nadane nadwyrężyć. Równie dobrze można by poddawać kwarantannie osoby, które są niemile widziane, tylko, żeby się ich pozbyć na jakiś czas. Myślę, że to, co mówię, jest tak wydumane, że... Raczej nie mam obaw.

**Opis pierwszej kategorii - pomysł 3.**

Aha, że po fakcie... To mogłoby być w porządku, bo ja jestem w stanie wrócić do domu, ściągnąć jakąś aktualizację informacji albo ewentualnie wpisać swoją lokalizację, gdzie przebywałam. Jestem w stanie powiedzieć, że to ciekawa aplikacja, szczególnie, że zakładam, że nie wymaga stałego połączenia. Wystarczy zasugerować systemowi, gdzie byłam i on ściąga mi informacje od innych użytkowników.

**Jak ta aplikacja wykrywa te osoby, które są zakażone?**

To pewnie trzeba by mieć tą drugą aplikację, która mierzy temperaturę ciała i jakieś parametry. Może wtedy. Że jakby one ze sobą współpracowały to miałoby sens.

**Czyli nie chciałabyś na bieżąco udostępniać swojej lokalizacji żadnej aplikacji?**

Tak. Nie mam niczego do ukrycia, ale nie lubię.

**A dlaczego tego nie lubisz?**

Nie ufam takiemu ciągłemu przepływowi danych, bo uważam, że może wyciągać coś, czego ja nie chcę ujawniać. Nie mówię, że mam coś do ukrycia, natomiast kiedy coś się dzieje bez mojej zgody, to jest to frapujące i trochę przerażające.

**To budzi strach i brak zaufania, że cały czas dane są pobierane?**

Tak.

**Ale ta aplikacja - pomysł 3, jest dla Ciebie do zaakceptowania?**

Jak najbardziej.

**Co byś zrobiła, gdybyś dowiedziała się od aplikacji, że byłaś w miejscu, gdzie można było się zarazić?**

Myślę, że trzeba się wtedy... Nie wiem, czy jest coś takiego, jak lekka kwarantanna. Jestem ciekawa, bo gdyby władze miały dostęp do takich informacji, to automatycznie taki człowiek znajdowałby się od razu na kwarantannie przymusowej. Najgorsze jest to, że taki człowiek bez kontaktów... Tak mi się przypomniało, bo koleżanka fizjoterapeutka mówiła, że robili im testy i mieli wyniki w ciągu 1 dnia. Ale tak realnie, na wyniki czeka się nawet tydzień. Więc czekam tydzień na wynik, czy mam koronawirusa. Potem okazuje się, że jednak mam i muszę siedzieć jeszcze 2 tygodnie w domu. Np. mój mąż bardzo stresuje się tym dziadkiem. Ja mu powiedziałam: kup ten test i zrób - na 100% mieliśmy koronawirusa. Nie ma możliwości. Od stycznia byliśmy 3 razy razem chorzy, bo wiadomo, że się zarażamy nawzajem i za każdym razem były te same objawy. Więc ja myślę, że któryś z nich był koronawirus. Ale mój mąż mówi, że kupię test, testy są nadzorowane, okaże się, że mam koronawirusa i jestem wyłączony z życia bez żadnej wiedzy, bez uprzedzenia na 3-4 tygodnie. I to jest trochę przerażające, bo przecież praca... Jak z tego wybrnąć? Kontrole, zobowiązania.

**Nie kupiliście testu?**

Nie. Jest to nieprzewidywalne, dlatego że ten test widzą wszyscy. I jak zrobimy ten test, to nie ma wyjścia - już wpada się w tą machinę.

**Ta aplikacja mogłaby ciebie informować, ale nie powinna tych informacji przesyłać do rządu, który by automatycznie wysyłał na kwarantannę?**

Szczerze mówiąc, tak obiektywnie, racjonalnie, to powinna informować. Żeby opanować pandemię i zachorowania, to trzeba takim osobom narzucać kwarantannę. Po to ona jest. Ale tak od strony życiowej, praktycznej, to wolałabym oczywiście, żebym mogła sama zadecydować. Ale są ludzie, którzy są nieodpowiedzialni i potem by chodzili i rozsiewali wirusa. Myślę, że te informacje powinny być przesyłane dalej i taki człowiek w otoczeniu powinien być poddany badaniu i wysyłany na kwarantannę.

**Nie uważasz tego za utratę prywatności?**

Nie. Wydaje mi się, że to taka sytuacja, w której dobro ogółu przewyższa prywatność.

**Do którego momentu powinno być takie coś utrzymane?**

Do momentu, w którym zagrożenie nie będzie już takie duże i realne. Nie znam się na tych parametrach. Rozumiem, że to może być też jakieś narzędzie do niepoprawnego sterowania ludźmi. Trudno jest w dzisiejszych czasach, szczególnie w Polsce, wynaleźć te prawdziwe informacje. I to mnie niepokoi. Ale może jakaś komisja...

**To niebezpieczeństwo jest teraz zagrażające?**

Myślę, że jest.

**Trudno określić ten moment, kiedy by nie było?**

Tak. Dlatego, że nie znam się na nauce o wirusach i nie jestem w stanie powiedzieć, kiedy ten wirus zaczyna wymierać.

**Opis pierwszej kategorii - pomysł 4.**

Myślę, że w porządku. Szczególnie, że monitoring jest teraz wszędzie.

**Czyli rozpoznawanie twarzy to nie jest coś, co twoją prywatność narusza?**

Wiem, że są takie systemy i robią to cały czas. Tak naprawdę my o tym nie wiemy, ale były historie tych porwań, kiedy oni byli w stanie naprawdę w ciągu bardzo krótkiego czasu określić, co się stało z jakimś dzieckiem. Bo wszędzie są monitoringi, które wprowadzone w odpowiedni system są w stanie tą twarz identyfikować. Uważam, że to nie jest jakiś wielki zamach na naszą prywatność. Wychodzimy gdzieś - to nie jest tak, że mamy monitoring w domu, tylko gdzieś w przestrzeni publicznej. Jest to jednak publiczna przestrzeń.

**Opis drugiej kategorii - pomysł 1.**

Bardzo w porządku. Myślę, że przydatne. Na razie chyba trochę nierealizowalne, bo to drogi interes.

**Ale nie masz obaw?**

Pracowałam jakiś czas temu w UPS. Byłam księgową. Ja pamiętam, że oni 2 lata temu już testowali takie rozwiązanie, że dron dostarcza paczki. Dlatego, że w Ameryce są te miasteczka mocno od siebie oddalone i to było rozwiązanie, mające zaoszczędzić czas kurierom. Stawiają drona na samochodzie, on sobie leci dostarczyć paczkę do farmerów, a kurier objeżdża innych ludzi w tej miejscowości. Także uważam, że jak najbardziej.

**Czyli to nie było w Polsce?**

To był amerykański filmik.

**Czyli ok, ale że za drogie, to nie ma tego w Polsce?**

Tak mi się wydaje. Nie jestem w stanie powiedzieć, jaki udźwig ma taki dron. Zakładam, że musi być odpowiednio lekki i silny, co musi być zbudowane z odpowiednich materiałów, które zakładam, że są drogie.

**Opis drugiej kategorii - pomysł 2.**

Jak najbardziej.

**Korzystałabyś?**

Nie korzystałabym, ale myślę, że dla wielu osób byłoby to bardzo dobre rozwiązanie. Jak byłam u tej kosmetyczki, to było tam włączone radio RMF FM. Jest taka akcja, że dzwoni się do radia i mówi się o swoim bohaterze. Był taki pan, który zadzwonił i powiedział: bardzo dziękuję mojemu zięciowi, nie lubię go, ale podczas kwarantanny przynosił mi jedzenie i picie do domu. Także zakładam, że jest to dobre rozwiązanie. Ten zięć nie musiałby przychodzić do teścia, który go nie lubi, bo być może któryś z sąsiadów byłby w stanie temu panu pomóc.

**Opis drugiej kategorii - pomysł 3.**

Zagadnienie sztucznej inteligencji jest bardzo złożone i dyskusyjne. Z tego opisu, to myślę, że bardzo ciekawe rozwiązanie technologiczne. Ale może naczytałam się za dużo opowiadań Flippa Dicka. Rozumiem, że takie rzeczy są w stanie się wyrwać może w dalekiej, niedalekiej przyszłości spod kontroli. Czytałam takie opowiadanie, że była wojna jądrowa i zbudowano maszyny, które pomimo działań wojennych, były w stanie do każdego człowieka żyjącego podstawowe produkty. I te maszyny miały swoje ufortyfikowane fabryki, w których produkowały te rzeczy. Wojna się skończyła, a te maszyny nie były w stanie się wyłączyć z trybu wojennego, a przyjęły monopol na produkcję. Ludzie próbowali przechytrzyć maszyny, aby przejąć znów władzę nad własnym życiem. I tak mi się skojarzyła ta aplikacja z tym opowiadaniem. Bardzo paralelne.

**Obawiałabyś się, że to się może wymknąć spod kontroli?**

W tej chwili, zupełnie racjonalnie, to nie. Ale myślę, że na dłuższą metę, to mógłby być problem. Te systemy się rozwijają. Ja niedużo o tym wiem, ale zakładam, że bym się bardzo zdziwiła, jakbym poczytała o takich rzeczach.

**Prezentacja aplikacji ProteGO Safe. Co myślisz o tej aplikacji? Co ci się podoba, a co budzi obawy?**

Kiedy mówiłaś o tych propozycjach technologicznych i byłam tam aplikacja, która monitoruje zdrowie, to ja byłam na nie. Ale jak to przeczytałaś, to jestem na tak. Uważam, ze to bardzo ciekawe rozwiązanie, potrzebne. Nie wiedziałam o tej aplikacji. Z tego, co widzę, ona nie wymaga stałego przyłączenia do sieci. Nie jest tak, że zbiera cały czas dane, tylko przechwytuje sygnał Bluetooth, a wiadomo, że to można wyłączyć. Co też zmniejsza jej skuteczność, bo ludzie już długo nie używają Bluetooth. Natomiast rozumiem, że jeżeli ktoś chce wziąć udział w tym projekcie, to włącza Bluetooth. Podoba mi się to, że w jednym miejscu mamy podstawowe informacje na temat koronawirusa potwierdzone. Bo w tym natłoku, gdzie nas karmią Internet i telewizja, trudno było wyłapać takie ogólniki. Także fajnie, że wszystko w jednym miejscu. Ta kontrola swojego stanu zdrowia to też bardzo ciekawa opcja. Szczególnie przekonało mnie zapewnienie, że będzie to służyć lekarzom. Rozumiem, że jeżeli mam tą aplikację, zapisuję to sobie, to nie są dane, które cały czas wypływają, tylko są w stanie... Z resztą tak naprawdę teraz wszystko jest w jakichś bazach danych. Nasze karty zdrowia też.

**Gdybyś pobrała tę aplikację, to dałabyś jej dostęp do Bluetooth?**

Chyba nie. Po pierwsze dlatego, że wydaję mi się, że tu ten Bluetooth tak wprowadzono prowizorycznie. Jestem przekonana, że chodziło tylko o to, że ludzie nie byli przyzwyczajeni do tego typu aplikacji, stąd ten Bluetooth, który daje im wybór. Natomiast w przyszłości, jeśli mielibyśmy jakieś pandemie, to na pewno będzie to zmienione na GPS. Jakby się chcieli bardziej przypodobać takim, jak ja.

**Co sądzisz o dzienniku zdrowia?**

Uważam, że dziennik zdrowia jak najbardziej. Szczególnie w tych początkowych etapach, kiedy potencjalność zarażenia jest największa. To by się przydało bardziej na początku pandemii, w marcu. Kiedy pogoda była nieprzyjemna i my przez długi czas nie wiedzieliśmy czy spotkaliśmy się z kimś zakażonym czy nie. Także myślę, że to daje bardzo dobry odczyt. I ta współpraca później z lekarzami.

**To jest rozwiązanie na te pierwsze etapy pandemii?**

Przy założeniu, że w kolejnych etapach ludzie przestrzegają zasad unikania koronawirusa.

**Ta aplikacja powinna być obowiązkowa?**

Myślę, że dobrowolna. Tzn... Jeżeli chodzi o jej cel, to powinna być obowiązkowa, bo daje bardzo dobrą podkładkę dla późniejszego leczenia i samokontroli. Ale nie wiem, jak trzeba by było zmusić ludzi, żeby wszyscy to ściągnęli. Natomiast myślę, że gdyby taka sytuacja się powtórzyła, to dlaczego nie, mogłaby być obowiązkowa.

**Czyli jest ta obawa, że mogłoby się to zamienić w GPS i pobieranie danych o lokalizacji?**

Danych ogólnie. To też jest tak, że teraz te karty zdrowia, informacje personalne, są na serwerach, w chmurach, ale są odpowiednio zabezpieczone. Tutaj, w telefonie nie ma się kontroli - przynajmniej ja nie wiem, jak to kontrolować.

**A czy to, że to aplikacja stworzona przez rząd, daje bezpieczeństwo?**

Raczej tak.

**Czyli problemem jest to, że jest na telefonie ta aplikacja, a nie kto ją zrobił?**

Tak myślę. Ale znowuż jest najpraktyczniejsza na telefonie. Z drugiej strony nie podajemy tutaj żadnych krytycznych informacji. Myślę, że w obecnej sytuacji ta aplikacja by się bardzo sprawdziła, natomiast tak perspektywicznie to zawsze może stać się narzędzie...

**Słyszałaś o tej aplikacji wcześniej?**

Nie.

**Prezentacja aplikacji Kwarantanna Domowa.**

Uważam, że to o wiele ciekawsza aplikacja niż ta poprzednia. Może nie w ten sposób, że ciekawsza... Ale czytam jej ideę i myślę, że w końcu znalazło się coś w dobrym miejscu w dobrym czasie. To jest trochę taka namiastka tych opasek lokalizujących. Uważam, że jeżeli osoba jest zarażona... W ogóle cieszę się, że jest to obowiązkowa aplikacja. Nie wiem, jak to rozwiązano w przypadku osób starszych albo takich, którzy nie mają telefonów. Natomiast zgadzam się z jej założeniami. To system, który daje możliwość rzeczywiście monitorować osobę, która siedzi w domu. Bo policja przyjedzie w określonej godzinie, w określony dzień. Wiadomo, że teoretycznie się nie wie, ale koleżanka była na kwarantannie i mówiła, że przyjeżdżali o tej samej godzinie. Tutaj mamy potwierdzenie, mamy jakąś namacalną rzecz, którą można monitorować cały przebieg, która sama nadpisuje kolejne informacje i która jest intuicyjna. To mi się bardzo podoba. Podoba mi się to, że ona dotyczy tylko określonego czasu. To nie jest tak, że ja muszę mieć tą aplikację codziennie, tylko na określony czas i to świadczy na jej korzyść.

**Co myślisz o tym, że jakaś aplikacja jest obowiązkowa?**

Nie mam z tym problemu. Jest to dla mnie trochę dziwne, bo nigdy wcześniej się nie spotkałam z aplikacją obowiązkową. Natomiast tak naprawdę, kiedy zakładamy konto w banku, to korzystanie z bankowości tylko przez komputer stwarza nam wiele niedogodności. I w takim wypadku taka aplikacja bankowa staje się wręcz obowiązkowa, bo inaczej trudno nam funkcjonować w dzisiejszych czasach z płatnościami telefonem, itd. Także jestem w stanie to zrozumieć. Uważam, że takie wyjątkowe sytuacje mogą też powodować takie wyjątkowe narzędzia. Tylko nie wiem, jak to działa u tych osób starszych albo u tych, którzy nie mają telefonu - czy mają jakąś alternatywę dla tego rozwiązania.

**Dalszy opis aplikacji.**

Śmieszna sprawa z tymi zdjęciami. Natomiast myślę, że bardzo dobrze dopełniają całości. Dlatego, że tak naprawdę z nimi jest powiązana lokalizacja. Dodatkowo zawiera informacje podstawowe i pomocne przy kwarantannie. Ja myślę, że bardzo ciekawe i bardzo potrzebne narzędzie. Nie wiedziałam, że rząd stworzył coś takiego.

**Twoi znajomi, którzy byli na kwarantannie, korzystali z tej aplikacji?**

Nie wiem. Koleżanka była na kwarantannie kilka dni po wybuchu w Polsce, więc może tej aplikacji nie było jeszcze. Wiem, że do niej policjanci przyjeżdżali.

**A to, że aplikacja nie zastępuje wizyt policji?**

Tak, to też jest ok. Jak mówiłam, tu nie chodzi o nas, tylko o ludzi, którzy są na zewnątrz. Wirus bardzo szybko się rozprzestrzenia, można przejść chorobę bezobjawowo. I ktoś, kto czuje się dobrze, myśli sobie, że nic się nie stanie, jak na chwilkę wyjdzie z domu. Myślę, że takie narzędzie, może nie terroru, ale nacisku, jest potrzebne w takiej sytuacji. To nie jest tak, że jest już na zawsze, tylko na określony czas, na te 2-3 tygodnie. Uważam, że naprawdę w tej sytuacji jest to jak najbardziej wytłumaczalne.

**A ta konieczność robienia zdjęć jest akceptowalna?**

Rozumiem, że chodzi im o to, że sprawdzają, czy ktoś nie wziął mojego telefonu. Np. pracuję w Niemczech i jak mam nie wrócić do pracy - zostawię córce telefon. I teraz telefon ściąga dane z domu, ale pan pracuje w Niemczech. Także myślę, że jest to bardzo dobre rozwiązanie i nie wiem, jak inaczej mieliby to rozwiązać, skoro to jest moje narzędzie mobilne.

**Myślisz o przyszłości po pandemii?**

To nie jest tak, że ja uważam, że już się wszystko skończyło. Tylko po prostu nie czuję już zagrożenia. Natomiast rozumiem, że są nadal zachorowania i nadal się pilnuję. Jeśli chodzi o przyszłość, to trudno mi sobie wyobrazić ten przyszły świat. Chodzi mi o ten moment przejściowy. To, co, nagle powiedzą, a to już nie nosicie maseczek. Nagle znikną wszystkie środki ochrony z kawiarni. Bardzo trudno mi to sobie wyobrazić, tak samo jak normalny powrót do pracy. Natomiast bardzo szybko przystosowaliśmy się do warunków domowych i myślę, że tak samo szybko przystosujemy się do normalnego życia.

**Myślisz o tym, jak będzie wyglądała przyszłość Polski?**

Zobaczymy, wybory chyba niedługo. Mam nadzieję, że rozwiną pewne rozwiązania, związane z obronnością zdrowotną. Myślę, że powinny powstać jakieś specjalne komisje, bo w sumie, grypa była zawsze i zawsze były jakieś epidemie grypy. Natomiast wydaję mi się, że jako że cały czas ta łatwość w podróżowaniu i w transporcie się zwiększa, to coraz częściej będziemy mieli do czynienia z wirusami z innych kontynentów i wydaje mi się, że trzeba by było zadbać bardziej o taką obronność biologiczną. Bo nikt nie był przygotowany na to. Uważam, że Polska sobie całkiem dobrze poradziła, ale nikt nie był przygotowany, a jest to przecież realne zagrożenie. Jesteśmy ludźmi, chorujemy, wirusy się przenoszą z człowieka na człowieka - to nie jest jakaś filozofia wielka. Brano pod uwagę, ale lekarze, ludzie zajmujący się wirusami wiedzą i wiedzieli, że do czegoś takiego dojdzie i to my byliśmy niedoinformowani - społeczeństwo. Myślę, że trzeba byłoby troszkę popracować nad uświadamianiem, nad podstawową higieną. Przyznaję, że u mnie w pracy zdarzało mi się przynajmniej raz na tydzień widzieć osobę, która była w toalecie i nie umyła rąk. Wydaje mi się, że jest to podstawowy odruch - bycie w toalecie i umycie rąk. A mimo wszystko są osoby, które o taką podstawową higienę nie dbają, to co mówić o wyższej. Też popatrzmy na Japończyków. Oni od kiedy pamiętam noszą te maseczki i chronią się nie tylko przed smogiem, ale też przed wirusami. Myślę, że to nie będzie w Polsce już takie dziwne.

**Czyli teraz już to nie będzie zaskakiwało, że ktoś chodzi w maseczce?**

Był pewien kontent ludzi, którzy nosili maseczki antysmogowe jeszcze przed koronawirusem. Jak się wchodzi na jakieś strony internetowe, do różnych sklepów, to są te maseczki antysmogowe, nawet w Medicine. I widziałam w tym roku może 2 osoby w maseczkach antysmogowych. Więc mnie to dziwi - niby normalne, a nikt nie nosi. Teraz będzie to już bardziej dopuszczane i ludzie będą normalnie chodzili w maseczkach.

**Zmieni się w jakiś sposób sytuacja społeczne? Jakichś grup?**

Wiadomo, że w tej chwili dotknięte są głównie te grupy osób, które z jakiegoś powodu straciły płynność finansową, czy mają zagrożenia takie ekonomiczne. Dla tych osób na pewno jest to duża zmiana. Co więcej, ja przed koronawirusem nie bałam się zmiany pracy. I rozmawiałam nawet ze znajomym - on też miał zmieniać pracę. A teraz rozmawiamy - kurczę teraz strach. Jednak coś tam siedzi z tyłu głowy i powstrzymuje nas przed radykalnymi, dużymi zmianami. Kojarzy mi się to z filozofią pokolenia moich rodziców. Mój tata ma 60 lat, mama jest nieco młodsza, ale mama pracuje 30 lat w tym samym zakładzie pracy. To samo tata Kuby - nie lubi swojej pracy, ale pracuje tam 30 lat. Dlaczego? Bo jest praca, a jak jest praca, to trzeba się jej trzymać. Ja pamiętam, że się podśmiewaliśmy, że my tak nigdy nie zrobimy, bo jest tyle pracy. Trzeba się rękami i nogami trzymać, żeby nie znaleźć pracy i tak było przed koronawirusem. A teraz może być problem i to zmienia moje nastawienie trochę. Trzeba być ostrożniejszym.

**Któreś z ograniczeń powinny zostać jeszcze utrzymane na dłużej?**

Szczerze, trochę się dziwię, że tak szybko pozwolili nie nosić maseczek na podwórku. I teraz naprawdę nikt nie chodzi w maseczkach. Dochodzi do takich sytuacji, że ludzie wchodzą do tej kawiarni i przecież nie będą, przechodząc obok stolików, wkładali znowu maseczki. Uważam, że już można było zostać przy tym chodzeniu w maseczce na dworze - mówię tutaj w mieście. W parkach, lasach niech sobie ściągają. Ale, żeby chociaż w tych głównych arteriach, w mieście, żeby te maseczki zostały, bo to wcale nie był problem, żeby chodzić w maseczce po dworze. Nie jest to wielkie wyrzeczenie, a wydaje mi się, że realnie, nawet jeśli nie powstrzymało rozprzestrzeniania się wirusów, a dawało ludziom jakąś pewność.

**To dlaczego to zostało zdjęte?**

Nie znam się na tych sprawach biologicznych - być może ten wirus sobie tak lata po podwórku, że nie ma sensu, żeby nosić maseczki. Z drugiej strony może mieli za dużo niedotlenień w szpitalach. Jestem ciekawa. Nie wiem.

**Ty planujesz jakieś zachowania utrzymać na dłużej?**

Jeżeli chodzi o higienę, to ja zawsze miałam bzika. Natomiast myślę, że teraz będę nosiła coś odkażającego ze sobą. Ja się zawszy brzydziłam jeździć tramwajami, w sensie siadać, a tam wcześniej siedział menel i potem ja mam tam usiąść? I zawsze stoję z tyłu i nie dotykam niczego. W sumie chyba niewiele się zmieni. Może ten płyn do dezynfekcji.

**Myślisz, że społeczeństwo zacznie bardziej dbać o higienę?**

Myślę, że troszeczkę tak. Widzę taki odruch, że jak ludzi wchodzą do galerii czy kawiarni i tam stoi płyn do dezynfekcji, to każdy klika. Także coś w tym jest.

**Spotkałaś się gdzieś z mierzeniem temperatury?**

Nie. Jak jechałam do domu pociągiem, to mąż mówi: ja cię szybciej odprowadzę na pociąg, na pewno będą ci mierzyć temperaturę. Oczywiście nikt mi nie mierzył temperatury, dworzec normalnie funkcjonował. Także nie spotkałam się.

**Myślisz, że w takich miejscach powinna być mierzona temperatura?**

Myślę, że tak, jeżeli jest zagrożenie. Bo np. zastanawiałam się, że jeżeli ja miałam jakiś ostry trening, a potem pojechałam na lotnisko, to wiadomo, że będę miała podwyższoną temperaturę. Albo jeśli kobiety są w ciąży, to mogą mieć podwyższoną temperaturę, to będę musiała okazywać jakieś dowody wtedy? Także myślę, że jeżeli jest taka potrzeba, jakieś podejrzenie, że coś się zaczyna dziać, będziemy mieli pandemię, to jest to wskazane i niech sobie mierzą temperaturę. Ale tak na co dzień, to nie wiem, czy to jest sens. Są ludzie, którzy po prostu mają wyższą temperaturę.

**Co się dzieje, jeśli ktoś na lotnisku ma podwyższoną temperaturę?**

Nie wiem. Słyszałam, że w wielkich korporacjach we Wrocławiu - np. Amazon, LG, nie chcieli odesłać ludzi na home office i mierzono temperaturę. Mój mąż pracował kiedyś w LG i rozmawiał z kolegą, który był tam w czasie pandemii i opowiadał, że po prostu to były żarty. Dlatego, że sprawdzali ich rozładowanymi miernikami. Albo stwierdzali: "nie wyglądasz na chorego, możesz przejść". Więc to też jest takie zagrożenie, że zacznie się takie rzeczy traktować po macoszemu. Nie wiem, co się dzieje z tymi ludźmi. Myślę, że zostają na jakieś testy, są uziemieni. Pamiętam, że jak moja koleżanka wracał ze Stanów już w czasie koronawirusa, to się bardzo stresowała, że utknie na lotnisku na długi czas. A jeszcze zestresowani, to też podwyższona temperatura dodatkowo.

**Masz jakieś obawy związane z ograniczeniem wolności czy nie?**

Nie przeszkadza mi to. Zakładam, że to by było tylko w takich miejscach publicznych, związanych z jakimś transportem - na stacjach, na lotniskach. Rozumiem, że jest w tym cel, żeby nie rozprzestrzeniać. Także nie miałabym z tym problemu.

**Wspomniałaś o drugiej fali, że może się wydarzyć. Myślałaś, żeby się przygotować?**

Nie.

**Dlaczego?**

Bo tak naprawdę cały czas przestrzegam pewnych zaostrzeń i nie musiałabym radykalnie wielu rzeczy zmieniać, żeby na nowo odnaleźć się w tej sytuacji pandemicznej. Myślę, że tutaj byłaby kwestia tych zakupów, ale skoro sobie radziliśmy wcześniej, to myślę, że to byłyby te same zasady - nic nowego.

**Jak rząd powinien zareagować?**

Myślę, że to powinny być znowu zaostrzenia, ale takie rozsądne. Nie mówię, że te, które były, nie były rozsądne, bo rozumiem, że w tamtej sytuacji nie do opanowania i bezprecedensowej trudno było podejmować inne decyzje, ale teraz wydaje mi się, że już moglibyśmy być nauczeni doświadczeniem. Już mamy aplikacje, już wiemy, że nie wszystkie działalności muszą zostać pozamykane. Ja bym myślała, żeby to się obracało wokół transportu, galerii handlowych i sklepów.

**Czyli to nie powinien być totalny *lockdown*?**

Nie. Bo to jest zgubne dla gospodarki. Teraz nam się będzie trudno dźwignąć, a co jeszcze po takim drugim przyłożeniu.

**Czego się najbardziej obawiasz w związku z 2 falą?**

Może trochę się obawiam tego, że będę się bała znów do domu pojechać. Chyba tego, Bo tak naprawdę nie czuję się... Mam pracę, pracuję z domu, więc pod tym kątem wcale nie muszę wychodzić tak naprawdę.

**Najważniejsze momenty w całej sytuacji dla ciebie.**

Najpierw wprowadzenie pierwszych zaostrzeń, home office, moje zachorowanie na coś - najprawdopodobniej na koronawirusa, potem przystosowanie się do tych cały czas zaostrzających się. Potem na pewno ta pierwsza Wielkanoc bez rodziny. Potem pierwsza fala odmrożenia zaostrzeń. Pierwsze spotkanie ze znajomymi, wypady do lasu. Potem 2 fala, wyjazd do domu, spotkanie ze znajomymi.

**A jeden najważniejszy moment?**

Jak słyszę koronawirus, to mam 3 migawki. Najpierw widzę siebie, jak siedziałam w połowie marca z koleżanką w Caffe Nero. Było chyba 4 klientów, w tym my. Już nikt nie wychodził. I słyszałyśmy informacje o tym, że Dino będzie zamykane, bo jakiś facet z koronawirusem jeździł. Druga migawka to jak zorientowałam się, że mam gorączkę, a to był piątek i miałam wyjść z koleżankami - to bardzo mocno pamiętam. I trzeci, to chyba jak siedzieliśmy sobie przy śniadaniu Wielkanocnym z komputerami i próbowaliśmy się ze wszystkimi skontaktować.
