# Supplementary material for: The challenges arising from the COVID-19 pandemic and the way people deal with them. A qualitative longitudinal study
Source: PLoS One. 2021 Oct 11;16(10):e0258133. doi: 10.1371/journal.pone.0258133 (PMC8504766; doi:10.1371/journal.pone.0258133)
Supplement: S1 Dataset — (ZIP) [file pone.0258133.s003.zip › Transcriptions/stage 5/4.5_M_32_couple, no children.docx]

**4.5_M_32_couple no children**

**Co się działo w ciągu ostatniego miesiąca?**

Bez większych zmian. Tyle, że zmieniła się pogoda i już człowiek korci, żeby coś pozmieniać, powyjeżdżać, coś porobić. W ten weekend byliśmy poza Warszawą ze znajomymi. Dwa noclegi na Mazurach, na rowerach. Ten wyjazd był chaotyczny, ostatecznie było mało rowerów, dużo się działo. Mamy też znajomych, którzy ze względów epidemii i dlatego, że mają remont w domu, żyją od dwóch miesięcy na działce. Wpadaliśmy też do nich. Spotkania rodzinne. To są weekendy, bo wtedy też z żoną mogę robić różne rzeczy. A od poniedziałku do piątku za wiele się nie zmieniło - sprawy domowo-organizacyjne.

**Czy podczas wyjazdów weekendowych, dostrzegasz obecność pandemii?**

Właściwie poza Warszawą się tego nie czuje. Mam takie wrażenie, że w małych miasteczkach...My kiedy wyjeżdżamy to po to, aby być bliżej przyrody. Po pierwsze - jest pandemia, więc nie ma co ryzykować. Po drugie - ileś rzeczy będzie zamkniętych, a po trzecie, jesteśmy spragnieni, żeby być w przestrzeni otwartej. Więc jesteśmy spragnieni przyrody. Na Boże Ciało wyjedziemy na 4 dni, ale znowu na Mazury, z moimi rodzicami i ich wnukami. Oni wynajęli jakiś domek i raczej nie będziemy widywać dużo ludzi. Będziemy robić grilla i jeździć nad jezioro rowerami, więc raczej tam też trudno o epidemię, nawet, gdyby tu była bardzo zauważalna. Byliśmy na weekend w Iławie -to miasto średniej wielkości. Nie czuć tam koronawirusa. Coś tam niby, ale nawet mieliśmy kontakt z policją - co prawda na świeżym powietrzu, więc też już nie ma obowiązku, ale panowie funkcjonariusze nie wyglądali, jakby się dla nich coś epidemicznie liczyło. Żadnych maseczek, rękawiczek, itd. W Warszawie czasami widać, że czasami mają w samochodzie, czy zawieszone na lusterku, czy na szyi, opuszczoną. To też pewnie takie udawanie, ale są przynajmniej pozory. A w tej Iławie nie widziałam nikogo w maseczce. Jeśli chodzi o nasze zachowanie - byliśmy z takimi znajomymi, którzy się nie przejmują, więc może my też się trochę nie przejmowaliśmy. Moja żona była temu przeciwna, bo uważa, że to zbyt duże folgowanie sobie. Ja - to nie jest tak, że ja nie wierzę w tego wirusa, bo myślę, że on jest - ale mam wrażenie, że trochę przestałem się przejmować. Mam wrażenie, że nikt nic już nie wie, jest straszny chaos, zmieniają się przepisy, podejście, interpretacje. WHO zmienia wytyczne, Ministerstwo Zdrowia też. Raz maseczki są dobre, raz złe. Raz minister zdrowia mówi, że będziemy je nosić ze dwa lata, albo do wynalezienia szczepionki, później w tym samym miesiącu znosi obowiązek, który zresztą myślę, że w miarę sensownie znosi, bo noszenie maseczki na pustych ulicach jest bez sensu. Ludzie tylko dmuchają w nie i... No właśnie. Poza tym, mam wrażenie, że rozmyła się sensowność. Ludzie je nosili - widziałem dużo takich scen - szli po dworze grzecznie, w maseczce, jakby to była choroba wietrzna, przenoszona przez kosmos, po czym spotykają znajomego na ulicy - niewygodnie się rozmawia, słabo się słyszy drugą osobę - więc ściągają maseczkę, pogadają chwilę, po czym znów ją zakładają i idą dalej. To jest totalnie bez sensu. To lepiej było od razu mówić, że można mieć tę maseczkę na szyi i dopiero jak się kogoś spotka, czy wchodzi do sklepu trzeba ją założyć. Mam wrażenie, że wszystko się zmieniało tryliard razy. Obostrzenia i nakazy. Nie wiadomo, jak wirus się rozwija. Epidemia w jednych krajach wybucha, w innych nie. Trochę nie wiadomo w końcu, jak z tym w końcu jest. Mam wrażenie, że lepiej, jakbyśmy funkcjonowali normalnie, natomiast z tyłu głowy - podoba mi się zostawianie tych maseczek na dłużej, żebyśmy pamiętali jeszcze, że jest epidemia. Jeśli rozmrażają gospodarkę, to ok. Natomiast z takim założeniem, że jak zrobi się źle, to znowu będą restrykcje, lockdown. Idealnie by było, gdyby było przewidziane, co działoby się, gdyby było coś. A u nas wszystko jest na biegowo i później będą dramaty, bo ktoś jednak zainwestował w biznes, albo czegoś tam nie zamknął.

**Które z zachowań związanych z pandemią pozostały w twoim życiu?**

Jak idę do sklepu, zakładam maseczkę, nawet, jeśli połowa ludzi już z nich nie korzysta. Choć w ostatnim czasie zdarzyło mi się już ze 2-3 razy wpaść po coś do Biedronki albo innych sklepów. Wcześniej się powstrzymywaliśmy - jeśli czegoś nie kupiliśmy, a nie było to dramatycznie potrzebne - trudno. Teraz miałem już takie sytuacje, jak na przykład, kiedy ostatnio wracaliśmy ze szwagierką z rowerów i padło hasło, że kolacja u nas - to wcześniej poszliśmy jeszcze po rzeczy na tą kolację. To było przekroczenie tej zasady jedne zakupy na tydzień. Nadal staram się robić zakupy rzadko, nie łazić ciągle do sklepu, ale zdarzają się już odstępstwa. Jak potrzebowaliśmy, byliśmy w Ikei. Zrobiliśmy tam dużo zakupów, bo kupiliśmy jeszcze rzeczy dla rodziny. Ale podejście się trochę zmieniło. Nie pojechałbym do Ikei na hot doga, czy tylko po to, żeby sobie pooglądać rzeczy, ale jeśli musieliśmy kupić konkretne rzeczy - pojechaliśmy. Nie poruszamy się komunikacją miejską. Ale też nie musimy. Gdybyśmy musieli, pewnie byśmy to robili. Mam wrażenie, że ten wirus występuje, może jeszcze człowieka trafić, ale Mazowsze się ostatnio tak uspokoiło i poza Śląskiem, reszta Polski też, że chyba tylko jakbym musiał wybrać się do szpitala, miałbym wrażenie, że mogę się tam od kogoś zarazić. Tam są w tej chwili największe ogniska koronawirusa.

**Uspokoiło, tzn.?**

Jest mała liczba przypadków chyba. Ja teraz już też przestałem to monitorować. Widzę, że nie ma wykładniczego wzrostu. Prawdopodobieństwo śmierci człowieka jest zawsze i teraz jest niewiele większe z powodu tego koronawirusa. A jeśli chodzi o zmieniający się świat, to było warte śledzenia, kiedy się rozpoczynało. Co to będzie, lockdown, kryzys, wojsko na ulicach, zamieszki, wszystko mogło się wydarzyć. Z różnych krajów było widać różne sceny. Ale patrzę teraz przez okno na własne osiedle i chyba nic takiego się nie zmieniło strasznie. Ja wiem, że są osoby, którym się zmieniło. Losowo niektórzy - przepraszam z wyrażenie - dostali po dupie. Nie było jasnych reguł. To nie było do przewidzenia, że np. ci, co pracują w IT nie dostali, a ci, co w usługach - jak jeden mąż. Jak ktoś miał internetowe usługi, to mu może kwitnie, a jak ktoś fryzjera - padło. Choć też wydaje mi się, że jeśli ktoś miał swoją stałą klientelę, to czarny rynek usług kosmetycznych kwitł.

**Są u ciebie jeszcze sytuacje, gdzie czujesz niepokój związany z koronawirusem?**

Na takim minimalnym poziomie cały czas. Że gdybyśmy się zarazili, może zarazilibyśmy też kogoś starszego z naszej rodziny, albo ktoś z rodziny nas. Ale raczej mała szansa, że byśmy umarli. Jeśli zobaczyć zgony w grupie wiekowej mojej i mojej żony, śmiertelność pewnie jest niska. Pewnie porównywalna do jeżdżenia samochodem lub czegoś podobnego. Są różne ryzyka, które oswajamy i stwierdzamy, że jesteśmy gotowi je ponosić. I wydaje mi się, że koronawirus też stał się tym w społeczeństwie.

**Jak to jest u twojej żony?**

Ona tak trochę ma - ja często to krytykuję - że jej postawy są bardziej emocjonalne, niż logiczne. W związku z tym, u niej to jest takie trochę chcę, a trochę się boję. Ona powiedziała jak wróciliśmy z tej Iławy, że tam żyliśmy, jakby epidemii nie było, a ona właśnie czyta wywiad z prof. Simonem - to chyba epidemiolog z Wrocławia, ma dużo pacjentów covidowych, zakaźnych, zajmuje się tym tematem - i on uważa, że to poluzowanie gospodarcze jest złe i doprowadzi nas na skraj katastrofy. Nie wiem, to jest ekspert i może ma rację. Ale może nie bierze też innych wymiarów pod uwagę. Zapytałem jej, że jak w takim razie chciałaby funkcjonować. Czy chciałaby nie robić takich wyjazdów. Ona powiedziała, że nie wie. Zapytałem jeszcze o coś innego, nie odpowiedziała mi. Jest trochę tak, że ludzie mówią, że szybka jazda jest niebezpieczna, a i tak wszyscy jeżdżą szybko. Ona powiedziała, że może nie należało iść do tej restauracji - swoją drogą, na świeżym powietrzu, bo była piękna pogoda - ale kiedy zapytałem, czy nie chciała tam iść, powiedziała, że właściwie to poszliśmy. Ani nie zaoponowała, ani nie sprzeciwiła się, nie zdecydowała się na nic innego. Trochę ta decyzja była może podjęta owczym pędem, bo ci znajomi to grupa bardzo mało przejmująca się, ale moim zdaniem trzeba by podjąć jakieś działania, jeśli człowiek chce zmienić swoje życie i tego koronawirusa bardzo mocno unikać. Jeśli chcemy się izolować, to może kupmy działkę za miastem. Ale jeśli chcemy siedzieć w bloku i robić to jeszcze tydzień, to nie ma to żadnego istotnego znaczenia. A jeśli mamy to robić jeszcze dwa lata, to wyprowadźmy się w góry, na Mazury, albo gdzieś indziej, gdzie nie ma ludzi. Bo poddawać się długotrwałej izolacji w Warszawie, to głupi pomysł.

**Dlaczego?**

Mieszkać w środku betonowej pustyni, dżungli, jak to nazwiesz - tworu, w którym jest zagęszczenie ludzi - nie wiem, ile jest w Warszawie ludzi na km2 - tysiąc? To jeżeli mamy nie korzystać w żaden sposób z miejskiej infrastruktury, tylko siedzieć w bloku, to naprawdę kupmy domek za miastem. Nawet na jakiejś rekreacyjnej działce. Wtedy zmieniajmy i się tego pilnujmy. Albo podejmujmy jakieś sensowne działania, ale róbmy to w sposób rozmyślny, logiczny, istotny statystycznie. A nie czujmy wyrzuty sumienia, że zjedliśmy frytki w knajpie w Iławie, bo akurat to jest wg nas z jakiegoś powodu bardzo niebezpieczne. Mam wrażenie, że nie ma w tym żadnej logiki.

**Uważasz, że twoje podejście jest racjonalne?**

Nie, ja jestem w tym zagubiony. Nie potrafię podjąć racjonalnej decyzji, więc już trochę to olałem. Uważam, że jest chaotycznie i nie wiem, co mam myśleć. I myślę, że wszyscy ludzie nie wiedzą, co myśleć. Dosyć typowo - ludziom zaczęło się wydawać, że tego wirusa nie ma, tylko dlatego, że nie znają nikogo, kto ciężko by go przechorował, czy umarł. Albo znają kogoś, kto miał pozytywny wynik testu, ale przeszedł go łagodnie. Więc ludzie to wyprą i powiedzą, że to nic groźnego.

**Czyli twoje podejście jest zagubione, ale nie emocjonalne?**

Tak myślę. Pewnie bym się trochę rozemocjonował, gdyby ktoś mi bliski walczył o życie. Ale nie wydaje mi się, żeby ta sytuacja była do uniknięcia. Wiem, że to brzmi trochę, jak pogodzenie się z beznadziejnym losem, ale... Ta sytuacja będzie trwać, ten wirus nie zniknie. Takie rzeczy w przyrodzie nie giną właściwie w ogóle. Zwłaszcza, biorąc pod uwagę proces jego rozprzestrzeniania się, inkubacji - nie można od razu odizolować chorych. W przypadku Eboli, SARSu możemy łatwo wyizolować pacjentów, w przypadku COVIDu nie. Zresztą, przy grypie też nie możemy, dlatego jest grypa szalejąca cały czas - jest wirusowa, więc nie ma na to lekarstwa. Więc to nie zniknie. To trochę tak, jak w latach 70. XX w. był gigantyczny program rugowania polio. Mówiono, że jeśli obejmie się szczepionkami 95% populacji świata, wirus zniknie. Tak pokazywały też modele matematyczne, symulacje. Ktoś chyba dostał za to nawet nagrodę Nobla, wpompowano w to miliardy dolarów. Później się okazało, że to nie do końca tak działa, bo kiedy przestają szczepić, to raz na 5 lat to polio gdzieś się pojawia. Okazało się, że w równaniu nie wzięto pod uwagę jakiegoś czynnika. Po korekcie, zmieniono strategię. Doszli do wniosku, że będą szczepić mniejszą liczbę ludzi i że ważniejsze będzie objęcie szczepieniami tej części populacji, która jest najbardziej narażona - np. lekarzy, pielęgniarki, nauczycieli - ludzi, którzy mieliby wysoką szansę zetknięcia i mogliby później roznosić chorobę. Więc kiedy sobie myślę o koronawirusie, myślę, że on nie zniknie. Być może zmutuje, być może jakieś populacje się na niego uodpornią, bo go przejdą. Część nie będzie go roznosić, bo umrze. Nie jest jasne, dlaczego w niektórych miejscach były większe wybuchy epidemii. Jest taka teoria, że być może osoby czarnoskóre są bardziej podatne, Chińczycy bardziej. Pytanie, dlaczego Włochy i Hiszpania eksplodowały tak strasznie, a inne kraje nie? Inny klimat, inna kultura? Była taka teoria, że we Włoszech dlatego, że starzejące się społeczeństwo. A może gdzieś indziej też takie jest? Naukowcy do tego dojdą, natomiast będzie to trwało i COVID-19 z nami będzie. Nawet ostatnio rozmawiałem ze znajomym stomatologiem - czy on jeszcze stosuje te maseczki, czy jest ubrany w kombinezon. Powiedział, że nie, bo dostaje już od nich duszności i powiedział, że się nic nie zmieni. I tak padło, że ciekawe jak zmieniłby się świat, gdyby śmiertelność wynosiła około 15%. Na co on stwierdził, że w historii były już wojny, ludobójstwa, wielkie epidemie i czy to komuś przeszkadzało? Po takich wydarzeniach świat i tak szybko wraca na dawne tory. Zmiany będą raczej niewielkie - może częściej będziemy myć ręce.

**Rozumiem, że pomimo tego starasz się zachować jakieś dawne zwyczaje, jak zakupy raz w tygodniu, ale nie zawsze ci to wychodzi.**

Miesiąc temu ruszyła gospodarka. Wszyscy to jakoś znoszą. Jak miną 2-3 miesiące i nie będzie nawrotu epidemii, to pewnie w ogóle mi przejdą te nawyki. Będę jeszcze obserwował na jesieni, czy coś się nie dzieje. Pewnie wszyscy będziemy, bo media będą o tym trąbić. Nawet ostatnio z kimś rozmawiałem i on stwierdził, że większość epidemii jest dwufalowa - Hiszpanka podobno też miała drugą falę. Nie wiem, czy tak jest, ale jeśli tak jest - i specjaliści też t zapowiadali - to jeśli to jest wirus grypopodobny, najprawdopodobniej możemy spodziewać się drugiej fali. Ja nie rozumiem do końca, czy możemy się jej spodziewać, ponieważ ten wirus jest podobny do innego wirusa, czy dlatego, bo jesienią zmienimy nasz sposób zachowania i zamiast po parkach i nad Wisłą, będziemy siedzieć w ciasnych pubach i galeriach handlowych, nie będziemy odpoczywać na wakacjach i będziemy rzadziej wietrzyć pomieszczenia, bo będzie nam zimno. Czy może o to, że jesienią będziemy osłabieni, więc będzie wyższa zachorowalność? Czy akurat pół roku po pierwszej fali jest druga, bo ludzie pilnują się jakiś czas, a później jest rozluźnienie, które pociąga za sobą drugą falę. Wszyscy mówią, że jesienią będzie druga fala. Jeśli druga będzie wyglądać tak, jak pierwsza w Polsce, to to nie była jakaś gigantyczna. Tak mi się wydaje. Pamiętasz, na którymś wywiadzie narzekałem, że nie widziałem statystyk śmiertelności rok do roku. W którymś momencie żona je znalazła - Polski i Włoch. Jakieś pół miesiąca czy miesiąc Włosi faktycznie mieli te statystyki zawyżone, Lombardia rzeczywiście skoczyła do góry. Natomiast Polska - i to jest abstrakcja - w marcu i kwietniu 2020 miała niższą śmiertelność, niż w tych miesiącach, w 2019. Ok, później wzrosła ta śmiertelność, bo zwariowali lub śmiertelność wzrosła od małej ilości ruchu - osoby, które pracowały zdalnie, wszyscy przytyliśmy, chyba, że ktoś jest fanem domowego fitnessu. Okazało się, że lockdown na miesiąc wpłynął na polepszenie stanu powietrza, mniejszą ilość wypadków na drogach. Epidemia okazała się uzdrawiająca dla statystyk. Zaskakujące.

**Wspominałeś, że byliście w restauracji. Jak to wyglądało? Były stosowane jakieś ograniczenia?**

Nie było żadnych różnic. Iława to turystyczne miasto nad jeziorem, większość lokali ma ogródki, większość miejsc dla klientów jest na zewnątrz. Nie siedzieliśmy w budynkach, na zewnątrz nie trzeba mieć maseczek. W restauracji, jedząc, zresztą też nie. Co prawda łamaliśmy ten przepis, że przy jednym stoliku nie mogą siedzieć osoby spoza jednego gospodarstwa domowego. Tak samo wynajęliśmy dwa domki w 10 osób i nikt nas nie pytał o to, czy jesteśmy rodziną. Pojechaliśmy na dwa samochody, razem robiliśmy grilla, jeździliśmy ze sobą na rowerach. Spędziliśmy ze sobą tyle czasu, że w restauracji nie miało to już znaczenia, a kontakt z kelnerem był bardzo mały.

**Uważasz, że to dobrze, że spędziliście razem ten czas?**

Nie wiem. Jak to zmierzyć? Nie mam zdania. Nie mam się za eksperta w każdej dziedzinie. Po prostu nie wiem. Tutaj należy postawić pytanie retoryczne, co jest lepsze - żeby gospodarka się pokruszyła, ludzie stracili pracę - tym bardziej, że gospodarka w tej chwili jest oparta na dużej konsumpcji. Poza tym, to też spowoduje pogorszenie stanu zdrowia wielu osób - nie kupią karnetu na siłownię, będą żyli w stresie, kupowali gorsze jedzenie. Nie pójdą do dentysty, bo pożałują pieniędzy - od tego dostaje się później Alzheimera w wieku starszym. Wszystko jest połączone. Dobrobyt idzie w parze z długością życia. To, że przestaniemy dziś produkować, może oznaczać, że skrócimy sobie życie. Poza tym, jak to traktować? Może poza długością życia jest jeszcze inny istotny walor? Jak to policzyć? Widziałem taki mem, kiedy to wszystko się zaczynało, ale jeszcze nie było paniki. To było we Włoszech lub w Anglii, taki stand przy wejściu do kawiarni, że oni nie stosują w tym lokalu żadnych maseczek i że tam przychodzi się umrzeć jak bohater - z piwem lub kawą w ręku. Komicznie trochę, ironizując, ale coś w tym jest. Z ilu rzeczy bylibyśmy w stanie zrezygnować? To pytanie, które zadałem żonie - jak dużo rzeczy chcesz poświęcić i na jak długi okres? Na co chcemy się zdecydować? I nie ma na to odpowiedzi.

**Już chyba otworzyli kina...**

Była śmieszna afera z koncertem transmitowanym z teatru. Jakieś disco polo chyba, czy inna muzyka biesiadno-ludowa. To była jakaś rocznica. TVP? Powstania PiSu? Nie wiem. To było trochę jak z marszem przedsiębiorców - który też jest jakiś prowokacyjno-głupkowaty, ale to inna sprawa. W każdym razie na marszu policja wypisuje mandaty za łamanie zakazu zgromadzeń. A w Wadowicach w ramach obchodów śmierci papieża spontanicznie gromadzi się tłum i wygląda na to, że zakazy zgromadzeń podczas epidemii dotyczą tylko tych, którzy są "nieprawomyślni". Jak ktoś jest prawomyślny i jest polskim patriotą, katolikiem i daj boże głosuje na PiS, zasady epidemii go nie dotyczą. I z tym teatrem tak było. TVP chyba wynajęła teatr, wiele ludzi miało się dobrze bawić. Wiele osób dostało w związku z tym piany na twarzy, zresztą słusznie, choć rząd tłumaczył się, że środki ostrożności zostały zachowane. Podobno tylko co trzecie miejsce było zajęte, czy coś takiego. Oprócz tego to podobno TV zorganizowała, nie rząd - choć wiadomo, jak to jest. Więc spektaklu nie można wystawić, ale już jak Maryla Rodowicz, czy inny Zenek śpiewa pieśni patriotyczne czy inne majteczki w kropeczki, to jest super. To ku chwale ojczyzny i epidemia tego nie tyka.

**Co to pokazuje?**

Wygląda na to, że część społeczeństwa będzie ponosić ogromne straty, zmieniać swoje życie przez to, że jest epidemia, tymczasem... Trochę upadł duch tego przedsięwzięcia - to trochę tak, że cały naród siedzi w domach, a Kaczyński jedzie na cmentarz. Teatry są zamykane, artyści teatralni nie mogą pracować, niektóre teatry upadają, ale jeśli jesteś kimś z PiSu, społeczeństwo nie pocierpi, jak zrobisz sobie koncert. Ludzie widzą polityków, którzy nie chodzą w maseczkach, tłumaczą się głupio, że oni zrobili sobie test lub coś w tym stylu, ale wy, zwykli obywatele, zwykłe szaraczki - noście maski. Mam poczucie, że jeśli wszyscy wokół nie będą o to dbać, to co mi z tego, że ja będę o to dbał? Nie mówię, że mam się od razu wystawiać na wielki ryzyko - nie chodzę do wielkich zgromadzeń, sklepów unikam - nie lubię ich, mogę ich unikać - ale na piwo z kolegami pójdę. Do parku, nad Wisłę, na bulwary - czemu nie?

**Rozumiem, że zauważasz nierówność w egzekwowaniu tych przepisów.**

Tak i ta nierówność jest zarówno w pionie - jak masz wyższą pozycję społeczną, jesteś bliżej koryta, to cię nie dotyczy, ale też w poziomie. Fryzjer jest pierwszą potrzebą człowieka, ale kino nie. Gdzie postawić granice? Dlaczego akurat w tym miejscu, a nie w tamtym? Trzeba by było jasnych decyzji, dobrej narracji, jakiegoś wytłumaczenia, planu - już na to narzekałem. W związku z tym, że jest jak jest... Ja rozumiem, że na początku był chaos. Ale wszyscy schowali się po domach. Ale wciąż jest tak ad hocowo. Kolejny przykład - czy wiesz, czy są już otwarte granice w Polsce?

**Nie mam pojęcia, jestem od tego odcięta.**

No właśnie, ale nawet, jakbyś czytała wszystkie newsy, nie dowiesz się tego. Minister ci tego nie powie, nikt. Wiesz, kto może ci to powiedzieć? Niemiecka kolej państwowa. Ogłosili datę, od której niemieckie pociągi będą jeździć do Polski. To znaczy, że niemiecka kolej się już dogadała z polską, prawdopodobnie wiedzą już, kiedy granice zostaną otwarte. Mądrze, że oni wiedzą to wcześniej, bo to jednak duża organizacja. Ale tam jest narracja, jakaś informacja w tych Niemczech. A u nas jest takie podejście - powiemy jak powiemy. Nie uważam, żeby Polska była najgorszym krajem na świecie. Ale mi by było trochę głupio mówić, słuchajcie, wszyscy robią, co chcą, ludzie nie chodzą w maseczkach, ale kina są zamknięte. Albo coś innego też.

**Jak ci się wydaje, jaki był ten klucz? Dlaczego w pierwszej kolejności fryzjerzy, później kina?**

Wydaje mi się, że ten klucz to było chyba mierzenie dwóch czynników. Jak to obserwowałem. Tam były chyba trzy wymiary. Pierwszy to było grupowanie się ludzi w danym miejscu - ile osób korzysta na raz - jaka jest ich liczba oraz bliskość. Dlatego fryzjerzy i tatuażyści byli napiętnowani, bo to blisko, dlatego zakazano im działalności. Drugi ważny wymiar to była chyba niezbędność do życia. Podejrzewam, że nawet, gdyby ludzie umierali na chodnikach z plamami na całym ciele, sklepy spożywcze byłyby pewnie otwarte, bo inaczej umieraliby też pewnie z głodu. Nie no dobra, jakby trzeba było chodzić wężykiem między trupami, to żywność rozdawałoby już pewnie wojsko czy jakaś policja. Jakieś racje żywnościowe. Ale sklepy generalnie były cały czas otwarte i nikt nie mówił, żeby je zamykać, nawet, gdyby było to jakieś bardzo niebezpieczne. Ale co to jest konieczne do życia? To było widoczne na przykładach mandatów - policjanci już sami nie wiedzieli, o jakie zasady chodzi. Zaglądali ludziom do siatek z zakupami, jak ktoś kupił samo piwo, wlepiali mu mandat, bo alkohol nie jest konieczny do życia, jak choćby chleb. Były takie pojedyncze przypadki. W związku z tym, ludzie zaczęli się już śmiać na FB, czy w innych miejscach, żeby pamiętać, że jak się idzie po browar, żeby kupić też bułkę. Trzeci wymiar, który był to mam wrażenie, że potrzeba polityczna pomieszana z losowością. Takie, to co teraz? Aha, kobietom poschodził manicure i mają strasznie długie odrosty. Dobra, to Mateusz, tych fryzjerów, otwieramy. Bo już strasznie ludzie bulgoczą. Ileś ograniczeń zluzowali przed wyborami, które się w końcu nie odbyły. Myślę, że teraz gdyby coś się miało dziać, to do tych wyborów 28 czerwca, choćby wszyscy górnicy w tym kraju zachorowali, to nie będzie żadnego lockdownu, żadnych obostrzeń dodatkowych. Bo mi się wydaje, że oni koniecznie chcą przeprowadzić te wybory.

**Aplikacje, rozwiązania technologiczne na czas pandemii. Czy coś słyszałeś?**

Tak. O tej jednej, która ma nas mierzyć, zachorowania. Taka trochę wzorowana na Chinach. Ona miała wejść, ale chyba nigdy nie wejdzie, bo epidemia się skończy, zanim rząd ją wypuści. Tam było tak, że chyba główny koder odszedł, bo była afera. Oni udostępnili do weryfikacji większość kodu - zresztą bardzo słusznie. Ja nie umiem programować, ale na tym Niebezpieczniku pisali o różnych rzeczach, które są błędnie robione w tej aplikacji. Część rzeczy, które były utajnione, ten główny programista, który odszedł z tego projektu, czy on odszedł jak dowiedział się, jakie są założenia - ujawnił. Okazało się, że nie do pogodzenia jest ochrona danych osobowych, zachowanie anonimowości z taką sensowną kontrolą. Ta chodziło chyba o przekazywanie ID telefonu, które można później połączyć z czymśtam. W każdym razie wychodziło na to, że jak już zainstalujesz tę apkę, państwo będzie miało o tobie wiedzę zawsze i wszędzie. A zdaje się, że i tak ta aplikacja słabo monitoruje to, czy się z kimś spotykasz, jak długą masz ekspozycję - czy jesteś zielony, żółty, czy czerwony. Bo tam miały być te flagi, jak mają Chińczycy. To oznaczenia, gdzie jak jesteś zielony, to jest ok, jak żółty to chyba masz się przebadać, uważać na siebie, a czerwony - wyszło, że zarażasz. To super rozwiązania, że robisz w laboratorium test i w pewnym momencie wyświetla ci się czerwona kropka w telefonie i już wiesz, że jesteś zarażony i nie możesz wychodzić z domu. Bo jak policja cię spotka i sprawdzi ci telefon, to już tylko doniesienia, kary. To by leczyło epidemię, tylko to jest straszna ingerencja państwa. To można potem do wszystkiego wykorzystać tak naprawdę.

**Jakie ty masz podejście do bezpieczeństwa podczas korzystania z różnych technologii?**

Nie mam 100% zaufania, wręcz zaufanie mam małe, ale niestety daję się przekupić wygodą. Latami używałem gmaila i nie używałem chroma - wtedy jeszcze nie był tak popularny. Wylogowywałem się z konta google chodząc po internecie. Sprawdzałem pocztę, wylogowywałem się z konta, po czym czyściłem sesję i dopiero używałem internetu. Z czasem, mam telefon na Androidzie, jestem tam ciągle zalogowany do gmaila. Korzystam często z google maps, w związku z czym mam tam zgody na używanie lokalizacji. Zresztą, jeśli masz je wyłączone, to Android i apki pytają cię o to non stop. Połowy rzeczy nie jesteś w stanie używać i prędzej czy później klikniesz "zezwól" - i później już ani razu o nic cię nie zapytają. Tam są na pewno jakieś funkcje, że jak korzystasz z lokalizacji w google maps, to część - przynajmniej tych z "googlowej rodziny" też ma do niej dostęp. Może to chodzi w tle. Więc ja mam poczucie dużej inwigilacji i sprzeciw wobec tego, ale dałem się przekupić wygodą - nawigacji, tego, że jak klikam coś w komputerze, to później mam to w telefonie - jejku, jakie to jest wygodne. Tak samo z FB. Messenger - już nie ma tego problemu, że coś jest tylko na komputerze, a coś tylko na telefonie - jak SMSy. Wydaje mi się, że te niebezpieczeństwa dzielą się na dwa nurty - pierwszy to kradzieże, wymuszenia, itd., drugi - ludzie wobec państwa. To tak, jak w USA podczas zamieszek niektórzy kradli dużo rzeczy. I okazało się, że Apple jest w stanie swoje ukradzione produkty namierzyć, bo ten sprzęt jest bardzo zintegrowany z telefonami, to są zamknięte środowiska, w przeciwieństwie do choćby Androida. Dlatego Apple ma dużą kontrolę nad tym, co się dzieje na iPhoneach. Poblokowali kradziony sprzęt, nie da się go włączyć i wyświetla się tylko komunikat, że nie można włączyć urządzenia, prosimy o zwrot do sklepu. Z jednej strony myślimy sobie - fajnie, ale ich załatwili. Z drugiej strony, już technologia skanowania twarzy jest bardzo rozwinięta. Kiedy nastąpi moment, że będziemy szli w demonstracji antyrządowej, jak w Hong Kongu, kamery zeskanują nam twarze i państwo powie na przykład, że tym, którzy szli w demonstracji, na miesiąc wyłączymy telefony. Wiadomo, część ruchów antyrządowych może też istnieć, ponieważ mogą komunikować się przez internet - pomarańczowa rewolucja, część arabskiej rewolucji wydarzyło się między innymi z jego pomocą. Wracając do tych aplikacji, ja się ich trochę boję. Może być kiedyś tak, że np. państwo będzie mogło dzięki nim łatwo namierzyć osoby, które przemieszczały się np. podczas godziny policyjnej, więc pewnie niosły jakieś tam opozycyjne ulotki - coś takiego. Więc inwigilacja jest duża. Fajne jest coś, co powoduje, że będziemy się chronić przed epidemią, ale jeśli ta epidemia nie jest aż tak duża, to czy chcemy oddawać resztki naszej kontroli?

**A jak duża jest ta epidemia?**

Nie no, nie wiem. Mam na myśli, że gdyby może była taka, jak w jakiejś apokalipsie zombie, to te apki może by mogły przywrócić jakieś normalne funkcjonowanie. Choć to bez sensu...

**Czyli jeśli jest taka epidemia, jaką mamy, to nie jest wystarczający powód, aby oddawać swoją prywatność dla tych aplikacji. A jeśli byłaby taka epidemia, typu atak zombie, to...**

To już by było za późno. Nie wiem, wiesz, musiałbym to przemyśleć. Ja nie ufam państwu. Uważam, że ono jest jednak ze swojej natury niebezpieczne dla obywateli. Musimy je akceptować, bo nie jestem anarchistą, państwa muszą być. Bo jeśli ich nie ma, one i tak są. Jeśli nie mielibyśmy państwa, mielibyśmy tu zaraz albo Niemcy, albo Białoruś. Natura nie znosi próżni i jakiś duży organizm musi funkcjonować. To tak, jak na rynku nie ma już małych sklepików, tylko są Biedronki, albo jakieś inne Carrefoury, bo nauczyliśmy się organizować i to sums sumarum to jest tańsze. Więc musimy akceptować państwa, choć one ze swojej natury są niebezpieczne.

**Pomysły na aplikacje****: Aplikacje analizujące dane osobiste (bieżące informacje o stanie zdrowia, historię przemieszczania się i kontaktów z innymi ludźmi) w celu monitorowania rozprzestrzeniania się koronawirusa.**

Bieżące informacje o moim stanie zdrowia - a skąd mój telefon ma je brać? Ja wiem, że one niedługo będą potrafiły jeszcze więcej... A, są smartwatche, one mierzą tętno i coś tam jeszcze. Być może niedługo będziemy mogli dokupić sobie jakiś malutki chipik, który wszczepimy pod skórę. Saturację - natlenienie krwi - chyba też mierzy się dosyć łatwo. W każdym razie może to jakoś tak będzie do zrobienia. Ale tak, żeby mieć telefon w kieszeni i on miał znać informacje o twoim stanie zdrowia, to raczej nie jest możliwe.

**Informacje o twoim stanie zdrowia to coś, czym trudno by było ci się dzielić z jakimś twórcą aplikacji? Czy to zbyt duża ingerencja?**

Żadną taką informacją nie chciałbym się dzielić. W przyszłości takie dane mogą co prawda pomóc w diagnozowaniu chorób w społeczeństwie. Dobra analiza na dużym zbiorze danych mogłaby dawać nam informacje ogólne o chorobach w populacji. Moglibyśmy zauważać niebezpieczeństwa z dużym wyprzedzeniem. Być może my wtedy też byśmy dostawali takie informacje w stylu, że warto by było się zbadać, bo ma się arytmię. To z jednej strony przekonywałoby mnie, aby takie dane przekazać, ale z drugiej strony i tak bym nie chciał, bo to jednak jakaś moja prywatność. Trudno byłoby mi to zważyć, ale ja osobiście byłbym za tym, żeby tych danych nie oddawać.

**Czyli w celu monitorowania rozprzestrzeniania się koronawirusa, nie chciałbyś takich danych oddawać.**

Nie chciałbym. Mam świadomość, że to pewnie kiedyś będzie, natomiast wolałbym nie.

**Aplikacje, które na podstawie danych lokalizacyjnych monitorują przestrzeganie kwarantanny domowej.**

Nie wiem, a co za problem zostawić telefon w domu? Sensowną alternatywą byłoby zakładanie tych niezdejmowalnych opasek, jak w więzieniach. Ale to już trochę głupio, nie? Niczemu nie zawiniłaś, przychodzi policja i mówi, że zaświeciłaś się na czerwono, bo jest pozytywny wynik na COVID-19, przyszliśmy z panem w pełnym skafandrze, założyć pani niezdejmowalny nadajnik. Może zdejmiemy go za dwa tygodnie. Głupio, nie?

**Dlaczego głupio?**

Duża niewygoda dla użytkownika. Duży nakład finansowy, kłopotliwość, ludzie by się tego wystraszyli. Ale przede wszystkim, to jednak kojarzy się z przestępcami. To jest całkowity brak zaufania do obywatela. Obrączkujemy cię, nie zrobiłeś nic złego, ale traktujemy cię jako potencjalnego przestępcę, że uciekniesz z kwarantanny. A sama aplikacja - co z tego? To jadę sobie na rower, albo pograć w koszykówkę na boisku. Większość ludzi przestrzega kwarantanny domowej. A jeśli komuś bardzo zależy na tym, żeby tego nie robić, to zostawi po prostu telefon w domu. To jest tylko utrudnianie tym, którzy i tak jej przestrzegają, bo oni będą musieli coś ściągać, gdzieś się logować. To pewnie nie będzie działać, coś ich źle zlokalizuje i wyjdzie, że i tak są przestępcami. Nie jestem do tego przekonany.

**Aplikacje oparte na automatycznej lokalizacji użytkowników, informujące ich, że znajdowali się w miejscach zagrażających zarażeniem się koronawirusem.**

Na jakiej podstawie będą wyznaczone miejsca zarażenia się koronawirusem? Gdyby to było na przykład tak, że robię sobie test na COVID i wychodzi pozytywny, to aplikacja skanuje np. tydzień wstecz te wszystkie trackery, które się przecięły. Ok, tylko to znowu by oznaczało, że musimy dawać te wszystkie informacje. To znaczy, że te miejsca zagrażające trzeba wyznaczać na podstawie wiedzy medycznej, więc wracamy do aplikacji nr 1. To znów jest bardzo duża ingerencja w prywatne życie. Nie mam takiego zaufania do państwa i rządu, żebym chciał, aby to funkcjonowało.

**Rozumiem, że wszystko rozbija się o to, jaki mamy rząd?**

Nie, gdyby to była PO, też bym nie chciał. Jako państwo i rząd - bym nie chciał, a że PiSowi nie ufam jeszcze bardziej, to tym bardziej.

**Monitoring z automatycznym systemem rozpoznawania twarzy, w celu szybkiej identyfikacji osób nieprzestrzegających zaleceń władz. Chyba o tym mówiłeś?**

No tak. To są funkcjonujące super rozwiązania. One są ekstra interesujące. Potęga tych automatów jest super, choć ja widzę w tym duże zagrożenie. To wszystko razem dawałoby dużą... Mam wrażenie, że półśrodki nie mają w tym przypadku sensu. Albo trzeba inwigilować dużą część społeczeństwa i możemy wtedy dobrze panować nad epidemiami, natomiast nie można trochę inwigilować. To chyba nie daje możliwości zbudowania sensownych modeli. I tu znów wracamy do tego, jak dużo swojej wolności byłabyś w stanie oddać w zamian za. Ja nie potrafię tego określić, to jest trudne i nie mam tego tak zupełnie przemyślanego. Z jednej strony, gdyby świat wyglądał inaczej - Polska byłaby trochę w próżni, wokół nas było dużo wolnego miejsca i dzikości, pozostała część świata byłaby jak Afryka - to wyrzućmy wszelką inwigilację, każdy człowiek powinien nosić przy sobie rewolwer i mieć wolność jak w amerykańskim westernie. Z drugiej strony, wszystkie te powiadamiania, alerty RCB, PESELe - dają nam pewną żywotność, moc. Myślę sobie o Izraelu - małe państwo otoczone wrogami. Tam wszyscy są przeszkoleni. I w przypadku ewentualnego zagrożenia, np. powodzi - mam wrażenie, że w Izraelu można by wszystkich zmobilizować w 3 godziny, gdzie u nas coś takiego zajęłoby miesiąc. W tym sensie te rzeczy dają nam pewną moc. Taki monitoring na przykład dałby nam brak epidemii, ale ja nie wiem, czy chciałbym się godzić na coś takiego. Trudno mi to powiedzieć.

**Drony dostarczające produkty medyczne i inne towary osobom potrzebującym.**

Kurczę, nie wiem dlaczego to muszą być drony. Mam wrażenie, że jesteśmy na tyle daleko z tą technologią, dronami, że może w Warszawie miałoby to sens, natomiast nigdzie indziej nie. Więc przy dronach znak zapytania. Ale system, gdzie robiłoby to wojsko, policja, harcerze, strażnicy miejscy? Apka, która pozwoliłaby mi zamówić pakiet wyrobów medycznych - płatnie, nie płatnie, może z jakąś subwencją państwową, że jeśli jestem poddany kwarantannie, mogę zamawiać jakieś mleko i ryż w gratisie, czy z dużym upustem. A kiedy jestem żółty, ale się boję, mogę sobie też coś tam zamówić. To uważam, że byłoby fajne.

**Dlaczego przy dronach znak zapytania?**

A dostałaś już kiedyś w Polsce coś dronem? Nikt nigdy jeszcze nie przetestował, żeby dron przewiózł coś z Marszałkowskiej na Nowy Świat i żeby ludzie się gapili, czy on nie spada im na głowy i czy nie zahacza o druty wysokiego napięcia, czy sygnalizację świetlną. Nie przetestowaliśmy w Polsce nawet jednego, jednej takiej usługi. To nie działa nawet w obrębie centrum, a nagle chcielibyśmy to zrobić, żeby państwo to zrobiło. Państwo nie jest jeszcze zaawansowane w takich rzeczach, to rzucanie się na głęboką wodę. Czy państwo, czy firmy prywatne to jeszcze pieśń przyszłości. Na początku te drony nie będą wozić zakupów z Biedronki za 100-200 zł. Ale są kurierzy, którzy wożą tak pojedyncze dokumenty - teczki z dokumentami. To na początku, po centrum Warszawy może coś takiego będzie latało. Ale to się wydarzy za 5 lub 10 lat. Raczej nie wcześniej. O ile nie za 50. Choć te 5-10 lat to będą pierwsze rzeczy. I wtedy moglibyśmy poszerzać grono odbiorców jakiegoś rozwiązania. Na chwilę obecną - nie ma szans.

**Aplikacje, w których użytkownicy mogliby informować się o tym, czego potrzebują i dzięki temu pomagać sobie nawzajem.**

To na przykładzie tej "Widzialnej ręki" na FB. To działa i jest bardzo fajne, oddolno-demokratyczne. To nie jest przez państwo. To jest tak sformułowane, że użytkownicy nawzajem informują się o tym, ze czegoś potrzebują i sobie pomagają. To mogłoby w jakimś sensie i zakresie działać też bez pandemii i dotyczyć różnych innych spraw.

**Masz tu jakieś obawy względem takiej aplikacji?**

Nadużycia mogą być zawsze, ktoś może się podłączyć i... W Allegro czy Uberze też są jakieś przestępstwa. Ktoś komuś przysłał cegłę zamiast telefonu, pojechał okrężną drogą, przysłał cegłę zamiast telefonu, itd. Ktoś może się podszywać pod kogoś innego, pod harcerza dostawcę mleka i ciasteczek, a później wejdzie staruszce do domu i ją okradnie. Zawsze będą takie rzeczy. Widzę ryzyko - należałoby wymyślić system oceniania, certyfikaty, tego typu rzeczy. Natomiast raczej bym pomyślał nad tym, jak zrobić, aby to było bezpieczne, niż dlaczego tego nie robić.

**Aplikacje sztucznej inteligencji, decydujące na podstawie zebranych danych, gdzie skierować największe środki i wysiłki do walki z pandemią.**

Brzmi spoko. Choć widzę zagrożenie błędnie podjętej decyzji - sztuczna inteligencja ma tę wadę, co normalna inteligencja. Jestem na tak, ale chciałbym, żebyśmy najpierw użyli biologicznej inteligencji. Bo to jest łatwiej uzyskać, niż sztuczną. Boję się, że to byłoby trochę marnowanie pieniędzy. Ale gdyby to było dobrze zrobione i dobrze działało, to ok.

**ProteGO Safe. Czy to ta aplikacja, o której słyszałeś?**

Nie pamiętam, jak ona się nazywała, czytałem o niej w jakiejś wczesnej fazie.

**ProteGo <czytanie informacji o apce, śmiech podczas czytania o module bluetooth>**

To trochę jak w Big Bang Theory - tam był odcinek, że oni sprzedawali jakieś zawieszki i tak myśleli, jak je ulepszyć, żeby sprzedaż wzrosła. I stwierdzili, że dodadzą bluetooth, bo z nim wszystko jest lepsze.

**Jest tu podejrzenie, że to może być nie koniecznie potrzebne, skuteczne?**

Nie wiem, nie znam się na tym, po prostu rozbawiło mnie, że jak ma bluetooth, to jest na pewno lepsze. Choć czytałem coś na Niebezpieczniku - ja się tym raczej mało interesuję, nie lubię, nie fascynuję się tym, choć to do mnie przesiąka i nie jestem w stanie od tego uciec - że tam są takie problemy, że wszyscy musieliby mieć ten bluetooth włączony i są jakieś minusy tego rozwiązania. To przekazuje jakąś dużą liczbę danych, tam trzeba chyba sparować urządzenia, więc musielibyśmy chyba być ciągle otwarci na parowanie urządzeń, wymianę informacji. To byłoby chyba niewygodne i coś tam jest chyba niebezpiecznego, choć nie pamiętam co. Bo teraz jest chyba tak, że to się defaultowo wyłącza, jest jakoś zaszyte w systemie, jeśli akurat z tego nie korzystasz. I tam ktoś to krytykował z powodów technicznych i niebezpieczeństwa użycia danych.

**<czytanie dalszego ciągu>**

Nie wiem, może to miałoby sens w Azji, oni są bardziej zdyscyplinowani, konkretni. U nas, nie wiem, nie potrafię się do tego odnieść, nie podoba mi się ten pomysł.

**Czyli wymaga to dużej dyscypliny?**

Tak, poza tym ja wolałbym to robić na kartce, a nie w jakiejś rządowej aplikacji. Wydaje mi się to skomplikowane i podejrzane. Nie podoba mi się. Nie widzę dla mnie, jako użytkownika, wartości dodanej.

**<czyta dalej>**

No dobra, jakby to dobrze działało, może byłoby spoko. Gdyby to było tak, że ludzie wypełniają tą ankietę regularnie i to na przykład w związku z tym, wysyłało ich na testy. Nie jesteśmy w stanie zbadać 100% społeczeństwa, ale możemy 15%. 5% to łatwo - lekarze, policjanci, górnicy...nie wiem, dlaczego górnicy, ale chyba górnicy są najważniejsi w tym kraju. W każdym razie badać ludzi, którzy mają duży kontakt z wirusem - to by było te 5%. A mielibyśmy pieniądze na przebadanie jeszcze 10%, niech oni wypełnią ankiety i wtedy uzyskamy grupę ludzi, którzy są w grupie ryzyka. Jakoś ich uszeregować i wtedy na kogo starczy pieniędzy, można by ich zbadać, ale nie robić tego tym sposobem zupełnie losowo. Pytanie, jak to robić. To znów mógłby być rodzaj przymusu, który mi się trochę nie podoba, bo nie lubię takich rzeczy, ale to mogłoby być skuteczne.

**<czyta dalej> (...) uzyskasz szybszą diagnozę (...)**

W przypadku COVIDu? Jaką szybszą diagnozę i odpowiedni schemat leczenia? Nie jestem ekspertem, ale jaki jest schemat odpowiedniego leczenia?

**<czyta dalej> (...) wiarygodne, aktualne informacje**

O, to jest piękne, jakby tam były wiarygodne i aktualne informacje, to ja bym instalował tę aplikację od razu. Natomiast porównaliśmy dziś dane - w związku z tymi górnikami na Śląsku, bo okazuje się, że oni są ważniejsi, niż lekarze - jakaś jastrzębska spółka porównuje na swojej stronie statystyki. One nijak mają się do tego, co jest podawane przez Ministerstwo. Ministerstwo podaje mniejszą liczbę dziennych zachorowań w skali kraju, niż ta spółka o swoich ludziach. Czyli te dane mają się jak pięść do nosa. Jeśli rząd nie jest w stanie skonfrontować, dobrze zebrać wszystkich danych, które raz dziennie podaje do opinii publicznej? Kopalnie są zarządzane przez państwo. Więc wychodzi na to, że państwo nie jest w stanie zrobić porządku w swoim ogródku. Dlatego ja nie wierzę, że oni w jakimś ProteGo Safe będą w stanie to zrobić, gdzie w grę wchodzi jeszcze uaktualnianie, technologia - o czym my w ogóle mówimy? Fajne, ale nie wierzę w to i mam na to dowód, dlaczego.

**Kwarantanna Domowa**

Ten opis mi mówi, że państwo jest podejrzanym tworem i muszę mu pomóc, stosując się do ZALECEŃ - czyli jest łagodnie, łagodnie - a później jest: "korzystaj z OBOWIĄZKOWEJ aplikacji". Fajnie jest, fajnie. Nie wiem, jak to jest technologicznie rozwiązane, kto w ministerstwie zbiera dane i jak to jest zrobione prawnie. Ale marketing zrobiony jest bardzo dobrze. To jest dobrze napisane, żeby odwrócić kota ogonem. "Nie dajmy się koronawirusowi", "razem", "czy pomożecie" - towarzyszu, po mo że my. "Aby się udało". I później jest, "korzystaj z OBOWIĄZKOWEJ aplikacji". Jest obowiązek oraz rekomenduje ją MC - wymusza ją. Nazywajmy rzeczy po imieniu.

**Czy jest tu coś, co ci się podoba/ wzbudza obawy?**

Wzbudza moje obawy to, że to nie będzie działać. Ale ma prawo. To, jeśli nie zostanie spartolone, ma prawo działać. Nie widzę tu tyle obostrzeń w sensie niemożności wykonania pewnych rzeczy. To nie jest oparte na nierealnych założeniach - z wyjątkiem jednego, który mnie ukłuł - mamy obowiązek korzystać z tej aplikacji, ale nie ma w tym kraju obowiązku posiadania telefonu komórkowego. Znam ludzi, którzy świadomie mają nie-smartfony. Mam nawet takich znajomych - mają nowe telefony, które nie są smartfonami - specjalnie - po to, żeby nie dało się zainstalować FB, Googlea, itd. On mówi, że go to wciąga i nie chce zajmować tak swojego umysłu. Ma tylko telefon i smsy.

**W takim razie ta poprzednia aplikacja jest oparta na nierealnych założeniach?**

Tak, tam było monitorowanie stanu zdrowia, kwestia problemu z bluetoothem - nie wiem dokładnie o co chodzi, kwestia trackowania danych - to duża baza, którą trzeba przechowywać. Tutaj grupa ludzi, więc i danych jest mniejsza. Ile osób jest objęta kwarantanną? 50 tysięcy? To nie jest 40 mln i państwo, policja, czy straż graniczna jest w stanie wymusić, żeby to przy nich zainstalować. Jesteśmy tu w stanie trochę skontrolować ludzi. Znowu - czy zarządzanie i kontrola mi się podoba? Nie wiem, ale to dotyczy tych, którzy muszą być na kwarantannie - państwo każe ci siedzieć w domu i jeszcze przyjeżdża to sprawdzać.

**Zainstalowałbyś tę aplikację, gdybyś był na kwarantannie?**

Nie wiem, być może próbowałbym się bronić. Może wyjąłbym kartę SIM i powiedział, że obecnie nie używam. Albo wygrzebał starą Nokię i powiedział, że mam taki telefon bez aplikacji. Rozumiem, że to służy temu, że ludzie mają siedzieć w domu. Ja wiem, ze ja bym nie wychodził. W związku z tym, nie widzę powodu, dla którego miałbym jeszcze dzielić się informacjami z państwem. Ja państwa nie lubię, więc może bym próbował tego uniknąć. A może nie, może bym się poddał? Nie wiem, na pewno szybko bym ją wykasował po zakończeniu kwarantanny.

**<ciąg dalszy opisu>**

A, tak, o tym selfie to było, coś nawet o tym czytałem. Jak to czytam, przypominam sobie, że miałem znajomego, który z tego korzystał. Robił sobie codziennie zdjęcia, coś tam jeszcze i na to narzekał. Że tego zdjęcia mu nie chciało przyjmować, coś takiego. Koleżanka, która jest lekarzem i już chyba dwa razy była na kwarantannie - choć nie była chora, a miała styczność - też musiała z tego korzystać. Nie wiem, czy to dobry pomysł. To tak, jak z odpowiedzią na pytanie "jak działać, co robić?" - jestem w tym na tyle zagubiony, że nie wiem. To jest tylko pomagaczka dla służb. I to jest dobrze wymyślone. Tu jest niby napisane "pomóż sobie, pomóż innym". Sobie w niczym tutaj nie pomagasz. Tu tylko pomagasz innym. Tu jeśli udowodnię, że siedzę w domu, nie przyjedzie do mnie policja - ma jedną osobę do odwiedzenia mniej.

**<ostatni fragment opisu>**

Nie wierzę, że to by działało, ale gdyby - byłoby ok. Nie wierzę, że to by działało, bo mamy na przykład sąsiadkę - starsza, schorowana kobieta. My i jeszcze jedni sąsiedzi jej trochę pomagamy. Raz w tygodniu przyjeżdżał do niej ktoś z hospicjum, jakiś personel medyczny, żeby sprawdzić, czy wszystko ok i ewentualnie wykonywał potrzebne zabiegi - na przykład raz na jakiś czas trzeba jej coś odessać z krtani, bo inaczej ciężko jej się oddycha, prawie nie może spać. I teraz, w czasie epidemii, hospicjum zawiesiło te wizyty. Skoro takie rzeczy zawodzą, że pani w jednym z ostatnich stadiów raka nie dostaje od państwa pomocy, to trochę nie wierzę w to, że klikniesz dwa razy w apkę i zaraz ktoś ci coś dostarczy. Może tak. Chciałbym, żeby to działało. Gdyby działało - super, ale mam wątpliwości. W pierwszym, wojowniczym odruchu powiedziałem, że bym to od razu szybko wyrzucił. Choć jak dłużej pomyślę, gdyby to miało oszczędzić pracy policjantom, może w czynie społecznym bym im tego oszczędził. Żeby oni już nie musieli wypalać państwowej benzyny, zużywać państwowego czasu. Kto wie, może by złapali w międzyczasie jakiegoś przestępcę, nie jadąc do mnie? Ale powinna być tu jakaś wymyślona gratyfikacja dla użytkownika. Że jak będziesz współpracować z Państwem, to cośtam. Wtedy by to mogło zadziałać. Takie win win. Państwo nie musi po ciebie wysyłać patrolu, to ty dostaniesz coś - choć nie wiem, co to by miało być. To powinna być nagroda, chciałbym, abyśmy odchodzili od tego schematu strachu przed państwem. Choć to trochę głupio, nie? Nagadałem tyle wcześniej, że dla mnie ważne jest bezpieczeństwo i prywatność, że czuję w państwie zagrożenie i że nie. A teraz mówię, że gdybym z tego tytułu dostawał dodatkowy przydział batoników na przykład, to bym to wszystko oddał i sprzedał. Może by wtedy tak było. Jakąś paczkę żywnościową? Pomyślałbym sobie, kurde, kilogram ryżu? Dwie paczki makaronu, czerstwy chleb i dżem? No nie wiem. Wartością dodaną jest, że policjanci mają mniej roboty.

**Z tego, co zrozumiałam, nadal uważasz, że jesteśmy w trakcie pandemii?**

Kurczę, nie wiem. Uważam, że to jest już taki stan przewlekły. W jakimś sensie tak, bo wirus nadal jest i pandemia znów może wybuchnąć, ale nie widzę na razie potrzeby jakichś radykalnych działań.

**Czy myślisz o przyszłości po pandemii?**

Przyszłość jest dzisiaj. Jeśli już, myślałbym, że tak, jak teraz, będzie w nieskończoność. Że wirus jest, może mutować i nie wiadomo, ile to będzie trwało. Może pół roku, może latami, może do wynalezienia szczepionki. Teraz dobrze by było, żeby ktoś, np. Sanepid, monitorował sytuację i jak znów zrobi się niebezpiecznie, to wtedy wprowadził znów obostrzenia. I powinien podać komunikaty, co robić w danej sytuacji, podać informacje o procedurach. Że jak sytuacja wygląda tak, to zachowywać się tak, że rozwój sytuacji jest monitorowany i gdyby było gorzej, to co będziemy robić i w jakiej kolejności. Np., że w pierwszej kolejności będziemy zamykać hotele i będą miały 4 dni od podania decyzji do wiadomości publicznej, na zastosowanie się do tego. Że później, w następnym kroku, jeśli byłoby gorzej, może być tak, że będziemy zamykać np. fryzjerów. I znów będą na to 4 dni. Chodzi o to, żeby dać ludziom czas na reakcję.

**Myślisz, że na drugą falę trzeba się jakoś przygotować?**

My obywatele? To trochę, jak z izolacją. Dla każdego jest to inny sposób trudności i dla niektórych może być to tak trudne, że nie będą w stanie się przygotować. Ci, którzy mogą i mają na to ochotę, mogą. Np. jeśli jesteś programistą, już dziś kup sobie działkę na Mazurach, czy innej wsi, zgromadź tam trochę zapasów i jak zacznie się epidemia, wyjedź tam. Wtedy ty i twoja rodzina będziecie bezpieczni i zredukujecie ilość możliwych transmisji w mieście. Na przykładzie korporacyjnej Warszawy, która uzdalniła się bardzo szybko. Natomiast jeśli ktoś pracuje tak, że jego praca i tak jest związana z kontaktem z ludźmi, jak ma to zrobić? Pani pracująca w Biedronce może co najwyżej kupić zapas papieru toaletowego i puszek.

**Uważasz, że w razie czego, powinniśmy mieć na powrót lockdown?**

Ta sytuacja, która go za sobą pociągnęła pokazała, że byliśmy wtedy nieprzygotowani - nie było maseczek, respiratorów, procedur - przede wszystkim. Nie było organizacji. Ale jeśli już i tak ich nie było, decyzje o zamknięciu wszystkiego były nie najgorsze. Daję takie słabe "tak". Wiem, że są ludzie, którzy stracili majątki, biznesy, mieli naprawdę różnego rodzaju kłopoty. Oni mogą się wściekać, natomiast nie wiemy co by było, gdybyśmy tego nie zrobili. Być może można by było dać jakąś korektę - moja i bardzo jaskrawa - po cholerę było zamykać te lasy? Dzisiaj na przykład byłem na spacerze z psem i dostrzegłem kolejny idiotyzm - koło nas jest przedszkole i oni mają tam plac zabaw, który jest cały ofoliowany. Czyli te dzieci siedzą w sali, bawią się razem i nie wierzę, że trzymają 2m odstępu - a jeśli tak, to bez sensu, po co w takim razie są w tym przedszkolu? To jakaś dziwna przechowalnia? Tymczasem na świeżym powietrzu mają wszystko zafoliowane. Zupełnie nie rozumiem tej idei.

**Są jakieś grupy, które zostały szczególnie dotknięte tą sytuacją?**

Wcześniej myślałem, że kryzys będzie dużo głębszy. Teraz sobie myślę, że będzie mniejszy, niż wszystkim się wydawało. Jeśli ktoś przepłynął z biznesem do dzisiejszego dnia, nie zatonął, to będzie dalej pływał. Tak mi się wydaje. Usługi, gastronomia, turystyka - wydawało się, że wszyscy padną. Zdaje się, że w związku z tym, że są zamknięte granice, nie da się już znaleźć noclegu nad morzem. Ja nie szukałem, ale wieści są takie, że wszystko jest pełne, a ceny zabójcze. Być może branża chce sobie odbić dwumiesięczne straty, być może wyczaili kąsek w związku z zamknięciem granic, które spowoduje, że osoby z zasobniejszym portfelem też pojawią się nad polskim morzem. Wszyscy mówią o kryzysie, a po Warszawie z roku na rok jeżdżą coraz lepsze samochody - nie mówię o klasie premium, a o parkingach pod blokami z wielkiej płyty. W Warszawie standardem zrobiły się zagraniczne wakacje. W związku z tym, mam wrażenie, że kryzys zatopił niektórych, a zbiednieli najbiedniejsi. Tradycyjnie jest tak, że w biednych uderzyło bardziej - również pod względem psychicznym. Bogatsi mają trochę więcej zasobów, większą płynność finansową, itd. Psychicznie oberwali mniej, bo byli zamknięci w 100m2 mieszkaniach lub domkach na przedmieściach, a nie 40m2 mieszkaniach, z dziećmi. Mają działki, domki nad jeziorem, wyjechali sobie - spoko, da się żyć. Jak nie można było wychodzić, ludzie z blokowisk mieli ciężej. Więc takie rozwarstwienie panuje. Jeśli ktoś miał pracę, którą można było uzdalnić, też go to tak nie dotknęło.

**Widzisz taką możliwość, że pewne z rozwiązań/ zachowań nabytych podczas pandemii zostaną z tobą na dłużej?**

Ze mną trochę jak z tymi nowinkami, przed którymi stawiam opór, ale one prędzej czy później do mnie docierają. Pewne, dość gwałtowne, zmiany nastąpiły. Rzadsze wizyty w sklepach, więcej zakupów online. Ze mną zostanie to na dłużej. Ale czy definitywnie - trudno powiedzieć. Jeśli wyniesiemy się z miasta, zmiany zostaną. Jeśli zostaniemy w Warszawie, żona wróci do biura, ja znajdę pracę stacjonarną, pewnie wszystko wróci za jakieś pół roku do normy. Ale jeśli wyniesiemy się za miasto, moja żona dogada się na pracę zdalną, to pewnie będziemy mniej korzystać z dobrodziejstw miasta - knajp, miejsc kultury.

**A takie mniejsze decyzje?**

Duże decyzje wpływają na małe. Jeśli nie podejmiemy dużych zmian, te małe zaczną wracać do poprzedniej formy.

**Czy któreś zalecenia/ obostrzenia powinny zostać na stałe?**

Nie potrafię powiedzieć, choć chyba bym wolał, żebyśmy powoli odmrażali jakieś kina, teatry. Może duże koncerty i mecze bym jeszcze trochę przetrzymał. A może stadiony z limitowaną ilością widzów? Ale to byłoby i tak bez sensu, bo i tak byłby to tłum. Z kolei robienie meczy na dużych obiektach, na znacznie ograniczoną liczbę widzów, też jest bez sensu.

**Do kiedy byś to wstrzymał?**

Nie wiem, może przeczekać jeszcze z pół roku? Jeśli październik-listopad to ma być druga fala, to do tego czasu może byśmy to jeszcze wstrzymali? Opener się odwołał, Woodstock chyba też - uważam to za dobre decyzje.

**Co myślisz o skuteczności takich rozwiązań, typu co któreś siedzenie w kinie/ na stadionie wolne?**

Jakąś skuteczność ma. To odległość x czas ekspozycji. Więc jeśli to 2m na świeżym powietrzu, to naprawdę dużo. Jeśli to metr w zamkniętej przestrzeni, to zdaje się, że po tygodniu w takim pomieszczeniu, jeśli jedna osoba jest chora, wszyscy są chorzy. Widziałem gdzieś takie case study. Przebadali to w gigantycznym biurowcu. I wyszło, że najbardziej zabójcze jest przebywanie w jednym pomieszczeniu. Więc jeśli ludzi rozsadzić i dać im 2-3 h spektakl, to nawet jak ich porozsadzamy, to część się zarazi. Powiedzmy, jak przyjdzie jedna chora, 4 się zarażą. Natomiast jak wejdzie więcej chorych osób, to to jest siedlisko zarazy. Nie wierzę w zupełne wygaszanie tego. Raczej musimy się nauczyć z tym żyć.

**Słyszałeś o mierzeniu temperatury w niektórych miejscach?**

To akurat jest fajne. Jedyny problem, jaki z tym mam, to to, że można ją mieć podwyższoną z różnych powodów. Jeśli to jest w pracy, w biurowcach - super. Najlepiej, jak by to robiły roboty, kamery, nie ludzie - wtedy się nie znudzi, nie spowszednieje. I gdyby codziennie np. przykładać głowę do jakiegoś czytnika, mieć np. 37 stopni, to wtedy odwracasz się na pięcie i wychodzisz. Szef nawet nie pyta, czy nie ściemniasz, tylko widzi, że Kowalski nie przyszedł, bo miał temperaturę, więc poszedł do lekarza, zrobić test, czy na kwarantannę na tydzień. Dobrze by było, żeby jeszcze było coś w stylu automatycznego zwolnienia i państwo by to jakoś dotowało, żeby zachęcać do takich zachowań, systemu automatycznej kontroli. Wtedy np. dla tych, którzy mogą, praca zdalna. Na lotniskach sprawa jest bardziej skomplikowana. Czy to, że mam podwyższoną temperaturę - a mogą być tego różne powody, mogę np. denerwować się lataniem - znaczy, że już nigdy nie polecę? To znaczy, że już np. nigdy nie polecę do rodziny, bo będę się denerwował, że się denerwuję? To sytuacja bez wyjścia. Ktoś może mieć lekką grypę, lekkie zatrucie. To nie wydaje się głupie, pod warunkiem, że np. byłyby wyznaczone jakieś strefy i umieszczalibyśmy tych ludzi strefami. Trochę to niesympatyczne, ale albo się godzisz, albo nie. Ja bym to przełknął. To takie, że nadal zgadzamy się cię przewieźć, dostaniesz batonika, wodę, ale będziesz np. siedział z tyłu samolotu, za szybą.

**Wydarzenia ważne dla ciebie**

Lockdown na początku. Taki surrealizm sytuacji, to, że miałem wrażenie, że wszystko się zmieniło, że świat się wali. No, nie wali się, ale tak, że to trochę jak wojna, atak na WTC - taki duży historyczny moment. Zastanawiałem się, czy to zmieni nasze życie. Teraz nie sądzę, że aż tak bardzo. Natomiast to zmieni świat. Po pierwsze to przejście do onlineu, po drugie zmiany polityczne - lockdown międzypaństwowy uświadomił wielu krajom, jak bardzo ich gospodarki nie są samowystarczalne. Łącznie z tym, że okazało się - co ukłuło naszą dumę narodową - że w Polsce leżymy, jeśli Chińczycy nam czegoś nie przyślą. To była dla nas taka szpileczka. Mocarstwa też to odczuły. USA, że wypchnęło tam tak dużą część produkcji, że już jest ciężko. Myślę, że świat się zmieni. Firmy były zmuszone przetestować online, w wielu miejscach okazało się, że ludzie pracują dobrze, nawet jeśli nie są kontrolowani na miejscu. Może będzie tak, że długofalowo ludzie wrócą do biur, ale może będą możliwe od tego odstępstwa. To super odstępstwo, o którym przed chwilą powiedzieliśmy, że jak jestem chory, pracuję z domu. Mówię o ludziach, którzy pracują na komputerach, żeby nie było wątpliwości. Dla mnie to są takie rzeczy - zmaganie ze zmianą, początkowym surrealizmem na początku. Druga rzecz - wkroczenie technologii online do wielu obszarów życia - praca, rozmowy z rodziną i znajomymi, nasze wywiady. Swoją drogą, dużo chętniej zgodziłem się na to, żebyśmy zrobili to z domu. Być może wasza metodologia bardziej lubi spotkania osobiste, natomiast dla mnie to jest zupełnie nieobciążające - pogadać sobie dwie godziny wieczorem przez internet. Dla mnie to też jest lepsze pod tym względem, że łatwiej mi przypomnieć sobie pewne rzeczy, o których mogę ci opowiedzieć, bo rozglądam się po mieszkaniu, wyglądam przez okno. Mogę ci coś pokazać, oprowadzić cię po mieszkaniu.
