# Supplementary material for: The challenges arising from the COVID-19 pandemic and the way people deal with them. A qualitative longitudinal study
Source: PLoS One. 2021 Oct 11;16(10):e0258133. doi: 10.1371/journal.pone.0258133 (PMC8504766; doi:10.1371/journal.pone.0258133)
Supplement: S1 Dataset — (ZIP) [file pone.0258133.s003.zip › Transcriptions/stage 5/14.5_M_55_couple, with children.docx]

**14.5_M_55_couple with children**

**Jak ci minął ostatni miesiąc?**

Wszystko dobrze w zasadzie. Pracy było trochę, bo trzeba było otwierać przedszkole, otwierać szkołę i poza tym wszystko normalnie w zasadzie. Cieszymy się wszyscy z tego, że jakaś normalność się powoli przywraca. Taki powrót do całkowitej normalności, jak przed pandemią, to prawdopodobnie długo jeszcze trzeba będzie czekać. Pewnie aż do momentu, jak jakąś szczepionkę wymyślą, jakieś tam ograniczenia cały czas będą. Można było się domyślić, że ograniczeń w nieskończoność trzymać się nie da, bo ludzie tego po prostu nie wytrzymają więcej i dlatego to jest oczywiste, że ograniczenia są znoszone. Wszyscy się też uczą jakoś tej choroby, tej pandemii, walki z nią, ale wszystko tak już normalnie powoli.

**Jak to wygląda w szkole, przedszkolu?**

Zainteresowanie ze strony rodziców posyłaniem dzieci jest niewielkie. Ludzie się cały czas jeszcze boją wysyłać dzieci do placówek. To nas trochę ratuje, bo gdyby zainteresowanie było większe, to są wprowadzone jakieś nowe normatywy dotyczące choćby liczby dzieci, które mogą być w placówkach i my nie bylibyśmy nawet w stanie przyjąć wszystkich dzieci, które są zapisane. Z tego co wiem, bo już takie nieoficjalne informacje są, zamierzają te normatywy w jakiś sposób zmienić. W jaki, to trudno mi powiedzieć, czy całkowicie się z nich wycofają, czy nie...Powinni się z nich wycofać, bo skoro można zrobić weselisko na 150 osób i to nie niesie za sobą żadnego ryzyka epidemiologicznego...Zakładam, że nie niesie żadnego, bo inaczej by nie pozwalali, prawda? [z sarkazmem].  Więc tutaj też nie powinno być żadnych ograniczeń. To jest nielogiczne. Ja wiem, dlaczego zostały wprowadzone te zgody na wesela takie wielkie - chcieli się swojemu elektoratowi przypodobać, bo gdzie się takie wielkie wesela organizuje? Głównie na wsiach, głównie jeszcze w Polsce wschodniej, czyli tam, gdzie jest elektorat PiS-u. Chcieli się przypodobać, bez zwracania uwagi, jakie to będzie miało skutki. Ja uważam, że takie wielkie imprezy, gdzie się tańczy, podskakuje wymienia się różnego rodzaju płynami fizjologicznymi, to one są dużo bardziej niebezpieczne niż to, że się dzieci ze sobą pobawią w szkole.

**Masz wrażenie, że to jest racjonalne, że rodzice boją się posyłać dzieci do szkół i przedszkoli?**

Wg mnie nie. To jest taka nadopiekuńczość, która w tej chwili jest bardzo w modzie. Panuje taka moda na nadopiekuńczość, może niektórym po prostu jest tak wygodniej, możliwe, że to dlatego, że się wakacje zbliżają, więc jeszcze przeczekamy to lato i damy sobie radę jakoś. Ja nie jestem epidemiologiem, ale uważam, że jakieś tam niebezpieczeństwo istnieje wszędzie, zawsze i w każdej sytuacji, ale też nie można wpadać w paranoję.

**Śledzisz dane epidemiczne teraz?**

Nie, przestałem już. Od czasu do czasu spojrzę, ale już przestałem się tym podniecać tak, jak to kiedyś było.

**Na ile twoje życie wróciło do normy sprzed pandemii?**

Nie wróciło, bo choćby to, że syn pracował, pracuje na lotnisku, a lotniska jeszcze cały czas nie wróciły i on siedzi tutaj w domu i w dalszym ciągu nie chodzi do pracy, córka chodziła do szkoły, nie chodzi. I to, że zwykle o tej porze to planowaliśmy już jakieś wyjazdy. Na razie jeszcze nie planujemy, chociaż jakoś tam się przymierzamy. Dopóki nie będzie ostatecznej decyzji co do tego jak będzie wyglądało podróżowanie...Nie tyle, czy można wyjechać z Polski, co normalnie wrócić. Wyjechać i tak można w każdej chwili, ale co będzie po powrocie? Poza tym żyjemy tak, jak przed pandemią. Gdzieś tam nawet na weekend jakiś wyjechałem. Ja dużo jeżdżę na rowerze i się ucieszyłem, że można jechać na rowerze nie mając żadnego namordnika wreszcie. Formalnie przepisy mówiły, że tylko po lesie można bez maski, co było bzdurne. Wczoraj sobie przejechałem 50 km i bardzo się ucieszyłem z tego.

**A spotkania ze znajomymi?**

Niby tak, jak przed pandemią, ale nie robiliśmy jakichś większych imprez jeszcze na razie. Mniej się ludzie ściskają ze sobą na powitanie...Zaczynają, ale z taką pewną rezerwą. Ta pandemia cały czas gdzieś tam jest i w różnych kontekstach się pojawia w rozmowach. Jedyne, co mnie trochę wkurza, to że u ludzi na ulicy widzę jakieś ogromne rozluźnienie. Ja rozumiem, że noszenie masek na ulicy i skuteczność tego jest wątpliwe, ale jak ja widzę, że wchodzę do sklepu czy do jakiejś knajpy i maski nie ma nikt, łącznie z obsługą, to już mnie to zaczyna niepokoić. Boję się, że to rozluźnienie będzie postępowało trochę za szybko. Wchodzę wczoraj do sklepu spożywczego i wśród kupujących maskę na twarzy miała może 1/3. Jest obowiązek noszenia maski w sklepie, a miała 1/3.

**A obsługa?**

Obsługa miała jakieś przyłbice. Ja na ogół też zakładam przyłbicę, bo w sklepie jest mi wygodniej - w masce mi okulary parują. Wczoraj naprawdę 1/3 miała maski i to w dwóch sklepach takich byłem, gdzie była taka sytuacja i to trochę mnie niepokoi, bo to chodzi o to, że ta maska nie chroni mnie, jak ja ją mam przed zarażeniem, tylko w sytuacji, gdybym ja miał tego wirusa, to ona ochroni innych. Może ludzie tego właśnie nie rozumieją.

**Coś jeszcze cię dziwi w zachowaniach innych?**

Przede wszystkim to. To jest takie nadmierne rozluźnienie, a szczególnie mężczyźni nie noszą tych masek, jak zauważyłem. To jest taki polski machoizm trochę, że co mi tam. Taki jestem odważny albo ja nie będę zapinał pasów, bo co mi tam.

**Emocje – zdjęcia**

1 - taki chaos związany ze zdejmowaniem ograniczeń. Brak konsekwencji. Chodzi głównie o otoczenie polityczne i to co słyszymy w wiadomościach. Chaos ze zdejmowaniem ograniczeń, totalny w polityce. ma wrażenie, że wszyscy mają jakąś ukrytą...Wszystkie ruchy, które są podejmowane przez rządzących i także przez opozycję - oni co innego mówią, a co innego mają na myśli. Jest niespójność w przekazie, jest brak logiki, jeżeli chodzi o zdejmowanie/ wprowadzanie ograniczeń, informują nas dosłownie w ostatniej chwili o różnych rzeczach. W czwartek powiedzieli, że od soboty nie będzie trzeba nosić maseczek. Dlaczego od soboty, a nie już od piątku? Co się zmieni między piątkiem a sobotą? Może to, że trzeba rozporządzenie opublikować. jedne ograniczenia się zdejmuje, innych nie. Szkoły i przedszkola - z tego najbardziej jestem niezadowolony, chociaż już napisaliśmy do rodziców przedszkola, że jesteśmy przekonani, że ograniczenia zostaną zdjęte od 1-go lipca i od 1-go lipca działamy tak, jak działaliśmy normalnie. W grę wchodzi też kwestia rozliczeń z rodzicami, bo teraz są jakieś trochę inne rozliczenia i zakładam, że od lipca już będą normalne.

**Jakie to są uczucia?**

Pewna taka niecierpliwość i rozdrażnienie.  Chciałbym jakieś plany wakacyjne zrobić i nie mogę, bo nie jestem jeszcze na 100% pewien czy nasz rząd zdejmie wszelkie ograniczenia, jeśli chodzi o podróżowanie. Są sygnały, że nie zdejmie, bo chcą wprowadzać jakieś tam bony urlopowe. Mam wrażenie, że oni by chcieli, żeby wszyscy ludzie wakacje w kraju spędzali, a jeszcze najlepiej tam, gdzie głosują na PiS. I to mnie tak właśnie niepokoi. Najbardziej mi w tym wszystkim przeszkadza takie poczucie zamknięcia. Nie tyle w domu, co w kraju. Taki brak wolności. To jest na poziomie raczej rozdrażnienia niż złości i taki niepokój, bo zachowanie rządzących jest bardzo nieprzewidywalne i mało racjonalne w tym wszystkim. Gdybym miał poczucie racjonalności w tych zachowaniach, to może bym się nie martwił. Skoro cała Europa zapowiada, że zdejmie ograniczenia od 1 czy 15 czerwca, to ja bym się nie miał czym niepokoić, bo skoro jesteśmy w UE, skoro wszystkie kraje zdejmują ograniczenia, to dlaczego Polska by nie miała ich zdjąć. Ale nie, bo oni właśnie mają zwyczaj robienia inaczej niż cała UE. To mnie drażni i niepokoi, bo nie wiem, jak będzie. Znajomi się już wybierają na nurkowanie do Chorwacji, a ja nie będę nic rezerwował, bo ja naprawdę nie wiem, co oni zrobią w ostatniej chwili. Nie mogę nic zaplanować, jestem w zawieszeniu. Nie martwię się jakoś o zdrowie, że zachoruję, bo jestem człowiekiem zdrowym i pewnie nie będę tego jakoś ciężko przechodzić. Zrobiliśmy sobie z żoną testy na przeciwciała i okazało się, że nie mamy przeciwciał. Szczerze mówiąc bardziej bym był zadowolony, gdybyśmy je mieli, bo to dawałoby nam pewien komfort osobisty, ale nie mamy. Zrobiliśmy te testy z ciekawości. Takie testy w warunkach domowych można zrobić.

7 - taka nuda. Tak się teraz czuję. Nic się nie dzieje, nic jakoś nie zmienia się w otoczeniu. Zwykle bardzo dużo się podróżowało, ciągle gdzieś były jakieś wyjazdy, ciągle nas nosiło, a teraz tego nie ma i to mi przeszkadza. takie trwanie w miejscu. Znam ludzi, którym to też bardzo doskwiera i rekompensują to sobie trochę takim jeżdżeniem po Polsce, zresztą ja też. Na weekend teraz byłem w Trójmieście, wczoraj na pół dnia pojechałem na rower, ale chciałbym pojechać nad ciepłe morze ponurkować, a trudno powiedzieć, kiedy to będzie możliwe.

**Jak teraz wyglądają zakupy u Ciebie?**

Nie wróciło do normy sprzed pandemii. Przeszedłem się po GH, żeby zobaczyć czy wszystkie sklepy są otwarte, ale nic nie kupiłem. Czy nie mam ochoty, może się jakoś obawiam podświadomie? Nie wiem. Chodzę i chyba będę chodził na zakupy tylko, jak będę czegoś naprawdę potrzebował. Raczej tego już nie będzie, że pójdę, przejdę się, zobaczę i jak będzie coś fajnego, to sobie kupię. Tego raczej już nie będzie, przynajmniej przez jakiś czas. I tak bardzo dużo kupuję online.

**Dlaczego?**

Bo w tych teraz sklepach jakoś jest inaczej. Jeżeli ja przed wejściem do każdego sklepu w GH muszę zdezynfekować ręce, to ja w czasie jednego pobytu ręce dezynfekowałem 20 razy. Ileż, do jasnej cholery, razy to można robić. Stoi gość i mówi, że proszę zdezynfekować ręce i ja te 20 razy to robię i one zaczynają mnie już po prostu boleć. W rękawiczkach pocą się ręce. Wszędzie jest ten płyn, chociaż wczoraj wszedłem do takiego maleńkiego sklepiku spożywczego koło domu - to ten sklep, gdzie nikt nie miał maski. Wisi napis, że proszę dezynfekować ręce, rozglądam się, gdzie jest płyn i nigdzie go nie ma. A tam akurat chyba powinienem dezynfekować, bo wchodziłem do sklepu z ulicy. Kupiłem 2 cukinie, ale uznałem, że to jest niepokojące zjawisko.

**Pytałeś, dlaczego nie ma płynu?**

Nie, nie chciałem się z nimi wdawać w dyskusję, ale poczułem się zaniepokojony, że nie mogłem tego zrobić. ja rozumiem, dlaczego przed wejściem do każdego sklepu, nawet w GH, trzeba te ręce zdezynfekować i wiem, że trzeba, tylko to właśnie, że trzeba spowoduje, że ja nie będę chodził do tych GH tak po prostu. Jeżeli bym chciał coś kupić w jakimś sklepie, to wszedłbym do tego konkretnego sklepu, w tym konkretnym sklepie bym te ręce zdezynfekował, kupił albo nie kupił i wyszedł. Takie chodzenie po całej galerii nie wchodzi w grę. Do GH poszedłem konkretnie po bieliznę. Bieliznę sobie kupiłem i przy okazji chciałem się przejść, żeby zobaczyć co tam jest, ale po kilku wejściach i kilku dezynfekowaniach rąk, po prostu przestałem to robić, bo już miałem tego dosyć. Do sklepu spożywczego chodzę z listą i zawsze tak było. W zakupach spożywczych właściwie wszystko wróciło do normy, ale to się nawet niewiele różniło w czasie obostrzeń.

**Mówiłeś, że brakuje ci restauracji?**

Tak. Otworzyli i poszedłem sobie na zupkę do Wietnamczyka, teraz też poszedłem na sushi, jak przejeżdżałem przez Elbląg. Było mniej ludzi, większe przestrzenie między stolikami. Doszedłem do wniosku, że restauracja, która najbardziej przestrzega tych ograniczeń, to jest McD. Tam wyraźnie są stoliki zaznaczone - tutaj nie siadaj, tutaj siadaj, tu tyle osób, tu tyle, posiłki są przynoszone do stolika. Bardzo poważnie do tego wszystkiego podeszli.  U Wietnamczyka w zasadzie bez różnicy, chociaż też chyba stoliki rzadziej stoją. Tam było najnormalniej ze wszystkich miejsc, ale też pani, która obsługiwała nie miała maseczki, co trochę mnie zdziwiło, ale stwierdziłem, że nie będę dyskutował z nimi.

**To był dobry pomysł, żeby otwierać restauracje?**

Ja uważam, że tak, oczywiście. Ja się tam czułem bezpiecznie. Jeżeli idę sam, nie stoję w tłumie innych ludzi, jak to zwykle było w McD. Teraz tego nie ma i nawet większy komfort mam. Chyba jednak mniej ludzi chodzi teraz do tych restauracji. Widać, że mniej jest osób.

**Zaraz otworzą nam kina, siłownie?**

Ja uważam, że z tymi siłowniami to jest...Wszystko trzeba w pewnym momencie otworzyć. Czy ja sam pójdę do siłowni? Nie wiem. Mam karnet wykupiony, pewnie zaczną znów nam pobierać miesięczną opłatę. Nie jestem pewien czy pójdę, bo tam tych osób sporo będzie i wiadomo, że jak się ćwiczy, jak jest pot...Poza tym przechodzenie z jednego urządzenia na drugie po kimś, kto tam ćwiczył...Ja jednak chyba nie pójdę. Z jednej strony nie boję się zachorowania, ale z drugiej wolałbym nie zachorować niż zachorować. Nie będę się sam o to napraszał. Czy do kina pójdę? Pewnie też nie, bo tam jednak jest sporo ludzi w jednym pomieszczeniu, a wentylacja tam też różnie wygląda. Z teatrem też pewnie poczekam. Nie wiem, zobaczę.

**Kino jest mniej bezpieczne niż restauracja?**

No tak, bo jednak nawet, jeżeli w tym kinie będzie siedziała co druga osoba, to i tak te odległości są bliższe, to jest mniej niż 2 m, jak jest w restauracji. To jest odległość jednego fotela. Sorry, ale w kinie ta odległość jest mniejsza i za mną będzie ktoś siedział, kto będzie coś tam wydzielał.

**A fryzjer?**

Na razie żona mnie tylko ostrzygła, ale jak będę potrzebował to pójdę.

**APLIKACJE**

**Słyszałeś o jakichś?**

Słyszałem, że są takie, że jak się je zainstaluję i ktoś inny je zainstaluje...No nie wiem...Że jak się u kogoś stwierdzi tego koronawirusa, to będzie można prześledzić jego drogę i jak się okaże, że ja byłem blisko tego człowieka przez jakiś czas, to będzie można mnie jakoś odizolować czy wysłać na kwarantannę, czy coś takiego. Ja takich rzeczy sobie dobrowolnie nie zainstaluję, ponieważ ja w ogóle bardzo niechętnie instaluję sobie jakieś rzeczy, które zbyt mocno śledzą jakieś moje poczynania czy to, gdzie ja się poruszam. Ja tylko słyszałem, że są takie aplikacje, ale nic konkretnie o nich nie wiem.

**Kategoria 1**

Ja do tej pory sobie na żadnym...Mimo, że mam smartfona z funkcją rozpoznawania twarzy i odblokowywania odciskiem palca, to ja z tego nie korzystam świadomie. Nie zainstalowałem sobie tego i nie zainstaluję. Nie jestem pewien, w jaki sposób te dane są przez kogoś gromadzone i wykorzystywane. Nawet, jeżeli oni tam piszą, że oni tego jakoś nie wykorzystują, nie gromadzą, nie nadużywają, to ja nie jestem tego pewien. Poza tym nie jestem pewien, czy za 3 miesiące regulaminu nie zmienią i zaczną to wykorzystywać i moje dane się gdzieś tam znajdą.

**Instalując jakąś aplikację w telefonie, sprawdzasz do czego ona ma dostęp?**

Tak. Ja mam sporo aplikacji, które mają dostęp do moich lokalizacji i z lokalizacją mam mniejszy problem. Problem mam z rozpoznawaniem twarzy i z odciskami palców. Z lokalizacją chodzi o równowagę wygody dla mnie i ryzyka dla mnie. Korzyści dla mnie jest dużo więcej, bo np. jak chcę kupić bilet na pociąg, to jak mam użytą lokalizację, to najbliższa stacja mi automatycznie wyskakuje i nie muszę jej wyszukiwać. Tak samo, jak zamawiam coś w internecie i wybieram najbliższy paczkomat. Nie widzę korzyści dla mnie z rozpoznawania twarzy i rozpoznawania odcisku palca. Znam wielu, którzy tak odblokowują telefon, a ja muszę wpisać kod PIN. To sobie wprowadzę i nie mus mój odcisk palca gdzieś krążyć w internecie.

**Co by się mogło złego stać?**

Ja mieszkałem w Moskwie kiedyś i tam jest oficjalnie wprowadzony system rozpoznawania twarzy. Jest całe mnóstwo kamer z tym systemem na ulicy i praktycznie, jeżeli władze mają ochotę rejestrować sposób poruszanie się kogoś, to wszystko tam jest. Mnie to trochę przeraża. Ja rozumiem, że są jakieś plusy tego, bo wyłapie się jakichś tam przestępców, tylko pytanie, kogo się uznaje za przestępcę. Kim innym jest przestępca w państwie demokratycznym, kim innym w państwie totalitarnym. Polska z obecnymi władzami zbliża się do bycia krajem totalitarnym. Nie wiem, czy to, że co jakiś czas na zamieszczam coś na FB nie sprawi, że ktoś będzie się chciał interesować tym, gdzie ja przebywam i dlaczego. Poza tym wszyscy pamiętamy, że Polska kupiła ostatnio od Izraela ten system. Jak on się nazywa...Taki, który umożliwia śledzenie każdego smartfona. Cała afera z tego była, oficjalnie polski rząd zaprzecza, ale tak naprawdę, to oni go kupili za bardzo duże pieniądze i nie wiem, dlaczego cisza o tym jest ostatnio, bo to po prostu jest skandal największy, jaki sobie można wyobrazić. Nasz rząd może bezkarnie i bez żadnych ograniczeń śledzić każdego obywatela bez jego wiedzy w dodatku. Nie wiem czy to już funkcjonuje, ale nie zostało kupione po to, żeby nie funkcjonowało, prawda?

**Myślisz, że system rozpoznawania twarzy też już funkcjonuje?**

Nie wiem, myślę, że jeszcze nie, ale może też w tym kierunku idą. Jeżeli władze same zainwestują, żeby móc rozpoznawać moją twarz, to trudno, ale dlaczego ja mam komukolwiek w tym pomagać. Takie aplikacje wzbudzają we mnie więcej obaw niż widzę w nich korzyści.

**Kategoria 2**

Założenia są piękne, w porządku. Może jak będę potrzebował, to coś takiego sobie zainstaluję. Na razie nie potrzebuję i nie chcę.

**Kwarantanna domowa**

To jest to, co jest prawnie obowiązkowe, dla wszystkich na kwarantannie. A jak ja nie mam smartfona, to co? Ja o tym myślę, że nawet jeśli trafiłbym na kwarantannę, to bym sobie tego nie zainstalował. Co to znaczy, że jest obowiązkowe? Na podstawie czego? Podstawę prawną poproszę. Jest coś takiego? Nie, ja czegoś takiego bym sobie na pewno nie zainstalował. Nie, nie, nie, nie. Piszą, że jest bezpieczne, a nie piszą w jaki sposób to jest bezpieczne, nie piszą jaka podstawa prawna, nie ma żadnej informacji dotyczącej przetwarzania danych i kto jest odpowiedzialny za przetwarzanie danych, i w jaki sposób ja mogę zarządzać tymi danymi po zakończeniu stosowania tej aplikacji. Dla mnie to w ogóle jest skandal, że coś takiego jest w państwie prawa oferowane i że ludzie są zmuszani, skoro piszą, że jest obowiązkowe.

**Gdyby udostępnili ci smartfona, wiedzę o tym, jak dane są wykorzystywane, udostępnili podstawę prawną?**

To wtedy ja bym robił wszystko, żeby jednak się z tego wykręcić. To jest coś, z pomocą czegoś jakieś władze będą mnie śledzić, a ja nie chcę, żeby jakiekolwiek władze mnie śledziły. Jak jestem na kwarantannie, to jestem. Jak chcą przysyłać policję, to niech ją przysyłają i dlaczego ja jeszcze mam na siebie nakładać jakieś kajdany? Po co? Oczywiście, że to nie powinno być obowiązkowe.

**Dla nikogo? A jeśli przyłapano kogoś na łamaniu kwarantanny?**

Dla nikogo, oczywiście, że dla nikogo. Na to są przepisy, można ich karać i uważam, że powinno się takie osoby karać. Oni piszą, że i tak można się spodziewać wizyty policji, to jeżeli tak jest, to niech sobie do mnie przychodzi policja.

**ProteGo Safe**

Od razu pierwsza rzecz nielogiczna - osoba podejrzewana o zachorowanie. W jaki sposób ona by miała się pojawić na ulicy? Osoba podejrzewana o zachorowanie powinna siedzieć w domu, a nie chodzić po ulicach.

**Może podejrzewana na podstawie swojego dziennika, taka pomarańczowa diodka się zapala, jeszcze nie czerwona, ale ty już jesteś informowany.**

I tutaj znów - ja nie wiem, kto przechowuje dane związane z tą aplikacją, a tutaj jeszcze są dane wrażliwe, bo dane o stanie zdrowia, które zgodnie z RODO podlegają szczególnej ochronie. Nie wiem, do kogo te dane trafiają, gdzie są przechowywane, jak długo, jaki ja mam wpływ na możliwość usunięcia tych danych. Normalnie, ja mam prawo żądać od administratora danych osobowych, usunięcia tych danych osobowych w każdej chwili. Ja tego wszystkiego nie wiem, więc musiałbym się z tym mocno zapoznać. Jeśli nie mam pewności, że te dane nie zostaną w pewnym momencie użyte przeciwko mnie...Ja np. napiszę, że mam 38 st. i mam kaszel, i czy nie skończy się to tym, że pójdę sobie na spacer i jakiś patrol mnie złapie i zaprowadzi do jakiegoś izolatorium. To jest dla mnie jakieś horrendum, taka aplikacja.

**Widzisz tu jakieś korzyści?**

Dla siebie? A jakie by to miały być korzyści? Nie widzę żadnych. Jedyne zyski, to zbieranie danych o stanie zdrowia obywateli przez władzę. Te dane później mogą być przeciwko tym obywatelom wykorzystane. Dla mnie to jest koszmar jakiś. Po rozmowie zobaczę, bo może tam są jakieś informacje o danych osobowych i ich przetwarzaniu. W Polsce, jeśli chodzi o przetwarzanie danych osobowych przez podmioty prywatne, to jest to bardzo szczegółowo regulowane, a jeśli chodzi o przechowywanie przez podmioty publiczne, to tutaj mam dużo mniej kontroli nad tym i dużo mniej wpływu. Jeżeli w to jest zamieszany jakikolwiek podmiot publiczny, to będę robił wszystko, żeby tych danych nie udostępnić.

**Jak to może być wykorzystane przeciwko obywatelowi?**

Może się zacznie jakieś kategoryzowanie obywateli na chorych, zdrowych, bardziej przydatnych, mniej przydatnych. Jeżeli ja bym wpisał, ile ja mam chorób, trafił do szpitala i trzeba będzie podejmować decyzję czy ten facet ma przeżyć, czy nie przeżyć, to sobie mogą pomyśleć, że ten facet i tak jest chory, i tak umrze, więc po co.

**Był taki pomysł aplikacji, którą trzeba by było zainstalować, żeby wejść do GH. Słyszałeś o tym?**

Chyba to ProteGo Safe w taki sposób też działa. Podejrzewam, że gdyby od tego zależało wejście do GH, to raczej w ogóle bym do niej nie wchodził. Bez wizyty tam da się przeżyć. Gdyby to dotyczyło wejścia do każdego sklepu spożywczego, to w tym momencie bym musiał, ale to byłby element represyjny. To wszystko służy zwiększeniu kontroli, odbiera prywatność. Dla nikogo nie powinno być obowiązkowe, a jeżeli będzie to powinno coś takiego być wprowadzane ustawą i ta ustawa by konkretnie musiała reglamentować przetwarzanie danych osobowych. Wszystko musiałoby być zgodne z przepisami ochrony danych osobowych, a szczególnie, jeśli chodzi o dane wrażliwe, którymi są dane o stanie zdrowia.

**Masz jakieś obawy na temat swojej przyszłości w kontekście pandemii?**

To, co mnie niepokoi, to ogólna sytuacja gospodarcza w Polsce. Ja działam w biznesie prywatnej edukacji i to jest rzecz dość droga. My już mamy do czynienia z sytuacjami, kiedy ludzie po prostu rozwiązują umowy z obawy na to, czy ich będzie stać na posyłanie dziecka do prywatnej szkoły/ przedszkola. Obawiam się, że w związku z ubożeniem ludności, będzie coraz mniej ludzi, którzy będą chętni na korzystanie z naszych usług. Niepokoi mnie sytuacja gospodarcza w kontekście pandemii, ale również w kontekście pozycji Polski w UE. Czy w dalszym ciągu obcokrajowcy będą chętnie w Polsce inwestować? Czy firny będą inwestowały w polską gospodarkę? Czy będzie taka działalność perspektywiczna, bo od tego też zależy ogólny poziom dobrobytu, rozwoju, dochodu ludzi. Nasza firma zdejmuje część dochodów od tej warstwy bogatszej społeczeństwa i jeżeli ludzi zamożnych, których stać na korzystanie z naszych usług będzie mniej, to nam też będzie gorzej. To mnie niepokoi.

**A jeśli chodzi o sytuację na świecie?**

Niepokoi mnie rozwój nacjonalizmów na całym świecie. Ogromny. A już teraz to, co się dzieje w USA, że są te bunty spowodowane rasistowskimi zrachowaniami policji, a Trump nie robi z tym nic, żeby jakoś załagodzić, tylko wręcz przeciwnie grozi, że wyśle wojsko, policję, że on to wszystko siłą zgniecie. Jeszcze bardziej antagonizuje społeczeństwo. On uważa, że im więcej konfliktów, im bardziej społeczeństwo będzie spolaryzowane, tym dla niego lepiej. W tym momencie ta biała większość może stawać się coraz bardziej protrumpowska.

Myślisz, że to jest w jakimś stopniu spowodowane pandemią, że się tak teraz tam dzieje?

Nie, chyba nie. To ma chyba inne tło.

**A obawy związane z pandemią?**

To właśnie te gospodarcze i to, że jakoś się kraje zamykają i również w UE. W czasie tej całej pandemii UE została jakoś tak na bok odstawiona. Każdy kraj wprowadzał swoje ograniczenia, każdy kraj się zamykał, układ z Schengen przestał nagle w przeciągu kilku dni istnieć. To, na co wszyscy pracowaliśmy latami. Każdy kraj zaczął swoje decyzje podejmować i to zamknięcie granic wewnętrznych to nie była żadna decyzja unijna. Każdy kraj sam z siebie zamykał granice. UE tutaj nie miała nic do powiedzenia i nie mogła nic powiedzieć, bo te kwestie związane z ochroną zdrowia nie zostały przeniesione na poziom unijny, a być może właśnie powinny być przeniesione, żeby zapewnić wprowadzanie tych ograniczeń czy jakichś kroków kolejny w jakiś bardziej skoordynowany sposób, a nie taki chaotyczny. Każdy kraj wprowadzał sam i to nie było w żaden sposób skoordynowane. Nawet z sąsiadami nie było uzgadniane to zamknięcie granic przez Polskę. Potem to się jakoś ustabilizowało i wyrównało, ale to, co się działo na granicach, że niemieckie służby przynosiły wodę Polakom, którzy czekali w kolejce, żeby wjechać do Polski...To był koszmar jakiś. Wg mnie to jest kolejne zadanie, które powinno być przekazane na poziom unijny - koordynowanie działań związanych z wystąpieniem pandemii/ epidemii na obszarze UE. To jest kolejny krok, który powinien nastąpić, tylko czy znów nacjonalizmy, które w prawie wszystkich krajach UE zaczynają wygrywać jednak nie będą stanowiły jakiegoś oporu przeciwko temu. Czy kraje nie będą uważały, że same dadzą sobie lepiej radę.

**A w kontekście przewidywanej przez niektórych II fali?**

Oczywiście, że to powinno być robione na poziomie UE, bo skoro mamy układ z Schengen, skoro mamy swobodę podróżowania i przemieszczania się, to w tym momencie wszelkie kroki powinny być podejmowane tym poziomie. My tak naprawdę jesteśmy takimi Stanami Zjednoczonymi Ameryki, z może większą trochę niezależnością poszczególnych stanów, ale tak naprawdę jesteśmy jednym organizmem i wszelkie działania powinny być podejmowane na poziomie UE. Tylko UE nie ma chyba nawet takiej instytucji, która by zarządzała kwestiami zdrowotnymi i to też nie zostanie wprowadzone tak szybko. To jest na pewno rzecz, o której należy poważnie pomyśleć. Holandia zamknęła granice, ale nie dla obywateli UE, a Polska zamknęła dla wszystkich. Brak skoordynowania doprowadzał do sytuacji koszmarnych - np. tranzyt, bo Polska nie pozwalała na tranzyt. Polacy latali przez Niemcy do Polski, a już Niemiec przez Polskę nie mógł.

**A jeśli chodzi o sytuację społeczną w Polsce, coś się zmieni?**

Mi się nie wydaje, żeby coś bardzo się miało zmienić. Na pewno ludzie będą się starali jakiś dystans społeczny od siebie utrzymywać, na pewno mniej chętnie będą chodzić na jakieś masowe imprezy. Ja i tak nie lubiłem chodzić na takie imprezy, więc tym bardziej nie będę, ale może będzie jakaś taka niechęć do większych zgromadzeń. Co społecznie? Trudno mi powiedzieć, bo jakoś nie myślałem o tym.

**Powinny być jakieś grupy szczególnie chronione?**

Nie bardzo sobie nawet wyobrażam, jak można np. te osoby starsze chronić, poza zachęcaniem ich do tego, żeby się same chroniły, czyli żeby same unikały kontaktów z innymi. Przymus tu będzie kontrproduktywny całkowicie. Chronienie dzieci...W jaki sposób? Znów pozamykamy przedszkola, żłobki, szkoły? Można wpajać w dzieci jakieś zasady higieny, żeby za bardzo się nie zbliżały do innych, w pewnych sytuacjach, żeby maseczki nosiły. Uczyć zasad utrzymywania większego dystansu społecznego. Może trzeba jakoś chronić, ale to powinno być bardziej na zasadzie dobrowolnej, na zasadzie uświadamiania, a nie na zasadzie wprowadzania ograniczeń. Osoby starsze może powinny móc sobie zamawiać zakupy do domu, które ktoś przywiezie i postawi. Tylko jak rozwiązać kwestie płatności? Ja sobie zamówię przez internet, ale nie wyobrażam sobie, żeby np. moja mama zapłaciła za cokolwiek online, tym bardziej, że jest prawie niewidoma. Poza tym taki kurier też może być potencjalnym zagrożeniem. Dobrze by było wprowadzić badania przesiewowe tak jak w Niemczech. Na to nigdy nie jest za późno i to byłoby logiczne, i dla wszystkich zrozumiałe, że każdy raz na miesiąc ma prawo bezpłatnie zrobić sobie test na koronawirusa. To jednak doprowadziłoby do wyłapania tych bezobjawowych nosicieli.

**Jakieś ograniczenia powinny zostać na dłużej?**

Powinno się bardzo poważnie zastanowić nad znoszeniem ograniczeń dotyczących wszelkich zgromadzeń publicznych. Szczególnie jakieś mecze, koncerty. Parę razy byłem na koncercie, gdzie jest wielka sala, ktoś śpiewa, ludzie się tłoczą w jednej sali, duszno. Dla mnie te wesela na 150 osób to jest jakaś porażka totalna. Ja wiem, że to jest jakaś polska tradycja, ale niech to będzie 50 osób, jakieś większe odległości. Te pleksi w sklepach są fajne i to mogłoby zostać. To, że się będzie dezynfekowało, czyściło - to też powinno zostać. Nie wiem na jak długo, bo zobaczymy czy i kiedy zostanie wynaleziona jakaś szczepionka. Nie wiadomo, czy szczepionka będzie szczepić trwale, czy przez rok, czy przez 3 lata. Za mało mamy jeszcze wiedzy, jeśli chodzi o naturę tej choroby.

**Czy jakieś środki ostrożności powinniśmy zaakceptować i uznać, że tak powinno być zawsze, bo np.  może być jakiś inny nowy wirus?**

Tak. To, co np. zostało wprowadzone w samolotach w tej chwili. Wszędzie zostały wprowadzone te filtry HEPA, które podobno 80% bakterii i wirusów zatrzymują. One i tak w większości samolotów już były, ale teraz zostały wprowadzone wszędzie po to, żeby można było normalnie latać samolotami. Mam syna pilota, który mi opowiadał, że LOT wprowadził, że ma być tylko 50% miejsc zajętych i jeżeli pilot widzi, że zostało zajęte więcej miejsc, to nie ma prawa wystartować. To jest przepis, który tylko Polska wprowadziła. W innych krajach tego nie ma. Skończy się to tym, że Polacy będą latać z Berlina, Bratysławy, Pragi. To jest bezsensowny przepis. Ktoś tego nie przemyślał i takie rzeczy też powinny być koordynowane na poziomie międzynarodowym.

**A mierzenie temperatury w rożnych miejscach publicznych?**

Taka bramka, która mierzy temperaturę może i ma jakiś sens. Mi już wielokrotnie mierzono temperaturę na lotniskach. Takie rzeczy były i niech sobie mierzą. To może sobie być.

**A ograniczenia w restauracjach?**

Mi to akurat nie przeszkadza, bo ja nie lubię tłumów i mi osobiście to pasuje, tylko dojdzie do tego, że te restauracje przestaną się prowadzącym opłacać. Doprowadzi to do podniesienia cen, do bankructwa niektórych restauracji. To, że w ogóle jakieś restauracje przeżyły, to już jest dobrze. Mi zawsze przeszkadzały stoliki za blisko ustawione w restauracjach i wolę jak te odstępy są większe i można swobodnie porozmawiać.

**Płyny i dezynfekcja rąk?**

Widząc, jak to już teraz wygląda w Polsce, to podejrzewam, że to zostanie bardzo szybko zarzucone i wg mnie niesłusznie. Mi zawsze bardzo przeszkadzało jak ktoś wychodząc z toalety nie mył rąk, a szczerze, to 50% facetów nie myje rąk wychodząc z toalety. To, że ludzie zostaną jakoś przyuczeni bardziej do zachowywania podstawowych standardów higienicznych, to tylko dobrze. To by przynajmniej zwiększyło bezpieczeństwo.

**Gdyby nadeszła II fala, to jak myślisz, co zrobi nasz rząd?**

Podejrzewam, że nie będzie już wprowadzał takich ostrych ograniczeń, bo podejrzewam duży opór przeciwko temu. Gospodarka nawet nie wytrzyma drugiego takiego ograniczenia. Tym bardziej, że widzimy po wielu przykładach, jak Szwecja czy nawet Białoruś, które takich ostrych ograniczeń nie wprowadziły, że sytuacja jakoś mocno się tam nie różni. W Szwecji jest wysoka śmiertelność, ale ona wynika prawdopodobnie z czego innego. Wynika z tego, że tam koronawirus zaatakował kilka ośrodków dla osób starszych. Jakieś ograniczenia na pewno będą wprowadzone, ale to już nie będzie na taką skalę, jak było teraz.

**Co ograniczą?**

Może znów te maseczki wrócą, może będą zachęcać do pracy online. Cały czas wielu pracowników pracuje z domu i to może zostać. Coraz więcej firm zacznie na to przechodzić. U mojego szwagra szef podzielił firmę na 2 zespoły i one się zmieniają co tydzień - tydzień w firmie i tydzień online. Chodzi o to, żeby te zespoły się ze sobą nie stykały i żeby nie było czegoś takiego, że cała firma zostanie zamknięta na kwarantannę i przestanie funkcjonować. Ja się szczerze mówiąc obawiałbym czegoś takiego, jak zamknięcie przedszkola czy szkoły na kwarantannę z powodu jakiegoś jednego zachorowania. Teraz jedna z nauczycielek zachorowała, ale na szczęście zachorowała w domu i nie pojawiała się w placówce, bo nauczała zdalnie. Ale wyobraź sobie, co by było, gdyby ona zachorowała w szkole. Wtedy szkoła byłaby zamknięta na kilka tygodni i pytanie, jak mają być prowadzone zajęcia, czy my mamy prawo wtedy pobierać czesne, itd. Ja się obawiam trochę tego przywrócenia normalności, ponieważ ryzyko, że jednak się ten koronawirus pojawi będzie większe. Podobno przechodzi to bezobjawowo i nadal prowadzi zajęcia zdalnie. Znam osobiście 2 osoby, które się zaraziły. Wiem, że istnieje ta choroba i każdego może dotyczyć.

**Czy powinno się jakoś przygotować na ewentualność II fali?**

A wiesz, to może być niezły pomysł. Nie myślałem o tym, ale dziękuję za sugestię, bo może rzeczywiście powinniśmy coś takiego mieć. Jak otworzą lotnisko, to Maurycy prawdopodobnie wróci do pracy. Siłą rzeczy będzie miał styczność z większą liczbą osób i jakieś tam większe narażenie na zachorowanie będzie miał. Poza tym, jak już pełną parą ruszy szkoła i przedszkole, to wtedy też to większe prawdopodobieństwo zakażenia jest. My mamy jakieś tam procedury na wypadek zakażenia w przedszkolu i szkole, ale też do końca nie wiem, jak wygląda kwestia prawna takiej sytuacji. Czy, jeżeli nas zamkną...W ogóle, to czy my mamy sami się zamknąć na kwarantannę? Kto wydaje tę decyzję? Próbowaliśmy się dowiedzieć i nikt nam nie jest w stanie udzielić odpowiedzi. To jest bardzo istotne w naszych relacjach z klientami - czy my się zamykamy na skutek decyzji organu państwowego, czy my się zamykamy sami. Jeśli się zamykamy sami, to nie mamy absolutnie żadnego prawa pobierać czesne od rodziców. Jeżeli zamknięcie następuje na skutek decyzji administracyjnej, to w tym momencie sprawa wygląda inaczej pod względem prawnym. Nikt nie jest w stanie udzielić mi informacji. My nie wiemy, jak się zachować, gdyby coś. Zachorowali górnicy w jakiejś kopalni, ale ta kopalnia cały czas działa i tej kopalni nie zamknięto.  Mi się wydaje, że to jest jakaś decyzja podejmowana indywidualnie w stosunku do każdego podmiotu czy do każdego obiektu, firmy, ale jaką to ma formę, to nie wiem.

**A ty prywatnie jakoś powinieneś się przygotować na ewentualność II fali?**

Testament mam napisać? Może powinienem.

**Myślałam o zabezpieczeniach, zapasach, itp.**

Można mieć w domu dużo ryżu, makaronu, ale ja to nie. Nie będę kopcował ziemniaków.  Też muszę się nad tym zastanowić, bo nie myślałem o tym. Ewentualność II fali na razie nie motywowała mnie do takich rozmyślań. To się raczej odsuwa od siebie niż o tym myśli.

**Jakie momenty były dla ciebie szczególnie ważne w całym tym czasie?**

Ciężko mi jest w tej chwili to sobie nawet przypomnieć jakoś. Taki moment przełomowy to był, kiedy zostały takie bardzo ścisłe ograniczenia wprowadzone - zakaz wchodzenia do lasu, 3 osoby na każdą kasę. To mnie jakoś tak przestraszyło...Trochę mnie to przestraszyło i jakoś tak doprowadziło do...Nie wiedziałem co dalej, nie wiedziałem jak długo nam przyjdzie w tym stanie funkcjonować, jak to wpłynie na funkcjonowanie firmy, czy nie stracimy dochodów, itd. To był ten taki przełomowy okres, a później było już jakoś luzowane. Z tych największych bzdur się powycofywali, jak zakaz wchodzenia do lasów czy te 3 osoby na kasę. Debilne ograniczenia. Sklepy przecież zaczęły pracować całą dobę, bo ludzie nie mieli możliwości w ciągu dnia się dostać do sklepu. To była jakaś taka ulga, ale na zasadzie, że no wreszcie zdjęli te debilne ograniczenia, a nie to, że ja się zacząłem cieszyć, że teraz jest już dobrze, bo dobrze wcale nie było.  Poza tym nic mi nie przychodzi do głowy.

**A przełomowe momenty w kontekście kraju?**

O tak, to te wybory, których nie było. Były wybory, których nie było i to rzeczywiście już śmiech na sali, parodia. Jeszcze jak ja obserwuję, jak to zostało odebrane na świecie, to już w ogóle wystawiliśmy się na pośmiewisko całego świata przez to. Poza tym to cały czas jakieś takie niezdarne kroki rządu na to wszystko podyktowane głównie polityką, a nie kwestiami epidemicznymi. Takich decyzji nie powinni podejmować politycy, tylko specjaliści. Politycy zawsze mają jakiś ukryty kontekst, który przeważa niestety. Uważam, że źle zarządzano, ograniczenia były nieprzemyślane i teraz też uważam, że luzowanie jest nielogiczne i totalnie niezrozumiałe - te wesela.

**Było jakieś działanie słuszne, dobrze i konsekwentnie zrealizowane?**

Nie. Może dobrze zrealizowana została kwestia organizacji tych szpitali jednoimiennych. One zostały w miarę dobrze zorganizowane, dla nikogo nie zabrakło respiratorów. Może dlatego, że nie było takich masowych zachorowań jakla we Włoszech, a może dlatego, że Polska miała trochę więcej czasu na przygotowanie się. Okazało się, że jest ich za dużo, ale lepiej, że ich było za dużo, niż jak by ich miało zabraknąć. Teraz wiadomo, że każdy szpital można w miarę szybko przebranżowić, wiadomo, jak to się robi. To daje jakąś gwarancję, że w przyszłości będzie pod tym względem lepiej. Respiratory jednak są potrzebne.

**Czy na tej samej zasadzie każdy w domu powinien mieć maski, rękawiczki i płyny?**

Tak, oczywiście. Ja na pewno będę miał. Ja używam jednak tych jednorazowych maseczek i mamy te płyny, rękawiczki, maseczki na jakiś czas.
