# Supplementary material for: The challenges arising from the COVID-19 pandemic and the way people deal with them. A qualitative longitudinal study
Source: PLoS One. 2021 Oct 11;16(10):e0258133. doi: 10.1371/journal.pone.0258133 (PMC8504766; doi:10.1371/journal.pone.0258133)
Supplement: S1 Dataset — (ZIP) [file pone.0258133.s003.zip › Transcriptions/stage 5/10.5_F_55_couple, no children.docx]

**10.5_F_55_couple no children**

**Co się u pani działo przez ostatni miesiąc?**

Działo się sporo, bo my jak chyba rozmawiałyśmy, to ja byłam po pierwszej wizycie mojej wnuczki, wtedy co tak mówiłam, że się tak rozpłakałam i że syn mi przywiózł wnuczkę, więc miałam ją samą. A potem ja sama zadecydowałam, że znowu chcę wziąć wnuczkę do siebie, tym razem już z tą starszą, z tą córką partnerki syna, Zosią. Były tutaj u mnie chyba z 5 dni, ciężko było.

**Obie wnuczki, tak?**

Obie, tak. To znaczy ta jedna to taka moja przysposobiona, bo, tak jak mówię, to jest córka partnerki syna, a na mnie mówi "ciocia-babcia". W każdym razie miałam je dwie. Wcześniej jeszcze byłam raz na działce z teściową.

**Pamiętam, że ostatnio wspominała pani, że planuje pani jechać.**

Tak, więc byłam tam, takie uruchomienie jakby działki, sprzątanie. Potem pamiętam, że wróciłyśmy z tej działko, to za dwa dni, to też przeżywałyśmy bardzo, bo miałyśmy badanie na przeciwciała COVID-a, licząc, że jesteśmy już dawno po Covidzie, ale niestety nie, nie mamy tych przeciwciał, czyli nie przechodziłyśmy tego. Mimo że sobie wszystko dopasowałyśmy, że wszystko by się zgadzało, no ale niestety nie. W każdym razie cała rodzina przeszła te badania i nikt nie chorował, więc wszyscy są zdrowi i oby tak dalej. Potem miałam właśnie znowu te dziewczynki. Potem, to wtedy pisałam do pani, bo miałam zakończenie tego swojego katalogu, więc dziękuję za udostępnienie mi jednego konta, bo to mi się liczyło. (Dygresja) Potem z kolei 23., w ten weekend majowy, pojechałyśmy na tę samą działkę, ale już z siostrą i koleżanką. Kiedy pierwszy raz pojechałyśmy z teściową na działkę, ja się wtedy pierwszy raz zobaczyłam z siostrą po tych kilku tygodniach, prawie dwóch miesiącach... Ponad dwóch miesiącach! Bo widziałyśmy się ostatni raz 13 marca, a tutaj jak się zobaczyłyśmy...No, dwa miesiące, koło 10 maja. Siostra też już jakby się przemogła, zaczęła też już jeździć do naszego taty, nie tylko już ja, ale też już siostra. Potem byłyśmy właśnie z siostrą i koleżanką na tej działce, a potem dojechała do mnie jeszcze teściowa, one sobie pojechały, ona dojechała. Robiłyśmy jeszcze tam kolejne zmiany, bo zmieniałyśmy kuchenkę na indukcyjną, nową pralkę na tej działce, więc było dużo takich prac. Było tylko pioruńsko zimno, ale ok. Wróciłyśmy tydzień temu w środę. W międzyczasie teraz na przykład w sobotę byłam na pierwszym spotkaniu tych koleżanek od siebie z pracy, byłyśmy w pubie "Lolek".

**Ale to takie spotkanie towarzyskie, tak?**

Tak, towarzyskie. Oczywiście przez cały czas w tym czasie wahały się te wszystkie historie z tą przyczepą nad morzem - czy będzie, czy nie będzie, jak to tam ma być w końcu. Ja jutro już wyjeżdżam do tej przyczepy. Najpierw ją tam będę... No to jest bardzo ciężka praca, ja już powoli...(Przerwa) Już jestem.

**Mówiła pani o tym, że z przyczepą jest dużo pracy.**

Bardzo, bardzo dużo. Jutro właśnie już planuję, tylko nie wiem, jak ja dzisiaj to wszystko, bo to muszę skompletować, wszystko zapakować, bo to mnóstwo rzeczy musi tam pojechać. Jadę sama na razie, już pomijając, co mnie tam czeka, bo to trzeba rozpakować całą tę przyczepę, poustawiać, umyć, zmienić zasłonki, firanki, uprać, no to jest na tydzień pracy.

**Pani jedzie tam przygotować przyczepę na sezon czy już tam zostać na dłużej?**

Tak. To znaczy, ja tam teraz w czerwcu będę trochę. Ja teraz przygotuję, potem na ten weekend długi mają przyjechać dzieci: syn z Kasią i dziewczynkami, no i jeszcze ten młodszy syn bez partnerki, bo ona z kolei zdaje wszystkie dyplomowe egzaminy na stomatologii, więc akurat nie może. Potem jak oni sobie pojadą, to ja znowu zostaję (...) z dziewczynkami sama w przyczepie. Jeszcze nie wiadomo, czy dojedzie do mnie na kilka dni teściowa czy nie, ale to się już okaże.

**Ludzie, którzy mieli rezerwacje, będą z nich korzystać?**

Tak, tak, oczywiście. Oni zaczynają od 27 czerwca.

**Wymieniłaby pani jeszcze jakieś inne ważne momenty?**

Jeszcze ważne coś, co się działo ze mną akurat, ja kiedyś mówiłam, że jestem depresyjna, że nie raz przechodziłam i cały czas te leki biorę, bo bez tego... Cały maj, można powiedzieć, upłynął pod kątem.... że bardzo bolał mnie brzuch z lewej strony i te wszystkie badania nie były tylko pod kątem COVID-a, ale wszystkie badania, które tylko mogły być, to zrobiłyśmy, no i które są dobre. Na początku maja doszedł też intensywny ból stopy, który ja już wielokrotnie miałam, że nie mogę chodzić. Więc według mnie to znowu się odbija cały ten stres, który był, no to ja teraz zaczynam mieć te wszystkie symptomy psychosomatyczne, które w końcu jakoś się wyciszą, ale tak to zwykle się odbywa. Zresztą moja synowa teraz w tym ostatnim czasie bardzo źle się czuła, do tego stopnia, że musiałam, no nie tyle co musiałam, ale załatwiłam im wizytę u psychiatry. Nie wiem, czy opowiadałam pani, że oni nie mogli sobie poradzić, a ja z dnia na dzień zadzwoniłam, umówiłam online i było. Nie wiem, czy to jest spowodowane tym stresem, który gdzieś nas jednak dopadł, w każdym razie ona się bardzo źle czuła, teraz powolutku zaczyna wychodzić, tak trochę lepiej, ale cały czas miewa te takie lęki, napady płaczu. Sama to widziałam, więc nie to, że to jest jakieś tam wymyślone, tylko nawet jak u nas byli, obserwowałam ją, widziałam, że się źle czuje i nagle przychodziła rozedrgana z płaczem. Ja to też bardzo przeżywam, więc gdzieś we mnie to się też jakoś kumuluje i być może właśnie te bóle brzucha, bo one nie były niczym spowodowane - nie zmieniłam ani diety, ani nic jakiegoś ważnego się nie zmieniło, natomiast ten ból był bardzo uciążliwy. Tak samo z tą stopą. Ja cały czas brałam oczywiście silne leki, Voltaren przeciwzapalny, przeciwbólowy, co też oczywiście zaczyna odbijać się na żołądku w drugą stronę, że zaczynam mieć jakieś kłopoty z biegunką itp., więc teraz odstawiłam te leki, bo stwierdzam, że to tak i tak boli mnie w głowie, a nie noga, więc już nie biorę tych leków.

**Ten stres był spowodowany sytuacją wokół pandemii?**

Ja myślę, że to tak wszystko się skumulowało, bo z powodu tego, to tam różne po kolei wychodziły jakieś: a to z tą przyczepą nerwy, a to z opiekunkami u dziadka, a to...

**Ale te wszystkie problemy były przez pandemię.**

Tak.

**Czy ten stres jest teraz mniejszy?**

Nie do końca, ponieważ oczywiście w którymś momencie, tak jak teraz na przykład możemy chodzić bez tych masek. Śmiałam się, że dzisiaj przyszedł kurier pierwszy raz bez maseczki i mówiłam: "O, wreszcie widzę pana twarz!", bo wcześniej nie widziałam. Nie do końca, bo na przykład niby ja jadę nad to morze, do tej przyczepy, jakby się nic nie działo, ale gdzieś tam z tyłu głowy zdaję sobie sprawę, że niech się tam gdzieś jakieś ognisko - tfu, tfu, tfu! - stworzy na tym kempingu, no to masakra, to w ogóle wolę nie myśleć, co będzie.

**Czyli myśl o wirusie cały czas jest?**

Tak, poza tym nadal to, co mnie bardzo tak martwi i niepokoi, to jak dalej potoczy się ta sytuacja z tymi opiekunkami, ponieważ ta, co teraz jest, co one się zdążyły wymienić na jeden dzień przed zamknięciem granic, jej się 8 czy 5 czerwca kończy wiza. Moja siostra już sprawdzała, że mimo że się kończy wiza, to skoro nie jest odwołany czas pandemii, to ona od ukończenia tej wizy ma jeszcze 30 dni i tam różne takie. Z kolei od innych osób wiem, że tak naprawdę można przekraczać tę granicę, bo można po prostu dojechać do granicy ukraińskiej i tam się na pieszo przekracza. I zaczynam się zastanawiać, czy oni na przykład jednak nie uznają, że ona jednak bezprawnie to sobie przedłużyła, że ją później deportują w momencie, jak przejdzie tę granicę, w związku z tym nie wiadomo, czy będzie otwarta w drugą stronę, czy przyjedzie ta druga. Dla mnie nadal to wszystko jest takie niejasne, niepewne. Już sam fakt, że ja wyjeżdżam na 3 tygodnie, no też się stresuję, bo to nie jest pod Warszawą, to jest ponad 400 km, więc - tfu! - niech się tam coś zadzieje z dziadkiem czy z tą opiekunką, to powrót jest możliwy, ale nie w 5 minut.

**Czy zauważyła pani istotne zmiany w swojej codzienności?**

Raczej nie. Jedyna zmiana to taka, że stwierdziłam, że już nie zamierzam farbować włosów, znowu zresztą je skróciłam, jak pani widzi, na króciusieńkie, no i że koronawirus uświadomił mi tak naprawdę, że to wszystko jest bez znaczenia.

**Dlaczego bez znaczenia?**

Bo to, czy będę miała piękne ufarbowane czy siwe swoje własne w momencie tych innych stresów, to jest nieistotne. A ponieważ ja jak mam długie włosy, to co - siedzę cały czas z wachlarzem, bo mam ciągle te uderzenia gorąca i w momencie, kiedy mam długie włosy, to po prostu mam całe je mokre i jest to obrzydliwe uczucie, więc stwierdziłam, że nie będę przysparzać sobie jeszcze większych cierpień z powodu tego, że mam długie włosy i się pocę. Najchętniej to bym się w ogóle ogoliła na łyso, ale aż tak daleko się nie posunęłam.

**Na ile pani życie wróciło do stanu sprzed pandemii?**

Dużo już wróciło. Owszem, bardzo pilnuję tego mycia rąk, staram się nigdzie czegoś tam nie dotykać, wiadomo, że zawsze mam tę maseczkę przy sobie, że jednak w sklepie ją zakładam, staram się nie stać blisko kogoś, gdzie ktoś kicha, prycha, to już w ogóle, więc coś w rodzaju takiej ostrożności większej.

**A spotkania z bliskimi wyglądają tak jak wcześniej?**

Tak, tylko się nie całujemy, nie przytulamy. Aczkolwiek zaczęła już przychodzić do mnie ta moja pani, która u mnie sprząta już od wielu, wielu lat, a że jeszcze teraz w niedzielę chyba miała urodziny, więc ja jej tam zawsze daję jakieś prezenty i wczoraj też jej nie poskąpiłam tych prezentów, tak sporo jej dałam i na koniec tak stwierdziłyśmy, a, niby nie wolno, więc buziaków sobie nie dałyśmy, ale tak się już przytuliłyśmy. Zresztą też ostatnio właśnie z jedną z koleżanek z pracy się spotkałyśmy przy siedzibie (miejsca pracy) i też tak dawno się nie widziałyśmy, i w którymś momencie, a, i też po prostu zrobiłyśmy te misiaczki, jak to się mówi, bo byłyśmy już utęsknione.

**Z czego to wynikało, że postanowiła pani spotkać się z koleżankami?**

Ponieważ stwierdziłam, że tak i tak już wszędzie wychodzę, spotykam się z całą rodziną już tak normalnie, ta izolacja już się skończyła wtedy i mamy taką grupę na WhatsAppie, i dziewczyny tam zaczęły, że koniecznie musimy się spotkać. Jedna z tych naszych koleżanek jest teraz w ciąży, równo za miesiąc ma termin, więc ona tak bardzo chciała, żeby jak najszybciej, żeby się jeszcze mogła dotoczyć, żeby to się jeszcze mogło wydarzyć.

**A jak do tego doszło, że zdecydowała się pani na kontakt z rodziną? Był moment, że wnuczka przyjechała...**

No to od tego momentu, potem już po prostu normalnie. Potem pojechałyśmy z teściową na tę działkę pierwszy raz, więc wiadomo, że już nie było żadnych maseczek ani nic.

**Jak wyglądają teraz pani wyjścia z domu?**

Ja rękawiczek nigdy nie nosiłam, bo uważałam, że to jest dla mnie głupi pomysł. Oczywiście staram się otwierać łokciem klamki, żeby tak bezpośrednio nie dotykać. Kiedyś też w sklepie byłam, śmiać mi się strasznie chciało, jak jakaś pani była w rękawiczkach, zerwała sobie torebkę na warzywa czy na coś i nie mogła w tych rękawiczkach jej otworzyć, więc zdjęła rękawiczki i tfu, tfu, tfu! Więc nie dość, że zdjęła maskę, tu napluła na rękę, otworzyła, po czym założyła rękawiczki, poszła dalej. Więc to takie dla mnie takie jest... Teraz na przykład w rodzinie mamy osobę na kwarantannie, bo mojej teściowej wnuczka przyjechała ze Stanów i mieszka u teściowej, teściowa mieszka u córki, a ta wnuczka mieszka tam, więc dla mnie to też jest takie... No bo oczywiście rodzice ją odebrali z tego lotniska, przywieźli jednym samochodem, oczywiście się tam nie witali, nie całowali, ale już ten kontakt był. Bo ja się pytałam, w jaki sposób odbywa się transport z takiego lotniska, ale to jeszcze na samym początku, jak moja przyjaciółka utkwiła w Egipcie wtedy i też oni wrócili z tego Egiptu, i musieli na kwarantannę. No i ja mówię: "Dobrze, Aguniu, i jak tam z tego lotniska?" - "No jak? Normalnie, wsiedliśmy do autobusu i pojechaliśmy". Więc dla mnie to wszystko jest takie... Oczywiście teraz policjant cały czas przychodzi do tej wnuczki, sprawdza ją, ale to jest na tej zasadzie, że codziennie przychodzą o tej samej porze, czyli tak i tak może wyjść. Znaczy, oni jej mówią, że oni nie wiedzą, o której przyjdą, ale przychodzą codziennie o tej samej.

**Jak wygląda teraz pani praca?**

Sprzed epidemii wróciło, natomiast u mnie nie wróciło, ponieważ, tak jak już opowiadałam kiedyś, ja pracowałam w tym (miejsce pracy)  i w tej chwili cały ten rok ja jestem w takim jakby dołku, ponieważ nie uzyskuję tych samych sprzedaży, które miałam rok temu, będąc w (miejsce pracy), bo jednak połowa mojej sprzedaży była stamtąd. Ja nie dochodzę do tego momentu, więc moje zarobki w tym roku są diametralnie mniejsze, nawet nie chcę mówić, boby się pani śmiała. Oczywiście ja cały czas....

**A liczba zamówień wróciła do tego, co było wcześniej?**

Katalog jest nie do porównania do katalogu - w jednym zamawiają, w drugim nie zamawiają, to tak różnie, nie można tego... Poza tym, tak jak mówię, dla mnie ten ostatni okres jest taki, że ja ciągle byłam czymś dodatkowo zajęta i przyznaję się szczerze, nie bardzo miałam czas, żeby się tak poświęcać. Bo ja powinnam dużo więcej w (pracy) robić, żeby mieć z tego większe korzyści, ale to jest ta praca online, to pisanie tych wszystkich postów, ja tego nienawidzę, nie cierpię. To już nawet nie o to chodzi, że ja jestem starej daty czy coś, ale dla mnie najważniejszy był ten taki... Ja miałam bardzo dobry kontakt z klientkami w (miejsce pracy), czy z klientkami, czy z przyszłymi konsultantkami, które na przykład namówiłam, żeby zostały, żeby też miały z tego jakąś korzyść, przyjemność i radość. Nie będę ukrywać, że miałam właśnie dobry kontakt i wszyscy mnie tak jakoś dobrze odbierali, i dla mnie to jest zupełnie co innego, niż pisanie postów online, czego ja nienawidzę, nie umiem. Dzisiaj mam mieć jakieś spotkanie, mam założyć jakąś grupę na Facebooku, nie mam pojęcia, jak się do tego zabrać. A teraz jeszcze dodatkowo większość jest na Instagramie, którego ja chorobliwie nie cierpię. Dla mnie Instagram to już jest w ogóle... No nie wiem, może jakby ktoś usiadł koło mnie i mi zaczął tłumaczyć krok po kroku, jak się to robi, to może by było inaczej, ale nie ma takiej osoby, a ja nie mam w sobie na tyle samozaparcia, żeby drążyć ten temat sama.

**Sytuacja w pracy nie wynika z samej epidemii?**

Nie, to nie jest akurat wina COVID-a. Już pomijając, że dzięki COVID-owi dostałam jedno postojowe, które znacznie mnie podreperowało. Mam nadzieję, że jeszcze drugie dostanę. Było zwolnienie z tego ZUS-u przez 3 miesiące, co też dla mnie jest bardzo istotne, więc akurat w tym przypadku można powiedzieć, że ten koronawirus wpłynął dobrze, pozytywnie.

**Osoby w pani otoczeniu wracają do normalności?**

Tak, wszystko to już jest oczywiście na innych zasadach, bo na przykład ostatnio też byłam u dentysty, nie będę ukrywać, po znajomości, bo mama partnerki mojego młodszego syna jest właśnie stomatologiem, ma swój gabinet i po powrocie z tej działki czułam, że coś tam jest nie za bardzo, a ponieważ bałam się, że jadę z dziewczynkami potem na długo, to wolałam to sprawdzić. Mama tej mojej Ani też długo nie pracowała, teraz już wróciła do pracy, ale jak ja ją zobaczyłam - od stóp do głów w tym kombinezonie. Mówi, że praca w takich warunkach, jest bardzo nieprzyjemna, cały czas gorąco i w ogóle, człowiek jest spocony, śmierdzący, jest okropnie. Ale wróciła do pracy.

**Jak się pani czuła, jak zobaczyła ją w tym kombinezonie?**

Bezpiecznie.

**Czyli to były pozytywne odczucia?**

Tak, dla mnie tak.

**Jest coś, co nadal przeszkadza pani w tej epidemii?**

No tak, denerwuje to, że nie wiadomo, jak długo potrwa. Bo teraz z kolei są te głosy, że to jest pełzająca epidemia, że to cały czas będzie się tak wałkować, tak trwać i trwać. Tak jak mówię, na chwilę obecną boję się, żeby nie zadziało się coś na tych kempingach, żeby nam tego z powrotem nie zamknęli. Już oczywiście nie mówiąc, że w tej sytuacji to już po prostu każdy ma takie same szanse: albo się zarazi, albo nie, jak widać wszędzie można się zarazić. Tak jak ostatnio czytałam, że u mnie tutaj na Mokotowie jakaś kobieta, która była w izolacji domowej z pozytywnym wynikiem COVID-a, poszła sobie na zakupy na bazarek. Jak wracała do domu, patrol policyjny ją zaczepił i pytają się, czy nie zna takiej i takiej osoby. Ona najpierw zaczęła mówić, że nie, że pewnie bierze kąpiel, a potem się przyznała, że to ona właśnie.

**Czyli ludzie tak się zachowują, że zarazić można się wszędzie?**

Dokładnie, choćby taki przykład, który właśnie podałam.

**Pokażę pani obrazki. Jak się pani czuła, gdy były u pani wnuczki?**

Byłam przede wszystkim bardzo zajęta. Byłam też trochę zestresowana, bo to jednak duża odpowiedzialność. Oczywiście oprócz tego, że byłam zmęczona, to byłam bardzo zadowolona, bo te dziewczynki są cudne, ta mała jest tak pocieszna i tak kochana. Z kolei ta starsza z częstotliwością 10 razy na godzinę mówi, jak ona mnie bardzo kocha i w ogóle, więc generalnie było mi fajnie, aczkolwiek po południu już zerkałam, która jest godzina, kiedy będą mogły już iść spać. Aczkolwiek ta starsza nie daje za wygraną, więc... Pomijając, że była tutaj u mnie, oczywiście też jakieś tam lekcje odrabiałyśmy, w międzyczasie raz przyszła teściowa, która z nią odrabiała, ja tutaj gotowałam, bo ta starsza dziewczynka ma teraz 10 lat i jest w takim wieku, że po prostu je na okrągło. Ona jest tylko głodna i głodna, nie mijają 2 godziny od posiłku, za chwilę jest znowu głodna, więc ja w tym czasie gotowałam, starałam się jednak, żeby coś tam zawsze było, a wieczorem ta starsza nie odpuszcza, więc albo oglądałyśmy do późna telewizję, filmy różne albo ja jej włączałam jej ulubione filmy przez internet z komputera. Wszystkie trzy spałyśmy w jednym pokoju, więc to było wszystko takie bardzo absorbujące. Jak oni już wyjechali, to ja byłam ledwie żywa.

**Była radość, ale było to też trochę męczące?**

Tak, dlatego troszeczkę się boję tego morza. Oczywiście tam są inne plusy, bo po pierwsze, jeśli tylko nie będzie padało, to cały czas jest się na powietrzu, piasek, babki, to, tamto, natomiast są to warunki kempingowe - do łazienki daleko, więc trochę się tego boję.

**Czy któryś obrazek pasowałby do wizyty pani wnuczek?**

Nie.

**Co musiałoby być na takim obrazku?**

Może jakbym się tak przyjrzała... Tutaj ten obrazek 15. jest to z jednej strony bardzo piękny obrazek, bo to taki jakby wodospad, tak?

**To jest fala uderzająca o brzeg.**

To nie. Jeśli byłby tam wodospad, toby to pasowało, że jest to coś pięknego, ale takie burzliwe, że coś tu się wokół tego dzieje, że to nie było siedzenie na tapczanie, to miałam na myśli.

**Jakie emocje towarzyszyły wizycie na działce?**

Generalnie moja teściowa jest naprawdę bardzo fajną babką. Oczywiście teraz trochę już zaczyna marudzić i narzekać na wszystko, czego ja jej nie omieszkuję powiedzieć. Ale generalnie było nam bardzo dobrze. Tam też było dużo spraw, bo trzeba było jeździć, załatwiać, pomagałam jej to wszystko sprzątać, układać. Ja się troszeczkę śmieję, że z moją teściową to jest coś takiego, że "idź tam, zostań tu", to jej się nie podoba, to... Człowiek nie jest w stanie w pewnym momencie wiedzieć, jaka będzie reakcja na coś, bo spodziewa się takiej, a jest zupełnie inna. Rok temu zrobiła nam potworną awanturę, że została zbita jakaś figurka Matki Boskiej ze Skępego, to jest taka Matka Boska Brzemienna i w tym roku, jak byłyśmy tam z koleżankami, to specjalnie pojechałyśmy do tego Skępego, znalazłyśmy ten klasztor i kupiłyśmy w dewocjonaliach tę figurkę, po czym ona: "Tak? O, a to dla mnie?". Tylko to jest wszystko takie... Nie to, że ona zapomniała czy coś, bo ona tak lubi konfabulować, trochę udawać, oszukiwać. Ja jej to zresztą zawsze mówię prosto w oczy.

**To było podobnie jak z wnuczkami - piękne momenty, ale burzliwie?**

Nie, nie, spokojnie, my się nie kłóciłyśmy.

**Jakiś obrazek do tego pasuje?**

Nie.

**Jak się pani czuła, gdy robiła pani badania?**

Początkowo te nadzieje wiązały się z tym, że jeśli już przeszłam, to być może jest to już za mną, że może rzeczywiście już drugi raz się na to nie choruje. Natomiast jak już nie przeszłam, to tak w pewnym momencie gdzieś to się tak troszeczkę oddaliło, w takim sensie, że oczywiście to zagrożenie jest, ale że to są jakieś typowe ogniska, że tak naprawdę taki przeciętny człowiek, jak nie ma żadnego kontaktu, to szansa na takie zarażenie jest niewielka, wtedy tak sobie myślałam. Oczywiście teraz, właśnie to, co opowiadałam, że przedwczoraj czytałam o tej kobiecie na bazarku, który jest koło mnie notabene, więc nagle okazuje się, że nie do końca, ale wtedy tak jakoś zaakceptowałam to, że ok, że nie ma, żyjemy dalej. Zresztą osoba, która robiła nam te badania, to jest z kolei mama Kasi, czyli partnerki mojego starszego syna i jej szpital (bo ona jest kierownikiem laboratorium), ten szpital został wyznaczony na te pierwsze badania przeciwciał COVID-a. Chyba nawet już to kiedyś mówiłam, że ona na przykład powiedziała, że jak była świńska grypa, to też mieliśmy w Polsce 800 czy 900 przypadków zgonów, tylko o tym się nie mówiło.

**Czyli zaakceptowała pani, że nie przeszła pani koronawirusa i dopowiedziała pani, że nie ma dużych szans, żeby się zarazić. Czy to jakoś panią uspokoiło?**

No chyba tak.

**Czy któryś obrazek pasuje do tej sytuacji?**

Co jest na 5.?

**To jest niebo z gwiazdami, kosmos, chaos.**

Nie, nie, ja jedynie bałam się w przypadku tych badań, czy inne wskaźniki nie są złe, dlaczego mnie ten brzuch cały czas boli, ale już jestem naprawdę skłonna uwierzyć w to, że to są takie jakieś nerwowe i stresowe. Nawet jak teraz o tym mówię i myślę, znów czuję, że mnie to boli z powrotem. Tak samo jak zapomnę o tej nodze, to idę normalnie i wiem, że ona mnie nie boli, ale jak za chwilę tylko coś, to natychmiast już kuleję i mnie boli. Całe życie z wariatami, pani Diano.

**Jak się pani czuła, jak się pani spotkała z koleżankami?**

W pierwszym momencie też nie bardzo mi się chciało iść, ale z tego względu, że to było w sobotę, a z piątku na sobotę w nocy miałam jakieś historie żołądkowe, że biegałam do łazienki, tak jakoś nie bardzo, z drugiej strony czy to jest jednak rozsądne, żeby się tak spotykać, więc delikatne obawy miałam. Ale poszłam, byłam.

**Mogła pani cieszyć się z tego spotkania czy cały czas był stres?**

Nie, potem jak już przyszłam, to było bardzo sympatycznie i fajnie.

**Czy jeszcze jakieś emocje pojawiały się w pani przez ten czas? Poczucie zagrożenia związane z tą sytuacją?**

Znaczy może nie tyle akurat z tą sytuacją, tylko ja tak chwilami, znaczy nie wiem, czy to jest akurat spowodowane koronawirusem czy po prostu upływem czasu, przemijaniem ogólnie, że ja mam chwilami takie momenty, kiedy tak się słabiej czuję i to są takie wyraźne, jak ja to wtedy określam, że muszę się na chwilę położyć, muszę się na chwilę schować. Nawet nie to, że spać, ale schować się, zamknąć oczy, poleżeć. Czasami to przechodzi, a czasami tak trochę dłużej się utrzymuje.

**Czy nadal radzi sobie pani z emocjami przez zajęcie się czymś?**

Tak, tylko z tą pracą to jest tak, że jak jest jakiś przymus zrobienia czegoś, to oczywiście tak, natomiast jeśli to jest szukanie sobie jakiegoś zajęcia, to czasami różnie to bywa. Bo wiadomo, że jak trzeba coś zrobić i wtedy to robię, to automatycznie zapominam i jest ok, ale jak wiem, że nie muszę, to tak różnie z tym bywa. Teraz na przykład ten wyjazd, ja z jednej strony bardzo się cieszę i chcę, ale tak naprawdę to on mnie bardzo tak trochę przeraża, bo ja już nie mam na to siły takiej. Zawsze tak to jechałyśmy z koleżankami, znaczy z koleżanką albo na dwa samochody, albo razem, natomiast teraz jadę zupełnie sama, ona dopiero przyjeżdża w piątek albo sobotę, bo ma przyczepę obok mnie.

**Jakie emocje pojawiają się u ludzi w pani otoczeniu?**

Moja siostra jest oburzona strasznie na córkę, która wysłała starszego synka do przedszkola. Zdenerwowała się, że jednak go wysłali, że ona znowu będzie się bała, już nawet nie chodzi o koronawirusa, ale oni też wszyscy bardzo ciężko chorowali, jak ten mały chodził do przedszkola i pozarażał ich wszystkich. Jak się okazuje, pewnie to nie był korornawirus, tylko te dziecięce wirusy i żołądkowe, i wszystkie. Więc to ją trochę złości i się odgrażała, że ona w takim razie znowu nie będzie do nich przychodzić, co oczywiście nie jest prawdą, bo już dzisiaj poszła tam. Moja teściowa, która zawsze w lecie jeździła na wczasy do ZAiKS-u, bo jej były mąż, znaczy mąż, który nie żyje już, był w Związku Artystów Polskich i teraz te wczasy znowu zostały jej przyznane, i też się tak bardzo zastanawia, czy ma jechać czy nie jechać, co z tym zrobić. Mojej siostry drugi wnuczek zawsze wyjeżdża na takie obozy judo i różne i teraz też nie wiadomo, czy ten obóz będzie czy nie będzie, czy pojadą. Mnóstwo jest takich niewiadomych, czy to tak już mamy po prostu wrócić do normalnego życia, a koronawirus jest z nami i będzie, będą ludzie chorować, ci, co się zarażą, niektórzy umrą, ale żyć należy normalnie czy dalej zachować tę daleko idącą ostrożność: nie wyjeżdżać, nie wychodzić, nie robić. Ja, jak widać, już raczej to robię, ale to nie oznacza, że ja zupełnie się tego nie boję. I to samo dotyczy teściowej czy mojej siostry.

**Powracanie do normalności pomaga znieść to zagrożenie koronawirusem?**

Tak, bo chwilami po prostu przestaje się o tym myśleć.

**Jak pani teraz robi zakupy?**

Nic tutaj się nie zmieniło, oprócz tego, że staram się oszczędniej zarządzać finansami, żeby nie było jakiegoś marnotrawstwa, aczkolwiek jak byłyśmy na tej działce z koleżankami, to a to kwiatki, a to coś tam, więc wydałam dużo pieniędzy, już pomijając te kuchenki, pralki, które akurat mąż finansował. Nie wiadomo, czy ta przyczepa nie będzie ostatni raz w tym roku, czy się zdecydujemy, żeby ją jeszcze w przyszłym roku stawiać, bo cena ma diametralnie wzrosnąć. A z kolei zakupiłam nową wykładzinę na podłogę przed przyczepę, kolejne garnki, które się zepsuły i gdzieś tak się zastanawiam, po co to było. Ale już zostało wydane, jest, więc już pojedzie jutro do przyczepy. Ale tak oprócz tego, to jakichś zbytnich nie robię zakupów, żebym dla siebie coś kupowała, to nie.

**Zakupy spożywcze są z taką samą częstotliwością?**

Z taką samą, ale to wynika z tego, że albo mnie nie było, albo wyjeżdżałam. Poza tym to taki w sumie wyszedł dobry sposób, że raz się te zakupy robiło większe i po prostu one były.

**To, że to zostało wynika z tego, że jest po prostu wygodnie?**

Tak.

**Stosuje pani środki bezpieczeństwa?**

W sklepie tak, zakładam maseczkę, czasami rękawiczki, jeśli jakieś warzywa czy coś. Ale nie we wszystkich sklepach są rękawiczki, a ja nie mam rękawiczek swoich, więc tam, gdzie nie ma, to nie ma.

**Nadal robi pani zakupy z listą?**

Tak. Ja muszę mieć listę, ja nawet teraz, jak się szykuję do tego wyjazdu, to też mam całą listę, żebym wszystko pamiętała, co mam zabrać, żeby czegoś nie przeoczyć, są jakieś sprawy do załatwienia, jakieś zakupy - ja mam dużo tych list.

**Ma pani poczucie, że do zakupów teraz trzeba się szczególnie przygotowywać, że to wyprawa?**

Nie. Wyprawa po zakupy w ogóle? Nie.

**Ostatnio było dużo napięcia wokół przedświątecznych zakupów, teraz już nie ma takich sytuacji?**

Nie, nie ma, bo już nie jest to wszystko tylko na mojej głowie, każdy już robi, teściowa już robi zakupy, moja siostra też już zaczęła część rzeczy kupować do naszego ojca, więc to już ze mnie zeszło, nie wszystko na mojej głowie.

**Czyli u nich wrócił do normy, więc u pani też jest spokojniej?**

Tak.

**Co pani sądzi o otwarciu kawiarni i restauracji?**

Sama nie wiem. To jest na tej zasadzie, że byłam w tym pubie "Lolek" i z jednej strony fajnie, że mogłyśmy się tam spotkać, aczkolwiek to prawda, że nie było tak, że każdy stolik, tylko że rzeczywiście były duże przerwy, ci kelnerzy, co nas obsługiwali, byli w maseczkach, rękawiczkach, każde sztućce, wszystko w pakiecikach jednorazowych. Znaczy nie tyle w jednorazowych, że sztućce jednorazowe, ale wszystko pozawijane. Jeśli ma się wracać do jakiejś normalności, to uważam, że to dobrze, no bo nadal przecież wszyscy gdzieś pracują, ludzie prowadzą restauracje, kawiarnie, zatrudniają innych i nadal jest to część gospodarki, więc to powinno wracać.

**Dlaczego zdecydowano się na otwarcie restauracji?**

Żeby wracało do normalności. Ja nie chcę tutaj już zagłębiać się w jakieś polityczne kwestie, że skoro wszystko jest otwarte, to mogą być i wybory, i to, i tamto. W innych krajach też już się powoli wszystko otwiera, wraca, aczkolwiek na przykład w Indiach nadal jest kolejny miesiąc totalnego lockdownu, oni tam mogą chodzić tylko do sklepu, ale to też poszczególne osoby z rodzin, a tak to są wszyscy zamknięci nadal.

**Czyli to jest kolejny krok, to naturalne, że się otwierają?**

Tak. Oczywiście można się spierać, dlaczego... Bo dla mnie na przykład siłownia jest takim miejscem, gdzie nadal nie powinno się chodzić. To nie jest kwestia, czy się je powinno otwierać czy nie, tylko nadal nie powinno się tam uczęszczać. Mój mąż, który jest zwolennikiem siłowni i ileś tam lat już chodzi, nawet on powiedział, że on do siłowni nie pójdzie, że to jednak jest coś innego siedzieć w restauracji, a być w siłowni, gdzie człowiek się męczy, sapie, dyszy, kicha, no to jednak te wyziewy są większe.

**Co pani sądzi o zabezpieczeniach w pubie?**

Ja tego do końca nie rozumiem. Nie tyle, że nie rozumiem, co dla mnie to nadal nie jest jakimś tam zabezpieczeniem, wystarczy, że któraś z nas byłaby chora, no to tak i tak wszystkie już polegniemy, więc nadal to jest takie...Nie tyle stwarzanie pozorów, co szukanie jakiejś drogi, że jednak w jakimś stopniu się ogranicza ryzyko zachorowania, tylko nie wiem, czy do końca rzeczywiście to, że każdy dostaje chlebek w torebeczce, co z tego, że dostajemy ten chlebek - my siedzimy razem wszystkie, a nie wiemy, kto ten chlebek pakował, ktoś go pakował. Tak jak na przykład ostatnio, jak byłam u fryzjera, to moja przyjaciółka prowadzi salonik, ja mówię, czy ma jakąś gazetę czy coś, ona mówi, że absolutnie, że teraz absolutnie żadnych czasopism, gazet ani nic. Ostatnio szłam po gazetę do kiosku, więc w tym momencie mówię, no dobrze, w kiosku są gazety, każdy je może kupić, sprzedawca je sprzedaje, więc gdzie tu jest jakaś logika do tego wszystkiego. My na przykład mamy katalogi, które rozdajemy i w tym momencie no co, jak wrzucę katalog do skrzynki, to ktoś mnie może pozwać, że mogę być chora i wrzuciłam katalog? Ale przecież saloniki z prasą wszędzie działają.

**Czy tłumaczy pani sobie inaczej ten brak logiki w zabezpieczeniach?**

No właśnie usiłuję sobie odpowiedzieć, ale nie zawsze właśnie jest to dla mnie przekonujące, że to ma sens.

**Czyli ta wizyta w pubie to nie do końca było bezpieczne?**

W ogóle nie było bezpieczne! Sam fakt, że wszyscy ludzie siedzieli, jedli, pili, no to tak naprawdę to było zdecydowanie się na tej zasadzie, że idziemy, ale to ryzyko jest, ono nadal jest. To nie to, że ja idę, bo bezpiecznie jest w pubie.

**Nadal to ryzyko jest.**

No oczywiście, tylko tak jak mówię, że albo po prostu wraca się do normalności w tym sensie, że nadal, jeśli są osoby chore, to ewidentnie trzeba je czy odizolować, czy nadal stosować kwarantanny. Aczkolwiek też ta kwarantanna jest dla mnie w pewnym sensie...Nie wiem, bo tak naprawdę każdy, kto przyjeżdża, powinien być w skafandrze odprowadzony do domu i wtedy ma sens, a tak, to co z tego, że przyleciał cały samolot ludzi, potem wszyscy wsiedli do autobusu, pojechali do domu i siedzą dwa tygodnie na kwarantannie.

**Czyli tutaj znowu nie ma tej logiki?**

Nie.

**Była pani ostatnio w galerii handlowej?**

Nie.

**Korzystała pani z usług fryzjera, czy kogoś jeszcze?**

Pedicure miałam, pedicure i włosy.

**Jakie były zabezpieczenia?**

O, na przykład jeszcze coś takiego! To był kolejny etap odmrażania, wtedy jeszcze restauracje były zamknięte, więc jak ja przyszłam do fryzjera, do tej mojej koleżanki, pytałam, czy ona zrobi mi coś do picia, a ona mi: "Nie mogę", że nie można podawać, mówi: "Ja ci dam szybko wody, ale masz wypić i wyrzucamy, jakby nic nie było". A w tym momencie idziemy do restauracji, gdzie normalnie wszyscy jemy, pijemy, a wtedy w tym zakładzie fryzjerskim nie można było nawet dostać filiżanki herbaty, podczas gdy w takim pubie "Lolek" wszyscy jedzą, piją herbatę, piją piwo i to jest podawane, więc nadal nie wiem.

**Czyli to też wydało się pani nielogiczne?**

Tak. Owszem, było coś takiego, że jak przyszłyśmy do tego "Lolka", to miałyśmy rezerwację na zewnątrz, nie w środku, bo myślałyśmy, że będzie ładna pogoda i będzie cieplutko i fajnie, ale ponieważ było bardzo zimno, to poprosiłyśmy o przeniesienie do środka i wtedy ten manager powiedział, że tak, oczywiście, ale każda z nas musi wziąć swoją podkładkę, swoje sztućce, które już dostałyśmy, że nie może to zostać już na tym stole. Więc dla mnie to też było takim ewidentnym, że skoro to już jest nasze, to my to musimy wziąć.

**Miała pani obawy w związku z korzystaniem z usług kosmetyczki i fryzjerki?**

Nie, bo ta moja przyjaciółka już do mnie wcześniej przyszła do domu i mnie ostrzygła, zresztą to była ta, która siedziała na kwarantannie po pobycie w Egipcie.

**Dlaczego jednak zdecydowała się pani spotkać z tymi koleżankami w pubie? Dlaczego nie w domu?**

No bo taką mamy tradycję, że zawsze się gdzieś spotykamy w jakimś miejscu, zawsze lubimy sobie coś zjeść, zamówić baniaczek piwa czy coś takiego, więc jakoś nigdy nie było tradycji, żeby się spotykać w domu.

**Czyli było ważne, żebyście panie kontynuowały tradycję?**

Tak, żeby się tak spotkać.

**Czy to był element powrotu do normalności?**

Nie mam jakiejś hierarchii powrotu do normalności. Może, że skoro można tak robić, no to ok, no to idziemy. Aczkolwiek tak jak mówię, z drugiej strony nadal to ryzyko wszędzie jest.

**Czyli robiąc różne rzeczy, świadomie decydujemy się na to ryzyko?**

Dokładnie.

**Czy słyszała pani o aplikacjach pomagających walczyć z wirusem?**

Coś tam co jakiś czas mi się pojawia, że "Bądź na bieżąco z informacjami", w telefonie na przykład.

**A takie aplikacje, które od pani pobierają informacje?**

Nie, nie słyszałam.

**Pierwsza kategoria aplikacji.**

Ale tę aplikację każdy może dobrowolnie sobie zainstalować, czy ona jest odgórnie?

**Dobrowolnie (...). To jest kategoria aplikacji, które zbierają dane.**

No ale to takie jakieś dla mnie inwigilowanie z jednej strony.

**Czy takie rozwiązanie jest potrzebne?**

Nie wiem, powiem szczerze, bo się nad tym...

**Oprócz inwigilacji jeszcze jakieś obawy się w pani budzą?**

No tak, że totalnie mnie ktoś kontroluje.

**I pani by to przeszkadzało?**

Tak.

**A widzi pani zysk czy raczej są to obawy?**

Raczej obawy.

**Opis drugiej kategorii, wyjaśnienie rozdzielania środków pomocowych przez sztuczną inteligencję.**

To oczywiście ma sens. Czy te aplikacje są w użyciu?

**Na świecie są używane. A co pani sądzi o dronach dostarczających produkty?**

Trudno mi to sobie wyobrazić, w jaki sposób miałoby się to odbywać.

**Podczas kwarantanny zamawiamy produkty spożywcze i dron nam to dostarcza, nie ma kontaktu z człowiekiem.**

Czyli mają zostać zachowane najwyższe środki ostrożności.

**Czy to jest potrzebne?**

Nie wiem, pani Diano, trudno powiedzieć.

**A jakieś obawy się w pani pojawiają?**

Generalnie, dlaczego mój mąż nie chce być na żadnym Facebooku ani nigdzie, bo on po prostu nie chce nigdzie udostępniać swoich jakichkolwiek danych i według mnie wszelkiego typu aplikacje to jest w jakimś stopniu zbieranie danych osobowych i tutaj to również polegałoby na tym zbieraniu danych. Jak starsza osoba, która nie ma nikogo ani internetu, to dla niej nadal jest to niedostępne, bo ona sobie tego nie założy, ani nie będzie umiała z tego skorzystać, nawet gdyby miała.

**Czyli to jest tylko dla pewnej grupy osób?**

Dokładnie.

**W tej drugiej kategorii też miałaby pani obawy ze zbieraniem danych?**

Oczywiście.

**Prezentacja - 1. aplikacja.**

Czyli to, że do wnuczki mojej teściowej policja przychodzi raz dziennie, to nie znaczy, że ona nie jest cały czas kontrolowana, tak?

**Musi mieć zainstalowaną tę aplikację.**

To już ma sens.

**Co sądzi pani o tej aplikacji?**

Bardziej to jakby do mnie przemawia. Ja nie wiedziałam, że to są takie aplikacje, że to trzeba w ten sposób wszystko zainstalować, zweryfikować, że taka osoba rzeczywiście... Dlatego dziwiło mnie to, że przychodzą o jednej porze, a co poza tym, że ta osoba dalej może sobie gdzieś tam wyjść. Z tego wynika, że jednak nie, że jest to kontrolowane. Według mnie to powinno tak być.

**Jakie dane zbiera ta aplikacja?**

Twarz, zdjęcie twarzy, numer telefonu, adres, gdzie się ta osoba znajduje.

**Jest coś, co się pani spodobało w tej aplikacji?**

To nie jest kwestia, czy mi się spodobało czy nie. Uważam, że ona ma sens, jeżeli rzeczywiście osoby przebywające na kwarantannie mają faktycznie być na tej kwarantannie i tego przestrzegać, to jak najbardziej.

**A coś budzi pani obawy?**
W tym momencie to nie tyle, że budzi obawy. Tu mamy konkretną sytuację, że ta osoba już jest na kwarantannie i jest objęta tym programem, tą aplikacją, więc jest to obowiązkowe. Zbieranie danych w tym momencie nadal jest, natomiast to już jest dla celów istotnych, żeby rzeczywiście epidemia nie rozprzestrzeniała się dalej, jeżeli ktoś przebywa na tej kwarantannie.

**Ta aplikacja powinna być obowiązkowa?**

Dla osób, które przebywają na kwarantannie? No skoro jest obowiązkowa, no to chyba powinna być. Tak, aczkolwiek to takie trudne wszystko jest, bo oczywiście każdy ma jakieś swoje poczucie sumienia i obowiązkowości, to też jest z drugiej strony taka kontrola, każdy, kto myśli logicznie, powinien sam wiedzieć, że należy przestrzegać tej kwarantanny. To też jest swojego rodzaju jednak taka jakby inwigilacja, że sprawdza, czy wychodzisz czy nie.

**Pobrałaby pani taką aplikację?**

Jeśli miałabym być na kwarantannie? A istnieje możliwość, żebym miała tego nie pobrać? Jeśli dotyczyłoby to mnie konkretnie, to ja tak i tak siedziałabym na tej kwarantannie, nie wychodziłabym, nic by mnie nie korciło, więc jeśli chciano by mnie sprawdzać o każdej porze dnia, to proszę bardzo, ale nie wiem, czy akurat mi ta aplikacja byłaby potrzebna. Może to jest na takiej zasadzie, że część osób chce to ominąć i nie chce się podawać rygorowi kwarantanny.

**Rząd powinien tworzyć tego typu aplikacje?**

Rząd nas tak i tak inwigiluje na każdym kroku i pod każdym względem, więc akurat tutaj w przypadku kwarantanny... To jest trudne pytanie, bo nie wiadomo, jak się odnieść do tego. Czy każdy rząd, czy ten rząd, czy ogólnie mając na myśli rząd...?

**Ogólnie. Może nie mieć pani zdania.**

Nie mam, szczególnie, że akurat właśnie nasuwają mi się zupełnie inne skojarzenia, chociażby te, które miały u nas miejsce, że zakaz wejścia na cmentarz, zgromadzeń czy coś, a wszystko to, co się odbywało, każdy dokładnie wie, o czym mówię.

**Prezentacja - 2. aplikacja.**

Ja raczej bym sobie takiej aplikacji nie zainstalowała. Dopóki nie ma bezpośredniego, konkretnego testu, który możemy wykonać, czy to z krwi, czy z wymazu z gardła, to jest to nadal wszystko takie gdybanie: a czy jesteś, a możesz, a nie możesz, a mógłbyś być, może to świadczyć. My idąc z teściową na to badanie przeciwciał, ja to powiedzmy, jeszcze się łudziłam, natomiast teściowa w zasadzie można było stwierdzić, że prawie była pewna, że to przechodziła i dopiero realny test wskazał, że nie.

**Aplikacja nie daje pewności?**

Nie.

**Czy coś mogłoby być potrzebne z tej aplikacji?**

Nie, nie wydaje mi się, chociażby z tego względu, że nawet jak ktoś zdecydowanie według niego miał objawy i dzwonił nawet na infolinię do Sanepidu, to w momencie, kiedy jakiś jeden czynnik się nie zgadzał, choćby ten, że nie był za granicą, co nie oznacza, że nie mógł złapać w warzywniaku na rogu, to taka osoba była od razu dyskwalifikowana, że ona mogłaby mieć tego wirusa. Ta aplikacja też mi tego nie potwierdza.

**Czemu więc służy ta aplikacja?**

Nie wiem, może skoro teraz wszyscy korzystają z różnego rodzaju aplikacji, to jest dalszy ciąg działań w naszym obecnym życiu, że w takim razie musi być też taka aplikacja, znaczy "musi" - że ktoś ją stworzył, na zasadzie, że będziesz czuł się bezpieczny czy tam...

**Ona tego poczucia bezpieczeństwa nie daje?**

Według mnie nie.

**Ona budzi jakieś obawy?**

Nie. Tylko według mnie ona nie jest po prosu nie wiadomo jak pomocna.

**Gdyby ta aplikacja byłaby polecana przez ministerstwo, postrzegałaby ją pani inaczej?**

Nie.

**Myśli pani o przyszłości po pandemii?**

Myślę w tym znaczeniu, że nie wiem, jak to będzie wyglądało, że cały czas jest to taką niewiadomą, z taką obawą lekką.

**Jest coś, co najbardziej zaprząta pani uwagę?**

Jest to, czy po prostu ten wirus już z nami zostanie, tak na zawsze, jak wirus każdej grypy sezonowej i jest to kolejne zagrożenie generalnie dla populacji, tak samo jak inne choroby, nowotwory i wszystko, z czym mamy do czynienia, tak samo to będzie jako jeden z dodatkowych czynników, który zagraża na zasadzie, że wszystko się może zdarzyć, można ulec wypadkowi na ulicy, można z różnych powodów chociażby dostać nowotworu czy w jakimś stopniu zarazić się gdzieś gruźlicą, co nadal jest jeszcze podobno spotykane. Zastanawiam się, czy po prostu on tak już będzie czy ta epidemia zniknie, jak większość epidemii, które miały miejsce na przestrzeni wieków.

**Jeżeli chodzi o przyszłość Polski, jest coś, czego się pani obawia?**

Nie tyle, że się obawiam, podejrzewam, że już nic nie będzie tak samo, chociażby nagle okazało się, że ileś tam tysięcy metrów kwadratowych powierzchni, które były wykorzystywane na biura itp., nagle okazują się zbędne, bo równie dobrze można pracować zdalnie.

**Czy o czymś jeszcze pani myśli?**

No, że oczywiście spowoduje to w wielu dziedzinach kryzys, nie każdy się szybko odbuduje, mnóstwo jednak firm się pozamykało i wręcz splajtowało, to będzie wymagało jakiegoś czasu, aby to zaczęło wracać, ale wydaje mi się, że to już nie wróci do takiego rodzaju konsumpcji, jak było przedtem.

**Jak ta konsumpcja może teraz wyglądać?**

Będzie niższy poziom tego, że to już nie będą tylko i wyłącznie te wyjazdy itp. na zasadzie czystej tylko i wyłącznie, no że każdy może sobie jednak zda sprawę z różnych rzeczy, chociażby z tego wpływu na środowisko. Ja przynajmniej co dwa lata jeździłam na jakieś wakacje, to teraz nie wiem, czy tak myślę o tym, żeby znowu gdzieś jechać, może mi to wróci, na razie nie mam, co myśleć, bo mnie nie stać w tej chwili na to, ale to już nie jest spowodowane koronawirusem.

**Nie myśli pani o wakacjach, bo te podróże już nie będą takie same?**

Tak.

**Jak jeszcze może się zmienić sytuacja gospodarcza?**

Myślę, że na pewno będą musiały być jakieś nowe rozwiązania, żeby ta gospodarka nadal się rozwijała, znaczy rozwijała - żeby uzyskiwała ten sam poziom, co miała. Nie wiem, czy to się uda od razu. Natomiast w jaki sposób do tego będzie się dochodziło, no to nie wiem. Na przykład mam takich znajomych, co prowadzą przedsiębiorstwo agroturystyczne, mają jakąś bazę klientów, teraz natomiast im się to zupełnie nie opłaca, bo żeby stosować się do wszystkich zaleceń, które są à propos wszystkich środków ostrożności, dezynfekcji, to oni w zasadzie nic na tym nie zarabiają, więc to się jakoś musi zmienić. Tak samo jak wszyscy deweloperzy, którzy budują biurowce, nagle to też się zmieni, bo one nagle będą siać pustkami, bo nie wszyscy wrócą do tych samych lokalizacji. Nie wiem, jak to będzie, na pewno czeka nas taka zmiana jakościowa. Nie wiem, czy dobrze umiem to pani przekazać, że już nie będzie najważniejsze, żeby były piękne wakacje, wspaniały samochód...

**Zmienią się wartości i priorytety?**

Tak.

**Czy któreś grupy społeczne będą mocniej dotknięte skutkami pandemii?**

Myślę, że na pewno, tylko tak jak mówię, nie umiem się tak zorientować konkretnie, ale chociażby na przykładzie nawet tej mojej przyjaciółki, która prowadzi ten salon, nagle okazało się, że w wyniku tej pandemii jeden fryzjer odszedł, pokazały się w nim cechy, których ona wcześniej nie doceniała, ale to wszystko w złą stronę ewoluowało. Powstają problemy, które teraz nagle wyszły na jaw.

**Czy któreś z obowiązujących ograniczeń powinny z nami zostać na dłużej?**

No ale co nam jeszcze zostało: maseczki w środkach transportu, w sklepach.

**Może coś zostało zniesione, a powinno zostać?**

Nie mam zdania na ten temat, bo w którymś momencie ten wirus, po tych badaniach, że nie mamy tych przeciwciał, nagle się okazuje... Znaczy ja się go nadal boję, ale ponieważ nie znam nikogo, kto jest chory ani kto był chory, nie wiem, czy nadal... Ja na pewno nadal będę przestrzegać higienę rąk, będę się starała bez względu na wszystko nosić maseczkę tam, gdzie będzie większe skupisko ludzi. Aczkolwiek nadal są zdania podzielone, że maseczka nie chroni tego, co nosi maseczkę, tylko chroni tego, co ewentualnie ma być zarażony, ale uważam, że jednak na pewno jakąś tam barierą jest.

**Czy te środki ostrożności powinny z nami zostać na zawsze?**

Ja nie myślę, że to powinno zostać z nami na zawsze, bo nie wiadomo, co będzie dalej z tym wirusem, czy on wygaśnie, czy on pozostanie z nami na zawsze, bo jeśli tak, to taką maseczkę każdy jednak zawsze powinien gdzieś mieć, jeśli się okaże, że to już tak będzie. Nie wiemy, jak to się teraz zakończy, bo wszędzie, gdzie już niby było dobrze, w Korei znowu są nowe zachorowania. Niektórzy twierdzą, że to będzie trwało dwa lata, ale potem jednak wygaśnie. Tak naprawdę teraz nikt tego nie wie, to jest sytuacja, ja nie mówię, że nowa na przestrzeni wieków, bo nie, bo przecież zdarzały się różne epidemie, natomiast na przestrzeni mojego życia to jedyna w swoim rodzaju, jeszcze czegoś takiego nie było.

**Czy któreś grupy powinny być szczególnie chronione?**

To już każdy indywidualnie musi do tego podchodzić, bo jeśli ktoś ma astmę, jest zdecydowanie słabszego zdrowia, mniejszej odporności, to wiadomo, że jest bardziej narażony, ale czy to jest związane z tym, że ma być w jakiś sposób chroniony? Trudno to określić, jak ma być chroniony. Co znowu ma być w jakiejś izolacji? Tak się nie da żyć.

**Czyli trzeba myśleć nie tylko o ochronie, ale też o jakości życia danej osoby?**

Dokładnie.

**Czy większe odległości między siedzeniami w kinach czy restauracjach to dobry pomysł?**

Generalnie uważam, że na razie, dopóki jest ta pandemia, to wszystko jest bez sensu. Nawet teraz, jak sobie tak uświadomię, że poszłam do tego pubu, to też to zrobiłam niepotrzebnie, jakbym to w tej chwili sobie analizowała, że to jest za wcześnie, ale tak jak mówię, ja tego nie wiem.

**Nie wiadomo, kiedy będzie za wcześnie?**

Tak.

**To jest takie pytanie: czy mogę iść teraz? A jeśli teraz powiem sobie, że za wcześnie, to za chwilę się okaże...**

Ale kiedy? Nie wiadomo kiedy!

**A mierzenie temperatury na lotniskach, kinach jest dobrym rozwiązaniem?**

Nie wiem.

**To buduje pani poczucie bezpieczeństwa?**

Nie.

**Słyszała pani o przewidywaniach, że może nadejść druga fala zachorowań?**

Tak. Właśnie nie wiem i obawiam się, czy ona będzie czy nie będzie, czy to będzie tak po prostu trwało i trwało cały czas, czy to się bardziej wyciszy, a potem powróci jako bardziej takie sezonowe zachorowania.

**A myślała pani o tym, żeby przygotować się na tę drugą falę?**

Nie.

**Czego dotyczą obawy w związku z drugą falą? Że znowu życie nie będzie normalne?**

Tak, że znowu zostanie wszystko przewrócone do góry nogami, że właśnie w tym momencie znowu nie wiem, czy znowu będziemy zamykani, czy będziemy izolowani, jak to wpłynie, czy będą otwierane szkoły czy nie będą, czy jednak będziemy musieli się po prostu uodpornić, a im te ograniczenia będą mniejsze, tym odporność populacji szybciej się ustali.

**Czy rząd powinien znowu wprowadzać ograniczenia przy drugiej fali?**

To takie jest gdybanie, nie wiadomo, jeśli na przykład druga fala będzie dużo silniejsza niż ta, to oczywiście, wiadomo, że znowu coś trzeba zrobić, natomiast jeśli to będą takie pojedyncze przypadki, nie że tysiąc osób dziennie, to czy to nadal ma trwać? Nie wiem tego. Co się dzieje we Włoszech? Oni przecież byli tak długo zamknięci i w ogóle, teraz... Co prawda ja przestałam już to kontrolować, czy tam nadal te zachorowania są i w jakim stopniu, wiem, że tam też już zaczynają wszystko otwierać, więc ludzie się cieszą. To jest taka sytuacja pierwszy raz na taką skalę takiej epidemii na całym świecie. Tak jak kiedyś wszyscy się bali potwornie AIDS i to nadal z nami gdzieś tam jest, już się pojawiły lekarstwa, nadal ludzie na to umierają, ale można to jakoś leczyć. Czy się tworzy szczepionkę na tego koronawirusa, czy ona będzie skuteczna? Nie wiem. Ta niewiadoma cały czas gdzieś z tyłu jest, natomiast ja się staram wrócić do jako tako normalnego życia, ale to nie znaczy, że ja się tego nie boję, nie obawiam, bo obawiam, ale najbardziej tej niewiadomej, co z tym będzie.

**Jakie były najważniejsze dla pani momenty w trakcie epidemii?**

Najbardziej sam fakt izolacji, to było takie najbardziej przykre, poza tym każdy się wtedy tego bał potwornie i w ogóle, i to, że nie możemy się spotykać ani z rodziną, ani z nikim. Ta izolacja to był przełomowy moment, że to się stało po razi pierwszy coś takiego.

**A późniejsze rozluźnianie izolacji też było ważne? Jaki to był moment, czy to było stopniowe?**

Jeden moment taki ważny, kiedy przywieźli mi pierwszy raz wnuczkę i wtedy ta izolacja z rodziną się zakończyła.

**A od tamtej pory pojawił się jeszcze jakiś ważny moment?**

Nie.

**A jakie momenty są ważne z perspektywy kraju?**

Najpierw zamknięcie szkół, kiedy dzieci nie poszły do szkoły, potem zamknięcie granic bardzo przeżywałam, ale związane to było konkretnie z moim przypadkiem, kiedy wymieniały się te opiekunki i nie potrafię nawet sobie wyobrazić, co by było, gdyby to się... Znaczy, wiem, co by było - gdyby ta jedna nie przyjechała, to ta druga by nie wyjechała pewnie. To najbardziej te chyba rzeczy.

**Szkoły, granice, a potem?**

Potem były tylko poszczególne dni, kiedy się obserwowało wzrost tych zachorowań, że coraz więcej, coraz więcej, to było takie bardzo depresyjne, stresujące. A potem po prostu nagle stwierdziłam, że ja już nie chcę tego... Jakieś informacje oczywiście do mnie docierają, ale to już nie jest tak, że ja to śledzę, że muszę obejrzeć, ile konkretnie dzisiaj, ile wczoraj, nie chcę nawet już.

**Czyli z perspektywy kraju ważne były te pierwsze przełomowe momenty?**

Tak.
