# Supplementary material for: The challenges arising from the COVID-19 pandemic and the way people deal with them. A qualitative longitudinal study
Source: PLoS One. 2021 Oct 11;16(10):e0258133. doi: 10.1371/journal.pone.0258133 (PMC8504766; doi:10.1371/journal.pone.0258133)
Supplement: S1 Dataset — (ZIP) [file pone.0258133.s003.zip › Transcriptions/stage 5/8.5_M_30_couple, no children.docx]

**8.5_M_30_couple no children**

**Nie widzieliśmy się przez miesiąc. Jak spędzałeś czas? Co robiłeś?**

Przez pierwszą połowę, czy nawet 2/3 miesiąca dalej pozostawałem w trybie zdalnym. Pracowałem z domu i w zasadzie wyglądało wszystko tak jak przez poprzednie dwa miesiące. A od ostatniej środy wróciłem do pracy stacjonarnej. I jeżdżę codziennie do pracy komunikacją miejską.

**A czym to było spowodowane, ten powrót do pracy?**

Dyrektywą naszej dyrekcji, która już nie pozwoliła na dalsze pełnienie pracy zdalnej, tylko w wyjątkowych okolicznościach. Więc już musiałem.

**A jakby była taka możliwość, to byłbyś chętny, żeby pracować dalej zdalnie?**

Tak, myślę, że przez jeszcze jakiś czas tak.

**Czy jeszcze jakieś inne ważne momenty, ważne sytuacje?**

Przełomowym momentem możemy nazwać, że część klubów sportowych zaczęła się otwierać, wiem, że siłownie nie, ale korty do badmintona czy tenisa tak. Z kortów do badmintona miałem już okazję skorzystać z czego się bardzo cieszę. I od wczoraj właściwie moja grupa kung-fu wznowiła treningi. Też oczywiście trochę w specjalnym trybie, bo raz że na świeżym powietrzu, dwa że na razie to bardziej takie treningi w formie rekreacyjnej, raczej polerowanie techniki bez ćwiczeń z partnerem, z zachowaniem większym odległości. No ale zawsze coś

**Byłeś już na treningu?**

Tak, wczoraj.

**A tak ogólnie na ile Twoje życie wróciło do stanu sprzed pandemii? Czy coś jeszcze pozostało z tych zachowań związanych z pandemią?**

Pozostało to, że dalej w sklepach zakładam maseczkę i większość osób to robi. Tak samo jak poruszam się komunikacją miejską to też w maseczce. Poza tym chyba w większym stopniu wróciło do normy. Choć są też pozytywy. Bo np. jest w pociągu którym jeżdżę jest znacznie mniej ludzi. Tak samo w tramwajach. Jak zostawiam samochód rano pod stacją kolejową, to też nie mam żadnych problemów z zaparkowaniem, a dawniej bywało ciężko.

**Do pracy wróciłeś bo był odgórny nakaz. A co jeszcze - co albo kto - wpływa na to, że pewne obszary wracają do normalności?**

W głównej mierze to wpływa chyba rząd i wydawane kolejne rozporządzenia o otwieraniu kolejnych gałęzi gospodarki, czy o tym czy np. trzeba czy nie trzeba nosić maseczki w miejscach publicznych. Czy np. dzieci będą mogły wrócić do szkół czy nie. No a jakie będą tego efekty społeczne czy epidemiologiczne to się okaże dopiero.

**Wróciłeś do pracy. Czy to jak pracujecie na miejscu różni się znacząco od tego, jak to wcześniej wyglądało?**

Nie, niespecjalnie. Poza tym, ze są jakieś dodatkowe wprowadzone rozwiązania sanitarne, jak płyny do odkażania przy wejściach do budynku, informacje typu, że w toalecie może przebywać jedna osoba tylko. Poza tym w większym stopniu to wróciło do normy.

**A jakieś wyjścia, spotkania z bliskimi, z rodziną to już ma miejsce czy nadal się wstrzymujecie?**

Z rodziną bliższą, z rodzicami, to się nie wstrzymujemy, natomiast z dziadkami jeszcze, ogólnie z osobami starszymi, się wstrzymuję. Ponieważ nie uważam, żeby to zagrożenie które było się istotnie zmniejszyło ostatnimi czasy, a jednak oni są najbardziej zagrożeni.

**A znajomi?**

Jeszcze nie było okazji, ale myślę, że się pojawią w najbliższym czasie. Wszyscy starają się zachować większy dystans od innych osób, ale skoro i tak się spotykamy w pracy, to nie ma powodu, żebyśmy się nie mieli spotkać po pracy też.

**Nie było okazji, nikt nie wychodził z takimi propozycjami? Czy nie było czasu?**

Chyba po prostu jakoś nikt nie wychodził z propozycją na razie.

**A jak to wygląda u Twoich bliskich i innych osób z Twojego otoczenia? Czy u nich takie zachowania się zmieniły?**

U mojej dziewczyny się nie zmieniło, ona dalej pracuje zdalnie. I na razie się nie zanosi, żeby w najbliższym czasie miała wrócić do pracy stacjonarnej. U moich rodziców w większym stopniu chyba wróciło to do normy, jeśli chodzi o mojego tatę to operacje już  - przynajmniej część -  jest przeprowadzana planowo. Jeśli chodzi o mamę, to jej się pojawiły chyba nowe możliwości pracy zdalnej - co chyba jest pozytywem w tej sytuacji. Ale raczej nie ma większych ograniczeń jeśli chodzi o pracę. A jeśli chodzi o znajomych, to chyba w większości jest sytuacja w miarę normalna. Taka mniej więcej jak przed tymi obostrzeniami. Ci którzy mogli częściowo pracować zdalnie, to dalej to robią. Ci którzy nie mieli takiej możliwości, to pełnią obowiązki w miejscu pracy.

**Ale z tego co rozumiem, to chodzi często w znacznej mierze o komfort, a nie o zagrożenie?**

Tak, zdecydowanie.

**Czy pozostało coś, albo pojawiło się coś co przeszkadza ci na chwilę obecną w tej sytuacji?**

Coś nowego? Nie wydaje mi się. Nie przychodzi mi nic do głowy.

**A coś co już było i cały czas przeszkadza?**

Cały czas mi przeszkadza, że siłownie są zamknięte. Ale poza tym to jest chyba jedyne ograniczenie, które bezpośrednio mnie dotyka i które by mi przeszkadzało w tym momencie.

**Powiedziałeś, że wróciłeś na kung-fu. A czy poza tym czy te plany, żeby w domu ćwiczyć? Czy udało Wam się znaleźć zamiennik dla tych obiektów sportowych zamkniętych?**

W moim przypadku to wyglądało tak, że im dłużej ten okres izolacji się przedłużał, im dłużej pracowałem zdalnie, tym w większy marazm popadałem i ciężej było mi się zebrać do czegokolwiek, więc tak jak jeszcze na początku tej izolacji jakoś próbowałem ćwiczyć, to potem już zupełnie nie.

Dopiero teraz do tego wracam powoli.

**Porozmawiajmy jak ostatnio się czułeś. [...]**

Częściowo jednak mimo wszystko towarzyszył mi pewien entuzjazm, że będę miał okazję do porozmawiania ze znajomymi na żywo. Częściowo towarzyszyło mi - nie wiem jak to nazwać - może jakiś rodzaj delikatnego zniechęcenia, to chyba najlepsze słowo jakie mi przychodzi do głowy. Spowodowane tym, że w zasadzie jadąc do tej pracy będę robił w tej pracy dokładnie to samo co bym robił w domu, bo dalej mam w tym momencie pracę tylko przy komputerze. Przy czym w zasadzie gdyby nie to rozporządzenie dyrekcji to mógłbym dalej pracować zdalnie, nie tracić 1,5 godziny dziennie na dojazdy, nie marnować paliwa czy nie marnować pieniędzy na kartę miejską. Więc pod tym względem to jest niepotrzebne trochę. Ale poza tym ten powrót do pracy z jakimś bólem się nie wiązał.

**Czy udało ci się przygotować obrazki?**

Tak, kilka znalazłem.

1. Obrazek przedstawiający to jak wyglądało moje utrzymywanie aktywności fizycznej podczas izolacji, a też częściowo to jak łatwo było mi się zebrać do jakiejś pracy podczas tej izolacji.

**Mówiłeś o marazmie. Czy jeszcze jakieś inne emocje w związku z tą trudnością zebrania się do czegokolwiek?**

Nie wiem. Nuda. Jakiś rodzaj zmęczenia, ale nie takiego wynikającego z przepracowania oczywiście, tylko raczej po prostu z trwania w tej sytuacji. Chyba głównie to

2. To też a ‘propos pracy zdalnej. Mój pies inaczej wygląda, ale też często się pojawiał czy podczas  wideo rozmowy służbowej  czy podczas pracy się nagle pojawiała głowa domagająca się głaskania. Więc to taki typowy obrazek dla mojego homeoffice.

**To ci przeszkadzało?**

Nie, przeszkadzać to nie przeszkadza. Raczej urozmaicenie.

3. Ten jest budzący trochę ambiwalentne uczucia. Ponieważ z jednej strony przedstawia dbanie o bezpieczeństwo, powiedzmy, społeczne, poprzez kontrolę temperatury. Z drugiej strony budzi nieprzyjemne skojarzenia właśnie z nadmierną kontrolą. Czy może się nasuwać skojarzenie z pistoletem, mimo że wiadomo że to raczej pirometr do mierzenia temperatury.

**A to było coś o czym dużo myślałeś ostatnio? Czy to było coś istotnego dla Ciebie?**

Nie, nie nazwałbym tego jakimś szczególnie istotnym. Ale po prostu takie skojarzenie z tą sytuacją.

4. To właśnie taki powrót do pracy - to mnie też skłoniło do zastanowienia czy i na ile się zmienią takie codzienne zachowania i codzienne interakcje międzyludzkie.

Czy odejdzie się od uścisku dłoni w stronę powitania łokciami, czy nie?

**I odeszło się?**

No ja na razie tego nie praktykuję, nie zauważyłem żeby w moim otoczeniu ktoś to praktykował. Ale zobaczymy co czas pokaże.

**A jak jest z podawaniem sobie rąk? Nie wiem, czy akurat w Waszym środowisku było to częste, ale wiem, że różnie to jest...**

Raczej w pracy rzadko z kim się witałem podając dłoń. Ze znajomymi, jako że ostatnio miałem kontakt bezpośredni dość ograniczony, to trudno mi powiedzieć. Ale raczej wszyscy unikają tego podawania ręki póki co.

Ostatnio jedną miałem sytuację, że ktoś dla mnie rękę wyciągnął. Wczoraj na treningu kolega, ale cała reszta tego unikała.

**Odpowiedziałeś na tą wyciągniętą rękę?**

Tak, tak. Jak najbardziej.

**Czy było coś jeszcze, odwołując się to tego, jak się teraz czujesz. Czy mógłbyś jeszcze opisać jak się teraz czujesz? Czy to jest zmęczenie? A może już coś zupełnie innego? Jak powinien wyglądać obrazek który dobrze oddawałby to jak się teraz czujesz?**

Teraz mam wrażenie że paradoksalnie od powrotu do pracy jestem mniej zmęczony. Może dlatego że jest więcej bodźców dookoła mnie, które jakoś mnie stymulują.

Tak jak 2/3 ostatniego miesiąca wiązało się z obrazem mnie leżącego na kanapie i pogrążającego się w apatii, to teraz trochę z tego wychodzę. I trochę się bardziej staję aktywny, społeczny na powrót.

**Wspominałeś wcześniej, że zagrożenie sytuacją nie minęło według Ciebie. A na ile czujesz się obecnie zagrożony? Czy coś wywołuje niepokój?**

Ja się czuję zagrożony dokładnie tak samo jak się czułem miesiąc temu. Bo nic się nie zmieniło jeśli chodzi o wskaźniki epidemiczne. Jak tam obserwuje regularnie wykresy przedstawiające krzywe zachorowań czy krzywe aktywnych przypadków koronawirusa to ten wzrost jest mniej więcej przez ostatni miesiąc stały. Więc dlatego nie czuję się jakoś bardziej zagrożony. Mam wątpliwości jak to nagłe odmrażanie kolejnych gałęzi gospodarki wpłynie długofalowo na stan zachorowań. Czy ta krzywa która jest na razie rosnąca, ale rosnąca liniowo czy nie zacznie rosnąć szybciej? Miejmy nadzieję, że się to nie przerodzi we wzrost logarytmiczny co by było katastrofalne. To jakieś moje wątpliwości. Trudno jest komukolwiek przewidzieć jak to się potoczy.

**A jeśli chodzi u bliskich to widzisz u nich nowe lub nasilające się emocje związane z sytuacją?**

U moich dziadków trochę widzę. Wiem, że u nich też na przedłużająca izolacja już się daje we znaki. Moja babcia już na przykład coraz częściej mówi, że ona sama pójdzie robić zakupy, że sama gdzieś pojedzie - czy taksówką czy autobusem nawet. W ich przypadku to jest już wyraźne zmęczenie tą sytuacją. I też takie zmęczenie pewnie tym, że  nie wiadomo jak długo trzeba będzie trwać w ten sposób. Ograniczając mocno kontakty z bliskimi - z dziećmi czy z wnukami.

 Ale jeśli chodzi o moich znajomych, to żadnych nowych emocji nie zauważyłem.

**A jak się odnosicie do tego że dziadkowie chcą coraz więcej rzeczy robić samodzielnie?**

Moim zdaniem jest to nienajlepszy pomysł. Ponieważ tak jak mówiłem to zagrożenie moim zdaniem się nie zmniejszyło wbrew  temu co w różnych mediach może się pojawiać i wbrew temu co można sądzić, gdy są wydawane kolejne rozporządzenia o zniesieniu obowiązku chodzenia w maseczce w miejscach publicznych. Co może dawać fałszywe poczucie bezpieczeństwa. Więc ja uważam, że rozsądniej by było jednak przynajmniej w przypadku osób starszych jak najdłużej jeszcze starać się wytrwać w tej izolacji. Aczkolwiek wiem, że na przykład moi rodzice też zaczynają mówić, że nie można wiecznie w takim stanie pozostawać, nie można się wiecznie izolować. Że kiedyś to się musi skończyć. Ja się odnoszę do tego dosyć niechętnie, ale coraz więcej osób zaczyna na to patrzeć przychylnym okiem.

**Jak teraz wyglądają u Ciebie zakupy?**

Takie większe zakupy domowe robię z podobną regularnością, tu się nie zmieniło. Natomiast jak wróciłem do pracy, to często w drodze do pracy odwiedzam sklep i sobie kupuję coś czy na śniadanie, czy na lunch. Więc moje wizyty w sklepie są częstsze teraz.

**Czy coś w tych zakupach domowych, większych się zmieniło? Z tego co pamiętam, to u Was dużych zmian nie było**

Nie było dużych zmian i nadal nie ma specjalnych zmian.

**A czy byłeś w ostatnim czasie w GH?**

Nie, nie byłem.

**A planujesz?**

Nie planuję.

**A czy w ogóle odczuwasz taką potrzebę?**

Nie, zupełnie nie.

**Restauracje i kawiarnie. Wspominałeś, że jeszcze nie miałeś okazji, ale co w ogóle sądzisz o tym, że zostały otworzone?**

Myślę, że zostały otworzone przede wszystkim dlatego żeby ratować gospodarkę i ratować tez biznesy poszczególnych ludzi. Bo ten przedłużający się lock-out myślę, że wiele osób mógł zrujnować lub doprowadzić do poważnych kłopotów finansowych. Więc jednak pod tym względem też się o nich trzeba zatroszczyć. Są też na pewno - mam nadzieję - że rządzący obserwują sytuację w innych krajach i to jak zmniejszanie restrykcji w innych krajach wpływa na wskaźniki zachorowalności i mam nadzieję, że właśnie pod wpływem tych danych decydują się na to otwieranie gałęzi w konkretnych terminach.

Sądzę, że mimo wszystko, mimo iż ryzyko rozwoju epidemii rośnie to wydaje mi się, że  jednak takie powolne otwieranie gospodarki to jest dobry krok. Tylko trzeba bacznie obserwować jak te wskaźniki zachorowalności się zmieniają.

**Ze znajomymi jeszcze nie byłeś, ale może - tego nie wzięłam pod uwagę - byłeś z dziewczyną?**

Tak, byliśmy w kawiarni. W zeszły weekend.

**Czemu zdecydowaliście się tam wybrać?**

Powód był dosyć specyficzny, ponieważ miałem problem z samochodem i się okazało, że muszę wyjeździć paliwo, żeby wymienić bak. Najłatwiejszym sposobem była wycieczka, więc pojechaliśmy do Kazimierza. I tam głównie chodziliśmy po wąwozach raczej unikając zbiorowisk ludzkich, ale też poszliśmy do kawiarni na kawę i gofry. Okazało się, że dzięki tej epidemii w Kazimierzu, który normalnie jest zatłoczony, to mimo weekendu było dość pusto i nie było problemu z unikaniem innych ludzi, z zachowaniem dystansu od nich.

**A jak było w tej kawiarni? Były jakieś zabezpieczenia?**

Był czynny tylko ogródek. I przy wejściu były środki do dezynfekcji. I to tyle.

**To według Ciebie jest wystarczająco? Czy coś byś jeszcze wprowadził, zmienił?**

Wydaje mi się, że w przypadku takiego miejsca to jest wystarczająco. Plus wszelkie kwestie ochrony osobistej - osoba obsługująca powinna tez być w jakiejś maseczce czy przyłbicy. I dezynfekować stoły i sprzęty. Takie wydaje mi się oczywiste w tej sytuacji rzeczy.

**I to miało miejsce?**

Tak, tak. W ogóle zauważyłem nawet przechodząc, mijając ogródki w kawiarniach że w większości miejsc pojawiły się informacje, tabliczki na stołach, że stół jest zdezynfekowany.

A zapomniałem, wracając byliśmy w McDonaldsie przy trasie. I tam podobnie to wyglądało - stoliki były dezynfekowane na bieżąco, obsługa w maseczkach, czynny tylko ogródek i środki do dezynfekcji rąk.

**Ostatnio otworzyli też zakłady fryzjerskie, salony kosmetyczne, kina, tego typu obiekty. Co o tym sądzisz?**

Myślę, że powód był dokładnie taki sam jak w przypadku restauracji. Raz że w skali makro gospodarka, w skali mikro to prywatne biznesy czy  kwestie osób pracujących w tych miejscach. No i tak samo uważam, że jest to po prostu kolejny etat, który w którymś momencie należy wprowadzić.

**A byłeś w którymś z miejsc tego typu?**

Jeszcze nie.

**Czyli dalej u fryzjera nie byłeś?**

Jeszcze się nie zebrałem. Nie otrzymałem sygnałów z otoczenia, że już coś powinienem zrobić ze swoją głową na przykład.

**Pytam, bo pamiętam, że wspominałeś że jak się idzie do fryzjera to jest tak ładniej :) a jak robisz to sam to nie jest tak idealnie**

Tak, ale też nie jestem osobą która by do tego jakąś ogromną wagę przykładała i wyczekiwała kiedy te salony fryzjerskie się otworzą.

**Teraz chciałabym porozmawiać o aplikacjach [...]. Czy w ogóle słyszałeś o różnych aplikacjach tego typu?**

Słyszałem na początku jak wprowadzano tę kwarantannę dla osób zakażonych dwutygodniową, że były plany wprowadzenia dla tych osób jakiejś obowiązkowej aplikacji monitorującej czy one pozostają w zamknięciu czy nie. Ale szczerze mówiąc nie wiem, czy to rozwiązanie powstało.

Chyba nie kojarzę żadnych specjalnych rozwiązań wprowadzonych przez rząd. Żadnych aplikacji. Ale może jak mi o jakiejś powiesz, to mnie oświeci.

**Ok, to przeczytam Ci parę pomysłów na aplikacje [...]. *Czyta wszystkie z pierwszej grupy.* Potem *czyta opisy z drugiej grupy.*Czy widzisz różnice w tych dwóch kategoriach i czy uważasz, że takie rozwiązania są potrzebne?**

Widzę różnicę i zdecydowanie sensowniejsze wydają mi się te rozwiązania z drugiej kategorii. W tej pierwszej kategorii jest to, może być to niestety duże pole - raz że do nadużyć, dwa że daje to, może powstać ryzyko, np. obejścia zabezpieczeń tych aplikacji i wycieku danych wrażliwych. Trzy, że nie bardzo wierzę w umiejętności polskiego rządu  do stworzenia sensownych aplikacji tego typu, pamiętając jakie były problemy z wdrożeniem tych rozwiązań informatycznych związanych z bazą pojazdów. Cztery, że wprowadzenie  takich rozwiązań które byłyby dla obywateli obligatoryjne mogłoby się  też nie sprawdzić, ponieważ wiele osób ma nadal problemy z obsługą takich rozwiązań. To tak na szybko, co przyszło mi do głowy.

Natomiast tej drugiej kategorii, to po pierwsze te rozwiązania nie potrzebowałyby takiej ilości wrażliwych danych, np. osobowych. Po drugie tak jak np. ta aplikacja gdzie miałoby  się zgłaszać czego się potrzebuje, to pozostawia dobrowolność dla użytkownika. Nikt nikogo raczej by nie zmuszał do korzystania z tego. Ten pomysł dostarczania leków czy jakichkolwiek innych produktów za pomocą dronów - też wydaje mi się bardzo sensowny.

**Powiedziałeś że obawy to głównie związane z wyciekiem danych wrażliwych. Czy jakieś jeszcze obawy?**

Oczywiście mogą też być obawy odnośnie nadmiernej kontroli obywateli przez rząd czy nadmiernej inwigilacji - takie rozwiązania kojarzące się jakimś Big Brotherem, Orwellem, czy systemami inwigilacji wprowadzanymi przez Chiny na przykład w ostatnim czasie. To tez może budzić obawy.

**To teraz bym chciała do tych aplikacji z polskiego podwórka przejść.**

**Kwarantanna Domowa. Co o niej myślisz? Do czego służy?**

Służy monitorowaniu osób objętych obowiązkową kwarantanną. Myślę, że mogłoby to być sensowne rozwiązanie gdyby było dobrowolne. Ponieważ nie wiem, jaki pomysł miało ministerstwo cyfryzacji na korzystanie z tej aplikacji przez osoby, które nie mają ani smartfon z androidem, ani iPhone ‘a. Czy zapewnią tym osobom te urządzenia? Po drugie czy osoby, które nie miały wcześniej żadnego smartfona to zostaną jakoś przeszkolone jak z tego urządzenia korzystać. Jak chociażby zmieniać z przedniego na tylny aparat. Plus wiem że w ipomeach jest z tym mniejszy problem, ale jeśli chodzi o androida to jest tak wiele różnych modeli z różnymi wersjami androida, że może być różnie ze stabilnością tej aplikacji w zależności od wersji systemu operacyjnego. Czy w zależności od różnych dodatkowych rozwiązań stosowanych przez producenta danego urządzenia. Więc uważam, że mogłoby to być sensowne rozwiązanie, ale takie dodatkowe. Dla chętnych. Mogłoby też trochę odciążyć  policję z kontrolowania tych osób.

**A czy są jakieś wzmianki w tym opisie, które budzą Twoje obawy? Poza problemami technicznymi**

Jest to dosyć ogólnie opisane, więc nie wiem, jak miałyby wyglądać te zadania.

**Zadania są takie same - musisz zrobić sobie selfie i system będzie porównywał sobie każde kolejne selfie.[...]**

A ok, zrozumiałem, że będą jeszcze jakieś dodatkowe zadania poza tym selfie.

To samo to rozwiązanie nie budzi moich specjalnych obaw, choć osoby z kolei bardziej techniczne mogłyby w dość łatwy sposób obejść te zabezpieczenia i spokojnie opuścić miejsce kwarantanny i byłoby to przez taką aplikację niewykrywalne.

**A czy gdybyś miał wybór pobrałbyś taką aplikację na swój telefon?**

Myślę, że w sytuacji obowiązkowej kwarantanny tak, bo wydaje mi się to mimo wszystko wygodniejszym rozwiązaniem niż meldowanie się dzielnicowemu. Czy taka bezpośrednia kontrola przez policję

**A tam też było napisane, że to nie jest zamiast, że to jest dodatkowo**

No tak, ale rozumiem, że powinno być tych kontroli mniej. Bo jeśli by ich nie było mniej, to nie widzę sensu tej aplikacji wtedy. To by mi się nie chciało w to bawić. Jeśli by ta aplikacja zdjęła przynajmniej częściowo ze mnie konieczność bezpośredniego meldowania się policji, to wtedy tak.

**A czy uważasz, że rząd powinien tworzyć takie aplikacje?**

Myślę, że tak. Że może to być w takiej sytuacji całkiem sensowne rozwiązanie.

**To teraz przejdźmy do drugiej.**

**ProteGo Safe. Co sądzisz? Czemu ona służy?**

Rozumiem, że w założeniu ta aplikacja ma ułatwiać korzystającym z niej osobom w zidentyfikowaniu jakiegoś bezpośredniego dla nich zagrożenia koronawirusem, czy przez kontakty z innymi osobami czy jakoś pomóc  w zdiagnozowaniu niepokojących objawów. Nie podoba mi się w niej tej element działający przez bluetooth, pozwalający wykryć czy potencjalnie mieliśmy kontakt z osobą zarażoną na przykład czy z jakimiś niepokojącymi objawami. Ponieważ to że znaleźliśmy się w zasięgu bluetootha z taką osobą wcale nie oznacza, że nam to bezpośrednio zagroziło. Bo bardzo dużo rozmaitych czynników może wpływać na ryzyko zakażenia. A poza tym też zasięg różnych urządzeń z bluetoothem jest bardzo różny. Natomiast ta opcja monitorowania objawów poprzez aplikację - myślę, że wielu osobom może ułatwić życie. Może też je zachęcić  do bezpośredniego kontaktu z lekarzem. Może je też zniechęcić do kontaktów z innymi ludźmi, jeśli takie objawy wystąpią. Więc ten aspekt może być pożyteczny.

**A czy sam pobrałbyś taką aplikację na swój telefon?**

Nie. Ja akurat bym nie pobrał. Wydaje mi się, że ta aplikacja w żaden sposób mi akurat nie pomogłaby w diagnozie objawów. Bez tej aplikacji mogę stwierdzić, co może być niepokojące, a co nie. Ale wiem, że nie wszyscy mają obeznanie w biologii, medycynie, czy związanych z tym sprawach.

**A czy uważasz, że rząd powinien tworzyć takie aplikacje dla obywateli?**

Tak, nie widzę przeciwwskazań, żeby to rząd tworzył takie aplikacje.

**To teraz chciałabym porozmawiać jeszcze o przyszłości. Czy myślisz w ogóle o przyszłości po pandemii? Jak postrzegasz ten czas? Czy to już jest teraz czy to będzie po..., Ewentualnie za rok wiosną? Jak myślisz o tym?**

Myślę o tym. Nie jestem w stanie przewidzieć jak długo to będzie trwało. Myślę, że nikt nie jest na tym etapie w stanie tego przewidzieć. Ponieważ te wskaźniki zachorowań mogą się bardzo dynamicznie zmieniać. Na razie dane z którymi się zapoznałem nie wskazują, żeby to miało mieć charakter sezonowy. Czy żeby miało się to sezonowo skończyć, czy żeby to się miała być druga fala sezonowa, tak jak pierwotnie na jesieni sugerowano - że na jesieni może być druga fala tak jak zwykle jest w przypadku grypy. Także bardzo trudno mi jest powiedzieć, jak ta przyszłość będzie wyglądała. To też może się zmieniać w zależności od tego, czy szczepionka powstanie czy nie. Czy jeśli powstanie to jaka będzie jej skuteczność. Jaka będzie jej dostępność.

**Czyli rozumiem, że myślisz o swojej przyszłości w związku z pandemią, ale nie jesteś za bardzo w stanie dojść do konkretnych wniosków ponieważ jest zbyt wiele zmiennych.**

Tak jest to dla mnie wielka niewiadoma.

**A czy jest taka rzecz, która jakoś najbardziej zaprząta Twoją uwagę w tym kontekście?**

Myślę, że główna kwestia to jest ta jak się będą kształtowały te wskaźniki epidemiczne. Czy one zaczną  maleć w Polce czy będą przez kolejne miesiące utrzymywały na w miarę stałym poziomie. Miejmy nadzieję, że nie. Czy nie wzrosną nagle jakoś gwałtownie.

**Czy masz może jakieś obawy w związku ze swoją przyszłością?**

Nie, ze swoją przyszłością bezpośrednio nie mam obaw.

**A czy myślisz może ogólnie o przyszłości Polski, albo szerzej przyszłości świata w kontekście pandemii?**

Tak. Czasem myślę, to właśnie jest związane z tym, że nie wiemy jak to się potoczy. Bo jeśli by znowu te wskaźniki w różnych krajach zaczęły rosnąć, no to może się to wiązać  z jakimś przedłużającym się kryzysem gospodarczym. Abstrahując od zdrowia i życia ludzi.

**Kryzys gospodarczy ewentualnie. A czy wydaje Ci się - wiem, że jest wiele rzeczy, które na to wpływa - czy według Ciebie coś się zmieni w związku z tą całą sytuacją?**

Wydaje mi się, że się zmieni. Zmieni się trochę przynajmniej w Europie wydaje mi się, że zmieni się podejście ludzi do elementów profilaktyki sanitarnej czy epidemicznej. Tak jak to już od lat, od czasów tej wcześniejszej epidemii SARS czy MERS w Azji, tam dla tych społeczeństw jest dużo naturalniejsze chodzenie w maseczce w miejscach publicznych w momencie jak tylko się pojawi jakieś większe zagrożenie wirusowe. Więc to może być jedna kwestia która się zmieni w naszym społeczeństwie. Kolejną może być podejście pracodawców czy całych firm do pracy zdalnej. Tu już widać, ze się zmienia i coraz więcej firm zorientowało się, że da się w ten sposób pracować, że w wielu sytuacjach może być to nawet korzystniejsze, ponieważ  wydajność pracowników nie spada, ale za to spadają znacznie koszty firm. To taki pozytywny aspekt tej epidemii. Może się też zmienić trochę podejście ludzi do podróżowania wydaje mi się

**W jaki sposób?**

Być może niektórzy będą mniej chętni do podróżowania przynajmniej przez jakiś czas. To może mieć pozytywny wpływ pod względem wskaźników epidemicznych, ale też negatywny jeśli chodzi o państwa czy regiony żyjące z turystyki. Już się słyszy o różnych włoskich regionach, które żyły głównie z turystów. Że jest tym ludziom mieszkającym tam bardzo, bardzo ciężko. Ponieważ ich dochody gwałtownie zmalały. To takie aspekty, które mi przychodzą do głowy póki co

**A czy masz jakieś przemyślenia, które dotyczą tego jak zmieni się sytuacja gospodarcza? Poza tymi regionami...**

Ogólnie sytuacja gospodarcza trochę się na pewno pogorszy. Na pewno wskaźniki takie jak PKB zmaleją, na pewno wzrosną wskaźniki bezrobocia. Wskutek tego pewnie też wzrosną nierówności społeczne, tak jak to na przykładzie Stanów Zjednoczonych doskonale widać, że bezrobocie gwałtownie rośnie, a jeśli chodzi o ten 1% najbogatszych to oni na tej sytuacji gwałtownie się bogacą. No to też może sprzyjać potem jakimś rozruchom, czy protestom w różnych krajach, np. w USA.

Trudno mi jednoznacznie określić jak ta sytuacja na świecie się zmieni. Ona się może jednocześnie zmieniać w bardzo wielu aspektach.

**A jeżeli chodzi o sytuację społeczną, której już dotknąłeś trochę, sytuację ludzi w ogóle. Czy według Ciebie  są jakieś grupy których szczególnie dotkną te zmiany?**

Nie wiem, jak nazwać... Znaczy tak, na pewno drobni przedsiębiorcy - im zwykle najciężej jest zareagować na takie zmiany gospodarcze. Tak samo w miarę świeże biznesy, które jeszcze nie zgromadziły jakiegoś kapitału, który by im się pozwolił zabezpieczyć na taką ewentualność czy które cały czas były na tym etapie dosyć agresywnego inwestowania - tak jak to często bywa, że przez pierwsze lata działalności raczej się zakłada, że taki biznes będzie przynosił straty,   po to żeby dopiero w kolejnych latach to obrócić w zysk. No i skutkiem problemów takich mogą mieć problemy ludzie zatrudnieni tam. Prawdopodobnie w największym stopniu osoby zatrudnione na niższych stanowiskach, zazwyczaj  w firmach niestety najłatwiej przeprowadza się redukcję takich osób. No ale na pewno w pewnym stopniu też ludzi na wyższych stopniach, specjalistów - to też może dotknąć.

**A jeśli chodzi o ograniczenia. Czy są według Ciebie takie, które powinny zostać z nami na dłużej?**

Tak, uważam, że na przykład obowiązek noszenia maseczek np. w sklepach. Czy obowiązek dezynfekcji rąk przed wejściem do sklepów czy miejsc publicznych, tak samo restauracji. To należałoby utrzymać, przynajmniej do póki sobie z tym wirusem nie poradzimy, np. szczepionką. Skutecznie. A ponieważ to są takie rozwiązania, które w wymierny sposób sprzyjają ograniczeniu rozprzestrzeniania się wirusa.

**Na początku mówiliśmy o dziadkach, którzy powinni być chronieni, ale z drugiej strony jest też jest coraz większe zrozumienie tego, że oni już tez mają dość tej izolacji. Czy są według Ciebie jakieś grupy które powinny być szczególnie chronione?**

Nie wiem jak rozumiesz szczególnie chronione - czy instytucjonalnie czy chronione w sensie samoizolacji?

**I tak, i tak. Czy jako społeczeństwo powinniśmy szczególnie dbać o seniorów, albo o dzieci albo o jakieś inne grupy społeczne.**

Wydaje mi się, że tak. Że przede wszystkim o te osoby, które są najbardziej zagrożone mówiąc wprost śmiercią z powodu koronawirusa. Czyli po pierwsze osoby starsze, po drugie osoby w z obniżoną odpornością czy  w wyniku chorób immunologicznych czy np. po chemioterapii. Na pewno te osoby trzeba szczególnie chronić. Aczkolwiek to raczej ten rodzaj ochrony się raczej odnosi do auto izolacji, a dwa, że raczej troski osób najbliższych. Bo nie wiem, jak na poziomie instytucji można byłoby to przeprowadzić.

**A gdzie według Ciebie przebiega ta granica żeby o kogoś dbać i go chronić, a tym, że ograniczamy mu wolność. Czy możemy ograniczać komuś wolność w trosce o jego bezpieczeństwo i życie?**

yyy, ograniczać wolności wydaje mi się, że nie. To zawsze powinna być mimo wszystko decyzja tej osoby. Co ona ze swoim zdrowiem postanawia robić - czy woli ryzykować, czy nie. Aczkolwiek myślę, że powinno się starać wspierać takie osoby w bardziej bezpiecznym postępowaniu, edukować. I właśnie może to jest ta rola instytucjonalna - żeby te osoby edukować. Tak samo osoby z najbliższego otoczenia powinny informować, że warto jednak te wszelkie względy sanitarne zachować.

**Mówiłeś, że warto by było, żeby ograniczenia, np. dezynfekcję rąk zostawić póki nie wynajdziemy szczepionki. A czy są pewne zachowania, ograniczenia, które powinny pozostać z nami na zawsze?**

Właśnie tutaj mam takie mieszane uczucia, bo z jednej strony te rozwiązania jak dezynfekcja rąk czy maseczki okazało się, że też bardzo skutecznie sprzyjają zahamowaniu rozprzestrzeniania się wirusa grypy czy innych wirusów. Dzięki tym środkom sezon grypowy wyjątkowo się skrócił w tym roku. Było wyjątkowo mało zachorowań. Natomiast jest to jednak pewna niedogodność, dodatkowe koszty na pewno. Pytanie jak długofalowo... Każda przesada w pewnym momencie może nieść jakieś negatywne skutki. Każdy kto kilka razy dziennie będzie alkoholem dezynfekował ręce, to skóra na rękach może zacząć pękać, wysychać i to też może być bezpośrednio problem większy dla tej osoby niż np. w normalnej sytuacji minimalnie większe ryzyko zachorowania. Natomiast być może warto by było - tak teraz mi to przyszło do głowy - te rozwiązania wprowadzać sezonowo, np. w momencie gdy ministerstwo podaje, że rosną wskaźniki zachorowań na grypę. To żeby zachęcać ludzi, żeby przed wejściem do sklepu te ręce zdezynfekowali. Czy żeby założyli maseczkę. Czy edukować o tym, że  w przypadku jak mamy jakikolwiek katar, kaszel, a nie zawsze możemy pozostać w domu w takiej sytuacji, żeby przynajmniej tę maseczkę w miejscach publicznych nosić.

**A co myślisz o ograniczeniach nowych? O jakich nowych ograniczeniach, w tych miejscach, które niedawno zostały otwarte słyszałeś?**

No wiem, że np. w salonach fryzjerskich są ograniczenia, chyba może być tylko 1 osoba w takim salonie w danym momencie. Że np. nie wolno podawać żadnych napojów. To mi się wydaje trochę na wyrost, patrząc na przykład na to, że równocześnie są otwarte restauracje czy kawiarnie. Ponieważ mimo wszystko wydaje mi się, że w takim salonie fryzjerskim łatwiej jest zachować higienę, łatwiej jest sprzęt zdezynfekować i jeśli jest możliwość podania napoju w takim miejscu nie uważam, żeby to zwiększało jakoś ryzyko zachorowania czy transmisji wirusa. Bardzo łatwo jest wszystko tam zdezynfekować i to ryzyko zminimalizować.

**Wróćmy na moment do mierzenia temperatury, które się pojawiło w Twoich obrazkach. Co sądzisz o mierzeniu temperatury w przychodniach, na lotniskach, w kinach i innych miejscach?**

Mam mieszane uczucia. Z jednej strony w przypadku osoby z ewidentnie podniesioną temperaturą to może mieć pozytywny efekt. Natomiast te termometry pozwalające z odległości zmierzyć temperaturę to raz że często są źle skalibrowane i ich odczyty są bardzo niepewne. Dwa że sama temperatura ciała niekoniecznie świadczy o tym, że jesteśmy zdrowi i może dawać nawet mylne poczucie tego, że nie jesteśmy zakażeni. Wiem, że na lotniskach mają wprowadzać taki przepis, że każdemu pasażerowi ma być obowiązkowo mierzona temperatura. Ale jak sobie pomyślę o tym, że np. musiałbym gdzieś lecieć albo po prostu bym zapłacił dużo pieniędzy za bilet lotniczy i miałbym jakiekolwiek obawy, że mogę mieć podniesioną temperaturę to wydaje mi się, że łyknąłbym tabletkę ibuprofenu, paracetamolu i temperatura była z powrotem w normie i nikt by tego nie wykrył. A ja potencjalnie mógłbym rozsiewać tego wirusa dalej. Więc to jest mocno niedoskonałe rozwiązanie. Aczkolwiek nie wiem, czy jest lepsze. Poza oczywiście edukacją społeczeństwa.

**A jeśli chodzi o te rozwiązania typu odległości między siedzeniami w samolotach, w kinach? W restauracjach? Co o tym sądzisz?**

To jest sensowne. To osłabia transmisję wirusa. Jakoś może zmniejszyć ryzyko, czy przynajmniej spowolnić rozprzestrzenianie się.

**Wprowadzono różne rozwiązania - jedne postrzegasz, jako bardziej sensowne, inne jako mniej. Według Ciebie dlaczego te wszystkie rozwiązania i ograniczenia są w ogóle wprowadzane?**

Dlatego, że jednak ten wirus cały czas jest zagrożeniem. Tak jak mówiłem wskaźniki zachorować nie zaczęły maleć, są dokładnie takie same jak miesiąc temu. Mimo to decydujemy się na otwieranie kolejnych gałęzi gospodarki. Więc trzeba w jakiś sposób próbować się zabezpieczać mimo wszystko. Jakoś minimalizować rozprzestrzenianie się wirusa na tyle na ile jest to możliwe.

**A jeśli chodzi o te rozwiązania, które według Ciebie są mniej sensowne i niekoniecznie mają realny wpływ na ograniczenie rozprzestrzeniania się koronawirusa. Jak to postrzegasz? Czy jest to w pewien sposób ograniczenie wolności naszej? Czy to jest za duża ingerencja państwa w życie obywateli? Czy inaczej?**

Nie wydaje mi się, żeby mierzenie temperatury było jakimś ograniczeniem istotnym wolności obywateli. Raczej są to względy sanitarne i zapewnienie bezpieczeństwa innym obywatelom. Wiem, że jest to rozwiązanie wadliwe, ale pewnie nie mamy lepszego. Ale może też istnienie takich rozwiązań, świadomość tego, że np. komuś przed wejściem do kina może być zmierzona temperatura i ktoś może zostać odesłany z kwitkiem być może wpłynie na świadomość takich osób i skłoni do zastanowienia się czy jeśli np. nie wszystko jest w porządku z moim zdrowiem, to czy warto się do tego kina wybierać. Może w ten sposób jakiś pozytywny efekt by to miało.

**Widzę że starasz się znaleźć pozytywy, pomimo tego pojawiło się to zdjęcie z mierzeniem temperatury. Więc próbuję zrozumieć Twój tok myślenia. Tam termometr przyrównałeś do pistoletu, a teraz jednak szukamy pozytywów w tym**

Jest to jakieś dodatkowe utrudnienie życia. Myślę, że nikt nie lubi być niepotrzebnie kontrolowany przez obce osoby i na pewno nie uważam, żeby to było rozwiązanie, które należałoby zachować na stałe. Natomiast wydaje mi się że może być warte rozważenia w sytuacji okresowego zwiększenia zagrożenia epidemicznego.

**Ok. to wróćmy jeszcze do ewentualnej drugiej fali prognozowanej. Wiem, ze słyszałeś o tym**

Jeśli chodzi o Polskę to nie uważam, żeby można było mówić o jakiejkolwiek drugiej fali. Ponieważ ta pierwsza fala cały czas trwa i się nie skończyła. Na tym mogę zakończyć

**Czy jest coś czego najbardziej się obawiasz - będę to nazywać drugą falą, aczkolwiek wiem, jak wygląda wykres zachorowań. Może lepiej: czy jest coś czego się obawiasz w perspektywie jesieni z koronawirusem?**

Zarówno w perspektywie jesieni, jak i lata jeszcze, obawiam się że ta liczba zachorowań może zacząć rosnąć szybciej. I znowu to o czym mówiłem przy poprzednich spotkaniach, że się obawiałem tego jak służba zdrowia sobie z tym poradzi. No póki co sobie  z taką, relatywnie niską jak na nasze społeczeństwo, liczbą zachorowań radziła. Pytanie jak sobie by radziła jakby ten przyrost zachorowań był znacznie większy. Tego się obawiam

**A czy myślałeś może żeby w jakiś sposób przygotować się na to co będzie?**

Nie, nie myślałem o tym. Nie wiem w jakim sposób ja mógłbym się na to przygotować. Natomiast to czego się jeszcze obawiam, to planując wakacyjne wyjazdy ewentualne to jest ta obawa, że wyjedzie się za granicę i nagle podczas wyjazdu w tym kraju czy w jakimś kraju pośrednim przez który trzeba przejechać, żeby wrócić do Polski te wskaźniki zachorowań wzrosną i ten kraj zamknie granice. I jak wtedy będzie wyglądał powrót do kraju. To moja obawa związana z ewentualnym planowaniem wakacji, które jeszcze na poważnie nie rozpocząłem. Ale powoli zaczynam o tym myśleć.

**A gdyby okazało się, że wskaźniki zachorowań nam wzrastają i to bardzo szybko, to jak myślisz, to jakie działania powinien podjąć rząd?**

To wszystko zależy od tego, jak szybko te wskaźniki rosną i od tego, jak w przypadku wzrostu zachorowań spadają możliwości radzenia sobie z tym szpitali. No i wtedy chyba jedynym rozwiązaniem dostępnym jest znowu zwiększanie na powrót lock-downu.

**Pod warunkiem, że szpitale nie będą sobie radzić tak? A póki będą miały moc przerobową odpowiednią to ten lockdown rozumiem nie byłby konieczny?**

No to wszystko kwestia zawsze tego... Trzeba rozważyć co będzie miało większe negatywne konsekwencje - czy ten lockdown i ograniczenia gospodarcze, ale to może mieć wpływ na życie również ludzi bezpośredni, czy właśnie skupienie się bezpośrednio na zdrowiu obywateli. I zmniejszaniu liczby zgonów w społeczeństwie.

**W jaki sposób bezpośrednio mielibyśmy się skupiać na stanie zdrowia?**

Tzn., tak jak każde państwo musiało sobie odpowiedzieć sobie na pytanie, czy ważniejsza jest gospodarka, np. PKB, czy zdrowie i życie obywateli. To oczywiście jest mocne, duże uproszczenie, ponieważ lockdown też może pośrednio wpływać na zdrowie obywateli. Chociażby przykładowo wzrost bezrobocia może wpływać na depresję obywateli, czy nawet na wskaźniki samobójstw podejrzewam. Ale podejście szwedzkie poskutkowało znacznie zwiększoną liczbą zgonów w stosunku do np. Polski. Jeśli by brać stosunek wielkości liczby zgonów do wielkości społeczeństwa.

**Tak na zakończenie. Podsumujmy 5 spotkań. Podaj proszę najważniejsze wydarzenia w trakcie trwania pandemii do tego momentu. Wydarzenia ważne dla ciebie najpierw.**

Nie wiem, czy będę w stanie je wymienić w dobrej kolejności chronologicznie. Na pewno lockdown - zamknięcie po pierwsze granic, po drugie restauracji i klubów sportowych, galerii handlowych, sklepów, jakiś gabinetów fryzjerskich. A potem plus te ograniczenia związane z nakazem noszenia maseczek w miejscach publicznych. Czy z zakazem w pewnym momencie wychodzenia z domu poza obowiązkami służbowymi. A potem kolejno łagodzenie tych restrykcji, np. zniesienie zakazu wychodzenia z domu, otwieranie restauracji, otwieranie sklepów. Otwarcie żłobków. Ostatnio zniesienie obowiązku noszenia maseczek w miejscach publicznych. No i otwarcie klubów sportowych, gabinetów fryzjerskich.

**Czy wszystkie te etapy były dla Ciebie przełomem i były dla Ciebie osobiście ważne?**

Jeśli chodzi o przełom, to na pewno największym przełomem był lockdown ogólnie. I potem drugim przełomem było - nie wiem  w którym miejscu umieścić zniesienie lockdownu. To taki płynący przełom. Myślę, że takim najbardziej przełomowym momentem było zniesienie obowiązku noszenia maseczek w miejscach publicznych. To taki symboliczny moment jeśli chodzi o lockdown.

**Dlaczego początek lockdownu było dla ciebie osobiście ważne?**

To była sytuacja w największy sposób zmieniająca życie całego społeczeństwa. I praktycznie każdego w jakiś sposób ta sytuacja dotyczyła. No i było to coś takiego z czym wcześniej nasze społeczeństwo nie miało dotyczenia

**Co wywarło na Tobie większe wrażenie - to że zamknięto granice państwa czy to że pozamykano różne lokale, instytucje?**

Wydaje mi się, ze w większym stopniu zamykanie lokali czy instytucji. Niż zamknięcie granic.

**Czy coś jeszcze poza maseczkami? Jakie emocje się z tym wiązały? Jak się z tym czułeś?**

Poczułem, że powoli zaczyna wszystko wracać do normy. Że trochę bardziej się społeczeństwo zaczyna zachowywać jak przed epidemią. Z jednej strony pojawił się delikatny optymizm związany z tym, że będzie można wrócić do codziennego życia, do spotkań ze znajomymi, ale z drugiej strony pewna obawa czy zniesienie restrykcji nie wpłynie na to, że wskaźniki zachorowań wzrosną i skutkiem tego trzeba będzie wrócić do lockdownu

**Czy coś dodać byś chciał jeszcze?**

to co uważam za istotne to w ostatnim czasie mam wrażenie że wzrosła liczba pojawiających się fejk niusów, teorii spiskowych związanych z koronawirusem. Kolejne strony twierdzące że wirus nie istnieje, że to spisek. I uważam, że z tym powinno się jak najsilniej walczyć. I na poziomie obywatelskim i na poziomie instytucji państwowych. Ponieważ to może mieć w przyszłości bardzo negatywne skutki dla społeczeństwa. I może sprzyjać rozwojowi czy tej epidemii czy jakiś przyszłych epidemii.
