# Supplementary material for: The challenges arising from the COVID-19 pandemic and the way people deal with them. A qualitative longitudinal study
Source: PLoS One. 2021 Oct 11;16(10):e0258133. doi: 10.1371/journal.pone.0258133 (PMC8504766; doi:10.1371/journal.pone.0258133)
Supplement: S1 Dataset — (ZIP) [file pone.0258133.s003.zip › Transcriptions/stage 5/3.5_F_54_single.docx]

**3.5_F_54_single**

**Co się działo w ostatnim miesiącu?**

No co było, mnóstwo rzeczy. Ze 30 dni minęło. No tak, Julka wróciła do domu.

**To duża zmiana.**

No, ale po dwóch miesiącach, to wie pani co, myślę, że już ona też powinna zacząć życie własne, chociaż tu było nam super. I nawet się nie znudziłyśmy za specjalnie. Chociaż ja myślę, że jej brakuje jednak takiego miejskiego życia. A mi się przyda trochę takiej samotności, bo ja się nieźle ze sobą czuję sama. Więc to jest jednak… Ale oczywiście bardzo mi było smutno, jak miała wyjechać. Ale to nie ostatni jej pobyt, więc… No i tak, co się zmieniła. Pora roku nam się trochę zmieniła jednak. Trochę się robi cieplej, skończyły się przymrozki w nocy, chociaż jest jeszcze chłodno. Wykonałyśmy z Julką kawał roboty w postaci pomalowania kuchni.

**Wow, to tego nie było. Zatrzymałam się na płocie dla psa.**

Tak, na kojcu. Kojec jest ma 20 na 3, ma 60 metrów kwadratowych. Taki jest kojec, tak że pies jest w kojcu. To znaczy w kojcu jest w nocy oraz kiedy mnie nie ma nigdzie na zewnątrz. Ale się bardzo uspokoił. Pies został wykastrowany więc chodził nam tu 10 dni w takim abażurze na głowie. Mnóstwo takich dużych projektów wykonałyśmy. Nie wiem, na jakim etapie byłyśmy, ale byłyśmy też na cmentarzach, więc wszystko ogarnęłyśmy, zrobiłyśmy nowe ławki, wszystko zostało pomontowane. No i tak, bo to było już po kojcu. No i tak. Także robiłyśmy dużo dużych rzeczy, trochę więcej małych rzeczy oczywiście.

**A jak teraz wyglądają dni? Jak taki dzień u pani wygląda?**

No teraz to wygląda dzień tak, że ja wstaję i idę pielić. Ponieważ mam zasiany kawał pola, mam takie poletko, na którym mam warzywa. Po prostu w związku z tymi wszystkimi projektami oraz w związku przede wszystkim z kuchnią, która nam zajęła ponad tydzień, bo to trzeba było wszystko wynieść a potem wnieść. Malowanie stanowi najmniejszy problem. To ponieważ to wszystko trwało, to nie chodziłyśmy na pole. Więc pole jest zaniedbane. Ale, mam taką rzęsę na tym polu. I muszę pracowicie znaleźć wśród tej rzęsy rośliny, które posiałam. Tak że ogólnie rzecz biorąc, teraz sobie biorę krzesełko, taki stołeczek malutki składany. I idę sobie, z dużym uwielbieniem ja to robię, ponieważ to jest taki moment takiego spokoju. Wie pani, takka praca, ona jest dość natrętna, w sensie taka upierdliwa. Ale ja chyba się kwalifikuję do takich zajęć. A mózg wtedy jest swobodny. Bo rozróżnienie powiedzmy jednego zadanego warzywa, nawet w formie mikroskopijnej od chwastu nie angażuje go specjalnie. Więc sobie tam myślę. Mi się zdarza też. I mam taki swobodny przepływ myśli. Takie oczyszczające, bym powiedziała. Jak się coś dzieje albo się nie dzieje, to człowiek tak siedzi i sobie tak myśli. A jeszcze ma ręce zajęte, co w moim przypadku jest ważne, bo ja muszę coś robić.

**A na ile to jest tak, że to życie jest zupełnie takie samo jak przed koronawirusem?**

Może ono tak powoli wraca do takiego stanu, mi się tak wydaje. Chociaż nie wiem, czy są przesłanki do tego, żeby tak miało być. Ale być może, rzeczywiście też się nad tym zastanawiałam, że ono już jest, no tak naprawdę prawie takie samo. Oprócz tego, że ci moi seniorzy są uwięzieni w tych swoich domach w dalszym ciągu i jeszcze nie mamy żadnych procedur, MOPS się jeszcze nie dorobił procedur w reżimie sanitarnym, w których można by było uruchomić tych moich seniorów, którzy tam dostają kota w tych chałupkach swoich. No to poza tym wraca. Ale ponieważ to jest istotny element mojego życia, ta praca z nimi, więc w pewnym sensie nie wróci. No na pewno się mniej przemieszczam. Bardziej jestem stacjonarna.

**Ale to wynika chyba z tego, że po prostu nie ma tych zajęć, a nie z tego, że się pani boi przemieszczać, bo koronawirus, tak?**

Nie, nie, no z tego, że nie ma tych zajęć, że to jest nieuruchomione. Bo jak bym miała wrócić do pełnego trybu, to musiałabym coś robić… A tak, to w ogóle straciłam, teraz to może jest trochę lepiej już, ale straciłam w ogóle takie poczucie dni tygodnia, jak była Julka. Naprawdę. Nie wiedziałam, że to sobota, bo zwykle jak dzieci przyjeżdżają, to powiedzmy na weekend. I ten poniedziałek, który zaczynał ten tydzień pracy od wyjazdu do Płocka i w ogóle. To teraz to po prostu ja nie wiem, co to jest za dzień na przykład. Robię zakupy wtedy, kiedy mi brakuje, a nie w poniedziałek. No, ogólnie rzecz biorąc taki…

**Czyli jeszcze to nie jest tak stan sprzed.**

Nie, nie, nie jest jeszcze stan sprzed. Ale myślę, że się powoli zbliżamy. Dzieci się przestały denerwować, jak Julka była, no to była, ale potem przyjechał Hubert z Magdą w weekend na przykład. I też chodziliśmy bez maseczek.

**A oni się bali wcześniej przyjechać? Bo ich długo nie było.**

Tak, ich nie było długo. Bo oni to się bali, że oni poroznoszą tę chorobę po prostu wszędzie tam, gdzie pójdą, to na pewno ją zaniosą. Nie wiem, na ile się bali o sobie, myślę, że bardziej o tych, do których jeżdżą. No, ale jakoś chyba się uspokoili, bo już wszyscy zaczęli chyba robić w miarę wszystko. Ja byłam w Warszawie. A, byłam u fryzjera przecież. Cholera, podstawa egzystencji. Więc jak byłam w Warszawie, to… No, są korki nawet. Więc myślę, że u was bardziej wróciło do… Nie, u nas w zasadzie ta norma występowała ciągle. Nosimy maseczki jeszcze tutaj. Chociaż teraz, jak został zniesiony obowiązek tych maseczek w sobotę, chyba od soboty, to w poniedziałek już na rynku to pojedyncze osoby widziałam w maseczkach. A tak, to gremialnie już bez maseczek, wszystkim się to znudziło po prostu.

**Ale pilnują tam u was na przykład w sklepach?**

A skąd. Znaczy w sklepach to nie wiem. Ale i w sklepie widziałam właśnie bez maseczek. Bo to wie pani, te przepisy są takie, że teraz kto i czego ma pilnować? U mnie to tu połowy z tego nie zrozumieją. Więc można bez maseczek, to się chodzi bez maseczek. A dalszy ciąg tego zdania, że jeżeli nie jest zachowane 2 metry, to trzeba chodzić w maseczce, to w ogóle… Wie pani, to trzeba w krótkich, żołnierskich słowach. Jest maseczka albo nie ma maseczki. A jak już są jakieś uwarunkowania albo jakieś warunki tego zajście, to myślę, że to jest na przykład błąd. Jeśli ktoś chciałby spowodować, żebyśmy coś robili tu na prowincji albo czegoś nie robili, to to trzeba w prostym komunikacie, jedno zdanie proste, niezłożone wykonać. Można bez maseczek, koniec. Oni nie czytają ciągu dalszego. Nawet, jak są 3 kropki gdzieś, to nie są zainteresowani, żeby to rozwinąć, żeby zobaczyć, co jest dalej. Przeczytali i dziękujemy. Także… No widziałam jeszcze parę osób, ale może to są te takie bardziej strachliwe. A ci, co nie noszą, to też uważają, że się stosują. Ona nie nosi, to nie nosi. No i tak. Zważywszy na niewystępowanie tej choroby u nas, to myślę, że nie jest tak źle. Damy radę.

**A jak Hubert z Magdą przyjechali, to mieli jakieś takie jeszcze, że nie wiem, nie przytulacie się, nie witacie? Nie wiem, jak się normalnie witacie?**

Przytulamy się. Nie, nie mieli. Ani nie mieli takiego zawahania. Tak że nie, wszystko było normalnie. Nie wiem, jak oni funkcjonują na zewnątrz, ale tutaj nie było takiego. Przyjechali, przytulaliśmy się, oni do psów, do wszystkiego. Magda jest psiara, więc tu się ładuje, jeśli chodzi o przytulanie zwierząt.

**A pani seniorzy, ma pani telefoniczny kontakt?**

Mam, tak.

**I co oni tam opowiadają?**

No, że siedzą w domu i na zakupy chodzą. Jedna z tych moich seniorek a to do parku idzie, a druga tam gdzieś chodzi koło domu, tak wychodzi na trochę, żeby rozprostować te nogi. I że już im się nudzi. I że tęsknią. No, jakoś sobie radzą, wie pani. Ale dla nich to jest trudne. Bo oni też nie są tacy, te zmiany u nich nie przychodzą tak szybko, więc oni się tak szybko nie przystosowują do takich nowych sytuacji. Tak samo jak będzie im trudno się przystosować do sytuacji, że mogą wychodzić. W sensie takim, że się nagle okaże, że ktoś tam się męczy, bo dawno nie wychodził, a ktoś tam coś tam. No, ale dadzą radę.

**A myśli pani, że powinny już te wasze zajęcia tego brydża wrócić?**

Wie pani, myślę, że one mogły trwać cały czas. Bo to jest taka dość hermetyczna grupa. Chociaż oczywiście, muszą dotrzeć jakoś na te zajęcia. Więc nieważne, jaka hermetyczna grupa, jaka jest, to i tak oni by się spotykali gdzieś z innymi. Ale myślę, że można wrócić. Zważywszy na procent czy na ilość zachorowań w Płocku i w powiecie płockim, no to myślę, że śmiało. Bo to jest oczywiste, że tam po prostu nie ma w ogóle nikogo chorego. Więc jak nie ma nikogo chorego w tym Płocku… No mamy ileś tam osób na kwarantannie chyba. Ale już ostatnio nie oglądam nawet tego. Bo mnie bardziej denerwuje niż wcześniej. I jakby nic nie wnosi. Bo już te liczby, no jedyne co, to czasem oglądam pana rzecznika, tego prasowego rządu, on jest taki papuśny i taki uśmiechnięty. I ogólnie rzecz biorąc, daje radę w tłumaczeniu tych bałwanów, więc… Ale z rzadka. Wie pani, są miejsca, gdzie nie jest bezpiecznie pewnie, tak? czyli takie miejsca, gdzie są bardzo duże skupiska ludzi, którzy pracują bardzo blisko siebie i taki mają charakter pracy. I tam jedna osoba wrzuca w to, gdzie prawdopodobieństwo, no nie wiem, ile tam, 1% czy 2% jest zachorowań, bo to trzeba jakoś w skali liczyć, no to myślę, że tam jest mniej bezpiecznie na pewno. A tak jak są powiaty na pewno, w których no można by uruchomić to wszystko, po prostu. Bo jakby nie ma się nawet od kogo zarazić. Jak by trzymać jeszcze takie, wie pani, wyizolowanie w takich większych komórkach. Tak jak byliśmy wyizolowani rodzinnie, tak może teraz jak by wyizolować te powiaty, gdzie nie ma chorych, no to być może to by… No nie wiem, ja już tak się łapię po prostu chyba wszystkich pomysłów, które by pozwoliły im wyjść z domu. Bo ja to wychodzę z domu. Ja mieszkam na dworze, po prostu. Tak naprawdę wchodzę do domu w celu, w celach. Żeby zjeść, napić się, pójść spać. A oni nie wychodzą w zasadzie. No chodzą teraz do sklepu. To jak mogą bezpiecznie być w sklepie, to na pewno mogą być na zajęciach.

**A dlaczego MOPS nie uruchamia tego jeszcze?**

Wie pani co, ja już próbowałam je zmotywować trochę, bo w Płońsku na przykład takie zajęcia dla seniorów zostały uruchomione. U nas w Płocku jest tak, że w tym MOPS-ie ta komórka, czy ten dział, czy nie wiem, jak to się nazywa tam organizacyjnie, do spraw tam… Oni się nazywają, no nieważne. Oni powstali z powodu takiego projektu unijnego dla ludzi starszych i wykluczonych. A teraz to się jakoś nazywa, że zostały pod to podciągnięte świetlice środowiskowe. I te panie z tego działu zajmują się młodymi i starymi. I te świetlice środowiskowe, które znajdują się w szkołach, ale są pod opieką MOPS-u organizacyjną i personelową, one musiały chyba najpierw, tak przynajmniej mówią, ja się na tym nie znam za bardzo, bo ja jestem wolontariuszem, więc na szczęście nie uczestniczę w żadnych zajęciach ich takich wewnętrznych. Ale że one musiały się zająć najpierw jakby procedurą i organizacją pracy w tych świetlicach. Od strony tam w rygorze i jak to tam pryskać i kogo pryskać. I że teraz, to tak już od poniedziałku, co był już, uruchomiły te świetlice, to się zajmą, będą myśleć o nas, o tych seniorach. No, zobaczymy. Mam nadzieję, że nie chcą nas przytrzymać do września. Bo myślę, że to byłby kiepski pomysł. Na pewno bezpieczniej byłoby ruszyć teraz po prostu. A poza tym oni potrzebują. To nie będą normalne wakacje i nic nie będzie tak naprawdę normalnie.

**Ale to znaczy, że pani by chciała prowadzić te zajęcia i w lipcu i w sierpniu na przykład?**

A ja zawsze tak prowadziłam. Bo robienie im przerwy na wakacje, to jest największy absurd świata. Dlatego, że po pierwsze tutaj ani nikt nie wyjeżdża za granicę specjalnie ani w ogóle. Bo to są starsi ludzie i ich na to po prostu nie stać zwyczajnie. Może jedną stać osobę, ale ich jest sporo. To jest po pierwsze. I jak ja nastałam tutaj, nie wiem, 4-5 lat temu, jak zaczęłam z nimi pracować, to wszystkie inne zajęcia organizowane przez ten MOPS dla seniorów, były przerywane z końcem roku szkolnego i zaczynały się we wrześniu.

**Jak dla dzieci.**

Tak. Ja rozumiem, że te panie, co tam pracują, mają dzieci. Ale to nie są panie w szkole, nauczycielki, które nie mają zajęć przez 2 miesiące, tylko to są panie, które pracują w MOPS-ie i ich obowiązuje zatrudnienie ja w innych biurach, urzędach, a nie jak nauczycieli. Więc ja im powiedziałam, zapytałam, czy ja mogę te zajęcia prowadzić. Bo co to dla nich za problem, skoro ja jestem, sama sobie otwieram. No to najpierw się zastanawiały, a później się okazało, że nie ma problemu. Ale po 2 latach, jak ja prowadziłam te zajęcia przez całe wakacje, to one w trzecim roku również te swoje zajęcia, które prowadziły wcześniej, czyli te śpiewy i coś tam, kółko krawieckie i jakieś, znaczy nie przerywało się na wakacje. Bo ja nie widzę powodu żadnego, sensownego. Oczywiście, jak ja gdzieś wyjeżdżałam, co się zdarzało rzadko, na tydzień, no to odwoływałam im jedno spotkanie czy dwa. 2 się nie zdarzyło chyba nawet. Poza tym wie pani, jak oni w ogóle gdzieś wyjeżdżają, to wyjeżdżają. To nie zmienia postaci rzeczy, że zawsze te 2 stoliki udało nam się skompletować. Nawet, jak nie wszyscy grali na okrągło, no to się tam zmieniali w kółku. No i teraz już są prowadzone cały czas, więc mam nadzieję, że panie nie wrócą do wcześniejszej tradycji, żeby nie było zajęć w wakacje. Bo skoro oni chcą, a ja mogę, no to…

**Ale też pani powiedziała, że teraz byłoby bezpieczniej im to zacząć niż we wrześniu?**

No wie pani, w obliczu tego, co opowiadają ci eksperci z tego WHO, że wrzesień, że może jesienią, że to może ze względu na lepsze warunki do roznoszenia wirusa… Nic nie wiemy tak naprawdę. Nie wiemy, czy to się skończyło, czy się kończy powiedzmy, czy wróci, czy to się usypia i się przegotowuje trochę ten wirus, ale przetrwa i… Myślę, że oni też tego nie wiedzą. Ktoś tam coś mówi i potem będzie mówił: „a nie mówiłem”? Ale tak naprawdę nie wiemy. Więc może teraz byłoby powiedzmy bezpieczniej. I wdrożyć wychodzenie jakieś tam w tym reżimie sanitarnym, cokolwiek by to miało znaczyć. Więc żeby jednak się przemieszczali w tych maseczkach, żeby z nich nie rezygnowali w czasie przejścia i coś tam. Nie wiem, czy będą w brydża grali w maseczkach, no zobaczymy.

**Żeby się przyzwyczajali do zajęć w tej nowej, innej rzeczywistości?**

Tak. Póki jeszcze jest w miarę… A jak wróci we wrześniu czy tam w październiku, no to już będziemy mądrzejsi. I oni też będą trochę bardziej oswojeni z tą sytuacją. Więc może też te instytucje nie będą takie wyrywne państwowe do tego, żeby znowu nas zamykać wszystkich w domach. Bo wiadomo, że jakoś wszyscy musimy sobie z tym poradzić. Najgorzej będzie miała służba zdrowia na pewno jak zwykle, bo oni zawsze mają przechlapane. Ale no co.

**A jak ci chłopcy, którym pani pomaga?**

Na razie jest cisza i spokój. Już wiadomo, że do końca roku to oni do szkoły nie wrócą. Pawełek będzie miał za miesiąc egzaminy, coś tam się uczy. Się uczy, ale samodzielnie. A ten drugi no to z tą mamą sobie jakoś radzą. Myślę, że nie ma ciśnienia, bo tak, oceny im się poprawiły. Bo rodzice wykonują część prac, tak dla świętego spokoju, a po drugie no jak tu komuś coś wytłumaczyć, jak się samemu ledwo to kuma. No, ale oceny się tam jakoś poprawiły, więc dzieci są chyba w miarę zadowolone. Rodzice też. Tutaj, bo myślę, że w Warszawie jest większy taki stres i to parcie na te oceny. Nawet nie tyle na tą wiedzę, tylko na te oceny i na to, do jakiej szkoły później pójdzie to dziecko i jaki będzie miało zawód. I najlepiej ile, jak ma 5 lat, ile będzie zarabiać, jak będzie miało 35. No, ale tak jest, musi pani sama przyznać, że jednak presja taka w mieście na pozycję i taką pozycję finansową, a w niektórych środowiskach na naukową pozycję, jest ogromna. A tutaj w zasadzie szczytem oczekiwań rodziców no to jest jakieś piętro wyżej nad nimi. Nie ma dużo takich, którzy uważają, że dziecko to się powinno uczyć po prostu nie wiadomo dokąd, byle się chciało uczyć. Nie, no więc tutaj kierowca TIR-a to już jest po prostu prawie profesor. Nie ma takiego ciśnienia, w związku z tym jest taka większa akceptacja tego. Może i dobrze, bo też i możliwości tych dzieci w większości nie są takie duże.

**A w ogóle się otworzyły świetlice w szkołach u was czy przedszkola?**

Na pewno się otworzyły szkoły. Ale na przykład nie ma dzieci zgłaszanych do tych szkół. W Warszawie nawet rozmawiałam z panią, z taką dziewczyną, która ma córkę w pierwszej klasie podstawówki, to mówi, że zostało zgłoszonych 7 chłopców a żadna dziewczynka. W związku z tym ona też tej swojej dziewczynki nie puściła do szkoły, no bo co będzie chodziła z siedmioma chłopcami? Ja mówię, wiesz, ale może jak byś puściła ją do szkoły, to może by inne dziewczynki też przyszły. A nie, nie, ona pierwsza to nie będzie. Ale u mnie na przykład też nie puszczają dzieci takich młodszych. One się otworzyły te szkoły teoretycznie, ale dopóki nie ma zgłoszeń. Nie wiem dokładnie, czy nie ma w żadnej klasie. Ale też rozmawiałam z taką mamą, ona ma sześciolatkę, która chodziła do tej takiej pierwszej klasy czy tam zerówki, ja już nie wiem, jak to teraz wygląda, to mówi, że ona jej nie puszcza, bo ona ma astmę. No dobra, no nie puszcza jej. No więc są niby otwarte, ale nie wiadomo, czy są otwarte. Ale w Warszawie to na pewno są.

**Ale myśli pani, że ludzie nie puszczają tych dzieci do szkoły, bo się boją? Czy dlatego, że nie mają takiej potrzeby? W sensie, że jest opieka dla tych dzieci i nie trzeba.**

Na pewno jest opieka dla tych dzieci. Nie ma problemu z opieką dla dzieci na wsi. Bo zawsze ktoś w domu jest. To są albo domy pokoleniowe albo… Tutaj na wsi nie ma kłopotu z opieką nad dziećmi. Jak jest jakiś dzień, czy tam były te weekendy, co tam jest ileś dni wolnych w trakcie, jak te majowe czy coś, że dyrektorzy szkół ogłaszają, że to są dni wolne, że je się odpracuje w inne dni, ale muszą teoretycznie zapewnić dzieciom opiekę w te dni w szkołach, prawda? Na okoliczność, gdyby rodzice na przykład nie mieli takich wspaniałych dyrektorów, którzy robią im wolne w te dni. I tutaj w zasadzie nie spotkałam się z tym, żeby takie dzieci szły do szkoły. Że przynoszą takie karteczki, czy dziecko będzie w tych dniach chodzić do szkoły, to jeszcze się nie spotkałam, żeby ktoś chodził. Zawsze ktoś jest po prostu w domu. A jak one tam, nie wiem, jak wyglądają te zajęcia, czy one się tam uczą, czy nie uczą. Nie wiem. Bo na początku słyszałam, że ponieważ nie ma obowiązku, tylko otwierane są te szkoły i jest możliwość oddania dziecka, no to rozumiem, że nie wiem, czy są prowadzone zajęcia takie regularne jak lekcje? Nie wiem. Nie mam bladego pojęcia. Ale tu już moim zdaniem wszyscy mają wakacje, takie psychiczne. Po prostu. Nie ma szkoły, no i super. Dzieci się cieszą, rodzice mniej, no ale też się cieszą. Dzieci w domu, nie trzeba ich rano budzić, wystawiać na te busy. Bo to trzeba rano dziecko obudzić, z godzinę wcześniej niż powinno wstać, bo musi pójść na ten bus, który je zabiera, bo to z tych wsi zwożą. Wie pani, niektóre dzieci mają lekcje na 8, a muszą wyjść za 15 siódma, stać na drodze już.

**No tak, bo on jedzie ten bus i po kolei…**

On jedzie, zawozi jedną partię dzieci, a potem jedzie po następne w inną stronę. Tych busów oczywiście jest parę, ale pomimo wszystko… No tyle dobrego, że te dzieci, jak niektóre są zabierane powiedzmy jako pierwsze, to w następnym semestrze będą zabierane jako ostatnie. Więc na przykład będą musiały być na drodze za 20 ósma, a nie za 20 siódma. Ale to i tak słabo.

**To jak byśmy miały ten miesiąc sobie wziąć, to ważne momenty, to było tak, był wyjazd Julki?**

Tak .To przed chwilą w zasadzie.

**Czyli to będzie kończyć ten miesiąc. A wcześniej takie ważne momenty? Myśmy się ostatni raz widziały zaraz po majówce. Co się takiego ważnego wydarzyło poza wyjazdem Julki?**

W zakresie koronawirusa?

**Nie wiem, czego. U pani w życiu albo w zakresie koronawirusa, coś zapadło w pamięć, coś nie zapadło.**

Na pewno w czasie tej wizyty zrobiłyśmy sporo takich rzeczy grubych, takich dużych projektów. To na pewno. To taki był okres, w którym nam się udało zrobić takie rzeczy, których w 3 dni się zrobić nie da. A jak się spotykamy na 2 tygodnie, to też jest tak naprawdę za mało czasu. No, więc na pewno tego czasu było dużo i w spokoju zrobiłyśmy takie duże rzeczy. Było super. W zakresie koronawirusa to nic mi się nie… Było miło, że otworzyli panią fryzjerkę (śmiech).

**Ale to trzeba było do Warszawy pojechać.**

No i ja tak jeżdżę do Warszawy, bo ja jestem niezwykle wierna. I chodzę do tej pani, ścięłam włosy mając lat 30 po raz pierwszy. I od tej pory chodzę do niej. To jest 24 lata. Tylko do niej. I mam takie głębokie przekonanie, że jak ona przestanie ciąć, to ja też przestanę obcinać włosy po prostu. Potem nauczę się je farbować samodzielnie, żeby nie chodzić. Więc jeżdżę do niej po prostu od zawsze. Z mojego punktu widzenia od zawsze. Tak że nie ma dla mnie takiego kłopotu, że ja jeżdżę do Warszawy w tej sprawie.

**A szybko się pani zapisała, jak tam otwierali?**

Ona ma taki jednoosobowy taki salon. Więc ona do mnie zadzwoniła, że wraca i sama zaproponowała mi. Usiadła sobie, że wraca od poniedziałku, bo już wiadomo było, że będą wracać. Zadzwoniła i poumawiała nas na jakieś tam godziny. Tak że… Jak ją usłyszałam, że to ona dzwoni, to byłam bardzo zadowolona (śmiech).

**Jeszcze przed podróżą, już będzie (śmiech).**

Mnie zapytała, czy jak ona by przyjechała do Warszawy do poniedziałku, to czy ja bym była zainteresowana. Ja mówię, że oczywiście. Ale muszę pani powiedzieć, że mi powiedziała, że są osoby, które jeszcze nie chciały. Że jeszcze jest parę klientek, które jej powiedziały na przykład, że no nie, że na razie one sobie tam same farbują, czy tam, wie pani, nawzajem sobie jakby w domu, radzą. No to one jeszcze i tak na razie pracują online w domu, nie wychodzą, to na razie niech te włosy odpoczną czy coś. Było część takich jak ja, że powiedziałabym, entuzjastycznie podeszłyśmy do powrotu do normalności. A było, jak rozumiem jest parę też takich osób, które się jeszcze obawiają. No sama mam koleżankę, którą już… Ja mówię dobra, już przyjedź do mnie albo już ja do ciebie przyjadę, wszystko jedno. Nie, bo ona jeszcze… Ale ona jest w ogóle strachliwa. Może strachliwa to złe słowo, ale bardzo przeczulona na punkcie zdrowia własnego i swoich najbliższych.

**A u tego fryzjera, jak pani pojechała, bo rozumiem, że pani pojechała zaraz, jak ona się otworzyła?**

Tak, tak, tak.

**Jak to wyglądało? Było to jakoś inaczej niż wcześniej, był ten rygor sanitarny?**

No tak. Było tak, że po pierwsze nie ma takiej możliwości, żeby się tam spotkać u niej. Czyli normalnie, bo ja nie lubię się spóźniać i się nie spóźniam, więc zawsze jestem wcześniej. A że jadę i wjeżdżam do Warszawy, gdzie są korki zwykle, więc mam taki… Zawsze jestem wcześniej, krótko mówiąc. No to jak nie byłam bardzo dużo wcześniej, żeby nie siedzieć jej nad głową, to wtedy szłam do niej z 15 minut przed. A teraz umówiłyśmy się tak, że ja przyjeżdżam na jakąś tam godzinę, a ona do mnie zadzwoni, jak poprzednia klientka wyjdzie. Że ona tam sobie zdezynfekuję coś. Ale przede wszystkim, że się nie możemy spotkać u niej. W sensie, że nie ma poczekalni. No i tak było, ja siedziałam sobie w samochodzie i ona zadzwoniła, że ja już mogę przyjść.

**To duży taki dyskomfort braku poczekalni, że trzeba w tym samochodzie odczekać?**

Mnie to tam za rybkę. Ja mam samochód, tak? Poza tym, jak się jedzie komunikacją miejską, to łatwiej przyjechać na czas. Ale jak bym miała chodzić pod blokiem, no to dzisiaj by nie było problemu. Ale jak na przykład pada deszcz, to trzeba by było z sobą coś zrobić. No nie wiem. Myślę, że to jest jakiś kłopot, ale myślę, że do przeskoczenia na pewno. Jest tak i jest po prostu. Nie miałam ciasteczek, tak jak zwykle ma jakieś ciasteczek.

**I kawki też nie ma?**

Nie, kawki też nie ma, w ogóle nie ma poczęstunku. Ale to mnie akurat nie… chociaż miała taki wazonik, taką salaterkę, taki pucharek, co były orzechy różne. To ja sobie zawsze garstkę tych orzechów. To teraz nie ma orzechów, bo nie można dotykać. Bo potem się to do buzi wkłada, to nie można. Jakoś nerwowo się rozglądała za jakimś termometrem. Bo mi opowiadała, że jak jakaś klientka przyjdzie, to żeby ona mogła jej pokazać, nie żeby zmierzyć temperaturę tej klientce, tylko żeby pokazać tej klientce, że ona nie ma temperatury, że jest zdrowa. Bo jednak spotyka ileś osób.

**A to nie wpadłam na to, że to w tą stronę. Bo ja myślałam, że to tym klientom trzeba tą temperaturę.**

No pewnie tak trzeba, ale ona miała takie przekonanie, że to ona powinna pokazać tym klientkom, że ona jest zdrowa i żeby ich nie zarazić. A poza tym to nic, bo strzyżenie w maseczce jest absolutnie niewykonalne. No więc ona miała taką przyłbicę. Taką, wie pani, pleksi przezroczystą. A poza tym to jedziemy. No i ja jeszcze dostałam taki pod spód, taki plastikowy fartuszek. Pelerynkę jak dostaję się u fryzjera, to teraz te pelerynki są jednorazowe, plastikowe, takie bardziej w charakterze śliniaka, bo sięgają mniej więcej do łokcia i do popiersia. Więc trudno uznać, że to przed czymkolwiek może ochronić. To znaczy nie wiem przed czym by mogło ochronić i co. Bo rozumiem, że to chodzi o to, żeby na tej pelerynce nie przenosiło się coś, te wirusy na tych starych pelerynkach. Ale ta nowa pelerynka w ogóle nie chroni ubrania przede wszystkim, ponieważ jest kusa. Więc i tak na tą pelerynkę, założyła mi tą pelerynkę, żeby się liczyło, sztuka była. Ale ani przed włosami ani przed farbą nie miała szansy mnie ochronić, więc dostałam jeszcze na to taką pelerynkę nieprzepisową.

**Czyli normalną, taką standardową, wielorazową.**

Tak, tak. Ale ona ma tych pelerynek tam tyle, że może je prać po każdym, a zresztą, bez przesady. No, ale dostałam cały ten zestaw plus tę pelerynkę jednorazową. Ona sama mówiła, że ciekawe, do czego to mogłoby służyć, ta pelerynka. Ewentualnie jako taki śliniak na przykład do jedzenia makaronu chińskiego w sosie sojowym. No może tak, bo myślę, że zasięg sosu sojowego większy nie jest.

**Ale farby na włosach jest.**

Myślę, że tak.

**Czyli bez maseczki pani siedziała. Nie było tak, że trzeba było farbę nałożyć i w tej maseczce z tą farbą siedzieć?**

No nie, bo jak? Przecież tu się farbuje, się tnie przy uszach, to jak z tą maseczką ciąć? No nie wiem, może tak się tnie. Ja nie wiem.

**Ja nie wiem, ja nie byłam.**

A ja też nie byłam w takim salonie, wie pani, ona prowadzi działalność własną i ma taki salon jednoosobowy. Który prowadzi, to nie jest sklep, w sensie bez witryny, bez ogłoszenia itd. Ma stałe klientki, do których przyjeżdża tutaj z Krakowa. Ona w ogóle jest w Krakowa. Tu jest tydzień czy 2 tygodnie w miesiącu. Więc nie widać jej jakby z ulicy czy coś. Nie wiem, jak wyglądają takie zakłady z ulicy. Mogłam się zapytać, bo mój syn był też u fryzjera. I to właśnie takiego tam jakiegoś, co ma taki salon, co stoi na parterze jakiegoś budynku w śródmieściu. Więc tam może te przepisy są bardziej ostre. I wszędzie są takie same, w sensie pewnie jakby zawodowo obejmuje ich to samo.

**No tak, ale ponieważ ona i nie widać, i ma te stałe klientki, to ona…**

Tak. Tak ma i jestem jej bardzo zobowiązana, że wycięła mi tą plerezę, bo już miałam loczki (śmiech).

**A na paznokciach też pani była?**

Tak!

**W tym samym dniu, czy była osobna wycieczka?**

Nie, w tym samym dniu. Już jak się umówiłam, to… A poza tym, wie pani, ja jestem stałą klientką i taką, bym powiedziała, z dość dużym stażem, to w obu miejscach cieszę się takim przywilejem, że jestem jedna z pierwszych, jeśli chodzi o umawianie, jako taki stały element wyposażenia. Więc w obu miejscach, ponieważ zawsze, 99% jeżdżę w środy do Warszawy, więc te wszystkie historie załatwiałam w środy mniej więcej o tych stałych godzinach. No i tutaj też panie już jak dzwoniły, to dzwoniły do mnie z propozycją terminu w środę.

**To pani manikiurzystka też dzwoniła sama?**

Tak.

**To pani rzeczywiście ma, że tak powiem….**

No, jak zamyka działalność, to też do mnie zadzwoniła, żeby odwołać wizytę. I że jak tylko się uruchomią, bo jeszcze wtedy nie było wiadomo, bo oni się zamykali na tydzień przed oficjalnym zamknięciem tych wszystkich miejsc, salonów. No i powiedziała mi, że zadzwoni, jak będą ruszać. No to ja grzecznie czekałam aż zadzwoni.

**A co pani sądzi o tym, że oni to już otworzyli? W sensie wtedy w połowie maja, te kosmetyczki, fryzjerów?**

Myślę, że musimy jakoś powoli wrócić do tej sytuacji. I tak mamy jakieś tam przepisy sanitarne. Na przykład u mojej kosmetyczki siedziałam w maseczce. Bo też ona nie obrabia twarzy, więc było łatwiej na pewno. No jakoś musimy to ogarnąć, przyzwyczaić się. Nie wyobrażam sobie takiego stanu, żebyśmy nagle się wycofali o ileś lat. Musimy się oswoić z sytuacją, jaka jest i postarać się przystosować jakoś, możliwie jak najbezpieczniej dla wszystkich. Ale też żeby się nie dać zwariować. Więc wie pani, tak naprawdę najważniejsze to jest chyba to, że oni zaczynają uruchamiać służbę zdrowia, która po prostu stanęła na głowie. Ja akurat jestem zdrowa szczęśliwie. I wokół mnie też nie ma osób, które są chore na jakieś choroby przewlekłe, wymagające opieki lekarskiej stałej. Ale w tej chwili to… Nie, no jakoś musimy zacząć normalnie funkcjonować. Rosną nam włosy, paznokcie, chorujemy, musimy pójść do pracy, do szkoły. Musimy teraz też mieć z tyłu głowy, że nie jesteśmy tacy bezpieczni, jak nam się wydawało. I że trzeba uważać, trzeba myć ręce. To nie jest jakiś duży… Dezynfekujmy je. Na pewno nam nie zaszkodzi. Może trochę wysuszy skórę, bo to na alkoholu są te sosy. Ale kupmy sobie krem z mocznikiem, będzie super.

**A jak teraz u pani wyglądają zakupy?**

Świetnie. Teraz już prawie w ogóle nie chodzę na zakupy (śmiech). Nie, no miałam takie wzmożone oczywiście, bo Julka wyjeżdżała, więc coś tam jej kupowałam na wynos. Przyjeżdżał Hubert z Magdą, więc też musiałam wykonać jakieś zgrubienie takie w lodówce, żeby tam było na rosołek i na coś. Jak mamusia gotuje takie dobre rzeczy i w ogóle.

**A, żeby wałóweczkę jeszcze przygotować.**

A no tak. I grilla robiliśmy, mamusia zawsze wydaje. Na początku nawet, jak Hubert był z Magdą, to tak nie za bardzo chcieli zabierać, bo oni tak samodzielnie i oni sobie radzą i w ogóle. Ja mówię, no dobrze, no trudno. I wszystko mają. A jak nie mają, to sobie kupią. No dobra. Ale teraz już zabierają, chętnie wezmą. Ja też ostrożnie przepytuję o każdą jedną rzecz, którą im wydaje, żeby nie było, że coś dałam, a nie miałam dać. Ale od kiedy wyjechali, czyli od poniedziałku, znaczy wyjechali w niedzielę, to jeszcze w sklepie nie byłam. Chociaż jakąś listę, tutaj coś tworzę. Nie mam kapsułek po prania na przykład oraz czegoś tam. Więc jakaś lista powstaje. Więc myślę, że dzisiaj, najdalej jutro, będę musiała odwiedzić jakiś sklep.

**Ale to nie tak, że do Płocka specjalnie?**

Nie, nie, nie. Nie, do Płocka to jeżdżę po takie jakieś bardziej… Byłam w poprzednim tygodniu przed wyjazdem Julki, jakoś na początku. Bo trzeba było kupić w Lidlu jakieś sosy, tydzień włoski był, to sery tarte. I takie tam historie.

**A jak pani była w tym Lidlu, to widać jeszcze tą pandemię, epidemię? Czy już w ogóle po zachowaniach ludzi nie widać?**

Po pierwsze nie ma już tych ograniczeń do ilości, znaczy są ograniczenia, ale one jakby już tych sklepów nie dotyczą, w takim sensie, że nie powodują kolejek przed sklepem. Po pierwsze wydaje się, chociaż część wózków przed sklepem, bo ja podeszłam do takiego… Wszyscy zawsze podchodzą do takiego sznura wózków, który jest najbliżej. Ja myślę sobie, dobra, ja już mogę podejść 2 kroki i pójść tam najdalej. I szarpię się z tym wózkiem, szarpię, a one były związane łańcuchem wszystkie. Żeby jakby ograniczyć liczbę osób wchodzących do sklepu. To ja tego nie zauważyłam, ale jednak te ograniczenia są. Chociaż nie powodują kolejek przed sklepem. Natomiast w sklepie to co można zauważyć, to to, że ludzie chodzą w maseczkach. No, jeszcze w poprzednim tygodniu trzeba było to robić, więc teraz nie wiem. I cały czas mi się wydaje, że mają więcej zakupów jeszcze w tych koszach. To znaczy myślę, że robią te zakupy rzadziej. Tak mi się wydaje. Bo w tych koszach jest, no jest po prostu dużo. Tak to kiedyś w tym Lidlu zwłaszcza, zresztą w Biedronce też, sporo było takich osób, które przychodziły i było widać, że mają 3 rzeczy. Z pracy gdzieś wychodziły. Widać było, że mają w ręku tam dwie bułeczki, jakieś tam picie i coś tam. I fajki albo batonik. A teraz w zasadzie, no może spotkałam taką panią jedną u nas w sklepie. Bo zawsze przepuszczam te osoby, bo zanim ja wyładuję z tego worka te karmy psie, kocie, to i tak mija trochę czasu. To jak za mną stoi pani, co ma 3 rzeczy czy pan, to przepuszczam. Bo się na pewno szybciej obrobią niż ja. To widziałam jedną taką. A tak to nie widzę dużo osób, które by miały taki… Jak już ktoś idzie, to ten koszyk usypany prawie. No i jednak podrożało sporo chyba. Bo tak jak patrzę, myślę, że jednak więcej wydaję. No może nie będę jeszcze takich wygłaszać opinii, bo jednak kupowałam dla większej ilości osób. Ale mi się wydaje, że podrożało w tych sklepach. Tu złotówka, tam złotówka, coś jednak chyba podorzucali. Ale nigdy nie śledziłam bardzo, więc głowy sobie nie dam uciąć.

**A przed wyjazdem Julki pojechałyście na jakieś zakupy do galerii handlowej?**

Nie.

**Nie? Bo myślałam, że tej tradycji musi stać się zadość i ta wizyta w Płocku.**

Nie, to nie jest taka tradycja, że zawsze jeździmy. Czasem nam się zdarzało. Ale nie, nie. Żadna z nas, Julka też nie szaleje. Że owszem, tu było o tyle atrakcyjnie w Płocku, że tu nie ma ludzi tak naprawdę w tych galeriach, nawet jak nie jest pandemia, po prostu nie ma. Ale nie, nie jechałyśmy.

**A co pani sądzi na przykład o tym, że już pootwierali restauracje, knajpy, gastronomię?**

No wszyscy musimy jakoś żyć. Ci, co prowadzą gastronomię też. I ci, co jedzą w gastronomii też. Nie wiem, co oni jedli w ogóle. Przecież sobie musieli sami robić, no na pewno, ale… Nie, no dobrze, wydaje mi się, że jakiś jest reżim, na coś uważamy. Oczywiście, że może się okazać, że popełniliśmy jakiś błąd. Ale z mojego otoczenia, nie tylko najbliższego, ale nawet myśląc o tej okolicy jak o powiecie powiedzmy, no to myślę, że nie ma zagrożeń. Myślę, że są jakieś może miejsca, które powinny się 3 razy zastanowić. Ale tych zachorowań jest chyba jednak coraz mniej. Nie wiem, czy my już jesteśmy na jakiejś krzywej spadającej czy…

**Nie wiem, bo ja nawet tego nie sprawdziłam.**

Ja też nie. Właśnie już ostatnio przestałam te krzywe oglądać i się tym tak interesować. Wiadomo, że to cały czas jest, tylko po jakimś czasie trochę się oswajamy z tym. Już to nie jest takie, człowiek się nie ekscytuje tymi krzywymi.

**Emocje – zdjęcia. Który obrazek pokazuje najlepiej ten moment, w którym pani teraz jest?**

Myślę, że tak naprawę to może żaden.

**A jaki powinien być?**

Ponieważ tak sobie myślę, że teraz jest tak… No tak nie wiadomo jak. To znaczy nie wiadomo jak, nie w sensie, że w ogóle... A może ta 8? To zostańmy przy takim obrazku. Bo to taki jest obrazek, wie pani… No jedziemy do przodu, ale tak naprawdę nie wiemy… Może ta mgła powinna być trochę dalej.

**Czyli to jest taka mgła w oddali, że my teraz widzimy, a potem trochę nie wiemy, co będzie za chwilę.**

Tak. A potem trochę nie wiemy. Ale musimy jakoś wrócić i się dostosować do tego. To myślę, że tak. No wie pani, takich emocji jakby dużych we mnie nie budzi tak sytuacja. Tak że jest jak jest, jakoś musimy się dopasować do tej sytuacji, bo ona się na pewno do nas nie dopasuje.

**A takie mam pytanie. Bo teraz jest dużo pomysłów na wprowadzanie takich różnych aplikacji albo rozwiązań technologicznych na czas pandemii. Czy słyszała pani w ogóle o jakichkolwiek aplikacjach związanych z pandemią, które ktoś wymyślił z okazji pandemii?**

Jedyną chyba, o której słyszałam, to jest taka do potwierdzania, że się jest w kwarantannie. Jakaś taka. Więc jedyną, o której słyszałam, choć nie mam bladego pojęcia, jak ona działa, ale rozumiem, że na zasadzie jakiegoś tam GPS i lokalizacji, potwierdzania, bo to mi ktoś mówił. Ja też jej ani nie widziałam ani nie bardzo się interesowałam, bo nigdy nie byłam w kwarantannie. Więc tylko o takiej słyszałam.

**Pokaże takie pomysły. Pierwsza rzecz, aplikacje analizujące dane osobiste, bieżące informacje o stanie zdrowia, historie przemieszczania się i kontaktów z innymi ludźmi, w celu monitorowania rozprzestrzeniania się koronawirusa. Co pani sądzi?**

Wie pani, no jak wszystko, co może być nadużywane, a może służyć. Jeżeli ono ma rzeczywiście na celu monitorowanie przemieszczania się czy rozprzestrzeniania się koronawirusa, czyli jakichś tam osób z kontaktu tak zwanego, no to super. Ale jak ktoś będzie sprawdzał, gdzie ja się przemieszczam w czasie wolnym, no to… To jak każdy kij, ma 2 końce.

**Ale myśli pani, żeby to dobry pomysł, żeby mieć taką aplikację i na przykład wprowadzać informacje, jak się pani czuje?**

Jak jestem chora, to może by było i dobrze. Jak już bym była z tym koronawirusem w domu i może bym się czuła bezpieczniej na przykład, jak bym wprowadzała codziennie rano temperaturę czy tam co 5 minut czy co godzinę czy cokolwiek. Żeby ktoś to monitorował. Albo nawet jakiś, nie wiem, komputer, który by analizował te dane i dał znać lekarzowi, że na przykład sytuacja jest poważna. Że wymaga jakiejś interwencji czy diagnostyki. To mi się wydaje, że na przykład do takiego celu diagnostycznego taka zdrowotna aplikacja byłaby sensowna. Nawet oglądałam, że takie mam zdaniem, bo oglądałam w międzyczasie, pewnie na jakimś tam NatGeoPeople czy coś takiego, taki program o takiej aplikacji… Teraz nie wiem, w jakichś krajach afrykańskich takie aplikacje, bo większość osób ma jednak telefon komórkowy. I takie aplikacje. A za to nie ma lekarza w promieniu 100 km czy iluś, żadnego lekarza. I wprowadzanie takich danych medycznych właśnie o stanie zdrowia powoduje, że część tych danych jest analizowanych przez sztuczną inteligencję po prostu, czyli przez komputer z jakąś tam ilością danych. I te dane są jakoś przesiewane. Oczywiście jest ryzyko, że ten komputer może się czegoś nie dopatrzeć też. Chociaż nie wiem, czy nie większe jest takie, że jakiś lekarz się nie dopatrzy. Ale myślę, że taka zdrowotna to jeszcze… Zwłaszcza, że oglądałam ten program właśnie ostatnio.

**Ale to miało być tak, że to lekarz ma dostęp do tych danych, tak?**

Tak. To akurat była taka aplikacja takiej sieci medycznej w Wielkiej Brytanii, ale oni jakby testują to w krajach afrykańskich, które się zgłaszają na ochotnika. Takie kraje, które mają bardzo złą, czyli ubogą opiekę medyczną. W sensie takim, że ani nie mają pieniędzy ani nie mają lekarzy, ani nie mają infrastruktury. I zgłaszają się na ochotnika. Bo każde testy nawet przeprowadzane na ich terenie, zawsze tą opiekę poprawiają. I to jest firma jakaś duża w Wielkiej Brytanii, która testuje teraz właśnie sztuczną inteligencję w zakresie pomocy lekarskiej diagnostycznej. Przy czym oczywiście… No tak. I tam jakieś były takie eksperymenty czy porównania, wychodzące nawet na korzyść tej sztucznej inteligencji czyli tych komputerów. Że jeśli chodzi o trafne diagnozy i wychwytywanie jakichś właśnie takich sytuacji zagrażających życiu. I wtedy to jest i tak weryfikowane przez lekarzy. To nie jest tak, że taki komputer stawia diagnozę, wypisuje receptę i pa.

**Rozumiem, że taka medyczna część tej aplikacji, OK. A to, że ta aplikacja miałaby to przemieszczanie się monitorować? W sensie sprawdzać, gdzie jesteśmy.**

Ja myślę, że ona w zamyśle…. W zamyśle chodzi o to, żeby stwierdzić, że jeżeli byliśmy chorzy czy jesteśmy chorzy, to z kim się w międzyczasie spotkaliśmy. I komu mogliśmy tę chorobę sprzedać. Że taki jest może dobry pomysł. Ale wiadomo, że dobrymi chęciami to piekło jest wybrukowane. Więc myślę, że historia przemieszczania się, to jednak… Bo o to tu chodzi, ale daje to pole do nadużycia. Gdzie ja jestem, z kim chodzę, do jakiego sklepu chodzę. Wie pani, no w dobie… No nie wiem. No ja bym nie chciała chyba, żeby ktoś wiedział, gdzie się przemieszczam.

**OK, jeżeli tam są tylko te dane medyczne, w sensie może pani sobie wpisywać w komputer czy w telefon, że się pani tu gorzej poczuła, tu coś tam. I potem żeby lekarz to analizował, to tak.**

Ja bym sobie sama pewnie nie wpisywała. Ale gdybym wiedziała, że te dane do czegoś mogą posłużyć, w sensie takim, że jeżeli ja rzeczywiście, ta sytuacja będzie coraz gorsza, to ktoś zareaguje, to myślę, że na to bym się zdecydowała na przykład i byłabym za tym. Natomiast... To nie chodzi o taką samokontrolę. Tylko chodzi o to, żeby jednak ktoś zewnątrz. Bo samokontrolę, możemy sobie tam pisać w notesiku czy coś i potem sami analizować dane. Ale to trzeba mieć jeszcze jakiś taki… Samokontrolę trzeba mieć, żeby to robić i samozaparcie (śmiech). A ja mam bardzo słabą silną wolę, więc…

**Następne: aplikacje, które na podstawie danych lokalizacyjnych monitorują przestrzeganie kwarantanny domowej. Chyba podobne do tego, o czym pani mówiła.**

No to właśnie to jest to chyba.

**Myśli pani, że to jest dobry pomysł?**

Wie pani co, myślę, że to jest pomysł… Myślę, że dla tych, którzy są na kwarantannie to jest dobry pomysł.

**Czemu?**

Po pierwsze policjanci, którzy jeździli, sprawdzali, czy ktoś jest na kwarantannie domowej, może mogłoby się zająć czymś innym na przykład. Jednak takie wszystkie pomysły, jak tych osób na kwarantannie są dziesiątki tysięcy, bo to taka jest skala, jedna osoba chora, a 60-70 na kwarantannie, jak mamy do czynienia powiedzmy z przedszkolem. A jak mamy do czynienia z zakładem pracy czy z kopalnią, no to już jest dużo więcej. Myślę, że te służby i tak w takich okresach mają trudno. Więc dopóki jest kwarantanna domowa i dopóki tą aplikację można sobie później usunąć z telefonu, to moim zdaniem to akurat jest całkiem w porządku. Bo ci policjanci powinni się zajmować tym, żeby nikt nie kradł, nie jeździł 200 na godzinę, tylko dlatego, że ich nie ma, bo oni sprawdzają, kto jest na kwarantannie, a kto nie.

**A taka: aplikacje oparte na automatycznej lokalizacji użytkowników, informujące ich, że znajdowali się w miejscach zagrażających zarażeniem się koronawirusem.**

No nie wiem. Myślę, że to chyba przesada już jest (śmiech). Tak naprawdę jak ktoś nas zlokalizuje, to zna naszą lokalizację. To jak to się będzie nazywać, to jest tylko zmiana nazwy na mniej… A co to znaczy na miejsce zarażenia się koronawirusem? Wszędzie mogliśmy się nim zarazić. Mogę się nim zarazić od kogoś, kto jeździ na rowerku. Myślę, że to by wprowadziło więcej zamieszania niż… Zaraz by się wszyscy chcieli zbadać.

**OK, czyli to jest tak, że… No dobra, zakładając, że mamy tą aplikację i ona mówi, słuchaj, mogłeś się zarazić w sklepie, w przychodni albo gdzieś. To myśli pani, że wtedy ludzie by się chcieli badać od razu?**

Myślę, że nie wiem, co by robili. Albo by znowu to olali, albo może by się chcieli zbadać na przykład, czy się zaraziłem czy nie. Myślę, że wprowadziłoby to więcej niepokoju i więcej obciążenia dla tej służby zdrowia, która i tak, myślę, ona najbardziej została, jej życie zostało wywrócone do góry nogami w tej szczególnej sytuacji. To jeszcze bardziej by się… Wie pani, ludzie się denerwują wtedy, mają objawy takie psychosomatyczne. Każdy katar i każdy ból gardła, na który normalnie by nie zwrócili uwagi, zaraz posiadają koronawirusa, dostają zaraz jakichś palpitacji serca. Myślę, że to akurat… Jeżeli ktoś wie, bo wiadomo, ktoś choruje i jest ileś osób tam z kontaktu, które powinny się ewentualnie obawiać albo może nie powinny, ale się obawiają. No to rozumiem, że są jakieś służby od tego, które nad tym panują. A jak ja mam dostać na komórkę coś takiego, to myślę, że to jest kiepski pomysł. W ogóle uważam, że taka automatyczna lokalizacja, to jak by jej nie nazwał, bo można to ubrać w ładne słowa, że to jest w sprawie, żebym ja wiedziała, że ewentualnie się mogłam zarazić, więc to jest dla mojego dobra. Ale tak naprawdę jest do powód również do jakichś nadużyć, do których już w tej chwili nie jesteśmy przyzwyczajeni.

**Następna: monitoring z automatycznym systemem rozpoznawania twarzy w celu szybkiej identyfikacji osób nieprzestrzegających zaleceń władz.**

No tak, a jeszcze następny punkt to będzie powrót komuny. Myślę, że to kiepski pomysł jednak (śmiech). To nawet nie brzmi dobrze. Wie pani, on jest tak samo kiepski niż inne, ale…

**Tutaj nawet nie ma ładnych słów.**

Tak. Zabrakło ładnych słów.

**To mam następny. Drony dostarczające produkty medyczne i inne towary osobom potrzebującym.**

No nie wiem. Zależy, nie wiem, czy mamy u nas takie strasznie niedostępne tereny.

**Że do takich niedostępnych terenów?**

Bo wie pani co, ja na przykład sąsiadom dostarczam potrzebne produkty. Oczywiście, no może jak byśmy mieli jakąś gorączkę krwotoczną albo, nie wiem, ebolę albo coś, co skutkuje śmiertelnością w 80% przypadków, no to może tak. Ale myślę, że dopóki możemy jakoś sensownie się zachowywać, to… No ja zanoszę sąsiadce produkty. Nie jestem dronem i robię to w sposób bezpieczny. Zostawiałam jej, żeby jej nie zarazić, bo ona starsza, więc ewentualnie gdybym ja miała te… I zostawiałam worek przy bramie no i tyle. Więc myślę, że to strzelanie z armaty do komara. Jak byśmy mieszkali w Chinach, gdzieś w niedostępnych terenach to może.

**To takie: aplikacje, w których użytkownicy mogliby informować się o tym, czego potrzebują i dzięki temu pomagać sobie nawzajem.**

No to chyba takie aplikacje to są jakieś. Są jakieś tam, świąteczna paczka, jest coś takiego? Wydaje mi się, że są jakieś, znaczy aplikacje albo to się gdzieś robi w ramach jakiejś innej aplikacji.

**A żeby w ramach pandemii na przykład coś takiego?**

Trudno mi powiedzieć. No nie wiem, kto by mógł z tych takich bardziej potrzebujących osób, czyli na przykład patrząc na koronawirusa, osoby starsze, to one myślę, że nie są w stanie opanować czasem wysyłania SMS, nie mówiąc o… No mają taką aplikację pod tytułem SMS, mogą poinformować, mają telefon. Więc myślę, że tworzenie takich aplikacji, jak nie ma takiej stuprocentowej izolacji… Nie mogę sobie, trudno mi sobie wyobrazić w obecnych czasach taką sytuację, że ktoś nie może się skomunikować przez telefon, czego potrzebuje na przykład.

**Aplikacje sztucznej inteligencji, decydujące na podstawie zebranych danych, gdzie skierować największe środki i wysiłki do walki z pandemią.**

Nie wiem, trudno mi powiedzieć. Rozumiem, że to jakby nie jest pytanie skierowane do nas, tylko do rządzących, czy oni panują nad tym, gdzie to skierować, czy nie.

**A myśli pani, że panują?**

Myślę, że panują. Myślę, że mają te dane, spływają im na bieżąco, ilości zachorowań. Myślę, że mają jakieś procedury, które ich informują o tym, skąd są na przykład zarażeni, jak podają czy podawali te dane o zakażeniach, to wiedzą, w jakim szpitalu, w jakim miejscu, wiedzą, gdzie jest ognisko choroby, gdzie się tam im rozprzestrzenia. No jakoś sobie radzą. Tak że myślę, że można spróbować, dlaczego nie, no może im to jakoś pomoże, trudno mi powiedzieć, na ile ich… Bo tak naprawdę to na podstawie czego podejmują takie decyzje, też jest w pewnym sensie sztuczną inteligencją. Bo to są jakieś programy, które im to analizują najprawdopodobniej. To już jest sztuczna inteligencja, jeśli się orientuję dobrze.

**(Prezentacja aplikacji kwarantanna domowa). Co pani o tym sądzi?**

Sądzę, że wprowadzono aplikację obowiązkową dla osób na kwarantannie.

**Ale dobrze, że to jest obowiązkowa aplikacja?**

Myślę, że dobrze. Skoro te osoby są na kwarantannie, no to myślę, że dobrze, że jest obowiązkowa. Jak by była dowolna, to w życiu nikt by z niej nie korzystał. Jak jest obowiązkowa, to się z tym nie dyskutuje. Są na kwarantannie, mają obowiązkowo. Nie jest tak, że jak nie zrobią zdjęcia, to od razu wysyłają do więzienia, tylko… Tylko ktoś przyjdzie i sprawdzi, czy nie łamiemy zasad kwarantanny, no więc. Myślę, że to ma sens. Przecież ja sama znam osoby, które były na kwarantannie, to dopóki nie wprowadzono tej aplikacji, to przychodził pan policjant. I kazał im, akurat oni na drugim piętrze mieszkają, więc mogli się wychylać przez okno. I żeby się pokazać, że są. A jak ktoś jest na 9 piętrze, no to się już nie wychyli. Bo ten policjant i tak nie wie, czy to on czy to nie on. Więc musi tam się pofatygować jakoś. No to jest pewnie i czasochłonne i niebezpieczne. Bo ktoś jest na kwarantannie to dlatego, że się albo kontaktował albo wrócił z zagranicy. Myślę, że to ma sens. Jeżeli mamy jakoś ogarnąć tą sytuację i nie mamy 10 osób na kwarantannie, tylko dziesiątki tysięcy albo setki może, to myślę, że to ma sens. Nie jest to jakaś straszna inwigilacja. Zwłaszcza, że jak rozumiem, można to później… To służy tylko w jednym celu. Potem można sobie to po prostu usunąć z tego telefonu i tyle.

**Ale tu napisali, że jednak policja i tak przyjeżdża.**

No właśnie mówię, może przyjechać tak jakby niezapowiedziana, to rozumiem. Oraz, że jeżeli się nie odpowie, nie wykona w ciągu 20 minut tego zameldowania się za pomocą tej aplikacji, to przyjedzie ktoś, żeby sprawdzić, czy nie łamiemy zasad kwarantanny. No wie pani co, jeżeli ktoś ma siedzieć w domu, no to ma siedzieć w domu. A nie wyjść na chwilę do sklepu albo na chwilę do sąsiadów albo na chwilę gdzieś. Na tym polega kwarantanna. Jest upierdliwa, ale no coś za coś. Takie są zasady.

**A ma pani jakieś obawy, które ta aplikacja jakoś budzi?**

No nie, dopóki ona nie jest… Dopóki to jest rzeczywiście używane, kiedy ktoś jest na kwarantannie, to jest w porządku. I jeżeli ja ją mogę usunąć po czasie kwarantanny, to mi się wydaje… No skoro mamy taką możliwość, to dlaczego z niej nie skorzystać? Skoro możemy, nie wiem, oglądać, czy mamy SMS-y, czy mamy telefony, które robią zdjęcia, no to dlaczego mamy sobie go nie zrobić? Rozumiem, że jak ktoś nie ma takiego aparatu, który robi zdjęcia, no to może będzie rozmawiał z tymi policjantami, czy oni nadal będą przyjeżdżać. No rozumiem, że nie idzie się do więzienia, jak się nie ma takiego telefonu. Są takie sytuacje, że ktoś może nie ma. Ale większość osób ma, to z tego korzystajmy. Bo myślę, że nie ma co się cofać do jakiejś tam technologii króla Ćwieczka, skoro jesteśmy w XXI wieku i możemy jakoś to ogarnąć.

**A na przykład taka sytuacja, ktoś wjeżdża do Polski, ma obowiązkową kwarantannę, bo wjeżdża. I mówi, że on nie chce na swoim telefonie zainstalować takiej aplikacji. Nawet jak ma możliwość robienia tych zdjęć. Co pani sądzi? Powinno się go zmusić?**

Nie wiem, jakie są procedury tak naprawdę, czy się powinno go zmusić. Myślę, że powinno się próbować go zmusić pod karą administracyjną najprawdopodobniej jakąś. Bo skoro są takie przepisy i skoro on wraca i wie o tym, bo jeżeli ktoś wraca z zagranicy do kraju, który jest zamknięty, którego, nie wiem, przestrzeń powietrzna jest zamknięta i nie przyjeżdżają żadne pociągu i nie może przekroczyć granicy jakby nieświadomy. Tylko decyduje się na powrót do kraju w sytuacji, kiedy cała Europa tak naprawdę jest zamknięta do poruszania się, bo to się teraz dopiero świeżo zmienia, no to myślę, że można go. I wiedział, że taki jest, korzysta z programu powrót do kraju czy lot do kraju, czy nie wiem, jak to się tam nazywało, wszystko jedno. No i taki jest warunek, po prostu. No może ja też nie chcę, ale taki jest obowiązek. Ja też nie chcę płacić podatków na przykład. Ale jest taki obowiązek w tym kraju, płacenia podatków. I można mnie do tego zmusić. Oczywiście, nikt mnie nie zmusi, żebym ja zapłaciła ten podatek. Ale mogę dostać karę administracyjną czy skarbową. I przyjdzie pan komornik i mi zabierze telewizor albo mi zlicytuje dom. Więc teoretycznie oczywiście nie można zmusić. Ale można próbować zobligować taką osobę. Albo ją ukarać.

**A myśli pani, że to jest prawnie, ta aplikacja jest zgodna z prawem?**

Nie wiem.

**Bo podatki to jakby rozumiem, w sensie jest w prawie naszym napisane, że trzeba płacić podatki. A zastanawiam się, czy to jest w prawie, że jest jakaś konsekwencja tego.**

No wie pani co, mamy jakiś stan epidemiczny ogłoszony. Na pewno są jakieś uregulowania wynikające… Bo normalnie, najprawdopodobniej w obecnych czasach nawet w naszym kraju, istnieją przepisy gwarantujące wolność osobistą. I stojące na straży niezależności. Ale w momencie zagrożenia epidemicznego, no są stany nadzwyczajne, którego nie mamy i są jakieś tam inne, ale na pewno każdy z tych stanów ogłaszany przez rząd czy prezydenta, ma swoje konsekwencje dla funkcjonowania państwa. I po to takie są przewidziane sytuacje, żeby pewne ograniczenia wprowadzić, swobód również, czy swobody przemieszczania się na przykład, no po to, żeby umożliwić, czy dla jakiejś tam siły wyższej. Oczywiście, jak wszystko, możemy nadużywać wszystkiego, również swobód osobistych możemy nadużywać. To, że możemy robić wszystko nie znaczy, że na przykład możemy kogoś zabić. Więc to, że mamy stan epidemiczny to na pewno wiąże się nie tylko z nazwą, ale również z jakimiś szczególnymi rozporządzeniami w zakresie na przykład swobody przemieszczania się osób chorych albo ograniczenia tej swobody. Albo możliwości zamknięcia miasta czy jakichś ośrodków, które… Nie znam się w ogóle na tym. Ale myślę, że te stany nie tylko dlatego po prostu się nazywają, bo mamy epidemię, to mamy stan epidemiczny, ale coś za tym idzie po prostu. Ale nie mogę pani odpowiedzieć na pytanie, bo nie wiem, czy…

**(Prezentacja aplikacji Protego Safe). Jakie wrażenie?**

Takie sobie (śmiech).

**To niech mi pani opowie o tym takim sobie wrażeniu.**

Myślę, że to… Cóż to może nam dać? Tak naprawdę chyba niewiele oprócz… Nie wiem czego tak naprawdę. Bo samokontrola stanu zdrowia i to, czy się spotkałam z chorym czy nie, no dobra, to już rozmawiałyśmy o tym, że mi się wydaje, że to jest kiepski pomysł. Bo samo spotkanie jeszcze, nie wiadomo, jak blisko to spotkanie i czy on na mnie napluł czy tylko przechodził drugą stroną ulicy na przykład. To myślę, że to jest kiepski pomysł. Dziennik zdrowia to bardzo fajnie, pod warunkiem, że ktoś to będzie analizował i sam monitorował. No ja sama nie jestem lekarzem. Mogę sobie napisać, że temperatura mi rośnie, potem 2 dni mi spadnie czy coś, nie będę tego przeglądać, bo oświrujemy wszyscy. Już nie mówiąc o tym, że ja myślę, że te wszystkie aplikacje są może bardzo świetne, ale dla osób młodych i w wieku średnim. Starsze osoby, myślę, że w ogóle nie mają szansy w ogóle żadnej korzystać z takich aplikacji. Po prostu nie dają rady, nie poradzą sobie ani z ankietami ani z czymś takim. Jeżeli coś nie jest powszechne, no to nie jest skuteczne. Większą kontrolę zyskuje, czy potencjalną, bo to myślę, że to nie jest tak, że to są aplikacje wymyślone po to, żeby nas kontrolować. Ja myślę, że to są aplikacje, które w ładnych słowach dają potencjalną możliwość jakiejś tam kontroli hipotetycznej, może większy opór nawet powodują, które ten okres komuny mają za sobą i pamiętają te próby… Czy nawet nie próby, tylko kontrole właśnie różnych sfer życiowych. Wtedy wprowadzaną bardziej powiedzmy chamsko, w sensie bardziej otwarcie. Bo nie było takich możliwości elektronicznych czy właśnie technologicznych. Ale myślę, że to budzi w nas taki, może nieuzasadniony niczym, ale taki lęk, że to potencjalnie może powodować później jakieś… Ktoś będzie wiedział, co ja robię. A ja sobie tego nie życzę, bo po prostu kiedyś ktoś mnie kontrolował i już bym nie chciała tego. Może młodzi ludzie na przykład podchodzą do tego zupełnie inaczej, mniej są podejrzliwi po prostu. Nie mają takich doświadczeń.

**A ten test oceny ryzyka, to jest czwarta kropka od góry. Co pani sądzi o takim teście?**

Kto to analizuje? My sobie sami to analizujemy? To tak jak psychozabawa w gazecie.

**System sprawdza, do jakiej grupy ryzyka kwalifikują się twoje odpowiedzi.**

Ale wie pani, co to znaczy, że system sprawdza. To znaczy, że jest jakaś pula odpowiedzi, no taka ankieta. To jest naprawdę, myślę, grube dość narzędzie. To myślę, że telefon do lekarza albo uruchomienie zakładów podstawowej opieki zdrowotnej dałoby lepszy test. Ankieta stworzona przez lekarzy. Mało mi się to wydaje tak naprawdę sensowne. Dopóki to będzie system taki zerojedynkowy, wie pani, bez… Gdyby to było naprawdę, tylko takich programów jest bardzo niewiele, ale takie naprawdę zaawansowane programy analizujące stan zdrowia, to być może tak. Źle się czuję… Moim zdaniem powinna być taka możliwość, ale nie w ramach jakiejś większej aplikacji. Powinniśmy mieć taką możliwość, żeby takie dane wprowadzić, jeżeli się czujemy chorzy, to w momencie pandemii powinniśmy, tak jak możemy online się połączyć z lekarzem na jakąś wizytę domową przez sieć, to powinniśmy móc wprowadzić jakieś dane. Ale znowu jest kwestia dostępu do takiej technologii. Wszystko, co nie jest powszechne, mi się wydaje takie no… No może i byłoby dobrze. Ja bym była zadowolona, gdybym miała taki system rzeczywiście godny zaufania w sensie przeprowadzonych jakichś prób. Jeśli to by był rzeczywiście na podstawie sztucznej inteligencji i taki zaawansowany system, który by coś mógł oceniać, no to super. I myślę, że przede wszystkim, sam fakt tego, że ta aplikacja jest dobrowolna mi się podoba. Ktoś by chciał, przeczyta to, przekona go, nie jest obarczone jakimiś obawami takimi bardziej globalnymi czy jakąś teorią spiskową, że ktoś tu go będzie sprawdzał, co on robi, to niech sobie to montuje. Ja na przykład bym sobie tego nie zamontowała. Wydaje mi się, że jestem dość sprawna, żeby ocenić, jak się będę źle czuła. I się gdzieś zgłosić w miejsce, gdzie mi ktoś pomoże. A z trzeciej strony jest cała grupa osób, które w ogóle nie będą miały do tego dostępu, bo są starsze.

**Na koniec chciałabym porozmawiać o tym, co będzie. Myśli pani w ogóle o przyszłości, bliższej, dalszej?**

Wie pani co, w pewnym sensie myślę o przyszłości (śmiech). Ale ta przyszłość to bardziej jest taka codzienność. Troszkę może w takiej innej wersji językowej. Ale no nie jest to jakieś, nie myślę o tej przyszłości jako o jakimś kolejnym etapie. Po prostu to jest ciąg dalszy. Mi się nie wydaje, żeby tutaj miały się jakieś… Na pewno coś się zmieni, po prostu. Będziemy ostrożniejsi troszkę, będziemy się zastanawiać może ze 3 razy zanim coś zrobimy albo czegoś nie zrobimy. I może część osób pozostanie w tych maseczkach, bo w nich zasiało to jakiś większy niepokój czy obawy. Myślę, że powoli wrócimy na tyle do normy, na ile się da. Z jakimś komponentem takiej ostrożności. Nie wydaje mi się, żeby miało być jakoś bardzo źle. Wracamy do normalności, myślę, że już wszyscy wiedzą czy widzą, że musimy wrócić do niej, do pełnej aktywności, do tego, żeby iść do pracy, żeby szkoły były szkołami, że ten eksperyment, żeby rodzice byli wszystkim, i rodzicem, i nauczyciel, i wychowawcą, i opiekunem, no to jest kiepski pomysł. Bo to jednak ten system edukacji jakoś tam działa. Może nie najlepiej, ale jakoś tam działa. I musimy wrócić do pracy i chorujemy też na inne rzeczy niż na koronawirusa, więc musimy jakąś uzyskać pomoc w zakresie itd.

**A ma pani poczucie, że sytuacja gospodarcza jakoś się zmieni?**

Myślę, że ona się na pewno zmieni. Moim zdaniem się poprawi. Że ta sytuacja takiej chwilowej zapaści… Myślę, że ten moment taki zdecydowanie gorszy, część osób pewnie przypłaci to jakimś takim bardziej gruntownym kryzysem, bo nie wszyscy są, szczególnie u nas takie mniejsze przedsiębiorstwa czy takie jednoosobowe czy rodzinne, jak tego nie przetrzymały, to mogą się nie podnieść. Bo one nie mają jakichś zapasów. Ale myślę, że dłuższy czas to potrwa, ale wrócimy na jakąś taką… Na pewno będzie lepiej. Ceny będą rosły powoli, tam paliwa, już rosną. To ja już widzę, że jak rosną ceny paliwa, to znaczy, że więcej osób korzysta z samochodów i można je podnieść itd. Więc od tego się wszystko zaczyna. Więc myślę, że idziemy w dobrą stronę, musimy jakoś się wydźwignąć. Chociaż tak jak tutaj, to nie widać było tego bardzo. Ale myślę, że w globalnej skali na pewno takie ochłodzenie wystąpiło. Nawet rząd zauważył mniejsze wpływy z PIT.

**Czyli jednak pani trochę gdzieś te media śledzi, skoro o tym PIT…**

No coś tam mi mignęło. Rano, raz na jakiś czas załączam sobie do kawy ten Polsat nieszczęsny. Chociaż ta pani, co mnie wkurza, jest coraz częściej, więc jak ją widzę, to ją wyłączam. A teraz już w ogóle nie będę oglądać, bo teraz bym oglądała tylko kandydatów na prezydenta, to jeszcze bardziej mnie wkurzy. Bo nie bardzo lubię, jak mi ktoś mówi to, co ja chcę usłyszeć (śmiech). Nie muszę tego słuchać, mogę sama sobie to powiedzieć przy pieleniu.

**A sytuacja społeczna, sytuacja ludzi? Czy ma pani poczucie, że są grupy, które w jakiś szczególny sposób odczują pandemię?**

Czy ja wiem, czy są, które odczują. Ale odczują negatywnie?

**No jakkolwiek, negatywnie, pozytywnie. W ogóle jakby…**

No nie wiem, no na pewno są. Na pewno są takie grupy, no nie wiem, kibice sportowi na przykład. Którzy nie mogą uczestniczyć na razie… Albo młodzi ludzie, którym w tej chwili trudno jest na przykład, czy trudno będzie wrócić na jakieś dyskoteki czy jakieś koncerty organizowane. Na pewno coś się pozmienia. Ale ja wiem, czy na trwale to… To życie się na pewno trochę zmieniło. Może, jak nam coś zabrali, to docenimy bardziej jak to wróci. Może nie będziemy tacy roszczeniowi jako ludzie po prostu. Sam fakt, że można to stracić szybko, zwykle taka wiedza przychodzi z wiekiem. A może teraz przyszła taka wiedza z pandemią, że ktoś jakaś refleksję będzie miał na ten temat. Chociaż na pewno nie wszyscy. Ale może część osób coś z tego wyniesie dobrego. Oprócz tego, że nam ogranicza się pewne rzeczy. Dobrze. Moim zdaniem to nie jest tak źle.

**A któreś ograniczenia powinny zostać na dłużej, tak profilaktycznie?**

Ja wiem? Nie mam bladego pojęcia, jak tam jest z tymi… Cały czas nie mam takiego bardzo jasnego zdania na temat tych maseczek. Czy one w ogóle pomagają i w czym. Bo co tutaj można by było… Wie pani, dystans społeczny zachować w metrze na przykład? Nie wiem, jak się zachowuje taki dystans. Myślę, że te maseczki jednak w czymś pomagają. Może nie plujemy tak na siebie, czy nie wydychamy jednak bezpośrednio na kogoś. Myślę, że to nie zaszkodzi. Z drugiej strony, jeżeli nie wszyscy będą to robić, tylko to będzie dobrowolne, nie wiem, na ile one nas chronią. Albo czy one chronią naszych… Jeżeli my nosimy maseczkę, to czy chronimy siebie, czy chronimy innych. To jest podstawowe pytanie. Jeśli chronimy siebie, to je nośmy, ci, którzy chcą się ochronić. Ale jeżeli chronimy głównie innych, a nie siebie, no to myślę, że to powinno być obowiązkowe wtedy.

**Żeby miało sens.**

Tak, żeby to miało jakiś sens.

**Bo teraz chyba jest tak, że w sklepie to chyba się powinno mieć tą maseczkę, tak mi się wydaje.**

No oczywiście, że tak jest. Ale jak już rozmawiałyśmy, pierwsze zdanie brzmi inaczej tych wytycznych. W związku z powyższym tej maseczki po prostu nie będą nosić. Ja tutaj miałam, nie wiem, ktoś tutaj był u mnie, jakaś sąsiadka. I były dwie osoby i zdania były 3. Jak Hubert przyjechał z Magdą, czy to znaczy, że trzeba nosić te maseczki, czy że ich nie trzeba nosić i gdzie je trzeba nosić i kto to będzie sprawdzał, czy pani jest od kogoś pół metra czy metr czy półtora metra. Myślę, że w tych przestrzeniach publicznych jednak dalej powinniśmy je nosić, jeżeli uważamy, że oni pomagają. Jeżeli takie są wytyczne Światowej Organizacji Zdrowia, że powinniśmy je nosić do momentu, kiedy nie będzie dostępna szczepionka, która będzie chociażby łagodziła skutki tego wirusa. Bo on pewnie, jak to wirus, nie bardzo się da zrobić szczepionkę eliminującą. Chociaż może to nieprawda, bo ospa to też wirus chyba. A dało się wyeliminować. Ale może on nie mutuje akurat. No nieważne. Jeżeli będziemy mieli szczepienie i będziemy mogli się jakoś inaczej chronić, no to w porządku. Ale chociażby dla takiego… Ja bym chciała na przykład się dowiedzieć, czy ta maseczka mnie chroni i kiedy ona mnie chroni. Czy wtedy, kiedy ja ją noszę, czy wtedy, kiedy inni ją noszą (śmiech). I czy ona mnie chroni, jak ja ją zakładam piąty raz czy nie. Czy ona mnie chroni tylko jak jest nowa na przykład. Tego wszystkiego nie wiemy.

**A myśli pani, że powinno się jakoś szczególnie chronić tych starszych albo dzieci?**

No dzieci to chyba właśnie jak się okazuje nie, bo one są jak zwykle odporniejsze, mają młode organizmy i mają przeciwciała własne i nasze i w ogóle. A osoby starsze, no pewnie powinno się jakoś je chronić, bo na pewno są bardziej podatne na taką sytuację. Więc…

**A jak taka ochrona mogłaby wyglądać?**

Powinny być na pewno obowiązkowo szczepione, jak tylko taka szczepionka się pojawi. Bo do tej pory te szczepienia są dobrowolne. Myślę, że oni w ogóle powinni zostać objęci… Dobrowolne choć chyba bezpłatne dla osób starszych. Bo mi się wydaje, że w zakresie tej podstawowej opieki zdrowotnej szczepienia są dla osób starszych na przykład przeciwko grypie. Ja myślę, że powinno się ich wszystkich szczepić przeciwko grypie obowiązkowo. Bo część w ogóle nie wie, że może być szczepiona, część lekarzy nie informuje. Część informuje, ale oni a, to może oni się zaszczepią następnym razem albo coś tam. Myślę, że tutaj jednak takie regulacje zastępujące… No, jak ktoś zdeklarowanie nie chce i podpisze oświadczenie, że on absolutnie świadomy tego, że takie są konsekwencje czy takie są możliwości, że on wie, że ma bezpłatnie, ale nie chce pomimo tego, no to nie można go zmusić. Ale myślę, że dla 90% a może więcej, strzelam, ale dla większości starszych osób, to że coś jest obowiązkowe, to oni by się temu poddali. I by byli na pewno bezpieczniejsi. Czasami trzeba pomyśleć za kogoś. Tak jak podejmujemy decyzje za dzieci, które nie są w stanie ocenić pewnych rzeczy. Tak myślę, że powinniśmy podjąć też decyzje za osoby starsze, które dla odmiany albo są skostniałe takie w swoich poglądach albo po prostu nie są w stanie już, bo dzieci jeszcze, a oni już ocenić sensowności czy konsekwencji pewnych działań. Bo po prostu już nie są w stanie sobie wyobrazić tego, co to może się stać. Bo on, a, kiedyś tak było i przeżyłem, to i teraz przeżyję. To taki argument koronny tych starszych osób. No ale świat idzie naprzód. Kiedyś nie byłoby możliwe takie przenoszenie choroby, bo nie było lotów samolotem, nie było czegoś. Więc myślę, że powinniśmy jednak nawet w imię ograniczenia takiej swobody… No wprowadzenie czegoś obowiązkowego to jeszcze nie jest ograniczenie swobody moim zdaniem. Pod warunkiem, że jest prawo do tego, żeby się odwołać od takiej decyzji. Że istnieją przeciwskazania na przykład do szczepień. Bo na pewno też takie są przy niektórych chorobach. Ale te osoby powinny być świadome jednak ryzyka. Myślę, że być może teraz byłby czas na to, żeby taki obowiązek wprowadzić. Bo jednak ta sytuacja koronawirusa pokazała, że to jest realne niebezpieczeństwo. Że ten koronawirus to jest taki trochę jak grypa. No oczywiście dużo groźniejszy, ale że grypa też potrafi zabić. Więc może się jednak zaszczepmy i zróbmy to obowiązkowo, a nie bezpłatnie, tylko dobrowolnie.

**Czyli można by wprowadzić na przykład to, że jednak dla osób starszych ta szczepionka jest, że tak powiem, rekomendowana, obowiązkowa.**

Obowiązkowa. Nie, wie pani, rekomendowana to ona jest i teraz.

**Obowiązkowa, dobra.**

Obowiązkowa to oznacza to, że lekarz pierwszego kontaktu czy też ostatniego kontaktu, musiałby powiedzieć: proszę pani, trzeba się zaszczepić, tu jest obowiązkowe szczepienie dla pani, bezpłatne, szczepimy. I nie proponujemy jej szczepienie, tylko ją informujemy, tak jak jej wypisujemy receptę, tak ją szczepimy. I jest siostra w każdym tym POZ i szczepi.

**A takie na przykład ograniczenie, że mówimy, słuchajcie, idzie ta druga fala. Ale już nie decydujemy się na takie totalne zamknięcie gospodarki, tylko na przykład osoby starsze izolujemy.**

No jak by szła rzeczywiście, no to może powinniśmy ich ochronić. Myślę, że musimy reagować w miarę sytuacji. Jak będzie szła druga fala taka, jak szła pierwsza, no to zamykajmy. Ochrońmy ich, skoro oni są bardziej podatni, a gorzej to przechodzą, to ich ochrońmy.

**Ale powinno to być prawnie rozwiązane, że oni po prostu są w domach. Czy coś takiego? Czy my po prostu im mówimy, słuchajcie, dla waszego bezpieczeństwa musicie zostać w domu.**

Nie, no myślę, że mówimy im. Bo teraz też im tak mówimy. To nie jest tak, że ich ktoś może aresztować na ulicy. Chyba, że już mamy tą pandemię i wtedy znowu wprowadzamy stan jakiś tam, wyjątkowy, nadzwyczajny czy któryś z nich. I znowu wykonujemy jakieś ruchy takie bardziej nerwowe. Oczywiście możemy im rekomendować zostanie w domu, a możemy ich też obowiązkowo w tym domu posadzić. Ale to jakby, no myślę, że to zależy od jakiejś takiej bardziej globalnej polityki. Nie możemy sobie sami ich tam posadzić w domu. Sobie sami to możemy im powiedzieć, wiecie co… A to jest nieludzkie, powiedzenie im, nie wychodźcie, nie spotykajcie się czy coś. Bo dlaczego niby? Możemy im powiedzieć, słuchajcie, to jest ryzykowne, więc powinniście nosić maseczki jednak, zwracać uwagę, nie stać komuś na głowie w sklepie i coś. Powinniśmy im przypominać też, bo myślę, że… Są takie reklamy, nawet ja je widuję, takie w sprawie właśnie tam myjemy ręce, jak dotknęliśmy, to nie dotykamy nosa i ust i coś. To myślę, że cały czas to powinno zostać. Bo to jest taka informacja, możemy też się uchronić przed innymi chorobami. Przed zwykłym przeziębieniem, które oni też gorzej znoszą.

**A na przykład to mierzenie temperatury na przykład u tego fryzjera? Czy teraz jak na lotniskach będą mierzyć temperaturę. Albo jak pani będzie szła do przychodni, to będą mierzyć temperaturę przy wejściu. Co pani o tym sądzi?**

Myślę, że to są ruchy takie trochę pozorowane. Bo jak ja idę do przychodni, no to idę, bo się źle czuję, tak? Bo coś potrzebuję. Oprócz sytuacji, kiedy potrzebuję receptę albo idę do psychiatry albo do okulisty, kiedy nie muszę mieć… Kiedy jak mam temperaturę, to może zarażę okulistę oraz wszystkich jego pacjentów. No to jak idę do pierwszego kontaktu, no to zwykle dlatego, że jestem chora. Mam temperaturę i co? Posadzą mnie gdzie indziej, tak?

**Nie wiem, tego właśnie nie wiem. Wiem, że teraz jest tak, że przy wejściu do przychodni jest mierzona temperatura.**

No to rozumiem, że jak ktoś ją ma, no to musi być jakaś konsekwencja tego. Że jak ktoś ma, to co? To idzie do domu? To by było głupie, bo zwykle idziemy do lekarza, bo mamy temperaturę i źle się czujemy. Też jest to jakaś duża grupa klientów przychodni.

**A na przykład te odległości? W kinie teraz co któreś miejsce będzie. Albo w samolocie chcą wprowadzić, że nie wszystkie miejsca, tylko co któreś, takie rozwiązanie.**

No wie pani co, no pod warunkiem, że wprowadzimy je również w kolejce do wejścia do samolotu oraz przy taśmie z bagażami. Bo możemy się nie spotkać w samolocie ale poczekać na bagaż… Nie wiem, albo maseczki. Trudno mi powiedzieć. Myślę, że ten dystans jest ważny. Bo na pewno te wirusy się nie transmitują na duże odległości. Więc tak jak ta Organizacja Zdrowia na początku czy w trakcie tej pandemii, mówili jej pracownicy czy jakieś osoby medialne z tej Organizacji, że na przykład w krajach afrykańskich, oni się tak bardzo tego wirusa nie boją, ponieważ tam dystans społeczny jest czymś, co występuje od lat. Ponieważ oni mają te różne choroby zakaźne, czy ebolę czy gorączki krwotoczne i to im się zdarza raz na jakiś czas, w związku z powyższym oni są przyzwyczajeni do dystansu społecznego. I tam jakby nie było sytuacji krytycznych. Bo na początku myśleliśmy, że te kraje, powiedziałabym, mniej rozwinięte, są w gorszej sytuacji. A okazuje się, że one są w lepszej sytuacji, bo one już takie przechodziły czy przechodzą na bieżąco taki obostrzenia w zachowaniu. W związku z powyższym są do tego przyzwyczajone. Więc myślę, że uczmy się, wprowadźmy taki dystans społeczny, bo to nas nie kosztuje. Wiadomo, że przy stole w domu usiądziemy z naszymi bliskimi razem. Ale w tym sklepie, tam gdzie się spotykamy, tam w ogóle nie kontrolujemy kto z kim i gdzie był. A nawet, jak byśmy go zapytali, to nie wiadomo, czy on wie. A jak wie, to co nam to da. Więc myślę, że są takie rzeczy, które niewiele kosztują nas wyrzeczeń. A które może dobrze by było wprowadzić. Nie widzieliśmy takiego powodu wcześniej, ale może taki teraz powód się pojawił. I to może całkiem dobrze zaskutkować na przykład właśnie nieprzenoszeniem się innych wirusów. Przecież te przeziębienia, które… Wiemy o tym, jak się przenosi przeziębienie. Jedna osoba jest zakichana, to za 2 dni pół biura ma katar. A za 3 dni ich rodziny mają katary. No i dopóki to jest katar i raz na jakiś czas któraś z tych osób zachoruje na jakieś tam zapalenie oskrzeli powikłane po tym przeziębieniu, no to jeszcze jest OK. Ale jak to jest koronawirus? No to może jak będziemy taki dystans społeczny trzymać, no to może będzie całkiem niegłupio. Może mniej będziemy chorować też na inne choroby.

**Dość dużo w mediach się mówi o tym, że na jesień może być coś. Nie wiadomo do końca czy będzie, ale może być. Jak wtedy powinniśmy zareagować, jak państwo, rząd, społeczeństwo?**

Nie wiem. Zależy pewnie od skali tego zjawiska. Jeżeli jesteśmy w stanie się znowu jakoś tam… Przede wszystkim będziemy wiedzieć, co nas czeka. Jednak to już nie będzie takie macanie w ciemnościach zupełnych, tylko będziemy wiedzieli, jaka jest skala, co daje izolacja. Myślę, że do rozważenia. Jeśli uda się na przykład nie dopuścić do takiego roznoszenia się tego wirusa. Jeżeli będzie szansa na jakieś rozwiązania w postaci szczepionek czy wizja będzie jakaś bliska, to być może trzeba się będzie jeszcze raz poddać takiej kwarantannie, w sensie kontaktów z wieloma osobami czy między krajami. Myślę, że w takiej skali jak się nie zaczniemy, to mamy raptem 2 czy 3 miesiące wakacji, czy tego lata, nazwijmy to, czy ciepłych miesięcy. Gdzie to się będzie, jak rozumiem, gdyby się miał realizować taki scenariusz, że to ze zmianą pogody, że te wirusy się będą lepiej roznosić. No to myślę, że jest dopuszczalny jakiś jednak rodzaj czy stopień odizolowania jednych od drugich, żeby się znowu nie powtórzyło… Ale będzie nam na pewno łatwiej. Łatwiej i to przyjąć, łatwiej wprowadzić, łatwiej jakoś stopniowo jakieś może ograniczenia. Nawet może nie żeby zapobiec w ogóle, tylko żeby ograniczyć rozwój tej choroby. Ale już mamy przetarte szlaki. Już coś będziemy wiedzieć, czy coś działa, co nie działa.

**Ale to znaczy, że znowu zrobić tą pracę zdalną, zamknięcie szkół?**

Nie wiem, jaka będzie skala zjawiska. Praca zdalna myślę, że w wielu miejscach jest prowadzona w dalszym ciągu, pomimo tego, że nie ma takiej potrzeby. Nagle się okazało, te biura muszą ruszyć, jeżeli będą ruszały w jakimś tam reżimie sanitarnym, to trzeba będzie rozrzedzić tych pracowników. No to część może zostanie w domach. Może to zmniejszy koszty jakieś. Może to nie jest takie głupie. Może się przekonamy nagle, że są rzeczy, które można zrobić, choć wcześniej myśleliśmy że nie można. Że musimy tego pracownika kontrolować i go widzieć. A teraz może jak on ma laptop firmowy, może nie musimy go widzieć, bo rejestrujemy, kiedy on się loguje a kiedy przestaje. I na pewno można sprawdzić ile czasu na przykład nic nie robi, nie dotyka klawiatury. Bo to na pewno można monitorować i zawsze tak było, zawsze można było. Więc może niektóre rzeczy po prostu, nie musimy ich rozkręcić na… No tam, gdzie nie można pracować online, w stolarni albo kopalni, w miejscach, gdzie produkujemy, no to pracujemy normalnie. A tam, gdzie możemy się ograniczyć, no to może to jest i dobry pomysł.

**A co na przykład ze szkołami wtedy? Bo też jednak to są skupiska, dużo tych ludzi w jednym miejscu.**

No jest dużo. Zwłaszcza, że jakby taka szkoła pociąga za sobą też i rodziny, mobilizację osób, które odbierają te dzieci i coś tam. No wie pani co, to wszystko moim zdaniem zależy od skali. Od tego, czy to wróci i w jakiej sile to wróci. No nie wiem, jakie są dane. Na pewno można przeanalizować dane, które dotychczas spłynęły, jeśli chodzi o zakażenia właśnie czy o zarażenia tym wirusem dzieci, o to, jak one przechodzą. Można zrobić na przykład może testy osób, które mają przeciwciała. Bo myślę, że można by było to wykonać. Te osoby, które mają przeciwciała, czy jeszcze raz będą chorować czy nie. Nie wiem, czy oni to wiedzą. My tego nie wiemy na pewno.

**A tak z perspektywy całego tego okresu, od momentu, kiedy to się wszystko zaczęło, jakie dla pani były takie najważniejsze, przełomowe wydarzenia, takie w trakcie trwania, które coś zmieniały? Albo u pani albo w perspektywie szerszej.**

Z mojej perspektywy najbardziej odczułam taką izolację fizyczną od rodziny na początku oraz od znajomych czy od tej grupy seniorów, którymi się zajmuję. To jest coś takiego, co najbardziej odczułam no i częściowo nadal odczuwam. Bo to jeszcze nie zostało zniesione. No i nie widuję się ze znajomymi. Takie ograniczenie fizycznych kontaktów z ludźmi.

**A teraz nie można ze znajomymi?**

No powoli można, no już dzieci przyjechały, pojechałam do fryzjera, więc to się uruchamia powoli. Ale na pewno to było, jeśli pani pyta o coś takiego, co najbardziej…

**To ta izolacja fizyczna.**

Tak, to na pewno to. No i co? I myślę, że może nieuzasadniony a może na szczęście nieuzasadniona oczywiście taka obawa przed skutkami ekonomicznymi. Być może uda nam się jakby zminimalizować te zapowiedzi. Mam nadzieję cały czas, że ekonomicznie też jest… Byłam zaniepokojona tym, jak to się będzie rozwijać, jak gospodarka globalnie, w ogóle jak sobie poradzi z taką sytuacją izolacji i wyciszenia. No może nie będzie aż tak źle. Może te 2 miesiące czy 3 to nie jest taka katastrofa straszna, jak się spodziewaliśmy.

**Ale tak w skali kraju?**

No tak, w skali kraju, nawet może w skali bardziej globalnej, w skali światowej.

**Że to jednak może nie będzie aż tak źle?**

Może nie będzie aż tak źle, tak. To była taka jedna z większych obaw moich, taki powód do zmartwień.

**Coś jeszcze? Bo perspektywa się zmienia w miarę, jak się zmienia sytuacja. I pewne rzeczy wydawały nam się na początku takie ważne, to przestają być ważne, a czasami niektóre zyskują tą ważność?**

Nie chyba nie.

**Dziękuję bardzo.**
