# Supplementary material for: The challenges arising from the COVID-19 pandemic and the way people deal with them. A qualitative longitudinal study
Source: PLoS One. 2021 Oct 11;16(10):e0258133. doi: 10.1371/journal.pone.0258133 (PMC8504766; doi:10.1371/journal.pone.0258133)
Supplement: S1 Dataset — (ZIP) [file pone.0258133.s003.zip › Transcriptions/stage 5/20.5_F_25_couple, with child.docx]

**20.5_F_25_copule with child**

**Co działo się przez ostatni miesiąc?**

Wszystko było normalnie. Tak, jak do tej pory. Miałam dużo więcej zajęć z racji tego, że ruszyły korepetycje i wszystko inne. Trochę więcej zamieszania, maturzyści, których przygotowywałam, piszą już w przyszłym tygodniu. Więc więcej pracy, a tak, chyba nic szczególnego się nie zmieniło. Widzieliśmy się też wreszcie z rodziną Michała - męża, to było tydzień temu, odwiedziliśmy naszego chrześniaka. W końcu go zobaczyliśmy. A tak, nic szczególnego.

**Przełomowe etapy, ważne sytuacje**

Kiedy zadzwoniła siostra Michała i nas wreszcie zaprosiła, żeby zobaczyć małego. On był miesiąc w szpitalu, potem jakieś półtora miesiąca w domu. Ale już chyba nadszedł ten czas, że można się spotykać, to znaczy ona chyba tak uznała, że to już. Więc byliśmy w odwiedzinach.

**Kiedy chrześniak był w domu, jego mama nie chciała, żebyście ich odwiedzali ze względu na koronawirusa, czy chodziło o coś innego?**

Ze względu na koronawirusa. A teraz już chyba uznała, że już można. Nie będę tego bliżej komentowała <z przekąsem>. W każdym razie dostaliśmy zaproszenie, pojechaliśmy. Był moment przełomowy, ona uznała chyba, że koronawirusa już nie ma. Albo nie wiem. Parę dni później mój teść miał urodziny i też cała rodzina została zaproszona na to wydarzenie i wszyscy się widzieliśmy.

**Na ile twoje życie wróciło do stanu sprzed pandemii?**

Trochę to wróciło do normy, byłam już nawet w restauracji raz. Też około tydzień-półtora tygodnia temu. To było w weekend. Trochę szkoda, że... To znaczy "szkoda" - ja wiem, dlaczego te placówki są jeszcze zamknięte - ale nie wychodzę z dzieckiem na place zabaw, czy te zamknięte place zabaw, w pomieszczeniach - chodzi mi o te wszystkie hula parki z kulkami, zjeżdżalniami. Ona bardzo woła i bardzo chce, bo kiedyś jeździliśmy bardzo często. Więc jedynie tego nie robię. No, na basen nie chodzimy - wiadomo, jest zamknięty. Ale większość rzeczy już robimy. Fajne jest, że można się już spotykać, że mogę pojechać do teściów, Helenka może widzieć się z braćmi i siostrami. Jest już ok.

**Kto/ co wpływa na to, że życie wróciło do normy i jest już "OK"?**

Wydaje mi się, że ja cały czas zachowywałam się dość normalnie. Jeżeli ktoś chciał się ze mną zobaczyć - mieliśmy taką jedną dziewczynkę z rodziny Michała, której mama miała podejście takie, jak ja - to my się widywałyśmy. Ona pozwalała córce bawić się z Helenką, nie widziała w tym żadnego problemu - bo skoro ani jedna, ani druga nie chodzą do przedszkola, tylko cały czas siedzą w domu, to normalnie się widywaliśmy. Ale z resztą rodziny mojego męża się nie widywaliśmy. Oni uznawali, że koronawirus, nie, i koniec kropka. Tydzień temu, czy dwa, oni chyba uznali, że koronawirusa już nie ma, choć ja nie widzę zbytniej różnicy - bo jest nadal i myślę, że będzie już z nami do końca. Ale już słyszałam jakieś pogłoski na tych urodzinach teścia, że komunię im przełożyli na wrzesień. Mieliśmy mieć komunię i tam właśnie jeden chłopiec ma ją przełożoną na wrzesień. No, ale ona nie wie, jak to będzie, bo pewnie będzie już druga fala koronawirusa, także... no.

**Jak myślisz, co wpłynęło na to, że oni stwierdzili, że koronawirusa nie ma?**

Nie mam pojęcia właśnie. Dla mnie to jest głupota, bo on w ogóle nie minął. Ja nie widzę różnicy między tym, co było tydzień temu, dwa tygodnie temu, albo tym, co jest dzisiaj. Dla mnie nie ma żadnej różnicy. Jest tak samo, tak? Rozumiem, na początku, pierwsze dwa tygodnie, kiedy ludzie wracali do domów, kiedy nie było wiadomo, kto jest chory i tak dalej. Ta dwutygodniowa kwarantanna jak najbardziej była w porządku i byłam za tym, sama się do tego stosowałam. Po prostu, żeby nie zarażać innych osób. Ale jeżeli minęły te dwa tygodnie - my byliśmy zdrowi i ktoś inny też, to ja nie widziałam problemu w tym, żeby się spotkać. No, ale każdy się jeszcze bał. Ja nie wiem, co się zmieniło przez te dwa miesiące. Ten koronawirus, jeżeli był, a myślę, że był i jest, no nie ma informacji żadnych takich, że on zniknął. Więc nie wiem, co skłania ludzi do takich zmian. Być może zmęczenie, być może im się już nie chce. Nie mam bladego pojęcia. Stosowałam się do obostrzeń, poza tym, wiele miejsc było zamkniętych, więc nawet, gdybym chciała, nie mogłabym z nich korzystać. Powtarzałam, że trzeba zachować ostrożność, trzeba uważać, ale dla mnie to była paranoja, o czym już wspominałam, że była otwarta Castorama i można było tam wejść, w środku było ze 200 osób, a nie można było się spotkać z rodziną. Ludzie wchodzili do sklepów, do Lidla, Biedronki, gdzie też było kilkadziesiąt osób w środku, każdy macał, dotykał produktów. Ja równie dobrze mogę się zarazić będąc na poczcie czy w sklepie. Dla mnie to była paranoja, że najbliższe osoby się od siebie odcięły, gdzie każdy się zna, wie, co robi ktoś inny - kto siedzi w domu, nie chodzi do pracy, kto z kim ma kontakt. Najbliżsi się od siebie odcięli, a byli tacy, jak moja bratowa, która wtenczas szła robić zęby innym ludziom. Dla mnie to jest hipokryzja. To taki zawód, gdzie ona na spokojnie mogła zrezygnować. A z nami się nie widywała - bo nie, bo koronawirus - a szła do pracy i robiła zęby obcemu człowiekowi, gdzie to jest kontakt ze śliną, ze wszystkim. I tym bardziej nie zna tego człowieka, to jest zwykły klient. Dużo było takich sytuacji dziwnych. Bo dla mnie jeśli ktoś się bał i dał się zastraszyć, a to było akurat bardzo łatwe do zrobienia, bo media huczały, itd., więc ludzie byli zastraszeni naprawdę na wysokim poziomie, to jak ktoś opowiada się za czymś, to powinien się do tego stosować. Czyli jeśli boję się, bo jest koronawirus i będę chroniła swoją rodzinę, to siedzę w domu za przeproszeniem na tyłku i nie widuję się z nikim. A nie jakaś taka selekcja i to jeszcze durna selekcja, bo ktoś wybiera sobie obce osoby, a nie na przykład moje dziecko, Helenkę, które siedzi w domu i nie chodziła do tej pory w ogóle do przedszkola. No to dla mnie to głupota.

**Co wpłynęło na to, że spotkania z rodziną wróciły?**

Dla mnie to jest głupie myślenie i durne, to w ogóle się nie trzyma kupy. Koronawirus był, jest i pewnie będzie. Pewnie nie pozbędziemy się go tak samo jak chorowania na katar i wszystkie inne rzeczy. Trzeba w tym jakoś normalnie funkcjonować. Myślę, że spotkania wróciły może dlatego, że rząd zaczął znosić te obostrzenia? Zakomunikował całej Polsce, że zaczynają się otwierać restauracje na przykład. To aha, skoro tak, to może mogę zacząć spotykać się ze znajomymi. Choć dla mnie to myślenie jest takie... No.

**Rozumiem, że widzisz w tym niekonsekwencję...**

No tak, niekonsekwencję właśnie. Bo rząd - no ja mam taką nadzieję, że po to - pozamykał to wszystko, żeby zahamować rozprzestrzenianie się tego wirusa. Im mniej będzie tych wszystkich spotkań, koncertów... Oczywiście się z tym zgadzam. Ale teraz otwierają to nie z tego powodu, że koronawirus zniknął, a dlatego, że gospodarka była zamrożona przez trzy miesiące. I trzeba nagle, może nie nagle. Trzeba po prostu nauczyć się z tym żyć i funkcjonować. Bo upadłaby cała gospodarka, poza tym, ludzie nie zarabialiby pieniędzy. Poza tym nie można żyć zamkniętym w domu przez ile - rok? Dwa? Dziesięć? Osiemnaście lat? Dla mnie to jest bez sensu. Oczywiście ten pierwszy czas jest dla mnie zrozumiały, ale wirus nie zniknął, ciągle mamy nowe zachorowania, dane podają.

**Mówiłaś, że byłaś w restauracji, widziałaś się ze znajomymi. Czym to jest spowodowane, że już robisz te rzeczy?**

To nie była moja inicjatywa. Mój mąż ma bardzo dużą rodzinę. Jest w tej rodzinie dziewczynka starsza od Helenki o rok i jej mama od początku mówiła, że nie ma problemu, że dziewczynki mogą się spotykać, pobawić. Spotykaliśmy się tylko z nimi. Ode mnie z rodziny nie widziałam się z nikim, zaczęli sami do mnie przyjeżdżać. Raczej znali moje zdanie na ten temat, nie chcieli do mnie wcześniej przyjeżdżać, więc ja w to nie wchodziłam. Powiedziałam, że rozumiem, w porządku i tak dalej. Ale przyszedł czas, że ktoś tam do mnie napisał, czy może wpaść na kawę. No ok, wpadnij. To było tak jakby z ich inicjatywy, a ja nie miałam nic przeciwko, bo skoro widzę, że ktoś siedzi miesiąc w domu, nie pracuje, nigdzie nie wychodzi, jest zdrowy, a ja tak samo, to raczej nie widzę przeciwwskazań do spotkania się. Tak samo jak z tym przyszłym chrześniakiem mojego męża - jeszcze nie było chrzcin. Nie można było do nich przyjeżdżać, przyjeżdżał do nas tylko teść i on był zły na całą tą sytuację. Ta córka mieszka z nim i on mówił, że nie wyobraża sobie sytuacji gdzie wnuczka - Helenka - nie może do niego przyjechać. Więc ja powiedziałam wtedy - tato, ja na siłę tam nie przyjadę i nie będę robiła z siebie debila, jeśli jestem tam niemile widziana. Jak miałabym się tam zachować? Tato tylko kiwał głową. Aż w końcu, półtora tygodnia temu zadzwoniła ta siostra Michała i zapytała, czy moglibyśmy wreszcie odwiedzić tego Olusia. Więc pojechałam, nie komentowałam, ok.

**Czy podczas tych spotkań lub w pracy, widzisz jakieś różnice w zachowaniach ludzi, tym, co wydaje się dla nich ważne?**

O boże, mnie to właśnie drażni. Rozmawiamy o głupotach, a nagle ktoś zacznie mówić o tej pandemii. To jest już nudne, rozmawiamy o tym od stycznia, tak? Bo w styczniu wydarzyło się to w Chinach, to znaczy dostaliśmy o tym informację w styczniu, czy tam w grudniu. To już jest po prostu nudne i denerwuje mnie, że ciągle jest klepane jedno i to samo i bardzo mnie to drażni, bo tak jak mówię, trzeba zacząć żyć normalnie i normalnie się zachowywać, a nie ciągle ten koronawirus i koronawirus. Ale no nie, chyba nic więcej się nie zmieniło. Ja udzielam korepetycji. Te spotkania wyglądają tak samo, jak wyglądały wcześniej. Mój mąż mówił, że nadal nie wchodzi na sklepy, tylko zostawia towar przed sklepami, a osoby pracujące na sklepie zabierają towar. Tak, to chyba tyle.

**Był czas, kiedy udzielałaś korepetycji online?**

Nie, online nikt nie chciał się spotykać. Tylko tyle, że te młodsze dzieci wysyłały mi prace domowe, żeby je odrabiać, bo rodzice nie nadążali z nauczaniem online. A z maturzystami po prostu się nie spotykałam i te spotkania wróciły od jakichś dwóch tygodni. Lekcji online nie było, bo domyślasz się, jakie podejście mają rodzice. Odrobić im pracę domową i tyle, nic więcej. Nie myślą o tym, by dziecko opanowało dany materiał, tylko aby mieć spokój od nauczycieli. Więc ja się nie wtrącam, każdy ma jakieś tam zdanie na ten temat. Ja proponowałam lekcje online, powiedziałam, że dla mnie to bez sensu jest odrabianie pracy domowej, wysłanie jej i tyle, bo to dziecko będzie miało teraz półroczne zaległości. Ono nic nie rozumie. Załóżmy, że poruszali trzy działy w ciągu tego ostatniego okresu. Ale skoro rodzice chcą po prostu odbębnić.. później będą płakać. Ale ja się nie wtrącam, bo musiałabym się ze wszystkimi kłócić. Rozmawiałam ostatnio online z koleżanką, która też udziela korepetycji. Ona mówi, że ma o wiele więcej uczniów. Mówi, że to tragedia, bo wszyscy codziennie wysyłają jej tylko i wyłącznie prace domowe. No więc paranoja - widać, że nie tylko ja miałam taką sytuację, a wszyscy chcieli tylko pooddawać, powysyłać gotowe prace i nic więcej.

**Czy jest coś, co ciągle przeszkadza ci w tej całej sytuacji? Albo może pojawiło się coś nowego?**

Nie, całe szczęście, znieśli już te maseczki na zewnątrz, co było też totalną paranoją, bo tak, jak mówię, wejść do sklepu w maseczce, ok, zrobić zakupy, wchodzimy do jakiegoś zamkniętego pomieszczenia, jest mało miejsca itd., no ale jak widziałam tych ludzi, którzy jeżdżą na rowerze w maseczkach, no to, no paranoja. Ostatnio nawet czytałam artykuł lekarza, że to przeokropnie wpłynie na nasze zdrowie, to, że my podczas wysiłku hamujemy sobie rozwój płuc, wydolność oddechową, itd. Tym bardziej, młodzi ludzie może jeszcze nie tak bardzo, ale starsze osoby, które mają problem z wejściem po schodach, a jeszcze muszą wchodzić z maseczką na buzi. To dla mnie było w ogóle bez sensu. Więc fajnie, że na ulicy można było poruszać się bez tego. Chociaż ja tutaj, u siebie na wsi, nie zakładałam maseczki. Ale jeśli musiałam załatwić coś w Radomiu, musiałam maseczkę zakładać. Ale była też sytuacja, że stałam w kolejce do salonu Orange. Nastałam się półtora godziny i półtora godziny musiałam spędzić w tej maseczce. To była tragedia. Ale to tyle chyba. Może przez to, że u mnie w domu rodzice też podeszli do tego w miarę racjonalnie, a może normalnie, choć może ktoś powie, że właśnie nieracjonalne. Oprócz tych pierwszych dwóch tygodni, staraliśmy się żyć normalnie.

**Pomyśl o ważnych momentach, które miały miejsce w ciągu ostatniego miesiąca. Jakie towarzyszyły ci wtedy emocje?**

Jak otworzyli lokale, oczywiście czułam się bardzo szczęśliwa. Bardzo, ale to bardzo. Z tego względu, ze jak już mówiłam, lubię chodzić do restauracji, a jeszcze otworzyli tę restaurację, którą najbardziej lubię w Radomiu. Akurat przyjechała jeszcze moja koleżanka z Łodzi, więc pojechaliśmy w czwórkę, jak normalni ludzie, i zjedliśmy sobie pyszną kolację. Więc było super. To był taki moment, w którym zaczęłam sobie zdawać sprawę, że jest normalnie. Nigdzie więcej nie wychodziłam... A, ostatnio był dzień dziecka, więc byłam z córką na lodach, na Żeromskiego - to jest taki długi deptak na starym mieście. Jak zobaczyłam te wszystkie dzieci, które biegały, wściekały się, bawiły na rowerach, wrotkach - pomyślałam, że wreszcie jest normalnie. I te dzieciaki były takie zadowolone, a jeszcze była ładna pogoda. Co do spotkań rodzinnych, nie jechałam na nie z radością, bo do każdego miałam w głębi duszy jakieś pretensje. Więc te spotkania nie były raczej takie super, wow, nie widziałyśmy się tyle, co u ciebie, kopę lat. Tylko raczej takie sztywne. Każdy wiedział, co zrobił, no bo sporo było takich sytuacji, przez które powinnam się teraz chyba nie odzywać do końca życia. Może przytoczę ci jedną, żeby było wiadomo, o co chodzi. Jest ta bratowa, która robi zęby. I ona robiła swojemu dziecku urodziny. Szymonowi, który właśnie miał mieć teraz tę komunię. I to jest Helenki ulubiony brat. Przeokropnie za nim tęskni, nawet świnki u nas na wsi nazwała jego imieniem. Codziennie do nich dzwoniliśmy. W urodziny złożyliśmy życzenia, a u nas zawsze wszystkie imprezy odbywają się rodzinnie. My nawet w weekendy jesteśmy całą rodziną. Więc myślałam, że pewnie nie robią urodzin. Co się okazało, wygadał się teść parę dni później, że urodziny jednak były. Byli oni - no ok, mieszkają razem. Był jeden z braci Michała, więc znów kompletnie z dupy - za przeproszeniem. Bo Michał ma pięciu braci, a był tylko jeden z nich. Plus ona zaprosiła sobie dwie koleżanki z dziećmi. No więc to jest taka właśnie konsekwencja. Wkurzyłam się bardzo, bardzo się zdenerwowałam, było mi ogromnie przykro. Najgorzej denerwuje mnie paranoja wśród ludzi. Teraz spotkaliśmy się na urodzinach teścia, ona tłumaczyła się, że z tą koleżanką utrzymywała jakiś tam kontakt i tak dalej. No to ja się pytam, jaki był problem utrzymywania kontaktu z nami. Jej tłumaczenia i to, że ona stwierdziła, że mniejszymi intruzami są dzieci znajomych, a nie jego siostra, z którą on od samego początku utrzymywał bliski kontakt, no to tragedia no. Teraz wypadałoby w ogóle o tym zapomnieć i nie wspominać, bo przecież była kwarantanna, ludzie kierują się różnymi emocjami, różnych rzeczy słuchają. Jedni myślą tak, a inni inaczej. Ale dla mnie to zawsze trzeba być człowiekiem i tyle. A to było dla mnie może nie to, że wredne, ale po prostu... No nie, nie będę mówić więcej na ten temat, bo się wkurzę. Więc kiedy widzę się z taką osobom, nie mam ochoty rozmawiać z nią, co się u niej działo przez ostatnie dwa miesiące. Więc te spotkania były po prostu sztywne i tyle. Ale sam fakt, że te osoby - bo tam było parę takich sytuacji - same przychodziły i chciały się jakoś na siłę tłumaczyć, myślę, że parę dni później, albo teraz zrozumiały, co zrobiły i jak to było odbierane. Ale no.

**Obrazki**

Pierwsze wyjście z koleżanką - 4. Ta moja koleżanka, z którą bardzo się lubimy, rzadko przyjeżdża do domu, z racji, że dopiero skończyła szkołę i ma teraz rok stażu jako lekarz. Na jej nieszczęście, a moje szczęście, złamała rękę i nie mogła pracować, więc zjechała do domu. Przyjechał jej chłopak, spotkaliśmy się razem, było fajnie. Bardzo lubię takie spotkania, do tego ta restauracja, którą otworzyli, jest też jej ulubioną restauracją. Zawsze tam się spotykamy. Było miło, bardzo sympatycznie, super.

Urodziny brata Helenki - 9. Tutaj jest to tornado, piorun. Takie emocje mi towarzyszyły, bardzo się zdenerwowałam. Dodatkowo było mi przykro, może bardziej ze względu na moją córkę. Bo ja to tam, czy ktoś mnie zaprosi, czy nie, to robi jak uważa, ale szkoda mi było dziecka. Że przez dwa miesiące codziennie dzwonimy na kamerce, tęskni za nim przeokropnie, a ktoś sobie stwierdził, że mniejszym intruzem będą dzieci znajomych.

Urodziny teścia - 15. Rozprysk fali, wody, z tego względu, że sporo spraw... Ktoś chciał wyjaśniać sporo spraw. W takim sensie, że tłumaczyć się, itd. Może nie spotkanie żywiołów, już tak poetycko nie będę mówić, ale myślę, że wiesz, o co mi chodzi. To były spotkania różnych poglądów, różnego myślenia, różnego przedstawiania sytuacji, które wydarzyły się przez ostatni czas.

**Jak powinien wyglądać obrazek, który opisywałby to, jak czujesz się w ciągu ostatnich kilku dni?**

Nie wiem, jak mógłby wyglądać taki obrazek. Ale ciągle katuję już męża, żebyśmy gdzieś pojechali. Może na ten weekend, może z dzieckiem, może bez. Bo baardzo chce mi się już wyjechać, ja też lubiłam wyjeżdżać, mieliśmy zaplanowanych kilka wyjazdów od lutego do teraz, ale one się niestety nie odbyły. Więc już wczoraj siedziałam na telefonie i szukałam czegoś, żeby przynajmniej na dwie noce pojechać i trochę odpocząć, zmienić klimat. Więc nie wiem, jaki to miałby być obrazek, ale ciągle myślę o tym, żeby gdzieś pojechać.

**Rozumiem, że nie czujesz się obecnie zagrożona sytuacją?**

No nie. Z każdym tygodniem czułam się coraz mniej zagrożona. U moich najbliższych, z którymi mieszkam wszystko jest naprawdę tak, jak było. Nie wiem, dlaczego, ale gdziekolwiek pojedziemy, każdy rozmawia o koronawirusie, a u nas się o tych rzeczach nie rozmawiało. Odkąd rodzice z babcią wrócili do domu z działki, życie wróciło do normalności. Ten koronawirus był pobocznym aspektem, na zasadzie przypominania mi, żebym wychodząc nie zapomniała rękawiczek, maseczki. To było tyle. My nie rozmawialiśmy o tym, że się boimy, jakie mogą być zagrożenia, co się może wydarzyć. Na początku tak, dlatego też rodzice i babcia się wyprowadzili. Ale teraz od dłuższego czasu jest normalnie. Nie widzę u nas w domu żadnych zachowań, które byłyby sprzeczne z tymi zachowaniami, które mąż, rodzice, czy babcia prezentowali wcześniej.

**Pamiętam, że u ciebie niewiele zmieniło się w kwestii zakupów, robiłaś je dość często w małych, lokalnych sklepach. To się utrzymało?**

Tak, to się utrzymało. Z tym, że jak ostatnio miałam trochę więcej na głowie - teraz miałam jeszcze egzaminy u siebie w szkole, więc teraz już bardzo dawno nie byłam na zakupach, przejęła to mama. Ja wychodzę rano, jadę na egzamin albo do pracy, mam jakieś korepetycje. Więc teraz zakupy robi mama, ale tak, jak robiliśmy je wcześniej, tu nic się nie zmieniło.

**Byłaś może w ostatnim czasie w galerii handlowej?**

Byłam. Najpierw byłam 1 czerwca z Helenką, żeby wybrała obie prezenty, a później byłam w piątek [jest środa], żeby kupić prezent dla teścia na te urodziny, o których mówiłam. Ogólnie jest pusto. Co innego jest, jak zobaczy się ludzi w maseczkach na zewnątrz. Ale jak się tam wejdzie, jest dużo osób w maseczkach i to ogólnie rzecz biorąc wygląda przerażająco. W każdym sklepie każą dezynfekować ręce, albo stoi jakaś osoba, która sama dezynfekuje - nie daje możliwości wyboru, czy mi się chce zdezynfekować ręce, czy nie. Na przykład w TK Maxxie tak jest. Na bramce stoi jedna osoba - oni się tam wymieniają, bo jak wchodziłam, była dziewczyna, a jak wychodziłam, chłopak - i trzeba pokazać ręce i oni psikają jakiś tam płyn, dezynfekują ręce. Jest sporo ludzi w galeriach. Jak na początku ktoś ode mnie był, to mówił, że jest bardzo mało osób, że każdy myślał, że ci ludzie rzucą się na galerię, ale okazało się, że jeszcze ludzie się bali. A już teraz, jak byłam w piątek i poniedziałek, to jest naprawdę bardzo dużo osób. Tylko z drugiej strony widać, że tego towaru jest mało. Bardziej starają się wyprzedawać to, co było. To jest zrozumiałe, ale na półkach jest pusto i przejrzyście. Tak, jak w TK Maxxie jest zawsze wszystko zawalone, tak teraz na półkach są prześwity, jest więcej wyprzedaży, niż jakiś tam rzeczy.

**Jest dużo pozamykanych sklepów? Da się coś takiego zauważyć?**

Są jeszcze pozamykane sklepy, ale nie wiem, z jakiego powodu. Słyszałam, że ze trzy sklepy u nas w galerii w ogóle - nie to, że splajtowały - ale zrezygnowały całkowicie. Gdzieś indziej będą się otwierać, czy coś. Ale nie wiem, czym to było spowodowane, tym przestojem związanym z koronawirusem, czy tak było w planach. Nie mam pojęcia. Ale na przykład Reserved - który jest dużą firmą i w tej galerii to duży, dwupoziomowy sklep i na pewno dużo zarabiał, jest zamknięte. Nie wiem, dlaczego. Nie przyszło mi nawet do głowy sprawdzić, dlaczego.

**Otworzono restauracje, kawiarnie - wiem, że tego ci brakowało, ale co o tym sądzisz, dlaczego te miejsca zostały otworzone?**

No bo trzeba było z czegoś żyć. Z jakichś pieniędzy trzeba żyć, a jakby nie patrzeć, te restauracje są jakąś sporą częścią naszej gospodarki. Włochy utrzymują się tylko i wyłącznie z restauracji i turystyki. Ja to się cieszę, w poniedziałek jeszcze miałam taką sytuację, że tak miałam ochotę usiąść sobie na dworze, wypić piwko na ogródku, pod parasolami, bo dawno tego nie było - człowiek nie wychodził. Tym bardziej nie doceniał takich wyjść, to była dla nas normalność. Tylko teraz byliśmy na Dniu Dziecka, więc jak Michał powiedział, że jak chcę, to żebyśmy poszli, to powiedziałam, że nie usiądę z dzieckiem i nie będę piła piwa pod parasolem. Ale dla mnie fajnie, ja nie widzę różnicy między tym, czy ja wejdę do Castoramy czy usiądę w restauracji. Jak ktoś ma zachorować, to zachoruje, jak nie, to nie, już tyle czasu minęło, nic w mojej najbliższej rodzinie się nie stało i ja też nie znam osoby, która by zachorowała. Tym bardziej uważam, że ja bym przeżyła z tym koronawirusem, takie jest moje zdanie, że jakbym zachorowała, to bym z tego wyszła. Nie jestem chora na nic innego, nie mam żadnych chorób współistniejących. Więc tak, jak i zapalenie płuc, tak. Słabsze osoby umrą..Żeby to nie zabrzmiało, jak jakaś ruletka. Ale traktuję to jak każdą inną chorobę, którą jeden człowiek przejdzie, drugi nie da rady i tyle, no.

**Czy w tej restauracji lub innych, były stosowane jakieś środki ostrożności?**

No ja byłam tylko raz, z Helenką lody wzięliśmy "na wynos", w rękę i na spacer. W restauracji byłam ze znajomymi. Poszerzyli tam patio, w środku było napisane, że chyba 8 osób... Nie pamiętam dokładnie, ale byłą jakoś tam ograniczona liczba osób. Co drugi stolik był wyłączony z użytku. My usiedliśmy na patio, bo tak nas pani przydzieliła. Na stolikach nie stało kompletnie nic - żadnych solniczek, pieprzniczek, oleju. Na blacie nie było nic, może z tego względu, żeby ludzie nie dotykali, nie macali palcami. Obsługa miałaby pewnie później więcej roboty z dezynfekcją. Zanim usiedliśmy, pani przy nas dezynfekowała cały stolik i krzesła. Ale mój mąż mówi, że nie zdezynfekowała menu, które nam podała. Może nie przy nas. W każdym razie Michał mówił, że miał zapytać jeszcze o tę kartę, ale ja już powiedziałam, żeby przestał. Kelnerki miały przyłbice. Ale już wtedy, to było półtora tygodnia temu, ludzie nie siedzieli w maskach. Ja nie wiem, jak to tam prawnie, ale nie było prawie osób w maseczkach.

**Twój mąż chciał zapytać o tę kartę, bo faktycznie go to interesowało, czy raczej chciał się pośmiać?**

Niee, on jest raczej takim czepliwym człowiekiem. Skoro zdezynfekowali wszystko inne, to chciał zapytać, czy też karty. Ale ja to, ja się nie czepiam, więc kazałam mu siedzieć cicho i się nie odzywać. Ale bardziej w żart to obróciliśmy. Choć pewnie gdybym nic się nie odezwała, to on by zapytał o te karty.

**Myślisz, że to bezpieczne, że takie miejsca są otwarte i na przykład taka karta mogła być niezdezynfekowana?**

No dobrzeee, ale to ja też mogę sobie iść, umyć ręce. No nie wiem, jest milion rzeczy. Gdybyśmy chcieli być bezpieczni, to powinniśmy zamknąć się w pokojach - każdy w osobnym - nakupować sobie konserw, umyć wszystkie konserwy, wyparzyć pojemniki i siedzieć teraz, tylko nie wiem jeszcze przez jaki czas, bo według mnie ten koronawirus nie zniknie i będą na niego zachorowania. Więc powinniśmy odciąć się od świata, tylko co to za życie? Więc pozostaje albo próbować żyć normalnie i co będzie to będzie... No, ja jakoś nie czułam się, będąc tam, niebezpiecznie.

**W ostatnim czasie umożliwiono też korzystanie z salonów kosmetycznych, fryzjerów, siłowni, kin. Czy byłaś w jakimś miejscu tego typu?**

U kosmetyczki byłam. Ale miało być otwarte od poniedziałku, ja byłam w czwartek w podziemiach <śmiech>. Śmieję się, że w podziemiach. Po prostu, ktośtam znajomy prowadzi i po prostu byłam u niej.

**Jak było?**

Normalnie. U nas jest mała miejscowość, każdy się w jakiś sposób zna. Zapytałam się, czy zrobią mi paznokcie, powiedziała, że tak, przyjedź, zrobimy. I przyjechała tam do salonu dziewczyna, która robi te paznokcie i mi je zrobiła.

**Były stosowane jakieś zabezpieczenia?**

Nie, nie stosowałyśmy żadnych zabezpieczeń.

**A co ogólnie sądzisz o stosowaniu środków ostrożności typu zostawianie co drugiego siedzenia pustego, zachowywania odległości, itp.?**

A muszę mówić? <śmiech>

**Jasne, że nie musisz, jeśli nie chcesz.**

Ogólnie rzecz biorąc, ja wierzę, że ktoś mądry to wymyśla i wymyśla to dla naszego dobra, a nie w innych celach. Więc jeśli takie rzeczy są potrzebne, tj. żeby utrzymać odległość, dezynfekować - fajnie, rozumiem to. I tyle. Ale chyba nigdy się nie dowiemy, czy tak powinno być, czy nie. Czy to nam pomogło, czy nie, czy to jest wymyślone, czy nie, co jest prawdą, a co kłamstwem. Dlatego ja staram się żyć normalnie i tyle. Tym bardziej, że mamy bardzo dużo znajomych, zarówno ja, jak i mój mąż mamy bardzo dużą rodzinę - i nikt nie zachorował. Nie znam ani jednego przypadku. Jeśli chodzi o kino, niedługo na pewno się wybiorę - może za dwa tygodnie, może później, zobaczymy, jak będzie z czasem - jeżeli ktoś mi powie, że mam usiąść co drugie miejsce, usiądę. Jak mi powie, że mam zdezynfekować, zdezynfekuję, itd. Ale ja osobiście, wewnętrznie, nie mam żadnych obaw. Dostosuję się, bo skoro ktoś mówi, że tak będzie lepiej, to niech tak będzie. Ale ja tak będąc w tym kinie nie będę drżała, jakby ktoś usiadł obok mnie i złapał mnie za rękę.

**Dobrze, że otwierają takie miejsca jak kina, siłownie...**

Kiedyś musieliby otworzyć. Tylko kiedy? Na co czekać? Ja uważam, że skoro wirus jest, to on nie zniknie, tym bardziej z dnia na dzień. Każdy już zapowiada tą drugą falę zachorowań no i tak będzie. Trzeba będzie nauczyć się z tym normalnie żyć. A w ogóle, jestem ciekawa właśnie, jak tam ta Szwecja. Bo nie interesowałam się tym. Przestałam się interesować czymkolwiek związanym z koronawirusem, a nie mam pojęcia jak wygląda sytuacja w Szwecji, o której mówiłyśmy.

**Z tego, co ostatnio czytałam, jest spora grupa społeczna, która nie popiera polityki związanej z koronawirusem. Śmiertelność jest dość wysoka, problem z zachorowalnością jest spory.**

Czyli tak, jak mówiliśmy, że pewnie będzie bardzo dużo osób chorować.

**Jest na pewno spora grupa społeczna, która nie zgadza się z tym, jak do sprawy podchodzi ich rząd.**

No to niech siedzi w domu. Przecież może siebie chronić, prawda? Przecież dali im wybór. Ktoś z wyboru mógł się nie restaurować, a nie... No, dobra, nie ważne. Wiesz o co mi chodzi? Tam nie było tak, że ktoś kazał im chodzić do restauracji, kazał im się widzieć z innymi ludźmi. Tylko mogli włożyć maseczki, rękawiczki i zachowywać się tak, jak tam było zalecane i tyle.

**Chciałabym teraz porozmawiać z tobą o rozwiązaniach technologicznych i aplikacjach stworzonych przez rząd na czas pandemii. Słyszałaś o tym cokolwiek?**

Ja ogólnie słabo się też znam na technologii, więc dla mnie te wszystkie aplikacje, itd. to jest czarna magia. W takim sensie, że w ogóle nie znam nowinek technologicznych. Ale na przykład ta aplikacja związana z kwarantanną - tam osoby, które były na kwarantannie miały ściągnąć aplikację i dawać znać przez aplikację, że są w domu. To taką aplikację chciałam ściągnąć mamie i w ogóle ona nie działała. Jej model telefonu nie odpowiadał aplikacji? A moja mama nie ma wcale starego telefonu. Chodzi mi o to, że ma normalnego Androida i normalnego smartfona. Próbowaliśmy pobierać to parę razy, ale wyskakiwał komunikat, że ta aplikacja nie pasuje do tego modelu telefonu - coś w tym stylu.

**Twoja mama miała obowiązek zainstalowania tej aplikacji?**

To chodziło o babcię - ja ci mówiłam, że na nią nałożono kwarantannę, ale informacja o tym przyszła do nas trzy dni przed zakończeniem tej kwarantanny.

**No właśnie, i musiała pobrać aplikację?**

Tak. I wtedy jak przyjechała policja i mama mówiła, że babcia jest na działce ze swojej dobrej woli, bo tak postanowiła. I ta policja powiedziała, żeby babcia ściągnęła aplikację, więc próbowaliśmy to zrobić, ale niestety nie dało się. Te próby były później, jak policja odjechała. Ale jak się nie udało, mama zadzwoniła do dzielnicowej i powiedziała, że nie ma możliwości ściągnąć tej aplikacji i dzielnicowa machnęła ręką na to. I byli u babci raz czy dwa razy. A tak, to ciągle przychodziły smsy z informacją, że babcia ma obowiązek pobrać aplikację, a jeszcze tego nie zrobiła.

**Ok, to do tej i jeszcze jednej aplikacji wrócimy za moment, a teraz przeczytam ci pomysły na różne rozwiązania, podzielone na dwie grupy. To są pomysły nie tylko z Polski, ale też z innych krajów. (...) Co o tym myślisz? Myślisz, że takie rozwiązania są potrzebne?**

To pierwsze to w ogóle śmiech na sali. W celu identyfikacji koronawirusa - na pewno, tak. Nie no, w ogóle bez sensu i szkoda słów. Ok, rozumiem tę aplikację z dronem - żeby podsyłać - to może w jakimś stopniu nam pomoże. Gdyby mieli nam na głowę spuszczać jakieś pomoce. Być może jeszcze to, żebyśmy mogli sobie nawzajem pomagać. U nas powstało bardzo dużo grup. Jest jakaś tam pomoc "Widzialna ręka", czy coś takiego i tam bardzo dużo osób dołączyło do tej grupy i ludzie w różny sposób sobie pomagali z różnymi sprawami, podczas tego koronawirusa. To chyba ze Szwecji przyszło do nas, "Study circle", bezpłatna nauka, gdzie każdy daje coś od siebie. To spoko. Ale to śledzenie? Chociaż i tak śledzą nas bez naszej wiedzy bardzo dobrze i skutecznie, no ale to już jest jakieś nasze zezwolenie, żeby stać się jakimiś tam pionkami, itd. W ogóle bez sensu i nie wiem, czemu to ma służyć, bo na pewno nie chodzi o koronawirusa.

**Co dokładnie budzi twoje obawy?**

To, że całą siebie oddajesz komuś. Dlatego też mój mąż wcześniej w ogóle nie używał karty, bo jego denerwuje to, że ktoś sprawdza, gdzie on robił zakupy, co kupił, ile pieniędzy wydał, itd. Przecież to wszystko, z tych informacji, ktoś ma bardzo dobre korzyści, tak? To identyfikowanie twarzy, gdzie się poruszaliśmy - to nie służy niczemu dobremu. Tylko będzie wykorzystywane przeciwko nam. W ogóle bez sensu. Uważam, że nie potrzebujemy takich rzeczy - jedyne co, to ta aplikacja, gdzie mamy się wymieniać jakąś pomocą, to ok. Na przykładzie "Widzialnej ręki" wiem, że to może działać sprawnie i wiele osób może być zadowolonych. Ale inne aplikacje raczej nie są nam potrzebne, na pewno nie do walki z koronawirusem. Jeśli jest jakaś zarażona osoba, ona raczej jest w stanie określić, gdzie ostatnio była i może sama o tym powiedzieć. Wydaje mi się, że nie ma potrzeby zatajania takich informacji, w takiej sytuacji, gdyby nastąpiła. Chociaż słyszałam - choć nie wiem, czy to nie był żart, że jakiś facet chodził po ulicy i pluł na ludzi i był później oskarżony o terroryzm.^^[[1]](#footnote-1)^^ I miał być oskarżony o terroryzm, bo mógł zarazić ludzi. Nie mam pojęcia, czy był zarażony, coś mi się tylko tak obiło o uszy, ze teraz plucie na kogoś może być uznane za terroryzm. Ale nie daję sobie uciąć ręki, że tak było naprawdę, bo to mi ktoś tylko powiedział. Ale uważam, że nie wszyscy chcą zabijać innych. Gdyby do mnie przyjechała policja i zapytała, gdzie byłam, to bym powiedziała, gdzie byłam. Moim priorytetem nie byłoby zarażenie jak największej liczby osób w taki sposób,aby nikt się o tym nie dowiedział. Starałabym się pomóc i wydaje mi się, że każdy normalny człowiek zrobiłby to samo. Więc nie widzę sensu w ogóle w śledzeniu ludzi, sprawdzaniu gdzie chodzą, jeżdżą, co robią. To już w ogóle jakieś dyktatorskie państwo... No nie. Chociaż i tak mamy, podobno.

**To teraz przeczytam ci trochę o aplikacjach z naszego kraju. ProteGO safe (...) Co myślisz o tej aplikacji?**

No właśnie nie wiem, co myśleć, czemu ta aplikacja ma służyć? Ja bym jej w życiu nie pobrała. Nie wiem w ogóle jeszcze po co ktoś to wymyślił. Ja tu widzę, że to jest Ministerstwo Sprawiedliwości i Zdrowia się pod tym podpisuje?

**Ministerstwo Cyfryzacji, we współpracy z Sanepidem i lekarzami.**

Nie wiem. Sprawdzenie mojego stanu zdrowia. Ja wiem, jak się czuję, jaki mam stan zdrowia. Nie potrzebuję ich odpowiedzi, ani testów. Objawy koronawirusa chyba już poznaliśmy do tej pory i wiemy, kiedy jesteśmy w grupie ryzyka, kiedy powinniśmy zgłosić się do lekarza. Więc w ogóle nie wiem kto ma to sprawdzać i w jaki sposób to sprawdzą. Jak będę miała 40 stopni gorączki, będę wiedziała, że coś jest nie tak, więc nie wiem po co mam to wpisywać w ten dziennik. Nie wiem po co to jest stworzone i w jaki sposób... Co, nagle internetowo mnie zbadają?

**No właśnie, pamiętasz, jakie informacje pobiera aplikacja?**

Po pierwsze wszystkie nasze dane, po drugie to, gdzie się znajdujemy, tak? Zrozumiałam, że jak pójdę do sklepu i będę miała tę aplikację, ona powie mi, że ktoś koło mnie stoi, który jest zarażony? No w ogóle super. Ta aplikacja nie jest mi w ogóle potrzebna, do niczego. Nikt nie jest w stanie nas internetowo zbadać, a tym bardziej nikt nie potrzebuje informacji o moim samopoczuciu i tym gdzie i z kim będę przebywać. To jest już dla mnie kierowanie człowiekiem, ukrócenie jego wolności, przestrzeni, czegokolwiek.

**Uważasz, że rząd powinien tworzyć takie aplikacje dla obywateli?**

Nie, w ogóle. Ja na polityce znam się mało, albo i w ogóle. Nie interesuje mnie to. Ale po samym przeczytaniu tego opisu, gdzie tam jest ciągle podkreślane, że to "dla twojego dobra", to takie wpływanie na twoją psychikę. To ma taki wydźwięk, że jak tego nie pobierzesz, to nie będziesz zdrowy. To takie wiesz, chwyty marketingowe. No to jest w ogóle bez sensu. Dla mnie już samo to, jak korzystamy z konta bankowego... Mąż ma konto firmowe i ogólnie wszystko jest do wglądu. Księgowa musi mieć do tego dostęp, bo ona drukuje druki, to idzie gdzieś dalej. Jak ktoś może patrzeć, na co ty wydajesz pieniądze? Dla mnie to już jest masakrą. Że wyświetla mi się na karcie, w jakim sklepie zrobiłam zakupy, za ile i którego dnia. Więc to już jest w jakiś sposób... No ale wiadomo, są i Facebooki, Instagramy, to tak można by było bez końca wymieniać. Ja, jako prosty, szary człowiek, który w ogóle nie zna się na polityce - po pierwsze stwierdzam, że do niczego nie jest mi to potrzebne, a po drugie, to na pewno nie jest dla mojego zdrowia. Nikt nie pomoże mi internetowo. Jak uznam, że coś jest nie tak, to pójdę do lekarza i mnie zbada. Nie wiem, jak samemu można określić, jakie jest samopoczucie, itd. To bez sensu jest.

**Kwarantanna domowa. Już trochę o niej mówiłyśmy, ale przeczytam ci jeszcze. (…) Co o niej sądzisz, czemu to służy?**

Ja uważam, że ta aplikacja jest tak samo niepotrzebna, jak i tamta. Z tego względu, że, tak jak oni mówią, to jest jakaś pomoc dla służb, itd., przecież to można tak ominąć, że hej. W takim sensie, jeśli ktoś byłby na kwarantannie, a łamałby prawo, to nawet korzystając z tej aplikacji, można to zrobić, ominąć. Na zrobienie zdjęcia masz 20 minut. No przepraszam cię bardzo. W tym czasie jesteś w stanie przemieścić się sporą część drogi. Można. I na pewno jest jeszcze wiele innych sposobów. Tak samo jak zrobienie zdjęcia selfie, itd. Równie dobrze selfie może zrobić sobie jakaś inna osoba, która siedzi w domu. I potem ta osoba może... Wiesz, o co mi chodzi? Gdybym ja była na kwarantannie, a na przykład zdjęcia za mnie robiłaby sobie moja mama, która siedzi w domu?

**No ale system będzie porównywał twarze.**

To moja mama będzie ciągle robiła sobie zdjęcia, od początku. To nie jest z dowodem tożsamości, itd. No nie ważne, na pewno da się to ominąć wieloma rzeczami. Tyle, że to nie jest w ogóle potrzebne. Wystarczy jedna niekontrolowana wizyta policjantów dziennie. Gdzie naprawdę, nie mają dużo do roboty. Wreszcie mogli coś zrobić przez te parę miesięcy - sprawdzać, czy ludzie są w domu. Więc wystarczy, że wiesz, że dziś odwiedzi cię policja, ale nie wiesz, o której godzinie. No to boisz się - ja bym się bała wyjść, bałabym się kary. Pomijając fakt, że gdybym miała kwarantannę, to bym się do niej stosowała. Chodzi mi o taką osobę, która nic sobie z tego nie robi i miałaby ochotę wyjść. To wydaje mi się, że automatycznie, wiedząc, że policja przyjedzie, siedziałaby w domu. No bo wiadomo, każdy boi się kary, boi się utraty pieniędzy. Więc ta aplikacja jest w ogóle nie potrzebna do niczego.

**Gdybyś była na kwarantannie, pobrałabyś aplikację?**

To, kiedy babcia miała ją pobrać, to był sam początek - pierwsze dwa tygodnie. Kiedy oni dudnili w TV, że jest ta aplikacja, ja nawet nie wiedziałam, jak ona wygląda. Nawet nie przyszło mi do głowy, żeby to sprawdzić na przykład na swoim telefonie. Więc kiedy policja przyjechała do babci i powiedziała, że jest taki obowiązek, to stwierdziłam, ze dobra, zobaczymy tę aplikację. Ale się nie dało tego pobrać. Zaproponowałam mamie, że przywiozę jej inny telefon, ale ona powiedziała, żebym dała spokój i skoro się nie da, to się nie da. I tyle, więc daliśmy sobie spokój, tym bardziej, że służby nie zareagowały jakoś znacząco - też stwierdzili, że skoro nie możemy, to ok. Jakbym ja miała pobrać, miałabym zepsuty telefon <śmiech>. I nie mogłabym też pobrać tej aplikacji. Robić zdjęcia o różnych porach dnia...

**Uważasz, że ta aplikacja powinna być obowiązkowa?**

Rząd w ogóle nie powinien stworzyć tej aplikacji, a już tym bardziej o obowiązkowości nawet nie mam mowy. Ale tak, jak mówię, ja się nie znam. Ja tylko mogę mówić, co myślę, bez żadnej wiedzy, kompetencji.

**Nie musisz się znać. Pytam o twoją opinię.**

Rząd nie powinien robić wielu innych rzeczy, a robi. Jedni się z tym zgadzają, inni nie. Tak było od zawsze. Trzeba zachować swój rozum i zdrowy rozsądek i się temu nie poddawać. Tak samo, jak jest boom - czy będą nas szczepić, czy nie. No w życiu jedynym nie pójdę i się nie zaszczepię. No nie wiem, no.

**Dlaczego?**

No bo nie. Nie jestem chora i się nie będę szczepiła przeciwko czemuś czego n... Chodzi o to, że ja tego nie znam. Nie znam tej choroby, nie wiem, co będzie w tej szczepionce, no nie zaszczepię się, ni ciula się nie zaszczepię, no nie wiem no. Tylko najgorzej się boję, że ludzie znowu ześwirują i na przykład nie będę mogła dać dziecka do przedszkola, bo jest niezaszczepione. Zaczną wprowadzać takie zaostrzenia, że nagle osoby, które są niezaszczepione, będą po prostu takim drugim sortem w społeczeństwie. Nie wiem, co będzie w tej szczepionce. Po coś wywołali tę wielką burzę o nic - naprawdę o nic - no, jakoś nie odejmując szacunku osobom, które zmarły, tak? Ale umierają ludzie na świecie i będą umierać. I umierają na różne choroby. I nie widzę potrzeby robienia z tego aż takiego szumowiska. Bo to jest po prostu jedno, wielkie szumowisko. Pomysł z tymi szczepionkami, że będą obowiązkowe, to jest jedna, wielka tragedia. No nie zaszczepię się! Tylko jestem ciekawa, co zrobią, jak zmuszą ludzi.

**A ogólnie się szczepisz?**

Ja byłam szczepiona przez rodziców, jak byłam mała. Wtedy były te normalne... Znaczy normalne - nie było tych 5 w jednym, 6 w jednym. Tylko te takie przeciw odrze, te, co dostaje się w szpitalu. Te, które powinno się dostawać. Moja córka też jest szczepiona. Jest szczepiona zwykłymi szczepionkami. Nie ma zrobionych szczepień ponadpodstawowych. Żyje, ma się dobrze i mam nadzieję, że tak będzie. Ale na pewno żadnych kombinacji. Teraz wymyślono nagle, że będą szczepić całe społeczeństwo. Nie. Gdybym to znała, wiedziała, a nie teraz będą co robić? Jakieś eksperymenty na 34 milionach ludzi? I skąd my wiemy, co się stanie za kilka lat? W takim sensie, nie jestem... No nie, nie będę nawet mówić. Ja nie wiem po co, czemu ma służyć ta szczepionka. Ja nie czuję się na tyle zagrożona, żeby wprowadzać coś do swojego organizmu. Czego nie znam, co nie jest w żaden sposób jakąś naturalną substancją. Zaraz powiesz, że jem chipsy <śmiech>. Ale wiesz, o co mi chodzi?

**Yhm.**

To jest tak samo, jak narkotyk. Nie znam, nie wprowadzę tego nigdy do swojej krwi. Tak samo, jak jest z tą szczepionką. W ogóle nie widzę sensu robienia sobie tej szczepionki, bo nie czuję się w żaden sposób zagrożona. Po drugie, nie wiem, po co ona ma być zrobiona. I tyle. Jeżeli będzie obowiązek, to tego nie zrobię. Tylko boję się tego, że jak ci wszyscy antyszczepionkowcy...Ja nie jestem antyszczepionkowcem! Zaszczepiłam dziecko tak, jak sama jestem zaszczepiona - podstawowymi szczepionkami. Oczywiście w ośrodku zdrowia byłam najgorszą matką, która zezwala na ból dziecka - przeokropny, rozumiesz. Bo pani doktor mi powiedziała "bo będę musiała ją szczepić 6 razy! 6 razy ją kłuć! A tak, byłaby ukłuta tylko jeden raz." Więc powiedziałam jej, że nie ukłuje jej sześć razy, tylko tyle, ile ja pozwolę. Po pierwsze maksymalnie może pani ukłuć w każdą inną kończynę - czyli cztery razy. Po drugie, ja zezwalam tylko na dwa ukłucia. A kolejne, kolejną serię - poproszę za tydzień czy dwa tygodnie. No, ale byłam najgorszą matką. Że skoro mam pieniądze, to przecież mogę iść do apteki i kupić tę szczepionkę za 200 złotych. I wpakować trzymiesięcznemu dziecku chemię. Gdzie nie wiem, jak ono zareaguje, bo różnie dzieci reagują. A później się okaże, że miała jakiś genetyczny problem i w zestawieniu z tą szczepionką, nagle dziecko jest autystyczne. I problemem nie jest szczepionka, tylko wada genetyczna. To ja się pytam, gdzie ludzie sprawdzili wady genetyczne przed podaniem szczepionki? To nie może być żadna loteria, że moje dziecko przeżyje, a twoje nie. Dlatego ja robiłam to stopniowo, odwlekałam w czasie, w takim sensie, że im starsze dziecko, tym bardziej odporne i lepiej będzie reagować na różne substancje z zewnątrz. Ale ma zrobione podstawowe szczepionki starą formą. Ja żyję 25 lat i nigdy mi się nic nie działo, a byłam szczepiona starym systemem i jest ok. A dzieciaki, które są szczepione nowym systemem - 5 w jednym, 6 w jednym - nagle się pojawia dużo problemów. No skądś się to wzięło. Ja nie mówię, że każda szczepionka powoduje autyzm, Aspergera, śpiączkę. Ja tego nie mówię, bo szczepionki przed różnymi chorobami są potrzebne, ale te modyfikacje, które wprowadzili - nagle stało się, że jest większa liczba zachorowań. No bo jest. Na różne choroby. I ja znów, jako prosty człowiek, nie dam mojemu dziecku sześciokrotnej dawki czegośtam, tylko rozłożę to w czasie. Ale już się śmiałam, bo przedszkola zaczęły informować, że dzieciaki nieszczepione nie będą mogły chodzić do szkoły. Podobno mamy wolność wyboru, słowa, życia, wszystkiego. A niestety jej nie mamy. Bo skoro rząd nie może zmusić rodziców do szczepień, to zmusi ich innymi sposobami. Właśnie takimi, że dziecko nie będzie mogło iść na basen albo do szkoły, bo jest nieszczepione. Choć to dla mnie też głupota. Przecież ono nie zarazi tą nieszczepionką innego dziecka. No, ale no. Nieważne już. Tak, jak mówię, rząd nie powinien robić różnych rzeczy, a robi - i tyle.

**Czy myślisz o przyszłości po pandemii?**

W ogóle nie określam już obecnego stanu rzeczy, jako życie w pandemii. Uważam, że trzeba żyć, jak się żyło, z tego względu, że to nie zniknie. Tak, jak inne wirusy nie znikają, tylko mamy co jakiś czas chore dzieci. Dla mnie już teraz jest normalnie. Może wreszcie ukrócą te negatywne wiadomości, które dostajemy każdego dnia - choć teraz jest ich chyba mniej. Kiedyś oglądałam wiadomości i mówię, boże, już o jakimś wypadku powiedzieli, o czymśtam. Już jakieś inne wiadomości wprowadzają do kraju, niż tylko te związane z koronawirusem. Więc wirus nie zniknie. Chyba, że rząd uzna, że już jest czas, żeby on zniknął, to wtedy powiedzą społeczeństwu, że zniknął. Ale wirusy nie umierają, tylko będą.

**Czy jest coś jeszcze, co w jakiś szczególny sposób zaprząta twoją uwagę?**

Obawiam się tylko jednego. Widzę, że można żyć sobie spokojnie, a ktośtam u góry pociągnie za taki sznurek, jakim teraz jest koronawirus, i nagle rozsypuje ci się życie. Nagle nie masz, gdzie iść do pracy, nagle wszystko zaczyna być zahamowane. Okazuje się, że nie stanowimy sami o sobie. Bo jeśli rząd pozamyka wszystkie sklepy, to my nie będziemy mieć gdzie kupić produktów. Bo nam zamknie. Nadal się sprawdza to, co mówiłam kiedyś. Jeśli mam swój ogródek, swoje zboże, świnki, krówkę - jestem samowystarczalny. Nie interesuje mnie, czy nagle produkty wzrosną sto procent i nie będzie mnie na nie stać. Jestem sam w stanie przeżyć, bez tego, co rząd zrobi na górze. Ale to jednak jest taki strach - miałam zaplanowane wakacje, były zaplanowane komunie, wesela. I nagle boom, coś się dzieje i ty nagle nie możesz zrealizować swoich planów, mimo, że byś chciała, albo nawet nie zgadzasz się z rządem, czy myślisz inaczej - jeśli się nie zgadzasz, dostaniesz karę. Nie jesteśmy wolnymi ludźmi, w żaden sposób - to mnie przeraziło. I to nawet nie chodzi o to, że jesteśmy zniewoleni przez jakieś nałogi, czy inne rzeczy, a przez tych ludzi, którzy nami kierują, czyli przez rząd. Jednak od nich bardzo dużo zależy.

**Czy myślisz o tym, czy coś na świecie lub w kraju się zmieni?**

Nie no, raczej nie myślałam. W ogóle się nad tym nie zastanawiałam.

**Czy myślisz, że ogólnie coś się zmieni?**

Mówiłam to już na początku - że możliwe, że ludzie zaczną doceniać to, co wokół nich - spacery, spotkania z bliskimi. Ale minie miesiąc - i tak się właśnie dzieje - zostały otworzone galerie i ludzie już nie idą do lasu i nie cieszą się z tego, tylko idą na zakupy do galerii. Więc wiedziałam, że to potrwa bardzo krótko i nie zmieni się nic w ich rozumowaniu.

**Czy masz jakieś przemyślenia dotyczące ewentualnych zmian sytuacji gospodarczej?**

Nie mam bladego pojęcia, jak to będzie. Wydaje mi się, że trzy miesiące to nie było tragedii. Bo to jakoś przez trzy miesiące było zamknięte, prawda? Myślę, że nie upadniemy jako państwo. Zostało wydane dużo kasy na te wszystkie dofinansowania. Nie mam pojęcia, jaki był budżet państwa - bo raz mówią, że pieniędzy nie mają, innym razem mają duże zasoby - więc nie wiem. Jeśli chodzi o moje najbliższe środowisko - u nas nic się nie zmieniło. Ja nie widzę strat związanych z wirusem. Akurat tak nam się poszczęściło, że mój mąż pracuje w takim miejscu, że nie mamy żadnych strat.

**Myślisz, że w kraju lub na świecie sytuacja ekonomiczna ulegnie zmianie?**

Nigdy się nad tym nie zastanawiałam i o tym nie myślałam.

**Ograniczenia - czy coś będziesz utrzymywać, czy któreś powinny zostać na dłużej?**

Jedyne, co utrzymuję, a czego nie robiłam wcześniej, to ciągłe mycie rąk. No, może nie ciągłe, tak, jak było to podczas pierwszych dwóch tygodni, ale ja często nie myłam rąk. Nie byłam taka obrzydliwa, żeby... No, może to nie jest kwestia obrzydliwości, może robiłam źle? To jakieś maniery i takie tam. Ale jak przychodziłam z dworu, potrafiłam zrobić sobie kanapkę i ją zjeść. Mój Michał jest zupełnie inny, on ciągle mył te ręce. Ale zdałam sobie sprawę, że jak wchodzę do siebie do domu skądś indziej, to powinnam te ręce umyć, a tego nie robiłam. Ale poza tym, nic innego chyba nie zostanie, poza tym zwracaniem uwagi na to, że jak wracam ze sklepu do domu, to powinnam te ręce umyć.

**Czy są jakieś grupy wiekowe/społeczne, które powinny być wg ciebie szczególnie chronione przed zarażeniem?**

Nie mam pojęcia, w jaki sposób mieliby być chronieni. W ogóle, ogólnie rzecz biorąc, powinniśmy dbać o osoby starsze, tylko jak dbać, żeby się nie zaraziły? To znowu trzeba by ich odizolować, a ja uważam, że wręcz przeciwnie - osoby starsze powinny być wdrażane do społeczeństwa jeszcze bardziej, a nie izolowane. Powinno być więcej klubów seniorów, takich aktywności. Uważam, że seniorów trzeba aktywizować. Widzę różnicę, jak babcia funkcjonuje z nami - młodymi ludźmi. Kiedy robimy obiad - robimy wspólnie, babcia na przykład zmywa, dostaje różne obowiązki, zajmuje się Helenką. Mam też jej obraz przed urodzeniem Helenki. Ona była zamknięta w pokoju i nie miała po co wstawać. Jej ciągle było niedobrze, bolała ją głowa, ciągle leżała. Wydaje mi się, że ona nie miała motywacji, a to to jest najgorsze. I wydaje mi się, że jej zdrowie by się znacznie pogorszyło, gdyby ona nie wstawała z tego łóżka, niż tak, jak teraz, gdzie chodzi na spacery, wychodzi na podwórko, codziennie rano się ubiera - teraz to ona się stroi na te dializy. Przez ostatnie dwa lata widzę, że ona bardzo odżyła. A wystarczyło dać jej obowiązki i jakieś tam zajęcia. Im więcej dajemy jej czynności do zrobienia, im bardziej jest nam potrzebna, tym lepiej się ona czuje. Więc nie wiem, jak można by było chronić tych seniorów, wdrażając ich równocześnie do społeczeństwa. Nie mam pojęcia. Izolacja to chyba najgorsze, co może być. Uważam, że lepiej umrzeć wcześniej, a żyć pełnią życia, niż żyć nijak, bez sensu, zamkniętym w pokoju i żyć 80 lat.

**Czy są wg ciebie działania/zasady związane z koronawirusem, które warto by wdrożyć do społeczeństwa już na stałe?**

Były rzeczy, które mi się nie podobały, ale było też i w drugą stronę, jak z tymi grupami pomocowymi. Nagle też okazało się, że można być dla siebie miłym, zorganizować posiłki dla lekarzy - chociaż są różne dyskusje, czy powinni je dostawać, czy nie. Ale ok, nagle są firmy, które potrafią dać kasę na służbę zdrowia - może też nie powinni na to, bo płacimy na to co miesiąc i każdy jest ubezpieczony i nie uważam, żeby znowu prywatny człowiek musiał ratować coś rządowego - nie tędy droga. Ale ludzie stali się sobie bardzo pomocni, nagle okazuje się, że sami możemy robić maseczki, nagle ktoś organizuje przyłbice, cośtam. Dużo osób potrafiło zrobić coś za darmo, od siebie, zmobilizować się. To było bardzo fajne. Nie chodzi mi, żeby dofinansowywać służbę zdrowia, bo absolutnie nie - ja w ogóle uważam, że powinny być te ubezpieczenia sprywatyzowane. Sama jestem ubezpieczona 25 lat, a chodzę do lekarzy prywatnie. Uważam, że jakbyśmy sobie sami odkładali te pieniądze na kupkę, mielibyśmy dużo więcej pożytku, niż ubezpieczając się. Ale chodzi mi o zachowania ludzi - oni faktycznie w dobie kryzysu potrafią się spiąć i dać dużo od siebie. Na co dzień tego nie widać, bo wtedy człowiek człowiekowi wilkiem, a nie odwrotnie. To było fajne - to była jedyna rzecz, która powinna zostać. Grupy wsparcia, takie tam. Zwłaszcza dla starszych osób, choć nie uważam, że powinno im się podstawiać pod nos, bo nie. Dopóki człowiek sam funkcjonuje, jest dużo lepsze, niż podstawienie pod nos. I czasami nam się wydaje, że to wyjście babci na zakupy jest takie męczące, to jest to jedyne przewietrzenie i jakaś aktywność w ciągu dnia.

**Co myślisz o nowych rozwiązaniach/ ograniczeniach w miejscach, które niedawno ruszyły?**

Nic o tym nie słyszałam.

**Są takie pomysły, żeby na przykład mierzyć temperaturę na wejściu do różnych instytucji/ placówek, np. na lotniskach, w przychodniach. Co o tym sądzisz?**

Niech sobie mierzą. Nie uważam, że to coś złego, raczej nikogo nie skrzywdzi. Tylko ktoś może być przecież chory na coś innego, nie koniecznie koronawirusa. Od zawsze dzieciaki zarażały się od siebie grypą, wirusami. Tak samo ospą, jak kaszlem, gorączką. To było od zawsze i nie rozumiem, dlaczego teraz jest taki boom na to. Ale skoro chcą mierzyć temperaturę - jasne, niech mierzą. Powinniśmy też sami jakoś się kontrolować. Oni zmieniają teraz cały porządek, regulaminy wszystkich miejsc ze względu na koronawirusa. Dla mnie to jest paranoja. Że ze względu na koronawirusa robią taki cyrk. A dlaczego nie zrobili go wcześniej, kiedy dzieciaki chorowały na grypę? Nie wiem, po co wprowadzają teraz te zmiany - cały czas chcę wierzyć, że dla naszego dobra. Nie wtrącam się, nie będę z tym dyskutować i walczyć. Jak mają mi zmierzyć temperaturę - niech mierzą. Jak mam usiąść co drugie siedzenie - siądę. To byłaby walka z wiatrakami - w ogóle nie będę tracić swojej energii do przekonywania kogoś do mojego zdania. Niech robią, jak chcą, mi nie będzie przeszkadzało, jak mi zmierzą temperaturę.

**Zastanawiałaś się nad skutecznością takich działań?**

Jeżeli wierzyć statystykom, u nas nie było najgorszej sytuacji z tym koronawirusem. Biorąc pod uwagę informacje, które zostały nam podane, ta liczba zakażeń nie jest duża, dziennie nie przybywa nam po kilka tysięcy, a po kilkaset przypadków. Więc to było pod kontrolą, nie mieliśmy sytuacji jak we Włoszech, Hiszpanii, Szwecji, czy Niemczech. A na ile jest w tym wszystkim prawdy to ja nie wiem i się tym nie przejmuję. Tak, jak u nas zrobili szpital jednoimienny, tylko dla osób z koronawirusem, i wiesz, co mówią lekarze i pielęgniarki? Że wreszcie odpoczywają na dyżurach. Dlatego te wszystkie posiłki dla tych lekarzy, to wszystko, co mówili w TV, a co jest naprawdę, to dwa różne światy. Jest u nas w rodzinie taka pani z dermatologii. Mówi, że jest 5 pielęgniarek na zmianie, a 3 pacjentów leży, bo np. mają oddział "ozdrowieńców", tj. mają już testy ujemne, ale muszą jeszcze leżeć dwa tygodnie, czy ileś, bo jeszcze w pocie, czy ślinie jest wydzielina i muszą zostać w szpitalu. Lekarze teraz jak idą na dyżur, to nie mają po co. I taka jest sytuacja u nas w Radomiu, gdzie jednak, podobno - wg wiadomości - kilkaset osób z tego szpitala miało wynik pozytywny. A pielęgniarki nie mają, co robić. Druga sprawa jest taka, że nie ma przyjęć poza koronawirusem. Brat Michała ma dość poważny problem z kręgosłupem, chciał dostać się na NFZ i musi szukać szpitala lub lekarza ok. 100 km od nas, bo nie pracują. Rehabilitacja, wszystko było wstrzymane, a lekarze nie mają co robić. I musiałby iść prywatnie, albo pod Kielcami na NFZ. W TV mówią, że lekarze są tak zapracowani, a ja rozmawiałam z kilkoma osobami z tego szpitala i mówią, że nie mają, co robić, a szpital jest jednoimienny, więc nie mogą przyjmować innych pacjentów. Więc nie wiem, jak tak bardzo stoją na tej pierwszej linii frontu, skoro się nudzą. Poza tym, od zawsze uważałam, że to jest ich zasrany obowiązek i tyle. Ja, jeżeli nie chciałam być lekarzem i nie chciałam ratować ludzkiego życia i mieć czyjeś życie ponad swoje, nigdy do takiego zawodu nie szłam. Tak samo jak strażacy, policjanci. To jest ich dobrowolny wybór. I to, że teraz są potrzebni, to jest naturalna rzecz i nie uważam, że trzeba ich teraz stawiać jakoś mega wysoko, bo to jest po prostu ich praca.

**Wspomniałaś, że już podczas spotkań rodzinnych mówiło się o drugiej fali zachorowań. Co o tym myślisz?**

Jak będzie, tak będzie. Szkoda, bo znowu będą odwołane imprezy i znowu będą jakieś niedogodności, ale osobiście znowu się tym nie martwię.

**Uważasz, że rząd powinien zareagować tak samo, w przypadku drugiej fali? Wszystko pozamykać?**

Nie wiem, naprawdę nie mam zdania na ten temat. Jak okaże się, że jest nawrót pandemii i zaczną wreszcie chorować ludzie z mojego okręgu, zobaczę to na własne oczy, to może zmienię zdanie. Na tę chwilę - ile jest teraz zachorowań? 20 tysięcy?

**Ponad 24 tysiące zarażonych, 12 tysięcy ozdrowiałych.**

No. Te liczby wydają się przerażające, ale jeśli porównać to do tego, ile mamy Polaków, ludności, to jest naprawdę bardzo mało. I tyle.

**Czy jest coś, czego się obawiasz w perspektywie drugiej fali zachorowań?**

Nie to, że obawiam, ale być może znowu będzie mnie męczyć, że wszystko pozamykają i nie będzie można się ruszyć z domu. Chodzi mi raczej o decyzje rządu, a nie moje zachowania.

**Myślałaś, żeby w jakiś sposób przygotować się przed nadejściem ewentualnej drugiej fali?**

Nie <śmiech>, nie myślałam, o tym. Na pewno nie będę się przygotowywać. Mam na wsi wszystkie zwierzęta. Mam króliki, świnki, kury. Jesteśmy samowystarczalni.

**Podsumowując nasze 5 spotkań. (...) Wymień wydarzenia ważne/ przełomowe dla ciebie i ważne z perspektywy kraju. Opowiedz, dlaczego postrzegasz je jako takie.**

Jakie trudne pytania. Przełomowe... Pierwszy, to kiedy wprowadzono stan pandemii w Polsce. Wtedy było wielkie boom, zamykanie granic. To się wiąże z pierwszym "pacjentem zero". Później, ważne wydarzenia... Później, to było całkowicie normalnie. Ale to może, że rodzice z babcią się wyprowadzili, zostaliśmy sami. Trzecie, jak wrócili. Zaczęliśmy żyć normalnie. Później, kolejne, to było spotkanie ze znajomymi w tej większej grupie - spotkaliśmy się w 16 osób. A później - kiedy zaczęliśmy się spotykać z rodziną Michała.

**Ok, pacjent zero - dlaczego to było przełomowe?**

Zmieniło się całe nasze dotychczasowe funkcjonowanie, zaczęli wprowadzać, zamykać sklepy, granice, zaczęło się wszystko zmieniać. Żyliśmy sobie normalnie i nagle, boom, musieliśmy zacząć przyzwyczajać się do nowych zasad.

**Wyprowadzka rodziców i babci**

Od zawsze mieszkaliśmy z nimi, wszyscy razem. To był pierwszy raz, kiedy zostaliśmy sami z mężem i córką w domu. Mama bardzo tęskniła za Helenką. Niby fajnie, bo sami, po swojemu, ale było ciężko, z racji tego, że byliśmy przyzwyczajeni, że jesteśmy wszyscy razem, a ich nie było.

**Kiedy wrócili - pamiętam, że to była duża radość**

Tak, to był moment, kiedy zaczęliśmy wracać do normalności. Nic nie działo się babci, miała negatywne testy. U nas nic się nie działo, zaczęliśmy znów normalnie funkcjonować.

**Pierwsze spotkanie w większym gronie znajomych**

My bardzo lubimy spotykać się ze znajomymi, zawsze spotykaliśmy się w dużych paczkach. To było pierwsze taki spotkanie po dłuższej przerwie. Na święta się nie widzieliśmy, a zawsze się widujemy wszyscy - wiadomo, najpierw z rodziną, później ze znajomymi. Mi bardzo brakowało takich kontaktów towarzyskich, a wtedy się spotkaliśmy, było bardzo fajnie, nawet nikt nie wspominał o tym koronawirusie. Było tak, jakbyśmy nie widzieli się dosłownie tydzień. Jedna z kolejnych normalności wróciła.

**Spotkanie z rodziną Michała**

To był przełom, bo oni bardzo ostro, kategorycznie do tego podeszli. To było pierwsze spotkanie po bardzo długim czasie. Ludzie się obudzili. Nie mam pojęcia, co nimi kierowało, bo jak mówiłam, sytuacja się w ogóle nie zmieniła. Nie wiem, nie pytałam, skąd takie decyzje, bo jak mówiłam, nie chciałam się denerwować.

**Chciałabyś dodać jeszcze coś, co wydaje ci się ważne, a o czym nie powiedziałyśmy?**

Tak, ostatnio widziałam się z taką znajomą. I ona mówiła, że w styczniu, jej siostra gdzieś wyjechała i na ten czas zostawiła swoje dziecko pod opieką dziadków. Ta mała zaczęła chorować, miała gorączkę, duszności. Zabrali ją do lekarza - to znana tutaj, około 60 letnia pediatra. Kiedy ona zobaczyła to dziecko, kazała im się odsunąć od dziewczynki, i sama zbadała ją, po czym przepisała jakieś lekarstwa. Ale - a to było w styczniu - powiedziała im też, że muszą natychmiast odizolować się od tej dziewczynki. On zapytał, co się dzieje, a ona odpowiedziała, że nic, że to taki paskudny wirus, ale oni, jako dziadkowie, muszą się od niej odizolować. Oni mówili, że będzie ciężko to zrobić, a ona powiedziała, żeby zrobili wszystko, żeby jednak to dziecko na czas choroby było daleko od nich. Nie mam pojęcia, co było dalej. Ale słyszałam też dużo takich informacji, że dużo dzieciaków na przełomie stycznia i lutego jeździło do lekarzy i lekarze nie wiedzieli, co im jest. Dawali antybiotyki na te gorączki, ale gorączka nie spadała, a po tygodniu, czy iluśtam, choroba przechodziła - jakby organizm sam zwalczył tę chorobę, a lekarze nie wiedzieli, co to jest. O tym już słyszałam wielokrotnie, od chyba wszystkich matek, które prowadziły dzieci do przedszkoli. Chcę przez to powiedzieć, że to się pewnie nie zaczęło w marcu, tylko wcześniej. I nie wiem, dlaczego,w marcu był boom, a nie było w listopadzie. No, to tyle chciałam ci jeszcze powiedzieć.

1. <https://www.fakt.pl/wydarzenia/swiat/koronawirus-usa-lizal-polki-w-sklepie-oskarzyli-go-o-terroryzm/czeplff>

   Jednak „tylko” lizał półki w Walmarcie. :) [↑](#footnote-ref-1)
