# Supplementary material for: The challenges arising from the COVID-19 pandemic and the way people deal with them. A qualitative longitudinal study
Source: PLoS One. 2021 Oct 11;16(10):e0258133. doi: 10.1371/journal.pone.0258133 (PMC8504766; doi:10.1371/journal.pone.0258133)
Supplement: S1 Dataset — (ZIP) [file pone.0258133.s003.zip › Transcriptions/stage 5/6.5_M_24_couple, no children.docx]

**6.5_M_24_couple no children**

**Poprzednio mówiłeś o rozmowie rekrutacyjnej, że prawdopodobnie wszystko poszło zgodnie z planem i mówiłeś, że przyszedłeś na rozmowę w maseczce, ale potem rekruter powiedział, żebyś ją zdjął i właściwie, jeśli chodzi o inne obostrzenia, to na tej rozmowie nic takiego nie było?**

Nic w zasadzie. Spotykałem się jeszcze z innymi pracownikami w placówce, też z nim i rozmawiałem bezpośrednio twarzą w twarz nie zachowując absolutnie odległości 2 m i w zasadzie tyle. To była taka formalność tylko i to na samym początku tylko w portierni, a później już żadnych obostrzeń i regulacji. Tylko mi pani rekruterka wspomniała, że było ryzyko w tej placówce, że zostaną odizolowani i wspominała, że ktoś im nawet pralkę miał dostarczać, ale ostatecznie nic z tego nie wyszło, izolacji nie było i wszystko było w porządku. Mieli mieć tę kwarantannę w laboratorium. Nie wiem, dlaczego nie w domu. Nie dopytywałem.

**Mówiłeś, że musiałeś uzupełnić jakąś kartę z informacjami na swój temat?**

Tak, to była informacja o tym, czy ja byłem poddawany testom, czy byłem chory...Nie pamiętam, co tam dokładnie było, ale na pewno, czy odwiedziłem jakiś kraj, w którym była pandemia, np. Włochy i Hiszpanię, czy moi bliscy też mieli wykonywane testy i chyba było tam jeszcze kilka innych opcji, ale nic szczególnie istotnego.

**Czy jeszcze coś ważnego wydarzyło się w ostatnim miesiącu?**

Spłonęło mojemu koledze mieszkanie. Na szczęście nic mu się nie stało takiego poważnego. W zasadzie nic mu się nie stało, jego dziewczynie też, tylko po prostu spłonęła cała kuchnia doszczętnie, mieszkanie było do odmalowania. Generalny remont trzeba było przeprowadzić i ten remont nadal jest w toku. Ja się udzielam dość mocno, jako że ten kolega mieszka koło mnie na Białołęce. W tym tygodniu jeszcze u niego nie byłem, ale myślę, że jutro, pojutrze może podejdę mu pomóc. I tyle. Jakoś staje na nogi powoli. To było bardzo dla mnie istotne i bardzo to przeżyłem. W zasadzie było tak, że ja wtedy byłem w Przemyślu i moja dziewczyna poszła na grilla z tym kolegą i jego dziewczyną, i siedziała z nimi do 3 w nocy. Parę godzin później dostałem telefon, że prawie się spalili. To było mocne dla każdego, ale najważniejsze, że żyją.

**Ty nadal pobierasz pensję ze swojej starej pracy?**

Tak, bo ja jeszcze nie składałem wypowiedzenia. Aktualnie jestem w trakcie rekrutacji. Dostałem w mailu informację, że to jest już ostatnia ścieżka rekrutacji i pani z HR mówiła wcześniej, że mam złożyć dokumenty, coś tam jej wysłać, ona wystawi raport i wtedy do mnie zadzwoni, i powie mi, że mogę już złożyć wypowiedzenie. Teraz czekam na ten telefon i informację, że mam już zielone światło.

**Chodzisz teraz do pracy?**

Nie, jeszcze nie.

**Słyszałeś, że macie wracać?**

Nic. Nie dostaję żadnych informacji, żadnego maila, żadnego telefonu. Nie mam też kontaktu żadnego z pracownikami, tyle, że mam nr telefonu i mogę zadzwonić, ale nie odczuwam takiej potrzeby specjalnie.

**Ale dostajesz wynagrodzenie?**

No tak. W zasadzie zawsze dostawałem je do 8-go. Mamy dzisiaj 9-ty i ja dalej go nie dostałem, ale w umowie jest, chyba że do 10-go mam dostać.

**Na ile twoje życie teraz wygląda tak jak przed pandemią?**

Trochę już wróciło...Trochę tak, a trochę nie, bo dalej siedzę w domu i nie pracuję poniekąd, ale powiedzmy już to wygląda tak, jak mogłoby wyglądać przed pandemią. Czyli już jestem w drodze rekrutacji do innej pracy, już normalnie można chodzić do sklepów, maseczek już nie trzeba nosić, itd. To jednak wraca o wiele szybciej do poprzedniego stanu niż mi się wydawało, że będzie wracało. Myślałem, że do końca wakacji albo i dłużej będziemy chodzili w maseczkach cały czas, a teraz widzę rozluźnienie pełną parą i nawet jak trzeba nosić maseczki w sklepie, to widzę, że dużo osób tego nie robi. Sam czasem nie zabieram, bo w sumie nikt się już do tego nie przyczepia i to też chyba nic nie daje już na dobrą sprawę. Rękawiczek nie trzeba też nosić w sklepach...Nie wiem, jak z tymi kawiarniami, bo ja też w sumie nie śledzę już informacji aż tak bardzo, ale z tego co wiem, to chyba baseny, siłownie, to się miało wszystko pootwierać na początku czerwca i tym bardziej mnie to dziwi, że dalej nie dostaję żadnej informacji ze swojej kawiarni, że będziemy mogli niedługo wrócić do pracy. Ja nie wiem nic, ale zakładam, że skoro otworzyły się zakłady fryzjerskie, baseny, siłownie, to kawiarnie też raczej. Restauracje przecież działają.

**Co myślisz o tym, że tak szybko to wszystko wraca do normalności?**

Z jednej strony dobrze, fajnie, bo ludzie też na pewno się trochę lepiej z tym czują, ja też się lepiej z tym czuję, jest większa swoboda zdecydowanie, ale mogą powrócić zachorowania i o tym się dowiemy zapewne w przeciągu kilku tygodni, czy to luzowanie wychodzi ludziom na dobre na zdrowiu, czy nie. Nie mam zdania, żeby stwierdzić, czy to jest dobre, czy nie, czy to za szybko, czy nie. Myślę, że to zachodzi jakoś tak naturalnie, bo rozumiem, że jest możliwość przywrócenia jakichś obostrzeń, jakby znowu wystąpiły jakieś zachorowania, więc wydaje mi się, że jest ok na razie

**Czy pewne zachowania, przyzwyczajenia zostały ci jeszcze z tamtego czasu?**

No tak. Jak jestem w sklepie albo w tramwaju, w komunikacji miejskiej, to siłą rzeczy staram się zachowywać dystans od ludzi. Mam świadomość tego, że może jeszcze warto zachować taki dystans. Nie zawsze się tego trzymam, ale mam gdzieś z tyłu głowy, że niedawno trzeba było tak robić i może nadal trzeba tak robić. To jest taka rzecz w zasadzie. Chyba nic więcej mi nie przychodzi do głowy. Ostatnio byłem w Lidlu i było bardzo dużo ludzi, w GH też...Ja już sam nie wiem, czy zachowywać ten dystans czy nie, ale mam świadomość, że całkiem niedawno każdy huczał, że trzeba go zachowywać, a teraz już widzę ogromne rozluźnienie i sam trochę wpadłem w ten nurt. Już sam nie wiem. Po prostu chyba jest mi to obojętne, ale mimo wszystko, jeśli o tym pamiętam, to staram się to stosować.

**A twoi bliscy, rodzice? Jak oni do tej sytuacjo podchodzą?**

Moi rodzice od samego początku podchodzili bardzo luźno. Tata był już o tyle spokojny o to, że miał wrażenie, że on już to przechorował, bo był mocno chory na początku marca, moja mama też zresztą. Oni byli w Afryce w lutym, więc nawet możliwe, że coś ich tknęło. Zresztą ja też byłem chory na początku marca. Miałem takie grypowe objawy. Oni bardzo luźno do tego podchodzili, ale jak były restrykcje, że trzeba było siedzieć w domu, to siedzieli w domu, ale nie, żeby się jakoś specjalnie bali o siebie. Moja babcia w sumie też dosyć luźno do tego podchodziła i dalej podchodzi. Obecnie jest w Bieszczadach ze znajomym.

**Jak był ten największy lockdown, to babcia siedziała w domu?**

Babcia wychodziła. Sporo chodziła, bo mówiła, że nie może usiedzieć. Oczywiście mówiłem jej, żeby w miarę możliwości siedziała w domu mimo wszystko, ale ona ma znacznie mniejsze mieszkanie niż my tutaj mamy, więc rozumiem ją, że ciężko było usiedzieć w miejscu. To też nie było tak, że ona chodziła jakoś super często na zakupy, tylko bardziej się przejść nad rzeką czy gdzieś po ulicach miasta.

**Emocje - zdjęcia**

1 - pasuje, bo jest coraz większy ruch na ulicach miasta i to się widzi, i w GH. jest coraz bardziej tłoczno. To jest pozytywne pod tym względem, że to jest znak tego, że już ma być normalnie, że jest już normalnie po części, ale z drugiej strony może to spowodować wzrost zachorowań ponownie, więc i tak, i nie.

2 - wspominałem o tych śmieciach jakiś czas temu i nie widzę już ich tak aż tak dużo, ale zdarzyło mi się też w jakąś gumę wdepnąć.

4 - teraz bym ją wybrał, bo zbliżamy się do siebie coraz bardziej mimo wszystko, jest bardziej tłoczno w galeriach. Ja już też nie mam w sumie żadnych oporów, żeby wsiąść do tej komunikacji miejskiej, pójść do znajomego. W ogóle już nie mam oporów przed wychodzeniem a wcześniej miałem. Mam to z tyłu głowy, że ten dystans niby powinien być zachowany, ale już się tak nie boję i nie zwracam na to uwagi aż tak bardzo. I też sobie myślę, że już minęło tyle czasu, tyle już wychodziłem na zewnątrz, tyle się spotykałem z ludźmi, że jeżeli miałbym zachorować, to bym już chyba zachorował. Już prawdopodobnie jednak zachorowałem, przechorowałem i mam jakąś odporność, ale nie musi tak wcale być i może mi się tylko wydawać.

11 - bo ostatnio było trochę burz.

**Czy myślisz teraz, że jesteśmy po pandemii/ w trakcie?**

Nie, jeszcze na pewno nie jesteśmy po pandemii, chociaż np. widziałem wczoraj, że w Nowej Zelandii od 17 dni nie wykryto żadnego przypadku i ozdrowiała ostatnia osoba zachorowana. Tam można pewnie powiedzieć, że jest już po pandemii, ale u nas jest jeszcze na to za wcześnie. Trzeba trochę odczekać, parę miesięcy może i jeżeli wtedy nie byłoby żadnych nowych przypadków i wszystkie osoby by wyzdrowiały, to można mówić, że jest już po pandemii. Póki ci jesteśmy powiedzmy na prostej do normalności, do wyzdrowienia, ale na pewno jesteśmy jeszcze w trakcie pandemii.

**Czy ty czujesz się tak, jakby było po, czy nadal jednak w trakcie?**

Nadal jeszcze czuję, że nie jest tak, jak było, więc skoro nie jest tak jak było przed nią, to znaczy, że jest jeszcze jednak pandemia.

**Czy jakieś sytuacje sprawiają, że bardziej się boisz, stresujesz?**

Nie, wydaje mi się, że nie.

**I nie szukasz już żadnych informacji?**

Nie, kompletnie nie.

**Wiesz, ile teraz jest osób zakażonych?**

Nie wiem. Pytasz o Polskę czy o świat? Na świecie jest chyba 5 mln, ale mogę się mylić, a w Polsce chyba 17000? Chociaż nie wiem, czy to nie za mało w zasadzie...Tylko 17000? Mogłem się pomylić. Nie śledzę już z własnej woli, żeby szukać informacji na temat koronawirusa. Przestało mnie to już w ogóle interesować praktycznie i żyję już swoim życiem, ale mimo wszystko dalej nie czuję, że jest do końca normalnie, ale jesteśmy na dobrej drodze.

**Spotykasz się ze znajomymi. To już są spotkania w większych grupach?**

To są raczej takie pojedyncze dalej, bo nie mam za wielu znajomych blisko tutaj na Białołęce. Cały czas wspominałem o tym koledze z dziewczyną, więc spotkania w 4 osoby. Za tydzień jadę na wesele i tam będzie prawdopodobnie 150 osób. Miałem cichą nadzieję, że się nie odbędzie żadne z tych wesel, ale ta dziewczyna, która ma mieć w przyszłym tygodniu to wesele, czekała do ostatniej chwili z ogłoszeniem czy będzie, czy nie i ogłosiła chyba tydzień temu, że ostatecznie się odbędzie, bo wtedy weszły te przepisy, że zgromadzenia do 150 osób maksymalnie. Stwierdziła, że to wesele się odbędzie i jedziemy.

**Jak to się będzie wyglądało?**

Nie wiem, nie mam pojęcia. Myślę, że max 150 osób i pewnie w jakichś odległościach od siebie będą siedziały. Formalnie będą miejsca przynajmniej, a potem to już wiadomo, że nikt nie będzie tego przestrzegał. Liczyłem, że się nie odbędzie, bo ja nie jestem jakimś super fanem wesel i zabawy tego typu. Jeżeli chodzi o wesela, to jest ok i akceptuję to pod tym względem, że można po prostu się napić i najeść dobrych rzeczy przeważnie, ale ja nie przepadam za muzyką disco polo a taka przeważnie niestety tam jest.

**Co z koncertami, które miałeś w planach?**

Jeden, który miał być jutro w Krakowie został już przeniesiony do Gdańska na przyszły rok. Trochę szkoda, że się nie odbędzie, ale fajnie pod tym względem, że w Gdańsku, bo bardzo lubię Trójmiasto i organizatorzy zachowali się na tyle fajnie, że osoby, które wykupiły bilety na dwa dni tutaj, będą miały bilety na 3 dni, bo ten nowy będzie trwał 3 dni. Wspominałem o Pol&Rock i już też wiem, że go nie będzie, tylko będzie jakaś największa domówka świata. Są jeszcze koncerty, które miały się odbyć w jesieni i tutaj nie wiem, bo sporo jeszcze nie jest odwołanych i całkiem możliwe, że się odbędą. Jeśli będą, to już bym poszedł na pewno. Kiedyś wspominałem, że bym się bał i teraz podczas wakacji też chyba jeszcze bum się bał iść, ale w sumie zmieniłem trochę zdanie i jakby coś się już odbywało na jesień, to nie miałbym żadnych oporów. Już teraz w sumie nie mam żadnych oporów, żeby chodzić w jakieś większe skupiska ludzi nawet.

**Zakupy już robisz tak jak przed pandemią?**

Tak. Planowanie jest dalej, bo jednak, żeby ugotować coś konkretnego na co mam ochotę, to muszę zrobić listę, ale żeby konkretne pory, to nie. Przeważnie to są soboty na większe zakupy, ale wczoraj np. musiałem się wybrać kawałek dalej na Modlińską po pastę do curry, bo akurat chciałem zrobić curry na obiad.

**A co z siłowniami?**

Myślę, że niedługo bym się wybrał. Nie chce mi się samemu, póki co. Mam jednego kolegę chętnego na to, ale on mieszka na Targówku i trochę mu się czasem nie chce ruszać z domu, bo on jeszcze gdzieś dalej jeździł. Też umawiałem się na siłownię z tym kolegą, któremu spaliło się mieszkanie, ale on z wiadomych względów nie będzie teraz chodził. Póki co mam wał nad Wisłą do biegania i jakieś ćwiczenia w domu.

**Masz jakieś obawy związane z bezpieczeństwem na siłowni?**

Nie, ale zastanawia mnie w sumie, jak to będzie wyglądało. Gdzieś widziałem jakieś wytyczne dla siłowni, że każdy sprzęt trzeba dezynfekować po użyciu, coś tam trzeba robić...Strasznie dużo tego było i zastanawiam się, ile z tego będzie przestrzegane. Czy każdy uczestnik będzie brał ten płyn do dezynfekcji, dezynfekował po sobie, trzymał się 2 m od innego uczestnika siłowni? Nie wiem, ale pewnie trochę się trzeba będzie stosować do tego, bo ktoś na pewno będzie tego pilnował.

**To jest potrzebne, żeby takie zasady wprowadzać?**

Hmm...No myślę, że lepiej wprowadzić niż nie wprowadzić i że to mimo wszystko może jakoś zwiększyć bezpieczeństwo, bo na pewno są ludzie, którzy jeszcze nie zachorowali i istnieje ryzyko, że mogą zachorować. Jeżeli to miałoby pomóc zapewnić większe bezpieczeństwo, to pewnie, czemu nie. Pandemia trwa, jeszcze się to nie skończyło i myślę, że takie kroki są jednak potrzebne.

**Słyszałeś co z kinami?**

Nie.

**Mają być otwarte, ale odstępy co jedno miejsce i chyba jeszcze co jeden rząd. Tak jest też chyba w samolotach. Co o tym myślisz?**

Myślę, że takie rzeczy są trochę dla zaspokojenia samych ludzi. Uspokojenia ludzi. I te procedury w siłowniach też. A jak to się w praktyce ma, to w sumie nie wiem. Czy to ma jakieś znaczenie wielkie, czy ktoś siedzi 2 m ode mnie, czy pół metra ode mnie. Tym bardziej, że jest to pomieszczenie zamknięte, nie ma świeżego przewiewu. To są chyba procedury dla psychicznego uspokojenia ludzi, ale nie działają w rzeczywistości tak jak powinny. Dezynfekowanie powierzchni już bardziej chyba. 31:41

**APLIKACJE**

**Słyszałeś o takich związanych z pandemią?**

Tak, w sumie coś mi się obiło o uszy. Dla osób... Nie wiem, czy to jest aplikacja, ale one chyba miały obowiązek zainstalować coś w swoim smartfonie, żeby śledzić ich lokalizację. Dla osób, które przebywają na kwarantannie. O tym to już słyszałem jakiś miesiąc temu albo nawet więcej. Myślę, że spoko w sumie. Jak już wiadomo, że osoba jest chora to faktycznie przydałoby się, żeby ją odizolować od reszty społeczeństwa i jeżeli taki ktoś ma taką aplikację w telefonie, to wiadomo, co się z nim dzieje. To jest ok, bo słyszałem o jakichś przypadkach, że ktoś z potwierdzonym koronawirusem sobie gdzieś wychodził, jak gdyby nigdy nic. To faktycznie mógłby być problem. Też rozumiem obawę przed utratą prywatności, jeżeli takie coś się ma, ale z drugiej strony mamy różne inne aplikacje w telefonie, które też pewnie nas śledzą, a nawet o tym nie wiemy, więc...

**Kategoria 1**

**Aplikacje analizujące dane osobiste (bieżące informacje o stanie zdrowia, historię przemieszczania się i kontaktów z innymi ludźmi) w celu monitorowania rozprzestrzeniania się koronawirusa.**

To jest aplikacja, którą ktoś mógłby sobie zainstalować, czy musiałby sobie zainstalować? To jest różnica, czy to jest osoba chora...Jak bym sobie raczej takiej aplikacji...No historia przemieszczania się...Niezbyt, niezbyt. Gdyby u mnie zdiagnozowano koronawirusa, to myślę, że bym się zgodził, ale tak, poza tym, to dobrowolnie bym się nie zgodził. Nie mam nic więcej do dodania. Po prostu nie chcę, nie miałbym ochoty, żeby ktoś miał wgląd do takich spraw, informacji. Ktoś może i tak już ma, ale przynajmniej nic mi o tym świadomie nie wiadomo.

**Ta aplikacja pobiera bieżące informacje o stanie zdrowia, historię przemieszczania się i historię kontaktów z innymi ludźmi. Czy czegoś szczególnie byś nie chciał udostępnić?**

Myślę, że historia przemieszczania się. W ogóle nie widzę potrzeby udostępniania tego komukolwiek, kontakty z innymi ludźmi też nie, informacje o stanie zdrowia w sumie też nie. Po co komu informacja o moim stanie zdrowia? Chyba, że byłbym chory na koronawirusa i wtedy to co innego. Tak to nie. Nie widzę żadnej potrzeby, żeby coś takiego robić.

**Aplikacje, które na podstawie danych lokalizacyjnych monitorują przestrzeganie kwarantanny domowej.**

Ok, to uważam za potrzebną rzecz, ale tylko w przypadku choroby zdiagnozowanej. Na pewno bym się zgodził na to, ale uważam, że w ogóle powinien wejść taki przepis. Jakaś ustawa dla osób, które są chore. Jeżeli tylko jest możliwość umieszczenia nadajnika GPS jakoś przy tej osobie, to powinna taka ustawa powstać.

**Aplikacje oparte na automatycznej lokalizacji użytkowników, informujące ich, że znajdowali się w miejscach zagrażających zarażeniem się koronawirusem.**

Hmm...Nie wiem w sumie. Ta automatyczna lokalizacja użytkowników...To jest takie wątpliwe, ale z drugiej strony miejsca zagrażające...Nie wiem. Tutaj nie mam trochę zdania. Chodzi mi o tę automatyczną lokalizację, bo ja, jak chcę sobie włączyć lokalizację w telefonie, to robię to manualnie, a tutaj coś, co mnie samo namierza...To tak nie wiem. Tu bym się trochę kłócił. Potencjalnie każde miejsce może być zagrażające zarażeniem się koronawirusem, bo wszędzie są ludzie, przynajmniej w mieście, więc chyba też nie. Raczej na nie. Jeżeli to jest dobrowolne to ok, ale ja bym się raczej nie zgodził, bo niepotrzebne mi są te rzeczy.

**Mógłbyś się np. dowiedzieć, że w miejscu, gdzie byłeś wczoraj, przebywała osoba, u której zdiagnozowano koronawirusa.**

Ok, to bardziej widziałbym to na zasadzie takiej mapki, że sobie pobierasz, ona jest aktualizowana na bieżąco, ale bez podawania twojej lokalizacji.

**Monitoring z automatycznym systemem rozpoznawania twarzy, w celu szybkiej identyfikacji osób nieprzestrzegających zaleceń władz.**

      To chyba też nie. Raczej zdecydowanie na nie. Po prostu nie. Z rozpoznawaniem twarzy to zawsze miałem jakieś obawy w sumie nawet, jeżeli chodzi o kamerki w laptopach, w telefonach. Też często się słyszy, że dobrze to zakryć jakąś taśmą, bo może się ktoś włamać do naszego urządzenia i nas po prostu obserwować i takie coś to już...To jest dość takie agresywne działanie. Ja bym się raczej nie zgodził, bo to też miałoby być dobrowolne?

**Nie wiem, jakby to miało działać.**

      Nie, to jest trochę bez sensu. Uważam, że to jest mocna ingerencja w prywatność, dane osobowe. Tak nie bardzo

**Kategoria 2**

**Drony dostarczające produkty medyczne i inne towary osobom potrzebującym.**

      Ok. To akurat super pomysł. Drony są dość nową technologią i pewnie, że tak. Uważam, że to byłoby dobre rozwiązanie, tylko też trzeba by było to mocno kontrolować, żeby nie zostały jakoś przechwycone albo, żeby nie wykorzystać takiego drona do jakichś niecnych celów, np. do stworzenia zagrożenia jakiegoś terrorystycznego. Może zamiast dostarczać materiały medyczne to dilerzy narkotyków by korzystali z takich dronów, więc to by trzeba było dobrze kontrolować po prostu

**Aplikacje, w których użytkownicy mogliby informować się o tym, czego potrzebują i dzięki temu pomagać sobie nawzajem.**

      Ciekawe, czemu nie? Myślę, że ok, ale nie wiem, czy trzeba by było dedykowaną aplikację do tego stwarzać. Można by było po prostu na jakimś forum, nawet na portalu społecznościowym zrobić grupę i może tam się wymieniać takimi informacjami, ale w sumie, jeżeli to by miało komuś pomóc w zrobieniu jakiegoś projektu takiej aplikacji i miałby coś dzięki temu osiągnąć, a jeszcze ktoś by na tym faktycznie skorzystał, to czemu nie.

**Miałbyś jakieś obawy, żeby konkretnie rządowi przekazywać jakieś swoje dane?**

      Tak. W sumie nie wiem czemu. Nie chcę być inwigilowany po prostu. Nie wiem, czy ma dla mnie różnicę, czy to jest aplikacja rządowa czy jakiejś prywatnej firmy. Chyba nie ma. Nie chciałbym być inwigilowany i tyle. Jeżeli chodzi o media społecznościowe, to też staram się podawać jak najmniej informacji, na FB nie wrzucam już zdjęć, itd. Uważam, że im mniej się podaje informacji w internecie, w sieci o sobie, tym człowiek powinien się poczuć bezpieczniej. Byłbym skłonny do tego, jeżeli faktycznie byłbym w kwarantannie, ale jak nie mam zdiagnozowanej choroby, to raczej nie.

**Aplikacje sztucznej inteligencji, decydujące na podstawie zebranych danych, gdzie skierować największe środki i wysiłki do walki z pandemią.**

To rozumiem, że też jakieś aplikacje pomagające rządowi?

**Nie wiem. To wygląda na jakąś rządową aplikację?**

Jeżeli dla rządu, to spoko, bo nie wiem po co komuś innemu miałoby to być potrzebne. Może też jakimś firmom prywatnym faktycznie albo jakimś fundacjom...No, to mogłaby być potrzebna rzecz.

**ProteGo Safe [pierwszy slajd]**

Nie słyszałem o tym. Pewnie jakaś ochrona, bo od "protection" i "safe". Nic więcej mi to nie mówi.

**[drugi slajd]**

Ostatni punkt jest całkiem fajny. Jeśli ktoś faktycznie się tym interesuje, to ma wszystko zebrane w jednym miejscu, nie musi szukać, tylko odpala w prosty sposób aplikację i ok.

Dziennik zdrowie, jeżeli komuś rzeczywiście chciałoby się go wypełniać...

**A tobie by się chciało?**

Nie. Ja wiem, jakie ja mam podejście do niektórych rzeczy czasami. Coś sobie powiem, że będę robił i robię to przez jakiś czas a potem robię to coraz mniej regularnie aż nawet całkiem przestaję. Druga sprawa, to technologia Bluetooth...To jest takie bardzo ogólne dla mnie i ja nie wiem, co się pod tym może kryć. Chętnie bym poznał, co się kryje pod tym więcej. Informowanie o spotkaniach z chorymi...No, ok. Byłoby wygodniejsze, gdyby to była jakaś mapka, bo wtedy wiem, że tu może lepiej nie iść albo iść tędy, albo tędy. To rozumiem, że byłoby w czasie rzeczywistym informowanie, więc trochę za późno jakbym podszedł do kogoś i nagle się dowiaduję, że to jest chora osoba. Nie wiem, czy to jest super potrzebna aplikacja. Ja bym się nią nie interesował na pewno. Nie powinna być obowiązkowa. Dobrowolna, absolutnie. Szczerze mówiąc nie wiem, czy jakakolwiek aplikacja powinna być obowiązkowa, skoro nie można zmusić nikogo do posiadania smartfona, który ma aplikacje, np. starsze osoby. Jeśli ktoś posiada, to pewnie trzeba to jakoś udokumentować, że ktoś posiada jakieś urządzenie zdolne do odpalania takich aplikacji i wtedy może ok, ale nie wiem, czy to nie za dużo papierkowej roboty byłoby przy czymś takim.

**Kwarantanna Domowa**

**[pierwszy slajd]**

O, obowiązkowa...

**[drugi slajd]**

W porządku. Myślę, że jest ok, bo ona nie jest aż taka inwazyjna w sumie. Podajesz lokalizację, ale odbywania kwarantanny. To wcale nie musi oznaczać, że ty mieszkasz w tym miejscu, że jesteś zameldowana, itd. Formularz, to rozumiem, że podaję jakieś swoje dane. Imię i nazwisko to na pewno, nr telefonu...Podaje się chyba to, co przy zakupach internetowych, tak mi się wydaje + geolokalizacja. Dla mnie to jest spoko, bo osoby objęte kwarantanną to jest troszkę inna sprawa. W porządku. Ja bym taką aplikację pobrał. Nie znam nikogo, kto był na kwarantannie i nie znam nikogo, kto był chory nawet.

**[trzeci slajd]**

To zrobienie sobie zdjęcia jest trochę bardziej inwazyjne. Hmm...Nie wiem. Ja np. nie lubię robienia zdjęć. Mogę mieć taki kaprys i tyle, ale rozumiem, że to jest obowiązkowe i jakoś trzeba to weryfikować, że faktycznie ta osoba to ta osoba i też nie mam w sumie pomysłu, jak inaczej by to można zweryfikować. Może rozmową przez telefon i jakimś analizatorem głosu, ale to też pewnie nie jest tak super dokładne.

**Nie lubisz sobie robić zdjęć, czy chodzi o prywatność z tą twarzą?**

Też prywatność, tak. Tak na dobrą sprawę, jak robię sobie zdjęcie i je gdzieś przesyłam, to moje zdjęcie już trafia pewnie do jakiejś bazy. Nie wiem, jak to potem może być dalej wykorzystane. Uważam, że robienie sobie zdjęcia tutaj jest najbardziej inwazyjne. Sms ok i jeszcze przesłanie lokalizacji. Przesłanie zdjęcia - miałbym największe opory, żeby tak robić, ale musiałbym być chory i być postawiony przed faktem, że mam taką aplikację, mam ją sobie zainstalować i ktoś m i każe albo mnie prosi. Wtedy pewnie myślałbym inaczej, ale jak teraz patrzę, to stwierdzam, że to jest inwazyjne i miałbym największe opory przed tym, ale chyba ostatecznie bym się zgodził, jeżeli byłbym chory i miał obowiązek korzystania z tego.

**Rząd powinien wprowadzać takie obowiązkowe aplikacje?**

Takie sprawdzające, czy ta osoba faktycznie się stosuje, to chyba powinien, bo to jest oszczędność i czasu i pieniędzy, jeżeli chodzi o służby. łatwiej jest zrobić aplikację niż wysyłać służby do tej osoby co jakiś czas. I to nie jest narażanie tych osób, które przychodziłyby sprawdzać taką osobę na kwarantannie, więc to też bezpieczeństwo. No, ale jednak też ingerencja w prywatność tej osoby, bo robienie sobie zdjęcia i to nawet nie jednego, tylko kilku zdjęć podczas kwarantanny...Pewnie idzie się przyzwyczaić do tego, ale miałbym opory.

**Jak ty myślisz o przyszłości po tej całej pandemii?**

Myślę, że ludzie w końcu o tym zapomną, że coś takiego w ogóle było, jak już faktycznie każdy wyzdrowieje. Ludzie się podniosą z tego oczywiście, jak najbardziej i myślę, że zapomną nawet już całkiem niedługo, może już nawet zapomnieli co nieco, że coś takiego jak koronawirus istnieje. Ja samo to hasło słyszałem dość dawno z ust czyichś. Już nie widzę aż tylu informacji w tym upday'u, o którym wspominałem wcześniej. Bardziej nie informacje na temat samego koronawirusa, ale już funkcjonowanie gospodarki, itd. po tym koronawirusie. Tam raczek skutki są pokazywane. Mi koronawirus nawet trochę pomógł, jeśli chodzi o pracę, bo dostałem ją w sumie właśnie dlatego, że jest koronawirus. Był tam potrzebny człowiek młody, który jest w stanie też przenosić jakieś ciężary nieraz i który ma wyższe wykształcenie w tym kierunku, więc byłem idealnym kandydatem. Jeżeli chodzi o moją przyszłość, to ona się nakreśliła całkiem fajnie i pozytywnie dzięki temu. Nie spodziewałem się tego absolutnie i jestem zaskoczony wręcz, że się tak udało zdziałać. Najciężej będzie miał sektor kultury, czyli właśnie koncerty, być może kina i artyści też różni muzyczni. Mimo wszystko oni głównie żyją z koncertów, jakichś tam wydarzeń, wydawania płyt i to też było utrudnione w czasie tej pandemii. Myślę, że ogromne straty niektórzy ponieśli, jeżeli chodzi o różne kluby i tego typu rzeczy. Często widziałem na FB, że zwracają się o pomoc do ludzi, bo mogą zaraz upaść. Ten sektor miał najtrudniej i on będzie najdłużej wstawał, ale też myślę, że w końcu się to odbuduje, bo ludzie tego potrzebują po prostu. Potrzebują rozrywki, koncertów, wydarzeń, kina. W końcu to wszystko stanie na nogi, ale nie wiadomo, jak długo to zajmie.

**Uważasz, że zbiórki na kluby to jest dobra rzecz?**

Pewnie, że tak. Żyjemy w takich czasach, że ludzie w ogóle nie boją się prosić o pieniądze w internecie. Powiedziałbym, że nawet to jest już naturalne, że rożni youtuberzy wrzucają już link po prostu, żeby można było wpłacać jakieś darowizny. Jeśli chodzi o kluby, to jest to dobre posunięcie i pozwalające im przetrwać, a są na pewno ludzie, którzy mogli sobie pozwolić, żeby wspomóc swój ulubiony klub albo swojego ulubionego artystę, albo swojego znajomego, który pracuje w branży muzycznej, etc.

**Czyli nie będzie jakichś dużych załamań rynkowych, gospodarczych, światowych?**

Pewnie już są, ale to wszystko w końcu wróci na dawne tory. Już tak było, że niektórzy przeżyli załamanie, bankructwa, itd., i to też zweryfikowało, kto jest najsilniejszy na rynku. Cóż, każdy będzie sobie musiał w końcu jakoś poradzić gorzej lub lepiej, ale myślę, że jakoś sobie poradzi w końcu. Mój kolega, któremu zamknęli firmę, któremu spaliło się mieszkanie na dodatek, to cóż...Lekko nie ma, ale żyje i ma uśmiech na twarzy, i daje radę, i wie, że się z tego podniesie. Jak on to zrobi, to zrobi to też wiele innych osób.

**Czy jakieś ograniczenia, zasady powinny być utrzymane dłużej albo zostać na zawsze?**

Na pewno fajnie by było, ale nie można tego traktować, jako nakaz, bardziej jako taki wewnętrzny przymus. Ludzie powinni o tym pamiętać i stosować się do tego w przyszłości, żeby właśnie ręce odkażać, po każdym przyjściu do domu i w każdej możliwej sytuacji te ręce umyć nawet i umyć je dokładnie a nie pobieżnie bardzo. Tak jak się widuje na tych wszystkich instrukcjach - przez 30 s, między kciukami, itd. To powinno zostać, ale ciężko to traktować, jako jakiś przymus, nakaz. Pewnie niektórzy będą się do tego stosowali. Jeśli chodzi o inne nakazy, to nie mam pomysłu za bardzo co mogłoby zostać.

**U ciebie coś zostanie?**

Tak, na pewno to mycie rąk, chociaż też nie wiem, na jak długo w sumie, ale na pewno będę miał taką świadomość przez najbliższy czas, że było coś takiego i warto dbać o tę higienę bardziej, lepiej. Co jeszcze?...Zakupy robię tak, jak robiłem, poruszam się też tak ja było. Chyba nic się u mnie takiego nie zmieni na dobre.

**Mówi się trochę o 2 fali?**

Tak, po tym odmrożeniu, po zluzowaniu tych obostrzeń, czyli te zgromadzenia do 150 osób, większa liczba ludzi w kościele. O tym słyszałem, ale nie, że ktoś o tym oficjalnie mówił tylko na zasadzie, że słyszałem i mi się obiło o uszy.

**Myślisz, że można się jakoś przygotować na kolejną falę?**

No tak. Myślę, że można już się przygotować na robienie większej ilości testów, skoro to jest już spodziewane, że może być ta 2 fala. Szycie maseczek. Te maseczki są sytuacyjne i jeżeli ktoś jest chory, to faktycznie pomaga, bo rozprzestrzenianie się, ale osobom zdrowym niekoniecznie. Można doszyć maseczki i tak się przygotować. Ja nie odczuwałbym potrzeby robienia zapasów jedzeniowych. Uważam, że jedzenia to na pewno nie zabraknie prędko, chyba że będzie jakaś wojna, ale to co innego. Ze swojej strony może wyposażyłbym się w więcej rękawiczek i w zasadzie tyle. Z tymi rękawiczkami faktycznie czasami był problem jak chciałem gdzieś kupić a wygodniej było mieć swoje, bo ja tych foliowych w sklepie strasznie nie lubię. One są niewygodne. Obecnie w ogóle nie odczuwam potrzeby, żeby kupować większą ilość.

**Czy myślisz, że rząd jakoś inaczej się powinien się zachować przy 2 fali czy znowu lockdown, zamykanie usług?**

No pewnie tak, bo jednak, mimo wszystko, sporo to dało, że właśnie było takie zamknięcie, że były zamknięte studia fryzjerskie, kina, ograniczenie osób w sklepach. To się chyba w pewnym momencie uspokoiło. Nie wiadomo, jak będzie teraz, ale myślę też, że już w tym momencie coś powinno zostać przez rząd omówione, przygotowane. Właśnie, co w razie, gdyby była ta 2 fala. Jak teraz znowu pomóc przedsiębiorcom, jeżeli zamkną im się firmy znowu. Takie finansowe plany, to myślę, że rząd powinien układać.

**Któreś decyzje inaczej powinny zostać podjęte?**

Tak, chyba stan klęski żywiołowej. W końcu nie wiem, czy został ogłoszony. Chyba nie. Wydaje mi się, że powinien zostać ogłoszony. Czemu, to teraz nie powiem, ale wiem, że to miało mieć jakąś ważną konsekwencję, że gdyby on zostało ogłoszony, to coś tam i to by gdzieś pomogło w jakimś sektorze albo właśnie przedsiębiorcom pomogło w czymś. Teraz, gdyby było znowu tak poważnie jak wcześniej, to od razy trzeba by to zrobić i tyle.

**Najważniejsze dla ciebie momenty przełomowe?**

Z tego co pamiętam, to wybory, które w końcu się nie odbyły, ale miały się odbyć i wielkie jazdy z tym były, co z tym zrobić. No i to kombinowanie PiS do ostatniej chwili, może trochę sprawdzanie wyborców, jak oni się zachowują. W sumie to było dość ważne i chyba cała Polska tym trochę żyła. Nie, że dla mnie to było jakoś wyjątkowo ważne, ale trochę to obserwowałem i nie dało się o tym nie wiedzieć. To bym uznał za kluczowy moment pandemii, a poza tym to było powiązane dość mocno z pandemią. Z ważnych rzeczy to zwolnienie się dziennikarzy z Trójki. To też bardzo istotne dla mnie. Lubię Trójkę, lubiłem, dużo jej słuchałem, lubiłem wielu tych dziennikarzy i to było takie nakładanie cenzury w mediach publicznych, a za komuny już nie żyjemy. To był bardzo duży cios. Dalsza destrukcja mediów publicznych. Co jeszcze?...Wprowadzenie nakazu noszenia maseczek. To był taki znak, że dzieje się, że dzieje się dużo. Chyba też w kwietniu, jak rząd ogłosił, że można chodzić do lasów, do parków. To było fajne, że jest już trochę luźniej i to też się dało odczuć. I chyba tyle w zasadzie. Zapadnie mi w pamięć na pewno to spalone mieszkanie, bo to podczas pandemii, że dostałem w końcu nowy sprzęt komputerowy, na który czekałem bardzo długo w sumie, że zaraz przed pandemią udało mi się zamieszkać w bardzo fajnym mieszkaniu, bo też często sobie myślałem, że ale by była lipa, jakbym nadal mieszkał w tamtym mieszkaniu. Nie było takie złe, ale mieliśmy patologiczną rodzinę zaraz obok. Patologiczni na maksa i do tego stopnia, że sprowadzili plagę pluskiew na blok.  I chyba tyle w zasadzie.
