# Supplementary material for: The challenges arising from the COVID-19 pandemic and the way people deal with them. A qualitative longitudinal study
Source: PLoS One. 2021 Oct 11;16(10):e0258133. doi: 10.1371/journal.pone.0258133 (PMC8504766; doi:10.1371/journal.pone.0258133)
Supplement: S1 Dataset — (ZIP) [file pone.0258133.s003.zip › Transcriptions/stage 5/17.5_F_35_single, with child.docx]

**17.5_F_35_single with child**

**Co się działo przez ostatni miesiąc?**

Wróciliśmy do normalności, ewidentnie. Przynajmniej u nas, ja wróciłam do normalności, znowu dużo pracuję, nie mam na nic czasu, dzień jak co dzień.

**Czyli przez ten ostatni miesiąc pracowałaś?**

Tak, cały czas.

**Czy były jakieś ważne momenty w międzyczasie?**

Nie, od momentu, kiedy oficjalnie otworzyli tę moją branżę, to dla mnie to już wszystko wróciło do normalności. Nawet już nie wiem, który jest etap, co tam otwierają, co można, czego nie można. Jeszcze tydzień temu chyba był taki moment, że te maseczki można ściągnąć, lokale pootwierali, naprawdę ja się czuję, tak jakby było normalnie, dzień jak co dzień.

**Zdjęcie maseczek i otwarcie lokali było ważne dla ciebie?**

Niby nigdzie... Chociaż nie, byliśmy na pizzy, ale tak jakby nie odczuwam braku tych lokali, tylko to był taki moment "ok, już jest normalnie", już nastał dzień, kiedy wróciliśmy do normalności.

**Czy pozostały jakieś zachowania związane z epidemią?**

U mnie absolutnie nic. Potem jednak się zorientowałam, że wchodząc do sklepu, to w ogóle maseczka potrzebna, o, Jezu, ludzie w maseczkach, a, to powinnam mieć maseczkę, dobra, powiem, że mam astmę - totalnie, jakby nie istniał jakikolwiek... To, co było miesiąc temu, jakby tego nie było.

**Czyli podczas zakupów też nie nosisz maseczki?**

Nie, tym bardziej, że mnie te maseczki na mieście i w sklepie denerwowały, nie noszę, mam błąd po prostu, absolutnie nie ma koronowirusa koniec, kropka.

**Co wpływa na to, że czujesz, że wróciłaś do normalności?**

Chyba to, że zaczęłam rozmawiać z ludźmi i kogo nie zapytam, ale naprawdę, kogo nie zapytam, ja nie wiem, gdzie był ten koronawirus, chyba tylko w telewizji i w szpitalach, bo nie znam osoby, która by mi powiedziała: "Moja babcia ciężko przeszła, mój teść ciężko przeszedł", bo ja nie mówię o młodych ludziach, że mają umierać, ale nikt absolutnie, ani jedna osoba, a mam mnóstwo klientek, nikt mi nie powiedział: "No ale wiesz, u mnie znajomy, bliski sąsiad, ktokolwiek miał koronawirusa i leżał tydzień w łóżku". Wszyscy albo mieli testy, ale mieli bezobjawowo. No to jak mieli bezobjawowo, to kogoś by zarazili pewnie, a tutaj się okazuje, że wszyscy przechodzą bezobjawowo albo w ogóle nie mieli kontaktu, tak jakby ten koronawirus jest tylko w telewizji. Znaczy, on pewnie istnieje, nie mówię, że nie, ale nie sądzę, że jest aż tak śmiercionośny jak nas nastraszyli, a poza tym miałam kontakt z pielęgniarkami i one same nawet w szpitalach mówią, że to nie jest tak straszne, jak nam to przedstawiają, więc... nie ma.

**To trochę uspokaja?**

Tak.

**To znajomi wyciągnęli cię na pizzę?**

Nie, byliśmy rodzinnie, w niedzielę się nam nudziło i tak, no dobra, to idziemy na pizzę, no okej, to było takie normalne. Szokiem było dla mnie to, że o godzinie 18 nie było miejsc w restauracji. I ja taka: "O, kurczę!" i musieliśmy zmienić lokal, bo tam, gdzie zawsze jeździmy, o 18 (!) nie było absolutnie pół stolika wolnego.

**A to było związane z tym, że stoliki są dalej poustawiane?**

Było tyle ludzi. Nic nie było mniej tych stolików, dalej było ciasno jak nie wiem, mnóstwo ludzi.

**Czyli w środku nie zauważyłaś żadnej zmiany?**

Nie. Oprócz karteczki, że można zdjąć maskę w momencie zajęcia miejsca - pff, okej...

**A w tym drugim miejscu?**

Też było normalnie. Była karteczka, że w środku może być 20 osób, my usiedliśmy na zewnątrz, bo akurat wyszło słońce, ale nawet po ludziach nie było widać, żeby się coś krępowali, żeby się jakoś odsuwali od siebie. Mam wrażenie, że te wszystkie wytyczne, które są w tych restauracjach, w sklepach, to są tylko dlatego, żeby nie dostać mandatu. Tylko po to, bo ktoś nam kazał, to zrobimy te żółte linie. Tam było jakieś miejsce wytyczone dla osób odbierających jedzenie na wynos, ale nikt się tego absolutnie nie trzymał, nikt.

**Czy pojawiło się jakieś spotkanie ze znajomymi?**

W takich małych gronach, typu znajoma wpadła na kawę, to tak, ale nie grillowaliśmy jeszcze, ale nie ze względu na koronawirusa, ale bardziej z braku czasu, nie ma okazji na razie, zaraz jest Boże Ciało, to pewnie będziemy gdzieś tam grillować. A, nie, przepraszam, byłam na jakimś grillu, dwa tygodnie temu. Ale to nie było w ogóle takie podejście na zasadzie "Oj, spotkajmy się, bo był koronawirus", tylko "Robimy grilla?", wiesz takie... Naprawdę, już widzę, że wszyscy zaczęli wracać do normalności. Są jakieś tam jednostki, nawet powiem ci, że mój syn... Kiedyś tam wygrali jakąś grę terenową z jakiejś tam szkoły tańca i pani z tej szkoły się do mnie odezwały, że może by to teraz zorganizować, dzieci byłyby pod ich opieką, no bo we wrześniu nie wiadomo, jak to będzie. Ja mówię, że nie wiem, bo nie wiem, jaka jest sytuacja w klasie, to na 25 dzieci troje rodziców mi odpisało, że nie, a reszta, że tak, jak najbardziej, super, ekstra, idziemy. Więc to przedstawia sytuację, jak ludzie do tego podchodzą.

**Na tym grillu też zachowywaliście się normalnie, jak wcześniej?**

Tak jest.

**A jak to wygląda u ciebie w pracy?**

Wytyczne wytycznymi, a życie życiem. Nic się nie zmieniło przed pandemią a teraz, takie same mamy środki dezynfekujące, takie same rękawiczki. Pracuję normalnie, nawet już klientki te, które były na początku, a przychodzą teraz, to już przychodzą bez masek. Bo na początku wchodziły w maskach na zasadzie "Muszę czy nie muszę?", teraz je ściągają. Przecież ja i tak pracuję w masce, więc dla mnie nie jest to nic nowego. Myją ręce tak jak zawsze, bo trzeba umyć ręce, jak się gdzieś wchodzi itd. Więc nic, naprawdę nic się nie zmieniło. Wytyczne mówią, że nie możemy dawać kawy i herbaty, ja daję. Bo możemy dawać wodę w kubku jednorazowym, a herbaty nie, dla mnie to jest totalna bzdura. Normalnie, naprawdę.

**Macie poczekalnię? Bo podobno nie można.**

Teoretycznie nie, ale mamy. Wiesz, u nas to jest jeszcze tak, że te klientki tak są zapisywane, zawsze tak były, żeby one nie czekały, bo nikt nie lubi czekać. Więc mamy tę poczekalnię, jak ktoś przyjdzie 5 min wcześniej, to przyjdzie.

**Ale może sobie usiąść czy tam są taśmy?**

Nie, mamy normalnie, mamy kanapę, siadają. Normalnie, no naprawdę, normalnie funkcjonujemy. Jeszcze, chyba jak poszła ta informacja, że można zrobić wesele do 150 osób, to sorry, z całym szacunkiem do rządzących, ale skoro na weselu przy 150 osobach ja się nie zarażę, a zarażę się u kosmetyczki, siadając na kanapie, ludzie, no, trochę zdrowego rozsądku.

**Czy zmieniło się coś u twojej rodziny i znajomych?**

Wszyscy funkcjonują już całkiem normalnie, moi rodzice normalnie funkcjonują, wiem, że zakładają maski, jak gdzieś dalej wychodzą, ale nie ma już takiej paniki. Moja siostra normalnie, mój brat ma do nas przyjechać, bo ja nie wiem, czy ci mówiłam, mój brat nie był u nas od początku koronawirusa, czyli od marca. Nie wiem do końca, bo w sumie z nim nie rozmawiałam, czy to było na zasadzie, że się boi, czy się boi o rodziców, czy mu się nie chciało. Mają przyjechać w weekend, więc pierwszy raz się tutaj pojawią. A tak to normalnie, już ja byłam u babci, moja mama była u babci, już jakoś tak stwierdziliśmy, że skoro przez te dwa miesiące, kiedy ja wychodziłam, pojawiałam się między ludźmi, gdzie moi rodzice byli w górach, bo oni pojechali w góry i wrócili, nikt nigdzie nie jest zarażony, to już się nie zarazimy chyba.

**Co to znaczy "gdzieś dalej"?**

Do sklepu jak jadą, na spacer nie zakładają.

**Zakładają maseczkę w domu czy dopiero w sklepie?**

W sklepie, tylko ją zabierają ze sobą. Tak trochę może z ostrożności, trochę, żeby mandatu nie zgarnąć, nie do końca zakładają tylko dlatego, żeby się nie zarazić i to tak jest pośrodku gdzieś.

**Czyli ty się wyłamujesz, oni się jednak tego trzymają?**

Całe życie byłam zbuntowana.

**Czy jest jeszcze coś, co ci przeszkadza?**

Nagonka w telewizji. Denerwuje mnie to, nie oglądam. Mówiłam ci zresztą, że ja wcześniej bardzo lubiłam być poinformowana, w tej chwili w ogóle nie oglądam wiadomości, drażnią mnie, denerwują mnie, mam wrażenie, że to jest propaganda i robią nam wodę w głowie. Jedynie informacje, które oglądam, to na Instagramie takiego Łukasza, który przedstawia suche fakty. Więc to jest jedyne, co ja oglądam, tak żeby zobaczyć, czy rzeczywiście ta pandemia jest czy jej nie ma, w sensie, czy aż tak umieramy czy nie i nic więcej nie oglądam.

**Instagram jest twoim źródłem informacji o świecie?**

Tak, ten chłopak wszystkie takie ciekawe informacje ze świata tam wrzuca, więc mam taką 5-minutową dzienną pigułkę, co się zadziało na świecie.

**Co czułaś, gdy można było zdjąć maseczki na zewnątrz?**

Że wreszcie będzie normalnie. Jeszcze miałabym jakieś racjonalne wytłumaczenie dla chodzenia w maseczkach w sklepie, byłabym w stanie sobie sama wytłumaczyć, po co je mamy, ale na ulicy, to przecież była paranoja, jak w kagańcach, ja nie mogłam znieść tego widoku. I te spojrzenia! Jak idziesz w maseczce, to patrzą na ciebie, bo idziesz w maseczce czy patrzą, dlaczego ją masz i ci ludzie tak dziwnie się wszyscy na siebie patrzyli, tych ludzi się w ogóle nie rozpoznawało, no tragedia ogólnie. Więc jak już pozwolili na ulicy zdjąć, to było takie "Dobra, już jest w miarę normalnie".

**A kiedy otworzyli lokale?**

Wiesz co, ja nie byłam, moja mama była z koleżankami. Ja nie byłam, nie było to dla mnie jakieś takie wow, teraz lecę po knajpach i przelecę wszystkie knajpy w mieście, natomiast sobie pomyślałam, Boże, wreszcie ludzie będą mieli pracę, wreszcie to zacznie działać.

**A twoja mama poszła od razu po otwarciu?**

A kiedy otworzyli? Oni otworzyli chyba tydzień temu i ona zaraz, bo ja wiem, w środę, bo ona była stęskniona koleżanek.

**Koleżanek czy wyjść z koleżankami, czy samych wyjść?**

Wyjść z koleżankami, bo one tam mają taką swoją grupę koleżanek, z którymi raz na jakiś czas wychodzą i myślę, że tego już im brakowało, żeby wyjść i posiedzieć, poplotkować.

**Czyli spotkanie w domu to nie jest to samo?**

Nie, ona, wiesz, musi wyjść.

**Jak się czułaś, jak wyszliście na pizzę i jak widziałaś, że jest normalnie?**

Tak się czułam, w ogóle nie rozmyślałam nad tym, że Boże, wreszcie nas wypuścili z domu, mogę wyjść na pizzę - nie, to było takie normalne. Czułam się najnormalniej w świecie, jak w każdą niedzielę, że gdzieś tam się zawsze wychodziło, czy na jakieś lody, czy na spacer, czy cokolwiek i to było takie normalne.

**Czy udało ci się przygotować jakieś zdjęcia?**

Utopiłam telefon w piątek i nie odzyskali mi w ogóle danych, nie mam nic. Wczoraj właśnie byłam, żeby mi naprawili, ale niestety procesor padł, więc to jest tragedia a nie koronawirus, bo mam tam wszystkie dane, to jest po prostu dramat w tej chwili. Więc nie mam nic. Może jak znajdę w międzyczasie, a mam też takiego złoma, więc ja nie wiem jeszcze, jak się nim posługiwać, jak znajdę to, co miałam, gdzieś mi się to pojawi na nowo, to ci wyślę na maila.

**Możesz opowiedzieć, co było na tych obrazkach.**

Nie pamiętam, bo ja jak coś przeglądałam, to robiłam screena albo zapisywałam. Pamiętam, że ze dwa lub trzy miałam, ale nie pamiętam.

**Wybierz obrazek, który opisuje, jak czułaś się w ciągu ostatniego miesiąca.**

To ja bym wzięła szóstkę. Bo jest tak spokojnie, tak normalnie, tu się w sumie prawie nic nie dzieje, czyli tak jak przez ostatni miesiąc u mnie w życiu nic się nie działo innego niż zwykle. Było tak na maksa normalnie, że ani żadnych decyzji nie musiałam podejmować, ani się nad niczym zastanawiać, wstawałam rano i wiedziałam, co mam robić przez cały dzień.

**Czy to związane było z nudą?**

Nie, z nudą nie, z taką stabilizacją, że wstaję rano, idę do pracy, wracam z pracy, jem obiad - wpadłam w tę swoją rutynę i chyba lubię rutynę, na to wygląda. I to mnie uspokoiło, że ja się nie zastanawiałam, co ja mam jutro robić, ja wiedziałam, co ja będę jutro robić, byłam w stanie zaplanować cokolwiek. Czuję się tak normalnie, jak w każdy inny dzień wcześniej.

**Jak wyglądałby obrazek przedstawiający to, jak się teraz czujesz?**

O, Matko Boska, nie mam pojęcia. Co tam mogłoby być? Cisza, spokój, jakieś pewnie słońce, taka stabilizacja. Nie mam pojęcia, jak bym to narysowała. Nic emocjonującego by tam nie było, nic by się nie działo.

**Nie czujesz się zagrożona i nie pojawiły się nowe źródła zagrożenia?**

Absolutnie, wręcz przeciwnie, wręcz wszelkie informacje, jakie dostaję z różnych źródeł, czy od klientek, czy od znajomych, czy od służby zdrowia, prowadzą mnie ku temu, żeby być spokojnym.

**A jakie są emocje u twoich bliskich?**

Oni są ku temu, że rzeczywiście można żyć spokojnie, ale nie są jeszcze tacy pewni. Ja z nimi trochę jeszcze muszę dyskusje przeprowadzać, bo są tacy na pograniczu. Wydaje mi się, że to przez ten natłok informacji, że z każdej strony dostajemy sprzeczne informacje i jak tacy dziadkowie siedzą, i tutaj obejrzą jedną telewizję, tu drugą, i jeszcze posłuchają sąsiada, to nie wiedzą... Dzisiaj będą myśleć: "Okej, rzeczywiście, no dobra, Martyna, masz rację, nie ma tego koronawirusa albo jest, ale nie jest taki śmiercionośny", ale zaraz wieczorem obejrzą wiadomości, w których powiedzą, że kolejne 200 osób jest zarażonych i oni: "No, widzisz, widzisz, jednak jest 200 osób zarażonych". Więc oni mają jeszcze huśtawkę emocji.

**Ty ich uspokajasz cały czas?**

Ja to tam im kręcę awanturę, a nie ich uspokajam, bo ja nie mogę tego słuchać! Bo ja jak słyszę, że jest 200 osób zarażonych, ja mówię: "A ile tych osób leży w szpitalu ciężko chorych? Mamo, ogarnij się!". My teraz tutaj mamy wojnę domową.

**Dla rodziców głównym źródłem informacji jest telewizja?**

Tak.

**Oni się od tego nie odcinają?**

Nie, nie odcinają się i nie są w stanie tak do końca tak jakby wyciągnąć informacje i je poskładać. Mi się wydaje, że my trochę inaczej odbieramy media, przesiewamy je i wyciągamy jakieś informacje dla siebie, a moi rodzice, szczególnie mama, to po prostu, jak jest napisane, to tak jest. To jest na takiej zasadzie... Przedstawię ci, jak moja mama odbiera informacje. Jak ja wrzucę zdjęcie na Facebooka sprzed 3 dni, powiedzmy, byłam nad jakimś zalewem, a siedzę w domu, to ona leci i mówi: "Jak jesteś nad zalewem, jak jesteś w domu?!". Ja mówię: "Mamo, tak działa internet, ja mogę teraz mówić, że jestem w Honolulu, a być u siebie na łóżku". Ona: "O, matko, ale jak to?!", czyli ona wszystko tak, jak jest napisane, jak jest powiedziane, tak ona to odbiera. No i mamy wojnę.

**Ciężko jest zachować spokój?**

Nie, bo to jest jak klepanie głupot, jakby powtarzanie niesprawdzonych informacji. Ja jej cały czas mówię: "Mamo, weź wyczytaj, czy to naprawdę jest prawda, czy to jest tak" - "Ale tak powiedzieli w telewizji!", ja mówię: "W TVN-ie ci powiedzą jedno, w Polsacie drugie, w TVP trzecie i co będzie prawdziwe?", no i to jest tak.

**Czy tobie łatwo jest zachować spokój?**

Nie, bardzo mnie denerwuje, bo nie da się jej wytłumaczyć, bo ja swoje... To też nie jest tak, że na siłę chcę jej powiedzieć: "Mamo, to nie jest prawda", tylko chcę jej wytłumaczyć, żeby pogrzebała i poszukała informacji, a ona twardo: "Bo w telewizji powiedzieli". Ja mówię: "Mamo, to nie znaczy, że jak powiedzieli, to tak jest". No i to jest wojna, ja jeden argument, ona drugi, ja jeden, ona drugi i tak się przebijamy, i tak średnio co trzy dni, jak pójdzie jakaś istotniejsza informacja w jakichś wiadomościach, no to jest wojna.

**Dlaczego twoi rodzice nie odcinają się od wiadomości?**

Bo to jest chyba to pokolenie, dla którego wiadomości były jedynym źródłem informacji albo sąsiad i nie chcą mieć alternatywy, nie umieją grzebać w mediach, chociażby w społecznościowych. I oni to tak odbierają, że skoro ktoś to tak powiedział, to tak jest.

**Dlaczego twoi rodzice nie odcinają się kompletnie?**

Nie mam zielonego pojęcia, bo myślałam, że się ludzie nie odcinają od wiadomości, że każdy chociaż cokolwiek słucha, że się nie da odciąć w tych czasach od wiadomości tak absolutnie, że nawet jak mówią, że się odcinają, to się nie odcinają, więc nie wiem. Po prostu pewnie nie potrafią. Dla mnie jest niewyobrażalne odciąć się tak totalnie od wiadomości, to prawie jak się na pustyni zamknąć.

**To jest dla ciebie niewykonalne czy ważne jest, żeby mieć dostęp do informacji?**

Dwa w jednym. Jest to dla mnie niewykonalne, bo no dobra, odetnę się od internetu, telewizji, ale jak mi sąsiad powie jakiegoś newsa, to co, udam, że nie słyszę? Niewykonalne, no i jak? No trzeba coś wiedzieć.

**Twoi rodzice myślą podobnie?**

Myślę, że tak. Znaczy, mi się wydaje, bo patrzę też na swoje rodzeństwo, my jesteśmy wychowani przy wiadomościach, że zawsze są oglądane. I to nie jest tak, że tylko ja oglądam - oni oglądają, moja siostra ogląda, mój brat ogląda, mój syn ogląda wiadomości! Dla nas to jest takie chyba normalne, dlatego dziwnie mi jest pomyśleć o tym, że ktoś nie ogląda wiadomości. My zawsze, my jesteśmy wychowani na wiadomościach, kiedyś to była 19 wieczorynka, 19:30 wiadomości i się szło spać.

**A teraz jeśli nawet nie wiadomości w telewizji, to jakiś zamiennik.**

Tak, coś muszę wiedzieć. I oni tak samo.

**Jak teraz wyglądają zakupy?**

Wróciliśmy do normalności, co nie jest dobre, bo znowu kupuję rzeczy przypadkowo, nie planuję i zaraz pewnie będzie marnowanie jedzenia, coś czuję. Totalnie wróciło do normalności: rodzice jadą, ja jadę, ja kupuję, oni kupują. W pewnym momencie nastał taki moment, że bezwiednie wróciliśmy do normalności, ale tak zupełnie.

**Jesteś w stanie określić ten moment i/lub jego przyczyny?**

Myślę, że były trzy momenty: jak mi pozwolili wrócić do pracy, więc ja już nie byłam w domu cały dzień i chcąc, nie chcąc, już nie mogłam pewnych rzeczy za nich zrobić czy tam pomóc rodzicom. Dwa, to był moment, kiedy dzieci mogły wyjść z domu. Dla mnie to też było ważne, bo młody naprawdę bałam się, że jakiejś depresji dostanie, bo już była tragedia z nim. Jednak kontakt z rówieśnikami w wieku 9 lat jest bardzo istotny i źle już z nim było, on się miotał strasznie w domu. Jak mógł wyjść z domu, to też było normalne: on jest na dworze, ja jestem w pracy, dziadkowie są w domu. No i zdjęcie maseczek to było kolejne. Te trzy momenty stopniowo spowodowały, że wróciliśmy do normalności, wszystko robimy normalnie.

**Byłaś ostatnio w galerii handlowej?**

Byłam, ale nie po spodnie, byłam, bo... A, jeszcze na jednej imprezie byliśmy! O, widzisz, zapomniałam. Córka koleżanki miała urodziny i byliśmy na grillu, no to byłam po prezent. I byłam w szoku. Bo było pusto. Podjechałam w sobotę 12-14 i zaparkowałam pod samym wejściem na parterze i taka mówię: "O, kurczę!". Normalnie o tej porze, bym musiała wjeżdżać na trzecie piętro, a to było totalnie pusto, natomiast to było jeszcze, jak trzeba było nosić maseczki i była jakaś ograniczona liczba osób w sklepie, to widziałam, że kasjerki próbują opanować liczbę ludzi na sklepie, ale nie są w stanie tego zrobić.

**W jaki sposób próbowały to zrobić?**

One jakoś liczyły. W jednych sklepach trzeba było brać koszyki i po liczbie koszyków, które zniknęły, było widać, ile osób może jeszcze wejść. Ja byłam w Empiku, to w Empiku stała kasjerka i próbowała to przeliczyć, ale ja widziałam, że jej to nie wychodzi, bo wiesz jak to w sklepie: tu wejdzie, tu wejdzie trzech, więc tak pro forma stała i liczyła. To byłam, ale byłam chwilę, z 15-20 min.

**Tylko po ten prezent jeden raz?**

Tak.

**Dlaczego otworzyli restauracje?**

Jakbym miała tak racjonalnie... W ogóle nie rozumiem toku myślenia naszych rządzących, nie pojmuję tego. Fajnie, że to otwierają, natomiast zastanawiam się, czy zamknięcie Polski na dwa miesiące, bo to był totalny paraliż kraju, rzeczywiście dzięki temu nie mamy tej pandemii czy tak naprawdę ten wirus nie jest tak śmiercionośny. Zastanawiam się, czy to otwarcie tych wszystkich restauracji, to było po to, że rzeczywiście wszystko się już uspokoiło i już wirusa nie ma, czy to są zagrywki polityczne, żeby wybory się odbyły, bo przecież teraz jest jatka o te wybory całe. Nie wiem, dla mnie, jako szarego człowieka, spoko, niech otwierają, niech ludzie mają pracę, jest lato, będziemy wychodzić, natomiast nie jestem do końca przekonana, że powody polityków były właśnie takie czy to nie bardziej chodziło o zagrywkę polityczną. Nie wiem, jednego dnia mam tak, że sobie myślę, że super, że to pootwierali, a drugiego dnia mam tak, że myślę, Boże kochany, dwa miesiące temu nas zamknęli na cztery spusty, a teraz nagle wirus w połowie marca przyjechał i w połowie maja powiedział: "Dobra, to ja idę gdzie indziej". I nie wiem, czy kiedykolwiek będzie taki dzień, że sobie stwierdzę, okej, było tak i tak. Myślę, że może dzieci naszych dzieci dowiedzą się, jaka była prawda w tym wszystkim.

**Masz wątpliwości co do tego, czy to jest bezpieczne?**

Mam wątpliwości co do tego, nie czy to jest bezpieczne, tylko czy to, że nas zamknęli na dwa miesiące, było dobrą decyzją.

**Czy to było potrzebne, w tym sensie?**

Chociaż dwa miesiące temu, ja wszystkim mówiłam, że bardzo dobrze, że nas pozamykali, że to jest dobre rozwiązanie. Natomiast zastanawiam się, z takiego medycznego punktu widzenia, czy wirus jest w stanie w dwa miesiące po prostu powiedzieć: "Dobra, sorry, człowieku, ja odchodzę"? Nie mamy na to lekarstwa, po prostu wszystkie kraje się otwierają, granice się otwierają, po prostu high life, wycieczki. Więc zastanawiam się tylko, czy te dwa miesiące temu ten paraliż był potrzebny. Oni tam w telewizji opowiadają, że te tarcze itd. i że tak wspomagają polską gospodarkę. Wystarczy porozmawiać z co drugim szarym człowiekiem, to wcale nie jest tak łatwo, ludzie naprawdę mają teraz problemy finansowe, naprawdę wiele firm się pozamykało, tych małych, bo te duże zawsze sobie dadzą radę. Tu mają problem. No właśnie w tę stronę, czy dwa miesiące temu ten cały paraliż miał sens, nie wiem.

**Czy miałaś okazję być u fryzjera, w kinie, na siłowni...?**

Nie, nie byłam. Na siłownię nie chodzę, więc w ogóle... W kinach nie byłam, natomiast bawi mnie to co drugie miejsce wolne. Nie rozumiem tego, totalnie tego nie rozumiem. Czy jeżeli będę siedziała co drugie siedzenie, to się nie zarażę, a jak usiądę obok kogoś, to się zarażę? Tu z jednej strony opowiadają, że wirus przenosi się na klamkach i wszystkim, a z drugiej każą się odsuwać od człowieka. Czuję się jak w jakimś matrixie. Jak był taki film "Truman Show", kojarzysz? To ja się tak czuję. Ktoś, coś tutaj nie gra.

**Dlaczego otworzyli takie miejsca?**

Bo się ludzie buntowali. Bo ludzie potrzebowali już normalności. Nie tylko ja zauważyłam, że wśród znajomych nie ma ludzi umierających, chorych. Poza tym gospodarka padła, skąd na to kasę brać, przecież w tej chwili to jest rozdawnictwo pieniędzy. Nieważne, że za chwilę będzie trzeba oddawać te pieniądze, bo są te pożyczki i subwencje z tej tarczy, przecież to ludzie dostawali miliony. Dobrze, część będzie musiała oddać, ale część odda tyko 25%, no to przecież państwo nie ma pieniędzy, żaden z rządzących nie wyjmie ze swojej skarbonki, oni muszą ruszyć gospodarkę, żeby to wszystko miało ręce i nogi. Więc myślę, że to poszło.

**Byłaś może u fryzjera?**

Oczywiście.

**I jak to wyglądało?**

Normalnie. Najzwyczajniej w świecie. Ja chodzę do małego fryzjera, taki wiesz, mały salon, tam jest jedna dziewczyna. Normalnie, miała wystawione maseczki, płyn dezynfekujący, którego nikt nie używał, tylko umyliśmy ręce i tyle.

**Ale założyłaś tę maseczkę i ona też ją miała?**

One sobie stały, gdyby ktoś chciał wejść i założyć, to żeby nie było, że nie ma. Normalnie było, naprawdę.

**I fryzjerka nie miała żadnej przyłbicy, niczego takiego?**

Nie. Z tego, co ją pytałam, to mówi, że mnóstwo fryzjerów jednak nie pracuje, pracują tam, gdzie mają duże salony i zakładają do zdjęcia na Instagramie. Więc ja mówię, no właśnie, mamy teraz dwie rzeczywistości.

**A myślisz, że to bezpieczne?**

No ja nie sądzę, żebyśmy się mieli zarazić, myślę, że jest ok.

**Tak samo jak w kinie, tak?**

Tak, dokładnie, jeżeli na wesele... Albo nosimy maseczki, bo się zarazimy wszędzie, albo nie nosimy maseczek, bo się nigdzie nie zarazimy. Nie widzę różnicy w tej chwili, czy ja się zarażę w kinie, na weselu czy u fryzjera, wszędzie dla mnie jest to samo.

**A to dobrze, że te maseczki są obowiązkowe tylko w pomieszczeniach zamkniętych?**

Biorąc pod uwagę kasjerki, czy panie w urzędzie, które mają do czynienia co 15 min albo i częściej z obcą osobą, to okej, niech ci ludzie zakładają maseczki i dla ich bezpieczeństwa niech one mają te pleksy, po co ma im człowiek, jak mówi, to plunie przez przypadek, więc okej. Ale dla mnie, jeżeli ja wejdę do sklepu na 15 min? Myślę, że ryzyko zarażenia jest minimalne. Wiadomo, jak przejdzie ktoś koło mnie i kichnie, ale to mnie może wszystkim zarazić. Nie wiem, czy oglądałaś, ale podawali statystyki, że rok temu o tej samej porze roku zmarło 3000 więcej ludzi, to są dane z GUS-u, a dwa lata temu 4000 więcej ludzi, więc ja się pytam, gdzie jest pandemia. Nie zgadzają mi się statystyki. Ja tu nie wyciągam jakichś pochopnych wniosków, tylko patrzę na liczby i coś mi się tutaj nie zgadza.

**A podawali wytłumaczenie czy tylko suche liczby?**

Gdzieś tam się jakiś lekarz wypowiedział, że niby teraz bardziej o siebie dbamy, jesteśmy ostrożniejsi itd. Fakt faktem nie było tam podanej przyczyny zgonów, natomiast... Ja nie mówię, żeby miało umrzeć więcej ludzi w tym roku, ale to nawet nie jest taka sama liczba ludzi.

**A przemawia do ciebie to wytłumaczenie tego lekarza?**

No nie, błagam. Jeżeli w tych danych były zawarte wypadki samochodowe, to okej, ludzie siedzieli w domach, nie jeździli, to nie było wypadków samochodowych. Ale na zawał, wylew, zapalenie płuc czy na raka oni i tak by umarli. Nic mi to nie działa. Więc mówię, dla bezpieczeństwa pań kasjerek okej, niech one mają te pleksy, natomiast ja nie czuję się jakoś bardziej zabezpieczona przez maseczkę.

**Czy słyszałaś o aplikacjach stworzonych na czas pandemii?**

A jak się nazywają, może coś kojarzę?

**Jedna to ProteGO, druga Kwarantanna.**

Kwarantanna to gdzieś słyszałam, ale nie wiem, jak to działa, nie interesowałam się tym, a to pierwsze to nawet nie wiem, co to jest.

**Prezentacja aplikacji. Co o nich sądzisz?**

Dla mnie to jest inwigilacja. To jest tak, jak kiedyś, za czasów komuny robili to nielegalnie i podsłuchiwali ludzi, teraz ludzie sami się dadzą kontrolować. Nie no, dramat jakiś, to jest w ogóle bez sensu. Aplikacja, która mi zbada stan zdrowia? Tere-fere kuku, mhm, jasne. Nie, nie przemawia to do mnie, a jeszcze robienie zdjęć, kontrolowanie, no po to są służby, niech przyjeżdżają i sprawdzają, jeżeli ta kwarantanna rzeczywiście ma być. Inwigilacja, nic innego.

**Któraś do ciebie bardziej przemawia?**

Nie, żadna do mnie nie przemawia. Ja o tej Kwarantannie gdzieś słyszałam, gdzieś tam właśnie było, że codziennie trzeba te zdjęcia wysyłać, no ja mówię: "Na pewno zdjęcie wysłane da coś, czy ktoś się nie przemieścił z kwarantanny". A teksty pod tytułem, żeby wszystkich namówić, że czym więcej nas będzie korzystać, tym lepiej, no nie, nie przemawiają do mnie.

**Jakie informacje pobiera aplikacja?**

Jeżeli wpiszesz tam wszystkie swoje dane, nie wiem, jakie trzeba, ale na pewno chociaż dane zdrowia, to to już jest dużo. Na pewno imię, nazwisko będą chcieli, jest geolokalizacja, więc dokładnie wiedzą, gdzie ty jesteś. Ja wiem, że te wszystkie aplikacje i tak... Nie muszą już nas podsłuchiwać, wystarczy, że się włamią do telefonu i będą wszystko o nas wiedzieć, więc to naturalne, ale... Zastanawia mnie na przykład to, że mogą wymyślić taką aplikację, gdzie ty podajesz wszystkie swoje parametry zdrowia i jakiś komputer wyliczy, czy jesteś zdrowa czy chora, a nie są w stanie wymyślić systemu, który funkcjonuje prawidłowo do obsługi lekcji online. Nie są w stanie wymyślić systemu, który jest w stanie połączyć wszystkie szpitale, żeby wyniki badań były przekazywane. Nie ma dla mnie sensu taka aplikacja.

**A czy widzisz jakieś zalety?**

No co ona mi poda? Ta Kwarantanna jest tylko po to, żeby służby miały kontrolę nad ludźmi na kwarantannie, tak? Ale ta pierwsza co? Poinformuje mnie, że obok mnie idzie ktoś, kto może mieć podejrzenie koronawirusa?

**Tak.**

Boże, ja to jestem skłonna pomyśleć, że oni chcą nas pokłócić! Bo to dojdzie do tego, że ja będę na jakiejś imprezie i zaświeci mi się... To jest tylko system, to jest tylko aplikacja, wpiszę jakieś dane i on wymyśli, że ja mam jednak potencjalnie koronawirusa i co? Ludzie to wiesz, jeden przejdzie i ucieknie, a drugi nie wiem, no różne mogą być reakcje ludzi. Naprawdę są bardziej śmiercionośne choroby i nikt się tym nie przejmuje, ludzie chodzą z HIV, ludzie chodzą z naprawdę śmiercionośnymi, a tu, kurczę, koronawirus, który niekoniecznie zabija wszystkich. Ciekawa jestem, jak to się rozwinie, czy rzeczywiście mnóstwo ludzi to zainstaluje. Nie widzę, że to są dobre przesłanki rządzących do tego, żeby tę aplikację instalować.

**Czyli nie widzisz dla siebie korzyści z korzystania z takich aplikacji?**

Nie, nawet bym się bała ją zainstalować.

**A czy uważasz, że rząd powinien tworzyć takie aplikacje dla obywateli?**

Jak pamiętam, była taka informacja, że w Hiszpanii chcą wprowadzić opaski, że jakiś kolor dla niezarażonych, jakiś kolor dla wyzdrowieńców, jakichś kolor dla turystów, to jest jakaś paranoja, chcą nas oznaczać, takie wytykanie palcami: ty jesteś taki, ty jesteś taki. Nie w tym kierunku powinien świat zmierzać, a to zmierza... Ktoś, nie wiem kto... Nad szarymi obywatelami tak jakby ktoś chciał przejąć kontrolę, żeby doskonale wiedzieć, kto, co, po co, gdzie kto jest, gdzie kto chodzi, kto jaką chorobę przebył. Potrzebujemy trochę prywatności. Nie chciałabym być kontrolowana z wszelkich stron, więc nie, nie podoba mi się to.

**Dwie kategorie aplikacji. Czy widzisz jakieś różnice, korzyści?**

Ta druga jest bardziej skierowana na pomoc obywatelom, a pierwsza to jest totalna kontrola. To jest totalne kontrolowanie pod zasłoną naszego dobra. Bo ta druga, no jest to dla mnie trochę bez sensu, ale okej, jeszcze jestem w stanie zrozumieć, że chcą tam komuś pomóc, natomiast wydaje mi się, że nie ma takiej potrzeby, że ludzie sobie dają radę, żyjemy w dobie internetów, telefonów, można zadzwonić do sąsiada, żeby ci mleko przywiózł. Natomiast pierwsza dla mnie to jest totalna inwigilacja. No mówiłam, że "Truman Show".

**Ta inwigilacja budzi obawy. Z czym konkretnie są one związane?**

Sam fakt, że ktoś chce mnie aż tak kontrolować. (Przerwa)

**A czy widzisz jakieś korzyści dla całego społeczeństwa?**

Nie, w ogóle nie widzę. Po co ja mam wiedzieć, że ktoś gdzieś, nawet jak był zarażony, to co? Teraz go wyalienujemy, obtoczymy jego dom żółtą taśmą? Jak w "Shreku" było, że z widłami pójdziemy? Nie dajmy się zwariować. To jak ktoś HIV-a będzie miał, to będzie to samo? Nie widzę absolutnie żadnej przesłanki do tego, żeby takie aplikacje były.

**Czy myślisz o przyszłości po pandemii? Czy to już jest teraz?**

Ja już żyję bez pandemii, dla mnie pandemia była i jej nie mam. Nie odczułam jej aż tak... Te dwa miesiące był ciężkie, ale już wszystko wróciło do normy, w pracy jest wszystko w porządku itd., więc nie mam też takiego poczucia, że... Pewnie jakby coś mi się w pracy popsuło czy gdziekolwiek, to pewnie byłoby inaczej. Na razie myślę, że u mnie będzie normalnie, zobaczymy tak naprawdę we wrześniu, bo mnie osobiście będzie interesowało, co się będzie działo w szkole. A jak będzie na świecie? No nie wiem, jak będzie na świecie. Pandemia pandemią, ale naprawdę są inne tragedie na świecie, a nie jakiś koronawirus. Trochę ten koronawirus jest taką zasłoną dymną przed innymi istotnymi rzeczami. Takie jakieś mam dziwne wrażenie, że ktoś chce nam coś przepchnąć. To jest taka zasada, że jak chcemy przepchnąć jakąś ustawę, to zróbmy bójkę, ustawę antyaborcyjną i wtedy wszyscy będą o tym, a oni po cichu przepchną pięć innych ustaw. I tutaj mam trochę tak, że tutaj trąbią o koronawirusie, ile ludzi umiera, a... Zobacz nawet na tym Instagramie, o Nowym Jorku trochę ktoś tam wspomniał, że ktoś tam umarł, że tam się Murzyni gdzieś awanturują, że jest jakaś strzelanina, no trochę jej jest, ale nie do końca. Tam jest ogień otwarty, czy wojny gdziekolwiek. Tu koronawirus, a naprawdę dzieci umierają z głodu. Coś mi tu nie pasi.

**Czy otwarcie szkół najbardziej zaprząta twoją uwagę?**

W tej chwili tak, bo tylko to mnie interesuje tak osobiście. Zastanawiam się, co będzie z wyborami, co się zadzieje, bo jest to taki wielki znak zapytania, ta kampania jest bardzo dziwna, tak jakby nie wiadomo, co tam się będzie działo, co ci z jednej partii politycznej wymyślą nowego, nie jest to taka czysta gra. Ja wiem, że polityka nie jest nigdy klarowna i transparentna i wiadomo, że to jest polityka, ale w tej chwili jest to bardzo nieczysta i nieprawa gra polityczna. I to mnie zastanawia, bo jednak ja już jestem w tym wieku, gdzie te wybory będą mnie bezpośrednio dotyczyć. Może jak miałam 16 lat, to miałabym to w nosie, jakbym miała 70, to też już bym miała, dobra, niebawem koniec, a teraz to jest istotne, kto wygra i co się będzie działo, i czy te wybory będą przeprowadzone zgodnie z prawem i konstytucją czy może niekoniecznie. To są dwie sprawy, które mnie teraz interesują.

**Obawiasz się, że te wybory nie będą zgodne z prawem?**

Bardzo, bo zagrywki już są wbrew prawu robione i wbrew konstytucji. Zresztą, cała ta pandemia była tak rozegrana, żeby nie musieć przełożyć wyborów, więc to też była zagrywka polityczna, pomimo że okej, zamknęli, czy dobrze, czy źle to już w sumie nie wiem, ale nie zrobili tego tak, jak być powinno, żeby po prostu ogłosić stan wyjątkowy. I o tym nikt nie mówił, i do tej pory są dziwne sytuacje, drukowanie kart wyborczych chociażby.

**A teraz wina opozycji, że wybory się nie odbyły.**

Któregoś dnia oglądałam wiadomości jedynej słusznej telewizji z ciekawości, to powiem ci, że ja się nie dziwię, że ludzie w to wierzą, bo tam są tak pięknie informacje przekazane, tak są zrobione wycinki z wypowiedzi różnych ludzi, że powiem ci, no alternatywna Polska. Tego się obawiam, co się zadzieje i jak ludzie się do tego... Czy ludzie pójdą głosować, czy tupną nóżką i powiedzą: "Ja nie idę!" i to będzie masakra.

**To będzie gorsza opcja niż głosowanie?**

Oczywiście, że tak. Szanujmy wybory innych. Jeżeli jest tak, że pójdzie znaczna większość i wybiorą daną partię, to okej, to jest nas większość, ale jeżeli pójdzie minimalna liczba osób, z czego 3/4 to będą zagorzali fanatycy, no to sorry. Więc ja czasami mówię, to idźcie i zagłosujcie przeciwko komuś, nie na kogoś.

**Takie mniejsze zło?**

Tak mi się wydaje, że chociaż takie mniejsze zło. Chociaż ja w tym roku po raz pierwszy mam kandydata, ja nie pójdę głosować na mniejsze zło, tylko ja pójdę głosować na niego, ale do tej pory to tak było, żeby wybierać mniejsze zło, bo nie było kandydata, który do mnie przemówił, taki, że ja po prostu szłam i mówiłam: "Tak, okej, je jestem za tym, mówi tak, że mnie przekonuje i mu wierzę". Zazwyczaj to było tak, że trochę mu wierzę, trochę polityk - musi się do koryta dobrać. A w tej chwili jest inaczej, ale nie każdy może mieć takie poglądy, wiadomo, więc niech idą... Jak im się coś nie podoba w kraju, to niech chociaż spróbują to zmienić, a nie siedzą i tupią nóżką.

**Wspomniałaś o zamieszkach w Stanach. Jak widzisz przyszłość świata?**

Obawiam się, bo to są Stany i tam jest Trump, a Trump jest nieobliczalnym człowiekiem. Co on zrobi? Nie wiem. Też nie śledzę jakoś nagminnie polityki Stanów, ale patrząc na to, co Trump czasami robił, to nikt nie wiem, o co chodzi temu człowiekowi. I nie wiem, co się zadzieje. Na pewno Stany mają ciężką teraz sytuację. Czy to się odbije na nas, gdzieś tam na świecie? Ciężko powiedzieć, bo to są zamieszki dotyczące ich kultury, ale czy się tutaj gdzieś wściekną dalej, nie wiem, zobaczymy, co się będzie działo. Obserwuję ich cały czas, bo jestem bardzo ciekawa, czy to się zamknie w obrębie Stanów czy wyjdzie gdzieś dalej.

**Jak się może zmienić sytuacja gospodarcza?**

Mam takie obserwacje, że już popadało dużo firm, że będzie teraz znowu taka duża przepaść między najbogatszymi i najbiedniejszymi. Już było tak w pewnym momencie, że byli albo bardzo bogaci, albo bardzo biedni, tacy, co sobie latają własnymi samolotami, tacy, co ledwo od pierwszego do pierwszego i trochę wyciągam wnioski, że nastąpi to samo, nie będzie tej takiej równowagi. Bo naprawdę już było spoko w Polsce. Wiadomo, że były przypadki biedy, ale jak się patrzyło nawet tak na znajomych, to każdy jakoś sobie fajnie radził, w miarę miał przyzwoitą pensję, pracę, jeden lepiej, drugi gorzej, ale było okej. I znowu zacznie się ta przepaść, że będą ludzie, którym się udało i będą dalej bogaci i będą ludzie, którzy stracili wszystko i będą teraz się odbudowywać, będą mieli problemy. I mi się wydaje, że znowu będzie to zróżnicowanie, a to nie jest dobre.

**To w Polsce, a na świecie?**

Nie mam pojęcia, co się zadzieje na świecie. Myślę, że może być podobnie. Oczywiście tam w telewizji opowiadają, że wszędzie jest taki super socjal i wszystkim tak pomagają, tylko nie Polsce, myślę, że to nie jest tak kolorowo, bo to wszędzie dobrze, gdzie nas nie ma. Natomiast nie wiem, jaka jest tak do końca sytuacja gospodarcza w każdym kraju, jak się tam obywatele odnajdują, ale nawet jak pytam tych znajomych rozproszonych po Irlandiach, Angliach, to mówią, że ok., że mają socjal, ale że też się boją o jutro, bo nie wiedzą, co będzie.

**Czy są jakieś grupy społeczne, których szczególnie dotkną te zmiany?**

Myślę, że takie małe przedsiębiorstwa, takie jedno-, dwuosobowe, usługowe. Nawet na przykładzie salonów kosmetycznych: jak jest duży salon, ma dużą bazę klientów, to on sobie jakoś poradzi, ale jeżeli dziewczyna miała salon, gdzie miała za wynajem 3000 i dłubała paznokcie, i zostawało jej na życie z 1500-2000, może mieć problem. Czy transport chociażby, jak ktoś miał masę autokarów, to on jakoś uciuła, trochę mniej, trochę zwolni ludzi, ale jakoś może się odbije, ale jak ktoś miał jeden autokar, to będzie kłopot. I tak mi się wydaje, że te małe przedsiębiorstwa, które są bardzo ważne w gospodarce, może tu być kłopot.

**Czy któreś z ograniczeń powinny być utrzymane dłużej?**

Z jednej strony myślę sobie, że dobra, wróćmy do normalności, bo skoro są kina pootwierane itd., to po co te obostrzenia, nie ma różnicy, ludzie się grupują teraz wszędzie. A z drugiej strony czasami sobie myślę, czy naprawdę trzeba robić wesele na 150 osób. I przez to, że te obostrzenia tutaj są, tutaj ich nie ma, to trochę głupieję. Nie jestem w stanie ci powiedzieć, czy ja bym coś zatrzymała albo jakieś obostrzenia skasowała. Nie wiem, naprawdę.

**Czy planujesz sama któreś z tych rozwiązań utrzymać na dłużej?**

Nie, bo ja już naprawdę, maseczki nie używam na mieście, ręce myję, tak jak myłam, w pracy środki zachowujemy, tak jak było, więc tu się nic nie zmieniło, młody mój biega na dworze z chłopakami bez żadnej maseczki...

**A gdybyś została zaproszona na wesele na 150 osób?**

Właśnie nie wiem, zastanawiałam się nad tym, nie mam takiego dylematu, nie mam takiego zaproszenia, więc nie muszę podejmować decyzji i ciężko jest mi się odnieść do tego. Pewnie byłoby to zależne od tego, na czyje to jest wesele. Jeżeli to byłoby jakiejś kuzynki, tobym się zastanowiła, czy jest sens iść i w ogóle. Ale jeżeliby to było wesele super znajomych czy kogoś bliskiego z rodziny, pewnie bym poszła, bobym powiedziała, że nie opuszczę takiej imprezy.

**Czyli nie ilość osób a bliskość powiązań by decydowała?**

Tak.

**A czy są jakieś grupy, które powinny być dalej chronione?**

Wiesz, kto powinien być chroniony? Służba zdrowia, bo bez nich to będzie klapa. Oni rzeczywiście powinni mieć te testy, bo podobno mają robione co tydzień, żeby nie doszło do paraliżu. U nas w Radomiu, to pewnie słyszałaś, bo wszędzie było, że w Radomiu było najwięcej zakażonych, bo tam były chyba trzy oddziały pozamykane, bo doszło do paraliżu, bo jeden od drugiego się pozarażał, nawet bezobjawowo, ale jak im test wyszedł dodatni albo mieli kontakt z osobą dodatnią, poszli na kwarantannę i nie miał kto pracować. Osoby starsze zawsze powinny być chronione, ich zwykły katar może dobić. To tak jak ze znajomymi rozmawialiśmy, jak kolega mówi: „Jeżeli osoba ma depresję i rzuca się pod pociąg, co ją zabiło: depresja czy pociąg?". I on do mnie mówi: "Jeżeli osoba starsza jest schorowana, jest po prostu stara, zarazi się grypą, umarła na starość czy na grypę?". Trochę to podobne. Więc osoby starsze powinny zawsze siedzieć w domu, a nie łazić po przychodniach i po kościołach i nie stać w kolejkach od 7 rano pod przychodniami tylko po to, żeby pogadać.

**A czy powinno się ich chronić kosztem ograniczania ich wolności?**

Nie, w ogóle nie powinniśmy ograniczać sobie wolności to raz, a dwa, jak ja patrzę na te starsze osoby, czasami są takie akcje gdzieś na Mazurach, w Warszawie "Paczka dla Seniora", jak te osoby są mega samotne. I one jeszcze zostały zamknięte na cztery spusty w domach, i one potrzebują, żeby po prostu z kimś pogadać. Serio? To już czasami sobie myślę, że lepiej, żeby sobie wyszły i pogadały z człowiekiem, i miały zdrowie psychiczne w normie, niż z tej samotności by umarły na fotelu w domu, nikt by tego nie zauważył, boby im serce przestało działać. Zamykanie ludzi, ograniczanie ich wolności jest czymś najgorszym, co może istnieć. Dlaczego karą dla największego zbrodniarza jest zamknięcie go w więzieniu? Dlaczego jeżeli karzesz dziecko, każesz mu usiąść w jednym miejscu? Albo mówisz: "Idź do swojego pokoju i tam się zamknij"? My nie chcemy być ograniczani i zamykani, więc nie.

**Czyli chronić, ale w inny sposób?**

Tak.

**Służbę zdrowia trzeba chronić przez zwiększanie testów i środków ochronnych?**

Tak, niech im państwo zagwarantuje te kombinezony, maski, niech mają, tak jak być powinno, niech mają robione testy, czy one są wiarygodne czy nie, nawet dla spokoju takiego, że wiedzą, że jest test, że nie są zarażone. Bo bez służby zdrowia będzie tragedia, po prostu.

**Czy są takie działania, które powinny pozostać z nami na zawsze?**

Myjmy ręce! Może ludzie zaczną myć ręce i po prostu będą mieli odruch wchodzenia do domu i do jakiegokolwiek lokalu, i będą myć ręce. A reszta? Nie wyobrażam sobie chodzić z ludźmi dwa metry od siebie.

**Dlaczego uważasz, że powinniśmy to zostawić?**

Bo nie myjemy rąk, a najwięcej bakterii i jakichkolwiek zarazków przenosimy na rękach, jakichkolwiek, ja już nie mówię o koronawirusie, ale różne syfy mamy na rękach. Dbajmy po prostu o higienę, najzwyczajniej w świecie. Skoro epidemiolodzy mówią, że wystarczy myć ręce mydłem z ciepłą wodą, to nie trzeba kupować środków za 50 zł i rękawiczek, idź, umyj ręce mydłem za złotówkę.

**Dbajmy w ten sposób o siebie? O innych?**

Ja zawsze powtarzam, bądźmy egoistyczni i myślmy o sobie, bo jeżeli będziemy myśleć o sobie... Ja nie jestem w stanie, czy ty, czy ktokolwiek, zadbać o to, żeby inni myśleli o tym, żeby nie zarazić innych, nie jesteśmy w stanie. Ale jeżeli każdy z nas by myślał o tym, żebym ja się nie zaraziła, żebym ja nie roznosiła, takie egoistyczne "ja", to jeżeli ja pójdę i umyję te ręce za każdym razem, wchodząc gdziekolwiek, to ja tego nie przeniosę. Jeżeli każdy by tak zrobił, to tych "ja" będzie miliony i nikt tego nie przeniesie, to jest bardzo proste. Egoizm jest czasami zdrowy.

**Co myślisz o mierzeniu temperatury na lotnisku?**

Jeżeli ta temperatura byłaby rzeczywiście wyznacznikiem zarażenia, to okej, ale to jest tylko podejrzenie. Możesz być podziębiona, możesz się zmęczyć, bo biegłaś na to lotnisko. To nie jest jednoznaczne, że człowiek jest zarażony, a mierzenie temperatury to jest takie: "O, Boże, ktoś ma 37,3!" i od razu jest, że jest chory na koronawirusa, a może po prostu spocił się. Więc nie wiem, czy to jest taki środek bezpieczeństwa. I teraz co, taki człowiek, który chce polecieć, będzie miał zmierzoną gorączkę i nie poleci, bo będzie miał 37,8? Coś z nim zrobią? Ja nie wiem, jakie są dalsze procedury. Tam, gdzie ja pracuję, jest też rehabilitacja i jest taka sytuacja, że rehabilitant mierzy temperaturę i w poniedziałek, oni w poniedziałek otworzyli, przyszedł facet 37,3. I co? Zmierzył go, nie przyjęli go. Nawet nie wiem, czy on po prostu nie przywiózł żony, ale jemu też zmierzyli... Nie, on miał być przyjęty na rehabilitację, nie przyjęto go. I co? No i nic, nikt nic dalej z tym nie zrobił. Z jednej strony rozumiem tego rehabilitanta, bo mógł się obawiać, że rzeczywiście zostanie zarażony, ale z drugiej nic z tym nie zrobił, nie było żadnych innych kroków, żeby go skierować na jakieś badania, czy jakaś procedura "Jedź do Sanepidu, zrobią ci test", nic. Ja rozumiem jakieś działania, które mają jakiś cel, czyli pójdziesz na lotnisko, zmierzą ci temperaturę, masz podwyższoną, idziesz na test, siadasz w domu, czekasz 3 dni na wynik testu, dobra. Ale jeżeli masz mierzoną gorączkę, która powoduje tylko to, że ty nie możesz wykonać jakiegoś działania, to po co to jest?

**No właśnie po co wprowadza się takie procedury?**

Dla zamydlenia oczu ludzkości? Że niby tak bardzo o nas dbają? Nie wiem. Takie mydlenie oczu, że tak bardzo o nas dbają, mierzą gorączkę, jak ktoś ma gorączkę, to nie wpuszczą na pokład samolotu. Ale ten człowiek wyjdzie z lotniska, pojedzie samochodem, pójdzie do marketu, cokolwiek zrobi. To nie jest działanie, które powoduje ograniczenie zarażalności, w ogóle.

**Społeczeństwo się na to nabiera?**

Mhm, nabierają się na to. Nabierają się ewidentnie, bo nie raz słyszałam, że teraz dają dzieci do przedszkola i mają mierzoną temperaturę, ja mówię: "No i co?". Jak będą miały podwyższoną, to nie mogą wejść na zajęcia, no i co? No i nic. I to jest takie "A, bo mają mierzoną", ale po co? Jeżeliby na przykład było, że takie dziecko ma potem pójść do lekarza, ma być przebadane - okej, jest jakieś działanie, jest jakiś cel, który ma wykluczyć zarażenie. A w tej chwili? Nie ma to celu.

**A to wywołuje w tobie jakieś obawy?**

Nie, trochę mnie to bawi, że ludzie nie analizują tego, nie zastanawiają się nad tym, po co to jest robione. Ktoś im powiedział, że będzie mierzona temperatura, jak masz podwyższoną, to masz podejrzenie koronawirusa i... dalej nie ma! Ale oni się nie zastanawiają. Więc mnie to trochę bawi. Mnie bawił trochę rehabilitant, który latał jak kowboj z tym termometrem i mierzył ludziom gorączkę. Teksas mamy w pracy.

**Czyli widzisz w tym jedynie mydlenie oczu, że państwo podejmuje odpowiednie środki?**

Tak, bo nie ma to dla mnie żadnej zasadności, żadnej.

**Co myślisz o tym, że może nadejść druga fala zachorowań?**

Mamy dwie opcje: albo zrobią powtórkę z rozrywki, ale to jest według mnie mało prawdopodobne, bo nie pozwolą, żeby gospodarka znowu podupadła, albo będą mówić, że nic się nie dzieje, że sobie super poradziliśmy, że maseczki nam rewelacyjnie pomogły, będziemy wiedzieć w małych gronach, że jest znowu zarażalność, będziemy mylić grypę z koronawirusem, ludzie będą panikować. Wrzesień, październik, listopad to są miesiące, kiedy dzieciaki bardzo chorują, więc pewnie ludzie będą panikować, będą rodzice, którzy będą na siłę robić testy, nie zdziwię się, jak zaraz będą gdzieś płatne, te, co za 500 zł można zrobić, bo już te za stówkę śmigają, a podobno są w ogóle niewiarygodne. Więc bardziej obstawiam tę wersję, że powiedzą, że opanowaliśmy wirusa, wirus nie przekracza polskich granic. Wiesz jak mnie to bawi? Mnie strasznie to bawi, że w połowie marca przyszedł minister zdrowia i powiedział: "Dzień dobry, dzisiaj mamy wirusa", a na koniec maja przyszedł i powiedział: "Dzień dobry, wirus już wyszedł z naszego kraju". To jest wirus, on nie wyjdzie! Myślę więc, że to będzie tak, że ten wirus będzie, zostanie z nami, będzie w jakimś stopniu śmiercionośny jak grypa, nie mówię, że to jest to samo, bo ma inne objawy, natomiast będzie to takie... Tylko że ludzie będą bardziej panikować, czyli będą chodzić na zwolnienia, jak będą mieli gorączkę. To akurat dobrze, bo w świadomości pracowników jest tak, że jak jesteś chory, to musisz iść do pracy, bo pracodawca będzie ci suszył głowę, że co, ty z katarem nie możesz przyjść do pracy. Więc teraz jak każdy będzie chory, pójdzie, położy się na tydzień do łóżka i jakoś to będzie.

**I uważasz, że to akurat bardzo dobrze?**

Tak, bardzo dobrze, bo ja zawsze chodziłam chora do pracy, do mnie przychodziły chore klientki, było to takie normalne, bo przecież jestem tylko przeziębiona. A wystarczyło 3 dni poleżeć w domu albo tydzień i masz z głowy, a tak to człowiek chorował 2-3 tygodnie. Więc to akurat będzie ok.

**Czy jest coś, czego najbardziej się obawiasz w przyszłości?**

Tego, że nie wiem, w jakim kierunku zmierza ten świat. Jakoś tak coś mi tu nie pasuje i w tych rządzących, i w tych politycznych zagrywkach, coś tu jest takiego... Coś się dziwnego dzieje. Trochę się boję, żeby się ustrój u nas w kraju nie chciał zmienić przez przypadek, bo gdyby mój ulubieniec wygrał, to obawiam się jakichś dziwnych akcji z jego strony, bo ma takie zapędy do władania narodem. Tego się bardziej boję, bo nie chciałabym zostać ograniczona, nie żyłam w czasach komunistycznych i nie chciałabym w nich żyć. Bardziej tego, nie boję się tych wirusów.

**Czy myślałaś o tym, żeby przygotować się do drugiej fali wirusów?**

Nie, bo jak będziemy dalej funkcjonować, to tak sobie myślę, że rzeczywiście zacznie się sezon grypowo-koronawirusowy (bo teraz to będzie sezon koronawirusa pewnie), to jeżeli będę się czuła osłabiona, z jakimś stanem podgorączkowym, czy młody, czy ktokolwiek w domu, to zostaniemy w domu. Że nie będzie na zasadzie takiej "Oj tam, weź sobie Ibuprom, jutro pójdziesz do szkoły, ja pójdę do pracy". Pewnie rozsądniej do tego podejdziemy, ale to chyba tylko tak.

**Jak w idealnym świecie powinien zareagować rząd?**

Ograniczenia może nie, ja to bym chętnie... Żeby oni zrobili z taką ręką na sercu badania ludziom, ludziom podejrzanym, z kontaktami, żeby wykluczyć to. I mi się wydaje, że to byłaby najbardziej sensowna droga, bo testy są robione albo i nie. Co ci mówiłam, że nawet ta położna, która była zarażona i leżała w szpitalu, nikt z jej rodziny nie miał zrobionego testu, a to by było miarodajne, tym bardziej, że badania mówią, że jak przechorujesz, to raczej nie masz reaktywacji. Niby tam są osoby, które po ozdrowieniu na nowo zachorowały, ale gdzieś tam debatują, że prawdopodobnie nie wyzdrowiały do końca, że testy były niewiarygodne. Myślę, że testy by nam dużo pomogły. Zastanawia mnie też to, że tutaj tak bardzo chcą wykryć tego koronawirusa, a czy ktokolwiek, bo ja pytałam oczywiście, jak miał podejrzenie grypy, to miał zrobiony test? Nie. A to też jest śmiercionośna choroba. Natomiast wydaje mi się, że jeżeli chcielibyśmy w idealnym świecie zapanować nad koronawirusem, to powinni nas przetestować po kolei.

**A inne ograniczenia powinny być przywrócone wtedy?**

Nie, absolutnie, bo nie da się tak funkcjonować. Kwarantanna powinna być zarządzona dla osób, które mają dodatni test, nie dla podejrzanych, bo miał kontakt. Jeżeli masz dodatni test, siedzisz w domu na L4 dwa tygodnie i zdrowiejesz. I tyle. Ale już nie z takim reżimem, że idąc do szpitala, nie możesz się wypisać na własne żądanie, że dopiero po trzecim czy drugim teście pozytywnym możesz wyjść itd. Nie, nie dajmy się zwariować. Tym bardziej, że nawet patrząc na liczbę zachorowań w Polsce, a ile osób jest hospitalizowanych, to ta liczba hospitalizowanych jest małym procentem i pewnie z tych osób hospitalizowanych tylko część jest ciężko chora. Po prostu, jeżeli ktoś ma koronawirusa dodatniego, niech siedzi w domu dwa tygodnie i się kuruje.

**Wymień przełomowe wydarzeń dla ciebie i dla kraju w trakcie pandemii?**

To na pewno moment, kiedy zamknęli szkoły, odgórne ogłoszenie, że zamykają szkoły, bo jest koronawirus, to był taki moment strachu. Na pewno to wtedy, jak zamknęli salony, czyli moje miejsce pracy, to był taki drugi bardzo znaczący dla mnie moment. Potem jak otworzyli, bo między zamknięciem a otworzeniem coś tam się działo, ale nie było na tyle istotne, bo czekałam, aż mi pozwolą wrócić do pracy. I teraz to odmrażanie gospodarki i pościąganie obostrzeń, takie dosyć szybkie wracanie do normalności, bo jest to dosyć szybkie wracanie do normalności jak dla mnie.

**Coś jeszcze?**

Nie, to byłoby dla mnie z takich najistotniejszych. To są te momenty, na które ja cały czas czekałam, od jednego do drugiego. Jak przyszedł koronawirus, czekałam, czy przyjdzie dzień, kiedy nie pozwolą mi iść do pracy, jak nie pozwolili, to czekałam na dzień, kiedy mi pozwolą iść do pracy i w sumie od momentu, kiedy pozwolili mi iść do pracy, to ja już na nic nie czekałam. Nie czekałam na żadne wystąpienie premiera itd., ale ten jeszcze ostatni, czwarty etap odmrażania gospodarki wpłynął na to, że wróciliśmy całkowicie do normalności.

**Dlaczego te momenty były przełomowe?**

Ten koronawirus był, o nim się mówiło, były nawet takie śmieszki-heheszki i nagle takie bum, zamykamy szkoły. I takie o, ho, chyba to nie jest takie śmieszne i chyba nie do końca to jest żart, skoro zamknęli szkoły na cztery spusty. I to był moment dosyć sporej paniki, że coś się złego dzieje w kraju. To było to i potem drugi, kiedy zamknęli mi pracę. Nie dość, że tutaj strach o zdrowie, bo ja nie wiedziałam, co to jest, czy to jest ściema, czy to jest prawda, czy to nas pozabija, czy będziemy jak za czasów grypy umierać na ulicy, czy jest to jakaś bujda na resorach, a dwa sytuacja finansowa. Ja nie wiedziałam, co będzie, ja nie wiedziałam, czy będę pracować, czy dostaniemy jakieś tarcze, czy ja wrócę do pracy, no nic nie wiedziałam. To nie był moment, że straciłam pracę i szukam nowej, tylko byłam w punkcie zawieszenia. No i tak zaczęło to wszystko wychodzić na prostą, jak pozwolili nam wrócić do pracy i się rozdzwonił telefon, czyli ja już tak odetchnęłam, że okej (zakłócenia) ...do normalności, jest praca, jak jest praca, są pieniądze, mamy za co żyć. Bo nie oszukujmy się, jak ktoś mówi, że pieniądz nie jest ważny, to zawsze mówię: "Przelej mi, jak tobie niepotrzebny", bo nie da się żyć bez pieniędzy w tym kraju.

**"Rozdzwonił się telefon" - masz na myśli oficjalne otwarcie czy podziemie?**

Oficjalne, bo to podziemie, to było takie: "Ile my będziemy mogły tak funkcjonować?". Bo możesz funkcjonować tydzień, dwa, ale co, pół roku? To było nierealne! Pół roku po cichutku, żeby nikt nie zauważył? Strach, że cię sąsiad gdzieś tam sprzeda. Praca w podziemiach była taka, że przeżyjemy, ale ja nie byłam spokojna psychicznie, bo nadal nie wiedziałam, co się będzie działo, czy nie przyjdzie taki moment, że będzie trzeba zamknąć, mnóstwo miałam myśli. Więc bardziej ten moment oficjalnego otwarcia, kiedy ja już tak oficjalnie: "Dzień dobry, zapraszamy". I to już nastał taki spokój, że wracamy do normalności. I teraz ten moment, kiedy młody mógł wyjść na dwór, on już wrócił do normalności, całej naszej rodzinie wróciło zdrowie psychiczne, że my już tutaj funkcjonujemy normalnie, głowy nam funkcjonują normalnie, że on wychodzi, ja wychodzę, chłop idzie do pracy, ja idę do pracy, dziecko lata po podwórku - tak powinno być i tak to teraz działa. Pomimo nawet lekcji online, ale nas nie męczą te lekcje online, więc spoko.

**Czy chciałabyś coś dodać?**

Na pewno to, że nie możemy wierzyć internetom i telewizji, bo w czasie pandemii zauważyłam, że bardzo manipulują ludźmi, a zastraszony naród bardzo łatwo się kontroluje, wystarczyło nas przestraszyć. Kiedyś, jak już wróciłam do pracy, usiadłam i myślę, Boże, ktoś stanął po drugiej stronie ekranu, powiedział mi, że mam siedzieć w domu, bo jest jakiś wirus, i ja to zrobiłam, i zrobiło to miliony ludzi. Tobym się tak zastanowiła, o co tu chodzi.
