# Supplementary material for: The challenges arising from the COVID-19 pandemic and the way people deal with them. A qualitative longitudinal study
Source: PLoS One. 2021 Oct 11;16(10):e0258133. doi: 10.1371/journal.pone.0258133 (PMC8504766; doi:10.1371/journal.pone.0258133)
Supplement: S1 Dataset — (ZIP) [file pone.0258133.s003.zip › Transcriptions/stage 5/18.5_F_48_couple, with children.docx]

**18.5_F_48_coule with children**

**Powiedz mi, co u ciebie się działo przez ten miesiąc.**

Co się działo? Raczej tak bez większych zmian. Znaczy bez większych zmian nie, bo wróciłam do pracy swojej. Powoli też, dzielę 2 miejsca teraz. Bo pierwsze te 2 tygodnie takie były rzeczywiście intensywne u mnie w pracy, musiałam nadrobić, stęsknione klientki przyszły.

**Ale musiałaś też przeorganizować swoje miejsce pracy jakoś bardzo?**

Nie. Nawet się zastanawiałam, co mam zrobić, jak te wytyczne wyczytałam. No to tak szczerze mówiąc oprócz dezynfekcji klamek, czego nigdy nie robiłam, to pozostałe rzeczy… A poza tym po tym, co przeszłam w sklepie i jak te restrykcje były wprowadzone, potem jak to wyglądało, to ja już się nie napinałam. Już miałam do tego luz. Podejście spokojne. Więc tam koleżanki panikowały, ale to też rozumiem, bo one znalazły się, nie wiem, 2 miesiące później w tej samej sytuacji, w której ja byłam. Ja może tak bez paniki, bo bardziej zadaniowo, natomiast one chciały wszystko sprostać temu, co tam te zalecenia były napisane. Ja stwierdziłam, że dobrze, spokojnie. Za tydzień wszyscy o tym zapomną i w ogóle szkoda czasu, pieniędzy i… Więc nic więcej nie wprowadziłam i takie niektóre rzeczy, jak były zalecane i widziałam czy w internecie, ale też widziałam wśród okolicznych salonów, no to taki przerost w ogóle wszystkiego. Ja też rozumiem, że każdy chciał się dostosować i tak sprostać temu. Więc no tak, tutaj jakby dzielę 2 miejsca. Ale też jakby moje przypuszczenia się potwierdzają, że to nie jest taka praca, jak była przed.

**A czym się różni?**

Jest mniej klientek, to zdecydowanie. Też bardzo tak ostrożnie zapisują się, dopytują. Więc zdecydowanie ta ilość się zmniejszyła. I ja to zakładałam. Bo jakby no z prostej przyczyny, po pierwsze nie chodzą do pracy, większość pracuje jednak zdalnie. Więc to tak między kobietami jest, że jedna drugą nakręca, to w dużej mierze. Oczywiście nie wszystkie, ale jest mała grupa, która robi dla siebie. I to się potwierdza teraz, że nie dla swojej przyjemności, nawet nie dla swojego męża, partnera, tylko właśnie dla tych koleżanek w pracy. No nie wiem, też nie wychodzą, nie spotykają się. Nie ma wyjść, nie ma imprez. Więc też jakby tak jak przewidziałam, że te stałe klientki, które przychodzą systematycznie, przychodzą i robią to dla siebie, z potrzeby wyglądu czy z takiej potrzeby, że muszą, bo nie wiem, czy mają problem czy ze zdrowiem i jest im trudno coś tam wykonać, to tak, to one się pierwsze zgłosiły i one przyszły. Natomiast kolejna grupa, no to mówię, jak się nie pracuje i nie chodzi, no to też… Myślę, że też takie względy finansowe też dużo… Bo sporo osób może nie straciło pracy, ale jakby ta wizja utraty pracy jest. Natomiast no jakby wynagrodzenie u większości, to tak praktycznie, nie wiem, czy kogoś spotkałam, u kogo by się nie zmniejszyło. Więc ten czynnik ekonomiczny myślę, że też ma znaczenie. Też nie wszyscy mają czas, bo jak mają te małe dzieci, takie w wieku szkolnym, to dużo czasu im zajmuje. Ja byłam na to nastawiona i z tym się liczyłam i to się sprawdziło. Tak myślę, że może być gorzej niż lepiej, taka moja wizja, że tak… Bo ten pierwszy taki wow to był, no bo wyjście z domu, że jakaś taka drobna przyjemność. To na takiej zasadzie jak przychodzą dziewczyny po porodzie i manicure jest ich pierwszą, wielką przyjemnością. Że wychodzą na pół godziny i są między ludźmi, robią coś dla siebie, tylko dla siebie. I to mniej więcej teraz tak wygląda. Potem, myślę, że może być różnie, ale no tak to jest.

**Czyli do tego powrotu sprzed pandemii, to się szybkiego nie spodziewasz?**

Nie. Może tak pesymistycznie… Znaczy to nie jest, że pesymistycznie, ja patrzę bardziej realnie niż tak, że mam kogoś tam przerażać. Tylko tak po prostu to według mnie działa. Z drugiej strony… W sklepie jest też mniejszy ruch. Znaczy to z kolei wróciło sprzed tej pandemii. Ale też obawiam się, że to się nie utrzyma. Bo jednak było to wielkie wow, ja pewnie o tym wspominałam, że ja patrzyłam z przerażeniem, jak ludzie wykupują jakieś ilości, w ogóle nie patrzą na ceny. No nie wiem, taką lekką ręką wydają te pieniądze. To teraz z kolei zaczęli już myśleć, przeliczać, dopytywać się. Nie wiem, no powiedzmy coś, że jest drogie, że pytają się o cenę, o, jest drogie. Ale ja sobie myślę, półtora miesiąca temu było jeszcze droższe. I nikt na to nie patrzył i kupował w większych ilościach. Więc tak jakby dopiero teraz ludziom się oczy otwierają. Podejrzewam, że może być jeszcze spadek. Natomiast no mówię, taka branża spożywcza też jest jakoś tam w miarę chroniona. Do momentu kiedy nie będzie jakiegoś zakażenia. Bo ja jakby cały czas jestem tego świadoma, że może się tak zdarzyć.

**A twoje życie w jakim stopniu wróciło do czasów sprzed pandemii, tak jak oceniasz swoje zachowania, swoje działania, jak to wygląda?**

Jeszcze nie wróciło. Bo ja cały czas jestem taka bardzo aktywna i mam dużo zajęć. Troszeczkę mam więcej oddechu, bo mówię, to się uspokoiło i jest spokojniej. Natomiast no jeszcze też nie wróciło do tego tak jak było. Mogę wyjść, jakieś takie atrakcje na zewnątrz, bo jakby z tego nie korzystam. Natomiast mówię, jeszcze jestem w takim… dużo pracy, dużo zajęć. Spokojniej, ale jednak jeszcze to nie jest to co przed.

**A spotkania ze znajomymi, z rodziną, jak to wygląda w tej chwili? To już jest tak jak przed pandemią czy też nie?**

Nie. To na zasadzie, nie wiem, z kim się spotkaliśmy? Z jednymi znajomymi, którzy przyszli do mnie. Były u mnie raz koleżanki. Była męża rodzina, ale oni od dłuższego czasu nie przyjeżdżali do nas. Zresztą u nich jakoś przed samą pandemią, bo to było tam koniec lutego, jakieś tam były pogrzeby i oni byli tak jakby w innych sprawach. No i oni zaproponowali, bo rzadko możemy się z nimi spotkać. Bo jakby też tak pracują zmianowo, że ciężko jest się umówić z nimi. Sami wyszli z inicjatywą, więc tak oni przyjechali do nas. Ale oni mieli takie nastawienie, że przecież i tak pandemii nie ma. Więc to tak. Bo powiedzmy ci znajomi, te moje koleżanki na zasadzie, że słuchajcie, trzeba powoli wracać do normalności, spotkajmy się, że tu w trójkę, tu w czwórkę się spotkaliśmy, tak jakby stopniowo, tak sobie dozując. No, ale no tak oni do tego podchodzą, tak podeszli, może jakoś bardzo się nie ściskaliśmy, nie witaliśmy. Chociaż oni byli bardzo…

**Czyli oni w ogóle się zachowują tak, jakby jej nie było?**

Tak.

**W ogóle uważają, że to była ściema?**

Tak, tak, że w ogóle to takie…

**A jak ty się z tym czułaś, że oni się tak zachowywali?**

Znaczy tak im było dziwnie (śmiech). Znaczy jakby to, że oni nie uznają i oni się tak zachowują, no to tak, ale jakby no też ten nasz obszar… No ja jakby tak z ostrożnością i takim dystansem, co by się nie witać i tam wujek taki rzucający się do całowania, to tak mu delikatnie rękę podałam. Ale bez…

**No to podałaś rękę, a potem skorzystałaś z pierwszej okazji, żeby umyć ręce? Jak to jest z tym kontaktem?**

Może aż tak to nie. Ale sam uścisk dłoni no to nie uważam, że aż tak bardzo im zagroził. Ale chciałam zachować dystans.

**Jest coś, co ci teraz wyraźnie przeszkadza w sytuacji, jaka jest teraz?**

Znaczy przeszkadza mi, ale to było i też na początku, takie podejście ludzi, takie totalne rozluźnienie. Że powiedzmy, nie wiem, możemy wychodzić bez maseczek, no to nagle większość idzie bez maseczek. Albo, nie wiem, będzie od jutra, to już właściwie dzisiaj zaczniemy chodzić bez tych maseczek, bo przecież to jest od jutra. Więc mi taka niefrasobliwość, jakby takie… No to mi przeszkadza. Takie, że właśnie coraz więcej i coraz bardziej odważnie ludzie mówią, że to jest wszystko takie wykreowane, że nie było żadnej pandemii, że to taka ściema, że to ktoś tym steruje. I coraz więcej takich głosów się pojawia. I tak bardziej odważnie ludzie zaczynają mówić, tacy są bardziej pewni siebie. Więc już jakby nagle zginął ten strach, ten czas takiej paniki i tego izolowania. Takie mam wrażenie jakby dla niektórych to był taki, nie wiem, jakiś taki sen w ogóle, coś takiego fikcyjnego.

**Coś nierealnego, zapomnijmy, to było dziwne, tak?**

To w ogół była pomyłka, to się nie liczy, to w ogóle… Wręcz mam wrażenie, że niektórzy tak się czują, że tacy oszukani, że się dali wkręcić w coś. Ale bardzo często to też tak jak sobie kojarzę, to takie osoby, które bardzo się tego bały. I tak się bardzo zamknęły na początku. A teraz nagle przeszły na drugą stronę.

**A ty co myślisz o tym? Że jak to jest?**

Jak to jest? Wirus jest, bo jest, no są ludzie chorzy, są ci, którzy umarli. No jest gdzieś, ja tego nie wypieram. Myślę, że trzeba trochę z taką pokorą do tego podejść. Bez jakiegoś takiego… Mówię no na zasadzie, że jak każda inna choroba, może nas spotkać. Ale tu wiemy mniej więcej, bo też co chwilę coś tam nowego, ale pewne zachowania możemy sobie zbudować i się ochronić. Tak jak, nie wiem, że podam tylko rękę, nie będę się z wujkiem… No nie wiem, czy to mi pomoże, zaszkodzi… Znaczy no nie zaszkodzi mi, a może pomóc. Na tej zasadzie. Więc taki niewidzialny wróg gdzieś istnieje, ale…

**Czyli, że pewną ostrożność trzeba zachować, te ręce dezynfekować, jak trzeba – założyć maskę itd. To o to chodzi?**

Tak, tak, tak. Widzę, że ludzie coraz... Znaczy to też nie wszyscy, ale rośnie ilość osób, które coraz rzadziej dezynfekują te ręce, tak jak w sklepie wchodzą. Większość już taka wchodzi jakby, właśnie jak przed. Że tam spokojnie wchodzę z ulicy, nie wycieram, nie dezynfekuję, nie mam maseczki i jest dobrze. Wręcz taka też arogancja, bo jednak te moje dziewczyny cały czas się boją. Może nie aż tak bardzo, ale widzę, że mają takie obawy, w ten sposób. I też nawet mnie się pytały, co mają zrobić, bo tam gdzieś w sklepie obok słyszały, że nie obsługują albo nie wpuszczają, jak ktoś nie ma maseczki. Jeszcze przed tym, zresztą no do tej pory w pomieszczeniach zamkniętych trzeba nosić. No i co one mają zrobić. Bo jakby zwracają uwagę czy proszę grzecznie, żeby założyć. I spotykają się właśnie z taką, to je chyba najbardziej, bo na zasadzie ktoś: ojejku, przepraszam, zapomniałam. Albo: ojej, nie mogę, bo mam astmę. Natomiast takie: ha, przecież nie ma epidemii. I taka ignorancja i wydaje mi się, taki brak szacunku dla kogoś. No OK, to jakby… Ale jestem na czyjejś przestrzeni w miejscu publicznym. I jakby z taką… No to je chyba najbardziej, że z taką kpiną trochę, z taką arogancją. Bardzo tam przeżywały, że jakaś pani, zwróciły jej uwagę, że nie ma maseczki, że prosiły, no ona nie ma. Ale oczywiście obsłużyły, bo nie miały jakby takiego sygnału, że mają kogoś wyprosić. Więc pani potem zbliżając, kartą płacąc, poprosiła koleżanka, że pani podejdzie bliżej, zbliży tą kartę, bo nie ma sygnału. Na co ona: no przed chwilą pani się tak mnie bała. No takie no niesmaczne w ogóle. No, ale każdy ma prawo się bać no.

**Czyli ludzie nie rozumieją tego, że ktoś się może bać.**

Znaczy tak, ja rozumiem, że ktoś może się nie bać. Tylko niech szanuje moje obawy. Więc to mi przeszkadza. To mi przeszkadza, to mnie drażni. Ale no co…

**Emocje – zdjęcia.**

Najbardziej ta 6, która już była poprzednio, że to chyba to jest takie najbardziej. Że jest to słońce, bo tak to widzę, że jest to słońce. Brakuje mi takiego… Bardziej takiego… Znaczy nie, może 13 jest taka optymistyczna.

**Opowiedz o tej 6 najpierw.**

To jest ten taki ciemny las, ja sobie to tak interpretuję. Że te takie zachowania, ta taka ludzka głupota nieraz. Ale jakby gdzieś życie się toczy dalej i te pozytywne rzeczy, że też są na przykład osoby, które no też mają takie same zdanie, że… Że pozytywnie w przyszłość, że coś się zmienia, spokojnie i bez paniki, bez takiego chaosu.

**Czyli tu jest element nadziei u ciebie, że będzie lepiej, że będzie spokojnie. A to twoje poirytowanie to jest tylko poirytowanie czy to jest złość, wkurzenie? Na ile to jest silne?**

Wkurzenie i złość była na początku. A teraz ja chyba się trochę też z tym może i oswoiłam, nie wiem, czy to dobre słowo, albo że bardziej pogodziłam. Że no, taka akceptacja, że są tacy ludzie, tak jest.

**To jest taka bardziej rezygnacja, że nic nie można zrobić, bezsilność?**

Tak. Bo ja nawet dziewczynom powiedziałam, że słuchajcie, wy dbajcie o siebie, stosujcie to, co stosujecie dalej. Natomiast no świata nie zmienimy. Bo jakby szkoda też, nie wiem, nerwów, czasu. Bo widać, że one też były tym przejęte i też były bardzo. Bo i jedna zmiana i druga o tym samym wspomniała.

**Domyślam się, że były podziwiane, że takie dzielne, i że pracują w tych warunkach. I nagle mają taką zmianę, że się z nich trochę ludzie śmieją, że one się boją?**

Tak, dokładnie. Mój mąż potem stwierdził, mówi tak, że wiesz, to tak jak z tymi pielęgniarkami. Że najpierw brawo bijemy a potem rzucamy jajkami w ich drzwi. Najpierw było dziękujemy, że macie otwarty sklep i jesteście. I możemy tutaj w bezpiecznych warunkach robić zakupy. A właściwie teraz to już tak nie jesteście do końca potrzebni.

**Już nie jesteście tacy dzielni, tylko po prostu nawet trochę przesadzacie?**

Tak.

**A ta 13, jakie to są uczucia?**

Tu jest dużo słońca i takich promieni, że to właściwie bardziej ta nadzieja, że takie ku lepszemu (śmiech). Chociaż nie wiem, czy ja tak w to wierzę, ale małymi kroczkami. Może w ten sposób.

**Czy to pasuje do tego, jak się czujesz w tej chwili? Czy do tego, jak się czujesz w tej chwili, to jeszcze coś innego powinno być tutaj?**

Myślę, że nie. Myślę, że mam w sobie duże pokłady spokoju i takiej… Na równi z rezygnacją jest jednak akceptacja i takie pogodzenie, że jakby ta złość już przechodzi w spokój.

**A jakieś obawy w tobie są? Niepokój, czegoś się w tej chwili obawiasz w związku z tą sytuacją, którą mamy?**

Znaczy ja cały czas się obawiam, że jednak może ktoś z nas być zarażony. Ale to jest cały czas na tym samym poziomie. Na takim samym, tak sobie myślę, chociaż z drugiej strony myślę, czy teraz bardziej nie jesteśmy narażeni, gdzie takie rozprężenie jest ogólne. Gdzie jednak ryzyko jest może trochę większe? Chociaż zawsze było, to tak z drugiej strony… No mówię, to jest ta obawa cały czas. Ale to jest cały czas to samo.

**Są jakieś takie sytuacja, w których jakoś bardziej cię dotyka taka obawa?**

Nie, myślę, że to jest na równi. No nie mam na wszystko wpływu. To, co robię, to tak jak robiłam, robię do tej pory. Obawą największą, jaką miałam, ale to też o dziwo, też mi przyszła taka, właściwie tu bardziej bezradność ogarnęła i taka bezsilność, jak moi rodzice wywinęli mi numer… Ale to też mi pokazało, że nie mam na wszystko wpływu, nie jestem w stanie ochronić wszystkich, wszystkiego. I po prostu nie da się. Bo moi rodzice, którzy w ogóle, no tak jak mówiłam, że ja tam im wszystko przywoziłam i wszystko załatwiałam. I rzeczywiście już byli tacy zmęczeni i ta moja mama z tym fryzjerem, jak usłyszałam, że idzie do fryzjera. I mówi, wiesz, ja przez 70 lat nie miałam takiej fryzury. I widziałam, że już jest taka zmęczona tym, taka przytłoczona, że tak jest jej smutno. I właściwie to mój tata nawet taki był, że mu loki się kręcą. Mówię, fajne masz loki. Nie, facet powinien być wystrzyżony. No dobrze. No więc tak powoli im tak poluzowałam. Ale to może to też jest właśnie obraz całego społeczeństwa, że poluzujesz trochę, to po prostu pójdą dalej. No więc, mówię, dobrze mamo, no to nie wiem, powiedzmy do nas, bo ludzie się rzucili do tych marketów dużych itd. To mówię, właściwie do nas do sklepu, ja uważam, że jest bezpiecznie. Założysz maseczkę, będziesz miała rękawiczki, jak ich zaopatrzyłam, mają wszystkie te płyny. Mówię, tam są godziny takie luźniejsze, to sobie przyjedź, zrób te zakupy. Do fryzjera też wiem, do kogo się zapisywała, też jakby na spokojnie. Tata też miał tam jechać. Powoli gdzieś tam sobie poszli gdzieś, nie wiem, a, pojechali sobie po ziemię do kwiatów. Ale też na wolnej przestrzeni, nie do marketu. Więc ja w ogóle spokojna, powoli moich rodziców jak dzieci, wypuszczam w świat. Po czym dzwonię i mam mówi… A to już klientka mi opowiadała, jak się zezłościła na swoich rodziców, gdzie tam ojciec z ostrą niewydolnością a mama wymykała się potajemnie do Biedronki i ona się o tym dowiedziała. Więc dzwonię do mamy, pytam się. Mama mi tak opowiada, że byliśmy tu, tu. No byliśmy w aptece. Bo nie mogłam zrozumieć. Ale mówi, ta pani w aptece to nie wiedziała, czy ten lekarz przyjmuje, bo wiesz, tata ma wizytę u neurologa. Ja tak właściwie nie do końca wiedziałam, że jak to pani w aptece ma wiedzieć jak neurolog przyjmuje. Ale mówię, w jakiej aptece? No w tej przy szpitalu. Ale mówię, to nie ma innej apteki? No nie, bo wiesz, jak pojechaliśmy zapisać się do fryzjera, to pojechaliśmy do tej apteki, właściwie już byliśmy blisko. No i poszliśmy się dowiedzieć. Ale gdzie się poszliście dowiedzieć? Bo telefonu przez tydzień mi nikt nie odbierał. Otóż poszli, mówi, wiesz, bo teraz wejście do szpitala to jest od strony tam, gdzie karetki wjeżdżają. Ja mówię, ale jak to weszliście? Tam ktoś was wpuścił? No tak. I pani w rejestracji powiedziała, że wysyłali SMS-y no i tam jest na lipiec. I moja mama wręcz taka dumna, że załatwili sprawę. Ja mówię, ale jak tam weszliście? Ale tam nikogo nie było. I mówi, wiesz, my mieliśmy maseczki, my mieliśmy rękawiczki. Ja mówię dobrze, a co zrobiliście z tymi rękawiczkami, jak wyszliście z tego szpitala. No zdjęliśmy, leżą w samochodzie. I ja po prostu… Znaczy ja nawet nie byłam zła. Ja byłam najpierw przerażona, a potem tak właściwie, tak ze mnie wszystko opadło, takie… Ale mówię, że jak weszliście, że po co? Bo normalnie w jakichś takich sytuacjach, jak nieraz jakieś takie rzeczy, które mnie wkurzyły wręcz, jak rodzice coś zrobili takiego głupiego, bo wchodzą chyba już w taki etap, to nie, ja potrafiłam nawet tak ostro powiedzieć coś. No może nie to, że krzyczeć, ale z takim zdecydowanym i tak ze złością powiedzieć. Ja po prostu nawet nie miałam siły na to.

**Jak myślisz, czy to jest tak, że my jakoś powinniśmy przymuszać te osoby, które są trochę bardziej zagrożone, czy w bardzo podeszłym wieku czy chore na coś innego i je po prostu siłą zatrzymywać? Czy niech się dzieje, co chce? Co powinniśmy zrobić?**

Znaczy chyba siłą nie bardzo. To chyba się nie sprawdza. Ja myślę, że bardziej tłumaczyć, ale też no tłumaczyć… ja mówię, to tak jak trochę z dziećmi. Że niby tłumaczymy, uczymy czegoś, mówimy. A właściwie i tak zrobią to, co chcą. Są w końcu wolnymi, dorosłymi ludźmi. Ale jest dużo takich sytuacji. Bo… To znaczy ja nie rozumiem tego. Jakby nie zdawali sobie sprawy? Ale przecież ci sami rodzice uczyli nas jakichś odpowiedzialnych postaw. Więc nagle to w ogóle pryska czar, ten autorytet to nagle, że jak to.

**Ale myślisz, że powinniśmy nadal pilnować swoich rodziców czy dzieci, żeby były bardziej ostrożne? Cały czas tak jest, że musimy tego pilnować?**

Znaczy nie wiem, czy musimy, tylko czy chcemy. Bo to też jest… Na ile to jest taka ochrona rodziców, a na ile to jest taka ochrona nas, że mamy trochę takie nasze poczucie winy, może jakieś takie, że my tak dbamy… Ile w tym jest takiej w 100% troski tylko o rodziców, a ile o nas samych.

**Ty myślisz, że robisz to trochę dla siebie, dla swojego spokoju?**

Ja myślę, że też trochę dla siebie. Z drugiej strony też, jeśli i oni będą zdrowi, to i ja będę zdrowa, spokojniejsza. Jakby to wszystko jest takie stabilniejsze. A jeśli oni zachorują czy coś im się stanie, czy to będzie ten wirus czy inny, no to ja też będę obarczona. Więc też o swój komfort dbam. No tak chyba jest. Tak mi się wydaje. I mówię, tyle osób i tak nagle ci, powiedzmy ja mieszkam blisko rodziców. Ale ci, którzy gdzieś dalej i nagle nakazują tym rodzicom, gdzie nie widują ich codziennie, no to też… No co, nagle tak? Przecież w międzyczasie, jak ich nie widują i nie było koronawirusa również różne dziwne rzeczy mogły się dziać. Chociaż ja na drugi dzień się mamy zapytałam i tak zadzwoniłam, jak minął dzień. I się jej pytam, mówię tak, no chciałabym się dowiedzieć, gdzie dzisiaj mieliście wycieczkę, jakie atrakcje sobie zapewniliście. I tak mówię, mamo, jak ty to zrobiłaś, dlaczego wy tam poszliście? I rzeczywiście mama chyba po tym czasie mówi, wiesz co, ja nie wiem, dlaczego ja to zrobiłam. Po prostu nie wiem. Bo tak ojciec wołał do tego lekarza, do tego lekarza. Ale przecież mógł iść sam. Ale wiesz, jaki on jest, że chodź ze mną. No rzeczywiście, no tak funkcjonują. Ona rozumie, że to było wbrew wszelakiej… Gdzie, przecież sama mówiłaś, że tam sąsiadki wujek, który jest lekarzem był na kwarantannie, bo był tam ten wirus na oddziale, no sama mi o tym mówiłaś. Ona nie wie, po prostu. Więc też tak to jest, że nie mamy na wszystko wpływu. I choćby nie wiem, jak byśmy chronili, no nie da się w 100%.

**A na siłę ich nie zamykamy po prostu.**

No nie, bo to jeszcze gorsze głupoty mogą wychodzić. I mówię, mamo, przecież ja bym zadzwoniła, nawet ja bym pojechała do tego szpitala, skoro była taka potrzeba. A, ty masz tyle zajęć, już nie chcieliśmy, już i tak tyle dla nas robisz. I w ogóle nie chcieliśmy ci zawracać głowy. Ja mówię, no świetnie, no w ogóle mi nie zawróciliście głowy, jak sobie pomyślałam, gdzie wy byliście. Trochę byłam też zła na całą jakby informację. No mój tata ma prawie 80 lat. On dostał SMS-a, on ma ten telefon. Ale jakby on mu coś pika i on nie biegnie i nie sprawdza każdego piknięcia. On jak do nas przyjeżdżał i mówi tak: zobaczcie, coś tam przyszło do mnie. I tak sobie myślę, że ktoś, kto wysyła takie wiadomości, pewnie robi to z automatu. Ale umówmy się, no jednak większość starszych osób korzysta z takich wizyt, jeszcze w szpitalu. No gdzieś trochę wyobraźni, że nie każda starsza osoba, pomimo że jest ten numer telefonu, że ona go odczyta.

**Czyli to też się rozluźniło, nie ma takiej troski? Nikt nikogo nie chroni jak gdyby w tej chwili.**

No tak, no trochę takiej wyobraźni, że to nie jest młodzież czy nawet w średnim wieku, że funkcjonują z tymi telefonami, są non stop, nie wiem… Zresztą też mama nieraz mówiła, że spotkała się, że w aptece, no to tu pani napisze maila, tu pani kod wpisze. No też nie dajmy się zwariować, że każda starsza osoba musi, a zresztą starsza, nie starsza, no nie każdy musi pisać maile. No nie jest to nic złego, ale że to jest nakaz. Kobietę 70+, no dobrze, jest dużo fajnych, aktywnych, które się chcą uczyć i uczą. Ale większość jest takich, które nie korzysta. Ale z kolei moja mama miała wizytę w innym szpitalu, dostała SMS-a. Natomiast panie z recepcji dotąd dzwoniły, aż się dodzwoniły. Czyli jakby można. Można. Bo chciały się upewnić, czy ta wiadomość jakby… Wiadomość była automatu, natomiast one jeszcze dopełniały takiej formalności, czy rzeczywiście została poinformowana, żeby była w domu. Łącznie z tym, żeby miała naładowany telefon. Czyli można pomyśleć, jak się patrzy na ten PESEL, że to nie jest rześka trzydziestolatka.

**Tylko trzeba chcieć.**

No właśnie. Więc tutaj też byłam trochę zła, że zupełnie ten system zawiódł.

**A jeśli chodzi o twoje wyjścia z domu na zakupy, to to wróciło do normalności zupełniej sprzed pandemii czy jest inaczej?**

Nie, nie wróciło. Pojechałam 2 razy do Leroy. Pierwszy raz pojechałam, tak jak mąż mi zalecił, czyli rano. I rzeczywiście fajnie, zrobiłam zakupy, było pusto, bezpiecznie.

**Bo było mniej ludzi, dlatego ci zalecił, żeby rano?**

Tak, tak. Bo sam coś odbierał, ale on zamawiał przez Internet i tylko odbierał, więc nie wchodził tam na tą salę sprzedaży. I mówi, jak pojedziesz rano, tam nie wiem, od 6 czy od 7 jest otwarte, pojedziesz po 7, to będą tylko ci, co chcą coś kupić, nie będzie spacerowiczów. Ale ponieważ ja tam jakiejś jednej rzeczy zapomniałam i pojechałam po południu i… Może to nie był strach, ale ja z przerażeniem patrzyłam, jak to wszystko wygląda, ile jest ludzi, jak oni się zachowują. Na takiej dużej przestrzeni, że jak są oddzielone od kasy, że co 2 metry linie, to rzeczywiście podchodzą, stoją co 2 metry. Ale tam, gdzie nie ma już linii, to stoją jeden na drugim. Dosłownie. Ja chciałam przejść alejką, stały 4 osoby, rodzina. I z daleka powiedziałam po prostu przepraszam. Powiedziałam to wcześniej, z odległości, żeby się przesunęli. Normalnie bym powiedziała przepraszam, podchodząc bliżej do nich. Natomiast nie było żadnej reakcji. No i nie wiem, czy to na takiej zasadzie, dobra, nic się nie dzieje, nic mi nie jest, to jest nieprawda. Liczy się ja i tylko ja, a inni to są, bo są. No nie wiem, jak zobaczyłam takich nawet, no znam, bo to jeszcze ze szkoły koleżanka, która szła z mężem za rączkę, spacerując między tymi regałami w Leroy. To ja po prostu mówię nie, no albo ja jestem dziwna, bo ja bym chętnie poszła na spacer do parku z mężem. Czy gdzieś, nie wiem, nawet ulicą, nie po jakichś halach budowlanych. Więc na szczęście weszłam po jedną rzecz. Odwołałam wszystkie moje negatywne do tej pory, że karty i płatność kartą jest fe i be. Komfort mi dało to niesamowity. Ponieważ, właśnie, większość stała do tych kas, do kasjerek. Natomiast tam, gdzie są samoobsługowe i tylko płatność kartą, było luźno. Więc ja po prostu podeszłam, zapłaciłam i wyszłam. I stwierdziłam, że tak, to jest ten moment, w którym powinnam przeprosić wszystkich, którym mówiłam, że tylko gotówka (śmiech). Tak. I nie podobało mi się to. I stwierdziłam, że będę takich miejsc unikać. Albo rzeczywiście przemyślę, w jakich godzinach mam korzystać.

**Ty miałaś maskę i rękawiczki na sobie?**

Tak.

**A ludzie obok też czy nie bardzo?**

To był jeszcze czas, kiedy trzeba było mieć. Więc tam mieli, gdzieś pod brodą, obok brody itd. No mówię, też teorie takie, że ona nie chroni, że jak nosimy długo, to jeszcze bardziej się rozchorujemy… No nie wiem. Znaczy ja tak nie uważam. No owszem, jak niektórzy mają takie maseczki, które wyglądają jak za przeproszeniem ścierka do podłogi, bo widzę takie, no to tak, można się rozchorować. Natomiast w momencie, kiedy ja zmieniam. Albo jednorazowe wyrzucam albo mam bawełniane i je kilka razy zmieniam w ciągu dnia. No przecież lekarz jak 8 godzin stoi w maseczce przy operacji, to byłby wiecznie chory.

**A pamiętam, że na początku mi mówiłaś, że jak wracasz do domu, to się od razu przebierasz w czyste ubrania, czy to nadal jest?**

Tak. Znaczy… Nie tak często (śmiech). Natomiast tak, tak, pilnuję, żeby te rzeczy takie, gdzie jestem w tych miejscach, właśnie czy w sklepie, no u siebie w pracy to mam zupełnie oddzielny strój, no to tak.

**A byłaś w jakiejś galerii handlowej czy nie?**

Nie. Podjechałam tylko do Janek, bo mam najbliżej, chciałam wejść do Media Markt. Czy tam jakiś Saturn, z tych takich AGD. Podjechałam i się przeraziłam, bo znalazłam ostatnie wolne miejsca, a parking jest tam olbrzymi. I po prostu weszłam, zobaczyłam, co miałam zobaczyć, wyszłam i pojechałam. Nawet nie pomyślałam, żeby iść i oglądać, nie wiem, szukać. Znaczy nie potrzebowałam nic. Ja cały czas funkcjonuję tak, że jak coś potrzebuję, to właśnie jadę, biorę, kupuję, oglądam. Natomiast no nie.

**Masz wrażenie, że to nie są do końca bezpieczne miejsca cały czas?**

Tak. Tym bardziej, że mówię, zachowanie dystansu, no ludzie tego nie przestrzegają, jakby nie rozumieli tego. Znaczy co mnie przeraża, że ludzie w ogóle nie słuchają, nie czytają. Bo na przykład chodzą w maseczkach i się zastanawiają, że… Pytają nas się dzisiaj, dzisiaj jest co, środa, maseczek nie nosimy na wolnej przestrzeni do niedzieli? Od soboty chyba. Natomiast ludzie totalnie dalej nie wiedzą, kiedy, gdzie mają zakładać te maseczki, kiedy zdejmować. I to naprawdę duża grupa ludzi. I to młodzi, w różnym wieku. I tak wchodzą, to jak to w końcu jest z tymi maseczkami? Czyli na to pytanie, właśnie, że proszę założyć. Albo dzisiaj weszła pani i mówi tak, oj, bo tak ci ludzie chodzą z tymi maseczkami, tak je zakładają. No mówimy, że tak, bo jak się wchodzi do pomieszczenia, gdzie są inni, to trzeba je założyć. Więc ja naprawdę jestem przerażona społeczeństwem. Bo naprawdę te komunikaty są dla mnie jasne. No dobrze, nie usłyszałam w telewizji, ale wszędzie się pisze. Nie wiem, są jakieś takie, pojawiają mi się (niezrozumiałe), w telewizji widziałam taki słupek, obrazki wręcz, że nie wiem, nie nosimy maseczki na wolnej przestrzeni, tam jakiś las jest pokazane. Naprawdę ja uważam, że jest to prostym, przejrzystym językiem powiedziane. A ludzie po prostu nie wiedzą. Aż się boję w takich… Znaczy boję. No to nie są dla mnie bezpieczne miejsca, bo po prostu ludzie nie wiedzą, jak się mają zachować.

**A restauracje i kawiarnie, byłaś już?**

Byłam w kawiarni na zewnątrz. Umówiłam się z koleżanką, miałyśmy iść na spacer. Ale ja mówię, wiesz co, ja bym sobie tak może wzięła kawę na mieście, bo już wiem, że jest czynna. I tak mówię, to ja sobie wezmę, kupię tą kawę i sobie pójdziemy na spacer. Jak podeszłam, to pan się pyta czy na miejscu, czy na wynos. Więc ja tak… a to ja mogę na miejscu? To ja mówię dobrze, to na miejscu, tylko że na zewnątrz sobie usiądę, przyszła ta moja koleżanka. Ale to był dosłownie chyba pierwszy dzień, jak można było w ogóle usiąść. Pan jeszcze mówi tak, jeszcze taka uwaga, że jak pani siedzi, to może zdjąć maseczkę przy stoliku. Też było to trochę komiczne, bo jakby te stoliki były przy chodniku. No i jakby tu zdejmuję maseczkę, ale muszę iść, ale jak wstaję i idę to mam maseczkę. Natomiast my wypiłyśmy tą kawę, kawa nie była za szczególna, ale ona smakowała najlepiej na świecie. To było niesamowite, że mówię do koleżanki, słuchaj, chodnik, ruchliwa ulica, nieważne, że smog był tutaj niedawno albo jest nadal. Ale po prostu siedzimy, pijemy kawę na wolności (śmiech). Więc to było bardzo przyjemne, tak cieszyło.

**Czułaś się tam bezpiecznie?**

Tak. Tak. No, właściwie było kilka stolików, my zajęłyśmy tylko 1. Więc nikogo nie było, jakby…

**Uważasz, że to jest OK, że otworzyli restauracje i kawiarnie, to jest w porządku?**

Znaczy myślę, że tak. Chociaż tak jak słyszę, że przy stoliku mogą siedzieć tylko członkowie rodziny albo osoby mieszkające razem, to nie bardzo wiem, jak to ma zweryfikować właściciel. Takie trochę jest takie… No sporo rzeczy takich, przerost.

**Mniej logiczne, rozumiem. Ale dezynfekowanie stolików, te takie środki ostrożności, które tam są ustalone, to jest OK?**

Znaczy i te odległości, myślę, że tak. Ilość osób na tej sali, no to tak, myślę, że jest to w miarę w porządku.

**A za chwilę nam otworzą jeszcze kina, chyba od szóstego. Kina, siłownie. Co o tym myślisz?**

Co ja o tym myślę? To znaczy tak. Myślę, że to w ogóle odwrotnie się wszystko działo, jakby było zamykane nie w tym momencie, co trzeba. A w tym momencie, gdzie te zachorowania są dużo większe i są jakby chyba cały czas na takim stałym poziomie dość wysokim, to otwiera się. Więc ja logiki tu nie widzę. Trochę też myślałam nad tym, co mnie się kiedyś pytałaś, to był szwedzki tak?

**Tak, model szwedzki.**

No nie wiem, czy rzeczywiście w ramach naturalnej selekcji, czy nie lepiej było to tak zostawić (śmiech). Może to miało sens, że może bardziej gdyby… Bo to trochę tak jest, że jak nas dotyka, to bardziej o tym myślimy, bardziej się pilnujemy, tak musimy dotknąć tego. Więc może gdyby było tak, że o, sąsiadka zachorowała, to ja będę ostrożny. Ale skoro jakaś pani na Śląsku zachorowała, to przecież jestem bezpieczny. Więc może faktycznie lepiej byłoby tak postępować.

**Nie znasz nikogo, kto zachorował?**

Znam. No ale jakaś tam męża z pracy, dalsi. Oni na początku, ona była chora. Ale przeszła to bardzo łagodnie. Natomiast nikogo nie znam. Chociaż mam klientki, które jak teraz analizują i mi opowiadały, no to jedna ewidentnie rozważa, żeby zrobić te testy na przeciwciała. Bo oni byli na feriach we Włoszech. Tam się trójka z tej pięcioosobowej rodziny pochorowała. Byli już tam we Włoszech u lekarza, byli tutaj u lekarza. Ten najmłodszy miał zapalenie płuc. Ona sama mówi, że tak się po prostu dziwnie czuła. Miała gorączkę, tak właściwie nie wie, co jej było. I dopiero teraz, jak analizuje, to mówi, ja nie wiem, czy ja nie przeszłam tego koronawirusa. Natomiast mąż z najstarszym synem, im zupełnie nic nie było. I jeszcze tam ktoś słyszałam, że też jakieś takie, że… Gdyby zrobić te testy, to też nagle może się okazać, że już dużo wcześniej były te zachorowania.

**A myślałaś, żeby sobie zrobić test na przeciwciała?**

Nie, jakoś na razie nie myślałam.

**Nie masz takiej potrzeby, żeby się dowiedzieć, czy przeszłaś czy nie?**

Nie. Jakoś nie.

**Chciałam jeszcze z tobą porozmawiać o aplikacjach, które powstały przy okazji pandemii. Słyszałaś coś o takich aplikacjach, które sobie można pobrać i one będą do czegoś służyć?**

Te, które nie wiem, że wchodzę do galerii handlowej i jeśli mam aplikację jakąś tam określoną, to mogę wejść, a jeśli nie to nie. To słyszałam.

**A jeszcze coś?**

Nie wiem, jakoś nie przychodzi mi nic na myśl. Być może, że słyszałam, ale nie... Ja z tymi rzeczami to tak jestem daleko.

**Są takie 2 rodzaje, które chciałam ci przedstawić, a potem pokażę ci konkretne (opis aplikacji). Co myślisz o takich aplikacjach?**

Zupełnie nie jestem tym zainteresowana. Uważam, że to jest w ogóle… Bo to jest ten rządowy, tak?

**Tak.**

Nie, zupełnie uważam, że to jest totalne wchodzenie buciorami w nasze życie. I niczemu to nie służy. Bo i co z tego, że ja się znajdę w obszarze, gdzie ktoś jest chory czy zarażony, jak i tak nie dostanę pomocy od państwa, takiej jaka powinna być. Więc ja nie wiem, czy to służy (niezrozumiałe) czy to takiemu stłamszeniu jeszcze bardziej i wejściu totalnie na moją taką suwerenność.

**Czyli to ingeruje w twoją prywatność i kontroluje ciebie, a nie służy, żeby ci pomóc?**

Tak.

**Widzisz w ogóle jakieś zalety takiej aplikacji tego typu?**

Powiem tak, gdyby ktoś jest na kwarantannie, musi być, bo tak jak słyszę, że ludzie uciekają, wychodzą itd. No to na pewno usprawniłoby. Tylko, że z drugiej strony, ja wiem? No to jest znowu takie przymusowe. Nie wiem, tutaj kogoś zatrzymujemy w domu, pilnujemy, żeby nie wychodził na kwarantannie, po czym kupujemy maseczki za jakieś koszmarne pieniądze, za moje pieniądze. Więc nie, to ja nie kupuję tego.

**A w ogóle zwracasz uwagę, jak używasz w telefonie jakichś aplikacji czy one mają dostęp do twojej lokalizacji? Masz swój odcisk palca w telefonie, rozpoznawanie twarzy w telefonie?**

Mam odcisk, mam lokalizację, że nieraz, że wybierz swoją lokalizację i korzystam z tego… No najczęściej, jeśli się przemieszczam i korzystam z mapy, z nawigacji. Natomiast mi się to nie podoba. Ja miałam taką sytuację, bo ja mówię, totalnie jestem nietechniczna i tam sobie klikam, oglądam, zupełnie nieświadomie w większości. Może właśnie po tym incydencie zaczęłam zwracać na to uwagę. Bo kiedyś jechałam z koleżanką do teatru i jechałyśmy samochodem, no i ja miałam włączony swój telefon, który leżał w torebce obok. Nawet chyba nie była włączona nawigacja, bo leżał sobie, bo znałam drogę. I nawet pamiętam, w którym momencie zaczęłyśmy rozmawiać o aborcji. No i taka dyskusja, wymieniłyśmy swoje zdania. I długo, bo to pamiętam, od Zachodniego do Śródmieścia, taki był odcinek, bo dokładnie pamiętam, w którym miejscu zaczęłyśmy rozmawiać. Jak weszłam do teatru i włączyłam sobie telefon i weszłam chyba na Facebooka, tak, dokładnie. I tam się pojawiają takie powiadomienia, proponowane dla ciebie. I ja patrzę, a tam na tym było, chrońmy dzieci nienarodzone, precz tam z jakimś... I ja pytam się tej mojej koleżanki młodszej, ja mówię wiesz co, dlaczego ja mam takie rzeczy, przecież ja nie wchodziłam na żadne strony. Nie czytałam żadnych artykułów, w ogóle totalnie jakby to była tylko ta rozmowa w trakcie naszej jazdy. A ona mówi, no tak, ona jakoś tam powiedziała, że nie masz wyłączonego tam właśnie tej nawigacji, tych funkcji w telefonie. I to jakby ściąga głos i analizuje.

**Rozpoznawanie głosu?**

Tak i o czym się rozmawia. Znaczy mówię, nie jestem techniczna i ja byłam przerażona.Bo ona mówi, bo to jakby wyłapuje tematy, o których rozmawiamy. Na takiej zasadzie, że powiedzmy wyszukuje coś w przeglądarce. Nie wiem, to mąż się nieraz śmieje, mówi tak, znaczy więcej on szuka, ale mówi, że na przykład widać, że obejrzałem sobie buty, szukam buty. I potem wszystkie reklamy tych butów czy czegokolwiek… To bardziej ja męża śledzę. Bo jak on chce coś do domu, jakiś remont robi, to ja już wiem, co on kupuje, bo mi się to pojawia. Więc ja myślałam tylko, że jak się wchodzi na konkretne strony, te konkretne linki. Natomiast tutaj my tylko rozmawiałyśmy. I ona mówi, że też rozmowa na określony temat… Znaczy dla mnie to jest science-fiction, natomiast to się działo. Ja jakby tego doświadczyłam. Ja się czułam obrzydliwie. Ja takie miałam wrażenie, jak ja bym tam stała przy tej Rotundzie rozebrana. Więc no nie.

**Po prostu śledzą?**

Tak, tak. Ale z drugiej strony, mówię, nie, bo ja po prostu nie mogłam zrozumieć, ja się wszystkich tych młodszych pytałam naokoło, czy to jest możliwe, może ja coś nacisnęłam. Ale z drugiej strony, dlaczego na przykład nie pojawiło się, nie wiem, tanie wyjazdy do Czech, zrób aborcję, tylko ochrona? Bo jakby, to oni mi też tłumaczyli, że to przetwarza jakiś system i określone grupy, nie wiem, jak to nazwać, że one wtedy te swoje…

**Określone słowa to wyzwalają, tak?**

Tak. Jakby ci, którzy są zainteresowani i powiedzmy w tym przypadku konkretnym negują takie zachowania, no to już one wtedy… Ale to jest straszne w ogóle.

**To jest straszne. Zacznę wyłączać telefon, jak mi to teraz opowiedziałaś.**

I ja od tamtej pory zaczęłam właśnie przyglądać się. Więc takie coś to nie.

**To opowiem ci teraz o drugiej kategorii aplikacji. To są takie pomocowe aplikacje (opis aplikacji). Co myślisz o tego typu aplikacjach?**

Kierują środki albo nie skierują, bo można to wykorzystać w dwojaki sposób.

**To znaczy?**

Bo zależy, co chcemy uzyskać (śmiech).

**Ale nie wzbudzają twojego zainteresowania, twojego zaufania takie pomysły?**

Znaczy to bardzo było fajne i ciekawe. Tylko ja nie wiem, czy my jesteśmy takim krajem rozwiniętym na tyle, żeby to nam się sprawdziło.

**A jakieś obawy z nimi związane masz?**

No to jest pełna kontrola nad wszystkim. To, co mówię, to nagle nie wiem, no dobrze, widzimy jakieś tam zachorowania czy ognisko, no kwestia czy chcemy, nie wiem, pomóc czy zataić. Co z tym zrobimy, z tą wiedzą. Tu jeszcze nad tym by mądry człowiek musiał sprawować władzę, żeby to miało sens. Fajne by było, rzeczywiście, usprawnia, zdecydowanie. Tylko w momencie, kiedy lekarze i pielęgniarki nie mają sprzętu w konkretnych miejscach i proszą o pomoc. Więc myślę, że tu żaden dron by nie pomógł, bo fizycznie nie dostały pomocy, tak po prostu, gdzie można by było. I wszyscy o tym wiedzą.

**Fajne, tylko nierealne jak rozumiem.**

Tak.

**Kwarantanna Domowa (prezentacja aplikacji)**

Znaczy może to by było i dobre. Trochę tak jak założenie opaski więźniom. I chyba bym wolała mieć taką opaskę niż wykonywać te wszystkie polecenia i udostępniać swój telefon, swoje zdjęcie. To takie właściwie bardziej więzienie niż taka troska o obywatela, tak bym powiedziała.

**Dlaczego opaska lepsza niż to?**

Bo opaskę mam na czas kwarantanny, potem ją zdejmuję i jestem wolnym człowiekiem. A tutaj tak naprawdę udostępniam swoje zdjęcie, korzystając ze swojego telefonu, chyba, że na ten czas będę miała jakiś oddzielny telefon. No to jakby te dane nie zginą.

**Jak już je wrzucisz w ten telefon, to one już tam będą?**

I ktoś, ten system gdzieś będzie miał, to tak jakby no one będą przechowywane, one nie znikną przecież. Tak jak wszystko, co wrzucamy do sieci.

**Widzisz jakieś dobre strony tej aplikacji?**

Myślę, że może sporo osób takich niepokornych, mniej zdyscyplinowanych może troszeczkę pohamować przed takimi pokusami, że otóż nie będę się stosować. Też rzeczywiście dla tych służb może być to pomocne, że jednak tych takich bardziej niepokornych szybciej wyłapią. No to mogą być takie plusy.

**A co myślisz o tym, że ona jest obowiązkowa?**

Obowiązkowa. Właściwie no to też… Nie wiem, czy powinna być obowiązkowa. Bo właściwie to jest, w formie, no dobra, obowiązkowa to jest nakaz, ale co dajemy w zamian za to? No nie, no mojemu tacie dać taką aplikację, gdyby był sam bez niczyjej pomocy. To by było niewykonalne. A poza tym to jest takie nakazywanie. To nie.

**Czyli lepiej opaskę na nogę?**

Tak (śmiech).

**Faktycznie, to rzeczywiście jest tak, że jak masz opaskę, to oni doskonale wiedzą, gdzie ty jesteś.**

Tak. I to wystarczy. I wtedy niech też ktoś się wysili, żeby mnie śledzić, gdzie jestem, gdzie się przemieszczam. Bo tak naprawdę to jest takie, ja przejmuję wszystkie swoje… Ja wszystko przejmuję, bo muszę się meldować, muszę mieć opaskę, muszę wszystko swoje udostępnić. A co dostaję w zamian?

**Czyli nie dość, że wszystkie twoje dane trafiają do systemu, to jeszcze nic z tego nie masz. A co powinnaś z tego mieć? Co mogłabyś z tego mieć?**

Znaczy bardziej mi chodzi o coś takiego, już abstrahując od tych, bo to mi koleżanka powiedziała, jej szef jest Serbem. U nich to odbywało się tak, że jeśli ktoś przyjeżdżał i tam była obowiązkowa kwarantanna, to polegało to na tym, że państwo zapewniało miejsca na kwarantannę. Czyli nie jechał ktoś do swojego domu, tylko rząd jakby wynajdował hotele, jakieś pensjonaty, prywatne mieszkania, w których te osoby, które przekraczały granicę, były na tej kwarantannie. Nie wiem, czy to było kontrolowane też przez taką aplikację, kto to nadzorował. Natomiast państwo zapewniło takie warunki, żeby się nie przemieszczać, żeby w komfortowych warunkach się to odbywało. I to miało sens. Bo jakby państwo nakazało kwarantannę, ale też od siebie coś dało. A tutaj to właściwie co? Tyle, że masz być posłuszny i tyle. No co w zamian mogę dostać?

**Czy to rozpoznawanie twarzy to jest coś, co cię też niepokoi?**

No myślę, że tak, bo to tak jak odcisk palca. Potem tak naprawdę na całym świecie można w jakimś momencie, najmniej spodziewanym dla mnie, wykorzystać moją osobę.

**Na przykład?**

No nie wiem, to bym już tam fantazjowała bardzo (śmiech).

**Protego Safe (prezentacja aplikacji). Co myślisz o tym?**

Jeśli ktoś bardzo tak się… Nie wiem, jeśli ktoś bardzo się boi, jest taki bardzo na punkcie swojego zdrowia, prawdopodobieństwa zachorowania jest przewrażliwiony czy bardzo o to dba. Jeśli bardzo tak się interesuje, jak to wszystko wygląda, skrupulatnie śledzi, będzie to przydatne. Ale z drugiej strony, jeśli bardzo się interesuje, bardzo śledzi, to rzeczywiście te wszystkie informacje może sobie znaleźć, tylko poświęcając więcej czasu.

**A jak myślisz, jakie ona wykorzystuje informacje o tobie?**

No tak naprawdę czym się interesuję. No dobrze, mam temperaturę, szukam lekarza. Jakiego lekarza, gdzie szukam. Rekomendacje zachowań, gdzie szukać… No tak naprawdę dużo się można o sobie... Jak często sprawdzam te dane, w jakich państwach, bo to rozumiem, że to całego świata dotyczy. Z których danych najczęściej korzystam. To wiele takich zachowań. Czy bardzo się boję, czy nie, czy sprawdzam raz w tygodniu czy 3 razy dziennie. To myślę, że to też jakiś tam obraz osoby buduje. To tak trochę jak przez takie linie, to mi się przypomniało, to terroryści wykorzystywali, śledząc jak na przykład ktoś zamawia bilet. I nie wiem, powiedzmy, że wybiera sobie dania wegetariańskie, to już dużo się można dowiedzieć o takiej osobie. Nie wiem, czy jakieś koszerne jedzenie, gdzie się przemieszcza. To mniej więcej na tej zasadzie to można wykorzystać potem no w trochę gorszy sposób.

**Czyli też masz wrażenie, że ona jest śledząca?**

Tak. Bo jak ktoś chce, to rzeczywiście można wiele wyczytać o danej osobie. Choćby to, czy jest bardzo wystraszona, czy jest taka skrupulatna, czy nie wiem, zainstalowała aplikację, zajrzała 2 razy i właściwie zapomniała o niej. No to myślę, że też wiele można przeanalizować i wiele się dowiedzieć o takiej osobie.

**A ta pierwsza informacja, że może cię poinformować o spotkaniu z osobami chorymi? Bo będzie wiedziała, gdzie byłaś i kogo mijałaś na ulicy.**

No tak, ale to tak naprawdę to wszyscy by musieli mieć tą aplikację, żeby to było takie miarodajne. A poza tym no dobrze, że spotkałam, to jeszcze nie znaczy, że zachorowałam czy się zaraziłam. A poza tym, czy ta wiedza jest aż… Nie wiem, to jak ktoś jest bardzo podatny, to może dostać jakiejś takiej schizy, że ciągle mija chorych, zarażonych i zaraz będzie chory. To tak jak ja słuchałam o tych chorych, to przecież bym zaraz była chora (śmiech).

**Nie miałabyś ochoty, jak rozumiem, mieć tego w swoim telefonie?**

Na dzień dzisiejszy nie widzę takiej potrzeby.

**A w ogóle uważasz, że to jest sensowne, żeby państwo tworzyło taką aplikację dla obywateli i rekomendowało ich zakładanie?**

Nie. Niech się zajmą czym innym lepiej.

**Czym?**

(śmiech) Ogólnie może niech się zajmą gospodarką, swoimi decyzjami. Niech zaczną od podstaw. Bo to jakby jest fajne dla kraju wysokorozwiniętego, gdzie status każdego mieszkańca jest na przyzwoitym poziomie. Gdzie ludzie nie walczą z bezrobociem, które za chwilę będzie. Myślę, że są ważniejsze sprawy niż... To takie może być ekstra, gdzie, nie wiem, ludzie są zadowoleni, mają jakiś komfort życia, stabilizację. To OK.

**Jak myślisz, w jakim państwie to by było możliwe, żeby coś takiego było?**

No nie wiem. W Luksemburgu? Myślę, że tam spokojnie by się tym…

**Czyli zaufanie obywateli do rządzących.**

Tak. Akurat znam osobę, która tam mieszka i wiem, jak funkcjonują, jak to wygląda od strony państwa. Chociażby jak państwo dba o to, żeby dzieci się dostały, jak mają zapewnioną opiekę. To myślę, że tak, można sobie dołożyć taką aplikację, czy będę w międzyczasie narażona na spotkanie kogoś chorego.

**A ta aplikacja, o której wspomniałaś, że od niej by zależało, czy cię wpuszczą do galerii czy cię nie wpuszczą do galerii. Taką aplikację byś sobie założyła?**

Myślę, że nie jestem taką fanką galerii i nie mam takiej potrzeby tam przebywania, że aż bym musiała się tak obnażać i tak wszystkim ujawniać. Nie jest to takie aż dla mnie ważne. Żebym musiała tyle do siebie dać, żeby dostać spacer po lśniących płytkach.

**Czyli raczej byś zrezygnowała z wchodzenia do galerii niż zakładała sobie tą aplikację?**

Dokładnie.

**A co ty myślisz, co to będzie dalej z tą pandemia, jak ty myślisz o przyszłości? Czy masz jakieś swoje osobiste obawy, dotyczące ciebie, twojej rodziny w związku z pandemią?**

Boję się… Znaczy boję się, mam takie obawy, że może to nawet nie wzrastać, ale być cały czas na takim wysokim poziomie, że nie będzie malało przez takie nasze zachowania, ludzi. Na pewno ten taki aspekt gospodarczo-ekonomiczny, myślę, że to się bardzo odbije na wszystkich, że będzie to miało swoje żniwo. Trochę mnie niepokoi takie strasznie. Bo dla mnie to jest straszenie tym wrześniowym wybuchem w ogóle podwójnej chyba tej epidemii. No to też tak… Zajmijmy się tu i teraz. Myślmy, usprawniajmy to, co się dzieje teraz, a nie myślmy, co będzie we wrześniu. Bo jakby wybiegamy do… No dobrze, teraz jest jak jest, no dobrze, tak dużo może nie umarło, może tak nie ma ten, ale co to będzie we wrześniu. Więc też taki… Nie załatwiamy tego tu i teraz, ale już myślimy, co będzie we wrześniu. No mówię, to jest straszenie. Ale też reakcja ludzi jest taka, że ojej, tak straszą, co to będzie we wrześniu. Ale ja nie widzę tam strachu takiego, przerażenia. Na zasadzie takiej ciekawostki.

Będzie czy nie będzie, pomylą się. To tak jak trochę z prognozą pogody, będzie upalne lato czy deszczowe. To ja wolę takie konkretne rzeczy niż… Ja rozumiem, że część jest poparta jakimiś tam badaniami, jakąś analizą. Ale też no róbmy tu i teraz, działajmy. Lepiej, gorzej. Znaczy starajmy się lepiej. A nie od razu, co to będzie we wrześniu.

**A jeśli chodzi o gospodarkę, to czego się obawiasz?**

No takiego braku stabilności. Że jednak, no mówię, sporo osób straci pracę, to będzie takie wszystko zachwiane. No mówię, wszystko to jest takie nierówne, to jest niebezpieczne. Bo i wszystkie wzrosty i spadki. A poza tym też takie nastroje wśród ludzi, to też nie wróży dobrze, no jest jakieś takie… Nawet wśród tych znajomych, którzy stracili prace, tam gdzieś mam 2 osoby. To też jest takie rozgoryczenie, taki żal do wszystkich i wszystkiego. I jakby to też oprócz tego ekonomicznego aspektu, to też potęguje jakieś zachowania. Tak że się robi mało komfortowo.

**Masz takie poczucie, że któreś grupy społeczne ucierpią bardziej niż inne?**

Któreś grupy, ja wiem? Pewnie tak jak zawsze, ci średni. Bo na zasadzie, że ci, którzy zawsze jakoś tam wysoko postawieni, to zawsze sobie poradzą. Ci z tych niższych, uboższych, no to jeszcze są zaopiekowani. Bo jak usłyszałam, że bezrobotni mają dostać podwyżkę, to już zupełnie tego nie rozumiałam. O ile ci, którzy stracili pracę, owszem, a i tak. No to ci, którzy byli do tej pory na bezrobociu to nie wiem, dlaczego. Bo skoro im tyle wystarczało i nie szukali i nie angażowali się, to właściwie po co im pomagać, jeszcze jakby utwierdzać ich w tym, że robią dobrze? To nie. Więc tak naprawdę ta taka średnia, ten środek jak zwykle…

**Ta średnia klasa, której u nas właściwie nie ma podobno, tak?**

No której nie ma, a która chyba tak utrzymuje to wszystko.

**A jeśli chodzi o to, co się będzie działo na świecie, czy tutaj też masz jakieś obawy?**

No, to jest zagadka. Bo rzeczywiście, no nie wiem, jak patrzę… Znaczy rzadko patrzę na Trumpa, bo jakoś nie bardzo mogę na niego patrzeć. I to, co się dzieje w Stanach. No w kraju, który jednak ma dużo do powiedzenia i ma wpływ na to, to tam jest taki chaos. Że boję się, że to się przeniesie na całą resztę. Te duże państwa, nie wiem, no Chiny czy... No jest to wielka zagadka. Bo tak naprawdę z tego może być wszystko. Ten brak takiej stabilizacji, takiej… Duży, duży rozstrzał.

**A czy myślisz, że jakieś działania związane z pandemią, jakieś obostrzenia, one powinny zostać na dłużej z nami?**

Jakieś obostrzenia. Ja już nie wiem, bo nawet jak są, to tak… Może niech ci ludzie myją te ręce, rzeczywiście (śmiech). Niech to będzie obowiązek. Ja już straciłam taką nadzieję, że nawet jak są te obostrzenia i czy one są, nie wiem, mniej przydatne czy bardziej, to ludzie i tak przez chwilę się tylko stosują. Więc to co mówię, ja zaczęłam myśleć właśnie o takim daniu swobody. Że na zasadzie, dobrze, wykończcie się sami. Może rzeczywiście to by zadziałało bardziej niż jakieś właśnie zakazy, nakazy.

**Ale czy jak był ten ostry nakaz, że wszyscy mają założyć maski, tak jak rozmawiałyśmy, to mówiłaś, że rzeczywiście wszyscy w tych maskach chodzą. Prawda?**

Tak. Chodzili. I było dobrze. Po czym, nie wiem, czy się zmęczyli tym, czy o co chodzi, że nagle im to przeszło.

**No właśnie, czy powinno się tak nakazać ostro coś na dłużej albo na zawsze?**

Znaczy nie umiem powiedzieć, co konkretnie. Ale myślę, że powinno to być na pewno przemyślane. Tak rozsądnie, żeby to miało sens. Bo to też były takie sprzeczne komunikaty z tymi maseczkami, że tak, pomagają, nie pomagają. Szkodzą, nie szkodzą, zabezpieczają, nie zabezpieczają. Więc tak naprawdę no taki chaos się wdarł. Gdyby rzeczywiście na spokojnie przeanalizować, co pomoże, co nie, to myślę, że… Tylko mówię, to by musiały być naprawdę takie przemyślane obostrzenia, które faktycznie by miały przełożenie w sytuacji. Że jakąś taką gwarancję, no może nawet nie gwarancję, bo nie ma tej gwarancji. Ale jakoś poparte badaniami, doświadczeniami lekarzy. Bo lekarze bardzo różnie się tam wypowiadają.

**A według ciebie co powinno zostać z nami na dłużej albo na zawsze? Może najpierw co na dłużej, powiedzmy do wynalezienia szczepionki. Na co się powinno zwracać uwagę?**

No ja myślę, że takie gromadzenie, te tłumy, takie zbiorowiska, typu jakieś takie mecze, duże imprezy. Tam, gdzie jest bardzo, bardzo dużo ludzi. Bo w takim skupisku jest większe prawdopodobieństwo, że ktoś jest zarażony czy przenosi tego wirusa. Ale z drugiej strony jak to teraz podzielić większe, mniejsze? Bo jak usłyszałam, że może być wesele do 150 osób, to się pytam, jaka jest różnica między 150 a 180? Bo ja już nie widzę żadnej.

**A co w ogóle myślisz o tym, że można zrobić wesele na 150 osób?**

Znaczy, to już mnie rozśmieszyło tak naprawdę. Bo bądźmy konsekwentni, albo są te zgromadzenia i wtedy zarażamy się. Albo ich nie ma, albo podawanie cyfr. To po prostu powiedzmy, róbmy wesela. A nie jakieś podawanie cyfry, konkretnej ilości osób. Równie dobrze można się wśród 30 zarazić a wśród 200 nie. Więc to w ogóle jakiś wymysł jak z tymi lasami, czy nie wiem, czym tam jeszcze.

**A gdyby to od ciebie zależało, to byś pozwoliła na takie duże wesela teraz?**

Teraz? Myślę, że nie. Dlatego, że my bardzo, przynajmniej większość społeczeństwa bardzo celebruje te wszystkie rodzinne takie uroczystości. To jest nieraz takie właśnie pokazanie się, takie zrobienie show za wszelką cenę. Bo tak jak słyszę, jak ludzie biorą kredyty na wesela, to też tego nie rozumiem. Więc jestem sobie w stanie wyobrazić, że po prostu kosztem zdrowia, nieważne, że tam przyniosę coś do domu, ale pokażę się i zrobię to wesele.

**Czyli zostawiamy na dłużej na razie brak zgromadzeń. Choć trudno jak rozumiem powiedzieć, co to znaczy duże.**

No właśnie. Bo zawsze ktoś będzie poszkodowany. Znaczy ja myślę, że te maseczki, ja będę się upierać, że one mają sens. Jednak przy tej drodze kropelkowej, przy takich ludziach… Nawet to, jak nie wiem, widziałam jak ludzie wcześniej, jak zaczęłam się zastanawiać, nie wiem, kaszlą, kichają. Nie zatykają buzi. No to czy on jest zdrowy czy chory, czy chory na wirusa czy na coś innego, to zawsze zabezpieczy. Nie wiem, mnie wdychając, no mówię…

**Ale zostawiłabyś je wszędzie czy w pewnych miejscach?**

Takich publicznych miejscach. Bo jadąc rowerem, to trochę śmiesznie to wygląda z tą maską. Bo naprawdę może bym musiała kichnąć na kolarza obok, to prawdopodobieństwo jakieś jest, ale no umówmy się. Nie, takie właśnie, że wchodzę do sklepu, jestem w przychodni. W takich miejscach, w przychodniach, w szpitalach, uważam, że tak. Zresztą to nawet ja miałam wizytę, zadzwoniono do mnie, bo ja przed samą pandemią miałam umówioną wizytę do lekarza pulmonologa zresztą, żeby było śmieszniej. I myślałam, że to już się w ogóle nie odbędzie. I zadzwoniono do mnie z tej przychodni. I zapytano, czy jestem zdrowa, czy nie gorączkuję. I zaproszono mnie na godzinę i żebym miała rękawiczki i maseczkę. Ja tak nawet przez moment stwierdziłam, mówię tak do męża, może ja tam nie pójdę? Bo jak on się płucami zajmuje, to on pewnie biega po tych szpitalach, konsultuje tych chorych. Ale mówię, no z drugiej strony skoro przyjmuje, no to też, mówię dobra, wróć, nie wymyślaj. I szczerze mówiąc w takich właśnie, że nie wiem i pielęgniarka miała maseczkę, były dezynfekowane, rzeczywiście bardzo pilnowali, żeby pojedyncze osoby wchodziły. I ten lekarz taki… To ani to nie było dla mnie uciążliwe. A poza tym miałam poczucie bezpieczeństwa. I ta maseczka tego lekarza, i te rękawiczki jego, moje, wszystkich po kolei. I ci ludzie czekający pod przychodnią, że proszę zaczekać, bo teraz pani wychodzi z badań, nie mijamy się. Super. A nieraz sobie siedziałam w przychodni, po prostu wszyscy się tłoczyli i chuchali na siebie, dmuchali, bo ja muszę tu do okienka po numerek. Więc to było naprawdę, takie warunki mi odpowiadają (śmiech).

**To może myślisz, że tak powinno już zostać zawsze? Niezależnie od tego, czy znajdą szczepionkę czy nie. Bo zawsze może być jakiś nowy wirus albo jakaś grypa bardzo zaraźliwa, do której już niby jesteśmy przyzwyczajeni.**

No, ostrożności nigdy za wiele. Ja mówię, to nikomu nie szkodzi, bo to nie jest uciążliwe. Takie, że nie wiem, wchodzę tam, gdzie są ludzie chorzy, mam rękawiczki i mam maseczkę. Idę do jakiegoś dużego sklepu i… To nie jest coś, przynajmniej dla mnie, bo dla kogoś taka aplikacja może nie jest uciążliwa i jest zadowolony, że może być taki właśnie, a może ta maseczka być dla niego uciążliwa. Dla mnie nie byłoby to uciążliwe, a daje taki chociaż, nie wiem, na ile wiarygodne, ale takie poczucie bezpieczeństwa.

**Czyli zostawiamy maseczki w pomieszczeniach, komunikacjach, przychodniach.**

Tak.

**Płyny do dezynfekcji też, rękawiczki też?**

Rękawiczki tak, znaczy płyn do dezynfekcji też. Naprawdę, to jest… Ja już mam taki odruch, że szukam i patrzę i nie wzbudza to we mnie jakiejś… Po prostu, czy ja umyję rękę, a potem ją zdezynfekuję. No tak jest… No mówię, daje mi to namiastkę bezpieczeństwa.

**A co z takim dystansem nakazywanym? Że stoliki rzadko w restauracjach czy w kawiarniach, że trzeba co drugie miejsce w autobusie i tam tylko określona liczba osób może wsiąść do wagonu metra czy do tramwaju, co o tym myślisz? To też dłużej czy już nie?**

Tutaj nie wiem. Nie mogę się wypowiadać, bo bardzo rzadko korzystam z komunikacji. I tak naprawdę nie wiem, jak to technicznie miałoby wyglądać. Ktoś mi tam mówił, że jakiś autobus u nas jeżdżący w okolicy, tam przewidziano 75 osób. No to dobrze, to niech będzie więcej tych autobusów, skoro ma tak być. A poza tym kto ma liczyć te osoby? Dystans fajnie, tylko czy jesteśmy w stanie to zrealizować? Bo nakazać, zakazać można, tylko jak to się ma do rzeczywistości.

**Nie ma tego jak sprawdzić, ile tam jest osób?**

No nie, ludzie się spieszą do pracy, do jakichś tam zajęć. I jak teraz mają oceniać, czy on wsiądzie czy nie wsiądzie, kto ma o tym decydować? Współpasażer? Nie wiem, jakiś kontroler. No, ale mówię, nie korzystam z komunikacji miejskiej. Więc równie dobrze mogłabym powiedzieć tak, niech w autobusie będzie co 3 miejsce wolne. Ale no czy to utrudnienie ludziom życia nie będzie gorsze niż nawet znajdą się obok siebie w maseczkach? Może lepiej w maseczkach i obok siebie niż sfrustrowani, awanturujący się przed autobusem.

**A co myślisz o tych miejscach w samolocie? Że po jednej osobie w rzędzie, tam 50% chyba tylko pasażerów może latach. Jakieś takie są przepisy w tej chwili.**

No nie wiem, czy linie lotnicze wytrzymają to finansowo. Albo ile by musiały kosztować bilety, żeby to się opłacało. Bo umówmy się, to musi się opłacać.

**No tak, ale to jest rozwiązanie, które dla ciebie sprawia, że ty się będziesz czuła bezpieczniej w samolocie? Bo tam i tak trzeba mieć maskę.**

Właśnie samolot jest o tyle, ja nawet tak myślałam, że w ogóle mnie nie ciągnie, żeby wsiąść do samolotu. O ile, nie wiem, do autobusu, pociągu nie miałabym takich oporów, natomiast samolot mnie zupełnie na tą chwilę nie interesuje. To jest jednak taka zamknięta puszka. I tak naprawdę przecież te bakterie, wirusy, one się przemieszczają, czy ktoś jest w pierwszym rzędzie, a ja w ostatnim, tak naprawdę to jest zamknięte. Ja wiem, że te wszystkie wentylacje itd. Ale na przykład mój mąż po każdym locie jakimś dłuższym, on jest chory, jest przeziębiony. Albo dostaje opryszczki. Czyli coś tam działa na niego tak, że taki lot samolotem, on zawsze z jakąś infekcją, z czymś wraca. Więc jest takie osłabienie organizmu i to jest takie miejsce, gdzie prawdopodobieństwo jest większe. No nie wywietrzy się tego. A to świeże powietrze myślę, że dużo daje (śmiech).

**Pojawiają się takie miejsca, właśnie na lotniskach, gdzie mierzą temperaturę. I nie wejdziesz, jeżeli ci pokaże, że masz za wysoką. To mierzenie może się pojawić w kinach, w innych miejscach publicznych. Czy to jest coś, co uważasz, że jest dobrym rozwiązaniem, że powinno tak być? Czy też tobie to przeszkadza i ci się nie podoba?**

Powiem tak. Znaczy nie wiem, na ile to jest miarodajne. A poza tym nie wiem, czy ktoś przypadkowy, bo dla mnie to jest przypadkowa osoba, która będzie mi mierzyła tą temperaturę, czy ja bym chciała, żeby ktoś taki właśnie, jeśli to nie jest lekarz, pielęgniarka, żeby mi mierzył tą temperaturę.

**Ale wiesz, to są takie bramki. Wchodzisz w taką bramkę i ona ma czujnik i ona odbiera twoją temperaturę.**

No na pewno robi jakąś selekcję. Tylko z drugiej strony jaka ta temperatura będzie mówiła o tym, że ktoś jest chory? Bo przecież są takie osoby, które bez gorączki chorują ciężko. A są takie, które na zwykłą anginę mają po 40 stopni.

**No tak, oczywiście tu są różnice indywidualne. Ale czy to by zwiększało twoje poczucie bezpieczeństwa? Ty byś się dobrze z tym czuła, gdybyś miała nad sklepem taką bramkę i wiesz, czy wchodzi ktoś z temperaturą czy nie?**

No, myślę, że to by taką selekcję zrobiło, że byłoby to bardziej komfortowe. Jeśli by to było takie rzeczywiście dobrze mierzone, taka wiarygodna ta temperatura.

**I wtedy byś się zgodziła, żeby i tobie mierzyć?**

Tak. Tak.

**Czyli zawsze kogoś tam wychwyci, może zwrócić uwagę?**

Chociaż to też z tym mierzeniem jest tak różnie. Bo ostatnio też moja chrześnica miała, pojechała ze swoim dziadkiem do lekarza. I też miała mierzone w szpitalu i nie mogła z nim wejść. On tak naprawdę nie dałby sobie rady wejść do tego szpitala. No i też ona miała podwyższoną temperaturę, ona czekała. Bo tak naprawdę ona się rozgrzała w samochodzie. I też jest ten moment, gdzie no tak naprawdę nie powinna być wpuszczona. Tam minimalnie ta temperatura jej spadła, jakoś tam ubłagała, że weszła z tym dziadkiem. No i też, no można kogoś zdrowego wyeliminować albo już spojrzeć, że jest podejrzany, a ktoś, kto tak… Ale z drugiej strony nie zaszkodziłoby. Znaczy nie przeszkadzałoby mi to aż tak, żeby się nie zgodzić.

**W porównaniu do odcisku palca, to rozpoznawania twarzy…**

To zdecydowanie tak (śmiech).

**A lokalizowanie też jest takie okropne jak to, że masz, gdyby ci kazali mieć cały czas włączoną lokalizację? Nie ma rozpoznawania twarzy, ale jest przypisana do twojego smartfona i zawsze można cię… Też nie?**

Nie. Nie. Nie.

**A dlaczego nie?**

Ja bym się tym źle czuła, że mnie ktoś śledzi.

**To daje poczucie śledzenia?**

Tak. A poza tym, no ja uważam się za osobę taką, która... Chyba, że zrobi mi się tak, jak mojej mamie, ale na tę chwilę nie, staram się być odpowiedzialna. I jakby nie narażać siebie i innych. Więc skoro bym miała siedzieć w domu, ja no czułabym się w obowiązku i bym siedziała w tym domu. To tak jak nagle teraz dostałam wezwanie z leasingu i z taką formą, prosimy o terminowe wpłacanie rat tam leasingu, z takim wytłuszczonym. I na przykład ja się poczułam, znaczy źle się z tym poczułam, bo płacę na czas skrupulatnie od 3 lat. I co, dlatego, że teraz coś się zachwiało, no to ktoś mnie upomina, żebym to robiła na czas? Jak robię to zawsze. Ja wiem, że to po prostu z automatu. Ale no nie.

**Tak, to jest nieprzyjemne.**

Tak. Ja otworzyłam tę korespondencję, więc mówię hello. Nie miałam żadnego opóźnienia, płacę skrupulatnie, że wszystkiego się wywiązuję, a nagle jestem o coś upominana. Więc no nie. To tak mniej więcej, jak się mówi, że Polacy to są pijacy. No nie, ja nie poczuwam się do tego.

**Gdyby była ta druga fala, to co się powinno zadziać w Polsce, co powinno się zrobić?**

Żeby powstrzymać tą pandemię, czy...?

**No w ogóle. Występuje nagle jakiś gwałtowny wzrost zachorowań, mówią epidemiolodzy, że to jest druga fala. I co wtedy?**

To jest ciężkie, bo my nie zdaliśmy egzaminu z pierwszej. Najlepiej by było wyciągnąć wnioski z tego postępowania teraz, tylko jakie? Bo tak naprawdę ja mam wrażenie, że my jesteśmy cały czas w takim chaosie.

**W jakim zakresie nie zdaliśmy egzaminu z pierwszej?**

To jest cały czas chaos. To jest tak, zakładamy maseczki, nie zakładamy, pomagają, nie pomagają. Dziś zakładamy, jutro zdejmujemy. Spotykamy się w grupach, nie spotykamy się w grupach. To jest takie wszystko… Nie wiem, chociaż żeby był 1 tor taki stabilny. No nie ma zakażeń, no są na Śląsku, ale generalnie wychodzimy z pandemii. No nie, nie wychodzimy. Więc… Znaczy to jest ciężkie, bo to jest ciężkie dla wszystkich, natomiast… No bo tak, z drugiej strony zatrzymać wszystko, no to jest ciężko, żeby tak stanęło. No nie mam pomysłu jak by to miało się… Ja myślę, że bardziej to tak indywidualnie, że taka samodyscyplina, taka większa ostrożność i tak od jednostek. Że właśnie nawet nie nakazami, tylko szanujmy jedni drugich. Dobrze, ktoś się nie chce spotykać, nie chce się całować, uszanujmy to. Nie wiem, trzeba nosić maski, dobra, nie lubię ich, niefajne, ale wchodzę, jestem wśród ludzi, zakładam i nie dyskutuje. Może takie bardziej spojrzenie samemu na siebie, tak indywidualnie.

**A myślisz, że rząd powinien odgórnie przywrócić jakieś ograniczenia, które już były? Są takie, które masz wrażenie, że sprawdzają się w takiej sytuacji i po prostu: no trudno, trzeba to zrobić?**

Na pewno, znaczy to akurat nie rząd, ale na pewno ograniczenie ilości osób przez taką pracę zdalną. Myślę, że to też ogranicza ilość osób, które się przemieszczają. Zresztą wiele firm i nawet wśród znajomych no ciężko, ciężko, najgorzej ci, którzy mieli jeszcze dzieci, powiedzmy ileś osób pracowało w jednym czasie. Ale sporo osobom się to sprawdziło. I wiele firm na tym też i skorzystało. No i to też ogranicza ilość osób przemieszczających się. I jakby tu nie ma nakazu, takiego przymuszenia siedzenia w domu, to wychodzi automatycznie. Bo pracuję z domu. Więc nie ma tej komunikacji, nie chodzę po drodze, tu kawiarnia, sklep. Tylko też mam ograniczony ten zakres przemieszczania się. Ale z takich obostrzeń… Ale chociażby te wizyty takie w szpitalach, że nie tłoczymy się, tylko umówione te wizyty pojedynczo. Chociaż ze służbą zdrowia to w ogóle trzeba się cieszyć, jak się nieraz dostanie do specjalisty. Ale można w tym kierunku.

**A szkoły, przedszkola zamykamy, nie zamykamy?**

Znaczy ja w ogóle nie rozumiem tego, że wracają teraz żłobki. Dlaczego nie starsi? Ja bym może… Te małe dzieci owszem, ale te starsze? Przecież one spokojnie mogą… Nie wiem, no taki dwunastolatek, przecież może chodzić w maseczce, on wie, jak umyć ręce. Ale jak wytłumaczyć trzylatkowi, żeby się z kolegą nie bawił albo ta ciocia go teraz nie przytula i chodzi w jakimś fartuchu? To jest w ogóle… Nie, myślę, że szkoły mogłyby funkcjonować.

**A sklepy inne niż spożywcze, fryzjerzy, kosmetyczki, manikiurzystki?**

To, co ja powiedziałam na samym początku, że gdyby to było zamknięte, że wszystko razem, że hamujemy tą epidemię, że dobra, wybucha, zwiększają się zachorowania, jest ich dużo, stopujemy wszystko, to jestem na tak. Tylko, że wszystko razem, a nie tak dokładamy, dokładamy.

**Nie było w tym logiki, więc nawet trudno sobie wypracować takie na to spojrzenie.**

Tak.

**A tak z perspektywy twojej, jak patrzysz na cały ten czas pandemii, to już od początków marca to było, to jakie dla ciebie są takie najważniejsze momenty, takie przełomy w tym czasie? Takie ważne dla ciebie momenty.**

Ja wiem? Ja żyję w takim biegu i tak dużo się dzieje, że ciężko wyłapać te momenty. Ale pomimo tego pędu, takiego biegu, to chyba dla mnie takim ważnym było takie wyhamowanie i takie zastanowienie się nad niektórymi rzeczami. Takie nawet z rodziną, że bycie razem, że jednak wśród tych najbliższych jest najbezpieczniej. Jest komfortowo, możemy się wspierać, można więcej rozmawiać. I to są te plusy takie, które… Zresztą ta weryfikacja, tak jak mówiłam od początku, tych znajomych, którzy się odezwali, którzy nie. Taka obserwacja, jak to w ogóle wygląda. Takie pomimo wszystko wyhamowanie. Bo mówię, pęd i dużo się działo, ale może się dziać w różnych warunkach. Ale no tutaj taki… To myślę, że to najwięcej.

**A gdybyś miała popatrzeć na takie przełomy tego, co się działo w kraju, to są takie momenty, które są dla ciebie ważne, istotne?**

To zamieszanie z wyborami. To było w ogóle jakiś… To było jakieś nieporozumienie, w ogóle takie odczepienie od rzeczywistości. To w ogóle za dużo zamieszania. No właśnie to też wprowadziło taki chyba chaos, że to jest ta pandemia czy nie? Bo skoro mówimy o wyborach, można odbyć w innym czasie, w innym miesiącu i nikt z tego powodu nie umrze, a tu można umrzeć i to nie jest ważne, no to to było chyba takie, tu zupełnie niezdany egzamin.

**Coś jeszcze ci przychodzi do głowy z takich rzeczy w kraju, które się działy, które masz wrażenie, że jak patrzysz, co się działo, to było bardzo ważne, taki przełom, etap, wydarzenie?**

Ja bym musiała pomyśleć, poanalizować, ja tak na szybko to nie (śmiech).

**A w ogóle jest tak, że czasem się zastanawiasz, że boże, jak to było, co to było po kolei? Przypominasz sobie ten czas, czy starasz się to jakoś wypchnąć z pamięci bardziej?**

Nie, przypominam sobie, przypominam, tak. Ten taki pęd, że jak to było i w ogóle jak to się dało radę. Że niby było takie straszne, ale pokonało się tę sytuację. Że jakie to było zamieszanie. Tak, bo nawet mówię, jak teraz takie rozluźnienie i ci ludzie się inaczej zachowują, to tak, to mi przychodzą takie myśli, że jak to inaczej wyglądało. Jak nawet widzę konkretne osoby, jak je obserwuję, one inaczej się zachowywały. No to też…

**Ale co masz na myśli? Bo ten pęd to rozumiem, że jak zamknęłaś swój salon, musiałaś do sklepu, a tutaj rodzicom jedzenie. Jak zaczynałyśmy rozmawiać, to to był ten pęd, w którym się znalazłaś.**

Potem była ta zadaniowość, takie zadania, zadania. A potem jakby… To też ludzie na nas wpływają, że jak zaczęli odpuszczać, rozluźniać, to też taki spokój bardziej zapanował, że można było w bardziej komfortowych warunkach pracować. I właśnie więcej było czasu na takie obserwacje, że ci ludzie już tak nie rzucają się na takie zakupy. Co jeszcze takiego… Strach różne też rzeczy potęgował. Bo albo taką złość i agresję jeden do drugiego. Albo z kolei taką życzliwość i chęć pomocy. A jakby teraz odeszło. I te takie aroganckie zachowania i te takie bardzo, chęć pomocy. Znaczy ja mówię, żadne skrajności mnie nigdy nie przekonują, to tak jak jest robienie paczek na święta bożego narodzenia do wszystkich domów dziecka, wszystkim pomagamy. A jak tak zawsze myślę, no dobrze, dam teraz paczkę ze słodyczami jakiejś tam rodzinie czy komuś choremu, ale on jest przecież biedny przez cały rok, a nie tylko na święta.

**Tak, ale my jesteśmy tacy dobrzy akurat tylko w święta.**

I wtedy tak się uszlachetniamy. I wtedy to też jest mniej więcej, że sobie robimy więcej, że tacy w tym okresie to nagle się wybieliliśmy.

**Czyli były takie skrajności, że był duży strach, ogromna życzliwość, to wszystko się przeplatało. A teraz wracamy do takiego czegoś płaskiego.**

Tak, tak. Taka stagnacja, jest jak jest. Gdzieś tam może ktoś się obawia, ktoś się boi taki… Ale już bez takich właśnie skrajności i już jakby…Już tym sąsiadkom mniej te zakupy się robi. Już tak się nie pomaga.

**Już każdy sobie.**

Już każdy sobie, tak. Ja miałam nawet nadzieję, że może jakieś wyciągnięcie wniosków, że właśnie że będziemy lepsi, że może coś. No nie, jak zwykle się pomyliłam. Już więcej klaksonów na ulicy słychać.

**Te marzenia o tym, żeby postać w korku… Jak się spełniły, to wraca irytacja na korek.**

Tak.

**Czy coś jeszcze chcesz dodać, co wydaje ci się ważne z perspektywy czasu?**

Nie. Znaczy ciekawe, nie wiem, czy to dobre słowo, że ciekawe, ale no takie interesujące doświadczenie, taki… No mówię, dla mnie taki moment do wyhamowania, do zastanowienia się nad wszystkim, co się wokół, jak… No właśnie to nawet to pokazanie, takie potwierdzenie, że nie na wszystko mamy wpływ, nie wszystko ochronimy. Że choćby nie wiem jak byśmy się chronili i jakie maseczki, to zawsze gdzieś coś się może przemknąć. I to też w pewnym sensie daje mi spokój. I takie no, żeby się tak nie napinać, nie złościć tym, nie nakręcać. Bo nie przeskoczymy. To tak jakby utwierdziło mnie w czymś, o czym wiedziałam, ale jednak jak się tak dotknie, przerobi, to…

**Że musimy się pogodzić z tym, że nie na wszystko mamy wpływ?**

Tak, tak. Że co możemy, co jesteśmy w stanie. Natomiast choćby nie wiem jakie tak sobie robił zabezpieczenia, to…

**Dziękuję bardzo.**
