# Supplementary material for: The challenges arising from the COVID-19 pandemic and the way people deal with them. A qualitative longitudinal study
Source: PLoS One. 2021 Oct 11;16(10):e0258133. doi: 10.1371/journal.pone.0258133 (PMC8504766; doi:10.1371/journal.pone.0258133)
Supplement: S1 Dataset — (ZIP) [file pone.0258133.s003.zip › Transcriptions/stage 5/1.5_F_25_single.docx]

**1.5_K_25_single**

**Co się u ciebie działo przez ostatni miesiąc?**

Jak widać, inne tło jest, bo już się przeprowadziłam. W niedzielę przywiozłam rzeczy i już się powoli urządzam, więc jest jakaś zmiana. Ale kolejna rzecz to szukanie pracy. Mam jedną rzecz na razie na oku i rozmowy. Może zadzwonić do mnie właśnie telefon w tej sprawie dzisiaj. Poza przeprowadzką to nic takiego się nie zmieniało, bo nie miało co. Już jestem w mieszkaniu, w Warszawie. Tylko teraz, wczoraj właśnie miałam takiego dołka z tą pracą po tej rozmowie. Tam nie do końca mi wszystko pasuje w tej pracy i ta świadomość tego, że tego wyboru nie ma dużego, bo ta rekrutacja jest cały czas wstrzymana w wielu firmach. Te rozmowy, które miałam na początku kwietnia, co miałam czekać aż się odezwą, to się okazało, że oni nikogo jednak teraz nie zatrudniają. I to się może zmieni za 2 tygodnie albo miesiąc, ale nie wiadomo. I to się może przedłużać w zależności od tego, jak długo będą w mocy obostrzenia a propos biur. Bo to jest największy problem.

**Kiedy się przeprowadziłaś?**

W niedzielę.

**A jak z codziennością? Coś się zmieniło?**

Jak byłam u rodziców to nie, wszystko było tak samo. Raz mnie odwiedzili znajomi. Acha i ja w międzyczasie byłam w Krakowie u mojej przyjaciółki. Ona jest z moich okolic i była akurat z mężem u rodziców i zabrali mnie, wracając. Także odwiedziłam ją na 2 dni w Krakowie. Tylko większość rzeczy zamknięta, więc zwiedzania za bardzo nie było. Pogodę też miałyśmy słabą, więc przespacerowałyśmy się raz przez Stare Miasto i tyle. Ale to bardziej było, żeby się z nią zobaczyć i odwiedzić. To jest ta, co 2 maja miała ślub i była online transmisja. Wesele już wiemy, że się odbędzie, bo oni je przełożyli na 3 lipca, tylko czekali z decyzją na to, co będzie ustalone w kwestii zgromadzeń. Ponieważ ta liczba jest podniesiona do 150 to wesele się odbywa.

**Planujesz iść na to wesele?**

Tak. Rozmawialiśmy, że pewnie okaże się, że starsza część rodziny zrezygnuje z przyjazdu. I pewnie skończy się tak, że to będzie taka młodsza część rodziny, najbliższa rodzina i przyjaciele. Aczkolwiek wesele jest w piątek i nie wiem, kto dojedzie. Szczególnie, że część ze strony mojej przyjaciółki będzie dojeżdżała z Warszawy, spod Warszawy, a wesele jest pod Krakowem, więc trzeba też dojechać. Pewnie się trochę ludzi wysypie z tego powodu.

**Jakie były ważne momenty przez ten miesiąc - powiedziałaś o wizycie w Krakowie, o spotkaniu z przyjaciółmi...**

Oni przyjechali do mnie. Ale ja też z raz byłam tutaj w Warszawie, tylko... To jest zabawne, bo ja dojeżdżałam pociągiem spod Warszawy pół godziny codziennie do pracy. A odkąd w kwarantannie siedziałam w domu i tego nie robiłam, to jak już jechałam do Warszawy, to potwornie mi się jechanie tym pociągiem dłużyło. Widziałam zmianę tych nawyków, że ta podróż była tak długa, że aż odechciało mi się gdziekolwiek jechać. Gdzie wcześniej jeździłam tak w jedną i w drugą stronę codziennie i to nie był problem. Tzn. był problem, ale mniejszy. Więc tak raz albo 2 byłam w Warszawie kogoś odwiedzić. A i byłam też z koleżanką pracy i jej znajomymi nad Wisłą. Trochę spacerowałyśmy nad Wisłą. To był weekend przed tym, jak otwierali restauracje. Większość była zamknięta, coś tam było pootwierane, ale wydawały tylko jedzenie na wynos. Dziewczyny chciały koniecznie coś pić. Ja nie miałam w planach picia, bo potem miałam prowadzić samochód. Ale one chciały wypić jakieś piwo, tylko nie kupiły go w sklepie, tylko myślałyśmy, że coś kupimy w lokalach nad Wisłą. Ale z racji tego, że trzeba brać na wynos i nie można zostawać w lokalu, to nie można sprzedawać alkoholu i wszystkie lokale miały tylko bezalkoholowe piwa, bo nie mogą wydać alkoholu, które ktoś ma pić na zewnątrz. W ogóle chyba cofnięto to przyzwolenie na picie na schodach na bulwarach. Więc nie znalazły alkoholu. Długo szukałyśmy jedzenia, ale w końcu udało nam się jedzenie upolować. W knajpie można było wejść i na wynos wziąć. Te wszystkie obostrzenia typu kolejka odpowiednia, punkty na podłogach poustawiane, żeby jak ktoś wchodzi i ludzie są w środku, to żeby stali odpowiednio. Ale już też widzę, że w sklepach na rękawiczki nikt nie zwraca uwagi. Jak byłam wczoraj w Leroy Merlin kupić rzeczy do domu i założyłam te rękawiczki, ale potem kupowałam śrubki na wagę. Wyszła mi ta naklejka i naklejkę musiałam nakleić na woreczek. Ale naklejka przykleja się do rękawiczek [śmiech]. Więc już myślałam, że zwariuję i byłam już tak zdenerwowana, że zdjęłam te rękawiczki i robiłam zakupy bez, bo nie dało się nakleić głupiej naklejki. Więc ludzie już przestają nosić rękawiczki albo nikt na to nie zwraca uwagi w sklepach.

**Wracając do tych ważnych momentów, czy jeszcze jakieś były?**

Nic takiego. W pewnym momencie mój brat wrócił już do swojego mieszkania, więc zostaliśmy z rodzicami. A tak to w sumie nie.

**Jak wygląda teraz Twoja codzienność?**

Jest to takie jeszcze wszystko niepewne, bo wciąż się tu urządzam. Ale o tyle się zmieniło, że zajmuję się tylko sobą. Nie mam psa, z którym trzeba wyjść. Byłam w sklepach takie rzeczy do domu kupić. Ale wciąż nie mam konkretnych zadań do wykonania w ciągu dnia, tylko ustalam sobie, że teraz pójdę do sklepu, a teraz zamówię jedzenie. Plus jest taki, że tak jak u nas nie dojeżdża nic z dowozu jedzenia, tak tutaj mam mnóstwo opcji też wegetariańskich i wszystko pod nosem.

**Masz takie poczucie, że to życie wróciło już do normalności sprzed pandemii?**

Nie. Brak pracy i brak konkretnych... Najgorsze jest to, że mam niby znajomych w Warszawie, ale ja nic nie robię, a oni pracują. I na razie się z nikim jeszcze nie umówiłam. I doskwiera mi ten brak spotykania się z ludźmi. Możliwości, że mogę znaleźć wydarzenie, wyjść i tam może kogoś poznam. Wczoraj rozmawiałam ze współlokatorką, że ja już chcę takie momenty, że idę w miejsce, gdzie jest dużo ludzi, których nie znam, więc jest potencjał poznania kogoś nowego. Strasznie mi tego brakuje. Mimo, że mam iluś znajomych, z którymi się mogę spotykać - to może zabrzmieć strasznie - ale nudno się w kółko z tymi samymi znajomymi spotykać. Kocham ich, ale potrzebuję jakiś nowych bodźców. I jeszcze dobiła mnie ta rozmowa o pracę a propos spotykania ludzi, bo okazało się, że to jest projekt na pół roku, co mi odpowiada, ale cały będzie zdalnie. Na żadnym etapie nie pracuję z biura - nawet za 4, 5 miesięcy. I to mnie zasmuciło, bo cieszyłam się, że może nawet nie na stałe będzie wracanie do biura, ale że w jakimś wymiarze mogłabym do biura pójść i będzie to okoliczność do poznania nowych ludzi. A tutaj skończyłoby się to na mailach i kilku Skype z ludźmi, więc to nie jest ten sam sposób nawiązywania relacji z ludźmi, co w biurze.

**Nie wypełniłoby to tej potrzeby relacji z innymi?**

Tak. I to mnie tak zasmuciło wczoraj, bo mam takie poczucie, że jestem, siedzę, mam kilku znajomych dookoła, ale wciąż nie mogę żyć normalnie.

**Są takie elementy, które już wróciły do normalności? Np. wyjścia z domu?**

Tak. Wyjście już jest coraz bardziej normalne, bo nie muszę mieć maseczki na zewnątrz.

**Nie nosisz jej teraz?**

Nie. Na świeżym powietrzu nie. W tramwaju, w autobusie czy w sklepie. W sklepach akurat słyszałam, że zwracają uwagę na maseczkę samą. W restauracji nie byłam jeszcze. Jak one się otworzyły, to w Nadarzynie nic mi nie zmieniało, bo nie miałam żadnych, żeby pójść, a w Warszawie jeszcze nie byłam.

**A spotkania ze znajomymi? Już są normalne?**

Nie no, na szczęście moi znajomi... Bo miałam też takich znajomych, którzy byli przewrażliwieni i nie chcieli się spotykać. I to było frustrujące, bo ja miałam takie, że daj spokój, przyjedź tutaj. Na szczęściu już im przeszło. Szykuje się impreza urodzinowa mojej współlokatorki w sobotę. Ale tam będą ludzie, których znam. A tak, to spotkania normalnie, tylko np. jedna moja przyjaciółka była na czas kwarantanny w innym mieście u rodziny. Ktoś inny też wyjechał, bo nie trzeba siedzieć w Warszawie. Więc ludzie się tak porozjeżdżali. Albo nie mają czasu, bo pracują. Więc jeszcze za mało jest tych spotkań dla mnie, nie wróciło to w pełni. A może też tak to odczuwam, bo normalnie miałam pracę, siłownię 3 razy w tygodniu, więc nie doskwierał mi brak spotkań ze znajomymi w tygodniu. Miałam ustalone tylko 1 albo 2, bo resztę dni miałam zajęte na pójście na siłownię czy pójście do lekarza albo załatwienie innej konkretnej sprawy. I tak naprawdę na spotkania ze znajomymi zostawiałam piątek wieczorem albo sobotę. Więc może bardziej mi doskwiera fakt tego, że nic się nie dzieje, bo nie jestem tak zajęta, jak normalnie bym była.

**A ludzie w twoim otoczeniu już wrócili do normalności?**

Do normalności o tyle, że np. większość wciąż pracuje zdalnie. Moja druga współlokatorka pracuje w kawiarni, więc ona chodzi normalnie do pracy. Ale oni się już normalnie spotykają z innymi znajomymi czy rodziną. Więc raczej wszyscy wrócili na tyle, ile mogli, do normalnego trybu życia.

**Coś jeszcze ci przeszkadza w tej sytuacji?**

Nuda w jakiś sposób. Nie potrafię się skupić na czytaniu. Próbuję się zmotywować do takich rzeczy, jak uczenie się języka, bo mam tyle czasu czy ćwiczenia. Nie mam tego balansu, że robię coś dla siebie - czytam, ćwiczę, ale też, że spotykam się ze znajomymi. Nie ma tego balansu, więc już te rzeczy zaczęły mnie nudzić. Wczoraj miałam takie poczucie, że mi się nudzi i coś powinno się zacząć dziać. I ja wiem, że praca, nawet jeżeli ona się znajdzie, to jeżeli to nie będzie coś fantastycznego i zbieżnego z kierunkiem, w którym chciałabym się rozwijać, to też będzie mnie nudziło po jakimś czasie szybko. Ten brak wydarzeń i spotkań prowadzi po jakimś czasie do takiej nudy.

**Radzisz sobie jakoś z tą nudą?**

Nie wiem, mam poczucie, że sobie nie radzę. Wczoraj zadzwoniłam do paru osób, porozmawiałam z nimi. Pomaga to, że moja przyjaciółka jest w pokoju obok teraz i mogę do niej wejść i możemy porozmawiać. W te dni, kiedy nie jest w biurze.

**Jak się czułaś przez ten miesiąc?**

Na początku to była ekscytacja tym, że jest nowy plan na przeprowadzkę, że coś się zmieni.

**Wyświetlę ci obrazki i poproszę, żebyś do nich się odnosiła.**

To na początku ta 13 na zasadzie, że jest gdzieś tam światełko w tunelu. Ale jakoś w drugiej połowie, jak otworzyli restauracje i zaczęłam słyszeć, że moi znajomi poszli do restauracji... Social Media też mają duży wpływ, bo jak słyszę, że moi znajomi jeżdżą po Polsce w różne miejsca. Ja nie bardzo mogę, bo muszę być w trybie oszczędnościowym teraz, a nie wydawania pieniędzy na wyjazdy. Nie mam też swojego samochodu. I doskwiera mi brak znajomych z samochodem, którzy byliby skłonni się wybrać gdzieś w Polskę. Więc widząc to na social media, to jest akurat negatywne działanie.

**Jakie emocje to w tobie wywołuje?**

Trochę żalu, trochę zasmucenia. Druga połowa miesiąca, jak otworzyli restauracje, hotele, jak zaczęli więcej jeździć po kraju to taka bardziej 10 - że ja siedzę dalej, już mi się nudzi w tym ogrodzie z psem. Mnie na razie nie stać, bo muszę być w trybie oszczędnym ani nie mam jak wybrać się i pojechać gdzieś. Najlepiej by było, gdybym miała ten samochód, mogła wsiąść i pojechać, gdzie mi się podoba. I urozmaicić sobie wyjazdami może nie za granicę, ale jednak wyjazdami. Ten wyjazd do Krakowa był taki pocieszający. I sam fakt jechania pociągiem był taki na zasadzie, że jadę przed siebie gdzieś [śmiech]. To były 2 dni, więc dość krótko. Ja lubię zmiany. Jestem osobą, która się szybko nudzi i ja potrzebuję tych zmian. Ja też mam taką zdolność adaptacji do różnych miejsc i lubię podróżować. Siedzenie w miejscu przez tak długi czas już wchodzi mi na głowę i jest mocno frustrujące.

**Od tamtej pory do teraz był taki moment z innymi emocjami?**

Nie. Ostatnie te 2 tygodnie były takiego siedzenia w Nadarzynie i nudy, że nawet nie wyjdę do restauracji, która jest otwarta.

**A to, jak się przeprowadziłaś?**

Jest trochę za krótko, żeby mówić. Wczoraj miałam dołek, a w niedzielę się rozpakowywałam, więc byłam zmęczona całą przeprowadzką. A wczoraj miałam zjazd humoru po tej rozmowie. Weszła mi wizja tylko zdalnej pracy. Też godziny pracy mi nie odpowiadają, więc parę rzeczy mnie rozczarowało. A mam poczucie małej możliwości wyboru i takie, że powinnam brać to, co jest, a nie wybrzydzać. Bo jest mało ofert, mało się odzywają, bo rekrutacje są poprzesuwane, porozciągane w czasie. Więc ciężko mi powiedzieć, bo wczoraj miałam dołka, więc też 10. Ale to jest wizja tej pracy, tej nudy i wszystko razem.

**A te momenty spotkań ze znajomymi - jak się wtedy czułaś?**

Hmm... To na pewno było urozmaicenie. Najlepsze było to spotkanie w Krakowie, bo to jest moja bardzo bliska przyjaciółka i się rozumiemy. A te osoby, z którymi się widziałam troszeczkę mniej, więc trochę mi brakuje nie tylko poznawania nowych ludzi, ale takich relacji z głębszym zrozumieniem. To jest trochę tak, że w tym czasie każdy jest zestresowany, więc trochę mniej mogę wymagać od znajomych, bliskich przyjaciół tej uwagi, wysłuchania, bo każdy jest zajęty swoimi problemami, związanymi z tą sytuacją. Brak mi takich bardzo bliskich spotkań czy relacji z ludźmi, z którymi się tak dobrze rozumiem. Bo ta jedna przyjaciółka mieszka w Krakowie, więc jest dalej.

**Czyli te spotkania dawały urozmaicenie, ale nie poprawiały mocno humoru?**

Tak.

**Czyli nadal w tle byłaby to ta 10?**

Nie wiem. Z tymi znajomymi to tak może bardziej ta 11, bo gdzieś była ta tęcza z tyłu, ale tak przez szybę.

**Czujesz się zagrożona tą sytuacją?**

Na razie wciąż to samo, bo tej pracy nie mam i nie wiem, jak szybko się znajdzie i jaka ona będzie. To jest to zagrożenie. Innego raczej nie odczuwam.

**A jeszcze jakieś emocje, jak irytacja, zdenerwowanie?**

Tak, jak najbardziej. Irytacja też na to, że ten rynek pracy wygląda jak wygląda i dla mnie to było ileś czasu czekania na coś, co było ekscytujące i zostało totalnie zmienione - wyjazd i całe plany ze studiami. I teraz muszę wrócić do jakiejkolwiek pracy i najlepiej szybko. I to może nie być do końca zgodne z chęcią tego, co bym chciała robić. Teraz mi zależy głównie na tym, żeby szybko zacząć pracować i żeby to były jakieś odpowiednie dla mnie pieniądze. Więc taka frustracja, że chciałam zmienić coś w sytuacji życiowej, a jest 2 kroki w tył, a nawet 3. Finansowo też nie jest lepiej, bo przez ostatnie miesiące gdzieś tam przejadałam te pieniądze, które miałam odłożone na studia i idą na życie też teraz. Ten miesiąc na pewno będzie droższy niż wcześniejsze 3, stąd też dobrze by było szybko zacząć pracować. To ogranicza mi możliwość wyboru - nie mogę wybrzydzać, popatrzeć gdzieś indziej za czymś odpowiedniejszym, bo muszę szybko zacząć teraz pracować za odpowiednią na mój budżet sumę pieniędzy. I np. w tym projekcie to by było pół roku pracowania w czymś takim, co sobie jest, tak, żeby zarobić. Ale dla mnie pół roku to jest długo. Po ostatnich 2 latach takiej pracy, że pracowałam, bo muszę, a nie, że robię coś pasjonującego dla mnie, to jest to frustrujące. Cała ta sytuacja cofnęła mnie w tym rozwoju o 3 kroki i zabrała mi fakt tego, że miałam odłożone pieniądze i mogłam iść na bezpłatny staż na 3 miesiące i się rozwinąć. A tak to jestem uwięziona w jakiejś sytuacji, że muszę coś zarobić, bo jakoś w tej Warszawie muszę mieszkać. Z drugiej strony wcześniej było tak, że pracowałam w tej Warszawie, żeby zarobić, ale był cały ten plus, czyli mam blisko do znajomych, mogę w każdej chwili wyjść, jest mnóstwo wydarzeń. Wychodzę na imprezę i wracam do Warszawy. To był jeden z tych powodów, dla których nie lubiłam mieszkać z rodzicami wcześniej. Ale teraz nie ma nawet tego plusa, bo imprez jeszcze nie będzie przez jakiś czas. I to sprowadza się do spotkań takich domówkowych ze znajomymi, co jest ok, ale wciąż niewystarczające.

**Jak sobie radzisz z tymi emocjami?**

Ja chyba jednak wpadam bardziej gdzieś tam w takie samoumartwiające się ciągi. W takich sytuacjach na pewno pomaga mi terapia i z terapeutą obgadujemy te kwestie tego, co się teraz dzieje. Aczkolwiek była też taka terapia, na której terapeuta mi nie pomógł, bo raczej zarysował taki scenariusz, że z tą pracą może być teraz ciężko. Terapia trwa już 5 miesięcy. Ostatnie 2 miesiące miałam tak, że ona była raz na tydzień, wcześniej raz na 2 tygodnie. Więc to jest coś takiego, że zaczęłam na nią czekać, bo ona mi pomaga. Terapeuta daje mi pewne narzędzia, żeby różne rzeczy przerobić, co jest fajne. Więc na pewno terapia pomaga. Swoich sposobów nie mam. Raczej wpadam w takie, że siedzę i się zamartwiam albo zasmucam. Ewentualnie dzwonię wtedy do kogoś porozmawiać z kimś albo z moją mamą. Tylko mojej mamie już brak sposobów na pocieszanie. Już jej się skończyły i nie wie, jak mnie pocieszać czasami. Czasami sobie myślę, że to jest kwestia mojego podejścia i może powinnam je zmienić. Ale z drugiej strony mam takie poczucie, że nie dam rady wciskać sobie pewnych rzeczy. Jak nie czuję się z czymś ok, to nie czuję i nie wiem, czy na siłę zmienianie takiego podejścia... Mogłabym myśleć bardziej pozytywnie, ale miałabym wciąż takie poczucie, że zakłamuję rzeczywistość, żeby tylko poczuć się lepiej. Bardzo dużo dokłada to, że przed całą tą sytuacją miałam problem, że nie wiedziałam, co chcę robić ze swoim życiem. Albo inaczej - wiem, ale mnie na to nie stać. Więc muszę wymyślić, jak robić coś z życiem, żeby jednocześnie mieć na utrzymanie i na życie i jednocześnie jakąś satysfakcję z tego, co robię. Dlatego myślę, że sobie z tym nie radzę.

**Jak to jest z emocjami ludzi w twoim otoczeniu?**

Wiele osób jest sfrustrowanych brakiem możliwości spotkania się z kimś normalnie. Miałam wśród znajomych taką sytuację, że z kimś nie chcieli się spotkać, bo ten ktoś się spotykał z dużą ilością ludzi, więc odwołali spotkanie. Ale to już jakiś czas temu, bo teraz już większość ludzi zaczyna się umawiać, spotykać. Ale z tego, co widzę, to jest bardziej domówka. Spotkanie w gronie znajomych rodziny, ok, ale wyjście na jakieś masowe imprezy czy kluby, to jeszcze za wcześnie na takie rzeczy. Maseczek raczej nie noszą. Więc takie powolne odtajanie widać wśród moich znajomych. Ale nie znam nikogo, komu by się nad wyraz zmieniło życie. Pracują, nikt chyba nie został zwolniony na razie. Większość zdalnie pracuje.

**A jak postrzegasz takie zachowanie, że ktoś odwołał spotkanie z osobą, która miała za dużo kontaktów społecznych?**

Nie wiem, ja bym tego nie zrobiła. Bo dla mnie dużo istotniejsza jest interakcja z kimś oprócz mojej rodziny niż cały wirus. Dla mnie to jest trochę przesada, ale to była ich decyzja, więc im tego nie komentuję. Każdy sobie radzi z tą sytuacją na swój sposób i jedni są bardziej przewrażliwieni, a inni mniej.

**Jak teraz wyglądają u ciebie zakupy?**

Wciąż sporo przez Internet, ale byłam w Arkadii wczoraj. I byłam wcześniej też, jak jechałam albo wracałam z Krakowa, to weszłam do Złotych Tarasów i nawet weszłam do restauracji. Faktem jest, że jest mniej siedzeń, mniej miejsca. Więc, jakby był tłum, to by nie było gdzie usiąść. Ale jest też mnie ludzi. I to jest plus paranoi niektórych dla mnie przynajmniej, że oni sobie siedzą w domu, a ja mogę wyjść i mam miejsce. Jest mniej ludzi na mieście, w centrach handlowych. Pociągi są puste, bo dzieci, młodzież, studenci i niektórzy ludzie, którzy pracują zdalnie, nie jeżdżą do pracy, więc to jest dla mnie plus. Bo faktycznie jest pusto bardziej w komunikacji. Złote Tarasy nie były jakoś zatłoczone. I pomimo mniejszej ilości stolików - bo trzeba było porozsuwać, pooznaczali stoliki, przy których nie można siedzieć, żeby były te 2 m, to wciąż jest miejsce, żeby usiąść, bo tłumów nie ma. Jedyne co, to był tłum kurierów z Bolta, Uber Eatsa z tymi swoimi plecakami w strefie gastro. Wczoraj byłam w  Arkadii, to już tak normalnie, taka normalna ilość w tych sklepach. Nawet w tym Leroy Merlin, to sporo osób było. Niektórzy mają rękawiczki, inni nie. Ja już zdjęłam, jak się przyklejałam do naklejki w pewnym momencie. Maseczki raczej wszyscy mieli wewnątrz. Będąc w sklepie człowiek czuje się normalniej, bo jedyne co jest inne, to stacje dezynfekujące, ludzie w maseczkach i porozsuwane stoliki w strefach gastro.

**A zakupy spożywcze?**

Tylko te 2 dni były, więc byłam wczoraj coś dokupić. Bo w niedzielę i wczoraj zamówiłam sobie jedzenie ze swoją współlokatorką, ale to tak na zasadzie, że dopiero się wprowadziłam, to sobie zamówię jedzenie, bo nic nie mam. Ale to jest na pewno droższe niż kupowanie, gotowanie czy nawet wyjście. Ja mam tutaj blisko dużo różnych restauracji, więc planuję wyjście do nich. Zakupy większe planuję dzisiaj, ale zrobiłam wczoraj online, ale to dlatego, że w sklepie wegańskim chciałam parę produktów kupić, których nie będę miała w sklepie stacjonarnym tutaj i są też w niższej cenie. Więc chcę wypróbować wegański sklep z żywnością, ale to jest nowość i sprawdzam sobie to rozwiązanie. A dzisiaj pójdę na takie większe zakupy. I raczej nie będę chodzić codziennie, postaram się maks co 2 dni. Myślę, że na obiady np. póki mam mnóstwo czau, to będę chodzić gdzieś nieopodal, bo mam mnóstwo lokali też wegetariańskich, wegańskich, więc mogę sobie bezmięsnie codziennie zjeść gdzie indziej.

**Dlaczego nie planujesz codziennie wychodzić do sklepu?**

Wynika to z ograniczania wydatków bardziej. Ja jak już idę do sklepu, to wiem, że kupię za dużo albo więcej niż powinnam. Więc im mniej do sklepu pójdę, tym mniej pieniędzy się wyda na rzeczy niepotrzebne.

**A jak wyglądają zakupy przez Internet?**

Ubrania już przestałam zamawiać. Zaczęłam trochę stopować z racji, że muszę teraz budżet planować i wydatki mi skaczą przez mieszkanie. Więc już nie mogę sobie tak folgować i kupować tylu prezentów. Tam kupiłam coś ostatnio, teraz czekam na 2 przesyłki. Ale nie ubrania. Takie rzeczy w stylu, np. wielorazowe płatki kosmetyczne, żeby zmienić z takich jednorazowych. A, dobra, wczoraj zrobiłam sobie jeden prezent. Ale tak już mówiłam sama do siebie zamawiając, że to już jest ostatni taki wydatek i już koniec takich pierdół. Kupiłam sobie kominek na kadzidełko - on robi taki dym i ten dym spływa jak fontanna. Ja lubię takie pierdółki. Ale to już był ostatni i już powiedziałam koniec. Podejrzewam, że to też było tak, że wczoraj miałam gorszy dzień i kupienie sobie czegoś miłego, przyjemnego dla oka i zapachu, będzie poprawą humoru. To nie jest chyba dobra cecha, że zakupy poprawiają mi humor, ale tak jest. Ale już mocno zaczyna mnie stresować ta myśl o tym, że trzeba budżet... Jakby to nie jest tak, że miesiąc i będę w dupie, ale stresuję mnie ta kwestia pracy, szczególnie z tym, że wiem, że nie mogę wybrzydzać.

**Dlaczego się zdecydowałaś, żeby pójść do galerii?**

W Złotych weszłam, bo chciałam sprawdzić konkretną rzecz w konkretnym sklepie, który był zamknięty. A w Arkadii to do Leroy po techniczne rzeczy - śrubek mi brakowało, żeby coś skręcić, taśm mocniejszych, itd.

**A jak już tam byłaś, to pochodziłaś sobie po sklepach?**

Nie. Ale to jest raczej moje zapobieganie wydawaniu pieniędzy.

**Były jeszcze jakieś środki ostrożności w galerii?**

Stacje dezynfekujące. Np. te takie wysepki na środku korytarza, to jeżeli miały więcej niż jedno wejście, to były poodgradzane, żeby tylko z jednej strony tam wchodzić. Bardziej ograniczona dostępność. Albo w niektórych miejscach poprzyklejane te taśmy, żeby pokazać, gdzie ma stać jedna osoba, a gdzie następna. Plus te pleksi, odgradzające sprzedawcę od ludzi. Tylko te pleksi są szerokości stanowiska, kończy się tu (pokazuje tuż nad głową) i to trochę śmiesznie wygląda, ale są te pleksi.

**Co sądzisz o tego typu zabezpieczeniach?**

Nie wiem, bo jeżeli ten wirus jest w powietrzu i z tego, co oglądałam z tym wirusologiem film, to oni nie wiedzą, na jaką odległość ten wirus się może przemieszczać. To, jeżeli jest stanowisko i ta pleksi ma 50 cm i 120 cm wysokości, to człowiek by sobie pomyślał, że powietrze tak cyrkuluje, więc obejdzie to pleksi. Więc ja sobie tak wczoraj pomyślałam, że to jest bardziej na zasadzie, żeby ktoś kogoś nie opluł mówiąc. Tylko od tego mamy maseczki. Jak to jest w powietrzu i się przemieszcza, to się wydaję jednak na zasadzie zabezpieczenia, które ma uspokoić ludzką psychikę, że coś zostało zrobione. Ale czy de facto dzięki temu jestem bezpieczniejszy, to nie wiem tego. Tego się nie dowiemy, bo z tego, co słyszałam, to nawet wirusolodzy nie są w stanie stwierdzić. To tak było rzucone, że metr czy 2 od siebie, bo wtedy jest mniejsze prawdopodobieństwo oplucia kogoś. Ale jeżeli wydychamy to powietrze, jesteśmy w zamkniętym pomieszczeniu, to to i tak dotrze do tej osoby. Jeżeli tak jest, jak oni mówią, że ten wirus może cyrkulować dłużej w tym pomieszczeniu, to się to powietrze wymiesza. Gdyby to miała być kwestia tylko opluwania, to tak, bo zostanie to zatrzymane przez pleksi. Ale jeżeli jest to kwestia drobinek będących w powietrzu - trochę jak kurz, który się unosi, to one sobie latają po tym pomieszczeni i to im nic nie da. Pytanie czy tak jest. Bo z tego, co słyszałam, to nawet wirusolodzy nie są w stanie stwierdzić, jak długo to się unosi i może być w powietrzu przenoszone.

**Ostatnio zostały otwarte restauracje, kawiarnie - co o tym sądzisz?**

Ja myślę, że to dobrze, bo jest to kwestia samej gospodarki. Jest to ta ogromna gałąź, która została mocno nadwyrężona samym faktem tego, że nie można było do restauracji pójść. Mój terapeuta twierdzi, że jedno to jest, że odmrażają, otworzyli, ale drugie to jest, że wciąż wielu ludzi nie pójdzie z wyboru czy ze strachu - tak jak wielu nie jeździ komunikacją miejską. Więc wcale nie jest tak, że restauracje odżyją czy też będą zarabiać tak, jak zarabiały, bo nie będą wypchane ludźmi. Raz, że muszą mieć ograniczoną liczbę miejsc, a dwa, że wiele ludzi nie pójdzie, bo musi oszczędzać, a to jest droższe niż ugotowanie w domu albo bo się boją. Jak otwierali restauracje, to się śmialiśmy ze znajomymi, bo gdzieś w zasadach było napisane, że stolik musi być dezynfekowany co 15 minut. I się zastanawialiśmy, czy jest tak, że jak ktoś siedzi i je bez maseczki, bo tego nie da się przeskoczyć - nie da się jeść w maseczce, to kelner będzie co 15 minut też w trakcie jedzenia podchodził i dezynfekował stolik? Zastanawialiśmy się, jak to wygląda. A nasza koleżanka pracuje w kawiarni, więc ją to też obowiązuje. I ta odległość 2 metrów to też jest czasem nierealne, bo kelner stoi 2 metry od klienta, jak zbiera zamówienie?

**Ty byłaś już w restauracji?**

Byłam w Złotych w strefie gastro w jednej z restauracji. Ale ona była wpół pusta.

**Czyli nie odczuwałaś tych ograniczeń, bo ludzi było mało?**

Tak.

**A w żadnej kawiarni nie byłaś?**

Nie, w kawiarni jeszcze nie.

**Korzystałaś już z usług fryzjerów, kosmetyczek, itd.?**

A właśnie, zapomniałam o tym, a to było bardzo ważne! Jak się otworzyli fryzjerzy tego 18 maja, to byłam w czwartek przed Krakowem jeszcze. Byłam u fryzjera, byłam na paznokciach - czyli to, na co czekałam najdłużej. I byłam na brwiach też. A propos tego. Byłam na brwiach i ja chodzę w takie miejsce w podziemiach. Tam koło McDonald's jest taka dziewczyna i ona ma taki swój mały kwadracik na te brwi i bardzo lubiłam do niej zawsze chodzić. I też się z nią lubimy, więc tak zastanawiałam się czy ona przerwa ten czas. I wchodzę i pytam jak tam, czy się udało. Mówi, że tak, ale jest padnięta, bo sama siedzi, bo nie ma z czego zapłacić dziewczynom, które u niej pracowały, więc sama teraz siedzi po 12h, żeby odpracować. Przez te miesiące 1/3 pensji męża szła na wszystkie opłaty za to miejsce. Na szczęście ma męża, który mógł ją wspomóc w tym. Ale opowiedziała mi sytuację, że jest u siebie i wchodzi klientka i mówi, że chce regulację brwi za pół ceny. A ona się pyta, dlaczego za pół ceny? I ta klientka mówi, że jest po koronawirusie i ona nie ma pieniędzy. I ona jej na to: ja mam taką propozycję, że ja pani zrobię brwi za całą cenę, bo ja też nie mam pieniędzy. I ta klientka stwierdziła, że jest bezczelna i wyszła. Właśnie opowiadała mi taką szokującą historię osoby, która żądała usługi za pół ceny, bo jej nie stać po koronawirusie. Oczywiście skomentowałyśmy to na zasadzie, że jak mnie nie stać na takie usługi, to na nie nie chodzę, jak rzęsy czy brwi. Bo jakim cudem ona sobie wyobraża, że ona jako przedsiębiorca nie ma problemów finansowych po niepracowaniu przez 2 miesiące. Ale przetrwała, na szczęście jej się udało, tylko na razie nie ma z czego zapłacić pracownikom, więc musi sama. Ale ma ruch, bo wiadomo, wszystkie panie z zarośniętymi brwiami wyległy teraz, żeby brwi robić. Paznokcie zrobiłam. Na paznokciach byłam jakoś wcześnie rano, więc było pusto - byłam jedyna. Ale tam wszystko normalnie. Tylko tyle, że się w maseczkach siedziało. I u fryzjera też w maseczce siedziałam.

**A na brwiach?**

Też w maseczce. Tylko, że np. w przypadku brwi - ja chodzę na nitkowanie i jest moment, w którym ja muszę przytrzymać powiekę i ją naciągnąć, kiedy ona robi pod brwią na dole. I dotykam oczu, momentalnie zaczynam łzawić, bo ten ból zaraz łzy wychodzą. Więc dotykam, ocieram te oczy. I tego dotykania oczu jest mnóstwo. Później zapłaciłam telefonem, ale mimo wszystko dotykałam np. klamki. A nie miałam czym zdezynfekować rąk pomiędzy dotykaniem oczu a wyjściem. To jest takie coś, co jakiś sanepid by się mógł przyczepić.

**To obniżało twoje poczucie bezpieczeństwa?**

Nie. To jest bardziej takie moje myślenie w kwestii tego, co ktoś mógłby powiedzieć albo przyczepić się. Bo jednak logicznie rzecz biorąc, skoro mamy nie dotykać ust, nosa jak jesteśmy gdzieś, bo wtedy możemy przenieść wirus poza siebie, a taka sytuacja tam zaistniała. Ja np. wątpię, że bym miała tego wirusa, więc się nie stresuję. A i jeszcze przy nitkowaniu, dziewczyny jedną nitkę trzymają w zębach, robiąc to. Więc ona musiała mieć w maseczce dziurkę, przez którą przełoży tę nitkę. Ktoś mógłby się przestraszyć albo nie chcieć tego zrobić. Ja nie mam takiego podejścia, ale myślę o co inni mogliby się przyczepić.

**Co sądzisz o otwarciu takich miejsc jak siłownie, kina?**

Kina, teatry, to będzie mniejsza ilość siedzeń. Będzie się porozsadzanym i trzeba będzie być w maseczkach. Kino, kiedy trzeba będzie być rozsadzonym, to nie jest dla mnie duża różnica z restauracją, w której trzeba zdjąć maseczkę, żeby zjeść. Wręcz w restauracji jest to bardziej, bo zdejmujemy maseczkę, plujemy, kichamy, dotykamy ust jedząc. A w kinach będzie tego dużo mniej, jeśli będzie się siedziało w maseczkach. No chyba, że w tej ciemności i kiedy, nie będzie żadnego pracownika na sali, ktoś będzie zdejmował maseczkę, bo będzie mógł.

**Te zabezpieczenia postrzegasz jako nielogiczne?**

Tak. Bo jakby chcieć się przyczepiać naprawdę do tych wszystkich obostrzeń, to nie moglibyśmy wracać do tego, co robimy. Ale ja jak najbardziej jestem za tym, że trzeba odmrażać gospodarkę i cieszę się na to, bo poprawia to też moje samopoczucie, życie i ja bym tego nie cofała. Ale logicznie rzecz biorąc to jest takie... Skoro już wprowadzacie obostrzenia tu, to jak tu trzeba zdjąć maskę w restauracji, to wszystko bierze w łeb i nie ma sensu. Więc ja bardziej widzę te obostrzenia na zasadzie, że coś tam zrobiliśmy, ile się dało, to działaliśmy, ale to wciąż nie jest na takim maksymalnym zminimalizowaniem sytuacji, które są potencjalną możliwością zarażenia się. Do tego musielibyśmy przestać żyć w ogóle na te kilka miesięcy, co nie jest realne i nie jest dobre dla nikogo. I z mojego punktu widzenia w tym momencie staje się wycofanie gospodarki, problemy ludzi i w niektórych przypadkach samobójstwa, problemy z tym, żeby teraz dostać się normalnie do lekarza. Wcześniej dostanie się do lekarza było ciężkie czasami i czekanie na różnych specjalistów. A teraz to jest dodatkowo dołożone tym, że niektóre części szpitali albo niektóre są powyłączane, a jak się gdzieś ten koronawirus pojawił, to trzeba było zamknąć i wszystkich na kwarantannę. Więc jeszcze trudniej się dostać do lekarza. Szczęśliwie nie mam potrzeby nic u lekarza robić, ale muszę zrobić kilka badań ginekologicznych w tym miesiącu i zastanawiam się, jak to będzie wyglądało. Jeszcze nie dzwoniłam się umawiać, ale zastanawiam się, jak to przebiegnie. Więc to jest takie troszeczkę na pozór. Z drugiej strony inaczej się nie da. Kina to dla mnie jest mniejsze zło niż restauracje.

**A siłownie?**

Dopóki nie będę miała Multisporta to nie, bo to będzie duży wydatek. Więc zależy, jak szybko znajdę pracę i jak szybko będę miała Multisporta z tej pracy.

**Słyszałaś o jakichś aplikacjach, które powstały na czas pandemii?**

Jedna to była kwarantanna, którą sama miałam, jak byłam na tej kwarantannie po powrocie zza granicy. Oglądałam jakiś film na YouTube o aplikacji... Nie pamiętam, jak ona się nazywała. Ale to była aplikacja, która miałaby śledzić nasz telefon poniekąd, rejestrować, że jeżeli ja mam aplikację i telefon znajomego ma aplikację, rejestrować pod jakimś numerem i widzieć, że np. mój telefon był blisko telefonu jakiegoś znajomego, więc spotkaliśmy się. I ona w ten sposób mogłaby notyfikować, że jeżeli ktoś był zarażony albo trafił do lekarza. I jeżeli jego telefon pojawia się i jest oznaczony jako, że był zarażony, to do wszystkich osób, którzy mieli z nim styczność trafia alert, że miałeś kontakt z kimś zarażonym. Tylko tam też była dyskusja na temat prywatności na zasadzie tego, że coś nam siedzi w telefonach i śledzi, gdzie jesteśmy, z kim jesteśmy, itd.

**Co ty o tym sądzisz?**

Przy moich rodzicach, którzy mają skłonność do teorii spiskowych i są anty technologii to ja też mam sceptyczne podejście do tego typu rzeczy. Bo wolałabym, żeby nikt niepożądany nie śledził, gdzie jestem i co robię. Rozumiem założenie, że chcemy wyeliminować rozprzestrzenianie się i ma to dawać tym, którzy nie chcieliby być potencjalnie zarażeni i iść spotkać się z rodziną, to daje im taką informację, że mogę mieć wirusa, bo miałam kontakt z kimś, kto był zarażony, więc nie pojadę się zobaczyć z babcią. Więc takie założenia są szczytne i sensowne. Ale z drugiej strony też dają taki strach, że co, jeśli ktoś inny ma też dostęp do tych danych, nie tylko ja. I zostaje mi uniemożliwione pójście do pracy. Zostaję odcięta od różnych rzeczy przymusowo, bo ktoś się dowie, że ja miałam kontakt z kimś albo jestem zarażona i np. tracę pracę z tego względu, bo nie mogę do niej iść. Chyba, że pracuję zdalnie, ale są różne warianty. Wpływa to też na życie moich współlokatorek, które pewnie też będą wtedy objęte kwarantanną razem ze mną. Nie mogę wyjść do sklepu... I to może być nie do końca w kwestii tylko, że to jest dla mojej informacji, że ja zdecyduję, czy chcę wyjść czy nie i czy chcę chronić innych. Tylko na zasadzie takiej koronawirusowej policji? Nie ma czegoś takiego, ale to trochę idzie w tą stronę, że będzie jakiś organ, który będzie pilnować, że np. ty nie możesz wyjść, bo jesteś nawet potencjalnie zarażony. Nawet nie to, że ktoś ma objawy i jest chory, tylko, że jest potencjalnie zarażony i dla bezpieczeństwa ma siedzieć i nie wychodzić. Mnie to przeraża o tyle, że ja - może to być samolubne - ale nie mam aż takiej woli siedzenia w domu przez kolejne 2 tygodnie, miesiąc, niewidzenia się z nikim już po tym, co było, o ile nie jestem rzeczywiście chora i nie czuję tego, tylko po to, żeby potencjalnie nie pozarażać reszty Warszawy. Albo to może być na zasadzie, że byłam w miejscu, gdzie ktoś był zarażony. Co innego to jest spotkanie z tą osobą, a to może też być rozsunięte na kwestię, że np. byłam w takim Leroy Merlin, który jest ogromnym sklepem i tam był ktoś zarażony. Tych sytuacji może być mnóstwo, że potencjał, że ja rzeczywiście mam ten wirus jest bardzo mały, ale dla bezpieczeństwa odgórnie zostanie mi nakazane robienie czegoś. Więc trochę się bym bała, że to idzie w taką stronę. I to jest już przerażające wtedy.

**Opis pierwszej kategorii aplikacji. Co o tym sądzisz?**              
Te 3 pierwsze przykłady to jest to, co już powiedziałam i jak ta kwarantanna domowa po powrocie. Dostosowałam się do tej kwarantanny, bo było to prawnie narzucone i kara mogła być nałożona.

**O kwarantannie domowej za moment konkretnie pogadamy, a teraz porozmawiajmy o takim szerszym opisie kategorii aplikacji.**

3 pierwsze przykłady to to, co już mówiłam, czyli ta inwigilacja i kwestia prywatności, bo zdrowie społeczne - wszystko rozumiem, ale ja jako osoba po środku w tych poglądach na wirusa, ale jednak troszeczkę bardziej w sceptyczną stronę w tej kwestii, jak bardzo panika została wywołana, to dla mnie jest to przesada. Tak duże wejście w naszą prywatność na rzecz zdrowia społecznego, gdzie z mojej perspektywy to zagrożenie nie jest wielkie, bo nie mam wśród swoich znajomych nikogo, kto jest zarażony, nikogo takiego nie znam, nikt mi nie umarł. Nie uważam też, że te liczby, które są zmarłych teraz w Polsce - bo w innych krajach, to też nie ma co porównywać np. USA, które są dużo większe i mają większe liczby - ale u nas te liczby nie przekraczają innych gryp czy innych przyczyn śmierci ludzi. Jednak z tego, co widziałam w Polsce, to są głównie ludzie z chorobami współistniejącymi albo starsi. A ostatnio zobaczyłam jakiś artykuł. Był taki moment, kiedy Kasia Kowalska zapostowała na Instagramie, że jej córka jest podłączona do respiratora. I zobaczyłam taki artykuł, że już wszystko ok, że ta córka ma bliznę na szyi po tracheotomii bodajże i artykuł mówił, że były podejrzenia, że to jest związane z koronawirusem, a ta choroba nie była w żaden sposób związana z wirusem, to było coś innego. A to, co się wokół tego wydarzyło i jak w wiadomościach było mówione o tym... I że ona wrzuciła ten post i mówiła "uważajcie, bo to jest coś na serio". To okazuje się być takim szumem medialnym, a dziewczyna miała zupełnie inny problem.

**Te aplikacje nie są potrzebne, bo zagrożenie nie jest tak wielkie?**

Z mojej perspektywy tak. Osoby, która uważa, że zagrożenie nie jest tak wielkie, żebyśmy czegoś takiego potrzebowali. A wchodzą [aplikacje] w taki aspekt, który daje kontrolę i gromadzenie takich danych, które nawet, jak ktoś nie jest wielbicielem teorii spiskowych, to widzi, że coś tu nie gra, żeby rząd mógł nas tak inwigilować. Czy rządy w każdym kraju. A jak powiedziałaś o tym rozpoznawaniu twarzy przez kamerę, to ja się uśmiechnęłam, bo to brzmi, jak to, co gdzieś w Chinach wprowadzają. Trochę jak Black Mirror i niektóre te scenariusze... I daje możliwość też inwigilacji w innych sferach, a nie tylko tego, czy ktoś przestrzega obostrzeń w sferach medycznych. W sklepach, jak ekspedienci zwracają uwagę, to ludzie założą maseczki. W centach handlowych są ochroniarze. W różnych miejscach są ochroniarze danych miejsc, którzy mogą pilnować czy nawet organizator danych miejsc powinien zatrudnić osobę, która zajmuje się w danym miejscu pilnowaniem przestrzegania zasad. W Krakowie weszłyśmy z przyjaciółką do Tigera i jedna z ekspedientem stała przy drzwiach i mówiła: proszę zdezynfekować ręce. I pilnowała, żeby każdy zdezynfekował ręce i założył rękawiczki.

**Czyli są inne wystarczające rozwiązania zamiast aplikacji?**

Dodatkowo też nie będą... Tak, jak mówiłam, że miałeś kontakt z osobą zarażoną, więc potencjalnie możesz zarażać. Ale to wciąż nie daje mi pewności, że mam wirusa i zarażam, jak nie mamy testów, żeby je zrobić. Po co robić taką panikę? Pytanie, czy to byłoby tak, że ktoś mógłby przymusowo powiedzieć: nie przychodź do pracy, nie możesz wyjść, bo tutaj mamy zaznaczone, że miałaś kontakt z kimś, kto jest zarażony. I ja nie mam żadnej kwestii wyboru, tylko jestem przymuszona i robi się taka koronawirusowa policja, co już jest trochę przerażające i dla mnie za bardzo wchodzi w moją wolność. Bo jak mamy zwykłą grypę i zarażamy, to też jest nasz wybór, czy przeleżymy w łóżku czy idziemy do pracy zarażać resztę. Większość ludzi tak zrobi, że zostanie w łóżku. Przyjmując, że ten koronawirus rozprzestrzenia się bardziej... Ale są te obostrzenia, ludzie pracują zdalnie, jest mniej ludzi, dzieci nie chodzą do szkoły, nie ma tylu ludzi w komunikacji dzięki temu. To już jest dla mnie pójście za daleko.

**Opis drugiej kategorii aplikacji. Co o tym sądzisz?**

To ma już większy sens. Na pewno gdzieś mi mignęło Instastory którejś z influencerek - wolontariuszki, która się zajmuje dostarczaniem posiłków ludziom starszym. I to bardziej, bo ci starsi się rzeczywiście boją, są w tej grupie ryzyka, więc jest im potrzebny ktoś, kto im zrobi zakupy, itd. Są to ludzie, którzy i tak mało wychodzą w celach praca, rekreacja. Oni wychodzą do sklepu, do kościoła czy na spacer.

**I ci ludzie mogliby korzystać z tych aplikacji?**

Tak. Jedyny problem polega na tym, że są to ludzie, którzy mają mniejszą umiejętność posługiwania się tego typu technologiami. Ale to jest chociażby ta inicjatywa ING, żeby uczyć starszych ludzi korzystać z technologii. Więc takie rzeczy jak najbardziej. Bo jeśli ci starsi z własnego wyboru chcą zostać w domu, chcą się mniej narażać i to miałoby być dla nich lepsze - szczególnie w miejscach jak na Śląsk, gdzie jest duży wysyp zarażeń, to to jak najbardziej ma sens i to nie jest inwigilacją, tylko niesieniem pomocy tym, którzy tego potrzebują. Którzy nawet by jej potrzebowali bez wirusa, bo po prostu mają trudność z wychodzeniem z domu.

**Miałabyś jakieś obawy w przypadku tych aplikacji?**

Zależy, jakie konkretnie informacje by potrzebowały. Bo one by potrzebowały tylko takie informacje, że ktoś zgłasza, że czegoś potrzebuje, żeby mu coś przywieźć.

**A gdyby potrzebowały do tego lokalizacji?**

Ale to tak samo, jak ja mam aplikację na dowóz jedzenia i ona ma moją lokalizację. Więc i tak te dane udostępniamy w tym momencie. Niektórzy by podnieśli głos, że nawet w aplikacji Uber Eatsa, udostępniając lokalizację, ktoś ją ma i ktoś może sprzedać te dane, gdzie jestem. Ale to jest czymś innym niż takie śledzenie otwarte, gdzie ja daję przyzwolenie na śledzenie tego, gdzie ja chodzę i z którym telefonem znajomego się zetknęłam. To jest coś innego. Bo to jest adres na zasadzie dowiezienia komuś jedzenia, a nie, gdzie ja poszłam i gdzie ja konkretnie byłam. To jest dla mnie różnica. Nie wiem, czy jakieś obawy...

**Prezentacja aplikacji. Kwarantanna domowa.**

A propos tego pierwszego punktu, że osoby objęte kwarantanną mają obowiązek z niej korzystać, to mnie tego nikt nie powiedział. Ja ją ściągnęłam z własnej woli, jak się dowiedziałam, że jest. I to było tak, że jak ja zaczynałam kwarantannę, to ona dopiero zaczynała normalnie działać. Jakoś tydzień przed tym, jak ja jej używałam były komentarze i widziałam, że ludzie mieli problem z jej używaniem, bo ona nie do końca działała. Ja już używałam, to działała mi ok, ale nikt mi nie powiedział, jak wysiadałam z samolotu, nigdzie nie było napisane, że ja mam obowiązek jej używać.

**Tu jest napisane, że przychodzi SMS z linkiem. Ty go nie dostałaś?**

Wiesz co, nie pamiętam w tej chwili. Wydaje mi się, że nie. A jeśli go dostałam, to już po tym, jak z własnej woli ją ściągnęłam.

**Być może oni dopracowali tę aplikacje z czasem?**

Na pewno, tak. Formularz lokalizacyjny wypełniałam na zasadzie, że policja może przyjeżdżać na mój adres. I tak było, przyjeżdżali codziennie. I oni też zapytali, czy mam aplikację, ale to już później.

**Te zadania na robienie "selfie" też miałaś?**

Tak. I teraz na wykonanie zadania ma się 20 minut. Zdarzyło mi się parę razy... Zazwyczaj dostawałam te powiadomienia 2, czasem 3 razy dziennie, żeby zrobić to selfie o różnych porach. Ja spałam do 9, więc jeżeli dostawałam to wcześniej, to spałam np. Najpóźniej dostałam chyba o 19 tą wiadomość. I to nie było tak, że jeżeli tego nie wykonałam, to zaraz później przyjeżdżała policja, bo aplikacja dawała znać, że mnie nie ma. Co było trochę stresujące, bo parę razy tego nie zrobiłam. Albo zostawiłam telefon na górze, a zeszłam na dół albo nie słyszałam. Jest takie powiadomienie, które jest tylko jednym sygnałem. SMS to taki krótki sygnał, którego możemy nie słyszeć. Jak dzwoni telefon to dzwoni przez jakiś czas, więc nawet, jak się nie zdąży dobiec, to się go słyszy. Parę razy zdarzyło mi się pominąć i stresowałam się, co będzie i czy to będzie wyglądało, jakbym nie była na kwarantannie.

**W opisie jest informacja, żeby mieć włączony dźwięk i sprawdzać sms.**

No tak, ale kto sprawdza SMS co 20 min. A to naprawdę przychodzi o takich porach w ciągu dnia, że człowiek musiałby sprawdzać co pół godziny telefon, czy nie ma SMS. Były takie momenty w ciągu dnia, że ja schodziłam na dół i jak mi się przypomniało "o, nie wzięłaś telefonu, jesteś już na dole z godzinę i mógł przyjść SMS" i tak było. Miałam też sytuację, że zrobiłam selfie i był jakiś error, że się nie zapisało. Jeżeli ja nie wykonałam zadania, to przychodził SMS, że nie zostało wykonane zadanie i będzie informacja zapamiętana, że nie wykonałam zadania w czasie. I raz mi się zdarzyło, że ja zrobiłam to selfie, tylko był jakiś błąd zapisywania i przyszła informacja, że nie wykonałam zadania. Ale w żadnym stopniu nie stało się tak, że to są podstawy do informacji, że ja nie byłam na kwarantannie, że wyszłam z domu. I to jest taki minus tego, że można zasnąć, że można nie słyszeć, bo się coś robi. Ludzie robią różne rzeczy podczas kwarantanny, żeby zająć czas i nie każdy będzie przy tym telefonie siedział.

**Co sądzisz o tej aplikacji?**

Było to ok. Ja korzystałam z tego, bo uznałam, że to będzie na pewno wspomożenie instytucji, ale policja przyjeżdżała codziennie mnie sprawdzać. Jakby ktoś chciał, to w nocy te wiadomości nie przychodziły, więc w nocy mógłby gdzieś wyjść. Jakby udało mi się wstrzelić, że wyszłam do sklepu, dostałam SMS i wracam zrealizować zadanie do domu, to w 20 minut spokojnie ze sklepu u mnie zdążyłabym wrócić. Nie robiłam tego, ale ktoś mógłby tak sobie skoczyć do sklepu, który jest blisko i 20 min nie robi różnicy, bo widzi, że ma tego sms, to się pospieszy, żeby to selfie zrobić. Bo lokalizacja była ważna w momencie wysłania selfie, a nie dostania SMS.

**Są takie obszary, gdzie można oszukać tę aplikację?**

Tak, ale tak samo jak policyjne patrole.

**Jakie informacje o użytkowniku pobiera ta aplikacja?**

Kurczę, ja nie pamiętam, co ja tam podawałam, ale nie za wiele tego było.

**Tu jest napisane, że geolokalizacja oraz system porównywania twarzy. Miałaś jakieś obawy w związku z tym?**

Trochę o tym nie myślałam. Trochę też - nie wiem czy prawidłowo - wychodziłam z założenia, że te informacje są zachowywane podczas 2 tygodni mojego bycia w domu, a potem anulowane. Jeżeli tak to wygląda, to na 2 tygodnie czasu kwarantanny, to sprawdzają, mają to rozpoznawanie mojej twarzy. Chociaż a propos rozpoznawania twarzy, to czasami się śmiałam, ale robiłam te zdjęcia w różnym świetle. Raz miałam makijaż, raz nie. Miałam różne fryzury. Moja cera była w różnym stanie i na tych zdjęciach czasami wyglądałam bardzo inaczej. Czasami kamera telefonu też potrafi, jak z różnego kąta się zrobi zdjęcie, też trochę zakłamać wygląd twarzy. Miałam poczucie, że wyglądam czasem zupełnie inaczej, jak robiłam te zdjęcia.

**A to i tak akceptowało te zdjęcia?**

Tak. Nie odrzuciło mi nigdy.

**Może to działa jak Face ID?**

Nie wiem. Ja miałam poczucie, że te zdjęcia są gdzieś... Ta aplikacja nigdy nie dała mi do zrozumienia, że tam jest jakiś program, który rozpoznaje twarze. Ja zastanawiałam się czy te zdjęcia są gdzieś przechowywane i jakiś człowiek je porównuje czy oni mają rzeczywiście program do rozpoznawania twarzy. Bo ta aplikacja wyglądała na tak prostą i w ogóle nie kojarzyła się z sytuacją, że jest to program rozpoznający twarze.

**Było coś, co ci się spodobało w tej aplikacji?**

Tam była możliwość poprosić o pomoc, żeby dać znać, że coś jest potrzebne. To spoko. Ja nie miałam takiej - znaczy nie miałam... Ostatnie 4 dni cała moja rodzina była na kwarantannie, ale tylko ostatnie 4 dni, tak się złożyło. Ale nie miałam potrzeby, żeby ktoś chodził na zakupy dla mnie, bo rodzice je robili. Więc ja tego nie używałam, ale jak najbardziej, dla ludzi, którzy byli sami to pewnie miało sens.

**Rząd powinien tworzyć tego typu aplikacje?**

Hmm... Z mojego punktu widzenia, policja i tak sprawdzała mnie codziennie, więc różnica dla mnie żadna. Były dni, kiedy żadnego zadania nie udało mi się wykonać, bo obydwa razy przegapiłam. Nie wiem, czy ta aplikacja działa w jakiś sposób bardziej na zasadzie, że ludzie się bardziej pilnowali, żeby nie wychodzić. Sam fakt tej kary - to było takie mobilizujące, żeby nie wyjść, bo jest ta wysoka kara. Nie wiem. To też w jakiś pozorny sposób daje takie myślenie ludziom, że rząd stworzył aplikację i jakoś nową technologią wspierają wszystko. Ktoś, kogo policja nie sprawdzała codziennie, to mógł mieć wrażenie, że coś robią, żeby sprawdzać. Tylko dla mnie fakt tego, że da się to obejść albo jak się miało szczęście, żeby wyjść z domu albo jak mi zdarzało się nie wykonać zadania, bo nie miałam telefonu pod ręką. Dla mnie ona nie zawsze była w pełni adekwatna.

**Dlaczego się zdecydowałaś, żeby ją pobrać? Skoro nie dostałaś sygnału, że trzeba.**

Myślałam, że będzie to pomocne i jest to bardziej... Nie było mi mówione, że jest to potrzebne i obowiązkowe, ale uznałam, że pewnie ważne jest, żeby ją mieć. Dopiero później, jak zobaczyłam, jak działa, to stwierdziłam...

**Ważne z jakiego punktu widzenia? Żeby prawnie nikt się nie przyczepił?**

Tak. Udowadniania, że moja lokalizacja telefonu i te zdjęcia, i że robiłam te selfie codziennie.

**Ta aplikacja powinna być obowiązkowa?**

Jak już ją zrobili, to tak. Jak każdy używa tego, to ma sens, a jakby każdy miał używać, jak mu się podoba, to to nie ma sensu. Tylko mało było informacji wokół tego. Bo to, co mnie martwiło, to, że oni zapisywali moje zdjęcia i ta geolokalizacja. Na żadnym etapie logowania się do tego nie było informacji, kto, gdzie i jak przechowuje informacje o mnie i kiedy one zostaną usunięte - czy po 2 tygodniach znikają. Bo aplikacja usunęła się sama z siebie po 2 tygodniach. Jak skończyła się kwarantanna, to ja jej nie usuwałam, ona sama się odinstalowała. I ja chciałabym mieć pewność, że w momencie odinstalowania, kiedy już nie trzeba było dalej przetrzymywać tych danych, że one zostają usunięte. Też to, co mnie martwiło, to fakt, że pominęłam zadanie, a policja nie przyjeżdżała za chwilę sprawdzić, czy jestem, to zastanawiało mnie, co daje im ta informacja, że nie wykonałam zadania. Czy to jest w jakikolwiek sposób podstawa prawna do przyczepiania się, że nie byłam na kwarantannie. Też mi tego nikt nie wyjaśnił i trochę mnie to stresowało. Nic się takiego nie wydarzyło, ale w takim razie, na ile jest to prawdziwe, skoro w momencie, kiedy nie uda się komuś wykonać zadania - nie wiadomo, czy dlatego, że nie było go w domu, czy dlatego, że spał, mył się czy nie słyszał telefonu. Dane tej aplikacji nie dają jasnego przekazu czy ktoś trzyma się tej kwarantanny czy nie.

**ProteGO Safe. Słyszałaś o niej?**

To jest ta, o której widziałam film.

**Prezentacja szczegółów nt. ProteGO Safe. Czemu służy ta aplikacja?**

Z tego, co tutaj jest zarysowane, to teoretycznie ma pomóc mi kontrolować moje zdrowie. Tylko pytanie, kto przechowuje te informacje i na ile może je później wykorzystywać.

**Jakie informacje ta aplikacja pobiera?**

Wszystko, co ja napiszę o swoim zdrowiu. A mam takie obawy przed podawaniem danych zdrowotnych - to są bardzo wrażliwe dane.

**Jest coś, co ci się spodobało w tej aplikacji?**

Wiarygodne, aktualne informacje nt. sytuacji związanej z pandemią - hmm... Ja miałabym wątpliwości, co do wiarygodności. Oficjalne i aktualne to może, ale co do wiarygodności to miałabym problem, czy są to te prawdziwe dane, bo są różne w różnych miejscach. Tutaj niestety spiski i różne teorie spiskowe moich rodziców, to zawsze mi w głowie siedzi. Ja nie mam tak, jak moi rodzice, czy mój tata, ale całe życie było mi mówione, że mam nie podawać nigdzie niepotrzebnie adresu. Mój tata jest taką osobą, że jak ktoś chce od niego numer telefonu, adres, mail i nie jest to np. bank czy instytucja, to zawsze jest awantura i dlaczego on ma coś podawać. Ja może nie mam aż tak, ale przez te lata takiego wychowania, jak mam podać gdzieś adres, to się 5 razy zastanawiam nad tym czy na pewno bezpieczne miejsce, żeby podać moje dane i jestem dość mocno przewrażliwiona na tym punkcie. Więc takie wpisywanie ich do jakiejś aplikacji...

**Ty raczej byś nie pobrała tej aplikacji?**

Nie. Ten film jak oglądałam, to że miałam taką myśl, że zastanów się 5 razy zanim ją ściągniesz, bo zbiera informacje o naszym położeniu i spotkaniach z innymi użytkownikami.

**Ten argument, że gdyby wszyscy mieli taką aplikację, to moglibyśmy zapobiec rozprzestrzenianiu się wirusa, do ciebie nie trafia?**

Nie, bo dla mnie nie jest to aż taki problem, żebyśmy musieli być inwigilowani w ten sposób.

**Myślisz o tym, jak twoje życie po pandemii będzie wyglądało?**

Jak najbardziej. Najbardziej interesuje mnie to, co będzie teraz. Bo teraz muszę znaleźć pracę, która potrwa parę miesięcy. Pytanie, kiedy to będzie "po pandemii" - czy to będzie już w przyszłym roku, czy w tym. Kiedy to tak będzie w pełni. Bo wiele głosów się pojawia, że ta norma nie powinna być taka, jak wcześniej. Albo jak ta norma będzie wyglądała. Więc to też jest pytanie, jak długo w restauracjach będzie to porozsuwane, kiedy się uzna, że to koniec. Czy ktoś nie uzna, że przyszła druga fala na jesieni, itd.

**Co najbardziej zaprząta twoją uwagę?**

Praca - kwestia, jak będzie wyglądał rynek pracy w obszarach, które bardziej mnie interesują, a są zastopowane rekrutacje czy jakieś nowe projekty, stanowiska, bo nie można na razie wrócić do biur i ta praca jest uniemożliwiona też w kwestiach podróży. Że turystyka czy mnóstwo miejsc pracy będzie się otwierało dopiero z powodu zamkniętych granic - chociaż jakieś loty są wznawiane od lipca. Bardziej kwestia pracy, tego, jak będzie wyglądał rynek. Ale ja czekam na koniec, bo to będzie też możliwość planowania podróży czy wyjazdów zagranicznych, co dla mnie jest istotne.

**Jakie zmiany mogą nastąpić w Polsce?**

Nie zastanawiałam się jakoś i nie do końca jestem sobie w stanie wyobrazić. Poza tym, że wciąż te środki ostrożności zostaną, co też będzie wpływało negatywnie na różne biznesy. Jedna rzecz, to jakaś recesja gospodarki. Inflacja, co już widać - już ceny produktów niektórych skoczyły w górę.

**A sytuacja na świecie?**

Bardzo jestem ciekawa, jak będą wyglądały lotniska, loty w najbliższym czasie. Jak bardzo wydłużą się kwestie przed lotem. Bo kwestia przechodzenia przez kontrolę bezpieczeństwa na lotniskach jest pokłosiem 11 września.

**Ta pandemia to moment trochę jak zamachy terrorystyczne w tym sensie, że dużo się może zmienić na lotniskach, w podróżach?**

Wpłynie na to. Kwestia bezpieczeństwa została wprowadzona i została na stałe, bo walka z terroryzmem nie jest kwestią zamkniętą. Tylko nie jest taką kwestią ciągłą jak pandemia. Jak już minie ostatecznie za 2 lata powiedzmy, bo wtedy już powinniśmy mieć szczepionkę i to się uspokoi. Albo wymyślą jakiś inny lek albo zwyczajnie się uodpornimy i zagrożenie pandemiczne minie. Wtedy to będzie mogło wrócić do normy, bo będzie można zejść z obostrzeń w stylu dezynfekcja rąk, powierzchni i maseczki. Ale dopóki to nie minie, to będą wprowadzali tego typu rzeczy. Czy sprawdzanie tej temperatury. Chociaż sprawdzanie tej temperatury dla mnie jest śmieszne, bo wykazuje niewiele, a jeszcze może błędnie wykazywać, bo temperaturę podwyższoną można mieć z wielu powodów.

**To jest niepotrzebne twoim zdaniem?**

Nie wiem czy tak w ogóle niepotrzebne, ale dość wątpliwe, bo mogę mieć wirusa, a nie mieć objawu podwyższonej temperatury, a mogę mieć podwyższoną temperaturę - bo ktoś tak ma jak lata, bo się stresuje, bo się przeziębił, co przy okazji latania jest często - a nie mieć wirusa. Albo np. mam tę temperaturę wyższą, bo mam moment w cyklu kobiety, że mam wyższą temperaturę np. 37,1 i nie wpuszczą mnie na pokład samolotu. Jest to frustrujące, bo niekoniecznie te dane są w pełni wykazujące, że ta osoba jest chora. najlepiej, jakby były jakieś szybkie testy, które można zrobić. Albo wprowadzą, jak już się mówi, że Bill Gates mówił o cyfrowych paszportach, czyli cyfrowym certyfikacie, że ktoś już przeszedł i jest odporny albo że jest zaszczepiony. Najbardziej by mnie martwiły kwestie tego, że bez szczepienia nie będzie można podróżować, bo ja jestem sceptycznie nastawiona do masowych, przymusowych szczepień.

**A w kontekście tej sytuacji gospodarczej o czym jeszcze myślisz?**

Kwestia takich miejsc, jak kluby albo organizacje eventowe, które żyją z tych zgromadzeń. Większość festiwali tego lata się nie odbędzie - zostały przełożone na za rok. Pytanie, jak sobie tego typu miejsca poradzą. Czy jakieś ciekawe miejsca nie upadną z powodu tego wszystkiego. Najbardziej mnie zastanawia moja sytuacja ekonomiczna, która wynika z tego, co się dzieje gospodarczo w kraju i tego, jak będzie wyglądał rynek pracy, bo to mocno wpłynęło. Nie jest on tak otwarty i ma troszeczkę innego rodzaju oferty niż mnie by interesowały z tego powodu.

**A myślisz o sytuacji społecznej?**

Nie myślę, bo myślę, że większość ludzi... Będą tacy, którzy będą przestraszeni, którzy będą się gdzieś tam wstrzymywać. Ale jest mnóstwo ludzi takich, jak ja albo takich, którzy nawet, jak bali się wcześniej, to będą już tak zmęczeni sytuacją i będą tak potrzebowali wrócić do normalności, że będą mieli gdzieś zagrożenie. To będzie w tym momencie silniejsze dla nas niż zagrożenie w momencie, kiedy my nie widzimy ludzi padających na ulicy, bo się duszą. Że nie ma takich przesłanek, że na korytarzach naszych szpitali leżą duszący się ludzie i umierają - nie ma czegoś takiego. To będzie dużo mniejszą kwestią w postrzeganiu ludzkim zagrożeniem niż to, że ludzie wariują z samotności i niemożności poznawania nowych ludzi. Introwertyków to pewnie nie dotyka aż tak bardzo, ale ekstrawertyczną część społeczeństwa na pewno. Będzie to wynikało z tego, że dla niektórych jest to kwestia pieniędzy i pracy bądź jej braku. Więc ja myślę, że otwiera się teraz biznesy i niektórzy krzyczą, że za wcześnie czy, że za wcześnie maseczki zostały odpuszczone. Więc będą i takie głosy, ale będzie część ludzi, która będzie wracała do normalności, bo będzie tego potrzebowała.

**Może to w szczególności dotknąć jakąś grupę?**

Na razie mam takie poczucie, że uczniów i studentów mocno dotyka. Faktem jest, że zobaczono, że zdalnie pewne rzeczy działają. Liczę na to, że jak już wszystko wróci do normalności i będzie można wrócić do szkół, itd., to będzie taka większa świadomość tego, że w pewnych sytuacjach wprowadzenie zdalnego nauczania jest możliwe i będzie na to większa otwartość. Na pewno nie całkowicie, bo kwestia tego, że dzieci chodzą do szkoły i się ze sobą socjalizują, jest bardzo ważna. To samo w pracy. Moja współlokatorka mówi, że oni mają zdalną w pełni do końca czerwca, ale już im zapowiedziano, że nie będzie powrotu do pełnej pracy w biurze, jak było wcześniej, bo nagle zobaczyli, że zdalna działa. Klienci się przekonali do tego, że zdalnie można zawierać umowy. Więc mam nadzieję, że ta zdalna praca zostanie doceniona trochę bardziej i będzie balans pomiędzy zdalną a niezdalną. To wpływa na obniżenie kosztów utrzymania biura, nie ma problemu, że jest za dużo ludzi, a brakuje sprzętu. Czy to wpłynie na ludzi? Na pewno mają problem starsi i schorowani, bo muszą na siebie bardziej uważać i jeszcze trudniej teraz.

**Czy oni powinni być chronieni w szczególny sposób?**

Jak np. tego typu aplikacje, co rozmawiałyśmy?

**Na przykład. Ale to też moje pytanie do ciebie - w jaki sposób mogliby być chronieni?**

Trochę wydaję mi się, że mamy za mało środków i możliwości na taką pełną ochronę, jaka powinna być tym osobom dana. Pewnie byłoby dla nich lepiej. Ale w zależności od ich woli, bo moja babcia, która ma 84 lata twierdzi, że nie boi się iść do sklepu, bo na coś trzeba umrzeć. Więc zależy jak dla kogo. Ale dla tych, którzy mają jakieś inne choroby, które w połączeniu z wirusem mogą być dla nich groźne, to jak najbardziej dobrze byłoby im umożliwić czy wspomóc ochronę. Z tym, że pytanie czy mamy odpowiednie środki.

**A takie przymusowe środki ochrony dla takich osób?**

Nie, bo jeżeli to starsza osoba, która naraża samą siebie i chce to robić, to nie można jej wchodzić w wolność. Tak, jak niepełnosprawni mają naklejkę na samochodzie i mogą stawać na miejscu dla niepełnosprawnych i nikomu innemu nie wolno tam stawać to jest jakieś ogarnięcie przestrzeni, żeby oni mieli trochę wygodniej. To możemy zrobić dla tych zagrożonych, którzy potrzebują pomocy z zakupami np. Pytanie, co mamy zrobić, jeśli oni nie mogą iść do pracy, a nie pracują zdalnie i nie mają się, z czego utrzymać. Bo czy mamy środki, żeby utrzymać iluś ludzi, którzy z powodu swojego zdrowia są bardziej narażeni na koronawirusa i nie chcą z własnej woli wychodzić, więc potrzebują pomocy rządu. Mamy na to środki? Wątpię. Czy będą takie programy? Wątpię. Byłby fajnie, jakby były i jak najbardziej byłaby to pomoc dla tych ludzi i uważam, że powinna taka być, skoro uważają się za bardziej zagrożonych. Też pytanie, jak by się to klasyfikowało, bo jest też ileś ludzi w społeczeństwie, którzy są hipochondrykami i będą twierdzić, że są bardziej zagrożeni, a niekoniecznie są [śmiech]. Byłoby dobrze, ale jak to jest na zasadzie, że jest zagrożenie i jak się bardziej boisz, to masz prawo się bardziej bać i uważam, że powinno się pomóc, skoro jest taka sytuacja. Są kwestie od nas niezależne, bo obostrzenia dookoła i to, że rynek tak wygląda, nie jest moją winą ani winą pracodawców, tylko odgórnych obostrzeń, więc w tej sytuacji trzeba sobie nawzajem pomóc, ale bez narzucania tego. Nie narzucałabym na pewno przymusowego siedzenia w domu czy kwarantanny komuś, kto jest chory. Jeśli nie chce się chronić, to niech się nie chroni - to jest jego sprawa.

**Które z ograniczeń powinny zostać dłużej? Może któreś na stałe?**

Żadne [śmiech]. Ja bym chciała, żeby żadne nie zostawały. Nie wiem. Najgorsze jest to, że teraz trudno nam stwierdzić, które z nich faktycznie działają. Dowiemy się za ileś lat, jak będą badania, żeby stwierdzić, jak sytuacja faktycznie wyglądała. Też pytanie, na ile które dane są prawdziwe. Mój tata, zanim u nas wprowadzili obowiązek maseczek, znalazł wykresy, że w miejscach, w których jest obowiązek maseczek, to zarażenia były w mniejszym stopniu. Z tego, co pokazują liczby w naszym kraju w kwestii zarażeń i śmierci, to wynikałoby z tego, że zamknięcie granic i zamrożenie kraju na trochę zniwelowało to, bo sytuacja u nas nie jest tak drastyczna, jak w innych miejscach. Czy to przez to? Prawdopodobnie, ale nie stwierdzimy tego. Może to pomogło. Może takie rozsuwanie stolików, większy dystans - ok. Ale na jesień, zimę, co wtedy? Wtedy trzeba będzie wrócić do środka restauracji. Wciąż wiele lokali będzie miało problem, jeśli nie będzie mogło mieć całego obłożenia stolików. Czy wróci druga fala i wymyślą kolejne zamykania. Nie wyobrażam sobie kolejnego zamykania gospodarki, bo to będzie już katastrofa totalna.

**Jak powinien rząd zareagować na drugą falę?**

Ja widzę to tak, że pierwsza była niezapowiedziana. Więc zamrażamy się, o chcemy, żeby wirus się nie rozprzestrzeniał. Było to na przesileniu wiosennym, czyli tym czasie, kiedy jest to czas różnych wirusów, które krążą i że lato ma sprawić, że wirus zniknie. Ponoć nie ma to nic do rzeczy, bo w ciepłych miejscach wciąż jest. Nie wiem, na ile to jest prawda. Ale jesień będzie kolejnym takim czasem, stąd mówiło się o drugiej fali na jesieni. Ale powodem, który dla mnie ma największy sens przy zamykaniu gospodarki... Bo z tego, co widzę, skoro na razie największym problemem jest bardzo łatwa zarażalność - nie śmiertelność, bo jest ona u nas niska - tylko zarażalność i chodziło o to, żeby jak najmniej ludzi się zaraziło w tym samym czasie, bo im więcej się zarazi, tym mniej dostanie pomoc medyczną, co wpłynie na większą śmiertelność. Gdybyśmy mieli dla każdego, pomimo ogromnego zarażenia, pomoc medyczną, to umarłaby jakaś minimalna ilość ludzi, którzy byli na tyle chorzy, że organizm nie przewalczył. Stąd zamrażanie gospodarki miało wypłaszczyć ten wykres zarażeń, żeby nie obciążać służby zdrowia. Więc jak mamy teraz parę miesięcy i wiemy, że może być druga fala, to rząd powinien ładować w służbę zdrowia i rozwiązać to tym, że mamy dużo środków i szczególnie ważne jest to, żeby były osobne przygotowane szpitale tymczasowe, żeby nie zamykać normalnych szpitali. A nie, że jest nagle kwarantanna pół budynku jednego szpitala i całego personelu.

Jeżeli mamy służbę zdrowia, która jest przygotowana na 50% społeczeństwa zarażonego - to jest ogromna ilość, nawet wątpię, żeby taka zaistniała. Teraz mamy kilkanaście tysięcy zakażeń?

**Ponad 20.**

Ok. Ale jaki to jest procent z 39 milionów? Więc jakby przygotować miejsca na tą ewentualną drugą falę, które by sobie z tym radziły. Wtedy nie musimy zamykać gospodarki. Ok, te maseczki i inne obostrzenia wprowadzą. Ale nie trzeba wtedy gospodarki zamykać.

**Dla ciebie to ok, żeby maseczki znowu wprowadzili?**

Ponoć miejsca, gdzie wprowadzili maseczki, mają mniej zarażeń niż tam, gdzie nie. Więc może. Ale czy na 100%? Nie wiem. Plus, jak się mówi, to muszą być specjalne maseczki, które zakrywają szczelnie nos i usta. Bo te normalne, które mają dziury przy nosie i z boku nie do końca. Więc może też wyposażyć ludzi w odpowiednie maseczki, a nie te najzwyklejsze.

**Ty się planujesz jakoś przygotować?**

Najlepiej to by się było przygotować finansowo, ale to ciężko. W kwestii maseczek - nie, na razie o tym nie myślę. Moje podejście jest taki - może błędne, może jak bym się zaraziła, to by się zweryfikowało - ale ja mam takie poczucie, że dużo daje to, że ktoś ma dobrą odporność. Ja choruję rzadko. Przechodziłam grypę, anginę w styczniu. Nie miałam kaszlu, więc wątpię, żeby była to korona. Grypa była wywołana dwoma weekendami imprez i wystawianiem się na wiatr i deszcz. Przedtem nie chorowałam z 2 lata. Więc raczej odporność zawsze miałam na wysokim poziomie. Nie mam alergii. Mam bardzo mało problemów zdrowotnych, jak przeziębienia czy łatwość nabywania chorób. Mam poczucie, że mam na tyle dobrą odporność organizmu, że on poradziłby sobie z koronawirusem i by mnie nie zabił. Może jestem w błędzie, nie wiem. Mam nadzieję, że się nie dowiem nigdy.

Dla mnie większym problemem i większy wpływ na moje życie jest kwestia zamknięcia gospodarki. Tego, że nie mam pracy, że nie mogę jej znaleźć, że nie mogłam poukładać tak życia, jakbym chciała.

**Podsumowanie. Najważniejsze momenty w tej sytuacji dla ciebie?**

Pierwsze to były kwestie, jak tydzień po moim wyjeździe do Londynu w Polsce zdecydowali o zamknięciu szkół i granic. To był pierwszy punkt. Później przełomowym punktem była kwestia odwołania tej rozmowy o pracę w Londynie i moi wynajmujący, którzy zaczęli mówić o wyjeżdżaniu i decyzja o moim powrocie. To najważniejsze - czyli decyzja o moim powrocie do domu. Dwa tygodnie kwarantanny w domu. Koniec kwarantanny i siedzenia w domu [śmiech]. A od końca mojej kwarantanny - to był początek kwietnia, czyli w tym momencie 2 miesiące temu, w ostatnich 2 miesiącach przełomem było pierwsze wyjście do Warszawy. I teraz otwieranie fryzjerów i restauracji i to, że już można było wyjść do różnych miejsc. Pojechałam do tego Krakowa.

**Dlaczego otwarcie fryzjerów i restauracji było ważne?**

Bo mogłam wyjść do fryzjera, na paznokcie. To było takie trochę poczucie normalności w tym, że można wyjść i te usługi zrobić.

**A po tym jeszcze coś?**

Teraz przeprowadzka.

**Najważniejsze momenty w tej sytuacji z perspektywy kraju?**

Pierwsze stwierdzone u nas zakażenia to był bodajże 4 marca. Później tydzień, w którym najpierw zamknęli szkoły i 3 dni potem granice. A później ustalenie tych 4 etapów rozmrażania gospodarki. 18 maja był trzeci, 4 maja drugi, więc tam jakoś w kwietniu, pod koniec kwietnia. 20 kwietnia. Trzeci taki największy. I teraz ten czwarty w sobotę.

**Dziękuję. Z mojej strony to wszystko.**
