# Supplementary material for: The challenges arising from the COVID-19 pandemic and the way people deal with them. A qualitative longitudinal study
Source: PLoS One. 2021 Oct 11;16(10):e0258133. doi: 10.1371/journal.pone.0258133 (PMC8504766; doi:10.1371/journal.pone.0258133)
Supplement: S1 Dataset — (ZIP) [file pone.0258133.s003.zip › Transcriptions/stage 5/16.5_F_36_couple, with children.docx]

**16.5_F_36_couple wih children**

**Co się działo w ciągu ostatniego miesiąca?**

Dużo rzeczy się działo. Już tak trochę życie wróciło do normalności. Może tak jeszcze nie do końca super normalnie jest. Już teraz regularnie widujemy się z rodziną, byłam u jednej znajomej. Już zaczynamy funkcjonować w miarę normalnie. ZOO mi otworzyli, więc już byliśmy 2 razy w ZOO. Tak czekałam na to ZOO, moje dzieci zachwycone. Z babcią chodzimy do ZOO, bo babci nie chce się już siedzieć w domu, a w sumie do tego ZOO jest tak fajnie, można sobie pospacerować, więc z nami chodzi. Teraz wyjechaliśmy na wakacje. Bez mojego męża - ja z moimi rodzicami i z dziećmi. Będziemy chyba 8 dni, wracamy przed długim weekendem. Mój mąż cały czas pracował zdalnie i tylko się modlił, żeby nie wrócić, bo mu się to zdalnie podoba. Modlił się, żeby nie musiał iść stacjonarnie i na razie chyba nie będzie musiał wracać do końca roku szkolnego. Dopiero od września pewnie coś tam wróci.  Do tej pory było tak wszystko online, a teraz już zaczynają przychodzić też ludzie do nas, do niego, już bezpośrednio do domu też, także trochę wróciło to, że się widuje z ludźmi.

**Na lekcje do niego przychodzą?**

Tak.

**Co wróciło/ co nie wróciło do normalności?**

Jak tak się chodzi po mieście, to nie jest normalnie. W takim sensie, że cały czas są te ograniczenia w sklepach, nie można wchodzić normalnie w ileś osób. Teraz jesteśmy tutaj, stołujemy się w restauracjach i też co 2 stolik np. Ten dystans społeczny jest taki nienaturalny dla mnie. Te maseczki, to już nie trzeba ich nosić, ale w sklepach ludzie noszą, co też nie jest do końca naturalne. To nie jest jeszcze taka normalność. Też nie jest normalne to, że dzieci do szkoły nie chodzą. Do końca wszystko nie wróciło. Ja już nawet nie wspomnę o tym całym bałaganie politycznym, który mamy, który nie jest normalny, bo sytuacja nie jest normalna. To jest dla mnie nienormalne jeszcze.

**Czy Ty już chodziłabyś wszędzie bez maski, gdybyś mogła?**

Nie wiem. Ja nie mam kompetencji, czy to jest tak, czy tak. Ja się na tym kompletnie nie znam i nie wiem, czy to jest dobre, czy złe. Rękawiczek nad morze nie zabieraliśmy, ale mamy maseczki, mamy płyny. Staramy się korzystać z tego. Jak wchodzimy do jakiegoś sklepu, to zakładamy maseczki.

**Gdzie teraz mieszkacie?**

Mamy taki apartament, wynajęliśmy sobie. To jest zarezerwowane w Airbnb. Dzwoniłam do pani właścicielki wcześniej i się pytałam, czy wszystko działa, ale bez przesady z dezynfekcją, bo i tak, ile ten wirus jest w stanie przetrwać na tych rzeczach, jak tu nie było przed nami nikogo. Bez przesady.

**Jest jakoś inaczej w Jastarni niż było w Warszawie, jeśli chodzi o wirusa?**

Sklepy też mają te wszystkie przegrody, w sklepach obsługują w maseczkach i to jest jak u nas, natomiast jeśli chodzi o ludzi, którzy wchodzą/ wychodzą z tych sklepów, to tutaj jest taki mniejszy ten...U nas to jeszcze przecież po ulicy chodzili w maseczkach nawet jak już znieśli ten rygor, a tutaj to na ulicy nie widziałam nikogo. W sklepach i restauracjach to pewnie się boją tych wszystkich kontroli sanepidowskich - mają te przegrody, mają maseczki, mają płyny. Wszędzie jest to samo.

**Jak wygląda to na plaży?**

Nigdy nie jeździliśmy w sezonie, więc chyba jest tak samo. Ludzi jest tak nie za dużo, chociaż w zeszłym roku byliśmy o tej samej porze Mielnie i w tygodniu ludzi na plaży chyba było mniej niż tutaj teraz, takie mam wrażenie. Teraz są rodziny z dziećmi, bo dzieci nie mają szkoły, więc rodzice też w innych momentach mogą z urlopu skorzystać. Nie ma problemu z odstępami na plaży na razie.

**Czy jest coś, co ci jeszcze teraz przeszkadza w kontekście pandemii?**

Nie, teraz to już się chyba przyzwyczaiłam trochę i nic mnie raczej nie wkurza. Tym bardziej, że te najgorsze rzeczy, które mnie wkurzały, to chyba już minęły - takie, że nie można nigdzie wyjść albo spotkać się z ludźmi. To już minęło. Jedyne, co chciałabym, żeby jeszcze przywrócili, to te place zabaw, bo jednak dzieciaczki uwielbiają te place zabaw i grzebanie się w tych wszystkich piaskach. To bym jeszcze chciała, ale też nie jest to takie, żeby się wkurzać, że tego nie ma.

**Emocje - zdjęcia**

Ten okres mi się już kojarzy z taką dziecięcą radością. Ja bym tu widziała zdjęcie mojej radosnej córki, która po prostu się chichra na widok morza, plaży, wszystkiego, że już wychodzimy z domu, że gdzieś jedziemy. To jest ten obraz, który mam w głowie i to jest obraz na teraz.

**A czas ostatniego miesiąca?**

6 - już wychodzimy z tego ciemnego lasu, idziemy do słoneczka, gdzie już jest lepiej, cieplej, weselej, jest pozytywniej. Ogólnie idziemy ku lepszemu, mam nadzieję.

**Jest coś, czego się teraz obawiasz?**

No obawiam się. Obawiam się bardziej już nawet nie tego koronawirusa, ale tych wszystkich głupich politycznych pomysłów na radzenie sobie z kryzysem, który nas czeka. Ja generalnie ma takie myślenie bardziej wolnorynkowe, więcej trochę wolności a mniej takiego państwowego interwencjonizmu, a niestety mam wrażenie, że idziemy w stronę tych wszystkich "plus", że jeszcze więcej państwa w naszym życiu. Jeszce więcej chcą nam dawać wszystkiego i nie wiadomo co jeszcze wymyślać, bo muszą się czuć potrzebni chyba. jak już słyszę te "wakacje+", "warzywniaki+" i coś tam jeszcze, to mnie to najbardziej przeraża szczerze mówiąc. To idzie w takim kierunku, że państwo będzie się zajmować rzeczami, którymi nie musi się zajmować, bo ludzie by się sami nimi zajęli, jak by im pozwolono się zająć. Ludzie są w stanie sobie zorganizować warzywa i owoce i państwo nie musi się zajmować sklepami warzywnymi. To jest absurdalne. Tak samo, jak jesteśmy w stanie sobie zorganizować wakacje. Oni chcą rozdawać te pieniądze i to mnie też przeraża, że oni uważają, że budżet państwa jest z gumy, co się wiąże z dodrukiem pieniądza, z wysoką inflacją i to odpowiada ludziom, którzy nie mają pieniędzy, nie mają żadnych oszczędności, a my mamy trochę takich rzeczy, więc mnie się to nie podoba. Mnie to denerwuje, mnie to wkurza. Bardzo mnie denerwuje to, że oni przekonują do siebie taki elektorat katolicki, co mnie strasznie denerwuje. Nie jestem w stanie zrozumieć, dlaczego, czym oni przekonują tych ludzi. Jest to dla mnie nie do pojęcia po prostu. Dyskutuję czasem z ludźmi na FB i ludzie nie są w stanie mi odpowiedzieć na takie pytania i to nie strasznie frustruje. Ja się trochę znam na temacie, więc jak się tak gada o polityce, to tak ludzie jeszcze odpowiadają i próbują...Ale jak już np. zacytuję fragment z JP II, to już nie ma odpowiedzi na to. I to taki właśnie jakby przeciwko tym wszystkim działaniom, które robi nasz rząd, bo JP II był takim bardzo wolnorynkowym papieżem. On sprzeciwiał się tym całym socjalistom, komunistom, więc jak ja piszę coś takiego, to ci ludzie milkną i kompletnie nie są w stanie odpowiedzieć na te pytania. Zastanawiam się też, gdzie tutaj my jako Kościół popełniliśmy błąd. Ja się czuję tym nauczycielem w Kościele i zastanawiam się jak edukujemy studentów, teologów, bo tu nawet teologowie piszą takie rzeczy, księża, czego kompletnie nie jestem w stanie zrozumieć i z tego wynika głównie moja frustracja. Zauważyłam, że jak piszę od siebie, to jeszcze ludzie są w stanie się ze mną kłócić, a jak już wspomnę tylko JP II, to już nikt nie jest w stanie nawet dyskutować.  Ten temat teraz mnie angażuje i ja się trochę dokształcam, bo ja mam inną specjalizację, jeśli chodzi o teologię, więc trochę sobie doczytuję.

**Powróćmy jeszcze na chwilę do twojego powrotu do stanu sprzed pandemii. Na ile twoje zakupy wyglądają teraz jak dawniej?**

No jeszcze nie są takie same, bo staram się robić zakupy tak raz na kilka dni, a nie tak, że codziennie, chociaż jest już trochę lepiej, bo jednego dnia sobie wyskoczę i zrobię większe zakupy w większym sklepie, a potem z dziećmi np. pójdę na bazarek po warzywa, owoce. To już tak bardziej normalnie jest, chociaż z dziećmi jeszcze do takiego...Raz pojechałam z nimi do Selgrosa, bo musiałam i nie miałam ich z kim zostawić.

**Miałaś wrażenie, że to jest bezpieczna sytuacja?**

No tak. Tam nie ma akurat specjalnie jakichś tłumów w tym Selgrosie, więc bez przesady. Do Biedronki bym jeszcze z nimi nie poszła może i tak unikam takich sklepów, gdzie są większe tłumy, bo w Biedronce zazwyczaj są takie tłumy dzikie przy tych kasach zwłaszcza.

**Lubiłaś kiedyś pochodzić sobie po sklepie i pooglądać. A jak jest teraz?**

Teraz jest tak różnie - czasami tak, czasami tak. Jak jadę sama i wieczorem robię te zakupy, to zwykle chcę jak najszybciej. Czasami przejdę, czasami nie przejdę i to zależy od tego, jaki mam dzień i czy mi się spieszy do domu, ale to już nie jest takie tylko zadaniowe wyjście do sklepu. Ja już nawet byłam w GH na zakupach ciuchowych. Któregoś ranka powiedziałam mężowi, że ja się zamykam w pokoju i ja muszę trochę odpocząć od dzieci. On na to, że ok i że mogę nawet wyjść. No to, skoro mogę wyjść, to idę do galerii. Jeszcze były te ograniczenia i w niektórych sklepach nie było przymierzalni, więc tam, gdzie były zamknięte przymierzalnie to tylko dla dzieci kupiłam parę rzeczy. Tam, gdzie były otwarte, to sobie ciuchy kupiłam. Bluzki sobie kupiłam.

**Było jakoś inaczej niż przedtem?**

Właściwie nie. Była tylko dezynfekcja rąk, a że pozwolili mierzyć, to kupiłam. Ludzi nie było prawie wcale, bo to był poniedziałek rano. Było spoko, ale bardziej mnie cieszyło, że mam czas tylko dla siebie niż sam pobyt w GH. Już mi było wszystko jedno, gdzie to było, bo fajne było mieć ten czas tylko dla siebie. Dopiero pootwierali te wszystkie sklepy, więc było dużo promocji, co też było fajne, więc sobie połaziłam, popatrzyłam, parę rzeczy dla dzieciaków pokupowałam.

**Miałaś jakieś obawy?**

Nie, niespecjalnie. Byłam bez rękawiczek i w tych odzieżówkach dezynfekowałam ręce przy wejściu.

**W Warszawie byłaś w jakieś restauracji/ kawiarni przed wyjazdem?**

Nie, my zamawialiśmy sobie do domu jedzenie.

**Jak tutaj jest w restauracjach?**

Jest inaczej, jest. Przede wszystkim są rzadziej stoliki albo co drugi stolik w ogóle tylko. Wczoraj byliśmy w takiej, gdzie kanapy są ustawione jedna przy drugiej i były takie ekrany oddzielające jedną od drugiej. W restauracji też nie działają te wszystkie samoobsługowe rzeczy, w sensie bar sałatkowy albo coś takiego. Poza tym normalnie w miarę, ok. Tutaj, w Jastarni ludzie się zachowują, jakby nic się nie działo, jakby nikt nie słyszał o tym wirusie, nikogo to nie dotknęło. Tutaj to jest dla nich legenda, która krąży. Może się trochę obawiają tych turystów, ale oni bez tych turystów, to nie żyją. Zakażenia to był bardziej Gdańsk, a nie tutaj na Helu.

**Fryzjer/ kosmetyczka, korzystałaś już?**

Nie, nie byłam. Nie, nawet jakoś tak...Raczej nie miałam czasu, nie chciało mi się tego wszystkiego załatwiać..

**To jest ok, że to jest już otwarte?**

Chyba tak. Moje koleżanki już były. Ja nie korzystam z tego tak na co dzień i jak nie muszę, to nie pójdę. Nie jest to dla mnie jakaś strata wielka i nie zastanawiam się nad tym specjalnie.

**Jak podeszłaś do tych poluzowań z maseczkami, restauracjami, zaraz otworzą kina, siłownie?**

Z jednej strony uważam, że dobrze, bo ile możemy żyć w zamknięciu? To jest niedobre dla naszej gospodarki, bo ludzie tracą pracę, tracą dochody, nie można tak żyć. I całe życie polega jakby trochę na ryzykowaniu. Wyjście z domu to zawsze jest ryzyko, wsiąście do samochodu to ryzyko i kurczę, nie można żyć całe życie w strachu przed tym, że ktoś się zarazi. No tak, może się zarażę, a może zginę w wypadku samochodowym, a może, no nie wiem...Nie możną tak żyć całe życie, bo co to jest za życie? Nie da się tak żyć. Z drugiej strony zdaję sobie sprawę z tego, że trochę chyba jest to podyktowane politycznym kalendarzem, bo zachorowań wcale nie jest mniej, itd., a są zdejmowane te wszystkie rzeczy. Może takie noszenie maseczek, które nic nie zmienia w gospodarce, może trzeba to było przytrzymać, ale ludzie byli już bardzo wkurzeni tymi maseczkami. Ja też trochę, bo bardzo źle mi się w tym oddychało, ale no nie wiem...Nie znam się na tym. Nie da się żyć będąc cały czas zamkniętym w domu. Ludzie muszą pracować i nie ma takiej opcji, żeby wszyscy pracowali z domu. Tak się nie da, bo ten segment usług...My jesteśmy już społeczeństwem wysoko rozwiniętym, więc mamy bardzo duży ten segment usług, których się nie da realizować siedząc w domu. To, co robi nasz rząd to jest jakaś paranoja. My po prostu nic nie wiemy, tak? Mamy jakieś dane pokazywane gdzieś tam, raz mówią tak, raz mówią inaczej, ten Minister Zdrowia raz mówi, że nie trzeba maseczek, potem, że trzeba i że wszyscy, potem, że będziemy te maski 2 lata nosić, potem, że już nie będziemy ich nosić za miesiąc. Przecież to, co on gada...Za każdym razem przecież mówi co innego. On to tłumaczy tam jakoś tak pięknie, ubiera to wszystko w słowa, ale to się trochę kupy nie trzyma i my tak naprawdę nie wiemy, na jakiej podstawie są podejmowane te decyzje, bo ta krzywa zachorowań jest cały czas jakby spłaszczona, ale cały czas jest, tak? On nie powie wprost, że to dlatego, że gospodarka, nie powiedzą, że my nie mamy pieniędzy i wy musicie pracować. Taka jest okrutna prawda. Wydaje mi się, że oni się nie chcą do tego przyznać, że nie mają pieniędzy po prostu na to, żeby ludzie siedzieli w domach cały czas. Wydaje mi się, że to z tego wynika, a na jakiej podstawie oni analizy robią, kto to robi, to nikt tego nie wie. Słyszałam raz, jak on powiedział, że to są prywatne osoby, które pracują na państwowych uniwersytetach. Albo pracują na państwowych uniwersytetach, albo są to prywatne osoby i robią badania na własną rękę. No nie wiem, nie wiem. On mówi, że nie poda nazwisk tych naukowców, którzy się tym zajmują, bo to są prywatne osoby. Jak oni pracują dla rządu...Przecież my nie mamy prywatnych uniwersytetów na takim poziomie, oni pracują na państwowych, więc kurczę, to nie są prywatne osoby. No bez przesady. Piastują jakieś ważne profesorskie stanowiska i dla mnie to jest jakaś paranoja i złości mnie to.

**APLIKACJE**

**Słyszałaś coś o takich stworzonych przy okazji pandemii?**

Ja tylko słyszałam o tym rządowym czymś, co miało śledzić ludzi. Tylko to. Słyszałam, że to była klapa straszna. To miała być aplikacja dla ludzi na kwarantannie, która wysyła polecenie " zrób zdjęcie" czy coś tam i miała śledzić ludzi, czy oni faktycznie siedzą na tej kwarantannie. Słyszałam, że straszna klapa z tego wyszła. ze nie ma w ogóle obowiązku posiadać smartfona, bo część ludzi nie ma smartfona, albo jak mają to nie potrafią go obsługiwać. Moi rodzice mają, ale nie za bardzo umieją. Moja mama tylko zdjęcia robi i odbiera telefony, ale aplikacji nie umiałaby w życiu w ogóle włączyć. Rozumiem, że część ludzi nie jest w stanie tego w ogóle robić. Słyszałam, że tam w ogóle jakieś błędy były w tej aplikacji. Nie wiem, ile w tym prawdy jest, bo nie znam nikogo, kto był na kwarantannie. Nie wiem, czy to w ogóle jeszcze działa i czy jest obowiązkowe, czy nie.

**Kategoria 1**

To jest pomysł rodem z Chin, bo oni to dobrze wdrożyli i oni to robią. Z jednej strony może się wydawać, że przy takiej pandemii, chorobach, itd., to może być bardzo sprytne, bo faktycznie jest w stanie wyłapać itd. Z drugiej strony to niestety daje ogromną władzę i ogrom informacji rządowi. Oni po prostu za dużo wiedzą o ludziach. Oni i tak już dużo wiedzą o ludziach, a to im daje kolejną wiedzę. To jest narzędzie inwigilacji po prostu, no jest.

**Kategoria 2**

Nie wiem...Musiałaby się bardziej wgłębić w temat. Jak takie pomocowe, to ok niby. Jak ludzie coś robią, żeby pomóc komuś drugiemu, to niech robią.

**Kwarantanna Domowa**

Ja się zastanawiam, jak ona może być obowiązkowa, skoro nie ma obowiązku posiadania smartfona?

**Załóżmy, że dostajesz smartfona na czas kwarantanny. Czy to coś zmienia?**

No...Wiesz, jak coś każą robić, to każą. Pytanie, na jakiej podstawie taka rzecz jest obowiązkowa. Rozumiem, że jest jakieś rozporządzenie, itd., chociaż w naszym państwie to nie wiadomo. Po drugie zastanawiam się, czy osoby starsze są w stanie to obsługiwać. Moi rodzice już nie są tacy smartfonowi i moja mama by już nie przeszła przez tę instrukcję, tak?

**A gdybyś ty znalazła się w kwarantannie?**

Na pewno by mnie to nie cieszyło. To jest takie inwigilacyjne bardzo. Rozumiem, że ta technologia trochę pomaga śledzić takie osoby na kwarantannie, ale ona jest taka bardzo inwazyjna. No, robienie sobie zdjęć i analizowanie twarzy, dawanie swoich danych do analizowania twarzy o rożnych porach dnia, o różnych tych...To jakby pomaga tym ludziom od uczenia się rozpoznawania twarzy poznawania coraz lepiej tych technik rozpoznawania twarzy. To mi się np. bardzo nie podoba, że ja muszę cały czas komuś udostępniać moje zdjęcia. I właściwie, gdzie jest regulamin tego, co się z tymi zdjęciami dzieje, do czego one są używane, kiedy one zostaną usunięte? Może to gdzieś jest? Ja bym się tym zainteresowała. Pytanie jak to jest w praktyce - czy to faktycznie działa, czy nie działa.

**Widzisz tu jakieś korzyści z instalowania takiej aplikacji?**

Nie. Ja nie wierzę w takie rządowe projekty. Nie wierzę w taką uczciwość tego i nie wierzę, że to działa faktycznie po to, po co zostało stworzone. Do końca w to nie wierzę.

**To po co to zostało stworzone?**

Właśnie dla picu, fotomontażu i nie wiadomo, dlaczego jeszcze. Żeby to PR-owo dobrze wyglądało, że jesteśmy tak przygotowani, że stworzyliśmy taką wspaniałą aplikację i z obaczcie, jaką mamy piękną aplikację. Ja nie wiem, czy to działa w ogóle. Czy ci wszyscy górnicy na Śląsku korzystają z tego? Nie do końca w to wierzę.

**Czy taka aplikacja powinna być obowiązkowa?**

W momencie, w którym jesteśmy teraz, na taką pandemię, jak mamy teraz, to wydaje mi się, że nie. My nie mamy aż tak wysokiej śmiertelności, żeby to było aż tak konieczne.

**Dlaczego to zależy od śmiertelności a nie od liczby zachorowań?**

Nie kontrolujemy wszystkich, którzy mają katar, grypę, itd., bo to nie są śmiertelne choroby. A poza tym, jak nie jestem właścicielem żadnego smartfona i co? Dadzą mi?

Gdybym miała wybór, to wolałabym kilka razy dziennie pokazywać się w oknie policjantowi niż mieć taką aplikację.

**ProteGo Safe**

Ja przede wszystkim nie mam zaufania do żadnego rządu. Uważam, że im mniej państwo o mnie wie, tym lepiej. nie wiem, czy ta aplikacja też jest inwigilująca, ale też nie wiem, czy ona w ogóle działa, czy ktoś z tego w ogóle korzysta...Ja nie słyszałam. Te wszystkie takie rządowe projekty, to mam wrażenie, że robią tak dla picu i nikt z tego nie korzysta.

**Załóżmy, że to działa?**

To jest bardzo odważne założenie, że działa i że działa dobrze. [chichot] Mi by się przede wszystkim nie chciało takich rzeczy robić. Pisać jakieś...Zapisywać, jaka ja się czuję? Nie, to w ogóle nie. Nigdy w życiu bym tego nie robiła. Może, jakby ta choroba była taka faktycznie, że...Że to jest jedyna rzecz, która mnie może przed tym uchroni, to może tak, ale mi się takich rzeczy nie chce robić.

**Czy jak pobierasz jakąś aplikację, to sprawdzasz, do czego ona ma dostęp w twoim telefonie?**

Czasami ma to dla mnie znaczenie. Generalnie ja nie używam za dużo aplikacji, staram się korzystać jak najmniej. Staram się unikać i czasami niestety się zgadzam. Mój mąż wyłącza te wszystkie lokalizacje, żeby go nie oznaczało, bo to faktycznie cię wtedy śledzi cały czas i on wie cały czas, gdzie ty jesteś.  On powiedział, że on tak nie chce. Mi jest czasami wygodniej z tego korzystać jak gdzieś jadę samochodem, itd. Jeżeli czuję, że większą mam korzyść z tego, to wtedy się zgadzam, ale były takie przypadki, że się nie zgadzałam i nie korzystałam. Jeśli chodzi o te mapy Google, to korzystam z tego.

**A jeśli chodzi o rozpoznawanie twarzy?**

Nie korzystałam z żadnych. Nie znam takich aplikacji i raczej bym się nie zgodziła. To są już takie dane wrażliwe, mam wrażenie i potem nie wiadomo...Pytanie, w jaką stronę to idzie i obawiam się, że to idzie w taką stronę, że nauczą się te systemy twoją twarz rozpoznawać i wszędzie będzie widać, gdzie ty jesteś i co robisz cały czas. Mam nadzieję, że się mylę i nie chciałabym, żeby tak było, ale nie wiadomo.

**Taki Wielki Brat nad nami?**

No tak. Moglibyśmy powiedzieć, że przecież nic złego nie robimy, jesteśmy dobrymi ludźmi, nie łamiemy prawa...W tym co teraz było, że siedzisz w domu i nie możesz wychodzić, to okazuje się, że to będzie wykorzystywane do tego właśnie, że mamy chorobę, nie możecie nigdzie wyjść i kurczę, gdzieś wyjdziesz i zaraz cię rozpozna jakaś kamera i mandacik albo coś innego. Pytanie, dokąd ten świat zmierza i niestety obserwując to, co się teraz dzieje na świecie, to my bardziej naśladujemy Chiny pod względem walki z koronawirusem niż mamy jakiś swój pomysł, jako świat Zachodu. Izolujemy, wprowadzamy restrykcje. Izolujemy w sensie takim zamordystycznym, w takim, że wyjdziesz, to dostaniesz mandat a nie w takim, że jesteśmy wolnym narodem, ale wiemy, że mamy taką prośbę. Nie, że żyjemy w tej wolności i sami odpowiedzialnie się zachowujemy. Nie, rozporządzenie bach walnijmy w was, wychodzicie - mandat, idziecie na rower - mandat. To co się działo na tym strajku przedsiębiorców, to bardzo tak rygorystycznie podchodzą do właśnie takich prób protestów np. wobec polityki jakiejś. To bardziej przypomina rozwiązania chińskie, metody chińskie tego reżimu w Chinach niż takie podejście wolnościowe, europejskie, gdzie my mamy trochę inną kulturę. Może w Polsce to niekoniecznie, ale chcemy być tym Zachodem a nie Dalekim Wschodem. Mamy takie ambicje przynajmniej.

**Obił mi się o uszy kiedyś taki pomysł, że być może, żeby wejść do GH, to trzeba sobie będzie zainstalować aplikację, żeby można cię było w razie czego odnaleźć, jeśli było tam jakieś zachorowanie. Zainstalowałabyś sobie taką aplikację?**

Nie, wolałabym nie wejść do galerii. Ja też słyszałam o tym pomyśle i uważam, że to jest kretyński pomysł. Starsi ludzie nie obczajają tych aplikacji i to by była tak duża dyskryminacja tych ludzi, że nikt normalny by chyba tego pomysłu nie zrealizował.

**Jak myślisz, jak będzie w przyszłości w kontekście pandemii?**

Raczej jestem optymistycznie nastawiona do życia.

**Co najbardziej zaprząta twoją uwagę?**

Mnie najbardziej martwi ten kryzys gospodarczy i to jak sobie z nim poradzimy, bo uważam, że na razie idziemy w złą stronę, jako kraj. Mamy taki mały kryzys polityczny też. Mam nadzieję, że niedługo ta cała partia, która tworzy rząd i tworzy władzę ustawodawczą przejdzie na śmietnik historii, bo to, co odwalili ostatnio, to w pale się nie mieści. Bardzo mnie boli to, co zrobili z telewizją, że ona jest bardzo upolityczniona i jak bardzo ten przekaz propagandowy po prostu jest, i jak bardzo ludzie słuchają tego i kupują to. W ogóle mnie boli to, że ta telewizja została tak potraktowana.

**Czy pandemia jakoś się odbije na sytuacji społecznej w Polsce?**

Pewnie jakoś się odbije, ale jak, to nie wiem. Nie mam takich socjologicznych przemyśleń. Jedyna nad czym się zastanawiam, to jak kościół przez to przejdzie. To mnie bardziej interesuje i ciekawi. Czy ludzie wrócą do kościoła? Z jednej strony "jak trwoga, to do Boga", ale chyba okazuje się, że trwoga wcale nie jest taka duża jak by się mogło wydawać, po drugie poszły te msze online, które są bardzo wygodne i się nie chce wychodzić z domu niektórym. W kościele za dużo ludzi jeszcze nie ma.

**Myślisz, że może być odejście od "praktycznego kościoła"?**

Tak. nie wiem, czy to mnie martwi do końca, ale ciekawe to jest dla mnie.

**A co będzie się działo na świecie? Gospodarczo, społecznie?**

No, to też jest strasznie ciekawe. Trochę zaczynają naśladować chiński model rządów, gdzie mamy władzę, która za bardzo decyduje i inwigiluje ludzi. Cały Zachód generalnie i chyba tylko te kraje skandynawskie tak się nie zbliżają do tego. Naśladujemy ten model chiński coraz bardziej, liczymy, że państwo się wszystkim zajmie i wszystkie nasze bolączki...Rządzącym to jest na rękę, bo władza kusi. Ciekawe, czy produkcja zostanie trochę wycofana z Chin i przekierowana na Europę Wschodnią, czyli tam, gdzie jest taniej trochę i bliżej niż Chiny, żeby nie było takich dramatów, że nie ma dostaw, itd. Ciekawe będzie co się stanie na linii USA - Chiny. Teraz USA mają trochę te wewnętrzne problemy i ciekawe jak oni sobie z tym poradzą. Może się wydawać, że te ich problemy nie są związane z pandemią, ale może jednak to jest też trochę tym podyktowane, bo to jest ta biedniejsza społeczność, Afroamerykanów chyba bardziej dotknął ten cały lockdown i też są bardziej sfrustrowani. Podobno też większa śmiertelność jest wśród czarnych ludzi na tego koronawirusa i to też pewnie z różnych przyczyn. Gdzieś to tam jest ze sobą powiązane i nie bardzo chyba można odciąć jedno od drugiego. Z drugiej strony Amerykanie wysłali teraz swoją rakietę w kosmos i będą o ten kosmos teraz walczyć. Chiny tak ucichły teraz trochę. Chyba czekają, jak się rozwinie sytuacja i co się dalej będzie działo. Na pewno czeka nas jakieś przetasowanie, a jak bardzo, to zobaczymy. Takie polityczne i gospodarcze przetasowanie. Zastanawiam się, na ile Europa przeniesie się z tą produkcją z Chin i z dalekiej Azji.

**Masz obawy, że tak będzie, czy nadzieję, że tak będzie?**

To są plusy i minusy. jak było wszystko w Chinach, to było taniej, jak się przeniesie to będzie bliżej i może w Polsce też coś się będzie działo, ale będzie drożej. My nie mamy takiej taniej siły roboczej.

**Obostrzenia - czy któreś z nich powinny z nami zostać na dłużej/ na stałe?**

Nie wiem, nie wiem. Na pewno te wszystkie apele o częstsze mycie rąk są dobre, ale myślę, że ludzie sobie nie zdają sprawy, jak bardzo te wszystkie zarazki się roznoszą przez te smartfony, przez te ręce i to akurat spoko. Niech to przejdzie do kultury i niech to ludzie robią z własnej woli, a nie z jakiegoś tam przymusu.

**Mamy nadal mieć płyn w sklepie, pleksi w sklepie?**

Nie wiem. To pleksi mi się osobiście nie podoba. Uważam, że ten dystans społeczny też nie leży w naszej kulturze polskiej, bo rozumiem, że gdzieś tam na północy...taki dowcip jest, że u Szwedów znieśli ten 2 m dystans i oni się ucieszyli, że znowu mogą mieć 5 m dystansu. My żyjemy bardziej w kupie i dla mnie to jest bardzo nienaturalne, że są takie kolejki rozciągnięte, że za tymi foliami wszystko. Mam nadzieję, że to zniknie.

**Jak się witasz z rodziną, z przyjaciółmi, to się ściskacie czy jeszcze trzymacie dystans?**

Nie, no gdzie trzymamy dystans...Bez przesady, to już jest normalnie.

Było jakieś poluzowanie, które cię zaniepokoiło, było na nie za wcześnie wg ciebie?

Nie wiem. Nie, ja nie mam jakichś takich...Nie umiem odpowiedzieć na pytanie.

**Co myślisz o mierzeniu temperatury w miejscach publicznych?**

Nie chciałabym. Wydaje mi się, że to za bardzo wkracza w jakąś taką moją intymność. Można mieć podwyższoną temperaturę też z innego powodu. Nie, nie wiem, nie znam się na tym, nie myślałam o tym.

**A odległości w kinie co 2 miejsce, w samolocie 50% miejsc, w metrze, itd.?**

Może to jakiś efekt przynosi, ale zastanawiam się, na ile te prywatne firmy to pociągną. Czy będzie ich stać na to, żeby np. tak latać. samoloty podobno już są droższe. Gospodarczo to nie jest...Tak, jak w tych restauracjach. W weekend zjedzie się pewnie sporo ludzi z Trójmiasta i już pewnie nie będzie tak łatwo wejść do restauracji. Zobaczymy.

**Gdyby przyszła 2 fala, to co rząd powinien zrobić?**

To jest bardzo trudna decyzja, zobaczymy. Ja nie wiem co powinni zrobić. Uważam, że my płacimy im za to, żeby oni myśleli za nas w takich sytuacjach, o ile są kompetentnymi osobami. Uważam, że te osoby nie są do końca kompetentne, które teraz nami rządzą, ale jak ja bym znała odpowiedź na takie pytanie, to ja bym poszła do tego rządu i tam zrobiła porządek. Nie znam. Obawiam się, że znowu wpadną na pomysł, żeby zamknąć wszystkich i nas po prostu nie będzie na to stać. To już będzie masakra. Obawiam się, że oni mogą to ukrywać - nie będą robić testów, będą starali się uciszyć ten temat jak najbardziej, bo nie będzie nas stać gospodarczo na to, żeby kolejne miesiące ludzie siedzieli w domach, szkoły pozamykane, itd. Zasiłki opiekuńcze przecież dla rodziców są, przecież ci ludzie nie pracują wtedy.

**A ty powinnaś się przygotować jakoś do tej ewentualnej 2 fali?**

Nie no...Jedyna zmiana, jaka w naszym życiu nastąpi, to że nasz syn się wybiera do przedszkola. Jak nie pójdzie do tego przedszkola, to trudno, bo ja i tak jestem w domu z naszą córką, więc jakoś to może nie wpłynie za bardzo na nasze życie jeszcze na tym etapie. Przeżyliśmy raz, to przeżyjemy drugi raz. Jak już człowiek coś doświadczył, to już ma doświadczenie. Już będę wiedziała, czego się mam spodziewać w związku ze sklepami, z robieniem zakupów, itd.

**Czy powinniśmy jeszcze chronić jakieś grupy - starszych, dzieci?**

No tak. Ja uważam, że te osoby same powinny...Właśnie te osoby starsze, te, które mają choroby współistniejące. Myślę, że one same jakoś się ograniczają, chociaż widziałam wiele takich filmów w internecie, że emeryci chodzą po bazarach na zakupy i mówią, że na coś muszą umrzeć. To też jest coś za coś. ja to rozumiem. Jedni zamykają się w domu, bo chcą jeszcze pożyć, a drudzy chcą jakby korzystać z życia, a nie siedzieć w domu, bo dla nich to nie jest życie.

**Dajmy im wybór?**

No tak, bo przecież to jest ich indywidualna sprawa, to jest ich życie, tak? Nie można za człowieka decydować, jak on ma żyć. Czy on chce siedzieć w domu i żyć 5 lat, czy on chce wyjść, łazić sobie po mieście i ryzykować to, że może zachorować. Każdy podejmuje za siebie odpowiedzialność. Chce się zamknąć, to niech się zamknie i to jest jego decyzja. Ludzie mają różne potrzeby - są introwertycy, którzy będą szczęśliwi w domu i są tacy, którzy wolą zaryzykować. To jest częsty dylemat u wielu ludzi - czy poddać się operacji i mieć 50% szans na to, że przeżyją, albo żyć z chorobą i być pewnym, że się przeżyje jeszcze rok, dwa.

**Najważniejsze momenty całego okresu pandemii?**

Pamiętam sam początek bardzo, kiedy był ten szok taki tego zamknięcia, niewychodzenia, niespotykania się, zamkniecie szkół. Potem pamiętam święta, że sami je spędziliśmy i nikt się z nikim nie spotykał, tylko przez te komunikatory. Te święta były takie dziwne faktycznie i będzie się je wspominać przez lata jeszcze. No i tyle. Potem za każdym razem była taka ulga, że to już można, że to już otwarte, a już możemy to, a jeszcze coś tam.

**A takie rzeczy ważne z perspektywy kraju?**

Utkwiło mi, że 10 maja nie otworzyli mi lokali wyborczych. To mi bardzo utkwiło i myślę, że to się zapisze w annałach historii. Czegoś takiego jeszcze nie było. No i codziennie właściwie jakiś cyrk polityczny tam.
