# Supplementary material for: The challenges arising from the COVID-19 pandemic and the way people deal with them. A qualitative longitudinal study
Source: PLoS One. 2021 Oct 11;16(10):e0258133. doi: 10.1371/journal.pone.0258133 (PMC8504766; doi:10.1371/journal.pone.0258133)
Supplement: S1 Dataset — (ZIP) [file pone.0258133.s003.zip › Transcriptions/stage 6/1.6_F_25_single.docx]

**1.6_F_25_single**

**Opowiedz, jak u ciebie wyglądały te ostatnie miesiące od naszego ostatniego spotkania, co się w ogóle u ciebie działo. Wiem, że masz pracę.**

Tak. No więc w czerwcu, jak kończyłyśmy, to nie pamiętam, czy już wtedy mogłam ci powiedzieć, że coś tam się dzieje, czy dopiero to się zadziało chwilę później. Bo to bardzo szybko się działo, cały proces rekrutacyjny dość szybko działał, ja tam szybko też robiłam badania. No i szczęśliwie się tak udało też z badaniami tymi pracowymi zrobić tak, że zaczęłam w połowie czerwca. O tym, że się wyprowadzam od rodziców, to już chyba ci mówiłam.

**Tak, rozmawiałyśmy, jak już byłaś w tym mieszkaniu.**

A, no właśnie. Więc sobie tutaj mieszkam, od początku czerwca zamieszkałam, a już od piętnastego miałam pracę, więc nie było najgorzej. No i lato w mieście było całkiem przyjemne. Też zresztą jakby wiesz, wszystkie obostrzenia gdzieś tam do tego początku lipca były znoszone, trochę łatwiej się funkcjonowało . Nawet miałam ze 2 wyjazdy czy 3 w lecie. Takie oczywiście po kraju. Chociaż nie, przepraszam, na jednym z wyjazdów wyskoczyliśmy na czeską granicę, bardzo blisko. Ale wyjazd zagraniczny zaliczony. Więc lato przyjemnie, można było więcej rzeczy zrobić, wyjść. Nawet jak nie gdzieś w środku, w miejscach, to wiadomo, na zewnątrz. Więc lato w mieście było dla mnie do zniesienia. Praca jest w porządku. Bardzo mnie zasmuciło, jak się dowiedziałam, że… Bo na razie to jest półroczny kontrakt. Czyli tak naprawdę to on mi się kończy za półtora miesiąca. Jest tam jakiś teoretycznie plan, że prawdopodobnie się przedłuży. No tylko pytanie, czy rzeczywiście będę przedłużać. Trochę się rozglądam może za czymś fajniejszym. Zobaczymy, jaka będzie sytuacja. Bo szukanie tej pracy od kwietnia do czerwca zajęło trochę czasu. I miałam tyle szczęścia, że akurat trafiłam na coś. Więc całość zdalnie. No jest to bardzo obciążające, jakby to siedzenie 8 godzin, 5 dni w tygodniu przed tym komputerem w domu, i pracowanie, i mieszkanie tutaj. Więc tak jak ja lubiłam pracować z domu, bo już wcześniej mi się zdarzało, to jednak był taki układ, że byłam 2 dni w biurze a 3 z domu. I wszystko inne było otwarte, normalnie życie wyglądało. Więc gdzieś tam to nie było takie uciążliwe, że pracowałam z domu. Obecnie, kiedy wszystko trzeba robić z domu, to moją taką odskocznią od tego na pewno była joga. Na siłownię zaczęłam chodzić na tydzień przed tym, jak je zamknęli. Zebrałam się w sobie, poszłam z Multisportu na siłownię, że będę przychodzić. Chodziłam przez tydzień, byłam 3 czy 4 razy w tym tygodniu poprzedzającym zamknięcie siłowni o 7 rano. Więc wstawałam z samego rana, szłam na siłownię, żeby potem wrócić do pracy. No i tydzień po takim właśnie zmobilizowaniu się, zamknięto mi siłownię. Wczoraj napisała moja instruktorka od jogi, że jogę też muszą zamknąć. Bo jeszcze przez ten tydzień teraz joga funkcjonowała, chyba się nie kwalifikowała czy coś takiego. Ale teraz joga też już jest zamknięta. Na pewno obecnie jest to mocno dobijające, bo przy braku fajnej pogody nie zawsze ma się ochotę na ten spacer, w celu tylko spacerowania i bycia na zewnątrz, wyjść. Tak że lato minęło całkiem przyjemnie. Bo mieszkałam tu, w sumie trochę wyjazdów było. Nad morzem byliśmy w połowie sierpnia. No to tłum ludzi, wszyscy siedzieli na sobie, więc jaka pandemia, o co w ogóle chodzi, gdzie pandemia?

**Nie było pandemii?**

Nie było. To był żart, dokładnie. No wiadomo, teraz okres ten grypowy plus to, że dzieci wróciły do szkół. Dla mnie to jest jakby pierwszy czynnik, na zasadzie, dlaczego najpierw nie zamknięto szkół, tylko siłownie? Zawsze jest tak, że to przedszkola i szkoły są tym takim ośrodkiem rozprzestrzeniania się jakiejś grypy. I jak się powie, że no w przedszkolu krąży, ten chłopiec, tamta dziewczynka chora, to każdy wie, że w jakimś momencie dojdzie do rodziny jakoś ten wirus przez te dzieci. Więc ja myślę, że oprócz sezonu grypowego, no to jeszcze dołożyło się do tego w obecnej sytuacji i tej ilości zakażeń drastycznie większej niż wcześniej ta szkoła. Ja siedzę w domu. Jeżdżąc na jogę, bywałam w tramwaju, ale to na 5 minut. Mój przejazd na jogę to jest 5 minut w tramwaju. Najdłużej, kiedy bywałam w komunikacji miejskiej, no to, jeżeli jechałam do rodziców, pół godziny w pociągu. Więc też nie bywam, no poza jogą, bo na jodze akurat w małym pomieszczeniu wszyscy oddychamy bardzo głęboko i głośno przez półtorej godziny, więc jest to wybitnie zaraźliwe miejsce. Ja ani nie czuję się źle, ani nie mam utraty smaku, węchu ani innych rzeczy, więc na razie myślę, że nic mi nie jest. Ale to grono osób, które są chore, które znam, się poszerzyło. Mam znajomego z wynikami pozytywnymi, jakieś tam znane osoby, które obserwuję z wynikami pozytywnymi testów. No więc gdzieś tam przy tej ilości już to jest tak, że to dociera do nas. Nikt nie umarł z mojego kręgu znajomych czy nie był w sytuacji, żeby było naprawdę źle, ale nie miał na przykład miejsca w szpitalu. Takie historie słyszę, że ludzie gdzieś tam bardziej w naszym wieku próbują się dostać do szpitala, no i nie ma miejsc. I kolejne pytanie, co rząd robił przez te miesiące? Bo kiedy była pierwsza fala i wszyscy zostaliśmy zaskoczeni, obostrzenia, bo nic innego jakby na szybko się nie zrobi. Ale logicznym było, że przygotowaniem się na obecną sytuację, nie powinno być nakładanie kolejnych obostrzeń, które są nie do zrobienia w tym momencie, żeby gospodarka już totalnie nie zdechła. Tylko na przykład, chociaż teraz się za to zabrali, tylko trochę późno, czyli na przykład otwieranie tego szpitala polowego na Narodowym. To takie plany i takie miejsca powinny być zorganizowane wcześniej. Bo słabym punktem jest tu brak personelu medycznego i brak miejsc w szpitalach. A nie to, że obostrzenia trzeba zrobić i zamknąć wszystkich w domu. Bo nie można zamrozić życia. Przypominam sytuację z wyborami, kiedy diametralnie zmieniało się podejście do wszystkiego.

**Czyli na to można było się przygotować wcześniej. Mówiąc o tym okresie 4 miesięcy wymieniłaś kilka takich najważniejszych momentów. Jak byś miała się zastanowić, które z tych momentów dla ciebie było najważniejsze w całym tym rozwoju tej sytuacji? Najpierw to było znalezienie pracy i przeprowadzka. Później wakacje? Podczas wakacji byś wskazała, że to był podobny okres? Że to lato w mieście, to podobnie się czułaś?**

Tak. Od czerwca teraz do października to to tak. To mniej więcej te 4 miesiące, czerwiec – wrzesień podobnie, tak. To znaczy trochę mnie z takich rzeczy dobijających to już nie kwestia około pandemiczna, tylko bardziej właśnie to, że sytuacja finansowa, która jest wynikiem trochę, czyli jednak jestem bardziej w tyle niż w sytuacji, w której chciałam być przed pandemią. To, że jednak plan mi się, ten półtoraroczny studiów, bo miałam we wrześniu zaczynać studia. Więc gdzieś tam we wrześniu sobie myślałam, że w pierwotnym planie zaczynałabym teraz tą magisterkę, a nic z tego. Więc gdzieś tam moja głowa zaczęła wędrować w inne rejony, na zasadzie planowania może gdzieś indziej studiów. Bo już wiem, że nie chcę wracać do Wielkiej Brytanii z racji kosztów. Że to jednak nie jest opłacalne aż takiego kosztu finansowego ponosić, więc gdzieś tam w inne plany poszłam. Ale poza tym, no to cały gdzieś tam… Takie, jeżeli chodzi o pandemiczne względy, no to tak samo się czułam.

**Czyli dopiero teraz ta druga fala to jest dopiero taki inny moment.**

Tak, tak, tak.

**A jak byś miała się zastanowić nad swoją codziennością, to życie wróciło już całkowicie do takiego funkcjonowania sprzed pandemii? Bo jednak pracujesz cały czas zdalnie. Ale to jest dlatego, że musisz pracujesz zdalnie, bo ta firma nie funkcjonuje na miejscu przez pandemię, czy oni w ogóle zawsze pracowali zdalnie.**

Nie, nie. Znaczy mój kontrakt, ponieważ on był w czerwcu zakładamy na pół roku, czyli do grudnia, było powiedziane, że on w całości jest zdalnie. Ponieważ jest krótki, ponieważ i tak początek byłaby praca zdalna z racji tego, że nikt nie pracował w biurze, no to założyli, że w ogóle nie będą się zajmować robieniem na miejscu w biurze, bo po co, jak jesteśmy na krótki projekt. No, on się przedłuża, ale oczywiście nie było takiego planu przed nim. Wszyscy, pozostała część warszawskiego teamu pracuje z domu. Pierwsze plany były, że może od nowego roku się wróci, bo do końca roku na pewno nie. Ale z tego co wiem, to też to odpada teraz. Więc oni wszyscy nadal pracują. Ale to rozwijała się sytuacja i to było zmieniane, przesuwanie tego terminu powrotu do biura to była kwestia sytuacji. Ale dla nas to i tak by się nie zmieniało. Czyli nawet, gdybym przedłużyła umowę, to ja wciąż będę siedzieć i pracować w domu. Tylko tyle, co pewnie można by zorganizować jakieś spotkanie teamu w biurze, czy bylibyśmy zaproszeni. Bo tak to ja tak naprawdę pracuję w bardzo małym gronie, więc reszty biura nie znam w ogóle. I nie poznam ich nigdy tak naprawdę prawdopodobnie. O ile nie przedłuży mi się ta współpraca z firmą na parę miesięcy dłużej. Czego na razie nie widzę, ale jeszcze nie wiadomo. Więc no nie. Bo to jest jednak zupełnie co innego, że w ogóle nie idę do biura, że w ogóle tych ludzi nie poznaję, no to jest tak, jakbym pracowała sama ze sobą cały czas praktycznie.

**Czyli to jest też taka sytuacja, która jest inna przez pandemię.**

Tak, tak, tak.

**A jeśli chodzi o jakieś zakupy, spotkania ze znajomymi czy właśnie wyjazdy. Czy już się działy normalnie?**

Prawie normalnie, tak. Zakupy… Znaczy nie powiem, przerzuciłam się na więcej zakupów online, prawdopodobnie z wygody. Ale przestałam też kupować… Znaczy jedyną rzeczą taką, którą przed pandemią i w ogóle nie lubiłam kupować online no to były ciuchy, bo jednak wolałam je przymierzyć przed zakupem. I dopiero w pandemii się było zmuszonym ewentualnie, a potem zwracać. Ale ja w ogóle przestałam kupować ubrania obecnie.

**Ale to dlatego, że nie potrzebujesz?**

Tak. Ja mam, nie wiem, że 2-3 rzeczy w szafie, które kupiłam albo na początku pandemii albo jeszcze chwilę przed nią, na zasadzie, że to będą rzeczy do pracy. I ja ich jeszcze ani razu nie miałam na sobie. Bo nie muszę. Ja mam jednego calla dziennie, takiego półgodzinnego z trzema osobami, z którymi pracuję. To są wszystko 3 kobiety, więc też jakby… To jest tak, że jak są mężczyźni w biurze, to jednak myśli się o tym, żeby wyglądać lepiej. Trochę jest coś w tym. Ponieważ ja gadam z trzema kobietami codziennie tylko przez pół godziny, ja się ani nie maluję, zakładam to samo codziennie do momentu aż trzeba wrzucić do prania. Chodzę w wygodnych ciuchach, ciepłych ciuchach. Spodni w ogóle czasami nie trzeba mieć. Zakładam je tylko, jeżeli jest mi naprawdę, nie wiem, zimno, bo otworzyłam okno, żeby przewietrzyć. Więc przestałam kupować ubrania. Co jest akurat przyjemne, że nie muszę wydawać na nie pieniędzy. To jest akurat całkiem pozytywny aspekt, że nie mam takiego parcia na kupowanie ciuchów. I ponieważ to mi odpadło, no to mogę zamawiać większość rzeczy online. I do sklepu myślę, żeby pójść tylko, jeżeli potrzebuję czegoś na cito, na zasadzie, że nie chcę czekać na tą przesyłkę, bo to jednak zawsze zajmuje trochę czasu, tylko potrzebuję czegoś na już, na jutro, to wtedy się wybieram do sklepu ewentualnie. Albo żeby, nie wiem, zakupy były rozrywką.

**A zakupy spożywcze?**

Ze spożywczymi przerzuciłam się na zamawianie. Bo wypróbowałam to raz. I na zasadzie takich cięższych rzeczy, kartony soku, kartony mleka, takie ciężkie rzeczy, których nie chce mi się nosić do domu. Ale nie warzywa i owoce. A warzywa i owoce zaczęłam kupować na, mieszkam 2 przystanki od Hali Mirowskiej, więc po warzywa chodzę sobie tam. Bo w tych sklepach oni pakują te warzywa w plastik. Więc chciałam unikać kupowania takich warzyw z plastiku. Ja sobie chodzę z moimi torbami na targ i warzywa, owoce na targu. Ale wszystkie inne jakieś takie produkty pakowane, jakieś ciężkie właśnie napoje zaczęłyśmy ze współlokatorką zamawiać sobie online. I to wtedy przyjeżdża. Przy pierwszym zamówieniu dostałam kupon na kilka następnych zakupów, żeby dostawa była za darmo. Więc przerzuciłam się trochę na zakupy też takie online. Ale powiedzmy, że jakieś takie, nie wiem, coś drobnego po drodze do domu czy tam coś na już, no to takie drobne zakupy i na targu.

**A myślisz, że z tym online to będzie taka zmiana trwała? Czy jednak brakuje ci trochę takich normalnych zakupów stacjonarnych?**

Nie. Bo to bardziej też było na zasadzie wygody, że zawsze jak muszę iść po te ciężkie rzeczy, to muszę je potem donieść do domu. A tutaj przyjeżdża mi z nimi pod drzwi. Więc w kwestii kupowania takich cięższych rzeczy, których nie chce mi się tachać do domu, to myślę, że to będzie… Zależy właśnie od tego, czy będę miała jakiś kupon na darmową dostawę czy nie, czy tej darmowej dostawy już nie będę miała. Bo tam jest tak, że chyba od jakiejś kwoty jest darmowa dostawa, w stylu 250 zł. My się dobieramy z moją współlokatorką na zamówienie, więc nie jest tak, że ja muszę wydać całą kwotę, tylko zbieramy ją razem. No ale jakbym miała zostać z tym sama, albo wypadałoby, że ja chcę akurat zakupy a ona nie i ja coś potrzebuję, to też zrezygnuję z tego, bo wtedy podnoszę koszt tych zakupów dostawą. Tylko wtedy sobie pewnie pójdę kupić te potrzebne rzeczy sama. Więc tak bardziej na zasadzie, jak będzie darmowa dostawa to tak, bo to nie podnosi jakby całego kosztu zakupów. Dlatego, że próbowałam sobie porównać te ceny i ceny produktów są w porządku. Nie wszystkich, ale generalnie te, co chciałam, nie są jakieś droższe, wychodzi na to samo. Ale kiedy trzeba dodać dostawę, jej koszt, no to już wolę pójść i kupić w sklepie za tą cenę i nie płacić za dostawę. Może to są jakieś grosze w podzieleniu na każdy produkt. Ale zawsze jest to bardziej oszczędne myślenie.

**Oszczędne i wygodne. A jak jest z tymi spotkaniami z ludźmi? Jak to u ciebie teraz wygląda?**

Przez lato było na pewno normalnie, przez lato wszyscy gdzieś tam odtajali. Mam taką jedną osobę, która jest bardzo zafiksowana na tym strachu przed pandemią i chorowaniem. Z którą widziałam się tylko raz przez całe wakacje. I jakby ona widzę, że unika kontaktu, chęci spotykania się. Oczywiście nie wiem, czy to jest tylko kwestia strachu przed zarażeniem czy może nie ma ochoty się ze mną widzieć, bo tak też może być. Ale jedną mam taką chyba, przy której mogłabym o tym pomyśleć. A reszta znajomych to tak normalnie, w zależności od tego, kto ma czas i kiedy. No ostatnio na razie nikt się nie wycofywał od spotkań z powodu pandemii, tym bardziej w obecnej sytuacji, gdzie są tłumy na ulicach. To jest bardziej kwestia tego, że… Ponieważ ludzie trochę mieli inny plan i im się nie zburzył plan życiowy… Ja mam tylko pracę. Jak teraz mi zabrali jogę i gdzieś tam siłownię, no to może biegałam trochę, ale staram się unikać biegania ze względu na problemy z kolanami. Dlatego bardziej wolałam joga, siłownia. Ja nie mam nic poza pracą do roboty powiedzmy. To jest bardziej kwestia tego, że wszyscy są trochę bardziej zajęci jeszcze innymi swoimi obowiązkami czy planami. A ja nie taki miałam plan, więc no nie do końca. A też od kilku miesięcy staram się wykombinować, znaleźć jakiś sposób takiej pracy dorywczej albo czegoś, co pozwoli mi jeszcze trochę dorobić, bo ten tryb oszczędnościowy mam troszeczkę wyższy z racji czterech i pół miesiąca nie pracowania przez pandemię, żeby poprawić sytuację finansową. I tak właśnie nie mogę wyjeżdżać za bardzo za granicę, robić żadnych bardzo kosztownych rzeczy na razie nie będę, więc musiałam się skupić na tym oszczędzaniu. Tylko jest trudno. Z taką pracą na pełen etat ciężko znaleźć, też oczywiście z sytuacją na rynku pracy, ciężko było znaleźć coś dodatkowego.

**A masz takie poczucie, że teraz jakby sytuacja wróciła do takiego stanu jak to było w marcu, kwietniu, na początku lockdownu? Czy czujesz, że to jest inny etap, inny moment?**

Inny etap z tego względu, że no wtedy… To był potwornie stresujący czas w Londynie dla mnie i powrót z Londynu. To jest jedno. I całe w ogóle to, że musiałam się pogodzić z faktem, że coś nie poszło, jakby bardzo mocno nie poszło wobec mojego planu. Na szczęście obecnie nie burzy mi to planów zbytnio poza chodzeniem na jogę i na siłownię. Więc to już tak mnie nie dotyka. I uspokajające jest to, że mam pracę. Nie stresuję się tym, że jej nie mam, tak jak wtedy. I dostaję też już propozycję następnych. Więc właśnie też a propos pracy i ewentualnego przedłużania umowy, no to dziś miałam rozmowę z inną firmą o pracę. No więc to jakby trochę jest inny nastrój z racji tego, że nie jestem u rodziców, mam pracę i ze względu na to, że następną też uda się spokojnie znaleźć pomimo sytuacji.

**A jak to wygląda u twoich bliskich, osób z twojego otoczenia? Czy oni funkcjonują teraz tak jak przed pandemią czy gdzieś tam wracają do takiego trybu zdalnego bardziej jak podczas tego lockdownu?**

Nie, no myślę, że większość albo cały czas miała zdalną. Bo takie duże firmy, korporacje z biurami no nie wróciły przez cały czas do biur. Jakby wszystkie te firmy, miałam jedną przyjaciółkę, w której kancelarii, ona najpierw miała pół na pół, a od jakiegoś czasu była w biurze, już pracowała. Ale na przykład teraz napisała mi, że przy sytuacji zrobili jej znowu pół na pół. Czyli tydzień w biurze, tydzień w domu. Ale to jest jedna sytuacja taka. Więc albo było tak samo przez całą pandemię, czyli z domu, albo wciąż jakby funkcjonuje wszystko, tak jak było. Moja mama, póki co chodzi jeszcze do szkoły. Bo do wakacji nie chodzili do szkoły, potem miała wakacje, wiadomo. A teraz jeszcze chodzą do szkoły, ale to też pytanie, kiedy właśnie…

**Czyli te klasy 1-3, tak?**

Nie, moja mama akurat powyżej, czyli tam 4 w górę, tylko że szkoła specjalna. A szkoły specjalne podchodzą pod jakieś inne rozporządzenia. I z niewiadomych powodów, jeżeli jest rozporządzenie, że klasy 4 w górę normalnych szkół masowych nie idą, to to nie dotyczy szkół specjalnych. Na jakiej zasadzie to jest podejmowane, to chyba też trochę bardziej zależy wtedy od dyrektora szkoły. Dlatego jeszcze chodzą. Tylko pytanie jak długo. Problem jest taki, że szkoła mojej mamy w ogóle jest nieprzygotowana na pracę zdalna. A w przypadku szkoły specjalnej i dzieci głuchych no to jest to dość istotne. Tak że tam jeszcze nie wiadomo, jak będzie.

**A co tobie teraz najbardziej w tej sytuacji przeszkadza? Tej spowodowanej pandemią.**

Na pewno siłownia i joga zamknięta, bo to był taki mój stały punkt programu w tygodniu.

**Masz jakąś alternatywę dla tego?**

Ćwiczenia tutaj w pokoju. Jeszcze problem dla mnie jest taki, że dla mnie pójście w miejsce jest motywujące. Ja mam duże problemy ze zmotywowaniem się sama sobie tutaj w pokoju. I dlatego zawsze wolałam iść na zajęcia jogi do tego studia jogi, gdzie chodziłam. Tak samo z siłownią. Dzisiaj właśnie mam otwartą zakładkę takich, są te platformy z treningami. Jak to nie będzie jakoś kosztowne albo będzie to, bo niektóre z nich można podpiąć pod Multisport, sprawdzę sobie te platformy online, czy to jakoś tam na mnie działa. Tylko znam siebie i wiem, że jak nikt nie patrzy i nie ma nikogo dookoła, to niestety z tą motywacją i takim… No więc powiedzmy, że mam plan uskuteczniać jogę w domu. Podejrzewam, że jak połowę planu swojego zrealizuję, to będzie dobrze.

**Czyli tak naprawdę głównie ta kwestia ćwiczeń.**

Tak. No i trochę restauracji czy jakichś kawiarni. Bo sobie właśnie myślałam, że mogłabym sobie ustalać jakieś dni w tygodniu, kiedy brałabym książkę. Chcę zmotywować się też do czytania książki. Bo czasami w domu jest mi się ciężko zmotywować, żeby tą książkę wziąć i poczytać. Więc miałam plan, żeby wziąć książkę, iść do jakiejś konkretnej fajnej kawiarni albo jakiejś innej, której jeszcze nie znam, usiąść tam, poczytać. No nie zrobię tego teraz, żeby to był sposób na wyjście z domu, bycie w innym otoczeniu, wypicie tam dobrej herbaty, kawy. I gdzieś tam ileś czasu przeznaczenie sobie na właśnie czytanie danej lektury. I może to jeszcze, jest teraz też niemożliwe.

**(Emocje – zdjęcia). Najpierw wspomniałaś o przeprowadzce, rozpoczęciu pracy, to był początek nowego etapu, później lat w mieście. I teraz to nadejście drugiej fali.**

Myślę, że przeprowadzkę i lato w mieście razem można by gdzieś tam podpiąć pod 13 i 6. Czyli właśnie samo lato i słońce, gdzie ja jestem raczej fanką bardziej właśnie późnej wiosny i lata jako moich ulubionych pór roku i tego czasu, kiedy lepiej się czuję. I takiego spokoju, że coś wraca na takie normalne tory, zaczyna się praca. Więc sytuacja finansowa troszkę się ustabilizuje. Więc 6 i 13 na zasadzie słonecznej, przyjemniejszej pogody co też wzmaga przyjemny nastrój i lepszy humor. I większego uspokojenia, jeżeli chodzi właśnie o myśl o przyszłości. Bo zanim tę pracę miałam, to raczej był to niepokój w tej kwestii. A obecnie to jest bardziej 9. Też jest różnica, gdybyś mnie pytała tydzień temu, jeżeli chodzi o teraz rozpoczęcie się tej drugiej fali a jak pytasz mnie teraz. Bo jeszcze tydzień temu to nie byłoby dla mnie aż tak stresujące, to było bardziej na zasadzie no jestem niezadowolona z powodu obostrzeń, bo mi uniemożliwiają chodzenie na zajęcia sportowe. Ale obecna sytuacja mnie jeszcze bardziej gdzieś wytrąciła z równowagi. To jest trochę połączone z moimi rodzicami, którzy, chyba już wspominałam, że są fanami teorii różnych spiskowych. No i oni mają różne ponure scenariusze, którymi nas raczą z bratem. A teraz spędziłam kilka dni, właśnie tych ostatnich od czwartku, w domu. Więc słuchając rodziców i widząc, co się dzieje, tak jak jeszcze tydzień temu te pomysły rodziców to bym tak skwitowała na zasadzie, dobrze, to wy sobie tak myślcie. I nie przerażałyby mnie aż tak bardzo. To widząc obecną sytuację, te wizje moich rodziców robią się trochę bardziej prawdopodobne. Wciąż są bardzo abstrakcyjne, ale niektóre z tych rzeczy robią się bardziej prawdopodobne. I zaczęły mnie trochę bardziej stresować. Bo gdyby to miał być tylko lockdown na zasadzie taki, jaki już mieliśmy, to nie byłoby to pozytywne wydarzenie. Ale jest to coś, co już znamy i jesteśmy z tym oswojeni, to byłoby do… Nie wiem, do przetrzymania, nazwijmy to. Ale w momencie, kiedy dzieje się to, co się działo wczoraj i jeszcze nie wiadomo, jak się będzie sytuacja rozwijała, to zaczyna to być coś nowego.

**Tu masz na myśli głównie protesty?**

Protesty, tak.

**A jeśli chodzi o te wizje twoich rodziców, to czego oni dotyczą?**

Moi rodzice wróżą jeden ze stanów wyjątkowych. Czyli wprowadzenie na przykład stanu wojennego. Bo jednak rozmowy o stanie nadzwyczajnym były prowadzone przy okazji trochę wyborów, przed tymi wyborami. I już zaczynały się rozmowy o tym, czy w przypadku pandemii wprowadzać któryś ze stanów nadzwyczajnych. Te dyskusje na temat stanu klęski żywiołowej itd. Obecna sytuacja w kraju jakby trochę bardziej wydaje mi się realny scenariusz stanu wojennego. Bo jakby obecnie to już wszystko jest w stanie się wydarzyć, z tym, co się wydarzyło w zeszłym tygodniu. Przynajmniej z mojego punktu widzenia. I to mnie wprowadziło w taki wręcz paniczny nastrój, takie ataki paniki w ostatni weekend. Gdzie u mnie to jest naprawdę rzadkie i bardzo mi się rzadko zdarzają takie rzeczy. Więc może nawet nie sam początek pandemii i większa ilość zakażeń, bo to będzie skutkowało jakimiś tam obostrzeniami i trudnościami. I tym, że może będzie się ciężej dostać do lekarza. Aczkolwiek no mam prywatną opiekę w celach innych niż koronawirus. Więc gdzieś tam mi to nie przeszkadza aż tak i byłabym to w stanie przetrwać. No tak inne już scenariusze typu jakieś… Znaczy moi rodzice mają jeszcze inne różne, dziwne wizje. Na przykład zamówili rolety antywłamaniowe na okna w domu i kupili dla nas wszystkich maski gazowe. Więc jakby… To są bardzo abstrakcyjne różne wizje. I mówię, normalnie skwitowałabym takim… My z bratem tak popatrzymy na siebie porozumiewawczo i z takim pobłażaniem, słuchając rodziców. Ale część z tego, co oni mówią, zaczyna dla mnie być bardziej realne w obecnej sytuacji.

**I to, jak powiedziałaś, to jest potęgowane, tak naprawdę głównie przez sytuację związaną z protestami, niekoniecznie przez samą pandemię.**

Tak, tak. Bo gdyby to była sama ta sytuacja, to myślę, że byłoby to za mało. Ale sytuacja polityczna obecnie już byłaby, uważam, pretekstem do tego, żeby za chwilę jednak wprowadzili stan wojenny. Bo w obecnym momencie nie zdziwiłoby mnie to. Bo to byłby jedyny sposób, żeby ludzi spacyfikować i żeby ludzie nie wychodzili.

**Wspominałaś o takich wręcz atakach paniki. Jeśli byś miała powiedzieć, że czujesz się zagrożona sytuacją, to zagrożona bardziej w związku z sytuacją społeczno-polityczną, a nie z samą pandemią?**

Tak. Nie boję się, broń boże, samego zachorowania. Bo tak jak mówię, moje podejście do tego, jak ja bym przechodziła chorobę, jest takie samo. Więc to się nie zmieniło. Połączenie obu sytuacji zaczęło być mocno stresujące.

**A robisz coś z tym, żeby sobie poradzić z tym stresem?**

Miałam takie poczucie, że tak jak normalnie gdzieś tam po prostu rozmowa z kimś bliskim byłaby mi potrzebna. Z racji tego, że tutaj się z mamą różnimy trochę poglądami, nie bardzo miałam w niej oparcie i nie czułam, że mogę z nią porozmawiać obecnie. Więc no gdzieś tam w niedzielę, wracając już od rodziców, musiałam się koniecznie spotkać z przyjaciółką, do której pojechałam wieczorem, żeby tylko chwilę się z nią zobaczyć. Bo potrzebowałam się komuś wygadać. Więc no spotkania z ludźmi. Różnie z tym bywa, bo niektórzy są zajęci albo się boją, albo nie wiem, tam ktoś jest na kwarantannie. Więc myślę, że najbardziej właśnie spotkania i rozmowa z przyjaciółmi. Ale poza tym to jest raczej… Nie, nie mam. Bo to jest dla mnie naprawdę obcy stan. I bardzo rzadko się tak czuję. Więc nie mam jeszcze jakichś takich sprawdzonych sposobów.

**A jak twoi bliscy? Jak w ogóle oni odczuwają tę sytuację? Czy jest ktoś, kto reaguje podobnie do ciebie, czy oni reagują inaczej?**

Nie, no myślę, że nie. Moi rodzice robią w domu bunkier, więc jak by rzeczywiście coś się działo, to ja przynajmniej wiem, że mam miejsce, które jest naprawdę nieźle zabezpieczone, zapasy żywności itd. Więc mam gdzie się chować, jak coś. Tylko wolałabym, żeby do tego nie dochodziło. Bo też gdzieś będąc w domu przez dłuższy czas miałam poczucie, że dobrze, że ja jednak nie mieszkam z rodzicami. Więc wizja dłuższego pobytu z nimi w domu z przymusu nie widzi mi się jakoś bardzo. Nie, no oni podchodzą do tego raczej na zasadzie takiej dziwnej ekscytacji. Bo to nie jest tak, że oni podchodzą do tych planów i wizji z przerażeniem, tylko… Nadzieją na to, że to ma odmienić to, jak świat obecnie wygląda. Że to ma być zmiana na lepsze generalnie później. Więc nie ma strachu w tych ich wizjach. Oni podchodzą do tego na zasadzie, szykujemy się i będziemy po prostu gotowi. No mój brat tak jak ja, pobłażliwie podchodzi do tych ich opowieści i raczej go to nie stresuje aż tak. Mnie to o tyle też stresuje, że ja mam ten plan, czy gdzieś tam rodzą mi się te plany kolejne wyjazdowe. Więc dla mnie wizja tego, że będzie jakiś kolejny rok, że ja wciąż nie będę mogła wyjechać na studia w tym przypadku, myślę tak o studiach. Trochę też pojawia się opcja, że mogłabym może w obecnej firmie być przeniesiona do biura w Amsterdamie. I gdzieś tam takie plany wyjazdowe do pracy na przykład na początku przyszłego roku bądź po wakacjach następnych może jakieś studia magisterskie właśnie w Holandii, pod tym kątem zaczęłam myśleć. Bo są też trochę tańsze niż w wielkiej Brytanii. I jakby to bardzo burzy mnie ten plan. Więc to mnie wytrąca o tyle z równowagi, że mi to mocno zaburza plany życiowe. I ja cały czas tkwię w sytuacji, z której nie jestem zadowolona, która miała być inna. I no męczy mnie ona, bo jest to już 10 miesięcy innej sytuacji. Jak to miałby być kolejny rok, gdzie ja gdzieś od trzech lat powiedzmy od powrotu z podróży, to chyba wspominałam, że no nie byłam w tym miejscu, w którym chciałam. I wyjazd do Londynu miał być tym początkiem zmiany mojej sytuacji i zostało to wszystko… Więc wizje, że miałyby się dziać naprawdę poważne rzecz, które nas by uziemiły i byłyby to daleko bardziej idące zmiany niż sama pandemia, no mocno mnie też wytrącają z równowagi. Na zasadzie ja już nie mam siły siedzieć w miejscu, ja potrzebuję iść dalej. Bo bardzo, bo też z moją osobowością mnie to męczy. Ale to się łączy z poznawaniem nowych ludzi, właśnie wyjeżdżaniem, mieszkaniem w innych miejscach, bo taki był mój plan. A ja od 10 miesięcy zamiast być w nowym miejscu i poznawać nowych ludzi, siedzę w domu i pracuję zdalnie przez 5 dni w tygodniu.

**A czy u twoich znajomych widzisz jakieś sposoby radzenia sobie z tą sytuacją, oni znaleźli jakieś sposoby odreagowania, jakieś inne nawyki? Oni jakoś się zabezpieczają?**

Nie, znaczy jakby mówię, to są tylko wizje moich rodziców, więc moi znajomi broń boże nie wiedzą o takich rzeczach. Nie, wątpię. Nie znam też nikogo, kto miałby aż tak zmieniony plan jak ja. Bo jednak ludziom się pozmieniały planów wakacyjnych, urlopowych. A nie życiowych, aż tak bardzo jak moje. Więc no nie.

**A ta znajoma, która unika spotkań, ona faktycznie nie wychodzi z domu?**

Ja wiem, że ona pracowała do lipca. I w lipcu wiem, że… Znaczy ja nie wiem, ponieważ nie mamy kontaktu. Ja też widząc jej taką oschłość w kontakcie, jest to efekt iluś takich miesięcy naszej takiej… może trochę odsuwania się w relacji? Nie wiem. Jeszcze sprzed pandemii. Więc ja przestałam się dopytywać. Ale z tego, co wiem, no ona jest typem introwertyka. Więc ona była w lockdownie przeszczęśliwa, że nie musi nigdzie wychodzić. Nie ma tak zwanej presji społecznej, żeby się spotykać, więc introwertyk siedzi szczęśliwy w domu. No ja mam odwrotnie. Ja umieram w takiej sytuacji. Tak że ja nie wiem, gdzie ona pracuje. Bo nawet jak pytałam w wiadomościach, to nie dostałam odpowiedzi. Więc nie wiem, czy pracuje czy co robi. Wiem, że pracuje jej chłopak, więc prawdopodobnie to jest jakieś ich źródło utrzymania. Więc podejrzewam, że raczej zamknęła się i nie za bardzo wychodzi.

**Ale uważasz, że taki strach przed tym zachorowaniem to jest uzasadniony, to jest racjonalny?**

W naszym wieku uważam, że nie do końca. Jak by jeszcze miała, nie wiem, często widywała się ze swoimi dziadkami, ale ma ich daleko i nie widuje się z dziadkami, więc… Więc dla mnie jest on troszkę przesadzony i śmieszny w naszym wieku. Bo nawet jeżeli miałaby zachorować, no to… Nawet uważam, że może lepiej byłoby to przechorować i potem mieć na to papier, co pewnie ułatwi podróżowanie, jak już się ma przechorowanego koronawirusa, bo nie trzeba będzie się szczepić. Tak jak niektóre kraje, już chyba Francja wprowadziła obowiązek szczepienia czy testów? Już nie pamiętam. To papier, że się przechorowało, będzie zwalniał na przykład z tego szczepienia, żeby gdzieś pojechać.

**Ale to tak ma być? Że zaświadczenie o przebyciu choroby będzie zwalniać z tego?**

Znaczy nie wiem. To się pewnie okaże, kiedy będą jakieś badania prowadzone. Różne takie są historie. Gdzieś tam jak czytałam jakieś te scenariusze, w stylu, że teraz w podróży różne kraje mogą wprowadzić obowiązek właśnie okazania testu. Co już się dzieje. Na przykład w ostatnich tygodniach jak się leciało do Grecji, to trzeba było mieć zrobiony test. Wiem, że właśnie we Francji to w ogóle będzie szczepienie. Ja nie jestem pewna, to są takie moje przypuszczenia, bo są o tym rozmowy. Ale nigdzie nie ma nic na pewno. To się na pewno będzie zmieniało pod względem wszelakich badań, które się będą ukazywały. I pytanie, kiedy będzie szczepionka. Wiem, że pobiera się tą krew od ozdrowieńców. I na tej bazie mają być prowadzone testy ewentualnych leków czy jakiegoś leczenia. Więc to wszystko pewnie będzie zależało od tych wyników, tej sytuacji, jeżeli chodzi o leki i szczepionkę. Ale tak czy siak myślę, że po prostu bycie ozdrowieńcem jest pozytywne i jest jakby lepszym pomysłem. Pytanie, czy ja nawet będę wiedziała, czy przechorowywałam. Bo nie zdziwiłabym się, gdyby się okazało, że już gdzieś tam wirusa miałam w organizmie i mam go zwalczonego. Ale po prostu o tym nie wiedziałam, bo po prostu nie miałam żadnych objawów.

**Planujesz sobie zrobić test na przeciwciała?**

Chciałam. Ja nie patrzyłam, ile one teraz kosztują. Pamiętam, że jeszcze w poprzednim lockdownie, w takiej gazetce naszej gminy, którą moi rodzice gdzieś tam regularnie do domu przynoszą, było, to był chyba właśnie kwiecień jeszcze albo maj, no jakoś tam początek, było ogłoszenie, że każdy, kto wracał z zagranicy czy był tam w obszarach czy miał kontakt z osobą zakażoną, jakakolwiek taka osoba, która przebywała na kwarantannie, no a ja byłam na kwarantannie po powrocie z zagranicy wtedy w marcu, kwietniu, że może nieodpłatnie właśnie mieć test na koronawirusa i test na przeciwciała. Ale nie zrobiłam tego. Nie wiem, czy tak nadal by to wyglądało, pewnie w obecnej sytuacji może być trochę ciężej się do nich dostać. Zależy, jeżeli to trzeba byłoby zrobić prywatnie, czy to kosztuje, czy można zrobić badanie na przeciwciała… Na przeciwciała bym zrobiłam. Bo gdybym wiedziała, że ja mam to, to oddawałabym krew. A dwa… No, wolałabym wiedzieć, że jeżeli już przeszłam, to przeszłam. Ja byłam oddawać krew we wrześniu. Tylko tak normalnie, regularnie. Ale oni chyba nie robią przy tej okazji testu koronawirusa, robią te wszystkie pozostałe. No, ale mówię, ja czułam się cały czas dobrze, nie miałam żadnych przesłanek, żeby… Miałam problemy z zatokami na początku sierpnia, przy okazji moich urodzin to było. Jakiś taki był chłodniejszy wieczór, w który się zgrzałam, będąc na takim szybkim marszu, szybkim spacerze wieczorem. Było chłodno, byłam za cienko ubrana, wróciłam do domu i napiłam się lodowatej wody z lodówki. Następnego dnia obudziłam się z bólem gardła i to tam przeszło mi w zatoki. Ale to wyglądało na takie klasyczne nieodpowiednie ubranie się plus nieodpowiednie picie, żadnych duszności. Więc poza tym przez cały ten okres nic mi się nie działo, nie czułam się źle.

**Porozmawiajmy trochę o obecnych ograniczeniach, obostrzeniach. Ostatnio zostały wprowadzone nowe obostrzenia. O jakich zmianach słyszałaś?**

Ja zawsze dostaję ten alert RCB SMS-em. I nie wiem, na czym to polega, że ja dostaję go zawsze, czasami nawet 2 dziennie. A mam znajomych, gdzie mówię, ja dostałam alert, tam opowiadam, co w nim jest, a te osoby w ogóle go nie dostają, więc nie wiem, na czym to polega. No więc zawsze wchodzę na tą stronę (niezrozumiałe), gdzie tam jest dokładnie napisane. No i co, nosimy maseczki na zewnątrz i wszędzie teraz. Chociaż to jest trochę śmieszne, żeby w każdym miejscu na dworze te maseczki mieć. Rozumiem oczywiście to jak było w pojazdach komunikacji miejskiej, sklepach, tak jak było. Zamknięte siłownie, teraz moją jogę też mi zamknęli od wczoraj. Restauracje najpierw do którejś godziny były otwarte, a teraz tylko na dowóz albo na wynos. Co jeszcze… No wiem, że w kinach i takich miejscach z widownią, no nie zamknęli tej kultury z powrotem na amen, tylko 25% miejsc, jakby 25% obłożenia. No znowu się tam zmniejszyła ilość w tramwajach i autobusach teoretycznych miejsc. Ale kto mógłby, byłby w stanie policzyć, ile osób jest w tramwaju? Aha, jestem dwudziestą szóstą osobą, to nie wsiadam. No jakby… Jest to śmieszne. Ja nie we wszystkie, bo tam są zakładki, jeżeli chodzi o różne dziedziny. No to wiem, że wydarzenia sportowe bez widowni. Chyba tyle pamiętam. Bo przeglądałam tak pobieżnie, na zasadzie co można, czego nie można, co będzie zamknięte.

**Co w ogóle myślisz o tych ograniczeniach? Bo wspominałaś, że te maseczki to jest nie bardzo, żeby w każdym miejscu na zewnątrz je nosić.**

Nie widzę powodu, dla którego ja na chodniku, gdzie czasami wychodzę z domu i zanim tam jakiegoś człowieka spotkam w ogóle na chodniku, no to mija kilka minut. I ja zazwyczaj wychodzę bez maseczki a zakładam ją dopiero, jak dochodzę do skrzyżowania, gdzie czekam z kimś na światłach, a potem idę do tramwaju zaraz. No i w sklepach wiadomo, w każdym budynku. Ale na zewnątrz cyrkulacja powietrza jest na tyle duża, że to trochę nie ma sensu. Oczywiście, to trzeba by pytać wirusologa, jest ileś teorii, każdy ma swoją o tym, jak to się rozprzestrzenia. Było ustalone te półtora metra, bazując na jakichś tam innych wirusach. Ale pytanie, czy rzeczywiście ten konkretny wirus jest w stanie się na większą odległość przenosić czy nie. Tego nie wiadomo. Jakby w ogóle za dużo rzeczy nie wiadomo, żeby cokolwiek w ogóle orzekać. A więc tak, maseczki to jest… Zresztą, no wiadomo, nie każdy zmienia je co chwila, to jest czasami ta sama maseczka przez cały dzień… Pojawiło się znowu w niektórych sklepach, że rękawiczki chcą z powrotem. Ale te rękawiczki to od początku też była głupota. Bo to, że mam rękawiczkę, nie znaczy, że nie podrapię się nosie albo nie włożę jej do oka nie myśląc, a potem czegoś dotknę. Więc nie chodzi o rękawiczkę, tylko ewentualnie… Ja pamiętam, że jak czepiali się, że nie noszę rękawiczek w sklepie, zakładałam rękawiczki i dezynfekowałam rękawiczki, a nie ręce. Co też miało chronić skórę przed tymi płynami. Bo ja może nie mam aż takich problemów. Ale znam osoby, które mają problemy skórne. I od używania tych płynów, które są jakimiś totalnie pewnie masowo produkowanymi płynami do dezynfekcji powierzchni płaskich, w tych wszystkich sklepach są rozcieńczone albo nawet nie, w tych wielkich butlach. No to nie są te żele antybakteryjne, zapachowe, które można było kupić wcześniej w aptekach. Co jeszcze…

**A co sądzisz o tych restauracjach, że teraz są znowu zamknięte i tylko na wynos?**

No tutaj jeszcze widzę jakiś sens taki, że no jednak restauracje były miejscem, w których trzeba było zdjąć maseczkę, bo nie można jeść z tą maseczką. I to były sytuacje potencjalnie większego zagrożenia naplucia jednak tych drobinek w oddychaniu, mówieniu, jedzeniu, żeby się wydostawały. Więc na pewno jest to tragiczne dla branży gastronomicznej. Ale tutaj jeszcze widzę jakikolwiek sens.

**A jeśli chodzi o szkoły? O to, że te klasy do trzeciej chodzą, a powyżej trzeciej już nie?**

Nie wiadomo, ile dzieci roznoszą ten wirus. Nawet, jak jakieś chorują i mają objawy, albo nie mają, no to jednak… Jeżeli to dziecko przechodzi bezobjawowo, no to to dziecko jest nosicielem i roznosi po rodzinie i wszystkich innych. Każdy, kto myślał, że dzieci będą przestrzegały wszystkich tych zasad, tych regulaminów, które tam im się dawało na początku roku szkolnego, z tymi maseczkami, ja słyszałam historię, że przychodzi Jasiu do domu i: Jasiu, a co ty masz za maseczkę, to nie jest twoja maseczka. No tak, wymieniłem się z Krzysiem. No jakby nie można tego odczekiwać od dzieci. I po to były całe wakacje, żeby przygotować te wszystkie miejsca. Oczywiście, rodzicom, którzy teraz muszą z dziećmi coś zrobić, będzie trudniej. Ale jak już mamy coś utrudniać, no to zostawiając dzieci w domu, żeby nie nosiło się to wszystko przez szkoły. Ja potem słyszę, ale chorują tylko nauczyciele. No tak, bo nauczyciele, u człowieka dorosłego objawy się pojawiają, u dziecka nie. Ale to, że nauczyciel to ma, może być kwestią tego, że dziecko mu to przyniosło skądinąd. Z domu czy od innych dorosłych.

**Czyli byłabyś raczej za tym, żeby szkoły zamknąć, skoro wszystko jest zamknięte, to szkoły też?**

Tak. Jakby pomijając wf wszystkie inne zajęcia są możliwe do przeprowadzenia zdalnie, jest to tylko teoria. Jest to trudne i ja jestem przekonana, że oświata nie jest do tego przygotowana odpowiednio. Ale na to były wakacje, żeby chociaż próbować jakoś tę sytuację gdzieś tam polepszyć. No więc uważam, że to było zaniedbane i było totalnie bez sensu pomysłem, żeby dzieci wracały do szkoły. Jeszcze pytanie, na ile to jest możliwe, żeby wszystkim umożliwić tę naukę zdalna. Bo w szkole u mojej mamy jest to duży problem. W szkole specjalnej. Ale szkoła specjalna, oni mają też troszeczkę mniejszą ilość uczniów. To nie jest masówka, to jest zupełnie inna proporcja, jeśli chodzi o ilość. Wielu uczniów też mieszka w internacie w szkole. Więc oni nie chodzą do domu, tylko oni wszyscy mieszkają w internacie i chodzą do szkoły. Więc trochę bardziej siedzą we własnym sosie, nie roznosi się to aż tak bardzo. Jak w szkołach masowych, gdzie każde dziecko idzie do domu i potem wraca. Te dzieci jeżdżą autobusami, te dzieci jeżdżą tramwajami, więc jakby wszędzie jest większy tłum przez to, tak?

**A co sądzisz o tym ograniczeniu dla osób starszych, ograniczenie wychodzenia z domu? Mówię ograniczenie, bo sama nie jestem do końca pewna, czy to jest zakaz czy rekomendacja, żeby nie wychodzić?**

Chyba to jest rekomendacja, bo wciąż, tak jak z maseczkami tam te mandaty chyba dostają, ale żaden z tych mandatów teoretycznie nie jest zgodny z konstytucją, więc wszystkie mandaty o nienoszenie maseczki można potem obalić i jest to wycofywane. No to chyba w kwestii powiedzenia starszej pani, niech pani siedzi w domu i jak ta pani wyjdzie, no to nie można jej za to ukarać. To też mocno uderza w konstytucję, podejrzewam (śmiech). Więc to chyba jest tylko zalecenie. No nie wiem, moja babcia ma 84 lata i od kwietnia… W kwietniu siedziała w domu. Ale pod koniec kwietnia, w maju już chodziła do sklepu. I chodzi do tej pory, nic jej nie jest. Więc to też zależy od organizmu, chorób współistniejących. Jak ktoś się bardziej boi o swoje zdrowie, na pewno lepiej, żeby został w domu. Nie powiem, mniejszy tłok w tramwajach, autobusach jak ludzie starsi siedzą w domu. Ale nie wiem, nie znam też statystyk z naszych szpitali, na ile ci ludzie, którzy tam zajmują te wszystkie łóżka i brak miejsc, to są ludzie starsi, młodsi, w średnim wieku. Na pewno jest większe zagrożenie, że te osoby będą potrzebowały więcej opieki i ewentualnego szpitala czy respiratora. Więc pewnie lepiej by było, gdyby zostali w domu. Ale mówię, nie jestem wirusologiem, znawcą ani lekarzem, ale sytuacja we Włoszech z marca, no to jest kwestia starszego społeczeństwa u nich. I też średnio odpornego społeczeństwa, które je pizzę i makarony cały czas, tak? Oczywiście mówię, to jest tak w mocnym takim uproszczeniu. Ale na pewno ich dieta jest mniej zdrowa niż, nie wiem, skandynawska. I organizmy Włochów są na pewno mniej odporne niż Skandynawów. I plus na północy tak statystycznie to społeczeństwo starsze. Więc pokazywałoby to, że rzeczywiście lepiej, żeby te starsze osoby… Bo ja nie wiem, jaki jest procent śmiertelności obecnie. Ale on wciąż nie jest rzeczywiście zatrważający. Problemem tutaj jedynie jest to, że są osoby, które naprawdę mocno przechodzą… Nie znam osobiście, ale słyszałam historię osób w moim wieku, które gorączkowały bardzo mocno przez tydzień, miały naprawdę problemy właśnie z oddychaniem. I na pewno jest to trochę przerażające, więc każdy wolałby być w szpitalu, na zasadzie, że jest w miejscu, które gdzieś tam jest w stanie ewentualnie mu pomóc niż w domu, bo może być za późno. A nie ma miejsc dla każdego, kto gdzieś tam trochę silniej przechodzi zarażenie. Bo słyszałam o sytuacjach, że para, jedna osoba przechodziła bezobjawowo albo nawet w ogóle nie wychodziło na teście, a druga przechodziła bardzo ostro. A więc tu problemem jest brak miejsc. I brak też personelu szpitalnego, bo lekarze są, z tego co się słyszy, wykończeni.

**A jeszcze chciałam dopytać o to zamknięcie siłowni, basenów. Co o tym sądzisz, czy to ma sens? Bo wspomniałaś, że na tej sali od jogi, faktycznie w tej małej przestrzeni przebywaliście i ćwiczyliście.**

Patrząc z punktu widzenia takiego, że oddychamy, tam nie ma opcji założenia maseczki, no to jest to narażające bardziej niż pójście do sklepu, uważam. Było nas tam zazwyczaj od 4 do 10 osób, no więcej to naprawdę… Znaczy raz się zdarzyło, żeby była taka grupa w stylu 10 osób. Zazwyczaj to jest dość nieliczna grupa. Jesteśmy w jakichś odległościach od siebie rozstawieni. Nie wiem. Patrząc z logicznego punktu widzenia, pewnie ma to jakiś sens. Nie wiem, na ile na siłownię, bo z kolei siłownia, na którą chodziłam, o tej 7 rano było tam dużo mniej ludzi niż na jodze na dużo większej przestrzeni. Więc też nie wiem, na ile by tam to miało wpływ. Ale teoretycznie jest to w takich przypadkach możliwe zawsze i wszędzie.

**A zakaz imprez? Zarówno tych rodzinnych, wesel itd. ale też organizacji eventów?**

No wesela to akurat było takie źródło zakażeń jeszcze przez wakacje, jak się odtajało i mogli wrócić do organizacji. No to jednak słyszałam bardziej w trakcie wakacji, że na jakimś weselu była korona, to teraz wszyscy w kwarantannie. No też jest pewnie logiczne, żeby takich imprez… Bo jednak impreza na zasadzie dużo ludzi w jednym miejscu, obcych sobie, ściśniętych na jakiejś mniejszej przestrzeni, no to potencjalnie to jest właśnie większym… Tak że na pewno ma to większy sens niż noszenie masek na ulicy, kiedy dookoła w promieniu 10, 15 czy nawet 20 metrów nikogo nie ma.

**A te ograniczenia w transporcie zbiorowym? Wspomniałaś o tym, że ciężko policzyć tych ludzi w tym tramwaju.**

Jeszcze nie zdarzyła mi się sytuacja, żeby ktoś wyrzucał, czy żeby ktoś to liczył. Więc to jest fikcja. To jest tylko żeby było, że powiedzieliśmy, ile ma was być w środku. Ale jestem przekonana, że niejednokrotnie w ostatnim tygodniu jechałam w tramwaju, w którym było więcej ludzi niż ten numerek na szybie.

**A które z tych obostrzeń mają realny wpływ na ograniczenia rozprzestrzeniania się wirusa twoim zdaniem? Wspomniałaś o tych imprezach, że to jest faktycznie tak, że to może mieć sens. O maseczkach mówiłaś, że niekoniecznie.**

No chyba imprezy. Bo nie wiem, czy jakakolwiek… Nie wiem, nie było sytuacji czy nie słyszałam o sytuacji, żeby na jakiejś siłowni była korona i wszyscy, którzy tam chodzą, nagle że siedzą w kwarantannie. Głównie słyszałam to o weselach. Więc pewnie te wesela.

**A szkoły? Mówiłaś, że raczej…**

No tak, to też według mnie w ogóle jest… Tylko to podejrzewam, że nie jest jakby widziane w ten sposób, bo po dzieciach tego po prostu nie widać. Dzieci pewnie dużo rzadziej mają te objawy, nie robi się dzieciom testów. I tylko jak któryś nauczyciel zachoruje, to dana szkoła idzie na kwarantannę.

**A ty przestrzegasz tych wszystkich ograniczeń? Czy jest tak, że czasami coś tam ci się zdarzy omijać?**

Poza tym, że nie noszę tej maski wszędzie, jakby jak nie ma ludzie dookoła, to ją zdejmuję, żeby odetchnąć, bo to zaczyna być męczące, jeśli przez cały czas mojego wyjścia mam ją na twarzy. Poza maseczką, że zdejmuję ją czasami… No obecnie nie zakładam tych rękawiczek sklepach, ja po prostu odkażam, jeżeli jest gdzieś tam płyn, to odkażam nim rękę. No na imprezy nie chodzę, bo ich zwyczajnie nie ma, takich masowych.

**A gdyby były, to miałabyś opory, żeby pójść?**

Nie wiem. To znaczy teraz z racji tego, że jednak widzę właśnie, że więcej ludzi w moim otoczeniu, których znam, gdzieś tam te wyniki ma pozytywne albo ma zakażenie stwierdzone, to tak bardziej na zasadzie, że jeżeli chciałabym potem jechać do rodziców, to miałabym obawy, jeżeli chodzi o zarażenie babci. Więc może na jakąś wielką imprezę masową, koncert czy coś takiego, gdzie jest dużo nieznajomych mi ludzi, niekoniecznie. Na imprezę w stylu domówkę i kilkoro ludzi czy jakaś taka grupa ludzi, których znam, czy znajomi znajomych to jeszcze tak. Ale takowych też nie ma raczej. Teraz nikt nie… Ostatnio miałam w połowie sierpnia imprezę urodzinową. Z przyjacielem, który obecnie wiem, że miał wynik pozytywny i jest właśnie na izolacji, bo nie ma objawów. Chociaż znaczy tak, ma objawy obecnie, po tym jak dostał wynik pozytywny. Bo jest to chyba trochę projekcja hipochondryczna, taką mamy teorię. No więc... Ale my wszyscy, którzy mieliśmy z nim kontakt, no na razie wszystkie moje współlokatorki, jedna bywała też u swojej rodziny, ja też byłam u swojej, nic u nikogo się nie dzieje. Więc podejrzewamy, że na nim się skończyło. A widziałyśmy się z nim 2 i pół tygodnia temu. Tak że myślę, że coś już by się działo, gdybyśmy coś miały.

**Czyli jest tak, że raczej przestrzegasz tutaj tych ograniczeń. Ale dlaczego ich przestrzegasz tak naprawdę? Co cię motywuje?**

Jeżeli chodzi o maseczkę to dla świętego spokoju. Rodzice mieli taką sytuację w Biedronce, czekając do kasy… Ja jak wychodzę na zewnątrz, to zakładam po prostu soczewki, żeby nie nosić okularów przy maseczce. Tata ma okulary, więc nie nosi soczewek. I zazwyczaj ściąga tą maskę pod nos. Szczególnie będąc w sklepie, wewnątrz. Oczywiście co jest totalnie bez sensu, wtedy maska nie ma żadnego, najmniejszego sensu. I jeżeli ktoś ma tą maskę tylko pod nosem albo w ogóle na brodzie. Najlepsi ludzie to są ci, którzy są w jakimś miejscu, mają maskę na twarzy, na tej brodzie. I ona nie zakrywa nic. To jest w ogóle hit. Na zasadzie, że mam maskę, żeby jak będzie, nie wiem, szła policja, to szybko naciągnę. Nie wiem, po co mają tą maskę na tej brodzie. Moja mama się śmieje, że to ocieplacz, taki podtrzymywacz brody. I rodzice mieli scysję… Młoda para, moi rodzice są w wieku 56 i 60 lat. Więc są bardziej w grupie ryzyka. I młoda para przed nimi zaczęła się czepiać, że tata ma tą maskę zsuniętą i tam była wielka awantura. Więc jak mama się w końcu… Oczywiście awantura wynikła z tego też, że mój tata jest taki dość konfliktowy. Bo wystarczyło, nie wiem, ja bym po prostu naciągnęła tą maskę na nos, powiedziała, odczep się już pan i kontynuowała zakupy. Mój tata niekoniecznie, więc była awantura. I oni zaczęli podburzać kasjera, żeby nie obsłużył, że on nie obsłuży moich rodziców jak tata tej maski nie… I cała oczywiście drama. I jakby no to jest trochę takie bez sensu, że z takich pierdół zaczynają się robić dramaty w sklepie. I to naprawdę nie ma znaczenia w tym momencie, czy tą maskę ma się pod nosem, na nosie czy na brodzie. Bo jeżeli ona jest noszona tak długo, jest to jedna maska na kilka dni. A ja osobiście przepieram sobie, mam tę maskę materiałową. Ale moi rodzice mają te maski chirurgiczne, gdzie noszą jedną taką chirurgiczną tą niebiesko-białą dopóki nie zostanie tam wymięta, że trzeba ją wyrzucić. Więc w żadnym stopniu nie pełni to swojej roli. I podejrzewam, że wiele ludzi robi tak samo. No w ilu samochodach widziałam wiszące maski na lusterku. Bo się je chwyta kiedy się wychodzi z samochodu. Żeby to działało, z tego co czytałam, te chirurgiczne maski trzeba by wymieniać co 2 godziny. No więc o czym my tutaj mówimy. No, więc ja to robię dla świętego spokoju, żeby właśnie takich sytuacji nie było.

**A powiedz mi, bo chyba na jednym z naszych pierwszych spotkań rozmawiałyśmy o pandemii tak w kontekście źródeł tej całej sytuacji i w ogóle postrzegania, skąd to się wzięło, jaką to ma rolę na świecie itd. Czy coś w twoim myśleniu na ten temat się zmieniło?**

Tak jak mówię, moi rodzice mają oczywiście tutaj, to jest wielki plan rządu globalnego, żeby ludzi zastraszyć i kontrolować itd. Znaczy jakby protesty, które się dzieją, pokazują, że trochę coś z tego nie wychodzi. Bo jak ludzie chcieli, to wyszli na ulicę i mieli w dupie pandemię. Z drugiej strony jest łatwiej rządzić ludźmi zastraszonymi i robić, co się chce z ludźmi, którzy się boją. Dodatkowo wprowadzać podziały w stylu plandemia i antycovidowcy, którzy maszerują przeciwko noszeniu maseczek. Gdzie no, ja też nie zgadzam się z tym, że to ma jakiś sens, nie zawsze to działa, jest to niekoniecznie działające, jest to mniej przyjemne. Ale nie będę z tego powodu robić marszu, że to uderza w moją wolność, noszenie maseczki. To już uważam za dziecinadę z kolei. Bo tak naprawdę nie wiemy, co działa, co nie działa. I jeżeli możemy coś zrobić, bo a nuż będzie działało i się dowiemy o tym za parę lat, jak będą wyniki badań, no to lepiej zrobić niż nie zrobić. I chociaż wątpię w użyteczność masek i jest to niewygodne, no to z dwóch rzeczy myślę, że mądrze jest jednak je nosić niż nie nosić. Nawet, jeżeli nie dla mnie samej, no to właśnie dla innych ludzi. No jest to jakiś egoizm i takie patrzenie tylko przez pryzmat swojej wygody na pewno tu wchodzi. Ja powiem tak, ja nie jestem aż taką fanką tych wszystkich teorii jak moi rodzice. Ale mam też takie podejście do życia, że jestem skłonna uwierzyć w wiele rzeczy. Że ani nie mówię nie, ale nie mówię tak jakichś tam tym teoriom moich rodziców. Bo tak naprawdę wielu rzeczy się nigdy nie dowiemy. Nigdy nie dowiemy się, skąd pandemia się wzięła i czy tak naprawdę była tylko przypadkiem, w stylu Chińczyk zjadł nietoperza, czy rzeczywiście była jednak czyimś planem, nie wiem, chińskim, rosyjskim czy innym, bo różne są teorie. Ja myślę, że tego nigdy nie będziemy wiedzieć.

**Sama także skłaniasz się w którąś stronę? Czy po prostu jesteś otwarta na te rozwiązania i tak przyjmujesz, że (niezrozumiałe).**

Ja ostatnio mam bardziej takie neutralne podejście do wszystkiego. Na zasadzie, jeżeli ja miałabym powiedzieć, że coś jest takie albo takie, jakby określać swoje zdanie, to musi to wynikać z tego, że ja coś widziałam na własne oczy. Pomijając wiarę i kwestie pobożności, takiej wiary duchowej, no to są inne kwestie. Ale na tym jakby wiara polega. Ale kiedy mówimy o chorobach, rzeczach medyczno-naukowych no to nie jest kwestia wiary, tylko jednak dowodów naukowych. Moi rodzice oczywiście wątpią w jakiekolwiek badania, bo to wszystko jest spisek. Więc ja na pewno nie jestem w stanie powiedzieć, że ufam każdej instytucji. Bo jest jednak połączenie polityczno-naukowo. Nawet czytałam ostatnio w książce obecnej, czytam taką historyczną książkę, w której mowa jest o tym, że pieniądze na badania i rozwój technologiczny na świecie brał się z tego, że ktoś miał z tego korzyści. Na początku w dużym względzie były to korzyści polityczne, nie tylko kwestie zdobywania, chociażby wyprawy różnych poszukiwaczy i odkrywanie nowych lądów, to było jedno, że kosztowności, które się z ameryk znosiło, ale jakby polityczne i władza, to zawsze się łączy. Więc nie można oddzielić nauki od polityki, bo to gdzieś tam zawsze ma… Jakby no chociaż w tym momencie. Pandemia wpływa na politykę, wpływa na to, co się w Stanach dzieje i obecne wybory. Pandemia też ma ogromny udział w tym. Więc tego nie można rozdzielać. Ale dopóki nie byłabym w stanie naocznie sama udowodnić i zobaczyć, no to nie będę w żadnym wypadku mówić, że coś jest takie, a tutaj inne. Więc bardziej to jest kwestia otwartości na te różne teorie. Sceptycyzmu, ale na każdą ze stron. Ja mogę być sceptyczna, jeżeli chodzi o różne udziwnione teorie spiskowe. Ale też sceptyczna, jeżeli to jest kwestia tylko przypadku i tego, że rzeczywiście przypadkiem wirus się z Chin wydostał od jakiegoś jednego nietoperza zjedzonego na targu. Aczkolwiek od iluś lat już przewidywano, że już czas na kolejny wirus, kolejną pandemię, bo coś musi się pojawić, czego jeszcze nauka nie zna, świat medycyny nie zna.

**A czy wśród tych teorii, bo ja teraz obserwuję takie postrzeganie, że tak naprawdę tej pandemii nie ma, że to jest po prostu sposób na kontrolowanie społeczeństwa. Czy wśród tych teorii gdzieś też pojawia się takie myślenie? Że pandemii w ogóle nie ma?**

Znaczy tak, tego, że choroby w ogóle nie ma, to myślę, że bym nie stwierdzała. Bo to widać po tym, że ludzie chorują. I wcześniej nie znałam tych ludzi, więc mogło być bardziej takie stwierdzenie, że nie znam nikogo, kto zachorował, więc w sumie z mojego punktu widzenia, to nie ma. Trochę to, że się tak rozluźniły wszystkie obostrzenia i ludzie w ogóle już przestali się zachowywać, jakby była jakaś pandemia w lecie wynikało z tego, że pomimo tego, co dochodziło do nas z Włoch, nikt nie umierał u nas na ulicach. Ani nawet na szpitalnych podłogach. Co pewnie jest w jakimś stopniu wypadkową lockdownu w kwietniu, maju i czerwcu. Ale pewnie tylko trochę. Pytanie, jak będzie teraz. Więc to nie jest kwestia może wiary… Choroba, powiedzmy sobie, że jest. Wirus gdzieś tam jest. To nie jest tak, że zaprzeczałabym w ogóle jego istnieniu. Ja bardziej uważam, że cała panika wywołana sytuacją została rozdmuchana do takich rozmiarów, że część ludzi zaczęła się bać. Więc już nawet sama panika… Pomijając lockdown i rzeczywiste zamykanie biznesów, sama panika wywołała to, że mniej ludzi kupowało bilety lotnicze albo chciało gdzieś wyjeżdżać. Branża turystyczna cały czas obrywa. Bo tak jak wewnątrz kraju mogła gdzieś tam odtajać przez wakacje, ta turystyka czy branża turystyczna, która się skupiała wyłącznie na wyjazdach zagranicznych dostała i obrywa cały czas. Więc sam strach, rozsiana informacja sprawiała, że mniejsza podaż na pewne rzeczy. Bo ludzie przestali kupować pewne wycieczki, chodzić do restauracji z powodu strachu. To jest jedno. No kolejna rzecz, ale to pewnie będzie widać dopiero już jak pandemia będzie się chyliła ku końcowi albo już się skończy, czyli rzeczywista śmiertelność w porównaniu, takie porównania teraz widzę, porównywanie obecnej śmiertelności albo obecnej ilości zakażeń do ilości zakażeń i śmiertelności w każdym sezonie grypowym. Te liczby są albo większe albo zbliżone. Ja osobiście nie patrzyłam na te statystyki, chciałabym sobie usiąść i sam gdzieś tam to policzyć. Ale widziałam jakiś post czyjś. No, póki widzę post i nie sprawdzę tego sama, to nie będę też tak zawierzać. Ale pytanie właśnie, czy ilość ludzi, którzy zarażają się taką grypą sezonową, nie jest taka sama. Śmiertelność, pytanie właśnie, na jakim poziomie jest śmiertelność przy okazji grypy sezonowej. Bo jeżeli jest to poziom podobny lub koronawirusa jest mniejszy w kwestii śmiertelności, to ta panika była potrzebna. Jedynym czynnikiem, który tu był inny, no to jest kwestia przechodzenia choroby i to, że rzeczywiście respirator w niektórych przypadkach byłby potrzebny. Albo, że jest to dużo cięższe przechodzenie choroby niż w przypadku gryp sezonowych. Szczerze mówiąc nie znam się też na grypach sezonowych, bo ja nigdy nie chorowałam na grypę sezonową. Nigdy się nie szczepiłam. Ale nikt z moich znajomych nie chorował, a tym bardziej nie umarł z powodu powikłań po grypie sezonowej. Ale pytanie, jeżeli jest to wszystko zbliżone, no to dlaczego panika jest do tego stopnia rozdmuchana. To oznaczałoby, że cała panika była niepotrzebna. I wtedy czemu nie mieliśmy paniki i zamykania wszystkiego w momencie, kiedy miesięcznie na grypę sezonową zachorowało kilkanaście tysięcy ludzi w ciągu miesiąca? Podejrzewam też, że to, że tyle ludzi teraz uderza przy okazji objawów koronawirusa do szpitala, też byłaby to mniejsza liczba niż w momencie, kiedy jest panika, że mam koronę, bo objaw jest, taki, powiedzmy ta utrata węchu, smaku. To chcę iść do szpitala, bo wiem, że to jest tak poważne, że aż muszę iść do szpitala, bo pandemia, ta choroba, od której teraz cały świat stoi. A gdyby tej paniki nie było, no to tak jak ktoś z normalną grypą nie idzie do szpitala, jeżeli naprawdę nie czuje się fatalnie, tak samo nie robiłby tego ktoś teraz, kto nie jest w takim stanie, że musi do tego szpitala iść.

**Ale to w ogóle uważasz, że ta obecna sytuacja jest poważna?**

W kwestii choroby? I zagrożenia dla ludzi?

**Tak, tak, tak.**

Musiałabym, szczerze mówiąc, ja w ogóle nie patrzę na te statystyki, nie wiem, ile jest zakażeń, nie wiem, jaka jest śmiertelność. Więc musiałabym sobie to sprawdzić, obliczyć. I jeżeli popatrzyłabym na te liczby, to jakoś mogłabym decydować. Biorąc pod uwagę śmiertelność, z tego co słyszałam i się ostatnio dowiadywałam, no to nie jest ona jakoś zatrważająca, więc nie. Na pewno jest duża… Jakby duże prawdopodobieństwo zachorowania obecnie. Tylko pytanie, kto jak przechodzi i czy w ogóle ma objawy i jak to przechodzi.

**Czyli tą powagę tej sytuacji oceniałabyś na podstawie statystyk po prostu śmiertelności, na ile to jest poważne zagrożenie. A jak oceniasz zachowanie ludzi obecnie? Czy wydają ci się te zachowania racjonalne, które obserwujesz gdzieś na ulicy czy znajomych?**

Pomijając moją koleżankę w moim wieku, która się zamyka w domu, co już dla mnie jest przesadne. Na zasadzie, jeżeli się chce chronić kogoś starszego, jeszcze bym to zrozumiała. Ale w naszym wieku jest to, myślę, przesadna panika. Tak jak mówię, o siebie się nie boję. Oczywiście odpukać, żebym potem nie żałowała. Myślę, że większość ludzi racjonalnie. Oczywiście takie awantury w stylu ta z moimi rodzicami, gdzie młodsze rodzeństwo moim rodzicom zwracało uwagę na maseczkę, są to takie niepotrzebne różnice i… No, ci ludzie, którzy jednak w tym tramwaju siedzą z tą maską na brodzie, to jest z kolei też bez sensu. Ale ile ludzi, tyle opinii, tyle różnych spojrzeń. I każdy ma swoją teorię. Jeden uważa, że to totalnie bez sensu, inny się boi i będzie krzyczał na każdego, kto nie nosi maski. Więc to wprowadza więcej sporów po prostu. Myślę, że większość ludzi jednak zachowuje się racjonalnie. Współczuję ludziom, którzy muszą w pracy nosić cały dzień maskę. I im się nie dziwię, jeżeli ją ściągają na jakiś czas albo pod nos. Ja nie zwracam ludziom uwagi, bo sama wiem, jakie to jest niewygodne. Jeżeli ja mam tą maskę i jestem w sklepie na chwilę, to mnie to w ogóle nie rusza. A gdybym musiała gdzieś siedzieć dłużej z kimś, kto nie chce tej maski nosić, no to też nie robiłabym z tego jakiejś afery, ale pewnie miałabym do tego jakieś tam już obiekcje. Bo według mnie największym zagrożeniem jest siedzenie w jednym pomieszczeniu, im mniejsze tym gorzej, przez długi okres czasu.

**Wspominałaś trochę o tym, jeśli dobrze zrozumiałam, że rząd mógł zrobić wcześniej coś, żeby się przygotować na obecną sytuację. Czyli twoim zdaniem można było temu zapobiec? Temu, co jest teraz, tak gwałtownemu przyrostowi zachorowań?**

Troszkę tak, zmniejszyć, bo….

**A co można było zrobić, żeby to zmniejszyć?**

Przede wszystkim nie posyłać dzieci do szkoły. A w kwestii tego, że no jest teraz sezon grypowy, więc tego się nie uniknie, że taka pogoda i taki czas, przygotować szpitale. Czy teraz albo ten stadion. Ale w Wuhan wybudowali szpital w 9 dni na początku pandemii. Ja nie mówię, że mieli wybudować jakiś szpital w 9 dni. Ale mieli całe lato na to, żeby przygotować jakieś polowe placówki na ten okres. Ja uważam, że zajęcie się… Znaczy na pewno to jest trudne, żeby znaleźć brakujący personel medyczny. Ale z tego, co widziałam czy gdzieś czytałam, to obecnie w ogóle przepychają jakiś taki przepis, żeby można było łatwiej zatrudniać medyków obcokrajowców. Nawet, którzy nie mają wizy czy coś takiego. Bo jest taki brak medyków, że nawet, jeżeli trafi się ktoś z wykształceniem medycznym, kto nie ma, nie wiem, wizy, normalnie miałby większy problem, żeby dostać pracę w kraju. Ułatwiają tym ludziom zatrudnienie. To pokazuje już desperację. A zaplanowanie tego, że hej, nie mamy personelu, trzeba coś z tym zrobić, można było zrobić wcześniej. No teraz zapłacą ponoć podwójnie lekarzom, no chwała im za to. Jakby chociaż tyle się tym ludziom należy. Jeszcze przy tym, jak na początku pandemii były całe historie w mediach o współlokatorach z budynków czy osiedli, którzy wypisywali jakieś dziwne rzeczy na drzwiach albo wręcz pisali jakieś pisma do spółdzielni, że oni nie chcą, żeby lekarz tam wracał. No to już jest w ogóle nie do pojęcia, to już pomijając to. Ja nie znam żadnego lekarza osobiście, który byłby jakoś tak mocno dotknięty sytuacją. Jedyne historie to w social mediach albo wiadomości czytałam. No, ale na pewno kwestia skąd weźmiemy więcej personelu medycznego, ratowniczego, pielęgniarek. Osobno placówki na koronę. Żeby też nie było sytuacji, w których ktoś idzie do jakiegoś szpitala. I jak jedna osoba z koroną się pojawia, to na 2 tygodnie trzeba zamknąć cały oddział szpitala i wszyscy pacjenci pozostali są dotknięci tym. Ja słyszałam, że opróżniali jakiś oddział szpitalny i jakaś kobieta, która szła na oddział onkologiczny szła, coś miała być przy nowotworach, była w szpitalu. Powiedziano jej, że trzeba opróżnić cały oddział, bo na koronę musi być miejsce. Więc ludzi z oddziału onkologicznego się wyrzuca. To jest w tym momencie horror. Też wiem, że śmiertelność jest podniesiona taka, że a: ludzie, którzy mają problemy innego rodzaju, nie wiem, krążeniowe, oddechowe, innego rodzaju problemy zdrowotne, nie idą do szpitala w odpowiednim czasie, bo się boją korony, więc unikają szpitala do ostatniego momentu. I jeżeli karetka nie zdąży na czas, to jest jakby już po wszystkim. Albo zwyczajnie nie ma dla nich miejsc, jeżeli takiego miejsca potrzebują z innym problemem. A więc tutaj rzeczywiście najsłabszym ogniwem jest służba zdrowia, co wiemy nie od dziś w różnych aspektach, a w obecnej sytuacji tym bardziej. Więc trzeba by było się skupić na tym.

**A jak byś miała tak ogólnie zastanowić się nad tymi decyzjami, działaniami rządu, to jak w ogóle je oceniasz? Czy one uspokajają ludzi i zapewniają bezpieczeństwo? Czy właśnie budują tę panikę i frustrację?**

Frustrację. No ludzie, w kwestiach gospodarczych wszystkie te biznesy, ludzie są wściekli. Jeżeli kogoś osobiście nie dotykał koronawirus w rodzinie czy osobiście, to dla niego już nie ma znaczenia, czy się zaraz czy nie zarazi, jakby ma w dupie obostrzenia, bo musi pracować, musi mieć na życie. W momencie, kiedy ktoś nie ma co do garnka za chwilę włożyć albo z czego zapłacić czynszu, to nie obchodzi go choroba, która jest taka trochę nie do końca wiadomo, czy rzeczywiście taka groźna, jak się wydaje. Czy ta panika była taka potrzebna. To jest jedno. Drugie to jest cała jednak ta, w social mediach i jakichś tam wiadomościach przez całe lato były te, ile było tych sytuacji, kiedy było jakieś obostrzenie, nie wiem, noszenie masek, niewychodzenie czy coś, a niektórzy politycy się nie stosują. Więc nie są przykładem. Kolejny aspekt. Jak to było w wakacje, mój ból jest większy niż twój, no więc… To na pewno nie jest dobrym przykładem. I wiele ludzi pewnie ma bardziej gdzieś obostrzenia, jeżeli coś takiego widzi. No nie wiem, czy fakt tego, że politycy mają szybszy dostęp do testów i opieki zdrowotnej…

**Czyli nie tylko same decyzje, ale też takie podwójne standardy podchodzenia do tych decyzji przez polityków.**

Tak.

**A skąd w ogóle teraz czerpiesz informacje na temat sytuacji?**

Wiesz co, żadne portale wiadomościowe. Wiadomości to tylko jak jestem w domu, to przez chwilę mi migną, ale ja już je wyłączam, bo nie jestem w stanie. Znaczy pomijając, że ja nie sprawdzam, nie sprawdzam poziomu zarażeń, ani jakby nie bardzo siedzę w temacie, bo zazwyczaj nie mam ochoty tego czytać. Wystarczy, że nie mogę iść na siłownię czy na jogę. Więc zdarza mi się jakiś artykuł na Facebooku przewinąć. I Instagram chyba. Co na pewno nie jest najlepszym źródłem tych informacji. Dlatego wszystko, co powiedziałam, że słyszałam albo widziałam, też podchodzę do tego z taką szczyptą jednak umiaru i dozą takiego… Że dobra, to widziałam na Instagramie, to widziałam na Facebooku, na ile to jest fake news nie wiadomo.

**Czyli to jest trochę tak, że jak ci coś mignie przy okazji, to gdzieś tam wpada, a nie wyszukujesz aktywnie.**

Tak. A a propos Facebooka właśnie 2 tygodnie oglądałam sobie 2 filmy gdzieś tam na raz, social dilemma a propos Google’a, Facebooka. I zaraz po nim jeszcze film o Cambridge Analitic i całej tej dramie tych wyborów 4 lata temu. Więc po obejrzeniu tego tak a propos fake newsów i jak można było, najgorszym przykładem było (niezrozumiałe) wyboru. Ale w przypadku Facebooka był przykład tej rzezi, tego ludobójstwa w Birmie. I to jest szokujący przykład tego, jak fake news doprowadził do realnego masowego zabójstwa. I to jest przerażające. Bo jeżeli coś takiego było możliwe… Oczywiście dotyczy to krajów, w którym mało wykształconym ludziom podało się Facebooka przed twarz. Trochę może jest to mniej takim zagrożeniem u nas. Ale mimo wszystko jakimś jest. Więc jeżeli coś takiego się widzi, no to na zasadzie takiej można ludziom wmówić wszystko. Pandemia to pic, pandemia jest naprawdę zagrożeniem, chowajcie się, nie chodźcie. Tutaj tylko zależy, kto jest po której stronie i które treści jemu się podsyła, bo on będzie skłonniejszy w nie uwierzyć. Dlatego mówię, nie biorę tych rzeczy, które widzę za pewnik, dopóki bym ich nie sprawdziła. A na razie nie sprawdzałam. Tak jak mówię, chciałam sobie sprawdzić te liczby w kwestii zarażeń i śmiertelności, porównać je z normalnymi statystykami grypowymi, ale jeszcze tego nie zrobiłam, bo nie zmieni to niczego poza gdzieś tam zaspokojeniem mojej ciekawości.

**A jak byś miała to sprawdzać, to gdzieś byś to sprawdziła? Te statystyki.**

Jest taka strona, którą poznałam przy okazji czytania takiej książki o statystyce, Gapminder. Więc oni podają tam różnego rodzaju statystyki. Pytanie, czy mieliby te. Podejrzewam, że światowa jakaś organizacja zdrowia, które mają, WHO czy CDC. Oczywiście moi rodzice w życiu nie uwierzyliby tym organizacjom, bo nie będą im wierzyć, z wiadomych względów. Ale na czymś trzeba to oprzeć. Więc myślę, że tym organizacjom… Można by było pewnie poszukać innych źródeł, ale tak samo jak te informacje z WHO czy CDC, każde inne mogą być zmanipulowane. Więc w sumie, z mojego punktu widzenia niczemu nie można tak naprawdę stuprocentowo uwierzyć. Mogłabym iść i sama policzyć, ale tego też nie zrobię.

**Czyli to jest tak, że te informacje nawet z WHO czy CDC, one nie do końca są przez ciebie postrzegane jako wiarygodne?**

Na pewno bardziej niż wszystko inne, niż jakiekolwiek inne źródło. Ale gdzieś ta doza i ten głos moich rodziców, że to też są organizacje światowe, finansowane przez konkretne, inne organizacje, kraje, rządy… Na pewno jest jakiś taki… Ziarenko mojej… Tylko to jest tak w porównaniu do innych źródeł, jest to ziarenko takiego, że tu może być coś zakłamane, nie wiem na ile, nie sprawdzę tego nigdy. A więc na pewno bardziej zawierzałabym tym organizacjom typu WHO, CDC czy jakimś nie wiem. U nas na pewno Ministerstwo Zdrowia ma też jakieś te swoje statystyki niż jakiemukolwiek innemu źródłu. Ale to ziarenko wątpliwości na pewno gdzieś tam jest.

**Czyli dobrze zrozumiałam, że teraz ogólnie mniej poświęcasz czasu na korzystanie z mediów informacyjnych niż wcześniej? W sensie niż…**

Nie. Wiadomości nie oglądałam w sumie wcześniej też za dużo. Nie, ja wręcz za dużo siedzę na Instagramie.

**Więcej niż wcześniej?**

Trochę tak, ale to nie jest kwestia szukania informacji, tylko to jest kwestia scrollowania Instagrama, bo muszę zająć myśli, odciąć się. To jest niewłaściwy sposób odstresowywania się.

**Bardziej kwestia zajęcia czasu?**

Tak. bo nie mogę teraz wyjść na dwór, bo jest zimno, nie mogę iść na siłownię, jogę. Wcześniej oczywiście mimo jogi i siłowni też siedziałam dużo na Instagramie. I był to taki zapychacz czasu, wyłączać mózgu. Obecnie, no jest on trochę od kilku dni bardziej stresujący niż cokolwiek uspokajający. Bo Instagram jest teraz pełen protestów przecież. Ale… Znaczy ja na początku pandemii wyłączyłam Facebooka na jakiś czas. Używałam tylko Messengera. I usunęłam aplikację Facebooka z telefonu. Więc na pewno scrolluję mniej. Bo fejs to w ogóle już jakby… (niezrozumiałe) i bezsensowne scrollowanie Facebooka w stylu nagle zawieszam się na filmiku o gotowaniu albo o ładnych pieskach. Więc odcięcie Facebooka było dobre. I udało mi się. Z Instagramem mam trochę większy problem, bo tam był taki czynnik, że jednak podglądam znajomych w ten sposób. I jest mi go trudniej odciąć.

**Ale tego Facebooka odcięłaś na początku pandemii na wiosnę, nie teraz?**

Nie, na wiosnę. Ja wyłączyłam fejsa całkiem, że jakby nawet nie było mnie widać. I po miesiącu, po dwóch go włączyłam. I wchodzę obecnie tylko poprzez przeglądarkę w komputerze. I też usunęłam sobie z paska moich zapamiętanych linków na Facebooka, więc muszę go wpisać. Więc to też jest takie, że jak ta ikonka tam była, to o sobie przypominała. A jak teraz jej nie ma, to nawet czasami po 2 dniach się reflektuję, o, bo miałam coś tam, jakieś powiadomienie dostać, jakieś konkretne odnośnie jakiejś tam grupy, która rzeczywiście mnie interesuje. I sobie przypominam, to sobie wchodzę. A nie jest tak, że wchodzę po prostu codziennie, żeby na tym fejsie posiedzieć. Więc jednak udało mi się troszkę spacyfikować i odciąć. Z Instagramem jest gorzej. Znaczy może jest gorzej dlatego, że jest mniej zajęć innych, które można robić. Ale nie dlatego, że nie wiem, szukam tam informacji o pandemii.

**Na koniec chciałabym z tobą porozmawiać o takiej perspektywie na przyszłość. Czy w ogóle zastanawiasz się nad tym, jak będzie wyglądała sytuacja po pandemii?**

Zobaczymy, co się wydarzy w Stanach przy okazji wyborów. Bo to, co się tam dzieje, to jest w ogóle... Stany to jest chyba ostatnie miejsce, w którym chciałabym być obecnie. Bo też sytuacja ta BLM-owa i wszystko to, co tam się wydarzyło przez ostatnie miesiące, to jest w ogóle… Pandemia to jedno, jakby wszystkie te głosy i teorie, że pandemia jest zaplanowana i takie tam to jedno. Ale chociażby ruch cały BLM-u. Filmy, które nagle stały się viralami za (niezrozumiałe) do tego, to nie były pierwsze rasistowskie filmy, jakie my widzieliśmy. Jakby tych filmików krążyło w sieci i to tak viralowo no już kilkanaście od dłuższego czasu. Filmik o chłopaku, który jest zastrzelony bez sensu czy bez konkretnego powodu przez białego policjanta. Ile tego było wcześniej? Jaki jest nagle powód w 20202 roku, żeby cały ruch BLM się przeradzał w protesty na ulicach? Podobne na naszych teraz, może nawet trochę większe. Tak jak mówię, nie jestem aż taką fanką teorii spiskowej jak moi rodzice, ale to nie wygląda jak przypadek. Że, nie wiem, to był kwiecień, to był maj? Kiedy się zaczął BLM, już nie pamiętam.

**Ja też już nie pamiętam. Ale chyba później. Bo gdyby to się zadziało jakoś wtedy, to pewnie byśmy rozmawiały o tym. Więc może wakacje.**

A w listopadzie wybory. Więc tu ewidentnie komuś zależało, żeby nagle cały dramat, który nie jest nowością, te wszystkie rzeczy, żeby nagle w 2020 roku, w czasie pandemii, którą z kolei obecny prezydent Stanów neguje, przerodziły się w takie akcje, które tam się działy. I tak samo będzie wyglądało, nie wiem, znaczy mniemam, że tak jak w lecie już sporo rzeczy wróciło do normy, to generalnie życie raczej wróci do normalności. Pytanie, co się pojawi, czy to będzie to lekarstwo z tego osocza ozdrowieńców, czy to będzie szczepionka? Szczepionka na pewno wywoła kolejne jakieś rozdwojenie, antyszczepionkowcy powiedzą, że ja się nie dam zaszczepić. I pozostali będą mówili, że są idiotami, że się nie dadzą zaszczepić. I będzie kolejna kłótnia. Więc ja liczę na to, że może jakieś lekarstwo jednak będzie, które nie będzie szczepionką. Chociaż z kolei tutaj antyszczepionkowcy pewnie też nie dadzą się nim leczyć. Nie wiadomo. Ja też nie jestem skłonna się szczepić. Ale coś trzeba zrobić pewnie.

**Ale dobrze rozumiem, że żeby ta sytuacja się skończyła tak naprawdę, to musi być albo lekarstwo albo szczepionka?**

Myślę, że tak, że jedno z dwóch, żeby to tak szybciej doszło do końca. Zobaczymy też, czy będzie trzecia fala. Teraz sezon grypowy, teoretycznie trwa od mniej więcej teraz, października, listopada. Do kiedy? Do kwietnia, do maja? Pandemia zaczęła się luty-marzec dla nas. I szczęśliwie poprzednie obostrzenia, wycofywano je w maju, w czerwcu. Bo najgorzej było koniec marca, kwiecień i maj, no i te doniesienia z Włoch itd. Pytanie, jak długo to teraz potrwa? Czy to będzie cały czas rosło do kwietnia, czy do końca roku trochę zmaleje. Jakby nie wiadomo, co będzie teraz z ilością zakażeń przy obecnym ich stanie. Czy już wszyscy przechorujemy za 3 miesiące i już będzie spokój, bo już przechorowaliśmy i już w sumie wszyscy czy większość społeczeństwa będzie ozdrowieńcami, no nie wiadomo, co się wydarzy i ile to potrwa. Więc to zależy jakby od tego. Aha, moi ukochani rodzice mają przesłanki o tym, że będzie kolejny wirus zmutowany, SARS-COV-21, więc niektórzy na przykład mają takie wizje. No, ja mam szczerą nadzieję, że się bardzo mylą. Myślę, tak mi się wydaje, że pomijając lotnictwo i podróże zagraniczne, pozostałe aspekty życia wrócą do normy, jak już będzie albo jakieś lekarstwo albo wszyscy przechorujemy. Więc zagrożenie nagle masową okupacją szpitali minie. Bo to o to chodzi. Albo, nie wiem, ci co mieli przechorować i wyzdrowieć, to wyzdrowieją, ci co mieli umrzeć, to umrą. Czyli podejście Szwedów na przykład, którzy obostrzeń nie mieli. Tylko pewnie w trochę mniejszym społeczeństwie jest to trochę mniejszy problem niż w naszym, 4 razy większym społeczeństwie niż szwedzkie. Chyba 4, nie wiem, ilu ich tam jest. Ale u nich chyba jest około 10 milionów obywateli. Więc mamy mniej więcej 4 razy więcej obywateli, więc to trochę co innego. Bo chyba oni mają jednak zbliżoną powierzchnię kraju do naszej, więc wiadomo, rozproszenie ludzi jest tam trochę większe. Nie przewiduję wielkich różnic w życiu poza podróżami zagranicznymi i obostrzeniami w kwestiach przekraczania granic. Tak jak teraz, niektóre kraje są wciąż zamknięte. Inne mają swoje listy czarne, czerwone, nie wiem, jakie te listy są. Krajów, z których można przyjechać, z których nie można przyjechać, z których trzeba mieć test negatywny, żeby wyjechać. Więc to pewnie będzie jeszcze trochę trwało na świecie, jeżeli chodzi o to. Aczkolwiek myślę, że nawet w kwestii podróżowania, to szczepionka albo lekarstwo może trochę zmienić i poprawić sytuację. Ale pewnie jeszcze długo pozostałości w stylu maseczki na lotnisku i gdzieś tam takie zachowywanie odległości, jak ktoś jest chory albo mierzenie temperatury. Tak jak obecnie przecież to, że przechodzimy przez całe sprawdzanie i security na lotnisku jest pozostałością ataków terrorystycznych, które jednak no nie dzieją się na co dzień. Więc kto wie.

**Więc część z takich rzeczy może też zostać po pandemii tak?**

Tak. Myślę, że nie zdziwiłabym się, gdyby miały maseczki zostać. Na zasadzie, mierzymy temperaturę, to ktoś z wyższą temperaturą musi mieć właśnie maseczkę albo odkażać dłonie, albo siedzieć gdzieś daleko. Albo w ogóle nie wejdzie na pokład. Chociaż no kiedy zagrożenie samym konkretnie tym wirusem minie, to mam nadzieję, że już nie. Ale tutaj może być na lotniskach… Też pytanie jak szybko wszystkie linie lotnicze będą się podnosiły. Aha, jeszcze jest jedna kwestia, czyli organizacja pracy w biurach. Bo to już tą dyskusję o tym, jak to będzie w post pandemicznym świecie wyglądało. Bo nagle niektórzy się zorientowali, że da się robić tę pracę z domu, którą wszyscy robili w biurze. I nie muszą jeździć do tego biura. I te wszystkie przepełnione biura, gdzie trzeba było wykupywać tyle powierzchni i ludzie się nie mieścili, nie było biurek dla ludzi w biurach, można zrobić tak, żeby było hybrydowo. Czyli będą ludzie przyjeżdżać tylko kilka razy w tygodniu, więc nie będzie żadnego problemu. Co dla mnie jest naprawdę odkryciem po prostu roku, że naprawdę? Da się robić te rzeczy zdalnie? To chyba było…. Znaczy wystarczyło pomyśleć (śmiech). Ale to jest w bardzo wielu firmach kwestia starych, to już rozmawiałam gdzieś tam z młodszymi ludźmi w firmie mojej czy poprzedniej, menadżerowie starej daty po prostu nie są w stanie się przerzucić. A to zazwyczaj oni siedzą w tych miejscach decyzyjnych, żeby takie zmiany zaprowadzać nawet w wielkich korporacjach.

**Ale to jak byś miała całkowicie pracować zdalnie na dłuższą metę, to byłoby to dla ciebie męczące?**

Znaczy całkowicie właśnie nie. Pytanie dla mnie to by było, na ile mogę się przemieszczać. Bo w przypadku obecnym ja mogę na przykład pojechać do rodziców pracować. I tylko mówię, OK, dzisiaj pracuję z tego i tego adresu. Bo w kwestiach ubezpieczenia, oni muszą wiedzieć, pod jakim adresem jestem. Więc muszę mojej menadżerce, będąc w kraju, mogę pracować gdziekolwiek, tylko muszę napisać, z jakiego adresu. Ale już w kwestii wyjazdu za granicę i na przykład pojechania sobie, nie wiem, na południe, gdzie jest ciepło i kiedy już będzie można i pracowania zdalnie stamtąd, to już nie jest tak hop siup, bo ja jestem w trakcie pracy, a ubezpieczenie moje normalne nie obejmuje bycia za granicą. Więc gdyby tak można było, że te ubezpieczenia są jakoś inaczej dograne albo można z firmą dogadać, że oni nie mają nic przeciwko, że ja pracuję z innych krajów również, to dla mnie to by było idealne, bo ja wtedy mogłabym łączyć podróżowanie z pracą. Ale w przypadku, w którym nie można, czyli i tak muszę brać urlop na czas wyjazdu zagranicznego, to raczej byłaby to dla mnie kwestia łączenia. Czyli 2-3 dni mogę przyjść do biura… W mojej poprzedniej firmie był taki system, ja w ogóle miałam zdalną i przychodziłam do biura, teoretycznie powinnam być w biurze 2 dni w tygodniu. Ale czasami byłam tylko 1. Jak już przychodziłam, to nie na całe 8 godzin, tylko na 5. A trochę z rana i trochę z wieczora pracowałam z domu. Taki akurat też miałam tryb pracy, że to tak mogło wyglądać. Więc bardziej coś takiego. Plus zaczęto, jak ja już odchodziłam z firmy, zaczęto wprowadzać coś takiego, że niektóre z biurek były tak zwanymi flex deskami. Czyli biurkami niezajętymi na stałe przez nikogo. Bo reszta pracowników, każdy musiał mieć swój komputer i swoje stanowisko, którego raczej nikt nie zajmował, kiedy go tam nie ma. Natomiast takie biurko stoi i miejsce się marnuje, kiedy ktoś nie przychodzi do biura. Więc te flex deski nawet miały takie swoje naklejki z numerem. I oni opracowywali jakąś aplikację, która miałaby te wszystkie numerki tych biurek. I można by w tej aplikacji sprawdzić, które są, albo ile miejsc dzisiaj jest wolnych. Czyli na przykład, gdyby wprowadzić system, że to nie jest tak, że każdy ma swoje biurko, tylko każdy ma jednak laptopa i komputer, z którym przychodzi albo jest w stanie zalogować się do swojego konta z każdego komputera. I ludzie mają aplikację, w której sprawdzają, OK, dzisiaj jest tyle ludzi w firmie, że dzisiaj nie będę szedł, bo nie ma miejsca. Jeżeli nie ma takiej potrzeby. Bo jeżeli ma się spotkanie, to wiadomo. I wprowadza się system, w którym można sobie zająć to biurko, przyjechać i tego dnia posiedzieć. Jak nie ma potrzeby, to się zostaje w domu. Pewnie i tak te biurka nie byłyby codziennie zajęte w jakimś dużym stopniu, bo większość ludzi nie miała potrzeby, że jest spotkanie, na którym musi być osobiście, to po co jechać do pracy?

**A w tym myśleniu o przyszłości jest coś, czego się szczególnie obawiasz?**

No, planów moich rodziców, w sensie ich wizji. Bo one są już nie do przewidzenia, są mocno… No nie wiem, po angielsku disturbing.

**Takie niepokojące?**

Rozpraszające, niepokojące, tak. Nie wiem teraz jak przetłumaczyć. No jakby ja jednak mam ten mój plan, że muszę, ja tutaj już nie wytrzymuję. Miało mnie tutaj nie być, więc chcę wyjechać. Póki co powiedzmy mam plany, jeżeli chodzi o osiąście w Polsce, gdzieś pewnie w przyszłości, jeżeli chodzi o rodzinę. Ale póki rodziny nie mam, nie mam ani partnera, ani męża ani chłopaka, jakby nic mnie tutaj nie trzyma poza tym, że obecnie pandemia. No to ja chcę pomieszkać w różnych miejscach na świecie, popodróżować, jeszcze popracować na świecie. To będzie moje dążenie, kiedy będzie taka możliwość. Myślałam, że może już od stycznia będę wyjeżdżała do tej Holandii, gdyby tam rzeczywiście była dla mnie oferta pracy w obecnej firmie. Obecnie myślę, że styczeń to jeszcze byłoby za wcześnie na wyprowadzkę do innego kraju. Bo w danej sytuacji, kiedy ja za 2 miesiące muszę się zbierać i wyprowadzać, no to nie zdążę. Bo musiałabym poczekać i mieć pewność, że sytuacja się ustabilizowała. Więc bardziej luty, marzec, może kwiecień byłby lepszym pomysłem. Ale pytanie, czy wtedy będę miała taką możliwość. Może być tak, że praca będzie na mnie czekała w styczniu, a w kwietniu już nie. No więc pytanie. Gdzieś tam trochę zaczęłam się mocniej bać tych scenariuszy moich rodziców, z powodu sytuacji politycznej, która obecnie się dzieje. Więc mam nadzieję, że to się nie przerodzi w nic gorszego niż tylko lockdown. Moją obecną nadzieją jest, żeby jedynym, co nas obecnie zatrzymuje, był jakiś lockdown w stylu tego… Znaczy obecny też nie jest tak mocny jak ten z wiosny. Więc możemy dojść do takiego lockdownu jak wtedy. Jak przy okazji Wielkanocy, kiedy jednak przed Wielkanocą za wyjazd z miasta bez konkretnego celu, usprawiedliwienia, były mandaty w wysokości 30 tys. złotych. I przy okazji Wielkanocy przecież oni prosili ludzi, żeby ograniczyli jeżdżenie. Za 2 miesiące mamy święta Bożego Narodzenia, znowu będzie to samo.

**Myślałaś już o tym, o świętach Bożego Narodzenia?**

No, szczęśliwie mój wyjazd do rodziców to jest trochę co innego jak wyjazd z Warszawy tak daleko. Więc jakby… ja też mam mniejszą rodzinę, to jest zawsze u nas pod Warszawą, więc nie powinnam mieć problemów ze spędzeniem świąt u rodziców, myślę.

**Więc będzie po prostu jak co roku, bo też w gronie rodzinnym spędzaliście święta?**

Tak. I moja rodzina jest mała, my nigdy nie robimy takich spędów rodzinnych większych, bo tak to u nas zawsze wyglądało. Więc tutaj jakby nic się raczej dla mnie nie zmieni.

**A co do Święta Zmarłych, planujesz odwiedzić…**

Znaczy ja raczej, nie wiem, czy wspominałam w naszych poprzednich rozmowach, razem z bratem jesteśmy wyznania protestanckiego. Ja od piętnastego roku życia, brat parę lat po mnie, a jest 4 lata młodszy. Więc ja też nigdy nie chodziłam z jakiejś własnej potrzeby na groby. To zawsze załatwiała mama na zasadzie tradycji i babcia. To mój brat zawoził babcię na grób jej mamy, czyli naszej prababci teraz w sobotę. Na zasadzie, no po prostu musiał tam babcię zawieźć. Ale nasz tata też nie przepada za chodzeniem na cmentarze. Więc jakby u nas w rodzinie nie ma takiego parcia, że to są zjazdy rodzinne.

**Czyli nie ma też spotkań z tej okazji.**

Nie, nie, nie, broń boże, nigdy u nas nie było spotkań z powodu wszystkich zmarłych. Znaczy u nas się to w ogóle rzadko na święta zdarza. Nie mam licznej i nie mam też takiej rodziny, która jest bardzo mocno blisko ze sobą niestety, więc to nigdy nie był u nas jakiś…

**A czy przez ten czas byłaś na jakiejś takiej imprezie rodzinnej, na jakimś weselu, na komunii?**

Na weselu. Na jednym weselu. Tam chyba wspominałam, że 2 maja była transmisja ślubu. To był ten ślub, który miał być w kwietniu, na który miałam lecieć właśnie z Londynu. Właśnie czekam na pieniądze za te bilety. Bo w końcu napisałam i mają mi odesłać pieniądze z LOT-u. W maju była transmisja. I oni wesele z tej połowy kwietnia przełożyli na 3 lipca. I wesele się wtedy odbyło. Było mniej osób, było widać, że jest trochę mniej licznie. Szczególnie, jeśli chodzi o rodzinę tą starszą. Ale na tym weselu byłam. Nie mieliśmy przypadku korony, więc szczęśliwie nikt z nas potem nie był na żadnej kwarantannie. Ale to było jedyne wesele, na którym byłam.

**Ale wyglądało wszystko normalnie?**

Tak. Znaczy była msza, na zasadzie takiego… Zrobili po prostu odnowienie przysięgi podczas tej mszy. I po tej mszy było już normalnie tak jakby wesele, które normalnie byłoby po mszy ślubnej, tam, gdzie miało być. Ja jechałam tam z przyjacielem, jechaliśmy samochodem akurat wtedy razem.

**Miałaś jakieś obawy?**

Nie.

**Czyli wszystko normalnie. A jeszcze wracając do tej perspektywy na przyszłość. Miałaś jakieś przemyślenia dotyczące, jak się zmieni sytuacja gospodarcza czy może sytuacja społeczna jakichś grup ludzi po pandemii?**

Nie myślę o tym na tyle, że ja wiem, że w mojej rodzinie się nic nie pozmienia. Bo tak, tata nie pracuje, wręcz dostał propozycję pracy od nowego roku znowu. W takiej firmie niemieckiej, z którą ma kontrakt. I jak są jakieś okazje na współpracę, to wtedy się do niego odzywają. Mama jest nauczycielką, więc ona dostawała wypłatę nawet, jak nie chodziła do pracy przez te pół roku praktycznie. Od marca do… Więc jakby jej wypłata może była minimalnie mniejsza, a może nawet nie. Nie pamiętam. Brat pracuje w dziedzinie IT, studiuje zaocznie IT, więc tutaj też nie ma się czym martwić. Trochę się martwiłam pracą, bo sytuacja w trakcie kwietnia, maja była ciężka. Ale to wynikało trochę z tego, że firmy były nieprzygotowane na procesy rekrutacyjne zdalnie. Obecnie firmy się już dostosowały, bo musiały przez 10 miesięcy. Więc już przeprowadzenie procesu rekrutacyjnego online nie jest problemem. I tak też dzisiaj miałam rozmowę telefoniczną. Cały proces w firmie obecnej był prowadzony telefoniczne, zdalnie. I wiadomo przez Zoomy, bluejeansy i inne. Więc trochę przestałam się już tym stresować. Poza tym mówię, na razie pracę mam, jest potencjał jej przedłużenia bądź odzywają się mnie do mnie inne firmy, więc na razie nie panikuję z braku pracy jeszcze. Zobaczymy, co będzie, czy przedłużę tą, czy dostanę nową. Podejrzewam, że gdzieś tam ta grupa i rozwarstwienie… Czytałam, że gdzieś w którymś kraju rozwarstwienie społeczne na zasadzie zamożności się zwiększa. Czytałam też artykuł, w którym było napisane, że Jack Bezos o tyle i tyle milionów mu się powiększył majątek. Ale jakby czemu tu się dziwić? E-commerce teraz właśnie rośnie w siłę. Ja pracuję w mediach i rozrywce, więc to też na razie nie będzie kulało. Więc trochę jestem spokojna, jeżeli chodzi o pracę. Najspokojniejsza będę, jak już będę miała coś nagranego na pewno od tego grudnia. Bo na razie stresuje mnie fakt, że w połowie grudnia mogę zostać bez pracy, jak moje plany nie pójdą pomyślnie. Może jest to przesadny jakiś tam strach czy panika. Ale na pewno jakieś rozwarstwienie społeczne się pojawi. No sporo branż i ludzi popadło w bankructwo. Na pewno trochę to potrwa zanim się ludzie z tego wytoczą. Pytanie, jaka będzie pomoc rządu? Bo niby jakaś tam była, ale jak to się ma do rzeczywistych potrzeb? Tak że zobaczymy. Ale nie myślałam o tym jakoś za bardzo, bo szczęśliwie mnie to aż tak nie dotyka.

**Czy chcesz coś jeszcze dodać?**

Nie, już chyba wszystko wyrzuciłam z siebie. Znaczy tak w ogóle to mogę ci powiedzieć, to jest jedna z rzeczy, które zauważam. Praca 5 dni w tygodniu 8 godzin. No, rozmawiam tylko ze współlokatorkami, ale też wszystkie pracujemy, więc mało. I mam tylko tego półgodzinnego calla dziennie. Więc ja tak mało mówię do ludzi, że z moją potrzebą dużej ilości mówienia i z moją osobowością, jak już się z kimś widzę, to też mój temperament, który jest normalnie raczej taki większy, sprawia, że ja tylko właśnie mówię szybciej, więcej, jakby tak bardziej nerwowo? Zauważam po sobie, że tak bardzo muszę powyrzucać z siebie jakieś słowa, że aż to wpływa na to, jak to mówię. I czasami mam wrażenie, że niektórych to przytłacza.

**OK.**
