# Supplementary material for: The challenges arising from the COVID-19 pandemic and the way people deal with them. A qualitative longitudinal study
Source: PLoS One. 2021 Oct 11;16(10):e0258133. doi: 10.1371/journal.pone.0258133 (PMC8504766; doi:10.1371/journal.pone.0258133)
Supplement: S1 Dataset — (ZIP) [file pone.0258133.s003.zip › Transcriptions/stage 6/14.6_M_55_couple, with children.docx]

**14.6_M_55_couple with children**

**Jak ci minęły ostatnie miesiące?**

Mieliśmy trochę pracy w firmie, ale zdążyłem być 2 x na nurkowaniu w Chorwacji. Jeden wyjazd był w lipcu, drugi w sierpniu, wszystko poszło dobrze. Jeszcze się wybrałem we wrześniu z córką do Holandii i do Niemiec samochodem. Pojechaliśmy odwiedzić znajomych i w ogóle tak się wyrwać. Teraz raczej się nigdzie wyrywać nie będziemy, chociaż teoretycznie mam zaplanowane safari nurkowe w listopadzie, tylko nie wiem, czy do niego dojdzie. W Egipcie. Jeżeli będzie możliwy przelot, to będę chciał polecieć.

**Przełomowe momenty?**

Nie, nic się ważnego nie działo, co by jakoś wpływało na moje życie. Wakacje były względnie normalne, bo zazwyczaj lataliśmy gdzieś dalej, a z uwagi na warunki pojechaliśmy tym razem samochodem w miejsce, które znamy, nurkuje się tam fajnie.

**Jaka jest twoja codzienność teraz?**

Wszystko jest w cieniu epidemii, ponieważ prowadzimy szkołę i 2 przedszkola i wszystko co się dzieje wokół epidemii ma na nas jakieś przełożenie. Zanim nastąpiło zamknięcie szkoły mieliśmy 1 przypadek koronawirusa u nauczyciela w przedszkolu, 1 przypadek u nauczyciela w szkole. Musieliśmy sami wrzucić na online 2 klasy. oczywiście zgłosiliśmy sprawę do Sanepidu, który nie raczył nam w ogóle odpowiedzieć, więc sami podjęliśmy decyzję. Do tej pory nam nie odpowiedział. Najpierw był do nich telefon, kazali nam wysłać wniosek, na wniosek oczywiście nie odpowiedzieli. Formalnie nie mogliśmy zrobić nauczania online bez zgody Sanepidu, ale mamy przecież jakąś własną odpowiedzialność przed ludźmi i nie będziemy ich narażać. Oczywiście to jest świetna wymówką, że złożyliśmy do Sanepidu, Sanepid nic nie robi, więc my nic nie musimy robić, ale to nie o to chodzi i dlatego sami podjęliśmy decyzję i poinformowaliśmy tylko Sanepid, że tak robimy. Nikt nie zareagował, ale oni nie wyrabiają i nie mają tam ludzi, którzy by potrafili takie rzeczy przerobić. Przez pół roku, które mieli, żeby się do tego przygotować nie zrobili nic. Wszyscy myśleli, że jakoś to będzie, a okazało się, że jakoś wcale nie jest. Jest jeszcze gorzej w tej chwili. O ile pół roku temu ta kwestia koronawirusa była taka jakaś abstrakcyjna, bo mało kogo to dotyczyło osobiście, to w tej chwili ludzie, których znamy zapadają na tę chorobę, niektórzy objawowo i wszyscy czujemy, że to jest gdzieś blisko.

**Czy coś w twoim życiu jest takie jak przed epidemią?**

Tak. Staram się parę razy w tygodniu zrobić te 40-50 km na rowerze...Bardzo dużo rzeczy jest tak, jak przed pandemią. Ja i tak pracowałem w domu, więc tu się niewiele zmienia w moim przypadku. Dla mnie osobiście, to ta epidemie poza jakimś brakiem możliwości spotykania się ze znajomymi...Formalnie możemy się spotykać, tylko ludzie nawet nie chcą się spotykać i chodzić na jakieś imprezy. Nawet na takie prywatne, domowe. Nie chcą.

**Ale spotykacie się, czy nie za bardzo?**

Tak, spotykamy się, ale to jest naprawdę w takich ograniczonych gronach osób. Znajomi nas zapraszali, żeby do nich na Mazury pojechać w weekend i to, że nie pojechaliśmy, to nie dlatego, że się boimy koronawirusa. Boimy się, ale nie w tej sytuacji, bo to nie byłoby spotkanie w wielkim gronie. Mieliśmy po prostu jakieś inne rzeczy na głowie.

**Poza tym, że mniejsza liczba osób na spotkaniu, to jeszcze jakoś się te spotkania zmieniły?**

Tak, oczywiście. Nie całujemy się na przywitanie, staramy się utrzymywać jakieś większe dystanse jeden od drugiego. Ja zresztą nigdy nie lubiłem, jak ktoś stoi za blisko obok mnie i coś do mnie mówi, bo ja zawsze wolałem utrzymywać większy odstęp niezależnie od wirusa. Taki mam charakter, ale w tej chwili jak widzę, że jakiś znajomy do mnie za blisko podchodzi, to ja się cofam i znów się cofam. To nie jest duża zmiana, bo w pewnym stopniu zawsze się tak zachowywałem, ale zauważyłem, że ludzie też raczej nie podchodzą zbyt blisko. Niektórzy nadal tak robią, ale może po prostu oni tacy są i robią to jakoś bezwiednie. Zresztą ja w tej chwili to nawet nie mam oporów, żeby powiedzieć, że chciałbym zachowywać trochę większy odstęp. Kiedyś nie wypadało tak powiedzieć, prawda? Teraz ok i nikt nie ma z tym problemu.

Znasz osoby, które w ogóle odmawiają spotkań?

Tak. Nasi znajomi powiedzieli, że on ma astmę, że teraz z nikim się nie spotykają, siedzą w domu, starają się jak najmniej mieć kontaktu i się nie spotykają.

**Co najbardziej ci teraz przeszkadza?**

Najbardziej to taki brak konsekwencji naszego rządu. Brak logiki w ich postępowaniu. Jeżeli się mówi, że trzeba przede wszystkim chronić osoby starsze i zamyka się restauracje, to jest to brak logiki. Bo gdzie chodzą ludzie starsi? Głównie chodzą do kościołów a do restauracji chodzą głównie ludzie młodzi. Ja nie mówię, że nie trzeba zamykać restauracji, ale uważam, że jest bardzo dużą uciążliwością, jeśli np. chodzisz do pracy i nie możesz zjeść lunchu, bo knajpa jest zamknięta. We Włoszech, które też są bardzo dotknięte epidemią restauracje są czynne do 18-tej i może to jest jakieś wyjście. A jeżeli już całkowicie zamykamy, to zamknijmy też kościoły, niech ten kler da ludziom dyspensę. I to jest najbardziej wkurzające, bo to nas wszystkich prędzej czy później dotknie. Poza tym, jeżeli mamy do czynienia z takim brakiem logiki we wprowadzaniu tych wszystkich obostrzeń, to ludzie przestają wierzyć w ich sens w ogóle i zaczynają kwestionować wszystko. Tak samo było z tym zakazem wejścia do lasu, który nie miał sensu epidemicznego, tylko był represyjny i ludzie zaczęli też olewać totalnie zakazy, które mają sens. To powoduje, że ludzie tracą zaufanie do rządzących, które i tak było nieduże. W tej chwili już totalnie mają to gdzieś i to jest niebezpieczne. Dzisiaj Morawiecki powiedział, że będzie jakiś program pomocy dla targowisk i bazarów. Kurczę, przecież targowiska i bazary nie zostały zamknięte. One działają i oni mają tam normalny biznes. Ja podejrzewam, że oni już w ogóle stracili kontakt z rzeczywistością. Może ktoś kiedyś chciał zamknąć targowiska, oni ich nie zamykają, ale mieli już ppt zrobionego. No kurczę, jaki sens? A może należy się spodziewać, że teraz zamkną targowiska? Tylko tego przecież się nie robi. To jest takie partactwo...Żeby nawet prezentacji porządnej nie umieć zrobić i nie przemyśleć jej wcześniej. Przecież jak ja bym takie gówno zrobił pracując w korporacji, to by mnie na kopach wywalili? A to jest rząd kraju europejskiego średniej wielkości.

**Emocje**

13 - to było w lecie. To była taka nadzieja na to, że będzie spokojnie, że jednak nie będzie jakichś kolejnych obostrzeń, że jednak w jakiś tam sposób z tego będziemy wychodzić.

15 - to, co się dzieje teraz. Spokojny wodospadzik, spokojne płynięcie wody, a na to z góry wtacza się wielka fala, która zaraz to wszystko zaleje.

2 - też pasuje do obecnej sytuacji. Tak się jakoś przykleiliśmy i nie możemy się odkleić.

**Jakie uczucia ci teraz towarzyszą?**

Jakaś taka niewiadoma co będzie dalej. Szczerze mówiąc trochę się boję, że jednak i mnie to będzie dotyczyło, a nie jestem osobą najmłodszą i nie chciałbym trafić na Stadion Narodowy. To jest coś, czego nie było poprzednio, bo służba zdrowia była w miarę wydolna, te oddziały zakaźne jakoś tam funkcjonowały i dawały pewność, że jak człowiek tam trafi, to będzie zaopiekowany. Jak trafisz na stadion to raczej...No dupa po prostu. Szanse, że się stamtąd wyjdzie żywym są niewielkie. Boję się też, że jednak zostanie wprowadzony lockdown i on będzie niestety bez jakichkolwiek. Programy pomocowe będą na pewno dużo mniejsze niż były, co może się przełożyć na naszą osobista sytuację finansową. Myśmy i tak stracili poprzednio, bo odmówiono nam wszelkich form pomocy, dostaliśmy odmowę pomocy z tarczy i jedynie dostaliśmy 50% ulgi w ZUS-ie, a straciliśmy dużo więcej przychodów. Obawiam się, że teraz może być jeszcze gorzej. Sytuacja może zagrażać również naszej osobistej egzystencji finansowej.

**Boisz się o swoje zdrowie, a o zdrowie innych?**

Moja żona jest młodsza, dzieci mam młode i zdrowe, także trochę mniej. Mama ma 90 lat, ale jest zdrowa i się dobrze izoluje. Najbardziej to ja się boję właśnie o siebie, ale mimo to wybieram się na to nurkowanie w listopadzie, chociaż szczerze mówiąc trochę wewnętrznie mam nadzieję, że to jednak będzie odwołane. Sam tego nie odwołuję, bo nie lecę tam sam. czytałem bardzo dużo publikacji, że w samym samolocie prawdopodobieństwo zarażenia się jest dość nieduże, a większość ludzi i tak tam będzie przebadana. Egipt jest bardzo bezpiecznym krajem na razie i w całej tej historii lotnisko jest najmniej bezpieczne.

**Dlaczego zacząłeś się bać o siebie? Mówiłeś poprzednio, że nawet jak zachorujesz, to najpewniej przejdziesz to lekko, że jesteś zdrowy, że dbasz o siebie...**

W 100% człowiek pewien nigdy nie jest. Rzeczywiście jestem zdrowy i nic mi nie dolega, ale niektórzy też są zdrowi, a mimo to przechodzą to bardzo ciężko albo nawet kończy się to bardzo fatalnie. Wiek też jest tu jakimś elementem zwiększającym poziom ryzyka. Tym bardziej, że jak patrzę na statystyki, to większość osób, które trafiały do szpitala w stanie ciężkim to były osoby powyżej 70-tki, a w tej chwili ten poziom wieku się obniżył. Ryzyko jest. Poza tym kiedyś człowiek miał gwarancję, że zostanie dobrze zaopiekowany, a w tej chwili przy tym totalnym rozpadzie służby zdrowia, to oni nie są w stanie tego opanować. Naprawdę nie chcę trafić na Stadion Narodowy i dla mnie to jest perspektywa najgorsza, jaką można sobie wyobrazić. Ja nigdy w życiu nie byłem w szpitalu. Byłem na jakiejś drobnej operacji 2-3 dni w prywatnym szpitalu, w prywatnym pokoju, z prywatną łazienką w warunkach bardzo luksusowych, a tu trafiasz na salę 100-osobową na stadionie. No sorry. I w tym momencie nie masz jakiejkolwiek szansy na indywidualne podejście, bo jak masz szczęście to do ciebie raz dziennie podejdzie jakaś pielęgniarka, która jest po kursie jednodniowym.

**Wiedziałbyś co masz zrobić, jakie są procedury?**

Zadzwoniłbym do lekarza i wysłuchałbym instrukcję. To jest jedyne co mogę zrobić. Nie do końca jestem pewien jakie są procedury, bo ja od ponad 20 lat nie uczestniczę w państwowej służbie zdrowia i leczę się wyłącznie w Medicover, a tu podobno Medicover nie jest w stanie dać nawet skierowania na test. Tym bym się nie przejął, bo bym sobie zapłacił 400 zł i zrobił sobie test prywatnie. Ja wolę zapłacić za test, który mi przywiozą taksówką do domu niż stać gdzieś kilka godzin w kolejce. Mnie akurat na to stać. Już robiłem sobie taki test, bo chciałem sprawdzić, jak system działa, bo wiem, że będę musiał zrobić test przed wylotem i tam są te 72 godz., więc chciałem wiedzieć, jak to wygląda. Poza tym to było akurat po moim powrocie z Holandii, gdzie spotykałem się jednak z wieloma osobami., bywałem w knajpach, itd.

**Jak się czułeś czekając na wynik?**

Jakoś tak zupełnie bez emocji. Ja byłem raczej pewien, że wynik będzie negatywny i taki przyszedł.

**Masz jakieś sposoby na radzenie sobie z niepokojem, który odczuwasz?**

Dzisiaj sobie np. pojadę nad Zalew Zegrzyński i z powrotem. To jest bardzo dobra metoda. Słuchawki na uszach, dobry audiobook i można jechać. To jest najlepsze. I praca, bo jak masz dużo roboty to się nie zajmujesz głupotami.

**Jak radzą sobie ludzie w twoim otoczeniu?**

Mam bardzo dużo różnych ludzi w otoczeniu. W najbliższej rodzinie mam człowieka, który jest antycovidowcem jeszcze cały czas, chociaż ostatnio rura mu zmiękła. On uważa, że to jest spisek elit, Billa Gatesa, itd. Od początku tak myślał i na początku było nawet jeszcze gorzej, ale teraz jednak mięknie mu rura i widzi, że to jednak istnieje. Kiedyś to w ogóle była zmyślona pandemia wg niego. Wszystkim nam chyba mięknie rura powoli, chociaż podobno są tacy, którym nie mięknie. Trudno mi powiedzieć, jak sobie ludzie radzą ze swoimi lękami. Prawdopodobnie te wszystkie jego poglądy są spowodowane po prostu lękiem i to jest jakieś takie zaprzeczenie.

**Sam byłeś w kwarantannie?**

Nie, nigdy. Te osoby, o których wiem, że chorowały, to nie są osoby, z którymi na co dzień przestaję.

**Czy coś się zmieniło w twoim stosunku do aplikacji covidowych?**

Na temat ProteGoSafe poczytałem sobie na niebezpiecznik.pl i wiem, że to jest aplikacja napisana na kolanie, bez zastosowania wymaganych procedur bezpieczeństwa komputerowego, internetowego. Nie ma sensu jej instalowanie i ja tego na pewno nie będę instalował. Nie może być obowiązku instalowania czegokolwiek. Zdaje się, że jest jakaś aplikacja do zainstalowania na kwarantannie, że trzeba sobie robić selfie i przesyłać, ale nawet jak ktoś nie zainstaluje, to przysyłają policjanta. Jak teraz jest pół miliona ludzi na kwarantannie, to nawet policja nie jest w stanie tego skontrolować. Tym bardziej, że teraz takie durne przepisy zostały wprowadzone. Kiedyś było tak, że jeżeli jakakolwiek osoba w rodzinie jest skierowana na kwarantannę, to cała rodzina musi być na kwarantannie. A teraz jedna osoba jest na kwarantannie, a cała reszta normalnie funkcjonuje i na kwarantannie nie jest. Bzdura totalna, ale z drugiej strony ja rozumiem, że jakby oni teraz całe rodziny zaczęli wysyłać na kwarantannę, to tych ludzi byłoby nie pół miliona a 2 miliony na kwarantannie, a ktoś musi pracować w tym kraju. Tylko w tym momencie zadajesz sobie pytanie, jaki w ogóle jest sens kwarantanny? Dziecko miało kontakt z chorym nauczycielem i jest na kwarantannie a jego rodzice nie.

**Czy orientujesz się w obecnych obostrzeniach, śledzisz zmiany?**

Kiedyś to śledziłem, teraz staram się śledzić, ale nie jestem w 100% pewien, że wiem wszystko. Wiem to, co dotyczy bezpośrednio nas jako placówki edukacyjnej. Nawet nie znam do końca tych przepisów dotyczących maseczek i nie wiem, czy jak jadę na rowerze przez puste pole, to muszę mieć maseczkę, czy nie. W lesie nie muszę mieć, w parku też nie, ale pole nie jest ani lasem, ani parkiem, więc teoretycznie chyba muszę mieć maseczkę. To jest znów totalna bzdura i większość krajów już odeszła od nakazu noszenia maseczek na powietrzu. Mówią tylko, że jeżeli idziesz zatłoczoną ulicą to załóż. Ja oczywiście stosuję metodę zdrowego rozsądku i jeżeli jadę przez Legionowo, gdzie widzę ludzi, to maseczkę zakładam, a jeżeli jadę przez pole, gdzie nie ma nikogo w pobliżu, to nie zakładam. Używam masek jednorazowych. Zakupy robię w Selgrosie i tam są takie rękawiczki jednorazowe przy warzywach i wtedy je zakładam. Takie zakładanie rękawiczek wszędzie dla mnie nie ma sensu.

**Które obostrzenie wydają ci się sensowne i podwyższające bezpieczeństwo?**

Przede wszystkim bardzo ważne jest noszenie maseczek we wszystkich pomieszczeniach zamkniętych i na zewnątrz tam, gdzie są skupiska ludzi. To jest bardzo ważne i dla mnie to jest podstawowe ograniczenie. Wiele krajów to stosuje i to już zostało naukowo udowodnione, że to rzeczywiście działa, chociaż cały czas słyszy się tych palantów, którzy krzyczą, że maseczki nie pomagają. Nawet jacyś lekarze durni tak mówią.

Uważam też, że zamknięcie szkół i wyższych uczelni ma sens. Jeśli chodzi o restauracje, to generalnie ma to sens, szczególnie teraz kiedy siedzi się w środku. Większość transmisji np. w Chorwacji była w klubach nocnych a nie w restauracjach, gdzie siedziało się na powietrzu. Teraz mamy taką porę roku, że może rzeczywiście jest sens ograniczyć pracę restauracji tylko do godzin dziennych. Nikt nie zakazuje podróży, więc jak jedziesz samochodem z Warszawy do Szczecina, to chcesz gdzieś zjeść, musisz gdzieś zjeść. Możesz zjeść w McD, ale to nie o to chodzi i skoro nie ma zakazu przemieszczania, to te osoby, które podróżują i muszą zjeść, to powinny mieć taką możliwość. To ograniczenie powinno być inaczej zrobione, ale jest jak jest i zamknęli wszystko.

**Siłownie i baseny?**

Byłem parę razy w siłowni zanim zamknęli, ale nie czułem się tam zbyt ok. Przez 4 miesiące byłem może 3 razy, a wcześniej chodziłem 2 x w tygodniu. Specjalnie mnie to nie ciągnęło i miałem dość duże obawy, żeby tam chodzić. Co do basenów, to nie wie, czy one są jakimś elementem transmisji. Powinniśmy wszyscy zakładać, że ci, którzy wprowadzają te ograniczenia, to je przemyślenia, ale ktoś ten zakaz wstępu do lasu też wymyślił i w tej sytuacji nie jestem taki do końca pewien. Często mam wrażenie, że część z tych zakazów jest wprowadzana po to, żeby pokazać, że rząd coś robi i tylko tyle.

**A co z klasami 1-3?**

Też jest sens je pozamykać i tak samo przedszkola, tylko gdyby pozamykano te młodsze klasy i przedszkola, to obawiam się, że budżet kraju by tego nie wytrzymał, bo trzeba by było płacić zasiłki rodzicom. Oni już w tej chwili widzą, że to było bardzo duże obciążenie dla budżetu i tych ograniczeń nie wprowadzają wyłącznie ze względów budżetowych. Po prostu nie ma na to pieniędzy. Podobnie jest w pozostałych krajach europejskich. Wszędzie przedszkola są czynne, młodsze klasy w szkołach są czynne. Nie wiem, na jak długi czas trzeba by je było zamknąć, żeby to zadziałało. W sytuacji wiosennej tak naprawdę pomogło to, że się robiło cieplej i ludzie mniej czasu spędzali w zamkniętych pomieszczeniach. Teraz zaraz będziemy mieli zimę, Polak bardzo przeciągów nie lubi i ludzie uważają, że największym wrogiem dla naszego zdrowia są przeciągi. I tak mieliśmy ze strony rodziców krytykę, że u nas w przedszkolu są okna pootwierane, bo dzieci się po przeziębiają, ale przecież my musimy otwierać te okna.

**Zakaz imprez?**

To jest słuszne. Od samego początku było wiadomo, że wesela i stypy były głównym elementem transmisji. Uważam, że w ogóle pomysł, że pozwolono w maju/ czerwcu robić wesela bez żadnych ograniczeń...Dla mnie to było po prostu chore, zwłaszcza, że wesele nie jest podstawową potrzebą życiową człowieka. Oczywiście marzeniem wielu jest mieć wesele, białą suknię, ale wesela się robi głównie w środowiskach wiejskich, bardziej w Polsce wschodniej niż zachodniej. PiS się po prostu chciał swojemu elektoratowi przypodobać. Nie jestem pewien czy teraz nie ma wesel. W czerwonej strefie chyba wolno do 50 osób. Nie jestem pewien jak to wygląda w tej chwili, bo jeśli można do 20 osób, to jest jeszcze ok. W domu możesz przecież zrobić sobie prywatną imprezę.

**Nie, teraz do 5 osób.**

Do 5-ciu? Czyli nie śledzę tych wszystkich ograniczeń, jak widać. Takie ograniczenie ma jakiś sens, ale kto zweryfikuje, ile osób się spotyka w domu? To jest chyba taka bardziej sugestia niż jakiś zakaz, który można wyegzekwować.

**Ochrona seniorów?**

Raz poszedłem do sklepu w godzinach dla seniorów, bo ja już mogę i takich kolejek jak były wtedy to dawno nie widziałem. Więcej nie będę w tych godzinach chodził. Bezpieczniej nie jest dzięki temu, absolutnie nie. Zakaz wychodzenia z domu jest, ale oprócz wychodzenia do pracy, oprócz pójścia w celu zaspokojenia podstawowych potrzeb i oprócz pójścia do kościoła, czyli praktycznie mogą wychodzić wszędzie. Kolejny zakaz totalnie na papierze. Gdyby jednocześnie zamknięto kościoły, to miałoby jakiś sens, ale nie można też kogoś pozbawiać prawa do przemieszczania się jakimś zwykłym rozporządzeniem. Wielu z nich to jedynie boi się kary boskiej i jeżeli im się powie, że będą mieli dyspensę, to do kościoła też nie pójdą.

**Czy jest coś w tych obostrzeniach, co uważasz za niepotrzebne?**

Noszenie maseczki, kiedy jadę na rowerze po pustym polu. Poza tym wprowadzono przepis, że nawet osoby razem zamieszkujące muszą w samochodzie być w maseczkach. To jest bzdurne. Może się z tego wycofali jakoś ustnie, ale rozporządzenie nie zostało zmienione.

**Czytałam, że się wycofali i że teraz w ogóle w samochodzie nie trzeba mieć maseczki.**

Czyli kolejne ekstremum, bo jednak noszenie maseczki ma sens. Oni to w ogóle wprowadzili po to, żeby ułatwić pracę policji. Policjant widzi, że jadą 2 osoby, nie mają maseczki, no to je zatrzymuje i karze. Chodzi o to, żeby mu zaoszczędzić czasu na wnikanie czy są to osoby razem zamieszkujące czy nie. Ale przepisy nie powinny być wprowadzane po to, żeby ułatwić pracę policji. One powinny mieć jakiś sens. Mówimy o jakimś ograniczaniu naszych swobód i ograniczyć je można, ale nie po to, żeby ułatwić pracę policji.

**Powinno się teraz wprowadzić jakieś bardziej radykalne ograniczenia albo np. stan wyjątkowy?**

Uważam, że powinno się wprowadzić stan wyjątkowy, bo wtedy byłaby czysta sprawa, jeżeli chodzi o wypłatę rekompensat firmom, które na tym straciły. Teraz jest bardzo niejasne, komu się te rekompensaty należą. Myśmy się w lipcu odwołali od odmowy dofinansowania do wynagrodzeń i jak na razie nic. Chyba w ogóle się tym nie zajęto jeszcze. Gdyby był stan wyjątkowy to by było złożenie wniosku do wojewody i tyle. I wtedy też nie byłoby w ogóle tej dyskusji na temat maseczek. Ja bym się wtedy podporządkował, zresztą i tak się podporządkowuję, ale nie byłoby dyskusji na temat zgodności tego z konstytucją. Teraz ludzie się odwołują od mandatów, bo przepis jest wprowadzony niezgodnie z prawem i większość mandatów jest anulowana, ale to bez sensu obciąża system sądowniczy.

**Nie obawiałbyś się nadużyć ze strony władzy?**

Oczywiście, że tak, szczególnie od tej władzy, ale oni i tak tej władzy nadużywają. Można się spodziewać tych nadużyć, ale gdyby oni chcieli je robić i im by na tym zależało, to już dawno by stan wyjątkowy wprowadzili. Oni go nie wprowadzają tylko ze względu na możliwość bardzo prostego dochodzenia odszkodowań.

**Czy ty coś byś jeszcze zrobił?**

Przede wszystkim zamknąłbym kościoły, zakazałbym wesel większych niż 20 osób. no i nie robiłbym takich głupot jak zaostrzenie prawa aborcyjnego, co wyprowadziło ludzi na ulicę. To będzie teraz trwało i trwało. Nawet, jeśli wszyscy na ulicach noszą maseczki to one jednak do końca nie chronią. Teraz sami nie wiedzą jak się z tego wycofać, nie mogą się przyznać, że mają Trybunał pod kontrolą. Największa głupota, jaką można było zrobić w tej sytuacji i uważam, że oni to rozwiążą właśnie w ten sposób, że brutalnie rozpędzą te demonstracje. Może wprowadzą najpierw coś w rodzaju stanu wyjątkowego. Ten wzrost zakażeń po demonstracjach będzie pewnie mniej więcej za tydzień.

**Co teraz myślisz o koronawirusie?**

Nic się nie zmieniło w moim myśleniu. To jest bardzo niebezpieczny wirus.

**Jak oceniasz obecne zachowania ludzi?**

Mało jednak wychodzę, czasami idę do sklepu, ale obserwuję, że jednak ludzie te maseczki noszą, ale bardzo często noszą je nieprawidłowo. Mam taki sposób, że jak w kolejce ktoś za blisko do mnie podejdzie to zaczynam kasłać. Działa od razu. Złości mnie jak ktoś ma źle założoną maskę i nawet zwracam ludziom uwagę. Jak wracałem z Niemiec zatrzymałem się w Burger King i weszło takich dwóch bez maseczek. jak zwróciłem im uwagę to mi powiedzieli, żebym spierdalał po prostu. I co miałem zrobić? Grzeczne zwrócenie uwagi nie poskutkowało. Mam jednak wrażenie, że osób, które ostentacyjnie nie noszą maseczek lub noszą je źle jest dużo mniej niż kiedyś.

**Spodziewałeś się, że będzie tak jak jest? Można temu było jakoś zapobiec?**

Wszyscy mówili, że będzie ta 2 fala, specjaliści tak mówili, ale ja jednak miałem nadzieję, że jej nie będzie. Nie wyobrażałem sobie, że ten wzrost będzie aż taki. Widać, że podobne zjawisko jest we wszystkich krajach i prawdopodobnie ma to dużo wspólnego z tym, że więcej czasu spędzamy w zamkniętych pomieszczeniach. Wszyscy mówią, że to w związku z tym, że było rozluźnienie latem, że jeździliśmy, że podróżowaliśmy i może tak, ale wg mnie to jest powrót do szkół, który miał znaczenie, uczelnie też na początku pracowały, okna pozamykane, zaduch i kilkaset osób.

**Kiedy poczułeś, że jednak idzie ta 2 fala?**

To było w połowie września. Ten nasz wyjazd do Holandii i Niemiec był taki na zasadzie, że jedźmy, bo zaraz wszystko pozamykają. I rzeczywiście to był taki ostatni moment, kiedy można było legalnie wjechać bez kwarantanny z Polski do Niemiec. Już wtedy Niemcy były zamknięte na Holandię, więc jadąc z Holandii do Polski mogliśmy tylko przejechać tranzytem. Nie zrobiliśmy tego i przenocowaliśmy w Niemczech, bo nie chciało nam się jechać całą noc. Różne kraje mają różne sposoby. W Niemczech każdy może pójść i zrobić sobie bezpłatnie test. Każdy.  I za 24h najpóźniej masz wynik. Z biznesowego punktu widzenia chyba taniej jest wykryć jak największą liczbę osób bezobjawowych. U nas ten test kosztuje 400 zł, ale podejrzewam, że państwo kupuje go dużo taniej. Wydanie 150 zł na osobę byłoby pewnie tańsze niż przerobienie tych stadionów. Brak jest jakiegokolwiek myślenia biznesowego. Niemcy nie mają tego problemu i dzięki temu nie ma aż takiej transmisji, bo ludzie się samoizolują. Teraz się okazuje, że Błaszczak jeździł po wszystkich szkołach wojskowych z bezobjawowym Covidem i teraz tam połowa składu ma Covida. No ludzie...

**Czy cokolwiek ten rząd zrobił dobrze?**

Dobrze, że nie zamknęli granic zupełnie, bo jak się okazuje, ta transmisja międzykrajowa jest niewielka. To znaczy niby nie są zamknięte, ale trochę są, bo do wielu krajów musisz albo test zrobić, albo idziesz na kwarantannę, ale jak gdzieś bardzo musisz pojechać, to sobie to badanie zrobisz i nie ma takiego poczucia jakiegoś zamknięcia, wyłączenia totalnego w swobodzie podróżowania. Ja nie wiem na jak długo, bo podejrzewam, że oni od 1.11. będą chcieli jakieś kolejne ograniczenia wprowadzić. Podejrzewam, że dadzą jeszcze ludziom pójść na cmentarze i koniec. Podejrzewam, że oni będą się starali zrobić coś w rodzaju lockdownu, ale będą to chcieli zrobić tanio, więc albo zrobią lockdown bez rekompensat, albo zamkną część branż bez zamykania szkół i przedszkoli, żeby tych zasiłków nie płacić.

**Coś jeszcze dobrze zrobili?**

Nie mam punktu, gdzie ja mógłbym ich pochwalić. No nie mam.

**Źródła informacji?**

Co pewien czas śledzę, ale to nie jest tak, że ja codziennie sprawdzam te statystyki i te krzywe. W momencie, kiedy mnie jakiś obszar zaciekawi, no to sprawdzę. Śledzę nadal rosyjskie media, bo mam tam kontakty. W momencie, kiedy poziom zachorowań w Polsce zbliżył się do poziomu Rosji, która jest krajem 10 razy większym od Polski, to w tym momencie mamy problem. Czysto statystycznie nawet patrząc.

**Sprawdzałeś, ile jest u nas dzisiaj zakażeń?**

Nie, jeszcze nie zdążyłem, więc nawet nie wiem. Ogólnie to akurat sprawdzam, ale dzisiaj jeszcze nie miałem kiedy.

**Dzisiaj jest ponad 16300.**

Tego się należało spodziewać, bo przecież zawsze po weekendzie tak jest.

**Jak oceniasz wiarygodność informacji? Komu wierzysz?**

Nie wiem czy oni tymi danymi statystycznymi manipulują. Myślę, że jeżeli manipulują to sztucznie obniżając liczbę testów. Jednak nie jest łatwo dostać się na ten bezpłatny test a nie każdego stać, żeby wydać 400 zł. Przez dłuższy czas chyba w ten sposób starali się to ograniczyć i to mnie najbardziej niepokoi - ta niedostępność testów i to staranie się ograniczania ich jakiegoś. Z punktu widzenia tego co oni wydają na walkę z pandemią w ogóle, to przetestowanie nawet wszystkich, to naprawdę nie byłyby duże pieniądze. Jak ja sobie robiłem prywatnie, to taksówką rano do mnie przyjechała z wymazówką i wynik miałem tego samego dnia o 21-szej i nie musiałem czekać kilku dni na wynik jak słyszę, że się dzieje.

Mam wrażenie, że jeśli chodzi o wiarygodność informacji, to teraz jest gorzej niż na początku pandemii. Dla mnie oni są coraz mniej wiarygodni. Mam wrażenie, że teraz oni mają więcej do ukrycia. Oni próbują zrobić tak, żeby nie siać paniki, żeby jakoś ludzi uspokoić, ale z drugiej strony to jest tak jak w tej historyjce, że nie ma co babci niepokoić, niech się babcia cieszy. Ludziom się jednak powinno mówić prawdę i jak jest, ale oni bardziej się swoją popularnością przejmują niż zdrowiem fizycznym i psychicznym społeczeństwa.

**Znalazłeś jakieś źródła, którym wierzysz bardziej niż innym?**

Nie. Jeśli chodzi o dane statystyczne to za bardzo nie ma innych źródeł, które mogłyby dać ogląd sytuacji. Nie ma żadnej równoległej statystyki. Są ludzie, którzy jakoś weryfikują te ogólne dane na podstawie danych lokalnych, które są publikowane lokalnie, ale to się mniej więcej zgadza. Za bardzo się tego chyba nie da zmanipulować i to by była już chyba zbyt duża kombinacja.

**Czy masz jakieś osoby, ekspertów, którym wierzysz bardziej/ mniej?**

Oni w sumie wszyscy mają ten sam przekaz. Nie słyszałem żadnego eksperta, który by kwestionował to co się dzieje, konieczność stosowania ograniczeń. Wszyscy są w miarę wiarygodni, żaden z nich nie mówi, że polityka naszego rządu jest super a przynajmniej takiego nie słyszałem, chyba że był urzędnikiem ministerstwa. Oni są w miarę wiarygodni, każdy z nich zwraca uwagę na nieco inne aspekty, ale w miarę wszyscy mówią to samo.

**Jak sobie wyobrażasz koniec pandemii?**

Ja jednak liczę na szczepionkę, że będzie. Jeżeli się pojawi jakaś szczepionka w Polsce to będę się starał od razu się zaszczepić. Wkurzyli mnie w tym roku, bo ja na grypę się szczepię od 25 lat i to był pierwszy rok, kiedy nie miałem możliwości zaszczepić się na grypę. Jestem wkurzony na to, bo uważam, że właśnie teraz trzeba się szczepić, a szczególnie jeśli szczepię się co roku i wiem, że to jest skuteczne, bo ani razu od 25 lat nie zachorowałem. Medicover nie był w stanie zapewnić wystarczającej liczby szczepionek. Próbowałem jakieś reklamacje pisać, jakieś pisma i sorry, ale nie ma.

**Szczepionka to koniec pandemii?**

Jeżeli będzie zaszczepione dużo osób, to będzie koniec przynajmniej w tych krajach, gdzie szczepienia będą powszechne, ale podejrzewam, że cały czas jednak będą na świecie obszary, gdzie ludzie się nie będą szczepić choćby ze względów finansowych. I tak zostaną tacy, którzy się nie będą szczepić, bo nie. Ostatnio wszędzie zaczęli publikować jakąś tam ustawę niby z 2010 r., że niby można kogoś zmusić do szczepienia, ale ta ustawa zawsze była. To jest po prostu kopia ustawy z lat 60-tych, która była wprowadzone w momencie, kiedy Polska walczyła z gruźlicą, z ospą. Zawsze można było ludzi zmusić do szczepienia, więc uważam, że akurat zmuszenie ludzi do szczepienia w momencie, kiedy mamy pandemię, która jest groźna ma sens. Zaszczepić siłą i tyle. Nie jestem aż takim wolnościowcem i uważam, że szczepić się trzeba, chyba że ktoś ma przeciwskazania medyczne. Właśnie po to, żeby ci ludzie, którzy mają obniżoną odporność byli zdrowi, to wszyscy inni się powinni zaszczepić.

**Jak myślisz, kiedy będzie ta szczepionka?**

Mówi się, że jakaś brytyjska jest już prawie gotowa i ma być już w styczniu/ lutym. Podobno jest bardzo dobra, ale jeszcze wyprodukować szczepionkę w takich ilościach to nie jest taka prosta rzecz. To jest jakaś hodowla biologiczna i proces, który wymaga czasu i mocy produkcyjnych. Nie wiem, kiedy ta szczepionka będzie dostępna, żeby zaszczepić wszystkich. Widząc co się dzieje ze szczepionką na grypę, można przypuszczać, że podobnie będzie ze szczepionką na koronawirusa. Można przypuszczać, że z tym koronawirusem będziemy żyć jeszcze kilka lat.

**A ten model szwedzki, odporność stadna?**

Jak widać odporności stadnej nie mają, bo wzrasta tam liczba zachorowań. To co jest teraz wprowadzone w Polsce to jest właśnie model szwedzki, bo przecież wszystko jest otwarte. Knajpy są zamknięte i to jest jedyne co nas różni od modelu szwedzkiego. Polaków niestety trzeba jednak brać za mordę, bo nie są tak zdyscyplinowani jak Szwedzi. Niemcy też się zupełnie inaczej zachowują i też bardziej dostosowują się do nakazów i zakazów i dlatego tam ta liczba zachorowań na 100 tys. jest o połowę albo jeszcze więcej niż połowę niższa niż u nas.

**Co sobie myślisz teraz o przyszłości świata?**

Jest to jakiś tam kryzys, jeden z tzw. czarnych łabędzi, które od czasu do czasu nawiedzają ziemię i trzeba sobie z tym jakoś poradzić. Będzie to dość długo trwało, zostanie na stałe w historii jako duży kryzys porównywalny może z grypą hiszpanką, która została trochę przyćmiona przez I wojnę. Czytałem, że na niż zmarło więcej osób niż było ofiar I wojny światowej. Świat jakoś z tego wyjdzie, tylko pytanie, kiedy. Na pewno to będzie miało bardziej długofalowe skutki, jeżeli chodzi choćby o coś takiego jak podróże służbowe. Ludzie się nauczyli załatwiać dużo spraw online i podejrzewam, że tak już zostanie. Podróży służbowych na pewno będzie mniej i to już zostanie. Prawdopodobnie w ogóle przez wiele lat ludzie będą mniej podróżować albo może będzie zupełnie przeciwnie, bo będą odreagowywać okres zamknięcia. Trudno powiedzieć. Byłem w Chorwacji w lipcu i w sierpniu. Jadąc tam w lipcu miałem wrażenie, że jest mało ludzi i na autostradach, i tam na miejscu, a w sierpniu już był tłok, bo ludzie już się zorientowali, że można i mniej się już obawiali. Szczególnie dużo było Polaków, którzy zobaczyli jaki tłok jest nad polskim morzem, a w Chorwacji wcale nie było lepiej. To chyba też było jakieś odreagowanie tego zamknięcia i ja też w podobny sposób zareagowałem. Jeszcze nigdy nie było czegoś takiego, żebym 2 razy w odstępie 3 tygodni pojechał gdzieś na urlop i to jeszcze w to samo miejsce.

**Myślisz, że to był błąd, że ludzie pozwolili sobie na to odreagowanie?**

Dla mnie to nie był błąd, bo pojechałem i nie zachorowałem ani nikt z moich znajomych nie zachorował. Ja podejrzewam, że nawet gdyby ludzie nigdzie nie jeździli, to tak czy tak stałoby się to, co jest teraz. To jednak chyba nie na skutek tych wyjazdów i podróży wzrosły zachorowania tylko to jednak były te szkoły, przebywanie w zamkniętych pomieszczeniach. Starałem się śledzić skąd był taki duży wzrost zachorowań w Chorwacji, i większość to były wesela i kluby nocne. Jak oni to zauważyli, to nocne kluby szybko zamknęli.

**Czy ludzie się zmienią? Jak się zmienią?**

Podejrzewam, że wejdzie w krew utrzymywanie większego dystansu. Podejrzewam, że my powoli będziemy zwiększać tę przestrzeń prywatna wokół siebie. Ku mojej ogromnej radości. Nie wiem na jak długo to zostanie. Nigdzie też nie jest powiedziane, że nie będzie kolejnej epidemii, bo przy tak dużej mobilności ludzi nie można tego wykluczyć. Może będzie nawet jeszcze bardziej zjadliwa niż ta. Nie wiem. Nie można wykluczyć, że w ludziach będzie większy poziom lęku, że staną się mniej beztroscy. Teraz wszyscy to przeżywamy, bo każdy z nas zna kogoś, kto to przeszedł a wielu zna kogoś, kto przeszedł to dość ciężko, więc na pewno nastąpi większa rezerwa. Poza tym gospodarka mocno ucierpiała i sporo ludzi ma jednak mniej pieniędzy. Nawet w Polsce. To też trochę czasu potrwa zanim to wszystko odbije się od dna.

**Które grupy będą najbardziej dotknięte zmianami?**

Ludzie, którzy działają w branżach najbardziej dotkniętych - tak jak ci moi znajomi mający sieć hosteli. Oni bardzo mocno dostali finansowo i już ich nie będzie stać np. na takie wyjazdy jak wcześniej, bo oni bardzo dużo podróżowali po świecie. Zamiast na pd. Afryki będą jeździć na Mazury. Już tak robią. My osobiście aż tak mocno finansowo nie dostaliśmy. Zarabiamy mniej, ale nie musimy aż tak bardzo się ograniczać, choć też nie wiem czy tak zawsze będzie. Gdyby zamknęli w tej chwili na dłuższy czas szkoły i przedszkola to musimy się liczyć z tym, że będziemy mieli niższe dochody i jak długo wytrzymamy też nie wiemy. Teraz nauczyciele w edukacji publicznej dostali podwyżki od września, więc my też musieliśmy podnieść wynagrodzenia i bardzo możliwe, że będzie nam trudniej przetrwać, bo mamy zwiększone koszty stałe.

Możliwe, że brak kontaktu osobistego, brak bezpośredniej wymiany kontaktu też będzie miał wpływ. To może mieć przełożenie i ja jakoś to widzę u swoich dzieci, a szczególnie u córki, która w zeszłym roku robiła maturę. Decyzja o tym na jakie pójść studia bardzo często wynika też z rozmów z kolegami, z dzielenia się doświadczeniami. Tego nie było i ona w sumie taka trochę jest zagubiona, nigdzie w tym roku nie poszła studiować, nie wiedziała, gdzie. Podejrzewam, że brak tej wymiany idei między ludźmi, między młodzieżą doprowadza do takiego ich zagubienia. W pewnym wieku autorytetem nie są rodzice tylko rówieśnicy i im więcej takich różnych wymian tym dla młodzieży lepiej. I dobrze, niech oni się od siebie uczą, ale tej wymiany brak.

**Co planujesz 1-go listopada?**

Nigdy nie chodziłem z tej okazji na cmentarze, więc dla mnie to akurat bez różnicy.

**A Boże Narodzenie?**

Ja byłem bardzo zadowolony w Wielkanoc, że nie musimy się spotykać w jakimś większym gronie i myśleć jak te święta zrobić. Jeżeli będzie tak samo, to ja się będę tylko cieszył. Ja nie przywiązuję żadnej większej wagi do takich tradycyjnych świąt. Może to o mnie źle świadczy a może jestem po prostu za mało polski w tym wszystkim. Na większą uroczystość rodzinną też bym nie poszedł. Żebym chciał spotykać się w większym gronie to naprawdę musiałoby nastąpić duże zmniejszenie zachorowań. Ja miałem taką sytuację w połowie września i byłem na takiej większej imprezie prywatnej. Po tej imprezie jakiś kaszelek mnie złapał i byłem przekonany, że totalny koszmar, że złapałem coś. Kaszel po 2 dniach przeszedł i było ok, ale jakiegoś tam stracha miałem.

**Idąc na imprezę też miałeś stracha?**

Miałem pewne obawy. Ta impreza była częściowo na zewnątrz i robiłem tak, że jak wchodziłem do pomieszczenia to zakładałem maseczkę, a jak wychodziłem to ją zdejmowałem i wszyscy się ze mnie śmiali. Byłem jedynym, który się tak zachowywał i rzeczywiście wszyscy patrzyli na mnie jak na jakiegoś dziwaka.

Ja rzeczywiście łapię się na jakiejś mojej niekonsekwencji, bo jak rzeczywiście nie mam gdzieś ochoty pójść, to zawsze mam super pretekst w postaci pandemii i że może lepiej się nie spotykać, ale jak mam ochotę iść, a tam akurat miałem ogromną ochotę, to poszedłem. Robiłem te numery z maseczką oczywiście, ale po prostu miałem ochotę iść na imprezę. Staram się zachować jak najwięcej elementów normalnego życia, bo przecież nie można się odizolować totalnie od wszystkiego. Chodzi o to, żeby ograniczyć ryzyko, ale całkowicie go nie wyeliminujesz. Im jednak tych kontaktów będzie mniej tym lepiej. Czysty rachunek prawdopodobieństwa. Może dotrwam do szczepionki.
