# Supplementary material for: The challenges arising from the COVID-19 pandemic and the way people deal with them. A qualitative longitudinal study
Source: PLoS One. 2021 Oct 11;16(10):e0258133. doi: 10.1371/journal.pone.0258133 (PMC8504766; doi:10.1371/journal.pone.0258133)
Supplement: S1 Dataset — (ZIP) [file pone.0258133.s003.zip › Transcriptions/stage 6/3.6_F_54_single.docx]

**3.6_F_54_single**

**Co się działo od czerwca?**

A czy pani mówiła, że ja mam zapisywać, co się działo? No nie wiem, pani mnie zaskoczyła pytaniem, nie wiem, czy było coś ważnego. Widocznie nie, skoro nie pamiętam. Były wakacje. Na wsi to jest dzień jak co dzień, czy wakacje, czy nie wakacje. Nie wyjeżdżaliśmy nigdzie, jeśli o to pani pyta, ale i tak nie wyjeżdżamy.

**Zajęcia seniorskie wróciły?**

Seniorzy wrócili, ale bardzo późno, bo wrócili od września, ponieważ jeszcze przy okazji postanowili nam zrobić ten MOPS, jakąś reorganizację i remont tego mieszkania, w którym gramy. Wrócili we wrześniu, a zginęli mi znowu od poniedziałku. Właśnie skończyliśmy. Ledwo co zaczęliśmy i już skończyliśmy. Słabo, bo oni byli stęsknieni i w ogóle, słabo.

**A jak komentowali powrót we wrześniu?**

Wrócili wszyscy, a oni wszyscy mają powyżej 70. Wrócili mi wszyscy, byli bardzo zadowoleni, że mogli wrócić przede wszystkim. A czy się boją? Może się trochę boją, ale sobie z tym radzą. Myślę, że teraz będą się coraz bardziej bać pewnie. Te informacje, chyba nie da się już ukryć, że mamy tego koronawirusa i to już nie jest 10 przypadków dziennie, tylko 20 000, to już robi jakąś różnicę, skala takiego zjawiska. Może mam jedną seniorkę, która się boi głównie o swojego męża, ale przychodzi. Przychodziła, bo teraz zamknęliśmy znowu wszystko.

**Z czego się bierze to, że mimo strachu przychodzą?**

Myślę, że to się bierze stąd, że oni potrzebują wyjść. Oni mieszkają w 30 m2 i dostają świra. Oni jak są nawet z partnerami życiowymi, to są z nimi od 40 lat, więc znają się jak łyse konie i się trochę nudzą.

**Czyli mają potrzebę zmiany otoczenia?**

No tak, potrzebę kontaktu. Stanowią dosyć zżytą grupę, poza tym stanowią taką grupę, która nie może np. zacząć grać online. Ani nie mają online, ani nie mają dodatkowych umiejętności, żeby sobie ten online załatwić.

**A gdyby mogli, toby dalej chodzili?**

Tak, myślę, że tak, chodziliby. Ta moja grupa by chodziła.

**Co musiałoby się stać, żeby sami z siebie zrezygnowali?**

Nie wiem, wolę nie myśleć, co by się miało stać. To są starsze osoby, oni już wiele przeżyli i myślę, że po prostu... Oni się zabezpieczają, uważają na siebie, ale też przychodzi pewnie taki moment, przynajmniej na większość z nich, nie jakichś panicznie bojących się o siebie, że oni po prostu nie boją się tego aż tak, nie taki diabeł straszny, tak mi się wydaje. Nie wiem, co by się musiało stać, pewnie dla każdego z nich coś innego, ale zasadniczo... Jakoś sobie z tym radzą.

**Jak teraz wygląda pani dzień?**

Nie wiem, w co ręce włożyć. Oczywiście brakuje mi tych poniedziałków i bardzo mi tam miło jeździć do nich i wozić im różne... Zawiozłam im na przedostatnie zajęcia dwie dynie ponad 20 kg i podzieliłam, bo to nie do udźwignięcia przeze mnie, nie mówiąc o nich. Tak że tych poniedziałków mi brakuje, ale robić to ja cały czas mam, bo to jest koniec jesieni, więc warzywa trzeba zabezpieczyć, a trzeba je zebrać, a jabłka z drzew zdjąć, winogrona zdjąć - ja mówię pani tylko o tym, co jest niezrobione, nie opowiadam o tym, co jest zrobione. Teraz na 1.11. postanowiliśmy rodzinnie, że nie będziemy się zajmować cmentarzami, nie będziemy po nich spacerować ze względu na zagrożenia covidowe i w związku z powyższym spotkamy się tutaj na wsi, więc jeszcze muszę zrobić jakieś zakupy, coś ugotować do jedzonka. Zbieram spady jabłuszkowe spod drzewek u siebie i nie tylko, u sąsiadów i robię z nich sok, nie nudzę się. Dobrze, że pani nie obejmuje tą kamerką reszty mieszkania, boby pani zobaczyła, co mam jeszcze w planach. Mam takie krzesło, na którym mam taką stertę do prasowania, już nie mówiąc o zlewie itp.

**Czyli roboty jest dużo, nie nudzi się pani?**

Nie, ja się nigdy nie nudzę.

**Jak wygląda spotykanie się z dzieciakami?**

Julka pracuje, więc nie przyjeżdża. Tzn. była w weekend np. 2 tygodnie temu, to nie jest tak, że ona w ogóle nie przyjeżdża, ale nie przyjeżdżają tak, jak przyjeżdżali. Teraz przyjadą wszyscy pewnie.

**Julka wróciła do tej samej pracy?**

Ona pracuje teraz w wioskach dziecięcych, SOS Wioski Dziecięce, w tej fundacji. Mówiąc szczerze, to nie wiem, czy przyjedzie ona, na pewno przyjedzie Hubert z Magdą, ale nie wiem, czy przyjedzie Julka, ponieważ ona od tygodnia bierze codziennie udział w protestach i demonstracjach, w związku z tym nie wiem, jaką decyzję podejmie, bo coś tam przebąkuje. Już nie wiem, czy ona się bardziej boi, że coś tam złapała przy tych i chce być taka dla mamusi zdrowa, czy bardziej chce chodzić w dalszym ciągu na te demonstracje, bo jednak są rzeczy ważne i ważniejsze, pod pewnymi względami zwłaszcza. Krótką mówiąc, ja rozumiem i też kiedyś miałam taki, wszyscy może mieliśmy albo powinniśmy mieć taki moment w życiu, żeby się nie zgadzać z tym, co się dzieje i jakoś to trzeba zademonstrować, bo inaczej te oszołomy po prostu nas pokonają. Więc tutaj nie wiem, ale tak, z dziećmi się spotykamy. Przeszło nam, dzieciom moim może głównie, taka obawa, że oni tu przywiozą jakieś coś tam.

**To Hubert chyba miał największą obawę?**

No i Hubert od tygodnia jest w domu po to, żeby przyjechać do mnie na święta.

**Czyli on nadal zapobiegawczo działa?**

On jest taki bardzo akuratny. Ja myślę, że najgorszą rzeczą w tej chwili, która by się mogła zdarzyć każdemu z nas, to jest zachorować. Na cokolwiek poważnego, z wyjątkiem kataru i alergii, ponieważ myślę, że to, co najbardziej kuleje w tej chwili, to jest opieka medyczna. Z różnych powodów, częściowo mi nieznanych, a częściowo takich rozpoznawalnych. Obawiam się, że konieczność skorzystania z opieki zdrowotnej byłaby w tej chwili najgorszym, co nas może trafić, tak sądzę.

**Czyli dobrze, że on tak chucha, przynajmniej nic nie przywlecze też innego?**

Sam się też nie zarazi niczym w związku z powyższym. Myślę, że dobrze, na pewno dobrze.

**A jak pani otoczenie z tym koronawirusem?**

Oni z koronawirusem, to teraz jakoś... Moja najbliższa sąsiadka, najstarsza z tutejszych, boi się i przestała wychodzić, robię jej zakupy znowu, gdzieś tam robi mi karteczki. Ale czy reszta się boi? To pani nie powiem, nie wydaje mi się. Myślę, że większość jednak... Chociaż może. Trudno mi powiedzieć, ja tutaj tych kontaktów też mam stosunkowo niewiele tak naprawdę, więc nie wiem, czy się bardzo boją, ale myślę, że bardziej niż kiedyś. Już w tej chwili mamy jakieś po raz pierwszy od początku tej pandemii, sytuacja jest taka, że to już nie są jakieś większe ogniska, tylko że każdy tam albo kogoś zna, albo... U nas w skali tych Staroźreb to tam nie wiem, ile mają tych mieszkańców, parę tysięcy, to mamy już jakieś zachorowania tutaj, a to jakiś tam rodzic dziecka ze szkoły, a to ktoś tam, nieważne, czy to wiadomo kto, czy nie wiadomo, ale już te przypadki się pojawiły lokalnie. To już jest blisko, więc ja myślę, że oni przynajmniej już wiedzą, że to jest. Jeszcze może nie jest tak... Przynajmniej część z nich myślę, że nie wie, czy to groźne i jak groźne, bo to zależy, jak te osoby chore to przechodzą, ale się zbliżamy. Jakieś pierwsze osoby dostały mandaty za chodzenie bez maseczki od pana policjanta, to już tak poważna sprawa. Chociaż maseczki, jak pani spojrzy, wszędzie zresztą, bo ja w Płocku byłam ostatnio, tam się spotkałam z dwoma czy trzema seniorami, bo im coś tam obiecałam załatwić, jakieś maseczki, przez które będą mogli oddychać, bo oni wszyscy noszą maseczki pod nosem. Regularnie, gdzie pani nie spojrzy, to te maski są na ustach i pod nosem, ten nos jest nieobjęty, bo oni po prostu nie mogą w tym oddychać. Oddychają, jak to samo powietrze wciągają, które wydychają, po prostu za chwilę (to jednak są starsze osoby) im się a to w głowie kręci, a to coś tam. Zresztą młodzi też mają te maski pod nosem, jeśli w ogóle je mają. W dalszym ciągu tutaj te przepisy są... Nie wiem, no dobra, noszą. Ostatnio pojawiły się takie śmieszne memy, że jeżeli przeżyłeś w tej jednej jednorazowej masce pierwszą i drugą falę pandemii, znaczy, że jesteś niezniszczalny, nieśmiertelny. I tak to można nazwać, bo te jednorazowe maseczki są noszone wielorazowo zdecydowanie.

**Ale widać, że jakoś to przeżywają? Choćby te szkoły znowu zdalne?**

Ci moi seniorzy to chyba nie bardzo. Ani seniorzy, ani tutaj w okolicy. Mam jedną mamę tych uczniów, których ja uczę po lekcjach, to oni są tacy trochę, chcą się upewnić, że nie stracą pomocy, chociaż nie wiem, czy to rozsądne. Myślę, że to już było grane. Na razie szczęśliwie jeszcze nie zamknęli nas całkiem. Ale myślę, że skutki dla gospodarki były tak opłakane i ten rząd, który rozdaje chętnie, tylko że nie ma co, to nie będzie jakiś bardzo skłonny do pozbawienia się znowu jakiejś części podatków, no bo z czegoś musi dawać, więc może nas nie zamkną wszystkich znowu w domach na jakiś czas, tylko będziemy mogli jeszcze wykonywać jakieś czynności. W bibliotece byłam przedwczoraj, jeszcze działa, chociaż w składzie osobowym ograniczonym.

**Możecie wejść do środka i wybierać z półek?**

Tak. A to jest bardzo skomplikowane. Wpierw nie mogliśmy, wpierw zamknęliśmy wszystko. Potem jak otworzyliśmy biblioteki, to mogliśmy sobie zamówić książkę i pani mogła pójść w półkę i przynieść nam tę książkę. Potem mogliśmy wchodzić po 2 osoby, obsikać się tym sosem od stóp do głów, pójść po książkę, przynieść sobie książkę i pani ją nam wydawała bezdotykowo, tzn. trzeba było ją otworzyć na tej naklejce i ona ją skanowała, i w ogóle nie macała, bo myśmy ją macali, a jak oddawaliśmy książki, to one szły na kwarantannę. Na kwarantannie były tydzień książki, a w supermarkecie jak pani wzięła do ręki słoiczek, obejrzała z 3 stron i potem go odłożyła, to on nie był na kwarantannie, ale w bibliotece te książki owszem. I teraz w bibliotekach to się nie zmieniło, oprócz tego, że... Zezwolono nam chodzić, ale to 2 osoby mogą być w bibliotece, co powoduje, że przed bibliotekami są takie kolejki jak przed bankami, przynajmniej w Płocku. Teraz znowu możemy i poszłam sobie, a pani mnie zapytała: "A dezynfekowała pani ręce?!", ja mówię: "No, oczywiście", bo nie dałam jej książki rękami i chodziło o to, czy ja na pewno je zdezynfekowałam po wejściu do biblioteki. Tak że ogólnie rzecz biorąc możemy chodzić do biblioteki, ale to jest utrudnione. Ale przynajmniej można pochodzić po półkach teraz.

**A fryzjer? Jeździ pani regularnie do Warszawy?**

Tak, na razie mam nadzieję, że mi się nie zamknie.

**Czy coś przeszkadza pani w sytuacji covidowej?**

Przeszkadza mi, że jest ten covid, ponieważ nie mamy na to żadnego wpływu i wiele od nas nie zależy, żeby nie powiedzieć nic i to trochę przygnębiające jest, że to cały jest, że teraz wraca, co się oczywiście dało przewidzieć i wszyscy tak mówili, że tak będzie, ale to, że to się sprawdziło, to nie jest fajne, bo to się ciągnie. To, co mi przeszkadza, to to, że nie mogę chyba czegoś zaplanować. Ja nie mówię, że ja bym chciała wyjechać na Gwadelupę, bo może bym chciała, ale nie za bardzo, ale bym chciała np. wiedzieć, czy będą święta, gdzie będą święta, że je spędzę, że chciałabym np. pojechać gdzieś na jakąś wycieczkę albo gdziekolwiek i nie wiem, bo tak naprawdę nie mogę zaplanować, co będzie za tydzień. I to mi przeszkadza na pewno, ale jak to mówił klasyk, nie ma co się przejmować tym, na co nie mamy wpływu. Łatwiej się mówić, niż się robi.

**Czyli brak kontroli, wpływu.**

Tak, zasadniczo tak, to jest taka bezradność. Nie wiemy, co zrobić i dupa, jesteśmy zawieszeni. Ja wczoraj przeżyłam koszmar, jak jechałam do Warszawy, koszmar po prostu, ponieważ utknęłam w korku wjazdowym na ponad 3 h z powodu protestu rolników. Wpierw wyjechałam z tego korka, cofnęłam się, próbowałam się przebić jakąś inną stroną, ale to mi się nie udało i musiałam się wrócić do tego korka, ale wróciłam do niego na początek, a nie tam, gdzie wstałam wcześniej. Więc w sumie zajęło mi dojechanie do Warszawy godzinę, a wydostanie się z tego korka i przyjechania na spotkanie ponad 3 h i po prostu - rzadko mam takie - byłam po prostu zdruzgotana, absolutnie nie wiedziałam, co zrobić, byłam zagubiona, bezsilna, bezradna i taka... Nie byłam wkurzona, tylko byłam po prostu zrozpaczona, że ja nie mogę sobie poradzić, że tkwię w tym, tak naprawdę w sytuacji bez wyjścia. Jedynym wyjściem, które dla mnie nie było wyjściem, to było wycofać się i wrócić do siebie spod tych Łomianek. Więc to jest takie uczucie, które akurat w tym korku było takie, powiedziałabym, namacalne, bo to się działo tu i teraz, ale myślę, że to jest takie uczucie, które w tej chwili, może dlatego mnie tak w tym korku to ścięło, że w makroskali to uczucie jest z nami cały czas. Może nie w takim natężeniu w związku z tym, ale to jest właśnie takie zagubienie, nic nie możemy zrobić. Jakoś na co dzień się żyje, co zrobić, ale taka perspektywa, że to nie jest tak, że się skończy za 2 tygodnie i przeżyjemy jakoś. Oczywiście, że przeżyjemy, ale czy to się skończy za 2 tygodnie, czy to w ogóle jesteśmy w stanie wskazać jakiś moment, kiedy to się skończy, to nie wiem. Bo jeżeli nie jesteśmy w stanie zapewnić wszystkim osobom, które chcą, szczepionki przeciwko grypie zeszłorocznej, ja nie mówię przeciwko covidowi, tylko przeciwko zeszłorocznej grypie, to znaczy, że my - i myślę, że w skali świata - po prostu jesteśmy absolutnie niewydolni w takim zakresie.

**Oprócz bezsilności, jakie jeszcze uczucia?**

Czy ja wiem... To jest takie smutne może trochę? Że tacy jesteśmy. Może trochę tak.

**A jest pani zmęczona tą sytuacją?**

No może tak, to dobre słowo, może jestem zmęczona trochę. Zmęczenie sytuacją, że to cały czas trwa.

**Pani myśli o tym covidzie na co dzień?**

Ja w dalszym ciągu nie oglądam telewizji za bardzo, a wróciły programy sportowe, więc już w ogóle nie oglądam tego TVN. Chociaż ostatnio ze 3 czy 4 dni, od czwartku to oglądam, jak są te protesty, coś tam patrzę w tę telewizję i się martwię o to dziecko swoje młodsze, które tam występuje. Ale już chyba... Chociaż trudno się odciąć od takich informacji całkiem. Jak była Julka w weekend, 1,5 tygodnia temu, to ona na bieżąco w tej komórce te wiadomości, jak siedzi w domu i pracuje, to ma ten TVN24 włączony ciurkiem, więc coś tam mi przemyci. Ale staram się nie myśleć o nim, udaje mi się, bo po prostu nie siedzę i nie myślę. Nie żebym w ogóle nie myślała, ale staram się tu i teraz się koncentrować, bo co możemy więcej wymyślić oprócz tego, że jesteśmy tym zmęczeni i bezradni.

**Ale to trzeba sobie znaleźć dużo zajęć, które zaprzątną głowę?**

No nie wiem tak naprawdę, czy trzeba. Moje zajęcia mi zaprzątają głowę, ponieważ one stoją w kolejce jakby, więc ja cały czas coś kombinuję, ja coś muszę zrobić, żeby jakoś je tam pogodzić. Poza tym myślę, że ja większość z tych zajęć lubię, więc tak doraźnie udaje mi się na co dzień, nie jest to coś takiego, co mi zajmuje, to myślenie o rzeczywistości czy covidowej, czy politycznej, która mówiąc szczerze chyba bardziej mnie martwi, więc jak już mam się czymś martwić, to się martwię tym fiutem chorym, który rządzi nami, a ten covid, moim zdaniem, oczywiście on jest pewnie groźniejszy w skali światowej, ale w naszej skali to nie jestem taka pewna. W związku z powyższym, ponieważ mam rzeczy, które lubię robić i je robię, i mam większego wroga niż covid, to może o nim akurat tak często nie myślę ostatnio.

**Przez ostatnie 4 miesiące był taki moment, że myślała pani, że to się zbliża ku końcowi?**

Chyba nie za bardzo, bo nam nie dawali takich, naukowcy, nie dawali nam wielkich szans, że to się skończ, cały czas była mowa o tym, że zbliża się jesień, zbliża się druga fala zachorowań i coś tam. Więc od strony takiej, powiedziałabym, wiedzy to myślę, że wszyscy wiedzieliśmy, że to przyjdzie. Oczywiście ja miałam nadzieję, że może nie przyjdzie to z taką siłą, ta skala zjawiska jest rzeczywiście zatrważająca w tej chwili. Miałam nadzieję np. na to, że będę się mogła zaszczepić przeciwko grypie zeszłorocznej, co w ogóle okazało się być absolutnie absurdalne, niemożliwe. Mi się to wydaje karygodne, że nawet osoby zagrożone się nie mogą, ani starzy się nie mogą, ani lekarze się nie mogą zaszczepić, nikt się nie może zaszczepić, bo ktoś tego nie naprodukował albo nie zamówił, albo już chuj wie co nie zrobił. To jest słabe, co tu dużo mówić.

**Skąd się wzięło to, że mamy aż tyle przypadków dziennie?**

Myślę, że się wzięło z tego, że przestaliśmy być czujni. Tzn. że na falę zachorowań zwykłych przeziębień nałożył się drugi wirus, wiadomo, że one zawsze chętnie atakują w sytuacji, kiedy ktoś już jest wzięty, że się tak wyrażę. Myślę, że się bardzo na to złożyło to, że uznaliśmy w skali globalnej, że wszystko będzie dobrze. To jest tak, że te media jakoś kształtują nasze pojęcie, więc jeżeli pan Morawiecki mówił, że koronawirus się skończy, bo już się kończy, no to się kończy i ludzie - oczywiście to nie jego wina - ale ludzie po prostu stracili czujność. Straciliśmy czujność, rozluźniliśmy taką dyscyplinę, którą jednak mieliśmy, tak że mniej się wychodziło, się uważało i te maseczki, i rękawiczki, i te ręce. Ja byłam świadkiem, miesiąc z temu może, jakiejś awantury w Biedronce, kiedy przyszedł jakiś młody chłopak, takie karczycho szersze od głowy i zwrócił uwagę jakiejś pani sklepowej, że on się spieszy i że może by kogoś jeszcze dodatkowego do kasy, a ta pani mu powiedziała, że może by maskę założył, no to zaczęło się takie agresywne dość zachowanie. Tak że on uważał, myślę, że część z nich w dalszym ciągu tak uważa, tylko że teraz, jako że mają taki bat nad sobą finansowy, i ta policja, i te mandaty, to może to trochę się poprawi, bo widzę, że ci ludzie chodzą w maskach w tej chwili. Co prawda ten nos na wierzchu, ale chodzą, więc myślę, że i ta młodzież... Co z tego, że... Teraz oczywiście od tygodnia mamy strefę czerwoną wszędzie i jesteśmy bardziej czujni, ale mamy po 20 000 zachorowań dziennie, a jeszcze niecały miesiąc temu, na pewno koniec września widziałam młodzież ze szkoły (tj. takiej szkoły ponadpodstawowej, czyli jakieś technikum czy coś) w Płocku przed szkołą, poza terenem szkoły, banda 50-60 osób, stojących w jednej kupie zwartej, palących papierosy. Po pierwsze, ja rozumiem, bo ja sama byłam w szkole i paliłam papierosy, to ja nie o tym mówię, tylko ta młodzież bez masek, w bliskości do siebie takiej, że tam jak jedna osoba miała, to całe 60 trzeba by było puścić na kwarantannę. I oczywiście, oni to przechodzą może bezobjawowo, bardzo proszę, ale ich rodzice, dziadkowie itd. już nie. Jeżeli możliwe jest zachorowanie bezobjawowe, to roznoszenie takiej choroby jest po prostu niekontrolowalne. Myślę, że te szkoły, choć to może brzmi absurdalnie, no bo na każdą rzecz można spojrzeć z różnych stron, ma różne aspekty, nie wiadomo, czy lepiej, żeby oni w ogóle nie chodzili do szkoły, nie wiem, co to by było wtedy, czy mają chodzić i siać tym towarem. Ktoś to roznosi, no my. Cały czas, myślę, że jesteśmy na takim niezbadanym gruncie. Dopóki nie będziemy mieli jakiejś broni, to te wszystkie działania pewnie trochę poprawią sytuację, ale na razie na to nie wygląda, im więcej jest tych przypadków, wiadomo, tym gorzej, one rosną po prostu geometrycznie, ta ilość zachorowań. Bo to nie jest tak jak na początku, że jedna osoba zaraża dwie, teraz jedna osoba zaraża nie wiadomo ile. Zobaczymy. Może jak wycięliśmy te szkoły znowu, przynajmniej te klasy starsze i ponadpodstawowe, i oni wszyscy się uczą w domach, może trochę się ta sytuacja poprawi, zobaczymy. Dopóki nie będziemy mieli szczepienia, to... I tak jeszcze będą sceptycy szczepień, ale przynajmniej ci, co się zaszczepią, podniesie się im odporność, choć to nie jest tak, jak wiadomo, przy wirusach, żeby można było się zaszczepić i koniec, nie ma takich, nie wiem, które to choroby, ospa to w sumie wirus, więc nie wiem, jak to jest z tymi szczepieniami. Też chyba nie jest tak, że jak ktoś raz zachorował na ten covid, to już na pewno never w życiu na niego nie zachoruje. Chyba to tak nie jest, bo on jak to wirus, on się po prostu mutuje i zmienia się, w związku z powyższym pewnie ten poziom przeciwciał (bo to robi szczepionka - podnosi poziom przeciwciał), więc ten poziom przeciwciał pewnie jest wyższy, ale to jeszcze nie znaczy, że się nie zachoruje.

**Dobry rozwiązaniem byłoby zamknięcie też młodszych klas?**

Chodzą i roznoszą, ale to zależy, jak na to patrzeć, czy patrzymy na to w skali funkcjonowania całego kraju, czy wyłączamy ten coivd jako jedyny problem. Oczywiście, jeżeli patrzymy na to wyłącznie pod kątem tego covidu, to na pewno byłoby dobrze zamknąć wszystko. Ale nie możemy tego zrobić, bo żeby małe dzieci zostały w domu, to rodzice muszą być w domu, to muszą dostawać zasiłki opiekuńcze i zaczynamy wpadać w to samo błędne koło, bo ci ludzie też jakoś muszą żyć, muszą spłacać kredyty. Zamkniemy wszystko, covid pewnie byśmy ograniczyli, ale jak się okazuje, go nie zlikwidujemy, potem każde rozluźnienie tej dyscypliny powoduje wzrost zachorowań. Myślę, że musimy jakoś patrzeć tak bardziej, jakby nie ma wyjścia, możemy sobie teoretyzować, ale tak naprawdę to jesteśmy bardziej złożonym mechanizmem czy organizmem i musimy i pracować... I tak myślę, że nie wróciliśmy do takiej aktywności, sposób działania jest inny, część osób jednak pracuje online, wszyscy ci, którzy mogą najprawdopodobniej. I o tyle jest nam łatwiej. Jesteśmy troszkę ograniczeni w takim poruszaniu się, mogliśmy trochę ograniczyć taką aktywność ruchową, przestaliśmy się może widywać z tyloma osobami, już nie chodzimy bez przerwy na jakieś imieniny, ja mówię w skali globalnej. Ale zamkniemy szkoły i co zrobimy z tymi rodzicami? Z drugiej strony jak nie ma klas 4-8 w szkole i nie ma szkół ponadpodstawowych, to zagęszczenie tych dzieci w szkole podstawowej też jest mniejsze, od tego zacznijmy, bo one już po tym korytarzu nie potykają się same o siebie. Chodzą tylko małe dzieci, to tak: mają jednego nauczyciela w zasadzie, jeśli byśmy odjęli im angielski i religię, gdzie te panie się pewnie mieszają, toby było jakieś zabezpieczenie. Na pewno jest to takie ograniczenie, na które możemy sobie pozwolić w tej chwili, bo taka jest konieczność, bo nie możemy tego wszystkiego zostawić po prostu i zamknąć. Ani nie możemy tego zostawić, jak jest, bo widzimy, co się dzieje, ani nie możemy wszystkiego zamknąć, bo wiemy, co się będzie działo za chwilę. Musimy się nauczyć z tym żyć, więc jakieś próby są wykonywane pogodzenia tego wszystkiego, zobaczymy z jakim skutkiem. Obyśmy się nie pochorowali w międzyczasie.

**Zna pani kogoś, kto zachorował?**

Nie, ale ja mam w otoczeniu lekarzy, którzy opowiadają mi, co się dzieje w tych szpitalach, jak wygląda sytuacja obstawy personelu, już nie mówiąc o tym chorym systemie, że do fryzjera może pani pójść oraz do manicurzystki, ale do lekarza nie może pani pójść i ma pani teleporady. Ja rozumiem, że można tele wypełnić receptę komuś, ale nie można tele spojrzeć pacjentowi do gardła, do nosa, do ucha, posłuchać serca itd. Zawód lekarza to jest trochę taki zawód jak policjanta czy wojskowego. Przyjmując taką odpowiedzialność, że się jest lekarzem i składa się tę przysięgę, to uważanie, że będziemy wypisywać przez całe życie tylko recepty, siedzieć w wygodnym fotelu i zaglądać pacjentowi ewentualnie do nosa albo i nie, jest jakimś nieporozumieniem po prostu. To są ludzie, którzy mają jakąś misję w pewnym sensie, nawet nie w pewnym, tylko po prostu, niosą pomoc innym, decydują się na taki zawód. Jest to zawód ryzykowny, oni o tym wiedzą, bo się spotykają z różnymi chorobami, niektóre są zakaźne, a to, co się teraz dzieje, to według mnie to jest naprawdę jakieś grube nieporozumienie. Oczywiście, pewnie można odsiać część osób, które nie potrzebują pójść do lekarza w sensie takim, że nie są chore, tylko potrzebują pójść po receptę, a może potrzebują pogadać w poczekalni czy coś, w porządku, wyznaczajmy godziny, nie wpuszczajmy wcześniej tych pań, żeby nie siedziały na kupach, ale to, że ci lekarze nie przyjmują pacjentów. Pan psychiatra, który udziela teleporady, z całym szacunkiem dla pana psychiatry myślę, że to jest naprawdę nieporozumienie, co on może zobaczyć u pacjenta. Ja rozumiem, że jak ma pacjentów dementywnych i rodziny przychodzą po recepty, to czy taka pani opowie o członku rodziny w gabinecie czy przez telefon, to może nie ma różnicy, ale jak on ma bezpośrednio wizytę z chorym, to ja sobie w ogóle tego nie wyobrażam. Ja sama uczęszczam do pana psychiatry i pobieram jakieś leki, a on mnie pyta przez telefon, czy ja się dobrze czuję: "Co tam u pani?"- "A w porządku" - "No to dobrze", to on mi wypisze receptę na 10 miesięcy. I mi wypisał receptę na 10 miesięcy. Oczywiście, że ja mogę, jak się słabo poczuję, zadzwonić do niego w międzyczasie i ustawić, może coś dostanę więcej, ale mi się to wydaje absolutnie absurdalne. To, że ci lekarze przyjmują w takim trybie, bo to tak naprawdę wszyscy przyjmują i dopiero jak on przez telefon stwierdzi, ale żeby to już się stwierdzało często - skąd. Np. takich seniorów - on ich w ogóle nie ogląda, przepisuje im te recepty, oni mają mnóstwo różnych chorób, a to cukrzyca, nadciśnienie, coś tam, coś tam, nie wiadomo, czy te leki działają, czy nie działają, ja nie wiem, czy on im robi jakieś badania. Tu patrzę po tych swoich, to nawet jedna pani się dostała do niego, bo powiedziała, że się źle czuje i coś tam, i ją serce boli, a serce to nie boli w ogóle, proszę pani. Ale dostała się do niego akurat, bo jej powiedział: "To niech pani przyjdzie, siostra zrobi EKG". Akurat był, przejrzał to EKG, nawet ją wysłał na jakieś echo serca i coś. Mówię pani, że to działa źle. Także nie chorujmy, bo nie ma ani opieki medycznej, ci lekarze w szpitalach, ten personel jest okrojony zdecydowanie. Jeszcze nowe szpitale powstaną, nie wiem, kto tam będzie przyjmował tych pacjentów, nie wiem. Nie chorujmy, żebyśmy nie musieli tego sprawdzić, bo jest słabo.

**Są jeszcze jakieś obostrzenia, które panią dotykają?**

Oprócz noszenia maseczki, to ja nie wiem, czy ja w ogóle podlegam jakimś... Podlegam niemożności spotkania się więcej niż 5 osób, to odwołali mi zajęcia z seniorami. Czy w rodzinie się mogę spotkać, czy nie mogę jakoś? Ja noszę przyłbicę, mam ją w samochodzie, więc jak wychodzę z domu, to wsiadam do samochodu, to bez mała ją zakładam, jeżeli jadę z kimś, to zakładam, jeżeli jadę sama, to nie zakładam, zakładam ją, jak wysiadam z samochodu. W sklepie mamy godzinę dla seniorów, 2 godziny dla seniorów, owszem, zdarzyło mi się, że mi to nie było na rękę. Bo pojechałam do Płocka do weterynarza na umówioną godzinę, na 10 i zabrałam psa sąsiada, i zresztą tego sąsiada, i myślę sobie, a dobra, to jak już wyjdę z tej przychodni, to pojadę na zakupy. Ale okazało się, że słabo jest, bo o 11 wyszłam i załatwiłam wszystkie sprawy, bo Płock jest mały, więc tam spotkałam tych seniorów, dałam im, co było dla nich, no i 11 bach, nie zrobię zakupów, bo seniorzy. No i co? No i nie zrobię, to zrobię później. Myślę, że te obostrzenia, przynajmniej w moim przypadku, jeśli są, to one mi nie przeszkadzają, a jeśli mi przeszkadzają, to myślę, że ich zastosowanie nie jest bezzasadne, w związku z powyższym jestem za.

**Teraz seniorzy nie powinni wychodzić z domu.**

Powyżej 70 r.ż., ale z wyjątkami, że idą do pracy albo że idą po zakupy, albo że idą się pomodlić. Chyba te 3. Myślę, że oni są największą grupą ryzyka, nie dlatego może nawet, że najczęściej chorują na covid, tylko że najczęściej chorują już na inne choroby i połączenie tego covidu z czymkolwiek innym powoduje, taki organizm jest po prostu dużo bardziej osłabiony i podatny na powikłania tego covidu czy na same skutki tego wirusa, i o to chodzi, to jest dla ich dobra. Mają furtkę: mogą z psem, mogą wyjść. Konieczności takie są na pewno, tak jak z psem wychodzą albo idą do pracy, no jak idą do kościoła... No oni też gdzieś muszą wyjść. Ja jestem akurat ateistką, ale z dużym szacunkiem dla ludzi wierzących, choć głównie dla wierzących a nie dla tych, co tam leżą krzyżem w kościele, ale powiem pani... No to jest jakieś miejsce, to jest jakaś furtka dla tych ludzi. Jeżeli oni w tej maseczce do tego kościoła sobie pójdą i w tej ciszy czy nie ciszy wysłuchają (miejmy nadzieję) jakiegoś mniej jadowitego księdza, to dla tych ludzi to też jest jakaś... To, że jest jednak takie miejsce, gdzie oni mogą wyjść. Krótko mówiąc, czego nie mogą zrobić: nie mogą sobie pójść z sąsiadem na ławeczkę, usiąść i pogadać na tej ławeczce. Nie wiem, czy oni tak strasznie często chodzili na te ławeczki ci seniorzy. Bo trzeba popatrzeć na to z tej strony, czego oni tak naprawdę nie mogą zrobić w stosunku do tego, co robili. Te zakupy, być może ograniczenie tych zakupów, chodzenia do sklepu, może i słusznie, bo to jest tak, że jednak wszyscy tam chodzą, dotykają, macają, stoją w kolejce. Tak jak u nas w tych warunkach takich wiejsko-małych miasteczek to oni w tym sklepie się spotykają, tam się prawie wszyscy znają, do tego sklepu przyjeżdżają i ci z takich mniejszych osad czy wsi, i ci, którzy mieszkają w tych większych, tam się spotykają, to sobie pogadają, to jak sobie pogadają, to naplują na siebie i coś tam. Też kiedyś, do cholery, muszą pogadać. Jakoś ich trzeba... Mnie to nie dotyczy w tym sensie, że ja jeszcze nie mam 70+, ale np. robię im zakupy, tej sąsiadce. Jej córka mówi: "Mamo, to nie wychodź, my przyjedziemy i coś", to jak ja już jadę na zakupy, to dzwonię do niej: "Zrób listę". Ta córka mieszka z 10 km czy więcej trzeba przejechać, niby to żaden problem dla nich na pewno, ale. Na pewno jest bezpieczniejsza, jak siedzi w domu, bo to nigdy nie wiadomo, nie wie pani, kiedy to się stanie. Już jakieś tam ograniczenie jest chyba w tych koszykach znowu, bo coś widziałam, na drzwiach sklepu mi mignęło, że coś tam 30, nie wiem, czy to na kasę, nie no, już teraz nie powielamy błędów, więc już wiemy, że pewnie na powierzchnię, na m2 sklepu czy czegoś.

**A zakaz imprez rodzinnych?**

My to nie odczuwamy, ale tutaj odczuwają głównie ludzie, którzy mieli pozaplanowywane jakieś wesela i takie historie, bo oni teraz też nie wiedzą, co robić. No bo te wesela, które miały się odbyć wiosną czy wczesnym latem, to zostały odwołane. To ich poprzepisywali na jakieś listopady, październiki, to jeszcze byli cały czas optymistycznie nastawieni, bo było lato, ale jak się zbliżamy teraz, to tak: podobno... Ja tutaj mam taką rodzinę, która właśnie odkłada ten ślub. I teraz przełożyli ich na któryś listopada, i oni mają ten ślub na 100 ileś osób, to już było wiadomo jakiś czas temu, że 100 ileś to nie, tylko 70 i teraz nie wiadomo, komu odmówić. Wszyscy mają te zaproszenia już, bo to miało być kiedyś i co teraz trzeba zrobić? Trzeba zadzwonić, że ty nie przychodzisz i ty nie przychodzisz, czyli kogoś trzeba potraktować odmownie, a tak naprawdę nie wiadomo, czy wystarczy potraktować odmownie 50 osób, czy nie trzeba potraktować odmownie 70, oni mają tu duże rodziny.

**Ale teraz to już w ogóle nie wolno żadnych wesel.**

Teraz już w ogóle nie wolno. To teraz przynajmniej może jest o tyle lepiej, że po prostu wszystkim sorry, ale nie, a ta dziewczyna mówi, że ona nie wie, bo jak ona nie może zaprosić, to kogo ona ma nie zaprosić. Sama ma dużą rodzinę, ten jej partner czy przyszły mąż może jakąś mniejszą, ale to jakieś ciotki, wujków, to w ogóle jest jakaś kaszana, jak się zaprasza na te wesela takie. 150 osób to sama rodzina. Ja już byłam na takim weselu, młodzi ludzi, w zasadzie znajomych tam... Głównie mają rodzinę i ta rodzina taka wyrąbiście wielka. Ale jak tam mama panny młodej ma siedmioro rodzeństwa, to może pani sobie to wyobrazić, ile oni mają, zwłaszcza, że oni te dzieci mają wcześnie, to w setki osób po prostu idzie. Ale teraz już na szczęście nie można w ogóle. Faktem o tyle może lepiej, że oni tutaj, przynajmniej w tych takich, jak ja tu widzę, może to tylko są ci moi ludzie, ale że to są takie wesela właśnie, że jest głównie rodzina, to nie są znajomi. Świadkami to jest rodzeństwo zwykle. Naprawdę, ja byłam na weselu tutaj jakieś 1,5 roku temu czy 2 lata temu w sierpniu, byłam zszokowana, bo to młodzi ludzie, żeby w ogóle nie mieli takiej grupy znajomych, że to są jacyś tam koledzy czy coś, tylko wszystko... Jak ten Staś mi opowiadał, kto tam siedzi przy tym stole, to po prostu: ten to mąż tego, brat tego tamtego, to jest syn siostry matki żony, ja pierniczę. Ja mówię, a gdzie są jacyś... U nas jak są wesela czy tam jakieś przyjęcia, to głównie są koledzy, znajomi. Jak Julka chodziła na jakieś wesela, to najbliższa rodzina oczywiście, ale głównie to dla tej pary młodej. A tutaj niech pani zapomni, para młoda ma przechlapane, bo on pije, ona biega i sprawdza, kto jest, kogo nie ma, tu trzeba roznieść jakieś flaszki, później trzeba każdemu wydać do domu jeszcze pół litra.

**Zakazali w ogóle wesel, więc odpadł im problem, kogo wybrać.**

To był największy problem moim zdaniem. I oni odwoływali w ogóle te wesela, bo musieliby kogoś pominąć. Już nie mówiąc o tym, że nigdy nie było wiadomo, jak już dojdzie do tego terminu, to jaka będzie sytuacja, więc teraz to może nawet jest jakby wszystkim bardziej na rękę, no z wyjątkiem organizatorów, którzy mają minus coś tam, tam jakiś zadatek przepadnie w końcu, a może nie.

**A jeszcze jakieś ograniczenia dotykają panią lub pani bliskich?**

Hubert jest z Magdą, oni są razem, lubią we własnym sosie, im jest dobrze, nie mają dzieci, to nie borykają się z takimi problemami ograniczeń takich, że siedzą w domu i po prostu mają wszystko na raz: i dzieci, i sprzątanie, tak że oni sobie jakoś radzą. Julka miała taki moment... Teraz jest tak naprawdę po raz pierwszy w tej pracy, pracuje online, a wcześniej pracowała też w ciągu... We wrześniu oni mieli jakąś taką akcję zbierania funduszy w tym SOS i puścili taką akcję reklamową na różnych programach telewizyjnych i wynikiem tej akcji były smsy, które ludzie, nie zrozumieli zresztą czy też zrobili złe założenie, ale wszystko jedno, ludzi wysyłali im smsy i oni do tych ludzi oddzwaniali. Żeby to ogarnąć czasowo, to pracowali na dwie zmiany. I ta zmiana, która pracowała na 9, to chodziła do pracy, a ta zmiana, która zaczynała o 12 i pracowała do 20, pracowała w domu. I ona była tą zmianą domową, bardzo chętnie się zgłosiła, bo uznała, że pośpi sobie i owszem, mogła pospać, tylko później w ogóle nie miała nic już z dnia, bo ledwo wstała, poszła, zrobiła sobie kawę, przetarła lewe oko i prawo oko i porozmawiała przez 8 h, i potem była 20. I w zasadzie dupa. Bo trzeba by było teraz jakoś się przygotować do wyjścia, wykąpać może, coś tam, a tu w ogóle po ptakach. Więc pracowała tak 2 tygodnie i powiedziała temu szefowi, że ona to już wolałaby wrócić jednak na tę dzienną akcję czy się z kimś zamienić, bo ona by już popracowała. Popracowała ze 3 tygodnie, jak ich teraz wrócili do domu już, jakoś od tygodnia jest home office. Na pewno bezpieczniej, ale...

**A jak ona znosi ten home office?**

Wolałaby chodzić do pracy, a z drugiej strony docenia to, że może wstać... Jak pracuje rano, od tej 9 do 17, to nawet chyba nie najgorzej. Jest z tym kotem swoim, więc jest w porzo, koleżanki tam jeszcze jakieś przychodzą, spotykają się w takim mniejszym wymiarze osobowym, a teraz to w ogóle protestuje, więc już wychodzi na okrągło. Nie wiem, może jakieś skutki tego będą opłakane.

**Czy teraz boi się pani?**

Dalej nie jestem wśród tych najbardziej się bojących, bo znam osoby bardziej się bojące, w mieście szczególnie, bo może tutaj na wsi to nie znam, ale w mieście mam część koleżanek, które naprawdę się boją i ograniczają taką aktywność do absolutnego minimum. Rezygnują z fryzjerów, z jakiś takich rzeczy, ze spotkań ze znajomymi, tak że nie, nie należę do tej grupy, bo myślę sobie, przynajmniej tak mi się wydaje, że staram się realnie ocenić niebezpieczeństwo. Dopóki jesteśmy jakoś tam, oczywiście to się może w każdej chwili okazać nieprawdą, jak np. zachoruję, to się okaże, że te ograniczenia, które ja wzięłam pod uwagę, były niewystarczające, ale myślę, że tyle możemy zrobić, ile możemy, na ile taka maseczka nam pomaga. Pewnie, gdybyśmy siedzieli wszyscy w domu, byśmy pewnie nie chorowali, ale trzeba pójść i do sklepu, ktoś musi te zakupy zrobić. Na pewno nie boję się jakoś strasznie, to nie znaczy, że się nie obawiam, że zachoruję. Jasne, że tak i staram się tego uniknąć, ale trzeba też w międzyczasie jakoś normalnie żyć w miarę i zachować jakieś zdrowie psychiczne, i próbować znaleźć jakąś równowagę w tym wszystkim. Zobaczymy, czy się uda, czy nie.

**W którym momencie pani poczuła, że robi się poważnie?**

Jak w połowie października chyba zdałam sobie sprawę z tego, że na początku października, czyli 2 tygodnie wcześniej, zachorowania dzienne to były 5 000, one rosły sobie, rosły, rosły po cichutku, ale jak dotarło do mnie, że na początku października, 3 czy 4 ilość zachorowań to 5 000, a 15 to było 10 000, a jeszcze się nie skończył październik, już mamy 20 000, tak że ta liczba przyrasta w taki sposób... Bo dopóki to przyrasta liniowo, w sensie takim, że przyrasta tak, jak nam przyrastało wiosną, czyli ileś tam przypadków dziennie, a tutaj jak nam się mnoży tak, że w ciągu paru dni nam się podwaja ta liczba, to zaczyna być tak naprawdę groźne, bo mi się wydaje, że nie kontrolujemy tego. Bo jak przyrastało tam po 200-300 osób dziennie i to było codziennie 300 plus/minus parę, to jest inna sytuacja, bo to jest wzrost liniowy, a jak pani idzie do góry parabola po łuku, to rzeczywiście statystycznie da się zauważyć, że to przestaje rosnąć liniowo, tylko zaczyna się ten wzrost taki znaczący, to się podwaja, potraja, to błyskawicznie idzie w tej chwili. Myślę, że to jest ten moment, kiedy ja sobie pomyślałam: no dobra, to już chyba się zrobiło poważnie teraz, teraz to już dotyczy na pewno nas wszystkich. Bo jednak ja tutaj miałam na tej wsi, szczególnie podczas tej pierwszej fazy pandemii, takie poczucie, że to jest tak daleko, a teraz myślę, że jest bardzo dużo tych zachorowań, a w związku z tym to się już po prostu wszędzie dzieje, to nie jest tak, że górnicy, bo górników mamy daleko, ale mamy kogoś w Staroźrebach, to już jest blisko. A w tych Staroźrebach, ja wiem, bo już wtedy o tym rozmawiałyśmy, że ci ludzie nie przestrzegają tych zasad. Myślę, że dlatego, że po prostu nie rozumieją, że dzieje się to, co tu się dzieje, znaczy, że oni coś robią, bo ktoś im tak powiedział albo tak zrozumieli, ale ponieważ nie rozumieją, dlaczego to się robi, nie myślą o tym, więc to nie jest związane ze zrozumieniem, tylko po prostu ktoś im tak powiedział, że tak trzeba robić. Chodzą w tych maskach, ale ten nos mają na wierzchu. Chodzą w tych maskach, bo trzeba, ale bez takiego... Jak ma pani zinternalizowaną, mówiąc paskudnym językiem, jakąś wiedzę, to nie jest w stanie jej pani zmodyfikować i dostosować do sytuacji - chodzą w maskach, a gdzie ta maska jest, to już oni tam nie doczytali tego i że to drogą kropelkową, i że coś tam. W związku z powyższym myślę, że rozpowszechnianie się tej choroby w takich warunkach jest po prostu szybsze czy łatwiejsze.

**Patrzy pani na wskaźniki, ile osób zmarło?**

Nie, nie patrzę. On chyba jest w miarę (tak jak patrzyłam na niego ze dwa razy, jak Julka była głównie albo jak słucham przez radio) to mi się wydaje, że procentowo to jest tyle samo co wcześniej. To oczywiście jest dużo więcej osób, bo mamy dużo więcej zachorowań, ale procentowo to jest ten sam poziom, więc co tutaj patrzeć. Można się epatować takimi liczbami, ale w dalszym ciągu jak sobie to porównamy wszystko do liczby mieszkańców i coś tam, no to tak zawsze jest się czym pocieszyć, nie ma co się tym nakręcać, na to nie patrzę. Patrzę jednak na to, że oczywiście, że tych osób zmarłych jest więcej, bo jest dużo więcej zachorowań, więc bardziej się martwię tym, że jednak ta choroba jest coraz powszechniejsza... (zakłócenia) Więc to już nie jest, że to tylko duże miasta albo duże skupiska ludzkie, albo coś tam. Zdarza się wszędzie, bo już po prostu roznosimy to wszędzie.

**Mówiła pani, że się rozluźniliśmy i stąd takie skutki - dało się namówić ludzi, żeby dalej trzymali ten reżim?**

Myślę, że administracyjnie by się dało, co nie zmienia faktu, że było trudno. Przyszło lato, wakacje i tak za bardzo nie można było wyjeżdżać, ale część osób wyjeżdżała. Pewnie by się dało, skutki covidowe może byłyby ciut lepsze, ale jednak tak naprawdę to nie wiem, bo latem chyba nie obserwowaliśmy jakiegoś wzrostu zachorowań, tylko one się zaczęły późnym latem czy wczesną jesienią. Myślę, że to nie jest jeden czynnik. Myślę, że tutaj zadziałało wiele czynników, być może można było wcześniej... Ale jakby człowiek wiedział, że się przewróci, toby usiadł. Nigdy nie wiadomo, kiedy jest to wcześniej, kiedy ten moment miałby nastąpić, bo toby trzeba było wyprzedzić taką sytuację. Teraz można powiedzieć, że jest musztarda po obiedzie, ponieważ to mleko się wylało. Mleko się wylało, próbujemy je zetrzeć, ale jak już go nie będziemy więcej gotować, to nie wykipi znowu może. Nie wiadomo, gdzie jest ten moment, nikt tego nie pokaże ani do przodu, ani wstecz takiego momentu, w którym trzeba to było np. zamknąć, żeby było lepiej. Bo tak naprawdę myślę, że nikt tego nie wie. Pewnie, że jakbyśmy usiedli i liczyli wstecz te wszystkie zachorowania, kiedy oni się pozarażali, to pewnie dałoby się może teraz post factum pokazać taki czas, kiedy byłoby lepiej np. w ogóle nie uruchamiać szkół albo coś. Ale co z tego, że możemy taki moment wskazać teraz, kiedy wcześniej nie mogliśmy tego zrobić i myślę, że nikt nie mógł. Jakbyśmy się słuchali tylko, tak jak rozmawiałyśmy już o tym chwilę temu, że jakby tylko wyabstrahować sobie sprawę tego coivdu, to ona by nie była taka bardzo może skomplikowana, ale nie żyjemy tylko z coivdem, tylko musimy pracować, musimy jeść, musimy płacić podatki, bo państwo musi utrzymywać służbę zdrowia, nauczycieli i Bóg raczy wiedzieć co. To nie jest organizm prosty, tylko bardzo, bardzo, bardzo złożony. Trzeba działać w zadanych warunkach. Oczywiście, że patrząc na to, epidemiolodzy krzyczą na alarm, no tak, ale ci epidemiolodzy też dostają jakieś papu i to papu ktoś produkuje itd.

**Patrzy pani, jak jest w innych krajach?**

Nie. Już przestałam patrzeć, jak jest w innych krajach, bo co nam to da. Nie wiem, jak jest w innych krajach, spodziewam się, że może lepiej, ale cóż nam to może pomóc. Myślę, że np., chociaż tak jak mówię, w ogóle nie wiem, jak jest w innych krajach, bo nawet na to nie patrzę, ale myślę, że może lepiej jest być w Niemczech, bo oni są w ogóle lepiej zorganizowani i mają dużo więcej pieniędzy publicznych, dużo większe rezerwy, których nie wydają na rzeczy, które my tutaj sponsorujemy, czyli całą tę taką - powiedzmy może bardzo nieładnie, ale może dosadnie - patologię społeczną, którą tu dotujemy bezustannie i im wszystko dajemy, no bo oni nic nie mają, bo muszą się napić, więc nic nie mają. I tam na pewno są szczepienia i coś tam. Myślę, że może są miejsca, gdzie jest lepiej niż u nas.

**Czyli Niemcy mogłyby być takim krajem?**

Myślę, że mogłyby być. Znając ich i nakłady, i to, jak reagowali na pierwszą falę pandemii, jak się zachowywali, ile robili badań itd. Ale co nam to da, skoro my nie jesteśmy w takiej sytuacji jak oni, ani gospodarczej, a w szczególności politycznej i finansowej, to co nam to da. Na pewno są też kraje, gdzie jest gorzej, mogę sobie wyobrazić. Nie wiem, jakie one są, ale myślę, że są. Mam nadzieję, że u nas nie jest najgorzej, nie, może nie, nie mam nadziei, to głupio zabrzmiało. Ale myślę, że są jakieś kraje, gdzie jest gorzej i też nie możemy się tym pocieszać, że tam jest gorzej, bo oni może mają jakieś inne uwarunkowania i jeszcze mniej pieniędzy, i jeszcze bardziej odjechanych przywódców, może być na pewno gorzej. Nie pociesza mnie w ogóle taka sytuacja, że ktoś głoduje, to nie jest dla mnie pocieszenie, że ja mam zapasów na rok, co z tego.

**Jak będzie wyglądać u pani Boże Narodzenie?**

Jeszcze nie mamy decyzji. Nawet wczoraj rozmawiałam o tym z moim bratem i myśmy tutaj jeszcze, rodzina moja, czyli ja z dziećmi, nie podjęliśmy jeszcze decyzji, co z tym robimy, bo nie znamy warunków brzegowych, nie możemy się wypowiedzieć na ten temat jeszcze, chociaż może i możemy powiedzieć, że nigdzie się stąd nie ruszamy, ale może nam się uda ruszyć. Szkoda by było, ale cóż. Tak że jeżeli chodzi o święta, to jeszcze nie wiemy, jeszcze czekamy. Na pewno nie idziemy na cmentarze, na pewno odmówiliśmy mojemu bratu wzięcia udziału w urodzinach jego środkowej córki, ona 1.11. się urodziła, zwykle jeździliśmy do Warszawy na jakieś spotkanie. W zeszłym roku też byliśmy, mój brat prowadzi szkołę taką, szkoła demokratyczna to się nazywa, na Ursynowie, gdzieś tam w Pyrach, wszystko jedno i tam nawet byliśmy w tej szkole, więc było tam sporo dzieci, rodzice, ktoś tam. A Julka dodatkowo potem bierze udział w takich urodzinkach, które sobie Ninka tam dla własnych koleżanek 5 czy 6 organizuje w domu i ona tam jeździ wtedy, i je tam czesze, maluje im paznokcie i coś tam. Więc ustaliliśmy, że nie będziemy brali udziału w tym zajściu w tym roku, czyli ani w Święcie Zmarłych w taki tradycyjny sposób, tylko po prostu pojedziemy sobie po, bo przed to chyba nam się nie uda, ale może jakoś po uda nam się, jak już z powrotem otworzą cmentarze dla ruchu samochodowego, bo to jest taki moment teraz... Aczkolwiek wczoraj jak się próbowałam wyciąć z tej blokady, to podjechałam nawet pod Cmentarz Północny i wydawało mi się, że jeszcze samochody stamtąd wyjeżdżały, więc wczoraj może zamiast stać w tym korku, mogłam przynajmniej buchnąć na cmentarz. Ale tego nie zrobiłam, no i trudno, powiedziałabym, że dla tych zmarłych z całą pewnością, z mojego punktu widzenia, jest wszystko jedno, jeśli chodzi o nas, to nie jest wszystko jedno, bo to mnie jednak, jakoś tam to taki dzień dla nas ważny rodzinnie. Ale są rzeczy ważne i ważniejsze. Chociażby to, że moje dzieci muszą na Cmentarz Północny dojechać środkami komunikacji, żeby nie jechać tam samochodem, boby musiały wyjechać o 5 rano. Ja akurat nadjeżdżam ze strony Gdańska, więc nadjeżdżam jakby po drodze i jestem w miarę bezkorkowo, oprócz tego, że stoję godzinę na Wójcickiego, no to poza tym jest super. No ale nie, i te znicze, i tych ludzi tam po prostu tryliard, w ogóle nie ma, o czym mówić. Tam zrobiłyśmy porządki na cmentarzu w maju z Julką, porobiłyśmy ławki nowe przecież i wszystko, to akurat mamy ogarnięte, a że nie zapalimy znicza, zawsze zapalamy na różnych grobach, więc może ktoś, kto będzie przechodził, zapali, a jak zapalimy tydzień później, to nie będzie jakiegoś problemu. A co ze świętami, to nie wiem, to rzeczywiście jest słabe. W zeszłym roku po raz pierwszy święta zrobił mój brat i bardzo bym nie chciała, żeby początek tej wspaniałej tradycji jakoś mu umknął, ponieważ poprzednich 30 lat święta robiłam wyłącznie ja, dla ogromnej rzeszy ludzi. I to wszystkie 3 dni świąt, bo to raz rodzina, potem znajomi i to znajomi w 2 turach kiedy jestem tu na wsi, bo się wszyscy nie mieszczą, więc w ogóle masakra. Więc jak już Wigilię przejął w zeszłym roku mój brat, a przebąkiwał o tym chyba z 5 lat już, więc jak w zeszłym roku to przebąkiwanie się zmaterializowało, to nie chciałabym z tego zrezygnować. A bardzo nam miło jest spotykać się razem, bo tak nie widujemy się bardzo często, przede wszystkim z tego powodu, że on jest młodszy ode mnie 10 lat, więc jest jakby na innym etapie życiowym. Nie wiem, co pani powiedzieć, po prostu nie wiem. Chciałabym się spotkać, ale na pewno nie będziemy ryzykować, jeżeli to będzie ryzykowne. A on na razie teraz też jest na zdalnym nauczaniu tych dzieci, ale jak to będzie do grudnia, kto to wie.

**Ma pani w głowie jakieś scenariusze?**

Jeżeli dalej będziemy mieć czerwoną strefę i ten poziom zachorowań się utrzyma, to myślę, że nie będziemy jechać, spędzimy to tutaj rodzinnie w takim sensie... Nie wiem, jak tam będzie z Wigilią, bo Hubert z Magdą teraz już zapowiedzieli, że od tego roku, to oni będą razem występować, tzn. ze wszystkimi tego konsekwencjami, czyli że będą albo tu, albo tam, ale dwuosobowo, tak że już ten model...

**W pakiecie już: albo wszyscy, albo nikt.**

Albo Julka tylko, Julka jest na razie w modelu pojedynczym w tym sensie, że ma jakiegoś chłopaka, ale to jeszcze nie jest taki chłopak świąteczny i te rzeczy. Ale jak nie przyjadą w Wigilię, to nie wiem, czy przyjadą w pierwszy dzień świąt czy w drugi, rozumiem, że będą teraz dzielić to, ale nie w taki sposób, że jedno tu, drugie tam, tylko że razem tu albo razem tam. Oczywiście, mnie zawsze brakuje tego kontaktu, może inaczej, nie tyle brakuje, co zawsze chętnie ich widzę u siebie i cieszę się, jak są, ale ja rozumiem, to są młodzi ludzie, którzy rozpoczynają czy rozpoczęli już jakiś czas temu własne życie i nie będę się czuła obrażona albo urażona, jak pojadą na Wigilię do tamtych rodziców. Bo z jakiego powodu ja bym miała się czuć urażona? Że nie wybrali mnie? Ale tam też są rodzice. I to jeszcze w takiej sytuacji, powiedziałabym w cudzysłowie może gorszej, bo Magda jest jedynaczką. Ja mam Julkę, a jak już będę bardzo smutna, to mam brata, który ma trójkę dzieci, więc mogę się jeszcze tam umieścić. Tak że scenariusz jest taki, że może być każdy scenariusz. Może gdybym ja mieszkała w Stanach, to sytuacja by była dużo gorsza, bo możliwość przejechania czy znalezienia się w innym geograficznie miejscu jest na pewno bardzo ograniczona, w związku z powyższym czekając z nadzieją, że będzie lepiej, nagle się okaże, że będzie gorzej, to byłabym na pewno w czarnej dupie, ale tutaj to jak ich nie zobaczę na święta, to ich zobaczę tydzień później. W tym roku wszyscy jak odpuściliśmy, nie byliśmy na urodzinkach, bo to był początek tej pandemii, kwiecień i było słabo wtedy, i spędzaliśmy urodziny przez telefon, no i co, i trudno, bywa i tak. Nie ma co dramatyzować moim zdaniem.

**Na Wielkanoc byliście w małym gronie?**

Tak, jakoś tak było. Tak, była Julka tylko, bo ona przyjechała w ostatni weekend marca, a Wielkanoc była w kwietniu, ale Hubert nie przyjechał wtedy. To tak było. Julka była do końca maja, czyli Wielkanoc spędziliśmy tutaj w domu z Juleczką. Ja jestem ateistką, dla mnie też te święta mają w zasadzie tylko wymiar rodzinny, nie mają wymiaru religijnego, w związku z powyższym też mi jest pewnie łatwiej, nie wiem, bo nie byłam w sytuacji opozycyjnej, ale też nie ma tak... Myślę, że ta Wielkanoc zwłaszcza dla katolików jest takim świętem bardziej uduchowionym może czy ważniejszym z punktu widzenia dogmatu wiary. Pewnie, że mi szkoda, ale jak się rodzinnie zobaczymy na sylwestra np. albo kiedy indziej, to się zobaczymy kiedy indziej.

**Jaka jest różnica w zobaczeniu się tydzień później?**

Jak będziemy się spotykać tylko rodzinnie, znaczy, jeżeli ja będę się spotykać tylko z dziećmi, to w zasadzie nie ma żadnej różnicy. Bo nawet te potrawy wigilijne, to dopóki jeszcze żyły starsze osoby w domu i dopóki byliśmy taką bardziej wielopokoleniową rodziną, mama Marka to dalej żyje, ale babcia Marka i ktoś tam, jak my się spotykaliśmy w gronie rodzinnym w dużo osób, jak moja mama żyła, to wtedy te potrawy wigilijne, zwłaszcza ze strony rodziny Marka, bo ja jestem z domu ateistka, więc u mnie tam nie było takich, więc ja robiłam tu jakiś pierdyliard potraw wigilijnych, których np. większość moje dzieci nie jedzą, bo nie jedzą buraków, grzybów, nie jedzą czegoś i czegoś. Więc o tyle się różni, że nie ma takich obostrzeń tradycyjnych, tak bym powiedziała. Jak jesteśmy sami, a zdarzyło nam się kiedyś być samodzielnie, bez mojego brata i jego rodziny albo bez mojej rodziny, już tutaj na wsi, tylko z dziećmi, to po pierwsze gotujemy to, na co mamy ochotę. Więc każdy się zgłasza, co by zjadł i robimy jakąś zupę, staramy się, żeby to było w miarę odświętne, ale tak bez przesady, np. zamiast barszczu, którego oni nie jedzą, gotujemy rosół, który jedzą chętnie. Ale jak się spotykamy z moim bratem i mój brat ma teściów, to tam ryba, tu coś tam, tu barszczyk, pierogi takie, srakie, owakie itd. Oraz mamy też dress code inny, a mianowicie mamy dress code dres, co najbardziej chyba cieszy mojego syna. Nie żeby on się jakoś w garnitur wbijał, nie, ale to mu robi dobrze, że on może usiąść przy tej kolacji wigilijnej czy w czasie tych świąt, tak jak chodzi cały dzień, że się nie trzeba przebrać, wymyć na świeżo podłogę, napluć na nią i ją wypucować, że mamy taki ogólnie rzecz biorąc luz. To nie znaczy, że nie ma takiego odświętnego charakteru, ale to nam pasuje. Zresztą jakieś serwetki z Mikołajem, to nie, że w ogóle absolutna abnegacja, że rozkładamy gazetę i na niej jemy, nie, ale taki mamy większy luz. To tym się różni. A jak będziemy u brata, to oni tam zrobią, w zeszłym roku o to zadbali, bo to po raz pierwszy u nich Wigilia, oni zadbali, żeby były tam jakieś właśnie potrawy wigilijne, zamówili nawet jakąś chyba rybę, bo ja się tam zgłosiłam, że ja im zrobię, co oni sobie zażyczą, coś im tam wykonałam nawet, ale jakąś rybę to we własnym zakresie. A też ich nie robili, bo oni mają inny stosunek do życia ogólnie, wyrażający się z grubsza w tym, że szkoda czasu na sprzątanie, gotowanie i takie rzeczy, dużo więcej czasu efektywnie i sensownie spędzają z własnymi dziećmi i to im odpowiada, i świetnie. Ale stanęli na wysokości zadania wigilijnego, bo jakaś ryba i po grecku, ale były też oliwki na stole, tak że taki był eklektyzm, jeśli chodzi o tradycję. Nie jesteśmy niewolnikami.

**Czyli trochę będzie szkoda, ale najwyżej spędzicie święta po swojemu?**

Tak, zdecydowanie, jakoś sobie poradzimy.

**Myśli pani o przyszłości po pandemii?**

Nie. Ja w ogóle nawet nie myślę o Bożym Narodzeniu, o Bożym Narodzeniu myślę, bo mnie pani wyrwała do odpowiedzi, a co dopiero o dalszej.

**Co musiałoby się stać, żeby uznała pani, że to koniec pandemii?**

Tak naprawdę myślę, że końca nie będzie, tzn. zawsze będziemy już teraz, nawet jak nam te zachorowania spadną do jakiejś tam trzycyfrowej liczby i przestaną być podawane przez media, bo pewnie taki moment nadejdzie, to myślę, że i tak z tyłu głowy będziemy mieli, że to już nigdy nie wróci do takiego stanu, że to się nic nie stało, bo to nam daje taką nauczkę, że stać się może coś, co jest absolutnie od nas niezależne. Nagle przychodzi na nas jak grom z jasnego nieba przysłowiowy i co? I musimy się z tym zmierzyć. Mam takich znajomych ze Śląska i tata tej mojej przyjaciółki, który urodził się przed I wojną światową, był lekarzem i w czasie II wojny, potem wszystko stracił za komuny, nieważne, w każdym razie on miał takie powiedzenie: "Dziecko, oby wam nie przyszło żyć w ciekawych czasach". Oczywiście, on myśląc o sobie, że żył na pewno w ciekawych czasach w tym sensie, że wiele się działo, ale to jest bardzo trudne i że tak naprawdę najlepiej jest, jak się nic nie dzieje specjalnego, bo wtedy możemy żyć każdy własnym życiem i nie mamy takich warunków narzucanych, tak jak teraz, przez sytuację: czy to wojna, czy coś, to jednak były dramaty życiowe. Ludzie nie tak sobie wyobrażali swoje życie i co, ktoś ich pytał o zdanie? Nie, po prostu. Więc myślę, że my też niestety żyjemy w ciekawych czasach i byśmy pewnie chcieli wszyscy, żeby one przestały być takie ciekawe. Może ja też bym chciała, żeby one przestały być takie ciekawe i żeby wróciła taka codzienność bardziej monotonna, ale w związku z powyższym bardziej kontrolowana przez każdego z nas, ale czy tak będzie, to nie wiem. Myślę, że teraz to i tak, przynajmniej nasze pokolenie będzie miało z tyłu głowy taki alarm, że to nie musi tak być, jak jest, łagodnie. Jeśli w ogóle wrócimy do jakiegoś spokoju.

**Czy zaczniemy traktować koronawirusa jak grypę?**

Może nie jak grypę, ale jako chorobę, jak już ogarniemy szczepionkę na tę chorobę, to wtedy podzielimy się na tych, co się będą szczepić i na tych, co się nie będą szczepić oczywiście, czyli zaczniemy powracać do normalności, czyli zaczniemy mieć własne zdanie. Te wirusy z grupy koronawirusy to są wirusy znane, te SARS-y i coś tam, te choroby gdzieś tam powracają, są jakieś zarzewia tych chorób, w które się wdraża szczepienia i w tych krajach, zwłaszcza trzeciego świata, to się dzieje. I oni z tym żyją na co dzień, nie tylko z tym, tylko z takimi chorobami, które nam się pojawiają okresowo i są zakaźne, więc jakieś tam te dengi czy ameby, czy inne diabły i będziemy musieli po prostu mieć z tyłu głowy, że są takie choroby, one się po prostu pojawiły i są. Jak się pojawiły bakterie paciorkowca czy zostały odkryte, to ludzkość musiała zacząć z nimi żyć po prostu. Z wiedzą o nich, bo wcześniej może po prostu nie byliśmy w stanie ich rozróżnić czy wyekstrahować, ale od kiedy wiemy, że są, to z nimi żyjemy. I tak samo będzie z tym covidem i on tam się będzie jakoś mutował, i będziemy musieli znaleźć na tę chorobę jakieś lekarstwo, lekarstwo w cudzysłowie oczywiście, czyli jakieś sposoby działania. Jak znaczna część osób będzie się szczepić, to może ta, która się nie będzie szczepić, też nie będzie tak narażona na tę chorobę, bo nie będzie tyle przypadków. Myślę, że ona już z nami zostanie. Myślenie magiczne, że to zniknie, już mamy, ja już mam za sobą, jeśli w ogóle tak kiedyś myślałam, to już jest to za mną.

**Jakie będą skutki covidu w kraju?**

Myślę, że skutki covidu będą na pewno mniejsze niż skutki polityczne. Jeżeli chodzi o skalę gospodarczą ze względu na covid, to w ogóle o tym nie myślę, bo to i tak jest ściśle związane, tego nie można, znowu wracamy do sytuacji, że tego nie możemy rozdzielić od sytuacji politycznej, ponieważ są osoby, które podejmują decyzję, co robić w związku z covidem i jego skutkami i od tego, jaką one decyzję powezmą, zależy to, jak to będzie wszystko wyglądać, więc tu myślę, że abstrahowanie od sytuacji politycznej jest nie na miejscu, jest bezcelowe. Myślę, że tak trochę jak po wojnie, bo to w pewnym sensie jest jakiś rodzaj takiej siły wyższej w takim sensie, że nie wiemy, co się stanie, więc tak jak po każdej wojnie jakoś będziemy dochodzić do równowagi przez jakiś czas na pewno. Może to porównanie do wojny jest nadużyciem, bo to na pewno nie jest taka skala zjawiska, ale w pewnym sensie może możemy się posiłkować takim porównaniem z braku innego, bo to też są i ofiary śmiertelne, i chore, i coś tam, w jakichś środowiskach. Jakoś na pewno się podniesiemy gospodarczo. Oczywiście, może nam być łatwiej lub trudniej, ale jakoś dojdziemy do siebie. Na pewno nie bez ofiar, w sensie gospodarczym też. Część osób, już najprawdopodobniej zamknęło swoją działalność, część firm ograniczyło mnóstwo rzeczy, wydatków, na które nie muszą wydawać, jakiś sponsoring różnych organizacji czy klubów, czy czegokolwiek. Mogę sobie wyobrazić, że jak brakuje kasy, to człowiek patrzy. Ja też patrzę, na co wydaję pewnie bardziej niż wcześniej. Takie życie.

**A skutki społeczne?**

Nie wiem, trudno mi powiedzieć. Co to znaczy społeczne? Myślę, że jak już wrócimy do takiej w cudzysłowie normalności, czyli przestaniemy chodzić w maseczkach i będziemy mieli lek, który nas uspokoi, czy go zażyjemy, czy nie, jeżeli będziemy mieli takie poczucie, że jesteśmy jakby zdrowotnie bezpieczni, to myślę, że społecznie to nie będzie jakichś skutków. Powoli zaczniemy, tak jak i gospodarka, podnosić się z tej takiej izolacji, zaczniemy wychodzić i jak nie będzie zachorowań, to ludzie jakoś wrócą. Przecież tego nie da się tak naprawdę oddzielić. Jeżeli gospodarczo, finansowo zaczniemy lepiej stać i tam odkuwać się w cudzysłowie, to i społecznie nie będziemy odczuwać takich skutków.

**A jest jakaś grupa, która może bardziej odczuć pandemię?**

Ja w ogóle obserwuję, że jest takie ogólne obniżenie nastrojów. To, że ta pandemia wróciła czy też że się nie skończyła, że się nasiliła znowu, że to jednak obniżyło nastrój wielu osób. I to nie tylko starszych osób. Bo być może wydawało nam się, że zmierzamy ku końcowi, a teraz ten koniec się nam zdecydowanie oddalił, mamy powtórkę z rozrywki, znów poszliśmy do góry, więc zanim spadniemy, to minie czas i pewnie dłuższy, niż minął itd. Więc myślę, że to może być taki skutek. Te osoby, które mają raczej skłonność bardziej do refleksji, to być może ten obniżony nastrój może się utrzymać i myślę, że to nawet nie chodzi o grupę czy wiekową, czy zawodową, czy coś, ale o sposób reagowania na takie sytuacje, że ta pandemia bardziej trwale robi taką rysę na psychice, człowiek jest bardziej depresyjny, smutny, że to wpływa na obniżenie nastroju i że to nie tak łatwo się podnieść. Bo możemy np. wziąć się za książkę i spróbować się podnieść, bo sobie coś poczytamy, ale tę książkę musimy wziąć albo taką, która nam podniesie ten nastrój, albo wręcz przeciwnie - wziąć taką, która jest zgodna z tą rysą, która powstała już na naszej psychice i będzie po prostu wtedy trudniej. Myślę, że może tak być, że część osób zniesie to gorzej, wyjdzie jakaś pokiereszowana z tego zajścia.

**Czy na koniec jeszcze jakieś przemyślenia covidowe?**

Nie, mam takie przemyślenia, że może nie będziemy się spotykać więcej z tym covidem. Nie, żebym nie lubiła z panią pogadać, ale to jest taki, dla pani pewnie też, choć pani zawodowo to robi, to może trochę inaczej, ale jak tak siadamy i gadamy o tym, to to już się staje takie bardziej realne i musimy rzeczywiście przemyśleć to, normalnie mogę spychać, zdmuchnąć sobie tę myśl, bo sobie znajdę inną, która jest równie atrakcyjna, a może atrakcyjniejsza, a tu jak tak siadamy i rozkminiamy tę sytuację, pani wypytuje...
