# Supplementary material for: The challenges arising from the COVID-19 pandemic and the way people deal with them. A qualitative longitudinal study
Source: PLoS One. 2021 Oct 11;16(10):e0258133. doi: 10.1371/journal.pone.0258133 (PMC8504766; doi:10.1371/journal.pone.0258133)
Supplement: S1 Dataset — (ZIP) [file pone.0258133.s003.zip › Transcriptions/stage 6/12.6_M_33_couple, with children.docx]

**12.6_M_33_copule with children**

**Co się działo od naszego spotkania?**

Wakacje całkiem spoko minęły. Byliśmy na kilku wyjazdach, na ostatnim we wrześniu. Ten temat całkowicie zszedł na 2 plan albo nawet na 3-ci. Dużo poświęcałem się pracy potem dużo z dzieciakami, ale ten rytm utrzymywałem cały czas, który miałem wypracowany. Rytm głównie pod dzieciaki i zajęcia dodatkowe. Jeśli chodzi o sam Covid, to bardzo oddziaływało to na nas na wiosnę, co zaskutkowało inwestycją. Kupiliśmy działkę i budujemy dom w okolicy jeziora. To na pewno jest taki pozytyw, który z tej sytuacji się rozwinął. Od 3 tyg., działa tam ekipa, która ten dom buduje. Praktycznie dom już prawie stoi, więc jesteśmy podekscytowani i cieszymy się z tego, że w przyszłym roku będziemy mieli gdzie pojeździć w razie jakichś lockdownów, czy gdyby inne kierunki były dla nas niedostępne. Zawsze będziemy miejsce, gdzie będziemy mogli sobie zmienić otoczenie. W maju podjęliśmy decyzję, żeby kupić działkę. Jak z tobą rozmawiałem to już miałem te plany, ale jeszcze trzymałem to dla siebie, bo nie wiedziałem, jak to wyjdzie, czy znajdziemy. Udało się znaleźć fajną działkę, w czerwcu udało się kupić, troszeczkę na niej popracowaliśmy w międzyczasie, czyli ogrodzenie, trawa. Potem planowanie budowy i budowa. Jak pierwszy raz opuściliśmy dom po zamknięciu na majówkę, to pojechaliśmy do teścia i tam taki pomysł się narodził. Traktujemy to jak inwestycję tak naprawdę.

**Jak wygląda teraz wasza codzienność?**

Jest inaczej ogólnie. Mamy teraz różne przygody z tym Covidem u nas w rodzinie, w domu, bo zawitało, zapukało też do mojego domu. Nikt sobie testów nie robił, natomiast ogólnie jakoś tak zawirowało teraz życie, bo jakieś objawy mieliśmy wszyscy w domu. Po kolei, bo najpierw dzieci, potem ja, potem żona, dziadkowie. Trochę taka ulga, bo niemożliwe, żebyśmy my mieli a dziadkowie nie, ale przechorowali swoje i widać, że wychodzą na prostą. Ulga, bo najbardziej od początku martwiliśmy się o dziadków. No i takie różne śmieszne sytuacje.

**Macie dużą pewność, że to był koronawirus?**

Raczej tak. Nie mieliśmy węchu, smaku i tu było takie zdziwienie z tego powodu.

Ważne, przełomowe momenty?

To właśnie decyzja i kupno tej działki i taka nadzieja na przyszłość, że będzie gdzie pojeździć. Unikaliśmy trochę imprez zbiorowych i po weselach nie chodziliśmy. Poza tym wszystko toczyło się normalnym nurtem.

**Z bliskimi i rodziną się spotykaliście?**

Tak, zdecydowanie. Blisko i często. Chwilowo odcięliśmy się od rodziny, bo zauważyliśmy u siebie objawy. Sami sobie nałożyliśmy kwarantannę.

**Czy są obszary życia, które wróciły do stanu sprzed epidemii?**

Chyba ciężko coś takiego w tym momencie znaleźć, bo dużo się zmieniło i to na każdym gruncie. Inaczej wygląda wyjście do kina, do sklepu, do szkoły, itd. ja teraz 3-ci tydzień jestem w domu, gdzie nie byłem w ogóle w biurze. Wiele rzeczy się zmieniło i obawiam się, że tak szybko to nie wróci do takiej normalności. Nawet wyjeżdżając na basen z dzieciakami to już jest taki odruch, że maseczka w kieszeni czy jakieś rękawiczki, żele. To już chyba taka norma się stała. Jest ta świadomość, że rzeczywiście ta 2 fala nadeszła i to moim zdaniem patrząc na okolicę i na ludzi, to taka dosyć wysoka jest ta fala. Chyba nie ma takiego obszaru, który by wrócił całkowicie do normalności, czy w firmie, czy w domu, czy gdziekolwiek. Zawsze gdzieś w tle jest Covid.

**Kiedy stało się jednoznaczne, że ta 2 fala już jest?**

Jakoś pod koniec września. Słyszałem już takie pierwsze doniesienia z przedszkoli i jestem [przekonany, że tylko dlatego pojawiła się ta druga fala, bo ruszyły szkoły. Jednak poszło to tak pionowo do góry, że mnie aż zaskakuje jak całe wakacje mógł być taki spokój...Wiadomo było, że są przypadki jakieś, ale wszystko do opanowania. Jak skończył się wrzesień, to już były doniesienia z okolicy, że tu ileś nauczycieli, tam iluś nauczycieli, część dzieci wysyłają do domu, część zostaje. Myślę, że to jest jedyny racjonalny powód tej 2 fali. Później to już się miesza z tymi przeziębieniami. My jesteśmy przekonani, że mamy Covida, ale testów nie zrobiliśmy, więc też nie wiadomo. Nie mieliśmy gorączki, duszności, kaszlu. Tak naprawdę to 2 dni gardło mnie bolało. Było mówione, żeby nie chodzić do przychodni, bo tam jest Covid i to zadziałało bardzo pięknie, bo chyba ostatnia rzecz o jakiej pomyślałem, to żeby się gdzieś zgłosić z bólem gardła. Później przyszedł brak węchu i teraz dopiero wraca. Szkoła i mieszanie się dzieciaków doprowadziło do tego, nabrało to wszystko tempa i w tym momencie Sanepid tego kompletnie nie ogarnia. Moi znajomi zrobili sobie test i sami dzwonili do Sanepidu co mają zrobić. Gdzieś tam w systemie kwarantannę, ale jedna osoba ma test pozytywny w domu i co z resztą osób? Nie było czegoś takiego jak na wiosnę, że 2 wynik pozytywny powodował jakąś interwencję czy wywiad ze strony Sanepidu. W tym momencie potrafią ludzie tydzień czekać w domu i nie ma żadnej informacji, co mają robić. Chciałbym być pewny czy mogę wrócić do biura i ja chyba po prostu przeczekam to jeszcze tydzień, żeby być na 100% pewnym, chociaż nie wiem, czy można być. Teraz dobrze się czuję i wszyscy w domu dobrze się czują i jakby mi teraz wyszedł wynik pozytywny, to sam bym nałożył na siebie kwarantannę jeszcze na tydzień, dwa, ale to już byłby chyba przerost formy nad treścią, bo ta kwarantanna cała już by się mijała z celem, itd.

**Śledziłeś też statystyki?**

Te ciągle zwiększające się statystyki to gdzieś trafiały do ucha, bo tak naprawdę to ja już dawno nie śledzę tych informacji covidowych tak jak to na początku było. Jakoś ten temat był poza moim zainteresowaniem. Docierało do mnie, że łamiemy kolejne bariery, były kolejne restrykcje wprowadzane. W tym tygodniu powinienem być na szkoleniu, ale Niemiec nie przyjechał, bo jednak on by musiał trafić na kwarantannę i robić sobie testy, więc zostało to przełożone. Widać, że to rzeczywiście jest w otoczeniu, że to się dzieje i tak naprawdę same statystyki były, są, ale już nie docierały do mnie w taki sposób, że przejmowałem się tym. Widziałem co się dzieje w najbliższej okolicy i nie musiałem patrzeć przez pryzmat całego kraju. U nas tego nie było praktycznie do połowy września albo jakieś pojedyncze przypadki i zaczęły si pojawiać nagle jak grzyby po deszczu.

**Zauważyłeś zmiany w zachowaniu osób z twojego otoczenia w ciągu ostatnich miesięcy?**

Nie. Chyba bardziej to, że każdy sobie jakoś z tym Covidem bardziej wyluzował. Zaczęliśmy się spotykać, jakieś wspólne ogniska. Mało osób wspominało o tym Covidzie i tak to wszystko przygasło troszeczkę. Każdy złapał do tego taki dystans. Chyba przez to, że były wakacje i ja rzeczywiście wtedy słyszałem o bardzo małej liczbie przypadków i ani u znajomych, ani nigdzie jakichś poważnych problemów z tym nie było. Każdy miał świadomość, ale już niekoniecznie się tak pilnował. Jak były jakieś imprezy okolicznościowe, jakieś wesela, na które nie musieliśmy iść to po prostu nie chodziliśmy. W grupie znajomych i z rodziną normalnie się spotykaliśmy.

**Nie chodziliście, bo baliście się zarażenia?**

Nie wiem. Może to była dobra wymówka, żeby nie iść? Ciągle były te różne ograniczenia w zależności od strefy i łatwo się można było wytłumaczyć, łatwiej było odmówić.

**Gdybyście bardzo chcieli to byście poszli?**

Myślę, że tak. Tutaj to bardziej był pretekst. U nas pójście na wesele też zawsze się wiąże z tym, że trzeba zaraz organizować opiekunkę dla dzieci...To uciążliwe jest troszeczkę.

**Czy masz wrażenie, że to zdystansowanie do koronawirusa nadal się utrzymuje?**

Nie, teraz się sytuacja zmieniła. Najbardziej to widzę po sobie. Na pewno znajomi mają podobne zdanie co ja, bo na szczęście nie spotkałem jakiegoś takiego ciężkiego przypadku zachorowania w moim najbliższym otoczeniu. Na 20 osób, które wiem, że chorują albo przechorowali, to było to podobnie jak u nas w rodzinie, że może 2 dni gorszego samopoczucia, a później mijało. Człowiek też może sobie pomyśleć, że to może być jakieś pompowanie bańki informacyjnej. Sam dużo ciężej przechorowałem grypę 2 lata temu, gdzie mnie zwaliło na 4 dni i straciłem chyba że 3 kg leżąc w łóżku. A tutaj połaskotało mnie troszeczkę. Duża ulga to dziadkowie, którzy mają pod 90-tkę. Oni chyba bez świadomości, że to może być Covid przechorowali sobie to. Taki nurt psychologiczny tu też wchodził w grę, żeby za bardzo ich nie uświadamiać.

**Jakie emocje ci towarzyszyły, jak zachorowałeś?**

Nie chciało mi się wierzyć troszeczkę, byłem zaskoczony, zdziwiony. W pierwszej kolejności pomyślałem, gdzie byłem przez ostatnie 48 godz., ale sobie dałem na wstrzymanie i poczekałem co się będzie działo. Trochę się też bałem, bo ja jestem przewlekle chory i mam astmę i nie wiedziałem jak to się rozwinie u mnie. Czy trafi mi na płuca, czy nie, czy będę właśnie miał te cięższy przypadek i potem bylem taki pozytywnie zaskoczony.

**Wiedziałeś co masz robić?**

Wiedziałem, że mam się izolować tak naprawdę. Mieliśmy różne zaproszenia w rodzinie i grzecznie odmówiłem. Powiedziałem, że jestem przeziębiony. Nie widywaliśmy się z rodziną i ze znajomymi. Z jakichś opcji tutaj wybieramy, bo na początku nie do końca byłem pewny czy to jest to. Te objawy covidowe przyszły jak ja już się dobrze poczułem. Trochę dmuchałem na zimne, ale potem się okazało, że to wcale takie zimne nie było. Ten schemat postępowania znałem też zachowań znajomych.

**Korzystałeś z jakiejś aplikacji?**

Nie. Zacząłem się interesować, ile trwa choroba, jakie są objawy, jak się może rozwinąć, ile czasu się zaraza, itd.

Czy w obecnej sytuacji coś jeszcze cały czas ci przeszkadza?

Tak, brak pewności czy to jest już ten moment, że mogę wyjść do ludzi czy nie.

**A w szerszym kontekście sytuacji pandemii w porównaniu do tego co było przed Covidem?**

Dla mnie to jest zupełnie inny świat teraz. Teraz trochę odetchnąłem, bo zetknąłem się z wirusem i osobiście jestem troszeczkę spokojniejszy. To jest zupełnie inna rzeczywistość teraz wyjść na ulicę, jechać gdzieś, robić zakupy, wszystko. Czekam tego momentu, kiedy wszystko wróci do normy. Jeszcze nie wróciło i obawiam się, że to musi potrwać.

**Co musiałoby się stać, żeby dla ciebie rzeczywistość wróciła do normy?**

Nie wiem. Impreza masowa bez ograniczeń? Gdyby nie było tych zachorowań, tej nagonki, tego szumu informacyjnego, maseczek? Coś, co było wcześniej. Obawiam się, że to się szybko nie stanie.

**A wynalezienie szczepionki mogłoby to pomóc?**

Ja się obawiam, że ten koronawirus po prostu przejdzie do normalności. On będzie już z nami i już nie będzie tak, że to trwa chwilę i później już tego nie ma. Być może ta pierwsza fala, która przejdzie przez wszystkich może wskazać osoby, które przechodzą to ciężej/ lżej, a być może to też jest uzależnione od tego kto jaką w danej chwili ma odporność. ja liczę na to, że organizmy się same nauczą tego wirusa i przeciwciała będą działać tak, że po prostu będziemy to przechodzić. To będzie cały czas gdzieś w tle, ale będziemy to przechodzić jeszcze lżej. Fajnie by było i to jest bardzo optymistyczna wizja, że rzeczywiście to znika za rok i wraca wszystko do normalności, ale to pewnie będzie już wędrowało po regionach jak grypa i może być tak, że nie pozbędziemy się tego.

**Emocje**

6 - działka i radość związana z tym zakupem. Taki moment, że będzie fajnie, itd. Takie wyobrażanie sobie jak będziemy tam spędzać czas, plany.

8 - wrzesień, bo całe wakacje były spoko. Wróciliśmy z urlopu i zaczęły się pojawiać jakieś przypadki. Znowu było coś takiego, że nie wiadomo co będzie za chwilę, że znowu zaczyna się coś dziać i tak naprawdę ta 8 mogłaby być i do teraz, ale kolejny ważny punkt to inny obrazek. Nie wiadomo co będzie za chwilę, pojawia się ta 2 fala, na pewno będą jakieś zawirowania i nic sobie nie poplanujemy tak normalnie. Stąd ta mgła.

2 - na dzisiaj. To jest związane z wirusem, że się przypałętał, przykleił. Taki pech, niefart. Chyba nie bez przyczyny porównałem tego wirusa do gumy, bo teraz traktuję go jako coś takiego bardzo błahego jak ta guma, że wkurzy, trochę coś może zamieszać, ale chyba najbardziej ten wirus rośnie w naszych wyobrażeniach, że to tak rzeczywiście może zagrozić, że nie wiadomo jak się na niego zareaguje, że jest ten strach o dziadków i rzeczywiście teraz przybrał formę takiej wkurzającej gumy, która się przypałętała. Trzeba posiedzieć w domu, odizolować się, ale za chwilę będziemy szli dalej. Nie zatrzyma nas ta guma.

**Jest jeszcze coś, co by dobrze oddawało twoje emocje z tego całego czasu?**

Moje emocje to najbardziej chyba taka obawa. Ja miałem największy dylemat, bo miałem jakieś kontakty z ludźmi. Co zrobić - czy tych ludzi poinformować, czy nie? Czy z przytupem mówię, że chyba mam Covid i wtedy testy, kwarantanna oficjalna, itd., czy nie. Były taki 1-2 dni takich wątpliwości, ale przekalkulowałem sobie to ryzyko. na ten moment sam nie wiem skąd to miałem i skąd to doszło, może się domyślam, ale stwierdziłem, że to nie jest tak, że jak ja się z kimś zobaczę, to on zaraz będzie chory. Jest wiele punktów, w których może dojść do zarażenia i niekoniecznie to muszę być ja, bo może to być nawet stacja benzynowa. Poza tym to nic nie zmieni i jeśli ci ludzie będą mieli objawy, to sami tak samo jak ja będą siebie obserwować i sami zdecydują co robić z tym dalej. Stwierdziłem, że nie będę niepotrzebnie wzbudzał paniki a poza tym ciężko się wychodzi przed szereg. Trzeba albo dużej odwagi...Ciężko jest stanąć i powiedzieć, że mam Covid i mogłem zarazić ciebie, ciebie i ciebie. Niech jedna osoba z nich będzie zarażona i ja się zastanawiam, czy ona później nie będzie miała do mnie jakichś problemów w przyszłości i zostanę zapamiętany jako ten, który zakaził Covidem. Stwierdziłem, że nie, bo musiałbym powiedzieć wszystkim albo nikomu i w sumie na razie jak obserwuję tych, z którymi miałem kontakt, to wszystko jest ok, 2 tyg. już minęły i chyba będzie ok. Nie zawirowały dzięki temu ich życia, bo mając taką wiedzę to oni też już by myśleli, czy się odizolować, czy czekać.

**Na ile czujesz się obecnie zagrożony sytuacją?**

Samym Covidem/ wirusem nie czuje się zagrożony. Jeszcze te emocje nie opadły po tym pierwszym zakażeniu i teraz zaczynają mi się pojawiać myśli jak to dalej działa. Przydałoby się znaleźć informacje czy to zależy od tego, w jakim momencie miałem kontakt z wirusem, czy teraz będzie już tylko lepiej, czy może się zdarzyć, że po jakiejś imprezie czy rajdzie po górach będę zmęczony i wtedy mnie będzie to mogło bardziej dotknąć, czy nie. To jest do przemyślenia i do sprawdzenia w przyszłości, natomiast w tym momencie czuje trochę ulgę, zadowolenie, że nie miałem z tym żadnego większego dyskomfortu, jeśli chodzi o zdrowie.

**Wśród rodziny, znajomych zauważasz jakieś źródła, które wywołują lęk?**

Nie, wręcz przeciwnie. Ludzie raczej się śmieją, że to jest tak nadmuchane. Nadmuchane, a to jest po prostu katar czy ból gardła i po wszystkim. Nadmuchane przez media. ja może w 100% nie podzielam tej opinii, bo zdaję sobie sprawę, że być może są ludzie tacy, którzy mogą to ciężko przechorować. Rzeczywiście 80% przechodzi to bardzo lekko czy bezobjawowo. Te statystyki, które pojawiają się w tym momencie o zakażeniach i ile osób choruje, to patrząc wokół mnie ja bym pomnożył jeszcze razy 4 i to i tak chyba jeszcze byłoby mało, żeby pokazać przez ile osób to rzeczywiście przeleciało. Jest masa ludzi takich, którzy widzą u siebie te objawy i to trwa tylko ze 2 dni lub całkiem bezobjawowo.

**O jakich zmianach w ograniczeniach słyszałeś i co o nich myślisz?**

Baseny nam zamknęli i to odczułem z dzieciakami. Normalnie to właśnie teraz zbieralibyśmy się na basen. Ograniczenia związane z 1.11., ale to za bardzo nas nie dotknęło, bo i tak siedzimy w domu jeszcze. Widzę, że ludzie bardzo na to reagują i komentują. No i to zamknięcie granic, bo to też jakoś oddziałuje na moją pracę. Próbujemy tarza skoordynować różne rzeczy i to jest przesuwane, przestawiane, lecą nam harmonogramy i stale trzeba kombinować i ustalać nowe terminy, które też są niepewne.

**Czy te obostrzenia są potrzebne?**

W tym momencie to co robi rząd jest bezsensowne, bo działają półśrodkami. Moja opinia jest taka, że każdy będzie musiał przejść tego Covida, nie będzie wyjątków. Jeśli rząd by chciał rzeczywiście to zahamować, zdusić to musiałby wprowadzić całkowity lockdown. Bezkompromisowy. Być może wyglądałoby to jak w kwietniu, że zakaz wychodzenia. Dla mnie jest bez sensu, jeśli klasy od 4 w górę mają zdalnie a 0-4 mają chodzić do szkoły. I co chwila jakieś grupy wyłączają, bo kwarantanna. Albo zamykamy szkoły, albo chodzimy dalej i przyjmujemy inną formę walki z wirusem. Inaczej to nadal się roznosi.

Ograniczenia w transporcie zbiorowym?

Dużo myślałem o tym, jakie muszą być warunki spełnione, żeby kogoś zarazić i myślę, że transport zbiorowy mógłby działać tylko na określonych zasadach tak jak to było wcześniej. Gdyby była te dezynfekcja robiona i te odległości to jak najbardziej. Maseczki. W tym momencie na to wszystko jest już moim zdaniem za późno. Przynajmniej tu u nas w regionie. być może gdzie indziej to jeszcze jest dobry moment. Moje okolice...Tu się dzieje po prostu i kto ma przechorować to przechoruje i później to wróci do normalności.

**Maseczki?**

Ta maseczka jednak przy takiej liczbie osób, które mogą być chore, rzeczywiście jest sensowna na ulicy. Tak, w jakimś sposób może pomóc, bo niesie się ten wirus w czasie oddychania czy rozmowy.

**Są jakieś obostrzenia, których nie przestrzegasz albo przestrzegasz tylko dlatego, że musisz?**

Chyba najbardziej to, że w moim mniemaniu powinienem być na kwarantannie a nie jestem. Powinienem być, ale nie chcę, bo ta kwarantanna nie działa tak jak powinna działać. Nieraz jest tak, że rzeczywiście nie da się zamknąć i korzystać z aplikacji i po prostu trzeba opuścić miejsce zamieszkania, żeby zorganizować jakieś jedzenie. Kwarantanna powinna działać tak jak działała w kwietniu. Ja podchodziłem do sprawy tak, że jeśli ktoś jest chory, to rzeczywiście sanepid zareaguje odpowiednio szybko, jest informacja z kim się człowiek spotykał, można te osoby wziąć na kwarantannę, żeby się obserwowały. Jeśli to działa szybko i sprawnie to ma to sens, ale jeśli to już nie działa, bo jest za dużo przypadków i Sanepid tego nie ogarnia, to ja sobie teraz testu nie zrobię, bo ja zostanę w domu np. na 10 dni kwarantanny w momencie, kiedy już będę zdrowy. To jest dla mnie bez sensu, żeby się gdzieś sprawdzać. Zastanawiam się tylko czy przez firmę nie zrobić sobie jakichś badań, że tego już nie mam, żeby móc wrócić do biura. Mam odgórnie narzuconą pracę zdalną, także mnie to bardzo nie boli, że siedzę w domu.

**Czy twoje myślenie o koronawirusie się zmieniło?**

Nie pamiętam co wtedy, mówiłem, ale chyba nie wyszukiwałem żadnych teorii spiskowych. Po prostu pojawił się wirus i się rozprzestrzenił w bardzo szybkim tempie. Minął rok rok zanim z Wuhan zawędrował do mojego domu.

**Co myślisz o zasadności wprowadzania restrykcji w Polsce i na świecie?**

Myśląc o tych osobach, którym ciężko to zakażenie przeżyć...Z jednej strony mamy gospodarkę i taki lockdown bardzo ją hamuję, i ludzi, którzy prowadzą własne firmy, i żyją z miesiąca na miesiąc, i utrzymują pracowników. To jest dla nich ogromny cios. Jakby Polska była krajem, który stać na wprowadzenie lockdownu, to powinni to zrobić i wprowadzić to kompleksowo. Ogłosić, że przez 2-3 tyg. zostajemy w domach, kto zachoruje to zachoruje, sprawdzimy, itd. To chyba nie na naszą kieszeń i w tym momencie musimy sobie jakoś radzić. Takie połowiczne lockdowny nie są już moim zdaniem skuteczne i na ten moment ja już bym żadnych nie robił. To już się dzieje i te liczby, o których słyszymy w TV są o jakiś miesiąc do tyłu w stosunku do rzeczywistości. teraz to jest kolejna i pewnie nieskuteczna próba, żeby to zahamować.

**Czy obecna sytuacja jest poważna?**

Ciężko mi powiedzieć. Ja mam już teraz taki pogląd, że to nie jest choroba, która jest bardzo ciężka i trzeba tylko niektórym osobom zapewnić jakieś wsparcie medyczne. Wszyscy się nastawiają na Covid a jest bardzo dużo osób, które chorują na inne choroby i tutaj tego wsparciu mogą nie dostać, bo brakuje personelu albo szpitale stają się jednoimienne. Tych miejsc brakuje, konsultacje zdalne nie zawsze są skuteczne, itd. Sytuacja jest poważna, bo Covid tyle namieszał w służbie zdrowia, że teraz ciężko ludziom uzyskać jakieś wsparcie w chorobie. Ja miałem ostatnio sytuację, że syn nabił sobie śliwę i miałem stres, bo jakieś 2 lata moje dziecko też uderzyło się w głowę, po 30 min zaczęło wymiotować, musieliśmy jechać na SOR, został na 2 dni, tomografię miał robioną, itd. W tym momencie ja sobie nie wyobrażam, żeby brać roczne dziecko, jechać na SOR, stać w tych kolejkach po 6 godz. i jeszcze nie daj Boże powiedzieć, że mamy jakieś objawy to wtedy już w ogóle nie wiadomo co by z nami zrobili. taka niepewność straszna, ale na całe szczęście nic się nie stało. To już jest problem i na pewno już nie jest tak jak było kiedyś. W jakimś serialu śpiewali, że omijaj szpitale i teraz tak się dzieje, że ostatnia ostateczność to udać się na SOR.

**Czy zachowania ludzi są adekwatne do sytuacji?**

Zachowania są różne i dużo czynników na to wpływa. Ja widzę po sobie, bo znając ten Covid, wiedząc jak on wygląda i czym się go je, i jak on działa, to podejmuje się pewne decyzje, które nie są zawsze uwarunkowane samym zachorowaniem tylko dochodzą jeszcze warunki zewnętrzne typu konsekwencji zgłoszenia tego gdzieś, czy zrobić badania i być na kwarantannie. Być może ta osoba jest blisko jakiejś grupy ludzi, którzy nie mogą sobie pozwolić na kwarantannę ze wglądu na interesy, jakiś mały sklepik czy wymiana opon. Z takimi przypadkami się spotkałem. Ci, którzy mogą iść na kwarantannę to mają niejako komfort być może. Jest bardzo dużo czynników, które naciskają na ludzi w różny sposób.

**Dało się jakoś zapobiec sytuacji, którą mamy?**

Nie, absolutnie nie. To po prostu będzie i każdy będzie musiał to przechorować. Chyba, że rzeczywiście człowiek się izoluje, ale u mnie w rodzinie to jest niemożliwe, bo w 5- osobowej rodzin nie jest możliwe, żeby się odciąć od świata i się izolować tak, żeby nie było możliwości zakażenia. Nie ma szans. Szkoły napędziły tę drugą falę, ona mam nadzieję przejdzie, ludzie się uodpornią i trochę to wygaśnie. Pewnie w okolicy Nowego Roku fala zacznie trochę spadać. Statystyki zawsze będą wysokie, bo to pewnie będzie region po regionie wędrować ten wirus i pewnie gdzieś za miesiąc to się ustabilizuje i zacznie spadać.

**Jakie emocje mogą wywoływać decyzje rządu, które nazwałeś półśrodkami?**

Punkt widzenia zależy od miejsca siedzenia. Wiadomo, że jak coś będzie uderzało w jakąś branżę, to ta branża będzie się buntować. Każdy patrzy pod swoim kątem. Każdy będzie próbował sobie zrekompensować straty i będą to krytykować. I dlatego też nie każdy tę kwarantannę będzie przechodził nawet jak będzie zarażony. Rządu nie stać, żeby wszystkich zadowolić finansowo z powodu strat. Na pewno uderzy to w gospodarkę i w ludzi.

**Skąd czerpiesz informacje?**

Chyba internet, smartfon.

**To są te same źródła co wiosną?**

Raczej te same, ale nie mam ostatnio czasu w ogóle śledzić, co się dzieje na świecie. Mam jeden portal, który czasem sobie przejrzę, ale na pewno codziennie nie przeglądam wiadomości. Na pewno omijamy TVP Info i TVN24 jak przerzucam kanały. Mniej więcej wiem co się dzieje, jeśli chodzi o te ustawy przepychane nocą, o te strajki, protest rolników. Jestem na bieżąco, ale raczej ze źródeł, które śledzę sobie na fb. Chyba głównie fb, chociaż to też nie jest chyba dobry pomysł, bo ostatnio obejrzałem dokument Dramat Społeczny i te informacje mogą dotyczyć bezpośrednio mnie. Polecam tak czy siak.

W wakacje z poszukiwaniem informacji było podobnie jak teraz. Też za bardzo nie szukałem wiadomości o tym co się dzieje na świecie. Głównie ze względu na to, że nie miałem czasu, nie żyłem już tym. Na początku, jak ten Covid się pojawił, to rzeczywiście te serwisy informacyjne były non stop gdzieś w tle, aż miarka się przebrała i nie włączyłem tego do dzisiaj. Obserwuję, ale nie zatrzymuję się, nie sprawdzam, jak to wygląda tak na prawdę w Polsce czy na świecie. Wiadomo, że jak się pojawi jakaś informacja, że coś na świecie się zadziało, to ja bardzo szybko sobie ją wyszukam gdzieś i przeczytam o co chodzi.

**Ten czas poświęcony temu jest mniej więcej taki jak przed pandemią?**

 To jest zdecydowanie mniej. Kiedyś lubiłem sobie usiąść, włączyć TVN24 czy TVP Info i po prostu posłuchać co się dzieje. W tym momencie omijam to szerokim łukiem. Same informacje covidowe już mnie za bardzo nie interesują, natomiast to, co się dzieje w Polsce i wszystko związane z protestami, z rządem, który wprowadza takie rzeczy jak niszczenie kompromisów...Aż się tego słuchać nie chce i omijam wszystkie serwisy informacyjne. Ja mam swoje zdanie na ten temat i nie daję się wciągać serwisom w tę ich propagandę. Nawet, jeśli któryś serwis się opowiada za moimi poglądami, to sam przekaz, który kieruje do ludzi to jest taka mowa nienawiści, że już sama forma tego zniechęca i nie oglądam.

**Skąd wiadomo, że dana informacja jest prawdziwa lub nie? Jak to weryfikujesz?**

Nie wiadomo tak naprawdę. Weryfikuję rozmawiając z ludźmi. Najbardziej wiarygodne dla mnie są informacje od ludzi a nie z mediów. Konsultuję się, rozmawiam, podejmuję jakąś dyskusję, ale z ludźmi, którzy nie są zafiksowani tym całym medialnym przekazem. Niektórym jak się powie Kaczyński, to z nimi już nie ma dialogu, bo to przechodzi w jakiś wyższy ton i nie ma to sensu. I z żoną dużo rozmawiam, ona też ma poglądy podobne. Nie ma świata czarno-białego. Czasem z żoną mamy różne zdania, ale jakoś umiemy o tym dyskutować i jakoś to wygląda normalnie. Nie kłócimy się przy stole.

**Co myślisz o przyszłości Polski?**

Niestety jest to bardzo skuteczne narzędzie rządzących. Pandemia się stała narzędziem i dopóki będzie trwała albo będzie podsycana medialnie, że trwa to niestety nie widzę świetlanej przyszłości. To pozwala rządzącym czuć się bezkarnie, bo zawsze mogą sobie wprowadzić jakiś stan wyjątkowy, zawsze mogą dowalić restrykcje. To jest uniwersalne narzędzie. Ludzie, którzy wyszli na ulicę protestować mogą zostać bardzo szybko zamknięci w domu ze względu na pandemię. Może być ograniczona ta gałąź gospodarki, która w tym momencie najmniej im opasuje. To jest najgorsze, że walka zamiast walki o ludzkie zdrowie to media i rząd coraz bardziej wykorzystują pandemię jako narzędzie polityczne. Nie mam zaufania, a na początku miałem i byłem zadowolony, że nasz rząd tak szybko zareagował na Covid. Podobała mi się ta reakcja rządu i że wyszliśmy przed szereg w Europie. Wtedy wydawało mi się to fajne i myślę, że na tamten moment było fajne. Teraz widzę, że oni już się sami gubią, że gdzieś za tym się kryją afery i moje zaufanie do rządu i jego decyzji jest teraz bardzo nieduże.

**A przyszłość świata?**

Myślę, że ktoś po prostu na tym zarobi, może siły się zmienią. Jak jest kryzys to trzeba go wykorzystać i ktoś na tym zarobi potężne pieniądze, co spowoduje, że być może siły polityczne się zmienią na świecie. Na pewno się coś zmieni. Mam nadzieję, że taki pozytyw z tego będzie, że już będziemy przygotowani na coś jeszcze gorszego, jeśli chodzi o takie wirusy i tego typu zagrożenia. Być może to jest taki na razie sygnał ostrzegawczy dla wszystkich, żeby ludzie bardziej umieli zabezpieczyć się przed tego typu katastrofami zamiast np. łożyć na zbrojenia. Troszkę nas wszystkich ta pandemia zaskoczyła.

**Są jakieś grupy, które szczególnie ucierpią/ zyskają?**

Szczególnie zyskają kraje, które będą sprzedawać szczepionkę. Lobby farmaceutyczne jest już chyba największe na świecie. Jest tak finansowane i ma taki obrót, że może wpływać na wiele rzeczy. Tutaj ktoś na pewno zarobi. Nie wiem kto, czy Rosja, czy Chiny, czy Stany. Na pewno nie Polska. Najwięcej straci gospodarka i te osoby, które prowadzą małe i średnie biznesy. Tam, gdzie jest kraj bardziej zamożny to może bardziej zrekompensuje te straty, ale przewróci to trochę rynek do góry nogami. Korporacje są bardziej bezpieczne tak jak Auchan w stosunku do małego sklepiku.

**Spotykałeś się z rodziną przy okazji 1.11.?**

Nie. Kościół też sobie zrobiliśmy na transmisji w TV. Będziemy chcieli przeżyć to święto w kolejną niedzielę i wtedy sobie pojeździmy po rodzinie, po cmentarzach. Przyczyną było to, że byliśmy chorzy. W takich małych miejscowościach cmentarze nie są tak pilnowane i jak byśmy chcieli to na pewno mogliśmy pójść.

**Poszlibyście, gdybyście byli zdrowi?**

Tak.

**Myślałeś już o Bożym Narodzeniu?**

Tak i jestem dobrej myśli. Spędzimy z rodziną. Podejrzewam, że do tego czasu to już każdy to przechoruje i już będzie wtedy po i będziemy mogli się normalnie spotkać. Może nie tak licznie jak zazwyczaj, że 20-25 osób, ale na pewno nie będzie jak w Wielkanoc.

**Obawiasz się, że rząd wprowadzi wtedy jakieś restrykcje?**

Nie wiem, dlaczego, ale zawsze jak są jakieś ważne święta dla Polaków i dla wierzących, to zawsze wtedy są wprowadzane jakieś największe obostrzenia. Może to jest jakaś moja teoria spiskowa. Ludzie jakoś nie podchwytują tej mojej teorii, ale nie wiem, czy to jest takie jakieś uderzenie w Kościół zaplanowane czy nie, natomiast obawiam się, że jakieś ograniczenia na pewno będą. Jestem nastawiony tak, że będą. Teraz też w ostatniej chwili zmienili z tymi cmentarzami i to jest takie nie fair i trochę dziwne. W piątek ogłosili, że zamykają cmentarze na 3 dni. Efekt był odwrotny do tego jaki pewnie miał w zamyśle rząd, bo każdy się w piątek rzucił pędem na cmentarz. Były takie tłumy jak przejeżdżałem obok...Nie fair wobec handlujących i producentów. Nie fair, bo na ostatnią chwilę a nie np. na 2 tyg. przed.
