# Supplementary material for: The challenges arising from the COVID-19 pandemic and the way people deal with them. A qualitative longitudinal study
Source: PLoS One. 2021 Oct 11;16(10):e0258133. doi: 10.1371/journal.pone.0258133 (PMC8504766; doi:10.1371/journal.pone.0258133)
Supplement: S1 Dataset — (ZIP) [file pone.0258133.s003.zip › Transcriptions/stage 6/13.6_M_46_couple, with children.docx]

**13.6_M_46_couple with children**

**Co się działo przez ostatnie miesiące?**

Od czerwca do momentu aż zaczęła się 2 fala nie działo się nic szczególnego. Pracowaliśmy w przyzwoitym wymiarze godzin, wróciły nam zabiegi planowe, wszystkie poradnie, gabinety działały. W momencie jak się zaczęła kolejna fala to się radykalnie zmieniło. Zostaliśmy szpitalem może nie jednoimiennym, ale przeznaczonym do leczenia koronawirusa i właściwie wszystkie oddziały, poza tym, na którym ja pracuję (kardiologia, onkologia), to reszta oddziałów przyjmuje pacjentów z koronawirusem. Można też u nas leczyć pacjentów bez koronawirusa.

**Praca wygląda tak jak wtedy na wiosnę?**

Nie. Moja praca teraz w ogóle nie wygląda, ponieważ ja sam mam koronawirusa i siedzę w domu. Mam do wtorku wolne z racji izolacji. Nam się pojawiły dodatkowe pomieszczenia z sąsiedniego oddziału. Zostały wyznaczone dodatkowe łóżka, takie boxy po 2 łóżka dla pacjentów z koronawirusem i z podejrzeniem, którzy czekają na wynik. Trochę dodatkowych pseudozabezpieczeń, izolacji, przejść foliowych i różnych takich rzeczy jest. Do tych pacjentów chodzimy w specjalnych ubraniach, wcześniej tego nie było, bo nie było takich pacjentów właściwie. Zdarzał się raz na 3-4 tygodnie taki chory. Teraz właściwie codziennie jakiś przyjeżdża z regionu.

**Jak się pan dowiedział, że jest pan chory?**

To było niezbyt oczywiste, bo miałem zapalenie zatok i objawy dla mnie niespecyficzne dla koronawirusa. Dopiero jak syn zaczął mocno gorączkować i jeden dzień miał taki do 40 st., żona straciła węch, to stwierdziłem, że zrobię test. Utrata węchu u mojej żony też nie jest niczym szczególnym, bo jej się to co jakiś czas zdarza przy okazji różnych infekcji. Dla mnie nic takiego się nie wydarzyło, co by mogło zwrócić uwagę, że mam koronawirusa tylko po prostu z przyzwoitości stwierdziłem, że skoro jest kilka takich objawów u moich bliskich, które mogą pasować do całości, to zrobiłem ten test.

**Formalnie jak to później wyglądało?**

Ponieważ z racji zawodu mam trochę świadomości to zadzwoniłem do lekarza 1-go kontaktu, on mnie wpisał na listę osób w izolacji, wystawił zwolnienie. W poniedziałek zadzwoniła pani z Sanepidu a wczoraj dostałem sms, żebym zainstalował aplikację Kwarantanna, co jest nieadekwatne do stanu, w jakim ja się obecnie znajduję, bo jestem w izolacji a nie na kwarantannie. Nie zamierzam jej instalować. Moja rodzina robiła testy wczoraj rano, więc dzisiaj pewnie mogą mieć wyniki.

**Jak długo pozostają państwo w izolacji?**

Zwykle to jest 10 dni, ale jeśli są objawy to lekarz 1-go kontaktu może wydłużyć czas tej izolacji. Jeśli pacjent jest bezobjawowy to 10 dni. Syn miał jeden dzień gorączkę, która ustępowała po lekach i na drugi dzień już się dobrze czuł. Żona tylko straciła węch, ale jej się to zdarza dość często i była osłabiona, ja przechodziłem zapalenie zatok, więc jakiś uciążliwy katar. Nawet nie kasłałem i gorączki też nie miałem.

**Byli państwo przestraszeni?**
Ja nie byłem, żona była zdenerwowana. Ją denerwowało, że będzie na kwarantannie, że są kolejki do badań, bo bardzo dużo u nas na podkarpaciu jest zachorowań. Ona wybrała się w poniedziałek po południu zrobić test i jej się nie udało, bo była taka duża kolejka, więc zrezygnowała. Wczoraj rano robiła badanie. Była poirytowana sytuacją, że ewentualna kwarantanna skaże ją na 20 dni izolacji od normalnego życia i mojego syna również. Mnie to nie denerwuje. Ja sobie zdaję sprawę z tego jak to wygląda.

**Pan sam zadzwonił do lekarza 1-go kontaktu...**

Ja sobie nawet sam test zleciłem, bo mam takie prawo jako pracujący w szpitalu. Wpisanie na listę do izolacji też było z mojej inicjatywy, bo jest to sprawa oczywista. Jeśli nie zrobi tego lekarz 1-go kontaktu, to zrobi to Sanepid, chyba, że mają taki natłok pracy, że nie zadzwonią, bo takie sytuacje też się zdarzają.

**Dlaczego zdecydował się pan nie instalować aplikacji?**

Jeżeli ona jest dla osób na kwarantannie a ja jestem na izolacji, to zainstalowanie tej aplikacji podejrzewam, że zrobiłoby mi problem, bo by się okazało, że ja bym wrócił po 10 dniach do pracy, a aplikacja dalej by sprawdzała, czy jestem w domu na kwarantannie. Pewnie musiałbym się tłumaczyć z tego i przez miesiąc nie mógłbym się wygrzebać, znając opóźnienia w przepływie informacji. Albo to będzie precyzyjnie skierowane albo dla mnie nie istnieje.

**Czy od czerwca do teraz były jakieś ważne momenty z pana perspektywy?**

Wydarzenia sprzed kilku dni związane z decyzją Trybunału, bo dla mnie jest to coś skandalicznego w odniesieniu do pandemii. To nie jest teraz czas na podejmowanie takich decyzji, a nasza władza się tym teraz zajęła. Dla mnie to jest prowokacja, ewentualnie po raz kolejny próbowali wykorzystać pandemię, żeby przeforsować pewne niewygodne dla ludzi decyzje. Wydawało im się może, że się uda, a okazało się, że się nie udało, bo ludzie wyszli z domów. Jest to narażenie społeczeństwa na zwiększoną ilość zachorowań idiotyczną decyzją. Należałoby zająć się rzeczami istotnymi a to nie było teraz najistotniejsze. To jest dla mnie najważniejsza rzecz, bo przeformatowania szpitala się spodziewałem i było dla nas oczywiste, że jeśli będzie 2 fala, to ona będzie większa i my będziemy obciążeni większą odpowiedzialnością. To co było u nas na wiosnę, to z różnych względów było dużo słabiej wyrażone. Raz, że mniejsza liczba zachorowań, dwa, że dużo mniejsza liczba testów. teraz w ciągu dnia się robi w województwie podkarpackim tyle testów dziennie, co kiedyś się robiło w ciągu kilku dni w całej Polsce. Myśmy nawet nie wiedzieli, że wokół nas są ludzie, którzy mają koronawirusa, a teraz wiemy. Teraz przed każdym planowym przyjęciem robimy ludziom test. Wszystkie operacje, które możemy ustalić na za np. 2 tygodnie traktujemy teraz jako planowe, mimo tego, że pacjent wymaga pilnego leczenia i powinny być jako pilne. Ci pacjenci mają robione testy i jeśli wymagają bezwzględnego leczenia to ich przyjmujemy, jeśli mogą zaczekać 2 tygodnie to czekają. Ale widać przynajmniej, ile testów jest robionych, bo jak ustalamy kalendarz i każdy ma ileś osób do obdzwonienia, to zdarza mi się w ciągu dnia zlecić 10 testów. A jest nas 8 na oddziale.

**Ale 2 fali środowisko się spodziewało?**

Tak. Nie wiedzieliśmy, że aż tak dużo zachorowań, ale wiedzieliśmy, że będzie. Nie było jakoś szczególnie rozmów o tym wśród kolegów, bo uważamy, że to bez sensu jest siać panikę. Tak miało być, więc jest. Ludzie będą się musieli zachowywać tak, jak mówią przepisy i tyle, a żadne szczególne przygotowania do tego okresu też nie miałyby sensu. Nie ma stanu klęski żywiołowej, nie ma klęski głodu, w sklepach jest zaopatrzenie, można sobie z tym poradzić.

**Wyjechali państwo na jakiś urlop przed 2 falą?**

Syn był na obozie tenisowym, a my pojechaliśmy na 4 dni na rowery w pobliże miejsca tego obozu. Chcieliśmy spędzić trochę czasu na świeżym powietrzu, ale była brzydka pogoda, więc nie wypaliło to. Korzystaliśmy z uroków podkarpacia i trochę pojeździliśmy na rowerze, byli u nas znajomi z Warszawy, część przyjechała z rowerami, więc pokazaliśmy im region i podobało im się. Wizyta znajomych trwała prawie tydzień, było 8-9 osób, więc było wesoło. Wakacje były u nas.

**Czy zanim okazało się, że ma pan koronawirusa, to życie już wróciło do normy? Było jak przed pandemia?**

Nie. Zostały mi 4 bilety na koncerty, które zostały odwołane, przeniesione. Z jednej trasy wykonawca w ogóle zrezygnował i sprezentował fanom wydawnictwo w postaci nagrań koncertowych. Zamiast biletu można było mieć coś takiego i to mi się bardzo spodobało. Lubię bardzo tę grupę i jestem regularnie na ich trasach, a tym razem mi się nie udało. Oni połowę trasy zrobili, a połowa im się wyłożyła przez pandemię. Nie zdecydowaliśmy się też wyjechać nigdzie za granicę. To nie wróciło do normy, bo nie było takiej dostępności jak byśmy chcieli do wyjazdów. Oczywiście nie poddajemy się i ja zarezerwowałem noclegi na marzec na wyjazd narciarski za granicę, więc mam nadzieję, że będzie ok.

**Spotkania ze znajomymi już wyglądały normalnie?**

Tak. Chodziliśmy do kina, do restauracji, więc częściowo to życie wróciło do normy. Wiadomo, że jak się idzie do restauracji, gdzie zawsze był tłum a teraz jest połowa miejsc wolnych, to nie jest to normalność, ale taka próba powrotu do jak najlepszego funkcjonowania.

**Na początku 2 fali jakoś państwo zmienili zachowania?**

Zgodnie z zaleceniami. Ja mam takie podejście, że jak najbardziej szanuję ustalenia, nie staram się tego łamać ani wymyślać swoich, bo to nie ma sensu. Nie jestem szczególnie strachliwy, jeśli chodzi o sprawy zdrowia. Te obostrzenia, które powinniśmy zachować to ja sam jak najbardziej szanuję i też mojej rodzinie zwracam uwagę na to. Maseczki jak najbardziej, unikanie wielkich zgromadzeń ludzkich też, aczkolwiek nie powstrzymuje mnie sytuacja od chodzenia do sklepu i w miarę normalnego funkcjonowania. Ludzie sami decydują czy mają przychodzić do mnie do gabinetu i ja go nie zamknąłem. Uważam, że zbyt wiele ludzi może na tym za wiele stracić bojąc się infekcji. Infekcja koronawirusem jest teraz taka popularna, że czy ci ludzie przyjdą do mnie czy nie, to pewnie nic to nie zmieni. Zarażą się od znajomych. Wczoraj mi żona przeczytała jakąś informację z internetu, że na podkarpaciu wykrywalność koronawirusa sięga między 60 a 90% u badanych. To jest straszliwie dużo. Fakt, że my kierujemy ludzi do badanie głównie takich, których podejrzewamy i tych przed leczeniem szpitalnym, ale jednak u tych, których kierujemy do szpitala mogłoby być mniej tych rozpoznań, a jest ich dość sporo. Jak patrzę na kalendarz, to tak ze 40% pacjentów nam odwołuje zabiegi, bo mają koronawirusa. Albo nie chcą, bo się boją, albo mają koronawirusa. Jest tego zdecydowanie więcej niż na wiosnę. Przy potwierdzaniu przyjęcia na tydzień przed zabiegiem, duża część z nich rezygnuje. Infekcja koronawirusem jest teraz powszechna.

**Co panu teraz najbardziej przeszkadza w obecnej sytuacji?**

Ogólna sytuacja to kolejny epizod różnych dziwnych posunięć władz, która zajmuje się nie tym czym powinna. My w szpitalu nie mieliśmy np. rękawic lateksowych tylko winylowe. Winylowe rękawiczki nie wiem do czego służą. Chyba do przenoszenia miotły czy czegoś. Takich rękawic w ogóle nie powinno być w szpitalu a nie ma innych. Są oczywiście jednorazowe, sterylne chirurgiczne, których używamy do operacji i drobnych zabiegów. Brakuje dobrych ubrań. Były takie, które były zatwierdzone przez WHO przy wirusie ebola a nie do pracy przy koronawirusie. Całe mnóstwo takich drobiazgów wypływa, a ktoś za to wziął grube pieniądze, sprzęt z Chin sprowadzał. Nie ma respiratorów w szpitalu. Co z tego, że mamy łóżka i chętny personel do leczenia, jeśli nie mamy sprzętu? Nawet prostych reduktorów tlenu nie ma. To kosztuje grosze i tego powinno być, ile dusza zapragnie, a takich rzeczy nie ma. Żeby chociaż co 2-gie łóżko miało. Takie rzeczy mi przeszkadzają. Oczywiście kłamstwa władz, propaganda sukcesu i cały ten goebbelsowski przekaz, który się leje z wiadomości. Oni nie widzą nic innego tylko sukces. Jest cały czas wszystko super. A to co się ostatnio zdarzyło i zachęcanie ludzi chyba do wojny domowej, bo jak to inaczej nazwać, to żałosne jest. I w imię jeszcze powoływania się na religię. Religia dla mnie jest tak osobistą sprawa, że nie powinien się w to mieszać rząd. Jest moją sprawa jak ja postrzegam religię. Ja szanuję dobra kultury, dziedzictwa narodowe z wartościami też kulturowymi, nienamacalnymi, takimi jak Program Trzeci, który szlag trafił, jak kościoły, które oprócz miejsca kultu są też jakimś dorobkiem naszej kultury i to są zabytki naszej kultury. Ludzie się wcale na to nie porywają, bo nikt nie chce niszczyć takich wartości, natomiast znowu zaczyna się szczucie ludzi przeciwko ludziom i obracanie prawdy w sposób, który miałby przynieść korzyść rządzącym. Nie ma zezwolenia na to. Dla mnie jako lekarza temat aborcji w trudnych sytuacjach jest tak bardzo indywidualną sprawą, że nakładanie na ludzi obowiązków w tym przypadku to jest coś niezwykle trudnego i to trzeba naprawdę mądrze zrobić, przemyśleć, skonsultować to z mądrymi ludźmi i posłuchać różnych stron. I przede wszystkim dać możliwość wyboru ludziom, bo mogłoby się okazać, że możliwość skorzystania z aborcji wykorzysta tylko garstka ludzi. Ja poza wszystkim jestem przeciwnikiem aborcji, ale uważam, że są sytuacje, kiedy zmuszanie kobiety do urodzenia dziecka jest nieludzkie i absolutnie jestem przeciwnikiem aborcji jako rozwiązania na trudną sytuację domową, itd. To nie usprawiedliwia. Natomiast zmuszanie kobiety do urodzenia zdeformowanego płodu, to naprawdę trzeba być strasznie okrutnym i to mi się nie podoba. To jest jedna z głównych rzeczy, która uważam, że się zmieniła w trakcie pandemii. Jak oni w ogóle mogli się zabierać za takie rzeczy teraz? Ktoś pomyślał sobie, że pewnie ludzie nie wyjdą, bo jest zakaz, bo złą sytuacją, bo się będą bali, to sobie teraz zrobimy to, co może by nie wyszło, bo by były protesty. Na szczęście okazało się, że to nie w ten sposób.

**Emocje**

6 - to ten pierwszy okres do połowy września. Całkiem przyjemnie, gdzieś tam słońce wychodzi zza drzew, nie wiadomo czy to wschód czy zachód słońca, ale jest. nie jest źle, trawa zielona, ładnie. daje wrażenie takiego w miarę spokoju. Może nie jest to idealna, czysta i jasna sytuacja, ale gdzieś tam wygląda jakiś promyczek. Las jest bardzo gęsty.

4 - początek 2 fali. Nic niestety się nie zmieniło, jeśli chodzi o dyrekcje i rządzą idioci, więc myśmy sami pewne rzeczy poustalali i porobili. Dla mnie to jest taki obraz, który najbardziej mi pasuje do naszego zaangażowania. Solidarność, współpraca, nikt się nie wycofuje, nikt nie zwiewa, mamy świetne grono pielęgniarskie, dobrych lekarzy u mnie na oddziale. Ludzie nie schowali się przed problemem, tylko wspólnie staramy się jakoś układać codziennie, żeby było najbardziej bezpiecznie jak można. Zdajemy sobie sprawę z obostrzeń, niektóre osoby już przechorowały, niektóre z ciężkim przebiegiem

16 - ostatni tydzień i to są nasi politycy. To decyzja Trybunału zachowania czołowych polityków obozu rządzącego.

**Ta 16 daje też poczucie zagrożenia?**

Chyba nie. To raczej gniew niż zagrożenie.

**Czy czuje się pan w jakiś sposób zagrożony obecną sytuacją? Czegoś się pan obawia?**

Na razie nie bardzo. Obawiam się trochę o stan bliskich, bo rodziców mam starszych, po 70-ce i teściowa też, więc oni mają większą możliwość na niezbyt korzystny przebieg zakażenia i chyba tyle.

**Jeśli chodzi o ten gniew, to czy ma pan jakieś sposoby, żeby sobie z nim radzić?**

Na razie pozwalam się emocjom wyszumieć. Po prostu wściekłość.

**Jakie emocje obserwuje pan w swoim otoczeniu?**

Na razie tylko emocje u żony, bo ona też jest zawsze zaangażowana bardzo mocno w takich sytuacjach. Jest też mocno zawiedziona, że nie może pójść na manifestację. Jak się okazało w niedzielę po południu, że mam test dodatni, to straciła możliwość wychodzenia z domu i nie poszła na manifestację, i to ją bardzo mocno zabolało. Dla niej to też są ważne rzeczy. Oglądamy wiadomości, słuchamy wiadomości, które mówią nam jak się teraz sytuacja kształtuje. To dla niej też myślę, że najważniejszy moment w tej chwili.

**Żona ma jakiś sposób, żeby sobie z tym radzić?**

Nie pytałem jej o to. Może czasami idzie poćwiczyć na dół. Myślę, że to jest bardzo dobre rozwiązanie dla problemu z nadmiernym napięciem jak człowiek pomacha siekierą albo pojeździ na rowerze i się dobrze zmęczy. W trakcie tych czynności oczywiście się intensywnie myśli i po godzinie takiego myślenia połączonego z wysiłkiem fizycznym robi się luźniej w głowie. Przynajmniej ja tak mam.

**Obostrzenia. O czym pan słyszał ostatnio, że się zmieniło?**

Zostały zamknięte siłownie, restauracje. To zapamiętałem. To jest ważne, że restauracje.

**Co pan sądzi o obecnych obostrzeniach? Czy to są dobre decyzje?**

Nie potrafię odpowiedzieć. jeszcze 2 tyg. temu mógłbym powiedzieć, że to są dobre decyzje, natomiast teraz mam wrażenie, że tempo rozprzestrzeniania się zakażeń jest tak duże, że nie ma to już większego wpływu. Ta garstka ludzi, która chodzi na siłownię nie zmieni dynamiki rozwoju pandemii nawet u nas w mieście czy w regionie. Nie ma to znaczenia. Takie mam wrażenie, ale nie wiem, czy rzeczywiście tak jest, bo nie jestem specjalistą i nie mam odpowiednich danych. To mogło mieć dużo większe znaczenie, kiedy ta dynamika była nieduża i w jakiś sposób wtedy jesteśmy w stanie kontrolować ludzi, którzy są zakażeni i nie, a w tej chwili tak naprawdę nie wiadomo. Sądząc po tym co się bada w testach, to tak dużo jest testów pozytywnych, jest mnóstwo osób bezobjawowych, które chodzą [po ulicy i zarażają wszystkich. Chyba takie ograniczenia niewiele zmienią. Ilość osób, która chodzi do restauracji, siłowni i jakichś obiektów sportowych nie jest aż tak wielka w porównaniu z tym, jak dużo ludzi się spotyka ze sobą np. w sklepie spożywczym.

**Lepiej nie wprowadzać w ogóle ograniczeń w takiej sytuacji?**

Kiedyś się nad tym zastanawiałem. Myślę, że teraz odizolowanie osób starszych mogłoby mieć znaczenie, żeby rzeczywiście ci ludzie, którzy są zdrowi, nie mają objawów zostali z dala od tego głównego nurtu przez jakiś czas. Trzeba by było pewnie zaproponować, żeby przez kilka miesięcy, i to by było na pewno trudne. Może to by jakoś zmieniło przebieg i zmniejszyło ryzyko narażenia się na infekcję dla tych osób. Jakiś matematyk kiedyś wymyślił model, przy pomocy którego można byłoby ograniczyć epidemię. Pozwolić, żeby ludzie, którzy są w miarę zdrowi, młodzi przyjęli na siebie tę fale zakażeń, doprowadzili do tego, że organizm wytworzy odporność przynajmniej na te parę miesięcy i to spowoduje, że dynamika zakażeń spadnie, i wtedy może kontakt osób starszych z tym społeczeństwem już zakażonym, gdzie ilość nowych zakażeń jest dużo, dużo mniejsza niż to było na początku, spowoduje, że nie będzie to na tyle groźne jak np. jest teraz bez żadnych ograniczeń. Nie wiem. Czy tak rzeczywiście jest, to musieliby mądrzy ludzie powiedzieć, a może nawet nie ludzie, a zespoły ludzi, którzy myślą o tym z punktu widzenia epidemiologii, biologii, ale też i jakiejś gałęzi matematyki, która się zajmuje przewidywaniem. To chyba miałoby jakiś sens, a zamknięcie tych kilku siłownie w Przemyślu...Jakie to ma znaczenie?

**A jeśli chodzi o nakaz noszenia maseczek?**

Śmieszny jest ten nakaz, ale może akurat trochę ograniczy. Maseczka nie zabezpiecza przed zakażeniem siebie tylko trochę zmniejsza ilość patogenów, które z siebie wydalamy. Ale nie należy twierdzić, że nosimy maseczki i super, wspaniale, to już nikogo nie zakażę. Zmniejsza ryzyko, zmniejsza możliwość, ale o ile? Nie wiem. 10%?15%?20%? chyba nie więcej. Nikt tego nie sprawdzi.

**Nauczanie zdalne od 4 klasy?**

To są żarty. To jest kolejny z tych żartów ekipy rządzącej. Szkoła jest świetnym miejscem, gdzie się tego wirusa roznosi. Dzieci nie chorują tak jak dorośli, nie mają objawów. Aż tak dużo dzieci nie ma objawów jak dorośli. Świetnie się tam wymienia pomiędzy sobą. Ja bym zostawił dzieci w szkole, jak już i tak zostaje to nauczanie początkowe. Może studenci nauka online jest ok. Dzieci od 4 klasy to niewiele zmieni. Ja nie wiem czy do nas do domu właśnie Mikołaj nie przyniósł wirusa, bo tam pani w świetlicy miała koronawirusa. Wstrzymano zajęcia na 3-4 dni i miał wrócić do szkoły, ale nie wrócił, bo już cała Polska stanęła, a on chodzi do 4 klasy. Najbardziej chyba niebezpiecznym źródłem jestem ja, bo w pracy stale mamy jakiegoś pacjenta z koronawirusem od mniej więcej miesiąca, więc ja też mam szansę złapać. te szkoły to trochę śmieszne. To są właśnie takie pomysły, żeby trochę zrobić, trochę nie zrobić, bo to już niewygodne i trzeba będzie jakieś zasiłki wypłacać rodzicom tych dzieci i to kłopot. Dla mnie uczeń 4 klasy też wymaga nadzoru rodziców i mam to na co dzień. W sumie może dobrze się złożyło, bo ten cały obowiązek spadłby na żonę, a tak to i ja z nim czasem siądę do tego komputera. Muszę przyznać, że się poprawił standard nauki, ale też Mikołaj zmienił szkołę i może dlatego wygląda to dużo lepiej. Jest to bardziej usystematyzowane, nauczyciele mają jedną platformę do tego, żeby się spotykać z uczniami. Rzeczywiście teraz te dzieciaki aktywnie uczestniczą i wygląda to całkiem, całkiem. W porównaniu z tym, co było na wiosnę, dużo, dużo lepiej.  Wynika to raczej z zaangażowania nauczycieli i zależało to bardziej od szkół a nie jakichś systemowych pomysłów.

**Jeśli chodzi o gastronomię, to ma pan podobne zdania jak o siłowniach?**

Tak. Na pewno powoduje to duże problemy dla właścicieli lokali. Oni się próbują ratować jedzeniem na dowóz i tak jak poprzednio staramy się coś zawsze kupić od tych naszych lokalnych producentów czy lokali gastronomicznych. Specjalnie, żeby im pomóc, ale na pewno jest to dla nich bardzo duży problem. Może to być większy problem dla nich niż korzyść z ograniczenia epidemii.

**Zakaz imprez rodzinnych, eventów?**

Wesela też były świetnym sposobem i po każdym weselu były jakieś skoki ilości zakażonych. Przynajmniej u nas. Normalne, bo duże skupisko ludzkie przez kilkanaście godzin, czarna owca jedna i mamy problem.

**Państwo mieli okazję być na jakiejś uroczystości rodzinnej?**

Mieliśmy komunię syna. To było na początku września i my zrobiliśmy w domu przyjęcie dla najbliższych, więc była najbliższa rodzina tylko i sąsiadka, która jest jak rodzina. Bez dużych hucznych imprez. Sama komunia, jako uroczystość w kościele też duża, bo było 20 dzieci, przyszła najbliższa rodzina i to się już zrobiło dużo ludzi. W kościele ludzie byli w maseczkach. Podejrzewam, że gdyby nie pandemia to byłoby jeszcze więcej ludzi. My nie robilibyśmy i tak większego przyjęcia.

49:51

**Ograniczenia w transporcie zbiorowym?**

Nie korzystam, nie potrafię powiedzieć, jak to wygląda u nas. Dla mnie kluczowe jest, czy my jeszcze w ogóle możemy wpłynąć jakoś na dynamikę pandemii czy nie. Jeśli mamy szansę, to takie ograniczenia jak najbardziej tak. Ma to znaczenie, bo jednak inaczej funkcjonują ludzie na siłowni, inaczej w restauracji, kinie czy nawet w sklepie. Komunikacja miejska, gdzie w szczycie wsiadają ludzie, to bywa tak tłoczno, że jest człowiek na człowieku i idealne warunki do szerzenia się epidemii. To stwarza rzeczywiście groźne warunki i trudno tam zapanować nad ludźmi. To może mieć sens pod warunkiem, że jeszcze możemy cokolwiek spowolnić. Mam wrażenie, że to już taka lawina jest u nas, że ludzie tylko czekają. Ja miałem takie przekonanie, że w sumie dobrze, że już się zaraziłem, bo ileż można czekać, skoro ja miałem przeświadczenie, że na pewno będę chory. Może dobrze to mieć za sobą a przynajmniej ten pierwszy epizod. Nie wiem czy będą i ile będzie kolejnych, bo z tego co mówią naukowcy, wcale nie jest pewne, że będziemy mieli jakąś dłuższą odporność. Może być tak, że zanim się pandemia skończy to niektórzy z nas kilka razy zachorują i tyle. Już się mówi o tym, że niektórzy ludzie chorowali po 2 razy. To już są fakty a nie domysły i jakieś podejrzenie.

**Co teraz pan myśli o koronawirusie? Czy coś w myśleniu o nim się zmieniło?**

Nie szukałem informacji, skąd się wziął. Doszły mnie ostatnio informacje od któregoś z kolegów, że w Wuhan powstawały laboratoria zajmujące się też wirusami, które chyba mieli zbudować i zarządzać nimi Francuzi. To może nie być prawda, ale że mieli to być Francuzi, którzy postawili tak wysoko normę bezpieczeństwa, że Chińczycy się na to nie zgodzili i zrezygnowali z francuskiej ekipy. Z laboratoriów zaczęły ginąć sprzęty, zwierzęta wynoszone przez pracowników. Wydaje się to trochę dziwne, bo gdziekolwiek by nie powstawały takie obiekty, to tam nie trafiają ludzie z ulicy i prości, więc można to w kategorii żartu potraktować. Ale takie pomysły są. Wydaje się dziwne, żeby przypadkowo powstał wirus, który będzie miał aż taką zjadliwość i tak szybko i fantastycznie się rozprzestrzeniał. Nie ma żadnych dowodów na to, że go stworzyli ludzie, ale nic też nie przemawia za tym, że nie. Na pewno wykorzystali ludzie fakt powstania takiego zagrożenia i tej pandemii. Wielu ludzi ją wykorzystało do zrobienia biznesu, do zrobienia różnych ruchów politycznych i przeforsowania zmian, które pewnie byłyby niemożliwe wcześniej jak była w miarę spokojna i stabilna sytuacja. Nie mam przemyśleń na temat pochodzenia i wydaje mi się, że to mogła być jakaś produkcja ludzka, ale czy to przypadkowo się wydostało, czy celowo, to tego nie potrafię powiedzieć. Ostatnio oglądaliśmy film Epidemia Strachu. Stary film, ale bardzo adekwatny do obecnej sytuacji. Chyba jest z 2010. Inny wirus i co innego atakuje, ale bardzo fajnie pokazana sytuacja, która nas właśnie spotkała.

**Ta sytuacja jest poważna? Należy się bać?**

Poważna, pewnie. Z różnych względów. Raz, że wirus jest zjadliwy i powoduje więcej...Wydawało mi się wcześniej, że może to nie jest aż tak, że nie będzie powodował aż tak dużych strat u ludzi. Nie mówię tylko o zgonach, ale też o kalectwie, o następstwie przechorowania, bo zmiany jakie się pojawiają np. w płucach, to dla mnie jako lekarza są czymś takim znaczącym. Nie ma aż tak dużo pacjentów po grypie z takimi powikłaniami. To nie jest skutek bezpośredniego działania wirusa tylko raczej odpowiedzi organizmu na obecność wirusa i reakcji zapalnej organizmu, która niszczy sam organizm. To jest taka autodestrukcja spowodowana wirusem. Poza tym politycy w różnych krajach wykorzystują pandemię do załatwiania swoich spraw. Nie potrafię przytoczyć żadnych przykładów oprócz naszego kraju, bo nie interesuję się polityką zagraniczną, ale uważam, że to jest świetny moment, żeby trochę na ludzi wpłynąć. Myślę, że bardzo to zmieni sposób myślenia niektórych ludzi, bo to nie ma tylko wpływu zdrowotnego, ale też taki socjologiczny aspekt ma ta pandemia. Ludzie się nagle znaleźli w sytuacji, która była znana z filmów popularnonaukowych. myśmy to oglądali z otwartymi ustami, że to ciekawe, niesamowite, wyłączaliśmy telewizję i życie toczyło się dalej poprzednim rytmem. Podróżowaliśmy, zwiedzaliśmy świat, loty wszędzie, połączenia wszędzie, można było przelecieć z Hanoi do Sajgonu za 20$. Ludzie już muszą zmienić myślenie, bo to przestanie być takie łatwe i takie dostępne. Teraz się okazuje, że ma się pieniądze i nie można polecieć, bo nie ma połączeń, bo są obostrzenia, bo coś tam. Ludzie zaczną inaczej patrzeć na świat. Nie tylko bezpośredniość pandemii nam szkodzi, nie tylko przechorowanie, ale też zmienia się sposób w jaki można np. planować wakacje, wyjazdy, spotkania z rodziną, która mieszka np. w Nowej Zelandii. Proste spotkanie, które kiedyś było banałem i kwestią kupienia biletów, to teraz będzie graniczyło z jakimś wyczynem logistycznym, bo trzeba będzie zaplanować 2 kwarantanny, trudny przelot, zapłacić za to kupę pieniędzy i jak się okaże, że trzeba podsumować koszt takiego spotkania, to on wyjdzie 20 razy większy niż było przed pandemią. Takie rzeczy też dochodzą. Będzie mniejsza swoboda, jeśli chodzi o interesy. Kiedyś się otwierało stronę internetową z koncertami i ja tak robiłem, szukałem i jeździłem. Jak był koncert w pobliżu to jeździłem i nie był to wielki problem, a teraz tego nie ma. Kupiłem bilety na koncert w lutym i nie wiem, czy on będzie czy nie. Kupiłem, bo to mój ulubiony zespół. Nie będzie, trudno. Takie rzeczy zaczną mieć znaczenie. Wiadomo, że nie w każdym środowisku i nie dla każdego to jest ważne, ale dla niektórych ludzi tak.

**Myśli pan, że to kiedyś wróci do normy sprzed pandemii?**

Myślę, że koncerty tak, podróżowanie nie. Podróżowanie chyba nie wróci już. Będzie to bardzo długo trwało.

**Czy ludzie zachowują się adekwatnie do sytuacji?**

Raczej tak. Ja miewam chwile kryzysu po 6 godz. przebywania w maseczce, bo mam wrażenie, że te gumki od maseczki przepiłują mi uszy. Używam jednorazowych maseczek, żeby je zmieniać i wyrzucać, ale te gumki po 6 godzinach ciągłego trzymania maski na twarzy...To po prostu boli. Ja wynalazłem wreszcie sposób, żeby maseczka nie trzymałą mi się już na uszach tylko na głowie, bo to było nie do wytrzymania. Ciągłe noszenie maski to można dostać szału. Tortura to jest. W ogóle nie myślimy o osobach, które pracują w sklepie w kategorii takich prozaicznych spraw. Rękawiczki i maska przez cały dzień w sklepie. Ta przyłbica jest dużo mniej skuteczna i mniej daje niż maska, ale oczywiście jest dopuszczalna, więc też można.

**Dało się zapobiec sytuacji, którą mamy teraz? Rząd mógł coś zrobić?**

Nie. Znaleźliśmy się w takim momencie rozwoju cywilizacji, że dużo z nas pracuje za granicą, mnóstwo rzeczy jest kupowanych za granicą i przewożonych. jedyne co mogłoby uchronić jakiś region to całkowita izolacja. I tak jest w jakichś krajach. Chyba na Czukotce nie ma żadnego zachorowania. Alaskę bym bardziej podejrzewał, że tam wirus szaleje jak wszędzie, bo tam też chcą mieć świeże truskawki, sałatkę, itd. i będą te towary ściągane, a z towarami podróżują ludzie.

**Drugiej fali też nie dało się zapobiec?**

Też nie. To znowu musiałaby być całkowita izolacja. W ogóle trzeba by było nie wychodzić z domu przez parę miesięcy. Żyć z jakichś zapasów, nie kontaktować się i może wtedy by się okazało, że ten wirus wymarł i należałoby wtedy też zostawić chorych samych sobie, czyli przyzwolić na to, żeby ludzie poumierali. Straszna rzecz, niewykonalne dla mnie. Może troszkę demonizuję, ale wydaje mi się, że na każdym etapie, nawet jeśli bardzo, bardzo pilnujemy, to zawsze jest ryzyko przeniesienia infekcji na inną osobę. Mimo strojów, zabezpieczeń, specjalnych procedur. My tymi wszystkimi rzeczami zmniejszamy ryzyko infekcji, ale nigdy nie stwarzamy 100% bezpieczeństwa.

**Można było się lepiej przygotować na 2 falę?**

Myślę, że tak - w sprzęt się zaopatrzyć odpowiedni. 70 mln za respiratory gdzieś tam poszło w świat. Nikt o tym nie mówi. Ileś tam pieniędzy poszło też na sprzęt, który nie miał atestu i okazało się, że ten wielki transport z maskami na początku też był nietrafiony, jeśli chodzi o atest. Nie ma to znaczenia tak naprawdę dla nas, ale może rząd powinien się bardziej przyjrzeć tego typu sytuacjom i się rzeczywiście przygotować. Takie reduktory do tlenu, że ich nie ma to jest śmiech. To są drobiazgi, które mogą być produkowane rzemieślniczo, ale należało to przewidzieć. Poszły miliony na respiratory, na wybory, a kupę sprzętu można było kupić za to. Poszło to w powietrze i nikt nic na ten temat nie mówi, ale nie mamy takich podstawowych rzeczy. To na pewno rząd mógł zrobić. Jeśli chodzi o szkolnictwo, to mam wrażenie, że to jest tak samo jak u nas w szpitalach, czyli jesteś dyrektorem szpitala to się martw. Masz leczyć pacjentów z Covidem, to się martw i tak samo jest chyba w szkołach. Każda szkoła sobie indywidualnie jakiś system wypracowuje, a to przecież powinno być przygotowane. komputery nie są w tej chwili drogie i można też było zadbać o to. Ja się dziwię, dlaczego moje dziecko ma 2 godz. zajęć dziennie z 5-ciu, które miał do tej pory. Przecież online też można 5 godz. poprowadzić, tym bardziej, że one są po 30 min, bo na tym się kończy darmowa sesja w Zoomie. Zajęcia są okrojone. Muszę przyznać, że niektórzy nauczyciele się przykładają i te dzieci chętnie uczestniczą, więc da się to zrobić. Trzeba było nad tym pomyśleć i zaproponować to nauczycielom. U nas też nigdy dyrekcja nie zawoła koordynatorów na spotkanie i nie powie, że mamy to i to, mamy takie propozycje i zróbmy tak. Zawsze mówią, że jest problem i będziecie sobie musieli poradzić. To mnie do szału doprowadza i mam wrażenie, że z nauczaniem online jest dokładnie tak samo. Nie było to przygotowane i zaplanowane. U mnie żona prowadzi zajęcia online i dziecko się uczy online, ale są rodziny, gdzie jest 2-3 dzieci w wieku szkolnym, a ja mam kolegę, który ma 7 dzieci i wszyscy też się uczą online. Oprócz dwójki najmłodszych, reszta się uczy. Skąd oni mają wziąć komputery? A rodzice też pracują i nikt ich nie zapytał, czy tam potrzeba cokolwiek. Nie wiem, jak oni to rozwiązują. Część wiem, że przez komórki, popożyczali od ludzi laptopy i tyle.

**Decyzje rządu związane z obostrzeniami uspokajają ludzi?**
Myślę, że tak. Ludzie sobie myślą (pomijam przedsiębiorców, bo dla nich to jest zawsze klęska), że jest gorzej, jest coraz więcej chorych, ale jest ograniczenie, więc ok, czyli nie jest to puszczone samopas. Coś tam robią. Mamy te maski, nie można wychodzić, teraz mamy te godziny dla seniorów, czyli coś tam myślą.

**Jak wygląda u pana śledzenie informacji. Sprawdza pan codziennie?**

[niezrozumiałe] Na początku tej 2 fali patrzyłem jak ta dynamika wygląda, ale jak zaczęło to rosnąć tak, to przestałem, bo to nie ma sensu. Telewizji unikam jak ognia, pooglądałem tylko w związku z ostatnimi wydarzeniami, ale tak to niechętnie. Uważam, że te popularne programy są nieobiektywne. TVP przechodzi już szczyty obłudy i zakłamania, natomiast opozycyjne media, TVN, wcale też nie są najlepsze. Są 2 różne przekazy czasem i chyba nie chce mi się tego oglądać. Szkoda mi czasu. Oglądam filmy, programy dokumentalne, ale niezwiązane z polityką, a informacje bieżące raczej z internetu. To są te same źródła co wcześniej, czyli specjalistyczne medyczne.

**Ile czasu pan poświęca na korzystanie z mediów?**
Chyba mniej niż kiedyś.

**Jak pan ocenia wiarygodność przekazu, który do nas trafia?**

Myślę, że jest bardziej wiarygodny niż na wiosnę. na wiosnę był bardziej manipulowany przez ekipę rządzącą. Wypowiedzi Morawieckiego na temat epidemii w trakcie wyborów to masakra przecież i ten okres po wyborach. Tamten okres kompletnie zakłamany i niewiarygodny a teraz jest lepiej. Teraz po prostu fala jest tak duża, że się nie da uniknąć już jak dużo ludzi już zrobiło te testy, a ponieważ testy są dostępne i można je łatwo zlecać, to więcej prawdy wychodzi.

**Po czym można poznać, że te informacje są wiarygodne?**

Na wiosnę, jak porównywałem ilość testów wykonanych w Polsce i w Niemczech i równocześnie słyszałem, że w Polsce jest mniej zachorowań i nasz kraj się uchował przed pandemia lepiej niż inne państwa, to było to śmieszne. Przecież jesteśmy porównywalni z innymi krajami i funkcjonujemy w życiu UE normalnie, z normalnym eksportem, wyjazdami ludzi, pracą w różnych częściach Europy i świata, więc tym samym i przepływ patogenów jest podobny. Nie ma się co łudzić i my nie jesteśmy wybitni, a raczej bym powiedział, że należałoby się u nas spodziewać większej ilości zachorowań niż w krajach wyżej rozwiniętych i bogatszych niż Polska. Nawyki higieniczne i kultura funkcjonowania są trochę inne. Moim zdaniem ludzi łatwiej wziąć w ryzy w krajach typu Belgia, Holandia, bo im jest bliżej do łatwiejszego sterowania, zaplanowania ich życia niż w Polsce. W Polsce każdy jest lekarzem, księdzem, piekarzem, mechanikiem, a ze szwagrem to już można wszystko zrobić, więc chyba mówienie, że w Polsce jest mniej zachorowań i jesteśmy wyjątkowi to bzdura. Dynamika wzrostu u nas jest taka jak we Francji, Hiszpanii, Niemczech. Mam wrażenie, że teraz ta liczba zachorowań w Polsce jest bardziej wiarygodna, bo jest zbliżona do tej w Europie. Chodzi o analogię do danych z krajów podobnych Polsce, bo nie mam innych danych, na podstawie których mógłbym coś sądzić. Mam dostęp do rejestru badań i wyników pacjentów, więc widzę, ile tych badań jest zlecanych. Jest tego bardzo dużo, więc chyba jesteśmy bliżej prawdy wreszcie. Nie jest to oczywiście idealny obraz, bo uważam, że mnóstwo osób chodzi po ulicy niezdiagnozowanych. Od wielu osób słyszałem ostatnio, że mieli objawy zapalenia zatok, które nie był dotąd podawane jako charakterystyczne, a okazuje się, że mieli potwierdzony dodatni wynik na koronawirusa, więc może się znowu coś tam pozmieniało. jakaś malutka mutacja. Ja nie miałem gorączki i moi koledzy z katarem, gdzie katar wcześniej też nie był wymieniany...Katar był objawem różnicującym i to się chyba już zmieniło.

**Po czym będziemy mogli poznać, że pandemia się skończyła?**

Naukowo to musi zmaleć bardzo ilość nowych zachorowań. Bardzo, bardzo, prawie do zera. Musi się wytworzyć odporność populacyjna, czyli to co mamy z grypa, bo grypy już się tak nie boimy. Może jak się pojawią szczepienia, to też będzie sygnał, że mamy to troszkę w ryzach. Oczywiście wiadomo, że szczepienie na grypę to jest szczepienie na ten typ wirusa, który był a nie na ten, który się pojawi, ale ma to nas uchronić przed ciężkim przebiegiem. Tu jest analogicznie. Gdyby dojść do takiego poziomu to będzie to sygnał, że trochę opanowaliśmy pandemię. Z punktu widzenia społecznego, to jak ludzie przestaną się bać i przestaną o tym mówić. Nikt o grypie nie mówi i ludzie nadużywają tego słowa dla nazwania zwykłych infekcji. Nikt się nie boi słowa grypa, a infekcja Covid to nadal strach i straszne wizje. Jak ludzie przestaną się bać, to też będzie sygnał, że jakiś ważny etap mamy za sobą.

**O jakich zmianach po pandemii pan myśli?**

Przychodzą mi do głowy podróże i stan środowiska naturalnego. Oczywiście też praca nad szczepieniami. Przestanie ludzi dziwić noszenie maski, bo mnie bardzo dziwiło to w Azji, gdzie to było bardzo popularne. Myślałem sobie, że to nienormalne i że po co oni te maski noszą i co to zmienia. teraz to mnie nie dziwi i myślę, że większość starszych osób po 70-ce, gdyby 5 lat temu im pokazać ludzi w maskach chodzących po Przemyślu, to byliby zdziwieni i pukaliby się w czoło. Teraz te maseczki są różne i niektóre ładne, ciekawe, bo ludzie zaczęli je traktować jako element garderoby. Ludzie już nie będą się dziwić odkażaniu rąk i ludzie teraz po prostu to robią, nie jest to nic dziwnego.

**A zmiany w środowisku?**

Myślę, że na plus. to mówią ludzie, którzy mają możliwość wyjechać w miejsca, gdzie kiedyś był duży ruch turystyczny i teraz ustał. Mówią, że przyroda eksploduje. W lutym byliśmy w Afryce, gdzie było dziko, ale ludzie, którzy tam byli po kilku miesiącach i znali te rezerwaty sprzed pandemii, to mówią, że teraz tam jest dużo więcej zwierzyny, że te zwierzęta przestały się bać. Przyroda ma szansę trochę odżyć bez turystyki. To będzie pozytywny aspekt.

**Czegoś się pan obawia w tej przyszłości po pandemii?**

Raczej nie. Myślę, że może będzie większy problem z podróżowaniem, będą wyższe ceny przelotów i też inne miejsca noclegów. Mnóstwo firm splajtowało i zanim to się odrodzi, to minie sporo lat. To się rodziło latami i długo się będzie odradzać, zwłaszcza w Afryce i w Azji.

**A sytuacja gospodarcza?**

Będzie gorsza, dużo gorsza. Mnóstwo ludzi straciło taki sposób zarabiania na życie, gdzie im wystarczało od 1-go do 1-go i mieli w miarę komfortowe życie i żyli sobie w miarę spokojnie. Przy dobrym zarządzaniu małą rodzinną firma żyli sobie w miarę spokojnie. Obowiązki wobec państwa to nic innego niż podatki, ubezpieczenie zdrowotne i może gdyby o tym zapomnieć, to te firmy mogłyby jakoś powrócić do funkcjonowania, Teraz najgorsze są tego typu rzeczy. Czynsze. Jeśli ludzie nie będą mieli za co żyć, nie zapłacą podatków, a jak nie zapłacą podatków to będzie gorsza sytuacja w kraju. To jest wszystko bardzo ściśle powiązane. Pewnie sytuacja gospodarcza też wróci do normy, ale zajmie to kilka lat, a jeszcze przy idiotycznych decyzjach dzisiejszego rządu...

**Są grupy, które mogą zostać szczególnie dotknięte?**

Drobni przedsiębiorcy. Zakład fryzjerski, mała restauracja, kawiarnie. Dla wielu to jest biznes życia, pracuje tam kilka osób, ludzie chcą pracować, mają pensje i jeżeli to runie, to odbudowa tak prosperującej firmy dla niektórych osób będzie trudna, jeśli stracą płynność finansową. Można zmniejszyć pensję pracownikom, jeśli się na to zgodzą...Przy pierwszej fali państwo dawało jakąś tam pomoc...Bardzo kiepsko było to wymyślone z przesunięciem płatności ZUS a nie ze zmniejszeniem czy jakimś ograniczeniem, czy możliwością zrezygnowania na jakiś czas. Jak państwo nie pomoże, to wielu ludzi będzie miało problem z powrotem do funkcjonowania jak przed pandemią.

**Myślał pan o Bożym Narodzeniu w tym roku?**

Jeszcze nie. Myślę, że będzie jak co roku. Wielkanoc była inna, bo był zakaz i rzeczywiście rodzice do nas nie przyjechali, ale Boże Narodzenie już chyba będzie w porządku. jest tak dużo zachorowań, że chyba rządzący pójdą po rozum do głowy i stwierdzą, że nic z tym nie zrobimy i koniec panowania nad pandemią. To pandemia panuje nad nami i tyle.

**Chciałby pan jeszcze coś dodać?**

Przeczytałem gdzieś, że zwiększyła się ilość rozwodów. Sam się nad tym zastanowiłem, że to też może mieć znaczenie. W kontakcie międzyludzkim ważne jest też funkcjonowanie w pewnych odrębnościach. Przymusowy pobyt razem przez dłuższy czas może być groźny, bo każdy troszkę swobody potrzebuje, takiego luzu i ma to bardzo duże znaczenie. Ta sytuacja teraz zmusza ludzi do przebywania razem czasem wbrew ich chęci, powstają napięcia, niespełnione oczekiwania i to może być groźne.
