# Supplementary material for: The challenges arising from the COVID-19 pandemic and the way people deal with them. A qualitative longitudinal study
Source: PLoS One. 2021 Oct 11;16(10):e0258133. doi: 10.1371/journal.pone.0258133 (PMC8504766; doi:10.1371/journal.pone.0258133)
Supplement: S1 Dataset — (ZIP) [file pone.0258133.s003.zip › Transcriptions/stage 6/15.6_M_43_couple, with children.docx]

**15.6_M_43_couple with children**

**Co się u pana działo od czerwca?**

Powróciliśmy do względnej normalności w takim funkcjonowaniu, oczywiście cały czas gdzieś tam w reżimie sanitarnym. Przetrwaliśmy wakacje, lato w całkiem dobrej kondycji, nawet z możliwością krótkiego wypadu w góry i nad polskie morze. Zaskoczyły nas Bieszczady, które były przeładowane, szczególnie miejsca turystyczne, zawsze te Bieszczady były najczęściej lekko pustawe, a teraz np. wycofaliśmy się z odwiedzin na zaporze w Solinie, bo nie szło wejść już na deptak. Taka ciekawostka. Co było wtedy zaskakujące, że ludzie chyba wtedy zapomnieli o tym, że jesteśmy jednak wciąż w okresie pandemicznym i w zasadzie ani dystansu, ani zabezpieczeń indywidualnych nie zauważyliśmy, byliśmy w zasadzie jedynymi, którzy posługiwali się maskami na tamten moment, to był sierpień. Z rzeczy życiowych przystąpiliśmy do pierwszej klasy szkoły podstawowej, przystąpiliśmy w znaczeniu jako rodzina, bo to jest zawsze wydarzenie też rodzinne. Syn poszedł po raz pierwszy do pierwszej klasy, do szkoły, więc przygotowanie tego chwilę czasu nam zajęło, bo to dosyć duża zmiana życiowa, ale zmiana zdecydowanie na plus. Syn się bardzo szybko zaadaptował, musiał wejść również w rygor prac domowych, co mu przysparzało na początku trochę trudności, bo jest to czas, który trzeba przeznaczyć jednak na jakąś tam formę pracy. Wcześniej aż tyle pracy nie wykonywał, takiej mentalnej w domu, poza zerówką, więc to była jakaś nowość. Póki co działamy w trybie stacjonarnym, to znaczy, że szkoła działa i lekcje się zwyczajnie odbywają, więc nie ma problemu. Jak będzie to wyglądało, zobaczymy jutro, pojutrze albo w piątek, zobaczymy, kiedy jeszcze władze centralne podejmą decyzję o zamknięciu. Póki co... Automat telefoniczny z Inspektoratu Sanitarnego, który informuje nas o tym, że jesteśmy na kwarantannie, nie wiem, czy pani wie, ale nie musi mieć pani decyzji, nie musi pani z nikim rozmawiać, jak odbierze pani numer warszawski, jakiś 48 22 102 chyba, to automat poinformuje panią, że jest pani na kwarantannie ze względu na kontakt. Ale to taka ciekawostka, która została wprowadzona na świeżo w kraju. Wracając: jesteśmy w trzecim miesiącu szkoły podstawowej, jest na razie ok, syn robi postępy, jest zadowolony z tego, jak realizowane są zajęcia dodatkowe, które formalnie nie mogą być realizowane ze względu na pandemię, ale jeżeli to wychowawca prowadzi te zajęcia, to one mogą się w tym samym gronie odbywać, więc tam jest jakiś hiszpański, jakiś klub odkrywców, jakieś dodatkowe zajęcia matematyczne. Z tego punktu widzenia jesteśmy zadowoleni. Musieliśmy się nauczyć kolejnej aplikacji, jaką jest Librus, czyli elektroniczny dziennik. Z racji tego, że jesteśmy obydwoje już na ten moment nie tyle może zacofani, co zatrzymaliśmy się około 10 lat temu w rozwoju cyfrowym, więc to też była jakaś ciekawostka dla nas. Z kolejnych zmian: musiałem przygotować całe miejsce pracy do tego, żeby zacząć jakoś funkcjonować w tym świecie, musiałem wymienić komputer, ponieważ jak się okazuje Microsoft Teams ma swoje wymagania, więc zakupiłem komputer stacjonarny, z którego jestem bardzo zadowolony. Musiałem też podciągnąć światłowód do gabinetu, w którym siedzimy, bo się okazało, że jednak sieć bezprzewodowa LTE operatora komórkowego jest niewystarczająca i te transmisje nie są satysfakcjonujące. No i musiałem podzielić stanowisko pracy na trzy ekrany, żeby móc pracować z trzema rzeczami na raz, więc do takich większych konferencji mam monitor 65 cali i na nim faktycznie dobrze widzę, przy takiej przekątnej ekranu to już jest coś.

**Czyli przygotował się pan do pracy zdalnej na dłuższą metę?**

Tak, zdecydowanie. To jest, tak jak widać w tle, gabinet też częściowo muzyczny i pokój zabaw, ale on służy przede wszystkim na ten moment jako miejsce pracy.

**Jak teraz wygląda pana praca?**

Uczelnia pracuje w trybie zdalnym, te zajęcia, które mogłyby się odbywać w kontakcie bezpośrednim, face to face, one zostały przesunięte u nas na drugą część roku akademickiego, na lato, tak powiedzmy sobie, bo rok akademicki trwa do końca lipca, żeby to faktycznie zrealizować, natomiast te, które są teraz, są przypisane do pracy zdalnej, w związku z tym też ta inwestycja w sprzęt. Trzeci tydzień się teraz rozpoczął, odkąd jestem w Centrum Powiadomienia Ratunkowego, czyli w Urzędzie Wojewódzkim, na numerze 112, jestem na pracy zdalnej. Taka była decyzja kierownictwa, żeby przesunąć nas. W trakcie wiosennego rzutu pandemicznego my nie mieliśmy tej pracy zdalnej, jeździliśmy normalnie do pracy, natomiast na ten moment widocznie kierownictwo stwierdziło już, że to ryzyko jest zbyt duże. Zresztą tam na numerze 112 są też daleko posunięte zmiany takie organizacyjne w pracy dla operatorów, dla tych, którzy odbierają te rozmowy, oni mają już teraz pokoje dwuosobowe, w których mają stanowisko pracy i dwa łóżka, w razie czego będą tam mieszkać i pracować w takich mini celach z dostępem do aneksów kuchennych i łazienek, ale będą po prostu pozamykani. Natomiast to, że jestem na pracy zdalnej, częściowo wynika z tego, że miałem jakąś infekcję o nieswoistym przebiegu i musiałem sobie zrobić trzytygodniową przerwę w pracy, zostałem o to poproszony ze względu na objawy, które generowałem, nikt nie chciał ze mną rozmawiać.

**Miał pan robiony test?**

Nie, nie robiłem testu. Miałem infekcję w pierwszym tygodniu października, która polegała po prostu na suchym kaszlu i dusznościach, taki sezon, to nie są duszności, które by mnie powaliły, ale np. widziałem przy wchodzeniu po schodach, że mam jakąś delikatną zadyszkę, więc ta wydajność układu oddechowego nie była 100% na pewno. Natomiast nie miałem absolutnie żadnych innych objawów i w trakcie teleporady lekarz rodzinny się pytał, czy mam gorączkę, czy straciłem smak, czy mam jakieś dolegliwości bólowe, mięśniowe, czy bóle głowy - ja takich objawów nie miałem, w związku z tym dostałem receptę, którą wykupiłem. Po tygodniu brania leków ponownie skontaktowałem się z lekarzem w ramach teleporady, ponieważ objawy w żaden sposób nie ustąpiły, ale w dalszym ciągu nie miałem gorączki ani utraty smaku, ani żadnych innych dolegliwości, więc została zmieniona diagnoza, dostałem inne leki. Po tygodniu byłem na kontroli osobistej, ponieważ to wskazywało na zapalenie oskrzeli być może czy zapalenie płuc, okazało się, że coś tam w tych oskrzelach jest, więc w trzecim tygodniu dostałem antybiotyk, przeszedłem cykl leczenia astmatycznego, z krótko działającymi β2-mimetykami. Później miałem podejrzenie jednak zapalenia zatok bocznych nosa, więc dostałem steroidy do nosa, a później dostałem antybiotyk i teraz jest mniej więcej piąty tydzień i jako tako już się czuję. Oczywiście mam jeszcze jakieś drobne objawy związane z kaszlem, ale jak mówią lekarze, to może jeszcze trochę potrwać, więc w teorii mógłbym wrócić do pracy, ale już teraz zapadła decyzja centralna, że jednak pracujemy zdalnie. Więc ja tak de facto jestem szósty tydzień tylko na pracy zdalnej. Przez te pierwsze 3 tygodnie nie mając pewności, co to jest, bo też podejrzewałem, że ze względu na dużą liczbę kontaktów różnorakich, że to może być taki nieswoisty przebieg koronawirusa, ale jednak nie było wskazania, wtedy jeszcze nie było możliwości zrobienia testów antygenowych, tych tańszych, stwierdziłem, że inwestowanie 500 zł w wynik, który jest często niemiarodajny albo nierozstrzygnięty, to nie jest dobra inwestycja, dlatego też poddałem się takiej częściowej izolacji rodzinnej, to znaczy, że więcej czasu spędzam w tym pomieszczeniu, w którym jestem teraz, mam tutaj węzeł sanitarny, mam łóżko, więc mogę tutaj spędzać całkiem dużo czasu. Odizolowałem się od świata zewnętrznego w zasadzie całkowicie i ok, przetrwałem bez większych problemów. Natomiast zauważałem też, że jednak pojawiało się jakieś osłabienie, ja to wiązałem z tą niewydolnością oddechową, ale jeszcze jedna rzecz, która przyszła mi do głowy, to jest obniżenie sprawności intelektualnej, tzn. ja zacząłem zauważać, że zarówno procesy poznawcze i wykonawcze nie są już takie wydajne, jak były w okresie przed infekcją, więc sam nie wiem, czy to był covid-19 czy nie, ale zauważam, że ta praca mentalna idzie mi zdecydowanie słabiej, mam większe problemy z doborem słów, częściej używam pętli fonologicznej, żeby sobie coś zapamiętać, ogólnie widzę taki delikatny zjazd, jeśli chodzi o sprawność intelektualną. Jak prowadzę zajęcia, to też mi to przysparza problemów. Czasami staram się używać prostych słów, zapominam fachowych terminów. Jest to faktycznie zauważalne, może jest to obszar do weryfikacji empirycznej w badaniach.

**Czy czuł pan strach, że to jednak może być koronawirus?**

Nie, ja na zasadzie refleksji post factum teraz dochodzę do wniosku, że to mógł być koronawirus, ale na tamten moment nie myślałem raczej tak, bo wskazania objawowe były bardzo jasne i czytelne. Teraz mówi się, że ten przebieg może być nieswoisty, że tych objawów może być więcej i że mogą być inne aniżeli te, które są najczęściej generowane przez osoby choroby. Teraz myślę, że to mogła być infekcja, ale niekoniecznie wtedy tak myślałem.

**Przed obecną izolacją życie wróciło do stanu sprzed epidemii? Co z ograniczaniem kontaktów?**

One były ograniczane, oczywiście że tak. Doszło do pewnego rozluźnienia, bo sam fakt, że byliśmy na wakacjach, że wyjechaliśmy gdzieś, ale wyjechaliśmy sami, mieszkaliśmy indywidualnie, to nie było też potrzeby ograniczenia. Byliśmy raz na basenie zamkniętym, na termach, to było takie jedno odstępstwo. Syn chodził na zajęcia karate, zajęcia sportowe, chodzi do szkoły. Nie prowadziliśmy bogatego życia towarzyskiego ani też zbytnio nie wychodziliśmy na żadne imprezy plenerowe, więc w tym zakresie było tego troszeczkę mniej. Relacje rodzinne chyba też trochę były przygaszone, no bo gdzieś tam poza osobistymi kontaktami z rodzicami (też rzadszymi niż wcześniej), to tak z pozostałymi członkami rodzin nie widzieliśmy się, np. z rodzeństwem, ja się nie widziałem ze swoją siostrą. Małżonka częściej się widuje z rodzeństwem, bo mieszkają po sąsiedzku, więc można się spotkać czasami gdzieś przypadkiem na ulicy, ale faktycznie mimo przywrócenia pewnych swobód, my do końca nie powróciliśmy do normalnego rytuału życiowego. Ale to też nie stanowiło znaczącego problemu. W miarę normalnie funkcjonowaliśmy, ja wróciłem do pracy do szpitala, zresztą też chyba rozmawialiśmy, jak ja pracowałem w szpitalu i pracowaliśmy tam do końca sierpnia, i później znowu mieliśmy przerwę, że tak powiem techniczną. I teraz 9.11. mamy wrócić z powrotem. Jak to będzie wyglądało, też jeszcze do końca nie wiemy. Ale to był taki powrót do normalności, z tym, że oczywiście wiadomo, że podstawowe środki zabezpieczenia indywidualnego: w trakcie wizyty z pacjentem, klientem zawsze w maseczce, to rzecz taka standardowa.

**Czy pana działalność gospodarcza wróciła do normalności?**

Wróciła do normalności razy 3, ponieważ musiałem nadrobić te szkolenia dla kierowców zespołu medycznego, których nie zrobiłem wcześniej, więc tego było naprawdę mnóstwo. Natomiast teraz 9.10. miałem już ostatnie zajęcia szkoleniowe i planowo nie miało być już ich więcej, więc na razie jestem wstrzymany, ale nie ze względów pandemicznych, tylko to tak już poukładał się grafik na ten moment, że akurat z tego obszaru nic więcej się nie dzieje. Przetarg wygrałem, bo jak się okazuje, byłem znowu jedynym, który był zainteresowany udziałem w przetargu, więc pewnie będę to realizował ponownie, jak cała procedura zostanie zakończona. Więc w tym zakresie nie widzę jakichś znaczących... Tak jak rozmawialiśmy na przełomie maja, czerwca i też kwietnia, to był zdecydowanie najlepszy rok finansowy od wielu, wielu lat i dalej to podtrzymuję. Teraz zobaczymy, jak to się będzie dalej rozwijało.

**Wydaje się panu, że życie podczas lata wróciło do normalności, a teraz przypomina to, co było na wiosnę?**

Myślę, że wygląda inaczej, bo chyba jednak tej wolności doznaliśmy latem i to całkowite zamknięcie będzie zdecydowanie trudniejsze dla wszystkich, ale też nie wiem, czy będzie technicznie wprowadzone, bo jednak z nadwyżki budżetowej zeszliśmy na zupełne zero, więc nie ma już pieniędzy na to, żeby nas zamykać i nam dopłacać, więc trzeba będzie poszukać innych rozwiązań. To, co się dzieje, w przestrzeni społecznej, politycznej, publicznej i ulicznej też wskazuje na to, że raczej jest to mało prawdopodobne. Ale widzę, że jednostkowo w moim otoczeniu ludzie jednak faktycznie, zarówno rodzina, jak i znajomi, zaczynają powoli zabezpieczać się na okoliczność częstszych pobytów w domu. Czyli faktycznie więcej pracy zdalnej, może niekoniecznie wszystko będzie zamknięte i będziemy zamknięci w czterech ścianach, ale jednak ta tendencja do izolacji czy do autoizolacji, takiej celowej, się pojawia. Chyba przywykliśmy do tego, że to tak musi wyglądać, przynajmniej może ta bardziej świadoma część społeczeństwa albo ta, która nie chce sobie i innym przysparzać problemów.

**Czy coś panu przeszkadza w izolacji lub skutkach pandemii?**

Chyba mój stan psychomotoryczny. Pewne osłabienie, które jest teraz aktualne, czyli mój stan zdrowia po prostu. Czy coś mi jeszcze bardziej przeszkadza? Może utrata motywacji do prac remontowych w domu, bo już mam po dziurki w nosie remontów, które częściowo sam wykonywałem, częściowo firmy, mam jeszcze coś tam do zrobienia, ale to już... Powiedzmy, że poczułem się gorzej, czyli od 6 tygodni w zasadzie nic nie zrobiłem i omijam to szerokim łukiem, więc jakieś rzeczy, które leżą, są do zamontowania albo wyniesienia, przeszkadzają mi, a jakoś nie widzę supermotywacji do tego, żeby się za to zabrać. Czy tak z elementów zewnętrznych? Może niekoniecznie powiązane z pandemią, ale przeszkadzają mi co poniektóre decyzje władz rządowych, działania albo brak działań, ale to jest bardziej forma refleksji nad tym, co obserwuję. Natomiast czy mi coś przeszkadza jako tako? Tak de facto chyba nie. Jakby nie widzę tego. No dobra, może faktycznie wolałbym pójść na siłownię. W ubiegłą sobotę założyłem koszulę i się okazało, że w miejscach, w których ona kiedyś była przyciasnawa, czyli w okolicach barków, zrobiła się luźna, natomiast zrobiła się ciaśniejsza nieco niżej, więc jakby ta tkanka mięśniowa spłynęła z okolicy obręczy barkowej w okolicę bioder. Nie wiem, jaki proces za to odpowiada, ale tak się stało, więc jakaś forma aktywności by się przydała. Więc może przeszkadza mi zamknięcie co poniektórych obiektów sportowych. Mogę zawsze wrócić do jakiegoś drobnego biegania, ale nigdy bieganie nie było ani moją domeną, ani moją ulubioną dyscypliną, ani rower, raczej traktowałem je uzupełniająco. A w domu mi się po prostu nie chce i nigdy mi się nie chciało ćwiczyć i zawsze jednak to wyjście, jakiś obiekt sportowy, to było odcięcie się od rzeczywistości, ze słuchawkami na uszach, coś w rodzaju medytacji albo uczestnictwa w Eucharystii, każdy ma swoją własną wersję. Więc tego może mi brakuje jedynie. Mój szwagier ma jakiś podstawowy zestaw i on normalnie, regularnie, 3-4 razy w tygodniu ćwiczy, trenuje. To jest oczywiście kwestia motywacji, nawyków, chęci, ja się przyznaję bez bicia, że mi się nie chce, bo to jest dom, bo ćwiczyłbym w miejscu, w którym pracuję, zawsze wolałem to wyjście, bo to też miało dla mnie dodatkowe znaczenie, właśnie to, że wychodzę, nie tylko samo ćwiczenie, ale też sam fakt wyjścia i tego mi brakuje. Da się w domu, oczywiście że się da, ale mi się po prostu nie chce. I nigdy mi się nie chciało.

**Jakie były najważniejsze momenty w rozwoju pandemii w ostatnich miesiącach?**

Mi się wydaje, ale to oczywiście są tylko i wyłącznie moje przypuszczenia, że wakacje były momentem, kiedy zaczęliśmy się crossować, wymieniać, jeździć po kraju, więc byliśmy mniej stacjonarni, co też umożliwiło pewnie w jakimś tam zakresie migrację patogenów, ale później kluczowym elementem był jednak moment powrotu wszystkich do aktywności zawodowej, bo o ile wakacje, urlopy czy lato częściowo spędzaliśmy w przestrzeni otwartej, na tyle jednak powrót wrześniowy do aktywności zawodowej, do szkół i później częściowy przynajmniej powrót studentów na zajęcia w październiku, ja myślę, że kluczowy był powrót do powakacyjnej normalności zawodowej, spędzanie dłuższych okresów w pomieszczeniach ze współpracownikami, gdzie tam wiemy już, że ta ekspozycja poniżej 15 min może wcale nie musi być aż tak bardzo groźna, ale powyżej 15 min ona faktycznie wzrasta. Jednak czasu spędzanego w biurach, klasach, miejscach pracy - tego czasu było zdecydowanie więcej aniżeli 15 min na jedną osobę, więc mi się wydaje, że to był ten element. Pewnie też ogólne jesienne pogorszenie stanu zdrowia, które się pojawia. Wiadomo, że covid nie jest jedyną chorobą czy jedynym zagrożeniem, nawet powiedziałbym, że jest marginalnym z punktu widzenia śmiertelności, ale zwykła grypa i przeziębienia też się po prostu pojawiają, jakieś tam drobne infekcje wirusowe czy bakteryjne z innymi szczepami. Pewnie to też w jakimś tam zakresie, pewnie to się nakładało, być może jedna infekcja na drugą, ciężko jest stwierdzić, ale tak przypuszczalnie, z mojej perspektywy jako człowieka, który się na tym w ogóle nie zna, ale postanowił się jednak wypowiedzieć, to tak to wygląda.

**Czyli od momentu powrotu mamy ten sam etap, który narasta?**

Tak, to się będzie rozwijało, tak jak mówię. Nie ma już ognisk infekcji, to się zdecentralizowało, infekcja się rozsiała ze względu na naszą aktywność po prostu. Z drugiej strony czy szło cokolwiek innego zrobić? No nie szło.

**Emocje.**

Dobrze, ja myślę, że w dalszym ciągu kluczowym elementem jest obrazek nr 6 i 13 ze względu na samą kolorystykę i odwołanie się do słońca, do światła, 6 do podążania w jakimś kierunku, słońce wskazuje w lesie jakiś kierunek, my też dążymy raczej ku pozytywom, nie mamy co do tego wątpliwości. I 13 myślę, że w bardzo podobnym tonie, że one raczej wskazują na pozytywne ujęcie całości. Trudno jest mi wskazać jakieś bardzo klarowne emocje, ale też na pewno nie jest to taka neutralność emocjonalna. Zamiast powiedzieć, że ok, jest dobrze, ja mówię, że na pewno nie jest źle, ja nie widzę żadnych emocji negatywnych, nie widzę żadnej złości w sobie, smutku, wycofania, towarzyszących tym stanom zachowań agresywnych, autoagresywnych, obniżenia nastroju, afektu, nawet powiązanych z jakimiś zmianami pogody, nie, czegoś takiego nie mam. Wydaje mi się, że ten moment do tzw. drugiej fali pandemicznej w tym stylu funkcjonowałem i w dalszym ciągu, jeżeli spojrzę na siebie dzisiaj, to wydaje mi się, że to będą te dwa same elementy, czyli ponownie w dniu dzisiejszym oświadczam, że jest to 6 i 13, ale też 11 przy okazji, ze względu na ten pozytywny aspekt tęczy, który się pojawia, "a po nocy przychodzi dzień, a po burzy spokój", jeśli można to tutaj ująć w słowa popkulturowej poezji. Jest ta szyba z kroplami deszczu, jest ta tęcza i to jest taki dzień jak dzisiaj, choć dzisiaj akurat bez deszczu, ale wczoraj było deszczowo. Deszcz zawsze jakoś tam ograniczał możliwości wychodzenia na zewnątrz i tak teraz chyba jest. Jest taki mniej lub bardziej symboliczny deszcz, ale jest ta tęcza za oknem.

**Z 11 wiąże się nadzieja?**

Zdecydowanie, oczekiwanie pozytywnego rozwiązania wielu spraw, może niezależnie od czasu pandemicznego. Oczywiście, że pojawiają się jakieś tam wątpliwości, ale one są powiązane raczej z obawą. Nie idę w kierunku lęku, ale jakieś delikatne obawy, myślenie już o poranku, tuż po przebudzeniu hipokamp od razu wrzuca w pole uwagi jakieś treści powiązane z aktywnością zawodową i to się wiąże z jakimś zaangażowaniem mentalnym, tzn. ja widzę, że to gdzieś powraca, takie persewerujące luminacje, to wraca jako niechciane, ja to przeżuwam, przetrawiam, rozpracowuję. To może być taki negatyw, ale to nie jest raczej powiązane z czasem pandemicznym, chyba że jest to może powiązane z moim stanem zdrowotnym. Jak jest obniżona sprawność intelektualna, to być może te mechanizmy obronne, żeby nie dopuszczać takich treści, są nieco, trochę słabsze. Mam do rozwikłania jakieś sprawy zawodowe i to wzbudza delikatny niepokój u mnie. Z tym że to raczej chodzi mi tutaj o decyzje, że będę musiał ograniczyć w jakimś zakresie, czyli z czegoś zrezygnować w swojej aktywności zawodowej, ponieważ nie jestem w stanie pogodzić wszystkich obowiązków. Więc ja nie obawiam się utraty pracy, ja się boję tego, że mam jej za dużo, a jestem coraz mniej wydolny, choćby też ze względu na wiek po prostu i tyle, bo zdawać by się mogło, że 43 lata to nie jest wcale aż tak dużo, ale to na pewno jest więcej niż 23. Więc taki element negatywny, ale on nie jest powiązany z pandemią albo nie jest wprost powiązany z pandemią. Ale na ten moment te 3 elementy wciąż są dla mnie wiążące.

**Czy pojawiają się w panu inne obawy?**

Tak, pojawiają się, ale one są niezbyt powiązane z okresem pandemicznym. Np. mam zamknięty samochód, który od kwietnia stoi w garażu i ani razu go nie odpaliłem i obawiam się, że jego kondycja będzie fatalna i że będę musiał włożyć dużo wysiłku w to, żeby on ruszył, ale też z drugiej strony nie chce mi się tego robić. Psuje się również mój samochód użytkowy, codzienny, na firmę i wiem, że to jest dobry moment, żeby kupić kolejny samochód, ale kiepski moment, żeby sprzedać samochód, więc gdzieś tam w minimalnym zakresie zastanawiam się, co zrobić z samochodem, który być może zaraz będzie niesprzedawalny albo sprzedawalny za niewielkie pieniądze. To są jakieś takie życiowe obawy, ale nie wiem, czy miałbym je wcześniej, przed okresem pandemiczym, po prostu podjąłbym działania i koniec.

**Jak pan sobie radzi z negatywnymi stanami?**

Styl zadaniowy, tzn., że trzeba siąść i podjąć decyzję. Oczywiście te decyzje (przynajmniej w obszarze zawodowym) wynikają z wielu różnych czynników, ja muszę najpierw poczekać, aż pewne rzeczy się zadzieją, żeby podjąć ostateczną decyzję. Ten czas czekania jest może bardziej obciążający, ale wiem, że to jest tylko i wyłącznie okresowe, że na pewnym etapie ten problem zostanie przeze mnie rozwiązany. Rzeczy, na które nie mam wpływu, czasami powracają do mnie, ale z drugiej strony nie jestem w stanie... Wtedy to może bardziej taka strategia unikowa, że zaczynam robić coś innego. Nic nie poradzę na to, że mi się nie chce kończyć remontu, ale też nic nie poradzę, że nie chce mi się jechać naprawiać starego samochodu, zabytkowego, po prostu odsuwam te myśli, zajmuję się czymś innym i czekam, aż powrócą po raz kolejny. Ale tak jest w tych mniej istotnych rzeczach, wydaje mi się, że może być jakieś unikanie, robienie czegokolwiek innego, gotowanie. W rzeczach istotnych po prostu czekam na moment, wiem, że decyzje muszę podjąć i ja te decyzje podejmuję, czyli działam zadaniowo.

**Pojawiły się nowe sposoby spędzania wolnego czasu? Pasje pomagają panu czy nie ma pan na nie czasu?**

Pewnie bym znalazł czas, tylko że troszeczkę ten czas się rozciągnął w takim znaczeniu, że jest go więcej, ale też tempo wykonywanych czynności jest nieco wolniejsze. Więc jeśli bym funkcjonował na takim etapie sprawności, wydajności jak przed okresem pandemicznym, pewnie bym znalazł czas, żeby zrobić więcej rzeczy dla siebie, ale też nie zawsze chce mi się sięgać po tę gitarę. Dawno nie sięgałem, trochę siedziałem ostatnio przy pianinie, trochę słuchałem muzyki. Ale czy coś nowego się pojawiło? Nie, powiedziałbym chyba nawet, że jakby bardziej myślenie o tych sprawach rodzinnych... O, moją nową rozrywką jest odrabianie zajęć domowych z synem, to jest jakieś novum, które się pojawiło. Mam już bardzo poważne objawy alergiczne na widok logotypu Netflix, więc już unikam, zdecydowany przesyt. W sumie zacząłem grać w jakąś grę strategiczną, taką wieloosobową. Jest to aplikacja na tablecie, uczestnictwo w wydarzeniach historycznych i rozbudowa swojej wioski, miasta, która nazywa się DomiNations. Może teraz już nie jestem jakoś tam aktywny, ale był taki moment, że codziennie wieczorem przynajmniej zaglądałem, żeby zobaczyć na jakim etapie rozwoju cywilizacyjnego jestem i ok, to mi jakoś sprawiało przyjemność. Teraz natomiast jestem zdecydowanie mniej aktywny, bo też jest trochę innej aktywności zawodowej, powiedzmy, że to był element wakacyjno-urlopowy, ale nie wycofuję się z tej aktywności, zamierzam swoją wioskę - czy już na ten moment może miasteczko - utrzymywać przy życiu. To jest jakiegoś rodzaju nowość. Gotowanie tak, remonty już nie, aczkolwiek będę musiał wrócić w ramach przyzwoitości, żeby to skończyć. Mniej chyba relacji... W mniejszym stopniu wykorzystujemy komunikatory do komunikowania się z otoczeniem, to jest taka zmiana, ale pewnie do tego będzie trzeba wrócić, być może jest jakiś przesyt. Czy coś nowego się jeszcze pojawiło? Nie no, pies jeszcze, pies jest też nowością od czerwca. Nie jest to szczęśliwie mój pies i ja pomijam jego obecność w domu, bo nie ja byłem zainteresowany posiadaniem psa, więc jest to pies mojego syna i mojej żony, ale tak, zdarza się, że mimo wszystko ja również wychodzę z tym psem albo uczestniczę we wspólnych spacerach, tzn., że mój syn i moja żona wyprowadzają psa i mnie razem.

**Jakie emocje obserwuje pan u osób w swoim otoczeniu?**

Widzę, że pojawiają się obniżone nastroje, szczególnie u tych osób, które mam w swoim najbliższym otoczeniu, tutaj mam na myśli rodziców czy teściów, czyli rodziców żony. Oni faktycznie są już gotowi na to, żeby się ponownie zamknąć, trochę powyjeżdżali na wakacje, teść powrócił do aktywności zawodowej, a teraz znowu widzę, że będą musieli się wycofać. Ale o ile przy pierwszym lockdownie zakładali jakiś powrót, to teraz widzę, że oni chyba stwierdzają, że to już jest taki stan stały i że być może trzeba będzie już przejść na pełną emeryturę, nie robić nic poza emeryturą, bo może wcale nie być takiej możliwości, żeby coś takiego robić dalej. W gronie znajomych czy rodziny, wśród osób z mojego pokolenia, może ciut młodszych nie widzę takich zmian. Oni się faktycznie być może szykują, może spędzają mniej czasu w przestrzeni publicznej, ale nie widać, żeby to było pójście w kierunku depresji egzogennej, czegoś, co przychodzi z zewnątrz i strasznie atakuje.

**A widzi pan ich sposoby radzenia sobie z tymi emocjami?**

U teściów? Bo mogę się raczej odnieść do nich tutaj. Teściowie odkryli Netflix, a teść zaczął kolekcjonować wina, które pija, sprowadza sobie różne wina i je degustuje. Nie wiem, czy to jest sposób radzenia sobie czy to raczej jest wejście już w nawyki emeryta, może bardziej w ten sposób, ale przy okazji pewnie w ten sposób też sobie radzą z tym, co się dzieje. Z tym że to nie jest jeszcze wysokie natężenie, bo oficjalnie nie jesteśmy jeszcze zamknięci, przynajmniej przez kilka dni, zobaczymy, co nam premier powie jutro, pojutrze, najdalej w piątek.

**Czy w pana otoczeniu pojawiły się zachorowania na koronawirusa?**

Zdecydowanie jest ich bardzo dużo. W zasadzie w każdym miejscu pracy, w rodzinie, na ulicy, tak, są zachorowania i są kwarantanny.

**Czy ta bliskość wirusa budzi w panu obawy?**

Nie.

**Jaki jest przebieg choroby u tych osób?**

W większości przypadków były to łagodne przebiegi, ale w dalszej rodzinie straciliśmy jednego członka rodziny. To nie jest bezpośrednio moja rodzina, krew z krwi, tylko w rodzinie żony daleki kuzyn teścia zmarł w szpitalu na Szwajcarskiej w Poznaniu, ale on odmówił podłączenia do respiratora, bo powiedział, że respirator to jest już droga tylko i wyłącznie w jedną stronę, nie chciał przedłużać i w zasadzie podjął decyzję o... Nie wiem, może liczył, że uda mu się wyjść bez tego respiratora, ale jak widać nie udało się, przynajmniej takie są relacje lekarza, który prowadził pacjenta. Starsi ludzie umierają, nie ma co ukrywać, straciłem dziadków, tutaj zmarł daleki kuzyn teścia, takie rzeczy się po prostu dzieją, w żaden sposób mnie to nie dotyka ani też nie mam żadnych związanych z tym obaw. Kuzyn mógł mieć w okolicach 70 lat.

**Te sytuacje nie budzą w panu obaw, nie są dla pana bezpośrednim zagrożeniem.**

Tak. Jeśli nie miałem, to być może będę miał, jeśli będę miał, to przebieg będzie taki albo taki, ale zakładam, że nie musi być wcale źle. Jeszcze tutaj mam grupę krwi, jak pokazują wstępne badania, że grupa krwi też ma znaczenie, akurat jestem w tej najbezpieczniejszej grupie krwi. Już pomijając jakieś dowody naukowe, po prostu się nie boję.

**O jakich obostrzeniach ostatnio pan słyszał?**

Ostatnim głośnym obostrzeniem było zamknięcie cmentarzy do dnia wczorajszego, dzisiaj są już otwarte, ograniczenie, zamknięcie imprez sportowych dla kibiców, osób oglądających, ograniczenia w miejscach rozrywki o wyższym poziomie kultury, typu kina czy teatr, jest to 50 % miejsc, ograniczenia w komunikacji miejskiej, zamknięcie siłowni przy jednoczesnym otwarciu szkół tańca i grupowych organizowanych zajęć sportowych, jak np. zajęcia karate dla mojego syna. Co jest też w pełni zrozumiałe, bo jednak na siłowni możemy się zarazić zdecydowanie bardziej, aniżeli tańcząc z cudzą partnerką na odległość 30 cm na sali, gdzie tańczy 40 par. Nie obostrzenia, ale sugestie pracy zdalnej w każdym miejscu, gdzie to się da. Też żadna nowość, czyli przesunięcie całej pracy administracji publicznej na pracę zdalną na najbliższe 2 tygodnie na tyle, na ile te obowiązki pozwalają i to już jest wytyczna realizowana we wszystkich urzędach. Czy my coś jeszcze mamy? No oczywiście wprowadzenie obowiązku noszenia maseczek w przestrzeniach otwartych, publicznych, w miejscach pracy i w środkach transportu, czyli w zasadzie wszędzie poza domem, z wyłączeniem aktywności sportowej na świeżym powietrzu, czyli biegać, jeździć na rowerze czy chodzić po lesie w dalszym ciągu można bez. Czy coś jeszcze? Nie przychodzi mi nic więcej na myśl na ten moment. Być może coś tam jeszcze jest... A, zamknięcie restauracji, pubów, z możliwością organizacji sprzedaży na zewnątrz, z dostawą albo z odbiorem osobistym, ale nie można usiąść i zjeść ani wypić.

**Co pan myśli o tych obostrzeniach?**

Moje stanowisko się nie zmienia. Mimo że jesteśmy bogatsi w doświadczenia wiosenne, to de facto wciąż nie wiemy, co jest dobre i co jest skuteczne. Więc lepiej robić to, co robią mniej więcej inni, aniżeli tworzyć swój własny model, który może się zakończyć w równym stopniu, z równym prawdopodobieństwem albo sukcesem, albo katastrofą. Całkowitego lockdownu nie będzie, ale to, co robimy, wydaje mi się, że w kilku procentach obniża rozprzestrzenianie się infekcji. W żaden sposób nas nie zabezpiecza w 100%, ale na pewno obniża i być może trochę spowalnia tempo wzrostu liczby zachorowań. No właśnie, wie pani, ile dzisiaj nam zachorowało?

**Nie.**

Ja też właśnie nie sprawdzałem. Zróbmy 5 min przerwy.

(przerwa)

**Na ile noszenie maseczek ogranicza rozprzestrzenianie się wirusa?**

Ma realny wpływ. W zależności oczywiście od tego, jaki model maseczki nosimy, ale ma realny wpływ, ponieważ jest taką fizyczną barierą, podstawową, która nawet w przypadku rozprzestrzenienia tego wirusa przez osobę zakażoną, jest on jednak o kilka % mniejszy. Nie zabezpiecza nas to w 100% w ogóle w żaden sposób, ale na pewno biorąc pod uwagę maseczkę plus dystans, to sumarycznie to na pewno nam coś tutaj daje. Oczywiście też zależy od maseczki, bo dostałem takie maseczki, które chronią z dużym prawdopodobieństwem mnie, natomiast wszystkie moje aerozole są wydalane na zewnątrz bez żadnych ograniczeń, więc to jest takie egocentryczne podejście do noszenia maseczki, bo ja mogę infekować, ale zmniejsza się ryzyko mojej infekcji. Na razie jeszcze z nich nie korzystałem, ale takie modele również są. Więc tak, jestem za.

**Rekomendacja izolacji seniorów - co pan o tym sądzi?**

Z racji tego, że jest to jedna z najbardziej narażonych grup, to ok, jestem też zdecydowanie za.

**A zamknięcie siłowni?**

Jest to zaskakujące w jakimś zakresie. Myślę, że to po prostu byłoby skuteczne w momencie, kiedy byśmy zamknęli większość tego typu instytucji, a nie tylko wybiórczo jakieś. W tym zakresie należało zostawić siłownie z ograniczeniami co do liczby osób ćwiczących i koniec. To by było wtedy skuteczne, na tym etapie takie wybiórcze traktowanie... Owszem, skoro zamknęliśmy część, a części nie, to wiadomo, że z tej części zamkniętej ludzie nie będą się infekowali, więc jest to jakiś zysk dla zdrowia publicznego, ale tutaj zdrowy rozsądek zaczyna się burzyć jednak mimo wszystko.

**Zamknięcie gastronomii?**

Tak, wydaje mi się, że to też jest skuteczne czy dobre z punktu widzenia gospodarczego, to jest osobna kwestia. Ale miałem też możliwość obserwowania, jak to wygląda w restauracjach, mimo zachowania dystansu, faktycznie wchodziliśmy w maseczkach, siadaliśmy i te maseczki były zdejmowane, biegali między nami klienci... Tzn. ja tutaj mówię z perspektywy takiej ogólnej, nie to, że byłem i użytkowałem restauracji, ale biegający kelnerzy między stolikami, którzy też zwiększają ruch powietrza. Można to było też próbować jakoś inaczej rozwiązać, że więcej jednak stolików na dworze, ograniczenie stolików wewnątrz, jakieś próby zadziałania w tym kierunku. Był to jakiś czynnik ryzyka, więc to zamknięcie z punktu widzenia zdrowia jest właściwe i wskazane, mimo że smutny los gospodarczy czeka pewnie przedsiębiorców, to z punktu widzenia zdrowia publicznego jestem na tak.

**Eventy?**

Zdecydowanie pójście w dobrym kierunku. Trzeba pamiętać o tym, że nawet te koncerty, które odbywały się na wolnym powietrzu, one najczęściej bardzo zagęszczały uczestników, gdzie były na salach zamkniętych, też była nieco wyższa temperatura, słaba wentylacja, więc też większa wilgotność, większa temperatura, ludzie się pocili, musieli obniżać temperaturę ciała poprzez pocenie się i też zwiększoną wydajność układu wydechowego, czyli częstsze, głębsze oddychanie. Nie ma co ukrywać, to jest siedlisko samego zła.

**Zamknięte cmentarze?**

Patrząc sobie jednostkowo na zamknięcie na ten czas, chyba zbyt wiele nie zmieniał tutaj, bo wszyscy się nagle rzucili w piątek po 15 i były straszne tłumy na cmentarzach, zresztą całkiem sporo ludzi było też wcześniej i teraz też będzie całkiem sporo. Może jedynie tylko to było dobre, że ludzie nie przemieszczali się znacząco po kraju, tylko raczej przebywali stacjonarnie. Wiadomo, że to są takie okazje, że teraz w tygodniu każdy pójdzie sobie indywidualnie na ten cmentarz, a tak wszyscy by się rodzinnie rzucali sobie na szyję, staliby dwudziestkami przy jednym grobie. Myślę, że to też jest pójście w dobrym kierunku, ale jeśli już, to trzeba było zamknąć być może te przestrzenie na dłuższy okres, ale to by znowuż wtedy pociągnęło za sobą konsekwencje zamknięcia parków i w ogóle wszystkiego, no więc naprawdę trudno jest mi to ocenić. Ja zachowam postawę neutralną względem zamknięcia cmentarzy, nie jestem w stanie tego rozsądzić, czy ja się z tym zgadzam czy nie. Z punktu widzenia gospodarczego znowuż działanie to zaowocowało nam chryzantemami we wszystkich przypadkowych miejscach miasta, które były ustawiane przez ludzi, ale co do wpływu na zdrowie nie wiem, nie potrafię tego ocenić.

**Zmiana w organizacji szkół?**

To chyba trzeba w szerszym kontekście rozpatrzeć. Dobre jest to, że dzieci młodsze chodzą, z punktu widzenia znowuż w bardzo szerokim kontekście, w modelu biopsychospołecznym, bo rodzice mniejszych klas mogą w miarę normalnie pracować, a nie zajmować się dziećmi. Wiadomo też, że dziecko nie nauczy się pisać przez komputer, przez nauczanie zdalne, to musi być praca indywidualna na miejscu, ten kontakt bezpośredni. Jeśli chodzi o młodzież starszą, ok, tutaj chyba było więcej jednak zachorowań, więcej dało się zauważyć tych zachorowań w przypadku szkół z dziećmi starszymi czy z młodzieżą aniżeli w nauczaniu początkowym. Zresztą moja szkoła funkcjonuje całkiem nieźle i nie ma tam żadnych przypadków, a po liceach się jednak zdarzały. Więc biorąc pod uwagę samo zadziałanie w tym kierunku, to zamknięcie tych starszych szkół na krótki okres, bo wiadomo, że nauczanie online to nie jest żadne nauczanie, to jest bzdura, my niczego nie uczymy, ja patrzę po swoich studentach, jak to w praktyce wygląda, że ja muszę się naprosić, żeby ktoś włączył kamerę czy mikrofon i mi odpowiedział, więc być może są zalogowani, ale nie są obecni tak de facto. Albo są dyżurni wyznaczani. Więc pod tym względem jest to słabsze, ale znowuż ograniczenie kontaktów jest dobre. Natomiast rozstrzygamy to dzisiaj w zdecydowanie szerszym kontekście, wszyscy ci, którzy nie chodzą do szkoły, mają na tyle dużo wolnego czasu, że zaczęli sobie traktować protesty również jako rozrywkę. Więc tak czy inaczej do jakichś kontaktów dochodzi. Samo ograniczenie kontaktów w szkołach jest ok, ale kontekst sytuacyjny nam mówi, że może lepiej było ich zostawić, bo wychodzi na jedno, a nie pojawiają się nam jakieś niepokoje uliczne, że tak powiem. Ale oczywiście widocznie była w tym jakaś strategia polityczna i ta decyzja musiała zapaść w tym momencie, ale chyba też nikt nie spodziewał się, że pojawią się aż tak liczne protesty. Wracając do krótkiego podsumowania, samo zamknięcie szkół dla dzieci i młodzieży starszej jest ok, a w tym aktualnym kontekście chyba niewiele zmieniło.

**Pan przestrzega tych ograniczeń?**

Tak, przestrzegam tych ograniczeń. Zastanawiam się, czy ja może coś złamałem kiedyś. Wczoraj wynosiłem na swojej posesji śmieci bez maseczki, ale byłam w obrębie własnego majątku, nieruchomości, więc zakładam, że nic wielkiego się nie wydarzyło.

**Czy teraz też przestrzega pan zasad ze względu na pełnioną funkcję w pracy?**

Tak, zdecydowanie tak, obywatelska przyzwoitość. Mimo świadomości, że środki zabezpieczenia nie są idealne, tak jak powiedziałem, niczego lepszego na ten moment nie wymyśliliśmy. Stosuję się i zamierzam się stosować.

**Czy coś się zmieniło u pana w spojrzeniu na przyczyny pandemii?**

Tak, w zasadzie odciąłem się od źródeł, nie myślę już o tym, co się wydarzyło. To zostało przedyskutowane, na samym początku może to miało jakieś znaczenie, robiło to jakieś wrażenie, na ten moment koncentrujemy się, ja się np. koncentruję na tym, żeby przetrwać w jak najlepszej kondycji, bez większych strat. Nie mam już żadnych refleksji na temat źródeł pandemii. Musztarda po obiedzie.

**Czy obecna sytuacja jest poważna?**

Tak, jest poważna.

**Z czego wynika ta powaga?**

Chociażby z tego, że mieliśmy 193 mld nadwyżki budżetowej, na ten moment mamy 0, czyli nie stać nas na kolejne poważne ograniczenia.

**Czyli chodzi głównie o straty finansowe?**

Tak, bo na ten moment nie doszliśmy jeszcze do ściany w opiece medycznej, więc póki nie będzie konfliktów etycznych: kogo podłączyć, a kogo odłączyć od respiratora, to nie jest jeszcze aż tak bardzo poważnie. Oczywiście nie zazdroszczę tym, którzy chorują na cokolwiek innego aniżeli infekcja, bo w zasadzie możliwe są jedynie teleporady, więc tutaj sytuacja... (zakłócenia) samej pandemii.

**Czy ludzie w pana otoczeniu zachowują się adekwatnie do sytuacji?**

Tak, z nielicznymi wyjątkami tak. W mojej szkole są osoby, które nie wierzą w istnienie koronawirusa i wbrew temu, co wszyscy mówią i sugerują, w dalszym ciągu dumnie chodzą całymi rodzinami, nie, przesadzam, to jest jedna rodzina, która chodzi bez żadnych ograniczeń i środków zabezpieczenia indywidualnego. Są oni też bardzo mocno przekonani, że głęboka wiara i praktyki religijne ustrzegą ich przed zachorowaniem. Ok, to jest ich obszar ideologiczny, ja nie będę rozstrzygał tych zachowań w kategorii grzechu czy nie, bo to nie jest do końca moja sprawa, ale ja zauważam taki fakt, to się dzieje, z tym, że osoby te nie są też, jak widzę, poddawane żadnemu ostracyzmowi, po prostu omija się je szerokim łukiem na chodniku i tyle.

**Czyli jednak ludzie omijają te osoby?**

Tak, ale to jak powiedziałem, to jest z perspektywy moje szkoły podstawowej jedna rodzina.

**Czy dało się zapobiec obecnej sytuacji?**

No pewnie, że się dało. Nie wiem, czy mogę o tym mówić z punktu widzenia pełnionych funkcji, ale myślę, że dobra, można powiedzieć. Jeśli środki od wielu, wielu lat przesuwane byłyby na opiekę medyczną i nawet organizacje działań Głównego Inspektoratu Sanitarnego, Sanepidu i nie byłyby to instytucje, w których brakuje komputerów i w których pracuje 6 ludzi przed okresem emerytalnym, bo Sanepid nigdy nie był do niczego tak de facto potrzebny, nie mielibyśmy teraz tego. Czyli dofinansowanie tych instytucji faktycznie by dzisiaj uprościło sprawę, a na ten moment w Ostrowie Wielkopolskim nie mamy w ogóle Sanepidu, bo 100% ludzi jest chorych. Niskie zatrudnienie powoduje, że dowiadujemy się o kwarantannie już w trakcie jej trwania, tak jak to było w przypadku mojej rodziny, gdzie oni mieli jakąś informację, że mieli kontakt, że dziecko miało kontakt w szkole, poszli na autoizolację i po 5 dniach zadzwoniła pani z Sanepidu i powiedziała im, że oni już od 5 dni są na kwarantannie, ale że nie miał kto zadzwonić, bo brakuje ludzi do pracy, w związku z tym oni już im zaliczają te 5 dni, ale ja mam jeszcze 5 dni wytrwać i to jest oficjalna decyzja administracyjna. Tutaj widać takie zaniedbania w wielu obszarach, ale jakby pandemia się na to nałożyła przypadkowo. Był też taki świetny mem, gdzie dwóch polityków przy stole rozmawia, jeden mówi do drugiego: "Wiesz, przez ten ostatni czas zajmowaliśmy się nie tymi meblami, co trzeba, bo zajmowaliśmy się stołkami, a nie łóżkami". I to też pokazuje troszeczkę, w którym kierunku poszliśmy. Patrząc szerzej, może niekoniecznie trzeba było inwestować w społeczeństwo w znaczeniu takiego socjalistycznego rozdawnictwa w postaci 500+, dodatkowych emerytur dla osób już pobierających te świadczenia, tylko trzeba było te środki pozostawić w budżecie, po prostu je zostawić w budżecie na czarną godzinę, która nie wiadomo co wtedy mogła oznaczać, ale to niestety teraz trochę pokutuje. I też brak systemowego zabezpieczenia opieki medycznej, w znaczeniu pozwolenie lekarzom na odjeżdżanie do innych krajów, gdzie lepiej zarabiają. Ja nie mówię, żeby ich tutaj blokować instytucjonalnie, konstytucyjnie czy przywiązywać ich na siłę jakimiś kontraktami za marne pieniądze, ale zakładam, że jeśli lekarz wyjeżdża do innego kraju, bo dostaje lepsze pieniądze, to znaczy, że w kraju trzeba mu zapłacić jeszcze lepsze i zostawić tych ludzi tutaj, na miejscu. To można było robić, ale to były działania systemowe, które wymagały takiego ogólnego działania od kilku lat. Natomiast my chyba potrzebowaliśmy też trochę oddechu jako społeczeństwo i to w tym okresie wakacyjnym, letnim faktycznie było. Może niekoniecznie trzeba było mówić, że już wygraliśmy z koronawirusem, że on już nie stanowi zagrożenia, tylko wciąż mówić, że oddychamy, musimy trochę odpocząć, ale że druga fala tak czy inaczej będzie, bo wszystko na nią wskazywało. W zasadzie nie było drugiej fali pandemii, tylko cały czas jest ta pierwsza, która się nasila w dalszym ciągu. Trzeba było dać trochę oddechu ludziom, ale trzeba było też myśleć, co by tutaj zrobić, żeby szpitale polowe były już gotowe 3 czy 4 miesiące temu, żebyśmy już mieli mechanizmy na zatrudnienie dodatkowych kadr medycznych czy też, żeby osoby te były zakontraktowane w jakiś tam sposób, że studenci kierunków medycznych... O, to o tym możemy też zaraz porozmawiać. To, co się dzieje na uczelni aktualnie, to też powinno już być zabezpieczone, czyli powinny być np. jakieś praktyki wakacyjne dla studentów wszystkich kierunków okołomedycznych, czyli również kosmetologii, zdrowia publicznego, elektroradiologii itp., żeby im zrobić już przeszkolenie podstawowe medyczne, żeby te osoby mogły w ramach zajęć dydaktycznych czy praktycznych w trakcie roku akademickiego trafić do szpitali w charakterze pomocy medycznej. To się dzieje teraz, ale wojewoda wystąpił do rektora o pełne listy studentów, nie wiem, chyba w zeszłym tygodniu dopiero. A, i wystąpił również wojewoda o listę absolwentów kierunków medycznych z ostatnich 5 lat. Te listy już dawno powinny leżeć u wojewody, z pół roku temu i wojewoda już powinien się zastanawiać, co z nimi robić, jak zagospodarować tych studentów i rozmawiać z rektorami uczelni medycznych, w jaki sposób zaliczyć im tę aktywność pozadydaktyczną na rzecz dydaktyki. To powinno już być wszystko dawno gotowe, przygotowane i powinno być tylko pstryknięcie palcem, wojewoda podpisuje i od jutra już nie mamy zajęć, są studenci przypisani do konkretnych szpitali, są przeszkoleni i wiedzą, co mają robić, wiedzą, jak pracować w charakterze sanitariuszy czy pomocy medycznych, czyli tego personelu medycznego dodatkowego i po prostu mieć już gotowe przypisane miejsca. To się dzieje dopiero teraz, czyli, moim zdaniem, pół roku za późno. To... (zakłócenia) wszystko wcześniej z punktu widzenia systemowego.

**Jakie reakcje wywołują w ludziach decyzje rządu?**

Frustrację chyba może jeszcze nie, oczywiście poza sferą taką etyczno-polityczną, czyli mówię tutaj o zerwaniu kompromisu aborcyjnego, to to doprowadza faktycznie do frustracji, co też widać przynajmniej u części społeczeństwa. Natomiast wydaje mi się, że decyzje, które są, generują raczej nastrój wyczekiwania w związku z tym, co się będzie dalej działo. Ale ja jeszcze nie widzę w szerszym kontekście takim społecznym jakichś niepokojów, owszem, tak jak mówię, teściowie, ale to jest mój przypadek indywidualny, że oni się jakoś szykują na to, że być może to może już być koniec całkowity, tzn., że oni już będą musieli zostać na emeryturach, że już nie będzie żadnej aktywności zawodowej. To to w sensie indywidualnym, ale tak ja nie widzę niczego takiego niepokojącego na ten moment w otoczeniu jeszcze.

**Czy poszukuje pan aktywnie informacji na temat pandemii?**

Raczej mam swoje rytuały. Jednym z punktów dnia jest prześledzenie danych z Ministerstwa Zdrowia, bo od tego też zależy nasza dalsza aktywność i polityka rządowa, jeśli dzisiaj nie przekroczyliśmy 20 000 przypadków, to jest duże prawdopodobieństwo, że jutro nas jeszcze nie zamkną. Czyli to jakby wpływa, więc ok, ja to śledzę, ale nie jestem przykuty do telewizora i mediów w żaden sposób i to też zawsze sugeruję i rodzinie, i studentom na zajęciach, że słuchajcie, musicie żyć normalnie, musicie planować swoje działania, musicie być aktywni, nie wiążcie się z mediami, bo to nie jest zbyt dobre, żeby jedyną treścią, która dociera, jedynym przekazem było tylko to, czy jest taka a nie inna liczba osób zarażonych. To jest informacja do pozyskania i trzeba iść dalej. Od niej też zależy oczywiście dalsze działanie, tzn. wiem, czy muszę po raz kolejny kupić 50 kg ryżu i 100 rolek papieru czy też nie. W tym sensie takim zadaniowym - tak, ale zbyt wiele więcej mnie nie interesuje. Owszem, docierają do mnie takie informacje, jak wczoraj jeden z analityków tak przypadkiem zauważył, że na konferencji premiera Morawieckiego wyniki zakażeń nie sumowały się do 100% i się okazało, że brakuje blisko 20% zakażeń u ludzi młodych. No i to teraz jest taka ciekawostka, ale to też tak z boku, ja aktywnie nie poszukiwałem tej informacji i nie szukam też w żaden sposób sensacji w tym wszystkim, ale faktycznie wygląda na to, że te 20% zakażeń u osób młodych zostało niestety zakamuflowane gdzieś w tych danych. Czyli one zostaną opublikowane za tydzień jako 20% nowych zakażeń u ludzi młodych i pewnie będzie to zrzucone na karb protestów. To są rzeczy, które, jeśli są prawdą, to we mnie uderzają, ale tak jak powiedziałem, nie szukam na siłę takich sensacji, to gdzieś tam mi przypadkiem taka analiza wpadła w ręce, bo staram się normalnie funkcjonować. Media społecznościowe... Logowałem się ostatnio na Facebooka, żeby zobaczyć profil społecznościowy szpitala, w którym składałem przetarg, żeby zobaczyć, co tam jest, ale tak, poza tym w żaden sposób nie śledziłem. Chyba od czerwca, tak jak się spotykaliśmy, nie logowałem się ani razu na mediach społecznościowych. Ja raczej korzystam już teraz tylko i wyłącznie, mam konto na Facebooku, ponieważ jest to droga dystrybucji linków do treningów karate, przynajmniej tak jeszcze było w okresie wakacyjnym, teraz te treningi odbywały się na żywo, ale od listopada mają wrócić online, więc znowuż się będę logował, ale tylko w celu pozyskania transmisji treningu. Nie, nie uczestniczę w tym świecie, wycofałem się z tego już dosyć dawno, wydaje mi się, że to już będzie więcej niż rok. Media społecznościowe jest to dzieło szatana, podtrzymuję swoje zdanie, życie toczy się zupełnie gdzieś indziej. Nawet media społecznościowe są dla mnie narzędziem, z którego korzystam w bardzo, bardzo ograniczonym zakresie, nawet zawodowym.

**Z jakich źródeł czerpie pan informacje, oprócz komunikatów MZ?**

Prawdę mówiąc, jeśli mam możliwość, to śledzę to na żywo w wersji audio-video, czyli oglądam któryś z dostępnych kanałów informacyjnych, trzech podstawowych, nie różnicuję tego, aczkolwiek są takie, które mnie mniej irytują i są takie, które mnie bardziej irytują, bo np. wolę kolor niebieski na pasku TVN24 aniżeli czerwony na pasku TVP Info, bo ten niebieski jest przyjemniejszy w odbiorze. A jeśli nie mam dostępu, to sprawdzam na stronie internetowej Ministerstwa Zdrowia bezpośrednio. W radio, czy kiedykolwiek w radio w samochodzie? Nie, w radio nigdy nie słyszałem, więc albo to jest telewizja, a jeśli nie mam możliwości, to najczęściej na smartfonie sprawdzam, to oznacza, że nie mam po prostu dostępu do mediów.

**Czy można wierzyć docierającym do nas informacjom?**

Z dużym prawdopodobieństwem tak, jest jakiś margines błędu, bo to są statystyki, wiadomo, że one przechodzą przez wiele rąk itd., więc jakiś tam pewnie jest plus minus błąd pomiarów w tych statystykach, ale zakładam, że one są w miarę rzetelne i wiarygodne. Oczywiście wiadomo, że to też można wykorzystywać do celów politycznych, tak jak powiedziałem pani przed chwilą o pewnych obserwacjach, które ktoś tam poczynił, że można również nimi jakoś tam manipulować, jeśli to się zadziało oczywiście. Ale tak, jeśli mamy tyle przypadków, to znaczy, że w tej okolicy mamy tyle przypadków i koniec. Mogą być tam jakieś wartości odstające i jakieś błędy pomiaru. Liczba to jest w miarę twarda dana i tyle.

**Czy zwiększył się pana czas korzystania z mediów?**

Trochę tak, wydaje mi się, że się zwiększył z racji tego, że miałem jednak te 3 tygodnie przerwy w aktywności zawodowej, jakiejś tam autoizolacji czy też nawet chwilowego zwykłego zwolnienia, siłą rzecz, kiedy człowiek nie jest w stanie zrobić wielu rzeczy, bo się gorzej czuje, to zwiększa się zainteresowanie i muzyką, i książką, i telewizją również.

**Czyli to wynikało bardziej z pana sytuacji niż potrzeby informacji?**

No nie, nie mam takiej potrzeby, tym bardziej, że pracuję w instytucji, która zapewnia takie informacje jeszcze przed podaniem jej do wiadomości publicznej. A że nie miałem kontaktów, byłem na zwolnieniu, więc te informacje faktycznie pozyskiwałem w inny sposób.

**Czy myśli pan o tym, jak będzie wyglądała przyszłość po pandemii?**

No tak, pojawiają się takie refleksje o tym, jak to będzie wyglądało. Nie są one może zbyt częste i zbyt głębokie, ale tak, pojawiają się. Na pewno wiemy, że ta pandemia będzie nam towarzyszyła. To jeszcze mogą być miesiące, a sam wirus będzie nam towarzyszył być może do jakiejś kolejnej poważnej mutacji albo jego całkowitego wyginięcia, co jest raczej mało prawdopodobne, więc będziemy go mieli cały czas w świadomości. Natomiast po okresie pandemicznym wiele rzeczy wydaje mi się, że się zmieni, ale słońce będzie świeciło w dalszym ciągu i wciąż będzie w nas wiele radości, optymizmu, szczeniackiego luzu, będzie tak w miarę normalnie, ale w przestrzeni gospodarczej na pewno zwiększy się ranga pracy zdalnej w ogóle, bo jest ona po prostu tańsza. Ludzie są wydajni, nauczymy się w końcu tak pracować, nauczymy się zarządzać własnym czasem w ten sposób, żeby ta praca zdalna i zwiększone przebywanie we własnym domu nie prowadziły do obniżenia nastroju i zaburzeń afektywnych. To widać już po uczelniach, że rektorzy mówią: "O, nauczanie zdalne jest fajne, bo jest 30% tańsze, w związku z tym możemy o 33% obniżyć wynagrodzenia pracownikom, którzy pracują zdalnie, bo dlaczego mają zarabiać więcej, skoro nie robią tego, co kiedyś i w ten sposób ich koszty są tańsze, nie trzeba ogrzewać sali i koszty internetu i prądu są przerzucone na pracowników, więc wow, to jest super". Więc to pewnie zostanie ze względów gospodarczych i ekonomicznych. Mam też nadzieję, że tak jak po zawaleniu się hali wystawowej w Katowicach w trakcie wystawy gołębi, że wtedy się okazało, że pogotowie ratunkowe jest niewydajne i że trzeba stworzyć nowy model i trzeba stworzyć ratownictwo medyczne jako takie i uporządkować to, i trzeba wykształcić ratowników medycznych, którzy będą wyręczali lekarzy i sanitariuszy w tych podstawowych czynnościach medycznych, tak samo ta pandemia wydaje mi się, że docelowo trochę zmieni myślenie na temat medycyny ratunkowej czy medycyny chorób zakaźnych, działań epidemiologicznych, czyli będzie wiadomo, że już w Sanepidzie nie będzie mogło być tak, że będę pracowały 3 panie w wieku przedemerytalnym, które miały do spółki 1 komputer z Windowsem '98, tylko że to wymaga jednak inwestycji i to musi wszystko hulać, i musi być nawet przeinwestowane na wypadek takich działań w przyszłości, nawet jeśli mielibyśmy na tym tracić czy to przeinwestować, to to musi działać po prostu i koniec. Tak wydaje mi się, że na tym poziomie systemowym to na pewno się pozmienia. Wydaje mi się też, że finalnie może być zmiana układu sił politycznych. Na pewno obóz rządzący garstką sił dociągnie ten kraj do końca pandemii, ale pod koniec tej walki tam już nie będzie ani motywacji, ani sił, ani nie będzie chętnych do tego, żeby rządzić dalej i w tę nową rzeczywistość popandemiczną wejdziemy z nowym układem sił. Mam też takie przeczucie. Nie jest to poparte żadnymi twardymi danymi, ale tak może być, więc być może po okresie pandemicznym będzie nami rządził ktoś inny. Czy to pójdzie w stronę radykalizmu z prawej strony czy z lewej strony, czy centrum, czy jakiejś dziwnej opcji prawicowo-lewicowej, nie mam bladego pojęcia, ale może być w tym zakresie zmiana. A tak, poza tym wydaje mi się, że zaczniemy żyć z większą fantazją, tzn., że część z nas przestanie odkładać pewne rzeczy na później, będzie więcej takiego hedonizmu w tym pozytywnym sensie. Tak jak ktoś ostatnio mi napisał: "Wiesz, wyciągnąłem najlepszą zastawę stołową, jaką mamy, bo kiedy będziemy z niej korzystać? Kiedyś? Zostawiać to na kiedyś? To jest zupełnie bez sensu. Zbije się, to się zbije, to się kupi nową, ale po co oszczędzać takie rzeczy". Wydaje mi się, że też w tym kierunku może pójść myślenie ludzi na przyszłość. Część może będzie starała się zaoszczędzić więcej środków finansowych czy bardziej zabezpieczyć na okoliczność takich właśnie sytuacji, a część stwierdzi, że hulaj dusza, piekła nie ma. Mamy wolność, nie wiadomo, jak długo będzie ona nam dana, może trzeba po prostu poszaleć i w końcu polecieć na tę Grenlandię z rodziną, żeby zobaczyć, jak tam jest, aniżeli odwlekać ten wyjazd już kolejny rok, jak to jest w moim przypadku.

**A zmiany w sferze społecznej? Jakaś grupa będzie szczególnie dotknięta?**

Nie wydaje mi się. Obniży się pogłowie na pewno osób starszych, umieralność w tej grupie z przyczyn naturalnych czy medycznych zawsze była wysoka, a teraz będzie jeszcze większa, bo mamy covid i też mamy obniżoną wydajność opieki medycznej, więc ta śmiertelność będzie większa, więc na pewno będzie ich mniej, fizycznie tych osób starszych będzie mniej. Prezes ZUS-u już pewnie odbija kolejnego szampana na ten moment, bo nie będzie musiał wypłacać środków. Wydaje mi się, że będziemy musieli nadganiać też z zakresem wiedzy ogólnej, kompetencji, umiejętności w związku z brakiem zajęć dydaktycznych w tradycyjnej formie. Może trzeba będzie zrobić tak, żeby powiedzieć, że ten rok akademicki i rok szkolny w ogóle się nie odbył i że wszyscy pozostają na tym samym poziomie, wciąż jesteśmy na drugim roku studiów, wciąż jesteśmy w trzeciej klasie szkoły podstawowej, i zacząć to wszystko od początku. Być może to też będzie taka grupa, która wchodzi w życiu, uczy się, kształtuje i kształci się, że to oni mogą być pokrzywdzeni, bo też może będą wiedzieć i umieć trochę mniej. Czy ktoś jeszcze... Gospodarczo oczywiście mali przedsiębiorcy, gastronomia, wiadomo, że to też będą grupy, które będą starały się odbić po tym okresie pandemicznym, co pewnie będzie prowadziło do nieco wyższych cen usług. Na pewno zmieni się też układ sił takich zawodowych, znaczy bardzo spora część ludzi pewnie będzie musiała zmienić pracę, bo być może dotychczasowe miejsca pracy nie będą już dostępne albo po prostu nie będą istniały. Nie wiem, jak będą wyglądały ruchy migracyjne po okresie pandemicznym, czy osoby z krajów wschodniej Europy w dalszym ciągu będą zainteresowane pracą u nas czy też już nie, zobaczymy. To są takie pytania, które się pojawiają, ale które jakby nie przysłaniają chyba w moim przypadku takiego bardzo słonecznego i pozytywnego podejścia do okresu popandemicznego.

**Czy pojawiają się u pana obawy w związku z przyszłością?**

Na pewno będziemy mieli kryzys gospodarczy spowodowany chociażby obniżeniem zasobów pieniężnych w kraju. Jednak państwo musi zainwestować w pandemię i to będzie na pewno jakiś tam negatyw.

**Po czym poznamy, że pandemia się skończyła?**

Po zniesieniu wszystkich obostrzeń. One nigdy nie zostaną zniesione do końca, ale jeśli zostaną zniesione z przyczyn oczywiście racjonalnych, opartych na dowodach, jeśli zostaną ściągnięte w 75%, to będzie to oznaczało, że to już mamy okres popandemiczny. Ja nie mówię tutaj o tych krótkich okresach, bo być może będzie znowuż okres wytchnienia letniego, coś takiego, być może część tych obostrzeń będzie zniesiona, ale to nie wchodzi w zakres mojego myślenia. Jeśli na stałe zostaniemy z tymi 25% obostrzeń, to będzie oznaczało, że koronawirus jest, ale jest po pandemii, możemy żyć w miarę normalnie.

**Jak w tym roku będzie u pana wyglądało Boże Narodzenie?**

Wie pani co, ja nie wiem, jak będzie wyglądała moja rzeczywistość po 9.11., jak ruszy przetarg w szpitalu, więc dla mnie myślenie o Bożym Narodzeniu to jest jak myślenie o XXII wieku. Bez znaczenia. Ostatnie Boże Narodzenie było całkiem ok, jeśli będzie kolejne takie, ja nie widzę przeciwwskazań. Gwiazdor i Mikołaj tak czy inaczej nie przyjdą do mnie, więc bez znaczenia.

**Czyli bardziej dla syna będzie trzeba zaplanować atrakcje?**

Tak, dokładnie.

**Czy chciałby pan coś jeszcze dodać?**

Jeszcze przeszła mi przez myśl jedna zmiana, ale to już taka zmiana zawodowa. Po długim okresie oczekiwania moja żona, która pracuje w sądownictwie, przeszła pozytywnie wszystkie etapy rozwoju zawodowego i Krajowa Rada Sądownictwa zawnioskowała do prezydenta RP o nominację sędziowską dla mojej żony, więc to jest też taka zmiana na duży plus, ale też wynikała z wielu działań podjętych przez ostatnie kilkanaście lat i tutaj też pewne wątpliwości i obawy, czy prezydent będzie miał czas i ochotę na tę nominację, bo ma inne sprawy na głowie. Ma czas do końca czerwca, więc zobaczymy, ale lepiej by było już mieć to potwierdzenie finalne wcześniej, aniżeli czekać, nie wiem, te 8 miesięcy, bo jest okres pandemii. To chyba tylko tyle, bo o tym też zapomniałem powiedzieć, co się zmieniło u nas, że żona jest z jednej strony już prawie sędzią, ale z drugiej strony jeszcze tym sędzią faktycznie nie jest w sądownictwie administracyjnym. Jest po zakończonych wszystkich etapach proceduralnych, ale brakuje tej wisienki na torcie, czyli tej imprezy u prezydenta w Pałacu Prezydenckim, czyli wręczanie tych tych, uściski, całuski, pamiątkowe zdjęcia, ale bez tego ani rusz. Czy to się wydarzy? No nie wiem, mam wrażenie, że z tym będziemy też czekać raczej do okresu letniego, żeby to ryzyko było mniejsze, czyli gdzieś tam jest strata kilkumiesięczna, też finansowa w tym wszystkim, nie ma co ukrywać. A tak poza tym? No nie wiem, zobaczymy, będziemy obserwowali rzeczywistość. Ja też z zainteresowanie wyczekuję, jakie będę decyzje władz uczelnianych i wojewódzkich, czy tylko studenci trafią do szpitali czy my pójdziemy razem z nimi do tych szpitali z racji tego, że nie będziemy prowadzili zajęć dydaktycznych, jaka będzie nasza ranga, bo niby mamy wykształcenie wyższe, spora część medyczne, pielęgniarskie, a ci, którzy nie mają lekarskiego, to tak jak w moim przypadku od 20 lat praca na uczelni medycznej być może będzie powodowała, że zostanę gdzieś tam przydzielony, chociażby do opieki nad studentami pracującymi w szpitalu, nie pod kątem merytorycznym, tylko bardziej organizacyjnym. Nie wiem, siedzę i czekam, zobaczymy, jak to będzie wyglądało. Tak jak pani mówiłem, dzisiaj nas jeszcze nie zamkną, bo liczba nie przekroczyła 20 000 zakażeń, więc kolejny dzień do realizacji zajęć. Zaraz zaczynam obrony prac magisterskich online, więc trzeba po prostu działać z optymizmem i planować, realizować to życie w takim zakresie, w jakim się da. To taki mój postulat ogólny rzucany w przestrzeń.
