# Supplementary material for: The challenges arising from the COVID-19 pandemic and the way people deal with them. A qualitative longitudinal study
Source: PLoS One. 2021 Oct 11;16(10):e0258133. doi: 10.1371/journal.pone.0258133 (PMC8504766; doi:10.1371/journal.pone.0258133)
Supplement: S1 Dataset — (ZIP) [file pone.0258133.s003.zip › Transcriptions/stage 6/18.6_F_48_couple, with children.docx]

**18.6_F_48_couple with children**

**Co się działo od czerwca, w czasie wakacji? Jak spędziłaś ten czas?**

Spokojnie, na wyjeździe, na działce, na którą i tak jeździmy co roku. Tylko był to dłuższy wyjazd. Nie były jakieś tam inne miejsca. A ja lubię to miejsce, więc mi to w ogóle nie było... Wręcz było mi to na rękę. I mówię, no praca, ten wypoczynek. Tak że tak spokojnie, stabilnie.

**Jakieś ważne, przełomowe momenty z tego okresu do teraz przychodzą ci do głowy?**

Ważne, przełomowe momenty. Znaczy tak ogólnie, tak?

**Takie wyznaczniki czasu z tego okresu.**

Ten okres wakacyjny, lipiec, sierpień to taki spokojny, spokojny, stabilny okres. Wrzesień właściwie też. Natomiast no tam ze zdrowiem we wrześniu, zaczęło mi szwankować i to jakby, to już taki nowy etap.

**Czyli do tego zdrowotnego momentu wszystko było spokojnie. Masz wrażenie, że coś zmieniło się w twoim życiu w ciągu ostatnich trzech miesięcy?**

Właściwie nie. Od tego momentu jak się widziałyśmy, to nie. Natomiast zmieniło się powiedzmy od tego marca i to tak sobie trwa.

**A cokolwiek w twoim życiu wróciło do stanu sprzed pandemii?**

Takie codzienne sprawy, domowe, praca, dom. To, co robię, jak spędzam… Znaczy nie, czas trochę inaczej spędzam. Ale jakby jest taka część, którą zawsze robiłam i która pozostała. Więc mówię, dom, praca, że chodzę, wracam. Wróciłam na zajęcia też, znaczy teraz z powrotem nie chodzę. Ale był taki moment, że właśnie powrót, ten wrzesień początek, koniec sierpnia to był powrót na zajęcia, takie joga, jakieś ćwiczenia. To był taki powrót. I teraz znowu się to zablokowało. Takie mam wrażenie, że tak jakbym cofnęła się do marca.

**No właśnie, czy to jest tak, jak byś cofnęła się do marca, czy jednak jest jakaś różnica?**

Ja podchodzę do Covidu i mój stosunek do Covidu takiego ogólnie, jako że jest, że jest to coś niebezpiecznego, że istnieje i jest to coś nowego, uczymy się tego, to jest niezmienne. Było tak właściwie do tego momentu teraz, zmieniło się o tyle, że ja się zaczęłam go bać. Mówiłam dobrze, no choroba jak każda inna, że trzeba ją leczyć, że to, że jest niebezpieczna. Natomiast w tym momencie, kiedy ze zdrowiem nie za bardzo, to ja się go zaczęłam bać. Tak faktycznie poczułam taki lęk… No, lęk.

**Jak zaczęłyśmy się spotykać, to mówiłaś o lęku i mówiłaś, że się przebierasz po każdym powrocie do domu...**

Tak. Tylko, że to jest inny lęk.

**Na czym polega ta inność?**

Tam to był powiedzmy sam lęk, a tu jest taki lęk ze strachem. Zdecydowanie bardziej się boję.

**Masz wrażenie, że to jest bardziej namacalne dla ciebie w tej chwili i jest bliżej, to o to chodzi?**

Tak. I jakby może mi faktycznie przeszkodzić w leczeniu. Że Covid jest bardziej niebezpieczny dla mnie teraz od mojej choroby.

**Czyli to jest kwestia twojego zdrowia. A zdrowia twoich najbliższych też?**

Teraz jest mojego. No i zaczynam to odczuwać, te moje obawy, ten lęk jakby realnie zaczynam odczuwać. Bo powiedzmy już mam dwie wizyty odwołane, ponieważ lekarze są na zwolnieniach. I jakby to mi się przesuwa (niezrozumiałe) jako choroby, tylko boję się, że Covid całkiem zamknie mi drogę do leczenia.

**Masz jakieś sposoby, żeby sobie radzić z tym strachem, tym lękiem?**

Staram się ograniczać kontakty. Zrezygnowałam z uczestniczenia w marszach. Chociaż jak się natknęłam na niego w mojej miejscowości, to bokiem, ale przeszłam, bo… Nie mogłam, po prostu szłam bokiem, gdzieś chodnikiem przy murze, ale szłam. Tak, żeby mieć trochę takiego dystansu, nie wchodzić w tłum. I nie wiem, do dużych sklepów i tak nie jeździłam, ale powiedzmy wcześniej nie miałam takiej obawy, że jak muszę wstąpić, to wstąpię. No teraz staram się nie.

**Ale chodzi mi o to, że jak cię dopada taki niepokój co z twoim zdrowiem, z leczeniem, to czy masz jakiś taki sposób swój, który ci jakoś pomaga, neutralizuje ten lęk. Co robisz wtedy?**

Albo zaczynam sprzątać. Ale już wysprzątałam to, co miałam wysprzątać. Biorę książkę, włączam sobie jakiś serial, film. Staram się zająć czymś innym. No książka, jakiś film, nie wiem, program rozrywkowy. Żeby zająć myśli zupełnie czymś innym.

**A zmienił ci się typ książek, po które sięgasz albo typ filmów, które oglądasz?**

Nie. Aby to był inny temat. Abym skupiła się właśnie na… Bardziej na przykład też zadzwonię do koleżanki, żeby z kimś porozmawiać, z jakąś znajomą. I wtedy pomaga.

**Czy gdyby nie dotyczyło to twojego stanu zdrowia i tego opóźniania leczenia, to czułabyś się tak samo, jak czułaś się w marcu? Czy jest coś, co i tak stanowiłoby różnicę?**

Myślę, że byłoby podobnie jak w marcu. Ponieważ wtedy też nie szarżowałam tak, z taką pokorą podchodziłam do tego, że jest… Nie na granicy, że… Zresztą to tak jak było, że zaczęłam nosić maseczkę, więc nie było to dla mnie jakimś problemem. Więc ja od początku miałam świadomość, że jest to coś niebezpiecznego. Patrząc, że gdzieś to jest daleko jak we Włoszech, no nie do końca realne, ale jest. Przyjmowałam to, że może to być u nas w większej skali. Więc myślę, że to by było na tym samym poziomie. Ale jest to nieznane.

**Nadal jest to nieznane. A jak obserwujesz swoje otoczenie, to jak ono funkcjonuje? Czy to też jest tak jak w marcu, czy jest jakoś inaczej?**

Jest jakoś inaczej. Jest większy lęk na pewno. Jedna koleżanka bardziej się boi o dzieci, o siebie. Ona ewidentnie więcej o tym mówi, większy jest niepokój. Druga troszeczkę bardziej tak zaczęła. Bo ta druga była z kolei tak nastawiona, że no dobrze, no jest, jest, no co tam, przecież nawet, jak ktoś zachoruje… Na zasadzie, no nie wiem, jak umrę, to umrę, tak bardziej lekkomyślnie. Teraz troszeczkę spokorniała. Ale tak troszeczkę. I dobrze jej z tym. Rodzice bez zmian. Znaczy też wrócili do tego marca, że jednak trzeba się znowu zamknąć, trzeba uważać, trzeba nie chodzić. Tu się nie zmieniło. Troszeczkę tak poluzowaliśmy spotkania towarzyskie, ale to na zasadzie takiej, że właśnie jak się spotykamy, to w czwórkę a nie w dziesiątkę. Jak było nas 6 osób i koleżanka miała dylemat, bo mieliśmy się w 6 osób spotkać. I najpierw to przedyskutowaliśmy. Na zasadzie, czy mi to nie przeszkadza, jak ona ma się zachować. Już bardziej tak do tego się podchodzi, nie takie spontanicznie, dobrze, wpadniemy, pobawimy się. Tylko jednak z taką świadomością, że jakieś zagrożenie czyha. Każdy jest ostrożny, każdy jakoś tam się zabezpiecza, ale nie ma tej gwarancji. Więc inaczej wygląda nawet takie planowanie spotkania.

**Ale jak się spotykacie w tej chwili ze znajomymi, to siedzicie w maskach czy w przyłbicach?**

Nie.

**A odległości się zwiększyły?**

Troszkę tak. Tak, bo nawet rzeczywiście u tej koleżanki inaczej był ustawiony stół a wcześniej siedzieliśmy troszeczkę w innej konfiguracji. No nie ma te półtora metra, no nie, jest może z półtora, bo tak siedzieliśmy nie stykając się.

**Rozumiem, że wobec tego nie ma ściskania na powitanie, tańców, itd. raczej też nie?**

Nie.

**Raczej wychodzicie do ludzi się spotkać czy ludzie do was?**

(śmiech) Teraz jest tego tak niewiele, że bardziej chyba wychodziliśmy. Faktycznie, chyba więcej wychodziliśmy. U nas jakieś takie pojedyncze. Ale mówię, tego jest tak mało. Aż naprawdę my się, nie wiem, po miesiącu widziałyśmy.

**A jak szłaś ostatnio na to spotkanie, to miałaś taką pokusę, żeby się wykręcić Covidem, żeby nie pójść, bo się boisz czy nie?**

Nie. Bardziej byłam zmęczona i takie miałam wahania, czy powinnam odpocząć, bo jestem zmęczona, czy powinnam iść, że jednak jakaś rozrywka i zajęcie myśli czymś innym będzie dla mnie lepsze. I wybrałam to, że jednak jak się czymś zajmę i się pośmieję z głupot to zdecydowanie lepiej. Ale taki miałam moment, co mam wybrać, ale bardziej pod takim kątem, jak ja się czuję, pomijając Covid.

**I rzeczywiście lepiej się czułaś jak wyszłaś?**

Tak.

**A spotkania z rodziną też są rzadsze czy nie?**

Rzadsze są. Rzadsze. Nie ma takich obiadków niedzielnych. Na chwilę posiedzimy, czy rodzice jak przyjeżdżają do nas, to powiedzmy, posiedzimy sobie, jakąś kawę wypijemy. A jakichś takich w większym gronie rodzinnym to nie.

**A z rodziną przytulacie się na przywitanie czy też nie?**

Nie.

**Jak się z tym czujesz? Bo rozumiem, że przed marcem tak nie było.**

No nie, nie. Jak się z tym czuję? Ja się chyba do tego przyzwyczaiłam. Po prostu teraz działamy inaczej. Trzeba się cieszyć tym, że się widujemy, że w ogóle jest ten kontakt. A że nie ma takiej… Ja sobie tak myślałam, że… Bo tam znajomi rozpaczali w wakacje, że nie można wyjechać, jakiś tam był bunt, że pojedziemy, a co to, może samochodem. Ojej, bo jakieś atrakcje, bo tanio. Nawet mąż już miał taki kryzys, że może jednak faktycznie gdzieś polecieć do jakiegoś ciepłego miejsca. Ja mówię, no ale utkniesz gdzieś. To nawet nie chodzi o to, że sama choroba, ale utkniesz gdzieś, w obcym kraju, no nie wiem, kwarantanna tam, tutaj, nie wiadomo. Czy nam jest to potrzebne. Czy naprawdę nie da się wytrzymać. No ja z tym nie miałam problemu. Po prostu tak jest, tak to przyjmuję. I, bo tutaj nawet ostatnio wspominaliśmy, że normalnie o tej porze byśmy planowali sylwestra. Więc nie ma tego. Tak sobie myślę, że… Ja to się w ogóle cieszę, że były takie lata, gdzie mogłam jeździć, poznawać. Że ja co roku chodziłam na sylwestra od tak naprawdę nastolatki. I nigdy go nie spędziłam w domu. I nie mam takiego żalu, że teraz tego nie będzie. Tylko bardziej to traktuję tak, że jak fajnie, że zawsze chodziłam, że zawsze miałam czas, zawsze miałam siły. Że tak korzystałam, że właściwie teraz, jak tego nie będzie, no to nie jest taka strata, że ojejku, że przecież ja w tamtym roku nie byłam i teraz znowu nie będę. Nie. Cieszę się z tamtych podejmowanych decyzji, że korzystałam to, co miałam zaoferowane. A teraz jest inaczej.

**A praca? Jak jest w pracy? Nadal jesteś i w sklepie, i w tym kosmetycznym biznesie?**

Nie. Wróciłam do swojej, do kosmetyki. W sklepie jestem tak sporadycznie, żeby tak po prostu pobyć, takie sprawy już bardziej papierkowe, faktury, coś porozmawiać, ktoś coś potrzebuje. Natomiast nie wiem jak, bo teraz teściowa trochę się wycofała. Chociaż ona nie musi być tam aż tyle. Ona sobie chodzi rano. Bo ma taki swój rytuał, że chodzi rano, musi tam swoim okiem tam popatrzeć. I to jej wystarcza. I potem tak naprawdę nie musi być, bo też nie ma takiej potrzeby, bo nie ma takiego ruchu i nawału pracy jak wtedy było. Już to jest wszystko spokojniejsze. Natomiast w swojej pracy w kosmetyce jest inaczej. Nie wróciła ta ilość klientek. W ogóle też rytm dnia inaczej wygląda. Kiedyś tak naprawdę, dziś jest piątek, to nie było możliwości, żebym ja była o 17 w domu. To nie było takiej opcji. Generalnie większość to były wieczory. Czwartek, piątek to był cały dzień do późnego wieczora. 21-22 wracałam do domu. Teraz tego nie ma.

**Nie ma imprez, na które się kobiety szykują, jak rozumiem.**

Te panie, które pracują zdalnie, to przychodzą w ciągu dnia. Bo biorą laptop, dobrze, nie mam teraz jakiegoś spotkania, konferencji, to mogę sobie posiedzieć godzinę. Mieszkają blisko, więc sobie w ciągu dnia. Więc tak naprawdę to też jest plus, że ja jestem wieczorami w domu. To jest ta różnica. Natomiast właśnie, nie ma tych imprez, nie ma wesel, nie ma jakichś wyjazdów, integracji, chrzcin, komunii. Imienin, właśnie też ludzie tak towarzysko nie wychodzą do restauracji. I są stałe klientki, które zawsze chodziły, bo miały taką potrzeby, robią to dla siebie. Natomiast nie ma tych, które rzeczywiście… Bo tak, znowu dzieci w szkole, tak naprawdę część straciła pracę albo mają zmniejszone, więc to jest przyjemność taka ekstra.

**Czyli finanse?**

To też. Ja tak na początku się śmiałam, bo to też było tak, że no tak, bo nie jeździmy, nie chodzimy do pracy, pracujemy zdalnie. I ja to tak się śmiałam, mówię, jak to pokazuje, że kobiety, dla siebie, dla męża nie zrobi, tylko dla koleżanki.

**Rozumiem, że praca zdalna obniżyła popyt i to nie wróciło. A czy jakiś zabieg jest szczególnie mało oblegany w tej chwili?**

Znaczy ja mam manicure, pedicure, podologia. Więc to są takie stałe zabiegi. Natomiast u koleżanki ewidentnie widać, że nie ma rzęs, doczepiania, takie zabiegi droższe, ekstra. Takie podstawowe zostały. Jakieś problemy z cerą, czyszczenie. Ale to naprawdę tak minimalnie.

**Czyli tego na pokaz już nie trzeba.**

Tak, tak, tak. Tylko to, co muszę już tak dla siebie, albo te brwi to widać, to zrobię. Natomiast już tam, czy skóra jest nawilżona czy nie, to już nie do końca… Znaczy to też, tak jak obserwuję, to się tak wśród kobiet nakręca. Bo jak się rozmawia i tak... Zresztą sama nieraz tak mam, że o czymś nie myślę, ale posłucham i o kurczę, a może ja też bym sobie tak zrobiła? To jest tak, że nie tylko swoja inicjatywa, ale nieraz tak stadnie idzie. Że koleżanka przedłużyła paznokcie, to ja też sobie przedłużę. Koleżanka zrobiła kolczyk w uchu, to ja też sobie zrobię. No a tego nie ma.

**To prawda, nie ma takiego indukowania się wzajemnego.**

Rzeczywiście fryzjer pozostał. I teraz jak zaczęły się te obostrzenia, jak zaczęła się zwiększać ilość chorych, to masowo rzeczywiście telefony się pojawiły, że właśnie henna no i fryzjer. Fryzjer koniecznie, żeby przygotować się, że teraz już nie damy się tak zamknąć niechlujnie w domu, tylko będziemy elegancko siedzieć.

**Pokażę ci te same obrazki, które już ci pokazywałam (emocje – zdjęcia).**

Ten okres wakacji no to jedynie z tych, to jeśli bym miała coś dopasować, to ta 13. Jest taka ciepła, taka letnia bardzo.

**Jakie to są uczucia, jakie to są stany, w których byłaś?**

To taka błogość, lekkość. To był w ogóle oddech, bo to na wolnej przestrzeni, na powietrzu, w ogóle nieograniczone żadnymi właśnie hotelami, pokojami.

**Błogość, przestrzeń, swoboda, tak?**

Dokładnie. Tak sielsko, anielsko, nic dodać, nic ująć.

**Jeszcze coś do tego czasu wakacyjnego czy tylko ta 13?**

Tylko, bo tutaj właściwie… Trochę bym 1 dorzuciła, bo takiej ilości osób, my mamy działkę na Mazurach i mamy ją od 25-26 lat. I takiej ilości osób, to ja nie widziałam. W okolicy, na działkach, nie widziałam przez tyle lat. Były różne okresy, było bardziej gwarno, mniej gwarno. Ale tak jak było w tym roku, no to nie było przez tyle lat. I mówię, samochodów zobaczyłam taki korek niemalże jak na Alejach Jerozolimskich.

**A ten moment zachorowania? Czy zdiagnozowania?**

No to ta 5 jest jakaś taka najczarniejsza chyba. Chociaż nie, może aż tak czarno nie jestem… Właściwie 10.

**I co to są za stany? Jakie uczucia?**

Samo to, że jest to czarno-biała grafika, więc taki jakby kontrast dla tej sielskiej, anielskiej 13. Robi się mroczno tak po prostu. To są takie… Uczucie trochę szoku, trochę niedowierzania. I takiej niewiadomej, bo właściwie te drzewa i to, co pomiędzy, to tworzą taką pustkę, taką niewiadomą. Bo tu nie ma takiej typowej… No tu jest burza, 9. Ale ta 9 z tą 10 mi się tak najbardziej. Bo jakby taki strzelił piorun i się taka wielka pustka w 10 zrobiła. Tak, faktycznie, od 9 bym zaczęła, że nagle w ten spokojny stabilny w miarę uderza ten piorun. I tutaj ta 10 to ten taki…

**Taki grom z jasnego nieba?**

Tak. Ten kolor z tej 10, to takie szaro-czarne.

**Ale nie takie czarne jak 5.**

Nie.

**A teraz? Jak myślisz o tym, jak się czujesz teraz?**

Ale też do obrazka mam się odnieść?

**Tak. Chyba, że nie ma pasującego obrazka.**

Ta 14 mi pasuje. Tak, 14 zdecydowanie. Dlatego, że ten Covid tak naprawdę związał mi ręce. I jakby on mnie blokuje, że nie mam tej przestrzeni, takiej swobody w działaniu. Że to jest ta blokada, która uniemożliwia mi dalsze leczenie i muszę się z tą pętlą mierzyć. Tak, to jest taka blokada. A tu jeszcze bym, nie ma takiego, jak mają więźniowie kulę u nogi. Iść do przodu, a mnie po prostu to trzyma i tą kulą tak naprawdę jest ten Covid.

**Czyli coś mocniejszego niż ta 2 z gumą do żucia.**

Ta 2 to jest w ogóle nic.

**Wymyśliłaś sobie jeszcze jakieś czynności, sposoby, które by ci jakoś…**

Spacer. Zdecydowanie takie wyjście na powietrze. Chociaż też to mi dobrze robi na głowę, mi się wietrzy głowa i super. Bo sobie muzykę i z kijkami, czy nawet tak z mężem powoli. No, z nim to muszę powoli chodzić. Wolę takie bardziej energiczne, bo wtedy lepiej mi się spala stres. (niezrozumiałe) też na powietrzu to jest fajne. Natomiast mi się też tak przyblokowało, żebym się na przykład nie przeziębiła. I tu mam takie właśnie, że kurczę, no poszłabym. Ale włącza mi się taki instynkt samoobronny, żeby się, właśnie, czegoś nie zrobić tak, żeby sobie nie zaszkodzić, jakby z taką ostrożnością. Ja nigdy taka nie byłam. Ale no dobra, nigdy też nie byłam w takiej sytuacji.

**A na przykład zaczęłaś wcześniej nosić czapkę niż zwykle byś nosiła, tego typu rzeczy też?**

No, może aż tak nie, ale powiedzmy, nie wiem… Patrzę, jaka jest pogoda. Żeby tak już całkiem spontanicznie nie wyjść. Że może jednak dopnę trochę tę kurtkę i założę sweterek. Tak się bardziej zaopiekowałam sobą.

**A jakieś suplementy, kiszonki? Bo są różne opowieści o tym, co się powinno robić.**

To znaczy jakby to już nie. Ja witamina C, a właściwie witaminę C to dopiero teraz i tak z takim na bakier. Ale na przykład to już wcześniej zaczęłam. Piję wodę z cytryną. Witaminę D3 i jakiś tam cynk, selen. Ale to biorę od dłuższego czasu. I jakby mam w głowie, że ta witamina D3 to jest to, co nam daje odporność. I tak sobie powiedziałam, no co mogę więcej zrobić? No mogę zadbać w ten sposób, że te swoje witaminy biorę, które brałam. I pilnuję, żeby ta witamina D3 była w moim składzie. Ale była od zawsze. Bo ja bardziej przypominam synowi, córce, mężowi, że pamiętajcie, żeby tą witaminę D3 sobie brać. Nie wiem, no kiszone buraki, ja to od czasu do czasu lubię, ale nie mam takiego zafiksowania, że muszę codziennie szklankę. Jak mi się zachce, jak mi się przypomni, to sobie pojem kiszonych ogórków. Ale bez takiego, że to jest taki mój podstawowy element. No nie.

**A jak się ma twoja obawa przed możliwością kontynuacji leczenia do obawy przed samym koronawirusem?**

Tak naprawdę to bardziej boję się koronawirusa. Bo powiedzmy to, że mi spowolni leczenie, to jest raz. Ale stres, który mam, też obniża odporność. I zdaję sobie sprawę, że w momencie, kiedy zachoruję, ja nie muszę koniecznie przejść, tak jak zawsze przechodziłam jakieś lekkie przeziębienie. Wtedy mogę być narażona na bardziej mocne objawy. Że właśnie nie ma tej pomocy, dostępu do lekarzy pierwszego kontaktu. Czy chociażby tych łóżek, respiratorów i co tam by było potrzebne. I to mnie tak właśnie ściska. No, bo jakby Covid spowalnia moje leczenie. Ale tu może okazać się, że to jest bardziej groźne niż ta moja choroba. Stąd mówię, dostałam jakiejś takiej… To taki lęk może. No nie wiem, czy z paniką. Ale ja nigdy aż tak nie reagowałam na jakieś takie trudne sytuacje i chorobowe czy w rodzinie czy. I właśnie mówię, to nie do końca dotyczy, że to jestem ja. Bo podejrzewam, że gdyby kogoś z moich najbliższych to dotknęło, to też bym miała te obawy tego Covida, żeby ktoś nie zachorował. Więc ta sytuacja ewidentnie to wygenerowała.

**Ale czy tutaj takie dojmujące jest, ta zapaść w służbie zdrowia i że możesz nie dostać pomocy czy samo to, że możesz ciężko przechodzić Covid?**

I to i to. Ja w ogóle przestałam oglądać telewizję. Przestałam. Tak naprawdę, ile jest zachorowań, dla mnie nie ma znaczenia, czy ich jest tysiąc czy 2 tysiące, to w ogóle mnie te cyfry… Bo tak naprawdę można zachorować jak jest 500 osób, 2 tysiące osób. Bo jeśli mnie to spotka, no mówię, no tak jak w marcu. Wtedy też mogłam zachorować, tylko byłam w innej sytuacji.

**To na ile śledzisz sytuację, to co się dzieje? Skąd czerpiesz informacje?**

To znaczy tak, ktoś, klientka przyjdzie i powie, dzisiaj było tyle zachorowań. Gdzieś tam ktoś jeździł w karetce ileś godzin. Mąż mi też opowiada. Ale tego jest tak dużo, że to można by było się tym sycić 24 na dobę.

**To jest bez przerwy w tej chwili, są statystyki pokazywane. Pamiętasz, ile było dzisiaj?**

Nie. Nie. Mi ktoś mówił i w ogóle… No mówię, nie, bo mnie to strasznie nakręca. A mi jest to niepotrzebne.

**To jak przychodzi do ciebie klientka i chce o tym rozmawiać, a ty nie chcesz tego słyszeć, to co robisz?**

To znaczy samo słuchać, kiedy to już jest takie przefiltrowane… To znaczy media chyba, ten głos lektora, te obrazki, wtedy jest tyle tych bodźców, że ja bardziej to przyswajam. Natomiast, jak to jest takie przefiltrowane to nie, to w takiej formie mogę przyjąć.

**A szukasz jakichś elementów optymistycznych czy ich raczej nie dostrzegasz w tej chwili, jak to jest?**

Ale optymistycznych…

**No takich, że może nie będzie tak źle z jakiegoś powodu.**

Trzymam się tego, że powiedzmy, że jeśli mazowieckie jest, że jest najwięcej zachorowań, to sobie tak tłumaczę, że gęstość zaludnienia jest duża i jakby siłą rzeczy te cyfry muszą być wyższe. I tu wtedy mówię dobrze, no jest dużo, ale to też z czegoś wynika. Że takie Podlasie nigdy nie osiągnie takiego wyniku, no bo nie.

**Jak sobie radzą inni ludzie z takimi strachami, lękami? Co obserwujesz, co robią, żeby sobie pomóc lepiej radzić?**

Na przykład słyszę tak jak, znaczy może ja jestem bardziej wyczulona, nie wiem, ale słyszę wśród klientów, jak rozmawiają, czy koleżanki, a to sobie lampeczkę wina wypijemy. Alkohol. Zdecydowanie słyszę, że alkohol. Zresztą obok mam sklep monopolowy taki… No, taki sobotnio-niedzielny. I nieraz tam wstępuję po wodę. Nie lubię tego sklepu, bo on jest dla mnie taki śmierdzący, taki typowy monopolowy. Ale nieraz po drodze wstępuję, jak wodę zapomnę sobie wziąć. Też jak patrzę, w jakich ilościach znika tam alkohol. Ten sklep jest od zawsze. Ale mam wrażenie, że to nie jest tak, że oni mniej przywożą. Bo to są stałe dostawy. Ale rzeczywiście tego alkoholu ubywa.

**Bardziej niż w marcu?**

W marcu nie chodziłam, ale myślę, że no, może tak być. Ale na pewno ten alkohol się pojawia, że ludzie tak właśnie, że drinka sobie wypiję, a jakieś winko. Nawet tak jak spotkałyśmy się raz z koleżankami, tak naprawdę to był wrzesień, koniec sierpnia, początek września. I nie spotykałyśmy się wcześniej i stwierdziłyśmy, że dobrze, no może spróbujemy wyjść i poszłyśmy do jakiejś pizzerii. I dziewczyny bardzo chciały, że… Zawsze szukałyśmy takich miejsc, żeby fajne jedzenie spróbować, no jakieś winko, drinki. Ale bardziej było nastawione na takie, żeby coś fajnego, coś innego do jedzenia. A tutaj właściwie one wszystkie tak się zgodziły, jedna z koleżanek prosiła, żeby było blisko domu, bo miała dzieci i nie miała z kim ich zostawić na dłużej. I rzeczywiście chyba też ten alkohol był taki, że a, żeby było wino.

**Jak myślisz, co daje alkohol?**

Ja wiem? No, jakieś pewnie rozluźnienie, zapomnienie. No nie wiem, mi w stresie alkohol nie pomaga, ja to jakoś tak inaczej (śmiech).

**Jeszcze jakieś zaobserwowałaś sposoby radzenia sobie u innych?**

Sporo osób ucieka w sport. Na przykład na moich zajęciach jogi była stworzona grupa taka weekendowa. Zresztą sama się na nią też zapisałam, bo miałam taką potrzebę, żeby chodzić więcej niż w tygodniu. I wiedziałam, że na pewno w weekend pójdę. To rzeczywiście było dużo więcej osób, żeby tak się fizycznie zmęczyć, nie wiem. Zresztą widzę na ulicy tu w mojej okolicy, że więcej biegających, na rowerach jeżdżących. Więcej.

**Masz wrażenie, że ucieczka w wysiłek fizyczny coś daje. I jeszcze coś ci przychodzi do głowy?**

No nie, teraz tak mniej się spotykamy, to może mniej takich…

**A negowanie pandemii, negowanie wirusa, spotykasz się z tym jeszcze?**

Tak. Mniej. Trochę tak już, bo ci, co tak całkiem negowali, tacy byli bardzo, troszkę mniej, bo chyba się wystraszyli. Ale też nie jest to na zasadzie, że kurczę, pomyliłem się, jednak jest to. Tylko no jednak, coś tam trochę bardziej, no faktycznie. Nie ma takiej odwagi powiedzieć, że no myślałem inaczej. Jeśli już jest, to stopniowo, nie ma takiego, że kurczę, jednak to się dzieje. Nie spotkałam się. Bardziej z takim łagodnym, że no jednak, no więcej, no faktycznie no chorują ci ludzie. (niezrozumiałe) to ludzie to wypierają.

**A sama znasz kogoś, kto zachorował?**

Tak. Moja koleżanka zachorowała z mężem, zachorowali. Jest teraz też w trakcie, bo też rozmawiałyśmy, korzystając z komunikatora, no bo wiesz, ja już jestem po teście, że ujemny, tam już minęło czasu, ale ja się nie czuję komfortowo, żeby z kimś przebywać. Że ona jeszcze ma obawy, że jeszcze to roznosi.

**A jak przechodzili? Lekko czy tak sobie?**

Takie przeziębienie ze stanem podgorączkowym. Natomiast, no ona się już śmiała, że mąż był ciężko chory, bo miał 38, 9 i kaszel. I był bardzo ciężko chory, no był umierający. Natomiast wystarczyło, że zadzwonił do swojej mamy i powiedział, że dzisiaj się słabo czuje, więc teściowa ją telefonami bombardowała, no zadzwoń po pogotowie, bo on się dziś źle czuje. Dotąd ją męczyła, że wezwała to pogotowie. Przyjechało pogotowie, stwierdziło, nie, no pan całkiem dobrze wygląda. Dostał zastrzyk w tyłek. I wyzdrowiał, już nie jęczał. Więc, no był chory, bo temperatura była, kaszel był, natomiast trochę dokładał do tego. Ale na przykład ona ma trójkę dzieci – żadne nie zachorowało. Tylko oni to przeszli.

**Może złapali od dzieci, ale dzieci same nie zachorowały?**

No też. Ale nie wiem, czy jest człowiek w stanie określić, jeśli był w różnych miejscach.

**A ty sama siebie podejrzewałaś kiedyś, że masz koronawirusa? Robiłaś sobie test albo byłaś na kwarantannie?**

Nie. Chociaż teraz właśnie, jak się pojawiło to takie, taki lęk o to, czy ja się dostanę do lekarza czy nie zachoruję, to mi się teraz bardziej tak… W pierwszym tygodniu w marcu, jak to się zaczęło, jak zaczęłam oglądać te wiadomości, mówiłam, że zaczęłam sobie mierzyć temperaturę, to mniej więcej miałam to samo teraz. Tylko nie było to związane z tym, że wzrosła ilość zachorowań. Tylko z tym, że jestem chora i czy ja dostanę się do lekarza. Na przykład dla mnie jest teraz bardzo stresujące, jak idę do przychodni czy tam do szpitala i ktoś ma mi zmierzyć temperaturę. Ja po prostu dostaję jakiegoś, no to jest nienormalne, ale ja tak czuję, że jakbym miała temperaturę. I ostatnio się śmiałam, bo wchodziłam 3 razy do szpitala. I już się śmiałam do tego pana ochroniarza przesympatycznego, że wie pan co, ten termometr mnie bardziej stresuje niż alkomat (śmiech). Bo nie prowadzę po alkoholu. A tutaj nie wiem. I jest to dla mnie stresujące. Ale mówię, to są jakieś urojenia, nie mające potwierdzenia… Bo oprócz jakiegoś stanu zdenerwowania no nie mam jakichś objawów, żeby mnie coś bolało, no kaszel, gardło, katar. Ale tak sobie myślę, że czy to nie jest Covid, że ja się dziwnie czuję.

**Czyli jak zakaszlesz albo jak zaboli cię coś w plecach, to masz takie od razu sygnały?**

Tak. Ale potem mówię, wróć, nie myśl.

**Zajmij się czymś innym?**

Tak, tak. I jak się zajmuję czymś innym, to mi to przechodzi.

**A w ogóle wiedziałabyś, jak się zachować, gdybyś rzeczywiście źle się czuła, miała gorączkę, to co byś zrobiła?**

Zadzwoniłabym do lekarza pierwszego kontaktu. I nie wychodziła z domu.

**Chciałabyś robić sobie test czy nie?**

Nawet mi proponowała, znaczy nawet jak rozmawiałam i z koleżanką, rozmawiałam też z panią doktor, pewnie ten test będę musiała zrobić, jeśli będzie ode mnie tego wymagał szpital, jeśli w ogóle się dostanę. Natomiast też mi lekarz zaproponowała, że mogę sobie zrobić teraz, żeby powiedzmy, nie wiem, uspokoić się, że nie mam. A jeśli mam, to jakby od dnia, kiedy zrobię ten test minie 10 dni i już będę wiedziała, od kiedy jestem chora. Żeby też skrócić ten czas oczekiwania. Ale nie zdecydowałam się. Stwierdziłam, że nie będę wychodzić przed szereg. Jeszcze jak posłuchałam o tych kolejkach, to stwierdziłam, że nie. Nie trzeba być nadgorliwym. Przemknęło mi, ale nie, nie. Wycofałam się z tego.

**Czyli to, czy byś robiła test, to by zależało od tego, czy lekarz ci każe to zrobić?**

Jeśli lekarz każe, no to muszę zrobić. Ewentualnie tak, jak już będę, że rzeczywiście temperatura, że jakiś kaszel, ból głowy, no już takie objawy, że coś mi jest. Natomiast samo to, że czuję, że tam dreszcz, temperaturę mam 36 i 7 no to nie.

**A korzystałabyś z tej aplikacji, o której rozmawiałyśmy, czy w ogóle myślałaś o tym, żeby ją sobie zainstalować?**

Nie, nie. Nie (śmiech). To ja sama siebie muszę kontrolować, nikt już nie musi.

**Czyli twoje poczucie bezpieczeństwa od tego by się nie poprawiło.**

Na pewno nie.

**A czy ty się dobrze orientujesz w tym, co wolno, czego nie wolno, jakie są obostrzenia, jakie są zakazy, jakie nakazy w tej chwili.**

Godziny dla seniorów, to doskonale wiem. Które są w ogóle od czapy, ale dobra, no takie są. No noszenie maseczek w przestrzeni publicznej i zamkniętych pomieszczeniach. W lesie nie trzeba nosić maseczki. Co tam jeszcze jest takiego? Nie wiem, czy coś jeszcze z takich, co mnie dotyczy tak bardzo. No dezynfekcja to jakby zalecana, nie jako jakieś obostrzenie. Nie znam dzisiejszego przemówienia i zaleceń, chyba cmentarze są zamknięte. To mi doleciało, bo jakoś tak słyszałam. No, 1-3 pracują, starsi są na zdalnym. No nie wiem, czy coś jeszcze powinnam.

**Od razu zaczęłaś, mówiąc o tych godzinach dla seniorów, że trochę bez sensu itd. Czy widzisz wśród tych obostrzeń takie, które twoim zdaniem są bezsensowne, nic nie dają. I takie, które są sensowne?**

Ja cały czas będę przy tym, że sensowne jest noszenie maseczek. Mamy dyskusję z mężem, bo on twierdzi, że w miejscach takich publicznych zamkniętych, takich jak komunikacja, sklep trzeba, natomiast na powietrzu nie. Ja uważam, że na powietrzu też, chociażby z takiej technicznej, że jak już mamy tą maseczkę, idziemy, to nie bawimy się nią, nie zakładamy, nie pamiętamy. Jak wchodzę do sklepu, to już mam tą maseczkę, a nie dopiero za drzwiami zakładam. Uważam, że noszenie maseczek jak najbardziej. Zachowanie dystansu też, gdybyśmy siebie pilnowali. Natomiast u nas nie ma chyba tego w kulturze, bo, to mi koleżanka ostatnio i właśnie takie słyszałam głosy, że ludzie bardzo agresywnie reagują. I nawet jak się, bo ona mówi, że stała w Lidlu, więc miała dużo zakupów, więc nawet ta odległość między tą panią przed nią, no nie była taka, że stała przy niej. I jaka już ta kobieta pakowała i ona przeszła krok dalej, zaczęła na nią krzyczeć, żeby nie szła za nią, nie podchodziła. I ona tak mówi, może faktycznie się zagapiłam, zamyśliłam, przepraszam. To słowo przepraszam w ogóle nie załatwiło, bo ona jeszcze się nakręcała. I właśnie dużo słyszę takich, że ludzie tak reagują, nawet jak ktoś się zapomni niechcący albo nie pomyśli, że jest 90 cm a nie półtora metra, to słowo przepraszam w ogóle nie ma znaczenia. W ogóle takie skrajności, że albo nie zwracamy w ogóle uwagi, albo jesteśmy nadgorliwi i zwracamy uwagę, ale nie przyjmujemy i już jest cała moja racja i ta frustracja przychodzi. Więc tutaj fajnie by było ten dystans zachować. No jest ciężko. Bo niektórzy zapominają i niektórzy w ogóle… to dla nich jest takie, jeszcze tą maskę tam sobie założy, żeby nie zapłacić tego 500 czy tysiąc czy ile tam jest…

**Co czujesz, jak widzisz, że ktoś nie ma maski? Chociaż teraz już rzadziej, jak rozumiem. Ale np. tak ostentacyjnie źle założoną?**

Denerwuje mnie to. Bo to jest takie… Znaczy ja mówię tak, mogę się z tym zgadzać, nie zgadzać. Ale szanujmy czyjąś przestrzeń. Bo jeśli ja uważam, że to jest głupie, nie wierzę w to, no, jest mi ciężko oddychać. Ale może komuś to pomoże, ktoś będzie miał z tego powodu lepszy komfort. No nie wiem, ja uważam, że to mi nie zaszkodzi, a może komuś pomóc.

**Zwracasz uwagę, żeby sobie naciągnęli też na nos na przykład czy nie?**

Po tym jak w sklepie musieliśmy zwracać uwagę i spotkaliśmy się z różnymi reakcjami, naprawdę nieraz chamskimi po prostu, to ja odpuściłam. Ja jak widzę kogoś takiego, ja się odsuwam. Ja sobie sama robię dystans. Bo to jest takie, że już doszłam, że świata nie zbawię, ludzie są okropni. Natomiast u siebie w pracy powiedzmy, ja nawet powiesiłam. Bo niektórzy przychodzili, że proszę założyć maseczkę, bo uważali, że przychodzą, że się znamy, to się znamy. Ale nie przebywamy ze sobą non stop, jest to dalej obca osoba. Że nie wiem, stała klientka, ale dalej jest to ktoś z zewnątrz. I tutaj zdecydowanie proszę o założenie maseczek. Natomiast teraz już tego nie ma. Natomiast no też różne były takie reakcje, że nie wiem, dziewczyna się zapytała, a to muszę założyć? Ja mówię, tak, ja bardzo poproszę. No ale to jakby u siebie to już mam takie…

**Czy to jest dla nich bardziej oczywiste w tej chwili, jak ta druga fala absolutnie przyszła?**

Tak. Ale dalej mam wrażenie, że u większości zadziałał nie strach tylko kary finansowe. Ja się z tym zgadzam i mówiłam od początku, że jak by było przez kieszeń, to by było od początku.

**Które obostrzenia jeszcze uważasz za sensowne?**

(śmiech) Które za sensowne…

**To przejdźmy przez różne obostrzenia. Maski sensowne, zdecydowanie, powiedziałaś. Rękawiczki, dezynfekcja?**

Tak.

**Jedno i drugie? I rękawiczki i dezynfekcja?**

Nie, no albo jedno, albo drugie. Niektórzy widzę, że rękawiczka, którą dezynfekują, ale może traktują to jako dłoń, że w tej rękawiczce wchodzą. Bo ja powiedzmy mam cieniutkie rękawiczki, które zdejmuję. Bo też różne są te do dezynfekcji, nieraz jak się dotyka, to bardziej można się zetknąć niż nie dezynfekując.

**Ograniczenia dla osób starszych? Godziny dla seniorów, zakaz wychodzenia z domu?**

To znaczy to ma sens. Tylko, że godziny dla seniorów, ale od 10 do 12 OK, ale tylko w tych godzinach. To ma sens i to jest fajne. Natomiast taka jak ostatnio i rzeczywiście doświadczyłam tego, bo bardzo mi zależało, żeby do 10 załatwić. Potem te dwie godziny zupełnie nie mogłam nic załatwić. A weszłam na pocztę, weszłam do apteki i byli mocno dojrzali klienci. I tak no powoli, spokojnie. A ja po prostu wiedziałam, że tak, tu muszę wysłać, bo urząd skarbowy. I muszę. I nie mam kiedy, i godziny pracy poczty, mam związane ręce. Potem idę do pracy, nie mam poczty w okolicy po drodze itd. A mam starszych przed sobą. Wchodzę do apteki i również. Mąż był umówiony na wizytę do lekarza, musiał mieć jakąś maść. I było za 10 dziesiąta. I stoją dwie starsze panie i one są na pogaduchach. A ja po prostu już aż, przy moim spokoju zaczęło mnie aż wbijać w podłogę, bo mówię tak, jeśli ja nie kupię teraz tego leku, a nie daj boże okaże się, że jego nie ma w tej aptece, ja nie mam czasu już szukać po 12 i mój mąż pójdzie na wizytę bez tego, co potrzebuje. Ale zapytałam się, czy mnie pani obsłuży, bo jest za pięć dziesiąta. No więc pani tłumaczyła, że tak, bo podobno to jak weszłam przed 10, to i tak mnie obsłuży, bo jakby byłam już.

**W aptece to w ogóle chyba mogą odstąpić od tego, jeżeli lek jest szybko, niezbędnie potrzebny.**

Natomiast panie też były, bo tam chwile tak sobie, że im to też tak dezorganizuje i też są umęczone tym po prostu już.

**Czyli OK, niech będzie od 10 do 12, ale niech potem nie łażą po sklepach.**

Tak. Żeby 9:30 nie było tej starszej osoby, że wchodzę i załatwiam. To wtedy w porządku. W domu niech sobie siedzą. Chociaż… No lepiej niech siedzą. Bo z kolei takie ograniczenie, no, spacer do parku, no różne pomysły mają seniorzy.

**Kościół. Zamknęłabyś kościół?**

Ja bym nie zamykała, tylko zachowała te odstępy.

**Przestrzegane, jak rozumiem.**

No tak. Tu mam taki dylemat, bo właściwie tych tłumów nie ma w kościele. Tak nawet jak obserwuję, bo tak naprawdę mamy na swojej ulicy też kościół. I on jest też duży, więc może… Ale nie ma takich tłumów, jak widzę, nawet wychodzą ludzie z kościoła. A może ktoś ma taką potrzebę, może dla kogoś jest to ważne, ta strefa taka duchowa, że musi być w tym kościele. No ja nie muszę iść do kościoła i nie mam z tym, ale… No tutaj mam takie… Nie zamykałabym. Znowu to jakby się też wyklucza, bo seniorzy w domu, ale tak naprawdę oni chodzą najwięcej do kościoła. Więc skoro jest otwarty, to jest pokusa, że pójdę. Więc mogli połączyć to faktycznie, kościół z seniorami.

**Ale to jest trudne, żeby tak odciąć człowieka od miejsca, które być może coś mu daje więcej?**

Tak. Bo powiedzmy, no nie wiem, jeden pójdzie do psychologa, drugi porozmawia z sąsiadką, a ktoś może potrzebuje. Ja to szanuję i jest to takie no…

**Zamknięcie szkół dla starszych dzieci i zostawienie klas 1-3 i przedszkoli działających. Co o tym myślisz?**

Tu są takie sprzeczne. Bo najpierw słyszę komunikat, że przenoszą małe dzieci, są nosicielami. I nagle one chodzą i się spotykają i się przemieszczają. Żeby zachować dystans i zalecić noszenie maseczek – młodzieży i starszym jest łatwiej. Słyszę wśród nauczycieli i rodziców, że ciężko, te maluchy przychodzą, bo to jest ich pani Zosia kochana. I nawet moje klientki nauczycielki mówią, no ja muszę podejść do tego dziecka. Nie wiem, długopis pokazać. No nie da się tak. Mamy maseczki, mamy przyłbice, ale nie da się tak, że z tymi dziećmi, nie podchodzić do nich. U starszych, wydaje mi się, że jest to łatwiej zorganizować.

**Ale wobec tego zamknęłabyś wszystkie szkoły, żeby to miało sens? Chodzi mi o to, czy to ma sens z punktu widzenia zastopowania. Czy to rzeczywiście wzrasta nam bezpieczeństwo od klas 4 w górę i studentów.**

Ja bym bardziej te młodsze zamknęła.

**A zamknięcie restauracji?**

Znaczy tak, te restauracje, które znam i wiem, jak funkcjonowały i te, które pozostały… Tak naprawdę nie było nigdzie słychać, że ktoś się zaraził w restauracji. No nie wiem, nawet w tych, których znam, jest odległość między stolikami. Są oczywiście kelnerzy w maseczkach. Ja bym osobiście restauracji nie zamykała. Z zachowaniem oczywiście odstępów. Bardzo fajnie byliśmy, pojechaliśmy w góry. I tam było rzeczywiście bardzo fajnie zorganizowane, że trzeba było wejść w maseczce, trzeba było przy, tam stała kelnerka albo menadżer, zdezynfekować dłonie, każdy, kto wchodził. Kolejny kelner prowadził do stolika, który był zdezynfekowany, przygotowany. I rzeczywiście to było widać. No i już potem przy konsumpcji… Ja czułam się bezpiecznie. Miałam ten komfort. Natomiast, jak wychodziliśmy, stała jakaś tam rodzina i bardzo się awanturowali, że ta maseczka, dlaczego. Dlaczego oni mają te ręce dezynfekować. Natomiast stanął pan, stanowczo powiedział: nie? Zapraszam kolejnych. I to mi się bardzo podobało. Bo widać, że oni dbali o to, żeby i oni się czuli bezpiecznie i klienci. Nie czułam tam, że mogę się…

**I nadal byś mogła chodzić w tej chwili, gdyby to było otwarte, tak?** **A zamknięcie basenów, siłowni czy tej jogi?**

I tu miałam dylemat. To znaczy ja byłam przeciwko, że jak to, na siłowni przecież dorośli ludzie, młodzi ludzie. Przecież sprzęty są oddalone od siebie. My miałyśmy też odstępy na tej jodze. Kameralnie, tam wszyscy przestrzegali, nie wiem, miałyśmy do dezynfekcji, każda ma swoją matę. Pokupowałyśmy sobie swoje sprzęty, jakieś paski, woreczki itd. I tam się czułam bezpiecznie. I tak, szkoła jogi, tam ten budynek był zarejestrowany jako klub sportowy, obiekt sportowy. Czyli wszedł automatycznie w to, że musi być zamknięty. No siłownie jak siłownie. Ale na przykład na ćwiczenia chodzę do takiego, to jest taki pruszkowski wynalazek, takie coś a la dom kultury. W ogóle duży obiekt, typu, że jest kilka sal, tu się odbywa balet dla dzieci, tu taniec towarzyski, obok ktoś gra w squasha, my robimy pilates. I wszyscy się spotykają w jednej szatni. I to jest otwarte. Bo w tytule ma jakąś nazwę. I mówię zaraz. Jest to tak niesprecyzowane. Ja się wycofałam z racji tego, że po prostu muszę unikać skupisk. I stwierdziłam, że to będzie moją ochroną, że jednak nie będę chodziła. Ale nawet, jak jeszcze nie wiedziałam, że będę wymagała leczenia, to nie podobało mi się to. Dlatego, że dobrze, weszłam w masce, tam większość weszła w masce, były zajęcia. Tu dzieci z karate wybiegły, tu wyszły panie z poprzednich zajęć, już nie założyły maseczek. A jak ja wyszłam z tych swoich zajęć, to byłam jedyną w maseczce. To działa. I tam można chodzić… Natomiast, jeśli chodzi o siłownie, to ja mówiłam no nie, sami młodzi przecież, dorośli, tam można to zdezynfekować. No można zachować te warunki. Do momentu, jak dzisiaj mi klientka opowiedziała, że ktoś tam z jej znajomych zachorował i zaraził się od brata jakiegoś, z rodziny. Który ma wynik pozytywny, zrobił sobie. Ale mówił, że on tak dobrze się czuje, że chodzi na siłownię. No i tu znowu stwierdziłam, tak jak byłam przeciwko, że powinny być otwarte, to jak usłyszałam tę historię i sobie tak pomyślałam, że kurczę, my chyba jesteśmy takimi, których trzeba krótko trzymać. No to ma to sens. Tylko no właśnie. Bo mówię, do tej pory twierdziłam, siłownie, no jak to, młodzi ludzie chodzą, no przecież zdrowi.

**To równie dobrze w sklepie może (niezrozumiałe). Do ciebie może przyjść klientka, która tak się dobrze czuje.**

Więc gdzie jest ta nasza jakaś odpowiedzialność. Ale z drugiej strony to by należało wszystko zamknąć.

**No właśnie dlatego pytam. Bo to, że akurat ten chłopak tak się dobrze czuje i chodzi na siłownię, to równie dobrze mógłby chodzić sobie do restauracji, do sklepu, na pedicure, gdziekolwiek.**

I tu mi się zburzył cały… Bo na przykład u dentysty, tak jak chodziłam i potem miałam iść, i on zadzwonił do mnie, że zadzwoniła jego pacjentka, że otóż ma Covid z mężem, a była u niego i go poinformowała. Więc on w tej sytuacji zrobił sobie test. I mówi do mnie, że on widzi test ujemny. Natomiast mówi, ale ja się źle czuję. Jemu też się wygenerowało, że on mówi, ja kazałem dziewczynom, żeby mi w gardło zaglądały, tam ta pomoc. Jego już gardło bolało. I niezależnie od tego, że mam ujemny, postanowiłem, że 2 tygodnie posiedzę w domu, zrobię kolejny test. Ja oczekuję, że tak działa społeczeństwo. Ale z drugiej strony, no tak, chodzą młodzi na tą siłownię. No to niech będzie otwarta. Tylko, że taki młody pójdzie do swojej babci i ją zarazi. Bo skoro on nie myśli. Ale myślenie widać nie jest mocną stroną. (ns) otwarte i niech sobie będą.

**I niech będą, niezależnie czy to ma wpływ czy to nie ma wpływu jak rozumiem?**

Bo ja sobie myślę, że taka restauracja na przykład… No z reguły jest to taki właśnie, no tak sobie myślę, dobrze… Ale że tej osobie, która to prowadzi, żeby utrzymać ten biznes, w który zainwestowała, w jej interesie jest, zresztą to mówili restauratorzy, żeby maksymalnie zadbać o wszystkie obostrzenia, żeby dezynfekować, przestrzegać odległości. Oni o to dbali. Siłownia jest bardziej takim… No, sieciówki to nie są jakieś prywatne biznesy, to też inaczej. To na zasadzie jak ja widzę, co się dzieje w Biedronce, a co się dzieje u mnie w sklepie. Że tu jest sieciówka, tam jest duży, prężny prezes, zastępca, udziałowiec itd. I właściwie czy im się zamknie jedna Biedronka czy dwie, oni tego nie odczują. A ja robię wszystko, żeby przetrwać i żeby to funkcjonowało, żeby pracownicy byli, żeby nie zachorowali. Więc patrzę od tej drugiej strony, że prywatna ta inicjatywa, no ludziom zależy, więc dostosowują się do tego. Nie patrząc, czy to jest mądre czy nie, że hektolitry spirytusu wylewamy na dłonie.

**A zakaz imprez, spotkania do 5 osób w tej chwili?**

Myślę, że to bym utrzymała. Bo jednak ewidentnie było widać, że po weselach były masowe zachorowania. To jest jednak taki… No wesele, wiadomo, że się tańczy, dotyka, oddycha inaczej, nie w maseczkach. Wesela, no mówię…

**Tak samo z dużymi eventami, targami, imprezami sportowymi?**

Na targach to jest też inna specyfika. Tam idą ludzie, którzy… No tam biznesy się załatwia. Żeby, nie wiem, moja firma działała, tamta firma działała, te kontrakty, nie wiem, zapoznanie się z ofertami. Myślę, że tam ludzie by bardziej odpowiedzialnie podchodzili do tego. I można stworzyć przestrzeń, można ograniczyć ilość osób. Tam się kontrahent z kontrahentem nie musi ściskać, całować i pić wódki. Można to załatwić z dystansem. A jednak wiem, że to pomaga, nie tylko mailowo, tak jak słyszę. Można to załatwić, bo słyszę od klientek, od znajomych. To bym zostawiła.

**Czyli takie rzeczy związane z przyjemnością, żeby pójść sobie potańczyć do klubów, w kontakcie z innymi ludźmi itd. to do zamknięcia. Ale jednak biznes, utrzymanie pracy i to wszystko, żeby przetrwać to jest ważna rzecz….**

Tak. Gdzie nie ma takiej spontaniczności, takiego luzu. Bo ludzie są w pracy. Nie wiem, nie musi jechać z firmy trzech przedstawicieli, może jechać 1. Już ograniczamy tą ilość. To nie muszą być tłumne, nie wiem, nie musi być 10 hostess. Na zasadzie, żeby to ograniczyć. Natomiast tam, gdzie jest alkohol, zabawa, rodzina, spontanicznie, to się wszystko rozluźnia i wtedy nawet ci, którzy bardzo pilnują dystansu, no to puszczają te hamulce.

**Ograniczenia w transporcie zbiorowym sensowne?**

Powiem tak: nie mogę się o tym wypowiadać, bo nie korzystam. Nie byłabym obiektywna, nie mam na ten temat zdania. Z jednej strony no dobrze, ale jak ci ludzie mają się przemieszczać? No więcej autobusów, OK, ale czy my tak jesteśmy przygotowani? Czy stać miasto na to, żeby zapewnić je. Bo to jeszcze musi być kierowca, jeszcze musi być mechanik, który to przejrzy, mnóstwo rzeczy. Ale z drugiej strony, jeśli wprowadza się w miarę możliwości zdalną pracę, ogranicza się ilość osób, które przemieszczają się, no też może to… Znaczy w tym momencie, kiedy młodzież nie jeździ do szkół, na uczelnie, no to tak naprawdę jest mniej tych osób, które podróżują. Ale patrząc na to, jak są obładowane środki komunikacji, to… No tak, ale też ci, którzy jeżdżą tą komunikacją narzekają na to, że właśnie, bo ktoś maseczki nie trzyma, bo ktoś rozmawia przez telefon. Bo komuś, jak się zwraca uwagę, no to też agresywnie reaguje, że też nieprzyjemne sytuacje. Brak nam chyba takiej samodyscypliny, takiej trochę pokory, że dobra, jestem w tym autobusie, trzymam tą maseczkę odtąd dotąd. Wysiadam, dobrze, jest powietrze, trochę ją obniżę. Nie ma takiej jedności, że ktoś też jest obok mnie.

**Pojawiają się takie bardzo sprzeczne opinie. Są ludzie, którzy mówią, zróbmy pełen lockdown, zamknijmy na 3 tygodnie czy ileś, różnie to mówią, ale niech to się wykisi w domach i już będzie wiadomo, kto jest chory, będziemy dalej funkcjonować. Inni mówią: ten lockdown bez sensu, to nic nie da. I tak to wybuchnie. Jakie jest twoje zdanie?**

Ja co jakiś czas zastanawiam się nad tym. Dla mnie w ogóle podstawą było jak było te pierwsze kilka zachorowań, dla mnie wtedy to było bez sensu zamykanie wszystkiego. I tak naprawdę teraz przydałoby się takie zamknięcie, jakie było w marcu. Teraz miałoby to sens. Teraz tak. Tylko, że nas już na to nie stać. Tamto było zrobione szybko, pochopnie, na pokaz, ze strachu, z interesu jakiegoś. I nie miało sensu. Natomiast przy tej ilości i z zachowaniem tak, jak było wtedy, uważam, że miałoby sens. Ale już jest jakby po.

**A te protesty, które się przetaczają przez ulice? Myślisz, że to będzie miało konsekwencje w postaci dużego wzrostu, nie wpłynie, bo się odbywa na powietrzu, czy np. nie wpłynie, bo to młodzi ludzie?**

Myślę, że w jakiejś części może wpłynąć, ale nie aż tak. Ja obserwując ten nasz protest, rzeczywiście w tym najbardziej skupionym byli młodzi ludzie. Ale oni mieli maseczki. Więcej ich widziałam w maseczkach niż w takich w średnim wieku, chodzących na co dzień po ulicy. Gdzie też się mijali ludzie. No jest to na powietrzu. Nie wiem, czy aż… Znaczy ja bym nie zrzuciła, że wzrost zachorowań jest przez to, że są protesty. To na pewno nie. Bo zachorowania zaczęły się dużo wcześniej. Te przesunięcia z robieniem testów, wyników, zanim ludzie się dostaną. Więc wpływ na zwiększenie tak, ale jakby to nie jest pierwszorzędne, że to od tego. To nie.

**A jak myślisz o tej drugiej fali, o tym koronawirusie w ogóle jako o wirusie, masz więcej pomysłów niż miałaś na temat tego, skąd on się wziął, dlaczego to tak wybuchło, czym w ogóle jest? Co teraz myślisz o koronawirusie?**

Jest to przerażające, że jest to coś, co się tak rozprzestrzeniło. Gdzie medycyna, chemia, biologia, nauka poszła do przodu. Jest dużo więcej narzędzi niż kiedyś, gdy była hiszpanka. Że to ma taki zasięg, takie zbiera żniwo i jest nie do opanowania. Mój umysł nie jest w stanie tego objąć jaka to jest skala. Jak dzisiaj słyszałam, że na ostatnich jakichś wyspach pojawiły się dwa zachorowania. Gdzie tam do tej pory nie było. To było jakieś jedyne miejsce na świecie.

**Tak? Już się pojawiły w tych dwóch ostatnich?**

Tak. (niezrozumiałe) stwierdzono u nich. Co prawda ten redaktor, bo to w radio słyszałam, powiedział, że nie, jeszcze jest Korea Północna, tam nie ma zachorowań, więc no to z takim przekąsem. Natomiast no mnie przeraża, że to jest coś takiego… No i właśnie, czy wymyślił to człowiek, czy natura sama to (niezrozumiałe). Słyszę różne pomysły, że to jakaś broń jest… Nie wiem. Bo z jednej strony świadoma jestem tego, że człowiek jest w stanie zrobić coś takiego i rozprzestrzenić. I wcale by mnie to nie zdziwiło. Z drugiej strony też natura jest taka, że też potrafi. I to jest możliwe, i to jest możliwe.

**A co cię najbardziej w tym przeraża? Bo mówisz, że to jest przerażające. To, że tyle osób zachoruje? To, że tyle osób może umrzeć? To, że ty zachorujesz? Że ktoś bliski zachoruje? Jaka jest gradacja tego strachu?**

To, że jest to taka ilość chorych jednocześnie na całym świecie. Że cała Europa jest z tyloma zachorowaniami. Potem, że jednak to żniwo umierających osób jest jednocześnie. Że to jednocześnie dzieje się w tylu miejscach. I jest to takie, no, jakby schemat jest też podobny. Że ilość tych zachorowań idzie w kolejnych państwach, wzrasta, wzrasta proporcjonalnie ta ilość zgonów. I jest takie realne zagrożenie dla mnie. Przy tak dużej ilości prawdopodobieństwo też wzrasta.

**Myślisz, że można było zapobiec jakoś temu, co w tej chwili w Polsce jest? Tej sytuacji aż takiego wzrostu liczby zachorowań?**

Myślę, że ten początek był takim… Nie wiem, zamknięte granice. Ale przyjeżdżały osoby i one tak naprawdę wjeżdżały do Polski bez jakichś tam… No kwarantanna. Tylko kwarantanna odbywała się w domu. A koleżanka mi opowiadała, jej szefem jest Serb. I u nich było tak, że on, jak pojechał do domu, musiał być na kwarantannie. Natomiast tam było tak, że on tą kwarantannę spędzał w hotelu. Za pobyt w tym hotelu, który był wynajęty przez państwo dla przyjeżdżających. I jakby hotel był ratowany, bo państwo opłacało. Natomiast on, żeby dostać się do domu, musiał być tam na kwarantannie. Natomiast u nas byli wpuszczani ludzie i przyjeżdżali. Syna kolega przyleciał ze Stanów. Przejechał jakoś tam przez Niemcy, dojechał. Jego kwarantanna miała się zacząć powiedzmy po iluś godzinach. I on wyznaczył miejsce, babci mieszkanie, że on będzie na tej kwarantannie. Natomiast on był 24 godziny na terenie Polski bez jakiejkolwiek kontroli, opieki. On mógł się widzieć z rodzicami, z siostrą. I pojechał na kwarantannę. No to było bez sensu. Bo on już był z tyloma osobami. Był taki cyrk. Po co angażować policję, bo oni go sprawdzali, czy on jest na tej kwarantannie, gdzie on już, tak naprawdę, jeśli miał przywieźć tego wirusa, to już go przywiózł i poczęstował.

**Czyli takie niekonsekwentne, nielogiczne działanie.**

Tak.

**I to by mogło zapobiec tej drugiej fali, gdyby w tym była taka konsekwencja?**

Nie, no już nie oszukujmy się, zapobiec to nic by nie zapobiegło. Ale ten chaos cały, tak jak powiedziałaś słusznie, niekonsekwencja w działaniach. I dalej jest chaos. Nie ma takich spójnych działań. Dobrze, to zrobiliśmy źle, nie powtarzamy tego. A tu zamykamy, otwieramy szkoły. Znaczy samo to, że mówiąc o tym, że będzie druga fala od marca do października… No jak wczoraj czy przedwczoraj, jadę samochodem i słyszę, że rząd debatuje, skąd wziąć łóżka. No myślałam, że po prostu… Zorganizowanie łóżek, miejsc do transportowania chorych, organizacja taka, to od marca można było tyle rzeczy zorganizować, takich podstawowych. Bo wiadomo, nie przeszkolimy anestezjologów. Ale takie podstawy nawet lokalowe, żeby wiedzieć, że z koronawirusem będą tu a nie tu. No budowanie łóżek na stadionie, gdzie mamy szpital na Działdowskiej, owszem, rozwalający się, stary, ale już z takim zapleczem, że mamy piętro, mamy pokoje. No dobrze, ja nie muszę być w luksusach i w nowym stadionie. Tylko tak, żeby ludziom, którzy pracują, obsługują, było łatwiej. Nawet zrobić tą segregację chorych. Chyba jeszcze jeden jest szpital zamknięty dziecięcy w Warszawie, który też by mógł posłużyć. No od marca można było to zorganizować. A nie mamy wzrost zachorowań, rząd debatuje.

**I co czujesz, jak coś takiego słyszysz?**

No po prostu wściekłość, że… brak słów.

**Masz wrażenie, że przez ten czas od marca, kiedy się okazało, że ten koronawirus jest, czy rząd zrobił coś dobrze? Masz za co pochwalić ich?**

Nie. Nie, to jest jedna, wielka porażka, jedno wielkie nieszczęście. Ich błędy i złe działania to są jedne za drugim. To jest jedna wielka katastrofa.

**Ale masz wrażenie, że można było przynajmniej temu paraliżowi szpitalnemu zapobiec, tak?**

Tak. Takie podstawowe rzeczy, które są, przez jakiś czas można zorganizować. No mówię, nie wyszkoli się anestezjologa. Ale jakieś takie służby medyczne, organizacja, że dobrze, jak mamy tysiąc zachorowań, to organizujemy taką grupę, jak mamy 20 tysięcy, to organizujemy taką. Jakiś schemat działania, plan A, B. A nie plan tworzy się dopiero teraz. No nie, tak chaotyczne działanie. Może ja jestem taka, że lubię wiedzieć, lubię mieć jakiś tam plan. Nie lubię bardzo mieć takiego ścisłego, ale jakiś chociaż scenariusz.

**Powiedziałaś, że ograniczasz korzystanie z mediów świadomie. Ale te konferencje premiera czy ministra zdrowia to oglądasz? Czy przynajmniej sczytujesz? Pamiętam, że mąż ci filtrował informacje w pewnym momencie, wtedy na początku.**

Nie. Słuchać tego, nie słucham. Wystarczy, że wysłuchałam Kaczyńskiego w sejmie. Ja naprawdę nie muszę dokładać sobie.

**Czyli to powoduje twoją wściekłość, mówiłaś, frustrację, co jeszcze?**

Po prostu jakieś najgorsze instynkty się we mnie wyzwalają, o które nawet nie podejrzewałam siebie. Po prostu jak patrzę na te twarze i ten ich bełkot w ogóle bez sensu, przerzucania… To nie, to dla mnie jest po prostu no *kosa.* Nie, w ogóle nie ma tam logiki, nie ma kultury nawet medialnej, takiej politycznej. Jeśli tam w ogóle jest jakaś… No nie, ja tego nie mogę słuchać.

**A jak oceniasz wiarygodność informacji, które są przekazywane przez media?**

Mój syn ma taki zwyczaj, ogląda wiadomości na Polsacie, ogląda TVN, a potem ogląda wiadomości na Jedynce.

**To ma świętą cierpliwość (śmiech).**

Ma silną psychikę, jest twardy. Ja kiedyś też się skusiłam, bo tak sobie dla takiego porównania, to jest przerażające. To są 2 światy. Ja mam wrażenie, że się cofnęłam, oglądając Wiadomości, jak byłam takim dzieckiem małym, Dziennik Telewizyjny jak był. I takie trutututu, siedzi tam dostojna pani i czyta wiadomości. Ja po prostu to, co słyszę, że jakby… Bo sobie porównywaliśmy tam, to było wcześniej, to porównywaliśmy sobie właśnie dany temat, jak było na Polsacie, na TVN-ie i potem w Jedynce.

**One są jedne po drugich? Jest tak, że Polsat, TVN i potem Wiadomości?**

Tak. Na Polsacie troszeczkę się nakładają, bo one są za dziesięć 19, więc te 10 minut, czyli te pierwsze, najważniejsze, bo potem to coraz lżejsze tematy.

**Potem są Fakty o 19, tak?**

Tak. I potem 19:30 są Wiadomości. Więc taka… I to jest przerażające, jak to jest różne. Bo no dobrze, są różne formy. Można coś inaczej. Ale to jest tak różne. Że w ogóle o jednym temacie, jak można inaczej powiedzieć, to jest aż niewiarygodne.

**A same twarde dane, statystyki, które są podawane?**

No tam są różne firmy badawcze robią dla nich.

**Czyli to też się różni, statystyki też się różnią, które są podawane?**

W TVP to tak. A nawet, jak jest na niekorzyść, to jest to tak powiedziane, że to zupełnie, to wyszło na dobre. To jest tak uwikłane, że to jest fenomen, że można tak to zamącić.

**A wobec tego są jakieś takie osoby, którym… Jak byś chciała sprawdzić coś i dotrzeć do rzetelnej wiedzy, to szukasz wypowiedzi tego człowieka, tego eksperta, nie wiem, tej stacji, tej gazety, tego radia, cokolwiek. Jakieś takie rzetelne źródło dla ciebie. Jest?**

Szczerze mówiąc to jest taki chaos medialny. Bo tak jak w TVN jest przekazywane. Tam z kolei jest za dużo, tam jest robione takie show. Tam jest to wszystko takie śliczne, zagraniczne z kolei. I mówię, tu jest taka sztywna dla mnie komuna. Tu jest takie wow, show, jest to jeszcze inaczej przekazywane. Że też nie do końca dla mnie są te wiarygodne źródła. Bo jak ja patrzę na Wiadomości i one dla mnie są właśnie takie toporne i takie… To druga strona, patrząc z kolei na takie właśnie błyskotliwe i takie bardzo medialne, takie z fleszami, też może się nie skusić na słuchanie tego. A dla mnie, jeśli tak naprawdę, no to Polsat. Że na zasadzie, że znajdę… Nie mam na to zupełnie czasu i to dalej mi mąż przekazuje, filtruje i czyta. Chociaż nieraz te jego poranne wiadomości, to mówię, już daj spokój, nie dobijaj.

**Czyli to się nie zmieniło. Bo tak samo było wtedy, że mąż czytał, mąż sprawdzał i robił ci kompendium wiedzy.**

Tak. Trzyma się głównie prasa i oni tam to. I to jest ciekawy artykuł, to jest ciekawy. Natomiast, gdybym chciała sama, szukam dziennikarza, jego jakby historia, co on… Gdybym tak chciała rzeczywiście zagłębić się w temat, to to by było takie…

**To, że każda stacja nadaje trochę inne informacje to jest jedna rzecz. Ale czy myślisz, że starają się manipulować ludźmi czy nie?**

No Jedynka zdecydowanie. To tych mistrzów manipulacji to ja znam.

**A TVN też manipuluje?**

Znaczy brakuje mi tak, że powiedzmy… Nieraz brakuje mi takiej krytyki, bo jakby opozycja też nie jest idealna. Żeby tak trochę też pokazać te błędy drugiej strony. Bo to nie jest tak, że są jedni winni. Tylko mówię, to jest takie otoczone tymi błyskotkami, że to tak się wszystko rozpływa.

**Czyli w zasadzie brakuje bezstronnego medium. Kogoś takiego, kto by obie strony tego sporu i te wszystkie rzeczy mierzył. A jak sobie myślisz o przyszłości, to jak to będzie dalej? Kiedy to się skończy z tą pandemią? Od czego to zależy?**

Teoretycznie… To znaczy najbardziej zależy od szczepionki. Tylko że szczepionka… No właśnie, to są lata badań. Zawsze tak, z tego co kojarzę, to jakieś 5 lat zawsze trwały te badania, żeby ta szczepionka była taka rzetelna. Na przykład mój mąż mówi, że jak tylko będzie wynaleziona szczepionka, to on się szczepi. Natomiast ja mówię no nie. Ja poczekam. Więc…

**Poczekasz na co? Aż co się zdarzy?**

Aż będzie to, no nie jakiś taki okres, nie wiem, szczepionka wchodzi na rynek w maju, a ja się w czerwcu szczepię. Zakładam, że niech minie rok, 2 lata.

**I o tym, że ona działa, co dla ciebie będzie świadczyło? To, że nikt po niej nie umrze, czy jakieś musisz mieć inne wskaźniki?**

No, że spadnie zachorowalność. Że nie będzie to się tak rozprzestrzeniało w takim tempie. Bo ewidentnie powinno spowolnić.

**Czyli ty się sama wcześniej nie zaszczepisz, ale chciałabyś, żeby inni to zrobili za ciebie.**

To znaczy, skoro są chętni. No, mój mąż jest chętny.

**Dzisiaj usłyszałam jeszcze, zanim zaczęłyśmy rozmawiać, poza zamknięciem cmentarzy, to że nasz premier powiedział, że już w styczniu jest szansa, że będziemy mieli szczepionkę w Polsce.**

Znaczy ja jemu nie wierzę, no ale dobrze. To mniej więcej tak jak z szczepionką na grypę, namawiali się szczepić, żeby była. I wszyscy szczepcie się, szczepcie. Po pierwsze nie ma szczepionki. To jak mam się zaszczepić?

**Nie udało ci się zaszczepić w tym roku?**

To znaczy ja się nigdy nie szczepiłam. I nie planowałam się szczepić. I zresztą tak jak rozmawiałam, bo mam koleżankę, która pracuje w firmie, która produkuje opakowania do leków i jakby współpracuje z tymi firmami, więc szczepionek nie było. W momencie, kiedy one nawet dotarły, było już za późno. Bo nawet ona sama rozmawiała z panią, która koordynuje te szczepionki. I pyta się tej kobiety, która pracuje przy tych szczepionkach. Że no mają, oni mają dostęp do szczepienia. Ale paradoksalnie oni mają podpisaną umowę z jakąś lecznicą. Gdzie jest, że obowiązkiem lecznicy jest zapewnienie szczepionek na grypę dla konkretnego zakładu, tej firmy. Oni nie mieli tych szczepionek. I musieli, żeby tamci wywiązali się z tej umowy, u konkurencji zamawiać. A poza tym ona mi tak wytłumaczyła, szczepionka dotarła, tak naprawdę była dostępna początek października, gdzie zaczął się wzrost zachorowań na Covid. I mówi, żeby teraz się zaszczepić na grypę, czyli wprowadzasz chorobę dla organizmu…

**Osłabiasz organizm?**

Osłabiasz organizm. Czyli trzeba się odizolować na 3 tygodnie. Więc mówi, jaki jest sens osłabiać się świadomie? I jeszcze zetknąć z Covidem. To lepiej się zetknąć z Covidem, jest większa szansa, że się łagodnie przejdzie. Więc szczepionka w styczniu na Covida, to niech się pan premier sam zaszczepi.

**Zobaczymy, czy się zaszczepi. Czyli skończy się, jak będzie szczepionka, jak ludzie zaczną się szczepić, jak będzie mniej zakażeń. W lekarstwo na koronawirusa nie wierzysz, że jakieś wynajdą?**

Znaczy, czy wierzę, tu wiara nic do tego nie ma (śmiech). Ja życzę tym naukowcom, bo na pewno tam się prześcigają te firmy. I ludzie na pewno są zaangażowani w to, żeby… I oby tak się stało. I jest też szansa, że wirus sam odpuści. Bo skoro nie wiemy tak do końca, skąd on przyszedł, co go spowodowało. Może zbierze swoje żniwo i odpuści?

**Czyli nic nie wiemy, jak długo i co będzie.**

Ja sobie nie stawiam takich jakichś wytycznych. No trzeba to po prostu przetrwać.

**A myślisz, czy coś się zmieni w związku z pandemią po pandemii? Czy nastąpią jakieś trwałe zmiany w Polsce, na świecie, w ludziach?**

W ludziach? Obawiam się, że nie.

**A co byś chciała, bo powiedziałaś z żalem, że obawiasz, się, że się nic nie zmieni?**

Ja zawsze mam taką nadzieję i tak sobie myślę, że jakieś trudne doświadczenia, takie ciężkie przeżycia powinny dawać ludziom do myślenia, trochę pokory, jakiegoś dystansu, wyhamowania. Ale jak słyszałam takie głosy, że no dobrze, no to zachoruję, no przecież to jest jak grypa. Już na wstępie nie ma takiego… No dobrze, takiego dystansu, no troszeczkę, no dobrze, jest jakaś choroba. To nie widzę… No właśnie, jak osoby chore idą i narażają innych. I myślą tylko o sobie. To jak myślimy tylko o sobie, żyjemy w swoim takim, czubek nosa jest wszystkim, co nas obchodzi, no to szansa na to, żeby się zmieniło… Może się zmienić u tych, których to bezpośrednio dotknie. To jest tak, że to też może sprowadzić na ziemię, że OK, biję się w piersi, trzeba tak tu inaczej patrzeć. Ale jak ktoś przejdzie obok, to myślę, że niewielki procent…

**Czyli nie sądzisz, żeby ludzie stali się ogólnie bardziej empatyczni, wrażliwi na innych? Jeśli chodzi nawet już nie tyle o postawy tego typu, ale jak myślisz o życiu ludzi za rok, za dwa, za 5 lat, czy tutaj jakieś zmiany będą?**

Myślę, że zapomną. Bardzo lubimy zapominać. Bo tak sobie też pomyślałam, teraz to jest takie… No też, żeby nie było krzywdzące, że przechodzą ludzie obojętnie, że właśnie tutaj, a co ja zarażę, nie zarażę. Bo z kolei też spotkałam się z dużą taką życzliwością i zrozumieniem, że jednak ludzie pomimo trudnej sytuacji chcą pomagać drugim. Koleżanka ostatnio mówi, podawała pani w urzędzie przez okno jakieś dokumenty. Tylko dlatego, że zapomniała, że się nie umówiła telefonicznie, przeczytała kartkę, że nie załatwi tego. I dzwoniła przez telefon i komuś tam tłumaczyła, że słuchaj, mam urlop, nie załatwię, bo się nie umówiłam. I nagle wychyla się pani przez okno i pyta się, pani ma jakieś dokumenty do zostawienia, to pani je poda. No mówi, kurczę, są jakieś tam właśnie wytyczne w urzędzie, ta kobieta słyszy, jak ja rozmawiam i jednak wyciąga. Mogłaby udać, że tego nie słyszy. Ja też miałam taką sytuację, gdzie no też musiałabym przejeżdżać z jednego miejsca do drugiego i generalnie twardy przepis mówi tak, że ja powinnam pojechać w inne miejsce. Ale też facet tak zupełnie bezinteresownie jakoś tu zadzwonił, tu zapytał, że udało mi się. No i jakiś taki jest odruch. Tylko obawiam się, że jak zapominamy, to tak jak cała historia nasza wygląda, że zapominamy i nie wyciągamy wniosków.

**Czyli, jeśli dobrze rozumiem, obawiasz się, że to straszne doświadczenie, które przechodzimy, niczego nas nie nauczy.**

No nie, no wojna też nas nie nauczyła. A była dużo straszniejsza od Covidu. I właśnie to mnie przeraża teraz, ta sytuacja, to co się dzieje, że nie wyciągnęliśmy wniosków, nie zdaliśmy egzaminu z demokracji. I zaczynamy powielać błędy sprzed lat. Też, że dzisiaj grupy, Narodowcy się jednoczą, to po prostu…

**Mój syn tam pojechał z dziewczyną.**

Moje dzieci są, więc też generalnie…

**Też tam są?**

Tak. Więc siedzę jak poparzona i… I dumna. Ale się boję. Po prostu się boje tych wściekłych, zamaskowanych ludzi, którzy… aby bić. O cokolwiek. Więc dobrze, że jestem zajęta (śmiech).

**Zamknęli cmentarze. Ale gdyby nie zamknęli, to miałaś zwyczaj 1 listopada jeździć na cmentarze i odwiedzać groby?**

Tak. Chociaż zdarzały się takie lata, że nie byłam. Nie wiem, może ze 3 razy nie byłam. Natomiast też nie podchodzę do tego tak, że… Znaczy ja na przykład pojechałam do swoich dziadków wcześniej. Na zasadzie, że nie chciałam przeciskać się między ludźmi na cmentarzu. Nie robi mi to różnicy, czy ja będę we wrześniu, październiku czy w listopadzie. Ja myślę o moich dziadkach, babci szczególnie, cały czas. I to, czy ja zapalę świeczkę dziś, jutro, za miesiąc czy za pół roku, to w ogóle nie ma…

**Nie planowałaś jutro i pojutrze?**

Nie, ja nie planowałam, ja się nie wybierałam. Mąż był, teść nie żyje, więc był, zawiózł. Ja nie będę i to mi w ogóle nie…

**A myślałaś już o świętach Bożego Narodzenia? Bo chodzą słuchy, że będzie tak jak w Wielkanoc, albo i gorzej z nimi. Czyli zakazy spotkań.**

Nie myślałam. Natomiast te święta wielkanocne ja dobrze wspominam. I nie widzę, żeby Boże Narodzenie miało być… Nam nie przeszkadza, że nie będzie biesiadowania jakiegoś.

**Czyli wielka uroczystość rodzinna na całą możliwą rodzinę nie jest ci potrzebna.**

Nie. Tak jak powiedziałam o sylwestrze, na który zawsze chodziłam od kilkudziesięciu lat też nie zrobi mi nic. Taki czas i po prostu.

**Masz jeszcze jakieś przemyślenia w związku z tematem, z pandemią, z przyszłością?**

Jest to na pewno trudny czas dla wszystkich. Na pewno czuję takie zmęczenie całą sytuacją. Takie po prostu, że to już jest takie męczące. Takie po prostu w całokształcie. Już nawet bez tych wahań takich, kiedy większy lęk, mniejszy, obawy, obostrzenia. Po prostu jest takie zmęczenie. Takie po prostu.

**Obniżenie nastroju też takie ogólne, czy to niekoniecznie?**

Nie, nie. Takie zmęczenie. Bo jak coś już się ciągnie i się wałkuje tyle czasu, to już takie jest no nużące. I to już męczy. Męczy, bo znowu coś od nowa i to samo. I nie widać tego lepszego, tylko jakby idzie w gorszą stronę.

**Dziękuję.**
