# Supplementary material for: The challenges arising from the COVID-19 pandemic and the way people deal with them. A qualitative longitudinal study
Source: PLoS One. 2021 Oct 11;16(10):e0258133. doi: 10.1371/journal.pone.0258133 (PMC8504766; doi:10.1371/journal.pone.0258133)
Supplement: S1 Dataset — (ZIP) [file pone.0258133.s003.zip › Transcriptions/stage 6/8.6_M_30_couple, no children.docx]

**8.6_M_30_couple no children**

**Co działo się u ciebie od naszego ostatniego spotkania?**

Jakoś w tym okresie, z tego, co pamiętam, wracałem z home office'u już do stacjonarnego trybu pracy i w tym trybie pozostaję cały czas. W międzyczasie odbyły się wybory, z różnymi problemami. Były wakacje, dzieci nie chodziły do szkół i przez chwilę był względny spokój z epidemią, bo tam cały czas, z tego co pamiętam, było koło kilkuset zachorowań dziennie, pewnie do jakichś 400. Potem we wrześniu uczniowie wrócili do szkół, w październiku wrócili studenci i powoli chyba z powrotem się zaczęła wtedy mniej więcej rozkręcać epidemia. Ludzie pewnie trochę się przyzwyczaili do tego życia z koronawirusem dookoła i stali się mniej ostrożni, więc pewnie też to się przyczyniło do nasilenia epidemii.

**Jak wyglądały twoje wakacje?**

Wyjechałem na tydzień w sumie tylko nad morze. Byliśmy w takim miejscu, że mogliśmy z łatwością unikać tłumu i innych ludzi. Ale z jakichkolwiek wyjazdów za granicę zrezygnowaliśmy. Były to plany wakacyjne zminimalizowane mocno.

**Z jakiego powodu zrezygnowaliście z wyjazdów?**

Głównie z powodu takiej niepewności, że nie było wiadomo, czy te granice zostaną otwarte, czy może jak wyjedziemy, to nie będziemy mogli wrócić, jak tu zarezerwujemy, to czy uda się te pieniądze odzyskać, a jeśli się nawet uda, to po jakim czasie, ile to będzie musiało być zamrożone, tak że stwierdziliśmy, że łatwiej będzie zorganizować jakiś po prostu spontaniczny wyjazd nad morze, a potem jeszcze wyjechaliśmy w połowie września w góry, jak już się trochę sezon turystyczny skończył.

**Czy wybieraliście miejsca z dala od ludzi?**

Tak, to było jedno z kryteriów i udało nam się znaleźć taki jeden mikrokemping przy szkółce windsurfingowej czy szkółce kiteowej, który był na skraju Chałup, więc ani na tym kempingu nie było dużo ludzi, bo 4 przyczepy na krzyż, na plaży to też był akurat taki odcinek, że nikt tam nie chodził, bo wszyscy się tłoczyli raczej bliżej centrum Chałup, więc tak, zdecydowanie to było jedno z istotnych kryteriów.

**Epidemia była w waszych myślach czy po prostu zawsze unikacie tłumów?**

Na pewno jedno i drugie, myślę, że żadne z nas nie lubi tłumów, ale pewnie jeszcze chętniej szukaliśmy takiego miejsca, jeszcze chętniej wybraliśmy takie miejsce na skraju, z dala od innych ludzi. I dlatego też unikaliśmy tych bardziej tłocznych miejsc na plaży czy w samym mieście, restauracjach itd.

**Czy baliście się zakażenia?**

W niewielkim stopniu pewnie tak, to cały czas się głównie w naszym przypadku chodziło o takie kwestie, żeby tego nieświadomie nie przenieść na kogoś z rodziny np., głównie chyba o to chodziło.

**Czy stacjonarny tryb pracy ci przeszkadza?**

To akurat wynika ze specyfiki mojej pracy. Mam takie okresy, że mam dużo pracy na komputerze i wtedy mogę to zdalnie wykonywać, a teraz od paru miesięcy głównie jednak siedzę w laboratorium i albo robię jakieś doświadczenia biochemiczne, albo jakieś doświadczenia ze zwierzętami, więc tego się zdalnie po prostu nie da zrobić, a terminy gonią, nie bardzo mogę przekładać to na przyszłość. Teraz np. widząc, jak szybko to się rozwija, staram się to przyspieszyć, bo nigdy nie wiem, czy nagle nie będzie jeszcze większy lockdown wprowadzony i czy nie będę po prostu zmuszony zawiesić te doświadczenia.

**Twoja codzienność, poza pracą, wróciła do stanu sprzed epidemii?**

Nie no, trochę się różni, widać, że cały czas jednak ludzie w miejscach publicznych te maseczki noszą, oczywiście ostatnio trochę jeszcze bardziej restrykcyjnie, niż to było np. w okresie wakacyjnym, gdzie można było obserwować takie lekkie rozluźnienie. Ale widać, że jednak cały czas ta epidemia jest w głowach ludzi.

**Poza maseczkami jest jeszcze coś, co odbiega od normy?**

W ostatnim czasie znowu ten lockdown na pewno odbiega od normy, bo np. większość siłowni jest zamknięta, restauracje działają tylko na trybie wydawania posiłków na wynos, więc to już się staje mocno widoczne i mocno dotkliwe.

**Jak wyglądają twoje spotkania z bliskimi?**

Przede wszystkim są mocno ograniczone. Jedna moja babcia sama wręcz unika spotkań i narażania się na jakikolwiek bliższy kontakt i ryzyko transmisji wirusa. Dziadkowie z drugiej strony może aż tak nie unikają, ale też spotykamy się na pewno trochę rzadziej i staramy się jednak zachować dystans, witać się z daleka wyłącznie itd. A jeśli chodzi o spotkania ze znajomymi, to ostatnio głównie się to ogranicza do spotkań ze znajomymi z pracy, z którymi i tak się widzę w pracy, więc ten kontakt jest trochę mniej ryzykowny, aczkolwiek oczywiście staramy się w miarę możliwości jakiś tam dystans zachować. Natomiast część innych znajomych w ogóle się trochę boi wirusa i boi się spotykać z kimkolwiek. Mocno są te kontakty ze znajomymi na pewno ograniczone.

**Czy do czasu zamknięcia gastronomii zdarzało ci się wychodzić ze znajomymi?**

Do kina tylko z dziewczyną chodziłem, natomiast ze znajomymi tak, parę razy się w tym okresie spotkaliśmy w jakiejś restauracji czy barze, ale zawsze to było na powietrzu, w jakimś ogródku restauracyjnym czy jakaś plaża nad Wisłą, chyba ani razu się nie zdarzyło, żebyśmy siedzieli w środku w jakiejś restauracji. No może na tym urlopie, jak byliśmy ze znajomymi, to mieszkaliśmy i tak w jednym domku i szliśmy do restauracji, razem siedzieliśmy przy jednym stole, ale to jedyna taka sytuacja.

**Czy siedzenia nad Wisłą było spowodowane wirusem?**

Jedno i drugie, raz, że przyjemnie jest oczywiście posiedzieć na zewnątrz, ale też pamiętam, że jak się umawialiśmy, jak zastanawialiśmy się, gdzie pójść, to jednak ta możliwość siedzenia na zewnątrz w tej sytuacji pandemicznej, zwłaszcza dla tych osób, które cały czas siedziały na home office'ie i nie kontaktowały się z innymi ludźmi, to oni czuli się z tym na pewno dużo swobodniej.

**Czy zdarza się wam chodzić do kina?**

Byłem 2 tygodnie temu ostatni raz w kinie i w tej sytuacji uważam, że jest to dosyć bezpieczne, bo poza nami w kinie były 3 osoby na całej sali, wiadomo, że sala jest klimatyzowana, więc ten obieg powietrza jest, można usiąść z dala od innych ludzi. Poszliśmy w niedzielę, więc też centrum handlowe całe było wyludnione, nie było mowy o jakimkolwiek kontakcie z innymi ludźmi, więc w takiej sytuacji uważam, że to było zupełnie bezpieczne. Problemem jest tylko brak interesujących filmów w kinach obecnie.

**Czy jeszcze zauważyłeś jakieś różnice w pracy?**

Od dłuższego czasu na wszystkich korytarzach instytutowych wszyscy się starają chodzić w maskach. Od pewnego czasu my już w samym laboratorium, na naszych wewnętrznych zakładowych korytarzach też staramy się te maski nosić. I teraz drugi tydzień mamy taki w pracy, że dyrekcja wydała zarządzenie w końcu o tym, żeby część pracowników, która może, żeby poszła na tryb zdalny pracy, więc dla niektórych pracowników jest to taki tryb pracy zmianowej, część osób przychodzi we wtorki i czwartki, część przychodzi np. w poniedziałki i środy. Tak to mniej więcej wygląda u nas teraz.

**To było zarządzone odgórnie?**

Odgórne było zarządzanie ostatnio, żeby kierownicy zakładów oddelegowali do pracy zdalnej wszystkich pracowników, których mogą oddelegować do takiej pracy. Jeśli nie mogą, to żeby tych pracowników w miarę możliwości rozdzielili, żeby np. nie siedzieli w czworo w jednym pokoju.

**Ta praca zmianowa wynikała z tego zarządzenia?**

Tak.

**Czy widzisz zmiany w zachowaniu bliskich?**

W trybie pracy np. u większości rodziny i znajomych to się nie zmieniło nic od paru miesięcy, bo praktycznie wszyscy, którzy byli w trybie zdalnym, to dalej są w trybie zdalnym, ci, którzy pracowali w takim trybie trochę mniej regularnym, jak moi rodzice np., to też mniej więcej tak pracują dalej, co mogą robić zdalnie, to robią zdalnie. A jeśli chodzi o ich nastawienie i jakiś poziom komfortu psychicznego związanego z tym, to na pewno pogorszyło się to w ciągu ostatnich paru tygodni, jak te zachorowania zaczęły iść w tysiące. Widzę, że część osób rzeczywiście mocno się przejmuje tym, mocno się denerwuje, ma duży stres z tym związany.

**Czym się to objawia?**

Takim ogólnym poddenerwowaniem. Nie wiem, jak mogę to inaczej nazwać.

**Czy są jakieś podobieństwa między tym, co jest teraz, a co było wiosną?**

Myślę, że wygląda to podobnie, tylko ten poziom stresu jest wyższy.

**Czy coś ci przeszkadza w tej sytuacji?**

Oczywiście, że np. noszenie maseczek jest średnio komfortowe, ale nie mogę zupełnie szczerze powiedzieć, żeby mi to jakoś przeszkadzało, bo po prostu na tyle rozumiem, że jest to potrzebne, że już przestało to we mnie wzbudzać jakieś negatywne emocje. Więc póki co dla mnie osobiście nie jest to jakieś dotkliwe. Współczuję ludziom, którzy pracują w gastronomii czy mają jakieś kluby sportowe, bo im musi być na pewno ciężko w tej sytuacji, ale na mnie bezpośrednio się to, póki co nie odbija.

**Emocje.**

11 - widzimy tutaj deszcz na szybie, a zza tej szyby przeziera tęcza, co wskazuje na to, że był to jakiś trudny okres, natomiast przyszła odwilż i jest przynajmniej na jakiś czas trochę lepiej. To by mi się kojarzyło z tym okresem powrotu do pracy stacjonarnej i z tym okresem, jak powoli ta krzywa zachorowań zaczęła się stabilizować.

Do wyborów to może 2, bo z tymi wyborami był straszny rozgardiasz i rządzący się nie mogli zdecydować czy robić te wybory, czy ich nie robić, w końcu się zdecydowali, że robić i że przecież nie ma żadnej epidemii, ale ogólnie trochę taki fuckup a z tymi wyborami moim zdaniem był, straszne zamieszanie i zdecydowanie niesmak po nim pozostał. Wtedy też mi towarzyszył niesmak na pewno, jakiś taki przykry obowiązek, przynajmniej dotyczący drugiej tury, bo byłem oczywiście zdecydowany na kogo głosować, ale powiedzmy, że nie był to kandydat moich marzeń nadal. Natomiast mimo wszystko jakaś nadzieja się z nim wiązała, że jednak będzie lepiej, ale na koniec się okazało, że jednak wyszło jak zwykle i jednak silniejszy niesmak pozostał.

Okres wakacyjny i względny spokój - 13. Taki sielankowy krajobraz, późno letnie zboże to tak właśnie kojarzy się ze spokojem, beztroską, takim okresem, kiedy na chwilę można było zapomnieć o tym wszystkim, co się dzieje albo próbować zapomnieć, albo starać się nie myśleć.

**I to faktycznie tak wyglądało?**

Nie do końca. Znaczy na pewno trochę byłem spokojniejszy, ale zdawałem sobie sprawę, że to nie jest tak, jak premier mówi, że pandemia została zatrzymana itd., bo jednak śledziłem codziennie te liczby zakażeń i widziałem, że to jednak nie maleje, ale albo się utrzymuje na stałym poziomie, albo wręcz rośnie. Spodziewałem się, jak skończy się powrót właśnie najpierw uczniów, potem studentów do szkół i powrót też pewnie bardziej zorganizowany ludzi do pracy po wakacjach, więc gdzieś tam z tyłu głowy miałem, że za chwilę będzie gorzej.

**Codziennie śledziłeś słupki zachorowań?**

Znaczy może nie aż tak, żebym codziennie to sprawdzał, ale na pewno raz w tygodniu co najmniej śledziłem takie podsumowanie tygodniowe na jednej ze stron, którą obserwuję, tam bardzo zawsze ładne są zestawienia tych danych i co jakiś czas tyle co w mediach czy gdzieś na Facebooku Ministerstwa Zdrowia widziałem informacje o tych zakażeniach.

**Dlaczego wydawało ci się ważne, aby to sprawdzać?**

Interesowało mnie to po prostu chyba. Wydaje mi się, że tu akurat taka zwykła ciekawość przeważała. Pewnie jakaś taka chęć skonfrontowania się z tym, czy moje przewidywania sprzed tygodnia czy kilku tygodni okażą się ostatecznie słuszne czy nie.

**Który obrazek pasuje do powrotu uczniów i studentów?**

Najpierw bym dał 10, a potem bym dał 16. 10 to takie właśnie wrażenie, że zachmurzyło się, nadciągnął taki niekorzystny front i może jeszcze się nie zaczęła ulewa czy jakaś burza, ale już widać, na co się zanosi i że raczej będzie gorzej. To odpowiada tym początkowym wzrostom zachorowań, gdzie to najpierw rosło stopniowo, ale jeszcze w miarę powoli, no ale potem w pewnym momencie zaczęło to przyrastać szybciej i to już kolejny obrazek.

**Jakie emocje towarzyszyły ci przed tą burzą?**

Jakieś takie poczucie nadciągającego zagrożenia, nie tylko związanego z samą chorobą, ale też z ewentualnym lockdownem, że może wrócić ta sytuacja z wiosny. Jakiś taki rodzaj niepewności, jak to dalej będzie wyglądało. Chyba głównie to.

**I ten moment, kiedy wrócili do szkół studenci?**

Może nie jestem w stanie powiedzieć, czy to akurat był ten moment, czy to się zbiegło w czasie z powrotem właśnie studentów czy nie, czy to w jakiś sposób nasiliło te zachorowania czy nie, może to była tylko koincydencja, aczkolwiek pewnie nie. Natomiast jeśli chodzi o ten okres wzrostu zachorowań, to jest właśnie ten obrazek, kiedy to gwałtownie przyspieszyło, te zachorowania zaczęły iść w tysiące i widać było, że sytuacja jest dużo poważniejsza, niż była kiedykolwiek, że nie zapowiada się, żeby to miało jakoś szybko przystopować, nawet gdyby wprowadzono jakieś ostre restrykcje. Plus jakaś taka wtedy była najbardziej widoczna cała ta opieszałość rządzących, to, że oni tak naprawdę przez ten okres względnego spokoju w żaden sposób się nie przygotowali na tę większą falę zachorowań. No i tutaj jakieś już rosnące poczucie zagrożenia z tym związanego.

**Kiedy poczułeś, że sytuacja robi się coraz bardziej poważna?**

Nie wiem, czy był jeden taki moment, przez pewien czas to przekonanie zaczęło się we mnie rodzić i rozwijać, tak to bym nazwał. Jak śledziłem doniesienia medialne i najpierw pojawiały się żółte strefy, potem pojawiało się coraz więcej tych czerwonych stref, potem cała Polska była żółta, potem cała Polska była czerwona, tak że to myślę, że taka była gradacja tego wszystkiego, nie jestem w stanie podać jednego momentu, kiedy stwierdziłem, że tak, to już jest to, teraz jest groźnie.

**Śledziłeś słupki zachorowań - czy była granica, kiedy zacząłeś się tym bardziej przejmować?**

Nie, nie wydaje mi się, żeby to była jakaś konkretna liczba.

**Jak czujesz się teraz?**

W ciągu ostatnich kilku dni jestem ogólnie poddenerwowany całą też sytuacją polityczną, bo to ze wszystkich stron, ze wszystkich mediów non stop jesteśmy tymi informacjami bombardowani, ale też sam to wszystko śledzę. Natomiast jeśli chodzi o samo zagrożenie epidemiczne, to jestem jeszcze trochę bardziej ostrożny niż byłem wcześniej. Przez ostatnie 2 tygodnie np. nie jeździłem komunikacją miejską. Raz, że z powodu wzrostu zakażeń, dwa, że właśnie jakieś 2 tygodnie temu złapałem lekkie przeziębienie i też starałem się i sam izolować od jeszcze jakichś ewentualnie innych wirusów, i też samemu minimalizować ryzyko, że nawet jeśli już nie mam objawów, to kogokolwiek nawet jakimś głupim przeziębieniem zarażę. Natomiast dzisiaj już np. do pracy pojechałem komunikacją miejską, tak żeby sprawdzić, czy może z okazji tego, że są szkoły zamknięte i coraz więcej firm przechodzi na tryb zdalny, to czy jest luźniej w tej komunikacji. Okazało się, że rzeczywiście jest luźniej, natomiast na wszelki wypadek wziąłem ze sobą jeszcze poza maseczką przyłbicę dla dodatkowej ochrony, ostatecznie jej nie założyłem, bo np. w pociągu w wagonie poza mną było z 5 osób, więc byłem o parę metrów oddalony od jakiejkolwiek osoby.

**Czy jesteś teraz ostrożniejszy niż wiosną?**

Myślę, że tak. Jestem bardziej ostrożny, większą uwagę przykładam do tego, żeby tę maskę mieć, mieć ją dobrze założoną, żeby np. w miejscach publicznych nie dotykać niczego, żeby dezynfekować ręce, jak np. wracam do domu albo jak wchodzę do pracy.

**Jakie jeszcze emocje ci towarzyszą?**

Często np. w miejscach publicznych czuję się zdecydowanie bardziej zirytowany, niż się czułem wcześniej, jak np. widzę kogoś, kto ostentacyjnie nie nosi maski albo ma maskę tzw. przeciwmandatową, która przed niczym nie chroni, bo jest zrobiona z jakiejś koronki, to mnie mocno irytuje. Poza tym może jest jakiś większy we mnie lęk przed zarażeniem się i to pewnie raz, że z tego względu, że bardzo bym nie chciał teraz musieć brać wolnego w pracy, bo deadliny mnie gonią, a też nie chciałbym w najbliższym czasie mieć nic wspólnego z polską opieką zdrowotną, bo ona już ledwo przędzie i różnie to może być z dostępem do niej.

**Który obrazek oddaje twoje obecne samopoczucie?**

Może 9. To mi się skojarzyło z takim zagrożeniem, które jest bardzo dobrze już widoczne, ale nie wiadomo, czy mnie bezpośrednio dosięgnie czy może uda mi się go uniknąć, natomiast ono tam cały czas na horyzoncie jest i trzeba przedsięwziąć jakieś środki, żeby w miarę możliwości go uniknąć.

**Czego obawiasz się w obecnej sytuacji?**

Obawiam się zdecydowanie zarażenia kogoś z rodziny. W jakimś tam minimalnym stopniu też się obawiam, że sam się zarażę i że może jakieś tam powikłania, które są mało prawdopodobne, ale jednak się zdarzają, że mogą u mnie wystąpić, więc ze względu na zdrowie też się staram unikać, w miarę możliwości minimalizować ryzyko zakażenia.

**Czy robisz coś, żeby radzić sobie z emocjami?**

Nie, myślę, że nie, nie jest to taki poziom lęku czy stresu, który ja musiałbym jakoś odreagowywać, który bym jakoś rzeczywiście odczuwał mocniej, nie, nie mam problemów ze snem, tak ogólnie nie czuję się zestresowany.

**A czy twoi bliscy stosują jakieś metody radzenia sobie?**

Nie zauważyłem.

**Czyli funkcjonują tak jak wcześniej?**

Są poddenerwowani, często zlęknieni, ale nie zauważyłem, żeby ktoś coś z tym robił, żeby jakoś próbował sobie z tym radzić.

**Jak twoi bliscy starają się odpoczywać w obecnej sytuacji?**

Moi rodzice relaksują się głównie w domu. W weekendy ewentualnie jadą na grzyby. To jest dla nich, wydaje mi się, taki sposób na odstresowanie się, ewentualnie tam w jakimś bardzo wąskim gronie, w takiej czwórce brydżowej się spotkają ze znajomymi raz na jakiś czas i sobie wieczorem pograją w brydża, dla nich to chyba byłyby główne sposoby na jakiś reset umysłowy i odstresowanie się. Znajomi moi: część np. chodzi na ściankę cały czas, niektórzy wiem, że np. w domu ćwiczą jakąś jogę, inni po prostu chodzą z psem do parku. To takie rzeczy, jakie mi przychodzą do głowy, jeśli chodzi o znane mi osoby.

**Czy te sposoby spędzania czasu odbiegają od tych sprzed epidemii?**

Myślę, że odbiegają u tych osób, które np. regularnie chodziły na siłownię, bo większość siłowni jest zamknięta i muszą sobie znaleźć jakąś inną formę aktywności, ale wydaje mi się, że dla większości, przynajmniej dla ludzi z mojego otoczenia, to się mocno nie zmieniło.

**Czy znasz kogoś, kto zachorował?**

Znam. Jakieś 2-3 tygodnie temu zachorował mój brat cioteczny. On jest 7-8 lat młodszy ode mnie, nawet ten przebieg u niego nie był jakoś specjalnie poważny, głównie jakieś tam nieprzyjemności typu utrata węchu czy smaku. Bardziej przez parę dni się wszyscy denerwowali, czy przypadkiem nie zaraził babci, której przywoził zakupy, aczkolwiek na szczęście zachowali najwyraźniej odpowiedni dystans i do tej transmisji wirusa nie doszło. 2 tygodnie temu kolega z zakładu poszedł na zwolnienie lekarskie. Tzn. najpierw wziął po prostu kilka dni wolnego, bo się kiepsko czuł, po paru dniach jak mu te objawy się pogarszały, to zdecydował się zrobić test na koronawirusa i niestety był wynik dodatni, więc od zeszłej środy mu jeszcze 10 dni nałożyli obowiązkowej izolacji. No i wiem, że parę osób od nas z instytutu, których może nie znam osobiście jakoś lepiej, ale oczywiście znam z widzenia, z widzenia kojarzę, też wiem, że poszło na zwolnienie, bo miało pozytywny wynik. A i jeszcze mój lektor od angielskiego też najprawdopodobniej ma koronawirusa, nie robił sobie jeszcze testu, ale ma wszystkie typowe objawy, więc to też jest najprawdopodobniej to. Więc tak, już dużo osób z mojego otoczenia ma tego wirusa.

**Czy jesteście z pracy kierowani na darmowe testy?**

Nie, z pracy niestety nie jesteśmy kierowani, co uważam, że jest głupie. Zwłaszcza akurat u nas w pracy, bo to nie byłby żaden problem, żeby po prostu zamówić odczynniki do tych testów, bo mamy sprzęt potrzebny do wykonania tych testów, każdy w zasadzie, kto u nas pracuje, ma umiejętności, żeby te testy przeprowadzić, więc moglibyśmy sobie nawet tutaj tylko na własne potrzeby te testy wykonywać za jakieś grosze, ale niestety nikt z tym nic nie robi. I też nie ma jakiejś takiej organizacji, żeby np. poinformować chociażby osoby z tego samego zakładu, że ktoś od nich ma koronawirusa i poszedł na zwolnienie i że może jednak warto objąć izolacją też inne osoby, które miały z nim kontakt. Tutaj niczego takiego nie ma niestety.

**Dowiadujecie się pocztą pantoflową?**

O tym koledze z zakładu tak. Jedyne co, to nasza pani dyrektor poinformowała oficjalnie mailowo wszystkich pracowników, że ma dodatni wynik, natomiast dalej pocztą pantoflową dowiedzieliśmy się o kolejnych osobach z różnych innych części instytutu, różnych innych zakładów, że tu 3 osoby, tam 2 osoby przechodzą.

**Czy ludzie starają się ukrywać, że zachorowali?**

Nie wiem, wydaje mi się, że przynajmniej te osoby, o których ja wiem, że chorowały, to nie starały się w żaden sposób tego ukryć, tylko wydaje mi się, że brak tutaj jest takiej odgórnej organizacji. Wiadomo, że nikt sam z siebie nie ma nawet możliwości, żeby wysłać informację do wszystkich ludzi, z którymi mógł mieć kontakt w pracy, to myślę, że odgórnie powinno być robione, czy przez kierownictwo danego zakładu, czy przez kierownictwo całego instytutu, natomiast takiej procedury u nas najwyraźniej nie ma. I nie wiem, z czego to wynika.

**Czy byłeś na kwarantannie?**

Nie.

**A z twojego najbliższego otoczenia ktoś był?**

Najbliższe otoczenie to właśnie ten kolega.

**Czy on wiedział, jak się zachować?**

Wiedział, jak się zachować, jest inteligentną, wykształconą osobą, więc wiedział, że należy zrobić sobie test i gdzie go zrobić, i poinformować osoby, z którymi mógł mieć kontakt, że jest w ogóle podejrzenie tej choroby i to zrobił.

**Czy te osoby korzystały z aplikacji "Kwarantanna domowa"?**

Nie wiem nic na temat tego, żeby korzystały i podejrzewam, że nie korzystały.

**Czy znasz kogoś, kto korzysta z tego typu aplikacji?**

Nie, nie słyszałem, żeby ktokolwiek z tego korzystał.

**Jesteś na bieżąco z wprowadzanymi obostrzeniami?**

Nie śledzę tego jakoś super poważnie. Wiem, że doszło do, chociażby z tych takich najbardziej widocznych rzeczy, to, co mówiłem o tych siłowniach czy restauracjach, ale wiem, że też było najpierw ograniczenie np. liczebności na weselach czy jakichś uroczystościach, czy to w żółtej strefie czy czerwonej, potem w ogóle obecnie zabroniono organizacji takich wydarzeń. Wprowadzili też obowiązek posiadania maseczki w samochodzie, jak się z kimś innym jedzie, oczywiście maseczki z powrotem we wszystkich miejscach publicznych, na ulicy chociażby, bo już przez pewien czas chyba nie było obowiązku noszenia maseczki na ulicy. To tyle, co pamiętam.

**Jak się czujesz z tymi obostrzeniami i czy one idą w dobrym kierunku?**

Tak jak mówiłem, to noszenie maseczki mi specjalnie nie przeszkadza. Nie jestem przekonany co do np. zamknięcia siłowni. Wydaje mi się, że lepszym rozwiązaniem było ograniczenie znowu liczby osób, które są wpuszczane na siłownie i jakiejś większe obostrzenia co do dezynfekcji sprzętu, ale to są jednak takie miejsca, gdzie raczej idą osoby zdrowe, które się dobrze czują, więc wydaje mi się, że to ryzyko transmisji wirusa tam jest mimo wszystko niższe niż chociażby podczas zakupów w sklepie. Tak samo, jeśli chodzi o restauracje, myślę, że też może lepszym rozwiązaniem by było najpierw jeszcze jakieś trochę większe ograniczenie liczby stolików czy liczby gości w restauracjach, czy jakichś odległości stolika od stolika, tak żeby wszyscy ci ludzie, czy prowadzący restauracje, czy pracujący w restauracjach - najczęściej niestety na jakichś śmieciowych umowach - po prostu mieli z czego żyć. Myślę, że to w tę stronę powinno było pójść i znacznie wcześniej trzeba było, moim zdaniem, wprowadzić tryb hybrydowy w szkołach np. Pamiętam, że w wakacje były takie pomysły, wychodzące od różnych ekspertów czy od społeczeństwa, żeby np. część uczniów wróciła do szkół 2 tygodnie wcześniej, część wróciła 2 tygodnie później, żeby tak się wymieniali, żeby trochę zmniejszyć to zagęszczenie w szkołach. Myślę, że to było dobre rozwiązanie, które niestety nie zostało zrealizowane.

**Co myślisz o maseczkach w przestrzeni publicznej?**

Wydaje mi się, że jest to takie mniejsze zło, że nawet jeśli czasem jest to nieuzasadnione, żeby nosić maseczkę na pustej ulicy, to może jednak łatwiej jest to po prostu kontrolować, bo może część ludzi np. potem zapomina założyć tę maseczkę, wchodząc do sklepu, autobusu czy pociągu, a tak mają, powinni ją przynajmniej mieć cały czas na twarzy.

**A ograniczenia dla seniorów?**

Rekomendacje, żeby nie wychodzić z domu, wydaje mi się, że są słuszne, bo oni są najbardziej zagrożeni, jeśli tylko mogą zminimalizować ryzyko zakażenia, to powinni to robić. Jeśli chodzi o godziny dla seniorów, to nie wiem zupełnie, czy to ma sens. Nigdy nie byłem w sklepie w czasie tych godzin dla seniorów, więc nie wiem, czy tych seniorów wtedy tam jest więcej czy mniej, czy te godziny się sprawdzają. Wiem, że np. jak jestem tutaj obok pracy rano w Hali Banacha, koło godz. 8-9, to wiem, że w tych godzinach jest mnóstwo seniorów, więc wydaje mi się, że to może być wątpliwe rozwiązanie. A może warto je jakoś przewalczyć.

**Zakaz organizowania imprez?**

To, wydaje mi się, jest konieczne, bo na weselach nie da się niestety, co by nie mówić, nie da się uniknąć bardzo bliskiego kontaktu z innymi ludźmi, nikt nie może tego zapewnić. Powiedzmy, jak to jest jakaś mała, tylko taka uroczystość rodzinna, 20-osobowa, to jeszcze ok i w takich samych warunkach myślę, że można by było zorganizować stypę. Znaczy, wiem, że można, bo my tak w wakacje musieliśmy zrobić, ale na pewno wyjątkowo nierozsądne by było organizowanie w tej sytuacji dużego wesela. I tak samo nierozsądne było organizowanie tych wesel w wakacje uważam. Te restrykcje zostały zdjęte, wiem, że dużo ludzi organizowało często bardzo duże wesela i moim zdaniem to było bardzo głupie. Moi znajomi zrezygnowali z wesela, do którego się przygotowywali przez rok i uważam, że to było bardzo odpowiedzialne zachowanie z ich strony.

**Organizowanie jakichkolwiek wesel było nieodpowiedzialne?**

Trudno mi nazywać 20-osobowy rodzinny obiad weselem, więc mając na myśli wesele, to chodzi mi o dużą imprezę na 50 czy więcej osób.

**Branża eventowa powinna zawiesić działalność?**

To też jest trudna sprawa. Jeśli chodzi o imprezy w jakichś klubach, tego typu koncerty czy w jakichś halach, to nie powinno być dopuszczone, bo tu raz, że jest bliski kontakt człowieka z człowiekiem, a dwa, że jest ograniczona przestrzeń. Natomiast jakiś czas temu się natknąłem na jakieś badania, mogę teraz złe liczby przytoczyć, natomiast to w Stanach chyba sprawdzali, jak tamtejsze protesty wpłynęły na zwiększenie liczby zachorowań i tam to był wzrost bodajże rzędu 3% tylko, więc to może świadczyć o tym, że imprezy na otwartym powietrzu, oczywiście z zachowaniem jak najlepszych środków ochrony osobistej, mogłyby być może funkcjonować, ale myślę, że to wymagałoby jeszcze dokładniejszego przyjrzenia się tym danym czy może zwiększenia liczby tych badań, żeby to w pełni racjonalnie ocenić.

**Ograniczenia w transporcie publicznym?**

Trudno mi powiedzieć, bo nie widziałem, szczerze mówiąc, żeby te ograniczenia były kiedykolwiek czy gdziekolwiek egzekwowane, bo przez całe wakacje czy jeszcze we wrześniu, październiku funkcjonowały te ograniczenia co do liczby pasażerów, natomiast wielokrotnie czy w tramwajach, czy w pociągach widziałem, że ta liczba pasażerów była wyraźnie większa niż dozwolona, nikt nic z tym nie robił, żaden kierowca, żaden konduktor. Co też trochę rozumiem, bo ludzie stoją na dworcu i wiadomo, że są zdeterminowani, żeby wsiąść do tego pociągu, jeżeli następny mają za godzinę. No i sobie wyobrażam, jak to są często, przynajmniej te pociągi, którymi ja jeżdżę, jakaś linia z Piaseczna do Mińska Mazowieckiego, no to jak ktoś się dosiada na jakiejś środkowej stacji, to jest duże ryzyko, że mógłby w ogóle nie wsiąść do żadnego z tych pociągów i nie dojechać. Nie wiem, na ile to jest możliwe do wyegzekwowania. Być może lepsze by było namawianie ludzi do rezygnacji z transportu zbiorowego w miarę możliwości niż wprowadzanie jakichś restrykcji.

**Zdarzyło ci się też złamać te obostrzenia w transporcie zbiorowym?**

Jest to bardzo prawdopodobne, nigdy nie liczyłem pasażerów w pociągu. Ja wsiadam na takich stacjach, które zwykle nie są jakoś mocno przepełnione. Jest taka stacja w połowie mojej drogi mnie więcej, gdzie często bardzo dużo ludzi się dosiada, ale tak, jest to bardzo prawdopodobne, że ja też złamałem ten zakaz czy to obostrzenie, bo nie wiem, czy to można zakazem nazwać.

**Czy zdarzało ci się łamać inne obostrzenia?**

Tak, zdarzało mi się np., idąc ulicą, cały czas mi się zdarza, że jak idę ulicą i nie ma żadnych ludzi dookoła mnie, to zdejmuję maseczkę, zakładam ją przed wejściem do tramwaju, metra, sklepu albo jak jacyś ludzie zaczynają się dookoła pojawiać.

**Załóżmy, że idziesz ulicą bez maseczki i w pewnym momencie ktoś zaczyna się do ciebie zbliżać: zakładasz maseczkę czy omijasz tę osobę?**

Jeśli jestem w stanie zachować dystans kilku metrów, to myślę, że nie zakładałbym w tej sytuacji. Natomiast mam taką drogę do pracy, gdzie jest chodnik szerokości 1,5 m, to w takiej sytuacji zakładam po prostu maseczkę odpowiednio wcześniej, jest to, wydaje mi się, taki proces niewymagający myślenia, jest to rodzaj odruchu już teraz chyba.

**Czy zmieniło się twoje myślenie dot. źródeł koronawirusa?**

Nie dokonałem żadnego przełomowego odkrycia niestety, jeśli chodzi o pochodzenie koronawirusa, więc chyba nie mam nowych wniosków, żadnych nowych informacji. To, co było wiadomo na wiosnę, to mniej więcej cały czas jest potwierdzane z tego, co wiem.

**Pojawiają się nowe teorie - czy teraz jesteś bardziej skłonny się nad nimi zastanawiać?**

Myślę, że jestem co najwyżej jeszcze bardziej sceptyczny co do jakichkolwiek doniesień i jak pojawiają się jakieś nowe doniesienia na temat koronawirusa, to musi być naprawdę duża siła tego dowodu, żebym wziął to pod uwagę, bo taka liczba fake newsów czy jakichś po prostu przekłamań medialnych i clickbaitów się pojawiała i pojawia cały czas, że we mnie chyba ten sceptycyzm tylko narasta.

**Czy obecna sytuacja jest poważna?**

Uważam, że jest bardzo poważna, ponieważ nasza służba czy ochrona zdrowia jest na granicy wydolności i jak liczba zachorowań będzie przyrastała w takim tempie, to za chwilę skończą się miejsca w szpitalach, dostępne respiratory, specjaliści umiejący obsługiwać respiratory i po prostu ludzie będą umierać tylko dlatego, że nikt im nie udzielił pomocy. Uważam, że sytuacja już teraz jest bardzo poważna.

**Czyli ta powaga sytuacji wynika ze słabego przygotowania a nie śmiercionośności wirusa?**

Wirus wydaje się, że jest dosyć groźny, zwłaszcza dla osób z grup ryzyka jest dosyć duże ryzyko groźnych powikłań, natomiast on się staje szczególnie groźny, jeśli w środku tych powikłań nie ma możliwości udzielenia odpowiedniej pomocy, jak właśnie bardzo często w przypadku tego wirusa konieczne jest podłączanie do respiratorów. Tych respiratorów, wiadomo od miesięcy, że mamy za mało i że brakuje też specjalistów do ich obsługi, i też już wiemy, że nasze władze nic z tym problemem od paru miesięcy nie zrobiły.

**Czy ludzie obecnie zachowują się adekwatnie do sytuacji?**

Coraz częściej tak. Coraz rzadziej widzę ludzi, którzy by nie mieli maseczki w miejscach publicznych, coraz rzadziej widzę ludzi, którzy mają te maseczki źle założone, coraz częściej widzę, że są to ludzie w porządnych maseczkach, czy to przynajmniej chirurgicznych, czy też jakichś wyższej klasy, a rzadziej w bawełnianych samoróbkach, więc wydaje mi się, że trochę ta odpowiedzialność ludzi wzrosła.

**Czy dało się zapobiec obecnej sytuacji?**

Tej obecnej na pewno się dało zapobiec. Pewnie nie dałoby się zapobiec wzrostowi zachorowań, ale na pewno dało się zapobiec tak szybkiemu, gwałtownemu wzrostowi zachorowań.

**Jak oceniasz decyzje podejmowane przez rząd?**

Jako fatalne, chaotyczne, niezorganizowane, nieprzemyślane, na ostatnią chwilę. Widać po prostu było przy każdej kolejnej konferencji premiera, że oni w ogóle wcześniej się nie zastanawiali, nie mieli żadnego planu, co zrobić, jak będzie 1000 chorych dziennie, co zrobić, jak będzie 10 000 zakażeń dziennie i że to wszystko było po prostu przygotowywane ad hoc.

**Jakie emocje wywołują w tobie takie działania?**

Złość, irytację, niechęć.

**Czy czerpiesz informacje z tych samych źródeł co na wiosnę?**

To są te same źródła, co korzystałem.

**Czy nadal aktywnie szukasz informacji o sytuacji na świecie?**

Myślę, że rzadziej.

**Skąd brałeś liczbę zakażonych?**

To jest taki bardzo sensowny moim zdaniem blog, który się nazywa "Fizyk wyjaśnia". Tam co tydzień pokazywały się takie zestawienia statystyczne, natomiast też te codzienne liczby zakażeń to np. z informacji zamieszczanych przez Ministerstwo Zdrowia czy ze strony, która się nazywa chyba "Worldometer", gdzie dla wszystkich państw są podane liczby zakażeń, zgonów itd., więc też tam patrzyłem co jakiś czas.

**Czy czas poświęcany na korzystanie z mediów się zmieniał?**

Nie, wydaje mi się, że nie. Raczej nie jest to coś, czemu ja bym dużo czasu poświęcał, raczej to są 2 kliknięcia, żeby spojrzeć na liczby i ewentualnie właśnie raz w tygodniu 3 min na przeczytanie tego dokładniejszego zestawienia, takiego tygodniowego podsumowania i analizy danych.

**Jak oceniasz wiarygodność mediów?**

Trudno powiedzieć, bo są media... Jako społeczeństwo teraz mamy wszyscy praktycznie dostęp do zarówno bardzo wiarygodnych mediów, jak i do totalnie niewiarygodnych mediów. Trudno mi powiedzieć. Te, powiedzmy, najpopularniejsze, mainstreamowe media są moim zdaniem średnio wiarygodne, głównie to wynika z tej pogoni za sensacją i takim gorącym tematem, to jest bardzo często istotniejsze dla nich niż przedstawienie wiarygodnych danych, ten sztandarowy przykład nowych odkryć amerykańskich naukowców, które się pojawiają co i rusz w gazetach, telewizji. Amerykańscy naukowcy co chwilę dokonują przełomowych odkryć, polscy naukowcy też co chwilę są o krok od wynalezienia lekarstwa na raka, ale zazwyczaj się okazuje, że to są jakieś zwykłe badania in vitro.

**Czy zmienił się twój sposób oceny prawdziwości informacji?**

Nie, już wcześnie w podobny sposób oceniałem, tzn. jak czytam jakąś informację, zwłaszcza taką, która jest dla mnie nowa, to patrzę przede wszystkim na źródła tej informacji. Jeśli to jest źródło takie jak rzetelne czasopismo naukowe, już najlepiej klasy jakiegoś "The Lancet", "Nature" czy tym podobnych czasopism, to już samo świadczy o tym... Oczywiście jeśli to jest coś, co budzi moje wątpliwości, to jeszcze sam otwieram sobie ten artykuł i czytam przynajmniej abstrakt i wnioski z tego artykułu, czy autor jakiejś informacji prasowej czegoś tam nie przekłamał czy nie zmanipulował. Ale jeśli ta informacja pochodzi z takiego czasopisma, to wiem, że to zostało zweryfikowane przez światowej klasy recenzentów i że to prawie na pewno jest wiarygodna informacja, na tyle potwierdzona, na ile tylko metodologia pozwala ją potwierdzić. Natomiast jeśli chodzi o źródła niższej klasy, to zawsze budzi we mnie jakąś wątpliwość, siła dowodu jest niższa.

**Czy myślisz o przyszłości po pandemii?**

Trudno też mi się wypowiedzieć, bo żeby myśleć o przyszłości, to trzeba widzieć, kiedy ta przyszłość popandemiczna może się zacząć.

**Co musiałoby się stać, żeby stwierdzić, że pandemia się skończyła?**

Jest kilka możliwości na pewno. Jedna możliwość np. jest taka, że doszło już do takiej liczby zakażeń, że jest jakaś wykształcona odporność zbiorowa w społeczeństwie i ta transmisja wirusa została zahamowana. Natomiast z tego, co wiem, coraz więcej danych się na razie pokazuje takich, że ta odporność stadna może nigdy nie nastąpić w przypadku koronawirusa, bo jest coraz więcej przypadków ponownych zakażeń i wszystko wskazuje na to, że ta odporność utrzymuje się raczej przez kilka miesięcy tylko, a nie trwa. Druga opcja jest taka, że może zostać wynaleziona szczepionka, ale po pierwsze ta szczepionka musi mieć dosyć dużą skuteczność, po drugie tej szczepionki trzeba wyprodukować na tyle dużo, żeby dostarczyć ją do pewnie 80-90% społeczeństwa, a po trzecie trzeba jeszcze te 80-90% społeczeństwa nią zaszczepić, co też nie będzie łatwe i nie będzie szybkie. Kolejna możliwość jest taka, że po prostu musimy się z tym pogodzić, że jakiś % społeczeństwa umrze i przetrwają najsilniejsi, ale oczywiście takiego scenariusza jak najbardziej staramy się uniknąć, bo to by była katastrofa. Kolejna możliwość jest taka, że przez następne lata będziemy żyć tak, jak żyjemy teraz, już zawsze będziemy nosić maseczki, wszystko starannie dezynfekować.

**Ludzie będą chcieli się szczepić?**

Podejrzewam, że pewnie jakaś połowa społeczeństwa będzie się chciała zaszczepić, a pewnie jakieś dobre kilkadziesiąt % się będzie mocno ociągać ze szczepieniem, żeby zobaczyć, jak to na innych podziałało, a pewnie też jakiś % społeczeństwa będzie aktywnie walczyć ze szczepionkami, bo to ich zdaniem odbieranie wolności, czipowanie, depopulacja etc.

**Czy zdarza ci się myśleć o przyszłości Polski?**

Zdarza mi się w ostatnich dniach coraz częściej myśleć o przyszłości Polski.

**Czy koronawirus dużo zmieni, jeśli chodzi o przyszłość Polski?**

Trochę na pewno. Na pewno ucierpi chociażby gospodarka, na pewno ucierpi bezpośrednio wielu ludzi, którym po prostu upadną biznesy czy stracą miejsca pracy, więc może też wzrosnąć poziom bezrobocia, ubóstwa. Oczywiście jak gospodarka kraju ucierpi, to nie wiadomo, jak to wpłynie na wszystkie programy socjalne, na finansowanie służby zdrowia. Trudno przewidywać, jak mocno na tym kraj ucierpi, w jakimś stopniu na pewno ucierpi.

**A na całym świecie dużo może się zmienić?**

Myślę, że w kontekście świata to wygląda podobnie, to są w gospodarce przynajmniej naczynia połączone, jedne kraje mocniej ucierpią z powodu handlu, inne kraje mocniej ucierpią z powodu turystyki, a jeszcze inne z powodu po prostu sytuacji wewnętrznej. Ale na pewno na światową gospodarkę też to wpłynie.

**Czy obawiasz się czegoś w związku z przyszłością?**

Tak ogólnie, jeśli chodzi o przyszłość świata, to w kontekście pandemii się nie obawiam. Może się obawiam tego, że inne sprawy schodzą na dalszy plan, jak jakieś reformy energetyki np., tego typu rzeczy. To myślę, że w takiej dalszej perspektywie dla świata może być bardziej istotne niż koronawirus.

**A sytuacja społeczna?**

Myślę, że jeśli to będzie się utrzymywało w takim stopniu, jak teraz jest, że jednak wirus krąży i ryzyko zakażenia jest duże, to mocno to wpłynie na społeczeństwo, na jakieś kontakty społeczne z innymi ludźmi, wydarzenia społeczne, kulturę jako taką i dostęp do kultury.

**Kultura będzie upadać?**

Po części pewnie tak, widać już zastój duży, jeśli chodzi o rynek filmowy, mnóstwo produkcji zostało wstrzymanych i przełożonych na nie wiadomo kiedy, chociażby przykład tego "Tenetu" Nolana, który wyszedł w miarę niedawno, miał być wielkim hitem, a z powodu pandemii okazał się klapą finansową, mimo iż obiektywnie jest to dobry film, który powinien był bardzo dobrze na siebie zarobić. Nie wiem, jak cała ta sytuacja wpływa na teatry np. czy opery, bo na pewno obecnie są restrykcje, jeśli chodzi o liczbę widzów, na pewno musi to wpłynąć też na przychody teatrów, mam nadzieję, że to się nie będzie wiązało z zamykaniem ich na trwałe. Więc w tej mierze na pewno na kulturę to wpłynie.

**Jakieś grupy społeczne szczególnie odczują pandemię?**

Myślę, że jest sporo takich grup. Chociażby wszyscy pracownicy ochrony zdrowia, czy to lekarze, czy sanitariusze, czy ratownicy, oni mają obecnie bardzo ciężką sytuację i też pytanie, jak długo oni będą w stanie tak pracować, w takim stresie i przy takiej ilości obowiązków, zachowując własne zdrowie. Na pewno to wpłynie też np. na nauczycieli, którzy też muszą w takiej mocno nietypowej rzeczywistości się odnaleźć. Podejrzewam, że dla wielu starszych nauczycieli to też może być duży problem, żeby prowadzić zajęcia w takim trybie zdalnym. A też pytanie jak to wpłynie na poziom nauczania i realizację programu. W kontekście uczniów i nauki zdalnej, na pewno to też mocno wpłynie na mniej zamożną część społeczeństwa, bo już na wiosnę było słychać o sytuacjach, że wielu uczniów nie miało dostępu do komputera czy do internetu czy po prostu był jeden komputer na całą rodzinę, a trójka dzieci potrzebowała dostępu do zdalnych lekcji, a rodzice potrzebowali komputera do swojej pracy zdalnej, więc ktoś musi ucierpieć zawsze.

**Planujesz obchodzić Święto Zmarłych?**

Nie planuję obchodzić w ogóle. Ja w ogóle nie jestem religijny, więc nawet jeśli miałbym potrzebę odwiedzenia cmentarza, to w żaden sposób data 1.11. nie byłaby dla mnie tutaj istotna, mógłby to być dowolny dla mnie dzień.

**A Boże Narodzenie?**

To w dużym większym stopniu. To jest taka dla mnie przede wszystkim rodzinna tradycja, że wszyscy się spotykają przy jednym stole, rozmawiają, dzielą prezentami. Na pewno też tutaj trzeba to będzie w jakiś sposób ograniczyć, przede wszystkim ze względu na bezpieczeństwo dziadków. Jeszcze chyba nikt z nas się nie zastanawiał, jak to w praktyce przeprowadzić, ale na pewno będzie to w pewien sposób przykre, na pewno mniej przyjemne niż w ubiegłych latach.

**Oprócz stypy, uczestniczyłeś w innych uroczystościach?**

Nie.

**Jak wyglądała ta stypa?**

Grono było mocno ograniczone do bliskiej rodziny, raczej bez znajomych. I to właśnie było pewnie niecałe 20 osób. Ale też staraliśmy się, ja w niewielkim stopniu, ale wiem, że część rodziny, która to organizowała, to bardzo dużą wagę przykładała do tego, żeby to była restauracja z dużym ogródkiem, żeby można tam było siedzieć na powietrzu, a nie w ciasnej sali, przede wszystkim też nie tłoczyć się i nie mieć kontaktu z innymi ludźmi. Była wybrana mała kameralna restauracja, niedaleko lasu, z bardzo dużym terenem zielonym.

**Czy pandemia przyniesie nam coś dobrego?**

Przyniesie na pewno dużo kompetencji cyfrowych, wiele ludzi pracuje zdalnie i czy pracownicy, czy pracodawcy się dowiadują i orientują, że praca zdalna bardzo często może być sensownym rozwiązaniem, czy w przypadku pandemii, czy nawet w normalnych warunkach. Podejrzewam, że nawet jak już pandemia kiedyś zniknie, to wiele firm pozostanie przynajmniej częściowo przy takim systemie pracy przynajmniej częściowo zdalnej. Dzięki temu też pewnie wiele nowych rozwiązań informatycznych powstało czy się rozwinęło. Mam nadzieję, że też zostanie ludziom taka świadomość, że istotne jest, czy w przypadku koronawirusa, czy w przypadku innych wirusów, dbanie o higienę rąk czy dbanie o to, żeby np. jak po prostu jesteśmy zwyczajnie przeziębieni, a musimy wyjść z domu, to żeby założyć maseczkę i ochraniać innych ludzi przed rozsiewanym przez siebie wirusem.

**Może chciałbyś coś dodać?**

Nie wiem, może powstrzymam się mimo wszystko od jakichś deklaracji politycznych, bo to nie miejsce.
