# Supplementary material for: The challenges arising from the COVID-19 pandemic and the way people deal with them. A qualitative longitudinal study
Source: PLoS One. 2021 Oct 11;16(10):e0258133. doi: 10.1371/journal.pone.0258133 (PMC8504766; doi:10.1371/journal.pone.0258133)
Supplement: S1 Dataset — (ZIP) [file pone.0258133.s003.zip › Transcriptions/stage 6/16.6_F_36_couple, with children.docx]

**16.6_F_36_couple with children**

**Jak ci minęły ostatnie miesiące?**

Były wakacje, trochę powyjeżdżaliśmy nad morze w czerwcu i w lipcu, w sierpniu pracowałam, więc siedzieliśmy w domu, od września nasz syn poszedł 1-szy raz do przedszkola, więc mieliśmy trochę zmian organizacyjnych w domu. Bardzo dobrze zniósł pójście do przedszkola. Ja się obawiałam trochę, ale naprawdę jest przeszczęśliwy i pokochał przedszkole. My też jesteśmy przeszczęśliwi z tego powodu, ale jeszcze z Monią siedzę.

**Jakieś ważne momenty z tego ostatniego czasu?**

Przełomowe było pójście syna do przedszkola i to, że już nie jest ze mną cały czas. I jeszcze po raz pierwszy, odkąd urodziłam Monię pojechałam sama bez dzieci na weekend. Miałam taki weekend tylko dla siebie, 2 noce. Fajnie tak się trochę wyrwać i oderwać od świata. Wakacje to też fajna sprawa i fajnie, że były. Już trochę minęło, już trochę się to zapomina. 2 tygodnie temu byłyśmy na tym weekendzie z koleżankami, więc to mam trochę bardziej na świeżo.

**Jak wygląda teraz twoja codzienność?**

Ogólnie chyba wszystko wróciło do normy sprzed pandemii nie licząc chodzenia w tych maseczkach w sklepach. teraz znowu są kolejne jakieś ograniczenia, więc znowu pewnie będzie się wracać do tamtego stanu. Generalnie chyba już tak mniej rygorystycznie, nawet przy takiej liczbie zachorowań się podchodzi do tego. Na pewno kontakty z ludźmi wróciły do normy i nie widzę tutaj jakiejś różnicy, wyjazdy też, bo w sumie wyjeżdżaliśmy i też tak jakby nie było tej pandemii. To nas nie ograniczyło, przynajmniej przez ten czas, który był do tej pory. Inaczej się robi zakupy, bo tam trzeba nosić te maseczki, ale do sklepów chodzimy, też robimy zakupy i stacjonarnie, i przez internet. Jak nam pasuje tak robimy. Do kościoła też chodzimy normalnie. Nie sądzę, żeby były w naszym życiu jakieś zmiany.

**Spotkania z ludźmi jak przedtem, czyli jak?**

Spotykaliśmy się w dużym gronie, były normalne spotkania rodzinne. To wszystko funkcjonowało. Teraz jest taka sytuacja, że moje dzieci są przeziębione, więc się nie spotykamy chwilowo, ale to też jest normalne, bo za każdym razem jak dzieci mają katar to ja nie wożę ich po ludziach. We wrześniu i w październiku jeszcze się spotykaliśmy i też w dużych grupach. Teraz miałyśmy ten weekend kobiecy i to była forma takich pseudo rekolekcji. One odbyły się w innym miejscu, bo okazało się, że tak, gdzie planowałyśmy, to nie mogłyśmy tego zrobić, ale to są takie rzeczy bardziej techniczne niż że nagle się nie będziemy spotykać w ogóle. Nie wie, bo może ktoś tam porezygnował, ale ja np. nie i te moje najbliższe koleżanki też nie zrezygnowały z tego.

**A jak funkcjonuje twoje otoczenie, twoja rodzina?**

Część funkcjonuje tak jak przedtem, ale są też tacy znajomi, którzy nie posłali dzieci do szkoły czy przedszkola. Zwłaszcza ci, którzy posyłali do prywatnych. Przeszli np. na edukację domową albo nie posłali dziecka do przedszkola, bo i tak siedzą w domu, więc po co płacić. Na ten rok tak zrobili. Na pewno, gdyby nie pandemia, to ludzie nie nosiliby tych maseczek, nie dezynfekowaliby i nie myli tak rąk, jak teraz to robią. Może trochę też się przyzwyczaili do tego. Nie widzę chyba innych zmian.

czy coś ci szczególnie teraz przeszkadza?

Że rosną teraz te zachorowania i że znowu są kolejne ograniczenia. Wolałabym jednak normalnie funkcjonować, żeby szkoły i przedszkola normalnie działały, żeby można było się normalnie spotykać.

**W czasie wakacji miałaś takie poczucie, że właściwie świat wraca do normy?**

W zasadzie tak, chociaż gdzieś tam była świadomość, że nie jest to do końca normalny czas i że to pewnie zostanie z nami i będzie ta 2 fala. To gdzieś tam wisiało. Była taka świadomość, że to gdzieś tam jest tylko ta świadomość nie wpływała tak na emocje w sensie, że się bałam poruszać, gdzieś wyjść czy cokolwiek.

**Stosowałaś się do ograniczeń, które były?**

Był obowiązek chodzenia do sklepu w masce i stosowałam się., bo jakby szanuję to, że ludzie mają swój biznes i po co robić ludziom kłopot. Bardziej z tego powodu, ale też rozumiem, że to zmniejsza w jakimś stopniu zakażenie. nie jestem ekspertem, nie wiem, może się mylę.

**Emocje**

Czas, który minął:

13 - tylko to mi się na razie kojarzy i pasuje. Jest taki bardzo optymistyczny i pasuje do wszystkich momentów z wakacji, które są właśnie takie beztroskie. To jest czas na odpoczynek, na czas z rodziną. Fakt, że jak Julian szedł do przedszkola, to miałam jakieś obawy, ale nic mi tu nie pasuje

15 - to chyba najbardziej odzwierciedla ten moment początku przedszkola. Są te fale, które idą i nie wiadomo czy się tego bać, czy nie, czy się rozproszą, nie rozproszą. Może być dobrze, ale nie wiadomo do końca.

Teraz:

13 - też to zdjęcie, bo ja jednak widzę to światło w tunelu. Wiadomo, że są pewne rzeczy, które mnie denerwują, powodują mój dyskomfort, ale życie toczy się dalej i przetrwamy to jakoś. Je generalnie jestem optymistyczną osobą.

**Jakie to są uczucia?**

Optymizm, nadzieja...

11 - to chyba jest lepsze na teraz, bo pada deszcz i jest niefajnie, ale tęczę widać za oknem i wiadomo, że ten deszcz się kiedyś skończy i będzie to słońce.

**Masz teraz jakieś obawy?**

Mam przede wszystkim taką obawę, że nas wszystkich ten lockdown gospodarczo dobije, bo jednak biznesy to są naczynia powiązane i jak jedno pozamykają, to zaraz ludzie nie będą mieli pieniędzy na inne. To mnie martwi i widzę po znajomych, że jest dużo gorzej w wielu branżach niż było rok temu. Ludziom się sypią biznesy. Martwi mnie też, że jak moi bliscy zachorują to może to oznaczać koniec ich życia, aczkolwiek taka obawa zawsze jest, bo mogą wsiąść do samochodu i zginąć w wypadku. Nie wydaje mi się, żeby ta choroba miała aż taka śmiertelność, żeby nie była porównywalna z innymi tragicznymi rzeczami, które zawsze mogą nas spotkać.

**Obawy teraz vs obawy wiosną?**

Teraz jest dużo mniejszy strach, jeśli chodzi o zdrowie, bo nie jest to tak śmiertelne jak się mogło wydawać na początku. Nie tak, jak myślałam o tym na początku, aczkolwiek to, że zapycha to służbę zdrowie to też jest niedobre. To może jest kwestią złego zarządzania a nie samego wirusa. Ludzie może umierają na zawały a nie na wirusa, bo nie mają dostępu do lekarza. Natomiast im dalej w las, tym skutki gospodarcze są coraz gorsze, więc ta obawa na pewno wzrosła. Pewnych chorób nie da się zatrzymać i ten lockdown niekoniecznie zatrzymuje to wszystko a decyzje o zamykaniu niektórych biznesów podejmują ludzie i to jest ludzka decyzja a nie siła wyższa.

**Czego dotyczą obawy ludzi w twoim otoczeniu, twoich bliskich?**

Ludzie w moim wieku, którzy mają dzieci, rodziny, biznesy, to mają podobne obawy jak ja. Moi rodzice bardziej natomiast obawiają się o swoje zdrowie, bo oni już nie muszą pracować. Tłumaczę mojej mamie, że my musimy pracować, bo inaczej nie będziemy mieli na życie. Jej jest wszystko jedno, bo i tak dostanie pieniądze i nie mogą nagle wszyscy w kraju przestać pracować, bo umrzemy z głodu. Lekarstwo tu może być gorsze od choroby niestety, a za dawanie lekarstwa jest już odpowiedzialny lekarz, czyli ci, co podejmują decyzje. Ci, co mają biznesy na pewno bardziej obawiają się o sytuację gospodarczą, finansową niż o swoje zdrowie.

**Masz jakieś sposoby na radzenie sobie z obawami, neutralizowanie ich?**

Na pewno, żeby zwrócić swoją uwagę na coś innego i zając się czymś inny, Jak tylko się przestanie siedzieć w komputerze i czytać te wiadomości, zacznie się bawić z dziećmi, czytać książkę czy coś, to od razu trochę jest inaczej. Także zracjonalizowanie sobie pewnych rzeczy, bo pewne rzeczy są przejściowe nawet i jeśli czeka nas większa bieda, to trudno. Nikt nie obiecywał nam, że będziemy zawsze bogaci, piękni, zdrowi i szczęśliwi. Trzeba sobie poradzić z pewnymi rzeczami i z tym co życie stawia przed nami.

**Znasz kogoś, kto chorował, był na kwarantannie?**

Ja nie chorowałam, nie byłam na kwarantannie, ale moja siostra teraz z całą rodziną wleciała na kwarantannę. Już przeżyła to 2 razy. Znam osoby, które chorowały. W lecie chorowała koleżanka mojej siostry. Na weselu zachorowała, a była panną młodą. Dzisiaj się dowiedziałam od rodziców, że ich sąsiedzi właśnie są chorzy. Nie słyszałam wśród znajomych o przypadkach cięższych ani żeby ktoś wylądował w szpitalu czy pod respiratorem czy umarł. W miarę lekki przebieg.

**Znasz procedury, wiesz co robić w razie czego?**

Ja osobiście to bym nie robiła nic i nawet nie mówiła o tym, bo podobno to są jeszcze gorsze konsekwencje. Teraz to chyba lekarze rodzinni na testy kierują albo samemu sobie zrobić test. Nie wiem, jak teraz jest z kwarantanną, bo przedtem wszyscy, z którymi się miało kontakt trafiali na kwarantannę, więc czasami można ludziom większa tym zrobić krzywdę niż jest z tego pożytek. Pewnie bym nie poszła sobie zrobić testów, męża raczej też bym nie wysłała. Jakby coś, to byśmy stwierdzili sami, że pewnie jesteśmy na to chorzy i sami byśmy się izolowali. Nie musiałby nam nikt kazać tego robić. Zazwyczaj jak jesteśmy chorzy to nie widujemy się z ludźmi i nie wychodzimy z domu. To jest raczej normalne zachowanie i nie musi mi nikt tego odgórnie narzucać. Wszystko zależy od ciężkości objawów, bo jeżeli mielibyśmy przechorować tak jak normalną grypę...Gdyby pojawiły się duszności, to pewnie trzeba by było, żeby jakiś lekarz zainterweniował, ale póki jest tylko gorączka, jakiś kaszel i nie ma duszności, to można to sobie samemu wyleczyć.

**Chodzi o to, żeby nie trafić do systemu, żeby nie stać w kolejkach, o coś jeszcze?**

O to wszystko razem. Nie potrzebujemy żadnego L4, więc nie potrzebujemy też zajmować nikomu miejsca w tych kolejkach do testu. Dodzwonić się do lekarza rodzinnego to też czasami graniczy z cudem. Jakbym miała gorączkę i kaszel, to zakładałabym, że to jest to i nie mam potrzeby robienia sobie testu, żeby mieć pewność.

**Gdybyś mogła bez kolejki, za darmo się testować raz w tygodniu, żeby wiedzieć?**

Nie ma nic za darmo. nawet jeśli jestem ubezpieczona i ktoś wyda na te testy moje pieniądze, to nie będzie potem na coś innego. Dla własnej ciekawości bym tego nie robiła i to jest marnowanie pieniędzy.
**Obecne obostrzenia**

Patrzę, wiadomo, że patrzę co tam wymyślą nowego, co kolejnego wymyślą do zamykania. Staram się też czytać o tym, żeby móc też funkcjonować na świecie, bo jak mi wprowadzili godziny dla seniorów, to dobrze jest o tym wiedzieć, bo to też mnie dotyczy. To ja mam wpływ na to, jakie jest moje bezpieczeństwo, a nie zasady, które są wprowadzane. Rozumiem, że jak jest restauracja otwarta i ja do niej pójdę, to idę tam na własne ryzyko. Wiemy, że ten wirus się rozchodzi jak ludzie się spotykają, ale z drugiej strony chyba nas nie stać na to, żebyśmy się nie spotykali, bo musimy pracować a dzieci muszą chodzić do szkoły, muszą się uczyć. Ten lockdown niby gdzieś tam to hamuje, ale właśnie są tego ogromne skutki i właśnie nie jestem pewna czy te wszystkie ograniczenia nie są gorsze niż choroba. Społecznie lockdown też bardzo wpływa. Teraz spotkania przez internet stają się już codziennością i nikt się nie dziwi. Na nas może nie wpływa to aż tak bardzo, ale na młodzież w znacznie większym stopniu. Oni są bardziej podatni na takie zmiany i jednak takie spotkanie online nie jest tym samym co spotkanie człowieka z człowiekiem. To może mieć duże konsekwencje i teraz może bardziej boimy się tego drugiego człowieka. Ludzie boją się dotknąć, zbliżyć się i to też jest takie bardzo negatywne. To jest taka zmiana w złą stronę.

**Ty też boisz się do kogoś zbliżyć?**

Nie.

**Jak się witasz z ludźmi?**

Tak samo jak się witałam. Z najbliższym gronem znajomych i przyjaciół, to za każdym razem jak się widzimy, to sobie dajemy buziaka, przytulamy się. Jak kogoś poznaję nowego to podaję mu rękę i nie mam z tym problemu. Nikt jeszcze mi nie odmówił podania ręki.

**Spotykasz się czasem z barierowymi gestami?**

Tak, ale to są raczej takie pojedyncze rzeczy. Mam taką jedną koleżankę, z którą się spotkałyśmy przypadkiem na mieście. Chciałam się z nią normalnie przywitać i ona, że nie, że może z daleka. No dobra. Tak mi się wydawało wtedy, że to jest jednak przesadzone. Jak się wychodzi na miasto, na ulicę i nawet ona była bez maseczki. Może to jest taki brak zaufania z jej strony do mnie? Jak ja bym była chora albo podejrzewała, że jestem chora to przecież bym sama blisko do niej nie podeszła i bym nie chciała jej pocałować. To jest normalne między znajomymi, że jak ktoś z nas jest przeziębiony, to trzyma się trochę z daleka.

**Co wobec tego z tymi ludźmi, którzy są bezobjawowi i podobno przenoszą wirusa?**

Nie wiem. Myślę, że można być bezobjawowym nosicielem, ale tego się nie da tak do końca...To trzeba by było być naprawdę konsekwentnym i naprawdę nikogo nie dotykać. Ja uważam, że to nie jest tego warte i wg mnie to jest za wysoka cena. Nie obraziłam się na tę koleżankę, nie chciała to nie. Ja nie chowam urazy. Okazywanie bliskości i w ogóle bliskość z innymi ludźmi jest dla mnie na tyle ważna, że nie chcę jej utracić na nie wiadomo jak długi czas. Ja jestem człowiekiem, który okazuje swoje uczucia przez dotyk i bardzo tego potrzebuję i względem najbliższych jak też względem przyjaciółek i koleżanek.

**Maski?**

Ja już się przyzwyczaiłam i nie sprawia mi to już takiego problemu. Chyba to jest sensowne, póki co. Póki to nie zostanie z nami już na zawsze. Noszę taką najzwyklejszą materiałową maskę, najcieńszą jaką znalazłam, bo jak jest trochę grubsza to już mi się źle oddycha. Biję się w piersi i moje sumienie podpowiada mi, że robię źle, bo nie zmieniam jej za często i też nie za często ją piorę, a podobno to jest siedlisko różnych bakterii. *[wybucha śmiechem]*. I ja się naprawdę biję w piersi i grzeszę, że tego nie robię *[nadal ze śmiechem]*. Noszę ją gdzieś w torebce, w kieszeni, ale mam teraz 2 maski, żeby zawsze jedna gdzieś była w torebce. Rękawiczek jednorazowych nie używam - tylko do warzyw i pieczywa jak zawsze było.

**Dezynfekujesz ręce?**

Tak, czasami. Czasami, bo jak idę do GH i mam przed każdym sklepem dezynfekować ręce to też jest bez sensu, żeby co 5-10 min.

**Nauczanie online?**

Generalnie słabo, że tak jest. Czy jest to sensowne? Trochę te szkoły już nie wyrabiają, bo nauczyciele też zaczynają chorować i być na kwarantannach. Operacyjnie już nie dają rady działać, ale nie wiem czy to cokolwiek zatrzyma i jakie będą tego skutki. Trudno mi powiedzieć. Myślę, że to komplikuje życie wielu ludziom. Zamknąć całkiem to na pewno nie. Rozumiem, że od 4 klasy, dlatego że te dzieci mogą już zostać same w domu. Siostra mi mówiła, że chyba te dzieci już można same zostawić, a tych mniejszych nie, więc ludzie nie pójdą wtedy do pracy, a jak nie pójdą do pracy, to...

**Zamknięcie restauracji?**

To głupie jest, bo naprawdę ludziom się zamyka coś i...Powinni chociaż jakieś odszkodowanie dostać za to. Nie można tak z dnia na dzień po prostu ludziom powiedzieć, że nie możecie pracować i radźcie sobie. To jest cios poniżej pasa. Poza tym czy to faktycznie jest miejsce, gdzie ludzie się zarażają? Nie wiem tego i nigdzie nie spotkałam się z żadnym badaniem, gdzie faktycznie dochodzi najbardziej do zakażeń. To jest takie zgadywanie trochę i zamykanie na wszelki wypadek. Wybieramy sobie jedną branżę na oślep i teraz będziemy eksperymentować na ludziach, tak? To jest takie okrutne moim zdaniem.

**Zamknięcie basenów, siłowni?**

Też bez sensu. Rozumiem, że obostrzenia, ale ludzie ich chyba przestrzegali tam, a baseny to chlor wszędzie, więc już w ogóle nie rozumiem. Trzeba powiedzieć ludziom, że korzystają na własna odpowiedzialność i już.

Imprezy masowe, targi?

Nie wiem czy zamknięcie tego coś zmieni. Rozumiem, że to są miejsca, że wystarczy jeden chory, który pochucha i potem wszyscy ci ludzie rozjadą się po Polsce, i. To jestem w stanie jakoś zrozumieć, ale czy skutki tego znowu nie będą za duże?

**Wesela, chrzciny, stypy, komunie?**

Nie wiem. To już chyba wtedy w ogóle trzeba zabronić spotkań. Dzisiaj mogę być w gronie 5 osób a jutro w gronie zupełnie innych 5 osób. Przecież to bez sensu. Nie wiem. To jest kwestia jakichś swobód obywatelskich, ale też i biznesów. Wiem, że na weselach akurat ludzie zarażali się od siebie faktycznie, bo tam się nie przestrzega tych norm typu maseczki. tak samo kluby nocne.  Wszystko się rozbija o to, czy ta choroba jest aż tak groźna, że trzeba z tego wszystkiego rezygnować. ja nie umiem sobie do końca odpowiedzieć na to pytanie.

**Komunikacja?**

Nie jeżdżę, nie wiem, jak to wygląda. Nie jeżdżę komunikacją, bo nie muszę.

**A gdyby wszystko puścić na żywioł i niech się dzieje co ma być?**

Muszę się zastanowić...Jakieś obostrzenia chyba są dobre, bo one nie wpływają tak na gospodarkę i nie aż tak bardzo na gospodarkę. MDD może rzeczywiście są w stanie zahamować rozwój choroby tak, że nie mamy przeładowania w tej służbie zdrowia. Ale czy przeładowanie wynika w tym momencie z wirusa czy z tego, że się nie przygotowaliśmy na to, na co mogliśmy się przygotować? Robiliśmy ten lockdown i ja się zgadzam, że ten czas nie został wykorzystany na przygotowanie się na to, że tych zachorowań będzie dużo więcej. Stosujemy teraz dokładnie tę samą metodę jak wtedy, która nie do końca ma jakieś uzasadnienie...Co uzasadnia, że jak zamkniemy siłownie, restauracje to będzie tych zachorowań mniej? Nie powinno się grać w ciuciubabkę. Nie ma planu. Rozumiem, że jest to po to, żeby rządzący dawali poczucie, że oni coś robią, że oni próbują jakoś ratować sytuację. Już słyszeliśmy, że zachorowań jest więcej, bo ludzie się nie stosują do przepisów. Zawsze państwo powinno być przygotowane na takie rzeczy, zwłaszcza jak już mieliśmy ostrzeżenia od marca, że może nas to spotkać. Uważam, że nie został wykorzystany ten czas i teraz znowu się zamykamy, żeby znowu nie doszło do tego zapchania. Tutaj jest błąd ludzki a nie sama kwestia natury, czyli wirusa.

**Co trzeba było zrobić?**

No właśnie - czy tego dodatkowego szpitala nie można było zbudować wcześniej? Dopiero jak już słyszy się, że nie ma miejsc, to oni to robią. Te procedury też tak wydłużają czas otrzymania pomocy, że są tragiczne i wozi się tych pacjentów po mieście. Procedury powinny usprawniać działanie a nie utrudniać. Mam wrażenie, że ktoś tam nie myśli logicznie. Polowe szpitale mogły być wcześniej.

**Czy sytuacja teraz jest poważna?**

Uważam, że tak. Nawet nie sam wirus jest tu taki groźny tylko np. wyrostek robaczkowy, zawał. Nie chcą cię przyjąć, bo nie masz testu, bo się boją. Te szpitale nie mają izolatek. To nie powinno się zdarzać. Zaniedbani są pacjenci niecovidowi, odwołane są te wszystkie operacje, że robią miejsce w szpitalach. Kiedyś te operacje trzeba będzie zrobić i odkładanie tego na później wydłuża kolejkę a część ludzi pewnie nie dożyje. Covid stał się taką najważniejszą chorobą, a jednak trzeba chyba trochę rozłożyć te siły. Nie mówię, że nie zajmować się pacjentem z Covid, ale też zajmować się innymi pacjentami. Zamiast wydawać pieniądze na ratowanie turystyki może trzeba było wydać na służbę zdrowia i nie zamykać turystyki. Nie kupować sobie głosów tylko kupować lekarzy.

**Czy w ogóle rząd zrobił coś dobrze?**

Chyba trudno mi będzie znaleźć cokolwiek. Jeśli chodzi o te początki, to można było powiedzieć, że ok, że jeszcze nie było wiadomo z czym mamy do czynienia, dane trzeba zebrać, itd. Nie widzę jednak, żeby była jakaś chęć zbierania tych danych. Nie wiemy czy lockdown działa.

**Wprowadzenie godziny policyjnej, stanu wyjątkowego, dodatkowe dyscyplinowanie obywateli jak w innych krajach?**

Trudno to porównywać, bo mamy inny system prawny. Gdyby nie było w systemie prawnym wpisane, że za stan wyjątkowy należą się odszkodowania, to już dawno ten stan wyjątkowy byłby wprowadzony. Rządu na to nie stać, a tak to mogą sobie zamykać biznesy i kto im co zrobi? To jest po prostu nieludzkie, że komuś się mówi, że od jutra już nie będzie zarabiał, bo ja ci zabraniam. Nie może tak być i takiej osobie należy się odszkodowanie bez dwóch zdań. Nawet, jeśli ta decyzja o zamknięciu biznesu jest słuszna. Być może dobrze by było, żeby stan wyjątkowy został wprowadzony, bo można by było pozwać państwo polskie o odszkodowanie a teraz nie można i uważam, że to jest niesprawiedliwe. Rządzącym budżet się nie dopina i pieniądze też się kończą. To też rozumiem. Niestety to jest konsekwencja tego, że obiecuje się dużo pieniędzy różnym ludziom i rozdaje, nie oszczędza. I pieniądze się kończą. Ja nie widzę, żeby rząd o nas dbał i mam takie uczucie od 20 lat, że jest wiele zaniedbań. To jaką mamy służbę zdrowia teraz to nie jest kwestia tylko ostatnich 6 miesięcy. To jest efekt zaniedbań wielu, wielu lat. Można było jednak cokolwiek zrobić. Jeśli teraz da się wybudować szpital polowy na stadionie, to dało się to zrobić również wcześniej i tyle.

**Czy protesty, które teraz się odbywają wpływają jakoś na twoje poczucie bezpieczeństwa?**

Nie, na moje poczucie bezpieczeństwa nie. Może będzie gorzej przez to, ale to nie przez protesty jest jak jest.

**Masz wrażenie, że ludzie zachowują się adekwatnie do stopnia powagi sytuacji?**

Zawsze jak się wyjdzie to się kogoś skrajnego spotka. Są i zamaskowani w rękawiczkach jak i...Ale większość ludzi chyba już się przyzwyczaiła do tych maseczek i do dezynfekcji rąk. Wiadomo, że czasami ktoś opuści tę maskę czy coś, ale ja staram się nie oceniać tych ludzi, że to jest jakieś zło, bo rozumiem, że są różne sytuacje i czasami nie można złapać oddechu czy coś i tyle. Co mogę zrobić, jak widzę człowieka bez maski? No nic. Mam się zdenerwować na niego, że chodzi bez maski? Nie, nie złości mnie to. Zakładam, że ma swoje powody, że tej maski nie ma, bo inaczej by ją założył.

**A ludzie negujący istnienie wirusa?**

Też rozumiem niektóre ich argumenty. Podzielam takie, że to jest bezprawne. Nie można rozporządzeniem wpisać, że trzeba zakrywać nos i usta, tak? jak będzie ustawa, to się to zmieni, ale do tej pory nie było. Nie podoba mi się ta hipokryzja rządzących. Oni doskonale wiedzą, że to nie miało podstawy prawnej, bo jakby miało to by nie musieli tego wprowadzać. To jeszcze bardziej podkopuje zaufanie. Ja naprawdę bardzo bym chciała, żeby oni zrobili coś dobrego i bardzo im tego życzę. Od tego też zależy wszystko co jest wokół nas, ale takie sytuacje podkopują moje nadzieje. Nie ma ich za co pochwalić.

**Jak wygląda twoje poszukiwanie informacji teraz?**

Nie, już nie sprawdzam. Jak mi się coś pojawia na stronie głównej to widzę, ale nie, że się interesuję codziennie, ile tam jest. Nie mam jakiegoś ulubionego i sprawdzonego źródła informacji czy eksperta. Generalnie zaglądam do różnych opinii i staram się sama z tego budować coś w głowie, co mi się kupy trzyma i jest w miarę logiczne. Nie zmieniłam tego co czytałam kiedyś i nadal korzystam z tych samych źródeł. Nadejście 2 fali nie spowodowało, że nagle zaczęłam bardziej szukać informacji. Chyba już jestem tym tematem zmęczona. Rozumiem, że jak będzie coś ważnego, to się jakoś przebije i gdzieś tam to znajdę na jakimś fb czy gdzieś. Świadomie nie poszukuję i nie przeszukuję co tam nowego na froncie pandemicznym.

**Masz wrażenie, że jesteśmy rzetelnie informowani o sytuacji?**

To dobre pytanie...Jeśli ktoś ogranicza się do jednego źródła to może być źle poinformowany i dlatego ja staram się zaglądać i patrzeć co ludzie z różnych stron mają do powiedzenia. Mam znajomych na fb, którzy mają różne poglądy i patrzę co inni mają do powiedzenia. Nie zamykam się w jednej bańce informacyjnej. To się u mnie nie zmieniło. Mam takie źródła, którym nie ufam od lat, np. nie ufam telewizji i nie oglądam jej. Nie mówię tylko o TVP, bo ja generalnie nie oglądam wiadomości ani na TVP, ani na TVN. Czytam sobie i lubię np. wykop.pl, bo tam są opinie z różnych perspektyw, które ludzie wrzucają i mają różne argumenty. Tam faktycznie są informacje, które są i sprzyjające władzy, i krytyczne dla władzy. ja też dużo słucham zagranicznych rzeczy i zagranicznych ekspertów. Słucham zazwyczaj jakichś Amerykanów na yt. To są ludzie z jakimiś tytułami naukowymi i fachową wiedzą.

**Kiedy będzie koniec pandemii?**

Nie wiem. Może kiedy przestaniemy się bać. Lekarstwo spowoduje, że nie będziemy się bać. Może jak będzie szczepionka, może jak będzie lekarstwo a może jak to po prostu zaniknie i nagle się okaże, że nie ma tych zachorowań, bo każda epidemia prędzej czy później wygasa, jak historia pokazuje. Czy za pomocą odporności stadnej, czy za pomocą leku, czy szczepionki. W końcu to wygasa.

**Zakładasz sobie w głowie jakąś perspektywę?**

Nie mam pojęcia. Sama epidemia to jedno, ale tutaj czynnik ludzki jest jeszcze nie do przewidzenia. Czy jakiś polityk nie wymyśli, żeby dla swojego dobra nie wprowadzić czegoś jeszcze? Może będą chcieli przedłużać to dla własnych korzyści?

**Widzisz ryzyko, że jesteśmy manipulowani tą sytuacją?**

Myślę, że jest takie ryzyko.

**Czym jest dla ciebie koronawirus teraz w porównaniu do tego, co myślałaś wiosną?**

Myślę, że jest naturalny a nie wywołany przez człowieka, chociaż...Nie, nie sądzę, aczkolwiek jestem skłonna zmienić zdanie jak ktoś mnie przekona. Na początku chyba myślałam, że jest bardziej groźny. Teraz wydaje mi się, że jednak nie. Więcej danych już też mamy.

**Mówiłaś, że obawiasz się o kwestie gospodarcze. czego jeszcze obawiasz się myśląc o przyszłości?**

Obawiam się, że zostanie to bardzo sprytnie wykorzystane przez ludzi, którzy mają niezbyt szlachetne motywacje, intencje. Mam wrażenie, że zgadzamy się teraz na ograniczanie naszych praw, coraz dalej przesuwamy tę granicę i obawiam się, że nie wrócimy do tego co było wcześniej. Zostaniemy z tym ograniczeniem praw. Część samostanowienia zostanie nam na długo albo na zawsze odebrana. Nie wiem, czy to jest prawda, że w ustawie covidowej jest nawet przymus tej szczepionki wpisany. Nie podoba mi się przede wszystkim to, że jeszcze nie ma szczepionki a już myślą o tym jak nakazać szczepienie. To nie powinna być taka droga. Może najpierw miejmy tę szczepionkę, upewnijmy się, że ona jest bezpieczna, upewnijmy się 10 razy czy to wszystko jest ok zanim zmusimy każdego obywatela, żeby ją przyjął. ja nie będę pierwsza, która się pójdzie zaszczepić. Rozumiem, że są szczepionki, które są sprawdzone od lat, od lat przyjmowane i już wiemy jaki one mają wpływ na populację, itd. Te wszystkie nowe szczepionki, nawet te sezonowe na grypę to one nie do końca...Mogą czasami tak osłabić organizm, że można czasem zapaść na inną chorobę. Mojej mama lekarka kazała się szczepić na grypę sezonową i mama zaczęła się bardzo źle czuć. Moja siostra, która jest farmaceutką kazała jej natychmiast to przerwać. To nie są od lat testowane te same szczepionki jak te, które dajemy dzieciom i żyjemy z nimi od nie wiem ilu lat. Najpierw trzeba to wszystko dobrze sprawdzić. Szczepionka szczepionce nie równa i trudno porównywać szczepionkę na grypę ze szczepionką na żółtaczkę, z którą żyjemy już od wielu lat.

**Czy myślisz, że może coś pozytywnego wyniknąć z pandemii? Czy coś zmieni się na lepsze? Coś na gorsze?**

Będziemy częściej myć ręce. Może niektórzy docenią, że mają bliskich, że mają rodzinę. Może zobaczą, że sami są potrzebni komuś. Najbardziej się martwię o młodzież, bo to jest grupa, która jest najbardziej podatna na wpływy. Walczyliśmy z tym, żeby oni nie siedzieli przed tymi komputerami, żeby wylogowali się do świata, a teraz każemy im siedzieć przed komputerem. To będzie problem, że to będzie ich świat i będą mniej znać ten świat realny niż ten w komputerze. Kontakty społeczne będą inne, bo inna jest komunikacja pisana niż rozmowa i kontakt. Nawet rozmowa online jest już inna. Faktycznie może zostać upośledzona umiejętność rozmowy 1 na 1, która jest konieczna do budowania trwałych związków, do budowania relacji z drugim człowiekiem. No i żeby nie zabili nam tej gospodarki.

**Jak planujesz spędzić 1.11.?**

Jeszcze nie wiem, bo dzieci mają katar. Zwykle jeździliśmy na cmentarz tego dnia. Czasami jeszcze jeździłam z rodzicami na dalsze groby, ale w tym roku nie pojedziemy. Nie mieliśmy zwyczaju spotykania się w większym gronie rodzinnym tego dnia, więc tylko te cmentarze były.

**Chodziły plotki, że może wprowadzą zakaz przemieszczania się tego dnia?**

No ale żeby to zrobić to potrzebny jest chyba stan wyjątkowy. Nie można ludziom zakazać przemieszczania się rozporządzeniem.

**Czy byłoby sensowne, żeby ludzie się nie przemieszczali?**

Na tym etapie to już chyba nie ma żadnego znaczenia. Miałoby to sens, jeżeli faktycznie moglibyśmy powiedzieć, gdzie są te ogniska, ale jeżeli nie wiemy to faktycznie trzeba by było wszystko zamknąć, zamknąć wszystkich w domach.

**Święta Bożego Narodzenia?**

Mam nadzieję, że już będzie dobrze, bo święta świętami, ale my mamy zaplanowany wyjazd sylwestrowy ze wszystkimi znajomymi...Uzgodniliśmy, że tak czy tak wszyscy jedziemy, więc mam nadzieję, że do tej pory już na te imprezy pozwolą. jak nie pozwolą to stwierdziliśmy, że i tak pojedziemy. Dopóki nie wprowadzą stanu wyjątkowego to nie mogą zakazać ludziom spotykania się w święta. Będą wchodzić do każdego domu i sprawdzać, ile jest ludzi?

**Ale ty byś spędziła wtedy święta tak jak Wielkanoc tylko we własnym gronie?**

Chyba na tym etapie już nie.

**A gdyby miała się teraz odbyć jakaś duża impreza rodzinna, wesele?**

No nie pojawi się, bo jest zakaz. W lokalu nie można zorganizować.

**A gdyby była w domu np. na 50 osób?**

To pewnie bym poszła.

**Czy jest jeszcze coś, co uważasz za ważne?**

Ja myślę, że trzeba zachować optymizm. nie wszystko od nas zależy i trzeba się z tym pogodzić. Wkurzanie się na rzeczy, na które nie mam żadnego wpływu jest bardzo destrukcyjne i staram się tego nie robić. Nie spotykam się raczej z ludźmi, którzy by jakoś bardzo panikowali z tego powodu. Z powodu choroby, a nie z powodu np. sytuacji materialnej, w jakiej się teraz znajdują. To faktycznie nie ma co nawet gadać, bo to są tragedie. Nie mam wrażenia, żeby w moim otoczeniu panował jakiś strach przed chorobą, nie ma takich ludzi, którzy jakoś bardzo by się bali. Poprzednio moja mama bardzo bała się spotykać, wszyscy w sumie baliśmy się spotykać i nie spotykaliśmy się. Mama źle to przeżyła psychicznie i teraz już nie ma czegoś takiego, że się boi spotkać z najbliższymi ludźmi, z dziećmi, z wnukami. Takie zamknięcie to jest za wysoka cena dla niej i jest już w stanie zaryzykować. Oczywiście do pewnego stopnia, bo jak dzieci mają katar, itd., to nie przyjeżdża. Tydzień, dwa wytrzyma, ale nie 3 miesiące.
