# Supplementary material for: The challenges arising from the COVID-19 pandemic and the way people deal with them. A qualitative longitudinal study
Source: PLoS One. 2021 Oct 11;16(10):e0258133. doi: 10.1371/journal.pone.0258133 (PMC8504766; doi:10.1371/journal.pone.0258133)
Supplement: S1 Dataset — (ZIP) [file pone.0258133.s003.zip › Transcriptions/stage 6/20.6_F_25_couple, with child.docx]

**20.6_F_25_couple with child**

**Jak minęły ci ostatnie miesiące?**

Chyba wszystko było raczej normalnie. Wróciły spotkania rodzinne, byliśmy na wakacjach za granicą. To był przełom sierpnia i września. U nas raczej wszystko było ok, nic złego się nie wydarzyło, było w porządku. Powiedzmy, że było normalnie oprócz tego ciągłego noszenia maseczek, które utrudniają życie.

**Na ile wyjazd na wakacje był normalny?**

Było bardzo normalnie. Jechałam do Eurocamp i tam nie było nakazu noszenia maseczek ani na plaży, ani na weselach i wszędzie normalnie się zachowywaliśmy.

**Dlaczego nie było nakazu?**

Nie wiem, nie mam pojęcia. Jak wchodziłam na recepcję to musiałam założyć maseczkę, ale u nas nie było stołówek, bo mieliśmy osobne domki, a w restauracjach było tak samo jak u nas, że nie trzeba było nosić maseczek. Nie trzeba było w parkach, na wszystkich przejściach, placach zabaw, na plaży.

**Jak postrzegasz obecną sytuację w porównaniu z początkiem roku?**

Różnica jest taka, że ludzie może są po prostu bardziej świadomi i już tak się zrobić nie dadzą, jak się dali zrobić na początku roku. I mam nadzieję, że takich osób będzie coraz więcej, bo dzięki temu...Rząd chyba doskonale wie, że jak wprowadzą takie restrykcje jak wtedy to ludzie się z tym nie zgodzą. To jest w tej sytuacji lepsze. Jeżeli chodzi o wzrost liczby zachorowań to nawet mi się nie chce dyskutować, bo są i zachorowania w mojej rodzinie bliższej i dalszej, i nie widzę żadnych różnic z innymi chorobami, które przechodzimy na co dzień.

**Co znaczy, że ludzie są bardziej świadomi?**

Że np. wiedzą, że koronawirus był, jest i będzie, i trzeba nauczyć się z tym żyć, a nie zamykać się w domu. Wtedy ludzie zamknęli się na jakiś miesiąc, nie spotykali się, nie widywało się rodziców. Przecież to jest nienormalne. Moja córka chodzi do przedszkola i katar, itd. jest cały czas. To jest zrozumiałe, bo mamy ten okres czasu, gdzie ludzie będą chorować. Zawsze jesienią ludzie chorują i tak jak ktoś ma grypę, tak też ma koronawirusa. U mnie nie było ani jednego poważnego przypadku, który zagrażałby życiu i zdrowiu. Ma moja siostra, która ma ok. 30 lat i przechodzi to jak normalną grypę. Mój znajomy miał koronawirusa i stracił węch i smak. Brat mojego męża ok. 40-tki ma koronawirusa i stracił tylko węch i smak, ktoś tak,jak zwykła grypa. Nie widzę w ogóle sensu robienia takich cudów i koniec, kropka.

**Zdarzyło ci się być na kwarantannie?**

Nie. Całe szczęście u mnie nikt z domowników nie chorował.

**Czy ta siostra to ta, która się bała?**

Właśnie śmiałam się ostatnio z tatą, że dotyka to tych ludzi, którzy najbardziej się pilnowali i najbardziej cudowali ze światem. U mojego męża z rodziny też z nimi się nie widywaliśmy, bo oni się bali najbardziej i potem też kontakt był znikomy, bo nadal się bali i teraz właśnie oni chorują. Siostra też była najbardziej przewrażliwiona. Ona nie jest w ogóle w grupie ryzyka, tylko jedni podeszli do tego bardziej normalnie a inni byli zastraszeni kompletnie.

**Co jeszcze różni obecną sytuację z tą z wiosny?**

Dla mnie jedyna różnicą jest to, że teraz kompletnie, ale to kompletnie nie boję się i jeżeli by wprowadzili jakieś restrykcje w sensie, że zakaz spotkań towarzyskich, to na pewno bym tego nie praktykowała. Jeżeli wprowadzą zakaz spotkań na święta, a pewnie tak zrobią, jestem o tym przekonana...Przed chwilą wyświetliła mi się wypowiedź wicepremiera...Jest wicepremier w ogóle czy nie ma?

**Jest.**

No to jego wypowiedź, że będzie jakaś restrykcja, jeśli chodzi o handel, różnych działalności gospodarczych nie pozamykają, itd., bo oczywiście koronawirus najczęściej jest rozprzestrzeniany na spotkaniach rodzinnych i tutaj trzeba się spodziewać zmian. Czyli nie trzeba się bać o swoja pracę, tylko trzeba się bać spotkań rodzinnych i spotkań z przyjaciółmi. I on to jeszcze ujął, że spotkania z ludźmi spoza pracy, czyli ze wszystkimi. Już wcześniej się spodziewałam, że na święta coś tam będzie wprowadzone, ale nie mam zamiaru się w ogóle do tego stosować i nie będę się stosować, bo te zachorowania będą i będą rosły, i będzie chorych coraz więcej, bo jest robione coraz więcej testów, ale trzeba z tym żyć i zachowywać się normalnie.

**Jak spędziliście 1.11.?**

U teściów nie byliśmy, bo brat jest chory, a rodzice widzieli się z tym bratem, więc prosili, żeby nie przyjeżdżać. Ja na cmentarzach nie byłam i mi to specjalnie nie wadzi, bo dla mnie nie ma znaczenia czy ja zapalę świeczkę 5 dni później, czy tydzień później. Poza tym o 15.30 chyba dopiero podali, że cmentarze będą zamknięte, a ja po drodze akurat mijałam cmentarz ok 17-tej i był taki natłok ludzi, że nawet nie dałoby się chyba przejść. Wszyscy, którzy usłyszeli, że jest zakaz to chyba wzięli świeczki i wiązanki, i zamiast się gromadzić 1.11., to się zgromadzili w piątek. Całkowicie bez sensu. Mi to nie zrobiło różnicy a poza tym staram się odwiedzać cmentarze regularnie.

**Co odróżnia twoją codzienność teraz od tej przed epidemią?**

Denerwuje` mnie tylko to, że trzeba już wszędzie nosić te maseczki. To jest bardzo, ale to bardzo uciążliwe, bo jest niewygodnie. Ja nie czuję w ogóle potrzeby ich noszenia, a po drugie nie wierzę w jakąkolwiek ich ochronę. Może zmniejsza się ryzyko zakażenia, ale nie wiem w jaki sposób mogę się zarazić wychodząc z samochodu i nie mijając żadnego człowieka. Nie wierzę też, że mogę się zarazić przez sytuację, w której mijam człowieka.

**A jeśli chodzi o maseczki w pomieszczeniach?**

Ja bym osobiście nie nosiła. Uważam, że te maseczki i tak nic nie dają. Może zmniejsza się jakby ryzyko. Ok, jeżeli się zmniejsza chociaż o 2% to ryzyko i ja mogę uchronić kogoś to będę nosiła, bo są ludzie, którzy się boją i widzę jeszcze, że są tacy co dramatyzują bardzo, ale to bardzo. Ostatnio byłam z ciocią w cukierni i była wywieszona kartka, że tylko 2 osoby mogą tam przebywać. Była jedna osoba tylko już przy kasie i weszłam z ciocią normalnie. Ta pani się odwróciła i powiedziała, że jesteśmy bardzo zacofane, bo jest restrykcja, że tylko 2 osoby mogą być i że w tym momencie ma nas pani wyprosić. Naskoczyła na nas tragicznie. Ja to w ogóle bym nie robiła problemu z tego, że jest 1 osoba więcej. Ta pani poszła pewnie potem do restauracji w niedzielę, gdzie pewnie było 30 osób bez maseczek i nie sprawiało jej to problemu. To była pani ok. 40-tki. Dla mnie to jest śmieszne, ale fakt, że tak zareagowała oznacza, że są ludzie, którzy się boją i chcą, żebym się stosowała do tych zasad, ja to szanuje i dlatego będę się stosowała do tych zasad.

**Jak to wygląda u twoich bliskich? Co wróciło do normy, co jest inaczej?**

Nie ma żadnych zmian. Nie mnie oceniać i pewnie się okaże. Jak mnie dotknie ta choroba i coś tam złego się wydarzy to może będę pluła sobie w brodę, chociaż wątpię. Oni zawsze podchodzili do tego z dużym dystansem i nawet w marcu, kwietniu czy w wakacje żyliśmy całkowicie normalnie. Mama się trochę przejęła, jak okazało się, że jej siostra ma koronawirusa. Ona troszkę gorzej przechodziła, bo przez 3 dni np. w ogóle nie mogła wstawać z łóżka, była bardzo wycieńczona, itd. Minął tydzień i wszystko wróciło do normy. Mama trochę się przejęła, zawoziła obiady i w ogóle. Powiedziała wtedy, że kiedyś to się nie słyszało, że choruje ktoś z bliskich, że to było bardzo odległe. Gdzieś był ten koronawirus, coś się słyszało, ale tak naprawdę go nie było. Tutaj jednak naprawdę jest bardzo dużo osób chorych, tylko chorych tak samo jak na inne choroby i dlatego może podchodzimy do tego bez żadnych nerwów, bez strachu.

**Emocje.**

16 - przez całe wakacje i do tej pory było dla mnie normalnie. Żyłam tak jak rok i 2 lata temu oprócz tego noszenia maseczek, ale chyba ważne jest to ostatnie wydarzenie, czyli protest. Jeżeli mam się opowiedzieć za którąś ze stron, to się nie opowiem za żadną ze stron. Tak samo jestem za życiem jak za prawem wyboru i byłam za tym protestem. Byłam za tymi dziewczynami, za sobą, za swoja córką do momentu, kiedy ten protest nie przerodził się w 2-gą dyktaturę, a w ogóle nie o to w nim chodziło.

**Jakie to były emocje? Dlaczego 16?**

Rząd, który wprowadził zakaz aborcji i zmienił przepisy w konstytucji wywołał pożar w całej Polsce i zrobił to celowo wg mnie, żebyśmy się sami chyba pozabijali. To jest nienormalne, żeby w takim okresie, gdzie każdy jest podenerwowany lekko mówiąc, gdzie każdy się boi o swoją przyszłość, każdy boi się o pracę i tego co będzie...Niektórzy się boją o zdrowie, niektórzy co będzie, jeżeli zamkną kolejne działy handlu, itd., a oni jak zwykle, trochę po cichu, za plecami koronawirusa zmieniają w ten sposób prawo. I jeszcze, gdyby oni zmienili coś innego, ale aborcja? Wcześniej byli geje, nie geje, lesbijki. Przecież to jest zawsze tak burzliwy temat, który zawsze, ale to zawsze dzielił Polaków i będzie dzielił. Ja jestem za życiem, spotkam się z kimś, kto jest za aborcją i przecież nie ma możliwości, że będzie ok i spoko. To zawsze będzie dzieliło i zawsze będzie wywoływało duże emocje, będzie wywoływało też gniew niektórych. I widać, co się działo na tych protestach. Ten protest był fajny tydzień temu, jak się zaczął, bo kobiety wyszły, przeszły, ok, spoko. Ale to, co się działo teraz... Miałam okazję rozmawiać z osobami ze służb, które obstawiały ten protest i była tam policjantka, która oczywiście była za kobietami, była za protestem, ale jak zobaczyła co się działo w piątek, to mówi, że to była po prostu patologia. Gdzieś się chyba pomyliło ludziom, w jakim celu wyszli na ulicę i to jest najgorsze. Zaraz się zacznie, że chrześcijanie i niechrześcijanie, zaraz będzie, że kościoły, księża i cała reszta.

**W tobie to też wywołuje złość?**

Temat we mnie nie wywołuje złości. Ja staram się iść na kompromis i ten kompromis odpowiadał mi bardzo. Wiem, że w innych osobach wywołuje złość podobnie jak poruszanie tematów politycznych na różnych zjazdach rodzinnych. Natomiast to, co zrobił ten rząd, że wzniecił ten pożar w nas wszystkich albo w większości z nas to już było chyba poniżej pasa, żeby akurat w takim okresie, bez żadnych negocjacji, bez zapytania. Denerwuje mnie, bo na początku dziewczyny używały wulgarnego języka a politycy mówili, żeby przecież ze sobą rozmawiać, żeby negocjować. To się pytam, kto z nami, kurwa rozmawiał wcześniej? No bez jaj. Nikt nie negocjował 10 dni temu, to czemu się dziwić takim reakcjom? Od zawsze ktoś nami kierował i będzie kierować, tylko ja staram się żyć po swojemu i zgodnie ze sobą. Dlatego mnie nigdy tematy polityczne nie interesowały.

**Jak w tym momencie się czujesz, czy któryś obrazek pasuje?**

 8 - no właśnie nie mam teraz już żadnych emocji. U mnie jest codzienność. Tu jest mgła jakaś i ewentualnie mogę mieć złe samopoczucie tylko ze względu na pogodę, bo nienawidzę deszczu, jesieni, pluchy. To jest mgła bez żadnych podtekstów. po prostu jesień.

**Ograniczenia. Jak się z nimi czujesz, co słyszałaś, jak to oceniasz?**

zamknęli restauracje i jest na wynos. Jak zamknęli je na początku roku to byłam bardzo zła, ale przez tyle miesięcy człowiek jest w stanie już przyzwyczaić się do pewnych rzeczy i ta informacja mnie w ogóle nie zaskoczyła. Wiedziałam, że w końcu zamkną. Będzie wzrost liczby zarażonych i zaczną zamykać.

**Skąd to wiedziałaś?**

Bo przecież tak zapowiadali, tak mówili. Przecież mówili to już w marcu, że na jesieni będzie 2-ga pandemia. Przez wakacje, gdzie ludzie sobie pozwolili na więcej...Przez te nasze piękne wakacje znowu wzrosła liczba zakażeń. Jak jest 2-ga fala i jak znowu rośnie liczba zakażeń, to są restrykcje i dlatego wiedziałam, że te restauracje będą zamknięte prędzej czy później. To jest dla mnie duży dyskomfort, że nie mogę zjeść w restauracji, bo bardzo lubię, ale nie wzbudziło to we mnie takich silnych emocji jak wtedy. Wiedziałam i byłam na to przygotowana.

**Czy powinno się podejmować takie działania?**

Oczywiście, że nie. Jeżeli zamykają, to powinni zamknąć wszystko, żeby to miało sens, czyli taki lockdown jak było na początku. Wtedy nie było spotkań rodzinnych, ludzie nie widzieli się nigdzie, szli tylko po bułki do sklepu i tyle. Rozumiem, bo to jest ograniczona jakaś aktywność społeczna i wtedy jest możliwość, żeby uchronić przed rozwojem tego "strasznego śmiercionośnego koronawirusa".

**Taki lockdown zahamowałby wzrost zachorowań?**

No chyba to jest logiczne i myślę, że tak, tylko teraz już nikt się temu nie podda. ludzie nie będą się do tego stosować i dlatego dla mnie zamykanie restauracji a zostawienie kina? Nie wiem, można dyskutować. Ja bym wolała zostawić restauracje a zamknąć sieciówki, ale oni raczej już patrzą, na ile jakaś firma udziela się w budżecie państwa. Nie wiem.

**Co powoduje, że ludzie nie będą chcieli stosować się do lockdownu?**

Bo mają już dość i nie chcą. Teraz w każdej rodzinie był jakiś przypadek i mam nadzieję, że mało było tych przypadków, gdzie ktoś naprawdę przechodził tego koronawirusa źle. Mam nadzieję, że nikogo młodego nie doprowadziło to do śmierci. W takim sensie, że wtedy bardzo współczuję. Myśląc logicznie i twardo stąpając po ziemi, to ja sobie zdaję sprawę, że jak moja babcia 80-letnia zachoruje, to może być źle, ale to jest tak samo, gdyby zachowywała na coś innego. Wiem, że mój teść jest po 2 zawałach o po udarze i wiem, że gdyby zachorował to też może być źle, ale też tak samo jak przy każdej innej chorobie. Dlatego ja nadal nie wiem, dlaczego ten koronawirus jest tak wyróżniany spośród innych chorób. Widzę wręcz osoby potrzebujące na tę chwilę pomocy, której nie mogą otrzymać przez "śmiercionośnego koronawirusa". To jest trochę nie po kolei.

**Ograniczenia dla osób starszych?**

Ja mam 2 babcie i one raczej już wymagają naszej opieki. Nie chodzą raczej same na zakupy, nie wychodzą sobie do koleżanek. Babcia i tak raczej nie wychodziła z domu. Mam w grupie panie, które tworzą kółka dla seniorów albo jakieś kluby i wiem, że to jest bardzo pomocne i dobrze, że ci ludzie wychodzą, fajnie, że ktoś się nimi zajmuje, że mają zainteresowania, że mają co robić w ciągu dnia. Gdyby ich zapytać, to wiem, że powiedzą, że to jest największa głupota ich zamknąć, bo wtedy senior się trochę cofa. Jak dziecko. Środki ostrożności powinny być jak najbardziej zachowane, ale wiem, że ci starsi ludzie tak jak przychodzili na te zajęcia, tak nadal przychodzą. Nie boją się, bo uważają, że wola czas spędzać fajnie, mimo tego, że mogą się zarazić niż mieliby siedzieć zamknięci w domu. Wtedy dopiero dostaliby depresji i wtedy dopiero byliby chorzy. Rozmawiałam o tym tak z miesiąc temu, jak był zjazd i wtedy chyba jeszcze nie było tego zakazu. Nie wiem, bo nie orientowałam się w tym.

**A godziny dla seniorów?**

Najbardziej nienormalne w ciągu dnia, czyli 10-12. Większość matek, jak zaprowadzi dziecko do przedszkola to ma czas do 12-tej i to jest jedyny czas, kiedy mogą zrobić zakupy. No ale mogą nic załatwić, bo są godziny dla seniorów. To jest najbardziej chyba debilne wymyślenie tych godzin. Rozumiem, gdyby to było od 7 do 9 albo 8-10. Nie oszukujmy się, ale każdy senior wstaje wcześnie i o 6 jest już na nogach, i ma czas, żeby coś z samego rana załatwić albo w godzinach popołudniowych senior też ma na to czas, a my niestety nie mamy. Godziny 10-12 to jest strzał w kolano. Ja wiem, że nie dotyka to osób, które pracują, ale jest bardzo dużo osób, które zajmują się domem i dziećmi o do południa to jest jedyny czas, kiedy można coś załatwić a teraz nie można załatwić nic.

**Nauczanie zdalne od 4 klasy?**

Taka sama sytuacja była przy 1 fali. Też pierwsze do szkoły wróciły najmłodsze dzieci, żeby rodzice mogli pracować. To jest logiczne. W ogóle to zdalne nauczanie to jest o dupę potłuc. Może jeszcze na studiach, bo tam i tak więcej się pracuje samemu. Nauczanie zdalne w szkole podstawowej jest w ogóle bez sensu i będzie jedna wielka ciemnota za rok, za pięć lat. Już są duże problemy. Ja wróciłam do korepetycji i teraz po tym poprzednim nauczaniu zdalnym jest jedna wielka tragedia. Dzieci nie potrafią nic, chociaż materiał niby jest przerobiony. Jeżeli to potrwa, a raczej na 2 semestr nikt nie wróci do szkoły, to po takim roku są ogromne straty. I wydaje mi się, że są nie do nadrobienia. Uważam, że szkoła powinna być stacjonarna.

**Baseny, siłownie?**

Na basenach szkółki chyba nadal prowadzą zajęcia. Wiem, że moi znajomi nadal chodzą na siłownię i to chyba działa na zasadzie, że przygotowują się do zawodów, bo na takiej zasadzie chyba mogą. Zamknięcie tego nie ma w ogóle sensu., bo sens miałby tylko typowy lockdown. Tak logicznie myśląc, bo nie chcę tego lockdownu, bo w ogóle uważam, że nie ma potrzeby. Jest dużo teraz takich jakby uchybień, więc ktoś sobie chodzi na siłownię. I dobrze, ja też bym chodziła, gdybym miała teraz na to czas. Osoby, które podtrzymują swoja sprawność niech sobie nadal ją podtrzymują. I super. Basen przecież bardzo dobrze wpływa na odporność człowieka i to zawsze było wiadomo. Niech ludzie sobie chodzą i używają sportu, Bez sensu, że pozamykali, ale to też nie był dla mnie szok, bo było wiadomo, że to zamkną.

**Imprezy rodzinne, wesela, stypy?**

Bardzo współczuję osobom, które na ten rok planowały wesela, bo to był jeden wielki cyrk. Koleżanka miała mieć w kwietniu wesele, przełożyła na listopad, bo uznała, że już na pewno wszystko będzie dobrze. 2 tyg. przed weselem nie wiedziała nadal co zrobić, bo ograniczyli wtedy liczbę osób do 75, a ona miała zaproszonych 150. Nie była w stanie wybrać, więc był wielki płacz, po czym za tydzień zakazali wesel w ogóle. Odbędzie się tylko ślub w kościele.

**To jest dobra decyzja rządu?**

Nie wiem. Wszyscy mówili, że to przez wesela się ludzie zarażali. Teraz będą się zarażać przez to, że wyszli na ulicę. No nie wiem. Nie mogę się do tego odnieść, bo dla mnie w ogóle to, że ktoś powiadamia o takich restrykcjach na 1-2-3 tygodnie przed...Jeszcze na początku było jakoś tak, że te wesela zaplanowane na 3 tyg. od decyzji, to mogły się jeszcze odbyć. To jest wielka tragedia dla ludzi, którzy mają mieć to wesele, bo to jest najważniejszy dzień w życiu i nie mogą sobie nic zaplanować. Na kiedy? Na maj? Raz będzie 150 osób, raz 75, raz w ogóle nie będzie można mieć tych wesel. Moja koleżanka była na weselu i 200 osób poszło na kwarantannę, a po tygodniu okazało się, że test był źle wykonany i ta osoba wcale nie miała koronawirusa. No nie wiem...

**Transport zbiorowy?**

A jakie są ograniczenia? Ciężko mi odpowiedzieć. W ogóle ciężko mi mówić co sądzę o restrykcjach, bo ja za nimi nie jestem, a jeżeli już to powinien być całkowity lockdown, bo takie pół na pół nigdy nie da dobrych efektów. Wirus był, jest i będzie, i trzeba nauczyć się z nim żyć a nie blokować społeczeństwo i zamykać ludzi w domach. Może dobrze, że jest ograniczona liczba pasażerów i może dla nich to dobrze. Mnie akurat to nie dotyczy.

**Co myślisz teraz o koronawirusie?**

Myślałam, że pochodzi z laboratorium i raczej do tej pory zdania nie zmieniłam, ale się w ten temat w ogóle nie zagłębiałam. W ogóle przez ten czas od naszego spotkania się tym nie interesowałam. Wtedy nawet starałam się żyć normalnie a teraz to jest w ogóle poza mną o poza tym jak funkcjonuję. Dla mnie to jest temat całkowicie uboczny.

**Czy obecna sytuacja jest poważna?**

Ta powaga na pewno jest przeceniana, ale to jest tylko moje zdanie. Pewnie to się kiedyś okaże - za rok, za trzy, kto miał rację. A może nigdy się nie okaże? Jest przeceniana i w ogóle jest niepotrzebna. Mam nadzieję, że się nie mylę.

**Jak oceniasz obecne zachowania ludzi?**

Biorąc pod uwagę ostatnie wydarzenia, to nie zachowują się adekwatnie do sytuacji, ale wcale im się nie dziwię. Do sytuacji, którą wprowadził rząd, bo to rząd dyktuje sytuację.

**A w stosunku do zagrożenia koronawirusem?**

Społeczeństwo też jest podzielone, bo jest dużo osób takich jak ja i mój mąż, i moja rodzina, ale są też tacy ludzie jak ta pani w cukierni. Zdania pewnie są podzielone i osoby, które wyszły na marsz to chyba raczej się nie bały koronawirusa. Ja też bym wyszła i ja też bym się nie bała. Teraz będzie tak, że wszystko jest przez te dziewczyny, bo nawet słyszę głosy teraz wokół siebie, że po co one wyszły? Teraz to przez nie będzie to i to, i wszystko pozamykają. Ja mówię, że pozamykaliby i tak, a teraz wszystko zrzucą na te kobiety i znowu będzie społeczeństwo złe na te kobiety, które wyszły a nie na rząd, nie na sytuację, którą stworzył rząd tylko na te biedne kobiety, które wyszły.

**Jakie emocje wywołuje w tobie taki schemat szukania kozła ofiarnego?**

Dlatego wybrałam obrazek z ogniem. jeden szary człowiek nie jest w stanie nic zmienić. Ja nie jestem w stanie zmienić rządu, polityki, tym bardziej, że się na tym nie znam, ale kto nie byłby u władzy, to zawsze ktoś będzie próbował nami kierować. Trzeba tu zachować logiczne myślenie. Nie można odbierać wolności wyboru.

**Jak oceniasz działania rządu?**

Nie wiem. Jak czytam argumenty za tym, że wszystko jest zaplanowane to wydaje mi się, że tak faktycznie jest, a jak zaraz przeczytam artykuł o tym, że to jest jak losowanie i oni nie wiedzą co faktycznie robią, to też wydaje mi się to prawdą. Nie wiem. Zawsze coś się robi pod przykrywką, zawsze będą zrzucać winę mną kogoś i będą wprowadzać kolejne zakazy wtedy, kiedy będą mogli zwalić winę. To się sprawdza i w to ja wierzę, bo widzimy to na porządku dziennym. Były różne prognozy na przyszłość i to co było mówione parę miesięcy temu teraz się sprawdza, więc jednak są jacyś mądrzy ludzie. Ja polityką się nie interesuję, więc różne sprzeczne ze sobą rzeczy czasem wydają mi się logiczne jak o nich czytam. Nie mam rozeznania i nie potrafię ocenić, ale większość działań jest na pewno bardzo mądrze zaplanowanych.

**Skąd czerpiesz informacje?**

Raczej ich nie czerpię, nie wyszukuję. Mam parę osób, z których ideologią się zgadzam i jeżeli oni jakiś artykuł udostępnią, to go czytam. Sama nie szukam, więc to są głównie media społecznościowe. Mało oglądam wiadomości, mało interesuję się wiadomościami ze świata. Na wiosnę sporo jeszcze było oglądania telewizji, ale po jakichś 2 tygodniach przestało się u nas ją oglądać. Tylko rodzice włączali TVP Info, żeby wiedzieć jaka jest liczba zachorowań i co się dzieje. To tylko rodzice. U mnie to się nie zmieniło, bo ja i przed pandemią bardzo mało interesowałam się i polityką, i sytuacją związaną z naszym krajem i ze światem. To jest stały niski poziom zainteresowania.

**Wiarygodność mediów?**

Nie wiem, komu można wierzyć. Raczej nie wierzymy w to, co mówią politycy. Jeżeli politycy mają udział w jakiejś stacji TV, to od razu to widać, w którą stronę idzie ten przekaz wiadomości i chyba nie ma u nas telewizji ani radia...No może jest radio Trójka, ale większość nie mediów nie jest obiektywna, bo są związane z jakąś tam partią. Każdą informację można przekazać na kilka różnych sposobów. nie wiadomo do końca, co jest prawdą, bo w większości przypadków to jest gra wpływów. Tak samo jest z liczbą zakażeń. Ostatnio mój wujek robił test razem z żoną. Za 2 dni zona ma wynik testu, a wynik wujka się zgubił. Lekarz mówi, że Ewa (ich córka) ma wynik negatywny tak samo, jak w czerwcu, a Ewa nie robiła testu w czerwcu. To jest w ogóle cyrk na kółkach. Jak te liczby mają być wiarygodne? A ile jest takich sytuacji, że ma się wynik pozytywny albo negatywny, mija parę dni i okazuje się, że test był źle zrobiony. Jak te liczby mają odzwierciedlać prawdziwe informacje?

**Co musi się zdarzyć, żeby pandemia się skończyła?**

Nie znam definicji słowa pandemia. Jestem pewna, że wirus nie zniknie. Nie ma możliwości, że z całego świata zniknie ten wirus tak jak nie znikają inne choroby i ludzie z tym żyją. Absolutnie nie uważam, że zniknie, jak wprowadzą szczepionki i nas zaszczepią. Wirus nie zniknie a pandemia już dawno powinna się skończyć, jeśli chodzi o samo sformułowanie, o obostrzenia, itd.

**Co musi się zdarzyć, żeby zniesiono wszystkie obostrzenia i żebyśmy żyli jak kiedyś?**

No właśnie nie wiem i chyba to jest najgorsze. Przeczytałam wpis takiego doktora, bardzo fajne zresztą i mądre, ale pisał, żeby się uzbroić w cierpliwość na około 2-3 lata. To jest niepojęte. Ale co za 2-3 lata się stanie takiego, że nie przedłuża na kolejne 2-3 lata? Może tego trochę się boję, że pewne obostrzenia zostaną na zawsze. Te maseczki...Może nie będzie ich na świeżym powietrzu, ale w pomieszczeniach być może zostaną na zawsze, może dezynfekcja rąk też zostanie na zawsze przy wejściu do sklepów. tak samo w przychodniach. Boję się, że te maseczki, dezynfekcja, rękawiczki mogą zostać. Dezynfekcja rąk mi tak nie przeszkadza i mycie rąk jest bardzo dobre, ale na początku ludzie poniszczyli sobie okropnie dłonie przez dezynfekcję. Jednak musi być ta flora bakteryjna na naszych rękach. Ta higiena osobista jest dla mnie ok, ale myślę, że takie niektóre elementy mogą zostać z nami na zawsze albo na bardzo długo.

**Przyszłość Polski i zmiany w ludziach?**

Nie wiem, co się może zmienić w ludziach. Uważałam, że jak minie miesiąc po zamknięciu lasów i ludzi w domach, to ludzie o tym zapomną. I właśnie mamy listopad i ludzie nie chodzą do lasu, nie cieszą się drzewkami, nie cieszą się naturą. Ludzie znowu są w galeriach. Są pewne zmiany, do których zmusza nas dana sytuacja, ale potem raczej wszystko się normalizuje i zostaje takie jak było. Teraz znowu nie ma czasu dla rodziny, a przecież wtedy ludzie zobaczyli, że to jest taka ważna wartość. Nic się nie zmieniło, znowu ludzie poszli do pracy, znowu trzeba zarabiać, znowu mamy ciężką sytuację w kraju, znowu boimy się o przyszłość, znowu trzeba robić zapasy i świat gna do przodu. Możemy, tylko czy jesteśmy w stanie się od tego odłączyć, jeżeli jesteśmy częścią jakiejś społeczności? Raczej ta społeczność nie pozwoli nam żyć całkowicie inaczej. Są pewne ramy, z których nie możemy się wychylać, bo niedługo nie będziemy mieć na chleb.

**Pandemia zmieni coś w sytuacji gospodarczej Polski?**

Na pewno już zmieniła, bo straty są na pewno przeogromne. I dlatego oni teraz też nie zamykają pewnych firm, bo wiedzą, że ludzie muszą zarabiać i muszą trafiać zyski do państwa. Państwo nie ma już pieniędzy, żeby pomagać. Nie mieli nigdy, ale nie są już w stanie dać teraz zwolnień z ZUS, po te 5 tysięcy dla działalności gospodarczych. Nie mają pieniędzy, więc nie zamkną tej pracy.

**Sytuacja gospodarcza na świecie się zmieni?**

Niektóre firmy i ludzie tracą, ale ktoś też dużo zyskuje. Na tym, że ja stracę, ktoś dużo zyska. Na świecie jest tak samo. A kto zyska, kto straci to nie wiem. Była jakaś równowaga i jakaś zmiana teraz nastąpi, ale u kogo?

**Są jakieś grupy, które będą szczególnie dotknięte zmianami?**

U nas już zamknęło się na zawsze parę restauracji, zamknął się jeden z fajniejszych klubów w Radomiu, bo też nie dali rady. Fryzjerzy i kosmetyczki raczej nie będą mieli problemów, bo zawsze będą zarabiać i jak nie w salonach to w domu, więc dadzą sobie radę. W marcu była już upadłość 2 innych klubów. Od marca straty są ogromne i co takim klubom pomoże 5 tysięcy?

**Brałaś udział w jakichś uroczystościach rodzinnych od czasu naszego ostatniego spotkania?**

Tak. Byłam na komunii, na chrzcinach i na takiej większej 40-ce. Nie byłam na żadnym weselu, ale tylko dlatego, że nie miałam okazji. Nie było niepokoju, Dla mnie dziwne było to, że goście byli bez maseczek, a obsługa musiała je nosić. To jest w ogóle bez sensu. Nie rozumiałam, dlaczego ta obsługa musi się tak męczyć, tym bardziej, że było bardzo gorąco.

**Jak się czułaś na tych imprezach?**

Bardzo dobrze, bo ja bardzo lubię przebywać z ludźmi. Zero strachu a wręcz fajnie, że można się było spotkać w większym gronie. W sierpniu były chrzciny, we wrześniu komunia, więc to i tak było już po wakacjach, gdzie w wakacje już mieliśmy sporo luzu, więc to nie było żadne wow, bo widziałam już tych ludzi wcześniej i wszystko dla mnie było normalnie. W majówkę jak spotkałam się ze znajomymi, to wtedy było wow po tak długim czasie. Tutaj już nie było takich emocji, bo wszystko znormalizowało się w wakacje. U nikogo nie zauważyłam żadnych obaw. Zauważyłam, że dużo osób odmawiało udziału w weselach, bo mówili, że się boją. Na ile się bali a na ile to była wymówka to jest inna sprawa. Jak ktoś nie chce uczestniczyć, bo ma jakieś obawy, to absolutnie to jest jego sprawa i takie decyzje trzeba szanować. jak ktoś już przychodzi, to nie wyobrażam sobie, żeby zachowywał się jakoś dziwnie.
