# Supplementary material for: The challenges arising from the COVID-19 pandemic and the way people deal with them. A qualitative longitudinal study
Source: PLoS One. 2021 Oct 11;16(10):e0258133. doi: 10.1371/journal.pone.0258133 (PMC8504766; doi:10.1371/journal.pone.0258133)
Supplement: S1 Dataset — (ZIP) [file pone.0258133.s003.zip › Transcriptions/stage 6/9.6_F_25_couple, no children.docx]

**9.6_F_25_couple no children**

**Teraz jesteś w domu u rodziców...**

Ze 2 miesiące mnie tutaj nie było, przyjechałam z mężem, bo rodzicom popsuł się piec i zostałam na kilka dni.

**Jak wyglądały ostatnie miesiące?**

Głównie praca, bardzo dużo pracy i nadal się to ciągnie. Tak naprawdę mało skorzystałam z lata, szybko przeminęło i byłam jeszcze na urlopie w Bieszczadach.

**Myśleliście o jakimś wyjeździe za granicę?**

Wcześniej, jeszcze przed marcem, ale potem musieliśmy zmienić plany, skończyło się na Bieszczadach i było bardzo przyjemnie. Znam osoby, które jeździły za granicę. Głównie to była Grecja. Oprócz tego, że trzeba było nosić maseczki w samolocie to wszystko funkcjonowało tam normalnym wakacyjnym tempem i sposobem. Gdybym wiedziała, że wyjadę i wpuszczą mnie z powrotem, to chyba bym się nie za bardzo obawiała wyjechać, szczególnie w wakacje, kiedy liczba zachorowań nie była tak wysoka jak teraz, o wiele niższa. Pojechaliśmy w Bieszczady, a ja chciałam wyjechać w Alpy, ale baliśmy się, że jak pojedziemy do Austrii czy do Niemiec to już nas nie wpuszczą z powrotem, bo w sumie trudno było przewidzieć, czy nagle nie zacznie się jakaś kolejna fala. Podeszliśmy tak asekuracyjnie. Pojechaliśmy w Bieszczady w lipcu i mam wrażenie, że ludzie byli wtedy głównie na Mazurach. Do tego mieliśmy fatalną pogodę. Było bardzo mało turystów i przez cały dzień nie spotykaliśmy nikogo.

**Jakieś przełomowe momenty?**

Te miesiące tak m i przepłynęły ciurkiem i tak naprawdę nie odnajduję tu żadnego punktu krytycznego.

**Jak jest teraz?**

Tak samo jak wcześniej, chociaż rzeczywiście od maja się bardzo dużo zmieniło, bo jednak o wiele swobodniej można już było funkcjonować. Człowiek się jednak bardzo szybko przystosowuje i ja się już przyzwyczaiłam do tego, że chodzi się w masce, już nie łapię się na tym, że zapomniałam maseczki. Cały czas ma się maseczkę, ma się jakiś żel antybakteryjny i weszło to już w krew. Szczerze mówiąc nie czuję się już jakby działo się coś dziwnego. Oczywiście teraz jest ten wzrost zachorowań i jak ta liczba się zwiększa to człowiek zaczyna trochę się niepokoić, ale mimo wszystko tak wyraźnie się nic nie zmieniło. Ja w sumie zawsze działałam zapobiegawczo, chociaż muszę przyznać, że jednak ostatnio staram się tak nie wychodzić. Widzę też tutaj w domu po rodzicach - tata dostał przykazanie od mamy, że ma iść na zakupy i zrobić zapasy, żeby już nie wychodziło za dużo osób i tak samo też kazałam mężowi. Kupili ileś tam mąk, ileś cukru, więc tak jak na kryzys. Ograniczamy po prostu kontakty z ludźmi, nie spotykamy się z tymi, z którymi nie muszę się spotkać. Wcześniej rodzice robili zapasy w marcu i chyba dopiero teraz im zeszły. Mama pracuje w służbie zdrowia i większość rodziny pracuje w służbie zdrowia, więc biorę poprawkę na to co przekazują media. Mam wiadomości z pierwszej ręki, wiem co się dzieje w szpitalach, że jest bardzo, bardzo dużo zachorowań, bardzo dużo śmierci. Moja mama pracuje w mniejszym szpitalu, ale przygotowujemy się po prostu na to, że zrobi się 2-gi marzec. Znowu będą zamknięte sklepy, będzie jakiś lockdown większy niż teraz.

**Kiedy zaczęłaś znowu przejmować się sytuacją?**

Tak i zapamiętałam dokładnie ten moment, bo ja się bardzo długo raczej nie przejmowałam, robiłam swoje i wszystko było w porządku. Mąż codziennie mówił, że "o, już mamy tyle zachorowań", a jednak nie wiem czy ty się spotkałaś z kimś chorym na koronawirusa, bo ja w życiu? Albo o tym nie wiedziałam.

**Tak osobiście to nie.**

No właśnie. Ja mam tak samo i dlatego może łatwiej mi to było ignorować, bo nie znałam nikogo osobiście. Koleżanka mi napisała, że ma koronawirusa, ale nie miała żadnych objawów tylko po prostu wyszedł jej pozytywny test i też biorę poprawkę na to, co ona mówi. Moment był taki, że mąż w pewnym momencie powiedział, że jak będzie tak dalej to my pewnie nie pojedziemy na Boże Narodzenie do domu. I ja wtedy, że jak to, że ja na pewno pojadę, że to niemożliwe. Mąż odpowiedział, że ja to może tak, ale on jeździ do pracy i będzie się bał, że coś przywlecze swoim rodzicom i to był dla mnie cios, bo ja się zaczęłam bać, że nie pojadę na święta do domu. Wielkanoc to pół biedy, ale Boże Narodzenie? No nie. To był ten moment 2 tyg. temu.

**A pamiętasz ilu było wtedy zakażonych?**

Nie, nie pamiętam.

**Czy czujesz, że twój stan wrócił do tego jak było w marcu/ kwietniu?**

Nie. Przyzwyczaiłam się do tego jak wygląda sytuacja i to już nie jest nowość. Człowiek już tego nie analizuje, nie zastanawia się nad tym tylko po prostu funkcjonuje w tej nowej rzeczywistości tak troszeczkę bezmyślnie.

**Twój maż pracuje poza domem, ty online, tak?**

Tak. była mowa o tym, że w biurze jest remont do sierpnia i potem może wrócimy, ale w lipcu już otrzymaliśmy informację, że do końca roku na pewno będziemy pracować w domu. Poza tym pracuje w takiej branży, że im się po prostu nie opłaca, żebyśmy wracali do firmy i mam nadzieję, że kiedy skończy się epidemia, ten najgorszy okres to nie będę musiała wracać do biura.

**Jak się czujesz z takim trybem pracy, z tym, że starasz się nie wychodzić z domu?**

Jeszcze 2 tygodnie temu jak miałabym iść na zakupy do GH, to po prostu założyłabym maseczkę i zrobiłabym to dla przyjemności. Teraz już bym tego nie zrobiła ze względu na liczbę zachorowań. Natomiast bardzo dobrze mi się pracuje z domu. mam o wiele więcej czasu, nikt mi nie przeszkadza i bardzo mi się podoba, że mogę sobie sama organizować czas po pracy. Mamy nawet taki nakaz wewnętrzny, żeby coś porobić ciekawszego po pracy niż zazwyczaj dlatego, że się nie było w biurze i człowiek się nie socjalizował.

**Tej socjalizacji ci nie brakuje?**

Nie. Pracuję z zespołem i cały czas ze sobą piszemy, rozmawiamy i to nie są tylko zawodowe sprawy. Zresztą z koleżankami z pracy się widuję i ze znajomymi spoza pracy też się widywałam do ostatniego czasu. Ostatni raz chyba byłam na urodzinach 2 tygodnie temu i teraz zakładam, że będziemy właśnie raczej uważać przez najbliższy czas.

**Gdzie teraz wychodzisz?**

Od tych 2 tyg. się ograniczam, bo wcześniej w ogóle i chodziłam na siłownię, na fitnessy. Teraz niestety nie mogę i moim priorytetem są zakupy spożywcze. Myślę, że spokojnie wyszłabym też do Rossmanna, gdyby mi czegoś zabrakło. Nie zamawiałam jeszcze niczego przez internet, więc zakupy rzeczy potrzebnych do domu.

**Emocje**

4 - kojarzy mi się na pewno z wakacjami, bo to był dla mnie czas wielu spotkań i zacieśniania więzi przyjacielskich i rodzinnych.

11 - dzisiaj. Może ze względu na pogodę, ale też ta 2 fala kojarzy się z jesienią, z takim deszczem i przede wszystkim patrzymy przez szybę. Może nie zamknięci w domu, ale raczej ograniczamy się na ten świat zewnętrzny.

**Jakie to są emocje, które teraz ci towarzyszą?**

Miałam dzisiaj straszny dzień w pracy, więc może nie będzie to obiektywne. Może nie rezygnacja, ale taka akceptacja?

**A wizja zbliżającego się lockdownu?**

No, to już było, więc już wiadomo na co się przygotować, na co zwracać uwagę. Martwię się tylko, jakie to wywoła skutki gospodarcze, żeby nie było takich wielkich problemów jak ostatnio. Zamykanie jakichś tam usług kosmetycznych to w ogóle jest bez sensu i niepotrzebnie takim przedsiębiorstwom odbierano pracę. Restauracje wszystkie się przerzuciły teraz na dowóz, więc myślę, że tu też nie ma takiej tragedii. Rozumiem zamykanie siłowni, chociaż jak chodziłam na zajęcia, to i tak wpuszczało się mniejszą liczbę osób, trzeba było się ustawiać w odstępach. kaszle się na się na siebie cały czas, ale było to rozwiązane dość sensownie i zajęcia, które wymagały bliskości były odwoływane.

**Już nie ma tej autostrady za mgłą?**

Tak. To już było i trzeba przez to przejść.

**Wtedy zaczynała się wiosna i to poprawiało nastrój?**

Tak. Jednak Wrocław jest taki dość duszący. Mieszkamy w centrum, nie mamy balkonu i jest po prostu brzydko. Przyjechałam do domu i pierwsze co zauważyłam to drzewa. jesteśmy z gór, więc piękne kolorowe drzewa, przyroda i nawet ten deszcz w górach wygląda inaczej. Samo to, że tutaj jest wiatr, a on jest taki energetyczny. We Wrocławiu tego nie ma, a tutaj człowiek może wreszcie poczuć, że żyje. to zależy, w jakim miejscu się tę jesień przeżywa.

**Nie odczuwasz takiego strachu przed zarażeniem się?**

Ja nie i jeżeli już miałabym się bać, to tego, że moi bliscy się zarażą.

**A jakie emocje towarzyszą teraz twoim bliskim?**

Chyba też spokojne. Jeśli chodzi o moja rodzinę, to oczywiście wszyscy się pilnują, ale nie ma jakiegoś przerażenia. A moja babcia to już jest taki pershing, że ona codziennie do sklepu będzie wychodzić. Jej maseczka nie przeszkadza i nosi na szczęście. Mówi, że jak ma umrzeć, to i tak umrze, więc dlaczego ma gdzieś nie pójść? W tym roku będzie miała 80 lat i jest po nowotworze, więc powinna się bać, ale nie. Z kolei od strony mojego męża, to oni się strasznie boją. Byłam u teściowej w niedzielę i oni są przerażeni, i jeszcze sami się nakręcają. Boją się też, bo teść opiekuje się ojcem, który ma raka płuc i każda infekcja dla niego jest niebezpieczna. Ale tyle czasu już trwa pandemia i wszystko jest w porządku, że mam nadzieje, że taka sytuacja się utrzyma.

**Jak się nakręcają?**

Jak teść nas żegnał, to mówił jaki jest przerażony, że musi do pracy chodzić, ale nie dziwię mu się, bo pracuje w szpitalu i akurat tam jest koronawirus w tym szpitalu. Teściowa jest bardziej rozsądna w tym temacie, ale też jak wcześniej jeździła raz w tygodniu do dziadków, to teraz jeździ raz na 2 miesiące, bo boi się, że coś im przyniesie. Mąż powiedział, że jak będzie miał zagrożenie, że coś przywiezie rodzicom, to nie pojedzie na święta do domu. U jego siostry w firmie dziewczyna się rozchorowała i w czwartek straciła smak. Tam się sytuacja jeszcze bardziej nakręca, bo wyszedł test niejednoznaczny. Oni od kilku dni siedzą całym zespołem w domu, bo nikt nie wie, czy się zaraził, czy ona ma tego koronawirusa czy nie, a pracują przy produkcji żywności i nie wiedzą, czy mają przyjść nadzorować procesy, czy nie mogą, więc jeszcze dodatkowo teść i teściowa się martwią. Oni się bardziej boją, ale jednak dziadek jest chory i teść też jest po zawale i pracuje w szpitalu, w którym jest koronawirus. Jest w grupie większego ryzyka. To wszystko wynika ze zwiększonej liczby zakażeń.

**Ty nie znasz nikogo, kto chorował?**

Nie znam, ale ja jestem pewna, że miałam na wiosnę.

**Wiesz co powinnaś zrobić, gdybyś miała jakieś podejrzenia?**

Zastanawiam się. Wtedy, gdy myślałam, że mam koronawirusa to rzeczywiście byłam przerażona i nie wiedziałam, gdzie się zwrócić. Myślę, że gdybym była w takiej sytuacji to po prostu odświeżyłabym swoje informacje - weszła na jakąś stronę rządową i sprawdziła, jak to wygląda teraz, bo jednak szpitale są przepełnione. Pewnie trzeba na jakąś infolinię zadzwonić i oni wtedy... Wątpię, żeby gdzieś się zgłosić, jakieś testy...Dzisiaj koleżanka mówiła w pracy, że u kogoś z jej znajomych wykryto w pracy i że Enel Med im te testy zapewniał tylko nie wiem, czy do domu im przywożą...Nie wiem.

**Rzeczywiście szpitale są przepełnione?**

Tak. Moja ciocia pracuje w 2 szpitalach i jeden z nich się przemienił w covidowy, więc został jej jeden i została poproszona, jako że jest samotna, żeby pracować na oddziale covidowym. Mówi, że na początku się bała, ale z drugiej strony tam, gdzie pracowała na internie i tak codziennie ktoś był z Covidem i człowiek nie wie, czy ma, czy nie ma. Mówi, że lepiej już się zabezpieczyć i wiedzieć, że ktoś jest chory i że trzeba się zachowywać inaczej.

**O jakich zmianach w obostrzeniach słyszałaś?**

Na pewno zamknęli siłownie, wprowadzili znowu godziny dla seniorów. Ja uważam, że zawsze powinny być godziny dla seniorów. Kultura nakazuje, żeby w ogóle puszczać seniorów zawsze, ale ludzie nie zawsze mają takie poczucie i zawsze mi jest przykro jak jakaś starsza pani gdzieś tam stoi, a tylko margarynę ma np. Gdyby zawsze był taki nakaz, to może inaczej by to wyglądało i trochę zmusiło jednak ludzi do działania. Chyba zgromadzenia są do 10 osób, zamknięte restauracje i tylko na dowóz. Nie przychodzi mi do głowy nic więcej.

**Co myślisz o tych ograniczeniach?**

Uważam, że są najsensowniejsze w tym momencie, żeby powstrzymać rozprzestrzenianie się. Po prostu ograniczenie kontaktów. Gastronomia to ok, że jest na dowóz. Mam kartę Multisport i akurat jak zaczęłam chodzić, to zamknęli mi siłownie. Uważam, że wystarczyłoby ograniczenie aktywności. Poza tym ktoś, kto ćwiczy regularnie ma matę w domu, więc spokojnie takie zajęcia mogłyby się odbywać online. Oni niby coś tam teraz wprowadzają...Samo zamknięcie siłowni jest w porządku. Myślę o takich zajęciach grupowych. Jak ktoś pójdzie podnosić ciężary to też napoci się na ten sprzęt i myślę, że sporo się tam mogło przenosić. Koronawirus to chyba najmniejszy problem.

**Brałaś udział w takich zajęciach online?**

Nie, ale zaczynam się zastanawiać. Muszę doczytać, czy tam, gdzie chodziłam są takie zajęcia. Dopiero 2 dni temu dostałam wiadomość, że nie pobiorą nam opłat za listopad, bo niby to ma być tylko przejściowe zamknięcie i mają nadzieję, że za miesiąc się otworzą.

**Konieczność noszenia w maseczkach wszędzie?**

To nie jest jakieś dotkliwe. Chyba już się przyzwyczaiłam przy pierwszej fali. Zresztą już jest zimno, więc ta maseczka robi się całkiem praktyczna. Używam maseczki a nie przyłbicy.

**Czy to wpływa na ograniczenie zakażeń?**

Na pewno ma dobry wpływ, ale czy wielki to bym polemizowała, bo jak się patrzy na ludzi to wszyscy mają poodsłaniane nosy. Jednak trudno jest oddychać, jak się ma też nos zasłonięty. Mniej bakterii uwalniają, ale mimo wszystko coś tam wciąż jest. Wiele osób te maseczki to chyba ze śmietnika wyciągnęło albo nie prało ich od początku pandemii i w tym bym widziała problem. Jeżeli ktoś jest rzeczywiście przejęty jak mój mąż i sprawdza przepuszczalność maseczki próbując zdmuchnąć zapałkę przez maseczkę i przejmuje się, żeby ona była wyparzona i czysta, to wtedy jest w porządku i myślę, że ma to realny wpływ. W większości przypadków ludzie zakładają po prostu tylko na usta, żeby nie dostać mandatu i wewnętrznie się poczuć troszeczkę pewniej. To minimalizuje ryzyko, ale to jest raczej takie średnie i nie zapobiega. Ja tego przestrzegam i piorę maseczkę. jak mój mąż wrzuca swoją maseczkę, to ja moją też wrzucam. W lecie to było rzadziej, ale teraz to po 2-3 przejściach ją wrzuca do prania. Kupiliśmy też jednorazowe i teraz raczej tych będziemy używali. Skorzystaliśmy, że jeszcze były tanie.

**Ochrona seniorów?**

My wszyscy mówimy babci, że nie powinna tak wychodzić, ale ona jest nie do przegadania. Rzeczywiście najbezpieczniejsze dla osób starszych jest siedzenie w domu, ale żal by było babci jakiejś zabronić wyjść na chwilę na spacer. Nawet dla zdrowia, żeby się wzmocnić. Moja mama w maju mówiła, że nie można się aż tak wyjaławiać, bo musimy potem być przygotowani na inne zagrożenia, na inne wirusy na jesieni. Spacery jednak wzmacniają i dotlenienie się jest zdrowe, więc takie rozprostowanie kości nie powinno być zabronione. Trudno potem jednak nadzorować takich ludzi, bo każdy to sobie inaczej interpretuje.

**Zakaz imprez rodzinnych, wesel?**

U nas było jedno wesele odwołane i zostało przełożone na przyszły rok. Z moich znajomych wszyscy byli w tym roku na jakimś weselu oprócz nas. Jednak zawsze tam jest sporo osób, których się nie zna. U nas w tym roku było sporo imprez rodzinnych, ale wtedy było mniej zachorowań. Nie pojechaliśmy na grilla do mojej przyjaciółki, bo jednak były tam osoby, których nie znamy i nie wiemy czym się zajmują czy są odpowiedzialne, z kim przebywają na co dzień. na imprezę rodzinną przyszliśmy, bo to są ludzie, z którymi obcujemy na co dzień i ufamy im na tyle, że wiemy, że jak ktoś by się źle czuł, to by nie przyszedł. Stosujemy kryterium zaufania. To był lipiec, sierpień, a teraz myślę, że długo nie będzie żadnych spotkań rodzinnych. Zakaz wesel jest zasadny. Jak ktoś jest zarażony i napoci się tam, nachucha, to z tego jest wielka część zachorowań. Rozumiem też ludzi, że mają potrzebę żyć normalnie, że wydali duże pieniądze na to wesele. To jest podejmowanie ryzyka i takie decyzje też wypływają potem na innych ludzi i gdzieś tam nawet na nas pośrednio. To jest znak zapytania i ten kij ma dwa końce.

Spotkaliście się z niezrozumieniem, jak nie pojechaliście na tego grilla?

Nie. Teraz takie zachowania są rozumiane.

**Co teraz myślisz o koronawirusie?**

Myślę, że będzie więcej takich koronawirusów i że trzeba się na to przygotować, że to jest dobra próba takiego kryzysowego zarządzania, że nie można zaprzeczyć, że istnieje, szczególnie, że ludzie cały czas umierają i to jest taki widoczny znacznik. Na pewno wiele w tym jest polityki i próby wykorzystania pewnych kwestii i tego koronawirusa. Dla każdej instytucji jest to wyzwanie i szansa na pokazanie, że jest dobre zarządzanie, że szybko się reaguje, że ma się jakieś osiągnięcia. Firma, dla której pracuję, pracuje nad szczepionka i to jest już w ogóle wyścig szczurów. Kto pierwszy ten lepszy i będzie bogaty. To jest sytuacja dla niektórych firm i organizacji, żeby na tym skorzystać.

**Twoje myślenie na temat koronawirusa się nie zmieniło?**

Chyba nie. Rozmawiałam wczoraj z rodzicami, że jesteśmy pokoleniem, które nie miało możliwości, na szczęście, doświadczać wielu kwestii, wielu takich problemów. Nie znamy bezpośredniej wojny, nie mamy konfliktów zbrojnych i słyszymy tylko o nich. Mama miała zajęcia na poligonie w szkole pielęgniarskiej, żeby umieć jak najszybciej reagować w sytuacjach kryzysowych. Ja się zastanawiam, czy moje pokolenie to umie. I nie, nie jesteśmy tego nauczeni. Nie ma już wojska obowiązkowego. Są jakieś zajęcia z przysposobienia obronnego, ale kto tego słucha? Gdyby doszło co do czego, to duża część młodego społeczeństwa miałaby problem z poradzeniem sobie w wielu sytuacjach.

**Nie jesteśmy gotowi na tę sytuację jako społeczeństwo?**

Trochę chyba tak, ale mam nadzieję, że to jest też przyczynek do tego, żeby zacząć się zastanawiać nad tym, co można zmienić i myślę, że ludzie zaczną się nad tym zastanawiać. Ja nie byłam nigdy w harcerstwie, ogniska bym nie rozpaliła. Brakuje nam wielu umiejętności, które wydają się nam zbędne, a w takim czasie jak dzisiaj nagle zyskują na wartości. Działanie w sytuacji kryzysowej. Mnie też brakuje takich umiejętności.

**Czy ludzie teraz zachowują się adekwatnie do sytuacji?**

Wcześniej też się nie zachowywali. Wiele osób przestrzega zasad i rozumiem, że jest też obecnie taka sytuacja, że trzeba wybierać jak reagować i jak się zachowywać w tłumie. Myślę o protestach, bo z jednej strony rozumiem, ale z drugiej ja bym się bała, że ja kogoś albo siebie zarażę i że będę wektorem. Ten kij znowu ma 2 końce. To jest troszeczkę nieodpowiedzialne, ale rozumiem. Nie potrafię nie usprawiedliwić tej sytuacji, że ludzie się decydują na łamanie obostrzeń dotyczących gromadzenia się.

**Ty nie chodzisz na takie zgromadzenia?**

Nie, bo jak już zaczęłam unikać, to już unikam wszystkiego. Nie chodzę na protesty ze względu na koronawirusa.

**Jak oceniasz działania rządu w sytuacji, jaką mamy?**

Te obostrzenia i zasady są wymyślone dość na prędcy i trochę się nie kleją do siebie. Jedna przeczy drugiej. Rozumiem, że realizowanie tych zasad jest trudne i trudne jest odpowiednie ich ułożenie tak, żeby zawrzeć wszystkie punkty a mimo wszystko pozostawić jakieś tam pole - tak jak np. z tymi biegaczami. Sport jest zdrowy i niech sobie biega, ale z drugiej strony ma nie nosić maseczki czy nosić maseczkę i się przyduszać? To jest pierwsza taka sytuacja w Polsce, więc już macham ręka na te przeczące sobie zaostrzenia i kieruję się swoim wyczuciem i swoją intuicją. Wydaje mi się, że człowiek potrafi odróżniać dobre od złego i mądre od głupiego.

**Nie obawiasz się, że twoja intuicja może doprowadzić do ukarania cię mandatem?**

Boję się. Ostatnio wysiadałam z auta pod domem i już było ciemno. Mąż włożył maseczkę i powiedziałam, żeby dał sobie spokój, ale głupio by było, gdyby mnie ktoś złapał tuż pod domem i włożyłam tę maseczkę.

**Duży wpływ na ciebie ma zachowanie twojego męża?**

No tak, bo ja dużo spraw trochę trywializuję, a on rzeczywiście się przejmuje, ale on to jest ten mózg naszego związku, a na mózgu warto polegać.

**Nadal informacje czerpiesz głównie od niego?**

Na temat świata to ja się też wczytuję, ale o koronawirusie to tak, głównie od męża. Teraz w domu mam też informacje z pierwszej ręki a poza tym tutaj jest też telewizor, więc można sobie poprzerzucać i sprawdzić co się mówi. Akurat na temat koronawirusa przekaz chyba jest spójny.

**Jaki obraz wysnuwa się z tych mediów?**

Tragedia i kryzys i dlatego też staram się tak nie nakręcać, bo mam nadzieje, że jeżeli będę przestrzegać zasad i uważać na siebie to jestem w stanie wyjść z tej sytuacji obronna ręką. A będę się stresować, jak się ewentualnie rozchoruję.

**Media nakręcają negatywnie sytuację?**

Bardzo mocno, natomiast nie jestem pewna czy to jest złe zjawisko, bo nie lubimy się podporządkowywać i zastanawiam się co by się działo, gdyby sytuacja nie była tak nakręcona. Wtedy ludzie chyba w ogóle by chodzili bez maseczek i się nie przejmowali. Teraz wszystkie media o tym trąbią na całym świecie, człowiek jest tym przytłoczony, ale jakby z większą chęcią się stosuje do tych wszystkich zaostrzeń. Na mnie taki nadmiar informacji nie ma dobrego wpływu, ale widzę zasadność.

**Myślisz, że dało się zapobiec 2 fali?**

Trudno mi powiedzieć. Wydaje mi się, że to jest naturalne zjawisko przy takich wirusach. Pewnie można było to w jakiś sposób zmniejszyć, ale też nie jestem pewna. Teraz ta liczba chorych zwiększa się wykładniczo, więc jest też o wiele większy wzrost. Główne ogniska koronawirusa są chyba właśnie z takich imprez rodzinnych, ale człowiek jednak potrzebuje normalnie funkcjonować. To nie jest tak, że ma na nas jakaś atomówka spaść, tylko jest jakaś choroba, która przygasa, potem jest trochę silniejsza i też rozumiem tę potrzebę socjalizacji. To jest też zdrowe dla psychiki, żeby nie zwariować.

**Może wszyscy powinniśmy się zarazić, żeby zbudować odporność?**

Na początku wypowiadał się specjalista i mówił, że i tak ok. 70% przejdzie koronawirusa objawowo lub bezobjawowo i kto wie, czy już wszyscy nie przeszliśmy?

**Myślałaś, żeby zrobić badanie i to sprawdzić?**

Chyba da się to sprawdzić, chociaż podobno i tak można się 2 raz zarazić. Myślałam o tym, ale kto wie, czy nie okaże się, że ja akurat teraz go mam?

**Jak widzisz przyszłość?**

Przy 1-szej fali wszystko jakby się wstrzymało, bo my też rozglądamy się za naszym własnym lokum i myślimy o kredycie. Do maja nie robiliśmy nic poza przeglądaniem ofert. W tej chwili życie ruszyło i mimo tej 2 fali my nadal szukamy. Życie już nie stoi. To były tylko 2 miesiące wstrzymania, a teraz wszystko toczy się dalej i ja też nie zamierzam dać sobie tak...Tak się wstrzymać.

**Co się zmieniło w twoim myśleniu?**

Wydaje mi się, że już trochę to znam, że to się powtarza. Nie ma sensu się tak zatrzymywać w miejscu i trzeba pozytywnie patrzeć w przyszłość a taką nadzieją, że będzie po prostu.

**Macie obawy związane z tym, że pandemia może jakoś wpłynąć na wasze sprawy mieszkaniowe?**

W lipcu widzieliśmy się z doradcą kredytowym, żeby zweryfikować rynek. Mieliśmy niepokojące informacje od znajomego, że 4 banki nie chciały mu z powodu Covid udzielić kredytu. Rzeczywiście sytuacja się zmieniła, że w najgorszym momencie 3 dziennie wnioski kredytowe do nich wpływały a normalnie jest 200, ale ludzie się zaczęli rozpędzać od maja, kiedy gospodarka wróciła na własne tory. Ludzie potrzebują miejsca, gdzie będą żyć tym bardziej teraz, kiedy my nie pracujemy w biurach. Bardzo się zmieniła sytuacja na rynku, mieszkania bardzo podrożały. Zawsze to była tragedia a teraz to chyba bez sensu jest kupowanie mieszkania i dlatego myślimy, żeby się budować. Okazuje się, że to wychodzi prawie tak samo finansowo. Doradca nas pocieszał, że już raczej nie dopuszczą do drugiego takiego lockdownu jak był i że uzyskanie kredytu może będzie nieco trudniejsze, ale nie jest to niemożliwe

**Czy ten lockdown może się różnić?**

Jednak ten pierwszy był zaskoczeniem dla wszystkich i przedsiębiorstwa po prostu stanęły. W tej chwili oni już nie mogą sobie pozwolić na taki przystanek. U mnie nic nie stanęło w pracy, ale mąż był chyba miesiąc na postojowym. Od strony ich klientów oni mieli cały czas zapotrzebowanie na produkt, który nie był produkowany. Chodzi o samochody. Ludzie cały czas potrzebują jeździć samochodami, mimo lockdownu. Już nie pozamykają 2-gi raz przedsiębiorstw i będą rozwiązania, które zapewnią jakąś alternatywę.

**Nie masz teraz takich obaw, że znowu wszystko się zatrzyma, że stracicie płynność finansową, itd.?**

Podświadomie zawsze jest taki strach, ale to mnie może spotkać zawsze i jakbym miała się nad tym cały czas zastanawiać, to nie podjęłabym w ogóle żadnej decyzji. Chcę normalnie żyć, chcę wpakować pieniądze w swoje własne lokum a nie w wynajmowane. Boję się też inflacji, tego co się stanie z pieniędzmi, które udało się nam odłożyć. Na razie się wstrzymujemy i nie podejmujemy pochopnych decyzji, ale myślę, że sytuacja mieszkaniowa długo nie wróci na miejsce. Prawie już kupowaliśmy, ale to był bliźniak. Rozmyśliliśmy się i ja poczułam ulgę. Nieruchomości wolnostojące bardzo spadły a mieszkania bardzo podskoczyły, więc tutaj wybór jest prosty.

**Bardziej patrzycie na to pod kątem tego jaka jest sytuacja na rynku, a nie ilu jest zakażonych na świecie?**

Tak, chociaż jednak ten okres, w którym tak bardzo zmieniło się moje postrzeganie to są dopiero 2 tygodnie, więc ja nie zdążyłam jeszcze z mężem dobrze przegadać sytuacji. Wydaje mi się, że teraz będziemy się starali o wiele rozsądniej wszystko rozegrać i może wstrzymamy się do końca roku i zobaczymy co będzie. To nie zmienia faktu, że cały czas szukamy, patrzymy, bo ten rynek cały czas działa i jest aktywny. Więcej to po prostu wymaga od nas świadomości i zdrowego rozsądku.

**A jeśli chodzi o twoją pracę?**

Mam umowę na czas nieokreślony od września i to jest dobra sytuacja. Lubię swoją pracę, ale uważam, że zarabiam za mało. trudno jest mi się wystarać o podwyżkę, mimo iż uważam, że na nią zasługuję. To jest też przez koronawirusa, bo powiedziała mi ostatnio moja superwizorka, że wstrzymują się z decyzjami finansowymi do końca roku. Taka jest polityka firmy. Z jednej strony może bym i chciała zmienić pracę, ale z drugiej strony wolę zostać tu, gdzie wiem, że jakoś mi idzie i wiem, że jest zapotrzebowanie na moje usługi. Mój mąż też powinien mieć przedłużoną umowę na czas nieokreślony w sierpniu i nie dostał tej umowy, bo polityka firmy w czasie Covidu jest taka, że nie zmieniają nikomu statusu umowy.

**Kiedy się skończy czas Covidu?**

Sama się nad tym zastanawiam i nie mam pojęcia. To pewnie będzie czas, kiedy będę musiała wrócić do biura, a nie chcę. Przypuszczam, że pewnie to będzie czas, kiedy wymyślą szczepionkę, więc może to być lato przyszłego roku? Czasem widzimy, że robią te badania, więc wiemy, że badania trwają.

**Jak planujesz spędzić 1.11.?**

Cała moja rodzina jest pochowana w jednym mieście w woj. lubuskim i moi rodzice pojechali na groby w zeszłym tygodniu, żeby uniknąć ludzi na cmentarzach. Mnie nie wzięli, żeby niepotrzebnie nie zwiększać liczby osób. Mama pojechała z tatą, bo jest bardziej doświadczonym kierowcą i bezpieczniej się z nim czuła na drodze. Wiele osób wybierało się w tym tygodniu, ale w mojej rodzinie te groby się odwiedza regularnie, a nie tylko 1.11., więc nie czuję jakiegoś przewinienia, że nie ma mnie tam teraz.

**Powinny zostać zamknięte cmentarze?**

Myślę, że to niegłupie jest, ale czy ludzie to zaakceptują? Myślę, że byłyby problemy, bo tak naprawdę my przychodzimy na ten cmentarz dla siebie a nie dla tych zmarłych. O zmarłych się powinno pamiętać cały rok i to jest miejsce, w którym tylko jest pochowane ciało. Wydaje mi się, że to nie jest ważne, kiedy mówimy o życiu, a jest tutaj duże ryzyko, że ktoś się zarazi, mimo, że to jest na dworze. To by nie był głupi pomysł, szczególnie w przypadku metropolii.

**A co z Bożym Narodzeniem?**

To mnie bardzo rusza niestety, bo nie ma nic przyjemniejszego niż święta w domu. Dodatkowo to jest czas, kiedy ma się kilka dni wolnego, jest śnieg i jest bardzo fajnie. Ja w tej chwili nie dopuszczam do siebie takiej myśli, że nie przyjadę na święta. Zobaczymy, jak się rozwinie sytuacja. Mam nadzieje i w tej chwili nie chcę nawet o tym myśleć. Wierzę, że przyjedziemy i wszystko będzie w porządku.

**Mąż powiedział, że nie pojedzie jak będzie jakieś zagrożenie. Co musi się stać, żeby on nie pojechał?**

Co jakiś czas mąż musi pojechać do pracy i bałby się pojechać, gdyby w tygodniu poprzedzającym święta musiał jeździć do pracy. Rzeczywiście nie wiadomo, kto tam przyszedł. I druga sprawa to czy nie zamkną miasta, czy nie będzie znowu zakazu przemieszczania się, bo tak chyba było na Wielkanoc. Jeżeli tego wszystkiego nie będzie to myślę, że nawet ten zakaz przemieszczania dałoby się przejść, bo jednak jedziemy do domu rodzinnego. To będzie zależało od tego, ile razy będzie w pracy przed świętami, ale zakładam, że to będzie na tyle dużo zachorowań nadal, że oni będą starali się to zminimalizować, więc mam nadzieję, że te święta będą normalne.

**Co by się musiało stać, żebyś ty nie pojechała do rodziny na Boże Narodzenie tak jak na Wielkanoc?**

Teraz inaczej już na to patrzę, bo na Wielkanoc moi rodzice byli trochę zdziwieni, że nie przyjechaliśmy, bo tak naprawdę, to ja się martwię o nich. Z drugiej strony zostawić męża samego w domu? No też nie. Nie mamy dzieci, więc jeszcze korzystamy z tej możliwości, że on może być ze swoimi rodzicami a ja ze swoimi i się rozdzielamy, ale nie chciałabym, żeby został sam we Wrocławiu. Myślę, że to będzie wspólna decyzja i jakaś suma wypadkowych grudniowych.

**Boże Narodzenie jest dla ciebie ważniejsze niż Wielkanoc?**

Z punktu widzenia religijnego Wielkanoc jest ważniejsza, ale po prostu lubię bardziej Boże Narodzenie i nie mówię o prezentach. Jest to po prostu magiczny czas. Szczególnie tutaj w górach, gdzie jest śnieg i inaczej się te święta odbiera. I to jest czas takiego leniuszkowania, wielkiego odpoczynku, ładowania baterii i ja na to czekam cały rok. Bardzo też lubię kolędy i bardzo przykro mi będzie, jeśli nie będę mogła pójść na Pasterkę. jestem dobrej myśli i myślę, że będzie dobrze z tym Bożym Narodzeniem.

**Gdyby było tak jak teraz, ale mogłabyś pojechać do rodziców, to zrezygnowałabyś z Pasterki?**

O, na pewno tak. To jest jednak bardzo dużo ludzi a odkąd wzrosły zachorowania to unikam tłumów. Wszystkich. Mam nadzieję, że zaostrzenia się dostosują do tych świąt, bo dla Polaków najważniejsze jest Boże Narodzenie i dużo osób nawet nie obchodzi Wielkanocy. Mam nadzieję, że to zostanie wzięte pod uwagę.

**Dyspensa z ograniczeniami na Boże Narodzenie?**

Nie wiem, jak można to rozegrać, ale może zniosą jakieś zakazy przemieszczania się przed świętami. Jestem ciekawa.
