# Supplementary material for: The challenges arising from the COVID-19 pandemic and the way people deal with them. A qualitative longitudinal study
Source: PLoS One. 2021 Oct 11;16(10):e0258133. doi: 10.1371/journal.pone.0258133 (PMC8504766; doi:10.1371/journal.pone.0258133)
Supplement: S1 Dataset — (ZIP) [file pone.0258133.s003.zip › Transcriptions/stage 6/10.6_F_55_couple, no children.docx]

**10.6_F_55_coulpe no children**

**Co się u pani działo przez ostatnie miesiące?**

Oj, dużo się działo. Wyjechałam nad morze, do przyczepy, byłam tam ze swoją wnuczką i tą jej starszą siostrą. Było bardzo fajnie, aczkolwiek bardzo ciężko, bo to pierwszy raz byłam tak sama z dziećmi. W międzyczasie oczywiście tu jakieś były problemy z moim tatą, moja siostra tu została, bo tak jak mówiłam, mój tata jest chory i jest z opiekunkami, ale jakoś tam udało się to wszystko ogarnąć. Potem w lipcu większość czasu byłam tutaj w domu w Warszawie. Potem w sierpniu też byłam z dziećmi nad morzem. I tak w zasadzie jakoś lato minęło.

**Udało się w końcu pani mieć gości w przyczepie?**

Tak, udało się, natomiast zakończenie tego sezonu było bardzo nieprzyjemne. Bo tam wszyscy generalnie wynajmują te przyczepy i wiadomo, że tak jest, ja nigdy nie miałam tam żadnych ani złych tych swoich ludzi, którzy ode mnie to... Z reguły zawsze to byli sami znajomi i to tacy bardzo zaufani, zaprzyjaźnieni. I w tym roku ja tam byłam bardzo długo przecież też, bo w sumie 2 tygodnie w czerwcu, potem 10 dni w sierpniu, natomiast nie chcieli mi podpisać umowy na przyszły rok. Wynaleźli sobie kilkanaście osób takich, których nie wiem, chcieliby się pozbyć z kempingu albo szukali miejsc w pierwszym rzędzie. Więc było to bardzo nieprzyjemne. W końcu podpisali, ale cały urok tego wszystkiego prysł, szczególnie tak jak mówię, 90% osób na kempingu wynajmuje swoje przyczepy, bo inaczej to by ten kemping w ogóle nie działał. I dlatego tak mi było przykro strasznie, bo to akurat pojechałam sama zamykać we wrześniu i taką miałam właśnie nieprzyjemność dużą. No ale zobaczymy, co będzie w przyszłym roku.

**To miało jakiś związek z pandemią?**

Nie. Znaczy, może inaczej: miało z pandemią, ponieważ w tym roku ten kemping ogłosił, że jeśli ktoś nie chce przyjechać ze względu na pandemię, to może zrezygnować, natomiast nie traci miejsca na przyszły rok. Dużo osób zrezygnowało m.in. też z korzystania w pierwszym rzędzie, bo tam jest to akurat dosyć istotne, gdzie się stoi. I na te miejsca mogli przyjeżdżać nowi ludzie, którzy chcieli. Jeśli się komuś spodobało, to np. ten ktoś chciał już sobie w przyszłym roku też rezerwować, a nie mógł tego zrobić, ponieważ to miejsce jest czyjeś teoretycznie, dlatego oni szukali, kogo by tutaj wysiudać z tych pierwszych rzędów. No więc takie to było dosyć nieprzyjemne, ale zobaczymy, jak to będzie w przyszłym roku.

**W te wakacje czuć było pandemię czy wszystko wróciło do normalności?**

To nie tak, że... Oczywiście, że było czuć, natomiast ja odkąd wyjechałam tam w czerwcu, bo ja wyjechałam już 5 czy 4 czerwca, potem 10 przyjechała cała rodzina i tam byliśmy wszyscy trochę, a potem ja zostałam z tymi dziewczynkami, bo ta starsza też już nie miała szkoły, tylko było wszystko zdalne, więc ja przez ten pobyt nad morzem można powiedzieć, że zapomniałam o pandemii. Tam w ogóle pod tym względem było super. Więc nie, nie odczuwałam nic. Co jakiś czas się gdzieś tam coś słyszało, że tutaj jacyś ratownicy byli zakażeni, a to w Juracie, a to ktoś tam na kwarantannie. Oczywiście, wiadomo, na kempingu te wszystkie aplikatory do dezynfekcji rąk i tam jakieś, ale tak to nie, w ogóle się tego nie odczuwało. Nikt tam nie chodził w maseczkach.

**Nawet w sklepach?**

W sklepach w mieście tak, jak najbardziej, ja zakładałam, ale było sporo osób, które w ogóle tego nie przestrzegały.

**Po powrocie w lipcu do Warszawy pandemia znowu zaczęła być widoczna?**

Nie. Ja tam troszeczkę też byłam jeszcze na działce w lipcu, może z 10 dni, może z tydzień. Też tam m.in. zostałam trochę z wnuczkami, znaczy z moją i z tą starszą przyszywaną tak zwaną. Nie, nie odczuwało się tego.

**Jakie były najważniejsze momenty w rozwoju pandemii od czerwca? W wakacje trochę ucichło...**

Nie to, że ucichło, tylko że ja po prostu, ponieważ ja byłam cały czas gdzieś tam zajęta, to ja tak z premedytacją to od siebie odsunęłam. Oczywiście wiadomo, że codziennie się słuchało, ile jest nowych zakażeń, więc jak już np. było 500, to już w ogóle myśleliśmy, że to już jest koniec świata. A w porównaniu z tym, co mamy teraz...

**Wróciło do pani myślenie o pandemii, gdy wzrost zakażeń był gwałtowny?**

Wróciło do nas dosyć wcześnie, ponieważ u mnie się sytuacja zmieniła diametralnie w domu z tego względu, że skoro dzieci poszły do szkół od 1.09., a moja synowa jest nauczycielką i ona też wróciła do pracy, a ponieważ tam też była taka sytuacja, że mój syn przygotowywał się, znaczy ten starszy, co ma córkę, przygotowywał się do takiego bardzo poważnego egzaminu, co prawda ten egzamin był teraz w październiku, ale on już od września, więc oni mieli u nas w większości przebywać, na zasadzie, że przyjeżdżali... A, i jeszcze w lecie, co jeszcze się działo, bo tego nie powiedziałam, że w lecie oni też u nas mieszkali, ponieważ cały lipiec był remont w ich mieszkaniu na Targówku, więc w ogóle wszyscy tutaj przyjechali. W każdym razie my się z mężem bardzo martwiliśmy, co będzie, jak się zaczną... Pozwoliliśmy im tylko dlatego mieszkać tutaj, ale jak się cokolwiek miałoby zdarzyć, że jakieś ogniska w szkole, czy że oni będą mieć jakiś kontakt, to natychmiast muszą wyjeżdżać, bo my nie możemy iść na kwarantannę. A jeszcze co było po drodze takiego też bardzo trudnego, ten wrzesień też był cały taki, bo z kolei znowu się zmieniały opiekunki u naszego taty. W lipcu jeszcze było kilka takich sytuacji... No to najpierw w czerwcu, co mówiłam, że tam tata gorzej się czuł, ja byłam wtedy nad morzem, moja siostra tutaj w Warszawie wzywała karetkę pogotowia i tam jakoś się tym zajmowała, natomiast potem była taka sytuacja też w lipcu, kiedy ja byłam w Warszawie i nikogo nie było z moich, czyli ani męża, ani jednego syna, ani drugiego, ani mojej teściowej, tylko ja i moja siostra, no i też właśnie tata bardzo źle się poczuł, zaczął wymiotować, ja też wzywałam karetkę i ta karetka go zabrała do szpitala. Trzymali go 36h na SOR-ze, nie przyjęli go do szpitala w ogóle i to był horror, który my przeżyłyśmy, bo nie można tam było przecież ani wejść, ani zobaczyć, co się z nim dzieje. Mimo że to było lato i było ciepło, to wiadomo, że wieczory są już chłodne. My oczywiście razem za tą karetką natychmiast dowiozłyśmy tam pieluchy, ubrania na zmianę, takie rzeczy. A jeszcze mój tata miał taki kaszel taki strasznie... I ci ratownicy medyczni z tej karetki bali się najbardziej tego, że jak będą wieźć tatę, to że zarzyga im całą karetkę, było stwierdzenie takie, więc jeszcze ojciec był owinięty w taki nasz koc, że jakby miał rzygać, to na ten swój koc. Mówię to dlatego, że potem, tak jak mówię, my zaraz za tą karetką pojechałyśmy z siostrą, zawiozłyśmy tam też ubrania. Cały następny dzień dzwoniłyśmy, co się dzieje i w ogóle, że jeszcze nie, jeszcze nic nie wiedzą, po czym wieczorem powiedzieli, że zrobili jakieś tam badania, ale że generalnie nic się tam takiego nie dzieje i że ojca wypisują. Transportem o 2 w nocy został przywieziony do domu, można powiedzieć, że golusieńki, w samym pampersie. To było straszne. Ja mówię, jak to, przecież tam były rzeczy, natomiast ci ludzie, którzy transportowali go, oni też są wynajmowani, bo to nie jest karetka, no też taki transport medyczny, powiedzieli, że oni nic nie wiedzą, oni dostali pacjenta, a to, czy pacjent miał na łóżku torbę... Mój tata jest taki trochę już nie tyle z demencją, co taki był otumaniony, nie bardzo wiedział, trudno było, żeby pilnował swoich toreb, podobno nawet nie wiedział, że to są jego i w ogóle. Nikt tam w tym szpitalu się niczym nie zajął, jak ja przyjechałam zaraz po te torby, jak oni go odwieźli, to "Tak, tak, rzeczywiście zostały torby". To było takie straszne, bo w tym momencie, jak nie ma się nad czymś kontroli w ogóle, to...

**Ten długi pobyt na SOR-ze pani taty był spowodowany koronawirusem?**

Trudno powiedzieć, przecież my tam nie mogłyśmy wejść, więc jeśli dzwoniłyśmy, to tylko mówili: "My tu mamy Meksyk i na razie nic nie wiadomo". Bo to na Stępińskiej, gdzie teraz w ogóle już nie przyjmują nikogo, zamknięty szpital.

**Samo to, że nie mogła pani tam wejść, było związane z pandemią.**

Tak. I proszę sobie wyobrazić, że oni go przywieźli w pampersie, pewnie musieli zmienić tego pampersa tuż przed jego wypuszczeniem, natomiast jak ja pojechałam po te rzeczy, które on miał, plus te rzeczy, które z niego zdjęli, to po prostu były w tak opłakanym stanie, wszystko było tak zasikane, brudne, śmierdzące, czyli oni go trzymali cały dzień tam w tych ubraniach, w tym przesikanym do cna kocu, a potem po prostu to wyrzucili gdzieś do jakiejś siatki, do takiego worka na śmieci mi to wszystko zawiązali, a ojca puścili w samym pampersie do karetki. To właśnie... tragedia.

**Czy pani codzienne życie wróciło do stanu sprzed pandemii czy wygląda jak w marcu?**

Teraz powoli zaczyna znowu tak wyglądać. Bo jeszcze co tam się dalej działo... W tym wrześniu to tak, jak mówiłam, potem musiały się wymienić opiekunki. I to też całe wakacje trwało, bo to już nie było na takiej zasadzie jak przedtem, że po prostu jedna przyjeżdża, a druga wyjeżdża, nie było problemu w ogóle, teraz musiałyśmy tutaj występować o umowę o pracę, że zatrudnimy tę osobę, wszystko to oczywiście są i dużo większe koszty, bo musimy płacić i ZUS, i to, i tamto. Zresztą za tę poprzednią opiekunkę, która tak normalnie wtedy jeszcze zdążyła się wymienić przed zamknięciem granic, to po prostu jej wykupywałyśmy takie ubezpieczenie zdrowotne, to też w obawie, bo nie wiadomo, co się będzie działo. Ona po pierwsze wtedy tak naprawdę to mogłaby tu jeszcze zostać i cały czas być, bo przecież ta pandemia nie jest jeszcze odwołana, a wszyscy ci, którzy tam do którejś daty przyjechali i nawet skończyła im się tam wiza czy coś, to mogliby dalej być, natomiast ona koniecznie chciała pojechać, bo była teraz w takim wieku emerytalnym i chciała sprawdzić, czy jej się tam należy ta emerytura, żeby załatwić jakieś formalności, dlatego koniecznie chciała tam jechać. Jak się później okazało, jak pojechała, nic się jej nie należy, więc to było po prostu też niepotrzebne, bo mogła jednak tutaj zupełnie spokojnie być. A ta druga wcale nie chciała przyjeżdżać, więc to też były nerwy i jeszcze dodatkowo jak ta druga... to też wszystko robiłyśmy w takim... Bo tak, z piątku na sobotę przyjechała ta jedna i w sobotę wieczorem ta pierwsza wyjeżdżała, ale ta, co przyjechała, to od razu trafiła na kwarantannę, tyle tylko, że była z ojcem na kwarantannie, więc najpierw po kryjomu ta pierwsza musiała wyjść, żeby wyjechać. Szczęśliwie w sobotę nikt tam nikogo nie sprawdzał, ale od niedzieli do tej drugiej zaczęli już dzwonić, sprawdzać ją, czy ona jest na tej kwarantannie i w ogóle, no więc po prostu przynosiłyśmy zakupy pod drzwi i tyle. To było we wrześniu.

**I ta pani, która przyjechała, była tak od razu sprawdzana?**

Tak, była. Oni jeszcze tam oczywiście jakąś tam aplikację kazali zakładać, ale ponieważ ona ma telefon starego typu, więc tam nic nie zakładała, więc po prostu do niej dzwonili. Bo jeśli się ma tę aplikację, to trzeba robić te zdjęcia, wysyłać, a ona nie, więc po prostu ją sprawdzali na tej zasadzie, że dzwonili, ale to tak nawet bardzo grzecznie dzwonili, pytali się, czy się dobrze czuje, kazali jej wyjść na balkon, pomachać, bo tam, powiedzmy, policjant na motorze stał pod klatką gdzieś, a potem zadzwonili, że jutro już ostatni dzień i że jeśli wszystko w porządku, no to już... Nie, to jak zadzwonili ostatniego dnia, że jeśli wszystko w porządku, to już od 12 dzisiaj wieczorem jej się kończy ta kwarantanna i może wychodzić. Więc to też były nerwy. Ja w międzyczasie miałam też dużo tę swoją wnuczkę, tak dzień po dniu, bo tam oczywiście ten mój syn... No tam jeszcze w wakacje miałam taką nieprzyjemną sytuację z moim synem, ale o tym, jeśli mogę, to nie chciałabym mówić. Natomiast bardzo dużo się zajmowałam w tym wrześniu, więc ten wrzesień też był ciężki i wcale... A tutaj zaczęły się z powrotem w tych szkołach... A, no i co się działo - byliśmy wszyscy chorzy. Do końca nie wiemy, czy to był koronawirus czy nie, bo jak ja się tak zajmowałam... Tzn. ja twierdzę, że to nie był koronawirus, aczkolwiek niektórzy chcieliby, żeby to tak było, ale nie. Ja zresztą na wiosnę miałam raz robione badania na przeciwciała, czy przypadkiem nie przechodziłam, ale nie przechodziłam. Potem coraz częściej zaczynało dochodzić do nas, że ktoś już w pobliżu tego koronawirusa ma, więc to już było takie, m.in. tego mojego drugiego syna dziewczyny tata i siostra rodzona już przeszli tego koronawirusa, też chorowali, więc my się wtedy też z nimi nie kontaktowaliśmy, mimo że oni tam nie mieli kontaktu, ale nieważne. W każdym razie najpierw zaczęła kaszleć moja najmniejsza wnuczka. Potem tak od niej po kolei wszyscy byliśmy chorzy, w sensie bardzo duży, uporczywy kaszel, u mnie w zasadzie tylko był ten kaszel, ale ja byłam chora prawie 3 tygodnie. Oczywiście miałam teleporadę i moja pani doktor powiedziała, że skoro ja to też złapałam od tej mojej wnuczki i ta starsza dziewczynka też tak długo, to nie jest to prawdopodobne, żeby to był koronawiurs. Poza tym ani nie straciłam węchu, ani smaku, ani nic takiego, ani nie byłam zmęczona, po prostu dręczył mnie ten kaszel. Kasię też, czyli synową, ale synowa od razu wzięła antybiotyk, więc jakoś łagodniej to wszystko przeszła, najciężej przechodził mój syn, który miał też gorączkę, ale tak jak mówię, też nie miał żadnych takich... Poza tym wszyscy się tutaj spotykaliśmy i ani mój mąż, ani nikt inny, nic żadnych nie było tak o... Więc jak rozmawiałam z tą swoją lekarką, to ona... (przerwa) Ja jeszcze miałam taki problem, ponieważ my byłyśmy umówione z naszymi koleżankami z Avonu, z pracy, że pojedziemy jedną odwiedzić do zupełnie innego miasta, 200 km od Warszawy i ja bardzo się denerwowałam, czy ja mogę jechać czy nie, stąd te teleporady. No i właśnie ta moja pani doktor powiedziała, że spokojnie, że to nie jest koronawirus, że to jest przeziębienie, że mogę jechać. Więc ja pojechałam i rzeczywiście też nikt tam nic z tych osób, co tam były, a byłyśmy tam w 10 osób, nikt się nie rozchorował, więc mniemam, że jednak nie mieliśmy tego koronawirusa.

**Jak państwo podejrzewali, że to może być koronawirus, to bali się państwo?**

To się tak zmienia, pani Diano, bo to raz się myśli, że jest ok, potem... Znaczy tak, im mniej się czyta o tym, to jest lżej, natomiast jak już coraz częściej się znowu zaczyna czytać o tych wszystkich przypadkach, to strach wraca. Moja teściowa w tej chwili też się izoluje zupełnie. Bo jeszcze całe lato to przychodziła tutaj do mnie, czy nawet we wrześniu, jak ja byłam z tymi dziewczynkami, to przychodziła, odwiedzała, natomiast teraz nie.

**Jakie elementy teraz przypominają początek lockdownu w marcu?**

M.in. np. w tej chwili, od zeszłego tygodnia też tak na wszelki wypadek dzieci u nas nie są. Z tego względu, że tam w szkole było bardzo dużo przypadków nauczycieli u tej Kasi, zanim akurat zamknęli te szkoły, no i tak po prostu dla bezpieczeństwa.

**A jak wygląda praca?**

Wszystko jest online.

**Czyli tak jak było?**

Tak jak było. Tam akurat chyba, jak my się już pożegnałyśmy wtedy w czerwcu, to tam potem była taka sytuacja, że był potężny atak hakerski na stronę Avonu i to skutkowało tym, że np. jeden katalog trwał 2 miesiące zamiast 3 tygodni, bo tam oni nie mogli wszystkiego zebrać do kupy. No i to teraz skutkuje tym, że też się po prostu jakieś zmiany duże robią, zmienia się ilość katalogów, no i co chwila zmiany w ogóle takie w firmie odgórne, na zasadzie, że lecą głowy, przychodzą nowi i tak cały czas. No, to tak się dzieje, nieciekawie generalnie.

**Pani mąż pracuje normalnie?**

Tak, pracuje, tam się jakoś to wszystko powoli, nie wróciło do stanu sprzed epidemii, ale trochę się w lecie ruszyło, natomiast teraz znowu zaczyna się zastój. Niby to jeszcze nie jest zamknięte, niby tego lockdownu nie ma, ale ludzie się boją już, też nie chcą przychodzić, nie chcą zarówno kursanci... Tak, że są tygodnie, że już np. nikt nie dzwoni, żeby się zapisać nawet. Też takie osoby, które tam pomagały mu w pracy, prowadziły te egzaminy, takie biurowe też, takie jakby sekretarki, to też jedna stwierdziła, że ona zostaje i pracuje, ale dwie powiedziały, że nie, że jednak od teraz się wycofują. Mąż pracuje, chodzi codziennie do pracy, bo nie jest zamknięta szkółka, jest nadal otwarta, ale jest już wyraźny spadek.

**Jak teraz wyglądają spotkania z bliskimi? Jest normalnie?**

Nie, właśnie tak jak mówię, do zeszłego tygodnia było wszystko normalnie, przynajmniej z moimi dziećmi, bo oni tutaj prawie cały czas też mieszkali, mieszkali od niedzieli do czwartku, potem na te 3 dni jechali do domu, więc w zasadzie cały wrzesień i październik tak to się odbywało. Natomiast odkąd zamknęli tę szkołę, odkąd wiadomo było, że tam jest więcej tych nauczycieli jest chorych, to od zeszłego tygodnie nie przyjeżdżają, stwierdziliśmy, że przynajmniej do niedzieli, jeśli nadal nic tam się nikomu nie będzie działo, to wrócimy z powrotem do tego. Ja do taty muszę chodzić, bo teraz, tu akurat tego jeszcze nie mówiłam, bo w zeszłym tygodniu była kolejna taka akcja, że się tata bardzo źle czuł, był nawet moment, że myślałam, że to już jest koniec. Znowu była wzywana karetka, ale tym razem ja już po prostu nie pozwoliłam go zabrać. Najpierw oczywiście też byłam porządnie ochrzaniona, dlaczego ja wzywam karetkę, jeśli nic takiego się nie dzieje, to, że nas nie poznaje, to nie jest powód. Powiedziałam, że zadzwoniłam do lekarza pierwszego kontaktu, który powiedział, że mam wzywać karetkę, po to, żeby sprawdzić parametry, że coś się dzieje. Oni teraz tak zrzucają jedni na drugich, że to powinien lekarz lecieć natychmiast w podskokach, a nie wzywać, no takie tam. Tata, jak to lekarka powiedziała, on miał taki jakby kolejny rzut miażdżycowy i tej swojej choroby, i ze starości po prostu już tak, więc był strach, że rzeczywiście 2 dni bardzo majaczył, bredził, nikogo nie poznawał, cały czas mu się coś wydawało, okropne to było. Teraz troszeczkę wrócił, przychodzi pielęgniarka, robi kroplówki, więc ja chodzę do taty, no bo chodzę, nie chcę tam ich straszyć, że dzieci do nas teraz nie przyjeżdżają dla bezpieczeństwa, bo nie chcę, żeby się tam... Natomiast cały czas siedzę tam u nich w maseczce, nie tak jak kiedyś, ale tata akurat nie bardzo sobie zdaje sprawę z pandemii, on nawet nie wie, że ta pandemia jest, jeśli nawet wiedział w marcu, to teraz tym bardziej nie wie. Więc jak się pyta, dlaczego jestem w maseczce, to mówię, że tak teraz trzeba i tyle. Więc ja do ojca chodzę. Oczywiście, że za każdym razem się denerwuję, że a co jeśli, ale tak wobec tego, jak dużo jest tych zachorowań, to teraz tak naprawdę wszędzie już się można zarazić, więc to nie jest tak... Natomiast my musimy z tym żyć, musimy do niego chodzić, chociażby tak jak w zeszłym tygodniu, zanim ta karetka, to on też zwymiotował, też trzeba go było przebrać, podnosić na łóżku, więc razem z synem byłam tam, bo dwie osoby już by sobie nie dały rady, no nie jest wesoło.

**Co pani najbardziej przeszkadza w pandemii w tym momencie?**

Sam fakt w ogóle istnienia tej pandemii, bo do mnie czasami wręcz nie może dojść, że tak jest i że nie wiadomo, jak się to skończy, czy ten wirus zostanie już z nami na zawsze, jak długo będzie trwała ta pandemia, bo to teraz tylko przecież są jakieś szacunki, niektórzy tam szacują, że to będzie trwało jeszcze rok, a niektórzy, że dwa lata, niektórzy, że pół roku, to jest takie trudne dosyć.

**Znowu taka niepewność?**

No ogromna.

**A ma pani sposoby, żeby sobie z tym radzić?**

Tak do tego zeszłego tygodnia to jeszcze nawet nie tyle, że miałam sposoby, żeby sobie radzić, tylko po prostu byłam bardzo zmęczona wszystkim, ogólnie, no bo i kłopoty z dziećmi, to jednak jest bardzo obciążające, jak 4 osoby mieszkają, mimo że ja ich przecież bardzo kocham i wszystko, ale to zupełnie co innego. Potem cała ta akcja była - wtorek, środa - z tym ojcem, więc też byłam ledwie żywa. I jeszcze co się nałożyło: dokładnie w tym samym dniu, kiedy była wzywana ta karetka, kiedy ja wzywałam karetkę, to było we wtorek, tydzień temu, a do kilku dni miałam jakieś takie dziwne telefony, że jak oddzwaniałam, to tam się włączała jakaś infolinia jakiegoś zakładu rehabilitacyjnego "Wigor". A jak moje dzieci były małe, to ja też z nimi chodziłam na różne rehabilitacje do takiej właśnie przychodni "Wigor", która jest tutaj na Ursynowie blisko. Tak się zastanawiam, po co oni tak do mnie dzwonią teraz z tej przychodni. A potem dopiero okazało się, jak jeszcze raz oddzwoniłam, że to dzwonią z domu pomocy, z tego takiego ośrodka opiekuńczo-leczniczego, takiego domu pomocy społecznej, tzn. to nie jest dom pomocy społecznej, tylko to jest właśnie taki mający podpisaną umowę z NFZ-em taki opiekuńczo-leczniczy, gdzie my 2 lata temu, jak tata tak strasznie, jak to się wszystko stało i tata zachorował, to my różnych opcji szukałyśmy i zanim ta opcja nastała, że są jednak opiekunki i że tata został w domu, no to najpierw nie wiedziałyśmy, jak to zrobić, to też jeździłyśmy właśnie po tych różnych domach opieki i m.in. w jednym z nich złożyłyśmy taką na oczekiwanie. I dokładnie w tym dniu, kiedy właśnie ojciec tak się strasznie źle czuł, nie poznawał w ogóle, oni zadzwonili, że mają miejsce, że mogą go przyjąć i to też z NFZ-u. Teraz taki ośrodek pobiera 75% emerytury i to bez względu na to, jak wysoka jest ta emerytura, czy ma się 1000 zł czy ma się 5000 zł. Pierwszy taki moment był, że może my go tam oddamy jednak, że sobie tu już nikt z tym nie poradzi i to też takie było straszne, bo całą noc płakałyśmy z siostrą, bo nagle sobie zdałyśmy sprawę, że jeśli go oddamy tam, to możemy go już nie zobaczyć, bo przecież tam nie ma też odwiedzin, tak jak i w tych szpitalach, i w tych ośrodkach teraz z racji tej pandemii. No więc dopóki jednak będziemy, dopóki te opiekunki też będą dawały radę, no i razem z nami, bo my tam musimy już jeździć pomagać, to niech będzie w domu. Mimo że ta moja lekarka pierwszego kontaktu mówiła, żebyśmy jak najbardziej załatwiały, bo sobie nie poradzimy. Ale nie byłyśmy w stanie, więc ja tam zadzwoniłam i po prostu zgodnie z prawdą powiedziałam, że mamy opiekunki, podpisaną z nimi umowę, one już nie muszą wiedzieć, co mamy z nimi podpisane, ale to też jest przecież rzeczywiście, bo teraz za miesiąc znowu mają się wymienić i tamta druga ma już wizę na pół roku, i też już na podstawie właśnie umowy o pracę, którą my jej damy. Z drugiej strony tamta też bardzo liczy, że przyjedzie, bo dla nich to jest jednak zarobek ogromny. No więc tata na razie jest... Tak jak mówię, do tej pory ten wirus oczywiście jest, natomiast tyle tu się dzieje u mnie w domu, że to wszystko razem się jakoś tak miesza, nie tylko wokół tego wirusa.

**Emocje**

 9 - te błyskawice, czyli że już się dzieje źle i dodatkowo jeszcze ta trąba powietrzna. Ten obrazek generalnie w tej chwili przedstawia moje emocje nie tylko związane z pandemią, tylko ogólnie z tym wszystkim, co się teraz u nas dzieje w kraju.

**Jakie emocje budzi ten obrazek?**

Po pierwsze takiego strachu, niepewności, złości, wściekłości, czy to wszystko nagle nie pójdzie w powietrze razem z tą trąbą.

**A okres letni?**

Zastanawiam się właśnie... Może 12. Ta 12 kojarzy mi się z takim lekkim... Bo mówimy o pandemii? Tak, to jest jakiś taki spokój, nic tutaj się takiego nie dzieje, ale tak jak mówię, odnoszę to do sytuacji związanej z pandemią, bo tak jak już słyszała pani, u mnie cały czas się dużo działo i różnych innych emocji niezależnych. Oczywiście pandemia miała na nie wpływ, chociażby ten szpital z ojcem, ten SOR i to wszystko, natomiast generalnie, gdyby... Chociażby i te wyjazdy, to wszystko inne było w takim... No ja nie myślałam wtedy o tej pandemii, może w ten sposób.

**Strach z obrazka z błyskawicą - przed czym to jest strach?**

Ten obrazek jakby był podzielony dla mnie. Ta błyskawica to jest ten strach bardziej związany z pandemią, bo ja się autentycznie zaczynam bać, że wcale nie jestem taka silna, w sensie organizm mój nie jest taki i że jeśli rzeczywiście mnie ten wirus dopadnie, to nie wiem, co będzie się działo. Po prostu się boję. I dlaczego ta błyskawica - no że to już się tak dzieje, jak taki grom z jasnego nieba, że już naprawdę zaczynamy, że już jest niebezpiecznie. Natomiast ta trąba powietrzna jest dla mnie dodatkowo, że jeszcze mimo że ta pandemia, to jeszcze w takiej sytuacji, właśnie w przypadku tej pandemii, jeszcze dzieją się takie rzeczy, które jeszcze dodatkowo mogą to wznieść w sensie nie optymistycznym, tylko tym negatywnym, że może się jeszcze dużo różnych dziwnych rzeczy zadziać.

**Ma pani jakieś sposoby radzenia sobie z emocjami?**

Wczoraj np. upiekłam po raz pierwszy ciasto drożdżowe ze śliwkami. Potem żałowałam, że w ogóle zaczęłam to robić, bo tyle było cackania się przy tym, ale gdzieś tam właśnie... Bardziej to było takie impulsywne, ponieważ zmieniłam kartkę w kalendarzu na listopad, a w kuchni mam taki kalendarz kuchenny i właśnie tam był obrazek, który tak bardzo do mnie przemówił, właśnie tego ciasta drożdżowego ze śliwkami i stwierdziłam, że w takim razie to zrobię. Dzisiaj byłam też u taty, zawiozłam im. Nie wyszło to jakieś doskonałe, ale jak na pierwsze ciasto drożdżowe, które robiłam, to nie było źle.

**Czyli znalezienie sobie zajęcia, też manualnego?**

Trochę tak. Oprócz tego akurat teraz znowu jest końcówka katalogu, bo w środę jest ostatni dzień, więc też już jestem taka mocno zajęta, jak to się mówi, pracowo, więc też... Natomiast przez to, że nie ma tych dzieci, to tak troszeczkę taka jestem bardziej może odprężona.

**Z dziećmi miała pani więcej pracy?**

To też, tak, oczywiście.

**Chodziło też o zagrożenie zarażeniem?**

Nie no, wtedy akurat jeszcze tak nie myślałam. Natomiast wszystko się na to składa. Ta mała krzyczy, ciągle coś chce, starsza kłóci się z mamą, bo nie chcą odrabiać lekcji albo nie tak jak sobie one życzą, cały czas taka nerwowa sytuacja. Tu pies, który za chwilę boimy się, że ugryzie tę młodszą, a ta z kolei jest w nim zakochana, co chwilę się nad nim nachyla i najchętniej by go za ogon podnosiła do góry. Poza tym też była taka sytuacja, że syn przez 2 tygodnie nie miał samochodu, więc ja dodatkowo musiałam ich wszędzie wozić, przywozić, odwozić, a to nie jest ulicę dalej, tylko ja mieszkam na Mokotowie, przy Woronicza, a oni na Targówku, prawie w Markach, więc to też, po prostu o tak było dużo.

**Jak znajomi i rodzina czują się w czasie pandemii?**

Ja w tej chwili znowu prawie nie mam z nikim kontaktu ze znajomych, bo ostatnie takie spotkanie z tymi znajomymi to to, co mówiłam, że wyjechałyśmy, spotkałyśmy się w 8 koleżanek, to to było dokładnie 2 czy 3 października. Od tamtej pory nie spotykałam się z żadnymi znajomymi. Z siostrą widzimy się cały czas z racji taty. Teściowa się izoluje, jeszcze dodatkowo 2 tygodnie temu miała operację na zaćmę i też jest w jakimś takim totalnym strachu przed tym koronawirusa, więc ze sobą się prawie... Jedynie czasami jej jakieś zakupy przywiozę czy coś. No to też się z nią nie widzę.

**A ludzie na ulicy?**

Po prostu tak jakby już wszyscy przywykli do tych maseczek, tak jakby kompletnie się już tym nie tyle nie przejmują, co że tak jest. Natomiast to, co ja np. widzę chociażby w swojej Biedronce, do której chodzę, do której mam najbliżej, to te wszystkie obostrzenia, że tam znowu ileś klientów na tyle i tyle m2 czy tyle do kas - absolutnie to nie jest przestrzegane.

**A na wiosnę było to przestrzegane?**

Było, tak, każdy krzyczał, pilnował swojej przestrzeni, żeby się za blisko nie zbliżyć, stały osoby, które liczyły osoby w sklepie i wpuszczały tyle, ile można, a teraz.

**O jakich nowych obostrzeniach pani słyszała?**

O zamknięciu tych wszystkich restauracji, siłowni, wszystkie te gastronomiczne punkty, że tylko na wynos, że dzieci mogą tylko w towarzystwie dorosłych do 12 lat, czy do 16, już sama nie wiem. To zdalne nauczanie, które jest w szkołach powyżej klas 3., tzn. od 4., bo to akurat tę naszą Zosię już obowiązuje. Zakładanie maseczek wszędzie, oprócz parków, dla mnie to jakieś głupie jest. Jak idę pustą ulicą, to muszę iść w maseczce, a w parku mogę ją zdjąć? Bez sensu. I tyle chyba.

**Co pani myśli o tych ograniczeniach?**

Dla mnie w tej chwili najbardziej uciążliwe jest chodzenie w maseczce na zewnątrz, ponieważ jest duża wilgoć, cały czas ta maseczka jest mokra, jest to okropne, nieprzyjemne, poza tym ja noszę okulary i cały czas mi parują szkła, nic nie widzę, to jest takie...

**Czy noszenie maseczek ma wpływ na ograniczenie zakażeń?**

Myślę, że tak, że jednak powinno się nosić te maseczki, mimo że nie jest to przyjemne.

**Ale nawet w parku?**

No właśnie dla mnie nie ma sensu o tyle, że jaka jest różnica między pustą ulicą a... Nie wiem, ale to też trudne pewnie byłoby takie, że ta maseczka powinna być w momencie, kiedy jesteśmy w jakiejś przestrzeni z ludźmi, natomiast człowiek idzie sam ulicą i nie widzi żywego ducha, to nie wiem, czy jest sens zakładania tej maseczki. Ale to pewnie byłoby trudne do egzekwowania, w którym momencie już jest ten niebezpieczny moment czy bezpieczny.

**Czyli łatwiej organizacyjnie narzucić wszędzie?**

Tak, aczkolwiek oczywiście... No ale maseczki są chyba wszędzie, wszyscy we wszystkich krajach.

**A zamknięte restauracje?**

Nie uważam, że powinny być zamknięte. Szczególnie, że to znowu uderza strasznie jednak w tę branżę gastronomiczną i uważam, że jak do tej pory, jeśli się rzeczywiście przestrzegało tych miejsc, że nie każde zajęte czy coś tam, to było dużo lepsze rozwiązanie, bo jednak można było jakoś funkcjonować, w sensie oni mogli, ta gastronomia, a nie było jakiegoś takiego dużego zagrożenia. Bo w tym momencie nadal chociażby, skoro to jest już zamknięte, to autobusy też nie powinny jeździć i tramwaje tak samo, bo przecież ci ludzie jednak...

**A co z siłowniami?**

Mój mąż jest bardzo przeciwny, że zamknęli mu siłownię, bo on strasznie długo się zmagał z tym, czy ma zacząć, jak już były otwarte na nowo, czy ma chodzić czy nie i w momencie, kiedy zaczął chodzić z powrotem, to po tygodniu mu zamknęli. Więc się wścieka ogromnie, szczególnie że mówił, że było super, w sensie, że było mało ludzi rzeczywiście i ta przestrzeń większa, i w ogóle. Natomiast ja siłowni generalnie nie kojarzę jako miejsce takie czyste i bezpieczne, więc sam fakt, że tam się jednak i prycha, i kicha, w sensie że człowiek się męczy i oddycha, i sapie, tak sobie wyobrażam tych panów na siłowni, więc dla mnie to jest takie ogólnie...

**Mniej bezpieczne niż restauracje?**

Tak. Ale to takie moje odczucie, może się tu mylę, nie wiem.

**A nauczanie zdalne powyżej 3. klasy?**

Ja w ogóle nie wiem... Albo szkoły powinny być zamknięte w ogóle, albo być otwarte, no jaki sens ma... Oni to tłumaczą, że jeden nauczyciel zajmuje się wszystkim w tych klasach 1-3 i że prowadzi wszystkie zajęcia, że się nie zmieniają między sobą. Natomiast taką decyzję uważam, że należało podjąć od razu od 1.09., a nie teraz, kiedy to już wszystko zostało wymieszane i kiedy tak i tak wszyscy się już, na około tyle co miały, to się już porozdzielały te wirusy i to wszystko.

**Zalecenie niewychodzenia z domu dla osób starszych?**

Według mnie to zalecenie ma związek z tym, że jak ci ludzie zachorują, zarażą się i zachorują, to większość z nich będzie to przechodziła ciężko, a nie ma miejsc w szpitalach. Uważam, że dla seniora jest to najgorsze, co może być, jeśli się go zamknie w domu i nie pozwoli się nigdzie wychodzić, natomiast to jest takie manipulowanie strachem, że nie powinien. Oni też to sobie mogą w ten sposób... Czego się boi najbardziej moja teściowa właśnie? Nie tego, że zachoruje, tylko tego, że trafi do szpitala, a w tym szpitalu jest, jak jest i ona akurat tego się najbardziej boi.

**Czyli izolacja może mieć złe skutki dla samopoczucia, ale może uchronić?**

Tak.

**Zakaz organizacji imprez?**

Nie wiem, co mam na ten temat sądzić, bo skoro tak i tak wszyscy się już... W tej chwili to to już dla mnie nie tyle, że nie ma sensu, co... To nie jest tak, że można teraz to zatrzymać i że się w ten sposób to ograniczy. Według mnie ten wirus jest już tak rozprzestrzeniony, że to w tej chwili już...

**Jakie emocje mogą budzić w ludziach te obostrzenia?**

Po pierwsze budzić niepokój, po drugie też... To jest takie wpływanie na takie ograniczenia wręcz wolności, tego, co możemy robić. Każdy jest na tyle chyba - powinien przynajmniej być - mądry sam dla siebie i stosować się do różnych nie tyle zaleceń. No że jeśli jest taka sytuacja, to sama nie będę się pchała gdzieś w jakiś tłum, czyli narzucanie takiego nie tyle rygoru, co takiej samokontroli, ale to wynikałoby z tego, że ja sama to tak mogę zrobić, natomiast nie takie narzucanie z góry, że tego nie można, tego nie można, tutaj tak. Ostatnia sytuacja z tymi cmentarzami to dla mnie po prostu coś strasznego.

**Że to już za bardzo wejście w wolność?**

Tak, ja oczywiście nie wybierałam się teraz na ten cmentarz właśnie m.in... Ale to nie znaczy, że ktoś specjalnie nie przyjechał po to... Już nie mówiąc o tych wszystkich ludziach, którzy z tego żyją.

**Czyli część tych obostrzeń ogranicza wolność a nie samego wirusa?**

Dokładnie.

**Pani przestrzega tych ograniczeń?**

Oczywiście chodzę w maseczce, bardzo dbam o to, żeby zawsze dezynfekować, myć ręce. Pierwsza rzecz, jak tylko przychodzę skądkolwiek, gdziekolwiek, to zawsze ręce. Te takie elementarne: staram się niczego gdzieś nie dotykać, jak mogę, to zawsze gdzieś tam łokciem czy coś. Ale zdaję sobie sprawę, że jak wchodzę do Biedronki chociażby, to tak i tak, tam jest tyle ludzi, co z tego, że oni są w tych maseczkach, każdy wszystkiego dotyka i w ogóle, staram się po prostu zminimalizować jakiekolwiek ryzyko, natomiast wiem, że ono jest.

**Czy coś się zmieniło w pani myśleniu na temat źródeł koronawirusa?**

Nie.

**Czy obecna sytuacja jest poważna?**

Bardzo. Bo już jest taka globalna, na całym świecie. Wynika to dla mnie z tego, że migracja obecnie i w ogóle przemieszczanie się wszystkich ludzi na całym świecie jest tak ogromne, że po prostu ten wirus jest już wszędzie. Ja do tej pory tylko czytałam o większych epidemiach, natomiast nigdy za mojego życia się to nie stało, a teraz uważam, że jest to jak najbardziej i też tak naprawdę nie wiemy, jak to się dalej skończy, jak to się dalej potoczy.

**Czy ludzie zachowują się adekwatnie do tej poważnej sytuacji?**

Generalnie myślę, że spora większość tak, że raczej przestrzega, przynajmniej teraz, ostatnio, w lecie zdarzało się, że widziałam kogoś w sklepie bez maseczki, ale teraz raczej tak.

**Czy dało się zapobiec obecnej sytuacji?**

Nie wiem. Nie wiem z tego względu, że mnie sam fakt rozprzestrzeniania się tego wirusa na całym świecie, mnie się to jakoś nie mieści w głowie. Ja nikogo tutaj teraz, broń Boże, nie bronię, że tak i tak nic nie można byłoby z tym zrobić, bo to nie o to chodzi, tylko... Nie wiem.

**Czy obecne działania rządu związane z pandemią są zasadne?**

To wchodzi już bardziej w taką ocenę polityczną rządu, a nie chciałabym się tutaj skłaniać do niczego. Generalnie sytuacja jest bardzo ciężka, natomiast mimo totalnego mojego braku jakiegokolwiek poparcia dla tego rządu, co jest, ja nie wiem, co można by było zrobić innego, żeby temu zapobiec, jeśli chodzi o pandemię koronawirusa. To nie jest tak, że o, mogliście zrobić to, to, to - nie wiem, co można by było zrobić.

**Czyli trudno oceniać, kiedy nie wiadomo, co można byłoby zrobić?**

Tak. Natomiast inne, że tak powiem, bez względu na opcję, co ja myślę, natomiast wprowadzenie teraz takiego kija w mrowisko, to, co oni zrobili, to już jest dla mnie niebywałe po prostu.

**Pogorszenie całej sytuacji.**

No tak.

**Śledzi pani informacje na temat pandemii?**

Trochę, ale to na zasadzie takiej, nie jak kiedyś, że tak czytałam i w ogóle, nie czytam tego, bo się denerwuję, więc wiem, że rosną z dnia na dzień. Najbardziej to chyba teraz uzależniłam się od telefonu i Facebooka, w telefonie tam można wszystkiego się nie tyle dowiedzieć, co cały czas jest o tym głośno, więc też się łapię na tym, że najczęściej w tym telefonie tak czytam, ale staram się nie za dużo.

**Mniej pani teraz czyta na ten temat niż na wiosnę?**

Tak. Na zasadzie takiej, że już o tych przyczynach, co, u kogo, dlaczego jedni tak, dlaczego tacy bardziej - już nie mogę tego czytać. Natomiast informacja, że z dnia na dzień zwiększa się ilość chorych, dociera zewsząd i jest, natomiast ja już się w to nie wczytuję, nie chcę już czytać, a co zrobić, żeby tak - nie.

**Czy źródła informacji się zmieniły?**

To, co miałam na myśli z tym Facebookiem, że to są jakieś tam po prostu relacje osób, które wrzucają coś na Facebooka, na tej zasadzie.

**Czy ogólnie czas przeznaczany na media zwiększył się?**

Nie, nie zwiększył się.

**Czy informacje na temat koronawirusa są wiarygodne?**

A propos wysokości zachorowań, nowych zakażeń itp.? Ja to przyjmuję, natomiast za chwilę zaraz od razu ktoś gdzieś dodaje, że wiadomo, że ilość zachorowań jest wielokrotnie wyższa niż to, co podają, nie wiem tego, wiadomo, że więcej się na pewno robi tych testów, więc stąd też... Zdaję sobie sprawę, bo też już dochodziły do mnie takie różne informacje, że część osób np. wie, że jest chora, ale się nie bada.

**Dlaczego się nie badają?**

Wczoraj wyczytałam, ktoś udostępnił taki post, że się nie badają, bo nie chcą narazić całej rodziny na bycie na kwarantannie, że sami się izolują, są w domu, łykają Gripex itp. i się w domu leczą, aż to przejdzie. Natomiast jeśli objawy są tak specyficzne, jak utrata smaku i powonienia, to nie ma co tutaj szukać jakichś innych powodów, a mimo to się nie zgłaszają ani do Sanepidu, ani nigdzie.

**Co panie sądzi o takim postępowaniu?**

To też jest takie trudne. Jeśli taki ktoś się izoluje i rzeczywiście nie naraża w ewidentny sposób innych ludzi, to rzeczywiście nie ma dla mnie powodu, żeby tam iść, natomiast to często też wynika z tego, co czytałam, że wielu ludzi nie stać na to, żeby pójść na kwarantannę, nie wiadomo, jak długo taka kwarantanna może trwać, bo często człowiek jest zdrowy, ale jak już się gdzieś tam raz zapisał, to oni potem go badają, sprawdzają i może wychodzić ten test nadal pozytywny, a mija już 6 tygodni od zachorowania i to jest takie... Dla takich ludzi jest to tragedia, bo nie wszystkich stać na takie...

**Jak poznać, czy dana informacja jest wiarygodna?**

Na pewno kto to podaje. Bo to Ministerstwo Zdrowia podaje te informacje, to zakładam, że jeśli z MZ, to jest to wiarygodne, natomiast jakieś takie inne źródła, nazwijmy to "Plotek coś tam, coś tam", no to na to w ogóle nie zwracam uwagi.

**Zastanawia się pani, jak będzie wyglądać przyszłość po pandemii?**

Nie tyle, jak będzie wyglądała po pandemii, tylko ja cały czas się zastanawiam, jak długo to będzie trwało. I to jest dla mnie takie bardzo przejmujące, bo trudno mówić o czymś, co będzie po pandemii, jak nie wiadomo, jak długo się z tym będziemy mierzyć.

**Słyszała pani jakieś prognozy?**

Podobno to ma trwać do końca przyszłego roku.

**Po czym poznamy, że pandemia się skończyła?**

Według mnie musi być zahamowany przyrost. Znaczy przyrost, no w ogóle muszą zanikać nowe zachorowania.

**Czyli nie do 0, tylko tendencja spadkowa?**

Mhm. Ja myślę, że ten koronawirus... Znaczy, ja nie wiem, czy ja myślę, tylko to, co czytam, że są takie przypuszczenia, że tak naprawdę nie będzie można go tak wytłuc do cna, tylko że on już zawsze będzie, tylko tyle że może powstaną szczepionki, które będzie można podawać, tak jak chociażby szczepionki przeciwko grypie. Ale czy ten koronawirus będzie też miał taką formę sezonową i czy to co roku będzie, ja nie wiem. Nie mam tutaj żadnej medycznej wiedzy na ten temat ani mikrobiologicznej, jak to się może dziać.

**Czy na dłuższą metę coś się zmieni?**

Myślę, że na pewno... Nie wiem jeszcze, w których branżach, natomiast większość chociażby sprzedaży, nazwijmy to sprzedaży, przeniesie się online tylko i wyłącznie.

**A w relacjach społecznych?**

Może jedynie to, ale to jest w tzw. optymistycznym wariancie, że jeśli się to już wszystko pokona i w ogóle, to znając skłonność polskiego narodu do nie tyle wyśmiewania się, bo to nie jest to słowo, ale do ujmowania pewnych rzeczy w żartobliwy sposób, to będziemy sobie wtedy wszyscy z tego mocno żartować.

**Czy zmieni się sytuacja gospodarcza?**

Na pewno się zmieni, trudno mi to określić w wielkości globalnej, bo chociażby np. tak jak teraz wszyscy bardzo się bali, że to źle wpłynie na różne dziedziny życia i gospodarki, to wiem, bo też już słyszałam takie różne skądinąd, że np. w jakiejś tam, przyjmijmy,  kancelarii obroty i zyski były dużo większe w tym roku, kiedy jest pandemia, niż były przedtem. Więc to też nie jest powiedziane... Na pewno się zmieni, ale to nie znaczy, że u wszystkich spadnie. Nic już nie będzie takie samo. Może ludzie się przebranżowią w jakiś sposób, ja nie mówię, że taka sprzedaż, która przejdzie całkowicie online, ma nie przynosić odpowiednich korzyści.

**W jakim sensie nic już nie będzie takie samo?**

Tak ogólnie miałam na myśli, nie tyle w gospodarce, ponieważ ta sytuacja wymusza znajdowanie różnych innych rozwiązań i może się okazać, że niektóre te rozwiązania potem będą lepsze i nie będzie powodów, żeby wracać do starych, jak się skończy pandemia.

**Czy kogoś szczególnie dotkną te zmiany?**

Na pewno gastronomia ucierpi bardzo. To jednak był ogromny rynek, a teraz chociażby po tym wiosennym lockdownie mnóstwo różnych firm po prostu się pozamykało. Tutaj mam na myśli te takie wyższe, znaczy różne, nawet sieciowe restauracje, wszystko to nie przetrwało. Co dalej... Generalnie handel, ja nie wiem, czy to będą książki czy to będą ubrania, ale część zdecydowanie przejdzie na tzw. online tylko i wyłącznie.

**A co z podróżami?**

To to już jest w ogóle według mnie... Już nie mówię o liniach lotniczych, które na pewno tracą, biura podróży... Mam sama koleżankę z Kanady, która zresztą teraz jest uziemiona w Polsce, bo nie może wrócić, bo Polska nie ma połączeń z Kanadą przynajmniej do 10.11. jako kraj jeden z bardziej zagrażających zarażeniem wirusem.

**To z inicjatywy Kanady czy Polski?**

Polski. Oczywiście ona może pojechać do Niemiec czy Holandii i polecieć stamtąd, ale ona akurat też ma bilet lotowski, jest w ogóle agentem turystycznym, więc to też tam zupełnie inaczej. Wiadomo, że jak będzie już bardzo chciała, to po prostu jakąś inną drogę znajdzie, żeby wylecieć. Tam u niej jeszcze dodatkowo sprawy rodzinne też się troszeczkę pogmatwały i ona też musiała tutaj zostać. Poza tym też nie bardzo chce, bo jak tam przyjedzie do Kanady, to też będzie na tej kwarantannie 2 tygodnie, czego też by nie chciała, no różne takie. Natomiast ona jest właśnie przykładem osoby, co prawda mieszka w Kanadzie, ale najlepiej też to obrazuje, podejrzewam, że w Polsce też ta cała branża turystyczna ma ogromne straty, a ona po prostu z dnia na dzień straciła pracę i to taką...

**Czyli branża turystyczna mocno ucierpi?**

Tak, bardzo, bardzo. Tak jak ona np. mówiła, że ona w styczniu i lutym tak dużo pracowała, teraz w tym roku, tuż przed tą pandemią, że ona np. w samym styczniu zarobiła tyle, ile czasami przez rok, ale potem okazało się, że totalnie to wszystko po pierwsze się zawaliło, po drugie to też tam nie jest dokładnie... Bo jeśli np. ona nie tylko sprzedaje wycieczki, wyjazdy, ale również bilety lotnicze, za które musiała wziąć pieniądze, a w momencie, kiedy to wszystko zaczęło być odwoływane i w ogóle, to ona też musiała robić jakie refundy. Koszmar po prostu, została bez pracy z dnia na dzień, a utrzymywała rodzinę z tej pracy przez prawie 30 lat.

**To stało się w Kanadzie, ale w Polsce pewnie mogło to wyglądać podobnie.**

Ale z tego, co wiem, tam np. Kanadyjczycy są bardzo bojaźliwi, jak nie, to już nigdzie nie jeżdżą, a u nas przecież wszyscy jeździli i do Grecji, i do Chorwacji, wszędzie. Jeszcze do tego stopnia, żeby na last minute pojechać teraz w październiku do Egiptu, na pewno słyszała pani. ITAKA wysłała grupę do Egiptu, świetny last minute i w ogóle i tam oczywiście u kogoś stwierdzono koronawirusa, zamknęli ich na 2 tygodnie w pokoju.

**Jak wyglądało Święto Zmarłych? Spotkała się pani z rodziną?**

Nie. Ze względu na pandemię. My od razu z siostrą postanowiłyśmy, że 1.11. nie pojedziemy, miałyśmy to zrobić w zeszłym tygodniu w poniedziałek, ale nie doszło do tego, a potem już zaczęło się dziać, jak się działo i teraz dopiero pojedziemy pewnie w czwartek.

**Myślała już pan, jak może wyglądać Boże Narodzenie?**

Powiem szczerze, że się jeszcze nie zastanawiałam nad tym.

**Czy sytuacja uspokoi się na tyle, żeby móc spotkać się z rodziną?**

Nie wiem, trudno mi to określić teraz.

**Miała pani okazję uczestniczyć w uroczystości rodzinnej?**

W weselu nie, ale konkretnie 4.10. byłam na komunii Zosi. Ona akurat miała taką komunię, że była sama, tzn. podczas takiej normalnej mszy dla dzieci. Wszyscy byli w maseczkach, część na zewnątrz, szczęśliwie taki był jeszcze ciepły, ładny wtedy dzień. Potem to się odbywało w restauracji. W restauracji były 3 duże rodziny komunijne, czyli my, potem inne stoły to jakaś tam inna i jeszcze jakaś tam inna.

**Liczba gości była ograniczona?**

Tam chyba była jakaś, ale u nas tak i tak było niewiele osób, tylko 12, więc nawet nie wiem, jaka była ta liczba ograniczeń, nie wiem.

**Maseczki były w kościele, a na samej uroczystości już nie?**

Tak.

**Miała pani jakieś obawy w związku z tą uroczystością?**

Nie, nie miałam tych obaw, poza tym to była taka sytuacja, że już trzeba było tę komunię odbębnić, brzydko mówiąc. Ja tam wcale niekoniecznie chciałam na to iść, ale ze względu na Zosię...

**Czyli obawy się nie pojawiły?**

Znaczy pojawiły się, z tego względu też ta liczba osób była taka mała, bo nie zostały zaproszone wszystkie tam babcie i jakieś inne, byli tylko tak naprawdę już bardzo, bardzo najbliżsi, z takiego bliskiego grona.

**Te osoby nie zostały zaproszone ze względu na wiek?**

Tak.

**Czy chciałaby pani coś dodać?**

Nie.
