# Supplementary material for: The challenges arising from the COVID-19 pandemic and the way people deal with them. A qualitative longitudinal study
Source: PLoS One. 2021 Oct 11;16(10):e0258133. doi: 10.1371/journal.pone.0258133 (PMC8504766; doi:10.1371/journal.pone.0258133)
Supplement: S1 Dataset — (ZIP) [file pone.0258133.s003.zip › Transcriptions/stage 6/4.6_M_32_couple, no children.docx]

**4.6_M_32_couple no children**

**Jak wyglądały u ciebie ostatnie miesiące?**

Dość dobrze, bo były wakacje, było ciepło, było miło. Mieliśmy sporo wolnego czasu i w związku z tym, że wszystko jest uzdalnione... Dziwne są te wakacje, bo trochę się baliśmy, trochę nie baliśmy, część rzeczy jest pozamykanych, inne rzeczy są otwarte, część ludzi w ogóle się nie przejmuje, inni się przejmują nadal - było tak dziwnie, ale w związku z tym, że my i tak lubimy spędzać outdoorowo czas, to po wyeliminowaniu... Przykrością był dla nas brak podróży zagranicznych. Nie wiem, czy pamiętasz, my jeździmy, wykorzystując nasz samochód, takiego mamy busika, w którym śpimy, jest kamperem takim małym. No to to nam trochę poodpadało i żeśmy po Polsce pojeździli i na Słowacji byliśmy, którą traktujemy trochę tak, że byliśmy 50 km za granicą. Bo oni mają po prostu góry, po których można chodzić z psem i są dość pustawe, w związku z tym byliśmy też właśnie na Słowacji i to była jakby cała zagraniczna wycieczka, to trochę taka za granicą, ale nie za granicą.

**Dlaczego postanowiliście nie jechać gdzieś dalej?**

Był w tym motyw finansowy, ale nie taki najistotniejszy, ale też chodziło o to, żeby nie wpaść w jakąś kwarantannę, raczej myśleliśmy o tym, może głupio, ale myśleliśmy o tym, że czasem jeszcze się ponastawiamy, spakujemy, poniesiemy jakieś wstępne koszty, pojedziemy gdzieś tam i np. się odbijemy. Było pytanie, czy Chorwaci będą wpuszczać, trochę co jest w tych krajach, trochę do tego dochodził też jakiś element takiego strachu związanego z tym, że gdyby człowiek zachorował albo coś się stało za granicą, to tam może nie najlepiej, bo nie mamy przetartych ścieżek, nie wiemy, jak działa system. Zawsze jest takie niebezpieczeństwo, ale tutaj było jakoś tak trochę... Jakby trzeba było, to pewnie byśmy pojechali, ale koniec końców nigdzie żeśmy się tak specjalnie nie wybierali. Myśleliśmy o Rumunii, bo to też chodzenie po górach. A, bo jeszcze jedna była istotna rzecz, że jak jedziesz za granicę, to - chyba że ktoś lubi plażować, to nie my - ale większość osób albo zwiedza, albo korzysta z jakiejś infrastruktury typu imprezy w hotelu, takie rzeczy albo nas nie interesują, albo odpadały, bo zwiedzanie miasta w tych epidemiach, jak nie wiadomo, co będzie pootwierane i tego typu rzeczy, to tak jakoś no nie. A jeśli chodzi o takie właśnie outdoorowe rzeczy, to myśleliśmy o Rumunii przez chwilę, bo tutaj tak jest po górach takich pustych, że pod względem epidemii to byłoby bardzo fajne, ale jakoś żeśmy się nie zdecydowali, na tej Słowacji byliśmy.

**Był taki moment, że w Rumunii było bardzo dużo zakażeń, różne państwa dookoła zamykały granicę z Rumunią i nie dało się tam wjechać.**

No właśnie, ale tak naprawdę jakby być mocniejszym, takim pewniejszym siebie i odważniejszym oraz nie mieć kilku ograniczeń, czyli np. pytanie, jaką kto ma sytuację. Dla jednego kwarantanna jest strasznym bólem, jak dostanie po powrocie do Polski, a dla jednego właściwie nie, to zależy od sytuacji materialnej, wykonywanego zawodu i jakichś jeszcze czynników, bo jak będziemy mieli parę z dziećmi i psem, którzy żyją w 40-metrowym mieszkaniu w środku miasta, to kwarantanna to jest dramat, a jak mieszkasz gdzieś na wsi, masz dom z dużym ogrodem i jeszcze pracujesz zdalnie albo jesteś emerytem lub rencistą, no renciści to nie wiem, czy jeżdżą na takie wakacje, ale jakimś emerytem czy kimś takim i dostaniesz 2 tygodnie siedzenia w domu, to pod warunkiem, że ci ktoś znajomy zrobi duże zakupy, to po prostu przesiedziałeś w domu i trudno. To wszystko bardzo zależy. My jesteśmy gdzieś tak pewnie po środku, bo nie lubimy być uwięzieni w domu, ale nie zniszczyłoby to nam życia jakiegoś w pracy, firmie, czymś takim, tak zawodowo by się to na nas nie odbiło, bo wykonujemy pracę zdalnie, jesteśmy w stanie.

**Odczuliście na wakacjach koronawirusa?**

Na Słowacji? Nie, w bardzo zbliżony sposób do Polski. Ja nawet nie wiem, jakie tam były przepisy, zachowywaliśmy się tak, jak wszyscy naokoło, a tam wszyscy naokoło zachowywali się tak, jak w Polsce, tzn. nosili maseczki  (ja nie wiem, czy na ulicach tam nie trzeba było, ale chyba nie), w sklepach ludzi nosili maseczki, to tak jak my i tak jak w Polsce jakaś dezynfekcja rąk, ale nie jakaś taka niesamowicie rozbudowana. Jeśli chodzi o granicę, to przejechaliśmy tak jak normalnie w Schengen, żadnej kontroli, nikt nic od nas nie chciał. Byliśmy w jednym miejscu, w którym było sporo ludzi, ostatniego dnia podjechaliśmy tam, bo zastanawialiśmy się, żeby pójść od strony słowackiej na Babią Górę, wracając do Polski, ale się strasznie zepsuła pogoda i jedliśmy sobie obiad, i czekaliśmy, zastanawialiśmy się, czy nam się jeszcze opłaca tam siedzieć, czy nie czekać, tam jakaś burza przechodziła. To było w takim schronisku, byśmy powiedzieli, chociaż to nie było w górach, tylko samochodem można było dojechać, ale jednak było na granicy parku krajobrazowego, to jednak schronisko i tam był jakiś bieg przez te góry, i dla dzieci, i dla dorosłych zrobiony, i taka impreza masowa. Tam rzeczywiście za dużo osób nie nosiło maseczek. Może czasami w jakimś pomieszczeniu, ale tak z przymrużeniem oka raczej to traktowali. Więc chyba taki podobny stan jak w Polsce. Bo myśmy jeszcze poza tym, że byliśmy tydzień na tej Słowacji, to byliśmy jeszcze tydzień między Gorcami a Beskidem Sądecki, tak to się chyba nazywa i jeszcze byliśmy parę pojedynczych dni, jakieś takie weekendy... A, i byliśmy na kajakach jeszcze. To też był taki wybór ze znajomymi, bo tam było parę osób, które się covidowo mocno przejmują, więc one chciały pojechać do miejsca, gdzie... Na Czarnej Hańczy byliśmy i spaliśmy na takich mikrokempingach, na których najczęściej w ogóle byliśmy sami. Przyjeżdża wieczorem tylko jakiś właściciel, zbiera opłatę i sobie jedzie, więc to też tylko tyle, że w 8 osób byliśmy w swoim sosie przez tydzień, ale to też na otwartym powietrzu, tak naprawdę mogliśmy się od siebie nie pozarażać. Wracam do pytania: na Słowacji było bardzo podobnie jak w Polsce.

**Oprócz wakacji były jakieś przełomowe momenty w ciągu ostatnich miesięcy?**

Czyli taki okres wakacyjny, bez tych ostatnich dni? Bo wzmożona, lawinowo rosnąca liczba zakażeń trwa od jakichś 2 tygodni mniej więcej w Polsce i mniej więcej tydzień temu pojawiły się pierwsze obostrzenia, znaczy zostały przywrócone czy pojawiły się nowe obostrzenia, bo cały czas mieliśmy te takie minimalne: w sklepach maseczki i w miejscach pracy chyba jeszcze mniej osób mogło siedzieć koło siebie. Ale to wszyscy się już trochę tak przyzwyczaili do tego i częściowo stosowali, a częściowo olewali, a od 2 tygodni jest coraz więcej rzeczy i rozumiem, że nie o tym na razie jeszcze chcesz mówić. To tam nie było żadnych przełomowych rzeczy związanych z epidemią. Tylko taka różnica (jeżeli szukasz różnic między epidemią i nie epidemią), że znalazłem pracę i cały proces rekrutacyjny był online, co pewnie przed epidemią nie miałoby miejsca, zarówno w sensie zwyczajów, jak i też... Po pierwsze dlatego, że przed epidemią to stanowisko pracy było niezdalne, teraz jest 100% zdalne i nie wiem, czy tak częściowo przynajmniej nie zostanie po epidemii, bo podobno są dość zadowoleni z tego, jak ludzie pracują w tej formule. Ale to był pewien sprawdzian umiejętności w ostatnim etapie rekrutacji i normalnie to byłoby na sali, bo to umiejętności szkoleniowe trzeba pokazać, że się umie poprowadzić grupę i tu robiłem to zdalnie, w związku z tym to było łoł, to było mocno inne niż to, co zwykle robię. To jest taka duża zmiana. Normalnie pewnie bym robił taką próbkę szkolenia na sali, miał flipchart, mógł coś przygotować, jakieś materiały itd., tu było 100 % zdalnie, więc to też na pewno było coś innego, co epidemia wymusiła. I to dziwne takie było dla mnie. Dobra, zrobienie tego wszystkiego było ok, ale dowiedzenie się przez telefon, że będę u nich zatrudniony i nikogo nie poznałem na żywo z tej firmy, a podpisali ze mną umowę, to było coś dla mnie tak dziwnego w ogóle. Myślałem, że będzie taki ostatni moment np., że jeszcze powiedzą: dobra, to zróbmy jeszcze takie ostatnie spotkanie, żeby szefowa mogła mnie poznać, ale nie, 3 rozmowy online, potem ta próbka szkoleniowa i mnie zatrudnili. To było ciekawe.

**Czyli nie było momentu, że pracowałeś w biurze?**

Nie.

**A ile już pracujesz?**

Miesiąc. Nominalnie od 1.10., ale też ze względów wdrożeniowych to się wszystko przesuwa, więc tak naprawdę tak aktywnie to dopiero teraz zaczynam.

**Czyli pewnie zmienił się twój rytm dnia?**

Tak, aczkolwiek myślę, że duże zmiany nastąpią dopiero teraz. Bo do tej pory różniło się to tylko tym, że musiałem zadbać o to, żeby o 9 pstryknąć komputer, bo zasadniczo tam pracujemy typu 9-17, to są takie korowe godziny. Na razie dostałem taką dużą partię materiałów do nauczenia się i przerobienia, jakieś certyfikacje itd., podszkolenie wewnętrzne, które właśnie zaczynam w przyszłym tygodniu. To też wynikało z tego, że musiałem dostać dostępy, a to jest jakaś cała długa procedura, duża korporacja itd. i w związku z tym to trwało strasznie długo. Nie wypadało np. tak, że jakby do mnie zadzwonili lub do mnie napisali, nie odpowiadać przez 2 h, bo to by było trochę tak, że nic nie robię, a już ten. W związku w tym musiałem odpalić komputer, być trochę tak pod mailem, ale miałem na tyle mało rzeczy do zrobienia, że mogłem sobie pozwolić na to, żeby spokojnie na pół godziny z telefonem w kieszeni wyjść na spacer z psem, zrobić obiad w międzyczasie, takie rzeczy. Więc zmienił się trochę ten tryb, ale myślę, że od przyszłego tygodnia intensywność będzie dużo, dużo inna, bo teraz będę miał od poniedziałku 10 dni 6-godzinnych szkoleń z jakimiś tam pracami domowymi czy jakimiś takimi jeszcze materiałami do przerobienia, testami, w związku z tym myślę, że to będzie zupełnie inaczej, niż teraz było.

**Twój tryb dnia jest taki jak przed pandemią? Jak on wygląda?**

U mnie to jest wszystko dziwne, bo funkcjonowałem ileś lat w trybie chodzenia do pracy do biura i to można było mówić o jakimś rytmie, jakichś regułach i zasadach. Jak w zeszłym roku się zwolniłem z pracy i pojechaliśmy w naszą podróż, potem wróciłem i wszystko uległo przemodelowaniu, po czym przyszła epidemia i znowu się przemodelowało. Potem po pierwszym lockdownie, gdzieś w czerwcu, wszystko się tak zaczęło otwierać powoli, myśmy się nie przejmowali, zrobiliśmy sobie wakacje, żeby jakoś odetchnąć, było przyjemnie i się nie przejmować różnymi rzeczami. W międzyczasie się rozgrywała pod koniec wakacji ta moja rozmowa rekrutacyjna, udało mi się dostać, co było w ogóle łoo, fajnie i teraz się to powoli zmienia... Chodzi mi o to, że jak pytasz, jak wyglądało wcześniej, to do czego się odnosisz: do sprzed roku, z początku epidemii, sprzed epidemii czy sprzed naszej podróży. Bo u mnie to jest tak trudno określalne, ale na pewno  nigdy nie pracowałem z domu, znaczy pojedyncze dni jakieś, jak trzeba było coś tutaj, hydraulik przychodził albo jakaś taka rzecz, to jakieś takie naprawdę pojedyncze. Albo np. jako przedłużenie, zdarzało mi się 2 maja, między 1 a 3 pracować z domu, bo byłem gdzieś na wyjeździe, wziąłem ze sobą komputer służbowy i szef się na to zgodził, to tak miałem kiedyś, ale jako takiej formy regularnej, a nie tylko takiego... Bo wiadomo, 2 maja, 28 grudnia to są takie sezony ogórkowe, nic się nie dzieje wtedy w firmach, wystarczy, że masz komputer, czasem zerkniesz na maila, czasem coś tam poklikasz, ale nie ma powodu, żeby... I tak w biurze połowy osób nie ma, więc to się czasami zgadzają, jak ci zależy, żeby pójść na zdalną pracę, ale tak, żebym ja pracował zdalnie z domu 100%, to dopiero teraz, ten miesiąc, a właściwie przyszły tydzień będzie takim początkiem.

**Jak się czujesz z tą perspektywą pracy zdalnej?**

Pomysł pracy zdalnej mi się bardzo podoba, ponieważ ja będę prowadził szkolenia dotyczące marketingu online i obsługi platform do ustawiania kampanii marketingowych. Takie szkolenia, właściwie takie wdrożenia, bo to będą wewnętrzne, to nie będzie sprzedawane na zewnątrz, tylko to są wewnętrzne dla osób z tej korporacji, żeby się mogły w tym wdrożyć... Z jednej strony te szkolenia fajnie jak są na sali, bo tworzy się grupa, poznaje się ludzi, łatwiej jest skupić uwagę, jeżeli biuro działało i będzie działać w przyszłości, to oni się do tego biura przyzwyczajają, więc to jest wszystko fajne. Nie wiem, czy taka forma też się nam jako takim po prostu biologicznym istotom, które jednak lubią być odbiciem rzeczywistości, będzie podobać, bo długie patrzenie się w monitor jest czasami trochę takie dziwne. Z drugiej strony wszystkie materiały, które mamy i które możemy im dać, wszystko, co ci ludzie będą robić w przyszłości, będzie na komputerach, więc jak są dobrze zrobione materiały, to oni mogą dużo więcej z tego wynieść, bo np. można zrobić tak, że rozsyłasz linki i im się otwiera jakaś prezentacja albo jakieś narzędzie do ćwiczenia i możesz im powiedzieć: "No to przeklikajcie sobie, zobaczcie to z lewej, to z prawej, to ustawcie tam". W ten sposób oni mogą zobaczyć te narzędzia na żywo, tak jak będą to mieli w przyszłości, tak jak będą z tego korzystać. Tak jak lekcje informatyki warto prowadzić na komputerach, a niekoniecznie lekcje informatyki warto prowadzić na kartkach i tablicy. Więc tutaj po prostu ta forma daje też dużo możliwości. Jeżeli chodzi o to, to uważam, że to jest nawet fajne, w sensie ma plusy, ma minusy, ale to nawet może być ok. Minusem jest dla mnie to, że fajnie jest, że nie muszę chodzić do biura, ale przydałby mi się pokój, który byłby gabinetem w domu, bo tak trochę z żoną tutaj mamy ciasno. Ale też sobie jakoś to rozwiązaliśmy przechodnio, bo niedaleko mieszka teściowa, ma wolny pokój i tam wstawiliśmy biurko i krzesło, żeby jak już oboje mamy takie dni, że gadamy i intensywnie pracujemy, to żebyśmy mogli nie siedzieć w jednym pokoju. Bo my mamy tylko sypialnię i duży pokój z taką lekko przechodnią kuchnią, to nie jest aneks, ale ta kuchnia jest taka półotwarta, w związku z tym jak jedno z nas nie jest w małej sypialni, nie leży na łóżku, to przebywamy w tym samym pomieszczeniu, jak pracujemy, więc jak oboje musimy rozmawiać, to to już jest za ciasno, więc tu by się nam przydało więcej przestrzeni. Fajnie jest też móc się czasami tak w ramach jakiejś socjalizacji zaprzyjaźniać z ludźmi, z zespołem, tutaj tak trochę człowiek siedzi, to jest takie dziwne, jak się jest nowym pracownikiem. Ale mam też te plusy. Teraz powiedziałem o minusach, a teraz plusy siedzenia w domu: mogę sobie pozwolić na to, żeby dużo mniej się przygotowywać przed wyjściem, przed rozpoczęciem pracy, nie muszę też dojeżdżać. To jest tak naprawdę mnóstwo plusów czasowych i takich czasami organizacyjnych. Najfajniej byłoby, gdyby to działało tak jak teraz, ale nie było epidemii i mógłbym czasami sobie przyjechać do biura, jak ja tego potrzebuję albo jak ktoś ode mnie tego potrzebuje. Czyli gdybym miał to zestawione elastycznie w miarę, moje biuro jest niedaleko, więc to też nie jest plus, ale np. mógłbym być może, gdybym wiedział, że będzie taki tydzień, co nie muszę być w ogóle w biurze, to mógłbym sobie pojechać gdzieś właśnie na działkę albo w góry, tylko żeby był internet w tygodniu, wtedy weekendami być poza Warszawą, to bardzo fajna, kusząca wizja dla mnie. Więc najfajniej byłoby pracować z domu, ale móc czasem przyjechać do biura, gdy jest taka potrzeba: albo ja mam tę potrzebę, albo moja firma ma potrzebę, żebym ja tam był, to to najlepszy układ, a taki, że jestem uwięziony w domu, to trochę jest takie rzeczywiście czasem kłopotliwe.

**Jak jest teraz u ludzi z twojego otoczenia?**

Mógłbym spokojnie wymienić takich, którzy od pierwszego dnia do dzisiaj mają na to totalnie wywalone, w sensie nie są osobami buntującymi się, nie mam w swoim otoczeniu prywatnie... Dobra znam jedną taką osobę z dalszej rodziny, która jest, jak to teraz nowe określenie się ukuło, jest foliarzem. Foliarze byli zawsze, ale to zyskało na popularności i to jest piękna nazwa zbiorcza na wszystkich takich z teoriami spiskowymi, czyli płaskoziemcy, antyszczepionkowcy, generalnie  osoby, które w jakimś obszarze, niekoniecznie we wszystkich, jakąś mainstreamową wiedzę odrzucają. To jest określenie pejoratywne. Bo można by jakoś inaczej o nich powiedzieć, pozytywnie: superświadomi, otwarci ludzie albo coś takiego, no ale nie, no to foliarze jednak. Ci moi znajomi tacy nie są, nie negują istnienia tego covida. Może ewentualnie na zasadzie, że jest trochę panika wzbudzona za duża i że śmiertelność nie jest aż tak wielka, w związku z tym nie należy się aż tak przejmować. Ale to są też osoby, które np. jeden kolega i tak codziennie jeździł, bo pracuje w jakimś tam specjalistycznym sklepie w centrum i on i tak codziennie jeździł metrem do pracy, w związku z tym mówi: "Kurczę, co ja będę się tutaj napinał, przejmował i nie wiadomo co". To są raczej ludzie, którzy ani nie mieszkają ze starszymi rodzicami, ani nie mają dzieci, więc ja rozumiem, że oni też mają takie poczucie trochę młodzieńczej nieśmiertelności, że to nie zagraża im itd. I nie są w tym jakoś bardzo samolubni, ale też z drugiej strony nie są tacy "Broń, Boże, żebym kogoś nie zaraził albo ktoś mnie". Ale mam też takich, co się bardziej przejmują. Większość naszych znajomych... Myślę o kimś, komu się zmieniła sytuacja zawodowa, parę osób jakimś utrudnieniom uległo, to mam tak: kolegę informatyka - zero zmian; drugi grafik komputerowy - utrudnienia w takim najcięższym lockdownie, bo tam niektórych rzeczy koncepcyjnych nie umieli dopiąć, jak nie siedzieli razem w pokoju, ale bez zmian; koleżanka, która pracuje w edukacji - totalnie się jej posypało na początku wszystko i ona chyba cienko przędzie, ale bez chyba jakichś wielkich takich problemów, bo w końcu jakieś im się obozy letnie (bo ona w takiej edukacji pozaszkolnej) chyba im się w końcu udały, jakieś tam żagle dla dzieci itd., więc to chyba ok. Parę osób pracuje w jakichś instytucjach publicznych, no to tam redukcji nie było żadnych, tylko tyle, że muszą nosić jakieś maseczki, a część pracowała częściowo zdalnie, więc nawet byli zadowoleni. Jeden kolega prowadzi biznes, to jemu się posypało tak na maksa, bo on wynajmuje pokoje dla studentów, no to on mówił, że jest masakra od września i że zastanawiał się, czy nie zbankrutuje. Ale chyba jakoś się tam jeszcze na powierzchni trzyma i rozumiem, że czeka, trzyma kciuki, żeby w końcu uczelnie wróciły do stacjonarnych zajęć, bo mówi, że od września, w którym zwykle mu się wszystko wynajmuje, to było kiepsko. Ale tak to nie pamiętam, żeby był ktoś, komu się tam tak posypało zupełnie, większość ludzi była w stanie jakoś obronić swoje przychody, albo przechodząc na zdalną pracę, albo nie przechodząc, ale mimo wszystko pracując.

**A twoja najbliższa rodzina?**

Nie ma za bardzo osób panikujących w moje całej rodzinie takiej najbliższej. Przez najbliższą rozumiem mnie i moją żonę, to jest taka naj, najbliższa, domownicy, a dalsza to bliscy, ale nie domownicy, to są rodzice i nasze siostry z rodzinami, i ewentualnie jeszcze moja ciocia i babcia. My wszyscy dość blisko siebie mieszkamy na Bielanach, więc dość utrzymujemy ze sobą kontakty, też tak się odwiedzamy często i spotykamy, i rozmawiamy itd. Więc tak jak mówię, to są moje siostry, siostra mojej żony, która zresztą wyprowadziła się teraz, bo pojechała... Tutaj trochę stresu i niedogodności spowodował nam ten covid, bo ona do Budapesztu się wyprowadziła i miało być, że nie będzie tak źle i że będzie dało się radę podróżować, my ją będziemy mogli czasem odwiedzić, czasem ona nas i tak wyszło średnio, bo na początku sierpnia się wyprowadziła i przyjechała we wrześniu raz tylko, a my nie daliśmy rady jej w końcu odwiedzić ani razu. To tak akurat minus. Ale w mojej rodzinie, czyli my, moi rodzice, moja babcia i moje siostry (siostra mojej żony też jest najbliższa, dużo gadamy przez Skype'a, dziewczyny się tam zdzwaniają na Messengerze), to tam nie ma nikogo takiego chyba bardzo panikującego. Trochę jesteśmy przejęci, bo się o rodziców martwimy. Babcia to już zapowiedziała (babcia ma 84 lata chyba), że ona się nie przejmuje, bo po prostu jak przyjdzie ten covid, to ona umrze i tyle. Babcia ma specyficzne podejście, zwykle ludzie tak gadają, a jak potem coś się dzieje, to są przerażeni, ja jestem przekonany, że moja babcia nie będzie, może w chwili śmierci tak, ale ona ma raczej specyficzne podejście do życia. I my na początku się przejmowaliśmy, chcieliśmy jej nie odwiedzać itd., a potem doszliśmy tak rodzinnie do wniosku, że ok, ale tak naprawdę jeśli pandemia potrwa jeszcze ze 2 lata i tam różne rzeczy będą, a ona i tak chodzi do sklepu czy na spacery, czy z koleżankami się jakimiś widuje, mniej trochę niż przed epidemią, ale jednak tak, to bez sensu, żebyśmy my... Bo co, jak przestaniemy się z nią widywać na najbliższe pół roku, to może się okazać, że już w ogóle się z nią, do końca jej życia, przestaliśmy widywać. Więc to jest trochę tak, że zakładając, że jest prawdopodobieństwo, że my ją akurat zarazimy, a nie ktoś inny, akurat my ją zarazimy i ona umrze, to wyeliminujemy to ryzyko i przedłużymy jej życie, ale ono straci sporo na jakości. Ona, jak przez miesiąc się tam nie widywaliśmy czy dwa, marzec-kwiecień czy kwiecień-maj, to tak bardzo podupadła na duchu, widać było, jak się z nią przez telefon rozmawiało, czasem mój ojciec bardzo rzadko wpadał, na jakiś spacer szli, to mówił, że babcia tak właśnie kiepsko bez naszego towarzystwa. W związku z tym tak teraz sobie myślimy, że to może trochę nie ma sensu, lepiej jest poodwiedzać. My się mało szlajamy gdzieś, pracujemy obydwoje z domu, robimy zakupy raz na jakiś czas, w wakacje widywaliśmy się sporo ze znajomymi, ale teraz trochę ograniczyliśmy, bo dużo jest tych zakażeń i w związku z tym szansa, że my akurat przyniesiemy jest mała. Największym prawdopodobieństwem jest rodzina mojej siostry, bo tam dzieci chodzą do szkoły, przedszkola i mój szwagier chodzi do biura, bo musi, nie codziennie, ale tak, z tej puli bielańskiej to oni. Jeszcze jest moja ciocia, z którą zwykle jesteśmy w miarę blisko, ona mieszka w Łomiankach, to jest kawałek dalej. Ona jest przedszkolanką, więc ona prędzej czy później trafi na covid, już miała w grupie chłopca, który jest teraz na kwarantannie, bo jego ojciec ma covid, więc to jest kwestia dni, może tygodni, aż się przypałęta.

**Mówiłeś, że nie masz w rodzinie osób panikujących...**

I nie dokończyłem tego, najbardziej panikująca jest moja żona. Ona przejmuje się najbardziej, najbardziej się denerwuje itd. I ona najbardziej się boi nawet nie o nas, bo wiadomo, że może być różnie, akurat na nas też może trafić, że akurat nasz organizm będzie sobie słabo z tym radził, ale najbardziej się martwi chyba o swoją mamę i moich rodziców, może o swoją siostrę trochę, chociaż ona jest zdrowa, więc nic jej nie będzie.

**Co dla ciebie znaczy osoba panikująca?**

To jest dobre pytanie. Dzisiaj się spotkałem, dosłownie przed twoim wywiadem wyszedłem na chwilę z domu, bo akurat do pobliskiego parku przyjechał mój kolega z 3-letnią córką. Bo tu jest staw jakiś i kaczki są, i szczury, i oni przyjeżdżają tutaj oglądać te szczury, wiem, to dziwne, ale tacy są. W Parku Olszyna, na Broniewskiego jest takie oczko wodne z takim mokradełkiem i tam dosyć duża rodzina szczurów żyje. Jak się rano tam przechodzi, to wtedy biegają dosyć licznie. Szczury są wszędzie tak naprawdę, tylko ich nie widzimy. Tam akurat sobie tak trochę w naturalnym środowisku żyją i oni przychodzą obserwować, jak te szczury, bo to są obrzydliwe, ale przeciekawe stworzenia. Więc spotkałem się z nim i właśnie się zdziwiłem, bo był jeden szczur, więc jego córce się tam zaczęło nudzić, ja mówię: "To co, tutaj jest plac zabaw obok, idziecie tam na plac zabaw?", on mówi: "Nie, my nie chodzimy na place zabaw w ogóle". Ja sobie myślę, kurczę, no rozumiem, ale mówię: "Tak teraz?", on mówi: "Nie, od marca nie chodzimy" i dla mnie to było takie na zasadzie, że... Bo oni są tacy przejmujący się i tak sobie myślę, kurczę, ja rozumiem, że np. na jakiś taki plac zabaw na Placu Wilsona, duży, dzieciaków dziesiątki pewnie tam są, zwłaszcza w sobotę, to można trochę nie chcieć, może tam być jakieś zagęszczenie, jakiś covid itd., ale taki plac w małym parku w brzydką pogodę, nie ma żadnych dzieci, tylko tyle, że musiałoby to dziecko akurat dotknąć jakiegoś zakażonego miejsca i wsadzić tę rękę później sobie do buzi. Niby jest jakieś prawdopodobieństwo, ale podobno zakażenie przez dotyk jest bardzo rzadkie. To jest tym ciekawsze, że oni tej dziewczynki nie posyłają do przedszkola teraz, ale to mówią, że z różnych przyczyn. Teraz to się trochę boją covidu, bo jest dużo tych zakażeń, ale wcześniej to życiowo robili coś innego i nie musieli, a i jeszcze mówi, że i tak zaraz zamkną przedszkola, więc oni chcą jej robić bigosu w głowie, żeby nie czuła, że jej się coś co chwila zmienia. A jeszcze za tam 3 miesiące chcą się wyprowadzić do innej dzielnicy, więc i tak by zmieniali przedszkole, więc mówią, że już tak po prostu ona przez pół roku nie będziemy chodzić do tego przedszkola. I to jeszcze bym rozumiał, że ona jest trochę w takiej bańce ochronnej, ją i siebie trzymają, ale oni z drugiej strony chodzą do pracy codziennie, znaczy na zmianę, bo dzielą się tak, żeby właśnie być w stanie się nią opiekować, a przynajmniej jedno z nich chodzi dużo do pracy, ona, bo on pracuje więcej zdalnie i też mają taki układ, że on pracuje na pół etatu, żeby właśnie się wyrobić z opieką nad dzieckiem. I wtedy sobie myślę, kurczę, to ona chodzi do pracy, pracuje w biurze w Centrum Nauki Kopernik, czyli jeszcze ma styczność z ludźmi, którzy mają styczność z mnóstwem innych ludzi, i nie chodzą na plac zabaw od pół roku? Oni nie są spanikowani w takim sensie, że to już jest taka uzewnętrzniona panika, że biegają, krzyczą i nie są w stanie opanować emocji, ale tak, dla mnie chronią się w zbyt dużym stopniu w miejscach, które nie mają takiego logicznego przełożenia.

**Patrzysz na prawdopodobieństwo zakażenia lub kontaktu z zakażonym i na podstawie tego podejmujesz decyzję.**

Tak się staram, choć czasami przyznam szczerze, że podejmuję takie - jakbyśmy rozmawiali o chorobach wenerycznych - zachowania niebezpieczne, ryzykowne. Np. było tak, że staraliśmy się nie chodzić na jakieś imprezy, ale nie jakoś tak strasznie i było pytanie, gdzie stawiać granicę, od kiedy jakaś impreza, spotkanie czy wspólny wyjazd jest już przesadą i należałoby nie jechać, i podjęliśmy kilka takich decyzji, które były takie właśnie, że trochę świadomie, a trochę z takiego ugięcia, że już chcemy nie odpuszczać. Ostatnio gdzieś ze znajomymi, już moja żona uważała, że tak trochę nie powinniśmy, ale ja jeszcze poszedłem sobie do kogoś na imprezę, na taką imprezę 30-latków: 10 osób pije piwo na kanapie, je pizzę i gada, to taka impreza. Ale byliśmy np. pod koniec sierpnia w Białowieży, w Teremiskach (to jest tam, gdzie Adam Wajrak mieszka, gdzie tę słynną puszczę wycinali), na takim, jak to nazwać, to się nazywało mikrofestiwal. Z racji epidemii był mikro, bo tego nie rozgłaszali nigdzie, taki dla tych aktywistów z Puszczy Białowieskiej, którzy jej tam bronili, oni tak we własnym gronie i nas ktoś tam (mamy znajomych) też zaprosił. Pojechaliśmy tam i tam się przewinęło przez ich taką świetlicę, obóz, stowarzyszenie, nie wiem, jak to nazwać, taki budynek, gdzie były wykłady tego dnia, różne rzeczy, przewinęło się tak pewnie z 60 osób. I tam gotowaliśmy wspólnie posiłek, byliśmy na takim spacerze ornitologicznym, przyrodniczym właściwie bardziej, po tym lesie, były jakieś wykłady, jakieś wspólne... To jest takie trochę merytoryczne, trochę też takie społeczne spotkanie tych ludzi. Bardzo spoko ludzie, oczywiście mnóstwo różnych takich dziwaków, ale nie tak wielkich, jak się spodziewałem. I jak wracaliśmy, to było tyle... Myśmy się nawet zastanawiali, kurczę, może trochę przegięcie, może coś. I z naszymi znajomymi przyjechał taki jeden gość, który się później nie zabrał z nimi z powrotem i myśmy go wzięli do samochodu, jechała nas tam w końcu szóstka w naszym siedmioosobowym samochodzie, i on się rozchorował. On mieszkał w pokoju obok nas w jakiś wynajętej agroturystyce. Więc on się rozchorował podczas tego wyjazdu i wracał już właściwie taki z gorączką i pokasłujący. I spędziliśmy z nim razem 2 dni, a potem wracał razem z nami samochodem. No i my tak właściwie, kurczę, może nie covid, ale już tak trochę w stresie. I tam właśnie była też ta ekipa z nami od tych, co te szczury, rozumiesz, to są ludzie, którzy chronią żubry, a w mieście oglądają szczury, to jest to samo towarzystwo. Oni jechali innym samochodem (ci, co oglądają szczury), ale z nami jechało ich rodzeństwo. I oni też są tacy trochę wydygani, nie dali po sobie poznać w samochodzie, ale jak on wysiadł, to potem okazało się, że dzwonili do nas i byli trochę przerażeni, że on na pewno z tym covidem i na pewno już będzie teraz źle. I my tak trochę, że nie ma czym się przejmować itd., ale jednak mimo wszystko gdzieś żeśmy sobie taką minikwarantannę zrobili, w sensie np. chodziliśmy po jakieś zakupy do warzywniaka lokalnego, ale się nie szwędaliśmy, nie spotykaliśmy się z nikim, nie wyłaziliśmy nigdzie przez kilka dni. Potem się okazało, że on poszedł na jakieś badania krwi czy czegoś tam, bo on miał tydzień wcześniej operację, miał jakieś usunięcie kamieni nerkowych czy czegoś takiego i okazało się, że go zakazili jakąś bakterią i on miał tę gorączkę i się źle czuł, bo po prostu zaraził się tą bakterią i organizm walczył, miał po prostu stan zapalny organizmu, dostał antybiotyki i mu przeszło. Okazało się, że to nie covid, ale było to właśnie takie zachowanie ryzykowne, w którym już wyglądało na to, że może właśnie żeśmy się złapali na zakażenie przez kogoś.

**Byłam zdziwiona, że twoja żona wzięła udział w tym wydarzeniu.**

Ja trochę też byłem zdziwiony tym, ale ona strasznie chyba chciała. Jak tam byliśmy na miejscu, to trochę zaczęła się denerwować, że to może jednak głupota, ja mówię: "Wiesz co, może tak, ale już jesteśmy tutaj, to się już wyczilujmy i już się po prostu cieszmy tym, bo już nic nie zmienimy za bardzo". Ale to też w sierpniu tych nowych zakażeń było tak mało, że to się wydawało chyba wszystkim, że to jakoś tak oswoiliśmy ten lęk, był nienarastający.

**Jak teraz się czujesz w kontekście koronawirusa?**

Trudno to oddzielić. Przede wszystkim jestem bardzo zły, żeby nie powiedzieć brzydziej, generalnie to jestem zirytowany, zły, ale bardziej na taką sytuację społeczną niż na sam covid. Poprzednio pamiętam, że opowiadałem ci dużo o polityce i że mnie te sprawy gdzieś tam rozgrzewają, i zaciekawiają, i w ogóle ten cały covid był interesujący. Z samym coivdem dużo więcej się nie dowiedzieliśmy, a na kwestie organizacji walki z tym covidem oraz kwestie polityczne już trochę nie mam siły, już mi ręce opadają i w ogóle... Już nie mam siły nawet o tym mówić, to jest po prostu... W związku z tymi protestami są wszystkie te nakładki na Facebooku: "Piekło kobiet" i "Protest", i "Ruch Ośmiu Gwiazd" itd. i jest taka nakładka też, która mi się strasznie podoba, taki tylko kwadracik na dole i napis w stylu "Mam tyle powodów do protestowania, że już nawet nie wiem, którą nakładkę mam wybrać". Ja mniej więcej się tak czuję, a nie chcę też się rozemocjonować tak mocno negatywnie, że stracę jakikolwiek rzeczowy ogląd sytuacji, a już jestem tak podkurwiony niemalże na wszystko, co się dzieje dookoła.

**Czyli to jest bardziej wkurzenie na decyzje rządu niż covid, ale to jest połączone...**

No tak, to jest właśnie połączone. To jest dużo negatywnych emocji, jest na pewno dużo złości, dużo irytacji, dużo frustracji, jest też trochę takiego zrezygnowania.

**Ta złość towarzyszyła ci przez całe ostatnie miesiące?**

Nie. W wakacje zrobiliśmy sobie wakacje. Po pierwsze w kwestii epidemii niewiele się działo, wszyscy tak odpuścili, zaświeciło słońce i wszyscy zapomnieli nagle o tym, że jest koronawirus. Może głupio, może dobrze, bo może trochę wszyscy odpoczęli i dzięki temu teraz przeżyjemy jakoś psychofizycznie ten nadchodzący najprawdopodobniej w najbliższych dniach lockdown, na to wszystko wygląda. I wtedy tak się niewiele działo, więc my też niewiele czytaliśmy, powiedzmy, że sprawdzaliśmy najświeższe informacje, ale tak... A teraz to co się dzieje, okazało się, że nic nie zostało zrobione, jeszcze odgrzewają jakieś sprawy pobudzające ogólną złość i zamiast się zajmować gospodarką i zwalczaniem skutków tego covidu, to... No nie, nie mam siły już nawet o tym mówić. Przecież to są debile, jełopy i w ogóle, no nie mogę po prostu tego znieść. Oni ogłosili teraz, przycięli w ostatniej nowelizacji ustawy, którą nie uważam za super rozwiązanie, bo to jest jak rzucanie ochłapów, ale powiedzieli, że rzucą ochłap, jakiś dodatek covidowy dla lekarzy i środowisk medycznych, po czym przycięli, tam nie wszystkim grupom dali i to jest jakieś takie, że w końcu niby dadzą, ale tak naprawdę tylko tym, którzy pracują przy czymś tam, w związku z tym część się będzie czuła oszukana i nawet już powiedzieli... A, więc się z tego wycofali, więc to jest fatalne, po czym jak to przycięli, to dzień później wyszło czy ogłosili, czy gdzieś tam ich przyłapali, że 307 nowych limuzyn zamierzają kupić. Toż to jest PR-owo debilne, to ludzi strasznie podwkurza. Czy teraz jest akurat konieczne, żeby kupować 307 nowych limuzyn? Gdzie oni chcą jeździć podczas tego covidu? Dokąd? Na spotkania z ludźmi? Ja tego nie rozumiem. Będą jakichś ambasadorów przyjmować? Jakieś pielgrzymki będą do innych krajów urządzać? O co chodzi? Brakuje karetek, a oni kupują limuzyny. Ja nawet nie wiem, jakie to są kwoty, może to są kwoty, które są nieznaczące, bo czasami jest tak, że podnosimy pensję prezydentowi i to, ile on zarabia albo jaki jest wydatek na jego biuro, to nie ma znaczenia w kontekście... Bo to jest tak, że prezydentowi podnieśli pensję z 10 do 15 000 i ludzi strasznie to denerwuje, ale to nie ma realnego przełożenia na to, czy lekarze, nauczyciele dostaną kliką złotych podwyżki, bo lekarzy jest 10 000 i każde 100 zł, to są miliony dla nich, a w przypadku prezydenta mówimy o kilkudziesięciu tysiącach rocznie, w związku z tym to jest nie ta skala. Ale z tymi limuzynami nie mam pojęcia, jaka to jest skala, nie przeliczałem tego, dokładnie nie sprawdzałem, ale jaka by nie była, no to kurczę... Długo by móc opowiadać. Z tymi zniczami teraz czy tam kwiatami to co zrobili, przecież to jest dramat.

**Masz jakąś hipotezę, dlaczego rząd to robi?**

Nie, nie jestem w stanie tego wytłumaczyć! Moim zdaniem to jest coś pomiędzy, bo tego nie da się wytłumaczyć tym, że oni są... Np. zawsze sobie wyobrażaliśmy, że oni mają inną wizję, że oni inaczej widzą świat, chcą inaczej zarządzać państwem, np. centralnie, a nie oddolnie, że wierzą w siłę państwa, nie w samoorganizację ludzi itd. Dodatkowo można było ich o pewnego rodzaju cynizm posądzać, że manipulują po to, żeby się utrzymać przy władzy, że są populistyczni. No ok, dobra, możemy tego nie cenić, ale można to zrozumieć. Jeszcze można było im zarzucać, że są po prostu złymi ludźmi. Natomiast niektóre decyzje teraz są w ogóle... Nie da się jednoczynnikowo tego wytłumaczyć, że ktoś jest głupi, zły i jakiś niestąpający po ziemi w ogóle, odrealniony zupełnie. I żaden z tych czynników: to, że taka jest ich polityka i sposób patrzenia na świat ani to, że mają jakieś wartości i w związku z tym coś, ani to, że po prostu nie potrafią sobie czegoś zorganizować - nie, wygląda na to, że po prostu... Nie, to jakieś pomieszanie wszystkiego. Przecież w czwartek jeden wicepremier - nie wiem, bo to Sasin, on chyba jest wicepremierem do spraw aktywów państwowych, on ma jakieś wysokie stanowisko, czy minister - jeden minister mówi, że na pewno nie zamknie niczego, następnego dnia wychodzi premier i mówi, że zamykają wszystkie te cmentarze. Przecież to jest jakiś obłęd. Nie jestem w stanie tego... I dlaczego, i po co? Z rozmrażaniem tej aborcji w tej chwili, czy im naprawdę o to chodziło, żeby tę aborcję, to było takie istotne i chcieli to tak przeforsować, nie wydaje mi się, ja tego nie rozumiem naprawdę.

**Czyli to jest poczucie niezrozumienia tej sytuacji, które skutkuje złością i bezradnością?**

A jak byś chciała to naprawić? Ja wiem, ty nie możesz mówić, ale ja zadaję retoryczne pytanie, sobie też zadaję pytanie, co można w tych różnych sytuacjach zrobić. W niektórych widzę, w niektórych się narzucają, np. z tymi cmentarzami powiedzieć 2 tygodnie wcześniej: "Słuchajcie, może zamkniemy", ale już ideałem byłoby powiedzieć, kiedy zamkniemy, w sensie, że jeśli będzie tyle zakażeń albo jeśli w danych województwach nastąpi coś, zacznie brakować miejsc, to przytniemy to, jak tyle, to przytniemy to. Albo niech ktoś wychodzi i chociaż powie, dlaczego to zrobiliśmy, czym się sugerowaliśmy, jaka liczba nas przekonała do tego, bo ja mam wrażenie, że to jest nadal wszystko takie z palca. Bardzo jest mi trudno, już mi ręce opadają, naprawdę, no po prostu to się skończy w końcu... A z drugiej strony oni mają wszystkie instrumenty, w związku z tym zastanawiam się, jak ta sytuacja może się potoczyć. Nikt nie ma na nich żadnej siły przełożenia. Jedyną możliwością jest tak naprawdę gigantyczny protest, strajk, który co? Jakiś przewrót ma nastąpić? To chyba musi się skończyć rozlewem krwi, bo jak inaczej to może się skanalizować w konstruktywne rozwiązanie - w żaden sposób, a przecież jak będzie rewolucja teraz jakaś, nieważne czy to będzie pucz wojskowy, czy jedni będą strzelać do drugich, czy coś, no to przecież nic mądrego, dobrego z tego nie wyniknie, to będzie tylko jakiś chaos i totalny bałagan. Więc ja sam nie widzę konstruktywnej metody, żeby to się rozwikłało. Jak ci protestujący wszyscy przegrają, to rząd uwierzy w to, że już może robić totalnie wszystko, znowu tylko trzeba wziąć na przeczekanie, raz krócej, raz dłużej. A jak wygrają protestujący, to jak oni mogę wygrać? Co może się takiego zdarzyć w tej chwili? Odwołają nieodwoływalny wniosek trybunału konstytucyjnego? No to jest niemożliwe. Powiedział, że taka ustawa jest niezgodna z konstytucją, znaczy co, konstytucję będą poprawiać? Jest taki bałagan, że nie będą w stanie tego przeprowadzić, nikt się na to nie zgodzi. Jedyne co, to mogą powiedzieć, że sorry, ten trybunał to jest jednak nielegalnie wybrany, to jednak 2 lata temu go żeśmy nielegalnie wybrali. Jak oni mogą to zrobić? Do tej pory szli w zaparte w mniejszych sprawach, a teraz się okazuje, że sorry, to jednak przez 2 lata was oszukiwaliśmy wszystkich i jednak w ogóle to się nie liczy, pobite gary. Nie da się tego odkręcić, naprawdę się nie da, nie wiem, jak to można by odkręcić w sposób taki, który byłby dla naszego państwa i społeczeństwa budujący. Po prostu zabrali nas wszystkich w taki kozi róg, że nie da się tego sensownie jakoś rozwiązać, ja przynajmniej nie widzę.

**Czy rząd mógł zapobiec drugiej fali?**

Nie. Wydaje mi się, trudno mi mówić na pewno, nie wiem, czy ktokolwiek, czy jest jakiś ekspert, który z czystym sumieniem mógłby powiedzieć, że on wie na pewno, jak jest i jak będzie, i było, i jakie w ogóle były możliwe opcje. Wydaje mi się, że to jest bardzo trudne, żeby tej drugiej fali nie było, wydaje mi się, że to było niepowstrzymywalne tak naprawdę. A jeżeli było powstrzymywalne, to czy chcielibyśmy otwartą dyskusję przeprowadzić, co trzeba byłoby zrobić, że powstrzymać to i jakiego typu koszty musielibyśmy ponieść jako społeczeństwo, jak przemodelować całkowicie swój sposób życia. To jest tak duża zmiana, że nikt by tego nie zrobił. Bo oczywiście, że my jesteśmy w stanie to zrobić. Jacyś tam w Korei Południowej czy w Chinach, czy gdzieś, to są w stanie to zrobić, to są zupełnie inne społeczeństwa, znaczy mniej lub bardziej totalitarne, a nawet te, które nie są totalitarne, to mają taki zupełnie inny sznyt, taki zupełnie inny sposób myślenia, w Europie to jest niemożliwe po prostu do zrobienia. W Ameryce wolność jest jeszcze ważniejsza i indywidualizm, i inaczej jeszcze zbudowane społeczeństwo, więc oni sobie radzą jeszcze gorzej. To, co zwykle jest ich plusem, to w przypadku takiej epidemii jest ogromnym minusem.

**Czy śledzisz teraz zakażenia?**

Nie, zakażenia to w ogóle staram się nie, ponieważ uważam, że tam podawane są liczby głównie... Po pierwsze nie do końca ufam tym statystykom, po drugie uważam, że w sposób taki głupowato-manipulacyjny są podawane te liczby często. Ani w ich rzetelność nie wierzę trochę, ani w to, że one są... Chyba podawałem kiedyś przykłady, teraz musiałby poprzypominać sobie, ale staram się omijać nawet wzrokiem te liczby, ile było tam zgonów dzisiaj, wczoraj, ile nowych zakażeń w ostatnim tygodniu. Zwykle każdy serwis internetowy ma jakieś swoje ulubione liczby, które w kółko podaje, tak żeby ludzie mogli je sobie porównywać niby, ale one są tak dobrane jakoś, żeby działać na emocje. To nie jest dziwne, bo media w ogóle tak działają, w związku z tym omijam je, bo mam wrażenie, że one są takie tylko, że jedyne, co mogę z nich wywnioskować to to, że spojrzę i się przestraszę. No to po co mam się przestraszyć albo się przejąć, to jest bez sensu, jeżeli to nie daje mi tak naprawdę żadnej dobrej informacji.

**Ale pamiętasz jakiś moment zmiany po wakacjach?**

Pamiętam taki moment, że właśnie ja nie śledzę i jakoś coś z żoną rozmawialiśmy o czymś tam, a propos chyba tych wybuchających protestów czy coś takiego i że ona powiedziała, że to wszystko przy 20 000 zakażeń czy 30, wymieniła jakąś liczbę i ja tak łoo, to już tyle mamy w tej chwili. Więc nawet nie byłem świadomy, że to aż tak lawinowo idzie. Ale mam taką świadomość, że staram się nie przywiązywać do tej liczby, bo po pierwsze nie wiem, kogo tam klasyfikują do tego, po drugie ta liczba tych nowych zakażeń jest jakoś proporcjonalna do ilości zrobionych testów, co jest logiczne. Więc to jest tak, że mówimy: "O, chyba się uspokaja", bo pokazuje, że 30% testów jest np. przez 3 dni robione. Z jakiegoś powodu, nie dlatego, że się skończyły albo cokolwiek, tylko dlatego, że np. my nie robimy żadnych testów przesiewowych, na żadnej grupie społecznej, na żadnej powtarzającej się grupie, nie robimy tak, żeby np. w ramach nawet... Nie mówię, żeby cały kraj, ale wybrać sobie populację jakoś i ich testować regularnie co tydzień, co miesiąc i na tej powtarzającej się w miarę grupie sprawdzać, ile tych ludzi tam w środku jest zakażonych. To by dało jakąś informację, czy idziemy w górę czy w dół. Bo tak jak my osoby, które mają objawy albo się kontaktowały z kimś, kto miał objawy, a jak zaczyna brakować testów, to tylko tych z objawami albo stwierdzonych, wysyłamy na testy, to znaczy, że tak naprawdę to służy tylko temu, żeby ewentualnie takie przeciwdziałanie: próbujemy tylko wyizolować tych, którzy będą chorzy. Ale to nie daje nam informacji, ile osób w społeczeństwie, w populacji jest chorych. Walczymy tylko z tymi, którzy aktualnie się objawili jako chorzy, natomiast jak ludzie chodzą po ulicach, nie mając specjalnych objawów np., to nie wiemy, ilu tak naprawdę jest tych cichych nosicieli.

**Myślisz, że można powiedzieć, że teraz jest druga fala?**

Mimo wszystko na to wygląda, że tak. Jeśli różnice były takie w stylu ludzie się tam lipiec-sierpień ekscytowali, że mieliśmy w zeszłym tygodniu po 1000 dziennie, a teraz mamy 1500, a może to błąd pomiarowy, trzeba by było myśleć nad tym, sprawdzać kilka liczb, sprawdzać, czy nie zmieniła się liczba testów, sprawdzać, czy nie zmienili czegoś w jakichś przepisach, bo oni też czasami... Od przyszłego tygodnia jest jakieś rozporządzenie, które zmienia to, że do liczby wykonanych testów zostaną doliczone pozytywne wyniki z testów, które ludzie wykonują prywatnie, bo one do tej pory były nieuwzględnione w statystykach, a teraz chyba wymusili jakimś rozporządzeniem czy czymś, żeby przesyłali informację o tych pozytywnych testach do Sanepidu czy gdzieś - zmienią metodologię zliczania generalnie. I wygląda na to, że też trochę zrobią tak, żeby wychodziło, że zakażonych jest dużo więcej i to trochę sugeruje, że rząd zamierza zmanipulować liczbami troszkę albo bardzo, żeby znowu był skok epidemii, znaczy skok tych zakażeń, żeby można było powiedzieć, że epidemia jest w jakimś krytycznym momencie. I tu można sobie domniemywać, jeżeli tak rzeczywiście... No można powiedzieć: "O, teraz dopiero pomyśleli, jak to zrobić, ktoś na to wpadł i uszczelniają sposób podawania informacji, żeby było lepiej". Dobra, możemy wierzyć w dobre zamiary i że akurat teraz się połapali, ale wygląda na to, że równie dobrze można powiedzieć, że mają jakiś w tym cel i dwa cele się narzucają od razu, tzn. pierwszy jest taki, żeby powiedzieć, że to z powodów protestów zwiększyła się liczba zakażeń i to właśnie opozycja morduje naród, i kobiety, i lewacy, i wszyscy tam cykliści, a drugi jest taki, że chcą to instrumentalnie potraktować, żeby powiedzieć: "Słuchajcie, jak teraz już was nie zamkniemy, to już zaraz będzie koniec" i w ten sposób rozwiążą sprawę jakichś protestów, bo wprowadzą tak jak w Czechach albo na Słowacji, już nie pamiętam, w którym kraju, jest godzina policyjna i jakieś tam bardzo duże kontrole itd. Czy to ma sens? Trudno jest mi powiedzieć. Natomiast chodzi o to, że mogą użyć tej wzmożonej epidemii do tego, żeby zapanować nad tym buntem społecznym, który im się zrodził.

**Który sami wywołali nie wiedzieć dlaczego w tym momencie?**

Tak. Właśnie nie wiem czemu. Z głupoty? Myśleli, że to im się tak upiecze? Czy może im rzeczywiście strasznie zależy na tej aborcji? Bo wydawało się bardzo długo, że Prawo i Sprawiedliwość, w sensie ta wierchuszka: Jarosław Kaczyński z paroma osobami - im prywatnie nie zależy bardzo jakoś na ograniczeniu tej aborcji i starali się, jak tylko mogli, unikać tego tematu. W sensie być może, gdyby nie mieli żadnego sprzeciwu społecznego, toby to zrobili, ale chyba wyglądało na to, że z koniem się kopać nie chcą. Ewentualnie takie bardziej jakieś odłamy katolicko-chrześcijańskie w samym PiS-ie to chciały, ale to była tam chyba nieduża grupa, tam przecież połowa tych pisiorów to są jacyś rozwodnicy, ja myślę, że tak naprawdę wewnętrznie to im to zwisa, że kalkulacja była dla nich ważniejsza. Więc dlatego nie jestem w stanie tego zrozumieć, bo wydawało się, że tutaj kalkulacja się absolutnie nie opłaca, w sensie, że można było ten temat mrozić i mrozić, i jeszcze mówić wszystkim, obydwu stronom: "Słuchajcie, nie wolno tego teraz tykać, bo jest epidemia, bo teraz nie czas na to, nie czas na to, nie czas na to". Wydawało się, że to naprawdę... No chyba że tam wewnętrznie mieli takie mocne głosy, które powiedziały, że jak tego nie ruszą, to dojdzie do rozłamu wewnątrz partii. Z zewnątrz trochę trudno wszystko wiedzieć.

**Czyli liczby zakażonych nie śledzisz...**

Wiem, jaka jest, bo chyba wczoraj oglądałem wykres jakiś, ale nie śledzę tego tak, że nie sprawdzam codziennie czy co drugi dzień. Staram się tak właśnie raz na 2 miesiące albo raz na miesiąc obejrzeć wykres, ale to taki trend.

**Gdzie oglądasz te wykresy?**

Najczęściej na TVN24.

**Jak się czujesz z ograniczeniami, które teraz obowiązują?**

A jakie są teraz ograniczenia? Na pewno trzeba nosić maseczkę na twarzy. Na pewno dzieci powyżej 4 lub 5 klasy podstawowej nie chodzą do szkoły i to mnie nie dotyczy bezpośrednio, bo ja nie mam dzieci. Wygląda na to, że te szkoły należałoby zamknąć, w sensie, że wygląda na to, że jeśli chcemy walczyć z epidemią... Chyba że przyjmujemy zupełnie inny model, co też można rozważyć, ale jeśli chcemy walczyć z epidemią, to chyba te szkoły trzeba by było zamknąć całkowicie, tylko zdaje się, że po pierwsze strasznie długo szli w zaparte, że nie będą ich zamykać, więc trochę to jest ciężko przyznać, a po drugie to chyba chodzi o kasę. Raz, że ludzie psychicznie to ciężko znoszą, bo z tymi dziećmi być 24h na dobę, to jednak ludziom jest ciężko, zwłaszcza jak dzieci nie mogą się bawić na podwórku, tylko muszą siedzieć w domu, jeszcze zdalne lekcje to jest naprawdę kłopot. A poza tym chyba chodzi o kasę, o te zasiłki opiekuńcze wypłacane przez ZUS dla rodziców, którzy się opiekują dziećmi podczas ich niechodzenia do szkoły. Więc to wiem. Maseczki na ulicach, w sklepach itd., w biurach pewnie też. Już nie było chyba ograniczeń liczby osób w sklepie, a teraz znowu są, bo widziałem, że się pojawiły w sklepach takie, że tam tu może przebywać tylko 7 osób, tutaj w sklepie może tylko 70. W kościołach chyba też są jakieś ograniczenia, ale zdaje się, że ona są nie bardzo super rygorystyczne, bo nigdzie nie widziałem kolejek. Pod apteką, ale pod apteką u nas na osiedlu czasami się tworzyły nawet przed epidemią kolejki, a teraz chyba po prostu do środka ludzie trochę mniej wchodzą, co w ogóle super, jak będzie zima, to słabo, ale tak ludzie w ogóle powinni... Wszyscy tam przychodzą po jakieś leki na grypę i stoją po 10 osób w tej małej aptece, to nawet spoko, że teraz się nauczyli stać na zewnątrz. Są jakieś ograniczenia liczby osób w sklepie, ale nie widziałem, żeby to było takie super, super znaczące, żeby ludzie jakoś tak, żeby to było bardzo doskwierające. Do Biedronki wchodzi nadal tyle, że i tak się robi ciasno. Nie wiem, od czego jest zależne, chyba od m2 powierzchni sklepu, nie? Bo jeszcze był taki moment, że od ilości otwartych kas czy coś takiego, ale to chyba od m2 sklepu. W komunikacji miejskiej na pewno są jakieś ograniczenia, ale one się chyba nie zmieniły, tak mi się wydaje. Nie wiem, bo nie jeżdżę teraz komunikacją miejską ze względów głównie covidowych. Nie musimy jeździć, więc zrezygnowaliśmy z biletów miesięcznych, a jak zrezygnowaliśmy z biletów miesięcznych, to na jakieś pojedyncze przejazdy po prostu bierzemy samochód.

**Zamknięcie gastronomii, siłowni, basenów itd.**

To, jeśli się mnie pytasz, czy to są słusznie, czy niesłusznie zrobione rzeczy, to jest jedna kwestia. Ale pytałaś, czy mi doskwierają, to ostatnio nie byłem, ale rzeczywiście basen by mi doskwierał, czasami chodziłem na basen. Na siłownię czy jakieś fitnessy to bardzo rzadko chodzimy, kiedyś tam dawniej to moja żona chodziła, ale teraz nie.

**A restauracje?**

My czasami jadamy jedzenie, głównie w dni tygodnia jak pracujemy albo coś robimy i nie mamy czasu albo wieczorami zamawiamy jakieś, ale bardzo rzadko wychodzimy, a teraz w covidzie to bardzo, bardzo rzadko, więc to też nie jest coś, co nam... Nadal możemy zamówić pizzę, odebrać sobie kebaby czy tam falafel raczej, bo jemy bezmięsnie, tam hindusa jakiegoś czy chińskie żarcie, czasem coś zamawiamy, czasem coś jemy, ale bardzo nas to nie... Jakby zamknięte były zupełnie na amen, to byłoby nam czasem przykro albo doskwierało. O, czasem tak imprezowo, że będąc gdzieś ze znajomymi, ale to już sezon też się kończy taki outdoorowy, że idąc gdzieś na mieście, nie wstąpimy do jakiejś kawiarni albo na jakieś piwo, to to może tak. Natomiast to jest taka rzecz, bez której spoko możemy żyć dłużej.

**Coś ci doskwiera w tej całej sytuacji?**

To nie są formalne... Nieformalnie to, że mieliśmy się jakoś z kolegami pospotykać i bym się chętnie na jakieś piwo ustawił czy coś, ale tak trochę jednak się izolujemy, w sensie, że tak staramy się za bardzo... A że pogoda jest kiepska, bo tak normalnie, to byśmy poszli nad Wisłę albo na jakieś ognisko, albo na rowery gdzieś, to to jest na świeżym powietrzu, to tak spoko, ale tak teraz rzeczywiście spotkać się w 5-10 osób, a najczęściej się spotykamy gdzieś... O, wczoraj, co prawda nie mogłem, ale pewnie właśnie bym nie poszedł, wczoraj koleżanka zapraszała nas z okazji jakichś tam urodzino-imienin na piwo do siebie, że tam parę osób przyjdzie, ja nie mogłem, bo coś robiłem wieczorem, ale moja żona powiedziała, że ona i tak by nie poszła i no nie zakaże mi, ale jakbym nie szedł, to lepiej. Ja jej przyznałem rację i mówię, że to ja też już odpuszczę w ogóle, bo się jeszcze zastanawiałem, że i tak wrócę po 21 do domu, to zmęczony będę, to już olewam. Ale nie mam zakazu od państwa, tylko to jest nasza wewnętrzna potrzeba, my nie chcielibyśmy teraz być chorzy po prostu, jak teraz można, to lepiej nie jeździć do szpitala.

**Czyli ze spotkaniami jest tak: spotykasz się ze swoją najbliższą rodziną...**

I tego bardzo długo bym chciał nie odpuszczać, w sensie nie blokować.

**Spotykasz się ze znajomymi w plenerze, pojedynczo. Większych spotkań w domu staracie się unikać.**

No właśnie chyba od tego tygodnia zaczniemy unikać na jakiś czas. Bo coraz więcej jest tego koronawirusa naokoło. W tej najbliższej naszej rodzinie się nie wydarzyło, w dalszej rodzinie np. mamy jedną gałąź, w której ktoś ze szpitala przyniósł i już w tej chwili połowa z nich jest na kwarantannie, i u nas właśnie chcielibyśmy uniknąć tego scenariusza jak najdłużej. Bo też doniesienia są różne, ale wygląda na to, że w służbie zdrowia też nie jest za ciekawie. Raz, żeby to odciążyć dla innych, a dwa, że samemu teraz tam trafić, to też, człowiek podobno 14h w karetce siedział, albo mu nie pomogą, albo przyniesie coś... Mieliśmy w tę niedzielę rodzinny obiad, w związku z tym, że cmentarze były zamknięte i co? Ktoś z nas przyniesie i potem rodziców sowich zakazimy, też wolałbym... Mam takie poczucie, że jak się zakażą za 3 miesiące albo za pół roku, to może wtedy będzie już trochę luźniej w szpitalu.

**Czyli wolicie nie spotykać się z ludźmi ze względu na to, jak funkcjonują szpitale, a nie ze względu na strach przed koronawirusem?**

Ja mam takie poczucie, że to jest trochę nieuniknione. Niech się mylę, ale mi się wydaje, że ta szczepionka to nie nadejdzie tak szybko i wydaje mi się, że prędzej czy później każdy z nas będzie musiał to przejść i rzeczywiście być może jest tylko lepiej to przechodzić w momencie, jak akurat nie ma zapaści w służbie zdrowia.

**W marcu-kwietniu robiłeś zakupy dla całej rodziny, jak jest teraz?**

Teraz tak nie robimy absolutnie, nie ma takiego pomysłu, dlatego że chyba aż takiego nie ma poczucia zagrożenia, mimo wszystko jest mniejsze, a po drugie, to trochę głupio mówić, ale dla mojej... Moja babcia to nigdy się nie zgadzała i zawsze mówiła, że nic nie potrzebuje i zawsze dopiero co była na zakupach, i nie dało rady na niej wymusić różnych rzeczy, ale widzimy, że dla tych... No, moi rodzice jeszcze są przed 60, więc głupio o nich tak mówić chyba, ale powiedzmy, że już seniorzy, głównie moja mama robi zakupy, moja teściowa i moja babcia, no to wszystko jest trudne.. Jak im zakazać chodzić na zakupy i to są takie ich sprawunki codzienne, w związku z tym to jest taka motywacja, żeby wstać, ruszyć się, wyjść z domu, one już są na emeryturach albo na jakichś przedemerytalnych wszystkich rzeczach, więc to jest taki ich naturalny rytm, sposób funkcjonowania, nie mają dużo jakichś takich alternatyw, nie wiem, jak to powiedzieć. Po prostu widzę, że jakbym robił za nie wszystkie zakupy, to po pierwsze to jest dla nas upierdliwe jakoś tam i zżera nam czas i energię, to jeszcze jest chyba trochę niepotrzebne, bo ja mam wrażenie, że one wtedy tak się zamykają w sobie i tak to wszystko służy depresji, i przybieraniu na wadze głównie, jak się siedzi w domu... Nie wiem, czy mnie rozumiesz, ale to jest wtedy taka atmosfera takiego bycia osaczonym. Np. moją teściową to bardzo dotknęło, tak że ja widziałem, że ona się robi taka wystraszona, spanikowana po tych 2 miesiącach siedzenia w domu. Mówiła nam potem przez telefon czy przez Messenger, że już właśnie sobie robi taki spacer wokół bloku. Jest ileś % społeczeństwa, które normalnie zupełnie funkcjonuje, a ona prawie przemykała w tym kwietniu czy maju wokół własnego bloku. Pójście do parku to nie było naprawdę, to tylko ci idioci z rządu to zamknęli te lasy, pójście do parku nie było jakimś mega niebezpiecznym momentem, powietrze tam nie było jakieś skażone w porównaniu do tego, co się w twoim bloku wydobywa z klatki wentylacyjnej. Przecież sąsiedzi, którzy oddychają tym samym powietrzem co my, też mogą mieć koronawirusa obok. Więc po prostu właśnie już miałem takie wrażenie, że tak wtedy zaczęło wchodzić na głowę, że każdy inny człowiek jest zagrożeniem, że świat zaraz się na mnie rzuci i to tak wchodzi na głowę, że właśnie - wiem, że mówimy o zakupach teraz, ale że właśnie staramy się mobilizować i pilnować, żeby przestrzegali jakichś zasad, już się też tego nauczyli w miarę. Nie wiem, co robi babcia. Babcia żyje swoim życiem i jak kamikaze jest gotowa po prostu tam do końca działać, a reszta wiem, że się przejmuje, dba o to w miarę i mam nadzieję, że są w miarę bezpieczni. W związku z tym wydaje mi się, że lepiej zostawić im ten rytm funkcjonowania. A poza tym wszystkie sklepy działają dosyć normalnie, jak była ta najściślejsza epidemia, to rzeczywiście tam było takie pytanie trochę, ile razy chodzić, czy się tam jeszcze coś dostanie, czy nie będzie, a zaraz się coś pokończy i było kupowanie takich dużych ilości na zapas przez chwilę, to wtedy też robiłem te zakupy, bo to po prostu duże ilości. Ale też się baliśmy bardziej, teraz już tak trochę jest bardziej spoko.

**Teraz robisz jakieś zapasy?**

Powtórzę mniej więcej to, co pewnie wtedy: my z natury rzeczy mamy trochę zapasów. To jest taki mniej więcej w miarę naturalny u nas stan, to nie jest jakieś takie wyjątkowe. I mamy taką jeszcze drugą szafeczkę. I oczywiście, na bieżąco kupujemy świeże warzywa, pieczywo. Nauczyliśmy się zamrażać pieczywo od pandemii, to już też opowiadałem chyba i to nam zostało, i chyba zostanie z nami na dłużej, bo to jest mega wygodne, w sensie, że idę raz po 8 bochenków chleba, raz na miesiąc czy raz na 2 tygodnie, i jest bardzo spoko.

**A lista została z wami?**

W takiej formie, jak była, czyli wypełniana bardzo dokładnie, szczegółowo i rozmyślnie na takiej zasadzie, że "bo nie można już pójść do sklepu po małe zakupy", to nie. Ale my zawsze byliśmy z listą i nadal z nią jesteśmy, ale takiego nie mamy strachu w tej chwili, może to się zmieni za chwilę, ale nie mamy strachu o przerwanie tego łańcucha dostaw, więc nie sądzę, żeby zabrakło teraz już papieru toaletowego czy makaronu, czy czegoś tam jeszcze.

**Wspomniałeś o lockdownie, że obiło ci się o uszy, że może się pojawić...**

Obstawiam tak. Skoro byli gotowi zamknąć te cmentarze, skoro populistyczna prawicowa partia jest gotowa zamknąć cmentarze, na które 1.11. uwielbiają chodzić jej wyborcy, ludzie często bardziej nastawieni tak tradycyjnie i przywiązani do takich bardziej namacalnych form wyrazu różnych rzeczy, a niekoniecznie opierający się tylko na tej symbolice i bardziej abstrakcyjnych formach, to jak oni byli gotowi w cudzysłowie zaatakować cmentarze, to znaczy, że inne rzeczy pewnie też są bardzo gotowi zrobić. Pytanie trochę o ekonomię: na co ich stać i kiedy ich ludzie zjedzą, bo strasznie się społeczeństwo wkurzy całe, od lewa do prawa, bo różne rzeczy zaczną padać i nawet jeżeli tylko przejściowo. To jest, tak jak już rozmawialiśmy też kiedyś: ludzie nie mają swoich oszczędności, w związku z tym szybko, łatwo część społeczeństwa może być naprawdę nad krawędzią i ja myślę, że oni to trochę liczą, że nawet jeżeli nie z powodów moralnych własnych, że to kurczę, to będzie na nich, jak zrujnują kraj itd., to raczej też na zasadzie kalkulacji, że "ha, no to już nas naprawdę dojadą kamieniami, butelkami z benzyną". A do tego jeszcze dochodzi to, co mówiłem o tej zmianie naliczania tych zgonów czy tam ilości zakażonych. I jeszcze jakieś inne przesłanki mówiące o tym, i liczby samych zakażeń rosną teraz bardzo mocno, więc coś jest na rzecz, coś może się wydarzyć. Myślę, że to jest bardzo prawdopodobne.

**Tylko nie wiadomo, jak ten lockdown będzie wyglądał.**

A ty wiesz? Chodzi o to, że nikt nie wie, bo przecież nikt nie ogłosił, nikt nie pomyślał, żebyśmy zarządzali krajem w ten sposób, żeby ktokolwiek mógł cokolwiek wiedzieć. Przecież w czwartek ci mówi minister, że nie zamkną cmentarzy, więc jeszcze jedziesz w nocy na giełdę kwiatową nabyć towaru i w piątek ci wyłączają te cmentarze, więc nikt nic nie wie. Żyjemy w takiej totalnej tymczasowości, nieprzewidywalności, jakby żyć w związku, w rodzinie z jakimś takim furiatem, który nie wiadomo, kiedy wybuchnie, to tak trochę jest z naszą władzą, w sensie nic nie wiesz, nie ma ustalonych żadnych reguł, więc możemy tylko spekulować. Moja spekulacja jest taka, jakbym miał stawiać pieniądze, musielibyśmy się założyć, to raczej bym się zakładał po stronie, że będą ograniczenia, pytanie jakie, jak mocne, jak dużo.

**Czy godzina policyjna byłaby dobrym rozwiązaniem?**

Nie wiem, chyba bez sensu, wydaje mi się, że nie. Bo w koronawirusie to mamy się nie grupować, to po 22 - nie wiem, od której godziny jest tam ta godzina policyjna, nawet niech od 20 będzie - to gdzie po 20 masz te skupiska ludzi, którzy się wymieniają koronawirusem? Chodzi o to, żeby do domów do siebie nie chodzili? Na imprezy? Lepiej chyba jest wyłączyć te punkty zbiorcze, jakieś sklepy, galerie handlowe czy inne takie rzeczy i to jest skuteczniejsze niż trzymanie ludzi na siłę w domach. To było pewnego typu uproszczenie, w które poszliśmy wszyscy, żeby nam było łatwiej o tym myśleć i żeby łatwiej było zarządzać, że dom jest bezpieczny, a reszta jest zagrożeniem, ale to nie tak. Skupiska zamknięte są największym zagrożeniem. Siedzenie w domu jest bezpieczne, ale nie znaczy, że wyjście na spacer z psem albo jazda na rowerze, albo - widziałem ostatnio, nawet zdziwiłem się, że taka aktywność, ale dla mnie ok w miarę, było takie ukłucie: o, dziwne zgrupowanie, ale potem sobie pomyślałem, a właściwie to pies to trącał, przecież się i tak nie pozarażają od siebie - grupa biegaczy, widziałem taką wczoraj w Ursusie. Stali w kółku i się ewidentnie rozgrzewali przed wspólnym bieganiem w środku nocy, w sensie to nie była noc, tylko 20, ale ciemno już, wszyscy w strojach sportowych i robili jakieś skłony, ewidentną rozgrzewkę przed wspólnym bieganiem. No i takie rzeczy, one są ciut bardziej niebezpieczne niż bieganie samemu, ale nie są dużo bardziej niebezpieczne niż zrobienie zakupów w Biedronce. A czy są potrzebne, bo w tej epidemii chcieliśmy też ograniczać te rzeczy, które... Nawet jeżeli coś jest niebezpieczne, ale konieczne, no to jest konieczne, musimy to robić - robienie zakupów. Ale jeżeli jest niekonieczne, a choć trochę niebezpieczne, to możemy to wyłączyć, nawet jeżeli nie wyłączamy czegoś bardziej niebezpiecznego, bo jest konieczne. I można powiedzieć, dobra, po cholerę ludzie będą biegać, teraz jest epidemia albo coś. No tak, ale ta epidemia już jest z nami pół roku, nie wiadomo, jak długo będzie, odporność ludzi, wszystkich jednostek po kolei, dbanie o układ odpornościowy jest właściwie priorytetową sprawą i to w ogóle szerzej nawet niż w epidemii. Bieganie, chodzenie na spacery, jeżdżenie na rowerze (może nie w smogu, ale na szczęście jeszcze nie ma na razie nad Warszawą) - powinniśmy o to dbać jak najwięcej. W związku z tym zamykanie ludzi w domach przy naszych standardach mieszkaniowych, żeby się ludzie tam kisili i jeszcze teraz będzie zimno, przestaną wietrzyć te mieszkania, obłęd. Godzina policyjna bez sensu, niech ludzie biegają po nocy po parkach, jak ktoś tak lubi. A nawet niech na piwo sobie pójdą na ławeczkę we dwóch, trzech. Nie wolno, bo to alkohol, ale epidemicznie to nie jest żadne zagrożenie, a buduje zdrowie psychiczne albo zdrowie fizyczne. Dla mnie godzina policyjna nie, nie widzę takiej konieczności. Dużo wcześniej bym wyłączył kościoły np. i to jest trudno zrobić, bo niektórym ludziom też to jest bardzo potrzebne, ale po prostu jest przyczepiona do tego siła polityczna, więc nikt tego nie ruszy.

**Skąd się wzięli teraz ci foliarze? Mówiłeś, że w rodzinie też masz.**

Tamta dziewczyna, to jest siostra cioteczna mojej żony, to ona jest już foliarzem od dawna, bo ona jest takim z medycyny alternatywnej, takim antyszczepionkowcem, nie płaskoziemcą, ale antycovidowcem może też jest, nie wiem, nie pytałem jej. Ona tam dziecka stara się nie zaszczepić i jakby co, to pewnie raka będzie leczyć cieciorką. Trochę to jest przerażające, trochę śmieszne a trochę straszne. Skąd to się wzięło? Ja sam się czasem zastanawiam, czy ja też gdzieś nie jestem, w którym momencie ktoś inny powiedziałby, że mój sposób patrzenia na coś jest właśnie foliarstwem, bo jest trochę tak, że nam się wydaje, no musi się nam tak wydawać, że to, co my myślimy, jest słuszne, bo jakbyśmy myśleli inaczej, to byśmy zmienili po prostu zdanie. Ale ogólnie wydaje mi się, że po pierwsze to jest kwestia edukacji i wykształcenia, i złego sposobu edukacji. To nie chodzi o to, że osobom, które uważają, że ziemia jest płaska, nikt nigdy nie powiedział, że ona jest okrągła, bo wszyscy mówili, tylko nikt nigdy nie powiedział ludziom, z czego to wynika, jak doszliśmy do takiego wniosku, jak można to zbadać, zmierzyć itd. To jest taka podstawa, taka gleba, na której wyrosło to foliarstwo, a potem tym zasianiem, tudzież podlaniem tego całego ekosytemu był internet i social media. Teorie spiskowe były zawsze, a teraz ich liczba jest ogromna i ilościowo jest też ich dużo, w sensie liczby ludzi, którzy w nie wierzą i różnorodność tych teorii spiskowych. I to wynika też z tego, że świat się po prostu zrobił bardzo skomplikowany. Mamy mnóstwo ludzi, którzy czują się ekspertami, a nimi nie są, mogą wyrażać swoje zdanie i szerzyć jakieś nieautoryzowane przez środowiska naukowe, każdy w internecie może się wypowiedzieć. Świat jest niezwykle skomplikowany, technologia daje dużo różnych możliwości, ludzie do końca tego nie jarzą, nie obczajają, starają się sobie to wytłumaczyć i potem jest taki problem, że czasami jakaś teoria jest chwytliwa - taka pseudoteoria, to nie są teorie, to są wytłumaczenia często - bo nasz umysł żąda prostych, szybkich rozwiązań, a nowoczesne rozwiązania, tak jak z tymi masztami 5G czy innymi rzeczami, się wymykają takiej prostej percepcji. I to chyba dlatego. Słaba edukacja, internet, każdy może się wypowiadać we wszystkim, plus tematów jest mnóstwo, ludzie mają czas, żeby się tym zajmować, więc to dlatego.

**Twoje przekonania dotyczące koronawirusa mogłyby zostać uznane za foliarskie?**

Nie, tak ogólnie raczej to była uwaga. Myślałbym pomyśleć nad tym, co jest takiego specyficznego, w co ja wierzę, a w co nie wierzą inni, wierzę albo co uważam. Na pewno coś by się znalazło, np. jeżeli chodzi o klimat albo przyrodę, tu mam swoje przekonania takie, które mnie interesują i przykładam do tego jakąś dosyć dużą wagę, a mam świadomość, że to może być nieprawda i że ktoś inny może uważać, że to, co ja bym powiedział np. o zmianach klimatycznych, jest właśnie jakimś lewicowym bełkotem, że te zmiany klimatyczne są, a ja nie jestem w stanie zagwarantować, że to jest prawda, bo może się mylę.

**Powiedziałeś, że szczepionka nie będzie szybko...**

Takie jest moje przekonanie, ale nie...

**... czyli jakimś rozwiązaniem jest, żeby przejść tę chorobę.**

To będzie taka konieczność, że nie unikniesz tego, bo zdaje się, że w końcu wyszło na to, że tak jak z grypą, to jest taki szczep wirusa, że zdaje się, że to nie jest jak świnka, że raz przejdziesz i po sprawie, masz odporność na całe życie, tylko zdaje się, że to jak z grypą, że można co roku przechodzić, więc odporność jest tylko jakaś tam czasowa, a jeszcze on pewnie będzie mutował ten wirus itd. Więc podejrzewam, że każdy z nas albo większość będzie musiała przejść tego koronawirusa. Nie że to jest sposób.

**Co musiałoby się wydarzyć, żeby móc powiedzieć, że to koniec pandemii?**

Dobre pytanie. Nie wiem. Bo to zależy co też rozumiem przez słowo "epidemia/pandemia". Epidemia od pandemii się różni chyba tylko zasięgiem, więc jeżeli mówimy o Polsce, to nie ma znaczenia, jakiego słowa użyjemy. Kiedy epidemia się skończy? Od strony formalnej wtedy, kiedy ją rząd odwoła, czyli właściwie może to nastąpić w dowolnym momencie, nawet w szczycie zachorowań, znając nasz rząd, czyli tu od strony formalnej możemy się spodziewać wszystkiego. Od strony takiej organizacyjnej... No bo możemy powiedzieć, że wtedy się skończy epidemia, kiedy się skończą zarażenia, ale one się prawdopodobnie szybko nie skończą, być może ten wirus, tak jak grypa, zostanie z nami na zawsze. Mogłoby się okazać, gdyby rzeczywiście był morderczy, to mógłby nas wytruć, tych, którzy są nieodporni, zostaliby tylko jacyś tam odporni, ale on taki morderczy nie jest, więc będzie się z nami kołatał. Więc wygląda na to, że... Myślę, że to będzie tak, że będzie jedna, druga fala jeszcze, potem część z nas poumiera, część się uodporni, tacy, którzy mają bardzo pechowy układ odpornościowy i akurat to ich wybije i potem to się będzie między nami kołatać, i będzie jak z grypą. Epidemie grypy przecież pojawiają się, są te fale zachorowań, które czasami osiągają duży rozmiar i wtedy ktoś już zaczyna je tytułować epidemią. Nie wiem, czy jest jakiś oficjalny próg, od jakiej ilości osób w populacji zarażonych jest to epidemia, ale myślę, że ona się skończy po prostu na wiosnę, tak podejrzewam. Ale zarażeni będą już z nami... No chyba że powstanie rzeczywiście szczepionka, która zakończy funkcjonowanie tego koronawirusa na zawsze, ale w związku z tym, że on mutuje prawdopodobnie, no to chyba nie, będzie jakiś następny, jak z grypą, na grypę też się szczepimy, ale te szczepienia nie wyeliminują całkowicie ani rodzajów... W sensie, bo jest ileś rodzajów, mutuje się ten wirus, a po drugie nie mamy szczepień na wszystkie rodzaje, tylko ewentualnie się szczepimy na jakiś rodzaj i on też przy innej odmianie grypy trochę nam pomaga, bo się te przeciwciała się tam wytwarzają czy coś takiego.

**Jak pandemia wpłynie na świat i Polskę?**

Trudno powiedzieć. Naprawdę nie wiem. Mam czasami wrażenie, że wszystko wróci do normy po prostu za jakiś czas, ale mam wrażenie, że też po prostu świat się cały czas zmienia, i technologia się zmienia, i nasz świat wygląda zupełnie inaczej niż 10-20 lat temu, więc mam wrażenie, że nawet jeśli on wróci do normy jakiejś, to on będzie i tak wyglądał inaczej, bo po prostu nastąpiło takie przyspieszenie niektórych rozwiązań: jakieś dostawy zdalne, praca zdalna, większa robotyzacja, więc to trochę będzie kolejny krok. Część rzeczy zostanie z nami na zawsze, na dłużej, ale to np. były rzeczy, które mi się wydaje, że w perspektywie 10 lat i tak by nastąpiły, tylko nastąpiły już. Wszyscy przecież mówili w końcu, że będą cyfrowi urzędnicy, już nie ma po co chodzić do urzędów i strasznie długo świat z tym zwlekał, i to się tak nie mogło dokonać, a tak to się nagle okazało, że w miesiąc pyk, pyk, pyk i wszyscy zrobili. Niektóre rzeczy z nami zostaną, inne nie, ale co to dokładnie będzie i ile... Czasami jak patrzę na to, co się dzieje, to jednego dnia mi się wydaje, że wrócimy prawie że do normalnego funkcjonowania, a innym razem sobie myślę, że to absolutnie już nigdy nie nastąpi i ileś rozwiązań z nami zostanie na długo.

**Co byś najbardziej nie chciał, żeby z nami zostało?**

Na kilku płaszczyznach można odpowiedzieć na to pytanie. Pierwsza jest taka, co uważam za ogólnie słuszne, a druga jest taka, która mnie dotyczy. Bo np. chodzi o to, że uważam, że nie fair albo niefajne jest zamknięcie siłowni i powinny być otwarte, bo to dobre dla ludzi, nie chciałbym, żeby fitnessy, siłownie, jakieś takie miejsca były zamknięte długo, niepotrzebnie długo i żeby poupadały, bo to jest ludziom gdzieś tam potrzebne i buduje zdrowie, i jest w ogóle fajne. A ja nie chodzę, więc dla mnie to oni mogą wszyscy w cholerę tam się zamknąć, ale czy ja bym chciał, żeby to z nami zostało, ten brak tych siłowni, no chyba nie. Więc teraz jest pytanie, czy np. mówimy o rzeczach, które są dobre lub złe, praktyczne i potrzebne ogólnie społecznie albo np. co ja bym chciał, żeby dla mnie nie zostało. Ja bym chciał najbardziej, żeby totalitaryzm z nami nie został, który się tutaj skrada chyba. O, to bym najbardziej chciał. Już i tak się mówi o jakiejś takiej recesji demokracji na świecie i żeby nie było tak (ogólnoświatowo i w Polsce również), żeby po tej epidemii nie został czasem z nami totalitaryzm jakiś. To bym chciał, żeby nie zostało. A druga, to żebym mógł sobie chodzić, spotykać się ze znajomymi na piwo, do nich do domu. I reszta ma już mniejsze znaczenie, właściwie to są chyba te dwie główne rzeczy.

**Czy myślałeś o tym, jak będą wyglądały tegoroczne święta?**

Mam nadzieję, że nie będzie... Jeśli musiałby już być taki lockdown od strony formalnej... Bo to są znowu dwie rzeczy: jedna to jest, jak to wygląda w kwestii przepisów i od strony formalnej, a druga to jest, co my osobiście robimy czy się nie przejmujemy albo jesteśmy gotowi przekraczać te obostrzenia lub czasami narzucać sobie większe, niż każe rząd. Bo na Wielkanoc można było się spotkać, wszyscy ze sobą mogli, ale myśmy tego nie zrobili. To nie było nielegalne, to było jakieś tam zalecenie, ale myśmy tego nie zrobili, bo się baliśmy o rodziców i w końcu tak wszyscy spędzali głupkowato święta osobno. I wydaje mi się, że strasznie już by musiało wejść nam na głowę i chyba nie nastąpią takie święta, żebyśmy spędzali je wszyscy w rodzinie osobno. Myślę, że będziemy je spędzać razem. I nie mam pewności co do tego, ale wydaje mi się, że my byśmy je spędzili razem, nawet gdyby rząd... No gdyby zapowiedział, że będą łapanki i będą rozstrzeliwali tych, którzy są w Wigilię nie u siebie w domu, to być może byśmy nie zaryzykowali, ale jakby było tylko tak, że tam jakiś mandat za to grozi, to wydaje mi się, że prawdopodobieństwo, że będą ścigać ludzi po domach, jest bardzo małe, więc nawet gdyby to było tak, że znowu odradzają albo zakazują, to pewnie i tak byśmy się spotkali, mniej lub bardziej. Więc myślę, że będzie w pewnym sensie normalnie, w sensie, że my się w tym naszym najbliższym gronie spotkamy. Zwykle spędzaliśmy święta tak, że Wigilia była w najbliższym gronie, a pierwszy, drugi dzień świąt się do jakichś wujków, ciotek jeździło trochę dalszych w odwiedziny. Tych odwiedzin będzie na pewno mniej. Albo my nie będziemy chcieli, albo ktoś z naszej rodziny nie będzie chciał nas przyjąć, tego typu rzeczy będą, ale to tak bardzo nie odczujemy tego. Mam nadzieję, mówię tu o sobie, ale też o mojej żonie, mam nadzieję, że szwagierka przyjedzie z tego Budapesztu, bo to pewnie byłoby dla nas przykre, gdyby jej nie było, a tak to myślę, że w miarę normalnie będą te święta przebiegać.

**Czy w ostatnich miesiącach brałeś udział w jakimś weselu itp.?**

Nie. Ale mieliśmy znajomych - i to nas zdziwiło - którzy chodzili na wesela. Nawet mam wrażenie, że niektórzy byli z tej grupy trochę tak jak ja, czyli w miarę analizujących lub tak jak moja żona, czyli trochę się bojących, łącznie z takimi osobami, które w służbie zdrowia pracują i uważają, że rząd tutaj niedobrze, niedobrze itd., po czym jak z tym AIDS - przespali się z nieznajomym.
