# Supplementary material for: The challenges arising from the COVID-19 pandemic and the way people deal with them. A qualitative longitudinal study
Source: PLoS One. 2021 Oct 11;16(10):e0258133. doi: 10.1371/journal.pone.0258133 (PMC8504766; doi:10.1371/journal.pone.0258133)
Supplement: S1 Dataset — (ZIP) [file pone.0258133.s003.zip › Transcriptions/stage 6/2.6_F_27_single.docx]

**2.6_F_27_single**

**Opowiedz, co się działo od czerwca u ciebie, jak ci minął ten czas.**

Od czerwca wielokrotnie myślałam sobie, że za szybko skończyłyśmy to. Dlatego, że działy się jeszcze rzeczy, których za bardzo nie pamiętam, ale które wydaje mi się, że jeszcze były istotne z punktu widzenia waszego powiedzmy. A o których nie miałyśmy możliwości porozmawiać.

**Może sobie coś przypomnisz.**

Nie jestem pewna, ale mogę próbować. Co się działo? Były wakacje, nigdzie nie wyjechałam na wakacje, byłam w hotelu tylko na weekend.

**Ale ty nigdy nie wyjeżdżasz na wakacje w wakacje?**

Zazwyczaj wyjeżdżam na wakacje. Ale no przede wszystkim trochę zapomniałam, że są wakacje, bo jednak to nie był taki… Zresztą tak naprawdę ja nie miałam nigdy czegoś takiego, że muszę wziąć urlop na wakacje, bo wcześniej pracowałam w przedszkolu, gdzie wakacje po prostu były. Wcześniej jeszcze byłam na studiach i gdzieś tam pracowałam, ale to było tak, że też mogłam sobie robić wakacje, kiedy chciałam. Więc tak naprawdę to były moje pierwsze wakacje, które spędziłam tak naprawdę w pracy. I to trzeba zaplanować sobie te wakacje. A też nie byłam do końca pewna, jak to wygląda. Zresztą wtedy, kiedy myślałam o wakacjach… Nie, nie myślałam. Jakoś po prostu o tym nie myślałam. Nie ze względu chyba na tego wirusa. Chociaż już pod koniec wakacji uznałam, że na pewno nie mogę pojechać nigdzie za granicę. Mimo, że otworzyli już granice i w ogóle, to i tak uznałam, że… Może nie ja, ale rozmawiałam z Filipem. I Filip powiedział, że wolałby nie jechać nigdzie za granicę. I w sumie nie pojechaliśmy.

**A dlaczego on by wolał nie jechać za granicę? Pamiętasz, jak on to argumentował?**

Bał się powrotu generalnie, że może być jakiś utrudniony czy coś. A wolałby nie być w takiej sytuacji, w której na przykład utknęliśmy gdzieś za granicą. I nie możemy pracować zdalnie, bo nie mamy laptopów, trzeba się martwić o ten powrót, kombinować, szukać. I czy to jest warte, żeby być parę dni gdzieś, na przykład we Włoszech, gdzie były tanie bilety, a bardzo dużo zakażeń? No raczej nie.

**To mieliście taką rozkminę wakacyjną. Co jeszcze się działo? Co jeszcze zapadło ci w głowę?**

Wiesz co, my wróciliśmy do pracy dość wcześnie. W czerwcu, wydaje mi się, ale to musiało być po tym, jak ostatnio rozmawiałyśmy. Na początku wrócili sami headzi, bo mieli tak jakby dać taki wzór innym, że oni chodzą, więc jakby… Bo bardzo naszemu zarządowi zależało, żeby jednak pracować nie zdalnie. Trochę ze względów finansowych, że bardzo im przeszkadzało płacenie za biuro, w którym nie jesteśmy. A drugi powód był taki, że po prostu łatwiej się jednak skomunikować i obgadać różne rzeczy twarzą w twarz niż na Zoomie. Więc pracowaliśmy z biura. No i długo nie wiem, co się działo, wydaje mi się, że zupełnie nic się nie działo. I jakoś miesiąc temu może, kiedy te… Aha, jeszcze mogę zaznaczyć, że… Nie, no byłam jeszcze gdzieś tam, byłam u koleżanki w Częstochowie, gdzieś tam zdarzało mi się. Faktycznie, byłam jeszcze u koleżanki w Częstochowie, to w ogóle tam nie myślałam o koronawirusie żadnym i w ogóle nie miało to żadnego wpływu. Byłam też na różnego rodzaju imprezach, byłam na mieście. Nie przejmowałam się absolutnie żadną pandemią. I właśnie czekałam, znaczy no czekałam, duże słowo, ale no czekałam na tysiąc dziennych zachorowań. I to było tak, że… Znaczy no w formie żartu czekałam, jakby tak samo podchodzę do tego, jak podchodziłam wcześniej, czyli, no nie wiem, w formie żartów. Bo nie wiem, co innego można zrobić niż się z tego śmiać. Nie można tego w żaden sposób zatrzymać. I czekaliśmy właśnie z takim kolegą, wysyłaliśmy sobie te statystyki jak było już prawie tysiąc czy coś. I właśnie w pewnym momencie wysłaliśmy sobie, o już, to jest ten moment. I od tego tysiąca zachorowań nie minęło długo, jak już było dwa. A od tego dwa już było nagle 10. I trochę mnie… Znaczy jak powiem przeraża, to uznasz to, że to mnie naprawdę przeraża, nie. Ale jest to trochę niepokojące, ilość zachorowań. Spodziewałam się tego dużo wcześniej tak naprawdę, bo wydaje mi się, ale też nie znam liczb, że powiedzmy, jak rozmawiałyśmy ostatnim razem, no to w Hiszpanii tyle było czy we Włoszech dziennych zachorowań. Więc spodziewałam się, że kiedyś to się może wydarzyć. Myślałam, że to się wydarzy wcześniej. No wydarza się teraz. Niepokoi mnie ilość, jakby różnice pomiędzy jednym dniem a kolejnym, kiedy to są naprawdę ogromne różnice. I kolejnego dnia można się spodziewać rekordu zakażeń. Zdarza mi się jeszcze patrzeć na te statystyki i tak sobie myśleć o tych liczbach tak naprawdę. Bo tam ilość uznanych za wyzdrowiałych osób to jest powiedzmy 100 tysięcy. Czyli tak naprawdę 10 ostatnich dni… Tyle osób, ile zachorowało w dziesięciu ostatnich dniach to jest tyle, ile wyzdrowiało od marca. Czyli jest to troszeczkę niepokojące, jakby ci ludzie w ogóle nie zdrowieli. Jeszcze mogę ci powiedzieć o obostrzeniach na przykład w mojej pracy. Na początku, chyba na początku wprowadziliśmy system zmianowy. Przez tydzień obowiązywał. A jeszcze w międzyczasie albo nawet wcześniej, wcześniej, zakupiliśmy dla wszystkich w pracy przyłbice i był obowiązek chodzenia w przyłbicy albo w maseczce po biurze. Nie tylko po korytarzach, ale też siedzenia przy biurku.

**Ale wy macie open space, nie?**

Tak, częściowo. Potem kolejny był tydzień pracy zmianowej. A potem kolejny tydzień był już całkowicie zdalnie. Znaczy nieobowiązkowy, ale…

**To znowu jesteście w domu.**

Tak. Tak, tak, tak. Od dwóch tygodni. Tylko, że w zeszłym tygodniu jeszcze przychodziłam do biura, bo prawda jest taka, że trochę wygodniej mi się pracuje dlatego, że teraz mam bardzo dużo obowiązków. Dlatego, że stała się jeszcze duża zmiana w moim życiu niezwiązana z koronawirusem, ale może warto wspomnieć. Moja szefowa i właściwie jedna osoba z mojego działu oprócz mnie odeszła z pracy i nie ma nowej osoby. Więc oprócz tego, że mam swoje obowiązki, to jeszcze mam obowiązki jej. I znalezienie kogoś na jej miejsce. Ale też dostałam awans i podwyżkę, więc… Ale w każdym razie mam dużo więcej pracy, dlatego wygodniej było mi pracować z pracy. Bo byłam na miejscu i jeszcze była wtedy nasza pani prezes, więc mogłam sobie z nią obgadać różne rzeczy. Czego nie wiedziałam na przykład, to mogłam po prostu twarzą w twarz z nią porozmawiać, bo ona też przychodziła. Ale teraz oni wyjechali na miesiąc do Dubaju, żeby nie być w Warszawie. Więc nie mam już żadnego powodu, żeby przychodzić. Może się wybiorę, bo na pewno wygodniej się pracuje z biura, ale na razie odpoczywam.

**A jak oni powiedzieli, że dobra, wracamy do biura, to co sobie pomyślałaś wtedy? Pamiętasz? Miałaś z tym problem, czy stwierdziłaś OK, no to wracamy.**

OK, wracamy.

**A jak teraz powiedzieli, że dobra, to system zmianowy a potem do domu, to co sobie pomyślałaś?**

Nie, jakby… Zarówno teraz jak i wtedy, wydaje mi się, że to była czymś uargumentowana decyzja. I ta gospodarka i to wszystko musiało wrócić do normalności, więc przyjęłam to za pewnik, że po prostu trzeba to zrobić, trzeba wrócić do biura. I dobrze, bo nie wyobrażam sobie sytuacji, w której cały czas się pracowało zdalnie, można byłoby oszaleć. I nie mówię tu o sobie, ale wydaje mi się, że ludzie mogliby na pewno oszaleć. A teraz jakby, no… Przyklasnęłam powiedzmy tej decyzji, bo to są już duże liczby i naprawdę dużo osób jest chorych. I jeszcze oprócz tego nie tylko dużo osób jest chorych, ale dużo osób jest chorych, ale nie wie o tym. W sensie, że jest tak, że… Z tego, co słyszałam, nie wiem, czy powtarzam głupotę czy nie, ale że nie wszystkie przypadki, które na przykład są gdzieś prywatnie badane, są wpisywane do tego wpisu. Albo ktoś na przykład sam sobie zdiagnozował koronawirusa. Znaczy zdiagnozował, nie ma smaku, nie ma węchu i ma gorączkę, więc może to oznaczać wszystko, ale to tak naprawdę ktoś ma koronawirusa, tylko po prostu nie poszedł do lekarza, tylko siedzi 2 tygodnie w domu. Więc tych osób może być zakażonych dużo więcej niż jest. Poza tym teraz też znam osoby, które są chore. I to nie jest tak, że sąsiad brata, tylko naprawdę znam osobiście te osoby. Więc też inaczej do tego podchodzę.

**Czyli coś się zmieniło w momencie, kiedy zaczęły chorować osoby z twojego otoczenia? Takie, które znasz z imienia, nazwiska. To o to chodzi, tak?**

To jest również taka sytuacja, bo kiedyś po pracy mieliśmy iść do Dzika. Nie poszłam, bo mi się nie chciało po prostu. Nie miałam żadnego wytłumaczenia, po prostu mi się strasznie nie chciało i poszłam robić coś innego. A oni wszyscy poszli i w poniedziałek wszyscy byli chorzy. A potem za parę dni stracili smak i węch. I siedzą w domu. I po prostu strasznie się cieszę, że nie poszłam z nimi. Poczułam trochę bardziej realność tego koronawirusa.

**A jak patrzysz na te liczby, mówisz, że one są już duże. Spodziewałaś się ich w pewnym momencie, myślałaś, że przyjdą wcześniej, przyszły teraz. Jakie to uczucia w tobie wywołuje, że to jest taka liczba? Teraz jest 16?**

No, albo 18. Niepokój. Trochę to jest takie niepokojące, to nie jest coś, co chciałabym coś z tym robić. To nie jest coś, że myślę o tym dużo. Ale jak o tym się dowiaduję, to czuję coś nieprzyjemnego. Zresztą ja też sobie zdaję sprawę z tego, że nawet jak zachoruję, to nie wydaje mi się, żeby coś się stało złego. Nie martwię się też o rodzinę ani nic takiego. Tylko po prostu to wszystko jest ciężką sytuacją. I to mnie po prostu martwi, taka ciężka sytuacja, nie tyle dla mnie, co po prostu dla wszystkich.

**Ale to chodzi o taką napiętą atmosferę wśród ludzi? Jak byś to, ta trudna sytuacja, to co to jest?**

Nie tyle napiętą, co w nie do końca swobodny sposób można żyć. Trzeba pamiętać o swoim zdrowiu. Chciałam powiedzieć, że o ograniczeniach, że ktoś nam coś narzuca, ale właśnie nie do końca. Po prostu trzeba pamiętać o tym, żeby uważać na siebie. Bo o ile te obostrzenia nie są tak drastyczne teraz jak w żadnym momencie wcześniej… Znaczy było bardziej drastycznie niż teraz jest. To po prostu teraz, wydaje mi się, że mam świadomość tego, że te obostrzenia są z jakiegoś powodu. I dużo bardziej się ich pilnuję. Nie narzekam. To znaczy nie narzekam, kupiłam sobie przyłbicę zamiast maseczki. I nie mam na co narzekać, bo jest mi w tym wygodnie, nie parują mi okulary. Mogę oddychać, więc totalnie się do tego przyzwyczaiłam. Do tego stopnia, że jak wychodzę z pokoju, to bardzo często wracam po przyłbicę na przykład, bo mi się przypomina, że trzeba wychodząc ją wziąć, a idę tylko do kuchni. Nie wiem, od czego zaczęłam, ale no może trzeba dbać o swoje zdrowie, coś takiego sobie właśnie myślę.

**Czyli ty nosisz przyłbicę. Zdecydowałaś, że przyłbica jest po prostu wygodniejsza niż maseczka.**

Tak, ale mam jednorazowe czasem sobie założyć.

**Jak jeszcze dbasz o sobie. Co w tym dbaniu jest dla ciebie ważne?**

Jeszcze do tej przyłbicy mogę się odnieść? Dlatego, że jak na przykład jestem w pracy, to wszyscy nie rozmawiamy z bliska. I jak wcześniej mówiłam, że nie rozmawiamy z bliska, to to jest zupełnie inne nierozmawianie z bliska niż wtedy. Bo wtedy mi się wydawało to dziwne. Ale teraz naprawdę nikt nie podejdzie blisko, tylko ktoś na przykład stoi na początku pokoju i mówi. Albo od razu zakłada maseczkę. Jak ktoś na przykład siedzi sam w pokoju, bo teraz pracuje sam i nie ma nikogo z jego zespołu, no to siedzi sam bez maseczki. Ale jak tylko wchodzę, pierwsze co robi, to zakłada maseczkę. I naprawdę ten dystans społeczny jest większy niż 2 metry na pewno. I też widać to naprawdę, że nawet osoby, które nie chciały nosić maseczek albo uważały, że to głupie, teraz po prostu bez dyskusji tę maseczkę noszą.

**Jak rozmawiałyśmy na wiosnę, to mówiłaś, że ten dystans, to bycie daleko od siebie jest takie nienaturalne. Bardzo ci wtedy to przeszkadzało. Pamiętam, że długo o tym rozmawiałyśmy, że to takie dziwne uczucie. A jak się teraz z tym czujesz, przyzwyczaiłaś się już do tego?**

Nie, teraz ciągle jest dla mnie dziwne. Ale teraz czuję taki respekt może do nas? Albo podziw, że mimo tych oporów i że dla nas to jest dziwne jakby pilnujemy tego. Bo tak naprawdę, jak jestem z koleżanką z pracy, mogłybyśmy równie dobrze stać obok siebie blisko, bo nikt nie widzi, bo nikt nas nie kontroluje. Raczej jest marna szansa, że ktoś z nas ma koronawirusa, szczególnie, że robiłam test. Ale po prostu pilnujemy tych zasad, bo po prostu tak trzeba.

**Twoja szefowa pojechała do Dubaju, to co ty sobie pomyślałaś, jak ona wyruszyła w podróż? Nie wiem, czy ona tam pojechała pracować…**

Ja ci mogę wytłumaczyć, co ja poczułam, bo bardzo długo o tym rozmawiałyśmy. Oni tam pojechali dlatego, że… Basia mi wytłumaczyła, że on nie lubi po prostu tej pogody, nie chce siedzieć w domu, nie lubi pracować z domu. Tam mamy też biuro… Nie mamy biuro, ale tam jest spółka dubajska, więc też ma tam co robić, klientów pozyskiwać czy coś takiego. Tam jest, wydaje mi się, że też mniej zakażeń. I woli być w miejscu, w którym jest ładna pogoda po prostu. A i tak tutaj nie zrobi wiele więcej niż zrobi w Dubaju. Jedyne co, to mówiła, że się martwi o rodziców trochę. Ale tak samo nie będzie mogła nic im pomóc w Warszawie jak tam.

**A co ty sobie pomyślałaś?**

Bardzo, niesamowicie się ucieszyłam, bo trochę obowiązków mi odeszło. Albo trochę komunikacja będzie opóźniona, zakrzywiona, więc będę miała czas, żeby robić te rzeczy, które naprawdę muszę, niż odpowiadanie na jej pytanie albo robienie tego, co ona chce. Więc naprawdę bardzo, bardzo się ucieszyłam. Bardzo się ucieszyłam, znalazłam im loty, ogarnęłam im test na koronawirusa tak, żeby wszystko się udało jak najszybciej. I jak wsiedli już do samolotu, to poczułam niesamowitą ulgę. Ale jest to związane z pracą, a nie z koronawirusem, więc nie wiem, czy odpowiedziałam na twoje pytanie.

**Zastanawiałam się, bo teraz spotykałam się z takimi reakcjami, że jak ludzie zaczynają podróżować, że to jest nieodpowiedzialne, bo to jest jednak przemieszczanie się.**

Ja absolutnie nie pomyślałam, że to jest nieodpowiedzialne. Właśnie pomyślałam sobie, że to jest… Nie, no nie odpowiedzialne, ale totalnie normalne, bo… Faktycznie, może się przemieścić, ale zrobiła ten test 96 godzin przed wylotem. I nie miała tego koronawirusa. I będzie tam. I tam będzie przynajmniej miesiąc. Więc to jest tak samo, jak by tutaj łaziła po Warszawie.

**A co cię skłoniło do zrobienia testu?**

Jak pracowaliśmy na zmiany, to prawda jest taka, że ja nie do końca pracowałam na zmiany. Dlatego, że wiedziałam, że coś sobie będę musiała zrobić w biurze czy coś. A i tak siedziałyśmy we dwie z Magdą, z którą i tak się spotykam prywatnie, same w pokoju. Więc za dużej ilości kontaktów z ludźmi nie miałam. I po prostu pracowałyśmy we dwie bez zmian. Zresztą to był jej ostatni tydzień, więc też dużo rzeczy musiałyśmy obgadać we dwie, musiała mi pokazać. Więc wygodniej było, żebyśmy pracowały we dwie z biura. A chłopak jeden ma koronawirusa z pracy. I po prostu wszystkie osoby z tej jego grupy czy te, które miały z nim kontakt, firma nam zapewniła te testy. I przy okazji powiedziałam, że ja, bo ja trochę łaziłam po tym biurze i czy mogę sobie zrobić też w takim razie. Bo to nawet nie chodzi o mnie, że się boję. Tylko po prostu boję się, że ktoś, kto widział mnie, że byłam w obydwu tych grupach naraz, będzie chodził i mówił, że mam koronawirusa i że wszystkich zaraziłam. I wtedy narażę całą firmę na kwarantannę. Dlatego uznałam, że bezpieczniej będzie po prostu ten test zrobić.

**Powiedziałaś, że w wakacje właściwie nie było, pojechałaś do Częstochowy do koleżanki. I były wakacje w hotelu. Na czym polegały twoje wakacje w hotelu?**

Byłyśmy z Dominiką w hotelu. Polega to na tym, że się jedzie do hotelu i się jest w spa.

**A, do spa pojechałyście, OK.**

Nie, bo wiesz co, bo chodzi o to, że na przykład… Bo nie wyszłyśmy z tego hotelu. Pojechałyśmy tam w jakiś tam dzień i wyjechałyśmy, opuściłyśmy teren hotelu, wracając do Warszawy. Więc tak naprawdę to po prostu byłyśmy w hotelu. Nie byłyśmy w żadnej miejscowości, bo nawet nie widziałyśmy nic w tej miejscowości, po prostu byłyśmy w hotelu.

**A gdybyś porównała swoją codzienność teraz do tego, co było przed pandemią, to jakie ty największe zmiany widzisz, które się utrzymały u ciebie?**

Przed pandemią w lutym?

**Tak. Jeszcze zanim to się wszystko zaczęło.**

Szczerze mówiąc nie pamiętam. Po prostu, tak jak już mówiłam ci ostatnio, ale po prostu bardzo płynnie przystosowałam się do tego wszystkiego. I zmiany ogromne, jeśli chodzi o każdy aspekt mojego życia. Ale czy na to miała wpływ pandemia, albo czy mi to jakkolwiek przeszkadza? Chyba nie.

**A jak tam z waszym gotowaniem?**

Przez długi czas gotowałam. Zdarzyły mi się co najmniej 3 tygodnie, kiedy codziennie przynosiłam sobie jedzenie do pracy. Zdarzyły mi się też więcej tygodni, kiedy z Magdą na zmianę przynosiłyśmy sobie jedzenie, w sensie jedna gotowała dla dwóch. I takich tygodni zdarzyło nam się jeszcze 3 co najmniej. Gdzie na przykład zamówiłyśmy zero albo jeden, to jest ciągle tak, że w ogóle jakby zero. Więc takich tygodni zdarzyło się bardzo dużo. I naprawdę przez długi czas tego pilnowałam. I to jest spoko, to gotowanie. Oszczędza się też bardzo dużo pieniędzy, jak się okazuje. Teraz trochę odpuściłam, ale dlatego, że… Nie, nie odpuściłam nawet. Nie. W ostatnim tygodniu tak, ale przez przypadek. Miałam jedzenie gotowe do pracy, tylko po prostu zamówiła mi moja szefowa właśnie ostatnio, bo zamawiała coś i się spytała, czy ja też chcę spróbować. Albo przed wyjazdem powiedziała, czy nie zjemy razem pizzy, bo byłyśmy tylko we dwie w biurze. Ale to nie było dlatego, że nie byłam gotowa. Byłam gotowa na swoje jedzenie.

**A jesteś zadowolona z tego, że utrzymałaś to gotowanie domowe?**

Tak. To znaczy były też dni, kiedy byłam wkurzona na to postanowienie. Bo nie chciałam łamać tego challenge’u. To nawet nie chodzi o to, że… Nie, po prostu nie chciałam, dlatego wracałam wkurzona, zmęczona do pracy. Dlatego, że w pierwszym tygodniu, jak nie było tej Magdy, no to jednak miałam bardzo, bardzo, bardzo dużo pracy. I wracałam zmęczona. I jeszcze trzeba było robić obiad. Potem mi się przypominało, że nie mam cebuli, to była jedyna rzecz, którą miałam kupić, wracając do domu. Nie kupiłam tej cebuli, więc musiałam wyjść. Potem trzeba było robić to jedzenie, nie chciało mi się tego robić. Byłam głodna. Ale musiałam albo poczekać albo zjeść coś z międzyczasie. I jest to trochę frustrujące. Ale da się to przeżyć, to jest też troszeczkę zajęcie sobie czasu. Tak, tak, jestem zadowolona z tego. Teraz troszeczkę czuję, że właśnie odpuszczam. Ale nie wiem dlaczego. Ale mam nadzieję, że…

**A brakuje ci wyjść do knajp albo takiego zamawiania?**

Brakuje mi tego, że te knajpy nie są otwarte. I to nawet nie chodzi o to, że chciałabym coś zjeść konkretnego, tylko po prostu… Gdzieś, nie pamiętam, dokąd my wyszliśmy, ale właśnie miałam ochotę sobie gdzieś usiąść, była ładna pogoda w sobotę. I chciałam gdzieś sobie usiąść i posiedzieć i coś zjeść. Nawet nie to, że byłam głodna, tylko po prostu tak, trochę mi brakowało. Tak ciągle, tak, brakuje mi takiej swobody po prostu. Najbardziej właśnie w tym wszystkim brakuje mi takiej swobody. To mi przeszkadza, że nie mam swobody. I jestem w stanie się przyzwyczaić, mogę gotować w domu, mogę zamawiać do domu. Ale po prostu chciałabym mieć wybór.

**Mówiłaś, że też trochę wychodziłaś, chodzicie do Dzika. Jak byś porównała takie swoje życie towarzyskie, na przykład wychodzenie, spotykanie się ze swoimi znajomymi z tym, co teraz i z tym co w zeszłym roku, jak to jest?**

No do miesiąc temu nie widziałam żadnej różnicy, absolutnie żadnej. Bardzo swobodnie sobie funkcjonowałam. A teraz nie wychodzę po prostu.

**Nie wychodzisz?**

Nie. Ale nawet nie ma gdzie, jest zimno. Ale no właśnie, też jest zimno, więc trochę też się nie chce. I też mam dużo więcej pracy, więc tym bardziej się nie chce. Naprawdę potrzebuję jednego dnia regeneracji. A jeżeli mam mieć kaca tego dnia, no to wolę go nie mieć.

**Co jeszcze? Jedzenie u ciebie było, te wyjścia towarzyskie. A na przykład jakoś więcej z rodzicami się spotykasz? Czy to jest tak jak było…**

(niezrozumiałe), że pytasz, bo tak. Znaczy w ogóle nie wiem, czy ci opowiedziałam, ale to jest istotne, że nie jeżdżę komunikacją miejską i nie chodzę pieszo, tylko mam hulajnogę.

**Wcześniej jeździłaś taksówkami.**

Tak. A teraz jeżdżę hulajnogą. Znaczy no teraz nie, bo jest zimno i teraz za bardzo nigdzie nie jeżdżę. Ale tak, teraz jeżdżę hulajnogą. I to mi otworzyło troszeczkę drogę do jeżdżenia do rodziców. Bo sobie tą hulajnogą można pojechać szybko, to nie jest długo, nie mam żadnego z tym problemu. Więc tak, z rodzicami widziałam się dużo razy. Znaczy no dużo to na przykład 3 albo 4, ale dla mnie to jest i tak dużo. Ostatnio się pokłóciłam z moją mamą, to też mogę ci powiedzieć, dlatego że moja mama bardzo chciała, żebyśmy poszli na cmentarz. Ja w ogóle nie chcę iść na cmentarz. Główny powód jest taki, że po prostu nie chce mi się tam iść. I w zeszłym roku nie byłam na cmentarzu. I mamie było bardzo przykro i czy pójdę w tym roku. No ja mówię, dobrze, pójdę. Potem się okazało, że to nie jest jeden cmentarz, tylko to są dwie niedziele z rzędu, więc mi się to nie podobało, bo się zgodziłam na jeden. W każdym razie spotkaliśmy się z babcią. I to mi się nie podobało, do mojej mamy w ogóle nie docierają te argumenty. Bo też nie do końca mam te argumenty. Bo nie mogę powiedzieć, że się boję, skoro się nie boję. Nie chcę okłamywać w taki sposób mojej mamy. Zresztą trochę ją okłamałam i potem złapała mnie wielokrotnie na tym, że gdzieś wychodzę a nie chciałam iść na ten cmentarz. Ale to jest po prostu niekomfortowe dla mnie, chodzić po dworze w tej przyłbicy. Ja wiem, że mówiłam, że to jest spoko. I to jest spoko, o ile muszę zrobić rzeczy, które naprawdę muszę zrobić albo chcę zrobić. A chodzenie po cmentarzu w przyłbicy to w ogóle nie należy do tych rzeczy. Ale widziałam się z rodzicami na pewno więcej… Więcej niż bym planowała na przykład. Właśnie wydaje mi się, że odkąd można było wychodzić, to wydaje mi się, że dużo więcej czasu spędzałam z rodzicami.

**A jak oni w tej całej sytuacji teraz się odnajdują?**

Moja mama super. Dlatego że spodobało jej się bardzo pracowanie zdalne. I nawet rozważa w ogóle niepracowanie, tylko bycie w domu, bo jej się tak bardzo spodobało. Ona zawsze o tym myślała. A teraz, jak zobaczyła na żywo przez długi czas, jak się fajnie siedzi w domu, to rozważa w ogóle niepracowanie już nigdy, tylko siedzenie w domu. Znaczy zajmowanie się domem.

**A co twoja mama robi?**

Coś z ubezpieczeniami. Pracuje w Avivie, ciężko powiedzieć, co ona tam może robić. Ale jakiś tam ma zespół, coś, tego typu. I spodobało jej się bardzo siedzenie w domu. A mój tata zawsze pracował dużo z domu, więc teraz nie robi mu to różnicy. No i tak normalnie. A nie, mój tata to w ogóle mówi, że denerwuje go bardzo ta pandemia.

**Ale denerwuje go co w tej pandemii?**

Chyba, że jest. Bardzo się buntuje przeciwko obostrzeniom, z tego co zauważyłam. Nie to, że nie nosi tej maseczki. Ale mówi, boże, ta maseczka… I trochę nie wierzy może w tą pandemię? Nie mogę powiedzieć, że nie wierzy, bo zdaje sobie sprawę. Zresztą on też chyba robił test albo zna kogoś, kto ma tego koronawirusa. Tak że nie jest tak, że nie wierzy, ale że jakby albo nie chce przyjąć do wiadomości albo po prostu już jest tym zmęczony.

**Masz jeszcze kogoś w swoim otoczeniu, kto jest zmęczony tą całą sytuacją?**

Nie, chyba aż tak jak mój tata to nie.

**A Filip jak w tym wszystkim teraz?**

Filip wrócił do pracy dużo później niż ja, chyba w sierpniu. I bardzo bał się tego powrotu. Pamiętam właśnie, że siedzieliśmy dzień wcześniej i on mówił, że nie wyobraża sobie w ogóle bycia w tym biurze. I siedzieć 8 godzin, to dla niego w ogóle coś, czego nie będzie w stanie zrobić. Ale po jednym dniu okazało się, że było wspaniale i kocha chodzić do biura. I mówi, że super, że z tymi ludźmi się widzieć i super z nimi rozmawiać. Czego ja w ogóle nie rozumiałam, bo już długi czas byłam w biurze wtedy. I bardzo mu się podobało. I nawet teraz, jak mogli pracować zdalnie, tak że mogli przychodzić albo nie przychodzić, to przychodził do biura.

**Powiedziałaś, że trochę jednak sprawdzasz te statystyki, zerkasz, ile tam ich jest. Jest tak, że w ogóle śledzisz w mediach te wszystkie doniesienia, gdzieś czytasz o tym?**

Tak, tak, tak. To znaczy czytam to, co jest napisane, może tak. Czytam to, co mi się pojawia. A jak mi się pojawia o koronawirusie, to o nim czytam. A jak mi się pojawia o protestach, no to o tym czytam. Jak teraz jest więcej o protestach, to niestety nie czytam o koronawirusie, bo nie ma żadnych informacji.

**Ale to jest tak, że na przykład jak Onet czy wirtualna, nie wiem, jakie strony przeglądasz, to jest tak, że wyłapujesz sobie, co politycy mówią, czy co ludzie mówią, czy co lekarze mówią? Jakieś masz takie konkretne rzeczy, które cię bardziej przyciągają?**

Nie, nie, nie. Mnie najbardziej interesują te dzienne statystyki. To jest rzecz, która mnie najbardziej interesuje. Bo mam jakiś taki ogląd tej sytuacji. Nie wiem, czy lekarze, politycy albo ludzie mogą mi powiedzieć coś więcej na ten temat. Ale sama jestem w stanie sobie ocenić, jak sprawa wygląda, jak widzę, ile osób zachorowało, ile osób wyzdrowiało, ile umarło. I w którą stronę to idzie. Ale wczoraj oglądałam i Wiadomości, i Fakty dla porównania. Więc mam jakąś świadomość tego koronawirusa.

**A w ogóle masz wrażenie, że w mediach jest dużo koronawirusa?**

Tak. Tak, na pewno ostatnio było, jak weszły te obostrzenia większe niż żółta strefa. Chociaż nie, właściwie Warszawa w żółtej strefie to już było takie raczej… ważna sytuacja. I od tej pory było bardzo dużo. A szczerze mówiąc muszę ci powiedzieć, że tak bardzo mnie nie interesowało koronawirus, że nie do końca wiedziałam, co to znaczy być w żółtej strefie jak Warszawa weszła to żółtej strefy. Jakby wiedziałam, że są jakieś strefy. Ale zupełnie nie wiedziałam, co to oznacza. I musiałam sprawdzić.

**I gdzie wtedy sprawdzałaś?**

Wpisałam w wyszukiwarkę żółta strefa Warszawa i przeczytałam.

**A poza tym, że denerwuje cię ten brak swobody, bo rozumiem, że to jest twój największy problem, że nie możesz zrobić tego, na co masz w danym momencie ochotę, bo są różne obostrzenia.**

Nie mogę zrobić czegoś spontanicznie, tak. I to nie chodzi o żadną konkretną rzecz, tylko… No nie mogę po prostu zrobić nic spontanicznie. Nie wiem, nawet tak abstrakcyjna rzecz jak wyjechanie gdzieś na weekend za granicę, wiąże się z tym, że nie mogę, bo muszę zrobić dużo wcześniej test. I to nie jest takie hop siup. Nie to, żebym to planowała. Ale właśnie chodzi o to, żebym mogła tego nie planować.

**Czyli ty byś chciała tak sobie myśleć, o, dobra, tani bilet, to polećmy na weekend. Tak?**

Tak.

**Wsiąść w samolot, polecieć i wrócić po 2 dniach. I mieć weekend gdzieś.**

Na przykład.

**A jeszcze coś jest, co tak ci… Nie chcę powiedzieć, że się nie podoba, to chyba jest złe słowo. Przeszkadza ci. Czy jeszcze coś przeszkadza ci w takiej codzienności?**

Głównie przeszkadza mi to i przeszkadzają mi też w sumie trochę decyzje rządu, trochę ich nie rozumiem… Nie wiem, dlaczego na przykład dzieci nie mogą chodzić bez opiekuna dorosłego od 9 do 16, czy tam do 17. I to tylko w dni robocze. Dlatego, że dzieci chodzą też w weekendy i też po 16 mogą to robić. Nie wiem, w jaki sposób to na przykład ma powstrzymać koronawirusa w jakikolwiek sposób. Może to chodzi o to, żeby dzieci były naprawdę na lekcjach zdalnych. Ale nie wiem, nie rozumiem tego. No, trochę tej decyzji rządu nie rozumiem, dlaczego nie można wprowadzić… Znaczy rozumiem, dlaczego nie można wprowadzić takich obostrzeń, jak były wcześniej, bo nie mamy na to możliwości gospodarczo. Ale dziwne są te rozwiązania. Dlaczego na przykład zamknięte są siłownie, a salony kosmetyczne już nie? Nie wiem. To mi trochę przeszkadza, że to jest trochę takie po omacku wszystko. I trochę też widzę, że bardzo zachowawcze są te decyzje. I jak na przykład podczas pierwszej fali koronawirusa rząd rozdawał pieniądze na prawo i lewo, wszystkim dawał pożyczki bezzwrotne, postojowe, zapomogę na coś, tak teraz też jest jakaś pożyczka, ale nie dla wszystkich, tylko na miesiąc, tylko jak wykażesz coś tam. Ja rozumiem, dlaczego tak jest, że to zmniejszy budżet zdecydowanie i już dużo pieniędzy zostało rozdanych. Ale trochę jestem może zła na to. Dlatego, że trzeba było patrzeć trochę bardziej w przód. Znaczy no nikt nie był w stanie tego przewidzieć. Chociaż może ktoś właśnie był.

**Jest jeszcze coś, co tak ci przeszkadza? Może być tak, że to ci przeszkadza, to cię złości. W takim codziennym funkcjonowaniu, jak sobie myślisz o takiej swojej codzienności. Masz takie coś, co sobie myślisz, kurde, już mogłoby nie być.**

Nie, no to ograniczenie swobody dotyczy wszystkiego zupełnie. Mogłabym powiedzieć, że już mogłabym nie nosić tej przyłbicy. No tak, ale to wiąże się z tym, że to nie jest swoboda i muszę w niej chodzić. Albo że te godziny dla seniorów. Chociaż to też się okazało, że to jest tylko w tygodniu, więc aż tak mi to nie przeszkadza. I co mogłoby nie być jeszcze? Jakieś są ograniczenia w sklepach. Ale w sumie nie jestem pewna, bo byliśmy w Złotych Tarasach i tylko do jednego sklepu musielibyśmy stać w kolejce. I po prostu zrezygnowaliśmy. Co jeszcze? No dzisiaj jeszcze byłam u okulisty, u optyka. I też musiałam czekać w kolejce, bo mogła być tylko jedna osoba. To mi też trochę przeszkadzało. Ale w sumie i tak, jak przymierzałam okulary, to miała zdjętą tą maseczkę. Więc nie wiem, trochę mi przeszkadza taka niekonsekwencja ludzi. Mimo, że mówiłam o tym, że respekt i w ogóle ludzie tego pilnują. No to nie w każdym miejscu da się tego pilnować. A, no i jeszcze byłam u lekarza, nie pamiętam jakiegoś, chyba dermatologa. I też w taki kretyński sposób się siedziało, czyli 2 metry od siebie. I nawet ta pani do końca nie wiedziała, co mi się stało z tym palcem, bo siedziała tak daleko. A, byłam też u ginekologa, ale było całkowicie normalnie.

**Emocje – zdjęcia.**

Ja chyba wiem, 2.

**Ale 2, to jest teraz, jak się czujesz?**

Tak.

**A jak byś pomyślała o momencie, kiedy się dowiedziałaś, kiedy zobaczyłaś, że stuknęło 10 tysięcy dziennie.**

I tysiąc i 10 tysięcy to jest to samo dla mnie. I może 1 albo 9. Bardziej 1. Albo 15. 15, bo tutaj ten komunikat mi zasłonił. 15.

**15. Na te liczby.**

Tak, tak. 15.

**To opowiedz mi najpierw o tym 15. Jak były te liczby, co to jest za emocja, jak ty się poczułaś wtedy?**

Że coś wyrywa się spod kontroli. I uderza ze zdwojoną siłą, na co nikt nie jest gotowy do końca. No właśnie miałam świadomość, że nie jesteśmy gotowi. Chociaż trochę też mam wrażenie, że ludzie, nawet nie tyle rząd, co ludzie wynieśli z pierwszego lockdownu powiedzmy, że teraz są trochę bardziej gotowi na to. No nawet to, że kupiłam sobie przyłbicę, a nie maseczkę. Bo wiedziałam, jak to bardzo denerwuje. Więc po prostu uznałam, że będzie mi wygodniej. A muszę się przyzwyczaić do chodzenia w ten sposób. Też wydaje mi się, że ludzie racjonalniej robią zakupy. Na przykład nie brakuje już rzeczy na półkach. Co tak naprawdę jest zaskakujące, bo powinno brakować jeszcze bardziej. Dlatego, że wtedy, w lutym, powiedzmy w marcu było dużo mniej zachorowań. Ta pandemia była dużo mniej spotykana na ulicy powiedzmy. Dużo trudniej było się zarazić niż teraz. A jednak ludzie pozamykali się w domach i wykupili totalnie wszystko. Co teraz jest to dziwne, że ludzie tak nie panikują. Ale nie panikują. Więc może dlatego, że są przygotowani na to. Znaczy nie to, że mają zapasy, tylko po prostu wiedzą, że te produkty ciągle będą i będzie się dało iść do sklepu. Ale w dalszym ciągu uważam, że 15, dlatego że coś się jednak wyrywa spod kontroli. I coś idzie do nas ze zdwojoną siłą. I może się stać coś niedobrego.

**OK, ale jak coś się wyrywa spod kontroli, to jak się ty wtedy czujesz? Jak określisz swoje emocje albo to, co tam w tobie siedzi w środku? Bo to nie tobie się wyrywa spod kontroli, tylko w ogóle się wyrywa.**

No właśnie to jest problem, że to się nie mi wyrywa i nic z tym nie mogę zrobić. Więc jak się czuję, no…

**Ale to jest tak, że masz takie poczucie bezsilności?**

Nie, nie. Ja nie uważam, żeby to w ogóle było moim obowiązkiem cokolwiek z tym robić. Moim obowiązkiem jest powiedzmy uważanie na siebie i uważanie, żebym nie zaraziła innych ludzi, to OK. Ale czy w jakiś sposób ja mam sama zwalczać koronawirusa? Nie wiem, nie czuję się w obowiązku jakkolwiek…

**Ale gdybyś miała znaleźć najbliższe uczucie temu, jak ty się czujesz w momencie, kiedy widzisz, że sytuacja wymyka się spod kontroli.**

Tak wewnętrznie czuję taki niepokój, coś tam może mnie uciśnie w żołądku. Może trochę się zastanowię. Ale to nie są jakieś takie silne emocje, które mną szargają. Dlatego, że po pierwsze wiem, że nic z tym nie zrobię. Znaczy no oprócz tego pilnowania siebie itd. A po drugie też nie widzę w tym jakiegoś zagrożenia. I na przykład jak poprzednim razem się troszeczkę martwiłam o ten kryzys gospodarczy, teraz też się martwię, ale teraz trochę inaczej. Bo wtedy też patrzyłam przez siebie na tą sytuację. A teraz na szczęście przez to, że jestem sama w pracy, jakby mam pewność stuprocentową, że nie zostanę absolutnie nigdy zwolniona. I nawet jak nasza firma upadnie, to jakby wiem, że Basia ma kolejny pomysł na biznes. I wątpię, żeby była taka sytuacja, że nie będzie chciała mnie zabrać ze sobą. Prawdopodobnie będę pierwszą osobą, którą weźmie ze sobą. Dlatego… Dostałam też podwyżkę taką, że w ogóle się nie musisz o pieniądze. I nawet przy obniżce pensji i tak będę mogła odkładać pieniądze. Więc jakby nie martwię się o ten kryzys przez to, że no mnie po prostu nie dotyczy. Naprawdę nie wiem, co by się musiało wydarzyć i co by musiało upaść, żebym czuła się personalnie zagrożona czymś.

**Czyli tak stabilnie finansowo i jakby tak życiowo się czujesz zabezpieczona. A powiedz mi o tej 2 w takim razie. Bo to jest to, jak teraz się czujesz.**

No takie… Wdepnęliśmy w gówno, coś takiego. Że można było patrzeć pod nogi i przygotowywać się… znaczy patrzeć pod nogi. Jeśli chodzi o wejście w gumę, no to nie jest do końca analogiczne, dlatego że nie możesz się uchronić przed tym bardziej niż patrzeć pod nogi. A wydaje mi się, że… No, mogliśmy patrzeć pod nogi po prostu. Bo, tak jak mówię, w wakacje nie czułam w ogóle, że jest jakikolwiek koronawirus, że trzeba w jakikolwiek sposób pilnować siebie albo innych. Ludzie jeździli na wakacje, robili różne rzeczy takie… No po prostu nie pamiętali o tym, że jest koronawirus w jakimkolwiek stopniu. Nie mieli na uwadze z tyłu głowy ani nic. Wydaje mi się, że nas zaskoczył ten wzrost zachorowań.

**Ale nas, czyli ludzi? Społeczeństwo?**

Tak. Nas czyli Polskę. Wydaje mi się, że nie byliśmy na to tak gotowi, jak moglibyśmy być. Dlatego teraz są przepychane jakieś ustawy, które powinny tak naprawdę wejść w życie dużo wcześniej. Jak ta, że nie trzeba być… Nie wiem dokładnie, ale chodzi o to, że osoba, która nie ma absolutorium czy nie ma czegoś medycznego… Czy ma dyplom, ale nie wiem co, może pomagać, czy tam może leczyć. Też branie teraz na siłę studentów piątego roku medycyny czy tam szóstego do przymusowej pracy w szpitalach… Wydaje mi się, że można było to zaplanować w jakikolwiek sposób inaczej. Ale właśnie też dlatego to jest to wdepnięcie w gumę, dlatego że teraz już się stało, to ciężko coś z tym będzie zrobić.

**Tak już się przykleimy do tej gumy.**

No tak. Ale też no taka analogia z tą gumą jest też taka, że tą gumę ciężko jest zdrapać. W sensie trzeba zamrozić i potem skrobać, ale to nie do końca wiadomo, czy to wyjdzie, to będzie brudne. A jeśli chodzi o gumę w ogóle na bucie, no to jak się przyklei i już potem się chodzi bardzo długo, no to jest problem już na całe życie. Znaczy no trzeba wyrzucić buty. A Polski się raczej nie da wyrzucić.

**Jak rozmawiałyśmy na wiosnę, to ty mówiłaś, że ty właściwie się nie boisz. W sensie nie miałaś w sobie lęku. On się w ogóle pojawił w jakimkolwiek momencie od tamtej pory?**

No przy tym wzroście zachorowań tak. Jeśli chodzi o chorobę, to nie, nie, nie. Chociaż trochę, jak ci moi znajomi byli w Dziku, a ja nie byłam, a potem oni, jedno z nich chyba przyszło do pracy czy coś. To sobie tak pomyślałam, może ja też jestem chora? I tak przy każdym jedzeniu myślę, czy na pewno mam smak. Mam, ciągle mam. Trochę się nawet rozczarowałam, że nie mam tego koronawirusa, szczerze mówiąc.

**Jak to?**

No, coś innego by było. Coś nie wiem, ktoś by mi może współczuł. To są takie jakieś egoistyczne bardzo powody, nic konkretnego. Albo mogłabym po prostu trochę odpuścić i nie pracować aż tak, bo mam koronawirusa.

**Ale masz takie trochę napięcie czekania, kiedy się zarazisz?**

Wydaje mi się, tak naprawdę, jak rozmawiałyśmy, że ja miałam już tego koronawirusa. Bo naprawdę wydaje mi się, to jest niemożliwe. Ja rozmawiałam z ludźmi z pracy, którzy tego koronawirusa mają, mimo że tam nie zrobili tych testów. Jakby są przekonani, że mają koronawirusa, ja rozmawiałam z nimi. Nie mieli maseczki. Bo pod koniec dnia, jak już nie było nikogo i siedziałyśmy we 3 powiedzmy, to ta dziewczyna, która ma tego koronawirusa teraz, zdjęła maseczkę, rozmawiałyśmy w normalny sposób. Nie jakoś super blisko siebie, ale też nie daleko. Siedziałyśmy przy jednym biurku powiedzmy, tylko po dwóch stronach. Czy to jest możliwe, żeby naprawdę się nie zaraziła? Albo mam jakąś super odporność albo już tego koronawirusa miałam wcześniej. Bo ciężko jest w to uwierzyć, że przy takiej ilość kontaktów z różnymi ludźmi, nie miałam tego koronawirusa. Szczególnie, że jak wchodzę do sklepu, to nie dezynfekuję tych rąk za bardzo. Nie pamiętam o tym.

**A skąd ci ludzie, bo mówisz, że oni mają koronawirusa, nie robili testu, ale wiedzą, że mają koronawirusa. Skąd oni to wiedzą?**

Mają gorączkę, nie mają smaku ani węchu. I też każda osoba, z którą rozmawiają. Na przykład chłopak tej jednej dziewczyny, też po jakimś czasie, po 3 dniach nie miał smaku ani węchu i też ma gorączkę.

**Czyli myślisz sobie, że musiałaś już mieć przy takiej ilości kontaktów?**

No, albo mam pecha albo właśnie farta.

**To masz pecha czy farta? To wolałabyś już mieć tego koronawirusa, już być po czy nie?**

Nie, to nawet nie chodzi o bycie po, tylko po prostu o fakt posiadania. Nie wiem, bardzo ciężko to jest wyjaśnić i bardzo głupio to brzmi, jak mówię to na głos. To znaczy takie wewnętrzne myśli. Ale no, coś byłoby to innego po prostu. Bo ja się w ogóle nie boję, że coś mi ta choroba zrobi. To jest jak każda inna choroba. A prawda jest taka, że ja też nigdy nie chorowałam na nic zupełnie. Ani nie miałam świnki, ani różyczki, ani ospy. Mój brat, mieszkając w jednym mieszkaniu, miał ospę. A ja nie miałam tej ospy. Więc nie wiem, nie miałam żadnej nigdy choroby. Więc nie wiem, jak to się czuje. Może po prostu chciałabym poczuć jakąś chorobę.

**Chciałabyś poczuć jakąś chorobę. OK.**

No tak, ale to jest do takiego stopnia, że to nie jest taka choroba, którą naprawdę nikt by nie chciał mieć. Tylko właśnie wiem, że coś takiego, co się ma i przechodzi zaraz po dwóch tygodniach.

**Czyli jak się dowiedziałaś, że znajomi, z którymi nie poszłaś do Dzika się źle czują, to miałaś takie dość ambiwalentne te odczucia? Z jednej strony się ucieszyłaś, że nie byłaś, a z drugiej tak…**

Nie, no ucieszyłam się z rozsądku i generalnie wiem, że dobrze nie mieć koronawirusa. Ale z drugiej strony coś nowego. Ale to też nie jest tak, że jestem osobą, która chce mieć koronawirusa. Nie, tylko po prostu coś by się zmieniło. Trochę też mogłabym odpuścić po prostu, bo mam koronawirusa.

**Czyli to byłby taki pretekst do zwolnienia na przykład? Takiego w sensie slow down, że nie muszę tyle pracować, będę odpoczywać?**

Tak.

**A jak w pracy się okazało, że ten chłopak ma i tam stwierdzili, że będą testować, to co sobie wtedy pomyślałaś?**

Wiesz co, okazało się, że on był w pracy od 10 do 12. I możliwe, że go widziałam z daleka. Co sobie pomyślałam? Że wiem, że będzie straszna panika w pracy, a wiem, że będą do mnie z tym przychodzić, ja nie mam nic do powiedzenia na ten temat. I nie lubię po prostu… O, na przykład denerwuje mnie trochę, jak ludzie panikują z powodu koronawirusa.

**To opowiedz mi o tym, jak cię to denerwuje. Co cię najbardziej w tym denerwuje? Jakie zachowania ludzi?**

Najbardziej takie właśnie zachowanie, że na przykład koniecznie muszą pracować z domu, bo się boją. Albo, że muszą mieć test, bo mieli kontakt z osobą, która miała kontakt z osobą, która ma koronawirusa. Nie, po prostu ja nie rozumiem tego strachu za bardzo. To mnie denerwuje, bo ja po prostu tego nie rozumiem. Nie jestem w stanie się postawić w ich sytuacji zupełnie. Znaczy w ich sytuacji jestem, tylko nie umiem poczuć tego strachu w żadnym wypadku. To jest dla mnie niezrozumiałe. Bo wiadomo, widać, ludzie umierają na koronawirusa. Ale też widać, ile osób siedzi w domu po prostu i potem wraca do funkcjonowania i się zupełnie nic im nie dzieje. I zwykłej grypy się ludzie tak nie boją.

**Czyli dla ciebie takim porównaniem czy to jest bardzo źle czy nie, to jest jakby trochę, że no umiera trochę osób, bo tam codziennie podają te liczby. Ale z drugiej strony masz takie poczucie, że jakby ileś osób choruje, nawet jeżeli nie ma tego testu, ale ma objawy koronawirusa. I potem jakby i tak dochodzi do siebie. I tych jest dużo więcej.**

No tak. Tak. Nie przytoczę żadnych statystyk, ale raczej to nie jest tak, że umiera połowa społeczeństwa lub połowa osób, która ma koronawirusa. Albo nawet jedna trzecia. Tylko dużo, dużo mniej. I to są zazwyczaj osoby starsze. Wiadomo, trzeba się bać o osoby starsze, jeżeli ma się kontakt z dziadkami albo z rodzicami. No to faktycznie warto na to zwrócić uwagę i się z nimi nie spotykać. Ale mam wrażenie, że te osoby nie boją się o siebie, tylko się boją właśnie… Znaczy się boją o siebie właśnie. I wydaje mi się, że to jest takie robienie z igły widły.

**Czyli to jest trochę tak, że to młodzi ludzie robią tak trochę niepotrzebnie zamieszanie wokół tego?**

Ja nie mówię, że wszystkie młode osoby, ale że są takie osoby.

**Bo chodzi mi o to, że twoja obserwacja bardziej dotyczy ludzi młodszych niż tych bardzo starszych, których rzeczywiście może to mocniej dotknąć, tak?**

Nie znam tych bardzo starszych.

**Ale dobra, masz takie poczucie, że gdyby to była pani, która ma 60-70 lat i by myślała w ten sposób, to sobie myślisz, że to jest jakoś usprawiedliwione, że ona tak myśli i ona się bardziej boi?**

Tak. Tak, tak, tak.

**Bo zagrożenie dla niej jest realne bardziej, bo więcej starszych osób umiera niż młodszych osób. OK, dobra. A myślisz, że takie chronienie tych starszych przez niespotykanie się czy to z rodzicami, czy to z dziadkami, czy to jest dobra droga?**

Przyznam ci się szczerze, że jest dla mnie wygodną drogą, dlatego będę ją w jakiś tam sposób popierać. I argumentować tym, że mi się nie chce z nimi spotkać tym, że się boję o nich. No, ale faktycznie, no słabo by było, gdyby moi rodzice zachorowali i umarli. Więc no tak. Ale czy to jest dobra droga? Tak, no nie widzę innej niestety. Niestety nie widzę innej drogi.

**A jak ci twoi znajomi, rozumiem, że ten chłopak z pracy, to miał test, tak? W sensie było wiadomo, że on ma tego koronawirusa i dlatego was przetestowali wszystkich?**

Tak.

**A na przykład kazali wam wtedy tą aplikację, namawiali do tych rządowych…**

Nie, bo to też nie były przetestowane wszystkie osoby, tylko osoby z jego działu, które z nim siedziały tego dnia. Jakby osoby, które siedziały z nim w pokoju jednego dnia. I ja, bo łażę po tym biurze cały czas i mogłam akurat mieć z nim kontakt. A jak nie z nim, to z innymi ludźmi. Bo bardzo dużo się przemieszczam po biurze. Ale nikt nikomu nie kazał ściągać aplikacji ani nikt nikomu nie kazał robić tego testu. Właściwie to była tylko sugestia, że jak chcecie, to możemy wam zapłacić za ten test. Ale jakby nie ma przymusu żadnego. I tyle. Absolutnie nikt nie kazał. Bo też moja szefowa jest taka, że ona bardzo nie chce siać paniki. Dlatego też… Trochę też podchodzi jak ja do tego pod tym względem, że właśnie nie nakłania do takich… Jakby zapewnia te wszystkie narzędzia, typu maseczki jednorazowe w biurze, czy jakiś płyn do dezynfekcji i to jest. Ale jakby nie mówi o tym głośno i nie krzyczy, że to jest, żeby właśnie nie wprowadzać takiej pandemicznej atmosfery może. I też była taka sytuacja, że jedna dziewczyna bardzo chciała, żeby dziewczyna na recepcji mierzyła rano temperaturę przy wejściu do budynku. I przez długi czas… Bo jakby przez te wakacje, faktycznie, teraz mi się przypomina, że były osoby, które miały kontakt z osobą, która miała koronawirusa. Albo były osoby, które… Był chłopak, u którego dziewczyny w biurze ktoś miał koronawirusa. Czyli daleko tak naprawdę od tej osoby zakażonej. Ale jednak było jakieś tam prawdopodobieństwo. I to na przykład było bardzo wyciszane u nas w pracy. W sensie, że bardzo nie mówiło się o tym na głos. Tylko te osoby w ciszy szły na zdalne. Żeby właśnie nie wprowadzać takiej atmosfery albo paniki. Bo są też osoby w pracy u mnie, które bardzo panikują. Ale w każdym razie jedna dziewczyna się o tym dowiedziała i zażądała tego mierzenia temperatury na dole. I przez długi czas zarząd właśnie, znaczy przez długi czas, 2 dni powiedzmy, nie chcieli tego robić. Bo to nie jest po pierwsze z jakimikolwiek zaleceniami. Znaczy z zaleceniami tak, ale z zarządzeniem żadnym nie jest, nie ma obowiązku. A to wprowadza taką atmosferę pandemii. Czego właśnie chcieliśmy uniknąć, mam wrażenie.

**Czyli to jest taka bardziej atmosfera pandemii, że się czuje taki lęk, strach ludzi, że oni się po prostu inaczej zachowują?**

Tak.

**A jak myślisz o tych osobach, które wprowadzają tą atmosferę i tak strasznie przeżywają, to oni mają jakieś cechy wspólne?**

Ja nie wiem, czy to są jacyś ludzie, czy to nie jest po prostu tak, że trochę to się nakręca samo przez siebie? Że powiedzmy jedna osoba faktycznie się naprawdę bardzo boi. Bo ja nie neguję, że ktoś się może bardzo bać. I ktoś się może bardzo bać i po prostu, nie wiem, coś opowie jakiejś innej osobie i ta druga osoba trochę się wkręci w to banie… A trochę po prostu na przykład uważa, że rozsądnym jest bać się, skoro ktoś się tak boi, no to może faktycznie ja też powinienem się też bać. I tak. Więc nie wydaje mi się, żeby one miały cechy wspólne, wydaje mi się, że to jest bardziej taka kula śnieżna.

**Czyli to takie nakręcanie się. Czyli ludzie nakręcają się, bo widzą kogoś, że się boi. Są jeszcze jakieś elementy, które wpływają na to, że to się szybciej nakręca albo mocniej? Masz takie poczucie, że są momenty, które przyspieszają to nakręcanie się?**

Nie, naprawdę nie. Naprawdę nie wydaje mi się, żeby to było coś takiego, że ktoś z rodziny kogoś ma koronawirusa i dlatego ktoś się boi. Tylko właśnie może dlatego, że nikt z rodziny nie ma. W sensie nie to, że im życzę, żeby ktoś z rodziny miał. Tylko chodzi o to, że nikt ze znajomych nie ma tego koronawirusa ani nic. Tylko to jest po pierwsze dla nich w ogóle nieznane. Że nie znają ani skutków ani tego, jak się czuje taka osoba. I na przykład w telewizji jakieś osoby z respiratorami. Albo że brakuje respiratorów. Albo że nie ma miejsc w szpitalach. Takie rzeczy. Ale wydaje mi się, że to jest bardziej nieznajomość.

**A masz wrażenie, że to, że właśnie media podają te informacje o braku miejsc w szpitalach, o tych respiratorach, to to wpływa na ludzi, którzy się boją bardzo?**

Nie jestem pewna, czy podają takie… Nie wiem, jak często podają te informacje. Myślę, że mogą wpływać. Bo nie wiem, co innego mogłoby wpływać.

**Chciałabym więcej pogadać o obostrzeniach, które teraz są. Powiedziałaś, że wprowadza rząd jakieś obostrzenia, ty nie do końca je rozumiesz. A jakie, że tak powiem, ci zapadły w głowę. Te dzieci 8-16 czy tam 9-16?**

Tak. Ja też nie wiem, ale o tym mowa. Uważam, że są w jakiś bardzo losowy sposób nakładane te obostrzenia. I naprawdę nie wiem, mam wrażenie, że po prostu ktoś, na przykład w rządzie mówi jedno zdanie, znaczy pół zdania, a druga osoba dokańcza i wtedy oni uznają OK, takie obostrzenie może wejść. Jakby jeżeli nie słyszą początku zdania. Powiedzmy, że ktoś w jednym pokoju mówi jedno, ktoś drugie i łączą to. Naprawdę, na przykład tego z tymi dziećmi nie jestem w stanie zrozumieć. Szczególnie, że moje siostry cioteczne, zobaczyłam na Instagramie, że jedna jest blisko mojego domu, więc się spytałam, co robi. Ona akurat była na buble tea, więc zeszłam do niej. No i była sobie z koleżanką sobie buble tea i połazić po mieście, poszukać vibe’u, pojeść frytki i tak łaziły. I jeżeli to w jakiś sposób rozprzestrzenia koronawirusa, to dlaczego to można robić w weekend? Nie jestem w stanie tego zrozumieć. Bo tak samo łażą, jak by łaziły od poniedziałku do piątku. I pewnie inne dzieci też. No nie wiem, nie znam innych dzieci. Nie wiem, one też za bardzo tego nie rozumieją. Ale one też w ogóle nie rozumieją tego zakazu. Bo ja się ich spytałam, one powiedziały, że nie rozumiem, dlaczego sama nie jestem w stanie się sobą zająć. Czy coś takiego. One wychodzą z takiego założenia, że to chodzi o opiekę. Że na przykład może się stać coś niebezpiecznego i będą potrzebowały mamy. Może tak one myślą.

**A poza dziećmi? Jakieś jeszcze ograniczenie, które zobaczyłaś, usłyszałaś, wiesz, że jest. Poza tym, że trzeba chodzić w maseczkach albo przyłbicach.**

A w ogóle to jest śmieszne, bo… Wiem, że trochę nie odpowiadam, ale może chcesz to usłyszeć. Pamiętam, że jak nigdzie nie trzeba było chodzić w maseczkach, był taki czas, nie wiem, z miesiąc temu albo kiedyś, to sobie z kimś rozmawialiśmy, z Filipem we dwójkę albo z kimś innym, ale były 3 osoby na pewno. I opowiadaliśmy, jak to było u nas. A, bo przyjechał do nas kolega z Londynu. I mu opowiadaliśmy i ja mówię, ej, a chyba było coś takiego, że cały czas trzeba było chodzić w maseczkach. I nie mogliśmy sobie w ogóle przypomnieć, czy trzeba było czy nie trzeba. Faktycznie, jednak okazało się, że trzeba było. Ale w ogóle dla nas to jest coś takiego, jak… No nie wiem, jakieś dziwne wspomnienie, którego w ogóle nie mamy. I faktycznie, było tak teraz, że trzeba chodzić w tych maseczkach. Ograniczona ilość osób w sklepie, ale też inaczej niż ostatnio. Bo ostatnio… Nie powiem ci, bo nie wiem tego, od czego to zależy, od ilości kas czy coś, tego nie wiem. Ale pamiętam, że były ogromne numery na każdych drzwiach sklepu, ile osób może wejść, ile osób nie może wejść. Teraz tego w ogóle nie widzę. Na pewno w Carrefourze i Żabce pod moim domem wiem, że coś takiego, były jakieś takie numery. Teraz w ogóle nie wiem, ile osób mogę wejść. Nie wiem, czy ktoś sprawdza to. Nie wiem, czy kiedykolwiek sprawdzał, wydaje mi się, że nie. Ale jakoś to jest też trochę takie właśnie zmniejszające atmosferę pandemii, nie ma tych numerów. No gdzieś tam są na pewno, ale nie jest to tak powszechne jak wszędzie. I może nie ma obowiązku takiego albo po prostu nikt tego nie sprawdza. I tak jak mówiłam, w Złotych Tarasach tylko do jednego sklepu nie mogliśmy wejść z powodu tych obostrzeń. I też nikt za bardzo, mam wrażenie, nie pilnuje ilości osób. Bo byliśmy w jakimś tam sklepie z butami. I było coś takiego, że są takie, przy wejściu był stoliczek, na którym były takie klipsy od butów, zapobiegające kradzieży. I każdy powinien wziąć jeden. I tych klipsów było tyle, ile może być osób na sklepie. Tylko, że byliśmy jedynymi osobami, które to wzięły. I nie mieliśmy z tego powodu żadnych… Znaczy no nikt nikomu nie kazał brać tych klipsów. Więc nie wiem, ile było osób w tym sklepie, czy za dużo, czy za mało. No nie wiem. Coś takiego jest, jeśli chodzi o te sklepy, ale nie mam pojęcia. I jeśli chodzi o sklepy spożywcze, całkowicie się przestawiłam na zamawianie zakupów online. Mimo, że tak jak ci mówiłam, uwielbiam chodzić po sklepach i patrzeć na rzeczy, no to jeśli chodzi o oglądanie rzeczy w internecie to jest trochę podobnie. Można sobie przejrzeć to wszystko bez chodzenia po prostu. Myślę, że nawet jak się skończy pandemia i do sklepu będzie… Nie, przepraszam, to jest w ogóle niezależne od pandemii, po prostu będę to robiła już zawsze.

**Ale to jest tak, że z jakaś regularnością zamawiasz sobie zakupy spożywcze do domu?**

Tak, co 2 tygodnie.

**Jakieś jeszcze obostrzenia, które zauważyłaś, albo tak cię dotyczą?**

Zamknięcie siłowni. Nie chodzę na siłownię, po prostu czytałam o tym w internecie, że ktoś tam się burzy. No nie dziwię się, bo jeżeli to jest czyjś zarobek, to faktycznie. Zamknięcie restauracji. W ogóle też widziałam śmieszną rzecz na Facebooku, że jakaś restauracja w Gdańsku czy w jakimś innym mieście zrobiła coś takiego, że pracownicy mogą spożywać posiłek w restauracji. Słyszałaś o tym?

**Nie. Opowiedz.**

Pracownicy mogą spożywać posiłki w restauracji, w swoim miejscu pracy. Dlatego oni zrobili coś takiego, że podpisują umowę z każdą osobą, która wchodzi do restauracji, umowę o dzieło. Gdzie chodzi o wykonanie, o napisanie oceny na Facebooku. Ocenienie posiłku na Facebooku. I podpisuje się taką umowę i można tam w tym lokalu zjeść. I dostaje się złotówkę za ten posiłek. I się złotówkę mniej płaci za ten posiłek.

**Czyli złotówka za opinię, za wykonanie pracy?**

Tak.

**Co sądzisz o takim podejściu?**

Dla mnie to jest trochę na krawędzi. Bałabym się, będąc właścicielem takiej restauracji, naprawdę bałabym się coś takiego zrobić. Dla mnie to jest niezgodne, takie omijanie prawa… Nie lubię czegoś takiego robić. Bo to jest takie publiczne… Nie, no mogłabym złapać prawo i nie boję się czegoś takiego. Ale takie jawne, publiczne, to jest dla mnie trochę za dużo. Ale w sumie, no nie wiem, najwyżej by zamknęli tę restauracji. No nie wiem, nie może być już gorzej chyba niż jest teraz tak naprawdę. Nie wiem, czy można iść do więzienia za otwarcie restauracji, raczej nie. Nie, uważam, że super pomysł, fajnie ktoś wymyślił. Cieszę się, że Polacy tak naprawdę, wydaje mi się, że młodzi, ale może wszyscy, są tacy właśnie pomysłowi. To mi się bardzo podoba, że na wszystko wynajdują jakieś fajne rozwiązanie.

**Ale to jest trochę obchodzenie boczkiem prawa.**

No tak, ale to jest tak samo, jak otwarte Żabki w niedzielę, bo to jest placówka pocztowa. I nikomu to nie szkodzi. I jakby no też nie wiem, czy… Czy to komuś szkodzi. Czy naprawdę jest tam szansa zarażenia się. No na pewno jest. Ale czy jakaś duża? Nie wiem, wydaje mi się szczerze, że to jest fajne. Po prostu wydaje mi się, że to jest fajne.

**Jakieś jeszcze obostrzenia? Knajpy zamknęli, w sensie, że można na wynos wziąć. Siłownie zamknięte. Te limity w sklepach. Coś jeszcze?**

Dzieci. I jeszcze…

**A skąd ty sądzisz o tym, że te dzieci poszły na zdalną, część dzieci nie chodzi do szkoły?**

Ja rozmawiałam z dziećmi, one chodzą do prywatnej szkoły. I tam te lekcje odbywają się. W sensie naprawdę odbywają się i nie są po łebkach za bardzo. Mam wrażenie, że jakoś to idzie. Natomiast rozmawiałam też chwilę przed tobą z Magdą, która rozmawiała ze swoją siostrzenicą. A właściwie z jej mamą. I ona opowiadała, że ci nauczyciele absolutnie nie są przygotowani do nauczania zdalnego. Ja się spytałam, jak to, przecież to już jest drugi raz, kiedy coś takiego robią, więc powinno to wyglądać lepiej. A ona powiedziała, że nie, dlatego że to są jakby inni nauczyciele. Bo tamci nauczyciele wzięli sobie urlopy jakieś tam, na dziecko, coś tam, zagrożenie życia, jakieś takie urlopy. Dlatego teraz to są ci nauczyciele, którzy wtedy mieli urlop. Dlatego w ogóle oni nic nie wiedzą, nie ogarniają. I finalnie mama tej dziewczynki siedzi z nią wieczorami. Bo jakby w rozwoju dziecka ważne jest to, żeby to dziecko nie zatrzymywało się z nauką. Szczególnie, jak ma, nie wiem, 10 lat. Chodzi o to, żeby codziennie jakaś stałość i uczyło się cały czas czegoś. Nie można robić dziecku przerwy od nauki na miesiąc. No więc ona siedzi z nią wieczorami albo w ciągu dnia tak naprawdę też. I bardzo dużo czasu jednak spędza nad tym. Chciałam powiedzieć, że marnuje, ale nie marnuje, tylko wykonuje jakby nie swoją pracę. Wykonuje właśnie i swoją, i nie swoją, i za kogoś.

**Czyli to trochę od rodziców zależy, na ile to dziecko nie straci, będąc w domu?**

Tak. Od ich możliwości, od ich czasu, od ich umiejętności. No nie każdy musi super umieć nauczać. I na przykład mój tata, jak coś pytałam, żeby mi wyjaśnił, to krzyczał, denerwował się, że ja nie rozumiem. I jestem pewna, że tak jest w większości polskich domów. Więc martwi mnie po prostu… Jakby rozumiem, dlaczego te dzieci nie chodzą do szkoły, dlaczego pracują zdalnie i w ogóle. Ale martwi mnie poziom tego. Bo nie wiem, bardzo głupio, żeby dzieci miały wyrwany rok edukacji z życia tak naprawdę. Ale nie mam rozwiązania.

**A co sądzisz o tym, że jest ten podział na te dzieci, które chodzą i te dzieci, które nie chodzą do szkoły?**

Wydaje mi się, że ten podział wynika z tego, że po prostu starsze dzieci mogą siedzieć w domu same, a młodsze nie. Chodzi o zapewnienie jakiejś tam opieki tym dzieciom. To jest dla mnie zrozumiałe. Ale no na przykład fajnie by było, tylko że nie wiem, na ile są możliwości w szkołach. Też dawno w szkole nie byłam, żeby były lekcje… Te, które są w szkole, były też transmitowane zdalnie. I wtedy rodzic by miał wybór czy posyłać dziecko czy nie, bez jakby uszczerbku na jego wiedzy. No i są to kwestie do rozwiązania, typu na przykład kartkówki… No nie wiem, trzeba byłoby to przemyśleć, ale ja nie zajmuję się tym zawodowo, nie wiem, nie mam żadnego doświadczenia w tym temacie. Ale coś takiego uważam, że byłoby fajnego wtedy. Naprawdę rodzice mieliby wpływ na to, w jaki sposób dzieci, czy chodzą czy nie, czy ktoś się boi, czy się nie boi.

**Jakieś jeszcze przychodzą ci obostrzenia, które mamy? Słyszałaś jeszcze o czymś?**

Chyba coś z kulturą związane, 25% publiczności. Coś takiego. No nie może być też żadnych jakichś takich zgromadzeń. O boże, zapomniałam ci powiedzieć najważniejszej rzeczy, że nie mogę zrobić urodzin przez koronawirusa i to mnie denerwuje. Wiedziałam z tyłu głowy, że coś jest, co mnie denerwuje, ale nie wiedziałam co. A są to właśnie moje urodziny.

**A jakie urodziny byś chciała zrobić?**

Wiesz co, ja za bardzo nie robię żadnych urodzin nigdy, ale właśnie teraz chciałam. Przez to, że są to 25 urodziny, przez to, że przez długi czas nie można było się spotykać. Że każdy ma swoje życie, po tym koronawirusie każdy chciał nadrobić w jakiś inny sposób swoje życie i spotkać się ze mną. A tak chciałam po prostu zaprosić jakichś ludzi do jakiejś knajpy. I bardzo, naprawdę bardzo chciałam to zrobić. Bardzo mi na tym zależało. Wiedziałam, że trzeba będzie wydać na to dużo pieniędzy i odłożyłam sobie te pieniądze. Znaczy może nie tyle odłożyłam, co pogodziłam się z myślą, że je wydam. Bardzo nie lubię wydawać oszczędności, ale pogodziłam się z tym, bardzo mi na tym zależało. Naprawdę niesamowicie mi na tym zależało. No i wiem, że teraz nie mogę. Jeszcze pamiętam, że... Bo ja długo gadam o urodzinach, bo to było dla mnie bardzo ważne, naprawdę. I to było jeszcze tak, że wtedy były restauracje otwarte normalnie. I ja napisałam do jednej, czy mogę, a oni powiedzieli, że właśnie nie robią rezerwacji, bo nie wiadomo, jak to będzie wyglądało. Potem były restauracje otwarte w godzinach jakichś tam, do którejś tam. I sobie wtedy myślałam, dobra, no to może w takim razie zrobię te urodziny w ciągu dnia. Ale potem się okazało, że nie mogę ich zrobić wcale.

**Ale to znaczy, że na przykład nie chcesz ich zrobić w domu, zaprosić ludzi?**

Myślałam o tym. Nie chcę za bardzo tego robić. No w ostateczności zrobię to, bo co mam innego? Mogę się obrazić i nie robić urodzin wcale albo zrobić je w domu. Tylko że zrobienie w domu też jest chyba trochę nielegalne. Ale czy policja będzie chodziła i liczyła osoby w domu?

**A jest nielegalne robienie w domu?**

No wydaje mi się, że zgromadzenia do 5 osób… A jakby to będzie zgromadzenie ponad 5 osób.

**Ale zgromadzenia publiczne chyba są do 5 osób.**

A prywatne mogą być duże?

**Nie wiem.**

Znaczy czy wolno czy nie wolno i tak pewnie zaproszę tych ludzi tutaj. Ale właśnie chciałam zaprosić tym ludzi gdzieś indziej. Jeszcze rozważałam zrobienie urodzin w biurze. Bo w biurze jest dużo miejsca, to nie będą urodziny w domu, tylko gdzieś indziej. Można tam przyłazić, bo i tak nikogo nie ma. Ale to też nie są wymarzone urodziny. Też zaplanowałam sobie urodziny po tym, wtedy jak już będzie można. Ale tak, to mnie bardzo zdenerwowało z koronawirusem. To mnie najbardziej zdenerwowało z tego wszystkiego.

**Czy jest jeszcze coś, co teraz się dzieje, co cię denerwuje? Albo jakieś obostrzenie, które albo cię denerwuje albo myślisz sobie, że jest bez sensu.**

Znaczy bez sensu właśnie wszystkie, bo są wprowadzone za późno. I właśnie tak bardzo randomowo. Nie jestem w stanie pojąć to, dlaczego można iść do kosmetyczki, a nie można iść na siłownię. Albo na przykład, no są w niektórych miejscach jakieś treningi indywidualne, które mogą być. Bo albo obchodzą prawo albo nie ma zapisane w ustawie, czy gdzieś to jest napisane, że tego nie może być. Jakby nie rozumiem, to jest w bardzo nieuważny sposób zrobione, niezaplanowane. No nie wiem, po prostu nie jestem akurat tego w stanie zrozumieć. Nikt nie ma żadnego planu moim zdaniem, to jest po prostu tak, jakby komuś przyszło do głowy w ostatniej chwili.

**A tobie się zdarzało na przykład sprawdzić, co jest dokładnie napisane o jakimś obostrzeniu, które cię bardziej interesowało?**

A z tego, co mówię, masz wrażenie, że nie?

**No właśnie nie wiem. Czy w ogóle weszłaś kiedykolwiek w jakieś obostrzenie, żeby sprawdzić, czy mogę to zrobić czy nie, zgodnie z prawem.**

Nie, nie, nie. Przeczytałam w nagłówku. Bo też głównym źródłem moich informacji o koronawirusie są nagłówki nawet, a nie same artykuły. Więc z nagłówków gdzieś przeczytałam, że nie można jeść i pić na ulicy. I powtarzam to, że nie można tego robić. Tylko, że robię to. Robię to, ale wiem, że nie można. Tylko, że też nie wiem, czy nie można. Jakby ktoś mnie zapytał, jesteś pewna? Nie, przeczytałam w nagłówkach. A, OK.

**Czy jeszcze jest coś, co robisz i myślisz sobie, że to może nie jest tak, jak powinno być, w sensie nie jest zgodne z tym, co jest napisane gdzieś?**

Też chyba za bardzo nie można palić na ulicy, zdarza mi się. Nie wiem, może raz mi się zdarzyło.

**Ale ty zdejmujesz przyłbicę do palenia?**

No tak. Zdejmuję. Ale dzisiaj jadłam kanapkę z Subwaya na ulicy i było bardzo dużo policjantów i widziało, jak to robię i nic mi nie powiedziało.

**Czyli czasami po prostu zdejmujesz sobie przyłbicę na ulicy i jak jesz, palisz, pijesz…**

No tak, ale no kurczę, to nie jest tak, że pluję na ludzi, którzy są wokół mnie. Tylko wiadomo, że jestem wtedy jakoś tam w miarę sama. To nie robię tego w tłumie, tylko jak idę, ale nie idą obok mnie na przykład ludzie.

**A masz takie poczucie, że jest jakieś obostrzenie, które według ciebie powinno zostać wprowadzone a nie zostało? Które by rzeczywiście zadziałało?**

Wiesz co, ja ci się przyznam, że ja nie wiem, gdzie ci ludzie chorują. Właśnie nie wiem, gdzie można zachorować. W sensie no wtedy w Dziku, jasne. To było oczywiste, że oni poszli do tego Dzika, mimo że tam nikt nie kontrolował ilości osób, ani nikt… Bez maseczki, ludzie krzyczeli, pluli na siebie, to zrozumiałe, że w jakichś klubach można było się faktycznie zarazić. Natomiast teraz jakby nie wiem… Znaczy teraz, wiadomo, na proteście na przykład można się zarazić. Ale teraz nie zbieramy żniw tego protestu, bo w piątek był ten protest. To w ogóle jeszcze nie są te osoby, które są chore.

**Czyli jeszcze nie zdążyli się zarazić, więc jeszcze nie ma ich w statystykach.**

Nie.

**To skąd te kilkanaście tysięcy?**

Nie wiem, naprawdę przyznam ci się, że ja się zastanawiam, gdzie oni się zarazili, bo ja nie mam pojęcia. Ja nie wiem. Ja trochę też żyję w bańce. Chodzę sobie do tej pracy, to sama siedzę w biurze, tą hulajnogą pojadę albo tą taksówką. I może nie wiem, jak się żyje. Ale ja po prostu nie mam pojęcia, gdzie ci ludzie się zarażają. I jak bym wiedziała, to może bym miała pomysł na jakieś obostrzenie. Ale tak absolutnie nie. No też widzę ludzi, którzy raczej chodzą w tych maseczkach i przyłbicach. Ale może to jest dlatego, że mieszkamy w Warszawie. Ale z drugiej strony nie, bo w województwie mazowieckim w ogóle też jest dużo zachorowań. Więc to nie jest tak, że żyję w centrum miasta i dlatego nie wiem nic na ten temat. Bo powinnam też wiedzieć. Ale nie wiem. Nie wiem, jakie są miejsca, w których można się zarazić koronawirusem, szczerze mówiąc. Może właśnie w tych, które są zamknięte.

**Ale nie jesteś przekonana do tego?**

Nie wiem, ja naprawdę… Można wszędzie się zarazić tak naprawdę, ale nie wiem.

**A masz takie poczucie, że sytuacja jest teraz taka poważna? W sensie, że już jakby jesteśmy na etapie poważnej sytuacji, takiej zagrażającej?**

Wiesz co, tak naprawdę, bo nie dość tego, że jest ten koronawirus, który był dla mnie ważny i na przykład jak się umawiałyśmy, to było dla mnie ważne i miałam dużo do powiedzenia. Ale przez to, że teraz są te protesty i w ogóle taka dziwna sytuacja w Polsce generalnie, to ten koronawirus zszedł u mnie na drugi plan. Ale w połączeniu razem z tą sytuacją w Polsce, która się odbywa powiedzmy od tygodnia, jest to dla mnie niesamowicie dziwna sytuacja. I jakby omijam ten temat polityczny, dlatego że rozmawiamy o tym koronawirusie. Ale jak by to połączyć razem, to jest to dla mnie… No, boję się trochę. Boję się, bo nie wiem w ogóle, co się dzieje. Nie byłam nigdy w takiej sytuacji. I nikt raczej nie był, gdzie ludzie w maseczkach chodzą strajkować. I to jest kombo dwóch tych rzeczy, które są… bardzo przerażające. I o ile koronawirus mnie osobiście aż tak nie stresuje, tak to, że ludzie od 5 dni strajkują na ulicach i jeszcze jest ten koronawirus, to już jest dużo.

**OK, czyli to połączenie ze strajkami, protestami.**

Tak. I wiesz, no wyszłam teraz jeszcze do Żabki chwilę przed rozmową z tobą i słyszałam, no też mieszkam w centrum, ale słyszałam strajki. No to jest dziwna sytuacja, po prostu nie do pomyślenia w ogóle. I jeszcze trzeba w tych maseczkach.

**A sam koronawirus przed strajkami, tydzień temu, miałaś takie poczucie, że to jest poważna sytuacja?**

Tak, ja cały czas mam poczucie, że to jest poważna sytuacja, jak widzę ilość zachorowań dziennie. Naprawdę ja mam poczucie, że to jest poważna sytuacja. Tylko przez to, że nie mam na nią wpływu i… Mam wpływ, wydaje mi się, że mały, chociaż tak naprawdę duży, skoro widzę powiedzmy dziennie 30 osób i żadnej z nich nie zarażę, no to jest całkiem spory wpływ. Ale tak, no wyrywa się spod kontroli. Tylko też nie wiem, kto tę kontrolę sprawuje tak naprawdę.

**Powiedziałaś na początku, jak rozmawialiśmy o twoich rodzicach, powiedziałaś, że twój tata ma tak trochę już, że on już tak do końca nie wie, czy jest ta pandemia, już jest zły na tę sytuację? Ty też tak odczuwasz czasami, że sobie myślisz, kto to w ogóle wymyślił ta pandemię, że to w ogóle jakby…**

Nie, nigdy nie wątpiłam w istnienie pandemii.

**Nie?**

Nie, ja bardzo wierzę w to, co mi podają media. No widzę, że te osoby są chore. Nie wiem, dlaczego mam nie wierzyć w tę pandemię. W jej jakby… w to, jak bardzo poważna jest ta choroba, w to mogę trochę wątpić. I nawet, jak wątpię też trochę w ten strach tych ludzi… No też uważam, że to jest troszeczkę robienie z igły widły, dlatego że no powiedzmy te pierwsze lockdowny i w ogóle, pojawiły się w sytuacji, kiedy tak dużo osób zachorowało, tak dużo osób nie umierało. I dlatego może ludzie uważają, że to nie jest aż takie ważne, bo… Nie, nie wiem. Nie wiem, zgubiłam się w tej myśli. Ale chodzi o to, że nigdy nie wątpiłam w istnienie tej pandemii, tylko po prostu uważam, że jest trochę rozdmuchana może. Była rozdmuchana wtedy, teraz nie.

**A co najbardziej było rozdmuchane? W którym obszarze masz takie poczucie jakby był przerost formy nad treścią?**

Właśnie w kwestiach takich gospodarczych typu zamykanie wszystkiego, co się tylko da. Nie, nie co się tylko da, po prostu zamknięcie wszystkiego. Zamknięcie ludzi w domach. Nie wiem, tylko że teraz tak sobie myślę, że tak naprawdę, no jeżeli wtedy ludzie by wychodzili z domów, no to może ta sytuacja byłaby wtedy, a teraz byłaby dziesięciokrotnie większa. Nie wiem. Martwi mnie tylko to, że nie jesteśmy w stanie sobie pozwolić na takie obostrzenia, które były wcześniej. Przez to, że już jest mniejszy budżet na to powiedzmy. A teraz by się przydały te obostrzenia jakby dużo bardziej.

**Czyli bardziej niż wtedy, kiedy było mało zachorowań to teraz trzeba by zamknąć.**

Tak.

**Masz takie poczucie, że nie robimy tego, że Polska tego nie robi, bo po prostu nas na to nie stać?**

Trochę tak. Zresztą tak jak ta pożyczka, która jest teraz, to też nie jest dla wszystkich. I trzeba tam spełnić jakiś szereg warunków. Których wtedy na pewno nie trzeba było spełniać. Bo postojowe nawet dostałam ja, a aż tak bardzo nie ucierpiałam na tej pandemii.

**A masz w swoim otoczeniu takich ludzi, którzy mówią, że żadnej pandemii nie ma?**

Nie. Tylko widzę ich w internecie, ale nie znam takich ludzi.

**A rozmawiałyśmy jeszcze, chyba rozmawiałyśmy na wiosnę o tym modelu szwedzkim, pamiętasz?**

Rozmawiałyśmy. I cieszę się, że mi o tym powiedziałaś, bo potem mogłam opowiadać ludziom, jak dużo wiem o świecie.

**A w ogóle sprawdzałaś od tego czasu cokolwiek, co się dzieje w innych krajach?**

W innych krajach tak. Znaczy czytam w internecie. Bo jest ten Łukasz z Konfliktów i Katastrof Światowych i on pisze rzeczy różne. A w ogóle to jeszcze on przestał pisać tak dużo o koronawirusie i to mnie wkurzało. I chodziłam i mówiłam, boże, dlaczego on już nic nie dodaje, skąd mam wiedzieć. A tak, to jest właśnie takie w bardzo przyjazny dla oka sposób podane informacje, które muszę wiedzieć. Znaczy które warto wiedzieć. Jeśli chodzi o inne kraje, widzę to tam, ale aż tak mnie to nie interesuje, dlatego że nie mam porównania. Bo wtedy nie śledziłam, więc teraz też za bardzo nie… Nie umiem się do tego odnieść po prostu. Nie wiem, czy 30… Też nie za bardzo wiem, ile osób mieszka tam. I no nie wiem wszystkiego o wszystkich państwach, więc nie jestem w stanie się w żaden sposób odnieść, czy to jest dużo czy mało. I też nie wiem, co te liczby oznaczają, jeżeli chodzi na przykład o życie w tym państwie. Bo na przykład wiem, że u nas 10 tysięcy zachorowań to jest zamknięcie wszystkiego. A tam jest może 30 tysięcy i wcale nie. Więc nie wiem. Dużo czasy by mi to zajęło, a nie jestem aż tak ciekawa, żeby to wiedzieć. Natomiast, jeśli chodzi o Szwecję, to swego czasu zwracałam uwagę na to, jak tam u nich. Bo zachowali się inaczej niż reszta świata. Ale wydaje mi się, tylko też nie jestem pewna, coś takiego zauważyłam, że chyba mają tych zakażeń mniej więcej co w Polsce, są na takim samym poziomie pandemii teraz. Ale nie powiem.

**Bo oni chyba tydzień czy 2 tygodnie temu znieśli część obostrzeń, które mieli.**

Ale to nie jest tak, że oni nie mieli obostrzeń?

**Oni mieli takie zalecenia. Mieli zalecenia, zalecali osobom starszym, żeby zostały w domu i ograniczali wychodzenie. I jedyny zakaz chyba, który mieli, to zakaz zgromadzeń powyżej 50 osób i chyba go znieśli. Teraz na razie proszą, żeby nadal nie robić bardzo dużych, ale już można. Co sądzisz o tym w ogóle, że Szwecja idzie nadal modelem innym jak wszystkie kraje?**

A na jakim etapie zachorowań, czy na jakim etapie pandemii są?

**Nie mam zielonego pojęcia.**

No właśnie, bo wydaje mi się, że na podobnym, co Polska. Nie dam sobie głowy uciąć, ale wydaje mi się, że tak. No jeżeli na podobnym, co Polska albo na niższym, to w dalszym ciągu są na plusie, a nie na minusie. Więc w dalszym ciągu uważam, że to jest bardzo dobry pomysł. Albo nawet, jeszcze muszę powiedzieć, że nawet jeżeli mają więcej zachorowań teraz, to porównując z Polską, mają na przykład teraz większy budżet na różnego rodzaju pomoc na lockdown teraz. Dlatego, że tamtych pieniędzy wtedy nie wydali.

**Bo ty cały czas operujesz tymi liczbami zachorowań, które oni podają…**

Bo nie wiem, czym innym mogę operować.

**A jak ty to rozumiesz, co to dla ciebie znaczy, że teraz jest 16 tysięcy, czy tam 17 czy 18. To jakich ludzi to jest 18 tysięcy?**

Nie wiem jakich. Ale to jest sytuacja 9 razy poważniejsza niż była ileś tam tygodni temu.

**Czyli to chodzi tylko o to, czy ta liczba rośnie.**

No tak. Tak, tak.

**A ty wiesz w ogóle, co oni za liczby podają?**

Tak (śmiech). Ale czy pytasz mnie, czy wiem, co oznacza ta liczba? No tak, że 18 tysięcy nowych osób ma koronawirusa. Znaczy no dostało pozytywny test.

**OK, dobra. Bo tak się zastanawiałam. Bo ludzie różnie rozumieją tą liczbę. A jak Łukasz przestał publikować, to szukałaś sobie innego, fajnego źródła?**

Nie. Bo ja ci się przyznam w ogóle, że jak Łukasz pojechał na wakacje, to było chwilę po tym, jak przestałyśmy rozmawiać, no to już uznałam, że nie ma koronawirusa. Bo on pojechał na wakacje, w ogóle mnie nie obchodzi jego życie, więc od followowałam go i w ogóle nie sprawdzałam. I cały czas go nie followuję, tylko po prostu teraz wchodzę. Ale jakby wtedy już uznałam, że nie żadnego koronawirusa, już nie trzeba się tym interesować, skoro nawet Łukasz już przestał publikować, to jest spokojnie. Ale potem z jakichś innych źródeł się dowiedziałam w takim razie, że jest ten koronawirus, że powrócił. No i już teraz zaczęłam sprawdzać.

**OK. Bo Łukasz był dla ciebie takim wiarygodnym źródłem. Uważałaś, że to, co on publikuje, to jest OK i jakby…**

To znaczy słyszałam, że on robi pewne pomyłki. Może być nie do końca wiarygodnym źródłem, ale jest w bardzo przystępny sposób mi podane. I dostarcza mi dokładnie tyle informacji, ile potrzebuję. Bo mi się wydaje, że on się nie myli w liczbach, tylko w czymś innym na przykład. Że na przykład ogląda konferencję i pisze. I na przykład coś nie do końca usłyszał, więc pisze coś, co nie jest do końca prawdą. To jest w dalszym ciągu dla mnie OK. Nie muszę oglądać tej konferencji, po prostu mam w pięciu punktach napisane, co to dla mnie oznacza. I tyle.

**A masz jeszcze jakieś inne źródło, takie mediowe, o którym myślisz, że jest rzeczywiście wiarygodne i że tam można sprawdzać, co się dzieje?**

No właśnie niestety mam ostatnio takie przemyślenia, że żadne takie źródła są za bardzo niewiarygodne. Przez to właśnie, że oglądałam te Fakty i Wiadomości zaraz po sobie. A jeszcze było o tych protestach, na których byłam. I jakby wiedziałam, bo tam byłam. I widziałam, że to nie do końca wszystko jest tak, jak było. I też no strasznie są te informacje tak… spropagowane? Tak się nie mówi, ale wiesz, o co chodzi.

**Ale wiem, o co chodzi.**

Więc nie do końca wiem, gdzie szukać informacji. Dlatego, że ciężko powiedzieć, które są prawdziwe… Nie, może są wszystkie prawdziwe tak naprawdę, tylko sposób przekazania jest inny. I nie do końca chcę właściwie narażać się na coś takiego, że ktoś mi poda swoją opinię i po prostu wtedy ją będę miała. A może się okazać na przykład głupia albo niesłuszna. I potem będę powtarzała głupoty.

**Jeszcze chciałabym z tobą pogadać trochę o przyszłości. Myślisz w ogóle o tym, kiedy to się skończy? Teraz w tym momencie.**

Tak. Słyszałam, że 6 stycznia.

**A czemu ta data?**

Nie wiem. Bo teraz w pracy mam takie sesje z kołczem raz na miesiąc. I właśnie trochę rozmawialiśmy o koronawirusie. Znaczy mnie to w ogóle nie przeraża, dlatego on bardziej chciał o tym rozmawiać, bo myślał, że mnie przeraża. Ale ja powiedziałam mu, że zupełnie nie, więc urwaliśmy temat. Ale on powiedział właśnie, że 6 stycznia.

**Ale dlaczego 6 akurat a nie 7 na przykład.**

Nie wiem. Nie pytałam, ale myślałam, że to jest jakaś oficjalna data, dlatego nie dopytałam. Ale potem poszukałam i nic nie było na ten temat nigdzie powiedziane, więc nie wiem, skąd on to wziął. Ale też powiedział, że wie, że będzie ciężko i że ludzie się od siebie oddalą. I że będzie dużo trudniej niż za pierwszym razem. I to mnie troszeczkę przeraziło. Tylko że przez to, że naprawdę to, jak on wypowiedział te słowa, bardzo mnie przeraziły. Bo uważam, że on może z jakiegoś powodu wiedzieć. Bo jest trenerem. No nie wiem, ma jakiś autorytet.

**A co cię najbardziej przeraziło w jego słowach?**

To, że będzie trudniej niż za pierwszym razem. Bo już się przyzwyczaiłam do tego pierwszego razu. I jeżeli na przykład teraz jest tak jak za pierwszym razem to spoko. Ale jeżeli ma być jeszcze jakoś gorzej, że na przykład… No, on powiedział, że oddalimy się od siebie, jeśli chodzi nie ja z nim, tylko o ludzi generalnie. I to mnie trochę przeraziło, bo w sumie może przez to, że to powiedział, ja to zaczęłam zauważać. A może faktycznie tak jest?

**A czułaś wcześniej to oddalanie się, obserwowałaś to u kogoś?**

Nie, ale akurat ja z nim rozmawiałam tego dnia jak… Dzień po konferencji chyba tej ostatniej. W piątek jakoś, no. Tydzień temu. I teraz zauważyłam to. Ale może mam więcej pracy? Nie, no ale właśnie nie ma takiej możliwości wyjścia sobie z kimś gdzieś, pogadania. Wiem, że to mi nigdy wcześniej nie przeszkadzało, ale teraz może mi przeszkadza, jak mi to powiedział. Nie wiem. Nie wiem, ale jest w sumie trochę dla mnie autorytetem. I tak to powiedział, że wie, że tak będzie, to może tak będzie.

**A już pomijając ten 6 stycznia, jak myślisz, kiedy ty poczujesz, że to już się zbliża ku końcowi albo że już jest koniec? Co musi się zdarzyć? Nie chcę daty, chcę wydarzenie.**

Nie wiem. Dlatego, że pamiętam jak ostatnim razem ci powiedziałam, że to wtedy, jak nie trzeba będzie nosić maseczki na ulicy. Coś takiego powiedziałam.

**No, coś takiego powiedziałaś chyba, tak.**

I jak sobie właśnie ostatnio nie miałam maseczki na ulicy, a widziałam, że jest dużo zachorowań, to sobie myślałam, ale ja byłam głupia, to wcale nie tak. Albo było też tak, że… Jeszcze trzeba było nosić… Nie, nie, nie. To raczej tak właśnie, że nie, to wcale nie o to chodzi. Więc właśnie nie wiem do końca, o co chodzi. Ale właśnie może wiem, dlatego że przeczytałam, oczywiście u Łukasza, że w Australii przez dobę nie zanotowano żadnego nowego przypadku. A myślałam, że raczej to nie jest możliwe. Myślałam, że to będzie zawsze ktoś chorował po prostu. A teraz, skoro w Australii… Nawet, jeżeli to jest jedna doba, a potem, jutro znowu ktoś zachoruje albo pojutrze, to wydaje mi się, że to jest właśnie już taka droga ku końcowi. Bo 18 tysięcy dziennie a zero osób, to jest jednak ogromna różnica. I oznacza może jakiś koniec.

**Czyli to muszą być dni, kiedy nikt nie będzie chorował, to znaczy, że już zbliżamy się ku lepszemu.**

Na przykład. Albo nie, no zbliżamy się ku lepszemu, to jest wtedy, kiedy powiedzmy przez tydzień będzie tendencja spadkowa. Nie przez tydzień. Może miesiąc. Bo chciałam powiedzieć tydzień, bo mam nadzieję, że to trochę szybciej skończy. Ale jednak wydaje mi się, że wyznacznikiem może być na przykład miesiąc.

**Że po prostu musi spadać liczba zarażonych.**

Tak.

**A masz poczucie, że szczepionka pomoże? Wierzysz w ogóle, że wynajdą tą szczepionkę?**

No ta szczepionka miała być chyba już dawno.

**I co sobie myślisz, że miała być dawno, ale jej nie ma. Przynajmniej chyba nie ma.**

No chyba nie ma. No nie wiem, jak będzie, to zobaczymy.

**A zaszczepisz się?**

Nie wiem. Nie wiem. No tak… Tak, ale…

**Trochę bez przekonania to tak. To jakie masz za, a jakie przeciw?**

Nie mam żadnych… No za to jest takie, że generalnie uważam, że trzeba się szczepić. I słusznym jest szczepienie się. Ale i tak z rozsądku i z tego, że to jest społecznie akceptowana decyzja. Ale dlaczego bez przekonania? Bo tak jak mówię, ta choroba nie przeraża mnie, nie wydaje mi się, żeby mi w jakimkolwiek stopniu zagrażała. No tylko właśnie z uwagi na tych innych ludzi, których mogłabym zarazić, no to faktycznie lepiej być zaszczepionym niż nie być. Ale OK, mogę to zrobić. Tylko właśnie, to będzie wymagało ode mnie zrobienia czegoś.

**To ty mi mówiłaś, że dobrze by było, żeby był jeszcze lek na to. Myślisz, że są w stanie wynaleźć lek na koronawirusa?**

Na bank nie ja to mówiłam. I nie, nie. Dobra, mogą wymyślać, tylko że jak już się nauczyliśmy, koronawirus sam się leczy. Dobra, tutaj akurat może wyjść moja niewiedza. Ale generalnie chodzi o to, że tego koronawirusa, on po prostu przechodzi po dwóch tygodniach. Nie wiem, może to jest coś głupiego, co mówię, ale wydaje mi się, że właśnie tak to działa. Znaczy w jakichś tam skrajnych przypadkach oczywiście nie wystarczy samo czekanie powiedzmy. Ale raczej to jest choroba, która przechodzi po prostu. Więc czy jest potrzebny lek? No pewnie tak, żeby 2 tygodnie nie leżeć w domu, tylko żeby krócej… Ale no nie jest niezbędny.

**A jak sobie myślisz o tej przyszłości, to myślisz sobie o przyszłości na przykład w kontekście... Masz w ogóle przemyślenia albo zastanawiasz się, jak to będzie na przykład z gospodarką w Polsce, jak to będzie z różnymi firmami? Z różnymi branżami, biznesami?**

No tak, myślę. No, ciężko będzie po prostu. Wydaje mi się, że będzie tym ludziom bardzo, bardzo ciężko. I nie jestem w stanie się postawić w takiej sytuacji, jak ci ludzie mogą być.

**A jak myślisz, kto najbardziej ucierpi tak finansowo?**

Wydaje mi się, że organizatorzy takich wydarzeń, których od początku roku nie mogło być. Na pewno nie wydaje mi się, żeby to była branża turystyczna, ktokolwiek z branży turystycznej. Znaczy pewnie trochę tak, ale nie najbardziej. Dlatego że z tego, co mi się wydawało w wakacje, bardzo dużo osób gdzieś wyjeżdżało. I wiadomo, to nie są takie zyski, które mieli po prostu przeciętnie. Ale może są w stanie nadrobić w jakikolwiek sposób braki. Restauracje to może być ogromny spadek ich zarobków przez to, że są zamknięte. A czynsz w dalszym ciągu trzeba płacić i takiego różnego rodzaju rzeczy. Ale właśnie takie jakieś wydarzenia, imprezy masowe, targi. Które polegają na tym, że ludzie się po prostu spotykają ze sobą.

**A z tymi knajpami to masz jakieś takie myśli czasami, żeby pójść jednak do tej knajpy i zamówić to na wynos i zabrać? Bo oni mogą na wynos sprzedawać. Żeby wesprzeć knajpy?**

Wiesz co, miałam takie przemyślenia ostatnio też. Bo mi wpada coś do głowy i sobie myślę, że ci powiem. Oczywiście nie zapisuję tego, na szczęście wraca. Mam takie przemyślenie, ale to jest tylko na podstawie mojego życia, że teraz ludzie nauczyli się gotować sobie samemu, przygotowywać jedzenie. I wydaje mi się, że teraz będzie tej branży gastronomicznej trudniej niż wcześniej. Bo wcześniej ludzie jeszcze troszeczkę nawoływali do wspierania, mieli oszczędności, chęć pomocy. I naprawdę chętniej zamawiali to jedzenie, żeby wesprzeć. Głównie żeby wesprzeć, a nie… No i też po to, żeby zjeść, bo potrzebowali zjeść. A teraz wydaje mi się, że sytuacja przyzwyczaiła ludzi do tego, że da się gotować samemu w domu, że jest na to czas, że nie trzeba cały czas zamawiać, że to wychodzi taniej. Wydaje mi się, że może być im trudniej. Mówię to na podstawie pewnie mojego życia i trochę moich znajomych. Więc nie wiem, jak dobrym odzwierciedleniem rzeczywistości jesteśmy, ale takie przemyślenie mam. A czy myślałam o tym, żeby iść, coś wziąć na wynos? Tak, myślałam. I nawet zamawiałam… Wczoraj zamówiliśmy coś. Więc to nie jest tak, że nie zamawiam. Ale zamawiam dużo mniej. Na przykład udało nam się ostatnie 2 weekendy nic nie zamówić, co wcześniej było niespotykane.

**Czyli taka branża bardziej eventowa od tych wszystkich dużych imprez to ucierpi, trochę knajpy ucierpią. Teraz bardziej niż na wiosnę, bo ludzie się nauczyli gotować i obrabiać w domu to jedzenie i szykować sobie obiady. Jakaś jeszcze, masz wrażenie, że komuś jeszcze będzie trudniej?**

Znaczy, tak jak powiedziałam, tej turystycznej branży trudniej na pewno niż kiedyś. No nie wiem. Nie mam innego pomysłu. Na pewno ktoś ucierpi bardziej jeszcze, ale nie wiem kto.

**A ten kołcz ci powiedział, że tak ludziom będzie trudniej, w sensie, że ludzie się oddalą od siebie. A masz poczucie, że jeszcze jakoś ta sytuacja wpłynie na ludzi?**

Wydaje mi się, że tak. Wydaje mi się, że może to być trochę za długo. I też mam takie poczucie, że jakby ten pierwszy lockdown powiedzmy się skończył jakoś tam i ludzie sobie pomyśleli i w sumie ja też, że super, przeżyliśmy to i już teraz można wyjść na prostą i wrócić do powiedzmy normalności. Której na przykład ja już nie do końca pamiętam, jak to było. A teraz niestety jest taka niespodzianka, że nie… I wydaje mi się, że też nagle przyszło to wszystko. I nie byliśmy tak samo gotowi jak wtedy powiedzmy. Tylko, że teraz mamy takie poczucie, że to już się skończyło, a teraz trzeba jeszcze raz do tego wracać.

**Ale jak sądzisz, co dla ludzi jest najtrudniejsze w tej sytuacji?**

Trochę zamknięcie w domu. Trochę taka świadomość, że to się nie skończyło i nie wiadomo, kiedy się skończy. Bo o ile za pierwszym razem nie wiadomo było, kiedy się skończy, no to już się skończyło i miało się świadomość, że to się skończyło. A teraz mamy tą wiedzę, że może wrócić do nas w każdym momencie. I to ze zdwojoną siłą tak naprawdę. Więc to jest taka troszeczkę obawa, coś nieznanego.

**Jakieś jeszcze myślisz, że ludzie będą mieli, nie wiem, obserwujesz u ludzi jakieś takie spadki nastrojów albo to, że się gorzej czują? Jakoś to widać albo mówią o tym?**

Wiesz co, no myślę, że to jest trochę przez pogodę też. I też się ja troszeczkę gorzej czuję. Ale no jest taka wszechobecna atmosfera właśnie koronawirusa, która powoduje po prostu takie gorsze samopoczucie. Plus jeszcze pogoda, to, że się robi wcześnie ciemno. To nie są takie rzeczy, które sprzyjają pozytywnemu samopoczuciu.

**A masz poczucie, że jakaś grupa jeszcze tak szczególnie narażona na te takie zmiany społeczne, to co się dzieje między ludźmi?**

Myślę, że dzieci. Dzieci właściwie wszystkie do liceum może… Nie wiem, nie wyobrażam sobie takiej sytuacji, kiedy przez prawie rok się nie widzi koleżanek, tylko rodziców albo rodzeństwo. Uważam, że to na pewno wpłynie jakoś na ich rozwój. I też niestety wydaje mi się, że to może być tak, że nauczy dzieci żyć w wirtualnym świecie. A o ile na przykład przed tą pandemią powiedzmy dzieci dużo czasu spędzały przed komputerem i przed telefonem, to teraz muszą jeszcze więcej. A właściwie wszystkie spotkania są zabraniane. Więc wydaje mi się, że to może ogromny wpływ… O ile na przykład my jesteśmy dorośli i możemy się z kimś spotkać na naszą własną odpowiedzialność powiedzmy, to dzieci po prostu nie mogą. I nie do końca w sumie wiedzą, dlaczego. Też nie wydaje mi się, żeby do końca rozumiały, dlaczego jest ten koronawirus albo jak to wygląda. I wydaje mi się, że może zrobić ogromną szkodę, nie tylko pod względem nauki, ale też pod względem kontaktów społecznych.

**Czyli dzieci są taką grupą narażoną na to, że po prostu gorzej się będą pod tym społecznym względem rozwijać, przez brak kontaktów z rówieśnikami po prostu.**

Tak.

**Czy jeszcze jest jakaś grupa, która też odczuje mocniej niż inni?**

Starsze osoby, wydaje mi się, że też. Przez to, że są bardzo samotne tak czy inaczej. A teraz nie mogą się spotykać powiedzmy ze swoją rodziną. Dlatego, że bezpieczeństwo, z powodów niebezpieczeństwa. I wydaje mi się, że też mogą to bardzo źle znosić. Szczególnie, jak komuś umrze mąż albo żona i ma dzieci albo wnuki, które się teraz nie spotykają z nimi, dlatego że boją się o nich. No to na pewno jest im dużo ciężej. I też nie mogą chodzić pewnie do kościoła. Albo może mogą… Nie, babcia mi coś mówiła, że ona nie może już chodzić do kościoła, bo nie wiadomo, czy ją wpuszczą. I nie będzie szła na marne.

**A masz wrażenie, że to, że tych starszych się nie odwiedza, że trochę się ich izoluje dla ich dobra, to jest rzeczywiście jakby finalnie dla ich dobra? Że oni nie ucierpią tak społecznie na tyle, żeby to równoważyło brak zagrożenia koronawirusem?**

Nie wiem, wydaje mi się, że się często nie myśli o samotności osób starszych niestety. I trochę się ich spisuje na straty. Że no dobra, i tak są starzy, to nie trzeba się z nimi spotykać. No nie wiem, nie wiem, ciężko mi powiedzieć. Ale właśnie jak się widziałam ostatnio z babcią, to ona sobie znalazła chłopaka w sanatorium generalnie. I to jest taki internetowy chłopak tylko, ale moja babcia jest strasznie podekscytowana tym chłopakiem i cały czas o nim mówi. W każdym razie babcia się mnie pytała, czy mam jakiegoś chłopaka, ja mówię, że nie mam. I babcia tak patrzy na mnie, mówi Ala, wiesz co, ja ci muszę powiedzieć, że ja codziennie płaczę, odkąd nie ma dziadka, to ja jestem taka samotna. Znajdź sobie kogoś, bo to nie można tak samemu. I nie wyobrażam sobie, że moja babcia z jakiegokolwiek powodu płacze. Zresztą wyobrażam sobie, że babcia nie płacze, tylko… Nie wiem, jakie mogą być zajęcia babci. Ale bardzo mnie uderzyło to, że babcia jest powiedziała, że jest taka samotna, że płacze. Więc na pewno, jeżeli mojej babci jest smutno, to innym babciom pewnie też. A moja babcia ma jeszcze tego chłopaka z internetu, a inne mogą nie mieć.

**Powiedziałaś, że na cmentarze nie chcesz jechać. W sensie tam, zgodziłaś się na jeden, uległaś mamie. Kiedy jedziecie? Byliście już?**

Już byliśmy. Wtedy się widziałam z babcią.

**A macie jakieś plany świąteczne na Boże Narodzenie?**

Nie wiemy chyba jeszcze. Ja nie pytałam, bo nie chcę wiedzieć.

**A normalnie jak spędzałaś? W zeszłym roku na przykład, pamiętasz? Byłaś u rodziców na święta?**

Tak, to akurat pamiętam. Zawsze tak samo, jesteśmy najpierw u jednej babci, a potem u drugiej w Wigilię. A potem w ten pierwszy i drugi dzień świąt to tak różnie, kto ma ochotę to organizować. Teraz nie wiem. Wiem, że na pewno jeżeli nie pojadę na święta do domu, to będzie mi smutno siedzieć samej w domu. Tak, to mimo, że nie chce mi się tam jechać, to wiem, że jakby będzie to dla mnie bardzo trudny wieczór, jeżeli będę musiała go spędzić sama mimo wszystko. Więc w sumie mam nadzieję, że odbędą się te święta, chociaż…

**A w jakiej sytuacji myślisz sobie, że będziesz musiała jakby nie jechać?**

Dobre pytanie.

**W sensie wiesz, kiedy będzie ten moment, kiedy sobie pomyślisz, nie, no to już nie jadę.**

Nie, ja nigdy nie pomyślę, że nie jadę, bo to nie jest zupełnie moja decyzja, czy tam będę czy nie. Tylko po prostu jeżeli moja babcia decyduje, że jest robiona Wigilia, to nie ma dyskusji i się tam jest. A jak babcia mówi, że nie będzie Wigilii, no to… Ale masz rację, to nie musi być wcale nie mój wybór, tylko mogłabym po prostu pojechać sama do babci i siedzieć z nią we dwie. Albo do rodziców chociażby.

**Bo mówisz: gdybym musiała zostać w domu, to będzie mi smutno. I zastanawiam się, co mogłoby cię zmusić do pozostania w domu?**

Tak, no masz rację, po prostu pojadę do rodziców, jeżeli babcia nie będzie robiła Wigilii.

**Ale ja nie chcę cię namawiać.**

Nie, nie, nie, nie. Ale jeżeli faktycznie wypowiadam takie słowa, że będzie mi smutno, to po prostu mogłabym się zdobyć, żeby mi nie było smutno.

**I wtedy się po prostu przełamiesz i pojedziesz, tak?**

Tak. Tak, tak, tak, tak.

**Ale rozumiem, że jeszcze planów nie ma, nie oznajmiła ci mama, jak spędzacie święta.**

Nie. Nie, no ale już teraz podjęłam decyzję, że najwyżej pojadę do rodziców.

**A w międzyczasie mieliście jakąś imprezę rodzinną przez cały ten okres, taki od wiosny do teraz?**

Nie. Nie, mimo że były 50-te urodziny mojego taty. Mimo, że… były urodziny mojego brata. Były też urodziny mojej siostry ciotecznej, mojej mamy.

**To dużo tych urodzin. I nikt nie robił?**

Nie.

**A czemu?**

Bo jest koronawirus. Nie wiem, nie wiem, ciężko mi jest to powiedzieć. To są raczej ich decyzje. Nie robili nic, ale wiem, że na przykład moi rodzice jeździli na działkę i zapraszali różne osoby. I te osoby, które właściwie byłyby na tych urodzinach, były na tej działce.

**A co sądzisz w ogóle o ludziach, którzy w tym czasie, teraz na przykład były te wesela, takie komunie. Dużo było na przykład we wrześniu komunii. Wszystkie się z maja przeniosły na wrzesień.**

Znaczy ja nic nie myślę, bo ja nie czuję potrzeby robienia ani komunii ani wesela. Komunię miałam, ale to nie był mój wybór. Jakbym teraz miała zrobić komunię, to bym na pewno tego nie zrobiła. Tak samo z weselem. Znaczy ja rozumiem, że jak dla kogoś to jest ważne, żeby wydać tyle pieniędzy i zarezerwować tę salę parę lat wcześniej tak naprawdę. I tę sukienkę i te przygotowania, jeżeli dla kogoś to jest ważne, to nie dziwię się, że zrobił to wesele.

**Ale myślisz, że w kontekście sytuacji, którą mamy w Polsce i tego, że były te zachorowania, to była dobra decyzja tych ludzi, żeby robić te wesela?**

Nie wiem. Nie wiem, jeżeli ktoś na przykład wziął kredyt na to wesele i zapłacił te wszystkie zaliczki, no to nie wyobrażam sobie sytuacji, w której ze względu na ilość zachorowań, mieliby to odwołać. Znaczy ja też nie wiem, ile kosztuje wesele. Wiem, że są jakieś takie super drogie wesela za 100 tysięcy na przykład. Albo nawet tańsze. Ale jeżeli ktoś wziął na to kredyt i jakoś nie ma na to pieniędzy, a coś takiego chce robić, no to nic dziwnego, że to zorganizował. Nie wiem, czy na weselach można się zarazić koronawirusem. W sensie, bo nie wiem, ile osób się zaraziło na weselu, a ile na przykład w pracy. To jest to, że ja po prostu nie wiem, gdzie ci ludzie się zarażają, więc nie wiem, co powinnam krytykować.

**Jeszcze masz jakieś przemyślenia koronawirusowe?**

Nie, właśnie chyba nie. Niczego nie zanotowałam, ale na szczęście dużo mi się przypomniało.

**Dziękuję.**
