# Supplementary material for: The challenges arising from the COVID-19 pandemic and the way people deal with them. A qualitative longitudinal study
Source: PLoS One. 2021 Oct 11;16(10):e0258133. doi: 10.1371/journal.pone.0258133 (PMC8504766; doi:10.1371/journal.pone.0258133)
Supplement: S1 Dataset — (ZIP) [file pone.0258133.s003.zip › Transcriptions/stage 6/5.6_M_39_single.docx]

**5.6_M_39_single**

*Rozmowa bardzo utrudniona przez chorobę Kuby. Po pierwszych kilkunastu minutach miałam wrażenie, że nie bardzo jest w stanie się skupić, czasami się “zawieszał”.*

**Co z twoim testem?**

Mam pozytywny. Tydzień byłem dosyć mocno chory a teraz jest już dobrze. Czasami mnie trochę jakby przytyka, czasami gardło, czasami muszę zakasłać, ale już jest lepiej. Nie byłem zdziwiony, że mam pozytywny test, bo u mnie połowa załogi miała pozytywny wynik. Ja wiem nawet po kolei kto się od kogo zarażał, bo to widać było po chorobowym. Ja taką samą chorobę przechodziłem w marcu i było identycznie. Uważam, że wtedy byłem niezdiagnozowany. Oczywiście ja nie do końca cały czas w te testy wierzę. Jak się czyta, to ta metoda PCR jest taka nie do końca wiarygodna i czasami przekłamuje.

**Co czujesz w związku z tym, że już wiesz, że to koronawirus?**

Ja wtedy chyba z miesiąc dochodziłem do siebie. Bardziej ta izolacja teraz mi dokucza. Siedzę od 1,5 tygodnia zamknięty sam ze sobą i jeszcze mam tak siedzieć do poniedziałku, czyli 2 tyg. w zamknięciu. Ja już od razu na kwarantannie zresztą zacząłem być chory, bo jak tylko wróciłem z pracy to byłem już sztywny.

**Musisz zrobić sobie kolejny test?**

Wg wytycznych na stronie gov.pl to już system sam mi nadał, że od dnia, kiedy próbka dojechała do laboratorium jest liczone 10 dni izolacji. System to wprowadza i teraz już nic wg wytycznych nie trzeba robić, chyba że pracuje się w służbie zdrowia. Moje szefostwo zarządziło, że skoro poszła taka zaraza to musimy robić sobie kolejny test, ale tego już nie zleca lekarz tylko zakład pracy i pod zakładem jest jakiś bus. Na czas robienia testu dostaje się podobno nieobecność usprawiedliwioną.

**Jak znosiłeś tę świadomość, że to koronawirus?**

Jak ja dostałem ten wynik, to ja już praktycznie byłem zdrowy. Robiłem go w środę, 6 godzin stania, chory, z temperaturą kurwa, wśród tych ludzi kaszlących. Tragedia. Jakieś 200 osób było. Stare babcie...Nie mogłem na to patrzeć tak mi ich było szkoda, tylko co? Mam 50 osób przepuścić? No sorry. Jak robiłem test to byłem chory w środę, a wynik był w niedzielę. W weekend tak mnie naparzało gardło, że bez konsultacji z lekarzem wprowadziłem ten antybiotyk, który miałem w marcu. Po prostu doczytałem, że to jest na dolne i górne drogi oddechowe.  Po 2 dniach była od razu bardzo duża różnica. Dzisiaj mówię jeszcze powoli i staram się jak najkrócej, żeby sobie nie podrażniać tego gardła.

**Jak ci minęły letnie miesiące? Co się wydarzyło?**

Wróciłem na trasę i trochę tego grania miałem po kraju. Organizowałem też swoje eventy. Nie wiedziałem, jak to wypali, ale to był strzał w 10-kę. Oczywiście zgodnie z przepisami. Na 1-szą imprezę mogłem 150 osób wpuścić, na kolejnej już mogłem wpuścić dużo więcej, bo tam chodziło tylko o to, że na jednego uczestnika ileś metrów. Ludzie wypisywali deklaracje covidowskie na wejściu. To było już legalnie, ale na świeżym powietrzu. Ta druga impreza to już na kilkaset osób. Czekałem na to bardzo. Nie dość, że ludzie byli bardzo głodni imprez, a w tym mieście to już w ogóle i pogoda dopisała, bo było w nocy ze 30 st. To przerosło moje oczekiwania. Oczywiście na wejściu był dezynfekator i te deklaracje. nakazane było mieć maseczki, ale nakazywać wtedy można było. Ludzie chcieli odetchnąć, żyć.

**Na czym polega ta deklaracja?**

Zatwierdzają regulamin m in. o dezynfekcji, o maseczkach, o bezpiecznej odległości.

**Co jeszcze się wydarzyło?**

W tym czasie bardziej ze wszystkim i się widywało, rozmawiało. Ludzie mówili, że mają dość i mają w ogóle gdzieś tę pandemię i nie wierzą w to.

**Zanim poszedłeś na kwarantannę, to jak wyglądała twoja codzienność? Czułeś, że coś nadchodzi, że może ta 2 fala?**

Ja przestałem w lato i już nawet na wiosnę czytać o tych zakażeniach, ale na pewno widać było, że to wszystko znowu idzie do lockdownu. Nagłaśniane to było.

**Pamiętasz ten moment, kiedy pomyślałeś, że to znowu się zaczyna?**

Może i był, ale ja jakoś nie zarejestrowałem tego. Po prostu widać było po imprezach, że zaczynają wprowadzać obostrzenia, że te żółte i czerwone strefy. Trzeba było mniej ludzi w tych klubach i na zewnątrz. Ja już musiałem potem jedną swoją imprezę odwołać. To było do przewidzenia. Jesienią ludzie zawsze chorują. Teraz pod każdą chorobę się podpina koronawirusa tak jakby innych nie było. Gdyby te testy były miarodajne i pewne w 100%, ale nie są. W tamtym okresie zaczęły się też rozmowy z przyjaciółmi na temat naszej gospodarki a raczej jej braku kurde, bo to jest dramat. Było wiadomo, że na jesieni obnażona zostanie już tak na dobre nasza służba zdrowia a nasza gospodarka to było wiadomo, co się wydarzy, bo to jest jak domino. Tyle branż przestało zarabiać, w tym moi znajomi, że współczuję im po prostu tego momentu, w którym są.

**Czy w twoim życiu codziennym coś wróciło do stanu sprzed pandemii?**

Co mogło wrócić do normalności...Boże, nie wiem. Człowiek już nawet nie myśli o tym co było kiedyś tylko stara się ułożyć jakoś do danej sytuacji i to jest chyba naturalne, że człowiek się musi przystosować.

**A jest coś, co ci szczególnie przeszkadza?**

Na pewno rząd, władza, która tylko dolewa oliwy do ognia wprowadzając ustawy/ nie ustawy i ludzi tylko jeszcze bardziej wkurzyli. Nie dość, że są ludzie wkurzeni na to co władza nasza wprowadza, to jeszcze są wkurzeni na to, że nie pracują, nie zarabiają, potracili majątki i są zdeterminowani` i wkurzeni podwójnie. Ja się nie dziwię, że w czasie pandemii ludzie wyszli na ulicę. Też byłem na strajku, ale potem się rozchorowałem. państwo też się nie przygotowało do ewentualnej 2 fali, a Morawiecki jak mówił, że już nie ma się czego bać, to zależało mu tylko na tym, żeby się wybory odbyły i żeby wygrali. To było jasne.

**Emocje**

3 - to zaczynanie, budowanie czegoś na nowo, przystosowanie się do obecnej sytuacji, taka trochę kalkulacja, w co warto teraz zainwestować i gdzie rozpoczynać jakieś nowe projekty.

6 - las, który stał się dla mnie taką oazą spokoju. Tam się wyciszam i relaksuję.

13 - taka wolność, bo w lecie człowiek w ogóle nie myślał o pandemii tylko robił na co miał ochotę.

12 - tak było do lipca, bo ja nie sądziłem, że w ogóle będę grał na jakichś imprezach. To jest taka stabilność. Praca, dom, jakiś las, jezioro i nic więcej.

**Jak jest teraz?**

4 - to czas strajków. Takie związanie międzyludzkie, że w słusznej sprawie ludzie się zebrali do kupy. Taka solidarność.

16 - to jest nasze państwo, nasza władza, która przez swoją głupotę i niekompetencję podpaliła ten kraj.

14 - to są moi znajomi, którzy mają powiązane ręce, są bezsilni i są w totalnej kropce, są bez pracy.

10 - to aura za oknem, bo jesień i ludzie teraz znowu będą pozamykani w domach.

1 - świat będzie znowu raczej bez samochodów, bez samolotów.

**Wracamy do tego co było w marcu czy będzie jakoś inaczej?**

Nie, no gorzej będzie. Wtedy wszyscy wiedzieli, że to jest chwilowe, że za chwile będzie wiosna i lato, a teraz się dopiero zaczęła jesień.  Ja się spodziewałem, 2 fali.

**Jest coś, czego się obawiasz?**

Tego, co właściwie się już dzieje, tego, że jest bardzo źle. Sytuacja gospodarcza w naszym kraju i nie tylko, ale i służby zdrowia. Kontakt z lekarzami jest tak mocno ograniczony, że koronawirus nam zablokuje dostęp do normalnej służby zdrowia w celach innych. To może być większe niebezpieczeństwo niż koronawirus.

**Jakie obawy są u ludzi w twoim otoczeniu?**

Każdy wie, że jest bardzo zły moment i nie mam takich znajomych, którzy uważają, że jest świetnie. Nawet jeśli u nich jest wszystko ok z pracą itd., to ktoś dookoła ma gorzej. mam kolegę, który od pół roku nie ma kontaktów z rodzicami. Tylko na odległość, przez płot pogada. Nie wnikałem, dlaczego i kto bardziej się boi, ale przyjęli taką formę komunikacji.

**A twoje kontakty?**

Jakoś tak się zrobiło, że człowiek blisko jakoś z innymi nie był. Potem było trochę bliżej i teraz prawdopodobnie pozarażałem wszystkich w domu. tata chory, mama chora, brat, dziewczyna mojego brata. Dramat.  Tata najgorzej to przechodzi i zobaczymy jak będzie. Na razie przeczekują i jak ich by zamknęli to teraz nie ma kto im przynieść jedzenia. Mama nadal pracuje, tata jest na emeryturze. Oni na razie uważają, że mają jakąś infekcję, ale wiedzą, że ja miałem test pozytywny.

**Kto teraz dba o ciebie?**

To różnie. Sąsiad, koleżanka, brat. Ja bardzo duże zakupy zrobiłem 2 dni przed tym jak się rozchorowałem, więc byłem zaopatrzony w prowiant.

**To nietypowe dla ciebie. Co spowodowało, że takie duże zakupy?**

Żeby rzadziej wychodzić do sklepu, Teraz robię rzadziej i nie takie krótkoterminowe.

**Pamiętam, że w marcu starałeś się jak najmniej do sklepów i na jak najkrócej. Teraz jest tak samo?**

Nie, aczkolwiek teraz jak wyjdę z zamknięcia to na pewno będę miał trochę z tyłu głowy, żeby omijać skupiska. Na pewno jestem w tym momencie trochę bardziej narażony na infekcje, bo jestem po antybiotyku i osłabiony po chorobie i mogę coś innego złapać.

**Czy przejście zakażenia daje ci jakieś poczucie bezpieczeństwa?**

To tak uspokaja człowieka, że przetrwałem i dam sobie radę.

**Co teraz myślisz o koronawirusie?**

On jest tylko jest jakąś odmianą grypy, która jest może bardziej zaraźliwa. Ja nie do końca twierdziłem, że go nie ma. On jest tylko ja go traktuje jak każdą inną chorobę. To się nie zmieniło i trzeba się nauczyć z nim żyć.

**Uważasz, że on jest demonizowany?**

Czasami nie wiadomo co o tym myśleć. Człowiek przechodzi ze skrajności w skrajność. Może jest, może trzeba uważać, może nie. Potem przychodzą wakacje i się nic nie dzieje...Nie wiem. Państwo to też...Takie ruchy, że na Narodowym zrobią szpital to tylko ludziom grają na psychice. To oznacza, że nasza służba zdrowia jest w totalnie złej kondycji. Jeśli jest tylu` zakażonych, którzy mają objawy i trzeba ich hospitalizować, to uważam, że taki szpital na stadionie jest trochę nie na miejscu.

**Dlaczego?**

Uważam, że tylu chorych nie ma nawet jeśli są z tymi pozytywnymi wynikami. Ci co są w szpitalu to muszą najwyraźniej być w szpitalu, ale ci, co mają dodatni wynik nie muszą.

**To ten stadion będzie dla kogo?**

No właśnie nie wiem.

**Co rząd powinien był zrobić?**

Nie okłamywać ludzi. to wszystko jest przekazywane tak jak im pasuje. jak trzeba było wygrać wybory to wirusa nie ma i jest w odwrocie.

**Powinni byli utrzymywać większe obostrzenia przez cały czas?**

Nas nie stać na to i żadnego z krajów. Gospodarka musiała ruszyć. Największe zaniedbanie to jest służba zdrowia. Powinni byli przystosować szpitale kupić respiratory. No nie wiem, skoro jest tak źle.

**A czy coś zrobili dobrze?**

No nie. Ja nie widzę pozytywów. Żadnych.

**Spodziewasz się, że ten lockdown będzie taki sam jak wiosenny czy jakiś inny?**

Co najmniej taki. Nie wiem czym jest kwarantanna narodowa. Może zamkną wszystko zostawiając apteki i sklepy spożywcze. Może zamkną granice, może godzina policyjna. Mojej pracy nie zamkną.

**Wiosną bałeś się, że cię mogą skoszarować?**

W tym momencie, to są wydziały, gdzie pracownicy przychodzą co 12 godz. do pracy, bo połowa załogi jest na kwarantannie albo na izolacji. Ja też byłem na nadgodzinach i pewnie jak wrócę to pewnie też będę. Generalnie jest zakład na to przygotowany, że nas tam zamkną. Zobaczymy, nie wiem.

**Śledzisz zmiany w obostrzeniach?**

Tak mniej więcej, żebym wiedział co mogę załatwić, a czego nie. A i zapomniałem o ważnym aspekcie, bo zamykają tyle rzeczy a nie zamykają kościołów. Skoro odsetek starszych ludzi, którzy to ciężej przechodzą albo umierają jest dużo wyższy, to czemu nie zamykają kościołów? Bo to jest ich elektorat.

**A te godziny dla seniorów?**

Jestem ciekaw, czy seniorzy rzeczywiście korzystają> Później chyba też mogą chodzić? jakie są te godziny?

**10-12.**

Dlaczego to ma ich chronić? Jakie tu ma znaczenie godzina?

**Powinno się ich bardziej zatrzymać w domach?**

W jakimś kraju już chyba jest izolacja starych ludzi. Nie pamiętam, gdzie, ale to podobno wcale im nic nie dało. Starzy ludzie nie chodzą na siłownie, raczej mało jeżdżą na rowerach, nie uprawiają sportu. Ich sportem jest iść do sklepu albo do lekarza i nie ma ich chyba aż tylu.

**Co myślisz o zamknięciu restauracji, siłowni, basenów?**

W zdrowym ciele zdrowy duch, więc siłowni się chyba nie powinno zamykać. Widziałem, że w siłowniach wszyscy byli w maseczkach. W restauracjach było widać, że co 2-gi stolik, więc to jest jakaś odległość. Są 2 strony medalu, bo mówimy o zdrowiu i o kwestii gospodarczej. Jeśli ludzie są świadomi zagrożenia to i tak mniej wychodzą, więcej zostają w domu. To wszystko się znowu sprowadza do wiarygodności tych wszystkich testów. Gdyby to było pewne to mielibyśmy jasny obraz.

**Jeśli nie jest pewne, to czy dobrze, że pozamykali właśnie to?**

Ciężko powiedzieć.

**A szkoły w trybie online?**

To ma sens, ale w przypadku pełnego lockdownu, bo jeśli go nie ma, to co da wysłanie dzieci na online? Co ci rodzice zrobią z tymi dziećmi? Z tego co gdzieś wyczytałem to nie ma znaczenia czy to są dzieci czy nie, jeśli chodzi o zarażanie. jakaś bzdura była na wiosnę, że dzieci nie zarażają, więc szkoły online mają sens w przypadku, jeśli rodzice są na lockdownie.

**A zakaz imprez to dobry ruch?**

Jak zamykamy, to zamykamy i już, więc dlaczego są równi i równiejsi? Dlaczego kościołów nie zamykają zamiast siłowni?

**Jak jest z twoim stosowaniem się do obostrzeń? Jak było latem, jak jest teraz?**

Maskę noszę, nie? W lecie nie nosiłem. Zakładałem w sklepie, żeby nikt się nie przyczepił.

**Jaką maskę nosisz?**

Mam takie z pracy albo mam taki komin, albo bandankę. Jakoś nie za często piorę i zmieniam. Jak mi się przypomni. Noszę, bo trzeba nosić. Z tego co wiem to te maseczki po 3 minutach są do niczego. Pewnie te moje domowe jeszcze szybciej, ale jak wszyscy to wszyscy. Może to w jakiś sposób powoduje zmniejszenie. Coś tam może pomaga.

**Wiesz jak się zaraziłeś?**

Mieliśmy szkolenie przy jednym stole a w pracy chodzimy bez masek.

**A jak jest w tym pracowym autobusie?**

Teraz już wszyscy są w maskach. Może się trochę przejęli sytuacją.

**Masz wrażenie, że ludzie zachowują się adekwatnie do sytuacji, która jest?**

Trzeba powiedzieć, że wszyscy noszą te maski wszędzie teraz.

**Czy któreś z obostrzeń dają ci zwiększone poczucie bezpieczeństwa?**

Obostrzenia mają chronić innych. Dezynfekcja rąk jak najbardziej, dystans też, ale ciężko czasami uniknąć. Las to był największy absurd.

**A jest teraz jakiś podobny absurd?**

W mniejszej skali, ale te kościoły.

**Robić lockdown czy nie?**

Nie wiem, musiałbym to jakoś bardziej przemyśleć.

**Czy szukasz informacji na temat tego co się dzieje?**

Nie. Ja nawet nie mam świadomości od wiosny, ile jest tych zakażeń. Wiem, że teraz jest dużo, że przodujemy w tym na świecie, ale nie szukam tego. czasami zrobię szybki research i wycofuje się, bo ja mam swoje życie, swoje sprawy i staram się omijać te tematy, dostosowywać. Robię jak można i jadę dalej. Nie mam na to wpływu.

**A co zależy od ciebie?**

Jeśli zakładam wszędzie maskę to mam jakiś wkład w to wszystko i tyle.

**Skąd przychodzą do ciebie teraz sygnały o tym co się dzieje?**

Fb, znajomi, Instagram.

**Twoim zdaniem jest dużo gorzej niż było wiosną?**

Tak. jest niepokój wśród ludzi i o gospodarkę i o zdrowie. Ja też mam większy niepokój niż miałem wiosną, ale boję się bardziej o znajomych, o rodzinę niż o siebie. O samego siebie raczej nie.

**Kiedy będzie koniec pandemii?**

Nie będzie. koronawirus będzie już zawsze i trzeba się nauczyć z nim żyć. Nie wiem co ze szczepionkami, ale na razie chyba jeszcze daleka droga. Nie wiem, czy one będą, czy to ma sens. My wiemy o nim jeszcze bardzo mało. Pół roku wiedzy na temat nowego wirusa to chyba mało. Nie wiem czy w ogóle powstanie coś takiego jak lek na wirusa, który się mutuje. Z tego co czytałem to nabycie takiej masowej odporności to są lata a nie miesiące.

**Jak teraz będzie wyglądał świat?**

Świat musi się dostosować do warunków, musi się dostosować gospodarkę. Mnóstwo branż poupada i nie wrócą już nigdy do takiej formy, jak np. branża eventowa, turystyczna, hotelarska, linie lotnicze, gastronomia. Wszyscy teraz będą mieli z tyłu głowy jak dostosować swój sposób zarabiania pieniędzy do takiej sytuacji.

**A społecznie coś się zmieni?**

Psychicznie podupadnie cały świat i psychiatrzy, psychologowie będą mieli pełne ręce roboty. Ludzie kłócą się w domu, tracą majątki, napięcie jest bez przerwy dookoła Już wskaźnik przemocy w rodzinie wzrósł, samobójstwa wzrosły. Niestety, ale to było do przewidzenia.

**A znasz ludzi, którzy sobie dobrze radzą pomimo tej sytuacji? Widzisz, że mają jakieś sposoby na to?**

Mało jest takich ludzi, którzy już przeszli z pkt. A do pkt. Może teraz więcej ludzie piją, żeby sobie radzić? Myślę, że wielu ludzi się za ruch w lecie, bo wiedzą, że na odporność dobry jest ruch.

**A u ciebie coś się zmieniło? Doszły jakieś nowe sposoby radzenia sobie lub pewne przestały działać?**

Staram się na ile mogę oszczędzać, czy może bardziej nie wydawać pieniędzy. Myślę 2 razy zanim coś kupię, bo wszystko podrożało. Nic nie doszło nowego. Muzykę nadal robię, ale nie ma teraz występów. Do streamingu pewnie wrócę jak wyzdrowieję. Myślę, że ci ludzie, z którymi byłem blisko teraz są jeszcze bliżej.

**Co myślisz o zamknięciu cmentarzy?**

Decyzja najgłupsza to zamykanie dzień przed. Ludzie cały rok czekali, żeby zarobić. Co najmniej 2 tyg. wcześniej powinna być taka informacja, bo czy się pójdzie w ten dzień czy w inny to nie o to chodzi.

**Jak myślisz co będzie z Bożym Narodzeniem?**

Nie wiem. Za daleko jeszcze do tego. I tak pójdę do rodziców.

**A powinien być stan wyjątkowy?**

Nie stać ich, ale jeśli tak już to poszło to dla dobra gospodarki, dla przedsiębiorców, żeby mieli z czego żyć, to owszem.

**Czy uważasz, że lockdown zmniejszyłby liczbę zakażeń?**

Tak.

**A jak na tę liczbę wpływają protesty?**

Ja protesty popieram. Ludzie po prostu mają coraz mniej do powiedzenia i to o to tu chodzi. Ludzie powinni mieć wybór i państwo nie powinno o tym decydować. Ludzie nie mogą teraz tego przeczekać, bo teraz albo nigdy. Oni wprowadzają coś tak jak chcą i dobrze wiedzą, kiedy to robią. Wzięli na pewno to pod uwagę, że ludzie się zbuntują i oni wtedy powiedzą, że liczba zakażeń to przez te strajki. Oni dokładnie to przekalkulowali i mają w dupie te zakażenia. Władza dba o siebie.
