# Supplementary material for: The challenges arising from the COVID-19 pandemic and the way people deal with them. A qualitative longitudinal study
Source: PLoS One. 2021 Oct 11;16(10):e0258133. doi: 10.1371/journal.pone.0258133 (PMC8504766; doi:10.1371/journal.pone.0258133)
Supplement: S1 Dataset — (ZIP) [file pone.0258133.s003.zip › Transcriptions/stage 6/7.6_M_28_couple, no children.docx]

**7.6_M_28_couple no children**

**Co się działo u ciebie od czerwca? Co zapamiętałeś?**

Przede wszystkim zapamiętałem, że do połowy września czułem się dużo luźniej niż wcześniej. W pracy za wiele mi się nie zmieniło, ale miałem zupełnie inne wakacje niż od 8 lat, bo zawsze cały lipiec i sierpień miałem wyjęty, więc wakacje zaczynałem koło 20.08. Tutaj było inaczej i były bardziej normalne wakacje w lipcu, ale tak nie do końca normalne. Udało nam się wyjechać w sierpniu samochodem do Słowenii, bo granice były jeszcze otwarte, zachorowania były w miarę akceptowalne przez rząd i inne rządy. Akurat jak wróciliśmy w niedzielę ze Słowenii, to w piątek zamknięto granicę w Słowenii. Na wakacjach było bardzo fajnie. Miałem wrażenie, że trochę bardziej się przestrzega zasad, restrykcji, itd. w Słowenii niż w Polsce. Byliśmy dobrze przygotowani, tzn. mieliśmy kilka maseczek na wyjazd, wszystkie dokumenty, ubezpieczenie na wszelki wypadek, ale jakiegoś takiego super lęku nie mieliśmy, że coś się może wydarzyć. Nie obawialiśmy się, że możemy być zakażeni i ponieść to gdzieś dalej. Było bardzo miło. Na początku lipca wróciły też jakieś spotkania ze znajomymi. Na pewno nie w takim stopniu jak rok wcześniej, ale wróciły. Takie spotkania raczej w małym gronie, na max 5-8 osób a nie na np. 50. Czuliśmy się zdecydowanie luźniej, tym bardziej że te spotkania były na świeżym powietrzu, więc nie czuliśmy super dużego zagrożenia, ale oczywiście też z zasadami zachowania bezpieczeństwa i dystansu. Oczywiście, na ile to było, możliwe, bo czasami jak się przebywa w takiej większej grupie to tak się do końca pewnie nie da. Zapamiętałem jeszcze z wakacji częstsze wychodzenie, większą swobodę i tak jak wcześniej czułem jakiś lek w głowie, czy coś takiego, to tutaj jakoś to minęło. Nie mówię, że czułem się w ogóle świetnie i luźno, natomiast na pewno lepiej niż wcześniej.

**Kiedy poczułeś, że zaczyna wracać brak luzu, że sytuacja się zmienia?**

O ile bardzo łatwo mi przyszło przyzwyczajenie, że trzeba nosić maseczki w sklepach, to później ten moment, że znowu trzeba nosić maseczki na ulicy. Ale to też działało zupełnie inaczej niż na samym początku. Na początku ta sytuacja była bardziej nieznana, niepewna, a teraz mimo tego, że liczba zakażeń jest dużo, dużo większa, obycie z tą sytuacją przez kilka miesięcy miało taki efekt...Nie podchodzę do tego olewająco i od razu się zastosowałem do tego, ale te pierwsze kilka dni po tej przerwie były takie inne. Jeśli trzeba je nosić to trzeba. Wydaje m i się, że już kilka dni wcześniej zanim był ten obowiązek to już myślałem, że jednak trzeba bardziej uważać i założyć tę maseczkę trochę częściej niż tylko w sklepie czy gdzieś w pomieszczeniu.

**Jak teraz wygląda twoja codzienność?**

Bardzo podobnie jak przed latem. Znów więcej siedzę w domu i znów wróciło takie poczucie, że lepiej znowu ograniczyć te wyjścia. Raczej wyjścia typowo zakupowe albo jak mam super ważną sprawę do omówienia z szefem to 3-4 razy się z nim widziałem w ciągu miesiąca, ale to też w takim mocnym dystansie i w maseczce nawet siedząc w kawiarni. Zmieniła się też swoboda pod takim względem, że miałem plany, żeby się spotkać z rodzicami czy z kimś tam i jednak w rozmowach stwierdziliśmy, że może warto poczekać, że teraz nie jest zbyt pewnie, że by to robić. Bardziej się czuje chyba zagrożony ja niż moi rodzice, bo mój tata pracuje z wieloma ludźmi, więc to bardziej chyba on mógłby mnie zarazić niż ja jego, mimo tego, że to ja żyję w wielkim mieście to chyba oni są trochę większym zagrożeniem niż ja. Mam mniej spotkań służbowych, więcej maila, więcej telefonu znowu. Podobało mi się, że w wakacje wystarczało spotkanie na 2-3 godz. i to zastępowało konwersacje w 30 mailach. To się trochę zmieniło.

**Co jeszcze jest podobne do codzienności wiosną?**

Chyba trochę szybciej znowu poczułem takie zmęczenie sytuacją. Wtedy to nastąpiło po miesiącu, dwóch. Dokładnie nie pamiętam, ale chwilę to trwało. Teraz jak było kilka miesięcy luzu, było lato, ciepło a teraz ciemno, coraz zimniej i jednak mniej chce się siedzieć w domu, mimo wszystko. Może to jesień, ale może też to ta sytuacja. Codzienność to też ostatnie 7 dni i byliśmy oboje na protestach 3 razy. Tego bym się nie spodziewał, że będą takie sytuacje się dziać.

**Teraz i na wiosnę - jakie różnice widzisz?**

Na pewno mniej strachu mimo wszystko. Może już pewne przyzwyczajenie do sytuacji i obycie się z nią, ale też mam wrażenie, że mam trochę większe zaufanie do siebie. Faktycznie uważam, tzn. jak były 1-sze tygodnie cały czas miałem z tyłu głowy, żeby tylko nie zapomnieć się zdezynfekować po przyjściu ze sklepu. Teraz już jestem o to spokojny i robię to automatycznie. Wiem jak się zabezpieczyć i jak się zdezynfekować później. Nie mam aż takiego wielkiego strachu, że mogę się zarazić, staram się uważać jak mogę. To się nie zmieniło, ale zmieniło się podejście do tego i jest bardziej automatyczne i weszło mi w nawyk.

**Masz jakąś listę rzeczy, których starasz się przestrzegać? Co robisz, jakie masz zasady?**

Nie spotykam się w większym gronie niż 3-4 osoby, nie chodzę do kina, do GH, do restauracji. Już teraz nie można, ale i tak w ciągu ostatnich dni nie chodziłem. Maseczka czy przyłbica przestały mi już mocno przeszkadzać. Nawet pod koniec lata byliśmy w kinie dwa razy i nie było problemu, żeby wysiedzieć w maseczce. Mam zawsze żel antybakteryjny i dezynfekuje się przed i po, unikałem skupisk ludzi. Unikałem, bo protestów trochę zmieniły sytuację, ale tam też staram się mocno uważać. Mam takie poczucie, że jeśli się zarażę to z czynników bardziej niezależnych niż zależnych ode mnie.

**Jest coś, co było na wiosnę, jakieś zachowanie, które było i już go nie ma, bo już np. obyłeś się trochę z tym?**

Miałem takie podejście, że może jakimś cudem lato zmieni sytuację epidemiczną. Nie wiem, dlaczego. Może były jakieś mity, że temperatura zabije wirusa, ale to szybko minęło. Mimo wszystko to lato i poczucie, że będzie trochę więcej swobody być może ze względu na wolne powietrze...To dawało taką większą nadzieję. nawet pamiętam jak kiedyś pokazywałaś mi zdjęcia to wybrałem słońce, bo jakaś nadzieja. Teraz nie mam chyba takiej potrzeby, żeby mieć nadzieję. Po prostu jest ta sytuacja. cały czas dochodzą do mnie słuchy o postępach w szczepionkach czy coś, ale wiem, że to jest raczej odległe i to potrwa. Wtedy liczyłem, że to może się jak najszybciej zamknie, że może w ciągu kilku miesięcy.

**Nie masz potrzeby mieć nadziei, bo się pogodziłeś z tym, czy myślisz, że już nie ma szans, żeby się skończyło?**

Chyba trochę to drugie. Jak będzie mogła się ta sytuacja jakoś rozwiązać to się rozwiąże. Nie staram się stawiać jakichś terminów, że np. żeby tylko wytrzymać do wiosny, bo wtedy może będzie wszystko w porządku. Tak właśnie robiłem wiosną. Teraz tak nie mam, ale może też dlatego, że zobaczyłem, że tak też się da funkcjonować, mimo obostrzeń i rezygnacji z czegoś i nie jest to aż tak wpływające na samopoczucie czy życie. Wiosną chyba bardziej to wszystko przeżywałem. być może dlatego, że to była nowa sytuacja. Może też pora roku wpływała na to, że zbliżające się lato dawało jakąś większą nadzieję. Teraz chyba tak realnie na to patrzę.

**Coś ci teraz przeszkadza w tej sytuacji?**

To co się dzieje ze strony rządu chyba najbardziej. Wiosną to nawet ci opowiadałem, że irytuje mnie fakt, że ludzie olewają te maseczki czy jakieś tam środki, a teraz będąc na protestach widzę, że każdy ma maseczkę i stwierdzam, że chyba ludzie sobie to wzięli do serca. Wzrost zakażeń temu przeczy, natomiast sytuacje, które się dzieją ze służbą zdrowia czy podejście rządu, który skupia się na rzeczach zupełnie innych niż pandemia, trochę mi psuje to wszystko.

**Za ten wzrost zakażeń odpowiadają zachowania ludzi?**

Nie do końca potrafię powiedzieć, bo z jednej strony nikt nie lata samolotem i nie rozpyla tego nad ludźmi, żeby się zarazili i to oni się personalnie zarażają. Może na to wpływać fakt, ze szkoły zostały otwarte. mnóstwo ludzi też wyjeżdżało. Nie do końca wiem. Jednak chyba to musi być w dużym stopniu wina ludzi. Tak czy tak. Wiem, że np. branża klubowa nie do końca przestrzegała wszystkiego, co musi być przestrzegane, więc nie potrafię powiedzieć czy to jest wina kilkorga ludzi, czy tych wszystkich, którzy chodzili na imprezy. Powinno być tam 150-200 osób, a ktoś potrafił wpuścić 1000. Z jednej strony ponoszą za to odpowiedzialność ludzie, którzy to umożliwiają, a z drugiej sami ludzie. Ja, gdybym widział, że jest tyle osób w pomieszczeniu to bym raczej z niego wyszedł, a ludzie chyba nie wychodzili. Być może ta odwilż obostrzeń tak wpłynęła na ludzi rozluźniająco. Pośrednio wydaje mi się, że rząd sobie z tym nie poradził i nie wiem, jak podzielić tę odpowiedzialność. Przede wszystkim każdy powinien uważać na siebie.

**Masz wrażenie, że ludzie sobie pozwalali na więcej niż było dozwolone?**

Wydaje mi się, że tak. Lato pod względem imprezowym było dosyć mocne. jakieś wspólne wyjazdy, wakacje. nie mam też pewności, czy ten wzrost zakażeń to nie jest efekt większej liczby testów niż w wakacje. Cała ta sytuacja stwarza wiele wątpliwości i dylematów. Nie wiem co jest prawdą, co jest czyja winą. Trochę się zacząłem w tym gubić.

**Emocje**

6 - wakacje - znowu muszę wybrać to słońce, ale nie dlatego, że to symbol nadziei tylko symbol takiego dobrego samopoczucia chyba. Większa swoboda. przestrzeń. Słońce, które oznacza, że jest milej i swobodniej, a z drugiej strony jest w ciemnym lesie i nie do końca rozświetla te wszystkie drzewa, i mimo wszystko nie jest do końca tak dobrze i fajnie, tylko jednak jakaś taka ciemność wokół tego słońca.

1 - wróciliśmy ze Słowenii i zamknęli granicę. Gdyby nie udało nam się wyjechać to chyba czułbym się jak w tym korku taki uwięziony lekko, taki bez możliwości wytchnienia. Jak pomyślałem sobie, co by było, gdybyśmy wyjechali tydzień później i musielibyśmy wracać, to czułbym się wtedy bardzo źle. Właściwie czułem się lekko zestresowany, bo w weekend, kiedy wracaliśmy to Austria chyba zamknęła możliwość wjazdu dla Czechów i pod koniec wyjazdu już chyba byliśmy dość mocno zestresowani, bo słyszeliśmy co się dzieje i spodziewaliśmy się, że właściwie mogą nam nawet zamknąć granicę, kiedy będziemy chcieli wrócić albo trafi nam się jakaś kwarantanna, itd. W pierwszej chwili przypomniał mi się ten stres, że najpierw obawa, że mogliśmy w ogóle nie wyjechać, albo musielibyśmy się wracać, albo już przy powrocie stanąć w wielkim korku lub mieć problemy z wjazdem. taki stres, którego już nie było, ale jednak gdzieś z tyłu głowy cały czas był.

**Jakiś następny ważny moment?**

Pod koniec sierpnia spędziłem weekend u rodziców i wracając zastanawiałem się czy to było mądre, bo oni mieli kontakt z wieloma ludźmi i potem jeszcze większy moment zagrożenia, kiedy się dowiedziałem, że mój przyjaciel, z którym się widziałem...Spędziłem z nim wieczór oglądając razem mecz, więc to trwało 3-2.5 godz. i okazało się, że musi iść na kwarantannę. Zrobił test i bardzo się bałem, że ja też mogę być zarażony, ale okazało się bardzo szybko, że nie był zarażony. Wtedy pomyślałem z sobie, że na co dzień w wielu sytuacjach uważam na siebie, a jest taki moment nieuwagi, zaskakujący czasami i mógłbym być już z koronawirusem.

**Któryś obrazek pasuje do momentu, kiedy jeszcze nie było wyniku testu?**

Chyba 2 - mam z tyłu głowy, że mogę się zarazić w jakiejś niewinnej sytuacji, ale jakby nie dokuczałem tego do głowy. Myślałem, że uważam na siebie, przestrzegam zasad i raczej powinno być ok, a tu jedno niewinne spotkanie i takie wejście jak w gumę.

**Jak teraz się czujesz?**

5 lub 7 - jakby długa droga przed nami i aura oddaje to, co się dzieje za oknem. Bardziej 7. Ja bardzo lubię jesień, ale taką, kiedy mogę przebywać swobodnie na zewnątrz po prostu, a teraz czuję, że nie mogę.

**Masz rytuały, bo miałeś różne na wiosnę?**

Lista zakupów jest nadal i to się nie zmieniło, ale to raczej z nawyku i to mi przychodzi już automatycznie. Z tym wychodzeniem co 3 dni to już tak nie do końca. Teraz jest troszeczkę luźniej i jak mi zabraknie czegoś to nie muszę czekać tych kilku dni, żeby znowu wyjść na zakupy tylko idę i kupuję. Te zakupy też nie są codziennie na pewno.

**Ta długa droga przed nami to jakie to emocje?**

Nie jestem może szczęśliwy z tego powodu, ale jestem raczej spokojny i czuję się dosyć stabilnie. Trzeba tę drogę pokonać i wytrzymać. Nie jest to moja ulubiona droga, ale też nie jestem codziennie bardzo zły. Właściwie w ogóle nie czuje złości tylko czuję, że musi upłynąć ten czas i muszę to jakoś znieść.

**To jest takie zadaniowe, że zacisnąć zęby i dać radę?**

Z jednej strony nie, bo nie staram się myśleć o jakiejś dacie, kiedy to się zmieni, ale też mam takie poczucie, że kiedyś to się musi wydarzyć po prostu. Nawet, jeśli to będzie rok, to na tyle się przyzwyczaiłem i przywykłem, że jestem w stanie to znieść.

Odrobinkę wyczekuję, ale wiem, że to szybko nie nastąpi.

**Jak sobie radzą twoi rodzice?**

Pracują i nie jest to praca, która można wykonać w domu, bo tata pracuje w fabryce, która produkuje meble, a mama dorabia sobie w bistro. Wiem, że uważają, ale chyba są bardziej zagrożeni nawet w takich przypadkach niezależnych od nich. mam większe opory, żeby się z nimi zobaczyć przez to.

**Gdybyś mógł im coś sugerować, to np. sugerowałbyś mamie, żeby zrezygnowała z tej dodatkowej pracy?**

No tak. Rozmawiałem z nią o tym i teraz sytuacja trochę się wyjaśniła, kiedy gastronomia ma tylko dowozy i wynosy. tamto miejsce było głównie dla uczniów liceów, więc teraz nie funkcjonuje. Mieliśmy takie rozmowy i mówiła, że czeka co się wydarzy, ale teraz mówi, że sama nie wie, czy wróci. Trochę ją namawiałem, nie robiła tego, jeśli nie musi. Tak na spokojnie.

**A jak twoje rodzeństwo?**

Siostra wróciła z Hiszpanii do Polski, bo tam zwolnili ją z pracy, więc trochę nie miała co tam robić. Ona mieszkała w Dublinie i pracowała dla Ryanaira w biurze, potem przeniosła się do Hiszpanii. Teraz pracuje dla Ryanaira irlandzkiego, ale w polskim biurze i niestety to biuro jest daleko, więc narzeka, że musi dojeżdżać, że pracuje z ludźmi. Czuje się trochę bardziej zagrożona i nie dziwię się. ona troszkę bardziej się boi niż ja. Brat teoretycznie jest dyrektorem finansowym w firmie, która ściąga jakieś duże urządzenia chłodnicze do fabryk, ale jednocześnie to jest tak mała firma, że czasami musi jeździć do tych kopalń czy fabryk, żeby coś tam nadzorować i on też czuje się troszkę bardziej zagrożony. Jak jeździ gdzieś dalej to nie zostaje nawet na noc, nie chodzi po restauracjach tylko raczej starał się ograniczać kontakt z ludźmi. Każde z nich czuje jakiś większy lęk niż ja, ale ja nie muszę pracować z ludźmi aż tak mocno.

**Masz znajomych, którzy boją się mniej niż ty?**

Tak naprawdę chyba nie. Znam osobę, która od marca w ogóle nie spotyka się z ludźmi. Bardzo rzadko się z kimś widzi i to tylko spacery w dużej odległości, a tak to tylko sklep i jakiś spacer krótki i bardzo cały czas się obawia. Było kilka ważniejszych okazji, żeby się zobaczyć, ale mówiła, że się boi, że nie chce ryzykować i już. Mam też takie grono znajomych, które pod względem imprezowym i towarzyskim tak luźniej podchodziło do tego, pojawiły się kluby, domówki, gdzie było dużo osób i nawet picie z jednej szklanki czy coś takiego. Mniej się przejmują, mniej się ograniczają.

**Masz kogoś w otoczeniu, kto zachorował, źle się czuł?**

Z bliskiego otoczenia nie znam nikogo, kto zachorował. Pod koniec lata z grona moich dalszych znajomych z branży muzycznej kilka osób zachorowało, bo to się roznosiło na imprezach. Z bliższego otoczenia kilka osób miało podejrzenie i oni sobie robili takie domowe kwarantanny zapobiegające, ale na testach było ok. Osoby, która zachorowała nie znam.

**Śledzisz liczbę zachorowań?**

No nie, właściwie nie. Ktoś tam czasami mi powie, napisze, ile tysięcy. Jak zaczęły się protesty to kilka razy oglądaliśmy TVN24 dłużej, więc tam pasek pokazuje. Właściwe nie wiem co o tym myśleć.

**Co myślisz o tym, że dzisiaj jest ponad 20000?**

Jestem zszokowany, że nie znam ani jednej z tych osób, które codziennie się zarażają.  Zaczynam się gubić, bo nie wiem jakim cudem to się tak szybko roznosi. To wychodzi tygodniowo ok 100-140 tysięcy osób. To jest ogrom.

**Szukasz jakiegoś źródła, które ci powie, dlaczego to tak wygląda?**

Właśnie nie. Od czasu do czasu coś mi wpadnie w oko w internecie i poczytam co mówi lekarz, natomiast większość komunikatów, które widziałem to były, że trzeba na siebie uważać, nosić maseczki, apel o rozsądek, o przestrzeganie restrykcji. Z tego się wielu konkretów nie dowiaduję.

**Chciałbyś, żeby ktoś ci wytłumaczył jak to wygląda z naukowego punktu. widzenia?**

Myślę, że chciałbym wiedzieć z ciekawości a nie z lęku. To właściwie dziwne, że tak to działa u mnie, ale może tak mi jest trochę prościej mimo wszystko, że nie czytam, nie szukam i nie interesuję się tym aż tak. Czy to będzie 18, czy 20 tysięcy...Może gdybym znał mechanizm, to byłbym, mniej zaskoczony, że to jest aż taka liczba, ale trochę bardziej jestem teraz myślami z protestami i z tym co się dzieje ze strony rządu niż z samą sytuacją koronawirusa. Nawet czytając komunikaty cały czas słyszę, że te protesty mogą tę liczbę zachorowań powiększać, ale nie sprawdziłem czy faktycznie tak się dzieje.

**Gdzie mógłbyś to sprawdzić?**

To jest dobre pytanie. Nie za bardzo wiem, jak to zweryfikować. Uważam, że część rzeczy jest mówiona z innych powodów niż z tych, o które powinno chodzić. Nie wiem czy takie informacje są jawne, że akurat tego i tego dnia tyle osób się zaraziło, bo było ognisko osób, które spotkały się na proteście. Nie wiem tego.

**Poza maseczkami, jakie jeszcze obostrzenia ciebie dotykają?**

Z godzinami dla seniorów nie mam problemu. Czasem chętnie bym wtedy wyszedł do sklepu, ale rozumiem to i wspieram to nawet, żeby te osoby miały 2 godz. dla siebie i były mniej zagrożone. Spotkanie do 5 osób - i tak nie sądzę, żebym w najbliższym czasie spotykał się w większym gronie w zamkniętym pomieszczeniu. Restauracje i tego typu miejsca - już wcześniej w zasadzie nie odwiedzaliśmy.

**Zamawiacie na wynos?**

Ostatnio nie, ale też mam taką myśl, że może trzeba będzie zamówić po to, żeby wesprzeć znajome i sprawdzone miejsca.

**A siłownia?**

Miałem plan, żeby po wakacjach wrócić. Wiosną przytyłem, teraz trochę schudłem. Już nie jest tak, że siedzę w domu i jem. To się zmieniło. Siłownia była w planach, ale to nie wyjdzie.

**A to, że seniorzy mają ograniczenia w poruszaniu się?**

Nie wiem co powiedzieć. Niestety część osób nie ma opiekunów czy kogoś zaufanego i nie wiem, jak to oceniać. Liczę, że ktoś życzliwy się znajdzie do pomocy, ale państwo tego nie gwarantuje.

**A to, że mają nie wychodzić, nie widywać się z ludźmi? Dobre rozwiązanie?**

Wydaje mi się, że są osoby, które bardziej cierpią z tego powodu, że muszą siedzieć same w domu niż to, że są wśród ludzi i mogą być zagrożone. To może ich bardziej dotknąć pomimo tego, że fizycznie będą mniej zagrożone. Skłaniam się, żeby mogli przynajmniej wyjść do parku. Psychicznie mogą bardzo źle znosić zamknięcie.

**Czy są jakieś jeszcze słuszne/ niesłuszne ograniczenia?**

Nie wiem. Wcześniej miałem taki moment, kiedy dowiedziałem się, że Dominika będzie musiała prowadzić stacjonarnie swoje zajęcia. Wiem, że to ćwiczenia i taka specyfika zajęć, ale byłem trochę zły z tego powodu i uważałem to za ryzyko. Raz, że zagrożenie, ale też nieprecyzyjność przepisów mogłaby być dla niej problematyczna, jeśli przyjdzie do niej student i powie, że nie założy maseczki, bo nie i każdy sąd mu przyzna rację, że nie. To się zmieniło na szczęście. Więcej nic mnie specjalnie nie oburzyło.

**A to, że starsze klasy nie chodzą do szkoły teraz?**

Nad tym też się zastanawiałem. Ciężko mi ocenić, bo nie mam dziecka, ale to jest chyba bardzo trudne dla rodziców, którzy pracują w domu, żeby siedzieć z dziećmi i dzielić się urządzeniami do pracy. To jest logistycznie bardzo trudne. To pewnie ogranicza zagrożenie, ale chyba właśnie największy problem mam z takimi ograniczeniami, z którymi ludzie muszą radzić sobie sami. Starsze klasy w domu, ale niech będzie jakaś pomoc w wypożyczeniu urządzeń. Tak samo z seniorami. Macie siedzieć w domu, ale nie będziemy się przejmować, że jesteście sami i nikt z wami nie zamieni słowa.

**A to, że 0-3 w szkole a reszta zdalnie?**

Nie wiem, jakie są kryteria do tego. To chodzi o to, że te dzieci do 3 klasy mniej roznoszą wirusa, są bardziej odporne? Chciałbym poznać powody tej decyzji i jakieś racjonalne argumenty. Przyczyny i skutki, bo nie do końca wiem, jaka jest logika i powód ku temu.

**Jest jeszcze coś czego nie rozumiesz w działaniach rządu związanych z koronawirusem?**

Nie wiem jakie jest podejście do kultu religijnego, bo w niektórych miejscach były mocne ograniczenia wcześniej a jednak kult religijny był traktowany inaczej.

**Dalej można chodzić.**

No właśnie. Pamiętam, że branża fitness miała apel, że dlaczego do kościoła można a do nich nie można. Tego trochę jednak nie rozumiem. Wydaje mi się, że w tej sytuacji nie powinno być równych i równiejszych. Dla niektórych ta siłownia jest miejscem, którego super potrzebują. Nie ma w tym logiki tylko po prostu jest taka decyzja.

**Jakie emocje w tobie wywołuje działanie rządu?**

Pamiętam, że byłem pozytywnie zaskoczony decyzjami rządu i szybkimi obostrzeniami, ale teraz mam wrażenie, że to już wymknęło się spod kontroli i mam znacznie mniejsze zaufanie teraz. Już nawet nie mówię o Trybunale, bo już znacznie wcześniej czułem, że to się zaczyna wymykać spod kontroli. Przez te letnie miesiące można było podjąć jednak jakieś kroki, żeby się zabezpieczyć.

**Jakich kroków byś oczekiwał?**

Na pewno pod względem służby zdrowia i sprzętu, którego brakuje. Już nie wspominając o pieniądzach wydawanych na co innego i o tych, które przepadły. To powinien być priorytet, tym bardziej, że mówiło się od dawna, że ta 2 fala na pewno przyjdzie i że latem to wszystko się nie skończy. Wszyscy zwiastowali, że będzie podobnie albo gorzej. Nie podoba mi się, że jak są podejmowane decyzje to nie są podawane konkretne argumenty czy przyczyny, tylko raczej to jest obchodzone taką PR-ową gadką, że tak trzeba i że prosimy, apelujemy, zachęcamy. To jest takie niejasne i nielogiczne dla wieku osób.

**Masz wrażenie, że rząd zadziałał za późno?**

To zabezpieczenie przed sytuacją, to że są rekordy zachorowań a w tym momencie rząd zajmuje się zupełnie innymi rzeczami. W czasie obrad sejmu jest tysiąc innych spraw a ta sprawa jest albo bagatelizowana, albo zrzucana, albo po prostu już są troszkę bezradni i nie chcą się do tego przyznać. Coraz mniejsze zaufanie mam do rządu przez to.

**Te liczby podawane są wiarygodne? Czy oni rzeczywiście wyłapują tych chorych?**

Nie wiem, bo z jednej strony słyszę, że testy mają duży margines błędu, że zdarza się, że po kilku dniach okazuje się, że jednak ktoś nie ma koronawirusa...Słyszałem, że nadal ciężko jest dodzwonić się do Sanepidu, czy żeby coś takiego załatwić, więc wydaje mi się, że to mogą być racjonalne liczby albo nawet mniejsze niż są.

**Ty byś wiedział, jaka jest ścieżka w razie złego samopoczucia?**

Szczerze mówiąc nie. Wcześniej się tym interesowałem, teraz o tym troszkę zapomniałem i pewnie bym od razu wchodził do internetu i szukał co trzeba zrobić. Czasami mam niestety taką myśl, że bardziej obawiam się nie tego, że będę chory i jakie skutki będzie miał wirus tylko tego, że właśnie będzie mi tak super trudno coś załatwić, że będę musiał zgłosić to, że jestem chory, ogarnąć to formalnie, urzędowo...

**To ważne, żebyś zgłosił?**

Kurczę, no...Wydaje mi się, że bym chciał to zgłosić mimo wszystko. Z poczucia obowiązku. Miałbym spokojniejsze sumienie. Tak samo poinformowałbym wszystkich ludzi, z którymi się widziałem w ciągu ostatnich dni, że jest taka sytuacja. Chciałbym być fair wobec nich i fair sam ze sobą. Zastanawiałem się, ile jest osób, które nie zgłaszają.

**Co byś chciał zgłosić?**

Właściwie to nawet nie wiem jak to teraz działa, bo wcześniej trzeba było zgłaszać, była obowiązkowa kwarantanna, przychodził pan policjant albo była ta aplikacja. Teraz nie wiem. Przy takiej skali to jest chyba nie do zrobienia, żeby policjanci sprawdzali...Jak jest dziennie 20000, to jest kilka tysięcy osób w Warszawie i okolicach...Nie wiem.

**Wiesz, ile osób jest na kwarantannie w tej chwili?**

Zupełnie nie.

**W zeszłym tygodniu był taki dzień, że było 350000 osób na kwarantannie.**

Ojej...To sprawdzenie i skontrolowanie są fizycznie nawet niemożliwe. I teraz wraca temat tego, że policja musi wychodzić na ulicę i pilnować porządku na protestach a nie pomóc w czymś innym nawet. To samo można odnieść do służby zdrowia, bo jednak cały czas karetki i ratownicy są na protestach a brakuje rąk do pracy przy zachorowania. Przecież ludzie, poza tym normalnie chorują, mają inne problemy zdrowotne. Zupełnie mi się to w głowie nie mieści.

**Masz wrażenie, że sytuacja jest już bardzo, bardzo poważna?**

Wydaje mi się, że tak. Parę miesięcy, gdybym pomyślał, że może być 20000 dziennie to byłem przerażony, spanikowany. Teraz to jest takie raczej, że może wolę o tym nie myśleć i się nie interesować i może przez to jestem tak trochę obok tego. Ale to też uświadamia, że to jest coraz bliżej, bo im więcej jest chorych tym większe zagrożenie.

**Zwracasz uwagę, ile osób umiera?**

No właśnie na to nie zwróciłem uwagi. Rzuciło mi się w oczy, że jak już było powyżej 15000 zachorowań to chyba ok. 500 osób zmarło dziennie. To już jest naprawdę pokaźna liczba. To jest poważne też z tego względu, że nie wiem, jak sobie radzi z tym służba zdrowia teraz. I o to się najbardziej obawiam, że sprzętowo czy ludzko to jest pewnie mega trudne.

**Zastanawiasz się nad tym jak będzie w przyszłości?**

Zastanawiam się, bo część osób usiłowała mnie przekonywać, że to jest kwestia otwarcia szkół i że to będzie spadać zaraz, ale powoli się już zaczyna kończyć ten okres 2 tyg. od otwarcia szkół i zamiast być lepiej to jest gorzej. Nie wiem jak na to patrzeć. 1.11. się zbliża, ale większość ludzi albo się nie wybiera na cmentarze, albo idzie tam w inne dni i w godzinach pracy, żeby było jak najmniej ludzi. Jak będzie Boże Narodzenie to zupełnie nie wiem.

**Rozmawiacie o tym co będzie na święta?**

Z rodzicami jeszcze nie rozmawiałem, ale coraz bardziej m i się pojawia, że jednak może trzeba będzie odpuścić spotkania rodzinne nawet w Wigilię. Wielkanoc to jest też mocno rodzinne święto, ale nie aż tak. Boże Narodzenie to jest najważniejsze święto rodzinne w całym roku... No nie wiem...Jest to przerażające i nie wiem, jak można nad tym zapanować. Rząd może nadal apelować i mówić, a ludzie...

**A powinni zamknąć cmentarze na 1.11?**

Wydaje mi się, że tak. Rozumiem i też często chodziłem na cmentarz w to święto i dla wielu osób jest to ważne tylko, że mam podobne odczucia jak do mszy świętej. To, co można oddać zmarłym na cmentarzu można też w domu skupiając się na tym. Albo można iść na cmentarz innego dnia. Zamknięcie to unikanie ryzyka. Cały czas ta strefa religijna jest bardziej pobłażliwie traktowana a to też może powodować ognisko i łańcuszek.

**A w przypadku Bożego Narodzenia?**

Tutaj byłoby ciężko na pewno. Może warto zamknąć cmentarze, żeby właśnie zamknąć ognisko. jeden z komunikatów, który czytałem to, że może dobry były lockdown na 14 dni, gdzie zamyka się totalnie wszystko właśnie po to, żeby to się przestało tak rozprzestrzeniać, żeby dać trochę odpoczynku służbie zdrowia. Może to by było jakieś wyjście?

**To by miało szansę pomóc?**

Nie wiem czy nie byłoby lepiej zrobić to już wcześniej niż teraz.

**Gdybyś teraz miał podjąć decyzję na temat świąt?**

To ja bym został w domu pewnie. Byłoby mi trudno, bo uwielbiam te święta, ale chyba wolałbym zostać. Nie wiem jak wiele osób by się zdecydowało na coś takiego, ale jeśli miałbym decydować za cały kraj to nie wiem. Na pewno zamknąłbym centra handlowe chociaż to duża strata dla gospodarki, ale tutaj chyba nie ma dobrych rozwiązań. Są złe albo jeszcze gorsze.

**A twoi rodzice jak się zapatrują na te święta?**

Jeszcze nie wiedzą. Wiem jedynie, że zrezygnowali z cmentarzy i już nie chodzą do kościoła od pół roku.

**A co myślisz o dalszej przyszłości? Kiedy to się skończy?**

Optymistycznie patrząc i zakładając, że będziemy mieć szczepionkę na początku roku, to dystrybucja, szczepienie może zająć co najmniej pół roku...Optymistycznie myśląc, chciałbym, żeby świat wracał do normy czerwiec/ lipiec albo po wakacjach dopiero. Chciałbym, żeby 2022 był już w miarę normalny na pewno, żeby wakacje a przynajmniej koniec lipca był już w miarę ogarnięty (z racji zawodowych).

**Kiedy będziesz miał poczucie, że to koniec?**

Myślę, że jak ta szczepionka będzie już trafiać do ludzi i będzie działać faktycznie, bo z tym też może być różnie. Spodziewam się, że będzie, ale nie wiem, kiedy będzie mogła trafić do ludzi w takiej ilości, żeby ktoś mógł zaszczepić tych ludzi, żeby dla wszystkich wystarczyło, żeby była dostępna, żeby nie kosztowała 1000 zł, więc chyba musi jeszcze trochę czasu upłynąć.

**A myślisz o przyszłości Polski?**

Wydaje m i się, że część osób poważnie już odczuwa tego skutki albo za chwilę odczuje. Głównie gastronomia, imprezy masowe, eventy, itd. Nie wiem, jak w innych gałęziach to się odbije. Prędzej czy później znacząca liczba osób odczuje tego skutki albo już odczuła i co będzie z przyszłością Polski to nie wiem.

**Jeszcze jakieś skutki widzisz? Poza gospodarczymi?**

Jednak śmierć wielu osób, co bardzo martwi, tym bardziej że czytam czasem opinie w internecie, że trzeba się bardziej troszczyć o tych młodych niż o tych starszych. Ci starsi i tak są wiekowi, więc jak zachorują albo umrą to już trudno. Ja w ogóle nie mam takiego podejścia. Nie nam oceniać, ile ktoś ma żyć. Jeśli ta osoba mogłaby pożyć nawet tylko kilka miesięcy dłużej, to i tak nie powinno się tego tak oceniać. Nie do końca też wiadomo, jakie skutki zdrowotne pozostawia wirus u tych, którzy przeżyli, bo jeszcze za wcześnie, żeby o tym mówić.

**Jeszcze coś będzie efektem pandemii?**

Kiedyś się z tego śmiałem, ale mówi się, że ludzie siedzą cały czas z domu i z tego powodu są rozwody. Byłbym skłonny w to uwierzyć, bo ludzie mają czasem różne charaktery i jak są cały czas ze sobą a wcześniej nie byli to taki może być skutek. Z drugiej strony, może ludzie docenią pewne relacje, ludzi wokół siebie. Nie wiem.

**Masz wrażenie, że jest jakaś grupa społeczna, która najbardziej odczuje zmiany, najbardziej ucierpi?**

Pod względem zdrowotnym to tacy, którzy już wcześniej chorowali na coś innego i teraz będą mieli gorsze skutki zdrowotne. Myślę, że są też ludzie, którzy zyskają na tym finansowo. Często spotykam takie podejście, że potrzebowaliśmy tego czasu, żeby coś zrozumieć albo coś zmienić w swoim życiu. Zupełnie tak nie myślę. Nie myślę o tym poetycko czy metafizycznie, że los mówi, że tak się musiało wydarzyć. Wszyscy mogą poczuć jakieś skutki. zastanawiam się nad dziećmi, które się uczą zdalnie czy nawet o studentach, bo wydaje mi się, że to jest zupełnie inne nauczanie. Ja nie wiem, czy chciałoby mi się uczyć w taki sposób jak miałem kilkanaście lat. Chodzenie do szkoły zapewnia jakąś systematyczność, jakiś schemat. Nie wiem czy teraz dzieci młodzież, studenci czują zapał, czy się uczą. Może potem odczują skutki tego, że jednak to nie była taka nauka jak ta stacjonarna. Ci wszyscy ludzie, którzy się musieli przebranżowić. Taki moment potrafi zniszczyć jakieś plany i to też może mieć skutki w psychice, złamać kogoś czy wpłynąć źle. Do wszystkiego to można chyba odnieść. Jednych może to wzmocnić w relacjach czy w czymś, innych złamać. Jeśli miałbym to odnieść do siebie, to ani to, ani to. Przynajmniej na razie. Nie czuje się tak, że coś musiałem zrozumieć i to coś mnie wzmocniło albo że jestem na tyle zły, smutny czy załamany, że będę o tym pamiętał na długo, że w tym okresie coś się zmieniło we mnie.

**Masz jeszcze jakieś przemyślenia?**

Takie, że tak szybko można było do tego przywyknąć, ale jednocześnie to jest na tyle zdradliwe i groźne, że wystarczy chwila nieuwagi i się wchodzi w gumę. To mnie najbardziej irytuje w tej sytuacji. Pewnie też dobrze byłoby wiedzieć, kiedy to się skończy, przynajmniej w przybliżeniu, a dynamika tej sytuacji jest taka, że nic nie wiadomo
