# Supplementary material for: The challenges arising from the COVID-19 pandemic and the way people deal with them. A qualitative longitudinal study
Source: PLoS One. 2021 Oct 11;16(10):e0258133. doi: 10.1371/journal.pone.0258133 (PMC8504766; doi:10.1371/journal.pone.0258133)
Supplement: S1 Dataset — (ZIP) [file pone.0258133.s003.zip › Transcriptions/stage 6/17.6_F_35_single, with child.docx]

**17.6_F_35_single with child**

**Jak wyglądały ostatnie miesiące?**

Pod koniec maja wróciłyśmy do pracy i potem już było normalnie. Pracy było...O Jezu...Życie jakby wróciło do normy. Młody siedział w domu, bo wakacje, ja strasznie dużo pracowałam i tak było do września. Ja się śmieję, że klientki się chciały "odgruzować", bo po 2-3 miesiącach kwarantanny był taki boom. Ja pracowałam po 10-12 godz, dziennie i nadrobiłam wszelkie zaległości siedzenia w domu. Zazwyczaj pracowałam ok. 8 godz. dziennie.

**Był jakiś wyjazd?**

Tak, wyjechaliśmy w góry. Mamy dom w górach i było analizowanie czy jedziemy gdzieś, czy do siebie. Jednak z tyłu głowy było, że nie pchamy się w kurorty, gdzie jest mnóstwo ludzi. Po pierwsze nie lubimy, jak jest dużo ludzi a poza tym po co mamy się pochorować. Niby nie wierzy w tego całego koronawirusa i uważam, że to jest nadmuchane po coś innego, ale z tyłu głowy jest, że nie będziemy się pchali w miejsca, gdzie nam każą nosić maski.

**Po coś innego, nadmuchane?**

Mi się fakty nie zgadzają i nawet dzisiaj rano czytałam ustawę. Ten internet huczy cały, że koronawirus a są przepychane ustawy, które już były a teraz jest o nich głośno. Jest koronawirus znany od lat, ale to i tak jest jakiś promil a nie dziesiątki ludzi umierających. Teraz przepchnęli tę ustawę antyaborcyjna, ludzie zaczynają się bić na ulicach. Ja to czekam aż dojdzie do rozlewu krwi. Teraz internet huczy, że ustawa antyaborcyjna jest po to, żeby przepchnąć ustawę o szczepionkach. Ona jest z 2008 roku i akurat ten artykuł nie jest zmieniony w ogóle i są to dosłownie 2 zdania. Zastanawiam się o co tu chodzi i po co jest ten zamęt informacyjny. Ja mam teorię, że dążą pomału do totalitaryzmu w kraju, żeby nami zawładnąć. Wirus istnieje, ale to jest zasłona dymna do czego innego. ja wiem, że to jest problem światowy, ale w większości krajów już wprowadzili jakieś godziny policyjne. Nie uwierzę w to, że koronawirus do 21 nie zaraża a po 21 zaraża. Jest to bzdura wierutna. restauracja o 19 jest spoko a o 21 już nie jest spoko? No nie. Dzieci powyżej 4 klasy mogą siedzieć w domach a młodsze nie mogą.

**Jakie są te decyzje, wg ciebie?**

One są w ogóle bezpodstawne i one nie mają na celu uchronić nas przed wirusem. My się przed nim nie uchronimy. On był co roku. Może nie ten, może nie taki zmutowany, ale zawsze wrzesień, październik, listopad to były zachorowania i to było normalne. Mam wrażenie, że teraz pół świata chce zmienić nastroje polityczne i ustrój polityczny w krajach. Po cichu.

**Czy ten wirus jest czy nie jest niebezpieczny?**

On jest niebezpieczny, ale tak samo jak grypa. Jak ktoś miał prawdziwą grypę a nie przeziębienie to wie jak to jest. Grypę prawdziwą miała garstka ludzi, a większość miała przeziębienia. Teraz większość ludzi przejdzie koronawirusa średnio, będą tacy co 2 tyg. nie wyjdą z łóżka i będą tacy, którzy trafią do szpitala. Zamykanie nas w domach nas nie uchroni, bo oni zamykają młodych ludzi, przedsiębiorców a to starzy ludzie są najbardziej narażeni i to ich powinni zamknąć, nakazać im siedzieć w domu i dbać o tych staruszków w rodzinie. A oni hulaj dusza, piekła nie ma. Idź na targ, to zobaczysz co się dzieje. Zamykają przedsiębiorców, którzy kasę kręcą dla tego kraju. Kto chodzi do restauracji? Młodzi ludzie, a oni zamknęli ludziom z dnia na dzień firmy i ci ludzie poupadali, bo oni już się nie podniosą drugi raz. I to nie są dyskoteki, bo to bym jeszcze zrozumiała, bo ok., tam jest tłum ludzi. Jeżeli zamykają restaurację, gdzie jest 20 stolików, to sorry. Serio?

**Ważne, przełomowe momenty?**

Sytuacja się cały czas zmienia, już miało być w porządku i znowu nie jest. Dla mnie takim momentem chyba było jak młody wrócił do szkoły, bo ja nie wiedziałam co będzie i jak to będzie. Bardzo była napięta atmosfera w szkole. Ta sytuacja skłóca ludzi. Ostatnio widziałam taki post, że wrzucili do słoika czerwone i czarne mrówki. Mrówki sobie dobrze żyły i człowiek zatrząsł tym słoikiem. Czarne myślały, że to czerwone spowodowały to trzęsienie, czerwone, że czarne i zaczęły się zabijać nawzajem. To samo dzieje się teraz wszędzie, bo są 2 obozy. Albo jesteś za czymś albo przeciwko czemuś. Nie możesz wierzyć w koronawirusa i uważać, że pewne decyzje są źle podjęte, bo albo możesz wierzyć, albo nie wierzyć. I w szkole to samo, że albo nosisz maseczkę, albo jej nie nosisz. Jest jakiś dramat. Ludzie się tak kłócą, tak sprzeczają. Ja się boje w pracy odezwać, bo nie wiem, na który front trafię. Nie ma w ogóle konstruktywnej rozmowy. I to jest taki wg mnie cel. Decyzja rządzących to jest jedno, ale ludzie nie czytają. Możemy się z czymś nie godzić i każdy ma do tego prawo, ale większość osób nie ma argumentów. Albo fake newsy puszczają. Szkołę otworzyli to najpierw nagonka, że po co, drugi obóz, że trzeba. Jak w miarę się uspokoiło to była afera, że dlaczego na basen i że tam się zarażą. Drugi obóz, że muszą, bo tam się uodparniają. Potem fake, że za tydzień zamkną szkołę, potem, że za 2 tygodnie. W świetlicy jak przy stoliku siedzi 2 dzieci to spoko, przychodzi 3-cie i muszą założyć maseczki. Są w tej samej sali, z tymi samymi wirusami przez 5 godzin, ale pani każe i nie ma dyskusji. Czara goryczy się przelała, jak usiedli w 5 osób z jednej przy stoliku i pani na nich nakrzyczała, że narażają ją na śmierć. Dzieci 10 lat. Co się dzieje? Przełomem w moim życiu była szkoła, bo ona mnie wykończy.

**Wydarzyło się coś jeszcze?**

Nie. Niby te żółte i czerwone strefy były, ale jakoś nie przykładałam do tego wagi. Wiem, że muszę chodzić w maseczce, itd., ale ja nie jestem jakimś typem biegającym po świecie, więc ja tę maseczkę zakładam tylko jak idę do sklepu, a tak to siedzę w domu albo u znajomych. jakoś mi to nie doskwierało.

**Jest jakoś inaczej niż było w maju/ czerwcu?**

Są bardzo takie 2 obozy. Ja też się przecież bardzo bałam i większość osób jednak ostrożnie podchodziła do tego koronawirusa. Ok, mamy siedzieć w domu to siedźmy. Zaczynając od wyborów, kiedy premier mówił, że nie ma koronawirusa, kiedy dali bony na wakacje i wypuścili w cały świat - jedźcie, hulajcie, bawcie się dobrze, a we wrześniu jest nagle po 16000 zarażonych dziennie, to jest taki konflikt między ludźmi. Wtedy był jakby jeden obóz. Może nie każdy się godził na decyzje rządu, ale się godził, że ok i musi tak być. Teraz są ci, którzy uważają, że mamy się zamknąć w domach i nosić maseczkę i są ci totalnie zbuntowani. Wewnętrzne konflikty się zaczynają, bo przecież ludzie wyszli na ulice. Jutro będzie chyba Warszawa zablokowana.

**Jak wygląda twoja codzienność?**

Ja chyba żyję normalnie. Z pewnymi rzeczami się pogodziłam i nawet ich nie analizuje. Jak mam iść do sklepu to zakładam maseczkę, nie walczę z systemem, myje ręce, chodzę do pracy, młody do szkoły jeszcze normalnie. U nas jest normalnie. Jakbym wyłączyła internet i telewizję, to uważałabym, że jest na świecie normalnie i zastanowiłoby mnie tylko to, że ludzie chodzą w maseczkach.

**Co wpływa na to, że u was jest normalnie?**

Chyba za bardzo się nie rozwodzimy nad tym koronawirusem. Oczywiście on jest co chwile przy jakiejś dyskusji, bo nie da się tego uniknąć, ale staram się przesiewać pewne informacje i jak czegoś nie jestem pewna to przegrzebać się, żeby chociaż trochę dowiedzieć się prawdy. I wtedy jest mi trochę lepiej. Ja lubię wiedzieć.

**Czy coś wróciło do stanu sprzed pandemii?**

Chyba nie i mi się wydaje, że już nigdy nie wróci. Ludzie będą się bali i nie będzie normalnie. Będą się bali kichania, prychania, zarazków, ludzi, ludzie będą sobie wrogami, nie będą przyjaźni wobec siebie. Teraz jak kichniesz, to naprawdę musisz się tłumaczyć, dlaczego albo jak masz katar. Może w jakich mniejszych gronach będzie normalnie, w rodzinach, ale te dalsze kontakty to nie będzie normalnie. Na świecie nie będzie normalnie, w pracy nie będzie normalnie.

**Jak wyglądają twoje spotkania z bliskimi?**

Normalnie, bez zmian. Już jest wszystko w porządku. Ze wszystkimi się widujemy, jemy obiady, spotykamy się tak jak kiedyś, jeżdżę do babci, bo stwierdziłam, że sorry, ma 90 lat i mam w nosie koronawirusa.

**To była twoja decyzja czy babcia też chciała?**

Babcia jest bardzo chora, więc ona już nie pamięta kto do niej przychodzi, kto nie. Pamiętam, że gdzieś w okolicach 26 maja stwierdziłam, że ja to mam w nosie i jadę, i od tamtej pory jeżdżę. Myje ręce, nie łażę po mieście, już bez przesady. Nie przyniosę tego koronawirusa w kieszeni. Nie muszę jej całować i tulić, ale mogę obok niej usiąść. U nas to już wróciło całkowicie do normy.

**Faktycznie unikasz całowania i tulenia?**

Tak. To jest zmiana. Z koleżankami też piąteczka i hej, nie całujemy się, ale mi to bardzo odpowiada, bo ja nie lubię jak mnie znajomi przytulają. Jest to nawet dla mnie bardziej komfortowe niż całowanie się ze wszystkimi.

**A wyjścia z domu?**

W wakacje dużo wychodziliśmy. Byliśmy tak spragnieni ludzi, że chyba nawet po to, żeby na nich popatrzeć, że oni są. Chodziliśmy nawet pod parasole, żeby wypić kawę, co raczej wcześniej nam się nie zdarzało. Teraz to się ukróciło. Myślę, że te spotkania teraz też będą takie w mniejszych grupach i to nawet ze względu na wygodę. Jak się szło do restauracji to nikt nie musiał nic szykować, a teraz któraś musi się naszykować, żeby przygotować tę posiadówkę, a że każda ma dzieci to już nie jest takie łatwe. Spotykamy się normalnie tylko w mniejszych grupach. Te grupy nie są spowodowane koronawirusem tylko logistyką. Ciężko jest wszystkich zebrać w jednym czasie, czasem nie da się ułożyć z zostawieniem dzieci.

**Jak to wygląda u osób z twojego otoczenia? Są jakieś zmiany?**

Łażą, jak łazili. O ile w 1-sze turze koronawirusa siedzieli wszyscy, to jak to wszystko w wakacje ruszyło, to jednak wszyscy zaczęli wychodzić, wyjeżdżać. Nawet my byliśmy teraz na weekendzie. Ludzie wyjeżdżają, rezerwują długie weekendy i jakby nie dopuszczają `tego, że mogą nie móc pojechać.

**Poziom lęku jest teraz u nich niższy niż wiosną?**

Zdecydowanie. Łączą puzzle. Zarażeń dookoła rzeczywiście jest mnóstwo i na każdym kroku ktoś jest zarażony, ale te osoby nie przechodzą tego bardzo ciężko. Jest garstka ludzi, która trochę mocniej to przeszła, ale zdecydowana większość przechodzi po prostu jak bardzo mocne zapalenie płuc, oskrzeli. Nie wiem, jak to nazwać. I po prostu chyba ludzie przestają się bać zarażenia.

**Coś nadal przeszkadza ci w tej sytuacji?**

Maseczki na dworze. To mnie denerwuje, bo ja nie rozumiem czemu na świeżym powietrzu, kiedy jest wiatr i krzyczą, żeby wietrzyć pomieszczenia, to ja mam chodzić w maseczce na dworze. Jeszcze rozumiem na jakichś przystankach.

**Jak sobie z tym radzisz?**

Noszę maseczkę i nie łażę po dworze, a jak łażę to w miejscach, gdzie nie ma policji. Lasy, parki, itd. po prostu nie wychodzę w miejsca uczęszczane, na jakieś wielkie ulice. Nie lubię jej nosić, ale jak już idę to wolę założyć niż się kłócić o mandat.

**Zakrywasz usta i nos?**

Tak. tego nie rozumiem. jak już masz założyć tę maseczkę, to załóż ją normalnie. Absolutnie tego nie pojmuję. Albo jej nie zakładaj albo ją załóż. Da się żyć w tej maseczce i się od niej nie umrze, chyba że 3 dni człowiek będzie w niej chodził nie zdejmując jej. Normalnie ją zakładam.

**Emocje**

15 - syn wrócił do szkoły. Nie wiedziałam co się będzie działo, byłam pełna nerwów, trochę obawiałam się konfrontacji i nie wiedziałam, jak mam rozmawiać, żeby nie zostać zaatakowaną. Starałam się być taka w miarę neutralna, bo nienawidzę się kłócić i każdy konflikt jest dla mnie wykańczający. Bardzo się tego obawiałam a wszyscy byli wzburzeni, naburmuszeni.  Bardzo się obawiałam, że co się nie powie, nie zrobi, to ktoś zacznie się awanturować. Pierwsze kilka tygodni to awantura była o wszystko.

8 - teraz się czuje, że nie wiem jak mam się czuć. Teraz wiem, że ta cała sytuacja idzie w jakimś kierunku, ale ja nie wiem, w którym. Nie boję się tak bardzo o swoją przyszłość jak w marcu, że ja nie wiedziałam co będzie grane, bo nie uważam, że nagle mi ktoś odbierze możliwość zarabiania, i dom, i pracę, i wszystko, ale ja nie wiem, w jakim to kierunku podąża i nie mam pojęcia co jest za tą mgłą.

**Jakie jeszcze emocje ci towarzyszą?**

Mam jeszcze taki wewnętrzny bunt, bo są te 2 poglądy i ludzie się strasznie będą kłócić. To widać na ulicach i chyba jeszcze będzie gorzej, tylko nie wiem po co ci ludzie chcą się kłócić. Przecież to jest bez sensu. ja chcę żyć spokojnie tak jak żyję, tak jak sobie pracuje, moje dziecko jest zdrowe, rodzice są zdrowi i ja tak sobie chcę żyć. Chciałabym mieć święty spokój.

**Na ile czujesz się zagrożona sytuacją?**

Mam z tyłu głowy, że mogą mi zabronić pracować bądź wprowadzą zakaz wychodzenia z domu i pozwolą mi iść do pracy, ale klientka i tak nie przyjdzie. Boję się tego, ale już nie tak jak kiedyś, bo ja wiem, że to się w końcu skończy. Ten lockdown nie może być bez końca. Miesiąc, dwa i to wróci do normy. Boję się tego zamknięcia znowu albo takich decyzji ograniczających wolność moją i mojej rodziny, ale wiem, że to musi mieć kiedyś koniec.

**Co jeszcze pomaga ci radzić sobie z tymi myślami?**

Że w końcu nam pozwolą wyjść. W marcu nas zamknęli i nikt nie wiedział, ile to będzie trwało i jak to się skończy z pracą i czy ja będę miała gdzie wrócić do pracy. Teraz wiem jaki był finał tamtej sytuacji i myślę, że tu będzie podobnie. oni nas w końcu wypuszczą a klientki wrócą ze zdwojona siłą. Niewiadoma jest najgorsza, a jak już wiesz to jest łatwiej.

**Jak to wygląda u twoich bliskich?**

My mamy takie podejście, że ten koronawirus jest bardzo nadmuchany i nie o to chodzi. Mój brat i bratowa się boją panicznie, ale moja bratowa zawsze była hipochondrykiem, więc to akurat nic nowego. Siostra wkręca się w jakieś historie, że są przepychane ustawy, że nam będą czipy wszczepiać, że nas teraz kontrolują, a mój tata się po prostu boi zarażenia, bo ma 66 lat. Ja nie rozmawiam o tym w domu, bo boję się, żeby oni na mnie nie naskoczyli. Siedzę cicho, kiwam głową i tyle.

**Jak oni sobie z tym radzą?**

Mama też jest taka raczej neutralna, tata jest panikarz. Ogląda wszystkie wiadomości, wszystkie liczby, ile było chorych, ile testów, ile osób umarło. jak mu kiedyś powiedziałam, że we wrześniu na grypę umarło więcej osób, to mnie spytał skąd to wiem, to mu powiedziałam, że z tych samych źródeł, z których on wie, ilu jest dzisiaj zarażonych. Moja siostra się wywnętrza na różnych grupach w internecie, wrzuca jakieś screeny ustaw, itd., a mój brat po prostu nie przyjeżdża i nie wychodzi z domu.

**Są u nich jeszcze jakieś emocje poza lękiem?**

Tata się denerwuje. jak chcesz mu przemycić jakąś informację niezgodną z jego poglądami, to od razu łapie nerwy. Moja siostra też, ale oni są podobni. Wczoraj miałyśmy mocną spinkę na temat tej ustawy o szczepieniach, że oni mogą nas zmusić. powiedziałam, że z ona jest z 2008 r i krzyk, że tak, ale ją znowelizowali. Zrobiłam jej screeny z dziennika ustaw, ale i tak uważa, że to jest fake. Reaguje na mnie agresja i nie docierają do niej żadne, ale to absolutnie żadne racjonalne argumenty. Nie mam pojęcia z czego to wynika. Ona zawsze była ciężkim charakterem i moja racja jest najmojsza. Nie wiem czemu się nie da, ale zawsze ciężko było ją przekonać, żeby zmieniła zdanie. Taki ma charakter.

**Znasz kogoś, kto zachorował?**

Tak, dużo osób. Jeszcze w marcu zachorowała położna i to był taki fenomen. potem długo, długo nic i długo miałam opinię, że jak może być koronawirus jak nikt koło mnie nie choruje, a jak chorują to bezobjawowo, a potem to już się sypnęło. Jak się zaczęło w październiku, to się sypali...Jedna dziewczyna u mnie w pracy się rozchorowała, ale była 3 dni chora i jej syn z objawami, ale nie zaraziła nikogo. To jest bardzo zabawne, bo oni mają 3 dzieci i tylko ona i jeden syn byli zarażeni. My kilka dni wcześniej byłyśmy u nich i żadna z nas też się nie zaraziła. Miała koronawirusa, bo nie miała węchu, a to taki główny objaw. Nie poszła na test. To było 3 tyg. temu i wtedy stwierdziłyśmy, że pójście na test wiąże się z tym, że nas udupią na kwarantannę, więc nie poszła. To by jej i tak nic nie dało, bo przecież leków i tak by jej nie dali, itd. Zadzwoniła do swojej doktorki, ona się bardziej młodym przejęła, ale dała leki standardowo używane przy zapaleniu oskrzeli i nic im nie jest. Ewidentnie miała, bo jak węchu nie miała...Nie poszła na test, bo byśmy wszyscy byli na kwarantannie.

**Ona sama podjęła tę decyzję czy wspólnie z wami?**

Ona już miała być na 1-szej kwarantannie w połowie września, bo u jej syna dziewczynka była zarażona. Te dzieciaki poszły na zdalne nauczanie i ta moja koleżanka nie odbierała telefonu, bo wiedziała, że zadzwoni Sanepid i żeby nie narzucił jej kwarantanny. Nie można tak robić, ale *[śmiech]* to było za zgodą pani Kierownik Sanepidu, bo ona jest jej koleżanką. Powiedziała, żeby nie odbierała telefonu, bo jak by miała się zarazić, to dawno już by się zaraziła. To naprawdę było śmieszne, bo w efekcie Sanepid zadzwonił do niej w niedziele rano, ona nie odebrała, a kwarantannę miała mieć do poniedziałku do północy. To w ogóle bez sensu było, więc ona tylko młodego zostawiła w domu a sama nadal pracowała. Takim tokiem myślenia, to jak ona miała kontakt z kimś, to my wszyscy byśmy byli na kwarantannie. No i nauczona doświadczeniem wiedziała, że test jej nic nie da poza podniesieniem statystyk na TVP, leków jej nie dadzą, nic jej nie dadzą, więc przesiedziała kilka dni w domu i tyle.

**Jak odnosisz się do takiego zachowania?**

Jeśli siedziała w domu i nie kichała, nie prychała na nas, to spoko, niech siedzi. test nic nie da oprócz tego, że nakaże zamknąć się w domach, masz kontrolę policji, masz ściągnąć jakąś durną aplikację, która ma cię kontrolować. Poza tym wynik testu jest teraz dostępny na pscjent.gov. Inne badanie nie są, a ten "śmiercionośny " koronawirus ma być ogólnie dostępny. Nie, spoko i jeżeli nikt nie prycha, nie chodzi i nie zaraża, to po co ten test robić?

**Tobie zdarzyło się być na kwarantannie?**

Nie, jeszcze nie. Wszystkim klientkom mówię, że absolutnie tu nie były, my się nie znamy. Chociaż teraz i tak bym nie była, bo tylko osoba zarażona jest na kwarantannie, tak? Jakoś coś zmienili. Nie, już wiem. Jak nauczyciel miał koronawirusa, to uczniowie byli na kwarantannach i musieli z nimi też być wszyscy domownicy. Teraz zmienili, że tylko ten uczeń, czyli tylko osoba z bezpośredniego kontaktu. Takiego też dostałyśmy cynka z Sanepidu.

**O jakich jeszcze zmianach w ograniczeniach słyszałaś?**

Że nie można kupować i sprzedawać alkoholu i narkotyków od 21 do 7 rano, ale na terenie Holandii. Mam 2 screeny zrobione z tego rozporządzenia, bo najpierw poszło bez tego, że to na terenie Holandii. Potem dopiero dopisali.

**A w Polsce?**

Baseny, siłownie są zamknięte, restauracje są zamknięte i jakieś są ograniczenia w liczbie osób na kasę znowu, ale tego nie odczułam. Chyba jest godzina dla seniorów, ale ja byłam wczoraj na zakupach i mnie wpuścili. Nie wiem, czy tam się nie zmieniło, że może tylko sugerują seniorom w tych godzinach. Wczoraj byłam o 11 na zakupach i dopiero się zorientowałam pod Biedronką, bo tam pan rozdawał jakieś numerki klientom. Wszędzie przedtem mnie wpuścili.

**Jak czujesz się z obostrzeniami, które są?**

Odczułam 2 - nie mogłam iść z dziewczynami do restauracji, a byłyśmy umówione i basen. Basen to mnie sfrustrował tak, że byłam jak wulkan. Myślałam, że tam wszystkich pogryzę. Najpierw poszła informacja, że zamykają, za chwilę, że dla szkół i uczniów baseny są otwarte. Świetnie. I teraz młody mógł jechać na basen ze szkołą w autokarze 30 osób, małe szafki i się nie zaraża, a na indywidualne zajęcia, gdzie ma swój tor wykupiony nie mógł, bo jego instruktorka nie ma szkółki. Ja jej płacę i wykupuję bilet, jako klient indywidualny, bo ona nie ma firmy jako szkółka i nie dało się tego obejść. No i nie chodzi. Chciałyśmy z koleżankami zrobić zajęcia zorganizowane i wykupić 3 tory, ale to chodzi tylko o to, że ona nie ma firmy, nie jest zatrudniona przez basen tylko też jest jako indywidualny uczestnik.

**Czy to faktycznie ma wpływ na ograniczenie epidemii?**

Żaden, absolutnie żaden. Jeszcze jestem w stanie zrozumieć, że sauna, jacuzzi. Ale jeżeli on idzie trenować na basen sportowy, to tam nikt nie będzie się chlupał w wodzie tylko pływał. Basen podnosi odporność. Coś mi tu nie działa.

**Ograniczenie wychodzenia z domu dla osób starszych ma realny wpływ?**

Może nie na ograniczenie epidemii, ale na to, żeby osoby najsłabsze się nie rozchorowały. Umierają ludzie 60+ patrząc na statystyki. To są osoby, które może zabić katar. Też jest 70-latek, który lata po świecie i jest ok i jest 70-latek z chorobami. jeżeli to jest osoba schorowana, bierze leki, z nadciśnieniem, to niech ona posiedzi w domu. Uważam, że mają rodzinę, a jak nie mają to są różne Caritasy, ktokolwiek kto może im dostarczyć jedzenie. Niech oni wychodzą sobie na spacer, ale niech nie ładują się do Biedronek i do kościołów.

**Odgórne narzucenie zostanie w domu jest tu w porządku?**

W ogóle ograniczenie komukolwiek wolności nie jest w porządku. Człowiek, jak mu coś zakazujesz, to jeszcze bardziej się wkurza i właśnie będzie to robił. łatwiej by było, gdyby komunikacja była taka, że bardzo was proszę, siedźcie w domu. Dobrze by było, gdyby się wypowiedział nie premier tylko jakiś autorytet w tej dziedzinie - lekarz, epidemiolog, który by nie narzucił pod sankcja kary tylko zasugerował i pokazał argumenty, dlaczego warto babciu z dziadkiem zostać w domu. I wydaje mi się, że gros tych dziadków by posłuchało. U nas za wszystko ma być kara, a od dawna jest udowodnione, że ludzie naprawdę wolą być nagradzani niż karani.

**Nauka zdalna powyżej 4 klasy?**

To jest temat rzeka. Nie wiem i nie mam pojęcia co by było dobre. Jak było zdalne nauczanie i te lekcje były prowadzone po łebkach, było źle. Jak było nauczanie stacjonarne, było źle. Jak jest zdalne, ale prowadzone zgodnie z planem, jest źle. Nie ma chyba dobrego wyjścia. Poza tym przez tę decyzję wyszedł konflikt między nauczycielami, bo teraz nauczycielki 0-3 się buntują, że czemu one mają chodzić do szkoły. Obserwując sytuację w szkole mojego Miłosza, to tam nic się nie działo. Było jedno zarażenie. Nasza pani dyrektor jest żoną wiceministra Skurkiewicza. On miał kontakt z tymi wszystkimi żołnierzami, zaraził się. Podobno bezobjawowo. Śmiałam się, że jak Trump się zaraził to i Skurkierwicz i Andrzej się zaraził, bo to nie może tak być, że tylko Trump. W dniu, kiedy Skurkiewicz miał podejrzenie, to ona od razu wyszła ze szkoły i zabrała 2 córki i od tamtej pory nie pojawiły się w szkole, ale nie miały żadnych objawów, nic. Media huczały oczywiście, że koronawirus w szkole w Radomiu. Dyrektorka sobie i dziewczynkom zrobiła test, chociaż nie miały objawów i oczywiście były zarażone. Media huczały, że 29 przechodzi na naukę zdalną i jest prośba o nauczanie hybrydowe, tylko nikt się nie wczytał, że chodzi o te 2 klasy córek. Burza była taka, że dramat. Nikt więcej się nie pozarażał, dzieci, które są "tykającymi bombami" nie pozarażały nauczycielek, nic się nie działo. Teraz w jednej z tych klas chyba jakaś nauczycielka ma objawy, więc na skalę szkoły, gdzie jest 700 osób mamy 3 osoby bezobjawowe i 1 nauczycielkę objawową. To jest dużo?

**Wszyscy powinni uczyć się stacjonarnie?**

Chyba tak. Tylko w takich rygorach jak były teraz, że nie wychodzili na przerwę. Niech siedzą w klasach, niech nauczyciel do nich przychodzi, w miarę możliwości niech wychodzą na dwór jak jest WF czy ze świetlicy.

**Zakaz organizowania imprez, eventów?**

Jeżeli chodzi o jakiś koncert to w porządku, rozumiem. W ogóle bez takich imprez można żyć, to może poczekać i to nie jest rzecz pierwszej potrzeby. A wykształcenie dzieci tak, bo rzeczywiście rośnie nam zacofany naród. Lepiej niech się zajmą nauka a eventy odłożą na później.

**Transport zbiorowy?**

A jak tam jest? Co 2-gie miejsce? Rozumiem, że to jest ok, żeby nie było tłoku, ale nie wiem kto i jak nad tym panuje i czy rzeczywiście jest tak, jak mówi ustawa. Bo może jest ustawa, żeby była, ale nikt jej nie przestrzega, bo nie ma za bardzo jak. Gdybym była zmuszona do korzystania z komunikacji to bym się nie bała. Pojechałabym normalnie. Moja mama jeździ codziennie i mówi, że spoko poza tym, że SM sprawdza maseczki. Jeździ już od września i nic się nie dzieje. Nie, nie bałabym się.

**Jest coś, czego nie przestrzegasz?**

Nie noszę maseczki w lesie. Mnie mało to wszystko dotyczy, bo nie jeżdżę autobusami, pociągami, nie chodzę na eventy, nie miałam zaplanowanego wesela ani żadnej imprezy. Do szkoły i tak nie wchodziłam od 3 lat - teraz jest zakaz. Mnie to naprawdę mało dotknęło.

**Dla tych rodziców, którzy wchodzili do szkoły, taki zakaz ma wpływ na ograniczenie rozprzestrzeniania się wirusa?**

Jest to jakiś pomysł, bo te szatnie nie są duże, to jest stara szkoła. Nie było jak przejść i dlatego zrezygnowałam z wchodzenia do szatni. To akurat jest ok, bo wreszcie jest pusta szatnia.

**Czy uważasz, że obecna sytuacja jest poważna?**

Jest bardzo poważna, bo sytuacja z koronawirusem doprowadza do wszystkich innych jakby to był zapalnik do tego wszystkiego, co się dzieje w kraju. Gdyby nie koronawirus to myślę, że dalej byśmy żyli w spokoju, a nie ma spokoju w kraju. Myślę, że będzie źle. Może mnie personalnie różne takie sytuacje nie dotkną, ale dla przedsiębiorców to może być nieciekawie.

**A jeżeli chodzi o samą chorobę?**

W ogóle nie jesteśmy do niej przygotowani jako społeczeństwo, ale też szpitale, lekarze. To wszystko leży, kwiczy i płacze. To jest dramat co się dzieje. W ogóle nie jesteśmy przygotowani. Nikt przez pół roku nie zastanowił się nad tym, żeby usprawnić system ochrony zdrowia. W ogóle. Zastanawiali się tylko nad tym jak ograniczyć rozprzestrzenianie się koronawirusa, ale nikt nawet nie wpadł na to, żeby naszykować szpitale, itd.

**Mówiłaś, że wiele osób przechodzi to łagodnie.  Czy rzeczywiście szpitale muszą być przygotowane?**

Tu bardziej mi chodzi o to, że robią szpitale covidowe na łapu capu, na zasadzie, że dzisiaj ty masz być szpitalem covidowym nie biorąc pod uwagę liczby mieszkańców, czy jest inny szpital, itd. Ograniczają, że lekarz pracujący w szpitalu covidowym nie ma prawa gdziekolwiek indziej pracować, a już nie daj Boże jak tam pójdzie zarażenie, to wszyscy lecą na kwarantannę. I tu się zrobił zastój. Radom ma niecałe 200 tys. mieszkańców. Jak było 100 zachorowań na Polskę to zamknęli jeden cały szpital. Mamy 2 szpitale. Ludzie nie mieli gdzie chodzić do lekarza. Nie daj Boże, żeby sobie wtedy złamać nogę. Dochodziło do absurdów. Potem decyzja, z dnia na dzień, że likwidujemy szpital covidowy i zostawiamy tylko jeden oddział. Na 200 tys. mieszkańców zostawili 20 łóżek i 3 respiratory. Tak jakby ze skrajności w skrajność poszli. Są osoby, które potrzebują pomocy lekarskiej i są też osoby, które nie potrzebują być w szpitalu, ale potrzebują pójść do lekarza. I znowu robi się korek w szpitalach. nadal ci lekarze pracujący w szpitalach covidowych nie mogą nigdzie indziej leczyć, są zablokowani. Nie wpadli np. na pomysł, żeby zamknąć mały szpital pod Radomiem, gdzie będzie 100 łóżek i wywożą teraz ludzi do Warszawy. Budują wielki szpital polowy. Jak wojna. Najpierw były namioty pod szpitalami dla tych co mieli objawy, tylko tam wszyscy siedzieli na kupie po kilka godzin. Nie przemyśleli racjonalnie, żeby znaleźć rozwiązanie, więc osoby z objawami Covid idą po prostu do POZ. Mam koleżanki, które tam pracują na rejestracji i mówią, że spoko, bo one mają pleksę, tylko to są przychodnie nowego typu, gdzie z jednej strony są chore dzieci, z drugiej przychodnia onkologiczna i obok przechodzi ktoś z Covid. O ile dla mnie ten wirus nie będzie śmiercionośny, to osobę z nowotworem zabierze w kilka tygodni. Mam wrażenie, że piszą jakieś ustawy na kolanie, podejmują decyzję przy herbacie - dzisiaj zamykamy to, otwieramy tamto, itd. U nas większość lekarzy i nie chcę oceniać, bo to kwestia sumienia, ale większość lekarzy zwolniła się ze szpitala, bo byli ograniczeni i nie mogli przyjmować w przychodniach. Z jednej strony jest bunt, bo przyjmują tylko prywatnie a nie na NFZ, ale nikt nie widzi drugiej strony, że oni się pozwalniali, bo ich ograniczyli. Są oczywiście tacy, którzy lecą tylko na kasę i teraz są żniwa, ale są tacy, którzy wiedzieli, że nie podołają, że mają dzieci albo że nie chcą tam pracować i chcą pracować w zwykłych przychodniach. Służba zdrowia jest naprawdę w tym kraju nieudolna.

**Czy ludzie zachowują się adekwatnie do sytuacji?**

Chyba tak, chociaż nie wiem...Niby się zachowują, ale się gdzieś buntują. Niby się godzą na pewne decyzje rządu, ale się buntują. Np. noszą maseczkę, ale na nosie już nie. Najbardziej ten bunt widać właśnie w tych maseczkach. Albo jej nie założą, albo założą nieprawidłowo. Wszystkie inne obostrzenia raczej stosują. Nie pójdą do sklepu jak są godziny seniora, nie zrobią imprezy, chociażby dlatego, że żaden lokal tego nie zrobi. Ludzie są zmuszeni respektować te prawa. Gdyby każdy zachowywał się rozsądnie, nosił maseczkę w sklepach i mył te ręce, to myślę, że jakoś by to funkcjonowało. Ja cały czas mam z tyłu głowy, że gdyby ten komunikat o maseczkach i myciu rąk była nam inaczej przekazany, to ludzie by inaczej do tego podeszli. Wirusolog powinien powiedzieć, że ta maseczka ma takie i takie zastosowanie, że bawełniana jest do bani, ale lepiej taka niż żadna, ale pierz ją. Wtedy jest szansa, że ludzie stwierdzą, że to nie jest takie złe dla nich. Tak samo mogło być z weselami, że nie róbcie wesel, starajcie się nie robić, itd. A oni blokowali najpierw na 50 osób, potem na 20, itd. Ludzie musieli kombinować i robili np. niby 2 imprezy. Polak potrafi kombinować. daj mu tylko zakaz a on go obejdzie. Szwajcaria tak zrobiła, że u nich chyba nie ma takich mocnych obostrzeń jak u nas tylko wszystko0 jest, że zróbcie to dla własnego dobra i to w miarę to funkcjonuje, a u nas muszą batem i to się odbija czkawką. Cokolwiek rząd nie postanowi to będzie źle. jak Szumowski powiedział, że maseczki są niepotrzebne to naród siedział i szyli na potęgę maseczki. I wszyscy nosili. Jak każą nosić, to mówią, że się poduszą i dostaną grzybicy płuc. Nie ogarniam tego kraju czasami i mentalności naszej.

**Czy masz te same źródła informacji co wiosną?**

Te same. Czasami jeszcze grzebię bardziej w ustawach. W marcu tego nie robiłam.

**Śledzisz liczbę zakażonych?**

Nie, ale nawet jak nie chcę to słyszę, bo trąbią to wszędzie. Pierwsze wiadomości w radio, jakie idą to liczba zakażeń, internet to samo, mam wyłączony telefon i wskakuje mi powiadomienie - dzisiaj 16000 zarażonych. Z lodówki wyskakuje, a nawet jak tej lodówki nie otworzę, to któraś klientka mi powie.

**Czy czas, który poświęcasz na śledzenie informacji jest podobny do tego na wiosnę?**

Jest zdecydowanie mniejszy. Skupiam się tylko na informacjach, których nie jestem pewna, że są prawdziwe. Oglądałam Dylemat Społeczny i on mi jeszcze zasugerował, żeby grzebać. Skupiam się tylko na tym, co mnie konkretnie interesuje.

**W wakacje ta ilość czasu była inna?**

Wtedy nikt nie oglądał wiadomości, nikt nie mówił o korona wirusie, o zarażonych. Nic się nie działo. Ja miałam wrażenie, że naprawdę zgodnie z obietnica premiera koronawirus poszedł od nas. Wiadomości były zajęte zupełnie czym innym. TVN jeszcze próbował przemycać informacje o korona wirusie, ale TVP zero, nic nie było.

**Kiedy to się zmieniło?**

Pierwsze uczucie, że znowu się zaczęło, to jak było rozpoczęcie roku online. Wtedy pomyślałam, że trzeba na nowo obserwować. no i u nas w Radomiu pierwsze zarażenie ucznia było 4.09. Był taki tydzień, dwa, że w Radomiu chorowały tylko szkoły i co chwile ktoś szedł na zdalne. Potem już media zaczęły, że jest 1000, 2000 zarażonych dziennie i te liczby już naprawdę były duże i robiły wrażenie.

**Jak oceniasz wiarygodność mediów?**

W ogóle nie są wiarygodne. Jeżeli ktoś chce wiarygodną informację, to naprawdę musi wygrzebać i też ja nie mam do końca pewności czy to jest prawda. Boję się tego, że wszelkie informacje są to może nie tyle fejki, co są podkoloryzowane. Ja szukam źródeł, ale nie w Wikipedii tylko jakichś istotnych źródeł i próbuje to jakoś przesiać przez swoje logiczne myślenie. Ale to, jeśli chodzi o jakieś ustawy albo informacje, które mogę sprawdzić, ale czy Trump jest zarażony, czy Duda - tego ja nie wiem. Nie mam pojęcia, czemu Duda się nie pojawia od tygodnia w mediach, nie mam pojęcia co się dzieje w Korei i czemu Kim Dzong Un się nie odzywa. Nie wiem co się dzieje i nie wiem czy zdjęcia tych szpitali polowych ze Stanów były prawdą czy nie prawdą. Mogłabym powiedzieć, że wiem tylko wtedy, gdybym tam była. Teraz nie wiem. Od lat w podobny sposób wyszukuje informacje i tu się nic nie zmieniło. Kiedyś było to łatwiejsze i kiedyś nie miałam aż takiego poczucie, że ktoś mnie okłamuje i że ja muszę dociec prawdy. Dawniej musiałam tylko potwierdzić daną informację. Teraz ja muszę dociec, czy to co mówią jest prawdą i to się zmieniło. Zmienił się sposób mojego myślenia.

**Z czego to wynika?**

Z natłoku informacji, są sprzeczne informacje, a wszystkie wyglądają na prawdziwe. Mam wrażenie, że kiedyś aż tak nie było. Kiedyś szybko można było dojść prawdy i wiedzieć, że sąsiadka spod 7 wali ściemę jak coś mówi. Dla naszych rodziców TV to była świętość i jak tam powiedziano, to tak musi być. 78-80-latek zawsze wierzy TV, bo tam musi być prawda, a my już jesteśmy w takich czasach, że tam już nie ma prawdy.

**Czy myślisz o przyszłości po pandemii?**

Mam takie dziwne wrażenie, że już nigdy nie będzie takiego momentu. Ja sobie nie wyobrażam, że Andrzej powie, że nie ma koronawirusa. Jedyne co może powiedzieć, to że w miarę go opanowaliśmy i wydaje mi się, że dużo wody musi się przelać, żebyśmy zapomnieli o tym co się działo. Chyba nie będzie tego "po pandemii". Może nasze dzieci, a my już nie.

**Co musiałoby się wydarzyć, żeby ta sytuacja się zakończyła?**

Musiałby ktoś powiedzieć, że sorry, nie ma żadnego koronawirusa, żartowałem. Nie wiem, nie mam pojęcia.

**Gdyby była szczepionka, lek?**

To byłby jakiś krok ku dobremu, tylko zanim się dowiemy, że szczepionka działa. to będzie kolejne 10 lat. No chyba, że to będzie lek, bo lek działa od razu. Poza tym myślę, że ludzie na szczepionkę zareagują...Nie będą się cieszyć. W Brazylii już jeden poddany szczepieniu zmarł. Poszła już informacja, oczywiście cichaczem. Oczywiście tak może się zdarzyć, ale wydaje mi się, że przy tych wszystkich fake newsach dużo ludzi się nie zaszczepi. sama nie wiem czy bym chciała się zaszczepić i wolałabym lek.

**Gdyby było mniej zarażonych to mogłoby to oznaczać koniec tej sytuacji?**

Teoretycznie bym do tego tak podeszła, ale boję się, że te informacje są trochę fałszowane. Nie znam celu tego i nie wiem czemu mieliby to fałszować i do czego to prowadzi, natomiast nie jestem pewna czy ja bym im tak uwierzyła, że już jest po wszystkim.

**Co myślisz o przyszłości Polski?**

Wojna będzie. Już jest wojna. Dramat będzie. Mam nadzieję, że tylko wojska nie wyjadą na ulice, natomiast wydaje mi się, że PiS dąży do totalitaryzmu. Robi to zgodnie z książką. Czytałam taki wpis politologa w marcu o tym jak delikatnie i nieświadomie wprowadzić totalitaryzm w kraju i to się dzieje. Boje się, że będzie godzina policyjna, że kobiety, które protestują zostaną zaraz spałowane, bo złamią prawo, bo nie można wychodzić. Rolnicy wyszli, przedsiębiorcy wychodzą. Dramat jest i po prostu wszyscy mają po kokardkę. Były pogłoski, że będzie 2 fala i że nas zamkną i wtedy mówiłam, że ludzie wyjdą na ulice i na to nie pozwolą, bo nie mają za co żyć. Widzą, że to nie ograniczyło rozprzestrzeniania się koronawirusa, itd. No i proszę. Bardzo źle się dzieje.

**Myślisz o przyszłości świata?**

Też się źle dzieje. Nie mam pojęcia kto tym steruje i o co tu chodzi. Nie chcę powiedzieć, że koronawirus nie istnieje, ale myślę, że on jest przykrywką. Ludzie troszkę zrobili sobie samowolkę i komuś się to nie podoba. Tylko kraje, które są niezależne od innych w miarę sobie radzą i one nie muszą wprowadzać dziwnych obostrzeń, godzin policyjnych. jak to w ogóle brzmi? Mamy XXI wiek i podobno demokrację i ktoś ma mi zabronić wyjść o 21. Chore. Kraje, gdzie była komuna wprowadzają to. Coś mi tu nie gra. Ja nie pamiętam tamtych czasów i nie pamiętam jak to zostało wprowadzone, ale jak tak czytam, to za dużo mi się tu zgadza. Ten koronawirus był takim zapalnikiem do tego, żeby ktoś zaczął panować, bo kocha władzę.

**Czego się obawiasz?**

Że moja wolność będzie zabrana, że ja nie będę mogła spokojnie iść na spacer, że mój syn nie będzie mógł gdzieś jechać na studia, że nie będę mogła firmy prowadzić, pracować. I tak nie jest to wolna amerykanka, ale było w miarę ok. Boje się, że odbiorą mi przyjemności, mną które ja ciężko pracuję. Nie wyobrażam sobie, że nie będę mogła pojechać na wakacje, bo ktoś mi powie, że nie bo lotów nie będzie z kraju albo zabronią mi przemieszczania się od którejś godziny. Boje się, że będzie nie bo nie. Moje klientki, które studiują za granicą będą tam walczyć o obywatelstwo i nie dlatego, że nie chcą być Polkami, tylko chcą mieć alternatywę na w razie czego. Paszport innego kraju może otworzyć im świat.

**Jak zmieni się sytuacja gospodarcza?**

Na pewno zostanie ograniczona. Ograniczyli działalność firm małych a duże nadal funkcjonują. Pozamykali małe firmy. Teraz już nie przetrwają i ci, wszyscy ludzie pójdą teraz do pracy na etat. Nie wiem po co, bo ekonomicznie mi się to nie zgadza. Dla państwa jest lepiej, żeby był przedsiębiorca, ale partia ma większą kontrolę nad pracownikiem niż nad przedsiębiorcą. Mam wrażenie, że to wszystko prowadzi do upadku gospodarki. tylko po co ma upaść, komu jest źle i na co? Nie rozumiem tego.

**A sytuacja społeczna?**

Przede wszystkim zabierany jest nam kontakt międzyludzki i zamykamy się w 4 ścianach. Nasze życie ma się przenieść do internetu. Ja nie wiem po co, bo nie znam racjonalnego powodu, ale do tego to dąży.

**Jakieś grupy społeczne szczególnie ucierpią?**

Na pewno osoby niewykształcone, niezaradne życiowo. One sobie nie poradzą i będą siedzieć i klepać biedę. tak samo było za komuny. Jak człowiek jest wykształcony, to ma lepsze perspektywy na życie. Może być mi ciężko zapewnić dziecku dobrą edukację, ale zrobię wszystko, żeby mu pomóc. Osoby, które tylko egzystują to mogą się pogodzić z tym, że żyją od 1-go do 1-go i będzie ok, bo one nie chcą nic więcej osiągnąć w życiu i jakby do niczego więcej w życiu nie dążą.

**Jak planujesz spędzić 1.11.?**

1.11. w tym roku nie ma. Nie planuje. zawsze było obiad u mojej babci, ale podupadła na zdrowiu, więc nie ma obiadu rodzinnego. koronawirus utwierdził rodziców w tym, żeby nie robić w tym roku tego obiadu. Na groby pójdziemy normalnie i jak zwykle późnym wieczorem, bo ja nie lubię tłumów od zawsze. Pójść pójdziemy i nie wyobrażam sobie, żeby nie pójść.

**A Boże Narodzenie?**

Mam nadzieję, że będzie normalnie i chyba ludzie nie pozwolą sobie na to, żeby nie pojechać na Boże Narodzenie. Wielkanoc jest w naszej tradycji, bo jest, ale bez Bożego Narodzenia sobie nie wyobrażam. Nie ma takiej opcji, żeby nie było choinki, żeby rodzina nie usiadła przy Wigilii. Musiałoby tu wojsko przyjechać i mi zabronić.

**A jeśli zrobią godzinę policyjną, itp. to będziesz się starała jakoś znaleźć sposób, żeby zorganizować te święta?**

Oczywiście, że tak. Nie wyobrażam sobie Wigilii bez stołu wigilijnego z całą rodziną. Nie ma takiej opcji. I wydaje mi się, że większość osób Wigilii nie wyobraża sobie bez stołu wigilijnego.

**Brałaś udział w dużych uroczystościach rodzinnych od czerwca?**

Nie, nic nie mieliśmy. Na szczęście. Ja bym pewnie chciała iść, ale presja wszystkich dookoła...Ja bym miała mętlik w głowie a tak nie musiałam podejmować żadnych decyzji. Ja bym raczej nie miała obaw, ale chodzi tu o presję społeczną. Dla mnie nie ma różnicy czy to jest wesele, kościół czy praca, gdzie codziennie widuje wiele osób. Nie ma różnicy.

Tylko spokój nas uratuje i ja jestem przerażona tym co się dzieje. Byle do piątku, bo w piątek mają coś wymyślić. Ten piątek będzie istotny, bo chodzą pogłoski, że chcą zamknąć naszą branżę. Jest to realne, że zamknie. Wszyscy mówią, że jak zamkną branżę beauty, to wszyscy i tak pójdą do pracy i mają to w nosie, ale może zrobić totalny lockdown, ale ja do pracy będę mogła iść, tylko do mnie nikt nie będzie mógł przyjść. To mnie tylko interesuje teraz.
