# Supplementary material for: The challenges arising from the COVID-19 pandemic and the way people deal with them. A qualitative longitudinal study
Source: PLoS One. 2021 Oct 11;16(10):e0258133. doi: 10.1371/journal.pone.0258133 (PMC8504766; doi:10.1371/journal.pone.0258133)
Supplement: S1 Dataset — (ZIP) [file pone.0258133.s003.zip › Transcriptions/stage 1/6.1_M_24_couple, no children.docx]

**6.1_M_24_couple, no children**

**Przedstaw się.**

Mam 25 lat. Obecnie siedzę w domu, ale jestem zatrudniony na umowę o pracę jako barista w Costa Coffee. Skończyłem studia pierwszego stopnia z technologii chemicznej i mam tytuł inżyniera, ale go jeszcze nie odebrałem, bo wybuchła pandemia. Byłem w trakcie szukania pracy w zawodzie, ale sytuacja jest jaka jest i skutecznie mi to przerwało poszukiwania. Mieszkam z dziewczyną i z dwoma kotami. Zainteresowania to koncerty muzyka, rock i metal. Sport, windsurfing, tenis. Pochodzę z Przemyśla.

**Powiedziałeś, że podczas epidemii nie pracujesz?**

Byłem raz w pracy, bo Costa miała taką inicjatywę albo dalej ma, przygotowania paczek dla lekarzy, dla 3 szpitali w Warszawie. I raz zostałem oddelegowany, żeby to zrobić. Ale dostaję wynagrodzenie za przestój.

**Pamiętasz, kiedy ta sytuacja się dla ciebie zaczęła?**

Tak. Ja to mniej więcej zacząłem odbierać jako bardzo poważną sprawę mniej więcej 14 marca mi się wydaje, gdzieś w połowie marca. Pamiętam jeszcze, że Dzień Kobiet całkiem miło spędzaliśmy z dziewczyną. Było coś tam słychać o tym koronawirusie, ale w ogóle się tym nie przejmowaliśmy, że to może dotrzeć do Polski. Pierwsza osoba zarażona w Polsce to już był sygnał, że jest poważnie. Zmiany praw, itd., to już wiedziałem, że coś się dzieje. Ale absolutnie sobie tego nie wyobrażałem w ten sposób, że może dojść aż do takiego wzrostu zachorowań.

**Co sobie myślałeś, jak były pierwsze zachorowania?**

Tak, myślałem sobie, że się tego nie spodziewałem i mówiłem sobie po prostu: *wow*. Nie spodziewałem się, jak to będzie dalej. I w ogóle nie wyobrażałem sobie, jak to wpłynie na życie zawodowe, na poszukiwanie pracy, na przyjazd do domu na święta chociażby.

**Czyli na początku słyszałeś doniesienia, ale nie spodziewałeś się, że to dotrze do Polski?**

To znaczy, być może się spodziewałem, ale nie brałem tego na poważnie. Ja żyłem sobie swoim życiem, a koronawirus żył sobie swoim życiem. I w zasadzie to tyle.

**I później to dotarło do Polski i też dotarło do Ciebie?**

Tak. Na początku pamiętam, że była cała panika, że ludzie zaczęli masowo chodzić do sklepów i wykupować makarony i papier toaletowy, itd. I stwierdziłem, że to jest ogromna głupota, bo tak naprawdę wykupują to, a ktoś może naprawdę potrzebować tych produktów. I tak było przez kilka pierwszych dni, a potem się to uspokoiło.

**Pamiętasz jakieś ważne momenty, które były ważne i zmieniły twoje podejście do sytuacji?**

Szczerze mówiąc, chyba nie.

**A miałeś wrażenie, że następują jakieś ważne zmiany?**

No jasne, tak jak mówiłem o prawie chociażby. Zmiana różnych praw, ludzie, którzy masowo zaczęli tracić pracę. Mam kolei, który założył firmę w lutym i zacząłem sobie o nim myśleć, jak on się będzie utrzymywał. Także zacząłem patrzeć bardziej globalnie w ten sposób na rynek pracy.

**Masz jakieś obawy w tej sytuacji?**

Wydaje mi się, że teraz jest tak, że firmy masowo zwalniają pracowników, ale jeżeli to się skończy - nie wiem, kiedy, pewnie jak pojawi się jakieś lekarstwo - to nagle zacznie się bum i firmy ponownie zaczną zatrudniać pracowników, nawet tych niedoświadczonych i tutaj jakoś widzę swoją szansę, że gdzieś mogę się załapać. Ale póki co, moją sytuację widzę tak, że po prostu siedzę w domu przez najbliższe dwa miesiące jeszcze prawdopodobnie.

**A skąd taka myśl, że to mogą być 2 miesiące?**

Przeczucie po prostu. Ale też, jak powiedziałem o lekarstwie... W zasadzie, to może nie być nawet dwa miesiące. Powiem tak, to jest optymistyczne podejście, ale oglądając jakieś kanały naukowe, ktoś mówił, że znalezienie szczepionki może potrwać nawet do roku/półtora, więc to jest właściwie najbardziej pesymistyczne podejście. Ale jeśli chodzi jeszcze o szukanie pracy, to jestem w na tyle komfortowej sytuacji, że jestem na umowie o pracę i dostaję to wynagrodzenie. Nie jest tak, że jakoś bardzo się denerwuje, ale powiedzmy, że mam już parcie na pracę w zawodzie, bo do tego cały czas dążyłem, kończąc studia, ale też będąc na studiach.

**Jeśli chodzi o obecną pracę, to nie masz obaw, że możesz stracić źródło utrzymania?**

Nie, myślę, że Costa Coffee jest na tyle dużą firmą, że może sobie pozwolić na zatrzymanie pracowników. Z tego, co mi wiadomo, pensje pracownicze wcale nie są częścią, na którą Costa wykłada najwięcej pieniędzy. Największe straty przynoszą braki dochodu z powodu zamkniętych kawiarni. Nie miałem żadnych przesłanek, żeby mnie to miało dotknąć w najbliższej przyszłości.

**Czy obawiasz się czegoś w związku z sytuacją?**

Tak, wiadomo, boję się o swoją rodzinę. Bardzo chciałbym, żeby stosowali się do zaleceń i utrzymywali izolację. Szczególnie boję się o swoją babcię, która jest już w wieku podeszłym, ma 76 lat. I wiadomo, o rodzinę najbliższą też, czyli mama, tata i brat. Chociaż oni mieszkają na wsi w domu jednorodzinnym, więc nawet jeżeli chcą się poruszać, to mogą wyjść, mamy sporą działkę. Z resztą Przemyśl jest na tyle małym miastem, że gęstość zaludnienia nie jest tak duża, jak w Warszawie. Chociaż też nie wiem, czy to jest dobry argument w zasadzie, ta mała gęstość zaludnienia. Bo tak naprawdę wszędzie jak pójdą, a ktoś będzie akurat, to może dojść do zarażenia. W każdym razie, boję się o swoją rodzinę, ale wierzę, że jak będą się stosowali, to nic strasznego się nie stanie. Boję się, że w tym najbardziej pesymistycznym scenariuszu, ta pandemia potrwa bardzo długo i będziemy musieli siedzieć w tych domach przez jeszcze co najmniej parę miesięcy. I minie dużo fajnych wydarzeń kulturalnych, dużo koncertów. I o to się też boję, ponieważ miałem kilka fajnych planów koncertowych na ten rok, ale już teraz wiem, że te na wiosnę się nie spełnią. To są też te obawy.

**Te wydarzenia na wiosnę już są odwołane?**

Tak, dokładnie.

**Na lato też masz jakieś koncertowe plany?**

Tak, też miałem. Jeszcze mam nadzieję, że będą, ale... Szczerze mówiąc nie wiem, czy to byłby w ogóle dobry pomysł uczestniczyć w takim wydarzeniu zbiorowym. Nawet za te 2-3 miesiące, jak to wszystko się skończy. Nie wiem, czy nie miałbym pewnych obaw. W pewnym momencie w marcu, jak to się zaczęło, to już zacząłem myśleć nad tym, że pewne wydarzenie, które miało być w Warszawie 18 kwietnia zostanie odwołane. Ale nawet, gdyby nie zostało, to prawdopodobnie i tak bym nie poszedł, bo wiadomo, nie byłoby to zbyt mądre i odpowiedzialne.

**Co by musiało się wydarzyć, żebyś nie miał obaw, żeby pójść na taki koncert?**

Szczerze mówiąc nie wiem, ciężko mi przewidzieć, jak to będzie wyglądało za 2 miesiące, ponieważ wzrost zachorowań cały czas następuje i nie wygląda, żeby to miało się zatrzymać. Chciałbym pójść, ale nie wiem, czy to będzie odpowiedzialna decyzja brać udział w czymś takim.

**Boisz się trochę o rodzinę, a o siebie samego?**

O siebie też jak wychodzę do sklepu, jak najbardziej. Niedaleko miejsca, w którym mieszkam jest Auchan, tam zazwyczaj się zaopatruje i ruch w tym sklepie jest zazwyczaj dość mocny Przy wejściu do sklepu trzeba minąć praktycznie zawsze kogoś, czy ochroniarza, czy osobę stojącą przy kasie i mimo, że przy wejściu stoi mydło do dezynfekcji rąk i rękawiczki, to i tak trzeba minąć tych ludzi i wtedy zazwyczaj się obawiam. Ale jeśli chodzi o krążenie po sklepie, to staram się zachować te 3-4 metry odległości zazwyczaj.

**Boisz się, że zachorujesz?**

Tak. To znaczy... Wiadomo, że nikt nie chciałby zachorować. Mówi się, że korona wirus jest największym zagrożeniem dla osób starszych, ale są też doniesienia, że osoby młodsze, które nie miały chorób współtowarzyszących też zachorowały i bardzo ciężko przechodziły chorobę albo nawet zmarły. Więc każdy człowiek jest indywidualnym bytem i nie wiadomo, jak to będzie działało na mnie, czy też na kogoś z mojego towarzystwa, na moją dziewczynę. Więc tak, boję się, że mogę w ten sposób się zarazić. Nie wiadomo, jakie to będzie miało konsekwencje długofalowe. Bo mówi się, że teraz można zachorować i przejść bezobjawowo albo się wyleczyć, ale nie wiadomo, co będzie za 2-3 lata, czy nie okaże się wtedy, że ktoś ma uszkodzone narządy wewnętrzne przez to.

**A boisz się o swoją dziewczynę?**

Tak. W tej chwili nie, bo siedzi w domu razem ze mną cały czas, ma *home office*. Ale był taki okres, kiedy pandemia się rozwijała u nas i ona jeszcze musiała chodzić do pracy na Domaniewskiej do Mordoru i nawet raz się okazało, że ktoś w budynku miał koronawirusa. I wtedy pracowników wysłali do domu, zdezynfekowali budynek, ale i tak później pracownicy musieli przychodzić. Więc, jeśli dzieją się takie sytuacje, to jestem niespokojny. Na szczęście tydzień później już każdy dostał *home office*.

**Skala lęku**

No właśnie, bo... Strach i lęk to są dla mnie dwie różne rzeczy. Okej, tu jest poziom lęku, więc powiedzmy, że 50.

**Dlaczego rozróżniasz strach i lęk?**

Nie mam pojęcia. To jest subiektywne odczucie. Musiałbym się zagłębić w definicje tych słów, ale są to jakoś dla mnie rozróżnialne słowa.

**Obrazki - który z nich najlepiej oddaje twoje emocje w tej chwili?**

Tak, myślę, że nr 10, ponieważ on jest w stylistyce czarno-białej, są drzewa, jest apokaliptyczny bym powiedział. I mam wrażenie, że tak to teraz wygląda, jakby było postapokaliptycznie, jak były jakieś doniesienia o zamykaniu miast, to wtedy się tak czułem. Jakbyśmy się przenieśli do świata postapokaliptycznego. Też nr 12, ponieważ siedzę w domu i gdybym siedział sam, to czułbym się sam jak kamień. O, nawet jest 4, więc jest tam ja, dziewczyna i koty.

**Czujesz, że ci to towarzystwo teraz pomaga?**

Zdecydowanie. Dobrze się dogadujemy, dobrze nam się siedzi, nie nudzimy się sobą, mamy czas na spędzanie czasu razem. Więc pod tym względem jest fajnie, ale wiadomo, że człowiek nie jest przystosowany do życia w zamknięciu cały czas, nie czuje się z tym zbyt dobrze i chciałbym gdzieś się wyrwać, ale nie uważam, żeby to było odpowiedzialne. Też jeżeli chodzi o wyjazd na święta do domu, to musiałem sobie go darować i uważam, że jest to odpowiedzialna decyzja.

**Co sprawia, że obserwujesz te postapokaliptyczne obrazy?**

Ludzie np. też w kombinezonach ochronnych - chyba to jest najbardziej oddającym obrazem to, że coś niepokojącego się dzieje. Jak gdzie wybucha bomba atomowa, to też pojawiają się ludzie w skafandrach i wiadomo, że dzieje się coś niepokojącego. Obrazy z mediów i wiadomości, które pokazywały ludzi w skafandrach były niepokojące i tak się właśnie czułem, jakbyśmy żyli w postapokaliptycznym świecie opanowanym przez zarazę.

**Pojawia się niepokój w związku z tym?**

Tak. Poziom na skali wcześniej, który zaznaczyłem, to bardziej chodziło mi o niepokój niż strach. Jest on potęgowany przez media, ogromne nagłówki, liczba ofiar, zachorowań, gospodarka upada - to wszystko podsyca nastroje.

**Coś jeszcze zwiększa u ciebie teraz niepokój?**

Nie, myślę, że nie. Ja staram się też nie oglądać telewizji za wiele, bo od pewnego czasu uważam, że tam nie ma za wiele wartościowych treści. Wiadomości są bardzo stronnicze zazwyczaj. Ale czasami mi się zdarza. I jak się to zaczynało w Polsce, czyli w połowie marca, to ewidentnie ludzie zaczęli panikować, wykupując artykuły, i mam wrażenie, że to też przez to, że media w ten sposób działały.

**A ty robiłeś zapasy?**

Nie, absolutnie. Ani leków, ani produktów. Uważam, że sklepy są na pewno na tyle dobrze zaopatrzone i są jakieś procedury albo były, kiedy te firmy zaczynały działać w Polsce, na różne sytuacje kryzysowe. I o to, że się skończy jedzenie w Polsce, to się nie bałem. Bardziej bałem się o to, że w danym momencie może brakować, bo ludzie zaczną wykupować i tak faktycznie było. Ja w ogóle nie szedłem do sklepu, żeby robić zapasy, tylko szedłem, bo chciałem sobie coś ugotować na obiad konkretnego. I powiedzmy, szedłem po makaron, a makaronu nie ma i tak to wyglądało przez kilka dni. Czy mięsa, i to zazwyczaj te produkty się rozchodziły w dwie godziny od dostawy.

**Czyli nie czułeś potrzeby, żeby robić zapasy, mimo, że widziałeś, że produkty się kończą?**

Nie, ponieważ wyszedłem z założenia, że może to być potrzebne niektórym potrzebującym ludziom bardziej. Widziałem, jak starsza pani wchodziła do sklepu sama i targała worek zakupów - brała to, co mogła. Nie chciałem dokładać cegiełki do tego, żeby ludzie było do tego zmuszeni.

**A dlaczego ludzie tak robili?**

Bo zaczęli panikować, po prostu. Bo obawiali się izolacji, że będą musieli siedzieć w domu i nie będą mogli wychodzić. I... Nie wiem w zasadzie. Nie mam pojęcia. Zaczęli robić zapasy na wszelki wypadek, na zaś.

**A ty się tego nie bałeś, bo miałeś wrażenie, że sklepy będą zaopatrywane?**

Też miałem przeczucie, że taki bum będzie na kilka dni i faktycznie po kilku dniach się to skończyło i wszystkie produkty były na półkach. Po prostu ta sytuacja była bardzo nowa dla ludzi i taki impuls nimi targnął, żeby to robić. I też jeszcze ważna sprawa, że to się zaczęło samo nakręcać. Kilka osób zobaczyło, że inni robią zakupy i też poszli robić zakupy. Każdy za kimś innym poszedł. I chcą nie chcąc, ja też musiałem w tym uczestniczyć poniekąd. Musiałem stać po 40 minut w jednej kolejce po produkty i też brałem takie, jakie były. Raz mi się zdarzyło kupić coś więcej, żeby nie chodzić do sklepu raz na dwa dni.

**Pamiętasz, co wtedy kupiłeś?**

Tak, bodajże kaszankę, bo kaszanka wtedy była, jakiś makaron, trochę więcej warzyw i chyba jakieś suche produkty typu płatki śniadaniowe, owsiane. O, z mlekiem też był problem, także mleko też wziąłem wtedy.

**Czy to, jak teraz robisz zakupy, wygląda inaczej niż przed epidemią?**

Tak, zupełnie inaczej. Chociażby to, jak wyglądają kolejki do kas. Odstęp między ludźmi, stoisko z płynem do dezynfekcji rąk przy wejściu, jedna kasa czynna samoobsługowa i jedna kasa, gdzie jest kasjer. Zazwyczaj było więcej kas czynnych. A w Żabce pod blokiem są te plastikowe ekrany i tego nie było wcześniej. Zakupy inaczej wyglądają. i wchodząc do sklepu człowiek, przynajmniej ja mam takie poczucie, że muszę tu spędzić jak najmniej czasu i wziąć to, co jest najbardziej potrzebne, najlepiej z listy i nie chodzić, nie przeglądać, tylko spędzić jak najmniej czasu i wyjść.

**Czyli robisz listę?**

Tak, zawsze. To znaczy, teraz zawsze robię, a wcześniej nie zawsze robiłem. Często kupowałem spontanicznie.

**Co zazwyczaj masz na liście?**

To jest różnie, to na co mamy ochotę z dziewczyną. Znajdziemy ciekawy przepis i to zrobimy, naprawdę nieregularnie. Czasem coś wegetariańskiego, czasem coś mięsnego. Bez jakiejś reguły.

**A jak teraz wygląda twój dzień?**

Mój dzień wygląda tak, że zazwyczaj chodzę spać trochę wcześniej teraz niż podczas studiów, bo wtedy się zdarzało chodzić spać i o 4/5 - dużo było nauki. Teraz zasypiam koło 22/23, czyli też wstaję siłą rzeczy wcześniej. Czyli powiedzmy budzę się o 7:00, jemy z dziewczyną śniadanie i ona siada do pracy zazwyczaj o 8/9. Biorę prysznic i dziewczyna w dni powszednie sobie pracuje, a ja sobie działam. Mamy większe mieszkanie teraz, dwa pokoje i można się oddzielić. Mój dzień wygląda w ten sposób, że biorę gitarę do ręki i spędzam z nią jakieś 3 godziny. Potem zazwyczaj coś czytam lub oglądam przez 2 godziny. Potem siadam do drugiego laptopa, takiego grata, który jest średnio sprawny i uczę się programowania w C++, bo i tak nie mam nic innego do roboty, a uważam, że to może się jakoś przydać.

**Zacząłeś teraz się tego uczyć?**

Przez epidemię stwierdziłem, że warto byłoby to opanować chociaż troszkę. A wcześniej miałem takie podejście... Ciężko mi to trochę wchodziło. I na studiach miałem taki jeden przedmiot, gdzie musiałem to zrobić i zostać oceniony za to i wtedy ciężko mi to wchodziło. Ale wychodzę z założenia, że jeśli człowiek nie musi czegoś zrobić, tylko coś chce, to łatwiej mu to przyjdzie i teraz jest trochę łatwiej. Więc przez epidemię stwierdziłem, że warto by było się rozwinąć chociaż trochę w ten sposób. Jeżeli chodzi o pracę i zatrudnienie, to biorę pod uwagę to, że może nie być dla mnie pracy w moim kierunku - technologia chemiczna, i nawet myślałem nad tym już zanim się obroniłem, że może być kiepsko. I przez pierwszy miesiąc od obrony szukałem pracy i dostałem jedną odpowiedź na ogłoszenie, ale pod Warszawą, więc musiałem zrezygnować, bo było za daleko. Więc boję się też o swoje zatrudnienie w mojej branży. Także wolę się jeszcze zabezpieczyć trochę w ten sposób, że może się nauczę tego programowania, bo jest dużo dobrze płatnych ofert pracy. Najchętniej myślałem, żeby połączyć informatykę z chemią. Póki co, nie wiem jeszcze jak, bo nie znam rynku pracy, ale jakbym się wdrożył, to wiedziałbym więcej.

**Wracając do twojego dnia...**

Kończę się uczyć programowania, dziewczyna kończy pracę o 16/17 i wtedy siedzimy razem, zazwyczaj coś oglądamy albo oddzielnie spędzamy czas, czy gotujemy obiad.

**Czyli nie jest także cały dzień leżysz i nic nie robisz?**

Nie, absolutnie, ja mogę tak działać przez 2-3 dni, ale na dłuższą metę się z tym bardzo źle czuję. Jak miałem bardzo dużo obowiązków na studiach, to każdy weekend super. Jak jeszcze się nie trzeba było uczyć czy na święta, to zawsze wtedy wykorzystywałem czas w ten sposób, że nie robiłem nic. Potrzebowałem się zregenerować w ten sposób, bo wiedziałem, że jak wrócę do nauki, to nie będzie na to czasu. Ale teraz, jakbym miał cały dzień leżeć, spać albo tylko coś oglądać, to nie bardzo.

**Czy jest coś, co ci szczególnie przeszkadza w codziennym życiu?**

To, że nie mam swojego komputera, bo nie ukrywam, że lubię też grać. Jest laptop, którego używamy, ale to jest laptop do pracy i pograć, to średnio. Gram często też online z kolegami i uważam to za fajne spędzanie czasu. Też pogłębianie więzi z kolegami w ten sposób. Przeszkadza mi to, że nie można pójść do kina, na basen, na siłownię, pojechać gdzieś. Raczej pojechać pewnie można gdzieś w ustronne miejsce, ale... Raz, że ja nie mam samochodu, dwa, że przemieszczanie się teraz to nie jest zbyt odpowiedzialna decyzja. Przeszkadza mi też to, że w mediach jest cały czas temat o koronawirusie i nie ma nic innego niż to, ale rozumiem, że tym żyje teraz cały świat. Jak mi przeszkadza, to wyłączam telewizor.

**Widzisz jakieś plusy tej sytuacji?**

Myślę, że plus będzie taki, że może się troszkę powietrze oczyści w ten sposób. Chociaż... Właśnie nie wiem, jak to rozumieć do końca. Bo niby ludzie powinni siedzieć teraz w domach i nie korzystać z transportu publicznego, więc teoretycznie powinni więcej jeździć samochodami, jeśli potrzebują. Ale właśnie pewnie nie potrzebują, więc nie jeżdżą i nie zatruwają środowiska spalinami. Jeżeli tak jest, to mógłbym to traktować jako plus tego. Czyli mniej szkodliwa ingerencja w środowisko. Być może mniej śmieci w lasach, górach, itd., bo może ludzie nie jeżdżą w takie miejsca. Chociaż widziałem, że jak był ostatni weekend i było ciepło, to było pełno ludzi na plaży nad morzem - co uważam za totalną głupotę. Plusem jest też to, że siedzę w domu i mam czas dla siebie. Mogę się uczyć programowania bez przeszkód, grać na gitarze i się rozwijać tutaj. Plusem jest to, że spędzam więcej czasu z dziewczyną, ale... Też fajnie, jak ktoś ma prywatność, więc po to mamy drugi pokój.

**Myślisz, że bez tego dodatkowego pokoju byłoby ciężko?**

Myślę, że tak. Wcześniej mieszkaliśmy w takim mieszkaniu, że nie można się było dzielić i sami sobie nawet powiedzieliśmy, że w tamtym mieszkaniu byłoby ciężko.

**Wspomniałeś o ludziach, którzy wychodzą na ulicę, zbierają się na plaży...**

To jest nieodpowiedzialne. Widziałem w wiadomościach w TVN ujęcia, to człowiek na człowieku. Ludzie jakby nie wzięli tego do siebie, że zaraża się drogą kropelkową i im bliżej jest człowiek drugiego człowieka, tym większe ryzyko. I w ogóle tego nie wzięli do siebie. Poza tym, z tego, co mi wiadomo we Włoszech właśnie tak to się zaczynało, że ludzie kompletnie zbagatelizowali problem, chodzili na mecze, zbierali się w pubach i dlatego tak to się rozwinęło u nich. Powiedziałem sobie wtedy, że mam nadzieję, że skoro u nas są podejmowane takie kroki, czyli odpowiednio wcześnie są zamykane szkoły, restauracje, kawiarnie, to może u nas tego nie będzie. Ale widząc takie obrazy, szczerze mówiąc, nie wiem.

**Czemu twoim zdaniem ludzie się tak zachowują?**

Bo nie mogą żyć w zamknięciu. I nawet jak są zakazy, grzywny, nawet niektórzy to przyznają, że wiedzą, ze jest choroba, ale oni nie mogą żyć w zamknięciu i ogólnie to panika za bardzo.

**A ty myślisz, że obostrzenia są adekwatne czy to jest za duża panika?**

Nie, myślę, że są adekwatne, jak najbardziej. Myślę, że zamknięcie szkół to był dobry pomysł i też działanie odpowiednio wcześnie, chociaż można było działać jeszcze wcześniej. Już jak były w ogóle pierwsze wzmianki o tym wirusie w Chinach, to już można było działać u nas, ale wiadomo, że jeszcze wtedy każdy to bagatelizował. Dopiero jak to się zaczęło szybko rozprzestrzeniać, to... Bo największy problem tego wirusa jest chyba taki, że on po prostu się za szybko rozprzestrzenia, lawinowo. I wtedy właśnie ludzie zaczęli panikować. Także myślę, że te obostrzenia są adekwatne.

**Jakie zachowanie obserwujesz wśród swoich bliskich?**

Szczerze mówiąc, jeśli chodzi o moją rodzinę, to rozmawiając z mamą, słyszę, że nie chce jej się siedzieć w domu, że często chodziłaby na rower. Mówiła, że była na tenisie z moim bratem i pusty kort. Wolałbym, żeby siedziała w domu, ale ja mogę powiedzieć jedno, a ona i tak zrobi swoje. A mój ojciec jest lekarzem z kolei i on bywa w szpitalu. Siłą rzeczy jest jakieś większe ryzyko w naszej rodzinie. Po prostu mówię, żeby uważali na siebie i wiem, że są to ludzie potrafiący myśleć i mam nadzieję, że zadbają o siebie w taki sposób, w jaki uważają, w jaki mogą. W odpowiedzialny sposób. A jeżeli chodzi o znajomych, to z tego, co mi wiadomo, trzymają się w izolacji i siedzą w domach.

**Ty teraz nie wychodzisz z domu za bardzo?**

Nie, tylko na zakupy.

**Czy jest w twoim otoczeniu ktoś, kto panikuje w tej sytuacji?**

Nie.

**Skąd się wziął koronawirus?**

Wziął się z tego, że... Z tego, co mi wiadomo, to on przeszedł od nietoperzy na łuskowce, a z łuskowców zarazili się ludzie i to prawdopodobnie wzięło się z tego targu w Wuhan. I tam było chyba kilkudziesięciu albo kilkunastu zarażonych i potem już dalej to poszło. Więcej ludzi się zaraziło. Ktoś przyleciał z tym do Europy. I wystarczyła jedna osoba, żeby nagle było kilkaset tysięcy zarażonych w Europie.

**Dlaczego tak szybko się rozprzestrzenił?**

Dlatego, że przenosi się drogą kropelkową. I dlatego, że ludzie mają tendencję do przemieszczania, nie lubią siedzieć zamknięci i stąd przemieszcza się ten wirus.

**Słyszałeś o innych teoriach, dotyczących tego, skąd się wziął ten wirus?**

Tak, pewnie. Np., że jest to wirus, mający przetrzebić populację ludzką i wytępić chorych, starszych ludzi, jakkolwiek strasznie by to nie brzmiało. Jeszcze jakieś teorie spiskowe typu, to z laboratorium w Wuhan uciekł jakiś wirus i to było niekontrolowane. Ale też słyszałem ciekawą teorię, ale właściwie nie spiskową, że po prostu ludzie, wyrzynając lasy deszczowe, mają taki kontakt z dziką naturą, jaki nie mieli powiedzmy 100 lat temu i siłą rzeczy mają kontakt z tymi dzikimi zwierzętami i mają większe ryzyko zarażenia się czymś takim. Więc nawet, jeżeli wirus nie pochodziłby bezpośrednio z tego targu, to mówiąc bardziej globalnie, ludzkość przez swoje działania, naraziła się na taki wirus.

**Czy twoim zdaniem ludzkość na to zapracowała?**

Myślę, że to jest przypadek. To znaczy przypadek... Tak, jak mówiłem na początku. Z nietoperzy na łuskowce, łuskowce trafiły na targ w Wuhan, itd... Nie uważam, żeby to była kara czy coś, bo takie pandemie już bywały w historii ludzkości.

**Czyli nie uważasz, ze w tych teoriach może być ziarnko prawdy?**

Powiem tak, w każdej teorii spiskowej gdzieś jakaś prawda może być. Poza tym często bywało tak, że teorie spiskowe stawały się prawdziwe, stawały się faktami - bywały takie przypadki. Więc nie mówię, że tak nie jest. Jakieś prawdopodobieństwo może być, jak najbardziej. Ale nie mówię, że tak jest na 100% i że jestem pewien.

**Czy tobie się wydaje, że Polska jest dobrze przygotowana na tę sytuację?**

Niezbyt. Jeśli chodzi o służbę zdrowia, wydaje mi się, że nie bardzo. Głównie przez doniesienia, o jakich słyszałem. Ale też nie moglem tego zweryfikować, bo każdy może sobie założyć fake konto na Facebooku i coś napisać, udostępnić. Żyjemy w takich czasach, gdzie fake newsów jest naprawdę dużo. Ale jakieś relacje do mnie dotarły, czy od rodziny, czy od znajomych, że były próby tuszowania na samym początku pojawienia się wirusa po to, żeby nie siać paniki. I jeśli chodzi o służbę zdrowia, to widać wszędzie, że brakuje maseczek. Ludzie się skrzykują, żeby kupować maseczki dla szpitali. Jurek Owsiak zamawia z Chin dostawę sprzętu medycznego. Uważam, że jeżeli chodzi o służbę zdrowia, to nie jesteśmy na to przygotowani.

**A w innych obszarach?**

Jeśli chodzi o gospodarkę, to pomoc dla przedsiębiorców, to wszystko jak to wygląda, to wszystko stoi na głowie i też nie jesteśmy do tego przygotowani. Pomoc dla przedsiębiorców jest za mała. Jakieś odraczanie rat czy spłat kredytów, to jest tak naprawdę zaciskanie pętli na szyi, że ta powiem. Bo co to da, że za 3 miesiące wszystko się skumuluje i ktoś będzie musiał zapłacić cała kwotę. Także ogólnie sytuacja jest nowa dla każdego i nie wiem, czy ktokolwiek był na to przygotowany.

**Czy można się było przygotować?**

Chyba nie. Myślę, że takiego czegoś ludzkość nie przeżyła w ostatnim okresie. Więc nawet wirus SARS był też w Japonii w latach 2003/2004 i było głośno o nim, ale był tylko w Japonii i zmarło chyba 900 osób tylko, a już w tej chwili nawet nie wiem, ile jest zgonów łącznie, ale myślę, że znacznie więcej. Ciężko powiedzieć, żeby ktoś był przygotowany.

**Czyli nie śledzisz na bieżąco tego, ile jest zgonów i zakażeń?**

Nie. To znaczy mam taką aplikację w telefonie. Nazywa się UpDay i tam jest zazwyczaj 10 głównych nowinek z dnia i można wejść w linki i przejść do artykułu. Często robię tak, że włączam tę aplikację i przewijam te 10 artykułów i pod spodem jest taki abstrakt zazwyczaj o tym, co jest w artykule. I czasami tam jest podane, ile jest zgonów. Ale akurat dzisiaj nie sprawdzałem, ile jest zgonów, ile zarażonych.

Ale wrażenie roi to na pewno, jak się usłyszy, że tego dnia zmarło 100 osób, a poprzedniego dnia tylko 5.

**To są artykuły z innych serwisów?**

Tak. Z tego, co mi wiadomo, to jest redakcja tego UpDay. Ja to miałem wbudowane w telefonie i to funkcjonuje w różnych krajach. Ta wersja, którą ja mam, jest polską wersją.

**Skąd jeszcze czerpiesz informacje?**

W zasadzie, Ministerstwo Zdrowia - tam są informacje na bieżąco podawane, ile zakażonych, ile zmarło. I telewizja - TVN24 głównie, TVN zwykły i czasami Polsat News.

**Mówiłeś też o kanałach naukowych na YouTube?**

Tak, tylko już przestałem to śledzić, bo ten koronawirus jest wszędzie i nie wiem, co mi to da, że jeszcze więcej się o nim dowiem. Tak, te kanały funkcjonują i wrzucają treści. Głównie Nauka. To Lubię - to Tomasz Rożek prowadzi. On tam często podaje dane statystyczne, np. kiedy szczepionka mogłaby być wynaleziona na to, kiedy teoretycznie pandemia mogłaby się zakończyć, dlaczego podejście Wielkiej Brytanii jest złe, jeżeli chodzi o koronawirusa. Bo oni przyjęli inną politykę niż my, czyli nie żeby się izolować, tylko zarazić jak najwięcej ludzi, żeby oni się uodpornili. Jeszcze Uwaga! Naukowy Bełkot - Dawid Myśliwiec. Pamiętam, ze oglądałem jeden dłuższy film na temat tego, czy ten wirus jest i skąd się wziął. Mówił, jak działa ten wirus. Takie nawet techniczne sprawy, odnoszące się do działania tego wirusa.

**Masz poczucie, że twoja wiedza na ten temat jest wystarczająca?**

Myślę, że nie. Ja myślę, że nawet naukowcy jeszcze nie do końca poznali… Chociaż pewnie już, jakby wiadomo, były badania na ten temat robione i strukturę tego wirusa poznano. Ale nie wiadomo wszystkiego, bo jakby było wiadomo, to byłby już lek na to. Więc o sobie na pewno mogę powiedzieć, że nie wiem wszystkiego.

**Dlaczego już teraz nie czytasz o tym?**

Szkoda mi na to czasu. Wolę pograć na gitarze, porobić coś innego. Jak będę miał taką potrzebę, to włączę jakiś film. Mam już swoje sprawdzone źródła i wiem, że to są rzetelne źródła i że ci ludzie zajmują się nauką na poważnie. Więc wiem, gdzie szukać.

**Skąd wiesz, że te źródła są rzetelne?**

Podświadomie jakby oceniam merytorykę tych kanałów. Widzę, w jaki sposób są zmontowane te filmy. Podają źródła, powołują się na badania naukowe niejednokrotnie. Także tak oceniam rzetelność źródła.

**Jak oceniasz wiarygodność informacji na ten temat w ogóle?**

Cóż… Na pewno jakieś łańcuszki na Facebooku - uważam, że to jest niewiarygodne. Tak jak wcześniej mówiłem o służbie zdrowia, że widziałem posty różnych osób, to nie wiem, czy to jest wiarygodne, bo każdy może założyć *fake* konto. Ale to było udostępniane przez moich znajomych, u których widziałem, że ma tego kogoś wspólnego w znajomych. Ale myślę, że nie wszystkie takie informacje są wiarygodne na Facebooku. Fejkowe artykuły bardzo łatwo stworzyć. Poza tym oglądam TVN24 i tam często się wypowiadają lekarze. Zakładając, że są to prawdziwi lekarze, to myślę, że też są wiarygodnym źródłem.

**Pamiętasz jakiś fejkowy artykuł, na który trafiłeś?**

Tak. Był taki gość - Jerzy Zięba. Ale z tego, co wiem pokasowali wszystkie kanały i starają się go wyłączyć z Internetu, to chyba ostatnim razem on mi się pojawił. To był człowiek, który mówił, żeby wlewać sobie dożylnie nadtlenek wodoru na koronawirusa. Ewidentnie niewiarygodne źródło.

**Dlaczego to jest niewiarygodne źródło?**

On już od pewnego czasu uprawiał pseudonaukę. Widziałem kilka jego filmów i on mówił niestworzone rzeczy, jak np., żeby wprowadzać metale ciężkie do swojego ciała. Studiowałem taki kierunek, i wiem, że wprowadzanie metali ciężkich do ciała nie skończy się dobrze. Więc chociażby na tej podstawie.

**Masz poczucie, że twój kierunek studiów coś ci daje w tej sytuacji?**

Nie za bardzo szczerze mówiąc.

Kiedy ta sytuacja się skończy twoim zdaniem?

Powołując się na doktora Rożka, szczepionka może być za rok albo nawet za półtora. Także to jest dość pesymistyczne podejście. Ale może się to też skończyć w momencie, kiedy ludzie oprzytomnieją, nie będą wychodzili na plażę, tylko będą siedzieli na tyłkach w jednym miejscu i wirus sam wymrze. Jest to teoretycznie możliwe, ale czy praktycznie, to nie wiem.

**Co to dla ciebie znaczy, że to się skończy?**

Przestaną być nowi zarażeni ludzie, a ci zarażeni wyzdrowieją, czyli badania wykażą, że w ich organizmie nie ma już wirusa.

**Masz jeszcze jakieś refleksje w kontekście tej sytuacji?**

Tak. Mam nadzieję, że ludzkość, rządy różnych państw wyciągną z tego konsekwencje, że w dobie takiego kryzysu trzeba się wcześniej zabezpieczyć na takie sprawy. I jeżeli chodzi o nasz rząd, to może zaczną bardziej się przyglądać przedsiębiorcom, bardziej im pomagać, itd. Taką mam nadzieję.

**Czy jakieś grupy ludzi twoim zdaniem mają szczególnie teraz utrudnioną sytuację?**

Tak, starsi ludzie, to na pewno. I młodzi przedsiębiorcy, z tego co mi wiadomo. Mówię na przykładzie mojego kolegi, który wyruszył z firmą w styczniu i teraz ma straty same, nie ma zamówień. Więc młodzi przedsiębiorcy, ludzie, którzy mają firmy od niedawna mogą być najbardziej poszkodowani. A wiadomo, że korporacje, jak chociażby Costa Coffee, jakoś to odczują, ale się utrzymają na rynku.

**Praca w korporacji daje ci poczucie bezpieczeństwa?**

Tak, zdecydowanie. Niestety nie zdążyłem przepisać sobie umowy i dostaję wynagrodzenie tylko za 3/8 etatu, ale zawsze jest to coś. Daje mi to poczucie bezpieczeństwa, że mam tę pracę i jakieś wynagrodzenie i dostaję dalej jakąś zapomogę od rodziców. Odetchnąłem z ulgą, bo dostałem jedną pracę w Lesznowoli, ale odmówiłem i cieszę się, że to zrobiłem, bo nie wyobrażam sobie dojazdu do pracy teraz 38 km w jedną stronę.
