# Supplementary material for: The challenges arising from the COVID-19 pandemic and the way people deal with them. A qualitative longitudinal study
Source: PLoS One. 2021 Oct 11;16(10):e0258133. doi: 10.1371/journal.pone.0258133 (PMC8504766; doi:10.1371/journal.pone.0258133)
Supplement: S1 Dataset — (ZIP) [file pone.0258133.s003.zip › Transcriptions/stage 1/20.1_F_25_couple, with child.docx]

**20.1_F_25_couple with child**

Mam 2l córeczkę i jestem w związku małżeńskim od 2 lat. Mieszkam jeszcze z rodzicami i teraz jeszcze z babcią w Jedlińsku.

**Czy wg pani coś zmieniło się w ostatnim czasie? Co najbardziej?**

Ogólnie, to zmieniło się chyba wszystko, a najbardziej to (i to mi najbardziej przeszkadza) możliwość spotkań z innymi osobami. Jest wszystko utrudnione - korzystanie z całej kultury, z najpotrzebniejszych rzeczy, czyli z robienia zakupów, ale chyba najcięższe jest to, że nie można się widywać z bliskimi i ze znajomymi.

**Kiedy dla pani sytuacja uległa zmianie?**

2 tyg. temu, jak wprowadzili kwarantannę w Polsce. Wcześniej zmieniała się sytuacja na świecie, ale nas to tak jakby nie dotyczyło do momentu, kiedy odkryto pierwszego przypadku w Polsce i wtedy został wprowadzony stan wyjątkowy. Wtedy dopiero to odczuliśmy, bo zostały wprowadzone jakieś restrykcje.

**Jakie były kamienie milowe rozwoju całej tej sytuacji?**

Ten moment, o którym powiedziałam był najbardziej istotny, bo teraz zmienia się sytuacja stopniowo, ale nie tak wyraźnie jak wtedy. Teraz, od 1 kwietnia weszło znowu jakieś zaostrzenie, ale to są już zaostrzenia tego, co było, więc nie jest to już tak bardzo odczuwalne. Mogliśmy być w 5 osób w sklepie, teraz są to 2 osoby i raczej ja nie czuję tej zmiany, bo u nas są małe sklepiki. Tak czy siak to już było od początku wprowadzone, że można było przebywać tylko pojedynczo w sklepie albo na poczcie. Nie korzystam z takich sieciówek, jak Biedronka, Lidl, bo one są w odległości 20 km ode mnie.

Te obostrzenia w małych sklepach były już wprowadzone od początku przez właścicieli.

**Od jak dawna to jest wprowadzone w małych sklepach?**

Dokładnie nie odpowiem, bo robimy zakupy na zmianę. Nie dopytywałam, jak to wyglądało wcześniej, ale byłam parę dni temu, w sobotę i już można było po 1 osobie w sklepie, a na poczcie 2 osoby, a ten przepis przecież wszedł od dzisiaj dopiero.

**Mieszkała pani z rodzicami, z babcią? Jak to teraz wygląda?**

Mieszkam z jedną babcią - mamą mamy, z rodzicami, z moim bratem i z moją rodziną. No i trochę się sprawa teraz skomplikowała, ponieważ moja babcia ma niewydolność nerek i musi jeździć na dializy 3 x w tyg. I to nie jest tak, że ona może sobie nie pojechać, bo po prostu musi. Mamy jeszcze parę innych miejsc, w których możemy mieszkać, więc rodzice wyprowadzili się z babcią w tamto miejsce i zostałam sama z mężem i bratem w domu z tego względu, że już pojawiły się jakieś sygnały dotyczące tego, że już może być chora, osłabiona po dializach. A wiadomo, że na dializach są sami schorowani ludzie, którzy najbardziej są narażeni na tę chorobę, na to, żeby złapać tego koronawirusa. Stwierdziliśmy, że warto się rozdzielić i zmniejszyć to ryzyko.

**W trosce o bezpieczeństwo babci?**

Raczej mojej córki, bo babcia jakby nie może się ustrzec od tego, bo ona musi jeździć do ośrodka na te dializy. Chodzi o córkę, bo jak są razem w domu, to nie da się zachować tego bezpieczeństwa. babcia spędzała z nią prawie cały czas i mówi, że nie może jej nie całować, jak ona jest blisko.

**Czy poza tymi wyjazdami babcia wychodzi z domu?**

Nie. Babcia nawet bała się wyjść na podwórko, bo myślała, że w powietrzu jest ten wirus. Starszy człowiek, on natury jeszcze nie rozumie, więc to, co my jesteśmy w stanie jej przekazać, to tyle wie. Przetłumaczyliśmy jej, że może być na podwórku, ale do sklepu, do koleżanek, innych córek nie chodziła.

Babcia bała się tego wirusa, a pani i pozostali członkowie rodziny?

Chyba największa obawa była związana z naszym dzieckiem, bo nie oszukujmy się - jak ktoś ma dziecko, to nie myśli o sobie, a raczej o dziecku. Tym bardziej, że napływały takie sygnały, że dla ludzi zdrowych w pewnym wieku to nie jest jednak takie groźne i większa jest szansa na wyleczenie osoby 20-30 letniej niż schorowanej babci albo małego dziecka, które tę odporność ma jeszcze nie do końca zbudowaną.

**Obawy o wasze zdrowie wam nie towarzyszą?**

Towarzyszą, ale to bardziej pod tym względem, że co będzie, jak będziemy musieli się rozdzielić, jak nie będziemy móc widzieć dziecka, itd. Raczej o sobie tu mało myśleliśmy z mężem. Obawa o rodziców, o dziecko, o babcię, ale no nie o nas. Obawy nie tyle przed samym wirusem, co raczej o konsekwencje z tym związane.

**Skala lęku.**

Myślę, że ok. 65 teraz. Wydaje mi się, że wcześniej bałam się o wiele mniej z racji tego, że nie wiedziałam, jak to się rozwinie w naszym kraju, chociaż reakcje służb państwa były bardzo szybkie. O wiele szybsze niż w innych krajach. No i tutaj się jakaś iskierka nadziei pojawia, że być może nie będzie takiej sytuacji, jak np. we Włoszech. Wcześniej było mniej zachorowań, teraz liczba zachorowań rośnie. Wcześniej nie było przypadków u nas w Radomiu i jeszcze czuliśmy to bezpieczeństwo, że tutaj nikt nie choruje. Potem pojawiły się te przypadki w Radomiu, potem okazało się, że kwarantannę mają nawet jakieś osoby u nas na wsi. Okazało się, że ten wirus już nie jest tak daleko od nas, tylko jest bardzo blisko nas no i w każdej chwili my niestety też możemy się zakazić.

**Czy coś jeszcze oprócz docierających informacji wpływało na zwiększenie/ zmniejszenie leku?**

Raczej spokojniej to już chyba nie będzie i raczej spokojniej to już się nie będę czuła. Zmienia się ciągle ta liczba zachorowań i osób, które są chore wokół nas. Na pewno informacje, które dopływają z tv i internetu mają wpływ. Niektórzy starają się uspokajać, pokazywać statystyki, na innych portalach ciągle bębnią, że jest tragedia i niestety nie będzie kolorowo. Teraz ciężko jest porównać te informacje - która jest prawdziwa, która jest fałszywa. Jakiś czas temu był sygnał, że jakiś naukowiec z Wrocławia opracował już jakąś bazę tej szczepionki. I wtedy wszystko fajnie, super, jest nadzieja, ale no niestety potem w tv to ucichło i nic już o tym nie mówią. On podobno wysłał swój patent do wszystkich krajów za darmo, do laboratoriów, żeby coś z tym robili. I wydaje mi się, że to jest rzetelna informacja a w tv nic o tym nie mówią i tak naprawdę nie wiadomo czy to może dawać nadzieję, czy nie.

Tv i internet są jedynymi źródłami informacji, bo jak inaczej możemy się dowiedzieć o sytuacji, która jest teraz? Tylko i wyłącznie z tv i internetu. Nie ma innej możliwości. To, co nam powiedzą politycy, bo to oni teraz występują w tv, albo Minister Zdrowia, to wypadałoby...Wypadałoby, bo ja nie mówię, że wierzę. Tylko oni teraz udzielają jakichkolwiek informacji i np. mówią, że jesteśmy bardzo dobrze przygotowani, już od dawna szykowaliśmy się na to, a u nas po paru dniach już brakowało całego sprzętu medycznego. Te informacje niekoniecznie są prawdziwe, ale tylko takie możemy teraz zdobyć.

**Co świadczy o tym, że informacja jest prawdziwa?**

Jak widzi się, że jest zgodna z faktami. Teraz jest bardzo ciężko to stwierdzić, bo podpisze się pod artykułem paru doktorów i wypadałoby stwierdzić, że jest jak najbardziej prawdziwa, ale nie wiem, co musiałabym robić, żeby sprawdzać te informacje, które podają w tv. na pewno jest z tym ciężko. Ja nie mówię o informacjach na Pudelku, bo wiadomo, że to jest komercyjny portal, ale np. na Wirtualnej Polsce pojawiają się codziennie informacje, więc uważam, że one są prawdziwe. Podają statystyki zakażeń, itd., ale nie mam dostępu do medycznych materiałów każdego szpitala, żeby to potwierdzić. Uważam, że powinni mówić prawdę, ale jak jest to ciężko stwierdzić. tak samo było z Chinami. Mówią, że w Chinach statystyki były mocno przerysowane w dół, jeżeli chodzi o liczbę zgonów i szary człowiek nie jest w stanie tego sprawdzić.

**Czyli uważa pani, że bardziej wiarygodne informacje będą na portalach, które uważa pani za godne zaufania?**

Tak, dokładnie.

Wirtualna Polska wydaje się pani wiarygodna. Jakie jeszcze są wiarygodne źródła informacji?

Oglądamy na okrągło, tzn. wcześniej jak byli rodzice, bo teraz telewizor jest wyłączony...Oglądaliśmy TVP Info, gdzie właśnie pojawia się Prezydent, Minister Zdrowia, itd. i są na bieżąco transmitowane wywiady z nimi. Teraz jest pytanie, na ile oni mówią prawdę. To źródło na pewno w jakiś sposób jest rzetelne i prawdziwe, bo to są wywiady na żywo i nie ma możliwości kłamania, ale teraz na ile oni mówią prawdę? Kółko się toczy i dlatego ja już teraz nie oglądam tv i staram się przestrzegać pewnych zasad, o których zostałam poinformowana albo przeczytałam na WHO, itd. Po co codziennie oglądać to samo i się stresować? Trzeba żyć dalej, a co będzie to będzie. Już nie ma odwrotu i nic więcej nie zrobimy.

**Teraz mniej czasu poświęca pani na słuchanie informacji, a jak było zanim nadeszła historia z koronawirusem?**

Ja ogólnie oglądam bardzo mało wiadomości i bardzo mało tv z racji tego, że mój mąż nie ogląda w ogóle. U nas telewizora nie było kompletnie oprócz tego, że przechodziłam przez salon i rodzice mieli włączony telewizor. Oglądałam bardzo mało. jak pojawiła się ta historia z koronawirusem to TVP Info było włączone non stop, więc z czystej ciekawości człowiek oglądał. Rodziców nie ma od niedzieli i nie oglądamy praktycznie w ogóle, oprócz jakichś bajek z dzieckiem i na tym koniec. Już naprawdę chyba nic nowego nie powiedzą. Poza tym teraz jak była ta zmiana od 1.04., to przychodzą mi alerty na telefon, że zaszła jakaś zmiana, ale to już jest od WHO jakaś ważna informacja i żadnych innych już raczej nie oglądam. Wchodząc na pocztę mam statystykę na WP, ile osób jest zarażonych no i na tym koniec. Ileż można oglądać to samo? Z zasadami bezpieczeństwa zapoznaliśmy się na samym początku i już nic nowego nie mogą powiedzieć, oprócz tego, że np. zostały zamknięte granice albo, że ludzie mają tylko 2 dni na wjazd. Mnie to nie dotyczyło, bo nikt z rodziny nie przebywał za granicą. Jakieś poszukiwania domów do kwarantanny to też są rzeczy, które mnie osobiście nie dotyczą. Dotyczyło mnie tylko to, czy mogę zrobić zakupy, czy mogę wyjść na spacer, itd. Ja nic więcej, jako ja, jako człowiek żyjący w społeczeństwie nie mogę zrobić. siedzimy w domu, nie spotykamy się ze znajomymi, ale cóż więcej? Nic więcej się nie zrobi

**Emocje - zdjęcia**

**14**

Tu mamy węzeł, supeł. Wybrałam dlatego, że jesteśmy właśnie w takiej sytuacji, że nic nie możemy zrobić. jesteśmy związani fizycznie na pewno, ale emocjonalnie także. Myślę, że wiele osób będzie miało teraz problemy psychologiczne, w sensie, że pogłębi się depresja, itd. Jeżeli chodzi o mnie, to chyba najgorsze jest to, że nie możemy robić tego na co mamy ochotę. Jest tu węzeł, ale równie dobrze mogłaby być klatka. Jesteśmy w przysłowiowej klatce i musimy poczekać na wyjaśnienie tej sytuacji, Najgorsze, że nie wiemy, ile będziemy czekać a już ograniczenia są takie wprowadzone, że obyśmy mogli jeszcze wychodzić na ten spacer i wyjść do sklepu, bo ja byśmy musieli siedzieć tylko w domu, to już w ogóle byłaby tragedia.

**Jakie konkretne emocje tu są?**

Przede wszystkim bezsilność. Chyba bezsilność jest najgorsza, że sami nic nie możemy zrobić. Stosować się do zaleceń i nic więcej. Sami nie jesteśmy w stanie zwalczyć tego wirusa, nie jesteśmy w stanie wsiąść w samochód, wyjechać i zostawić tego problemu, bo jest to problem globalny a nie tylko w naszym kraju.

**Jak te bezsilność i reszta tych emocji mają się do tego, co czuła pani wcześniej? Czy te emocje zmieniały się w różnych momentach ostatnich tygodni?**

Na samym początku, jak rząd wprowadził kwarantannę, to jeszcze widzieliśmy się z teściami i nie było to dla mnie problemem. Ta osoba zarażona była z Zielonej Góry, daleko, u nas nie było jeszcze żadnego przypadku. Oczywiście to jest takie głupie myślenie, bo nikt nie zrobił sobie jeszcze testu. Potem wraz z napływem tych informacji i ostrzeżeń, wraz z pojawiającymi się kolejnymi przypadkami już wizyty zostały zaprzestane. Straszyli nas, więc coś zrobiłam, ale chyba dobrze zrobiłam, że przestałam się kontaktować z kimś. Te wiadomości straszą, ale może też dogłębnie uświadamiają i taki jest w tym cel, żeby ludzie zaczęli się bać a nie olewać tak, jak ja w tych 2-3 pierwszych dniach. Uważam, że to nie było prawidłowe. To, że ktoś jest zdrowy, to nie znaczy, że jest zdrowy, tylko po prostu nie ma wykonanego testu. Testów jest robione bardzo mało niestety, więc wykrywalność też jest bardzo mała. To, co podają statystyki trzeba pewnie pomnożyć x 2 lub 3.

**Co w obecnej sytuacji jest dla pani największym wyzwaniem?**

Chyba brak kontaktów z innymi ludźmi. Jestem osobą bardzo towarzyską i nie potrafię żyć sama ze sobą niestety i nawet tylko z córką i z mężem, więc my non stop jesteśmy i jednych, drugich, trzecich, ktoś nas odwiedza, ktoś przyjeżdża. Dla nas ta sytuacja jest bardzo, ale to bardzo nienormalna. Można pobyć sobie samemu 2-3 dni, ale nie 3 tyg. czy ileś, żeby nie spotkać się z rodziną, nie móc normalnie zjeść obiadu u teściów, że moja córka nie mogła spotykać się ze swoją siostrą, bratem. Chyba to jest najcięższe.

**Co pani robi, żeby sobie z tym radzić?**

O rany, nic nie robię [śmiech] Jak mam sobie radzić? Nie wiem, jak mam sobie radzić. Często rozmawiamy na Messengerze, codziennie rozmawiamy ze wszystkimi. Chociażby z racji tego, żeby moja córka chociaż zobaczyła innych. Niby ma tylko 2l, ale codziennie rano ona się ubiera i mówi, że jedzie do babci Tereski albo siedzi w oknie i czeka na Szymona, na Marysię i wymienia z 15 osób, które pamięta. Każe mi się chować i mówi, że znajdzie ją ciocia Iga. Ona kompletnie nie wie, dlaczego ona nie może wychodzić i dlaczego nikt do nas nie przyjeżdża. Tłumaczę jej, że wszyscy są chorzy, idą do doktora, biorą leki, bo tylko to jest dla niej wytłumaczenie. My dorośli potrafimy jeszcze coś zrozumieć, a ona kompletnie nie wie, dlaczego nagle zostaliśmy tak całkowicie sami i nie może się z nikim pobawić.

**Czy to tłumaczenie dziecku jest trudne?**

Bardzo trudne, bo to się aż serce kraje. Jest mi jej szkoda. Ja mogę się nie spotkać z koleżanką i sobie to odpuścić, bo jestem dorosła i dam sobie radę, a dziecka po prostu jest szkoda, bo ona nie rozumie, nie wie. Jak ja jej powiem dzisiaj, że ktoś jest chory, to ona zapyta mnie za 3 godziny znowu. Dzień w dzień się ta sama sytuacja powtarza, że ona chce gdzieś jechać albo na kogoś czeka.

**Proszę sobie przypomnieć ten szał zakupowy jakiś czas temu. czy zrobiła pani wtedy jakieś dodatkowe zakupy?**

Na początku, jak była ta cała sytuacja z zakupami, to oczywiście się śmiałam. Bardzo się śmiałam i mówiłam, że wszyscy powariowali, ale mój mąż musiał iść do sklepu po jakąś głupią rzecz i mówił, że naprawdę są puste półki, że naprawdę nic nie ma. Wtedy pomyślałam, że my też chyba musimy zrobić te zakupy, z racji tego, że jak wszyscy inni wykupią...No, ale mama kupiła jakiś zapas jedzenia na 2-3 dni. To nie było 20 kg mąki, tylko jeden makaron, jedna mąka, itd. Oczywiste jest, że sklep nie nadążał się zatowarowywać i wystarczyło, że minął ten szał, minęły 2-3 dni i przyjechał kolejny transport. Mój mąż pracuje w firmie transportowej i akurat wozi mięso, więc miał dostęp dla najbardziej dla nas potrzebnej żywności, czyli do mięsa, więc byliśmy spokojni.

**Czy ta sytuacja wpłynęła na pani zachowanie, jako konsumenta?**

Tak, bardzo dużo teraz zamawiam przez internet. Niestety. Jest dużo wolnego czasu, nie można pójść do galerii, do sklepu. Czasami z nudów nawet. Śmieją się wszyscy, że już wiosennych rzeczy nie trzeba kupować, bo się nie wychodzi, a letnich tym bardziej, bo nie wiadomo w co się zmieści człowiek. Ale zamawiam, zamawiam sporo rzeczy. Z nudów, bo się siedzi i ogląda te rzeczy, torebki, itd. Pewnie jak bym była w sklepie, to bym nie kupiła tych rzeczy, a tak to codziennie coś się zamawia niestety. Kurierzy pracują normalnie. Jakby kurierzy nie pracowali to by nie było problemu i człowiek by pieniędzy nie wydawał.

**Mama wtedy zrobiła zakupy na kilka dni, a państwo też?**

Nie, raczej normalnie. Teraz też staramy się raz na 2 dni chodzić do sklepu, więc kupuję na bieżąco. Jedynie zrobiłam zapas mleka córce, bo teraz się myśli o dziecku a nie o sobie. Gdyby coś tam się wydarzyło, to żeby miała to mleko i jakieś jogurty, żeby ona raczej była zabezpieczona w takie rzeczy, które się szybko nie popsują a i tak je wykorzystam później. Mleko modyfikowane w proszku kupiłam i zamiast 1 kupiłam 3. tak samo 3 kaszki i tyle. Robimy takie zakupy na 2, maksymalnie 3 dni.

**A marki wybierane wcześniej i teraz. Czy coś się zmieniło?**

Raczej te same, nic nie zmieniłam. Jest bardzo dużo promocji teraz, ale akurat są promocje w tych sklepach, w których kupowałam lub zamawiałam wcześniej, więc wszystko to samo.

**Są jakieś produkty, na które bardziej teraz zwraca pani uwagę? Tak, jak papier toaletowy dla niektórych?**

Nie, na to nie zwracam uwagi. nawet nie kupiłam żelu antybakteryjnego i żadnych takich rzeczy. Mieliśmy maseczki, rękawiczki - to wszystko jest, ale bez zatowarowywania się po prostu. Żelu nie kupiłam, bo można myć po prostu ręce w domu, jest spirytus jak trzeba by było i nie potrzeba płacić 30 zł za małą buteleczkę, choć podobno już są w sklepach normalne ceny. jakoś nie miałam na to szału. Jak wychodzę do sklepu, to zakładam rękawiczki, potem je wyrzucam i tyle.

**A te maseczki i rękawiczki?**

U nas była taka sytuacja, że u taty pracuje dużo pań z Ukrainy i jak się zaczęła ta sytuacja w Polsce, to poprosił jedną, która umiała szyć i po prostu uszyła. Przy okazji jeszcze uszyliśmy do Ośrodka u nas, do Straży. Z tym nie miałam problemu i nie kupowałam.

**Od jakiegoś czasu są kolejki przed sklepami. Jak się pani do tego odnosi?**

Jest to denerwujące, ale wiadomo, że każdy chce coś tam sobie kupić. Jest to ograniczenie osobowe i niestety tego się nie uniknie. Wiadomo, że mnie denerwuje jak stoi 20 osób przed pocztą i muszę czekać godzinę, ale wiem, że tak musi być i nie zwracam na to uwagi. Gdyby była taka kolejka 2 miesiące temu, to bardzo bym się denerwowała. Teraz to stało się normalne. Mam w głowie, że tak teraz musi być i tak jest bezpieczniej, i to nie jest wina ludzi, którzy pracują w środku. Takie są rozporządzenia, trzeba się dostosować i tyle. I jak wiem, że jadę na zakupy, to zajmie mi to 1.5 godz. a nie 20 min jak dawniej.

**Skąd wziął się problem związany z koronawirusem?**

Z jakiegoś laboratorium, ale ja nie wiem, bo nie jestem na tyle mądra. Ja to widzę tak, że to powstało w laboratorium i całkowicie nie przez przypadek. Od dawna było mówione, że III wojna to będzie wojna w rękawiczkach. Nie sądzę, że to jest ta III wojna, tzn., mam nadzieję. Jeszcze nie wiadomo co się stanie, jak ten wirus dojdzie do Afryki, bo tam nie ma żadnych zabezpieczeń i niestety tam medycyna jest bardzo słaba, więc na pewno bardzo dużo osób umrze. Jeszcze nie wiemy, jaka będzie skala tego całego problemu, bo to jest za wcześnie, o wiele za wcześnie, żeby o tym mówić. Są takie czasy, że teraz nie trzeba zabijać na ulicy. Wystarczy mieć fartuch i te białe rękawiczki, żeby coś takiego zrobić. Wydaje mi się, że znikąd to się nie wzięło, z powietrza to się nie wzięło, z natury to się nie wzięło. I tyle. Jestem apolityczna, więc ja się kompletnie nie interesuję, co się dzieje i mnie to w ogóle nie interesuje, chociaż wiem, że moja teściowa jest niekoniecznie prawidłowa, ale jestem tego zdania, że ja, jako szary człowiek nie jestem w stanie nic zrobić i nie mam na to wpływu niestety tak jak 3/4 ludzi na świecie. Światem rządzą pieniądze wysoko postawieni ludzie i tyle. Ja się w politykę nie zagłębiam, na wybory nie chodzę. Uważam, że każdy człowiek powinien dbać o siebie a nie myśleć, co będzie miał, jak się zmieni władza, jak się zmieni rząd. Powinniśmy sobie zapewnić bezpieczeństwo i swojej rodzinie poprzez ciężką pracę. Uważam, że jeżeli człowiek ciężko pracuje to zawsze będzie miał. Z takich kombinowań, cudowań, 500+? To jest na chwilę. Nie interesuję się, nie znam się na polityce, myślę przede wszystkim o sobie i swojej rodzinie. Był przykład pana z jakiegoś państwa, który miał owce, miał krowę, miał kury, miał zboże, itd... Jego dzieci się nawet chyba uczyły w domu. I on powiedział, że jego właśnie nie interesuje sytuacja na świecie. On, bez względu jaka będzie władza na świecie, to dla niego jajko, mleko od krowy, mięso ze świni, którą ma, będą miały taką samą wartość. Dba tylko o siebie i czy zmieni się waluta, czy inflacja itd., to na niego nie ma to wpływu, bo on stanowi sam o sobie. Ja jestem trochę tego zdania. Podobno to jest zła postawa, bo trzeba się troszczyć też o swoje państwo, ale...Państwo za bardzo się o mnie nie troszczy i ja za bardzo państwa nie interesuję i dlatego ja też...Szanuję państwo, jak najbardziej, ale gdyby była wojna, to ja bym uciekła.

**Co wg pani sprawiło, że ten wirus zaczął się tak szybko rozprzestrzeniać?**

Nie wydaje mi się, że tak szybko. Jednak ten wzrost liczby osób był bardzo mały na początku, ale było też bardzo mało wykonanych testów i dlatego ciężko coś stwierdzić po tych statystykach. Wydaje mi się, że te kontakty między ludźmi, że były przez jakiś czas otwarte granice, że ludzie wracali z Niemiec, z Włoch, itd. Lądowały potem samoloty z ludźmi, którzy byli na wycieczkach, bo sobie skorzystali z fajnej okazji cenowej i pojechali na wycieczkę a potem płakali, że nie mają jak wrócić. Dla mnie to już w ogóle beznadziejność i głupota ludzka, i powinni tam zostać. To, że nagle osoby, które mieszkają od 15 lat we Włoszech zaczęły wracać do Polski. Przez 15 lat ich krajem były Włochy a teraz będzie Polska, bo się zaczęło źle dziać, więc w ogóle tego nie popieram I wydaje mi się, że nie powinny te samoloty u nas lądować, że nie powinny były być otwarte granice, że od razu powinny zostać zamknięte i nie powinien Prezydent sprowadzać Polaków do domu.

**Można było coś zrobić, żeby zapobiec epidemii?**

Nie mogliśmy nic zrobić, bo to nie było od nas zależne. Może ta 1-sza osoba...Nie wiem, nie będę tutaj gdybać. Wydaje mi się, że nie mogliśmy zrobić nic. Mogliśmy wcześniej zamknąć granicę, nie sprowadzać kolejnych ludzi, nie powinien być w ogóle organizowany ten Lot do Domu. Na ten czas powinni byli zostać tam, gdzie byli, a zwłaszcza osoby, które skorzystały z jakiejś fajnej promocji i poleciały do Tajlandii. Albo tak, jak moja siostra, która poleciała do Wietnamu. Na wycieczkę pojechała, gdzie tam już była masakryczna sytuacja. Moja siostra cioteczna. Dla mnie to jest ludzka głupota. I potem ona stamtąd wracała tutaj do Warszawy i pracuje jeszcze w policji, gdzie ma jednak styczność z wieloma osobami w biurze, itd. Co więcej mogliśmy zrobić? Nic. U nas w Polsce dość szybko były wprowadzone różne zakazy i nakazy, więc myślę, że więcej nie. No oprócz tej młodzieży, która tam się grupowała na tych wałach, dużo osób siedziało na plaży razem, ale wydaje mi się, że i tak to w bardzo małym stopniu zaszkodziło. Nie tak, jak we Włoszech, gdzie ludzie balowali całe noce. Po Włochach można się było spodziewać, bo niestety taki mają temperament i oni inaczej by na pewno nie zrobili.

**Sądzi pani, że jako kraj jesteśmy przygotowani na to, co się dzieje?**

Mówią, że jesteśmy, ale wcale nie jesteśmy. Już 2-3 dni po tym jak u nas w Radomiu wykryli tego koronawirusa, to już brakowało na oddziałach masek, przyłbic, okularów ochronnych, fartuchów, itd. A podobno mieli być przygotowani. I teraz prywatni ludzie szyją na potęgę wszystko za własne pieniądze. Nie zobaczą za to nawet grosza, ale po to, żeby jakoś pomóc. To powinno zapewnić państwo, ale nie zapewniło. Dlatego, jak sami się o siebie nie zatroszczymy, to niestety nic to nie da. Oczywiście jedyną osobą w Polsce, która myśli jest Jurek Owsiak, którego bardzo mocno wspieram i popieram. I oczywiście on, jako jeden z nielicznych przeznaczył dużą sumę pieniędzy na zakup tych respiratorów i całego sprzętu. To on teraz myśli jak z Chin sprowadzić jakieś kolejne rzeczy i już tam z MON...Ma jakiś samolot wylądować, ale to oczywiście Owsiak musi robić brudną robotę a oni nie mogli tego zrobić wcześniej. Oczywiście też szanuję sportowców, którzy jakieś swoje prywatne pieniądze wyłożyli, ale chodzi mi o taką organizację i jedną z większych jest właśnie WOŚP, która dużo robi a tak dostanie po dupie na pewno

**Rozumiem, że widzi pani dużo uchybień rządu?**

Uważam, że rząd bardzo dobrze zareagował w takim sensie, że wprowadził nakazy, zakazy, zamknięcie granic, później spotykanie ze znajomymi, sklepy, galerie, itd. Super, ale takie przygotowanie techniczne? Jest bardzo słabe, bo brakuje po prostu sprzętu a sprzęt jest teraz najważniejszy. Samo robienie testów. Trąbi się, że Niemcy mają taką słabą sytuację, ale maja taką sytuację, bo wykonują 3000 testów dziennie. Dlatego mogą więcej wykryć. My się cieszymy, że mamy mniej, a tak naprawdę jest robione 300 testów. Chodzi i o skalę, że tych testów brakuje, nie ma, a o sytuacji w Chinach wiedzieliśmy już bardzo dawno i wydaje mi się, że mogli przeznaczyć na to pieniądze zamiast na co innego i jednak ten sprzęt po prostu kupić.

**A jako Europa jesteśmy przygotowani?**

Oczywiście wszyscy mogli się lepiej przygotować, ale niestety profilaktyka jest u nas bardzo słaba, ponieważ nadal ludzie nie rozumieją, że lepiej wydać pieniądze na profilaktykę niż na leczenie. Nie wiem, czy akurat na koronawirusa można się było przygotować. Mogłaby być też pandemia spowodowana szczurami, że byłby ogrom szczurów na świecie i byłyby potrzebne całkowicie inne środki bezpieczeństwa. Nagle potrzebne by były trutki i nagle też by tego brakowało. Niestety nie jesteśmy w stanie zabezpieczyć się na wszystko, ale jeżeli była taka sytuacja w Chinach, to państwa mogły już zacząć coś robić a nie czekać. Chiny też tą informację podały na pewno za późno, bo pierwsze przypadki były już w listopadzie, gdzie była cisza u nas,

**Czy te decyzje, które podejmuje nasz rząd wpływają jakoś na poziom lęku, który odczuwają ludzie?**

Nie wiem. To chyba zależy od danej osoby i jej pewności emocjonalnej. Im bardziej rząd mnie straszył, tym bardziej ja się doprowadzałam do porządku. A ktoś może popadać w większą bezradność i depresję. To jest sprawa indywidualna - czy ktoś wszystko bierze do siebie, czy tak jak ja, że zobaczę liczbę zgonów, ale nie siedzę w kącie i nie płaczę tylko wracam do swojego porządku dziennego. Jak ktoś jest słabszy psychicznie albo ma jakieś jeszcze inne problemy np. ze starszymi osobami, ma dziecko niepełnosprawne, gdzie musi dojeżdżać do szpitali, to jest to całkiem inna sytuacja. Ja nie muszę jechać do szkoły, na studia, do pracy, do szpitala. Mam dziecko przy sobie w domu i żyję.

**Jakieś pani przemyślenia, refleksje w ostatnim czasie?**

Jedyne co uważam, co na pewno jest bardzo złe, co wprowadził rząd, to jest oddzielenie matki od dziecka w szpitalach. To jest bardzo ciężka sytuacja i nie wyobrażam sobie, gdybym była na miejscu tej matki. Mojego męża siostra urodziła wcześniaka w szpitalu, jak właśnie pojawił się koronawirus. I wyszło rozporządzenie, że niestety matki wychodzą po porodzie a dziecko zostaje. To by była naturalna sytuacja, gdyby można było to dziecko odwiedzać, a tu niestety Aneta musiała wrócić po tygodniu a dziecko jest w szpitalu. I jedyne informacje jakie ma to o zmieniającej się sytuacji zdrowotnej, zrobione zdjęcie raz na 3-4 dni albo i nie. A dziecko będzie tam musiało spędzić jakieś 1,5 miesiąca. Ona jest naprawdę w okropnym stanie i to chyba tylko druga matka może zrozumieć jakie to są uczucia. Wg mnie powinno być to miejsce dla matki i oczywiście matka nie może opuszczać oddziału. Fundacja chce coś tam zdziałać, tylko jak teraz miałby wyglądać ten powrót matki do dziecka. Jakby miały wyglądać badania? Gdyby mogła od razu zostać, to byłaby inna sytuacja. To jest bardzo przykre i bardzo trudne.

**Czy to co się teraz dzieje odciśnie się jakoś na społeczeństwie?**

Mam nadzieję, że ludzie wreszcie docenią rzeczy, które mają. mam znajomych, którzy zazwyczaj spędzają weekendy w klubach, pubach a teraz się cieszą, że wyszli do lasu i że mogą do niego wyjść. Ja mam nadzieję, że ludzie wreszcie docenią, co tak naprawdę jest w życiu ważne - to, żeby odwiedzić mamę, dziadka, pobyć z przyjaciółmi, wyjść na spacer. To naprawdę są takie rzeczy, które powinny cieszyć, a nas niestety już nie cieszyły. Wręcz przeciwnie - w ogóle tego nie robiliśmy. Teraz się okazuje, że oglądanie serialu na Netflixie nie jest już takie interesujące, jak trzeba to robić codziennie przez 3 tygodnie, bo się nie ma co robić. A kiedy to było fajne. Tak zostać z Netflixem na 3 dni? Teraz już nawet przeglądanie telefonu się nudzi. Mam nadzieję, że mentalność ludzi się zmieni i docenią to, co jest naprawdę ważne. Teraz jak niektórzy nie mogą się zobaczyć np. ze swoimi rodzicami, to może zdają sobie sprawę, że niewłaściwie wykorzystywali ostatni czas a nie wiadomo czy to nie był ostatni czas. Bo nie wiemy kto zachoruje i jak się sprawa potoczy. To mam nadzieję skłoni ludzi do myślenia, żeby posegregować swoje wartości i czy te pieniądze są ważne, czy ta praca jest taka ważna, czy może ten spacer z żoną i z dzieckiem jest bardzo atrakcyjny.

[umawianie kolejnego spotkania]

Tylko proszę mi przypomnieć, nawet smsem na jakąś godzinę przed, bo ostatnio bardzo mi się mylą dni tygodnia. Wcześniej nigdy mi się nie myliły dni tygodnia, bo mam bardzo uporządkowany każdy dzień i wypchany miałam na maksa. Ja wszystko wiedziałam co, o której godzinie i nie mogłam sobie pozwolić, żeby dzień pomylić. Teraz niestety, jak się siedzi w domu i robi prawie, że to samo...Teraz nie mam nic zaplanowanego i każdy dzień wygląda prawie tak samo i dlatego te dni się po prostu mylą.

**Teraz pracy jest dla pani więcej czy mniej?**

O wiele mniej. Ja udzielam np. korepetycji z matematyki i wcześniej musiałam spędzić czas na dojeździe do ucznia, przestudiowanie 20 zadań i wrócenie do domu, co mi zajmowało 3 godz. Teraz wysyłają mi zadania zdalnie i oni rozwiązują swoje, ja im wysyłam poprawione. O wiele skraca mi się czas mojej pracy. Ze studiami tak samo. Wcześniej trzeba było być na zajęciach, a teraz robię jedną pracę w pół godziny i mam z głowy. mam o wiele więcej czasu, tylko nie ma jak z tego czasu korzystać.
