# Supplementary material for: The challenges arising from the COVID-19 pandemic and the way people deal with them. A qualitative longitudinal study
Source: PLoS One. 2021 Oct 11;16(10):e0258133. doi: 10.1371/journal.pone.0258133 (PMC8504766; doi:10.1371/journal.pone.0258133)
Supplement: S1 Dataset — (ZIP) [file pone.0258133.s003.zip › Transcriptions/stage 1/2.1_F_27_single.docx]

**2.1_F_27_single**

**Czy możesz powiedzieć mi coś więcej o sobie?**

Studiowałam psychologię, ale niestety nie udało mi się obronić; ale nie potrzebuję tego więc nie mam przymusu. Pracuję teraz w administracji, pracuję właściwie w pojedynkę. Jestem osobistą asystentką pani prezes. To dla mnie super praca, bo polega na realizowaniu tasków niemożliwych, ogarnięcia czegoś, pojechania gdzieś - cały czas mogę być nabodźcowana, świetnie się w tym odnajduję. W wolnym czasie, którego mam niestety dość mało, głównie leżę i oglądam telewizję. Głupio mi się do tego przyznać, to dlatego, że nie miałam telewizji przez długi czas. Poza tym bardzo lubię spacery. Ćwiczyłam też jogę, w dalszym ciągu medytuję. Poza tym maluję. Nie profesjonalnie, ale to dla mnie coś jak medytacja, tyle że palcami.

**Kiedy wyprowadziłaś się od rodziców?**

W styczniu zeszłego roku, czyli na piątym roku studiów.

**Mieszkasz sama?**

Nie, mieszkam z moim przyjacielem.

**Opowiedz mi teraz trochę o początku aktualnej sytuacji. Kiedy pierwszy raz pomyślałaś, że coś się zmienia? Kiedy zaczęło się to dla ciebie?**

Kiedy pomyślałam, tego nie wiem... nie wiem w dalszym ciągu czy sobie to uświadamiam. Pierwszy raz weszło to do mojego życia w pracy. Miałam wysłać koleżankę na konferencję do Singapuru (zajmujemy sie robieniem kampanii i mamy biuro w Singapurze). Powiedziała "nie wiem czy pojadę, bo jest tam jakiś wirus". Właściwie ona wprowadziła mnie w ten temat. Ona codziennie tym żyła. W końcu nic z tego wyjazdu nie wyszło. Druga koleżanka planowała dłuższe wakacje w Azji, miesiąc chyba lub trzy tygodnie. Ona szóstego lutego wyjeżdżała więc było to jakoś wcześniej. Na bieżąco śledziliśmy temat, bo pracujemy w małej grupie i żyliśmy tą sytuacją. W końcu koleżanka pojechała i nie było jej miesiąc czy trzy tygodnie. Widzieliśmy na instastories jak chodzi w maseczce, ale w dalszym ciągu wydawało nam się to dość abstrakcyjne. Potem ona wróciła i ja pojechałam na urlop na Cypr i tam nawet mowy nie było; ktoś powiedział, że jest tam za gorąco na koronawirusa. Wróciłam do Polski i wtedy zaczęło być o tym głośniej, ale jeszcze na luzie. Chyba jeszcze nikt nie był wtedy w Polsce chory. Tydzień później wróciłam do pracy i był już boom, wszyscy o tym mówili i wszyscy o tym wiedzieli. Dwa tygodnie temu już pierwsze firmy szły na prace zdalną, ale nasza prezes mówiła, że chce tego uniknąć. W środę jeszcze pracowaliśmy normalnie, zaopatrzyliśmy się w żele i inne środki. W czwartek podjęła decyzję, że od piątku pracujemy zdalnie i od tego czasu pracujemy w domu. Śmieszna sytuacja - nie tyle śmieszna - po prostu budynek, w którym pracowaliśmy do tej pory, wyburzają, więc musieliśmy jeszcze zrobić przeprowadzkę. Właściwie te dwa tygodnie byłam cały czas w biurze, bo trzeba było to ogarnąć.

**Były jeszcze takie momenty, kiedy dowiedziałaś się o czymś istotnym i wpłynęło to na ciebie?**

Chyba parę dni temu szłam do pracy pieszo Nowym Światem, nie było nikogo i przejeżdżał radiowóz z megafonem. Faktycznie byłam jedyna na Nowym Świecie i poczułam się jak w grze komputerowej, poczułam niepokój. Zachowuję wszystkie środki ostrożności - maseczka, żele - ale przez to, co zobaczyłam, dziwnie się poczułam. Co jeszcze... ja może nie, ale przyjaciel z którym mieszkam prosi mnie żebym chodziła z nim do sklepu, bo on się po prostu boi. On nie lubi zmian, jak widzi ludzi stojących daleko od siebie to źle na niego wpływa - chodzimy więc razem. Co jeszcze... fajna sytuacja, można o tym wspomnieć. Byliśmy razem w sklepie i zachowywaliśmy się normalnie, żartowaliśmy. Ale jak teraz wychodzimy to wiemy, że robimy zakupy, żadnych żartów, i wracamy. Wydaje mi się, że nie robimy tego świadomie, to odpowiedź na to co się dzieje wokół. Mam wrażenie, że coś sie zmieniło. Kilometrowe kolejki przed biedronką, ludzie stojący co parę metrów to rzecz, której nigdy nie widziałam, strasznie dziwnie się czuję. Nie tak, że się boję, to po prostu dziwne.

**Nie boisz się, ale jest to dziwne. Wytłumacz mi, co to znaczy?**

Po prostu mam wrażenie, że nigdy czegoś takiego nie czułam. To całkowicie nowe, ale nie jak nowy sport czy miejsce, tylko jest w tym nutka niepokoju. Może dlatego, że każda inna rzecz, jaką robiłam, była już sprawdzona przez kogoś, a ta nie. Nie mam żadnej pewności.

**Pokażę Ci zdjęcia. Mówisz, że to nie lęk, ale "coś innego", inne uczucie. Które z obrazków najlepiej pokazują twoje emocje? Co na nich widzisz?**

Osiem albo dziewięć... osiem. To droga, którą trzeba iść, ale nie wiadomo, co jest dalej. Inni wiedzą co tam jest, ale ja i ludzie obok mnie nie.

**Obrazek jest metaforyczny. Czego się spodziewasz po tej mgle na obrazku, ale w swoim realnym życiu?**

Boję się tego, że upadnie moja firma i większość miejsc pracy. To jest niepokojące. Zresztą widzę, ile osób straciło pracę w mojej pracy. Wydaje mi się to okropne, bo teraz nie znajdzie się szybko pracy. Jak ktoś nie ma oszczędności, to nie wiem co zrobić. To jest dla mnie niepokojące, nikt nie ma nad tym kontroli.

**Czy jeszcze któryś z obrazków pasuje do twojego samopoczucia?**

Nie. Patrzyłam na dziewiątkę bo nie byłam pewna co tam jest, ale ona mnie na pewno nie opisuje.

**Dobrze, mamy już ujęte te etapy, w których czułaś zmianę. W których z nich zaczęła pojawiać się obawa, niepokój o to, co będzie?**

Zapomniałam o ważnym momencie, kiedy obniżono u nas pensje. Zdałam sobie wtedy sprawę, jak bardzo nas wszystkich to dotyka. To jest trochę przerażające; to nie firma nie ma pieniędzy, ale nikt ich nie ma.

**Zaczęłaś sie wtedy stresować?**

Możliwe, ale ja nie jestem tym bardzo zestresowana. Czuję się jak w grze komputerowej. To nie emocja. Wiem, że nikt nie żył w taki sposób w jaki teraz żyjemy. Jest inaczej.

**Opowiedz, jak teraz wygląda twoje życie.**

Przez to, że jestem asystentką i pracuję zdalnie, jest dość ciężko. Non stop jeżdżę do niej. Musiałam zrobić biuro tutaj, czyli przywieźć drukarkę i dokumenty do domu. Wstaję rano o godzinie rozpoczęcia pracy, co jest super, czyli koło 10-10.30. Mogę bardziej planować dzień. Kończy się tym, że dużo później wracam i jestem bardziej zmęczona, ale to już mój wybór. Rzeczy wychodzą nagle - podjechać po podpis, pojechać coś załatwić. Moje życie na co dzień tak wygląda, ale zaczyna się w biurze i kończy się w biurze. Teraz zaczyna się i kończy w domu, a czasami bardzo późno gdzieś na mieście.

**Czy dużo więcej pracujesz?**

Tak, ale myślę, że to kwestia przeprowadzki, a nie epidemii. Ale gdybyśmy pracowali w biurze, byłoby łatwiej dopilnować wszystkich spraw. Przez to, że ludzie nie przychodzą i nikt ich nie pilnuje, wychodzi tak, że muszę pakować rzeczy za innych.

**Jak spędzasz czas po pracy?**

Od siedmiu dni kończę o 20 więc wracam i kładę się spać... trudno mi powiedzieć co bym robiła, pewnie oglądalibyśmy we dwójkę jakiś film i szli spać. Ale ostatnio jestem tak wyczerpana, że po prostu nie mam siły robić nic.

**Ale już się spakowałaś? Firma juz przeniesiona?**

Firma jest już w nowym biurze, tylko ludzie z tamtego biura też nie chcieli się sprawnie pakować i musiała to robić jedna dziewczyna. Jak nasze rzeczy przyjechały, jeszcze nie byli gotowi. Teraz trzeba patrzeć na ludzi od przeprowadzki - żeby wszystko było na właściwym miejscu. Więc jeszcze nie zakończyliśmy przeprowadzki, jeszcze ze dwa tygodnie.

**Co jeszcze się zmieniło w związku z epidemią? Poza przeprowadzką.**

Właśnie wydaje mi się, że gdyby nie przeprowadzka, to bym żyła normalnie. Ale od wczorajszych obostrzeń nie byłam jeszcze na zewnątrz, więc nie wiem, jak to wygląda. Ale pewnie i tak będę robiła zakupy w małym sklepie na dole. Wiem, że mam siedzieć w domu i nie wychodzić na imprezy więc po prostu tego nie robię.

**Przeszkadza ci to?**

Wiadomość tak, ale w praktyce myślę, że nie. W pierwszy weekend miałam straszną ochotę wyjść, nudziło mi się. Ale czy bym to zrobiła gdybym mogła? Raczej nie.

**Myślisz, że to dlatego, że nie mogłaś wyjść?**

To taki zakaz, który po prostu chce się złamać. Jak trzeba siedzieć w domu to będę siedziała, poza pracowymi rzeczami.

**A czy są jakieś pozytywne zmiany?**

Chciałam o tym powiedzieć, że widzę wśród znajomych z pracy, że stoimy dalej od siebie, rzadziej się dotykamy. Po prostu to się dzieje. Jak koleżanka coś ode mnie odbierała i zeszłam do niej na dół, to się później dowiedziałam, że bardzo daleko od siebie stałyśmy, nie tak jak normalnie.

**Jak się z tym czujesz? Z tym, że ludzie zachowują się w ten sposób?**

Szczerze mówiąc to się cieszę. To znaczy, że są świadomi tego, co się dzieje. Mają chęć chronienia się, a zasady zostały wpojone. Ale wolałabym być już bliżej ludzi. Wydaje mi się, że ja też stoję daleko, ale wolałabym stać bliżej. Nie dlatego, że potrzebuję bliskości, tylko nie chciałabym mieć tego poczucia, że coś jest dziwne.

**Czyli to jest kwestia tej dziwności sytuacji, odrealnienia. To miałaś na myśli mówiąc o grze komputerowej?**

Tak, tak, tak.

**Jest coś jeszcze?**

Nie pijemy z jednego kubka, nie wymieniamy sie piciem. To są drobne rzeczy. Mycie rąk sześćset razy w każdym momencie, w sensie dezynfekowanie. Nawet nie przywitałam się z rodzicami kiedy się spotkaliśmy. Wydaje mi się, że nigdy tego nie robiłam, ale dopiero teraz to zauważyłam. Dopiero teraz było dla mnie ważne, że się nie przywitaliśmy.

**Jak możesz podsumować tę sytuację, czym ona jest dla ciebie? Epidemia, stan wyjątkowy, jak byś to nazwała?**

Nie wiem zupełnie, mimo tego, że znam sytuację.

**A co sobie myślisz o tym? Jak się w tym odnajdziemy, co będzie?**

Zastanawiam się. Ciężko mi uwierzyć, że to szybko się skończy. Nie ma pewności. Zastanawiam się, czy damy radę wyjść z tego szybko, czy to kwestia kilku miesięcy. Chiny są wyżej technologicznie niż Polska. To może być bardzo długo, tego się boję. Gospodarka nie będzie funkcjonować tak, jak powinna. Oglądam wiadomości w TVP, głównie dla beki, i tam wszystko jest bardzo łagodnie pokazane. To trochę pokrzepiające. Pozostaje mieć nadzieję i tyle.

**Przygotowaliśmy prostą skalę. Dotyczy ona lęku - od 0 do 100. Gdzie byś siebie umieściła?**

Czterdzieści.

**A co się składa na ten lęk na "40"? Jakie są jego elementy?**

Pustki na ulicach. To nowa sytuacja i coś niepokojącego, że nie ma ludzi. Poza tym krzyczące radiowozy, to też uspokajające, bo kroki zostały podjęte. Natomiast bardziej wzbudza lęk. To jest już naprawdę jak z gry komputerowej. Poza tym to ile osób straciło pracę, to że firmy upadają. Tak, to jest to.

**Boisz sie w tej sytuacji?**

Nie. Nie boję się o siebie ani rodziców, chociaż wiem, że nawet osoby młode już umarły. Jest w porządku. W tej całej sytuacji o chorobę boję się najmniej.

**Zastanawiałam się, na ile w twoim lęku jest obawa przez zachorowaniem. To się nie pojawia w twojej opowieści.**

Nie, absolutnie, zapomniałam w ogóle o tym. Była taka sytuacja, że byłam przed okresem i sprawdzałam gorączkę, bo ludzie mówili, że należy ją sprawdzać. Byłam zestresowana, ale nie tym, że umrę albo coś, ale że będę musiała siedzieć w domu.

**Dobrze, nie boisz się choroby, ale jednak myjesz ręce i przestrzegasz innych zasad.**

Bo wiem, że trzeba. Ale nie jest to kwestia wewnętrzna strachu przed chorobą.

**Ale jak to jest - robisz tak, bo inni tak mówią, czy dlatego, że to coś daje?**

Hmm... wiem, o co chodzi, ale nie przeszło mi przez myśl, że będę chora. Na wszelki wypadek myję te ręce i chodzę w rękawiczkach.

**Ale na wypadek czego to robisz?**

Tak naprawdę nie robię tego dla siebie. Przeczytałam, że młodzi przechodzą to zwykle bezobjawowo. Raczej boję się, że może kogoś nieświadomie zarażę. Nawet nie będę o tym wiedziała, a ktoś przeze mnie umrze. Tego chciałabym się ustrzec.

**Teraz próbuję zrozumieć dlaczego podejmujesz to działanie.**

Mama mi tak mówiła, ludzie mówią, więc czemu miałabym tego nie robić? Noszenie masek i rękawiczek trochę karykaturyzuje tę sytuację. Przez to robi się jeszcze dziwniej.

**Zakładasz maseczkę wychodząc do sklepu?**

Tak, kupiłam, bo po prostu chciałam ją mieć. Chciałam mieć maseczkę i chodzić w maseczce. Tyle.

**A osoby z twojego otoczenia? Np. Twój przyjaciel?**

Pije od kilkunastu dni bez przerwy. On pracuje w Medicoverze, ale na facebooku. Ma dużo pracy i musi ciągle odpowiadać na wiadomości o koronawirusa. To są rzeczy, które musi konsultować i które muszą być zrobione na teraz. Raczej zupełnie nie wychodzi z domu. Nie wiem, czy ktoś u nas był od tego momentu.

**Coś jeszcze zmieniło się w waszym funkcjonowaniu?**

Tak, moim zdaniem żyjemy ze sobą lepiej niż kiedykolwiek. Znamy się już 8 lat, mieszkamy razem od roku. Teraz spędzamy czas cały czas razem, nie wiem czy to kwestia lęku i braku innych możliwości.

**Nie jesteście zmęczeni swoją obecnością?**

Mam takie momenty, ale krótkie. O dziwo jesteśmy ze sobą dużo bliżej i widzę, że inni też tak mają. Moja szefowa i jej była dziewczyna mieszkają razem i też mam wrażenie, że lepiej dogadują się ze sobą niż gdyby nie było tej sytuacji.

**Jak myślisz, skąd to się bierze? Na przykład te twoje koleżanki. Musiały się nie dogadywać wcześniej, skoro się rozstały.**

Przychodzi mi na myśl, że ludzie potrzebują oparcia, bo się boją. Wydaje mi się, że po prostu potrzeba towarzystwa.

**A twoi rodzice, jak się odnaleźli w tej sytuacji?**

Nie wiem... zaskakująco dobrze moim zdaniem. Rozmawiają ze mną więcej. Siedzą w domu i nie narzekają. Nie zauważyłam, żeby się bali lub żeby coś było nie tak.

**Masz wśród znajomych jakąś osobę, która się boi, okazuje to?**

Wydaje mi się, że moja szefowa - prezes. Ona się generalnie nie boi. Kiedy koronawirus był już wszędzie, chciała jeszcze jechać do Dubaju, ale to się rozmyło. Ona była we Włoszech wtedy, kiedy to się rozprzestrzeniło. Ale od kiedy wprowadziła pracę zdalną, już w ogóle nie wychodzi poza dom. Widzę, że boi się, ale może tego, że jej firma upada. Widzi tylko męża, mnie i panią sprzątającą. Gdyby była to tylko obawa o konsekwencje to widywałabym ją w biurze, a tak nie jest. Może bać się zarówno wirusa, jak i konsekwencji tej sytuacji.

**Umiesz odtworzyć, jak Twoje obawy ewoluowały?**

Na początku prawie mnie to nie obchodziło. Uświadomiłam sobie, jak szybko to się przemieszcza. Fajnie, że świat jest tak rozwinięty, że ludzie tak się przemieszczają. Ale z drugiej strony nie jest to komfortowa sytuacja. Uspokajało mnie to, że długo nie było tego w Polsce. Dalej mówię, że nie jestem przerażona. To taki lęk, który mam z tyłu, ale nie towarzyszy mi na co dzień. Z każdym dniem zdaję sobie sprawę, że wpływ epidemii jest coraz większy.

**Kiedy twoja szefowa wprowadziła pracę zdalną, pomyślałaś sobie, że trzeba się przygotować?**

Właśnie nie. Jacyś ludzie robili zapasy, ale czułam to tak jak zakupy przed wyjazdem na wakacje. Jak nie kupię, to nic się nie stanie. Dalej tak żyję, sklepy są otwarte, jest jedzenie w domu. Jedzenie można też zamawiać. Nie martwię się w ogóle zapasami.

**Masz wrażenie, że ten stan epidemii wpływa na twoje zachowania jako konsumenta?**

Obawiam się, że nie.

**Dlaczego "obawiasz się"?**

Może powinno jakoś wpłynąć - żeby kupować mniej lub odpowiedzialnej, żeby nie wyrzucać. Ale tego chyba nie robię. I nie chciałabym robić. Chciałabym żyć cały czas tym samym życiem, kupować głupoty. Chciałabym ogólnie kupować mniej rzeczy zbędnych. Ale to nie dotyczy tej sytuacji, bo to cząstka tego, co miałam codziennie. Taka stałość, nawet jak inni panikują.

**Zrobiłaś jakieś zapasy kosmetyczne lub inne? Takie, że masz więcej, niż zwykle.**

Nie. Tampony, ale to dlatego, że mama mi powiedziała. Moje zakupy nie mają tu nic wspólnego z wirusem. Z Filipem kupiliśmy więcej, ale to dlatego, że spędzamy więcej czasu w domu - na przykład kapsułki do zmywarki.

**Jecie na mieście czy zamawiacie jedzenie?**

Zamawiamy. Na mieście nie i tego brakuje. To fajne, bo można spotkać się z ludźmi.

**Zamawiacie tyle samo jedzenia co dawniej?**

Dzisiaj siódmy raz z rzędu zamawiałam jedzenie, bo pracuję, a Filip chyba je normalnie. Może trochę więcej gotuje. A nie, przepraszam, jemu mama przywiozła jedzenie.

**Jak to wygląda wśród znajomych? Korzystają częściej z cateringu i podobnych usług?**

Wydaje mi się, że ci, którzy mniej zarabiają, więcej gotują. Muszą oszczędzać. Ale gdyby było ich stać to by zamawiali.

**Co jest dla ciebie największym wyzwaniem w sytuacji epidemii?**

Nie widzenie ludzi, nie podawanie ręki... to jest dla mnie problem. Brak naturalnego kontaktu.

**A to, że nie możesz pójść do knajpy lub kina?**

Do kin nie chodziłam, to nie problem. Z knajpami się pogodziłam. Chodzi o bycie z kimś w jakimś miejscu, a tego nie mogę. Na początku jeszcze można było przez przyzwolenie "w głowie". Teraz juz tego nie ma, po prostu nie.

**To zakaz?**

To jest u ludzi w głowie. Wiem, że policja nie przyjdzie jak zaproszę koleżanki. To jest w głowie.

**Co spowodowało, ze jeszcze tydzień temu nie mieliśmy tego w głowie, a teraz już mamy?**

Liczba zachorowań, obostrzenia. Nie chodzi nawet o zakaz, ale to, że władze podejmują już jakieś środki. To, że nie można wejść do Żabki w kilka osób. Nawet jeśli nikt nie złapie za rękę, to teraz warto przemyśleć, czy na pewno chce się to robić.

**Czy coś jeszcze jest wyzwaniem poza trudnością ze spotykaniem?**

Nie, myślę, że dobrze się przystosowałam. Nie boję się specjalnie. Zakupy robię w sklepie na dole, to Carrefour. Teraz nie kupuję codziennie, ale nie wiem, czy to nie kwestia pracy i powolnej obsługi.

**Porozmawiajmy o kolejkach. Jak je odbierasz?**

Odrzuca mnie widok długich kolejek przed sklepem. Nawet, jeśli wiem, że nie stoi w nich więcej osób niż normalnie.

**Czyli mamy następujące elementy "gry komputerowej": pusty Nowy Świat, kolejki przez sklepami, stanie daleko od siebie... co jeszcze?**

Radiowozy, które krzyczą, że nie wolno wychodzić z domu. Wiadomości na TVP, które wyglądają jak z Korei Północnej. Cisza jest jeszcze elementem tej gry. Wiele razy mieliśmy tak z Filipem. Wychodziliśmy, a było jakbyśmy wracali z imprezy nad ranem.

**Czy są jeszcze jakieś elementy, które budują ten nastrój gry?**

Teraz więcej nie przychodzi mi do głowy. Nie jestem często poza domem. Jeżdżę do pracy, na poczcie byłam raz czy dwa...

**Pogadajmy o tym, skąd się ten koronawirus wziął. Słyszałaś, czytałaś coś?**

Tak, od zwierząt na targu w Chinach. Słyszałam, że od nietoperza, ale wydaje mi się, że to jest żart. To nie jest potrzebna mi wiedza więc ją wyrzucam. Zainteresowałam się, ale żeby powtórzyć...

**Ale nie było to coś, co ci zapadło w pamięć?**

Wiem o tym targu w Chinach i zwierzętach, ale co do jakiejś genetyki tego wirusa to nie mam pojęcia.

**A jak myślisz, co sprawiło, że wirus zaczął się tak rozprzestrzeniać?**

Technologia, to jak jesteśmy mobilni, to jak łatwo można kombinować. Wówczas nie myślałam jeszcze, że koronawirus będzie w Polsce. Wtedy jakaś pani, która była chora, wzięła tabletki przeciwgorączkowe, oszukała kontrolę na lotnisku i napisała o tym na facebooku. Ludzie są sprytni, łatwo jest oszukać i można się wszędzie szybko przedostać.

**I co pomyślałaś o tej pani?**

Ciężko było mi w to uwierzyć, jak można być tak nieodpowiedzialnym. Później nie mogłam już znaleźć tej informacji. Niesamowita historia, pewnie cały samolot był zarażony. Wydaje mi się, że nie zdawała sobie sprawy, do czego się przyczynia.

**Wydaje ci się, że można było zapobiec rozprzestrzenianiu wirusa?**

Wydaje mi się, że nie. Późno się wszyscy zorientowali, na jaką to było skalę. Gdybyśmy wcześniej zamknęli granice to może... ale wydaje mi się, że nie.

**Myślisz, że jako kraj jesteśmy przygotowani?**

To jest bardzo trudne pytanie, o tym dużo myślę. Wydaje się, że jesteśmy przygotowani, że wszystko idzie ładnie. Kwarantanna, akcja zostań w domu... ale nie jestem w stanie przewidzieć, czy to naprawdę działa, czy też mamy mało testów i nie wiemy, czy ludzie są chorzy. Nie wiem, co rząd mógłby innego zaproponować. Trzeba było po prostu zrobić to wcześniej, ale kiedy?

**Rozumiem, że zgadzasz się z wprowadzanymi zmianami, ale co twoim zdaniem można było zrobić wcześniej aby poprawić sytuację w jakiej jesteśmy? Jak to widzisz?**

Można było wcześniej zamknąć granicę. Wydaje mi się, że wirus bardzo się rozpowszechnia na lotniskach, w samolotach... ale i tak można wrócić do domu, do Polski. Ale nie wiem. Dlatego nie pracuję w rządzie, bo nie wiem. Nie wychodzenie z domu to też dobry pomysł, ale ludzie i tak wychodzą na zakupy i wtedy można się zarazić. Trudno mi odpowiedzieć, nie zajmuję się tym.

**A poszczególne działania rządu? Na przykład zamknięcie szkół.**

Mam brata, który chodzi do szkoły. Ludzie, którzy pracują zdalnie, też mają dzieci. Wiem o zamknięciu szkół. Uważam, że nie jesteśmy jako Polska przygotowani na nauczanie zdalne. Nie uczy się tak, jak powinno. Może coś się zmieniło od kiedy skończyłam liceum, ale jak wiele? W innych krajach może to być bardziej rozwinięte. Poza tym, co jest niepokojące, w niektórych rodzinach jest jeden komputer na dwójkę dzieci. To dobre rozwiązanie jeśli chodzi o zdrowie, ale nie o edukację. Zdrowie jest jednak ważniejsze.

**Później wprowadzano inne obostrzenia. Czy któreś z nich zapadły ci w pamięć? Jak je oceniasz?**

Zakaz zgromadzeń, to że nie można było pić nad Wisłą - to bardzo dobra decyzja prezydenta Warszawy. Ograniczenia w sklepach to też dobra decyzja. Widzę, że faktycznie jest to pilnowane. Zgadzam się z każdym obostrzeniem, mimo że wprowadzają trochę strachu - ale to też dobrze. Dla mnie może to być zaostrzone jeszcze bardziej, dlaczego nie skoro to powstrzyma wirusa? Tak samo z zamykaniem placów zabaw, parków. Jak siedzieć w domu to siedzieć w domu.

**Jak myślisz, ile ludzie wytrzymają w domu? Ile ty wytrzymasz?**

Ja wytrzymam tyle ile będzie trzeba. Ja jestem naprawdę dobra w czelendżach. Ale wydaje mi się, że ludzie pozwalają sobie na coraz więcej. Ludzie trochę odpuszczają, ale może znowu panuje lęk po wprowadzeniu nowych obostrzeń. Może dobrze, żeby rząd wprowadzał stopniowo coraz więcej lęku u społeczeństwa.

**Więc myślisz, ze trzeba ludzi trochę postraszyć?**

Tak, w takiej sytuacji tak. Jeśli chodzi o wychowanie dzieci to absolutnie nie, ale narodu tak. To kwestia całego narodu. To nie jest głupie strasznie, naprawdę wszyscy mogą być chorzy, choć mam wrażenie, że mnie to nie dotknie.

**Powiedziałaś o sobie. Co myślisz o tym, jak twoi rodzice albo Filip odnajdą się w sytuacji epidemii?**

Nie wiem, ale trzeba to zrobić. Oni zdają sobie sprawę. Gorzej, gdy ktoś nie zdaje sobie sprawy, wtedy może nie wytrzymać. Po to jest właśnie polityka strachu.

**Skąd czerpiesz informacje o tym, co się teraz dzieje?**

Z instagramu chłopaka, który nazywa się Łukasz - "konflikty i katastrofy światowe". To jest chłopak, który założył fanpage na facebooku. Zrobił to dawno, kiedy były jakieś może zamachy... on już długo to prowadzi i ma strasznie dużo followersów. On to robi też na instagramie, czyli łatwo i przyjemnie można sobie to zobaczyć. Zawsze jak widzę jego update'y to oglądam w pierwszej kolejności.

**Co daje ci wiedza o epidemii w liczbach? Czy brakowało by ci czegoś, gdybyś nie sprawdziła jego wiadomości?**

Świadomość, że coś wiem. Ale niczego by mi nie brakowało, gdybym nie kliknęła. Jak widzę, że jest, to po prostu chętnie obejrzę. To w jakiś sposób ważne. Tak jak story jakiejś bliskiej koleżanki na instagramie. Poza tym oglądam TVP i TVN. Czasami też coś usłyszę w radiu w taksówce, jeżdżę tylko taksówkami. Ale u tego chłopaka Łukasza wszystko jest, nie trzeba szukać.

**Jakie informacje zapamiętałaś spośród tego, co widzisz np. na Facebooku?**

Ja już wszystko wiedziałam od tego Łukasza. A nie, jednego nie wiedziałam i to przeczytałam na Facebooku. Jakieś dziecko wypadło z okna i ono miało koronawirusa. Rodzice byli pijani i oni też mieli koronawirusa. Myślę, że to prawda.

**Czy teraz więcej korzystasz z mediów, więcej czytasz?**

Nie, nawet mniej. Nie mam na to czasu.

**Śledzisz memy, social media związane z koronawirusem? Co o nich myślisz?**

Pojawiają mi się, ale nie śledzę. Dobrze jest żartować bo nic innego nie można robić. Jeden się modli, drugi robi memy. Treści z koronawirusem traktuję jak wszystkie inne. Jak widzę, to się skupiam, ale nie szukam tego.

**U mnie koronawirus zdominował feed. Jak to wygląda u ciebie? Przeszkadza ci to?**

U mnie też. Nie przeszkadza mi to, to jest ważne, to jest numer jeden. To dobrze. To znaczy taka jest sytuacja. Po prostu mi to nie przeszkadza.

**Wierzysz w statystyki, które są pokazywane?**

Ja niestety wierzę we wszystko w internecie, nie dopuszczam, żeby to było przekłamane. Filip mówi, że to reżyserowane, ale ja nie chce myśleć, że jest inaczej.

**Wiesz ilu ludzi jest chorych w Polsce albo na przykład Hiszpanii? Myślisz, że to wiarygodne dane?**

W Polsce 2500, w Hiszpanii jakoś 100 000 lub 90 000. Myślę, że to wiarygodne dane. Ale w Polsce mogą po prostu nie być sprawdzone, bo nie ma testów. Nie byłam nigdy w takim szpitalu więc nie wiem. Nie wiem czy to jak testy ciążowe, które można kupić i sobie w każdym momencie sprawdzić. Ale wiem, że kiedyś czekałam w kolejce w NFZ, że leżałam na korytarzu. Wydaje mi się, że jest więcej, bo nikt tego nie sprawdził. To jest bardzo złe.

**Czy to by coś zmieniło, gdybyś dowiedziała się teraz, że np. nie 2500 ale 25000 osób choruje w Polsce?**

Tak, to już duża liczba. Pomyślałabym "O KURCZĘ", "KURDE" i nic więcej. Gdyby to była nagła zmiana to bym się wystraszyła, ale jeśli stopniowa, to nie. Taka jest kolej rzeczy.

**Wyobraź sobie, że dostajemy duży pakiet testów i liczba zdiagnozowanych rośnie do 50 000.**

Wtedy bym się przestraszyła. Może przez pół godziny chodziłabym i wiedziała, że coś mnie męczy z tyłu głowy. Ale jeśli bym wiedziała, że ktoś te testy zrobił, a nie zachorował nagle, to nie zmieniłoby to chyba zbyt wiele.

**Czy natrafiłaś ostatnio na jakąś informację na temat koronawirusa, o której pomyślałaś, że na pewno nie jest prawdą?**

Właśnie nie. Słyszałam o tych fake newsach, że są, ale nie dotarły do mnie. Ubolewam nad tym, że żadne fake newsy do mnie nie dotarły. Słyszałam, że zamkną Warszawę, ale od razu wiedziałam, że to jakaś bzdura. Ale później ludzie zaczęli mówić, że mają znajomych w rządzie i że to stąd. Niestety nie docierają do mnie fake newsy, też dlatego, że nie rozmawiam z ludźmi o koronawirusie.

**A o czym rozmawiacie?**

Z rodzicami o tym jak sie czuję. Poza tym o tym co robiliśmy, co w pracy. Trzeba się skupiać bardziej na tym, co jest w pracy. Dział prawny długo pracuje. Dużo tych rzeczy muszę robić ja. Z Filipem może czasem rozmawiamy o koronawirusie: "wiesz ile osób zachorowało" albo "wiesz, nie można wychodzić" - "spoko".

**Znasz jakieś osoby, które buntują się przeciw środkom zaradczym wobec epidemii?**

Nie. Kurcze, nie rozmawiam. Nie wiem, skąd oni mogą mieć takie informacje. Nie rozmawiam z ludźmi poza tymi, z którymi rozmawiam na co dzień. Zresztą dlaczego miało by mnie obchodzić ich zdanie tak naprawdę.

**Jak rozumiem, w waszych rozmowach pojawiają się różne tematy, jak na co dzień?**

Jestem pewna, że w każdej rozmowie padło coś na temat koronawirusa, na przykład "nie podawajmy sobie ręki". Ale nie są to przemyślenia, dzielenie się swoimi uczuciami na ten temat. To raczej jak "o, pada śnieg w kwietniu" kiedy pada śnieg w kwietniu. Dziwnie, co?

**Zdarzyło ci się czytać coś bardziej naukowego? Od Sanepidu albo WHO?**

Nie, u tego Łukasza właśnie jest wszystko podsumowane. Nie potrzebuję sprawdzonych źródeł bo ufam, że on ma sprawdzone źródła. To taka dawka informacji jakiej potrzebuję, żeby wiedzieć co się dzieje na świecie.

**Na koniec zapytam, jakie są twoje przemyślenia o przyszłości, o tym co będzie w ciągu najbliższych dni. Masz wrażenie, że coś się zmieni?**

Przez to, że siedzę u tego Łukasza na tym instagramie, widzę mniej więcej z jakim tempem to się rozpowszechnia. Wstaję i zawsze u niego już jest relacja. Na początku nikt nie był chory, później dziesięć kolejnych osób, teraz nowe zachorowania pojawiają się w setkach. Myślę, że tego będzie jeszcze więcej. 2500 chorych to nie jest ogromna liczba w skali kraju. Nie wiem, co mogło by być w ogóle inaczej. To jest to uczucie gry komputerowej, że nie wiem, co będzie dalej. Zupełnie nie wiem. Pytałaś o te etapy, to są właśnie te etapy przejścia gry. Checkpointy. Pierwsze zachorowania, check, nie można wychodzić z domu. I tak dalej. Teraz jest troszeczkę inaczej, bo ludziom kończy się kwarantanna, więc będzie mniej policji. Ci ludzie nie mogli wychodzić, ale tak bardzo nie mogli wychodzić, może teraz zaczną.

**Jak sądzisz, ile osób może być na kwarantannie?**

Nie wiem, ale od znajomych wiem o 12-15 osobach. Więc może... 100 000 osób jest na kwarantannie? Wydaje mi się, że wszyscy ludzie, którzy pracowali za granicą, teraz wrócili.

**Jak sądzisz, dlaczego oni wrócili?**

Jeżeli studiują to mogła być kwestia rodziców. Ci ludzie, których ja znam ze studiów za granicą, są utrzymywani przez rodziców. Czemu oni podjęli taką decyzję, nie wiem. Może taki okres lepiej przeżyć z rodziną. Na pewno nie jest to kwestia zaufania do władzy. Widziałam zdjęcia z akcji "lot do domu", przedstawiały setki ludzi na lotnisku. To świetna okazja do rozpowszechniania wirusa. Ten wirus to nie jest coś, co można było zatrzymać. Może dobrze, że wrócili, przynajmniej spędzą czas z rodziną, a nie sami.

**Czy masz jeszcze jakieś przemyślenia, o których nie powiedziałaś?**

Wydaje mi się, że nie. Mam takie przemyślenie, że ludzie wariują pod tym względem, że muszą robić rzeczy, których nigdy nie robili i jeszcze muszą się tym chwalić. Na przykład oglądać spektakle teatralne. Widzę na instagramie, że ludzie, którzy wolą iść na wódkę, wstawiają informacje, że oglądają spektakl. To mnie doprowadza do szału, bo nie zaczynają się tym interesować naprawdę. Albo ćwiczenia przez internet. Nic do tego nie mam, spoko inicjatywa, ale dziwna sytuacja. Ludzie koniecznie muszą się spotkać wieczorem w internecie, żeby się napić. Nie wiem po co. Może to chęć uświadamiania, ale coś takiego też jest.

**A robisz tak?**

Nie, z ludźmi z pracy rozmawiałam dwa razy, ale zawsze ktoś nie mógł. To się już uspokoiło. Poza tym tylko jeden znajomy mieszka sam, pozostali spędzają czas z kimś. Miałam się umówić, ale nie wyszło. W sumie nie.

**Czy zauważyłaś jeszcze jakieś nowe rodzaje aktywności?**

TikTok. Nie wiem, czy wiesz, co to jest.

**Wiem, że nawet Duda zrobił.**

Ja wiedziałam od dość dawna, bo moje siostry są fankami. Jeździłam z nimi na zlot fanów TikToka. Oglądałam to żeby się z nich śmiać, bo to debilne, co te dzieci robią w internecie. Teraz wszyscy oglądają i nagrywają TikToki. TikTok na tym bardzo dużo zarobi. No i super, że ktoś miał taki pomysł.

**Coś jeszcze?**

Fajnie, że się pojawiają takie obywatelskie pomoce starszym albo przedsiębiorcom, kupowanie bonów na kawę w przyszłości czy cośtam. Fajnie, że ktoś wspiera, ja osobiście nie.
