# Supplementary material for: The challenges arising from the COVID-19 pandemic and the way people deal with them. A qualitative longitudinal study
Source: PLoS One. 2021 Oct 11;16(10):e0258133. doi: 10.1371/journal.pone.0258133 (PMC8504766; doi:10.1371/journal.pone.0258133)
Supplement: S1 Dataset — (ZIP) [file pone.0258133.s003.zip › Transcriptions/stage 1/18.1_F_48_couple, with children.docx]

**18.1_F_48_couple with children**

**Powiedz coś o sobie?**

Mąż, 2 dorosłych dzieci (20 i 26). Pracuję - prowadzę swoją działalność gospodarczą, zajmuję się domem, staram się trochę aktywności fizycznej. Prowadzę z mężem sklep i również zajmuję się podologią i manicurem w salonie kosmetycznym. Syn mieszka z nami, córka oddzielnie.

**Kiedy to wszystko się dla ciebie zaczęło?**

Te pierwsze sygnały, takie delikatne, to zaczęły się z takiej przyjemnej strony, bo szukaliśmy urlopu na wakacje. Od kilkunastu lat mamy planowane wakacje listopad/ grudzień, a w ubiegłym roku zupełnie nam to nie szło i się nie udało. Pod koniec stycznia zaczęliśmy intensywniej się tym zajmować, śledzić sytuację na świecie i patrząc realnie, że jednak coś się dzieje, że to się rozprzestrzenia. Patrzyłam na Chiny, te Włochy się już pojawiły, ta Azja, że jednak coś się dzieje. I stwierdziliśmy, że jednak jakiś wirus, coś, że może odpuścimy i zobaczymy jak to się rozwija, bo jednak jest to jakiś problem. To był ten pierwszy raz przy zupełnie przyjemnych tematach, ale jednak zastopował nas w działaniach.

Potem jakieś pierwsze zakażenia, jakieś relacje telewizyjne i tak powoli docierało tak bardziej realnie.

**Były jakieś ważne, kluczowe momenty rozwoju tej sytuacji?**

Tak bardziej mnie postawiło, że to się dzieje, to wiążę to z moją pracą, jako kosmetyczka, gdzie używam maseczek jednorazowych. To jest taka rzecz, którą mam zawsze i pani w hurtowni mi powiedziała, że może powinnam jeszcze kupić maseczki. Powiedziałam, że jeszcze mam i nie potrzebuję większego zapasu. Ona na to, że teraz ten wirus i oni teraz więcej sprzedają tego. Powiedziałam, że ok., że wobec tego poproszę. I tutaj tak już...Chociaż to jeszcze nie było takie, że to się dzieje, tylko, że zaczęło czegoś brakować i że bardziej się zaczęły sprzedawać tego typu rzeczy do dezynfekcji, te maseczki. Powiedziałam sobie wtedy, że jednak coś jest na rzeczy.

**Jakie emocje to w tobie wzbudzało?**

Pierwsze było takie zaskoczenie i że to trochę może przesada, ale potem, kolejnym razem jak już rzeczywiście stwierdziłam, że dokupię te maseczki, bo gdzieś z tyłu głowy...Tych informacji było już więcej. Wtedy z kolei taka bardziej złość mnie ogarnęła, bo okazało się, że maseczki zdrożały, że pani mi mówi, że ktoś im wykupił i sprzedaje 2 x drożej na allegro. Wtedy się zezłościłam, że coś się dzieje, a już ktoś na tym zarabia. Wtedy to już przeszło do złości.

**Jakiś kolejny moment?**

Takich rzeczy, co bym bardziej zapamiętała...No, ale to już chyba po 4 marca to już bardziej było intensywne i już to się bardziej nakręcało. A potem to było chyba ze 2 tygodnie temu, jak już ogłoszono...Pamiętam, że to była środa i że będą szkoły zamknięte. I wtedy rzeczywiście to było takie...Ja wtedy do końca nie miałam kontaktu, bo byliśmy wtedy na pogrzebie, ale już mówiono o tych zasadach bezpieczeństwa, więc rzeczywiście na tym pogrzebie o całowaniu z rodzina na przywitanie nie było mowy. Jakoś wszyscy do tego podeszli poważnie - jakiś uścisk dłoni jedynie. Wieczorem jeszcze pojechałam do sklepu i zobaczyłam, że pod CH jest więcej samochodów, ale mnie to nie zdziwiło, bo była 17.30, ludzie wracają z pracy...I ja jeszcze cała taka w nieświadomości weszłam, żeby kupić sobie mleko migdałowe...Weszłam, spojrzałam - nie ma tego mleka, ale nie ma też żadnego innego nie ma. Byłam zupełnie jakby odizolowana od tego i w całej swojej naiwności stwierdziłam, że ok., że po prostu wykupili ludzie. Poszłam dalej, kupiłam bułki, a jak doszłam, żeby kupić mięso, to zobaczyłam, że jest pusta lada, jakieś pojedyncze rzeczy. Zdziwiłam się, że tak mięso wykupują, a pani za ladą mówi, że tak, że w magazynach zaczyna brakować. Brakuje to brakuje - ja dalej tak naiwnie i wzięłam jakieś 4 udka...Ja się śmieję jak o tym opowiadam, bo ja musiałam strasznie komicznie w tym tłumie wyglądać, bo jeszcze sobie kupiłam żonkile i patrzę, a znajomy, którego zobaczyłam ma 4 opakowania papieru toaletowego. Te takie wielkie. Mówię: "To pan tyle papieru kupuje?". A on do mnie takim poważnym tonem: "To pani nie ma???" Ja dopiero wtedy się rozejrzałam, że ludzie stoją w kolejkach, że są załadowane wózki - jakby świątecznie, nawet plus i że każdy ma ten papier toaletowy sławny. A ja z tymi żonkilami, z bułeczkami i w ogóle nie pasuję do tego otoczenia. Pomyślałam sobie, że ludzie tak wykupują, ale przecież ja nic nie potrzebuję, żeby sobie kupić więcej. Wyszłam ze sklepu i zadzwonił mąż, że z naszego sklepu jest wszystko wykupione z takich podstawowych rzeczy. I wtedy właśnie pomyślałam sobie, że coś się dzieje jednak.

**Co to było za uczucie?**

Takiego zaskoczenia, że coś się dzieje, ale...Ja widziałam wśród tych ludzi panikę, natomiast na pewno mnie to nie dotknęło. Ja miałam odczucie, że przesadzają, że właściwie po co gromadzić takie rzeczy jak mamy wszystko wokół siebie. raczej ze spokojem do tego podeszłam.

**A potem?**

Potem było tak, że u mnie w pracy klientki zaczęły odwoływać wizyty, ale to też był taki niewielki procent. Stopniowo to zaczęło narastać, ale w sklepie i męża zaczęło być więcej pracy i tutaj już się zrobił niepokój, bo ja zostaję bez tej swojej kosmetyki...No ok., to jeszcze nie ma tragedii, ale tutaj jakby trzeba pomóc, bo teściowa ma 70 lat i niekoniecznie, żeby w tym sklepie była - sklep mąż przejął po rodzicach. Jedna z pracownic jest po operacji, więc nie pracuje, zwiększa się ruch, zwiększa się tempo pracy. I tu zaczął się taki niepokój, jak to wszystko zorganizować, jak ogarnąć, co zrobić.

**Skala lęku.**

Obecnie zatrzymałabym się tu (63). W tym sklepie, w środę zatrzymałabym się na 20.

**Jak teraz wygląda twoje życie w obecnej sytuacji?**

Ja bym to nazwała takim wyzwaniem. tak naprawdę wszystko się zmieniło. Nie pracuję na co dzień tam, gdzie pracowałam. Teraz doszły mnie informacje, że wszystkie salony kosmetyczne zostaną zamknięte, ale ja już tę decyzję podjęłam 2 tygodnie temu. Zmieniły się moje godziny pracy, ponieważ zastępuję teściową i pomagam mężowi, więc wstaję dużo wcześniej. O 5 jestem już na nogach. Tu jestem bardziej aktywna i spędzam ten czas w sklepie pilnując i pomagając. Jestem w takim centrum tego wszystkiego. Też, ponieważ rodzice są starsi i schorowani, zresztą oboje po nowotworze, więc zamknęłam ich w domu. Sprawunki, lekarstwa - tym się musiałam zająć i tego się nauczyć. Może tak do końca nie narzekam teraz. Narzekałam, bo wystałam się w kolejkach, nie wiedziałam, który lek mam kupić, dlaczego taki mam zamiennik i czy to jest dobrze, i musiałam stać 2 raz, bo się kontaktowałam z lekarzem. Ale plusy w tym też widzę. Rodzice są bardzo samodzielni i nigdy nie potrzebowali tyle mojej pomocy, ale teraz przejęłam te wszystkie ich zakupy, apteki, lekarzy.

**To jest plusem?**

W pewnym sensie tak, bo okazuje się, że kiedy miałam więcej czasu...Może czasu więcej nie, ale było to spokojniejsze życie, ustabilizowane, przewidywalne, to ja nie byłam 2 x dziennie u rodziców. A teraz okazuje się, że w tym nadmiarze pracy, w takim zmęczeniu, bo jednak jestem zmęczona i niewyspana. Okazuje się, że jednak znajduję czas i mogę. To jest plus, ale też trochę dostałam takiego pstryczka w nos, że może wcześniej mogłam więcej i częściej. Sprawia mi też przyjemność, że mogę tam być choć przez chwilę, bo przecież nie przesiaduję u nich. To są takie przyjemne momenciki w ciągu dnia. Wchodzę do nich do przedpokoju, podaję rzeczy, jakieś odbieram, ale staram się jak najmniej tam przebywać i jak najdalej. Mamie musiałam też wytłumaczyć, że przychodnia jest zamknięta i że ja to wszystko teraz za nią załatwię. To się zmieniło zdecydowanie, bo mieszkamy 3 km od siebie a potrafiłam tydzień, dwa nie wstąpić. Teraz jestem codziennie i w całym tym zamieszaniu, chaosie jestem w stanie do tego się zmobilizować.

**Co jeszcze zmieniło się teraz w twoim codziennym życiu?**

Weekendy zdecydowanie się zmieniły. Ja jestem osobą, która musi ciągle coś robić, gdzieś muszę coś nowego zaczynać, działać, więc te weekendy też były takie, że a to gdzieś wychodziliśmy, to jakieś plany, to znajomi i to się odcięło. Siedzimy w domu. Po prostu sobota, niedziela jesteśmy w domu. To też w sumie odbieram jako plus. Może gdybym siedziała cały tydzień w domu i weekend również, ale ja z kolei doceniam ten czas, że właśnie nie muszę się na jakaś imprezę szykować, nie tego, że szybko, bo do kina, bo tu, bo bilety, bo czas, bo...Ja po prostu siedzę w domu. Ja chyba tak ogólnie zawsze wśród tych złych rzeczy staram się znaleźć te plusy. Nie celebrować tych minusów. Staram się. Ok., jest źle, przepadło mi jakieś wesele, jakieś urodziny, coś. Dobra, uwielbiam to, ale nie ma, to jest coś innego, co jest też fajne.
**Gdzie jeszcze znalazłaś te plusy?**

Takie zatrzymanie, że mogę się zatrzymać i tak na spokojnie. Nie pędzić. Większość wpadła w takie coś, że pędzi, że ta gonitwa, bo praca, bo więcej, bo szybciej. Ja też w to weszłam, dostosowałam się, ale nie jest to takie do końca moje, więc takie wyhamowanie i możliwość takiego zatrzymania się tu i teraz jest dla mnie atutem.

**Masz poczucie, że ta refleksja wpłynie na twoje życie później?**

Myślę, że tak, tzn. chciałabym. Chciałabym, żeby móc to docenić, ale nie mam pewności, że nie wrócę do poprzedniego trybu, jak to wszystko już przejdzie. Ale są jeszcze wspomnienia, do których można wracać, odnieść się i sobie hamować. Może nie tak intensywnie, ale jednak wyhamować.

**Co przeszkadza ci teraz najbardziej?**

Chyba ta intensywność pracy, ten natłok pracy i klientów w sklepie. Jest jednak tata troska też o pracowników, o taki byt, o to, co będzie. Taka niepewność w sensie, że np. robimy zaopatrzenie i czegoś brakuje. Trzeba się do tego dostosować. Ja jakby nie mam z tym problemu, bo czy ja zjem mąkę taką czy taką, czy zjem ryż, czy makaron...Nie ma dla mnie to znaczenia, natomiast widzę, że ludzie się robią nerwowi. I to, co mnie złości, to że " Jak to? Czegoś nie ma?" albo, że jest nie takie. Ludzie dalej żyją w takim komforcie, że musi być. Ale to zawsze mi chyba przeszkadzało.

**Wskazałaś powyżej 60 na skali lęku. Czego dotyczą twoje obawy?**

Najbardziej zdrowia - mojego i moich bliskich, a potem to się rozszerza na dalsze otoczenie. A jeszcze nie powiedziałam, że miałam takie 2 kryzysy. I to były takie kryzysy, że nawet zmierzyłam sobie temperaturę. [śmieje się] To był taki i lęk, i panika, i strach. Było wszystko. Wtedy skoczyłoby na tej skali na jakieś 80.

**Co to były za sytuacje?**

Zaczęłam słuchać tych wszystkich informacji, przekazów w tv, oglądać te relacje z Włoch, te trumny, że to się dzieje. Zaczęłam słuchać w tv, jakie są objawy i dostałam niemalże kaszlu. Stwierdziłam, że się trzęsę, że chyba mi coś jest, więc zmierzę sobie temperaturę. I w ubiegłą sobotę też tak miałam, ale to też było po całym takim tygodniu, gdzie normalnie przeczytam i wysłucham tylko jakichś informacji, a tu usiadłam i zaczęłam słuchać, i słuchać. Stwierdziłam, że jest mi zimno i w ogóle, zmierzyłam temperaturę, ale potem stwierdziłam, że nie, że ja jestem po prostu zmęczona i słucham tego, i że to w ogóle nie jest to. [śmiech]. To były takie momenty przez oglądanie tv.

**Starasz się unikać oglądania?**

Tak. Włączam na chwilę, żeby wiedzieć. Czytam to, co jest na pasku i koniec. Wolę zdecydowanie przeczytać sobie takie informacje niż obejrzeć na ekranie.

**Zmieniłaś jakieś swoje codzienne przyzwyczajenia, nawyki?**

W rękawiczkach w swojej pracy kosmetycznej pracowałam zawsze, położyłam te rękawiczki w sklepie i dziewczyny mają. Dla mnie używanie rękawiczek w pracy to norma, ale przedtem jak pomagałam w sklepie, to ich nie używałam, teraz używam. Maseczek nie noszę. Mycie rąk, o którym wszyscy krzyczą, to ja już mam taki nawyk, który wynika z pracy i od dziecka byłam tego uczona, że w domu też trzeba myć te ręce i pilnować tego. Moja mama zawsze dbała o te rzeczy. Zmieniło się to, że wracając do domu zdejmuję całe ubranie i wrzucam do pralki. Też znalazłam plus tego, bo nie jest to fajne, jak się 3 x dziennie przebieram, bo mam pranie, które muszę zrobić i wysuszyć. Znalazłam tu plus, bo stwierdziłam, że ja w końcu wiem, po co mam tyle szmat w szafie i mogę sobie pozwolić na to, że zmieniam 2 x dziennie spodnie i wrzucam do pralki.

**Ta twoja obawa o zdrowie? Opowiedz o tym.**

Ja nie boję się najbardziej choroby, jako choroby, tylko bardziej boję się tego, że nie będzie pomocy, że nie dostanę się do lekarza, że nie będzie tego testu. To są moje obawy, bo choroba, jeśli nas spotyka, jaka to by nie była choroba, to jest i leczymy ją. Bardziej boję się, że nie będzie tej możliwości leczenia. Ten lęk jest bardziej na tym u mnie skupiony niż na samej chorobie.

**Pokażę ci teraz kilka zdjęć. Które z nich oddają twoje emocje w tej chwili?**

Na pewno 6, bo najbardziej przyciąga moją uwagę i 1.

**6**

To jest las, taki trochę ponury, bo ten boczny cień padający, ale to słońce przebijające się przez te drzewa to jest taki promyk, że to minie. jestem w tym lesie - taka przytłoczona w tej pustce i to mija, bo gdzieś tam jest to słońce. taka nadzieja, która się w tej pustce pojawia. Ciężko jest, mroczno, ale słońce gdzieś pomiędzy tymi drzewami się przejawia. Te moje emocje tutaj są takie zbilansowane, dlatego, że taka jestem osadzona w tym a to słoneczko jest takie...Takie to jest pół na pół - i pozytywne, i negatywne.

**1**

To jest ten pęd i tu po prostu widzę mój dzień. Dużo się dzieje, szybko z każdej strony, mnóstwo rzeczy, mnóstwo jakichś spraw. Takie tu i teraz. To jest ani miłe, ani niemiłe. Ja lubię, jak się dużo dzieje. Może nie takie rzeczy, ale ja jestem zadaniowcem, więc w takim pędzie ja się też odnajduję. to jest pęd, ale taki do ogarnięcia. Chyba bardziej pozytywnie, bo ten pęd bardziej mnie napędza niż hamuje.

**Gdybyś miała opisać zmiany w emocjach, które zachodziły w ostatnim czasie?**

Zanim nastała ta sytuacja, to ten bieg i chaos nieraz mnie złościł, nieraz mnie denerwował, a teraz jakby z większą pokorą to przyjmuję. Jakichś wielkich zmian w moich emocjach w ciągu tych 3 tygodni nie ma. Jest to jakby na jednym poziomie. Weszłam w daną sytuację i jestem tu i teraz. Oprócz tych dwóch chwilowych skoków paniki, to myślę, że nie. Bardziej chyba zmęczenie mi dokucza.

**Czy to, że jesteś teraz tak zajęta przeszkadza ci/ pomaga?**

Bardzo pomaga. [śmiech] Zdecydowanie. Teoretycznie jestem w stanie sobie wyobrazić, że jestem zamknięta w domu i nie mogę wychodzić, ale to co sobie wyobrażam a rzeczywistość...To mogłoby być całkiem inaczej. Myślę, że przez jakiś czas bym wytrzymała, tylko jaki to byłby czas? Na pewno ja bym sobie wynajdywała zajęcia, bo nie wyobrażam sobie, że siedziałabym i nic nie robiła. Pewnie nadrobiłabym czytanie książek, o którym marzę, a zupełnie teraz nie mam na to czasu. Myślę, że ciężko by mi było. Na pewno ciężko, bo ja jednak jestem takim typem aktywnym.

**Czy obecna sytuacja ma wpływ na twoje podejście do zakupów?**

Raczej nie. Zdecydowanie zaczęłam gromadzić rękawiczki, ale to dlatego, że muszę je mieć dla pracowników. Tu zdecydowanie zaczęłam robić zapasy. Poza tym chyba nic się nie zmieniło, ale to może dlatego - i nawet ludzie się ze mnie śmieją, że ja zawsze mam zapasy. Nie myślałam, żeby zacząć kupować coś innego. Staram się o tym myśleć tak, żeby tu też nie przesadzać. Czy ja zjem ryż, czy makaron, to nie ma dla mnie znaczenia. Ja strasznie nie lubię chodzić na zakupy, wokół siebie też nie mamy żadnego sklepu, żeby można było wyjść w każdej chwili, więc ja lubię mieć wszystko pod ręką w domu. Przysłowiowy papier toaletowy czy mleko. Takie rzeczy ja mam zawsze w takich ilościach, że je po prostu mam. Dużo gotuję i wymyślam sobie te rzeczy, więc ja muszę mieć wszystko tu i teraz, żebym mogła sobie poszaleć w kuchni. Nie kupiłam nic więcej niż zwykle. Był taki moment, że poszłam do Rossmanna, bo skończył mi się dezodorant i tam też wszyscy kupowali jakoś więcej wszystkiego - tabletki do zmywarki, coś tam. I wpadła taka młoda dziewczyna z telefonem przy uchu. Przybiegła i mówi do słuchawki: "Jest! Jest aspiryna! Ile wziąć opakowań?" I ja miałam taki moment, że tak stałam i myślałam, że ja też muszę chyba kupić aspirynę, że powinnam, ale za chwilę pomyślałam, że po co? Przecież ja jej nie używam. Ale było tak, że jakby poddałam się, że ktoś, że takie zainteresowanie, że jest ten produkt taki nieosiągalny i przez chwilę pomyślałam, że wobec tego ja sobie też kupię. Sama się z siebie śmiałam. Nie, u mnie się tu nic nie zmieniło.

**To, że macie sklep zmienia jakoś twoje podejście?**

Myślę, że może częściowo daje mi to jakiś komfort, ale myślę, że jakby nie było tego sklepu, to też by się raczej to u mnie nie zmieniło. Nie wszystko mamy w tym sklepie i słyszałam, że ludzie wykupywali np. kurczaki i tego typu rzeczy. Ja zamrażalnik mam zawsze pełen, więc nie miałam takiej potrzeby, żeby teraz coś więcej. Staram się ograniczać pieczywo i mogę żyć bez pieczywa, ale bardzo mnie zaskoczyło, że ludzie zaczęli kupować 20 bochenków chleba na raz. Też nie miałam potrzeby, żeby kupić do domu ten chleb na zapas, bo stwierdziłam, że to już jest totalne szaleństwo. W czasie wojny nawet pracowały piekarnie i to jest niemożliwe, żeby tak wszystko totalnie stanęło, żeby chleba nie było. Ja nie jestem taką osobą, że muszę np. jeść krewetki i muszę koniecznie jeść je teraz. Nie będzie tego, co sobie wymarzę, to zjem chleb z dżemem i też będzie dobrze. Nie wyobrażam sobie, że mogłoby dojść do takiej sytuacji, że nie ma totalnie nic. Takiej wersji nie przyjmuję, a jest tyle zamienni9ków, że spokojnie przetrwam.

**Czy jest coś, co dla ciebie jest teraz dużym wyzwaniem?**

Największym jest poranne wstawanie. Jestem zmuszona rano wstawać, być gotowa i funkcjonować, i to jest dla mnie duże wyzwanie.

**A ograniczenie np. spotkań towarzyskich, wyjść?**

To jeszcze nie jest wyzwaniem. Na razie cieszę się tym małym gronem. Brakuje mi takiego wyjścia na powietrze, takiego długiego spaceru. Nawet w niedzielę miałam taką chęć, żeby się wymknąć i przejść, ale jednak nie, bo i tak cały tydzień wychodzimy...Ale takiego bycia na świeżym powietrzu to mi brakuje.

**Z twoich obserwacji, jak radzą sobie ludzie w twoim otoczeniu?**

Myślę, że radzą sobie coraz gorzej. Pierwszy tydzień to był taki boom na zakupy. Był rzeczywiście większy popyt, ludzie robili te zakupy, ale na takiej zasadzie, jak robilibyśmy zakupy na święta, że posiedzimy w domu, że sobie pogotujemy, że tak odpoczniemy. Bardziej tak wakacyjnie. Potem zaczęło się robić spokojniej po tym pierwszym zachłyśnięciu i kupieniu sobie litrów mleka, kilogramów mąki, itd. Teraz, już pod koniec ostatniego tygodnia i ten tydzień, ludzie są zdecydowanie bardziej nerwowi, bardziej niemili dla siebie, tak bardzo szorstko do siebie podchodzą, szybciej się irytują na takie błahe rzeczy. Z drugiej strony widzę coraz większą dyscyplinę, żeby nie podchodzić, żeby się nie nachylać, żeby wyjść, bo jest kartka, że tylko 3 osoby...Widzę, że ta świadomość wzrasta. Nie wiem, czy to jest świadomość, czy lęk. To jest chyba tak pół na pół. Ludzie są już chyba zmęczeni tym siedzeniem w domu, tym słuchaniem. Ja, gdybym siedziała w domu, to na pewno bym nie słuchała, bo wiem, że to źle na mnie działa. Były takie sytuacje, że jak był ktoś kaszlący, to klienci zdecydowanie reagowali, prosili, żeby wyjść, żeby tu nie stać?

**A twoje najbliższe otoczenie? Jak oni sobie radzą?**

Moi rodzice myślę, że dobrze. Mama jest takim typem kanapowca, więc jej to tak bardzo może nie robi różnicy. Może bardziej, że do sąsiadki by poszła, z kimś by się spotkała na ploteczki, ale ma telefon i korzysta z kontaktu ze światem w ten sposób. Tata gorzej, ale on to się wymknie na podwórko, samochód umyje...Myślę, że moja mama ma teraz święty spokój, że jej nie wyganiam na spacery.

Teściowa gorzej to znosi, bo ona była cały czas aktywna zawodowo pomimo wieku, ten sklep to jest jej całe życie. Zdecydowaliśmy, że ona teraz nie będzie tam chodziła i ona rzeczywiście źle to znosi. Widzę, że jest i smutna, i taka zamyślona, mówi, że co ona ma robić, że już nie wie, że już oczy ją bolą od czytania. Jak ogląda tv, to też się źle czuje, bo patrzy co się dzieje, itd. Ona jest taka przygaszona i gdyby jej pozwolić, to na pewno by biegła do tego sklepu przed nami. Została odcięta od tego, czym żyła. My jej przynosimy jakieś faktury, żeby miała jakieś zajęcie...Staramy się w miarę możliwości, żeby z nią więcej rozmawiać, ale też nie bardzo mamy możliwości. Zaproponowała, że wobec tego obiady będzie gotować - mieszkamy w jednym domu, ale to są oddzielne gospodarstwa. I dla mnie to też jest nowa sytuacja, bo ja jestem taka Zosia Samosia i bardzo chętnie, ale nie, żebym codziennie chodziła do niej na obiady. Jeśli ona ma się lepiej poczuć, to oczywiście też mogę tu odpuścić.

Mamy ciocię, która z kolei całkowicie się zamknęła i boi się wszystkiego. Zastanawiała się jak do listonosza wyjdzie emeryturę odebrać i cały plan sobie opracowała. Wymyśliła, że otworzy wcześniej furtkę, że założy rękawiczki, będzie miała swój długopis, połozy pieniądze i dopiero wtedy zdejmie rękawiczki. Jak przyjeżdżają córki, to nawet nie wchodzą do mieszkania - każe im zostawiać zakupy, rozmawia tylko z okna do furtki.

**Jak reagowałaś na wiadomości o ludziach, którzy wychodzą z domów mimo zalecenia do pozostania?**

Mnie to złości. Jest taka a nie inna sytuacja, to się jednak dzieje, napływa. Sytuacja jest poważna. Złości mnie to też dlatego, że mam do czynienia z lekarzami, z pielęgniarkami i widzę jak oni się zachowują po dyżurze w szpitalach, w których nie ma nawet tego wirusa. Przychodzą kobiety na zakupy i mówią, że coś jeszcze dokupią, bo idą na dyżur i nie wiedzą czy i kiedy wyjdą. Widzę żywą osobę, która jest w takiej sytuacji a nie na ekranie i do mnie to dociera, że to jest prawdziwe. Lekarz po dyżurze przysyła kogoś, żeby odebrał zakupy, żeby nie wchodzić do sklepu, żeby też chronić nas. I potem widzę, że ktoś bawi się na bulwarach? To mnie złości. Czy ludzie nie wiedzą, czy nie chcą tego wiedzieć, nie zdają sobie sprawy? Nie ma mojej zgody na takie sytuacji i sama trzymam się restrykcji.

**Jak myślisz, skąd się wziął koronawirus?**

Dobrze, że nie mam czasu zastanawiać się nad tym. Nie mam swojego zdania. Te wszystkie informacje, które docierają i które słyszę, że ktoś specjalnie, że ktoś wypuścił, wyhodował...Nie wiem, nie mam pojęcia. Słucham, ale nie utożsamiam się z żadną opinią i żadna mnie jakoś nie przekonuje bardziej. Od tego są eksperci a takie spekulacje, że ktoś gdzieś usłyszał, widział...To nie dla mnie.

**Myślisz, że można było jakoś zapobiec epidemii?**

U nas w kraju można było. Nawet ten napływ osób wracających z zagranicy. Można to było to już wcześniej. Zachęcić wcześniej do powrotu. Wcześniej informować, mówić więcej o tym, że w razie, gdyby to do nas przyszło to lepiej nie zostawać za granicą i wracać. Za mało było edukacji i mówienia, żeby nie jechać tam i tam. Ja zamknęłam swój salon już 2 tygodnie temu, ale dopiero dzisiaj pojawia się taki oficjalny komunikat, że salony fryzjerskie i kosmetyczne będą zamknięte. W środowisku, czy na jakichś forach było wiele dylematów i wiele osób, które skusiły się, bo klienta namawiała, bo znajoma, bo przecież sterylnie. A to nie o to chodzi, bo sterylnie jest u nas zawsze. Chodzi o to, że były takie pokusy, żeby jednak nie zamykać, żeby przyjmować te klientki. Były takie sytuacje, że nie chodzę do pracy, to wymknę się na godzinę na manicure. Właściciele i pracownicy byli w rozterce, co zrobić. Rozmawiałam z koleżankami, że gdyby była jasna decyzja, że zamykamy, to w 100% by się zamknęli. Trzeba było wcześniej to wszystko zamknąć, bo w takiej sytuacji zrobienie sobie manicure czy jakiegoś zabiegu na twarz, naprawdę nie jest rzeczą niezbędną. Bez tego można żyć. Zakaz nieraz jest potrzebny i w mojej branży było to potrzebne dużo wcześniej. Myślę, że równo ze szkołami trzeba było zamknąć. To był taki moment na to.

**Czy wtedy w styczniu, gdy szukaliście dla siebie wyjazdu, było wg ciebie za mało informacji?**

Tak, zdecydowanie trzeba było więcej o tym mówić. Dochodziły jakieś sygnały, le to tak daleko, itd. Nawet mój mąż, który wszystko tak wnikliwie czyta i interesuje się ogólnie sytuacją na świecie, mówił, że tej jakiś wirus i też nie miał jakichś informacji konkretnych. Myślę, że za mało groźnie to było przekazywane i powinno było być przejrzyściej. Więcej informacji po prostu.

**Jak Polska jest przygotowana na to, co się dzieje?**

Nie jesteśmy przygotowani. Nie ma odpowiedniej ilości testów, nie ma maseczek i ubrań ochronnych dla personelu medycznego. Trzeba to było zabezpieczyć wcześniej. Rząd powinien skupować maseczki, rękawiczki. Jak to się stało, że spekulanci odsprzedawali te produkty za kosmiczne pieniądze? I oni potrafili wyprzedzić sytuację a rząd nie wiedział? Ja sama się zbuntowałam jak zobaczyłam na allegro maseczkę, którą kupowałam po 20-30 zł za opakowanie, za 200 zł. Potem były naciski, żeby to ukrócić i już teraz nie wolno, ale to też było za późno, bo ludzie w panice i w niewiedzy kupowali. Mnie aż krew zalewała, że to się dzieje. Ucięto to, ale wszystko robią za późno. Nie ma sprzętu dla lekarzy, ubrań ochronnych. To nie są drogie rzeczy, jeśli to państwo zamawia i takie rezerwy powinny być. Tak naprawdę jesteśmy na początku epidemii, a już sobie nie radzimy.

**Co jeszcze można było zrobić?**

Zająć wcześniej konkretne stanowisko, a nie takie półśrodki. Konkretne decyzje.

**A w porównaniu z Europą?**

Staram się bardzo mało tych informacji oglądać i one są takie szczątkowe, ale to co Włosi robili - chodzili po imprezach, spotykali się. Rzeczywiście zamknięcie restauracji było u nas sensowne. Tam oni sobie zupełnie nie poradzili i my obserwując to wszystko powinniśmy byli wcześniej zamykać. Ja bym wprowadziła kary pieniężne za przemieszczanie się. Zdecydowanie. jednak jesteśmy takim narodem, że jak przejdzie przez kieszeń, to człowiek zrozumie. Jak zapłaciłam mandat, to do dziś pamiętam, w którym miejscu zapłaciłam. A już ci, którzy są na kwarantannie, to dla mnie jest w ogóle nie do pomyślenia, że wychodzą.

**Czy wprowadzanie obostrzeń cię uspakaja czy raczej wprowadza niepokój?**

Chyba uspokaja. Gdyby zrobiono wcześniej takie zdecydowane cięcie, że zamykamy wszystko na 2 tyg., to ileż więcej osób można by uchronić. 2 tygodnie odcięcia od wszystkiego, od usług, od restauracji. Gdyby zostały tylko piekarnie, to każdy z nas jest w stanie przeżyć i myślę, że to by w dużej mierze spowolniło. Nie zatrzymało, bo to się chyba nie da, ale na pewno ograniczyłoby rozprzestrzenianie się. Ja jestem za drastycznymi cięciami. Gdyby zamknięcie miasta miało zatrzymać głupotę ludzką, to nie wiem, czy nie byłabym za tym.

Zamkniecie Helu, gdzie mówiono, że ostatnio ruch był taki jak w wakacje, moim zdaniem ma sens. Niech przemieszczają się tylko miejscowi a nie turyści. Skoro duża część ludzi nie jest w stanie dostosować się do obostrzeń, to może jest to sposób. Gdybym nawet była odcięta na 2 tygodnie a kogoś innego by to zahamowało i nie rozprzestrzeniała się ta choroba, to nie czułabym się jakoś bardzo pokrzywdzona. Trzeba ponosić jakieś straty.

**A 4 tygodnie, 6 tygodni?**

Nie. Dlatego mówię o 2 tyg., że to by było takie zatrzymanie, żeby tę kulę śnieżną wyhamować. Dłużej to może fizycznie nie byłoby możliwe i ludzie by się może zbuntowali. Gdyby to było zaplanowane, konkretne decyzje podawane, to też chyba łatwiej by było się społeczeństwu dostosować. Tak naprawdę, to mamy chaos. Od jutra będą nowe obostrzenia i też nie wiemy jak się dziś zachować, i co nas czeka jutro. Brakuje zaplanowanej strategii.

**Gdzie szukasz informacji, z jakich źródeł korzystasz?**

Najczęściej z wiadomości w TVN, chociaż Polsat to filtruje jakoś tak bardziej przystępnie, więc jakiś pasek na Polsacie też. GW, jeśli znajdę czas, ale tam to zdecydowanie wolę jakiś felieton przeczytać i takie relacje z życia ludzi niż taką czystą politykę i te inne. Radia słucham i RFM mam ostatnio nastawiony, ale to są takie szybkie i krótkie informacje. Właśnie takie szybkie i krótkie mi wystarczają. Mąż zdecydowanie codziennie musi z GW.

**Które źródła uważasz za najbardziej wiarygodne?**

Chyba jestem za GW, bo jednak to jest takie przemyślane, to jest słowo pisane a jednak w tych wiadomościach jest szybko, spontanicznie, jakieś są urywki, migawki, a tutaj można się skupić. Zakładam, że ktoś to przemyślał na spokojnie, napisał, zredagował, itd. W serwisach informacyjnych to zwykle jest migawka, jakiś urywek czegoś. Tu jakaś płacząca matka, krzyczące dziecko i to jest taka gra na emocjach. Próbuję czasem tv publicznej, ale nie daję rady. Nieraz nawet z ciekawości, żeby popatrzeć i mieć jakieś porównanie, ale mój maż jest wielkim przeciwnikiem. Trudno idzie z tymi stacjami i tu się można zdenerwować. [śmiech]

**A źródła internetowe?**

Na FB mam jakieś grupy - mam naszemiasto, jakieś warszawskie informacje, jakiś Onet, ale staram się ograniczać to. Onet to też...Unikam, bo to jest też takie na szybko, pojawiają się reklamy, a ja jestem taka ciekawska, że zaglądam do komentarzy. Wtedy mogę się i zdenerwować, i zezłościć i stracić czas. Głównie stracić czas. Tam każdy pisze, co chce i jak chce, a potem idzie na bulwary.

**Zdarza ci się wyszukiwać jakieś konkretne informacje?**

Tak, wtedy na wyszukiwarce wpisuję np. koronawirus objawy i korzystam z takich źródeł, jak np. Wikipedia czy jakieś medyczne strony. Fajne są te ulotki, że kaszlesz, kichasz, masz temperaturę, ale te dokładniejsze informacje to przez wyszukiwarkę. Szukam źródeł popartych jakimś autorytetem a nie dziennikarza z Faktu czy Pudelka. Próbuję się odcinać od nadmiaru informacji.

**Czy w tej chwili mniej korzystasz z mediów niż wcześniej?**

Może rzeczywiście tym momentem zmiany była ta panika. Zmierzyłam sobie temperaturę, stwierdziłam, że się dobrze czuję i stwierdziłam, że nie mogę tak słuchać tego, bo to mi szkodzi. Mój mąż to wszystko ogląda, ale on potrafi to jakoś lepiej filtrować i tak nie przyjmuje do siebie, albo o tym nie mówi, bo tak też może być. Ja jestem bardziej podatna na takie rzeczy, że gdzieś tam umarło już ileś osób, zachorowało...

**Sprawdzasz statystyki?**

Na pewno nie biegnę rano, żeby od razu sprawdzić. Albo teściowa powie, bo ona siedzi, ogląda i to jest jej...Może nie zadanie, tylko ma wtedy poczucie, że ona też coś robi, ogląda statystyki i nam przekazuje. Przez weekend np. zupełnie tego nie śledziłam. Gdzieś jakaś migawka, gdzieś widzę cyfrę i tyle. Na pewno nie siadam i nie patrzę, ile teraz mamy cyferek. Na początku też tego nie robiłam.

**Jak radzą sobie osoby z twojego otoczenia?**

Ja mam zajęcie, ja się skupiam na takich pracach tu i teraz, ale część osób skupia się na tym, żeby przetrwać to, że siedzą w domu. Na tym poziomie zużycie energii jest chyba podobne. Mam koleżankę, która sama wychowuje małe dzieci. Ona jest dzielna i głośno mówi, że wszystko będzie dobrze, itd., natomiast mam wrażenie, że to jest takie zaklinanie. Ja, z racji tego, że mam wspierającego męża już nawet mam łatwiej.

Mój syn ma edukację online i jest zachwycony, ale on ma to bardzo dobrze zorganizowane.

**Co znaczy, że jest wspierający?**

Uspokaja mnie jak oglądam albo przefiltruje mi te informacje na zasadzie, że ty tego nie oglądaj, ja ci opowiem. Ja się też zawsze boje, że zaśpię i tu też mówi, że spokojnie, że przypomni a nawet jak godzinę później przyjadę, to nic się nie stanie. To jest takie poczucie bezpieczeństwa, że jest ktoś obok, że nawet jak coś mi nie wyjdzie...

**Czy masz wrażenie, że ludzie od ciebie też potrzebują wsparcia?**

Ja chyba zawsze mam trochę taką rolę. Na pewno w pracy, bo tam są ludzie w różnych sytuacjach i bardzo różnie reagują. Ja trochę to próbuję takim żartem rozładować, bo rzeczywiście mają ciężko. Są zmęczeni, z kartkami robią te zakupy, bo na 3 domy, bo co tej babci kupić. Ludzie są obciążeni i zdecydowanie to jest bardzo ciężki moment. Denerwują się i też trochę odpowiadam za to, żeby ktoś kaszlący jednak nie stał w sklepie. Trochę próbuje to żartem, że np. może zrobimy pakiet startowy koronawirusa. Myślę, że to pomaga. Mnie to chyba też pomaga. Może to też ja się tak bronię przed tym.

**Nie pojawiła się wśród twoich obaw sytuacja gospodarcza?**

Ja jestem świadoma tego, ja wiem, że to wszystko runie. To jest jak domino i to dotknie każdego mniej lub więcej. Nie muszę tu czytać diagnoz ekonomicznych. W pierwszym momencie był we mnie też taki lęk, ale ja jakby na spokojnie jak usiądę i przeanalizuję...Tu pomaga mi też mąż, który mówi, że to jest tak, to jest tak, to przesuniemy, to zrobimy tu i tu.

**Czy jest coś jeszcze co ciebie porusza, dotyka?**

Bardzo mnie zirytowało, nawet wkurzyło, jak usłyszałam po 3 dniu jak dzieci nie poszły do szkoły...Te lamentujące matki, że jak one wytrzymają z dziećmi, jakie to jest straszne. Nie bardzo to mogłam zrozumieć. Nawet w sklepie to słyszałam od klientek, że o Boże, że z tymi dziećmi. Koleżanki mówiły, że idzie zwariować, bo dzieci skaczą, jedna wywiozła do dziadków, bo mają małe mieszkanie i nie wytrzymają. Mnie to z jednej strony zdenerwowało, że jak tak można, z drugiej przeraziło trochę. Ja rozumiem 2 tygodnie, ale po 3 dniach być zmęczonym własnym dzieckiem i być tak nieszczęśliwym wręcz, że ono skacze, krzyczy? Przecież to są ich dzieci a nie jakaś tragedia, że musi się z nimi siedzieć. Przecież jest tyle fajnych rzeczy do zrobienia z nimi. Jak to jest, że własne dzieci tak przeszkadzają? Potem sobie pomyślałam, że rzeczywiście te dzieci mają teraz tyle zajęć, tak mało czasu się z nimi spędza, że takie 3 pełne dni mogą być męczące.
