# Supplementary material for: The challenges arising from the COVID-19 pandemic and the way people deal with them. A qualitative longitudinal study
Source: PLoS One. 2021 Oct 11;16(10):e0258133. doi: 10.1371/journal.pone.0258133 (PMC8504766; doi:10.1371/journal.pone.0258133)
Supplement: S1 Dataset — (ZIP) [file pone.0258133.s003.zip › Transcriptions/stage 1/16.1_F_36_couple, with children.docx]

**16.1_F_36_couple with children**

Mam prawie 36, mieszkam z mężem i 2 dzieci (3.5 i 1.5). Teraz jestem na wychowawczym i pracuję trochę dorywczo. Z wykształcenia jestem teologiem i afrykanistą, a pracuję...To jest praca biurowa, dział finansowy. dorywczo piszę artykuły i robię korekty i redakcję artykułów naukowych.

**Od kiedy poczułaś, że coś się zmienia, że coś się dzieje w związku z koronawirusem?**

Nie jestem w stanie dokładnie powiedzieć. Rozmawialiśmy o tym z mężem i byliśmy przygotowani na to, że może się zacząć coś dziać...Zaczęliśmy myśleć o tym co kupić, itd., i wydaje mi się, że zaczęliśmy myśleć o tym na tydzień zanim zamknęli szkoły. O tym, że zamknęli szkoły dowiedziałam się w sklepie, jak akurat byłam na takich dużych zakupach. Dowiedziałam się od ludzi, którzy stali w kolejce, ale my już wiedzieliśmy wcześniej, że to jest ostatni moment, żeby zrobić takie duże zakupy, że już potem może nie być takiej okazji.

**Te pierwsze sygnały, to było co?**

Chyba jakieś po prostu informacje z newsów, ze świata. Wiedzieliśmy od dłuższego czasu o tym, co się dzieje w Chinach, bo też moi rodzice mieli akurat jechać do Chin. Na początku lutego odwołali im tę wycieczkę. Już wtedy wiedzieliśmy, że coś tam się dzieje, ale nie wiedzieliśmy, na ile to przyjdzie do Europy. Po feriach to już były te historie, że ci, co wracają to już mogą być chorzy, bo we Włoszech już są ludzie chorzy...Moja siostra akurat wróciła też z Włoch...Wiedzieliśmy, że coś się już dzieje, nie wiedzieliśmy na jaką skalę.  Wiedzieliśmy, że w Chinach zamykają ludzi na kwarantannę i wiedzieliśmy, że jak my będziemy potrzebowali czegoś takiego, że jak my będziemy chorzy, to musimy mieć pewne rzeczy - mieć pieluchy dla dzieci chociażby. Wiedzieliśmy, że to będzie taki czas, że nie będziemy mogli liczyć na naszych rodziców, bo to są starsze osoby i nie chcielibyśmy ich na takie rzeczy narażać. Pieluchy, jakieś jedzenie i takie podstawowe rzeczy, żeby nie być zależnym od innych ludzi. To było zanim jeszcze były pierwsze zachorowania w Polsce.

**Czy były jakieś punkty przełomowe, ważne momenty rozwoju tej sytuacji?**

Na pewno zamknięcie szkół, bo mój mąż pracuje też jako nauczyciel i dla nas to był taki moment, że wiedzieliśmy już, że się dużo zmienia. On już przestał chodzić do pracy i pracuje z domu, więc to też dużo zmieniło. Przestaliśmy się też spotykać z ludźmi. jak już zamknęli te szkoły i powiedzieli, że trzeba się dystansować...Przestaliśmy się spotykać z rodziną też i to było takie najbardziej...

**Wiedzieliście wcześniej, że zamkną szkoły?**

Mąż pracuje w prywatnych szkołach i nie miał wcześniej takich informacji. Nie zastanawialiśmy się nad tym, bo my nie mamy dzieci w wieku szkolnym i nie dotyka nas to tak bardzo.  Był to taki moment, że coś tam się zaznaczyło, ale na moje życie to jakoś nie wpłynęło, poza tym, że mąż przestał chodzić do pracy. Miałam wrażenie, że zamknięcie szkół to jest dobra decyzja, że to może zatrzymać jakoś tę epidemię u nas. Ja się kompletnie na tym nie znam, to jest w ogóle nie moja dziedzina, ale słucham różnych opinii.  Moje subiektywna opinia była, że to jest dobra decyzja.

**Jak nazywacie tę sytuację?**

Epidemią, pandemią. Z dziećmi to trochę inaczej, bo mamy 3-latka w domu i zawsze mówimy mu, że jak przejdzie koronawirus. W domu jest nazwa koronawirus, bo my to tak zdziecinniamy między sobą, bo wiemy, że on słucha.

**Czyli mamy odwołaną wycieczkę do Chin, zamknięcie szkół. Jeszcze jakieś momenty istotne?**

Ja mam wrażenie, że co drugi dzień się coś zmienia [śmiech] i coraz jest dziwniej. Po pierwszych 3 dniach już przestałam mieć jakiekolwiek oczekiwania, co się będzie działo następnego dnia. Jeszcze pamiętam, że bardzo duże wrażenie na mnie zrobiło, jak powiedzieli, że zamykają granice. Nie wiem, czy to było jak zamknęli szkoły, czy później. Miałam wrażenie, że nagle jesteśmy w zupełnie innym kraju niż wcześniej, że poszłam spać w innym kraju a obudziłam się w innym. Dla mnie to był duży szok. Nie wiem, dlaczego. Jakoś wydawało mi się, że świat jest taki bliski, że nie potrzeba wiele zrobić, żeby pojechać. Mam zaplanowane wakacje za granicą, mam rodzinę za granicą i nagle się okazuje, że nie pojedziemy nigdzie na wakacje, że nie widziałam się z tą rodziną od 10 lat i nie wiadomo, kiedy się z nią zobaczę. Nagle to nie jest takie proste wyjechać gdzieś za granicę. Nagle jesteśmy w innej rzeczywistości i trzeba obie zupełnie inaczej poukładać świat w głowie, inaczej go odczytać, inaczej zacząć funkcjonować w tym świecie. Przez kilka pierwszych dni jakoś nie byłam w stanie przestać o tym myśleć. Ten świat jest po prostu inny niż ten, który znałam. Przyzwyczaiłam się do świata, w którym nie ma problemu z wyjściem do sklepu, z wyjazdem za granicę, nie ma problemu z niczym tak naprawdę i wszystko jest na wyciągnięcie ręki.

**Jaki przymiotniki pasują do tego świata teraz?**

Zamknięty, klaustrofobiczny i wszystko stało się nagle odległe, niedostępne.

**To powoduje obawy, niepokój?**

Trochę tak. To powoduje taką niewiadomą, że taki nieprzewidywalny jest ten świat teraz i nie można nic zaplanować na dłużej. Już część planów trzeba było poodwoływać, bo np. mieliśmy święta spędzić z rodziną w innym mieście. Trzeba się z tym oswoić a ja się jakoś nie oswoiłam do końca.

**Skala lęku**

Teraz to ja tak ok 30 bym wzięła.

**Czy to się jakoś zmieniało w czasie?**

Na początku było gorzej. Po tym zamknięci szkół to było ok 50-60. teraz mam wrażenie, że już się przyzwyczaiłam do tych zmian i wiem, że one będą a wtedy one tak zaskoczyły. One były pierwsze, one były duże i teraz już więcej scenariuszy się pojawia, więcej jest dywagacji na ten temat. tamto było takie większe.

**Czego obawiałaś się wtedy, czego teraz?**

Zmieniło mi się poukładanie świata i każda taka niewiadoma. Nie rozumiałam tego świata, który jest wokół a teraz już się trochę przyzwyczaiłam, bo to już 3 tyg. I przyzwyczaja się do tych coraz to nowych ograniczeń, bo teraz to są takie mniejsze kroczki. Pierwszy był taki duży. Już pewne schematy sobie wypracowałam w sensie robienia zakupów, siedzenia w domu. Te nowe ograniczenia już nie zmieniają tak codziennego życia i nie uderzają tak bardzo.

**Czego dotyczą twoje obawy?**

Najbardziej się boję tego, że ktoś z moich bliskich zachoruje i może się to dla niego skończyć źle.

**A o swoje zdrowie?**

No jakoś mniej [śmiech]. Nawet gdzieś przeczytałam, że ludzie się bardziej boją, że ktoś bliski zachoruje niż, że oni sami. Jak ktoś nie żyje, to o tym nie wie. Jak czytam o tym wirusie, to jestem chyba w tej grupie mniej zagrożonej niż np. moi rodzice i teściowie.

Jeśli chodzi o obawy o przyszłość, to obawiam się o sytuację gospodarczą, która może z tego wyniknąć i myślę, że wyniknie, Czeka nas jakaś recesja i wiadomo, że to też wiąże się z jakimiś obawami, ale na to akurat byliśmy przygotowani. Już z mężem byliśmy przygotowani, że przyjdzie kryzys gospodarczy, że przyjdzie wcześniej czy później i na to byliśmy psychicznie przygotowani. Kryzys był zapowiadany przez różnych ludzi, którzy się znają na finansach. Niektórzy już wróżyli, że w poprzednim, niektórzy, że w tym roku będzie taki kryzys poważniejszy jak w 2007-2008, że polityka wszystkich państw do tego prowadzi. To nas nie zaskoczyło. Wiedzieliśmy, że to kiedyś nastąpi, ale dotąd zawsze było to kiedyś, a teraz wiemy, że to już, że już będzie na pewno. Już jest początek.

**Zrobiliście coś, żeby się do tego przygotować?**

Tak. Wiedzieliśmy, że to będzie kryzys finansowy i przygotowaliśmy się do tego finansowo. Zadbaliśmy o to, żeby mieć zabezpieczenie.

**Co się zmieniło teraz w twoim życiu?**

Dużo się zmieniło, tak. Mimo, że ja generalnie jestem w domu z dziećmi, bo oni nie chodzą do przedszkola i żłobka, to jednak my dużo chodziliśmy w różne miejsca i codziennie gdzieś z domu wychodziliśmy.  Codziennie wychodziliśmy na spacer i to zawsze było połączone z jakimiś zakupami, bo ja nie lubię spacerów bez celu. Jeździliśmy do dziadków albo oni do nas, do siostry, która też ma dzieciaki, do koleżanek, syn chodzi na zajęcia rytmiczne i to też odpadło. Planowałam zacząć chodzić od wiosny często do ZOO, bo mamy blisko. Na teatrzyki chodziliśmy...A teraz siedzimy w domu.

**Kiedy przestaliście wychodzić?**

Tego dnia, co zamknęli szkoły, to ja jeszcze byłam z dziećmi na zakupach i to była masakra, bo wszyscy wtedy ruszyli na zakupy, a ja z tymi dziećmi...To była środa i chyba jeszcze w piątek byłam z dziećmi na zakupach, bo mąż jeszcze pojechał do pracy w piątek. Na początku myślałam, że to nie będzie tak, że z dziećmi nie będzie wolno do sklepu iść. Po weekendzie okazało się, że jednak nie, że dzieci do sklepu w ogóle nie wpuszczają. Nie wiem, czy to jest oficjalne, ale nie powinno się z nimi chodzić, bo one wszystkiego dotykają. Może to nie było takie zakazane, ale to przyzwolenie społeczne na łażenie z dziećmi po sklepach było bardzo niskie już. I faktycznie nie widziałam już nigdy ludzi z dziećmi w sklepie od tamtej pory i od tamtej pory chodziliśmy już tylko na takie krótsze spacery z dziećmi. Teraz wychodzimy, ale tak nie codziennie. Sama nie wiem, bo jednego dnia uważam, że ok, drugiego, że nie ok. To są tylko takie spacery wokół osiedla, żeby trochę się przeszli. Z małą nawet bez wózka.

**A kontakty towarzyskie teraz?**

Z koleżankami to i tak częściej do siebie pisałyśmy niż rozmawiałyśmy i to się akurat nie zmieniło, natomiast z siostrą i rodzicami widywaliśmy się i rozmawialiśmy telefonicznie a teraz spotykamy się na Skype'ie. To ze względu na dzieci, bo jak one widzą swoich dziadków czy braci ciotecznych, to łatwiej im nawiązać konwersację niż przez telefon.

Oprócz tego, że siedzę w domu to niespecjalnie się coś zmieniło w moim życiu. Zrobiłam sobie też osobiste postanowienie związane z tą pandemią. Jestem z wykształcenia teologiem, robię doktorat z teologii i mam trochę wiedzy na ten temat. Śledzę różne rzeczy na FB i jestem też związana z niektórymi mediami katolickimi, bo piszę dla nich artykuły, itd. Na stałe współpracuję z miesięcznikiem Misjonarz i trochę współpracuję z takim portalem Misyjne Drogi. Na ich FB czasami widzę jakieś głupie komentarze i ponieważ wiem, że redaktorzy, którzy tam pracują na nie reagują, bo im nie wypada, więc ja tam czasami komentuję. Jak ktoś pisze jakieś rzeczy niezgodne z nauką kościoła, to ja staram się to skorygować. Może uda mi się przekazać jakąś wiedzę o tym, co faktycznie kościół mówi na pewne tematy. Tutaj się zwiększyła moja aktywność, bo stwierdziłam, że może jednak trzeba się podzielić swoją wiedzą, doświadczeniem.

**Te ograniczenia dotyczące kościoła, świąt. Jak to odbierasz?**

To nic nie zmienia w moim życiu.

**Co z rzeczy, które się zmieniły najbardziej ci przeszkadza?**

Teoretycznie mogłabym spotkać się z rodzicami i siostrą, ale wiem, że nie powinnam i oni też czują, że nie powinni. To mi bardzo przeszkadza. Przeszkadza mi, że tych dzieci nie mogę zabrać na zajęcia, do ZOO, nie mogę wyjść z nimi po świeże pieczywo, tylko jem czerstwy chleb sprzed kilku dni. Oglądanie zewnętrznego świata przez szybę. Nie doceniało się tego co się miało i dopiero teraz człowiek zaczyna doceniać to wszystko, co było taką oczywistością. Ja się czuję strasznie ograniczona tym, że wychodzę na zakupy tylko 2 x w tygodniu, bo mam swoje upodobania konsumenckie i czuję, że one są po prostu strasznie ograniczone. A jak widzę kolejkę gdziekolwiek, to dostaję po prostu...Ja nie pamiętam za bardzo kolejek, które były za komuny. Trochę pamiętam, ale ja tego nie doświadczyłam empirycznie. Teraz jak widzę kolejkę i widzę, że muszę stać w tych kolejkach, to strasznie mnie to denerwuje, strasznie.

**A może są też jakieś korzyści z obecnej sytuacji?**

Jeszcze nie widzę korzyści. Nie, jest jedna korzyść - jednak jak się chodzi mniej po sklepach, to się mniej wydaje pieniędzy. Nie chodzi się po restauracjach, gotuje się w domu i po prostu wydaje się mniej pieniędzy.

**Zdjęcia - emocje**

**8 i 4**

**8**

To jest metafora naszego życia. Jedziemy wszyscy tą samą drogą, widać troszeczkę, ale dalej już nie wiadomo co jest. I tak musimy jechać do przodu, bo nie ma wyjścia, ale co tam dalej i kogo będziemy mijać, to nie za bardzo widać. Mam nadzieję, że za tą mgłą się przejaśnia. Teraz życie jest takie szare, nie ma kolorów, które powinno mieć. Jest ograniczone, nie ma tych radości, które dają te kolory. Generalnie ja jestem optymistką, ja myślę, że będzie dobrze i widzę słońce za tą mgłą. Wiem, że mgła to jest tylko to, co przysłania słońce i że ono tam jest. Za tą mgłą są kolory.

**4**

Bo jednak rodzina jest najważniejsza teraz. Ja tu widzę rodzinę, obrączki, widzę starszą rękę, dziecięcą rękę. Wszyscy się trzymają, wspierają i to jest właśnie taki czas. Ważne jest takie wsparcie rodzinne. Widzę to moją rodzinę, moich rodziców przede wszystkim.

**Czy twoje emocje zmieniały się w czasie w ostatnich tygodniach?**

Z jednej strony jest lepiej, bo człowiek się oswoił z warunkami w jakich żyjemy. Z drugiej strony jest trochę gorzej, bo już mnie trochę rozsadza w tym domu. Nie lubię aż tyle siedzieć w domu, Młodsza córka jeszcze jest za mała i nie wiele rozumie, ale tego starszego też już trochę roznosi, chciałby pojechać do dziadków, wyjść gdzieś, pojechać do swoich braci ciotecznych. Codziennie widzę w nim większą frustrację i mnie też to już denerwuje. On też to przezywa, że widzi dziadków na tym Skype'ie, nie chce się z nimi rozstać, cały czas mówi, że ich kocha, że tęskni. Mam wrażenie, że też staje się taki bardziej nieposłuszny w tym domu. Jemu się już nie chce tego domowego przedszkola robić. Lubi te swoje książeczki z naklejkami, labiryntami, ale dzisiaj jak usiadł do tego, to powiedział, że już go to nie cieszy. To mnie też już denerwuje, że nie mogę zapewnić mu innych rozrywek. Robienie 3 tyg. tego samego zaczyna go nudzić. Nie dziwię się, bo mnie też już zaczyna to nudzić. To jest taka frustracja.

**Jakie masz sposoby radzenia sobie z tą sytuacją?**

Jeszcze chyba nie mam sposobu. Na razie ta sytuacje nie wpłynęła bardzo głęboko na moje życie wewnętrzne, żebym musiała sobie jakoś specjalnie radzić z tym. Dzieci oglądają zdecydowanie więcej bajek. Zawsze miałam zasadę, że tylko wieczorem przed spaniem, a teraz trochę więcej im puszczam i faktycznie na to bardziej pozwalam.

**A twój sposób spędzania czasu? Coś się zmieniło?**

Nie, ja chyba robię to samo, co dotąd robiłam.

**Mówiłaś, że zmieniło się w sferze zakupów?**

Tak, to się bardzo zmieniło. Tego dnia, co zamknęli szkoły miałam już przygotowaną listę zakupów, bo wcześniej ustaliliśmy z mężem. Faktycznie wtedy zrobiłam takie większe zakupy z myślą o tym, że możemy być my zamknięci w domu. Kupiłam więcej takich rzeczy z długim terminem ważności. Ja już kiedyś robiłam takie większe zakupy, ale potem się od tego odzwyczaiłam. Bardziej lubię kupić mniej niż...Wyniosłam z domu, bo moi rodzice tak funkcjonują, że nakupują dużo i trzymają to wszystko w szafach i to zjadają co jakiś czas. Ja to trochę wyniosłam z domu i tak robiłam na początku, jak się wyprowadziłam, że miałam poupychane dużo rzeczy w domu. Potem stwierdziłam, że to jest bez sensu, bo przecież wszystko jest. Wiadomo, że jak się coś trafi, jak jest promocja, to kupię więcej, ale to nie jest tak, że muszę mieć i się trochę od tego odzwyczaiłam. Teraz na tej liście były właśnie przede wszystkim takie rzeczy w puszkach, kupiłam więcej makaronów, bo wiedziałam, że trzeba więcej rzeczy takich, żeby przeżyć. Bo to są rzeczy na przeżycie a nie na to, żeby jeść tak normalnie.

Puszki, makarony, co jeszcze wydawało ci się niezbędne?

Jeszcze te rzeczy dla dzieci - pieluchy, chusteczki te mokre, bo wiedziałam, że muszę je mieć. Chociaż tak naprawdę to nie jest takie do końca niezbędne, ale jednak człowiek się przyzwyczaja do tego. Papier toaletowy! Ale to chyba każdy. [śmiech] I właśnie nie wiem, dlaczego. To jest pierwsza rzecz, którą zawsze wszyscy wykupują.

**Ile tego papieru wzięłaś?**

Ja nie wzięłam jakoś dużo, bo ja zawsze kupuję takie duże paczki, bo mamy swój ulubiony i teraz mamy może z półtorej paczki i to starczy pewnie na 1.5 miesiąca na 2 osoby.

**Ten papier był na liście zakupów?**

Tak, bo to była taka lista na przeżycie.

**Na jaki czas na przeżycie?**

2 tyg. mniej więcej.

**Co tam było takiego, czego normalnie byś nie kupowała, czego kupiłaś istotnie więcej?**

Ja kupiłam takie rzeczy, które wiem, że i tak zjemy. Nie kupowałam żadnych takich rzeczy, że jak nie będę mogła wyjść z domu, to potem wyrzucę. Wszystko to, co my jemy - makaron, sosy, pomidory w puszce. Ja z tego w końcu zrobię sos.  Może nie zjemy tego wszystkiego w 2 tyg., może na pół roku się to rozłoży, jak raz na tydzień zrobię ten makaron. Nie było tam żadnych rzeczy, których nie kupuję na co dzień, może poza owocami w puszce. Kupiłam kilka puszek z owocami, bo dzieci lubią owoce, a świeże szybko się psują.

**A kupiłaś jakieś rzeczy pod wpływem impulsu albo dlatego, że inni to kupują?**

Nie.

**Zwykle robisz zakupy z listą?**

Nie.

**A jak teraz wyglądają u was zakupy?**

Teraz jeżdżę sama po zakupy i jeżdżę do jednego sklepu 2 x w tygodniu. Staram się wybrać taki sklep, gdzie kupię wszystko, czego potrzebuję na 3-4 dni. Nie liczę tu pieczywa, bo mamy taki mały sklep osiedlowy, gdzie pieczywo można kupić.

**Zachowujesz specjalne środki ostrożności idąc do sklepu?**

Tam są te rękawiczki zazwyczaj. Nie korzystam z własnych rękawiczek, tylko biorę te do pieczywa. Ostatnio w Selgrosie używałam żelu, który tam jest, bo pomyślałem, że pewnie strasznie zasyfiony jest ten wózek, więc wzięłam na ręce i nawet sobie tę rączkę wytarłam. Nie mam maseczki, nie mam jak jej sobie uszyć, więc nie mam. Nie mam dyskomfortu z tego powodu.

**Wspominałaś, że kolejki cię irytują?**

Przez ostatni czas robiłam zakupy w Lidlu, bo tam wszystko jest i są warzywa, i owoce, i kupię tam jakąś wędlinę, i mięso, ale już chciałam pojechać do innego sklepu po rzeczy, których w Lidlu nie ma. I pojechałam do Selgrosa. Tam w środku oni mają takie chłodnie i wpuszczają tylko odpowiednią liczbę ludzi a ponieważ to jest półhurtownia, więc są ludzie, którzy kupują po 100 kg ziemniaków i zajmuje im to trochę czasu. Pod tymi chłodniami były po prostu kolejki i ja stałam do jednej chłodni w kolejce, do drugiej, wyszłam z tej chłodni mięsnej i kapnęłam się, że miałam tam coś jeszcze kupić. Jak pomyślałam, że mam drugi raz stać w tej kolejce, to już myślałam, że po prostu się rozpłaczę [śmiech]. A jeszcze widziałam, że są kolejki do kas i wiedziałam, że jeszcze kolejna kolejka mnie czeka. Jak wychodziłam z Selgrosa, to jeszcze była kolejka na zewnątrz do Selgrosa, więc całe szczęście, że przyjechałam wcześniej, bo jeszcze tam bym stała. To nie na moje nerwy, strasznie mnie to złości i powiedziałam, że już nie pojadę do Selgrosa. Będę jeździć do Lidla, ale też nie wiem jak to teraz będzie, bo są nowe obostrzenia i jeszcze po nich nie byłam. Następną wycieczkę mam dopiero w piątek, ale jeszcze wprowadzili te godziny dla seniorów, a ja zwykle ok 10-tej jeździłam, więc jeszcze te godziny będę musiała zmienić. Chyba muszę jechać rano, bo po południu już nic nie będzie, bo wykupią mi te najlepsze kąski. Nie wiem, zobaczę. Muszę jeszcze z mężem pogadać jak to zrobić, ale to ja u nas robię zakupy, bo on musi zostać z dziećmi. Po pierwsze mój mąż nie jeździ samochodem a po drugie to ja gotuję, ja wiem, co potrzebuję. Nie da się za bardzo zrobić listy co kupić, bo ja przychodzę, a tu tego nie ma, tamtego nie ma. Najgorsze było jeszcze to, jak zamknęli te szkoły i w 2 dni ludzie wszytko wykupili. Ja poszłam w piątek, bo chciałam kupić kawałek mięsa na obiad i nic nie było. [śmiech] To był też dla mnie niezły szok.

**Samo przebywanie w sklepie jakoś się zmieniło?**

Jest inaczej, dlatego, że jestem sama na zakupach i inaczej to zupełnie wygląda. Generalnie wolę zakupy bez dzieciaków. W sumie ja i tak zawsze zerknę na wszystko co jest w sklepie, bo może jakaś okazja, może coś się trafi.

**Kupujesz to samo co wcześniej, jecie tak samo jak wcześniej?**

Generalnie to się nie zmieniło, trudniej mi jest tylko zaplanować wcześniej. Planuję w sklepie bardziej - zobaczę jakie jest mięso i do tego dobiorę resztę. Chociaż...Wcześniej też tak robiłam. Patrzę co jest w sklepie i kupuję to, co jest ładne, co jest tańsze, w promocji. Tu jest tak samo.

**Jaka sfera życia wydaje ci się teraz największym wyzwaniem w związku z ograniczeniami, które wprowadzono?**

Najbardziej mnie denerwuje to, że nie mogę się spotkać z rodziną i nie widujemy się tak jak zwykle.  Moi rodzice też dużo pomagali mi przy dzieciach i jak oni przychodzili, to ja np. mogłam gdzieś wyjść. Sama. Nagle sobie zdaję sprawę, że nie mogę zostawić tych dzieci z rodzicami, żeby chociaż chwilę móc odpocząć od nich. Ja nie mam jak wyjść i odpocząć od dzieci. Nawet jak zostawię je z mężem, to wychodzę tylko do tego sklepu na godzinę i to nie jest żaden odpoczynek dla mnie, bo mój mózg pracuje, muszę zaplanować na 4 dni, czego wcześniej nie robiłam. Nie było tak nigdy, że jak zapomnę czegoś kupić, to już mogiła. [śmiech] Nie mam gdzie oderwać się od...Nie lubię mówić, że "odpocząć od dzieci", ale jest potrzeba, żeby się gdzieś tak na chwilę wyrwać, zrobić coś dla siebie, zmienić to otoczenie. Tego mi brakuje.

**Gdzie byś poszła, gdyby jutro znieśli wszystkie ograniczenia?**

Pojechałabym do rodziców albo do siostry.

**Jak radzą sobie ludzie w twoim otoczeniu? Rodzina, znajomi?**

Trudno mi powiedzieć. Każdy chyba inaczej przeżywa. Dziadkowie bardzo przeżywają, że nie mogą się zobaczyć z wnukami. Oni też chyba bardzo zdają sobie sprawę, że jak zachorują, to są na tej liście najbardziej zagrożonych i że mogą nie zobaczyć ich już nigdy więcej. To bardzo ich dotyka. Ci moi znajomi, którzy mają dzieci szkolne, to przeżywają teraz bardzo domowe nauczanie. Żyją bardzo tą szkołą i tym, że muszą dzieciaki uczyć sami i sami się tego uczyć jak ich uczyć. To jest zupełnie inny tryb tego życia codziennego. To dotyczy głównie kobiet, bo faceci to już zaczynają się zastanawiać co dalej z pracą, itd. Taka obawa o byt.

**Udało ci się zaobserwować jakieś zachowania, sposoby, które pomagają?**

Chyba nie. Nie szukam czegoś takiego i nie zwracam może na to uwagi. Wydaje mi się, że wszyscy sobie jakoś radzą. Każdy chyba ma teraz lepsze i gorsze dni, i chyba ja też tak mam.

**Jak myślisz, skąd się wziął koronawirus?**

Nie wiem, nie mam pomysłu. Ja sądzę, że takie rzeczy się zdarzają. Zdarzają się różne klęski, choroby, epidemie i tyle. Ja temu nie dopisuję żadnych nadprzyrodzonych przyczyn. Nie wydaje mi się również, że to jakiś spisek, jakież zaplanowane działanie. Są choroby na świecie i czasami zdarzają się takie, które są trudniejsze. Zdarzały się w przeszłości, mamy szczepionki teraz na choroby, które kiedyś zabijały dziesiątki tysięcy ludzi. Trochę się znam na Afryce i tam cały czas się zdarzają epidemie. Są np. takie lokalne epidemie odry. To, że tyle lat żyliśmy w Europie bez żadnej epidemii/ pandemii, to było po prostu nasze szczęście, ale nikt nam tego szczęścia nie obiecywał, że będzie nam dane na zawsze.

**Można było tej epidemii jakoś zapobiec?**

To jest kompletnie poza jakąś moją kompetencją, żeby to ocenić.

**Jak Polska jest przygotowana do walki z epidemią?**

Fatalnie. Służba zdrowia jest w fatalnym stanie od lat. Każdy Polak, który siedzi teraz w domu to chyba tylko boi się, żeby nie iść do lekarza, bo o ile czekał na specjalistę ileś lat, to teraz będzie jeszcze gorzej.

**Jakieś państwo w Europie radzi sobie lepiej niż inne?**

Trudno powiedzieć, bo ja tylko czerpię wiedzę z tego, co przeczytam w inernetach. Na podstawie tego trudno powiedzieć, co jest prawdą do końca, co nie jest prawdą. Wiadomo, że we Włoszech jest kiepsko. Niemcy mają chyba niską śmiertelność, ale czy oni liczą wszystkie te ofiary? Nie wiadomo. Są kraje bogatsze od Polski i one na pewno będą sobie lepiej radzić. Takie kraje, jak Niemcy - są bogatsi, mają więcej lekarzy.

**Jak nasz rząd radzi sobie z tą sytuacją?**

No źle. Nie mam kompetencji, na ile te wszystkie ograniczenia są dobre czy złe. Oceniam bardziej to, jak to jest robione. To jest jakieś przegłosowywane nocą, jakieś spec ustawy, które udają coś, są ograniczenia przemieszczania się, o których czytam, że są bezprawne, bo powinny być ograniczone ustawą a nie rozporządzeniem. Uważam, że nie powinno się tak robić. Myślę, że sytuacja będzie w Polsce coraz gorsza, a politycy nadal próbują coś ugrać w polityce, zamiast działać na rzecz ludzi.

**Niepokoją cię te decyzje?**

Mam sprzeczne uczucia, bo z jednej strony uważam, że to co robią, robią źle, a z drugiej strony im szybciej to wszystko walnie, jak oni zrobią coś źle, jak to się wszystko szybciej rozwali, to może szybciej się pozbieramy.

**Czy jakieś decyzje zwiększyły twoje poczucie bezpieczeństwa?**

Nie, nie przychodzi mi nic takiego do głowy...To zamknięcie granic było dobre, faktycznie. Miałam nadzieję, że może to się uda zdusić u nas w kraju, żeby nie przynosić z zewnątrz, ale jednak chyba nie.

**Skąd czerpiesz wiedzę?**

Nie oglądam tv, korzystam tylko z internetu. Patrzę na informacje z TVN24. Patrzę też na to, co ludzie piszą na FB, ale tam to jest takie bardzo subiektywne i do końca nie da się tego sprawdzić, ale to takie ciekawostki. No i korzystam z portalu Wykop. Tam sobie ludzie wrzucają różne informacje z różnych źródeł. I też na YT sobie słucham różnych ludzi, ale zazwyczaj anglojęzycznych.

**Co sprawia, że uznajesz jakieś źródło za wiarygodne?**

No właśnie mam z tym coraz większy problem. Z rozróżnianiem co jest prawdą, co nieprawdą.

**Zmienił się czas, który poświęcasz na siedzenie w internecie?**

Nie, jest tak samo. Czasami słucham też audycji radiowych na YT, ale to też się nie zmieniło. Chyba Radio Wnet najbardziej. Serwisów informacyjnych nie słucham i o tym co się dzieje w main streamie wiem z TVN24. Przeglądam nagłówki i czytam, jak mnie coś zainteresuje. Tak, żeby wiedzieć, co się dzieje na świecie.

**Masz jakieś zwyczaje, że coś najpierw otwierasz, czegoś słuchasz?**

Tak i to się nie zmieniło. Zawsze jak wstaję, to przeglądam co się dzieje na świecie, czy ktoś coś dom nie napisał. Najpierw zazwyczaj TVN24 i na Wykop zaglądam co się pisze poza main streamem. Nie pojawiły się u mnie jakieś nowe źródła. Generalnie lubię wiedzieć co się dzieje i to też się nie zmieniło. Posłuchałam sobie rozmów z epidemiologami, bo ja jestem fanką długich form. To było bardzo ciekawe.

**Jakaś prasa?**

Papierowej nie kupuję w ogóle i ja wolę słuchać niż czytać, więc nie.
