# Supplementary material for: The challenges arising from the COVID-19 pandemic and the way people deal with them. A qualitative longitudinal study
Source: PLoS One. 2021 Oct 11;16(10):e0258133. doi: 10.1371/journal.pone.0258133 (PMC8504766; doi:10.1371/journal.pone.0258133)
Supplement: S1 Dataset — (ZIP) [file pone.0258133.s003.zip › Transcriptions/stage 1/5.1_M_39_single.docx]

**5.1_M_39_single**

Mieszkam i urodziłem się w Płocku, pracuję w (miejsce pracy) w (branża) a poza pracą jestem muzykiem, DJ, producentem muzycznym, artystą. W (miejsce pracy) kieruję skomplikowanym procesem technologicznym - wyrabiam ropę naftową. Wolałbym być tylko artystą, ale stety, niestety mam też pracę. W tej chwili raczej stety i to się chyba dobrze składa, że ona jest, bo mam w tej chwili z czego żyć.

**Kiedy dla ciebie zaczęła się sytuacja z koronawirusem?**

Znajomi na początku uznawali mnie za panikarza, a ja od razu wiedziałem, że to się przeniesie do Europy, jak tylko to poszło poza strefę Wuhan. Wiedziałem, że się rozprzestrzeni, ale nie wiedziałem, że ten wirus jest taki agresywny. Wiedziałem, że to jest początek tego wszystkiego jak zaczęło się to rozprzestrzeniać na inne kraje azjatyckie a tak na dobrą sprawę, jak wylądowało to we Włoszech. We Włoszech był taki dzienny przyrost, że coś niesamowitego. Pamiętam to jak dziś - taki mój wpis na FB, bo wszyscy sobie jaja robili o makaronie, który ludzie zaczną wykupywać, itd. Natomiast ja napisałem i pamiętam jak dziś, że było 230 osób zarażonych we Włoszech, w Polsce jeszcze nie było nikogo, a ja napisałem, że czy tego chcemy, czy nie, to ten wirus jest już w Europie i jest już w Polsce, i to jest tylko kwestia czasu, kiedy się ujawni. Co niektórzy uznali mnie za może nie pesymistę...Oni uważali, że więcej jednak ginie ludzi w wypadkach, na raka, na powikłania pogrypowe. Ja odpisałem, że z powodu żadnej grypy ani wypadków nie będzie odwoływane Euro ani igrzyska. I tak będzie właśnie. 24 lutego o tym napisałem.

**Jak się czułeś z tymi przewidywaniami?**

Wiedziałem, że będzie poważnie, bo nawet jeśli te kilkanaście osób jest tak nagłaśniane. Pamiętam, jak była epidemia świńskiej grypy, SARS i jakoś to się w ogóle żadnym echem nie odbiło w moim życiu. Jeśli ludzie panikowali przy kilkunastu osobach to wiedziałem, że to będzie coś poważnego. Nie zbierał się rząd jak ktoś był chory na świńską grypę. Pewnie było tych przypadków tyle samo, co teraz, ale jednak...

**Poważnie, to znaczy jak? Co to dla ciebie oznaczało?**

To było w momencie, kiedy ludzie sobie robili jaja z wykupywania makaronu i chodziło mi o to, że to...To jeszcze nie był u mnie strach, strach się gdzieś po drodze przewijał...Byłem przekonany, że nie powinno się z tego robić jaj, tylko trzeba zastanowić się nad jakimiś środkami ostrożności. Moi przyjaciele mieli wtedy lecieć do Azji. Ja byłem w Azji w zeszłym roku i ja wiem, jakie tam są obostrzenia jak nie było tej choroby, jakie kontrole tam są, więc domyślałem się, co tam jest teraz. Miałem też kontakt z koleżanką, która mieszka w Singapurze i wiedziałem, że życie wygląda tam w miarę normalnie na co dzień, ale lotniska są puste. Nie wiem co czułem do końca, tylko to, co pamiętam.

**Jakieś ważne, przełomowe momenty?**

Na przełomie styczeń/ luty wykupiłem samolot do Dublina, bo mam tam przyjaciela i często tam grywam w klubie. Miałem już wykupiony bilet i taka mi myśl przeszła, że albo teraz albo coś czuję, że to się już nie wydarzy. Polecieliśmy z przyjaciółmi, granie było 29.02, wróciłem 1.03 i na lotnisku już parę osób miało maseczki, trochę widziałem, że obsługa ma jakiś płyn. Trochę zasiany był taki niepokój w Modlinie. Ja też sobie jeszcze wtedy takie podśmiechujki robiłem, że na lotnisku w Dublinie wiadomo, którzy to Polacy, bo tylko Polacy noszą maseczki.

**Kolejne momenty?**

Tydzień później grałem imprezę w Płocku i wisiała na włosku, bo był jakiś 1-szy niepotwierdzony przypadek gdzieś pod Płockiem, ale postanowiliśmy zaryzykować. Ta impreza wypaliła a przypadek okazał się negatywny w dniu imprezy, więc odetchnęliśmy, że nie robimy błędu, bo ludzie mogliby wpaść już w jakąś panikę i zostać w domu. To było 7.03. i potem to już poszło jak domino. Ja 14.03 dostałem temperatury i już 3 tydzień nie mogę wyjść z tej choroby. Czuję się dziwnie. Przez 5 dni miałem temperaturę i do dzisiaj się czuję dziwnie. Cały czas mam jakby nieżyt dróg oddechowych. Po drodze był wewnętrzny niepokój, gdy dopadła mnie temperatura i dostałem jakichś takich...Myślę, że to są sprawy nerwicowe, że takich ataków paniki dostawałem, bo się przestraszyłem, że to może być koronawirus i że zostałem z tym sam. Podjąłem decyzję o samoizolacji i od 13.03 przez 2 tyg. nie wychodziłem nigdzie. Rodzice mi przynosili lekarstwa i jedzenie na wycieraczce. Dla bezpieczeństwa, bo tata jest w 1-szej grupie ryzyka. jest po 2 zawałach, cukrzyca, nadciśnienie. Tfu, tfu, lepiej dmuchać na zimne. 2 wizyty lekarskie miałem przez telefon, dostałem e-recepty, brat mi wykupił. Teraz była 1-sza wizyta taka fizyczna u lekarza. Poszedłem w maseczce + taki komin jeszcze założyłem, oddałem krew, bo przy okazji jeszcze choruję na tarczycę, więc i odporność niska, no i żeby mnie osłuchał ten lekarz. Powiedział dzisiaj, że nic niepokojącego nie słyszy, co oczywiście jest dobrą rzeczą, ale ja nadal czuję się tak jakoś dziwnie. Na pewno przez ten cały okres człowiek się denerwował tą sytuacją. Inaczej by było, gdybym ja był na kwarantannie, ale bym był zdrowy. Jeżeli to nie jest koronawirus, to mój organizm wybrał sobie najgorszy z możliwych terminów. Zgłaszałem do Sanepidu, że byłem w Dublinie, ale uznali, że minęło 13 dni od wylądowania i się nie kwalifikuję do testu. Nie wiem, jak będzie do piątku, bo do piątku mam jeszcze chorobowe. Gdyby zrobienie testu było proste, to ja już dawno bym go sobie zrobił, ale to trzeba się udać samemu na oddział zakaźny. Tydzień temu było tak, że robią wymaz i to idzie do Warszawy i trzeba ileś tam czekać. Nie wiem, jak jest teraz, bo to się z dnia na dzień pogarsza. W Płocku nie ma miejsca, gdzie można zrobić testy. W tej chwili jest ok 30 takich placówek w kraju i najbliższa od Płocka jest Warszawa. Na początku w 24h był wynik, tydzień temu 2-3 dni, teraz nie wiem.

**Skala lęku**

Na tę chwilę to jest 30, bo czuję się już lepiej. Było gorzej, jak czułem się gorzej i wtedy przynajmniej 50 by było na pewno.

Lęk, obawa były wtedy, gdy dostałem temperatury. Bałem się, że jestem chory, wiedziałem, że mam problemy z odpornością i taki natłok myśli przyszedł. Od razu się zastanawiałem, z kim się ostatnio widziałem, dzwoniłem do tych osób, żeby się obserwowały i na razie z nikim nie obściskiwały.

**Czego teraz dotyczą twoje obawy?**

Jest niepokój związany z tym, że ja jestem taki niedoleczony nadal. Myślę, że gdy się poczuję lepiej, to będę lepiej spał, bo bardzo źle sypiam i to na pewno przez nerwy. Odczuwam to w ten sposób, że dostałem skurczów łydek a one są wtedy, kiedy jest brak magnezu. Powodem może być sport, kawa, alkohol i stres. Nie piję kawy od 3 tyg., nie piję alkoholu, nie biegam, więc to jest na 100% stres.

**Czyli jest taka obawa tu i teraz o zdrowie, a są też jakieś inne?**

O pracę się nie obawiam, bo firma, w której pracuję na pewno się nie zamknie. To będzie chyba ostatnia firma, która się zamknie w tym kraju, bo musi być zachowana ciągłość, bo to jest firma strategiczna i państwowa. Natomiast dochodzą do mnie głosy, że możemy zostać na stałe w pracy, bo możliwe, że nas skoszarują. Nikt o tym nie pisze, ale w (miejsce pracy) zostało zakupione 600 łóżek polowych na wypadek, gdyby poszła taka epidemia, że pracowników trzeba by było zatrzymać w pracy. Zostalibyśmy skoszarowani jak wojska rezerwy. Ja podpisywałem papiery, które powodują, że ja będę musiał się stawić w tej pracy. Nie będę wtedy z wyboru wziąć sobie np. urlopu. Nie wiem czy nie będzie to miało miejsca, jak nasz rząd wprowadzi stan wyjątkowy...Tego się też obawiam, bo jak mam to jakoś przetrwać, to już wybieram dom, w którym już jak większość nie wytrzymuję, ale wolę w domu niż w pracy. Co z tego, że nam dadzą łóżka, jedzenie, ciuchy? To będzie jak więzienie. Więzienie o lekkim rygorze. Nie znam do końca kruczków prawnych, kiedy każą nam to zrobić - czy jak będzie stan wyjątkowy, czy może jak w firmie będzie koronawirus i będą chcieli przeprowadzać kwarantannę na terenie firmy...Tego nikt głośno nie powie. My się domyślamy i wiemy, że pod stołem podpisywane są jakieś papiery, możliwe, że i wojsko obstawi firmę. Uważam, że jest to prawdopodobieństwo 50 na 50, że tak się stanie. Jestem pesymistą, jeśli chodzi o rozwój tej epidemii. I realistą, bo to jest jak każda grypa. Ona nie znika, ona jest cały rok, więc ten wirus nie zniknie. Nie ma szans, że on się skończy. Dopóki nie będzie szczepionki a przede wszystkim leku. I kto się przede wszystkim będzie chciał szczepić? To też jest pytanie.

**A ty jak myślisz?**

W tym momencie do zwykłych szczepionek ludzie podchodzą tak, że...Ci tzw. antyszczepionkowcy. Nie mam pojęcia jak to się tutaj rozwinie. Nie wiem, czy sam bym się zaszczepił teraz, bo najpierw bym spojrzał na rozwój wypadków chyba. Co się dzieje z ludźmi, którzy się zaszczepili. Ja jestem jeszcze młody, ale jak zareagują na to starsi. Nikt nie wie jakie są powikłania po tym wirusie i nikt nie wie, jakie będą powikłania po tej szczepionce. Nikt nic nie wie i wszystko to jest po omacku. Czytałem wczoraj wypowiedź doktora, który jest ordynatorem na oddziale zakaźnym - jeśli jesteś zdrowy i silny to przeżyjesz a reszta? Wszyscy jesteśmy zagrożeni. Nie tylko starsi, bo młodzi też. Odporność jest kluczową sprawą w tym momencie i ja się właśnie u siebie trochę tym martwię, bo jest słaba przy mojej niedoczynności tarczycy. Mam nadzieję, że teraz wyjdą mi badania dobrze i będę wiedział coś więcej.

**Jesteś zamknięty w domu od 13.03.Jak sobie z tym radzisz?**

Już byłem gdzieś tam 2 x w lesie, byłem na takich zakupach dużych i raz byłem u rodziców, ale nie zbliżaliśmy się do siebie. Byłem na niedzielnym obiedzie, bez przytulania i w ogóle. Mama na mnie wymogła, że bardzo tęskni. Ja nie chciałem i powiedziałem, że może najlepiej, żebyśmy się zobaczyli dopiero w święta.

**Co najbardziej ci teraz przeszkadza?**

Brak możliwości zrelaksowania się + dochodzi to, że jeszcze trochę czuję się chory. Gdyby nie to, to pewnie dzisiaj też bym był w lesie, bo jest słońce. Myślę, że wiele osób się pod tym podpisze, bo myślę, że wiele osób miałoby ochotę się upić w tym momencie.  Chociaż raz. Tak, żeby człowiek na chwilę przestał o tym myśleć, mógł się pośmiać z przyjaciółmi, posiedzieć, pobawić się. To mi jest potrzebne. Wszyscy wiemy, jak teraz będziemy to doceniać, kiedy to wszystko wróci. Jeżeli to wróci...

**Masz poczucie, że ta sytuacja jakoś cię zmieni?**

Myślę, że wiele osób ta sytuacja zmieni i że konsekwencje tego napięcia na wielu wyryją na wielu osobach duży wpływ plus to, że stracą pracę. Przez brak finansów małżeństwa się porozpadają. Niektóre małżeństwa są fikcyjne a jeszcze jak mają spędzić ze sobą 24 na dobę przez 7 dni w tygodniu, to też idzie tu ku zagładzie. Takie rzeczy nie przetrwają i myślę, że będzie mnóstwo bankructw, samobójstw. Jedynym plusem jest to, że teraz nie wchodzimy w jesień, w zimę, bo to by jeszcze gorzej nas nastrajało. Ludzie będą się izolować w lecie, a największym rarytasem będzie uciec do lasu do domku. W tym roku nie będzie nic lepszego. Dzisiaj już trzeba by było zaklepywać sobie taki domek na odludziu.

**Czy znajdujesz jakieś pozytywne aspekty dla siebie z ostatnich tygodni?**

Takie przemyślenia ma chyba każdy z nas. Chodzi o to, że ludzie się jakoś do siebie teraz zbliżają. Troszczą się o siebie, zadzwonią, zapytają, jak ktoś to znosi. Myślę, że za chwilę będą sobie chcieli jeszcze bardziej pomagać, bo słyszę już, że moi znajomi tracą pracę. Najprostsze przysługi będą teraz na wagę złota. Nasz rząd, nasze państwo nam nie pomoże. To my sami musimy sobie poradzić. Zauważam wzrost życzliwości międzyludzkiej.

**Jeszcze jakieś plusy?**

Niektórzy poświęcają więcej czasu dla rodziny, więcej czasu poświęcają dzieciom.

**A plusy dla ciebie. Czy ty coś takiego znalazłeś?**

Jest takie powiedzenie, że uważaj o co prosisz, bo może się wydarzyć. Oprócz tego, że pracuję na zmiany, co bardzo męczy, to moja pasja muzyczna też powoduje zarywanie nocy w klubach, mnóstwo godzin w samochodzie, w samolotach. To wszystko bardzo mnie męczy i trochę ostatnio moja gwiazda przygasła i wtedy pomyślałem sobie, że w końcu to jest czas, żeby odpocząć, zwolnić trochę, bo w moim wieku zawał to jest koniec. W naszym wieku ludzie nie przeżywają zawałów, więc pomyślałem, żeby zwolnić, żeby zająć się życiem prywatnym bardziej. Nie mam żony, dzieci, chciałbym, ale nie potrafię sobie ułożyć życia prywatnego i może bym w końcu je ułożył? Myślałem, że jak zwolniło to będzie na to czas, ale potem znowu to przyspieszyło, mnóstwo propozycji współpracy i występów...I jak ta sytuacja nastąpiła, to znowu pojawiła się myśl, że w końcu odpocznę. Psychicznie, bo uważam, że zmęczenie psychiczne jest dużo gorsze niż fizyczne. To jest taki chciany/ niechciany plus tej sytuacji.

**Masz poczucie, że odpocząłeś psychicznie?**

Jak to się wszystko zaczynało, to pomyślałem, że to wszystko będzie w dobrą stronę szło, ale zaraz choroba i cały ten misterny plan się zwalił.

**Zdjęcia - emocje**

**11**

To jest strzał w 10, jeśli chodzi o sytuację, w której człowiek w tym momencie jest. Człowiek jest zamknięty i jednocześnie widzi świat, który jest na wyciągnięcie ręki, ale w tym momencie jest niedostępny, bo jest epidemia. Idzie wiosna, więc barwy jakieś kolorowe. Człowiek jest dobrej myśli, że będzie lepiej, bo musi, ale jednocześnie ten deszcz zatrzymuje człowieka...Ale widać to słońce, więc człowiek wie, że w końcu przejdzie ten deszcz. Człowiek wie, że może, ale jeszcze trzeba poczekać.

**Ty właśnie tak się czujesz, że wiesz, że to się musi skończyć tylko trzeba poczekać?**

Tak, to są chyba moje uczucia w tej chwili. To są odczucia i chyba też najbardziej to się odnosi do obecnej sytuacji.

**Przypomnij sobie swoje emocje w ostatnich tygodniach? Jakie były, jak się zmieniały?**

1-szy tydzień to był wysoki pik stresu. Od 13 do 20 marca to był taki tydzień, że bardzo źle to na mnie wszystko wpłynęło i potem zaczynało to tak powoli schodzić. Dzisiaj już jestem taki bardziej...Oczywiście wywoływało we mnie stres pójście do przychodni, ale podobno do niewoli też można się przyzwyczaić. Podobno. Czuję się taki trochę w potrzasku. Nie nazwałbym tego byciem zrezygnowanym. Teraz jest mniej negatywnych emocji niż na początku, ale to nie znaczy, że jest więcej pozytywnych. Raczej to jest taka płaska, może lekko wznosząca się linia, jeśli chodzi o pozytywne emocje. Na pewno takie piki pozytywności to były moje 2 wypady do lasu. mam takie swoje miejsce, że wchodzi się głęboko w las a potem są pagórki, które działają przy chodzeniu jak cardio na serce, więc to bardzo pomogło mi się odprężyć - las sam w sobie, ale jeszcze chodzenie pobudziło krążenie, lekka zadyszka i poczułem, że żyję. I jeszcze była ze mną dziewczyna, więc jej obecność też na mnie dobrze wpłynęła. Zaczynamy się spotykać. Nie wiem co tam będzie z tego, ale została na noc, więc to też na mnie tak dobrze wpłynęło. Serotoniny jakby człowiek dostał. Znajomi nawet następnego dnia powiedzieli, że wreszcie odżyłem trochę. Oni chyba myśleli, że ja jestem jakimś hipochondrykiem albo, że się zamartwiam. Łatwo tak powiedzieć osobie, która jest zamknięta w domu z kimś kogo kocha. Taka osoba nigdy się nie postawi na miejscu osoby, która żyje samotnie, sama w 4 ścianach. Tym bardziej, że nie mogłem się z nikim widzieć przez 2 tyg., bo byłem chory. Oni nie rozumieli tego. Nie dość, że sam, to jeszcze chory zostałem z całym tym problemem.

**Wymyśliłeś jakieś sposoby radzenia sobie z tą trudną sytuacją, w której się znalazłeś?**

Na początku myślałem, że będę robił muzykę, która mi zawsze daje przyjemność, ale byłem bardzo słaby, więc nie mogłem tego robić. Potem, jak wróciła energia to trochę porobiłem, ale z muzyką jest jak z weną. To jest z lodami waniliowymi, które uwielbiam, ale chcę je jeść, jak mam na nie ochotę a nie dlatego, że mam litr lodów w lodówce. Niech sobie leżą. Robienie muzyki mnie relaksuje, bo się odcinam dzięki temu. Włączam sobie też muzykę w tle i układam sobie puzzle. Totalnie jestem wtedy odklejony, gdzieś tam odlatuję. Skupiam się na wynajdowaniu elementów, w ogóle mózg się na chwilę od...Gdzieś tam wąsko idzie. Zawsze to lubiłem i to wpływa tak na mnie, żeby nie myśleć o złych rzeczach, złych emocjach.

**Coś jeszcze ci pomagało?**

Jesteśmy dorośli, więc chyba to jest logiczne, że na pewno seks. Jak byłem odizolowany, to jest to wstydliwe, ale też na pewno ludzkie...Pornografia. Chyba dla większości ludzi w tym momencie wielką rolę grała, tym bardziej, że serwisy pornograficzne zrobiły darmowe premium z okazji koronawirusa. Na większość ludzi, którzy są sami to chyba dobrze wpływa. Przynajmniej na chwilę człowiek się może odciąć.  Ostatnio czytałem artykuł - rozmawiano z ludźmi, którzy jakiś czas spędzili w całkowitej izolacji. Człowiek w stacji badawczej, człowiek w kosmosie...To mi dało do myślenia, żeby narzucić sobie jakiś rygor dnia codziennego. Te dni są teraz podobne. Dzień Świstaka się na to mówi, ale to jest zawsze takie zapełnienie tego dnia, żeby człowiek trochę mniej o tym myślał. Nawet nie, żeby były pozytywne emocje, ale żeby chociaż tych negatywnych było mniej, takie odizolowanie się od tych złych. To może być posprzątania, umycie naczyń, ugotować coś, przewietrzyć mieszkanie, zmienić pościel, uprać ciuchy. Uważam, że ten koronawirus będzie trwał i trwał, więc trzeba przewidywać pewne sprawy. Są różne streamy, więc postanowiłem kupić kamerę i właśnie dzisiaj przyszła. Myślę, że za tydzień zacznę ze swoim kanałem i że to też świetnie na mnie wpłynie.

**A jak sobie radzisz z zakupami?**

Nie robię żadnych niepotrzebnych. Kupiłem kamerę, bo to wpłynie na mnie pozytywnie i to jest zakup długoterminowy a nie zachciewajka na tę chwilę. Kupuję teraz tylko rzeczy 1-szej potrzeby na przetrwanie - środki czystości, jedzenie.

**Był taki dzień, że wszyscy rzucili się do sklepów? A ty?**

Nie wpadłem w coś takiego. Zrobiłem zakupy na ok 3 dni. Obrałem taki system, żeby na większe zakupy wychodzić raz w tygodniu. U mnie nie było reguły z zakupami, bo często jadałem na mieście, trochę u rodziców, często w pracy.

**A jakieś zapasy zrobiłeś?**

Zanim się ten szał zaczął, to wyprzedziłem to wszystko trochę i po prostu kupiłem zgrzewkę wody, zgrzewkę papieru toaletowego, ale nie 5 zgrzewek. Wiedziałem, że te towary będą, tylko będzie brakować tych, które są bardziej niszowe. Pomyślałem, że mogą z półek zniknąć takie rzeczy, bo ludzie raczej będą kupowali to, dzięki czemu mogą przeżyć. Ten papier toaletowy dla ludzi był na samej górze potrzeb i ja nie wiem czemu, nie rozumiem tego do dziś.

A kupiłeś jakieś rzeczy, bo bałeś się, że za chwilę może ich nie będzie?

Ja tak zrobiłem z moim piwem, bo piję tylko jeden rodzaj piwa, które jest rzadko kiedy. Stwierdziłem, że jak zamkną tę fabrykę, bo idzie koronawirus. Wiadomo, że Tyskie, Lech - tego będzie w bród zawsze, bo to są przemysłowe piwa. Ja piję takie kraftowe piwo z Tarnowa, więc wykupiłem wszystkie i tego wszystkie to było chyba 6 sztuk.

**Pamiętasz w którym momencie to zrobiłeś?**

Jak się pojawił 1-szy przypadek koronawirusa w kraju, ale nie od razu, bo wszyscy się wtedy rzucili, więc poczekałem i kupiłem parę dni później. I wiadomo - poszedłem tuż przed zamknięciem hipermarketu, żeby się w ten tłok nie wpieprzać. W ogóle taką technikę obierałem przy tych wszystkich zakupach, żeby iść w te mniej uczęszczane miejsca albo na samo zamknięcie.

**Jakieś jeszcze środki ostrożności zachowujesz?**

Bez maseczki i bez komina już nie wychodzę. Przed chorobą chodziłem w kominie. Rękawiczek nie mam, ale zawsze mam przy sobie płyn dezynfekujący. Omijam ludzi, trzymam bezpieczną odległość, ale to chyba wszyscy taki nawyk mają. Staram się jak najmniej przebywać i chodzić do sklepów. Jeżeli czegoś naprawdę nie potrzebuję, to nie idę i trudno.

**Jak radzą sobie ludzie w twoim otoczeniu z obecną sytuacją?**

To zależy w jakiej kto jest sytuacji psychicznej, fizycznej, materialnej. Moi rodzice to wielka ostrożność. Mój tata jest bardziej taki, że czerwona lampka mu się świeci cały czas, mama jest trochę bardziej wyluzowana, ale jednak są w takiej jakiejś gotowości cały czas.

**A znajomi?**

Ta świadomość wzrastała stopniowo. Najpierw sobie jaja robili, później sceptycznie a teraz bardzo do niektórych to dotarło i podchodzą poważnie. Niektórzy wchodzą do domu i spryskują sobie buty nawet. Większość już chyba traktuje tę sytuację poważnie.

**Od którego momentu? Co się takiego stało, że to nabrało powagi?**

To się chyba rozpędziło, jak pozamykali szkoły.

**Jak myślisz, skąd się wziął koronawirus?**

Jest kilka wersji, a która jest prawdziwa to nie wiadomo. Albo rzeczywiście wypłynął ten wirus, albo USA przyłożyło do tego rękę, albo sobie Chiny same to zrobiły. Ten koronawirus już był wcześniej i to nie jest tak, że on się nagle pojawił...Nie wiem. Nie zastanawiam się kto i przez co, tylko o skutkach człowiek myśli i jak to wszystko w ogóle zatrzymać.

**Można było zapobiec tej epidemii?**

Raz był taki przypadek, że 8000 zachorowało w Chinach i zatrzymali to. Podobno, bo czy to jest prawdziwa historia to nie wiem. Jeśli wtedy się dało zatrzymać, to może i teraz też, tylko wymknął się jakoś. Jest mnóstwo rzeczy, które się wydarzyło na plus dla Chin dzięki temu wirusowi. Gospodarczo będą jeszcze silniejsi. Dokładnie wiedzieli co się wydarzy, bo mieli już podobne przypadki. Byli przygotowani na reakcję łańcuchową i umieli ją dostosować pod siebie. Obcokrajowcy zaczęli uciekać z Chin, akcje firm, które miały swoje siedziby w Chinach spadły po prostu do poziomu bezcen i oni je wszystkie po prostu powykupowali. Wyprosili kapitał zagraniczny ze swojego kraju i wprowadzą tam swój. Początkowo myślałem, że Stany mogą się cieszyć, że Chiny mają problemy. Trump powiedział i przynajmniej jeden to mówi, że na tyle na ile mogli to zatrzymali i jeżeli umrze 100-200 tysięcy w samych Stanach to, że to będzie sukces. Ja uważam, że umrze więcej, że z pół miliona tam umrze co najmniej.

**A jak Polska jest przygotowana?**

W ogóle nie jest i nie była. Jeżeli w szpitalach po kilku dniach nie mają maseczek, to jest to już żenada. Żaden kraj nie jest i nigdy nie będzie przygotowany na taką skalę. To są też wszystko polityczne sprawy i dla mnie zawsze było żenujące szarpanie Jurka Owsiaka za WOŚP i teraz role się trochę odwróciły. On jednak miał jakiś wpływ i znaczenie przez ten czas.

**Czego nie zrobiono?**

Zbyt późny lock down.  To powinno być zrobione zaraz po pierwszych przypadkach. Powinni byli wtedy zrobić chociaż tyle, co było do przedwczoraj. Teraz te 2 osoby na kasę czy coś, to już...Zamknięcie szkół, co ma wylądować niech wyląduje, co ma odlecieć niech odleci, Polacy do kraju, kwarantanna przymusowa. Na pewno w milionach sztuk maseczki i milionach litrów płyn dezynfekujący. Wiem, że jak wrócę do pracy, to dostanę litr płynu i 2 maseczki - dla mnie i dla członka rodziny.

**Te wczorajsze obostrzenia?**

Bezsensowne, bo już dawno powinien być wprowadzony stan wyjątkowy. Jeżeli jest stan wyjątkowy, to koszty epidemii przechodzą na państwo a nie na przedsiębiorców. U nas jest wszystko do góry nogami. Zamykanie lasu? ja byłem w lesie i naprawdę każdy zachowuje dystans. Ci, co mają rozum, to dla ich wszystko może być otwarte, ale tych ludzi z rozumem. Niektórzy są po prostu bezmyślni. Już żadne obostrzenia nie robią na mnie wrażenie. W Irlandii nie mogą się poruszać dalej niż na 2 km. jakby tak było to przeżyję.

**Jak Europa sobie radzi? Jest jakiś przykład godny naśladowania?**

Niektórzy chyba kopiują chyba Koreę czy Japonię. Bardzo restrykcyjnie odszedł Wietnam - nawet dzisiaj sprawdzałem. Tam jest 300 osób zarażonych. Przekraczało się granicę, wojsko, przymusowa kwarantanna, ale nie w domu tylko w namiotach i tyle. W Europie nie wiem i żadne liczby nic nie powiedzą, bo wszystko jest przekłamane. Albo nie są robione testy, albo tu jest taki natłok tych zarażonych. Na pewno większość krajów sobie radzi lepiej od Polski, bo pomagają a nie rzucają betonowe koło ratunkowe swoim obywatelom rządzący. U nas jest gorzej z tym.

**Jakie są twoje źródła informacji?**

Nie mam tv, więc moim źródłem jest internet. Wchodzę raczej na rzetelne strony. Jest taka światowa strona worldmeters.info, która ma teraz zakładkę o koronawirusie. Tam jest cały czas odświeżane, dane są ze wszystkich krajów. Uważam, że jest bardzo rzetelna. Jest też taki dokument w Excelu zrobiony przez polskich naukowców. Na podstawie danych z MSZ robią symulacje zachowanie się wirusa, są prognozowane dane. Świetnie i na bieżąco oni to zmieniają. To, że ktoś to zrobił? Szacunek. To jest polskie źródło i o Polsce.

**Coś jeszcze?**

Słucham wypowiedzi lekarzy. Raczej opinie wyrabiam sobie na podstawie kilku źródeł i wtedy wyrabiam sobie zdanie co jest fake'iem albo naginaniem prawdy.

**Po czym można to poznać?**

Informacja musi mi się sprawdzać w wielu niezależnych źródłach, żebym uznał, że jest prawdziwa.

**Duże portale jak Onet, Interia, WP?**

Raczej jak ktoś na FB coś wrzuci, bo sam nie wchodzę na takie strony. Nie chcę się też już nakręcać, już wystarczy. Trzeba siedzieć w domu i starać się być zdrowym, bo co ja więcej mogę?  Teraz tych rzeczy jest tak dużo i tyle już wiem, że czasem tylko wchodzę gdzieś. Uważam, że nie ma już co za dużo czytać, bo to w niczym nam w głowie już nie pomoże i żebyśmy bardziej się nie denerwowali.

**Wobec tego korzystasz teraz bardziej z rozrywkowych treści?**

Filmy pomagają.

**Sprawdzasz dane codziennie, np. rano?**

Nie sprawdzam nic. W połowie dnia może czasem gdzieś zerknę.

**A radio?**

Nie mam.

Ludzie starsi są naszpikowani wiedzą z tv i z radia. Wpaja się im przekaz władzy.
