# Supplementary material for: The challenges arising from the COVID-19 pandemic and the way people deal with them. A qualitative longitudinal study
Source: PLoS One. 2021 Oct 11;16(10):e0258133. doi: 10.1371/journal.pone.0258133 (PMC8504766; doi:10.1371/journal.pone.0258133)
Supplement: S1 Dataset — (ZIP) [file pone.0258133.s003.zip › Transcriptions/stage 1/11.1_M_35_couple, with child.docx]

**11.1_M_35_couple with child**

**Przedstaw się.**

Mam na imię Oskar i mam 35 lat. Mam spore doświadczenie i prywatnie i służbowo. Interesuję się polityką - lubię wiedzieć, co mnie otacza. Jestem bardziej przedsiębiorcą. Jestem w związku partnerskim z jednym dzieckiem. Teraz mieszkamy na wsi i mamy ten komfort, że możemy wybrać, czy mieszkamy na wsi czy w mieście. Ze swojego domu na wsi mamy 20 metrów do stacji benzynowej, więc mamy wszystko pod ręką. My prowadzimy tą stację benzynową. Ostatnie dwa tygodnie mieszkaliśmy na wsi i nasze informacje ze świata zewnętrznego to były albo plotki znajomych albo to, co przeczytaliśmy na telefonie. Telewizor mamy wyłączony. Jest włączany jedynie przez dzieciaka do bajek. Wczoraj pojechaliśmy do Warszawy sprawdzić, jak mieszkanie i cały dzień miałem włączone kanały informacyjne. Więc dwa różne światy. Tutaj mamy życie naprawdę spokojne, to świat zamknięty na wsi. Oprócz plotek ludzi w sklepie nie ma ogólnego spojrzenia na to, co się dzieje globalnie na świecie i w Polsce. My tu żyjemy naprawdę spokojnie. Staramy się, żeby dziecko nie wychodziło w ogóle, nawet na stację benzynową, to my w trójkę wychodzimy i sobie wieczorami robimy ogniska za domem. Ostatnio malowaliśmy płotek w ogródku. Tylko kwestie na stacji benzynowej, które albo dotyczą tego, co wprowadza rząd albo nasi pracownicy mają lęki, jeśli chodzi o wirusa i sposób wykonywania pracy. Ale dla nas to codzienność i my nie robimy z tego wielkiego *wow*.

**Co się zmieniło w Warszawie przez dwa tygodnie, kiedy ciebie nie było?**

Te narzucone zasady przez rząd są chyba wykonywane. Moje ulubione lokale w okolicy są zamknięte, jest tylko na wynos. Mam część znajomych, prawie wspólników, którzy przebranżowili się, zamknęli lokale i robią na wynos. Są różne zmiany. Ja na przykład pierwszy raz się spotkałem z tym, że u nas były place zabaw pozamykane taśmami. Dobrą chyba praktyką było to, że mamy wszystko otwarte na oścież, żeby nie używać drzwi, klamek. Takie rzeczy, których tutaj nie miałem przez te dwa tygodnie. Teoretycznie mniej ludzi na ulicy, w sensie pojazdów, ale samych pojazdów na parkingach jest sporo. Nawet się zastanawiałem, czy ludzie powyjeżdżali gdzieś na wieś, ale nie, widzę, że ludzie siedzą w domach. Tydzień temu obejrzałem nagranie dronem [Warszawy] i apokaliptycznie to wyglądało. No, ja teraz byłem tylko 24 godziny tam. Ja jestem osobą, która widzi więcej pozytywów niż negatywów, więc ja sobie w ten sposób obserwuję.

**Co sprawiło, że teraz zdecydowaliście się wyprowadzić na wieś?**

W Warszawie jest jakby stagnacja. My w Warszawie bardzo dużo korzystamy z wieczornych wyjść na piwo. Nie siedzimy w domu w Warszawie i jeśli mamy siedzieć w domu, to na wsi naprawdę przyjemniej się siedzi w domu. Mamy tu swoje ogromne podwórze, sadek, w którym możemy robić ogniska, ale też prowadzimy stację benzynową i tu trzeba reagować na te obostrzenia rządu. Trzeba też reagować na klientów, pracowników z zagranicy, którzy wrócili na wieś. To dobrze jest, jak się jest na miejscu. Zazwyczaj było tak, że tylko moja partnerka tu przyjeżdżała i pilnowała. Więc ten czas wykorzystujemy też na modernizację stacji. Jest nam wygodniej. Patrząc przez pryzmat tego, że mamy siedzieć w domu w Warszawie i być zamknięci w 4 ścianach, to są plusy bycia na wsi w tej sytuacji. Może też taka głupia i naiwna myśl, że jest mniejsza populacja, więc mniejsze ryzyko rozprzestrzeniania się tego wirusa.

**Pamiętasz moment, kiedy dla ciebie sytuacja zaczęła się zmieniać?**

Hmm... Moja mama jest bardzo mocno związana ze szkolnictwem w Polsce i dużo wcześniej miałem informacje typu, że szkoły wprowadzają zajęcia wśród uczniów podstaw higieny i już w tym momencie wiedziałem, że te szkoły będą zamykane. Więc już poczułem, że jest grubo, że to nie przelewki. I to był tylko i wyłącznie ten moment. Od tego wszystko później poszło lawinowo i ruszyło z impetu. Jak nagle dziecko, któremu musiałem liczyć sekundy podczas mycia zębów, wraca ze szkoły i samo myje ręce przez 20 sekund, to już jest jakiś postęp. I to jest jeden z plusów tej całej sytuacji.

**A jaki był kolejny ważny moment?**

Nie wiem, co było w kolejności, czy pierwsze obostrzenia rządu wobec restauracji czy to już było... W momencie, kiedy zamknęli szkoły, my z dnia na dzień się spakowaliśmy i przyjechaliśmy na wieś. Więc ja już zupełnie zacząłem funkcjonować w innym świecie. Wydaje mi się, że to były jakieś obostrzenia rządu i te informacje, że pierwsze osoby śmiertelne i z dnia na dzień te informacje, które dostawałem na telefonie z Google, że zwiększa się ta liczba osób. Ja na to bardzo spokojnie patrzę. Nie jestem na tyle osobą zafokusowaną, że każdą informację chłonę i myślę "o jejku, jejku" albo "co będzie jutro". Nie, ja raczej na spokojnie. Wszystko, co się dzieje wokół, że w Stanach zmienia się sytuacja, że w Azji jest tak, a nie inaczej - ja to przyjmuję, natomiast ja jestem osobą, która bardziej zastanawia się nad konsekwencjami gospodarczo-politycznymi niż nad tym, że za dwa dni ta sama historia będzie zaraz w Mazowieckim.

**Powiedziałeś, że odczułeś zamknięcie restauracji. A co jeszcze, z ograniczeń wprowadzonych przez rząd, odczułeś?**

Jeśli chodzi o mnie samego, to chyba mnie to trochę męczy, ale to ze względu na to, że nie mogę wyjść, spotkać się z kimś. Specyfika bycia na wsi... Można posiedzieć weekend, ale jako osoba, która dużo siedzi na komputerze i pracuje przez Internet, nagle nie mam zasięgu na wsi, to jest to dla mnie dyskomfortem trochę. Natomiast idzie się do tego przyzwyczaić. Osobiście to odczułem, natomiast mam takie kwestie osobisto-służbowe. Był taki moment, że na stacji benzynowej trzeba było uspokajać pracowników, bo był moment, że dziewczyny naprawdę były przestraszone samą pracą i co to będzie. Że są narażone, bo na stacji jest przemiał ludzi różnego typu i z różnych stron. Doszło do takiego momentu, że musiałem szukać firm, które zrobią potrzebne rzeczy i okazało się, że firmy, które normalnie chętnie wzięłyby to zlecenie od ręki, zaczęły robić problem z przyjazdem. Przykładowo, zamówiłem tydzień temu pleksy z firmy reklamowej i nie dostałem odpowiedzi, bo nigdzie nie ma. Poza tym, nie mogę ściągnąć chrześniaka do siebie, bo często go do siebie zabieramy i próbowałem namówić rodziców, żeby mi go dali, ale powiedzieli nie. Poza tym, na tę chwilę nie wpływa to na mój budżet. Nawet obroty przez to wielkie bum były dużo większe. Fakt, że sami musieliśmy jeździć po hurtowniach i kupować w różnych miejscach produkty, bo miało brakować, ludzie fiksowali. A, że chcieliśmy wykorzystać to i pewnie wiele firm wykorzystywało taką potrzebę ludzką. I tyle. Prywatnie naprawdę, najbardziej to jest ten relaks i spokój. Bo służbowo, jeśli chodzi o przedsiębiorczość, to na tę chwilę odbieram jakby to była codzienność problemów, które wiążą się z prowadzeniem działalności. Może za miesiąc, jakbyśmy musieli się zamykać czy coś, to będę myślał o dużo większych problemach. Natomiast na tę chwilę ja tego nie widzę. Jestem dość twardym zawodnikiem, jeśli chodzi o narzucanie sobie... Też się łapałem na tym, że ludzie się nie witają. Dla mnie to było takie chore, ale okej. Ale to bardziej nie ze strachu przed tym, że się zarażę, tylko z szacunku do tej drugiej osoby, musiałem sobie nagle przypominać, a no tak, nie witamy się. Dzisiaj musiałem odebrać z hurtowni dwie rzeczy, to moja kobieta mi powiedziała, weź no załóż rękawiczki, jak wychodzisz z tego samochodu. To założyłem rękawiczki.

**Dlaczego je założyłeś?**

Dostosowujemy się do tego, co naprawdę może pomóc. Biorąc pod uwagę, co się dzieje w USA, zero mieli stosunku do tego, co powinni robić, no i są efekty. U nas fajnie to gra, sprawdza się stare powiedzenie, że nasz naród potrafi się połączyć w ciężkich momentach i widać, że w większości to gra. Zakładam, bo powinno być to normą. Bo oprócz mojego "mam to gdzieś" są też inni ludzie dookoła. Tym bardziej ludzie, którzy ze mną żyją. Chodzi głównie o nich, ale też o innych.

**Jak wygląda teraz wasze życie w tej chwili? Co się zmieniło?**

Jeśli chodzi o życie prywatne, to jest największy plus z tego życia prywatnego, to jest... No, mogą być różne konsekwencje tego, bo są różni ludzie i różne rodziny. Niektórzy się mogą w rodzinach pozabijać, jak są zamknięci. Ale ja mam cały czas wiarę w to, że wielu ludzi się zbliży do siebie. To jest dużo czasu spędzania ze sobą. To jest uczenie się... Bo to nie jest, jak normalnym dniu, 3 godziny dziennie, tylko tu już siedzimy ze sobą i spędzamy czas. Spędzać z kim czas 3 godziny dziennie, a 12 godzin dziennie, to już może być trudne, ale uczymy się. To jest bardzo duży plus. Mi jak np. rodzice mówili, łącznie z moją kobietą, że kurczę, ile ci nauczyciele zaczynają zadawać, co się dzieje, mówię: ekstra! Niech oni teraz sami posiedzą tyle godzin, co w szkole, to zobacz, ile wyniosą z samych siebie. Problemem nie jest to, ile nauczyciele zadają, tylko, że teraz rodzice muszą z nimi siedzieć. Wcześniej to było śniadanko rano, ubieranie się, wyjazd do szkoły i basta, nie mam dziecka. A teraz to jednak wymaga poświęcenia. Ale ja uważam, że to jest na plus.

**Myślisz o tym w kategoriach poświęcenia?**

Myślę, że dla niektórych tak.

**A dla ciebie?**

Dla mnie nie, bo ja widzę to na duży plus. Ja rozumiem w taki sposób tą sytuację, że dla mnie to jest fajne. Tym bardziej, że ten efekt, o którym myślałem, jest bliski finału. Dla mnie to nie było poświęcenie, ale znam ludzi osobiście, którzy naprawdę siedzą w korporacji po 12/16 godzin i ich pierwszym, podstawowym elementem życia jest praca i ta praca zdalna to nie jest wszystko, co dla nich znaczy słowo praca i oni naprawdę, pewnie mają teraz ciężko posiedzieć z dziećmi tyle godzin, a może nawet ze swoimi partnerami. Mam świadomość, że to dla niektórych to może być kwestia poświęcenia.

**A jak wy teraz organizujecie sobie dzień?**

Okazało się po tych kilku dniach, że nasze dziecko i odrabianie lekcji zdalnie lepiej mu wychodzi, kiedy ja jestem daleko, bo przy mnie, to w ogóle nie chce mu się uczyć i jest tak rozkojarzony, że robi wszystko, żeby tego nie robić. Więc ja sobie siedzę, coś sprzątam, ogarniam. Wieś ma taki aspekt, że tu jest zawsze co robić. Mogę wyjść, posprzątać, coś pograbić. Każdy ma swoje zajęcie. Czasem idę na stację benzynową coś pomóc, coś zadecydować. Teoretycznie spędzamy ze sobą więcej czasu, ale praktycznie każdy... W naszym przypadku się okazało, że my sobie nawzajem nie przeszkadzamy. To nie jest dla nas takie załamujące i nie widzimy takiej różnicy, bo jeśli ja sobie siedzę na komputerze i coś robię, to tak było. Maro coś sobie robił, oglądał czy się bawił, ja siedziałem przy kompie i pracowałem, a Ewelinka robiła coś innego, dokumenty, itd. Naprawdę, oprócz tego, że nie wychodzimy w ogóle na zewnątrz, to to nie ma tak wielkiego znaczenia. Jak patrzę na innych, to niektórzy mają z tym ogromny kłopot, są zmęczeni tym, można powiedzieć.

**Co robicie wspólnie w tych dniach?**

Powiem ci, że pierwszy raz ten chłopak malował płot. Taką podstawową rzecz. Nigdy w życiu by tego nie robił. A byliśmy w trójkę, kupiliśmy farby, wałki, ubraliśmy się w stare ciuchy i sobie malowaliśmy w trójkę płot - rzecz, która nie byłaby realna, żebyśmy robili wspólnie przynajmniej przez najbliższe kilka lat. Robimy sobie ogniska. Siedzimy do tej 11 przy ognisku i sobie gadamy o różnych rzeczach, nawet głupich. Na pewno jest więcej rozmów, bo to samo z siebie wynika. My ogólnie, mamy telewizory w domach, ale mogłoby ich nie być. Natomiast teraz potrafimy w trójkę położyć się i oglądać coś. Może trudno z doborem filmu, żeby każdy był *happy*, ale wybieramy, że jeden film jest dla tej osoby, drugi dla tej i nie ma spinki. Powiem ci też takie rzeczy... No, może w Warszawie się gdzieś ganialiśmy, ale ostatnio grałem w zbijaka z dzieciakiem. Zaczynamy się odnajdywać w tej sytuacji i robimy coś. Gdyby było cieplej, to jeszcze bardziej byśmy wykorzystywali spędzanie czasu na zewnątrz. Ja nie chcę też, żeby nagle się okazało, że dziecko po miesiącu w domu obejrzy 1000 nowych filmów, bo też nie o to chodzi. Robimy tyle dla nas normalnych rzeczy i może się okazać, że o wielu nie mówię, bo ich nie widzę. Ostatnio się udało, że w trójkę sprzątaliśmy i to też szok, bo u nas jest ciężko ze sprzątaniem przez którąkolwiek osobę.

**Jakich rzeczy wam brakuje?**

Brak możliwości spotykania się z ludźmi. Marek jest jedynakiem, więc my staramy się choćby w weekendy załatwiać mu kogoś do zabawy, żeby nie był sam jako dzieciak, żeby miał wspólne zabawy. Tu jest blokada. Nie możemy pojechać wspólnie do galerii. Zdarzało się, że jechaliśmy tylko po karty Pokemon dla małego do galerii, a teraz tego nie robimy. Ogólnie nasz tryb życia się tak mocno nie zmienił. Mamy blisko rodzinę tutaj i z jednej i z drugiej strony i tą rodzinę ciężko namówić, żeby się spotkać. Czy coś jeszcze mi przeszkadza? Na razie jeszcze nie. Nie mam tak istotnych rzeczy, żeby mój stan emocjonalny negatywnie się zmienił. Już trochę długotrwałe spędzanie czasu na wsi jest męczące, ale w mieście w tej sytuacji też bym tak miał, więc trzeba się dostosować.

**Skala lęku**

30. Tylko ze względu na bliskich, a nie na siebie.

**O co się boisz w kontekście swoich bliskich?**

Boję się o moją Ewelinkę, bo nasz sposób codziennych spraw, jeśli chodzi o prowadzenie działalności, wymaga od nas kontaktu z ludźmi i różnorodnego kontaktu z różnymi ludźmi. Nawet taka prosta rzecz, jak liczenie gotówki, która była w rękach wielu ludzi, to może wprowadzić jakiś lęk. Boję się o bliskich, o własną mamę, która jest w wieku osób 70+, starych palaczy z problemami swojego wieku, mieszkająca niestety z moją siostrą i z jej rodziną, gdzie oni na tę chwilę normalnie pracują w dużych firmach. 10, jeśli boję się o samego siebie - może to po prostu zwyczajny brak odpowiedzialności, a 20 ze względu na bliskich. Albo jestem egoistą, bo myślę o bliskich i o tym, co by było, gdyby coś im się stało *[śmiech].*

**Czy ten lęk się zmieniał w zależności o d rozwoju tej sytuacji?**

Narastało, aczkolwiek widzę, że to jest sinusoida. Był taki moment, kiedy zacząłem widzieć u ludzi lęk i strach, zaczęło mnie to zastanawiać i może mi się przez to trochę podniosło. Natomiast ludzie zaczęli się też przyzwyczajać, jest mniej tego lęku i ja sam w sobie nie mam tak wielkiego. Tydzień temu ten lęk był nieco większy. Ale to też był napływ informacji, zdarzeń.

**Czy to, że nie oglądałeś telewizji, sprawiło, że mniej się zacząłeś bać?**

Myślę, że to jest bardzo duża zależność, bo jak ludzie sobie włączą jakiekolwiek stacje telewizyjne i nakręcanie na korona wirus albo oszukane wybory, to... Jak siedzisz między ludźmi dłuższy czas, to w jakiś sposób wpływa na ciebie - albo tok myślenia albo jako samo istnienie problemu. A my tutaj sucho - albo czytamy informacje z Internetu albo ich nie czytamy. Dostajemy info, 3 wyrzucam, a 1 czytam. Moje informacje o korona wirusie, to jest: czytam rano, dostaję informację, ile zakażonych, ile umarło, po czym idę na stację i słyszę plotki. Więc na pewno, gdybym siedział tak jak moja mama, która non stop mówi, że u niej prawie 24h jest włączona jakaś stacja informacyjna i non sto albo programy, albo informacje wokół wirusa. To jest nakręcanie siebie nawzajem niepotrzebne. Wystarczyłoby tylko uczyć od dzieci po dorosłych - bo okazuje się, że dorośli też nie potrafią myć dobrze rąk i w niektórych miejscach się normalnie zachowywać. My jesteśmy zamknięci i dostosowujemy się do zasad: mycie rąk, częstsze mycie twarzy, z dala od osób, troszkę dalej niż zazwyczaj, niewitanie się z osobami, wprowadzamy na stacji zasady, które nakazuje rząd i dalej żyjemy. Natomiast, jak ja bym słuchał cały czas o tym samym, no to chyba lęk byłby większy niż 30. Ja mogę być złym przykładem, bo widzę, jak ludzie na wsi rozmawiają między sobą, to powiem ci, że te rozmowy też są ciekawe.

**Opowiedz mi o tym, jak oni rozmawiają.**

Oni opowiadają o tym, ile osób umarło, o lekach i sposobach higieny - już słyszałem milion rozwiązań, mają ich chyba więcej niż lekarze. Osoby, które są chore, to już koniec z nimi, bo nie ma tego leku. Są sytuacje, że osoby z zagranicy wchodzą i pytają, kogo mogą pocałować. To jest w ogóle takie skupisko nieświadomości - mówię o różnych aspektach. Fajnie się tego słucha do momentu, aż pracownik nie zaczyna trząść rękami i mówić: "Oskar, boję się bo...". Podam ci przykład. W sklepie na wsi obok facet wszedł do sklepu i zaczął się naśmiewać i kasłać, to dziewczyna potrafiła wpaść w panikę, rozebrała swoje ubranie sklepowe i wyskoczyła ze sklepu w panice. Jest ciekawie.

**Skąd ich zachowanie się bierze?**

Dla nich jest ważniejsze to, co usłyszą między sobą niż to, co usłyszą w telewizji. To, co usłyszą w telewizji każdy przerabia na swój sposób i rozmawiają między sobą. To rozumowanie tego, co usłyszeli w telewizji jest różnorodne, ale i tak najważniejsze dla nich jest to, co powiedziała ich koleżanka lub kolega. Nie mówię o młodzieży, bo młodzież ze wsi, np. przyszedł chłopak młody i powiedział, że dla niego to wszystko pic na wodę i bujda. Ja nie wiem, z czego to się bierze. Nie chce nazywać ludzi z takich środowisk o mniejszym intelekcie, bo to są specyficzni ludzie. Wczoraj się nad tym zastanawiałem, czemu nie ma tak dużo informacji na temat zachorowań, zakażeń w rejonie naszych gór, tej południowej Polski. Gdzie ludzie żyją w swoich środowiskach i sposób leczenia wirusa, to jest odkażajmy się, ale nie w znaczeniu rąk, tylko alkoholu. Mężczyźni i kobiety są z natury twardsi i jest inne postrzeganie tego. Zobaczyłem osobę na stacji benzynowej po dwóch tygodniach w masce, to wszyscy twierdzili, że to szajbus jakiś. Przez telewizor mówią: "nie wychodźmy z domu", a widać te osoby, że wychodzą. Jak mówią, żeby nie wchodziło do sklepu więcej osób niż po 3 do kasy, to przez połowę pierwszego dnia faktycznie ktoś otworzy drzwi, popatrzy, ile jest osób, natomiast w drugiej połowie dnia dzień wraca do normalności. Chodzą nawaleni starsi panowie. Kobiety starsze spotkają się w sklepie, siedzą we 3 koło siebie i gadają o Bóg wie czym. Życie wraca do normy po 6 godzinach *[śmiech].*Chyba, że my faktycznie jesteśmy w takim rejonie, gdzie niedaleko nas, kilkanaście kilometrów, jest kilka osób zarażonych, jedna czy dwie osoby zmarły. Dużo osób wróciło z zagranicy, nic nie robiąc sobie z tego. Ja na szczęście mam kontakt z osobą reprezentatywną na wsi, czyli z sołtysem i się pytałem, jaka jest współpraca między sołtysem a Urzędem Gminy i cieszy mnie to, że ludzie zaczęli mówić o takich osobach. Ja powiedziałem wprost: jeszcze raz mi przyjdą na stację benzynową, to dzwonię na policję i do widzenia. Dla mnie to jest nieistotne, kto to jest, po prostu dzwonię na policję i do widzenia. Przyjedziesz i kupisz rzeczy na dwa tygodnie albo nie przyjedziesz, bo się obrazisz do końca świata i jeden dzień dłużej. Niektórzy zaczynają tego pilnować. Trafiają się osoby na tyle kompetentne, że tłumaczą tym ludziom przed sklepem i proszą o wsparcie służb porządkowych, Straży Pożarnej. Więc nie jest tak, że wrzucamy do jednego wora wieś i tępaki żyją i tak swoim życiem. Absolutnie nie można tak tego określać, ale jest inaczej. Jak zobaczyłem na placu taśmy u nas, to zrobiłem wielkie oczy, że tu jest już taki postęp, a my na wsi jesteśmy kilka dni w plecy. Ja mam też taki obraz, że 500 metrów ode mnie jest altana zrobiona przez gminę i tam były schadzki. Więc nie spodziewałem się, że na placu zabaw [w Warszawie] zobaczę taśmę.

**Pokażę ci teraz obrazki. Które z nich najlepiej oddają twoje emocje?**

5. To jest zbiór informacji. Gdzieś widać skoncentrowane, informacje, wydarzenia, milion dookoła osób. Coś się gdzieś dzieje, nie mamy na to wpływu.

**To jest chaos?**

Obecnie tak. Taki mały chaos, bo nic nie jest zapewnione, co będzie jutro. Może chaos to za mocne. Patrzę ogólnikowo, a patrząc na siebie, to 2.

**2**

Gdzieś mi się przykleiła guma i jestem trochę ubezwłasnowolniony. Coś się ciągnie za mną brzydkiego. Gdzieś mam dyskomfort w stopie.

**Wracając do 5, jak się czujesz z tą niepewnością?**

Na razie chyba wszystko sobie układam w głowie. Ten procent niepewności, który posiadam, to jeszcze jest ok, jeszcze jest w normie. Natomiast ogólnie scharakteryzowałem tę sytuację ogólnie, naszą wieś, Polskę, jak to jest na tę chwilę o tej chorobie. Milion gwiazdeczek i nie wiemy, co jest dalej. W mojej skali jeszcze niewiadoma jest okej.

**Jest tutaj zdjęcie, które pokazuje, jak się czułeś w pierwszym momencie tej sytuacji? Kiedy syn wrócił i mył dłużej ręce.**

1. Ja interesuję się polityką też globalną i relacjami między państwami. Sytuacja ogólna, jaka jest na świecie, ta globalizacja, jeśli chodzi o ilość nas na świecie i to, jak brakuje nam takiego zachowania higieny własnej. Niby udajemy społeczeństwo coraz bardziej świadome, to ta higiena wcale się nie... Patrząc na ludzi w Warszawie, których znam, higiena jest na jednym poziomie. Patrząc na wieś, ta higiena jest spadkowa. My powinniśmy tę higienę coraz bardziej rozwijać, bo jest nas coraz więcej na tym świecie i różnych brudnych syfów. Więc jak ja wreszcie zobaczyłem, że ktoś wpadł na pomysł, żeby zrobić zajęcia dzieciakom, co uważam, że powinno być przynajmniej raz w miesiącu, nawet na ocenę, żeby i wpoić, że to jest naturalne. Pomyślałem sobie: "O, wreszcie mamy efekt globalizacji, że gadamy o higienie". Dużo gadamy o higienie, ale nic z tym nie robimy. Ja cały czas uważam, że albo III wojna światowa albo koronawirus to jest selekcja naturalna, która powinna być. Bo albo uczymy się, że nie zaśmiecamy tego świata, w którym żyjemy i siebie... Innego wyjścia nie będzie. Więc, jak dziecko zaczęło myć ręce, to pomyślałem: wreszcie!

**Czy w kolejnym momencie, kiedy zamknęli szkoły, zacząłeś się przygotowywać do tej sytuacji?**

Nie. Nie musiałem zareagować, bo ludzie, którymi się otaczam, są ludźmi, można powiedzieć, intelektualnymi, więc nasze rozmowy były takie, że razem decydujemy o czymś, nie spotykamy się. Jedyna moja myśl była taka, żeby kupić młodemu nowy tablet, żeby lepiej mu się uczyło.

**A kupiłeś?**

Nie. Jeden tablet leży i to niezły, a on siedzi na starym laptopie. I jeszcze to, co ekstra wynikło z tego wszystkiego, to, że wreszcie w Polsce dzieci, chociaż nie tylko dzieci, ale też nauczyciele, zaczęli uczyć się normalnego istnienia w dzisiejszym świecie. Na mnie to nie wpłynęło o tyle, że nie pomyślałem sobie: "O Kurde! Muszę kupić pół tira papieru toaletowego i 40 litrów wody". Ja się śmiałem, bo jeszcze byłem w Warszawie, w momencie, kiedy ludzie w Makro, w Selgrosach, w galeriach kupowali od groma papieru toaletowego. Słuchajcie, jak będzie faktyczny problem, to nie zabraknie papieru wam, a nawet jak go nie będzie, to najważniejsze, żeby wam nie zabrakło wody. Bo nawet jak nie będzie papieru, to skorzystacie wody i się umyjecie. A papier jest i będzie. Ja jestem zwolennikiem myślenia, że powinniśmy się pozamykać jako miasta, jako centra skupiskowe. Zamykanie lokali, restauracji niekoniecznie może było po kolei, jak odizolowanie się pomiędzy populacjami. Ale mi chodzi też o przedsiębiorstwa. Bo jeśli byśmy szczelnie zamknęli miasta, to te przedsiębiorstwa na rynku by dalej sobie działały.

**Skąd się u ludzi wzięło to, że kupowali więcej?**

Tylko i wyłącznie wpływ człowieka na człowieka. Budowanie lęku pomiędzy sobą. My uwielbiamy nakręcać się... Nie wiem, kto wpadł na pomysł, że już jest koniec świata. Ja tego nie rozumiałem. Na szczęście mam znajomych, którzy też nie rozumieli, ale mam też znajomych bardzo mocno intelektualnych, którzy już myślałem, że pojadą w góry na biwak i będą tam siedzieli z dzieciakami przez najbliższe 2-3 miesiące, bo się będą bali wyjść na powietrze. Nie mam pojęcia, skąd się to bierze. Też byłem w szoku, jak dziecko w technikum/liceum tłumaczyła swojemu ojcu, będąc w Selgrosie, mieli pełen wózek papieru toaletowego i podeszli do mydeł, których nie było już. I ta dziewczyna mówi: "A, pani nam tłumaczyła, że można kupić spirytus i glicerynę i to też jest ok". W 1-8 klasach wprowadzili zajęcia z higieny, a myślę, że powinni uczyć tego od przedszkola do studiów. Ktoś będzie rozsiewał błędne informacje i są one powielane. Gdyby w dwóch największych elektrowniach w Warszawie był koronawirus, odłączają nam prąd, jesteśmy odcięci od świata, jedyne co nam zostanie, to woda, tylko i wyłącznie zimna. Kurczę, ja nie czaję myślenia... Brak logiki w tym, że ludzie myślą, że z dnia na dzień coś będzie zamknięte. To też da fajne uwarunkowania globalne dla przedsiębiorstw. My z perspektywy małego przedsiębiorstwa widzieliśmy wzrost, więc ci globalni to na pewno mega mieli wzrost.

**Jak u was teraz wygląda robienie zakupów?**

Tak samo plus to, że jemy rzeczy robione przez siebie. U nas wieczorem było hasło albo gdzie idziemy zjeść albo skąd zamawiamy. Nikt nie miał czasu i ochoty, żeby spędzać czy w kuchni. Chyba, że jako przyjemność, żeby wspólnie. A tak, to telefon... Do tego stopnia, że w okolicy nas znają i wiedzą, gdzie mają podjechać, jak dzwonimy. Ale wiemy, że lokale teraz dostarczają, a z tych usług nie korzystamy. Ja uważam, że to jest zbyt duża możliwość przeniesienia tych zarazków przez kuriera albo dostawcę. A kupowanie, to my idziemy na stację, bierzemy dla siebie produkty, wiemy, co za 2 dni dojdzie na stację, więc nie wykupujemy się papierami toaletowymi. Nie byliśmy w tej grupie ludzi, którzy.... Fakt faktem, zaczęliśmy się zastanawiać co i jak, bo jak jednego dnia ci ucieka kilka rodzajów konserw ze sklepu, to pomyśleliśmy: Boże, zaczyna się bum. Zadzwoniłem do znajomych, którzy mają restaurację, pytać, jak oni się zatowarowują, na co oni, że na razie spoko. Teraz chłopak, który zamknął i daje tylko na wynos, mówił, że jeszcze nie miał takiego obrotu. Jest ten towar, nie brakuje go. Ludzie patrzą na drugich ludzi i widzą sąsiadkę obładowaną zakupami, myślą, że pójdą, bo zaraz zabraknie. Ja nie słyszałem, żeby ktoś z rządu powiedział - to też może być gospodarczo celowe, słuchajcie, koniecznie wykupujcie rzeczy, bo tego nie będzie. To był wymysł międzyludzki.

**Skąd się biorą ci pierwsi ludzie, którzy zaczynają robić zapasy?**

Niestety, ale jestem też zwolennikiem teorii spiskowych. Może nie zwolennikiem, ale wiele teorii spiskowych przeanalizowałem i jestem bliższy powiedzeniu, że to był spisek niż że nie.

**Opowiedz mi o tym.**

Nasz rząd nie wspominał absolutnie o takich rzeczach, może nie chciał wprowadzać paniki. Ja, jakbym był producentem papieru toaletowego albo producentem żywności z długą datą ważności, to sam bym to nakręcił. To czysty biznes. Tak, jak Chińczycy pod Warszawą skończyli szyć ciuchy z metką Adidas, tak teraz szyją maseczki. Gdzieś tam każdy widzi biznes. Tu się nakłada bardzo dużo czynników i grup społecznych. Są grupy społeczne interesów i widzą w tym interes. Są grupy zwykłych ludzi, którzy się boją i ich obecny lęk wynosi 95. To normalni ludzie, wykonujący swoje obowiązki, ale są bardziej wrażliwi na zewnętrzne historie. Nie chciałbym powiedzieć, że to stricte przedsiębiorcy. Ciężko to określić. Myślę, że kilka grup musi w tym brać udział różnego pochodzenia, żeby to miało tak szybki efekt. Nie chcę precyzować, że to grupy biznesowe ani że to brak świadomości ludzi. Na pewno przesada pustych półek to jest nie tylko świadomość ludzka. Nasz kraj ma rezerw żywieniowych na półtora roku - oficjalnie. Nieoficjalnie, myślę, że jakbyśmy przez 4 miesiące nie produkowali, to byśmy przeżyli. Nie wiem, gdzie jest źródło tego nakręcenia społecznego. W dzisiejszym świecie ciężko to sprecyzować, bo jesteśmy w dobie Internetu, więc ciężko sprecyzować zarodek. Może być bardzo dużo źródeł jednoczesnych, bo każdy ma swoje argumenty za.

**Co wiesz o koronawirusie, o jego pochodzeniu i rozprzestrzenianiu?**

Ja mam taką wiedzę, że podobno znaleźli osobę w Azji, która wypuściła takie coś. Z drugiej strony, czy ja się mam zastanawiać, czy to przez to, że mysz kopulowała ze szczurem albo z wężem i powstał wirus. Żyjemy na takim świecie, tak go zabrudziliśmy, że takie wirusy, to mogą codziennie powstawać i codziennie mutować. To będzie i od tego nie uciekniemy, to jest naturalne. Zastanawiam się, w którym momencie znajdzie się ten złoty środek i firma farmaceutyczna będzie go przez 2 miesiące sprzedawała w kolosalnych pieniądzach, a później sprzeda licencje i wszystkie inne farmaceutyczne firmy będą to sprzedawać. Bo to też będzie odpowiedź na to, jak to było naturalnie wytworzone, a jak celowo. Nie wiem. Bardzo fajnie, że ludzie zaczęli się nad tym zastanawiać, nad higieną, nad zagrożeniami. Nie będę się zastanawiał nad tym, co było źródłem, bo uważam, że codziennie przy dzisiejszym trybie życia, może być inny zarodek innej bakterii i byłoby to sfokusowanie się na szukanie winnych. Dziwne jest to, że nagle ten wirus w przeciągu kilku dni pojawia się globalnie, czyli staje się pandemiczny. To dziwne, że ani go wcześniej nie było, ani go nie stwierdzano i nagle się rozrasta. Rozumiem, że jest inny sposób podróżowania po świecie, jest to szybsze i ten efekt może być szybki. Natomiast my żyjemy w takim świecie i musimy się do tego przyzwyczaić. Albo się będziemy pilnować normalnie na co dzień i takie coś się nie będzie rozwijało. Mamy prosty przykład, że po dwóch tygodniach od stwierdzenia tego wirusa u nas, my mamy taki stan zachorowań, bo wprowadziliśmy szybko obostrzenia, natomiast w Hiszpanii jeszcze w tamtym tygodniu przeganiali ludzi z imprez. Problem jest w nas samych.

**Myślisz, że dało się zapobiec pandemii?**

Myślę, że nie, natomiast na pewno można by było zmniejszyć skalę, gdyby nasza higiena osobista była mocniejsza na co dzień. Bo *de facto*, to mycie rąk powinno być za każdym razem, kiedy wracamy do domu, kiedy dotykamy obcej rzeczy. Może wtedy ta skala byłaby mniejsza. Ale rozprzestrzeniać się będzie. Przykład - na lotnisku więcej bakterii jest w pojemnikach, do których wkładamy rzeczy do prześwietlenia niż w toaletach. No jaki my mamy wpływ na to? To inaczej, teraz włodarze wszystkich krajów powinni myśleć nad takimi zabezpieczeniami międzykontynentalnymi. Teraz jestem na etapie tego, że wyrzucamy koszyki w naszych sklepach, bo to nosicielstwo zarazków. Pracownicy dezynfekowali koszyki i to było dwa tygodnie temu. Reagowaliśmy sami, nie czekaliśmy, aż coś powie rząd. Staramy się podchodzić do tego logicznie. W Leroy Merlin, jakiś mądry dyrektor jeszcze przed obostrzeniami, wprowadził zasadę, że każdy wózek po użyciu przez klienta, przechodził dezynfekcję. Super sprawa. Gdyby tak było robione naturalnie, że stawiamy takie i takie wymogi przy sklepach, przy lotniskach, to już byłoby zmniejszenie tego. Kiedy tramwaj czy autobus w Warszawie był odkażany. Niech mi pokażą czy w tym, w tamtym roku był jakiś autobus odkażany. Więc może faktycznie było potrzeba takich rzeczy, żeby zaszła jakaś reakcja. Autobus powinien być optymalnie raz na miesiąc odkażony ze wszystkiego. Żyjemy w aglomeracjach różnych ludzi i naturalnie społeczeństwo się wymienia nie tylko już miastami, krajami, tylko też kontynentami. Więc gdzie to było wcześniej? Teraz się obudzili? Fajnie, musiało coś takiego tąpnąć, żeby ludzie się zastanawiali. Może taksówki, kina, ubery, też powinny robić takie rzeczy. Nie tylko umycie toalet, ale dezynfekcja miejsc, w których spędzamy czas. Kiedy jakieś biuro było zamknięte w Warszawie na Mordorze i było odkażane biuro. U nas na osiedlu raz w miesiącu jest dezynfekcja garażu i my do tego jesteśmy przyzwyczajeni.

**Które kraje mają dobry system radzenia sobie w tej sytuacji?**

Jak zobaczyłem, jak w Azji wjeżdzają wozami wojskowymi strzelają prosto w ludzi odkażaniem, to bym się nie zgodził, natomiast bardzo mi się podobało, że są kraje, w których na ulicy stoją jednostki i przechodnie podchodzą w kolejce do osób i są pryskane torebki, komórki i myte są ręce. I to jest naturalne i osoba dalej idzie. Chce - podejdę do kolejki, a nie chcę, to ominę. Są kraje, w których łatwo jest dać obostrzenia, bo dzieci dawno miały naukę zdalną. Ja się cieszę, że my też zaczynamy się uczyć takich rzeczy. Latem w Polsce są parawany z wodą. Niech zrobią takie parawany w miastach ze środkiem odkażającym. Są kraje, które robią ciekawe rzeczy, ale to się wiąże z chrzanionymi finansami.

**Co sądzisz o obostrzeniach rządu? Np. o tym, że dzieci poniżej 18 r.ż. nie mogą być na ulicy bez opiekunów?**

Nie mogli sobie dać rady. Ja sam widziałem, że dzieciaki się spotykają. To jest wymuszenie na rodzicach odpowiedzialności. No kurde, w dobie Internetu chyba nie ma dla nich problemu pogadać na Hangoucie. A te dzieciaki się przeganiało. U nas na wsi pani sołtys poprosiła, żeby policja jeździła i kontrolowała, czy chodzą dzieci. Super, bo te dzieciaki mogą być zdrowe, a mogą przenosić. Bo są to zdrowe organizmy. Dla mnie wszystkie obostrzenia oprócz wsparcia dla przedsiębiorstw są dobre. Niestety dzieciaki w pewnych względach nie mają odpowiedzialności i powinno się to zrzucić na rodziców. Skoro narzucili nam, że ja nie mogę iść na koncert, to dlaczego ci młodzi się spotykają w grupkach kilkuosobowych. Jak usłyszałem w radiu, że facet się przebrał za żonę, żeby udawać ją przed policją, która sprawdzała, czy ona jest na kwarantannie, to jest cyrk. Polska jest takim narodem, który genetycznie jest nauczony kombinować. Natomiast powinni wprowadzić to, że nie można wchodzić do sklepu. Markety powinny być zamknięte, niech dadzą się teraz nakręcić małym sklepikarzom. Te globalne firmy i tak mają pieniądze na to, żeby stagnacja była przez kilka miesięcy. Dam ci przykład - byłem na stacji benzynowej w Warszawie i pracownica powiedziała do klienta, który podszedł bez rękawiczek, że na tej stacji nie obsługują ludzi bez rękawiczek. Facet nic nie powiedział, ale gdyby mówił coś negatywnego, to sam bym mu zwrócił uwagę i powiedział: "Facet, idź doczytaj, co się dzieje i ustosunkował się do większości". Fajnie, że ci ludzie pracujący pilnują siebie.

**Skąd czerpiesz informacje o tym, co się dzieje?**

Nie włączam telewizora. Jedynym moim źródłem jest Google. Korzystam tylko z powiadomień, które przychodzą. Włączyłem też powiadomienia RCB, z których korzystałem w górach i one przychodzą. To są najbardziej statystycznie, ale nie gospodarczo i politycznie o tym, co się dzieje na świecie wokół mnie, jeśli chodzi o zagrożenia. Nie wchodzę na jakieś *strcite* telewizyjne albo prowadzone przez jakąś korporację serwisy, bo nie są one rzetelne, są ukierunkowane i należy pamiętać, że dużo statystyk, które dostajemy nie do końca są pełne. Ludzi umiera więcej, ale nie stwierdzają koronawirusa, nie mają czas ich badać. Zarażonych jest jeszcze więcej. Szkoda im wydawać pieniędzy na badania osób nieżyjących.

**Komu szkoda?**

Mam takie informacje od ordynatorów, że jeśli ktoś nie przyjechał ze wskazaniami, że może być chory, a był leczony na przewlekłą chorobę, to oni nie badają tej osoby na koronawirusa i trzeba wiedzieć, że te statystyki nie są pełne. Szkoda zarządowi danego szpitala przeznaczać na to pieniądze. Oni mają dużo ważniejszych rzeczy. Badania nie są pełne, trzeba mieć taką świadomość. Też nie może być tak, że w niektórych województwach nikt nie jest zarażony. Nie mamy rzetelnych badań.

**Gdzie jeszcze widzisz powody, dla których nie mamy rzetelnych badań?**

Może nas informują, ale ja nie oglądam ministra zdrowia, czy UE ma jakiś wspólny system badań. Czy przyjęliśmy system wewnętrzny i badamy na swój sposób. Powinien być ogólnoświatowy i w kolejnych krajach automatycznie wdrażany. Podejrzewam, że te systemy się różnią. Inna jest służba zdrowia w Polsce, a inna chociażby we Włoszech i ja nie twierdzę, że w Polsce jest gorzej niż tam. Bo tam losują, kto ma przyjść do szpitala na dzień dzisiejszy. To jest okrutne, żeby ktoś nie był przyjęty do szpitala.

**Dlaczego we Włoszech jest taka zła sytuacja?**

Myślę, że brak odpowiedzialności ludzi i ze względu na różnorodność społeczeństwa. Brak odpowiedzialności jednostkowej. Tam jeszcze chcieli robić mecze piłkarskie, bo nie wyobrażali sobie życia bez tego. Rząd nie był na to przygotowany, bo pierwsza fala uderzeniowa na Europę. My mieliśmy to szczęście, że mogliśmy się na kimś wzorować i patrzeć na błędy innych. Teraz się dziwią, że w Chicago jest taka sytuacja. My jednak szybko się dostosowujemy. Tutaj naprawdę, czapki z głów dla ogółu, bo my się szybko dostosowujemy.

**Skąd wiesz, że to, co wyskakuje na Google, jest wiarygodne?**

Ja nie mówię, że jest. Jak zbiorę z 6 różnych źródeł informacje, to sobie je uśredniam. Nie przywiązuję do nich jakiejś ogromnej... Statystycznie na to patrzę. Mam świadomość, nie przywiązuję do nich wielkiej uwagi. Nikt mi nie powiedział, jaki jest klucz, czy my mamy wspólny klucz z Hiszpanią i z Włochami. Nikt nam tego nie mówi. Każdy kraj radzi sobie teraz sam. Może nasz klucz jest błędny, jest dużo więcej chorych i żyjemy w nieświadomości. Albo nasz klucz jest dużo lepszy i ci wszyscy, to są chorzy na grypę zmutowaną. Nie mam zielonego pojęcia. To wszystko to jest taka jedna wielka mgła. Wiemy jedno, jest jakiś wirus, wirus, który potrafi zabić, który szybko się mutuje i tak naprawdę tyle wiemy. I nie wiemy, czy powstał z X miejsca, czy jego przyczyną było XY, czy jego przyczyną jest naturalny albo nienaturalny czas. Nie wiem. Nie ma co się nad tym zastanawiać. Nie mamy na to wpływu i co możemy robić, to tak naprawdę siebie i swoich bliskich uczulać i nie skamlać, że źle, że coś robią, bo na razie jest fajnie, że robią obostrzenia, bo na razie efekt rozrastania się tego dziadostwa jest mniejszy. Więc nie jest źle. Jeśli chodzi o przedsiębiorczość, to uważam, ze nie powinno być żadnej pomocy dla przedsiębiorców. Zauważ, że ja patrzę z perspektywy, że moja kobieta i ja jesteśmy przedsiębiorcami i teoretycznie powinno być w to mi graj, natomiast uważam, że to jest błąd. Te pieniądze powinny być wykorzystywane na służbę zdrowia, na takie rzeczy jak dezynfekcja. Na rozwój naturalnych zachowań higieny. W żadnym wypadku to nie powinno iść na przedsiębiorców i mówię to z pełną świadomością. Polska nie powinna ratować przedsiębiorstw, bo nie powinniśmy się na tym skupiać. Natomiast wszystkie obostrzenia związane z ludźmi jak najbardziej. Nawet ja bym wprowadził więcej.

**A gdzie byś szukał informacji, gdybyś chciał wiedzieć więcej?**

Patrząc na Polskę, szukałbym informacji u źródeł. My mamy informacje wcześniej od osób pracujących w sanepidzie, od ordynatora szpitala. To, co słyszymy w telewizji nie do końca jest stanem faktycznym. Czyli u takich źrodeł. Globalnie, to tylko i wyłącznie statystyki. W jednym kraju informacje napierniczają na drugi kraj, a w swoim kraju starają się to [przedstawić], że jest w miarę ok. Każda informacja jest nie do końca rzetelna i jeśli naprawdę więcej chcemy wiedzieć, to trzeba to wyśrodkować. Instytucje rządowe podają statystyki na stronach swoich i porównywać to można z tymi stronami, które podają informacje i to uśrednić. I tylko w ten sposób. Bo jeśli faktycznie interesuje mnie, jak jest sytuacja na świecie, to po prostu uśredniać. A skoro mamy trochę więcej czasu posiedzieć w domu i w Internecie, to możemy to uśredniać. Nie musimy na pewno słuchać kolegi, bo jest moim kolegą od lat i on usłyszał od swojego kolegi. To powoduje wypaczenie błędów i tej sytuacji. Są naprawdę urzędy kryzysowe. W Stanach to działa dobrze, ale u nich to działa od kilkudziesięciu lat i się z tego rozliczają. I one są w miarę rzetelne. Natomiast jakbym popatrzył na polską instytucję od spraw kryzysowych, to bym się zastanowił, na ile te statystyki są sprawdzone. Nikt nie jest w stanie tego powiedzieć, ale na razie to my wszyscy żyjemy w jakiejś chmurze. I czy to jestem ja czy ty, to rząd jest tak samo w chmurze. Tak naprawdę nikt nic jeszcze nie wie. Po pierwsze nie mamy na to wpływu. A po drugie jedyne, co możemy zrobić, to dbać bardziej o siebie i naszych bliskich.

**Chcesz coś jeszcze dodać?**

Musiało takie coś być, musiało powstać. Ja uważam, że ogólnie jest dużo więcej plusów, a to było naturalne, że to się wydarzy, tylko kwestia czasu.
