# Supplementary material for: The challenges arising from the COVID-19 pandemic and the way people deal with them. A qualitative longitudinal study
Source: PLoS One. 2021 Oct 11;16(10):e0258133. doi: 10.1371/journal.pone.0258133 (PMC8504766; doi:10.1371/journal.pone.0258133)
Supplement: S1 Dataset — (ZIP) [file pone.0258133.s003.zip › Transcriptions/stage 1/14.1_M_55_couple, with children.docx]

**14.1_M_55_couple with children**

Mieszkam w domu z żoną i 2 dorosłych dzieci. Razem z żoną prowadzę własną firmę i w tej firmie zajmuję się głównie prowadzeniem takich spraw administracyjnych - księgowość płace, itd.

**Od kiedy dla ciebie zaczęła się sytuacja koronawirusowa?**

Ona się zaczęła w zasadzie koniec stycznia, początek lutego, ponieważ planowaliśmy wyjazd do Afryki i już zaczęliśmy lekko odczuwać takie napięcie z tym związane. Czuliśmy, że mogą być pewne ograniczenia w podróżowaniu, ale jeszcze wtedy nie zostały wprowadzone. Odczuliśmy to fizycznie jak wlatywaliśmy do Namibii, że wszystkim mierzono temperaturę, trzeba było jakieś kwestionariusze wypełniać. To był pierwszy taki moment. Poza tym zaczęły się już pojawiać różnego rodzaju ostrzeżenia, napisy, ale to były takie w zasadzie takie bardziej sygnały niż jakiekolwiek problemy. Cała podróż była bez problemu. Powrót do Polski był 23 lutego, czyli też jeszcze nie było żadnych ograniczeń, wszystko było w porządku. Później jeszcze też chodziliśmy do jakichś restauracji i w zasadzie było wszystko w porządku. Od 6 do 8 marca jeszcze polecieliśmy do Londynu, a to było tydzień przed wprowadzeniem tych wszystkich ograniczeń. W Londynie zauważyliśmy, że było już dużo mniej ludzi na ulicach. W restauracjach, do których wcześniej zwykle nie dawało się wejść gdzieś w China Town nie było żadnego problemu, żeby wejść z ulicy. Stoliki? Proszę bardzo, wolne. Od razu można było zauważyć, że nie ma żadnych turystów z Chin, a ich w Londynie było bardzo dużo zawsze i oni robili część tłumu. W zasadzie, oprócz tego nie było żadnego problemu. Kilka dni później - my prowadzimy szkołę i 2 przedszkola, dowiedzieliśmy się, że musimy wszystko pozamykać.

**Momenty, które dla były dla ciebie przełomowymi, znaczącymi?**

To właśnie była decyzja o zamknięciu szkoły i przedszkoli, bo to zaczęło nas bardzo, siłą rzeczy absorbować zawodowo. Jeżeli chodzi o takie ograniczenie, że restauracje są zamknięte czy sklepy pozaspożywcze są zamknięte, to my specjalnie i tak nigdy nie byliśmy zakupowiczami i chodzenie do galerii handlowych specjalnie nas nie absorbowało. Chodziliśmy raczej z konieczności. Mieszkamy poza centrum Warszawy, mamy obok duży las, więc ograniczenia w wychodzeniu, czerpaniu świeżego powietrza osobiście nas za bardzo nie dotyczą. Nawet teraz, bo do tego lasu możemy i tak swobodnie wyjść.

**Co zmieniło się teraz w twoim życiu?**

Rzeczywiście dużo więcej siedzę w domu, nie spotykam się z ludźmi, nie spotykam się z rodziną. Poza tym bardzo często gdzieś się wyjeżdżało na weekend i siłą rzeczy tych wyjazdów nie ma. Poza tym od czasu ogłoszenia tych ograniczeń już miałem zaplanowane 2 wyjazdy - służbowy do Rosji i prywatny do Izraela. Siłą rzeczy te wyjazdy nie doszły do skutku, więc jednak dotyczy nas to.

**A jeśli chodzi o takie codzienne życie?**

Ja i tak dużo czasu spędzałem w domu, bo pracuję w domu, więc tutaj u mnie nie ma tak naprawdę żadnej różnicy. To nie jest tak, że chodziłem do pracy a teraz mam home office, bo ja zawsze miałem home office. To, że dzieci są w domu cały czas to też jest różnica, bo syn chodził do pracy. Pracował na lotnisku i teraz siłą rzeczy nie pracuje i siedzi w domu. On pracuje w firmie, która się zajmuje obsługą pasażerską na lotnisku, a skoro nic nie lata, więc nie ma kogo obsługiwać. Oficjalnie jest na urlopie, bo nawet nie ma jak zdalnie pracować. Siedzi i nic nie robi.

**Jak to znosi?**

Nie wiem, trudno powiedzieć. On ma komputer, gra po prostu non stop w jakieś tam gry komputerowe, czyli wykonuje to, co zalecają władze - siedzi w domu. Wcześniej wychodziłby na leniucha a teraz wychodzi na odpowiedzialnego obywatela.

**Czy coś ci szczególnie teraz przeszkadza?**

Tak, przeszkadza mi brak swobody podróżowania. I to bardzo. Uważam, że jest to dla mnie osobiście bardzo duże ograniczenie, ponieważ dla mnie swoboda podróżowania i swoboda przemieszczania się jest istotną rzeczą. To, że nie mogę sobie wyjechać gdzieś za granicę czy nawet po Polsce sobie za bardzo pojeździć nie mogę dla mnie jest poważnym problemem. Czuję się ograniczony w swoich prawach, jakiś taki zamknięty. Boję się, żeby te ograniczenia, żeby ta sytuacja z wirusem nie była pretekstem do pozbawienia nas możliwości podróżowania również w przyszłości. Boję się, że granice już zostaną zamknięte. Na zawsze.

**Kiedy taka obawa się u ciebie pojawiła?**

Od momentu, kiedy zostały zamknięte granice. Po części wiedząc, jakie zapędy ma władza w Polsce, a po drugie to nie chodzi tylko o zapędy naszej władzy, ponieważ podobnego typu tendencje występują również w innych krajach. I obawiam się, że skutki polityczne tego będą takie, że się kraje po prostu zaczną zamykać i zaczną się w Europie rządy bardzo autorytarne. To już widzimy na Węgrzech.

**A masz też jakieś obawy dotyczące teraźniejszości?**

Moje obawy są głównie skierowane w przyszłość. Gdyby się okazało, że za te 4 miesiące liczba zachorowań zacznie spadać, została znaleziona jakaś tam...Czy społeczeństwo nabierze jakiejś odporności, bo w pewnym momencie pewnie wszyscy to odchorują tak czy inaczej i nabiorą jakiejś odporności...Gdyby się okazało, że te wszystkie ograniczenia będą znoszone, łącznie ze swobodą podróżowania, to super, ale boję się, ze tak nie będzie, ze rządzący będą tę atmosferę strachu próbować podgrzewać i mówić, że na wszelki wypadek my wprowadzimy te ograniczenia, bo nigdy nic nie wiadomo i to jest wszystko dla waszego dobra i dla waszego bezpieczeństwa. A większość ludzi im w to uwierzy. Zrobi się getta z każdego kraju, Ja jestem w tym wieku, że ja wiem, jak te ograniczenia wyglądały w praktyce kiedyś i uważam, że ta swoboda, to co było do tej chwili, to jest ogromne osiągnięcie, do którego nasz kraj doszedł i uważam, że byłoby tragedią, gdyby ta swoboda zniknęła.

**A masz jakieś obawy, lęki związane bezpośrednio z koronawirusem?**

Nie, nie mam żadnych. Naprawdę nie mam. Ja się nie boję tego, że zachoruję. Czasami myślę, że nie byłoby fajnie, gdyby człowiek zachorował, ale to nie jest strach, który mnie paraliżuje w jakiś sposób. Uważam, że te ograniczenia, które sobie narzucam... Staram się unikać kontaktu, nie jeżdżę, nie odwiedzam ludzi, staram się unikać wyjść, gdzie byliby inni ludzie - nawet do sklepu chodzę przed 22-gą, kiedy już tam naprawdę nie ma ludzi i jest pusto. Uważam, że te ograniczenia, które sam sobie wprowadziłem są dla mnie w miarę bezpieczne. To samo robi moja rodzina i staramy się izolować. Uważam, że przez to trzeba przejść i nie czuję jakichś strasznych lęków przed tym, co będzie. Boję się też o biznes. O ile my jako szkoła i przedszkola nie jesteśmy tymi, którzy stracili wszystkie dochody, ponieważ w dalszym ciągu szkoły częściowo dostają dotacje budżetowe, więc my te dotacje mamy dostawać cały czas. Spadły nam przychody w jednym z przedszkoli, ponieważ skoro nie świadczymy usługi to teoretycznie rodzice dzieci mogliby w ogóle nam nie płacić. Zaproponowaliśmy im podpisanie porozumienia z czesnym obniżonym o połowę, ale to i tak jest obniżenie o połowę naszych dochodów. Jeżeli chodzi o szkołę, to nie ma żadnego powodu do obniżania czesnego, ponieważ szkoła prowadzi zajęcia online, ale już dostajemy sygnały, że niektórzy już próbują nie płacić. A niektórych może być nawet wkrótce nie stać, żeby wysyłać dzieci do niepublicznej szkoły, bo zostaną pozbawieni źródeł dochodu. Ogromne grupy ludzi samozatrudnionych czy specjalistów jakichś. Ludzie mają np. zakład kosmetyczny i wysyłają dzieci do naszej szkoły. I w tej chwili przychody zero, więc oni też muszą myśleć co najpierw zrobią. Już mamy kilka wypowiedzeń umowy z końcem roku szkolnego. Boimy się o spadek dochodów, bardzo możliwe, że jako spółka będziemy mieli w tym roku stratę, ale jakąś tam poduszkę finansową mamy na przetrzymanie jakiegoś okresu, jakichś kredytów nie mamy, itd. Nie mamy takiej obawy, że zaraz zbankrutujemy i nie będziemy mieli co jeść, bo tak nie będzie. Jeszcze mamy pewne obawy, że jeśli wpływ tego kryzysu na gospodarkę się przedłuży, to nie jestem pewien czy rządzący nie zaczną wprowadzać jakichś drastyczniejszych kroków. Czy nie zaczną np. konfiskować lokat bankowych, czy części lokat bankowych. Takie przypadki już mieliśmy w UE - w 2013 r na Cyprze wprowadzono jednorazowy podatek od lokat w wysokości 47%. Nie jest powiedziane, że coś takiego nie nastąpi w Polsce i w innych krajach europejskich.

**Skala lęku.**

Taki zgeneralizowany lęk czy niepokój to tak na 50%.

Bardziej się boję o przyszłe konsekwencje gospodarcze i finansowe obecnej sytuacji i kryzysu. Zarówno na gospodarkę kraju, jako całości - wiadomo, że im lepsza sytuacja kraju, tym chętniej ludzie wysyłają dzieci do prywatnych szkół, bo mają więcej pieniędzy i również taka sytuacja, co będzie ogólnie z gospodarką. Czy czasem nasz rząd nie będzie się musiał uciec do drastycznych kroków, takich o których mówiłem, czyli np. konfiskowanie oszczędności. Jeśli chodzi o te obawy, to na tej skali zaznaczyłbym 80%.

Jest jeszcze ta obawa przed ograniczeniem naszych swobód w przyszłości i to jest jakieś 60-70%. Te moje inne, prywatne obawy o swoje własne zdrowie i swój własny dobrostan to jest jakieś 20-30%.

**Czy widzisz jakieś pozytywne strony, jakieś korzyści, które mogą płynąć z obecnej sytuacji?**

Żadnych, dla mnie nie ma absolutnie żadnych. Nie wiem, jakie by ktoś mógł widzieć tu korzyści dla siebie. Chociaż...Te video spotkania ze znajomymi spowodowały, że np. z pewną grupą znajomych ze studiów spotkaliśmy się online, a pewnie w normalnej sytuacji nawet by nam to do głowy nie przyszło. To jest jedyna taka niewielka korzyść - odświeżenie jakichś znajomości.

**Emocje - zdjęcia**

**8 i 16**

**8**

To jest droga, jedziemy, tylko nie widzimy dokąd, bo jest mgła i bardzo możliwe, że za tą mgłą jest jakieś niebezpieczeństwo i bardzo możliwe, że nie będziemy mieli możliwości zareagować w porę. Te czasy pokazały, że wszelkiego rodzaju planowanie możemy sobie wsadzić w pewne miejsce. Nie jesteśmy w stanie zaplanować niczego i bardzo możliwe, że to, co będzie w przyszłości nas bardzo mocno zaskoczy. To absolutnie nie są pozytywne emocje.

**16**

Jest niszczone coś lub niszczy się coś, co było do tej pory podstawą naszego rozumienia świata. To na czym opieraliśmy swoje założenia, czyli jakieś poczucie bezpieczeństwa...Rzeczywiście, my od dłuższego czasu żyliśmy z takim poczuciem bezpieczeństwa i przynajmniej u nas w Polsce nie ma żadnych wojen i większych zagrożeń. Wszyscy żyjemy w poczuciu dużego komfortu i nagle to zaczyna się walić. Dosłownie walić i dość szybko to następuje. Takie podstawy egzystencjalne naszego życia nagle płoną. Podejrzewam, że ten pożar nie będzie ugaszony. Być może przyczyny będą ugaszone, ale skutki będą bardzo niszczycielskie, ponieważ nie jest powiedziane, czy to się nie przerodzi np. w jakieś bunty społeczne. Już widzimy, że sporo ludzi traci pracę, oszczędności i niedługo w ogóle mogą zostać pozbawieni środków do życia. I tu nie mówię tylko o Polsce, bo czy te bunty nie naruszą jakiegoś status quo w Europie i na świecie. Historia pokazuje, że wiele konfliktów było spowodowane warunkami naturalnymi. Imperium Rzymskie upadło na skutek ochłodzenia klimatu, czyli zmiany klimatyczne spowodowały zmiany porządku światowego. I czy tutaj też nie będzie czegoś takiego? Biorąc pod uwagę, jak szybkie jest teraz życie, to te zmiany też mogą być szybsze niż kiedyś to następowało. Kiedyś to mogły być stulecia a teraz może zająć kilka lat.

**Czy twoje emocje jakoś ewaluowały w czasie?**

Na początku to był lekki niepokój, bo ja dużo podróżuję i pamiętam, że chyba w 2008 była epidemia SARS w Azji Środkowej i też było to mierzenie temperatury, chodzenie w maseczkach. Takie akcje już były na świecie i ja na początku myślałem, że to będzie kolejna tego typu akcja. Już to widziałem, już to przeżyliśmy. Teraz widzę, że sprawa jest dużo poważniejsza i z tego zacząłem sobie zdawać sprawę dopiero jak zamknięto szkoły, granice.

**Obecne obostrzenia dotyczące wychodzenia z domu, spotykania się - jak sobie z nimi radzisz?**

Jakoś specjalnie z tego powodu nie cierpię, ale to wynika z tego, że ja nie jestem jakąś super towarzyską osobą. Bez tego daję sobie radę i to nie jest coś, czego mi jakoś specjalnie brakuje. Gdyby to były tylko takie ograniczenia, to w ogóle by, mnie to nie obeszło. My się spotykamy online, dużo częściej rozmawiam przez telefon, do siostry dzwonię i godzinę na telefonie siedzimy. Ten brak możliwości spotkania osobistego...ja wcześniej też tak specjalnie do spotkań osobistych nie dążyłem. Fajnie, że przyjdą do domu znajomi i napijemy się wina, ale już nauczyliśmy się pić wino online. To akurat zupełnie mnie nie obeszło.

**Powiedziałeś, że teraz do sklepu chodzisz późnym wieczorem. Czy coś jeszcze zmieniło się w sferze zakupów?**

Sporo rzeczy zamawiam online, ale i tak dużo rzeczy zamawiałem online. W zasadzie nic się nie zmieniło, ale może się zmieni, bo podobno jeszcze jakieś ograniczenia zostały wprowadzone. Od 10 do 12, to musiałbym mieć 65 lat a jeszcze nie mam.

**A pamiętasz ten moment, kiedy był taki run na sklepy?**

Tak, to było, jak ogłosili, że szkoły będą zamknięte i wszyscy pojechali do sklepu. Ja nie poszedłem do sklepu. Poszedłem wieczorem przed 22 - mam Selgrosa obok i nikogo nie było, w sklepie wszystko było. Widać było, że półki z mąką zostały opustoszone. A, wiem co jeszcze się zmieniło. Widać w sklepie, że jest o wiele węższy asortyment niektórych towarów. Ja np. piekę chleb i mąka, którą zwykle używałem - jej po prostu nie można kupić w Selgrosie, więc zacząłem zamawiać mąkę online - taką, która jest mi potrzebna do pieczenia chleba, bo to jest żytnia określonego typu i jej po prostu nie ma. Ale można ją jeszcze cały czas zamówić na Allegro, więc zamówiłem jej więcej. Piekę chleb 2 x w tygodniu od wielu lat a teraz częściej, bo wszyscy jesteśmy w domu i wszyscy go jemy.

**Zrobiłeś zapasy czegoś jeszcze?**

Kawy jeszcze teraz kupiłem sporo, bo my pijemy taki rodzaj kawy, który nie jest oficjalnie importowany i nie ma go oficjalnie w sklepach. To jest kawa, która jest np. w sklepach w Niemczech i można ją kupić w Polsce z prywatnego importu. Ja zawsze ją kupowałem na Allegro i 2 tyg. temu zamówiłem 10 opakowań tej kawy, a teraz zamówiłem jeszcze 20, bo sobie pomyślałem, że jak nie jeżdżą tak ludzie do Niemiec, to niedługo zapasy tego mogą się skończyć. Nie mamy tutaj w domu zapasów papieru toaletowego i takich rzeczy. ja zakładam, że to będzie zawsze. To nie jest tak, że my chodziliśmy wcześniej codziennie do sklepu. Większe zakupy robiło się raz na tydzień i teraz też tak będziemy robić. Tutaj się nic nie zmieniło.

**Czy masz wrażenie, że dobrze sobie radzisz z sytuacją, która jest teraz?**

Tak. Mój obecny nastrój jest ok. Nie mam depresji jakiejś, chociaż może specjalista powinien to ocenić.

**A jak radzą sobie ludzie z twojego otoczenia?**

Moja rodzina chyba sobie radzi z tym dobrze. mam znajomych, których ta sytuacja dotknęła bardzo mocno zawodowo, bo akurat działają w branży turystycznej i praktycznie z dnia na dzień zostali pozbawieni całkowicie źródła dochodu. I zostali z ogromnymi kosztami stałymi związanymi z działalnością. Prowadzą 2 hostele i hotel w Warszawie i nagle zero przychodów a koszty na poziomie kilkuset tysięcy zł miesięcznie. Na razie zwolnili ludzie i tyle. Mogłem ich jedynie obserwować online i widać u nich bardzo duże napięcie. Jeszcze u jednego ze znajomych jest to związane z tym, że przez to, że choruje chłopak, to ta choroba jeszcze go umieszcza w kręgu podwyższonego ryzyka. U niego widać, że on to wszystko bardzo osobiście przeżywa i on się stara maksymalnie izolować od otoczenia, bo jakby jemu coś się stanie, gdyby zakontraktował tego wirusa, to jest bardzo mocno zagrożony. Mnie to nie dotyczy, bo ja jestem zdrowy, mnie nic nie dolega. Wszystkie te rzeczy, o których mówią - choroba płuc, cukrzyca, nadciśnienie, choroby serca...Ja tego nie mam, więc jakieś ryzyko zawsze jest, ale akurat o siebie się nie obawiam. A u niego jest ta sytuacja i ja u niego widzę bardzo wysokie napięcie. Oni to tak trochę przykrywają, bo oni zawsze byli bardzo weseli, dowcipni, ale też widzę, że oni po prostu nie chcą na ten temat rozmawiać. Ucinają te rozmowy i próbują od tego się odciąć. Powiedzieli, że oni i tak na ten temat tak dużo myślą, że z przyjaciółmi to już chcą porozmawiać o czym innym, pogadajmy sobie o muzyce, np.

**Obserwujesz jakieś zmiany zachowań wśród ludzi?**

Ja mieszkam w takim miejscu, że my się tu wszyscy znamy z sąsiadami. Kiedyś na spacerze rzeczywiście stało się bliżej a teraz rozmawiamy w odległości 10 m. Nawet nie 2 tylko 10. Widzę, że ludzie rzeczywiście na poważnie te ograniczenia biorą i słusznie, że to tak jest.

**A jak ty idziesz do sklepu, to się zabezpieczasz?**

Tak. Maseczki sobie jakieś chałupnicze kupiłem na Allegro. Beznadziejne i żałuję, że je kupiłem. Zakładam, ale tak bardziej symbolicznie je zakładam, bo to chyba warto. Może coś tam uchroni...Może tak, może nie. niewygoda jest taka, że jak mam maseczkę, to mi okulary parują. I wtedy ręką do oczu...To może lepiej nie zakładać wcale. Rękawiczki mam takie nitrylowe w domu. Kupiliśmy niedawno setkę takich rękawiczek i rzeczywiście, jak idę do sklepu, to zawsze mam przy sobie. Zawsze mam przy sobie jak wychodzę z domu, bo rzeczywiście nie wiadomo czy nie będzie takiej sytuacji, że trzeba będzie założyć.

**Jak myślisz, skąd się wziął koronawirus?**

Ja jestem bardzo daleko od jakichś konspirologii. Wiem, że teraz jest mnóstwo takich teorii, np., że to Chińczycy po to, żeby supremację gospodarczą osiągnąć, itd. Pierdoły. Ja uważam, że ten wirus po prostu się pojawił, wymknął się spod kontroli. Chińczycy nie chcieli i na początku nie wolno było o tym mówić. Oni sami w porę nie ostrzegli, że coś takiego się pojawiło. I to są fakty. A jakichś spiskowych teorii, że jakiś żydowski spisek, żeby zniszczyć świat...Czasem słyszę takie rzeczy od wydawałoby się inteligentnych ludzi. To jakaś bzdura totalna. Tak samo nie mam jakichś swoich własnych teorii czy te kroki, które teraz zostały wprowadzone - czy one są słuszne, czy nie. Uważam, że skoro cały świat takie kroki wprowadza, to one są słuszne. Trzeba słuchać specjalistów. Jeżeli WHO, która naprawdę jest chyba jedynym autorytetem, jeśli chodzi o takie sytuacje, rekomenduje, żeby zostawać w domu i maksymalnie ograniczać kontakty międzyludzkie, to znaczy, że tak trzeba robić. Tylko w wielu przypadkach, a w Polsce na pewno, jest to ogólny pretekst do ograniczenia swobód obywatelskich. Zresztą widać, że pod pretekstem wprowadzania ustawy walczącej z koronawirusem, wprowadza się przepisy, które z walką z koronawirusem nie mają nic wspólnego. I są wprowadzane, bo można i może ludzie nie zauważą, a jak zauważą to nie będą mieli nic przeciwko temu, bo uznają, że tak trzeba, że to w trosce o nasze dobro.

To, że wirus został wyprodukowany i wymknął się spod kontroli to też jest konspirologia. Ja w to nie wierzę.

**Można było coś zrobić, żeby się tak nie rozprzestrzenił?**

Nie znam się na tym i nie chcę się wypowiadać. Nie wiem. Bardzo możliwe, że można było coś zrobić na wcześniejszym etapie. Już w listopadzie czy na początku grudnia chińscy lekarze próbowali sygnalizować, że coś jest nie tak, że wirus się pojawił i kazali im się zamknąć a nawet niektórzy zostali za to ukarani, że opublikowali takie informacje. Ja uważam, że można było szybciej ostrzec np. WHO, że takie ryzyko jest. Może wtedy by się przedsięwzięło jakieś kroki bardziej ograniczające. W sytuacji demokratycznych społeczeństw trudno byłoby rządom wprowadzać jakieś ograniczenia, kiedy nic się nie dzieje. Nikt jeszcze nie zachorował, to dlaczego my mamy wprowadzać jakieś ograniczenia? Tak robili w Wlk. Brytanii, w Stanach, więc bardzo możliwe, że to wcześniejsze ostrzeganie nie miałoby większego wpływu. Poza tym ludzie mogliby nie zrozumieć tego. Ja też myślałem, że to będzie tak jak z SARS, że to zostanie ograniczony tylko do Azji.

Kiedy poczułeś, że sytuacja jest poważna?

W zasadzie już jadąc do Anglii 6 marca już czułem, że może nie powinienem, tym bardziej, że planowaliśmy tam pójść do teatrów - mieliśmy 2 spektakle zarezerwowane, a wiadomo, że tam ludzie siedzą obok siebie. Zastanawiałem się, ale polecieliśmy. A potem to już tak poleciało wszystko jak wróciliśmy z tej Anglii...

**Jak oceniasz przygotowanie Polski do tego, co się dzieje?**

Przygotowanie jest bardzo kiepskie, zwłaszcza w porównaniu z tym, co robi się w innych krajach. Nie mówię o Włoszech. Mówię szczególnie o Niemczech, o Francji, Szwajcarii. Dla mnie modelowym przykładem są Niemcy i to, w jaki sposób sobie z tym radzą. Mają bardzo dużą liczbę zachorowań, ale też bardzo dużo ludzi testują i wcześnie izolują te osoby, które przechodzą test pozytywnie. Wiemy wszyscy o tym, że nosicieli potencjalnych, którzy zarażają, może być dużo więcej. Każdy z nas może być nosicielem nie mając żadnych objawów. Gdyby mi zrobiono test, wykryto wirusa i stwierdzono, że trzeba mnie izolować, to ja bym to w pełni zaakceptował. Ale przynajmniej bym wiedział, dlaczego. Takie bezsensowne wkładanie ludzi na kwarantannę w Polsce nie robiąc im żadnych testów...Masz siedzieć w domu 2 tyg. i tyle. Przynajmniej w połowie tej kwarantanny powinno się komuś takiemu test zrobić, czy jest zachorowanie, czy nie ma. Tymczasem ludzi wypuszcza się po 2 tyg. kwarantanny i nie robi się im tego testu. Już nie mówiąc o tym, że jeżeli mówi się o tym, że te maseczki pomagają, to niech one będą, do cholery jasnej dostępne dla wszystkich. A to, że nie są dostępne, że musimy sobie na Allegro takie chałupnicze badziewie kupować...To też świadczy o tym, że nie jest ten kraj do tego przygotowany. tym bardziej, że tych maseczek i odzieży ochronnej brakuje również dla służby zdrowia. To już jest skandal. Albo to, że się pracowników służby zdrowia nie bada raz na jakiś czas, że im się nie robi testu. Z tego co widać, osobami, które w tej chwili rozprzestrzeniają tę chorobę i zarażają, w dużej części są pracownicy służby zdrowia. Pytałaś, jakie są konsekwencje... Ja miałem zaplanowane lekarskie badania profilaktyczne. Nie są to jakieś bardzo pilne rzeczy, ja je wszystkie poodwoływałem, ale głównie dlatego, że nie chcę chodzić do POZ. Uważam, że mogę nawet kilka miesięcy poczekać bez tych badań i nic się nie stanie. Jest sytuacja z moją mamą, która miała mieć operację na oczy - ma 90 lat i już prawie nie widzi. Ta operacja została odwołana - formalnie z powodu choroby lekarza, ale nie jestem pewien, czy nawet gdyby termin został przywrócony, to czy ja bym rekomendował mamie pojechać na tę operację. Nie wiem, co mam zrobić w tej sytuacji, bo jeśli przywrócą termin i mama powie, że chce, to ja powinienem pojechać do Koszalina i ją przywieźć?

**Widzisz jakieś dobre działania podejmowane przez rząd?**

Widzę, że narzucenie ograniczeń w kontaktowaniu się między ludźmi jest jak najbardziej słuszne. Słuszna była decyzja o zamknięciu szkół, o zamknięciu GH, o zamknięciu wszelkich siłowni, zakładów fryzjerskich, tylko że trudno mi powiedzieć... Te ostatnie ograniczenia, żeby liczba osób w sklepie to była liczba kas x 3...Czy to jest akurat słuszne? To powinno być zależne nie od liczby kas, ale od powierzchni sklepu. Nie może być np. większej liczby osób niż ileś tam na m2 czy na 10m2. Zależność od liczby kas to jest debilizm, moim zdaniem. ja sporo lat byłem w zarządzie rosyjskiego Selgrosa i trochę się znam na handlu detalicznym i uważam, że uzależnienie od liczby kas to jest najbardziej debilne kryterium, jakie można było sobie postawić.  Poza tym wprowadzenie tego ograniczenia, że od 10 do 12 mogą robić zakupy tylko osoby powyżej 65 r.ż. To wyobraź sobie takiego Selgrosa, który w tych godzinach mógłby się praktycznie zamknąć, bo tam nie będzie nikogo w tych godzinach. To jest kolejna bzdura. Ja rozumiem, że jakieś małe sklepiki na osiedlach czy w Żabkach. Ale taki sklep wielkopowierzchniowy jak Selgros czy Auchan, żeby się w tych godzinach zamknął i był tylko dla 65+? To jest totalna bzdura.

**A zamykanie lasów, zakaz spacerów nad Wisłą?**

Tu akurat wiem, o co chodzi. Akurat w ostatni weekend jechałem rowerem nad Wisłą i uważam, że wprowadzenie takiego zakazu, żeby w ogóle tam nie chodzić...Uważam, że to jest przesadzony zakaz, bo nie widziałem, żeby tam szczególnie gęsto było. Było sporo rowerzystów, ale ci rowerzyści zachowują od siebie odległość. Było trochę ludzi na spacerach, ale były to rzeczywiście rodziny i jedni od drugich zachowywali dosyć duże odległości, więc ryzyka dużego nie było. Jestem w stanie zrozumieć, że będzie coraz cieplej i jednak będzie taka tendencja, żeby tam wychodzić. To ograniczenie jestem w jakimś stopniu w stanie zrozumieć. tego ograniczenia ze sklepami nie. Poza tym mówi się, że chodzi o rozrzedzenie liczby kupujących. To niech pozwolą handlować w niedzielę. Jeżeli się markety budowlane zamyka w sobotę, to jest to bzdura, bo przez to więcej osób pójdzie w tygodniu i będzie większe skupienie w krótszym czasie. Dla mnie to jest ograniczenie nielogiczne i ono nie zostało w żaden sposób skonsultowane ze specjalistami. Uważam, że wprowadzając ograniczenia, jeśli chodzi o handel, powinno się spytać profesjonalistów, czyli handel, co wy proponujecie w celu ograniczenia tego natłoku ludzi w sklepach. I oni sami by mogli zaproponować rozwiązania, które byłyby dobre, ale może nie byłyby zgodne z partyjną ortodoksją, czyli np. tym, że jest zakaz handlu w niedzielę.

**Czy jakieś decyzje były wg ciebie konsultowane ze specjalistami?**

Mam wrażenie, że nie i że we wprowadzaniu tych ograniczeń biorą udział wyłącznie urzędnicy a oni są niestety bardzo oderwani od rzeczywistości. Sam Selgros ograniczył liczbę wózków do 150 i rzeczywiście widać było, że np. w sobotę przed południem to tam była kolejka przed sklepem. Wieczorem nie było problemu. Handel sam potrafi się samoograniczyć - ten taki rozsądny handel, czyli profesjonalne sieci handlowe, które są zarządzane przez specjalistów.  Ja się boję, że te ograniczenia, które wprowadzono doprowadzą do tego, że ludzie będą się ustawiać w kolejce, stać w tej kolejce, a ona przecież też jest zagrożeniem jakimś. Nawet lepiej by było, żeby ci ludzie się rozproszyli po dużym sklepie. W Selgrosie, jeszcze zanim to stało się prawem, już były rękawiczki i płyny dezynfekujące. Oni sami myślą, co robić. W takich czasach jest tendencja do nadregulowania wszystkiego.

**Decyzja o zamknięciu granic była słuszna?**

Tak, tylko ona została wprowadzona nie od tej strony co trzeba. Dopuszczenie do tego, żeby przed granicami gromadziły się tłumy i żeby się czekało po dwadzieścia parę godzin to był skandal, bo właśnie takie decyzje doprowadzają do rozprzestrzeniania się zachorowań. Jeżeli już, to bądźmy konsekwentni i każdego, kto przekracza granicę kierujmy na kwarantannę, ale jednocześnie róbmy mu testy. Bzdurnym ograniczeniem było niepozwalanie na tranzyt przez Polskę. Litwini nie mogli do siebie do domu wrócić. Wszyscy jesteśmy w UE i nagle jeden kraj nie pozwala ludziom przez swoje terytorium przejechać? A co by było, gdyby jakikolwiek kraj wprowadził takie ograniczenie wobec obywateli polskich? Od razu mielibyśmy skandal, a jak my coś takiego zrobimy, to wtedy jest dobrze, bo my się bronimy. Niemcy do tej pory nie wprowadziły takiego ograniczenia.

**A coś jeszcze powinno być zrobione?**

No wreszcie wprowadzono to, że jak ktoś jest na kwarantannie, to jest na kwarantannie a nie, że mieszka w domu z całą rodziną i jedna osoba jest na kwarantannie a reszta rodziny sobie wchodzi, wychodzi. Teraz, jak nie można takiej osoby odizolować w domu w oddzielnym pokoju z łazienką, to cała rodzina musi być na kwarantannie. To jest słuszne.

**Skąd czerpiesz informacje?**

Szukam informacji i jest to może nawet taka trochę obsesja w tej chwili. Ciągle szukam tych informacji przez co nie mogę za bardzo skupić się na czymś innym. Myślałem, że sobie np. siądę i poczytam książkę w spokoju, ale nie, bo ciągle włączam te wiadomości, szukam jakichś opinii w internecie na ten temat. Przez to człowiek się nie może skupić na innych czynnościach.  Postanowiłem sobie, że może ja po prostu przestanę się tym wszystkim przejmować i rzeczywiście wezmę tę książkę, a co będzie to będzie. Mam problem ze skupieniem się nad książką.

**Jakich informacji szukasz?**

Patrzę na statystyki, na krzywe zachorowań, bo próbuję to sobie jakoś statystycznie, matematycznie wyjaśnić, skąd to się bierze. Już nawet widzę, że jeszcze tydzień temu byliśmy tam, gdzie Włochy 3 tyg. wcześniej, a teraz już jesteśmy tam, gdzie Włochy 4 tyg. wcześniej, czyli u nas to narasta słabiej niż we Włoszech. Patrzę też cały czas na informacje gospodarcze - jak te kursy walut się kształtują...Nie jestem pewien czy dane statystyczne, jeśli chodzi np. o inflację, są rzetelnie podawane i to nie trochę niepokoi. Boję się, że nawet te rzeczy będą u nas trochę zmanipulowane politycznie, żeby nie straszyć ludzi i będą nam mówili, że jest lepiej niż jest. To jest całkiem możliwe, bo GUS to jest agenda rządowa praktycznie, NBP nie spełnia kryteriów niezależności Banku Centralnego a to te 2 instytucje podają główne dane i obie mogą manipulować. To mnie też bardzo niepokoi, bo dla osób, które są w biznesie, zarządzają firmami te dane o sytuacji makroekonomicznej są bardzo istotne.

**Które źródła informacji uznajesz za wiarygodne?**

Bardzo rzetelna jest GW. Ja ją zawsze czytałem i uważam, że jest dużo bardziej czytelna niż cokolwiek innego. Ze stacji tv to TVN czy Polsat. Polsat w miarę rzetelnie próbuje wszystko podawać. TVP Info oglądam, żeby wiedzieć co oni tam mówią i czasami jestem przerażony no i jeszcze źródła zagraniczne. Mieszkałem parę lat w Rosji, więc media rosyjskie i staram się te niezależne media rosyjskie śledzić. W Rosji wprowadzili to samo co w Polsce teraz i nawet jeszcze bardziej próbują inwigilować ludzi. Nie możesz wyjść bez jakiejś specjalnej apki z kodem. W Moskwie są takie zarządzenia, bo oni tam wprowadzili ograniczenia regionalne i to w Moskwie jest całkowity lock down.

**Jakie strony w internecie?**

GW, bo mam subskrypcję, jeszcze Rzeczpospolita, Polityka + te rosyjskie i więcej już nawet nie mam czasu. Muszę się z tym opanować, bo nie można cały czas siedzieć i tylko wiadomości śledzić. Doszedłem do wniosku, że w tej chili to już jest za dużo. Poświęcam na to znacznie więcej czasu niż wcześniej.

Dla rozrywki jakieś filmy oglądamy i to wszystko. Bo co jeszcze pozostało? Można pójść pobiegać, bo akurat tu, gdzie mieszkam, to mogę.

**Jak sądzisz, jak długo to będzie trwało?**

Wiem, że szkoły nie otworzą się przed czerwcem. Jestem przekonany, bo to byłoby barbarzyństwo i zresztą podejrzewam, że rodzice by nie posłali dzieci nawet jakby pozwolono. Podejrzewam, że zacznie się latem uspokajać wszystko. W Chinach już w zasadzie to opanowali, ale tam zastosowali tak drakońskie kroki i dość szybko...Nie wiem czy w tych naszych ustrojach by przeszło. Obawiam się, że pozwolą rozjechać się ludziom na Wielkanoc, bo my jesteśmy takim krajem katolickim, przywiązanym do wartości rodzinnych. To będzie katastrofa. Przed Wielkanocą powinno się wręcz zamknąć miasta. To powinni zrobić. Po prostu nie wyjedziesz i koniec. Uważam, że to może tak na miesiąc się powinno wprowadzić. Nie można dopuścić do tego, że Polacy się rozjadą na święta. W rozporządzeniu jest napisane, że liczba osób w kościołach, na pogrzebach - 5, ale do 11.04. Od 12.04 - 50 osób. To jest wg mnie skandal. I to jest taka niekonsekwencja. Jestem przekonany, że specjaliści z MZ chcieli, żeby te ograniczenia były, ale przecież te rodzinne wartości, ta Wielkanoc...Po prostu nóż się otwiera. Dla mnie zamykanie Parków Narodowych to jest totalna bzdura. Już co jak co, ale wyjście na świeże powietrze i spacerowanie po lesie nie sprzyja rozprzestrzenianiu się choroby.
