# Supplementary material for: The challenges arising from the COVID-19 pandemic and the way people deal with them. A qualitative longitudinal study
Source: PLoS One. 2021 Oct 11;16(10):e0258133. doi: 10.1371/journal.pone.0258133 (PMC8504766; doi:10.1371/journal.pone.0258133)
Supplement: S1 Dataset — (ZIP) [file pone.0258133.s003.zip › Transcriptions/stage 1/9.1_F_25_couple, no children.docx]

**9.1_F_25_couple, no children**

**Czy możesz opowiedzieć mi o sobie?**

Mam 25 lat, 26 w maju - jeżeli dożyjemy (śmiech). Skończyłam filologię polską, najpierw byłam na edytorstwie, a potem na krytyce literackiej. Pracuję jako księgowa. Mieszkam we Wrocławiu, a pochodzę z Karpacza - to taka mała miejscowość pod Jelenią Górą. W wolnym czasie sporo czytam, ale najwięcej oglądam seriali. To nie jest raczej powód do dumy, ale zapytaj o jakikolwiek serial, a powiem, na ile gwiazdek go oceniam. Poza tym trochę ćwiczę, ale nie przemęczam się. Życie jest wypełnione raczej praca-dom-praca-dom. Pracuję w nieciekawych godzinach, od 9 do 17, niewiele da się zrobić. Rano zwykle staram się iść na siłownię, teraz poćwiczyć w domu. Po południu normalnie chodziło się na zakupy. Zostało tylko oglądać "Jak poznałem waszą matkę" po raz piąty. Teraz mój tryb dnia wygląda tak, że wstaję o 7:20, ćwiczę sobie, o ósmej wstaje mój mąż, który też pracuje z domu, bo na ósmą ma do pracy. Robię śniadanie sobie i jemu. O 9 zaczynam swoją własną pracę i pracuję do 17. Ostatnio nie za bardzo chce mi się oglądać seriale więc czytam. Tygodniowo czytam 6 książek więc jestem dumna z siebie, ale nie są grube. Zamówiłam dużo z wydawnictwa Vesper i po kolei czytam sobie klasykę typu Adwokat Diabła, Psychoza. Na razie wynajmujemy mieszkanie. Mieliśmy kupować, ale ze względu na to co dzieje się teraz jest problem i czekamy.

**Czy pamiętasz moment, kiedy to wszystko się dla ciebie zaczęło?**

Powiem szczerze, że z początku nie brałam tego poważnie. Ale jest taki moment. Przychodziłam do pracy i koleżanki o tym ciągle rozmawiały, że niedługo do Biedronki nie będziemy mogły pójść. No i w pewnym momencie przyszła koleżanka i mówi "Boże, mój Paweł kazał nam jechać do sklepu i kupować po kilka litrów mleka i wody". Zadzwoniłam do rodziców i sądziłam, że będą podzielać moje zdanie. Są rozsądni i mama jest pielęgniarką. I ona mówi, że zaczyna się. Że nie chce nas straszyć, ale żebyśmy nie szli do kina. Od tego momentu zaczęłam myśleć, że coś się dzieje. To był marzec, ten tydzień, kiedy dopiero wprowadzono obostrzenia. Pamiętam, że w piątek miałam próbny dzień home office'u. To był taki pierwszy rzut tych informacji, chyba środek marca. Chyba dwa tygodnie temu. Nagle zaczęłam pracować w domu, raczej z myślą, że to próba. Okazało się, że mamy zostać od poniedziałku w domu. Zaczęto wprowadzać obostrzenia, że nie można wychodzić i trzeba mieć swoje własne rękawiczki. Wszyscy nagle zaczęli interesować się tematem. Rozmawiałam z koleżanką, która miała brać ślub 11 czerwca, no i mówi, że nie bierze bo sala weselna odwołała wszystkie śluby. Powiedzieli "to może 12 listopada" a ona odpowiedziała "to mam gości z grobu na wesele zabrać?". Na początku w ogóle mnie to wszystko nie obchodziło, ale teraz jestem drugi tydzień na home office. Jeszcze dwa razy byłam z przyjaciółkami na kawie. Zrobiłam zakupy w Biedronce. Rozlałam tam zupę i kupiłam trzy chleby tostowe, bo może lepiej się zaopatrzyć.

**Czy pamiętasz jakieś rzeczy, sytuacje, decyzje, które wpłynęły na ciebie?**

Myślę, że dobrze zrobiliśmy. Rodzice już robili zakupy. Z mężem też tak zrobiliśmy, ale powiedzieliśmy sobie, że zrobimy je ze względu na potrzeby, a nie wirusa. To nam pomogło, bo w pierwszym tygodniu nie musieliśmy już robić zakupów. To było rozsądne, ale rozsądne nie były te dwie kawy - bo się rozchorowałam potem i bałam się, że to koronawirus. Kaszlałam i znajome bały się ze mną wyjść. Zostałam w domu, a w sobotę miałam gorączkę i inne objawy jak przy koronawirusie. W niedzielę zadbałam o siebie, bo jak mam iść do szpitala, to przynajmniej wyglądać dobrze. Ale wydobrzałam i zrobiłam sobie małą kwarantannę, przez półtora tygodnia nie wychodziłam z domu. Mieszkam w bloku, w którym są prawie tylko starsi ludzie, więc lepiej zachować ostrożność. To było trudne, nie mogłam wytrzymać w domu. Bałam się wtedy, że mam koronawirusa, ale nie byłam pewna co z tym zrobić. Czy mam dzwonić do wydziału epidemiologicznego? Co się wtedy stanie? A może powinnam od razu jechać, a nie dzwonić. Nie wiem jak się to załatwia. Koleżanka, która leciała z USA, chyba z San Francisco i do Drezna pojechał po nią jej tata. Byli w kontakcie, ale na kwarantannę wrzucili tylko ją. Bała się myć włosy, bo wtedy może przyjechać policja, a ona nie podejdzie do okna.

**Czyli raczej nie wiedziałaś, co masz robić, niż bałaś się?**

Tak, i przez tę dezorganizację właśnie się bałam. Przez niewiedzę. Cieszę się, że w Polsce coś się robi, tym bardziej, że mamy niższy stopień zachorowalności niż w innych krajach. Jak widzę, że ludzie z dziećmi chodzą za oknem albo dziewczyna jeździ na rolkach...

**Czy teraz się boisz?**

Od kiedy jestem w domu to nie śledzę, bo się niepotrzebnie nakręcam. Najlepiej po prostu prowadzić higieniczny tryb życia, tylko bardziej. Jak wynoszę śmieci to idę w rękawiczkach. Staram się nie wychodzić, bo nie jesteśmy w stanie rozpoznać, czy jesteśmy nosicielami. Ja tu nie widzę wielkiego bezpośredniego zagrożenia i nie boję się, że skończy się to apokalipsą. Ale dzisiaj bardzo mocno poczułam skutki gospodarcze czy związane z wykonywaną pracą. Jestem księgową więc jeszcze jest jakiś popyt i raczej nie stanie nam firma, no ale u męża zmniejszyli już wszystkim wymiar etatu na 80% i wielu pracowników nowozatrudnionych przechodzi na jakieś postojowe. Tutaj widzę realny skutek. Wcześniej te zakłady pracy i instytucje nie miały gotowych rozwiązań i one zaczynają funkcjonować. Dzisiaj się trochę zmartwiłam swoją pracą, ale chyba po prostu w sobie, tak emocjonalnie. Mam dużo pracy więc myślę, że byli by idiotami gdyby mnie zwolnili.

**Pokaż mi na skali, jaki poziom lęku związany z koronawirusem odczuwasz?**

Myślę, że tak 45.

**Co jeszcze sprawia, że się boisz?**

Myślę, że duży procent to lęk o mamę. Dziwnie się tam rzeczy mają. Pytałam, czy nie przeszkadza jej maseczka, po czym ona powiedziała, że nie mają maseczek. Jak ktoś kaszle to odwraca głowę. Moja mama pracuje w takim sprywatyzowanym szpitalu, głównie zajmują się tam chorobami dróg oddechowych, więc mama martwi się o swoich pacjentów. Jak złapią koronawirusa to koniec z nimi. Mają też pacjentów NFZetowych, wizyty często są odwoływane. Mam nadzieję, że te 30 lat to się zahartowała. O siebie nie martwię się, zaletą mojego wieku jest raczej silny organizm. Bardziej martwię się, że mogę zarazić kogoś nie wiedząc o tym. Chyba szacuje się, że nawet 70% populacji może złapać i po prostu przechodzić wirusa w mniej lub bardziej zjadliwy sposób.

**Które obrazki najlepiej opisują to, jak się teraz czujesz?**

Dwójka, bo ta cała sytuacja z koronawirusem wydaje mi się kuriozalna. Nie wiem dlaczego takie skojarzenia, ale te różowe buty i guma to sytuacja trochę komiczna, ale też niezbyt przyjemna. W ten sposób widzę tego koronawirusa, trochę. Nie mówię teraz o zgonach i innych negatywnych aspektach, ale to dla mnie takie niespodziewane i takie dziwne. Nigdy bym nie pomyślała, że będę w takiej sytuacji. To po prostu dziwne jak ten obrazek.

**Co jest najdziwniejsze w tym, co się teraz dzieje?**

Zaczynam się czuć, jakbyśmy byli bohaterami Walking Dead czy jakichś tam postapokaliptycznych wizji. Cała codzienność się zmieniła, nawet ta rutyna w pracy wygląda inaczej, bo zmieniają się wszystkie zasady. Wszyscy pracują z domu. To jak z filmu bo zawsze jest jakaś niedowierzająca, potem choruje, jest czas stagnacji i wszyscy mówią "spokojnie, ogarniemy to, mamy dobrą służbę zdrowia" i nagle 1/4 minęła i widzimy tylko trzaskające drzwi w opuszczonym szpitalu i krew na ścianach. Teraz jesteśmy w etapie stagnacji, ale nie jesteśmy w stanie powiedzieć jak to się dalej potoczy. Ja na początku marca widziałam się z kuzynem, który studiuje biologię. Opowiadał nam jak funkcjonują wirusy i śmialiśmy się wszyscy z tego, a teraz siedzę i się martwię jak bezpiecznie wyjść do sklepu. Dla mnie cała sytuacja jest naprawdę abstrakcyjna. Ta rutyna pracy trzyma mnie jeszcze przy jakichś realnych odczuciach. Tak się wszystko zmienia, nawet z pracą męża, nie pomyślałabym, że coś takiego się stanie.

**Co robisz, aby radzić sobie z tą sytuacją?**

Raczej nie zagłębiam się w to, co się dzieje. Jak wydają zaostrzenia czy komunikaty to słucham, ale niepotrzebnie o tym nie czytam. Staram się czerpać przyjemność z siedzenia w domu. Zaczęłam bardzo dużo czytać, ale też mniej dbam o siebie. Wcześniej musiałam wracać z pracy, a teraz mam ten czas na przykład na umycie okien nową maszynką do mycia okien. Raz wpadłam w panikę i zaczęłam myć klamki, ale ogólnie jestem pedantką i bardzo dbam o czystość. Przetarłam wtedy wódką bo nie miałam nic innego. Tylko mąż mnie uświadomił, że ona nie ma dość procentów. Teraz jak wyjdziemy co 3-4 dni na zakupy to klamkę przetrę, ale raczej płynem i ciepłą wodą. I oczywiście nie wychodzimy jeśli nie jest to konieczne. Duże zakupy robimy raz w tygodniu i raczej zastanawiamy się wtedy, co chcemy jeść przez tydzień. Ewentualnie do paczkomatu. Wcześniej nie planowaliśmy, bo oboje jedliśmy w okolicy pracy. Teraz musimy się żywić z zamrażarki i lodówki. Dla męża najważniejsze jest mięso, a ja polegam na chlebie i zupie, bo nie chce mi się gotować. Schodzi u nas bardzo dużo mleka no i woda. Ewentualnie makaron, bo jest najprostszą rzeczą.

**Czy teraz więcej kupujesz online?**

Ja wolę zakupy online i zawsze kupowałam online, ale teraz jedyne moje wyjścia są warunkowane zakupami lub wyjściem do paczkomatu. Skorzystałam też z promocji w Mohito. Ale kupiłam dużo czekolady i lodów więc nie wiem, czy te spodnie po kwarantannie będą dobre. Normalnie tyle ich nie jadłam, bo w pracy nie jem lodów i czekolady. W domu mogę sobie na to pozwolić, także dlatego, że na czekoladę mam uczulenie i mnie wysypuje. Tutaj nikt nie widzi.

**Widzisz jeszcze jakieś pozytywy?**

Na pewno jest czyściej. Jak pracowałam poza domem to rzadziej robiłam generalne porządki. Też kwiatki nam odżyły trochę. Chyba chciały towarzystwa. Kwiatami zajmuje się mąż. Poza tym więcej rozmawiam z mamą. Mamy dobry kontakt, ale mamy już poukładane życia. Role się odwróciły bo ja się martwię o nich, a nie oni o mnie. Teraz to się znowu odwróciło.

**Czy z innymi ludźmi utrzymujesz teraz kontakty online?**

Miałam takie postanowienie, żeby odpisywać wszystkim na Facebooku, ale nie udało się. Ja mam z tym problem, bo uważam, że lepiej się spotkać. Na początku miałam wzlot, ale teraz już zaliczyłam upadek i mam sporo zaległości. Dzisiaj miałam nawał rozmów. Zaczęło się od 17, zadzwoniła przyjaciółka od ślubu w listopadzie, potem zadzwoniła mama, potem zadzwonił kolega i powiedział, że idzie do paczkomatu jutro i będzie nam machał. Kontakty nawet się jakoś zaciskają pod tym względem, że miło jest, kiedy się wie, że ktoś się o nas troszczy i się interesuje. Takie miłe to jest.

**Radzicie sobie ze spędzaniem całego czasu razem?**

Zależy dla kogo wersja. Jak się pyta siostra męża, to mówię "kochana, masakra". Jak się pyta mama to "mamo, raj". A szczerze mówiąc to jest w porządku i nie mam z tym problemów.

**Czy coś cię męczy?**

Najbardziej to, że jest przyjemnie na zewnątrz, a nie mogę wyjść. Ja jestem wierząca, a zbliża się wielkanoc i w ogóle tego nie czuć. I nie mogę wyjść. W klepie widzi się te króliczki i kurczaczki, tandeta, ale jakie to ładne. Inaczej człowiek by sobie kupił krzyżówki, a tak to jak dzikus. Brakuje mi tego realizowania potrzeb. Czułabym się samolubnie gdybym wyszła po jakąś zachciankę.

**Macie jakieś plany na spędzenie świąt?**

W zasadzie na pewno trzeba by było trochę u rodziców i u teściów, ale teść zajmuje się swoimi rodzicami w podeszłym wieku. Nie chcemy ryzykować. Natomiast mama pracuje w szpitalu, więc nie chce nas narażać. Może zrobimy jakieś śniadanie przez skype'a. Nie wiem czy oglądałaś "Jak poznałem waszą matkę" i tam Marshall ma takie gumowe ręce i jedzą kolację.

**Mówiłaś, że brakuje ci tego czasu świąt w tradycyjnej formie - jak się z tym czujesz?**

Bardzo mi nieprzyjemnie. Kiedyś nienawidziłam tego, ale teraz bardzo lubię, bo wtedy mogę śpiewać i nikt nie słyszy jak śpiewam, bo jest tyle ludzi. Podoba mi się ten rytm, że rano są przygotowania, a potem chodzi się wieczorem na mszę. Śpiewy mi się podobają. Wszystko tam jest w punkcik. Wielkanoc to wiosna, zielono, ładnie, mama się tak nie denerwuje jak na Boże Narodzenie. Zawsze czekałam na wielkanoc i teraz widzę, że będzie problem. Nie przeżywam, takie to wszystko jest pozbawione sensu jakieś.

**Co się musi wydarzyć, żebyś uznała, że już możesz pojechać do rodziców?**

Myślę, że musiałaby bardzo spaść liczba zachorowań. I czekałabym na zielone światło od władz.

**Jakie obserwujesz postawy wobec tej sytuacji?**

Zacznę od koleżanki z pracy. Jak pracowałyśmy razem przy biurku, to ona miała tego chłopaka, który jej powiedział, ubrał się w moro i powiedział, jedziemy na zakupy. Jak ja się z niej śmiałam. A okazało się, że on był najmądrzejszy z nas wszystkich. Oni zrobili wielkie zakupy na dwa tygodnie przed wybuchem wielkich kolejek w sklepach. Bardzo się stresowali jak wszystko zaczęło eskalować 3 tygodnie temu, to okazało się, że babcia i ciotka tego chłopaka w moro były w szpitalu i obsługiwał je lekarz, który wrócił z Włoch, gdzie był na nartach. On zajmował się tą babcią i tą ciotką. Ale nic im się nie stało. Wiem też, że ta koleżanka bardzo się denerwuje mamą, która miała problemy zdrowotne przed koronawirusem. Chciała by pomóc mamie w domu, ale obawia się ją teraz odwiedzać. Natomiast ta koleżanka od wesela podchodzi do tego raczej racjonalnie, czyli jak my.

**Jakie podejście jest racjonalne?**

To nie panikowanie, ale zachowywanie umiaru. Jeśli coś się stanie i będę musiała iść do apteki, to nie będę zgrywała bohaterki tylko pójdę. Ale jak pomyślę "napiłabym się winka" to tego nie zrobię.

**Jakie masz podejście do ludzi, którzy spędzają czas na zewnątrz?**

Gdyby tego nie zabronili to ja bym była za - dlatego, że uważam, że to bardzo zdrowe. Na rolkach mniej, bo się człowiek jeszcze przewróci, ale jak ktoś biega to jest sam. Zazwyczaj biegają w maseczkach. I to jest dobre, ale skoro to jest zabronione to dlaczego mam ryzykować? Jak ktoś umie jeździć na rolkach to rolki też są zdrowe. Myślę, że wprowadzono zakazy dlatego, że ludzie nie rozumieją, że sport to nie wychodzenie z rodziną. Ja chyba zawsze trochę usprawiedliwiam ludzi, ale na pewno jest we mnie jakaś niezgoda kiedy widzę grupę młodzieży, która odbywa spotkanie towarzyskie. Jak emerytki wyjdą to jeszcze, niech sobie siedzą na ławce razem. Ale dlaczego młodzież nie mogła się w domu spotkać? Jeszcze siedzą i wszystkich denerwują. Może właśnie im zazdroszczę, że ja jestem za racjonalna i nie wyszłam sobie też ze znajomymi.

**Co wiesz o pochodzeniu wirusa?**

No oczywiście, że Donald Trump, żartowałam. Ogólnie słyszałam parę teorii i nie uważam ich za niemożliwe, ale na 100% z tego co zawsze, czyli z brudu, niedopatrzenia. Jak wszystkie inne wirusy. To jest jakaś odzwierzęca choroba. Wiem, że to ludzki wirus, ale ta mutacja jest zwierzęca. Ktoś nie zadbał o zwierzątko i nasze strefy się połączyły i powstał ten wirus. Po prostu brak higieny w kontaktach ze zwierzętami zarażonymi. Raczej to możliwe, że to Chiny, wiadomo, to społeczeństwo jest rozwarstwione i ludzie mieszkają tam z kurami w niektórych miejscach. To ta racjonalna odpowiedź. Ale nie wydaje mi się też niemożliwa opcja, że to z jakiegoś laboratorium. W sensie oczywiste jest to, że każdy kraj ma jakieś instytucje zajmujące się sprawami epidemicznymi, gdzie się bada choroby. Może się komuś coś wymknęło, ale to bardzo mało prawdopodobne. Wzięło się z niedopatrzenia i z brudu. A co do teorii to słyszałam, że to próby Chińczyków. Nie no, innych nie słyszałam. Że Chińczycy chcą zawładnąć światem, albo Trump, haha. W te spiski nie wierzę bo raczej jestem zdystansowana do teorii spiskowych.

**Skąd wiesz, że to jest teoria spiskowa?**

A to jest ciekawe pytanie. Wydaje mi się, że one się mocno wymykają takiej przyziemności. Kto wie, pewnie kilka teorii spiskowych jest prawdziwych, ale zwykle są takie głupie. Że Chińczycy i władza nad światem? Za długo żyję, żeby mi się wydawało, że żyję w kiepskim serialu.

**Czy uważasz, że tej epidemii dało się zapobiec?**

Pewnie i tak i nie, bo wszyscy wiedzą, jak sprawa ma się w Chinach. Oni szybko ogarnęli się z wirusem, ale to chyba też po części ich mentalność. Jak się za coś wezmą to się staje hurtowe. My jesteśmy sami winni, w sensie Europejczycy w ogóle, bo to my przywoziliśmy śmieci do tych biednych Chin. Kto by pomyślał, że z tego się jakieś choróbsko weźmie. To oczywiste. Poza tym wiemy jak tam ludzie żyją i jak rozwarstwione jest to społeczeństwo. Wszyscy wiedzą o obozach.

**Czyli co trzeba było zrobić, aby temu zapobiec?**

Chyba nie jestem w stanie podnosić tutaj takich dywagacji, ale wątpię, aby zainteresowanie społeczne wzrosło. To by już cokolwiek zmieniło, gdyby ludzie się interesowali. Mówiłam o tych obozach - w dzisiejszych czasach rzecz niepojęta, a one tam są. Trudno mi powiedzieć, czy to jest politycznie i życiowo możliwe, żeby cokolwiek w tych Chinach zmienić. Są silnym państwem i mogą stanowić zagrożenie.

**Masz poczucie, że Europa jest gorzej przygotowana?**

Europa to nie była w ogóle przygotowana. Ja też nie wierzyłam, że ten wirus przeniknie do Europy. Przy tym jak wygląda komunikacja w dzisiejszych czasach, to jest oczywiste, że wcześniej czy później jakaś osoba zarażona przejedzie albo przyjdzie. Chiny na pewno zajęły się tym bardzo dobrze, ale ten naród jest inny, ci ludzie są karni i żyją po wojskowemu. Kwarantannowy mores im nie przeszkadza, oni potrafią odnaleźć się w tych realiach. My jesteśmy niesforni. Wiem że Niemcy na początku "ahaha nic się nie dzieje", a potem masakra, bo dużo zgonów, dopiero teraz się wzięli. O Włochach każdy słyszał, bo też byli nieprzygotowani. My pewnie zrobiliśmy wszystko co w naszej mocy, ale są ludzie, którzy normalnie mają pracę, która polega na tym, że zajmują się przewidywaniem tego, co by się stało, gdyby epidemia się wydarzyła. Może te rozwiązania powinny zafunkcjonować wcześniej i może powinny być bardziej restrykcyjne.

**Czy rozwiązania stosowane u nas są adekwatne?**

Są. Zaostrzenia, o których słyszymy, sprawiają, że ja inaczej patrzę na sytuację. Kiedy trzeba było tylko unikać skupisk to nie przejmowałam się. Ale jak ktoś powie, że trzeba rękawiczki, że wszyscy poważnie, żeby nie wychodzić, to człowiek zaczyna się sam w sobie kulić i dostosowuje się. Więc dobrze, że się zaostrza ta polityka.

**Jak reagujesz na kolejne zakazy?**

Trochę się stresuję, jakiś stopień jest dodany. Po drugie coraz bardziej staram się je przestrzegać. Bo te pierwsze to tak na spokojnie. Siedziałam w domu, bo bałam się, że coś by mi się stało czy kogoś bym zaraziła. Ale gdybym była zdrowa to bym nie miała oporów, żeby wyjść z domu po zbytek. Obostrzenia sprawiają, że biorę sprawę na poważnie. Dostosowuję się do warunków i nie chcę nikomu zagrażać.

**Śledzisz nowe obostrzenia?**

Raczej tak, ale tylko na tym kończę. Ja wiem o tym wprowadzonym dwa dni temu, jeśli są inne to o nich nie wiedziałam. Dwa dni temu wprowadzono, że dzieci poniżej określonego roku życia nie powinny wychodzić, że zakaz sportu na powietrzu. Stwierdziłam, że jak już biegaczom zabraniają wychodzić, to przerażające. Byłam pewna, że w święta już się wszystko skończy, a wszystko działa tak, jakby sytuacja miała się przedłużyć. Na przykład mówiła mi dzisiaj koleżanka (ta od wesela), jej mama jest nauczycielką, i ona już dostała normalnie polecenia dotyczące przeprowadzenia rady pedagogicznej końcowej przez internet. To mnie przeraziło, ale rozumiem, że muszą wydać takie decyzje. Może to się skończyć za tydzień, ale oni muszą swoje zrobić.

**Jak myślisz, kiedy to się skończy?**

Nie mam pojęcia. Myślałam, że do wielkanocy. Potem, że maj to maks. Ale teraz w pracy mówią, że maj-czerwiec. Tutaj słyszę, że rada pedagogiczna ostateczna ma być przez internet. Ta sama koleżanka mówiła mi, bo ona pracuje na uniwersytecie, że w ogóle mają iść rozporządzenia dla studentów, że oni sesję będą mieli w takim trybie eksternistycznym zaliczać. Nikt nie jest w stanie powiedzieć ile to będzie trwało.

**Skąd bierzesz informacje o tym, co dzieje się na świecie?**

Ja mam zawsze w ogóle problem z sortowaniem tych informacji. Trudno znaleźć źródło, które nie będzie stronnicze, a ja bym chciała znać same fakty. Dlatego przeglądam popularne portale jak Onet, ale tam zazwyczaj bzdury. Trzeba uważać na clickbaity. Najlepszy clickbait jaki widziałam był o tym, jak X wylał wiadro pomyj za szyję Kożuchowskiej. Uważam bardzo na Onet i WP. Staram się czytać różne strony, a potem jakby wypadkową traktuję za coś, czemu można uwierzyć. A najlepszym źródłem jest i tak mój mąż, bo on wszystko sortuje i mi mówi. Koleżanek nie słucham, staram się raczej odcinać od tego. Mój mąż sprawdza dużo różnych źródeł.

**Czy masz jakieś nowe źródła, których nie znałaś przed epidemią?**

Nie, chociaż zainteresowała mnie strona - nie mam pojęcia jak się nazywa - ale jak rozmawiałam z mężem o wirusie to on mi pokazał, to jest chyba amerykańskie więc mi się czytać nie chce, że tam jest rozpiska krajów z koronawirusem i wszystkie informacje. To było ciekawe, bo dowiedziałam się, że Polska nie jest w takiej złej sytuacji, bo Niemcy i Włosi są w gorszej. Nie wiem jak to się nazywa, ale to ciekawa strona, czyste liczby.

**Czy korzystasz teraz więcej z mediów?**

Raczej tak, ale jak pracowałam jeszcze w biurze to traciłam dużo czasu na bzdury, na przykład po pracy wybierałam przez 10 minut brokuła w Biedronce. Teraz nie muszę się malować, nie muszę się ubierać, to już bardzo dużo czasu jest na plus. Teraz mogę sobie przejść kilka centymetrów po skończeniu pracy i poczytać. Człowiek ma też mniejsze wyrzuty sumienia jak posiedzi dłużej. Też nie mam wyrzutów sumienia pić codziennie wino, przecież nie idę do biura. Nie żebym się zaniedbywała, ale moja skóra się cieszy, paznokci nie będę komentowała, bo chciałabym, żeby jakaś pani mi pomogła. Ja nie umiem w paznokcie. Kiedyś ciocia mi powiedziała tak: Aniu, musisz dbać o paznokcie, bo ludzie patrzą na ręce. Dlatego chodzę na zabiegi i czuję się bardzo dobrze. A teraz nie mogę chodzić. Dwa dni temu wydano zaostrzenie, że należy zamknąć salony kosmetyczne.

**Jak oceniasz wiarygodność informacji, które do ciebie docierają?**

Wierzę w liczby. Jeżeli podają liczby to zakładam, że etyka dziennikarska nakazuje im, żeby chociaż liczby się zgadzały. Natomiast w takie insynuowanie, wymyślanie co będzie, w to nie wierzę. Czekam, aż to się skończy i sztab naukowców wywiedzie wnioski. Bardzo nie lubię takich niesprawdzonych niepewności. A najlepiej, jak się podadzą na jakiś zagraniczny licznik albo zagraniczną agencję prasową, która w jakiś sposób ma renomę, to jest dobra liczba wtedy. Z dystansem podchodzę do ilości zachorowań i wierzę tylko tej tabelce, o której mówiłam, że tam jest zestawienie. Zakładam, że tamte liczby są zgodne. Dużo się dzieje i nie jestem pewna, czy polskie media nadążają za tym. Zastanawiam się, skąd oni biorą te informacje, muszą mieć jakiś kontakt w jakiejś agencji, która im to daje. Komu ona to daje, czy ona to publikuje? Czy ona daje określonym jakimś w ustawie dziennikarskim podtypom? Nie mam pojęcia jak to działa. Ta nieznajomość źródeł tak mnie dystansuje od informacji.

**Gdy podejrzewałaś, że masz koronawirusa, to gdzie szukałaś informacji?**

Oczywiście, ze w internecie. Na Pudelku nie sprawdzałam. Czytałam stronę rządową i ona odsyłała do kolejnej strony rządowej. Nie zaspokoiła tak naprawdę mojej ciekawości. Pod względem konstrukcyjnym uważam, że była zrobiona świetnie. Bardzo dobrze jak na to, że musiała być zrobiona bardzo szybko. Były podane numery. Ale informacji było za mało, a może ja źle szukałam.

**Co myślisz o informacjach z mediów społecznościowych?**

W ogóle nie wierzę. Chyba, że już to coś wiem, wtedy wierzę. Nie zatrzymuję się nad postami typu "zamykają Dino" tylko czekam, aż wyda oficjalny komunikat władza. Ale okazało się, że obie koleżanki, które mówiły o tym zamknięciu się Dino, to sprawa sprowadziła się do jednego pana. Nie wiem, jak się nazywa ten zawód, że się jeździ po firmach i się prezentuje towary. I on jeździł po Dinach w jakimś powiecie. Więc jest możliwe, że zamknięto tylko te Dina, w których on był.

**Co byś zrobiła, gdybyś teraz chciała znaleźć informację o koronawirusie?**

Wstyd powiedzieć, że bym męża zapytała. Pewnie bym weszła na Google i wpisała coś w stylu "koronawirus wydarzenia kalendarium" i otworzyłabym kilka różnych stron, które wzbudzają moje zaufanie. Nie wiem, czy Onet może je wzbudzić. Pewnie te najwyżej wywindowane, co nie jest takie dobre, bo oni za to płacą. I stworzyłabym taką wypadkowa informację z nich wszystkich.

**Czy koronawirus pokrzyżował wam plany zakupu mieszkania?**

Są dwie szkoły - jedna, że ta bańka pęknie, ale ja jestem za drugą szkołą, mam dystans. Na pewno zmieni się sytuacja na rynku, ale nie chcę się nastawiać pozytywnie. Nie wiem, co nam przyniosą następne 2-3 miesiące. Dlaczego mam się przejmować, jak potem mogę dowiedzieć się, że ceny jednak poszły w górę. Teraz jest wszystko zatrzymane. Ja czekam do września na umowę na czas nieokreślony, bo dopiero wtedy mogę myśleć o kredycie na dobrych warunkach, ale teraz sytuacja się zmieniła i nie jestem w stanie powiedzieć, co będzie we wrześniu. Patrzę też po znajomych. Ta koleżanka od ślubu, to oni też kupują mieszkanie na kredyt i wpłacili zadatek. Ale nie dostali kredytu, bo banki są zdystansowane, tak jak ja. I dostali odpowiedź odmowną.

**Co oznacza to "zdystansowanie"?**

Może trochę się w środku boję, dlatego mówię sobie, że trzeba być bardzo mocno obiektywnym i staram się utwierdzać w tym obiektywizmie. Najłatwiej o niego z dystansu. Robię to tak, że najpierw wyobrażam sobie siebie tutaj, potem w tym okropnym bloku, potem we Wrocławiu, potem w dolnośląskim i okazuje się, że jestem taka mała i nieznacząca, że moje decyzje nie mają wpływu na nic. I wtedy udaje mi się złapać ten dystans. Łatwiej mi się wtedy kierować umysłem, a nie uczuciami. Ta wielka dla mnie decyzja nie jest wielka w skali globalnej. Tysiące podejmują takie decyzje. Zazwyczaj korzystam z tej metody. Teraz stosuję ją prawie na co dzień. Wcześniej robiłam to, gdy na przykład ktoś mnie w pracy zdenerwował. Ostatnio rozmawialiśmy o tym mieszkaniu i z jednej strony słyszę, potanieją. I ja tak, zobaczymy, tak się zdystansowałam i stwierdziłam - właśnie. Zobaczymy.

**Czy masz jeszcze jakieś ważne przemyślenia?**

Chyba nie. Myślę, że pomyślę o takich rzeczach za godzinę. Będę wtedy miała takie filozoficzne myśli.
