# Supplementary material for: The challenges arising from the COVID-19 pandemic and the way people deal with them. A qualitative longitudinal study
Source: PLoS One. 2021 Oct 11;16(10):e0258133. doi: 10.1371/journal.pone.0258133 (PMC8504766; doi:10.1371/journal.pone.0258133)
Supplement: S1 Dataset — (ZIP) [file pone.0258133.s003.zip › Transcriptions/stage 1/4.1_M_32_couple, no children.docx]

**4.1_M_32_couple, no children**

**Powiedz coś o sobie.**

Mam 32 lata, mieszkam w Warszawie, mam wykształcenie wyższe techniczne. Nie mam dzieci, mam żonę. Mój obecny oficjalny status zawodowy jest taki, że jestem bezrobotny, ale nie jest to związane z koronawirusem. W czerwcu ubiegłego roku złożyłem wypowiedzenie z pracy i pojechaliśmy z żoną na kilkumiesięczne wakacje. Od września zajmowałem się pracami dorywczymi i obecnie od stycznia zacząłem szukać pracy. Byłem na kilu rozmowach kwalifikacyjnych, ale wciąż szukam, zastanawiam się, co chciałbym robić. Teraz jest koronawirus, więc mam trochę wymówkę, żeby nie szukać. Pewnie w najbliższym czasie byłoby i tak ciężko coś znaleźć. A wcześniej zajmowałem się marketingiem online.

**Kiedy obecna sytuacja się dla ciebie zaczęła?**

To zależy, jak na to spojrzeć. Przełomowym punktem był moment - to był czwartek lub piątek - w którym było jakieś wystąpienie prezydenta lub premiera o tym, że wprowadzają te pierwsze rzeczy - wtedy chyba nie było jeszcze mowy o izolacji społecznej, ale, że wprowadzają pierwsze rzeczy online, że będą jakieś ograniczenia. Powoli powstawała panika, że będzie szedł ten wirus, więc ludzie zaczęli się rzucać do sklepów. Potem władza zaczęła powoli, ale nieśmiało dawać komunikaty, że oni wprowadzą jakieś obostrzenia. I u mojej żony w firmie były wytyczne, że wszyscy będą pracować zdalnie. Ona w środę zbierała rzeczy z firmy, żeby mieć to, czego będzie potrzebować na kilka najbliższych tygodni. Ale ona i tak często pracuje z domu, więc dla nas to nie jest jakaś sytuacja zupełnie nietypowa. A ja w czwartek robiłem większe zakupy na takiej zasadzie, że i tak nam się skończyło trochę rzeczy w domu, ale kupowałem trochę też rzeczy ekstra, bo nie wiadomo było, co to będzie. I to był punkt, w którym dla mojej rodziny, dla mojego gospodarstwa domowego to się zaczęło. Moja rodzina trochę później zaczęła się przejmować taką izolacją społeczna. I dla mnie to był ten moment. Ale te doniesienia, co się dzieje we Włoszech i świadomość tego, że to do nas przyjdzie, powstała gdzieś wcześniej. Nie potrafię dokładnie powiedzieć, kiedy, ale pewnie jakoś przed marcem. Zwłaszcza, że mamy znajomych, którzy przebywali długo we Włoszech. I było tak, że już się zaczęło coś mówić - to nie była jeszcze ta poważna sytuacja w Bergamo, Mediolanie, itd. - ale już się bali, że zamkną im lotniska i wracali trochę w pośpiechu całą rodziną z długiego pobytu. My wtedy, może nie tak, żebyśmy się jakoś strasznie martwili, ale zastanawialiśmy się, że to ich dotyczy. I byliśmy na bieżąco, bo to już zaczęło nas dotykać. Tym bardziej, że tam był taki temat, że być może trzeba będzie im jechać pomóc. Więc dla mnie ten moment przełomowy to było 3-4 tygodnie temu, gdzieś na początku marca, gdy myśmy się zamykali w domach. To był początek tej akcji #StayAtHome. Ale gdzieś wcześniej też o tym mówiliśmy, rozmawialiśmy i gdzieś to się pojawiało.

**A dlaczego właśnie ten punkt był dla ciebie przełomowy?**

Bo zmieniliśmy trochę organizację funkcjonowania. To był pierwszy moment, w którym zaczęliśmy myśleć o tym, że - bez paniki szczególnej, bo wiedzieliśmy, że to... Oczywiście, są pojedyncze osoby, które mogą mieć pecha, czyli możesz akurat w najbliższym sklepie, na ulicy, w tramwaju, spotkać kogoś, kto właśnie chory wrócił z Chin, Włoch, Niemiec, czy skądkolwiek. Natomiast mieliśmy taką świadomość, że jak nie jesteśmy osobami, które podróżują, są ciągle gdzieś w centrum, tylko gdzieś mieszkamy sobie na bocznych obszarach Warszawy i też jesteśmy dość mocno w stanie obniżyć tą ilość kontaktów społecznych, np. zawodowo. Ja w tej chwili nie pracuje, a gdybym pracował tak, jak wcześniej, to też, z tego co wiem, cała moja poprzednia firma przełączyła się na online. Firma, w której pracuje żona, przełączyła się w 4 dni na online, bo to są takie branże, które mają taką możliwość. Więc tylko tyle, że myśmy na początku zrobili trochę większe zakupy, ograniczyliśmy jeżdżenie komunikacją miejsca i chodzenie do kawiarni, galerii i takich rzeczy. I dlatego to był ten ważny moment, bo, bez paniki, ale zaczęliśmy inaczej funkcjonować. Idąc za tym, co było w komunikatach od rządzących, że każą coraz bardziej uważać, śledziliśmy te statystyki i zastanawialiśmy się, jak będzie wielki przyrost i kiedy nastąpi apogeum chorowania. Natomiast cały czas to było w atmosferze braku paniki, ale uważania na siebie.

**Jakie były ważne momenty w rozwoju tej sytuacji?**

Pierwsze sygnały o tym, że będzie jakiś rodzaj *lockdownu* - zamykanie granic, zamykanie się w domach, być może jakiś zakaz zgromadzeń, mówiło się o zamkniętych szkołach - to są takie pierwsze oznaki, że coś się będzie działo i to już wpływa na twoje życie mniej lub bardziej. Na nasze nie wpływało w sposób drastyczny, ale zmieniliśmy kilka naszych przyzwyczajeń w ciągu tych kilku pierwszych tygodni. Raczej bym powiedział, że to jest zmiana w stylu wspinania albo staczania się po równym terenie. To znaczy, że naokoło nas i my sami też coraz bardziej staramy się uważni. Tych kontaktów na początku obcięliśmy do połowy, a teraz już pewnie wyeliminowaliśmy 3/4 albo nawet 90%. Na początku jeszcze spotykaliśmy się, może nie w wielkich gronach, ale np. z rodziną rodzicami, siostrą, babcią i pojedynczym kolegą - tak, żeby mieć jakieś kontakty społeczne, ale takie w małych grupach, w otwartej przestrzeni. A teraz już powoli dochodzimy do tego momentu, że bardzo na to uważamy i właściwie nie mamy większych kontaktów. Dlatego mówię, że to jest takie stopniowe. Coraz mniej tych kontaktów, dokładniejsze mycie rąk. My raz dziennie - może to jest takie przesadzone - ale płynem do dezynfekcji przemywamy klamkę zewnętrzną i wewnętrzną od dziwi wejściowych. Mamy psa i dosyć często wychodzimy nadal z psem, mimo wszystko, ale tych wejść/wyjść do domu jest sporo i te rzeczy, których dotykamy po wejściu do domu przed umyciem rąk raz dziennie dezynfekujemy.

**Te zmiany wynikają z obostrzeń rządu czy to są wasze wewnętrzne decyzje?**

To, że rząd coś zmienia, wprowadza, wpływa na nasze nastawienie psychiczne, natomiast prawie w ogóle nie... Jesteśmy dwa kroki przed tymi zmianami, mam wrażenie. Np. jeśli oni mówią, że od jutra co drugie miejsce w autobusie musi być wolne - dla mnie to trochę dziwna regulacja, która jest trudna do egzekwowania i wymaga dużo dobrej woli od obywateli - to mnie to nie dotyczy, bo ja od dwóch tygodni nie jeżdżę autobusem. Jeśli oni mówią - proszę się nie spotykać, zamykamy kawiarnie, kina - to ja już nie byłem od dwóch tygodni w żadnych. Nic, co na razie zostało wprowadzone, poza tym, że się nie spotykam ze znajomymi w grupach kilku, kilkunastuosobowych - to jest coś, co powstało razem z decyzją rządu. Reszta rzeczy, mam wrażenie, że my jesteśmy ostrożniejsi, ale też dlatego, że nas to dużo nie kosztuje. Jesteśmy ostrożniejsi niż rząd. Jesteśmy w komfortowej sytuacji.

**Skąd wiecie, co trzeba robić?**

Wydaje mi się, że jest mnóstwo rzeczy, które należą do wiedzy ogólnej - ale może tutaj tak nie jest. Po pierwsze pojawiło się dużo artykułów i śledziliśmy, co się dzieje w Chinach, w Wuhan, w Hubei, i też z ciekawością ja patrzyłem, jak sobie radzi rząd chiński z tą sytuacją. Człowiek widzi, jakie tam są zalecenia. Czytaliśmy o tym, jak przenosi się ten wirus, jak szybko, jakie są objawy, jakie są zalecenia WHO. Tych informacji jest bardzo dużo, one są wałkowane w kółko, więc człowiek wyrabia sobie jakąś wiedzę. Wydaje mi się, że ja mam taką, może bardzo ogólnikową wiedzę o tym, jak się rozprzestrzeniają epidemie jako elementy systemu złożonego. To było związane trochę z moim tokiem studiów - miałem zajęcia z modelowania rozszerzania się epidemii. Więc mam świadomość tego, że izolacja społeczna jest dobra zarówno dla całego społeczeństwa, jak i dla mnie, to znaczy chroni mnie, że my tutaj nie będziemy zarażeni i nie będziemy musieli się udawać do szpitala, w którym prawdopodobnie w szczycie zachorowań nie doznamy dobrej opieki. A jakbyśmy byli w bardzo ciężkim stanie, to być może umrzemy. A dodatkowo pomagamy innym przez to, że siedzimy w domu, że nie zarazimy kogoś, albo nie rozniesiemy dalej tej epidemii, nie zajmiemy komuś informacji w szpitalu. To jest miks wiedzy i docierających informacji. Tej wiedzy dałbym tak 20/30%, a jednak większość jest informacji. Gdyby u nas panowała Ebola, SARS, choroba wściekłych krów, czy jakaś inna, to, jak się nie jest epidemiologiem, to trzeba by było za każdym razem się dowiedzieć, jak konkretnie działa ten wirus. W jednym przypadku nie jedliśmy wołowego mięsa, w innym unikamy ptactwa, a teraz musimy unikać ludzi akurat.

**Jaki masz stosunek do tej sytuacji?**

Rozbiłbym to na wiele poziomów. Stosunek do tego, że występuje epidemia - tak, epidemia jest naturalną rzeczą na świecie. Teraz jest większe wyzwanie, ponieważ jesteśmy zglobalizowani i te choroby się bardzo mocno przenoszą. Jesteśmy jak w wielkim mrowisku, w którym się bardzo wiele dzieje, więcej niż w jakimś średniowieczu i myślę, że to jest wyzwanie. Po drugie, my w Europie czy w Ameryce, żyjemy w bardzo dużym dobrobycie i jesteśmy przyzwyczajeni, że jesteśmy szczepieni, nie ma polio i tego typu rzeczy, nie umieramy z głodu, prawie nikt nie umiera, bo ma chorobę płuc, czy się zatruł czymkolwiek. Jesteśmy dobrze odżywieni, dobrze leczeni, mamy szczepienia, więc trudno nam jest pokiwać głową i powiedzieć, że po prostu ileś osób umrze i tak już jest. Myślę, że 300 lat temu było trochę łatwiej. No, może nie łatwiej, bo ludzie byli przerażeni, ale dla nas to jest takie zaskoczenie, odzwyczailiśmy się od tego, że są sytuacje wyjątkowe i nagle nam się zamyka ulubiona kawiarnia albo nie możemy wyjsć z domu. Takiej sytuacji w Polsce nie było już od... Ja mam 30 lat i ze swojego życia nie pamiętam tego typu rzeczy. Ostatnie to było pewnie w stanie wojennym, czyli 1981. Czyli 40-latkowie nie pamiętają takiej sytuacji, a 50-latkowie pewnie też, bo jak ktoś miał 5 czy 7 lat to niewiele pamięta. I to jest dla nas zaskoczenie duże, a to jest trochę naturalna rzecz.  Jakie jest moje nastawienie dalej do tego? Uważam, że jesteśmy nieprzygotowani jako kraj, ale też jako te wszystkie kraje z klubu Europy, Ameryki, Australii, Nowej Zelandii - tych, które nieszczęścia zwykle omijają, bo są zamożne, dobrze przygotowane, mają różne służby, są higieniczne i nawet terrorystów w miarę szybko wyłapujemy. A tu nagle się okazało, że jednak nie do końca jesteśmy przygotowani. Być może nawet jest trochę tak, że kraje, które są biedniejsze, też będą się z tym zmagać, bo mają różne niedobory, itd. Natomiast mają to opracowane, a my trochę zapomnieliśmy, jak to jest radzić sobie z takimi sytuacjami, jak głód, wojny, epidemie. My jako Polacy też nie jesteśmy przygotowani, bo uważam, że Polska jako kraj jest słabo zorganizowana.

**Czujesz jakieś zagrożenie w tej sytuacji?**

Trochę tak, ale nie jakieś bardzo wysokie. W skali od 0 do 10...

**Skala lęku**

Przed koronawirusem byłbym na 10, bo zawsze człowiek się czegoś boi, a teraz jestem na 20.

**Przed czym jest ten strach/lęk w twoim przypadku?**

Po pierwsze, istnieje małe prawdopodobieństwo, bo ja i moja żona jesteśmy zdrowi, większość osób w mojej rodzinie też, że mi się coś stanie. Jest szansa, że się nie zarażę, jest szansa, że będę tym cichym nosicielem, a nic mi nie będzie, a nawet jak przejdę, to w związku z moim wiekiem, jest duża szansa, że jak już się zarażę i coś mi będzie, to ja z tego wyjdę. W związku z tym, powiedzmy, mam 1/100 albo 1/10 procenta szans, że umrę. Zagrożenie jest wielkie, ale ta szansa jest mała, więc się jakoś bardzo nie boję. To tak samo, jak codziennie może mnie potrącić samochód, spaść samolot którym lecę albo cokolwiek innego - to jest jakiś lęk, człowiek uważa, żeby mu bagażu nie ukradli na lotnisku, nie chodzi wieczorem po najgorszej dzielnicy miasta sam. I tutaj też - robię, co mogę. Siedzę w domu, nie mam za dużo kontaktów społecznych i muszę liczyć na to, że się nie zarażę i nie jestem przerażony. Trochę się martwię o członków mojej rodziny, bo tu skala rośnie. Jest kilka osób i wystarczy, że zabije jedną, to będzie mi też przykro. I trochę się o nich boję, bo nie wszyscy są tak dobrego zdrowia. I to są te główne rzeczy, które napawają mnie lękiem. Trzecia rzecz, to jest w małym stopniu, bo tutaj jest duże prawdopodobieństwo, ale mniejsze zagrożenie. To znaczy, że sytuacja ekonomiczna mojego gospodarstwa domowego ulegnie pogorszeniu. Np. będę miał trudniej znaleźć dobrą pracy albo nie będę mógł się realizować w takim kierunku, w jakim chcę, bo będę musiał wybrać jakąś pracę, która mnie mniej interesuje, ale jest za lepsze pieniądze i pozwala nam się utrzymać. Ale to mało wpływa, bo ni sądzę, żebym nie miał, co do garnka włożyć w przeciągu pół roku… właściwie nigdy - pół roku, roku.

**To są obawy o to, co jest teraz, czy o to, co będzie w przyszłości?**

Obydwie rzeczy są w przyszłości. W tej chwili nie zachoruję, bo nie wychodzę. A ekonomicznie w tej chwili sam koronawirus nie obniżył naszych przychodów.

**Ten strach o bliskich jest większy niż o siebie samego?**

Bo zdaję sobie sprawę, że prawdopodobieństwo jest większe. Nie umrą wszyscy, ale nie przejdą tego bardzo ciężko, ale komuś się może to nieszczęście przytrafić. Mam 2 starsze osoby w najbliższej rodzinie w wieku 60+, dwie 50+ w tym jedna średniego zdrowia i jeszcze mam ciocię, która ma problemy typu astma - nie jakieś silne, ale jest w grupie ryzyka. O nią też się trochę martwię. Ona wychowuje dwójkę dzieci i to byłaby ogromna strata i trudna sytuacja, gdyby coś złego jej się stało.

**Jakiego określenia używasz na tę sytuację, kiedy rozmawiasz z bliskimi?**

Czasami mówię w obecnej sytuacji albo w stanie epidemii. Zależy, do czego to się odnosi. Jeśli mówię o sytuacji zdrowia albo poczynaniach rządu, które mi się nie podobają, to mówię o jakimś bałaganie, o burdelu albo coś w tym stylu.

**Spójrz teraz na obrazki. Które z nich najlepiej oddają twoje emocje w tej chwili?**

Hmm... Trudne to jest. Nie wiem. Jest mi trudno odpowiedzieć na to pytanie. To zależy o jakim aspekcie tej sytuacji pomyślę. Nie widzę niczego takiego, co by miało w sobie element złości. Pożar ewentualnie jest taki, bo szkoda lasu, który płonie. Np. 9 to coś strasznego, przykrego - jakiś żywioł, trochę jak ta epidemia. Ale każdy z nich jest dosyć spokojny, ma w sobie coś kojącego. Ja jestem dość spokojny, ta sytuacja nie wpłynęła na moje życie jakoś dramatycznie, nie boję się kryzysu gospodarczego tak strasznie. Ale jestem zirytowany sytuacją polityczną w naszym kraju i to, że rząd nie chce wprowadzić tego stanu wyjątkowego, przełożyć wyborów, itd., to strasznie mnie to wkurwia. I nie ma tutaj obrazka, na którym widziałbym złość. O, 15 ma taką falę rozbryzgującą się. To może to. Są ludzie, którzy poświęcają się mocno, jacyś lekarze, pielęgniarki, część społeczeństwa siedzi w domu, itd, a ileś osób u steru marnotrawi różne wysiłki ludzi. I to mnie denerwuje. 12 mi się też podobała, bo była takim - nie lubię takich obrazków - ale te kamienie są takim zen wewnętrznym. Różne rzeczy być może się dzieją - wieje wiatr, deszcz pada na te kamienie i one sobie prawdopodobnie będą i tak stały i to ich nie wzrusza. I to jest taki element, jak się czuję. Na zasadzie, takiego stoicyzmu trochę, że naokoło rzeczy się dzieją, ale one po prostu są i pytanie, jak do nich podejdziemy. Może tak ludzie nie myślą, ale ja sobie tak myślę, że jak sobie człowiek planuje życie na 60 czy 70 lat, to trudno nie spodziewać się, żeby nigdy się nic nie zawaliło, nie zniszczyło. W związku z tym, jak przychodzi taka epidemia, a póki co dla mnie nie jest dramatyczna, to właściwie nie widzę powodu, żeby aż tak bardzo... Cieszę sie, że mogę podejść do tego ze stoickim spokojem.

**A kiedy ta sytuacja byłaby już dramatyczna?**

Być może w moim odbiorze osobistym byłaby już dramatyczna, gdyby wielu moich bliskich, znajomych, przechodziła przez tą chorobę. Gdyby leżeli w szpitalu albo w domu, nikt by im nie mógł pomóc i by umierali. Nie wiem, jak będzie za chwilę, ale wydaje mi się, że w tym momencie służby jakoś działają mimo wszystko.

**Wracając do tych obrazków, ty jesteś tym kamieniem, jak na obrazku?**

Chciałbym być. Mi jest łatwo, nie mam dzieci. Jakbym miał na moim metrażu jeszcze dwójkę dzieci, to być może już nie byłbym tym kamieniem zen. Wydaje mi się, że wszystkim nam ta cierpliwość w obecnej sytuacji jest potrzebna. Trzeba siedzieć w domu, może będzie chudszy okres. Ja wiem, że wielu osobom się teraz wali świat, jak np. ktoś zainwestował w biznes pół roku temu duże pieniądze, a teraz się skrócił, bo to jest hotel np. Są branże, które cierpią. Jeśli ktoś dokonał dużej inwestycji zaraz przed epidemią, to ja go rozumiem, że jemu nie jest tak łatwo. Ja jestem w takiej sytuacji, w której na to zen mogę sobie pozwolić.

**Ten spokój wynika z tego, jaką jesteś osobą, ale też z tego, że sytuacja ci na to pozwala?**

Tak. Ale częściowo, ja mam też świadomość, że jestem osobą uprzywilejowaną. Że jestem wykształcony, miałem możliwość wsparcia rodziców i poukładania sobie życia w odpowiedni sposób. Natomiast uważam, że na jakimś poziomie zrobiłem to dobrze i jestem w miarę przygotowany na tego typu sytuację. Być może nawet gorzej w sytuacji dużego prosperity funkcjonowałem gorzej - nie osiągnąłem szczytu kariery, nie żyłem pełnią życia, podróżując. Są takie migawki czasami. Ludzie na Instagramie wrzucają, jakie robią fantastyczne rzeczy. Może nigdy aż tak niesamowicie nie funkcjonowałem, ale z drugiej strony mamy oszczędności, które, nawet, jeśli oboje stracilibyśmy pracę i nie mieli dochodów, to musielibyśmy obniżyć standard życia, ale bylibyśmy w stanie funkcjonować z zaoszczędzonych środków przez jakiś okres - 3 miesiące, pół roku, może nawet rok. Dlatego cieszę się, że mogę mieć trochę zen.

**A z czego wynika ta złość, o której mówiłeś?**

Po pierwsze, uważam, że jesteśmy nieprzygotowani na to jako Polska i że zmarnowaliśmy czas, kiedy było wiadomo, że ten wirus do nas dotrze i trzeba będzie wprowadzić działania epidemiczne. Po drugie, teraz uważam, że bardzo duża część społeczeństwa w jakimś stopniu poświęca się i organizuje i oddolnie ludzie robią często lepszą robotę niż rząd. Ludzie zrzeszają się, szyją maseczki, przywożą jedzenie do szpitali. To jedzenie dla szpitali, to jest bardziej symboliczne, ale myślę, że mimo wszystko, dzięki temu ratownicy mogą się poczuć docenieni przez zwykłych ludzi. A potem słyszymy, że rządowe działania są źle wykorzystane albo wręcz czasami dobre działania są blokowane. Przykład z ostatnich dni - samolot zakupiony przez WOŚP został przyblokowany z jakiegoś powodu. I to wygląda na sprawę polityczną. WOŚP jest na cenzurowanym i w związku z tym, żeby nie było sukcesu Owsiaka, przyblokują przywiezienie respiratorów, maseczehcz, czy czegoś takiego - nie wiem, co tam dokładnie jest. Albo mu to zarekwirują, jak to przywiezie, bo jest ta ustawa, że nie wolno wwozić/wywozić takich rzeczy i one trafią do rezerw. Mam mniejsze zaufanie do rządu niż do Jerzego Owsiaka i uważam, że Jerzy Owsiak z WOŚPem rozdysponuje te środki, na które sam zebrał pieniądze od ludzi, lepiej niż rząd.

**Czy zacząłeś tę złość odczuwać teraz czy ona się pojawiła wcześniej w jakimś konkretnym momencie?**

Ona jest gdzieś cały czas w związku z tym, że ja źle oceniam działania rządzącej partii. Ta złość na ich działanie jest od dawna, ale gdzieś tam w tle. Ale a propos obecnej sytuacji to zdenerwowanie wskoczyło na wyższy poziom, bo ja już jestem zirytowany ich działaniami z poprzednich okresów rządu. W pierwszej chwili, jak zdecydowali o zamknięciu szkół, to uważałem, że to jest dobra reakcja. I był taki moment, kiedy sobie pomyślałem, że to jednak są skurwysyny, ale się dobrze zachowali. A teraz coraz częściej, mimo, że nadal dobrze tamte działania oceniam i zrobili, to co należało, to bardzo negatywnie oceniam i bardzo mnie to denerwuje, że są takie pojedyncze przesłanki, składające się na obraz tego, że rząd próbuje nie mówić o tym, jakie są niedobory, trudne sytuacje. Próbują przemilczeć różne błędy, niepowodzenia. Tam była ta sytuacja z pielęgniarką, którą zwolniono za to, że napisała na Facebooku, że w jej szpitalu brakuje maseczek i strojów ochronnych. Potem było to pismo, które podpisał chyba minister albo vice minister, że prosi, żeby nie wypowiadali się lekarze, tylko osoby uprawnione. Wyglądało na to, że wypowiadać się mogą tylko osoby, które są rzecznikami z ramienia partii, które będą opowiadać tylko o tym, jak jest fantastycznie. Może być w tym element próby skontrolowania tego, żeby nie było paniki i tak mogą to interpretować osoby, które ufają rządowi, że oni chcą, żeby był spójny przekaz, żeby ludzie czuli się bezpieczni, itd. - może tak. Natomiast ja im nie ufam i uważam, że to jest gra polityczna. I ten dodatkowy aspekt, że nie chcą przełożyć wyborów prezydenckich, że Andrzej Duda, jako jedyny z kandydatów ma możliwość prowadzenia kampanii wyborczej, a inni nie. Mam wrażenie, że jest to bardzo sprytnie wykorzystywane i że duża część aparatu rządzącego nie skupia się na działaniach antyepidemicznych, ale też na tym, jak to zrobić, żeby dobrze wypaść, ugrać jakiś kapitał polityczny, żeby ludzie widzieli w nich bohaterów narodowych. Jak robią to tylko na zasadzie białego PRu,  że Andrzej Duda chodzi po szpitalach, ma zdjęcia z zakasanymi rękawami jak amerykański prezydent, to jest w porządku. Ale jak już zakazuje i wyrzuca ludzi z pracy i próbuje ukrywać różne rzeczy - knebluje usta lekarzom, dziennikarzom, itp., to to już jest dla mnie ta czarna strona. Tak nie wolno robić, to jest łamanie zasad gry i na to jestem wściekły. Jesteśmy w specyficznej sytuacji. Część osób umiera i będzie umierać i można byłoby spróbować, żeby ludziom ulżyć i żeby śmierci było mniej - i na tym skoncentrować swoje działania. Poza tym, mnóstwo osób coś daje od siebie, są akcje społeczne i ogólne poczucie, że należy nie łamać zasad. 100% tego nie robi, ale na pewno więcej niż połowa. Część zmieniła nawyki konsumpcyjne i jeździ rzadziej do sklepów, robi zakupy online.

**Jakie podejmowałeś działania, żeby sobie radzić z tą sytuacją?**

Większość działań polega na braku działań. Musiałbym spojrzeć w kalendarz. Od którego my tak w domu siedzimy? No w lutym niemożliwe, że już. Ale jakoś od 6 marca? *[Głos żony w tle z podpowiedzią, że od 11 marca]* Naprawdę? Tylko tyle? Rzeczywiście, 3 tygodnie. Myślałem, że 4 tygodnie.

**Masz poczucie, że to trwa dłużej?**

Tak, mam wrażenie, że to już trwa miesiąc. Miałem wrażenie, że to był początek marca. I wracając do tych działań, to 11 marca pojechałem robić większe zakupy z myślą o tym, że zaraz będą jakieś zmiany, krótsze godziny otwarcia sklepów, może czegoś będzie brakować, itd. Zrobiliśmy większe zakupy.

**Czy to były zwyczajne zakupy, tylko, że większe, czy coś się zmieniło?**

Nie, zwyczajne zakupy, tylko trochę większe. Pamiętam, że we wtorek wieczorem wracałem z basen i weszliśmy z siostrą do Biedronki po jedną rzecz i pomyślałem, że może zrobię większe zakupy, ale było tuż przed 22:00. Spojrzałem na półki i powiedziałem: wszystkie produkty są na półkach, nie ma paniki, nie ma problemu. Więc stwierdziłem, że zrobię jutro albo po jutrze na spokojnie, jak nam się skończą rzeczy. I jak w środę albo czwartek poszedłem do sklepu, to się okazało, że nie ma połowy rzeczy na półkach. Więc kupiłem to, co było. I w piątek poszedłem jeszcze dokupić pare rzeczy, takich, które kupujemy nie w Biedronce i przy okazji też takich, których w Biedronce nie było, czyli ryż i chyba pomidorów w puszce nie byłem w stanie w Biedronce kupić. Pojedynczych rzeczy brakowało. I od tego momentu przez ostatnie 3 tygodnie chodzę do sklepu raz w tygodniu. Teraz pewnie jeszcze rzadziej będę to robił, raz na 10 dni. Robię jedne zakupy z dużą, rozbudowaną listą. Raz jadę do dużego sklepu - do Biedronki albo czegoś podobnego i raz w tygodniu chodzę do piekarni po świeże pieczywo. Jak uznam, że to jest dodatkowe ryzyko, to zrezygnuję z tej piekarni i zacznę kupować niedobre pieczywo z Biedronki. W piątek, 13 marca, nie poszliśmy na imprezę na urodziny koleżanki i od tego momentu już nie widywaliśmy się ze znajomymi. Ona w końcu je odwołała i się spotkaliśmy na Messengerze. W 2 weekendy 14-15.03 i 21-22.03 jeszcze funkcjonowaliśmy normalnie w rodzinie. Widywaliśmy się z rodzicami, teściami, dziadkami. Natomiast od tego tygodnia już powoli staram się też tutaj, żeby tego kontaktu było mało. Widujemy się czasami, żeby wymienić jakież rzeczy, jak paczki, zakupy, itp. Dzisiaj poszedłem do teściowej po kamerkę i tyle, że podała mi przez próg i pomachaliśmy sobie, żeby na siebie nie chuchać, nie dmuchać. Wieczorami jeżdżę na rowerze jak jest ciepło. I nawet jeździłem z kolegą, uznając, że odległość duża zachowana i to jest ok. On jest programistą i też właściwie nikogo nie widuje, więc szanse, że y jesteśmy zarażeni są bardzo małe, a przynajmniej jest jakiś kontakt z kim innym niż własna żona. Dosyć dużo gram w planszówka generalnie, a teraz się przełączyłem na granie ze znajomymi online. Są takie rozwiązania, jak Kurnik, itp. i można grać.

**Zawsze robiłeś zakupy z listą czy to się teraz pojawiło?**

Zakupy z listą staram się robić, ale od kiedy mam więcej czasu, to częściej chodziłem do sklepu tak na zasadzie, żeby coś dokupić na bieżąco, jakbym zapomniał. Jak oboje z żoną pracowaliśmy na etatach i czasu było mniej, to się bardziej skupialiśmy na tym, żeby zaplanować obiady na najbliższe dni, bo nie chciałoby nam się iść po jedną rzecz. Lista zawsze funkcjonowała, ale jak miałem więcej czasu, to było do niej luźniejsze podejście.

**A pojawiły się jakieś produkty, których wcześniej nie kupowałeś?**

Nie. Kupiliśmy trochę rzeczy takich długoterminowych, gotowych lekko, jak np. tortellini, że jakby nam zabrakło i już nie było albo jakby już strzelali na ulicy do ludzi, to żeby można było jeszcze ograniczyć wychodzenie z domu. Ale to bardziej na zasadzie, że albo jak będziemy leniwi albo jak się rozchorujemy i nie będziemy chcieli gotować. I to sobie leży, tego nie ruszamy, a tak to jadamy to, co do tej pory jadaliśmy. Wydaje mi się, że w porównaniu do innych ludzi, my zawsze mieliśmy w domu jakieś zapasy. Nie z lęku, ale też z takiej wygody, że jak nie chce nam się iść do sklepu, to puszki z pomidorami i ciecierzycą są i zawsze można coś zrobić.

**Czyli nie czujesz, że twoje zachowania konsumencie się teraz bardzo zmieniły?**

Dotyczące żywności nie. Ale wszystkie wyjścia do pubu, knajpy, itd. - to się ucięło. Mamy więcej czasu, jesteśmy we dwójkę, więc fajnie razem pogotować i jemy mniej na mieście. U nas jedzenie na mieście było takim społecznym elementem spotkań ze znajomymi - teraz tego nie ma. Więc dużo mniej pieniędzy idzie na knajpy i piwo w pubie.

**Co jest dla ciebie największym wyzwaniem w tej sytuacji?**

Ja jestem człowiekiem rodzinnym i dość towarzyskim i mi tego brakuje dość mocno, takiej swobody spotkań i swobody poruszania się. Natomiast jest to do przeżycia. Nie cierpię na pewno, dlatego, że jesteśmy z żoną w domu i już nie możemy na siebie patrzeć - to nie jest dla nas chyba problem. Dla nas problemem będzie, jeśli możliwość spacerowania byłaby mocno ograniczona. Bo naszym dziennym rytuałem są spacery z psem. To może być zarówno dla nas, jak i dla psa kłopot, jak nie będzie można sobie spokojnie 4 razy dziennie wyjść na zewnątrz albo pojechać gdzieś dalej. My często w weekend pakujemy psa do samochodu i jedziemy do Łomianek albo do Puszczy Kampinoskiej, gdzieś na łąkę. I jak te rzeczy zostaną uregulowane i zabronione, to coś, co będzie na mnie wpływać bardzo negatywnie.

**Czy przygotowujecie się jakoś na taką możliwość, że nie można byłoby wyjść z psem?**

Takiej możliwości, żeby higienicznego wyjścia z psem nie było, to chyba nie. Może być kłopot, gdybyśmy zostali poddani tej przymusowej kwarantannie, bo teoretycznie nie wolno wyjść na zewnątrz nawet z psem i idea byłaby taka, żeby nikt nie przychodził psa wyprowadzić. To jest taka jedyna rzecz, na którą nie jesteśmy przygotowani. Pewnie, gdyby ta choroba dotknęła kogoś z mojej rodziny, np. babcię czy teściową, czyli takie osoby, które potrzebowałyby więcej pomocy, to pewnie bym jechał np. do szpitala. U nas nie ma dzieci i jestem tylko ja i żona i jesteśmy zmotoryzowani, więc pewnie byłbym taką pierwszą osobą, która jechałaby do szpitala, żeby kogoś zawieźć albo odwiedzić. Wolałbym, żeby nie zajmowali się tym moi rodzice, bo są bardziej zagrożeni i mają kontakt ze swoimi wnukami. Dwie rzeczy, które mam nieobcykane to: co zrobić z psem, jak nas zamkną na amen i co zrobić, jak ktoś z moich bliskich będzie chory i trzeba będzie jechać do szpitala albo pomagać. O, żona mi podpowiada jeszcze jedną zmianę - nie chodzimy do kościoła. Na mnie to wywarło mniejszy wpływ.

**A jak twoi bliscy radzą sobie w tej sytuacji?**

Nie spotkałem się z czymś takim, żeby ktoś powiedział, że to głupie gadanie i on się nie stosuje albo jest strasznie wściekły albo przerażony strasznie. Jedna sąsiadka na osiedlu jest mocno przestraszona, a poza tym większość naszych bliższych znajomych tak spokojnie do tego podchodzi. Jeden kolega powiedział, że się w ogóle nie przejmuje, chodzi do pracy, tylko go wkurza, że mu się życie socjalne wyłączyło i nie może wyjść na piwo. Drugi powiedział, że właściwie to okazja, żeby samemu przetestować pracę zdalną przez miesiąc jako taka rzecz na przyszłość czy zespół się dogada, itd. *[Głos żony w tle]*. A faktycznie, żona mi tu podpowiada, bo ja pomyślałem o swoich znajomych, a mamy też wspólnych. Żona ma 3 przyjaciółki i każda z nich jest w dużo trudniejszej sytuacji niż my. Jedna jest w kończącej się ciąży i będzie niedługo rodzić i martwi się, jak to będzie. Wie, że są obostrzenia w szpitalach i jej mąż nie będzie mógł do niej wejść. Poza tym jej mąż jest ratownikiem medycznym, więc w ogóle się martwili. Oni założyli po prostu, że on będzie chory na korona wirusa i trudno. Natomiast on zaplanował, że zanim ona będzie miała termin porodu, to on będzie miesiąc na kwarantannie. Więc on już miesiąc z nią nie mieszka i ona sama siedzi w domu. On mieszka z jej rodziną, w której wszyscy są medykami i zakładają, że będą chorzy. Oni się martwią, ale są twardzi psychicznie. Mamy koleżankę, która bardziej to przeżywa, bo siedzi z dzieckiem, a mąż jest policjantem i na nich wpływa to nieodprowadzanie dziecka do żłobka, bo mają kłopot ze zdalną pracą. Trzecia koleżanka się martwi, bo jest matką samotnie wychowującą dziecko i jest lekarzem. U nich w szpitalu jest słaba jest sytuacja. To jest dla nas przykład informacji z pierwszej ręki. Na przykład były transfery między oddziałami i na jakimś oddziale były już objawy, to ordynator powiedział, że nie ma powodu testować tych pielęgniarek, które tam były. Więc ona pracuje z osobą, które mają ogromne prawdopodobieństwo zarażenia wirusem. I jak będzie na kwarantannie, to jej dziecko pozostanie bez opieki. I ona się tym denerwuje.

**Obserwujesz wśród swoich znajomych zachowania, które oceniasz negatywnie?**

Wśród znajomych chyba nie. Słyszałem tylko o takich osobach. Ciocia ma sąsiadów, którzy są na kwarantannie po powrocie z zagranicy i jak przychodzi policja, to są w domu, po czym jak odjeżdża radiowóz, to jadą na zakupy. Raczej nie są zarażeni, ale od tego jest kwarantanna, żeby przestrzegać tej ochrony. I to jest takie negatywne zasłyszane zachowanie i pewnie jest ich tam sporo. Pytanie, ile negatywnych zachowań spowoduje przekroczenie punktu krytycznego, że epidemia się będzie rozwijać, bo pojedyncze będą zawsze.

**Skąd czerpiesz informacje o tym, co się dzieje?**

Z serwisów informacyjnych internetowych. Wchodzimy na: Gazeta.pl, TVN24, DoRzeczy, czasem na TVP, ale to rzadko, dzisiaj chyba Polsat przejrzałem. Raczej czytujemy dogłębniej te antyrządowe, nie prawicowe, jak TVN24, czy Gazeta, ale też zaglądamy na te po drugiej stronie, żeby mieć szerszy obraz. A i Facebook - czytamy co się dzieje w social mediach. Wiadomo, że to nie daje pełnego obrazu dokładnych danych, jest mnóstw *fake newsów*, itd., ale np. filtrujemy te treści w ten sposób, że wiem, że jak moja koleżanka, która jest lekarzem, napisze post o tym, co się dzieje w szpitalu albo da komentarz pod postem innej lekarki to oznacza, że ten post jest prawdziwy. Większe prawdopodobieństwo, że to nie jest ściema czy manipulacja.

**Czy te wiadomości, które znajdujesz w serwisach typu Gazeta.pl, uznajesz za prawdziwe?**

Wierzę bardziej chyba TVN24 i się przyzwyczaiłem do niego. Na Wirtualnej Polsce - może na samej, to nie, ale chyba Money.pl należy do tej spółki, i to uważam za w miarę sensowne. Też te Onetowe. Lubię czytać czasami Gazetę.pl, bo oni mają bardziej, nie newsowe artykuły, tylko dłuższe analizy, natomiast uważam, że Gazeta.pl jest czasami narwana i z nimi się trochę tak... W sensie mocno krytycznie podchodzę do tego, co tam jest napisane. I lubię czasem poczytać też tę prorządową część i sprawdzić, co oni tam piszą, żeby zobaczyć, co inni ludzie czytają oraz czy ja nie zamykam się w bańce informacyjnej. Tak do końca w 100% to chyba nie wierzę nikomu.

**Mógłbyś rozwinąć kwestię wiarygodności przekazu w mediach społecznościowych?**

Jeśli to jest informacja przekazana przez osobę, co do której wiadomości mam pewność to wierzę w to. Te wiadomości mogą też być uwiarygodnione, czyli np. tej osoby nie znam i odnoszę się sceptycznie, to jeśli ktoś mi to uwiarygodni i mam z trzech źródeł taką samą informację, to już jest to dla mnie jakiś rodzaj uwiarygodnienia. Idealnie, jak daną informację potwierdzają przeciwstawne opiniowo dzienniki. Na przykład, jeżeli na TVN24 napiszą, że jest fatalnie i kraj umrze zaraz, to nie wiem, ale jak już na TVP napiszą, że jest bardzo źle, to już wiem, że naprawdę jest źle.

**Czy w taki sam sposób podchodziłeś do różnych informacji przed epidemią?**

Tak, tak, stanowczo. Mam świadomość, że informacje, z którymi się stykamy, bardzo często mogą być nieprawdziwe i manipulujące, z różnych przyczyn. Taka najbardziej spiskowa jest taka, że ktoś ma w tym interes, żeby przedstawiać w jakiś sposób. Poza tym wszyscy grają na emocjach i chcą większej oglądalności, a wiadomo, że negatywne newsy się sprzedają lepiej, itd. Tworzy się taka tendencja do przesady. Nie ma parytetu dobrych informacji.

**Słyszałeś o jakichś spiskach, związanych z koronawirusem?**

No pewnie, że tak. Nie siedzę w tych teoriach bardzo mocno, ale słyszałem. Teoria nr 1 - ten wirus jest specjalnie wypuszczony przez Chińczyków, że testowali jakąś broń i to się wydostało z laboratorium. Może i tak - kto to tam ich wie. Więc, że to nie jest przypadek. Druga, to że Amerykanie spuścili na Chińczyków, żeby ich osłabić. Że ktokolwiek, Bill Gates z Warrenem Buffetem, chcą zdepopulować planetę - to takie, jak o płaskiej ziemi albo o szczepionkowcach.

**Traktujesz te teorie na równi z teoriami o płaskiej ziemi?**

Raczej tak. No to, że ten wirus mógł się pojawić, że ktoś niechcący coś wypuścił z jakiegoś laboratorium chorób zakaźnych i że Chińczycy to zatuszowali, to jest możliwe, nawet bardzo możliwe. Ukraińcy budowali elektrownię i akurat im wybuchła. Rzeczy się zdarzają. Ale taka bardzo teoria spiskowa, że to specjalnie, to nie.

**Skąd się wziął koronawirus?**

Ja bym się trzymał tego, że jest to jakiś rodzaj mutacji wirusa odzwierzęcego. Była jakaś teoria, że był owad, od którego się to przeniosło na nietoperze i stamtąd wyewoluował i to się przeniosło na ludzi. Możliwe też, że to było badane - i to jest druga opcja, że było badane w laboratorium i się komuś wymsknęło. Też bym się nie zdziwił, bo to nie musi być w złej wierze.

**Czy aktywnie poszukujesz informacji o koronawirusie?**

Do pewnego stopnia tak. To jest mój zwyczaj od wielu lat, że codziennie robię sobie prasówkę. Jak mam mniej cierpliwości to trwa tak 15 min., a tak z reguły przy porannej kawie poświęcam taką godzinę na przejrzenie różnych informacji. Teraz to trochę ograniczam, nie chcę mi się już, bo tego jest za dużo. I jest non stop tylko jeden temat wałkowany w dodatku często w nakręcający emocje sposób. Więc trochę się z tego wyłączam, żeby nie oszaleć.

**Czy poświęcasz więcej czasu na czytanie informacji niż przed epidemią?**

Nie, tyle samo. Na początku, jak *lockdown* się zaczynał, to wszyscy się interesowali, bo nowość i zupełna zmiana sytuacji w kraju. Więc wtedy czytaliśmy więcej, patrząc, co robi nasz rząd i co robią inni. Teraz widać, że to postępuje, ale w sposób opanowany, więc nie sprawdzam co pół dnia, ile osób się zaraziło, a ile umarło.

**W którym momencie to się uspokoiło?**

Dla mnie tak z tydzień temu. Pierwsze dwa tygodnie były takie, że nie wiadomo było, czy to bardzo szybko nie eskaluje. Jak przedłużyli do świąt, to miałem poczucie, że przedłużamy działania, które znamy, więc nic gwałtownego się nie zmienia. Pewna rutyna wchodzi. Przez ostatnie trzy tygodnie działam według opracowanego schematu, więc dopóki on się nie zmieni gwałtownie: nie muszę mieć przepustki do sklepu, nie muszę mieć przy sobie cały czas paszportu albo dopóki nie odbiorą mi psa. Nie wiem, co mogłoby się wydarzyć takiego. Rutyna uspokaja panikę.

**Czy korzystanie z mediów powoduje niepokój?**

Tak, na pewno. Media w dużej mierze zrobione są w ten sposób, że mają tę warstwę informacji, ale też sprzedają emocje. Więc, jak się ma cały czas dostęp do informacji, to człowiek cały czas tym żyje. Ile może być nowych informacji na temat koronawirusa? Niedużo. Więc zdrowo jest to ograniczyć, bo później człowiek nie dostaje już informacji, tylko emocje.

**Czy pojawiły się jakieś nowe źródła, z których wcześniej nie korzystałeś?**

Trafiłem na coś takiego *Epoch Times*, czy coś takiego. To jest polskojęzyczna strona o tematyce dalekowschodniej. Trafiłam tam przez Facebooka, gdzieś to się pokazało. I ta strona jest ewidentnie antychińsko-komunistyczna, czyli skierowana przeciwko chińskiej partii komunistycznej. Mam wrażenie, że tam może być dużo teorii spiskowych, więc podchodzę do tego ostrożnie. Ale tam jest dużo informacji o tym, jak ta sytuacja była rozwiązywana w Chinach i też dużo informacji o nadużyciach rządu Chińskiego. Nie mam pewności, czy to nie jest jakaś strona dla płaskoziemców - mam nadzieję, że nie. I tu jest na przykład taki artykuł: Chiny rozpoczynają globalną kampanię dezinformacyjną, która ma na celu zrzucenie na innych winy za wirusa. To brzmi jak teoria spiskowa.

**Dlaczego to brzmi jak teoria spiskowa?**

Wszystkie informacje o szpiegach, tajnych operacjach, o tym, że ktoś próbuje coś ukryć, itd. są podejrzane. Ja nie wierzę w większość teorii spiskowych, ale zdziwiłbym się, gdyby wszystkie były nieprawdziwe. Jak myślę sobie o chińskim totalitarnym rządzie, to bardzo możliwe, że to robią. Ale po przeczytaniu jednego artykułu ze średnio znanego źródła, to nie wiem, czy bym w to wierzył. Trafiłem tam na taką jedną fajną informację o tym, że Chińczycy tuszują prawdziwą liczbę ofiar tego wirusa. Tam były zestawione dane z chińskiego Ministerstwa Zdrowia, ile umarło osób na koronawirusa, z danymi z telekomunikacji, ile zmalała liczba kart sim.

**Dlaczego bardziej wierzysz w ten konkretny artykuł?**

Dlatego, że było odwołanie do zestawienia dwóch liczb z oficjalnych statystyk, które były sprzeczne. To trochę tak, jakby ktoś zatuszował jedną liczbę i zapomniał o drugiej. To do mnie przemówiło. Do artykułów z tego portalu podchodzę z zainteresowaniem, ale sceptycznie. Takie kraje, jak Chiny czy Rosja, są to dla mnie kraje o niskiej wiarygodności i możliwe, że informacje są zmanipulowane.

**Co sprawia, że nie jesteśmy dobrze przygotowani na tę sytuację jako Polska?**

W długofalowej perspektywie wiemy, że nasza służba zdrowia jest niedofinansowana i jest tam dużo problemów. Czyli już na starcie mamy do czynienia z organizacją, która jest w wielu miejscach niesprawna: niedobory lekarzy, pielęgniarek, kadra starzejąca, niewykwalifikowana. Po drugie, działania epidemiczne, czyli zapasy, plany strategiczne - mam przekonanie, że ich nie ma albo są słabe, tylko na papierze. Jestem przekonany, że u nas jest dość kiepsko. Dodatkowo, informacje, że taki stan może się u nas pojawić były 2 miesiące temu i mam poczucie, że zostało to przegapione, chociażby tym, że długo szły uspokajające komunikaty od rządu, a potem się okazało, że po tygodniu czy dwóch są niedobory i problemy organizacyjne. I nie ma rozwiązań, izolujących osoby podejrzane o zachorowanie. Nie ma izolatek, punktów przyjęć, gdzie ludzie są odosobnieni. Uważam, że to bylibyśmy w stanie zrobić lepiej. Jeżeli w połowie marca wszyscy połapali się, że to idzie wielkimi krokami, to od połowy stycznia były pierwsze raporty dokładne na ten temat.

**A jeśli chodzi o Europę, to masz podobne wrażenie, jeśli chodzi o przygotowanie do epidemii?**

Tak, zwłaszcza, że na Włochów padło, że u nich się to wysypało losowo. Na to się nałożył ich południowy temperament, że żyją bardziej socjalnie, wychodzą, przytulają, jest dużo więcej dotyku, pocałunki na przywitanie, itd. i u nich poszło szybciej. Zdziwiło mnie, że w Wielkiej Brytanii i chyba Szwecji też powiedziano, że nie będą robić tak radykalnych ruchów, jak zamykanie granic, po czym nastąpiła gwałtowna zmiana polityki. Tak samo w przypadku USA - Trump długo mówił, że ten koronawirus to mrzonka i teraz zmienili politykę.

**A gdzie byś tu umiejscowił Polskę na tle innych państw pod względem przygotowania do epidemii?**

Hmm... ciekawe pytanie. Myślę, że jesteśmy tak samo kiepsko przygotowani, jak większość. Nie uważam, że odstajemy mocno w dół, natomiast w stosunku do niektórych mieliśmy więcej czasu i wydaje mi się, że niektórzy sobie z tym za chwilę lepiej poradzą. Wierzę, że Niemcy są dobrzy organizacyjne i wprowadzą takie rozwiązania, które mniejszym kosztem i szybciej ich wyprowadzą z tej sytuacji. Z dużym zainteresowaniem będę śledził to, co w Szwecji się wydarzy, bo oni nie zdecydowali się na radykalne ruchy. Z tym, że mają trochę łatwiej, bo mają wydolniejszą służbę zdrowia, są bardziej zdyscyplinowani i nie dotykają się tak, jak Włosi.
