# Supplementary material for: The challenges arising from the COVID-19 pandemic and the way people deal with them. A qualitative longitudinal study
Source: PLoS One. 2021 Oct 11;16(10):e0258133. doi: 10.1371/journal.pone.0258133 (PMC8504766; doi:10.1371/journal.pone.0258133)
Supplement: S1 Dataset — (ZIP) [file pone.0258133.s003.zip › Transcriptions/stage 1/10.1_F_55_couple, no children.docx]

**10.1_F_55_couple, no children**

Mam 55 lat, mieszkam w Warszawie. Pracuję, możesz to zrobić - jestem liderem (miejsce pracy). Od 12 lat jestem Ambasadorką (miejsce pracy), od 3 jestem liderem. Mieszkam z mężem i z psem.

**Pierwszy moment, kiedy sytuacja się zaczęła, gdy coś się zmieniło?**

Trudno ustalić, kiedy był ten pierwszy moment. Jak zaczęły napływać te informacje z Chin. Mimo, że tam się zaczęło dużo wcześniej - w grudniu, to powiem szczerze, że ja w grudniu nawet o tym nie słyszałam. Ominęła mnie zupełnie ta wiadomość. Można powiedzieć, że na początku lutego poczułam, że nadchodzi jakaś zmiana i to może dotknąć Polskę. Co prawda wtedy tak tego jeszcze nie odbierałam, to było daleko. To akurat zaczęło się już chyba wtedy we Włoszech, te pierwsze przypadki. Nie było to jeszcze takie bardzo niepokojące, ale zastanawiające, że co się dzieje?

**Pamięta pani takie najważniejsze etapy rozwoju tej sytuacji w Polsce?**

Pamiętam od momentu, jak zamknęli szkoły. To była chyba środa 11 marca. Od czwartku jeszcze dzieci mogły chodzić do szkoły, natomiast już od poniedziałku były szkoły zamknięte. Ten moment pamiętam dobrze. To jest związane z tym, że pomagam synowi w przywożeniu przyszywanej wnuczki. 2-3 razy w tygodniu czasami ją odbierałam ze szkoły albo zawoziłam.

**Jakieś inne ważne etapy?**

Ta szkoła to był taki główny moment, że coś się zaczyna, że to już nie są żarty, że coś się już dzieje.

**Jak się pani czuła w tym momencie, gdy zamknięto szkoły?**

Uważałam, że to dobrze, że tak powinno zostać to zrobione. Natomiast zaraz potem zauważyłam, że jest takie rozluźnienie ogólne, że wszyscy sobie chodzą, spacerują. Bardzo mnie to denerwowało, że to nie są wakacje tylko powinno się jednak zostać w domu. Po prostu. Bardzo mnie kiedyś uderzyło i to tak najbardziej, bo mnóstwo jest tych filmików na FB...Ja akurat wpadłam na taki filmik z dziećmi - 3 młodych chłopców ok. 10 lat. Szkolnych, czyli zamiast być w szkole, to biegają sobie po dworze i mają frajdę. I dokuczają Wietnamce wyzywając ją bardzo brzydkimi słowami i oczywiście, że ma koronę, i żeby natychmiast - nie będę używała tych niecenzuralnych słów, ale żeby natychmiast wyjeżdżała. Jeszcze dodatkowo rzucają w nią jakimiś patykami a na koniec rzucają w nią lodami. To było dla mnie tak okropne...Pod tym względem, że tymi dziećmi nagle nie ma się kto zająć, że są tak puszczone na żywioł i po drugie, że teraz taka fala hejtu się zacznie w różnych innych sferach. Przy tym ona nie była Chinką tylko Wietnamką, ale to już jest wiadomo...

**Czy wtedy, gdy zamknęli szkoły, obawiała się pani tej sytuacji?**

Nie, jeszcze nie. Teraz się boję straszliwie.

**Skala lęku**

To się zmienia, w ciągu dnia nawet. Teraz, myśląc o sytuacji ogólnej w kraju, to tak na 80.

**Jak wygląda pani życie codzienne teraz?**

Zmieniło się dużo. Mój syn razem z partnerką, ich dzieckiem i jej starszą córką u nas pomieszkiwali przez 3-4 dni w tygodniu.14 marca oni wyjechali i od tamtej pory jesteśmy sami z mężem, bo nie zgodziliśmy się na to, aby oni wrócili do nas tutaj ze względów bezpieczeństwa dla wszystkich, więc jesteśmy sami z mężem. Jeszcze na początku marca brałam udział w jakichś swoich spotkaniach w Avonie, w różnych szkoleniach, 3 marca byłam na takim bardzo spektakularnym szkoleniu dla makijażystów. potem mieliśmy takie spotkanie mniejsze, natomiast teraz wszystko jest już tylko w internecie, po drugie ja też zawsze miałam i mam klientki, do których jeździłam i teraz jest ich znacznie mniej. Mniej jest tej pracy. Bez porównania. Każdy się boi, ja też się boję. Nawet jak ktoś coś zamawia i ja tę paczkę organizuję, to wymieniamy się niemalże na klatce. Zostawiam, ktoś potem podchodzi.

**Co pani teraz najbardziej przeszkadza?**

Może nie tyle to, co się zmieniło w moim życiu, ale ta niepewność, co będzie dalej. To tak najbardziej i po prostu się boję o różne rzeczy. Ja z siostrą mamy starszego tatę, bardzo chorego, niewychodzącego od kilku lat i to też jest dla nas ogromnym stresem W tym tygodniu, kiedy zamknięto szkoły, zaraz później granice...O, to zamknięcie granic było też takim ważnym etapem. Nam pomagają przy tacie 2 panie z Ukrainy, które się wymieniają i to był taki stres mega, bo one się zdążyły wymienić w piątek a w sobotę zamknięto granicę. Ja nie wiem, co by było, gdyby tego się nie udało zrobić.  Mniej mi przeszkadzają te zmiany, które nastąpiły a bardziej ten niepokój o to, co się wydarzy w przyszłości.

**Są jakieś pozytywne strony tego czasu, tych zmian?**

A można tu znaleźć coś pozytywnego? Może widzę, a może to mi się wydaje...Do tej pory życie było cały czas w takim ogromnym pośpiechu. My z mężem generalnie bardzo dobrze żyjemy, ale gdzieś po tylu latach tak to się wszystko rozszerza...Teraz tak jakby więcej tego porozumienia jest. ta relacja z mężem jest lepsza. Może on zresztą był zawsze taki, tylko nie było na to jakiegoś takiego...Teraz jesteśmy po pierwsze cały czas sami, po drugie tylko prawie ze sobą rozmawiamy w domu, aczkolwiek mąż cały czas wychodzi do pracy.

**Jaki jest pani stosunek do tego co się dzieje?**

Przede wszystkim nie mogę uwierzyć, że coś takiego się zaczęło na świecie. Nie tylko w Polsce, ale w ogóle. Zewsząd dochodzą różne teorie spiskowe kto komu i za co tego koronawirusa wystawił. Czy to było specjalnie, nie specjalnie? Podejrzewam, że my - tacy przeciętni, szarzy ludzie się tego nigdy nie dowiemy. Czy to tylko zemsta natury, czy jakiś ludzki czynnik miał miejsce, czy czegoś po prostu nie dopatrzono i ten wirus się jakoś uwydatnił, wyciekł? Nie wiem. Nie jestem w stanie sobie w ogóle wyobrazić, że to w ogóle ma miejsce, aczkolwiek natychmiast wróciłam do danych historycznych o różnych epidemiach. To się działo, zawsze się działo, więc dlaczego nie miałoby się zadziać teraz? Dotyczy to mojego życia, życie trwa tyle ile trwa, były okresy dłuższe...Ale jest to takie nie do uwierzenia.

**Czego najbardziej się pani obawia?**

Tego, że...Już zaczynam płakać...Każdy teraz, nawet jeżeli nie dotyka to go osobiście w sensie takim, że z grona rodziny, znajomych jeszcze nikogo nie ma zarażonego...Z tego co się dzieje wynika, że gdzieś każdego nas to w jakimś stopniu dotknie. I tego się boję. Tego, że ktoś nie przeżyje i nie wiem kto to może być - czy to będę ja, czy to będzie ktokolwiek inny, czy tata, jeśli w jakiś sposób zostanie zawleczony koronawirus do domu. Nie wiem. Po prostu tego się boję.

**A jeśli chodzi o sytuację gospodarczą?**

Oczywiście to też mnie niepokoi, ale ja jakoś całe życie jestem pod kloszem mojego męża. No nie wiem...Może jestem w tej komfortowej sytuacji, że nigdy na swoje barki nie musiałam brać tego ciężaru utrzymania i wszystkiego, że zawsze to jakoś będzie. Różne tutaj były też kryzysy finansowe w rodzinie, ale zawsze jakoś się z tego wychodziło. Raz lepiej, raz gorzej, ale nie było jakiejś takiej sytuacji maksymalnie tragicznej. Tego rodzaju obawy schodzą na dalszy plan w tym momencie. Na pewno coś się zmieni, ale dla mnie to co będzie dalej jest takim późniejszym procesem. Na razie boję się tego, co jest teraz. Tego, że chorujemy, że możemy zachorować. Już od jakiegoś czasu nie mogę słuchać tych wiadomości, nie włączam, bo jak tylko słyszę, ile jest tych zgonów we Włoszech, w Hiszpanii w ciągu jednego dnia, to jest to dla mnie nie do wyobrażenia

**Zdjęcia – emocje**

W tej chwili to 8. na tym obrazku jest droga, która gdzieś tam prowadzi, natomiast dalej jej już nie widać. Nie wiadomo, co dalej jest, ta mgła to przesłania. Kojarzy mi się z taką niewiadomą. Nikt teraz nie wie, jak to dalej się wszystko będzie układać pod względem zachorowalności, jak to się będzie wszystko toczyło. Te badania nad tym wirusem trwają i to jest wszystko tak bardzo po omacku. Niby wszystko wiadomo, ale okazało się, że najpierw starsi ludzie chorują, potem jednak, że wszyscy i takie to jest...taka duża niewiadoma.

**Z jakimi emocjami ten obrazek się kojarzy?**

Takiego lęku. Co będzie? Negatywny stan.

**Skąd się biorą u pani te emocje?**

Źródłem jest sama sytuacja, która zaistniała, czyli po prostu rozprzestrzenianie się tej pandemii. To jest powodem, że się boję.

**A emocje, które miała pani przed pandemią. Też miała pani uczucie lęku o takie sprawy, które się dzieją na co dzień?**

Uczucie leku to nie jest dla mnie nic nowego. Ja jestem osobą bardzo lękliwą, zmagającą się od wielu lat, chociaż teraz to jest może wyciszone bardzo, ale zmagam się z dużą depresją, więc lęk nie jest mi obcy. Taki nawet wręcz nieuzasadniony. Lęk to jest lęk i czasami leku nie można wręcz...Taka panika, lęk...Czasem nawet trudno go zobrazować, dlaczego on jest. Jakieś tam podstawy u tego leżą, samej depresji, natomiast oczywiście, że lęki kiedyś miewałam bardzo często. Ten lęk teraz jest inny i to jest zupełnie co innego. Jest taki nazwany, konkretny, wiadomo o co chodzi, przed chorobą. Ten lęk jest uzasadniony i ja mogę nazwać, dlaczego on jest. Wiem, czego ja się boję?

**Jak te uczucia zmieniały się w czasie wraz z rozwojem sytuacji?**
Na początku jak zamknęli szkoły, granice to gdzieś tam ciągle jeszcze nadzieja. Taka głupia nadzieja, że to do nas nie dojdzie. Potem to było takie obserwowanie, czekanie na każdą wiadomość następnego dnia czy coś się zmienia. Potem coraz więcej, coraz więcej. Potem zaczęły dochodzić już dużo gorsze wiadomości z całej Europy, nagle te Stany, które tak wybuchły. Mam bliską przyjaciółkę, która mieszka w Kanadzie, więc tam oni te zmiany wprowadzają bardzo powoli. Tak jakby nikt się tym nie przejmuje. Co jeszcze mnie bardzo uderzyło - Indie. Dodatkowo przeżyłam to tak bardzo osobiście, ponieważ ja w styczniu byłam w Indiach. Wróciłam tuż przed tym, jak to wszystko zaczęło się dziać. I mam kontakt z przyjaciółką, która jest Hinduską, mieszka w Bombaju. Od niej wiem, że najpierw ta godzina policyjna, teraz są na 3 tyg. zamknięci w domu wszyscy, tylko jej brat i mama mogą wychodzić po jakieś rzeczy. I to tak nagle dochodzi tak z boków. Co mnie jeszcze bardzo w tym momencie denerwuje - my zostaliśmy w domu, nie pozwoliliśmy synowi przyjechać. On oczywiście bardzo chciał tak jak zawsze, ale powiedziałam, że nie, że nie ma mowy, bo jeśli ktoś ma zachorować to lepiej jednostkowo niż.…Bo wiadomo, że to potem przejdzie na wszystkich. Tam są małe dzieci. Jedni mówią, że małe dzieci nie chorują a tu nagle półroczne dziecko umarło w Stanach dzisiaj. Stwierdziłam, że niech siedzą u siebie. Ale, co zaczęło mnie bardzo złościć? Ja wychodzę, bo muszę wyjść z pieskiem. Uważam, że 3 razy to jest wystarczająco. Jestem wczesnym rankiem, w ciągu dnia i wieczorem. Nie ma sensu wychodzić z nim częściej, choćby dlatego, żeby się nie narażać. A wychodząc z tym psem widziałam całe tabuny tych ludzi i to były całe rodziny, z malutkimi dziećmi. Nagle wszyscy okazuje się, że uprawiają sporty, jeżdżą na rowerach, rolkach. Oni robili wrażenie, jakby byli na wakacjach i to mnie tak bardzo złościło. Może ja też jestem jakimś potencjalnym zagrożeniem dla kogoś, bo tego nikt nie wie, ale dla mnie to było nie do pomyślenia, kiedy ja już wiedziałam, że np. w Indiach zostali zamknięci ludzie w domach. Już nie mówiąc o tych wszystkich grillach, o tych, co sobie jadą za miasto, bo muszą sobie pochodzić. To mnie wścieka, że ludzie nie widzą tego realnego zagrożenia. Przecież nie po to nie chodzą do pracy...I to mnie tak złości...Oni bardzo lekceważą sytuację. Jeżdżą sobie po tych plantach warszawskich. Dla mnie to jest głupota. jak musiałam zawieźć klientce zamówienie, to całą drogę się zastanawiałam, jak ja mam się zakryć, mimo, że jestem zdrowa. Ale wejdę na to 4 piętro bez windy i będę zdyszana, więc jak tu się po prostu zasłonić. A oni wszyscy tak...

**Jak pani sądzi, dlaczego oni chodzą po ulicach, mimo tych ograniczeń?**

Może nie zdają sobie sprawy po prostu albo są...trudno to nazwać...Głupi, mało inteligentni. Może to są młodzi ludzie, którzy nigdy...Ja jeszcze pamiętam stan wojenny, ale może oni wyrośli w innych warunkach i im się wydaje, że ich to nie dotyczy? Brak życiowego doświadczenia, a w końcu każdego to gdzieś też dotknie. Jak byłam młoda to wiedziałam, że ludzie chorują, że są nowotwory, ale myślałam, że nas to nie dotknie, bo ten rak to jest gdzieś. Potem okazało się, że na raka zachorowała nasza mama i po 1.5 r zmarła, więc nagle okazało się, że to dotyka też nas. Najbardziej się, właśnie boje tego, że ten koronawirus gdzieś wdepnie w nas z jakiejś strony i nawet nie będziemy wiedzieć skąd, I właśnie przez tych ludzi, którzy tak się rozprzestrzeniają, to nawet nie wiadomo gdzie, zwłaszcza, że się mówi, że część ludzi może przechodzić bezobjawowo.

**Wracając jeszcze do emocji. One narastały wraz z rozwojem sytuacji czy od początku czuła pani pewien lęk i on została właściwie taki sam?**

W tej chwili to jest tak, że są takie huśtawki nastroju bardzo. Nawet w ciągu dnia. Oczywiście bywały takie dni gorsze, że już wszystko źle. Potem gdzieś tam jakaś iskierka, że może jednak nie będzie tak źle. I tak jest do tej pory. Poza tym sam fakt, że mam mniej tych zajęć, mniej takiej pracy, że coś muszę, to też bardziej się człowiek nad tym zastanawia i to przeżywa. Wczoraj można powiedzieć, że przez cały dzień nie pomyślałam o koronawirusie, bo miałam ważne wydarzenie. Musiałam prowadzić szkolenie online pierwszy raz w swoim życiu, więc od rana byłam tym zestresowana i reszta zeszła na plan dalszy.

**Jak pani radzi sobie z tą sytuacją na co dzień? Jakie działania pani podejmuje?**

Na pewno znowu zaczęłam gotować obiady dla męża, bo wcześniej nie było na to czasu. Obiecuję sobie, że wysprzątam cały dom, ale jeszcze do tego nie doszło. Na razie zaczynam się pogrążać w chaosie domowym, bałaganie. Może gdzieś tam nastąpi ten moment, że się przełamię. Póki co jest tak, że nic nie muszę. Do dzisiaj nie posprzątałam jeszcze biurka pi tym wczorajszym live. Coraz większy zaczyna być dla mnie stres, bo coraz mniej jest w lodówce i wiem, że muszę w końcu iść do sklepu. Jeszcze tydzień temu chodziłam. Oczywiście zakładałam maseczkę, rękawiczki a teraz boję się wychodzić. Wczoraj zamówiłam dla całej naszej rodziny maseczki z tym filtrem i mają przyjść dzisiaj albo jutro. Wielorazowego użytku, które się dezynfekuje, więc mam nadzieję, że to mnie też trochę uspokoi. Cały czas do tej pory napotykam sprzeczne sygnały, bo nie wiadomo czy te maski nosić, nie nosić, kto ma je nosić, czy tylko chorzy a zdrowi nie powinni. Bo jeśli zdrowi noszą, to sobie więcej szkody mogą narobić, a z kolei jak ktoś nosi maskę, to inni myślą, że jesteś chory.

**Znalazła pani jakieś nowe hobby, odkryła coś nowego?**

Nie, na razie nie, aczkolwiek mam to w planie. Zawsze bardzo lubiłam szyć i w tej chwili też sobie obiecuję, że wyjmę to szycie, że uszyję pościel dla maleńkiej wnuczki.

**Wróciła pani do gotowania. Co pani to daje? Czerpie pani z tego przyjemność?**

Nie, po prostu bardziej chcę, żeby mąż był zadowolony i nie mam już teraz argumentu, że nie mam czasu tego robić. Teraz wypada. Gdybym rzuciła się chociaż do mycia wszystkich okien, to mogłabym powiedzieć, że nie miałam siły, ale tak się nie dzieje, więc przynajmniej muszę ugotować.

**Jak zmieniły się pani zachowania zakupowe?**

Staram się chodzić rzadziej do sklepu. Ostatni raz w sklepie byłam w sobotę, a mamy czwartek. Wcześniej codziennie wstępowałam do sklepu.

**Robiła pani dodatkowe zakupy w związku z epidemią?**

Tak. Najpierw mąż mówił, że trzeba zrobić zakupy, ja, że absolutnie nie ma takiej potrzeby. Potem młodszy syn, który też nie mieszka z nami zadzwonił do ojca, że muszą zrobić zakupy i oni razem z dziewczyną pojechali po te zakupy, przywieźli. My mamy łóżko na takich wyższych nogach, więc pod tym łóżkiem jest dużo miejsca. Cała powierzchnia pod tym łóżkiem jest zakupami zastawiona. Potem mąż zdenerwował się na mnie, że ja też nie chcę jechać i zrobić zakupy, więc musiałam pojechać i kupić takie podstawowe - puszki, jakieś takie rzeczy. Mąż powiedział, że nawet, jeśli nie będziemy musieli tego wszystkiego zjeść, to nie szkodzi, oddamy do schroniska czy gdzieś, ale jednak, żeby to było. Ja nie chciałam tego robić, bo to we mnie wzbudzało taką panikę. Na początku jak te granice, to był taki moment, że w sklepach już nic nie było, no ale potem to wszystko wróciło i znowu jest w sklepach. Był taki moment, że się wchodziło i pusta Biedronka, nie było nic.

Jak się pani wtedy czuła?

Takie bardziej niedowierzanie. To nie było takie lękliwe, tylko no jak to?

**Co państwo kupiliście? Puszki i ci jeszcze?**

Syn z dziewczyną kupili różne rzeczy. Makarony, kasze, ryże, dżemy, słoiki z sosem pomidorowym, groszki, kukurydze, papier toaletowy, chusteczki wyciągane.

**Te same marki co zwykle czy jakieś inne?**

Te same.

**Jak się pani czuje z tym, że ma pani zapasy?**

Ja uważałam, że w obecnym stanie gospodarczym jednak nie zabraknie żywności w sklepach, aczkolwiek nie wiem jak to dalej będzie. We Włoszech był też dramat, bo zakupy tylko przez internet i dowożono raz na 2 tyg., w sklepach nie było nic. Nam jeszcze daleko jest do tego scenariusza włoskiego, ale ja nie wiem czy to się nie wydarzy.

**Kiedy był moment decyzji, że robicie zapasy?**

Pierwsze były zrobione jeszcze pod koniec lutego i wtedy to syn zrobił zakupy. Oni nie mieszkają z nami, ale u siebie nie mają miejsca i dlatego przywieźli do nas. Ja te swoje zapasy zrobiłam ok 17 marca.

**Teraz planuje pani zrobić kolejne zapasy czy mniejsze zakupy?**

Takie na 4-5 dni, żeby nie musieć znowu iść do sklepu.

**Zakupy przez internet. Coś tutaj się zmieniło?**

Nie. Ja generalnie zakupów przez internet prawie nie robię. Zdarza się, ale rzadko. Ostatnio były te maseczki, jeszcze muszę ten płyn. Ostatnie większe robiłam w grudniu na święta.

**Tych zapasów suchych produktów ma pani na 4-5 dni czy na dłużej?**

Ja takich zapasów nie robiłam, bo to, co już mamy w szafkach to i tak wystarczy na wiele dni. Nie będę kupować nowych, dopóki nie zużyjemy tego co mamy. Starczyłoby nam tego na 2 miesiące.

**Co jest teraz największym wyzwaniem? Coś sprawia pani szczególną trudność?**

Na pewno więcej jem, co mi się nie podoba. W codziennym funkcjonowaniu to nie, ale może jestem teraz taka bardziej rozmemłana, bo wcześniej bardziej się musiałam jakoś zorganizować a teraz po prostu mi się nie chce. Nic nie muszę.

**A brak kontaktu ze znajomymi, rodziną?**

Problem odczuwam, bo bardzo tęsknie za wnuczką. Ona roczek skończy w kwietniu. Z jednej strony, to można powiedzieć...Może to nie było dla nas udręką, ale jak 4 osoby mieszkają razem z nami w ciągu tygodnia, to też takie...My już przez wiele lat mieszkaliśmy sami a tu sytuacja się zmieniła od września. Ludzie mówią, że przynajmniej mam spokój. Oczywiście jak siedzieli nam na głowie, to marzyłam, żeby sobie wreszcie pojechali i teraz rzeczywiście nie siedzą mi na głowie, ale trochę brakuje mi tego. Smutno bez tej wnuczki. Ale oni też trudno się ze sobą dogadują czasami, więc może to też dobre jest dla nich, żeby sobie posiedzieli sami. Niech się tam poukładają trochę.

**Kontaktuje się pani z rodziną przez komunikatory?**

Tak, oczywiście. Kontaktu osobistego to nie zastąpi. Rzadko z tego korzystamy. Najczęściej rozmawiamy przez telefon albo wymieniamy się smsami. Raz tydzień temu rozmawiałam online z synową i dziewczynkami.

**A spotkania towarzyskie? Tu odczuwa pani trudności, zmiany?**

Nie. Zmiany są o tyle, że człowiek z nikim się nie spotyka. Ja nie byłam towarzysko bardzo zaangażowana, więc nad tym się teraz nawet nie zastanawiam. Cały czas mamy kontakt telefoniczny.

**Brak dostępu do kina, kultury?**

W kinie byłam na początku lutego a przedtem chyba nie byłam z rok, więc to nie problem.

**Czy teraz jakoś inaczej spędza pani czas z mężem?**

Mąż tak samo pracuje jak pracował, ale częściej jesteśmy tylko my sami, a ponieważ mój mąż zawsze dużo mówił, to teraz musi mówić do mnie.

**Jak wygląda teraz pani typowy dzień?**

Nie mam takich stałych punktów. Mam takie zadania na dzień - dzisiaj np. porządek w rzeczach z Avonu, potem ugotuję obiad, wiadomo, że muszę wyjść z psem. Poza tym nadal jeździłam w różnych sprawach - z zamówieniami, odebrać psa od fryzjera...Wczoraj cały dzień był pod kątem szkolenia. Ustalam jakiś cel, zadanie i realizuję je albo nie.

**Takie zadania jakoś pomagają przetrwać ten czas?**

Tak, bo jeśli się czymś tak zdecydowanie zajmę, to wtedy nie myślę, nie włączam tv. Potrafię się wręcz czasami wyłączyć, ale to jest trudne i chwilowe. Jak nie myślę o tej sytuacji to czuję się lepiej. Zdecydowanie.

**Jak pani otoczenie, bliscy radzą sobie w tej sytuacji?**

Siostra bardzo słabo sobie radzi. Ona od 14 marca nigdzie nie wychodzi i jest sama w domu. Domyślam się, że jest jej smutno, często płacze. Też są takie momenty, że troszeczkę lepiej sobie radzi, a potem...Młodszy syn bardzo dużo pracuje, bo ma i zdalną pracę, i czasem musi pojechać do pracy. Ten drugi, to oni są po prostu w domu i się tam docierają. Drugi syn musi się też cały czas dużo uczyć. Obaj są na aplikacji adwokackiej, więc obaj są też zajęci w domu. Moja siostra też wynajduje sobie jakieś zadania - powyciera kurze, a to sobie pośpi, poogląda coś w tv. Nie widuje się ze swoimi dziećmi, ale też gotuje i czasami zięć przyjeżdża i mu wystawia do zabrania. Zupki, rosołki dla wnuków.

**Jak pani sądzi, dlaczego syn zdecydował się zrobić zapasy?**

Uległ też chyba takiej panice po prostu. Nie mam pojęcia czy to go uspokajało.

**Skąd się wziął koronawirus?**

Koronawirusy są i to jest jeden z wielu, które są. Te wirusy były przenoszone przez zwierzęta w chinach, chyba przez nietoperze i ludzie przez jakąś nieuwagę...Z tego co ja wiem, to na targu w Wuhan. Został zawleczony przez sprzedawców. Nie chce mi się wierzyć, że można sprzedawać mięso nietoperzy. Jedzą psy, to może...

**Wspominała pani, że są różne teorie?**

Tych teorii spiskowych pojawia się mnóstwo. Że zostały zakażone wojska amerykańskie, które przyjechały do Chin, żeby ich zarazić. Inne, że były badania nad tym wirusem w Wuhan i to w jakiś sposób wypłynęło. O tyle mnie to niepokoiło, że już wcześniej niektórzy o tym dawali znać, że istnieje ryzyko takiej epidemii i to było bagatelizowane.

**Są jacyś winni tej sytuacji?**

Nie umiem na to odpowiedzieć. Winnego najlepiej znaleźć, żeby się uspokoić, że ktoś jest winny. Nie wiem.

**A dlaczego zaczął się tak szybko rozprzestrzeniać?**

Bo jest bardzo zaraźliwy, a przede wszystkim dlatego, że ruchy ludności na całej ziemi są takie, że po prostu...To przemieszczanie się z państwa do państwa. Stąd miedzi innymi.

**Można było jakoś temu zapobiec?**

Nie wiem. Podobno były wzmianki o tym już w grudniu. Ja nie słyszałam. Gdybym wtedy wiedziała, to w życiu bym się nie zdecydowała na wyjazd do tych Indii, a ja byłam w tych Indiach zupełnie nieświadoma. Dopiero jak wróciłam do Polski zaczęło do mnie docierać, że coś się dzieje. Nikt chyba nie wie, jaka była prawdziwa przyczyna pojawienia się tego wirusa. ja na pewno tego nie wiem. Gdyby ktoś to wiedział, to pewnie dałoby się jakoś uchronić przed tym. Są teorie, że nie szanujemy natury, że to się gdzieś odbija, że dlatego tak mutują te wirusy, że w końcu za to też człowiek musi zapłacić. Dla mnie to są takie teorie, które mają nas w nie tyle uspokoić, co usprawiedliwić, że coś takiego się dzieje, że tak wygląda świat, takie jest życie. To są teorie, które mają ludziom pomóc. Może są ludzie, którzy wiedzą, jak było naprawdę, a to są tylko teorie.

**Wydaje się pani, że rządy, ludzie wyżej postawieni wiedzą coś więcej?**

Myślę, że tak.

**Czy jesteśmy przygotowani, jako świat na zmierzenie się z pandemią?**

Nie.

**Można było przygotować się lepiej?**

Należałoby być zawsze przygotowanym na coś takiego, ale to dopiero doświadczenia nam to pokazało. To jest pierwsza sytuacja na taka skalę od epidemii hiszpanki, mimo, że były te SARS, ptasie grypy, świńskie grypy, ale one się tak nie rozprzestrzeniały?
**Jak radzi sobie Polska? Jest przygotowana?**

Nie umiem tego ocenić. Z tego co czytam, słyszę, to często są jakieś braki odpowiedzialności osób za to co się dzieje np. w Szpitalu Bródnowskim. To jest trudne, bo ta zaraźliwość jest tak ogromna...Nie każdy wie, że jest zarażony, może zarażać innych i w tym momencie rzeczywiście należałoby wszystko zamknąć, żeby to się nie rozprzestrzeniało. Osoby z personelu medycznego, które miały kontakt też powinny być w kwarantannie do momentu wyniku, bo one same nie wiedzą. A dużo się czyta o przypadkach, że te ogniska gdzieś w szpitalach powstają i są roznoszone również przez personel medyczny. Strasznie nie chciałabym się znaleźć w sytuacji, że mogłabym kogoś zarazić. Straszne to dla mnie jest. Taka odpowiedzialność, że ja kogoś, ktoś kogoś, a ten ktoś może umrzeć. Każdy człowiek może się czuć współodpowiedzialny.

**Jak ocenia pani działania i decyzje polskiego rządu?**

Ja im nie zazdroszczę tego działania, bo znajdują się 1-szy raz w takiej sytuacji i każdy rząd byłby oceniany za to, co zrobi. Uważam, że dobrze robią to co robią, tylko jest kwestia na ile wystarczy nam szpitali, środków wspomagających...My jeszcze nie wiemy co nas czeka, bo jesteśmy dopiero na samym początku i jak to się rozwinie nikt nie wie.

Decyzja o zamknięciu szkół, restauracji była dobra.

**A podjęta w dobrym momencie?**

Jak już wiadomo było, że we Włoszech jest taki wysoki stopień zakażeń...Wiadomo, że Polacy uwielbiają jeździć do Włoch, wszystkie te ferie. To jest takie gdybanie, ale może już wtedy trzeba było zamknąć, zrobić taką blokadę, żeby ci, którzy już przyjechali dalej tego nie roznosili. Nie wiem, czy to był odpowiedni moment, czy nie powinni byli zrobić to wcześniej, ale dobrze, że w tym momencie a nie później.

**A kolejne obostrzenia były słuszne?**

Tak

**Te decyzje panią uspokajały czy wręcz przeciwnie?**

Oczywiście, że uspokajały, tylko potem się słyszało, że ileś osób nie przestrzega tej kwarantanny i znowu to wywoływało we mnie taką złość, że nadal narażają, że ludzie nie podchodzą do tego poważnie.

W moim środowisku mam osoby, które wróciły z zagranicy i poddały się absolutnie kwarantannie. Opowiadały mi, że przyjeżdża policja i sprawdza, więc część na pewno potraktowała to bardzo poważnie. Ja też mogłabym powiedzieć, żeby dzieci przyjechały i siedziały razem z nami, ale nie, absolutnie. Moja teściowa, która mieszka blok dalej - też się nie widujemy i zakupy przez taką kratę jej wystawiamy. Część na pewno to przyjęła i stosuje się do zasad. Śmieszyło mnie, że spacery są określane jako niezbędne, bo jak wszyscy wyjdą na taki spacer...

**Jak zamkniecie szkół, granic oddziaływało na ludzi? Na ich samopoczucie?**

Nie spotkałam się, żeby ktoś to skrytykował. ja też uważam, że tak trzeba było zrobić.

Jedyne, czego się bałam i tak też było w przypadku mojej siostry, że co zrobimy, jeśli nie będzie tej możliwości, żeby przyjechała opiekunka. Tutaj się po prostu bałyśmy. Czasami ktoś mówi, że po co UE? Wystarczyło, że pojawił się wirus i wróciły granice.

**Na ile czuje pani, że ludzie, jako jednostki mają wpływ na tę sytuację?**

Pojedyncze jednostki to tylko to, że zostaną w domu, że nie będą się bez potrzeby szwendać po ulicach, że zastosują się do tych podstawowych zaleceń. Chociażby to mycie rąk, dezynfekowanie. Zastosowanie się do zaleceń i potraktowanie tego poważnie.

**Skąd pani czerpie informacje?**

Głównie przeglądam Onet i TVN24. Mam FB, ale mało jestem aktywna. Ja nie cierpię tych wszystkich mediów społecznościowych. nie tyle nie cierpię, co trudno mi jest się tam znaleźć z moją pracą, a wiem, że powinnam i to jest dla mnie taką gorzką pigułką nie do przełknięcia. Ja tego nie czuję, nie umiem, nie lubię, nie chcę. To jest już dla młodych i ja się w tym nie czuję dobrze.

**Czy Onet i TVN24 to źródła, z których korzystała pani też przed epidemią?**

Tak.

**A czas poświęcany na korzystanie z mediów?**

Nie spędzam teraz na tym więcej czasu. Na początku tej epidemii może więcej, bo człowiek ciągle tego słuchał i łaknął tej wiedzy, o co chodzi. W tej chwili staram się informacje o koronawirusie tak raz, dwa razy dziennie się o tym dowiedzieć. Nie mogę już tego czytać tak jak wcześniej, bo powoduje to we mnie od razu takie...Wiem, że jest z dnia na dzień coraz więcej i w ogóle.

**Jak ocenia pani wiarygodność tego, co do nas dociera?**

Chciałabym wierzyć, że one są rzetelne, ale nie mam najmniejszej pewności, że tak jest. Te nazwy się pojawiają, bo COVID to jest choroba, którą wywołują, ale jest jeszcze...Tak, SARS VO2. Jak czytam na Onecie czy na FB, bo tam też czytam, jak ktoś coś opublikuje...Jak czytam, że ktoś pisze "rozprzestrzenianie się koronawirusa COVID19", to mnie to zaczyna denerwować. W jakim sensie mogą być wiarygodni, skoro nie wiedzą, że COVID to jest choroba, którą powoduje wirus a sam wirus to jest SARS VO2. Równie dobrze te wszystkie informacje...Niekoniecznie są wiarygodne. Jak widzę błąd, to od razu zaczynam wątpić w wiarygodność.

**Po czym można poznać, że informacja jest wiarygodna lub nie?**

Przede wszystkim to przez kogo jest przekazywana. Jest np. strona Medonet i ewidentnie tam najwięcej tych błędów się pojawia. ja się zastanawiam czy tam nie ma nikogo, kto może się zająć taką kwestia sprawdzenia, czy te wiadomości nie zawierają błędów? Ja już nie mówię o tych informacjach od rządu. Ja już nie wiem, co jest prawdą a co nie jest. Moja siostra zawsze twierdzi, że internet zawsze kłamie. Ja do tego staram się tak nie podchodzić, ale nie możemy wierzyć...Na pewno w tv i kanałach informacyjnych jest więcej tych wiarygodnych informacji. Można się spierać czy to jest TVP czy TVN24, czy jeszcze jakiś inny komunikator. Uważam, że w tv informacje są bardziej wiarygodne niż w internecie. W internecie każdy może podać jakąś swoją informację a w tv jest jakiś profesjonalista, który to przygotowuje. Jeżeli ktoś jest ekspertem to możemy bardziej mu wierzyć niż temu co jest w internecie.

**Czy Onet i Medonet to są różne portale?**

Teraz właśnie się zastanawiam. Być może to jest jakoś razem połączone. Generalnie korzystam z Onetu a na Medonecie zobaczyłam te błędy.

Coś jeszcze chciałaby pani dodać od siebie?

Głównym przemyśleniem jest taka nadzieja, żeby się udało jakoś z tym zawalczyć, ale tak jak na tym obrazku - nie wiem, co tam jest dalej za tą mgłą.
