# Supplementary material for: The challenges arising from the COVID-19 pandemic and the way people deal with them. A qualitative longitudinal study
Source: PLoS One. 2021 Oct 11;16(10):e0258133. doi: 10.1371/journal.pone.0258133 (PMC8504766; doi:10.1371/journal.pone.0258133)
Supplement: S1 Dataset — (ZIP) [file pone.0258133.s003.zip › Transcriptions/stage 1/19.1_F_39_couple, with children.docx]

**19.1_F_couple, with children**

**Ile masz lat, czym się zajmujesz?**

Mam 37 lat. Pracuję w księgowości, jestem samodzielną księgową. Mieszkam w małej miejscowości pod Warszawą, mam dwoje dzieci, męża. I pracujemy zdalnie w tym momencie, obydwoje z domu.

**A w wolnym czasie co lubisz robić?**

W wolnym czasie lubię jeździć na rowerze, chodzę na basen, czytam książki, spędzam czas z dziećmi.

**Powiedziałaś, że masz dwójkę dzieci?**

Tak, mam dwie córki. Jedna skończy w tym roku 7 lat, a druga 12.

**Chciałam się dowiedzieć, jak sobie myślisz o tej całej sytuacji, która się teraz dzieje w Polsce, kiedy dla ciebie to się wszystko zaczęło?**

Dla mnie to był 12 marca bodajże, kiedy ogłoszono, że szkoły będą zamknięte, to była środa. I od czwartku miały być zamknięte szkoły. I w tym momencie, tego samego dnia, dyrektorka finansowa, która jest jakby moją przełożoną, zadzwoniła do firmy, żeby zorganizować dla mnie komputer, bo trzeba wziąć pod uwagę to, że będę chciała pewnie zostać z dziećmi. A w sumie z mojego zespołu finansowego tylko ja i właśnie ta dyrektorka mamy dzieci. Więc ona tak podeszła, no, tak fajnie podeszła do tematu. Bo miło mi było, że o mnie pomyślała. Że wie, że też się boję. I że jest taka możliwość. Pozostałe dziewczyny nie mają jeszcze dzieci, więc tak inaczej może trochę na to patrzą. I udało się zorganizować komputer, mimo że opieka nad dziećmi mogła być jeszcze zapewniona w czwartek i piątek, to ja już zostałam w domu. Zostałam z moimi dziećmi w domu, mój mąż też już był w domu. I wtedy się klarowało jakby, co tam będzie dalej. Bo wstępnie to miał być czas pracy zdalnej na te 2 tygodnie. Potem się jeszcze jakoś inaczej wyklarowało. Tak że praktycznie od samego początku ta sytuacja już tak była ze mną związana.

**Od 12 marca to już, zaraz, to już 3 tygodnie chyba od tamtego czasu minęły, prawda?**

Tak, tak, ostatnio liczyła. Chyba dzisiaj jest dwudziesty pierwszy dzień, coś takiego.

**Czyli to było ważne, ta informacja o ogłoszeniu zamknięcia szkół, ze względu na twoją pracę. I to było z tego względu dla ciebie jakieś takie ważne. A czy pamiętasz jakieś inne takie ważne momenty w tej całej sytuacji, które nastąpiły albo przed tym 12 marca albo od 12 marca do teraz? Jakieś takie punkty przełomowe.**

Właściwie to nie. Bo tak naprawdę to jest ogólnie takie hasło, że to się dzieje, jest duży szum. Ale ja raczej, mimo że mam Facebooka, Instagrama i czasem poczytam gazetę.pl, to raczej staram się jakoś nie zaśmiecać sobie głowy tymi wszystkimi informacjami. Wokół mnie, też w mojej rodzinie, wśród znajomych też nie było żadnego takiego przypadku. Więc no tylko tyle, że jesteśmy zamknięci w domu. A poza tym to jakoś nie było takich punktów przełomowych, nic takiego jakiegoś drastycznego.

**To właśnie o tym, skąd w ogóle bierzesz informacje na temat tej całej sytuacji to też na pewno będę chciała z tobą trochę później porozmawiać. Więc dobrze, że zaczęłaś o tym mówić. A czy w ogóle, bo ten 12 marca był jakiś taki przełomowy dla ciebie. A czy przed 12 marca w ogóle coś słyszałaś o tej całej sytuacji, interesowałaś się?**

Tak. Ale to wszystko na zasadzie żartów. Po prostu wróciliśmy z ferii, zaczęły się wtedy te informacje, że we Włoszech coś tam się dzieje. I najczęściej wtedy jakieś osoby w kręgu, w pracy, w kręgu zawodowym zaczęły wracać z tych ferii. I były hasła w stylu: wrócili z Włoch, ciekawe, czy się nie zarazili, nie podawaj mu ręki. No, na tej zasadzie. Ale to raczej takie żarty. Więc to tak nie docierało. My jesteśmy firmą francuską, podlegamy pod firmę francuską, więc jakieś tam zarządzenia były, maile od centrali, żeby maseczki kupić, płyny dezynfekujące. Ale to jeszcze wszystko tak naprawdę na spokojnie. Widać było w pracy, że częściej ludzie myją ręce. To na tej zasadzie. Nic tak więcej się nie działo, tylko tyle. Tak delikatnie praktycznie. No później, od tego momentu, kiedy zarządzili te zamknięcie szkół, to może tak bardziej poważnie się zaczęło o tym myśleć. I chyba ludzie tak zaczęli poważniej traktować.

**Czyli to jest tak, że ludzie dookoła ciebie mieli takie podejście trochę na zasadzie żartu. I ty też miałaś takie podejście wtedy do tego?**

Starałam się nie przejmować tą sytuacją całą, tą epidemią. No i dopóki nie było w Polsce pierwszego przypadku, to też miałam raczej takie luźne podejście. A jeszcze były komentarze w stylu, że to jest podobne do grypy, na grypę umiera u nas więcej osób, więc nie powinniśmy popadać w jakieś skrajności.

**Czyli ten pierwszy przypadek też pamiętasz, że o tym usłyszałaś, o tym pierwszym przypadku?**

Tak. Tak jakby wszyscy wyczekiwaliśmy, że w końcu będzie ten pierwszy przypadek. Bo codziennie, jak się rozmawia w pracy, bo głównie w pracy się o tym mówiło, to każdy, wiadomo, że tam coś przeczyta, coś powie dalej. I czekaliśmy, że pewnie to nastąpi. Tylko nie wiadomo było kiedy.

**Czyli trochę takie oczekiwanie na to.**

Tak. I też ta moja właśnie dyrektor finansowa już sama mówiła, bo ona ma praktycznie w identycznym wieku dzieci, powiedziała, że jak chyba dojdzie to do Polski, to ona się z dziećmi zamyka w domu. No, ja tak sobie myślałam, że no tak, ona się może zamknąć, ale tutaj pracownicy niższego szczebla nie mają pola manewru, tak? No, jak widać potoczyło się tak, jak się potoczyło.

**Czyli teraz pracujesz zdalnie.**

Tak.

**I jak w ogóle twoje życie teraz wygląda, takie codzienne?**

Raczej taki harmonogram jakiś stały jest, można powiedzieć. No śpię do oporu, można powiedzieć, do tej ósmej. Bo od ósmej zaczynam pracę. No to to się zmieniło, że mogę w ostatniej chwili wstać, włączyć komputer i praktycznie w tym czasie mogę sobie robić śniadanie, kawę. To siadamy sobie z mężem, robimy sobie śniadanie, takie fajniejsze. Dziewczyny wstają troszkę później, bo zazwyczaj 9. No, starsza córka ma te video lekcje, więc ona czasami dołączy o tej ósmej. Pracuję, wydrukuję jakieś materiały dla mojej młodszej córki. Ona jest w zerówce, więc dostajemy mailowo. I coś tam spróbuję jej wytłumaczyć, omówimy jakiś tam temat, dam jej jakąś pracę, ona coś robi. W pierwszy tydzień przyznam się, że pomyłam okna (śmiech). W trakcie pracy. Tak, więc to było takie szaleństwo, że o, wolne, wolne. Znaczy no wolne, no wiadomo, jakie wolne. Ale jednak nie takie, że siedzę przy biurku non stop. Więc sobie tak po jednym oknie dziennie myłam. Taką sobie przerwę robiłam od biurka. No, w trakcie też jemy obiad. No wiadomo, do tej szesnastej to jest ta praca. Ciągle podchodzę, sprawdzam maile, jak mam, wiadomo, takie bardziej terminy jakieś, to staram się więcej czasu przy tym komputerze siedzieć ciągle. W ciągu dnia moje dziewczyny wychodzą na dwór, na rolki. Mieszkamy w domu, więc mogą wyjść na ulicy. Tylko z okien nadzorujemy, czy wtedy nadzorowaliśmy, czy nikogo tam nie ma w pobliżu. Jak ktoś szedł, to w taką boczną uliczkę przy domu wjeżdżały. A po południu wszyscy wychodziliśmy na dwór. I jeszcze wieczorem ja z mężem sami na spacer. Mamy takie tereny bardziej, jakieś łąki, takie jeziorko blisko jest, więc się przeszliśmy. No i praktycznie tyle. No nigdzie więcej się nie ruszam, więc tak wygląda dzień. Teraz może trochę inaczej, bo te zaostrzenia są. Więc tak wiadomo, że trzeba nigdzie się tam do sklepu, w jedną osobę chyba bodajże, jakoś tak teraz jest, że nie pójdzie się we dwie.

**Czyli teraz jest inaczej pod względem zakupów, ale pod względem tego wychodzenia, jeżdżenia na rowerze, tych spacerów zostało tak jak było?**

No nie wychodzimy na te spacery gdzieś daleko. Ale mamy możliwość na podwórku i na ulicy przed domem. Praktycznie nikogo nie ma na ulicy. Ludzie, widzę, że się stosują do tej kwarantanny. No my też chcemy się stosować. I myślę, że od początku tak rygorystycznie raczej. Więc nie ma takiej możliwości, żeby z kimś się tutaj stykać pod tym domem na ulicy. Więc staramy się. Bo można zwariować, tak cały czas w domu będąc.

**I z tego wszystkiego jak byś powiedziała, co ci najbardziej przeszkadza w tej sytuacji?**

Najbardziej to, że nie mogę pokonać jakiejś odległości większej. Że nie mogę sobie po prostu wsiąść na rower i przejechać, gdzie chcę. No tak pobyć sama, może też to. Że jesteśmy cały czas wszyscy razem. No jest, staram się doszukiwać takich aspektów pozytywnych tej strony, że no mogę tak z dziećmi trochę więcej pobyć, że tak może, jak to w tych memach krążyło, że się zaprzyjaźniamy. No i to jest może pozytywne takie. Ale ogólnie to też jest potrzebny taki czas dla siebie. A nie ma w sumie takiej możliwości.

**Czyli brakuje ci trochę tego, żeby pobyć sama ze sobą.**

Tak, tak. No i mam wrażenie, że jak się pracuje w domu, to nie ma oddzielenia tej strefy prywatnej od służbowej. Bo ja nawet po południu tutaj… No wiem, że jest trudna sytuacja, nie wiadomo, jak się to skończy, czy wszyscy utrzymamy pracę, więc też staram się być taka dyspozycyjna i w każdej chwili pomóc, jak jest potrzeba. Więc nie jestem taka totalnie odcięta od tej pracy. Nie wiem, jak to dalej będzie. Teraz jeszcze, no momentami tak sobie myślę, że to jest takie trochę denerwujące. Ale na dłuższą metę to może tak przytłoczyć, myślę.

**No właśnie, masz jakieś takie obawy co do tego, co się dzieje teraz?**

Staram się właśnie nie myśleć jakoś tak bardzo naprzód, tylko robić to, co robię. Że mam jeszcze co robić, mam jakieś tam dokumenty. Bo jak to w księgowości, jak spływają dokumenty, no to ja tam sobie jadę, jechałam raz w tygodniu z samego rana, żeby tak z nikim za bardzo nie mieć kontaktu, zabierałam segregator, bo blisko mam pracę, zabierałam segregator z dokumentami i tyle. I sobie tak księgowałam. No, ale wiem, że coraz mniej spływa tych dokumentów. I może być taki moment, że się skończy. Więc nie wiem, jak to dalej będzie. U mnie opcja jest taka, żeby jeszcze pójść, na ten zasiłek, na tą opiekę na dzieci.

**I na czym to polega?**

To polega na tym, że ZUS płaci 80%. Także, no mniej wynagrodzenia się dostaje. No i to tyle. I tam jest, nie wiem, ile tam teraz tej opieki teraz jest. Chyba to trochę wydłużyli. Bo to normalnie ma się chyba 60 dni na dziecko, jeśli dobrze pamiętam? W ogóle na dzieci, ile by się nie miało tych dzieci. Ale ze względu na to, że ta przerwa szkolna jest, no to oni chyba to jakoś trochę wydłużają, żeby jakoś tak to ogarnąć, tymi limitami dni.

**Czyli boisz się, tak jakby twoje obawy, lęki wynikają z tego, że możesz po prostu mieć problem z pracą, tak?**

Tak. Boję się tego, że nagle się okaże, że jest mało tej pracy i będą jakieś zwolnienia. Że nagle się okaże, że z zespołu pięcioosobowego wystarczą na przykład 3 osoby. Które będą… Bo na pewno to nie będzie może teraz od razu odczuwalne, ale może za 2 miesiące? Nie wiadomo. Mogą się jakieś zlecenia dla firmy pokończyć, może nie będzie aż takich przychodów. Nie wiadomo, jak to będzie.

**Czy jest jeszcze coś takiego, czego się boisz teraz?**

Nie wiem w sumie, czy to można uznać, że się boję. No, że nic nie będzie takie samo (śmiech). Tego się może boję, że nic nie będzie takie samo.

**To znaczy?**

Że to się będzie przeciągać. Nie wiem, że może ten koronawirus się nie skończy. No, ja nie czytam, ale słyszę, mój mąż sporo czyta. Może to będzie dłużej trwało. No, to wtedy to już… Nie wiem, nie chcę wybiegać tak bardzo do przodu, bo to wtedy to człowiek za dużo myśli i się tylko nakręca niepotrzebnie. Może jeszcze być może będziemy pracować zdalnie, tak?

**A jeśli chodzi o jakieś takie obawy związane ze zdrowiem?**

Nie boję się. Ja się tego nie boję. Jedynie może boję się o mojego męża, bo on ma taką obniżoną odporność, jest chory na łuszczycę. Takie osoby są bardziej narażone. Więc też przez to tej kwarantanny tak bardzo przestrzegamy. Bo mi może nic nie być, dzieciom może nic nie być, a jemu nagle się to wszystko tak rozkręci, że skończy się to źle.

**Czyli twoje obawy w większości dotyczą przyszłości, tak? Nie tego, co się teraz dzieje.**

Tak. Bardziej tej odległej może przyszłości. Teraz robię to, co mogę i jakoś tam funkcjonujemy.

**(Skala lęku).**

Myślę, że 60.

**60 i czujesz, że ten lęk się zwiększył od tego powiedzmy 12 marca albo spadł?**

No, może troszeczkę.

**Troszeczkę zmniejszył czy zwiększył?**

Myślę, że trochę się zwiększył. Przez to, że widzę, że te obostrzenia są takie, że jest więcej tych zachorowań.

**Czyli z jednej strony, że jest więcej zachorowań, z drugiej strony, że jest więcej obostrzeń. To też sprawia, że się trochę bardziej boisz, tak?**

No tak, tak, tak. Bo już takiej wolności nie ma. Jak człowiek nie czuje takiej wolności, no to jest przytłaczające.

**A są jakieś takie konkretne z tych obostrzeń, które pamiętasz, które czujesz, że najbardziej na ciebie oddziałują czy wpływają?**

Najbardziej to, że nie można się poruszać swobodnie, że trzeba zostać w tych domach. No ja mam ten plus, że jestem w domu. Ale i tak, jak wspominałam wcześniej, że nie mogę się po prostu oddalić gdzieś na dalszą odległość. Tylko praktycznie w konkretnym celu. No i na krótko, załatwić i wrócić. Tak jak te zakupy czy apteka.

**Mówiłaś, że brakuje ci takiej swobody, żeby po prostu sobie wsiąść i gdzieś pojechać daleko.**

Tak. No rowerem 2 godziny gdzieś tam… No, fajnie tak. No i to, że zaraz się zrobi wiosna, zaraz się zrobi fajnie. No i to jakoś tak człowiek czeka, przynajmniej ja tak mam, że cały rok pracuję, pracuję, czekam na te wakacje. I w wakacje sobie już, 2 razy jedziemy na urlop taki dłuższy, spędzamy czas z dziećmi. I jest fajnie, jest ta fajna temperatura, fajna pogoda. A w tym roku myślę, że tego nie będzie.

**Rozmawialiście już jakoś o tym urlopie?**

No właśnie mamy zaplanowane urlopy, w sensie wypisane wnioski urlopowe, te harmonogramy poakceptowane w pracy i ja, i mój mąż. Ale nie wiem, ja już liczę na straty te wakacje. Bo mi się wydaje, że nic z tego nie będzie. A jeśli wypuszczą nas z tych domów, to po prostu będzie takie wariactwo, bo my zazwyczaj na wakacje jeździmy pierwszy urlop taki za granicę, a drugi nad polskie morze. Będzie takie wariactwo, że tak, za granicę się nie pojedzie, bo cały czas człowiek jest nieufny i wtedy na pewno… Nie sądzę, że to wszystko tak wygaśnie. A w Polsce, jak tak każdego wypuszczą, to ja nie wiem, co się będzie działo. Wszyscy po prostu… No wyobrażasz sobie, co się będzie działo (śmiech). Wszyscy będą chcieli żyć. I każdy tak będzie szukał rozrywek. Będą tłumy, będzie, nie wiem, ceny może jakieś wywindowane. No, też każdy będzie chciał sobie odbić to, ten kryzys.

**Czyli na straty już spisałaś raczej wakacje tegoroczne.**

Chyba tak. Chyba trzeba jakoś racjonalnie podejść do tego, może coś innego zaplanować.

**A pamiętasz, kiedy właśnie zaczęłaś o tych wakacjach myśleć, że ich nie będzie w tym roku?**

Niedawno. Wcześniej jeszcze tak brałam pod uwagę. Dopiero, jak zaczęli przedłużać te izolacje, tą pracę z tego domu. Po tych pierwszych 2 tygodniach przedłużyli. I potem jeszcze tam. No i zadzwonili do mnie z pracy, że jeszcze tam 2 tygodnie, że do świąt prawdopodobnie A teraz już u mnie w pracy mówią, że dłużej. Więc teraz już tak zaczęłam myśleć. A poza tym druga sprawa, że u mnie w pracy zaczynają wysyłać ludzi na urlopy takie przymusowe. No, właściwie proszą, ale to jest jednak przymusowe. Jakoś próbują ratować to. Jak ktoś nie ma co robić, to wysyłają. Więc też nie wiadomo, jak to dalej będzie. Męża też już proszą, żeby wykorzystywać.

**I ty też już miałaś taką rozmowę o tym, żeby te urlopy wykorzystywać?**

No nie, ja na razie nie, bo ja mam jeszcze co robić. A jeszcze mam tę opcję takiej opieki na dzieci. To jest takie L4, coś w tym stylu. Że opiekuję się dziećmi, które są w domu. Więc jeszcze to. I w zależności od tego, jak długo będą te szkoły, to tak długo będę mogła chyba z tego korzystać. Nie wiem, to zależy jak tam rząd to poustala dalej. No, jeszcze z jednej obawy, o której mogę powiedzieć to to, że te dzieciaki, no są poza szkołą. To jednak te lekcje video trwają krótko. No i z tego, co widzę, to jedni nauczyciele się bardziej angażują, a inni po prostu kończą przed czasem i niewiele powiedzą. Tak jak na przykład matematyka, która jest ważnym przedmiotem. To tutaj nauczycielka się nie przykłada akurat moim zdaniem, bo mam akurat okazję teraz właśnie to zobaczyć, jak to wygląda.

**12 lat to jest która klasa?**

Ona jest w 6 klasie, bo ona poszła o rok wcześniej jako sześciolatka.

**I teraz możesz sobie zaobserwować, jak te lekcje wyglądają teraz. I mówisz, że nie do końca ci się to podoba, jak są prowadzone.**

No nie do końca.

**O co się obawiasz w kontekście tych lekcji? Że są źle prowadzone?**

Obawiam się o to, że oni nie przerobią tego materiału. Znaczy nie omówią tego materiału, który powinni omówić do końca roku. I jak to się jeszcze będzie może przeciągało, to nie ma szans tego przepracować jakoś. Mogą dostawać jakieś prace dodatkowe, które ona robi, jakieś prezentacje, wypracowania, różne rzeczy. Ale to nie jest to samo, niż jak poszliby do szkoły i po prostu odbyło się to tyle, ile trzeba. Tym bardziej, że plan lekcji jest taki, że jest 7 lekcji przypuśćmy, a jest video lekcja z dwóch danego dnia. No to też tak nie za ciekawie. Jeszcze ona ma pływanie, bo ona chodzi do klasy pływackiej, więc codziennie miała 2 godziny pływania. No to to gorsze, bo forma spadnie. Tego się nie da zdalnie zrobić.

**Czyli po prostu boisz się o jakieś takie zaległości, które będą miały.**

Tak, a to jest szósta klasa, później to moment, minie. Jeszcze w tamtym roku był przecież ten strajk nauczycieli, więc też praktycznie trochę tam uciekło. No i to mi się nie podoba. To mi się nie podoba, właśnie ta cała edukacja, to wszystko.

**A jeśli chodzi o młodszą córkę?**

Ona jest w zerówce. No, dla niej też fajnie byłoby, jak by miała jakiś kontakt z rówieśnikami. Bo widzę, że zaczęła tak lgnąc do starszej córki. Przytula się tam ciągle do niej, chodzi do pokoju. Cały czas no tak wzorem dla niej jest, bo widzę, że ją naśladuje, zaczyna mówić podobnie. No, to też fajnie byłoby, jak by miała ten kontakt z dziećmi innymi. Druga sprawa, że rozruszać by się mogła. Bo te dzieciaki jednak biegają. To, że wyjdzie 2 raz na dwór, no, pojeździ trochę, tam pobiega. Ale to nie to samo. Oni nawet na korytarzu przecież biegają, jest jakiś tam ciągły ruch. Druga sprawa, to poćwiczyłaby trochę, może tak bardziej dyscyplina jest w szkole i te dzieci jedno drugie mobilizują może i pani mobilizuje do tego, żeby tam czytała czy robiła jakieś zadania. Bo ja może nie mam aż takiego chyba wpływu na nią. Mam wrażenie, że inaczej się traktuje mamę niż nauczyciela. Coś tam robimy, ale jak ona mówi: nie, nie chcę już tego robić, nie lubię, nie podoba mi się, no to ja odpuszczam. Bo czasami trochę tak, wiadomo, namówię, ale nie zawsze. Jak mam ważną pracę, teraz w tym momencie muszę maila jakiegoś tam przeczytać, coś zrobić, no to odpuszczę. No i to tak właśnie wygląda. Więc pewnie też nie dopilnuję tego idealnie.

**No właśnie, bo teraz też jesteś trochę w takiej roli wychowawczyni czy nauczycielki dla swoich córek.**

Tak, tak.

**I jak się z tym czujesz?**

No, na pewno jest to dużo pracy. Ale oni tam są po to. Więc oni, wiadomo, że jakoś tam się w tym sprawdzają, a ja to co? Tyle, ile mogę, w jakimś tam ułamku tego całego czasu.

**(Emocje – zdjęcia). Chciałabym, żebyś wybrała jeden obrazek, który odzwierciedla najlepiej to, jak się teraz czujesz.**

**7**

**Dlaczego ten obrazek wybrałaś?**

No, bo to taka właśnie, no nie wiadomo, co będzie dalej. Taka nie kończąca się ta droga. I wokół tak smutno, pusto, te barwy takie szare. Taki jak myślę o przyszłości, że nie wiadomo, co będzie. Tak jak o tych wakacjach, że nie wiadomo, czy będą. No i w sumie człowiek taki samotny bez ludzi i bez kontaktu z ludźmi, tylko najbliżsi. A jednak ludzie są potrzebni teraz, tak widzę. Że to jednak te interakcje takie są ważne.

**To też jest coś, co teraz jakoś tam zrozumiałaś bardziej, że potrzebujesz tych ludzi jednak?**

Tak. Tak, tak. No i też trzeba zatęsknić za sobą, za mężem, za mną. To też jest potrzebne. No, odpoczynek od siebie, taka izolacja, że potem fajniej wrócić do siebie. To wszystko jest potrzebne. Bo ludzie, jak są cały czas ze sobą, to też potrafią trochę się denerwować wzajemnie. I dlatego ktoś inny też jest potrzebny, te inne grupy. Żeby tak jakby na inne tematy porozmawiać.

**Czyli taka izolacja i niepewność tego, co tam jest na końcu tej drogi.**

Tak, co będzie dalej. No tak, teraz święta za tydzień i też nie wiadomo, jak to będzie wyglądało.

**No właśnie, macie jakieś plany?**

No my zazwyczaj wyjeżdżamy wszyscy na święta do mojego teścia, do moich dziadków. Właśnie z Dianą spędzamy święta. Ale w tym roku no to nie jest to możliwe. Tak jak my raczej stosujemy się do tego wszystkiego, więc nie będziemy nigdzie jeździć, nikogo narażać. Śmiałam się, że też może połączymy się video, w video rozmowie i postawimy kamerkę na końcu stołu i tak będziemy rozmawiać. Zrobimy śniadanie albo obiad.

**Ale to też jest tak, że to jest jakieś ważne dla ciebie to święto, żeby wyjechać, spotkać się z rodziną zwykle?**

Znaczy, odkąd się urodziłam co roku, 2 razy w roku, bo i na Boże Narodzenie i Wielkanoc, zawsze wyjeżdżamy na święta. Więc to jest dziwne, żeby nie wyjechać.

**No właśnie, jak teraz z tym się czujesz, że będziesz musiała zostać?**

No dziwnie się czuję. Bo to nie będzie dla mnie takie typowe święto. To będzie taki bardziej zwykły dzień, bym powiedziała. No i to, że nie można pójść do kościoła. A jeszcze te święta się tak inaczej przezywa, bo one są takie raczej refleksyjne. No i fajnie pójść na tą drogę krzyżową ten tydzień przed. I no ogólnie jest taki klimat inny. Myśli się o sobie, o swoim życiu, o wszystkim. Człowiek się trochę zmienia, próbuje się zmienić, jakoś tak poprawić. A teraz nawet nie było takiej okazji przez cały ten post. Jakoś tak nie poczułam tego. No, w niedzielę sobie włączamy mszę świętą w telewizji albo w radiu. I wszyscy siadamy, słuchamy. Albo chociaż jest cicho, wiadomo, ta młodsza córka tak nie usiedzi, więc sobie tam chodzi, no, ale słyszy. A teraz nie ma takiej możliwości nawet, żeby wyjść. I to też jest takie dziwne.

**Czyli zostajecie, będziecie świętować… Właśnie, czy zostajecie w domu i będziecie świętować w domu? Czy będziecie normalnie dzień jak co dzień?**

Będziemy świętować, będziemy świętować na pewno.

**No i mówisz, że to, że nie można pójść do kościoła też jest jakoś takie…**

No, dla mnie tak. Bo tam najczęściej, jak ma się zły dzień, jak coś się dzieje, no to wystarczyło pójść po prostu i sama ta świadomość, że tam się było, to już tak działało, że jakoś tak psychicznie lepiej było. Więc to jest, mi tego brakuje.

**A czy z tych obrazków jest jakiś taki, który bardziej by pasował do tego 12 marca, kiedy dopiero się ta cała sytuacja zaczęła? Czy to byłby ten sam?**

Może ta 1.

**1 to są te autobusy, jakiś taki korek. Czemu ten obrazek bardziej?**

Kojarzy mi się z takim bałaganem, chaosem, nie wiadomo, co dalej. I w ogóle, co robimy, takie szaleństwo, wszyscy podejmują jakieś decyzje. Tak, dużo dyskutowania, wyznaczania jakichś zadań, celów. Tak, żeby ogarnąć to, żeby jakoś wszystko dobrze działało. Tak mi się kojarzy ten dzień.

**No właśnie, bo to też jest to, o co chciałam zapytać. I chciał cię zapytać, czy jakoś się przygotowywałaś do tego? Podejmowałaś jakieś działania?**

No, może tydzień wcześniej byliśmy na takich zakupach większych.

**Tydzień wcześniej to znaczy tydzień przed…**

Przed jeszcze tym dwunastym. No i tak, po prostu kupiliśmy jakieś pięciolitrowe takie bańki wody ze 3, trochę makaronu, trochę mąki. Tak jak to ludzie mówili, że a, kupić to, to, to. No to tam troszkę się tego kupiło, żeby było. Wiadomo, że to się zużywa. I mąkę, i cukier, takie rzeczy, więc to nie zaszkodzi, może sobie leżeć. Więc tak się przygotowałam jedynie. Tylko w ten sposób. A, jeszcze zamówiliśmy sobie jakiś tam Apap w aptece internetowej, takie rzeczy. I tyle. I właściwie tyle.

**Czyli zrobiliście takie większe zakupy. I mówisz, że wtedy kupowaliście jakieś po prostu takie większe zgrzewki wody, jakieś większe liczby takich produktów, które się zużywają po prostu?**

Tak. Tak, a które się przydadzą zawsze. I z nich coś można zrobić. Wiadomo, że takie pewniaki.

**A jeśli chodzi o te zakupy w aptece, powiedziałaś, że Apap. Czyli to takie codzienne lekarstwa?**

No takie przeciwbólowe, przeciwgorączkowe. Apap, Ibuprom. No, bo już jakieś tam krążyły informacje, mój mąż już to wszystko wyczytał, więc on się tym zajął.

**Czyli to w kontekście jakby walki z tym koronawirusem te lekarstwa?**

No chyba tak. Myślę, że tak. Tak. No, on chyba też się boi trochę o swoje zdrowie. Więc tak myślał też o sobie.

**Czujesz w ogóle, jak teraz robisz zakupy, bo rozumiem, że robisz zakupy cały czas jakieś?**

Mój mąż jeździ. I nie chce, żebyśmy z nim jeździli. Mnie nie chce też zabierać. No, żeby jakiegoś ryzyka nie zwiększać.

**I od jakiego czasu tak jest, że jeździ sam?**

No już chyba jak… No, ten pierwszy tydzień taki. Nie od tego 12 marca, tylko tam kilka dni później, to od tego czasu. I raz w tygodniu. Najczęściej rano. To jest albo piątek rano albo w sobotę z samego rana, jak tylko sklep jakiś ma się otwierać, to stara się, żeby jak najmniej ludzi było. I też bardzo tak zachowuje te warunki higieny. Rękawiczki. No i nas też pilnuje bardzo. Już moje dziewczyny mają nawyk taki, że przychodzą z podwórka, myją ręce. I pryskamy sobie tam tymi odkażaczami.

**A ty w ogóle teraz robisz jakiekolwiek zakupy? Wliczając online.**

Byłam w paczkomacie, byłam wieczorem w sklepie, jak jeszcze na te spacery tak sobie chodziliśmy we dwoje. No to tak weszliśmy na chwilę, bo mówię, że może jakieś warzywa kupimy, a akurat nie było tak ludzi za bardzo. Też zobaczyłam, czy nie ma tych ludzi. Bo jakiś taki lęk się rodzi w człowieku, że o, już ta epidemia jest, no to nie wiadomo, gdzie i od kogo można się w każdej chwili zarazić. No jakieś takie nie wiem, takie nerwowe to jest może, taka paranoja czy coś, nie wiem. Tak że byłam tam, przy tym paczkomacie, właśnie w sklepie po warzywa. I rano któregoś dnia w osiedlowym sklepie po pieczywo. Ale widziałam, że na przykład jak weszłam jedną alejką, a inna osoba była w sklepie, to ona weszła drugą alejką, żebyśmy się minęły. Pieczywo też starałam się tak higienicznie brać. Ale widziałam, że ludzie normalnie niektórzy, bez rękawiczek, bez folijki, pieczywo normalnie bierze ręką do reklamówki albo praktycznie się ociera o człowieka. Tak że różne zachowania w ogóle widziałam. I to też takie jest denerwujące. Bo można uważać samemu, ale ktoś inny na przykład traktuje to, tak można powiedzieć olewczo. Że nic mu nie będzie. Ale nie myśli o innych, no nie?

**Czyli widzisz takie różne zachowania, że niektórzy rzeczywiście przestrzegają tych zasad, starają się unikać tego kontaktu, a inni wręcz przeciwnie.**

Tak. Dokładnie. Na przykład znajoma mówi, że a, ona to w ogóle… Bo my chcieliśmy dokupić jeszcze te środki takie odkażające, teraz już nigdzie nie można tego kupić. I znajoma mówi, że a, ona to się niczym nie pryska. I nic jej nie jest. I nawet spotyka się z ludźmi. Ale powiedzieliśmy jej, że to nie jest poważne zachowanie, bo przecież sama możesz nic nie mieć, żadnych objawów, ale przeniesiesz na kogoś, a ten ktoś inny przeniesie na babcię na przykład. I ludzie sobie z tego nie zdają sprawy kompletnie. Bo o chorobach się też nie opowiada. Tak jak mówią, że niektórzy mają dodatkowe choroby, ci, co poumierali, że jakieś dodatkowe obciążenia i dlatego zmarli, bo był słabszy organizm. No to o tym się też często nie wie, czy osoba, z którą tam się spotkałaś akurat nie ma czegoś takiego.

**Czyli można kogoś innego zarazić, samemu nawet nie mając jakichś objawów, tak?**

Dokładnie.

**Czyli na jakieś tam zakupy ci się zdarzało chodzić. I kupowałaś jakieś pieczywo…**

Tak, warzywa. Świeże rzeczy, tak.

**Czy wasze w sumie zakupy, takie codzienne, które robicie, czy tam co kilka dni jakoś się zmieniły? I kupujecie inne rzeczy?**

Nie wiem, czy się zmieniły. Chyba nie, chyba podobnie.

**Trochę mówiłaś o tym, że to, czego ci najbardziej brakuje, to taka możliwość wyjścia, pojechania sobie gdzieś dalej. Chodzi mi po prostu o to, co jest dla ciebie wyzwaniem największym w całej tej sytuacji? I mam wrażenie, że trochę o tym powiedziałaś. Chciałam teraz tylko potwierdzić, czy dobrze zrozumiałam to, co jest dla ciebie największym wyzwaniem.**

Nie wiem sama, czy to można nazwać wyzwaniem, to co powiedziałam. Nie wiem, trudno mi powiedzieć. No może to, że to przebywanie takie ciągłe razem to też jest wyzwanie. No, bo jest. No naprawdę nie można sobie znaleźć kąta, bo zaraz przypałętają się pacholęta (śmiech). Żeby po prostu funkcjonować normalnie, nie być nerwowym, nie krzyczeć. No wiadomo, nie zarażać jakimś tam swoim zniecierpliwieniem z tego tytułu, że tak jest się w kółko w tej grupie.

**Próbujesz sobie jakoś z tym radzić?**

No ja zaczęłam po prostu ćwiczyć godzinę dziennie. Chociaż tyle. Bo nie jeżdżę na tym rowerze, nie wychodzę na te większe spacery. I widzę, że… No fajnie tak. Mam taką małą trampolinkę, włączam sobie muzykę, hantle, gumy od córki pożyczyłam. Tam rozkładam jej matę, w jej pokoju się rozkładam. I one już wiedzą też, że ja sobie tam ćwiczę. No zawsze to trochę energii wyjdzie. Jest to potrzebne. I może sylwetka się poprawi do lata (śmiech).

**Czyli ten ruch codzienny sobie zastępujesz tym ćwiczeniem w domu.**

Tak.

**Mówiłaś o tej twojej znajomej, która nie za bardzo się tym przejmuje, co się dzieje teraz dookoła. Jak sobie myślisz o osobach w twoim otoczeniu, jakimś bliskim znajomym, to co widzisz u nich? Jak oni się zachowują, co oni robią?**

No w większości to raczej każdy się tak stosuje do tych zaleceń kwarantanny i siedzą w domu. Niektórzy młodsi ode mnie bardzo się denerwują, bo na przykład wzięli kredyty na mieszkanie i nie wiadomo, co dalej. Koleżankę mam taką w pracy, która jest akurat na zastępstwo w tym momencie w naszym zespole za dziewczynę, która jest w ciąży. Więc też jeszcze będzie półtora roku tu pracować. Ale boi się właśnie, jak to będzie, bo niby jest świeżym pracownikiem i właśnie ten kredyt ma na głowie. No, to praktycznie w większości to są takie osoby, które się raczej stosują. I widać, że trochę się denerwują.

**Czyli nie masz za bardzo takich osób w twoim otoczeniu, oprócz tej jednej znajomej, o której powiedziałaś, które zupełnie jakoś lekceważą to, co się dzieje.**

Nie, nie, nie. Nie, tylko ona właśnie. I dlatego ją tak zapamiętałam.

**I mówiłaś, że to, co myślisz o tym, to że to jest niepoważne, chyba tak powiedziałaś.**

No jest niepoważne, tak. Uważam, że tak. No wiadomo, że się nie uniknie tego zakażenia, tak mi się wydaje. Ale chociaż może w tych szpitalach nie będzie takiego przeludnienia. Jak będą ludzie w złym stanie, to może jakoś dadzą radę wyleczyć tych ludzi, jak ktoś już trafi, że będzie tych łóżek wystarcza ilość, tych respiratorów. Bo ich brakuje.

**Chciałabym też, żebyśmy trochę porozmawiały o tym całym koronawirusie, tak zupełnie luźno. Bo bardzo dużo się mówi o tym wszędzie tak naprawdę teraz. A jak tobie się to wydaje, skąd się to wzięło? Skąd się wziął ten koronawirus?**

No chyba z tego Wuhan.

**Z Wuhan, OK, ale jak on się zaczął w ogóle rozprzestrzeniać na całym świecie?**

Aż tak się nie interesowałam. Tylko po prostu, jak to było poza Europą, to mnie to tak nie interesowało. Dopiero, jak się we Włoszech zaczęło to dziać i zaczęły te osoby wracać z tych ferii i tak u nas się zaczęło mówić, to może tak trochę bardziej zaczęłam się interesować. Więc może o Europie bardziej mogę coś powiedzieć. A już tam poza nie za bardzo.

**Czyli zaczęłaś się interesować, jak we Włoszech była sytuacja, że był ten wirus. Myślisz, że w ogóle na którymkolwiek etapie było tak, że można było zapobiec tej całej pandemii?**

Populacja ludzi jest tak duża, że nie wiem, czy dałoby radę zapobiec temu. Przecież teraz jest tak, że możemy się tak przemieszczać szybko, te samoloty, to wszystko. Wydaje mi się, że nie. A jeszcze jak oni mówią, że to tak łatwo można się zarazić, przez dotyk. Słyszałam o przypadku, ktoś znajomy właśnie opowiadał, że w Radomiu w bloku chłopak wrócić z Londynu bodajże. I okazało się, że był chory. I jakaś pani, która chyba 2 piętra niżej mieszkała, okazało się, że też jest chora. I podobno dotknęła klamki. I na tej klamce to się utrzymywało i ona przez to się zaraziła. To jest moment i nawet można nie mieć świadomości. A jeszcze przecież były momenty, kiedy nikt nie dezynfekował tych klamek i niczego.

**Czyli można się nawet zarazić, dotykając czegoś, czego dotknęła ta osoba zarażona wcześniej, tak?**

Tak, tak. Tak słyszałam.

**A wy dezynfekujecie jakieś powierzchnie w domu?**

My jedynie klamki trochę przecieramy, te wejściowe. Ale w domu nie, tylko po prostu ręce sobie, myjemy ręce tymi mydłami antybakteryjnymi. A potem tych alkoholem czy czymś tam z alkoholem sobie spryskujemy. Mój mąż się zna, ja się nie znam, jak to się nazywa. Tak że tyle.

**No właśnie, mówiłaś, że macie te środki do dezynfekcji różne. Potem, że też chcieliście je kupić, ale już ich nie było nigdzie.**

No właśnie podobno nie ma nigdzie. Sprawdzaliśmy w internecie.

**Czyli kupiliście je specjalnie w związku z epidemią na samym początku?**

Tak, ale niedużo. Tak, tak, tak trochę. A potem zaczęli ludzie tak szaleńczo wykupować. W ogóle był taki moment chyba, że od razu jak ogłosili, że te szkoły zamykają i w sobotę chyba chcieliśmy jakieś... Mąż chyba… Nie, jeszcze wcześniej, zanim szkoły. To mąż kupił dziewczynom takie żele antybakteryjne do szkoły, żeby sobie tam ręce przecierały. No i jakoś za 2 dni, już chyba na półkach ktoś wrzucił jakieś zdjęcie gdzieś, że w ogóle puste półki, że żele wykupione, tam jakieś mydła. No bardzo tak się ludzie rzucili. Taka panika się zaczęła robić.

**I pamiętasz, co sobie myślałaś, jak zobaczyłaś te zdjęcia z pustymi półkami?**

No, że jakieś wariactwo. Od razu mi się skojarzyło z tymi latami PRL-u, co te półki takie puste były. Jeszcze te memy o tym papierze toaletowym, przecież ciągle, że zabraknie i w ogóle. No to normalnie mi się z tym skojarzyło, że wraca wszystko chyba (śmiech). Miałam takie myśli, że pozamykają te sklepy, że chyba trzeba robić jakieś zapasy, bo wiadomo? Może jakieś strefy porobią, jak będzie dużo tych zakażeń? Nie wiem, na przykład z Łomianek nie będzie można się wydostać i co? I wtedy ani nie dowiozą towaru ani nic. No miałam takie myśli, takie trochę dziwne.

**I teraz nie masz już takich myśli, że mogą zamknąć…**

No nie. Bo widzę, że wszystkie takie miejsca, gdzie jest zagrożenie, że ludzie się przemieszczają, czyli galerie handlowe, że są pozamykane, tam te sklepy odzieżowe. Ale spożywcze i apteki, które są najważniejsze, no to jednak to jest otwarte. Tylko te zasady jakieś tam pozmieniali, tak?

**Czy jakby to miejsce, w którym mieszkasz, to że mieszkasz jakby pod Warszawą, to daje ci jakieś poczucie bezpieczeństwa? Albo wręcz przeciwnie, że się obawiasz z tego powodu, że mieszkasz tu, gdzie mieszkasz?**

Nie, nie, obawiam się. Uważam, że w bardzo dobrym miejscu mieszkam. Jeśli chodzi o te sklepy, no to jest duży wybór sklepów. I właśnie tuż po zamknięciu szkół otworzyli Lidla w Łomiankach, więc pierwszy Lidl, więc akurat zaopatrzenie jest bardzo dobre. I można było wszystko dostać. I świeże warzywa i wszystko świeże. I widzę, że się starają z tymi dostawami. Poza tym, no mamy ten dom z tym podwórkiem, no to jest dla mnie największy plus. Nie wyobrażam sobie, w bloku byśmy zwariowali na pewno z dziećmi. Bo przecież dzieciaki muszą wyjść. I samemu też się chce wyjść, tak? Z psem się pobawimy, to też tak inaczej, więcej czasu się tam spędzi. I teraz jeszcze słyszałam, bo obok mamy szpital dziecięcy w Dziekanowie Leśnym, mają przekształcać na szpital zakaźny. No to też jest rzut beretem, nie daj boże, jak by coś się działo. Więc uważam, że najważniejsze rzeczy, no mamy, tak?

**No właśnie, bo to, że będzie szpital zakaźny, to jest dobre dla ciebie?**

Tak, uważam, że tak. Dlatego że jest mało tych miejsc, są przeludnione. No i nawet nie wiadomo, gdzie się udać. No niby są jakieś tam ścieżki wytłumaczone, co należy robić, gdzie się udawać i tak dalej. Ale, no wiadomo, że jak jest mało szpitali na jakąś tam ludność, to trudno się dostać i tych łóżek więcej pozajmowanych. A jak ja mam tutaj rzut beretem, no to tak się bardziej może bezpieczniej się czuję psychicznie.

**Że w razie czego to masz gdzieś blisko takie miejsce po prostu.**

Tak.

**Powiedziałaś, że macie psa, tak?**

Tak, mamy.

**I macie z psem jakiś problem teraz?**

Nie, nie, nie. Nie, nie mamy problemu.

**Chodzi mi właśnie o jakieś wychodzenie na spacer czy coś takiego.**

Nie, nie, nie, to nie jest problem. Więcej się z nim bawimy, więcej spędzamy czasu. Bo on jest na podwórku. Bo to jest spory pies, seter irlandzki. Ale właśnie dzięki temu, że tak się spędza nie gdzieś tam poza domem zupełnie ten czas, tylko tutaj w obrębie domu, to też pies na tym chyba korzysta, bo więcej się z nim bawimy, dzieciaki tak bardziej lgną do niego.

**Czyli tak naprawdę bardzo doceniasz to, że masz rzeczywiście dom z ogródkiem, bo bez tego myślisz, że by było bardzo ciężko.**

Tak. No myślę, że tak. Wyobrażam sobie ludzi, którzy są w bloku. I tak jak koleżanka mówiła, że wyjdzie na balkon trochę, tak? No to nie wyobrażam sobie tego.

**I też w takim otoczeniu mieszkacie, że wszystko jednak tam jest. I mówisz, że jakoś nie zauważyłaś, żeby na przykład w Lidlu czy w sklepach jakichś były braki ogromne, tak?**

No nie zauważyłam, nie.

**Wracając jeszcze do tego, bo zaczęłam mówić o tym, czy mogliśmy zapobiec jakoś tej epidemii, powiedziałaś, że nie za specjalnie, bo ludzie tutaj cały czas się gdzieś przemieszczają. I że trudno by było temu zapobiec. A jak ci się wydaje, na ile Polska jest teraz przygotowana na tę sytuację?**

No nie wiem, oni chyba teraz robią wszystko, żeby wyglądało, że jesteśmy przygotowani. Ale według mnie to chyba robią za mało tych testów. Nie wszystkim robią testy. Z tego, co też słyszałam wśród znajomych, męża kolega wrócił z Egiptu i od razu go wzięli na czternastodniową kwarantannę. No i całą rodziną byli zamknięci w domu, czteroosobowa rodzina. Po tych 14 dniach bodajże przyszli zrobić test. Tylko jemu i żonie. Dzieciom nie. Bo jak ich wyjdą pozytywnie, no to wiadomo, że dzieciom też. No to takie głupie podejście. Więc mi się wydaje, że mają mało tych testów. I mało ich pewnie robią. A może dlatego mało robią, żeby w tych statystykach to wyglądało, że aż tak dużo tych zakażeń nie ma. A jak to się tak szybko rozprzestrzenia, to mi się wydaje, że jest dużo więcej.

**Czyli powinno być więcej na pewno testów robionych.**

Tak.

**I że jakby nie do końca ufasz tym statystykom, które są.**

Nie ufam im, tak, nie ufam. Myślę, że to ma dobrze wyglądać przed wyborami.

**No właśnie, co myślisz o tej całej sytuacji teraz z wyborami?**

Bez sensu. Bez sensu i nie wiem, jak to rozwiązać. Nie wiem, czy jak nikt nie pójdzie głosować, to pójdą tylko ci, którzy są za PiS-em i znowu wyjdzie na to, że Duda wygra? Nie wiem, nie wiem, naprawdę. A inni się zachowają rozsądnie, ale no w sumie na niekorzyść. No, bo jak wygra Duda, no to… Nie wiem, naprawdę. Nie wiadomo, jakie rozwiązanie jest dobre.

**Czyli podsumowując, trochę tak uważasz, że nie jesteśmy przygotowani za bardzo, tak?**

Tak. Uważam, że nie jesteśmy.

**Ale czy w ogóle było tak, że można było się przygotować? Jak gdyby Polska czy mogła się przygotować na to?**

Chyba nie. Patrząc na inne kraje też, to chyba nie.

**Nie dało się jakoś zapobiec, przygotować.**

No chyba nie. Po prostu za szybkie jest to tempo tych przyrostów, tych zachorowań. Ale może, jak byśmy nie przestrzegali kwarantanny, byłoby jeszcze szybsze i byłoby jeszcze gorzej? I byłoby to widoczne, że jeszcze bardziej jesteśmy nieprzygotowani? Nie wiem. Mogło być gorzej.

**Masz takie poczucie, że w Polsce ludzie generalnie przestrzegają tych zasad, których trzeba przestrzegać?**

Znaczy u mnie wokół na osiedlu wszędzie widzę, że tak. Widzę, że przestrzegają. A dalej się nie ruszam, więc nie wiem (śmiech). Ale widziałam, że jak zamknęli szkoły, to trochę nie denerwowało, że dzieci w grupach sobie jeździły. Bo akurat któryś weekend był taki ciepły. Więc na deskorolkach, rowerami, jakieś grupki. I trochę mnie to denerwowało, że my tutaj pilnujemy, żeby nasze dzieciaki nie miały kontaktu z innymi, a ktoś sobie po prostu, takie ma podejście.

**Czemu to jest takie denerwujące?**

No, dlatego że powinniśmy może jakoś tak solidaryzować się. I skoro mówią, że można uniknąć takiego tempa wzrostu tych zachorowań, przestrzegając te zasady, no to chyba po coś to zostało zrobione. A nie, żeby sobie robić wakacje.

**A twoje dzieci w ogóle jak na to reagują, że nie mogą spotykać się. Trochę mówiłaś o tej młodszej córce, że potrzebuje kontaktu z innymi.**

Młodsza, no widać, że częściej przyjdzie, zaczepia nas, zagada z nami. Widać, że nie usiądzie, nie wyizoluje się w swoim pokoju i tam się bawi, czy coś robi. Tylko tak kontakt łapie. I na dole mieszka moja mama, mamy oddzielne wejścia, mieszka moja mama z siostrą i jej mężem, i tam dwoje dzieci. Więc ten Szymon jest starszy od mojej Asi o rok. Więc tam będzie 8 lat teraz miał. Więc oni jak wychodzą w południe na dwór na ulicę, to sobie razem jeżdżą. No to niby jakiś tam ten kontakt jakiś jest. A moja starsza córka, jak na początku usłyszała, że będziemy teraz pracować zdalnie, to powiedziała, że ona się wyprowadza z domu, bo ona nie wytrzyma (śmiech). Bo ona myślała, że… W ogóle, jak ja przyszłam z laptopem do domu i z segregatorem, to mówię, że ja teraz z domu będę pracować, bo szkoły zamknięte. A ona: co? To ja myślałam, że ja sobie odpocznę (śmiech). Ale potem jej przeszło, już się wszyscy przyzwyczaili.

**Czyli widzę, że wszyscy, bo trochę mówiłaś o sobie tak, że na początku myślałaś, że to takie wolne trochę będzie.**

Tak, no trochę tak myślałam, tak.

**I córka trochę też tak myślała. Ale już teraz się okazało, że jest trochę inaczej jednak.**

W ogóle zanim video lekcje się zaczęły, to dostawali na Librusie dużo wiadomości, co zrobić. A tu prezentacja z historii, ileś stron, a tu z angielskiego, tu to, a tu to. To było rozbicie takie… Znaczy oni to dostali tak, że na ileś dni do przodu. A ona wszystko porobiła chyba w 2 dni i siedziała do oporu, bo ona chciała mieć dalej wolne. Kolejne dni będzie miała wolne, więc wszystko poprzesyłała, porobiła. A później się okazało, że pani od matematyki powiedziała, że nie, ona już anuluje te wszystkie zadania, które zadała, bo rozpisała je na video lekcje, i będą omawiać na video lekcjach. A ona mówi, że już wszystko zrobiła (śmiech).

**I teraz jakby jest tak, że przyzwyczaiła się do tego, że jednak nie odpocznie sobie od rodziców i czuje się teraz lepiej? Czy jakoś gorzej, też jej przeszkadza ta sytuacja, czy nie za bardzo?**

Ona jest, ma taki swój świat, trochę czyta. Tam wiadomo, że na tych komunikatorach trochę tam z koleżanką pisze, z chłopakami. Myślę, że jakoś się oswoiła z tym, nie przeszkadza jej chyba. No mówię, mamy takie stałe jakieś zajęcia w ciągu dnia. Więc jakoś to chyba wpływa dobrze na wszystkich, że wiadomo, jakiś taki stały harmonogram. Bo ta praca, tutaj obiad, to, to, to, wyjście na dwór. A dla siebie też mają czas taki, więc…

**Ten harmonogram to jest coś, co wprowadziliście tak oficjalnie, czy jakoś tak naturalnie…**

Nie, on naturalnie się jakoś tak pojawił, wszedł w życie.

**Bo wcześniej, jak pracowaliście normalnie, to ten taki harmonogram jakiś mieliście, taki codziennego dnia?**

Znaczy wszystko jakoś tak… Ten dzień był krótszy chyba. Bo wracało się najczęściej, no, jak ja o 16 wyszłam z pracy, no zanim tam czy jakieś tam zakupy zrobiłam, odebrałam córkę, to około tej 17 się wracało. Później mąż wracał. No, starsza córka już była w domu. Więc obiadokolacja, coś takiego, to już było po 17. No i praktycznie, no bardzo krótki był ten dzień. I to jakoś tak wszystko szybko leciało. No tutaj też mam wrażenie, że mi szybko płynie czas, naprawdę szybko. No nie wiem, uważam, że chyba tak pożyteczniej może się spędza go. Bo wiadomo, o 16 można tą klapę komputera zamknąć, no tam czasem się tego maila jeszcze sprawdzi. Ale dzieciaki są pod ręką, porozmawiać można.

**A z tą rodziną, która mówiłaś, że tam mieszka na dole, to też jakoś się teraz spotykacie, spędzacie razem czas?**

Zachowujemy dystans. Może to jest śmieszne, ale jednak zachowujemy dystans. Mój szwagier pracuje zdalnie. Siostra pracuje w laboratorium, rzut beretem od domu. Ale jest tam odizolowana i bardzo tak mają wszyscy… No tam jest ona chyba na dyżurze ze swoim kierownikiem na zmianę, zamieniają się. I wszystko dezynfekują. Wiem, że dezynfekują, nikogo nie wpuszczają do środka. A nie mogą zamknąć tego laboratorium. A moja mama pracowała na noce. I jeździła na Wawer do takiej firmy produkcyjnej. No i to też tak trochę się denerwowaliśmy, żeby się z nią może za bardzo nie spotykać. Albo jak szła na dwór, to mówiliśmy dziewczynom, żeby się nie zbliżały za bardzo do babci. Wiadomo, że nikt tego teraz nie traktuje w kwestiach obrażania się, czy tam nie wiadomo czego. To są względy bezpieczeństwa. Więc to było na tej zasadzie, że po prostu ta odległość i tyle. Teraz moja mama sobie wzięła miesiąc bezpłatnego urlopu, czyli nie dostanie pensji w kwietniu. Ale mówi, że boi się. Bo były momenty, kiedy na przykład, jak jechała do pracy wieczorem, to było mało ludzi w autobusie. Ale rano, jak się wszyscy zebrali, a jeszcze rozkłady jakieś takie były inne, bardziej świąteczne chyba, to mówi, że było tyle ludzi, że nie było możliwości, żeby jakąś odległość zachować. No i że bała się po prostu. No my też się baliśmy. I o nią, i w ogóle wszyscy, tak? Więc uważam, że dobrze zrobiła. Człowiek przez miesiąc może jakoś z głodu nie umrze, jakieś tam ma się oszczędności i każdy może pomóc, tak? A to bezpieczeństwo jest ważne.

**Czyli dla bezpieczeństwa teraz już zrezygnowała twoja mama z tej pracy. Ale nadal jakiś tam dystans zachowujecie teraz?**

No, nie chodzimy tam do siebie wzajemnie po domach. Tylko po prostu tyle, co na powietrzu się bardziej spotykamy, rozmawiamy. Na tej zasadzie.

**I też mówiłaś o tym, że ta praca zdalna, bo i ty pracujesz w domu i twój mąż pracuje teraz w domu, jak wam się pracuje razem ze sobą? Pracujecie w tych samych godzinach?**

Tak (śmiech). No mamy taki stół, 2 metry. Więc on jest na jednym końcu stołu, ja na drugim końcu stołu. Na początku, to on się tam denerwował, że go rozpraszamy i że on to nie wytrzyma tak siedzieć w domu. Zasłonką nas jakoś przegrodził, tak od okna przeciągnął, do stołu przyczepił, żebyśmy się nie widzieli. Ale teraz to już wszyscy jakoś, widzę, co on robi mniej więcej. I on widzi, jak ja pracuję. I nie przeszkadza mi to. On to ma ciągle te telekonferencje, więc w kółko na słuchawkach, też coś ustalają, dzwonią i tak dalej. No więc moje dziewczyny stwierdziły, że co tata robi w pracy? Zuzia dzisiaj mówi, że ona chciałaby mieć taką pracę jak tata, bo tata tylko ciągle gada, żartuje sobie przez telefon, nic nie robi (śmiech).

**Czyli mogły zobaczyć, jak wygląda praca rodziców teraz.**

Tak, dokładnie.

**I wybierają pracę taty jednak, nie pracę mamy.**

Znaczy moją też. Bo weszłam w sumie na chwilę zobaczyć jakieś wiosenne ubrania, bo mówię, że muszę tej młodszej córce zamówić jakieś buty przez internet. Nie lubię przez internet, bo to często trzeba zwracać, ale jak nie ma innej opcji… No i liczę na to, że może się w końcu wyjdzie gdzieś w tym ubraniu, więc te nowe buty się przydadzą. No i weszłam tam na chwilę. No to też mówią, że a my to mamy takie prace, że ja to sobie ubrania oglądam, a tata to gada przez telefon i sobie żartuje. Że one to chciałyby mieć takie prace. Ja mówię, tylko nie powiedzcie komuś kiedyś przypadkiem, że mama to ma fajną pracę… Bo różnie to bywa, przyjdą do mnie do pracy i coś palną, że mama to ma fajną pracę (śmiech).

**Mama w pracy ogląda sobie sukienki.**

Dokładnie.

**Ale właśnie, czyli kupujesz ubrania jakieś na wiosnę, licząc, że będzie można wyjść w nich gdzieś jeszcze?**

No tak, tak. No, trochę schodzi z tym oglądaniem. Więc ja tak nie lubię, bo wiem, że internet to jest taki pożeracz czasu. Więc czasem sobie chwilkę wejdę, tak coś zobaczyć. Porównać ceny czy jakieś tam promocje są, żeby tam nie przepłacać. No i zamówiłam tam jakieś spodnie tej młodszej córce, jakąś taką cienką kurtkę. No moja starsza córka to Bershkę lubi, więc… Ale ja tak nie mam orientacji w tych rozmiarach w tej Bershce. A ona jest taką nastolatka, to jeszcze nie kobieta, więc niektóre mogą być takie niepasujące do końca. Więc mówię, jak tylko otworzą galerię, jedziemy do Bershki. I kupujesz sobie, co tam chcesz. I już wymyśliłyśmy, co tam chce sobie kupić. A mąż mówi: jak ci ludzie wszyscy pojadą do tych galerii, co tam się będzie działo! (śmiech) Jak ich wypuszczą z tych domów. No, bo tak może być.

**Czy myślisz o tym, że pojedziesz do tej galerii, pojedziecie na te zakupy, to masz w głowie gdzieś jakiś taki czas, kiedy to może być?**

Nie, nie mam. Kompletnie nie mam. No, dochodzą do mnie ciągle te głosy… U mnie jest niewiadoma, ale u męża już tam jakieś procedury wprowadzają. I a to jakaś koleżanka, która jest bliżej jakiegoś dyrektora słyszy, że do końca maja to na pewno nie wrócą. A to ktoś tam mówi, że w czerwcu. A Zuzia dziś mówiła, że szkoły to mają być przez całe wakacje jeszcze zamknięte. I nie będzie wakacji, dopiero we wrześniu. Więc jest taki szum w ogóle tych różnych informacji, nie wiadomo komu wierzyć. Ale biorę to pod uwagę, że do końca roku szkolnego to nie wiadomo, czy w ogóle wrócą do szkoły. A jak oni nie wrócą, to tym samym ja.

**Chciałam zapytać o te zakupy przez internet. Czyli przed całą epidemią nie lubiłaś za bardzo przez internet kupować. Ale nie lubiłaś kupować dla siebie, dla dzieci czy w ogóle nie za bardzo?**

Znaczy ja czasami kupowałam buty dla siebie. Ale w większości przypadków, może w 70% zwracałam. Zawsze jakiś tam rozmiar niedopasowany, tu ciasne, tu coś tam. Ale zazwyczaj ja na takich stronach, gdzie jest darmowa przesyłka i ten zwrot jest darmowy. To na takich, więc tam nie traciłam. A dzieciakom, to już wolę pojechać, mamy galerię taką małą w Łomiankach, tam mamy najważniejsze sklepy, Smyk, Reserved, H&M, więc tam się wszystko praktycznie kupi. Więc już wolę tak pojechać, przymierzą szybko, oblecimy sklep. I wiem co i jak. Nie muszę biegać później, zwracać. Dlatego z dziećmi lepiej stacjonarnie. No sama, to tam czasami właśnie coś tam się kupi.

**I teraz mówisz, że jesteś zmuszona trochę do tego, żeby te zakupy robić dla dzieci też przez internet. Tak? Bo powiedziałaś, że zamówiłaś w końcu coś dla córki.**

Tak, tak, tak. No takie najpotrzebniejsze rzeczy, które wiadomo, że są potrzebne. Jak wiem, że wyrosła z jakiejś wiosennej kurtki, a ta kurtka jednak będzie potrzebna, to wiadomo, że muszę ją kupić. Sobie już jakiejś sukienki, którą bym chciała czy coś, to stwierdzam, że nie ma sensu, bo nie wiadomo, tak? Może w zimę stąd wyjdziemy. Więc nie ma sensu. No na tej zasadzie.

**Czyli dla siebie wiosennych rzeczy nie kupujesz na razie, bo nie wiadomo, czy w ogóle…**

Na razie nie. A i tak sporo ubrań mam, więc na razie nie potrzebuję.

**Czyli te ubrania przez internet to taka konieczność, którą czasem trzeba, jeśli to jest ważne, bo to dla dzieci.**

Tak, dokładnie.

**A jest jeszcze tak, że coś teraz przez internet kupujesz prócz ubrań?**

Oglądałam jakieś takie świąteczne, wielkanocne zajączki, takie rzeczy. Ale też nie mam siły… Znaczy nie mam siły, nie mam czasu może zbytnio, żeby tak usiąść i… No, bo to trochę trzeba poświęcić, poszukać tam, pogrzebać, żeby coś kupić i zamówić. Dobrze, że jest ta opcja z paczkomatem. W sumie nie byłoby źle tak zamówić. No muszę usiąść i zamówić, bo w sumie już niewiele czasu zostało do świąt. To jedynie takie rzeczy oglądałam, nie zamawiałam.

**Czyli jakieś takie dekoracje do domu. A wcześniej takie dekoracje kupowałaś przez internet czy stacjonarnie?**

Stacjonarnie, bo lubiłam tak pochodzić, porównać sobie. A przez internet często kosmetyki, do włosów najczęściej, takie profesjonalne bardziej. I pewnie będę coś tam zamawiać w międzyczasie, jak się coś pokończy.

**Mówisz, że paczkomat jest OK. Dlaczego ta metoda jest dla ciebie najlepsza?**

Dlatego, że nie ma tej styczności z drugim człowiekiem. No i można aplikacją w telefonie się połączyć, więc jest bezpiecznie.

**Po prostu bezkontaktowy taki jakiś odbiór tych paczek.**

Tak.

**Mówiłaś trochę o tym przed chwilą, że czujesz, że masz taki szum informacyjny z różnych źródeł. No to właśnie, skąd ty w ogóle teraz bierzesz informacje różne o tym, co się dzieje na świecie?**

Ja w ogóle o tym nie czytam, zero. Tylko to, co mój mąż powie. On jest w sumie takim przekaźnikiem, mógłby wszystko powiedzieć i mu uwierzę. No tak, także tylko do niego. Na przykład, nie wiem, wstajemy rano i on tam mówi, a dzisiaj doszło ileś tam ofiar, a ileś tam zarażonych. Ale to jest za mało. I praktycznie on mnie takimi swoimi poglądami tak zaraża. Tak że jego jakiś tam punkt widzenia słyszę. I myślę podobnie. Raczej sama nie czytam. Bo nawet nie wiem co za bardzo.

**Czy zawsze tak było, że twój mąż był twoim głównym źródłem informacji o świecie?**

Znaczy on mi dużo mówił, ale ja dużo też czytałam sama. A teraz, jak jest ten temat, to kompletnie nie. Kompletnie. Już wolę czasami obejrzeć jakieś wiadomości, czy jak jest ta konferencja taka. Ale to też bardzo rzadko, może 2 razy w tygodniu. To tylko tyle. Ale nie czytam, nie czytam, naprawdę. Nie chcę sobie głowy jakoś tak zaśmiecać.

**Czyli mówisz, że nie czytasz, bo po prostu nie chcesz tym sobie zaprzątać głowy?**

Tak.

**Jeśli chodzi o telewizję, oglądasz czasem wiadomości i te konferencje, gdzie tam są ważne…**

Tak, TVP Info. Jak tam jakieś zasady wprowadzali, to patrzyliśmy, co się dzieje.

**Czyli to nie jest tak, że szukasz, wyszukujesz informacje, co tam się dzieje i ile jest ofiar?**

Nie, nie. Nie.

**A twój mąż, wiesz, skąd czerpie te informacje, które ci podaje?**

Znaczy jest jakaś taka strona, jakaś taka ciekawa mapa. I on tak mi opowiadał. Jest taki cały wykres, nie wiem. Pokazywał mi, jak to jest fajnie zrobione. I w ogóle, że to jest wiarygodne. Jeszcze jakaś taka amerykańska też strona. Więc na takich raczej. No już takich konkretnych, bym powiedziała, chyba sprawdzonych informacjach bazuje.

**Czyli ty jakby wierzysz w to, co on ci mówi?**

Tak. Ale też to jest wysłuchanie na zasadzie powiedział i tyle. Ale nawet, jak mi powie, to ja nie jestem w stanie powiedzieć, ile my mamy ofiar śmiertelnych dzisiaj albo zarażeń. Bo naprawdę kompletnie nie myślę o tym.

**Bo też chciałam zapytać, na ile wy w ogóle rozmawiacie o tym tak na co dzień z mężem. O tej całej sytuacji, o tym koronawirusie i w ogóle.**

No trochę tam rozmawiamy. Rano, jak wstaniemy, on tam sprawdzi jakieś wiadomości, to się zawsze wymienimy. Jak gada na telekonferencjach ze znajomymi, to też słyszę, co tam, jak się wymieniają, jakimi argumentami i co tam kto powie ciekawego. No tyle, co znajomi też, jak ktoś miał jakąś styczność czy właśnie tą kwarantannę czy coś, to też jak tam to jest traktowane. U nas w rodzinie chłopak, który jest właścicielem apteki, okazało się, że przyszła do niego pracownica powiedzieć o pogrzebie mamy, wziąć urlop, a była chora na koronawirusa. No i on musiał się zgłosić do Sanepidu, żeby zrobili mu test. I opowiedział nam, jak to wyglądało wszystko. Więc też z tej ręki mam jakieś tam informacje.

**Czyli masz takie informacje z pierwszej ręki też, jak wygląda ten cały proces.**

Tak.

**I co myślisz? Że ten proces jest prosty, trudny?**

Chaos. Jest jeden chaos moim zdaniem. Tak jak właśnie ten kuzyn pojechał, musiał pojechać sam, żeby mu pobrali tam, nie wiem, co oni pobierają, krew? Chyba krew, tak? Do tego badania. Więc musiał pojechać sam, nie przyjeżdżała żadna karetka, żadne tam służby, nic takiego. Więc też narażał pewnie jakieś inne osoby, jak by się okazało, że jest chory. Finalnie się okazało, że jest zdrowy. No i na wynik tego czekał bodajże chyba 50 godzin. A mówili, że to tak szybko. Więc też długo, tak? I do tego czasu musiał być sam w kwarantannie. Zamknął się w tej aptece, żeby do tego czasu nie mieć kontaktu ze swoją rodziną. Więc taki chaos, mam wrażenie cały czas.

**Masz takie poczucie, że ty byś wiedziała, co zrobić w takiej sytuacji?**

Nie. Kompletnie nie, kompletnie. Już kiedyś sobie gdzieś zobaczyłam, ktoś opublikował na Facebooku, co trzeba zrobić po kolei. I jak zamykają w tej izolatce, co trzeba lepiej mieć, bo tam wielu rzeczy brakuje. I zaczęłam sobie coś tam spisywać. Ale zajęłam się czymś innym i tam przerwałam to. No i nie spisałam do końca. Ale na tą chwilę to nie wiedziałabym co robić naprawdę. Mam wrażenie takie, że bym się nigdzie nie dodzwoniła, bo by były jakieś przeciążone linie. Bałabym się może trochę ruszać z domu, żeby może stan się nie pogorszył czy coś. Z drugiej strony bałabym się być w domu, żeby nie narażać reszty. No i wiadomo. Taki bałagan taki trochę.

**No tak, bo też mówiłaś o tym, że twój mąż choruje i przez to ma obniżoną odporność.**

Tak.

**Więc to też pewnie sprawia, że pewnie jeszcze bardziej byś nie wiedziała co robić w tej sytuacji.**

Myślę, że bym pewnie spanikowała, jak by przyszło co do czego.

**Czyli nie pamiętasz, co sobie tam spisałaś, co trzeba mieć…**

Znaczy, zaczęłam mu coś tam opowiadać. Pamiętam, że ktoś pisał, żeby wziąć cienki koc, jakieś prześcieradło, poszewkę na poduszkę, bo tam zamykają, jest tylko jakieś fizelinowe jakieś prześcieradełko. Żeby wziąć sobie jakąś kanapkę, herbatę, jakiś mały czajnik elektryczny, klapki, jakieś tam środki higieniczne. No, takie tam różne rzeczy. No i mówię, że ładowarkę do telefonu. A on mówi, że przecież w izolatce nie można mieć żadnej elektroniki. Ale ja mówię, ale to jest pierwsza izolatka, w której tam trzymają bodajże 48 godzin i dopiero później jak się okaże chyba ,że pozytywny, to cię biorą do następnej. No i tak wymieniamy się poglądami. Coś tam ja przeczytam, coś tam on.

**Ale to czemu w tej izolatce nie można mieć elektroniki?**

Znaczy w tej już, gdzie jest stan pogorszony i te respiratory, ta cała… tak mi się wydaje. Bo tam, jak widać jakieś kadry w wiadomościach, to ci ludzie są nieprzytomni, tak? W takich kolejnych…

**Znaczy w telewizji, tak?**

Bo to może nie jest izolatka, te kolejne to może nie jest taka izolatka, tylko już taka sala, gdzie tam leży tak więcej osób. Przynajmniej takie widziałam kadry już gdzieś tam, w wiadomościach.

**Czyli nie czujesz się za bardzo przygotowana do tego?**

No nie. Nie wiem, czy da się przygotować do tego. Bo to w sumie w takiej sytuacji to my jesteśmy zdani na tych lekarzy. No, myślę, że to jest w ogóle teraz taki moment, że w ogóle nie daj boże, żeby się coś innego przytrafiło. Jakiś zawał, udar czy cokolwiek, nie daj boże. Bo nie wyobrażam sobie.

**Ale jakby lekarzom w tym momencie ufasz, w tej sytuacji teraz?**

Znaczy o to leczenie tego koronawirusa czy w ogóle? O to leczenie. Znaczy, no ja im ufam. Tylko bardziej nie ufam temu systemowi całemu. Że to jest przeciążone. Że może się okazać, że będzie… No, że nie dadzą rady. Jak ilość jakaś duża pacjentów, natłok, to nie dadzą rady. To tego się boję, temu nie ufam. Że może, że są procedury jakieś, ale nie zadziałają wszystkie na czas. Bo za dużo będzie chorych. Tego się najbardziej boję.

**Też mówiłaś o tym, że starasz się za bardzo nie czytać za dużo tych informacji. Ale z tego, co mówisz, to tak mam wrażenie, że mimo wszystko różnymi kanałami one jakoś do ciebie docierają?**

No tak, no coś tam dociera, tak.

**Masz takie wrażenie, że ten czas, który poświęcasz na różne media, czy to jest telewizja czy internet, czy jakieś facebooki, czy ten czas jest teraz mniejszy czy większy?**

Mniejszy. Chyba mniejszy. Myślę, że mniejszy.

**Czyli on jest mniejszy niż ten czas przed koronawirusem?**

Tak, tak.

**I co u ciebie wywołują te informacje wszystkie? Jak zaczynasz przeglądać internet, jakieś strony internetowe, Facebooka? To co ci to robi, że nie chcesz tego czytać?**

Dla mnie najważniejsze informacje, które wiem ,no to wiem, jaka jest sytuacja. Że jest zła. Wiem, że mamy być na kwarantannie. Wiem, że to powoduje, ma powodować to, że będzie w mniejszym stopniu przybywać chorych, że o to chodzi. I to jest dla mnie najważniejsze. A to, ile tych osób zmarło, ile jest chorych, to jest dla mnie niewiarygodne, co podają, więc nie będę o tym czytać. Bo znowu w szpitalu w Zielonej Górze czy gdzieś ktoś zmarł, a tutaj to coś tam, a tu to ktoś rozniósł chorobę niepoważnie, bo nie pomyślał o czymś tam. No to o tym nie chce mi się czytać. Bo to jest chaos. I po co mi o tym czytać, tak? No wiem, że nie panują nad tym, bo jest dużo tego. I może ciężko tak zapanować. Więc dlatego.

**Czyli chodzi po prostu o to, że te najważniejsze rzeczy to wiesz. I już nie chcesz więcej tego czytać, bo nie do końca wierzysz w to, co oni tam podają.**

Tak. To jest tylko sensacja, tak? Że tutaj się ktoś zaraził w ten sposób, a tu to robił, a tam to siedzą w parku wszyscy zamiast być na kwarantannie. To takie są sensacje. Najważniejsze wiadomości podadzą na konferencji, co mamy robić. I się stosujmy do tego i tyle. Żebyśmy jakoś nie zagrażali innym.

**Czyli uważasz, że te informacje płynące z mediów są raczej niewiarygodne. A wiarygodne są te konferencje.**

Jeśli chodzi o tą liczbę chorych, o to wszystko.

**Wiem, że powiedziałaś, że starasz się o tym nie czytać, ale czy masz jakieś takie źródła, na których wiesz, że możesz polegać? Że one są wiarygodne, że jak będziesz potrzebowała jakiejś informacji, to tam ją znajdziesz i będziesz mogła w nią uwierzyć?**

Które rozwieją jakieś moje te wątpliwości, które mam?

**No, na przykład.**

Znaczy, jeśli chcę coś w ogóle tak się dowiedzieć, to sobie włączę Fakty. Czasami TVN24. I TVP Info jak są te konferencje. I tyle.

**Ale z Facebooka cały czas i z Instagrama powiedziałaś, że korzystasz.**

Tak, korzystam, ale nie odpalam tych linków, które mi tam wyskakują. Bardziej to takie luźne rzeczy. W sensie znajomemu się dziecko urodziło albo tam… No, najczęściej to się wszyscy zarzucamy memami jakimiś, związanymi z koronawirusem. To jest szaleństwo. Bo i moja córka starsza też nam ciągle coś pokazuje. I mój mąż, i ja, i cała rodzina sobie przesyła, wszyscy znajomi. I jest tylko śmiech z tego. Tak rozładowujemy może emocje.

**Czyli jakby, że te memy są tym sposobem, żeby trochę się tak…**

Tak, tak. Na początek to były memy związane ze zdalną pracą męża i żony, albo z tym, że dzieci są w domu i co z nimi zrobić. No różne, różne.

**A teraz jakie memy są najczęściej?**

Teraz różne (śmiech). Na przykład ostatnio widziałam mema, że nowe meble są w Ikei, trumna do złożenia. Ale to też, czarny humor, ale się śmiejemy z tego. Nie wiem, może będzie tak, że ten świat wyginie, nie wiem, nie mam pojęcia (śmiech). No staram się aż tak może nie myśleć negatywnie. Ale wiem, że ten rok będzie ciężki. I nie będzie taki, jak pozostałe lata. Żeby można było sobie coś planować. Nie wiemy, jaki będzie.

**Powiedziałaś, że o tej przyszłości jest trudno mówić i myśleć, tak jak zresztą o tym obrazku rozmawiałyśmy. Masz jakieś takie myśli co do tego, jak to wszystko może się skończyć?**

Nie wiem. My z tego, co tak rozmawiamy w domu, no i też w pracy tam moja przełożona coś mówiła przez telefon, że jak będzie ten przyrost taki maksymalny tych chorych i nagle okaże się, że zaczną spadać te zachorowania, to chyba wtedy będzie się tak trochę kończyło. No i może wtedy jakieś decyzje dalsze będą podejmowane. Nie wiem, czy od razu wtedy ludzi wypuszczą do normalnego… (niezrozumiałe) … No te warunki różne, te zasady, że jakoś będzie ich coraz mniej może wtedy. Ale nie wiem, kiedy to nastanie. Słyszałam o… że teraz jakoś w ciągu, od weekendu mówili, że w ciągu 7-10 dni chyba będzie największy wysyp tych zachorowań.

**Teraz, tak?**

W ciągu 7-10 dni, tak od weekendu mniej więcej. Czyli wydaje mi się, że przed Wielkanocą, na Wielkanoc.

**I myślisz, że z czego to wynika, że teraz ma być taki wysyp?**

No nie wiem, może z tej zarażalności? Bo to chyba tak są jakieś tam prognozy takie, jakieś modele matematyczne, które wyliczają. I chyba na tej zasadzie to jest jakoś liczone.

**Czy masz jeszcze jakieś przemyślenia, refleksje, cokolwiek dotyczącego tej sytuacji obecnej? O czym tak sobie myślisz?**

No, dla mnie rodzina jest najważniejsza. No i widziałam taki fajny filmik, bardzo mi się podobał, anglojęzyczny, gdzie jakaś dziewczyna, taka lektorka, były fajne takie kadry z całej planety w ogóle, te pożary takie w tej Amazonii, że po to był ten koronawirus, żebyśmy tak zobaczyli, że ważna jest ta rodzina. Żebyśmy spędzali więcej czasu razem, żeby nie zżerał nas ten stres. Nasza planeta krzyczała, żeby jakoś tak… No, wiadomo i nie zadeptać jej, i jakoś tak ją chronić. Bo już naprawdę i te pożary, i powodzie, i jakieś huragany. I to tak mi się podobało bardzo. Że, nie wiem, wstrzymał się ten ruch uliczny i powietrze było bardziej czyste. Fajny był ten film.

**Czyli, że są jakieś takie pozytywy tej całej sytuacji, które można dostrzec.**

Tak naprawdę to gonimy cały czas wszyscy.

**Czyli widzisz pozytywy dla planety, ale też dla swojej rodziny, że możecie razem jakoś…**

Tak. Wiesz, co wczoraj przeczytałam? Przeczytałam, że w Tatrzańskim Parku Narodowym zwierzęta zaczęły wychodzić w takie miejsca, gdzie ich dawno nie widziano. Bo wiesz, ludzie zadeptują te Tatry, jest ich dużo. I nawet pojawił się niedźwiedź albinos. Niesamowite, co? I to daje do myślenia, takie wiadomości. Takie to chętnie kliknę i przeczytam. To jest takie pozytywne. Więc może po coś to było wszystko.

**Czyli szukasz bardziej takich pozytywnych informacji?**

Tak, tak.

**Jak by ci się udało znaleźć ten filmik, to podeślij mi go na maila. A te memy skąd bierzecie?**

Oj, to wszystko jest, mówię ci, poczta taka (śmiech). Znajomi, rodzina i wszyscy, i to krąży. Niektóre to wracają jeszcze do nas jeszcze od kogoś innego. Tego jest tyle, ludzie mają taką wyobraźnię, że ja jestem w szoku w ogóle, co wymyślają.

**I nikt się jakoś nie obraża za te memy w otoczeniu?**

Nie, nie, nie.

**Dziękuję bardzo.**
