# Supplementary material for: The challenges arising from the COVID-19 pandemic and the way people deal with them. A qualitative longitudinal study
Source: PLoS One. 2021 Oct 11;16(10):e0258133. doi: 10.1371/journal.pone.0258133 (PMC8504766; doi:10.1371/journal.pone.0258133)
Supplement: S1 Dataset — (ZIP) [file pone.0258133.s003.zip › Transcriptions/stage 1/7.1_M_28_couple, no children.docx]

**7.1_M_28_couple, no children**

**Maćku, powiedz mi kilka słów o sobie - co robisz, czy pracujesz?**

Mam 28 lat, jestem już po studiach i pracuję. Zajmuję się szeroko rozumianą komunikacją, pracuję dla jednego z największych festiwali muzycznych - (nazwa). Jestem głową działu komunikacji, który zajmuje się PR i marketingiem.

**Co robisz w wolnym czasie?**

Dużo oglądam, trochę interesuję się sportem, trochę czytam. Temat podróżniczy nie jest mi obcy, w ciągu roku staram się zaplanować sobie kilka miejsc.

**Odnośnie obecnej sytuacji w Polsce - kiedy poczułeś, że coś się zmienia?**

Pierwszy impuls odczułem pod koniec lutego, kiedy dochodziły informacje, że sytuacja się rozwija. Bardziej bezpośrednio odczułem to, gdy moja dziewczyna Dominika wracała z USA. Zaczęliśmy obawiać się o możliwe problemy z powrotem oraz o to, czy sama się nie zaraziła. To był pierwszy poważny moment, przełom lutego i marca.

**Pamiętasz jakieś konkretne wiadomości, komunikaty z tamtego okresu?**

Pamiętam, że wirus zaczął przedostawać się z Azji do Europy i na inne kontynenty. Wtedy sytuacja zaczęła nabierać rumieńców, zaczęła coraz bardziej nas dotykać. Nadszedł moment zastanowienia, że to jest poważne.

**Jakie jeszcze momenty pamiętasz?**

Jak zaczął się marzec sam poddałem się małej kwarantannie - przez to, że ona wróciła i obawialiśmy się, że mogła się zarazić. Wtedy nie było to jeszcze tak dosadnie nazwane. Po chwili wszedł w życie nakaz zamknięcia szkół. Od tego momentu stało się to jeszcze poważniejsze.

**Dlaczego zamknięcie szkół zwróciło twoją uwagę?**

Kurczę, jeśli chodzi o Polskę, to za swojego życia nie pamiętam takiego momentu, aby zamknięto szkoły poza feriami, wakacjami czy świętami. To było zaskoczenie, wcześniej bym się tego nie spodziewał.

**Czy jest jeszcze jakiś moment zwrotny?**

Na pewno pierwsze zarażenia w Polsce. Później pierwsza osoba, która zmarła. Sam rozwój sytuacji to już stała obserwacja, nie jestem w stanie wskazać wyróżniających się momentów.

**Co pomyślałeś, kiedy pierwsza osoba zmarła?**

Kurczę, pomyślałem chyba, że sytuacja jest poważna - ale to już wiedziałem wcześniej. Ale może zacznie się zmieniać, rozwijać. Skoro ktoś umiera, to na pewno jeszcze więcej osób zwróci na to uwagę i pojawią się dalsze środki ostrożności.

**Co myślisz o sytuacji we Włoszech? Robiła na tobie wrażenie?**

Zrobiła na mnie ogromne wrażenie. Z przeżyć osobistych mieliśmy zarezerwowany wyjazd wielkanocny do Włoch. Jeździmy tam kiedy możemy, przynajmniej raz w roku. Właściwie nie było dużej różnicy w myśleniu Polska - inny kraj europejski. To było w pobliżu, obok nas.

**Kiedy zacząłeś obawiać się tej sytuacji?**

Wydaje mi się, że to było wtedy, kiedy Dominika wracała ze Stanów. Wtedy nie było to jeszcze tak poważnie odbierane w Europie i w Polsce. Ale już przez to, że Dominika była na innym kontynencie, obawiałem się tego, co będzie, gdy ona się zarazi. Byłem świadomy, że jeśli ona się zarazi, to i ja. To było dość stresujące, ale nie do tego stopnia, żebym uciekał z domu albo mówił "ty wracasz, a ja wyjeżdżam".

**A następny moment, kiedy poczułeś, że się stresujesz?**

Myślę, że ten moment zamknięcia szkół. To nie była ogromna różnica, ale samo to, że podejmowane są coraz poważniejsze środki. Jeszcze to, że musiałem zobaczyć się z rodzicami w pierwszym tygodniu marca. Oni nie mieszkają w Warszawie i zacząłem zastanawiać się, czy moja wizyta nie jest ryzykiem dla nich. Mają ponad 60 lat, a to może oznaczać duże ryzyko.

**Jak wygląda twoje życie w tym momencie?**

Sam tryb mojego życia nie zmienił się jakoś super mocno. Pracuję zdalnie, więc mój tryb dnia jest podobny. Kiedy się obudzę to zaraz siadam do pracy - jestem przy komputerze i telefonie. Zmieniło się to, że nie mogę wyjść z domu aby odpocząć po pracy. Nie mogę pospacerować, pójść do kina, spotkać się z kimś. Na pewno to się zmieniło, że nie spotykam się z innymi, tego mi brakuje. Poza tym co jakiś czas monitoruję tę sytuację - w Polsce i na całym świecie.

**Czego ci najbardziej brakuje w odpoczynku po pracy, za czym tęsknisz?**

Przede wszystkim za świeżym powietrzem. To mnie bardzo relaksowało. Wolę nie spacerować w takiej formie, jak wcześniej. Na pewno mocno ograniczyłem wychodzenie. Robię zakupy dwa razy w tygodniu, unikam okazyjnych wyjść.

**To znaczy, że planujesz lepiej te wyjścia? Jak się do nich szykujesz?**

Tak, czasami sporządzam listę zakupów przez jakieś dwa dni. Staram się przewidywać to, czego może mi się zachcieć.

**Bardziej brakuje ci samego wychodzenia czy kontaktu z innymi ludźmi?**

Brakuje mi obu rzeczy, które łączy poczucie, że mogę zrobić cokolwiek kiedy chcę. W takim rozumieniu brakuje mi wolności.

**Jesteś w stanie zastąpić brak spotkań narzędziami online lub telefonem?**

Tak, to się na pewno pojawiło. Paradoksalnie mam lepszy kontakt z osobami, z którymi w normalnej sytuacji kontaktu bym pewnie nie miał. Włączyło mi się, że może warto się odezwać, zapytać co słychać. Na pewno więcej telefonów, pojawiły się jakieś połączenia z kamerą. Staram się to jakoś zastępować.

**Jak wygląda twój kontakt z rodzicami?**

Rozmawiamy koło dwa razy w tygodniu, wcześniej to było raz. Zwykle rozmawiałem z mamą, teraz dzwoni też tata, który zwykle tego nie robił bo wolał spotkać się rzadziej, ale na żywo.

**Czy inni ludzie też inicjują kontakt?**

Trochę tak jest. Też dostałem kilka telefonów, których nikt się nie spodziewał, z pytaniami "co słychać", "czy wszystko w porządku". Nawet właściciel mieszkania, które wynajmuję, pytał ze dwa razy czy niczego nam nie potrzeba. Ludzka uprzejmość.

**Czy widzisz jakieś pozytywne strony tego, co się teraz dzieje?**

W sumie to, o czym już powiedziałem. Ta sytuacja dała impuls, żeby przypomnieć sobie o niektórych, wznowić relacje, dowiedzieć się czegoś więcej o sobie. Mój kot też się cieszy, że ludzie są z nim w domu.

**Jaki jest twój stosunek do tej sytuacji w sensie ogólnym?**

Myślę, że kwarantanna jest dobra, może jest jej nawet za mało. Może mocniejsze zakazy ułatwiłyby poradzenie sobie z tą sytuacją wokół nas.

**Jak sądzisz, na jakie zakazy mógłbyś się zgodzić ty lub społeczeństwo?**

Na przykład to, co dopiero teraz wyszło oficjalnie od rządzących - że domownicy osoby na kwarantannie też są izolowani. To się kłóciło wcześniej z logiką. Może śpią w oddzielnych pokojach i starają się unikać siebie, ale jednak korzystają z tej samej kuchni, łazienki. Poza wyjściem do sklepu mój kontakt ze światem to widok z okna czy przejażdżka samochodem, jednak widać było, że nie wszyscy traktują sytuację poważnie. Oceniam po tym, jak widzę dziesięć osób idących obok siebie albo grupę rowerzystów, osoby w parku czy gdzieś tam. Nie wszyscy traktują problem tak jak powinni.

**Myślisz, że zakaz powinien być większy, obejmować działanie fabryk, firm, przedsiębiorstw?**

Pod względem zagrożenia tak, ale wiem, że to trudne jeśli chodzi o sytuację gospodarczą, która dotyka ludzi tracących źródła dochodu.

**Co jeszcze myślisz o zakazach, na przykład o obostrzeniach w sklepach?**

Uważam, że to jest dobre. Wszystko, co może pomóc, jestem absolutnie za. Niektóre zakazy powinny być zaostrzone dużo wcześniej. Wydaje mi się sensownym zarządzenie, że seniorzy mają dla siebie 2 godziny w sklepach, to są osoby najbardziej zagrożone. Wcześniej było widać, że takich osób pojawia się sporo. Te osoby może po prostu muszą częściej wychodzić, bo nie są w stanie wnieść większych zakupów, kupić jednorazowo leków. Poza tym takie rzeczy jak wyeliminowanie rowerów miejskich. Z komunikacji miejskiej za bardzo nie korzystam i nie wiem jak to jest weryfikowane, ale odseparowanie od siebie osób też może wyjść na plus. Widzę też, że coraz więcej osób nosi maseczki i rękawiczki, coraz więcej osób zdaje sobie sprawę z tego, jak można się zarazić.

**Na ile ty czujesz się zagrożony, boisz się?**

Tak, boję się. Nawet kiedy wracam ze sklepu i zamykam drzwi, to później też zdarza mi się sprawdzić czy nie zapomniałem - i wtedy uzmysławiam sobie, że dotykam tego, co dotykałem zanim umyłem ręce po powrocie ze sklepu. Wystarczy chwila nieuwagi.

**Jak określasz siebie na tej skali (0-100)?**

Myślę, że 70.

**Co się składa na ten wynik? Lęk ma różne elementy składowe.**

Boję się takich momentów, kiedy na przykład dotykałem rzeczy w sklepie i muszę odebrać telefon. Czuję, że to zagrożenie jest wszędzie na zewnątrz. Czuję się w miarę bezpiecznie w domu. Staram się unikać potencjalnych zagrożeń, ale wiem cały czas, że to jest obok. To nie paniczny lęk, ale staram się być realistą - to jest obok.

**Czy jest jeszcze coś, czego się boisz?**

Samo to, że ja się zarażę, sprawia, że zarażę Dominikę. Staram się to maksymalnie ucinać. Wiem, że to może się zacząć ode mnie, ale też może być odwrotnie, choć to nie jest moja pierwsza myśl. Chyba nawet bardziej się boję, jak ona wychodzi.

**Czego boisz się najbardziej w kontekście jej zarażenia?**

Boję się tego, że nie wiem, jak by to zniosła. Różni ludzie znoszą to inaczej - bezobjawowo, objawowo. Obawiam się, co może się jej stać, bo nie wiem. Oczywiście boję się też o innych przyjaciół, o rodzinę. Niektórzy nadal muszą chodzić do pracy i są w kontakcie z innymi. Szczególnie boję się o starszych. Moi rodzice mieszkają z 80 letnią babcią, bardzo bym nie chciał, żeby miała jakiekolwiek ryzyko. Boję się tego, że nie wiem, co się wydarzy dalej, ile to potrwa i z jakimi skutkami. Nie wiadomo, jak to dotknie nas wszystkich. Nawet moi najbliżsi znajomi obawiają się o swoją pracę, sytuacja idzie do przodu. O swoją aż tak się nie boję, bo mój festiwal odbywa się pod koniec lipca. Wiem, że jak się nie odbędzie, to firma nie upadnie, bo ma inne projekty. Nie jestem aż tak zmartwiony jeśli chodzi o moją sytuacje zawodową. Najbardziej boję się o najbliższych.

**Czy myślisz o konsekwencjach dla kraju, gospodarki?**

Jestem świadom wpływu na gospodarkę. Od sytuacji zawodowej najbliższych, przez sytuację przedsiębiorców w ogóle. Nie mówię o Polsce, boję się o wszystkich ludzi, których to dotknie. To sytuacja nad którą ciężko zapanować. To po prostu się dzieje. Nikt nie spodziewał się tych wydarzeń, ludzie nie są przygotowani, nie mają oszczędności.

**Które ze zdjęć najlepiej oddają twoje emocje związane z tą sytuacją?**

Myślę, że 11. Myślę, że jeszcze 13. Zwróciłbym jeszcze uwagę na 14 i 1.

**Opowiedz mi o zdjęciu 11.**

Ono odzwierciedla w dużej mierze moją obecną sytuację. Mój kontakt ze światem to często widok przez okno. Też symbolika tego zdjęcia, deszcz. Też symbolika tęczy, że to się zmieni. To zdjęcie mnie trochę uspokaja. To, że podejmuję takie działania jak niewychodzenie z domu, też mnie trochę uspokaja. Czuję się bezpieczniej.

**Opowiedz o zdjęciu 13.**

To dla mnie wyraz małej nadziei, ucieczki od tego. Miałem dwa razy okazję wyjechać z miasta w trakcie tego czasu, totalnie uciekam w stronę natury, odcięcia.

**Opowiedz o zdjęciu 14.**

Na dwa sposoby. Jeden to uścisk, ograniczenie wolności. Z drugiej strony zacieśnienie więzi, wzmocnienie relacji, wsparcie.

**Jeszcze zdjęcie 1.**

Zastanawiałem się, czy wybrać to zdjęcie, ale zwróciło moją uwagę. To wyraz paniki nad całą tą sytuacją, wyraz niepokoju. Nie zakładam takiej sytuacji, że będzie trzeba uciekać z miasta, ale mała obawa jest.

**Jak czułeś się wtedy, gdy sytuacja dopiero się zaczynała?**

Wtedy nie wiedziałem, że ludzie będą się tak wspierać. Do opisania tamtego momentu wybrałbym to zdjęcie, o którym mówiłem, że mnie uspokaja, że siedzenie w domu jest najlepszym środkiem. Obawa pewnie też podobnie, to wszystko co się po kolei dzieje nie powodowało we mnie narastających lęków.

**Jakie działania podjąłeś, żeby zniwelować stres związany z powrotem Dominiki ze stanów?**

Przede wszystkim czytałem na ten temat, dlaczego to takie ryzykowne i kto jest zagrożony. Wtedy miałem obawy czy wyjechać do rodziców choćby na chwilę. Nie robiłem zapasów, nie byłem jedną z tych osób. Monitorowałem poważnie całą tę sytuację.

**Myślisz, że robienie zapasów to ważny element przygotowań?**

Myślę, że jestem w stanie zrozumieć, że niektórzy tak uważają. Ja nie podzielam tego poglądu. Niektórzy ludzie tak reagują, myślą, że bardziej kontrolują sytuację.

**A jak ty kontrolujesz tę sytuację?**

Ostatnio skupiłem się na kontroli informacji. Staram się nie czytać o tym przez cały dzień tylko 2-3 razy dziennie. Były dni, kiedy byłem na takim stand-by i to nie było dobre, stres przez to wzrastał. Ograniczam wyjścia z domu i myślę, że to najlepsze co mogę teraz zrobić. Nawołuję bliskich do tego, aby unikać kontaktu z innymi.

**Czy zamawiacie jedzenie z dostawą?**

Tak, zdarza nam się. To jest jakiś kontakt więc może to być jakaś forma zarażenia. Nawet jak otwieram pudełko z pizzą to staram się myć od razu ręce. Pojawia się myśl, że trzeba z tym uważać, ale z drugiej strony zamawianie jedzenia to jakiś sposób na umilenie czasu.

**Jak jeszcze umilasz sobie czas?**

Więcej gotuję, to odpręża. Gotuję w inny sposób niż wcześniej, robię wielką fajna kolację, robię drinka. Odpręża mnie też słuchanie muzyki. Takie podstawowe rzeczy. Także wiadomości od bliskich. W weekend dużą przyjemnością jest wyjazd za miasto jeśli jest możliwe. Oczywiście z wyborem miejsc, gdzie nikogo nie ma. Przyjemność sprawia też myślenie co zrobię, jak to się zakończy.

**W takim razie co zrobisz, jak to się zakończy?**

Na pewno spotkam się z przyjaciółmi i skończę zakrapianą imprezą w plenerze, na pewno pójdę do kina w jak największej częstotliwości. Może uda mi się w końcu wyjechać dalej, bo w tym momencie nasze plany się nie spełnią.

**Kiedy twoim zdaniem ta sytuacja się skończy?**

Zależy co. Jeśli chodzi o kwarantannę to liczę, że do lipca uda się opanować sytuację żebyśmy mogli swobodniej się poruszać. Nie wiem co z sytuacją na granicach, ona też się pewnie mocno uspokoi lub zmieni. Podejrzewam, że nie będziemy sobie tak swobodnie wędrowali przez najbliższy czas, powiedzmy pół roku. Sam nie wiem.

**Jakie jeszcze działania ułatwiają ci funkcjonowanie w tej sytuacji i które z nich byś doradził innym?**

W tym kontekście doradziłbym szukanie czasu wolnego od zmartwień, nie śledzenie sytuacji non-stop. Radzę robić to, na co się po prostu ma ochotę. To trudne pytanie, nie wiem czy chcę dawać komuś rady jeśli do końca nie wiem, czy sam sobie z tym radzę. Doradzałbym po prostu cierpliwość, uwagę i ostrożność. Cały czas jestem dobrej myśli, że to się skończy. Staram się mówić wielu osobom, że może warto poczekać. Jak ktoś ma rodzinę, to może warto wykorzystać ten czas. To zawsze okazja, żeby pobyć z kimś. Staram się szukać pozytywów w tym wszystkim.

**A jakie znalazłeś pozytywy tej sytuacji?**

Hmm, pozytywem jest to, że nadal czuję się dobrze... nie wiedziałem jak to będzie, ale czuję się całkiem dobrze. Nie panikuję, nie mam super negatywnych emocji, nie denerwuję się, nie krzyczę.

**Jak twoim zdaniem radzą sobie inni?**

Znoszą to różnie, aczkolwiek moje najbliższe grono przyjaciół czy rodziny postępuje zazwyczaj podobnie jak ja. Też starają się robić sobie małe przyjemności lub wrócić do starych znajomości. Ale też stresują się mocno. Ja mam trochę inna sytuację jeśli chodzi o samo przebywanie w domu; pracuję tak od kilku lat i odnajduję się w tym. Wiele osób ma problem z długim funkcjonowaniem w domu, nie mogą odnaleźć skupienia i miejsca do pracy. Ludzie narzekają na samodyscyplinę, dom nie jest dla nich miejscem do pracy. Mnie to nie dotyczy. Natomiast w kontekście stresu wszyscy jesteśmy na tym samym poziomie.

**Jakie jeszcze problemy obserwujesz?**

Sama sytuacja zawodowa. Ludzie nie wiedzą czy dostaną 100%, 60% czy 30% pensji. Niektórzy od dawna chorują, a teraz mają problem z dostępem do leków, bo one po prostu nie przylatują do Polski, podobnie z dostępem do służby zdrowia w innych sprawach niż koronawirus.

**Myślisz, że ludzie się teraz nudzą?**

Niektórzy tak, szczególnie ci, którzy mieszkają sami. Inni zupełnie odwrotnie - ci, którzy siedzą w domach całymi rodzinami i nie mogą znaleźć miejsca, żeby odpocząć. Słyszę od ludzi z pracy, że nie mają komfortu, czasu dla siebie. Część osób zaczyna już korzystać z rzeczy pojawiających się online jak joga, jakieś ćwiczenia. Nie wiem czy nazwałbym to nudą, może raczej znużeniem, brakiem dodatkowych aktywności.

**Co wiesz o pochodzeniu koronawirusa?**

Wiem, że pochodzi z Wuhan, tam się to zaczęło. Rozprzestrzenił się przez brak reakcji na początku, ale teraz Chiny poradziły sobie bardzo dobrze i nie mają praktycznie nowych zachorowań (takie dochodzą informacje). Także przez nieuwagę ludzi i lekkomyślność. Czyta się o lekceważeniu zagrożenia przez osoby, które nie stosowały się do zalecanych środków i tak to później zawędrowało do innych państw.

**Czy sądzisz, że można było zapobiec rozprzestrzenianiu się epidemii?**

Wydaje mi się, że można było podjąć próby. Nie zakładam powodzenia, ale wydaje mi się, że można było spróbować. Szczególnie we Włoszech, gdzie to wszystko urosło do strasznych rozmiarów. Może ciężko obwiniać ludzi za to, jacy są. Oni kulturowo nie mogą siedzieć w domach, ale dalej uważam, że to lekkomyślność. Co do Polski to wydaje mi się, że można było zrobić więcej. Mam nawet takie głosy od moich znajomych. Nawet Dominika powiedziała, że tylko raz dostała jakąś kartkę z informacjami o objawach. Miała trzy połączenia, a tylko po jednym dostała taką informację. Przy tym ile osób lata, nie dziwię się, że to się rozprzestrzeniło.

**Czy słyszałeś, czym naukowcy lub politycy tłumaczą ekspansję wirusa?**

To pewnie dlatego, że mnóstwo ludzi miało ze sobą kontakt. Dzisiaj wyczytałem taką analizę, że wśród osób zaszczepionych na grypę jest mniejszy procent zarażonych niż na przykład we Włoszech lub w USA. Czytałem też jakieś niestworzone historie, że ktoś stworzył wirusa, żeby zahamować gospodarkę na świecie. To jakieś spiski i zmowy, przeczytałem to na facebooku. Dziwne miejsca w internecie.

**Czy uważasz, że jesteśmy przygotowani na epidemię w sensie globalnym?**

Wydaje mi się, że nie. Tak długo nie było na świecie aż tak paraliżującego wirusa, że nie było założonych funduszy na rzecz pandemii czy epidemii. Nie było środków higieny czy bezpieczeństwa. O higienę na pewno można było bardziej zadbać. Było mnóstwo komunikatów na początku. Było ich chyba 6 czy 8 na infografikach, które pouczały jak dokładnie myć ręce. Ja byłem zaskoczony, że dla kogoś może to być niewiadoma, żeby na przykład myć ręce przed posiłkiem.

**Czy jest jakieś ograniczenie rządowe, z którym nie zgadzasz się?**

Chyba nic takiego nie przychodzi mi do głowy. Raczej powinniśmy robić więcej. Na myśl przychodzi mi tylko ograniczenie przebywania osób poniżej 18 roku życia bez opiekuna na ulicach. Ciężko uogólnić ten zakaz w kontekście odpowiedzialności za kogoś. Dyskusyjne może być też to, że dwie osoby nie powinny przebywać obok siebie nawet idąc na głupie zakupy. Teraz już nie chodzimy oboje na zakupy, stosujemy się. Ale ciężko to robić idąc na przykład razem do auta. Te zakazy dają duże pole do interpretacji. Jest też mowa o tym, że można wyjść na chwilę z, powiedzmy, psem, a po tym wrócić. Dla jednej osoby chwila to 5 minut, dla innej spacer mający 30 minut; 3 km lub 500m. To pozostawia duże pole do popisu. To, że ograniczane jest wejście do lasów i parków. Jako użytkownicy lasów nie mieliśmy pewności jak z tym jest, czy wszystkie są pozamykane, jakie są restrykcje i ograniczenia. Różnie można to interpretować. Dla niektórych niejasna była też zasada dotycząca seniorów, że nie tylko mają dwie godziny na wyłączność, ale też mogą przyjść później - jak to jest z tym później? Ja nie mam z tym zupełnie problemu, ale niektórzy chyba nie do końca jasno rozumieli, że mogą też przyjść w każdym innym momencie.

**Czy wraz z wprowadzaniem kolejnych restrykcji twój stres rośnie?**

To powoduje taki lęk, że skoro wprowadzają kolejne, to zaraz będą jeszcze kolejne. Dotychczasowe restrykcje nie stresowały mnie, raczej wprowadzały niepewność co dalej, czy będą mocniejsze, czy będą negatywne skutki. Dla mnie to zawodowy stres, bo mojej pracy dotyka zakaz organizowania imprez masowych.

**Myślisz, że te ograniczenia zmniejszają ilość zakażeń?**

Mam nadzieję, ale nie jestem przekonany. To nie ze względu na same restrykcje, ale zachowania ludzi. Gdyby od początku traktowali to poważnie, to nie trzeba by było wprowadzać kolejnych.

**Myślisz, że są takie grupy, które szczególnie wyłamują się z wprowadzanych zakazów?**

Początkowo wydawało mi się, że młodsi ludzie po zamknięciu szkół nie do końca zrozumieli ten zakaz - że to nie wolne, ale że mają siedzieć w domach. Widziałem takie grupy, które się przechadzały. Nie jestem w stanie jakoś konkretnie sklasyfikować.

**Opowiedz mi skąd bierzesz informacje na temat aktualnej sytuacji.**

Korzystam właściwie z tych samych źródeł co wcześniej - głównie onet.pl i TVN24. Nie złapałem się na jakiejś niewłaściwej informacji. Jest taki profil Łukasza Boka, który przekazuje informacje. To są te źródła, na których się najbardziej skupiam. Nie włączam ich w tle, raczej zaglądam co jakiś czas. Na pewno patrzę na liczbę zachorowań i zgonów; bardzo lubię Włochy więc przyglądam się, co się u nich dzieje. Patrzę jakie są nowe rozporządzenia, newsy gospodarcze i nie tylko. Taki ogólny przegląd ważnych informacji, które pokazują się na paskach i nie tylko.

**Co dają ci informacje o zachorowaniach i zgonach?**

Głównie to, czy zmienia się tendencja. Na przykład to, czy zakazy dają jakiś efekt, choć w krótkim czasie raczej nie mogą dać efektu. Według prognoz czeka nas jeszcze większy boom na zachorowania. Ale to jest trudne pytanie, właściwie nie wiem. To mnie wcale nie uspokaja. Staram się wierzyć, że w końcu ludzie faktycznie zostaną w tych domach, to zahamuje, zadziała i zacznie spadać. Zaczniemy opanowywać sytuację. Teraz to pewnie niemożliwe więc nie wiem dlaczego. Efekt restrykcji będzie widzialny dopiero za jakiś czas.

**Czy teraz korzystasz z mediów częściej, niż przed sytuacją z koronawirusem?**

Nie oglądałem tak często TVN24 na przykład. Przegląd informacji porannych zawsze starałem się sprawdzać w internecie. Ale teraz trochę więcej, częściej patrzę na informacje ogólne niż na przykład na dział kultura lub sport. Odkryłem tego Łukasza, który, jak się okazało, już wcześniej był dobrym źródłem. Dużo osób spośród moich przyjaciół o nim mówiło, że jest taki gość, który co chwila wrzuca aktualizacje z różnych krajów i sam stwierdziłem, że to jest dobre źródło.

**Czy trafiasz na informacje, które uznajesz za niewiarygodne?**

Myślę, że to się łączy z poglądami politycznymi. Źródła w stylu TVP Info, ogólnie serwisy informacyjne w TVP z dość odważnymi teoriami w stylu "opozycja naraża kraj na niebezpieczeństwo" - to z automatu nie wchodzę. Oczywiście niektórzy ludzie na Facebooku wrzucają różne newsy u siebie i nie traktuję ich jakoś poważnie. Jak ktoś coś wrzuci i napisze "za chwile szczepionka na koronawirusa" to nie traktuję tego poważnie jak nie mam poważnego źródła.

**Czym jest dla ciebie źródło poważne?**

To takie źródło, które znam od lat lub które potwierdzają też inne źródła. Nie zawsze szukam źródła, które ma informacje jako pierwsze, tylko raczej jak kojarzę czy znam dziennikarzy, lub gdy ktoś inny to potwierdzi. Zdarza mi się wchodzić na strony źródłowe typu WHO, ale stwierdziłem po kilku wizytach, że to bez sensu. Przekaz, jaki mamy w mediach, jest wystarczający jeśli zgadza się z tymi danymi. Jeśli chodzi o Sanepid to go nie sprawdzałem - moja mama jest jego pracowniczką, więc od niej mam informacje co się dzieje.

**Rozmawiasz z mamą na ten temat?**

Tak, opowiada mi o różnych rzeczach. Nie dzwoni codziennie ani konkretnie w tym celu, ale przy okazji mówi mi o tym. Czasami mówi o rzeczach, których nigdzie nie przeczytałem, ale raczej jej wierzę. Miałem tak raz czy dwa, że zadzwoniłem do niej dopytać o maseczki czy rękawiczki. Nie często, ale to się zdarza.

**Masz jeszcze jakieś przemyślenia związane z tą sytuacją?**

Też nazywam to sytuacją. Zazwyczaj nazywam ją słabą, straszną, ciężką, trudną, kiepską, średnią. Jeśli chodzi o inne przemyślenia... w pewnym momencie dużo o tym myślałem, teraz przechodzi to w przyzwyczajenie, rutynę, ale cały czas jest z tyłu głowy. Dwa razy coś mi się przyśniło na ten temat. Moim główny przemyśleniem, tym co mnie stresuje, jest to, jak się zmieni świat - gospodarczo, pod względem nastrojów i samopoczucia, organizacji i różnych struktur, podróży, organizacji pracy, nowej przyszłości. To nawet nie sytuacja teraz, ale to, co będzie później. Czy zaczniemy się do tego przygotowywać? Czy coś się zmieni pod tym względem? No i kiedy to się może realnie zakończyć? Czy to będzie tak, że będziemy mieli szczepionkę i wirus zniknie, czy będzie to taka powracająca choroba jak grypa? Czy ten wirus przejdzie do codzienności? Nie wiem jak się wszyscy odnajdziemy w tej sytuacji.
