# Supplementary material for: The challenges arising from the COVID-19 pandemic and the way people deal with them. A qualitative longitudinal study
Source: PLoS One. 2021 Oct 11;16(10):e0258133. doi: 10.1371/journal.pone.0258133 (PMC8504766; doi:10.1371/journal.pone.0258133)
Supplement: S1 Dataset — (ZIP) [file pone.0258133.s003.zip › Transcriptions/stage 1/1.1_F_25_single.docx]

**1.1_F_25_single**

**(Informacje organizacyjne. Prośba o przedstawienie się respondentki)**

Nazywam się Kasia i mam 25 lat. Obecnie zajmuję się poszukiwaniem pracy, bo epidemia zamieszała mi mocno w planach życiowych. Studiowałam projektowanie biżuterii, pracowałam w wielu miejscach, trochę podróżowałam, dużo się przemieszczałam. Plan był taki, że miałam wyjechać do Londynu na pół roku, znaleźć tam pracę i odłożyć pieniądze na studia, a następnie rozpocząć studia związane z projektowaniem w Birmingham. Plan był, żeby zacząć studia od września. Obecnie to duży znak zapytania, skłaniający się ku temu, że nie zacznę tych studiów we wrześniu, bo finansowo w tym momencie nie jest tak, jak zaplanowałam. Coś jeszcze dodać?

**Mieszkasz obecnie sama czy z kimś?**

Ponieważ ja się wyprowadzałam [do Londynu] to nie wynajmowałam mieszkania i w tej sytuacji dość niezaplanowanej wróciłam z powrotem do rodziców, więc mieszkam w domu rodzinnym, gdzie mamy jeszcze babcię. Ale dom jest na tyle duży, że nawet na tej kwarantannie, że mogę być odseparowana i mieć swoją łazienkę, co jest całkiem pozytywne. Bo tak dyskutowaliśmy o innych ludziach, że jak ktoś ma małe mieszkanie na kwarantannie to to może być problem. Więc tu akurat mam duże szczęście, że ja mogłam spokojnie wrócić do domu, nawet nie martwiąc się o to, że muszę się z babcią stykać. Teraz już spędzam czas z rodzicami, bo wszyscy czujemy się dobrze, a nikt z nas nie wychodzi od mojego powrotu. Na początku nie chciałam wyjść, dopóki się babcia nie schowała do pokoju. Więc [mieszkam] z rodzicami, babcią i psem.

**To jest tymczasowa sytuacja, że mieszkasz z rodzicami?**

To zależy, jak szybko znajdę pracę i jak szybko będę mogła wychodzić, bo na razie są różne głosy, że zaostrzą wszystko. Więc jeśli nie będę miała pracy, to wyjazd, żeby oglądać mieszkanie, nie wiem, czy będzie wystarczającym powodem, żeby wyjść. Ale ja nie lubię tu mieszkać na obecnym etapie życia ze względu na dojazdy, bo od Warszawy nie jest tak daleko, ale dojazdy są uciążliwe. Nawet jakbym finansowo była w stanie opłacić te studia, to nie wiem, jak to będzie wyglądało we wrześniu z lotami. Także na razie studia odkładam na jakieś dalsze lata, aczkolwiek mam poczucie, że im dalej z wiekiem, coraz mniej człowiek ma motywację do takich rzeczy. Więc sytuacja jest dla mnie przejściowa, ale nie wiem jak długo.

**Dzięki za podzielenie się tymi informacjami. Mam prośbę. Spróbuj przypomnieć sobie, kiedy obecna sytuacja rozpoczęła się w Polsce. Pamiętasz taki pierwszy etap?**

Nawet sprawdzałam dokładnie, bo miałam bardzo intensywny czas myślenia nad tym, co zrobić, będąc w Londynie. Ja do Londynu leciałam 2 marca. Pierwszy przypadek pacjenta zero u nas w Polsce to był 4 marca. I w sumie jedynymi przesłankami o tym, że to może nie był dobry pomysł, było to, że w tygodniu przed moim wylotem, w ostatnim tygodniu lutego, moja mama zaczęła coś mówić, że myślą o zamykaniu granic, ale to się wydawało takie - zamkną, no i co? To było takie na zasadzie, dobra, to niech zamkną Azję, ale po co u nas to zamykać? Ale w sumie to już był moment, kiedy we Włoszech się coś działo, bo w weekend przed wyjazdem miałam panieński przyjaciółki i grupa znajomych odwołała swój wyjazd na narty do Włoch z powodu wybuchu już we Włoszech. Ale to było wciąż na zasadzie takiej, że większość grupy odwołała, bo się bali, ale ta osoba, która była z nami na panieńskim była rozczarowana i stwierdziła, że ona by i tak pojechała, gdyby nie to, że większość grupy stwierdziła, że nie. Po dwóch tygodniach okazało się, że bardzo dobrze, że nie pojechali, bo mogliby w ogóle nie wrócić.

**Powiedziałaś, że pacjent zero i to, że twoi znajomi zmienili plany to były pierwsze momenty. A czy to był taki moment, że czułaś, że dla ciebie coś się zmienia?**

Nie, bo u nas nic konkretnie się nie działo. Ja poleciałam 2 marca, to był poniedziałek, no a u nas te pierwsze pomysły, zamknięcia szkoły, to było tydzień później - poniedziałek/wtorek. A od zamykania szkół, pubów, restauracji, przeszli w 3 dni tak naprawdę do zamknięcia granic. W jeden tydzień to się wszystko wydarzyło i to był mój drugi tydzień pobytu w Londynie. I to się wszystko działo strasznie szybko. W pierwszym tygodniu ja jeszcze miałam rozmowy o pracę. Plan był taki, że w pierwszych tygodniach znajdę tę pracę, żeby te pół roku tam być. Zaaplikowałam, żeby mieć cokolwiek, takie kelnerowanie, bo koszty życia w Londynie są duże. I miałam mieć taki trening do takiej pracy kelnerskiej, dorywczej, 16 marca w poniedziałek. I to był poniedziałek po tym, jak u nas zdecydowali o zamknięciu granic. W Wielkiej Brytanii wciąż nic się nie działo. Oni tam się strasznie ociągali z podejmowaniem jakichkolwiek decyzji. W momencie, kiedy u nas wymyślili zamykanie szkół i w przeciągu 3 dni zamykanie granic, w Londynie Johnson mówił jakieś bardzo dziwne rzeczy. Wyglądało to tak, jakby oni stwierdzili, zobaczymy, przeczekamy, ile osób umrze i się zarazi. I tak naprawdę u nich zaczęły się obostrzenia jakiekolwiek tydzień po nas, a takie porządne 2 tygodnie później.

**I to był taki moment, że ty poczułaś, że coś się zmienia?**

Najgorszy moment to był 13 marca, piątek, niecałe 2 tygodnie po moim przyjeździe. 16 miałam mieć ten trening do pracy i ponieważ to jest kelnerowanie i przemysł turystyczny, oni odwołali ten trening. Więc my i inne kraje zaczęły zamykać granice, a Londyn i Wielka Brytania jeszcze nic nie robili sami wewnętrznie, ale wiadomo, że to, że inne kraje zamykają granice, oddziałuje chociażby na przemysł turystyczny w innych. Więc oni już zaczęli się wycofywać z kosztów, bo fala tego, że nie będzie takiej potrzeby, sprawiła, że oni już odwołali ten trening. Ja i tak zestresowana tym, że trochę nie wyszło mi znalezienie takiej pracy, jak chciałam, w momencie, kiedy praca taka dorywcza została odwołana, to już mnie bardzo zestresowało. I to pokazało, że to przetrwanie w Londynie skończy się tym, że wydam wszystkie pieniądze, jakie mam odłożone na studia. Ja miałam opłacone mieszkanie na trzy miesiące, do maja. Także miałam już opłacone to mieszkanie, ale wiadomo, że to jedzenie kosztowałoby dużo więcej niż u nas. Mieszkałam u rodziny w jednym pokoju i wiadomo, nie jest to moja rodzina, dużo mniejsza przestrzeń. Tu u siebie mam osobny pokój na poddaszu domu i to jest jak osobne mieszkanie, tylko do kuchni muszę zejść na dół. Tu mam dużo więcej swojej przestrzeni. Gdybym tam mieszkała, pracowała, wychodziła ze znajomymi, to byłoby to jak najbardziej do przeżycia, ale w momencie, jak ja tam siedziałam cały dzień, wychodziłam tylko do sklepu i nie było nic innego do roboty. Znajomi przestali wychodzić. Więc ja cały ostatni tydzień siedziałam z tą rodziną w mieszkaniu i to już zaczęło być bardzo uciążliwe psychicznie, bo czym innym jest siedzenie tu w zamknięciu, gdzie wszystko jest znane i moje - mam więcej swobody, a czym innym siedzenie z nieznaną rodziną. Cały czas myślałam o tym, co z pracą i że tam wydaje dużo pieniędzy. Rodzice zaczęli przekonywać mnie do powrotu już po tym trzynastym, kiedy odwołali mi rozmowę i ja się tym mocno zestresowałam. Ale ja mówiłam, że nie, że może jeszcze przeczekam. Ale im dalej w las, tym bardziej było widać, że rynek pracy mocno oberwie, cała gospodarka wszystkich krajów - Europejskich i nie tylko. Więc coraz bardziej widać było, że prognozy na zaczęcie jakiejkolwiek pracy robiły się coraz gorsze. I przez ten ostatni tydzień jeszcze aplikowałam i miałam nadzieję, że może coś by z tego wyszło. W ciągu tego ostatniego tygodnia, ta rodzina zaczęła coś mówić o ich wyjeździe do ich rodziny na Bahamy. Sytuacja była mocno niepewna w Wielkiej Brytanii. Z tego co mi rodzice mówili, u nas, pod Warszawą nie było aż tak tragicznie z tym panicznym kupowaniem. To, co działo się w Wielkiej Brytanii, było dość przerażające. Większość półek w sklepach było puste.

**Czyli to, że obserwowałaś, że półki są puste, w jakiś sposób potęgowało twoje emocje?**

Ja nie zamierzałam kupować takich ilości, bo wiedziałam, że to się zmarnuje, bądź nie ma takiej potrzeby. Sklepy są wciąż otwarte, nie zamknęli ich i można normalnie dostać jedzenie. Zajmuje to więcej czasu, ale każdy ma go więcej, jeśli nie chodzi do pracy. U mnie w domu do sklepu chodzą tylko rodzie. Babcia ma zakaz, mimo, że ona nie boi się wirusa. Taka niepewność, co ludzie wymyślą - a widziałam sceny, gdzie ludzie się zabijają w sklepach tam [w Wielkiej Brytanii]. Myślę, że to wynikało z tego, że u nas były jasno wytyczne, postawione przez rządz bardzo jasno i wszyscy wiedzieli, co się dzieje. W Wielkiej Brytanii było strasznie dużo dezinformacji. Wszyscy się przestraszyli zamknięcia sklepów, dlatego zaczęli tak panicznie kupować. Tam te informacje były takie jakieś, no nie wiem… Raz, że nie było takich wytycznych, co zrobimy, a dwa, że było bardzo dużo fejkowych informacji. Artykułów widziałam dużo dziwnych bardzo, że zamykają sklepy, bądź nie zamykają. Wiadomo, że nie mogliby tego zrobić tak dosłownie, żeby nikt nie mógł kupić, bo ludzie muszą coś jeść.

**Powiedziałaś o kilku ważnych momentach w rozwoju tej sytuacji. Powiedziałaś, że takim pierwszym momentem rozwoju sytuacji w Polsce był ten pacjent zero. Jak ty się wtedy czułaś? Czy czułaś już jakieś zagrożenie tą sytuacją?**

Nie.

**Czyli dla Ciebie ten moment, kiedy miałaś odwołany trening w Londynie, to był ten moment, że poczułaś zmianę?**

Tak. Do momentu odwołania tej rozmowy ja w ogóle nie dopuszczałam myśli, że że to dojdzie do takiego stopnia, że będą musieli wprowadzać te obostrzenia i tak mocno gdzieś tam zmieniać to nasze życie codzienne.

**A jakbyś miała opowiedzieć własnymi słowami o rozwoju tej sytuacji w Polsce, to jakie były etapy rozwoju tej sytuacji z twojej perspektywy?**

To był ten środek tygodnia tego pierwszego marca - pacjent zero. Usłyszałam, że jest pacjent, ale że go szybko izolowali i że on przyjechał z Niemiec. No i później następny poniedziałek - zamknięcie szkół. Koniec tygodnia - zamknięcie granic. I to tak w sumie poszło w Polsce dosyć szybko - od zamknięcia granic tak naprawdę do tego weekendu tam 14-15 [marca] mniej więcej sytuacja była już stabilna.

**Powiedziałaś, że jak był pacjent zero, to nie czułaś szczególnie zagrożenia sytuacją. A jak zamknęli szkoły, to jak się wtedy czułaś?**

Na pewno zdawałam sobie sprawę, że to, co się dzieje jest coraz poważniejsze, ale było to dla mnie logiczne. Szkoły są pewnie jednymi z największych i najbardziej regularnych zgromadzeń. I tak, jak nam dorosłym powie się: myjcie ręce, uważajcie na kichanie, kasłanie, to dzieci nie będą na to zwracały takiej uwag i mogą dużo łatwiej to poprzenosić. Tym bardziej, że mówiło się, że to jest tylko zagrożenie dla starszej populacji, co też podobno nie jest prawdą. Więc tak naprawdę informacji jest dużo sprzecznych. Ale mówiło się o tym, że dzieci mogą przenosić wirus bezobjawowo z racji tego, że odporność mają większą niż ludzie starsi. Wydawało mi się to logiczne i jakaś rzecz, która temu zapobiegnie. W ogóle mi nie przychodziło do głowy, że zamkną granice i inne miejsca typu restauracje. Jest to w jakiś sposób logiczne i teraz, jak o tym myślę, to się zastanawiam: kurczę, z jednej strony można było to przewidzieć, jak się pomyśli o tym logicznie, a z drugiej, człowiek nie dopuszczał myśli, że tak bardzo drastyczne kroki, oddziałujące na gospodarkę będą podjęte.

**A uważasz, że te decyzje uspokajały ludzi czy mogły potęgować panikę?**

Myślę, że paniczne kupowanie było jak najbardziej dowodem na to, że nie uspokajają nikogo. Na pewno wynika to też z fejkowych informacji, które nie wiem kto, ale na pewno ktoś fabrykował i wrzucał. W stylu: zamkną sklepy, więc trzeba dużo kupić i ci, co uwierzyli, zajęli się panicznym kupowaniem. Myślę, że zamknięcie szkół na 2 tygodnie brzmi na zasadzie: a ferie robią i to ma pomóc, i to pewnie nie jest aż tak stresujące. Myślę, że to, co było dużo bardziej stresujące, to jest zamknięcie granic i ograniczenie w jakimś stopniu wolności. Moi rodzice pamiętają czasy, kiedy nie było tak łatwo dostać paszportów i wyjechać za granicę. Nikt z nas nie pamięta takiej sytuacji, żebyśmy nie mogli swobodnie wyjechać. Już pomijam Shengen i otwarte granice w Europie, ale sam fakt tego, że można kupić bilet lotniczy i polecieć gdzieś w każdym momencie. Przynajmniej dla mnie to jest mocno ograniczające naszą wolność.

**Powiedziałaś o ważnych etapach w Polsce, że był to pacjent zero, później zamknięcie szkół i zamknięcie granic. Czy mogłabyś też opowiedzieć o etapach rozwoju tej sytuacji z twojej perspektywy? Może właśnie, kiedy podjęłaś decyzję o powrocie i jak to wyglądało?**

Nie zmieniałam planów i nie czułam zagrożenia sytuacji u nas do momentu zamknięcia granic. To był pierwszy moment, kiedy zaczęłam się stresować o sytuację, jak to będzie wyglądało. Trochę uspokajało mnie to, że Wielka Brytania nic nie robi, więc może u nich to się nie odbędzie tak, jak u nas. Do momentu zamknięcia granic miałam takie, że szukam pracy i wszystko będzie okej. W trakcie, kiedy my zamykaliśmy granice, to zamykały je też inne państwa. Wtedy bardziej to oddziałuje na Wielką Brytanię. U mnie ten stres największy o sytuację i o to, czy to w ogóle będzie możliwe, żeby dostać pracę, zaczął się wraz z zamknięciem granic u nas i w innych państwach Europy. Ale jak rodzice przekonywali mnie, żebym wracała, to mówiłam nie, nie chcę wracać, bo miałam zupełnie inny plan. Plan był taki, że na półtora roku wyjeżdżam do Wielkiej Brytanii - pół roku w Londynie i rok w Birmingham, i nie wracam tutaj przynajmniej jeszcze ponad rok. A tym bardziej, że złożyłam wypowiedzenie i skończyłam pracę w Warszawie 28 lutego. Więc jest to powrót do domu bez pracy, nie mamy możliwości wychodzenia - jedno to jest to, że policja sprawdza, czy jestem w domu, ale to są te dwa tygodnie. Teraz odliczam dni, kiedy będę mogła wyjść pobiegać czy do sklepu. Wracanie tutaj w obecnej sytuacji nie było zbyt zachęcające. W Londynie szukałam pracy takiej, jaką chciałam i było to trudne. Później okazało się, że muszę zmniejszyć oczekiwania i kiedy nawet o taką pracę było trudno. Później stwierdziłam, że szukam dalej zdalnych prac, ale różne możliwości się wykruszały z powodu pandemii. Dla mnie momentem decyzyjnym było to, że ta rodzina zaczęła myśleć o wyjeździe. Chcieli zostawić mnie w tym mieszkaniu, żebym tym mieszkaniem się opiekowała, a później mogła mieszkać tam za darmo. W normalnej sytuacji byłoby to kuszące, ale w sytuacji, kiedy ja miałam zostać w tym mieszkaniu zupełnie sama i nie miałabym się do kogo odezwać… Tutaj mam rodziców chociażby. To przestało mi się podobać i zaczęło mnie mocno stresować.

**Czyli to, że ta rodzina zaczęła mówić o wyjeździe sprawiło, że też pomyślałaś o powrocie?**

Zostanie w tym mieszkaniu samej, stresując się tym, że dużo rzeczy tam się psuło i zostałabym z popsutym mieszkaniem sama. Dodatkowo bez perspektywy pracy i to mogłoby potrwać najbliższe 3-4 miesiące - nie wiadomo, ile to potrwa. Na początku myślałam, że będą to tygodnie, bo wszyscy robili przestój na dwa tygodnie i wszyscy myśleli w kwestii tygodni. Teraz wiadomo, że nie będzie to kwestia tygodni, a miesiąca, dwóch samego odizolowania się, a co dopiero z sytuacją gospodarczą i na rynku pracy - to może być nawet do końca tego roku. Nie wiemy. Ja ostatnio oglądałam film z 3 scenariuszami, jak to może wyglądać.

**A jak wygląda teraz twoje życie codzienne i co się zmieniło w porównaniu do tego, co było wcześniej? Rozumiem, że kwarantanna może być dla ciebie zupełnie innym doświadczeniem.**

Rzecz w tym, że ja ten ostatni tydzień tam już nigdzie nie wychodziłam - nie widziałam się z nikim, wychodziłam tylko, żeby kupić jedzenie. I też rodzice wysłali mi moje rzeczy pocztą, więc musiałam zamówić kuriera i spakować te rzeczy. To była duża operacja z tym powrotem dla mnie, dlatego też tak to odsuwałam. Ale poza wychodzeniem raz dziennie, starałam się wyjść na spacer. Jedyna różnica teraz jest taka, że nie mogę wyjść na spacer do lasu i nie mogę wyjść pobiegać, czy do sklepu. Jest to śmieszne uczucie na zasadzie, że ja wiem, że nie mogę opuścić domu pod groźbą kary 30 tysięcy zł. Mam ogród, więc mogę wyjść do ogrodu z psem. Współczuję ludziom w bloku - albo tylko balkon albo wcale. To jest do przeżycia, bo wiem, że to tylko do niedzieli będzie trwało. A i tak nie za bardzo jest się z kim spotkać.

**Co w tej sytuacji ci najbardziej przeszkadza? Zaczęłaś mówić o tym, że to dziwne uczucie, że nie wychodzisz z domu, ale z drugiej strony nie tak bardzo inne od tego, jak było przez ostatni czas.**

W Londynie było to bardzo uciążliwe, bo byłam w stresującej sytuacji. Siedzenie cały dzień bez niczego do robienia, mając stresujące myśli na głowie, to jest to nieciekawa sytuacja. Kiedy człowiek ma coś, co zajmuje mu czas, to… A kiedy nie mogę nic zrobić, nie mogę znaleźć pracy… Ja, kiedy jestem zestresowana, to lubię robić rzeczy, które przybliżają mnie do celu, a nie siedzieć i czekać, więc to było bardzo ciężkie. Tutaj o tyle jest to łatwiejsze, że mam wszystkie swoje rzeczy. W domu mogę wymyślić sobie więcej rzeczy do robienia niż tam. Mama podrzucała mi książkę, mogę zająć się trenowaniem naszego psa i wyjść do ogrodu - mam więcej możliwości zajęcia czasu niż tam. Na pewno to, że nie mogę spotkać się ze znajomymi jest bardzo irytujące. Bardzo irytująca jest niemożność wychodzenia, żeby poznać nowych ludzi. To jest coś, na co się nastawiałam w Londynie, a co się wywróciło do góry nogami. Ja mam tak, że jeżeli siedziałam w domu rodzinnym, np. przez tydzień i nie mogłam wyjść do znajomych, to zaczynałam, potocznie mówiąc, wariować - nie działa to na nie dobrze. Na razie ten czas odczuwam trochę jako przymusowe wakacje, tylko, że nie mogę wyjść z domu. Bycie u rodziców i to, że nie muszę nic robić, kojarzy mi się z wakacjami. W poprzedniej pracy miałam kilka dni zdalnych, więc gdybym miała nadal pracę, to niewiele by się zmieniło, bo i tak musiałabym wstać i siedzieć przy komputerze. Przyjemne było to, że każdego dnia nic nie muszę - postanowiłam podelektować się tym faktem, że nie muszę nastawiać budzika, mogę wstać o 10:00. Tak wyglądał ostatni tydzień. Teraz zaczęłam już bardziej robić konkretne rzeczy. Między innymi, dlatego chciałam umówić się na to badanie, żeby mieć stały punkt w kalendarzu. Jutro mam rozmowę o pracę - to z takich pozytywnych rzeczy. Po rozmowie z moim terapeutą w sobotę, zmobilizowałam się. Terapię zaczynałam w innych celach, ale w kontekście obecnej sytuacji, zaczęliśmy z terapeutą rozmawiać o tym, jak przeorganizować sobie życie najbliższe. To pomogło mi wejść w myślenie konkretne i zaczęłam robić więcej rzeczy - zrobiłam porządki, przeczytałam książkę. Powoli zaczynam wracać do trybu, że coś mam zaplanowane i coś robię. Mam nadzieję, że ten tydzień będzie tak wyglądał. Poprzedni tydzień dałam sobie bez poczucia winy na to, żeby poleżeć, obejrzeć seriale, czy nawet kolorowanki dla dorosłych w stylu mandali. Więc zaczęłam robić takie dziwne rzeczy, na które nie miałam czasu, a które zawsze chciałam robić - coś w rodzaju terapii sztuką. Dałam sobie poczuć, że nic nie muszę i jest fajnie. Wcześniej miałam dużo pracy i było to psychicznie męczące i miałam takie myśli, że chciałabym takiego czasu, że nic nie muszę. Jest to w pewien sposób przyjemne, że mam dłuższy czas, że nic nie muszę. Nawet rodzice nie wyrzucą mnie na spacer z psem, bo nie mogę wyjść z domu.

**Jaki generalnie jest twój stosunek do tej sytuacji? Jak się czujesz?**

Hmm… Wzbudza on na tyle dużo niepokoju, że nie wiemy, kiedy się skończy. Nic nie wiadomo, bo do pierwsze takie w historii współczesnego świata zdarzenie. Teorie są różne - czy to jest przez kogoś wymyślone, czy nie jest, czy to jest przypadek, czy ktoś to zaplanował… Nie wiem. Słucham tych historii i ani nigdy nie jestem za czymś, ani przeciw czemu, dopóki sama nie widzę.

**Do tych teorii jeszcze wrócimy. Wspomniałaś o niepokoju. Pokażę ci teraz skalę poczucia lęku i mam prośbę, żebyś się do niej ustosunkowała.**

To znaczy tak… Ja lęk trochę bardziej odczuwam jako naprawdę takie zagrożenie bezpośrednie życia, czy też sytuacje jakieś bardziej poważne. Więc, jeżeli mówimy o lęku, to powiedziałabym, że między 20 a 10. Jeżeli miałby to być wyższy poziom, to raczej jest to niepokój na zasadzie, że nie wiem, co będzie i nie podoba mi się to. Ale nie jest to bezpośrednio, na razie przynajmniej, zagrażające. Niepokój byłby wyżej, a jeżeli chodzi o lęk, to nie jest to aż tak wysoko. Lęku jako takiego nie mam. Wiem, że to się w jakiś sposób rozwiąże i zakończy, tylko pytanie - kiedy. I ten niepokój jest o to, jak długo to potrwa i jak bardzo na nas się to odbije.

**A czujesz się zagrożona tą sytuacją w jakimś stopniu?**

Bezpośrednio na razie nie. Zagrożona to brzmi bardzo drastycznie. Bardzo dużo zależy od tego, kto jakich wiadomości słucha i co ogląda. Ja nie oglądam wiadomości i już przestałam je czytać, bo raz, że są sprzeczne między sobą, a dwa, że nie wpływają dobrze na psychikę - i tak nie zrobię nic z tym, co się dzieje. Nie zależy to w żaden sposób ode mnie. Wolę zaakceptować sytuację, myśleć, co mogę zrobić i zająć się swoimi planami. Na razie wolę myśleć, że to się, prędzej czy później, skończy i trzeba zaplanować, co dalej i przetrwać w mniej lub bardziej produktywny sposób to, co się dzieje. Myślę, że jeszcze nie wydarzyły się rzeczy, które odczułabym jako zagrożenie. Zagrożeniem byłoby, gdyby wprowadzili jeszcze większe obostrzenia na jeszcze dłużej, np. na zasadzie, że nie można wyjść do sklepu. Rodzice mają dosyć spore zapasy jedzenia i zawsze mieli je w domu, bo są zwolennikami teorii spiskowych, więc zapasy na apokalipsę są w domu już od jakiegoś czasu. Te zapasy wystarczyłyby na pół roku. Więc, gdyby to była sytuacja, w której zapasy zaczęłyby się kurczyć, albo byłby problemy z dostawą prądu czy gazu… Mamy Internet i przepływ informacji, prąd, gaz. Niczego nie wyłączają. Nie jest to anarchia i koniec świata, tylko ograniczenie pewnych wolności naszych. Wciąż nie jest to zagrażające. Gdyby nie mogli nam dostarczyć prądu, wody, byłoby to zagrożenie, bo to już dużo znaczniej wpływa na byt. Dopóki, nudny, bo nudny, ale byt mam wciąż taki, jak zazwyczaj, nie czuję się zagrożona.

**Jest coś, czego się obawiasz w tym momencie?**

W tym momencie obawiam się, nie o jakieś tam zachorowanie, tylko o sytuację ekonomiczną, finansową. Szczęście w nieszczęściu jest takie, że mam na tyle odłożonych swoich pieniędzy, że mogę przeżyć kilka miesięcy nie pracując i jakoś to będzie. Problem jest taki, że wolałabym, żeby było, tak jak ja bym chciała, a nie „jakoś to będzie”. Ale zobaczymy, jak sytuacja się rozwinie. Niepokój największy jest o sytuację finansową i jak będzie z pracą. Jest mi mocno przykro z powodu tych studiów. Na razie nie jest to totalnie przesądzone, ale raczej nie będzie mnie stać na studia po tej całej sytuacji. Nie wiadomo też, jak to będzie z podróżowaniem we wrześniu.

**A czy obawiasz się o swoich bliskich?**

Nie. Myślę, że rodzice też są zabezpieczeni. Mój tata nie pracował ostatnio od dłuższego czasu, więc jego to nie dotyka. Mama jest nauczycielką, więc wiem, że dostanie trochę mniejszą pensję w tym miesiącu, ale wciąż pensja będzie. Na razie nie wiadomo, jak będzie wyglądał kwiecień. Na razie wiadomo, że dostanie mniejszą pensję marcową i jeśli nie wróci do szkoły w kwietniu, to też mniejszą pensję za cały kwiecień. Ale, jeśli to będą dwa miesiące, to rodzice nie odczują tej sytuacji drastycznie. Mój brat z kolei jest w fenomenalnej sytuacji, bo pracuje w IT i w tym momencie ma mnóstwo zamówień, bo sieci są obciążone i wszystkim zależy na szybszym Internecie.

**Wspomniałaś o tym, że niepokój dotyczy głownie sytuacji finansowej, a samej choroby się nie obawiasz. Mogłabyś to rozwinąć?**

To znaczy… Informacje są sprzeczne - mówiło się, że to dotyczy starszych, a młodzi bezobjawowo. Ja mam takie przekonanie, że jakbym to przechodziła, to bezobjawowo albo to jakoś przetrwam, bo cechuje się wysoką odpornością organizmu i bardzo rzadko choruję. Wiadomo, że w tym momencie dość mocno stresowałoby mnie to, żebym nie przekazała tego babci. Babcia ma 84 lata i mamy pokoje daleko od siebie, ale wciąż - to ten sam dom. Babcia twierdzi, że nie boi się wirusa i na coś trzeba umrzeć, ale wiadomo, że to tylko jej gadanie. Rodzice też cechują się raczej dobrym zdrowiem i rzadko chorują.

**Pokażę ci teraz kilka zdjęć. Które z nich oddają twoje emocje w tej chwili?**

Myślę, że nr 7, który jest spójny z nr 8, ale przy nr 7 widać dużo więcej przestrzeni, a mniej więcej tak teraz wyglądają różne miejsca. Nr 4 kojarzy mi się z tym, że sporo ludzi organizuje się do wsparcia. I jak zwykle, w momentach kryzysowych, solidarność Polaków się uwidacznia i widać, że dużo ludzi zaczyna się wspierać. Poza tym 12 trochę przykuło moją uwagę, na zasadzie, że nic nie trzeba. To jest taki trochę zen - mam czas na zajęcie się sobą czy też niezajmowanie się sobą, tylko lenistwo. To czasami też jest dobre. Ja nie umiem odpoczywać, kiedy z tyłu głowy wiem, że mam coś do zrobienia i ta sytuacja jest pierwszą, że wiem, że ja nie mam nic do zrobienia.

**4**

Tutaj pierwsze skojarzenie, to była solidarność i chęć niesienia bezinteresownej pomocy.

**7**

Puste ulice kojarzą mi się z niepokojem, na zasadzie, że wywiało wszystkich z miasta. Po Warszawie teraz nie chodziłam, ale opowiadali mi znajomi. Kiedyś byłam w Rotterdamie latem i wszyscy byli na plaży, a w mieście nie było ludzi. Chodziliśmy po pustych ulicach normalnego miasta i przyprawiało mnie to o uczucie niepokoju, że skoro nie ma ludzi na ulicach, to coś jest nie tak. Więc tutaj widzę ten niepokój, niepokój o przyszłość.

**12**

Spokój i brak gonitwy. Prawdziwe odpoczywanie, beż żadnych obowiązków.

**Która z emocji dominuje?**

Jako że nie wychodzę, to nie angażuję się też w pomoc za bardzo. Myślę, że ta 12 najbardziej na ten moment. Bo ten niepokój na razie jest o sytuację finansową, ale jakby co, to mam wsparcie rodziny, więc jeszcze nie jest to aż tak dominujące. 12 najmocniej, potem 7 i na końcu 4, bo ja to obserwuje, ale nie jestem w stanie się sama zaangażować.

**Skąd się biorą te emocje?**

12 z faktu, że nic nie muszę, muszę siedzieć w domu i nie mam innego wyboru niż odpoczywanie. Wynika to z tego, że nie mogę wyjść do świata zewnętrznego. Niepokój rośnie, kiedy słucham bądź czytam wiadomości, ale staram się już tego nie robić i unikam. A ta solidarność, jeśli natknę się na jej przejawy w social media.

**Jak te emocje zmieniały się w zależności od etapów rozwoju sytuacji?**

Teraz jest relatywnie najlepiej z całego tego czasu, odkąd się zaczęło. Dla mnie największym stresem było to, że byłam w Londynie i nie wiedziałam, co z pracą i co dalej. W momencie, kiedy w Polsce zaczęło się coś dziać i w Europie, a w Wielkiej Brytanii jeszcze nie, ten stres zaczął rosnąć. Kulminacją był dzień, kiedy odwołali mi ten trening. Utrzymywało się to przez tydzień aż do decyzji o moim powrocie. Teraz już czuję ulgę - odzyskałam część pieniędzy za mieszkanie, mam pieniądze na teraz, mieszkanie u rodziców mnie nic nie kosztuje. Teraz nie wydaje pieniędzy i jestem w domu bezpiecznie ze swoimi rzeczami i to jest ulga. Uspokoiłam się w domu, bo tu czuje takie ogólne bezpieczeństwo i byt jest na lepszym poziomie.

**Jak radziłaś sobie w tej kulminacyjnej sytuacji dla ciebie, kiedy odwołali ci trening z pracy? Jakie działania podjęłaś?**

Ja bym powiedziała, że sobie raczej nie poradziłam z tą sytuacją, bo byłam w bardzo kiepskim stanie psychicznym. Pomagało mi to, że miałam zaplanowaną rozmowę z terapeutą. I rozmowy z moimi przyjaciółmi, którzy byli w stanie mnie wesprzeć telefonicznie czy też przez wideorozmowy. Jeszcze w niedzielę spędziłam cały dzień ze znajomymi w Londynie i to było odstresowujące. Ale to był ostatni moment, kiedy mogłam gdzieś wyjść i z kimś się zobaczyć. Później… nie powiedziałabym też, że było to radzenie sobie, ale starałam się zająć czas chodzeniem na spacery. A tu w domu już nie musiałam szukać sposobów na to, bo mam więcej zajęć, mam psa, wysyłam CV online.

**A jak spędzasz czas, kiedy jesteś w domu?**

To są takie dość głupie rzeczy, na które się nie ma czasu normalnie. Jak byłam mała, to mama dawała mi mandale do kolorowania i teraz to znów robię i jest to przyjemne.

**A co daje ci ta czynność poza przyjemnością?**

Jest to odstresowujące i jest to rodzaj medytacji. Jeśli chodzi o kostkę Rubika np., to też od miesięcy myślałam, że chcę się tego nauczyć. Mam problem ze zmuszeniem się do czytania książek, mimo że generalnie to lubię. Z tyłu głowy mam, że powinnam to zrobić. A od tej soboty… wysyłam te CV i jutro mam rozmowę o pracę. Sam fakt, że ktoś się odezwał, podnosi na duchu i daje jakąś perspektywę. Wczoraj już obliczyłam sobie wstępny potrzebny budżet, gdybym chciała się wyprowadzić i zaczęłam przeglądać oferty mieszkań na maj. To napędza u mnie myślenie i planowanie, co jest fajne. I *binge watching* na Netflix, co robiłam też, jak miałam dużo zajęć. A teraz nie muszę się czuć winna z tym, że oglądam, bo nie mam nic innego do zrobienia. Na oglądaniu seriali najszybciej mija czas. Ale teraz też czasami oglądam coś, co daje jakiś aspekt naukowy, poszerzenie ogólnej wiedzy.

**Co daje ci kontakt ze znajomymi i rozmowa z terapeutą poza wsparciem?**

Wtedy, jak byłam w Londynie, to byłam w innej sytuacji, a teraz jestem w takiej samej sytuacji, co moi znajomi, więc teraz już mniej są w stanie mnie pocieszyć.

**Jak zmieniło się twoje zachowanie jako konsumenta?**

Na kwarantannie wychodzić nie mogę, ale zostają zakupy online. Zrobiłam jedne zakupy, które już zwracam. Idzie paczka z dwiema sukienkami i torebką. Oszczędzanie to nie jest moja mocna strona i kiedy odblokowały mi się finanse przeznaczone na Londyn, a w najbliższym czasie nie planuję większych wydatków, bo mieszkam u rodziców, to wtedy kupuję. I jeszcze jak te jedne buty zwrócę, to kolejne mam w planach, więc ja na razie rozrzutnie. Aczkolwiek trochę się powstrzymałam. Te ciuchowe zakupy myślę, że już zrobiłam. Ale skończyły mi się suplementy i witaminy, więc zamówiłam. Jeszcze z takich rzeczy przyjemnościowych, to ja jestem fanką kominków z olejkami eterycznymi, więc zamówiłam sobie kolejną porcję. Mama chciała jeden, więc dla niej zamówiłam jeden, a przy okazji kilka dla siebie.

**To są głównie zakupy przyjemnościowe?**

Trochę tak, takie na poczucie się lepiej w obecnej sytuacji, bo nic innego nie mogę.

**Mówiłaś, że rodzice mają zapasy żywności. Mogłabyś to rozwinąć?**

Jeśli chodzi o suche produkty - kasze, makarony, to mają w większej ilości, raz, żeby po prostu były, a dwa, ze względu na ich apokaliptyczne przewidzenia. I tak jest od kilku lat, nie było to związane z obecną sytuacją. A te wszystkie świeże rzeczy, typu chleb, warzywa, wędlina, kupują w normalnej ilości.

**Czy te zapasy dają ci poczucie bezpieczeństwa?**

O tyle, że na dany moment, kiedy ludzie zaczęli panicznie kupować, ja miałam poczucie, że nie muszę się tym stresować, ani sama iść kupować, bo mamy to w domu. To nie jest kwestia tego, że ja się boję, że nie starczy, bo sklepy są otwarte. To jest bardziej poczucie bezpieczeństwa, że nie trzeba iść do sklepu i z tymi ludźmi się bić i nie zagraża mi to, że dla mnie nie wystarczy. To jest okropne dla tych starszych ludzi, bo czasem się widzi zdjęcia starszych osób w sklepie, że nie ma dla nich produktów, bo idioci wykupili i nie zostawili nic innym.

**Wracając jeszcze do zakupów online, czy one się zmieniły w stosunku do tego, co było wcześniej?**

Jeśli chodzi o to, co kupuje to nie, tylko zmieniło się w ilości wydawanych pieniędzy. Gdybym miała w Londynie perspektywę, że oszczędzam na studia, to nie kupiłabym dwóch torebek i sukienki. Ale w momencie, w którym plan się zmienił, to stwierdziłam, że dla uprzyjemnienia tego, że będę musiała siedzieć w Warszawie, kupię sobie dwie sukienki i torebkę.

**A jakie to marki?**

Zamówiłam w H&M sukienkę, która wyświetliła mi się na Facebooku i wpadła mi w oko. Kosmetyczne rzeczy i suplementy, to takie, których używam od dłuższego czasu i mam sprawdzone.

**Co jest dla ciebie w tym momencie największym wyzwaniem? Co sprawia trudność?**

Zakupy nie, bo to, czego potrzebuję, to mogę zamówić. Tylko to, co jest dla mnie trochę niewygodne to to, że wcześniej zamawiałam zwykle do paczkomatu, a teraz nie mogę wyjść po to. Teraz muszę zamawiać kuriera i jak przychodzi, to tata się czepia, że wydaję pieniądze na głupoty.

**Brakuje ci takich aktywności, jak chodzenie do kina czy muzeum?**

Do kina i muzeum nie chodzi się codziennie. Wcześniej nawet, jak miałam ochotę pójść na wystawę, to nie miałam czasu. Świadomość, że teraz nie mogę, jest średnio przyjemna, ale nie odczuwam tego na co dzień. W przypadku filmów zostaje nam Internet i różne platformy i niektóre filmy, które miały być w kinach, przeniosły się na te platformy. Niektóre muzea zaczęły robić oprowadzanie online, więc też można skorzystać. Ja z tego nie korzystałam na razie i myślę, że oglądanie sztuki schodzi na dalszy plan w obecnej sytuacji. Nie boli mnie to jakoś bardzo na dany moment.

**Jak twoje otoczenie radzi sobie z obecną sytuacją?**

Brat i spora większość znajomych może pracować zdalnie. Mam przyjaciółkę, której mąż pracuje w Brukseli, a ona sama była zabiegana i po ślubie bardzo mało ze sobą mieszkali, a w tej sytuacji to pozytywne, że w końcu mogą spędzić razem trochę czasu. Moja inna przyjaciółka też pracuje zdalnie, ale jej doskwiera fakt niemożności spotkania się ze znajomymi, ze mną, czy też pójścia na randkę. Mojemu bratu to też doskwiera, ale on może studiować i pracować zdalnie. Sporo ludzi jest nie dotkniętych tą sytuacją o tyle, że bardzo im to nie zmienia funkcjonowania. Moja sytuacja jest o tyle niefortunna, że ja nieszczęśliwie postanowiłam tuż przed tą sytuacją, szukać sobie nowej pracy. Ale nie obwiniam siebie, bo nie byłam w stanie tego przewidzieć. Nie mam nikogo, kto byłby w trudnej sytuacji, wszyscy pracują.

**Czy jest coś, co twoi znajomi robią, a co odebrałaś negatywnie? Czy widziałaś przejawy paniki?**

Nie, raczej nikt nie panikuje. Niektórzy źle znoszą niemożność wychodzenia i odczuwają frustracje. Ale nie postrzegam tego negatywnie, nie widziałam wpadania w panikę. Mam wrażenie, że wpadanie w panikę u nas dzięki radykalnym, ale dość jasnym wskazaniom rządu, było dużo mniejsze niż w Wielkiej Brytanii.

**Wiesz, skąd się wziął koronawirus?**

Ja wiem, co się mówi. Skąd się wziął? Myślę, że nikt z nas się na pewno nie dowie *[śmiech]* i to będzie tajemnica po wsze czasy. Pierwsze informacje były z tego targu dzikich zwierząt w Wuhan, że ktoś coś zjadł. Mówiło się o nietoperzach, ale też o jakimś innym gatunku. Oglądałam film, w którym była mowa o tym, że ten poprzedni SARS z 2008, tak jak świńska grypa i ptasia grypa, to są wirusy od zwierząt. A teorie spiskowe, czyli laboratorium amerykańskie, które znajduje się w Wuhan. Były też teorie o tym, że to Brytyjczycy, bo ich działanie było opóźnione względem Europy i powstała teoria, że coś wiedzą. Słyszałam też teorię, że to UFO robi na nas testy.

**Są jakieś elementy lub całe teorie, które cię przekonują, że można w nie uwierzyć?**

Ja mam takie podejście, że uwierzyć można we wszystko. Nie skłaniam się ku danej teorii, dopóki nie byłabym w stanie zobaczyć na własne oczy. Mam rodziców, którzy są krytyczni względem wielu rzeczy. Nie są stuprocentowymi antyszczepionkowcami, ale np. są przeciwni szczepionkom wszystkim naraz i jest to coś, ku czemu też się skłaniam, że lepiej jest szczepionek nie robić niż robić, jeśli nie ma takiej potrzeby. Nie jestem lekarzem ani chemikiem, więc nie sprawdzę, co jest w tych szczepionkach. To jest kwestia wiary/niewiary, czy są tam metale ciężkie, itp. Jedna z większych teorii to jest to, że ogólnoświatowa panika doprowadzi do tego, że każdy da się zaszczepić na koronawirusa, jak już ta szczepionka powstanie. Może to być taka jedna teoria, do której skłaniam się najbardziej, bo jest masa ludzi wprowadzona w taki stan, w którym będą skłonni zgodzić się na coś, na co nie zgodziliby się w innych warunkach. Mam dość wysoki sceptycyzm, jeśli chodzi o masowe, przymusowe szczepienie. Tak jak mówiłam, ja nie odczułam sama zagrożenia chorobą, bo nie mam nikogo znajomego, kto by zachorował.

**Czy można wskazać, że ktoś jest winny obecnej sytuacji?**

Jeśli to miałby być przypadek i kwestia Wuhan, to jest to spowodowane tylko różnicami kulturowymi, ponieważ Chińczycy jedzą inne zwierzęta niż my i nie jest to prawnie kontrolowane. Jestem skłonna uwierzyć w niektóre teorie, że ktoś to zaplanował i miał na celu wywołanie ogólnoświatowej paniki, która doprowadzi do masowych szczepień. Moi rodzice są zwolennikami tych teorii, dotyczących nowego porządku świata, depopulacji, itd. Czy to jest prawda? Nigdy nie powiem na 100%, że to jest prawda. Ale sam fakt, że wirus wybuchł w grudniu/styczniu, a ich chiński rok, który jest dużym świętem, zaczął się pod koniec stycznia i można się dopatrywać celowości. Chiński nowy rok jest najlepszym momentem na rozprzestrzenianie się wirusa po kraju, bo oni wszyscy spoza kraju wracają na ten czas do rodziny i wewnątrz kraju na czas święta rozjeżdżają się z dużych miast do mniejszych miejscowości. Ja to dopuszczam, filtruję i twierdzę, że coś może być prawdą, a może to być tylko przypadek tego nieszczęsnego nietoperza. I na pewno sceptycznie podchodziłabym do obowiązkowych szczepień na koronawirusa. Może nie jest to mądre, ale uważam, że mam odporność własną na wysokim poziomie i wolałabym ją nabyć poprzez zarażenie się, przejście wirusa bezobjawowo i uodpornienie się niż szczepienie się.

**Czy twoim zdaniem można było zapobiec tej sytuacji?**

Zamykając, czy też nie tworząc takich targów żywymi zwierzętami. A zrobili to dopiero teraz, jak wywołali ogólnoświatową pandemię.

**A świat według ciebie jest przygotowany na pandemię?**

Myślę, że nie bardzo. U nas zadziałano dość wcześnie, ale nasze zarażenia rosną i będą rosły. Prawdopodobnie większy sukces byłby odniesiony, gdyby te działania wprowadzono dużo wcześniej. Te choroby wcześniejsze, jak świńska czy ptasia grypa, nie dotknęły nas w taki sposób, że mówiło się o przeciążonym systemie zdrowia. A teraz rysuje się taki obraz: „siedźcie wszyscy w domu, bo umrzecie” albo „siedźcie w domu, bo będziemy mieli szpitale i podłogi zasłane trupami, tak jak rzekomo to jest we Włoszech”. Podobno Chiny ukrywały długo, jak wygląda ta sytuacja. Ja nie interesowałam się sytuacją w Chinach, dopóki to nie wybuchło u nas.

**A jeśli chodzi o Polskę, to nasz kraj jest przygotowany na tę sytuację?**

Myślę, że nikt nie był w stanie być przygotowany, bo jest to pierwsza taka rzecz. Ale z drugiej strony coś tam wiedzieliśmy o sytuacji w Chinach od dwóch miesięcy. Dla mnie to wyglądało tak, że: no dobra, w Chinach się to przewali, biedni Chińczycy. Ale to, co można było zrobić, to zamknąć Chiny, żeby nikt nie wyjeżdżał. Jeżeli u nich to wykryto listopad/grudzień, u nas do stycznia nie było dużo informacji, ale chyba oni już wiedzieli, że to jest ten rodzaj wirusa, który się tak przenosi. Oglądałam program ze specjalistą z CDC, który mówił, że największy problem z wirusem jest taki, że on się przenosi przez małe cząsteczki, których te maski zwykłe nie blokują, więc sam fakt oddychania czy mówienia w pomieszczeniu, może sprawić, że kogoś zarazimy. Logiczne wydaje się, że jeżeli jest to tego rodzaju rzecz, to pierwsze, co trzeba zrobić, to zapobiec wyjeżdżaniu ludzi i zamknąć to w Chinach w środku.

**Jak oceniasz w tej sytuacji działania polskiego rządu?**

Zrobili to bardzo szybko i bardzo sprawnie. Zamknęli granice jako jedni z pierwszych, razem z Czechami i Słowacją, w odpowiedzi na Włochy, ale dopiero, jak sytuacja zaczęła się robić poważna.

*[Policja, sprawdzająca czy respondentka jest na kwarantannie, przerwała rozmowę]*

Fajne jest to, że oni [policjanci] faktycznie sprawdzają, czy ktoś jest w domu. I jak ktoś mieszkałby sam, to pytają, czy zrobić zakupy i czy czegoś potrzeba. I to jest akurat ogarnięte fajnie i ta aplikacja też działa dobrze. To są, myślę, te pozytywne działania: ta aplikacja, to, że policja kontroluje codziennie. Z mojego punktu widzenia, podczas gdy byłam w Londynie, polski rząd zadziałał szybko i sprawnie. Zamknięcie szkół i większych zgromadzeń na pewno było dobre. Reszta ograniczeń jest średnio wygodna i coraz mniej mi się podoba, że to tak długo trwa, ale na pewno jest to konieczne. Więc generalnie raczej pozytywnie rozpatruję działania rządu. Niektórzy mówią, że jest to zbytnia panika, ale w tym momencie jest to panika na całym świecie.

**Uważasz, że działania polskiego rządu powodują zwiększenie paniki, czy wręcz przeciwnie, uspokojenie ludzi?**

W Londynie chaos i dezinformacja były dużo większe. Johnson powiedział, że trzeba przygotować się, że w tym roku umrze więcej obywateli niż zazwyczaj. Dla mnie jest to niewyobrażalne, żeby premier powiedział coś takiego. W skrócie oznacza to tyle, że nie będziemy robić nic i jak umrzecie, to umrzecie. Więc w tej sytuacji jakiekolwiek działania rządu byłyby uspokajające. Z mojego punktu widzenia sytuacja w kraju była niepewna i stresująca, ale informacje były dostarczane. W Internecie były *fakenewsy*, że zamkną sklepy, ale to nie była kwestia rządu. Rząd wyjaśnił i poinformował, uspokajając obywateli, że nie zamknie sklepów. Więc była panika na początku, a rząd uspokoił. W Wielkiej Brytanii były tylko zalecenia, żeby nie chodzić w miejsca publiczne, ale nie zamknęli pubów. Tylko, że u nas, jak ktoś lekceważy zagrożenie i nawet chciałby pójść do pubu, to tego nie zrobi, bo jest zamknięte wszystko. A w Wielkiej Brytanii połowa stwierdziła, że ich to nie obchodzili i wychodzili, bo mogli, bo było otwarte. Więc porównując do działań brytyjskiego rządu, decyzje rządu polskiego były pozytywne. Nie wiem, na ile nasza sytuacja jest faktycznie lepsza niż w innych krajach, a na ile to kwestia mniejszej ilości testów. Inne mam podejście do kwarantanny w domu, a inne bym miała, gdyby mnie przymusowo zabrali do jakiejś izolatki.

**Skąd czerpiesz informacje na temat obecnej sytuacji?**

Ja nie mam konkretnego źródła. Moi rodzice siedzą na alternatywnych źródła informacji, jak Podziemna TV, itp. To, co słyszę, to jest albo wersja rodziców albo bardzo masowe media. Najczęściej przewija mi się Facebook, bo na niego wchodzę i robię inne rzeczy i jak mi się przewiną wiadomości, to przewiną. Wiadomości głównego nurtu przewiną mi się tylko wtedy, kiedy przeskakuje po kanałach w poszukiwaniu filmu. Coś do mnie dociera, ale staram się nie słuchać tych informacji i nie przyswajać, bo są zazwyczaj mocno negatywne.

**Zdarza lub zdarzało ci się aktywnie poszukiwać informacji?**

Jak byłam w Londynie, to tak, żeby rozeznać się w sytuacji. Nie zwracam zbytnio uwagi, kto jest autorem. W masowej telewizji jest kwestia strachu i przerażających nagłówków. Czasami nagłówek jest przerażający, a jak się wejdzie, to okazuje się, że nic takiego. Strach i panika bardzo sprzyjają kliknięciu, ale to w mediach zawsze było, że nagłaśnia się tragedie i problemy, żeby się klikało. Nie bardzo wiem, któremu dostarczycielowi informacji można wierzyć. Nie mam nikogo takiego, kto byłby dla mnie bardziej wiarygodny niż ktoś inny. Jest taki lekarz - Dr Mike, którego śledziłam i on robił filmy bardziej uspokajające. Jest z wykształcenia lekarzem i ma większe prawo się wypowiadać i może mieć większą wiedzę. Czym innym jest rozgłaszanie informacji przez laików, którzy nic o tym nie wiedzą, a czym innym przez lekarza. Wiadomo, że jest strona WHO i nasz rząd ma wytyczne w zgodzie z WHO, więc powiedzmy, że temu można wierzyć. Ale oglądałam też wywiady z inspektorem CDC i on mówił, że nie wszystko jest potwierdzone, np. nie wiadomo tego, na jaką odległość wirus się może przenieść.

**Czy pamiętasz, gdzie szukałaś informacji, będąc w Londynie?**

Po prostu wyszukiwarka Google i hasłowo, klikając w artykuły. Najczęściej szukałam informacji, dotyczącej konkretnego kraju, np. Włoch, jak stamtąd dochodziły informacje.

**Czy twoim zdaniem czas, jaki poświęcasz na korzystanie z mediów, się zmienił?**

Szukałam dużo więcej, kiedy byłam w Londynie, kiedy sytuacja się rozwijała i chciałam wiedzieć, co mam robić. Po powrocie przestało mi być to potrzebne, a że te informacje są stresujące i nie wygląda to kolorowo, to jest to mało pocieszające. Wolę słuchać takich informacji, że to potrwa do świąt i może z początkiem maja będzie można wrócić do normalnego życia, bo taka wizja jest dla mnie do przetrwania. Jakby to miało trwać dłużej, to mocniej by mi zaczęło doskwierać.

**To oznacza, że teraz korzystasz mniej z mediów niż przed epidemią?**

Nie, bo ilość siedzenia na Instagramie jest na pewno dużo większa. Siedzę na social mediach więcej niż zwykle. Zawsze siedziałam dużo i uważałam, że marnuję dużo czasu, a że teraz wiem, że mogę, to sobie pozwalam. Na Facebooku, jeśli widzę te artykuły, to czasem kliknę, ale jak widzę, że jest to coś bardzo negatywnego, to wyłączam. Albo czasem nie klikam, tylko jest to kwestia tego, że znajomi coś publikują. Większość influencerów przełączyła się na akcje, jak ludziom uprzyjemnić czas. I są to fajne rzeczy, podbudowujące i jest to pozytywne, ale to też mocno przypomina o tym, jak sytuacja wygląda. I zaczyna mnie to męczyć. Wtedy przełączam się na Netflixa i jest to plus odmóżdżającej rozrywki, że skupiając się na niej, można zapomnieć o sytuacji na świecie.

**Po czym poznać, że dany przekaz jest wiarygodny lub nie?**

Bardziej wiarygodny jest dla mnie ktoś, kto jest specjalistą w danej dziedzinie, np. ten inspektor CDC i lekarze, wirusolodzy, którzy się wypowiadają. A poza tym, to nie wiem, nie mam na to klucza i nie określam sobie, że ten jest bardziej, a ten mniej wiarygodny. Ale też zdarza się, że jeden wirusolog mówi jedno, a drugi coś innego.

**Są jakieś źródła informacji, które są dla ciebie najbardziej wiarygodne?**

Nie. To jest tak, że przejrzę artykuł i taki, i taki i jak są sprzeczne to przyjmuję do wiadomości, że są różne informacje. Wolę mieć oba na względzie niż jeden konkretny, bo jest to w tym momencie kwestia wiary, nie sprawdzimy tego.

**Wspomniałaś wcześniej o stronie WHO. Korzystałaś z niej?**

Nie, ja tam nie wchodziłam. Tylko wiem, że rząd stosuje się do tych zaleceń.

**Dziękuję. Z mojej strony to wszystko.**
