# Supplementary material for: The challenges arising from the COVID-19 pandemic and the way people deal with them. A qualitative longitudinal study
Source: PLoS One. 2021 Oct 11;16(10):e0258133. doi: 10.1371/journal.pone.0258133 (PMC8504766; doi:10.1371/journal.pone.0258133)
Supplement: S1 Dataset — (ZIP) [file pone.0258133.s003.zip › Transcriptions/stage 1/13.1_M_46_couple, with children.docx]

**13.1_M_46_couple with children**

**Na początek chciałabym, aby się Pan przedstawił.**

Nazywam się Grzegorz, mam 46 lat. Mieszkam w niedużej wsi koło Przemyśla. Jestem lekarzem, jestem chirurgiem. Pracuję w szpitalu, mam też własną praktykę. Interesuję się muzyką, uprawiam sporty, jeżdżę na nartach, jeżdżę na rowerze, żegluję. Co jeszcze...

**Wspomniał Pan o żonie.**

Tak, mieszkam z żoną i z młodszym synem. Wspólnie w domu. Mamy nieduży teren wokół domu, ogród.

**Czy pamięta Pan pierwszy moment, kiedy obecna sytuacja w Polsce się zaczęła?**

Tak.

**Jaki to był moment?**

To była sytuacja, kiedy u nas w szpitalu zaczęto mówić o tym - ja pracuję w szpitalu wojewódzkim w Przemyślu - że na świecie pojawiła się epidemia. I że nie wiadomo, jak to będzie wyglądało, bo na razie ma to charakter epidemii, które pojawiały się kilka lat wcześniej. Ale dopóki nie będziemy wiedzieli jak rzeczywiście ten wirus się zachowuje, jaki przebieg ma infekcja, to właściwie na razie nic nie można powiedzieć, ale pewnie trzeba będzie się zachowywać jak do tej pory, czyli spokój, bez paniki, bez jakiegoś nadmiernego zaangażowania się. To jeszcze był moment, kiedy mówiono o epidemii w Chinach. Jeszcze nie było u nas.

Ale u nas w szpitalu już się o tym mówiło, i temat był aktualny.

**A czy dla Pana osobiście to też był taki moment, że Pan sam poczuł, że coś się zmienia? Że to nowa sytuacja?**

Nie. Ponieważ tego typu epidemie - może nie na taka skalę - ale zdarzały się w ciągu kilkunastu lat wcześniej, ich było kilka, więc ja to osobiście potraktowałem jak jedną z tych epidemii. Które oczywiście budzą zainteresowanie i naukowców i środowiska medycznego i lokalnie stwarzają zagrożenie dla populacji. Ale nie były to epidemie i o takim dużym zasięgu i dużym wpływie na sytuację wokół mnie. Więc też to potraktowałem to jako jedną z tego typu epidemii.

**A kiedy był ten moment dla Pana osobiście,  że Pan poczuł, że sytuacja jest inna?**

Jak się zaczęły masowe zachorowania we Włoszech. Masowe zachorowania i rosnąca liczba zgonów związana z tym. Na małym obszarze Włoch, mnóstwo zachorowań i spory odsetek zgonów.

**I wtedy myślał Pan, że to się może przenieść do Polski?**

Ja byłem pewien, że to się przeniesie. Że jak coś takiego się dzieje w jednym z krajów Unii Europejskiej, no to wiadomo że to trafi do nas. Polska przecież nie jest odizolowana. Żyjemy tak samo jak inni obywatele Unii.

**A jak miałby Pan swoimi słowami opowiedzieć o głównych etapach rozwoju tej sytuacji w Polsce? Jakie były kamienie milowe rozwoju tej sytuacji? Jak to z Pana perspektywy wyglądało**

Dla mnie - jak to się rozwijało? Czy moją opinię co się działo w kraju?

**Obie rzeczy, ale jakby Pan mógł zacząć od Pana - jak to było z Pana punktu widzenia.**

Dla mnie to wyglądało w ten sposób, że myśmy akurat z żoną i z synem byli na urlopie. Byliśmy 3 tygodnie od początku lutego, więc wylatując na urlop oczywiście mówiło się o zagrożeniu. Natomiast to nie była jeszcze sytuacja, która byłaby na tyle istotna, żeby się zmieniało zachowanie ludzi, czy na lotnisku, czy w miejscach, gdzie ludzie się gromadzili w większych grupach. Natomiast po przylocie na miejsce, gdzie spędzaliśmy urlop myśmy po raz pierwszy wypełnili - to było 5 lutego - deklaracje, w której pytano nas o to, czy mieliśmy kontakt z osobami zainfekowanymi wirusem. Czy z osobami chorymi objawowo. I to był pierwszy moment, kiedy ja też miałem jakąś informację, że cokolwiek się wokół mnie zmienia w związku z panującą epidemią. Ponieważ przez 3 tygodnie przebywaliśmy w terenach z dala od cywilizacji i docierały do nas informacje  przez Internet, ale byliśmy zaaferowani zupełnie czymś innym, więc nie było to aż tak istotne. Ja dalej miałem wrażenie, że jest to jedna z tych epidemii, która przebiegnie lokalnie i w miarę spokojnie uda się ją opanować i wyciszy się. Natomiast już wracają z urlopu, 24 lutego, w samolocie żeśmy dostali informacje i ankietę w której należało opisać nasz obecny stan zdrowia i ewentualnie informacje epidemiologiczne, gdzie moglibyśmy powiedzieć cokolwiek na temat ewentualnego kontaktu z osobą zainfekowaną. To rozdała załoga samolotu. To już taki istotniejszy moment, bo linie lotnicze europejskie, poważne więc to już informacja, że  to się dzieje więcej niż to co było do tej pory. Bo nigdy takiej ankiety nie wypełniałem w samolocie. Oprócz tego na lotnisku - to też rzecz, która mnie nie zaskoczyła, bo to jeden z częstszych widoków na lotniskach w krajach, gdzie występują różnego rodzaju epidemie i choroby zakaźne - mierzenie temperatury i ocena podróżnych kamerą na podczerwień, gdzie się tą temperaturę ciała w sposób zdalny sprawdza, nie tylko mierząc.

A później dla mnie taki istotny moment, to był fakt, że myśmy mieli wyjechać na jeden z weekendów w połowie marca na weekend w Bieszczady i okazało się, że miejsce do którego jedziemy, będzie dla nas niedostępne - to miejsce noclegowe - ponieważ nasze Państwo przygotowuje się - tak to muszę oględnie powiedzieć - do takiego dużego ruchu transgranicznego na granicy polsko-ukraińskiej. Ponieważ duża liczba Ukraińców ma wracać z Unii Europejskiej, zwłaszcza z Włoch, wracać do kraju w związku z tym co się dzieje we Włoszech. Więc to też już taka dla mnie informacja, że nie jest to sprawa błaha, ponieważ się angażuje w to straż graniczną, wojsko. I jest wzmożona ochrona na granicach. Więc też coś ważnego.

A później się zaczęły zmiany u nas w szpitalu.

To był też okres licznych zachorowań na infekcje para-grypowe,   bo to nie miało objawów typowej grypy z bólami mięśniowymi, kostnymi, itd. Ale infekcje z wysoką gorączką taką ok 39-40 stopni. Takie dość masowo przebiegające infekcje - w szkole np. w klasach dwudziestoosobowych pół klasy nie było, albo więcej dzieci w związku z tymi zachorowaniami. Ale to było traktowane jako infekcje grypowe, czy para-grypowe. Znaczy ludzie mówili "grypa", ale często ludzie mówią grypa na infekcje które nią nie są.

Więc to też trochę było powiedzmy dla nas... Z jednej strony zwracało uwagę, że coś się dzieje w społeczeństwie, że jakieś wirusy się pojawiły - bo wiadomo, że co roku występują te zachorowania, tak sezonowo. Ale żeby aż tak dużo ludzi było chorych, u mnie w pracy też koledzy chorowali - więc tego wcześniej nie było. I w tle ta epidemia koronawirusem.

Później się w pracy pojawiły dość szybko informacje o tym, że będziemy musieli zredukować przyjęcia planowe i zmiana charakteru pracy. I właściwie tyle

A jednocześnie oczywiście działania naszego Państwa. W mojej opinii takie bardzo zachowawcze - oczywiście jest infekcja, jest epidemia, ale u nas to w dziwny sposób było tych zachorowań dużo mniej niż w innych krajach Unii, więc jakoś tak śmiesznie. Zważywszy na to co się działo z tym powrotem obywateli Ukrainy do kraju, gdzie to naprawdę jest też duży czynnik ryzyka, taki epidemiologiczny. I epidemiologicznie ważny czynnik - tego typu sytuacje jak przejazd kilku tysięcy czy kilkunastu tysięcy osób, które potencjalnie mogą być zainfekowane, to powinno być ważne. A też było to potraktowane dziwnie, zwłaszcza w moim mieście. No i różnego rodzaju takie zachowania.

Myśmy wiedzieli bardzo szybko w naszym środowisku lekarskim, że te dane które są przedstawiane, najprawdopodobniej nie odpowiadają... rzeczywistości. Ale widać było, że ta epidemia w Polsce też się rozwija. Bo w różnych miejscach się pojawiały zachorowania, nawet w takich obszarach, gdzie początkowo ich nie było ludzie chorują. I właściwie tyle.

Dla mnie osobiście takie duże, ważne momenty to były działania Państwa, które w pewnym momencie zakazało gromadzenia się ludzi. Myśmy musieli pozamykać nasze gabinety - znaczy nie musieliśmy, ale świadomość zagrożenia jakie może nieść ze sobą dalsze funkcjonowanie miejsc typu gabinet lekarski czy zakład fryzjerski, to kazało nam te gabinety pozamykać, bo pacjenci przyjeżdżają z różnych regionów i zachowanie izolacji jest właściwie niemożliwe, bo się trafiają nawet osoby z promienia do 100-150 km. To było dla mnie bardzo ważne. Myśmy w prywatnej praktyce jeszcze przeprowadzili operację w połowie marca, w 3 tygodniu. A później postanowiliśmy zawiesić już. A nie przepraszam, to był ostatni weekend lutego, a później już zawiesiliśmy działalność.

**Powiedział Pan teraz o tych momentach ważnych z perspektywy kraju i o tym, że decyzje rządzących nadawały te różne momenty. Ale z Pana perspektywy to te doświadczenia wokół podróży (...). Jak w tych momentach - tych Pana osobistych - jak Pan się czuł? Czuł Pan jakieś zagrożenie? Czy to były jakieś inne elementy?**

Może nie zagrożenie, natomiast dla mnie to jest zawsze moment do zastanowienia się, jak ta sytuacja będzie się rozwijała? Czy my jesteśmy właściwie przygotowani do zetknięcia się z taką epidemią? Jakie to będzie miało konsekwencje? Bo dla mnie fakt wystąpienia samej choroby, epidemii z racji zawodu, nie jest dla mnie osobiście straszny, czy przerażający. Ja wiem, że muszę się w takiej sytuacji znaleźć. Jest oczywiście strach o najbliższych. Ja jestem w grupie ludzi, którzy są w szczególny sposób narażeni na infekcje w związku z tym, że pracuję w szpitalu publicznym i wiadomo, że trafiają tam pacjenci w różnym stanie. Więc zacząłem się martwić o najbliższych, czy nie przyniosę do domu infekcji. Natomiast dla mnie jest to jeden z elementów życia codziennego, zawodowego, więc mnie to nie przeraża, ja się tego nie boję. Natomiast zastanawiam się nad konsekwencjami.

I nad sposobem, jak sobie z tym poradzić.

**A jak wygląda Pana obecne życie, w tym momencie? Czy ono jest różne od tego, co było wcześniej?**

Zdecydowanie różne. Różne jest z powodów zawodowych, to o czym wspomniałem, że musiałem zawiesić działalność prywatną. To się wiąże z jednej strony z większą ilością wolnego czasu, z drugiej strony ze znacznie mniejszym przychodem. Wiadomo, że to ma wpływ na funkcjonowanie w domu. Oprócz tego - na razie o tych zawodowych aspektach mówię - zmieniono nam sposób pracy na oddziale chirurgii naczyniowej. To też wywiera wpływ  na moje codzienne życie. Pracowałem do tej pory w taki sposób typowy, 7h 35min, od 7 rano, do 14.35. A w tej chwili mam zmienioną pracę na model 12 godzin. Przychodzimy co drugi dzień do pracy. Zespół został podzielony na dwa mniejsze, po to żebyśmy się ze sobą nie stykali. Te grupy pracują razem, się nie mieszamy dyżurując czy pracując w ciągu dnia - dwa oddzielne zespoły lekarskie. I to ma też spore znaczenie. Wiadomo, że w codziennym życiu dochodzą dodatkowe odmienności - mamy syna, który ma 10 lat, jest w 3 klasie szkoły podstawowej. Wiadomo, że on do szkoły nie chodzi. Więc lekcje są prowadzone online, więc też trzeba go przypilnować. Zupełnie inaczej funkcjonuje się jeżeli dziecko zamiast 6 godzin w szkole spędza je w domu i w tym czasie ma się uczyć. Dziecko sobie kojarzy dom z odpoczynkiem, z relaksem i zabawą a nie ze szkołą. Chociaż i tak świetnie uważam zareagował i odpowiedział na te zmiany nasz syn. Natomiast jest to dla nas dodatkowy obowiązek, oprócz tego co było do tej pory. Odpadła mi część obowiązków zawodowych, natomiast doszły tego typu sytuacje.

**A co Panu w tej sytuacji najbardziej przeszkadza?**

Niepewność. Bo nie wiem, jak długo będzie epidemia trwała, nie wiem jak długo to się rozwinie. Mam świadomość katastrofalnych skutków gospodarczych. Mam znajomych, którzy mają różnego rodzaju działalności - mają zakłady fryzjerskie, hotele, hostele, gabinety lekarskie, sklepy. I wszyscy na tym cierpią. Niektórzy - tak jak powiedziałem, to katastrofa jest dla nich, bo jeżeli ktoś ma noclegi to w obecnej sytuacji nie jest w stanie w ogóle nic zarobić. Patrząc na to co dzieje się u nas w kraju dla mnie jest - to jest istotne i trudne, że  widząc co się  dzieje u nas w kraju ja nie jestem pewien co będzie z moimi znajomymi. Ja dopóki mam etat w szpitalu, to jeszcze się nie martwię o przyszłość. Tą finansową. Natomiast moi koledzy znajdują się w niezbyt dobrej sytuacji. I nie wiem, jak to będzie funkcjonowało u nich dalsze życie. W społeczeństwie. I to jest dla mnie trudne. Widzę jakiś - to jest oczywiście moja opinia, aczkolwiek wiele osób z mojego otoczenia to podziela - że zamiast żebyśmy w jakiś sposób byli otoczeniu ochroną ze strony Państwa, to próbuje się załatwiać jakieś inne interesy osobiste niektórych osób. I generalnie partii politycznych, a nie znaleźć się we właściwym miejscu i zacząć się zastanawiać, co zrobić z krajem który jak się skończy epidemia będzie w ruinie gospodarczej. No i tyle. I pomoc ludziom, bo ludzie potrzebują pomocy i w tej chwili, ale ta pomoc trochę inna. Ale trzeba się będzie zastanowić nad odbudową Państwa, bo ludzie potracą źródła utrzymania, dochodu i to nad czym pracowali wiele lat i co dobrze funkcjonowało to się w ciągu paru miesięcy rozleci. I to mnie martwi. I to mnie w jakiś sposób trochę przeraża.

**A czy widzi Pan jakieś pozytywne strony tego okresu?**

Oczywiście że tak. Z pozytywnych stron, to może taki troszkę ironiczne, ale świat zwolnił. Ludzie się trochę bardziej zastanawiają nad tym, co mają zrobić. Tak jak sobie wcześniej deptali po piętach, goniąc po sklepach, to teraz to jest troszkę, dużo wolniej i w bardziej przemyślany sposób. Cieszy mnie to, że się ludzie dostosowali do zaleceń, rzeczywiście to się widzi. Ludzie nie zachowują się arogancko, wypełniają te zalecenia naszych władz, ale też WHO. Chyba z większym spokojem zachowują się tak na co dzień - to co ja obserwuję u moich kolegów w pracy. To jest taki troszkę wolniejszy tryb życia, i zastanawiają się nad swoim życiem, nad tym jak funkcjonują z rodziną. Więcej czasu poświęcamy teraz sobie, bo siłą rzeczy będąc w domu - ci którzy nie mogą wykonywać swojej pracy zostają w domu - więc poświęcają sobie trochę czasu. Nie zawsze myślę jest to tak pozytywne jak by to mogło być, gdyby nie było nad nami zagrożenia i tych przykrych konsekwencji epidemii, że ludzie przebywając w domu tak naprawdę tracą dochód i jest to przymusowe, a nie dobrowolne. Lepiej byłoby to zrobić dobrowolnie, aczkolwiek spędzamy z dziećmi więcej czasu.

**A czy u siebie też Pan obserwuje te zmiany - że wolniej?**

Oczywiście. Ja w tej chwili co drugi dzień wolny - znaczy wolny od obowiązków zawodowych, od pracy w szpitalu. Oczywiście do gabinetu też nie chodzę. Ale to jest czas kiedy ja pomagam synowi się uczyć, pomagam w lekcjach. Robię z nim zadania, prace plastyczne i on nie jest całkiem wolny [czas]. Natomiast  co drugi dzień nie ma mnie w pracy i to jest naprawdę zupełnie coś innego. Moja praca jest mocno absorbująca i ja to odczuwam - ten dzień przerwy od pracy zawodowej jest dla mnie dobrze odczuwalny.

**Chciałabym wrócić do tego, co Pan powiedział o niepokoju i zagrożeniu Wspomniał Pan, że pojawia się strach o bliskich. Skala lęku**

Tak na 30.

**Z czym się wiąże ten strach o bliskich? Jak Pan się czuje w tej sytuacji?**

Moi rodzice mają ponad 70 lat. Są w niezłej kondycji fizycznej i nie mają większych problemów ze zdrowiem. Natomiast wiadomo, że wiek jest istotnym czynnikiem ryzyka powikłań przy infekcji tym wirusem. Więc boję się, że po ewentualnym kontakcie - a tak naprawdę zdaję sobie sprawę z tego, że prawdopodobnie prawie wszyscy będziemy mieli kontakt z wirusem - i oczywiście tylko część z nas zachoruje, a część poradzi sobie z tym bezobjawowo. Ale prawdopodobnie wszyscy będziemy mieli kontakt. Więc boję się, żeby to dla moich rodziców i dla mamy mojej żony nie była to sytuacja, kiedy oni rzeczywiście zachorują. Bo te powikłania są groźne. Jest nienaturalna dla nas odpowiedź organizmu na infekcję u tych osób, u których ona się rozwija pełnoobjawowa choroba. Sam wirus może nie wywołuje spustoszenia, ale reakcja organizmu i tego się obawiam. A wiem, że ponieważ pracuję z szpitalu i stykam się z różnymi ludźmi, my nie jesteśmy w stanie w niektórych przypadkach stwierdzić, czy ten pacjent jest zarażony czy chory, bo trafiają pacjenci w ciężkim stanie, nieprzytomni i ani nikt wywiadu nie zbierze, wykonanie testu trwa dłuższy okres - to nie jest kwestia kilku sekund, a my mamy parę minut aby człowiekowi życie uratować, więc po prostu stosując środki ochrony przystępujemy do działania. To są sytuacje, których ja wiem, że nie uniknę w mojej pracy- musiałbym się zwolnić, a to chyba nie o to chodzi, bo jakbyśmy wszyscy uciekli to byłby problem. Ale wiem, że nie mogę wykluczyć, że w trakcie takich interwencji ja się mogę zarazić i nawet nie wiedzieć o tym, że jestem nosicielem wirusa i zarazić inne osoby.

**Czy są jeszcze jakieś elementy, które (poza chorobą) potęgują ten strach?**

Oczywiście ta niepewność finansowa. Bo od 20 lat pracuję w tym szpitalu i wiem, jakie tam różne dziwne rzeczy miały miejsce i też się obawiam tego, że w pewnym momencie dyrekcja może powiedzieć "proszę państwa, przechodzimy na pracę w trybie dyżurowym, czyli będzie lekarz dyżurny, ordynator i może dyżurny który będzie miał dyżur telefoniczny i tyle bo mało operujecie bo pacjentów jest mało. I reszta nie będzie pracować, tylko będzie pan miał 3, 4  dyżury w miesiącu. I dostanie pan pieniądze za okres pobytu w pracy". Tak naprawdę my ruszamy czasami całą ekipą do działania bo tak trzeba, a później jest 3 dni spokoju, a potem znowu jest ciężki przypadek. Liczę się z tym, że mogą być różne dziwne ruchy w naszym szpitalu, bo wiadomo że pieniędzy na ochronę zdrowia zawsze brakowało w naszym kraju i będzie brakowało. Więc to mnie też martwi. A ponieważ nie jestem w stanie w inny sposób generować dochodu, to jest to też czynnik istotny.

Tak, to nie jest strach o finanse. To bardziej niepewność. Ale gdzieś tam w głowie tkwi taka myśl, która nie ustępuje, że może być różnie.

**Obrazki.**

Numer 1.

Numer 7.

Numer 4.

**Proszę opowiedzieć o 1.**

Widzę korek. Dla mnie tego typu sytuacja to jest oczekiwanie, coś się zatrzymało w miejscu, ja nie jadę, czekam. Wokół mnie ludzie mają dokładnie to samo co ja. Nie wiem, co będzie dalej. Wszyscy utknęliśmy i na razie się nie porusza nic. Trochę natłok myśli i właściwie tyle

**A 4?**

To są nasze działania w pracy. To jest dla mnie zachowanie ludzi. Rzeczywiście widzę, że społeczeństwo fajnie odpowiedziało na apele rządowe i stanęli na wysokości zadania. Nie torpedują tych działań. Ludzie się starają pomagać. Mamy lokalne inicjatywy, że ktoś potrzebuje pomocy, to można pomóc - że ktoś ma handel, gastronomię, czy coś to ludzie prowadzą lokalne akcje, że można jedzenie zamówić, kupić coś. Pewnie jest tego więcej niż było kiedyś, z większym zaangażowaniem takim ukierunkowaniem na konkretną osobę. Kiedyś się chodziło towarzysko, a teraz też po to żeby pomóc. Chodzi się np. po chleb do kogoś, albo kupić kawę. Kiedyś to było przy okazji, a teraz jest to przemyślane.

**A jeśli chodzi o 7?**

Tu jest trochę szaro, i tak to w tej chwili dla mnie wygląda. Nie ma za bardzo miejsca na jakąś radość. Jest trochę pusto, bo rzeczywiście wokół nas jest pusto. I tyle. I zobaczymy co będzie dalej.

**A który z tych obrazków pokazuje emocje pozytywne, a które negatywne?**

Pozytywne to 4.

1 - jest dla mnie oczekiwaniem. Powiedziałbym, że to obojętne, ale może trochę negatywne.

A 7 trochę negatywna. Bardziej niż 1.

**A która z emocji tych jest u Pana dominująca w tym momencie?**

Niepewność to nie jest emocja.

**Ale czy wokół tej niepewności nie powstają jakieś emocje, np. niepokój?**

W związku z tą sytuacją,   to chyba najbardziej ten niepokój. Ale ja nie mogę powiedzieć, ze jestem stale zaniepokojony.

**Bo to się przeplata  z innymi odczuciami?**

Oczywiście.

**Co wpływa na te emocje?**

Wpływa sytuacja. Znowu nawiążę do tego, co już mówiłem, że w mojej opinii epidemia została wykorzystana przez naszych rządzących do załatwiania swoich spraw. Ja widzę, że my jesteśmy jako ludzie marginalizowani, w konfrontacji z tym, że trzeba wybory przeprowadzić. Przepraszam, że tak dosadnie powiem, że to jest jedna z rzeczy, która mnie bardzo mocno męczy i zamiast żeby ktoś mówił, jakie mamy propozycje i plany wyjścia z bardzo trudnej sytuacji - trudnej gospodarczej. I zamiast pomyśleć o ludziach, jak im zapewnić przyszłość- tym którzy stracili, tracą i stracą źródła utrzymania to się mówi o głosowaniu. I się przemyca jakieś dodatki dziwne w sejmie, po to żeby umożliwić przeprowadzenie wyborów. W tym momencie jest to skandaliczne. To jest coś okropnego. Ja z natury jestem liberalnie nastawiony do życia i ludzi, ale tu się  po prostu nie da spokojnie. To jest coś okropnego. Są  ludzie zostawieni samym sobie i niech się martwią. Są jakieś propozycje pomocy, ale małej garstce ludzi. To są propozycje dla ludzi, które tak naprawdę nie za bardzo obciążą Państwo, a będzie można powiedzieć, że przecież pomogliśmy ludziom. A jak to jest mały czy duży przedsiębiorca, to jak straci źródło dochodu to tak samo będzie cierpiał. Jak ktoś zatrudnia 1000 osób, to nie znaczy że sobie poradzi. Jak sobie ma poradzić, jak utrzymuje ileś osób.

Dostrzegam też to co się dzieje pozytywnego [działania rządu]. Mamy zaangażowane osoby, które chcą coś zmienić. Może niekoniecznie z samego topu elit politycznych. Ale są ludzie, którzy chcą pozmieniać - naukowcy, którzy będą mieli jednak jakiś wpływ pomimo polityków. Nie jest całkiem źle, ale trzeba się zastanowić czy rzeczywiście forsować niektóre pomysły w tym momencie, czy to jest ważne. Moim zdaniem nie. Są rzeczy ważniejsze.

Kolejna rzecz, która budzi we mnie nie tylko niepokój, ale i wściekłość. Takie emocje jak złość. Ja mogę to podejrzewać, bo ja dowodów nie mam natomiast moim zdaniem społeczeństwo jest okłamywane. Jeśli chodzi o ilość zachorowań i sam przebieg badań - u ilu osób te badania są wykonywane, u ilu osób one nie są wykonywane, tam gdzie powinny być. To budzi moje zastrzeżenia i przeświadczenie że celowo się zaniża dane o ilości zachorowań, bo jest to świetny sposób na manipulowanie nastrojami społecznymi. Powiedzieć, że jest wspaniale. Że w krajach sąsiadujących z Polską jest  dużo gorzej, a u nas jest świetnie, bo żeśmy sobie poradzili. No przepraszam, ale takie bajki to można opowiadać, ale nie ludziom z branży. Epidemiologia jest bezwzględna i tyle. To budzi u mnie emocje.

**A te emocje, jak one się zmieniały w kontekście tych ważnych etapów, o których Pań wspomniał?**

Wściekłość mi narosła na działania naszej ekipy rządzącej na pewien poziom i ona się utrzymuje cały czas. Niepewność - myślę, że też na tym samym poziomie, bo na razie powiedzmy, że ta sytuacja nie jest bardzo dramatyczna. Mamy zaopatrzenie w sklepach, mamy paliwo, jest prąd nie ma przerwy w dostawie. Jeszcze się możemy przemieszczać, to nie jest tak bardzo rygorystycznie zakazane jak w niektórych krajach gdzie trzeba specjalnych pozwoleń, żeby opuścić dom i ludzie muszą się logować w serwisach, które im wydają pozwolenie. Na Cyprze chyba jest taka sytuacja, że trzeba uzyskać smsa z pozwoleniem na opuszczenie miejsca zamieszkania - żeby wyjść do sklepu, do lekarza, gdziekolwiek. W sklepie też można dostać wszystko u nas, pomimo krótkich epizodów takiej lekkiej paniki Polaków, którzy wykupowali makarony, kasze, ryż i papier toaletowy. To się wszystko uspokoiło.

Przy zachowaniu tych reguł zmniejszających ryzyko kontaktu z osobą zakażoną, powiedzmy że się da w miarę normalnie żyć. Ja mówię tylko o sobie, bo nie mówię o osobach, które straciły źródło utrzymania Bo to zupełnie coś innego.

Moje emocje się utrzymują na jednym poziomie, ale widzę, że będziemy mieli zachorowania. Początkowo ich nie było w Przemyślu, w tej chwili mamy już osoby, które mają chorobę rozpoznaną, i potwierdzoną obecność wirusa. Wiemy o tym, są w szpitalu, wymagały leczenia. Tego się spodziewałem, więc nie ma jakiś wielkich zmian.

Oczywiście w związku z tym,   myśli podjęli jakieś kroki. Ja się postanowiłem nie spotykać z rodzicami - ograniczam się do zrobienia im zakupów jeśli tego potrzebują i przez drzwi. Telefon i tyle. Tak, żeby zmniejszyć ryzyko infekcji.

**Chciałabym porozmawiać trochę o sposobach radzenia sobie. Jak to było na początku?**

Na początku ta sytuacja nie wymagała jakiś działań, dopóki nie potwierdzono zachorowań w kraju, dopóki się sytuacja nie zmieniła na tyle, że była potrzeba ograniczenia kontaktu z innymi osobami. Myśmy wiedzieli, że jeśli ktoś przyjeżdża z zagranicy i jest potencjalnym nosicielem wirusa, więc ludzie też podchodzili do tego odpowiedzialnie. Ale też mam z lokalnych informacji takie które mówią, że nie wszyscy się dobrze zachowywali. Ale na początku nie trzeba było nic robić tak naprawdę.

**A kiedy zaczął się ten moment, kiedy trzeba było podjąć jakieś działania?**

Dla mnie pierwszą zmianą to był moment, kiedy zawiesiliśmy funkcjonowanie gabinetu. Bo doszliśmy do wniosku też przy okazji tych operacji, które wykonywaliśmy u pacjentów - bo w prywatnej praktyce operujemy - więc rozmawiając  z pacjentami, żeby potwierdzić zabieg zaczęliśmy wypytywać, czy oni rzeczywiście tego potrzebują, czy koniecznie chcą żeby ten zabieg przeprowadzić, że może warto się zastanowić, żeby przełożyć na inne czasy, jak to zagrożenie będzie mniejsze. Myśmy nie mieli wtedy zachorowań w Przemyślu, ale pojawiały się informacje o tym, że są na terenie województwa pierwsze zachorowania - to chyba 2 osoby były. I myśmy sugerowali pacjentom, żeby się zastanowili i może zrezygnowali z tego zabiegu. I część osób zrezygnowała. Wiadomo, że to dla pacjenta czasem jest trudne, bo sobie niektórzy ustawiają tak funkcjonowanie w pracy i w domu, niektórzy się opiekują członkami rodziny, więc tego typu sytuacja musi być wcześniej zaplanowana [tu o tym, jak pacjentom było trudno]. To samo było z przyjęciami diagnostycznymi.

**Jak Pan spędza czas mając mniej obowiązków zawodowych? Jak Pan spędza czas?**

Wiadomo, że odpadły nam spotkania ze znajomymi. Całkowicie właściwie. Oczywiście mamy dostęp do Internetu, więc mamy szanse zrobić sobie  wideo-konferencję, co nam się zdarzyło. I było to całkiem fajne.

Aczkolwiek nie ma szans, żeby to zastąpiło normalny kontakt. Ponieważ lubię jeździć na rowerze, a mieszkam w takim miejscu, gdzie mieszkam na skraju wsi i wjeżdżam do lasu, więc nie jest to problem. Nie jest to zabronione, mogę sobie  na ten rower pojechać i też byliśmy rodzinnie - bo i żona jeździ i syna wdrażamy w temat. Nie chodzę na treningi, na które chodziłem, na spinning - to odpadło. Wpiąłem sobie jeden z rowerów w trenażer i jeżdżę. I tyle.

Wokół domu robiłem robię tyle samo, co robiłem wcześniej. W domu robię, to co robiłem.

Wyjeżdżaliśmy z rodziną, ponieważ mieszkamy w pobliżu Bieszczadów i dla nas to 1,5 godziny drogi, to już od czasu jak się zaczęła epidemia to nie mogliśmy pojechać, bo baza noclegowa odpadła. Pewnie w tym czasie byśmy już 2-3 razy w Bieszczadach byli. To jest na pewno strata odczuwalna.

Oczywiście cała ta sfera spotkań towarzyskich to jest istotna - nie chodzi o duże spotkania, ale czasem wychodziliśmy do restauracji, na obiad, kolację. I to jest odczuwalne.

**Co dają te aktywności sportowe?**

My się z kolegami śmiejemy, że sobie głowę wietrzymy. Relaks po bardzo dużym obciążeniu jakie mamy w pracy. Dla mnie to taka możliwość odłączenia się od myślenia o pracy, o konsekwencjach jest bardzo ważna. Ja muszę mieć trochę relaksu, a rower to umożliwia. Można się zmęczyć fizycznie, a to daje odpoczynek psychiczny.

**Czy podczas epidemii ta jazda na rowerze spełnia jeszcze inną funkcję?**

To tak samo. Poza tym, to jest kwestia utrzymania w miarę przyzwoitej kondycji fizycznej.  Bo to też chodzi o odpowiednie traktowanie swojego organizmu - ja jestem w takim wieku, że muszę o to zadbać.

Poza tym jazda w tych okolicach daje też efekt estetyczny, bo jazda w piwnicy z komputerem, to czysto treningowo. I to nie daje takiego relaksu, jak jazda na świeżym powietrzu z pięknymi widokami.

**A jeśli chodzi o te zdalne spotkania ze znajomymi. To jak Pan się dzięki temu czuł?**

Troszkę radości. Wymiany poglądów. Bo znajomi z Warszawy. I my tu jesteśmy  w Przemyślu i okolicach trochę z dala od głównego nurtu. Więc świadomość tego, co się dzieje - to co mówiłem wcześniej o zachowaniach naszego rządu i Państwa nie jest to u nas tak bardzo odczuwalne jak w dużych miastach. I opinie ludzi,  którzy prowadzą różnego rodzaju działalności, mnie pozwoliły jeszcze bardziej dokładnie przyjrzeć się problemowi. Bo ta wymiana poglądów na temat sytuacji jest bardzo ważna, i ona jeszcze bardziej  dosadny sposób pokazała niektóre niekorzystne konsekwencje tej sytuacji.

**Co jeszcze jest wyzwaniem dla Pana w tej sytuacji jeśli chodzi o ograniczenia?**

Wczoraj z żoną zastanawialiśmy się, czy wolno nam po wczorajszych aktualizacjach zaleceń, czy wolno nam będzie wsiąść na rower i pojechać z synem do lasu. Bo te informacje, które są na stronach rządowych przekazywane, nie są precyzyjne. Więc to jest zawsze furtka do tego, żeby można było patrząc z mojej strony naciągnąć prawo jeśli by ktoś chciał od strony obywatela, a z kolei od strony władz, żeby ukarać obywatela za coś co niekoniecznie jest precyzyjne, a ktoś sobie tak to zinterpretuje. Dla mnie niemożność precyzyjnego odbioru komunikatów jest niedobra. Nie lubię tego - to jest dla mnie też uciążliwość. Bo chciałbym wiedzieć, czy wolno, czy nie wolno. Niektóre sprawy są dość jasno określona: nie wolno wchodzić do parku,   nie wolno wchodzić na bulwary, promenady. Nie spotykamy się w restauracjach, barach, kawiarniach, itd., ale niektóre sprawy nie są tak oczywiste.

Utrudnieniem bardzo dużym jest to że nie ma zajęć w szkołach dla dzieci. Bo w jakiś sposób musimy się do tego przystosować i zająć się synem w tych godzinach, kiedy on powinien uczyć i tego przypilnować. Do tej pory robili to nauczyciele, teraz musimy to zrobić my. A ja nie jestem nauczycielem, tylko lekarzem. Wiadomo, że poziom opieki jest bardzo różny od tego, co moje dziecko ma na co dzień  - chodzi mi o taki poziom profesjonalizmu jeśli chodzi o poprowadzenie zajęć. Nawet jeśli on ma materiały z intranetu, to  ktoś kto jest nauczycielem zrobi to w zupełnie inny sposób. Inaczej dziecku wytłumaczy - ja jak nie potrafię  najprawdopodobniej. A może potrafię i robię to lepiej? Ale wydaje mi się, że nie. I to jest duże obciążenie. Tym bardziej może dla mnie to, że ja mam pracę, ale żona ma możliwość pracy w domu i też przypilnuje. Ale gdybyśmy razem pracowali, np. tak jak to często bywa w szpitalu, że jest małżeństwo lekarzy i oboje idą do pracy, to jest problem z dziećmi. Akurat ja nie mam z tym kłopotu. Tak się sytuacja ułożyła - i to jest niezwykle wygodne. Ale gdybyśmy razem chodzili do pracy, to byłby spory problem, co dalej? Bo ja postanowiłem, że do dziadków nie jeździmy. Mój syn się z dziadkami też nie widuje. A pomagali nam bardzo, więc to jest duże obciążenie.

**A jeśli chodzi o elementy rozrywki - kino, teatr?**

Tak, myśmy chodzili do kina. Mam w szufladzie trzy bilety na koncerty, które poodwoływano. Zobaczymy co będzie - na razie się tym nie przejmuję. Poczekamy, zobaczymy. Organizatorzy tych wszystkich imprez zobowiązali się do zmiany terminu. Niektóre terminy już mam podane, aczkolwiek uważam, że z jednego koncertu dalej nic nie wyjdzie. Bo ktoś ustalił na czerwiec. Ale to według mnie nie zafunkcjonuje.

**A jak radzą sobie inni?**

Mogę powiedzieć o ludziach, których najczęściej spotykam, czyli koledzy z pracy. Muszą sobie radzić tak samo jak my. Mam koleżankę, która pracuje ze mną na oddziale. Jej mąż jest lekarzem, pracuje na oddziale pediatrii w innym mieście i oni mają spory problem z dziećmi. Co zrobić jak oboje są w pracy? Też sobie muszą radzić i sobie radzą, ale jest to duże obciążenie.

**Czy wśród Pana otoczenia obserwuje Pan jakieś przejawy paniki?**

Tą panikę czasem widać. Bywa tak. Pierwsze objawy to te masowe zakupy takich produktów - suchy prowiant, to katastrofa, to było tak żałosne. I ma kolegów, którzy ulegli tej panice. I wysyłali wiadomości "kupuj makarony", bo nie wiadomo co będzie. No tak naprawdę to nie wiadomo, co będzie ale to było ze 3 tygodnie temu i to się szybko uspokoiło. Po 2 dniach już nie było problemu z towarami.

W pracy też mam sytuacje kiedy osoby z pozoru przygotowane do tego typu sytuacji jak epidemia i z wykształceniem medycznym powinny troszkę inaczej reagować. A reagują paniką. I wariują. Powinny wiedzieć, bo są przeszkolone i są to fakty ogólnie znane, że pewnego rodzaju zachowania są nieistotne z punktu widzenia epidemiologicznego. Nie powinno się ich wdrażać, bo są tylko przejawem strachu, nieuzasadnione zupełnie z medycznego punktu widzenia. A te osoby się upierają, żeby coś tam zrobić. Ciężko im wytłumaczyć. I dopiero relacja przełożony-podwładny może to rozwiązać. Bo mówi się "zakaz tego i tego". I dopiero to działa - w sensie wykonania polecenia i dopasowania się do sytuacji, natomiast podejrzewam, że w głowie dalej jest bałagan.

**Jak zmieniły się Pana zachowania jako konsumenta? Czy Pan kupuje inaczej?**

Pewnie, że tak. Rzadziej chodzę do sklepu. Kupujemy więcej rzeczy na raz, ale rzadziej, bo to żeby mniej przebywać w sklepie. Może to nie było bardzo częste, ale zdarzały mi się sytuacje, kiedy sobie szedłem do sklepu, aby sobie pooglądać różne rzeczy. Miałem zamiar kupienia czegoś i nie tylko przeglądanie Internetu, ale też wyjście do sklepu. Po to, żeby pooglądać, wziąć do ręki - to tego nie ma w tej chwili. Jest wyjście w określonym celu.  Rzadko miałem takie momenty, żeby sobie pochodzić po sklepach, ale bywało. Teraz w ogóle tego nie ma.

Nie robię zapasów.

**A zakupy online?**

Z racji na charakter mojej pracy, ja dużo zakupów robię online. Problemem mogą być drobne rzeczy, które do tej pory kupowałem w sklepach na przykład ze sprzętem gospodarstwa domowego - mogłem podejść kupić ładowarkę, rozgałęziacz, kabel, czy coś takiego i to było dostępne na wyciągnięcie ręki. Przejrzałem 6 produktów i po takie drobiazgi czasami mi się zdarzało pojechać do sklepu wracając z pracy. Większe zakupy, poważniejsze, przemyślane najczęściej robię przez Internet, ponieważ ciężko mi inaczej - jak chcę coś konkretnego, to najczęściej tego w sklepie nie ma, tak żeby pooglądać. I mogę te produkty odebrać kiedy chcę, bo korzystam z paczkomatu. Zakupy online są dla mnie wygodne od bardzo dawna i to się nie zmieniło.

**Zakupy rzadziej, ale czy produkty się zmieniły?**

Nie.

**Skąd się wziął koronawirus?**

Teorie spiskowe, tak? Skąd się wziął? Nie chciałbym wyrażać bardzo pewnych opinii, bo ja w ogóle nie mam pewności, bo ten typ wirusa funkcjonuje na świecie. Od zawsze. Jest to jeden z rodzajów, typów i tyle. I to czy on jest bardziej zjadliwy, czy mniej to zależy od mutacji jakie przejdzie wirus. Więc patrząc na to z racjonalnego, medycznego punktu widzenia to nie mamy do czynienia z niczym nowym i niczym dziwnym. Tego typu infekcje były i będą. Akurat ten typ wirusa powoduje bardzo duże spustoszenie z racji odpowiedzi na infekcję. Oczywiście możemy podejrzewać, że ktoś próbuje manipulować gospodarką, ale tak naprawdę tego typu opinie łatwo sformułować, dopasowując je do sytuacji podać różne rzekomo mądre uzasadnienie. Myślę, że nie chciałbym się wypowiadać na ten temat. Nie mam dość informacji. Opieranie się tylko i wyłącznie na opinii Internetu, bez znajomości faktów, czyli struktury wirusa, sekwencji nukleotydów w kwasie, jest bezpodstawne. Muszę popatrzeć na to z punktu widzenia naukowego i zgodzić się z tym, co zostanie opublikowane - to może być manipulacja, ale może to też być prawda. I tyle. Tu się musi nauka wypowiedzieć. Porównać wirusa z tym, co było do tej pory. I to naukowcy mogą powiedzieć, czy mamy tam jakieś modyfikacje, które są w kodzie genetycznym. Czy one są prawdopodobnie skutkiem mutacji, która u wszystkich wirusów ma miejsce, tak jak z wirusem grypy [tu o grypie]. Mogło stać się tak, że on ten typ wirusa tak zmutował, że stał się bardzo groźny.

Nie jestem zainteresowany aż tak dociekliwym badaniem tematu.

**A czy dało się zapobiec epidemii?**

Nie. Uważam, że jest to naturalne następstwo globalizacji. Mamy wspaniałe czasy, bo każdy może sobie wsiąść w samolot i polecieć  gdzie chce. Ceny biletów lotniczych stały się na tyle przystępne dla sporej części społeczeństwa, że latamy po całym świecie. Towary produkowane są przez kilka krajów na świecie. I te towary się rozpowszechnia na cały świat. Z jednego punktu, z Azji. Więc nie da się zapobiec. Jak ktoś jeździ po świecie i widzi wszędzie te same marki, te same reklamy - mamy w sklepach te same produkty. Jakby ktoś chciał, to może kupować na całym świecie takie samo jedzenie, mniej więcej. Mamy globalizację, przepływ czynnika ludzkiego i towarów. I nie da się zapobiec rozpowszechnianiu się tak agresywnego czynnika jakim jest ten obecny wirus.

**A czy świat jest przygotowany?**

Nigdy nie będzie przygotowany. Epidemia to jest coś, co jest moim zdaniem zaskakujące, ogromna ekspansywność, która jest nie do opanowania przez ludzi w chwili kiedy powstaje. No i skutki. Bo epidemię możemy mieć, natomiast w zależności od tego, jaki jest to czynnik, takie wywołuje skutki. I my się możemy tym przejmować, albo nie. Ale tych epidemii gdyby się zastanowić z takiego naukowego punktu widzenia nad czynnikiem który je wywołuje, to pewnie jest ich całe mnóstwo. Tylko one nie były istotne, więc nikt o nich nie mówił. A gdyby zbadać ludzi i sprawdzić, jak się rozprzestrzeniają różnego rodzaju drobnoustroje, to by się okazało, że mieliśmy pandemię za pandemią.

Ponieważ te drobnoustroje nie wywoływały efektów, to nich o nich nie mówił, nikt nie badał.

Nie jesteśmy przygotowani na żadną tego typu epidemię. Możemy mówić o lokalnych epidemiach grypy, nawet i chorób innych zakaźnych, które występują w Azji i Afryce. Ale tego typu zachorowania są nie do ogarnięcia.

**Nie do końca da się przygotować?**

Nie da. Przygotowanie ogólnie do masowych zachorowań - jakieś tego typu struktury są przygotowywane w każdym państwie, ale w starciu z już konkretnym patogenem i konkretnym zagrożeniem my się musimy dopiero do tego przystosować. I zgromadzić środki, przeformatować funkcjonowanie niektórych struktur, żeby odpowiedzieć na epidemię. To jest za każdym razem zaskoczeniem.

**A czy można wykonać jakieś kroki, które sprawiają, że jest się lepiej lub gorzej przygotowanym?**

Ja się nauczyłem, że w medycynie nie ma czegoś co zabezpiecza. Można traktować każdego typu  ryzyko w charakterze tylko i wyłącznie statystycznym. Czyli coś zmniejsza ryzyko wystąpienia następstw. Ale nigdy nie zapobiega. Nawet szczepionki nie zapobiegają, tylko zmniejszają ryzyko.

To jedynie wypełnianie zaleceń, które wszyscy powinniśmy już znać. To zmniejszy ryzyko infekcji u osób. Nasz stan zdrowia, który sobą prezentujemy też ma przecież znaczenie. To czy nasz układ odpornościowy jest w dobrej kondycji, czy nie. To ma znaczenie jak my przechodzimy infekcję. W tym przypadku też.

Ale jeżeli  jest wysoce zakaźny czynnik, to głównie zalecenia epidemiologów. Ograniczenie kontaktu i czekamy na to, aż naukowcy wyprodukują albo lek, albo szczepionkę. Bądź czekamy aż wirus zmutuje i przestanie być tak agresywny.

**Decyzje polskiego rządu - jak generalnie ocenia Pan?**

Myślę, że oni balansują między sytuacją kiedy zostaną oskarżeni o to, że nic nie robią... Może jako rząd chcą być postrzegani  jako dobry rząd, który pomaga ludziom, dba o to, żeby ograniczyć rozprzestrzenianie się epidemii, zapewnić ludziom poczucie jakiegoś minimum bezpieczeństwa, że ludzie nie są zostawieni samym sobie. Że rząd próbuje dać poczucie bezpieczeństwa ludziom, którzy tracą miejsce uzyskania dochodu. Ja to mówię z punktu widzenia rządu - że oni chcą tak funkcjonować. Bo z punktu widzenia społeczeństwa jest to zupełnie coś innego. A pomiędzy tym, żeby - ja przepraszam, ale to jest tak dla mnie widoczne, że nie mogę inaczej - a przeprowadzeniem wyborów prezydenckich. Takie mam wrażenie, że rząd się tak próbuje zachowywać - powiedzieć społeczeństwu, że oczywiście my się o Was martwimy, przygotowaliśmy tarczę ochronną dla przedsiębiorców, dbamy o was, zapewniliśmy środki odkażające, rękawiczki, maseczki, i takie inne rzeczy, a jednocześnie prą po trupach do celu, żeby przeprowadzić wybory. No jakim prawem zabrania się konsultantom wojewódzkim, specjalistom różnych dziedzin wypowiadania się na temat sytuacji epidemiologicznej? Przecież konsultant wojewódzki jest to osoba, która został wybrana przez wojewodę do reprezentowania tego województwa przed wszystkimi mieszkańcami pod względem merytorycznym. To jest ktoś kto ma, albo powinien mieć największą wiedzę na ten temat, powinien umieć odpowiedzieć na pytania, zareagować, zaproponować właściwe leczenie. Wskazać, gdzie się udać, co zrobić . Sprawuje jakąś pieczę nad specjalistami z tej dziedziny na terenie województwa. Więc jeśli ktoś taki ma zakaz wypowiadania się publicznego, a powinien w telewizji lokalnej, radio w sposób rzeczowy opisać sytuację województwa z danej dziedziny i tyle. I uważam, że społeczeństwo ma pełne prawo oczekiwać od konsultanta woj. rzetelnych informacji, móc się do niego zwrócić w razie wątpliwości i problemów. To są sytuacje  z mojego podwórka. A jeśli państwo zabrania wypowiedzi, to co się zaczyna dziać? Albo ukrywają przed ludźmi, rząd ukrywa, że ich działania są nieadekwatne, więc ewentualne opinie konsultantów będą psuły linię działania rządu, więc się tego obawiają. Gdyby grali  w otwarte karty nie byłoby tego problemu. Dlaczego tylko minister ma prawo się wypowiadać na temat sytuacji w kraju? Przecież minister nie wie, co się dzieje na Podkarpaciu. On zna statystki, natomiast poszczególnych sytuacji lokalnych znał nie będzie. Zna je konsultant wojewódzki, bo po to jest.

Następna sprawa. Tarcza? Jak ktoś mądry, kto prowadzi działalność gospodarczą przeczyta - nie trzeba wielkiej mądrości, trzeba przeciętnej inteligencji, żeby przeczytać co rząd przygotował dla ludzi, to traci się animusz i chęć funkcjonowania w takich układach. Bo wystarczy popatrzeć na to, co robią rządy innych krajów, które się znalazły w trudnej sytuacji. U nas się próbuje powiedzieć, że bierze się ludzi w opiekę, a to nie jest prawda. Tak naprawdę w dalszym ciągu obowiązek niektórych płatności zostaje na ludziach, tego się nie likwiduje tylko się przesuwa. Jeśli rząd przesuwa termin płatności składki ZUS dla większych przedsiębiorstw, o 3 miesiące, to jakie to ma znaczenie? Jeśli duży hotel, który ma 1000 miejsc noclegowych przesunie sobie składki zusowskie na za 3 miesiące, to on przez te 3 miesiące i tak ludzi nie będzie przyjmował. To są działania moim zdaniem marketingowe - rząd próbuje pokazać się w jak najlepszym świetle, natomiast to są ruchy pozorowane i ma to służyć zamknięciu ust społeczeństwu, które mówi "wy nic nie robicie", a rząd mówi "nieprawda, zrobiliśmy, to, to i to"  i tak naprawdę z tego nic nie wynika. Elektorat obecnego rządu funkcjonuje w inny sposób, więc ci ludzie, jeżeli usłyszą że rząd zrobił tyle dobrego dla przedsiębiorców to powie "ale przecież zrobili, to co się czepiacie, zamknijcie się, bo mogliście nic nie dostać, a dostaliście" - co też w Polsce jest strasznym stwierdzeniem "dostać coś od rządu". Ja tak postrzegam niestety. Z punktu widzenia mojego to są ruchy pozorowane. Ja jestem zatrudniony na kontrakcie w szpitalu, więc dla mnie zarobek jest wtedy kiedy pojawiam się w szpitalu. Jak się nie pojawiam, to nie dostaję za to pieniędzy. To jest zapłata za godzinę. To nie jest etat jak w biurze. W gabinecie tak samo. Ja straciłem obecnie dość sporo miesięcznego dochodu. I propozycje rządu w żaden sposób mnie nie dotyczą, oni mi nie pomogą. Ja się muszę sam zastanawiać, więc jeśli miałem kredyty, zobowiązania w stosunku do kolegów, rodziny - to to jest moja sprawa, żadne działania i ustawy mnie nie pomagają. A ja nie jestem dużym przedsiębiorcą. Ja mam samozatrudnienie.

Natomiast z punktu widzenia merytorycznego jako lekarz widzę oszustwo w statystykach ilości zachorowań. Rozmawiając ze znajomymi wiem, że celowo jest ograniczona ilość przeprowadzanych testów. Mnie zastanawia dlaczego osobom, które są na kwarantannie tych testów się nie wykonuje. Bardzo łatwo później pokazać statystykę, że u nas jest tylko tyle zachorowań. Skąd wziąć rzetelną ilość? Rzetelne dane dotyczące ilości zachorowań, nosicielstwa, jeśli się wykonuje mało testów? Ich będzie mało, bo się mało testów wykonuje. Porównajmy się z Niemcami, z Islandią, która jest na samym szczycie. I wtedy będziemy mogli powiedzieć, ile jest rzeczywiście zachorowań. Bo jak myśmy z kolegami obliczali ze 3 tygodnie temu, ile u nas może być zakażonych to nam wyszła ilość ponad 20-krotnie większa. Biorąc pod uwagę ilość wykonywanych wtedy testów. Teraz jest trochę więcej, więc trochę to inaczej wygląda. Ale uważam, że rząd działa nierzetelnie i celowo manipulując danymi próbuje u ludzi wzbudzić poczucie względnego bezpieczeństwa po to, żeby przeprowadzić nadchodzące wybory.

**A myśli Pan, że działania rządu uspokajają społeczeństwo, czy wręcz przeciwnie?**

Zdecydowanie w pewnych warstwach uspokajają. Bo ja to słyszę od ludzi "no może w Niemczech, we Włoszech tyle zachorowań, ale na szczęście ta Polska taka trochę inna". I to są stwierdzenia które u mnie budzą wściekłość, ale też niepokój, że ludzi mogą w ten sposób myśleć. Ludzie, którzy teoretycznie są wykształceni, ale to postrzeganie rzeczywistości jest u nich mocno zwichrowane przez - powiedzmy - wpływ działań rządu.

Jako lekarz oczekiwałbym, że premier stanie odpowiedzialnie przed narodem i powie "proszę państwa, mamy w tej chwili cel nadrzędny, czyli ograniczenie ilości zachorowań i w jak najlepszy sposób poradzenie sobie  z pandemią, przesuwamy wybory, odsuwamy na plan dalszy interesy polityczne i skupmy się na tym, co jest najważniejsze dla nas jako dla narodu, dla Polaków i odsuwamy spory i problemy polityczne na plan dalszy, a zajmujemy się rozwiązaniem sytuacji epidemiologicznej w kraju". A tego nie ma. Jak pani popatrzy na relacje z konferencji prasowej, gdzie minister zdrowia się wypowiada a obok stoi premier, to można - no, uważne oko zobaczy o co chodzi. To mi się bardzo nie podoba.

**Media. Skąd Pan czerpie informacje?**

Z portali medycznych. Portal "medycyna praktyczna", oprócz tego Ministerstwo Zdrowia. Do tego dochodzą różnego rodzaju informacje z WHO.

Dla mnie medyczne portale to są portale, które funkcjonują od wielu lat - właściwie od kiedy zacząłem funkcjonować zawodowo w Internecie, to te źródła już istniały i korzystam nieprzerwanie z tego. Rzetelne, poparte badaniami. Medycyna praktyczna jest wydawnictwem, które kiedyś funkcjonowało w formie klasycznych magazynów, a teraz świetnie też funkcjonuje jako portal do korzystania na co dzień. Tam są wytyczne, są wyniki badań - dla mnie to rzetelne źródło. W pojęciu medycznym funkcjonuje coś takiego jak evidence base medicine. I dla mnie chodzi głównie  o to - o medycynę opartą na faktach, a nie opinie nie wiadomo kogo. I to się liczy. Ja sobie nie mogę pozwolić na dywagacje.

**Aktywnie?**

Tak.

**Czas spędzany na korzystaniu z mediów? Czy większy niż przed epidemią?**

W niektórych aspektach tak. Do tej pory się  ze znajomymi nie widywałem online, natomiast jeśli chodzi o medycynę, to chyba nie więcej. Zawód mnie zobowiązuje do tego, że ja jestem stale w kontakcie z różnego rodzaju źródłami. Też charakter pracy wymaga` ode mnie stałego szkolenia, więc jestem kilka lub kilkanaście razy w roku na różnego rodzaju szkoleniach, najczęściej typu warsztatowego.  I one są zawsze połączone z częścią internetową (albo przygotowanie, albo po warsztatach i po konferencji). Nie ma szans funkcjonować bez źródeł internetowych

**Programy informacyjne w telewizji?**

Unikam jak mogę. Sam nie włączam. Czasami z żoną filmy, jakieś programy. Czasem zdarza nam się na dyżurze pooglądać i wtedy wiadomości. Ale ja nie włączam.

**Media społecznościowe?**

Tak. Korzystam

**Czy tam się pojawiają jakieś komunikaty na temat koronawirusa, pandemii?**

My korzystamy z mediów społecznościowych do porozumiewania się. I jest to często wykorzystywane medium do wysyłania informacji na szybko, w trakcie prowadzenia zabiegu - żeby się na przykład skonsultować z przedstawicielem technicznym. Jako telekonferencja.

**A przeglądanie mediów społecznościowych pod kątem informacji, które ktoś zamieścił?**

Nie, to nie ten kierunek. Media społecznościowe to rozrywka i przede wszystkim komunikacja z grupą znajomych, a nie miejsce do poszukiwania informacji o problemach medycznych.

**Po czym poznać, że medium jest wiarygodne?**

Po publikacjach naukowych. Jeżeli szukam sposobu na rozwiązanie problemu, pojawia się pacjent z rzadką chorobą a ja mam wątpliwości co do sposobu leczenia, nie mam pewności, to szukam konkretnych, najnowszych danych i wytycznych w formie np. zaleceń Polskiego Towarzystwa Chirurgii Naczyniowej. Tam mam statystykę, autorów, datę opublikowania. Wszystkie dane, które potwierdzają, że artykuł ma charakter pracy naukowej. To nie może być opinia grupy na Facebooku.

**A czy jakiś przykład mediów, które są kompletnie niewiarygodne?**

Facebook. To nie jest niewiarygodne, ale mamy tam natłok informacji i trzeba wybrać coś właściwego i tam jest ułamek promila informacji wiarygodnych w zalewie takich, które mi nie są potrzebne.
