# Supplementary material for: The challenges arising from the COVID-19 pandemic and the way people deal with them. A qualitative longitudinal study
Source: PLoS One. 2021 Oct 11;16(10):e0258133. doi: 10.1371/journal.pone.0258133 (PMC8504766; doi:10.1371/journal.pone.0258133)
Supplement: S1 Dataset — (ZIP) [file pone.0258133.s003.zip › Transcriptions/stage 1/15.1_M_43_couple, with children.docx]

**15.1_M_43_couple with children**

**Proszę powiedzieć coś o sobie.**

Nazywam się Marcin, mam 43 lata, mieszkam i pracuję w Poznaniu. Pracuję na (nazwa miejsca pracy) w Poznaniu, ale współpracuję też z innymi uczelniami. Prowadzę własną działalność gospodarczą. Jestem psychologiem w służbie cywilnej, współpracuję z (nazwa miejsca pracy) i mamy na ten moment dużo pracy. Mieszkam z rodziną - z żoną i synem, który ma 6,5 roku.

**Czy pamięta Pan pierwszy moment, kiedy ta sytuacja się w Polsce zaczęła? Kiedy Pan zauważył, że dla Pana coś się zmieniło?**

Pierwsza sytuacja, którą kojarzę, to połowa grudnia ubiegłego roku, kiedy pojawiły się pierwsze naukowe doniesienia na temat koronawirusa. Pojawiły się dywagacje na temat tego, czy i w jaki sposób wirus będzie nam zagrażał. Wtedy pierwszy raz o tym usłyszałem i nie wpłynęło to na mnie emocjonalnie - było to przyjęcie istnienia pewnego faktu.

**Czy pamięta pan pierwszą sytuację, która miała na Pana taki wpływ?**

Tak - było to zamknięcie przedszkola i konieczność pogodzenia obowiązków zawodowych z opieką nad dzieckiem oraz przejęciem obowiązku nauczania tego dziecka, ponieważ jest w zerówce. Wiązało się to z koniecznością dostosowania planu pracy i zmiany grafików, żeby pogodzić z żoną obowiązek opieki nad dzieckiem.

**Jakie z Pana perspektywy były ważne momenty rozwoju obecnej sytuacji w Polsce?**

Na pewno organizacyjne przygotowania instytucji, w której pracuję, do potencjalnej kwarantanny, ponieważ sam uczestniczyłem w tych przygotowaniach. Wtedy pojawiła się taka możliwość, że któregoś dnia pojadę do pracy i już tam zostaniemy zamknięci na kwarantannie jako służba cywilna. To była sytuacja, która wiele zmieniła z mojej perspektywy. Z perspektywy kraju, nie wiem, czy jest taki element, który bym wskazał jako krytyczny lub kluczowy. Funkcjonuję w tej rzeczywistości służby cywilnej koronawirusa, więc o części informacji wiem więcej, więc one mnie nie zaskakują w mediach. Jest to część mojej zawodowej rzeczywistości. Staram się unikać przekazów medialnych, które w dużej mierze są ukierunkowane na to, żeby w jakimś zakresie zarządzać naszym postępowaniem, czy też myśleniem. Ja te informacje posiadam z ramienia funkcji, które pełnię. Natomiast myślę, że w obiegu społecznym te pierwsze obostrzenia, które się pojawiły około 10 dni temu, mogły być taką obciążającą sytuacją, która części ludzi zapadła w pamięć.

**A jakie były ważne momenty w tej sytuacji z perspektywy pana bliskich?**

Ponownie to ten moment, kiedy dziecko przestało chodzić do przedszkola i zaczęło funkcjonować w innych realiach codziennych. Na początku była to fascynacja możliwością zostania w domu i nadmiarowego odpoczynku, a następnie możliwość wykonywania wszystkich prac wspólnie z dzieckiem w domu. Natomiast, jeśli chodzi o małżonkę, to nie wydaje mi się, że było to coś ponad zamknięcie przedszkola. Ważnym wydarzeniem było też wprowadzenie obowiązku autoizolacji osób starszych, ponieważ moi teściowie przebywają w takiej formie autoizolacji i starają się unikać kontaktów zewnętrznych. Więc z perspektywy tej dalszej rodziny, dla osób w okolicach 70 roku życia jest to duże wyzwanie. Widać też postępujące zmiany emocjonalne: wycofanie, utratę radości życia - jest to faktycznie zauważalne. U moich rodziców akurat tego nie zauważam, ale tam jest inna sytuacja, bo mój ojciec jest osobą niepełnosprawną ruchowo, przywiązaną do łóżka, więc dla niego sytuacja nie uległa zmianie - i tak z tego łóżka nie wychodził. Mama natomiast stara się pracować na tyle, na ile jest w stanie, więc też mocno nie odczuła zmiany sytuacji. Reasumując, w szerokim kręgu rodzinnym największą zmianę zauważam w sytuacji, zachowaniach i postawach swoich teściów, którzy są zamknięci w domu.

**Czy widzi pan jakieś większe zmiany w swoim życiu? Jak teraz wygląda pana życie codzienne?**

Zmieniło się o tyle, że muszę ujmować w planach zawodowych opiekę nad dzieckiem na przemian z małżonką. Z racji ograniczeń gospodarczo-ekonomicznych musiałem zrezygnować z planów, związanych z prowadzeniem działalności gospodarczej. Szereg planów został przesunięty na bliżej nieokreśloną przyszłość. Na razie jest to okres miesiąca, a jak to będzie realnie wyglądało, to zobaczymy. Musiałem przejść na system szkolenia online. Prowadzę zajęcia elektronicznie, wykorzystując systemy do e-learningu. Oczywiście więcej czasu spędzam w domu, aczkolwiek jeżdżę do pracy, ponieważ jest to mój obowiązek z racji zatrudnienia w Wydziale Zarządzania Kryzysowego. Fizycznie jeżdżę do pracy na dyżur. Na pewno mam bilans energetyczny na plusie, ponieważ spędzanie większej ilości czasu w domu sprzyja też jedzeniu. Natomiast niemożność skorzystania z oferty klubów sportowych też wpływa na zwiększenie masy własnej i to niestety nie mięśniowej.

**Co w tej sytuacji panu najbardziej przeszkadza?**

Niedostatki organizacyjne, technologiczne w organizacji pracy zdalnej. Niektóre narzędzia nie działają w systemie operacyjnym, w którym pracuję. W zasadzie, to nic więcej. Jedynie te problemy organizacyjne - dziecko w domu, trzeba to jakoś zorganizować i problemy z pracą zdalną. To są takie główne bolączki, które mi doskwierają. Natomiast w sensie takim emocjonalnym, odczuwanego stresu, napięć, lęków, czegoś takiego nie ma.

**Skala lęku.**

10.

**Czy widzi pan jakieś pozytywne strony tego okresu?**

Zdecydowanie. Nadrobiłem bardzo wiele zaległości domowych - rzeczy, z którymi borykałem się od dawna, albo takie, których nie planowałem robić, np. sprzątanie kotłowni czy też pielęgnacja zieleni wokół domu. W tym zakresie osiągnąłem duży sukces. Satysfakcja z wykonywanych prac domowych przysparza mi radości. Nadrobiłem też zaległości w zakupach online. Były to sprzęty i techniczne rzeczy, których nigdy nie miałem czasu zamówić, czy też odebrać od kuriera. Jest też więcej czasu na rozbudowę relacji z rodziną, z dzieckiem. Przykład - nigdy z moim synem nie graliśmy wspólnie w gry komputerowe, bo ja zakładałem, że nie jest mi to potrzebne do niczego, a teraz gry komputerowe, logiczne sprawiają nam dużo radości i frajdy.

**Jakie jest pana stosunek do tej sytuacji. Czy czuje się pan zagrożony?**

Nie, nie czuję się w żaden sposób zagrożony.

**Czy jest coś, czego się pan obawia?**

Chyba nie. Może ewentualnie, nie tyle, że się obawiam, ale rozpatrując mało prawdopodobną przyszłość, problematyczna mogłaby być sytuacja, kiedy moja mama by zmarła z racji zakażenia lub chorób współtowarzyszących i pojawiłaby się konieczność zorganizowania życia ojcu, który jest osobą sparaliżowaną i który mieszka 160 km ode mnie. Pojawiłby się problem, co zrobić z ojcem, czy oddać go do domu opieki społecznej czy zabrać go do siebie i w jaki sposób przeorganizować własne życie. To jest jedyny element, który, może nie tyle doprowadza do myślenia lękowego, ale czasami takie rozważania na temat technikaliów, jak można by to zorganizować, jeśli by się to wydarzyło, pojawiają się - może dwukrotnie przez ten okres przeszło mi to przez myśl. Natomiast z racji tego, że to się nie dzieje i jest mało prawdopodobne, nie zaprząta to moich myśli głęboko. Ale jak zostałem pociągnięty do odpowiedzi, to znalazłem coś, co mi przyszło do głowy.

**A jeśli chodzi o skutki finansowe lub gospodarcze, to czy jest coś, czego w tej kwestii się pan obawia?**

W zasadzie nie. Powiedziałbym, że nawet upatruję w tym jakiejś szansy, bo widzę pewne podobieństwo gospodarczo-ekonomiczne z przełomem 89/90 roku, kiedy system się zmienił. Wiadomo, że obecna sytuacja nie będzie miała aż tak dużego wpływu, ale bardzo wiele rzeczy gospodarczych i ekonomicznych trzeba będzie zacząć od początku i teraz trzeba się zastanowić, na ile i w jaki sposób można na tym zyskać. Z czysto przedsiębiorczego punktu widzenia. Jeśli jest chaos, to w tym chaosie zawsze ktoś zyskuje. W tym galimatiasie, dramacie i tragedii wielu osób, myślę, że można też odszukać jakiś pozytywów. Poza tym ta sytuacja na pewno uporządkuje wiele rzeczy, jak chociażby kontrakty w piłce nożnej, które były absurdalnie wysokie i nie odzwierciedlały realiów rynkowych. W skali mikro, dysponuję jeszcze jakimiś zasobami finansowymi w postaci oszczędności. Wiele rzeczy da się zorganizować, jak np. zawieszenie rat leasingowych za samochód. Jestem pracownikiem sfery budżetowej, więc nawet, jeśli uposażenia byłyby pomniejszane, to w moim przypadku one zawsze będą. Kuleje jedynie ta sfera prywatnej działalności gospodarczej, ale nie mam obaw gospodarczych, ani też społecznych. Ewentualnie, to, na co można zwrócić uwagę, to pewnie problemy z obniżonym nastrojem części ludzi w kwarantannie, co może w sferze ogólnospołecznej będzie zauważalne. Takie rzeczy, jak więcej prób samobójczych, więcej form agresji domowej, również powiązanej ze spożywaniem alkoholu, czy też, jak pokazują dane statystyczne, w ostatnim czasie zwiększona liczba pożarów, w których zginęło więcej osób niż z powodu koronawirusa.

**Pokażę panu teraz kilka zdjęć. Które z nich oddają pana emocje w tej chwili?**

Wybrałem 3,4,6, 12.

**3**

Jest to kawałek sztucznej nawierzchni do biegania. Kojarzy mi się to dwojako. Po pierwsze jako start do czegoś, co następuje. Mam tutaj na myśli rozwój epidemii, który dotyka mnie też w sferze zawodowej. I też mamy kolejne pokazane cyfry 8,7,6,5 i to jest forma odliczania do tego *peaku*, który będzie. Jest on nie do uniknięcia i musimy być na to gotowi. I właśnie ta gotowość, bo też musimy być przygotowani. Jeśli chodzi o jakieś stany, to wyraża on gotowość i oczekiwanie. Emocje raczej nie, raczej wyraża stan neutralny.

**4**

Bliskość, porozumienie, współpraca. Chciałbym to porównać do sfery rodzinnej, bo kontaktów jest więcej. Musimy współpracować, żeby tę sytuację dobrze znieść. Ale też z drugiej strony, z wykorzystaniem mediów społecznościowych, Messengera, nawiązujemy dużo więcej kontaktu z członkami rodziny, z którymi wcześniej mieliśmy zdecydowanie mniej tego kontaktu. Też z bliższą rodziną, jak szwagrowie, wykorzystujemy komunikatory, że porozmawiać i te więzi bardziej zacieśniać. Z jednej strony to jest taka sfera rozrywkowo-hedonistyczna. Z drugiej strony to też organizacja pomocy rodzicom i starszym członkom rodziny - od błahych rzeczy, jak wysłanie kuponu lotto, po ważniejsze rzeczy, jak zakupy. Wraz z członkami rodziny musimy skoncentrować się na pracy dla tej senioralnej części naszej familii. Tutaj na obrazku jest porozumienie, współpraca, ale też jedność. Są to skojarzenia zdecydowanie pozytywne, budzące ufność, nadzieję i dające radość.

**6**

Coś, co jest jasne i wyznacza punkt dążenia, do którego zmierzamy - pójście ku słońcu. Dążenie ku pozytywom w najbliższej przyszłości. Może niekoniecznie antycypacyjnie, że tu zaraz się wydarzy coś niesamowitego, ale docelowo myślę, że wyjdziemy obronną ręką z tego w perspektywie długofalowej. Myślę tu zarówno o sferze rodzinnej, jak i ogólnospołecznej. To pozytywny stan - powtórzyłbym ufność, nadzieję, nastrój oczekiwania, dreszczyk emocji odnośnie do tego, co będzie w przyszłości, na ile będzie to pozytywne i na ile uda się to wykorzystać.

**12**

Skojarzyła mi się z symboliką rodziny. Wszyscy są razem i są blisko siebie. Cztery kamienie, niezależnie od symboliki, kojarzą mi się z bałwanem, który zrobiliśmy w trakcie ferii zimowych, w trakcie wyjazdu na narty. Był on równie nieforemny i nieidealny jak te kamienie. Jest to element powiązany z rodziną, która przebywa razem, bardzo blisko siebie w pozytywnym sensie.

**Która z emocji jest dominująca?**

Element wspólnego przebywania całej rodziny, czyli 12, które kojarzą się z ciepłem, z budową relacji, z własną satysfakcją. Czasami, powiedziałbym nawet, że z uczuciem przyjemności, że wróciłem z dyżuru, że nic się nie wydarzyło i możemy być razem, że nie mam obowiązków zawodowych i w zasadzie mogę oddać się gotowaniu albo naprawianiu samochodu lub zabawek dla dziecka - odczucia zdecydowanie pozytywne. To coś, co bym nazwał poczuciem ulgi, to znaczy, że jestem już po pracy i mogę skoncentrować się na tym, co chcę.

**Co wpłynęło na te emocje?**

Predyspozycje osobowościowe, dostęp do rzetelnej wiedzy na temat sytuacji i przekonanie o tym, że kontroluję to, co się dzieję - mam poczucie kontroli. Ale też możliwość wyboru - z jednej strony konieczność zajmowania się dzieckiem, a z drugiej możliwość robienia wielu rzeczy, na które do tej pory nie było czasu - to jest budujące i bardzo pozytywne. Patrząc sobie na moje własne życie z przeszłości, to generalnie mam bardzo optymistyczne nastawienie do rzeczywistości, ale także nie koniecznie pozwalam płynąć temu życiu, tylko staram się dokonywać dobrych wyborów i być konsekwentnym w realizacji swoich planów rodzinnych i zawodowych.

**Jak na pańskie emocje wpływa sytuacja zewnętrzna?**

Ze względu na mniejszą ilość pracy i możliwość realizacji zadań, których wcześniej nie miałem okazji robić, powiedziałbym, że mój nastrój jest dużo bardziej pozytywny teraz, w tej sytuacji kryzysowej społecznie niż np. miesiąc temu, kiedy tej pracy było zdecydowanie więcej i brakowało czasu na życie prywatne i przyjemności prywatne. Sytuacja zewnętrzna w mojej mikroskali spowodowała nawet wzrost emocji pozytywnych.

**Jak pana nastrój zmieniał się w czasie, w zależności od rozwoju obecnej sytuacji?**

W pewnym zakresie początki były… frustrujące? Nie, frustrujące to jest za duże słowo. Ale wymagały zaangażowania intelektualnego, mentalnego w reorganizację życia. Natomiast w żaden sposób nie zdezorganizowało to mojego funkcjonowania ani nie obniżyło mojego nastroju. Nie zarejestrowałem żadnych obniżeń nastroju w typie depresyjnym ani takich negatywnych zjawisk. Oczywiście, mogły się pojawić chwilowe odczucia typu złość, bo np. dostałem jakieś narzędzie do e-learningu, które nie działało. Ale na ten moment jest to już bez znaczenia. Idąc dalej, wzrost zaangażowania w aktywność domową powodował więcej emocji pozytywnych, ale widzę, że to się ustabilizowało. Zakładam, że szczyt emocji pozytywnych mogę mieć już za sobą i teraz następuje powrót do życia, kiedy trzeba zrównoważyć pracę na rzecz domu i rodziny z aktywnością zawodową. Okres fascynacji sytuacją prawdopodobnie też już minął. Myślę, że już tego skrajnego hedonizmu już nie będzie.

**Jak pan sobie radził z sytuacją w momencie, kiedy poczuł pan, że coś się zmienia? Czy podjął pan jakieś działania, które odbiegałyby od tego, co pan robił wcześniej?**

Nie sądzę. Myślę, że były to działania typu: muszę rozwiązać problem. Czyli było zadanie, które trzeba było rozwiązać. Dla przykładu, miałem zaplanowane inaczej dyżury i w związku z nagłą koniecznością podzielenia się opieką nad dzieckiem z żoną, byłem zmuszony zadzwonić do kierownika i zmienić godziny dyżuru. Przełożeni rozumieli tę sytuację i nie stanowiło to problemu. Oczywiście były też takie działania aprowizacyjne, czyli, nie szalejąc oczywiście, trzeba było kupić dwie dodatkowe rolki papieru toaletowego, karton wina, zamrozić trochę chleba, trochę białka zwierzęcego. I to tyle - to były rzeczy wymagane przez sytuację. Działanie zadaniowe. Nie rwałem sobie włosów z głowy, nie zdezorganizowałem się.

**Co się zmieniło w zachowaniach pana jako konsumenta?**

Może minimalnie zwiększyłem zapasy domowe. Zawsze jakieś miałem, ponieważ rzadko robiliśmy zakupy, kupowaliśmy większe ilości różnych rzeczy. Poza tym kupiłem 3 pudełka klocków LEGO na zapas, czego wcześniej nie robiliśmy w rodzinie. Tankując samochód, zatankowałem 10 litrów dodatkowego paliwa do kanistra. Nie wiem, czy jest to racjonalne, bo koszt paliwa zaczął maleć, więc chyba straciłem na tym interesie, ale okej, to było działanie, które zdawało mi się właściwe w tamtym okresie.

**Jakie dodatkowe produkty kupił pan w formie zapasów?**

Głównie produkty spożywcze. W niewielkim zakresie kasze i makarony. Mam zaprzyjaźnioną rolniczą spółdzielnię koło Poznania i oni mają sklep online, więc zamówiłem różne warzywa i przetwory w słoikach, więc powiedzmy, że podwoiłem zamówienie, jakie zwykle składałem. Zazwyczaj kupowałem 15 słoików za 100 zł, a teraz kupiłem 30 słoików za 200 zł. Natomiast rzeczy zwyczajne codziennego użytku kupiłem w Lildlu.

**Czy były to jakieś konkretne marki produktów?**

Spółdzielnia Rolna Dąbrówka, produkty własne Lidla, ale to kupowałem od zawsze. Nawyki się nie zmieniły.

**A jeśli chodzi o chemię do domu - tego też kupował pan więcej?**

Tak, kupiłem płyn do dezynfekcji sprzętu elektronicznego, do laptopa, klawiatury i telefonu. Kupiłem to na bazie doświadczeń zawodowych, ponieważ u nas w pracy wprowadzili obowiązek używania takich produktów i postanowiłem kupić też do domu. Kupiłem też takie ogólne płyny do dezynfekcji i to jest takie nowum, którego wcześniej nie kupowaliśmy.

**Wcześniej wspomniał pan o zakupach online. Czy w tym zakresie się coś zmieniło w związku z epidemią?**

Kupuję więcej i takie rzeczy, które miałem w planach, ale nie mogłem tego zrealizować, czyli np. 40 metrów sztucznej trawy, zakupione online i przywiezione przez kuriera. Po to, żeby w końcu zrobić boisko do piłki nożnej, żebyśmy czasem z synem pokopali. To są tego typu dość nietypowe rzeczy, które gdzieś w głowie kiełkowały i teraz jest możliwość realizacji tego.

**To miało dla pana głównie funkcję przyjemnościową?**

Nie jestem fanem piłki nożnej, ale zabawy z piłką z synem lubię. Ale też druga sprawa, jest to tańsze niż płytki czy glazura w garażu.

**A wracając do zapasów, o których pan wspomniał, z jakiego powodu pan się zdecydował, żeby je zrobić?**

Na wypadek trudnych do przewidzenia zdarzeń, które w mojej opinii są mało prawdopodobne, ale zawsze należy sobie pozostawić pierwiastek niepewności co do przyszłości. Stąd też te dodatkowe produkty. Na wszelki wypadek, kiedy nie da się w 100 procentach przewidzieć przyszłości. Służyło to zabezpieczeniu na wszelki wypadek, gdyby miało się coś negatywnego wydarzyć, np. że spadłbym ze schodów i złamał nogę. Przygotowanie na coś, co jest trudne do przewidzenia i kontrolowania, ale w jakimś niewielkim zakresie jest możliwe.

**Obecnie robi pan zakupy spożywcze rzadziej?**

Najczęściej robiliśmy zakupy ran na dwa tygodnie i teraz mniej więcej te dwa tygodnie mijają, więc na ten moment częstotliwość jest taka sama. Ale może za tydzień, jak będziemy rozmawiali i będzie to trzeci tydzień, a my nie pójdziemy na zakupy, to wtedy będę mógł powiedzieć, że częstotliwość będzie rzadsza.

**Czy teraz pan ma więcej czasu wolnego niż wcześniej?**

Pytanie, co dokładnie oznacza czas wolny. Jeśli mówimy o czasie na realizację swoich bardzo indywidualnych potrzeb, to chyba mniej. Natomiast zdecydowanie więcej czasu jest na sprawy rodzinne. Nacisk został położony na czas wolny spędzany wspólnie z rodziną, a niekoniecznie na rozwój indywidualny. Czytam mniej, słucham mniej muzyki, zdecydowanie mniej czasu spędzam ze znajomymi.

**Jak ten czas spędzany z rodziną zmienił się w porównaniu do tego, co było przed epidemią?**

Głównie działania domowe powiązane z reorganizacją domu. Można posprzątać, ale też zrobić rzeczy, których się wcześniej nie robiło. Jak budowa bazy na środku salonu z mebli. Bardziej w tym kierunku. Trochę wspólnej zabawy, ale też porządkowania domu. Sprzątanie kotłowni potrafi też zaangażować całą rodzinę. To zmiana i ilościowa, i jakościowa.

**Czy w porównaniu do pierwszego momentu obecnej sytuacji dla pana, czyli jak syn przestał chodzić do przedszkola, do teraz coś się zmieniło?**

Taki euforyczny stan z początku, że można robić dużo nietypowych rzeczy, teraz się ustabilizował i powróciliśmy do zakresu obowiązków domowych. Skoro jest czas i mamy możliwości, to możemy coś zrobić. Powróciło to do optymistycznej normy, ale już bez szaleństw.

**Co jest dla pana w obecnej sytuacji największym wyzwaniem?**

Hmm… Chyba zmiana trybu aktywności fizycznej. To doskwiera. I też pewnego rodzaju nasycenie rzeczami, na które nie było wcześniej czasu, czyli np. człowiek już nie jada wszystkich rzeczy, które na początku jadał, bo nagle była możliwość gotowania. Czyli taki przesyt. Człowiek się nasycił wieloma rzeczami: stanem euforycznym, jedzeniem, możliwością gotowania, wypicia dwóch lampek wina. Teraz niektórych rzeczy mam już pod korek, ale tych pozytywnych.

**Wspomniał pan o aktywności fizycznej. Czy mógłby pan porównać swoją aktywność fizyczną sprzed epidemii do obecnej aktywności?**

Teraz nie uprawiam sportu w domu, chyba, że jakieś pojedyncze ćwiczenia z sześciolatkiem, ale to bardziej w formie zabawy. No i ta aktywność dotyczy noszenia różnych rzeczy, sprzątania. Nie jest ukierunkowana sportowo. A wcześniej byłem osobą dosyć aktywną sportowo. Od 2 do 4 razy w tygodniu byłem bardzo aktywny w różnych obszarach: siłownia, rower, basen, bieganie. Wyzwaniem będzie dla mnie powrót do starych nawyków tej aktywności. Z natury jestem dość stabilny, co znaczy, że będę musiał ponownie zacząć przekonywać siebie, żeby być aktywnym fizycznie, żeby powrócić do takiego momentu, kiedy będę już odczuwał satysfakcję z tego. Ze względów zdrowotnych i mojej tendencji do zwiększania masy ciała, zmuszałem się do tej aktywności fizycznej. Kiedy wypadam z rytmu, powrót bywa trudniejszy. I to jest takie moje osobiste wyzwanie.

**A jeśli chodzi o inne obszary zmian, jak np. spotkania ze znajomymi, to jak sobie pan z tym radzi?**

Na ten moment nie odczuwam wielkiego braku, bo ten czas jest stosunkowo krótki. Nie prowadziłem wybitnie bogatego życia towarzyskiego. To było 1-2 spotkania w miesiącu, w związku z tym, to jeszcze nie zmieniło mojego postrzegania tych sytuacji. Natomiast mam pod opieką w pracy 70 osób, w związku z czym tych relacji międzyludzkich jest w dalszym ciągu bardzo dużo. Nie jestem odcięty od świata. A druga sprawa, wykorzystujemy wszelkiego rodzaju komunikatory, żeby podtrzymywać relacje rodzinne. Syn też dzwoni do swoich kuzynów i rozmawiają.

**A kwestia braku dostępu do kultury: kina, teatry, czy też restauracje, to odczuwa pan brak tych obszarów?**

Z racji aktywności zawodowej to w zasadzie tylko w okresie letnim mam na tyle dużo czasu, żeby pozwolić sobie na wyjścia do restauracji. Ale 2-3 dni przed odwołaniem wszystkich imprez miałem okazję być na kabarecie, więc też nie odczuwam tego w sposób znaczący. Może bardziej będzie mi brakowało koncertów, ze względu na to, że jestem pasjonatem muzyki. Z drugiej strony, jest tyle możliwości… Odkryłem, że jest Netflix, na co wcześniej nie miałem czasu. Odkryłem, że są gry komputerowe, w które mogę z synem pograć. Więc dostępu nie brakuje. Powiedziałbym, że może nawet ciut mniej czytam niż wcześniej, a teraz zmęczenie fizyczne powiązane ze zmiennością sytuacji powoduje, że mniej się chce czytać - szybciej zasypiam. Poza tym, nie zauważyłem jeszcze znaczącej zmiany, nawyki się nie zmieniły.

**Czy w kwestii zakupów widzi pan trudności lub wyzwania?**

Nie.

**Mówił pan o pozytywnych stronach bycia w domu i spędzania czasu z rodziną. A czy w tym obszarze widzi pan także jakieś trudności?**

Siedzenie w domu oznacza siedzenie i człowiek z nudów częściej zagląda do lodówki i zwiększa masę ciała, co jest negatywne. A poza tym, to nie, jeszcze nie. Nie przeczę, że w przyszłości będzie to możliwe. Skoro nasyciłem się winem i gotowaniem dobrych rzeczy, to być może siedzeniem w domu też się w końcu nasycę.

**Jak pana otoczenie radzi sobie z obecną sytuacją?**

Mieszkam w specyficznej dzielnicy - jest to osiedle ludzi starszych, i zauważam to, że ludzie się izolują, jest mniejszy ruch samochodów. Widać, że część sąsiadów w ogóle nie wychodzi z domu, a część kręci się wokół domu, organizując sobie zajęcia, jak to na Poznaniaków przystało, żeby mieć ten niemiecki ład wokół domu. Jak już wspomniałem, moi teściowie, którzy mieszkają w pobliżu, też się izolują. Na początku wychodzili, a na ten moment siedzą tylko w domu i korzystają z tarasu i części ogrodowej, nie pojawiając się w przestrzeni publicznej. U rodziców wielkich zmian nie ma. Współpracownicy uczelniani są sfrustrowani systemem online. Współpracownicy z zarządzania kryzysowego nie mają czasu myśleć o tym, ponieważ na ten moment pracują tyle, na ile są w stanie, a czas poza pracą przeznaczają na sen.

**Czy obserwuje pan jakieś objawy narastającego lęku w otoczeniu?**

Chyba jedynie u teściów, którzy wyrażali obawy, że nie wiedzą, co będzie, że opieka medyczna nie funkcjonuje na tyle dobrze, na ile powinna. Wywodzę się z rodziny lekarskiej, więc też docierają do mnie bezpośrednie negatywne informacje spoza rzeczywistości medialne, czyli np., że inaczej wygląda rzeczywistość medialna, a trochę inaczej rzeczywistość szpitalna. Wiem, że są jakieś osoby chore, które nie pojawiają się w statystykach. W przypadku teściów niepokój bierze się stąd, że mają wiarygodne źródła informacji, że te perspektywy nie są tak świetlane. Mają ponad 70 lat, więc są w grupie ryzyka i nie wychodzą z domu. A przebywanie stale w domu przez dwie osoby może doprowadzić do takiej sytuacji, że już mruganie oczu jest strasznych hałasem. Zakładam, że poziom frustracji i lęku narasta, ale też poziom zrezygnowania. U siebie widzę jakiś napęd do działania, a widzę, że moi teściowie już się wycofują. Ale np. moja sąsiadka się nie wycofuje i pracuje z grabiami na zewnątrz.

**A czy ten rosnący niepokój teściów jest w pana opinii racjonalny, uzasadniony?**

Wydaje mi się, że na ten moment już nie do końca, bo jeżeli są odizolowani i nie nawiązują kontaktu z otoczeniem, to prawdopodobieństwo zarażenia jest bliskie zera. Oprócz tego lęku jest frustracja, powiązana z tym, że cały czas się siedzi w jednym miejscu i zmęczenie się pojawia - fizyczne i może sensoryczne tym telewizorem, tym, że w kółko to samo - na śniadanie to samo, na obiad to samo. Bardzo schematyczny tryb funkcjonowania, co może być dużym obciążeniem.

**Jak się panu wydaje, skąd się wziął koronawirus?**

No właśnie, bardzo trudne pytanie *de facto*. Może podzieliłbym to na pewnego rodzaju formy przypuszczeń. Jest to prawdopodobnie jedna z form dość agresywnych mutacji szczepów wirusowych, które były już wcześniej. Nie ma tutaj żadnej tajemnicy, że materiał genetyczny SARS jest tu obecny. Powiedzmy, że jest to 90% prawdopodobieństwa. Natomiast przyznaję się też bez bicia, że, powiedzmy, w 10%, mógłbym też rozważyć element spiskowej teorii dziejów. Zakładając, że jest to naturalnie funkcjonujący wirus, modyfikujący się wewnętrznie, genetycznie, ale też w tym całym działaniu może była też ludzka ręka. Ten wirus mógłby być również wsparty działaniami, nacelowanymi na rozwój chociażby broni biologicznej. Nie jest to pierwsza tego typu sytuacja na świecie, gdzie w tej sytuacji wirus kontrolowanie, bądź też niekontrolowanie wymknął się spod kontroli w określonym celu. Zjawiska, które się pojawiają w ogólnoświatowym rozprzestrzenianiu się wirusa mogą wskazywać, że mogło być to działanie celowe. Nie w znaczeniu wypuszczenia, tylko przygotowywania tego wirusa w warunkach laboratoryjnych.

**Jeśli to byłoby działanie celowe, to kto mógłby być za to odpowiedzialny?**

Nie jestem w stanie powiedzieć. Jest to poza moją sferą wiedzy.

**A co pana zdaniem sprawiło, że ten wirus zaczął się tak szybko rozprzestrzeniać?**

Łatwość transmisji poprzez drogę kropelkową, ale też przemieszczane się ludzi z wykorzystaniem środków transportu, jak samolot czy powroty do krajów macierzystych. Dotyczyło to całego świata. Więc też paniczne ruchy migracyjne spowodowały, że transmisja wirusa była większa.

**Wydaje się panu, że można było zapobiec tej sytuacji?**

Żeby w ogóle nie doszło do epidemii, to wydaje mi się, że nie, bo od momentu ekspozycji na wirusa do wyrazistych objawów mija od kilkunastu do 21 dni, więc siłą rzeczy, ludzie zupełnie nieświadomie zaczęli rozprowadzać wirusa. Zupełnie uniknąć się nie dało. Natomiast kwestie organizacyjne, być może sprawniejsze działanie, czy też bardziej restrykcyjne decyzje administracyjne by spowodowały, że byłoby mniej ofiar śmiertelnych. Szczególnie w krajach basenu Morza Śródziemnego.

**Jakie to mogłyby być decyzje? Mógłby pan podać jakieś przykłady?**

Odniósłbym się znów do sytuacji włoskiej i hiszpańskiej. To głównie wprowadzenie pewnych ograniczeń, związanych z uczestnictwem w życiu publicznym i społecznym, czyli obniżenie liczby kontaktów. Chociażby zamknięcie restauracji, czy dobitne sugestie pozostawania w domu od władzy. Czyli to, co my mądrzejsi już o pewne doświadczenia innych, wprowadziliśmy.

**Czy uważa pan, że obecnie świat jest przygotowany na zmierzenie się z tą pandemią?**

Bardzo trudne pytanie, wręcz filozoficzne. Myślę, że mierzy się z tą pandemią - nie ma specjalnego wyboru. Korzystamy z tego, co wiemy i z tego, co mamy i staramy się przetrwać sytuację w jak najlepszym stanie. Nikt nie rozłożył rąk. Wykorzystywane są wszystkie możliwości techniczne i ludzkie, które mogą spowodować, żeby tych zarażeń było jak najmniej.

**A czy uważa pan, że Polska jest przygotowana do tej sytuacji?**

Tak, wydaje mi się, że Polska jest przygotowana. Oczywiście, siłą rzeczy, znając liczbę łóżek szpitalnych, czy też respiratorów, jeśli doprowadzimy do sytuacji skrajnego rozlewu zakażeń, mogą się pojawić te wątpliwości natury etyczno-moralnej: kogo podłączyć pod respirator, a kogo skazać na śmierć. To jest możliwe i prawdopodobne, ale nie jest pewne.

**Co sprawia, że Polska jest przygotowana?**

Przede wszystkim to, że sami staliśmy się społeczeństwem dość rygorystycznym i staramy się przestrzegać zasad unikania kontaktu. Znacząco zmalała nam liczba imprez również nieformalnych, organizowanych przez ludzi. Poprzez element strachu, staliśmy się dość karni i rygorystyczni. Ale są również te uregulowania administracyjne, wsparte siłami porządkowymi - policją, wojskiem obrony terytorialnej, to taka motywacja zewnętrzna do pozostania w domu. Ale wydaje mi się, że to my sami jesteśmy odpowiedzialni za to, co się nie dzieje.

**A jak ocenia pan decyzje polskiego rządu w sprawie epidemii?**

Hmm… Zakładam, że kroki mogłyby być jeszcze zdecydowanie bardziej restrykcyjne.

**Co pan rozumie przez restrykcyjność decyzji?**

Większość firm czy instytucji powinna ograniczyć liczbę pracowników, siedzących na miejscu - to ostatnie ogniwo, jakie nam pozostało. Jednak widać, że ludzie jeżdżą do pracy. Ten ruch poranny zmniejszył się, ale on w dalszym ciągu jest. I wydaje mi się, że nawet wprowadzenie większej liczby patroli policyjnych, takich wyłącznie prewencyjnych, byłoby w stanie powściągnąć te ruchy. Nawet biorąc pod uwagę moje doświadczenie zawodowe z ostatnich dni, pojawiają się zapytania na linii 112: „Czy mogę jechać na ryby”. To są zachowania, niekoniecznie na ten moment potrzebne, są gdzieś realizowane. Być może, gdyby na ulicach było więcej służb mundurowych, niwelowałoby to tego typu działania czy pytania o to pod nr 112.

**Jak pana zdaniem decyzje rządu rzutują na nastrój społeczeństwa?**

Myślę, że ograniczenia, które są wprowadzone, są w szerokim zakresie akceptowane.

**A myśli pan, że mogą one uspokajać społeczeństwo czy też powodować zwiększenie lęku?**

Wydaje mi się, że możemy to podzielić na ludzi w okresie adolescencji, wczesnej dorosłości, środkowej dorosłości i być może częściowo też późnej dorosłości - tutaj jest mniej napięcia i - *de facto* strzelam - ale wydaje mi się, że u ludzi w tym wieku, gdzie jest największe zagrożenie, tego lęku i napięcia jest najwięcej. Ale może to być reakcja na obecną sytuację w ogóle, a nie wynikająca bezpośrednio z działań rządu. Czym innym jest kwestia polityki - tutaj ze względu na swój zawód, nie wypowiadam się w sferach politycznych.

**Skąd pan czerpie informacje na temat epidemii?**

Z danych, które napływają do Wydziału Bezpieczeństwa i Zarządzania Kryzysowego Wojewódzkiego Urzędu Wielkopolskiego, czyli w moim miejscu pracy.

**Czy są to dane, do których tylko pan ma dostęp ze względu na swój zawód, czy są one ogólnodostępne?**

To dane, które są później przekazywane do opinii publicznej. Różnica jest jedynie taka, że ja wiem trochę wcześniej, jak one wyglądają i o decyzjach rządowych też jesteśmy informowani na chwilę przed tym, jak to jest ogłoszone publicznie. Natomiast, ja nie oglądam programów informacyjnych. W Internecie też stosunkowo rzadko czytam informacje na temat koronawirusa, ponieważ je i tak posiadam i filtr medialny nie jest mi do niczego potrzebny. Te dane czasami się różnią. To jest temat główny w mediach, nośny i łatwo go skomercjalizować na zyski z reklam.

**Czy czas, jaki poświęca pan na korzystanie z mediów, zmienił się w stosunku do sytuacji sprzed epidemii?**

Zdecydowanie się zmniejszył czas korzystania z mediów linearnych: z telewizji i z portali informacyjnych też. Zacząłem trochę bardziej korzystać z Netflixa, a informacje o sytuacji mam ze źródeł wewnętrznych. Poza tym, nie ma transmisji sportowych na żywo, w związku z czym nie ma, czego oglądać.

**Korzysta pan z mediów społecznościowych?**

Posiadam konta, ale na Facebooku pojawiam się bardzo rzadko - raz na 3 miesiące, i to głównie ze względów pracowych, kiedy trzeba coś udostępnić. Korzystam z Messengera i WhatsAppa do komunikacji. Trend, który daje się zauważyć, to mnóstwo memów, które przychodzą, ale jest tego na tyle dużo, że nie sposób się wszystkim zainteresować, więc większość tego usuwam. Tak, żebym wolicjonalnie zalogował się na Facebooka i śledził, co tam się dzieje, np. w kontekście koronawirusa, to nie.

**Czy pana zdaniem przekaz, który jest obecny w mediach na temat koronawirusa, jest wiarygodny?**

Jeśli chodzi o twarde dane, to tak, jest on wiarygodny. Trudno jest aż tak bardzo przekłamać rzeczywistość, jak jest to możliwe np. w Korei Północnej. Ale to, co się daje zauważyć, to pewne opóźnienia w przekazywaniu informacji. Pierwsza ofiara koronawirusa w Poznaniu zmarła dwa dni wcześniej niż podały to media. To jest zauważalne, ale tylko przez osobę, która jest w tym zakresie zorientowana. Natomiast nikt nie przekłamuje aż tak bardzo rzeczywistości. Media pokazują te dane i te informacje, do których mają dostęp. Być może bardziej chodzi o manipulowanie tymi danymi w obszarze zarządzania państwem. Media żywią się tym, produkują wiele treści na ten temat i są to treści wtórne, powtarzane do znudzenia. Ale wydaje mi się, że tam braku wiarygodności nie można zarzucić.

**A jak ocenia pan wiarygodność przekazu w mediach społecznościowych?**

Ciężko mi się do tego odnieść, bo sam nie korzystam. Ale media społecznościowe działają szybciej i są w stanie podać pewne dane szybciej. To wiele też zależy od motywacji osoby, która kreuje daną wiadomość. Jeśli ktoś chce być uczciwy i rzetelny, to bardzo szybko jest w stanie przekazać informację rzetelną. Ale są też ludzie, którzy modyfikują rzeczywistość we własnym interesie, starając się zapewnić większy zasięg tworzonych treści. Są to jednak tylko moje rozważania teoretyczne - nie mam wielu doświadczeń w tym zakresie.

**Jak można poznać, czy informacja jest wiarygodna czy też niewiarygodna?**

Jeśli dany przekaz jest powtarzany przez kilka stacji telewizyjnych bądź radiowych, jest on dość prawdopodobny. W mediach społecznościowych chyba nie jesteśmy w stanie tego zweryfikować. Poza tym, mamy szereg doniesień, które będą dopiero wymagały potwierdzenia, jak np. informacja, że teina zwiększa odporność na koronawirusa - nie wiadomo, będziemy w stanie stwierdzić to za pół roku po przeprowadzonych badaniach. Jeśli medium powołuje się na wiarygodne źródła i żadna ze stacji telewizyjnych czy radiowych nie dementuje doniesienia, to możemy przyjąć, że dana rzecz się wydarzyła. Jeśli replikowalne badania coś potwierdzają, to też można to uznać za wiarygodne.

**Które źródła uznaje pan za wiarygodne, a które nie? Mógłby pan podać przykłady jednych i drugich?**

Nie ufałbym wszystkim doniesieniom z mediów społecznościowych. To jest taki kanał, że w prosty sposób można wygenerować wiadomość nieprawdziwą i ją rozprzestrzenić. Czym innym jest jakość komunikatu i sposób jego podania - każdy komunikat można zmanipulować na wiele różnych sposobów. Mamy też rożne grupy kapitałowe, powiązane z różnymi opcjami politycznymi w Polsce i każda z nich ma swoją narrację i swoją bańkę informacyjną. W jakimś zakresie wszyscy podają wiarygodne informacje, ale wszyscy podają je w inny sposób, uwypuklając różne szczegóły. Tyle mogę powiedzieć, zachowując neutralność polityczną.

**Dziękuję. To wszystko z mojej strony.**
