# Supplementary material for: The challenges arising from the COVID-19 pandemic and the way people deal with them. A qualitative longitudinal study
Source: PLoS One. 2021 Oct 11;16(10):e0258133. doi: 10.1371/journal.pone.0258133 (PMC8504766; doi:10.1371/journal.pone.0258133)
Supplement: S1 Dataset — (ZIP) [file pone.0258133.s003.zip › Transcriptions/stage 2/8.2_M_30_couple, no children.doc]

**8.2_M_30_couple, no children**

**Co zmieniło się od zeszłego tygodnia?**

Nic się nie zmieniło, chyba jest podobnie, jak było, odczuwam podobne emocje, postrzegam sytuację tak, jak ostatnio.

**Obrazki**

9. Ta zaraza kojarzy mi się z jakąś klęską, nieurodzajem - jak przy burzach, trąbach powietrznych. To dosyć ponury obraz. Gdybym miał nazwać konkretne emocje, byłby to jakiś rodzaj zagrożenia, strach przed bezpośrednio widocznym zagrożeniem. Na obrazku to jest dużo bardziej nasilone, niż u mnie, w związku z koronawirusem, ale to było moje pierwsze skojarzenie, w miarę pokrewne.

**Jakie czynniki wpływają na te emocje?**

Sytuacja w Europie związana z koronawirusem na razie specjalnie się nie poprawia. Cały czas przybywa chorych, liczba zgonów rośnie. Niestety ta krzywa wciąż nie chce się wypłaszczyć i widzimy, że to obciąża i będzie obciążać gospodarkę. Oprócz tego, niepokój wywołuje sytuacja polityczna, choćby ta dotycząca zbliżających się wyborów, które nie wiadomo czy, a jeśli tak, to w jakiej formie się odbędą. W związku z tym pojawiają się jeszcze próby wprowadzania autorytarnych zmian w prawie, najróżniejszych pakietów antykryzysowych, itp.

**Co robisz, aby radzić sobie z tymi emocjami?**

W stosunku do zeszłego tygodnia, nic się u mnie nie zmieniło. Zachowuję te same środki ostrożności, tj. zachowuję odpowiedni odstęp, a przed i po zakupach odkażam ręce płynem, który jest dostępny w sklepie. Ograniczyłem kontakty ze znajomymi. Nadal wychodzę jednak na spacery z psem i zamierzam to robić. Jeśli będę musiał przemieszczać się komunikacją miejską, gdzie mógłbym mieć problem z zachowaniem odpowiedniego odstępu, założyłbym maseczkę.

**Jak się z tym czujesz?**

Dobrze, uważam, że te działania są słuszne, że tak należy postępować, aby zmniejszyć ryzyko zarażenia siebie i innych.

**Jak postrzegają sytuację Twoi bliscy?**

Myślę, że w nich stale narasta lęk. Nikt nie spodziewał się, że to będzie się tak długo rozwijać, więc mam wrażenie, że u nich to poczucie zagrożenia mogło odrobinę wzrosnąć. Choć w ich zachowaniach nie zaobserwowałem żadnych zmian. Wielu moich znajomych jest poddenerwowanych tą sytuacją. Muszą odwoływać plany, jak choćby wesele lub mają problemy związane z brakiem pracy. To są jak na razie najbardziej dotkliwe i namacalne skutki tej sytuacji - na szczęście na razie nie znam nikogo, kto byłby chory. Niektórzy wspominają też, że przez siedzenie w domu trudno jest im się zmobilizować do bardziej produktywnych działań.

**Czy w stosunku do poprzedniego tygodnia, pojawiły się u Ciebie jakieś nowe zachowania? A może czegoś jest mniej?**

Nie, jest dokładnie tak, jak tydzień temu. Wychodzę i planuję wychodzić z psem tak samo często, jak to dotychczas robiłem, a spotkania ze znajomymi ograniczyłem już dużo wcześniej. W zasadzie to tyle, co mogłem zrobić.

**Czy pojawia się coś, co zaczyna Ci doskwierać?**

Brakuje mi tych kontaktów twarzą w twarz, głównie tego. Jednak spotkania i rozmowy online to nie jest to samo. Takich ludzkich interakcji trochę mi brakuje.

**Co sądzisz o wprowadzonych w ostatnim czasie obostrzeniach?**

Jeśli chodzi o zakaz wychodzenia z domu osób niepełnoletnich, uważam, że jest przesadą - nie wiem, dlaczego ktoś, kto ma 16 lat, miałby nie móc wyjść na spacer ze swoim psem. Tak samo pojawiło się obostrzenie, że dwie osoby, nawet te mieszkające razem, nie mogą razem wyjść na spacer, a jeśli to zrobią, musi je dzielić odległość 2 m. To jest absurdalne. Rozumiem, że powinno się zachować odstęp między obcymi ludźmi, ale nie pomiędzy osobami, które ze sobą mieszkają. Z tego co kojarzę, zmieniono też, na czas świąt i okres późniejszy, ograniczenia związane z liczbą osób przebywających w kościołach. Myślę, że jest to działaniem mocno przeciwskutecznym, jeśli chodzi o walkę z koronawirusem. Także biorąc pod uwagę ostatnie obostrzenia, myślę, że nie wniosą zbyt wiele dobrego, a mogą nawet przyczynić się na szkodę zdrowia i bezpieczeństwa - tu mam na myśli liczbę zgromadzonych w kościele.

**Starasz się stosować do tych regulacji?**

Jeśli chodzi o zachowania 2 m w stosunku do domowników, nie stosuję się do tego. Chodzimy razem z dziewczyną na spacery z psem i nie uważam, żeby to w jakikolwiek sposób zwiększało ryzyko zachorowania dla nas, czy kogokolwiek innego. Jeśli chodzi o kościół, akurat nie mam potrzeby chodzenia do niego, więc te zmiany nie dotyczą mnie bezpośrednio.

**A jak to wygląda w Twoim otoczeniu?**

Nie znam akurat nikogo, kto miałby poniżej 18 lat i wychodziłby sam. Ludzie w większości starają się zachowywać odstęp, choć widzę, że raczej nie stosują tej zasady wobec domowników, tym zakazem się raczej nie przejmują. Teoretycznie przepisy nie zabraniają uprawiania sportu na zewnątrz, choć widzę teraz znacznie mniej osób biegających, czy jeżdżących na rowerze. Uważam, że jeśli ktoś uprawia sport w pojedynkę, nie dotyka przedmiotów w otoczeniu na zewnątrz, na przykład ławek, przez co mógłby potencjalnie rozsiać wirusa, to nie jest zagrożeniem dla innych, a dba o własne zdrowie, co jest nawet wskazane.

**Jak odnosisz się do wprowadzonego w ostatni czasie wyłączenia z użytku terenów zielonych?**

Uważam, że nie jest to potrzebne. Nie wiem, czemu ma służyć. W lesie ludziom jest łatwiej zachować odstęp, niż tak, jak teraz - idąc po ulicy, kiedy nie ma dostępu do miejsc z dużą ilością przestrzeni. Nie wydaje mi się też, żeby to było skuteczne.

**Co wpływa na małą skuteczność takich działań?**

Jest grupa ludzi, którzy uważają, że wszystkie te zakazy są pozbawione sensu - z różnych powodów, np. uważają, że mają świetną odporność i wirus ich nie dotknie, albo nie przejmują się i nie myślą o tym, czy mogą się zakazić. Jest też mniejsza grupa ludzi - na szczęście mniejsza - uważająca, że wirus nie istnieje i jest to spisek. Natomiast ci, którzy łamią zakazy świadomie, nie dostrzegają sensowności niektórych z tych zakazów. Niektóre przesłanki mogą być racjonalne, mam też nadzieję, że moje przesłanki w tym przypadku taki są, a niektóre mogą wynikać z niewiedzy.

**Czy zaobserwowałeś w ostatnim czasie u innych zachowania, które wydają Ci się dziwne?**

Wśród znajomych nie, jednak wśród obcych widzę, że coraz więcej osób w sklepach nosi maseczki; jednak są one noszone często w bardzo śmieszny sposób - np. nie zakrywający nosa, albo tyko zawieszony na szyi, nie zakrywający w ogóle ust. Zdarza się też, że ktoś idzie w maseczce, jednak po chwili ją zdejmuje, ponieważ utrudnia mu mówienie. Więc zaczyna już mówić, czy krzyczeć wręcz, bez maseczki. Widuję też ludzi samotnie podróżujących w maseczkach, wewnątrz własnych samochodów. I o ile w niektórych przypadkach takie zachowanie może jest uzasadnione, to wydaje mi się, że takich uzasadnionych przypadków do noszenia maseczki w takiej sytuacji jest jakiś promil.

**Czy zdarza Ci się robić zakupy przez internet?**

Tak. Stoi za tym wygoda i cena - chyba nawet w większym stopniu, ale wygoda też jest istotna. Przed wybuchem epidemii zdarzało mi się robić zakupy spożywcze przez internet z dostawą do domu lub paczkomatu około raz na dwa miesiące. W obecnej sytuacji chciałbym robić ich w ten sposób więcej, jednak dostępne terminy są już bardzo odległe. Byłoby to ograniczenie ryzyka związanego z wirusem oraz wygoda - nie musiałbym wsiadać w samochód, stać w kolejce przed sklepem, robiąc zakupy, przestrzegać wielu środków ostrożności. Wystarczyłoby rozpakować przywiezione zakupy. Nie byłbym jednak zainteresowany zakupami "przez telefon", ponieważ wydaje mi się, że ciężko byłoby mi wybrać w ten sposób towar. Jeśli chodzi o pozostałe zakupy przez internet, zazwyczaj kupuję w sieci te rzeczy, które w sklepie są dużo droższe i trudniej dostępne - chyba nie ma jakiejś specyficznej grupy produktów, które nabywam w ten sposób. Teraz to może się zmienić, ponieważ otwarte pozostają już chyba tylko sklepy spożywcze i drogerie, co może spowodować, że niektóre towary będą dostępne tylko i wyłącznie przez internet. Ostatnio na przykład kupiłem kawę i filtry do niej, zamówiłem też suplementy diety - zdarza mi się zamówić białko, ostatnio też witaminę D. Jeśli chodzi o dostawę, wybieram taką opcję, która w danym czasie jest wygodniejsza. Teraz, z racji tego, że częściej jestem w domu, wolę wybrać kuriera. W innym przypadku, aby mieć większą elastyczność, zamawiałem produkty do paczkomatu.

**Jak wygląda Twoja dieta w porównaniu do tego, co jadłeś przed edpidemią?**

Jedzenie powinno sprawiać mi przyjemność, nie lubię monotonii w diecie, staram się urozmaicać to, co przygotowuję w domu. Dieta trochę mi się pogorszyła - jem obecnie więcej junk foodu. Za to ogólnie jem trochę mniej, ponieważ ciężko mi się zmobilizować w domu do jakichkolwiek ćwiczeń, a mam tak, że jak nie ćwiczę, spada mi łaknienie, więc spożywam przez to mniej kalorii. Trochę przypadkiem zniknęło też z mojego jadłospisu mięso. Od jakiegoś czasu, gotując w domu, nie jadłem go. Jednak kiedy kupowałem jedzenie w pracy i dostępna była jedynie opcja mięsna, jadłem taki posiłek 4-5 razy w tygodniu. To było głównie z lenistwa - kiedy gotowaliśmy w domu to zazwyczaj tak, żeby mieć obiad ja dwa dni. Gdybym brał go do pracy, nie starczyłoby na wspólny posiłek po powrocie, więc musielibyśmy gotować częściej. Poza tym z życiem w niewielkim mieszkaniu wiąże się też to, że nie za bardzo jest miejsce na przechowywanie większych ilości jedzenia, gdybyśmy chcieli robić je rzadziej, lecz w dużych porcjach. Nie mówiąc o tym, że prawdopodobnie miałbym dość jedzenia tej samej potrawy przez kilka dni. Obecnie nadal nie kupuję mięsa, przez co prawie zniknęło ono z mojego menu.

**Czy coś się zmieniło jeśli chodzi o sposób przygotowania posiłków?**

Oboje z dziewczyną pracujemy teraz zdalnie, więc prawie każdy posiłek jemy wspólnie, chyba, że któreś z nas ma w tym czasie np. konferencję. Przed epidemią jedliśmy wspólnie zazwyczaj tylko obiad. Spędzamy w kuchni podobną ilość czasu, jak przed epidemią - nadal staramy się gotować na dwa dni, a oboje jemy teraz nieco mniej, tak o jeden posiłek mniej w ciągu doby.

**Czy zamawiacie jedzenie do domu?**

Tak, mniej więcej raz w tygodniu, kiedy stwierdzimy, że nie chce nam się gotować. Wydaje nam się to zupełnie bezpieczne, tym bardziej, że w większości przypadków możemy zapłacić przez internet, a dostawa może przebiegać bezkontaktowo. Zresztą, koronawirus nie przenosi się drogą pokarmową. Wolimy wtedy jednak skorzystać z oferty jakiejś lokalnej restauracji, niż dużej sieciówki. To dla nas istotne, ponieważ restauratorzy mają obecnie trudny czas - lokale są pozamykane, a dostawy stały się jedyną szansą na utrzymanie się na rynku.

**Płatności**

Na pewno więcej teraz płacę bezgotówkowo. Dotychczas i tak używałem jej bardzo mało, aczkolwiek zdarzało mi się. Dotykanie terminala ma dla mnie niewielkie znaczenie - oczywiście, jeśli mam możliwość tego nie robić, to dobrze, jednak nie mam z tym problemu. Po prostu, dla bezpieczeństwa, umyję później ręce.

**Czy byłeś w sklepie po tym, jak wprowadzono limit klientów? Opowiedz o tym.**

Robimy zakupy z tą samą częstotliwością, jak podczas naszej ostatniej rozmowy, czyli raz w tygodniu. Zdarzyło mi się już być w sklepie po wprowadzeniu limitu klientów, jednak nie jest to dla mnie problem. Jest to dobrze skoordynowane, ludzie zachowują odstępy, czekają, nie denerwując się. Nie spotkałem się, aby ktoś nie stosował się do tych zaleceń. Przy wejściu był płyn do dezynfekcji rąk i rękawiczki. Na pewno w tej całej sytuacji przyjemne jest to, że kiedy już znajdę się w sklepie, w środku ludzi jest mniej, nie muszę też stać w kolejce do kasy. Robimy listę zakupów, tak, jak przed epidemią, jednak wtedy zdarzały mi się też bardziej spontaniczne zakupy, bez listy, kiedy na przykład miałem chwilę wracając z pracy. Teraz takie sytuacje nie mają miejsca, ponieważ pracuję z domu. Przed epidemią starałem się unikać pór, kiedy spodziewałem się w sklepach największego ruchu, tj. ok. 16-19. Obecnie biorę pod uwagę godziny zakupów dla seniorów - uwzględniam około dwugodzinny margines od momentu otwarcia sklepów dla wszystkich, ponieważ spodziewam się w tym czasie większej liczby ludzi. Poza tą poprawką, jadę na zakupy kiedy akurat mam chwilę i jest to dla mnie wygodne.

**Czy zdarza Ci się kupować rzeczy, które poprawiają Ci humor?**

Czasami mi się to zdarza, aczkolwiek są to raczej drobiazgi kupione przy okazji - ciastka, lody, tego typu rzeczy. Miewam tak, że parę dni chodzi za mną ochota na jakiś produkt i to są takie momenty, kiedy w końcu to kupuję. Obecna sytuacja raczej nie wpłynęła w tym obszarze na moje zachowanie - nie kupuję ich ani mniej, ani więcej. Na święta nie planuję kupować niczego specjalnego. Gdybym miał to zrobić, pewnie byłaby to spontaniczna decyzja i byłyby to drobne rzeczy spożywcze typu piwo kraftowe - na co dzień piję tańsze, ale bardzo lubię te kraftowe i zdarza mi się kupować je dla przyjemności, lubię też próbować tych, których jeszcze nie miałem okazji pić - czekolada, czy lody. Jednak jeśli chodzi o produkty takie jak czekolada, czy lody, podejrzewam, że byłyby to produkty ze średniej półki. Droższych rzeczy raczej nie kupuję spontanicznie, staram się, żeby moje wybory były przemyślane.

**Jak planujesz spędzić zbliżające się święta?**

Pewnie spędzę je z dziewczyną i rodzicami, jednak nie będziemy spotykać się z dziadkami, czy dalszą rodziną. Nie planujemy raczej dużych przygotowań - pewnie ugotujemy i pomalujemy kilka jajek. Pewnie zrobię zakupy dokładnie w ten sam sposób, co dotychczas - kupując jedynie więcej jajek i jakieś farby do nich. Poza tym nie planuję kupować niczego szczególnego. Jeśli chodzi o jedzenie, tym zazwyczaj zajmowali się moi rodzice i wiem, że w tym roku to też nie będzie na mojej głowie. Aspekt kościoła, czy święconki nie jest dla nas istotny. To dla nas bardziej czas, który możemy spędzić we wspólnym gronie, niż jakieś wielkie duchowe przeżycie. Chociaż z Wielkanocą najbardziej kojarzą mi się mazurki, które zawsze piecze z tej okazji mama. To chyba rzecz, której byłoby mi najbardziej brak, gdyby te święta miałyby się zupełnie nie odbyć. Myślę, że gdyby był to mazurek ze sklepu, nie miałbym w związku z tym tylu pozytywnych emocji, wspomnień. Ale nie przewiduję chwilowo trudności z tym związanych. :)
