# Supplementary material for: The challenges arising from the COVID-19 pandemic and the way people deal with them. A qualitative longitudinal study
Source: PLoS One. 2021 Oct 11;16(10):e0258133. doi: 10.1371/journal.pone.0258133 (PMC8504766; doi:10.1371/journal.pone.0258133)
Supplement: S1 Dataset — (ZIP) [file pone.0258133.s003.zip › Transcriptions/stage 1/17.1_F_35_single, with child.doc]

**17.1_F_35_single with child**

**Co zmieniło się dla Pani w ostatnim czasie?**

Syn jest w drugiej klasie szkoły podstawowej, ale jest bardzo samodzielny. Nie doświadczam nadmiaru obowiązków szkolnych syna – wczoraj udało mu się zrobić wszystkie zadania w 2,5 godziny. Mieszkamy w domku, razem z dziadkami, więc w razie czego oni też pomagają w opiece. Dzięki temu dotychczas, pomimo zamknięcia szkół, nadal istniała dla mnie możliwość chodzenia do pracy. To jednak zmieniło się wczoraj, razem z rządowym nakazem zamknięcia salonów.

**Jak to na Panią wpłynęło?**

Wcześniej to był wybór, czy chodzić do pracy, czy nie. Teraz ten nakaz pozostania w domu wywołuje duży lęk o przyszłość. Nie wiadomo, jak długo salony będą musiały być zamknięte, ile to potrwa i czy po tym wszystkim klientki wrócą. Możliwe, że przez ten czas, kiedy nie będą mogły korzystać z salonów, stwierdzą, że nie muszą tego robić. Nie wiadomo, jak będzie przez to wyglądała przyszłość, jeśli chodzi o sytuację materialną. Ten nakaz zamknięcia salonu był punktem zwrotnym, wywołującym u mnie duży niepokój i lęk o przyszłość.

**Ten lęk, jak rozumiem, jest bardziej związany z sytuacją materialną, niż z samym wirusem?**

Głównie. Raczej nie boję się wirusa, staram się po prostu zachować ostrożność, dbać o siebie. Na zakupy noszę rękawiczki – nie pamiętam dokładnie, kiedy zaczęłam to robić, to było jakoś po tym, jak zamknęli szkoły. Dezynfekuję też ręce – noszę ze sobą żel, czego dotychczas nigdy nie robiłam. Boję się za to o dziadków, o ich ewentualne zarażenie. Dlatego to ja robię zakupy, zajmuję się wszystkim. Oni siedzą teraz w domu i nigdzie nie wychodzą.

**A gdyby miała Pani określić poziom swoich obaw na dzień dzisiejszy, na skali 0-100?**

50.

**Rozumiem, że wczorajsze wydarzenia są dla Pani znaczące. Czy jest Pani w stanie określić jakieś punkty zwrotne, kamienie milowe w całej tej sytuacji, która dotychczas miała miejsce?**

Zanim wirus dotarł do Polski, miałam nadzieję, że to nas jakoś ominie. Jednak kiedy zmarła pierwsza osoba w Radomiu, poczułam, że to dotyka nas jakoś bardziej bezpośrednio. To jest inaczej, kiedy ludzie umierają gdzieś na świecie, czy nawet w kraju, ale w innym mieście. To jest bardziej normalne, bardziej do zaakceptowania. Radom jest dosyć mały, większość mieszkańców się zna. Ten moment, kiedy umarł ktoś z naszego bliższego otoczenia, był bardziej osobisty. Poczułam, że to dotyka nas to personalnie.

**Czy to zmieniło coś w Pani najbliższym otoczeniu?**

Kiedy zamknięto szkoły, to był taki pierwszy moment, po którym zaczęłyśmy się z koleżankami z pracy zastanawiać, jakie działania podjąć, aby ochronić siebie i swoje rodziny. W naszym zawodzie to normalne, że nosimy maseczki i rękawiczki. Jednak wcześniej klientki mogły przychodzić jedna po drugiej, na tak zwaną zakładkę. Postanowiłyśmy wprowadzić minimum 40 minutowe odstępy pomiędzy kolejnymi osobami. W tym czasie wietrzyłyśmy pomieszczenie. Każda klienta dostawała od nas maseczkę. Stwierdziłyśmy, że jeśli one nie chcą się chronić, my musimy – tak trochę egoistycznie – zadbać o siebie i własne rodziny. Myślę, że gdyby któraś klientka odmówiła założenia maseczki na czas zabiegu, odmówiłybyśmy przyjęcia jej. Ale zakładały maseczki chętnie, bez problemów. Do wczoraj miałyśmy wybór. Przestrzegając pewnych zasad, mogłyśmy zarabiać. Teraz wszystko stanęło pod znakiem zapytania.

**Jakie są największe wyzwania w obliczu takiej sytuacji?**

Jeśli chodzi o ograniczenie kontaktów za znajomymi, to na obecnym etapie nie jest to bardzo dokuczliwe. Wszyscy pracowaliśmy, więc dotychczas te spotkania też często odbywały się przy pomocy telefonu, komunikatorów. Nie było bardzo wielu okazji żeby wychodzić gdzieś wspólnie. Za to denerwuje mnie trochę, jak teraz ktoś zabrania mi wychodzenia z domu. Oczywiście rozumiem powagę sytuacji, jednak takie obostrzenia wywołują we mnie jakiś taki wewnętrzny opór. Co innego, kiedy nie wychodzę z domu – z czym zresztą nie mam problemu – bo jest to mój wybór. Z kolei takie restrykcje wzbudzają we mnie jakiś rodzaj buntu, choć oczywiście rozumiem ich powody. Jesteśmy jeszcze w tej komfortowej sytuacji, że mieszkamy w domku, więc mamy jakiś tam skrawek własnej zieleni. Ale ostatnio rozmawiałam z siostrą, że ta sytuacja jest trochę szalona – mieszkamy na tej samej ulicy, a nie widujemy się teraz, podajemy sobie jakieś tam rzeczy przez płot, żeby ograniczyć ewentualny kontakt.

**Za moment pokarzę Pani kilka obrazków. Proszę się im przyjrzeć i wybrać jeden, który najlepiej oddaje emocje, które towarzyszą Pani w tym momencie.**

7.

**Dlaczego akurat ten?**

To jest droga, która nie wiadomo dokąd prowadzi. Wokół jest spokojnie, ale nie wiadomo, co się później wydarzy. Te emocje, które mi towarzyszą, to taka niewiadoma, niepewność.

**Co ma wpływ na odczuwane przez Panią teraz emocje?**

Na pewno właśnie te kolejne wprowadzane zarządzenia. One mają bezpośredni wpływ na moje życie. Dlatego zaczęłam oglądać na przykład wystąpienia premiera, czego wcześniej nie robiłam.

**Jak w takim razie ocenia Pani decyzje i działania rządu podjęte w związku z obecną sytuacją?**

Trochę nie rozumiem, dlaczego zamiast zamknąć wszystko w jednym momencie, to było tak rozciągnięte w czasie. Był czas, że nie można było się spotykać ze znajomymi, ale na paznokcie można było przyjść. Od wczoraj salony są zamknięte, a na przykład sklepy takie jak OBI, czy Castorama wciąż są czynne, a jest w nich mnóstwo ludzi. Możliwe, że lepszym rozwiązaniem byłoby wydanie decyzji, żeby zamknąć wszystko w jednym momencie, choć też nie wiadomo, jakie przyniosłoby to skutki. Nie wiadomo, jak będą wyglądały działania rządu, jeśli chodzi o prowadzenie działalności. I nie mam na myśli jednorazowych zapomóg, a na przykład wydłużenie terminu zapłaty lub anulowanie obowiązku zapłaty chociaż części składek, jak składka ZUS.

**Czy według Pani jesteśmy przygotowani na zaistniałą sytuację?**

Nikt chyba nie jest przygotowany, żadne państwo na świecie. Na to chyba nie dało się po prostu przygotować. Oczywiście, są podejmowane pewne działania, ale wydaje mi się, że nikt nie mógł przewidzieć, jak to wszystko się potoczy. Choć oczywiście ogromnym problemem jest brak sprzętu w szpitalach, takiego jak respiratory, maseczki. Wszystkie działania były podejmowane trochę po omacku, nie wiadomo było, jakie będą ich konsekwencje i czy dzięki nim, da się powstrzymać wzrost zachorowań.

**Skąd czerpie Pani informacje dotyczące obecnej sytuacji?**

Głownie z internetu. Ważne jednak, żeby nie były to opinie, a dane, z których sama będę mogła wyciągnąć wnioski. Mam zaobserwowane różne profile na Instagramie i Facebooku. To w większości osoby z wykształceniem w danej dziedzinie – profesorowie, lekarze, pracownicy danych branży, studenci. Na przykład jest taka studentka medycyny, która studiuje we Włoszech i obserwuję ją na Instagramie.

**Czy znalazła Pani w ostatnim czasie jakieś nowe źródła informacji?**

Tak, były polecane przez osoby, które obserwuję już od dłuższego czasu. Ale znalazłam w ten sposób jeden, czy dwa profile, nie więcej.

**Jak dużo czasu poświęca Pani na śledzenie bieżących informacji?**

Zdecydowanie więcej, niż przed pandemią. Jednak to ważne, aby informacje pochodziły od autorytetów. W sieci krąży dużo fakenewsów – jest wiele osób, które mówią, że coś słyszały, choć nie są pewne, czy to prawda. Nie bardzo rozumiem, po co w takim przypadku dzielą się takimi informacjami. Wielu dziennikarzy wyciąga na przykład błędne wnioski z tego, co przeczytali lub usłyszeli. Dlatego też sama oglądam choćby wystąpienia premiera – postanowienia wprowadzane przez rząd dotyczą mnie bezpośrednio, więc chcę mieć na ten temat własne wnioski, własną opinię. Ważne jest, aby opierać się na rzetelnych informacjach, czyli takich, ze sprawdzonych profili, portali. Dlatego zamiast czytać opinie z wątpliwych źródeł, jak na przykład Pudelek, wolę sama wyciągać wnioski i sprawdzać na przykład stronę ministerstwa, czy WHO.

**Jak Pani myśli, skąd w ogóle wziął się ten wirus?**

Zmutował. Nie dało się chyba z tym nic zrobić. Choć może poprzez wcześniejsze zamykanie granic? Ale nikt chyba nie wiedział, jak to wszystko się potoczy. Nikt nie mógł się na to przygotować. Są różne teorie, ale ja myślę, że to nie tak, że ktoś to zrobił specjalnie. Bardzo smuci mnie to, jak ludzie szukają winnego. Nie wiem, dlaczego to robią, choć historia się powtarza – tak było też podczas epidemii hiszpanki, która też była odmianą grypy, czy podczas II wojny światowej, gdzie winny był Hitler. To co się stało teraz, że profesor popełnił samobójstwo przez falę hejtu – nie potrafię powiedzieć, dlaczego ludzie to robią. Ale widzę, że jak ktoś jest chory, to go za to obwiniają.

**O co obwinia się takie osoby?**

No właśnie o to, że są chore. Na przykład ten kierowca z Radomia. Biedny człowiek, on nawet mógł nie wiedzieć, że był chory, ten wirus może przecież nie wywoływać objawów. On umarł, a ludzie obwiniają go o to, że mógł kogoś zarazić, że pracował. Z drugiej strony, jak ktoś nosi maseczkę, to też można się spotkać z innym traktowaniem takich osób. Traktuje się tych ludzi, jakby od razu byli chorzy i winni.

**Dlaczego według Pani wirus się tak szybko rozprzestrzenia?**

Ponieważ ludzie się przemieszczają i tego nie da się zatrzymać. Ta fala powrotów do Polski, możliwe, że nie była dobrym rozwiązaniem, ale kto wie, czy z drugiej strony coś by dało to, jakby jej nie było. Nie wiadomo, czy ktokolwiek był w stanie to wszystko zatrzymać, wychwycić odpowiedni moment na przykład w Chinach. To tym bardziej trudne, że można przechodzić infekcję prawie bezobjawowo.

**A czy jeśli chodzi o wizyty w sklepie, czy coś się dla Pani zmieniło?**

Pojawiamy się w sklepie dopiero, jak już naprawdę wykorzystamy wszystko, co mamy w domu. To jest dla nas duża różnica, bo wcześniej pojawiałam się tam praktycznie codziennie, po kilka rzeczy. Teraz robię zakupy na kilka dni. Marnujemy zdecydowanie mniej żywności – okazuje się, że się da! To jest dla nas nowość, wcześniej jakoś nie zwracałam na to uwagi, pojawiałam się w sklepie często i często zdarzało się wyrzucać w domu resztki, niektóre produkty.

**Zrobiła Pani zapasy?**

W momencie tego całego szału akurat nie musiałam iść do sklepu, ale wiedziałam, że produkty będą, że to po prostu sklepy nie nadążają z zatowarowaniem się. Nie doświadczam braku produktów. Robię zakupy w Lidlu, tak, jak robiłam to wcześniej i tam naprawdę wszystko jest. Nie robiłam dodatkowych zakupów, nie zwracałam też w tej całej sytuacji szczególnej uwagi na konkretne produkty, które muszę mieć na zapas.

**Czy zmieniło się coś w kwestii wybieranych przez Panią produktów, ich marek?**

Wybieram te same marki, te same produkty, co wcześniej. Nie tyle ze względu na wirusa, co przez większą ilość czasu wolnego, zaczęłam jednak piec chleb. Mam Thermomix, stwierdziłam, że to dobra okazja. Teraz w ogóle można spędzić więcej czasu z rodziną, więcej w kuchni. Poeksperymentować, na co kiedyś nie było tyle miejsca. Jedyny problem jest w tym kontekście z dostępnością drożdży, ale znalazłam już piekarnię, w której będę mogła je kupić. Niestety, to będzie 1 kg blok, więc część drożdży będę musiała zamrozić. Chleba piekę taką standardową porcję z przepisu, tam wychodzą dwa bochenki. Jak się skończy, wtedy piekę drugi, nie mrożę go.

**A jak odnosi się Pani do kolejek, które ustawiają się od jakiegoś czasu pod sklepami?**

Nie jest to przyjemne, ale rozumiem, czemu służy. Poza tym okazuje się, że jak się pójdzie na przykład rano, to uda się zrobić zakupy bez stania w kolejce. A w sklepie, jak już mówiłam, bywam teraz rzadziej.
