# Supplementary material for: The challenges arising from the COVID-19 pandemic and the way people deal with them. A qualitative longitudinal study
Source: PLoS One. 2021 Oct 11;16(10):e0258133. doi: 10.1371/journal.pone.0258133 (PMC8504766; doi:10.1371/journal.pone.0258133)
Supplement: S1 Dataset — (ZIP) [file pone.0258133.s003.zip › Transcriptions/stage 1/12.1._M_33_couple, with children.doc]

**12.1_M_33_couple with children**

Pracuję jako inżynier, jestem kierownikiem zespołu przygotowania danych aplikacyjnych w firmie, która współpracuje z (nazwa firmy) Modernizujemy linie kolejowe. Jestem ojcem trójki dzieci, mężem. Z zainteresowań - od ostatnich dwóch lat - wędkowanie, obejrzenie fajnego serialu lub filmu, bo czasami nie starcza czasu wieczorami, żeby obejrzeć coś dłuższego. Te dni jednak wyglądają dość intensywnie - właśnie ze względu na pracę, która jest oddalona od mojego miejsca zamieszkania, muszę wcześnie wyjeżdżać. Do tego jeszcze dzieciaki. Mieszkam na wsi, w domu jednorodzinnym. Mieszkamy naszą pięcioosobową rodziną z dziadkami w bardzo podeszłym wieku. Mamy sporo terenu na zewnątrz, także zawsze jest co robić. Lista zadań, które sobie stawiam jest zawsze długa i tak naprawdę nie kończąca się.

**Kiedy zaczęła się dla Ciebie ta sytuacja? Kamienie milowe**

Kiedy wirus pojawił się w Chinach. Już wtedy z tyłu głowy pojawiła się myśl, że coś się zaczyna dziać, takiego globalnego i nie są to żarty. W tamtym czasie otaczały nas fale memów, obserwowałem tę sytuację jeszcze z przymrużeniem oka, choć beczka już wtedy się paliła. Tak naprawdę już wcześniej miałem świadomość, uważałem, że coś takiego może nastąpić. Jakiś czas temu, nie pamiętam dokładnie kiedy, mieliśmy przecież problem z Ebolą na kontynencie afrykańskim. To też, już wtedy, troszeczkę dawało do myślenia, że coś się może wydarzyć. Natomiast dopóki tak naprawdę nie zapukało do Europy, to było zupełnie co innego. Gdzieś tam się działo, nas nie dotyczyło. Zagrożenie było tam, natomiast nie tutaj. W tym momencie mamy tak skonstruowany świat, że człowiek nie utożsamia się z jakąś tragedią, mającą miejsce w innym regionie, bo tego jest dużo - na przykład właśnie Ebola, czy konflikty zbrojne. Nie wiem, jakim trzeba by było być człowiekiem, żeby z każdym takim wydarzeniem się utożsamiać. Jeśli chodzi o obecną sytuację wiedziałem, że coś takiego jest, ale ja na przykład do pewnego czasu nie zgłębiałem tego tematu, nie angażowałem się specjalnie. Krok bliżej do takiego poważniejszego myślenia, to był moment, kiedy wirus pojawił się we Włoszech, zaczął się tam rozprzestrzeniać. Miałem wtedy kontakt z osobami, które przebywały we Włoszech, z kolegami z pracy, którzy mają tam rodziny. Wtedy zaczęło się to bardziej na poważnie. Następnie od momentu Włoszech, to było już tak naprawdę obserwowanie, jak to się rozlewa wszędzie. Ja na przełomie lutego i marca jeszcze miałem zabieg, byłem hospitalizowany. Będąc w szpitalu, pojawiały się już przypadki, że jest podejrzenie, że ktoś z tego regionu jest zarażony. To nie były jeszcze przypadki potwierdzone, ale podejrzenia już się pojawiały. A od momentu wybuchu epidemii w Polsce, zmieniło się dużo. Od kiedy rząd wprowadził hasło "zostajemy w domu", mamy problem, pojawiły się pierwsze ograniczenia, to już wtedy zaczęliśmy to traktować dosyć poważnie. Bardzo poważnie, nawet bym powiedział.

**Co się zmieniło?**

Pierwszą taką odczuwalną zmianą, była dla mnie informacja, że nie pracujemy w biurach, tylko zdalnie. To zmieniło mi całkowicie tryb życia. Dotychczas musiałem wstać o 5:30, o 6 wyjechać, bo mam 100 km do pracy. Zanim dojechałem, była już 7:30. Wracałem tak naprawdę wieczorem do domu. Dla rodziny było już tego czasu mniej. Natomiast w momencie, kiedy dostaliśmy informację, że pracujemy zdalnie, to cały ten rytm, wypracowany przez lata, uległ zmianie. Zacząłem wstawać później, jeść śniadanie z rodziną, później pracowałem, tak jak do tej pory to robię, w odosobnieniu. Natomiast po pracy jestem od razu w domu. To była pierwsza duża zmiana, jeśli chodzi o moje życie i to, co przyniósł nam koronawirus.

**Jak postrzegasz tę zmianę?**

Ciężko powiedzieć. Na pewno w każdej sytuacji można znaleźć jakieś zalety. Mój syn, który ma już teraz 9 miesięcy, pokazuje już na pokój w którym pracuję, wie, że tata tam siedzi, woła. Widzę też dużo pozytywów biznesowych, na przykład w firmie, w której pracuję. W tym momencie gospodarka jest w zawieszeniu, więc pewne opóźnienia, które mieliśmy wygenerowane, mamy szansę teraz nadrobić. Nie gonią nas tak terminy, możemy więc spokojnie wyjść na prostą, dopracować produkt, który zostanie finalnie oddany w ręce klienta.

**A co jest największym wyzwaniem?**

Utrzymanie zdrowia, utrzymanie tego stanu rzeczy, jaki jest teraz. Słyszy się o zgonach, to są tragedie w każdej rodzinie. Oby - mam nadzieję i wierzę całym sobą - że to nas nie dotknie. Jest ta obawa o zdrowie i bezpieczeństwo rodziny; to rośnie od dzieci, domowników, po trochę dalszą rodzinę, znajomych i tak dalej. W tym tygodniu dowiedziałem się, że to miejsce, gdzie ten wirus dotknął moją rodzinę najbliżej, to moja ciocia. Dosyć bliska rodzina, to żona siostry mojego brata, mieszkają niedaleko nas. Ona pracowała na poczcie, ale w tej chwili nie pracuje, bo jest na kwarantannie - zresztą z całą rodziną. Nie ma objawów, ale miała kontakt z listonoszem, który miał pozytywny wynik testu.

**Poziom lęku**

Na chwilę obecną 35/40. Ale to jest uzależnione od czynników zewnętrznych. Jakiś czas temu miałem taki peak. Spływało dużo informacji, do tego dochodzi jeszcze fakt, że jestem przewlekle chory - mam astmę oskrzelową, więc jest to zwiększone ryzyko. Miałem tak, że obudziłem się w nocy i miałem taki moment, że czułem się, nie wiem jak to nazwać, czy przerażony... Sen wymieszał mi się z jawą. Śniło mi się, że choruję, leżę gdzieś. Podzieliłem się tym z żoną i to nie było dobre, bo każdy w moim najbliższym otoczeniu zaczął wtedy panikować. Ale ten fakt, że jestem mężem, ojcem, to widzę, że to, co czuję, mówię, bardzo oddziałuje na moje otoczenie. I wtedy postanowiłem, że to ja muszę dawać przykład takiego pozytywnego myślenia i wszedłem w tę rolę bezproblemowo. Jakoś sobie z tym poradzimy. Natomiast to ja muszę dawać ten przykład hartu ducha w tej sytuacji i w taką rolę wszedłem. I teraz, w tym momencie, można powiedzieć, że nie boję się niczego. Jeżdżę na zakupy, nie dopuszczam do siebie tego lęku. Jestem na takiej granicy. Te 35% to jest taki moment, że czuję to zagrożenie, ale ono na mnie nie oddziałuje tak, żeby się bać i na nie specjalnie reagować.

**Pamiętasz z czym był związany ten peak lęku, o którym wspomniałeś, jeśli chodzi o informacje, które do Ciebie docierały?**

Najbardziej przerażało mnie to, że w tych doniesieniach medialnych, które się pojawiały, że chyba wtedy poznałem działanie tego wirusa - co on atakuje dokładnie. Wcześniej coś tam tylko słyszałem, że zapalenie płuc, coś tam. To było chyba bezpośrednio związane z moją chorobą. Kiedy dowiedziałem się, że praktycznie każdy przypadek śmiertelny miał choroby współistniejące i że to atakuje właśnie układ oddechowy. Do tego miałem swego czasu moment, że media non stop u mnie pracowały. Ja sobie pracowałem przy biurku, a nad nim miałem włączony telewizor i w tle było włączone TVP Info, czy TVP 24. Cały czas jakoś ładowałem się tymi emocjami, które się wylewały z tego kontentu. Gdzieś przez chwilę złapałem jakieś przeziębienie, coś mnie tak tutaj uciskało w okolicy klatki piersiowej. I wtedy właśnie tak obudziłem się w nocy i pomyślałem, kurczę, może ja rzeczywiście mam tego wirusa, może rzeczywiście ja teraz zarażam. Natomiast chwilkę mi z tym zeszło, zanim sobie z tym poradziłem, wyszedłem z tego marazmu. Ja to próbowałem żonie wytłumaczyć, co się wtedy stało. To było tak, jakbym nie do końca się obudził, ktoś by mnie kocem przykrył i próbowałbym włączyć normalne myślenie - garść jakichś faktów, nie wiem. Nie potrafiłem sobie z tym poradzić i jakoś przeżywałem tę sytuację, ale jeszcze tej samej nocy powiedziałem sobie, że więcej nic już z tym nie zrobię, idę spać. Obudziłem się z zupełnie innym myśleniem. Natomiast samo to, że powiedziałem żonie o tej sytuacji, to zasiało jakieś ziarno takiego przestrachu. W tym momencie, myślę, że każdy ma takie podejście, nie tylko w sprawach takich, związanych z zagrożeniem, ale ze wszystkim, to znaczy - dopadają człowieka myśli pozytywne, że będzie dobrze i tak dalej, a za jakiś czas, dopadają negatywne. To nie jest tak, że można wyrobić sobie jedno stanowisko i go trzymać. Wystarczy, że ktoś coś powie, ktoś z zewnątrz, z okolicy. U nas w okolicy był ten kierowca, który rozwoził pasażerów, był zarażony i zarażał dalej; zmarł. To od razu człowiek reaguje tak, że 10 km dalej ktoś mieszkał, facet 40 lat i nie żyje. Są takie momenty. Natomiast w tym czasie, staram się temu tak nie poddawać, nie przeżywać. Bardziej już próbuję załagodzić, stłumić na przykład obawy żony - dzieci są jeszcze za małe, żeby to zrozumiały.

Widzę swoją rolę w tym wszystkim. To ja jestem skałą, która jest twarda. Jak ja zaczynam być miękki, to idzie później po wszystkich. Nie wiem, czy sam się do tego przekonałem - czy musiałem w ogóle, ale mam do tych lęków dosyć spory dystans w tym momencie.

**Jak sobie z tym wszystkim radzisz?**

Dbam o bezpieczeństwo, nie lekceważę tych wszystkich zasad bezpieczeństwa. Nawet jak wyjeżdżam na zakupy, mam maskę, rękawiczki, żele - wszystko co mogę zrobić w trosce o zapewnienie sobie bezpieczeństwa, robię. Ale to jest tak, że staramy się w domu funkcjonować normalnie. Nie oglądamy na ten moment mediów. Nie wiem, ile jest na ten moment zarażonych, ile zgonów. Mam w tym momencie wyłączony telewizor. Czasami włączam go wieczorem, na chwilę. Zobaczymy, jaka jest sytuacja. Teraz tak naprawdę nawet w mediach społecznościowych zawsze te najważniejsze informacje docierają. Była ostatnio konferencja premiera i ministra zdrowia, to obejrzałem, bo to były ciekawe informacje, które też wpływają na organizację życia. Natomiast nie mamy już włączonych informacji w tle, nie ładujemy się tą negatywną energią.

**Co działo się w Twoim dalszym otoczeniu, w obniesieniu do kamieni milowych?**

Ciężko powiedzieć, bo nie mam takiego kontaktu z ludźmi. Mogę liczyć tylko na kontakt telefoniczny. Ewentualnie mogę powiedzieć jakoś o sąsiadach. U nich zauważyłem, że też są świadomi zagrożenia, natomiast mam wrażenie, jak ostatnio rozmawialiśmy gdzieś tam zza drogi, jakby próbowali opowiedzieć, jak to wygląda z ich strony. Oni mają działalności gospodarcze, nie mogą sobie pozwolić na to, żeby się odizolować. Jeden sąsiad jest kierowcą autobusu, drugi prowadzi kilka sklepów, więc ciągle robi zakupy po hurtowniach. Myślę, że mają tę świadomość, natomiast nie widzę u nich jakiegoś lęku. Robią swoje. Może na swoich wewnętrznych przemyśleniach bardziej okazują taki lęk, ale z punktu widzenia zewnętrznego obserwatora - mamy sytuację, do której trzeba się dostosować i robimy swoje. A jeśli chodzi o pracę i o ludzi z pracy, z którymi się kontaktuję - nie było raczej takich rozmów, że może dojść do rozprzestrzenienia się tego wirusa, że ludzie zaczną umierać w otoczeniu, które znamy. Raczej też każdy robi swoje i nie ma takiego odczucia paniki. Bardziej rozmawia się na temat koronawirusa w kontekście biznesowym, tak, jak mówiłem. Że skoro on jest, to na przykład możemy zagrać w określony sposób. Nie widzę, żeby ludzie panikowali.

**Obrazki; który najlepiej opisuje to, co czujesz w chwili obecnej?**

3 mi się podoba, przykuwa moją uwagę. Kojarzy mi się ze sportem, z pokonywaniem swoich słabości, takiego lenia. Ostatnie moje pół roku wyglądało dosyć specyficznie. Zacząłem trenować, inaczej się odżywiać, zrzuciłem 20 kg. Mam teraz tak, że brakuje mi tego sportu. Obrazek skojarzył mi się z walką - wewnętrzną, z leniem, kiedy siedzi się na kanapie i myśli, czy iść, czy nie iść pobiegać. Dlatego mi się to tak kojarzy, bo w tej sytuacji, w której jestem, muszę cisnąć. To ciśnięcie sprawia satysfakcję. Tak samo, jak po ukończonym godzinnym treningu na bieżni jest ta satysfakcja. Być może ta postawa, którą teraz przyjąłem, to jest jakaś forma mobilizacji samego siebie i czerpania z tego satysfakcji. Jak widzę, że rzeczywiście wprowadzam jakiś spokój, jest to dla mnie satysfakcja. Jeśli chodzi o taką postawę, zawsze człowiek myśli jeszcze o tym, co by było w jeszcze gorszej sytuacji. Nie ma chyba człowieka, który nie myśli, że będzie gorzej, a później może być jeszcze gorzej. W głowie różne rzeczy się przewijają i zawsze jest to pytanie, jak ja poradzę sobie z sytuacją, jak zareaguję. Przerabiam też dużo takich książek, czy słucham teraz o Witoldzie Pileckim. I to są ludzie, którzy znaleźli się w takich sytuacjach, gdzie zastanawiam się, co ja bym zrobił, jak bym zareagował. To jest tak, że człowiek pyta się, czy byłbym bohaterem? No, na pewno, tak. Tymczasem prawda jest taka, że jest tyle czynników, że nie wiadomo, jak się zareaguje. A ten obrazek kojarzy mi się z taką determinacją, konsekwencją i czerpaniem z tego satysfakcji.

**Co pomaga Ci się mobilizować?**

Dawanie z siebie wszystkiego, co mogę - w domu i w pracy. Nie raz by się usiadło, obejrzało coś w telewizji. Ale cały czas mam coś do roboty. I ta praca myślę, że pomaga ogólnie ustabilizować emocje. Takie dawanie z siebie otoczeniu - mam tu na myśli dom, dzieci. Działamy normalnie. Jakieś odstępstwa od normy mogłyby mieć wpływ na dzieci, żonę, dziadków- ogólnie najbliższe otoczenie. Więc teraz nie zmieniamy nagle wszystkiego, dzieci nie mogą mieć bałaganu w pokoju, bo jest koronawirus. Nie, działamy, sprzątamy. Jak patrzę na sąsiadów, oni też robią swoje. Trzeba robić swoje. Trzeba dbać o swoje bezpieczeństwo, ale tutaj nie ma żadnych ustępstw, jedziemy z tym. Jest taka sytuacja, trzeba być elastycznym, dostosować się do niej i cały czas kontynuować. To się niedługo skończy i będziemy mieć dużo więcej możliwości.

**Podjęliście jakieś nowe działania, żeby dostosować się do tej sytuacji?**

Tak, tak, jak wspominałem, cały dzień wywrócił nam się o 180 stopni. Dzieci chodziły do przedszkola. Teraz są w domu. Nieraz dają popalić, widzę, ze czasami robią już takie rzeczy, że po prostu... Pontony na głowie, bieganie, itd. Ale wcale się im nie dziwię, bo u nas dzieciaki miały bardzo intensywny tryb życia. I to nie ze względu na nas, od razu się wytłumaczę (śmiech). Córka pozapisywał się na chyba wszystkie możliwe zajęcia. Po przedszkolu, w poniedziałek miała trąbkę. We wtorek tańce, w środę, ze swoim bratem jeździła na piłkę nożną. W piątki ich zabierałem na basen. W czwartki znowu piłka. I normalnie, cały dzień, po prostu energia. Natomiast teraz wszyscy jesteśmy w domu, trzeba się do tej sytuacji dostosować. Tak jak kiedy mówiłem o pozytywach - mogę zjeść ze swoją rodziną śniadanie, obiad - to jest takie fajne. Ale wiadomo, też trzeba jakoś te negatywy zaakceptować, że trzeba w tym domu przebywać, nie można pojechać sobie na plac zabaw. Chociaż i tak nie mamy źle, bo mamy miejsce do zabawy przed domem, na zewnątrz. Ale brakuje choćby tego, żeby tak normalnie spotkać się z rodziną. Myślę, że każdy musi się dostosować w ten sposób i to wymaga jakiejś pracy wewnętrznej. Żona też tak naprawdę spełnia się w pracy. Teraz jeździ tylko na godzinkę do dwóch. Część rzeczy załatwia zdalnie, natomiast dzieciaki dają jej nieźle popalić. Ja jeszcze mam taką odskocznię, że usiądę tu - popracuję 8 godzin, później się wymieniamy. Jak to dzieciaki - ciężko nad nimi zapanować i każdy musi się dostosować do tej sytuacji.

**Czego Ci najbardziej brakuje?**

Spotkań - ze znajomymi, z rodziną. Ogólnie nasz dom, to jest taki dom, gdzie co weekend, w niedzielę jest pełno ludzi. Już nawet nie bawimy się w planowanie, jak w piątek jadę na zakupy, kupuję dwa razy więcej karkówki, dwa razy więcej kiełbasy, bo wiem, że to pójdzie, zawsze ktoś przyjedzie. Tak się u nas zawsze działo. Dzieci są już przyzwyczajone, że jak się im mówi, że mają sprzątać w pokojach, pytają, kto przyjedzie. I zawsze ktoś był - rodzin bliższa, czy dalsza, znajomi. Tego mi najbardziej brakuje.

**A czy zrobiłeś jakieś zakupy związane z wirusem?**

Nie, robię zakupy normalnym trybem - tak, jak do tej pory. Ale to tez jest uwarunkowane tym, że moi rodzice mają sklep i na ich magazynach stoją całe palety produktów. Nie mamy tego niebezpieczeństwa, że jakby nagle zamknęli sklep - Auchan, Lidl, którykolwiek, to nie będziemy mieli produktów do życia na najbliższe miesiące. Nie czułem potrzeby robienia zakupów dla siebie, ale pomagałem organizować dostawy tu, do sklepów rodzinnych. Moi rodzice mają sklep i żony rodzice również. Wiedziałem, że był taki szał zakupowych - w hurtowniach brakowało mąki, makaronów, ryżów. Ale nie robiłem zakupów takich, które by u mnie gdzieś przeleżały. Dalej robię zakupy raz w tygodniu - to są wielkie torby, ale uwarunkowane zmianą systemu odżywiania, o której wspominałem. Mamy listy zakupów, a to, co kupimy, przeważnie zjadamy.

**Jeśli mielibyście przeżyć na tym, co macie, jak długo by to było.**

Na tydzień mamy tych zakupów bieżących i pewnie na kolejny tydzień starczyłoby tego, co gdzieś tam jest pomrożone, co jest pod ręką.

**A jeśli chodzi o marki produktów, które wybierasz? Coś się zmieniło?**

To są te same marki. Już wcześniej starałem się wybierać produkty polskie, ale nie jestem też aż tak radykalny, że nie kupię na przykład śliwki z Hiszpanii. Jeśli chodzi o koronawirusa - nic się nie zmieniło.

**Słyszałam, że wiele osób piecze teraz chleb samodzielnie. Jak to u Was wygląda?**

(śmiech) Dobre pytanie! Moja żona pracuje w piekarni, u swoich rodziców, także z tym nie mamy problemów.

**A jak odnosisz się do zjawisk, które pojawiają się w sklepach? Zauważyłeś jakieś zmiany w zachowaniu ludzi?**

Może nie tyle ludzi, co zauważyłem odgórnie nałożone restrykcje, jak 15 osób na sklep. Ludzie zaczęli używać rękawiczek. Ja tam jeszcze wcześniej wparowałem w masce i rękawiczkach - ludzie wtedy jeszcze tego nie mieli swego czasu. Nie czułem się jakoś dziwnie, nie zwracałem na to uwagi. Nie wiem, jak ludzie mnie postrzegali, czy nie zasiałem paniki. Ale widzę, ze teraz dużo osób w sklepach korzysta z masek, trzyma jakiś dystans. Są zmiany organizacyjne - kolejki przed sklepami, przy podejściu do kasy trzeba zachować odpowiednią odległość. Staram się dostosować. Noszę maskę i rękawiczki, zwracam uwagę, żeby się do kogoś nie zbliżać i ogólnie trzymać dystans. Ale to są ogólne zalecenia rządu, lekarzy. A tak z własnych przemyśleń - staram się nie mieć kontaktu. Nie zaglądać do tej samej lodówki. Jak już ktoś przy niej jest, czekam. Jak on odejdzie, podejdę ja.

**Kiedy zacząłeś nosić maskę i rękawiczki na zakupy?**

Może zacząłbym wcześniej, ale problem jest taki, że one nie są dostępne. Być może są do zamówienia przez internet, ale w pewnym momencie żona uszyła i zaczęliśmy te maski nosić. To było jak dosyć mocno przyspieszyła ta liczba zachorowań, przekroczyliśmy 500 osób zarażonych. Do tego dochodziły wszystkie informacje pozarządowe - że rzeczywiście zarażonych jest więcej, że nie jest tak pięknie, jak jest pokazane w telewizji, w szpitalach jest duży problem, umiera dużo ludzi, a statystyki są zakłamane. Spotkałem się z głosami, że te statystyki podają stan na miesiąc wcześniej - to działa z takim opóźnieniem. Ze względu na to, żeby zwiększyć bezpieczeństwo - w trosce głównie o dzieci i dziadków - zacząłem chodzić w masce. Nie mam wielu kontaktów z zewnątrz, od kiedy pracuję zdalnie to tyle, co pojadę na zakupy, albo na chwilę gdzieś. Ale zawsze jak wychodzę, staram się zakładać maskę.

**Jak myślisz, skąd wziął się problem wirusa?**

Jest wiele teorii, choćby spiskowych. Według mnie, prędzej czy później, to musiało nastąpić, patrząc choćby na historię. Hiszpanka, dżuma - co jakiś czas ludzkość z tym walczy. Natura zrobiła nam psikusa, to jest dla mnie naturalne. Nie jest to coś, co wywróciło mi światopogląd. Takie rzeczy się zdarzały i pewnie będą się zdarzać.

**Dlaczego wirus rozprzestrzenia się w tym tempie?**

Jest to kwestia globalizacji, że świat stał się na tyle mały. Od ogółu do szczegółu - w skali światowej - globalizacja i to, że wirus szybko przenosi się z człowieka na człowieka. Drugą rzeczą jest świadomość ludzi. O ile ja staram się nie zbliżać do ludzi, tak bardzo dużo jest jeszcze takich osób, które albo nie zdają sobie z tego sprawy, albo lekceważą to. Spotkałem się z przypadkiem, gdzie uczennica będąca na kwarantannie jest w domu, lecz pozostali domownicy funkcjonowali normalnie. To jest moim zdaniem skrajnie nieodpowiedzialne. Myślałem, że to będzie dla ludzi logiczne, a okazało się, że nie. Choć w tym momencie nowe regulacje zabroniły na szczęście takich zachowań, w takim przypadku już wszyscy domownicy objęci są kwarantanną.

**Jesteśmy przygotowani na to, co się stało?**

Nie, myślę, że nie. Z drugiej strony, to nie jest sytuacja, która cały świat wprowadziła w stan szoku. Mieliśmy do czynienia z epidemiami, jak choćby SARS. Te wydarzenia powinny dać do myślenia krajom - był sygnał, żeby się przygotować, a to też zostało zlekceważone. Według mnie nie jesteśmy na tyle przygotowani, żeby powiedzieć "tak, poradzimy z tym sobie". Nie ma sprzętu, ludzi, miejsc, gdzie można udzielać pomocy. Więc nie jesteśmy przygotowani - ani jeśli chodzi o populację ludzką, ani o kraj. Gdybyśmy byli, w każdym domu, w apteczce znalazłoby się obowiązkowo kilka masek, żeli do dezynfekcji, rękawiczek. Powinno się uświadamiać ludzi. Można by wprowadzić do edukacji elementy, które pokazywałyby, jaki będzie świat w przypadku pandemii i co należy wtedy robić. Żeby ludzie nie dowiadywali się teraz, kiedy to się już dzieje, żeby częściej myć ręce, czy używać tych żeli dezynfekujących. Powinno się ich uświadamiać o tym wcześniej. Mówić o tym, jak przenoszą się wirusy. Być może w ten sposób uświadamiać.

**Kto powinien to robić - rząd, a może powinny to być inicjatywy oddolne?**

Myślę, że po części rząd, po części powinno to zostać wprowadzone do programu nauczania. Natomiast to, co się dzieje teraz, oddziałuje na rodziców. Nauka mycia rąk i tego typu rzeczy, to jest też kwestia rodziców. Po tym, co dzieje się teraz, takie zachowania typu trzymanie dystansu, mycie rąk, pozostaną nam na długo w pamięci.

**Według Ciebie wirus zmieni społeczeństwo?**

Tak, zdecydowanie. Tak, jak mówił mój tata - przeżył stan wojenny, był na Ukrainie, kiedy był wybuch w Czarnobylu, ale mówi "takiego czegoś, to jeszcze nie widział". W takim sensie, żeby wydarzenie zmieniło na taką skalę tyle rzeczy. To z tych wszystkich katastrof jest dla niego największy szok. Z tych wszystkich katastrof ta jest tak rozprzestrzeniona, tak wygląda.

**Mówisz, szok, czy były tam jeszcze inne emocje?**

Nie wiem, mówimy tu o rozmowie telefonicznej i emocjach mojego taty. On niewiele daje po sobie poznać. Ale też, robi swoje. Jeździ, załatwia sprawy, nie daje po sonie poznać, że w jakiś sposób się boi. To, co mi powiedział, powiedział raczej w tonie "kurczę, to, co się teraz dzieje, nigdy czegoś takiego nie widziałem". On jest 60 letnim facetem, raczej nie boi się. Choć może wszedł w taką samą rolę, jak ja - niedaleko pada jabłko od jabłoni.

**Jak oceniasz obecne działania rządu w tej sytuacji?**

W porównaniu z innymi państwami myślę, że rząd w Polsce zachował się wzorowo, wprowadzając szybko odpowiednie regulacje. Nie ma rządów idealnych, które robiłyby wszystko wzorowo. Ale to jest na pewno ogromy plus, który może później nawet maskować ich późniejsze niedociągnięcia. W innych krajach rządy nie podeszły tak do sprawy i tam widać teraz tego efekty - mam tu na myśli Włochy, czy Hiszpanię. Rząd musi wziąć na siebie tę odpowiedzialność i nasz akurat - przynajmniej w teorii - dobrze zadziałał. W teorii, bo na razie nie znamy jeszcze dalszych skutków tych działań.

**Czy zmieniły się Twoje nawyki, jeśli chodzi o korzystanie z mediów?**

Zazwyczaj, kiedy pracowałem w biurze, nie miałem możliwości oglądania informacji w tle. To się pojawiło podczas pracy zdalnej, w domu. Dwa tygodnie przed tym przejściem na pracę zdalną, byłem na zwolnieniu lekarskim, ze względu na zabieg, o którym wspominałem. Wtedy odpoczywałem, nie pracowałem, spędzałem czas z rodziną. Jednak moje nawyki co do mediów się zmieniły. Wcześniej informacje czerpałem głównie z telewizji. Teraz prawie wcale nie włączam tych kanałów, bo można się nimi zdołować; sytuacja związana z wirusem wciąż jest numerem jeden. Bardziej widzę to, co pojawia mi się na telefonie, hasła. Mniej się zacząłem tym interesować, mniej tego oglądać. Staram się od tego odcinać. Na ten moment nie wiem, ile jest zarażonych, ile zgonów. Co jakiś czas dostaję tylko te informacje, które mnie bezpośrednio dotyczą na ten moment.

**A jeśli chodzi o rzetelność informacji, które do Ciebie docierają?**

Informacje z mediów publicznych, z tego, co słyszałem od zweryfikowanych źródeł - znajomych lekarzy, pielęgniarek, ludzi, którzy pracują w szpitalach akademickich - ta sytuacja, statystyki, wyglądają zupełnie inaczej, niż się podaje. Dlatego straciłem zaufanie do tego, co w ogóle pokazują media. Co kraj, to obyczaj - w Niemczech jest dużo lepiej rozwinięta służba zdrowia, robią więcej testów, więc maja więcej potwierdzonych zakażonych. Widziałem pytania kierowane do ministra zdrowia - to wszystko jest wymijające, statystyki są zdecydowanie zaniżane. To też przyczyniło się do tego, że nie oglądam tych danych - są fałszowane na czyjeś potrzeby, więc po co. Bardziej skupiam się na tym, co mnie bezpośrednio dotyczy, czyli wiadomości dotyczące na przykład jakichś ograniczeń.

**Co świadczy o tym, że informacja jest rzetelna?**

To zależy też od informacji, które napływają do nas od osób, które znamy, ufamy im, a są one bezpośrednio powiązanie z wydarzeniami, mają dostęp do pewnych informacji - jak na przykład znajomi lekarze. Dlatego takie informacje wydają mi się bardziej wiarygodne.

**A czy są jakieś media, którym można zaufać?**

Wydaje mi się, że najbardziej obiektywną jednostką w Polsce jest RadioZet. Tam jest atakowana jedna i druga strona. To radio wzbudza we mnie zaufanie - czy to właśnie w radiu, czy na profilu, w mediach społecznościowych, te informacje traktuję jako rzeczywiste. Natomiast mając na względzie, że oni opierają się na tych danych, które podaje rząd. TVP i TVN ścierają się, atakują wzajemnie. To sprawia, że nawet ten strzępek informacji, który mógłby być wartościowy, tonie w tym całym bełkocie.
