# Supplementary material for: The challenges arising from the COVID-19 pandemic and the way people deal with them. A qualitative longitudinal study
Source: PLoS One. 2021 Oct 11;16(10):e0258133. doi: 10.1371/journal.pone.0258133 (PMC8504766; doi:10.1371/journal.pone.0258133)
Supplement: S1 Dataset — (ZIP) [file pone.0258133.s003.zip › Transcriptions/stage 1/8.1._M_30_couple, no children.doc]

**8.1_M_30_couple, no children**

**Proszę sobie przypomnieć, kiedy zmieniła się obecna sytuacja w Polsce? Kiedy to wszystko się dla Pana zaczęło?**

Zaczęło w znaczeniu obostrzeń rządowych, czy zaczęło, w znaczeniu: kiedy pojawił się wirus i zachorowania?

**Subiektywnie, dla Pana. Czy właśnie kiedy pojawił się wirus, poczuł Pan, że coś się zmieniło, czy to obostrzenia wprowadzone przez rząd spowodowały, że zaczął postrzegać Pan sytuację inaczej?**

Pamiętam, że o wirusie mówiło się już w styczniu, jednak wtedy nie było go jeszcze w Polsce. Przypadki, jeśli dobrze pamiętam, pojawiły się jakoś pod koniec lutego. W pierwszej połowie marca pojawiły się obostrzenia i zaczęło się coś realnie zmieniać, w życiu. Dla mnie zaczęło się coś zmieniać w okolicy 10-12 marca.

**Z czym to było związane?**

Po pierwsze z informacjami o większej liczbie przypadków i taką widoczną, rosnącą świadomością ludzi wokoło. Można było zauważyć, że ludzie się zdecydowanie inaczej zachowują – stali się ostrożni, chodzą w maseczkach, starają się jak to tylko możliwe zachowywać odległość od innych, zakładają rękawiczki. I przez to też własne nastawienie się zmieniało.

**A gdyby miał Pan wymienić kamienie milowe rozwijającej się sytuacji, co by to było?**

Pierwsze to byłoby na pewno pojawienie się wirusa w Polsce – pierwszy odnotowany przypadek. Drugim pewnie by były pierwsze obostrzenia wprowadzane przez rząd. I myślę, że te obostrzenia wyznaczałyby kolejne kamienie milowe. Kolejne informacje o tym, co od strony prawnej się zmienia. To są jedyne momenty, które mogą wyznaczyć precyzyjnie punkt, w którym coś konkretnie się zmieniło.

**Czy Pana życie w tym momencie się jakoś zmieniło?**

Już od dwóch tygodni siedzę na home office. Akurat teraz robię dokładnie to samo, co bym robił w pracy, więc nic na tym nie tracę. Za to na pewno zaoszczędzam trochę czasu na dojazdach, kupowaniu jedzenia w pracy. Więc jeśli o mnie chodzi to jest całkiem, jak na razie, wygodnie.

**Czy jest coś, czego się Pan w tym momencie obawia? A może jest zupełnie odwrotnie?**

Zarażenia bliskich, przede wszystkim dziadków. I to jest ten minus, że jest konieczne ograniczenie kontaktów i prawdopodobnie osobne spędzanie świąt. Ja sam, jeśli chodzi o moje zdrowie, się nie obawiam. Wydaje mi się, że mam dosyć dobrą odporność – rzadko łapię jakiekolwiek infekcje i wydaje mi się, że nawet gdybym ja sam tego wirusa złapał, to przeszedłbym go raczej dosyć łagodnie. Aczkolwiek dla mnie największe ryzyko, zagrożenie jakie widzę, wiąże się z rozsiewaniem tego wirusa przeze mnie. To jest główna przyczyna, dla której staram się inaczej zachowywać – żeby po prostu innych nie zarażać, a nie konkretnie ze względu na swoje zdrowie, choć w minimalnym stopniu też na pewno na to uważam.

**Za moment pokażę Panu skalę lęku. Proszę się jej przyjrzeć i opowiedzieć, gdzie umiejscowiłby Pan siebie.**

Myślę, że zaznaczyłbym 30.

**A teraz pokażę Panu zestaw obrazków, proszę wybrać taki, który najlepiej opisuje Pana nastrój i odczucia w tym momencie.**

*Pokazano miniaturki wszystkich obrazów.*

11 może?

*Pokazano obrazek 11 w powiększeniu.*

**Proszę o nim opowiedzieć. Co przedstawia, jaka emocja jest z nim powiązana?**

Wcześniej nie widziałem tej tęczy w tle, więc to trochę zmienia kontekst, ale widzę wodę na szybie i rozmyty krajobraz w tle za szybą.

**W takim razie uznajmy, że tęczy nie ma.**

Nie wiem, czy potrafię konkretnie nazwać tę jedną emocję, w każdym razie wiąże się to dla mnie z takimi myślami jak spokój – bo na tym obrazku nic się nie dzieje – ale ten deszcz na szybie sprawia, że nie jest tak kolorowo i wprowadza taki smutniejszy nastrój.

**Co wpływa na Pana emocje, na to, jak się Pan teraz czuje?**

Myślę, że przede wszystkim to, że siedzę dłuższy czas w domu na tym home office'ie, cały czas słucham o kolejnych informacjach związanych z zarazą, z kolejnymi obostrzeniami – przede wszystkim to.

**A jak wyglądają te emocje, odczucia w porównaniu do tego, co czuł Pan wcześniej? Czy zmieniały się znacząco w momentach, o których mówiliśmy wcześniej?**

Na początku było mniej takich negatywnych odczuć, ten wirus nie budził jeszcze takiego lęku, jak teraz. Z czasem, wraz z obostrzeniami i obserwacją sytuacji pewien poziom lęku narastał. Nie tylko związanego z zachorowaniami, ale też wpływem całej tej sytuacji na gospodarkę, rynek pracowniczy, takie rzeczy.

**Proszę przypomnieć sobie moment, w którym poczuł Pan, że coś się zmienia. Czy podjął Pan wtedy jakieś działania, aby radzić sobie z zaistniałą sytuacją?**

Był to moment 2-3 dni przed tymi pierwszymi rządowymi restrykcjami, kiedy już jednak były zachęty do tego, aby unikać tłoku, zbędnych kontaktów z innymi ludźmi. Wtedy postanowiłem, że skoro mam taką możliwość, to na początek, na 2 tygodnie przechodzę na home office.

**Dlaczego było to dla Pana ważne?**

Przede wszystkim dlatego, żeby nie przyczyniać się do rozsiewania tego wirusa i swoimi kontaktami z innymi ludźmi nie narażać osób, które są mi bliskie.

**Czy oprócz tego, że postanowił Pan zostać w domu, podjął Pan jakieś inne działania?**

Ograniczyłem spotkania ze znajomymi. Do sklepu jadę wtedy, kiedy rzeczywiście kończą mi się produkty spożywcze i po prostu muszę coś kupić do jedzenia – wydaje mi się, że głównie to.

**Jeśli dobrze rozumiem, postanowił Pan pojawiać się w sklepie tylko jeśli zajdzie taka konieczność. Czy coś jeszcze uległo zmianie, jeśli chodzi o Pana zachowania konsumenckie?**

Nie, chyba tylko to.

**A kiedy podjął Pan decyzję o pojawianiu się w sklepie rzadziej? Czy to było jeszcze przed, czy już po wprowadzeniu pierwszych rządowych obostrzeń?**

Ograniczyłem te wizyty jeszcze przed pierwszymi obostrzeniami, ponieważ już wtedy zaczęła się straszna panika i ludzie tłumnie jechali do sklepów zrobić zapasy. Ja, jako że zawsze stroniłem od tłumów, postanowiłem wtedy wstrzymać się z zakupami tak długo, jak to było możliwe. Więc to było moje pierwsze działanie, choć z innych przyczyn, niż bezpośrednie zagrożenie wirusowe, które oczywiście było jeszcze większe, jak ludzie tłoczyli się w sklepach.

**A czy sam zrobił Pan jakieś dodatkowe zakupy?**

Nie, zrobiłem takie zakupy, jakie zawsze robiłem, żeby wystarczyło na kilka dni.

**Czyli rozumiem, że jeśli chodzi o ilość kupowanych przez Pana produktów, nic się nie zmieniło, w porównaniu do wcześniejszych zachowań, sprzed sytuacji związanej z wirusem?**

Nie zmieniło się. Może tyle, że jak już jadę do sklepu, kupuję dwa bochenki chleba, zamiast jednego, po to, żeby być znów w tym sklepie nie za parę dni – tylko po chleb – a za te 4, czy 5 dni. Ale jeśli chodzi o 90% produktów, to nic się nie zmieniło.

**A jeśli chodzi o produkty, które Pan wybiera, ich marki. Czy to są wciąż te same produkty, które kupował Pan wcześniej?**

Tak, tu się nic nie zmieniło.

**Może w takim razie pojawiły się jakieś inne zachowania, nie koniecznie związane z zakupami?**

Może mycie rąk stało się bardziej odruchowe, od czasu pojawienia się tej zarazy? Chyba tyle. Poza tym, moje zachowania się utrzymują.

**A jeśli chodzi o to mycie rąk – teraz coraz więcej osób zakłada na zakupy maseczki, rękawiczki. Czy Pan też takie działania stosował/ stosuje, a może zamierza zacząć?**

Nie, nie stosuję. Jeśli chodzi o maseczkę, ja na razie żadnych objawów nie mam – nie kaszlę, nie kicham. W sklepie po prostu utrzymuję dystans między innymi ludźmi, więc tą drogą kropelkową – nawet jakbym był nosicielem bezobjawowym – to nie mam szans nikogo zarazić. Ręce przy wejściu i wyjściu ze sklepu odkażam płynem, który tam jest dostępny. Ale jakbym na przykład musiał pojechać gdzieś komunikacją miejską, gdzie byłbym w zdecydowanie mniejszej odległości od innych ludzi, to też założyłbym tę maseczkę, żeby jakoś minimalnie zmniejszyć to narażenie. Aczkolwiek uważam, że jeśli zachowuje się od ludzi odległość, nie kaszle, nie kicha i oczywiście dba się o to, żeby nie dotykać swojej twarzy, to ta maseczka nic w takiej sytuacji nie poprawia.

**Rozumiem. Wcześniej nie było jeszcze tych kolejek przed sklepami, które teraz zaczynają się nam pojawiać i wygląda na to, że będą coraz większe, ponieważ wprowadzane kolejno obostrzenia są coraz bardziej restrykcyjne. Jak czuje się Pan w obliczu takiej sytuacji, jak by się Pan do tego odniósł?**

Trochę się obawiam, że będę musiał po prostu trochę dłużej poczekać przed sklepem, aż będę mógł wejść i że ta wycieczka po zakupy przedłuży się o 15-20 minut. Ale poza tym nie odczuwam ani większego lęku z tym związanego, ani też nie czuję się przeciwnie – bardziej spokojny, bardziej bezpieczny. Poza pewną niedogodnością związaną – być może – z koniecznością dłuższego oczekiwania przed sklepem, nie odczuwam na razie zmian.

**Przejdźmy teraz do nieco bardziej ogólnego spojrzenia na sytuację. Pewne rzeczy się zmieniły – pozostanie w domu to wcześniej był w większym stopniu wybór, teraz to raczej konieczność. Co obecnie jest dla Pana największym wyzwaniem?**

Szczerze mówiąc, ja żadnych wyzwań na razie nie widzę. Jak wychodziłem z psem na spacery, tak dalej wychodzę. Jeśli będę musiał pojechać do pracy, żeby coś fizycznie załatwić, to też pojadę – nadal wolno mi to zrobić. Nie odczuwam na razie konieczności bywania w sklepach odzieżowych, sportowych – jakichkolwiek innych, niż spożywcze, więc to mnie nie dotyka. Brakuje mi jednak obiektów typu siłowni, ścianek wspinaczkowych, klubów sportowych – to jest coś, co mnie najbardziej dotyka w tej sytuacji.

*Propozycja respondenta, żeby przejść na „ty”.*

**A jak myślisz, skąd wziął się cały problem? Czy są jacyś winni? Dlaczego wirus się rozprzestrzenia? Jak to postrzegasz, zarówno w skali globalnej, jak i lokalnej?**

Wirus się rozprzestrzenia, ponieważ jest niezwykle zaraźliwy – to jest pierwsza przyczyna. Drugą jest to, że przynajmniej przez kilka dni, a u niektórych zarażonych nawet cały okres, kiedy wirus pozostaje w organizmie, przebiega bezobjawowo, więc oni nawet nie zdając sobie sprawy z tego, że są nosicielami, mogą tego wirusa rozsiewać. Plus to, że na początku, zanim zaczęto wprowadzać te obostrzenia, oczywistym było, że jak ludzie zaczęli zbierać się w większe grupy, tempo rozsiewania wirusa rosło wykładniczo. Wraz z wprowadzaniem ograniczeń, można było trochę zahamować przyrost chorych, ale też na razie wydaje się bardzo prawdopodobne, jeżeli nie pewne, że ta liczba jeszcze przez dłuższy czas będzie rosła.

**Jaki to może być czas?**

Jeśli dobrze pamiętam, te szacunki matematyczne pokazywały, że niezależnie od obostrzeń sanitarnych, krzywa zachorowań będzie rosła przez jeszcze kilka miesięcy.

**Czy wydaje Ci się, że można było zrobić coś wcześniej, żeby sytuacja nie wyglądała tak, jak teraz?**

Na pewno można było wcześniej wprowadzić te obostrzenia, choć to oczywiście zawsze jest trochę wróżenie z fusów i każdy jest mądry po szkodzie. To, co można było przewidzieć ze strony rządzących to to, że potrzebne będą środki ochrony, takie jak maski, rękawiczki, fartuchy, filtry do respiratorów. I to można było z dużym wyprzedzeniem zamówić, bo ten wirus szalał w Chinach cały styczeń i był na to czas. To nieprzygotowanie ochrony zdrowia jest według mnie ogromnym zaniedbaniem rządu.

**Powiedziałeś już, co sprawiło według Ciebie, że wirus rozprzestrzenia się. A skąd według Ciebie on się wziął?**

Wziął się z innego szczepu koronawirusa, który zmutował. Z tego, co na razie wiadomo, co jest najbardziej prawdopodobne i na co wskazują dane naukowe, był to wirus występujący u zwierząt i został przeniesiony na ludzi.

**A czy wiesz, czym naukowcy, politycy, inni ludzie tłumaczą to, jak szybko rozprzestrzenił się ten wirus?**

Wydaje mi się, że wiem. Teraz wirusy będą rozprzestrzeniać się szybciej ze względu na bardzo dużą migrację ludzi po świecie. Bez zamknięcia granic nie sposób kontrolować przemieszczania się ludzi, a nawet zamykając granice jest to utrudnione, bo jednak jakiś transport i wymiana międzynarodowa musi być. Do tego przemieszczanie się ludzi wewnątrz kraju ciężko jest zablokować i kontrolować. Plus to, o czym wspominałem wcześniej – wysoka zaraźliwość tego szczepu wirusa.

**Czy myślisz, że można było zapobiec epidemii?**

Wydaje mi się, że nie. W momencie, kiedy wirus zmutował, zaraził odpowiednio wysoką liczbę ludzi, to już było prawdopodobnie niemożliwe do zatrzymania. Bardzo trudno było wyłapać ten ostatni moment, kiedy można było jeszcze zatrzymać to w małej grupie ludzi.

**Jak myślisz, czy jako kraj jesteśmy przygotowani na taką sytuację?**

Przykład Polski, Włoch, czy Hiszpanii pokazuje, że nie jesteśmy przygotowani. Niestety, ochrona zdrowia jest za mało wydajna i nie radzi sobie z taką liczbą zachorowań.

**Czy w związku z tym uważasz, że podejmowane w Polsce działania są słuszne?**

W tej sytuacji to jedyne wyjście, ale to nie leczy tych wszystkich zaniedbań, które już powstały, związanych choćby z tymi maseczkami, o których ciągle się mówi. Uważam to za skandaliczną sytuację, że lekarze nie mają podstawowych środków ochrony osobistej.

**A czy sam masz w domu niektóre z tych środków?**

Ja mam, tuż przed tymi obostrzeniami miałem możliwość wziąć kilka maseczek ze swojej pracy – jestem doktorantem w Instytucie Medycyny Doświadczalnej i Klinicznej, zajmuję się neurochemią.

Jeszcze wtedy był to produkt ogólnodostępny, więc nie doświadczyłem braku rękawiczek, czy maseczek. Teraz jednak widzę, że ich dostępność jest niska, a jeśli produkty się pojawiają, ceny są co najmniej kilkukrotnie wyższe, niż jeszcze dwa miesiące temu. Na przykład za paczkę rękawiczek, 3-4 miesiące temu płaciłem 15 zł, a teraz one są sprzedawane po 50-60.

**Zaniedbania o których mówisz są długo- czy krótkoterminowymi uchybieniami?**

Zarówno ten, jak i poprzedni rząd jest odpowiedzialny za to, że szpitale i cała ochrona zdrowia była przez lata niedoinwestowana. Uważam, że zdecydowanie za niski procent PKB był przeznaczany na ochronę zdrowia. Te maseczki i inne środki ochrony zdrowia to już takie ostatnie, bardziej widoczne zaniedbania, ale problem na pewno zaczął się znacznie wcześniej i był dużo głębszy.

**Czy Europa jest według Ciebie przygotowana na zaistniałą sytuację?**

Nie wiem, czy możemy zbiorczo traktować Europę. Jest w niej mnóstwo krajów, które radzą sobie z tą sytuacją i takich, które sobie z nią nie radzą. Na przykład Niemcy radzą sobie relatywnie dobrze, a właśnie Włochy, czy Hiszpania – bardzo źle. Ale gdybym miał podać jedną konkretną wartość, powiedziałbym, że Europa nie jest przygotowana. Są wskaźniki, abstrahując już od liczby chorych, że cała Europa będzie przez prawdopodobnie kilka lat znowu w recesji i jest to jeden z większych kryzysów – również finansowych. Dlatego wydaje mi się, że Europa nie jest przygotowana.

**Co sprawia, że dany kraj jest przygotowany?**

Po pierwsze są to sprawnie działające służby – na pewno bardzo wydajna służba zdrowia. Szybko i skutecznie wprowadzane procedury – na przykład kwarantanny, czy testów laboratoryjnych. Plus oczywiście dostęp do odczynników, do przeprowadzania tych testów i wszystkich niezbędnych akcesoriów medycznych.

**Co można by zmieniać, aby tak było?**

Należałoby zacząć od dofinansowania całej ochrony zdrowia, tak, żeby zatrzymać w Polsce jak najwięcej lekarzy i pielęgniarek. Zadbać o to, aby w szpitalach nie brakowało respiratorów, ale też - co ostatnio okazało się problemem – żeby w szpitalu nie brakowało personelu przeszkolonego do obsługi tego sprzętu. I oczywiście zadbać o dostępność wszelkich materiałów ochronnych, aby chronić zarówno personel, jak i pacjentów – nie tylko koronawirusowych, ale też innych. W szpitalach cały czas będzie mnóstwo pacjentów przebywających tam z różnych przyczyn, dla których tym bardziej groźny może być ten wirus.

**Z tego co powiedziałeś, zrozumiałam, że ważne są dla Ciebie procedury. Jak w związku z tym oceniasz te wprowadzane obecnie w naszym kraju?**

Procedury związane z kwarantanną i izolacją są dobre, ale brakuje mi wielu innych, związanych na przykład z pobieraniem próbek, przeprowadzaniem testów diagnostycznych, miejscem

przeprowadzania tych testów, czy dostępnością odczynników do nich. Tak więc uważam, że wprowadzone procedury są w większości niezłe, ale nie wystarczające.

**Według Ciebie, aby były one wystarczające lub przynajmniej lepsze, potrzebne było lepsze zaplecze finansowe, czy dobrze to zrozumiałam?**

Przede wszystkim chodziłoby o większą liczbę ośrodków wykonujących testy. Zaczyna się to wprowadzać na większą skalę, ale tu znów są ogromne zaległości, zaniedbania – to powinno być robione już od dawna. Teoretycznie każda uczelnia przyrodnicza w Polsce ma takie zaplecze plus mnóstwo placówek chociażby PANowskich mogłoby takie testy przeprowadzać, jakby tylko ktoś dostarczył im próbki i odczynniki. Ale te rozwiązania nie zostały wprowadzone i dopiero teraz pomału się je wprowadza.

**A jeśli chodzi o źródła informacji, z których korzystasz, czy coś się w tym aspekcie zmieniło?**

Nic się nie zmieniło – radio plus internet.

**Czy aktywnie poszukujesz aktualnych informacji?**

Przez większość czasu nie szukam aktywnie, bo jestem nimi zalewany. Aktywnie szukam, jeśli coś mnie zainteresuje, np. laboratoryjny sposób przeprowadzania tych testów – wtedy ja aktywnie zaczynam szukać i sprawdzać.

**Czy odkryłeś może jakieś nowe źródła informacji, z których wcześniej nie korzystałeś?**

Nie, nie wydaje mi się.

**A jak ci się wydaje – czas, który obecnie poświęcasz na śledzenie mediów jest porównywalny do przeznaczanego na nie dotychczas? A może coś się zmieniło?**

Odrobinę większy, choćby z tej przyczyny, że nie tracę czasu na dojazdy, więc mam więcej czasu wolnego dla siebie.

**Jednak rozumiem, że to nie tyle chęć znalezienia informacji na temat obecnej sytuacji, co większa ilość czasu wolnego jest tego przyczyną?**

Tak.

**A jak oceniasz wiarygodność mediów, do których mamy dostęp jako społeczeństwo?**

Jeśli chodzi o zdecydowaną większość mediów, to oceniam bardzo negatywnie. Niestety, większość krzyczących newsów wymaga zweryfikowania i zazwyczaj po ich zweryfikowaniu okazuje się, że są to informacje albo zupełnie fałszywe, albo trochę przekłamane, albo po prostu są niewłaściwie wyciągnięte wnioski z badań, które autor przejrzał i nie zrozumiał. Więc w przypadku znacznej większości portali ta wiarygodność w mojej ocenie jest dosyć niska. Z telewizją praktycznie nie mam styczności, więc nie sądzę, żebym mógł rzetelnie wypowiedzieć się na jej temat.

**A jeśli chodzi o tę wiarygodność, skąd wiadomo, że dana informacja jest prawdziwa lub nie? W jaki sposób i jak często je weryfikujesz?**

Po pierwsze patrzę na źródło – czy już na pierwszy rzut oka wydaje mi się wiarygodne, czy ono jest w ogóle podane. Jeśli nie ma podanego źródła informacji, z gruntu zakładam, że nie jest ona wartościowa. Jeśli jest podane, zwracam uwagę, czy ktoś powołuje się na konkretną publikację, czy na przykład na autorytet – lekarza, profesora, epidemiologa. Ewentualnie, jeśli odniesienie nawiązuje do zagranicznych mediów, trzeba z kolei sprawdzić te zagraniczne media. Ale nawet jak to jest to najbardziej rzetelne podane przeze mnie źródło – publikacja naukowa, warto sprawdzić, czy informacje trzymają się kupy, ewentualnie spojrzeć jeszcze do bibliografii. Jeśli z kolei jestem w stanie ocenić na pierwszy rzut oka, że informacje nie trzymają się kupy, z dużą dozą prawdopodobieństwa mogę stwierdzić, że ktoś wyciągnął z artykułu naukowego błędne wnioski.

**Jeśli chodzi o źródła, z których korzystasz, uważasz, że one w większości podają rzetelne informacje, czy jest z tym pewien problem?**

W większości mam do nich zaufanie, były przeze mnie wcześniej sprawdzane. Ale też zdarzają się wpadki, wyjątki.

**A czy mógłbyś podać źródła, które są według Ciebie wiarygodne i można na nich polegać?**

Obserwuję sporo różnych, zazwyczaj niewielkich, profili na Facebooku prowadzonych przez naukowców lub moich znajomych zajmujących się nauką – to są zazwyczaj wiarygodne źródła. Często linkują do wiarygodnych treści. Jeden, który przychodzi mi teraz do głowy to „Biokompost”, czy „Węglowy Szowinista”. Jest jeszcze „Nauka, głupcze”, powiedzmy, że „To

tylko teoria” - aczkolwiek to często wymaga sprawdzenia. Strona PAN. Z większych portali zazwyczaj OKO.press było jak dotąd wiarygodne – aczkolwiek nie śledzę ich na bieżąco, raz na jakiś czas przeczytam coś i ich strony.

**Czy myślisz, że wirus coś zmieni? Czy w momencie wygaszenia liczby zachorowań, wszystko po prostu wróci do normy?**

Wątpię, że zachowania społeczne, związane na przykład z higieną, ulegną zmianie. Obawiam się cięcia i ograniczania różnych programów pomocy społecznej, fali zwolnień pracowników na rynku, powrotu do większej liczby umów śmieciowych – myślę, że to będą bardzo odczuwalne efekty.

**Czy można było zrobić coś, aby nie dotknęło nas to gospodarczo w takim stopniu?**

Myślę, że znacznie wcześniej można było wprowadzić na przykład podatki progresywne, które zmniejszyłyby rozwarstwienie społeczne, które w wyniku kryzysu społecznego będzie się pogłębiać. Takie ogólne zmiany, które należałoby oczywiście wprowadzać dużo wcześniej i myślę przyłożyłoby się to też na wzrost PKB, nasza gospodarka byłaby silniejsza. Gdyby wcześniej przeprowadzić te reformy mające na celu wprowadzenie euro, też najprawdopodobniej nie odczulibyśmy tak mocno tych wahań finansowych.

**A jak odnosisz się do obecnych działań rządu związanych z gospodarką?**

Na razie mówi się o rozwiązaniach, które w mojej ocenie będą bardzo złe, jak ograniczenia kodeksu pracy, czyli to zwiększenie wymiaru godzinowego etatu i tygodnia pracy, ograniczanie urlopów, programów socjalnych. Moim zdaniem będzie to dla społeczeństwa zdecydowanie na minus.
