# Supplementary material for: The challenges arising from the COVID-19 pandemic and the way people deal with them. A qualitative longitudinal study
Source: PLoS One. 2021 Oct 11;16(10):e0258133. doi: 10.1371/journal.pone.0258133 (PMC8504766; doi:10.1371/journal.pone.0258133)
Supplement: S1 Dataset — (ZIP) [file pone.0258133.s003.zip › Transcriptions/stage 1/3.1_F_54_single.doc]

**3.1_F_54_single**

Mieszkam na głębokiej mazowieckiej wsi już od 8 lat, na stałe w zasadzie. Zajmuję się tutaj przede wszystkim współpracą z MOPSem w Płocku i z GOPSem u siebie, w mojej gminnej miejscowości, gdzie prowadzę zajęcia z seniorami. Jestem wolontariuszką w obu tych instytucjach. Prowadzę głównie zajęcia z seniorami, ale też zajęcia wyrównawcze, wizualne, z dziećmi – tutaj u siebie, na wsi.

**Czego dotyczą te zajęcia z seniorami?**

Po pierwsze są to zajęcia z brydża. Prowadzę z nimi takie koło brydżowe raz w tygodniu. Natomiast okazjonalnie, również w grupach – wtedy dużo większych – zajęcia plastyczne. Ale to już nie są zajęcia cotygodniowe. Tam jest taka grupa, która jest pozostałością po projekcie unijnym w MOPSie. To są zajęcia dla ludzi starszych i wykluczonych – tak to się kiedyś nazywało. MOPSowi udało się pozyskać pieniądze na 3 lata. Kiedy dotacje z Unii się skończyły, nie chcieli [MOPS] zostawić tych ludzi, więc stworzono nową komórkę. Nie wiem, jak wygląda to organizacyjnie, ale jest taka grupa seniorów i połączono ich jakoś ze świetlicami.

Płock w ogóle jest miastem starych ludzi, bo tak naprawdę tam dla młodzieży i dla młodych ludzi propozycji wielu nie ma. Ani pracy, ani niczego tak naprawdę. Więc ten Płock jest taki dość prężnie działający na rzecz starszych ludzi i w tej chwili tam obserwuję wręcz konkurencję ośrodków zajmujących się, czy robiących rzeczy dla starszych osób. Co zresztą bardzo dobrze robi tym starszym osobom, bo oni mają naprawdę dużo propozycji wielu miejsc, w których się mogą spotykać. Bo trzeba pamiętać, że takie spotkania, jeśli to ma być coś, z czego mogliby skorzystać, a nie tylko wykład na jakiś temat, nie mogą być jednorazowo organizowane dla bardzo dużych grup.

**Kiedy dla Pani zaczęła się sytuacja związana z koronawirusem?**

Ja nie oglądam telewizji, bo jeżeli ją oglądam, to jedynie programy sportowe – tylko one mnie nie denerwują. Więc tak naprawdę jeśli chodzi o media, to początek na pewno nie był dla mnie z nimi związany. Oprócz informacji, które gdzieś tam do mnie docierały chociażby przez telefon – nie jestem odizolowana całkiem – zwłaszcza od znajomych, którzy wiedzą, że ja nie oglądam żadnych wiadomości, dla mnie takim początkiem był komunikat o zawieszeniu wszelkich zajęć. Jeśli dobrze pamiętam, to było 9, czy nawet 10 marca. W MOPSie miała zostać zawieszona wszelka działalność zwyczajowa, dzieci miały przestać chodzić do szkoły od środy, czy tam czwartku. To był taki moment, kiedy zaczęłam myśleć o tym, że to jednak coś poważnego. Do tej pory mieliśmy jednego pana, który przyjechał z Niemiec i był w szpitalu, w Zielonej Górze. To wszystko oczywiście brzmiało mało śmiesznie, ale dla mnie również mało groźnie. Więc myślę, że to ten moment początku izolacji, tego, że coś się jednak dzieje poważnego.

**Jakie uczucia wtedy Pani towarzyszyły? Jakie były Pani pierwsze myśli?**

Trudno mi powiedzieć, trudno mi to sobie przypomnieć. Na pewno jestem za takimi rozwiązaniami. Myślę, że pomimo tego, że jestem absolutnie nie za obecną sytuacją polityczną, to myślę, że musiałam sobie pomyśleć, że to dobre rozwiązanie, słuszna decyzja. Na pewno nie wzbudza to we mnie jakiejś paniki. Jestem raczej nie strachliwa i myśląca racjonalnie. Nie przypomnę sobie pierwszej myśli, choć na pewno nie była to panika.

**A kolejny moment, kiedy znów poczuła Pani, że coś się zmienia? Był taki?**

Nie, to się nie zmienia, a pogłębia. Ta izolacja, czy też środki ostrożności się zwiększają. On block oceniam je jako słuszne. Z mojej perspektywy zmieniło się to, że zaczęłam oglądać wiadomości. Następnie zmieniło się to, że moje dzieci zauważyły, że ja oglądam wiadomości. W sensie takim, że ja się denerwuję, rośnie mi poziom agresji przez to pieprzenie w telewizji. Ale nie ze względu na koronawirusa, a z tych samych powodów, przez które zwykle nie oglądam wiadomości. Więc teraz już znowu przestałam je oglądać. Z punktu widzenia zewnętrznego świata, czyli tego, co się dzieje na zewnątrz, nie mogę powiedzieć, że jestem przerażona. Ale na pewno doceniam powagę sytuacji. Widzę, co się dzieje we Włoszech, widzę co się dzieje w ogóle na świecie i myślę sobie, że nie jestem optymistką. Mam pełną świadomość tego, że nas to po prostu nie ominie. Być może jesteśmy w stanie jakoś łagodniej przez to przejść. Może dlatego, że mamy mniej kontaktów i jesteśmy bardziej homofobiczni. I dlatego, że nasz rząd po prostu wie, że jesteśmy nieprzygotowani, więc musiał nas odizolować. Myślę, że na pewno słusznie, choć moim zdaniem to jest tak naprawdę złudne, że u nas jest lepiej, niż gdzie indziej. Za chwilę będzie jeszcze gorzej. Na pewno nie jesteśmy jeszcze w momencie, gdzie liczba chorych utrzymuje się w miarę na stałym poziomie, ani sytuacja się nie poprawia. Oczywiście, to minie, bo kiedyś musi.

Mam taką refleksję. Mieszkając na głębokiej wsi, rozmawiam z różnymi ludźmi – moi znajomi mieszkają głównie w dużych miastach, nie tylko w Warszawie. Sytuacja w mieście i tu, jest zupełnie inna. Rzeczywistość, w której żyjemy tutaj na wsi, jest po prostu zupełnie inna. Myślę, że powoli to dociera również do mieszkańców wsi, ale tu jesteśmy jakby odizolowani również na co dzień.

**Jakie są największe różnice, między miastem a wsią?**

Mieszkając w dużym mieście wydaje nam się, bo ja też w tym mieście przez kilkadziesiąt lat mieszkałam, że na tej wsi to są tacy ludzie jak my, tylko trochę im jest gorzej i mają trochę mniejsze możliwości. Myślimy, że oni generalnie funkcjonują tak, jak my. Obserwując to wszystko przez z te lata, od kiedy mieszkam na wsi i dzielę z nimi rzeczywistość, w której żyją, jestem zdania, że to jest zupełnie inny świat. Tu jest świat bez polityki. W którym ważne jest to, żeby mieć co włożyć do garnka. To jest świat, w którym poziom potrzeb, w stosunku do miejskich potrzeb, jest zredukowany do absolutnego minimum. W związku z tym, również poziom oczekiwań jest zredukowany do minimum. Trudno to wyrazić, choć wielokrotnie o tym myślę. Mieszkając w mieście, czy rozpoczynając swoją przygodę z prowincją, wydawało mi się, że możemy im tutaj pomóc. W takim sensie, żeby oni mogli sobie sami zapewnić rzeczy, które my mamy, tylko z naszą pomocą. Tymczasem oni w ogóle tutaj o takich rzeczach nie myślą. Nie potrzebują żadnego kina, teatru, książki. To jest dość niesamowite. Nie chcę powiedzieć, że to jest bieda. Dlatego, że z ich punktu widzenia to nie jest bieda. Choć z naszego punktu widzenia – tak. Znam rodziny, w których pod dywanem, czy tam pod płytą, na podłodze jest po prostu klepisko, nie ma betonowej wylewki. My, w mieście, nawet nie zastanawiamy się nad takimi rzeczami, czy to się dzieje, czy nie. I to nie są domy jakichś tam stuletnich osób, które nic już sobie nie zmienią, bo tak wolą. To są domy ludzi młodych, którzy z pokolenia na pokolenie rosną w takich warunkach – moim zdaniem zmniejszonych oczekiwań wobec życia. To pewnie sprawia, że oni mogą w ogóle jakoś funkcjonować. Bo jakby tak wejść tutaj, do takiego domu, jak ja wchodziłam do nich – 10, czy 12 lat temu, kiedy zaczynałam tu powoli przyjeżdżać, to naprawdę trudno było sobie wyobrazić, że komuś może nie przeszkadzać to, że żyje w takich warunkach.

**Jak taka rzeczywistość na prowincji wpływa na to, jak ludzie postrzegają sytuację związaną z koronawirusem?**

Oni w ogóle jej nie postrzegają. Rozmawiałam ostatnio z młodym człowiekiem. Jest tu część osób młodych, które zostały. Tutaj szczytem dobrej pracy jest kierowca tira. Część osób pracuje za granicą. Młodzi chłopcy w szczególności przy pracach fizycznych – jako spawacze, murarze, monterzy jakichś instalacji. Pracują w Niemczech, w Czechach i przyjeżdżają na weekendy. Przynajmniej większość z nich nie zdaje sobie za specjalnie sprawy, że przez takie działania zagrażają swoim rodzinom. Nie zdają sobie sprawy, że mogą zostać zarażeni i przechodzić infekcję bezobjawowo. Dla nich, skoro to jest bezobjawowo, to znaczy, że tego po prostu nie ma.

Niedawno pomagałam takiemu młodemu człowiekowi, który właśnie zjechał z Czech. To było jeszcze przed zamknięciem granic i przyjechał na weekend. No, ale już nie wyjechał. Jednak nie poddał się żadnej, nawet dobrowolnej, kwarantannie. Pomimo tego, że ma chorą matkę i żonę w ciąży oraz półtoraroczne dziecko. Nie przyszło mu to do głowy chyba. Wczoraj pomagałam mu poprawić CV, ponieważ już nie wróci do tych Czech, ze względu na jakieś kłopoty na granicy. Stwierdził, że poszuka czegoś na miejscu. Zasugerowałam, że teraz może taka nie najlepsza sytuacja do tego szukania pracy, na co on mówi, że to wszystko ściema jest. Dopytałam, co jest ściemą. Odpowiedział, że ten wirus, że najpierw mówili, że go nie będzie, potem mówili, że coś tam jest. On na początku to się nawet przejął, ale teraz mówi, że to ściema. Powiedziałam, chłopie, ty sobie włącz na przykład TVik i obejrzyj, co się dzieje we Włoszech, jak przed tymi cmentarzami po prostu korowody tych samochodów pogrzebowych, tych trumien i nie ma nawet gdzie tych zwłok trzymać... Ale nie, on ma teraz takie zdanie, że to jest ściema. A to jest młody człowiek.

Rozmawiałam też ze starszą sąsiadką. Oglądała jakieś wiadomości i była mocno zmieszana, poruszona. Prosiła, żebym zrobiła jej zakupy, jak będę jechała i że ona zrobi mi listę. Była mocno poruszona, ale nie na tyle na przykład, żeby jej syn, który jeździ na tirach, a ma 50 parę lat i pojechał do Austrii, a później coś przywiózł, nie przyjechał po tym do niej w odwiedziny, bo ona zrobiła już kartoflaka, czy babkę ziemniaczaną, czy cokolwiek. No, no nie mają wyobraźni moim zdaniem.

**Ta wyobraźnia jest ważna?**

To tak w ogóle jest kwestia wyobraźni. To jest osobna rzecz, ale ona z czegoś powstaje. Nie rodzimy się z bujną wyobraźnią. Musimy coś czytać i wiadomo, ona jakoś się wtedy kształtuje. Chyba że jesteśmy Anią z Zielonego Wzgórza. No, ale oni tu nie mają żadnej wyobraźni, umiejętności wytworzenia możliwych scenariuszy. To jest tutaj na takim absolutnie podstawowym poziomie – jest informacja i albo się można przestraszyć, albo nie. A jak damy 4 sprzeczne informacje, to już taki młody człowiek za dużo ma tego przetwarzania, więc po prostu najchętniej się z tego wyłącza. Tak, ściema i dziękuję bardzo. No i on już nie będzie więcej o tym myślał. W związku z tym, skoro sobie taki przepis na to wyrobił, to jest to ściema.

Niektórzy na przykład mówią o tym, ale to tak jakby ich to nie dotyczyło. Jest taka rozmowa, temat dyżurny. Coś trzeba o tym powiedzieć, bo rzeczywiście ktoś tam umiera i w ogóle, i tyle ludzi. Na przykład liczby w telewizji robią wrażenie, jak je oglądają. To wtedy: „tyle ludzi”! Ale u nas te 30, czy tam nie wiem, jaka jest aktualna liczba, to w ogóle nie robi żadnego wrażenia. W ogóle nas nie dotyczy, ich nie dotyczy. Gdyby to coś się stało gdzieś u nas. Gdyby był jakiś przypadek, że w Dupie Wołowej, gdzie pani doktor wróciła z Włoch i zaraziła dwie wsie, zanim doszła do wniosku, że jednak może jest chora. Moim zdaniem wtedy zachorowalność byłaby 90%, bo ci ludzie nie przestrzegają żadnego... Ale wtedy to by ich dotyczyło, oni by byli w centrum tych wydarzeń. Dopóki tak się nie stanie – i oby się nie stało – oni nie mają takiego wyobrażenia, scenariuszy. U nas tego na szczęście nie ma, więc nie ma problemu i taka jest konkluzja.

Oni się cieszą, że benzyna jest tańsza o złotówkę, czy półtora złotego za litr. To są w większości rolnicy. Muszą wyjechać na pola, więc to robią. Z zachowaniem zasad bezpieczeństwa, ale nie dlatego, że oni świadomie to robią, tylko dlatego, że w takim ciągniku siedzi jedna osoba. W związku z powyższym, w miarę bezpiecznie przemieszczają się po polu.

A teraz na przykład zwalniają z KRUSu. I oni nie wiedzą, czy mają płacić, czy nie, czy jakiś wniosek napisać. Ale za bardzo się tym nie przejmują.

No nie ma problemu. Jest problem, bo ciężko robi się zakupy. Bo teraz „wymyślili sobie”, że będzie trzeba stać pod sklepem. Mamy w takiej innej miejscowości nieduży supermarket, tam jest 20 wózków i do sklepu nie może wjechać więcej osób, niż jest wózków. Pozostali czekają. Ale nie w metrowych odstępach, tylko rozprawiają razem nad tym, kto to w ogóle wymyślił, że nie można wejść.

Akurat wczoraj byłam w jakiejś takiej pechowej godzinie, że w trakcie zakupów musiałam wyjść ze sklepu, bo miała być jakaś tam przerwa technologiczna, nie wiem. Jak się zmienia zmiana chyba. Nie wiem, czy oni tam wtedy dezynfekują lady, czy coś tam, wszystko jedno. I jest godzinna przerwa. I to jest dla tych ludzi skandal. Jak taka pani przyjechała ze swojej wsi 5 km, to jak teraz ma wrócić do tej swojej gminy, żeby znowu potem przyjechać do innej miejscowości. Ile to paliwa trzeba zużyć do tego celu? A jeszcze w międzyczasie nic nie można zrobić. Bo to jest miejsce, w którym i tak nic nie można zrobić, ale tak to przynajmniej można było pójść do szmateksu na przykład i pogrzebać w ciuchach, a teraz jest zamknięte.

I tak jest na tych głębokich wsiach. W Płocku, do którego zdarza mi się raz na jakiś czas pojechać, jest tak może trochę jak w Warszawie. Chociaż myślę, że ci ludzie nie mają aż takiej świadomości. Ale choćby przed aptekami są kolejki z odpowiednimi odstępami. U nas na wsi, gawiedź stoi w kupie i rozprawia. Także, gdyby którykolwiek z nich był zarażony, to nie ma nie ma możliwości, żeby się pozostała część nie zaraziła.

Z mojego punktu widzenia, na przykład dobrymi posunięciami są obostrzenia takie jak obowiązek chodzenia w sklepie, w rękawiczkach. Jak coś jest obowiązkowe, to to wykonają, zwłaszcza, jak ktoś im zwróci uwagę. Pomarudzą sobie, ale zrobią. To się wszystko zmienia, ale ten strach przed zaborcą, panem, czy jak go nie nazywać, jest silny. Ważne, żeby ludzie mieli świadomość, ze ktoś patrzy, w przeciwnym razie i tak zrobią po swojemu. Jak na przykład na stacji benzynowej, gdzie trzeba tankować w rękawiczkach. Dopóki ktoś nie zwróci im uwagi, to tego nie zrobią. Ale jak wyjdzie ktoś z obsługi – pomarudzą, ale założą rękawiczkę. Inna kwestia, że później nie będą potrafili jej odpowiednio zdjąć i wyrzucić.

**A jak Pani reaguje na tą całą sytuację?**

Jeżeli chodzi o to, że siedzę w domu, sama, bez wychodzenia i bez takich rzeczy, to ja to lubię i wcześniej również robiłam. Poza dniami, kiedy wychodziłam, czy do uczniów, czy to do tych seniorów, to pozostałą część tygodnia spędzałam w domu. To mnie w ogóle nie denerwuje, nie nudzi. W dalszym ciągu mam kolejkę rzeczy do zrobienia i takich, których i tak nie zdążyłam zrobić. Natomiast doskwiera mi na pewno to, że nie widuję dzieci. Obydwoje, dorośli już, pracując zdalnie, siedzą w domu. Postanowili, że zanim się ze mną spotkają, muszą co najmniej dwa tygodnie zostać w domu, bo jedno jedno i drugie pracuje w mieście i mieli styczność z ogromną liczbą ludzi – córka pracuje w centrum handlowym, a ja jestem z grupy ryzyka. Poczułam się z 15 lat starsza, niż jestem. Na początku uznałam, że przesadzają trochę, ale może i nie przesadzają, bo rzeczywiście mają dużo większe, niż ja, prawdopodobieństwo kontaktu z wirusem. Ale już tęsknię do nich. Córka, która mieszka sama z chorym kotem, przyjedzie do mnie w piątek, bo uznała, że już tę kwarantannę odbyła. To już będzie prawie 3 tygodnie, kiedy ona nie wychodzi, więc prawdopodobieństwo, że coś przyciągnęła, nie jest takie duże. Ona przyjedzie, bo myślę, że ona sama dostaje już świra sama ze sobą. Jakieś koleżanki robiły jej zakupy, bo uważała, że miała kontakt z jakąś koleżanką, która najprawdopodobniej jest chora. No, ale nie dowiemy się tego tak naprawdę oprócz tego, że ona nie jest chora osobiście. Więc teraz już zdecydowała, że w piątek do mnie przyjedzie.

No trochę mi doskwiera, tak naprawdę, brak kontaktu z bliskimi. Nie mam dużej grupy znajomych, zwłaszcza takich, z którymi się spotykam. Teraz, ponieważ izolacja jest trochę wymuszona, chętnie bym się z nimi spotkała, zagrała w brydża, co nam się ostatnio nie udało – mieliśmy taki pomysł, ale nam nie wyszło, z powodu braku czwartej osoby do gry. A teraz nie wiadomo, kiedy uda nam się spotkać.

Jak wychodzę, zachowuję się dosyć racjonalnie. W samochodzie mam rękawiczki, ubieram je. Mam to przemyślane. Zdejmuję je po wyjściu ze sklepu, nie używam ich już później, nie dotykam niczego. Myję ręce, ale to nie jest dla mnie nowość i nie zdzieram sobie skóry do krwi. I to tak naprawdę tyle. Przyjmuję to wszystko z jakimś takim... Myślę, że są mądrzejsi od nas, ode mnie na pewno. I mam nadzieję, że wiem co robią lub przynajmniej wybierają mniejsze zło.

**Czyli nie tyle samotność, co brak swobody jest problemem?**

Tak, nawet pojawiały mi się takie myśli, żeby się z kimś spotkać, nawet tu lokalnie z moimi seniorami, których wspieram. Ale później włącza się w głowie taki kontroler, i myślę sobie, a w dupie, wiadomo z kim oni się tutaj spotykają i czy ktoś myśli o nich, żeby ich nie zarazić? Na początku wydawało mi się, że to i tak nie dojdzie na tę zabitą dechami wieś, ale teraz wolę nie kusić losu. Nie podejmuję pewnych działań, żeby nie kusić złego. Parę dni temu się nawet pocieszałam, bo trochę martwię się o gospodarkę i kondycję finansową kraju, że ludzie przeżyli wojnę i przez 5 lat działy się straszne rzeczy, a się podnieśliśmy. Zdaję sobie sprawę, że to są jakieś koszty, dla niektórych nawet ogromne. Ale jakoś damy radę.

**Czy są jakieś pozytywne strony tej sytuacji?**

Na pewno staramy się je znaleźć. Jak rozmawiałam z moim synem, on jest teraz zadowolony, bo dużo czasu spędza z dziewczyną, z którą mieszka. Ona nie mówi za dużo, a to mu pasuje. Ale razem na przykład gotują. Więc to, że spędzamy więcej czasu z najbliższymi – to jest jakiś plus, choć mnie nie dotyczy. Ale to też różnie bywa, bo to zależy od tego, w jakich relacjach jesteśmy z tymi najbliższymi – czasami to, że spędzamy z nimi więcej czasu, może nas bardziej wkurzać, niż robić nam dobrze. Część osób spędza teraz więcej czasu z dziećmi, więc też potrafi ich to dużo kosztować. Zwłaszcza, że niektórzy muszą być teraz jeszcze nauczycielem, wykładowcą i innymi. Możliwe, że jak to wszystko się skończy, docenimy takie małe rzeczy, będziemy potrafili się nimi cieszyć. Przynajmniej te osoby, które mają świadomość, czym grozi ta sytuacja. Może nie będzie trzeba wyjechać na wakacje nie wiadomo gdzie. Chociaż to raczej dotyczy ludzi miejskich, bo na wsi ludzie nie wyjeżdżają. Tutaj nikt nie jeździ do Turcji, Hiszpanii, czy w takie kraje. Jak jeździli nad morze na trzy dni, to tak dalej pewnie będą robić. Trudno siedzieć w ich skórze i stwierdzić, czy to im coś da. Właściwie nawet nie chciałabym siedzieć w ich skórze.

**Skala lęku**

W obecnej sytuacji, w granicach 30. Trochę boję się o bliskich, trochę o moją sytuację materialną. Los moich dzieci nie jest mi obojętny, a one są bliżej tego wszystkiego – strefy zero, więc jest większa szansa, że zachorują. Ja dostaję alimenty od męża, więc jego kondycja finansowa jest dla mnie ważna. Cała sytuacja polityczno-gospodarcza jest niepokojąca. Jeśli na czele rządu stałaby Pani Merkel, martwiłabym się mniej, bo miałabym świadomość, że to jest bardziej racjonalne. Nasza rzeczywistość ekonomiczna jest taka, że jakoś się podniesiemy, ale nasza ekonomia jest bardzo powiązana z polityką. Możliwe, że dotychczas mogłam się od tych rządów odciąć, ale teraz okaże się, że już nie jestem wystarczająco daleko na tej mojej wsi, że ich decyzje wpłyną także na moje życie.

**Boi się Pani, że zachoruje?**

Raczej nie, choć jak zachoruję, to raczej wyzdrowieję, bo takie są statystyki. Choć prawdopodobieństwo, że zachoruję jest chyba jeszcze mniejsze.

**Proszę się zastanowić, jakie emocje Pani towarzyszą w tej sytuacji. Czy jest tu jakiś obrazek, który by do nich pasował?**

14 – on pasuje do tego, co się teraz z nami dzieje. To jest węzeł, który jakoś trzeba rozwiązać, jest symbolem obecnej sytuacji. I jeszcze 8 – taka droga we mgle. Nie bardzo wiadomo, co nas czeka. Nie widzimy tego, co będzie dalej i na przykład nie wiemy, jak sobie poradzić z tym węzłem. Ale mgła też kiedyś się skończy, nie jesteśmy w czarnej dupie. Jak już poruszamy się drogą, jesteśmy na niej, to widzimy to wszystko lepiej, niż patrząc z perspektywy komputera. Jakoś damy radę. A ja na przykład świetnie rozwiązuję węzełki.

**Kto powinien rozwiązać ten węzeł?**

On się z czasem sam rozwiąże. My podejmujemy jakieś czynności, które mają w tym pomóc, ale nikt z nas personalnie nie jest w stanie tego zrobić. Tak, jak nie jest w stanie rozdmuchać tej mgły. To po prostu za jakiś czas się stanie. Część rzeczy na pewno możemy robić my – zachowywać się dość rozsądnie, na tyle, żeby nie zaciskać tego węzła, choć większość nie zależy od nas. Musimy na pewno uważać. Jak jest mgła, należy zachowywać się rozsądnie, zwolnić, żeby się nie rozbić. I to od nas w pewnym sensie zależy, czy przeżyjemy, czy nie. Ale ona po prostu kiedyś minie. Nie jestem wirusologiem, choć nawet nie wiem, czy bym chciała, i niewiele ode mnie zależy.

**Jak się Pani z tym czuje, że tak niewiele od nas zależy?**

Niewiele od nas zależy w sytuacji indywidualnej walki z koronawirusem. Jeśli chodzi o nasze życie, jest nieco inaczej. To jest tak samo, jak zwykle, więc czuję się tak samo – na co dzień też niewiele ode mnie zależy. Jak to, co dzieje się na Wiejskiej, czy w Chinach – tam jeszcze mniej ode mnie zależy. Nie postrzegam tej sytuacji jako szczególnie innej w zakresie kontroli. To, na co mam wpływ, nadal kontroluję, czyli własne działania. Nie mogę mieć wpływu na działania innych, a jakie one są, to już Pani wie.

**Czy te zachowania innych Panią denerwują?**

Nie, po prostu wiem, jakie tutaj są realia. Co zrobić, tutaj po prostu tak jest. Staram się nie denerwować czymś, na co nie mam wpływu. Staram się też unikać takich sytuacji, w których to obserwuję. Dlatego też unikam oglądania wiadomości. To może jest postawa mało obywatelska, ale myślę sobie, że nie ma sensu denerwować się czymś, na co nie mam wpływu. Mogę powiedzieć takiej pani, nawet ze dwa razy próbowałam, nawet w trosce o siebie, że nie powinniśmy stać tak w kupie. I ta pani na mnie fuka, trochę się może oddali, ale później i tak pcha się do tego sklepu. Więc nie denerwuję się, bo nic, zwłaszcza na plus, to nie wnosi. Coś czasami powiem, ale nie występuję w pozycji mentora. I tak jestem tu postrzegana inaczej – pani z miasta przyjechała.

**Nadal jest Pani dla nich tą miastową?**

Myślę, że tak. Ja jestem trochę ich, bo im pomagam, robię tutaj różne rzeczy, z których się cieszą. Ale jednak przyjechałam z tego miasta, więc jak trzeba coś zrobić – PIT rozliczyć, czy coś napisać, zwracają się do mnie. W związku z tym, znoszę później do domu dużo jajek, bo nie chcę za to pieniędzy, więc oni się odwdzięczają tym, co mają, czyli głównie jajkami.

**A jakie emocje obserwuje Pani u ludzi w swoim otoczeniu, na wsi?**

Ja się teraz z nimi raczej nie spotykam, tylko rozmawiam przez telefon. Oni poza tym są w ogóle kiepscy w okazywaniu emocji. Nikt ich tego nie nauczył i oni nie bardzo wiedzą, jak to się robi. Tak, jak Pani mówiłam, ta jedna sąsiadka, która posłuchała sobie telewizji, zadzwoniła do mnie i powiedziała, że była „silnie poruszona, silnie zdenerwowana”. Choć oni też tak po prostu mówią. Myślę, że ona po prostu była poruszona tym, że tyle osób umiera, choć to jest daleko. Jeśli chodzi o większość, to myślę, że oni leją na to. Ewentualnie denerwują się, że czegoś nie można kupić, albo nie wiedzą, co będzie, bo ich dzieci nie chodzą do szkoły. Nie doceniają powagi sytuacji, bo to ich nie dotyczy, nie ma namacalnego wymiaru. To nie dzieje się w ich otoczeniu, więc to też jest inaczej. Samo to, że jest zimno na zewnątrz, też wpływa na tę sytuację, bo oni przez to oglądają telewizję. Gdyby było ciepło, oni w ogóle już by nie wiedzieli, co się dzieje. Oczywiście, nie mogę wypowiadać się za wszystkich, ale tutaj, na głębokiej mazowieckiej wsi, która jest na tyle blisko Warszawy, ze każdy, kto miał jeszcze jakieś predyspozycje do zmiany swojego życia, wyjechał, tak to wygląda. Jest tu na przykład dużo osób nietrzeźwych cały czas, którzy nie wiem, czy cokolwiek wiedzą na temat koronawirusa, nie mówiąc już o ich możliwościach wymówienia tej nazwy.

**Jak emocjonalnie reagują na tę sytuację Pani dzieci?**

Mój syn jest dość racjonalny. Ale na początku tych obostrzeń był taki moment, kiedy on miał zjazd emocjonalny i się denerwował, nie mógł spać, miał jakąś gonitwę myśli, wszystkim się okropnie przejmował. Głównie kondycją gospodarki. Choć może nawet nie tyle własną sytuacją, co położeniem ojca, który ma własną firmę, a oprócz utrzymania mnie i córki, która zaczęła dopiero pracę, na swoje barki wziął jeszcze utrzymanie nowej rodziny. Syn po rozmowie z ojcem bardzo się zmartwił. Choć po rozmowie ze mną i z dziewczyną, po kilku dniach przemyślał to sobie, jakoś zracjonalizował i wrócił do dobrej formy. Myślę, że w tym momencie jest w dobrej kondycji. Stosują wszystkie zasady, ale w jego przypadku można by myślę zdecydowanie mówić o lęku jednorazowym, ale potężnym. Córka jest na pewno bardziej niestabilna emocjonalnie. Ona przeżywa emocje mniej pod kontrolą rozumu. Jest bardzo zorganizowana, potrzebuje tego. Miała taki gorszy dzień, jak jej koleżanka miała temperaturę i rozmawiała z SANEPIDem. Nie dowiemy się, co to było, bo po trzech dniach gorączka przeszła. Po tym incydencie, córka miała lepszy nastrój. Teraz myślę, że doskwiera jej samotność. To nie tak, że ona nie potrafi być sama, ale myślę, że ona ma już po kokardę. Jakiś czas temu byłam w Warszawie, podrzuciłam jej zupę. Wyściskałyśmy się i było cudownie. Wie pani, jak jest źle, potrzebna jest mama. Ale niedługo się zobaczymy, bo skończyła się ta jej kwarantanna, którą ona sobie sama narzuciła. W piątek przyjedzie i będzie zdalnie pracować stąd.

**Jak usłyszała Pani o zamykaniu szkól, zaczęła Pani jakieś przygotowania?**

Ja nie mam zbyt dużej styczności ze szkołami, poza tymi dziećmi, którym pomagam. Choć to jest takie pomaganie, że ja im nie bardzo mogę pomóc, co nie jest dobrą wiadomością. To jest niezwykle trudne, jak na przykład mam ucznia z 5 klasy, który nie umie czytać, liczyć. Jest to po prostu dość specyficzny rodzaj pomocy. Na początku nawet się trochę ucieszyłam, pomyślałam, że to trochę jak ferie. Ale jak teraz są te lekcje online, gdzie oni mają sobie o czymś przeczytać, to w ogóle nie ma opcji, żeby oni coś zrozumieli. Więc mam takich dwóch uczniów i jeżdżę do nich. Ale nie przejmuję się za bardzo, że oni czegoś nie potrafią. Ta pomoc ogranicza się raczej do tego, żeby pomóc im funkcjonować w szkole, żeby w ogóle przez nią przeszli. Choć oni i tak są przepychani z klasy do klasy, skoro w 5 klasie jest uczeń, który nie potrafi czytać. Mi raczej chodzi o to, żeby ktoś go kiedyś za coś pochwalił. To nie jest nauczanie na ich poziomie, ale on może być szczęśliwy na inny sposób.

**A czy pojawiły się u Pani jakieś zmiany konsumenckie? Może zrobiła Pani jakieś zakupy na zapas?**

Nie, choć zastanawiam się na przykład, dlaczego ludzie kupili papier toaletowy. Zastanawiałam się nad tym z moim synem, stwierdziliśmy, że to może dlatego, że to objętościowo jest dużo, za stosunkowo niewielkie pieniądze. Bo przecież to nie jest schorzenie, które wywołuje biegunkę. Jeśli chodzi o moje nawyki, zazwyczaj raz w tygodniu, w poniedziałki, robiłam zakupy w Płocku, później ewentualnie drobne rzeczy na bieżąco. Miałam listę, taki porządek w tych zakupach. Teraz nie jeżdżę już do Płocka. Dużo jedzenia dostaję, jak te jajka. Dostaję też mleko, z którego robię sery, ale to nie w związku z koronawirusem – robiłam tak wcześniej. Teraz nie jeżdżę już do Płocka, robię zakupy bliżej, ale cierpię w związku z tym, bo nie mogę sobie kupić dobrego chleba. Nie to, żeby w Płocku był znów taki świetny, ale kupuję w Lidlu, taki, żeby miał przynajmniej jakiś smak chleba. Zrobiłam zapasy, bo kupiłam dwie kostki drożdży, a zazwyczaj mam jedną. Potrafię zrobić chleb sama i planowałam to, ale na razie jeszcze, w czasie koronawirusa, tego nie zrobiłam.

**Czy ludzie w Pani otoczeniu robią zapasy? Jak to wygląda?**

Mam tutaj znajomego, który w naszej miejscowości ma sklep. Jak zaczęto zamykać szkoły, ludzie wykupili makaron, choć z nieznanych mi powodów. Tutaj przecież podstawą wyżywienia są ziemniaki. Makaron wykupowano w miastach, było też o nim dużo w relacjach z Włoch, więc to chyba była przyczyna i makaron znikał też z hurtowni. Byłam też jakiś czas temu w Auchan, w Płocku. Tam jest taki kącik konesera makaronów, co mnie zawsze niezwykle bawi. I wtedy cała ta półka, łącznie z kącikiem, była pusta. Pomimo tego, że w kąciku te makarony mają głównie wysoką cenę. Ale teraz nie robię w Płocku zakupów, i nie robię zapasów. Ale teraz jest już chyba zwyczajnie, nie ma już pustych półek.

Tutaj w okolicy, zakupy zazwyczaj też robiło się w poniedziałki, ze względu na targ, który wtedy rozstawiał się w jednej z pobliskich miejscowości. Teraz targu nie ma, policja zabroniła. Choć nie wiem w sumie dlaczego, bo widziałam w zarządzeniach, że pan minister pozwolił na to, aby trzy osoby stały przy jednym straganie. W każdym razie oni zawsze robili część zakupów na targu, część w sklepach i zazwyczaj tak raz w tygodniu. Więc wydaje mi się, że te zakupy łącznie nie są wcale większe, niż wcześniej. No, poza tym momentem, kiedy był szał zakupowy, który już minął.

**Nie robi Pani zapasów, ale ile byłaby Pani w stanie przeżyć na tym, co Pani ma?**

Ja mam bardzo dużo przetworów. W tym momencie około dwustu słoików i to tak jest końcówka. Mam sad, kawałek ogródka i świra na punkcie przetwarzania. Jakbym nie mogła wyjść, może nie byłabym bardzo zadowolona, to z miesiąc? Ale jakby mnie Pani zapytała przed koronawirusem, byłoby podobnie. Tym bardziej, że ja mąką kupuję w młynie, a tam sprzedają najmniejsze worki po 5 kg.

**Skąd wziął się koronawirus?**

Poczytałam w internecie, gazecie. Wiem, że to grupa wirusów, że to jedna z nielicznych jadowitych odmian tego wirusa. Że jest zaraźliwy, ale nie bardziej, niż wirus odry i ospy. Więc jest umiarkowanie zaraźliwy. A śmiertelność jest spowodowana głównie zapaleniem płuc, które wywołuje. A wiem to, bo znajomy lekarz wyjaśnił mi ten mechanizm – z tymi cofającymi się rzęskami. Więc wiem sporo, warto znać swojego wroga.

**Co Panią skłoniło do tego, żeby się tego dowiedzieć?**

Ja co prawda nie szczycę się małą wiedzą na tematy polityczne, ale też się tego jakoś bardzo nie wstydzę. Mam do tego pełne prawo i dobry powód. Z kolei ignorancja w temacie koronawirusa, nikomu chwały nie przynosi. Jest to realny wróg, sytuacja, która dzieje się tu i teraz, dotyczy nas wszystkich. Trzeba go poznać, wiedzieć, czy mamy na to jakiś wpływ. Im więcej wiemy, tym bogatsi jesteśmy o to czy i jak możemy się chronić. Mam takiego znajomego, który informacje czerpie tylko poprzez czytanie nagłówków na wątpliwych portalach, typu Fakt. I on już sobie nie zadaje trudu, żeby cokolwiek więcej przeczytać. On podaje jakieś informacje, ale nie jest w stanie powiedzieć, skąd coś wie. Więc uważam, że coś na ten temat trzeba wiedzieć, a nie polegać tylko na tych nagłówkach.

**Jak Pani myśli, dało się zapobiec światowej epidemii?**

A jakie to ma znaczenie, co ja na ten temat myślę. Myślę, że się nie dało. Może wcześniej, jak nawet ten SARS, wirus z tej samej grupy, który dało się kilka lat temu ograniczyć do krajów azjatyckich. Ale teraz, kiedy zna się skalę zjawiska tranzytu, myślę, że nie dało się temu zapobiec. I tak myślę, że jest w miarę nieźle, jak na to, co się dzieje, że jakoś nad tym panujemy. Bez ograniczeń w skali świata, liczba ludności zmniejszyłaby się na pewno nie tylko o 4%. To jest zależne też od wydolności służby zdrowia i udaje się nam jakoś nad tym panować, dzięki tym ograniczeniom. Zarażalność jest wysoka, ale na szczęście śmiertelność już nie aż tak.

**Wie Pani mniej więcej, jakie są na ten moment statystyki w Polsce?**

Mniej więcej wiem, choć dziś nie zdążyłam obejrzeć Polsat News. To na wczoraj było coś w granicach 2 500, 2 771 chyba. I 0,1% śmiertelność. Ale z tego powodu, że my nie mamy w ogóle prawdziwych danych. Problem jest taki, i to wiem na podstawie wypowiedzi wirusologów, że u nas robi się za mało testów. Zgony, które kwalifikujemy jako umierających na koronawirusa są tylko wśród osób, u których wykryto koronawirusa za życia. To, że mamy różnice pomiędzy krajami w liczbach zachorowań, to błąd pomiaru, nie mamy wspólnych danych, nie liczymy stosunku przeprowadzanych testów, w stosunku do liczby mieszkańców.

**Myśli Pani, że jesteśmy gotowi na radzenie sobie z tą sytuacją?**

Są kraje w lepszej i gorszej sytuacji. Ale chyba nikt nie jest i nie będzie gotowy. Tak naprawdę prawdopodobieństwo wystąpienia takiej sytuacji w ogóle jest nieduże. Ale skoro już się w niej znaleźliśmy, spróbujmy się czegoś nauczyć. Można na przykład przyłożyć większą wagę do tego, żeby każdy potrafił obsłużyć się online. Może raz na jakiś czas powinniśmy wprowadzać elementy choćby nauczania zdalnego. Ale gdyby chcieć się do takich rzeczy w pełni przygotować, chyba stracilibyśmy z horyzontu bieżące sprawy, życie.

**Media – dlaczego nie TVP?**

Pamiętam socjalizm i nie chciałabym znów tego przeżyć. A w TVP znów jest to samo i to jest dla mnie żenujące, to jest groza. TVN jest dla mnie zbyt amerykański. Superstacja, którą kiedyś oglądałam, zmieniła się – poziom dziennikarstwa pozostawia dla mnie wiele do życzenia. W Polsacie jakoś da się to przeżyć. Choć jak widzę Morawieckiego, to też ciężko mi to oglądać. Jedynym wyjątkiem jest, jak występuje z ministrem zdrowia – on nie jest taki do bólu socjalistyczny. I wtedy stwierdzam, że może warto ich posłuchać, bo oni są tego wszystkiego najbliżej, zarządzają tym wszystkim. Mam duży problem, że nie mogę teraz oglądać sportu, bo wydarzenia się nie odbywają, pokazują same powtórki, a ile można je oglądać. Więc oglądam Animal Planet, Viasat Nature, Geo People, tego typu programy.

**A czego chce się Pani dowiedzieć, oglądając Polsat?**

Chcę być w miarę na bieżąco. Nawet tę godzinę dziennie. Jaki jest rozwój sytuacji pandemicznej – czy rośnie, czy maleje. To jest trochę propaganda, bo ja wiem, że jak oni mówią, że kontrolują sytuację, to i tak jej nie kontrolują. Ale to i tak działa trochę uspokajająco, że może u nas nie jest tak źle. A oprócz tego, słucham wiadomości dotyczących restrykcji, co wolno, czego nie. Bałam się ostatnio, że zamkną Warszawę. Byłam gotowa wsiąść w samochód i przyjechać po córkę wcześniej, bo gdyby ją tam zamknęli, to ona by chyba oświrowała w tym lokalu.

**A pozostałe media? Portale internetowe, gazety?**

Nie, jestem nie internetowa, wkurza mnie to. Mam wrażenie, że jak mam tam tak wiele możliwości znalezienia informacji, to to jest paradoksalnie strata czasu. Tam chcąc przeczytać artykuł na jeden temat, można przeczytać o tym samym na 17 różnych stronach, tylko z innym komentarzem. I ta pokusa jest dla mnie nieodparta, więc czytam je wszystkie, bo myślę, że może to wniesie coś nowego. A często nie wnosi i mam później poczucie straconego czasu, że w tym czasie mogłam pójść na spacer do lasu, z psem.

**A gazety?**

Moja sąsiadka – dawniejsza – w Warszawie, czyta Wyborczą. I ja te wszystkie gazety od niej dostaję, bo one są świetne na rozpałkę do pieca. Więc wkładając je do pieca, czytam nagłówki. Jak któryś mnie zainteresuje, zabieram taką gazetę do lokalu i przeglądam. Nie jestem w ten sposób z informacjami na bieżąco, mam jakieś dwa tygodnie opóźnienia. Ale właśnie w taki sposób, z pewnym opóźnieniem, ale jednak, dowiedziałam się na przykład, że mój znajomy, profesor Uniwersytetu Warszawskiego, został pobity. Więc tak wygląda mój kontakt z gazetami. We środę byłam w sklepie i jako dodatek do Gazety Wyborczej był Poradnik koronawirusowy. Więc kupiłam. Czytam jeszcze Dziennik Gazetę Prawną. Mam drugą znajomą, która w pracy prenumeruje tę gazetę. Robią tam jakieś prasówki, a resztę, która jest już im nie potrzebna, dostaję ja.

**A ten Poradnik? Jaki on jest?**

To jest 100 pytań o koronawirusa. Ja nie wiem, kto wymyślił te pytania – ludzie, czy redaktor. Część jest nieprzydatnych, ale część potrzebnych – jak się zakaża, itd.. To nie jest opracowanie o koronawirusie, ale takie pytania i odpowiedzi. Jest to krótkie, można się z tego może dowiedzieć więcej, niż z litego tekstu. Kilka stron, dobra forma. Choć i tak nikt nie czyta Wyborczej Jak idę rano do sklepu, leżą ze cztery gazety. W ten chwili tak naprawdę w ogóle nie kupuje się gazet. Ja nie kupuję gazet, a ludzie, którzy kiedyś tego nie robili, tym bardziej tego nie robią. Najbardziej poczytną gazetą jest chyba program telewizyjny, najczęściej widuję go w koszykach ludzi, w sklepach. Jeśli ktoś ktoś chciałby przekazać coś dużej grupie osób, powinien to chyba zamieścić właśnie tam. Ja go co prawda nie używam, ale większość ludzi – tak. Moja teściowa, która ma 94 lata, używa programu telewizyjnego. I podkreśla sobie, co będzie oglądać, a na czarno zakreśla, zamazuje, czego na pewno, absolutnie nie będzie oglądać.

**Co sprawia, że dane media/informacje są wiarygodne?**

To nie jest tak, że ja im ufam, bądź nie. Z założenia wiem, że nie wszystko, co jest podane, jest sprawdzone i prawdziwe. Tak, jak nie każda informacja jest dla opinii publicznej, żeby choćby nie wzbudzać paniki. Media „rządowe” są dla mnie absolutnie niewiarygodne, ponieważ dla mnie to jest i była propaganda sukcesu – koronawirus nie jest tu wyjątkiem. Pozostałe kanały są w miarę niezależne. W miarę, bo są zależne od osób, które kierują nimi finansowo. Ale w zakresie podawanych przez nie informacji „społecznych”, są w miarę bezpieczne, jeśli chodzi o indoktrynację. Politycznie ja ich w ogóle nie słucham. A wybór pomiędzy tym, dlaczego Polsat, a nie TVN, to już są kwestie tylko estetyczne. To nie jest tak, że TVN jest mniej wiarygodny. Chodzi bardziej o sposób prezentowania informacji. Była jakiś czas temu taka krótka migawka na Polsacie, w jednym z programów informacyjnych, o ZOO gdzieś pod Łodzią, które boryka się obecnie z dużymi problemami finansowymi. W tym materiale ludzie nie wypowiadali się jakoś bardzo emocjonalnie, jednak zachęcali do zakupu biletu, który będzie ważny do końca 2021 r., a wesprze ZOO finansowo, w tym trudnym czasie. Więc chciałam wejść na stronę tego ZOO, żeby kupić taki bilet. Jednak pierwsze, na co natrafiłam, to był artykuł TVN dotyczący tego, że na skutek transmisji materiału na ich stacji, wszyscy zaczęli się logować i kupować bilety. I to było przedstawione tak, że to wszystko zasługa TVN. Takie działania nie wzbudzają we mnie zaufania, bo przecież wiadomo, że to nie tylko zasługa TVN. Choć ogólnie cieszę się, że – jak tam również przeczytałam – w ciągu dwóch dni, udało się zebrać odpowiednią kwotę, żeby pomóc.

Więc wybrałam akurat Polsat. Co też nie oznacza, że oni nie robią podobnych rzeczy, jak TVN – pewnie robią i też nie mam co do tego szczególnych złudzeń.
