# Supplementary material for: The challenges arising from the COVID-19 pandemic and the way people deal with them. A qualitative longitudinal study
Source: PLoS One. 2021 Oct 11;16(10):e0258133. doi: 10.1371/journal.pone.0258133 (PMC8504766; doi:10.1371/journal.pone.0258133)
Supplement: Attachment — Submitted filename: Response to Reviewers.docx [file pone.0258133.s004.docx]

Dear Editor and Reviewers,

Thank you for your valuable time and useful contribution to our article titled “The challenges arising from the COVID-19 pandemic and the way people deal with them. A qualitative longitudinal study”. We appreciate the inputs you have given, and we are sure that it will definitely help improve our manuscript.

We have separated our responses to the reviewers’ comments according to several categories in order to achieve an integrated approach in our responses. Because some of the reviewers' comments address the same issues, we present them together. However, additionally in brackets next to each comment there is information as to which reviewer is its author. Reviewers' comments are highlighted in light yellow, and our responses are in light green. Information about changes made (line and page numbers) refers to the "Manuscript" file, without tracking changes.

**General comments:**

- (Editor) Please ensure that your manuscript meets PLOS ONE's style requirements, including those for file naming.

The revised manuscript has been prepared according to PLOS ONE’s style requirements.

- (Editor) We note that you have indicated that data from this study are available upon request. PLOS only allows data to be available upon request if there are legal or ethical restrictions on sharing data publicly.
- (Reviewer #2) Generally, interview data can be anonymized by appropriate redaction of names and other sensitive material. If this is not possible, the code sheets showing the extraction of themes from the interviews would be the next best thing.

We have decided to make the full dataset (transcriptions) available. We would like to point out that the interviews were conducted in Polish and transcriptions were also done in Polish. Making the data available in English would require translating this vast amount of data from Polish to English, which would require a huge financial cost that we are unable to afford.

- (R #2) There are some problems of English grammar, although these are not any worse than many late drafts by authors whose first language is English.
  E.g.: lines 101, 104-107, 219, 634.

We corrected the errors listed and checked the entire document for linguistic correctness. The entire manuscript was also proofread by a professional translator. We would also like to point out that the quotes were translated by the professional translator. In the original (Polish) quotations, we kept the natural statements of the respondents, which often contain language errors. These errors were also preserved in the translation. We added an appropriate footnote in the text (page 18), which informs the reader that the errors in the quotations are intentional and result from the desire to provide literal statements of the respondents.

- (R #2, R#1) Table 1. Use of undefined acronym (DINKS).

The acronym DINKS stands for Double Income, No Kids, a couple living together, both working and having an income, but not having kids. We realized that the use of this term is limited, so we decided to eliminate it altogether and changed the abbreviation DINKS to “couple, no children” (Line 292).

- (R #3) This is a long article and many parts can be condensed and tightened up. For instance, the description about interviews on page 14 is redundant. Some other themes also seem redundant in some ways. Ways of dealing and change of lifestyle may be considered to be combined.

Thank you for this comment, it is completely valid, and its implementation will definitely affect the overall quality of the article. Accordingly, the manuscript has been shortened - part of the method description and numerous citations from the description of the results have been removed. However, we decided not to link coping methods with a lifestyle change. While it may seem that ways of coping and lifestyle change are the same themes, they have different backgrounds. Lifestyle change can result from a variety of conditions (internal and external, and may not be volitional), while ways of coping are actions that a person takes intentionally, in response to events that occur. The response to a problem is crucial to us, so combining these two threads into one may cause us to lose important data.

**Introduction**

- (R #1) Please add a reference for making those statements (L. 34, 51,54, 131, 136)
- (R #1) L 43 This study is not the first with a qualitative methodology, please revise the whole manuscript.
- (R #1) L 54 which reactions are going to be reported? reactions in people, policies, government ?
- (R #1) L 99 include the name of the main author of the 3C model
- (R #1) L 186 please provide the first author of this model
- (R #1) L 199-201 This sentence is no needed: "However, it needs to be noted that Polizzi and colleagues (12) did not validate the model in an empirical study but based their recommendations on theoretical considerations and conclusions from previous research."
- (R #1) L 224-226 The aims or research questions should be in the same line as the results structure:
  A) COVID 19 Challenges and how to cope with them over time.
  B) How the psychological principles for natural disasters (3c's) could be a reference model to analyse the COVID 19 reactions?
- (R #1) The authors do not mention here in these research questions the 3C's models which should be taken into consideration.
- (R #1) L 219 an "s" is missing previous to reactions.

Changes have been made to the manuscript according to the comments above (we added, or corrected references, corrected language errors, clarified unclear wording).

- (R #1) L 209-211- It seems that some other qualitative studies have been done on COVID 19. Then please review the writing of the whole manuscript as it may not be the first qualitative study. Although, authors could point out their main contribution comparing their study with the previous study: it is a longitudinal study.
- (R #3) The authors claimed that none of the studies have employed qualitative methods, but is there any literature of quantitative study on similar topics, as this may serve as a baseline for this study.

We updated the literature review and clarified that we were referring to the lack of longitudinal qualitative research on the topic of pandemic COVID-19 - not qualitative research in general. Thank you for this comment, as it is a key value of our article that was not visible enough due to unclear wording.

- (R #1) L 66-88 Authors describe the 6 stages and the research questions could fit better at the procedure subsection in the methods section. I would suggest describing the evolution of the COVID19 different stages in Poland should be detailed as the reader could understand better the study.

Thank you deeply for this comment. We agree that the context of different stages of changes in Poland is extremely important in understanding the problems people are facing and we have prepared such a table in the first version of the text. We decided not to include it in the article because it was already lengthy, and the table was extensive (the changes in Poland took place almost daily). According to the comment, we decided to include the full table with the description of changes in the appendix to the article (supporting information – S1 Table).

- (R #1) Still in this part, I would suggest authors linking the natural disaster situation and this health pandemic situation.

We have made the changes in accordance with the comment above (Lines 172-173).

**Method:**

- (E) In order to improve reporting, in your methods section, please provide additional information about the participant recruitment method, including a description of any inclusion/exclusion criteria that were applied to participant recruitment and a description of how participants were recruited.
- (R #2) First, the authors do not describe how the subjects were recruited, aside from saying it was a purposive sample. This is inadequate. Were there prior relationships between the subjects and the investigators?

Information about the recruitment process was added to the method section (Lines 277-285). In addition to demographic criteria intended to ensure that the sample subjects were as diverse as possible, an additional criterion was to have a permanent Internet connection and a computer capable of online video interviewing. Study participants were recruited using the snowball method. They were distant acquaintances of acquaintances of individuals involved in the study. None of the moderators knew their interviewees personally, and no relationship was established with the subjects prior to the interview. The first contact with the respondents was a phone call to schedule the first interview, followed by an email consent to participate in the study, with a detailed description of the study and the data processing and usage.

- (R #1) L 228 Please omit the word research and insert Methods
- (R #1) The divide the content into these subsections:
  Design
  Participants
  Tools
  Procedure
  Data analysis

The structure of the “Methods” section was changed according to the above comments.

- (R #1) L 245 google meets is an online video platform not for chat (writing). The authors should clarify this point.
- (R #1) L 246 it is strange to say "use a video camera" when the computers have it already and in google meet you only have to "open" it.

We’ve changed the wording to clarify the information regarding the online platform (L 269).

- (R #1) L 276 up to here there were in-depth- interviews…now you write here semi-structured interviews. Please define which was taken into consideration.

All interviews we conducted were carried out using the individual in-depth interview method. The information about structuring refers to the very course of the interviews (so they did not have a loose structure, but were partially structured - following a pre-prepared scenario, but allowing for some changes in order to follow the respondent). We have made changes in the text to clarify this point (L 302).

- (R #1) L 580 please check the spelling as "wanin" is not a word.

The word waning, which we used in the text, comes from the term wane, which means to become weaker (please find the meaning here - <https://dictionary.cambridge.org/dictionary/english/waning>). In our opinion, this word conveys the sense we wanted to express, so we decided to keep it in the text.

- (R #1) L 821 Should state Table 3 now is Table 2 written.

We corrected the table captions - there are three tables in the manuscript that were incorrectly numbered and had incorrect references in the text.

- (R #1) L 300-305- after the procedure the data analysis subsection should be mentioned.
- (R #1) How did you find an agreement about the units of contents between the 5 researchers?
- (R #1) Did you divide them into dimensions after or previously? To facilitate the analysis. Which criteria were taken?
- (R #1) Did you find supra or subordinate factors in the analysis?

We have expanded the data analysis section and included the above information. We especially thank you for this comment, as the description of the analysis process will further enrich the presentation of the research process and the data obtained.

- (R #1) How many pages were analyzed from the transcription?

We’ve provided the information about the transcription in the text - the final page count of the transcript is approximately 1800 pages of text (L 327-334). The results presented below are only a portion of the total data collected during the interviews. While there are about 250 pages of the transcription directly related to the topic of the article, due to the fact that the interview was partly free-form, some themes merge with others and it is not possible to determine the exact number of pages devoted exclusively to analysis related to the topic of the article.

- (R #3) All the interviews were transcribed, but there is no description about the ways for ensuring data accuracy and translation. (I suppose the interview was conducted in Polish?)

We expanded the information about the transcription process in the data analysis section (L 325).

- (R #2) The Interviews were conducted online by 5 female interviewers with experience in Psychology. Does the gender of the interviewers make any difference? The educational background and occupations of the interviewees were included in the demographics, but how this factor may affect the results is not explained.

Thank you for this comment, which highlights the important issue of moderator influence on the results of the study. We have addressed this issue in the discussion section (L 833-839).

- (R #1) The question is why this model could be adequate for analyzing the reactions during health pandemic situation?

Thank you for pointing out that the connection between the model mentioned above and the results of our study is not clear. We have reworded the text to make it clear why this model is adequate to explain people' reactions during the COVID-19 pandemic. COVID-19 is undoubtedly an unprecedented, unusual situation. However, scientists have attempted to create theoretical models that systematize the reactions to difficult and unusual situations. One of such models is the model of 3Cs created by John Reich. This model accounts for the general rules of resilience in situations of stress caused by crises, such as natural disasters. Polizzi and colleagues reviewed this model from the perspective of the current COVID-19 pandemic. The authors claim that natural disasters and COVID-19 pandemic have much in common and therefore, the principles of resilience in natural disaster situations can also be used in the situation of the current pandemic. For this reason we believe that the model of 3Cs may be particularly adequate for analyzing the situation of COVID-19 pandemic.

- (R #1) L 254 Please take into account this matter in the limitations sections as those with less than higher education may be in a worse financial situation so this study may be biased in that way.

Thank you for this comment. The relationship between education and income in Poland is not as strong as in Western countries, nevertheless the demographic specificity of the sample could have had an impact on a certain bias in the results. A comment on this topic was introduced in the limitation part of discussion (L 826-832).

- (R #1) Table 1- Please make a support sentence of why dividing the sample into more or less than 40 years old. One participant with 40 is young or old?
- (R #3) The authors interviewed 20 participants, six times each, and totaling 120 interviews. The interviewees range from 25-55. Why were participants divided into two age cohorts on the basis of age 40? There were 6 out of 20 interviewers who are over 40. The dividing of the age groups needs clearer reasoning. Particularly, most of citations used as evidence are based on groups under 40 years old.

The division into the groups below and above 40 years of age was not a criterion for selecting respondents for the study and designing the study scheme, but it was introduced at the stage of constructing the table with the description of the respondents. We thought that this method would make it easier to describe the quotes. As we can see from the comments of the reviewers, this is unnecessary data reduction, and it introduces more misunderstandings and confusions. Therefore, in the current version of the article, we have changed the description of respondents with their exact age, and this is also how we describe the citations.

- (R #1) It is an inductive, deductive or mixed approach.

Information about the approach was added to the manuscript (L 340-342).

- (R #1) How many units of content did you find?
- (R #1) Table 2. Could this table be organized with the units of content found in each challenge and ways to cope?

Our analysis proceeded in a traditional manner (without the use of software to support qualitative analysis). Therefore, we used a broader, more contextually based unit of analysis rather than a narrowly focused level of analysis (e.g., sentences).

As far as the table is concerned, it is an adequate idea and we also realize that it is often presented this way in describing the results of qualitative research. However, we decided to leave the quotes in the text rather than in the table - for the simple reason of the length of the article (the entire table with the respondents' statements included is very large and unreadable).

- (R #2) Second, more needs to be said about the development of the data themes. The five challenges surely do not capture all the richness of 120 interviews. It would be helpful to hear about variability within the major themes as well as about what minor themes were identified.

Thank you for this observation. While we absolutely agree that there were many themes that emerged throughout the study (we have annotated more accurately that the interviews covered a broad area of issues regarding functioning during the COVID pandemic), we chose to include only those themes in the article that dealt directly with difficulties and ways of coping. In our article, we focused only on what was its main topic, and we avoided the issues that were not directly related. We are constantly convinced that the introduction of new threads will, firstly, extend a long article, and secondly, "blur" the main results of the study.

**RESULTS**

- (R #1) L 693 This model has been explained before in the introduction please do not repeat the information in the results section.

Repeated information about the model was removed from the results section.

- (E) Please include a copy of Table 3 which you refer to in your text on page 32.

We corrected the table captions - there are three tables in the manuscript that were incorrectly numbered and had incorrect references in the text.

- (R #3) The authors used quotations of the interviewees to support the thematic points. Some point has 2 or 3 citations, some used only one. As this is already a fairly long article, one more representative citation for each point might be enough? Or, the responses can be integrated into the description of the findings using quotation.
- (R #3) Most of citations used as evidence are under aged 40 groups. This requires some further discussion and explanation.
- (R #3) The authors may consider restructure the results section and tighten up some findings.

Given the length of the article, we decided to reduce the number of citations - at this point, one citation is presented per topic. Thank you very much for this comment, which has helped us to tighten up the manuscript.

Most of the quotes come from people under 40, which corresponds to the structure of our research sample, where people under 40 accounted for 70% of the respondents. In this version of the article, we decided to show the exact age of the respondents. Most of the citations still come from people under 40 years of age due to the sample structure. Nevertheless, in the new version of the article, some quotations have been replaced to reflect the greater demographic diversity of the respondents and their experiences.

- (R #2) There are appropriate qualitative statistics for testing most, if not all, of the conclusions the authors wish to highlight.

We understand the need to present some statistics in our study. We recognize that such methods exist, and we agree that there are valuable uses of numbers even in qualitative research, and that it can enhance the validity of the study. However, our approach to methodology is purely qualitative. We do not see the need or necessity to present the results of statistical analyses on such a small sample. Our thinking about the study and results from the beginning of the process is qualitative, looking at the broad context of the data rather than its linear relationship. Therefore, we made a conscious decision not to use statistics in presenting our results.

- (R #2) I recommend a table or tables showing the emergence of the major themes (concerns) across time and across subject characteristics. This would provide a framework for presenting and testing statements such as the one identified above.

While we agree that presenting changes in reactions of individual respondents would be an interesting concept, in our study we rather focused on looking for some patterns emerging in the whole sample. In our opinion, presenting difficulties and their changes in individuals will not contribute significantly to the results we describe. We present a general pattern and show some changes specific to particular groups of respondents (such as those with children) in the results section. Presenting individual changes would change the direction of the article entirely.

**DISCUSSION**

- (R #1) L 824 Please separate Discussion and conclusions

Discussion and conclusions sections were separated.

- (R #2) Third, the conclusions go beyond the data presented in the results. Statements such as (line 719) “The analysis of the results showed that the first solution (full submission to orders) occurred more often in people with higher COVID-19 anxiety.” are unsupported by data presented in the results. Worse, we are not even told how anxiety was measured. The conclusion is peppered with unsupported statements of this sort, which are testable, but no results have been shown.

Thank you for these observations. We absolutely agree that some of the themes raised in the results section and discussion do not reflect the data presented. We have decided not to briefly present these types of results (for example, those regarding changes in emotions, which are extremely interesting and relevant, but complex) and to focus on the issue of difficulties and how people deal with them, and to describe emotions in a separate article. However, we decided to keep the parts of the respondents' statements where they explicitly talk about being afraid of something (for example, coronavirus infection). However, we rephrased some terms to avoid talking about anxiety or other specific emotions.

- (R #1) Within the discussion authors should:
  First paragraph: relate the main results with the aims-research questions of the study.
  Second: detail and compare each result with previous results
  Third: add the limitations of the study
  Fourth: add some practical implications of the study
  Fith: the final paragraph should be the conclusions.
  Right now the discussion is too long in terms of length and the authors within 3 pages only have 8 references. It could be shortened and more focused on the contribution of the paper.

Thank you for this attention. The discussion was rewritten with the sections suggested. We have included additional subsections to make this section clearer to read. Thank you for this comment, as we completely agree that the summary and discussion should be more structured.

- (R #3) Do different age, occupation, lifestyle, even occupation have any impact on how the interviewees experience different restriction.

Our approach to qualitative research conducted on such a demographically diverse sample (e.g. age range 21-60, lack of repetitive jobs) does not allow us to draw conclusions about differences between subgroups. On the other hand, we observed differences between people whose professional situation changed or not during the pandemic (e.g. they switched to remote work or job loss) or differences depending on the approach to the pandemic (greater vs. less sense of threat). Such differences observed in the results are discussed in the article.

- (R #3) The limitation of this study needs to be described, such as the representativeness of the respondents

A comment on the limitations of the research sample was added in the discussion section. Furthermore, the summary and discussion have been rewritten and a separate subsection on limitations has been added.

- (R #3) Compared with the long presentation of results, the discussion is quite brief. Some parts require further explanation. For instance, p.40 “the study allowed deep understanding to be gained of the difficulties….” is not clear.

We agree that in the previous version of the manuscript the discussion was not relevant to the content of the article. Therefore, the summary and discussion have been rewritten, and we have included some additional issues (limitations, practical implications).

- (R #3) The paper ends abruptly. It requires a conclusion section to summarize the findings and its contribution/ or significance.

Conclusion section and comments on the significance of the findings were added to the discussion.
