# Supplementary material for: The challenges arising from the COVID-19 pandemic and the way people deal with them. A qualitative longitudinal study
Source: PLoS One. 2021 Oct 11;16(10):e0258133. doi: 10.1371/journal.pone.0258133 (PMC8504766; doi:10.1371/journal.pone.0258133)
Supplement: Attachment — Submitted filename: Response to Reviewers.docx [file pone.0258133.s005.docx]

Dear Editor and Reviewers,

Thank you once again for your valuable time and useful contribution to our article titled “The challenges arising from the COVID-19 pandemic and the way people deal with them. A qualitative longitudinal study”. The two most important changes we have made are the removal of the part of the description of the results presenting them in the light of the 3C theory and the significant shortening of the whole article. Please find our answers to the reviewers’ comments below.

Reviewer #1:

- Still I would suggest authors to shorten the length of the article. Almost fifty pages is too much for a research article. I would suggest to review each part and try to shorten some pages in each section. For example introduction is 5 pages and literature review another 5 pages. Research articles are normally around 3 pages for the whole introduction.

*This is the main change we have made in the revised article. We have significantly shortened the text, as suggested by both reviewers.*

Reviewer #2:

- Table 1 would be more informative if it showed the distribution of subjects over demographic characteristics, rather than listing each subject.

*In our view, presenting the demographic characteristics of each respondent adds value to the text and shows the diversity of the respondents. However, in line with previous suggestions regarding the length of the text, we have decided to move the table to an appendix.*

- The reporting of the results I find less satisfactory. The methodology describes extracting themes from the interview data. The results report on challenges. Are the five challenges the only themes that were extracted? Are the challenges composed of clusters of themes? Do the challenges capture all the themes identified?

*As presented in the text, the manuscript contains only part of the results, as it would be impossible to present all the findings from such a large number of interviews. For example, we do not describe specific changes in emotions here, and some residual information on this topic was removed from the text after the previous round of reviews because it would have been misleading throughout the text.*

- By focusing on challenges, are the authors not talking about successful coping behaviors?
- For example, the subjects’ reactions to the limitations on travel are uniformly portrayed as negative (limitations on freedom, etc.) did none of the subjects perceive this as an appropriate public health measure that gave the subjects some control over the situation?
- My greatest concern here is that the process of focusing on concerns has caused the authors to lose sight of successful coping behaviors.

*We understand where these doubts come from, but we cannot write about the positive aspects of the changes described, as they did not appear in our study. The participants perceived these limitations in an unambiguously negative way - perhaps a study conducted over a different period or one in which the participants retrospectively evaluate certain changes would have provided different results. However, in this paper, we describe the experience at the time of the study.*

- The interpretation of the results in the context of Reich’s framework is totally baffling in that it does not appear to refer to the results at all. There is one mention of one of the challenges in this section. Table 3 refers to some things that might be themes, but without a proper reporting of the themes, we don’t really know and are unable to judge its completeness. Without a better tie to the data, this section should be deleted.

*After careful consideration, we decided to comply with the above comment and remove the section presenting the results in the light of Reich's theory.*
